
JOURNAL INFORMATION
==============================
NLM Title Abbreviation: Innov Aging
Journal ID: Innov Aging
NLM Journal ID: 101703706
Journal ID: innovateage

Innovation in Aging

EISSN: 2399-5300
Publisher: Oxford University Press

ARTICLE INFORMATION
==============================
PMCID: PMC9770770
PMID: 36570860
DOI: 10.1093/geroni/igac059
Article ID: igac059
Article version: 1
Subjects: Full GSA 2022 Abstract Book Pdf, AcademicSubjects/SOC02600

Full GSA 2022 Abstract Book PDF

Publication date: 2022 Nov
Electronic publication date: 2022 Dec 20
Volume: 6
Issue: Suppl 1
Issue title: Program Abstracts from The GSA 2022 Annual Scientific Meeting, “Embracing Our Diversity. Enriching Our Discovery. Reimagining Aging.”
First page: NP
Last page: NP
Copyright: © The Author(s) 2022. Published by Oxford University Press on behalf of The Gerontological Society of America.
Copyright year: 2022
License: This is an Open Access article distributed under the terms of the Creative Commons Attribution License (https://creativecommons.org/licenses/by/4.0/), which permits unrestricted reuse, distribution, and reproduction in any medium, provided the original work is properly cited.
License URL: https://creativecommons.org/licenses/by/4.0/

Page count: 921

==============================

ISSN 2399-5300 | ONLINE
Vol. 6 | No. S1

### INNOVATION IN AGING®

An Open Access Journal of
The Gerontological Society of America

**Program Abstracts from The GSA 2022 Annual Scientific Meeting, "Embracing Our Diversity. Enriching Our Discovery. Reimagining Aging."**

OXFORD
UNIVERSITY PRESS

THE GERONTOLOGICAL SOCIETY OF AMERICA®

### INNOVATION IN AGING
### An Open Access Journal of The Gerontological Society of America

**Steven M. Albert, PhD, MS, FGSA**
**Editor-in-Chief**
University of Pittsburgh, USA
E-mail: smalbert@pitt.edu

**Karen J. Jung, MSW**
**Managing Editor**
The Gerontological Society of America
1220 L Street NW, Suite 901
Washington, DC 20005, USA
Email: ia@geron.org

**Christine E. Bishop, PhD, FGSA,**
**Associate Editor, Economics of Aging**
Brandeis University, USA

**Howard B. Degenholtz, PhD, FGSA,**
**Associate Editor, Long-Term Services and Supports**
University of Pittsburgh, USA

**Nancy W. Glynn, PhD, FGSA,**
**Associate Editor, Epidemiology of Aging**
University of Pittsburgh, USA

**Min-Ah Lee, PhD,**
**Associate Editor, Relationships & Well-being**
Chung-Ang University, South Korea

**Karen D. Lincoln, PhD, MSW, MA, FGSA, Associate Editor, Mental Health and Social Determinants of Health**
University of Southern California, USA

**Lena K. Makaroun, MD, MS, Associate Editor, Geriatrics**
**VA Pittsburgh Healthcare System, USA**
University of Pittsburgh, USA

**Richard Pak, PhD,**
**Associate Editor, Technology**
Clemson University, USA

**Alison Phinney, PhD, RN,**
**Associate Editor, Qualitative Research**
University of British Columbia, Canada

**J. Tina Savla, PhD, FGSA,**
**Associate Editor, Methodology**
Virginia Tech, USA

**Jennifer Tehan Stanley, PhD, FGSA,**
**Associate Editor, Psychological Sciences**
University of Akron, USA

**Roland J. Thorpe, Jr., PhD, FGSA,**
**Associate Editor, Minority Health/Health Disparities**
Johns Hopkins Bloomberg School of Public Health, USA

**Elaine C. Wiersma, PhD,**
**Deputy Associate Editor, Qualitative Research**
Lakehead University, Canada

### PROGRAM, PUBLICATIONS, AND PRODUCTS COMMITTEE

**Elham Mahmoudi, PhD, Chair**
University of Michigan, USA

**Rozalyn Anderson, PhD, FGSA**
University of Wisconsin–Madison, USA

**Deb Bakerjian PhD, APRN, FAAN, FAANP, FGSA**
University of California, Davis, USA

**Kalisha Bonds, PhD, MSN**
Emory University, USA

**Kirsten Corazzini, PhD, FGSA**
University of Maryland School of Nursing, USA

**Kara B. Dassel, PhD, FAGHE, FGSA**
University of Utah College of Nursing, USA

**Debra Dobbs, PhD, FGSA**
University of South Florida, USA

**Patricia Heyn, PhD, FGSA, FACRM**
Marymount University, USA

**Shannon Jarrott, PhD, FGSA**
The Ohio State University, USA

**Jung Kwak, PhD, MSW, FGSA**
The University of Texas at Austin, USA

**Karen D. Lincoln, PhD, MSW, MA, FGSA**
University of Southern California, USA

**Joann Montepare, PhD, FAGHE, FGSA**
Lasell University, USA

**Brianna E. Morgan, MSN, CRNP, ACHPN**
University of Pennsylvania, USA

**Tina M. K. Newsham, PhD**
University of North Carolina, Wilmington, USA

**Blanka Rogina, PhD**
University of Connecticut, USA

**Kaipeng Wang, PhD, MSW, MAS**
University of Denver, USA

**Fellow of The Gerontological Society of America (FGSA)**

### OUP PRODUCTION DEPARTMENT

**Rebecca Dowdy**, *Journal Manager - Production*

INNOVATION IN AGING
An Open Access Journal of The Gerontological Society of America

### EDITORIAL BOARD

**Kathleen Abrahamson, RN, PhD, FGSA**
Purdue University, USA

**Tamara A. Baker, PhD, FGSA**
University of North Carolina at Chapel Hill, USA

**Scott R. Beach, PhD, FGSA**
University of Pittsburgh, USA

**Julie Blaskewicz Boron, PhD, FGSA**
University of Nebraska, Omaha, USA

**Sara J. Czaja, PhD, FGSA**
Weill Cornell Medicine, USA

**Adam Davey, PhD, FGSA**
University of Delaware, Newark, USA

**Nancy R. Gee, PhD**
Virginia Commonwealth University, USA

**Megan Gilligan, PhD, FGSA**
Iowa State University, USA

**Ellen L. Idler, PhD, FGSA**
Emory University, USA

**Mary Ann Johnson, PhD**
University of Nebraska Lincoln, USA

**Giyeon Kim, PhD, FGSA**
Chung-Ang University, South Korea

**Amanda N. Leggett, PhD, FGSA**
Wayne State University, USA

**Hui Liu, PhD, FGSA**
Michigan State University, USA

**Mariana López-Ortega, PhD, MPP**
National Institute of Geriatrics, National Institutes of Health, Mexico

**Meghan K. Mattos, PhD, RN**
University of Virginia, USA

**Anne Collins McLaughlin, PhD**
North Carolina State University, USA

**David A. Nace, MD, MPH**
University of Pittsburgh, USA

**Marcia Ory, PhD, FGSA**
Texas A&M, USA

**Karl Pillemer, PhD, FGSA**
Cornell University

**Karen A. Roberto, PhD, FGSA**
Virginia Tech, USA

**Laura P. Sands, PhD, FGSA**
Virginia Tech, USA

**J. Jill Suitor, PhD, FGSA**
Purdue University, USA

**Robert Joseph Taylor, MSW, PhD**
University of Michigan, USA

**Jasmine L. Travers, PhD, RN**
New York University Rory Meyers College of Nursing, USA

**Noah J. Webster, PhD, FGSA**
University of Michigan, USA

**Richard Winett, PhD**
Virginia Tech, USA

**Jeremy B. Yorgason, PhD, FGSA**
Brigham Young University, USA

**Anna Zajacova, PhD, FGSA**
Western University, Canada

**Fellow of The Gerontological Society of America (FGSA)**

### Abstracts

Program Abstracts from The GSA 2022 Annual Scientific Meeting,
"Embracing Our Diversity. Enriching Our Discovery. Reimagining Aging."

Abstracts are arranged numerically by session and in the order of presentation within each session.
Abstracts are published as received.

### SESSION 1000 (PAPER)

### ALZHEIMER'S DISEASE AND RELATED DEMENTIAS (HS)

### C. DIFFICILE INFECTION AS A MAKER FOR DYSBIOSIS IS LONGITUDINALLY ASSOCIATED WITH COGNITIVE DECLINE AND DEMENTIA

Nicholas Resciniti, *University of Southern California, Los Angeles, California, United States*

Disruption of the gut microbiome (dysbiosis) is associated with cognitive decline and dementia; however, there is a lack of findings demonstrating how dysbiosis is longitudinally associated with cognitive outcomes. This study aimed to quantitatively assess whether there is an association between Clostridium difficile infection (CDI) as a marker for dysbiosis and cognitive decline and dementia. We used 6,147 older adults from the Health and Retirement Study and the Centers for Medicare and Medicaid Services from 1999-2012. Outcome variables included cognitive scores (0-27) and dementia (8 or less). The exposure was time-varying ICD-9 CDI. Generalized estimating equation were used for the main analysis. Having at least one CDI infection was associated with -2.85 (B = -2.85; 95% CI: -4.43, -0.26) at 2000 (baseline) and at 2002 (B = -2.85; 95% CI: -4.43, -0.26), 2004 (B = -2.85; 95% CI: -4.43, -0.26), 2006 (B = -2.85; 95% CI: -4.43, -0.26), and 2008 (B = -2.85; 95% CI: -4.43, -0.26), after adjusting. Those having at least one CDI had 6.62 greater odds (OR = 6.62; 95% CI: 1.68, 26.05) of dementia at 2000 and was associated with time points 2002 (OR = 4.12; 95% CI: 1.48, 11.52) and 2004 (OR = 2.57; 95% CI: 1.14, 5.81). This study indicated that those with a CDI had a greater cognitive decline and a greater probability dementia. These findings provide evidence that dysbiosis has a larger impact on cognitive outcomes closest to the initial disruption and is less impactful further on.

### DECIPHERING RACIAL AND ETHNIC DISPARITIES IN DEMENTIA AND COGNITIVE FUNCTION: A POLYSOCIAL SCORE APPROACH

Chenkai Wu[1], Yongjing Ping[2], Xi Chen[3], Michelle Odden[4], and Matthew Prina[5], *1. Duke Kunshan University, Kunshan, Jiangsu, China (People's Republic), 2. Duke-NUS, Singapore, Singapore, 3. Yale University, New Haven, Connecticut, United States, 4. Stanford University, Stanford, California, United States, 5. King's College London, London, England, United Kingdom*

The burden of dementia has been increasing rapidly in the US and huge racial/ethnic disparities exist. We examined whether social environment, measured in a comprehensive way (polysocial score approach), could modify the racial and ethnic differences in dementia. Data are from the Health and Retirement Study; 12,836 adults aged at least 65 years were included in the analysis. We assessed cognitive function by a modified version of the telephone interview for cognitive status. We included 24 social factors from five categories (economic stability, neighborhood environment, education, community/social context, and healthcare system) and used forward stepwise regression to screen for important ones. Polysocial score was created using 11 social factors and was classified as low (< 25), intermediate (25-31), and high (32+). We used the multivariable Poisson regression to estimate the incidence rate of dementia by three polysocial score categories and evaluate the interaction between race/ethnicity (non-Hispanic white, non-Hispanic Black, and Hispanic) and the polysocial score. Higher polysocial score is associated with a lower dementia rate in the overall sample and each racial/ethnic group. We found an additive interaction between race/ethnicity and polysocial score categories. In the low polysocial score group, Whites and Hispanics had substantially higher dementia rates than non-Hispanic Blacks (difference was 20.3 and 15.8 per 1,000 persons-years, respectively). These differences significantly attenuated and were no longer significant in the high polysocial score group (4.9 and 5.5 per 1,000 persons-years, respectively). The polysocial score approach offers a new opportunity to explain the racial/ethnic disparities in cognitive function among older adults.

### FUNGAL INFECTIONS, USE OF ANTIFUNGAL AGENTS, AND THE RISK OF ALZHEIMER'S DISEASE

Arseniy Yashkin, Igor Akushevich, Anatoliy Yashin, Galina Gorbunova, and Svetlana Ukraintseva, *Duke University, Durham, North Carolina, United States*

Alzheimer's Disease (AD) is a complex neurodegenerative disorder leading to progressive cognitive decline and death. Accumulating evidence suggests that common adult infections, such as recurrent fungal infections, may play a major role in AD development. In this study we used administrative claims data, 1991-2017, from a 5% sample of U.S. Medicare beneficiaries age 65+ to study the potential relationship between fungal infections, use of antifungal drugs and AD onset. In unweighted analysis using the Cox model, we found that after accounting for demographic and health-related differences, the presence of fungal infections,

© The Author(s) 2022. Published by Oxford University Press on behalf of The Gerontological Society of America.

independent of treatment increased the risk of AD [Hazard Ratio(HR):1.98; 95% Confidence Interval(CI):1.89-1.92]. The strength of the effect did not change when the population was restricted to Medicare beneficiaries with Medicare Part D (prescription drug) coverage in 2006+ (HR:1.99; CI:1.97-2.01). We then split the latter group into yearly split-episode data and calculated the medication possession ratios (MPR) of three major groups of antifungal agents: imidazole, triazole and polyenes and re-estimated our models. Although the effect associated with fungal infection did not change, higher MPRs of imidazole (HR:0.61; CI:0.48-0.76) and especially triazole (HR:0.37; CI:0.24-0.58) were found to be protective. In contrast, higher MPRs of polyenes were associated with increased risk (HR:1.32; CI:1.08-1.63). In summary, fungal infections were found to be strongly associated with AD risk with the effect being strongly modified by use of common antifungal treatments. More research is required to better define the biological mechanisms engendering these associations.

### GENETIC ARCHITECTURE OF ALZHEIMER'S DISEASE RISKS

Abanish Singh[1], Konstantin Arbeev[2], Anatoliy Yashin[2], and Igor Akushevich[2], *1. Duke University School of Medicine, Durham, North Carolina, United States, 2. Duke University, Durham, North Carolina, United States*

More than 6 million people in the US live with Alzheimer's disease (AD) and related-dementia. There is a racial disparity in the prevalence of disease. However, its full genetic architecture and complex biological mechanisms are not yet understood. This study aimed to identify AD's full genetic architecture. Hypothesizing that a large part of AD could be due to the complex interplay of multiple genes that may influence it directly or through comorbidity-risks, we performed a series of race-stratified GWASs on AD outcome and an array of correlated comorbidity-risks -- ADRD, depression, ischemic-stroke, cerebral-hypoperfusion, heart-failure, and cerebral-thromboembolism -- derived from Medicare records of HRS dataset's White and Black samples. We prioritized AD GWAS signals based on their co-associations with comorbidity-risks. In White samples, we observed well-known genome-wide significant AD associations with SNPs in APOE, TOMM40 genes. In addition, we identified that 19 genic-region SNPs from the top 25 SNPs were mapped to 13 genes and that at least 5 genes were known for their association with AD or other neuropathic, neurological disorders. About one-third associations were replicated in Black samples. From the top 25 SNPs in Black samples, each of the 16 genic-region SNPs was mapped to a unique gene, which included 6 genes that were known for their association with other neurological, neuropathic, or psychiatric disorders. The genes resulted from the two races differed. These findings suggest that AD may result from a complex interplay of multiple genes, comorbidity-risks, and other brain-related disorders, which may differ for the two races.

### GEOGRAPHIC DISPARITIES IN INCIDENCE AND MORTALITY OF ALZHEIMER'S DISEASE

Igor Akushevich[1], Arseniy Yashkin[1], and Julia Kravchenko[2], *1. Duke University, Durham, North Carolina, United States, 2. Duke University School of Medicine, Durham, North Carolina, United States*

Our estimates showed that geographic patterns of incidence rates extracted from Medicare data and mortality rates calculated using Multiple-Cause-of-death data are significantly different. The significant gap in mortality rates between the East and West parts of the U.S. present in death certificate data is not observed when Medicare data is used. In addition, high incidence rates of Alzheimer's Disease (AD) identified in the stroke-belt states when using Medicare data is not fully reproduced when death certificates are used. Therefore, we used 5%-Medicare data linked to the National Death Index to resolve this inconsistency in findings using both types of data for the same individuals. We found that the excess rates of AD in the stroke-belt states cannot be attributed to higher proportions of vulnerable populations as the associated differences hold across race- and gender-specific subgroups: 1,717 (1,704-1,731) vs. 1,280 (1,275-1,286) for Whites; 2,052 (2,017-2,086) vs. 1,821 (1,795-1,848) for Blacks; 1,538 (1,520-1,556) vs. 1,156 (1,140-1,164) for males; and 1,878 (1,861-1,894) vs. 1,383 (1,376-1,391) for females. We evaluated the sensitivity and specificity for having AD as an underlying cause of death in all these regions and developed a predictive model that predicts occurrence of AD in death certificate based on information in individual Medicare records. Our findings show that underrepresentation of AD in death certificates is a strong contributor to geographic disparities in AD mortality. Predictive quality of occurrence of AD in death certificates can be improved by advancing the predictive model and using a broader set of predictors from Medicare records.

### SESSION 1010 (SYMPOSIUM)

### ANTECEDENTS, OUTCOMES, AND CORRELATES OF METABOLIC HEALTH IN OLDER AGE: A MULTIDISCIPLINARY PERSPECTIVE

Chair: Johanna Drewelies Co-Chair: Elisabeth Steinhagen-Thiessen Discussant: Jeremy Walston

Metabolic syndrome (MetS) is a cluster of risk factors for cardiovascular disease associated with reduced physical fitness, higher disease burden, and impaired cognitive functions in late life. Thus investigating antecedents, outcomes and correlates of metabolic health is a priority in order to promote healthier lifestyles and successful aging. This symposium compiles four empirical interdisciplinary studies that examine the role of metabolic health/risk on different timescales. Employing advanced modeling approaches to data obtained in large-scale studies, these reports will shed light on potential antecedents, correlates, and consequences thereof. First, Kalyani and colleagues will report data from the SPRING (Study of Physical Resilience IN Geriatrics) study on the interrelation between metabolic syndrome, glucose intolerance, and physical resilience.Second, Buchmann and colleagues examine the association of the metabolic syndrome and Lipoprotein(a) finding that hormonal aspects and in particular menopausal alterations seem to moderate theassociation between MetS and Lp(a). Third, Demuth and colleagues show how metabolic health is related to specific DNA methylation age acceleration as derived from five different epigenetic clocks, namely PhenoAge, and GrimAge. Fourth, Duezel and colleagues examine the neural correlates of metabolic risk in a sample of older adults finding that lower metabolic risk is linked to greater GMI in the prefrontal cortex. Conjointly, findings demonstrate the multifaceted nature of metabolic risk across adulthood and across different timescales. Walston will critically discuss the contributions from an aging perspective and discuss implications for future research.

### ASSOCIATIONS AMONG LATENT FACTORS OF METABOLIC LOAD, BRAIN VOLUME, AND COGNITION IN HEALTHY AGING

Sandra Duezel[1], Johanna Drewelies[2], Ulman Lindenberger[3], Elisabeth Steinhagen-Thiessen[4], Ilja Demuth[5], and Simone Kühn[3], *1. Max-Planck-Insitute for human developement, Berlin, Berlin, Germany, 2. Humboldt University Berlin, Berlin, Berlin, Germany, 3. Max Planck Institute for Human Development, Berlin, Berlin, Germany, 4. Charité - Universitätsmedizin Berlin, Berlin, Berlin, Germany, 5. Charité Universitätsmedizin Berlin, Berlin, Berlin, Germany*

Metabolic syndrome (MetS) refers to risk factors for cardiovascular disease associated with reduced physical fitness, higher disease burden, and impaired cognitive functions. Available evidence indicates that metabolic functioning is related to brain structure at the level of specific indicators, such as hypertension and voxel-based morphometry, respectively. However, attempts to relate metabolic load to brain morphology at the construct level using structural equation modeling are scarce. Here, we used confirmatory factor analysis to: (a) examine level and change associations among latent factors of metabolic risk, regional grey-matter integrity (GMI), and cognition; (b) test whether these associations differ by sex. Analyses were based on a sample of 1,100 healthy adults (52% female) aged 60 to 88 years from the Berlin Aging Study II, and included MRI data for a sub sample of 341 (37 % female) individuals. Metabolic risk was defined by waist circumference, triglycerides, fasting blood glucose, and high-density lipoprotein; regional GMI by mean diffusivity, magnetization ratio transfer ratio, and VBM-based volume estimates; and cognition, represented by the three latent constructs episodic memory, working memory and fluid intelligence. Initial analyses indicate that individuals with lower metabolic risk show greater GMI in prefrontal cortex. Also, we found that greater prefrontal GMI was associated with higher fluid intelligence and working memory in men only. Additionally, we investigated the effects of previous levels of metabolic risk and cognition on subsequent changes in both domains over time. We highlight the benefits of latent factors for establishing brain-behavior relations, and discuss putative physiological substrates of individual differences in GMI.

### LP(A) AND METABOLIC SYNDROME IN OLDER PEOPLE

Nikolaus Buchmann[1], sabine schipf[2], Till Ittermann[2], Elisabeth Steinhagen-Thiessen[3], Ilja Demuth[4], and Marcello Markus[5], *1. Charité - University Medicine Berlin, Berlin, Berlin, Germany, 2. Institute for Community Medicine, University Medicine Greifswald, Germany, Greifswald, Mecklenburg-Vorpommern, Germany, 3. Charité - Universitätsmedizin Berlin, Berlin, Berlin, Germany, 4. Charité Universitätsmedizin Berlin, Berlin, Berlin, Germany, 5. Department of Internal Medicine B, University Medicine Greifswald, Greifswald, Germany, Berlin, Mecklenburg-Vorpommern, Germany*

Background: Although an inverse association between type II diabetes mellitus and Lipoprotein (a) [LP(a)] has already been well researched, there is sparse data on the association between metabolic syndrome (MetS) with Lp(a). MetS is highly prevalent in older people and insulin resistance in MetS might link Lp(a) with MetS. Thus, we analyzed the association between Lp(a) with MetS in two large cohorts the Berlin Aging Study II [BASE-II] and the Study of Health in Pomerania [SHIP-0]

Methods: Complete cross-sectional data was available for 5,743 BASE-II and SHIP-0 participants (48.7% men; age 58 [20-85] years). MetS was defined according to modified criteria of the AHA/NHLBI 2009 definition. The association between MetS with Lp(a) was examined by median regression adjusted for age, sex and study and models were stratified by gender and menopause.

Results: MetS was prevalent in 27.6% (n=1,573) participants. We found an inverse association between MetS with Lp(a) in the whole study sample (β=-11.9 [95% confidence interval (95% CI) -21.3 to -2.6]) as well as in men (β=-16.5 [95% CI -28.6 to -4.3]) and in postmenopausal women (β=-25.4 [95% CI -46.0 to -4.8]). In contrast to this, in premenopausal women a positive association between MetS and Lp(a) (β=39.2 [95% CI 12.3 to 65.9]) was evident.

Conclusion: Postmenopausal changes in hormone metabolism impact both MetS and Lp(a). With respect to the ongoing development of Lp(a)-lowering drugs, their use must be examined, particularly in old age and in subjects with MetS.

### METABOLIC SYNDROME, GLUCOSE INTOLERANCE, AND PHYSICAL RESILIENCE

Rita Kalyani[1], Ravi Varadhan[1], Qian-Li Xue[2], Frederick Sieber[1], Philip Imus[1], Brian Buta[1], Karen Bandeen-Roche[3], and Jeremy Walston[2], *1. Johns Hopkins University School of Medicine, Baltimore, Maryland, United States, 2. Johns Hopkins University, Baltimore, Maryland, United States, 3. Johns Hopkins Bloomberg School of Public Health, Baltimore, Maryland, United States*

When confronted with a major physical stressor, some older adults are able to recover to baseline with minimal changes, while others undergo irreversible declines. The ability to adapt and recover well from physical stress is called physical resilience and may be impeded by altered glucose-insulin dynamics. Among 55 participants in the pilot phase of the Study of Physical Resilience IN Geriatrics(SPRING), we examined associations between metabolic syndrome and/or diabetes status to physical resiliency recovery parameters after the clinical stressors total knee replacement and bone marrow transplant. These parameters included health outcomes, functional recovery, and pain in the weeks after the intervention. Additionally, we hypothesized that less resilient individuals would have abnormal glucose and insulin responses to a 75-gram oral glucose tolerance test at 0, 30, 60, 120 minutes and tested this in a subset (n=40) without known diabetes. Exploratory analyses of this pilot study will be presented during this session.

### RELATIONSHIP BETWEEN METABOLIC HEALTH AND 5 EPIGENETIC CLOCKS IN THE BERLIN AGING STUDY II (BASE-II)

Ilja Demuth[1], Sandra Düzel[2], Valentin Vetter[3], Johanna Drewelies[4], Yasmine Sommerer[5], Elisabeth Steinhagen-Thiessen[3], Denis Gerstorf[6], and Lars Bertram[5], *1. Charité Universitätsmedizin Berlin, Berlin, Berlin, Germany, 2. Max Planck Institute for Human Development, Berlin, Berlin, Germany, 3. Charité - Universitätsmedizin Berlin, Berlin, Berlin, Germany, 4. Humboldt University Berlin, Berlin, Berlin, Germany, 5. University of Lübeck, Lübeck, Schleswig-Holstein, Germany, 6. Humboldt Universität zu Berlin, Berlin, Berlin, Germany*

The diagnosis of metabolic syndrome (MetS) relies on cut-off values for five cardiovascular risk factors, three of which must be met to make the diagnosis. While this approach has certain advantages in the clinical setting, it only allows a binary classification relying on cut-off values. Thus, individuals receiving the same MetS diagnosis may differ in terms of disease severity. We have recently developed a continuous measure of the metabolic load (MetL) that was calculated as a latent factor from indicators that are used to diagnose the MetS enabling a more differentiated assessment. DNA methylation age acceleration (DNAmAA) is a widely accepted biomarker of aging which can be derived from a variety of epigenetic clocks. However, it is still unclear which aspects of aging these biomarkers represent best. Aim of the current study was to evaluate whether and how the MetS and MetL relate to DNAmAA derived from five epigenetic clocks (7-CpG clock, Horvath's clock, Hannum's clock, PhenoAge, and GrimAge). MetS was diagnosed according to the AHA/IDF/NHLBI criteria (2009) and the latent MetL factor was extracted from a confirmatory factor analysis considering the same diagnostic parameters. DNAmAA estimated from PhenoAge and GrimAge were significantly associated with MetS (beta=0.036 and beta=0.063, both $p<0.05$) and MetL (both beta=0.009 and $p<0.05$) in regression analyses adjusted for age and gender including 1,100 participants from the follow-up assessments of the Berlin Aging Study II (BASE-II, mean age 75.6 ±3.8 years). The results will be discussed with respect to clinical relevance in the context of current literature.

### SESSION 1020 (SYMPOSIUM)

### BEHAVIORAL AND SOCIAL IMPACTS ON DAILY VARIABILITY IN HEALTH AND FUNCTIONING IN OLD AND VERY OLD AGE

Chair: Anna Jori Lücke Co-Chair: Oliver Schilling
Discussant: Scott Hofer

In recent years, research has shown that people experience substantial variability in domains such as cognition, health, or social interactions from day-to-day or even moment-to-moment. This variability carries relevant information above and beyond an individual's mean levels of functioning, revealing, for instance, potential risk factors for healthy aging. Thus, aging research increasingly examined such variations in older adults' daily lives, aiming to further the understanding of aging processes with insights into short- and long-term predictors of daily health and functioning. In this symposium, we introduce research using repeated daily life assessments from older participants to elucidate behavioral and social impacts on variations in working memory performance, pain and self-rated health, as well as social interaction quality. Luo et al. show that diverse daily activities were linked with higher working memory performance one the same day. Regarding long-term prediction, Schilling et al. found that patterns of alcohol consumption across two decades were only weakly predictive of subsequent short-term variability in daily working memory performance. Turning from cognitive functioning to health, Lücke et al. observed bidirectional links of variations in daily sleep quality with variations in daily pain and health perceptions across several days. Finally, Hülür et al. addressed the role of communication technologies for older persons' social interactions and found that daily social interaction quality differs with the interaction modalities. Scott Hofer will discuss the implications of the presented findings for our understanding of variability in everyday functioning in old age, considering challenges and opportunities for future research in this field.

### LONG-TERM ALCOHOL CONSUMPTION AND STABILITY OF DAILY WORKING MEMORY PERFORMANCE IN OLD AGE

Oliver Schilling[1], Denis Gerstorf[2], Anna Jori Lücke[3], and Martin Katzorreck[4], *1. University of Heidelberg, Heidelberg, Baden-Wurttemberg, Germany, 2. Humboldt Universität zu Berlin, Berlin, Berlin, Germany, 3. Heidelberg University, Heidelberg, Baden-Wurttemberg, Germany, 4. University of Leipzig, Leipzig, Sachsen, Germany*

Mixed evidence of associations of alcohol consumption with cognitive aging suggested that low to moderate alcohol consumption predicts more favorable cognitive outcomes than abstinence, whereas higher consumption operates as risk factor for cognitive decline. Daily short-term fluctuations of cognitive performance have also been established as risk factor for subsequent cognitive decline. Bringing these two lines of research together, our study analyzed associations of long-term trajectories of alcohol consumption with ambulatory assessments (7 days, 6 beeps per day) of working memory (WM) performance in participants (N = 155, aged 66-69 and 86-89) followed-up from a long-term (>20 years) longitudinal aging study. Overall, the findings do not support the "risk-view", because long-term alcohol consumption patterns were not found to be predictive of either individual levels or intra-individual momentary fluctuations of WM performance. Follow-up analyses will examine the combined effects of alcohol consumption with further risk factors, such as long-term declines in health.

### BIDIRECTIONAL LINKS OF DAILY SLEEP QUALITY AND DURATION WITH PAIN AND SELF-RATED HEALTH IN OLD AGE

Anna Jori Lücke[1], Cornelia Wrzus[2], Ute Kunzmann[3], Denis Gerstorf[4], Martin Katzorreck[5], Christiane Hoppmann[6], and Oliver Schilling[7], *1. Heidelberg University, Heidelberg, Baden-Wurttemberg, Germany, 2. Ruprecht Karls University Heidelberg, Heidelberg, Baden-Wurttemberg, Germany, 3. Leipzig University, Leipzig, Sachsen, Germany, 4. Humboldt Universität zu Berlin,*

*Berlin, Berlin, Germany, 5. University of Leipzig, Leipzig, Sachsen, Germany, 6. University of British Columbia, Vancouver, British Columbia, Canada, 7. University of Heidelberg, Heidelberg, Baden-Wurttemberg, Germany*

Sleep and physical well-being (e.g., pain, self-rated health) are closely linked, but the temporal ordering, especially regarding day-to-day variations, is not well understood. Furthermore, sleep quality and duration are only moderately correlated and may differ in their association with physical wellbeing. Using data from 123 young-old (66-69 years, 47% women) and 47 old-old adults (84-90 years, 60% women) who rated sleep quality and duration as well as pain and self-rated health on seven consecutive days, we examined bidirectional links between sleep and physical well-being. Supporting our hypotheses, results showed that after longer and better than usual sleep, participants reported better self-rated health; only better sleep quality significantly predicted lower pain. In turn, both lower pain intensity and increased self-rated health predicted better subsequent sleep quality, but not duration. We discuss conceptual and practical implications of our findings.

### PERCEIVED QUALITY OF DAILY SOCIAL INTERACTIONS: THE ROLE OF INTERACTION MODALITY

Gizem Hueluer[1], Minxia Luo[2], Birthe Macdonald[2], and Carlotta Grünjes[1], *1. University of Bonn, Bonn, Nordrhein-Westfalen, Germany, 2. University of Zurich, Zurich, Zurich, Switzerland*

Older adults increasingly use digital technologies to communicate with others. In the present study, we examine the role of interaction modality (face-to-face, telephone, digital) for perceived quality of social interactions. We use data from 118 participants (age: M = 72 years, SD = 5, range = 65 to 94; 40% women), who reported on their social interactions over 21 days in an event-contingent experience sampling study. Relative to face-to-face interactions, participants reported feeling more accepted and calmer, but also less happy in telephone interactions. They perceived telephone interactions as more meaningful, but also as less pleasant. Relative to face-to-face interactions, participants felt less accepted, less close to their interaction partner, and less happy in digital interactions and they perceived digital interactions as less pleasant. In summary, our findings suggest that the modality of daily social interactions is related to their quality. We discuss implications of these findings for future research.

### DAILY ACTIVITY DIVERSITY AND DAILY WORKING MEMORY IN COMMUNITY-DWELLING OLDER ADULTS

Minxia Luo[1], Robert Moulder[2], Laura Breitfelder[3], and Christina Röcke[4], *1. University of Zurich, Zurich, Zurich, Switzerland, 2. Institute of Cognitive Science, Colorado University Boulder, Boulder, Colorado, United States, 3. University Research Priority Program (URPP) Dynamics Of Healthy Aging, University Of Zurich, Zurich, Zurich, Switzerland, 4. University of Zurich, Dynamics of Healthy Aging & Center for Gerontology, Zurich, Zurich, Switzerland*

Research has shown that diverse activity engagement has positive effects on cognitive functioning in older age. However, it is unknown whether the positive effect holds within persons across days and across people. We examined daily within-person association between activity diversity and working memory in older age and effects of potential moderators therein. We examined 16-day smartphone-based ambulatory assessment data from 150 older adults (aged 65+). Participants reported their present activities and completed working memory tasks seven times per day. Within persons, higher daily activity diversity was positively associated with higher daily working memory. Moreover, the prior day's activity diversity led to that day's higher working memory, but not vice versa. We did not find any moderating effects of age, education, or fluid and crystallized intelligence. Our results strengthen the evidence on the beneficial effect of activity diversity on cognitive performance. Results are discussed in the context of cognitive reserve theory.

### CAREGIVER HEALTH LITERACY AS A MODIFIABLE TARGET TO PROMOTE OLDER ADULT HEALTH

Rachel O'Conor, Morgan Eifler, Lauren Opsasnick, Laura Curtis, Julia Yoshino Benavente, Lee Lindquist, and Michael Wolf, *Northwestern University, Chicago, Illinois, United States*

Many older adults receive assistance in managing chronic conditions. Yet complicating the utility of caregiver support is whether caregivers have sufficient skills to aid in a patient's self-care. Health literacy (HL) is as an important determinant of older adults' health outcomes, but few studies have examined caregiver HL and patient outcomes. We interviewed 162 patient-caregiver dyads during an ongoing cognitive aging cohort study to examine associations between caregiver HL, measured using the Newest Vital Sign, and older adults' health outcomes. Physical function and mental health symptoms were assessed using PROMIS short form assessments. Patients' also self-reported emergency department (ED) visits and hospitalizations over the past 12 months. Chi-square and t-tests were performed, as appropriate. Patients were on average 73 years old and managing 4 comorbidities. The majority were female (70%), identified as Black (35%) or White (60%). Caregivers' mean age was 64 years; half were female (56%) and had limited HL (48%). Limited caregiver HL was associated with poorer physical function (M=43.0 (8.5) vs. M=46.0 (9.1), p=0.05), greater comorbidities (M=4.0 (1.9) vs M=3.3 (1.8), p=0.02) and more ED visits in the past year (36.7% vs. 19.3%, p=0.01). No differences by caregiver HL were observed for patients' mental health or hospitalization. Findings suggest that caregivers with limited HL are caring for medically complex patients, and further research should examine whether limited caregiver HL leads to poorer self-management of chronic conditions. The development of HL training for caregivers may better equip them to assist older adults and improve older adult health.

### EXPLORING EXPERIENCES OF PAIN MANAGEMENT AMONG FAMILY CAREGIVERS OF COMMUNITY-DWELLING OLDER ADULTS WITH DEMENTIA

Hui Zhao[1], Pamela Kulbok[2], Ishan Williams[3], Carol Manning[2], and Rafael Romo[4], *1. James Madison University, Harrisonburg, Virginia, United States, 2. UVA,*

*Charlottesville, Virginia, United States, 3. University of Virginia, School of Nursing, Charlottesville, Virginia, United States, 4. Dominican University of California, San Rafael, California, United States*

Pain is often under reported and under-treated in older adults with dementia. Formal caregivers receive training and resources to develop their pain management skills; yet family caregivers (FCGs), who bear the brunt of responsibility for pain management among community-dwelling older adults with dementia have largely been omitted from research. We conducted a qualitative descriptive study to gain a deep understanding of FCGs' experience in pain management. 25 adult FCGs of community-based older adults with dementia were living in central Virginia were interviewed. Participants were 29 to 95 years old, predominantly white, married, female, and high school graduates. Four themes emerged around exploring FCGs' pain management experience and each theme included sub themes: 1) Values: family caregivers make values-based decisions that rely on a diverse range of beliefs towards opioids and non-pharmacological approaches. 2) Barriers: pain management was hampered by patient-related factors (comorbidity, complexity of care) and FCG-related factors (lack of training and resources). 3) Support: FCGs perceived greater competence when well supported by professional caregivers (doctors, social workers) and family members. 4) Adaptation: FCGs employed many strategies to support themselves and build a sense of self-efficacy that can either inhibit or facilitate effective pain management for their loved-ones. Adaptation and support from professional or formal caregivers greatly improved FCGs' perception of competence in pain management, suggesting research and development of interventions targeting FCGs is warranted.

### FACTOR STRUCTURE OF PRE-LOSS GRIEF-12 IN CAREGIVERS OF PEOPLE LIVING WITH DEMENTIA

JuYoung Park[1], and James Galvin[2], *1. Florida Atlantic University College of Social Work and Criminal Justice, Boca Raton, Florida, United States, 2. University of Miami School of Medicine, Miami, Florida, United States*

While several studies on post-loss grief have been conducted, little research has examined assessment and treatment for pre-loss grief in family caregivers of persons living with dementia (PLWD). A total of 699 caregivers of PLWD were recruited through relevant dementia associations. The recruitment e-mail provided information about the study and instructions on how to participate by a link to the online survey. Confirmatory factor analysis (CFA) was performed to test the fit of the data from the caregivers and to assess the factor structure of the Prolonged Grief-12 (PG-12) to evaluate pre-loss grief accurately by identifying relevant items and eliminating items that are not appropriate for caregivers of PLWD to assess pre-loss grief accurately. CFA was conducted via the Full Information Maximum Likelihood estimation method to test the unidimensional model of the PG-12 in the study population. The initial model was modified to develop a better-fitting model and to detect misfitting parameters in the PG-12 by deleting irrelevant items for caregivers of PLWD. The adjusted dementia-specific 10-item version (PG-10-D) had significantly improved fit indices with RMSEA = .064, CFI = .972, and GFI = .963. An overall assessment of fit indicated that the model adequately approximated the data. Factor loadings ranged from 0.53 to 0.85. This study suggests that the dementia-specific, unidimensional PG-10-D, modifying the original PG-12, may be useful and parsimonious in assessing and quantifying pre-loss grief in caregivers. Further research is required to establish psychometric properties, including factorial validity and reliability, factorial invariance analysis and further factor analysis.

### POSITIVE ASPECTS OF CAREGIVING AND LOWER RISK OF FRAILTY AND SLEEP DISRUPTION IN THE NATIONAL STUDY OF CAREGIVING

Alexandra Wennberg[1], Loretta Anderson[2], Lenis Chen-Edinboro[3], Annachiara Cagnin[4], and Lorenzo Pini[5], *1. Karolinska Institutet, Stockholm, Stockholms Lan, Sweden, 2. University of Maryland, Baltimore, Reisterstown, Maryland, United States, 3. University of North Carolina Wilmington, Wilmington, North Carolina, United States, 4. Università degli Studi di Padova, Padua, Veneto, Italy, 5. University of Padova, Padova, Veneto, Italy*

Older adult caregivers have compounded risk for adverse health outcomes; however, evidence investigating the association between the spectrum of the caregiving experience and frailty has been limited. In the National Study of Caregiving, we examined the cross-sectional association between caregiving experiences and both frailty and sleep disruption outcomes among caregivers. We included 621 caregivers aged 65+ who completed a phone interview, including 36 items about caregiving. Using principal component analysis, we identified three caregiving components: general burden, financial burden, and positive emotions. Frailty was assessed via low energy, shrinking, weakness, reduced activity, and poor self-rated health. Sleep disruption was assessed with two questions regarding sleep interruption and trouble falling back asleep. In age- and sex-adjusted ordinal regression models, general burden was associated with frailty (OLO=1.21, 95% CI 1.12, 1.30) and trouble falling back asleep (OLO=1.08, 95% CI 1.00, 1.17); financial burden (OLO=1.15, 95% CI 1.02, 1.31) was associated with frailty. General (OLO=1.99, 95% CI 1.38, 2.88) and financial (OLO=1.98, 95% CI 1.25, 3.14) burden were associated with sleep interruption in the fully adjusted model. Positive emotions were associated with lower odds of both frailty (OLO=0.86, 95% CI 0.74, 0.99) and sleep interruption (OLO=0.75, 95% CI 0.58, 0.98). Caregiver burden was associated with greater risk of frailty in age- and sex- adjusted models and sleep interruption when further accounting for sociodemographic and health factors. Positive emotions of caregiving were associated with decreased risk of these outcomes.

### SESSION 1040 (SYMPOSIUM)

### CAREGIVER PARTICIPATION IN VIDEO VISITS: CHALLENGE OR OPPORTUNITY?

Chair: Megan Gately Co-Chair: Lauren Moo

Rapid expansion of telehealth in response to COVID revealed a digital divide for many patients, particularly older adults. Given the technical complexity of video visits (which may include downloading novel software and enabling a camera and microphone), video visits may be out of reach for older patients with less technological experience or with age and condition-related changes such as sensory loss or cognitive impairment. Involving caregivers in video visits (particularly technical set-up) may not only increase patient access but also enhance clinical care by allowing for collaboration with family. Caregivers may themselves benefit from video visits, given that video offers increased options for caregiver support. Though caregivers are often identified as critical components to older adults' accessing telehealth and may also benefit from telehealth services, caregivers' own technical needs are not well-understood. This symposium discusses caregivers' involvement in telehealth from multiple perspectives. The first presentation includes findings from a national clinician survey about caregivers' support role in occupational therapy video visits, including barriers and benefits (Gately et al). The second presentation includes findings from a regional survey of interprofessional clinicians about telehealth modalities to provide dementia family caregiver support during COVID-19 (Quach et al). The third presentation includes family caregivers' technology assistance requirements before and during a virtual, seven-session group skills training program, including benefits of individualized assistance (Moo et al). The fourth presentation includes caregiver perspectives about tele-geriatrics visits, highlighting caregivers' support role and enhancements of video versus phone (Boudreau et al).

### CAREGIVER CONTRIBUTION TO OCCUPATIONAL THERAPY VIDEO VISITS

Megan Gately[1], Dylan Waller[2], Emily Metcalf[1], and Lauren Moo[1], *1. VA Bedford Health Care System, Bedford, Massachusetts, United States, 2. VA Portland Health Care System, Portland, Oregon, United States*

Caregivers' role facilitating older adults' participation in diverse health care services delivered using video telehealth (i.e., live sessions) is not well-understood. This study surveyed occupational therapy (OT) practitioners across Veterans Health Administration (VHA) about caregiver participation in VA Video Connect (VVC), VHA's videoconferencing platform. 293 OT practitioners participated in the survey, with 47% reporting that caregivers participated in VVC often. The foremost reported patient factors necessitating caregiver participation in video visits were patient lack of technical skills (76%) and cognitive impairment (72%). Barriers to caregiver participation in video visits included poor connectivity and caregivers' own age or health related impairments, while benefits included increased collaboration with family (87%). This study enhances our understanding of caregivers' participation in video telehealth, highlighting factors driving caregiver participation and suggesting strategies to optimize this service delivery format for older adults.

### FACTORS INFORMING CLINICIAN CHOICE OF TELEHEALTH MODALITY TO SUPPORT DEMENTIA CAREGIVERS

Emma Quach[1], Emily Franzosa[2], Christine Hartmann[1], and Lauren Moo[1], *1. VA Bedford Health Care System, Bedford, Massachusetts, United States, 2. Icahn School of Medicine at Mount Sinai, New York City, New York, United States*

In summer 2020, 68 clinicians in the New England area caring for Veterans with dementia as part of a specialty team were surveyed regarding caregiver support services, with a 46% response rate (n=31). When faced with the need to abruptly discontinue in-person dementia support services, the majority of respondents offered caregivers support via telephone rather than video telehealth. Only 4 of 31 (13%) mentioned offering video visits for the first time to replace face-to-face visits. Clinician choice of modality largely reflected shifts among preestablished communication modalities/patterns and were influenced by clinician perception that older patients and their caregivers would prefer the telephone. Clinicians without experience using video telehealth with older adults were unlikely to offer video visits despite evidence that many older adults are willing and able to participate. Assessing caregiver wish/ability to participate in video visits may inform and shape clinician choice of telehealth modality.

### TECHNICAL SUPPORTS TO BRIDGE THE DIGITAL DIVIDE FOR OLDER ADULTS PARTICIPATING IN TELEMEDICINE

Lauren Moo[1], Andrew Nguyen[2], Kendra Pugh[3], Jaye McLaren[3], Steven Shirk[3], and Maureen O'Connor[3], *1. VA Bedford Health Care System, Bedford, Massachusetts, United States, 2. Bedford VA Research Inc, Boston, Massachusetts, United States, 3. VA Bedford Healthcare System, Bedford, Massachusetts, United States*

Older adults' relative lack of technological literacy is a barrier to telemedicine, including participation in virtual caregiver support programs. Details of technology assistance required before and during a virtual, seven-session group skills training program for family caregivers were recorded. A majority of participants, all older adults, had difficulty using videoconferencing technology that ranged from finding meeting links in emails to difficulty with basic device use. Personalized support was provided to each caregiver as needed. None required any support by the final session. Those with initial technology challenges felt a sense of accomplishment and increased tech comfort by the end of the sessions. While some older adults had difficulty using telemedicine technology, they were eager to learn and educable with personalized help. Infrastructure to provide this personalized help is necessary to support older adults' desire to engage in telemedicine and to reduce the digital divide.

### PERSPECTIVES OF CAREGIVERS OF RURAL, OLDER VETERANS ON TELEGERIATRICS CARE

Jacqueline Boudreau[1], Jennifer Conti[1], Meaghan Kennedy[1], Camilla Pimentel[2], William Hung[3], Lauren Moo[1], and Eileen Dryden[1], *1. VA Bedford Health Care System, Bedford, Massachusetts, United States, 2. VA Bedford Healthcare System, Bedford, Massachusetts, United States, 3. Icahn School of Medicine at Mount Sinai, New York, New York, United States*

Telemedicine is critical to extending healthcare's reach to rural older adults with complex medical needs, yet concerns remain about feasibility and acceptability for this population and their caregivers. We interviewed 30 rural Veterans ≥65 years old and/or their caregivers (n=21) about their experiences with video or telephone visits as part of an evaluation of Virtual Geriatrics, a network of Veterans Affairs tele-geriatric care hubs. Interviews were recorded, transcribed, and analyzed using rapid qualitative analysis. Caregivers deemed telemedicine a convenient option that prevented burdensome travel to remote specialists, facilitated caregiver involvement in visits, and matched quality of in-person visits. Caregivers often managed technology, enabling their loved one to participate in video visits. Telephone visits, while convenient, sometimes caused missed physical cues and hearing challenges which led providers to lean on caregiver communication. Our findings suggest telemedicine is feasible and acceptable for delivery for geriatrics care among rural adults and their caregivers.

### SESSION 1050 (SYMPOSIUM)

### COGNITION IN CONTEXT: INVESTIGATING THE ROLE OF BUILT, SOCIAL, AND NATURAL ENVIRONMENTS IN COGNITIVE AGING

Chair: Jessica Finlay Co-Chair: Philippa Clarke Discussant: Lilah Besser

While a growing body of evidence points to potentially modifiable individual risk factors for Alzheimer's Disease and Related Dementias (ADRD), the contexts in which people develop and navigate cognitive decline are largely overlooked. Geographic variation in ADRD rates suggest that environmental risk and protective factors may be important in cognitive aging and dementia caregiving. Community hazards are often heavily concentrated in underserved and underrepresented neighborhoods. This symposium aims to identify specific built, social, and natural environmental features associated with cognitive aging outcomes. The papers provide much-needed evidence on the role of neighborhoods and community networks for cognitive health and well-being among diverse older adults. First, Godina finds significant associations between neighborhood greenspace and microstructural indicators of brain health. Second, Westrick investigates the role of neighborhood disadvantage on long-term memory aging of older adults with and without a cancer diagnosis in later-life. Third, Finlay presents a new concept of Cognability to demonstrate which constellation of positive and negative neighborhood features may contribute most to healthy cognitive aging. Fourth, Nkimbeng identifies community networks and resources needed to inform dementia education and support care among African immigrants. Fifth, expert discussant Besser will share how these findings may inform upstream health promotion and reduce ADRD risk. She will discuss critical future research directions and methods to investigate environments of cognition. The symposium advances research assessing contexts of aging, and may inform public health and policy efforts to ameliorate community barriers and create more equitable opportunities to promote healthy aging in place.

### NEIGHBORHOOD GREENSPACE AND GRAY MATTER MICROSTRUCTURAL INTEGRITY: THE HEALTH ABC STUDY

Sara Godina[1], C. Shaaban[2], Caterina Rosano[3], Howard Aizenstein[4], and Andrea Rosso[3], *1. University of Pittsburgh School of Public Health, Pittsburgh, Pennsylvania, United States, 2. Department of Epidemiology, University of Pittsburgh School of Public Health, Pittsburgh, Pennsylvania, United States, 3. University of Pittsburgh, Pittsburgh, Pennsylvania, United States, 4. Department of Psychiatry, University of Pittsburgh School of Medicine, Pittsburgh, Pennsylvania, United States*

Neighborhood greenspace is positively associated with cognition and macrostructural indicators of brain health. Microstructural damage may be a sensitive marker for dementia-related neurodegeneration that precedes such macrostructural changes. We aimed to examine cross-sectional associations between neighborhood (census tract) greenspace and gray matter (GM) microstructure in 265 community-sampled adults (mean age=83, 57% women, 39% Black). Linear mixed effects regression models tested associations between quartiles of percent greenspace derived from the normalized vegetation index and mean diffusivity (MD) quantified using magnetic resonance imaging with diffusion tensor. Greater greenspace was related to higher MD in 4 regions: left calcarine (Q3 vs. Q1 st$\beta$=0.38, p=0.0348), left thalamus (Q2 vs. Q1 st$\beta$=0.35, p=0.0443; Q3 vs. Q1 st$\beta$=0.49, p=0.0061), and bi-lateral precuneus (left Q2 vs. Q1 st$\beta$=0.46, p=0.0096; right Q2 vs. Q1 st$\beta$=0.36, p=0.0385). The relationship between greenspace and cognition may be through paths other than GM microstructure; future research should explore other potential mechanisms.

### DOES NEIGHBORHOOD DISADVANTAGE ALTER MEMORY AFTER A CANCER DIAGNOSIS? A US HEALTH AND RETIREMENT STUDY

Ashly Westrick[1], Monica Ospina-Romero[2], Philippa Clarke[1], and Lindsay Kobayashi[1], *1. University of Michigan, Ann Arbor, Michigan, United States, 2. University of Wisconsin, Madison, Wisconsin, United States*

We aimed to determine the influence of neighborhood socioeconomic status (NSES) on long-term cancer-related memory decline of older adults. Incident cancer diagnosis and memory were assessed in the U.S. Health and Retirement Study (N=15,074, 1998-2016). Proportion of female-headed households with children, households with public assistance income, people with income below poverty, and proportion 16+ years unemployed was categorized into NSES tertiles. Linear mixed-effects models compared the standardized memory trajectories by cancer status and NSES. Cancer-free individuals living in more disadvantaged neighborhoods had worse mean memory function at age 75 and steeper memory declines than participants from less disadvantaged neighborhoods. An incident cancer diagnosis was associated with an acute memory drop at diagnosis for those living in the least disadvantaged neighborhoods. Cancer survivors had better memory prior to but not after diagnosis compared to cancer-free individuals across NSES. These findings could inform future interventions to promote cancer survivor's long-term aging.

### COGNABILITY: AN ECOLOGICAL THEORY OF NEIGHBORHOODS AND COGNITIVE AGING

Jessica Finlay[1], Esposito Michael[2], Kenneth Langa[1], Suzanne Judd[3], and Philippa Clarke[1], *1. University of Michigan, Ann Arbor, Michigan, United States, 2.*

*Washington University in St. Louis, St. Louis, Missouri, United States, 3. University of alabama at Birmingham, Birmingham, Alabama, United States*

This paper presents a new theoretical concept, Cognability, which aims to conceptualize how supportive an area is to cognitive health among aging residents. Cognability incorporates a both positive and negative neighborhood features related to physical activity, social interaction and cognitive stimulation in later life. We analyzed data from the Reasons for Geographic and Racial Differences in Stroke Study, a national sample of older Black and white US adults (n=21,151; mean age at assessment=67; data collected 2006–2017). Generalized additive multilevel models examined how cognitive function varied by neighborhood features. Access to civic and social organizations, recreation centers, fast-food and coffee establishments, arts centers, museums, and highways were significantly associated with cognitive function. Race-, gender-, and education-specific models did not yield substantial improvements to the full-model. Cognability advances ecological theories of aging through an innovative "whole neighborhood" approach. Findings may inform community interventions and policy to support healthy aging in place.

### FACTORS INFLUENCING WHERE AFRICAN IMMIGRANTS PLAN TO SEEK CARE FOR A RELATIVE WITH DEMENTIA

Manka Nkimbeng[1], Wynfred Russel[2], Tetyana Shippee[1], and Joseph Gaugler[1], *1. University of Minnesota, Minneapolis, Minnesota, United States, 2. African Career Education And Resources Inc. (ACER), Brooklyn Park, Minnesota, United States*

Many African immigrants did not hear about dementia until they migrated to the United States, which limits understanding of the disease and awareness of community resources. We examined the relationship between being a caregiver, care recipient country of residence, and seeking care using data from 152 African immigrants. Most participants (90%) were caregivers, 12% of the care recipients resided in Africa, and 59% reported that they would seek care within the formal health system. After controlling for age, gender, income and participant place of birth, caregivers were more likely (aOR: 2.31, 95% CI 0.37, 13.39) to seek care from community networks alone, as were those providing care to a recipient in Africa (aOR: 1.94, 95% CI 0.56, 6.71), but these were not statistically significant. These findings which trend toward preferences for seeking care through informal community networks (friends, religious organizations etc.), can inform dementia education and outreach for this community.

### SESSION 1060 (SYMPOSIUM)

### COVID-19 RECOVERY: LESSONS LEARNED AND POLICY ACTIONS FOR THE FUTURE

Chair: Christine Mueller Discussant: Kirsten Corazzini

Focusing on Covid-19 recovery, this symposium will feature a selection research studies that highlight lessons learned that are applicable across the continuum of care for older adults, their families and care partners. The collection of presentations bring perspectives from local to global. Each of the presenters will highlight implications for health and aging policy.

### IMPACT OF COVID-19 MITIGATION POLICIES: LESSONS LEARNED FROM US AND MIDDLE-HIGH INCOME COUNTRIES

Preeti Zanwar[1], Vinod Joseph KJ[2], Zaliha Omar[3], Flavia Santos[4], Evguenii Zazdravnykh[5], Patricia Heyn[6], and Arokiasamy Perianayagam[7], *1. Jefferson College of Population Health, Philadelphia, Pennsylvania, United States, 2. International Institute for Population Sciences, Mumbai, Maharashtra, India, 3. School of Health Sciences, Fujita Health University, Toyoake, Aichi, Japan, 4. University College Dublin, Dublin, Dublin, Ireland, 5. National Reserach University, International Center of Health Economics, Policy, and Management, St. Petersburg, Leningrad, Russia, 6. Center for Optimal Aging, Marymount University, Fairfax Station, Virginia, United States, 7. National Council of Applied Economic Research, New Delhi, Delhi, India*

The COVID-19 pandemic has been a natural global epidemiological experiment unique to our century and a massive shock to older adults and to systems that care for them. There was a lack of a global unified plan to mitigate and control the spread of COVID-19. Several middle-or-high income nations struggled to control the viral spread resulting in increased mortality due to a combination of lack of public health measures and existing disparities which were magnified during the pandemic. The purpose of this review by a team of international experts is to (1) to examine reasons for the varied COVID-19 responses within U.S. and among other middle-or-high-income countries and the emergence of variants and vaccine inequities, and (2) to examine the country specific burden of cultural/structural/political determinants on access to care and mortality among older adults in various settings.

### INFECTION PREVENTION CHALLENGES AND OPPORTUNITIES IN SMALL LONG-TERM CARE FACILITIES

Carolyn Ham, *Washington State Department of Health, Shoreline, Washington, United States*

Small, individually owned long-term care facilities experienced unique challenges to accessing infection prevention knowledge and resources during the COVID-19 pandemic. Three data sets were analyzed: 1) Multi-state qualitative interviews with public health and regulatory staff in spring 2021; 2) Online survey of Washington state adult family home providers on infection control knowledge and practices in fall 2020; 3) Non-regulatory Infection Control Assessment and Response evaluations conducted between March 2020 and January 2021. Consistent findings across datasets were ongoing difficulty obtaining personal protective equipment (PPE), inability to implement isolation precautions and high vulnerability to staffing shortages. Small facilities showed strengths in consistency of leadership and engagement with public health outreach. Facility size is a key factor in infection prevention disparities for older adults living in residential care. Promising policy interventions are prioritization of small facilities for PPE distribution and increasing the availability of public health infection prevention expertise.

### PREDICTORS OF NURSING HOME COVID-19 CASES: A COMMUNITY VULNERABILITY APPROACH

Matthew Peterson[1], and Larry Lawhorne[2], *1. UNCW College of Health and Human Services, Wilmington, North Carolina, United States, 2. Wright State University, Fairborn, Ohio, United States*

This study examined facility and community factors that were related to incident COVID-19 cases in nursing facilities. N=12,473 US nursing facilities were included in this study. Data from June 2020 - January 2021 from several sources were combined to create a dataset that included facility and community factors. Results indicated that higher staff shortages, poorer facility rating, for-profit ownership, proportionally more Medicaid and non-white residents were all significantly associated with higher COVID case rates over 8 months (all $P < 0.0001$). Community level predictors of higher cases included urban setting and higher Social Vulnerability Index (SVI). SVI was the strongest predictor of COVID case counts. This study assists in determining critical facility and community factors that predict increasing COVID burden in nursing facilities. Particularly, SVI is an important factor in determining facility and public health policy, and for targeting resources in large scale health crises such as the COVID-19 pandemic.

### IMPACT OF SOCIAL ISOLATION ON OLDER ADULTS IN NURSING HOMES DURING THE PANDEMIC LOCKDOWN: INFORMING POLICY

Rebecca Koszalinski[1], Diana Sturdevant[2], Patsy Smith[2], Julie Myers[3], Debara Yellseagle[2], Brenda Olmos[2], and Molly Netter[2], *1. OUHSC, Oklahoma City, Oklahoma, United States, 2. University of Oklahoma HSC, Oklahoma City, Oklahoma, United States, 3. University of Oklahoma Health Sciences Center, Oklahoma City, Oklahoma, United States*

Purpose: Describe outcomes of an equity-centered community design (ECCD) project examining the impact of COVID-19 restrictions and social isolation on nursing home (NH) owners, administrators, community leaders, clergy, and urban homeless shelter staff. • Methods: 3-phase, ECCD project. Key informants (KI) were recruited through snowball sampling if experienced in caring for older adults (OA) in NH, in the community, or were engaged in spiritual care of OA.• Results: Perceptions of fear, anger, frustration, sadness, and concern. Categories of Lost Communication and Socialization Strategies, Lost Freedom, Residents' Dying Alone and Inability to Pause to Grieve, Helplessness and Hopelessness, Inability to Take Time to Grieve, and Behavior Changes. KI offered New Ways of Communicating, and interventions and solutions were generated in Phase III.• Implications. Understanding of Stakeholder experiences and perceptions is important to achieving inclusive, equity-centered solutions. Future policy decisions must consider what matters most to those most directly impacted by those decisions.

### SESSION 1070 (SYMPOSIUM)

### ESPO AND ACADEMY FOR GERONTOLOGY IN HIGHER EDUCATION SECTION SYMPOSIUM: TEACHING STRATEGIES FOR INTEGRATING DIVERSITY IN AGING EDUCATION PRACTICES

Chair: YanJhu Su Co-Chair: Shan Qu Discussant: Joann Montepare

This symposium intends to echo one of the core values for the GSA 2022 Annual Scientific Meeting — embracing diversity. In an increasingly humanistic society, addressing and accommodating diversity is a constant topic in the field of education. Diversity includes many different factors: race, ethnicity, gender, sexual orientation, socioeconomic status, ability, age, religious or political beliefs. There is no longer just one type of student in the classroom. Therefore, it is very important to adjust our teaching methods to accommodate the diverse backgrounds of students and to help students understand their different fellow classmates. In accordance with the AGHE gerontological education competencies, these authors will provide insightful teaching strategies for integrating diversity in aging education practices. The first speaker will introduce a new pedagogy in promoting learning in diverse classrooms — Sharestart. The second speaker will focus on building community-based partnerships to promote diverse, intergenerational service-learning opportunities for interprofessional gerontology students. The third speaker will discuss their experience on fostering connections across generations in the LGBTQ+ community through intergenerational conversations. The fourth speaker will present humanist values on understanding aging diversity. Finally, the fifth speaker will discuss curriculum mapping as a strategy to teach diversity in gerontology programs.

### SHARESTART: A NEW PEDAGOGY TO PROMOTE LEARNING IN DIVERSE CLASSROOMS

YanJhu Su, *University of Massachusetts Boston, Boston, Massachusetts, United States*

Diverse classrooms are nightly valued. The role of teachers is not limited to imparting knowledge. Nor are students only limited to gaining knowledge from their teachers. In this symposium session, the speaker would like to introduce a new pedagogy — Sharestart, which is specifically designed for comprehensive student learning. This pedagogy was proposed by a senior high school teacher in Taiwan. It is a long-term and stable teaching method that integrates a diversity of abilities by enabling students to self-learn, read, think, discuss, analyze, summarize, express and compose in every class session. In this symposium presentation, the speaker will introduce the core values of Sharestart pedagogy, discuss the advantages and disadvantages, and present an application scenario applying the Sharestart pedagogy.

### REDUCING AGEISM IN THE LGBTQ+ COMMUNITY THROUGH INTERGENERATIONAL CONNECTION

Carrie Andreoletti, Christina Barmon, Andrea June, and Michael Bartone, *Central Connecticut State University, New Britain, Connecticut, United States*

Intergenerational interaction has the potential to reduce ageism and increase feelings of generativity in both younger and older adults. To expand aging education beyond our aging and gerontology classes, we collaborated with our campus LGBT Center and community partners to host several intergenerational conversations between younger and older LGBTQ+ adults and allies. The goal was to foster connection across the generations in the LGBTQ+ community through discussions of topics of mutual interest (e.g., ageism, identity & language). Participants in the LGBTQ+ conversations reported that they valued the opportunity to talk with members of the community from different generations and that the conversations changed their views of one another in a positive way. We will discuss the strengths and challenges of our program, ideas for future programs and research, and suggestions for integrating discussion of LGBTQ+ aging into the classroom.

### BUILDING COMMUNITY-BASED PARTNERSHIPS TO PROMOTE DIVERSE, INTERGENERATIONAL SERVICE-LEARNING OPPORTUNITIES

Jason Garbarino, and Janelle Sarnevitz, *University of Vermont, Burlington, Vermont, United States*

The next generation of healthcare providers require experiential learning opportunities which incorporate participation from historically marginalized, diverse populations. A longstanding interprofessional gerontology service-learning program with a measured ability to positively influence student perspectives working with older adults while reducing rates of social isolation among older participants, underwent recent changes in the recruitment of older adults to ensure increased participation from historically marginalized populations. Building partnerships with community-based organizations serving BIPOC, LGBTQ+, and rural older adults played an integral role in building a more diverse participant group for shared, intergenerational learning. Opportunities for student reflection via group debriefs and individual, written reflections promote a greater understanding and preparedness to work with diverse older adult populations.

### UNDERSTANDING AGING DIVERSITY THROUGH HUMANIST VALUES OF AGING: LOVE, HOPE, PEACE, AND STEWARDSHIP

Stephen Fogle, *University of Nebraska at Omaha, Omaha, Nebraska, United States*

Humanist values of aging are relevant for understanding diversity in gerontological education because they are manifested across all cultures and ages. This paper proposes attention to four humanist values of aging: Love, Hope, Peace, Stewardship. This paper seeks opportunities for students to understand aging diversity in half century context: from traditional frames of race and sexuality to global conversations on language and geography. Drawing from stories about older adults this paper gathers diverse opportunities for understanding of humanist values of aging. Selections strive toward expanding representation of aging diversity and accessibility for students. Teaching with stories of love, hope, peace, and stewardship as humanist values of aging matches AGHE initiatives for incorporating humanities into gerontological education and opens opportunities for recognizing diversity representation in our society for all ages. Understanding aging diversity through stories of older adults engaged with humanist values of aging promises enriching educational experiences for lifelong learning.

### CURRICULUM MAPPING AS A STRATEGY TO TEACH DIVERSITY IN GERONTOLOGY PROGRAMS

Darren Liu[1], and Betty Burston[2], *1. West Virginia University, Morgantown, West Virginia, United States, 2. University of Nevada Las Vegas, Nevada, Las Vegas, Nevada, United States*

Curriculum mapping is a process where instructors, program leaders and other instructional designers come together to prepare learning objectives for students throughout a learning journey. The concepts of diversity may be introduced at all levels of a gerontology curriculum. A curriculum that desires to integrate diversity education is especially imperative as it needs to be built upon a guiding principle of a didactic program within which its individual courses, learning objectives, and assessments all together serve the same purpose. This presentation provides insight to the curriculum mappings for diversity education in gerontology programs. While the current programs introduce diversity topics such as race, racism, implicit bias, etc., how and when each topic being introduced in bringing about maximum learning outcomes may be related to a well thought out curriculum mapping. Specifically, this session will go over a few examples of methods for the mappings of diversity topics in different courses.

### SESSION 1080 (SYMPOSIUM)

### FORGING AND SUPPORTING INTERGENERATIONAL CONNECTIONS: THE BREADTH OF INTERGENERATIONAL SCHOLARSHIP

Chair: Cal Halvorsen Co-Chair: Ling Xu Discussant: Amy Eisenstein

Research on intergenerational connections is wide ranging, highlighting the various ways that people from across the life course interact for mutual and community benefit. Intergenerational connections occur through both formal (e.g., community service programs) and informal (e.g., family interactions) means. Studies have shown that intergenerational connections can lead to an assortment of physical, social, psychological, and interpersonal health and well-being outcomes for younger and older people alike. This symposium will highlight the breadth of research on intergenerational connections, bringing together scholars who have used primary and secondary data to better measure, understand, and support them. The first presentation will report the results of a randomized controlled trial in Tarrant County, Texas that trained college students to connect with older adults experiencing cognitive impairment through reminiscence and digital storytelling. The second presentation will describe how intergenerational transfers of emotional and instrumental support relate to self-rated health among older Chinese adults living in Honolulu while considering the roles of gender and resilience. The third presentation will address an important question within intergenerational scholarship—how to measure intergenerational connections—by reporting the results of the newly developed and validated Intergenerational Contact survey among an online sample of younger and older adults. The fourth presentation will describe the pilot-year evaluation of the Gen2Gen Innovation Fellowship, a cohort model that supports and connects leaders of intergenerational initiatives throughout the U.S. To conclude, our discussant will place these diverse studies into context and note places for continued innovation in intergenerational scholarship to better inform the field.

### REMINISCENCE AND DIGITAL STORYTELLING TO IMPROVE WELL-BEING OF OLDER ADULTS WITH COGNITIVE IMPAIRMENT

Ling Xu[1], Noelle Fields[2], Kathryn Daniel[1], Brooke Troutman[3], and Daisha Cipher[2], *1. University of Texas at Arlington, Arlington, Texas, United States, 2. The University of Texas at Arlington, Arlington, Texas, United States, 3. Air Force Academy, Air Force Academy, Colorado, United States*

To address the growing concern about loneliness and diminished well-being among persons with cognitive impairment, an intergenerational intervention based on reminiscence and digital storytelling was offered by trained college student volunteers to older adults living in the community. A randomized controlled trial was used to assess the effects of the intervention. Younger and older adult participants were randomly paired and assigned to reminiscence (n=20) or control (social wellness, n=16) groups. Data were collected at baseline, mid-intervention, and at the end of the intervention. Friedman tests for non-normally distributed outcome variables and one-way repeated measures ANOVA for normally distributed outcome variables were conducted. Results showed that emotional loneliness, total social and emotional loneliness, quality of life, and positive affect significantly improved among older adults in the reminiscence group, especially between baseline and posttest. Results suggest that weekly intergenerational engagements with young adults benefit older adults with psychological well-being.

### INTERGENERATIONAL TRANSFERS AND HEALTH IN CHINESE OLDER ADULTS: DO RESILIENCE AND GENDER MATTER?

Sizhe Liu[1], Wei Zhang[2], Keqing Zhang[2], and Bei Wu[3], *1. Shanghai University of Finance and Economics, Shanghai, Shanghai, China (People's Republic), 2. University of Hawaii at Manoa, Honolulu, Hawaii, United States, 3. New York University, New York, New York, United States*

Emotional and instrumental supports from adult children have been shown to increase positive outcomes among older adults. In this study, we examined the association between intergenerational transfer and self-rated health as well as the mediating and moderating roles of resilience and gender, respectively, using data from Chinese older adults in Honolulu (N=400). We found that the impact of emotional and instrumental support varied by gender. Further, we found that resilience significantly mediated the positive effect of provided economic support on health of older men (resilience mediated 25.1% of the total effect) as well as the positive effect of received emotional support on health of older women (resilience mediated 35.3% of the total effect). Our study highlights the importance of considering resilience and gender differences when examining intergenerational transfers and health among Chinese older adults. The design of a culturally tailored policy would help promote the health of Chinese older adults.

### MEASURING GENERATIONAL DIFFERENCES IN INTERGENERATIONAL CONTACT WITH A NEWLY VALIDATED SCALE

Shannon Jarrott[1], Shelbie Turner[2], and Karen Hooker[3], *1. The Ohio State University, Columbus, Ohio, United States, 2. Weill Cornell Medical College, Pitman, New Jersey, United States, 3. Oregon State University, Corvallis, Oregon, United States*

2. Intergenerational contact influences attitudes about aging, but measures vary in breadth, depth, and psychometrics. We developed and validated the Intergenerational Contact survey of key contact dimensions using online samples. Here, we address: how do young and older adults differ in location and ratings of intergenerational contact? Young (n=433) and older adults (n=286) reported intergenerational contact settings and rated 18 items regarding contact (e.g., "I have something to offer younger/older adults.") with family and non-family members. Neighborhoods were a common setting for interacting with family and non-family members of different ages. Young adults reported higher negative contact ($p<.001$) and lower positive contact ($p<.001$) with related and unrelated older adults than older adults reporting having with younger adults. Developmental theory may partially explain these differences; we address next steps to explore how differences may affect interest in or avoidance of intergenerational contact, associated impacts, and potential interventions.

### PILOTING AN INTERGENERATIONAL ECOSYSTEM: THE GEN2GEN INNOVATION FELLOWSHIP AND RESULTS FROM YEAR 1

Cal Halvorsen[1], Eunice Lin Nichols[2], and Janet Oh[2], *1. Boston College, Allston, Massachusetts, United States, 2. Encore.org, San Francisco, California, United States*

Intergenerational service has a long history in the U.S., highlighting the positive health and well-being outcomes of bringing the generations together. In recent years, private foundations have increased their focus on intergenerational initiatives, yet the creation of an "intergenerational ecosystem" that is designed to spark, grow, and support new intergenerational initiatives has not been nationally tested. In response, Encore.org launched the Gen2Gen Innovation Fellowship in 2020 to pilot and hone a 9-month fellowship model that supports a diverse group of leaders of intergenerational initiatives. To assess the impact of the Fellowship, we employed a mixed-methods approach with subjective and objective data sources, including interviews and monthly and post-Fellowship surveys. Among the first cohort of 15 Fellows, results suggest that the cohort-style model encouraged peer-to-peer support and that the Fellowship positively influenced their intergenerational efforts. We conclude by describing lessons learned to inspire future efforts to strengthen the intergenerational ecosystem.

### SESSION 1090 (PAPER)

### GLOBAL RESEARCH IN DEMOGRAPHY AND HEALTH OUTCOMES, MIGRATION, AND DISCRIMINATION

### COHORT EFFECTS IN MULTIMORBIDITY AMONG OLDER US ADULTS: DIFFERENCES BY RACE/ETHNICITY AND NATIVITY

Nicholas Bishop[1], Steven Haas[2], and Ana Quiñones[3], *1. University of Arizona, Tucson, Arizona, United States, 2. Penn State, University Park, Pennsylvania, United States, 3. Oregon Health and Science University, Portland, Oregon, United States*

Multimorbidity (≥ 2 co-occurring chronic health conditions) affects > 50% of US adults aged 65 and older. Building on emerging work suggesting greater risk of multimorbidity in more recent cohorts of aging Americans, we examine heterogeneity in cohort-patterns of multimorbidity by race/ethnicity and nativity. Observations were drawn from the Health and Retirement Study (1998–2018) including adults aged 51 and older across 7 birth cohorts (born < 1924, 1924–1930, 1931–1941, 1942–1947, 1948–1953, 1954–1959, and 1960–1965). Multimorbidity was measured using a sum of 9 possible chronic conditions (heart disease, hypertension, stroke, diabetes, arthritis, lung disease, cancer, high depressive symptomatology, and cognitive impairment). Linear mixed models adjusting for age, period, and cohort effects tested whether cohort-specific multimorbidity risk varied for US-born White, US-born Black/African American, US-born Hispanic/Latino, and foreign-born Hispanic/Latino older adults. 30,607 adults contributed 158,727 total observations, grand mean age was 65.40 (SD=10.07), and mean multimorbidity was 2.18 (SD=1.60). US-born Black/African Americans had the greatest multimorbidity burden regardless of cohort. US-born Hispanic/Latinos experienced the highest cohort-related increases in multimorbidity, followed by US-born Black/African Americans. For example, compared to US-born Hispanic/Latinos born 1931–1941, US-born Hispanic/Latinos born 1954–1959 had 1.03 (CI: 0.78;1.28) more expected chronic conditions. Foreign-born Hispanic/Latinos had the smallest cohort-related increases in multimorbidity. Our results document heterogeneity in rising rates of multimorbidity across cohorts, suggest that US-born minority adults in Baby Boom cohorts may be at especially high risk of multimorbidity, and confirm protective effects of immigrant status among Hispanic/Latino older adults.

### DOES DISCRIMINATION MODERATE THE RELATIONSHIP OF AGE, GENDER, AND RACE WITH HEALTH OUTCOMES IN OLDER ADULTS?

Meghan Hendricksen[1], Jeffrey Stokes[2], Sarah Forrester[3], and Beth Dugan[2], *1. Marcus Institute for Aging Research, Roslindale, Massachusetts, United States, 2. University of Massachusetts Boston, Boston, Massachusetts, United States, 3. University of Massachusetts Medical School, Worcester, Massachusetts, United States*

Discrimination and experiences of prejudice are a social determinant of health, with potential consequences at the individual, interpersonal, and systemic levels. This study aimed to investigate the relationship between discrimination experienced by older adults and health outcomes. Data were drawn from 13,290 participants from the Health and Retirement Study, who completed the Leave Behind Questionnaire in 2012 or 2014. Logistic regressions were conducted to examine relationships between age, gender, and race/ethnicity and self-reported health (SRH), risk of stroke, and heart attack. Interaction terms were tested for everyday discrimination and age, gender, and race/ethnicity, then for health discrimination and age, gender, and race/ethnicity. Results found associations between age, gender, and race and SRH, stroke, and heart attack. Everyday discrimination moderated the association between gender and risk of heart attack, where differences in men and women's risk of heart attack were exacerbated at higher levels of discrimination. Similarly, everyday discrimination moderated the association between race and risk of heart attack, where differences in white and non-white participant's risk of heart attack were exacerbated at higher levels of discrimination. Experiences of discrimination in healthcare moderated associations between gender and both SRH and heart attack. Reduction in experiences of discrimination in all settings, but especially in healthcare, should be a top priority. Future work in the field of research around discrimination and health outcomes should address the mechanisms that are a product of historical systemic racism, ageism, and misogyny that impact the everyday lives of older adults, people of color, and women.

### HOPELESSNESS AMONG MIDDLE-AGED AND OLDER ADULTS: A COMPARISON OF NATIVE WHITES, NATIVE MINORITIES, AND IMMIGRANTS

Man Guo, Yi Wang, and Kara Carter, *University of Iowa, Iowa City, Iowa, United States*

Sense of hopelessness is closely linked to poor physical/mental health and elevated suicidal risk. The aging and immigration processes put middle-aged and older immigrants at a greater risk of feeling hopeless. However, we know little about hopelessness among this population. This study asks two questions: do middle-aged and older immigrants have higher levels of hopelessness compared to native-born Whites and native-born ethnic minorities? If so, what factors contribute to such differences? Data was from the 2018 psychosocial assessment of the Health and Retirement Study (N = 5,534). ANOVA was used to compare levels of hopelessness among three groups of middle-aged/older adults (50 yrs+): native-born Whites (n =3,603), native-born minorities (n = 1,209), and immigrants (n = 722). Linear regressions were used to examine the association between nativity/race and hopelessness, with five sets of explanatory variables (i.e., SES, health, social support, social engagement, and neighborhood characteristics) entered in the models individually and then collectively. The findings showed that middle-aged/older immigrants had the highest levels of hopelessness, followed by native minorities, and then native Whites. Controlling for each set of the explanatory variables respectively reduced the group differences between native Whites and native ethnic minorities, but not between Whites and immigrants. When controlling for all the explanatory variables, the levels of hopelessness no longer differed significantly between immigrants and native Whites. Findings suggest that immigrants' multiple disadvantages in personal, family, and social lives may contribute to their heightened levels of hopelessness.

Interventions are sorely needed to protect against hopelessness for this population.

### MIGRATION ACROSS THE LIFE COURSE AMONG OLDER ADULTS FROM PUERTO RICO

Maricruz Rivera-Hernandez, *Brown University School of Public Health, Worcester, Massachusetts, United States*

The prior literature regarding migration among Hispanics to US states has largely focused on younger migrants and often excluded people from Puerto Rico (e.g., Hispanic Paradox; Bostean, 2013). However, older Puerto Ricans appear to be moving to the US mainland (US Census Bureau, 2020, 2021). Therefore, the primary objective of this research was to examine migration among Medicare beneficiaries from Puerto Rico. Using data from Medicare, our results showed that migration trends among Medicare beneficiaries from Puerto Rico have increased over the past decade, including those with special needs. There were 5,593 (1%) Medicare beneficiaries who migrated in 2009 compared to 12,382 (~2%) beneficiaries who migrated in 2018. Among those who migrated, 3,679 were 65 and older in 2009 compared to 8,805 in 2018. The majority of older adults who moved to US states were female (59%), about 75 years old and were enrolled in Medicare Advantage. Destination states among Medicare beneficiaries from Puerto Rico included Florida, New York and New Jersey (comparable to those reported for the general population of Puerto Rico in the US Census). This research has policy implications. Although people from Puerto Rico have different reasons for migrating, those with long term care needs may have multiple varying reasons to move to the mainland. However, migration to the US may have additional challenges for people with high needs (Mellgard et al., 2019). For instance, Medicare coverage/network may not transfer to the US and plan switching/disenrolling may not be a straightforward process for people leaving Puerto Rico.

### TELEPHONE FRAUD AGAINST OLDER ADULTS IN CHINA: EVIDENCE FROM THE CHINA HEALTH AND RETIREMENT LONGITUDINAL STUDY

Rong-Fang Zhan[1], Elias Mpofu[2], and Cheng Yin[1], *1. University of North Texas, Corinth, Texas, United States, 2. University of North Texas, Denton, Texas, United States*

Background: Falling victim to telephone fraud is a devastating crisis for older adults because they can lose their life savings, adversely affecting their well-being. This study aimed to identify risk factors associated with telephone fraud among Chinese older adults. Method: A case-control study was conducted using the fourth wave of the 2018 China Health and Retirement Longitudinal Study data on older adults 60 years and above (N = 445 with telephone fraud experience; 9,994 with no fraud and other fraud experience). Control variables for the logistic regression analysis included the individual's demographics of gender, residential location, marital status, and educational level.

Results: Older adult males (OR = 1.424, 95% CI = 1.156-1.755, p = 0.001), urban residence (OR = 2.257, 95% CI = 1.706-2.986, p < 0.001), urban-rural integration zone (OR = 2.322, 95% CI = 1.654-3.260, p < 0.001), received higher education [OR = 1.704, 95% CI = 1.211-2.397, p = 0.007]), and higher pension classification (OR = 1.352, 95% CI = 1.220-1.500, p < 0.001) were associated with increased risks of telephone fraud.

Conclusion: Telephone fraud affects older adults in China at the upper rather than lower end of the socio-economic gradient, suggesting vulnerability from their use of digital technologies compared to those on the lower ladder of the socio-economic gradient. Findings highlight the need for fraud education of older adults with access to the digital broad band to reduce their risk for telephone fraud.

### SESSION 1100 (SYMPOSIUM)

### HEALTHY LEISURE: ADVANCING UNDERSTANDING OF LEISURE DECISIONS, ACTIVITIES, AND HEALTH CONSEQUENCES

Chair: Claire Smith Co-Chair: Soomi Lee Discussant: Allison Bielak

Leisure activities promote healthy aging, yet aging poses new challenges to engagement in active, meaningful, health-promoting leisure. This stalemate wherein people need to but struggle to engage in healthy leisure as they age calls for further research describing what healthy leisure entails, decision processes predicting it, and long-term consequences over the lifespan. This symposium brings together five rigorous studies aimed at understanding diverse aspects of leisure – how adults make daily leisure decisions, the health-promoting or demoting consequences of specific leisure activities as a person ages, and unique challenges faced by vulnerable populations. Paper 1 describes how daily energy and affect conjointly influence the types and variety of leisure activities midlife adults choose. Paper 2 identifies specific leisure activities that protect against cognitive decline in older adults, with consideration of vulnerable subgroups (illiterate, rural residents). Paper 3 investigates how seven leisure activities longitudinally predict cognitive impairment in older adults and differences by life course SES. Paper 4 identifies social interactions with friends as a key aspect of healthy leisure that longitudinally associates with cognitive function in older adults. Paper 5 focuses on a vulnerable population, older breast cancer survivors, and examines whether participating in cognitively stimulating leisure activities is associated with cognitive function (vs. non-cancer controls). These papers use different samples and datasets to describe the nature, causes, and consequences healthy leisure by specific population groups. The discussant, Dr. Allison Bielak will integrate key findings from these studies, discuss their theoretical and methodological contributions, and consider opportunities for future research.

### DECISIONS, DECISIONS: CHARACTERIZING WORKERS' DAILY DECISION PROCESSES DURING LEISURE TIME

Claire Smith, and Soomi Lee, *University of South Florida, Tampa, Florida, United States*

Decisions during adulthood set the foundation for healthy aging, but descriptions of healthy and unhealthy decision processes are missing. We extracted latent profiles of daily decision resources (energy and affect) and linked them to daily leisure activity. Diary data was collected from working adults (N=83; Mage=37 years) over the ten workdays (N=693).

We identified three daily decision profiles consistent with the Decision Triangle – (1) logical (energetic, unemotional), (2) automatic (less energetic, unemotional), and (3) visceral (unenergetic, highly emotional) – and one additional profile, (4) mild visceral (moderately unenergetic, moderately emotional). Daily logical decision-making related to more "want" leisure activities (i.e., aligned with desires/interests) and the greatest variety in leisure activities. Automatic engaged in the most chores. Visceral engaged in the fewest social activities and least variety in leisure activities. Our findings advance understanding of specific decision processes during leisure, which may have consequences for health and well-being as a person ages.

### SOCIAL INTERACTION WITH FRIENDS AND COGNITIVE FUNCTION: BENEFITS OF SPENDING TIME TOGETHER AND GIVING A HELPING HAND

Sae Hwang Han[1], Yee To Ng[2], and Julia Tucker[3], *1. University of Texas at Austin, Austin, Texas, United States, 2. UT Austin, Ann Arbor, Michigan, United States, 3. The University of Texas at Austin, New Braunfels, Texas, United States*

Despite the burgeoning literature linking social integration and cognitive function, studies that focus on the potential cognitive benefits associated with friendships are limited. Using eight waves of nationally representative data from the Health and Retirement Study (1998-2012), we investigated whether two distinct forms of social interaction with friends—(1) getting together for a chat/social visit and (2) providing informal helping to friends—were associated with cognitive function (assessed with a modified version of the Telephone Interview for Cognition Status) among individuals aged 50 and older (person N = 29,951) over a 14-year observation period. Multilevel models revealed robust evidence for within-person linkages between both forms of social interaction with friends and better cognitive function. The study findings are discussed in the context of declining social interaction with one's friends reported over the past several decades in the United States, which are partly driven by increasing leisure trends characterized by in-home entertainment.

### IMPACT OF LEISURE ACTIVITIES ON COGNITION IN OLDER BREAST CANCER SURVIVORS AND NONCANCER CONTROLS

Cassidy Doyle[1], Brent Small[1], Arielle Schwarzberg[1], Tim Ahles[2], Judith Carroll[3], and Jeanne Mandelblatt[4], *1. University of South Florida, Tampa, Florida, United States, 2. Memorial Sloan Kettering Cancer Center, New York, New York, United States, 3. University of California, Los Angeles, Los Angeles, California, United States, 4. Georgetown University, Washington, District of Columbia, United States*

Older adults represent the largest segment of cancer survivors and may be particularly susceptible to poor cognitive outcomes. This project examines the impact of leisure activities on cognitive performance in non-metastatic breast cancer survivors and healthy controls from the Thinking and Living with Cancer (TLC) Study. Additionally, this project investigates if genetic polymorphisms (APOE, COMT, BDNF) limit the benefits of leisure activities on cognitive performance. A battery of neuropsychological tests was used to create composite scores for learning and memory (LM) and attention, processing speed, and executive functioning (APE). Leisure activity participation was associated with better LM and APE composite scores. Age differences in LM and APE were not statistically significant among the most active participants. Regarding genetic polymorphisms, persons with APOE-ε4 exhibited greater cognitive performance benefits compared to non-ε4 carriers. Future research should examine the extent to which leisure activities can buffer longitudinal declines.

### LEISURE PARTICIPATION AND COGNITIVE IMPAIRMENT AMONG HEALTHY OLDER ADULTS IN CHINA

Wei Zhang[1], Qiushi Feng[2], Joelle Fong[2], and Huashuai Chen[3], *1. University of Hawaii at Manoa, Honolulu, Hawaii, United States, 2. National University of Singapore, Singapore, Singapore, 3. Xiangtan University, Xiangtan, Hunan, China (People's Republic)*

Leisure participation is beneficial to various health outcomes. This study examined a comprehensive list of leisure activities in relation to incidence of cognitive impairment among healthy older adults (65+) in China. Using data from the 2002-2018 Chinese Longitudinal Healthy Longevity Survey, we found that most of the leisure activities were protective of cognitive decline and three leisure activities (watching TV, doing housework, and playing cards/mah-jong) stood out as the most important ones. Additionally, our results revealed subgroup variations in the association between leisure participation and cognitive function: Leisure activities such as reading newspapers/books (not significant for the illiterate), gardening (not significant for the illiterate), and regular exercise (not significant for the rural residents) had different effects across different demographic social groups. Our findings suggest that intervention programs designed to prevent cognitive decline for older adults should consider subgroup and cultural variations in order to yield the best outcomes.

### LEISURE ACTIVITIES AND COGNITIVE IMPAIRMENT: HOW IS IT MODIFIED BY LIFE COURSE SES AND AGE AMONG CHINESE OLDER ADULTS?

Rongjun Sun, *Cleveland State University, Cleveland, Ohio, United States*

Recent research shows that not only do life course socioeconomic status and engaging in leisure activities have independent effects on cognitive performance of older adults, but also there are significant interaction effects between them. What is less clear is whether these effects, both separate and interactions between them, vary by age among older adults. We use data from the Chinese Longitudinal Healthy Longevity Survey 2005-2014 and a Generalized Linear Mixed Model to examine these relationships. Results show that engaging in leisure activities is significantly associated with cognitive impairment even into very old ages (85+), although such associations are partially absorbed by life course SES. Furthermore, interaction effects of leisure activities and life course SES are also detected across all age groups, although they vary by specific activities. Virtually all interaction effects point to one direction: individuals of higher life course SES enjoy extra benefits from engaging in leisure activities.

### SESSION 1110 (SYMPOSIUM)

### CHARTING THE POST-COVID-19 COURSE IN NURSING HOMES THROUGH STAKEHOLDER ENGAGEMENT AND ADVOCACY

Chair: Thomas Caprio Discussant: Brian Lindberg

Nursing homes sustained a disproportionate number of negative effects from the COIVD-19 pandemic including strained resources, significant decline in workforce capacity, high prevalence of infections and mortality among older adults, and a need for ongoing support and timely information related to COVID-19 vaccination and boosters. The HRSA-funded Geriatric Workforce Enhancement Programs (GWEPs) have responded to the growing need to support nursing homes in the recovery phase of the COVID-19 pandemic. Several of these GWEPs undertook new efforts under supplemental federal funding to conduct outreach and partner with nursing home stakeholders including direct care workers, residents, families, and community-based organizations to address gaps in education and training, to promote age-friendly care, and to address social determinants of health. In this symposium GWEPS from different geographic areas share their experiences in developing nursing home focused interventions, engaging feedback to refine curricula, and identify areas to advocate for future policy considerations to support and enhance nursing homes post-pandemic.

### AGE-FRIENDLY CARE: A FRAMEWORK TO ADDRESS HEALTH DISPARITIES IN NURSING HOMES

Leland Waters[1], Diane Berish[2], Annie Rhodes[1], and Michele Bellantoni[3], *1. Virginia Commonwealth University, Richmond, Virginia, United States, 2. Pennsylvania State University, University Park, Pennsylvania, United States, 3. Johns Hopkins University, Baltimore, Maryland, United States*

Three Geriatrics Workforce Enhancement Programs (Johns Hopkins, Penn State and Virginia Commonwealth) within Health and Human Services Region-3, implemented a project providing tailored educational content designed to meet the regional needs of nursing home staff, residents, and carepartners within the context of the COVID-19 pandemic. Semi-Structured, recorded focus groups were conducted with direct care workers, and family and resident counsels. All groups were interviewed about familiarity and comfort with the 3Ds (Dementia, Delirium, and Depression) as well as what mattered most to them as workers, residents and care partners. The overall goal was to enhance development of the nursing home workforce thus creating a meaningful clinical improvement in care. We aim to go beyond the improvement of routine clinical care and address the long-standing behavioral and mental health disparities observed in this resident population. Policy implications around education, engagement of workforce, resident, and carepartner voices will be discussed.

### STRATEGIES FOR SUPPORTING NURSING HOMES IN THE PANDEMIC RECOVERY AND BEYOND

Marla Berg-Weger, *Saint Louis University - - St Louis, MO, Saint Louis, Missouri, United States*

Best practice strategies are needed to address current and ongoing COVID-19 challenges for nursing home (NH) staff, residents, and families, including vaccine-related issues and staff, resident, and family mental health. Saint Louis University and University of Iowa Geriatrics Workforce Enhancement Programs along with Show-Me ECHO have engaged NH, community, and clinician stakeholders to identify and prioritize gaps in education/support needs, target audiences, and delivery methods. We conducted interviews with stakeholders at all levels of NH staff, community professionals, family members, and professional organizations to identify and prioritize needs. Guided by these findings and advisory groups at partner organizations, the collaborative developed outreach and curriculum strategies targeted to prioritized needs. This presentation emphasizes strategies for stakeholder engagement, identifying education/support needs, and outreach to NH workforce and care partners, and experiences and lessons learned in increasing staff vaccination rates will be shared. Implications for future policy and advocacy are highlighted.

### PROMOTING RESIDENT SAFETY THROUGH TRAINING FOR CERTIFIED NURSE AIDES, RESIDENTS, FAMILIES, AND OMBUDSMEN

Sarah Ross[1], Diana Cervantes[2], Jennifer Severance[3], and Janice Knebl[4], *1. University of North Texas Health Science Center, Fort Worth, Texas, United States, 2. UNTHSC, Fort Worth, Texas, United States, 3. UNTHSC-TCOM, Fort Worth, Texas, United States, 4. University of North Texas Health Science Center-TCOM, Fort Worth, Texas, United States*

Preventing COVID-19 infections and ensuring resident safety in the nursing home (NH) setting requires collaboration among NH leadership, staff, residents, and their advocates including families, caregivers, and nursing home ombudsmen. To address this multi-collaborative need, the University of North Texas Health Science Center Geriatric Workforce Enhancement Program developed training in two focus areas. The first focused on resilience training for Certified Nurse Aides (CNAs) with the United Way that addresses factors influencing well-being, economic stability, mental health, and other social determinants. The second focused on training residents, families, ombudsmen, and other resident advocates to become active partners with NH leadership and staff in the prevention and control of healthcare acquired infections. To ensure training reflects the needs and preferences of key NH stakeholders, we developed a Nursing Home Advisory Council in partnership with state Ombudsman Programs in Texas, Louisiana, and New Mexico.

### ADDRESSING THE NEEDS OF NURSING HOMES USING MULTIPLE APPROACHES TO EDUCATION AND SUPPORT

Sam Cotton[1], Anna Faul[1], Pamela Yankeelov[1], Barbara Gordon[1], Justin Magnuson[2], Cris Henage[3], Jennifer Hubbard[4], and Ellen Roberts[5], *1. University of Louisville, Louisville, Kentucky, United States, 2. University of Louisville Trager Institute, Louisville, Kentucky, United States, 3. Assistant Director of Education and Training, Chapel Hill, North Carolina, United States, 4. UNC Chapel*

*Hill School of Medicine, Chapel Hill, North Carolina, United States, 5. Division of Geriatric Medicine, UNC, Chapel Hill, North Carolina, United States*

At the University of Louisville and University of North Carolina Geriatric Workforce Enhancement Programs (GWEPs), we have collaborated to develop and implement a multi-faceted program to training to address the pressing challenges within NHs and foster community. We have created a Project ECHOs series for NH administers and nursing staff to foster support, while providing education on critical topics including age-friendly healthcare, vaccine hesitancy and infectious disease control, and ways to address health disparities. Additionally, we have developed videos for CNAs and direct support staff. For NH residents and families, we are designing education and offering services to address the disparities related to mental health needs. The UofL and UNC GWEPs mission is to provide a more holistic approach to NH care and opportunities for support and community. Implications for changes in policy and future practice within NHs and the use of collaborative approaches will be discussed.

### SESSION 1120 (SYMPOSIUM)

### PHYSICAL ACTIVITY AT THE INTERSECTIONS OF AGING AND DISABILITY

Chair: Annalisa Na Discussant: Annalisa Na

Physical activity is beneficial for older adults to maintain health and help manage chronic diseases but relies on routine participation. Some older adults continue with physical activity behaviors as they age, whereas others are negatively affected by impaired functional abilities, physical disabilities, and unmet psychosocial needs. This symposium presents quantitative and qualitative data on physical and psychosocial factors that can influence physical activity among older adults with varying levels and types of physical abilities. The first presentation will focus on aging into the disability associated with knee osteoarthritis, the leading cause of mobility decline among older adults that generally onsets after the fifth decade of life. The second presentation will focus on aging with a disability, specifically how health perspectives evolve when aging with a spinal cord injury. The third presentation will highlight how the COVID-19 pandemic has influenced outdoor physical activity among older adults when indoor activities became limited. The fourth presentation shifts perspectives to clinicians, specifically physical therapists, to explore the needs for addressing physical activity in the clinic. The fifth presentation outlines a physical activity intervention program and its implementation for community-dwelling older adults. Together, these presentations will provide practical insights for designing a person-centered program to improve physical activity for older adults including those aging into disability or aging with disability.

### IMPACT OF TOTAL KNEE ARTHROPLASTY ON PHYSICAL ACTIVITY

Annalisa Na[1], Olivia Rosenstein[1], Tony Chao[2], Chad Davenport[3], Karen Chapman[4], Ronald Lindsey[4], and Zbigniew Gugala[3], *1. Drexel University, Philadelphia, Pennsylvania, United States, 2. University Of Texas Medical Branch, Galveston, Texas, United States, 3. University of Texas Medical Branch, Galveston, Texas, United States, 4. University of Texas Medical Branch at Galveston, Galveston, Texas, United States*

Knee osteoarthritis (OA) pain often challenges the physical activity necessary for managing life-threatening chronic diseases. Standard severe knee OA treatment of total knee arthroplasty (TKA) and physical therapy (PT) is effective at improving pain and function but whether such benefits translate to physical activity is unclear. We enrolled 22 participants with severe knee OA scheduled for a TKA (Age,mean±SD=69.0±5.8y, female=63.6%) and assessed pain (i.e.,Numeric Pain Rating), physical function (i.e.,Knee Osteoarthritis Outcome Score), and physical activity (i.e.,activity monitors and journals for 7-days) before and 1-month, 3-month, and 6-month after TKA. Using paired-wise ANOVA, pre-to-postTKA pain and function improved but not physical activity. Using regression analyses, outpatient PT sessions during first two months post-TKA were positively related to 3- and 6-month physical activity (r=0.51-070, P=0.003-0.029). Standard TKA and PT for severe OA improved pain and function but not physical activity. However, early post-TKA PT and physical activity relationships are promising, warranting exploration.

### PERSPECTIVES ON HEALTH AND AGING WITH SPINAL CORD INJURY: A QUALITATIVE DESCRIPTIVE STUDY

Laura Baehr[1], Kathleen Fisher[1], and Margaret Finley[2], *1. Drexel University, Philadelphia, Pennsylvania, United States, 2. Drexel Univeresity, Philadelphia, Pennsylvania, United States*

Age of spinal cord injury (SCI) onset and life expectancy have increased over the last half century, but minimal inquiry on health and aging lived experience perspectives are available. The objective of this study was to begin to examine this through a qualitative descriptive design. Nine group interviews were conducted with individuals living with SCI (n=24) 22-76 with injury duration 3-47 years. Participant descriptions on health and aging were thematically analyzed. Health maintenance was related to physical routine to prevent secondary health conditions, injury acceptance, and engagement with disability networks. Aging outlook was connected to fear of dependence and lack of education on aging with SCI. These findings demonstrate that clinicians and researchers should investigate issues beyond routine self-management to support life with SCI. Personal considerations based on life stage when injured warrants investigation. Advocacy for peer-support is imperative at all life stages given its positive impact on health.

### OUTDOOR PHYSICAL ACTIVITY AMONG OLDER ADULTS DURING THE COVID-19 PANDEMIC

Justine Sefcik[1], Amina Panicker[2], Danielle Katsev[2], Annalisa Na[1], Sherry Greenberg[3], and Rose Ann DiMaria-Ghalili[4], *1. Drexel University, Philadelphia, Pennsylvania, United States, 2. Drexel Univeresity, Philadelphia, Pennsylvania, United States, 3. Monmouth University, Marjorie K. Unterberg School of Nursing & Health Studies, West Long Branch, New Jersey, United States, 4. Drexel University, College of Nursing and*

*Health Professions, Philadelphia, Pennsylvania, United States*

There is a gap in understanding how the current pandemic is affecting older adults' outdoor physical activity. This study aimed to explore older adults' perceptions of their outdoor physical activity during the current pandemic. A qualitative descriptive approach was taken with a conventional content analysis. Participants were primarily recruited through ResearchMatch. Eighteen community-dwelling older adults were individually interviewed from geographical locations across the United States; 61.1% female, 88.9% White, mean age 76.4 (range 68-92), 5 ambulated with a cane or walker. We identified an overarching theme of Benefits and Motivation in which older adults conveyed wanting to maintain and improve their health and used the outdoors to continue physical activity since indoor activities decreased during the pandemic. Walking was expressed as the most frequent outdoor physical activity. Implications of these findings will be discussed which include supporting community improvements to facilitate older adults' ease of maintaining a walking routine.

### FROM KNOWING TO DOING: WHAT IS NEEDED TO SUPPORT PATIENTS IN CHANGING PHYSICAL ACTIVITY

Anne Thackeray[1], Tonua Hamilton[1], Janice Morse[1], Rachel Hess[1], Molly Conroy[2], and Julie Fritz[1], *1. University of Utah, Salt Lake City, Utah, United States, 2. University of utah, Salt Lake City, Utah, United States*

Physical therapists often treat pain and functional limitations associated with chronic musculoskeletal conditions common in aging adults. While patient report improvement after physical therapy, these results do not translate to sustained physical activity. This is a lost opportunity to support aging adults in adopting behaviors proven to improve quality of life and reduce comorbidity burden. We conducted semi-structured interviews with 30 physical therapists to understand how they support adoption of physical activity and identify what is needed to improve uptake. Physical therapists endorse physical activity as essential in the management of MSK conditions. Eliciting motivation, addressing psychosocial needs, and empowering patients to actively engage in solutions were identified as significant challenges in the effort to change physical activity. At the clinician level, physical therapists identified the need for improved skills in motivational interviewing and person-centered communication. Improved coordination with mental health providers and community resources were identified as environmental needs.

### COACH2MOVE: THE STORY OF IMPLEMENTING A PERSON-CENTERED PHYSICAL THERAPY APPROACH IN CLINICAL PRACTICE

Ward Heij[1], Lieke Sweerts[2], Bart Staal[2], Philip van der Wees[2], Ria Nijhuis-van der Sanden[2], and Thomas Hoogeboom[2], *1. University of Utah, Salt Lake City, Utah, United States, 2. Radboud university medical center, Nijmegen, Gelderland, Netherlands*

Coach2Move is a person-centered physical therapy approach to promote physical activity among community-dwelling older adults, which consists of motivational interviewing and other motivational strategies. Coach2Move has been implemented in the Netherlands with 52 physical therapists and 294 patients in 16 practices. Implementation has led to better outcomes (increased physical activity, improved functional mobility, and lower levels of frailty) in fewer treatment sessions compared with usual care physical therapy treatment. Implementing was carried out using the following steps: 1) an e-assessment to determine the baseline level of knowledge regarding Coach2Move of the participating therapists; 2) two education days in Motivational Interviewing and Coach2Move specific motivational strategies; 3) three unique peer-assessment meetings and 4) a retest of the e-assessment one year later. We will discuss our rationale for selecting implementation strategies and which barriers and facilitators we have experienced in implementing successful scientific research in daily clinical practice.

### SESSION 1130 (SYMPOSIUM)

### PSYCHOSOCIAL RESILIENCE, FAMILY SUPPORT, AND COGNITIVE STATUS: FROM EVIDENCE TO ACTION

Chair: Hanzhang Xu Co-Chair: Bei Wu Discussant: Man Guo

Increasing evidence suggests the important role of social connections and family support in maintaining optimal cognitive status among older adults. This symposium includes four studies from China and the U.S with a focus on generating actionable evidence to inform the development of strategies that target psychosocial resilience and family support to promote cognitive health. Using data from the 2006, 2010, and 2014 waves of the Health and Retirement Study, the first study assessed the impact of social isolation on cognitive function, and how sleep disturbance mediated the association on cognitive decline. The study findings suggest addressing sleep disturbance might be a viable way to mitigate the negative effect of social isolation on cognitive function. Companion piece includes another HRS-based study that assessed the impact of loneliness on psychological resilience and cognitive health in later life. Findings from this study show loneliness is indirectly associated with baseline cognitive status and accelerated cognitive decline through deteriorating phycological resources. The third study used a prospective longitudinal design and applied group-based trajectory modeling to identify distinct family functions among 170 Chinese stroke survivors. Four family function trajectories were identified; healthy and stable family function was associated with better cognition and quality of life. Lastly, the fourth study aimed to use an experienced-based co-design approach to develop a cognitive training intervention to promote cognitive health in older Chinese immigrants in the U.S. This approach allows researchers to engage end-users early and to optimize the development of a culturally and linguistically relevant cognitive training intervention.

### COGNITIVE TRAINING FOR OLDER CHINESE IMMIGRANTS IN THE US: AN EXPERIENCE-BASED CO-DESIGN APPROACH

Hanzhang Xu[1], Eleanor McConnell[1], John myers[2], and Bei Wu[3], *1. Duke University, Durham, North Carolina,*

*United States, 2. Duke University School of Nursing, Durham, North Carolina, United States, 3. New York University, New York, New York, United States*

Older Chinese Americans face many socioeconomic barriers including limited English proficiency, low educational levels, and limited access to care. These socioeconomic disadvantages not only contribute to an increased risk of developing dementia but also worsen inequitable access to effective strategies to promote cognitive health. Cognitive training is shown to be beneficial to maintain or enhance cognitive function. However, most prior interventions were tested exclusively in non-Hispanic Whites. To address this gap, we aim to adapt empirically supported cognitive training activities into a culturally and linguistically relevant mHealth cognitive training intervention. The adaptation process of the cognitive training includes focus groups (n=6/group) with older Chinese Americans (3 groups) and adult children (2 groups) to adapt cognitive training components to our target population. We will then organize an experience-based co-design workshop to further refine the intervention. Engaging end-users early will optimize the development of a culturally and linguistically relevant cognitive training intervention.

### SOCIAL ISOLATION, SLEEP DISTURBANCE, AND COGNITIVE HEALTH: A LONGITUDINAL MEDIATION STUDY

Xiang Qi[1], Yaolin Pei[1], Susan Malone[2], and Bei Wu[3], *1. New York University, New York City, New York, United States, 2. New York University, College of nursing, New York City, New York, United States, 3. New York University, New York, New York, United States*

Research indicates that social isolation is associated with dementia, but the role of sleep on this association is less known. We aimed to examine the impact of social isolation on cognitive function, and how sleep disturbance mediated the association. Data came from the 2006, 2010, and 2014 waves of the Health and Retirement Study. Participants aged 50+ who completed the Psychosocial and Lifestyle Questionnaire in 2006 were included (n=5,036). Measures include the Social Isolation Index, modified Jenkins Sleep Scale, and Telephone Interview of Cognitive Status. Cross-lagged panel models were used in the analysis. After controlling socio-demographics, lifestyle, and loneliness, social isolation predicted subsequent sleep disturbance ($\beta$=0.05; P<0.01), which in turn predicted worse cognitive functioning ($\beta$=-0.02; P<0.01). The reverse pathways from cognitive function to social isolation were also statistically supported. Public health initiatives could reduce sleep disturbance by facilitating social integration and participation in community activities, thereby protecting against cognitive decline.

### LONELINESS AND ACCELERATED COGNITIVE DECLINE: MEDIATION EFFECTS OF RESILIENCE AND PURPOSE OF LIFE

Yaolin Pei[1], Xiang Qi[1], Sammy Wang[2], and Bei Wu[3], *1. New York University, New York City, New York, United States, 2. Rory Meyers College of Nursing, New York City, New York, United States, 3. New York University, New York, New York, United States*

Drawing from the Conservation of Resources Theory, we aim to understand the implications of loneliness on psychological resources (i.e., resilience and purpose in life) and cognitive health in later life. This study utilizes data (2006-2018) from the Health and Retirement Study to examine pathways, both direct and indirect through psychological resilience and purpose in life, from loneliness to cognitive trajectories over time. Respondents reporting higher levels of loneliness had worse initial cognitive function ($\beta$=-0.43; P<0.01) and accelerated cognitive decline ($\beta$=-0.05; P<0.01). Feeling lonely is associated with reduced resilience ($\beta$=-0.23; P<0.01) and purposed in life ($\beta$=-0.17; P<0.01) which, in turn, are associated with worse cognitive health. Finally, pathway analyses confirm that loneliness is indirectly associated with initial cognitive health and accelerated cognitive decline through deteriorating phycological resources. Positive psychological interventions can be beneficial by promoting resilience and purpose in life and subsequently improve cognitive health.

### THE TRAJECTORY OF FAMILY FUNCTION OF STROKE SURVIVORS AND ITS IMPACT ON COGNITIVE IMPAIRMENT: A LONGITUDINAL STUDY

Juan Li[1], Zhijian Liu[2], Fanfan Li[2], Jing Wang[3], and Bei Wu[4], *1. Huashan Hospital affiliated to Fudan University, Shanghai, Shanghai, China (People's Republic), 2. Naval Military Medical University, Shanghai, Shanghai, China (People's Republic), 3. University of North Carolina at Chapel Hill, Chapel Hill, North Carolina, United States, 4. New York University, New York, New York, United States*

This study examined the trajectory of family function of Chinese stroke survivors and its impact on cognitive impairment.We conducted a prospective longitudinal study and assessed family function of 170 stroke survivors and their family caregivers by Family Assessment Device(FAD) during acute stage and at 3, 6 months after onset via face-to-face follow-ups interview in Nanjing, China from January to October, 2020.Group-based Trajectory Model was applied to analyze trajectories of family function. Family function deteriorated with time. Four patterns of FAD trajectory (A: dysfunction-deterioration-improvement; B: severe dysfunction-stable; C: healthy function-rapid deterioration; D: approximate healthy function-stable) were identified. Healthy and stable family function was associated with better cognition and quality of life ($\beta$=-0.216,p<0.05, $\beta$=-0.159, p<0.05, respectively). Future studies are needed to further explore the reason why family function deteriorated and the linkage between family function and stroke survivors' outcomes.

### SESSION 1140 (BIOLOGICAL SCIENCES INVITED SYMPOSIUM)

### SYSTEMS BIOLOGY OF AGING

Chair: Timothy Rhoads

Program Overview: Aging is a complex process; numerous aspects of cell and animal physiology change with age and pinpointing a single causal mechanism has proven difficult. A systems-level approach, which attempts to integrate multiple perspectives of aging into a more cohesive understanding, addresses this challenge by leveraging the explosion in recent years of molecular and biological "big data." In this session, speakers will describe their efforts using systems biology approaches to achieve a multivariate assessment of the mechanisms of aging. Dr. Gladyshev will speak on recent work to understand the systemic mechanisms that underpin lifespan control. Dr. Levine moves the focus to epigenetic measures of aging, an emerging biomarker. Dr. Kane will describe efforts to examine frailty phenotypes and their biological underpinnings, as well as sexual dimorphism during aging in mice. Dr. Rhoads will describe the broad landscape of RNA processing, a mechanism with a recently described association with aging and delayed aging by caloric restriction, in multiple organisms and tissue contexts, as well as engagement of this mechanism during different aging interventions. Together our speakers advance our understanding of the multifactorial nature of aging biology and set the stage for the development of interventions that target all aspects of the aging process.

### RNA PROCESSING MECHANISMS DURING AGING AND CALORIC RESTRICTION

Timothy Rhoads, Josef Clark, and Rozalyn Anderson, *University of Wisconsin-Madison, Madison, Wisconsin, United States*

Caloric restriction (CR), a reduction in caloric intake without malnutrition, was first described in the 1930s and is a robust model to extend lifespan. While the specific mechanisms that produce delayed aging are still unknown, work has demonstrated the importance of metabolism – dysregulated mitochondrial metabolism is an aging hallmark, while CR upregulates energy metabolism. Crucially, transcriptional regulation plays a key role in implementing this metabolic program although details are still unclear. Splicing factors that are required for the beneficial effects of dietary restriction in nematodes have been identified, and large-scale exon usage changes have been observed across the metabolic network engaged by CR in various tissues from non-human primates, suggesting a translational mechanism that might contribute to how CR delays aging. We will discuss the landscape of RNA processing across multiple organism, tissue, and intervention contexts and provide a broad perspective of how RNA processing contributes to enhanced longevity.

### INVESTIGATION OF BIOLOGICAL DETERMINANTS OF FRAILTY IN MALE AND FEMALE MICE

Alice Kane[1], Patrick Griffin[2], Matthew Arnold[3], Maeve McNamara[2], Daniel Vera[4], David Vogel[4], and David Sinclair[3], *1. Institute for Systems Biology, Seattle, Washington, United States, 2. Harvard Medical School - Blavatnik Institute, Boston, Massachusetts, United States, 3. Harvard Medical School, Boston, Massachusetts, United States, 4. Volo Foundation, Jupiter, Florida, United States*

Frailty is quantified as the accumulation of health deficits over an organism's lifetime and gives a measure of the overall health of an organism. Preliminary studies have found an association between frailty and some of the hallmarks of aging including inflammation and senescence. However, the molecular underpinnings of frailty, and whether the mechanisms of frailty are distinct from or overlap those of aging is unknown. Previously we developed clocks based on frailty assessments that accurately predict age and lifespan in older male mice. Here, we expand these clocks to predict age, lifespan and frailty itself in younger, and also female mice. Additionally, we incorporate molecular measures including blood cell composition, plasma metabolomics, PBMC DNA methylation and stool microbial diversity measures into the clocks. These clocks provide important tools for the field, and also provide an indication of the molecular underpinnings of frailty and sex differences in these.

### SYSTEMS AGING AND REJUVENATION

Vadim Gladyshev, *Brigham and Women's Hospital, Boston, Massachusetts, United States*

What is aging? When does it begin? How to control lifespan? Can we rejuvenate organisms in addition to slowing down the aging process? There is no consensus on these questions, but recent developments in the field may allow to address these questions. In particular, DNA methylation of defined sets of CpG dinucleotides emerged as a critical and precise biomarker of the aging process. Multi-variate machine learning models, known as epigenetic clocks, exploit quantitative changes in the methylome to predict the age of bulk tissue with remarkable accuracy. Additionally, the first epigenetic aging clock that works at the level of single cells has been developed. Together with advances in genomics, transcriptomic longevity signatures and intervention strategies, these tools support quantification and manipulation of the aging process. Moreover, these tools may also be used to assess the possibility of age reversal. Several types of rejuvenation have been described, including the recently discovered process of early embryonic rejuvenation, culminating in ground zero, marking the beginning of organismal aging.

### SYSTEM SPECIFIC AGING SCORES: A STATE OF THE ART AGING CLOCK BUILT USING AGING SCORES FROM DIFFERENT BODILY FUNCTIONS

Raghav Sehgal[1], Albert Higgins-Chen[1], Margarita Meer[2], and Morgan Levine[2], *1. Yale University, New Haven, Connecticut, United States, 2. Altos Labs San Diego Institute of Science, San Diego, California, United States*

Aging is a highly heterogeneous process at multiple levels. Different individuals, organs, tissues, and cell types are innately diverse and age in quantitatively different manners. Epigenetic clocks have been developed to capture overall degree of aging and typically report a single biological age value. However, single measures fail to provide insight into differential aging across organ systems. Our aim was to develop novel systems-specific methylation clocks, that when assessed in blood, capture distinct aging subtypes. We utilized three large human cohort studies and employed both supervised and unsupervised machine learning models by linking DNA methylation to lower dimensional vectors composed of system specific clinical chemistry and functional assays. In doing so, we were able to develop 11 unique system-specific scores–heart, lung, kidney, liver, brain, immune, inflammatory, hematopoietic, musculoskeletal, hormone, and metabolic. We observe that in independent data, the specific systems relate to meaningful outcomes–for instance the brain score is strongly associated with cognitive functioning; musculoskeletal score is strongly associated with physical functioning; and the lung score is strongly associated with lung cancer. Additionally, system scores and the composite systems clock outperforms presently available clocks in terms of associations with a wide variety of aging phenotypes and conditions. Overall, our biological systems based epigenetic clock outperforms presently available epigenetic aging clocks and provides meaningful insights into heterogeneity in aging.

### SESSION 1150 (SYMPOSIUM)

### THE ART OF BALANCE: EMPOWERING DEMENTIA CARE PARTNERS TO OPTIMIZE THEIR WELLNESS, RESILIENCE, AND HEALTH

Chair: Tatiana Sadak Discussant: Soo Borson

By 2050, 140 million people will be living with dementia (WHO, 2021), most of whom will be cared for at home by family members, relatives, partners, friends, and neighbors, supplemented by help from paid caregivers. Because of their critical role in managing health and wellness for people living with dementia (PLWD), caregivers face unique stressors and challenges that can give rise to negative physical and mental health consequences. This symposium aims to strengthen our understanding about how to empower care partners to optimize health and wellness for themselves and PLWD in everyday living and during periods of health crisis. The first presentation characterizes behaviors care partners adopt that promote resilience in the face of dementia-related health crises (Zhou et al); the second describes facilitators and barriers to adoption of self-care behaviors that can promote resilience (Sadak et al). We then introduce a technology-enabled pilot intervention to strengthen care partners' self-care behaviors (Ishado et al.) and a physical activity intervention to promote maintenance of function (Li et al.). We conclude with a panel discussion of cross-cutting implications of these approaches for future research, clinical care, and policy.

### DEMENTIA CARE PARTNER SELF-CARE: A FRAMEWORK TO ILLUSTRATE MECHANISMS OF FACILITATORS AND BARRIERS

Tatiana Sadak[1], Susie Cho[2], Emily Ishado[1], and Soo Borson[3], *1. University of Washington, Seattle, Washington, United States, 2. UW, Seattle, Washington, United States, 3. USC, Santa Ana, California, United States*

Dementia care partner (CP) self-care is an important intervention target to enhance resilience and reduce risk of negative physical and psychological outcomes, such as illness, functional decline, depression and anxiety. Yet few studies have explored CP perceptions of what helps vs prevents them from consistently engaging in self-care activities. This exploratory study examined data from 100 semi-structured CP interviews on the barriers and facilitators to self-care. Inductive thematic analysis identified multiple facilitators and barriers that can be characterized as actual (did affect self-care) or potential (would affect self-care). CP capacity to participate in self-care activities was influenced by internal (e.g. setting boundaries) or external (e.g. social support) factors. We developed a framework to illustrate these mechanisms and guide interventions for optimizing self-care behaviors.

### MANAGING YOUR OWN WELLNESS: A PILOT INTERVENTION TO IMPROVE DEMENTIA CARE PARTNER SELF-CARE

Emily Ishado[1], Sarah McKiddy[1], Soo Borson[2], and Tatiana Sadak[1], *1. University of Washington, Seattle, Washington, United States, 2. USC, Santa Ana, California, United States*

Engagement in regular self-care activities could mitigate and reduce the risk of negative health outcomes in dementia care partners (CP), yet many report that they do not prioritize wellness-related activities for themselves. We conducted a pilot of a virtual technology-supported intervention with 19 dementia CP to evaluate usability, feasibility, acceptability, and preliminary effectiveness of cultivating daily self-care habits and creating a wellness routine based on personalized self-care goals. Case studies and inductive thematic analyses suggested that the intervention could help CP meet their self-care activity goals, improve their sense of well-being and capacity to provide care. After establishing the initial feasibility and usability of the intervention and the online goal-setting and tracking platform, we collaborated with scientists specializing in Human-Centered Design and Engineering to develop a prototype of a mobile application that will optimize portability, utility, and support testing of this intervention.

### VALIDATING A BEHAVIORAL FRAMEWORK OF RESILIENCE IN DEMENTIA CARE PARTNERS: AN ABDUCTIVE THEMATIC ANALYSIS APPROACH

Yuanjin Zhou[1], Dilara Hasdemir[1], Emily Ishado[2], Soo Borson[3], and Tatiana Sadak[2], *1. University of Texas at Austin, Austin, Texas, United States, 2. University of Washington, Seattle, Washington, United States, 3. USC, Santa Ana, California, United States*

One barrier to developing resilience-enhancing interventions for dementia care partners is a lack of an objective measure to capture resilience-related behaviors. To validate a behavioral framework of resilience in dementia care partners for future measure and intervention, we conducted semi-structured interviews with 26 care partners who experienced challenges created by a health crisis of their care recipients. We used abductive thematic analysis to identify 14 resilience-related behaviors care partners adopted to recover from care challenges. These behaviors included four problem-response behaviors, three help-related behaviors, three self-growth behaviors, two compassion-related behaviors, and two learning-related behaviors. Twelve care partners reported full recovery from the effects of their challenging experiences. Ten care partners reported partial recovery in the face of PLWD's declining health and their own unmet needs, and four care partners felt not at all recovered. This finding suggests new intervention targets for enhancing care partner resilience.

### INVESTIGATING PHYSICAL EXERCISE-RELATED MEDIATING VARIABLES IN THE RDAD-NW TRANSLATIONAL STUDY

Jingyi Li[1], and Barbara Cochrane[2], *1. University of Washington, Sammamish, Washington, United States, 2. University of Washington School of Nursing, Seattle, Washington, United States*

Declining physical function significantly worsens quality of life in dementia care dyads. Caring for people living with dementia (PLWD) at home is both physically demanding and emotionally stressful. Physical exercise is strongly recommended as a way of sustaining or improving overall function in dementia care dyads. However, little is known regarding the impact of home-based dyadic exercise interventions. We present results from a secondary analysis of data from a multicomponent translational study, Reducing Disability in Alzheimer's Disease – North West (RDAD-NW). We report results from an examination of the mediating role of exercise on the trajectory of change in physical function over time for both PLWD and their care partners.

### SESSION 1160 (PAPER)

### TRANSITIONAL CARE

### CARE COORDINATION, HEALTH OUTCOMES, AND HEALTHCARE UTILIZATION AMONG ADULTS WITH MULTIMORBIDITY

Briana Mezuk[1], Weidi Qin[2], Linh Dang[2], and Rodlescia Sneed[3], *1. University of Michigan, School of Public Health, Ann Arbor, Michigan, United States, 2. University of Michigan, Ann Arbor, Michigan, United States, 3. Wayne State University, Detroit, Michigan, United States*

Care coordination is a vehicle for improving patient-provider and provider-provider communication to improve outcomes and reduce unnecessary healthcare utilization, particularly for adults with multimorbidity. However, the clinical effectiveness of coordination at the population level remains unknown. This study examined the association between experiences of care coordination with subsequent health and healthcare outcomes among US adults over age 50. The analytic sample (n=695) included respondents from the Health and Retirement Study who had at least two chronic conditions, completed an Experimental Module on Coordinated Care in 2016, and were re-interviewed in 2018. Three domains of care coordination were examined as predictors: perceptions of coordination; using tangible supports (e.g., seeing a care coordinator); and using technical supports (e.g., patient portal). A range of outcomes related to health (i.e., self-rated health, functioning, pain) and healthcare (i.e., medication adherence, visits, hospitalizations, care satisfaction) were assessed in 2018. Weighted linear and logistic regression models, adjusted for demographic and socioeconomic characteristics, were fit for each lagged outcome. Higher engagement with tangible supports was positively associated with subsequent hospitalization (OR: 1.08, 95%CI: 1.01-1.15), greater pain (OR: 1.11 , 95%CI: 1.03-1.20), and marginally worse self-rated health (B=-0.02, $p < 0.063$). Better perceptions of coordination were also positively associated with care satisfaction (B=0.03, $p < 0.020$). Care coordination was not associated with functioning, adherence, or number of medical visits. Findings indicate the salience of tangible support for coordination among older adults with multi-morbidity, and that positive perceptions of coordination contribute to healthcare satisfaction.

### PATIENT AND CAREGIVER PERCEPTIONS OF MOBILITY AFTER DISCHARGE FROM SKILLED NURSING FACILITIES TO HOME

Ayomide Bankole[1], John Preisser[2], Ying Zang[3], and Mark Toles[4], *1. University of North Carolina-Chapel Hill, Chapel Hill, North Carolina, United States, 2. University of North Carolina- Chapel Hill, Chapel Hill, North Carolina, United States, 3. University of North Carolina at Chapel Hill, Chapel Hill, North Carolina, United States, 4. UNC Chapel Hill, Chapel Hill, North Carolina, United States*

Functional outcomes of older adults in post-acute care is poorly understood. The purpose of this study was to describe skilled nursing facility (SNF) patient or caregiver perceptions of patient mobility and factors associated with patient mobility in 30 days after discharge from SNF to home. We conducted a secondary analysis of data from a stepped-wedge cluster randomized trial of the Connect-Home intervention set in 6 SNFs in North-Carolina. The sample included SNF patients (N=249) and their caregivers (N=249). In 30 days after discharge, SNF patients or their caregivers (as proxies) rated patient mobility using the Life-Space Assessment (LSA). LSA total-scores (range= 0-120), with higher scores indicating greater mobility. Linear mixed models, with random effects to adjust for clustering in SNFs, were used to describe associations between LSA total-score and patient, caregiver, and health-system factors. Participants were typically female (63%), White (74%), and average age of 76.3 years. In 30 days after discharge, LSA total-scores ranged from 0-90 with a mean score of 22 (SD=16.01). Most (85%) required caregiver and/or assistive devices to transfer inside and outside of their homes. In the mixed models, lower LSA scores were associated with older age [-0.232(0.108), $p < 0.05$], lower cognition score [1.182(0.384), $p < 0.005$], diagnosis of dementia [-6.863(3.057), $p < 0.05$], lower baseline mobility in the SNF [0.296(0.142), $p < 0.05$] and longer SNF stay [-0.200(0.068), $p < 0.005$]. Low LSA scores in this sample suggest the need for caregiver support to continue rehabilitation and promote functional ability at home. Studies are needed to augment transitional care and address these care needs.

### PROVIDING INPATIENT CARE BEYOND HOSPITAL WALLS: GEOGRAPHIC FACTORS IN ACUTE HOSPITAL CARE AT HOME WAIVER PROGRAMS

Emily Franzosa[1], Ksenia Gorbenko[1], Abigail Baim-Lance[1], Gabrielle Schiller[2], Heather Wurtz[1], Sybil Masse[1], Katherine Ornstein[3], and Bruce Leff[4], *1. Icahn School of Medicine at Mount Sinai, New York City, New York, United States, 2. CUNY School of Public Health, Astoria, New York, United States, 3. Johns Hopkins, University, Baltimore, Maryland, United States, 4. Johns Hopkins University School of Medicine, Baltimore, Maryland, United States*

The Centers for Medicare and Medicaid Services' (CMS) Acute Hospital Care at Home waiver offers hospital-level reimbursement to provide acute hospital-level care in patients' homes for the first time. While this initiative may make acute care at home more financially viable for health systems, it also requires aligning Hospital at Home (HaH) operations with inpatient, rather than outpatient, regulatory requirements. We aimed to understand how participating HaH

programs adapted to these requirements. We conducted semi-structured interviews with multiple leaders from 14 HaH waiver programs (n=18 clinical/medical, operational and program directors) varying in size, urbanicity, structure, and region, examining data through thematic analysis. Both urban and rural participants described geographic effects of waiver requirements. For instance, to ensure response to patient emergencies within 30 minutes, programs contracted with paramedic services to expand service areas, added program locations or moved primary locations to other system hubs. Programs maximized staff capacity across service areas by "leasing" staff from other home-based programs, focusing on urban hubs with more staff, balancing in-person visits with remote monitoring, and providing "hybrid" in-person/video appointments. However, travel time, length of acute care visits, staffing shortages, the need for new skills (e.g., acute care nurses, dietitians) and limited state scope of practice regulations, particularly for paramedics, limited the area and populations served. Adapting to waiver requirements required significant efforts to address staffing, logistical and regulatory challenges. Future waiver improvements should explicitly consider the unique resources needed to expand hospital-level care in geographically diverse ambulatory environments.

### THE PARADOXICAL EXPERIENCES OF INFORMAL CAREGIVERS DURING THE TRANSITION FROM HOME TO A NURSING HOME

Lindsay Groenvynck[1], Bram de Boer[1], Audrey Beaulen[1], Erica De Vries[1], Jan Hamers[1], Theo van Achterberg4, Judith Meijers[1], and Hilde Verbeek[1], *1. Maastricht University, Maastricht, Limburg, Netherlands, 2. KU Leuven, Leuven, Vlaams-Brabant, Belgium*

The transition from home to a nursing home is a complex and emotional process, especially for older persons with dementia. The informal caregiver usually plays a central role in this care process, which is often fragmented. Therefore, this study aims to analyze the experiences of informal caregivers of older persons with dementia during this transition.An interpretative phenomenological design was used to analyze secondary data. In-depth interviews were conducted with informal caregivers, in the Netherlands, between February 2018 and July 2018. The study identified three, interwoven paradoxes influenced by the healthcare system and the healthcare professionals providing care. The first paradox described the initial negative emotions related to a nursing home move. Those emotions are a paradox to the feelings of relief and acceptance later in the transition process. The second paradox was related to a prospective need to postpone the transition for as long as possible and a retrospective need for a timely transition plan. The third paradox defines an internal struggle for the informal caregivers of wanting to remain involved while simultaneously experiencing a need for distance from care responsibilities. This study identifies a fine line between optimal and fragmented transitional care. The results can motivate informal caregivers to start planning the move. Similarly, it allows healthcare professionals to provide tailored support. Future research should focus on defining these paradoxes and their link with the healthcare system to determine if the transition from home to a nursing home can be optimized.

### THE ROLE OF SUBJECTIVE AGE IN PREDICTING POST-HOSPITALIZATION OUTCOMES

Anna Zisberg[1], Nurit Gur-Yaish[2], Efrat Shadmi[3], Ksenya Shulyaev[3], Juliana Smichenko[3], and Yuval Palgi[3], *1. University of Haifa, Israel, Haifa, Hefa, Israel, 2. Oranim Academic College of Education, Tivon, Hefa, Israel, 3. University of Haifa, Haifa, Hefa, Israel*

Subjective age contributes to a range of health and functional outcomes in older adults. Most of the evidence comes from studies in community dwelling older adults. The current study explores whether younger subjective age serves as a protective factor against hospital associated physical, cognitive, and emotional decline. This paper is a secondary analysis of a subsample (N=250) from the HoPE-MOR (Hospitalization Process Effects on Mobility Outcomes and Recovery) study for which subjective age was assessed at the time of hospital admission and outcomes were measured one-month post-discharge. Psychological and physiological subjective age was measured as a person's report on the degree to which they feel older or younger compared to their chronological age on a 5-point Likert-type scale. Measures of independency in Activities of Daily Living, Life-space mobility, cognitive function and depressive symptoms, were based on participants' assessment at admission and one-month post-discharge. In a sample of acutely ill participants, age 77.5±6.6, those with younger psychological subjective age had a significantly lower odds for poorer mental (OR=0.66, 95%CI 0.45-0.97), functional (OR=0.62, 95%CI 0.43-0.90) and cognitive state (OR=0.60, 95%CI 0.36-0.98), and better life-space mobility (OR=0.67, 95%CI 0.47-0.95). Findings were significant after controlling for numerous demographic, functional, cognitive, emotional and chronic and acute health predictors. Physiological subjective age was not significantly related to post hospitalization outcomes. Psychological subjective age could serve as a relatively simple parameter to identify older adults who are at risk for poor hospitalization outcomes for consideration of inclusion in preventive in-hospital and post discharge interventions.

### SESSION 1170 (SYMPOSIUM)

### USING RESEARCH TO ADVANCE THE DEMENTIA-FRIENDLY COMMUNITY MOVEMENT AT THE LOCAL, NATIONAL, AND INTERNATIONAL LEVEL

Chair: Clara Scher Co-Chair: Emily Greenfield Discussant: Joseph Gaugler

It is estimated that more than 55 million people worldwide are living with dementia. To address the social and health needs of individuals living with dementia and their care partners, researchers, policymakers and advocates have championed dementia-friendly communities (DFCs) as a population-level response. DFCs promote the well-being of those living with dementia, empower all members of the community to celebrate the capabilities of persons with dementia, and encourage individuals living with dementia to engage in their communities. The objective of this symposium is to describe the ways in which research can help to advance the dementia-friendly movement at the local, national, and international levels. First, Scher & Greenfield will describe the dimensions of implementation of DFCs in Massachusetts with implications for program monitoring and process evaluation. Second, Epps & colleagues will discuss the process of developing a person-centered tool to evaluate the impact of dementia-friendly programs in faith-based communities. Third, Somerville & colleagues will present findings from a study of community and organizational factors related to dementia-friendly readiness in community-based senior centers. Finally, Sun & colleagues will discuss the barriers and facilitators to implementation of DFCs in the USA during the COVID-19 pandemic. Taken together, these studies demonstrate the utility of quantitative and qualitative research methodologies to elucidate how and to what extent DFCs are implemented. Findings have implications for examining the population health impact of DFC efforts, as well as for attending to issues of health disparities and aging equity in the uptake, implementation, and sustainability of DFC initiatives.

### DIMENSIONS OF IMPLEMENTATION FOR DEMENTIA-FRIENDLY COMMUNITY INITIATIVES

Clara Scher, and Emily Greenfield, *Rutgers, The State University of New Jersey, New Brunswick, New Jersey, United States*

Dementia-friendly community (DFC) initiatives are a community-level approach to support individuals living with dementia and their care partners. DFC initiatives engage in a range of activities including raising awareness about dementia, enhancing local services, and advocating for inclusive public facilities and spaces. There is significant variation in the implementation of DFC initiatives worldwide which creates challenges for both process and outcomes evaluation. We used data from qualitative interviews with DFC leaders in Massachusetts to develop a framework to assess the implementation of DFC initiatives. Qualitative descriptive analysis yielded distinct dimensions such as robustness of multisectoral action teams, diversity of strategies for outreach to older adults, and degree of focus on coordinating care. Building on the tradition of using radar charts to evaluate DFC initiatives, we visualized the shape of implementation across the various dimensions. This framework can help to advance research, practice, and policy as part of the DFC movement.

### DEVELOPING A MEASURE OF COMMUNITY-LEVEL DEMENTIA FRIENDLINESS IN FAITH COMMUNITIES

Fayron Epps[1], Alexis Bender[2], Janelle Gore[2], Grayson Gunn[1], Dana Markert[1], and Miranda Moore[1], *1. Emory University, Fairburn, Georgia, United States, 2. Emory University, Atlanta, Georgia, United States*

As the prevalence of dementia rises, modifying built and social environments of persons living with dementia (PLWD) becomes increasingly essential. Although there is a call to rigorously evaluate dementia-friendly community initiatives, no known comprehensive evaluation tools exist to assess small communities (churches and community centers). Therefore, we are developing a person-centered Dementia-Friendly Community Assessment tool. This tool will consist of new and validated scales to assess the level of dementia-friendliness among churches enrolled in a dementia-friendly congregation program based in African American communities. A draft of a theory-informed survey instrument is being shared with stakeholders (church leaders, caregivers, and PLWD) during focus groups who will provide feedback on their understanding and relevance of questions on the instrument. The tool will then be modified based on feedback and the final instrument will be piloted with broad church membership. Our aim is to enable programs to quantify their dementia-friendly community initiative's impact.

### PERCEPTIONS OF DEMENTIA FRIENDLY COMMUNITY STAKEHOLDERS IN THE US: CHALLENGES AND STRATEGIES DURING COVID-19

Fei Sun[1], Ha-Neul Kim[1], Opur Fredrika[1], and Christian Conyers[2], *1. Michigan State University, East Lansing, Michigan, United States, 2. University of Michigan, Ann Arbor, Michigan, United States*

This study examines the challenges faced by Dementia Friendly Communities (DFC) during COVID-19 pandemic and the strategies used to address these challenges from the perspectives of DFC stakeholders in the U.S. Data were collected in 2020 through an online survey of 183 stakeholders (Mage =35.3, SD=8.8, 43.6% being female) involved who were in DFC design, implementation, or evaluation. Three challenges rated most critical by participants included limited funding (40.4%), difficulties to provide services due to policies to contain COVID-19 (35.7%), and lack of staffing (29.3%). Three rated most important strategies included seeking funding and government support (31.1%), developing partnerships and relationships with multiple sectors (29.1%), and recruiting persons with dementia and family caregivers as advocates (27.5%). Communities demonstrated resilience during the COVID-19 pandemic to implement DFC- related activities. To sustain DFC, enhancing national awareness, acquiring additional funding, and firm cogency from staff members, local/state government, and local communities are needed.

### SENIOR CENTER INVOLVEMENT WITH DEMENTIA-FRIENDLY COMMUNITIES: COMMUNITY AND ORGANIZATIONAL FACTORS

Ceara Somerville[1], Clara Scher[2], Caitlin Coyle[1], Emily Greenfield[2], and Ayse Akincigil[2], *1. University of Massachusetts Boston, Boston, Massachusetts, United States, 2. Rutgers, The State University of New Jersey, New Brunswick, New Jersey, United States*

As local hubs for aging services, senior centers are well-positioned to engage in dementia-friendly community (DFC) work. Yet centers vary in their engagement, especially as the DFC concept has been introduced only recently in the US. Using a mixed-methods approach, we drew on data from a survey of senior centers in Massachusetts, the US Census, and qualitative interviews with senior center staff to examine factors associated with DFC engagement. Centers that reported greater engagement were in municipalities with higher proportions of older residents from vulnerable groups (e.g., adults ages 80+, limited English proficiency, with a disability, living alone). They also reported greater programmatic, social service, funding, and staff capacity. Qualitative findings elucidated how senior center leaders drew on intrapersonal, interpersonal, organizational, and community assets to support local DFC efforts. We discuss implications for policies and practices to cultivate senior centers and other community-based organizations as leaders and partners toward DFCs.

### SESSION 1180 (PAPER)

### AGING IN PLACE AND COMMUNITIES

### A QUALITATIVE STUDY EXPLORING OLDER ADULTS' PERCEPTIONS, FACILITATORS, AND BARRIERS IN PLANNING FOR AGING AND FRAILTY

Erica Frechman[1], Cathy Maxwell[2], Mary Dietrich[3], Harleah Buck[4], Bethany Rhoten[5], and Amanda Davis[6], *1. Atrium Health Wake Forest, Concord, North Carolina, United States, 2. Vanderbilt University, Hermitage, Tennessee, United States, 3. Vanderbilt University, Nashville, Tennessee, United States, 4. Csomay Center for Gerontological Excellence, Iowa City, Iowa, United States, 5. Vanderbilt University School of Nursing, Nashville, Tennessee, United States, 6. Mercer University, Atlanta, Georgia, United States*

Aging in place is often a desired aspect of the aging trajectory but too often older adults and society in general, do not consider the factors involved in planning to make this a reality. Planning for aging and frailty entails a comprehensive life course approach to planning for one's aging process in five unique domains: communication/socialization, environmental, financial, physical care, and cognitive status. In order to promote upstream planning, there is a need to explore the lived experiences of older adults through understanding their viewpoints on planning, and what may promote or hinder this process. The purpose of this study was to explore how people perceive the concept of planning for aging and frailty and to identify facilitators and barriers involved in planning efforts. Using semi-structured questions, 20 community-dwelling older adults (aged 50-80) were interviewed. In-vivo coding and descriptive content analysis was utilized and revealed 17 codes within categories, including six codes and three categories in the perception domain (internal, external, future-oriented), seven codes and three categories in the facilitators domain (internal, external, systems), and four codes and two categories in the barriers domain (internal, systems). The depth of older adults' personal identities, life experiences, and the impact of societal influences emerged within the categories. Planning for aging and frailty is an unique concept that promotes planning through awareness and acceptance of the normal aging process. Future research through intervention development is needed to educate and empower older adults, optimize planning, and navigate barriers to planning.

### COMMUNITY CONTEXT OF OLDER ADULT CARE: A CASE STUDY DURING THE COVID-19 PANDEMIC

Lisa Skemp, *Loyola University, Chicago, Chicago, Illinois, United States*

Introduction: Expectations for older adults (OA) to live in the community and prevent costly long-term care assume OAs' informal network members are available, able, and willing to fill this need. Yet, little is known about the processes whereby OAs construct care networks, especially during COVID-19.

Methods: A longitudinal case study of one OA male who participated in the ethnographic community Older Adult Care study in one urban Chicago neighborhood is described. The OA male described his network on three occasions: 2/2018, 1/2021 and 9/2021. The care networks were described by size, density, and transitivity. Data analysis was performed using the R programming language. Adjacency networks were constructed using the network package, then visualized using the sna package.

Results: The OA's network went from 23 members prepandemic in time one to 13 in time two and 8 members in time three. As network size contracted, the network density increased from 25% in time two to 46% at time three, indicating a more interconnected network. Clustering varied over time and was at its lowest in time 2 (27%) and increased by time 3 (67%). Friends and church connections were 72% of his network in time one, whereas 71% of his network were family and neighbors in time three.

Conclusions: Our OA's large, relatively disconnected social network tightened to fewer, more closely connected members during COVID-19 months. Contextual variables (environment, health guidelines, fear, pets) influenced the networks. It is essential to understand OA care networks to promote healthy aging in community.

### EARLY DETECTION AND MANAGEMENT OF FRAILTY IN PRIMARY CARE: VALIDATION OF THE EFI-CGA WITH ELECTRONIC HEALTH RECORDS

Xiaowei Song[1], Grace Park[2], Barry Clarke[3], and Kenneth Rockwood[3], *1. Fraser Health, Surrey, British Columbia, Canada, 2. Fraser Health Authority, Surrey, British Columbia, Canada, 3. Nova Scotia Health, Halifax, Nova Scotia, Canada*

Background: Frailty is common in older adults and associated with many adverse outcomes. To promote early detection and management of frailty outside specialized geriatric services, we developed an electronic Frailty Index based on a Comprehensive Geriatric Assessment (eFI-CGA) in electronic health records. Here, we compare the eFI-CGA assessments between family physicians (FP) and geriatricians (GM).

Methods: Data from community-dwelling older adults were collected as part of the collaborative effort between Fraser Health and Nova Scotia Health to validate the eFI-CGA. The eFI-CGA was created following a standard procedure based on understanding deficit accumulation. A FP and a GM assessed each patient independently. Characteristics of the eFI-CGA were examined for each physician group using descriptive statistics and correlation analysis. FP-GM inter-rater reliability was tested using Cohen's Kappa.

Results: The first 30 cases were aged 80.8±5.2 years; 7% were women; with 12.9±2.8 years of education; 17% lived alone. Mild cognitive impairment or dementia was present in 20% participants. The mean clinical frailty scale (CFS) was 3 and the mean eFI-CGA was 0.20 by both FP and GM ratings. The CFS and eFI-CGA were closely correlated (r=0.76 for FP and r=0.71 for GM, p<.001). The eFI-CGA also showed an age correlation (r values >0.37, p values <.050). The average intraclass correlation coefficient was 0.79 for CFS and 0.90 for eFI-CGA (each p<.001).

Conclusion: Frailty data collected in primary care are highly comparable with geriatrician assessments. Ongoing work will test the generalizability of these findings using a larger sample with follow-up and outcomes evaluations.

### PLAN: PREPARING AND LIVING FOR AGING NOW; A DESCRIPTIVE STUDY INVESTIGATING READINESS TO PLAN FOR AGING AND FRAILTY

Erica Frechman[1], Cathy Maxwell[2], Mary Dietrich[3], Harleah Buck[4], and Bethany Rhoten[5], *1. Atrium Health Wake Forest, Concord, North Carolina, United States, 2. Vanderbilt University, Hermitage, Tennessee, United States, 3. Vanderbilt University, Nashville, Tennessee, United States, 4. Csomay Center for Gerontological Excellence, Iowa City, Iowa, United States, 5. Vanderbilt University School of Nursing, Nashville, Tennessee, United States*

Population aging and the universality of the aging process offers individuals and society the opportunity to consider strategies to optimize older adults' well-being and proactively prepare across their aging trajectory. Understanding aging through a holistic lens is essential to promote healthy aging in light of the risk for increasing chronic conditions, frailty, and disability that affect older adults. Planning for aging and frailty encompasses five domains that entail change as individuals age (communication/socialization, environmental, financial, physical care, cognitive status), and provides foci for a proactive approach to planning across life course. The aims of this research were to examine the stages of change through the Transtheoretical Model within the domains of aging, experiences (personal & experiences with others), and associations between contextual factors and stages of change for readiness to plan for aging and frailty. Using a cross-sectional design, 252 community dwelling-adults (aged 50-80) completed a survey on planning for aging and frailty. Results revealed a distribution of stages of change in readiness across domains of planning for aging and frailty with highest levels of planning in the financial domain (68.7%), and lowest levels in cognitive domain (28.2%). Participants reported increased experiences with others compared to personal experiences. Factors most indicative of planning include: older age, marital status, living situation, social support, and vulnerability. Planning for aging and frailty is an innovative concept that takes a comprehensive approach to the aging process. Future study is needed to promote planning across one's life course and develop interventions focused on enhancing well-being.

### SUBJECTIVE COGNITIVE DECLINE AS A PREDICTOR OF INCIDENT HOMEBOUND STATUS AMONG OLDER ADULTS

Julia Burgdorf[1], and Jennifer Reckrey[2], *1. Center for Home Care Policy & Research, VNS Health, New York, New York, United States, 2. Icahn School of Medicine, The Mount Sinai Hospital, New York, New York, United States*

In the US, over 2 million older adults are homebound, meaning they never or rarely leave their home. Being homebound increases risk for negative outcomes, including depression and mortality. Dementia diagnosis is a risk factor for becoming homebound, but over half of those with dementia do not receive a formal diagnosis. Subjective cognitive decline (SCD)—self-reported increase in memory loss over a given timeframe—is an early indicator of dementia that can be easily determined during routine medical encounters. SCD screening could be a valuable tool for identifying older adults at risk of becoming homebound—a critical first step towards supporting these individuals and their caregivers—but its predictive value is not yet known. We examined a nationally representative sample of 4,914 (weighted n=34,381,060) community-living older adults who responded to the 2016 and 2017 National Health and Aging Trends Study (NHATS). We modelled incident homebound status as a function of SCD using weighted, multivariable logistic regression while adjusting for older adult sociodemographic characteristics, health status, and caregiving support. In adjusted models, those who reported SCD experienced 43% greater odds of becoming newly homebound from 2016-2017 (aOR: 1.43; p=0.03). This relationship persisted among individuals without a formal dementia diagnosis (n=4,735); among these individuals, those who reported SCD experienced 50% greater odds of becoming newly homebound (aOR: 1.50; p=0.02). Findings suggest the potential value of SCD screening as an efficient method of identifying older adults who may benefit from resources that reduce the risk and/or mitigate the negative effects of becoming homebound.

### SESSION 1190 (PAPER)

### CARE AT HOME

### CHARACTERIZING HOME AND COMMUNITY-BASED SERVICE ENROLLEES IN MINNESOTA'S MEDICAID WAIVER PROGRAM

Justin Blackburn[1], Sharon Baggett[2], Ellen Burton[3], and Yonda Snyder[4], *1. IU Richard M. Fairbanks School of Public Health at IUPUI, Indianapolis, Indiana, United States, 2. University of Indianapolis, Indianapolis, Indiana, United States, 3. University of Indianapolis Center for Aging & Community, Indianapolis, Indiana, United States, 4. Sage Squirrel Consulting, LLC, Bloomington, Indiana, United States*

Medicaid waivers allow states' provision of home and community-based services (HCBS), leading to variations in design and delivery. States monitor expenditures, but cannot easily anticipate growth. Understanding the needs of this population can aid in early identification and improve service delivery. We conducted a Partitioning Around Medioids cluster analysis of first-time enrollees in Minnesota's Elderly Waiver HCBS program during 2019 to identify trajectories of entry and characterize activities of daily living (ADL) and instrumental activities of daily living (IADL) needs at enrollment. Administrative data collected via long term care consultation assessments provided enrollment, ADL/IADL needs, living arrangements, and other clinically relevant information that was linked to other sources including Minnesota health care program enrollment and utilization, Minimum Data Set skilled nursing facility (SNF) assessments, and calls to the Senior LinkAge Line (SLL)—a free long-term care counseling service. Of 5,284 first-time enrollees, most had prior engagement with state programs—nearly two-thirds had called the SLL, 36% had a SNF stay, and 56% had prior Medicaid enrollment. We identified six clusters representing three levels of living arrangements and two levels of need: 1) lived alone, low needs (32%), 2) lived with others, moderate needs (20%), 3) congregate living, moderate needs (14%), 4) congregate living, high needs (14%), 5) lived with others, high needs (11%), and 6) lived alone, high needs (9%). Lacking caregivers and prior Medicaid were possible exacerbating reasons for HCBS enrollment. The magnitude of the differences between clusters highlights the constellation of factors leading to enrollment in HCBS.

### HOME HEALTH AIDE CONTINUITY AMONG HOME-BASED LONG-TERM CARE CLIENTS

Jennifer Reckrey[1], David Russell[2], Mei-Chia Fong[3], and Katherine Ornstein[4], *1. Icahn School of Medicine, The Mount Sinai Hospital, New York, New York, United States, 2. Appalachian State University, Boone, North Carolina, United States, 3. Visiting Nurse Service of New York, New York, New York, United States, 4. Johns Hopkins, University, Baltimore, Maryland, United States*

Many older adults with functional impairment rely on paid caregivers (e.g., home health aides) to remain living at home. Continuity in the provider of healthcare services is known to impact care-recipient outcomes in settings like primary care; however, limited quantitative research has explored variability in provider continuity among home-based long-term care services. We conducted a retrospective pooled cross-sectional study using a secondary analysis of managed long-term care and home care agency records in order to: 1) describe home health aide continuity among a population of older adults receiving Medicaid-funded home-based long-term care using the Bice-Boxerman index, and 2) identify factors associated with greater home health aide continuity using multivariate regression. Among 3,864 older adults who received claim-paid home health aides services between January 1, 2018 and March 10, 2020, average home health aide continuity scores were lower (i.e., worse continuity) as care hours increased: 0.71 among those receiving < 3 service hours/day (n=1221), 0.62 among those receiving 3-7 service hours/day (n=1622), and 0.41 among those receiving >7 service hours /day. Among those with the highest care hours (>7 hours /day), increases in the level of ADL impairment and cognitive impairment were significantly associated with decreases in continuity scores. While clients with the highest care needs are the most dependent on their home health aides and may benefit most from stable paid caregivers, home health aide continuity scores are the lowest among this group. Future work will explore the impact of home health aide continuity on long-term care-recipient health outcomes.

### UNCOVERING PET OWNERSHIP BENEFITS, CHALLENGES, AND RESOURCES: A SURVEY OF PROFESSIONALS WORKING WITH OLDER ADULTS

Jessica Bibbo[1], Justin Johnson[1], Jennifer Drost[2], and Margaret Sanders[3], *1. Benjamin Rose Institute on Aging, Cleveland, Ohio, United States, 2. Geriatric Research Summa Health, Akron, Ohio, United States, 3. Northeast Ohio Medical University, Rootstown, Ohio, United States*

The human-animal bond can play a vital role in the lives of older adults, including those living with dementia. This study used the stress process model to identify the benefits, challenges, and resources associated with pet ownership professionals had encountered in their work with older adults (OA), persons living with dementia (PWD), and care partners (i.e., caregivers, CP). An interdisciplinary (e.g., social services, healthcare) sample (N=360, 89.97% female, Mage=53.22, SDage=11.84) completed an on-line survey addressing pet ownership issues encountered in their work. A conventional content analysis was conducted to analyze the open-ended items asking the benefits and challenges they had encountered, as well as whether and if they had seen pets shape the relationships between OA/PWD and CPs. The final open-ended item asked for any other experiences or thoughts they would like to share on the issue of pet ownership in older adulthood. Preliminary analyses indicated the central benefits of pet ownership for OA and PWD was companionship. Common challenges were providing basic pet care (e.g., feeding, managing waste) and the financial aspect of ownership. Results indicated that family/caregiver assistance was necessary to keep a pet in an OA/PWD's home and pets could be a source of caregiver burden. Pets provided OA a sense of purpose. Caring for the pet and the bond shared with the pet were also important resources for OA. Pets provided a focus of attention and were a source of engagement for PWD. Pets were a resource for the professionals to build rapport with their clients.

### UNDERSTANDING SPENDDOWN TO MEDICAID IN ASSISTED LIVING: SURVEY RESULTS FROM PARTICIPANTS/REPRESENTATIVES IN MINNESOTA

Sharon Baggett[1], Ellen Burton[2], and Justin Blackburn[3], *1. University of Indianapolis, Indianapolis, Indiana, United States, 2. University of Indianapolis Center for Aging & Community, Indianapolis, Indiana, United States, 3. IU Richard M. Fairbanks School of Public Health at IUPUI, Indianapolis, Indiana, United States*

The state of Minnesota has seen substantial growth in the use of Medicaid in Assisted Living and wanted to better understand how applicants spend down to Medicaid in order to identify potential upstream interventions to prolong the spend down period. As part of a mixed methods research effort, 231 new Medicaid participants or their proxies, 167 in AL and 64 at home, completed a telephone survey on their services use prior to Medicaid application, prior planning or thinking of how to pay for services once personal resources were exhausted, and experience of spenddown once in AL. Reaching these eligible participants required a unique, multi-step outreach process; given the abilities of participants to respond, the majority of surveys were completed by proxies. Among those in AL, 40% reported planning in advance about how to pay for services once needed; 58% knew of government programs to assist and 80% reported their plan included applying to the programs at some time. Thirteen percent (13%) of those in AL reported getting help from the Medicaid waiver at the time of move in; another 25% said they paid for themselves for less than one year before getting state support. More than one-third (34%) who paid for their services for some period of time after move in said the period was shorter than expected; another 41% said about as expected, and for 25% the period was longer than expected. Open-ended comments indicate a move to memory care was a key factor in spend down to Medicaid.

### YEARS WITH AND WITHOUT HOME-BASED SERVICES FOR THE 70+ POPULATION IN NORWAY: TRENDS OVER THREE DECADES, 1995–2019

Milan Chang Gudjonsson[1], Mona Michelet[2], and Bjørn Heine Strand[3], *1. Norwegian Institute of Public Health, Reykjavik, Iceland, 2. Vestfold Hospital Trust, Oslo, Oslo, Norway, 3. Norwegian Institute of Public Health, Oslo, Oslo, Norway*

Introduction: Life expectancy (LE) is increasing worldwide, while there is a lack of information on years of home-based formal care use among the aging population. The current study examined the trend of LE for formal care use among Norwegian older adults over three decades 1995-2016 in Norway.

Methods: A total of 25,263 participants aged 70+ were included in the Trøndelag Health Study (HUNT) survey 2 (1995-97), 3 (2006-08), and 4 (2017-19). Participants reported the use of formal care services including practical help and home nursing. The prevalence of both service uses was standardized to the Norwegian population by age, sex, and education using post-stratification weights. LE was estimated using National mortality data by age, sex, and education combined with the formal service use data using the Sullivan method to estimate expected years with and without basic services and nursing services in Norway.

Results: During 1995/97-2017/19, the service use decreased from 22.6% to 6.2% for practical help and 6.4% to 5.5% for receiving home nursing. LE at age 70 from 1995 to 2016 increased 3.4 years in men, and 2.4 years in women. Expected years receiving practical help decreased by 1.1 years (2.6 to 1.5 years) in men and 1.6 years (4.5 to 2.9 years) in women, while LE for home nursing increased from 0.7 years (0.7 to 1.4 years) in men and 1.3 years (1.4 to 2.7 years) in women.

Conclusions: Years receiving home nursing increased during 1995-2016, while years receiving practical help care decreased in the older Norwegian population.

### SESSION 1200 (BIOLOGICAL SCIENCES INVITED SYMPOSIUM)

### CHROMATIN STATE AND MODIFICATIONS IN AGING

Chair: Simone Sidoli

Program Overview: In this symposium, the speakers will discuss how genomic stability and chromatin state are affected during aging using complementary perspectives, model systems and technology. Methods such as structural genomics, genome-wide mapping, super-resolution microscopy, mass spectrometry, and enzymology are utilized to describe hallmarks of aging in models such as C. elegans, mice, and 2D vs 3D human cell culture. Dr. Lakadamyali applies innovative super-resolution microscopy to visualize and quantify the spatial organization of chromatin with nanoscale spatial resolution in single cells, revealing how disordered patches at the nucleosomal level correlate with cell phenotype. Dr. Sen will present a multi-omics approach on murine models to reveal that aged cells organize a specific type of heterochromatin unable to self-interact over long distances. These findings expose that global chromatin alterations pose a barrier and promote a cellular state that is refractory to injury sensing and repair. Finally, Dr. Sidoli will describe how mass spectrometry is applied to investigate combinatorial histone modifications that benchmark regions of decondensed heterochromatin, and show the enrichment of unusual histone modifications in a cohort of centenarian patients.

### MODELING REACTIVATED HETEROCHROMATIN USING AN AGING 3D CELL MODEL

Simone Sidoli, Stephanie Stransky, Ronald Cutler, Sarah Graff, and jennifer Aguilan, *Albert Einstein College of Medicine, New York, New York, United States*

Chromatin state and dynamics is modulated by chromatin interacting proteins, in particular histones and their post-translational modifications. We investigate the chromatin-bound proteome in aging to understand how DNA readout is misregulated in senescent cells. We focus on domains of reactivated heterochromatin, i.e. chromatin domains undergoing anomalous decondensation and decorated by histones co-modified with markers of condensed heterochromatin and active transcription. We optimize our mass spectrometry methods 3D cell models, and then apply them on B-cells retrieved from Ashkenazi Jews donors. In this cohort, we particularly focus on offsprings of exceptional longevity, i.e. individuals in their 70s with projected longer lifespan due to their centenarians ancestors. Preliminary data on 60 individuals revealed the enrichment of unusual histone modifications in the OPEL group, the group we utilize as indicator of projected longer lifespan. We have then applied biochemistry techniques to identify the role of these unusual modifications on chromatin readout.

### THE DYNAMIC GENOME-CHALLENGES AND OPPORTUNITIES FOR HEALTHY AGING

Peter Adams, *Sanford Burnham, San Diego, California, United States*

Chromatin is a key determinant of cell phenotype and function. Therefore, chromatin stability over the lifespan is presumably a pre-requisite for maintenance of cell and tissue phenotype and function, and hence healthy aging and longevity. However, chromatin is not a static fixed structure, but is a dynamic and plastic “breathing” assembly. This dynamic chromatin likely represents a challenge for a cell to achieve phenotypic stability, healthy aging and longevity. As a dynamic and plastic entity, chromatin is prone to change or drift, a process likely exacerbated by intrinsic cellular processes and extrinsic/environmental influences. We have proposed that cells possess mechanisms of chromatin homeostasis, or chromostasis, that preserve chromatin integrity, suppress phenotypic instability and so slow the pace of aging. Implications of dynamic chromatin for healthy aging and longevity will be discussed.

### SUPERRESOLUTION IMAGING OF CHROMATIN IN HEALTH AND DISEASE

Melike Lakadamyali, *University of Pennsylvania, Philadelphia, Pennsylvania, United States*

Super-resolution microscopy has been playing an instrumental role in providing new insights into how the genome is folded and packaged inside intact nuclei in single cells. I will present our work on using super-resolution microscopy to visualize and quantify the spatial organization of chromatin with nanoscale spatial resolution in single cells. Our work has revealed that at the nucleosomal level chromatin is a disordered fiber composed of groups of nucleosomes packaged at varying densities, which we named nucleosome clutches. Despite the heterogeneity of nucleosome clutch organization, the size and packing density of nucleosome clutches is cell-type specific and correlates with cell fate. Our recent results also show that nucleosome clutches and chromatin nano-structure can be remodeled via chemo-mechanical cues. In particular degenerative chemo-mechanical cues during disease lead to aberrant chromatin nano-structure and loss of mechano-epigenetic memory, potentially leading to alterations in cell phenotype.

### EPIGENETIC MECHANISMS OF TISSUE AGING

Payel Sen[1], Na Yang[2], James Occean[2], Daniel Melters[3], Changyou Shi[2], Lin Wang[4], Simone Sidoli[5], and Yamini Dalal[3], *1. National Institute on Aging, NIH, Baltimore, Maryland, United States, 2. NIA, Baltimore, Maryland, United States, 3. NCI, Bethesda, Maryland, United States, 4. NIH, Bethesda, Maryland, United States, 5. Albert Einstein College of Medicine, New York, New York, United States*

Epigenetic alterations are one of the key hallmarks of aging. How epigenetic changes broadly affect euchromatin-heterochromatin balance, three-dimensional chromatin conformation, histone composition and global transcriptome in aging have not been addressed. Using murine liver as a model organ and an integrated multi-omics approach, we find that aged cells assume a repressed chromatin state characterized by condensed chromatin, broad heterochromatinization, global suppression of the transcriptome, and inability to self-interact over long distances. Our findings reveal that global chromatin alterations pose a barrier and promote a cellular state that is refractory to injury sensing and repair.

### SESSION 1210 (PAPER)

### COVID-19 I

### FAMILY CAREGIVING FOR OLDER ADULTS IN LONG-TERM CARE DURING THE PANDEMIC: STRESS, SOCIAL SUPPORT, AND ADAPTATION

Geunhye Park, Erin Robinson, and Gashaye Melaku Tefera, *University of Missouri, Columbia, Missouri, United States*

Older adults residing in long-term care (LTC) are especially vulnerable to the COVID-19 pandemic. Federal and local health officials have issued strict visitation guidelines, including family caregivers. Given that family caregivers are essential in the well-being for older adults in LTC, these measures have had an enormous impact. As little is known about the experiences of family caregivers, the purpose of this study was to explore how the COVID-19 pandemic impacted family caregivers' roles, mental health, and adaptation. Semi-structured interviews (N=25) were conducted with family caregivers of older adults in LTC (Mean age= 59.7; 92% female) via phone/Zoom. An interview guide led the question asking process and participants were asked open-ended questions about the impact of COVID-19 related protocols on their caregiving, mental health, and sources of social support. Interviews were transcribed verbatim and analyzed in Nvivo, guided by Grounded Theory methods. The majority of participants (76%) identified as a child of their care recipient. Findings highlight that most participants experienced numerous changes to their caregiving tasks, such as assisting with activities of daily living (ADLs), limited monitoring for their loved ones, and a reduction of social support provided to the care recipient. Family caregivers also reported other changes in their roles that resulted in increased stress and mental health concerns. These concerns were discussed in detail, including ways in which family caregivers adapted to their new roles and managed their stress. Findings from this study will inform interventions geared to better support family caregivers, particularly during times of crisis.

### FAMILY CAREGIVING IN LTC SETTINGS DURING THE PANDEMIC: COMMUNICATION CHALLENGES AND LEVERAGING TECHNOLOGY

Gashaye Melaku Tefera, Erin Robinson, and Geunhye Park, *University of Missouri, Columbia, Missouri, United States*

COVID19 related lockdown and protocols caused disruptions in family caregiving for older adults living in LTC settings. However, there is a paucity of research on the challenges and experiences of family caregivers in maintaining their caregiving role during the pandemic. Hence, this qualitative study explores family caregivers' communication challenges and the role technology played in performing their caregiving roles. One-on-one in-depth interviews (N=25) were conducted with family caregivers (Mean age= 59.7; 92% female; 76% child) via phone/Zoom. Interviews were transcribed and thematically analyzed using Nvivo12. Findings demonstrate that family caregivers of older adults in LTC settings experienced severe communication barriers with staff at those facilities in the early onset of the pandemic, including delays of important information about their care recipients. Participants highlighted high staff turnover, inadequate training, staff unfamiliarity with technology, and poor internet connections as perpetuating communication barriers. During this time, their older care recipients experienced visual and hearing impairments that affected their ability to communicate, as well as cognitive decline. Despite this, family caregivers were able to successfully utilize various forms of technology to continue providing care supports and social support to their loved ones. Although participants relied on phone calls and email communications, they also used other platforms including Zoom, FaceTime, Nixplay, and TextNow. Participants used devices including landline phones, cellphones, computers, tablets, Ipads, and walkie-talkies to execute their communication. Implications of this study suggest that improving access and utilization of technology in LTC settings can enhance family caregiving during unprecedented events like the COVID19 pandemic.

### PREVALENCE AND PREDICTORS OF INCREASED VERBAL/PHYSICAL CONFLICT DURING COVID-19: CLSA FINDINGS

Gloria Gutman[1], Mojgan Karbakhsh[2], and Heather Stewart[3], *1. Simon Fraser University, Vancouver,*

British Columbia, Canada, 2. Simon Fraser University Gerontology Research Centre, Vancouver, British Columbia, Canada, 3. Simon Fraser University, Surrey, British Columbia, Canada

Child and spousal abuse rates have been shown to increase during various types of disasters. This study sought to determine the prevalence and determinants of older adults' experiences of increased verbal or physical conflict (+VPC), as a proxy for elder abuse, during the COVID-19 pandemic. Data are from the Canadian Longitudinal Study on Aging (CLSA), a cohort study of 51,338 Canadians aged 45–85 at enrollment (2012-15) with follow-up every 3 years until 2033.. We analyzed data of participants aged 65 or above at follow-up1 who took part in a COVID-19 sub-study (n=24,306). Experiencing +VPC was the main outcome variable; explanatory variables included gender identity, sexual orientation, age group, race/ethnicity, educational attainment, marital status, household income, working status, living alone, social support availability, cohesion with community, self-rated physical and mental health, anxiety, depression, and previous history of elder abuse. The overall weighted prevalence of +VPC was 7.4%. Gay/bisexual men, 55-64 age-group, not living alone, low social support, poor social cohesion, low self-rated health, poor mental health, and past history of psychological or physical abuse were all significantly associated with +VPC. Weighted multivariable logistic regression revealed gender, not living alone, higher scores of depression and anxiety, and past history of psychological abuse to be independent predictors of +VPC. Implications for post-pandemic recovery and for prevention strategies during future disasters include targeted outreach programs for the most vulnerable group which includes males, persons age 55-64, those with self-rated poor mental health and/or history of elder psychological abuse.

### THE IMPACT OF SOCIAL INEQUALITIES ON DIGITAL ENGAGEMENT OF OLDER ADULTS DURING THE COVID-19 PANDEMIC

Andraž Petrovčič[1], Darja Grošelj[1], Štěpán Žádník[2], Katja Prevodnik[3], and Bianca C. Reisdorf[4], *1. University of Ljubljana, Faculty of Social Sciences, Ljubljana, Ljubljana, Slovenia, 2. Masaryk University | Faculty of Social Studies, Brno, Jihomoravsky kraj, Czech Republic, 3. University of Ljubljana, Ljubljana, Ljubljana, Slovenia, 4. University of North Carolina at Charlotte, Charlotte, North Carolina, United States*

The COVID-19 pandemic has had profound implications on how older adults engage with the digital world. While there is converging evidence that during the pandemic the number of online newcomers has increased the most among older adults, digital inequalities have become even more condensed among the socially disadvantaged groups in this period. Importantly, such patters of age-related digital exclusion were not observed only in terms of self-reliant internet use but also in older adults' ability to access internet resources indirectly by asking others to act on their behalf – a practice known as use-by-proxy. Drawing on the van Dijk's resources and appropriation theory, we aim to understand which categorical and resource inequalities determine the direct and indirect forms of internet use among older adults during the pandemic. Therefore, in November 2021 a telephone survey (N = 701) was carried out among individuals aged 65+ in Slovenia. Three logistic regression models were fitted to explore the association between sociodemographic characteristics of respondents and their access to material and social resources with internet use as well as with availability and activation of use-by-proxy. The results suggested that both categorical and resource inequalities are predictors of internet use. Conversely, availability of use-by-proxy is strongly associated only with social resources, while use-by-proxy activation depends on material resources and household composition. Overall, we argue that social inequalities affect also the indirect forms of internet use in later life which means that the ability to compensate by help for digital inequalities is also stratified unevenly.

### DEMYSTIFYING NIH PEER REVIEW: YOUR APPLICATION FROM SUBMISSION TO SCORE

Elia Ortenberg[1], Karen Roberto[2], Dana Plude[3], Janetta Lun[1], and Helena Gabor[4], *1. National Institutes of Health, Bethesda, Maryland, United States, 2. Virginia Polytechnic Institute & State University, Blacksburg, Virginia, United States, 3. National Institute on Aging/National Institutes of Health, Bethesda, Maryland, United States, 4. Center for Scientific Review/National Institutes of Health, Bethesda, Maryland, United States*

What happens to applications after they are submitted to the National Institutes of Health, and how can you better prepare for the process of peer review? The Center for Scientific Review (CSR) works closely with the 24 funding institutes and centers at the National Institutes of Health that provide funding support for projects of high scientific merit and high potential impact. CSR conducts the first level of review for the majority of grant applications submitted to the NIH, which includes 90% of R01s, 85% of Fellowships, and 95% of Small Business Innovation Research (SBIR) applications as well as many other research and training opportunity activities. In this capacity, CSR helps to identify the most meritorious projects, cutting-edge research, and future scientists who will advance the mission of the NIH: to enhance health, lengthen life, and reduce illness and disability. The purpose of this symposium is to provide an overview of 1) what happens to NIH applications before, during, and after peer review at CSR; 2) new and current peer review policies and practices that impact application submission; and 3) strategies for developing a strong NIH grant application. Peer review is the cornerstone of the NIH grant supporting process, and demystifying the process can help attendees understand what's inside the "Black Box" and the criteria used to identify the most meritorious applications.

### AFTER REVIEW AND WAYS TO GET INVOLVED

Elia Ortenberg, *National Institutes of Health, Bethesda, Maryland, United States*

After the review meeting, the SRO prepares the Summary Statement, which is the primary outcome of peer review. Program staff and Advisory Councils at the Institutes use this official document to make decisions about which applications to fund. How can you best prepare for the next steps for your application? Understand the qualities of a competitive NIH application, talk to Program staff, and take advantage of the opportunity to become a peer reviewer yourself. From the field, apply the tips shared by applicants and reviewers about what they do and what they look for in a well-written application. Receiving the summary statement can be daunting and discouraging; but by de-mystifying the process and getting involved, you can help position your application towards maximum success.

### THE PARTNERSHIP BETWEEN THE CENTER FOR SCIENTIFIC REVIEW AND THE NIH INSTITUTE

Dana Plude, *National Institute on Aging/National Institutes of Health, Bethesda, Maryland, United States*

The playing field for NIH funding is highly competitive, and having a great idea is only the start of the process. There are many funding mechanisms for early career investigators, trainees, and research institutions. Understanding about different application types, who to talk to about your application at different stages of development, finding the right review panel, and learning about the policies and procedures pertaining to review are important first steps in preparing an application. In recent years, the Center for Scientific Review has undergone changes and updated its structure, policies and practices to stay aligned with its mission. In this presentation, attendees will learn about the roles played by CSR and the NIH Institute as applications move from review to funding.

### THE STUDY SECTION AND THE ROLE OF THE SCIENTIFIC REVIEW OFFICER

Janetta Lun, *National Institutes of Health, Bethesda, Maryland, United States*

Applications reviewed by the NIH Center for Scientific Review go through a process that starts with assignment to a review panel and ends with the generation of a priority score and a written evaluation (Summary Statement). This process is overseen by several key players, the most important being the Scientific Review Officer, who is the designated federal official overseeing all aspects of Level 1 review. Their role is to recruit and enlist expert reviewers to evaluate the scientific and technical merit of an application and assess its potential contribution and impact to the relevant research field. The goal of Level 1 review is to ensure that applications are expertly and fairly evaluated in accordance with the NIH policies of rigor, reproducibility, transparency and research integrity. In this presentation, attendees will learn about the how study sections are formed, how reviewers are involved and conduct their evaluations, and the guiding principles that underlie Level 1 review.

### INSIDE THE REVIEW MEETING

Helena Gabor, *Center for Scientific Review/National Institutes of Health, Bethesda, Maryland, United States*

What happens to applications on meeting day? What do study section members discuss? Review of applications are evaluated for their scientific and technical merit, and this is reflected in the reviewers' written assessment of the project's overall impact. In addition, reviewers score five core criteria and additional review criteria as defined in the funding opportunity announcement. The panel's initial assessment determines which applications will be discussed in full at the meeting. For each that is discussed, reviewers briefly present the strengths and weaknesses of the application and discuss the factors that inform their overall assessment. At the end of the discussion, a final impact score is given by all eligible members of the panel, which is reported on the summary statement. Every application undergoes a similar process that ensures that fair and consistent consideration is applied to all applications. At the end of the presentation, attendees will learn how the foundation and pillars of the NIH peer review process are put into practice through scoring, discussion, and potential impact on the field.

### SESSION 1230 (SYMPOSIUM)

### DIFFERENCES IN THE EXPERIENCES AND BEHAVIORS OF OLDER ADULTS IN NSHAP DURING THE COVID-19 PANDEMIC

Chair: Louise Hawkley Discussant: Dawn Carr

This symposium uses data from the National Social Life, Health & Aging Project to examine how and for whom older adults' social relationships and experiences changed with the advent of the COVID-19 pandemic. Zhang et al. examine video call usage among older adults with hearing impairment and how this might mitigate loneliness. They find that although this population reported greater loneliness during the pandemic, video calls attenuated this relationship in a dose-response manner. Wilder et al. evaluate 2015 resilience and socioeconomic status as predictors of older adults' reports that the pandemic led to a positive change in their lives. Results revealed greater odds of reporting a positive impact of the pandemic for those with higher education, adjusting for resilience. Compernolle et el. assess differences by cognitive status in changes in mental health from pre- to during the pandemic. Higher 2015 cognitive functioning was associated with greater pandemic loneliness, with decreased pandemic emotional support partly explaining this association. Copeland & Liu investigate the role of 2015 personal networks in receiving instrumental and emotional support during the pandemic, finding that larger and denser pre-pandemic confidant networks each predicted higher odds of receiving various types of support during the pandemic. Wong et al. explore whether and for whom relationship quality changed since the pandemic started. Two-thirds of partnered respondents reported unchanged relationship quality, and Black respondents were more likely to report improved relationship quality. These investigations highlight sub-group differences in older adults' changes in experiences, behavior, and well-being since the pandemic began.

### VIDEO CALLING REDUCES LONELINESS FOR HEARING IMPAIRED OLDER US ADULTS DURING THE COVID-19 PANDEMIC

Amanda Zhang[1], Kristen Wroblewski[2], Martha McClintock[2], Louise Hawkley[3], and Jayant Pinto[2], *1. University of Chicago Pritzker School of Medicine, Chicago, Illinois, United States, 2. University of Chicago, Chicago, Illinois, United States, 3. NORC at the University of Chicago, Chicago, Illinois, United States*

Older adults with hearing impairment (HI) experience communication challenges that may result in increased loneliness exacerbated by social distancing during COVID-19. Video-calling may mitigate isolation-related loneliness. We addressed this hypothesis in the National Social Life, Health, and Aging Project (NSHAP), a longitudinal, nationally representative study of home-dwelling older US adults. Hearing ability was determined by interviewer-rating and loneliness/video-calling use by self-report. Fewer older adults (≥55 years) with HI (n=221) reported regular use of video-calling during the pandemic vs. those without HI (n=2337) (14% vs. 30%, p<0.001). Older adults with HI reported greater loneliness during COVID-19 (p=0.031), with video calls mitigating this relationship through a dose-dependent effect in adjusted analyses (interaction p=0.001). Video-calling decreases HI-related loneliness, despite potential barriers those with sensory impairment face using this technology. Promoting greater adoption of video-calling could be a strategy to protect against HI-related loneliness in aging.

### DOES RESILIENCE OR SES PREDICT OLDER ADULTS FINDING A SILVER LINING DURING THE COVID-19 PANDEMIC?

Jocelyn Wilder[1], diane Lauderdale[2], Laura Finch[3], and Louise Hawkley[3], *1. NORC, Chicago, Illinois, United States, 2. University of Chicago, Chicago, Illinois, United States, 3. NORC at the University of Chicago, Chicago, Illinois, United States*

The COVID-19 pandemic has stretched our limits physically, mentally, and economically; however, some older adults report that it led to a positive life change. We used data from the NSHAP COVID-19 supplement to understand whether resilience and SES (both measured in 2015-16) are predictors of older adults' likelihood of endorsing a positive change during the pandemic. Additionally, we examined whether resilience and SES predict specific positive changes described in open-ended responses. Higher education significantly predicted reporting a positive change during the pandemic. Adjusting for resilience (OR 1.0, p= 0.185), the odds of endorsing any positive change were 1.9 times higher for those with an associate degree (p=0.027) and 3.3 times higher for those with a bachelor's degree or higher (p<0.001) compared to those who did not complete high school. Specific positive change domains also differed by SES. Additional research is needed to understand how SES and benefit-finding contribute to well-being.

### SOCIAL RESOURCES, COGNITION, AND MENTAL HEALTH DURING THE COVID-19 PANDEMIC

Louise Hawkley[1], Laura Finch[1], Linda Waite[2], and Ellen Compernolle[1], *1. NORC at the University of Chicago, Chicago, Illinois, United States, 2. National Opinion Research Center (NORC at Chicago), Chicago, Illinois, United States*

This study assesses the extent to which changes in mental health among older adults from pre- to during the pandemic varied by cognitive functioning and the role that decreases in social resources played in this association. We use data from the National Social Life, Health, and Aging Project (NSHAP)—a population-based panel study of older U.S. adults that has surveyed respondents every 5 years since 2005—and the NSHAP COVID-19 supplement, conducted between September 2020 and January 2021 (N=2,672). Results from linear regression models suggest that (1) higher cognitive functioning in 2015 was associated with greater loneliness ($\beta$=-0.03; p<.05) during the pandemic; (2) this association is explained in part by a decrease in emotional support during COVID-19 ($\beta$=0.94; p<.001); and (3) cognitive status did not moderate links between social resources with happiness nor loneliness. Results emphasize the importance of social resources for older adults' mental health, regardless of cognitive ability.

### WHO GETS HELP? A NATIONAL LONGITUDINAL STUDY OF PERSONAL NETWORKS AND PANDEMIC SUPPORT AMONG OLDER ADULTS

Molly Copeland, and Hui Liu, *Michigan State University, East Lansing, Michigan, United States*

Personal networks are a key component in the provision of social support for older adults. Such support is particularly critical during the COVID-19 pandemic, when traditional avenues of social engagement or assistance are disrupted. Here, we use nationally representative data from the National Social Life, Health, and Aging Project that assesses older adults' pre-pandemic personal networks and receiving instrumental help and emotional support during the pandemic. We find that larger pre-pandemic confidant networks predict higher odds of receiving needed help and support, higher odds of receiving help and support more often than before the pandemic, and lower odds of being unable to get help. Denser pre-pandemic networks also predict higher odds of receiving instrumental help and support during the pandemic, while having a greater proportion of kin in pre-pandemic networks predicts higher odds of receiving pandemic help for non-white older adults only. Together, results suggest that features of older adults' pre-pandemic confidant network structure and composition can provide underlying conditions for receiving social support during the pandemic.

### SESSION 1240 (SYMPOSIUM)

### DIVERSE CONTEXTS, UNIQUE EXPERIENCES: IMPACT OF SOCIAL ISOLATION ON PSYCHOSOCIAL FACTORS AND COGNITION

Chair: Jess Francis

The aging population is not a monolithic group. Older adults' experiences of and behaviors within their social networks are as varied as their diverse backgrounds and lifestyles. Although the threat of social isolation is particularly salient among older adults, the psychosocial and cognitive impact can differ along demographic and socioeconomic lines. The objective of this symposium is to highlight how different older adults are effected by their social network characteristics and structure, and how they mobilize their psychosocial resources to protect against loneliness, depression, and threats to cognition. First, Hong & Mejia examine how older men and women compare in their use of information and communication technology (ICT) as each influenced the experience of loneliness during the COVID-19 pandemic. Francis & Brauer also look at ICT use as they assess demographic and socioeconomic differences in social isolation, perceived mattering, and Facebook use among single older adults who are childless and live alone. Oh & Chopik explore uncoupled older adults along demographic lines in a 10-year longitudinal study of single people, their perceptions of change, satisfaction with singlehood, and major life events. Further exploring the theme of well-being during the COVID-19 pandemic, Cooper, Ajrouch, & Antonucci assess cognition, depressive symptoms, and their association with race among a sample of Whites, Blacks, and Arab Americans. Finally, Lee & Colleagues round out this symposium by sharing results of their study in which they examine the coping styles of non-Hispanic Whites and non-Hispanic Blacks and the impact on cognition.

### SOCIAL ISOLATION, LONELINESS, AND MATTERING AMONG OLDER ADULT FACEBOOK USERS FROM DIVERSE BACKGROUNDS

Jess Francis, and Simon Brauer, *University of Michigan, Ann Arbor, Michigan, United States*

Older adults who are childless and live alone are at risk for social isolation and loneliness. Studies show Facebook communication to be associated with feelings of mattering – the feeling that we are significant to others - a protective psychosocial resource. Despite being at similar stages in life, older adults, their technology use, and their experience of social isolation can vary greatly. This study explores how older adults from different demographic and socioeconomic backgrounds use Facebook and how that use is associated with perceptions of mattering, loneliness, and social isolation. This study employed online survey methodology among a sample of older adult Facebook users (n = 517; Mage=70; 50% Female; 92% White). Preliminary results show significant differences among income level, age, and gender regarding Facebook communication and mattering among older adults who are childless and live alone. We discuss our full results in tandem with qualitative data from our sample.

### NAVIGATING LIFE UNCOUPLED: CHANGES IN SINGLEHOOD SATISFACTION IN RESPONSE TO MAJOR LIFE EVENTS

Jeewon Oh[1], and William Chopik[2], *1. Syracuse University, Syracuse, New York, United States, 2. Michigan State University, East Lansing, Michigan, United States*

Experiencing challenging events without a partner who can be supportive can be hard and change perceptions of the singlehood experience. We conducted growth curve analyses predicting intercepts and slopes of singlehood satisfaction from ten major life events (MLEs). Single people (N = 5,191; Mage= 46.98, 60.1% women) were followed longitudinally over 10 years (2008-2017) and provided information on MLEs and singlehood satisfaction. People were fairly satisfied with singlehood (Mintercept=6.52/10), and satisfaction increased over time (Mquadratic slope=.01). Some difficult events (e.g., becoming ill and losing parents) were associated with declines in singlehood satisfaction. However, responses to MLEs were overall complex—unemployment was related to lower levels of satisfaction but unrelated to changes in satisfaction. Seemingly positive events (e.g., buying a house, getting a job) were also related to lower levels of satisfaction. Results for each MLE will be discussed in line with additional qualitative data on single people's perceptions of change.

### COPING STYLES AND COGNITIVE FUNCTION IN OLDER NON-HISPANIC BLACK AND WHITE ADULTS

Ji Hyun Lee, Ketlyne Sol, Afsara Zaheed, Emily Morris, Lindsey Meister, Jordan Palms, Alexa Martino, and Laura Zahodne, *University of Michigan, Ann Arbor, Michigan, United States*

Coping styles refer to cognitive and behavioral patterns used to manage the demands of stressors. This study aimed to characterize associations between coping styles and cognitive functioning across non-Hispanic Black and non-Hispanic White older adults. Cross-sectional data comes from the Michigan Cognitive Aging Project (N=457; 53% non-Hispanic Black). Coping styles are measured with COPE inventory. Global cognition was a composite of five cognitive domain factor scores derived from a comprehensive battery. Results show that Black older adults reported more emotion-focused coping than Whites, but there were no differences in problem-focused coping. Less emotion-focused and greater problem-focused coping were each more strongly associated with better global cognition among Black older adults, who showed disproportionately worse cognitive performance in the context of less adaptive coping. Coping style may be a particularly important psychosocial resource for cognitive health among Black older adults who may have less access to compensatory resources than Whites.

### SOCIAL RESOURCES AND STRATIFIED EXPERIENCES OF ICT USE AND LONELINESS DURING THE COVID-19 PANDEMIC

Sungjae Hong, and Shannon Mejía, *University of Illinois at Urbana-Champaign, Champaign, Illinois, United States*

Limits to in-person social interaction increased the risk of loneliness during the Covid-19 pandemic. This study examines how gender and previous information communication technologies (ICT) experience could stratify how ICT-mediated social interactions related to loneliness during the COVID-19 pandemic. We employed National Health & Aging Trends Study data collected in 2018 and 2020-2021 (N=2,962; 27% age 85+; 57% women; 77% white) to examine how the email and video call use before and during the COVID-19 pandemic was associated with loneliness and how the degree of impact differed by gender and previous ICT experience. In total, 35% reported feeling lonely some days or more frequently during the COVID-19 pandemic. Preliminary analysis showed older adults using video calls during the COVID-19 pandemic were 1.24 times more likely to report loneliness. This association was stronger among men than women and those who did not use ICT preceding the pandemic than those who used.

### DEPRESSION, SOCIAL RELATIONS, AND COGNITIVE HEALTH DURING THE COVID-19 PANDEMIC

Jasmine Cooper[1], Toni Antonucci[1], and Kristine Ajrouch[2], *1. University of Michigan, Ann Arbor, Michigan, United States, 2. Eastern Michigan University, Ypsilnati, Michigan, United States*

This study examines cognitive abilities, number of people in a social network, and presence of depressive symptoms among the "youngest old", "middle old", and "oldest old", by ethnicity. A sample of 600 people; 200 Blacks, 200 Whites, and 200 non-white Arabs were recruited for the survey. Those with missing data were omitted from analysis, which left 435. Data were collected via a computer assisted telephone survey. Data were analyzed using two-way ANOVAs. We found significant effects on cognition by ethnicity $f(2,430)=2.71$, $p<0.05$ and age group $f(2,430)=11.24$, $p<0.001$. There was a significant effect of the number of people in social networks on cognition across all groups f(2,430)=10.4, p<0.01. The presence of depressive symptoms was not a mediator. There was no significant interaction between ethnicity and age group, or ethnicity and social network structure.

### SESSION 1250 (PAPER)

### EDUCATION, CARE, AND WELL-BEING

In this sessions, various ways aging education in the classroom and beyond can be tailored to support care-focused activities to enhance well-being are discussed.

### INFORMAL STUDENT CAREGIVERS: BALANCING CAREGIVING RESPONSIBILITIES AND ACHIEVING ACADEMIC SUCCESS

Gretchen Tucker[1], Dana Bradley[2], Roberto Millar[3], Claudia Thorne[4], and Christin Diehl[5], *1. University of Maryland, Baltimore, Columbia, Maryland, United States, 2. University of Maryland, Baltimore County, Baltimore, Maryland, United States, 3. The Hilltop Institute, Baltimore, Maryland, United States, 4. Coppin State University, Baltimore, Maryland, United States, 5. The Hilltop Institute, Baltimore, Maryland, United States*

According to the American Association for Retired Persons (AARP), there are five million student caregivers in the United States. Seven out of ten student caregivers say their caregiving roles affect their academic achievements. An informal (unpaid) student caregiver may provide care for a spouse, family member (excluding child care), friend, or neighbor. Student caregivers often encounter obstacles in balancing their caregiving responsibilities while trying to achieve academic success. The goal of this mixed method study is to understand the experiences of informal (unpaid) student caregivers. The aim of this study was to expand on a pilot study to increase our understanding of what challenges students face while being a caregiver and what resources are most helpful to reach their academic goals. The study was a mixed methods study consisting of a survey, which was sent to both undergraduate and graduate students, followed by one-on-one interviews with students who wanted to provide additional information about their experiences. Data was collected from two universities in the Baltimore metropolitan area, one of which is a designated historically black university and the other a minority serving university. We present findings from this research with a focus on identifying challenges and opportunities for universities to serve informal student caregivers from diverse backgrounds. More broadly, this research will contribute to the universities' understanding of student caregivers and help identify new resources to assist students in achieving their academic goals while being a caregiver.

### INFUSION OF AGE-FRIENDLY PRINCIPLES INTO CURRICULA USING INTERACTIVE MODULES

Sherry Greenberg[1], Shayle Adrian[2], and Riad Twal[2], *1. Monmouth University, Marjorie K. Unterberg School of Nursing & Health Studies, West Long Branch, New Jersey, United States, 2. Seton Hall University, South Orange, New Jersey, United States*

This presentation highlights the development of Age-Friendly care interactive modules to prepare the future healthcare workforce to care for older adults in Age-Friendly Health Systems. To date, there is a gap in the educational preparation of nurses, advanced practice nurses, and other health professionals to work in Age-Friendly Health Systems upon entry into practice. This project, funded by a faculty innovation grant at Seton Hall University, provides undergraduate and graduate nursing and interprofessional students with the background knowledge to care for older adults in Age-Friendly Health Systems. The aim of this project was to develop five interactive modules to embed in undergraduate and graduate nursing and interprofessional curricula focusing on the provision of care to older adults using the evidence-based 4Ms Framework: What Matters, Medication, Mentation, and Mobility. Faculty collaborated with instructional designers in development of the modules using Articulate 360 to embed them in curricula. The first module focuses on the background of the Age-Friendly Health Systems movement, how health systems may become an Age-Friendly Health System, and the evidence for the 4Ms Framework. The second module focuses on assessment and act on strategies related to What Matters to older adults including advance care planning strategies. The third module covers crucial information related to Medication such as avoiding potentially inappropriate medications, deprescribing, and antibiotic stewardship. The fourth module focuses on Mentation and covers assessment and act on strategies for depression, delirium, and dementia. The fifth module focuses on Mobility such as promoting mobility and decreasing fall risk.

### TEACHING MEDICAL STUDENTS TO INCORPORATE THE 4MS OF AGE-FRIENDLY HEALTH SYSTEMS ACROSS THE CONTINUUM OF CARE

Jennifer Severance[1], and Sarah Ross[2], *1. UNTHSC-TCOM, Fort Worth, Texas, United States, 2. University of North Texas Health Science Center, Fort Worth, Texas, United States*

High quality and equitable health care for older adults involves patient-centered approaches that can improve the patient's engagement, decision-making, and health outcomes. To educate future health professionals about patient-centered care across the continuum of care, a HRSA-Geriatric Workforce Enhancement Program created a four-week online elective course for third and fourth-year medical students using the Age-Friendly Health Systems 4Ms framework. Faculty identified geriatric tools and best practices in the 4M's areas of What Matters, Mobility, Mentation, and Medication to develop self-directed asynchronous learning modules. Students were instructed on incorporating the 4Ms into patient assessments, care planning, interprofessional practice, and process improvement in different settings of care. Assignments included quizzes, case studies, discussion forums, and a patient experience interview with an older adult or caregiver. At course completion, students self-assessed their knowledge and skills and rated the course materials and assignments using five-point Likert-type items. Ninety-six students participated in the course between July 2020 and March 2022. Ninety-eight percent of respondents (n=80) felt the assignments were helpful, and 100% agreed the course improved their knowledge about Age-Friendly Health Systems and different care settings and processes. Also, 100% felt the content was applicable to their future practice and 96% would recommend the course. Qualitative thematic analysis of open-ended questions showed preferences for interactive elements, such as case presentations and guided interviews with older adults, as well as ways to improve student engagement. Educating students about Age-Friendly Health Systems can prepare future health professionals for collaborative patient-centered geriatrics care across a variety of settings.

### THE RESPONSIBLE DESIGNER: USING A HUMAN-CENTERED DESIGN APPROACH TO CREATE AN ASSISTIVE TECHNOLOGY FOR AD

Kimberly Mitchell, and Xiaopeng Zhao, *University of Tennessee, Knoxville, Tennessee, United States*

Our interdisciplinary team of designers, computer scientists, and engineers have begun the development of a low-cost social robot called FRED (the Friendly Robot to Ease Dementia). FRED has a conversational capability which will allow the device to be used as assistive companions for people with AD, engaging conversation, assisting with reminders, playing simple games and music, and relieving caregivers of some of their responsibilities. FRED was created using 3-d printing technology and a single board computer based on raspberry Pi. Interaction between human and the system was implemented using the Mycroft open-source voice assistant. The overall cost of the robot is estimated to be less than $300, rendering it possible for wide distribution among the public. Our presentation will share results from our participatory focus group which helped assist in the design and interactions of FRED. This human-centered design approach included neurologists, healthy older adults, as well as ADRD patients and their caregivers. A common language of important terms (e.g., feedback, interface, training, cognitive assessment) to aid in initial conversations were implemented. A thinking-aloud protocol was adopted once we had a prototype of FRED, where users were able to express freely any problems and/or concerns during interaction. Insights from the thinking-aloud results were used to improve the user interface design to enable the users and robot to interact and collaborate in an effective, natural way. Feedback from the focus group were carefully documented and the results will be shared during our presentation.

### GERIATRIC PRACTICE LEADERSHIP INSTITUTE—AN INTERPROFESSIONAL TEAM APPROACH USING THE IHI AGE-FRIENDLY MODEL

Janice Knebl[1], Jennifer Severance[2], Sara Murphy[2], Kathlene Camp[2], James Roach[3], and Thomas Fairchild[4], *1. University of North Texas Health Science Center-TCOM, Fort Worth, Texas, United States, 2. UNTHSC-TCOM, Fort Worth, Texas, United States, 3. TCU - Neeley School of Business, Fort Worth, Texas, United States, 4. UNTHSC, Fort Worth, Texas, United States*

According to the Institute of Medicine, immediate steps must be taken to educate and train both the current and future health care workforce to work collaboratively in addressing the diverse needs of the growing older adult population.1 Most healthcare professionals had very little education or clinical training in the care of older adults nor the most effective ways to work as a clinical team. The Geriatric Practice Leadership Institute (GPLI) is a collaboration between two universities providing inter-professional teams of early and mid-career professionals the skills and knowledge needed to leverage leadership skills to effectively work within interdisciplinary teams to provide age-friendly care to older adults. The GPLI incorporates the Institute for Healthcare Improvement (IHI) Age-Friendly Health Systems 4Ms' Framework into the training. The GPLI is an on-line, team-based program which engages 5-7 teams each session. Module topics include Age-Friendly Health Systems, organizational culture, leading self, leading inter-professional teams, and quality improvement. Additionally, teams select and completes a quality improvement project based on the Age-Friendly Health Systems 4Ms and submits a final report and presentation. The teams are also assigned a coach for support. Continuing education credits and a micro-credential are available to participants. The GPLI has trained over 175 health care professionals during the past 7 years with teams representing ambulatory to emergency responder organizations. The GPLI has been funded by the Health Resources and Services Administration (HRSA) Geriatrics Workforce Enhancement Program grant (numberU1QHP2873), which currently covers all costs for participants.

### SESSION 1260 (SYMPOSIUM)

### ELECTIONS MATTER: AGING POLITICS AND POLICY IN THE BIDEN ERA

Chair: Edward Miller Co-Chair: Janelle Fassi Discussant: Marc Cohen

The 2020 election of Joe Biden to the presidency has shifted the foundations of the American political system after four years of conservative policy leadership. Combined with a slim Democratic majority in Congress, the policies, priorities, and proposals of the Biden administration have important ramifications for all spheres of American life. Nowhere is this clearer than with older Americans who disproportionately supported Donald Trump's reelection but are among those who benefit most with enactment of the Democratic party agenda. Between 2010 and 2050 the proportion of the U.S. population aged 65 years or older is projected to increase from 13% to 20%. Population aging creates opportunities and challenges for older adults, their families, and society in general. The Biden administration has chosen to meet these challenges, in part, by seeking to expand the social safety net. By contrast, the Trump administration largely supported Republican priorities to draw back on the federal government's commitment to programs intended to support an aging population. This panel examines aging politics and policy during the first two years of the Biden administration. It reports on older adults' voting patterns and the role of issue framing in limiting public support for proposals such as Medicare for All. It analyzes provisions in the American Rescue Plan Act and Build Back Better Bill to bolster formal care and services for older adults, as well as federal actions to support family caregivers. Edward Miller serves as panel chair; Janelle Fassi serves as co-chair and Marc Cohen as discussant.

### ENHANCING FEDERAL REVENUE FOR HOME AND COMMUNITY-BASED SERVICES UNDER THE AMERICAN RESCUE PLAN ACT

Edward Miller[1], and Lisa Beauregard[2], *1. University of Massachusetts Boston, Boston, Massachusetts, United States, 2. Massachusetts Executive Office of Elder Affairs, Boston, Massachusetts, United States*

The American Rescue Plan Act (ARPA) includes a one year 10 percentage point increase in the Federal Medical

Assistance Percentage for Medicaid-funded home and community-based services (HCBS). The goal is to strengthen state efforts to help older adults and people with disabilities live safely in their homes and communities rather than in institutional settings during the COVID-19 pandemic. This presentation provides a detailed description and analysis of this provision, including issues state governments need to consider when expending the additional federal revenue provided. It also draws lessons from the Affordable Care Act's Balancing Incentive Program to suggest insights for the potential of ARPA to promote further growth in Medicaid HCBS programs. The presentation concludes by highlighting the importance of instituting strategies and processes for maximizing enhanced federal matching funds under ARPA in preparation for subsequent availability of substantial additional federal resources targeting Medicaid HCBS under other proposed initiatives.

### WHAT WAS IMPORTANT TO OLDER VOTERS IN THE 2020 ELECTION? A LOOK AT COVID-19, MISINFORMATION, AND POLICY

Elizabeth Rickenbach[1], Janelle Fassi[2], and Kevin Doran[3], *1. St. Anselm College, Manchester, New Hampshire, United States, 2. University of Massachusetts Boston, Goffstown, New Hampshire, United States, 3. st. Anselm, Manchester, New Hampshire, United States*

High older voter turnout rates, a growing aging population, and organizations that serve the interests of older adults have historically contributed to the importance of older adults for elections. Since 2010 older voters have tended to vote Republican, with White older adults typically preferring Republican candidates, and Black and Hispanic older voters typically preferring Democratic candidates. In the 2020 election, the 65+ group of voters showed the same Republican candidate favorability but followed a slow downward trend from recent elections. Grounded in a demographic, economic, and generational context, and considering theory and research from gerontology, political science, psychology, and sociology, this presentation will explore older voter turnout and candidate choice in the 2020 presidential election. The focus will be on considering past trends and public polling data to examine three key issues in relation to the behavior of older voters in 2020: COVID-19, presidential candidate platforms, and misinformation and the media.

### MEDICARE AND THE DYNAMICS OF US HEALTH REFORM POLITICS

Michael Gusmano[1], and Alan Monheit[2], *1. Leigh University, Bethlehem, Pennsylvania, United States, 2. Rutgers University School of Public Health, Piscataway, Massachusetts, United States*

Since the 2020 election, President Biden and Congressional Democrats have largely abandoned proposals for creating a "Medicare for All" system or adding a "public option" to the Affordable Care Act (ACA) marketplaces. This reflects a lack of sufficient enthusiasm, within the Congress or the public, for either option. These options are viewed by many current Medicare beneficiaries as a potential threat to their existing benefits. Indeed, framing the ACA and other efforts to expand insurance coverage as a threat to Medicare has become something that health reform opponents have done routinely since the 2010 midterm election. We will apply behavioral economics principles including loss aversion, hyperbolic discounting, and status quo bias, to explain why it has been easy to demonize reform options such as these. In addition to presenting data from public opinion polls, we will examine how Congressional and other party leaders have framed these choices for the public.

### THE IMPLICATIONS OF THE BUILD BACK BETTER BILL FOR AGING HEALTH POLICY

Pamela Nadash, *University of Massachusetts Boston, Boston, Massachusetts, United States*

The Build Back Better Bill passed by the U.S. House of Representatives (HR 5376) included ambitious healthcare and long-term care provisions that would have greatly benefited older people, although the Bill itself represents a considerable scaling down of the Biden Administration's original proposals. This presentation reviews key provisions of the Bill that would impact older people: paid family and medical leave, efforts to strengthen the direct care workforce, and investments in home and community-based services. In addition, the Bill expanded Medicare coverage to include hearing, dental, and vision coverage, and expanded tax credits for the medical expenses associated with family caregiving. Although the House bill failed to make it through the Senate intact, some of the provisions are likely to have staying power. This presentation reviews the Bill's progress and assesses implications for future efforts to expand access to these benefits.

### FEDERAL ACTIONS TO SUPPORT FAMILY CAREGIVERS UNDER THE BIDEN ADMINISTRATION

Eileen Tell, *ET Consulting, LLC, Belmont, Massachusetts, United States*

Family caregivers are the glue holding together the delivery and financing of long-term care. Replacing family care with paid care would cost roughly $470 billion each year. But family caregivers are struggling. They face many challenges – most notably the financial stress and the need for services and supports. Other challenges include lack of respite care, need for caregiver training, and lack of access to quality paid workforce. In order to address these challenges, Congress authorized the RAISE Family Caregiving Advisory Council. The RAISE (Recognize, Assist, Include, Support and Engage) Family Caregivers Act directs the Secretary of Health and Human Services to develop a national family caregiver strategy. This session presents the findings of two years of focus groups and interviews with family caregivers and hundreds of stakeholder organizations that support them, providing concrete input to the Biden administration on how to deliver on the broad objectives of the RAISE Act.

### SESSION 1270 (SYMPOSIUM)

### ENGAGING ADULTS LIVING WITH COMMUNICATION CHALLENGES IN RESEARCH: EXAMPLES AND PRACTICAL TIPS

Chair: Natalie Douglas Discussant: Roger O'Sullivan

This symposium contends that the engagement of older adults living with a variety of communication challenges in research about interventions designed to support them results in better translation and adoption of research findings. While engaging older adults in research often takes time, effort, and systematic tools, the benefits have been recognized by multiple stakeholders including policy makers, funding agencies, practitioners, older adults, and their families. Adults living with communication challenges may have difficulty expressing or comprehending language; however, that does not preclude their engagement in research activities, especially when this research involves interventions that concern them. This symposium will illustrate three exemplars of engaging adults living with communication challenges in research activities across international initiatives. First, qualitative data from 25 interviews from a project designed to engage persons living with stroke-based aphasia in friendship programming with be discussed. Then, mixed methods data from a community-based approach to support adults living with stroke-based aphasia, mild cognitive impairment, and traumatic brain injury will be described including the process of developing and using a multi-stakeholder advisory board. Finally, involving adults living with Frontotemporal Lobar Degeneration (FTLD) in research with a focus on building trust, staying connected between meetings, being transparent about expectations and applying a narrative approach will be described. Our discussion led by Dr. Roger O'Sullivan will focus specifically on the ethical implications of engaging adults living with communication challenges in research.

### GENERIC AND TAILORED INTERVENTIONS FOR COMMUNITY OLDER ADULTS WITH CHRONIC COGNITIVE COMMUNICATION DISORDERS

Ladan Ghazi Saidi, *Universoty of Nebraska at Kearney, Kearney, Nebraska, United States*

Many Community-Dwelling Older Adults (CDOA) live with debilitating chronic cognitive and communication disorders including Aphasia, Mild Cognitive Impairment, and Traumatic Brain Injury, and no longer receive healthcare services. This portion of the symposium will discuss why engaging CDOA and their caregivers with developing and selecting interventions for their cognitive communication disorders is important. Further, we discuss a community-based approach to make generic as well as tailored speech-language pathology services available to CDOA free of charge. The process of developing and using a multi-stakeholder advisory board of clinicians, researchers, CDOA, as well as a diverse advisory board of caregivers will be described, and challenges will be discussed. Further, we will present and discuss some relevant data.

### INVOLVING PEOPLE WITH A FRONTOTEMPORAL DEMENTIA DIAGNOSIS IN DEMENTIA RESEARCH

Martina Roes[1], Marie-Marleen Heppner[1], Sonja Teupen[2], Jan Oyebode[3], Claus Heislbetz[4], Vanaditz Goetz[5], Lara Pivodic[6], and Lauren Massimo[7], *1. German Center for Neurodegenerative Diseases (DZNE), Witten, Nordrhein-Westfalen, Germany, 2. German Center For Neurodegenerative Diseases (DZNE), Witten, Nordrhein-Westfalen, Germany, 3. Bradford University, Bradford, England, United Kingdom, 4. Hans-Weinberger-Akademie der AWO e.V., Muenchen, Bayern, Germany, 5. Hans-Weinberger Akademie der AWO e.V., Muenchen, Bayern, Germany, 6. Vrije universiteit Brussel, Ixelles, Brussels Hoofdstedelijk Gewest, Belgium, 7. University of Pennsylvania, Philadelphia, Pennsylvania, United States*

Frontotemporal Lobar Degeneration (FTLD) is a form of dementia that is characterized by prominent and gradual changes in behavior and language, whereas memory is relatively preserved (therefore different from Alzheimer dementia). In our FTD project we analyze care services for people with FTLD in Germany and we are working together with persons with FTLD and their partners as patient advisors. Our presentation will reflect on how we communicate (e.g. easy Language), how we communicate with the dyads (e.g. balance within the dyads), how we prepare for and engage during meetings as well as how this influences our research project. In summary, building trust and staying connected between meetings, being transparent about expectations and applying a narrative approach seems to be a way to successfully involve people with FTLD and their partners as advisors in dementia research.

### FRIENDSHIP IN APHASIA

Natalie Douglas, *Central Michigan University, Mount Pleasant, Michigan, United States*

People living with aphasia (PLWA) have identified friendship as a key area of need, and any programs or interventions designed to support this priority should have the input of PLWA at the forefront. This portion of the symposium will outline steps that were taken to engage PLWA in the development of evidence-informed interventions and programming to support friendship for PLWA, despite the challenges PLWA face in communication. The process of developing and using a multi-stakeholder advisory board of clinicians, researchers, PLWA, and family members will be described. Data highlighting key themes from interviews of PLWA, their significant others, and their friends will be presented including reciprocity, persistence, and communication strategies. Finally, communication techniques to engage PLWA in research activities as informed by PLWA themselves, clinicians, and researchers will be provided.

### SESSION 1280 (PAPER)

### FALLS AND BALANCE RECOVERY

### CHRONIC PAIN AND CIRCUMSTANCES OF FALLS IN COMMUNITY-LIVING OLDER ADULTS

Yurun Cai[1], Suzanne Leveille[2], Ling Shi[2], Ping Chen[2], and Tongjian You[2], *1. University of Pittsburgh, Pittsburgh, Pennsylvania, United States, 2. University of Massachusetts Boston, Boston, Massachusetts, United States*

Chronic pain is a risk factor contributing to mobility impairment and falls in older adults. Little is known about the patterns of fall circumstances among older adults with pain. This prospective cohort study described frequencies of fall circumstances (i.e., location, activities, and self-reported causes of falls) and examined the relationship between chronic pain and fall circumstances among 765 community-dwelling older adults (mean age=78.1, 63.9% women) in the MOBILIZE Boston Study. Pain severity, fall occurrence, and fall circumstances were recorded using monthly calendar postcards and fall follow-up interviews during a 4-year follow-up. Descriptive analyses summarized frequencies of fall circumstances. Generalized estimating equation (GEE) models examined the relation between monthly pain ratings and circumstances of the first fall in the subsequent month. Among 1,829 falls, 965 (52.8%) falls occurred indoors and 804 (44.0%) falls occurred outdoors, 60 (3.2%) falls with missing location information. Commonly reported activities and causes of falls were walking (915, 50.0%), slips/trips (943, 51.6%), and inappropriate footwear (444, 24.3%). GEE models suggested that compared to fallers without pain, fallers with moderate-to-severe pain had around twice the likelihood of reporting indoor falls (adj. OR=1.93, 95%CI:1.32-2.83), falls in living/dining rooms (adj.OR=2.06, 95%CI:1.27-3.36), and falls due to health problems (adj.OR=2.08, 95%CI:1.16-3.74) or feeling dizzy/faint (adj.OR=2.10, 95%CI:1.08-4.11), but they were less likely to report falls while going down stairs (adj.OR=0.48, 95%CI:0.27-0.87) or falls due to slips/trips (adj.OR=0.67, 95%CI:0.47-0.95) in the subsequent month. Future studies may investigate whether better pain management and tailored fall prevention in elders with chronic pain could lead to fewer falls.

### DOES PERTURBATION-BASED BALANCE TRAINING ON COMMERCIAL TREADMILLS IMPROVE BALANCE RECOVERY IN OLDER ADULTS?

Justin Whitten[1], Bryant Oleary[2], Dawn Tarabochia[2], and David Graham[2], *1. Montana State University, BOZEMAN, Montana, United States, 2. Montana State University, Bozeman, Montana, United States*

Background: Perturbation-based training (PBT) is a balance training method that causes a trip like event requiring a rapid step response to regain balance. There are numerous examples in the literature demonstrating the effectiveness of PBT but the need to use an expensive treadmill in a scientific laboratory limits the general applicability of PBT as a community-based intervention. A possible solution is to rapidly stop the treadmill belt during walking using the e-brake as the perturbation event. Importantly this could be performed on a commercially available, lower cost treadmill. Therefore, the purpose of this study was to evaluate the effectiveness of a commercial treadmill during PBT.

Methods: Seventeen participants completed either 9 weeks of PBT or conventional balance training based on ACSM guidelines. During an initial and final testing session participants balance recovery performance was evaluated. Participants were released from a forward static lean angle and asked to recover with a single step, during this test their movement was recorded and subsequently used to determine the Margin or Stability pre- and post-training. Participants were tracked for 6 months following the intervention and falls were recorded on a weekly basis. Results and Summary: There was no difference in balance recovery performance between groups following the training intervention and there was no difference in fall rate between groups in the 6-month follow-up period. We conclude that overall using the e-brake of a commercial treadmill is ineffective as a PBT strategy as it elicits no greater benefit than conventional exercise training.

### PREVENTING FALLS WITH PERTURBATION-BASED BALANCE TRAINING: HOW LARGE ARE THE HIP JOINT CONTACT LOADS, AND ARE THEY SAFE?

David Graham[1], Justin Whitten[2], Bryant Oleary[1], Rod Barrett[3], and Dawn Tarabochia[1], *1. Montana State University, Bozeman, Montana, United States, 2. Montana State University, BOZEMAN, Montana, United States, 3. Griffith University, Gold Coast, Queensland, Australia*

Perturbation-based training (PBT) is a balance training method presenting a high-challenge to balance which is extremely effective when compared to conventional training approaches. Common PBT methods use rapid treadmill belt translations with varying numbers of perturbations (20-1400 perturbations over 1-8wks). Importantly, the joint loads experienced during a stumble may be high enough to feasibly fracture bone (up to 12.7 Body Weights). However, the contact loads experienced during PBT are unknown. Because of increasing the prevalence of PBT it is necessary to specifically evaluate the range of joint loads experienced during PBT. Twelve participants completed a single PBT session of 24 perturbations. During both training and testing, participants movements were measured using a motion capture system which tracks body movements and records the forces under the feet. Hip joint contact loads were determined using Computational Musculoskeletal Modelling utilizing open-source software OpenSim. These techniques estimate the magnitude and pattern of force development of individual muscles and subsequently estimate the internal loads experienced by the hip joint.Hip joint contact loads were 4.90 ± 1.27 BW which is substantially lower that those previously reported by Graham et al. (2016) and Bergman et al. (2004) and is lower than the 5.5BW spontaneous fracture load boundary estimated by Schileo et al. (2014). Comparing the initial perturbation to the final perturbation revealed a 22% reduction in contact loads. We conclude that PBT performed using rapid translations on a treadmill are likely safe but suggest caution for individuals with poor bone mineral density or reduced neuromuscular function.

### PREVIOUS FALLS AND FEAR OF FALLING ON FUNCTIONAL LIMITATIONS: A LONGITUDINAL STUDY

Kehan Liu[1], Wenting Peng[1], Christina Miyawaki[2], Chunxiao Li[1], Yu Zheng[1], Siyuan Tang[1], and Minhui Liu[1], *1. Central South University, Changsha, Hunan, China (People's Republic), 2. University of Houston, Houston, Texas, United States*

Previous falls and fear of falling (FoF) are risk factors that affect older adults' daily activities. However, it remains unclear about their combined effects on functional limitations. Using Round 1 (R1) and Round 2 (R2) data from the National Health and Aging Trends Study, we examined whether falls and FoF in R1 independently and jointly predict functional limitations in R2 and the moderating role of demographic factors in this relationship among community-dwelling older adults aged 65 years and older. Previous falls and FoF were ascertained by asking participants whether they had fallen down in the last year and worried about falling in the last month. Functional limitations included any difficulties with mobility, self-care, or household activities. Poisson regression models were used to analyze data. Of 5,956 participants, 16.4% had falls only, 14.3% had FoF only, 14.5% had both, and 54.8% had neither. In the full adjusted model, those who experienced concurrent falls and FoF had a higher risk of functional limitations than those without falls and FoF (Mobility: Incidence risk ratio, IRR=1.44, 95% CI: 1.33-1.57; Self-care: IRR=1.29, 95% CI: 1.20-1.38; Household tasks: IRR=1.32, 95% CI: 1.21-1.44), as well as those with falls only (Mobility: IRR=1.32, 95% CI: 1.21-1.44; Self-care: IRR=1.26, 95% CI: 1.17-1.35; Household tasks: IRR=1.18, 95% CI: 1.08-1.29) and FoF only (Mobility: IRR=1.38, 95% CI: 1.27-1.51; Self-care: IRR=1.26, 95% CI: 1.17-1.35; Household tasks: IRR=1.31, 95% CI: 1.20-1.43). The findings suggest that strategies to improve falls and FoF together could potentially help prevent functional limitations.

### THE VALUE OF BALANCE CONFIDENCE IN OLDER ADULT FALL RISK ASSESSMENT

Tiffany Hughes[1], Edmund Ickert[2], Garrett Kellar[2], Lucy Kerns[2], and Cara Berg-Carramusa[2], *1. Youngstown State University, Beaver, Pennsylvania, United States, 2. Youngstown State University, Youngstown, Ohio, United States*

Fall risk assessment traditionally focuses on objective physical performance. Balance confidence, a subjective measure of physical function, may provide important information to better predict fall risk and guide assessment and intervention strategies. This study examines the associations of balance confidence congruency with physical performance measures and fall occurrence. One-hundred-fifty-five community-dwelling adults aged 60 and over completed a comprehensive fall risk assessment including physical performance tests (timed up and go, 4-stage balance, 30-second chair stand, and 4-M gait speed), activities-specific balance confidence (ABC) scale, and self-reported falls in the past year. Four groups were created based on congruency between balance confidence (low vs. high) and each physical performance measure or overall fall risk category (at fall risk vs. not at fall risk, based on the STEADI tool kit). Poison regression analyses, adjusted for age and gender, tested the association between group membership and number of falls in the past year. Participants with high balance confidence and at fall risk based on 4-stage balance performance (Estimate=0.88, $p < 0.001$), or high balance confidence and at fall risk following the STEADI screening algorithm (Estimate=0.69, $p = 0.003$) were at increased risk of more falls compared to participants in the group with high balance confidence and not at fall risk. These results suggest that older adults who overestimate their balance relative to their physical performance may be at increased fall risk, and that participant subjective reporting of physical performance should be paired with objective physical performance measures to better identify older adults at fall risk.

### SESSION 1290 (SYMPOSIUM)

### GENERATIONAL TRANSMISSION OF SOCIAL RELATIONS: FINDINGS FROM MULTIPLE US LONGITUDINAL STUDIES

Chair: Rita Hu Co-Chair: Toni Antonucci Discussant: Shevaun Neupert

This symposium provides diverse findings documenting the long reach of social relations over generations. Ali and Rohner examine data from 41 adult offspring showing that recalled perception of rejection of parents during childhood are associated with fewer positive caregiving behaviors and social interactions with their now aging parents. Using three waves of longitudinal data over 23 years, Manalel, Cleary & Antonucci examine changes in composition, proximity, and contact frequency in social relations among 193 participants who were 8-12 years old at wave 1 (1992). Findings indicate increased diversity from wave 1 to 2 and increased stability from Wave 2 to 3, reflecting normative life transitions. Gender and race differences were also evident. Suitor, Gilligan, Frase & Stepniak examine 725 adult (aged 30-60) children's experience of their mother's advice concerning experienced depression and whether these differ by race, age, and gender. While there were no age differences, men, regardless of race and black daughters receiving advice had higher levels of depression but this had little effect white daughters. Finally, Hu and Antonucci use the Social Relations Study to examine the longitudinal association between social ties and self-esteem. They examined 553 people who were 13-77 at Wave 1 in 1992. Findings indicate that network closeness matters with increases in weak and close, but not closest network size related to increase in self-esteem 23 years later. In sum, this symposium offers multiple and diverse perspectives of generations in social relations and their association with well-being over the life span.

### THE LONGITUDINAL ASSOCIATIONS BETWEEN SOCIAL NETWORK STRUCTURE AND SELF-ESTEEM

Rita Hu, and Toni Antonucci, *University of Michigan, Ann Arbor, Michigan, United States*

Based on the convoy model of social relations, the current study used Latent Growth Curve Modeling to examine the associations between overall network, closest, close and weak social tie trajectories across the lifespan and self-esteem later in life. Participants (N = 553) aged 13 to 77 in Wave 1 (1992) were surveyed again in 2005 (Wave 2) and 2015 (Wave 3). The overall network size increased significantly across the lifespan ($\beta = 0.56$, SE = 0.01, $p < 0.001$). The closet tie size trajectories were not significantly associated with self-esteem 23 years later. The growth of the close tie size was not significantly associated with self-esteem later. Weak-tie size growth was also significantly associated with higher self-esteem later ($\beta = 0.14$, SE = 0.00, $p < 0.05$). The findings highlight social network's effects on self-esteem across the lifespan, as well as the critical role weak social ties play in development.

### AS YOU SOW, SO SHALL YOU REAP: ADULTS' MEMORIES' EFFECT OF PARENTAL ACCEPTANCE REJECTION IN CHILDHOOD ON AGING PARENTS

Sumbleen Ali, and Ronald Rohner, *University of Connecticut, Storrs, Connecticut, United States*

Little is known how adults' memories of parental acceptance-rejection in childhood influence their behavior toward their aging parents. Grounded in interpersonal acceptance-rejection theory (IPARTheory), this study attempts to better understand how early parent-child relationships affect adult offspring who provide care to their parents in later life. Data were collected from 41

adult offspring. Findings revealed that adults who felt rejected by their parents in childhood reported fewer positive caregiving behaviors toward their now aging parents, were less satisfied with social activities with their parents, spent less time with them or visited them less frequently, and revealed less overall concern for their aging parents. Results were consistent with IPARTheory's expectations that if parents reject their children, then parents place their own dependent old age at the risk of counter rejection: As you sow, so shall you reap. Such findings may help researchers, clinicians, and practitioners better understand the well-being of aging adults.

### DEVELOPMENT OF SOCIAL CONVOYS: TRAJECTORIES OF CONVOY STRUCTURE AND COMPOSITION FROM CHILDHOOD THROUGH ADULTHOOD

Jasmine Manalel[1], Jennifer Cleary[2], and Toni Antonucci[2], *1. Hunter College, New York, New York, United States, 2. University of Michigan, Ann Arbor, Michigan, United States*

This study examines stability and change in social convoys from childhood to adulthood and variation in trajectories of convoy characteristics by gender and race. Multilevel models for convoy structure and composition were estimated using longitudinal data spanning 23 years. Respondents included a sample of 193 children aged 8 to 12 in Wave 1 (1992) who were surveyed again in their 20s (Wave 2) and 30s (Wave 3). Changes in composition, proximity, and contact frequency were observed at each wave. Between Waves 1 and 2, changes reflected age-normative trends toward network diversification typical of the transition to adulthood. Between Waves 2 and 3, changes were consistent with those expected as young adults settle into stable roles. We also identified convoy characteristics that differed between men and women and between Black and White individuals, emphasizing the importance of personal characteristics to fully understand form and function of social relations across the lifespan.

### "DID I ASK FOR YOUR ADVICE?": EFFECTS OF ADVICE FROM PARENTS ON ADULT CHILDREN'S DEPRESSIVE SYMPTOMS

J Jill Suitor[1], Megan Gilligan[2], Robert Frase[1], and Catherine Stepniak[1], *1. Purdue University, West Lafayette, Indiana, United States, 2. Iowa State University, Ames, Iowa, United States*

Studies of intergenerational relations typically conceptualize advice as a positive dimension of exchanges between parents and adult children. In contrast, we suggest that advice may often be a source of interpersonal stress and thus have detrimental effects on psychological well-being. Further, we suggest that these effects vary by race and gender. To address these questions, we use data from 725 adult children regarding advice from mothers, collected as part of the Within-Family Differences Study. Multilevel regression analyses revealed that the impact of receiving advice varied by the combination of gender and race. Among sons, receiving advice predicted higher depressive symptoms, regardless of race. However, receiving advice predicted depressive symptoms among Black but not White daughters. Taken together, these findings question whether advice is a positive dimension of intergenerational interaction and suggest that mothers' advice may have a negative impact on adult children's depressive symptoms, particularly for sons and Black daughters.

### SESSION 1300 (SYMPOSIUM)

### GRAY DIVORCE: AN INCREASINGLY COMMON PATH TO UNCOUPLING IN LATER LIFE

Chair: Torbjörn Bildtgård

Late life divorce is rapidly increasing in large parts of the Western world, in what has been described as a "gray divorce revolution". In the US the incidence of gray divorce doubled between 1990 and 2010, in Sweden it has more than doubled since the millennium and in Israel it has almost doubled since 1996. Still, gray divorce remains rather invisible both in family sociology, which mainly focuses divorce at earlier ages, and in gerontology, which still tends to view widowhood as the single path to late life uncoupling. This symposium introduces current research on gray divorce and addresses a range of questions, such as: Why do people divorce late in life? How does gray divorce affect later life, including economy, health, support networks and relationships to adult children? How do cultural values shape gray divorce? What is the difference between his and her gray divorce? The presenters represent different countries (the US, Israel and Sweden) and approach the topic of gray divorce with both quantitative and qualitative data.

### THE EXISTENTIAL CONTEXT OF GRAY DIVORCE

Torbjörn Bildtgård[1], and Peter Öberg[2], *1. Stockholms University, Stockholm, Stockholms Lan, Sweden, 2. University of Gävle, Gävle, Gavleborgs Lan, Sweden*

In later years divorce rates for older people has increased in many parts of the Western world in what has been described as a "grey divorce revolution". In Sweden divorce rates for people 60+ have more than doubled since the millennium. The purpose of this paper is to study the reasons older people attribute to their late life divorce. Qualitative interviews were carried out with Swedish men and women aged 62–82 who after the age of 60 had divorced from a cross-gender marital or non-marital cohabiting union (n=37). The interviews covered themes regarding the divorce process, including reasons for divorce, experiences of divorce and life as a grey divorcee. The results were analysed using principles from Grounded Theory. The results revealed four different types of divorce narratives: 1) Incompatible goals for the third age, 2) Personality change caused by age related disease, 3) A last chance for romance, and 4) Enough of inequality and abuse. A central insight generated by the study was the importance attributed to the particular existential conditions of later life in the divorce decisions. The results are discussed in relation to theories of the third age as a time of self-fulfillment, where the partner can either be part of or an obstacle to that project.

### THE DAWN OF A SOCIETY OF DIVORCEES: CHANGING PATTERNS OF LATE LIFE CIVIL STATUS

Peter Öberg[1], and Torbjörn Bildtgård[2], *1. University of Gävle, Gävle, Gavleborgs Lan, Sweden, 2. Stockholms University, Stockholm, Stockholms Lan, Sweden*

Half a century ago Lopata used the concept “society of widows” to describe the gendered reality of late life singlehood, where widowed women were excluded from coupled social life, depended on a community of other widows for social integration, and refrained from initiating new relationships due to “sanctification” of their former husbands. We use Swedish, American and EU census data 1970-2020 and a national survey to Swedes 60-90 years old (n=1225; response rate 42%) to illustrate a substantial change in the demographic landscape of late life civil status. More people enter later life as divorcees or become divorced at a high age. Among Swedes 60+ divorcees outnumber widowed people, and the incidence of late life divorce has more than doubled since the millennium in what has been called the “grey divorce revolution”. Many other Western countries follow the same demographic trend, posing important questions about the transformation of unmarried later life. We conclude by proposing the concept “society of divorcees” for this new demographic landscape of late life singlehood, argue that research is needed to capture this new reality, and discuss the implications of this change for access to social support later life.

### ADULT CHILD PROXIMITY AND REPARTNERING AMONG PARENTS AFTER GRAY DIVORCE

Susan Brown, I-Fen Lin, and Kagan Mellencamp, *Bowling Green State University, Bowling Green, Ohio, United States*

The rise in gray divorce is spurring growth in repartnering, which occurs more frequently after divorce than widowhood. Roughly 40% of men and 25% of women form a co-residential union following gray divorce. Drawing on the 1998-2014 Health and Retirement Study, we assessed whether parent-adult child proximity (living within 10 miles) was associated with men’s and women’s repartnering after gray divorce. Men’s relationships with their children tend to erode following gray divorce, and thus we anticipated that the association between proximity and repartnering was negligible. In contrast, women’s ties to adult children are largely resilient following gray divorce, which led us to expect that having a child nearby reduced chances of repartnering. Our analyses revealed that both men and women with a proximate child less often repartnered than those with no children nearby. Repartnering seemingly functions as an alternative means to “doing family” for those lacking nearby children.

### THE MEANING OF FAMILYHOOD FOR GRAY DIVORCE IN ISRAEL

Chaya Koren[1], Yafit Cohen[2], Naor Demeter[2], and Michal Egert[2], *1. University of Haifa, Haifa, Hefa, Israel, 2. School of Social Work University of Haifa, Haifa, Hefa, Israel*

Israel is a society that values familyhood alongside self-determination. Gray divorce rates in Israel are low, yet they have nearly doubled since 1996 from 2% to 3.65%. Little is known about grey divorce in modern societies and even less in societies located between tradition and modernity such as Israel. Deriving from data analysis, our aim is to present the meaning of familyhood for gray divorce in Israel from the experiences of individuals and couples who divorced at age 60+ and their adult children, using a life course perspective. 72 in-depth qualitative interviews were audio recorded and transcribed verbatim with divorced men, women, and their adult children, analyzed as individuals, dyads, and family units based on principles of dyadic interview analysis. Findings include three themes: (a) The value of familyhood as a divorce delay. (b) Between couplehood dissolution and familyhood preservation. (c) Gray divorce shaping a new familyhood. Implications are discussed.

### RELIEF, REGRETS, AND REINVENTION: LIFE AFTER GRAY DIVORCE

Jacquelyn Benson[1], Landon Olivia[2], Allison Donehower[2], and Caroline Sanner[3], *1. Washington University in St. Louis, Columbia, Missouri, United States, 2. University of Missouri, Columbia, Missouri, United States, 3. Virginia Tech, Blacksburg, Virginia, United States*

“Gray divorce,” or divorce which occurs in later life, is rapidly becoming more common in the United States. The purpose of this grounded theory study was to examine the lived experience of getting divorced in mid to later life. Data address the following research questions: 1) What are the divorcees’ expectations for the process of divorce and post-divorce life? 2) How do life phase factors and family relationships shape the divorce experience? Participants included 41 heterosexual men and women who legally divorced at the age of 55 or older and between 1-7 years from the time of the interview. Participants divorced from first and higher order marital unions. They included self-identified initiators, non-initiators, and co-initiators of the divorce. Results suggest that gray divorce is a complex experience marked by shifting feelings of ambivalence and certainty that are influenced by such factors as health and social networks, especially family.

### SESSION 1310 (PAPER)

### HOSPICE AND MEDICAL AID IN DYING PRACTICES: EXAMPLES FROM STATES AND NATIONALLY

### A NATIONWIDE EXAMINATION OF MEDICARE PART B UTILIZATION DURING HOSPICE ELECTION

Thomas Christian[1], and Michael Plotzke[2], *1. Abt Associates, Cambridge, Massachusetts, United States, 2. Abt Associates, Saint Louis, Missouri, United States*

This research characterizes trends in two hospice-specific modifiers: (1) “GV” indicating services for the terminal/related conditions by an attending physician not an employee of the hospice and (2) “GW” indicating physician services unrelated to terminal/related conditions. We identified Part B (carrier/physician supplier) claims during hospice elections in Federal Fiscal Year (FY)2020, and replicated an Office of Inspector General (OIG) approach calculating potentially “questionable” Part B claims, where the physician and diagnosis codes match between the hospice and Part B claims (without a GW modifier listed). Using logistic regression, we calculated adjusted odds ratio (AOR) and 95% confidence intervals (CI) to characterize this billing. Overall, $372.8 million in physician services occurred during hospice elections in FY2020. Of this, two-thirds ($247.8 million) included a GW modifier, one-quarter ($86.5 million) a GV modifier, $2.2 million both modifiers, and $40.8 million neither modifier. Replicating the OIG methodology, we calculated $19.4 million (5.2%) as "questionable". Beneficiaries electing hospice for 180+ days were three times more likely (95% CI 2.99-3.12) to have questionable billing as a beneficiary electing hospice 14-29 days, and facility residents were more likely to have questionable billing, relative to beneficiaries in their own homes. Questionable billing rates were also highest in the northeastern quadrant of the country. Lastly, we found ten percent of physicians accounted for almost three-quarters of all questionable billing. CMS should further monitor physician services during hospice to maintain the integrity of the benefit and ensure beneficiaries receive adequate care.

### AN EXAMINATION OF MEDICATION UTILIZATION DURING MEDICARE HOSPICE ELECTION: A NATIONAL STUDY

Thomas Christian[1], and Michael Plotzke[2], *1. Abt Associates, Cambridge, Massachusetts, United States, 2. Abt Associates, Saint Louis, Missouri, United States*

In addition to drugs received through the Medicare hospice benefit, each year Medicare hospice beneficiaries receive nearly $700 million in medications through Medicare Part D. Little is known about the nature of these medications. To explore this issue, we identified all beneficiaries who were enrolled in Part D and elected hospice during Calendar Year (CY) 2020. We cross-walked hospice dates of service to Part D medication fill dates. We also collected information on drugs hospices themselves provided and voluntarily reported on hospice claims. Nationally, 1.2 million out of 1.8 million beneficiaries had some drugs provided, through hospice alone (406,423), Part D alone (412,810), and also from both sources (348,659). Medications from Part D tended to be more expensive, upwards of $40 per fill, compared to medications from hospices, which were about $15 per fill or less. Moreover, we found that 98,561 of beneficiaries (8.4%) received the exact same drug from both sources. Across diagnoses, the daily rates of pre-hospice Part D utilization were nearly identical to Part D utilization after electing hospice, and there was no relation to prior Part D use and hospice election duration. Categorically, the greatest amount of Part D expenditures during hospice are for diabetic therapies ($100.2 million), followed by anticoagulants ($97.8 million) and bronchodilators ($57.2 million). CMS should continue to monitor the provision of medications during hospice to maintain the integrity of the benefit and to ensure beneficiaries receive adequate care.

### CHALLENGES ASSOCIATED WITH AND LESSONS LEARNED FROM AGGREGATING MEDICAL AID IN DYING DATA IN THE UNITED STATES

Molly Nowels, and Elissa Kozlov, *Rutgers School of Public Health, Piscataway, New Jersey, United States*

Nine U.S. jurisdictions have legal Medical Aid in Dying (MAID) and produce publicly available data on the use of MAID. We performed a retrospective observational cohort study of MAID data from Oregon, Washington, California, Colorado, Washington DC, Vermont, Hawaii, New Jersey, and Maine. We investigated the total number of deaths from self-administered lethal medications, number of prescriptions for MAID written, characteristics of MAID users and requesters, and challenges of aggregating these data. Over the last 22 years of publicly available data from all states or jurisdictions that have legalized MAID, demographics data was collected for 1,949 people who died by MAID. Persons using MAID were more likely to be non-Hispanic white (95.6%), have some college education (72.2% vs. 26.2%) and a cancer diagnosis (74.0% cancer, 10.9% neurological illness, 15.1% other). Patients who died from MAID prescription ingestion were more likely to have insurance (88.9% insured vs 11.1% uninsured or unknown). Aggregating data came with multiple challenges including states collecting data on different populations (deaths vs prescriptions), with different variable categories, and for different time intervals for reports. More research is needed to better understand why MAID is currently being accessed by an overwhelmingly white, educated, and insured population. This research also highlighted the need for a national research agenda on Medical Aid in Dying. As more states plan to adopt MAID legislation, having standardized data collection elements will help to elucidate how these policies are being implemented and accessed.

### HOSPICE UTILIZATION AMONG MEDICAID-ONLY AND DUALLY ELIGIBLE DECEDENTS IN CONNECTICUT

Julie Robison[1], Deborah Migneault[2], Noreen Shugrue[3], Doreek Charles[3], Bradley Richards[4], Dorothy Wakefield[3], Ellis Dillon[3], and Dawn Lambert[4], *1. UConn Health, Center on Aging, Farmington, Connecticut, United States, 2. UCONN Health, Farmington, Connecticut, United States, 3. University of Connecticut, Farmington, Connecticut, United States, 4. State of Connecticut, Hartford, Connecticut, United States*

Individuals with Medicaid as their health care payer source may be either "Medicaid-only," or "dually eligible," i.e., qualifying for both Medicaid and Medicare. Medicare included hospice as a full Medicare benefit in 1982; Hospice is an optional Medicaid benefit for states; Connecticut added the Medicaid hospice benefit in 2010. This study explores hospice use in Connecticut's Medicaid program by comparing hospice use and length of hospice enrollment by Medicaid-only vs. dually eligible and by diagnosis for decedents who died between 2017 and 2020 and had a hospice-appropriate diagnosis. This analysis of 2,990 Medicaid-only and 24,881 dually eligible decedents finds that dually eligible decedents had a significantly ($p<.001$) higher rate of hospice use (48.1%) compared to Medicaid-only decedents (29.9%). Medicaid-only decedents received hospice for a median of 12 days vs. 13 days for the dually eligible ($p=.12$). Dually eligible decedents consistently received hospice more often than Medicaid-only decedents across all diagnoses (cancer ($p<.05$) circulatory ($p<.001$), dementia ($p<.001$), respiratory ($p<.001$) and stroke ($p<.001$)). Dually eligible decedents had significantly more days of hospice than Medicaid-only decedents for subgroups with a circulatory ($p<.05$) or stroke ($p<.05$) diagnosis, but Medicaid-only decedents with a dementia diagnosis had more days of hospice than their dually-eligible counterparts. Additional multi-variate analysis will demonstrate how other factors, like age, may account for differences. Recommendations include encouraging physician use of hospice screening tools and developing education interventions with physicians and patients to increase understanding of the availability and benefits of hospice with particular focus on Medicaid-only patients.

### RURAL HOSPICE SOCIAL WORK: SUPPORTING PRACTITIONERS IN END-OF-LIFE WORK WITH LIMITED RESOURCES

Brandi Felderhoff[1], Angela Alvarado[2], and Valerie Alvarez[3], *1. Texas Woman's University, Denton, Texas, United States, 2. Kindred at Home, Hospice Division, Dallas, Texas, United States, 3. Perimeter Behavioral Hospital, Arlington, Texas, United States*

Rural areas and the agencies, including hospice, that serve them face immense challenges in terms of accessibility, and service delivery. Small hospices face an enigmatic combination of higher operating costs and lower reimbursement payments, forcing higher caseloads on staff, and straining already limited available resources. Multiple cost benefit studies indicate that Medicare hospice reimbursement rules are well suited to the expense structure of large volume hospices, usually in urban, population dense areas; however, it is not clear that they apply as abundantly to smaller volume, rural hospices. This study sought to garner a deeper understanding of the roles and challenges required for rural hospice social work practice. Individual interviews with 9 rural hospice social workers across organizations in Texas and New Mexico were conducted. A maximum variation sampling technique was used to purposefully sample social workers from hospice agencies in areas deemed as rural by the Association of Rural Communities in Texas, with fewer than 200,000 occupants in their counties. Using emergent thematic analysis, key themes materialized including the challenge of dual relationships, required tasks beyond the scope of practice, issues of autonomy, and meeting them (patients/families) where they are at. Results demonstrate the complexities of rural hospice social work practice, the culture of rural communities, and the need for research into evidence-based intervention strategies specific to rural hospice social work, that will guide practitioners through navigating these challenging conditions.

### SESSION 1320 (SYMPOSIUM)

### EXPERIENCES OF LESBIAN, GAY, BISEXUAL, TRANSGENDER, AND/OR QUEER (LGBTQ) OLDER ADULTS WITH SERVICE PROVIDERS

Chair: Rajean Moone Co-Chair: Barbara Gordon
Discussant: Rajean Moone

Many lesbian, gay, bisexual, transgender, and/or queer (LGBTQ) older adults have experienced a lifetime of oppression and discrimination that often results in significant health, economic, and social disparities in comparison to their peers. Historically labeled as illegal, immoral, and/or sick, LGBTQ older adults often fear, hide, and distrust health care and other social services providers. This symposium will highlight contemporary research on the experiences and perceptions of LGBTQ older adults in relation to service providers. The first presentation will contextual factors that influence the healthcare utilization of LGBTQ middle-aged and older adults. The second presentation will explore older adult couples' experiences of minority stress with service providers and effects on their relationships. The third presentation will explore explore the beliefs, experiences, and needs related to housing and housing discrimination in LGBTQ older adults living in coastal North Carolina. The fourth presentation will explore panel data of older LGBTQ adults residing in the U.S South and examine the effects of access to an LGBTQ affirming provider on health service utilization and aging outcomes.. The final presentation will explore the association between increased awareness, understanding, and belief in the Undetectable equals Unstransmittable campaign and having an LGBTQ affirming care provider. The symposium will conclude with a discussion on policy and practice impacts for aging services and health care providers.

### EXPLORING HOUSING AND HOUSING DISCRIMINATION FOR LGBT OLDER ADULTS IN COASTAL NORTH CAROLINA

Noell Rowan[1], and Haley Norris[2], *1. University of North Carolina Wilmington, Wilmington, North Carolina, United States, 2. Liberty Senior Living, Wilmington, North Carolina, United States*

This study explored the beliefs, experiences, and needs related to housing and housing discrimination in LGBT older adults living in coastal North Carolina. Surveys were administered to staff (N = 138) of various types of older adult living communities (including long term care, public housing, and specific units of care including memory care and skilled nursing) to understand the status of discrimination, any integrative activities and human diversity/cultural sensitivity training offerings for staff. Findings indicate that nearly 80% of the participants received no training in the specific needs of LGBT communities and less than 10% provided inclusionary marketing/advertisement. No prior studies to date have been conducted on housing needs or discrimination of LGBT older adults within this coastal region. Implications include extensive need for cultural sensitivity education to plan for and improve housing experiences for LGBT older adults.

### LGBTQ+ AFFIRMING CARE AND UNDETECTABLE=UNTRANSMITTABLE: EVIDENCE FOR OLDER GAY AND BISEXUAL MEN IN THE US SOUTH

Ellesse-Roselee Akre (she/her)[1], Tara McKay[2], Jeff Henne[3], Adam Conway[2], Isabel Gothelf[2], and Nitya Kari[2], *1. Geisel School of Medicine at Dartmouth, Lebanon, New Hampshire, United States, 2. Vanderbilt University, Nashville, Tennessee, United States, 3. The Henne Group, San francisco, California, United States*

One of the most significant innovations in HIV prevention is the use of HIV treatment to prevent HIV transmission. This information has been disseminated as the "Undetecta ble = Untransmittable" (U=U) message. Despite evidence of effectiveness, U=U awareness, belief, and understanding remain limited in some communities. In this study we examine whether having an LGBTQ affirming healthcare provider increases U=U awareness, belief, and understanding among midlife and older gay and bisexual men in the US South, an understudied and underserved population and region where new HIV infections are increasing. We use data from the Vanderbilt University Social Networks Aging and Policy Study (VUSNAPS) on sexual minority men aged 50 to 76 from four Southern US states collected in 2020-2021. We find that only one in four men report prior awareness of U=U, but awareness is higher among HIV-negative and HIV-positive men who have an LGBTQ-affirming provider. Having an affirming provider significantly increases U=U belief and understanding, improves risk perception accuracy, and increases the likelihood of having ever tested for HIV among HIV-negative men. Improving access to LGBTQ affirming healthcare may improve U=U awareness, belief, and understanding, which could help to curb HIV transmission in the US South.

### LGBTQ+ OLDER ADULT COUPLES' EXPERIENCES OF MINORITY STRESS BY SERVICE PROVIDERS

Sara Bybee[1], Kristin Cloyes[2], Kathi Mooney[3], Katherine Supiano[1], Brian Baucom[4], and Lee Ellington[5], *1. University of Utah, Salt Lake City, Utah, United States, 2. Oregon Health & Science University, Portland, Oregon, United States, 3. University of Utah College of Nursing, Salt Lake City, Utah, United States, 4. University of Utah Department of Psychology, Salt Lake City, Utah, United States, 5. University of Utah and Huntsman Cancer Institute, Salt Lake City, Utah, United States*

This study explored LGBTQ+ older adult couples' experiences of minority stress with service providers and effects on their relationships. Twelve LGBTQ+ cancer patient-partner couples (N = 24) completed surveys assessing demographics, stress, and health, and participated in dyadic semi-structured interviews. Descriptive statistics summarized demographic characteristics. Interview data were content analyzed to identify sources of minority stress. Participants were aged 50.9 years on average (SD = 9.9, R =32-70), mostly white (21, 87.5%), and had been together for 19.1 years (SD = 9.9, R = 9-44). Common minority stress sources included derogatory language, belittling comments, heteronormativity, and cisnormativity in routine healthcare, cancer care, and legal services. Couples attributed relationship strength and durability as mitigating negative effects of stress; they described feeling closer, stronger, and more confident in their relationships. Some couples denied experiences of minority stress by service providers and these couples ascribed their equitable care to their geographic location.

### EFFECTS OF DISCRIMINATION/VICTIMIZATION ON HEALTHCARE UTILIZATION VIA HEALTHCARE BARRIERS

Krystal Kittle[1], Kyungmin Kim[2], Karen Fredriksen Goldsen[3], and Kathrin Boerner[4], *1. University of Nevada Las Vegas, Las Vegas, Nevada, United States, 2. Seoul National University, Seoul, Seoul-t'ukpyolsi, Republic of Korea, 3. University of Washington, Seattle, Washington, United States, 4. University of Massachusetts Boston, Boston, Massachusetts, United States*

Aging lesbian, gay, bisexual, and transgender (LGBT) adults are a health disparate population with unique healthcare challenges. Using data from the Aging with Pride: National Health, Aging, Sexuality/Gender Study (NHAS; N = 2,560), we examined contextual factors that influence the healthcare utilization of LGBT middle-aged and older adults. Causal indirect, direct, and total causal effects based on counterfactuals were computed to assess mediational links between discrimination/victimization and healthcare utilization via healthcare barriers. Discrimination/victimization had an indirect effect on health screenings via fear accessing health services both inside and outside of the LGBT community, financial barriers to care or medication, and being uninsured. Discrimination/victimization also had an effect on routine checkups and having a regular provider, via fear seeking health services outside of the LGBT community, financial barriers and being uninsured. Findings can be utilized in LGBT cultural competency trainings for health and human service providers serving aging LGBT people.

### PROMOTING HEALTHY AGING THROUGH LGBTQ+ AFFIRMING CARE

Harry Barbee, Tara McKay, Nathaniel Tran, and Judy Min, *Vanderbilt University, Nashville, Tennessee, United States*

This study examines variation in health service utilization and aging outcomes among older LGBTQ+ adults by access to an LGBTQ+ affirming health care provider. Primary survey data (n=1128) come from the Vanderbilt University Social Networks, Aging, and Policy Study, a panel study of older LGBTQ+ adults residing Alabama, Georgia, North Carolina, and Tennessee. Respondents with an LGBTQ+ affirming health care provider were more likely to seek preventative care, including routine checkups, colorectal screenings, flu shots, and HIV tests. Respondents with an LGBTQ+ affirming health care provider also reported higher control over their mental health and lower levels of cognitive decline. Overall, this study suggests that increasing access to LGBTQ+ affirming care could reduce health disparities among aging LGBTQ+ populations.

### SESSION 1330 (SYMPOSIUM)

### MISSION AND VISION OF THE MULTISITE VA ELIZABETH DOLE CENTER OF EXCELLENCE (EDCOE) FOR VETERAN AND CAREGIVER RESEARCH

Chair: Ranak Trivedi

Enabling Veterans to remain safely in their homes is a Department of Veterans Affairs (VA) priority. Informal caregivers play a critical role in supporting Veterans with functional impairments through their own efforts and the use of home and community-based services (HCBS). The Elizabeth Dole Center of Excellence (EDCoE) for Veteran and Caregiver Research was created in 2018 as part of the VA Choose Home Initiative to expand VA capacity to deliver integrated, Veteran- and caregiver-partnered, data-driven approaches to care through a set of complimentary projects. The EDCoE is a virtual center, with its multi-disciplinary team spread across the country at 5 different VA Medical Centers. The team also collaborates with other VA caregiver researchers. Finally, the EDCoE is closely networked with national, regional, and local operational partners, allowing for rapid dissemination of findings. In this symposium, we will first discuss the mission and vision of the EDCoE. We will then discuss multiple projects that are led through the EDCoE. Paper 1 (San Antonio) will identify measurement gaps to guide monitoring and improving HCBS. Paper 2 (Miami/Canandaigua) will describe a national survey of 8000+ Veterans and 3500+ caregivers. Paper 3 (Palo Alto) will describe caregivers' barriers to accessing VA and non-VA HCBS. Paper 4 (Salt Lake City/Canandaigua) will describe the results of latent trajectory analyses of administrative data to identify groups of Veterans who use different combinations of HCBS and institutional services. Our results are used by VA operational partners and policymakers to improve services for Veterans and informal caregivers.

### UNMET NEEDS AND PERCEIVED BARRIERS TO ACCESSING HCBS AMONG CAREGIVERS OF VETERANS OF ALL ERAS

Ranak Trivedi[1], Victoria Ngo[2], Trevor Lee[3], Marika Humber[3], Rashmi Risbud[4], Shreya Desai[5], Josephine Jacobs[2], and Dolores Gallagher-Thompson[6], *1. Stanford University, Palo Alto, California, United States, 2. VA Palo Alto Health Care System, Menlo Park, California, United States, 3. VA Palo Alto Health Care System, Palo Alto, California, United States, 4. University of California, Davis, Davis, California, United States, 5. Veterans Affairs Palo Alto Health Care System, Menlo Park, California, United States, 6. Stanford University, Stanford, California, United States*

VA has several HCBS to offset caregiver burden, facilitate caregiving, and enhance Veterans' home-based care, but they remain underutilized. We aimed to describe: the unmet psychosocial and HCBS needs of caregivers, barriers to accessing services, and gaps in available programs. Twenty-three caregivers participated in a 1-hr semi-structured interview (62.9□13.5y; 74.0% women; 47.8% White; 17.4% Hispanic; 65.2% spouses). Caregivers provided 7.3 hrs of daily care (SD=5.5 hrs, Range=1-24); most had provided care for 1+ year. Barriers to accessing HCBS included: a) disagreement with Veterans regarding service preferences and needs; b) lack of awareness of VA and non-VA programs; c) delays in obtaining services; and d) emotional toll of caregiving on personal health and relationship with the Veteran. The VA may need to invest in advertising existing services, develop strategies to match caregivers with available services when needed, and enhancing mental health and relationship quality for Veterans and caregivers.

### CAREGIVER-SPECIFIC QUALITY MEASURES FOR HCBS: STAKEHOLDER PRIORITIES AND ENVIRONMENTAL SCAN

Lauren Penney[1], Erin Finley[2], Julie Parish Johnson[1], Jacqueline Pugh[3], Luci Leykum[1], and Polly Noel[1], *1. South Texas Veterans Health Care System, San Antonio, Texas, United States, 2. VA Greater Los Angeles Healthcare System, Los Angeles, California, United States, 3. South Texas Veterans Health Care system, San Antonio, Texas, United States*

Although informal family caregivers are increasingly recognized for their essential role in helping older and/or medically-complex adults live in the community for as long as possible, their priorities and perspectives have not been well-integrated into assessments of home- and community-based services (HCBS). Our aim was to identify measurement gaps to guide quality monitoring and improve HCBS. Caregiver concerns and quality measurement priorities were identified during a multi-level stakeholder engagement process (34 Veterans, 24 caregivers, and 39 facility leaders, clinicians, and staff) across four VA healthcare systems. We conducted an environmental scan and scoping review of national quality measure sets for HCBS, comparing caregiver-specific items against stakeholder-identified concerns and priorities. Only five of eleven non-VA measure sets and three of four VA measure sets included caregiver-specific items; these did not encompass the full range stakeholder-identified concerns and priorities. Measures that emphasize caregivers can help healthcare systems monitor and improve HCBS quality.

### MEASURING UNMET NEEDS OF HIGH-NEED, HIGH-RISK AMERICAN VETERANS AND THEIR CAREGIVERS USING A PROSPECTIVE SURVEY

Stuti Dang[1], Sandra Garcia[2], Richard Munoz[3], Polly Hitchcock Noel[4], Marianne Desir[1], Jared Hansen[5], Benjamin Brintz[6], and Orna Intrator[7], *1. Miami Veterans Affairs Healthcare System, Miami, Florida, United States, 2. University of Miami, Miami, Florida, United States, 3. Florida International University, Miami, Florida, United States, 4. South Texas Veterans Health Care System, San Antonio, Texas, United States, 5. VA Salt Lake City, Salt Lake City, Utah, United States, 6. VA Salt Lake City Health Care System, Salt Lake City, Utah, United States, 7. Canandaigua VA Medical Center, Canandaigua, New York, United States*

Success in delaying long term institutionalization (LTI) depends on creating means to adequately support each Veteran's needs. To better understand the unmet needs of Veterans, we identified a random sample of 20,000 Veterans from five VA sites. Veterans were stratified into low-, moderate- or high-risk tiers using a measure of predicted 2-year probability of LTI. Veterans and their caregivers were asked to complete separate surveys to assess demographic, physical, psychological, and social domains, unmet needs, and experience with HCBS and caregiver support programs. Responses were received between July-Dec 2021 from 8056 Veterans (80.3+/-9.8y; 94.0% men; 82.6% White; 8.9% Hispanic) and 3579 caregivers (71.1+/-13.1y; 75.1% women; 80.5% White; 15.1% Hispanic; 57.1% spousal) responded by mail (96%) or online (4%). Both Veterans and caregivers endorse complex Veteran unmet needs spanning medical, psychological, and social domains. Survey results will be used to inform HCBS policy to support aging Veterans and their caregivers.

### GERIATRIC AND EXTENDED CARE TRAJECTORIES AND TRANSITIONS AMONG VETERANS WITH LONG-TERM CARE NEEDS

Erin Bouldin[1], Benjamin Brintz[2], Jared Hansen[3], Randall Rupper[1], Rachel Brenner[1], Orna Intrator[4], Bruce Kinosian[5], and Mary Jo Pugh[1], *1. University of Utah, Salt Lake City, Utah, United States, 2. VA Salt Lake City Health Care System, Salt Lake City, Utah, United States, 3. VA Salt Lake City, Salt Lake City, Utah, United States, 4. Canandaigua VA Medical Center, Canandaigua, New York, United States, 5. University of pennsylvania, Philadelphia, Pennsylvania, United States*

We aimed to identify clusters of geriatric and extended care services used by Veterans, describe transitions between clusters, and identify factors influencing transition. We explored services across the continuum of care from clinic to home-based and institutional care services. Analyses include 104,837 Veterans Health Administration (VHA) patients 65 years and older, and used VHA and Centers for Medicare & Medicaid Services (CMS) data from FY15-FY17. Using latent class and latent transition analyses, we identified 5 latent classes: 1) No Services, 2) CMS Services, 3) Home-Centered Interdisciplinary Care, 4) Personal Care Services, and 5) VHA-CMS Care Continuum. Veterans most commonly transitioned from the CMS Care Continuum class to No Services. Veterans also frequently transitioned into the VHA-CMS Care Continuum class over time. Identifying service patterns can inform service delivery, program development, and future resource allocation to better meet aging Veterans' needs.

### SESSION 1340 (SYMPOSIUM)

### NOVEL APPROACHES TO MINIMIZE SOCIAL ISOLATION AND MAXIMIZE PARTICIPATION IN OLDER ADULTS WITH DEMENTIA

Chair: Juleen Rodakowski Discussant: Juleen Rodakowski

Older adults experiencing cognitive decline, including those with Alzheimer's disease and related dementias, are at-risk for social isolation and participation restrictions, especially given the COVID-19 pandemic. Previously examined research has primarily focused on reducing problematic behaviors and remediating cognition for individuals with cognitive impairments. In this symposium, we will discuss novel intervention approaches and under-studied aspects of lifestyle behaviors that minimize social isolation and maximize participation for older adults with Alzheimer's disease and related dementias. Information presented in this symposium is intended to inform future intervention research to achieve an optimal quality of life for those with cognitive impairments. The first presentation will describe an analysis of lifestyle behaviors and cognition in an older population. The second presentation will discuss the development of an intergenerational, active music intervention that is delivered virtually to individuals with dementia and their caregivers. The third presentation will describe the effects of an adaptive horseback riding program that is becoming increasingly prevalent and improves the quality of life of persons living with Alzheimer's disease and related dementia.

### ADAPTIVE HORSEBACK RIDING: AN INNOVATIVE PROGRAM FOR IMPROVING THE QUALITY OF LIFE OF PERSONS LIVING WITH DEMENTIA

Beth Fields, *University of Wisconsin-Madison, Madison, Wisconsin, United States*

More than 5 million Americans living with Alzheimer's disease and related dementia (ADRD) have likely experienced forced social isolation due to the COVID-19 pandemic and subsequent stay-at-home mandates and public closures. Forced social isolation negatively impacts the quality of life of persons living with ADRD by worsening behavioral and psychological symptoms. Given continued pandemic restrictions and poor outcomes, now is the time to develop and support programs designed to improve quality of life through meaningful interactions that occur in natural environments. This symposium will introduce 'Riding in the Moment', an adaptive riding program that improves the quality of life of persons living with ADRD. Program participants are provided a safe, social, and supportive natural environment where they can ride, groom, and care for horses. The experiences constructed from Riding in the Moment seek to impact the current public health crisis by reducing social isolation for a growing and vulnerable population.

### DEVELOPING A MUSIC PROGRAM ON ZOOM FOR OLDER ADULTS WITH DECLINING COGNITION: A PROGRAM DEVELOPMENT REPORT

Jennie Dorris[1], Heather DiCicco[2], James Becker[1], and Juleen Rodakowski[1], *1. University of Pittsburgh, Pittsburgh, Pennsylvania, United States, 2. BriTE Wellness Inc, Pittsburgh, Pennsylvania, United States*

The coronavirus pandemic upended in-person programming for older adults with cognitive decline, presenting an urgent need to explore options to offer virtual programming. This program development report describes the adaptation of a music program for older adults with memory loss from in-person to a digital format. The objective was to develop a music program that was both engaging for the older adults, acceptable for the music instructor, and clearly defined for future research and implementation. This report describes the content of the music program and the systematic process of its development, including stakeholder interviews, acceptability surveys, fidelity reviews, and the developed instructor manual. With these structures in place, future research could begin to discern if digital music programming can support older adults in the same way that has been demonstrated in-person, potentially offering a new way to support the cognitive and social needs of a vulnerable population.

### DECLINE OF AN ONLINE COGNITIVE COMPOSITE ASSOCIATED WITH MODIFIABLE LIFESTYLE FACTORS

Chao-Yi Wu, Kirsten Wright, Nathan Hantke, and Jeffrey Kaye, *Oregon Health & Science University, Portland, Oregon, United States*

Voluntary, self-administered online assessment platforms have gained popularity in monitoring cognition and lifestyle behaviors. This study used the AARP Staying Sharp online brain health assessment platform to estimate the progression of a cognitive composite and correlate it with six modifiable lifestyle behaviors (physical activity, diet, sleep, stress, social activity, and brain engagement) in 147,939 participants (68.6 ± 7.0 years old; 70% female). A cognitive composite was created using attention, immediate and delayed recall, and working memory tasks and lifestyle behaviors were collected by self-report. A lower frequency of exercise ($p=0.01$), a higher frequency of sitting ($p=0.03$), a lower frequency of getting in touch with family and friends ($p=0.04$), and the inability to manage stress ($p=0.02$) were associated with a decline in the online cognitive composite. A self-administered online assessment platform may be useful in informing lifestyle modification interventions for risk reduction of cognitive impairment and dementia.

### SESSION 1350 (SYMPOSIUM)

### PROMOTING HEALTHY AGING OF WORLD TRADE CENTER RESPONDERS

Chair: Katherine Ornstein Co-Chair: Fred Ko

By 2030 the majority of World Trade Center (WTC) rescue and recovery workers (general responders) will be aged 65 and over and at risk for age-related conditions and consequences. Despite the US government's commitment to support WTC responders who have exposure-related health conditions (e.g., cancers, PTSD), little research to date has focused on age-related issues in this population. Because WTC responders were exposed to high levels of toxicants and intense psychological trauma in the emergency response and cleanup following the 2001 WTC disaster—hazards that can accelerate the aging process – they are likely at increased risk for premature aging and associated age-related syndromes (e.g., functional decline, falls). An improved understanding of how aging affects the health of WTC responders is critically important to improving their clinical care, health outcomes, and overall quality of life. In this symposium we will present 3 studies that have shed light on the aging of WTC responders. First, we assess frailty among responder using a frailty phenotype. In the next study we examine the extent of polypharmacy among WTC responders and associated factors. Finally, we examine trajectories of frailty in this population over 2 decades of follow up using a validated clinical frailty index. This work represents the first examination of frailty and aging among WTC responders and will be an area of growing research as the population ages.

### FRAILTY TRAJECTORIES AMONG WORLD TRADE CENTER GENERAL RESPONDERS

Fred Ko[2], Katherine Ornstein[1], Nicolo Foppa Pedretti[2], Elena Colichino[2], ghalib Bello[2], and William Hung[2], *1. Johns Hopkins, University, Baltimore, Maryland, United States, 2. Icahn School of Medicine at Mount Sinai, New York, New York, United States*

As the WTC cohort ages, it is important to examine whether there are differences in frailty trajectories over time in the cohort and examine clinical risk profiles of subpopulations with different trajectories. We used longitudinal data collected annually from the WTC data center and included all individuals with 3+ Clinical Frailty Index (FI) measurements after 9/11 to examine frailty trajectories using latent class growth mixture models. These patterns of change of Clinical-FI all showed an increasing trend over time, but there were three distinct patterns with different rates of increase. These were associated with distinct profiles of characteristics including sociodemographic, occupational and exposure level. WTC responders with higher age, working in construction, and higher-intermediate WTC level of exposure had higher rate of increase of Clinical-FI over time. While WTC responders with younger age, working in protective environment, and with higher education had lower rate of increase of Clinical-FI over time.

### FRAILTY AND AGE-RELATED CONDITIONS AMONG WORLD TRADE CENTER GENERAL RESPONDERS

Katherine Ornstein[3], Fred Ko[1], Amy Park[1], Ahmad Sabra[1], Katelyn Ferreira[1], William Hung[1], Roberto Lucchini[2], and Erin Thanik[1], *1. Icahn School of Medicine at Mount Sinai, New York, New York, United States, 2. Florida International University, Miami, Florida, United States, 3. Johns Hopkins, University, Baltimore, Maryland, United States*

As WTC exposure may precipitate frailty, we examined patterns of frailty in the general responders cohort. We used two methods to assess frailty: a Clinical Frailty Index (FI) developed using a deficit accumulation approach utilizing clinical assessments performed during routine annual visits to a WTC clinical program in New York City; and a frailty questionnaire (5-point FRAIL scale) collected from a subsample of responders. Using a Clinical FI cutoff score of 0.25, over 25% of the 7,679 participants, median age 58.3 years (IQR 9.3), who had any visit to the WTC clinical program from 2017 to 2019, were frail. In a subsample of 100 participants with additional frailty assessment (FRAIL scale), we found that 27% were pre-frail, 5% were frail, 14% had recent falls, 9% had ADL dependencies; 34% had mild cognitive impairment. These data suggest that frailty and its related conditions are prevalent among the cohort despite younger age.

### POLYPHARMACY AND FRAILTY AMONG WORLD TRADE CENTER GENERAL RESPONDERS

William Hung[1], Ghalib Bello[1], Fred Ko[1], Ahmad Sabra[1], Erin Thanik[1], Roberto Lucchini[2], and Katherine Ornstein[3], *1. Icahn School of Medicine at Mount Sinai, New York, New York, United States, 2. Florida International University, Miami, Florida, United States, 3. Johns Hopkins University, Baltimore, Maryland, United States*

Polypharmacy in older adults is associated with increased risk of adverse events such as falls, adverse drug reactions and functional decline. WTC responders suffer from conditions related to WTC exposure and are at higher risk of polypharmacy. We examined patterns of medication use among WTC general responders aged 50 and over who had at least one visit from 2017 to 2019 (n=7,679); 56.1% took 5+ medications and 22.5% took 10+ medications. Factors associated with polypharmacy (taking 5+ medications) include age (OR 1.08, $p<0.001$), female sex (OR 1.33, $p<0.001$), obesity (1.90, $p<0.001$), fair/poor self-rated health (OR 1.32, $p<0.001$) and limitations on performing moderate activity (OR 1.52, $p<0.001$). Frailty, as measured by the deficit count in the Clinical Frailty Index, is associated with polypharmacy after adjusting for other covariates (OR 1.23, $p<0.001$). Addressing polypharmacy by improving medication use may yield health benefits for this rapidly aging population at risk for adverse outcomes.

### SESSION 1370 (SYMPOSIUM)

### SIGMA PHI OMEGA PRESIDENTIAL SYMPOSIUM: ITS ROLE IN ADVANCING THE FUTURE OF GERONTOLOGISTS AND HEALTH PROFESSIONALS

Chair: Diane Martin Co-Chair: Katarina Friberg Felsted

Sigma Phi Omega, The International Academic Honor and Professional Society in Gerontology (aka Sigma Phi Omega), was established in 1980 to recognize excellence of those who study gerontology and aging, and the outstanding service of professionals who work with or on behalf of older persons. The formation of this society provided a much-needed link between educators, practitioners, and administrators in various settings where older persons are served. Through the efforts of the international office and executive board officers, Sigma Phi Omega builds avenues to further their members' academic and professional gerontological excellence. The goals of Sigma Phi Omega are achieved primarily through activities of local chapters at higher education institutions worldwide. Sigma Phi Omega chapters serve as links within their respective communities to promote interaction between gerontology educators, students, alumni, and local professionals. This international organization has a laser focus on excellence within gerontology and health professions education. In this session, the first presentation will provide the history of Sigma Phi Omega and its outreach efforts; the second presentation will focus on the future goals of Sigma Phi Omega to expand its role as an international honor society in preparing gerontologists, service providers and health professionals working with or on behalf of older adults; and the third presentation will focus on the SPO Chapters and their relationship with the piloted Gerontological Society of America Student Chapters.

### SIGMA PHI OMEGA CHAPTERS AND THEIR COLLABORATION WITH THE PILOTED GSA STUDENT CHAPTERS

Marilyn Gugliucci, *University of New England College of Osteopathic Medicine, Biddeford, Maine, United States*

Networking is the action or process of interacting with others to exchange information and develop professional or social contacts. The newly piloted GSA student chapters are encouraged to build relationships that can lead to networking and collaboration. While it is important to maintain a unique Sigma Phi Omega institutional chapter identity, the GSA student chapters also focus on the field of aging within a Higher Education Institution. Sigma Phi Omega collaborating with GSA Student Chapters is mutually beneficial in expanding learning, exploring new ideas, building professional connections, creating innovative projects, and gaining insights to other opportunities for professional and personal growth. This session will provide guidance on how to foster collaboration and growth for each of these chapters while honoring respective chapter missions, requirements, and educational experiences.

### SIGMA PHI OMEGA'S FUTURE

Diane Martin, *University of Maryland, Baltimore Graduate School, Baltimore, Maryland, United States*

Sigma Phi Omega (SPO) is well established within the United States but as The International Academic Honor and Professional Society in Gerontology, it is time to intentionally expand globally in preparing gerontologists, service providers, and health professionals in the field of aging. This session will build on the platform provided by Dr. Katarina Friberg Felsted, Sigma Phi Omega immediate past president, and encourage discussion with and among attendees on ideas and creative approaches for advancing Sigma Phi Omega at colleges and universities worldwide. Broad input and feedback for mapping the future of Sigma Phi Omega across the national and international arenas provides opportunities and prospects that advances the fields of gerontology and geriatrics. The key is upholding Sigma Phi Omega's mission and vision that supports aging through the work our members do with or on behalf of older adults.

### SIGMA PHI OMEGA: HISTORY AND ITS OUTREACH EFFORTS

Katarina Friberg Felsted, *University of Utah, Salt Lake City, Utah, United States*

Sigma Phi Omega, The International Academic Honor and Professional Society in Gerontology (aka Sigma Phi Omega), was established in 1980 to recognize excellence of those who study gerontology and aging, and the outstanding service of professionals who work with or on behalf of older persons. Its mission and vision was thoughtfully crafted to support the endurance and outreach of Sigma Phi Omega within the United States and globally. This session will share the structure and function of Sigma Phi Omega and its accomplishments. This foundation is an essential platform for the launching of the organization's strategic plan as we move forward in the field of aging.

### SESSION 1380 (SYMPOSIUM)

### STIGMA IN THE CONTEXT OF ALZHEIMER'S DISEASE AND RELATED DEMENTIAS (ADRD) WITHIN RURAL AND UNDERSERVED POPULATIONS

Chair: Elizabeth Rhodus Co-Chair: Steffi Kim Discussant: Fayron Epps

Stigma in the context of Alzheimer's disease and related dementias (ADRD) is associated with a higher prevalence of depression, anxiety, social isolation, and poorer caregiver health. This is particularly true for underserved and rural communities; however, little is known about the sources of stigma and implications of stigma within these communities. This symposium explores sources of stigma along with implications of stigma in rural and/or underserved communities and introduces novel interventional considerations for addressing stigma. The first presentation by Rhodus and colleagues highlights implications of stigma in rural Appalachian communities as it relates to ADRD healthcare service and research participation. Next, Sabat and colleagues present findings of a recent intervention program, "Respite for All," specifically, implications of this program for caregivers' perception of stigma, as well as the person living with ADRD. This symposium also includes presentations focused on Alaska Native (AN) experiences with stigma and ADRD. Kim discusses findings of a community-based participatory research project using mixed-method to explore structural stigma in rural communities and needed initiatives for familial care partners. To conclude the program, Crouch and Rosich present results of a grounded theory, exploratory study aimed to understand the cultural practices and values that compose AN Elder beliefs and perceptions of ADRD, including stigmas. This symposium will conclude with a discussion on how researchers may begin to integrate approaches to address stigma in rural and underserved communities in order to enhance care utilization and quality of life for older adults caring for and living with ADRD.

### STIFLED UTILIZATION OF DEMENTIA-RELATED HEALTHCARE SERVICES DUE TO STIGMA IN RURAL APPALACHIA

Elizabeth Rhodus[1], Allison Gibson[1], David Gross[2], Rob Sprang[1], Kelly Parsons[1], Julia Johnston[1], and Gregory Jicha[1], *1. University of Kentucky, Lexington,*

*Kentucky, United States, 2. St. Claire Healthcare, Morehead, Kentucky, United States*

Residency in rural Appalachia is linked with heightened morbidity and mortality due to a myriad of conditions, many of which are associated with increased risk and prevalence of Alzheimer's disease and related dementias (ADRD). Despite this, access to and utilization of dementia-specific healthcare services in the region are limited. This study presents community-based stigma associated with enrollment in healthcare clinical research offered in rural Appalachia. Additional data from focus groups with care partners of people with memory impairment in rural Appalachia discuss implications of stigma in their communities. Findings elaborate on recruitment challenges associated with terminology, such as caregiver and dementia, as well as availability of diagnosticians. This study illustrates unique characteristics needed for community-based education programs tailored to the culture and customs of rural regions in order to increase utilization of healthcare for older adults at risk or living with ADRD.

### SOURCES OF STIGMA AND THEIR IMPACT ON ALASKA NATIVE ADRD CAREGIVERS' WELLBEING

Steffi Kim, *University of Minnesota, Minneapolis, Minnesota, United States*

Challenges such as isolation, scarce resources, and limited knowledge of the disease are often the result of stigmatizing experiences from multiple systemic sources. No studies have investigated the impact of sources of stigma on the quality of life in Alaska Native (AN) ADRD caregivers. This exploratory, mixed-method study within a community-based participatory research framework assessed the experience of family stigma among 40 AN caregivers of people with ADRD across Alaska by administering a measure of systemic stigma and describes the impact of stigmatizing experiences on AN caregivers' quality of life to develop preliminary data-driven stigma-reducing initiatives. AN caregivers completed the Family Stigma – Alzheimer's Disease Scale (FS-ADS), assessing caregiver stigma, layperson stigma, and structural stigma. Quality of Life was assessed with the Goodness of Life for Every Alaska Native (GLEAN) scale. Preliminary data on structural stigma and its impact on caregiver quality of life will be presented.

### THE RESPITE FOR ALL PROGRAM DECREASES STIGMA AND CAREGIVERS' ANXIETY WHILE INCREASING THEIR CONFIDENCE

Steven Sabat[1], Abigail Howell[1], and Daphne Johnston[2], *1. Georgetown University, Washington, District of Columbia, United States, 2. First United Methodist Church, Montgomery, Alabama, United States*

Feelings of burden and stigma are associated with social isolation (Adelman, et al., 2014) which is considered a public health concern (Brodaty & Donkin, 2009; Tatangelo, et al., 2018a). The stigma associated with a dementia diagnosis, care partners' burden, and diminishing financial resources have been found to be significant contributors to increased social isolation (Lee, et al., 2021; Sun, et al., 2021; Hung, et al., 2021). Care partners report significant anxiety in connection with behavioral manifestations of dementia attracting negative attention (McHugh, et al, 2012; Sanders, et al, 2008) or judged by the general public (Lee, et al., 2021), and those with high levels of grief commonly report experiencing social isolation (Sanders, et al, 2008). The Respite For All program for people living with dementia has led to decreases in stigma and caregivers' anxiety while increasing their confidence. We will discuss the nature of this program and present supporting data.

### TOWARD EQUITABLE AND DESTIGMATIZING DEMENTIA: ALASKA NATIVE POPULATIONS AND CULTURALLY BASED INTERVENTIONS

Maria Crouch[1], and Rosellen Rosich[2], *1. Yale School of Medicine, New Haven, Connecticut, United States, 2. University of Alaska Anchorage, Anchorage, Alaska, United States*

Alaska Native (AN) people's incidence and prevalence of Alzheimer's Disease and Related Dementias (ADRD) are projected to disproportionately increase in contrast to the U.S. population. This is alarming given that AN peoples experience health disparities exacerbated by prejudice, stigma, and systemic and structural inequalities. Twelve semi-structured interviews with AN Elders assessed the culturally derived meanings of memory function, loss, decline, and disease. Qualitative analyses observed eight culturally grounded themes and five interrelated and nested subthemes elucidating both the resilience and the stigmas, racism, and barriers faced by AN peoples: (1) Historical Trauma; (2) Oppression; (3) Distrust of Western Medicine; (4) Social Justice; and (5) Walking in Two Worlds. En masse historical and contemporary oppression, particularly within Western medicine, both contextualizes the present and points to the ways in which the strengths, wisdoms, and balance inherent in AN culture are imperative to the holistic health and healing.

### SESSION 1390 (PAPER)

### DEPRESSION, DEMENTIA, AND OUTCOMES

### ALZHEIMER'S DISEASE AND COMORBIDITIES: A COMPLEX INTERPLAY IN THE CONTEXT OF AGING

Konstantin Arbeev, Olivia Bagley, Arseniy Yashkin, Hongzhe Duan, Vinit Nalawade, Igor Akushevich, Svetlana Ukraintseva, and Anatoliy Yashin, *Duke University, Durham, North Carolina, United States*

There is evidence on high prevalence of comorbidity in people with dementia and on associations between comorbidities and progression of Alzheimer's disease (AD). Comorbidities accumulate with age and age is also a major risk factor for AD. Repeated measurements of comorbidity provide possibilities for gaining more knowledge about dynamic interconnection between comorbidities and AD development in the context of aging. We constructed the comorbidity index (CMI) for participants of the Health and Retirement Study aged 66+ years using data on onset of diseases from linked Medicare service use files (6,830 participants, 3,829 females, 3,001 males). We performed the joint analysis of longitudinal measurements of CMI and data on onset of AD and survival since onset of AD using the approach (the stochastic process model) that allows decomposing the overall association of trajectories of CMI with respective time-to-event outcomes into several aging-related characteristics represented by the model's components and evaluated indirectly from the data. We found that, overall, CMI is significantly (p< 0.0001) associated with increased risk of onset of AD and decreased survival chances for persons with AD and that this association can be decomposed into associations of AD outcomes with different aging-related components with differentiated impact of genetic and non-genetic factors (such as APOE, polygenic scores, sex, birth cohort). In particular, age patterns and time trends in such components contribute to trends in AD prevalence so that taking into account the age dynamics and time trends in comorbidities (represented by CMI) is essential for forecasting future trends in AD prevalence.

### PROGRESSIVELY LOWERED STRESS THRESHOLD FOR BEHAVIORAL SYMPTOMS OF DEMENTIA: A DYNAMIC STRUCTURAL EQUATION MODEL

Carolyn Pickering[1], Maria Yefimova[2], Danny Wang[1], Christopher Maxwell[3], and Rita Jablonski[4], *1. The University of Alabama at Birmingham, Birmingham, Alabama, United States, 2. Stanford Health Care, Stanford, California, United States, 3. Michigan State University, East Lansing, Michigan, United States, 4. University of Alabama at Birmingham, Birmingham, Alabama, United States*

The progressively lowered stress threshold model suggests that due to impairments in coping, persons living with dementia have a reduced threshold for stress and respond with more behavioral symptoms of dementia as stress accumulates throughout the day. While the propositions of the model have not been evaluated, this model serves as the basis of non-pharmacological interventions for behavioral symptom management aimed at modifying the environment to reduce stressors. These interventions have mixed success, which may be due to traditional longitudinal measurement models that don't account for the dynamic temporal nature of behavioral symptoms. This paper evaluates the progressively lowered stress threshold conceptual model as an explanation for behavioral symptoms of dementia and tests several of its hypothesized propositions using an intensive longitudinal design. A sample of N=165 family caregivers completed brief daily diary surveys for 21 days (n=2841) reporting on behavioral symptoms of their care recipients. Dynamic structural equation modeling was used as the analytic technique to examine the impact of caregiver and care recipient environmental stressors on the diversity of behavioral symptoms of dementia (number of different symptoms) to account for the nested data structure and autoregressive relationships. Results show direct relationships between environmental stressors and diversity of behavioral symptoms of dementia that same day and the following day. Findings provide support for the progressively lowered stress threshold model. Further, findings suggest an extension to the conceptual model is warranted given evidence of an exposure/recovery trajectory and the lagged effects of stress exposure on behavioral symptoms of dementia presentation.

### SYMPTOM BURDEN AND QUALITY OF LIFE FOR NURSING HOME RESIDENTS WITH DEMENTIA: UPLIFT TRIAL EARLY DATA

John Cagle[1], Jessica Orth[1], Todd Becker[1], Peiyuan Zhang[2], Mary Ersek[3], Wanzhu Tu[4], Alex Floyd[5], and Kathleen Unroe[6], *1. University of Maryland, Baltimore, Baltimore, Maryland, United States, 2. University of Maryland Baltimore, Baltimore, Maryland, United States, 3. University of Pennsylvania, Philadelphia, Pennsylvania, United States, 4. Indiana University School of Medicine, Indianapolis, Indiana, United States, 5. Indiana University, Indianapolis, Indiana, United States, 6. Indiana University Center for Aging Research, Regenstrief Institute, Inc., Indianapolis, Indiana, United States*

Communication difficulties in nursing home (NH) residents with dementia make valid assessment of symptoms and quality-of-life (QOL) challenging. Thus, researchers and clinicians frequently rely on proxy-based measures. The End-of-life Dementia-Comfort Assessment in Dying (EOLD-CAD) and two single-item QOL measures (7-point item; 5-point item) have been used in several studies, though evaluation of their psychometric properties is limited. We used baseline data from an ongoing multi-site randomized trial (UPLIFT) to describe symptoms and QOL and examine the measures' validity and reliability in 138 residents with moderate to severe dementia living at 16 facilities. Descriptive data and assessments of convergent validity and inter-rater reliability are provided. Based on assessments by 134 staff and 45 family, physical symptoms, physical distress, and emotional distress were reported as infrequent by staff and family; indications of well-being were more frequently observed. Median QOL was the same for staff and family observers (4="Life is so-so" [7-point item]; 3="Fair" [5-point item]). Inter-observer assessments of resident QOL (staff vs. family) were correlated (7-point item: r=0.47, ICC=.643; 5-point item: r=0.48, ICC=.645, p<.05 for all). Seven of 18 EOLD-CAD symptoms were significantly positively correlated. ICC values varied between high or moderately high: shortness-of-breath (ICC=.74), choking (ICC=.65), gurgling (ICC=.81), agitation (ICC=.51), fear (ICC=.46), crying (ICC=.65), peace (ICC=.57), and care resistance (ICC=.68) (p<.05 for all). Choking and gurgling were the most prominently reported symptoms by both groups.Early findings provide a contemporary assessment of QOL and symptoms among NH residents with dementia. Measurement properties affirm general reliability and validity of study instruments.

### SESSION 1400 (PAPER)

### DIVERSITY, EQUITY, AND INCLUSION IN FORMAL AND FAMILY CARE PROVISION

### "A GLARING OMISSION": BARRIERS TO INCLUDING LONG-TERM CARE RESIDENTS' VOICES IN THE NEWS MEDIA

Laura Allen[1], Dana Bradley[2], and Liat Ayalon[3], *1. Bar-Ilan University, Edgewood, Kentucky, United States, 2. University of Maryland, Baltimore County, Baltimore, Maryland, United States, 3. Bar Ilan University, Israel, Ramat Gan, HaMerkaz, Israel*

News media stories on residential long-term care (LTC) are mostly negative in tone, and the increase in news coverage of residential LTC during the COVID-19 pandemic intensified the pessimistic tone with panicked and accusatory reporting. Although there has been an increase in the amount of news coverage on residential LTC over the last two years, the perspectives of the residents themselves have been largely overlooked and excluded. The aim of this study is to identify and understand the barriers and embedded institutional logics in the news reporting process of LTC that prevent residents from being included and interviewed. Using a purposive sampling strategy, the researchers conducted 26 semi-structured interviews with LTC administrators (n=7), journalists on the topics of aging and LTC (n=7), and older residents living in LTC settings (n=12) in the United States. Thematic analysis is applied to the data to qualitatively extract the guiding logics frameworks of each stakeholder group. Findings are grouped into the following four themes: (1) residents are not considered "experts" by reporters and are therefore not a prioritized source of information; (2) facility administrators fear for the facility's public image & reputation and do not enthusiastically cooperate with inquiring reporters; (3) facilities were in physical lockdown for several months during the COVID-19 pandemic, and (4) residents feel forgotten and unheard. Implications of this study point to the social exclusion of older residents living in LTC and reductive reporting that fails to capture their nuanced and dynamic lived experiences.

### IMPROVING SERVICES TO LGBTQ+ OLDER ADULTS: NEEDS OF MICHIGAN LONG-TERM CARE PROVIDERS

Anne Hughes, Linda Keilman, and Leo Kattari, *Michigan State University, East Lansing, Michigan, United States*

Long term care (LTC) providers need information about providing competent care to LGBTQ+ older adults. Providers who are not educated can inadvertently provide biased care. Our research identified preferences for education among providers of LTC services in Michigan (MI). In this descriptive cross sectional study we used an online survey to collect data from MI facilities (N= 429). Survey items included facility characteristics, diversity training history, perceived need for training on LGBTQ+ older adults, barriers to training, interest in additional training on LGBTQ+ older adults and LTC, and training preferences. Results were obtained from 71 facilities. Thirty-seven percent of responses came from direct care workers, 63% from administrators. There was good support for diversity training, with 24% stating diversity training was "somewhat important" and 74% stating it was "very important". A majority (63%) had had some diversity training in the past year. Most (72%) endorsed the need and desire for more training on LGBTQ+ aging. More content on transgender older adults and concerns such as room assignments, dementia, and use of pronouns were identified. Barriers to training included: cost, availability of trainers with the appropriate expertise, ability to reach large numbers of employees, staff turnover, bias and ignorance among staff and residents, and need to provide rationale for this type of training. Most endorsed a mixed type of training and a training length between 1 and 3 hours. Diversity training is critical to LTC and needs to be expanded to include needs of the aging LGBTQ+ community.

### OLDER BLACK LESBIANS' NEEDS AND EXPECTATIONS IN RELATION TO LONG-TERM CARE FACILITY USE

Meki Singleton[1], Mary Anne Adams[2], and Tonia Poteat[3], *1. University of Southern California, Los Angeles, California, United States, 2. ZAMI NOBLA: National Organization of Black Lesbians on Aging, Atlanta, Georgia, United States, 3. University of North Carolina Chapel HIll, Chapel Hill, North Carolina, United States*

The older adult sexual minority (SM) population encompasses a vast array of individuals from diverse backgrounds. However, there is a dearth of gerontological research that focuses on the experiences of SM subgroups, particularly older Black lesbians. The purpose of this study was to explore older Black lesbians' needs and expectations in relation to the utilization of long-term care (LTC) facilities. We conducted secondary data analysis using data from 14 focus groups (n=100) that discussed health and aging with older Black lesbians. Transcriptions were analyzed in NVivo 12 using content analysis and structural coding. Three major themes were identified in relation to needs and expectations for LTC facility use: (1) consideration of or plans established to utilize a LTC facility, (2) concern for care facility environment, and (3) a desire to build one's own community instead of LTC use. Within these themes, prominent topics included having to rely on LTC due to a lack of family or social support, the possibility of being isolated and stifling their lesbian identity and creating communities of mutual support to avoid facility-based care. These findings illustrate how older Black lesbians are planning for a potential need for LTC, their concerns about utilizing LTC, and alternative approaches to avoid LTC use. There remains a continued need for LTC communities that are inclusive and supportive of SM older adults as well as more SM-only communities where older adults can live openly and authentically.

### RACIAL AND ETHNIC DIFFERENCES IN PARTICIPANT EXPERIENCE OF HOME- AND COMMUNITY-BASED SERVICES

Howard Degenholtz, *University of Pittsburgh, Pittsburgh, Pennsylvania, United States*

It is widely recognized that racial and ethnic minorities experience lower quality of care and worse health outcomes across a wide range of health services and long-term care settings. However, less is known about racial and ethnic differences in home and community-based services (HCBS). This study used the HCBS version of the Consumer Assessment of Health Providers Survey (CAHPS) to examine participant experience of care in a large, statewide Medicaid program. The CAHPS-HCBS is a validated measure of quality of HCBS that supplements measures of health outcomes and provides policy makers with critical data on system performance. As part of a larger evaluation of Pennsylvania's transition to Managed care for users of HCBS, representative samples of older adults were interviewed before and after the program was implemented. This study takes advantage of the phased implementation of managed care to estimate the causal effect of the program on differences in participant experience by racial and ethnic group. In general, non-Hispanic whites rated their overall satisfaction with HCBS lower than non-Hispanic Black and Hispanic people. The implementation of Managed care was associated with improvements in non-Hispanic Black and Hispanic ratings of personal care and service coordination, but not that of non-Hispanic whites. One implication of these findings is that managed care organizations can have positive impact on participant experience. The CAHPS-HCBS instrument is publicly available and can be used by state agencies to monitor racial and ethnic disparities in participant experience.

### UNDER THE SAME ROOF: GRANDPARENTAL CO-RESIDENCE AND CO-PARENTING PATTERNS IN MODERN CHINESE FAMILIES

Shuai Zhou[1], Chang Liu[2], and Xue Bai[2], *1. The Hong Kong Polytechnic University, Hung Hom, Kowloon, Hong Kong, 2. The Hong Kong Polytechnic University, Hong Kong, Hong Kong*

This study aimed to examine the role of grandparental co-residence in shaping coparenting patterns in the Chinese family context. Using a sample of 5,795 children (Mean age = 6.74 years; SD = 3.82) from the China Family Panel Studies 2012-2018, we offered a novel typology of six coparenting patterns based on daytime and nighttime child-care information: a) grandparental care only (15.4%); b) grandparent-parent coparenting (10.9%); c) parental care only (44.5%); d) grandparent-private coparenting (4.2%); e) parent-private coparenting (16%); and f) private care only (e.g., paid nanny, nursery schools/kindergartens, and others, 9.1%). Fixed-effects multinominal logistic regression was applied to investigate the effects of grandparental co-residence on daytime and nighttime childcare, and on daily coparenting patterns over time, controlling for a set of child and parental characteristics and family conditions. Grandmother co-residence contributed to both daytime and nighttime grandparenting, while grandfather co-residence increased the likelihood of grandparenting and private care during the daytime. In terms of daily coparenting, grandmother co-residence was associated with increased probability of grandparent-parent coparenting as well as grandparent-private coparenting, while grandfather co-residence merely increased the likelihood of parent-private coparenting. It also found that child's experience of separation with mother during the last year and mother's employment were two key determinants of coparenting behavior in Chinese families. Findings suggest that co-residing grandparents, particularly grandmothers, may function as a kind of supplementary care resource in response to the lack of maternal childcare resulted from migration and labor force participation.

### SESSION 1410 (SYMPOSIUM)

### EARLY-LIFE EDUCATION AND COGNITIVE AGING: DOES CONTENT, LOCATION, AND HISTORICAL CONTEXT MATTER?

Chair: Jacqui Smith Discussant: Laura Zahodne

Life course and cognitive aging theories about the sources of subgroup and individual differences in susceptibility to cognitive impairment frequently highlight the significance of early-life education, defined in research by total years of school and highest degree. A more nuanced approach could shed light on why and for whom education matters for brain aging processes. For example, one year of school in the 1930s may not be the same as one year in the 1960s, or in different school types and states in this historical period. In addition, post- high school training and degrees differ in content. Each paper in this symposium uses early-life education history and late-life cognitive data from the Health and Retirement Study (HRS). Speakers discuss what guided their selection of specific education content, location, contexts and analytic strategy. Williams-Farrelly asks how individuals characterized by privileged high school educational experiences in the period from 1930 to the 1960s benefited in terms of social mobility and later-life cognitive functioning. Nkwata, in contrast, focuses on early-life education experiences that potentially limit the skills acquired together with exposure to family adversity and stress. Yu utilizes sequence analysis to identify and examine the association between late-life cognition and variations in patterns of K-12 education defined by duration and order of racial/ethnic composition. Walsemann and colleagues ask if state-level differences in the timing and implementation of school desegregation policies explain Black-White disparities in the transition to cognitive impairment in the US South. The session concludes with an integrative discussion by Zahodne.

### SOCIOECONOMIC PRIVILEGE IN CHILDHOOD AND LATER LIFE COGNITION

Monica Williams-Farrelly, *University of Michigan, Ann Arbor, Michigan, United States*

Prior studies have demonstrated the effects of early socioeconomic status on later life cognition; yet the degree to which this effect operates directly, or indirectly through adult factors, is not well documented. Drawing from accumulation theories, we use longitudinal data from the Health and Retirement Study and its Life History Mail Survey (N=11,761) to examine the extent to which childhood socioeconomic privilege protects against later life cognitive decline and whether this effect is direct or indirect through measures of adult socioeconomic position. Analyses reveal that, among childhood privilege measures, consistent attendance in private school, participation in college preparation, foreign language, and multiple clubs in high school were most protective of later life cognition. The effect of cumulative childhood privilege on later life cognition is both direct and indirect through increased wealth and education credentials and the strength of this relationship is particularly robust for those demonstrating upward social mobility.

### EARLY-LIFE EDUCATIONAL EXPERIENCES, TOXIC STRESS, AND RISK OF NEUROCOGNITIVE IMPAIRMENT AMONG US ADULTS OVER AGE 50

Allan Nkwata, *University of Michigan, Ann Arbor, Michigan, United States*

Increasing evidence suggests that the risk of cognitive impairment in later life in the US has been declining in recent years. However, factors associated with this decline are not fully understood. This study aims to examine whether educational history, including college attainment and adverse childhood experiences (ACEs) are associated with neurocognitive impairment (NI) among midlife and older US adults. We will use data from the Health and Retirement Study (HRS) along with information on respondents' early educational experiences collected in the 2015 and 2017 HRS Life History Mail Survey to examine whether school context, educational content, academic ability, and ACEs are associated with NI. We will farther explore ACEs as potential modifiers/ mediators of the relationship between educational history and NI in our sample. Multivariable logistic regression models will estimate odds ratios (ORs) and 95% confidence intervals (CI)

with adjustment for demographic factors, socio-economic status (SES) and lifestyle factors.

### TRAJECTORIES OF K-12 SCHOOLS IN THE 1930S-1960S AND COGNITIVE AGING: VARIATION BY RACIAL COMPOSITION

Wenshan Yu, and Jacqui Smith, *University of Michigan, Ann Arbor, Michigan, United States*

Besides information about the highest degree, little information about early-life education is available in population surveys. We identified K-12 education trajectories among older adults in the Health and Retirement Study and examined their association with cognitive function. Drawing on 2017 Life History Mail Survey (n = 4,325), we used sequence analysis to determine and classify trajectories of school majority race. We identified five clusters: 1) mostly White schools (n = 3,027), 2) mostly Black schools (n = 673), 3) mostly Hispanic schools (n = 499), 4) partly missing on majority race (n = 267), and 5) mostly Mixed-race schools (n =113). Adding the cluster variable significantly improved the model regressing cognitive function on race, gender, number of years in schools, birth cohort, number of years since starting schools, and school locations (F=12.066, p<0.001). Future research should study heterogeneity of K-12 education using information other than the highest degree.

### STATE-LEVEL DESEGREGATION EFFORTS IN THE U.S. SOUTH POST-BROWN AND DEMENTIA RISK AMONG BLACK AND WHITE OLDER ADULTS

Katrina Walsemann[1], Mateo Farina[2], and Jennifer Ailshire[2], *1. University of Maryland, College Park, Maryland, United States, 2. University of Southern California, Los Angeles, California, United States*

Although education is a key protective factor against dementia, older U.S. adults experienced vastly different educational contexts. One of the most consequential legal decisions impacting educational contexts was Brown v. Board of Education, which declared ‘separate but equal’ schools unconstitutional. School desegregation in the U.S. South was inconsistent, however, and many states delayed implementation for years. State resistance to school desegregation created school environments for Black children that were stressful and discriminatory. Thus, state variation in exposure to school desegregation may serve to differentiate individuals in their risk for dementia. Our project links historical data on state-level school desegregation efforts to the Health and Retirement Study, a nationally representative sample of U.S. adults over 50. We determine if state-level variation in the timing and completeness of school desegregation explains Black-White disparities in transition to cognitive impairment and dementia among older adults who attended school in the US South post-Brown.

### SESSION 1420 (PAPER)

### EDUCATION AND INNOVATION IN HEALTH PROFESSIONS

In this session, gerontology educators describe varied ways to engage students in health professions programs in aging-focused training and teaching efforts.

### A MIXED METHOD APPROACH TO UNDERSTANDING NURSING STUDENTS' SELF-EFFICACY TO CARE FOR OLDER ADULTS IN GHANA

Diana Abudu-Birresborn[1], Martine Puts[2], Lynn McCleary[3], Vida Yakong[4], Charlene Chu[2], and Lisa Cranley[2], *1. University of Toronto, New York, New York, United States, 2. University of Toronto, Toronto, Ontario, Canada, 3. Brock University, St. Catharines, Ontario, Canada, 4. University for Development Studies, Tamale, Northern, Ghana*

To identify students’ motivation, gaps, and opportunities to design effective teaching strategies, it is necessary to examine nursing students' understanding of their self-efficacy to care for persons 60+ years. A mixed-method approach was employed. In phase 1, 170 second-and third-year nursing students from two public Nursing Colleges in Ghana were recruited. A cross-sectional survey included the General Self-efficacy to Care for Older Adults’ scale (GSE-COA), the Kogan’s Attitudes towards Old Peoples scale, and the Knowledge about Older Patients Quiz. Data were analyzed using descriptive statistics in SPSS IBM 26. The results of phase 1, informed the selection of students for phase 2. In phase 2, 17 students were purposefully selected for in-depth semi-interviews. Qualitative data were analyzed using thematic analysis. Both results were then integrated, interpreted, and presented jointly. Students’ mean age was 21yrs (SD=3.73), with 91 (54%) females. Most students 140 (71.0%) had lived with/were currently living with an older adult. The majority 164 (97%), had higher scores for GSE-COA (mean= 107, SD=14.29), indicating high self-efficacy. Qualitative results showed that students’ high self-efficacy was due to their familiarity with older adults at home and their perceived competence in routine nursing care. Students who demonstrated a high sense of self-efficacy were confident and perceived caring for older adults as a duty and responsibility, had experience/exposure to and were comfortable working with older adults. The findings demonstrate an opportunity to design effective teaching strategies to develop and sustain students’ interest and motivation in the care for older adults in Ghana.

### CROSS-DISCIPLINE COLLABORATIVE INTERNSHIPS TO REDUCE FEAR OF FALLING CONTRIBUTE TO AGE-FRIENDLY INITIATIVES

Andrea June, and Allison Seifert, *Central Connecticut State University, New Britain, Connecticut, United States*

The Age Friendly University (AFU) initiative provides a vision to inspire institutions of higher education to respond to the interests and needs of an aging population to promote well-being and quality of life. At Central Connecticut State University (CCSU), we have utilized multiple strategies to engage collaborators within our university and our community to further age-inclusive efforts. The purpose of this presentation is to describe the ongoing collaboration, through the Matter of Balance program, with the Connecticut Healthy Living Collective, an emerging hub of state, regional and local agencies and organizations dedicated to healthy aging in communities throughout our state. The Matter of Balance program is an empirically supported community-based treatment to reduce fear of falling among older adults. We will first discuss the pilot internship program offered within the Department of Psychological Science that trained students as program facilitators which was cut short in Spring 2020 due to COVID 19. We will then describe "Version 2.0" of the collaboration which was offered in the Spring 2022 semester. This time, it was grounded in a gerontology internship course that expanded to include research, faculty, and student collaboration with the Department of Physical Education and Human Performance. In addition to providing these overall descriptions, the presenters will review the strengths and challenges of each approach, share results about the students' experiences, and highlight the multidimensional value this intentional effort offers.

### FIRST-YEAR OCCUPATIONAL THERAPY STUDENTS KNOWLEDGE OF AGING; SUGGESTIONS FOR CURRICULUM REQUIREMENTS

LaVona Traywick[1], Brittany Saviers[2], Terry Griffin[3], and Teressa Brown[1], *1. Arkansas Colleges of Health Education, Fort Smith, Arkansas, United States, 2. University of Central Arkansas, Conway, Arkansas, United States, 3. Kansas State University, Manhattan, Kansas, United States*

There is a rising shortage of allied healthcare professionals, including occupational therapists, to meet the current and expected needs of the senior adult population. To educate occupational therapy students, there are national standards that all programs have to meet. Inconsistency occurs because there is not a set national curriculum. It is assumed that students will enter their respective occupational therapy programs with a base knowledge of aging due to prerequisite requirements. With IRB approval, that assumption was measured over four consecutive years at one prominent OT program. 192 first-semester occupational therapy students were administered the Facts on Aging Quiz along with additional questions regarding birth year and anticipated employment. Results showed that first-year occupational therapy students' knowledge of aging was poor (67.9% mean) regardless of their age or population work preference. Only 11.5% stated geriatrics was their preferred population with which to work; most students stated pediatrics. Statistical tests indicated a trend of decreasing mean scores of the cohorts. If this trend of decreasing gerontological literacy exists in occupational therapy, other health care disciplines may be experiencing similar fates. It appears there is a considerable gap between the needs of society and the knowledge base/desired population work preference of those entering healthcare education. Strategies to address that gap need to be addressed to prepare occupational therapy students and other health care practitioners to best meet the needs of the current population. Based on these study results, more emphasis needs to be placed on gerontological literacy for new occupational therapy students.

### PREPARING THE NURSING WORKFORCE TO CARE FOR OLDER ADULTS IN THE 21ST CENTURY

Deborah Brabham, *Florida State College at Jacksonville, Jacksonville, Florida, United States*

Nurses entering the workforce may have limited education in gerontological nursing. Therefore, many nurses are unprepared to provide quality care to older adults. An unprepared nursing workforce could negatively influence older adults' health outcomes. This study aimed to determine differences in senior nursing students' knowledge, attitudes, and perceived competency about older adults based on enrollment in a bachelor of science in nursing degree (BSN), associate's degree nursing (ADN), and practical nursing (PN) program that offers geriatric content in curricula. Albert Bandura's social cognitive, triadic reciprocal determinism model provided the theoretical framework to underpin this research study. A non-experimental, descriptive survey design included a convenience sample of students enrolled in a BSN, ASN, and PN program. A total of 178 students participated in the study. Palmore Facts on Aging Quiz 2, Kogan's Attitudes Toward Old People Scale, and the Hartford Geriatric Nurse Competency tool was used to collect data. From the results, it is clear that students enrolled in BSN, ADN, and PN programs demonstrated limited knowledge about facts on aging. Students' attitudes toward older adults were positive, and a correlation was found between knowledge and attitudes. Students perceived competency about older adults was high but purely subjective. Students' preference to work with older adults post-graduation in the PN group was higher than students in the BSN and ADN groups. These findings underscore the need to systematically design an evidence-based curriculum inclusive of geriatric content across (BSN, ADN, and PN) programs to prepare the nursing workforce to care for older adults.

### STEPS TOWARD RAISE: CO-DESIGN OF COMPETENCY-BASED EDUCATION TO ENGAGE FAMILY CAREGIVERS AS PARTNERS

Jasneet Parmar[1], Sharon Anderson[2], Wendy Duggleby[1], Michelle Lobchuk[3], Elisabeth Drance[4], Tanya L'Heureux[5], Johnna Lowther[6], and Kim Crowder[1], *1. University of Alberta, Edmonton, Alberta, Canada, 2. Faculty of Medicine & Dentistry University of Alberta, Edmonton, Alberta, Canada, 3. University of Manitoba, Winnipeg,, Manitoba, Canada, 4. University of British Columbia, Vancouver, British Columbia, Canada, 5. Department of Family Medicine, University of Alberta, Edmonton, Alberta, Canada, 6. Caregivers Alberta, Edmonton, Alberta, Canada*

Meeting the needs of a growing population of older people living with complex conditions is highly dependent on healthcare providers partnering with family caregivers (FCGs). FCGs provide 90% of the care yet are marginalized within healthcare systems. Educating healthcare providers to support FCGs is a necessary step towards addressing the inconsistent system of supports for diverse FCGs throughout variable care trajectories. Current research suggests that co-design benefits stakeholders, produces superior outcomes, and facilitates moving knowledge efficiently into healthcare practices. Currently, moving best practices into healthcare is a time-consuming process (10-17 years). This presentation will discuss a feasible three-phase co-design process that included 120 multilevel interdisciplinary stakeholders including FCGs, educators, researchers, not-for-profit and healthcare providers/leaders, educational designers, and policy influencers/makers: 1) Developing relationships and insights; 2) Translating insights into education design; and 3) Planning the implementation, spread, and scale-up. The research tools used included literature reviews, qualitative and survey research on specific topics, consultations (symposia, modified Delphi process, co-design meetings), and mixed methods evaluation. Three modules, Foundational, COVID-19, and Advanced have been developed. Learners report high satisfaction, relevance, and significant knowledge gains upon completion. This successful education co-design required three critical elements: 1) an engaged co-design team led by people knowledgeable about healthcare and FCGs; 2) team access to collaborators/staff with the appropriate theoretical, research, and facilitation skills; and 3) an educational design team to bring stakeholders' ideas to life. Leveraging stakeholders' insights are a critical step towards the RAISE act goal of educating healthcare providers to include FCGs as partners-in-care.

### SESSION 1430 (SYMPOSIUM)

### EMPOWERING BEHAVIOR CHANGE TO SUPPORT BRAIN HEALTH AMONG OLDER ADULTS

Chair: Sarah Lock Co-Chair: Duke Han

This symposium will provide highlights from the Global Council on Brain Health's (GCBH) body of work on behavior change. This work is focused on developing a better understanding of (a) how to persuade and motivate people to engage in sustained healthy behaviors, (b) how to change policies within local communities to support individuals' ability to make healthy choices, and (c) how to optimize conditions for brain health so all can thrive. The GCBH is an independent collaborative of scientists, clinicians, scholars, and policy experts convened by AARP to provide evidence-based advice on what people and professionals can do to maintain and improve brain health. The Council translates scientific research into actionable recommendations aimed at helping to drive behavior change in individuals across communities and cultures. Experts were brought together to build consensus around a range of factors at the individual and societal level that influence individual behavior and decision-making. This presentation will also draw upon lessons learned from the field of health promotion and examples from communication campaigns around heart health will be discussed as they relate to brain health. Nationally representative surveys, fielded by AARP Research including diverse respondents of adults 40+ and health care professionals diagnosing and treating dementia, found that misperceptions and stigma about dementia are commonplace and hinder efforts to address brain-healthy behaviors. Physicians who used to treat dementia patients, now themselves living with dementia, discuss how championing change, providing hope and refuting stigma can even help those living with a dementia diagnosis.

### HEART-HEALTHY LIFESTYLES REDUCE THE RISK OF COGNITIVE DECLINE

Sarah Lock[1], and Kristine Yaffe[2], *1. AARP, Washington, District of Columbia, United States, 2. University of California, San Francisco, San Francisco, California, United States*

The Global Council on Brain Health (GCBH) convened experts from around the world to examine the impact of cardiovascular risk factors on brain health. GCBH issue experts carefully considered how high blood pressure, diabetes and stroke, as well as lifestyle choices such as diet and exercise, influence brain health in adults age 50 and over. The GCBH concluded that the best evidence to date shows us "what's good for the heart is good for the brain." Their consensus puts powerful tools in the hands of adults wanting to protect their brain as they age. The GCBH adopted 10 recommendations for individuals to incorporate into their lives to keep heart and blood vessels healthy and reduce the risk for cognitive decline and dementia. These recommendations and 16 practical tips are provided in the final report. Liaisons from numerous civic and nonprofit organizations with expertise in heart and brain health helped develop these recommendations for adults 50+ and their health care providers.

### BOTH HEALTH CARE PROFESSIONALS AND PATIENTS HARBOR MISPERCEPTIONS ABOUT DEMENTIA

Laura Mehegan, and David Parkes, *AARP, Washington, District of Columbia, United States*

Healthcare providers underestimate the willingness of adults to engage in a healthier lifestyle to potentially slow the progress of the disease and the willingness of patients to participate in research. Few adults recognize the impact lifestyle modifications have on the risk for cognitive decline and dementia, but some significant differences exists among perceptions amongst diverse communities. While most adults are willing to modify selected brain-healthy behaviors, relatively few currently engage in brain-healthy behaviors all or most of the time. Numerous discrepancies exist between the realities of dementia and overall feelings about a diagnosis. Among the more startling findings is 48% of adults believe they will likely have dementia — far more than will actually develop it. Health care providers substantially overestimate the worry that adults age 40 and older would feel if they had dementia. While one in five adults (19%) said they would feel ashamed or embarrassed if they had dementia, a staggering seven in 10 providers (69%) said their patients would feel ashamed or embarrassed. These negative perceptions by healthcare providers carry over into the interactions they have with patients when dealing with cognitive function. Nine in 10 adults age 40 and older (91%) want to be told of a dementia diagnosis, but only 78% of providers said they always tell patients the truth. There is a recognition by everyone that early diagnosis is beneficial, but most adults over 40 are not aware there are treatments available for dementia. More than half of adults do not know that dementia cannot be cured.

### PHYSICIANS LIVING WITH DEMENTIA CHAMPION CHANGE, PROVIDE HOPE AND REFUTE STIGMA

Arnold Beresh,[1] Brenda Roberts,[2] Lisa Dedden Cooper,[3],
*1. National Council of Dementia Minds, White Lake, Michigan, United States*
*2. National Council of Dementia Minds, Elwell, Michigan, United States*
*3. AARP Michigan, Owosso, Michigan, United States*

This section of the symposium will show how to empower behavior change amongst the medical profession and the people they serve. Health care professionals who were diagnosing and treating dementia patients who are now themselves living with dementia will share insights into what needs to change by sharing "What I Wish I Knew Then." We have asked three people from the National Council of Dementia Minds to join us for the symposium. Dr. Arnold Beresh, Brenda Roberts, and Lisa Cooper to speak on What I Wish I Knew Then. This part of the presentation is designed to provoke discussion on how best to engage doctors to support better brain health for older adults in the future.

### SESSION 1440 (SYMPOSIUM)

### ESPO AND BUTLER-WILLIAMS SCIENTIFIC SYMPOSIUM: CAREER DEVELOPMENT TO PROMOTE DIVERSITY, DISCOVERY, AND AGING

Chair: Kalisha Bonds Johnson Co-Chair: Brianna Morgan Discussant: Patricia Jones

The NIA's Butler-Williams Scholars Program and GSA's ESPO Section are united in providing career development opportunities in a manner that promotes leadership, diversity, and inclusivity. This year's theme challenges our emerging scholars to embrace diversity and discovery while thinking—or rethinking—about the perspectives of older adults. Disparities in health associated with race/ethnicity, experience, sociocultural and socioeconomic factors, as well as access to and provision of health care are chief concerns of our aging population. GSA's early career professionals and 2021 alumni of the prestigious NIA Butler-Williams Scholars Program address these issues. Dr. Matthew Farina will present on the importance of identifying life course pathways in understanding the lived experiences of older adults from underrepresented racial groups. Dr. Kacie Deters will discuss how both demographics and genetic factors contribute to cognitive performance. Dr. Mirna Arroyo-Miranda will present findings on social isolation and cognition in Hispanic/Latino older adults. The final speaker, Dr. Jamaine Davis will present on the genetic factors that may contribute to dementia in African American older adults. The featured talks by rising stars deepen our understanding of the influence of diversity and key discoveries so we can reimagine aging research.

### DIFFERENCES IN LIFE COURSE PATHWAYS TO DEMENTIA INCIDENCE FOR BLACK AND WHITE OLDER AMERICANS

Mateo Farina, *University of Southern California, Los Angeles, California, United States*

Black Americans are 2-3 times more likely to have dementia than White Americans. Studies have evaluated how differences in exposures to life course risk factors, such as level of education, may be contributing to this disparity. When using this approach, substantial reductions in cognitive health inequality across race were accounted for, but not completely explained. However, this research assumes an underlying similarity in life course pathways to cognitive impairment risk across race groups. We used longitudinal data from the Health and Retirement Study (2000-2016) to evaluate life course pathways to dementia risk across race groups. We find substantial differences across race groups. Dementia risk for older black adults is greatly shaped by southern birth and education. For older white adults, adulthood factors such as wealth, health behaviors, and cardiometabolic conditions has a greater role. Our findings support calls for a closer examination of within group analysis among minoritized populations.

### VARIABLE PREDICTORS OF COGNITIVE PERFORMANCE WITHIN ETHNIC/RACIAL GROUPS USING A COMPUTERIZED COGNITIVE BATTERY

Kacie Deters, *University of California, Los Angeles, Inglewood, California, United States*

The Computerized Cognitive Composite (C3) may provide an efficient assessment of early cognitive impairment to inform early interventions and screening criteria for Alzheimer's disease clinical trials. The C3 battery, which includes the CogState Brief Battery, has components assessing memory, reaction time and aspects of executive function. The battery has little demand for spoken language and may reduce the sociocultural biases of traditional paper-and-pencil tests. The goal of this project was to determine demographic and genetic predictors of cognitive performance on the C3 battery across different ethnic/racial groups. We examined 4,026 cognitively normal participants (self-identified non-Hispanic Black, Asian, or White; Hispanic) at baseline visit from the Anti-Amyloid Treatment in Asymptomatic Alzheimer's Disease (A4) clinical trial. Linear models were performed to examine the association of C3 and years of age, years of education, gender/sex, and Apolipoprotein E (APOE) genotype, within ethnic/racial group. We found variation in demographic and genetic risk factors that predicted cognitive performance within ethnic/racial groups. These findings highlight the importance of within group analysis to identify risk factors for cognitive impairment.

### UNDERSTANDING CULTURAL DIFFERENCES OF SOCIAL ISOLATION AND ITS EFFECTS ON COGNITIVE FUNCTION OF HISPANICS/LATINOS

Mirna Arroyo-Miranda[1], Andrea Ochoa-Lopez[2], and Luis Medina[2], *1. University of Houston (Collaborative on Aging Research and Multicultural Assessment), Houston, Texas, United States, 2. University of Houston, Houston, Texas, United States*

Social isolation negatively impacts cognitive health. Although the mechanism is not fully understood, cultural values such as familismo, may help partially explain this susceptibility. Our work sought to understand how cultural differences in objective and perceived social isolation affect cognitive performance of Hispanic/Latinos (H/L) compared to non- H/L Whites. In our first study, using the TARCC dataset, objective covariates of social isolation (i.e. marital status, number of children, number of siblings, living situation), significantly explained a higher percentage of the variance in composite cognitive scores of H/Ls, compared to non-H/L Whites. In our second study, using the HABS-HD dataset, personal and health characteristics influenced the effect of perceived social isolation on cognitive status among older adults. Furthermore, compared to non-H/L Whites, fewer factors accounted for greater variance in cognition of H/Ls. Results suggest enhancing social connectedness of H/L older adults could be a modifiable factor for prevention of cognitive decline.

### A MOLECULAR LENS FOCUSED ON UNDERSTANDING ALZHEIMER'S DISEASE HEALTH DISPARITIES

Jamaine Davis, *Meharry Medical College, Nashville, Tennessee, United States*

Alzheimer's disease (AD), the most common cause of dementia in older adults, disproportionally affects African Americans (AA) with an incidence rate as much as three times higher, compared to other racial/ethnic groups. Multiple factors contribute to this racial disparity however, an in-depth understanding of the biological or genetic contributions does not exist. Compelling evidence indicate that genetic variants of the lipid transport protein, ABCA7, is more strongly associated with AD in African Americans. To understand how ABCA7 contributes to AD on the molecular level, we used a combination of structural and cell biology techniques. Our results suggest that the ABCA7 variant (T319A) reported to confer risk in AA, may contribute to AD by reducing the levels of phosphoinositol bisphosphate (PIP2), a phospholipid reported to be decreased in the AD brain. These results provide a framework for targeting mechanisms that increase PIP2 levels as an effective strategy mitigating AD disparities.

### SESSION 1450 (SYMPOSIUM)

### GIVING AND GETTING ACROSS THE GENERATIONS: NEW INSIGHTS INTO INTERGENERATIONAL TIES

Chair: Deborah Carr Discussant: Deborah Carr

Population aging raises debates about who will care and provide for older adults, while shifting economic and family patterns suggest that young adults may require ongoing support from their (grand)parents. These five papers use diverse methods and data to shed new light on upward and downward intergenerational exchanges. Fingerman and colleagues use data from a small racially diverse sample of young adults (ages 18 to 29) providing care to grandparents. They document psychological benefits yet potentially harmful life course disruptions (e.g., education). Wiemers and co-authors use the Health and Retirement Study Core and COVID-19 Module to document how adult children's transfers of time, money, and coresidence changed in response to the COVID-19 pandemic. Minoritized and lower SES older adults were less likely to receive help from family members, due to children's constraints. Whitworth documents disparities in the types of support college students receive from midlife parents, using the Panel Study of Income Dynamics Transition to Adulthood Supplement. Higher SES parents tend to provide financial support, whereas lower SES parents provide coresidence. Silverstein et al. use multigenerational data from Longitudinal Study of Generations. They find religiosity of parents during the offspring's adolescence affects their cognitive and behavioral religiosity in later life. Avni uses cross-national attitudinal data from the International Social Survey Programme (ISSP) and finds that Americans believe family should be responsible elder care, whereas Israelis and Germans believe the government should provide care. The discussant highlights implications of the study results for research and policies regarding intergenerational relations.

### CAREGIVING FOR AN OLDER RELATIVE IN YOUNG ADULTHOOD

Karen Fingerman[1], Shiyang Zhang[1], Zexi Zhou[1], Yee To Ng[1], Kira Birditt[2], and Steven Zarit[3], *1. The University of Texas at Austin, Austin, Texas, United States, 2. University of Michigan, Ann Arbor, Michigan, United States, 3. Pennsylvania State University, Pittsburgh, Pennsylvania, United States*

Caregivers are typically viewed as midlife or older individuals, but nearly 25% of self-identified caregivers are young adults (aged 18 to 29). We report findings from a small study of Hispanic/Latinx, African American, and Asian American young adult caregivers, using: quantitative and open-ended assessments and weekly diary surveys. We assessed caregiving tasks young adults provide, rewards, and detriments of providing care. In open-ended responses, young adults described their caregiving roles as emotional as well as hands on, and reported filling in for other caregivers who could not help. They reported goal disruptions (e.g., education, romantic ties) associated with caregiving, but felt they were repaying debts to their grandparents through caregiving. Diary data document vicissitudes in weekly caregiving experiences and well-being. Discussion focuses on the unique implications of caregiving ties between young adults and their grandparents when the grandparent incurs physical or cognitive health impairments.

### TRACING THE RELIGIOUS LIFE COURSE: INTER- AND INTRAGENERATIONAL SOURCES OF LATER-LIFE RELIGIOSITY IN BABY BOOMERS

Merril Silverstein, Joonsik Yoon, RianSimone Harris, and Woosang Hwang, *Syracuse University, Syracuse, New York, United States*

The development of religiosity in later life has its origins in earlier phases of the life course, yet few studies have investigated the contribution of early religious exposure to religious beliefs and behaviors in old age. This investigation uses multigenerational data from the Longitudinal Study of Generations taken from 385 baby boom children, 16-26 years of age, and their parents in 1971, and follows the children's religious orientations into midlife and old age. Guided by intergenerational transmission and path dependency perspectives, we found that parents' religious intensity in 1971 strengthened their children's cognitive and behavioral religiosity in later life as mediated by children's early and midlife religiosity. Our results demonstrate both intergenerational transmission followed by temporal stability in religious belief and practice. Evidence suggests that parental influence creates religious momentum in their children that carries from adolescence/young adulthood through the unfolding of their lives into old age.

### CROSS-NATIONAL DIFFERENCES IN ATTITUDES TOWARD FAMILY AND GOVERNMENTAL SUPPORT FOR ELDER AND CHILD CARE

Elinore Avni, *Boston University, Boston, Massachusetts, United States*

Population aging in wealthy western nations has raised concerns about who will provide care to older adults. At the same time, the rise of single parenthood and dual-career families has heightened the need for childcare. As governments and families face challenges in meeting these dual needs, this study compares responses to the question of "who should primarily provide" eldercare and childcare across three countries: the US, Germany and Israel. Analysis of 2012 International Social Survey Programme data reveals that while persons in the US endorse family as care providers to both older adults and children, Israelis endorse government as eldercare providers yet family as the source of childcare provision. German respondents prefer both government and family as childcare providers, yet believe the government should provide eldercare. The paper discusses how cross-national differences in attitudes toward care are associated with cultural and socio-economic characteristics, and highlights implications for policy and practice.

### FINANCIAL AND TIME HELP FROM ADULT CHILDREN DURING THE COVID-19 PANDEMIC

Emily Wiemers[1], I-Fen Lin[2], Janecca Chin[2], and Anna Strauss[1], *1. Syracuse University, Syracuse, New York, United States, 2. Bowling Green State University, Bowling Green, Ohio, United States*

Pandemic-induced challenges to health and economic well-being for older adults likely increased the need for help from adult children, disrupted the help children provided for needs unrelated to COVID-19, and changed interactions among time help, financial help, and shared housing. This paper uses data from the Health and Retirement Study Core and COVID-19 Module to assess whether adult children's transfers of time, money, and coresidence with parents responded to the pandemic-related challenges older adults faced. Because of unequal health and economic impacts of the pandemic, non-White and less-educated older adults, and those living in areas hard-hit by the pandemic may have been less likely to receive help from family members, their children may have been less able to substitute financial support for time or in-kind help, and older adults may have experienced greater disruptions in existing help arrangements so we examine differences by socioeconomic status, race-ethnicity, and local pandemic severity.

### MID-LIFE PARENTS' MATERIAL SUPPORT OF CHILDREN IN COLLEGE: A TYPOLOGY AND VARIATION BY SOCIOECONOMIC STATUS

Tanya Whitworth, *Boston University, Boston, Massachusetts, United States*

Many imagine the "typical" college student to be 18-22 years old, attending a four-year institution, living on campus, and receiving financial support from parents. This perception does not align with actual patterns. While some parents facilitate this "traditional college experience" for their children, others substitute co-residence for or combine co-residence with financial support; still others provide no material support at all. Using a diverse sample of U.S. college students from the Panel Study of Income Dynamics Transition into Adulthood Supplement (n = 1,579), this paper demonstrates variation in material support provided by parents. I employ a latent class approach to categorize students into assistance "types," and then use multilevel multinomial logistic regression models to predict assistance types from parents' socioeconomic status (SES). Lower SES parents have a higher probability of providing co-residence, while higher SES parents have a higher probability of providing financial support to students living away from home.

### SESSION 1460 (SYMPOSIUM)

### RECOMMENDATIONS TO IMPROVE NURSING HOME QUALITY: A DISCUSSION OF THE 2022 NASEM REPORT

Chair: Jasmine Travers

Every year approximately four million persons receive care in approximately 15,000 nursing homes across the U.S. The costs of this care exceed $168 billion per year and are projected to grow to $274 billion by 2024. Nursing homes have long been plagued by problems with quality of care.In 1986, the Institute of Medicine released, Improving the Quality of Care in Nursing Homes, a landmark report that fundamentally changed the nation's approach to nursing home operation and regulation. However, three decades later, significant challenges with nursing home quality remain, many of which were brought to light during the COVID-19 pandemic. As a result, in 2020, the National Academies of Sciences, Engineering, and Medicine (NASEM) convened a panel of 17 experts in nursing home care to examine our nation's approach to nursing home care, including clinical care, staffing, financing and payment, and regulation, with the goal of making recommendations for improving the quality of care in today's nursing homes and ensuring the safety and well-being of nursing home residents and staff. Expert members represented areas of diversity, policy, regulation, education, technology, quality measurement and reporting, and clinical practice. The release of this report is April 2022.In this symposium, four committee members will present the main findings and recommendations from the forthcoming and highly-anticipated report, with a particular focus on care delivery, workforce, quality assurance & policy, and equity. During this session, an emphasis will be placed on how the presented recommendations can be incorporated into policy and practice.

### LAYING THE REPORT FOUNDATIONS: A CONCEPTUAL MODEL OF NURSING HOME QUALITY OF CARE

Debra Saliba[1], Mary Ersek[2], and Marilyn Rantz[3], *1. UCLA & GLAHS VA, LOS ANGELES, California, United States, 2. University of Pennsylvania, Philadelphia, Pennsylvania, United States, 3. University of Missouri Sinclair School of Nursing, Columbia, Missouri, United States*

The committee began its work by developing a conceptual model for achieving resident-centered care. The model draws on existing evidence about the multiple dimensions of resident quality of life and the complexity of nursing home care and environments. Dr. Debra Saliba, a professor in UCLA's Borun Center and Los Angeles VA GRECC with expertise in long-term care policy and delivery, will describe this model and how it guided the committee's recommendations. She also will discuss how each presentation fits within the model's domains

### ACHIEVING QUALITY PATIENT-CENTERED CARE IN THE NURSING HOME SETTING

Colleen Galambos, *University of Wisconsin-Milwaukee, Milwaukee, Wisconsin, United States*

In 1987, The Nursing Home Reform Act was enacted as part of the Omnibus Reconciliation Act of 1987 (OBRA 87). At that time, the Health Care Finance Administration (now Centers for Medicare and Medicaid Services) issued comprehensive regulations and survey processes to "ensure that residents of nursing homes receive quality care that will result in their highest practicable physical, mental, and social well-being." Despite this landmark legislation, nursing homes struggle to provide quality care, and are additionally challenged by natural disasters and pandemics. This presentation will report on recommendations that examine, structures, policies, and care models that promote change and innovation, with a focus on safety, environmental modifications, patient centered approaches, and quality care. Dr. Colleen Galambos, a professor at University of Wisconsin-Milwaukee, with expertise in nursing home care delivery and quality improvement will present the care delivery recommendations from the NASEM report.

### EXPOSING THE REALITIES OF THE WORKFORCE IN NURSING HOMES

Christine Mueller, *University of Minnesota, Roseville, Minnesota, United States*

For decades, staffing in nursing homes has been highlighted as being inadequate and directly affecting the quality of care received by residents. The COVID-19 pandemic, occurring during the committee's deliberations, further highlighted and exacerbated the multiple workforce challenges in nursing homes. The committee's recommendations related to the nursing home workforce will be presented. Dr. Christine Mueller, a professor at University of Minnesota and with expertise in staffing and quality of care, will discuss strengthening the workforce to meet complex and varied resident needs that align with the committee's recommendations.

### ACHIEVING EQUITABLE QUALITY OF CARE FOR NURSING HOME RESIDENTS

Jasmine Travers, *NYU, New York City, New York, United States*

Inequities in care delivery within nursing homes are pervasive across the United States. Racial and ethnic minority residents disproportionately reside in nursing homes of poorer quality, lower staffing, and fewer resources when compared to their White counterparts. Dr. Jasmine Travers, a health services researcher at New York University and with expertise in disparities and long-term care, will present on recommendations to achieving equitable quality of care for nursing home residents.

### SESSION 1480 (SYMPOSIUM)

### LEVERAGING COMMUNITY-BASED KNOWLEDGE TO ADDRESS THE NEEDS OF OLDER ADULTS FROM RACIAL/ETHNIC MINORITY POPULATIONS

Chair: Catherine Garcia Co-Chair: Su-I Hou

Racially and ethnically minoritized older adults constitute the fastest-growing demographic in the United States. Yet, they are largely underrepresented in population-based studies of aging despite NIH policies encouraging inclusion since the mid-1980s. The resulting and continued exclusion of racially and ethnically diverse populations in aging research limits efforts to elucidate and better understand the determinants of health and healthy aging to improve the lives of minoritized older adults, their families, and/or communities. To enhance the inclusion of racially and ethnically minoritized older adults in recruitment, survey development, analysis, and dissemination efforts, this symposium focuses on leveraging community-based knowledge that centers the lived experiences of community members that are needed to improve health outcomes and equitable changes, especially with the ongoing COVID-19 pandemic. The first paper in this symposium highlights how Community-Based Participatory Advocacy is used in the Dine community, which includes working with indigenous older adults and decolonizing methodologies to facilitate community healing. The second paper focuses on developing culturally informed instruments to collect data on dementia care needs and resources among African immigrants. A third paper focuses on critical lessons learned in Project RAMA that provided crucial insights for a new initiative focused on Hispanic immigrant families affected by rheumatoid arthritis in Washington, DC, including recruiting through embedded community clinics and integrating community needs into the study design. The final paper discusses the meaningful inclusion of Asian American older adults regarding their demographic diversity and prioritizing engagement of community members and community-based organizations in the survey research process.

### LEARNING FROM ELDERS: WORKING WITH INDIGENOUS INTERGENERATIONAL MENTORS TO ADDRESS COMMUNITY NEEDS

Karen Kopera-Frye, Karen John, and Robynn Frank, *New Mexico State University, Las Cruces, New Mexico, United States*

Tribal Critical Race Theory (Brayboy, 2005) supports the use of decolonizing methodologies such as Community-Based Participatory Research when collaborating with Indigenous communities. This paper highlights the underlying processes in working with a Dine community on an intergenerational health project. COVID had culturally disruptive effects, e.g., social isolation, on New Mexico's Dine community. This project describes what can be best thought of as Community-Based Participatory Advocacy (Kopera-Frye, John, & Frank, 2021). Navajo students interviewed 13 area chapter elders on how COVID has impacted the community, particularly with Indigenous Ways of Knowing (IWOK). Thematic analysis indicated themes of loss, stress and social isolation, and health worker effects. Resilience was indicated in response to positive outcomes from COVID including a collaboration and coming together of community. Open dialogue workshops are ongoing to facilitate community healing from COVID. The results highlight the critical necessity of starting with the community elders and using decolonizing methodologies.

### A COMMUNITY-UNIVERSITY PARTNERSHIP TO ASSESS AFRICAN IMMIGRANT FAMILIES' EXPERIENCES WITH DEMENTIA

Manka Nkimbeng[1], Christina Rosebush[1], Kwame Akosah[1], Hawking Yam[1], Wynfred Russel[2], Elizabeth Albers[3], Tetyana Shippee[1], and Joseph Gaugler[1], *1. University of Minnesota, Minneapolis, Minnesota,*

*United States, 2. African Career Education And Resources Inc. (ACER), Brooklyn Park, Minnesota, United States, 3. University of Minnesota School of Public Health, Minneapolis, Minnesota, United States*

African immigrants are a fast-growing segment of the U.S. Black population, but the dementia care needs and resources of this population are not fully understood. We will describe the process of developing culturally informed instruments to collect data on dementia care needs and resources among African immigrants. Working together with a diverse project advisory board, a guide was developed and used to conduct community conversations about experiences with dementia/memory loss. Qualitative findings from these conversations were used to inform the development of a survey for quantitative data collection. Despite the challenges of conducting research during a global pandemic, having trusting relationships with a partnering community organization and project advisory board facilitated the successful development of instruments to conduct preliminary dementia care research in an underserved population. We anticipate that survey results will inform interventions that increase education, outreach, and access to dementia care and caregiving resources for this population.

### THE TIES THAT RECRUIT, RETAIN, AND BIND: ENGAGING HISPANIC IMMIGRANT FAMILIES IN THE TIME OF CRISIS

Jielu Lin[1], Anna Wilkinson[2], and Laura Koehly[1], *1. National Institutes of Health, Bethesda, Maryland, United States, 2. University Of Texas School of Public Health, Austin, Texas, United States*

In 2008, we launched Project RAMA (Risk Assessments for Mexican Americans) in Houston, Texas, seeking to understand how multigenerational Mexican immigrant families communicate about familial risk for complex disease. Several lessons were learned. First, our community advisory committee endorsed research goals. Second, we listened to the community with regards to immigration concerns and structural racism. Finally, in the summer and fall of 2008, Hurricane Ike struck the region. Because our team provided support and resources to families in need, we had a higher participation rate post-disaster. Pausing recruitment and postponing assessments led us to unexpectedly discover a long-term intervention effect that was not originally hypothesized. These lessons guide a new initiative focused on Hispanic immigrant families affected by rheumatoid arthritis in Washington DC. We discuss how we address challenges in the on-going project during the Covid pandemic, including recruiting through embedded community clinics and integrating community needs into study design.

### COMMUNITY-ENGAGED STRATEGIES FOR RECRUITMENT OF ASIAN AMERICAN OLDER ADULTS

Lan Đoàn[1], Simona Kwon[2], Chau Trinh-Shevrin[1], and Stella Yi[1], *1. NYU Grossman School of Medicine, New York, New York, United States, 2. NYU School of Medicine, New York, New York, United States*

Asian Americans one of the fastest growing older adult populations in the U.S., and are emerging as a high-need, lower-income population. Inclusion of Asian Americans in research is critical given anti-Asian rhetoric and hate crimes targeting Asian American older adults and because Asian Americans are the fastest growing racial/ethnic group in the U.S. We focus on recruitment strategies used during the implementation of 11 primary collection data efforts, including national and regional community health resources and needs assessment surveys, launched during the COVID-19 pandemic (starting May 2020). Unique recruitment challenges included the heterogeneity of language, culture, and sociodemographic characteristics of participants, digital literacy, and survey fatigue. Effective recruitment facilitators included: prioritizing community engagement at all research stages; aligning the research purpose with community priorities; recruitment through community-based organizations and bilingual community health workers; translating survey instruments; and regularly scheduled meetings with community-based organizations to discuss the survey progress.

### SESSION 1490 (SYMPOSIUM)

### LIFE AFTER LOSS: EMERGING INSIGHTS ON GRIEF ADJUSTMENT AND HEALTH IN LATE LIFE

Chair: Emily Mroz Co-Chair: Sara Hackett Discussant: Deborah Carr

Recent scholarship recognizes that grief adjustment is not a staged, linear process. In late life, the loss of close others is common and dynamic. However, little is known about the 'ripple effects' from loss experiences. As people are living longer, it is important to gain insight regarding how loss is processed, carried, and used for self-direction. This symposium responds to GSA's 2022 meeting theme of "Reimagining Aging" by presenting emerging research which reimagines grief adjustment across diverse populations. To begin, Sara Hackett, PhD will share her work on the continuing bonds of older conjugally bereaved women and how they shape perceptions about repartnering. Following, Emily Mroz, PhD will present on affective sequences in final memories from past spousal loss and demonstrate how sequences predict future caregiving confidence in these widowed older adults. Danielle McDuffie, MA will add to this rich discussion by shedding light on predictors of bereavement and grief outcomes specific to Black adults. Last, Holly Prigerson, PhD will outline unmet psychological needs of family caregivers of patients who die in ICU settings and present her work on EMPOWER, an intervention aimed to reduce psychological distress and foster caregiver adjustment across the loss experience in this setting. Stephanie Wladkowski, PhD, our discussant, will tie these perspectives together, facilitating an important dialogue regarding classic and newly-considered impacts of grief in late life. Further, she will discuss how through more closely examining peoples' experiences during and after loss, we can recognize late-life bereavement as shaping self-perceptions, relationships, and mental health.

### THE GUIDING POWER OF LOSS REFLECTION: FINAL MEMORIES AND FUTURE CARE

Emily Mroz, *Yale University, New Haven, Connecticut, United States*

The life story framework emphasizes that past loss experiences guide current attitudes and behaviors. Final memories from the death of a spouse promote post-loss meaning-making. Through reliable narrative analysis, this study delineates positive and negative affective sequences in final memories from spousal loss in older adults (N = 65; Mage = 81.59). It also examines reported confidence in successfully caring for another seriously ill close other the future. Results demonstrate that 62% of participants narrated their final memories with affect, and 38% told semantic, affectively neutral narratives. Additionally, those who narrated positive or neutral final memories exhibited more confidence in future caregiving than those who narrated negative memories, t = 2.24, p < .05. As care systems are increasingly reliant on family members to provide car for the seriously ill, links between past loss experiences, current caregiving confidence, and future care mastery emerge as areas of research prioritization.

### CONTINUING BONDS OR UNBREAKABLE VOWS?: CONJUGALLY BEREAVED WOMEN'S PERCEPTIONS ABOUT REPARTNERING

Sara Hackett, *University of South Florida, Tampa, Florida, United States*

The death of a romantic partner is regarded as one of life's most traumatic events. Despite established benefits from repartnering, women are typically less likely to repartner than men. Women oftentimes live longer than men and carry their grief into old age, but little is known about their integration of their loss into their lives going forward. Twenty conjugally bereaved women (Mean age = 78) participated in one 90-minute semi-structured interview to discuss their grief and their perceptions of their futures without their deceased partner. Thematic analysis revealed that participants viewed the deceased as an integral member of their present-day social convoy. Furthermore, participants expressed that they were averse to entering new romantic relationships because of either allegiance to the deceased, hesitancy about their age, or disinterest in caregiving for a subsequent partner. By exploring the reasons behind women's resistance to repartnering, interventions may be developed to better support them.

### PSYCHOLOGICAL RESPONSES TO IMPENDING AND PAST LOSS OF A LOVED ONE

Holly Prigerson, *Weill Cornell Medicine, New York City, New York, United States*

Psychological responses to an impending or recent death have received minimal attention in the research literature. I will present data to demonstrate the high levels of psychological distress (e.g. symptoms of peritraumatic stress, anxiety, depression and grief) reported by caregivers of a loved one with a life-threatening illness. I will report on studies that have examined how grief symptoms change over time, or not, as in the case of symptoms of Prolonged Grief Disorder (PGD) – a new addition to the Diagnostic and Statistical Manual of Mental Disorders -5-Text Revision (DSM-5-TR). A case will then be made for addressing psychological struggles of caregivers of patients who are dying in the intensive care unit (ICU). I will then present a conceptual model and manual of our psychosocial intervention (EMPOWER), which we designed to reduce symptoms of PGD and posttraumatic stress disorder (PTSD) by targeting symptoms of experiential avoidance and promoting adaptive coping.

### HOW DO INTERPERSONAL FACTORS AFFECT GRIEF AMONG BLACK MIDDLE- TO OLDER-AGED ADULTS?

Danielle McDuffie, *The University of Alabama/ Durham VAMC, Tuscaloosa, Alabama, United States*

This talk will explore: 1) the conceptual importance of select interpersonal factors inherent to Black culture and bereavement, and 2) the impact of these factors on grief in a Black bereaved sample. 103 Black adults aged 45+ were administered items assessing cultural (communalism, experienced racial violence) and bereavement-specific (expectancy and suddenness of loss, closeness to the deceased, anger) factors. Using linear regressions, closeness (p<.001), suddenness (p=.020), anger (p<.001), and communalism (p<.001) predicted reported levels of grief. This study identifies factors which substantially dictate the experienced levels of grief of bereaved Black adults in the latter half of life. With cultural factors notably impacting the Black bereavement experience, individuals seeking to work with this population must adopt strategies to keep bereaved Black adults connected to their communities throughout their loss. Resources to support Black adults in maintaining healthy continuing bonds with the deceased through abrupt or unsettling losses also appear paramount.

### SESSION 1500 (SYMPOSIUM)

### LIVED EXPERIENCES OF OLDER CHINESE IMMIGRANTS IN AFFORDABLE HOUSING DURING THE PANDEMIC: STRESS AND RESILIENCE

Chair: Kexin Yu Co-Chair: Iris Chi Discussant: Fei Sun

Older immigrants in affordable housing have unique sets of challenges during the COVID-19 pandemic posed by limited English proficiency and resources. The aggregated living condition could increase their risk of being exposed to the contagious disease. This symposium reports empirical findings of the social Network of Immigrant Chinese older adults in affordable Housing Environment (NICHE) project, which focused on the influence of COVID-19 on underprivileged Chinese older immigrants. We conducted 27 semi-structured interviews with foreign-born older Chinese immigrants (mean age 78.1, 69.23% female) in an affordable housing in Los Angeles to learn about their lived experiences and coping strategies during the COVID-19 pandemic. The first presentation focused on the changes in older Chinese immigrants' social life after the onset of the pandemic and aimed to understand the pandemic's impact on their depressive symptoms and loneliness. The second presentation describes older Chinese immigrants' perceived pandemic-related stressors and resilience across phases of the pandemic, including at the beginning, after they got vaccinated, and the rising of delta variant. The participants explained what supportive services had been helpful and what support they wished they could have over the past two years in the pandemic. The third presentation reports the experience of being discriminated against during the pandemic, the Chinese older adults' attitudes towards these discriminatory events, and coping strategies. Together, these three presentations will depict the lived experience of Chinese immigrants over two years during the pandemic and discuss intervention strategies and policy considerations for preparing for future crisis like the COVID-19 pandemic.

### SOCIAL NETWORK AND MENTAL HEALTH OF OLDER CHINESE IMMIGRANTS IN AFFORDABLE SENIOR HOUSING DURING THE COVID-19 PANDEMIC

Kexin Yu[1], Jiaming Liang[2], Yi-Hsuan Tung[2], Mutian Zhang[3], Luyan Yang[2], Shinyi Wu[2], and Iris Chi[2], *1. Oregon Health & Science University, Palo Alto, California, United States, 2. University of Southern California, Los Angeles, California, United States, 3. University of Southern California, Los Angele, California, United States*

Chinese older immigrants who live in senior housing communities are at high risks of experiencing discrimination and social isolation during the COVID-19 pandemic. This study examines how and to what extent the pandemic has affected this population's social network and mental health. Participants reported a decrease in social contact with their family and friends. Before the pandemic, many paid regular visits back to the home country and could not do so in the past two years. The loss of connection left some feeling despaired and expressed uncertainty on whether they could ever go back "home" before death. Participants also reported being in a low mood and feeling bored constantly. Participants reported resilience generated from their religious beliefs, having neighbors as role models, and wisdom learned from past life experiences. Knowledge produced in this project can inform the planning for responding to future crises in affordable senior housing.

### STRESS AND RESILIENCE ACROSS PHASES OF COVID-19: A QUALITATIVE STUDY WITH OLDER CHINESE IMMIGRANTS

Yi-Hsuan Tung[1], Kexin Yu[2], Jiaming Liang[1], Shinyi Wu[1], and Iris Chi[1], *1. University of Southern California, Los Angeles, California, United States, 2. Oregon Health & Science University, Palo Alto, California, United States*

The COVID-19 pandemic has challenged older Chinese immigrants' lives in physical, psychological, social, and spiritual aspects. This study employed a socioecological perspective of resilience to examine how older Chinese immigrants perceived and navigated through pandemic-related adversities. We conducted a time-bound retrospective qualitative investigation to capture participants' lived experiences between December 2019 to August 2021. Three phases of the pandemic-related adversities were identified, including uncertain threats and psychological impacts at the beginning, unmet needs and fatigue at 2nd and 3rd wave of infections, and benefit-risk balance after vaccinated. Despite adversities, the integration of strengths, opportunities, and social services at the individual, interpersonal, and neighborhood levels allows participants to appraise and individualize their problem-focus coping (e.g., risk mitigation), selective engagement (e.g., maintaining habits through other means), or emotion-focus coping strategies (e.g., acceptance). Findings highlight the importance of personal and community resources in fostering resilient responses.

### DISCRIMINATION AND COPING AMONG OLDER CHINESE IMMIGRANTS DURING THE COVID-19 PANDEMIC: A QUALITATIVE STUDY

Jiaming Liang[1], Kexin Yu[2], Yi-Hsuan Tung[1], Choi Ha Li[1], Shinyi Wu[1], and Iris Chi[1], *1. University of Southern California, Los Angeles, California, United States, 2. Oregon Health & Science University, Palo Alto, California, United States*

Discriminatory events against Asians, especially Chinese, became rampant during the COVID-19 pandemic. It is difficult for older Chinese immigrants to effectively protect themselves from racism-related attacks due to their personal and social disadvantages. This study explored older Chinese immigrants' experience of discrimination and coping strategies, as well as factors that influence their perceptions, attitudes, and coping preferences. Among 27 interviewees, 11 experienced discriminatory incidents themselves or known people around had been discriminated against during the pandemic. Thematic analysis revealed negative psychological impact of discrimination risk or experience. Most participants tended to adopt disengagement coping styles, such as avoidance, rationalization, and reducing social participation. Three primary influencing factors are: (1) perceived unkindness from government and public opinions; (2) concern for own health; (3) limited acculturation. Our findings suggest needing efforts to protect the safety of older Chinese immigrants, and raise their awareness and ability to defend themselves from racism and discrimination.

### SESSION 1510 (SYMPOSIUM)

### NORTH AMERICAN CONTEXTS USING COMPUTERIZED NETWORKS TO IMPROVE CARE DELIVERY FOR OLDER ADULTS

Chair: Gregory Alexander

Evidence shows that technology provides a means to improve communication and quality of care, through greater efficiencies in information management. However, research demonstrates that technology use does not consistently improve care. Therefore, there is a continuing need globally to evaluate and discuss the impact of technology in clinical care, especially in settings where older people have higher care needs. The panel includes five interdisciplinary experts with backgrounds in care delivery systems for older adults, engineering, informatics, health systems, quality improvement, mobile health, medicine, and nursing from the U.S. and Canada. This expert panel has three objectives: 1) Describe informatics research initiatives using computerized networks to improve care delivery for older adults, 2) Contrast information technologies used to manage information in health systems caring for older adults, and 3) Explain opportunities to improve health information technology systems used in care delivery for older adults.

### THE ASSOCIATION BETWEEN NURSING HOME IT MATURITY AND UTIS AMONG LONG-TERM NH RESIDENTS

Gregory Alexander, *Columbia University, New York, New York, United States*

Prof. Alexander will present a study linking national nursing home (NH) health information technology (HIT) to quality. This research explores relationships between NH HIT maturity and UTIs examined during a repeated cross-sectional study over 4 consecutive years (2013-2017). HIT maturity represents the diversity of technology use in NHs. Researchers conducted bivariate and multivariate regressions using MDS 3.0 resident assessments and post stratified survey weights controlling for NH characteristics. Our sample included 816 NHs. These NHs had 219,730 regular NH resident assessments within 90 days of a survey, representing 80,322 unique NH residents. Of these assessments, 4.1% recorded a UTI. In the multivariate analyses, administrative HIT capabilities was associated with lower odds of UTI (AOR: 0.906, 95% CI: 0.843, 0.973), controlling for covariates. Integration of administrative HIT systems may relieve the burden of tracing UTIs, data documentation and record retrieval affording NH staff time to focus their efforts on clinical care.

### WHAT IS THE DIGITAL MATURITY OF LONG-TERM CARE HOMES IN ONTARIO? A SURVEY PROTOCOL

Ramtin Hakimjavadi, *University of Ottawa, Ottawa, Ontario, Canada*

Ramtin Hakimjavadi will discuss the barriers and facilitators of implementing a health information technology (HIT) maturity survey in the Canadian context. Alongside Dr. Liddy's team, he is supporting the adaptation of the survey into a Canadian context, followed by a pilot study to distribute the survey to select nursing homes. Drawing from this work, Mr. Hakimjavadi will highlight the barriers and facilitators associated with the process of implementing the HIT maturity survey. Expected challenges include adjusting the survey format for respondents with limited resources, identifying a central contact within each nursing home, optimizing the survey user interface to limit response burden, and developing recruitment strategies to increase response rate. These challenges and the strategies to overcome them, impressions of the current state of HIT in Ontario nursing homes, and proposed action items to trend digital maturity in these settings over time will be discussed.

### A NATIONAL SNAPSHOT OF INFORMATION SHARING DURING CARE TRANSITIONS BETWEEN HOSPITALS AND SNFS

Julia Adler-Milstein, *University of California, San Francisco, San Francisco, California, United States*

Prof. Adler-Milstein will present national survey results about skilled nursing facilities (SNFs) information sharing with referring hospitals. The survey, which garnered a 53% response rate, found significant gaps in information sharing. Of 471 hospital-SNF pairs, 64 (13.5%) reported excellent performance on 3 dimensions of information sharing (completeness, timeliness, and usability), whereas 141 (30.0%) were at or below the mean performance. Having a hospital clinician on site at the SNF was associated with more complete (odds ratio, 1.72; P = .03), timely (odds ratio, 1.76; P = .02), and usable (odds ratio, 1.64; P = .04) information sharing. Hospital accountable care organization participation was associated with more timely information sharing (odds ratio, 1.88; P = .02). Despite significant federal investment in hospital health IT infrastructure, we conclude that hospitals are not tailoring information sharing processes to ensure that SNFs receive timely, complete, and usable information.

### ECONSULT IMPROVES ACCESS TO SPECIALIST ADVICE FOR PRIMARY CARE PROVIDERS

Celeste Fung[1], and Clare Liddy[2], *1. University of Ottawa, Department of Family Medicine, Ottawa, Ontario, Canada, 2. University of Ottawa, Ontario, Ontario, Canada*

Dr. Liddy will present an overview of the Champlain BASE™ eConsult service, with a focus on its implementation in long-term care (LTC). eConsult is a secure web-based tool that enables providers to communicate electronically with specialists concerning a patient's care. Evaluation of over 100,000 cases has consistently demonstrated positive impacts on access to care: PCPs receive responses in a median of 0.9 days, and two-thirds of cases are resolved without the patient needing a face-to-face specialist visit. A cross-sectional analysis of eConsult cases sent from LTC settings in 2018 demonstrated similar response times and referral avoidance to the larger sample (a median response time of 0.6 versus 0.9 days; proportion of cases resolved without a specialist visit 70% versus 67%). These findings, coupled with eConsult's proven success at improving access to and equity of care, demonstrate the importance of supporting eConsults expansion.

### BARRIERS AND FACILITATORS FOR ECONSULT: IMPROVING ACCESS TO SPECIALIST ADVICE IN LONG-TERM CARE

Celeste Fung, *University of Ottawa, Department of Family Medicine, Ottawa, Ontario, Canada*

Long-term care (LTC) residents continue to receive most of their specialist care outside of the home. These visits present significant risks and burdens for residents. Such risks can be mitigated by eConsult, which facilitates timely, resident-specific, and practical recommendations between primary care providers (PCPs) and specialists to support clinical decision making, reduce unnecessary transfers, and positively impact resident care and quality of life. During focus groups in LTC homes where eConsult is used, PCPs, senior leadership, and a nurse champion recognized eConsult as having great value for residents, especially those with challenges travelling outside of the home. However, they also described LTC homes as under resourced, with minimal IT infrastructure and coupled with time constraints present barriers to eConsult adoption in LTC. Enabling factors (engaging clinician champions, establishing delegates, integration into existing workflows) similarly identified as supporting eConsult implementation more broadly were discussed.

### SESSION 1530 (PAPER)

### SUPPORTING DIRECT CARE PROVIDERS

### BETTER NP PRACTICE ENVIRONMENTS REDUCE HOSPITALIZATION DISPARITIES AMONG DUALLY ENROLLED PATIENTS

Jacqueline Nikpour[1], Heather Brom[2], Aleigha Mason[1], Jesse Chittams[1], Lusine Poghosyan[3], and Margo Brooks Carthon[1], *1. University of Pennsylvania, Philadelphia, Pennsylvania, United States, 2. Villanova University M. Louise Fitzpatrick College of Nursing, Villanova, Pennsylvania, United States, 3. Columbia University, New York, New York, United States*

Adults dually-enrolled in Medicare and Medicaid experience twice as many hospitalizations and higher rates of ambulatory care-sensitive conditions (ACSCs) – such as coronary artery disease [CAD] and diabetes, compared to Medicare-only patients. Nurse practitioners (NPs) are well-positioned to address care needs of dually-eligible patients, yet NPs often work in unsupportive clinical practice environments. The purpose of this study was to examine the association between the NP primary care practice environment and disparities in all-cause hospitalizations between dually-eligible and Medicare-only patients with ACSCs. Using linked secondary cross-sectional data from the Nurse Practitioner Primary Care Organizational Climate Questionnaire (NP-PCOCQ) and Medicare claims files, we examined 189,420 patients with CAD and/or diabetes (19.1% dually-eligible, 80.9% Medicare-only), cared for in 470 practices employing NPs across four states (PA, NJ, CA, FL) in 2015. After adjusting for patient and practice characteristics, dually-eligible patients in poor practice environments had the highest odds of being hospitalized compared to their Medicare-only counterparts (OR 1.60, CI: 1.49-1.71). In mixed practice environments, dually-eligible patients had approximately 48% higher odds of a hospitalization (OR 1.48, CI 1.31-1.68), while in the best practice environments, dually-eligible patients had approximately 37% higher odds (OR 1.37, CI 1.21-1.57, p < .001). As policymakers look to improve outcomes and reduce costs among dually-eligible patients, addressing a modifiable aspect of care delivery in NPs' clinical practice environment is a key opportunity to reduce hospitalization disparities. Yet further efforts are needed to address remaining disparities by meeting patients' health-related social needs, such as poverty and access to care.

### CARE WORKERS' ADMINISTRATIVE BURDEN IN SWISS NURSING HOMES: A MULTICENTER CROSS-SECTIONAL STUDY

Dietmar Ausserhofer[1], Waltraud Tappeiner[2], Heike Wieser[2], Lauriane Favez[3], and Franziska Zúñiga[3], *1. College of Healthcare Professions Claudiana, Bozen-Bolzano, Trentino-Alto Adige, Italy, 2. College of Healthcare Professions Claudiana, Bolzano, Trentino-Alto Adige, Italy, 3. University of Basel, Basel, Basel-Stadt, Switzerland*

Apart from 'direct' resident care, care workers in nursing homes also perform tasks that are related to organizational or management activities. 'Indirect' care activities, such as administrative tasks, are often considered as burdensome, as they increase overall workload and keep care workers away from caring for their residents. So far, there is little investigation on care workers' administrative burden. The multicenter cross-sectional Swiss Nursing Homes Human Resources Project (2018) study included a convenience sample of 118 Swiss nursing homes and 2207 care workers (i.e., registered nurses and licensed practical nurses). Care workers completed questionnaires assessing the administrative burden, staffing and resource adequacy, leadership ability, implicit rationing of nursing care and care worker characteristics and outcomes. For the analysis, we applied 2-level binomial generalized linear mixed models. Overall, 73.91% (n=1561) of care workers felt strongly or rather strongly burdened, with one third (36.6%, n=787) reporting to spend 2h or more during a "normal" day performing administrative tasks. Ratings ranged from 42.6% (n=884; ordering supplies and managing stocks) to 75.3% (n=1621; filling out the patient's medical record). One out of four care workers (25.5%, n=561) intended to leave the profession, whereby care workers reporting higher administrative burden (OR=1.24; 95%CI: 1.02-1.50) were more likely to leave the profession. This study provides first insights on care workers' administrative burden in nursing homes, which can inform the development of interventions to reduce the workload related to 'indirect care activities' and to improve care workers' job satisfaction and retention in the profession.

### EMBRACING SPIRITUALITY IN THE WORK OF FRONTLINE NURSING HOME STAFF

Ruth Palan Lopez[1], Meghan Hendricksen[2], Ashley Roach[3], Anita Hendrix Rogers[4], and Susan Mitchell[2], *1. MGH Institute of Health Professions, Sharon, Massachusetts, United States, 2. Marcus Institute for Aging Research, Roslindale, Massachusetts, United States, 3. OHSU, Portland, Oregon, United States, 4. University of Tennessee Martin, Parsons, Tennessee, United States*

Good end-of-life care for nursing home (NH) residents and their families provides for spiritual support, however, the role that spirituality plays in the work of NH staff has not been well described. To understand more about spirituality and NH staff, we examined interview data generated from the Assessment of Disparities and Variation for Alzheimer Disease Nursing Home Care at End of Life (ADVANCE) study, a large qualitative study conducted in 14 NHs to examine differences in end-of-life care for residents with advanced dementia. We examined semi-structured interview data from nurses (Registered Nurses and Licensed Practical Nurses) (n=45) and certified nursing assistants (CNAs) (n=25) who described the role of spirituality or God in response to questions around their experience caring for residents with advanced dementia. Data were analyzed using the constant comparative method. Most participants were female (93%). Thirty-four identified as Black, 29 as white, and 7 as other racial groups. The average age was 47.8 years, and the average years of experience was 16.2 years. Participants reported several religious affiliations including Baptist (23); Agnostic/Atheist (2), Catholic (7), Methodist (4), Muslim (1), Other/Unaffiliated/Refused (18), Pentecostal (4), and Christian (9). We found that for many nurses and CNAs, God played an important role in their work including providing meaning and purpose, providing them with resilience to cope with frequently losing residents that they consider "family." The voices of the participants were powerful and provide important insights into potential strategies to retain and support these essential, front-line providers.

### STRATEGIES TO ADDRESS COVID-19 VACCINE HESITANCY AMONG OHIO NURSING HOME STAFF

Miranda Kunkel, Robert Applebaum, and Matt Nelson, *Miami University, Oxford, Ohio, United States*

Background: Nursing home staff members unvaccinated against COVID-19 pose an ongoing public health risk considering their frequent contact with older adults, a population especially vulnerable to the virus. In order to address vaccine hesitancy and foster vaccine confidence effectively among nursing home staff, evidence-based health communication strategies are necessary.

Methods: Using a dataset composed from three sources of Ohio nursing home data, the research questions guiding this study are as follows: 1) What is the relationship between engaging in various strategies to address vaccine hesitancy and vaccination rates among staff?; 2) Does hiring infection preventionists affect vaccination rates among staff? Multivariable linear regression techniques were used to assess all variables.

Results: Peer counseling and providing time off to the staff for vaccination statistically significant strategies to reduce vaccine hesitancy among staff. Compared to facilities that did not engage in peer counseling, those that did saw an average increase of 3.6% of their staff vaccinated. Compared to facilities that did not provide time off to their staff for vaccination, those that did saw an average increase of 4.2% of their staff vaccinated. Discussion: Peer counseling should be pursued as a viable strategy for fostering vaccine confidence among nursing home staff and further studied in more diverse populations. Implications for nursing home staff acceptance of facility-wide initiatives will be discussed.

### STRATEGIES TO IMPLEMENT AGE-FRIENDLY CARE FOR OLDER ADULTS ACROSS CONVENIENT CARE CLINICS

Heba AlDossary[1], Anna Bender[2], Megan Foradori[2], Anne Pohnert[3], Robin Hughes[2], Sherry Greenberg[4], and Mary Dolansky[5], *1. Frances Payne Bolton School of Nursing at Case Western Reserve University , Cleveland, Ohio, United States, 2. Case Western Reserve University, Cleveland, Ohio, United States, 3. CVS Health MinuteClinic, Woonsocket, Rhode Island, United States, 4. Monmouth University, Marjorie K. Unterberg School of Nursing & Health Studies, West Long Branch, New Jersey, United States, 5. Frances Payne Bolton School of Nursing at Case Western Reserve University, Cleveland, Ohio, United States*

A growing proportion of the population in the United States are older adults, and they are frequent users of convenient care clinics. This is particularly true for older adults from systematically marginalized communities who may be at greater risk for chronic health conditions and unmet healthcare needs. These trends render convenient care clinics critical sites to reimagine the provision of care for older adults. To improve the quality of care the CVS MinuteClinic implemented the Institute of Healthcare Improvement's Age-Friendly Health Systems 4Ms Framework (assessing What Matters, Medication, Mentation, and Mobility). Research to evaluate two interventions (reflective learning and virtual clinic participation) to improve healthcare providers' (HCP) uptake of the 4Ms was implemented. After the interventions, HCPs were provided a self-administered online survey to capture qualitative reports of reflections on learning and implementing the 4Ms. A total of 32 providers from both groups completed the survey. Using a codebook thematic analysis approach, facilitators and barriers for implementation were identified as were HCPs' innovative approaches to consistently and effectively implement the 4Ms. Facilitator themes were the presence of resources (e.g., peer coaches) and the perceived value of the 4Ms model. Barrier themes included time, documentation issues, workflow challenges, HCP stress, and HCP comfort. Innovation themes included designing efficient workflows, scaffolded 4Ms learning, and collaboration. These findings underscore the supports/innovations to amplify and challenges to address to support HCPs in learning and implementing the 4Ms to improve the quality of care for older adults.

### SESSION 1540 (SYMPOSIUM)

### SUPPORTING FAMILY CAREGIVING FOR PERSONS WITH DEMENTIA FROM CULTURAL AND LIFE-COURSE PERSPECTIVES

Chair: Jing Wang Co-Chair: Bei Wu

Providing person-centered and culturally sensitive support for persons with dementia and their family caregivers is critically important. This symposium focuses on understanding and supporting decision-making and family caregiving through a lens of a life course perspective. The first study explored Black and White family caregivers' experience of adjusting to their new role as caregivers of persons with dementia during the COVID-19 pandemic. The pandemic added to the complexity of their challenges and demonstrated the need for person-centered support. The second study provided insights into Chinese American family caregivers' attitudes toward and the preference of shared family decision-making on tube feeding for their family members with dementia. Almost all the participants considered pain and discomfort relief as a priority in decision making. The third study synthesized the literature on decision aid interventions for family caregivers of older adults with advanced dementia regarding tube feeding. The study found that culturally sensitive and family-centered decision aid interventions are needed to support the decision-making of tube feeding. The fourth study used data from two national databases and identified positive aspects of caregiving. It highlighted the significance of tailoring support for family caregivers based on their caregiving responsibilities and care recipients' cognitive status to enhance the positive aspects of caregiving. The last scoping review identified and synthesized existing technology-based interventions to address social isolation and loneliness among informal dementia caregivers. The results suggested that technology-based interventions could reduce feelings of loneliness and improve caregiver well-being.

### THE ATTITUDES OF CHINESE AMERICAN FAMILY CAREGIVERS OF OLDER PEOPLE WITH DEMENTIA TOWARD TUBE FEEDING

Yaolin Pei[1], Xiang Qi[1], Zhen Cong[2], and Bei Wu[3], *1. New York University, New York City, New York, United States, 2. School of Social Work, Arlington, Texas, United States, 3. New York University, New York, New York, United States*

The aim of this study is to examine the attitudes toward feeding in Chinese American dementia family caregivers. 53 Chinese American dementia caregivers were interviewed in 2021-2022. The survey included their anticipated decisions for tube feeding if their care recipients are at the end of life, goals of care, and knowledge of tube feeding. 32.7% of them would choose tube feeding for individuals with dementia at the end of life; however, 53.9% are confident that the patient would not choose tube feeding if patients are able to make decisions. 92.3% prepared to relieve pain and discomfort as much as possible. 42.3 % of them thought tube feeding are recommended to use if oral feeding difficulties arise. More than 60% thought tube feeding can improve survival and nutrition status. More education is needed for the decision-making regarding tube feeding at the end of life for Chinese American dementia caregivers.

### POSITIVE ASPECTS OF CAREGIVING IN DIFFERENT CAREGIVER GROUPS

Mengyao Hu[1], Dena Schulman-Green[2], Emma Zang[3], and Bei Wu[1], *1. New York University, New York, New York, United States, 2. Rory Meyers College of Nursing, New York University, New York City, New York, United States, 3. Yale Sociology Department, Yale University, New Haven, Connecticut, United States*

Previous studies have disproportionately focused on caregivers' negative experiences while overlooking the positive aspects of caregiving (e.g., quality of caregiver – care recipient relationship, meaningfulness of caregiving, and family cohesiveness) especially for caregivers of older adults with cognitive impairment. Therefore, we aim to identify how positive aspects of caregiving varied by care recipients' cognitive status (e.g., normal, mild cognitive impairment, dementia) and caregivers' relation to care recipients (e.g., spouse, adult child, other family member). We applied multilevel mixed-effects models on pooled three-wave data from the National Study of Caregiving and the National Health and Aging Trends Study (N = 2,717). The findings suggested that dementia and spouse caregivers had worse relationship with their counterparts. Overall, future research needs to study caregiver's experience integratively and focuses on caregiver's individual need. Policy makers need to fulfill caregiver's demands by establishing socially supportive programs.

### DECISION-AID INTERVENTIONS TO SUPPORT DEMENTIA CAREGIVERS' DECISION-MAKING ABOUT FEEDING OPTIONS

Xiang Qi[1], Yaolin Pei[1], Dena Schulman-Green[2], Mengyao Hu[3], Kaipeng Wang[4], and Bei Wu[3], *1. New York University, New York City, New York, United States, 2. Rory Meyers College of Nursing, New York University, New York City, New York, United States, 3. New York University, New York, New York, United States, 4. University of Denver, Denver, Colorado, United States*

We sought to provide an overview of the literature on decision aid interventions for family caregivers of older adults with advanced dementia regarding decision-making around tube feeding. The process was guided by Arksey and O'Malley's methodological framework. Six publications reporting on four unique decision aid interventions were included. All the interventions targeted caregivers of older adults with advanced dementia. Three of decision aids were culturally adapted from existing ones. The Ottawa Decision Support Framework and the International Patient Decision Aid Standards Framework were used in these studies. Interventions aimed to improve decision-making regarding tube feeding for caregivers through static delivery methods. Caregivers rated these decisions as helpful and acceptable. Reduction in decisional conflict and increase in knowledge were consistently found among dementia caregivers, but no intervention effects were found on preferences for use of tube feeding. Culturally adapted decision aids effectively improve decision-making regarding tube feeding among the target population.

### BECOMING A DEMENTIA CARE PARTNER DURING THE COVID-19 PANDEMIC

Hanzhang Xu[1], Laura Fish[2], Raman Nohria[2], Jacob Christy[2], Margaret Falkovic[2], Matthew Dupre[1], Ruth Anderson[3], and Truls Østbye[2], *1. Duke University, Durham, North Carolina, United States, 2. Duke University School of Medicine, Durham, North Carolina, United States, 3. UNC Chapel Hill, Chapel Hill, North Carolina, United States*

Persons with newly diagnosed Alzheimer's disease and related dementias (ADRD) and their care partners confront multiple challenges. These challenges have been even greater during the COVID-19 pandemic, where supportive resources often are limited or even discontinued. We conducted semi-structured interviews with 21 care partners of persons who were recently diagnosed with ADRD (2019-2020) to explore their lived experiences of adjusting to the new role. Directed and conventional content analyses were used and were informed by the life course theory. Care partners perceived difficulty in accessing medical and social services for their loved ones, particularly during the pandemic. Despite experiencing distress, some care partners chose not to seek help for fear of contracting COVID-19. This study provides insights on the unmet needs of care partners during a pandemic and highlights that effective, long-term strategies are needed to continue providing person-centered care to persons with ADRD and their families.

### REVIEW OF TECHNOLOGY-BASED INTERVENTIONS ADDRESSING SOCIAL DISCONNECTION AMONG DEMENTIA CAREGIVERS

Weiyu Mao[1], Xiang Qi[2], Iris Chi[3], and Bei Wu[4], *1. University of Nevada, Reno, Reno, Nevada, United States, 2. New York University, New York City, New York, United States, 3. University of Southern California, Los Angeles, California, United States, 4. New York University, New York, New York, United States*

Social disconnection is a major public health concern. Informal dementia caregivers are particularly vulnerable to social isolation and loneliness, as the majority are older adults and at elevated risk of adversity. Technology-based interventions could offer accessible, affordable, and convenient solutions. Using a scoping review approach, we aimed to identify and synthesize existing technology-based interventions to address social isolation and loneliness among informal dementia caregivers. In a systematic search across six databases within the last ten years, ten eligible studies were included. The intervention type, format, and duration varied widely. The results suggested that technology-based interventions could reduce feelings of loneliness and improve caregiver well-being. The findings offer opportunities to use technology to reduce or prevent social isolation and loneliness. Investigating the role of technology in interventions addressing social isolation and loneliness among informal dementia caregivers could potentially overcome barriers to low uptake of services and improve sustainability of the interventions.

### SESSION 1550 (SYMPOSIUM)

### TECHNOLOGY-BASED INTERVENTIONS AND ASSESSMENT FOR OLDER ADULTS WITH COGNITIVE IMPAIRMENT

Chair: JuYoung Park Co-Chair: Lillian Hung

During the COVID-19 pandemic, older adults with cognitive impairment experienced social isolation, stress, and challenges to stay healthy at home or in a long-term care facility. Technology-based interventions and assessment can be valuable in managing dementia at home before a crisis situation occurs, which can lessen caregiver burden and stress and improve quality of life for older adults with cognitive impairment. In the symposium, specific technology-based interventions (telepresence robot, online chair yoga, exergames, virtual cycling, video-conferencing platforms)

and assessment (IOM2 biofeedback device) were used for older adults with cognitive impairment. We cultivated a novel interdisciplinary approach to emerging clinical entities of technology-based intervention and assessment for older adults with cognitive impairment. In the symposium, we will present a variety of technology-based clinical interventions. Our first study explored the experiences of virtual family visits in four Canadian long-term care homes, using a telepresence robot. Online survey, interviews, focus groups, and observations were conducted to explore the experience. The second study assessed feasibility of a remotely supervised online chair yoga (CY) intervention for older adults with dementia in Florida and explored the preliminary effects of CY on psychosocial outcomes in this population. The third study evaluated the ease of use and quality of cardiac data using IOM2 biofeedback device for older adults with dementia. Cardiac rhythms were analyzed from pulse data measured using the IOM2 biofeedback device (UNYTE). The fourth study was a scoping review to analyze evidence about online group-based exercise programs.

### FEASIBILITY OF REMOTELY SUPERVISED ONLINE CHAIR YOGA INTERVENTION FOR OLDER ADULTS WITH DEMENTIA

JuYoung Park[1], Marlysa Sullivan[2], Keri Heilman[3], Jayshree Surage[2], Hannah Levine[4], Lillian Hung[5], and María Ortega[6], *1. Florida Atlantic University College of Social Work and Criminal Justice, Boca Raton, Florida, United States, 2. Maryland University of Integrative Health, Laurel, Maryland, United States, 3. University of North Carolina School of Medicine Psychiatry, Chapel Hill, North Carolina, United States, 4. Florida Atlantic University, Charles E. Schmidt College of Medicine, Boca Raton, Florida, United States, 5. University of British Columbia, Vancouver, British Columbia, Canada, 6. Florida Atlantic University Christine E. Lynn College of Nursing, Boca Raton, Florida, United States*

This study assessed the feasibility of a remotely supervised online chair yoga (CY) intervention for older adults with dementia, exploring preliminary effects of CY on chronic pain, mobility, risk of falling, sleep disturbance, autonomic reactivity, cardiac rhythms (using IOM2 biofeedback device), and loneliness in this population. Using a one-group pretest/posttest design, a home-based CY intervention was delivered remotely to a group of 10 older adults with dementia who were socially isolated due to COVID-19. The online intervention was conducted twice weekly in 60-minute sessions for 8 weeks; data were collected virtually at baseline, mid-intervention, and post-intervention. Results indicated that online CY is a feasible approach for managing physical and psychological symptoms in older adults with dementia, based on retention (70%) and adherence (87.5%) with no injuries or other adverse events during the intervention. Senior-friendly videoconferencing should be available so that more older adults can gain access to the online intervention

### USING TELEPRESENCE ROBOTS TO SUPPORT FAMILY VIRTUAL VISITS DURING THE COVID-19 PANDEMIC

Lillian Hung, *University of British Columbia, Vancouver, British Columbia, Canada*

In this project, virtual family visits were made to residents of four Canadian long-term care homes using a telepresence robot. Guided by the Consolidated Framework of implementation Research (CFIR), we conducted an online survey, interviews, focus groups, and observations to explore the participants' experience. Analysis identified three themes: (a) relative advantage (easy to visit), (b) capacity for change (readiness and organizational support), and (c) cultural safety (champions leading the way during challenging times). Preliminary results indicate that staff, residents, and families accepted the robot for easy connection but had concerns about privacy issues. Future research should apply inclusive methods to bring relevant stakeholders together to explore user experiences fully: who is affected in what ways and the benefits, risks, and burdens of emerging technologies.

### TECHNOLOGY AND GROUP EXERCISE INTERVENTIONS FOR PEOPLE WITH DEMENTIA OR MILD COGNITIVE IMPAIRMENT: A SCOPING REVIEW

Hannah Levine[1], Paavan Randhawa[2], JuYoung Park[3], and Lillian Hung[4], *1. Florida Atlantic University, Charles E. Schmidt College of Medicine, Boca Raton, Florida, United States, 2. Langara College, Vancouver, British Columbia, Canada, 3. Florida Atlantic University College of Social Work and Criminal Justice, Boca Raton, Florida, United States, 4. University of British Columbia, Vancouver, British Columbia, Canada*

We conducted a scoping review to analyze evidence about online group-based exercise programs. We searched six electronic databases and conducted team analysis with patient and family partners. Of 1,166 screened articles, the final review included 8 publications. Results identified three types of technology-based group exercise interventions for people with dementia or mild cognitive impairment (MCI): (a) exergames, (b) virtual cycling, and (c) video-conferencing platforms. These studies used psychosocial, physical function, biomarker, and cognitive outcome measures. The review identified three key impacts: (a) feasibility and accessibility; (b) physical, psychosocial, and cognitive benefits; and (c) adaptations necessary for persons with dementia or MCI. Over all, technology-based group exercise interventions were found to be accessible, feasible, and acceptable to persons with dementia or MCI. However, a "one-size-fits-all" game approach often did not work, suggesting that exercise interventions should be adaptable to meet the various needs of individual participants.

### SESSION 1560 (SYMPOSIUM)

### THE BOLD PUBLIC HEALTH CENTERS OF EXCELLENCE ON DEMENTIA: MOBILIZING PUBLIC HEALTH ACTION TO ADDRESS DEMENTIA

Chair: Matthew Baumgart Discussant: Nora Super

Under the BOLD Infrastructure for Alzheimer's Act, passed by Congress in 2018, the Centers for Disease Control and Prevention (CDC) established three Public Health Centers of Excellence (PHCOEs) to address Alzheimer's disease and related dementias. Awarded in the Fall of 2020, the three PHCOEs are: (1) Dementia Caregiving; (2) Dementia Risk Reduction; and (3) Early Detection of

Dementia. Each PHCOE is charged with identifying, translating, and disseminating research findings and evidence-based public health best practices in their particular area of focus – and then working with state, local, and tribal public health agencies across the country to undertake dementia-related activities in their communities. This Symposium will feature an overview by Dr. McGuire of the BOLD Act and CDC's efforts to address Alzheimer's and related dementia, followed by presentations on the work and the plans of each of the PHCOEs. Dr. Gaugler will discuss the Caregiving PHCOE's efforts to support unpaid caregivers of those living with dementia, including how best to meet the caregiving needs of diverse communities. Mr. Baumgart will present the Risk Reduction PHCOE's findings on the state of the evidence on risk reduction and how that evidence is being translated to public health action. Dr. Chodosh/Dr. Borson will review the plans of the Early Detection PHCOE to increase awareness of the value of earlier detection and the best ways of conducting detection strategies. Finally, Ms. Super will discuss how this public health work complements the work of the aging community in addressing Alzheimer's and related dementias.

### THE POSSIBILITY OF PREVENTION: THE PUBLIC HEALTH CENTER OF EXCELLENCE ON DEMENTIA RISK REDUCTION

Matthew Baumgart, *Alzheimer's Association, New York, New York, United States*

While we may not be able to predict with certainty whether any particular individual can prevent dementia, we are now able to talk about dementia risk reduction at the population level. The Public Health Center of Excellence on Dementia Risk Reduction reviews and synthesizes the state of the science on modifiable risk factors for cognitive decline and dementia. The Center translates that science into public health action – identifying interventions and best practices to address those risk factors – and develops tools and resources for public health agencies. The Center disseminates this information and works with public health to undertake community-level actions to address risk factors. The Center also works with public health agencies to: (a) address the social determinants of health with respect to dementia risk; (b) build capacity to enable smaller agencies to engage in risk reduction activities; and (c) partner with health systems to advance risk reduction.

### BUILDING OUR LARGEST DEMENTIA (BOLD) INFRASTRUCTURE FOR ALZHEIMER'S ACT: CENTERS OF EXCELLENCE

Lisa McGuire, *CDC, Atlanta, Georgia, United States*

While public health experts cannot yet say how to prevent Alzheimer's disease and related dementias (ADRD), emerging science indicates that ADRD may be slowed through the implementation of risk reduction strategies, early diagnosis, and better education and training of front-line health care professionals. The Building Our Largest Dementia (BOLD) Infrastructure for Alzheimer's Act was introduced into the Senate (S. 2076) on November 6, 2017 and signed into law on December 31, 2018 (P.L. 115-406) amending the Public Health Service Act (Section 398A; 42 U.S.C. 280c-3-4). CDC's Alzheimer's Disease and Healthy Aging Program (AD+HAP) was authorized to implement the activities outlined in BOLD which were designed to create a uniform national public health infrastructure. This presentation will provide an overview of BOLD and the demonstrate how BOLD Program and Public Health Centers of Excellence recipients are working together to build a uniform national public health infrastructure.

### SUPPORTING DEMENTIA CAREGIVERS: THE PUBLIC HEALTH CENTER OF EXCELLENCE ON DEMENTIA CAREGIVING

Joseph Gaugler, *University of Minnesota, Minneapolis, Minnesota, United States*

The Public Health Center of Excellence on Dementia Caregiving has established a robust, national network of 17 organizations to jointly develop and disseminate information, tools, and resources to support families, friends, and other unpaid caregivers of people living with dementia. The Center is establishing partnerships with local, state, tribal, and other public health organizations throughout the United States to provide technical assistance on the sustainability of dementia caregiving support programs as well as the cultural adaptation of messaging and tools to best meet the needs of diverse communities The Center also has a robust stakeholder engagement process so that the voices of caregivers and public health providers are incorporated in all Center efforts. The Center also collaborates with the Public Health Centers of Excellence on Risk Reduction and Early Detection to develop and refine cross-cutting resources and tools to further meet the complex public health challenges of dementia.

### PROMOTING EFFECTIVE DIAGNOSIS: THE PUBLIC HEALTH CENTER OF EXCELLENCE ON EARLY DETECTION OF DEMENTIA

Joshua Chodosh, *New York University Grossman School of Medicine, New York City, New York, United States*

The Public Health Center of Excellence on Early Detection of Dementia brings together a broad coalition of stakeholders to assure widespread awareness of why early detection of dementia matters. The Center assesses and disseminates the best evidence supporting early detection and how to accomplish it – the first step on the pathway to high quality, effective diagnosis and health care for people living with dementia. The Center's advisory group represents key state and county Departments of Health, healthcare systems and primary care providers, community organizations, and professional societies. The Center identifies effective materials and marketing strategies for increasing awareness about cognitive impairment, the value of earlier detection, and how best to conduct detection strategies. The Center works closely with state, county, and tribal public health agencies in dissemination, and seeks to establish collaborative linkages with healthcare systems and their primary care providers to build strategies for post-detection diagnosis and care.

### PUTTING IT TOGETHER: THE COMPLEMENTARY EFFORTS OF PUBLIC HEALTH AND AGING TO ADDRESS DEMENTIA

Nora Super, *Milken Institute Center for the Future of Aging, Santa Monica, California, United States*

The Public Health Centers of Excellence are part of a larger effort by the Centers for Disease Control and Prevention (CDC), through the BOLD Infrastructure for Alzheimer's Act and the Healthy Brain Initiative, to advance cognitive health and dementia as public health issues – and to expand the public health response to the Alzheimer's crisis. These efforts are designed to complement, not supplant, the long-standing work of the aging community to help people with dementia and their families. This presentation will discuss how the new and expanded public health work corresponds with the efforts of the aging community and will illustrate the unique roles of the public health and aging communities in the fight against Alzheimer's. In addition, the presentation will review initiatives – such as the Milken Institute's Alliance to Improve Dementia Care – that seek to bring both communities together on a common dementia agenda.

### SESSION 1570 (SYMPOSIUM)

### ARTIFICIAL INTELLIGENCE AND ROBOTIC APPROACHES TO SUPPORTING OLDER ADULTS

Chair: Walter Boot

Emerging technologies in the domains of artificial intelligence (AI) and robotics hold tremendous potential for supporting the health, wellbeing, independence, and quality of life of older adults. This session presents a sampling of research examining the promise of these technologies and barriers necessary to overcome in order for these technology-based solutions to be implemented, accepted, and adopted. This session will start with K. Trainum presenting a broad overview of the potential of robots to benefit individuals living in senior-living facilities, and a summary of the literature to date. L. Vergara will describe findings related to older adults' attitudes toward a socially assistive robot and implications for the design and implementation of artificial intelligence through socially assistive robots in the home environment. M. Han will present a study examining older adults' preferences for digital conversational agents that can be used to help monitor health, and factors that influence older adults' intention to adopt digital conversational agents. Finally, S. Zhang will discuss the potential of artificial intelligence approaches to maximizing adherence to technology-based cognitive assessment and training through a just-in-time adherence support system tailored to the motivations of the individual. An overarching theme of the session is the need for a careful, thoughtful, user-centered design approach to ensure the success of emerging technology-based solutions to support the health and wellbeing of older adults.

### ROBOTS IN SENIOR-LIVING FACILITIES: A SYSTEMATIC LITERATURE REVIEW

Katie Trainum[1], Elliott Hauser[2], and Bo Xie[1], *1. The University of Texas at Austin, Austin, Texas, United States, 2. University of Texas at Austin, Austin, Texas, United States*

Robots are promising technologies for improving both the care of older adults living in facilities and the job satisfaction of caregivers, but concerns about their efficacy, ethics, and best practices remain. To inform this ongoing debate, we examined the literature on robots utilized in senior-living facilities. Three rounds of searching and screening were conducted in February 2022, following PRISMA guidelines. Keyword searches produced 666 non-duplicate articles from 5 relevant databases. We screened titles and abstracts against inclusion/exclusion criteria, resulting in 127 articles. Full-text screening resulted in 78 articles for analysis, published between 2002-2022 and represent 17 countries. Most studies demonstrated promising effects of robots, however there was a lack of high-quality studies, overrepresentation of female participants, and focus on companion robots over service robots. Few studies addressed the effects of robots on caregivers or controlled for novelty effects. Research addressing these gaps should combine robotic and gerontological expertise.

### UNDERSTANDING THE ROLE OF A SOCIALLY ASSISTIVE ROBOT TO SUPPORT AGING IN PLACE

George Mois[1], Lizandra Vergara[2], Afnaan Afsar Ali[3], Mimi Trinh[4], and Wendy Rogers[3], *1. University of Illinois Urbana-Champaign, Urbana Champaign, Illinois, United States, 2. Federal University of Santa Catarina, Florianópolis, Santa Catarina, Brazil, 3. University of Illinois Urbana Champaign, Champaign, Illinois, United States, 4. University of Illinois Urbana-Champaign, Champaign, Illinois, United States*

Communities may lack the infrastructure and required resources to support healthy aging and aging in place. Advancements in technology development such as socially assistive robots and artificial intelligence present new opportunities to support and meet the needs of older adults. For example, Misty is a socially assistive robot designed to allow customizability and adaptability to support and meet diverse user needs pertaining to tasks and activities. Our research aim is to understand if Misty can facilitate social interactions with older adults, control the home environment, and provide reminders. We are exploring older adults' attitudes towards Misty through demonstrations of these activities. Through this research we provide insights pertaining to the facilitators and barriers in the acceptance and use of a socially assistive robot like Misty, to support healthy aging. Furthermore, our findings will provide guidance for design and implementation of artificial intelligence through socially assistive robots in a home environment.

### REFRAMING AGING: INTRA- AND INTERGENERATIONAL DIGITAL CONVERSATIONAL AGENTS TO SUPPORT OLDER ADULTS

Molly Han, and Cameron Piercy, *University of Kansas, Lawrence, Kansas, United States*

Digital conversational agents (DCAs) have become extraordinarily ubiquitous. Researchers envision the prospects of using DCAs to monitor health among older adults. However, older adults show hesitation to engage with DCAs. It is possible older adults prefer receiving human assistance rather than getting help from a machine. Another potential explanation is that communicative cues of DCAs such as voice need to be further optimized to invoke behavioral engagement. To understand how DCAs can better support older adults, we develop an experiment with three scenarios in which an agent (a human, an embodied DCA, a mixed presence of human and DCA) shares active aging information. We manipulate the agent's voice in terms of age (older voice, younger voice). We investigate how the interplay of agent categories and intragenerational/intergenerational voice cues affect older adult participants' evaluation of information and intention to adopt DCAs. Our study will contribute to DCAs design for older clients.

### THE DEVELOPMENT OF ADHERENCE SUPPORT MESSAGES FOR AN AI-BASED REMINDER SYSTEM

Shenghao Zhang, Michael Dieciuc, Andrew Dilanchian, and Walter Boot, *Florida State University, Tallahassee, Florida, United States*

Artificial Intelligence (AI) holds tremendous potential for aiding in the customization and tailoring of interventions. This talk will focus on the initial development of an adherence support system to maintain older adults' engagement with at-home cognitive training and assessment delivered via mobile devices. As part of the NIA funded Adherence Promotion with Person-centered Technology (APPT) project, a reminder and adherence support system is being developed to automatically detect adherence lapses and provide just-in-time reminders with tailored messaging to facilitate reengagement. The results of an initial study (N=40) will be presented in which participants were delivered reminders via SMS text messaging that were either tailored or not tailored to their self-reported motivation to engage with the intervention. Tailored messages were perceived as more motivating, and qualitative data informed additional ways in which messages might further be tailored based on individual preferences to support adherence.

### SESSION 2000 (SYMPOSIUM)

### A POLICY- AND PRACTICE-ALIGNED RESEARCH AGENDA FOR ENGAGING AND SUPPORTING DEMENTIA CAREGIVERS IN CARE DELIVERY

Chair: Catherine Riffin Co-Chair: Francesca Falzarano Discussant: Sara Czaja

The Cornell Institute for Translational Research on Aging (CITRA) Research-to-Practice Consensus Workshop Model is an evidence-based method for generating practice- and policy-informed research agendas on aging-related topics. The model aims to bridge the gap between research-based knowledge and practice-based insight by involving multidisciplinary stakeholders in all aspects of the agenda-setting process. Using the CITRA model as a guiding framework, we convened an NIA-funded Conference on Engaging Family Caregivers of Persons with Dementia in Healthcare Delivery, with the goal of generating actionable research priorities that will lead to improvements in dementia caregiver identification, assessment, and support in health and long-term care settings. Conference attendees were multidisciplinary thought leaders representing five stakeholder groups: family caregivers of persons with dementia, healthcare providers, researchers, payers, and policy advocates. This symposium will describe the CITRA model, using our caregiver conference as an example, and provide practical guidance on how to use the model to promote cross-disciplinary dialogue and integrate research, policy, and practice perspectives. The first presentation will provide an overview of the CITRA model and its 5-step method (Pillemer). Subsequent presentations will describe the model's application to the topic of improving caregiver identification and support in care delivery (Griffin) and discuss how novel technology-based adaptations to the model helped to facilitate hybrid participation of virtual and in-person attendees (Falzarano). The final presentation will delineate the major priorities that resulted from the conference, discuss ongoing and future dissemination activities, and offer practical suggestions for leveraging the CITRA model in future consensus efforts (Riffin).

### A FRAMEWORK FOR IDENTIFYING MAJOR RESEARCH PRIORITIES: THE CITRA RESEARCH-TO-PRACTICE CONSENSUS WORKSHOP MODEL

Karl Pillemer, *Cornell University, Ithaca, New York, United States*

The CITRA Research-to-Practice Consensus Workshop Model is an evidence-based method for identifying research priorities on aging-related topics. Its major goals are to promote knowledge translation and equal-status dialogue among stakeholders from multidisciplinary backgrounds. In this presentation, Dr. Pillemer, co-creator of the CITRA model, will provide an overview of the 5-step method: (1) Identifying a topic that is both an important challenge in aging-related policy or practice and one on which there is scientific evidence; (2) Selecting a panel of multidisciplinary experts from relevant stakeholder groups; (3) Preparing a non-technical background document that summarizes available research findings; (4) Convening multidisciplinary experts in a consensus conference involving panel presentations, discussion of the background document, and consensus activities; and (5) Preparing a final consensus report and disseminating the findings to relevant policy organizations, practice groups, and funding agencies. Common challenges and practical tips for executing each of the 5 steps will be addressed.

### CONVENING A NATIONAL CONSENSUS CONFERENCE TO PROMOTE DEMENTIA CAREGIVERS' ENGAGEMENT AND SUPPORT IN CARE DELIVERY

Joan Griffin, *Mayo Clinic, Rochester, Minnesota, United States*

The goal of the 2021 Conference on Engaging Family and Other Unpaid Caregivers of Persons with Dementia in Healthcare Delivery, funded by the NIA, was to establish a policy- and practice-aligned research agenda for enhancing dementia caregivers' engagement and support in health and long-term care settings. Using the CITRA model as a guiding framework, Dr. Griffin will discuss important planning decisions leading up to the event, including the formation of a Steering Committee, selection of expert panelists and conference participants, and preparation of the non-technical background report. Her presentation will also describe the format of the consensus conference itself, focusing on the structure of the panel presentations and consensus activities, including small group breakout sessions in which conference attendees were charged with generating research recommendations; the process for voting on and prioritizing the identified recommendations; and method for refining and organizing the recommendations into major priority areas.

### LEVERAGING TECHNOLOGY TO OPTIMIZE THE ENGAGEMENT OF VIRTUAL PARTICIPANTS IN A NATIONAL CONSENSUS CONFERENCE

Francesca Falzarano[1], and Lilla Brody[2], *1. Weill Cornell Medicine, Douglaston, New York, United States, 2. Weill Cornell Medicine, New York, New York, United States*

The COVID-19 pandemic has accelerated the uptake and use of technology in hosting virtual and hybrid (combining virtual and in-person) meetings and events. In this presentation, Dr. Falzarano will describe the ways in which the 2021 Conference on Engaging Family and Other Unpaid Caregivers of Persons with Dementia in Healthcare Delivery leveraged videoconferencing systems (e.g., Zoom) and other web-based platforms (e.g., Qualtrics) to enact a hybrid event. Specifically, she will discuss the various components involved in the event's planning and execution, including the appointment of a virtual liaison and audiovisual technicians to enable seamless integration and participation of both in-person and virtual attendees. She will also discuss how videoconferencing technology was used to facilitate the delivery of virtual panel presentations; strategies for immersing virtual attendees in both large group discussions and small group breakout sessions; and the process by which virtual attendees participated in the priority vote.

### ENGAGING AND SUPPORTING FAMILY CAREGIVERS IN CARE DELIVERY: RECOMMENDATIONS FROM A NATIONAL CONSENSUS CONFERENCE

Catherine Riffin, *Weill Cornell Medical College, New York, New York, United States*

This presentation, by Consensus Conference Director, Dr. Riffin, will describe the major priorities identified by the 2021 Conference on Engaging Family Caregivers of Persons with Dementia in Healthcare Delivery. Recommendations centered on the need for multidisciplinary collaborations that attend to (1) Identification and assessment of dementia caregivers in care delivery settings, (2) Reimbursement and financing for caregiver assessment and support, (3) Caregiver training and support across the care continuum, (4) Healthcare provider education on family-centered care, and (5) Technology innovations that support dementia caregivers. Across all areas, there was consensus regarding the need to leverage implementation science to promote the uptake of evidence-based interventions in clinical practice and use community-driven approaches to engage stakeholders from traditionally marginalized groups in future and ongoing initiatives. Findings are being used to inform federal agencies and foundations about high-priority areas, provide guidance to national committees, and accelerate programmatic research on caregiver engagement and support.

### SESSION 2010 (SYMPOSIUM)

### ADVANCING COMMUNITY-BASED SOCIAL INNOVATIONS FOR AGING THROUGH ENGAGED RESEARCH

Chair: Emily Greenfield Discussant: Suzanne Kunkel

Community-based social innovations (CBSI) for aging are programs, initiatives, and other structures that support healthy aging and aging in community in ways that complement traditional health and human service systems. This symposium features research on several manifestations of CBSIs in the US. The first paper orients to innovation among aging services agencies, drawing on data from local Councils on Aging in Massachusetts to examine variation in their levels of engagement with age-friendly community (AFC) work. Findings elucidate the importance of community context and organizational capacity for deepening involvement in AFC work over time. Addressing innovation within communities as a whole, the second paper applies social network analytic methods to examine the constellations of connections among AFC core teams and their key partners across nine localities in New Jersey. Focusing on housing, the third paper presents findings from an evaluation of a national demonstration program to enhance the capacity of service coordinators within senior housing communities. This paper implicates the important role of services staff in systems innovation. Finally, with attention to creating age-friendly communities, the fourth paper presents findings from a project in Ohio that equipped older adults with a tablet-based transportation "diary" app. The findings demonstrate ways in which this technology facilitated deeper insight on users' experiences in terms of their mood, with implications for transit design and programs. Collectively, the studies make evident the importance of leveraging the tools of research alongside engaged partnerships to strengthen the knowledge base for continued innovation in community supports for aging.

### EVALUATING AGE-FRIENDLY COMMUNITY INITIATIVES FROM A SOCIAL NETWORK PERSPECTIVE

Emily Greenfield, and Althea Pestine-Stevens, *Rutgers, The State University of New Jersey, New Brunswick, New Jersey, United States*

Age-friendly community initiatives (AFCIs) as community interventions systematically cultivate inter-organizational partnerships toward making policies, systems, and environments more supportive of healthy aging, aging in community, and aging equity. Building from a multi-year, university-community partnership to spur the development of AFCIs in New Jersey, we drew on social network theory and research methods to examine the networks of AFCI core teams and their key partners across diverse community settings. Data collection involved administering questionnaires to both AFC core teams and their key organizational partners. We report on the presence and strengths of dyadic relationships that comprise the networks, analyzed by sector, organizational function, and geographic service area of each network node. We discuss implications of the findings concerning policies and practices around sustainability planning for AFCIs as well as future directions for using network theory to understand AFC progress at various levels of geographic scope.

### AGE-FRIENDLY TRANSFORMATION: AN EXAMINATION OF COMMUNITY MECHANISMS INFLUENCING INVOLVEMENT

Caitlin Coyle, and Ceara Somerville, *University of Massachusetts Boston, Boston, Massachusetts, United States*

Age-friendly community initiatives (AFCIs) have become key policy efforts aimed at improving quality of life for older residents, but there is limited evidence about the process. This mixed methods study draws on survey and demographic data from 350 municipalities in Massachusetts to characterize communities by these categories: 1) not interested in AFCIs (n=109); 2) interested in learning more about AFCIs (n=84); 3) planning for age-friendly action (n=71); and 4) maintaining an AFCI (n=86). Interview data from key-informants contextualize the process of developing an AFCI. Thematic analyses suggest that progression through AFCIs is self-defined by the accumulation of momentum. Communities committed to AFCIs have higher proportions of vulnerable residents (e.g., living with disability, living alone, non-English speaking). Municipal resources (e.g., budget, aging services) correlate with more advanced stages of AFCIs. Implications of the variability across AFCIs, including the effort required for moving from concept to execution of AFCIs, will be discussed.

### LESSONS LEARNED FROM AN ENHANCED SERVICE COORDINATION MODEL IN SENIOR HOUSING

Alexandra Hennessa, and Robyn Stone, *LeadingAge, Washington, District of Columbia, United States*

Residents of affordable housing communities are growing older, and new residents are moving in at older ages. As these residents age, their need for services and supports increase. Approximately 5,200 housing communities subsidized by the U.S. Department of Housing and Urban Development (HUD) have a service coordinator on-site to help connect residents with needed services and resources. In 2021, the LeadingAge LTSS Center @UMass Boston (LTSS Center) conducted a process evaluation of the LSA Senior Connect Model, which was designed to build the capacity of service coordinators to better meet the needs of aging residents. The study activities included a review of program-related documents, analysis of resident assessments, interviews with staff, resident focus groups, and cost analysis. We will summarize the model's framework and tools, and discuss lessons learned from the process evaluation. Lessons for policy and practice will also be discussed.

### TRANSPORTATION AND MOOD: THE ROLE OF TRIP CHARACTERISTICS

Holly Dabelko-Schoeny[1], Noelle Field[2], Anthony Traver[3], Ian Murphy[3], and Katie White[3], *1. Ohio State University, Columbus, Ohio, United States, 2. University of Texas -- Arlington, Arlington, Texas, United States, 3. The Ohio State University, Columbus, Ohio, United States*

Safe and affordable transportation has a positive impact on the health and well-being of older adults. What is less understood are which factors influence these outcomes. To examine the impact of trip characteristics on the mood of older adults, residents in three neighborhoods in Franklin County, Ohio (n = 32) were provided tablets and used an app (MyAmble) to document their travel. During a 14-day period, 1,190 trips were recorded; 71% of which were completed by car. Participants reported 72% of the trips improved their mood. Perceived importance of the trip, challenges associated with the trip, and trip destinations to social activities and to employment/education explained 33% of the variance in mood. Challenges associated with the trip was the strongest predictor of impact on mood. Identifying trip characteristics that impact mood provides new insights for the design and implementation of travel interventions for older persons.

### SESSION 2020 (SYMPOSIUM)

### BEFORE IT WAS COOL: THE AGING NETWORK'S ROLE IN ADDRESSING SOCIAL NEEDS AND REDUCING DISPARITIES

Chair: Traci Wilson Discussant: Suzanne Kunkel

Decades before social determinants of health (SDOH) became such a focus of attention across health and social care, Area Agencies on Aging (AAAs), Title VI Native American Aging Programs, and their Aging Network partners had developed local systems of coordinated services and supports to assess and address health-related social needs. Every service and program provided by an AAA or Title VI program addresses a need related to SDOH risks. The Older Americans Act mandates that funded services such as congregate and home-delivered meals, support for caregivers, transportation, and housing supports are targeted to older adults with the greatest economic or social need. Using the foundational study on "ACL's Impact on the Social Determinants of Health" as a framework, this symposium will demonstrate how OAA-funded programs map to SDOH pathways. Presenters will describe specific programs and new practices within the Aging Network that identify and address social needs of older adults from historically marginalized and underserved communities. These initiatives, many of which were born through COVID response, include analyses and partnerships to advance health equity and care integration. Presenters will describe how Aging Network practices and partnerships have altered community environments to make community living accessible to older adults who have been traditionally underserved, and current workforce challenges that threaten continued progress. The session will conclude with an interactive discussion about gaps, strategies, and next steps to reinforce, articulate and demonstrate the value of Aging Network services in addressing SDOH risks and needs.

### "NO TIME TO OVERTHINK IT, JUST DO IT": AREA AGENCIES ON AGING AND INCLUSIVE COMMUNITY ENGAGEMENT

Traci Wilson, and Elizabeth Blair, *USAging, Washington, District of Columbia, United States*

AAAs have always been deeply engaged in addressing the health-related social needs of older adults from historically marginalized and underserved communities. As a confluence of COVID and social injustices moved issues of racial equity and inclusion to the forefront of national conversation, AAAs refocused their efforts to identify and address health inequities related to the social determinants of health (SDOH). One result of the COVID-19 pandemic and the urgency to reach and vaccinate older adults was that AAAs quickly formed new partnerships with culturally specific organizations. These relationships have developed and resulted in expanding culturally responsive service delivery. Presenters will describe this and other findings from a mixed methods study about AAA initiatives that seek to improve access and equity, such as inclusive community needs assessments, equity analyses that leverage census data to identify areas of greatest SDOH disparities, and dashboards to compare service recipients with community demographics.

### OLDER AMERICANS ACT PROVISIONS AND SOCIAL DETERMINANTS OF HEALTH: INTERSECTIONS AND IMPACTS

Shannon Griffin[1], Robbie Skinner[1], Suzanne Kunkel[2], Elizabeth Blair[3], Lois Simon[1], Robert Graham[2], Traci Wilson[3], and Kristen Hudgins[4], *1. Insight Policy Research, Arlington, Virginia, United States, 2. Miami University, Oxford, Ohio, United States, 3. USAging, Washington, District of Columbia, United States, 4. Administration for Community Living, Washington, District of Columbia, United States*

Older Americans Act (OAA) programs and services are strongly linked to social determinants of health (SDOH) and integral to addressing social needs among older adults and individuals with disabilities and their families. Supported by the Administration for Community Living, researchers examined the intersection of OAA-funded programs and services under Titles III, VI, and VII and their impacts on SDOH. This study used an integration of extant literature; secondary data; and interviews with ACL office leadership, AAAs, Title VI program staff, and other local service providers to examine existing social needs among older adults, equity prioritization within OAA-funded program development, and impacts of OAA-funded programs and services. Study findings highlighted relationships between ACL and Aging Network entities, best practices for and process challenges of transforming grant funding into recipient services, and OAA-funded program areas for possible future development.

### AAA WORKFORCE CHALLENGES AND THEIR IMPACT ON OLDER ADULTS: RESULTS FROM A NATIONAL POLL AND AGENCY INTERVIEWS

Elizabeth Blair, and Traci Wilson, *USAging, Washington, District of Columbia, United States*

Area Agencies on Aging (AAAs) play a critical role in addressing social needs that enable older adults to live independently. In 2022, over 90% of AAAs reported that the number of consumers seeking services and the complexity of consumer needs have increased. At the same time, the nation faces a shortage of direct care workers. Existing AAAs workforce challenges, such as worker shortages and staff burnout, have been exacerbated by the COVID-19 pandemic. We will share the results of a USAging poll of AAAs on the challenges facing their agency and provider workforce, reductions in volunteer staffing, and the impacts of workforce shortages on clients and services provided. Top impacts include clients not receiving the frequency of services needed, if at all. Services most impacted include personal care, respite, and transportation. Presenters will conclude by sharing innovative solutions that AAAs have developed to address these challenges.

### COMMUNITY-BASED ORGANIZATIONS CONTRACTING WITH HEALTH CARE PARTNERS TO ADDRESS SOCIAL DETERMINANTS OF HEALTH

Robert Graham, and Isha Karmacharya, *Miami University, Oxford, Ohio, United States*

Community Based Organizations (CBOs), such as Area Agencies on Aging, play a vital role in their communities by offering a range of services that address the health-related social needs of older adults and people with disabilities. As health care entities (HCEs) become more aware of the effects of unmet social needs on health outcomes, there has been a significant increase in the number of HCEs contracting with CBOs to provide a wider range of services and care coordination for older adults and people with disabilities. Using findings from four waves of the national Aging and Disability Business Institute's CBO-Health Care Contracting Survey (2017–2021), presenters will describe trends in CBO/HCE contracting, including increased diversification of partnerships with health care payers and providers, services provided, and populations served. Results suggest that more CBOs are forming integrated networks to improve contracting with HCEs, and that assessment for SDOH is the most common service.

### SESSION 2030 (SYMPOSIUM)

### BUILDING BRIDGES: AN AGE-FRIENDLY CONFERENCE FOR THE GEROPSYCHOLOGY WORKFORCE

Chair: Brian Carpenter Co-Chair: Jennifer Moye
Discussant: Joseph Dzierzewski

As in other disciplines, there is a high demand for psychologists who have specialized training in aging, but the demand far outstrips the supply, with only 1% of clinical/counseling psychologists identifying aging as their area of focus. The field of geropsychology has held a series of training conferences, the last in 2006, to define a training model and aging-related competencies. Fifteen years later, the field gathered again for a conference focusing specifically on the pipeline, with the goals of 1) understanding the recent shortage of applicants for positions in academic settings, 2) addressing underrepresentation of individuals from diverse racial and ethnic backgrounds across clinical and academic geropsychology, and 3) implementing concrete solutions. In this symposium we describe our two-day, four-hour virtual national conference held in 2021, attended by more than 150 psychologists nationwide, including the structure and outcomes of the conference and the progress of several ongoing working groups. The first paper summarizes quantitative and qualitative findings of a pre-conference survey on perceptions of the geropsychology workforce. The second paper describes a career pathways webinar aimed at graduate students, interns, and fellows to attract students to diverse careers in aging. The third paper describes discussions about the impact of the pandemic on geropsychology training. The fourth paper presents a survey and efforts of a working group focused on post-licensure training. This presentation offers a possible model for others considering ways to galvanize interest and training in aging.

### PERCEPTIONS OF THE GEROPSYCHOLOGY WORKFORCE

Jennifer Moye[1], Flora Ma[2], Nicholas Schmidt[3], Hannah Heintz[4], Rebecca Allen[5], and Brian Carpenter[6], *1. VA Boston Healthcare System, Boston, Massachusetts, United States, 2. Stanford HealthCare Psychology, Palo*

*Alto, California, United States, 3. Boston VA Healthcare System, Boston, Massachusetts, United States, 4. VA Boston Healthcare System, Jamaica Plain, Massachusetts, United States, 5. The University of Alabama, Tuscaloosa, Alabama, United States, 6. Washington University in St. Louis, St. Louis, Missouri, United States*

There is a longstanding shortage of teaching faculty and clinicians trained in psychology and aging (Moye et al. 2019). Further, there are few marginalized group members in the geriatric workforce. To better understand this issue, a survey was distributed to psychology and aging listservs in preparation for the "Building Bridges" conference. Problems noted by respondents (N=275) included fewer applicants for aging-related positions (42%), decreased interest in aging by students (32%), loss of aging-related positions (18%); 24% thought workforce problems are worse than 5 years ago. Similar themes emerged in qualitative comments including: (1) lack of applicants/ interest, (2) lack of or decline in the training/ education opportunities, (3) lack of finances/ funding/ resources, (4) lack of professional positions, and (5) positive experiences/ actions/ change. Themes specific to marginalized group members to support diversity, equity, and inclusion include mindful commitment, education (e.g., mentorship), and recognizing not doing enough.

### BUILDING BRIDGES POST-LICENSURE: DEVELOPING GEROPSYCHOLOGY CONSULTATION SERVICES

Erin Emery-Tiburcio[1], Erin Kube[2], Cecilia Poon[3], and Ann Steffen[4], *1. Rush University Medical College, Chicago, Illinois, United States, 2. VA Salt Lake City Healthcare System/ VISN 19 Clinical Resource Hub, Salt Lake City, Utah, United States, 3. Nebraska Medicine, Omaha, Nebraska, United States, 4. University of Missouri-St. Louis, St. Louis, Missouri, United States*

More than one third of psychologists report frequently treating older adults, while only 1% consider themselves geropsychologists. Continuing education opportunities in geropsychology have historically been scant, with even fewer opportunities for expert case consultation in work with older adults. To identify interest in geropsychology consultation, the Building Bridges Post-Licensure (BBPL) group conducted an informal survey of licensed psychologists via listservs. Results among 80 respondents indicated strong interest in group consultation regarding foundational knowledge, assessment, and intervention. This presentation will describe the BBPL group's efforts to examine effective models of consultation, develop a consultation model for geropsychology, and partner with both professional and community-based organizations to develop the infrastructure for geropsychology consultation.

### BUILDING BRIDGES TO FUTURE LEADERS: A CAREER PATHWAYS WEBINAR

Meghan McDarby, *Memorial Sloan Kettering Cancer Center, New York, New York, United States*

Educational webinars can improve attitudes toward careers in aging. We developed a 6-session webinar series about careers in geropsychology, detailing unique career pathways (e.g., academic, private practice, VA). Our evaluation of the series included trainees from graduate programs and postdoctoral fellowships. Participants rated their attitudes toward each career pathway at pre- and post-session. At post-session, participants reported significantly increased interest in careers in aging ($t(83) = 4.72$, $p < .00$). They also reported significantly increased understanding of the type of training required for careers in aging ($t(77) = 8.10$, $p < .00$). Importantly, at post-session, participants reported stronger beliefs that they could be successful in a career in aging ($t(84) = 3.86$, $p < .00$) and that they would have good work-life balance ($t(87) = 9.34$, $p < .00$). Results suggest that a webinar series may increase student interest in and understanding of unique career pathways in geropsychology.

### THE UNIQUE IMPACT OF THE COVID PANDEMIC ON PREDOCTORAL GEROPSYCHOLOGY TRAINING: RECOMMENDATIONS FROM THE FIELD

Candice Reel[1], Hannah Bashian[2], Julia Boyle[3], Mary Jacobs[4], and Michelle Mlinac[2], *1. The University of Alabama, Tuscaloosa, Alabama, United States, 2. VA Boston HCS, Boston, Massachusetts, United States, 3. New England GRECC, Boston, Massachusetts, United States, 4. Tuscaloosa VA Medical Center, Tuscaloosa, Alabama, United States*

The COVID-19 pandemic impacted predoctoral psychology training at the graduate, practicum, and internship levels including a greater reliance on telehealth and evolving learning needs. However its unique impact on geropsychology training has not been explored. The Building Bridges workgroup for predoctoral training faculty levels, we describe opportunities and barriers to address evolving geropsychology training needs during the pandemic as determined through working group discussion. Negative impacts to training identified include: decreased opportunities for 1) face-to-face patient care and 2) telehealth care due to disparities in telehealth access and utilization in older adults. Other impacts on the geropsychology pipeline include declining opportunities to see older adults at practicum sites. Conversely, increased media attention to the impact of COVID on older adults' physical and mental health may lead to graduate students' having greater interest in geriatric mental health and reinforcing a geropsychology career. Recommendations for training programs to address the long-term ramifications of the pandemic will be offered.

### SESSION 2040 (SYMPOSIUM)

### EDITORS INSIGHTS ON PUBLISHING OPPORTUNITIES FOR THE GLOBAL GERONTOLOGICAL COMMUNITY

Chair: Edward Miller Co-Chair: Elizabeth Simpson

Global aging has proceeded at an unprecedented and accelerating rate. The aging of the population creates both opportunities and challenges for older adults, their families, and society in general. Importantly, there is substantial variation in the effects of and response to global aging both within and across nations depending, in part, on prevailing cultural expectations and values, political and economic imperatives, and social and demographic characteristics. Thus, while some regions and countries have responded with innovative policies and programs to better enable the growing cohort of older adults to remain active and engaged in the community, other regions and countries have struggled with their response or barely begun to plan for the rising population of older adults. This symposium assembles editors at five leading gerontology journals to demonstrate the role that peer-reviewed scholarship can play in disseminating knowledge that informs gerontological research, policy, and practice internationally. Editors include: Edward Alan Miller, PhD, FGSA, Journal of Aging & Social Policy; Jeffrey Burr, PhD, FGSA, Research on Aging; Julie Robison, PhD, FGSA,, Journal of Applied Gerontology; Sandra Torres, PhD, FGSA, Ageing & Society; and Julie Hicks Patrick, PhD, FGSA, International Journal of Aging & Human Development. Each presenter will review the scope, content, and focus of their journals and the role and opportunities for international scholarship.

### THE ROLE OF GLOBAL SCHOLARSHIP IN THE JOURNAL OF AGING & SOCIAL POLICY

Edward Miller[1], Elizabeth Simpson[1], Michael Gusmano[2], and Pamela Nadash[1], *1. University of Massachusetts Boston, Boston, Massachusetts, United States, 2. Leigh University, Bethlehem, Pennsylvania, United States*

Policymakers, practitioners, and researchers need a balanced, thoughtful, and analytical resource to meet the challenge of global aging at a rate that's historically unprecedented. The Journal of Aging & Social Policy (JASP), which was founded in 1989, serves this role by drawing contributions from an international panel of policy analysts and scholars who assume an interdisciplinary perspective in examining and analyzing critical phenomena that affect aging and the development and implementation of programs for older adults from a global perspective. Study settings extend beyond the United States to include Europe, the Middle East, Australia, Latin America, Asia, and the Asia-Pacific rim. This presentation will document the scope, content, and focus of JASP, including the rise of international submissions, which now account for approximately half of articles published. Opportunities for publishing in JASP will be discussed; so too will strategies for navigating the peer-review process successfully.

### RESEARCH ON AGING: THE INTERNATIONAL VIEW FROM THE EDITORS' DESKS

Jeffrey Burr[1], Chagmin Peng[1], and Kyungmin Kim[2], *1. University of Massachusetts Boston, Boston, Massachusetts, United States, 2. Seoul National University, Seoul, Seoul-t'ukpyolsi, Republic of Korea*

We review the scope, content, and focus of the peer-reviewed journal, Research on Aging (SAGE), publishing its 45th volume this year. We will discuss how scholarship produced from researchers around the globe has changed over the years. Data on submissions, acceptance rates, and the important role of an international editorial board will be presented. The review process will be described, along with suggestions on how to increase chances of success when submitting original research. Although Research on Aging is sometimes considered to focus primarily on social gerontology, the scope in recent years has widened considerably, with manuscripts in aging studies published from such fields as economics, psychology, demography, public health, and public policy, as well as from sociology, and social work, among others. One of several special issues forthcoming in the journal will be described to demonstrate the possibilities for international impact.

### THE JOURNAL OF APPLIED GERONTOLOGY: AN INTERNATIONAL PERSPECTIVE

Julie Robison, *UConn Health, Center on Aging, Farmington, Connecticut, United States*

The mission of applied gerontology is to bridge science and practice to benefit the health and well-being of older people, their families, their communities, and other contexts. The Journal of Applied Gerontology features original investigations or meta-analyses/systematic reviews with significant clinical, policy, and/or practice implications. This presentation will provide insights from the Journal of Applied Gerontology and its commitment to publish and disseminate scholarship with international application. Following an overview of the growing internationalization of peer-reviewed submissions to the Journal of Applied Gerontology on a variety of topics and from a range of perspectives, the presentation will highlight opportunities to apply gerontological scholarship to aging contexts worldwide. Concluding comments will examine how outlets for dissemination and authors themselves can better position their work to enhance their influence on aging in an international context.

### TIPS FOR TIMELY (AND POSITIVE) REVIEWS: THE INTERNATIONAL JOURNAL OF AGING & HUMAN DEVELOPMENT

Julie Hicks Patrick[1], Abigail Nehrkorn-Bailey[2], and Danielle Nadorff[3], *1. West Virginia University, Morgantown, West Virginia, United States, 2. University of Wisconsin-Green Bay, Green Bay, Wisconsin, United States, 3. Mississippi State University, Mississippi State, Mississippi, United States*

For more than 40 years, under the leadership of four editors and two publishers, The International Journal of Aging and Human Development (IJAHD) has featured multidisciplinary scholarship related to aging processes and older adults. With the publication of eight issues a year, with over 800 pages of scientific content, the IJAHD places emphasis upon psychological and social studies of aging and the aged. However, the Journal also publishes research that integrates observations from other disciplines that illuminate the "human" side of gerontology. A more recent focus includes midlife development, as well. About half (47%) of the publications in the IJAHD are from international colleagues. This presentation will discuss tips for both international and US-based scholars for ensuring timely reviews and positive decisions for manuscript submissions, including such areas as key words, suggesting unbiased reviewers, formatting, writing mechanics, clearly-articulated methods, and a sound theoretical basis.

### PUBLISHING IN INTERNATIONAL JOURNALS AND THE EXPECTATION IT PLACES ON OUR WRITING: TIPS FROM AGEING & SOCIETY

Sandra Torres, *Uppsala University, Sweden, Uppsala, Uppsala Lan, Sweden*

Understanding the differences between academic writing genres is crucial to nailing down the craft that is writing for peer-review journal publication. Based on the experience of Ageing & Society, this presentation will bring attention to the different readers that peer-reviewed journals cater to and how these differences impact how we craft manuscripts about the research we conduct. Using this as a starting point, as well as allusions to how data collected in specific national settings can be reported so that it can be used to contribute to ongoing international debates in social gerontology, this presentation offers the Editor-in-Chief's top 10 tips for publishing in international peer-reviewed journals. By bringing specific attention to the routines that Ageing & Society utilize, and the expectations that its editors place on the different sections of a manuscript, this presentation aims to give attendees valuable insights into how to write for peer-reviewed outlets.

### SESSION 2050 (SYMPOSIUM)

### ENGAGING METHOD AND ANALYSIS TO DISCOVER AND REIMAGINE POSSIBILITIES FOR INDIVIDUALS AGING WITH AND INTO DISABILITY

Chair: Kelly Munly Co-Chair: Ted Ng Discussant: Lieke van Heumen

This symposium engages diverse methods and analyses to discover and reimagine possibilities for individuals aging with and into disability. The presentations advance an understanding of the diversity of service/care needs as well as of social capital, with exploration into assistive technologies, educational attainment, and assessment of successful aging. In the first presentation, the authors delineate key informant interview data identifying capacity-building needs in professional skills, organizational operations, service/care models, and public policies to effectively serve older adults aging with and into disability. The second presentation offers a content analysis of how researchers conceptualize, measure, and apply social capital to developmental disability throughout the life course, reviewing peer-reviewed articles from 5 disciplines, across more than 20 years. In the third presentation, using National Health and Aging Trends Study data, the authors investigate the longitudinal associations between disabilities, assistive technologies, and subjective well-being among older adults. In the fourth presentation, the author uses multivariate regression analysis to analyze National Health Interview Survey (NHIS) data, building on prior research analyzing the association between educational attainment and the perceived need for future help with activities of daily living (ADLs), often measuring disability in the literature. In the fifth and final presentation, the authors use the National Health & Aging Trends Study data to understand successful aging from the perspectives of those who experience disability, integrating subjective components of self-rated health and well-being, and providing insights on subjective perceptions on successful aging among older adults with disabilities.

### INCREASING SOCIAL CARE CAPACITY TO MEET THE NEEDS OF PERSONS AGING WITH DISABILITY

Michelle Putnam[1], Lieke van Heumen[2], Lydia Odgen[1], Caitlyn Coyle[3], and Kerri Morgan[4], *1. Simmons University, Boston, Massachusetts, United States, 2. University of Illinois at Chicago, Chicago, Illinois, United States, 3. University of Massachusetts, Boston, Boston, Massachusetts, United States, 4. Washington University School of Medicine in St. Louis, St. Louis, Missouri, United States*

This national study presents key informant interview data (N=28) collected 2017-2018 identifying capacity building needs in professional skills, organizational operations, service/care models, and public policies to effectively serve older adults with long-term serious mental illness, intellectual/developmental disabilities, physical and sensory disabilities. A multi-university, interdisciplinary team collected data through phone interviews, coded, and analyzed transcripts thematically and using content analysis. Key informants worked in national affinity organizations, state-level organizations, academia, and social care organizations. Findings are broad in scope, highlighting variances in rural/urban areas, state government dispositions, workforce characteristics, social and health care infrastructure, beliefs about disability and chronic conditions, and other factors. Findings will informed the development of a larger survey study aiming to articulate an agenda for building capacity to meet the needs of aging with disability populations.

### THAT'S WHAT FRIENDS ARE FOR: SOCIAL CAPITAL, DEVELOPMENTAL DISABILITY, AND THE LIFE COURSE

Jessica Hoyle, Jan Warren-Findlow, Lauren Wallace, James Laditka, and Sarah Laditka, *University of North Carolina at Charlotte, Charlotte, North Carolina, United States*

Social capital, resources from reciprocal relationships, helps us get by as we age. People with developmental disability have service needs that persist across the life course, which social capital can help address. However, social structures, communication problems and smaller social networks limit social capital for some people with developmental disability. We studied how researchers conceptualize, measure, and apply social capital to developmental disability research throughout the life course, reviewing peer-reviewed articles across 5 disciplines, from 2000 through February 2022. Of 673 studies, 71 met criteria. Studies used a common definition of social capital but no common measures. Fourteen studies focused on parents or other caregivers. Few included older adults with developmental disability as research participants. Results indicate a need to better understand social capital in the lives of people with developmental disability and how social capital resources can support and improve lives of people with developmental disability as they age.

### USING ASSISTIVE TECHNOLOGIES AS A MEASURE TO SUPPORT WELL-BEING IN LATE-LIFE DISABILITIES

Tai-Te Su[1], and Shannon Mejía[2], *1. The University of Illinois Urbana-Champaign, Champaign, Illinois, United States, 2. University of Illinois at Urbana-Champaign, Champaign, Illinois, United States*

In the United States, the trends of disabilities among older adults have been largely stable over the past decade. However, the impact of disabilities on health and quality of life remains substantial and requires continued research.

Along with a set of compensatory strategies, assistive technologies play a promising role in augmenting individuals' capacity and reducing environmental demands in daily activities. Using data from the five survey rounds of the National Health and Aging Trends Study (2015–2019), we aimed to investigate the longitudinal associations between disabilities, assistive technologies, and subjective well-being among older adults. A multi-class hierarchical spectrum was constructed to capture the state of disability and assistive technology use. Overall, results showed that subjective well-being decreased progressively along the spectrum. Additionally, assistive technologies were found to differentiate the associations between disabilities and well-being outcomes. Discussions focused on the insights and implications for successful accommodation to disabilities in later life.

### EDUCATION AND PERCEIVED FUTURE NEED FOR ADL HELP

Julia Finan, *Syracuse University, Syracuse, New York, United States*

Extant literature suggests that adults with higher education are more likely to avoid poor health outcomes and to rate their health as better than individuals with less education. The current study builds on prior research by analyzing the association between educational attainment and the perceived need for future help with activities of daily living (ADLs), often measuring disability in the literature. 2011-2014 National Health Interview Survey (NHIS) data for adults in the United States age 40 to 65 (N=55,166) were analyzed using multivariate regression analysis. Among non-Hispanic Whites, increased years of education predicted stronger anticipation of the need for future ADL assistance. For non-Hispanic Blacks, this relationship was reversed at the some-college level. Education was not predictive of perceived future ADL assistance need for all other racial-ethnic groups. Results of this study suggest education has a unique impact on anticipation of future need for ADL assistance among non-Hispanic Whites.

### CONCEPTUALIZING SUCCESSFUL AGING IN THE PRESENCE OF DISABILITY

Madina Khamzina, and Wendy Rogers, *University of Illinois Urbana Champaign, Champaign, Illinois, United States*

With the rapid demographic changes, it is predicted that every fifth person in the U.S. will be age 65 or older in the next decade. The quality of life and successful aging of older adults, who now live longer, is an important research area from societal and public health perspectives. Rowe and Kahn (1997) defined successful aging as having a low probability of disease and disability, high cognitive and physical functioning, and active engagement with life. A prominent limitation of this model is that it initially neglects essential components of aging and factors that are beyond an individual's choice and control, such as health declines and disabilities. We used the National Health & Aging Trends Study data to understand successful aging from the perspectives of those who experience disability integrating subjective components of self-rated health and well-being. These data provide insights on subjective perceptions on successful aging among older adults with disabilities.

### SESSION 2060 (SYMPOSIUM)

### ENSURING A KNOWLEDGEABLE GERIATRIC WORKFORCE: INTERDISCIPLINARY EDUCATION IN LONG-TERM SERVICES AND SUPPORTS

Chair: Gina Tucker-Roghi Co-Chair: Gail Towsley
Discussant: Linda Edelman

In this symposium, we describe five interdisciplinary educational programs to enhance the geriatric workforce in long-term services and supports (LTSS): Skilled nursing facilities (SNFs), assisted living, and home health and hospice. The interdisciplinary teams that came together to offer the programs leveraged technology to maximize access to experts in both the curriculum development process and the delivery of the educational programs for a wide range of target learners. The first program, an interdisciplinary telementoring series on dementia care for therapists in SNFs, was developed by a team of experts from occupational, physical and speech therapy. The program included asynchronous online didactic content and case-based learning relevant to therapists. The second program engaged faculty from seven graduate programs: Pharmacy, Physical Therapy, Occupational Therapy, Dentistry, Social Work, Nursing and Physician Assistant to co-create an interprofessional LTSS graduate certificate in Gerontology. The certificate encompasses asynchronous online courses to enable flexibility across programs and cover content specific to LTSS. In the third program, LTSS nurses completed an online educational course to better prepare them to care for complex older adults, improve leadership skills, become more resilient, and complete QAPI projects. The fourth program focused on utilizing interdisciplinary perspectives to educate LTSS direct care staff on the unique needs of LGBTQ older adults. The fifth program offered a virtual telehealth clinical experience to prepare physical therapy students for practice in a SNF setting. The curriculum included eight virtual modules and four telehealth patient encounters that exposed students to interdisciplinary teams in the SNF context.

### INTERDISCIPLINARY TELEMENTORING FOR THERAPISTS IN SNFS TO IMPROVE DEMENTIA CARE KNOWLEDGE AND SKILLS

Gina Tucker-Roghi, *Dominican University of California, Santa Rosa, California, United States*

Occupational, physical, and speech therapists possess knowledge and skills that have the potential to reduce the burden of care and improve the quality of life of skilled nursing facility (SNF) residents living with dementia. Despite this opportunity to impact the well-being of residents with dementia, many therapy practitioners lack specialized training on the management of dementia. Therapy practitioners (n=31) from 22 SNFs participated in eight weekly interdisciplinary educational sessions using the Project ECHO (Extension for Community Health Outcomes) tele-mentoring model. An interdisciplinary team of therapy experts collaborated on the development of the curriculum, which included didactic content on dementia-care best practices as well as case-based learning. This presentation describes the process of developing an interprofessional tele-mentoring therapy education program, challenges encountered during implementation, and strategies to retain learners. Evaluation data from participants will be shared and implications for future tele-mentoring interprofessional educational offerings for therapists will be discussed.

### DEVELOPING AN INTERPROFESSIONAL GRADUATE CERTIFICATE IN LONG-TERM SERVICES AND SUPPORTS

Gail Towsley[1], Leigh Elrod[2], Nicholas Cox[3], Alberto Varela[4], and Linda Edelman[5], *1. University of Utah, Salt Lake City, Utah, United States, 2. University of Utah Physician Assistant Studies, Salt Lake City, Utah, United States, 3. University of Utah College of Pharmacy, Salt Lake City, Utah, United States, 4. University of Utah School of Dentistry, Salt Lake City, Utah, United States, 5. University of Utah College of Nursing, Salt Lake City, Utah, United States*

The demand for long-term services and supports (LTSS) is increasing and the capacity and competencies of the interprofessional LTSS workforce need to be expanded to provide value-based, person-centered care. The purpose of this presentation is to describe the process of developing an interprofessional LTSS graduate certificate in Gerontology. We broadened an existing nursing graduate certificate to encompass content related to skilled nursing facilities, assisted living and home health and hospice agencies. We will discuss: 1) how we engaged faculty from seven graduate programs: Pharmacy, Physical Therapy, Occupational Therapy, Dentistry, Social Work, Nursing and Physician Assistant to co-create the certificate; 2) challenges and benefits to developing a certificate; and 3) the 15-credit hour certificate components. Our faculty partners were committed to offering a LTSS focused graduate certificate which includes didactic courses and clinical hours. Four students have enrolled in the LTSS graduate certificate in Gerontology since implementation in Spring 2021.

### TRAINING TO SERVE: INTERDISCIPLINARY EDUCATION ON LESBIAN, GAY, BISEXUAL, TRANSGENDER, AND/OR QUEER AGING

Rajean Moone, *University of Minnesota, St Paul, Minnesota, United States*

Contemporary research details the significant health, economic, and social disparities experienced by lesbian, gay, bisexual, transgender, and/or queer (LGBTQ) older adults in comparison to their peers. Fundamental to overcoming these disparities is service provider understanding of the unique needs and challenges faced by LGBTQ older adults. This presentation will focus on the development and implementation of the research-based, interdisciplinary curriculum Training to Serve (TTS). TTS is designed to provide LGBTQ age-sensitivity training to service providers and since its inception over 15,000 providers have been trained. Evaluation data from participants will be presented. The framework for the development of the curriculum, based on interprofessional education, adult learning, and minority stress theories, will be shared. Implications for future training development and translation to electronic modules will be discussed.

### IMPACT OF AN ONLINE COURSE ON GERIATRICS AND QUALITY IMPROVEMENT SKILLS FOR NURSES IN LONG-TERM SERVICES AND SUPPORTS

Jacqueline Telonidis[1], Kirstie Savage[2], Nanci McLeskey[2], and Linda Edelman[2], *1. University of Utah, Salt Lake City, Utah, United States, 2. University of Utah College of Nursing, Salt Lake City, Utah, United States*

Nurses working in long-term services and supports (LTSS) settings need training in geriatrics and quality improvement (QI). Our Geriatric Workforce Enhancement Program (GWEP) created an online Nurse Residency Program for nurses from other LTSS settings. Nurses (n=7) who completed the program showed statistically significant improvements in confidence in performing geriatrics skills ($t = -3.12$, $df = 6$, $p = 0.01$), attitudes about community-centeredness ($t = -2.14$, $df = 6$, $p = 0.04$), self-efficacy to engage in the treatment and assessment of depression ($t = -3.06$, $df = 6$, $p = 0.01$), and dementia ($t = -2.04$, $df = 6$, $p = 0.04$). Open-ended, satisfaction responses revealed improved self-efficacy with conducting a QI project. Empowering nurses in LTSS settings may improve quality of care, and decrease burnout and turnover. Future revisions will make the course both asynchronous and applicable to other professions in order to build stronger interprofessional LTSS teams.

### VIRTUAL TELEHEALTH CLINICAL EXPERIENCE TO PREPARE STUDENT PHYSICAL THERAPISTS FOR PRACTICE IN A SNF

Kathleen Manella[1], Kai Williams[2], and Jon Anderson[3], *1. Nova Southeastern University- Tampa Bay Regional Campus, Indian Rocks Beach, Florida, United States, 2. Ensign Services, Inc, Cypress, Texas, United States, 3. Ensign Services, Inc, San Antonio, Texas, United States*

Student training was suspended in most post-acute settings in the United States due to the COVID-19 pandemic. Through a collaborative partnership between a university and a healthcare organization we offered a virtual telehealth clinical experience to prepare student physical therapists for practice in a skilled nursing facility (SNF) setting. 35 students engaged virtually for 80 hours with two clinical instructors. The curriculum included eight virtual learning modules along with four telehealth patient encounters that exposed students to interdisciplinary teams in the SNF context. Students completed a retrospective pre/post Self-Perception of Learning Survey. Results demonstrated students' self-perception of learning was significantly increased implying an improved ability to practice in the SNF setting. This approach advanced student learning in patient care management, interprofessional practice, and telehealth practice in the SNF setting. Future work is planned to expand the program to include 20 physical therapy, occupational therapy, and speech and language pathology students.

### SESSION 2070 (SYMPOSIUM)

### ESPO AND BEHAVIORAL AND SOCIAL SCIENCES SECTION SYMPOSIUM: TAILORING INTERVENTIONS TO REACH AND MEET THE DIVERSE NEEDS OF DIVERSE CAREGIVERS

Chair: Kylie Meyer Co-Chair: Sara Hackett

Family caregiving cuts across populations, making caregivers a highly diverse population in terms of culture, family organization, care situations, and more. A 2021 report from the National Academies of Sciences Engineering and Medicine reinforces the need to develop and test tailored interventions in order to successfully reach and support family caregivers. Yet, there is limited practical guidance to help researchers to develop intervention programs tailored to the diverse needs of family caregivers. This symposium endeavors to address this gap by sharing accounts from researchers who have effectively tailored existing interventions to meet the diverse needs of diverse caregivers, as well as those who collaboratively worked alongside family caregivers in order to build a tailored program from the ground up. To begin, Kristin Cloyes, PhD MN RN will describe a study where she examines alignment between LGTBQ+ hospice family caregivers and other members of the hospice care teams, as it relates to support and communication needs. Next, Jung-Ah Lee, PhD, RN, FGSA, FAAN, will describe the experiences of racially and ethnically diverse caregivers who received a community health worker intervention to access resources tailored to their language needs. Jaime Perales-Puchalt, PhD, MPH, will describe the results of CuidaTEXT intervention study, which used tailored Short Messaging Service-based text message to deliver support to Latin family caregivers. Lastly, Ishan Williams, PhD, FGSA, will present on findings from research on how community engagement as well as culturally informed intervention can improve the representation of family caregivers, especially African American caregivers, in caregiving research studies

### ALIGNMENT OF COMMUNICATION AND SUPPORT NEEDS BETWEEN LGBTQ+ FAMILY CAREGIVERS OF HOSPICE PATIENTS AND PROVIDERS

Kristin Cloyes[1], Maija Reblin[2], Megan Thomas Hebdon[3], Miranda Jones[4], and Lee Ellington[5], *1. Oregon Health & Science University, Portland, Oregon, United States, 2. University of Vermont, Burlington, Vermont, United States, 3. University of Texas at Austin, Austin, Texas, United States, 4. University of Michigan Department of Psychology, Ann Arbor, Michigan, United States, 5. University of Utah and Huntsman Cancer Institute, Salt Lake City, Utah, United States*

Effective and equitable end-of-life care requires alignment between the support and communication needs of hospice family caregivers and the knowledge and competence of hospice care team members. We investigate alignment between LGBTQ+ caregivers and hospice providers in a secondary analysis of data collected in three studies: 1) Surveys identifying predictors of hospice providers' (N=122) attitudes toward LGBTQ+ caregivers and patients; 2) focus groups assessing hospice interdisciplinary care team members' (N=48) knowledge, training, and opinions regarding LGBTQ+ family caregiver and patient communication and care needs; and 3) in-depth interviews with LGBTQ+ hospice family caregivers (N=20). Synthesis of key findings show how alignment is impacted by lack of systematic, inclusive orientation and gender data, provider religiosity, expectations of disclosure, cisheteronormative assumptions about the universality of EOL experiences, and caregivers' experiences of minority stress. We discuss implications for developing interventions to improve provider communication, empowering caregivers, and addressing hospice-specific sources of minority stress.

### A COMMUNITY HEALTH WORKER–LED HOME VISIT INTERVENTION TO SUPPORT DIVERSE DEMENTIA FAMILY CAREGIVERS

Jung-Ah Lee[1], Eunae Ju[2], Eilleen Sabino-Laughlin[3], Lisa Gibbs[4], and Adey Nyamathi[3], *1. University of California, Irvine, Irvine, California, United States, 2. University of California Irvine, Irvine, California, United States, 3. UC Irvine, Irvine, California, United States, 4. University of California, Irvine, Orange, California, United States*

Diverse family caregivers of persons with dementia (PWD) are often faced with challenges in obtaining necessary resources due to limited English-proficiency, accessing available resources, and stigma. Diverse community health workers (CHWs) provided dementia family caregivers culturally and linguistically appropriate education, compassionate listening during a 3-month home-visit intervention. 25 participants were recruited from communities in California: Race/Ethnicity 32% Korean, 32% Vietnamese, 16% Latino, 20% White; 76% Female; 44% Spouse; Age=62.6 (28-83 years); 44% work; English proficiency=2.9 of 5. Recruitment is ongoing. The key themes from the exit interview with participants included (a) accepting the role of a caregiver, (b) better understanding and patience with the PWD, and (c) ongoing support from CHW. The overall satisfaction on the intervention was 4.7/5. The CHW-led home-visit intervention was well-received by ethnically diverse dementia family caregivers. Participants reported the usefulness of education, community resources, and compassionate support delivered in their homes over 3 months.

### DEVELOPING AND TESTING THE FEASIBILITY OF A TEXT MESSAGE DEMENTIA CAREGIVER SUPPORT INTERVENTION FOR LATINOS

Jaime Perales Puchalt, *University of Kansas Alzheimer's Disease Research Center, Kansas City, Kansas, United States*

Latinos experience several disparities in dementia including poor caregiver mental health and caregiving support access. Latinos' ubiquitous cell phone ownership and use of text messaging may offset those disparities. During this session, we will describe the development and feasibility testing findings of CuidaTEXT. CuidaTEXT is a tailored Short Messaging Service-based text message intervention to support Latino dementia family caregivers. This intervention was developed using mixed-methods research. This research resulted in a bilingual six-month intervention with automatic daily messages for all participants that address dementia education, self-care, social support, end-of-life, care of the person with dementia, behavioral symptoms, and problem-solving strategies. Participants who need more in-depth support, can use two functions: 1) every time they send a keyword-driven message (e.g., STRESS), they will receive an automatic message to support them with that topic, 2) for any other message they send (live chat), a coach will respond to their specific needs.

### AMPLIFYING COMMUNITY ENGAGEMENT TO INCREASE REPRESENTATION AND APPLICABILITY OF CAREGIVING RESEARCH

Ishan Williams[1], Randy Jones[2], and Travonia Brown-Hughes[3], *1. University of Virginia, School of Nursing, Charlottesville, Virginia, United States, 2. University of Virginia School of Nursing, Charottesville, Virginia, United States, 3. Hampton University, Hampton, Virginia, United States*

Alzheimer's disease and related diseases (ADRD) disproportionately affect persons of African American ethnicity, yet persons who identify as Black/African American are consistently and markedly underrepresented in Alzheimer's research. Prior research suggests that a complex array of factors, from mistrust in medical research to non-inclusive recruitment approaches, have led to the disparity. With the growing rates of ADRD among racial/ethnic groups in the US, it is imperative that research scientists develop interventions and clinical research that are culturally informed and meaningful to the lives of diverse caregivers. The goal of our research is to demonstrate the importance of community engagement and culturally informed interventions, and to offer best practices to advance the science of caregiver recruitment, which may ultimately improve overall representation across racial/ethnic caregiver groups. Research findings will highlight the variety of recruitment strategies used to build trust and more sustainable relationships with diverse communities often underrepresented in research.

### SESSION 2080 (PAPER)

### GLOBAL PERSPECTIVES ON COGNITION AND OUTCOMES

### AGE AT CARDIO-METABOLIC DISEASE ONSET IN A COHORT OF MIDLIFE WOMEN: SYSTEMATIC EXCLUSION MISESTIMATES THE MAGNITUDE OF RACIAL DISPARITIES

Alexis Reeves[1], Michael Elliott[2], Tene Lewis[3], Carrie Karvonen-Gutierriez[2], William Herman[2], and Sioban Harlow[2], *1. Stanford University, School of Medicine, Berkeley, California, United States, 2. University of Michigan, Ann Arbor, Michigan, United States, 3. Emory University, Atlanta, Georgia, United States*

Cohort studies of aging recruit participants at an age before most of the population experiences the study outcome, to document its natural history. The age of study commencement is usually based on "normative" aging among Whites. However, "weathering" can cause accelerated health declines in minoritized populations compared to Whites due to cumulative experience of multiple forms of marginalization. Thus, considering if weathering among minoritized individuals could affect selection into cohort studies is necessary to effectively estimate and understand racial/ethnic disparities in aging and disease. Using the Study of Women's Health Across the Nation (SWAN), a multi-ethnic longitudinal cohort, and its cross-sectional screening survey, we examine the effects of selection on the racial/ethnic differences in the age of onset of 4 cardio-metabolic outcomes (hypertension, isolated systolic hypertension, insulin resistance and diabetes). Selection at study commencement (left truncation and left censoring) had greater effects on outcomes with earlier age at onset (hypertension) and right censoring had greater effects on outcomes with later onsets (metabolic). Full adjustment led to an average 20-year decrease in predicted median age of onset for all groups across the 4 outcomes and tended to decrease the predicted disparity in age at onset. However, significantly earlier onset of each outcome for Black and Hispanic women compared to Whites remained. Not considering the full extent of selection bias in cohort studies can misinform our understanding of aging and disease, especially for minoritized populations who have higher prevalence of these leading causes of morbidity and mortality earlier in life.

### ASSOCIATION OF MID UPPER ARM CIRCUMFERENCE AND COGNITION: A POPULATION-BASED COHORT STUDY

David Lynch[1], annie Green Howard[2], Hsiao-Chuan Tien[3], Shufa Du[2], Bing Zhang[4], Huijun Wang[5], Penny Gordon-Larsen[2], and John Batsis[2], *1. UNC, Chapel Hill, North Carolina, United States, 2. University of North Carolina at Chapel Hill, Chapel Hill, North Carolina, United States, 3. UNC Chapel Hill, Chapel Hill, North Carolina, United States, 4. National Institute for Nutrition and Health Chinese Center for Disease Control and Prevention, China, Beijing, China (People's Republic), 5. National Institute for Nutrition and Health Chinese Center for Disease Control and Prevention, Beijing, Beijing, China (People's Republic)*

Introduction: Evidence suggests a positive association between muscle mass and cognitive impairment exists. Mid-upper arm muscle circumference (MUMC) is a simple measure that may provide prognostic information on cognitive status.

Methods: We included adults aged ≥55 years from the China Health and Nutrition Survey 1997-2018 with MUAC and triceps skinfold (TSF) measurements at each visit. Cognition was estimated based on a subset of the modified Telephone Interview for Cognitive Status (TICS, 0–27). Sex-stratified linear mixed-effects models accounting for within-individual and within-community correlation assessed the association between MUAC and the ratio of MUAC:TSF with TICS across age. We tested whether the rate of cognitive decline by age differed by quartiles of MUAC and MUAC:TSF in separate models. In cases of no statistical differences in cognitive declines by age, we tested whether overall cognitive function was associated with quartiles of MUAC and MUAC:TSF across all ages.

Results: Of 5,964 adults (53% female, age 62.4±6.4), mean MUAC was 26.6±3.74 and 26.2±3.9 cm, mean MUAC:TSF ratio was 2.9±1.6 and 1.94±1.1, and baseline TICS was 15.4±6.1 and 13.2±6.4 for men and women, respectively. MUAC was not associated with the rate of cognitive decline. Lower MUAC was associated with higher overall cognitive function scores for men ($p=0.01$) and women ($p=0.05$). For men and women there was no association between MUAC:TSF ratio and either cognitive decline or overall function.

Conclusion: MUMC can be a marker to predict overall cognitive function across this period in the lifecycle, suggesting that declining MUAC may help predict lower overall cognitive function

### DRINKING WATER QUALITY, DYSLIPIDEMIA, AND COGNITIVE FUNCTION FOR OLDER ADULTS IN CHINA

Xi Pan[1], Ye Luo[2], Dandan Zhao[2], and Lingling Zhang[3], *1. Texas State University, San Marcos, Texas, United States, 2. Clemson University, Clemson, South Carolina, United States, 3. University of Massachusetts Boston, Boston, Massachusetts, United States*

The current study aimed to examine the associations between drinking water quality and cognitive function and to identify the direct and indirect effects of drinking water quality and dyslipidemia on cognitive function among older adults in China. Data for the study were selected from China Health and Retirement Longitudinal Study (CHARLS, 2015) and 4,951 respondents aged 60 and above were included. Dyslipidemia was measured by self-reported dyslipidemia diagnosis and lipid panel. Three composite measures of cognitive function included mental status, episodic memory, and global cognition. Mixed effects models were conducted to assess the associations between drinking water quality or dyslipidemia and cognitive function. The mediation effects of dyslipidemia were examined by path analyses. Results showed that exposure to high quality drinking water was significantly associated with higher scores in mental status, episodic memory, and global cognition ($B = 0.31$, $p< 0.001$ for mental status; $B= 0.22$, $p< 0.05$ for episodic memory; $B = 0.53$, $p < 0.01$ for global cognition). Elevated blood triglycerides was associated with higher scores in mental status and global cognition ($B = 0.21$, $p< 0.05$ for mental status; $B = 0.32$, $p< 0.05$ for global cognition). Self-reported dyslipidemia diagnosis was a suppressor, which increased the magnitude of the direct effect of drinking water quality on mental status and global cognition. Findings of the study suggest that improving drinking water quality could be a potential public health effort to delay the onset of cognitive impairment and prevent the dementia pandemic in older people.

### IMPACT OF GENDER ON ASSOCIATION BETWEEN RACE AND DISABILITY: THE CALIFORNIA HEALTH INTERVIEW SURVEY (CHIS)

Lien Quach[1], Christine Vu[2], Isabelle Tran[3], Noah Peeri[2], and Uyen-Sa Nguyen[4], *1. Massachusetts General Hospital, Newton, Massachusetts, United States, 2. University of North Texas Health Science Center (UNTHSC), Fort Worth, Texas, United States, 3. Texas Christian University School of Medicine, Fort Worth, Texas, United States, 4. University of North Texas Health Science Center (UNTHSC), School of Public Health (SPH), Fort Worth, Texas, United States*

Women generally have higher prevalence of disability than men in later life. Moreover, Blacks and Hispanics usually have higher prevalence of disability than Whites. Little is known about the impact of gender on the association between race and disability. We used 2015-2016 CHIS data, restricted to adults ≥ 65 years old (n=15,044). Disability was classified as present or absent based on responses on questions related to "to physical, mental, and emotional conditions." Race was classified as: White, Black, Hispanic, Asian, and Other. We estimated sex- and race-specific proportions and 95% confidence intervals (CI) and used sex-specific multivariable logistic regression to examine the associations between race and disability adjusting for age, education, marital status, cigarette smoking, arthritis, hypertension, and diabetes, mental distress, and walking for work or pleasure. All analyses accounted for complex sampling weights. Approximately 52% of women and 47% of men had disability, while 48% of White, 48% of Black, 60% of Hispanic, 43% of Asian, and 50% of Other race responded as having disability. Adjusting for covariates, Hispanic women had 67% higher odds of having disability compared with White women (OR= 1.67, 95% CI= 1.07–2.60), but there were no differences in male counterparts (OR=1.03, 95% CI=0.68-1.56). Compared with White men, men of Asian or Other race had lower odds for disability, while associations were in the opposite direction in female counterparts; however, associations were not statistically significant. Further research is needed to understand higher prevalence of disability among older minority women compared with White women.

### BIOLOGICAL RISK FACTORS FOR DEMENTIA AND COGNITIVE FUNCTION AMONG OLDER INDIANS: FINDINGS FROM LASIDAD

Qiao Wu[1], Eileen Crimmins[2], Mateo Farina[2], Jung Ki Kim[2], and Xinyao Zhang[2], *1. University of Southern California, LOS ANGELES, California, United States, 2. University of Southern California, Los Angeles, California, United States*

A number of clinical and exam-generated biomarkers have been associated with dementia. We assess the relative importance of 41 biomarkers using a novel dataset from an understudied population – a national sample of older Indians. The value of our data includes the biomarker extensiveness, the validated classification of dementia, and the relatively lower average education of the population. We use both traditional social science methods based on biological theories and agnostic machine learning algorithms to examine how biomarkers explain variance in dementia diagnosis and cognitive functioning. Comparing different approaches shows how to best characterize the influence of biology and how to trim and combine biomarkers. The six approaches used in our study include: (1) 41 individual biomarkers; (2) identification of subsets of biomarkers with elastic net; (3) support vector machine learning; (4) factor analysis; (5) principal component analysis; and (6) factor classification based on a theoretical approach. Preliminary results show that all the biomarkers or a reduced set of biomarkers identified by elastic net do the best job at explaining variability in dementia outcome, but the biomarkers chosen as most important by elastic net do not match well our understanding of biological mechanisms. Traditional social science approaches (e.g. factor analysis and principal components approach) provide better understanding and interpretation of the relative importance of biological systems as well as the association between biomarkers and cognition. These results are informative for others collecting and analyzing biomarker data in population samples.

### SESSION 2090 (PAPER)

### HEALTHCARE WORKFORCE DEVELOPMENT AND OUTCOMES

### ADOPTION OF SNFISTS BY US NURSING HOMES DID NOT REDUCE REHOSPITALIZATION RATES AMONG POST–ACUTE CARE PATIENTS

Hye-Young Jung[1], Seiyoun Kim[2], and Kira Ryskina[3], *1. Weill Cornell Medical College, New York, New York, United States, 2. University of Pennsylvania, Philadelphia,, Pennsylvania, United States, 3. University of Pennsylvania, Philadelphia, Pennsylvania, United States*

SNFists, physicians and advanced practitioners who focus their practice in nursing homes, have grown in prevalence in recent years. In this retrospective cohort study, we measured the role of nursing home adoption of SNFists in rehospitalization rates. We used 100% Medicare facility and professional claims for hospitalized fee-for-service beneficiaries discharged to nursing homes in 2012-2019. We defined SNFists as generalist physicians and nurse practitioners with at least 80% of their Medicare Part B claims for nursing home care. All nursing homes that did not have a SNFist in 2012 were followed through 2019. We used an event study approach with staggered SNFist adoption to estimate the effect of a nursing home adopting at least one SNFist on the facility's unplanned 30-day rehospitalization rate, adjusted for patient case mix, facility and market characteristics. Models included facility and year fixed effects. Standard errors were clustered at the facility level. Of the 6,616 nursing homes in the sample, 77% adopted SNFists by 2019. Nursing homes that adopted SNFists did not experience a statistically significant change in rehospitalization rate compared to those that did not adopt: rehospitalization rates decreased by 0.02 percentage points (95% CI -0.35 to 0.32; p=0.93) one year after SNFist adoption and by 0.15 percentage points (95% CI -1.01 to 0.70; p=0.73) six years after adoption, compared to the baseline difference between the groups in the year before adoption. In sum, despite the wide adoption of SNFists by US nursing homes, we did not observe an associated decrease in rehospitalization rates.

### EXPLORING THE IMPLEMENTATION OF FOUR TRANSITIONAL CARE INTERVENTIONS FOR OLDER ADULTS: A COLLECTIVE CASE STUDY

Amal Fakha[1], Merel Leithaus[1], Bram de Boer[1], Theo van Achterberg[2], Jan Hamers[1], and Hilde Verbeek[1], *1. Maastricht University, Maastricht, Limburg, Netherlands, 2. KU Leuven, Leuven, Vlaams-Brabant, Belgium*

Four transitional care interventions, aiming to improve transitions between hospital and home settings for older adults, were implemented in Belgium. These interventions are complex in nature and their implementation in practice is often challenging. This study explores the implementation of these interventions from the perspectives of healthcare professionals involved in the process, and by investigating three implementation aspects: influencing factors, strategies used, and outcomes. A retrospective qualitative collective case study was conducted using semi-structured interviews with thirteen healthcare professionals. Thematic analysis was used, and the Consolidated Framework for Implementation Research guided the final data interpretation. Key findings indicated that implementation barriers were mostly at the organizational level (i.e., resources, structure, networks & communication), while facilitators were at the individual level (i.e., personal attributes, champions). The prominent strategy used to implement these interventions was engagement and additional strategies were suggested for the future sustainability of the interventions (i.e., building strategic partnerships, developing policies to support transitional care). The overall implementation was perceived as favorable with high adoption as a key outcome. This study highlights the crucial role of healthcare professionals, being motivated and committed to fostering the implementation of interventions in transitional care. It is important to use implementation strategies targeting factors at both the individual level as well as the organizational level for implementing transitional care interventions in the future.

### GERIATRICIAN CO-MANAGEMENT AND EDUCATION OF JUNIOR DOCTORS IMPROVE THE CARE OF HOSPITALIZED OLDER SURGICAL PATIENTS

Janani Thillainadesan[1], Sarah Aitken[2], Sue Monaro[2], John Cullen[2], Richard Kerdic[2], Sarah Hilmer[3], and Vasi Naganathan[2], *1. Department of Geriatric Medicine and Centre for Education and Research on Ageing (CERA), Concord Hospital, Concord, New South Wales, Australia, 2. Concord Hospital, Concord, New South Wales, Australia, 3. Royal North Shore Hospital, St Leonards, New South Wales, Australia*

We evaluated changes in processes of care after the introduction of a novel model of care for older vascular surgery inpatients. This model, called Geriatrics Co-management of older vascular surgery patients (GeriCO-V) embedded a geriatrician into the vascular surgery team who provided proactive geriatrics assessment of patients and education for junior surgical doctors. A pre-post study of the GeriCO-V model comparing prospectively recruited pre-intervention (n=150) and post-intervention (n=152) cohorts of consecutively admitted vascular surgery patients aged ≥65 years at an acute care academic hospital. Education of junior surgical doctors was embedded in the new model of care and included role modelling, provision of delirium and frailty screening lanyards and mobile Apps for 'just-in-time' learning, and a Wiki page of tips on assessing older patients. We measured processes of care by review of medical charts. After implementing the novel geriatrician and education embedded model of care, there was a significant increase in several processes of care by the junior surgical doctor: screening for cognition (8% vs 76%, p<.001) and delirium (2% vs 69%, p<.001), documentation of functional history (34% vs 76%, p<.001), medications (53% vs 74%, p<.001) and treatment preferences (5% vs 46%, p<.001) and prescribing of pharmacological venous thromboprophylaxis (93% vs 99%, p=.03) and co-prescription of laxative with opioid (60% vs 81%, p=.002). A collaborative model of care that embeds proactive geriatrician care and education of junior surgical doctors improves the quality of care for older vascular surgical patients.

### SWISS NURSING HOMES' ENGAGEMENT IN QUALITY IMPROVEMENT: A MULTICENTER CROSS-SECTIONAL STUDY

Lauriane Favez[1], Michael Simon[2], Christine Serdaly[3], and Franziska Zúñiga[1], *1. University of Basel, Basel, Basel-Stadt, Switzerland, 2. The Institute of Nursing Science, Basel, Basel-Stadt, Switzerland, 3. serdaly&ankers, Conches, Geneve, Switzerland*

Many publications have focused on quality improvement interventions in nursing homes but the self-initiated daily involvement of nursing homes in quality improvement activities has rarely been investigated (e.g., conducting surveys, using data to set quality aims and track quality of care over time, conducting quality improvement projects). Such activities can be carried out by nurses in expanded roles with competences beyond those of regular registered nurses (e.g., advanced studies, Bachelor/Master's degree), but evidence is lacking. We aimed to described the involvement of nursing homes and of nurses working in expanded roles in quality improvement activities in Swiss nursing homes. A cross-sectional multi-center study (2018-2019) using survey data from a convenience sample of 115 Swiss nursing homes and 104 nurses in expanded roles was used and descriptive statistics applied. Most nursing homes reported carrying several quality improvement activities (median = 8 activities out of 10 surveyed). The group of nursing homes working with a nurse in an expanded role (n = 83) were more engaged in quality improvement than the other group (median = 8 versus 6.5) Higher-educated nurses participated more in a majority of quality improvement activities than lower-educated nurses (Bachelor/Master-educated nurses: median of 13 out of 17 activities surveyed, versus diploma-educated nurses: 10), except for clinical teaching. This study provides insights into nursing homes' involvement in daily quality improvement and suggests that, depending on the targeted quality improvement needs, nursing homes use different profiles and education levels of nurses in expanded roles.

### SESSION 2100 (SYMPOSIUM)

### CHANGING THE NARRATIVE ON NURSING HOME CARE

Chair: Patricia D'Antonio

Redesigning our nursing home care system will require substantial policy change, and we know that policy change needs an effective framing strategy. This panel will include speakers who have been studying public mindsets and opinions about nursing home care and its implications for advocacy communications. We will also talk about the implications of this research for the larger reframing aging initiative.

### SESSION 2130 (SYMPOSIUM)

### INTERDISCIPLINARY DEMENTIA CARE WORKFORCE TRAINING AND QI IMPLEMENTATION: LEARNINGS FROM 25 HOSPICES

Chair: Abraham Brody Discussant: Joseph Gaugler

To build and sustain a dementia-capable clinical workforce and care delivery organizations, it is essential to systematically disseminate evidence-based, interdisciplinary dementia care programs. The outbreak of COVID-19, clinical staff shortages, and competing priorities experienced by healthcare organizations including staff vaccination and other regulatory requirements, pose significant challenges to the continuation of agency-wide, interdisciplinary dementia care workforce training and quality improvement. In this symposium, we present our experience Implementing Aliviado Dementia Care, a comprehensive, interdisciplinary dementia care workforce training and quality improvement program amid COVID-19 across 25 U.S. hospice agencies, as part of an embedded pragmatic clinical trial. Aliviado Dementia Care consists of interdisciplinary dementia care workforce training, treatment algorithms, assessments, care plans, caregiver education, and clinical workflow changes. The symposium begins with a discussion of the training, knowledge improvement, turnover, and replenishing strategies of hospice champions during COVID-19. We then discuss how we tailored dementia care training for each of the skilled hospice disciplines (i.e., medicine, nursing, social work, spiritual care) and provide two case studies elucidating how additional tailoring was performed to fit local agency culture and needs during COVID-19. Furthermore, we discuss how we developed and iterated training for home health/hospice aides, the usability and training results, and specific considerations for training aides during COVID-19. Lastly, we conclude the symposium with a discussion of salient strategies and technologies that help sustain our nationwide implementation despite the ongoing COVID-19. Throughout each of these abstracts we also discuss how COVID-19 affected, and created differential implementation of components of the intervention.

### NATIONWIDE ALIVIADO CHAMPION TRAINING: KNOWLEDGE IMPROVEMENT, CHAMPION TURNOVER, AND LESSONS LEARNED

Tina Sadarangani[1], Aditi Durga[1], Ariel Ford[2], Donna McCabe[3], Tessa Jones[1], Kimberly Convery[1], and Shih-Yin Lin[1], *1. New York University, New York, New York, United States, 2. New York University Rory Meyers College of Nursing, New York, New York, United States, 3. New York, New York, New York, United States*

Aliviado Champions are hospice employees selected by their agency to lead the local implementation of the Aliviado program. Accordingly, training and retention of champions are critical to hospices' success (or failure) of program implementation. As of February 2022, we have trained 308 champions across 19 agencies; 94% of the champions agreed all learning objectives were met. A total of 144 champions (97 nurses, 27 social workers, 16 chaplains, and 5 providers) completed pre- and post-training dementia knowledge surveys, demonstrating significant improvements in dementia knowledge, attitudes, and confidences in 7 out of 10 domains post training ($p<0.005$). The champion turnover rate during COVID-19 (March 2020-Feb 2022) was 11%. In addition, we share our experience adapting the originally 2 full-day, in-person Aliviado Champion Training into a Zoom-based format in response to COVID-19 including strategies to prevent Zoom fatigue. We also discuss strategies to address turnover and rapidly onboard new champions during COVID-19.

### INTERDISCIPLINARY DEMENTIA CARE WORKFORCE TRAINING DURING COVID-19 AND TWO HOSPICE CASE STUDIES

Rebecca Lassell[1], Aditi Durga[1], Shih-Yin Lin[1], Tessa Jones[1], Ariel Ford[2], and Abraham Brody[3], *1. New York University, New York, New York, United States, 2. New York University Rory Meyers College of Nursing, New York, New York, United States, 3. HIGN at the NYU Rory Meyers College of Nursing, New York, New York, United States*

Hospice is a care model characterized by interdisciplinary team-based, person and family-centered care. To optimize agency-wide interdisciplinary team-based hospice dementia care, at least two levels of tailoring of the dementia care workforce training are imperative, first, by discipline, and second, by hospices' local culture and needs. As of February 2022, a thousand and one skilled hospice interdisciplinary team members (not counting champions) across 18 hospice agencies have completed their discipline-specific Aliviado dementia care training, including 56 providers, 763 nurses, 129 social workers, and 53 chaplains. In this presentation, we describe how we tailored dementia workforce training for skilled interdisciplinary team members (first level tailoring), as well as provide two case studies elucidating how we performed further tailoring of the program for two large hospice agencies (average daily census: 354 and 868, respectively) in two different states to meet their local needs (second level tailoring), and lessons learned.

### ALIVIADO HOME HEALTH AND HOSPICE AIDE DEMENTIA CARE EXPERT PROGRAM IMPROVES AIDE DEMENTIA KNOWLEDGE

Shih-Yin Lin[1], Aditi Durga[1], Ariel Ford[2], S. Raquel Ramos[2], Michele Crespo-Fierro[3], Tina Sadarangani[1], and Abraham Brody[4], *1. New York University, New York, New York, United States, 2. New York University Rory Meyers College of Nursing, New York, New York, United States, 3. New York Univeresity Rory Meyers College of Nursing, New York, New York, United States, 4. HIGN at the NYU Rory Meyers College of Nursing, New York, New York, United States*

Most persons living with dementia receive home health or hospice aide services during their hospice stay. To equip aides with essential knowledge and skills to effectively communicate with and care for persons living with dementia, we developed a 17-video dementia care expert program for aides, available in both English and Spanish. The objective of this presentation is two-fold: first, describe the development and refinement of the Aliviado aide dementia care expert program, including topic selection, usability testing, and cultural tailoring; and second, discuss aide knowledge improvement, along with special considerations for training aides during COVID-19. The Aliviado dementia care expert program for aides has been completed by 414 aides across 15 hospices as of February 2022; of which, 384 aides (93%) completed a dementia knowledge assessment both before and after the training and demonstrated significant knowledge gains with moderate-to-large effect sizes ($p<0.0001$; Cohen's $d=0.63$).

### STRATEGIES AND TECHNOLOGIES TO SUSTAIN NATIONWIDE IMPLEMENTATION OF ALIVIADO DEMENTIA CARE

Abraham Brody[1], Aditi Durga[2], Ariel Ford[3], Rebecca Lassell[2], and Shih-Yin Lin[2], *1. HIGN at the NYU Rory Meyers College of Nursing, New York, New York, United States, 2. New York University, New York, New York, United States, 3. New York University Rory Meyers College of Nursing, New York, New York, United States*

It is well documented that knowledge improvement alone is not sufficient to sustain systems level changes. Through supporting dementia care workforce training and the embedding of Aliviado program tools (treatment algorithms, assessments, care plans, and caregiver education materials) into hospices' clinical workflow across the U.S., we learned that both technologies and human support strategies are essential for lasting changes. In this presentation, we first discuss our experience leveraging a powerful customer relationship management software (Salesforce) to assign, manage, and encourage completion of the interprofessional Aliviado Dementia Care training for thousands of active users across the nation. We then share how we provide additional human support and implementation tools in the face of COVID-19, which can inform implementation of similar trials, including office hours, training reports, Plan-Do-Study-Act worksheets, symptom management cheat sheets, champion calls, and electronic health records integration meetings with each agency to locally tailor and embed Aliviado tools.

### SESSION 2140 (SYMPOSIUM)

### KEY PREDICTORS OF COGNITIVE AGING

Chair: Nicholas Turiano Co-Chair: Julie Blaskewicz Boron

Understanding who will experience cognitive dysfunction and dementia is as important as identifying methods to combat such deterioration. This symposium seeks to highlight new advances in understanding cognitive deterioration and promote resilience in adulthood. Odd and colleagues utilized a national sample (2,643 adults aged 34-85) to show that 9-year decreases in executive function and episodic memory predicted increased risk of dying. Use of a brief cognitive assessment administered via telephone was unique – a method that may assist clinicians administering cognitive screenings to older adults in isolated areas. Graham and colleagues utilized a daily diary approach in 116 older adults (aged 60-90) to show that greater daily fluctuations in mindfulness were associated with higher episodic memory and executive functioning. Further, mediational evidence suggested that on days when mindfulness was greater, individuals perceived a younger subjective age. Willroth and colleagues provide evidence that higher scores on eudaimonic well-being in older adults (n=349) predicted greater cognitive resilience. Specifically, even though some participants had neuropathological burden (e.g., increased beta-amyloid, neurofibrillary tangles), they did not exhibit pronounced cognitive declines. Using data from 14 longitudinal studies, Yoneda and colleagues examined the impact of physical activity on cognitive impairment and death. Multi-state survival analyses demonstrated engagement in more physical activity reduced risk of cognitive impairment and death. This symposium suggests that examining changes in cognition, incorporating subjective and objective indices of cognitive impairment, utilizing long-term longitudinal and daily diary designs, and testing key modifiable behaviors is crucial to understanding and promoting optimal cognitive functioning in adulthood.

### CHANGE IN EPISODIC MEMORY AND EXECUTIVE FUNCTIONING PREDICTS MORTALITY RISK

Kaleena Odd[1], Julie Boron[2], Jacob Alderson[3], Margie Lachman[4], and Nicholas Turiano[3], *1. University of Nebraska Omaha, Omaha, Nebraska, United States, 2. University of Nebraska, Omaha, Nebraska, United States, 3. West Virginia University, Morgantown, West Virginia, United States, 4. Brandeis University, Waltham, Massachusetts, United States*

The current study explored whether the Brief Test of Adult Cognition via Telephone (BTACT) assessment could be used to predict longevity in a national sample of adults from the Midlife Development in the United States (MIDUS) study. Specifically, we examined whether 9-year changes in episodic memory (EM) and executive functioning (EF) predicted all-cause mortality risk (2004-2018). The sample included 2,643 participants (MAge=45.87; 92.23% white; 107 deceased) who completed the BTACT twice: between 2004-06 and between 2013-2017. Using change scores, decreases in EM (HR= 1.45 [1.09-1.93], p=.01) and EF (HR=1.585 [1.17-2.14], p<.001) increased the hazard of dying (controlling for age, gender, race, education, and self-rated health). Results suggest the BTACT is sensitive enough to detect health-consequential decreases in EM/EF. Future research should consider the BTACT as a viable assessment tool for older adults who may not have easy access to cognitive screenings.

### WELL-BEING AND RESILIENCE TO DEMENTIA-RELATED NEUROPATHOLOGY

Emily Willroth[1], Bryan James[2], Eileen Graham[3], Alifiya Kapasi[4], David Bennett[2], and Daniel Mroczek[3], *1. Washington University in St. Louis, St. Louis, Missouri, United States, 2. Rush University, Chicago, Illinois, United States, 3. Northwestern University, Chicago, Illinois, United States, 4. Rush Alzheimer's Disease Center, Chicago, Illinois, United States*

Not all older adults with dementia-related neuropathology in their brains experience cognitive decline or impairment. Instead, some people maintain relatively normal cognitive functioning despite neuropathologic burden, a phenomenon called cognitive resilience. Using a longitudinal, epidemiological, clinical-pathologic cohort study (N=349), the present research investigated associations between well-being and cognitive resilience. Consistent with pre-registered hypotheses, higher eudaimonic well-being (i.e., Ryff Psychological Well-being Scale) and higher hedonic well-being (i.e., Satisfaction with Life Scale) were associated with better-than-expected cognitive functioning and less-than-expected cognitive decline relative to one's neuropathological burden (i.e., beta-amyloid, neurofibrillary tangles, Lewy bodies, vascular pathologies, hippocampal sclerosis, and TDP-43). The association of eudaimonic well-being in particular was present above and beyond known cognitive resilience factors (i.e., socioeconomic status, education, cognitive activity, low neuroticism, low depression) and dementia risk factors (i.e., ApoE genotype, medical comorbidities). This research highlights the importance of considering eudaimonic well-being in efforts to prevent dementia.

### PHYSICAL ACTIVITY, COGNITIVE HEALTH, AND LONGEVITY IN OLDER ADULTHOOD

Tomiko Yoneda[1], Andrea Piccinin[2], Nathan Lewis[2], Jamie Knight[2], Jinshil Hyun[3], Luca Kleineidam[4], Scott Hofer[2], and Graciela Muniz Terrera[5], *1. Northwestern University, Evanston, Illinois, United States, 2. University of Victoria, Victoria, British Columbia, Canada, 3. Albert Einstein College of Medicine, Bronx, New York, United States, 4. University of Bonn, Bonn, Nordrhein-Westfalen, Germany, 5. Ohio University, Athens, Ohio, United States*

Research suggests that physical activity (PA) protects against cognitive impairment in older adulthood; however, the extent to which PA engagement throughout older adulthood influences the simultaneous risk of transitioning to cognitively impaired states and death is unclear. Applying coordinated data analysis across 14 studies, multi-state survival models (MSM) were independently fit to data from fourteen longitudinal studies (NTotal=52,039). PA was synthesized across studies using the metabolic equivalent of task approach. Adjusting for demographics and chronic conditions, meta-analytic results indicated that engaging in more PA was associated with a reduced risk of transitioning from no cognitive impairment to mildly impaired cognitive functioning (HR=0.94, 95%CI's=0.89,0.99) and death (HR=0.72, 95%CI's=0.65,0.80). Based on MSM estimates, multinomial regression models computed total life expectancy stratified by national PA recommendations. Meta-analytic results indicate a positive linear effect of PA, such that more PA is associated with longevity. This presentation will include discussion of knowledge translation efforts.

### WITHIN-PERSON RELATIONSHIP BETWEEN MINDFULNESS AND COGNITION IS MEDIATED BY SUBJECTIVE AGE

Lyndsey Graham, and Shevaun Neupert, *North Carolina State University, Raleigh, North Carolina, United States*

Cognitive functioning fluctuates daily throughout adulthood. Lapses in mindfulness can have cognitive consequences, which may be impacted by how old a person feels each day. Subjective age was examined as a mediator in the within-person relationship between mindfulness and cognition. 107 younger adults (aged 18-36, M = 19.96) and 116 older adults (aged 60-90, M = 64.71) completed reports of mindfulness and subjective age and tests of inductive reasoning and episodic memory for 8 consecutive days. Within-person multilevel mediation models indicated that daily subjective age mediated the relationship between daily mindfulness lapses and both indicators of daily cognition across ages. However, the mediation effect was stronger for younger adults on inductive reasoning but was stronger for older adults on episodic memory. These results show that daily changes in subjective aging are an important mechanism for daily cognition, with differential impact based on age and cognitive component.

### SESSION 2150 (PAPER)

### LONGITUDINAL, INTERVENTION CLINICAL TRIALS, AND QUALITATIVE STUDIES TO EXAMINE CAREGIVER OUTCOMES AND EXPERIENCES

### CAREGIVER SUBGROUP ANALYSES FROM AN EMBEDDED DEMENTIA CARE EFFECTIVENESS TRIAL

Richard Fortinsky[1], Dorothy Wakefield[2], Lily Zhong[2], James Grady[2], Catherine Piersol[3], Julie Robison[4], and Laura Gitlin[5], *1. University of Connecticut Center on Aging, Farmington, Connecticut, United States, 2. University of Connecticut, Farmington, Connecticut, United States, 3. Jefferson College of Rehabilitation Sciences, Philadelphia, Pennsylvania, United States, 4. UConn Health, Center on Aging, Farmington, Connecticut, United States, 5. Drexel University, Philadelphia, Pennsylvania, United States*

Efficacious dementia care interventions for family/informal caregivers are increasingly being tested for effectiveness in “real world” service delivery settings. We conducted an effectiveness trial in which we embedded the Care of Persons with Dementia in their Environments (COPE) program into Connecticut’s publicly-funded Home and Community-Based Services program. COPE is designed to build dementia management skills in caregivers of people with dementia in the home setting. In published results from this trial with the full study cohort, caregivers who received COPE, compared to controls, experienced improved well-being due to the dementia management skills they learned (perceived well-being), but they did not experience reduced levels of distress due to dementia-related behavioral and psychological symptoms (distress). For this presentation, we determined COPE effects for selected caregiver subgroups on perceived well-being and distress. Regarding caregiver sex, we found that COPE effects were statistically significantly positive on both outcomes for females (both $p<.05$) but on neither outcome for males. Controlling for sex, we also found positive COPE effects on both outcomes for daughters (both $p=.03$) but on neither outcome for spouses or sons. For perceived well-being, we found positive COPE effects for White ($p<.001$) but not for Black caregivers. For distress, we found positive COPE effects for caregivers living apart ($p=0.03$) but not for those living together with people with dementia. Findings suggest that male, Black, and co-residing caregivers may need more support from COPE, and more broadly demonstrate the value of subgroup analyses in offering greater precision when embedding nonpharmacological interventions in effectiveness trials.

### GENDER DIFFERENCES IN CAREGIVING PRACTICES AND MARITAL RELATIONSHIPS OF SANDWICH GENERATION COUPLES IN MUMBAI

SRUTHI ANILKUMAR HEMALATHA, SEKHER T.V., and NAWAJ SARIF, *International Institute for Population Sciences, Mumbai, Maharashtra, India*

Over the next few decades, the most important demographic trend for the Asia Pacific region will be population aging. The increasing aging population in India and the fact that the economic dependence of the older adults is high and the cost of education and child care is on the rise pose serious challenges to generations of individuals and couples who are expected to take care of both their children and their parents. This study's primary objective is to comprehensively understand the effects of giving care to their children and their parents on various aspects of the life of sandwich generation couples. The study uses primary data of 300 multigenerational households and 100 two-generation/one-generation households in Mumbai. Specifically, the study attempts to understand the effect of giving care to two generations on the marital relationship of the caregiving couples. Additionally, the study also explores gender differences in caregiving practices. Results from bivariate and multivariate analyses show that providing simultaneous care to two generations significantly impacts the marital relationship of the sandwiched couples as opposed to couples staying in one/two-generation households. Results also show considerable gender differences in caregiving roles and time spent on caregiving. Women spend more time assisting in household activities, while men are more likely to provide monetary support. Thus, extensive research on the sandwich generation in India is necessary to ensure the socio-economic well-being of the couples, the welfare of their children and parents, and the physical and psychosocial health of these couples.

### THIS TOO SHALL PASS: WEATHERING THE STORM AS OLDER FEMALE FAMILY CAREGIVERS FOR THOSE WITH AD/ADRD DURING COVID-19

Candace Harrington[1], and Dean Witt[2], *1. University of Louisville, Louisville, Kentucky, United States, 2. University of Louisville, Harrodsburg, Kentucky, United States*

Background:

Older female caregivers of persons with AD/ADRD are under-represented, under-reported, and understudied. Purpose: This qualitative study aimed to understand how COVID-19 affects older female caregivers' lived experience, ongoing capacity, and willingness to provide care for their loved one(s) with AD/ADRD. Specific Aims: Aim 1: Explicate older female caregivers' lived experience in the context of caring for family members with AD/ADRD during COVID-19. Aim 2: Elucidate how COVID-19 affected older female caregivers' relationships with their family members with AD/ADRD. Method: 172 units of meaning were extracted from 327 pages of transcripts and 972 minutes of interviews with urban (n = 10) and rural caregivers (n = 10). Thematic analysis was then conducted.

Results:

Respondents, recruited with purposive and snowball sampling, saw hastened AD/ADRD progression in their family member(s) attributed to social isolation. For many, long-term placement was not an option for financial reasons. Respondents providing full-time caregiving depended heavily on their care recipients' financial resources for basic living expenses, reporting placement in long-term care would leave them at risk of homelessness. Black caregivers expressed an "unspoken" cultural taboo about placement. For all, caregiver disability was the only impetus for placement. Respondents in rural communities more often reported faith-based gratefulness, moments of joy, relational harmony resulting from "being stuck together", and less availability of resources allowing virtual support. Urban caregivers reported more social isolation, less awareness of resources, more intentional family member distancing, and higher pandemic-related distress. Implications: These findings have broad economic, social, policy, research, and practice implications.

### USE OF DIGITAL TECHNOLOGY SOLUTIONS TO SUPPORT CAREGIVING BY FAMILY CAREGIVERS OF OLDER ADULTS

Rahul Malhotra, Nur Diyana Azman,
Veronica Shimin Goh, and Abhijit Visaria, *Duke-NUS Medical School, Singapore, Singapore*

Caregiving-relevant information and services are increasingly available online. Greater understanding of the extent and purpose of their use by family caregivers of older adults and specific caregiver sub-groups that are more or less likely to use them can inform both policies related to and the content of such solutions. We investigated the extent to which family caregivers of older persons in Singapore use digital technology solutions such as the internet and apps, and the purposes for which they use them. Information on digital device use was collected from 278 caregivers. Of them, 139 caregivers gave detailed information on how they had used the internet or apps to support caregiving in the last six months. Most (89%) caregivers used digital devices regularly (mostly smartphones (87%)). Digital device use was associated with caregiver age, ethnicity and education. Common generic online activities included sending instant messages (77%) and surfing websites (64%). While 54% had used the internet to support caregiving (most common purpose: search for information on care-recipient's health conditions), 43% had used apps to do so (most common purpose: coordinate care with family members or other caregivers). Such use was associated with caregiver age, education and care-recipient health. While use of digital devices and generic online activities are common among caregivers, their use of the internet or apps to support caregiving is less common. Information-seeking and coordination are indicative of avenues in which digital technology solutions can complement 'physical' channels of communication and support with and for caregivers, and be further expanded.

### SESSION 2160 (SYMPOSIUM)

### LUCIDITY IN DEMENTIA: EMERGING CONCEPTS AND DATA

Chair: Andrea Gilmore-Bykovskyi Discussant:
Basil Eldadah

Episodes of lucidity among people with advanced Alzheimer's disease and Alzheimer's disease related dementias (AD/ADRD), reportedly evidenced by an unexpected return of meaningful communication or connection, are an area of growing interest. Event transient recovery of abilities in the setting of advanced AD/ADRD are potentially significant for caregivers and the person living with dementia; as well as for shaping our understanding of AD/ADRD more broadly. Despite the potential significance of these events, existing evidence is largely comprised of anecdotal reports and retrospective/case reports. Recently, the National Institute on Aging funded six studies to advance empiric studies on episodes of lucidity in advanced AD/ADRD. This symposium will provide an update on progress and findings across these funded studies. The first presentation focuses on acceptability and feasibility of a prospective observation study focused on characterizing potential observable indicators of episodes of lucidity in individuals with advanced AD/ADRD near end of life encompassing audiovisual observation, informant field interviews and case reviews. The second presentation will provide an overview of a 3-phase mixed methods study focused on development of preliminary types of lucid episodes. Collectively, presentations demonstrate a variety of approaches to investigating episodes of lucidity and shed light on emergent methods for operationalizing these events. Implications of various conceptualizations for EL and conceptual decisions across studies will be reviewed.

### FEASIBILITY OF A LONGITUDINAL AUDIOVISUAL OBSERVATION PROTOCOL TO CHARACTERIZE LUCIDITY IN ADVANCED AD/ADRD

Andrea Gilmore-Bykovskyi, Laura Block, Clark Benson,
Jess Fehland, Meghan Botsch, Kimberly Mueller, and
Alison Coulson, *University of Wisconsin-Madison, Madison, Wisconsin, United States*

Episodes of lucidity (EL) among individuals with advanced dementia, which are predominantly reported near end of life, are clinically and potentially epidemiologically significant events. Audiovisual observation offers a valuable approach to studying EL, providing opportunities to characterize verbal/non-verbal features of EL as well as their surrounding contexts. Approaches to capturing and characterizing audiovisual data and potential verbal/non-verbal indicators of EL near end of life are lacking. We determined the acceptability and feasibility of a multi-faceted observational study protocol among people with advanced dementia near end of life incorporating longitudinal audiovisual observation, informant field interviews/logs, and case review of high likelihood EL events. Five eligible individuals were enrolled, yielding a 100% enrollment/retention rate, 54 observations/140 hours of observation across participants. Task load and usability ratings indicate study procedures with iterative refinements were feasible; surveys and qualitative appraisal from participants and staff endorse high rates of acceptability and feasibility.

### A METHODOLOGICAL APPROACH TO IDENTIFY EPISODES OF LUCIDITY AMONG PEOPLE WITH ALZHEIMER'S DISEASE

Kyungmin Kim[1], Theresa Frangiosa[2], Virginia Biggar[2],
Dawne Finnie[3], Lauren Bangerter[4], Joseph Gaugler[5],
Maria Lapid[3], and Joan Griffin[3], *1. Seoul National University, Seoul, Seoul-t'ukpyolsi, Republic of Korea, 2. UsAgainstAlzheimer's, Washington, District of Columbia, United States, 3. Mayo Clinic, Rochester, Minnesota, United States, 4. United Health Group, Edina, Minnesota, United States, 5. University of Minnesota, Minneapolis, Minnesota, United States*

People with late-stage Alzheimer's disease and related dementias (ADRD) who are assumed to have lost coherent cognitive capacity may exhibit unexpected episodes of lucidity (EL). Given the transient nature and lack of scientific explanation of the phenomenon, EL is under-investigated and poorly understood. To better understand this phenomenon, we set out to develop an operational definition of EL. Based on survey data from former and current family caregivers participating in UsAgainstAlzheimer's A-LIST® (N = 480), we defined four EL typologies. We then interviewed 25 caregiver respondents about their experiences and used analyzed qualitative data to refine the preliminary typologies. Finally, we conducted a Delphi consensus panel with clinicians, researchers, and health care providers with medical, pharmacological, and clinical expertise to describe potential explanations for EL to help further refine the typologies. Next we will test the validity of these typologies in a prospective, demographically diverse sample of current family caregivers.

### APPLICATION OF QUALITATIVE METHODS IN THE DEVELOPMENT OF AN INFORMANT-REPORTED MEASURE OF LUCIDITY

Mildred Ramirez[1], Jeanne Teresi[2], Julie Ellis[3], Paloma Gonzalez-Lopez[4], Stephanie Silver[4], Usama Bhatti[5], Davangere Devanand[6], and Jose Luchsinger[7], *1. Hebrew Home at RiverSpring Health, Riverdale, New York, United States, 2. Research Division, HHAR, Riverdale, New York, United States, 3. La Trobe University, Nursing and Midwifery, Bundoora, Victoria, Australia, 4. Research Division, Hebrew Home at Riverdale by RiverSpring Health, Riverdale, New York, United States, 5. Research Division, Hebrew Home at Riverdale by RiverSpring Living, Bronx, New York, United States, 6. Columbia University Irving Medical Center, New York, New York, United States, 7. Department of Medicine, Columbia University, New York, New York, United States*

The aim was a qualitative evaluation of a lucidity measure for front-line staff or family informants. A sequential approach to item development and evaluation was followed: refinement of the operationalization of the construct; review of seminal items, modification, and purification; confirmation of the feasibility of reporting methodology. Modified focus groups were conducted with 20 staff and 10 family members who participated using a web-based survey. Sample themes included: reaction when hearing the term; words that come to mind; description of and first reaction to referenced or observed "lucidity" events. Data were extracted from Qualtrics for analysis using NVivo. Semi-structured cognitive interviews were conducted with10 health professionals working with older adults with cognitive impairment. An external advisory board reviewed the clarity, breadth, and scope of the conceptual definition and item content. Suggestions for item modification derived from the focus groups and cognitive interviews resulted in the final lucidity measure.

### IS THERE A DIFFERENCE BETWEEN TERMINAL LUCIDITY AND PARADOXICAL LUCIDITY?

Andrew Peterson[1], Justin Clapp[2], Kristin Harkins[2], Melanie Kleid[2], Emily Largent[2], Shana Stites[2], and Jason Karlawish[2], *1. George Mason University, Fairfax, Virginia, United States, 2. University of Pennsylvania Perelman School of Medicine, Philadelphia, Pennsylvania, United States*

Recent arguments in aging and dementia research suggest that "terminal lucidity"—defined as unexpected communication or connectedness occurring shortly before death—is distinct from "paradoxical lucidity"—defined as an episode of communication or connectedness in a person who is assumed to have lost these capacities due to progressive neurodegeneration. We disagree with this distinction and argue that terminal lucidity is a special subtype of paradoxical lucidity. We suggest that specifying the relationship between terminal and paradoxical lucidity is important for investigating the underlying mechanism of lucidity in dementia.

### APPROACHES TO INVESTIGATING LUCIDITY IN ADRD: A PRELIMINARY RESEARCH FRAMEWORK TO GUIDE OPERATIONAL HARMONIZATION

Andrea Gilmore-Bykovskyi[1], Joan Griffin[2], Kimberly Mueller[1], Sam Parnia[3], and Ann Kolanowski[4], *1. University of Wisconsin-Madison, Madison, Wisconsin, United States, 2. Mayo Clinic, Rochester, Minnesota, United States, 3. NYU Grossman School Of Medicine, New York, New York, United States, 4. Penn State University, State College, Pennsylvania, United States*

Episodes of lucidity (EL) in Alzheimer's disease and Alzheimer's disease related dementias (AD/ADRD), also termed "paradoxical lucidity," have garnered increasing attention as an important area of research. Efforts to study lucidity suffer from a lack of clear definitional criteria, inconsistent conceptualization and diverse approaches to operationalizing features of these events. To advance systematic investigation of EL in AD/ADRD, there is a need for clarity and precision in labeling event attributes, markers, and specific measurement strategies that enable operational harmonization across distinct approaches to investigating the relatively broad and nascent phenomenon. To that end, we propose a preliminary research framework to guide harmonization of approaches to investigating EL in AD/ADRD. Our goal is to provide a systematized approach to characterizing operational decisions that facilitates comparability across different methodological approaches and measurement strategies while allowing for exploration of multiple definitional criteria, measurement standards, and interpretive approaches.

### SESSION 2170 (PAPER)

### PAIN, PHYSICAL ACTIVITY, AND FUNCTION

### EXPLAINING THE RACIAL AND ETHNIC DIFFERENCES IN ADL DISABILITY AMONG OLDER ADULTS: A POLYSOCIAL SCORE APPROACH

Chenkai Wu, and Junhan Tang, *Duke Kunshan University, Kunshan, Jiangsu, China (People's Republic)*

Disability in activities of daily life (ADL) is prevalent among older Americans. Racial and ethnic disparities in functional ability in old age continue to be a public health concern. We examined whether social environment, measured in a comprehensive way (polysocial score approach), could modify the racial and ethnic differences in ADL disability. Data are from the Health and Retirement Study; 5,925 older adults initially free of disability were included. Six ADLs were considered: bathing, eating, using the toilet, dressing, walking across the room, and getting in/out of bed. We included 24 social factors from five categories (economic stability, neighborhood environment, education, community/social context, and healthcare system) and used forward stepwise regression to screen for important ones. Polysocial score was created using 13 social factors and was classified as low (0-19), intermediate (20-30), and high (31+). We used the multivariable Poisson regression to estimate the risk of incident disability by three polysocial score categories and evaluate the interaction between race/ethnicity (non-Hispanic Whites and Others) and the polysocial score. A higher polysocial score is associated with a lower disability risk among non-Hispanic Whites and Others. We found an additive interaction between race/ethnicity and polysocial score categories. In the low polysocial score group, non-Hispanic Whites had a 4.7% lower risk of disability than the Others, while the difference significantly reduced to 2.4% and 2.6% in the intermediate and high polysocial score group, respectively. The polysocial score approach offers a new opportunity to explain the racial/ethnic disparities in functional capacity among older adults.

### GROUP-BASED TRAJECTORY MODELING OF NURSING HOME RESIDENT PAIN SCORES

Connie Cole[1], Susan Hickman[2], Justin Blackburn[3], Janet Carpenter[4], and Chen Chen[4], *1. University of Colorado Anschutz Medical Campus, Kendallville, Indiana, United States, 2. Indiana University School of Nursing, Indianapolis, Indiana, United States, 3. IU Richard M. Fairbanks School of Public Health at IUPUI, Indianapolis, Indiana, United States, 4. Indiana University, Indianapolis, Indiana, United States*

Up to 80% of older adults living in a nursing home (NH) experience pain and up to 32% have substantial pain. Pain in NH residents is associated with poor quality of life, higher likelihood of depression, and decreased life satisfaction. Pain in NH residents has often been studied using a cross-sectional approach, which fails to consider the temporal nature of pain. Therefore, the purpose of this analysis was to identify and characterize clinically meaningful, dynamic pain trajectories in NH residents using data from the Minimum Data Set. A retrospective longitudinal analysis was conducted using group-based trajectory modeling with pain scores from admission to discharge or a maximum of 28 assessments. We identified four distinct trajectories: 1) consistent pain absence (48.9%), 2) decreasing-increasing pain presence (21.8%), 3) increasing-decreasing pain presence (15.3%), and 4) persistent pain presence (14.0%). Relative to residents' in the consistent pain absence trajectory, the likelihood of being in the persistent pain presence trajectory was more than twice as high for those living in a rural versus (AOR 2.7, CI 2.2-3.4, p<.001), over 4 times higher for those with hip fracture (AOR 4.3 CI 2.6-7.0, p<.001), nearly 3 times higher for those with a fracture other than hip (AOR 2.9, CI 2.0-4.1, p<.001), and almost twice as high for those with contracture (AOR 1.7, CI 1.4-2.1, p<.001). Using residents' characteristics associated with persistent pain such as hip fracture or contracture may improve care planning based on early identification or risk stratification and can improve mitigation of persistent pain.

### RACE AND ETHNIC DIFFERENCES IN PHYSICAL ACTIVITY, OSTEOPENIA, AND OSTEOPOROSIS: RESULTS FROM NHANES

Elizabeth Vasquez[1], Md Towfiqul Alam[2], and Rosenda Murillo[3], *1. Albany University (SUNY), Rensselaer, New York, United States, 2. University of North Carolina at Greensboro, Greensboro, North Carolina, United States, 3. University of Houston, Houston, Texas, United States*

Introduction: Osteopenia and osteoporosis are common age-related disorders with enormous health and economic consequences to older adults and society. Physical activity (PA) is an important modifiable risk factor for bone mineral density (BMD). This study aims to determine whether current physical activity is related to osteopenia and osteoporosis (based on BMD) in a racial/ethnically diverse sample of older adults.

Methods: Femoral bone BMD data from the National Health and Nutrition Examination Survey (NHANES 2009-2010, 2013-2014, 2017-2018) was obtained for 3,331 adults 60-80 years old. Self-reported PA was categorized into high, moderate, and low. Linear regression models that accounted for the complex survey design of NHANES examined the association between PA and BMD for each race/ethnic group.

Results: Non-Latino blacks (blacks) and Latinos reported low levels of PA when compared to Non-Latino whites (whites) (40.7%, 38.2% and 32.4% respectively; p< 0.0001). Further, blacks and Latinos had a lower prevalence of osteoporosis (5.6%, 6.4% and 9.0% respectively; p< 0.0001), but have similar prevalence of normal BMD and osteopenia categories when compared to whites. There was a 0.03 g/cm2 difference in BMD between those in the high PA versus the low PA (p< 0.0001).

Conclusion: Our findings indicate that despite lower levels of activity, black and Latino older adults were less likely to have osteoporosis. High levels of activity were significantly associated with higher BMD after controlling for confounders. Considering the prevalence and burden of osteopenia and osteoporosis and projected increases of the older population we need more research evidence supporting the role of PA.

### TESTING THE MODERATING EFFECTS OF DEPRESSIVE SYMPTOMS ON A PHYSICAL ACTIVITY INTERVENTION

Emily Smail, Christopher Kaufmann, and Todd Manini, *University of Florida, Gainesville, Florida, United States*

Depressive symptoms affect the physical and cognitive health of approximately 20% of older adults. These symptoms have strong, bidirectional ties with physical activity levels and mobility disability. Physical activity has a positive impact on mood and depression and is highly recommended for symptom management across all ages. However, it's unclear whether elevated depressive symptoms interfere with potential benefits that physical activity has on other health outcomes like mobility loss. The Lifestyle Interventions and Independence for Elders (LIFE) Study randomized over 1,500 older adults (aged 70+) to either a physical activity (intervention) or successful aging (control) program with an average of 2.2 years of follow-up. Our analysis used Cox proportional hazards models to evaluate whether elevated depressive symptoms (measured using the Center for Epidemiological Studies-Depression (CESD)-11 scale with a cutoff score of 16/22 points) moderated the relationship between intervention status and the primary outcome (incident major mobility disability, objectively measured as the ability to walk 400 meters). In a secondary analysis of 1,534 older adults (Mage = 78.8, 66.7% female), we confirmed significant main effects of both the physical activity intervention and elevated depressive symptoms on incident major mobility disability but found no evidence of moderation (interaction p-value=0.989). Results indicate that the benefits of the intervention were comparable between those with and without significant depressive symptoms at baseline, supporting the inclusion of individuals with depression in clinical trials. In addition to potential symptom relief, promoting physical activity in older adults with depression is important for protecting against mobility loss.

### SESSION 2180 (BIOLOGICAL SCIENCES INVITED SYMPOSIUM)

### PRESIDENTIAL SYMPOSIUM: NUTRITION, METABOLISM, AND AGING

Chair: Rozalyn Anderson

Aging is associated with increased risk for a host of non-communicable diseases and disorders including diabetes, cardiovascular disease, cancer, and neurodegeneration. Although the underlying basis for this shared risk as a function of aging is not known, numerous diseases of aging have an established metabolic component. Data from preclinical and basic research indicate that the pace of aging is malleable, and studies in short-lived species have shown the ability of genetic and nutritional interventions to not only positively impact longevity but also to prolong health into older age. This symposium, “Nutrition, Metabolism, & Aging”, features internationally renowned aging research scientists whose work focuses on how nutrition and metabolism intersect with aging biology. We will hear from John Speakman from the University of Aberdeen who will present his research on “Metabolism and Extended Longevity”; Leanne Redman from the Pennington Biomedical Research Center whose work in humans will be described in her talk on “Dietary Interventions for Healthy Aging”; Holly Brown-Borg from University of North Dakota will discuss endocrine modulation of aging in her talk “Somatotropic Signaling in Health and Longevity”; and Valter Longo from the University of Southern California who will discuss the latest findings from his research in mice and humans in his talk “Periodic Fasting in Aging and Disease". Attendees will learn about nutrition and metabolism and how these cues are interwoven to regulate aging, impact health, and enhance longevity.

### SOMATOTROPIC SIGNALING IN HEALTH AND LONGEVITY

Holly Brown-Borg, *University of North Dakota, Grand Forks, North Dakota, United States*

The endocrine system is highly integrated and regulates growth, reproduction, metabolism, and stress responses that influence aging. Reduced growth hormone (GH) signaling extends health and lifespan in part, by altering metabolism maintaining enhanced insulin sensitivity and defense mechanisms. Diet composition affects metabolism and GH status integrates these nutrient signals, modulating metabolic responses that result in age-related disease susceptibility. Two pathways affected by somatotropic signaling include methionine and lipid metabolism. GH appears to regulate oxidative defense and the methionine pathway via enzymes that affect S-adenosyl-methionine, glutathione, DNA methylation, and detoxification activities. We also have evidence that GH deficient mice escape fatty liver disease when fed high-fat diets. Together our work and others indicate that GH plays a significant role in an organism’s ability to respond to nutrients and cellular stressors by regulating factors that counter stress, modulating metabolic responsiveness to nutrients, and detoxification of endogenous and exogenous compounds.

### PERIODIC FASTING MIMICKING DIET, LONGEVITY, AND DISEASE

Valter Longo, *Leonard Davis School of Gerontology, Los Angeles, California, United States*

Chronic dietary interventions have been known for decades to help prevent disease and extend longevity, yet most are difficult to adopt especially long-term. Brief periods of a diet that mimics fasting by regulating key starvation response genes including IGF-1, TOR-S6K and PKA lasting between 4 and 7 days and followed by long periods on a normal diet, are emerging as potentially effective pro-longevity interventions. These periodic fasting mimicking diets (FMD) provide low calories, sugars, and proteins and high levels of unsaturated fats. In mice, 4 day bi-monthly cycles of the FMD started at middle age extend longevity, reduce tumors by nearly 50%, reduce inflammatory diseases and increase cognitive performance at old ages. In humans, 3 monthly cycles of a 5 day FMD reduce markers or risk factors for aging, diabetes, cancer and cardiovascular disease including cholesterol, blood pressure, CRP, IGF-1, and fasting glucose, particularly in subjects with elevated levels of these markers at baseline. Here I will present our most recent mouse and clinical studies indicating that FMDs can help reverse insulin resistance, reduce risk factors/markers of aging and age-related diseases, and lower biological age.

### DIETARY INTERVENTIONS FOR HEALTHY AGING

Sai Krupa Das[1], Leanne Redman[2], *1. Tufts University, Boston, Massachusetts, United States*
*2. Pennington Biomedical Research Center, Baton Rouge, Louisiana, United States*

Aging is associated with a host of cellular and molecular changes that cumulatively result in a progressive decline in metabolic and physical function, development of chronic disease, and increased risk of mortality. Dietary interventions targeting these age-related changes have been shown to attenuate the aging process and improve healthspan, i.e., the length of time individuals are disease- and disability-free. Calorie restriction (CR) is one such intervention that has been shown to be effective in reducing disease risk and improving multiple markers of biological aging. The biological mechanisms mediating the observed benefits of CR are not fully understood but possibly involve changes in energy metabolism, oxidative damage, insulin sensitivity, inflammation, and function of both the neuroendocrine and sympathetic nervous systems. Despite the benefits of CR, sustained adherence remains a challenge. An alternative dietary approach with potential for impact on markers of aging and disease risk is intermittent fasting (IF), which represents a broad class of meal-timing interventions that involve alternating periods of eating and extended fasting. One novel form of IF called time-restricted eating (TRE) involves eating within a 10-hour period and fasting for the rest of the day. TRE appears to improve adherence to IF and especially improve cardiometabolic health in humans. Studies in rodents and pilot studies in humans have found that TRE reduces body weight and hunger, improves insulin sensitivity, lowers blood pressure, reduces inflammation and oxidative stress, improves circadian rhythms, and, in rodents only, extends lifespan. With feasibility data demonstrating reasonably high adherence rates, TRE is a promising strategy to improve markers of healthspan.

### SESSION 2200 (PAPER)

### RESILIENCE

### DISRUPTED ACTIVITIES AND CAREGIVERS RESILIENCE: LONGITUDINAL PATTERNS AND ASSOCIATIONS

Jeong Eun Lee, and Natasha Peterson, *Iowa State University, Ames, Iowa, United States*

PurposeRelatives are often caregiving for patients with advanced cancer and carry a heavy burden. Self-care and resilience-building activities offer building blocks to enhance their wellbeing and burden-bearing capacity. This study assessed the longitudinal patterns of disrupted activities of caregivers and their association with resilience reported by caregivers. MethodsData came from the National Study of Caregiving, a nationally representative study of caregivers. We analyze outcomes for caregivers for three waves of NHAT (2011-2017), estimating associations of disrupted activities and positive appraisals on 1) caregiving burden 2) resilience. Caregivers reported positive appraisals and the number of activities that have been interrupted due to their caregiving responsibilities. ResultsMultilevel models with heterogeneous within-person variance were fit to test the hypotheses on associations between disrupted activities, positive appraisals, and fluctuations in resilience and caregiving burden. Our analysis confirmed increasing disrupted activities among caregivers over time. When interrupted activities were higher than average, caregivers reported lower levels of resilience and higher levels of caregiving burden, suggesting that disrupted activities can contribute to caregivers. The reverse pattern was found for positive appraisals. ConclusionMore positive appraisals and lower levels of disrupted activities were associated with lower caregiver burden and higher levels of caregiver resilience. This study points to the critical value that caregiver self-care and positive appraisals are essential to bolster caregiver resilience and counteract caregiver burden. Future studies should illuminate the causal relation. This study will broaden our understanding of caregivers' self-care needs and provide practical implications for supporting caregivers.

### MEASURING RESILIENCE ACROSS THE LIFE COURSE: FINDINGS FROM HRS AND ADD HEALTH

Miles Taylor, Tyler Bruefach, and Dawn Carr, *Florida State University, Tallahassee, Florida, United States*

The study and measurement of psychological resilience (PR) is increasingly of interest to interdisciplinary audiences focused on successful aging across the life course. Though existing nationally representative datasets have not directly measured PR previously, recent advances have produced two novel measures showing promise among older and younger adults, respectively. We examine whether these measures capture similar internalized resources and if they effectively measure PR across race/ethnicity, gender, SES, and age. Analyses use two PR measures: the Simplified Resilience Scale (SRS) created in the 2006/8 Health and Retirement Study (N=14,064), and the Add Health Resilience Scale (AHRS) created in Wave 4 (2008) of the National Longitudinal Study of Adolescent to Adult Health (N=4,936). Both scales reflect Wagnild and Young's (1993) conceptualization of PR as an internalized, multidimensional psychological resource that is protective for health and wellbeing. Confirmatory Factor Analyses suggest that both measures capture a single construct of PR well, on average (one-factor structure; CFI >.95, RMSEA <.06). These measures also perform similarly across social groups; we observed configural, metric, and at least partial scalar invariance across race/ethnicity, gender, education, and age. Assessing the validity of these measures across diverse US populations at various life stages is critical to understanding how PR shapes aging processes over the life course, and for promoting successful aging throughout mid and later life. Future research can advance understanding of PR and its measurement by leveraging intersectional combinations of social statuses including race/ethnicity, gender, education, and age.

### RESILIENCE AND MULTIFACTORIAL STRESSORS AMONG OLDER ADULTS DURING THE COVID-19 PANDEMIC: A QUALITATIVE STUDY

Melissa Hladek[1], Deborah Wilson[2], Sabrina Shofner[1], Alden Gross[3], Karen Bandeen-Roche[3], and Nancy Schoenborn[1], *1. Johns Hopkins University, Baltimore, Maryland, United States, 2. Johns Hopkins University School of Nursing, Baltimore, Maryland, United States, 3. Johns Hopkins Bloomberg School of Public Health, Baltimore, Maryland, United States*

The COVID-19 pandemic represents a complex stressor that is experienced differently across individuals and age strata. The present study explored perceptions and experiences of older adults within the domains of health, social interactions, finances and care of existing chronic medical conditions; and strategies used to cope with these stressors. We recruited 30 people (mean age 81.4 years) stratified by frailty status to complete semi-structured interviews about what changes to the above domains had occurred and what coping strategies were utilized. Using inductive and deductive coding techniques, thematic analysis revealed three overarching themes. The first was Pandemic Experience, which was perceived as stressful, especially in the domains of social isolation from friends and family and concern for others' well-being. The second domain was Resilience where participants reported highly adaptable and creative ways to connect with others and viewed the pandemic from a lens of lifetime experience, which acted as a stress buffer. The third theme was Silver Linings where participants reported unexpected renewal like reconnecting with family and friends in more meaningful ways and reconnecting with nature. We found no meaningful distinction in experience by frailty status and explore reasons for this. Policy implications including internet access and training and societal aging biases are discussed in the context of aging and coping theories.

### RESILIENCY AMONG WHI WOMEN IN THE 80+ COHORT BY RACE AND NEIGHBORHOOD SOCIOECONOMIC STATUS

Jessica Krok-Schoen[1], Michelle Naughton[2], Crystal Cene[3], Aladdin Shadyab[4], Sparkle Springfield[5], Timiya Nolan[1], Ashley Felix[1], and Rebecca Jackson[1], *1. The Ohio State University, Columbus, Ohio, United States, 2. The Ohio State University Comprehensive Cancer Center, Columbus, Ohio, United States, 3. University of North Carolina, Chapel Hill, North Carolina, United States, 4. University of California at San Diego, La Jolla, California, United States, 5. Loyola University, Maywood, Illinois, United States*

Resilience, an individual's ability to successfully adapt to adversity, is a multifaceted outcome that may be affected by individual and community factors. A comprehensive examination of resilience by race and neighborhood socioeconomic status (NSES) among women aged 80+ is needed to better understand longevity in diverse populations. Women aged 80+ in 2011, in the Women's Health Initiative (WHI) study were included. Resilience was measured using the 3-item Brief Resilience Scale, with higher scores indicating better resiliency. Descriptive statistics and multivariable linear regression examined the association of demographic, psychosocial, and health variables with resilience by race (White, Black, Asian) and NSES. The majority of participants (n=29,367, median age=84.0) were non-Hispanic White (91.4%), and had multimorbidities (66%). There were no significant differences by race on mean resiliency scores (p=0.06). Mean resilience was higher among women with higher NSES (low NSES=3.94±0.83, moderate NSES=3.95±0.82, high NSES=4.00±0.81; p< 0.001). Optimism (p< 0.001), social support (p< 0.01), and physical/mental symptom burden (p< 0.05) were significant correlates of resilience among Asian, Black, and White women. Self-rated health (p< 0.001), depressive symptoms (p< 0.001), optimism (p< 0.001), social support (p< 0.001), physical/mental symptom burden (p< 0.001), and body mass index (p< 0.001) were significant correlates of resilience across women with low, moderate, and high NSES. Age was significantly associated with resilience among women with moderate (β=-0.004, p=0.019) and high NSES (β=-0.005, p=0.045). This study found several common correlates of resilience across race and NSES among women aged 80+ in the WHI. Future research to enhance resilience, such as through psychosocial and behavioral interventions, is warranted.

### ROLE OVERLOAD AND SLEEP DISTURBANCE AMONG CAREGIVERS OF PERSONS WITH DEMENTIA: THE MODERATING EFFECT OF RESILIENCE

Elliane Irani, Fei Wang, Stephanie Griggs, and Ronald Hickman, *Case Western Reserve University, Cleveland, Ohio, United States*

Sleep disturbance is a significant sleep health problem among caregivers of persons with dementia and could exacerbate numerous adverse health outcomes. Resilience is one's ability to recover from challenges and is a protective factor that is posited to blunt the effect of caregiving stress on sleep disturbance. Therefore, the purpose of this study was to examine if resilience moderates the association between perceived role overload and sleep disturbance using data from the 2017 wave of the National Study of Caregiving. The sample consisted of 480 informal caregivers (mean age = 61.77 years, SD = 13.69 years) of persons with dementia in the U.S. Participants completed self-report questionnaires about their sociodemographic and health characteristics, perceived role overload, resilience (i.e., adjusting to change and recovering from hardship), and sleep disturbance (i.e., frequency of trouble maintaining sleep). Data were analyzed using multiple regression with interaction terms to evaluate the moderation effect of resilience. Higher perceived role overload was associated with higher sleep disturbance, while controlling for caregivers' age, race, gender, education, and self-rated health. The association of perceived role overload with sleep disturbance was attenuated among caregivers with higher levels of resilience (b = -.029, 95% CI: -.058, -.001). This study highlights the stress-buffering and sleep health benefits of resilience in the context of dementia caregiving. Among caregivers of persons with dementia, approaches to improve their ability to recover, resist, and rebound during caregiving challenges may mitigate stress and optimize their sleep health.

### SESSION 2210 (SYMPOSIUM)

### RURAL SYSTEM-BASED COPING STRATEGIES FOR ADVANCING RESEARCH AND HEALTHCARE WHILE TRANSITIONING TO THE NEW NORMAL

Chair: Joan Ilardo Co-Chair: Angela Zell

The pandemic brought about many changes at a system level that were necessary to facilitate continuity of research and the practice of healthcare. Rural and underserved populations were and continue to be the hardest to reach both before and during the COVID-19 pandemic. Therefore, the innovative coping strategies that were implemented during the pandemic are essential to continue as we transition into the endemic stage and realize our new normal as researchers and practitioners. This symposium will highlight adaptations made to research and practice during the COVID-19 pandemic that will apply to future system-level strategies. Drs. Wiese and Park will present results from an online exercise intervention for rural older adults that are at risk of cognitive decline. Drs. Ilardo and Zell will present a roadmap inspired by the pandemic to promote food equity. Drs. Zell and Haque will address rural solutions to polypharmacy issues using an interdisciplinary team approach. Dr. Monaghan-Geernaert will discuss important implications that international caregivers have in fulfilling caregiving gaps in the US, followed by Dr. Freeman et al.'s discussion of rural, long-term care facilities (LTCF) experiences during the pandemic. These system-based coping strategies have policy implications for rural healthcare as we transition to the new normal.

### PANDEMIC-INSPIRED COLLABORATIVE ROADMAP TO PROMOTE RURAL FOOD EQUITY ACROSS THE LIFESPAN

Joan Ilardo, and Angela Zell, *Michigan State University, East Lansing, Michigan, United States*

Early during the pandemic, access to food by residents across the lifespan was problematic in many communities.

We observed well-intentioned responses by community organizations but a lack of centralized coordination across sectors. Most organizations were not aware of the efforts and capabilities of others causing duplication or gaps in services. Our team created a user-friendly, evidence-based roadmap to guide communities through developing and sustaining effective collaborative partnerships for food and nutrition-related problems to address together. We will describe elements of an effective, efficient roadmap development process using currently available evidence-based resources and creating evidence-informed resources when we identify gaps. Expected outcomes are: 1) format of the final roadmap; 2) types of groups willing to use it; 3) how well the roadmap was implemented; 4) feasibility of continued use of the roadmap by groups over the long term; and 5) potential to expand roadmap use to other communities.

### TEAMS AND TOOLS: ADDRESSING POLYPHARMACY USING THE ARMOR TOOL IN A RURAL NURSING CARE FACILITY

Angela Zell, and Raza Haque, *Michigan State University, East Lansing, Michigan, United States*

Federal guidelines by the Centers for Medicare and Medicaid Services around the use of an interdisciplinary team (IDT) approach have been in place for years. However, use of evidence-based tools to address polypharmacy have not been specified. A study was conducted to evaluate the implementation of the ARMOR (Assess, Review, Minimize, Optimize, Reassess) tool to address polypharmacy in a rural nursing care facility. The tool provided a stepwise approach to a standardized process for the IDT to discuss individual resident care plans including psychotropic medications, psychosocial concerns, falls, quality of life, functional status and other factors in their weekly meetings. Surveys and a focus group were used to measure attitudes and skills of the IDT in relation to the use of the evidence-based tool in the IDT process. Data collected presents an overall positive attitude for and improvement in skills in the IDT with the use of the ARMOR tool.

### DIGITAL LEARNING AND ONLINE CHAIR YOGA FOR RURAL UNDERSERVED OLDER ADULTS AT RISK OF COGNITIVE DECLINE

Lisa Wiese[1], and JuYoung Park[2], *1. Florida Atlantic University, Boca Raton, Florida, United States, 2. Florida Atlantic University College of Social Work and Criminal Justice, Boca Raton, Florida, United States*

We conducted a randomized control trial to test the feasibility of an online chair yoga intervention among rural older adults at risk for cognitive decline in an underserved, racially/ethnically diverse community. Participants were randomly assigned to either chair yoga (n=15) or computer brain games (n=15). Prior to the 12-week intervention, a computer training program was provided to all participants by local high school students, who followed a previously tested curriculum, and mentored the older adults in attending sessions. Digital literacy and cognitive, physical, and psychosocial measures were collected at baseline, 3 weeks, and post-intervention, with 3 and 6-month follow-up. We assessed students' attitudes toward older adults pre/post-intervention. Both student and resident samples were 98% minority (African American, Latino, and Afro-Caribbean), with residents' average age of 67.5 (SD = 8.3), years lived rural 38 (M = 11.5), and initial digital literacy of 48.5%. Additional outcomes will be detailed in this presentation.

### THE EFFECTS OF COVID-19 ON THE MIGRATION OF HOME HEALTHCARE WORKER

Pamela Monaghan-Geernaert, *Northern State University, Aberdeen, South Dakota, United States*

Home health caregivers provide a vital role in allowing people to age in place. Women, and in particular immigrant women, have become the largest provider of home health care in Western Industrialized Countries. Global push-pull factors affecting caregivers were greatly disrupted with the emergence of the global pandemic – COVID-19. The pandemic highlighted how fragile the system is and how the easily the 'grey economy' can be compromised. This research looks at border access, immigration restrictions and other factors which impeded the migration of caregivers. Poor countries suffered greatly with restrictions on exporting women who use cross national moves as a means to generate income for families in their homeland, and rich countries faced extreme shortages. The results were devastating for all parties – caregivers and care recipients alike.

### ASSESSING THE IMPACT OF COVID-19 ON THE HEALTH AND WELLNESS OF THE LONG-TERM CARE WORKFORCE IN RURAL AND NORTHERN AREAS

Shannon Freeman[1], Davina Banner[1], Hui Jun Chew[1], Tammy Klassen-Ross[1], and Piper Jackson[2], *1. University of Northern British Columbia, Prince George, British Columbia, Canada, 2. Thompson Rivers University, Kamloops, British Columbia, Canada*

There is growing recognition that the mental health and wellbeing of the LTCF workforce have been disproportionately impacted by COVID-19. Therefore, we sought to describe the experiences and challenges LTCF employees faced during COVID-19 in rural and northern communities and highlight factors affecting their ability to be resilient and provide high quality care. We conducted 53 qualitative interviews using zoom with LTCF care providers (care aides, nurses, social workers), staff (food service workers, recreation providers), and management between November 2021 and February 2021. Data was transcribed and thematic analysis undertaken. We will describe participants experiences stratified by LTCF employee type and highlight similarities and differences in participants experiences across geography and facility type (freestanding vs. co-located in hospital) and describe factors affecting well-being, job satisfaction, and retention. We will share an inventory of programs and strategies participants found useful to mitigate negative effects on their mental health and well-being.

### SESSION 2220 (SYMPOSIUM)

### SELF-PERCEPTIONS OF AGING IN THE CONTEXT OF CHALLENGING EXPERIENCES: PATTERNS OF RISK AND RESILIENCE

Chair: Hannah Giasson Co-Chair: Rachel Koffer
Discussant: Jacqui Smith

Self-perceptions of aging have important implications for health and well-being in later life. Early life experiences, cultural and societal notions about age, and one's present health and situational context may contribute to one's expectations and perceptions of their own aging (e.g., Levy, 2009; Diehl et al., 2014; 2021). However, self-perceptions of aging may also predict people's responses in the face of current or future challenges. This symposium takes a closer look at self-perceptions of aging in the context of different types of life challenges. Hu and Larkina discuss early life informal caregiving experiences as antecedents of negative self-perceptions of aging in later life. Koffer and Giasson discuss how ten-year longitudinal associations between subjective age and future loneliness differ among current caregivers and non-caregivers. Mejia and colleagues discuss the role of self-perceptions of aging in adaptation to life following a fall, highlighting potential protective effects of positive self-perceptions of aging and sense of control. Finally, Giasson and colleagues discuss positive self-perceptions of aging as predictors of preventive health behavior and resilience in the context of the COVID-19 pandemic. Dr. Jacqui Smith will conclude the session with an integration of common themes, practical implications, and future research directions that emerge from the four studies.

### POSITIVE SELF-PERCEPTIONS OF AGING AS PREDICTORS OF COVID-RELATED PREVENTIVE BEHAVIOR AND RESILIENCE

Hannah Giasson[1], William Chopik[2], and Alejandro Carrillo[2], *1. Arizona State University, Tempe, Arizona, United States, 2. Michigan State University, East Lansing, Michigan, United States*

Individuals have faced extraordinary challenges throughout the COVID-19 pandemic. Psychosocial strengths may promote individuals' resilience during this time. Positive self-perceptions of aging (SPA) have been found to predict a variety of health and well-being indicators. We examined SPA as a predictor of COVID-19-related behavior, adaptation, and resilience in a sample of 3,620 adults (Mage=65.88; 61.1% women; 65.4% white) from the 2016 and 2020 waves of the Health and Retirement Study. Linear regression results revealed that more positive SPA in 2016 was associated with more preventative health behavior ($\beta$=.03, p=.04), a higher likelihood of staying at home ($\beta$=.07, p<.001), less worry ($\beta$=-.27, p<.001), less stress ($\beta$=-.24, p<.001), less loneliness ($\beta$=-.27, p<.001), and greater resilience ($\beta$=.20, p<.001) during the first year of the pandemic (2020). Associations held after controlling for demographic covariates. Findings support SPA theories, suggesting linkages between SPA and adaptive behaviors and outcomes in the face of external challenges.

### SUBJECTIVE AGE AND FUTURE LONELINESS: COMPARING CAREGIVERS AND NON-CAREGIVERS ACROSS TEN YEARS

Rachel Koffer[1], and Hannah Giasson[2], *1. Arizona State University, Phoenix, Arizona, United States, 2. Arizona State University, Tempe, Arizona, United States*

Experiences that make age salient can shape how old or young a person feels relative to their chronological age. Caregiving for a loved one may contribute to subjective age as well as the linkages between subjective age and well-being. We examined longitudinal associations between subjective age and future loneliness ten years later among 2,557 caregivers and non-caregivers (Baseline Mage=55) in the Midlife in the United States Study (2004-2014). Linear regression results indicated an interaction between caregiver status and subjective age ($\beta$ = 0.60, 95% CI=[0.14, 1.06]), such that among non-caregivers, older subjective age predicted greater future loneliness, but among caregivers, younger subjective age predicted greater future loneliness. Associations hold after controlling for age, baseline loneliness, and caregiver status at follow-up. Future subjective aging research should examine the unique context of caregiving to further understand the meaning of subjective age and how it predicts important well-being outcomes among caregivers.

### POSSIBLE SELVES THROUGH CAREGIVING: CAREGIVING HISTORY'S EFFECTS ON SELF-PERCEPTIONS OF AGING

Rita Hu, and Marina Larkina, *University of Michigan, Ann Arbor, Michigan, United States*

Most studies examine the consequences of self-perceptions of aging (SPA). Little is known about the antecedents. The possible selves framework suggests that earlier life experiences can shape people's scenarios about their own aging. We examine the association between early-life informal caregiving experiences and SPA later in life using data from the HRS Retrospective Life History Mail Survey and the Psychosocial and Lifestyle Questionnaire ( N = 2,556, Mage = 72.9). Participants reported up to five periods of unpaid caregiving ($\geq$ 6 months) with family members. SPA was measured by the HRS 8-item scale. Step-wise linear regression revealed that compared with people who had not been caregivers, early-life caregivers reported more negative SPA later in life ($\beta$ = 0.20, 95%CI[0.12, 0.29]). The association holds after controlling for health-related and demographic covariates. Future research and interventions should focus on the consequences of early-life caregiving on the caregiver's scenarios about their own aging.

### SUBJECTIVE EXPERIENCES OF AGING AND PHYSICAL ACTIVITY IN THE YEARS THAT PRECEDE AND FOLLOW A FALL

Shannon Mejía[1], Tai-Te Su[2], and Faith-Christina Washington[1], *1. University of Illinois at Urbana-Champaign, Champaign, Illinois, United States, 2. The University of Illinois Urbana-Champaign, Champaign, Illinois, United States*

Falls are the leading preventable cause of death and disability in older adulthood. Subjective experiences of aging could facilitate fall prevention and adaptation to post-falls life. We use data from the 2008-2018 waves of the Health and Retirement Study to follow self-perceptions of aging (SPA), health-domain control (HDC), and physical activity (PA) in 12,000+ adults (Mage=69.09; 59% women; 83% white) to examine trajectories of subjective experiences and health behaviors preceding and following a fall. In total, 57% experienced falling. Both SPA and HDC were lower among fallers. Spline growth models showed that HDC, SPA, and PA significantly decreased over time. Additionally, the rate of decline in HDC and PA increased following the fall. After falling, the protective effect of HDC amplified, while positive SPA dampened change in PA. Our study illustrates the importance of subjective experiences of aging on adaptation and recovery in the context of falling.

### SESSION 2230 (SYMPOSIUM)

### THE FAMILY-CENTERED FUNCTION-FOCUSED CARE INTERVENTION: IMPLEMENTATION AND MEASUREMENT CONSIDERATIONS

Chair: Marie Boltz Discussant: Jane Flanagan

Family care partners can play an important role in promoting the functional recovery of hospitalized older adults living with dementia. Family-centered function-focused care (Fam-FFC) aims to create an “enabling” milieu for the person with dementia through, staff education, unit-based champions, environmental and policy assessment/modification, and individualized goal setting. In this patient/family-centered care approach, nurses purposefully engage family care partners in the assessment, decision-making, care delivery and evaluation of function-focused care during hospitalization and the 60-day post-acute period. Staff are provided with techniques to support positive interaction with patients and families and respect the role of care partners as advocates. The goal is to promote functional recovery and well-being in the patient, and prevent unnecessary long-term care admissions, while improving care partners’ preparation for caregiving and well-being. Fam-FFC was developed with the input of patients, families, nurses, and rehab therapists. This symposium focuses on key factors associated with the implementation of Fam-FFC, and relevant measures. After an introductory overview of Fam-FFC, the nature of staff/patient interaction during hospitalization, including the influence of gender, will be discussed in the first presentation. Two presentations will address the planning for transition to the post- acute setting, including the intrinsic factors associated with preparation for caregiving, in contrast to the decision to seek long-term care. The final presentation will describe the approach to promoting fidelity to the Fam-FFC intervention, within the context imposed by COVID-19 restrictions. Our symposium will conclude with a discussion of future direction for policy, practice, and research.

### FAMILY PREPAREDNESS AT HOSPITAL DISCHARGE OF THE PERSON LIVING WITH DEMENTIA

Marie Boltz[1], Irene Best[2], and Ashley Kuzmik[3], *1. Penn State, Pennsylvania State University, Pennsylvania, United States, 2. Pennsylvania State University, University Park, Pennsylvania, United States, 3. Penn State University, University Park, Pennsylvania, United States*

Family care partners face increased and more complex caregiving demands at the time of hospital discharge for persons living with dementia. This study examined family care partners’ needs for preparation and factors associated with the degree of preparation for caregiving. Care partners who were younger (t=3.26, p=.001), demonstrated anxiety (t= 2.6, p=.010) and depression (t =4.6, p <.001), and were caring for a person in pain (t=2.3, p=.023) had lower scores on the Preparation for Caregiving Scale. Care partners described the least preparation to care for the patients’ emotional needs (M= 2.8, SD=1.2) and deal with the stress of caregiving (M= 2.7, SD=1.2). Content analysis of nurses’ transitional care notes converged with these findings, while also describing a lack of preparation to advocate for information from medical providers. Findings suggest the need to attend to the psychological and informational needs of care partners, while considering patient well-being and comfort.

### GENDER DIFFERENCES IN INTERACTIONS AND WELL-BEING AMONG HOSPITALIZED PATIENTS LIVING WITH DEMENTIA

Anju Paudel[1], Marie Boltz[2], and Barbara Resnick[3], *1. Penn State Ross and Carol Nese College of Nursing, State College, Pennsylvania, United States, 2. Penn State, Pennsylvania State University, Pennsylvania, United States, 3. University of Maryland, Baltimore, Maryland, United States*

While the incidence of dementia is generally higher in women compared to men, gender differences in interactions and well-being in dementia is still unclear. This study examined gender differences in interactions and well-being among hospitalized patients living with dementia. A total of 140 hospitalized patients (53% female and 47% male) were included in the analysis. On average, the participants were 81.43 years old (SD= 8.29), had positive interactions with staff based on higher scores on Quality of Interaction Schedule, QUIS (5.81, SD= 1.36), and fair emotional well-being based on lower scores on Cornell Scale for Depression in Dementia, CSDD (7.79, SD= 5.59). Although men seemed to have more positive interactions (male=6.07, SD=1.13; female=5.59, SD=1.51) and greater wellbeing (male=7.52, SD=4.77; female=8.03, SD=6.25) than women, there were no statistically significant gender differences observed in linear models with appropriate covariates. Future work should continue to explore gender differences in interactions and well-being.

### PREDICTORS OF THE DESIRE TO INSTITUTIONALIZE IN CARE PARTNERS OF HOSPITALIZED PERSONS WITH DEMENTIA AT DISCHARGE

Ashley Kuzmik, *Penn State University, University Park, Pennsylvania, United States*

Hospitalized persons with dementia are more likely to be discharged to long-term nursing home care as compared to persons without dementia. The desire to seek this care is due in large part to the inability of family care partners to continue to provide care at home. This study aimed to identify predictors of desire to institutionalize (DTI) in care partners of persons with dementia at hospital discharge. A stepwise multiple linear regression was done to examine the dyad factors among 434 patient and care partners associated with DTI . Lower preparedness for caregiving (p < .001), higher caregiver strain (p = .039), cohabitation (p < .001) and care partner race (White; p = .016) were associated with DTI. Findings underscore the need to assess family preparedness for caregiving, strain, and living situation upon admission, and suggest the need to examine racial differences in the desire to seek nursing home care.

### INTERVENTION FIDELITY IN THE FAMILY-CENTERED FUNCTION-FOCUSED CARE INTERVENTION

Barbara Resnick, *University of Maryland, Baltimore, Maryland, United States*

This session will provide a description of the treatment fidelity (TF) plan from the Family-centered Function-focused Care (Fam-FFC) trial. Components of the TF plan, measures, procedures for implementation, and findings will be presented, and discussed within the context of the COVID-19 pandemic. The components of the Fam-FFC TF plan and results include: 1) Delivery based on completion of the steps in Fam-FFC ; 2) Receipt based on evidence of Staff knowledge of Fam-FFC (percentage of nursing staff that demonstrated test scores above 80%); 3) Enactment based on achievement of goals using the Goal Attainment Scale ; completion of the Fam-Path Audit of bedside goals and treatment plans, post-acute follow-up and plan update ; and evidence of Fam-FFC based on the Fam-FFC Behavior Checklist (80% staff performance of Fam-FFC). The TF plan demonstrated evidence of delivery, receipt and enactment of study activities. Findings will be used to develop an implementation trial.

### SESSION 2240 (AWARD LECTURE)

### MAXWELL A. POLLACK AWARD FOR CONTRIBUTIONS TO HEALTH AGING PRESENTATION AND LECTURE

Chair: Philip Rozario

The Maxwell A. Pollack Award for Contributions to Health Aging Lecture will feature an address by the 2021 Pollack Award recipient Namkee G. Choi, PhD, FGSA, of the University of Texas at Austin. This session will also include the presentation of the 2022 Maxwell A. Pollack Award to recipient Nancy Morrow-Howell, MSW, PhD, FGSA, of Washington University in St. Louis. The Maxwell A. Pollack Award for Contributions to Healthy Aging Award recognizes instances of practice informed by research and analysis, research that has directly improved policy or practice, and distinction in bridging the worlds of research and practice.

### ADDRESSING THE MENTAL HEALTH NEEDS OF HOMEBOUND OLDER ADULTS IN AGING SERVICE SETTINGS

Namkee Choi, *University of Texas at Austin, Austin, Texas, United States*

Despite significant and urgent mental health needs among low-income homebound older adults, the existing mental health service systems' reach for these older adults is almost nonexistent. Given the current and projected geriatric mental health workforce shortages, innovative approaches are needed to better serve these underserved older adults. This presentation will focus on a series of randomized clinical trials that tested acceptable and feasible mental health service delivery models for homebound older adults who are served by aging service network agencies that provide hot meals and case management. Findings from the real-world effectiveness trials of tele-delivered behavioral activation treatment for depression and loneliness by bachelor's-level lay counselors who are embedded in aging service agencies will be shared. Ways to promote a wider adoption of scalable and sustainable mental health service delivery models for homebound older adults will be discussed.

### SESSION 2241 (BIOLOGICAL SCIENCES INVITED SYMPOSIUM)

### HUMAN AGING INTERVENTIONS

Chair: Daniel Parker

Biological aging is the greatest risk factor for multiple non-communicable diseases affecting multiple organ systems. Evidence from animal studies suggest that the rate of biological aging is modifiable and that slowing the rate of biological aging may decrease the incidence of age-related morbidity and mortality. Based on these findings, several approaches are currently being studied to slow the rate of biological aging in humans. This symposium features internationally renowned aging research scientists whose work focuses on clinical interventions to modify biological aging in humans. We will hear from Rajagopal Sekhar from the Baylor College of Medicine who will present his research on "Improving oxidative stress, mitochondrial dysfunction, inflammaging, aging hallmarks and muscle strength in older adults: the novel role of GlyNAC and the 'Power of 3'"; Jamie Justice from the Wake Forest School of Medicine will present her work on "Response to dietary restriction interventions in older adults: biomarkers of cellular senescence and biological aging"; Reem Waziry from the Columbia University Mailman School of Public Health will present her work "Does Caloric Restriction Slow the Process of Biological Aging in Non-Obese Healthy Adults? Evidence from the CALERIE™ Trial"; and Daniel Parker from the Duke University School of Medicine will present his work "Does APOE genotype moderate the impact of diet modification and exercise training on age-related outcomes?". Attendees will learn about recent advances in interventions targeting human aging.

### IMPACT OF CALORIE RESTRICTION ON PLASMA ALZHEIMER'S DISEASE BIOMARKERS IN HEALTHY YOUNG AND MIDDLE-AGED ADULTS

Daniel Parker, P. Murali Doraiswamy, William Kraus, and Kim Huffman, *Duke University School of Medicine, Durham, North Carolina, United States*

Midlife cardiometabolic risk factors are associated with an increased risk of Alzheimer's dementia (AD). Moderate calorie restriction (CR) in healthy, non-obese young and middle-aged adults improves cardiometabolic risk factors. Plasma concentrations of amyloid β oligomers (Aβ-42 and Aβ-40) and total tau are emerging biomarkers of AD pathology. Our objective was to determine the impact of two years of CR in healthy young and middle-aged adults on Aβ-42, Aβ-40, and total tau in the Comprehensive Assessment of Long term Effects of Reducing Intake of Energy (CALERIE) Study. Participants were randomized 2:1 to 24 months of CR (prescribed as 25% reduction in baseline calorie requirements) versus an ad libitum (AL) diet. We quantified plasma Aβ-42, Aβ-40, and total tau using the ultrasensitive single molecule array (SIMOA) technology at baseline and two years in a subset of CALERIE (N=133). We used linear regression to evaluate the impact of CR, including age, sex, and presence/absence of the APOE-ε4 risk allele as covariates. We hypothesized that there would be differential CR effects based on APOE-ε4 carrier status; to test this, we included an interaction term. As compared to AL, there was a trend towards decreased Aβ-40, controlling for age, baseline Aβ-40 concentrations, and APOE-ε4 carrier status ($\beta=-12.59$, 95% CI[-27.14, 1.96], $p=0.093$) with 12% (average achieved) CR. The CR*APOE-ε4 carrier status interaction term was significant at a pre-defined threshold of $p<0.10$ ($p=0.062$).

Stratified by APOE-ε4 carrier status, CR was associated with a decrease in plasma Aβ-40 (β=-33.72, 95% CI[-65.16,-2.09], p=0.041). In conclusion, moderate CR in healthy, non-obese young and middle-aged adults impacts plasma biomarkers of AD risk, primarily in APOE-ε4 carriers.

### SUPPLEMENTING GLYNAC IN AGING IMPROVES GLUTATHIONE, MITOCHONDRIA, AND AGING HALLMARKS: A RANDOMIZED CLINICAL TRIAL

Rajagopal Sekhar, Premranjan Kumar, Chun Liu, James Suliburk, Jean Hsu, Farook Jahoor, Charles Minard, and George Taffet, *Baylor College of Medicine, Houston, Texas, United States*

Oxidative stress (OxS), mitochondrial dysfunction and aging hallmarks are important contributors to aging, but effective solutions to correct these defects in older adults (OA) are lacking. Via earlier translational studies we discovered that supplementation of GlyNAC (combination of glycine and N-acetylcysteine) improves/corrects these defects. We conducted a double-blind, placebo-controlled (RCT) in 24 OA (mean age 71y) to definitively determine the effects of supplementing GlyNAC vs. isonitrogenous placebo (alanine) for 16-weeks on intracellular glutathione (GSH), OxS mitochondrial function, inflammation, insulin-resistance, endothelial function, physical function, body composition and multiple aging hallmarks. 12 YA (mean age 25y) served as young controls and received GlyNAC for 2-weeks. Subjects were studied before receiving supplementation study, and after receiving supplementation for 2-weeks (OA, YA) and 16-weeks (OA). The RCT found that compared to YA, the OA had severe GSH deficiency (red-cells, muscle), mitochondrial dysfunction, OxS (TBARS, F2-isoprostanes), diminished physical function (gait-speed, muscle strength, exercise capacity), elevated waist-circumference and systolic blood pressure, and multiple hallmarks defects of aging (affecting mitochondrial function, mitophagy, nutrient sensing, inflammation, insulin-resistance, genotoxicity, stem-cells and cellular senescence). GlyNAC supplementation for 2-weeks rapidly improved several defects, and further improved/corrected multiple defects after 16-weeks. No improvements were seen in YA receiving GlyNAC, or in OA receiving the alanine placebo, suggesting that protein supplementation per se in OA does not improve defects. The results of this RCT provides proof-of-concept that GlyNAC supplementation improves/reverses GSH deficiency, mitochondrial dysfunction, OxS, inflammation, physical function/strength and multiple aging hallmarks. GlyNAC could be a novel, simple and safe nutritional supplement to improve/reverse age-associated defects and promote health in aging humans.

### CIRCULATING BIOMARKERS OF CELL SENESCENCE IN A RANDOMIZED TRIAL OF CALORIC RESTRICTION WEIGHT LOSS

Jamie Justice[1], Iris leng[1], Nathan LeBrasseur[2], Natalia Mitin[3], Stephen Kritchevsky[1], barbara Nicklas[1], and Jingzhong Ding[4], *1. Wake Forest School of Medicine, Winston-Salem, North Carolina, United States, 2. Mayo Clinic, Rochester, Minnesota, United States, 3. Sapere Bio, Durham, North Carolina, United States, 4. Wake Forest University, Winston Salem, North Carolina, United States*

There are no circulating markers exclusive to cell senescence for clinical trials in middle-aged/older adults. In this study we evaluated T-cell expression of tumor suppressor protein p16INK4a and plasma senescence associated secretory proteins (SASP)-factors in the context of an 18-week randomized trial of caloric restriction (CR) compared to a weight stable Control. The analysis includes 55 middle-aged/older adults (75%Women; 33%African American; 57.6±5.8 years) with obesity and prediabetes. We measured mRNA expression of select senescence and apoptosis gene transcripts (p16INK4a, p21, BCL2L, BAK1) in peripheral blood CD3+ T-cells (quantitative-RT-PCR) and a panel of 27 plasma SASP proteins (Luminex/multiplex; ELISA). Weight loss in those randomized to CR was -10.8±0.9 kg compared to +0.5±0.9 kg in Control. Using mixed models, T-cell expression of senescence-biomarkers p16INK4a, p21, BCL-2L, and apoptosis-marker BAK1 were not different between CR and control at 18 weeks (time × treatment adjusted for age, race, and sex, all p>0.05). We explored associations between biomarkers with weight loss; moderate spearman correlations (rs=0.28 to 0.64) were observed between weight loss and change in plasma SASP-factors (Activin A, I-CAM, MMP1, PAI-1, TNF-RI, TNF-RII, VEGF, uPAR, p< 0.05 all), but not T-cell biomarker expression. We evaluated associations between biomarkers and found change in T-cell p16INK4a was associated with change in plasma SASP-factors, Fas (rs=0.51), osteopontin (rs=0.34), myeloperoxidase (rs=0.39), and MMP2 (rs=0.40) (CR+Control combined). Among middle-aged/older persons with obesity undergoing 18-weeks CR, changes in T cell p16INK4a expression were correlated with reductions in key SASP factors, and weight loss was associated with lower pro-inflammatory SASP profile.

### DOES CALORIC RESTRICTION SLOW THE PROCESS OF BIOLOGICAL AGING? EVIDENCE FROM THE CALERIE TRIAL

Reem Waziry, *Columbia University, New York, New York, United States*

Calorie restriction (CR) slows aging and increases healthy lifespan in model organisms. We tested if CR slowed biological aging in humans using DNA methylation analysis of blood samples from N=197 participants in the Comprehensive Assessment of Long-term Effects ofReducing Intake of Energy (CALERIE™) randomized controlled trial. We quantified CR effects on biological aging by comparing change scores for six epigenetic-clock and Pace-of-Aging measures between n=128 CR-group and n=69 ad-libitum-control-group participants at 12- and24-month follow-ups. CR effects were strongest for DunedinPACE Pace of Aging (12-month Cohen's d=0.3; 24-month Cohen's d=0.2, p<0.01 for both), followed by DunedinPoAm and the GrimAge epigenetic clock, although effects for these measures were not statistically differentfrom zero (p>0.08). CR effects for other epigenetic clocks were in the opposite direction (all p>0.15). CALERIE intervention slowed Pace of Aging but showed minimal effect on epigenetic clocks hypothesized to reflect longer term accumulation of aging burden.

### SESSION 2245 (SYMPOSIUM)

### THE CANNABIS AND OLDER PERSONS STUDY: EXPANDING OUR MINDS THROUGH INTERDISCIPLINARY RESEARCH

Chair: Brian Kaskie Discussant: Benjamin Han

The Cannabis and Older Persons Study has examined the increasing use of cannabis among Americans over 60 years old since 2016. This year's symposium presents varied methodological approaches researchers have used to better understand the harms and benefits associated with cannabis use among older Americans. Divya Bhagianadh examines the association between cannabis legalization policies across the Untied States and corresponding use of end of life programs and services. Julie Bobitt analyzes interviews provided by older adults in Illinois and defines how attitudes held by medical doctors and other clinical care providers are critical to shaping cannabis use among older adults. Brian Kaskie considers the role of cannabis policy clusters. Jacobo Mintzer reviews findings from a controlled trial providing two cannabinoids to treat agitation among persons with Alzheimers in hospice. Thorsten Rudroff examines cannabis use among a sample of older persons at risk for falls. These studies reflect how cannabis use among older persons continues to grow and diversify, and how researchers have use different approaches to advance scientific understanding of determinants and outcomes, both desirable and undesirable, associated with cannabis use among older adults. This symposium offers policy makers and health care leaders a balanced perspective. On one side, we discuss how prevention and treatment efforts for substance use disorder, including cannabis use disorder, must increase proportionally to the increasing number of older cannabis users. On the other side, we consider how more than 5 million older Americans, especially those with pain, find some benefit in using cannabis.

### OLDER ADULTS' PERCEPTIONS OF CANNABIS AND OPIOIDS USE

Julie Bobitt, *University of Illinois at Chicago, Chicago, Illinois, United States*

Older adults in the U.S. are increasingly using cannabis as a method to manage pain. Some studies have linked increased cannabis use with an increase in prescription opioid use while others have suggested that older adults may be using cannabis as a way reduce/replace opioids. From April 2018 - January 2019 we conducted 12 focus groups throughout Illinois with 82 cannabis users aged 60+. To examine the relationship between cannabis and opioid use we used an inductive thematic analysis to code and theme the focus group transcripts. We found three themes 1) medical culture around opioids influences cannabis use; 2) past negative experiences with opioids influences cannabis use, and 3) aversion to ever trying opioids out of fear of an anticipated harm that may be brought about by opioid use such as overdose. In this session we present these findings and discuss cannabis use relative to opioids by older adults.

### MEDICAL MARIJUANA LAWS AND OUT OF POCKET EXPENSES AT END OF LIFE

Divya Bhagianadh, and Kanika Arora, *University of Iowa, Iowa city, Iowa, United States*

Resource intensive and costly End-of-Life (EOL) care is a significant healthcare policy concern in the U.S. In addition to the high Medicare spending, out of pocket (OOP) expenses are also high during EOL adding considerable stress during terminal days. The Medical Marijuana Laws (MMLs) is a significant policy in this context. Previous studies have shown increased use of MM among older adults, better pain management, influence on site of death as well as evidence of reduction in Medicare expenditure on drugs in states with MMLs. In this study, we explore the association between MML and OOP expenses during EOL using data from the Health and Retirement Study exit and core interviews from 1995 to 2018. We use a difference in differences (DD) and event study models to examine this question. We find evidence of increased OOP expenses on drugs and doctor visits with the effects concentrated among the early implementing states, among decedents who are White and among cancer patients. Despite its growing acceptance in palliative medicine, affordability of MM could pose a significant hurdle to terminally ill patients especially since MM and related costs are not covered by insurance.

### CANNABIS USE AND RISK OF FALLING IN OLDER ADULTS

Thorsten Rudroff, *University of Iowa, Iowa City, Iowa, United States*

The prevalence of cannabis use has significantly increased among US adults ≥ 50 years. However, the effect of chronic cannabis use on fall risk in older adults is unclear. A series of investigations were conducted to examine the intersection between cannabis use and fall risk in older adults. The findings indicated that: 1) Older (≥ 50 years) chronic cannabis users have a higher fall risk and walking impairments, 2) cannabis users might have a discrepancy between perceptual and physiological fall risk, and 3) chronic use of Δ-9-tetrahydrocannabinol (THC) might have negative influences on inhibitory control and brain activity. Future mechanistic (e.g., neuroimaging) investigations of the short- and long-term effects a variety of cannabis products (e.g., THC/CBD ratios, routes of administration) on cognitive/motor function, and fall incidence in older adults are suggested.

### POTENTIAL BENEFITS OF THC AND CBD IN THE TREATMENT OF HOSPICE-ELIGIBLE DEMENTED AND AGITATED PATIENTS

Jacobo` Mintzer, *University of South Carolina, Columbia, South Carolina, United States*

Today, half of the patients suffering from Alzheimer's disease (AD) will use hospice care in the last days of their life. Most of them will present with moderate to severe agitation. In the absence of evidence-based guidelines, Hospice care eligible patients with Agitation and Alzheimer's disease (AD) or other types of Dementia (HAD) are treated with a combination of antipsychotics, benzodiazepines, and opiates, which generate a variety of side effects. Two cannabinoids, tetrahydrocannabinol (THC) and cannabidiol (CBD), appear to be promising therapies for agitation in HAD with minimal side effects. Specifically, we suggest that a combination of THC and CBD oils have enhanced synergistic effects while maintaining a low side effect profile that the combination may provide. The evidence for this hypothesis will be discussed during the symposium.

### SESSION 2250 (PAPER)

### CAREGIVING

### A DUTY TO CARE: MALE PERSPECTIVES ON THE CAREGIVER ROLE FOR PERSONS WITH ALZHEIMER'S OR RELATED DEMENTIA

Michael Bueno[1], and Jo-Ana Chase[2], *1. University of California, Irvine, Placentia, California, United States, 2. University of Missouri, Columbia, Missouri, United States*

The population of family caregivers (FCGs) of persons with Alzheimer's Disease and related Dementias (ADRD) is growing, as is the proportion of males taking on this traditionally female role. Most caregiving research has mainly focused on females. Although female caregivers have reported more negative outcomes, men still report significant levels of burden. With the aging population and increased need for caregivers, there is a gap in knowledge exploring the male caregiving experience. Understanding male caregiving experiences can inform clinicians on developing future strategies to tailor support for this underrepresented group. The purpose of this qualitative descriptive study was to explore the experiences of male FCGs of people with ADRD. The Caregiver Identity Theory (CIT) was used to guide the study exploring participants' perception of self-identity within their caregiving relationship and self-identity as a male. Eleven male caregivers, recruited through social media and community resources, were interviewed by telephone or Zoom. Interviews were recorded, transcribed, and analyzed using thematic analysis. Four major themes emerged highlighting males' struggles with the unfamiliar caregiving role and changing identity; their acknowledgement of personal growth and discovery through caregiving, challenges in finding the "right" kind of support, and perceived reshaping of masculinity through the caregiving role. Male caregivers express unique experiences as FCGs suggesting future research is needed to explain gender differences in caregiving and identify additional factors that influence male caregivers' experiences. Furthermore, findings indicate clinicians should tailor support strategies for male FCGs' as they fulfill this potentially unfamiliar role.

### CAREGIVER STRAIN AMONG AFRICAN AMERICAN AND HISPANIC MALE CAREGIVERS WITH CHRONIC CONDITIONS

Matthew Smith[1], Chung Lin Kew[1], Tiffany Washington[2], Caroline Bergeron[3], Ashley Merianos[4], Ledric Sherman[1], and Kirby Goidel[1], *1. Texas A&M University, College Station, Texas, United States, 2. University of Georgia, Athens, Georgia, United States, 3. University of Ottawa, Ottawa, Ontario, Canada, 4. University of Cincinnati, Cincinnati, Ohio, United States*

Caregiving strain often stems from caregivers' unmet needs and is a risk factor for physical and psychological ill-health. This study aims to identify factors associated with caregiver strain among middle-aged and older African American and Hispanic male caregivers living with one or more chronic conditions. Data were collected from 431 male caregivers using a web-based survey (55% African American, 45% Hispanic). Linear regression models were fitted to assess factors associated with caregiver strain, which was measured using caregiving difficulty items from Behavioral Risk Factor Surveillance System. On average, participants were age 54.9(±9.51) years, they self-reported chronic conditions were 3.74(±2.62), and their caregiver strain was 14.7(±7.30). Among African American caregivers, higher caregiver strain was positively associated with living with children below age 18 ($\beta$=0.14, P=0.045) and feelings of social disconnectedness ($\beta$=0.16, P=0.018) and depression ($\beta$=0.15, P=0.035). Conversely, caregiver strain was negatively associated with having insurance coverage ($\beta$=-1.34, P=0.028) and disease self-management efficacy ($\beta$=-2.26, P=< 0.001. Among Hispanic caregivers, higher caregiver strain was negatively associated with age ($\beta$=-0.28, P=< 0.001) and positively associated with feelings of social disconnectedness ($\beta$=0.16, P=0.041). Findings suggest African American and Hispanic males with chronic conditions have differing caregiving experiences. Compared to Hispanic men, contributors to caregiving strain among African American men were multifaceted and associated with financial resources, household dynamics, mental health, and the ability to self-manage their chronic conditions. While bolstering social connectedness may offset caregiver strain, tailored mental health and disease-management programming are needed to meet the specific needs of African American and Hispanic male caregivers.

### CONTEXTUAL FACTORS ASSOCIATED WITH DECREASED DAILY DEPRESSION AND ANXIETY AMONG DEMENTIA CAREGIVERS

Frank Puga, Abigail Poe, Meghan Rafford, Danny Wang, and Carolyn Pickering, *The University of Alabama at Birmingham, Birmingham, Alabama, United States*

Family caregivers of individuals with dementia (IWDs) have an increased risk of developing depression and anxiety. Little is known about daily protective factors that mitigate this risk. The purpose of this study was to identify everyday coping strategies used by family caregivers that reduce the daily odds of experiencing depression and anxiety-related symptoms. Daily diaries were used to examine whether pleasant non-care activities with the IWD, social connection, and exercise were associated with a decrease in depression and anxiety-related symptoms. A national sample of ADRD caregivers (N=165) completed diaries over 21-days (n=2,841). Participants were asked about their daily experiences as caregivers, coping strategies, and mental health. Data were analyzed using mixed-level modeling. Depression and anxiety symptoms were endorsed by 141 (85.45%) and 155 (93.94%) participants, respectively. Social connection was associated with a decrease in the daily odds of depression (OR: 0.71, CI: 0.54 – 0.94, p=0.016) and anxiety symptoms (OR: 0.74, CI: 0.56 – 0.97, p=0.032). Caregivers were also less likely to endorse depression-related symptoms on days when engaging in a pleasant non-care activity with the IWD was reported (OR: 0.70, CI: 0.52 – 0.93, p=0.015). Finally, anxiety-related symptoms were less likely to be endorsed on days when caregivers reported engaging in exercise (OR: 0.65, CI: 0.49 – 0.86, p=0.003). The results from this study help elucidate coping strategies that may mitigate the risk of depression and anxiety associated with caregiving. These findings inform potential components for interventions to help support the health and well-being of family caregivers of IWDs.

### EXPLORING THE ASSOCIATION BETWEEN FAMILY DYNAMICS AND PREPAREDNESS FOR FAMILY CAREGIVING AT THE END OF LIFE

Aimee Fox[1], Julia Sharp[2], and Christine Fruhauf[2], *1. Kansas State University, Manhattan, Kansas, United States, 2. Colorado State University, Fort Collins, Colorado, United States*

The transition to providing end-of-life (EOL) care to a family member can be challenging for family caregivers as they face difficult decisions regarding medical course of treatment, increasing caregiving responsibilities, and anticipatory grief. Low preparedness for EOL caregiving is associated with higher levels of caregiver strain, increased levels of depression and anxiety, and complicated and prolonged grief after the death of the family member. Despite the breadth of caregiving research, little is known about how family relationships and interactions relate to caregiver preparedness for EOL caregiving. Thus, the purpose of this study was to explore the association between family caregivers' family dynamics and their perceived preparedness for the transition to EOL caregiving. A sample of 173 family caregivers were recruited to complete an online, self-report survey. A structural equation model was used to analyze the association between family dynamics and caregiving preparedness. Most caregivers reported balanced family cohesion (75.1%) and family flexibility (75.7%), but low family communication and low family satisfaction. In addition, 20.2% of caregivers reported being not at all prepared for the transition to EOL caregiving. Although there was a lack of meaningful association between family dynamics and preparedness for EOL caregiving, it may be that family relationships and interactions grow more complex as families age and individuals take on new and different roles (such as family caregiving) within the family system. This study demonstrates the need for future research to develop new measures to explore how aging family dynamics relate to family caregiver experiences and outcomes.

### FAMILY CAREGIVER SOCIAL CONNECTEDNESS: TECHNOLOGY USE ACROSS GENERATIONS DURING THE COVID-19 PANDEMIC

Janet Pohl[1], Jude Kolodisner[2], and David Coon[1], *1. Arizona State University, Phoenix, Arizona, United States, 2. Arizona State University, Tempe, Arizona, United States*

During the COVID–19 pandemic, maintaining connectedness was difficult for caregivers. Family caregivers represent multiple generations whose experience with and use of social technology to maintain connectedness can vary and differentially impact critical health outcomes. The aims of this study were to examine caregiver connectedness and technology preferences across three generations of caregivers who provide care to older adults with chronic illnesses. The semi-structured focus-groups/interviews conducted in August of 2020 with family caregiver participants including Millennials (n=6), Generation X (n=5), and Boomer (n=8). Two researchers analyzed the transcribed content via thematic analysis. Similarities and differences across generations were assessed via comparative analysis. The themes that emerged from the data were: (1) Millennials (a) Altered stage of life, (b) Altered connectedness, (c) Need others to understand, (d) Stay away from social network sites; (2) Generation X (a) Altered connectedness, (b) Need others to understand, (c) Burden, (d) Fear-of-failure; and (3) Baby Boomer (a) Altered connectedness, (b) Technology builds connectedness, (c) Information seeking. All generations expressed alterations in connectedness with caregiver role. Millennials and Generation X caregivers emphasized need for others to understand that caregiving altered their lives with unique responsibilities. Technology use differed across the generations, with Millennial texting for confidential communications. Millennial and Generation X caregivers do not use social media due to envy of others' fun. Baby Boomers expressed increased connectedness with the use of Zoom. Understanding the variation in the experience of caregiver connectedness and technology use by generation may identify targets for future caregiver connectedness intervention studies.

### SESSION 2260 (PAPER)

### INNOVATION IN GERONTOLOGY EDUCATION

In this session, gerontology educators describe a range of unique strategies for extending students' aging education and faculty development.

### EXPERIENCES AND RECOMMENDATIONS FOR STARTING A UNIVERSITY GERONTOLOGY RESEARCH LABORATORY

Britteny Howell[1], and Jennifer Peterson[2], *1. University of Alaska Anchorage, Anchorage, Alaska, United States, 2. University of Alaska Fairbanks, Fairbanks, Alaska, United States*

Although founding and directing an independent research laboratory is often expected of faculty at American universities, there are several barriers to successful completion of this important task. There is little guidance in the literature regarding exactly how to go about starting a research laboratory. The guidance that does exist for faculty often focuses on running research labs in the "hard sciences," such as biomedical science and engineering, leaving social and behavioral scientists out of such considerations. Additionally, smaller or teaching-focused universities often have little infrastructure or support for starting a research lab, so faculty at these institutions may not know where to begin. These barriers are significant concerns for junior faculty, who are often unprepared for the realities and challenges of starting a successful research lab while obtaining other milestones required of promotion and tenure. We present two examples of recently-formed gerontology research laboratories begun by junior faculty, one in the psychology department of a research university and one in public health at a teaching-focused university. Our case studies present the audience with specific examples, lessons learned, and guidance for starting their own gerontology research laboratory in higher education, as well as recommendations on maintaining its functioning during a global pandemic (COVID-19).

### PASSING THE TORCH: EXPLORING THE SUSTAINABILITY OF AN INTERPROFESSIONAL GERIATRICS FACULTY DEVELOPMENT PROGRAM

Kimberly Davis[1], Sarah Marrs[2], Ishan Williams[3], Kristin Zimmerman[4], Constance Coogle[2], Pamela Parsons[2], Patricia Slattum[2], and Leland Waters[2], *1. Virginia Commonwealth University/ School of Nursing, Richmond, Virginia, United States, 2. Virginia Commonwealth University, Richmond, Virginia, United States, 3. University of Virginia, School of Nursing, Charlottesville, Virginia, United States, 4. Virginia Commonwealth University, School of Pharmacy, Richmond, Virginia, United States*

Faculty development programs (FDPs) are an effective, evidence-based method of promoting the knowledge, skills and self-efficacy of faculty. However, the implementation and sustainability of curricula are rarely reported. Furthermore, the challenges to sustaining programmatic implementation of interprofessional FDP curricula in academic and clinical settings over time have yet to be extensively evaluated. Using dynamic sustainability as a framework, we aimed to assess the evolving barriers and facilitators that influence the implementation and sustainability of the geriatrics curriculum Capstone projects designed by faculty scholars in our FDP. We planned to report on projects that were and weren't successful. A survey, sent to 115 faculty scholars from eight different cohorts who completed our 10-month FDP, recruited faculty participants and set the stage for qualitative data collection to help us better understand the sustained impact of the program. Thematic analysis of virtual interviews with 17 Scholars revealed several key factors facilitating and hindering the implementation and dynamic sustainability of curricular projects. Three major themes and sub-themes were identified as follows: Project Implementation (Supportive Factors, Hindering Factors and Filling in Gaps in the Field); Pedagogical Development (Enhancement of Skills and Culture Change); and Sustainability Impact (Project Sustainability, Career Development and Passing the Torch). Supporting these factors through skills development may favorably impact project sustainability and thus the aspects of workforce development that catalyze practice change. Implementation of geriatrics-focused FDPs provides an evidence-based approach to sustainability. Further study of the ongoing barriers and facilitators to sustainability is encouraged.

### SPIRITUAL DEVELOPMENT AND AGING HUMANITIES: READING HESSE'S STEPPENWOLF THROUGH MOODY'S FIVE STAGES OF THE SOUL

Stephen Fogle, *University of Nebraska at Omaha, Omaha, Nebraska, United States*

This paper explores Herman Hesse's Steppenwolf through Harry Moody's Five Stages of the Soul. Steppenwolf speaks to gerontological education objectives as it deals squarely with humanizing old age though, "deeply lived spiritual events which [the main character] has attempted to express by giving them the form of tangible experiences." (Hesse, 1963, 21) Five Stages of the Soul is a seminal conceptual model for understanding spiritual development across the life course: particularly in old age. It documents lived experiences in five stages: call, search, struggle, breakthrough, and return. This paper follows Steppenwolf's main character, Harry Haller, though each of these five stages. In doing so, this paper positions aging and spiritual development center stage in a tale featuring themes of enduring struggle such as personal identities, war & violence, sex, magic, and technological advancement. The plot line of Steppenwolf matches Five Stages of the Soul with enlightening consistency. Originally published a century ago, Steppenwolf enjoyed resurgent popularity among the baby boom cohort of older adults in the United States and globally during the 1960s. Today, Steppenwolf retains relevancy for understanding aging and spiritual development in lived contexts of social chaos and uncertainty. Steppenwolf is a classic and engaging piece of literature for contemporary readers of all ages. This paper helps students and teachers of gerontology apply a conceptual model of aging to a relevant case study. The paper contributes to incorporating literary masterpieces into gerontological curricula. Finally, this paper sheds light on the humanity of older adults engaged in spiritual development.

### TEACHING THE INTERSECTION OF AGING AND DISABILITY FOR FIRST-YEAR STUDENTS

Kristine Mulhorn, *Drexel University, Wallingford, Pennsylvania, United States*

The honors college at Drexel included their Freshmen Seminar in a recent effort to offer various courses on the theme of aging across the campus. Usually, the themed courses are for juniors and seniors who can choose electives. This year, the theme of aging was interwoven into two freshmen seminars with a maximum of 20 students. The purpose of this course was to examine the complexity of these two concepts and recognize possible avenues for study, research, and careers based these perspectives. Disability is not only a way of describing how someone lives with biological or physical difference, but disability can be a social identity, and a group recognized in recent civil rights legislation. Aging is not only at the individual level, but an ongoing demographic change that is happening throughout the world. Students reflected on online quizzes on ableism and ageism to gain insights about their own biases and the biases within their chosen career fields. Through class discussions and reflections, students achieved course objectives: 1) describe how society can better prepare individuals to fully participate in society throughout life; 2) recognize key factors affecting those with disability and aging with disability as they navigate education, health services, housing and work; 3) synthesize literature on aging and disability to make recommendations for improving social participation for those either aging with disability or aging into disability; and 4) describe public policy challenges for those over 50 and those who identify as disabled.

### THE JOY AND CHALLENGES OF STORY CREATION WITH TIMESLIPS: STUDENT FACILITATOR PERCEPTIONS

Emily Ihara, Emily Perez, Kendall Barrett, Megumi Inoue, and Catherine Tompkins, *George Mason University, Fairfax, Virginia, United States*

Creative arts interventions for people living with dementia have been shown to improve mood, emotions, communication, and relationships for older people living with dementia and their care partners. Previous research demonstrates that TimeSlips, a creative storytelling intervention, provides a "failure-free" environment and an opportunity for individuals to use their imagination. Because it does not involve memory, people living with dementia are encouraged to contribute and interact, thus creating an environment that focuses on dignity and strengths rather than deficits. This case study explores the student facilitators' experiences of running TimeSlips sessions in different levels of care. The two facilitators ran continuous sessions over six months – with an individual at home, group sessions in memory care, and group sessions in assisted living. The facilitators journaled about their experiences after each session and the same picture prompts were used across the different types of sessions. Each journal entry was coded by two independent researchers using grounded theory principles. Through the coding, it was clear that the facilitators needed to use different skills to engage participants in storytelling based on their level of care. Themes that emerged include joy of connection, playfulness, and engagement. In addition, specific skills are needed to accommodate some behaviors of people living with dementia (such as aggressive behavior toward others in the group) and how to distract from those behaviors to continue with the storytelling. The benefits and challenges of each modality and gaps that may need to be addressed for student facilitators will be discussed.

### SESSION 2270 (SYMPOSIUM)

### COGNITIVE AGING ACROSS NATIONAL CONTEXTS: EVIDENCE FROM THE HARMONIZED COGNITIVE ASSESSMENT PROTOCOLS

Chair: Lindsay Kobayashi Co-Chair: Alden Gross
Discussant: Kenneth Langa

The global burden of dementia is rapidly rising and shifting to low- and middle-income countries. The triangulation of evidence across country contexts is essential for unlocking the causes of dementia and reducing its global burden. The Harmonized Cognitive Assessment Protocol (HCAP) is a recent innovation administered in the US Health and Retirement Study and several of its International Partner Studies. For the very first time, these HCAPs provide high-quality data for cross-national comparisons of later-life cognitive function that are sensitive to linguistic, cultural, and educational differences across diverse country contexts. However, despite the common HCAP protocols, human cognitive function does not lend itself to direct comparison across diverse populations without careful consideration of necessary test adaptations. This symposium presents results from analyses of the HCAP data in the US, England, Mexico, South Africa, China, and India, highlighting cross-national differences in later-life cognition identified using the HCAP data, and presenting key methodological concerns for cross-national comparisons of cognitive aging. First, Zhang will present findings comparing education gradients in later-life cognitive function across countries. Next, Cho will present longitudinal data comparing the relationships between short-term changes in household wealth in later-life and subsequent cognitive function across countries. Third, Avila-Rieger will present findings comparing sex/gender disparities in later-life cognitive function in the US and India and how they differ by education. Finally, Nichols will conclude the session by discussing differences in the measurement of cognition for the assessment of dementia across countries and implications for data interpretation and the design of future instruments.

### EDUCATION GRADIENTS IN LATER-LIFE COGNITIVE FUNCTION ACROSS LOW-, MIDDLE-, AND HIGH-INCOME COUNTRIES

Yuan Zhang[1], Brendan O'Shea[2], Xuexin Yu[2], Tsai-Chin Cho[3], Kenneth Langa[2], Alden Gross[4], and Lindsay Kobayashi[2], *1. University of North Carolina at Chapel Hill, Chapel Hill, North Carolina, United States, 2. University of Michigan, Ann Arbor, Michigan, United States, 3. University of Michigan School of Public Health, Ann Arbor, Michigan, United States, 4. Johns Hopkins Bloomberg School of Public Health, Baltimore, Maryland, United States*

Education is positively related to cognitive function. However, educational gradients in cognitive function may vary across older populations with different educational compositions and physical and social environments. We conducted one of the first cross-national comparative studies on educational differences in later-life cognitive function using harmonized data. Multivariable linear regressions were employed to estimate the association between education according to International Standard Classification of Education (ISCED) categories and cognitive function for adults ages 60+ from the United States, England, Mexico, South Africa, India, and China. Cross-country differences were tested using fully interacted models. Controlling for demographics and parental education, we found significant educational gradients in cognitive function in low- and middle-income countries; however, in high-income countries, only those with upper secondary education and above had a consistent cognitive advantage over those with primary education. This study suggests substantial country-level differences in cognitive benefits of educational attainment.

### SHORT-TERM WEALTH CHANGES AND SUBSEQUENT COGNITIVE HEALTH AMONG OLDER ADULTS IN CHINA, ENGLAND, MEXICO, AND THE US

Tsai-Chin Cho[1], Xuexin Yu[2], Alden Gross[3], Yuan Zhang[4], and Lindsay Kobayashi[2], *1. University of Michigan School of Public Health, Ann Arbor, Michigan, United States, 2. University of Michigan, Ann Arbor, Michigan, United States, 3. Johns Hopkins Bloomberg School of Public Health, Baltimore, Maryland, United States, 4. University of North Carolina at Chapel Hill, Chapel Hill, North Carolina, United States*

Household wealth is positively associated with later-life cognitive health, but little is known about the effects of changes in wealth over time and whether they differ across populations. In this study, we evaluated the within- and between-country relationships between short-term changes in household wealth and subsequent cognitive function among adults aged ≥65 years in China, England, Mexico, and the US. We used sampling-weighted, multivariable-adjusted linear models to estimate the relationships between household wealth change over 3- to 4-year periods and subsequent harmonized general cognitive performance factor scores using HCAP measures. We found that short-term decreases in household wealth were associated with poor subsequent cognitive health in the US and China, but not in England or Mexico. The observed associations were weaker in Mexico than in the US. In summary, macro-level social and economic structures may modify the association between wealth changes and cognitive health, although further investigation is needed.

### DIFFERENCES IN THE MEASUREMENT OF COGNITION FOR THE ASSESSMENT OF DEMENTIA ACROSS GEOGRAPHIC CONTEXTS

Emma Nichols[1], Derek Ng[1], Shabina Hayat[2], Kenneth Langa[3], Jinkook Lee[4], Andrew Steptoe[2], Jennifer Deal[5], and Alden Gross[1], *1. Johns Hopkins Bloomberg School of Public Health, Baltimore, Maryland, United States, 2. University College London, London, England, United Kingdom, 3. University of Michigan, Ann Arbor, Michigan, United States, 4. University of Southern California, Los Angeles, California, United States, 5. Johns Hopkins University, Baltimore, Maryland, United States*

Most cognitive assessments have been developed in high-income countries but are subsequently used in diverse contexts. Differences in culture and context may affect performance of cognitive items. We used the Harmonized Cognitive Assessment Protocol surveys in the US, Mexico, India, England, and South Africa (combined N=11,364) to quantify associations across countries between cognitive items and cognitive impairment status using age- and gender-adjusted logistic regression. Associations were stronger in the US (Median Odds Ratio [OR] across items=0.17) and England (Median OR=0.19), compared to South Africa (Median OR=0.23), India (Median OR=0.29), and Mexico (Median OR=0.28). Items assessing memory (e.g. delayed recall tasks) had the most consistent associations of the largest magnitudes across contexts. Transporting cognitive items among countries and cultures warrants caution. We identified items that performed well either in individual contexts or across the range of contexts considered; this information can be used to guide the design of future instruments.

### CROSS-NATIONAL VARIABILITY IN SEX/GENDER DIFFERENCES AND LATER-LIFE MEMORY: INDIA AND THE UNITED STATES

Ashly Westrick[1], Justina Avila-Rieger[2], Alden Gross[3], and Lindsay Kobayashi[1], *1. University of Michigan, Ann Arbor, Michigan, United States, 2. Columbia University Medical Center, New York, New York, United States, 3. Johns Hopkins Bloomberg School of Public Health, Baltimore, Maryland, United States*

Little is known about the extent of sex/gender differences in later-life cognitive health in low-income countries such as India. We compared sex/gender differences in later-life memory, overall and by educational attainment, across men and women aged ≥65 years in India and the United States. Data were from Harmonized Cognitive Assessment Protocols (HCAP) in the population-representative Longitudinal Study of Aging in India (LASI; N=4,096;) and U.S. Health and Retirement Study (HRS; N=3,345). Multiple-group models estimated interactions between sex/gender and educational attainment on harmonized episodic memory scores across countries. In the U.S., women had a memory performance advantage compared to men across all education levels. In India, men had a memory performance advantage compared to women overall, interactions revealed that this advantage was only present among those with no formal education. Among those with at least lower secondary education, women demonstrated an advantage that increased with increasing education.

### SESSION 2280 (SYMPOSIUM)

### DYADIC AND LONGITUDINAL STUDIES OF CLOSE RELATIONSHIPS: NEW INSIGHTS INTO HEALTH AND WELL-BEING ACROSS ADULTHOOD

Chair: Gloria Luong Co-Chair: Christine Fruhauf

This symposium includes 5 papers using intensive, longitudinal, and/or dyadic data on social relationships, revealing novel insights into health and well-being across adulthood. Fruhauf and colleagues conducted a feasibility and acceptability to test their Merging Yoga and self-management to develop Skills (MY-Skills) intervention, which targets caregiving dyads (caregivers and care receivers) dealing with persistent pain. Participants completed group interventions meeting twice a week for eight weeks, with each two-hour session including self-management education followed by yoga. A priori benchmarks for feasibility were met for the dyads, suggesting that these interventions may lead to improved well-being for caregiving dyads. Turner and Hooker conducted a 30-day microlongitudinal study of middle-aged daughters who are caring for their parents with dementia. On days when caregivers had more positive appraisals of caregiving than their own average, they had greater physical activity goal pursuit, independent of that day's negative appraisals. Luong and colleagues also leverage an intensive longitudinal study spanning 3.5 month that prospectively tracked older adults and their close partners as they transitioned into senior housing facilities. Well-being varied by marital status, and changes over time varied by age. Weidmann & Chopik examined older romantic couples from 36 nations and found that between-partner correlations between depressive symptoms, self-rated health, and cognitive performance diffed across cultures. Finally, Purol et al. found that among middle-aged and older couples, similarity effects of personality facets on health and well-being were relatively small in magnitude, suggesting personality similarity among couples may be overstated in the literature.

### INDIVIDUALS AND THEIR CAREGIVERS WITH PERSISTENT PAIN: A FEASIBILITY AND ACCEPTABILITY STUDY

Christine Fruhauf[1], Arlene Schmid[1], Aimee Fox[2], Jennifer Dickman Portz[3], Julia Sharp[1], Heather Leach[1], and Marieke Van Puymbroeck[4], *1. Colorado State University, Fort Collins, Colorado, United States, 2. Kansas State University, Manhattan, Kansas, United States, 3. University of Colorado Anschutz, Aurora, Colorado, United States, 4. Clemson University, Clemson, South Carolina, United States*

Persistent pain interventions targeting the caregiving dyad (i.e., caregivers and care receivers) are scarce. Thus, the purpose of this pilot study was to assess the feasibility and acceptability of the Merging Yoga and self-management to develop Skills (MY-Skills) intervention for caregiving dyads experiencing persistent pain. MY-Skills is a group intervention and was delivered in-person or online (due to COVID-19) twice a week for eight weeks, with each two-hour session including self-management education followed by yoga. Benchmarks for feasibility were set a prioi and included: recruitment, attrition, attendance, safety, acceptability/satisfaction, and study completion. Thirteen participants (caregivers n=7, care-receivers n=6) completed the in-person intervention and 18 individuals (9 dyads) completed the online version. Feasibility benchmarks were met, except for recruitment, where >1000 individuals were screened for eligibility. Interventions may lead to improved wellbeing, yet further research is needed to establish efficacy of health-related outcomes for the caregiving dyad experiencing persistent pain.

### POSITIVE APPRAISALS OF CAREGIVING SUPPORT DEMENTIA CAREGIVERS' PHYSICAL ACTIVITY GOAL PURSUIT

Shelbie Turner[1], and Karen Hooker[2], *1. Weill Cornell Medical College, Pitman, New Jersey, United States, 2. Oregon State University, Corvallis, Oregon, United States*

Middle-age daughters caring for parents with dementia have multiple roles and often neglect their own health. In addition to reporting negative appraisals of caregiving dementia caregivers also report positive appraisals, which may support their health behavior. In this study we examine how positive and negative caregiving appraisals interacted to predict physical activity goal pursuit among 27 daughters of parents with dementia who participated in the ACHIEVE Study, an NIH-funded 30-day study on caregiving (Noccasions=622). On days when caregivers had more positive appraisals than their own average, they had higher physical activity goal pursuit, even after controlling for that day's negative appraisals ($\beta$=0.31, SE=0.09, p=0.001). Moreover, the association between positive appraisals and goal pursuit was not dependent on that day's negative appraisals (p=0.42). Supporting adult-daughter caregivers to feel positively about their role as a caregiver on a day-to-day basis may support them to stay on track with their physical activity goals.

### DYADIC CHANGES IN WELL-BEING ACROSS THE TRANSITION TO SENIOR HOUSING FACILITIES: A PROSPECTIVE LONGITUDINAL STUDY

Gloria Luong, Andrea Russell, and James Miller, *Colorado State University, Fort Collins, Colorado, United States*

Previous research examining the transition into senior housing has focused primarily on individuals and has used cross-sectional, retrospective designs after older adults have already moved into the facilities. These designs cannot disentangle changes over time and how partners influence this process. The Relocation and Transitional Experiences (RELATE) study leverages a prospective longitudinal design that tracked 157 participants (97 who participated in the study as part of a dyad with a romantic partner or other close partner (family member)) prior to moving into senior housing and across 3.5 months after the move. Participants ranged between 40-95 years of age. Results showed that those who were unmarried reported fewer depressive symptoms, but also less social support and lower life satisfaction than married participants. Changes over the 3.5 month period in depressive symptoms, social support, and rumination also varied by age. This study suggests partners may influence one another's developmental health trajectories.

### LINKS BETWEEN DEPRESSIVE SYMPTOMS AND SELF-RATED AND COGNITIVE HEALTH IN OLDER COUPLES AROUND THE WORLD

Rebekka Weidmann, and William Chopik, *Michigan State University, East Lansing, Michigan, United States*

Mental and physical health are important concerns in late adulthood. To gain a deeper understanding of the associations between depressive symptoms and self-rated and cognitive health as they occur contextualized within romantic relationships and across different cultures, we examined these links in older romantic couples of 36 nations. To that end, we used data from North and South America, Europe, and Asia with a total sample of 47,396 romantic couples. Between-partner correlations of depressive symptoms, self-rated health, and cognitive health demonstrated substantial variations across cultures. Further, we found that the depressive symptoms in one partner were linked to worse self-rated health and worse cognitive performance in both partners. Age, education, and functional difficulties partly accounted for these associations. These findings were also characterized by cross-cultural variation. Hence, depressive symptoms represent a potential risk factor for health in couples.

### A MULTIPRONGED APPROACH TO MODELING DYADIC SIMILARITY AMONG MIDDLE-AGED AND OLDER ADULTS

Mariah Purol[1], Rebekka Weidmann[1], Louis Hickman[2], Jeewon Oh[3], and William Chopik[1], *1. Michigan State University, East Lansing, Michigan, United States, 2. University of Pennsylvania, Philadelphia, Pennsylvania, United States, 3. Syracuse University, Syracuse, New York, United States*

One surprising finding in the field of dyadic influence is the minimal influence that couple personality similarity has for life and relational well-being. Most research has focused on pairing of symmetric psychological characteristics on a broad, trait level (i.e., one person's extraversion with their partner's extraversion) across romantic partners. However, many more cross-trait combinations are possible, and facet-level traits may provide additional information that is traditionally lost. Among 1,366 middle-age and older couples, we examined similarity effects of personality facets on health, life, and relational well-being using difference scores, response surface analyses, and machine learning approaches. Across analytic and operational approaches, partner and similarity effects were relatively small in magnitude, although additional variance was explained for some outcomes (e.g., relationship satisfaction) than others (e.g., memory, chronic illnesses). Our findings constitute one of the more comprehensive examinations to date and suggest even the modest effects of personality similarity may be overstated.

### SESSION 2290 (SYMPOSIUM)

### EPIDEMIOLOGY OF FALLS: RACIAL/ETHNIC CONSIDERATIONS

Chair: Wenjun Li Discussant: Su-I Hou

Prevention of falls is important to independent living and good health in older age. This symposium brings together four studies on epidemiology of falls among community-dwelling older adults. The first study examined location and activity-specific fall rates among 388 older adults in Massachusetts. Compared to Asians, annual rates of indoor and outdoor falls and falls while walking were substantially higher among non-Hispanic White and Hispanics. The second study evaluated racial/ethnic differences in fear of falling, depression, lack of physical activity and negative self-perceptions of aging in 121 low-income older adults. The highest levels of fall risk were found in Hispanics and Asian Americans, fear of falling in African Americans, and depression in Hispanics. The third study assessed the relationship between objectively measured sedentary bout frequency with recurrent falls (2+ falls/year) among 2,918 men aged 79.0±5.1 years attending the Osteoporotic Fractures in Men Study (MrOS) Year 7 (2007-2009) visit. The analysis found a likely U-shaped association between sedentary bout frequency and recurrent falls risk, with the least and most active men are at higher risk. The fourth study investigated the association between fear of falling and falls risk among Chinese and non-Chinese older adults in Singapore using data collected via an electronic survey between January and February 2022. The analysis did not find significant ethnic differences in fear of falling. Although limited by sizes, these studies found racial and ethic differences in fall rates, physical activity, fear of falling and other risk factors, which are of great interest for further investigations.

### RACIAL DIFFERENCES IN LOCATION AND ACTIVITY-SPECIFIC FALLS IN MASSACHUSETTS, US

Wenjun Li[1], Linda Churchill[1], Annabella Aguirre[1], Anthony Clarke[1], Elizabeth Procter-Gray[1], Kevin Kane[1], Sarah Berry[2], and Marian Hannan[3], *1. University of Massachusetts Lowell, Lowell, Massachusetts, United States, 2. Hebrew SeniorLife / Harvard Medical School, Boston, Massachusetts, United States, 3. Hebrew SeniorLife, Roslindale, Massachusetts, United States*

Prevention of falls is important to independent living and good health in older age. Circumstances of falls may vary by older person's sociodemographic attributes, health conditions and living environment. Health Aging and Neighborhood Study (HANS) examined rates of location and activity-specific falls among 388 community-living older adults living in Central Massachusetts between 2018 and 2020. Data on falls were collected using monthly calendar, and information on falls was collected by trained staff via telephone interviews with the participants. The follow-up time ranging from 11.9 to 24.4 months. In total, 431 falls were reported, of which 97% had information on location of fall, and 95% had information on activity at time of fall. Annualized rate of any fall differed significantly by race/ethnicity. Asians had the lowest rate (0.26/year). Rate of Hispanics was approximately twice higher (0.55, p=0.04) and non-Hispanic Whites three times higher (0.84, p<0.001). For indoor falls, Asians reported significantly lower rates than Hispanics (0.10 vs. 0.44, p=0.005) or non-Hispanic Whites (0.10 vs. 0.36, p=0.009). Greater outdoor fall rate was associated with male gender (p=0.04); younger age (p=0.03); Asian (p=0.02) and, especially, non-Hispanic White race (p<0.001, with Hispanics as referent); higher income (p<0.001); and more years of education (p=0.001). Falls while walking were significantly associated with non-Hispanic White race (p<0.001) and Hispanic ethnicity (p=0.001). In summary, rates of any, indoor or outdoor falls vary significantly by race. The underlying mechanisms should be further investigated.

### FALL RISK, FEAR OF FALLING, PHYSICAL ACTIVITY, AND SELF-PERCEPTIONS IN ETHNICALLY DIVERSE LOW-INCOME OLDER ADULTS

Ladda Thiamwong[1], Rui Xie[1], Nichole Lighthall[1], Joon-Hyuk Park[1], Victoria Loerzel[2], and Jeffrey Stout[1], *1. University of Central Florida, Orlando, Florida, United States, 2. College of Nursing, University of Central Florida, Orlando, Florida, United States*

Falls and fear of falling (FOF) are the leading cause of disability, and hospitalization in ethnically diverse low-income older adults. The present study included an ethnically diverse sample of low-income older adults (N=121) to examine ethnic and income-based group differences in risk factors for falls (high FOF, depression, avoidance of physical activity) and the role of negative self-perceptions of aging in driving these risk factors. Results revealed the highest levels of fall risk among Hispanics and Asian Americans, FOF among African Americans, and depression among Hispanics. Accelerometer-based physical activity measures confirmed that low-income older adults had lower levels of moderate to vigorous physical activity (MVPA) compared with their high-income counterparts (p=0.04). Further, financial difficulty was related to MVPA (p<0.001), depression, anxiety, and fall risk (p=0.01). Critically, we also found a strong relationship between negative self-perceptions of aging and FOF (p = 0.002) across groups.

### THE ASSOCIATION BETWEEN OBJECTIVE MEASURES OF INTERMITTENT SEDENTARY BREAKS WITH RECURRENT FALL RISK IN OLDER MEN

Lauren Roe[1], Stephanie Harrison[2], Peggy Cawthon[3], Kristine Ensrud[4], Kelley Gabriel[5], Deborah Kado[6], and Jane Cauley[1], *1. University of Pittsburgh, Pittsburgh, Pennsylvania, United States, 2. California Pacific Medical Center, Research Institute, San Francisco, California, United States, 3. California Pacific Medical Center Research Institute, San Francisco, California, United States, 4. University of Minnesota Medical School and Minneapolis VA Health Care System, Minneapolis, Minnesota, United States, 5. Department of Epidemiology, The University of Alabama at Birmingham, Birmingham, Alabama, USA., Birmingham, Alabama, United States, 6. Stanford University School of Medicine, Palo Alto, California, United States*

BACKGROUND: We assessed the relationship between objectively measured sedentary bout frequency with recurrent falls (2+ falls/year).

METHODS: The sample included 2,918 men aged 79.0±5.1 years attending the Osteoporotic Fractures in Men

Study (MrOS) Year 7 (2007-2009) visit. Sedentary bout frequencies were defined as the average daily number of transitions out of 5+ minutes sedentary (<1.5 METS). Falls were self-reported from mailed questionnaires. Generalized Estimating Equations estimated the odds of recurrent falls.

RESULTS: Over four follow-up years, 1,025 (35.1%) men had recurrent falls. Compared to men with <13.6 transitions from sedentary (Quartile (Q)1), the odds of recurrent falls among men with 13.6-<17.0 transitions (Q2), 17.0-<20.4 transitions (Q3), and 20.4-<34.6 transitions (Q4) were 0.82 (95%CI: 0.66-1.01), 0.79 (95%CI: 0.64, 0.99), and 1.01 (95%CI: 0.81, 1.27), respectively, after adjustment.

CONCLUSIONS: A U-shaped association may exist between sedentary bout frequency and recurrent falls risk. The least and most active men are at higher risk.

### FEAR OF FALLING, FALL RISK, AND ETHNICITY AMONG OLDER ADULTS IN SINGAPORE: AN ELECTRONIC SURVEY

Wayne Chong[1], Tharshini Lokanathan[2], and W. Quin Yow[2], *1. Nanyang Technological University, Singapore, Singapore, Singapore, 2. Singapore University of Technology & Design, Singapore, Singapore*

This study investigated the association between fear of falling and falls risk among Chinese and non-Chinese community-dwelling older adults in Singapore. Preliminary data were collected from individuals who will turn 60 years old and above in 2022 via an electronic survey between January and February 2022. The majority (61%) of the 128 Chinese participants had no falls risk and low fear of falling as compared to other combinations of falls risk (absence, presence) and fear of falling (low, moderate, high), Pearson Chi Square (2) = 8.03, p = .02. Among the 16 non-Chinese participants, no dominant falls risk-fear of falling combination was found, Pearson Chi Square (2) = 3.47, p = .18. Multinomial logistic regression analyses found, however, that in the presence of falls risk, ethnicity did not predict moderate fear of falling, B = .24, p = .70, nor high fear of falling, B = -.37, p = .74.

### SESSION 2300 (SYMPOSIUM)

### FOSTERING MULTIDISCIPLINARY SOLUTIONS IN AGING: THE RESEARCH CENTERS COLLABORATIVE NETWORK

Chair: Stephen Kritchevsky Co-Chair: Odette van der Willik

The NIA supports the Research Centers Collaborative Network (RCCN) to build collaborations between scientists from the 6 NIA-sponsored center programs: Alzheimer's Disease Research Centers, Centers on the Demography and Economics of Aging, Claude D. Pepper Older Americans Independence Centers, Nathan Shock Centers of Excellence in the Basic Biology of Aging, Resource Centers for Minority Aging Research, and Roybal Centers for Translational Research on Aging. RCCN's premise is that researchers from different disciplines are most likely to collaborate when they are addressing common problems. To provide a forum for collaborative exchange, the RCCN has convened 6 workshops on topics that cross-cut the concerns of the various NIA center programs ranging from sustaining behavior change in older adults to measuring biologic age. After each Workshop the RCCN awards pilot funds related to the theme. This symposium will present key learnings from the workshops and present work of four RCCN pilot teams from the third and fourth workshops which focused on resilience and reserve, and life course perspectives on aging. Dr. Ramos will discuss how measurement of psychological resilience may predict physical resilience in older patients with lung cancer, while Dr. Reid will discuss positive affect as a source of resilience for older adults with chronic pain. Dr. White will discuss association between perceived discrimination trajectories and multimorbidity burden among middle-aged and older black adults, while Dr. Brooks will discuss development of a resilience index in chronic musculoskeletal pain. Dr. Kritchevsky will also summarize lessons learned across the first 6 workshops.

### ENHANCING RESILIENCE IN OLDER ADULTS WITH ADVANCED LUNG CANCER

Katherine Ramos, *DUKE UNIVERSITY MEDICAL CENTER, Durham, North Carolina, United States*

For older adults with advanced cancer, the negative impacts on their emotional and physical health are prevalent, often jeopardizing their capacity to effectively cope and exhibit resilience. Research on multidisciplinary interventions that promote resilience in older adults facing advanced lung cancer has received limited attention. To enhance how healthcare systems/teams may improve oncogeriatric care, we present study findings from a theory-driven behavioral intervention pilot (Self-System Therapy; SST) designed to target patient self-regulation (e.g., behavioral activation, promotion-focused goal setting) to enhance psychological and physical resilience. A total of 15 older adults with late-stage lung cancer completed 6–8-weeks of SST all delivered via videoconference. Self-report, accelerometry, and exit interview data will be presented along with prior pilot data of 30 older adults (total N = 45). Preliminary findings suggest that participants found the use of videoconference feasible and acceptable; with a 95% adherence rate, 7% attrition rate, and 95% satisfaction with SST.

### DISCRIMINATION TRAJECTORIES, RESILIENCE CHARACTERISTICS, AND MULTIMORBIDITY AMONG MIDDLE-AGED AND OLDER BLACKS

Kellee White[1], Kaitlynn Robinson-Ector[2], Melissa wei[3], Tianzhou Ma[2], Bethany Bell[4], and Ana Quinones[5], *1. University of Maryland College Park School of Public Health, College Park, Maryland, United States, 2. University of Maryland College Park, College Park, Maryland, United States, 3. David Geffen School of Medicine at UCLA, Los Angeles, California, United States, 4. University of Virginia, Charlottesville, Virginia, United States, 5. OHSU-PSU School of Public Health, Portland, Oregon, United States*

Discrimination is a chronic psychosocial stressor that can accelerate aging. While strong empirical evidence demonstrating linkages between discrimination and adverse health outcomes, the role of discrimination in multimorbidity burden has received less attention. Prior research on discrimination and health largely uses a single, static, cross-sectional measure to predict health at a second time point, that may mask significant heterogeneity in the dynamic nature of repeated exposures to discrimination. Characterizing longitudinal patterns of discrimination may be a better predictor of health risk and provide insight on resilience processes that influence aging-related outcomes such as multimorbidity. However, this relationship is not well understood. We investigate the association between discrimination trajectories, resilience characteristics, and multimorbidity burden among a sample of middle-aged and older black adults. Specifying discrimination trajectories and resilience characteristics that differentially predict multimorbidity burden may inform the design of culturally-relevant interventions to delay the development and progression of multimorbidity.

### PROTECTIVE RESILIENCE INDEX FOR SUCCESSFUL AGING IN MUSCULOSKELETAL PAIN (PRISM): PRELIMINARY FINDINGS

Amber Brooks[1], Kimberly Sibille[2], Emily Bartley[3], Cynthia Garvan[4], and Angela Mickle[5], *1. Wake Forest School of Medicine, Winston-Salem, North Carolina, United States, 2. University of Florida, Gainesville, Florida, United States, 3. Department of Community Dentistry &Behavioral Science, Gainsville, Florida, United States, 4. Department of Anesthesiology, Gainesville, Florida, United States, 5. Physical Medicine and Rehabilitation, Gainesville, Florida, United States*

Chronic musculoskeletal pain is a highly prevalent condition in older adults and is associated with a decrease in function, biological dysregulation and cellular aging, and increased risk of morbidity and mortality. Our previous work shows that a resilience index comprised of measures of recognized biobehavioral and psychosocial protective factors based on clinically validated norms (waist hip ratio, tobacco use, social support, perceived stress, optimism, positive affect, negative affect-low, active coping) was positively associated with telomere length, lower levels of clinical pain and pain-related functional decline which, also correlated with pain-related brain structure. We will report the initial findings from our multi-centered, NIA RCCN funded study, Protective Resilience Index for Successful Aging in Musculoskeletal Pain (PRISM), which seeks to refine and validate a resilience index that is predictive of biological and pain-related outcomes. This work will lay the foundation for a resilience index that can be used in everyday clinical practice.

### A PILOT RANDOMIZED CLINICAL TRIAL TO PROMOTE POSITIVE AFFECT IN PATIENTS WITH FIBROMYALGIA

Cary Reid[1], Anthony Ong[2], Judy Moskowitz[3], Mubarak Sanni[1], Patricia Kim[1], Elaine Wethington[4], Elizabeth Addington[3], and Erica Sluys[1], *1. Weill Cornell Medicine, New York City, New York, United States, 2. Cornell University, Ithaca, New York, United States, 3. Northwestern University, Chicago, Illinois, United States, 4. Cornell, Ithaca, New York, United States*

Fibromyalgia syndrome (FMS) is a chronic musculoskeletal condition characterized by widespread pain, impaired physical functioning, and deficits in positive affect (PA). Standard behavioral therapies for FMS focus on reducing negative affect (NA) and yield only modest benefits. We examined the feasibility, acceptability and preliminary efficacy of a 6-week, online, PA-enhancing intervention, entitled: Lessons in Affect Regulation to Keep Stress and Pain UndeR control (LARKSPUR). We enrolled participants from one large health system in New York City and ResearchMatch. Ninety-five FMS patients were randomized to LARKSPUR or a neutral emotion reporting arm. Results to date indicate that LARKSPUR is feasible: recruitment (82%), retention (93%); and acceptable: mean satisfaction score on 1-10 scale (9.4, SD=1.2). Planned efficacy analyses will evaluate treatment effects on pain intensity and interference, depressive symptoms, and stress appraisals. If effective, LARKSPUR will lay the foundation for understanding how behavioral treatments for FMS can be optimized through PA enhancement.

### SESSION 2310 (PAPER)

### GENERATIVITY

### ALASKA NATIVES AND GENERATIVITY: BENEFITS AND CHALLENGES OF GENERATIVITY IN A RAPIDLY CHANGING ARCTIC

Jordan Lewis, *University of Minnesota Medical School, Duluth, Minnesota, United States*

Generativity is not often associated with successful aging, but aligns with the cultural values and practices of Alaska Native Elders. This study explored the concept of generativity, why elders share their knowledge, how it makes them feel, and what they want everyone to know. This study interviewed 108 Alaska Native Elders across Alaska, ages ranging from 48 - 93 years old, and willing to share their knowledge of successful aging. Employing content analysis, we explored themes related to each research question on generativity. Elders felt motivated, happy, and good about sharing their knowledge, but only when others listened. Their motivations were to teach the real history of Alaska, leave a community or family legacy, and help youth engage in healthy behaviors. Knowing cultural values, your history, practical skills, and how to listen to your Elders were things they want to share with youth and fellow Elders. Participants also shared the challenges to being generative, including technology, lack of interest by others, and age differences in experiences and knowledge sought. We learned the importance of addressing the generative mismatch between generations and to improve quality of life for Elders and help youth age successfully, we need to create culturally meaningful activities to bring all generations together. Programs to teach youth to be healthy and develop a healthy identity through cultural practices and values, and provide Elders outlets to share and improve their quality of life will ensure cultural survival and foster and strengthen the cultural practice of Indigenous cultural generativity.

### COVID CHALLENGES TO FULFILLING GENERATIVITY: PITFALLS AND BRIDGES FOR OLDER VOLUNTEERS TO RE-ENGAGE COMMUNITY

Kimberly Shay, *Wayne State University, Farmington, Michigan, United States*

The ongoing COVID pandemic undermines the social resources known to preserve health and well-being among older adults. This deeply affects developmental tasks, as traditional paths to fulfilling generativity are impaired. Generativity, a later life stage of seeking to nurture and contribute to the next generation, is accessible through volunteering. This pandemic has revealed gaps in our knowledge of the challenges and adaptations to generativity and meaningful social engagements for older adults. To address this, the author conducted an ethnographic study at four key sites of volunteer engagement by older adults from 2021 to 2022, utilizing a community-based study of older volunteers. Methods included participant-observation at public events and in-depth interviews with 14 key informants. This was a novel study period, post-vaccine, when hopes were raised, but not always achieved for return to generative activities. Analyses revealed three key themes: threats to the foci and spheres of generative expression (Rubinstein, et al, 2015) enacted through volunteering; difficulties and successes creatively crafting new modes to reestablish prior social ties; and threats to full-adult personhood. These themes were linked to altered capacities, experienced during quarantine, to participate with and contribute to the community. Fulfilling later life generativity is intrinsically linked to the ability to participate in community social life, but the pandemic limited opportunities and prompted new challenges and solutions. This paper highlights the need for research to prepare for threats from future disasters and pandemics that challenge meaningful participation and generativity for older adults.

### DO INTERNALIZED AGE STEREOTYPES MEDIATE THE RELATIONSHIP BETWEEN VOLUNTEERING AND SELF-EFFICACY AMONG ADULTS 50+?

Andrew Steward[1], and Leslie Hasche[2], *1. University of Wisconsin-Milwaukee, Milwaukee, Wisconsin, United States, 2. University of Denver, Denver, Colorado, United States*

Volunteering is associated with increased self-efficacy among older adults, and self-efficacy has been shown to mediate the relationship between ageism and health. A growing, compelling body of literature based in stereotype embodiment theory identifies significant, negative public health impacts of internalized age stereotypes. Yet, little research explores whether volunteering may both reduce internalized ageism and enhance self-efficacy as people age. This cross-sectional study examined whether internalized age stereotypes mediate the relationship between volunteering and self-efficacy for adults 50+. A convenience sample of volunteers (n = 165) 50+ years of age residing in the U.S. Mountain West was recruited. A 15-minute, online survey was utilized. The independent variable was number of volunteer hours per week (mean = 6.45, SD = 5.38). The dependent variable was self-efficacy measured by five items from the five-point, Likert-type general self-efficacy scale ($\alpha$ = .83; mean = 4.81, SD = 1.08). Drawing from the self-stereotypes of aging scale, the indirect effects of five internalized positive (e.g., "wise" and "capable") and five negative (e.g., "grumpy" and "helpless") age stereotypes were tested. Results indicate that increased internalized positive, but not negative, age stereotypes partially mediated the relationship between volunteer hours and self-efficacy while holding constant age, gender, race, self-rated health, functional limitation, education, employment, and previous volunteer experience. Although positive age stereotypes have long been considered a form of ageism, the results of this study suggest that internalized positive age stereotypes may function as a form of esteem to promote enhanced psychosocial health as people age.

### EXAMINING THE PROCESS OF COMMUNITY-DWELLING OLDER ADULTS CREATING A LEGACY OF VALUES

Sarah Neller[1], Kristin Cloyes[2], Linda Edelman[3], Bob Wong[4], and Gail Towsley[4], *1. The University of Tennessee, Knoxville, Knoxville, Tennessee, United States, 2. Oregon Health & Science University, Portland, Oregon, United States, 3. University of Utah College of Nursing, Salt Lake City, Utah, United States, 4. University of Utah, Salt Lake City, Utah, United States*

Creating a legacy of values (e.g., legacy letter) is a way to promote generativity by communicating emotional and supportive instruction (e.g., values, life lessons) to others. This study aimed to a) understand the process of how older adults create a legacy of values, b) identify contextual factors surrounding creation, and c) describe older adults' experiences of creation. We recruited community-dwelling older adults who had previously created a legacy of values to participate in semi-structured interviews. Interpretive description guided data collection and analysis. We initially coded the transcripts deductively, based on the literature, and inductively; data were organized into categories (i.e., by contextual factors and process steps) and analyzed using constant comparison to identify patterns. Participants (n=16) had a mean age of 72.3 years (SD=5.8, range=65-83), most were female (n=14), and all had children and identified as non-Hispanic White. Motivations for creation included aging, transitions, or events (e.g., travel). Participants tailored the contents (e.g., life lessons) and format to recipients, revised their legacy of values over time, and recommended the process to others. The creation process was non-linear, iterative, and influenced by participants' personal and familial contextual factors. Synthesis of these findings resulted in developing the Process Model for Creating a Legacy of Values, which depicts the iterative and interconnected nature of the content, process, and experience of creating a legacy of values. Our findings indicate older adults are seeking resources to create their legacy of values in a self-directed way, and that it is an acceptable, accessible, and relevant process.

### HOW TO PUBLISH: GUIDANCE FROM GSA'S JOURNAL EDITORS

Suzanne Meeks[1], Rozalyn Anderson[2], Steven Albert[3], Derek Isaacowitz[4], and Karen Jung[5], *1. University of Louisville, Louisville, Kentucky, United States, 2. University of Wisconsin, Madison, Madison, Wisconsin, United States, 3. University of Pittsburgh, Pittsburgh, Pennsylvania, United States, 4. Northeastern University, Boston, Massachusetts, United States, 5. The Gerontological Society of America, Washington, District of Columbia, United States*

Each year the GSA publications team sponsors a symposium to assist authors who wish to publish in GSA's high impact and influential journals. The first part of the session will include five brief presentations from the editors of The Gerontologist, Innovation in Aging, and the Journals of Gerontology Series A and B plus journal managing editors. We will integrate practical tips with principles of publication ethics and scholarly integrity. The topics will be as follows: (1) Preparing your manuscript: strong and ethical scholarly writing for multidisciplinary audiences, (2) common problems that affect peer review, (3) addressing translational significance and fit to journal expectations, (4) transparency, documentation, and Open Science; and (5) working with Scholar One. Following these presentations, we will hold round table discussions with editors from the GSA journals portfolio. At these round tables, editors will answer questions related to the podium presentations and other questions specific to each journal. Intended audiences include emerging and international scholars, and authors interested in learning more about best practices and tips for getting their scholarly work published.

### PREPARING YOUR MANUSCRIPT: STRONG AND ETHICAL SCHOLARLY WRITING FOR MULTIDISCIPLINARY AUDIENCES

Suzanne Meeks, *University of Louisville, Louisville, Kentucky, United States*

This presentation will emphasize the importance of plain, good writing. Editors read 10 or more manuscripts per week with pressure to reject 80-90% of them. If the point and contribution are not clear in a quick scan of the paper, it will not be reviewed favorably. I will provide tips for writing that are commonly violated in submissions, provide references for additional writing support, cover expectations for language consistent with GSA's Reframing Aging initiative, and discuss some common publication ethics issues that arise during the review process, including author contributions and embedding your scholarship in the context of prior work.

### COMMON PROBLEMS THAT IMPACT PEER REVIEW

Rozalyn Anderson, *University of Wisconsin, Madison, Madison, Wisconsin, United States*

This presentation will review the most common issues that affect how reviewers see a manuscript submission. These include clarity, use of figures, and attention to existing research, especially establishing the significance and novelty of the work, and how to frame a narrative. I will also address responding to peer review. The focus will be on the biological science perspective (Journals of Gerontology Series A), but these issues are relevant to all submissions to GSA journals.

### DEFINING TRANSLATIONAL SIGNIFICANCE IN GERONTOLOGY

Steven Albert, *University of Pittsburgh, Pittsburgh, Pennsylvania, United States*

Innovation in Aging requires a statement from authors on translational significance. This requirement forces authors to consider the implications of their research for changing some component of aging. How does the research address a challenge posed by aging bodies, minds, relationships, or societies? The editorial board has developed criteria for assessing translational significance. Translational research must meet at least one of three criteria. It (i) must predict or explain a health or behavioral outcome, (ii) be advanced enough in deployment or development to assess these effects, and (iii) have a clear pathway to large-scale program delivery or change in clinical practice. The criteria rule out some kinds of submissions, such as scale development, single-case studies, or reviews of literature. We use these criteria to structure each article's required translational significance statement. Rethinking translation may help focus research across the full set of GSA journals.

### TRANSPARENCY, DOCUMENTATION, AND OPEN SCIENCE

Derek Isaacowitz, *Northeastern University, Boston, Massachusetts, United States*

Some GSA journals are especially interested in promoting transparency and open science practices, reflecting how some subdisciplines in aging are moving toward open science practices faster than others. In this talk, I will consider the transparency and open science practices that seem most relevant to aging researchers, such as preregistration, open data, open materials and code, sample size justification and analytic tools for considering null effects. I will also discuss potential challenges to implementing these practices as well as reasons why it is important to do so despite these challenges. The focus will be on pragmatic suggestions for researchers planning and conducting studies now that they hope to publish later.

### GSA MANAGING EDITORS' PERSPECTIVE ON SUBMISSION DOS AND DON'TS

Karen Jung, and Kathleen Jackson, *The Gerontological Society of America, Washington, District of Columbia, United States*

In this presentation, the managing editors of GSA's peer-reviewed journals will discuss how the editorial offices operate and their roles in the publishing process. The topics will include how to navigate the ScholarOne submission system, why it is important to read the Instructions to Authors, and how authors can work with the editorial offices to increase the visibility and impact of their published articles.

### SESSION 2340 (SYMPOSIUM)

### IT'S A COOL LITTLE TOOL: PROVIDER PERSPECTIVES ON IMPLEMENTING PAL CARDS DURING COVID-19

Chair: Katherine Abbott Discussant: Natalie Douglas

This symposium describes the implementation of a person-centered care (PCC) communication tool in nursing homes. PCC is a philosophy that recognizes "knowing the person" and honoring individual preferences. The communication tool is based on an assessment of NH resident likes and dislikes via the Preferences for Everyday Living Inventory (PELI). The PELI is an evidenced-based, validated instrument that can be used to enhance the delivery of PCC. The Preferences for Activity and Leisure (PAL) Card was developed to communicate important resident preferences across care team members. From July 2020 to July 2021 we lead a Quality Improvement Project (QIP) approved by the Ohio Department of Aging providing virtual coaching to providers who created 15-20 PAL Cards for their residents. Our first presentation describes the QIP where n=16 started implementation and n=11 communities completed the project during the height of the pandemic. Providers were non-profit (50%) with an average star rating of 3.29 (SD 1.33). Feedback from n=68 direct care staff on PAL Card usage are reported. The next four presentations describe implementation of PAL Cards from the perspective of the provider champions who contributed to n=66 monthly interviews. Interviews were recorded, transcribed verbatim, and coded in Dedoose using the Consolidated Framework for Implementation Research as an a priori coding scheme. We present barriers and facilitators related to the domains of Inner Setting, Characteristics of the Individual, Characteristics of the Intervention, and Process. The Discussant, Dr. Natalie Douglas will discuss the implications of initiatives to address the quality of resident care.

### DIRECT CARE STAFF PERSPECTIVES ON THE IMPACT OF PAL CARDS IN CARE DELIVERY

Katherine Abbott[1], Alexandra Heppner[1], Megan Kelley[1], Kamryn Kasler[1], Miranda Kunkel[1], and Kimberly Van Haitsma[2], *1. Miami University, Oxford, Ohio, United States, 2. Pennsylvania State University, University Park, Pennsylvania, United States*

This was the third time replicating the Preferences for Activity and Leisure (PAL) Card quality improvement project, but the first conducted entirely during the height of the pandemic. Nursing home providers attempted n=174 PAL Cards and completed n=166 (96%). Feedback from surveys with n=68 staff who came in daily contact with residents (e.g., 26% CNAs, 10% housekeepers, 6% maintenance, 9% dining) were collected by project champions. Staff had worked on average 6 years, indicated that they remember being been told about PAL Cards (81%), noticed them (94%), reported that the information on the PAL Card helped them start a conversation with a resident (79%), and helped them provide care (59%). PAL Cards are a feasible method to communicate important resident preferences to staff who come in daily contact with residents and may not have access to electronic health records. Recommendations for practice will be discussed including staff recommendations for improvements.

### EXAMINING THE NURSING HOME INNER SETTING CHARACTERISTICS THAT LEAD TO THE SUCCESSFUL IMPLEMENTATION OF PAL CARDS

Kamryn Kasler[1], Megan Kelley[1], Miranda Kunkel[1], Reese Moore[1], Faith DeVengencie[1], Alexandra Heppner[1], Kimberly Van Haitsma[2], and Katherine Abbott[1], *1. Miami University, Oxford, Ohio, United States, 2. Pennsylvania State University, University Park, Pennsylvania, United States*

Implementation efforts of evidence-based practices focusing on the nursing home setting remain understudied. The purpose of this study was to explore the role of the Consolidated Framework for Implementation Research (CFIR) inner setting domain on the implementation of PAL Cards. Monthly qualitative interviews (n=50) with project champions (n=16) were audio-recorded, transcribed, checked for accuracy, and coded using the domain "Inner Setting" from the CFIR in Dedoose. Major themes emerging from the data included networks and communication (e.g., including PAL Card information in employee newsletter) among staff, compatibility of the PAL Cards (e.g., aligns with community values and fits into existing workflows), and available resources (e.g., adequate time and staff to implement the QIP). High-quality communication channels to educate and collaborate were shown to be an integral component of successful implementation. Implications for policy and practice will be discussed.

### THAT'S JUST MY SUPERPOWER: THE ROLE OF NURSING HOME PROVIDER CHAMPIONS IN IMPLEMENTING A PERSON-CENTERED PROJECT

Miranda Kunkel[1], Megan Kelley[1], Reese Moore[1], Kamryn Kasler[1], Chloe Arrasmith[1], Alexandra Heppner[1], Kimberly Van Haitsma[2], and Katherine Abbott[1], *1. Miami University, Oxford, Ohio, United States, 2. Pennsylvania State University, University Park, Pennsylvania, United States*

Champions are crucial to successful intervention implementation; however, as an area of research champions have only recently begun to gain empirical focus. The purpose of this study was to identify the barriers and facilitators regarding champion qualities using the domain "Characteristics of the Individual" from CFIR. Qualitative interviews were conducted with nursing home provider champions (n=16) who led efforts to create PAL Cards for 15-20 residents and participated in a total of n=50 monthly interviews that were audio-recorded and transcribed verbatim. The following themes regarding characteristics of the individual emerged: champions' investment in their community, capacity and existing workload, and willingness to ask for help. While some champions saw the value of the PAL Cards, they lacked the bandwidth necessary to widely implement the intervention in their community. Champions who recruited a supportive team and valued person-centered care exhibited success in implementing PAL Cards.

### IT'S A VERY MANIPULATABLE TOOL: CHARACTERISTICS OF PAL CARD USE THAT IMPACT SUCCESSFUL IMPLEMENTATION

Reese Moore[1], Megan Kelley[1], Miranda Kunkel[1], Kamryn Kasler[1], Julia Baker[1], Alexandra Heppner[1], Katherine Abbott[1], and Kimberly Van Haitsma[2], *1. Miami University, Oxford, Ohio, United States, 2. Pennsylvania State University, University Park, Pennsylvania, United States*

Understanding the barriers and facilitators of an intervention can inform implementation efforts. The purpose of this study was to understand the characteristics associated with the PAL Card intervention that led to successful implementation in nursing home (NH) settings. Qualitative telephone interviews were conducted with n=11 NH champions who completed the PAL Card QIP. Interviews were recorded, transcribed verbatim, and coded using the CFIR Intervention Characteristics domain in Dedoose. Three main themes regarding the intervention characteristics emerged including, relative advantage (i.e., advantage of using PAL Cards versus an alternate intervention), adaptability (i.e., how well the PAL Cards can be altered to meet community needs), and complexity (i.e., perceived difficulty of PAL Card usage). Participants voiced the simplicity and benefit of PAL Card implementation within their communities for both staff and residents alike. Implications for policy and practice will be discussed.

### UNDERSTANDING PROCESSES NEEDED TO SUCCESSFULLY IMPLEMENT PAL CARDS IN NURSING HOMES.

Megan Kelley[1], Miranda Kunkel[1], Reese Moore[1], Kamryn Kasler[1], Lexi Talmage[1], Alexandra Heppner[1], Kimberly Van Haitsma[2], and Katherine Abbott[1], *1. Miami University, Oxford, Ohio, United States, 2. Pennsylvania State University, University Park, Pennsylvania, United States*

Background: The CFIR Process domain can be used to evaluate practices that are associated with successful or unsuccessful implementation. The purpose of this study was to understand the processes that led to successful implementation of the PAL Card QIP during the height of the pandemic from the provider champion's perspective.

Methods: Qualitative interviews with n=11 champions who completed the PAL Card QIP were audio recorded, transcribed verbatim, and coded using the CFIR Process Domain.

Results: Major themes regarding the process of implementation included engaging (e.g., staff and family), executing (adapting to different situations), planning (e.g., having a clear plan of implementation), and reflecting and evaluating (e.g., taking time to evaluate the project). Discussion: Providers articulated strategies that contributed to successful implementation during a global pandemic, which can help inform future quality improvement projects. Implications for practice and policy will be discussed.

### SESSION 2350 (SYMPOSIUM)

### MAKING CLASSROOMS MORE AGE-FRIENDLY: STRATEGIES FOR INTERGENERATIONAL EXCHANGE

Chair: Joann Montepare Co-Chair: Kimberly Farah
Discussant: Lisa Borrero

Aging populations are reshaping how we think about teaching and learning in higher education. As a result, educational opportunities for intergenerational exchange are on the rise with the growth of the Age-Friendly University (AFU) initiative. Endorsed by GSA's Academy for Gerontology in Higher Education, the AFU principles call for promoting intergenerational exchange to facilitate the reciprocal sharing of expertise between learners of all ages. Age diverse classrooms and learning spaces have distinctive needs and dynamics that instructors, and students, will need to learn how to navigate. This collaborative symposium, that brings together members of the Age-Friendly University (AFU) and Intergenerational Learning, Research, and Community Engagement (ILRCE) Interest Groups, will explore evidenced-based practices that contribute to successful experiences in and beyond the classroom. Montepare and Farah will provide an overview of the AFU initiative and intergenerational classroom issues. Jarrott and colleagues will address how technology and other strategies can prepare individuals for intergenerational learning experiences, including when circumstances limit in-person opportunities, such as during COVID. Dauenhauer and Heffernan will share insights from faculty about ways to develop and sustain a lifelong learning program that incorporates intergenerational interactions in the classroom experience. Leedahl will discuss strategies for developing age-friendly intergenerational internship experiences with community partners. Borrero, co-convener of the ILRCE Interest Group, will serve as the discussant.

### THE AGE-FRIENDLY UNIVERSITY (AFU) INITIATIVE AND THE CALL FOR MORE AGE-FRIENDLY INTERGENERATIONAL CLASSROOMS

Joann Montepare, *Lasell University, Newton, Massachusetts, United States*

Changing age demographics are reshaping societies and challenging our living, learning, and work communities to consider how they can respond to more age-diverse, older populations. The pioneering Age-Friendly University (AFU) initiative, endorsed by GSA's Academy for Gerontology in Higher Education (AGHE), calls for institutions of higher education to respond to these shifting demographics through more age-friendly programs, practices, and partnerships. Moreover, the 10 AFU Principles recommend a set of specific educational options including the promotion of intergenerational learning to facilitate the reciprocal sharing of expertise between learners of all ages. This presentation provides an overview of the AFU initiative with a focus on strategies for intergenerational teaching and learning. It sets the stage for the other presentations by describing core questions and considerations educators should take into account when mounting intergenerational practices in and beyond the classroom.

### EVIDENCE-BASED INTERGENERATIONAL PRACTICES SUPPORT AFU PRINCIPLES

Shannon Jarrott[1], Jill Juris[2], and Rachel Scrivano[1], *1. The Ohio State University, Columbus, Ohio, United States, 2. Appalachian State University, Boone, North Carolina, United States*

Theory and research informed a framework of best intergenerational practice that has proven effective in the field of community programs. Reflecting a number of AFU principles, this set of proven practices can promote positive intergenerational contact in college classrooms as well. Here, we present evidence-based and promising practices salient to the AFU, offering examples from the classroom and suggesting low- and high-tech solutions. For example, the intergenerational practices of offering meaningful roles and engaging youth and older adults in a novel activity can support AFU principle 4 reciprocal sharing of expertise between learners of all ages. Stakeholders, including instructors, university students, older adult learners, and community partners, benefit from preparation for the distinctive needs and opportunities of intergenerational learning experiences in higher education. Effective implementation of intergenerational strategies helps both younger and older learners to reimagine aging, offering the potential to address a critical need of individuals and communities.

### PROMOTING AND SUSTAINING A COLLEGE-WIDE LIFELONG LEARNING PROGRAM: FACULTY INSIGHTS AND EXPERIENCES

Jason Dauenhauer, Kristin Heffernan, and Afeez Hazzan, *State University of New York at Brockport, Brockport, New York, United States*

Research has identified the benefits and challenges of creating multigenerational classrooms for the purpose of intergenerational learning in higher education. However, much less is known about the systems of support needed at the organizational level to promote intergenerational learning within lifelong learning initiatives. With a focus on formal and informal organizational structures, this session describes insights from faculty who opened their courses to older adults as part of a college-wide course auditing initiative. Many did not understand their own role in the program and offered suggestions to address these issues. Recommendations include ways to acknowledge the work of faculty who participate in the program, and to engage more professors and older adults in the community to ensure program success. To create an age-inclusive, and age-friendly university, formal structural change led from the top-down is as important, if not more important, as creating informal networks that start at the faculty level.

### BEYOND THE CLASSROOM: A STUDENT INTERNSHIP MODEL THAT BUILDS AGE FRIENDLINESS AND CAREER READINESS

Skye Leedahl, Kristin Fratoni Souza, and Alexandra Morelli, *University of Rhode Island, Kingston, Rhode Island, United States*

For seven years, the University of Rhode Island (URI) Engaging Generations Cyber-Seniors Program has utilized university student mentors through an internship format and integration within service learning courses. In 2019, community interest in the program significantly increased as did student interest in gerontology internships. This led us to develop a robust internship program focused on building age and digital inclusivity across campus and the state of RI. The program integrates a three-pronged approach where students complete field hours within the URI Cyber-Seniors program, focus on enhancing the Career Readiness Competencies employers seek in graduates today as determined by the National Association of Colleges & Employers, and complete the components necessary to earn the Geriatric Education Center Interprofessional Teamwork in Geriatrics and Gerontology Certificate. This process has enabled 350 students from different majors, including Pharmacy and Human Development & Family Science, to complete internship experiences ranging from 30 to 210 hours.

### SESSION 2360 (AWARD LECTURE)

### MARGRET M. AND PAUL B. BALTES AWARD PRESENTATION AND LECTURE

Chair: Tamara Baker

The Margret M. and Paul B. Baltes Award Lecture will feature an address by the 2021 Baltes Award recipient, Laura B. Zahodne, PhD, of the University of Michigan. This session will also include the presentation of the 2022 Margret M. and Paul B. Baltes Award to recipient Eric S. Kim, PhD, of the University of British Columbia. The Margret M. and Paul B. Baltes Foundation Award in Behavioral and Social Gerontology recognizes outstanding early-career contributions in behavioral and social gerontology. The award is generously funded by the Margret M. and Paul B. Baltes Foundation.

### BIOPSYCHOSOCIAL RISK AND RESILIENCE PATHWAYS IN DEMENTIA INEQUALITIES

Laura Zahodne, *University of Michigan, Ann Arbor, Michigan, United States*

In the United States, racial/ethnic inequalities in Alzheimer's disease and related dementias persist even after controlling for socioeconomic factors and physical health. Persistent and unexplained disparities suggest: (1) there are unrecognized dementia risk factors that are socially patterned and/or (2) known dementia risk factors exhibit differential impact across social groups. This talk will present data from multiple longitudinal studies of brain and cognitive aging to support each possibility. On average, marginalized racial/ethnic groups are more likely than non-Latinx Whites to experience structural and interpersonal discrimination, social and economic constraints, as well as barriers to accessing high quality education. However, these same groups also show evidence of greater psychosocial resilience that is linked to better late-life cognitive health. This talk will demonstrate how specific psychosocial factors can contribute to or offset dementia disparities, illustrate major challenges to this work, and introduce new data collection efforts to advance the field.

### SESSION 2380 (SYMPOSIUM)

### NEIGHBORHOOD QUALITY AS IT RELATES TO HEALTH AND WELL-BEING IN OLDER AFRICAN AMERICANS

Chair: Regina Wright Co-Chair: Alyssa Gamaldo
Discussant: Anna Lee

With the burgeoning older adult population, there will be an increased demand for neighborhood environments that are conducive to successful aging. For example, the need for affordable and usable housing developments for older adults that provide greater opportunities for social engagement, social services, and convenience to neighborhood resources (e.g., grocery stores, healthcare) will continue to rise. Several initiatives have sought to develop age-friendly neighborhoods, which focused on improving accessibility and affordability of community resources. However, limited effort has focused on the health and cognitive effects of neighborhood-level socioeconomic disadvantage, with respect to neighborhood income, education, employment, and housing quality. This symposium will include presentations from two studies that explored how neighborhood disadvantage (measured by the Area Deprivation Index) relates to health and cognition. The objectives of the proposed symposium are the following:

(1) discuss how neighborhood disadvantage relates to subjective and objective measures of physical health; and (2) discuss how neighborhood disadvantage relates to cognitive functioning. Allan and colleagues explored the association between neighborhood disadvantage as it relates to changes in self-reported health and objective measures (i.e., blood pressure) of health among older Black adults. Wright and colleagues explored the association between neighborhood disadvantage and measures of subclinical cardiovascular disease in older Black and White adults. Aiken-Morgan and colleagues examined associations between neighborhood disadvantage and cognitive functioning among Black older adults. Lastly, McCain and colleagues explored associations between neighborhood disadvantage and mobility among Black older adults.

### EXAMINING LINKAGES BETWEEN NEIGHBORHOOD DISADVANTAGE AND SUBCLINICAL CARDIOVASCULAR DISEASE

Regina Wright[1], Alexa Allan[2], Alyssa Gamaldo[3], Adrienne Aiken Morgan[4], and Anna Lee[5], *1. University of Delaware, Newark, Delaware, United States, 2. The Pennsylvania State University, University Park, Pennsylvania, United States, 3. Pennsylvania State University, State College, Pennsylvania, United States, 4. University of North Carolina at Chapel Hill, Chapel Hill, North Carolina, United States, 5. North Carolina A&T State University, Greensboro, North Carolina, United States*

Limited research has examined the extent neighborhood disadvantage relates to subclinical CVD such as carotid atherosclerosis, arterial stiffness, and endothelial dysfunction which are key, understudied CVD risk markers. Our objective was to examine associations between neighborhood disadvantage and subclinical CVD and whether associations vary by age, sex, race, and education. The analysis included 165 Black and White older adults (mean age=64.5y). Neighborhood disadvantage was characterized using the Area Deprivation Index (ADI). Multivariable regression analysis, adjusted for age, sex, race, education, and depression, showed that national and state ADI were significant, inverse predictors of carotid atherosclerosis (near wall IMT; $p=.02$) and state ADI was a significant, inverse predictor of flow-mediated dilation ($p=.04$). A significant interaction between state and national ADI and education predicting carotid IMT emerged, but the simple slopes were non-significant ($p=.02$, .03). Future research should consider the role of neighborhood as a predictor of early cardiovascular risk.

### NEIGHBORHOOD DISADVANTAGE AND COGNITION AMONG AFRICAN AMERICANS IN THE BALTIMORE STUDY OF BLACK AGING

Adrienne Aiken Morgan, *University of North Carolina at Chapel Hill, Chapel Hill, North Carolina, United States*

Previous research has suggested an important and understudied association between neighborhood disadvantage and cognitive functioning among older African Americans. Data from the Baltimore Study of Black Aging (N=602) was utilized to examine the associations between Area Deprivation Index (ADI) and cognition. Multiple stepwise regression models, controlling for age, sex, education, and income, showed participants who scored better on measures of immediate memory, vocabulary, reasoning, and everyday cognition had lower ADI (less disadvantaged neighborhoods; $p < .05$). Interestingly, individuals living with higher ADI had better attention/working memory scores and better self-reported memory ($p < .05$). ADI was significantly associated with improvements or declines in performance, depending on the cognitive ability (e.g., learning and memory, working memory, global mental status, and visuospatial skill, $p < .05$). These findings suggest the importance of accounting for neighborhood context in African Americans' cognitive aging research.

### AREA DEPRIVATION INDEX EFFECTS ON LONGITUDINAL REPORTS OF PHYSICAL HEALTH AMONG OLDER BLACK ADULTS

Alexa Allan[1], Alyssa Gamaldo[2], Regina Wright[3], Adrienne Aiken Morgan[4], Anna Lee[5], Jason Allaire[6], Roland J. Thorpe, Jr.[7], and Keith Whitfield[8], *1. The Pennsylvania State University, University Park, Pennsylvania, United States, 2. Pennsylvania State University, State College, Pennsylvania, United States, 3. University of Delaware, Newark, Delaware, United States, 4. University of North Carolina at Chapel Hill, Chapel Hill, North Carolina, United States, 5. North Carolina A&T State University, Greensboro, North Carolina, United States, 6. North Carolina State University, Raleigh, North Carolina, United States, 7. Johns Hopkins University, Baltimore, Maryland, United States, 8. University of Nevada, Las Vegas, Las Vegas, Nevada, United States*

Despite the association of neighborhood quality with poorer adult health, limited research has explored the association between neighborhood disadvantage, e.g., Area Deprivation Index (ADI), and older Black adults' health, prospectively. The Baltimore Study of Black Aging ($n = 424$) was utilized to examine the association between ADI and prospective physical health. Multiple regression analyses, covarying for age, sex, education, and income, showed that living in a neighborhood with greater disadvantage was significantly associated with decreasing average heart rate from wave 1 to wave 2 (approximately 33 months apart) ($p < .05$). This result was specifically significant for women living in neighborhoods with greater disadvantage. Additionally, findings indicated that living in a neighborhood with greater disadvantage was significantly associated with increased difficulty with activities of daily living ($p < .01$). More research is needed to examine the implications of neighborhood context on older Black adults' health prospectively.

### ASSOCIATIONS BETWEEN NEIGHBORHOOD DISADVANTAGE AND MOBILITY LIMITATIONS AMONG AFRICAN AMERICANS

Dextiny McCain[1], Adrienne Aiken Morgan[2], Alexa Allan[3], Alyssa Gamaldo[4], Regina Wright[5], Roland J. Thorpe, Jr.[6], Jason Allaire[7], and Keith Whitfield[8], *1. North Carolina Central University, Durham, North Carolina, United States, 2. University of North Carolina at Chapel Hill, Chapel Hill, North Carolina, United States, 3. The Pennsylvania State University, University Park, Pennsylvania, United States, 4. Pennsylvania State University, State College, Pennsylvania, United States, 5. University of Delaware, Newark,*

*Delaware, United States, 6. Johns Hopkins University, Baltimore, Maryland, United States, 7. North Carolina State University, Raleigh, North Carolina, United States, 8. University of Nevada, Las Vegas, Las Vegas, Nevada, United States*

Research has shown that socioeconomic factors influence physical mobility; however, less is known about the influence of neighborhood disadvantage on mobility among African American older adults. Data from the Baltimore Study of Black Aging (N=602) were used to examine associations between neighborhood disadvantage, as measured by the Area Deprivation Index (ADI), and self-reported mobility limitations. Two-step logistic regression showed that participants residing in more disadvantaged neighborhoods, based on national ADI, had higher odds of mobility limitations, but those in less disadvantaged neighborhoods, based on state ADI, had higher odds of mobility limitations (national: OR = 1.02 (95% CI: 1.00–1.03); state: OR = 0.78 (95% CI: 0.65–0.92)). Stratified models showed the association was significant only within women (national: OR = 1.02 (95% CI: 1.00–1.04); state: OR = 0.77 (0.63-0.95)). These findings suggest that neighborhood disadvantage is an essential consideration when examining mobility limitations among African Americans.

### SESSION 2390 (SYMPOSIUM)

### RECRUITMENT AND ENGAGEMENT OF LGBTQIA+ OLDER ADULTS INTO AGING RESEARCH

Chair: Jason Flatt Co-Chair: Krystal Kittle Discussant: Jason Flatt

Lesbian, gay, bisexual, transgender, queer, intersex, and/or asexual (LGBTQIA+) older adults are a growing population that has been traditionally underrepresented in aging research. Due to stigma and discrimination, many LGBTQIA+ may be reluctant to disclose their sexual orientation and gender identity, which makes it difficult to recruit and engage LGBTQIA+ populations in aging research. In this symposium, we explore current efforts to recruit LGBTQIA+ older adults via social media, snowball sampling, or other novel methods, as well as ways engage LGBTQIA+ subgroups (e.g., caregivers, racial/ethnic minorities, transgender, intersex and other underrepresented groups). Two presentations focus on the recruitment of gender minorities including transgender and intersex older adults. Ethan Cicero will present a systematic review that highlights currents efforts to recruit older gender minority populations include those who identify as racial/ethnic minorities. Next, Nick Lampe will share methodological approaches for effectively recruiting and engaging transgender, non-binary, and intersex (TNBI) older respondents. Then Austin Oswald will highlight barriers and facilitators to engaging LGBTQ+ elders of color in participatory action research during the Covid-19 pandemic. Kasim Ortiz will examine correlates of survey response participation among older sexual minorities (LGB+) using internet-based sampling via the Census Household Pulse Survey. Finally, Krystal Kittle will highlight a three-stage, pilot intervention that aimed to recruit LGBTQ+ caregivers during COVID-19 pandemic restrictions. Jason Flatt, an expert in LGBTQIA+ aging research will facilitate a conversation about these results and place them in the context of current efforts to recruit and engage LGBTQIA+ populations in aging research.

### EQUALITY IN CAREGIVING: RATIONALE AND DESIGN FOR AN INTERVENTION ON CAREGIVER MASTERY AMONG LGB CAREGIVERS

Krystal Kittle[1], Kelly Likos[2], Danielle Verble[2], Jason Flatt[3], Joel Anderson[4], and Whitney Wharton[5], *1. University of Nevada Las Vegas, Las Vegas, Nevada, United States, 2. Emory University, Atlanta, Georgia, United States, 3. University of Nevada, Las Vegas, School of Public Health, Las Vegas, Nevada, United States, 4. College of Nursing, University of Tennessee, Knoxville, Knoxville, Tennessee, United States, 5. Emory University, Nell Hodgson Woodruff School of Nursing, Atlanta, Georgia, United States*

Compared to heterosexual and cisgender caregivers, lesbian, gay, bisexual, transgender and queer (LGBTQ+) caregivers face unique challenges (e.g., physical, emotional, financial strain). The initial purpose of this pilot study was to assess an existing evidence-based intervention, the Savvy Caregiving Program, according to context-specific challenges faced by LGBTQ+ caregivers of people with Alzheimer's disease and related dementias (ADRD), improve caregiver mastery and mood, while reducing caregiver burden and physiological stress biomarkers. Given the inability to perform research in-person due to COVID-19, we redirected our research efforts. Trial modifications included: national vs local participation, shift to social media recruitment, virtual SCP classes and focus groups, addition of cross sectional health and caregiving data collection for all LGBTQ caregivers, and removal of biomarker collection. We will present survey results from a national sample of 49 LGBTQ caregivers. Descriptive statistics and non-parametric tests were used to assess differences between ADRD and non-ADRD caregivers.

### RECRUITMENT OF TRANSGENDER, NONBINARY, AND GENDER DIVERSE OLDER ADULTS IN HEALTH RESEARCH: A SYSTEMATIC REVIEW

Ethan Cicero[1], and Jason Flatt[2], *1. Emory University, Atlanta, Georgia, United States, 2. University of Nevada, Las Vegas, School of Public Health, Las Vegas, Nevada, United States*

Despite the urgency reported by the National Institutes of Health and National Academy of Sciences to advance health knowledge about transgender, nonbinary, and gender diverse (TNGD) older adults, TNGD populations remain underrepresented in aging research. This review examines TNGD older adult recruitment approaches in health-related research. PubMed, CINHAL, and PsychINFO databases were searched using key terms/subject headings related to TNGD and aging/older adults to identify health-related, U.S.-based empirical studies published since 2011. Twenty-four studies met inclusion criteria (quantitative=15, qualitative=8, mixed-methods=1). Most (n=13) focused recruitment efforts on the broader LGBTQ community, whereas 9 studies (qualitative, n=5) specifically targeted TNGD older adults. One study that recruited from Amazon Mechanical Turk, but most TNGD-specific recruitment efforts used community-engaged approaches, including snowball and purposive sampling via listservs; groups, organizations, and events serving the transgender/nonbinary community; and outreach through researchers' personal and professional networks. Novel approaches are needed to increase TNGD representation in aging research.

### RECRUITING AND ENGAGING TRANSGENDER, NON-BINARY, AND INTERSEX OLDER ADULTS IN AGING RESEARCH

Nik Lampe, *Vanderbilt University, Nashville, Tennessee, United States*

Aging research often erases sex and gender variation, thus ignoring the experiences of older transgender, non-binary, and intersex (TNBI) populations. At the same time, researchers have faced unique methodological challenges with recruiting and engaging with TNBI older patient populations in sexual and gender minority (SGM) aging research. Using data from 51 semi-structured individual interviews with TNBI older Americans, I explore methodological approaches for effectively recruiting and engaging TNBI older respondents. I describe the importance of disclosing researcher positionality and understanding community discourse (e.g., intersex status as a sex identity vs. a medical condition) when recruiting and engaging with TNBI older adults. Finally, I share why some traditional recruitment approaches in SGM aging research (e.g., purposive/snowball sampling techniques) may not be as effective towards TNBI older adults while offering some alternative techniques for meaningful recruitment and engagement opportunities.

### BARRIERS AND FACILITATORS OF PARTICIPATORY ACTION RESEARCH WITH LGBTQ+ ELDERS OF COLOR DURING COVID-19

Austin Oswald, *Goldsen Institute of the University of Washington , Seattle, Washington, United States*

Participatory action research (PAR) is a promising approach to engage older adults in self-determined research that influences policies and practices; however, the voices of older adults are very often missing from most PAR projects. The purpose of this paper is to reflect upon the challenges and opportunities of engaging lesbian, gay, bisexual, transgender, and queer (LGBTQ+) elders of color in PAR during the Covid-19 pandemic. Data were generated through interviews with LGBTQ+ elders of color and their caregivers and analyzed thematically. Findings illustrate several barriers and facilitators of conducting PAR during the pandemic. Barriers included feelings of distrust toward research(ers), pandemic related issues and illnesses, and competing priorities and interests. Facilitators included leveraging community connections and participating in community-based activities. Study findings have the potential to advance a critical turn in gerontology that is committed to the liberation of marginalized older adults in aging research, policies, and practices.

### ASSESSING NONSAMPLING BIAS ERROR AMONG SEXUAL MINORITY OLDER ADULTS IN THE CENSUS' HOUSEHOLD PULSE SURVEY

Kasim Ortiz, *University of New Mexico, Albuquerque, New Mexico, United States*

Previous research shows data quality and nonsampling bias error reduce accuracy of COVID-19 vaccination coverage in the Census' Household Pulse Survey. However, it is unclear how these issues relate to older sexual minority populations. Moreover, previous research has shown that sexual minority older adults survey nonparticipation rates are similar to their heterosexual older adult peers. Yet, it is less clear whether racial/ethnic variability in nonparticipation rates and patterns of missingness among sexual minority older adults exist. The current investigation utilizes repeated pooled data from the Census' Household Pulse Survey (N=329,078) to fill these gaps. Findings reveal nonsampling bias error contributing to inflated racial/ethnic inequities in vaccination coverage among older sexual minorities, with distinct racial/ethnic variability in missingness patterns among sexual minority older adults. The paper concludes with recommendations for improving survey-based research and details suggestions to improve recruitment strategies that minimize nonsampling bias.

### SESSION 2400 (SYMPOSIUM)

### SOCIOEMOTIONAL EXPERIENCE IN CLOSE PERSONAL RELATIONSHIPS AND TRAJECTORIES OF COGNITIVE AGING

Chair: Sara Moorman Discussant: Lucas Hamilton

Linked lives is a key tenet of the life course framework: Individuals age and develop in the company of a social convoy, or core set of relationships. The quality and quantity of relationships with friends and family are well-known predictors of physical and mental health outcomes, with research on how relationships affect cognitive health just beginning to blossom. This symposium presents four sociological studies of how positive and negative experiences in central, long-term personal relationships – marriages, parent-adult child relationships, and friendships – relate to cognition and the development of cognitive impairment over long periods of the life course, using data from the Health and Retirement Study (HRS) and the Wisconsin Longitudinal Study (WLS). Stokes, Prasad, and Barooah find that experiences of loneliness in marriage are negatively related both to one's own cognition and to the spouse's cognition. Herd and Sicinski also highlight potential negative and gendered aspects of marriage, showing no differences in cognitive performance between married and single men, while married women's cognition is not as strong as single women's cognition. In parent-adult child relationships, Zhang and Liu demonstrate that social support has stronger positive effects, and social strain, stronger negative effects, for mothers as compared to fathers. Moorman and Pai examine friendships with non-kin, and find benefits of emotional and instrumental support to cognition in the long term. Discussant Lucas Hamilton will provide perspective from psychology, addressing ambivalence in relationships and the potential for bidirectional associations between social experience and cognitive function.

### SOCIAL SUPPORT FROM FRIENDS AND INCIDENCE OF COGNITIVE IMPAIRMENT A DECADE LATER

Sara Moorman[1], and Manacy Pai[2], *1. Boston College, Chestnut Hill, Massachusetts, United States, 2. Kent State University, Kent, Ohio, United States*

We inquire how diverse forms of social support from non-kin are associated with cognitive aging across ten years. We analyze data from 4,687 participants in the 2011 and 2021 waves of the Wisconsin Longitudinal Study (WLS), a cohort study of Wisconsin adolescents from the birth cohort of 1939 and their siblings. Net of sex, educational attainment, income, smoker status, depressive symptoms, self-reported health, diabetes, heart disease, high blood pressure, high cholesterol, and stroke incidence, each additional domain in which participants perceived that they had instrumental support available from friends in 2011 was associated with a 0.07 standard deviation increase in cognitive function (i.e., TICS-m score) a decade later (p = .001). Moreover, participants who had a non-kin confidante in 2011 had significantly lower odds of mild cognitive impairment (MCI) in 2021 (OR = 0.57; p = .006). Robust social engagement may help protect and maintain older adults' cognitive skills.

### DYADIC LONELINESS AND COGNITIVE HEALTH AMONG OLDER MARRIED COUPLES: LONGITUDINAL EVIDENCE FROM THE HRS

Jeffrey Stokes, Anyah Prasad, and Adrita Barooah, *University of Massachusetts Boston, Boston, Massachusetts, United States*

Loneliness is associated with numerous poor health outcomes, including mortality. Additionally, loneliness is not merely an isolated individual's experience; rather, loneliness occurs regularly even among the married, and can affect both spouses' health. We analyze 3-wave dyadic data from the Health and Retirement Study (2008-2018; N = 7,907 dyads) to determine (a) whether loneliness is associated with participants' own and/or their partner's verbal fluency and episodic memory over a nearly decade-long period, and (b) whether these measures of cognitive health predict older spouses' own or their partner's loneliness over the same period. Results indicated that (1) loneliness, episodic memory, and verbal fluency were all "contagious" within couples, such that baseline levels of each predicted participants' own and their partner's values at follow-up; (2) participants' own loneliness was associated with poorer verbal fluency and episodic memory at follow-up; and (3) neither participants' own nor their partner's cognitive functioning predicted future loneliness.

### RELATIONSHIP QUALITY AND OLDER PARENTS' COGNITIVE TRAJECTORIES: A NATIONAL LONGITUDINAL STUDY IN THE UNITED STATES

Yan Zhang[1], and Hui Liu[2], *1. University of Wisconsin-Madison, Madison, Wisconsin, United States, 2. Michigan State University, East Lansing, Michigan, United States*

This study aims to assess the impact of the relationship quality between parents and children on parents' cognitive function in later life, with an additional focus on variation by parents' gender. We analyze data from a nationally representative longitudinal panel survey of participants age 50 and older. We employ latent growth curve models (LGCM) to estimate how changes in parent-child relationship quality are related to cognitive trajectories over time. Maintaining frequent contact with children and receiving more support from children are associated with a slower rate of cognitive decline for older parents whereas experiencing relationship strain with children is associated with a faster rate of cognitive decline for older parents. These associations are stronger for mothers than fathers. This study highlights the importance of the "linked lives" of aging parents and their children. The findings have implications for the development of interventions and strategies to protect cognitive function in later life.

### GENDER, MARITAL STATUS, AND COGNITIVE HEALTH IN LATE LIFE: IS MARRIAGE MORE PROTECTIVE FOR MEN?

Pamela Herd, *Georgetown University, Washington, District of Columbia, United States*

While marital status appears to be protective for one's health, there is also evidence of gender differences in its protective influence. In short, men appear to differentially benefit from marriage compared to women. A growing body of work on later life cognitive functioning and dementia also finds protective effects for those who are married. But there is less evidence as to whether those patterns differ by gender. Using the Wisconsin Longitudinal Study, a nearly full life course longitudinal study, we find evidence that while there are no differences for men, married women, as compared to their unmarried counterparts, have lower levels of cognitive functioning at ~age 80. Differences in underlying health, educational attainment, and adolescent cognitive functioning do not explain the pattern. Similar to broader health, women do not appear to benefit from marriage in late life. Indeed, we find evidence of cognitive benefits of being single for older women.

### SESSION 2410 (SYMPOSIUM)

### THE CHALLENGE OF ASSESSING AND MEASURING SOCIAL INCLUSION AND EXCLUSION IN LATER LIFE

Chair: Charles Waldegrave Discussant: Marja Aartsen

Social exclusion is a serious problem that can lead to diminished well-being, health problems, premature death and increased societal costs. Depending on the definition used, 10 to 30% of the older adults experience social exclusion, and many have been confronted with prolonged isolation during the pandemic. Constructing measures for social inclusion and exclusion is a challenging yet important endeavor, and various approaches exist. For example, EU-policy makers define social exclusion mainly in terms of poverty and lack of labor market participation However, a too narrow definition of social exclusion leaves large groups of people unattended leading to sub-optimal understandings of social exclusion and ineffective interventions. This symposium brings together scholars from different cultures. The first paper discusses newly developed social and well-being scales, that more adequately address cultural notions of exclusion and discrimination experienced by older Māori (indigenous New Zealanders). Based on unique data from time-diaries kept by older women from several European countries and the U.S, the second speaker discusses how increased time spend alone is key factor behind widows' reduced well-being. The last study finds a northwest to southeast gradient in objective exclusionary states, with the rates in southeast Europe to be pronounced among older women.

### CO-CREATING WITH OLDER PEOPLE CULTURALLY SENSITIVE INCLUSION/EXCLUSION MEASURES

Charles Waldegrave[1], Chris Cunningham[2], Catherine Love[3], and Monica Mercury[1], *1. Family Centre Social Policy Research Unit, Lower Hutt, Wellington, New Zealand, 2. Research Centre for Maori Health and Development, Wellington, Wellington, New Zealand, 3. Family Centre Social Policy Research Unit, Wellington, Wellington, New Zealand*

This paper demonstrates a research approach to co-creating social and wellbeing indicator scales through a qualitative methodology, and then applying them in survey format to develop quantitative statistical correlation and regression analysis. We have been co-creating with older Māori (indigenous New Zealanders) a range of scales that more adequately address their notions of wellbeing, social connection, loneliness, discrimination, abuse, and neighbourhood wellbeing than the standard universal scales commonly used in most research. They are designed to be much more inclusive of participants' worldview and provide more authentic and accurate evidence for policy making and service provision. They draw on the knowledge and experience of the participant communities and reflect their understanding of the indicators. We compare the results with the more common universal indicators and highlight both the universal aspects of each indicator and the culturally specific features to provide more fine-tuned results for policy making and service provision.

### HOME ALONE: WIDOWS' WELL-BEING AND TIME

Michal Myck[1], Maja Adena[2], Daniel Hamermesh[3], and Monika Oczkowska[1], *1. Centre for Economic Analysis, CenEA, Szczecin, Zachodniopomorskie, Poland, 2. Wissenschaftszentrum Berlin (WZB), Berlin, Berlin, Germany, 3. Barnard College, New York, New York, United States*

We provide a comprehensive picture of well-being in widowhood. Most importantly, our analysis accounts for time use in widowhood, an aspect which has not been studied previously. Based on data from several European countries we trace the evolution of well-being of women who become widowed by comparing them with their matched non-widowed 'statistical twins' and examine the role of an exceptionally broad set of potential moderators of widowhood's impact on well-being. We confirm a dramatic decrease in mental health and life satisfaction after the loss of partner, followed by a slow recovery. An extensive set of controls recorded prior to widowhood, including detailed family ties and social networks, provides little help in explaining the deterioration in well-being. Unique data from time-diaries kept by older women from several European countries and the U.S. tell us why: the key factor behind widows' reduced well-being is increased time spent alone.

### GENDERED PATHWAYS AND OUTCOMES OF EXCLUSION FROM SOCIAL RELATIONS IN OLDER AGE

George Pavlidis[1], Thomas Hansen[2], Marja Aartsen[3], and Andreas Motel-Klingebiel[4], *1. Linkoping University, Linkoping, Ostergotlands Lan, Sweden, 2. Norwegian Institute of Public Health, Oslo, Oslo, Norway, 3. OsloMet Oslo Metropolitan University, Oslo, Akershus, Norway, 4. Linkoping University, Norrkoping, Ostergotlands Lan, Sweden*

Exclusion from social relations (ESR) in older age is associated with worse quality of life and adverse mental health outcomes. Recent evidence suggest that the perception of solitude and the evaluation of existing social networks modify the disadvantages of ESR among excluded older persons. This presentation takes a gender perspective on the topic, bringing together the quantitative evidence of the GenPath project. The results of the project suggest that over and above loneliness and objective ESR indicators, the perception of solitude and the evaluation of existing social relations modify the gendered risks of depression in older age. The findings also reveal a northwest to southeast gradient in objective ESR states, with the rates in southeast Europe to be pronounced among older women. It is concluded that objective and subjective factors at the micro and macro levels may shape gendered disadvantages among older persons who are challenged by ESR states.

### SESSION 2420 (SYMPOSIUM)

### THE COVID-19 EFFECT: ADAPTING FAMILY CARE TO A PANDEMIC CONTEXT

Chair: Amanda Leggett Discussant: Laura Gitlin

The COVID-19 pandemic carries risk for severe complications and mortality among older adults, placing their family caregivers in key support roles in helping their care recipients stay safe, maintain function, and abide by preventative recommendations. Yet such preventative safety precautions, changes to social support structures, and impacts on healthcare access may pose challenges with which caregivers must cope. This symposium considers how family caregivers adapted their care practices and adjusted to care-related challenges during the COVID-19 pandemic. First, Dr. Amanda Leggett presents data on pandemic-specific care-related challenges and supports experienced by dementia caregivers and their association with well-being and stress process outcomes. Ms. Sara Masoud shares mixed-methods data from persons living with dementia, caregivers, and healthcare professionals on their health and quality of life during the pandemic. Mr. Jiaming Liang presents dyadic findings from the National Study of Caregiving on persons living with dementia and their caregivers' perceptions of COVID-19 and personal and social COVID-specific preventative behaviors. Finally, Dr. Sheria Robinson-Lane offers a diverse caregiving perspective by presenting dyadic qualitative data on COVID-19 patients who were intubated in the hospital and their family caregiver, and offering themes on how caregivers adjusted to their new care role. To conclude, our discussant Dr. Laura Gitlin will offer insight on cross-cutting implications across studies and offer perspective on how lessons learned through pandemic caregiving may inform the field more broadly and enhance caregiver well-being beyond pandemic contexts.

### PANDEMIC-SPECIFIC SUPPORTS AND CHALLENGES: ASSOCIATIONS WITH DEMENTIA CAREGIVER WELL-BEING

Amanda Leggett[1], Hyun Jung Koo[2], Cathleen Connell[2], Laura Gitlin[3], and Helen Kales[4], *1. Wayne State University, Ypsilanti, Michigan, United States, 2. University of Michigan, Ann Arbor, Michigan, United States, 3. Drexel University, Philadelphia, Pennsylvania, United States, 4. University of California, Davis, Davis, California, United States*

This study examines the prevalence of pandemic-specific care supports and challenges (e.g., increased support from family and friends, difficulty accessing respite care, confusion on public health guidelines) and associations with stress and well-being among 100 family caregivers for persons living with dementia interviewed in 2021. Pandemic care challenges were common- 52% reported a decrease in support from family and friends, 43% had difficulty accessing medical care, and 31% had difficulty getting needed in-home and out-of-home services. Accounting for demographics and the care context, difficulties accessing various types of respite care (e.g., paid respite care, respite from family/friends) were associated with caregiver stress, burden, and less positive affect. Pandemic supports, including increased support from family and friends and receiving information on COVID-care were associated with greater positive affect, but not caregiver stress. While care-supports enhanced well-being, efforts to help caregivers take breaks from caregiving may have significant impact on reducing stress.

### UNEXPECTED CAREGIVING: LIFE AFTER A COVID-19 INTENSIVE CARE UNIT HOSPITALIZATION

Sheria Robinson-Lane[1], Florence Johnson[2], Amanda Leggett[3], Alicia Carmichael[4], Natalie Leonard[4], and Richard Gonzalez[2], *1. University of Michigan School of Nursing, Ann Arbor, Michigan, United States, 2. University of Michigan, Ann Arbor, Michigan, United States, 3. Wayne State University, Ypsilanti, Michigan, United States, 4. University of Michigan Institute for Social Research, Ann Arbor, Michigan, United States*

The coronavirus pandemic has led to an exceptional number of critical care hospitalizations followed by extended recovery periods that necessitate familial support. Using a qualitative descriptive approach, this study aimed to examine the strategies used by families to adjust to the caregiving role. Semi-structured interviews of patients who had been recently discharged from the Intensive Care Unit (ICU) (n=16) along with their family caregivers (n=16) were thematically analyzed. Three major themes were identified that highlight how family caregivers adapt to the caregiving role following an ICU COVID-19 related hospitalization including 1) engaging the support of family and friends, 2) shifting responsibilities to accommodate caregiving, and 3) managing one's emotions. Additional themes more specifically related to managing COVID-19 care included: 1) managing infection control, 2) care recipient's need for independence, and 3) managing support services. Flexibility and sufficient support allowed family caregivers to manage their new caregiving role and function optimally.

### THE IMPACT OF THE COVID-19 PANDEMIC ON DEMENTIA CARE: A MULTISTAKEHOLDER PERSPECTIVE

Sara Masoud[1], Carole White[1], Ashlie Glassner[1], Shanae Rhodes[1], Mayra Mendoza[1], and Kylie Meyer[2], *1. UT Health San Antonio, San Antonio, Texas, United States, 2. Case Western Reserve University, Cleveland, Ohio, United States*

Persons living with dementia (PLWD) and family caregivers are particularly vulnerable to the effects of the COVID-19 pandemic. A multi-methods study was conducted to describe the impact of the pandemic on dementia care from the perspectives of stakeholders, including PLWD, family caregivers, and health and social care professionals (HCPs).The study was conducted using a community engaged approach. Cross-sectional surveys were conducted with PLWD (n=27), family caregivers (n=161), and HCPs (n=77), followed by focus groups and interviews with a subsample of survey participants (n=55). Participants reported declines in health and quality of life for PLWD and family caregivers. Participants experienced delayed or cancelled dementia care attributed to the pandemic. Most reported telehealth and tele-support were effective alternative models to care. The pandemic impacted the quality and accessibility of dementia care. Results highlight opportunities to improve quality of care through addressing inequities and identifying approaches to address isolation and virtual care.

### ASSOCIATIONS BETWEEN COVID-19 PERCEPTIONS AND PREVENTIVE BEHAVIORS IN DEMENTIA CAREGIVING DYADS

Jiaming Liang, Maria Aranda, and Yuri Jang, *University of Southern California, Los Angeles, California, United States*

This study adopted a dyadic perspective to examine how the perceptions of COVID-19 (i.e., anxiousness & hopefulness) of dementia caregiving dyads are associated with their engagement in personal (e.g., washing hands, wearing mask) and social (e.g., avoiding physical contact and going restaurants/bars) preventive behaviors. Multiple cross-sectional Actor-Partner Interdependence Models (APIMs) were estimated using data from the 2020 NHATS/NSOC COVID-19 Supplements (N=1565). In the anxiousness models, participants' own feeling of anxiousness was associated with their own engagement in personal preventive behaviors (actor effects), and the perceived anxiousness of PLWD was associated with personal preventive behaviors of caregivers (partner effect). In the model on social preventive behaviors, both actor and partner effects were found on dementia caregiving dyads. No effect was found in the models on hopefulness. Our findings extend understandings of mutual influence within the caregiving dyads and demonstrate the possibility of developing interventions for caregivers to promote PLWD's health behaviors.

### SESSION 2430 (SYMPOSIUM)

### THE DAILY LIFE OF HEALTHY AGING: CONSIDERING PERCEPTIONS, APPRAISALS, AND CONSEQUENCES

Chair: Patrick Hill

For decades, researchers have shown how individuals deal with both stressful and positive events is central to our understanding of healthy aging. Multiple studies have demonstrated that reactivity to daily events shapes our health and wellbeing, with the potential for compounding effects to cause greater hindrance by older adulthood. This literature also notes that individuals differ significantly in their appraisals, resources, and experience with stressors, leading to the need for multimethod research into the role that daily events play in adult development. The current series of talks will investigate these topics using a variety of methodologies, while considering multiple perspectives on the study of stress and wellbeing. First, Megan Wilson will consider the deleterious outcomes associated with a specific form of daily stressors, everyday experiences of discrimination, focusing on how daily discrimination may negatively impact adults' sense of purpose in life. Second, Eric Cerino will discuss a collaborative effort that compares approaches to studying control beliefs at the dispositional and daily level, showing that researchers may benefit from considering daily stressor control as a construct unique from general beliefs. Third, Jessica Maras will also consider the role of perceptions, by examining how cognitive appraisals of stressful events impact future health outcomes. Fourth, Patrick Klaiber will present meta-analytic work that combines daily studies to consider whether the benefits of positive events on wellbeing differ by age. Taken together, these studies provide new insights into the daily life of healthy aging, and how it differs based on individual differences and stressor type.

### CUMULATIVE DAILY DISCRIMINATION AS A RISK FACTOR FOR REDUCED PURPOSE

Megan Wilson[1], Lydia Ong[2], Gabrielle Pfund[3], Anthony Burrow[4], and Nancy Sin[2], *1. Washington University in St. Louis, St. Louis, Missouri, United States, 2. University of British Columbia, Vancouver, British Columbia, Canada, 3. Northwestern University, Chicago, Illinois, United States, 4. Cornell University, Ithaca, New York, United States*

Past research shows that discriminatory experiences may reduce sense of purpose among older adults, though these associations are inconsistent across groups. Work is needed both to understand the nuanced role discrimination plays on individuals' sense of purpose, whether it leads to feeling derailed from life goals, and if effects differ for younger versus older adults. The current study asked 354 American adults (age 19-74) to complete daily surveys over the course of two weeks, to examine whether experiencing discrimination during that two-week period led to a decline in purposefulness. Overall, findings suggest marked stability in sense of purpose during this short timeframe. However, greater daily discrimination predicted decreases in sense of purpose, and increases in derailment over the two weeks. While these associations were similar across age, older adults did report less discrimination at baseline. Findings will be discussed with a focus on successful aging among marginalized groups.

### AGING-RELATED CHANGES IN DAILY STRESSOR CONTROL AND GENERAL PERCEIVED CONTROL ACROSS THE LIFESPAN

Eric Cerino[1], Susan Charles[2], Jacqueline Mogle[3], Jonathan Rush[4], Jennifer Piazza[5], Margie Lachman[6], and David Almeida[3], *1. Northern Arizona University, Flagstaff, Arizona, United States, 2. University of California, Irvine, Irvine, California, United States, 3. The Pennsylvania State University, University Park, Pennsylvania, United States, 4. University of Victoria, Victoria, British Columbia, Canada, 5. California State University, Fullerton, Fullerton, California, United States, 6. Brandeis University, Waltham, Massachusetts, United States*

Perceived control is an important psychosocial resource for healthy aging. Using data from the National Study of Daily Experiences (N=2,021, M=55.82 years, SD=10.35, 57.27% Female), we examined aging-related changes in stressor control across 10 years and compared these trajectories with measures of general control (mastery, constraints). Over 8 consecutive days in waves conducted in ~2008 and ~2017, people reported their perceived control over four types of stressors (arguments, avoided arguments, work, home). Longitudinal analyses revealed declines in stressor control across 10 years ($p<.001$), driven by declines in home stressors specifically. The rate of decline did not depend on baseline age. General control trajectories showed unique patterns of age differences in aging-related change such that declines (less mastery, more constraints) were steeper among older adults ($p<.001$). Results suggest that stressor control is a distinct domain of control beliefs with aging-related declines that differ based on type of stress experienced.

### THINK OF THE SITUATION IN A POSITIVE LIGHT: COGNITIVE REAPPRAISAL, AFFECTIVE REACTIVITY, AND HEALTH

Jessica Maras[1], and Kate Leger[2], *1. University of Kentucky, Lexington, Kentucky, United States, 2. The University of Kentucky, Lexington, Kentucky, United States*

Cognitive reappraisal is an emotion regulation strategy that benefits health and well-being, but it is currently unclear why this relationship exists. The current study examined how adults react affectively to daily stressful events as a mediating pathway between cognitive reappraisal and health and well-being outcomes across a 20-year period. Participants (N = 1,814) completed waves 1-3 of the Midlife in the United States survey series and the National Study of Daily Experiences, an 8-day daily diary. Results found that negative affective reactivity to daily stressors mediated the relationship between cognitive reappraisal and future health outcomes including the development of chronic conditions, functional limitations, and affective disorders. Exploratory analyses revealed that specific negative emotions, including being nervous and irritable explained this relationship. Findings from this study suggest that strengthening cognitive reappraisal skills may be a good way to reduce affective reactions to daily stressors and enhance long-term health and well-being outcomes.

### AGE MODERATES THE LINK BETWEEN DAILY POSITIVE EVENTS AND WELL-BEING: A META-ANALYSIS

Patrick Klaiber[1], Lydia Ong[2], and Nancy Sin[2], *1. University of British Columbia, Department of Psychology, Vancouver, British Columbia, Canada, 2. University of British Columbia, Vancouver, British Columbia, Canada*

Daily positive events are linked to better health and well-being, and this association might be stronger for older adults due to age-related changes in motivations and emotion regulation. We therefore investigated whether age moderated the links between positive events and health-related outcomes, using data from a meta-analysis of 142 studies spanning 50 years of research. Multilevel meta-analytical moderation analyses revealed stronger correlations of positive events with better well-being (i.e. higher positive affect and life satisfaction), mental health, and lower disability in older compared to younger samples (p's < .05). No age moderation was found for links of positive events with social connection, health behaviors, physical symptoms, perceived stress, self-reported health, and inflammation. Findings suggest that although daily positive events were protective for health and well-being for all adult age groups, these experiences may be especially beneficial for positive well-being and mental health in late life.

### SESSION 2440 (SYMPOSIUM)

### THE MORE THINGS CHANGE, THE MORE THEY STAY THE SAME: CHANGE AND CONTINUITY IN ENDURING SOCIAL TIES

Chair: Athena Koumoutzis Co-Chair: Kelly Cichy

Enduring social ties with family and friends hold important implications for health and well-being across adulthood. Social relationships are simultaneously sources of support and strain, and both positive and negative aspects of relationships change daily and over time. This symposium explores continuity and change in relationship strains (i.e., conflicts, support needs) experienced in the context of enduring social relationships, particularly in response to anticipated and emerging needs for support in later life. First, Meinertz, Gilligan, and Suitor use qualitative data from spousal dyads to compare mothers' and fathers' explanations of which adult child they prefer as their future caregiver. Next, using longitudinal data across two waves, Bui, Kim, and Fingerman investigate how different types of past support exchanges between parents and adult children are associated with older parents' care receipt and expectations. Third, Koumoutzis, Cichy, and Kinney explore the association between change in parental disability and adult children's intergenerational ambivalence (i.e., both positive and negative sentiments), including the extent to which adult children's stress and reward appraisals mediate the link between parental disability and ambivalence. Kyungmin and colleagues explore how older adults (age 62-76) felt burden in their relationship with their very old parents (age 81-101) and what factors are associated with feelings of burden across two cultural contexts, the U.S. and Korea. Lastly, using ecological momentary assessment, Birditt and colleagues examine longitudinal trajectories of negative ties (i.e., irritating, demanding) and the links between daily positive and negative social interactions and emotional well-being.

### CHANGE IN PARENTAL DISABILITY AND INTERGENERATIONAL AMBIVALENCE: THE MEDIATING ROLE OF APPRAISAL

Athena Koumoutzis[1], Kelly Cichy[2], and Jennifer Kinney[1], *1. Miami University, Oxford, Ohio, United States, 2. Kent State University, Kent, Ohio, United States*

Adult children often report intergenerational ambivalence (i.e., positive and negative sentiments toward their parents) that may be exacerbated when parents need increasing support. Evaluations or appraisal of providing support may mediate the links between stress and outcomes. Using structural equation modeling, we assessed the relationship between change in parental disability and intergenerational ambivalence through adults' perceptions of the stress and reward of providing help to parents. Participants included 369 adults (32% Black, 68% White) who provided information on 478 parents from Waves I and II of the Family Exchanges Study. The association between change in parental disability and intergenerational ambivalence was explained through stress appraisal; greater parental disability led to higher stress appraisal which led, in turn, to greater intergenerational ambivalence. The model did not significantly differ by race. Results show that stress, rather than reward, appraisal is an essential factor in determining relationship quality as parental care needs emerge.

### FEELINGS OF BURDEN AMONG OLDER CHILDREN OF VERY OLD PARENTS IN THE US AND KOREA

Kyungmin Kim[1], Yijung Kim[2], Joohyun Kim[1], Kathrin Boerner[3], Daniela Jopp[4], and Gyounghae Han[1], *1. Seoul National University, Seoul, Seoul-t'ukpyolsi, Republic of Korea, 2. The University of Texas at Austin, Austin, Texas, United States, 3. University of Massachusetts Boston, Boston, Massachusetts, United States, 4. University of Lausanne, Lausanne, Vaud, Switzerland*

Very old adults (80+) are the fastest growing population worldwide. Children of the very old adults may see their prolonged relationship with parents as a benefit (e.g., longer time together) but also as burden (e.g., prolonged responsibility in their own late life). Using a sample of older children (N = 219) from the Boston Aging Together Study and Korean Aging Together Study, we investigated the factors associated with older children's (aged 62–76) reports of burden in their relationships with very old parents (aged 81–101), focusing on how family relations were imbedded in different cultural contexts. Overall, American older children showed lower levels of burden, compared to Korean older children. The factors associated with burden differed by country; support given to parents and relationship quality were associated with American older children's burden, whereas support received from parents, familism, and negative relationship quality were associated with Korean older children's burden.

### INTERGENERATIONAL SUPPORT EXCHANGES AND OLDER PARENTS' CARE RECEIPT AND EXPECTATIONS

Cindy Bui[1], Kyungmin Kim[2], and Karen Fingerman[3], *1. University of Massachusetts Boston, Boston, Massachusetts, United States, 2. Seoul National University, Seoul, Seoul-t'ukpyolsi, Republic of Korea, 3. The University of Texas at Austin, Austin, Texas, United States*

Distinguishing between support and care, this study investigated how different types of past support exchanges with children were associated with older parents' care receipt and expectations. Older parents (N=190; Mage=79.98) reported on tangible, non-tangible, and childcare support exchanges with each of their adult children (N=709; Mage=52.69) in two waves of the Family Exchanges Study (2008 and 2013).

Multilevel, within-family, logistic regression models were estimated. Parents with functional limitations more likely received care from children whom they received more tangible support from at the prior wave. Parents without current limitations more likely named children whom they previously provided childcare support to and received more tangible support from as their expected future caregiver. These findings emphasize continuity in the transition from receiving tangible support to receiving and expecting care from adult children. The importance of older parents' childcare support given to adult children also highlights reciprocity in intergenerational care exchanges.

### CHRONIC NEGATIVE SOCIAL TIES: IMPLICATIONS FOR EXPOSURE AND REACTIVITY TO DAILY SOCIAL INTERACTIONS

Kira Birditt, Angela Turkelson, Richard Gonzalez, and Toni Antonucci, *University of Michigan, Ann Arbor, Michigan, United States*

Negative relationships (i.e., irritating, demanding) predict poor well-being and daily processes may account for these links. This study examined longitudinal trajectories of negative ties and their links with daily social interactions and well-being. A total of 169 individuals ages 33 to 91 reported negative relationship quality (spouse/child/friend) in 1992, 2005, 2015, and 2018 and completed 4-5 days of surveys every three hours regarding positive and negative social interactions and negative affect. Latent class growth models revealed two trajectories of negative relationships (moderate-stable and low-decreasing). Individuals in the moderate-stable trajectory reported more frequent daily interpersonal tensions but no link with positive interactions. The link between negative relationship trajectories and daily negative affect was moderated by daily positive interactions such that the association was reduced when individuals had positive interactions. Thus, negative ties may increase exposure but not reactivity to daily tensions and daily positive interactions buffer the negative tie-well-being link.

### THE ROLE OF GENDER IN FATHERS' AND MOTHERS' PREFERENCES FOR FUTURE ADULT CHILDREN CAREGIVERS

Naomi Meinertz[1], Megan Gilligan[2], and J Jill Suitor[3], *1. Iowa State University, Fairfax, Iowa, United States, 2. Iowa State University, Ames, Iowa, United States, 3. Purdue University, West Lafayette, Indiana, United States*

Previous caregiving research has focused on a single care recipient; however, no research has explored the potential simultaneous care needs of fathers and mothers in the same family. Drawing from gender role theory, we use qualitative data from 76 spousal dyads from the Within-Family Differences Study to compare fathers' and mothers' explanations of which adult child they prefer as their future caregiver. In 72% of families, fathers and mothers did not share the same care preferences suggesting that care preferences are dispersed among multiple adult children within the same family. In families in which fathers and mothers shared care preferences, 75% chose the same daughter. Additional findings showed that fathers' explanations for a preferred caregiver aligned with mothers' explanations; however, differences were identified based on children's gender. These findings shed light on the similarities and differences in parental care preferences and underscore the influence of gender in family care networks.

### SESSION 2450 (SYMPOSIUM)

### UNDERSTANDING MOBILITY, HEALTH, AND WELL-BEING OF OLDER ADULTS USING SENSING TECHNOLOGIES

Chair: Christina Röcke Co-Chair: Minxia Luo Discussant: Hans-Werner Wahl

Mobility has been identified as one important ingredient to older adults' health and well-being and is considered a high priority in the global agenda of healthy and active aging. However, mobility is still a relatively understudied concept in aging research. This symposium, including three empirical studies and one concept paper, presents how different sensing technologies can be utilized to examine mobility, health and well-being in older adults. Using infrared motion sensors and contact sensors, Wu and colleagues examine indoor mobility and show its associations with physical, cognitive, and mental health in community-dwelling older adults living alone. Luo and colleagues use a custom-built mobile GPS sensor and a smartphone-based ambulatory assessment to examine daily mobility and well-being in community-dwelling older adults. They find that a day with larger life space area, more time spent in passive transport modes, and higher number of different locations is associated with higher daily life satisfaction. Similarly, using a GPS sensor combined with a smartphone-based ecological momentary assessment, Kamalyan and colleagues examine life-space mobility, social interactions, and well-being in older adults with and without HIV. They show that prior day's at-home time is negatively associated with current day's happiness and that prior day's social interactions diminishes this association. Jansen presents a project combining sensor-based movement data, GPS-based geolocation data, and experience sampling to investigate relations between life-space mobility and social participation and the role of cultural and climatic differences across several European countries. Hans-Werner Wahl will discuss all papers from an ecological and contextualized aging perspective.

### GPS-DERIVED DAILY MOBILITY AND DAILY WELL-BEING IN COMMUNITY-DWELLING OLDER ADULTS

Minxia Luo[1], Eun-Kyeong Kim[2], Robert Weibel[2], Mike Martin[1], and Christina Röcke[3], *1. University of Zurich, Zurich, Zurich, Switzerland, 2. University of zurich, Zurich, Zurich, Switzerland, 3. University of Zurich, Dynamics of Healthy Aging & Center for Gerontology, Zurich, Zurich, Switzerland*

Mobility as a multidimensional concept has rarely been examined as a day-to-day varying phenomenon in its within-person association with older adults' daily well-being. Using a custom-built mobile GPS sensor („uTrail") combined with a smartphone-based ambulatory assessment, this study examined associations between daily mobility and daily well-being in community-dwelling older adults. Analysis included 947 days' data from 109 Swiss older adults aged 65 to 89 years. Multilevel modelling showed that, within persons, a day with larger life space area, more time spent in passive transport modes, and higher number of different locations was associated with higher daily life satisfaction, but not daily positive or negative affect. Follow-up analysis showed that daily maximum distance from home was positively associated with daily life satisfaction, providing a first indication that exposure to non-habitual environments might be a candidate mechanism to explain effects of mobility. Results are discussed in the context of healthy aging research.

### WEEKLY MOBILITY AT HOME AND ITS HEALTH AND ENVIRONMENTAL CORRELATES IN OLDER ADULTS LIVING ALONE

Chao-Yi Wu[1], Hiroko Dodge[1], Christina Reynolds[1], Lisa Barnes[2], Lisa Silbert[1], Miranda Lim[1], Jeffrey Kaye[1], and Zachary Beattie[1], *1. Oregon Health & Science University, Portland, Oregon, United States, 2. Rush Alzheimer's Disease Center, Rush University Medical Center, Chicago, Illinois, United States*

Mobility features are important for health. In this study, we quantified in-home mobility and explored potential health and environmental correlates. Participants included community-dwelling older adults living alone (n=139). Two indoor mobility features were developed (frequency; interdaily stability) using passive, room-level (bathroom; bedroom; kitchen; living room) infrared motion sensors, and contact sensors attached to homes' egress doors at entryways. Existing data on gait and cognition, weekly online surveys assessing mood (blueness; loneliness) and pain severity, and publicly available locality-based atmospheric (daylight hours) data were leveraged. On average, higher indoor mobility frequency was associated with faster gait speed (p=0.04), less time out-of-home (<.001), and less hours of daylight per week (p=0.003). Lower interdaily stability was associated with mild cognitive impairment (p=0.04), less time out-of-home (p=0.01), more severe pain (p=0.02), and loneliness (<.001). Pervasive sensing technology can be used to delineate multi-domain health in older adults who live alone.

### CONNECTING MOBILITY AND SOCIAL PARTICIPATION IN OLDER ADULTS: A QUESTION OF CULTURE AND CLIMATE?

Carl-Philipp Jansen, *Robert-Bosch-Hospital Stuttgart, Stuttgart, Baden-Wurttemberg, Germany*

The main aim of the project is to investigate the relationship between life-space mobility and social participation in older adults (65+) taking into account cultural and climatic differences across several European countries. A mixed-methods approach will be applied, combining sensor-based movement data, GPS-based geolocation data, and in-situ participant-reported information by use of experience sampling. This will allow a deeper insight into the connection of mobility and social participation taking into account different environmental and socio-cultural realities. Linkage of seasonal climate across different climate zones can help to extrapolate multi-faceted transformation of future mobility behavior of older adults under pressure of more extreme weather and climate conditions. This is of clinical relevance as there may be no "one size fits all" answer on how to best adapt to this pressure and it can be used to steer uptake and maintenance of health-enhancing mobility, social participation, and sustainable quality of life.

### SOCIAL INTERACTIONS REDUCE ASSOCIATION BETWEEN LIFE-SPACE AND DAILY HAPPINESS IN OLDER ADULTS WITH AND WITHOUT HIV

Lily Kamalyan[1], Jiue-An Yang[2], Caitlin Pope[3], Emily Paolillo[4], Laura campbell[5], Bin Tang[1], Colin Depp[6], and Raeanne Moore[7], *1. University of California San Diego, San Diego, California, United States, 2. Qualcomm Institute/Calit2, San Diego, California, United States, 3. University of Kentucky, Lexington, Kentucky, United States, 4. University of California San Fransisco, San Francisco, California, United States, 5. University of California San diego, San Diego, California, United States, 6. Department of Psychiatry, University of California San Diego, La Jolla, California, United States, 7. UC San Diego, San Diego, California, United States*

Objective: We used global positioning system (GPS) derived indicators and ecological momentary assessment (EMA) to assess real-time relationships between life-space, mood, and social interactions among older adults with and without HIV.

Methods: Participants (People with HIV [PWH] n=54, HIV- n=34) completed smartphone-based EMA surveys assessing mood and social interactions four times/day for 14-days. Participants' smartphones were GPS enabled. Mixed-effects models analyzed concurrent and lagged associations among life-space and mood.

Results: PWH spent more time at home (79% vs 70%, z=-2.08; p=.04) and reported lower mean happiness (3.2 vs 3.7; z=2.63; p=.007). More daily social interactions were associated with higher ratings of real-time happiness (b=0.12; t=5.61; p< 0.001). Prior day social interactions (b=0.15; t=7.3; p<.0001) and HIV status (b=-0.48; t=-2.56; df= 1026.8; p=0.01) diminished the effect of prior day time spent at home on happiness.

Conclusions: Interventions targeting social interaction may help increase positive mood in isolated older adults.

### SESSION 2460 (SYMPOSIUM)

### UNPACKING MILD COGNITIVE IMPAIRMENT/ YOUNG ONSET DEMENTIA IN THE WORKPLACE: EMPLOYER VERSUS EMPLOYEE

Chair: Philip Taylor Co-Chair: AnneMarie Levy Discussant: Allison Gibson

The symposium includes four studies exploring the impact of mild cognitive impairment and/or young onset dementia on the workplace, and area of increasing interest due to the aging workforce and government interest in prolonging work life. The papers adopt a rare, multi-level approach by examining both employee and employer perspectives, and how the interaction of both individual and organizational factors impact their self-reported outcomes. Following the paper presentations, Dr. Allison Gibson will lead a paper discussion. The goal of the symposium is to foster a research conversation amongst the presenters and symposium participants interested in exploring overlaps, potential for multidisciplinary collaboration and future research into resolution of workplace and workforce optimization with worker satisfaction that are identified with progressive cognitive impairment.

### THE DIMENSIONS OF YOUNG ONSET DEMENTIA (YOD) AND WHAT THESE MEAN FOR EXTENDED WORKING LIVES

Philip Taylor, *Federation University Australia, Victoria, Victoria, Australia*

Drawing from a review of the literature and international statistical sources, this paper identifies the global prevalence of young onset dementia (YOD), its complex aetiology and its social and economic costs. YOD is often defined as dementia affecting those aged 65 or under, although definitions vary. Prevalence estimates also vary but recent evidence suggests a rate in the region of 119.0 per 100,000 population for those aged 30-64, and this is expected to increase markedly in the coming decades. Research identifies that the causes of YOD vary greatly among younger people. Individuals experiencing YOD face a complex range of psychological, familial, and socioeconomic challenges, while often being active in the labour market at the time of diagnosis. Costs of YOD cannot be easily disentangled from those for dementia generally but include: the direct costs of the 'resources' drawn on by the person with dementia; indirect costs, usually considered in terms of the value of lost productivity by the person with dementia or their carer, and less tangible costs, such as in terms of psychological wellbeing, social exclusion and time costs. The paper concludes by considering the potential significance of YOD for the future labour market in the context of ongoing efforts to prolong working lives.

### REVIEW OF WORKPLACE-RELATED GUIDANCE PRESENTED BY ORGANIZATIONS DEALING WITH DEMENTIA OR MILD COGNITIVE IMPAIRMENT

James Carino[1], Philip Taylor[1], and Damian Morgan[2], *1. Federation University Australia, Victoria, Victoria, Australia, 2. James Cook University, Townsville, Queensland, Australia*

A range of organisations in English-speaking countries have prepared guidance materials for individuals who are facing concerns regarding dementia while employed. Some of these organisations have also prepared guidance materials for employers to assist them in engaging employees facing this condition. These are available on organisation websites and may be presented as a downloadable booklet, guide or one or more web pages, aiming to inform about the issues that need to be considered. Information of this type is becoming more important in assisting both parties as dementia is being detected and diagnosed earlier, and individuals need to consider their options regarding their future and their ability to continue to work. This presentation reports on a systematic analysis of around guidance items appearing on these websites. The topic areas, content, and approaches used to present this information to its intended audience were reviewed and compared. Analysis showed important gaps in content coverage from one guidance to another. No guidance presented by any of these organisations covers all areas identified that may potentially interest either employees or employers and in most cases it is not presented coherently for these groups. Employee topics that would benefit from further development include awareness of the condition, the importance of diagnosis and support, use of technology to assist in continuing to be functional and productive, and steps in transition from work to other activities. Employer topics that could be developed further include building awareness amongst employers and the workforce, identifying dementia in the workplace, engaging the wider work group, and positively managing employee separation from work.

### DE-RISKING THE EMPLOYER-EMPLOYEE RELATIONSHIP WHEN WORKERS HAVE MILD COGNITIVE IMPAIRMENT OR YOUNG ONSET DEMENTIA

Josephine McMurray[1], Arlene Astell[2], AnneMarie Levy[1], Jennifer Boger[3], and Catherine Burns[3], *1. Wilfrid Laurier University, Waterloo, Ontario, Canada, 2. University of Toronto, Toronto, Ontario, Canada, 3. Waterloo University, Waterloo, Ontario, Canada*

The employee-employer relationship is one that is well established in law and involves a contract between a worker who performs the work, and an employer who determines when, how and where the work is done. Many countries have introduced legislation that helps guide employee-employer behavior when workers' performance of their duties is disrupted by illness or disability, such as mild cognitive impairment or young onset dementia (MCI|YOD). At any one time, about 8-12% of the workforce is off due to occupational or non-occupational work injury or illness with direct disability and absence costs projected about 7% of payroll in 2000. Drawing from data gathered in a systematic literature review and case study, this paper determined that uncertainty and risk influence employer-employee relationships when workers are identified with MCI|YOD. Employers' industry context, size and corporate values influence their "integrated risk management" approach to workers with MCI|YOD, and early intervention is considered key to managing financial and liability risk. In contrast, progressive onset, delayed diagnosis, stigma, privacy concerns, workplace uncertainty, and lack of information may delay employee disclosure, a common employee risk mitigation strategy. We conclude by considering strategies to de-risk relationships between workers with MCI|YOD and employers using dual process theory to understand intuitive and rational decisions in the context of the disability management process.

### TRANSITIONS EXPERIENCED BY PEOPLE DIAGNOSED WITH DEMENTIA WHILE IN THE WORKFORCE

Jennifer Boger[1], Sheida Marashi[1], Anna Mäki-Petäjä-Leinonen[2], Mervi Issakainen[3], Ann-Charlotte Nedlund[4], Charlotta Ryd[5], Louise Nygard[5], and Arlene Astell[6], *1. Waterloo University, Waterloo, Ontario, Canada, 2. Insitute of Law & Welfare, Joensuu, Lappi, Finland, 3. Institute of Law & Welfare, Joensuu, Lappi, Finland, 4. Linkoping University, Linköping, Ostergotlands Lan, Sweden, 5. Karolinska Institutet, Solna, Sodermanlands Lan, Sweden, 6. University of Toronto, Toronto, Ontario, Canada*

Dementia is a progressive, irreversible neurological disorder that causes changes in cognitive function and behaviour. While at least 5% of people who develop dementia every year are under the age of 65, dementia in the workplace is currently not well recognised or supported. The changes associated with dementia present multiple challenges for individuals who wish to continue with their employment. Many lose their positions before receiving a diagnosis, whilst others take sick or disability leave or early retirement. The process of understanding what is happening and coping with this new situation is highly individualistic and involves several transitions. The MCI@work project is an international initiative taking place in Canada, Finland, and Sweden where we examine these transitions through the personal narratives of individuals who have either currently or recently gone through the experience of developing dementia whilst in the workforce. From these data, we have developed a framework for understanding the transitions experienced by people who develop dementia whilst in the workforce. The aim is to assist individuals and their employers to better understand the needs of people living and working dementia as well as engage in appropriate actions that support choices and dignified transitions either within the context of employment or out of the workforce.

### SESSION 2470 (SYMPOSIUM)

### USING INTENSIVE LIFESPAN DATA TO GAIN DEEPER INSIGHT INTO COPING AND EMOTION REGULATION PROCESSES

Chair: Claire Growney Co-Chair: Jennifer Bellingtier
Discussant: Carolyn Aldwin

Effective coping and emotion regulation are important for well-being across the lifespan. Successful maintenance or improvement in these processes are often invoked as explanations for age-related stability or enhancement of well-being. In this symposium, we leverage intensive data to gain a deeper understanding of how individuals manage daily emotions and stressors, critically examining evidence for age-related differences and similarities. Growney and English used experience sampling to examine interpersonal emotion regulation in adults aged 25-85, finding a negative association between age and interpersonal emotion regulation strategy use, but evidence suggesting more effective interpersonal emotion regulation in older age. Bellingtier and colleagues present evidence for age similarity in flexible emotion regulation strategy use across hassle domains in an experience sampling study of adolescents and adults aged 14-88, noting that hassle domain differentiation was associated with emotion regulation strategy use. O'Brien and Neupert used daily diaries to examine associations between daily stressor appraisals and affect in adults aged 60-90, identifying daily negative self-views of aging as a moderator which may be particularly consequential in older adulthood. Cerino and colleagues used data from the National Study of Daily Experiences to examine relationships between perceived stressor control and daily affect, highlighting differing findings across domains of interpersonal stressors. Finally, a discussion will center on the value of considering both age-related similarities and differences, age-relevant factors for successful emotion regulation and coping (e.g., negative and positive aspects of social relationships, views of aging), diverse contexts in which these processes occur, and statistical considerations with micro-longitudinal approaches.

### MOMENTARY INTERPERSONAL EMOTION REGULATION IN AN ADULT LIFESPAN SAMPLE

Claire Growney, and Tammy English, *Washington University in St. Louis, St. Louis, Missouri, United States*

Older adults are theorized to maintain emotional well-being by drawing on their available resources, such as social partners who support their emotional goals. In the present study, adults (N = 290) age 25-85 completed an experience sampling procedure (6x/day for 10 days) in which they reported their current emotions and use of interpersonal emotion regulation (IER) strategies. Unexpectedly, older age was associated with lower overall use of IER strategies. Results from multilevel models indicate that within-person associations between IER strategy use and positive affect were strongest among older adults, with older adults experiencing highest positive affect on occasions where they report engaging in IER. Between-person associations between IER strategy use and negative affect were only present among younger and middle-aged adults, indicating that younger individuals who use IER more on average tend to have higher negative affect. Findings highlight the role of social partners in older adults' emotional wellbeing.

### MOMENTARY EMOTION REGULATION ACROSS HASSLE DOMAINS

Jennifer Bellingtier[1], Gloria Luong[2], Cornelia Wrzus[3], Gert Wagner[4], and Michaela Riediger[1], *1. Friedrich Schiller University Jena, Jena, Thuringen, Germany, 2. Colorado State University, Fort Collins, Colorado, United States, 3. Ruprecht Karls University Heidelberg, Heidelberg, Baden-Wurttemberg, Germany, 4. Max Planck Institute for Human Development, Berlin, Berlin, Germany*

Adapting emotion-regulation strategy use to flexibly match contextual features of a hassle is thought to aid in effective coping. We investigated if hassle domains are a pertinent feature for understanding emotion-regulation strategy selection in the everyday lives of adolescents through older adults. Participants, ranging from 14 to 88 years old (N = 325), completed an experience sampling study of approximately 9 days over a 3-week period. At each momentary assessment, participants reported on their affect, hassles, and emotion-regulation strategies. Our findings indicated that strategy use varied by domain. For example, distraction was most common in the health domain, whereas emotion expression was least likely to be used at work or school. More strategies were used when hassles were associated with multiple domains. However, greater domain differentiation was not associated with reduced hassle reactivity. Findings were similar across ages suggesting domains may similarly relate to strategy selection across the lifespan.

### DAILY STRESSOR APPRAISALS AND SELF-VIEWS OF AGING PREDICT SAME-DAY AFFECTIVE RATINGS

Erica O'Brien[1], and Shevaun Neupert[2], *1. Pennsylvania State University, University Park, Pennsylvania, United States, 2. North Carolina State University, Raleigh, North Carolina, United States*

Prior work suggests that more frequent or higher exposure to stressors relates to less positive affect and more negative affect in daily life. Limited knowledge exists about whether subjective appraisals of such stressors (i.e., perceived negative impacts on daily routine, personal health and safety, and finances) also have negative links to daily well-being. This study examines this link using data from an 8-day daily dairy study (n=675 days) in an online sample of older adults (n = 110 people, ages 60-90). We also explored potential psychological moderators particularly relevant to the experience of aging (i.e., self-views of aging, S-VOA). Results from multilevel models indicate that people reported more negative affect and less positive affect on days with more negative appraisals, especially on those days when they also had more negative self-views of aging. These findings highlight S-VOA as psychological resources that help people cope with stressful events in everyday life.

### DAILY ASSOCIATIONS BETWEEN STRESSOR CONTROL AND AFFECT VARY AS A FUNCTION OF STRESSOR TYPE

Eric Cerino[1], Susan Charles[2], Jacqueline Mogle[3], Laura Klepacz[4], Jennifer Piazza[5], Jonathan Rush[6], and David Almeida[3], *1. Northern Arizona University, Flagstaff, Arizona, United States, 2. University of California, Irvine, Irvine, California, United States, 3. The Pennsylvania State University, University Park, Pennsylvania, United States, 4. Department of Psychological Sciences, Northern Arizona University, Flagstaff, Arizona, United States, 5. California State University, Fullerton, Fullerton, California, United States, 6. University of Victoria, Victoria, British Columbia, Canada*

Perceived control is an important psychosocial correlate of emotional well-being. Using data from the National Study of Daily Experiences (N=1,797, M=55.82 years, SD=10.35, 57.27% Female), we examined how self-reported control over different types of stressors (arguments, avoided arguments, work, home, network) was associated with negative affect (NA) and positive affect (PA). Over 8 consecutive days in waves conducted in ~2008 and ~2017, people reported their daily NA, PA, and control over stressors they had experienced. Within-person associations revealed lower NA on days when stressor control was higher than usual (p<.001), driven by control over arguments, avoided arguments, and work stressors specifically. PA was higher on days when individuals perceived greater control over avoided and actual arguments (ps<.001), but lower on days when individuals perceived greater control over network stressors (p<.01). Results suggest the facilitative role of control over daily stress for emotional well-being depends on the type of stressor experienced.

### SESSION 2480 (SYMPOSIUM)

### WHY LOOKING AT THE FUTURE? FROM PSYCHOLOGICAL TO CONTEXTUAL MOTIVATORS

Chair: Yaeji Kim-Knauss Co-Chair: Frieder Lang
Discussant: Christiane Hoppmann

Human beings can represent future events, anticipate future consequences, and act in light of those representations to achieve the most favorable outcomes in the future. Although future-oriented thoughts or behaviors concern distant and delayed rewards than the present and instant ones, their roles in people's well-being and successful aging have been well-reported. Therefore, what motivates or differentiates such future-oriented thoughts and behaviors has been a central focus in developmental psychology. With a particular interest in future-oriented phenomena regarding age and aging, we look at the roles of psychological or contextual factors that drive views on aging and old-age preparation. Cohn-Schwartz et al. examine how having contacts with older adults benefits self-views on aging via changes in aging stereotypes. Park & Hess explore how importance attached to functioning and perceived control over functioning in different domains predict old-age preparation and compare patterns across different age groups. Fung et al. propose that perceived control, self-relevance, and responsibility for old-age preparation could mediate the well-reported cultural differences in old-age preparation. Rupprecht et al. investigate the adaptivity of approach and avoidance motivation in old-age preparation across different life domains, cultures, and age groups. Kim-Knauss & Lang looks at how the experience of social restrictions during the pandemic functions as a 'wake-up call' and thus induces people to engage in old-age preparation. Taken together, we suggest that various psychological appraisals and contexts shape future-oriented thoughts and behaviors, but these may vary across cultures, age groups, and target domains.

### PANDEMIC-RELATED SOCIAL RESTRICTIONS INCREASED THE URGENCY AND ENGAGEMENT IN OLD-AGE PREPARATION

Yaeji Kim-Knauss, *Friedrich-Alexander University Erlangen-Nuremberg, Nuremberg, Bayern, Germany*

We investigated whether people who perceive more restrictions on social contacts during the pandemic set an earlier deadline to prepare for social connectedness in old age (i.e., the latest still good age to start preparation) than they had perceived in the pre-pandemic time. We also looked at whether this change in the deadline induces the engagement in the preparation concerning the same domain. We first fit the data obtained from 356 German adults in 2018 and 2020 (aged 22–95 years) to a change score model. The deadline for preparing for social connectedness in old age was set about 23 years earlier in 2020 compared to that reported in 2018. We found that perceiving more social restrictions during the pandemic predicted this shorter deadline, which in turn, induced greater engagement in the preparation. A possible consequence of the pandemic may be related to an increased motivation to prepare for old age.

### BENEFICIAL EFFECTS OF CONTACT WITH OLDER ADULTS FOR AGE STEREOTYPES AND SELF-VIEWS OF AGING

Thomas Hess[1], Sylvie Graf[2], Shyhnan Liou[3], Clara de Paulo Couto[4], Helene Fung[5], Jana Nikitin[6], Klaus Rothermund[4], and Ella Cohn-Schwartz[7], *1. North Carolina State University, Raleigh, North Carolina, United States, 2. Czech Academy of Sciences, Brno, Moravskoslezsky kraj, Czech Republic, 3. National Cheng Kung University, Tainan, Tainan, Taiwan (Republic of China), 4. Friedrich Schiller University Jena, Jena, Thuringen, Germany, 5. The Chinese University of Hong Kong, Hong Kong, Hong Kong, 6. University of Vienna, Vienna, Wien, Austria, 7. Ben-Gurion University, Beer-Sheva, HaDarom, Israel*

Intergenerational contact is beneficial for improving attitudes toward older people, including age stereotypes (AS). To date, however, research on the topic has focused on younger adults (intergenerational contact), overlooking the possible perks for older adults themselves (contact with same-age peers). The current study investigated the association between contact with older adults and views of the self in old age (VSOA) among younger and older adults in a domain-specific way. The sample comprised younger (39-55 years, n = 1,012) and older (65-90 years, n = 1,344) adults from the Ageing as Future international study. Findings indicated that contact with older adults was related to more positive VSOA and this was partly mediated by AS. These relations were stronger for older adults, indicating that interactions with other older adults may help favorably shape how older adults view their ingroup and aging. Beneficial effects emerged mostly in the friends and leisure domains.

### SELECTIVE ENGAGEMENT IN PREPARATIONS FOR OLD AGE: DETERMINANTS OF MOTIVATION

Jeongsoo Park, *Ajou University, Suwon-si, Kyonggi-do, Republic of Korea*

Preparations for old age in general are beneficial for one's adjustment in later life. Using Selective Engagement Theory (SET) as a conceptual framework, we examined how the importance attached to functioning, as well as perceived control over functioning in different domains (e.g., family, social relations, finances, health, etc.) predicted engagement in preparing for old age five years later. Two-wave data was obtained from Ageing as Future Study. The sample consisted of N = 1,255 aged from 30-85 in the US (n=315), Hong Kong (n=317), and Germany (n=623). Consistent with SET, ratings of importance were strongly predictive of subsequent preparations and more predictive than perceived control, with evidence in several domains of functioning that this was particularly true for older adults. These findings highlight the interaction between personal goals and resources in determining older adults' willingness to prepare for old age.

### RELEVANT YET UNCONTROLLABLE: PERCEIVED CONTROL AS A MEDIATOR OF CROSS-CULTURAL DIFFERENCES IN OLD-AGE PREPARATION

Helene Fung[1], Nicole Long Ki Fung[1], and Dwight Cheuk Kit Tse[2], *1. The Chinese University of Hong Kong, Hong Kong, Hong Kong, 2. University of Strathclyde, Glasgow, Scotland, United Kingdom*

Previous studies have shown that there are cross-cultural differences in old-age preparation rate (e.g. Kornadt et al., 2019). Drawing from the transactional stress-and-coping model (Lazarus & Folkman, 1984), we proposed that perceived control, self-relevance and responsibility for old-age preparation could mediate the cultural differences in old-age preparation. We recruited a sample aged 18 to 96 from Germany (N=366, Mage=52.63) and Hong Kong (N=252, Mage=57.47) to complete two online questionnaires across two years. Compared with German adults, Hong Kong adults prepared less (b=-2.159, p<.001), had lower perceived control (b=-0.899, p<.001) and responsibility (b=-0.713, p<.001), yet similar level of self-relevance over preparation. Preparation at time2 was related to self-relevance (b=1.004, p<.001) and control (b=0.785, p<.001) at time1. The cultural differences in preparation at time2 were only mediated by perceived control at time1 (indirect effect=0.706, p<.001). Findings highlight the importance to enhance individual perceived control over old age in promoting society-wide old-age preparation.

### PREPARING FOR A GOLDEN AGE? APPROACH AND AVOIDANCE MOTIVATION IN THE CONTEXT OF OLD-AGE PREPARATION

Fiona Rupprecht, and Jana Nikitin, *University of Vienna, Vienna, Wien, Austria*

When it comes to old-age preparation, individuals may be motivated by positive outcomes they wish to approach (e.g., social connectedness) or by negative outcomes they wish to avoid (e.g., loneliness). We expected approach motivation to be adaptive in younger ages, when resources and possibilities for old-age preparation should be plentiful. For older adults, whose resources and time for (continued) old-age preparation are limited, the maintenance- and loss-oriented perspective of avoidance motivation may however be the more adaptive one. Using data from 2054 individuals aged 18 to 96 years and representing five cultures, we adopted a domain-specific, cross-cultural, and age-differential perspective on our research question. Results indicate that individuals tend to be both approach- and avoidance-motivated when it comes to old-age preparation and confirm the age-differential adaptivity of approach and avoidance motivation in terms of both, actual preparatory behavior and psychological well-being.

### SESSION 2490 (SYMPOSIUM)

### A PUBLIC HEALTH APPROACH TO ALZHEIMER'S DISEASE RISK REDUCTION: WHERE WE ARE AND WHERE WE'RE HEADED

Chair: John Omura Co-Chair: Eva Jackson Discussant: Kelly O'Brien

A growing body of evidence has identified potential modifiable risk factors for Alzheimer's disease and related dementias (ADRD). In 2021, the National Plan to Address Alzheimer's Disease (National Plan) included a new goal to promote healthy aging and address risk factors to help delay onset or slow progression of ADRD. Applying a robust public health approach to ADRD risk reduction can help achieve meaningful progress at the population level. The activities outlined in the Building Our Largest Dementia (BOLD) Infrastructure for Alzheimer's Act (P.L. 115-406) are designed to create a uniform national public health infrastructure with a focus on various issues including risk reduction. The purpose of this session is to illustrate a public health approach to ADRD risk reduction, including its current status along with future directions and priorities. An overview of the National Plan's new goal regarding ADRD risk reduction (McGuire) and data highlighting the current burden of key modifiable risk factors in the United States along with important disparities (Omura) will be presented. Holt will describe how ADRD risk reduction is integrated into the work of BOLD funding recipients, and Head will present experiences implementing public health activities that support ADRD risk reduction in the field along with successes and lessons learned. Finally, priorities and future directions for a public health approach to ADRD risk reduction will be presented from the perspective of the CDC's Building Our Largest Dementia Infrastructure Public Health Center of Excellence on Dementia Risk Reduction (Baumgart).

### THE EVOLUTION OF A PUBLIC HEALTH APPROACH TO ADRD AND THE ROLE OF RISK REDUCTION

Lisa McGuire[1], and Eva Jackson[2], *1. CDC, Atlanta, Georgia, United States, 2. Alzheimer's Association, Chicago, Illinois, United States*

CDC, through the Healthy Brain Initiative (HBI) and Building Our Largest Dementia (BOLD) Infrastructure Act, is working to advance cognitive and brain health as integral components of public health practice, to keep older Americans healthy and independent as long as possible. HBI promotes brain health as part of public health practice and BOLD strives to build a uniform public health infrastructure. Both HBI and BOLD focus not only on people with cognitive decline or dementia but also their health care providers and caregivers. Recently, the 2021 Alzheimer's Disease National Plan added Goal 6, Accelerate Action to Promote Healthy Aging and Reduce Risk Factors for Alzheimer's Disease and Related Dementias. This presentation will describe the evolution of public health's role with respect to brain health and caregiving and how the national priority on risk reduction and healthy aging can be beneficial to the health and well-being of older adults.

### MODIFIABLE RISK FACTORS FOR ALZHEIMER'S DISEASE: CURRENT STATUS AND OPPORTUNITIES FOR ACTION

John Omura, *CDC, Atlanta, Georgia, United States*

In 2021, the National Plan to Address Alzheimer's Disease included a new goal to address risk factors for ADRD. The Behavioral Risk Factor Surveillance System (BRFSS) assesses several modifiable risk factors in its core survey and subjective cognitive decline (SCD), which may be an early indicator of developing ADRD, in the optional Cognitive Decline module. To assess the current status of modifiable risk factors in the US and identify opportunities for public health action, data from the 2019 BRFSS were examined. Prevalence of eight modifiable risk factors for ADRD and proportion of respondents with total number of risk factors was estimated among respondents aged ≥45 years, overall, by SCD status, and by selected demographic characteristics. Findings can inform strategies and priorities to support the National Plan's new goal to reduce modifiable risk factors to help delay onset or slow progression of ADRD and its symptoms.

### BUILDING OUR LARGEST DEMENTIA (BOLD) INFRASTRUCTURE FOR RISK REDUCTION

Heidi Holt[1], Laura Whalen[1], and Lisa Garbarino[2], *1. CDC, Atlanta, Georgia, United States, 2. Centers for Disease Control and Prevention, Atlanta, Georgia, United States*

The Building Our Largest Dementia (BOLD) Infrastructure for Alzheimer's Act (PL115-406) supports public health agencies to develop a uniform dementia infrastructure across the US. Applying a robust public health approach to ADRD that emphasizes data driven decision-making and action along with primary, secondary, and tertiary prevention strategies (e.g., risk reduction, early diagnosis, linkages to treatment and care, and the prevention and management of comorbidities leading to preventable hospitalizations and poor health outcomes) for persons living with dementia and their caregivers. Recipients of the BOLD Public Health Programs funds are expanding jurisdiction Dementia coalitions, updating, or creating Dementia Strategic Plans, and implementing strategies from those plans that address a wide variety of life-course strategies for brain health and dementia, including risk reduction. This presentation will explain how risk reduction is integrated and provide examples of several activities being planned and implemented by recipients.

### GETTING TO BOLD USING SYSTEMIC EDUCATION, EVIDENCE, AND NETWORKS: HELPING GEORGIANS B-SEEN

Elizabeth Head, *Georgia Department of Public Health, Atlanta, Georgia, United States*

The complex nature of Alzheimer's disease and related dementias (ADRD) demands a comprehensive public health approach. Georgia is Building Our Largest Dementia infrastructure using Systemic Education, Evidence, and Networks (B-SEEN), with a vision for every Georgian – professional, patient, and care partner – to B-SEEN. The strength of Georgia's B-SEEN project is the existing infrastructure. Leveraging this established network, Georgia has engaged in population-based efforts to increase impact in the dementia risk reduction, early diagnosis of ADRD, prevention and management of comorbidities and avoidable hospitalizations, and caregiving. These outcomes are being achieved by stakeholders disseminating evidence-based programs personalized to their community needs and the B-SEEN team leading coordinated activities that address dementia and support the promotion of brain health. This presentation will describe how ADRD risk reduction is integrated into a 159 county, de-centralized state and provides examples of several activities being implemented via Georgia extension, faith-based organizations, and dedicated partners.

### PUBLIC HEALTH PRIORITIES AND FUTURE DIRECTIONS FOR ALZHEIMER'S DISEASE RISK REDUCTION

Matthew Baumgart, *Alzheimer's Association, New York, New York, United States*

Reducing risk for diseases and chronic conditions is a fundamental priority of public health. Since 2007, the Alzheimer's Association has partnered with the CDC on the development of a series of Public Health Road Maps to guide the public health community in addressing cognitive health. In addition, the Alzheimer's Association's Public Health Center of Excellence on Dementia Risk Reduction, funded by the CDC, provides guidance on how public health can address the risk factors for cognitive decline and dementia. With recent advancements in the science on dementia risk factors, we can now identify targets for public health action. The addition of a national goal to address dementia risk factors underscores the urgency to act. This presentation will offer a perspective on how public health can move forward, through prioritization and action, in addressing risk factors for cognitive decline and dementia, including by addressing social determinants of health and health equity.

### ACCELERATED AGING AMONG ADULTS LIVING WITH CEREBRAL PALSY

Mark Peterson[1], Sudarshan Dayanidhi[2], Patrick McPhee[3], and Heidi Haapala[1], *1. University of Michigan, Ann Arbor, Michigan, United States, 2. Shriley Ryan AbilityLab/Northwestern University, Chicago, Illinois, United States, 3. McMaster University, Hamilton, Ontario, Canada*

Cerebral Palsy (CP) is the most common pediatric-onset physical disability, with an estimated prevalence ranging from 2.6-3.1 cases per 1,000 live births in the United States. There is a lack of clinical follow-up for individuals with CP after they transition from pediatric to adult primary care, and insufficient surveillance to track patients with CP longitudinally. Despite the shortage of research to examine the natural history of CP and chronic disease trajectories in this population, a range of secondary conditions arise at an accelerated rate as compared to the adult population without CP, prompting the widespread notion and clinical hypothesis that patients with CP are prone to accelerated aging. These factors further worsen functional status and quality of life, as well as lead to decreased independence. Despite the well-established interrelationships between physical and mental health disorders in the non-CP older adult population, the extent to which, mechanisms underlying, and time course associated with the development of these chronic conditions among adults living with CP has received little empirical attention. The proposed course will build upon our ongoing work by highlighting new findings from three centers doing research pertaining to aging with CP, and will cover novel mechanisms of musculoskeletal pathophysiology in CP, risk factors of and unique CVD profiles among adults with CP, and new findings related to health trajectory differences of adults with CP from clinical and population-representative cohorts. We will also provide insights into the pathophysiologic mechanisms linking early frailty and long-term health outcomes among persons with CP.

### SKELETAL MUSCLE MITOCHONDRIAL PHYSIOLOGY IN CHILDREN WITH CEREBRAL PALSY: CONSIDERATIONS FOR HEALTHY AGING

Sudarshan Dayanidhi, *Shriley Ryan AbilityLab/Northwestern University, Chicago, Illinois, United States*

During healthy aging, there is an overall decline in mitochondrial activity and abundance, increase in mitochondrial DNA mutations, increase in oxidative stress, and reduction in overall muscular capacity. Individuals with cerebral palsy (CP) have significantly increased energetics of movement, reduced endurance capacity, and increased perceived effort. We will cover the results of recent work in muscles in ambulatory children with CP that show a marked reduction in mitochondrial function. Muscles show that mitochondrial protein content and DNA copy number are lower, suggesting a reduction in mitochondrial abundance, along with a reduction in markers for mitochondrial biogenesis. Gene expression networks are reduced for glycolytic and mitochondrial pathways and share similarities with gene networks with aging and chronic inactivity. Given the importance of mitochondria for energy production and changes with aging, ongoing efforts are needed to assess changes in mitochondria across the lifespan in people with CP.

### THE FORMULA FOR CARDIOVASCULAR HEALTH AND WELL-BEING IN ADULTS WITH CEREBRAL PALSY

Patrick McPhee, *McMaster University, Hamilton, Ontario, Canada*

Persons with cerebral palsy (CP) have mobility limitations, heightened sedentary behavior, and are at increased risk for cardiovascular disease (CVD). Dr. McPhee will cover his innovative work to develop a core outcome set for cardiometabolic disease risk assessment in adults with CP, and will further discuss recent findings from ongoing work pertaining to novel CVD risk indicators showing accelerating cardiovascular aging in CP. Dr. McPhee will also present recent work on the role of exercise, sleep, and healthy nutrition in the context of healthy aging for individuals with CP across the lifespan.

### NAVIGATING THE PATHWAY TO CARE IN ADULTS WITH CEREBRAL PALSY

Heidi Haapala, *University of Michigan, Ann Arbor, Michigan, United States*

As individuals with CP age, they face unique challenges which complicate their ability to access and receive appropriate health care. These problems exist at the level of the health care system, the clinician, and the individual. At the system level, there is an inadequate number of professionals who are informed of and interested in the care of adults with CP. Adult caregivers are often not knowledgeable about and may feel less competent about patients with CP. Differences in the physiologic development of individuals with CP render well-established clinical protocols for risk screening of chronic diseases less effective. Dr. Haapala runs an adult CP clinic and will present her clinical experience with treating complex patients with CP. She will present specific information pertaining to her work in studying and treating chronic overlapping pain in adults with CP, as well as her efforts to reduce polypharmacy and opioid dependence in her patients.

### SESSION 2501 (BIOLOGICAL SCIENCES INVITED SYMPOSIUM)

### ESPO AND BIOLOGICAL SCIENCES SECTION SYMPOSIUM: HIGHLIGHTING THE FUTURE LEADERS OF GEROSCIENCE

Chair: Matt Yousefzaheh

### EARLY-LIFE PROTEIN TRANSLATION SPIKE DRIVES AGING VIA JUVENILE HORMONE/GERMLINE STEM CELL SIGNALING

Harper Kim[1], Danitra Parker[1], Madison Hardiman[1], Erin Munkácsy[2], Steven Austad[3], Yi-dong Bai[2], James Mobley[1], and Andrew Pickering[4], *1. University of Alabama at Birmingham (UAB), Birmingham, Alabama, United States, 2. University of Texas Health San Antonio, San Antonio, Texas, United States, 3. University of Alabama at Birmingham, Birmingham, Alabama, United States, 4. University of Alabama at Birmingham, Hoover, Alabama, United States*

Protein translation (PT) is high in early-adulthood across invertebrates, rodents, and humans but sharply declines thereafter. It has been implicitly assumed that elevated PT at young ages is beneficial to health and PT ends up dropping as a passive byproduct of aging. However, whether this holds true and how dynamic fluctuations in PT over time impact aging remain unknown. In Drosophila, we show that a transient PT spike in early-adulthood exerts long-lasting negative impacts on aging trajectories and proteostasis in later-life. Conversely, blocking the early-life PT spike robustly improves life-/health-span and prevents age-related protein aggregation. Further, greater early-life PT rise strongly predicts shorter future lifespan across fly strains and is observed in neurodegenerative disorders long before symptoms/pathologies appear. Proteomics-guided investigations revealed that during the early-adulthood PT rise, juvenile hormone triggers proteostatic dysfunction and drives aging via aggregation-prone large lipid transfer proteins. The early-life PT spike also transcriptionally represses stress responses essential for proteostasis maintenance and drives aging via germline stem cell signaling. Our findings suggest that PT is thereby suppressed after early-adulthood as an adaptive response to alleviate proteostatic burden, slow down aging, and optimize life-/health-span. We thus propose that the rise and fall in PT over time impact aging in the opposite direction from what was previously assumed. Our work provides a novel theoretical framework for understanding how lifetime PT dynamics regulate the onset of aging. Further, our study provides a foundation for future research, including whether high early-life PT spike is an early biological event driving neurodegeneration/age-related diseases.

### A FAT-PROMOTING BOTANICAL EXTRACT ARTEMISIA SCOPARIA EXERTS GEROPROTECTION IN C. ELEGANS

Bhaswati Ghosh[1], Hayden Guidry[2], Maxwell Johnston[1], and Adam Bohnert[1], *1. Louisiana State University, BATON ROUGE, Louisiana, United States, 2. Louisiana State University, Baton Rouge, Louisiana, United States*

Like other biological processes, aging is not random but subject to molecular control. Natural products that modify core metabolic parameters, including fat content, may provide entry points to extend animal lifespan and promote healthy aging. Here, we show that a botanical extract from Artemisia scoparia (SCO), which promotes fat storage and metabolic resiliency in mice, extends the lifespan of the nematode Caenorhabditis elegans by up to 40%. Notably, this lifespan extension depends significantly on SCO's effects on fat; SCO-treated worms exhibit heightened levels of unsaturated fat, and inhibiting Δ9 desaturases, which oversee biosynthesis of monounsaturated fatty acids, prevents SCO-dependent fat accumulation and lifespan extension. At an upstream signaling level, SCO prompts changes to C. elegans fat regulation by stimulating nuclear translocation of transcription factor DAF-16/FOXO, an event that requires AMP-activated protein kinase under this condition. Importantly, animals treated with SCO are not only long lived but also show improved stress resistance in late adulthood, suggesting that this fat-promoting intervention may enhance some aspects of physiological health in older age. These findings identify SCO as a natural product that can modify fat regulation for longevity benefit and add to growing evidence indicating that elevated fat can be pro-longevity in some circumstances.

### FUCOIDANS ARE NOVEL SENOTHERAPEUTICS THAT ENHANCE SIRT6 AND DNA REPAIR ACTIVITY

Lei Zhang[1], Brian Hughes[2], Gregory Tombline[3], Luise Angelini[2], Matt Yousefzadeh[2], Vera Gorbunova[3], Laura Niedernhofer[2], and Paul Robbins[2], *1. University of Minnesota, Saint Paul, Minnesota, United States, 2. University of Minnesota, Minneapolis, Minnesota, United States, 3. University of Rochester, Rochester, New York, United States*

With age, senescent cells accumulate in various tissues where they contribute to loss of tissue homeostasis, aging, and age-related diseases through their inflammatory senescence-associated secretory phenotypes (SASPs). Senotherapeutics able to selectively eliminate senescent cells, termed senolytics, or suppress the detrimental SASPs, termed senomorphics, have been demonstrated to improve age-associated comorbidities and aging phenotypes. To discover novel senotherapeutics translatable to promote healthy longevity, we conducted a drug screening of diverse natural products based on the characteristic senescence-associated β-galactosidase activity. Several fucoidans from different brown seaweed were found to exhibit potent senotherapeutic activity. Fucoidans are long-chain sulfated polysaccharides found in various species of brown algae including seaweed. The best senomorphic fucoidan was able to suppress senescence in cultured senescent fibroblasts, in ex vivo human tissue explants, and in vivo in mouse models of natural and accelerated aging. Specifically, fucoidan reduced markers of cellular senescence and SASP in senescent mouse and human cells. Acute treatment of the fucoidan in naturally aged mice reduced tissue senescence, especially in the kidney and lung. Chronic treatment of the fucoidan in Ercc1-/Δ progeria mice attenuated composite aging symptoms and extended healthspan. Interestingly, preliminary mechanistic studies demonstrated that fucoidan can improve non-homologous end-joining-directed DNA damage repair and increase the mono-ADP-ribosylation activity of SIRT6, suggesting a relationship between cellular senescence, DNA repair, and SIRT6 signaling pathways. Collectively, fucoidans were identified as novel senotherapeutics with translational potential for reducing cellular senescence, ameliorating age-associated phenotypes, and extending healthspan as well as able improve DNA repair pathways through modulation of SIRT6 activity.

### MOSAIC CHROMOSOMAL ALTERATIONS AND LONGEVITY

Anastasia Leshchyk[1], Qingyan Xiang[1], Stefano Monti[1], Thomas Perls[1], Stacy Andersen[2], Anastasia Gurinovich[3], Zeyuan Song[1], and Paola Sebastiani[3], *1. Boston University, Boston, Massachusetts, United States, 2. Boston University School of Medicine, Boston, Massachusetts, United States, 3. Tufts Medical Center, Boston, Massachusetts, United States*

Mosaic chromosomal alterations (mCAs) are structural alterations that are associated with mortality, age, cancer, cardiovascular disease, and diverse infections. The distribution of mCAs in long-lived subjects and individuals with familial longevity is not well described. We applied MOsaic CHromosomal Alteration (MoChA) caller on genome-wide genotype samples of 2025 centenarians, their siblings, and offspring and 273 unrelated controls from the New England Centenarian Study (NECS) and 3642 subjects with familial longevity and 920 controls from the Long-Life Family Study (LLFS). MoChA utilizes a Hidden Markov Model to detect mCA-induced deviations in allelic balance at heterozygous sites with Log R Ratio and B-allele frequency (BAF) with phased genotype information. We analyzed somatic mCAs in samples with genome-wide BAF phase concordance less than 0.51, LOD score greater than 10, and estimated cell fraction less than 50%. The results in the two studies showed that autosomal mCAs spanning over 100 kbase pairs increase with older age until approximately 102 years. However, the prevalence of the subjects with mCAs tends to plateau after that age, suggesting that the accumulation of mCAs is less prevalent in long-lived subjects. We also found that offspring and siblings of centenarians accumulate less autosomal mCAs (fixed-effect meta-analysis for NECS and LLFS: RR=0.78, p=0.033) compared to unrelated controls. In addition, consistent with results from other studies, mCAs are associated with increased risk for mortality (HR=1.08, p=0.02) and sex (Male RR=1.37, p=4.15e-05), and impact incident events of cancer, dementia, diabetes, and cardiovascular diseases even at extreme old ages.

### SESSION 2510 (PAPER)

### ADRD RISK

### ALZHEIMER'S DISEASE RISK REDUCTION HEALTH COACHING: ALCOHOL EFFECTS

Faika Zanjani[1], Annie Rhodes[1], and Brian Battle[2], *1. Virginia Commonwealth University, Richmond, Virginia, United States, 2. VCU, Richmond, Virginia, United States*

Lifestyle risk reduction at the community-level, is currently considered an effective method to decrease Alzheimer's disease (AD). As part of the Virginia Commonwealth University iCubed Health and Wellness in Aging Core, diverse older adults (60+) in Richmond, VA, with incomes below $12,000/year and managing either diabetes/cardiovascular symptoms, were offered weekly lifestyle telephone-health coaching for 12-weeks, providing education, support, and monitoring for AD lifestyle risk in 2020-21. The study sample (n=40, mean age 68 years (range: 60-77 years) was 88% African American/Black (n=35), 100% Non-Hispanic, 45% males (n=18)), 60% reporting memory problems and 53% reporting any alcohol consumption. Thirty-nine (95%) of subjects successfully participated in coaching sessions; on average 91.9% (11) sessions were completed. Participants provided positive anecdotal feedback and the need for continued coaching during COVID. Average drinks per day decreased across the study period (F=7.44; p=.01) and alcohol risk, defined as more than 1 drink/day, decreased (F=3.46;p=.07). Drinking at baseline was associated with differential change in nicotine dependence (F=14.00 ;p=.02), depression risk (F=3.20;p=.09), light physical activity (F=4.52;p=.05), and cognition (COGTEL) (F=6.35;p=.02). Drinking between-subject effects indicated poorer level differences for smoking risk (F=5.68;p=.02), physical inactivity risk (F=4.66;p=.04), and total health behavior risk (F=14.54;p=.001), but higher cognition-scores (F=3.18;p=.11) for drinkers. In conclusion, there may be a paradoxical health effect for alcohol, with associations for negative health behaviors, but positive cognitive functioning. In conclusion, this preliminary work creates the impetus for future large-scale AD risk reduction investigations to improve the lives of AD-risk, low-income, diverse older adults reporting alcohol consumption.

### ARE RECENTLY INCARCERATED OLDER ADULTS MORE LIKELY TO DEVELOP DEMENTIA THAN THE NEVER INCARCERATED?

Amy Byers[1], Richard Fortinsky[2], Brie Williams[1], W. John Boscardin[1], Yixia Li[3], and Lisa Barry[2], *1. University of California, San Francisco, San Francisco, California, United States, 2. University of Connecticut Center on Aging, Farmington, Connecticut, United States, 3. San Francisco VA Medical Center, San Francisco, California, United States*

Increasing numbers of older adults are reentering the community following incarceration (i.e., reentry). Yet, their risk of developing dementia and mild cognitive impairment (MCI) is unknown. We leveraged a national cohort of veterans who experienced reentry at age ≥ 65 years (N=5,920) and compared their risk of dementia or MCI to an age- and sex-matched never incarcerated sample (N=29,600). Those in the reentry sample were incarcerated for ≤10 consecutive years, experienced reentry between 10/01/2012 and 9/30/2018, and did not have a pre-incarceration MCI or dementia diagnosis per VA and CMS healthcare records (N=5,920). Fine-Gray models, controlling for race and pre-incarceration chronic conditions, serious mental illness, traumatic brain injury, and posttraumatic stress disorder, derived hazard ratios (HRs) and 95% Confidence Intervals (CIs). Samples were 99% male with average age 70 (±4.3) years. Reentry adults had a higher proportion of non-Whites (29% vs. 17%; p< 0.001) and more chronic conditions (2.1[±1.8] vs. 1.6[±1.6] p< 0.001). MCI incidence did not differ between reentry and never incarcerated groups (3.1% vs. 2.6%; HR=1.03; 95%CI 0.86-1.24). However, risk of any dementia was higher in reentry older adults (7.2% vs. 5.0%; HR=1.27; 95%CI 1.12, 1.44), as was risk of specific subtypes, e.g., vascular dementia (1.9% vs. 1.2%; HR=1.37; 95% CI 1.06-1.75) and frontotemporal dementia (0.2% vs. 0.1%; HR=2.3; 95% CI 1.2, 4.3). Those reentering the community in late life following incarceration may be a group that is especially at risk of developing dementia. These findings raise awareness of the need for appropriate transition planning for this vulnerable group.

### DEMENTIA TRAJECTORIES, MEDICAID COVERAGE, AND HEALTHCARE SERVICES USE

Wassim Tarraf[1], and Marc Garcia[2], *1. Wayne State University, Detroit, Michigan, United States, 2. Syracuse University, Syracuse, New York, United States*

The evidence base linking dementia risk trajectories to transitions in Medicaid coverage and levels of health services is underdeveloped. We use Health and Retirement Study (2007/08-2015/16) data on adults 70-years and older (Unweighted N=8,227) at baseline to test how longitudinal dementia classifications (Langa-Weir), over 8-years, influence change in Medicaid coverage, nursing-home use (NHU) and inpatient hospitalizations (IH). We fit Joint Growth and Discrete-time Survival Mixture models to generate longitudinal risk classifications for dementia accounting for survival, and generalized linear models to test associations of these classifications with change in Medicaid coverage, NHU, and IH. Average baseline age was 78.4 years (SD=7.1), 3-in-5 were female, 1-in-4 had less than high school education, and 4-in-5 were non-Hispanic Whites. A three-class solution (C1=high dementia prevalence and mortality risk (8.6%), C2=low prevalence (68.3%), and C3=accelerated dementia prevalence and mortality risk (23.1%)) provided the best fit to the data. We observed substantial increase in rates of for Medicaid coverage for C1 (21%; 95%CI=[18-25] to 51%[36-65] among survivors) and C3 (12%; 95%CI=[10-14] to 32%[27-37]), but not C2 (6% to 8%). NHU also accelerated substantially from 10 to 37% in C1 and 7 to 38% in C2. Rates of inpatient hospitalizations remained stable over time for all groups, with C1 and C2 being more likely to be hospitalized and have multiple re-admissions. Estimates were differentially attenuated through adjustment to covariables. We report important longitudinal dementia risk classifications, profile their socioeconomic and health attributes, and identify differential associations with critical health policy outcomes (Medicaid coverage and healthcare utilization).

### DO PERSONALITY TRAITS INFLUENCE THE ASSOCIATION BETWEEN DEPRESSION AND DEMENTIA IN OLD AGE?

Khuzeman Abbasi1, Ujunwa Ebili[2], Henry Egbuchiem[3], AFOLABI OREKOYA[4], Olawale Olanisa[5], Abimbola Arisoyin[6], and Adeleye Adaralegbe[7], *1 Vanderbilt University Medical Center , Glen Burnie , Maryland, United States, 2. Emel Specialists and Pediatric Center, Festac Town, Lagos, Nigeria, 3. The Everest Foundation, Los Angeles, California, United States, 4. Wellspring Medical Center, Riverdale, Georgia, United States, 5. IQVIA Biotech, Morrisville, North Carolina, United States, 6. TCA health, Lagos, Lagos, Nigeria, 7. University of Connecticut, Storrs, Connecticut, United States*

Depression and personality traits are independent predictors of dementia or cognitive impairment. Despite the well-established relationship between these two psychosocial factors and dementia, no research has been documented on how personality traits can influence dementia in older adults exhibiting depressive symptoms. This study explored the influence of personality traits on the association between change in depression and dementia in old age. We used a population-based longitudinal cohort study involving two waves of data collected 5 years apart, containing 2210 American older adults, from the National Social Life, Health, and Aging Project to explore if personality traits influence how change in depression predicts the development of dementia. We used multivariate logistic regression to examine the relationship between depression and dementia at T2 while adjusting for sociodemographic characteristics, comorbidity index at T1, and baseline dementia. Change in depression increased the likelihood of dementia at T2 by 4.2% (AOR = 1.04, p = 0.019) in the co-variate adjusted model. Personality traits, overall, did not influence how depression predicts the development of dementia. However, agreeableness individually nullified the effect of depression on the development of dementia, whereas extraversion was the only personality trait that significantly predicted dementia. We recommend the promotion of prosocial behaviors in old age as these appear to be protective. In addition, early life education and a strong social support can keep the depression-dementia spectrum at bay in old age.

### FOOD INSECURITY AND COGNITIVE TRAJECTORIES IN COMMUNITY-DWELLING OLDER ADULTS

Boeun Kim[1], Sarah Szanton[2], Roland J. Thorpe, Jr.[1], Deidra Crews[1], and Laura Samuel[1], *1. Johns Hopkins University, Baltimore, Maryland, United States, 2. Johns Hopkins University School of Nursing, Baltimore, Maryland, United States*

Food insecurity, defined as limited access to nutritionally adequate and safe foods due to social and economic conditions, has adverse effects on physical health and well-being. However, it remains unclear whether food insecurity accelerates cognitive decline in older adults. This study examined the association of food insecurity with cognitive decline. We analyzed data from 4,508 community-dwelling participants in the National Health and Aging Trends Study (NHATS) collected from 2011 to 2020, a prospective cohort study of a nationally representative sample of Medicare beneficiaries ages 65 years and older. Food insecurity was measured using 5 items within functional, social support, and financial limitations domains. Immediate and delayed recall were assessed by a 10-item word-list memory task (range: 0—10). Executive function was evaluated by the Clock Drawing Test (range: 0—5). Each year's cognitive functions were linked to the prior year's food insecurity values. Weighted linear mixed effect models were fitted. Prevalence of food insecurity at baseline was 3.5% (95% CI: 2.9 — 4.3). Persons with food insecurity were more likely to have Black race or Hispanic ethnicity, low income, and less than high school education. Food insecurity was associated with faster decline in executive function accounting for demographic and socioeconomic characteristics: the average difference, over 1-year, in executive function score between people exposed to and not exposed to food insecurity was 0.038 points (95% CI: -0.072 — -0.003). Food assistance programs or increasing healthy food access may reduce food insecurity and delay cognitive aging in community-dwelling older adults.

### SESSION 2520 (SYMPOSIUM)

### ADVANCING RESEARCH ON PERSON-CENTERED COMMUNICATION IN DEMENTIA CARE

Chair: Clarissa Shaw Co-Chair: Carissa Coleman
Discussant: Marie Savundranayagam

Communication is an essential component to achieving person-centered care. However, few measures exist for measuring person-centered communication and methods vary widely on measuring communication. The purpose of this symposium is to provide an overview of methods and recent findings related to research on person-centered communication. Four presentations will highlight a variety of methods and a variety of populations. Presentation one compares the congruence of communication by hospital nurses in a simulated care setting to communication during actual care encounters with hospitalized patients with dementia. Presentation two evaluates the impact of validation by family caregivers to persons living with dementia (PLWD) using a time sequential analysis of second-by-second coding of the caregiver's communication and the PLWD behavioral response. Presentation three introduces the Changing Talk Scale (CHATS) as a tool to measure gains in knowledge of person-centered communication in the nursing home setting. Presentation four determines the impact of person-centered care versus task-centered care on mealtime behaviors in nursing home residents with dementia using a psychometrically established behavioral coding scheme and time-sequential approaches. Communication strategies to use with PLWD frequently lack sufficient evidence to be deemed effective or person-centered. Advanced methods are needed to understand what constitutes person-centered communication based on behavioral responses and other health outcomes off PLWD. This symposium provides an overview of these methods with formal and informal caregivers and PLWD in varied care settings including the community, hospitals, and nursing homes.

### ESTABLISHING THE VALIDITY OF SIMULATION TO CAPTURE ELDERSPEAK COMMUNICATION IN DEMENTIA CARE

Clarissa Shaw[1], Katie Knox[2], Heather Bair[2], Erica Watkinson[2], Delaney Weeks[2], and Paige Hanson[2], *1. University of Iowa, Iowa City, Iowa, United States, 2. University of Iowa College of Nursing, Iowa City, Iowa, United States*

Using simulation may be an innovative approach for evaluating dementia care in research and education. The purpose of this study was to determine if communication in the simulated environment is congruent to communication in the naturalistic environment. Nursing staff (n=10) were each audio-recorded caring for an actual hospitalized person living with dementia (PLWD), and in three simulations evaluating the use of a manikin, young nursing assistant, and trained older adult standardized patient acting as the PLWD. The audio-recordings were coded for elderspeak (i.e., infantilizing) communication. Reactions to each simulation were gathered and qualitatively analyzed. The amount of elderspeak and neutral communication in the actual care encounters did not significantly differ (p>.05) from the communication in any of the three simulations indicating that simulation may be a valid method to capture elderspeak by nursing staff. The nursing staff indicated that the standardized patient provided the most realistic simulation.

### RESPONSES OF PERSONS LIVING WITH DEMENTIA TO VALIDATING COMMUNICATION BY FAMILY CAREGIVERS: A SECONDARY ANALYSIS

Kaitlyn Campbell[1], Carissa Coleman[2], and Kristine Williams[3], *1. University of Pennsylvania Hospital, Kansas City, Kansas, United States, 2. University of Kansas, Kansas City, Kansas, United States, 3. University of Kansas Medical Center, Lawrence, Kansas, United States*

Validation is a person-centered approach for communicating with people living with dementia (PLWD). This study evaluated how PLWD respond to caregiver communication that includes or fails to include validation. This secondary analysis of homecare videos (N=41) of family caregivers interacting with a PLWD during daily care used behavioral coding of validating communication (affirmation, acknowledging emotions, encouraging emotional expression, verbalizing understanding) in relation to responses of PLWD (resistiveness to care, apathy, or cooperation). A 10 second time-lag sequential analysis identified an 11% probability of a cooperative response when caregivers communicated using affirmations. Caregiver verbalizations of understanding resulted in a 6% probability and silence had an 8% probability of cooperative responses. Use of validating communication did not result in a significant probability of negative PLWD responses (apathy or resistiveness to care). Use of validating communication strategies may assist caregivers to achieve goals of care without negative responses from PLWD.

### THE CHANGING TALK SCALE (CHATS): A TOOL TO MEASURE GAINS IN KNOWLEDGE OF PERSON-CENTERED COMMUNICATION

Yelena Perkhounkova[1], Clarissa Shaw[1], Maria Hein[2], Carissa Coleman[3], and Kristine Williams[4], *1. University of Iowa, Iowa City, Iowa, United States, 2. University of Iowa College of Nursing, Iowa City, Iowa, United States, 3. University of Kansas, Kansas City, Kansas, United States, 4. University of Kansas Medical Center, Lawrence, Kansas, United States*

Assessment of knowledge gain from educational interventions is common in health care research. However, validity of tools developed to measure knowledge gains is often overlooked. For our current pragmatic clinical trial, we developed two 13-item parallel forms of the Changing Talk Scale (CHATS) to measure gains in knowledge of effective and person-centered communication due to the Changing Talk Online (CHATO) communication education for nursing home staff. This has been a multi-year, multi-study process of writing and editing test items in accordance with CHATO learning objectives and results of psychometric analyses of pilot data. Items were revised based on difficulty and discrimination. The tool has improved from an initial Cronbach's alpha of .27 (pre-education) and .14 (post-education) to .63 (pre-education) and .64 (post-education).

CHATS provides an exemplar of how measuring change in knowledge is a complex process that needs to be rigorously completed to accurately capture outcomes.

### TEMPORAL RELATIONSHIPS OF PERSON- AND TASK-CENTERED DEMENTIA CARE AND MEALTIME BEHAVIORS: SEQUENTIAL ANALYSIS

Wen Liu[1], Yelena Perkhounkova[1], Maria Hein[2], and Roger Bakeman[3], *1. University of Iowa, Iowa City, Iowa, United States, 2. University of Iowa College of Nursing, Iowa City, Iowa, United States, 3. Georgia State University Department of Psychology, Atlanta, Georgia, United States*

Person-centered mealtime care is highly recommended in dementia care. While current research examined associative relationships between person- and task-centered care and resident mealtime behaviors, few studies evaluated their temporal associations. Videotaped mealtime observations (N=160) involving 36 staff and 27 residents (53 staff-resident dyads) in 9 nursing homes were coded. Staff person-centered and task-centered approaches were conceptualized as antecedents of resident positive behaviors, functional impairments, and resistive behaviors using 5-, 10-, and 15-second time windows. Immediately after staff person-centered approaches, resident positive and resistive behaviors were more likely (p range=<.001–.29) and functional impairments less likely (p range=<.001–.62) with diminished effects in time. Immediately after staff task-centered approaches, resident positive behaviors were less likely (p range=<.001–.09). Person-centered mealtime care should be individualized, context-based, and resident-oriented. Resident resistiveness to care may be behavioral responses to person-centered care indicating mismatch to individual preferences and needs, warranting adequate awareness and appropriate assessment.

### SESSION 2530 (PAPER)

### COMMUNICATION STRATEGIES AND SENSORY FUNCTION

### IMPLEMENTATION OF MULTIMODAL INTERVENTIONS FOR CAREGIVERS OF OLDER ADULTS WITH VISUAL IMPAIRMENTS

Afeez Hazzan, Pamela Haibach-Beach, and Lauren Lieberman, *State University of New York at Brockport, Brockport, New York, United States*

There is a paucity of research on the impact of caregiving on the quality of life of unpaid caregivers for older adults aged 60 years or older with visual impairments (VI). The purpose of this study is to test multimodal interventions to improve quality of life and well-being in unpaid caregivers of older adults with VI. The objectives were: (1) to implement multimodal interventions targeted towards improving the quality of life of family caregivers of older adults with a VI; (2) to evaluate the efficacy/effectiveness of the interventions in improving the quality of life of older adults with a VI. The outcomes of interest include: quality of life, health, stress, burden, problem-solving, and barriers. For this study, a 10-week virtual intervention was implemented with 12 caregivers and eight older adults with visual impairments, for a total of 20 participants. The intervention was held for one hour for 10 consecutive weeks and included activities such as tai chi, yoga, meditation, music, nutrition, and other de-stressing techniques. Participants completed a series of questionnaires before and after the intervention period. These questionnaires include: Satisfaction with Life Scale, Living Arrangement and Indicators of Social Interaction survey, Caregiver quality of life (EQ-5D), the Perceived Change Index, and the Geriatric Depression Scale. Findings from the data analysis show excellent caregiver engagement throughout, including participation in the pre and post surveys. Results show the benefits of multimodal interventions for caregivers as well as older adults with VI. Future studies should focus on interventions that are most promising.

### STRATEGIES FOR EFFECTIVE COMMUNICATION USING PERSONAL PROTECTIVE EQUIPMENT IN NURSING HOMES: A QUALITATIVE STUDY

Clarissa Shaw[1], Kristine Williams[2], Carissa Coleman[3], and Paige Wilson[4], *1. University of Iowa, Iowa City, Iowa, United States, 2. University of Kansas Medical Center, Lawrence, Kansas, United States, 3. University of Kansas, Kansas City, Kansas, United States, 4. The University of Kansas Medical Center, Kansas City, Kansas, United States*

Care of persons with living with dementia is complicated by using personal protective equipment (PPE). Masks and other facial coverings although protective, prohibit visualization of lip-reading and nonverbal cues and obscure identity. The purpose of this qualitative study was to determine best practices for effective communication while using PPE from the provider (n=10), resident (n=5), and family perspective (n=4). The 19 participants were recruited from four states. Qualitative content analysis identified two major themes: (1) challenges experienced during nursing home pandemic care, and (2) the communication strategies to overcome PPE challenges. Although our interviews were not focused on experiences outside of using PPE, all participants voiced trauma surrounding the lockdown for residents and separation from their families. All participants expressed pessimism surrounding the PPE, but this was also met with feelings of normalization surrounding its use. The communication strategies discussed focused on emphasizing (1) verbal and nonverbal communication skills, and (2) person-centered communication. The general communication strategies recommended were consistent with expert opinions such as using written messages and gestures, using loud speech, using clear speech, identifying self, and varying approaches based on person and context. Person-centered approaches centered on the staff and resident "knowing each other." Specifically, knowing the resident is essential to understanding their personal barriers and facilitators to successful communication and knowing each other in a personal and trusting manner helps overcome barriers caused by PPE. This study provides practical suggestions for best practices in communication and reinforces the need for person-centered communication focused on psycho-emotional care.

### VERBAL COMMUNICATION BETWEEN NURSING HOME STAFF AND RESIDENTS WITH DEMENTIA AND APATHY: LANGUAGE ANALYSIS

Ying-Ling Jao[1], Yo-Jen Liao[2], Diane Berish[1], Marie Boltz[3], Wen Liu[4], Jacqueline Mogle[5], and Helen Kales[6], *1. Pennsylvania State University, University Park, Pennsylvania, United States, 2. Pennsylvania State University, Pennsylvania State University, Pennsylvania, United States, 3. Penn State, Pennsylvania State University, Pennsylvania, United States,*

*4. University of Iowa, Iowa City, Iowa, United States, 5. The Pennsylvania State University, University Park, Pennsylvania, United States, 6. University of California, Davis, Davis, California, United States*

Communication is fundamental in daily caregiving activities in nursing homes and impacts the quality of care. This study examined the verbal communication of nursing home staff and residents with dementia and apathy during daily caregiving activities. This study used a repeated measures design. Participants included 13 residents with dementia and apathy and 13 staff from two nursing homes. A total of 39 videos were recorded to capture staff-resident interactions during caregiving activities (3 videos for each resident). All video-recorded communication was transcribed and segmented into phrases. Bivariate correlations were used for preliminary analysis. The average length of each interaction was 7.5 minutes (range=1.5-12.5). On average, staff verbalized 46.6 words (range=9.3-121.9) with 11.3 phrases (range=3.7-23.4) per minute. Residents verbalized an average of 18.9 words (range=0-83.8) with 4.8 phrases (range=0-17.1) per minute. Unadjusted correlations demonstrated that residents' phrases per minute were significantly negatively associated with apathy (r=-0.36, p =0.0239), while being significantly positively associated with age (r=0.42, p=0.0071), cognitive function (r=0.42, p=0.0114), caregivers' words per minute (r=0.44, p=0.0055), and phrases per minute (r=0.42, p=0.0077). After adjusting for clustering by residents, caregivers' words and phrases remained significantly positively associated with residents' phrases. Findings suggest that higher resident apathy and cognitive impairment are associated with fewer verbalizations. When caregivers verbalize more phrases and words, residents are also likely to use more phrases. Findings identify the high-risk population for lack of verbal expression and emphasize the importance of staff verbal communication to promote verbal communication in nursing home residents with dementia and apathy.

### VISUAL IMPAIRMENT PREDICTS PHYSICAL FUNCTION DECLINE: THE HEALTH, AGING, AND BODY COMPOSITION (HEALTH ABC) STUDY

Atalie Thompson[1], Michael Miller[2], Christopher Webb[3], Jeff Williamson[2], and Stephen Kritchevsky[2], *1. Atrium - Wake Forest Baptist Health, Winston-Salem, North Carolina, United States, 2. Wake Forest School of Medicine, Winston-Salem, North Carolina, United States, 3. Atrium Health Wake Forest Baptist, Winston Salem, North Carolina, United States*

The relationship between visual impairment (VI) and decline in physical function with age is poorly understood. We constructed separate linear mixed models to evaluate the relationship of self-reported (visual function question (VFQ) score) or performance-based (visual acuity (VA); log contrast sensitivity (LCS); stereoacuity (SA)) VI with change in performance on the Short Physical Performance Battery (SPPB) over 8 years in 2219 Health ABC participants. Mean age was 75.5 years (range 71-82); 52.4% were female, and 37.4% were black. For all measures of visual function, better vision was associated with loss of approximately -0.3 SPPB units/year which was similar to the unadjusted change in SPPB over time (-0.328 units/year 95%CI (-0.35, -0.31)). Participants with LCS ≤1.3 log units experienced 58% faster rate of decline versus those with better LCS (test of difference in slopes p< 0.0001). Those with poor VA ≥20/50 showed a 50% greater decline in SPPB (p=0.0029), and those with low SA ≤85 arcsec demonstrated a 33% faster decline (p< 0.001) relative to those with better visual function. Compared to the slope at the mean VFQ score, a 1 standard deviation lower score was associated with 23% greater decline in SPPB (p< 0.0001). The difference in SPPB slopes remained significant across VI measures after adjusting for longitudinal decline associated with age, sex, and black race (all p< 0.05). Both self-reported and performance-based VI predicted faster declines in SPPB over time. Whether older adults with VI might benefit from targeted intervention to prevent declining mobility function remains to be evaluated.

### FUNCTIONAL HEARING AND VISION IMPAIRMENT AND SOCIAL ISOLATION OVER 8 YEARS IN COMMUNITY-DWELLING OLDER ADULTS

Alison Huang[1], George Rebok[1], Nicholas Reed[1], Jonathan Suen[2], Bonnielin Swenor[2], Thomas Cudjoe[3], and Jennifer Deal[2], *1. Johns Hopkins Bloomberg School of Public Health, Baltimore, Maryland, United States, 2. Johns Hopkins University, Baltimore, Maryland, United States, 3. Johns Hopkins School of Medicine, Baltimore, Maryland, United States*

One in four older adults experience social isolation. Sensory impairment is a potentially modifiable risk factor for social isolation. Little is known about the long-term impact of sensory impairment on social well-being. This study quantifies the longitudinal associations between hearing, vision, and concurrent hearing and vision impairment (dual sensory impairment) and social isolation over 8 years among older adults. Data were from the National Health and Aging Trends Study (NHATS), a nationally representative longitudinal study (2011- 2019) of U.S. Medicare beneficiaries. Social isolation was measured by a composite that incorporated four domains: living arrangement, core discussion network size, religious services attendance, and social participation. Baseline hearing and vision were measured by self-report. Associations between sensory impairments and odds of overall social isolation over 8 years were assessed using multivariate generalized logistic mixed models adjusted for demographic and health characteristics. Among 5,552 participants, 18.9% reported hearing impairment, 4.8% reported vision impairment, and 2.3% reported dual sensory impairment. Over 8 years, hearing impairment was associated with 28% greater odds of social isolation. Specifically, participants with hearing impairment were more likely to live alone and limit engagement in social activities. A similar pattern of association was observed for dual sensory impairment; however, estimates did not reach statistical significance. No association was observed between vision impairment and social isolation. Older adults with hearing impairment may be a clinically important subgroup to monitor for social isolation. Interventions for increasing social support and social participation may be especially valuable for older adults with hearing impairment.

### SESSION 2540 (SYMPOSIUM)

### CREATIVE QUALITATIVE RESEARCH METHODS WITH LESBIAN, GAY, BISEXUAL, TRANSGENDER, AND QUEER (LGBTQ+) OLDER ADULTS

Chair: Austin Oswald Co-Chair: Sara Bybee Discussant: Austin Oswald

The voices of lesbian, gay, bisexual, transgender, and queer (LGBTQ+) older adults are very often overlooked in research contexts. Creative qualitative methods have been utilized to study populations who have been neglected, empowering marginalized communities, and fostering equitable research processes and outcomes (Archibald & Blines, 2021; Jen & Paceley, 2021; McGarry & Bowden, 2017). This innovative symposium explores creative qualitative methods of data collection and analysis, such as creative writing and poetry, which have been employed in research about LGBTQ+ aging and also describes how each method may provide a unique contribution to the research process and literature. The first presentation describes the process of facilitating a weekly creative writing group with LGBTQ+ older adults and how creative writing can facilitate the retelling of life events and reimaging of new futures. The second presentation describes the process of analyzing pieces of creative writing in order to elucidate the potential and possibility of queer futurities and their implications for research on aging trajectories and imaginings. The third presentation details how found poetry created from dyadic semi-structured interviews sheds new light on the relationships of LGBTQ+ couples facing cancer. Through these three presentations, we will illustrate how creative methods contribute strengths of generating evocative and poignant narratives, illuminating not-yet-possible futures, and inspiring equally creative interventions. The overall objective of this symposium is to explore creative qualitative research methods for their utility in research with LGBTQ+ older adults, ultimately fostering more inclusive and nuanced research processes and products.

### IMAGINING QUEER FUTURES BEYOND BOUNDARIES: A NARRATIVE ANALYSIS OF CREATIVE WRITING

Sarah Jen[1], and Rebecca Jones[2], *1. University of Kansas, Lawrence, Kansas, United States, 2. Open University, Milton Keynes, England, United Kingdom*

Scholars have called for "queering aging futures" beyond normative assumptions or scripts (Sandberg & Marshall, 2019), which is well-aligned with queer theory's Cruising Utopia which suggests "cruising ahead" toward a queer utopian future that is not yet possible (Muñoz, 2009). Due to emphasis on form rather than content, narrative analyses enable the reimagining of queer futures not bound by material realities. This study presents a narrative analysis of 40 pieces of creative writing in Bi Women Quarterly (BWQ) that examine aging. Authors used writing to queer stories of relationships, activism, and aging. Many used incoherent, non-linear, and dreamlike or omnipotent storytelling to queer narratives, allowing them to "cruise" across time and versions of themselves, imagining futures that were new and unscripted. Narrative analysis allowed researchers to examine choices authors made in taking agency through storytelling. Findings indicate that queer people are well positioned to queer expectations of successful old age.

### USING FOUND POETRY TO EXPLORE SEXUAL AND GENDER MINORITY COUPLES' EXPERIENCES FACING CANCER

Sara Bybee[1], Kristin Cloyes[2], Kathi Mooney[3], Katherine Supiano[1], Brian Baucom[4], and Lee Ellington[5], *1. University of Utah, Salt Lake City, Utah, United States, 2. Oregon Health & Science University, Portland, Oregon, United States, 3. University of Utah College of Nursing, Salt Lake City, Utah, United States, 4. University of Utah Department of Psychology, Salt Lake City, Utah, United States, 5. University of Utah and Huntsman Cancer Institute, Salt Lake City, Utah, United States*

This study explored how relationships of sexual and gender minority (SGM) couples change through the cancer experience. Twelve couples (N = 24) completed surveys assessing demographics and dyadic semi-structured interviews. Thematic analysis was used to analyze interview transcripts. Participants had been together for 19.1 years on average (SD = 9.9, R = 9-44) and commonly described dyadic strength and durability as a result of cancer. Using in-vivo language extracted from the theme dyadic strength and durability, a found poem was constructed depicting how couples saw themselves as two-person teams united against any external stressors. When SGM couples experienced cancer together, it resulted in feeling closer to one another, like they could handle anything that came their way, and assured them that they would stay together regardless of any future hardships experienced. Creative qualitative methods revealed SGM couples facing cancer felt like unyielding, impenetrable, eternal duos with which to be contended.

### CREATIVE WRITING AS A TOOL FOR LESBIAN, GAY, BISEXUAL, TRANSGENDER, AND QUEER (LGBTQ+) HISTORY AND FUTURE MAKING

Lujira Cooper[1], and Austin Oswald[2], *1. Services and Advocacy for GLBT Elders, New York, New York, United States, 2. Goldsen Institute of the University of Washington , Seattle, Washington, United States*

The shift toward embracing creative methods in qualitative research opens new possibilities for gerontologists and older adults to explore the nuances of aging and its affective undertones. This paper describes the process of facilitating a weekly creative writing group for lesbian, gay, bisexual, transgender, and queer (LGBTQ+) older adults and their subjective experiences. Various creative writing practices (e.g., poetry, fiction, short story, biography) facilitates the retelling of life events and reimaging of new futurities. Done in community, it creates opportunities for social connectedness, collective meaning making, and psychosocial and instrumental support. Creative writing is a useful method for describing the LGBTQ+ aging experience not fully realized in gerontology. Our findings demonstrate the utility of creative methods in describing and re-imagining LGBTQ+ aging histories and futures. We argue for more creative methods that re-present the complexities of LGBTQ+ aging.

### SESSION 2550 (SYMPOSIUM)

### DIVERSITY OF EXPERIENCES OF AGING AMONG BIRTH COHORTS AND RACIAL/ETHNIC GROUPS IN NSHAP

Chair: Linda Waite Discussant: Amelia Karraker

Aging, the process of getting older, takes many forms. This symposium uses data from the National Social Life, Health, and Aging Project to study changes and differences between groups in the experience of older adults. Dale and coauthors look at the implications for future mortality of failing to respond to a follow-up interview in an ongoing survey. Those who drop out of NSHAP are more similar to those who died than to those who are reinterviewed, suggesting that survey dropout carries important information about health that has been ignored to this point. Choi and Waite compare social networks and social support for the Silent Generation cohort, born 1920-1947, to the Baby Boom cohort, born 1947-1965, and finds that the BB cohort has fewer kin in their networks and receive less support from family and friends than the older cohort, suggesting that they are more disconnected. Piedra and Iveniuk compare social networks of White, Black and Hispanic older adults. They find that Hispanics are initially more likely to have restricted networks, showing low social connectivity but increase in network diversity over time, suggesting resilience, whereas White and Black move toward lower connectivity as they get old. Iveniuk and Gupta compare marital histories of older adults in different racial and ethnic subgroups, with Blacks least and Whites most likely to be married at older ages, and Hispanics more likely to be recently widowed. These presentations point to the diversity of pathways through aging, and their consequences.

### IMPLICATIONS FOR MORTALITY RISK: CONSEQUENCES OF SURVEY NONRESPONSE IN HOME-DWELLING OLDER ADULTS

Marianne Razavi[1], William Dale[2], Colm O'Muircheartaigh[3], L.Philip Schumm[3], Ashwin Kotwal4, and Linda Waite[5], *1. City of Hope, Duarte, California, United States, 2. City of Hope Comprehensive Cancer Center, Duarte, California, United States, 3. University of Chicago, Chicago, Illinois, United States, 4 University of California, San Francisco, San Francisco, California, United States, 5. National Opinion Research Center (NORC at Chicago), Chicago, Illinois, United States*

Nonrespondents generally suffer from worse health outcomes than respondents. Are they unwilling or unable to respond? Our aim was to address this issue. Data (N=3,130) from 2010-2015 waves of National Social Life Health and Aging Project (NSHAP, W2, W3) was used. Four groups of participants were considered based on their response status at W3: alive, incapacitated, deceased, and nonrespondents. Nonrespondents represented cases with no information at W3, beyond their disability and death information. General linear models were used to compare group means at baseline (W2) in terms of mortality risk (Lee index) or cognitive impairment (MOCA), adjusted for demographic variables. Like the deceased or incapacitated groups, the nonrespondent group displayed significantly worse outcomes (Least Squares Means) than the alive group: Lee index alive=5.82, deceased=9.66, incapacitated=8.29 and nonrespondents=7.80; MOCA alive=21.57, deceased=19.79, incapacitated=19.19 and nonrespondents=19.84. Being a nonrespondent likely indicates incapacity, not reluctance to responding. Earlier follow-up surveys could optimize response rates.

### COHORT DIFFERENCES IN SOCIAL TIES TO FAMILY AND FRIENDS

Won Choi[1], and Linda Waite[2], *1. University of Chicago, Chicago, Illinois, United States, 2. National Opinion Research Center (NORC at Chicago), Chicago, Illinois, United States*

Dramatic changes in family life may have altered the structure and quality of social ties to family and friends. However, little is known about whether and how social relationships vary between older adults from different cohorts. Using data from the National Social Life, Health, and Aging Project, we compared social network composition and social support between older adults at ages 57 to 67 from the Silent Generation cohort (N=2,316) and the Baby Boom cohort (N=1,500). Compared with the Silent Generation cohort, the Baby Boom cohort had significantly higher odds of not listing any kin in their core discussion network. There were no cohort differences in proportion of friends in the network. The Baby Boom cohort also reported lower levels of family and friend support than their counterparts. Results suggest that the Baby Boom cohort is more socially disconnected from friends and particularly family compared with the Silent Generation cohort.

### THE SHIFTING SOCIAL NETWORKS OF OLDER ADULTS: AN ANALYSIS OF VARYING TYPOLOGY BY RACE/ETHNICITY

Lissette Piedra[1], and James Iveniuk[2], *1. University of Illinois at Urbana-Champaign, Urbana, Illinois, United States, 2. NORC at the University of Chicago, Chicago, Illinois, United States*

We explored whether network groupings change over time, and how they vary by race and ethnicity. We draw upon data from 6,489 respondents, collected across three rounds of National Social Life Health and Aging Project (NSHAP). Using Latent Class Analysis to identify groupings of social-relational characteristics, we arrive at: A ‘restricted’ class with overall low social connectivity, an ‘enriched’ class with strong connections across all domains and a ‘diverse’ class showing the greatest network range. In the first round, 49% of Hispanic respondents were in the ‘restricted’ class, compared to 24% of non-Hispanic Whites, and 37% of non-Hispanic Blacks. By the third round, the percentage of Hispanic respondents in the ‘restricted’ class dropped but rose for all other groups. We speculate that, over time, context-specific factors may be contributing to network resilience among Hispanics and network vulnerability among non-Hispanic Blacks.

### MARITAL SEQUENCING ACROSS THE LIFE COURSE: RACE AND COHORT INTERSECTIONS

James Iveniuk, and Riddhi Gupta, *NORC at the University of Chicago, Chicago, Illinois, United States*

In this paper, we undertake an analysis of marital/cohabitation sequences over a person's entire life course, examining intersections between race and cohort. We draw upon data from a nationally representative survey of older Americans (collected in 2005/2006; N=3005). Using optimal matching and cluster analysis, we find three clusters: those who have been without a partner for many years (10%), those who recently lost a spouse (27%), and those who are still married (62%). All three clusters tended to marry young; only 2% never married or cohabited. Non-Hispanic Black respondents were far less likely than Non-Hispanic White respondents to be in the still-married cluster. The oldest Hispanic respondents were also more likely to be in the recently-unpartnered cluster, compared to younger Hispanic respondents. Cluster membership was also associated with being married ten years later (2015/2016; 1535 retained), with the longstanding-unpartnered cluster less likely to be married, compared to the recently-unpartnered cluster.

### SESSION 2560 (SYMPOSIUM)

### EMBRACING DIVERSITY BY FOSTERING STRONG COMMUNITY CONNECTIONS

Chair: Robyn Golden Co-Chair: Erin Emery-Tiburcio
Discussant: Rani Snyder

Age-Friendly Health Systems are essential; however, most health and healing take place outside of the walls of a hospital or physician's office. Thus, it is imperative that health systems foster strong community connections, in particular with older adults from underserved communities who experience multiple barriers to quality, age-friendly health care. At Rush, there are several interdisciplinary initiatives designed to ensure that the 4Ms reach beyond the medical center and impact historically marginalized communities. In this symposium, we present three of these innovative approaches to bringing the 4Ms to into the community. Each presentation will describe the program and community served, explore how the 4Ms concepts are integrated, and present outcomes data on the program impact to date. Facilitating Caregiver Health and Wellness: Age-Friendly Health System Caring for Caregivers (AFHS-C4C) is an initiative to expand the age friendly health system to include caregivers. CATCH-ON Connect enables older adults to do What Matters to them by expanding virtual access to older adults, often from underserved communities. Finally, Rush@Home addresses the 4Ms by removing barriers to care and enabling older adults with medically complex needs to receive care in their home. The interactions of these programs will be discussed briefly by the session chair, along with service and policy implications of multiple programs addressing the 4Ms of an Age-Friendly Health System within one community.

### FACILITATING CAREGIVER HEALTH AND WELLNESS: AGE-FRIENDLY HEALTH SYSTEM CARING FOR CAREGIVERS (AFHS-C4C)

Robyn Golden[1], Diane Mariani[1], Leslie Pelton[2], Teresa Moro[1], and Ellen Carbonell[1], *1. Rush University Medical Center, Chicago, Illinois, United States, 2. Institute for Healthcare Improvement, Boston, Massachusetts, United States*

Family caregivers of older adults are vital partners in health care. The health care system is uniquely positioned to connect with caregivers and help address their own needs. The Age-Friendly Health System Caring for Caregivers (AFHS-C4C) model focuses on achieving health system change by identifying and incorporating caregivers into standard health care practices while assisting caregivers with their own health and well-being. Created at Rush University Medical Center, this model was tested in six AFHS. At Rush, caregivers (n=322) were mostly female (74%) and African American (38%) or white (37%). Between baseline and follow-up, statistically significant ($p<.05$) decreases were observed in caregiver burden (BSFC: 18.51 to 13.82), depression (PHQ-9: 8.40 to 4.09), and anxiety (GAD-7: 7.73 to 5.15). Application of this program at additional sites has yielded important lessons learned about engagement, training needs, and sustainability. These lessons will be discussed in the context of scaling and spreading interventions nationally.

### INCREASING TECHNOLOGY ACCESS TO FOR UNDERSERVED COMMUNITIES: CATCH-ON CONNECT

Erin Emery-Tiburcio[1], Siqi Wang[2], Steven Reaves[2], and Salvador Castaneda[2], *1. Rush University Medical College, Chicago, Illinois, United States, 2. Rush University Medical Center, Chicago, Illinois, United States*

Many older adults from underserved communities do not have access to technology or a reliable internet connection. Even when access is not an issue, older adults often find technology challenging. CATCH-ON Connect provides community-dwelling adults age 65+ with cellular-enabled tablets with pre-installed apps to enable them to reduce social isolation and engage in telehealth visits. This program also provides personalized technical assistance and education about accessing electronic health records and the 4Ms. For example, participants are encouraged to address the 4Ms during telehealth visits. Among 139 participants enrolled, 51% were non-Hispanic Black, 30% non-Hispanic white, 13% Hispanic, and 7% other. Willingness to engage in telehealth appointments increased 9% ($p<.05$), discussions with primary care providers about mobility increased by 13% ($p<.001$), and loneliness scores decreased by 0.6 ($p<.05$) in the first three months of participation. Implementation and policy implications for ongoing technology device and training services will be discussed.

### EXPANDING HOME-BASED PRIMARY CARE FOR MEDICALLY COMPLEX PATIENTS: RUSH@HOME

Walter Rosenberg, Nathaniel Powell, Elizabeth Davis, Leticia Santana, and Robyn Golden, *Rush University Medical Center, Chicago, Illinois, United States*

Medically complex older adults from underserved communities experience multiple barriers to obtaining necessary health care and services. Often these individuals are largely unable to leave their homes due to a lack of community resources facilitating travel to and from provider appointments. Rush@Home is a home-based primary care program that focuses on the 4Ms by incorporating patient navigation and social work services for medically complex older adults. The majority of the patients enrolled in the Rush@Home program identify as Black and/or Latino (>50%) and reside on the West Side of Chicago which is historically underserved in terms of health care access. To date, Rush@Home has enrolled n=234 participants with n=176 patients who are currently active in the program. Among participants there less than a 10% readmission rate and an ED visit rate of approximately 5%. This presentation will discuss the Rush@Home data and the importance of community engagement.

### SESSION 2570 (SYMPOSIUM)

### ESPO AND SOCIAL RESEARCH, POLICY, AND PRACTICE SECTION SYMPOSIUM: WHAT AROUND THE PERSON MATTERS: REIMAGINING ENVIRONMENTAL JUSTICE AND AGING

Chair: Kexin Yu Co-Chair: Sarah Dys

From Lawton's seminal Ecological Theory of Aging (ETA) to the recent development of Wahl's COntext Dynamics of Aging (CODA), conceptual and empirical work has repeatedly shown that the living environments fundamentally influence health and wellbeing in later life. The CODA framework posits five correlated contexts that can predict developmental outcomes in aging: physical, social, service, socioeconomic, and technological contexts. The ongoing COVID-19 pandemic further manifested the lack of environmental justice for marginalized and minoritized older adults, calling for reflection on the paradigm for ecological aging research, practice, and actionable policymaking. This SRPP/ESPO symposium featured emerging and established scholars' work that shed light on reimagining environmental justice for older adults with diverse abilities, backgrounds, and resources. Panelists will share stories of their professional development journeys, highlighting empirical evidence with under-researched, systematically excluded populations and examining new directions in aging and environment research. Topics include accessible and affordable housing, built environment, neighborhood contexts, conceptualizing the community, and the experiences of those most likely to bear the burden of precarious housing in later life. This symposium will hold the space for emerging scholars to learn and discuss short- and long-term practice and policy priorities for promoting environmental justice for older adults and provide tools to conceptualize their research informed through ecological and equity-centered perspectives.

### CONTEXTS OF COGNITIVE DECLINE: MAPPING NEIGHBORHOOD OPPORTUNITIES AND BARRIERS FOR HEALTHY AGING IN PLACE

Jessica Finlay[1], Robert Melendez[2], and Philippa Clarke[1], *1. University of Michigan, Ann Arbor, Michigan, United States, 2. Social Environment and Health Program, Institute for Social Research, University of Michigan, Ann Arbor, Michigan, United States*

Stark geographic variation in later-life health outcomes suggests that local built and social environments are critical in shaping disease and disability, physical and cognitive function, and engagement in everyday life among older adults. This paper presents a community-engaged mapping project aiming to depict the uneven distribution of hazards and amenities relevant to cognitive aging across the United States. Living in neighborhoods with opportunities for social interaction (e.g., coffee shops, senior centers), intellectual stimulation (e.g., museums, libraries) and physical activity (e.g., parks, walkable streets) may slow rates of cognitive decline and reduce risk for Alzheimer's disease. We assembled a community advisory board to translate research findings into a pilot website and interactive map that assesses neighborhoods for cognitive aging resources and amenities. The objective is to increase public awareness and inform public health and policy efforts to ameliorate community barriers and create more equitable opportunities to promote healthy aging in place.

### TOWARD STRUCTURAL CHANGE: ADVANCING PRAXIS ON AGE-FRIENDLY CITIES AND COMMUNITIES

Emily Greenfield, *Rutgers, The State University of New Jersey, New Brunswick, New Jersey, United States*

The global age-friendly cities and communities (AFCC) movement has inspired leaders across the public, private, and academic sectors to re-imagine how environments can better support long and healthy lives. There is growing recognition, however, of structural and other constraints that impede the translation of AFCC aspirations into systematic, comprehensive, impactful, and sustainable action. This paper will describe the importance of researchers' engagement in AFCC praxis to better support the movement toward structural change for aging equity and healthy aging. The paper will present a case example of academic researchers' long-standing involvement in collaborative efforts toward the development and sustainability of age-friendly community initiatives in New Jersey. The case will emphasize the importance of creating synergies across research, teaching, and service activities; cultivating coordinated AFCC efforts across the mico, meso, and macro levels; and developing sustainable structures for deliberate inter-organizational and multi-sectoral partnerships toward short- and long-term goals.

### A JOURNEY INTO HOW PLACE MATTERS

Terri Lewinson, *The Dartmouth Institute for Health Policy and Clinical Practice, Lebanon, New Hampshire, United States*

It is now well known that housing is a significant determinant of health and that systematic inequities shape the influence of home for many people. In this paper, I will present my research program designed to incrementally understand housing-health experiences of low-income residents in hotels, assisted living facilities, and senior housing apartments using qualitative, community-based, and biomedical approaches in Metropolitan Atlanta. Focused through an ecological lens, I will share insights from pedagogical and policy experiences that contributed to (and detracted from) the promotion of environmental justice for marginalized communities using inclusive strategies that were respectful of population identity. In this talk, I will discuss how institutional influences shaped opportunities for me to advance work on healthy housing as a social justice issue and detail my current project exploring air quality in a nontraditional home setting.

### SESSION 2580 (PAPER)

### HEALTHCARE UTILIZATION AND OUTCOMES

### COST OF US EMERGENCY DEPARTMENT AND INPATIENT VISITS FOR FALL INJURIES IN OLDER ADULTS: 2016–2018

Lisa Reider[1], Jason Falvey[2], Safiyyah Okoye[3], and Joseph Levy[1], *1. Johns Hopkins Bloomberg School of Public Health, Baltimore, Maryland, United States, 2. University of Maryland Baltimore, Baltimore, Maryland, United States, 3. Johns Hopkins School of Nursing, Baltimore, Maryland, United States*

Falls are a leading cause of injury among older adults. While numerous studies have estimated the economic burden of falls, how health care spending varies by sociodemographic and injury factors is not well understood. The purpose of this study was to describe the average annual frequency of emergency department and inpatient visits and associated costs for fall injuries among older adults in the United States and identify factors associated with higher cost using data from the 2016-2018 National Inpatient Sample and National Emergency Department Sample. The study cohort included encounters with an ICD-10 external cause of injury code for fall (W00-W19). Number of visits was computed using survey weights. Direct visit cost was estimated from charges, applying cost-to-charge and professional fee ratios. On average, an estimated 2.7 million (95%CI: 2.5-2.9) fall-related ED visits and 1.1 million (95%CI: 1.1-1.2) inpatient visits occurred annually. The annual average cost was $1,105 (95%CI: $1,083-$1,127) per ED visit and $18,047 (95%CI: $17,905-$18,189) per inpatient visit totaling $22.9 billion annually. Higher inpatient cost was associated with age (65-74: $20,258 vs 85+: $16,183), gender (men: $19,541 vs. women: $17,181), and race (White: $17,570 vs. Black/Hispanic: $19,602); higher ED cost was associated with age (65-74: $1,009 vs. 85+: $1,198) and dementia diagnosis ($1,369 vs. $1,073). Fifty five percent of inpatient and 25% of ED visits were for fracture which had higher cost compared to sprains, dislocations, and superficial injuries. Results indicate growing number of fall-related admissions and costs underscoring need for targeted prevention and intervention strategies.

### FORECASTING THE REGIONAL DISTRIBUTION OF HOME CARE PATIENTS USING BIG DATA OF INSURANCE CLAIMS IN JAPAN: 2015 TO 2045

Yasuhiro Nakanishi[1], Yuichi Nishioka[2], Yukio Tsugihashi[2], Tomohiro Kakinuma[1], Tatsuya Noda[2], Tomoaki Imamura[2], and Manabu Akahane[1], *1. National Institute of Public Health, Wako, Saitama, Japan, 2. Nara Medical University, Kashihara, Nara, Japan*

Regional distribution of home care patients and future demand in Japan are unknown. This study aimed to reveal the actual situation of home care patients by region and forecast demand up to 2045. Linked complete health and long-term care insurance claims data on Nara Prefecture (around 1% of the total population and area of Japan) patients aged 75 years or older who received planned and/or urgent medical treatment by physician home visit between April 2015 and March 2016 were extracted and analyzed by sex, age group, and municipality. We calculated the proportion of home medical care utilization and projected the number of home care patients for every five-year period up to 2045 across five administrative areas of the medical service in Nara Prefecture. Data on 12,656 patients, including 1,455 aged 75–79 years, 2,753 aged 80–84, 3,854 aged 85–89, and 4,594 aged 90 years or older, were extracted. The current proportion of patients receiving home medical care (unadjusted for age) by medical service administrative area showed a difference of up to 1.6 times for those aged 90 years or older. Results of forecasting showed a marked increase in the number of patients aged 90 years or older, with overall numbers continuing to increase up to 2040, reaching a maximum of around 25,759 then decreasing thereafter. The future increase in home care patient numbers could vary by age and area, and our findings suggest that public health policy based on the future demand in each area will be required.

### HOW ARE MULTIPLE CHRONIC CONDITION COMBINATIONS DIFFERENTIALLY ASSOCIATED WITH HEALTH SERVICE USE?

Aaron Ogletree[1], and Benjamin Katz[2], *1. National Institutes of Health, Washington, District of Columbia, United States, 2. Virginia Tech, Blacksburg, Virginia, United States*

A growing body of literature describes important advances in the study of chronic conditions, most notably a paradigm shift from the study of individual chronic conditions to the study of multiple chronic conditions (MCCs). Despite these advances, little research has explored MCC combinations, and almost no published research has explored how MCC combinations are related to health service utilization. Using data from the 2018 wave of the Health and Retirement Study, we categorized 16,447 older adults into one of 32 groups using self-reports of five prevalent chronic conditions: arthritis, diabetes, heart problems, hypertension, and respiratory problems. ANOVAs assessed associations between MCC combinations and two self-report measures of health service use: (1)number of medical visits, including emergency room visits, clinic visits, and house calls; and (2)total out-of-pocket costs for major medical expenses. Results show that older adults with more conditions had a greater number of medical visits ($p< 0.0001$). The pattern between total number of conditions and out-of-pocket costs is less clear, though still varied significantly ($p< 0.01$). Findings demonstrate variability in outcomes among MCC combinations with the same total number of conditions. Those in the Heart-Hypertension-Respiratory group averaged 9 medical visits while those in the Arthritis-Diabetes-Heart group averaged 16. Adults in the Diabetes-Heart group averaged $2,546 in out-of-pocket costs, which is nearly double the costs reported by the Diabetes-Hypertension group ($1,254). Findings highlight complex associations of MCC combinations with health service use, and can inform resource allocation, policy priorities, and care planning among providers serving older adults with MCCs.

### POPULATION TRENDS IN HEALTHCARE USE BY MEXICAN ADULTS AGED 60 AND OLDER WITH AND WITHOUT COGNITIVE IMPAIRMENT

Brian Downer, Jose Eduardo Cabrero Castro, and Rebeca Wong, *University of Texas Medical Branch, Galveston, Texas, United States*

Government policies that have greatly expanded health insurance coverage in Mexico have taken place in the context of rapid population aging and an increasing number of older adults living with cognitive impairment. We used data from the Mexican Health and Aging Study to investigate population-level trends in self-reported healthcare use by cognitive status in 2001, 2012, 2015, and 2018. Healthcare measures included having an outpatient procedure, any doctor visits, staying >1 nights in the hospital, and screenings for high cholesterol, diabetes, and hypertension. All outcomes were dichotomized as yes/no. The sample sizes included 6179 (2001), 8924 (2012), 9429 (2015), and 8916 (2018) participants aged 60 and older who completed a direct interview (total N=33,448). Participants with cognitive impairment were identified using five cognitive assessments (2001 n=1000; 2012 n=1273; 2015 n=1467; 2018 n=1372). Generalized estimating equations that adjusted for demographic characteristics and self-reported health conditions were used. The adjusted odds of having spent >1 night in the hospital, outpatient procedures, any doctor visits, and preventive screenings were significantly higher in 2012, 2015, and 2018 than in 2001 regardless of cognitive status. Overall, participants with cognitive impairment had significantly higher adjusted odds for >1 nights in the hospital (OR=1.31, 95% CI=1.20-1.42), but significantly lower odds for any doctor visits (OR=0.81, 95% CI=0.75-0.88), outpatient procedures (OR=0.70, 95% CI=0.57-0.85), and preventive screenings for high cholesterol (OR=0.75, 95% CI=-.70-0.81), diabetes (OR=0.78, 95% CI=0.72-0.85), and hypertension (OR=0.76, 95% CI=0.70-0.82). These results are important to understanding the healthcare needs of Mexico's growing older adult population.

### THE LONGITUDINAL RELATIONSHIPS BETWEEN SOCIAL ISOLATION AND HEALTH OUTCOMES: THE ROLE OF PHYSICAL FRAILTY

Fereshteh Mehrabi, and François Béland, *University of Montreal, Montreal, Quebec, Canada*

Social isolation is a public health issue that is linked to poor health outcomes. However, the mechanisms underlying this association remain unclear. The main objective of this study was to explore whether changes in frailty moderated the relationship between changes in social isolation and changes in health outcomes over two years. We examined the mediating role of changes in frailty when the moderation hypothesis was not supported. A series of latent growth models (LGMs) were used to test our objectives using data from three waves of the FRéLE study among 1643 Canadian community-dwelling older adults aged 65 years and over. Missing data were handled by pattern mixture models with the assumption of missing not at random. We measured social isolation through social participation, social networks, and social support from different sources of social ties. We assessed frailty using the Fried frailty phenotype. Our moderation results revealed that high levels of changes in social participation, support from friends, nuclear, and extended family members, and social contacts with friends were associated with greater changes in cognitive and mental health among frail older adults with diminished physiological reserves compared to robust older adults. Additionally, changes in frailty mediated the effects of changes in social participation and social contacts and support from friends on changes in chronic conditions. This longitudinal study suggests that frailty moderated the relationships between social isolation and mental and cognitive health but not physical health. Overall, social support and strong friendship ties are key determinants of frail older adults' health.

### SESSION 2590 (SYMPOSIUM)

### IMPLEMENTATION AND IMPACT OF MANAGED LONG-TERM SERVICES AND SUPPORTS IN PENNSYLVANIA

Chair: Howard Degenholtz

In 2018, Pennsylvania began implementing a mandatory Medicaid managed care program called Community HealthChoices (CHC). CHC replaces the 1915(c) aging waiver and 4 other waiver programs that covered adults with disabilities and people with acquired brain injury. The new program covers people receiving long-term services and supports (LTSS) in both nursing homes and home and community-based settings as well as people dually eligible for both Medicaid and Medicare. The program is administered by 3 managed care organizations (MCOs) that are obligated to coordinate with Medicaid Behavioral Health, Medicare Advantage and D-SNP plans. The MCOs are incentivized to serve people with LTSS needs in community-based settings by increasing access to HCBS and supporting transitions from nursing homes back into the community. This symposium will present findings from a comprehensive, mixed-methods evaluation of the program. Drawing from our qualitative interviews with key stakeholders and examination of program materials, we will describe some of the challenges of the implementation process. Next, we will share findings from interviews with cohorts of participants conducted before and after implementation of the new program. Next, we will present findings from analysis of Medicaid claims data to examine changes in use of home and community-based services as well as overall rebalancing. Finally, we will present both qualitative and quantitative data on person-centered service planning- a critical requirement for HCBS programs.

### EFFECT OF COMMUNITY HEALTHCHOICES ON PARTICIPANT QUALITY OF LIFE AND PSYCHOLOGICAL WELL-BEING

Howard Degenholtz, *University of Pittsburgh, Pittsburgh, Pennsylvania, United States*

A stratified random sample of participants was interviewed in each region of Pennsylvania during each phase of the implementation. In addition, comparison groups were interviewed from the third implementation region. This allowed us to draw causal inferences regarding the effect of the program on participant quality of life and psychological well-being. The sample was stratified to represent: people age 21-59 and those over age 60 who receive home and community based services (HCBS), plus people age 21 and older who are dually eligible for Medicaid and Medicare but do not use long-term services and supports. We found that engagement in preferred activities both inside and outside the home increased among people who used HCBS and those who did not. In addition, psychological well-being improved slightly while the prevalence of depressive symptoms declined. In general, the implementation of Community HealthChoices appears to have led to improvements in several measures of well-being.

### THE EFFECT OF COMMUNITY HEALTHCHOICES ON USE OF HOME AND COMMUNITY-BASED SERVICES

Keri Kastner, *University of Pittsburgh, Pittsburgh, Pennsylvania, United States*

Medicaid claims data for the years 2013 to 2020 were analyzed to examine use of common categories of home and community-based services (HCBS) as well as the proportion of people living in nursing homes as opposed to receiving long-term services and supports in the community. There was a long-term trend prior to Community HealthChoices of a shift the locus of LTSS away from nursing homes. The implementation of managed care to deliver LTSS continued, but did not appear to accelerate this trend. However, MLTSS did appear to control to growth in hours of personal care per person both in the aggregate and longitudinally within the same individuals over time. There were decreases in the use of adult day services and home delivered meals. However, the decline in home delivered meals was more than offset by an increase in uptake of the supplementary nutritional assistance program (SNAP).

### IMPLEMENTATION OF THE COMMUNITY HEALTHCHOICES PROGRAM

Teresa Beigay, *University of Pittsburgh, Pittsburgh, Pennsylvania, United States*

The Community HealthChoices program was implemented in three phases, starting in 2018 in the SouthWest region of Pennsylvania, followed by the SouthEast in 2019 and the remainder of the state in 2020. Interviews were conducted with participants in each region during the first several months of each implementation. Focus groups were conducted in the months immediately following each phase. We reviewed all participant materials and public education events in each region. The percentage of participants who reported having received information about the new program increased in each phase. This is consistent with our observation that more public education events were held in 2018 and 2019 than in 2017. However, satisfaction with information did not improve over time. Focus groups revealed a mixed picture of successes (new benefit cards were received and claims were paid) and challenges (providers were not aware of the program; selecting primary care providers was confusing).

### THE EFFECT OF COMMUNITY HEALTHCHOICES ON PARTICIPANT SATISFACTION WITH HOME AND COMMUNITY-BASED SERVICES

Todd Bear, *University of Pittsburgh, Pittsburgh, Pennsylvania, United States*

A stratified random sample of participants was interviewed in each region of Pennsylvania during each phase of the implementation using the Consumer Assessment of Health Providers Survey – Home and Community Based Services (CAHPS-HCBS) version. In addition, comparison groups were interviewed from the third implementation region. These data were combined with surveys conducted by the three Community HealthChoices managed care organizations (MCOs). Data were weighted to create geographically representative estimates of participant experience across a range of composite measures. We found that participant ratings of their personal care attendants declined on three out of four measures, however measures of medical and non-medical transportation improved as a result of the implementation of Community HealthChoices. There were notable differences between people of different racial and ethnic groups, with non-Hispanic whites consistently reporting lower levels of satisfaction with person care.

### THE IMPACT OF COMMUNITY HEALTHCHOICES ON PERSON-CENTERED SERVICE PLANNING

John Yauch, *University of Pittsburgh, Pittsburgh, Pennsylvania, United States*

Person-centered service planning (PCSP) is a required component of home and community-based services programs that operate under Medicaid waivers. CMS requires that states have processes in place to assure that participants and their family members (as desired and appropriate) are involved in decisions about their care. This mixed-method study combines interviews with participants and qualitative service coordinators regarding the PCSP process. Participant interviews using the Consumer Assessment of Health Providers Survey – Home and Community Based Services version found that the measure of "choice over services" improved slightly however, the "planning your care" measure was unchanged. Interviews with service coordinators found that the introduction of managed care had led to a system that was overly bureaucratic and focused on medical rather than social needs. This led to a perception that the service plans neglect aspects of daily living that are important for a good quality of life.

### SESSION 2600 (SYMPOSIUM)

### IMPLEMENTATION SCIENCE FOR THE SCALE-UP, SPREAD, AND SUSTAINABILITY OF ASSISTIVE TECHNOLOGIES FOR HEALTHY AGING

Chair: Shannon Freeman Discussant: Simon Carroll

In this symposium we present papers from four key stakeholders on a Michael Smith Foundation for Health Research (MSFHR) Implementation Science Team (IST) that is investigating the application of an emerging approach, the non-adoption, abandonment, scale-up, spread, and sustainability (NASSS) framework (Greenhalgh et al., 2017, 2018), to help predict and evaluate the success of technology-supported health and social care programs in British Columbia (BC). Specifically, our team's intention is to apply the NASSS framework to answer the following research questions: 1) Can the NASSS framework be used to enhance the effectiveness and impact of innovative ATs through improving the equitable scale-up, spread, and sustainability of these technologies for older adults? 2) What are the most important factors in the technology development and implementation process that contribute to equitable scale-up, spread, and sustainability of ATs for older adults? And 3) How can we improve transdisciplinary and intersectoral collaborations to enhance and improve the equitable implementation of ATs for older adults? Based on discussions from team, advisory, and expert panel meetings, a Rapid Realist Review (RRR) of the literature, and an analysis of data collected to date, the speakers will respond to these questions.

### ASSESSING INNOVATIVE ASSISTIVE TECHNOLOGIES FOR OLDER ADULTS; A KNOWLEDGE AND TECH DEVELOPER'S PERSPECTIVES

Robin Syme, *University of Victoria, Victoria, British Columbia, Canada*

Objective: CanAssist is a University of Victoria organization that has been developing assistive technologies (ATs) for almost two decades aimed at developing client-centred broad-impact solutions that address unmet need and help people improve their independence and quality of life. CanAssist's interest and involvement in this study is predicated on our belief that their approach to technology development align with the criteria needed for determining better tools for evaluating assistive technologies need to be developed and implemented. This is critical to our goal of providing successful customized technology solutions to sustain our clients' independence and autonomy.

Methods: From the beginning of the project, as a Research User Co-Lead, CanAssist has actively participated in regular advisory committee and expert panel meetings along with several other research activities to co-create all dimensions of the study.

Results: The results from the Rapid Realist Review and preliminary analyses of the interview data with older adults and caregivers have validated the need for more appropriate assessment/evaluation tools to address the varied AT needs of older adults and their caregivers. In particular, the study has provided opportunities for our staff and clients to examine and discuss important factors/processes for successful AT development and implementation.

Conclusions: As a key partner on this implementation science team, CanAssist will use the study's findings to provide information to our development and management teams on how to appropriately scale-up, spread, and sustain the use of ATs in the health and social care system.

### A RAPID REALISTIC REVIEW TO INFORM AN IMPLEMENTATION PROJECT; ASSISTIVE TECHNOLOGIES WITH CO-DESIGN AND OLDER ADULTS

Matilde Cervantes Navarrete, *University of Victoria, Victoria, British Columbia, Canada*

Objective: This presentation reports on a rapid realist review of participatory co-design approaches to developing assistive technologies with older adults. It provides an evidence synthesis of the key mechanisms, contexts and outcomes that drive success and failure in the use of participatory co-design in this field. This KISSS-AT sub-project, was undertaken to help inform stakeholder partners and the wider gerontechnological community of the key challenges and opportunities, that participatory co-design offers the field.

Methods: We conducted a rapid realist review (Saul et al., 2013). We identified 1060 citations from databases (AgeLine, BSC, CINAHL, MEDLINE, PycINFO, Sociological Abstracts and Web of Science), and 936 from hand journal searches (Ageing and Society, CSCW, Gerontechnology). We screened 311 full-text articles, with 19 articles for extraction. We extracted an additional 9 articles in order to capture post-search publications and a few articles identified through additional snowball citation searches. We analyzed the data for context-mechanism-outcome configurations (Pawson, 2015; Wong et al., 2013) that we found were relevant to our initial program theory, and used analytical induction to test emerging themes with our data set.

Results: We identified two key theoretical dimensions (1. Epistemological 2. Ethico-political) of participatory co-design with older adults, as part of our program theory, along with several C-M-O-Cs related to each dimension.

Conclusions: We found that paying attention to the underlying dimensions of participatory co-design, and the key mechanisms and contexts that support its successful implementation is fundamental to realizing the promise of this approach to gerontechnology development and implementation.

### ASSESSING INNOVATIVE ASSISTIVE TECHNOLOGIES (ATS) FOR OLDER ADULTS: AN EXECUTIVE SPONSOR'S PERSPECTIVE

Manik Saini, *British Columbia Ministry of Health, Victoria, British Columbia, Canada*

The BC Ministry of Health (BC MoH)'s Health Technology Assessment unit is part of the Partnerships and Innovation Division. Our interest in this project concerns the status of ATs and the health technology assessment (HTA) process. The role of AT implementation as a key feature of the potential for new ATs to maximize health impact for older adults is something the Ministry sees as critical to meet the goal of enhancing healthy ageing and sustaining independence and autonomy.

Methods: From the beginning of the project, as Executive Sponsor, the Partnerships and Innovation Division helped to co-create the research program and played a key role in developing partnerships with health authorities and other stakeholders. With monthly meetings and several other research activities, the project has provided multiple opportunities for genuine policy engagement.

Results: The results so far have seen the project extend the scope of its partnerships with key stakeholders in BC. The Ministry was on a local reference group of knowledge users for a rapid realist review of participatory co-design, and Mr. Saini is a co-author on a publication based on that work. The project received additional funding from the BC MoH, and recently the Ministry collaborated on a successful University of Victoria Research Acceleration Fund grant application.

Conclusions: The BC MoH sees this as a key implementation science project, which will provide guidance to the HTA process, as it inevitably confronts increased demand for the public provision of ATs for older adults, particularly vulnerable and disadvantaged groups.

### A KNOWLEDGE USERS' PERSPECTIVE; CONSTRUCTING MEANINGFUL PARTNERSHIPS AT THE IMPLEMENTATION SCIENCE PROJECT

Kahir Lalji, *United Way British Columbia – working with communities in BC's Interior, Lower Mainland and Central & Northern Vancouver Island, Burnaby, British Columbia, Canada*

Objective: United Way of British Columbia (UWBC) mission is to strengthen community capacity to address social issues for British Columbians. We use the Community-based Seniors' Services (CBSS) sector framework to promote healthy aging within the community. Our mission in Population Health is to keep older adults active, connected, and engaged. Assistive technology is very relevant to connect and support older adults. Over the past few years, the development of innovative assistive technologies (ATs) has gained significant momentum and is viewed as an important potential contributor to enhancing healthy ageing and living in place.

Methods: This project uses a Community-Based Participatory Action Research approach focusing on broader stakeholder engagement to enlist with a large array of knowledge users in participatory, collaborative action research processes. As a knowledge-user for this project, we have participated in monthly advisory committee and expert panel meetings and have participated on key team discussions.

Results: We have actively facilitated the development of effective and meaningful partnerships between the academic research community and UWBC and its partner organizations. This partnership has strengthened the capacity for implementation research and the potential for improved outcomes vis-à-vis our healthy ageing mandate in BC.

Conclusions: The UWBC will benefit from the outcomes from this study. We aim to disseminate the research results across our vast network of community organizations through the Collaborative Online Resources Education (CORE) initiative. Additionally, through our work with policymakers, we will support the equitable deployment of ATs to older adults and their caregivers in BC.

### SESSION 2610 (SYMPOSIUM)

### INNOVATIONS IN AGE-FRIENDLY UNIVERSITY (AFU) CAMPUS PRACTICES: ENGAGING COMMUNITY

Chair: Ramraj Gautam

The Age-Friendly University (AFU) global network adopted 10 AFU principles, and AGHE endorsed the AFU principles in 2016. Institutions of higher education are initiating several innovative age-inclusive approaches to teaching, research, and community engagement. This symposium features AFU partner institutions that will discuss their approaches, guided by the AFU principles, for engaging the older adult community to foster age-inclusivity at universities. Guided by the AFU Principle 8 (access for older adults to the University's range of programs) Elfenbein (University of North Georgia) will discuss the design and implementation of the University of North Georgia (UNG) Personal Enrichment, Action and Knowledge Series (PEAKS) program, a novel university and community coalition activity for older adults from the region. The program aims to develop a future life-long learning program. Gautam, et al. (University of Massachusetts Lowell) will discuss their initiative to engage the university's own retired community (AFU principle 9) in promoting intergenerational activities benefiting both students and the University. Melillo, et al. (University of Massachusetts Lowell) will share the qualitative study findings exploring the perceptions of emeritus professors regarding AFU principles 4 and 9. Clark, DiMaria-Ghalili and Gitlin (Drexel University) will discuss the design, implementation, and challenges of launching a pilot grant program at the University that focused on faculty, staff, and students, and integrated the 10 AFU principles. Townsend, et al. (University of Massachusetts Lowell) will highlight the Age Friendly Communities (AFC) and AFU initiatives involving graduate students working with 25 Action Group community-residing adults 50+ (AFU Principle 7 and 10).

### AN AGE-FRIENDLY UNIVERSITY INITIATIVE: EMERITI PROFESSORS' INTEREST IN INTERGENERATIONAL ACTIVITIES

Ramraj Gautam, Karen Devereaux Melillo, Sarawut Sritan, and Montri Khumrungsee, *University of Massachusetts Lowell, Lowell, Massachusetts, United States*

The Age-Friendly University (AFU) Climate Survey (in collaboration with UMass Boston) in 2019 identified the need to engage the University's own emeriti professors; this need reflects the AFU Principle 9. The objective of this study was to explore emeriti professor's perspectives and areas of interest in intergenerational activities. After IRB approval, an online Qualtrics survey was sent to 315 emeriti professors. Eighty-six (27.3%) participants responded to the survey. About 60% reported interest in guest lecturing either online or face-to-face, and 48% reported interest in sharing retirement experiences with students. In addition to descriptive statistics this paper will present the analysis of open-ended questions relating to intergenerational education, research, service activities and their interest in establishing an emeriti association as a venue to connect with the University. The AFU principles will be discussed in the context of engaging emeriti professors in intergenerational relationships benefiting both students and the University.

### EMERITUS PROFESSORS' PERCEPTIONS: QUALITATIVE RESEARCH EXPLORING AGE-FRIENDLY UNIVERSITY PRINCIPLES

Karen Devereaux Melillo, Ramraj Gautam, Sarawut Sritan, and Montri Khumrungsee, *University of Massachusetts Lowell, Lowell, Massachusetts, United States*

In 2020, the UML AFU campus launched the AFU Inventory and Climate Surveys (in partnership with UMass Boston). The results identified a need for campus improvement related to "involving retired faculty in University activities' (Principle 9) and "promoting intergenerational learning to facilitate the reciprocal sharing of expertise between learners of all ages" (Principle 4). An IRB-approved follow-up survey invited respondents to participate in one-hour virtual qualitative interviews to explore the research question: What are the perceptions of Emeritus Professors regarding AFU Principles 4 and 9? Emeritus Professor interviews (n=19) were held in 2021. Four researchers completed the content analyses, which revealed six major themes: promoting intergenerational learning activities; considering emeritus group; being proactive; supporting continued UML relationships; meaning of emeritus and preferred engagement; actual and perceived benefits. The findings will be discussed as to how AFU principles can benefit both Emeritus Professors and students through understanding of this untapped resource.

### THE AGE-FRIENDLY DREXEL PILOT GRANT PROGRAM: A GRASSROOTS APPROACH TO THE AGE-FRIENDLY UNIVERSITY MOVEMENT

Katherine Clark[1], Laura Gitlin[2], and Rose Ann DiMaria-Ghalili[3], *1. Drexel University - College of Nursing and Health Professions, Philadelphia, Pennsylvania, United States, 2. Drexel University, Philadelphia, Pennsylvania, United States, 3. Drexel University, College of Nursing and Health Professions, Philadelphia, Pennsylvania, United States*

In January 2022, Drexel University's AgeWell Collaboratory launched the Age-friendly Drexel Pilot Grant Program to catalyze university-wide knowledge about and projects that will establish our campus as a lifelong community. This suite of funded initiatives includes faculty, staff and students from seven different colleges, each focusing on different components of the 10 AFU principles. We sought out projects that: create learning environments that disrupt ageism; highlight the intersectionality of aging, race, ethnicity and health disparities; foster intergenerational connectivity; and innovate through research. Awarded projects are now increasing access to campus buildings through a design charrette; inventorying university wellness and recreational services to be offered to older community members, alumni, and retirees; developing novel intergenerational courses and experiences abroad through virtual reality, memoir writing and product design; adding older adults to existing community engagement programming; and addressing the technology divide. This presentation will cover the design, implementation, and challenges of this program.

### AFU INNOVATIONS: PEAKS (PERSONAL ENRICHMENT, ACTION, KNOWLEDGE SERIES)

Pamela Elfenbein, *University of North Georgia Institute for Healthy Aging, Oakwood, Georgia, United States*

With careful consideration, including SWOT analysis, demographic projections data, and community input, we envisioned and designed PEAKS – Personal Enrichment, Action, and Knowledge Series. In creating PEAKS our mission was to provide engaging programming designed for the older adults. Our vision was to establish the University of North Georgia, an AFU, as the region's premiere educational and activity resource for older adults in our region. In January of 2021 PEAKS began as monthly series of virtual programs that attracted our intended constituents. During the summer programming paused. During this pause, community members approached the university and asked for PEAKS to expand to include in-person experiences during the summer months. This presentation follows our journey as we research, create, and facilitate the PEAKS programs, both virtual and in-person. We also share our research focusing on the development of PEAKS and goals and the degree to which we achieve them.

### AGE-FRIENDLY LOWELL: INTERGENERATIONAL CONNECTION THROUGH EXPERIENTIAL LEARNING OPPORTUNITIES

Michelly Townsend[1], David Cornell[2], Andrew Hostetler[2], Robin Toof[2], Karen Devereaux Melillo[2], and Sabrina Noel[2], *1. University of Massachusetts Lowell, Wakefield, Massachusetts, United States, 2. University of Massachusetts Lowell, Lowell, Massachusetts, United States*

The rapid growth of the aging demographic requires students entering the workforce to be adept at engaging with older adults in a professional capacity. The Age Friendly Community (AFC) and Age Friendly University (AFU) initiatives present an opportunity for researchers and students to work alongside older residents to improve Age Friendly practices, policies, and infrastructure in their local communities. Age Friendly Lowell developed an Action Group (AG) of 25 Lowell residents aged 50y+, who provide feedback and guidance for 12 graduate students, as they develop measurement tools for a comprehensive community assessment. Students work with AG members to identify, pilot test, and adapt evaluation tools and learn innovative strategies for recruitment. This experiential learning structure is mutually beneficial, as residents lend their expertise, while students learn to echo those concerns throughout their work. This structure serves as a unique and effective model for other communities pursuing AFC and AFU designations.

### SESSION 2620 (SYMPOSIUM)

### INNOVATIVE APPROACHES TO EVALUATING THE LIFESPAN ASSOCIATIONS OF STRESS, HEALTH, AND WELL-BEING

Chair: Lewina Lee

Despite widespread agreement on the importance of stress in health and aging, the mechanisms by which psychosocial stressors influence emotional and physical health are not fully understood. Thorough operationalization of stressor exposure and stress response can contribute to a "common language of stress" (Epel et al., 2018) across disciplines, and a lifespan approach can inform the developmental timing of stress-health mechanisms. Guided by these considerations, this symposium presents five studies led by early-career researchers to delineate stress-health associations across the lifespan. Dr. Olivia Atherton will leverage father-offspring data over 25+ years to examine domains of early life stressors that are susceptible to intergenerational transmission and modifiers of such transmission. Drawing from daily diary bursts embedded within a longitudinal study, Dr. Emily Willroth will report on the role of affective reactivity to daily stressors as potential mediators of the association between childhood psychosocial stressors and all-cause mortality risk. Dr. Meaghan Barlow will present on a novel emotion construct – emotion globalizing – by considering the extent to which emotional response to daily stressors influences global assessment of well-being and age differences in these processes. Dr. Soomi Lee will consider job characteristics linked to 10-year stability and change in sleep health profiles in a national adult sample. Dr. Victoria Marino will illustrate a novel approach to assess the flexibility with which individuals select strategies for coping with stressors and describe its association with mortality. Altogether, this symposium contributes evidence on how psychosocial stressors may shape health and well-being across the lifespan.

### TO WHAT EXTENT IS THERE INTERGENERATIONAL CONTINUITY IN EARLY-LIFE STRESSORS?

Olivia Atherton[1], Eileen Graham[2], Avron Spiro[3], Marc Schulz[4], Robert Waldinger[5], Daniel Mroczek[2], and Lewina Lee[6], *1. University of Houston, Houston, Texas, United States, 2. Northwestern University, Chicago, Illinois, United States, 3. Boston University, Jamaica Plain, Massachusetts, United States, 4. Bryn Mawr College, Bryn Mawr, Pennsylvania, United States, 5. Harvard University, Boston, Massachusetts, United States, 6. Boston University School of Medicine, Boston, Massachusetts, United States*

Prior work has investigated the correlates and consequences of early life stress within a person's lifetime, but less is known about whether early life stressors are sustained across generations. Using multi-generational data from 1,312 offspring and their fathers (N = 518 families), we examined the extent to which there is intergenerational continuity in childhood social class, childhood home atmosphere, parent-child relationship quality, and childhood health, as well as whether person-level and family-level factors strengthen (or weaken) intergenerational continuity. Results suggest notable intergenerational continuity in childhood social class, but no continuity in childhood home atmosphere, parent-child relationship quality, or childhood health. Moreover, the intergenerational continuity of early life stressors was modified by father education level and education mobility, such that low education level conferred risks, and upward education mobility conferred benefits, for offspring adverse experiences. We discuss broader implications of the findings for future research, clinical interventions, and social policy.

### EARLY-LIFE STRESSORS, ADULT AFFECTIVE REACTIVITY TO DAILY STRESSORS, AND MORTALITY RISK

Emily Willroth[1], Jing Luo[2], Eileen Graham[2], Mina Antic[3], Maria Lopes[4], Avron Spiro[5], Daniel Mroczek[2], and Lewina Lee[6], *1. Washington University in St. Louis, St. Louis, Missouri, United States, 2. Northwestern University, Chicago, Illinois, United States, 3. Massachusetts General Hospital and Harvard Medical School, Boston, Massachusetts, United States, 4. Massachusetts General Hospital, Boston, Massachusetts, United States, 5. Boston University, Jamaica Plain, Massachusetts, United States, 6. Boston University School of Medicine, Boston, Massachusetts, United States*

Exposure to stressors in childhood is theorized to sensitize individuals to stressors experienced later in life. One way that stress sensitization might manifest is through greater affective reactivity to daily stressors. In turn, greater affective reactivity to daily stressors has been associated with poorer health and increased mortality risk. The present pre-registered investigation tested greater affective reactivity to daily stressors in later life as a potential mediator of the association between early life stressors and mortality risk in a sample of 144 men from the VA Normative Aging Study. Partially consistent with our hypotheses, greater early life psychosocial stressors were associated with greater positive (but not negative) affective reactivity to daily stressors in later life. However, neither early life psychosocial stressors nor affective reactivity to daily stressors were significant predictors of mortality risk. We will discuss implications of these findings for theories of stress experience across the lifespan.

### AGE DIFFERENCES EMOTION GLOBALIZING: AN EXAMINATION OF BOUNDARY CONDITIONS

Meaghan Barlow[1], Emily Willroth[2], Carsten Wrosch[3], Oliver John[4], and Iris Mauss[4], *1. Wilfrid Laurier University, Waterloo, Ontario, Canada, 2. Washington University in St. Louis, St. Louis, Missouri, United States, 3. Concordia University, Montreal, Quebec, Canada, 4. UC Berkeley, Berkeley, California, United States*

Emotion globalizing is an individual difference variable referring to the extent to which daily variations in an individual's current emotions spillover into their evaluations of life satisfaction. The present research sought to: 1) extend the conception of emotion globalizing to stressor-related emotions, 2) examine age differences in these processes, and 3) differentiate associations with evaluations of life satisfaction and day satisfaction. To do so, we used daily diary data from two adult lifespan community samples [Study 1: N = 133 females, age range = 23-78; Study 2: N = 137, age range = 18-95]. Multilevel models revealed older (compared to younger) adults exhibited less negative (but not positive) emotion globalizing and stressor-related emotion globalizing. No age differences were revealed in the association between stressor-related emotions and day satisfaction. These findings support and extend assumptions underlying the mechanisms of emotion globalizing and inform theories of emotional aging.

### MID-LIFE JOB STRESS AND SUPPORT PREDICT SLEEP HEALTH TRAJECTORIES THROUGHOUT ADULTHOOD

Soomi Lee[1], Claire Smith[1], Tammy Allen[1], Meredith Wallace[2], David Almeida[3], Orfeu Buxton[3], Sanjay Patel[4], and Ross Andel[1], *1. University of South Florida, Tampa, Florida, United States, 2. University of Pittsburgh, Pittsburg, Pennsylvania, United States, 3. The Pennsylvania State University, University Park, Pennsylvania, United States, 4. University of Pittsburgh, Pittsburgh, Pennsylvania, United States*

Good sleep is necessary for healthy aging, but it may be threatened by work stress. This study connected midlife job characteristics to trajectories of sleep health profiles (within-person configurations of key self-reported facets: duration, regularity, sleep onset latency or SOL, insomnia symptoms, feeling unrested, and napping) over one decade. A working adult sample (N=847, Mage=45) of the Midlife in the United States study provided data on sleep and job characteristics in 2004-2006 (T1) and 2013-2016 (T2). Four sleep profiles were consistently identified at both time points: (1) good sleepers, (2) irregular but sufficient, (3) short sleepers, and (4) long SOL/insomnia. Higher job demands at T1 predicted a transition from good or irregular/sufficient sleep at T1 to long SOL/insomnia at T2. Higher workplace social support at T1 predicted maintenance of good or irregular/sufficient sleep over time. Attention to job demands and workplace social support may help promote sleep health.

### COPING VARIABILITY AND ITS ASSOCIATION WITH ALL-CAUSE MORTALITY

Victoria Marino[1], Avron Spiro[2], and Lewina Lee[1], *1. Boston University School of Medicine, Boston, Massachusetts, United States, 2. Boston University, Jamaica Plain, Massachusetts, United States*

Coping strategies – cognitive, behavioral, and emotional tactics used to manage stressors – are associated with morbidity and mortality. Between-strategy coping variability, defined as (un)evenness in usage across coping strategies, may reflect context-specific coping and account for additional variance in health outcomes beyond mean strategy use. This study examined prospective associations of mean coping strategy use and between-strategy coping variability with time to death in 823 men from the Normative Aging Study. In Cox proportional hazard models, 1-SD higher in mean usage of positive action, negative action, prayer, withdrawal, and substance use strategies was associated with 17-32% greater risk of all-cause mortality over 27 years, after adjusting for baseline demographics, health status, and depression. Contrary to prior research, mortality risk did not differ by coping variability. We will consider findings within the stress and coping framework and discuss implications for biobehavioral pathways linking coping to all-cause mortality.

### SESSION 2630 (AWARD LECTURE)

### M. POWELL LAWTON AWARD PRESENTATION AND LECTURE

Chair: Tiffany Washington

The M. Powell Lawton Award Lecture will feature an address by the 2021 recipient David L. Roth, PhD, FGSA, of Johns Hopkins University. This session will also include the presentation of the 2022 M. Powell Lawton Award to recipient Jon Pynoos, PhD, FGSA, FAGHE, of the University of Southern California. The M. Powell Lawton Award is presented annually to an individual who has made outstanding contributions from applied research that has benefited older people and their care. The Lawton Award is generously funded by the Polisher Research Institute of Abramson Senior Care.

### FROM STRESSED CAREGIVERS TO HEALTHY CAREGIVING: THE IMPLICATIONS OF BALANCED PERSPECTIVES AND UPDATED FINDINGS

David Roth, *Johns Hopkins University, Baltimore, Maryland, United States*

Research on family caregiving continues to evolve and stress process models are now frequently balanced by perspectives of benefits emanating from prosocial behaviors including caregiving and volunteering. Initial findings that caregivers have elevated inflammation levels and shorter life expectancies than non-caregivers have been contradicted by numerous more recent findings from larger, population-based, epidemiological studies. In many ways, the caregiving literature shows a bias pattern that is sometimes found in other areas, where initial studies with relatively small samples and alarming results are widely cited, whereas subsequent studies with larger samples and contradicting results are given much less attention. A minority of caregivers are highly stressed, but most caregivers are resilient and face other challenges besides stress-related health problems. Caregivers are the backbone of long-term care, and interventions and policy initiatives to support caregivers are vital, but should be based on replicable findings of verifiable challenges to vulnerable caregiving subgroups.

### SESSION 2640 (SYMPOSIUM)

### METHODOLOGICAL APPROACHES TO GERONTOLOGICAL CANCER RESEARCH

Chair: Sean Halpin

The wide range of gerontological cancer research necessitates a variety of methodological approaches. In our symposium, we bring together researchers who represent varied approaches to studying multiple cancer types—with a focus on demonstrating how to apply different methods. First, Ye, will discuss the use of a unique cross-sequential design to facilitate comparison between health change in long-term older cancer survivors and demographically-matched older adults with no history of cancer. Zanwar, will present disparities in cancer screening using secondary nationally representative complex survey data, provide examples of survey data that can be utilized in aging and cancer prevention and control research, and present challenges and opportunities for using survey data. Von Ah, will discuss research methods pertaining to a series of non-pharmacological clinical trials and offer insight to reducing barriers and improving acceptability to technology-based intervention programs in older breast cancer survivors. Next, Castaneda will present quantitative (i.e., group trajectory modeling, conditional Poisson regression) and qualitative approaches to understand the role of monoclonal gammopathy of undetermined significance in healthcare utilization and progression to multiple myeloma. Last, Halpin will discuss the use of naturally occurring data such as participant observation and audio recordings to evaluate education for patients with multiple myeloma preparing for autologous stem cell transplant. Understanding how a variety of methodological approaches are applied to gerontological cancer research will help facilitate a broader understanding of the tools available for these studies.

### DIFFERENCES IN HEALTH TRAJECTORIES BETWEEN CANCER SURVIVORS AND NONCANCER OLDER ADULTS

Minzhi Ye[1], Eva Kahana[2], Gary Deimling[2], Adam Perzynski[3], and Kurt Stange[4], *1. Kent State University, Cleveland, Ohio, United States, 2. Case Western Reserve University, Cleveland, Ohio, United States, 3. MetroHealth and Case Western Reserve University, Cleveland, Ohio, United States, 4. Case Western Reserve University Center for Community Health Integration, Cleveland, Ohio, United States*

The current study addresses how cancer and aging influence older adults' health trajectories differently. The unique cross-sequential design allowed the study to compare the health changes between long-term (5 years +) older cancer survivors (breast, prostate, and colorectal cancer) and demographic-matched older adults without a history of cancer in the same geographic area within the same period by merging two longitudinal studies. The study also captured comprehensive information on health disparities over time. General linear models were employed. The findings showed that neither previous cancer experience nor aging affects health trajectories in later life. Conversely, comorbidities, being African American, female, having less than a college degree, and living alone significantly decreased the health trajectory in later life for all older adults. Moreover, when compared to other groups, older African American cancer survivors reported low scores in self-reported health after controlling for other conditions.

### ASSESSING CANCER SCREENING DISPARITIES USING SECONDARY SURVEYS: CHALLENGES AND OPPORTUNITIES

Preeti Zanwar, *Jefferson College of Population Health, Philadelphia, Pennsylvania, United States*

According to the WHO, cervical cancer is the fourth common cancer in women, with 90% each of the 604,000 new cases and 342,000 deaths in 2020 occurring in low-and middle-income countries. Cervical cancer can be cured if diagnosed early and treated promptly. Cervical cancer screenings by Pap-tests are evidence-based secondary prevention which are important in diagnosis and for receiving timely treatment for pre-cancerous lesions. I will present intersectional disparities in compliance with the U.S. Preventive Services Task Force guidelines for Pap testing in age-eligible women with disabilities by race/ethnicity using nationally representative Medical Expenditure Panel Survey. I find overall the proportion of women current with Pap testing is significantly lower among women with versus without disability. Additionally, I will provide example of other survey data such as the Behavioral Risk Factor Surveillance System that can be used for cancer screening and prevention and opportunities and challenges for using survey data.

### RANDOMIZED CONTROLLED TRIALS FOR CANCER-RELATED COGNITIVE IMPAIRMENT IN OLDER CANCER SURVIVORS: LESSONS LEARNED

Diane Von Ah, *OSU, Columbus, Ohio, United States*

Breast cancer survivors (BCS) face a myriad of late and long-term symptoms including cancer-related cognitive impairment (CRCI). In fact, up to 75% of the 3.8 million BCS report concerns with memory, processing information speed, and decision-making. It is hypothesized that a subset of vulnerable BCS incur 'accelerated aging' resulting in CRCI with older BCS at greatest risk. CRCI has many downstream negative effects on everyday functioning and health-related quality of life. Despite considerable need, there are currently no effective treatments which have been sufficiently validated for CRCI. Cognitive training, which is based on the principles of neuroplasticity (brain's ability to reorganize and form new neural connections to accomplish tasks), may be a therapeutic option. Clinical trials from our lab and others offer insights into the needs of BCS with CRCI and considerations (facilitators, barriers, acceptability and satisfaction) for older BCS will be highlighted to address this potentially debilitating symptom.

### THE ROLE OF A MONOCLONAL GAMMOPATHY OF UNDETERMINED SIGNIFICANCE DIAGNOSIS IN HEALTHCARE UTILIZATION

Maira Castaneda-Avila[1], Kate Lapane[2], and Mara Epstein[3], *1. University of Massachusetss Chan Medical School, Shrewsbury, Massachusetts, United States, 2. University of Massachusetts Chan Medical School, Worcester, Massachusetts, United States, 3. Meyers Health Care Institute, Department of Medicine, UMass Chan Medical School, Worcester, Massachusetts, United States*

Monoclonal Gammopathy of Undetermined Significance (MGUS) is an understudied precursor of multiple myeloma (MM), the second most prevalent hematologic malignancy in the US. MGUS is incidentally diagnosed, and its significance is unclear as only 1% per year transition to MM. MGUS is highly prevalent among adults aged ≥ 50 years. In this presentation, we will review mixed-method approaches. Using healthcare claims and electronic health records from patients in central Massachusetts, we applied group-based trajectory modeling and conditional Poisson regression. These analyses were complemented by a qualitative analysis of in-depth interviews with providers and MGUS patients. Together, these methodologies provided a comprehensive evaluation of the impact of MGUS on healthcare utilization in older adults. The qualitative analysis provided a better understanding of the patient and provider factors influencing healthcare utilization after an MGUS diagnosis. The presentation will highlight how the use of these methodologies provide different perspectives among understudied premalignant conditions.

### USING NATURALLY OCCURRING QUALITATIVE DATA IN GERONTOLOGICAL CANCER RESEARCH

Sean Halpin[1], and Michael Konomos[2], *1. Evidera, Decatur, Georgia, United States, 2. Emory University, Atlanta, Georgia, United States*

Cancer therapies for older adults have accelerated at breakneck speed in the last few decades, necessitating evaluation of their delivery and uptake to ensure patients receive their maximum benefit. Among the vast array of evaluation tools available, those utilizing naturally occurring data—data produced without intervention from a researcher—are a powerful but underused tool. In this presentation, we will review two methods for examining naturally occurring data, participant observation and conversation analysis (CA), in an educational intervention study of multiple myeloma patients receiving autologous stem cell transplant. First, we will review how participant observation of nurse-led education visits (n=70) was incorporated to iteratively improve video-based education. Next, we will review use of CA in reviewing audio recordings containing reference to the education videos of 12 nurse-led education visits (1011 minutes of audio). Ultimately, understanding the purposes of and ways of using naturally occurring data have potential for improving the evaluation of patient education.

### SESSION 2650 (SYMPOSIUM)

### NARROWING THE DISPARITIES GAP IN LTSS: POLICY AND PRACTICE

Chair: Rita Choula

Access to and the quality of long-term supports and services (LTSS) are not equitable for all older Americans.

Disparities have been documented qualitatively and quantitatively for marginalized racial and ethnic communities and LGBTQI+ communities but the specific causes of gaps in equity differ by community, locality, and state. To be effective, policy solutions must be grounded in the lived experiences of Black, Latino, Asian American and Pacific Islander, and LGBTQI+ older adults in those communities. This symposium showcases how community-based research can be employed to understand the root causes of inequities in LTSS access and care affecting older adults of color and LTGBTQI+-identifying older adults and presents community-grounded policy solutions to remedy those inequities. Papers 1 and 2 use participatory research methods to understand barriers to equitable LTSS care access and quality and develop locally grounded solutions to those barriers. Caldera (Paper 1) shares results from a study of Cook County, IL nursing home residents and their caregivers focused on racial and ethnic disparities in access to and experiences with nursing home care. Hado (Paper 2) presents findings from research in Georgia and New York examining disparities in access to and experiences with HCBS for older racial and ethnic and LGBTQI+ communities. Fashaw-Walters (Paper 3) discusses the how systemic racism is at the root of inequities in LTSS access for communities of color and shares actionable recommendations aimed at ending racial and ethnic inequities in LTSS policies.

### UNDERSTANDING RACIAL AND ETHNIC DISPARITIES IN NURSING HOME CARE IN COOK COUNTY, IL

Selena Caldera, *AARP, Washington, District of Columbia, United States*

Nursing facility data from the Illinois Department of Public Health reveals significant racial disparities in access to high quality nursing homes (NH) for older Black and Latino Illinoisans. While half of all Illinois NH residents live in a 1- or 2- star rated nursing home, 68% of Black NH residents live in such facilities. This study seeks to understand racial and ethnic disparities in access to, quality of, and experiences with care in Cook County, Illinois NHs and develop community-identified solutions to close quality, access, and equity gaps. We employ key informant interviews in a two-stage process that begins by developing a current state analysis of the experience with care through interviews with community stakeholders, including advocacy groups, policy and community leaders and public agencies. Those findings then guide interviews with older Black, Latino, and Chinese NH residents and their caregivers where we identify community-grounded solutions to closing equity gaps.

### NARROWING RACIAL AND ETHNIC DISPARITIES IN HOME AND COMMUNITY-BASED SERVICES

Edem Hado, *AARP, Washington, District of Columbia, United States*

Older adults overwhelmingly prefer to age at home and in their community, yet research shows uneven access to quality home and community-based services (HCBS), especially among diverse racial and ethnic groups. Our research in Georgia and New York employs participatory research methods, including key informant interviews and focus groups conducted in 2021 and 2022, to identify root causes of disparities in access to, quality of, and experience with HCBS for older racial and ethnic and LGBTQI+ communities. Some root causes identified include: complexity in and lack of funding, tension between equal versus equitable service provision, and meaningful community outreach, particularly in Latino communities. We then identify scalable opportunities policy and programmatic interventions to improve care and service equity for older adults in those communities.

### ADDRESSING SYSTEMIC RACISM ACROSS LONG-TERM SERVICES AND SUPPORTS

Shekinah Fashaw-Walters[1], Tetyana Shippee[2], and Jasmine Travers[3], *1. University of Minnesota School of Public Health, Minneapolis, Minnesota, United States, 2. University of Minnesota, Minneapolis, Minnesota, United States, 3. NYU, New York City, New York, United States*

Long-term services and supports (LTSS) are some of the most racially segregated healthcare services in the U.S. today. Marginalized users (e.g., Black and Latino older adults) have disproportionate access to high-quality care and subsequently report poorer health outcomes when compared to White users. It is important to acknowledge racism as a fundamental cause of these inequities to LTSS access. As the U.S. works to expand LTSS, it is critical to diversify and strengthen the LTSS workforce, increase Medicaid reimbursements along with efforts to improve accountability and transparency, reconsider payment models and the use of public reporting, improve quality metrics, implement effective support systems for patients of color, expand access to care, and increase promotion of integrated care. Health equity researchers, Drs. Tetyana Shippee, Shekina Fashaw-Walters, and Jasmine Travers will share 7 actionable evidence-based recommendations for LTSS policy change aimed to dismantle racism and advance health equity.

### SESSION 2660 (SYMPOSIUM)

### PHYSICAL AND MENTAL HEALTH OUTCOMES AMONG OLDER MILITARY VETERANS

Chair: Scott Landes Co-Chair: Janet Wilmoth

Older veterans are a unique health population, with physical and mental health outcomes impacted by the positive health aspects of military social capital as well as the negative aspects of military-related hazards. This symposium focuses on physical and mental health outcomes among older military veterans both before COVID-19, and during the COVID-19 pandemic. Three studies address veteran health outcomes pre-pandemic. Two of the pre-pandemic studies focus on veteran-only samples in order to determine whether aspects of marital quality predicted levels of loneliness, and risk factors for trauma re-engagement among those with medical illness. The third pre-pandemic study examines whether the increased mortality risk observed among older veterans compared to nonveterans varies by combat status. Two studies address veteran health outcomes during the COVID-19 pandemic. The first uses a sample of older veterans with PTSD who were surveyed pre-pandemic and during the pandemic in order to ascertain the mental and physical health impact of the pandemic. The second COVID-19 study uses data from a study of Veterans Affairs' Home

Based Primary Care (HBPC) providers to learn about the best practices discovered to ensure COVID-19 vaccination uptake by some of the most vulnerable older veterans. Results from each of these studies will shed light on policies and practices needed to ensure the best physical and mental health outcomes for older military veterans.

### MORTALITY RISK AMONG OLDER VETERANS AND NONVETERANS: THE IMPORTANCE OF COMBAT STATUS

Scott Landes[1], and Jennifer Piazza[2], *1. Syracuse University, Syracuse, New York, United States, 2. California State University, Fullerton, Fullerton, California, United States*

Over 17.4 million Americans have served in the U.S. armed forces. Although long-term mortality risk is reported to be higher among older veterans than nonveterans, research does differentiate whether there is variation by combat status. This study examined later-life mortality rates among nonveterans, noncombat veterans, and combat veterans. Data were from Wave 2 of the Midlife Development in the United States Survey (N = 4,633). Participants included 3832 nonveterans, 584 noncombat veterans, and 217 combat veterans. Mortality rates did not differ when comparing nonveterans and noncombat veterans. Combat veterans, however, had a higher risk of mortality than did than nonveterans. Combat experience is a determinant of long-term mortality risk among veterans. Future studies should account for combat status when comparing health and mortality between veterans and nonveterans. Because of their heightened mortality risk, combat veterans should be provided with additional services during and after their time in the armed forces.

### EXPLORING MULTIDIMENSIONAL RESILIENCE IN OLDER VETERANS WITH PTSD DURING THE PANDEMIC

Katherine Hall, *Durham VA Health Care System, Durham, North Carolina, United States*

The COVID-19 pandemic and restrictions for physical and social distancing has affected all older adults, but one segment that has unique needs and experiences is older veterans with PTSD. This presentation will explore how PTSD symptomology, and psychosocial functioning in this population have changed compared to pre-pandemic findings. The impact of the pandemic on daily life activities and functional impairment will also be explored. Participants recruited to a wellness clinical trial for older veterans with PTSD at two different timepoints (pre-pandemic, n=54; post-pandemic, n=28) completed PTSD and mental health assessments, and a physical performance battery testing strength, mobility, balance, and aerobic endurance. PTSD symptoms, specifically avoidance and hypervigilance were markedly lower in the post-pandemic sample. Higher levels of physical impairment were observed in the post-pandemic sample, suggesting a need for targeted outreach and health promotion programs among older adults with PTSD symptoms during the pandemic.

### MARITAL QUALITY AND LONELINESS AMONG AGING COMBAT VETERANS: THE MODERATING ROLE OF PTSD SYMPTOMS

Christina Marini[1], Jeremy Yorgason[2], and Anica Pless Kaiser[3], *1. Adelphi University, Garden City, New York, United States, 2. Brigham Young University, Provo, Utah, United States, 3. VA Boston Medical Center, Jamaica Plain, Massachusetts, United States*

Loneliness is a robust predictor of aging veterans' health. Even married older adults may experience loneliness if their relationships are of poor quality. We therefore examined facets of marital quality as predictors of loneliness within a sample of aging veterans: (1) companionship (relationship promotes connection to spouse) and (2) sociability (relationship promotes connection to others). We further evaluated whether veterans' PTSD symptoms moderated these associations. We utilized two waves of data from 269 Vietnam-era combat veterans (M age = 60.5, SD = .73) collected in 2010 and 2016. Upon controlling for baseline loneliness, demographics, and chronic conditions, higher companionship and sociability each predicted lower subsequent loneliness. We detected interactions between companionship and PTSD subclusters. For example, companionship protected against loneliness only for veterans with low and moderate (but not high) avoidance. Findings highlight nuances in how marital quality predicts aging veterans' loneliness, some of which are dependent on PTSD symptoms.

### DISCUSSING AND DELIVERING THE COVID-19 VACCINE: EXPERIENCES IN VA'S HOME BASED PRIMARY CARE PROGRAM

Tamar Wyte-Lake[1], Suzanne Gillespie[2], Chelsea Manheim[3], Aram Dobalian[4], and Leah Haverhals[5], *1. Veterans Emergency Management Evaluation Center (VEMEC), US Department of Veterans Affairs, North Hills, California, United States, 2. VA Finger Lakes Health Care System, Canandaigua, New York, United States, 3. Rocky Mountain Regional VAMC, Aurora, Colorado, United States, 4. Veterans Emergency management Evaluation Center, North Hills, California, United States, 5. VA Eastern Colorado Health Care System, Rocky Mountain Regional VA Medical Center, Denver, Colorado, United States*

COVID-19 vaccines represented a new type of vaccination with specific logistical challenges for distribution and administration. As the Department of Veterans Affairs (VA) embarked on a campaign to vaccinate its Veterans, homebound Veterans presented unique challenges. Therefore, we studied how VA's Home Based Primary Care (HBPC) teams vaccinated Veterans to inform best practices for future vaccination efforts for similar vulnerable populations. From March-May 2021, we fielded a survey to HBPC staff at 145 VA programs nationally, gathering insights from 73 teams. Findings highlighted the importance of vaccine communication and education; the need to prioritize HBPC Veterans receiving vaccines; ironing out vaccine distribution and administration logistics; and that internal and external structures were required to safely vaccinate Veterans in-home.

### ADDRESSING TRAUMA IN OLDER VETERANS IN HOME-BASED PRIMARY CARE

Jennifer Moye[1], Hannah Bashian[2], Hannah Heintz[3], Jennifer Daks[3], Lola Baird[4], Anica Pless Kaiser[5], Kelly O'Malley[1], and Anna Etchin[5], *1. VA Boston Healthcare System, Boston, Massachusetts, United States, 2. VA Boston HCS, Boston, Massachusetts, United States, 3. VA Boston Healthcare System, Jamaica Plain, Massachusetts, United States, 4. Boston VA Medical Center, Jamaica Plain, Massachusetts, United States, 5. VA Boston Medical Center, Jamaica Plain, Massachusetts, United States*

As Veterans age, confronting illness and role changes, they may experience later adulthood trauma reengagement (LATR). To better understand this process in medically ill Veterans, we completed six focus groups with 21 VA Home Based Primary Care (HBPC) mental health clinicians in rural and urban settings; 14 completed a follow-up survey. Thematic analysis of focus groups confirmed the LATR model with unique presentation and treatment challenges related to medical, physical, and cognitive changes. In surveys, the most challenging symptoms were: intrusive symptoms, dissociative reactions, and psychological/physiological reactivity (79%). In addition to focus group themes, surveys described challenges related limited social engagement, Veteran ambivalence, and lack of empirically supported treatments appropriate to HBPC. Results of these focus group and survey data provide key insights into the unique presentation of trauma in aging medically ill Veterans. Ongoing efforts to adapt materials and resources for this population will be described.

### SESSION 2670 (SYMPOSIUM)

### SENSORY LOSS AND BRAIN HEALTH: SPOTLIGHTING JUNIOR INVESTIGATORS IN THE SENSORY HEALTH INTEREST GROUP

Chair: Jennifer Deal Discussant: Heather Whitson

Sensory loss (hearing, vision, touch, smell, taste) is common in older adults, with over 2/3 of adults over age 55 experiencing loss in multiple senses. Sensory loss has been consistently linked to dementia and cognitive decline in epidemiologic studies. However, research gaps exist, with some senses having been studied more widely than others. Given differing underlying anatomy and physiology of the senses, the nature of the pathways linking sensory loss to dementia may vary and some have yet to be elucidated. For some, it may be that they are both caused by a common underlying biology (e.g., neurodegeneration). Alternatively, potential causal mechanisms include increased cognitive load, changes brain structure/function, social isolation and/or reduced activity. Additionally, gaps exist in how sensory loss may impact the lived experience of people with dementia. This session will address these research gaps by highlighting the work of outstanding junior investigators in the GSA Sensory Health Interest Group. The association between touch and dementia will be reported, a relationship which hitherto has not been well described. We will present on the relationship between olfaction and white matter integrity in the brain and describe how sensory function predicts levels of Alzheimer's disease biomarkers over 10 years. We will present evidence for associations between audiometric hearing, structural brain volumes, white matter integrity, and cognitive test performance on speech-in-noise processing and will conclude by describing how hearing loss may impact patient-provider communication among older adults with dementia.

### TOUCH SENSATION IS AN UNDERSTUDIED PREDICTOR OF DEMENTIA RISK IN OLDER ADULTS

Willa Brenowitz[1], Nathaniel Robbins[2], Elsa Strotmeyer[3], and Kristine Yaffe[1], *1. University of California, San Francisco, San Francisco, California, United States, 2. Geisel School of Medicine, Dartmouth, Lebanon, New Hampshire, United States, 3. School of Public Health, University of Pittsburgh, Pittsburgh, Pennsylvania, United States*

Few studies have focused on touch sensation as risk factor or marker of dementia, although other sensory impairments are associated with cognitive decline. We studied touch sensation as measured by peripheral sensory nerve function; impairment was defined as insensitivity to 10-g monofilament or vibration detection threshold >130µm of the toe, in 2,174 Black and White participants (52% women; 35% black, aged 70-79 years) from Health, Aging, and Body Composition Study who were ambulatory and without dementia at enrollment. Incident dementia over the following 11 years was determined based on medical records, cognitive scores, and medications. Impaired touch sensation was associated with a 1.63-fold higher risk of dementia (95% CI 1.21, 2.19) after adjustment for demographics, health behaviors, and health conditions. Associations persisted even after additional adjustment for hearing, vision, and smell (HR: 1.45; 95%CI 1.09, 2.03). These findings highlight the underappreciated association between poor touch sensation and risk of dementia.

### OLFACTORY IMPAIRMENT AND MICROSTRUCTURAL INTEGRITY OF THE BRAIN IN THE ARIC STUDY

Srishti Shrestha[1], Xiaoqian Zhu[1], Kevin Sullivan[2], Beverly Gwen Windham[2], Jennifer Deal[3], Honglei Chen[4], Michael Griswold[5], and Thomas Mosley[2], *1. University of Mississippi Medical Center, Jackson, Mississippi, United States, 2. UMMC-The MIND Center, Jackson, Mississippi, United States, 3. Johns Hopkins University, Baltimore, Maryland, United States, 4. College of Human Medicine, Michigan State University, East Lansing, Michigan, United States, 5. The MIND Center at UMMC, Jackson, Mississippi, United States*

We examined cross-sectional associations between microstructural integrity of the brain and olfaction in 1417 participants from the ARIC Study who completed MRI scans in 2011-2013 (mean age=76±2 years, 41% male). Microstructural integrity was measured by two diffusion tensor imaging measures, fractional anisotropy (FA, higher=better) and mean diffusivity (MD, higher=worse), and olfaction by a 12-item odor-identification test. In multivariable linear regression models, higher FA in several regions was associated with better olfaction, with the strongest association in the stria terminalis [β:0.333 (95%CI:0.188, 0.478) per standard deviation (SD) higher FA]. Higher MD was associated with lower olfaction in almost all regions, but associations were strongest for some temporal sub-regions [for example, hippocampus, β:-0.796 (95%CI: -0.942, -0.651) per SD higher MD]. Our findings suggest that neuronal microstructural integrity is an important predictor of olfaction; this may also have important implications in understanding early dementia neuropathology as olfaction is affected very early in dementia.

### TEMPORAL ASSOCIATIONS OF SENSORY FUNCTION AND BLOOD-BASED NEURODEGENERATIVE AND ALZHEIMER'S DISEASE BIOMARKERS

Natascha Merten[1], Adam Paulsen[2], Richard Chappell[3], Yanjun Chen[2], Karen Cruickshanks[4], Corinne Engelman[5],

Laura Hancock[6], and Carla Schubert[2], *1. School of Medicine and Public Health, University of Wisconsin-Madison, Madison, Wisconsin, United States, 2. Department of Ophthalmology and Visual Sciences, School of Medicine and Public Health, University of Wisconsin-Madison, Madison, Wisconsin, United States, 3. Department of Biostatistics and Medical Informatics, School of Medicine and Public Health, University of Wisconsin-Madison, Madison, Wisconsin, United States, 4. McPherson Eye Research Institute, University of Wisconsin-Madison, Madison, Wisconsin, United States, 5. Department of Population Health Sciences, School of Medicine and Public Health, University of Wisconsin-Madison, Madison, Wisconsin, United States, 6. Department of Neurology, School of Medicine and Public Health, University of Wisconsin-Madison, Madison, Wisconsin, United States*

Sensory changes are risk factors of neurodegeneration and Alzheimer's Disease (AD) but their relations with emerging blood-based biomarkers of neurodegeneration and AD are rather unknown. We assessed long-term temporal relationships of sensory functions and blood-based neurodegenerative and AD biomarkers.This study is based on 10-year follow-up data of n=1529 (primarily middle-aged) Beaver Dam Offspring Study participants. We conducted pure-tone audiometry, visual acuity testing, and quantified serum neurofilament light chain (NfL), total tau, and amyloid beta. Linear mixed-effects and linear regression models were used to determine longitudinal associations and the effect sizes of temporal effects.Preliminary analyses revealed that NfL increased slightly more slowly per year with every 1SD better hearing (-0.3%/year[-0.5%,-0.1%]) and vision (-0.2%/year[-0.4%,0.01%]). Effect sizes of baseline sensory function effects on NfL levels 10-years later were 3-4 times larger than effects of the opposite direction. Longer follow-up is needed to confirm whether hearing changes occur before blood-based NfL changes.

### ASSOCIATIONS OF AUDIOMETRIC HEARING, BRAIN MRI, AND COGNITIVE PERFORMANCE WITH SPEECH-IN-NOISE PERFORMANCE

Kening Jiang[1], Josef Coresh[2], Kathleen Hayden[3], Clifford Jack, Jr.[4], Thomas Mosley[5], James Pankow[6], Frank Lin[1], and Jennifer Deal[2], *1. Johns Hopkins Cochlear Center for Hearing and Public Health, Baltimore, Maryland, United States, 2. Johns Hopkins University, Baltimore, Maryland, United States, 3. Department of Social Sciences and Health Policy, Wake Forest School of Medicine, Winston-Salem, North Carolina, United States, 4. Mayo Clinic, Rochester, Minnesota, United States, 5. UMMC-The MIND Center, Jackson, Mississippi, United States, 6. University of Minnesota, Minneapolis, Minnesota, United States*

Speech-in-noise performance is fundamental to daily communications and comprehensive characterization is needed. We studied 590 dementia-free participants aged 70-84 years, including 428 hearing-impaired participants from the Aging and Cognitive Health Evaluation in Elders (ACHIEVE) study baseline (2018-19), which is a randomized controlled trial partially nested within the Atherosclerosis Risk in Communities (ARIC) Study, and 162 normal-hearing ARIC Visit 6/7 (2016-17/2018-19) participants. The Quick Speech-in-Noise (QuickSIN) test was used to measure speech-in-noise performance. Predictors included (1) Four-frequency better-ear pure-tone average (PTA); (2) Magnetic resonance imaging (MRI) measures (total and lobar volumes, diffusion tensor imaging, white matter hyperintensities); (3) Global and domain-specific (language, memory, executive function) cognitive performance. All predictors were standardized to Z-scores. We used multivariable-adjusted linear regression, adjusting for demographic and disease covariates. PTA and cognitive performance, but not MRI measures, were independently associated with speech-in-noise performance, with PTA explaining the largest variance, indicating the promising role of hearing aids.

### DEMENTIA AND HEARING LOSS: A DOUBLE HIT ON PATIENT-PROVIDER COMMUNICATION?

Danielle Powell[1], and Nicholas Reed[2], *1. Johns Hopkins University, Baltimore, Maryland, United States, 2. Johns Hopkins Bloomberg School of Public Health, Baltimore, Maryland, United States*

Hearing difficulty may adversely impact patient-provider communication and be exacerbated by the presence of other conditions like dementia. We examined the association between reported Alzheimer's disease/dementia and reported difficulty communicating with providers. Using the 2019 Medicare Current Beneficiary Survey, we included participants were aged ≥65 years who reported functional difficulty hearing. Exposure was the presence of reported Alzheimer's disease/dementia. Our outcome is reported difficulty communicating with medical providers. Multivariate logistic regression was used for association between the added presence of dementia and reported difficulty communicating with healthcare providers. Among 5,535 beneficiaries reporting hearing difficulty, diagnosis of Alzheimer's disease or another dementia showed 2.76(95%CI:1.97-3.87) times greater odds of reporting difficulty communicating with providers compared to not reporting dementia. In summary, older adults with reported hearing difficulty and dementia may have increased difficulty communicating with medical providers. Findings suggest hearing management may aid in improving health outcomes for adults with dementia.

### SESSION 2680 (PAPER)

### SUPPORTING PERSON-CENTERED CARE

### ASSESSING PREFERENCES FOR COMMUNICATING WITH TECHNOLOGY: A PERSON-CENTERED APPROACH TO CARE MANAGEMENT

Andrea Sillner[1], Marie Boltz[2], Logan Sweeder[3], and Kimberly Van Haitsma[4], *1. Pennsylvania State University Ross and Carol Nese College of Nursing, University Park, Pennsylvania, United States, 2. Penn State, Pennsylvania State University, Pennsylvania, United States, 3. The Pennsylvania State University, State College, Pennsylvania, United States, 4. Pennsylvania State University, University Park, Pennsylvania, United States*

Technological advances, such as telehealth, have been used to manage the multiple chronic conditions that impact over 25% of the US adult population. Technology-assisted communication (TAC) can help to bridge the gap in effective management of health conditions in the community by patients, informal caregivers, and healthcare providers, while emphasizing person-centered care. The purpose of this project was to develop a new theoretically-derived and evidence based subscale for the Preferences for Everyday Living Inventory (PELI) that addresses preferred TAC approaches for community-dwelling adults over the age of 50 years in the context of multiple chronic conditions (N=297). Results indicated that over 60% of older adults are satisfied with technology-based healthcare communications. In general, older adults in the sample are satisfied with all domains of technology-assisted communication that are asked within P-TAC, including timing, sending and receiving of information, and content of communications. Almost 80% (N=234) indicate that they are satisfied with the content of TAC. This research has lead to the development of assessment items that will allow providers to better assess and then integrate patient preferences for technology communication strategies into plans of care. Potential benefits of understanding preferences for TAC include alignment of chronic care management with preferred strategies which may lead to improvement care congruence and improved healthcare outcomes for the older adult.

### HEALTH LITERACY AMONG OLDER ADULTS IN SWITZERLAND: CROSS-SECTIONAL EVIDENCE

Clément Meier[1], Sarah Vilpert[1], Carmen Borrat-Besson[1], Gian Domenico Borasio2, Ralf J. Jox[3], and Jürgen Maurer[1], *1. University of Lausanne, Lausanne, Vaud, Switzerland, 2, Lausanne University Hospital and University of Lausanne, Lausanne, Vaud, Switzerland, 3. Lausanne University Hospital and University of Lausanne, Lausanne, Vaud, Switzerland*

Despite being widely regarded as a major cause of health inequalities, little is known regarding levels of health literacy among older adults in Switzerland. To fill this gap, this study assesses health literacy and its associations with individuals' social, regional, and health characteristics in a nationally representative sample of adults aged 58 years and older in Switzerland. We use data of 1'625 respondents from a paper-and-pencil self-completion questionnaire that was administered as part of wave 8 (2019/2020) of SHARE in Switzerland. Health literacy is measured using the short version of the European Health Literacy Survey questionnaire (HLS-EU-Q16). We use multivariable regressions to explore how respondents' sociodemographic characteristics are independently associated with health literacy. Overall, 6,8% of the respondents had inadequate health literacy, 24,6% problematic health literacy, and 68,6% sufficient health literacy. There were significant associations between health literacy and individuals' gender, education, economic situation, and self-rated health. Women had higher levels of health literacy than men ($p < 0{,}001$). Moreover, a higher education level ($p < 0{,}001$), fewer financial difficulties ($p < 0.01$), and higher self-rated health ($p < 0{,}001$) were positively correlated with adequate/higher levels of health literacy. One-third of older citizens have difficulties managing health-related issues in Switzerland. These findings call for targeted interventions, such as using simplified health or eHealth information tools, improved patient-provider communication, and shared decision-making, promoting lifelong learnings activities and health literacy screening for older patients to increase low health literacy and mitigate its consequences, thereby alleviating remaining social health inequalities in the Swiss population.

### MULTILINGUAL THEMATIC ANALYSIS USING VARIED TRANSLATIONS AND LINGUISTIC VALIDATION OF CODES: ADVANCING INCLUSION

Orly Tonkikh[1], Elena O. Siegel[1], Anna Zisberg[2], Nurit Gur-Yaish[3], Ksenya Shulyaev[4], Amos Rogozinski[4], and Heather Young[1], *1. University of California Davis, Sacramento, California, United States, 2. University of Haifa, Israel, Haifa, Hefa, Israel, 3. Oranim Academic College of Education, Tivon, Hefa, Israel, 4. University of Haifa, Haifa, Hefa, Israel*

Despite the widely acknowledged cultural diversity among older adults and family caregivers, the representation of people from different linguistic backgrounds in a single study is rare. Single language is a common inclusion criterion, limiting the diversity of samples. Performing cross-cultural qualitative research using several languages within a study requires a systematic data harmonization approach to assure the trustworthiness of the analysis. This paper describes strategies used to establish trustworthiness in the multi-lingual thematic analysis of a dyadic qualitative descriptive study of older adults hospitalized in general hospitals and family members who accompanied them during the hospitalization. Participant interviews (n=22) were conducted in English, Hebrew, and Russian according to individual preferences. Four of the dyads (8 participants) were interviewed in different languages. Based on the template analysis approach, we performed multiple multi-lingual translations and linguistic validation of an inductively identified high-level coding scheme. Each linguistic validation process included a reconciliation of two forward translations and harmonization using back translation, performed for each pair of languages. We describe the rationale for decisions regarding the translation process (the timing of translation after establishing a high-level coding scheme, using a hermeneutic translation approach to achieve conceptual equivalence, variability in the socio-demographic context of the translators, recruitment of both translators and linguists). This study provides principles of a feasible systematic approach to overcome linguistic barriers in caregiving research, providing an avenue for inclusive research among multi-cultural and multi-lingual study samples.

### OLDER ADULTS' PERSPECTIVES OF INDEPENDENCE THROUGH TIME: RESULTS OF A LONGITUDINAL INTERVIEW STUDY

Emily Taylor[1], Julia Frost[2], Victoria Goodwin[2], Susan Ball[2], and Andrew Clegg[3], *1. University of Exeter, Truro, England, United Kingdom, 2. University of Exeter, Exeter, England, United Kingdom, 3. University of Leeds, Leeds, England, United Kingdom*

Understanding how older people experience, and adapt to maintain, independence through time has implications for person-centred care. Quantifiable measures can provide a gauge of change in practice. However, little is known about how older people themselves perceive independence through time, or whether measures used are commensurate with what matters to older people. This study aimed to identify whether and how older adults assimilate their perceptions of independence in response to change through time. Two semi-structured interviews were conducted longitudinally, one year apart, to explore the views of 12 community-dwelling older adults, aged 76-85 years. A constructionist approach using dramaturgical and descriptive codes, facilitated the data interpretation. Sixteen analytical questions guided exploration of participants' perceptions of independence through time. Interview participants felt that common interpretations of independence underestimated, and omitted, important aspects of their experience through time. Some participants questioned the value of instruments that were insensitive to individual values and context. Changes in life trajectories required participants to adapt the form, or means of obtaining independence. The impact of change on participants' sense of independence was value-dependent, informed by the function a participant ascribed to maintaining independence. This study builds on the understanding of independence as a complex and multifaceted construct. The findings challenge the congruence of common interpretations of independence with older people's views, showing areas of commonality and discrepancy. Exploration of independence in terms of form and function provides important understanding about how continuity of function takes precedence to form in determining the maintenance of independence through time.

### RE-EXAMINING PATIENT-CENTERED CARE THROUGH DESIGN PROCESS

Kristine Mulhorn[1], and Shushi Yoshinaga[2], *1. Drexel University, Wallingford, Pennsylvania, United States, 2. Drexel University, Philadelphia, Pennsylvania, United States*

Service design is the adoption of design process to healthcare and other service sectors. This was a transdisciplinary research project in which investigators were faculty members from Graphic Design and Health Administration. In addition, two student research assistants were recruited from undergraduate Graphic Design and Health Science majors. The objective of service design is to involve consumers, designers, and businesspeople in an integrative process, which can be applied to post-acute rehabilitation hospital settings focusing specifically on the experience of those who are 65 and older. The aim of this pilot study was to explore "designing with people rather than just for them", an approach to improve the patient experience. Our first step involved on-site interviews. The patient narratives raised challenges and positive aspects of their interactions with the facility. On our initial site visit, we interviewed five members of clinicians and administrative staff. During the two follow-up visits, our student research assistants interviewed seven patients. Based on our staff member interview findings, we developed a revised set of questions for patients. The questionnaire was divided into three sections related to appointments: pre-arrival, during the visit, and after their appointments. Interview results were summarized in a visual data format and collaborative recommendations were made during the final presentation such as interior layout, wayfinding, online portal and their functionalities. Our findings also confirmed that the interior signage created confusion, promoting frequent questions to staff. These results will engage stakeholders and contribute to a co-designing process that will ultimately improve the patient journey.

### SESSION 2690 (SYMPOSIUM)

### TALKING ABOUT LONELINESS: QUALITATIVE INSIGHTS FROM OLDER ADULTS: IMPLICATIONS FOR RESEARCH, POLICY, AND SERVICES

Chair: Roger O'Sullivan Discussant: Gerry Leavey

The very personal and complex nature of loneliness is too rarely articulated in research papers. Each presenter in this interdisciplinary and international symposium presents insights into loneliness and /or social isolation that can help bridge this gap. Victor (Social Gerontology) using open ended responses from the 2018 BBC Loneliness Experiment, presents how 1480 older people describe loneliness and highlights the need to give more attention to existential loneliness. O'Sullivan (Public Health) presents the results of 18 life story interviews with older adults attending a mental health service. The analysis identified three different typologies of loneliness with specific recommendations for training and services. Phone-based support programs are increasingly being used as a solution for those experiencing loneliness. However, less is known about what aspects are most helpful. Perissinotto (Geriatrics and Palliative care) presents results from 38 qualitative interviews with a focus on barriers and facilitators to implementing a phone-based support intervention, particularly for older adults experiencing loneliness. Cudjoe (Medicine) presents qualitative data from older adults (English, Spanish and Mandarin speaking), living in non-profit affordable housing in 22 different states. Drawing on experiences of their social connections during the COVID-19 pandemic, the paper gives voice to the implications of the loss of common facilities, and opportunities to socialize with other residents, and the increased role technology plays in staying connected. Our discussant, Prof Leavey, a leader in the field of mental health, will reflect on the major themes emerging from these multidisciplinary perspectives, especially what they mean for public health and services.

### LONELINESS ACROSS THE LIFE COURSE; LIFE STORY INTERVIEWS WITH MENTAL HEALTH SERVICES USERS

Roger O'Sullivan[1], Annette Burns[2], Gerry Leavey[3], Jeannette Golden[4], Dermot Reilly[4], and Brian Lawlor[5], *1. Institute of Public Health in Ireland, Belfast/Dublin, Ireland, 2. Institute of Public Health, Dublin, Dublin, Ireland, 3. Ulster University, Belfast, Northern Ireland, United Kingdom, 4. Saint James' Hospital, Dublin, Dublin, Ireland, 5. Trinity College Dublin, Dublin, Dublin, Ireland*

Introduction: The complexity of loneliness and its negative impact on our health and wellbeing is well established. However, the qualitative experience of loneliness over the life course is poorly understood. Method: This presentation, based on 18 life story interviews, with a sample of older adults, who were attending a mental health service and objectively defined as lonely, provides an insight into the personal experiences of loneliness as well as the situations and factors associated with loneliness across the life course.

Results: The analysis identified three distinct typologies of loneliness; those who experienced (1) chronic loneliness across their life (2) those whose loneliness became chronic after a transition e.g. bereavement (3) those whose loneliness remained situational/transitional.

Conclusions: The insights are important to inform both general loneliness services and policy as well as specialist mental health services and training. The presentation demonstrates the importance of a life course approach to addressing and understanding loneliness.

### HOW DO LONELY OLDER PEOPLE TALK ABOUT LONELINESS? PRELIMINARY ANALYSIS OF THE BBC LONELINESS EXPERIMENT

Christina Victor[1], Manuela Barreto[2], and Pamela Qualter[3], *1. Brunel University London, London, England, United Kingdom, 2. University of Exeter, Exeter, England, United Kingdom, 3. University of Manchester, Manchester, England, United Kingdom*

Three types of loneliness, social, emotional and existential, are identified in research, policy and practice. Do these categories reflect the language used by older adults to describe their experiences of loneliness? We use data from the 2018 BBC Loneliness Experiment and focus upon lonely adults aged 60 and older, living in the UK and with a maximum score of 9 on the UCLA loneliness scale. 1619 participants meet these criteria, 1480 provided a response to the question ''What does loneliness mean to you?" Participants ages ranged from 60-94; 90% aged 60-74 and 38% male. Free text answers ranged from 1-189 words, included both subjective (feeling alone) or objective (being alone) words and described social (no one to talk to), emotional (lack of closeness) and existential (lack of purpose) loneliness. Lonely older adults 'talk' about the three different types of loneliness singly or in combinations when explaining what loneliness means to them. We conclude that:- (a) existential loneliness merits more attention as it is less prominent in research compared with other types of loneliness and (b) lonely older adults describe different types of loneliness in the same answer.

### EVALUATING A PHONE-BASED SUPPORT PROGRAM TO ADDRESS LONELINESS: LESSONS LEARNED FROM PARTICIPANTS AND STAFF

Carla Perissinotto, Ashwin Kotwal, Soe Han Tha, Katrina Hough, and Bri Matusovsky, *University of California, San Francisco, San Francisco, California, United States*

Introduction: Phone-based support programs are increasingly being used as a solution for those experiencing loneliness. However, less is known about what aspects of these support programs are most helpful to participants and even less known about the staff and volunteer who work in these programs.

Methods: Mixed methods structured surveys of participants (N=247 baseline, N=147 follow-up), and in-depth qualitative interviews of both participants (N=15, and staff=23).

Results: We present the results from the qualitative interviews with a focus on what barriers and facilitators are to implementing a phone-based support intervention, particularly for older adults experiencing loneliness. Preliminarily, 77% of staff and volunteers felt more connected themselves through their role in the phone-support program. 100% of staff also believe the callers feel less lonely as a result of using the line, and 80% feel they create a meaningful relationship with callers. Themes included and overall sense of need for expansion of these services, while better understanding the optimal length and frequency of calls. Consistent with staff responses, amongst callers, 90% fell more socially connected because they use the telephone support line. Similarly, to staff and volunteers, participants felt their needs were met during calls, but wished the length of calls could be flexible. This demonstrates that the line is beneficial to both participants and staff. There is high satisfaction on the quality of the calls and the connections made, but emerging themes suggest a need to scale services to meet demand at all hours and allow for flexibility in length of calls.

### GAINS AND LOSSES OF OLDER ADULTS LIVING IN SUBSIDIZED HOUSING DURING THE COVID-19 PANDEMIC

Marcela Blinka[1], Suzanne Grieb[2], Katherine Runge[3], Laura Andes[3], Carl Latkin[4], Cynthia Boyd[4], and Thomas Cudjoe[5], *1. JHSOM, Baltimore, Maryland, United States, 2. BEAD CORE, Baltimore, Maryland, United States, 3. Mercy Housing, Denver, Colorado, United States, 4. Johns Hopkins University, Baltimore, Maryland, United States, 5. Johns Hopkins School of Medicine, Baltimore, Maryland, United States*

Social isolation is prevalent among community dwelling older adults. Low income older adults living in subsidized housing may have increased risk for social isolation. To examine resident experiences and perspectives relating to their social connections during the COVID-19 pandemic, we conducted semi-structured interviews with 13 older adults (62+) who are English, Spanish, and Mandarin speaking recruited from a large non-profit affordable housing organization with communities in 22 states. Twelve housing communities were identified based on distributions of socio-demographic factors and prevalence of self-reported social isolation l in the housing community's annual survey of residents in order to maximize site diversity. We used qualitative thematic analysis methods to examined participants' views about their social connections before and during the COVID-19 pandemic, as well as their personal and the housing community's strategies to mitigate experiences of social isolation. Emerging themes include loss of common facilities and opportunities to socialize with other residents due to COVID-19 restrictions, and increased use of technology to stay connected.

### SESSION 2700 (SYMPOSIUM)

### THE EXPERIENCE OF THE COVID-19 PANDEMIC AMONG OLDER ADULTS IN PUERTO RICO

Chair: Denise Burnette Discussant: Yamile Martí Haidar

Older age groups and racial and ethnic populations are more likely to have medical conditions that increase their risks for illness, hospitalization and death from COVID-19. Puerto Rico has a high prevalence of acute and chronic health conditions and the lowest percent of people reporting good / excellent health of all U.S. states and territories. The effects of poor health on the island have been compounded in recent years by economic and political instability and by major natural disasters; yet, as of February 2022, 95% of persons aged 65 and older had received at least one vaccination and 88% were fully vaccinated. This symposium comprises five papers based on a study of personal, social, physical health, and mental health factors associated with COVID-19 in a community sample of 213 adults aged 60 and over in Puerto Rico. The first paper will introduce the study, including its purpose, theoretical framework, and methodology. Next, we will address methodological and ethical challenges of conducting research with older adults during the pandemic and strategies we used to overcome these. The third paper will report on the core focus of the project: to assess the knowledge, attitudes, and practices of older Puerto Ricans toward COVID-19. The fourth paper will focus on mental health effects of loneliness and loss, two of the most challenging exigencies of the pandemic for older adults. Finally, we will consider participants' responses to open-ended questions about how they have coped with the pandemic and made meaning of their experiences.

### OVERVIEW OF COVID-19 STUDY WITH OLDER ADULTS IN PUERTO RICO

Thomas Buckley[1], and Denise Burnette[2], *1. University of Pittsburgh, Pittsburgh, Pennsylvania, United States, 2. Virginia Commonwealth University, Richmond, Virginia, United States*

This presentation will provide an overview of our study with older adults in Puerto Rico during COVID-19. From January through December, 2021, we conducted telephone and face-to-face interviews with a nonprobability sample of 213 adults aged 60+ in 21 of the island's 78 municipalities. Average age of participants was 71.9 (SD=8.8), 55.9% were female and 55.4% had annual household incomes under $12,500. About two in five older adults reported feeling lonely (39.4%) and poor/fair health (39.9%). Fully 65.3% were overweight or obese, 59% reported at least one chronic disease (59.4%) and 25.8% had delayed medical care during COVID-19. KAP index scores were reasonably high and 97.2% of participants were either vaccinated or planned to be. We will also discuss the development, translation and adaptation of the data collection instrument and the important social, economic and political backdrops of successful public health measures during the pandemic in Puerto Rico.

### METHODOLOGICAL AND ETHICAL ISSUES OF RESEARCH WITH OLDER ADULTS IN PUERTO RICO DURING COVID-19

Sunghwan Cho[1], David Camacho[2], Thomas Buckley[3], Julia Vazquez[4], and Kiara Carrasquillo-Sánchez[5], *1. Virginia Commonwealth University, Richmond, Virginia, United States, 2. University of Maryland, Baltimore, Norwalk, California, United States, 3. University of Pittsburgh, Pittsburgh, Pennsylvania, United States, 4. University of Maryland, Baltimore, Baltimore, Maryland, United States, 5. Seneca Family of Agencies, Oakland, California, United States*

Engaging diverse groups of older adults is essential to addressing health disparities. In this presentation, we will describe how we used our research team's networks and knowledge of the target population to engage key stakeholders in our KAP project. We will address our partnerships with clergy, NGO staff and administrators, housing authorities, colleagues at Puerto Rican universities, and older adults who serve as informal community and institutional gatekeepers. We will discuss how these partnerships facilitated our ability to identify and access study sites and participants; conduct telephone and in-person interviews; ensure data quality through training and monitoring; and, assure safety through adherence to COVID-19 protocols. Finally, we will describe key cultural and ethical issues of conducting research with older adults through community partnerships during a pandemic. The presentation has implications for developing beneficial partnerships with local community leaders and enhancing the representation of diverse groups of older adults in research.

### KNOWLEDGE, ATTITUDES, AND PRACTICES TOWARD COVID-19 AMONG OLDER ADULTS IN PUERTO RICO

Todd Becker[1], Thomas Buckley[2], and Denise Burnette[3], *1. University of Maryland, Baltimore, Baltimore, Maryland, United States, 2. University of Pittsburgh, Pittsburgh, Pennsylvania, United States, 3. Virginia Commonwealth University, Richmond, Virginia, United States*

To better understand the dynamics of their health behaviors during the pandemic, we examined older adults' COVID-related knowledge, attitudes, and practices (KAP). KAP theory postulates that individuals acquire knowledge about a health condition which influences their attitudes and beliefs and that these, in turn, lead to health practices. We used hierarchical regression to examine the influence of knowledge and attitudes (Step 1) on practices, controlling for health and relevant covariates (Step 2). The Step 1 association between increased knowledge and better practices ($B = 0.14$, $p = .046$) became nonsignificant in Step 2. Greater worry about contracting COVID-19 remained significant throughout (Step 2: $B = 0.15$, $p = .043$). We further explored subgroup differences within KAP measures via bivariate analyses. For instance, women had significantly higher overall knowledge ($p = .013$), while men had significantly better overall attitudes ($p = .044$). We will discuss implications of such subgroup differences for practice and policy interventions.

### DOES LOSS OF A LOVED ONE TO COVID-19 MODERATE THE ASSOCIATION OF LONELINESS AND MENTAL HEALTH AMONG OLDER PUERTO RICANS?

Matthew Morgan[1], Seon Kim[2], Todd Becker[3], and Thomas Buckley[4], *1. Virginia Commonwealth University, Richmond, Virginia, United States, 2. Virginia commonwealth university, Richmond, Virginia, United States, 3. University of Maryland, Baltimore, Baltimore, Maryland, United States, 4. University of Pittsburgh, Pittsburgh, Pennsylvania, United States*

Loneliness is a well-established risk factor for poor mental health. During COVID-19, loneliness and mental health have been exacerbated by widespread disease-related mortality, suggesting that loss of a loved one may influence this relationship. Using data from 187 older adults in Puerto Rico, we assessed the association between loneliness and mental health and the potential moderating role of loss. Moderated multivariable linear regression results indicated that loneliness was significantly, positively associated with mental health ($B = 1.58$, $p < .001$). Although loss due to COVID-19 was not significantly associated with mental health ($p = .473$), it did moderate the relationship between mental health and loneliness ($B = -1.00$, $p = .048$). The lack of significant association of loss and mental health contrasts with previous research on COVID-19 and warrants further investigation. Nevertheless, the statistically significant interaction suggests that grief should be considered when assessing individual and community support during the pandemic.

### LIFE LESSONS: THE EXPERIENCES OF OLDER ADULTS LIVING IN PUERTO RICO DURING COVID-19

Humberto Fabelo[1], Matthew Morgan[1], Julia Vazquez[2], and Todd Becker[2], *1. Virginia Commonwealth University, Richmond, Virginia, United States, 2. University of Maryland, Baltimore, Baltimore, Maryland, United States*

What life lessons emerge from a group of older Puerto Ricans concerning their experiences with COVID-19. We will review study participants' reports of how they spent their time during the pandemic, what they found most difficult to cope with, and life lessons they learned as a result. We will use Erickson's life course theory of psychosocial development to frame their responses to these questions, including the crises (and strengths) of generativity versus stagnation (care) and ego integrity versus despair (wisdom). We will report evidence of care and wisdom as core ego strengths resulting from their lived experience. We will also review how isolation, sense of community, and key mental and physical health factors seemed to influence how their responses suggest coping strengths. We will discuss our findings in the context of social and cultural norms of current cohorts of older Puerto Ricans, highlighting the salience of community and family relations. (150 words)

### SESSION 2710 (SYMPOSIUM)

### THE HEALTH OF THE DIRECT CARE WORKFORCE: EVIDENCE AND IMPLICATIONS ACROSS LONG-TERM CARE

Chair: Kezia Scales Discussant: Kezia Scales

Direct care workers play a key role in supporting the health and wellbeing of older adults and people with disabilities across care settings—yet their own health risks are largely overlooked. The four papers in this symposium address this critical knowledge gap. First, McCall and colleagues will present a comparative analysis of the health status, health insurance coverage, and healthcare experiences of direct care workers across long-term care using National Health Interview Survey data. Next, Lee et al. will present the trends and characteristics of occupational injuries and illnesses among California's long-term care workers from 2019 to 2020 using California Workers' Compensation data, assessing the impact of COVID-19 on their occupational health. Sterling will characterize the physical and mental health of the direct care workforce before and during COVID-19 using data from the CDC's Behavioral Risk Factor Surveillance Survey, as well as drawing on qualitative and survey-based studies of unionized, agency-employed home care workers in New York. Sterling will also present findings from a pilot feasibility study of an intervention aimed at improving home care workers' well-being. Finally, Quinn et al. will synthesize findings on home care workers' occupational hazards—including needlesticks, musculoskeletal strain, violence and infections—and examine how preventing these risks can improve safety for both workers and clients. The discussant will draw out themes and implications from across these complementary studies, highlighting the importance of safeguarding direct care workers' health as a key step toward improving care quality and outcomes for older adults and people with disabilities.

### CARING FOR CAREGIVERS: HEALTH AND HEALTHCARE EXPERIENCES OF DIRECT CARE WORKERS

Stephen McCall, *Altarum, Saint Petersburg, Florida, United States*

Direct care workers play a significant role in supporting the health of older adults and people with disabilities across long-term care settings—but as a primarily low-income, women of color workforce, they likely face significant health risks themselves. To build understanding on the health of this essential workforce, this paper examines direct care workers' health status, health insurance coverage, and experiences utilizing, accessing, and paying for healthcare services using pooled 2014 to 2018 data from the National Health Interview Survey. We find direct care workers have worse health, lower rates of insurance coverage, poorer access and utilization, and greater cost barriers compared to other healthcare workers. We also observe disparities within the direct care workforce according to industry, gender, and race/ethnicity. This study helps inform efforts to support the health of direct care workers and stabilize the critical services they provide.

### THE PHYSICAL AND MENTAL HEALTH OF HOME HEALTHCARE WORKERS: WHAT DO WE KNOW AND WHAT CAN WE DO ABOUT IT?

Madeline Sterling, *Weill Cornell Medicine, New York City, New York, United States*

This presentation will use data from the 2014-2018 CDC's Behavioral Risk Factor Surveillance Survey to characterize the physical and mental health of home health care workers compared to other similar frontline, low-wage worker groups, before the COVID-19 pandemic. Next, using primary survey data collected in partnership with the 1199SEIU Training and Employment Fund, the health of the workforce during the COVID-19 pandemic will be presented, as well as the workforces' needs. The survey includes data from over 250 home health care workers employed in New York, NY who provided care during the pandemic. Finally, data from a pilot feasibility study of an intervention aimed at improving home care workers' well-being will be presented.

### IMPACT OF COVID-19 ON OCCUPATIONAL INJURY AMONG LONG-TERM CARE WORKERS IN CALIFORNIA

Soo-Jeong Lee, *University of California, San Francisco, San Francisco, California, United States*

Long-term care is the industry with the highest occupational injury and illness rate in the United States. During the Coronavirus Disease 2019 (COVID-19) Pandemic, healthcare workers have been affected physically and psychologically by increased workload and stress. Such increased physical and psychological demands may increase the risk of injury among long-term care workers. The aim of this study is to assess the impact of COVID-19 on occupational injury or illness incidence among long-term care workers. Analyzing California Workers' Compensation (WC) data reported in 2019 and 2020, the study will report the number and rate of WC claims among California long-term care workers by month and examine changes over time during 2019-2020. The study will also characterize WC claims during 2019-2020 by demographic (age, gender), occupational (occupation, job tenure), and injury or illness characteristics (cause of injury, nature of injury, affected body part) and identify characteristics with greater changes during COVID-19.

### HEALTHY AGING IN PLACE REQUIRES A HEALTHY HOME CARE WORKFORCE: THE SAFETY AND HEALTH OF PAID CAREGIVERS

Margaret Quinn[1], Pia Markkanen[2], Susan Sama[2], Catherine Galligan[2], John Lindberg[3], and Rebecca Gore[2], *1. University of Massachusetts Lowell/College of Health Sciences, Lowell, Massachusetts, United States, 2. University of Massachusetts Lowell, Lowell, Massachusetts, United States, 3. University of Massachusetts Lowell,, Lowell, Massachusetts, United States*

Home care (HC) aides provide health and personal care services that enable older adults to live at home as they age. Aides are increasingly in demand as our population ages rapidly. Yet there is an aide shortage due in part to instabilities in HC work organization and to occupational safety and health (OSH) hazards, resulting in aides losing work time or leaving their jobs. Aide injuries and illness interrupt the continuity of care delivery and HC hazards can put clients and family caregivers at risk. This research synthesizes recent findings of aide OSH studies in order to identify risks and preventive interventions to improve both HC aide and client safety. Mixed methods were used to characterize aide OSH hazards including needlesticks, musculoskeletal strain, experiences of violence, COVID-19 and other infections and harmful disinfection practices. Improving aide OSH can contribute to improved care services enabling older adults to live at home.

### SESSION 2720 (SYMPOSIUM)

### THE ROLE OF CAREGIVERS DURING DISASTERS

Chair: Tamar Wyte-Lake Co-Chair: Leah Haverhals

The COVID-19 pandemic laid bare the critical role caregivers play in supporting the older adult population, and how easily care structures can fall apart under the stress of a disaster event. As our population rapidly ages, it is imperative to better understand how to support caregivers to ensure relationships between caregivers and older adults remain robust and guarantee everyone's safety. Therefore, this symposium focuses on the roles of formal and informal caregivers during disasters, primarily the COVID-19 pandemic. Dr. Richard Chunga will present findings from a survey of homecare aides, exploring drivers of high turnover rates and describing how employers can improve job satisfaction. Dr. Lindsay Peterson will describe barriers and facilitators to disaster preparedness among caregivers, using interviews with caregivers from diverse backgrounds in Florida. Ms. Jessica McLaughlin (PhDc) will share experiences of informal female caregivers, derived from interviews across the United States (US). Chelsea Manheim (LCSW) will describe adaptations to care provision for rural Medical Foster Home (MFH) Veterans from interviews conducted with MFH care providers from across the US. Dr. Carrie Wendel-Hummell will share data around the strengths and challenges of the self-directed care model for home-based long-term care, drawing on interviews with consumers, caregivers, workers, and providers in Kansas. As a whole, these presenters will provide insights into experiences of caregivers as they navigated challenges associated with the COVID-19 pandemic and generate forward thinking on how to inform future disaster response. Sponsoring SIGs: Paid Caregiving, Family Caregiving, Assisted Living, Disaster and Older Adults

### FACTORS ASSOCIATED WITH HOME CARE AIDES' JOB SATISFACTION DURING COVID-19

Richard Chunga[1], Verena Cimarolli[2], Natasha Bryant[2], Kathrin Boerner[1], and Robyn Stone[2], *1. University of Massachusetts Boston, Boston, Massachusetts, United States, 2. LeadingAge, Washington, District of Columbia, United States*

The COVID-19 pandemic has pronounced already high turnover rates among home care aides (HCAs). High turnover negatively affects home care quality. Job satisfaction among HCAs is a major driver of turnover, but little is known about factors associated with HCAs' job satisfaction during COVID-19. Using survey data, the purpose of this study was to identify variables associated with job satisfaction among HCAs during the pandemic (N=114). Correlational analyses show that lower job satisfaction is associated with experiencing financial hardship, lack of PPE and employer protocols/guidance, understaffing, lower quality of employer communication related to COVID-19, and HCAs' lower perceived preparedness to care for clients with COVID-19. In a regression analysis, experiencing financial hardship and lower quality of employer communication remained significant predictors. Findings underscore the importance of employer supports in HCAs' job satisfaction and provide important lessons for how employers can support HCAs during the pandemic and beyond.

### BARRIERS AND FACILITATORS OF DISASTER PREPAREDNESS FOR CAREGIVERS OF PERSONS WITH DEMENTIA

Lindsay Peterson, Debra Dobbs, Sara Hackett, and William Haley, *University of South Florida, Tampa, Florida, United States*

Emergency preparedness for disasters such as hurricanes is critical. A common feature of disasters is their disruption of daily life, which is magnified for persons living with Alzheimer's disease and related dementias (ADRD). It is critically important to understand more about disaster preparedness for those with ADRD and the informal caregivers responsible for their safety. We conducted individual interviews between April and September, 2021. The sample included 50 family caregivers of persons with dementia (11 Hispanic, 12 Black, 27 White), who were asked about their disaster experiences, concerns about future disasters and level of preparedness. Interviews were transcribed and coded using a team coding approach. Initial analysis identified three main themes, 1) caregivers attitudes about the importance of disaster preparedness, 2) what makes it difficult to prepare, and 3) facilitators of preparedness. Results have the potential to guide program development to improve preparedness among diverse caregivers of persons with ADRD.

### THE ROLE OF WORKPLACE POLICY ON EMPLOYED FEMALE CAREGIVERS DURING THE CORONAVIRUS PANDEMIC

Jessica McLaughlin, *Knoebel Institute for Healthy Aging , Denver, Colorado, United States*

The coronavirus pandemic has indelibly impacted society over the past two years. This qualitative study explores how working female caregivers, in particular, experienced the pandemic and how the workplace supported them during this time. Findings from interviews with 29 working female caregivers revealed that many caregivers were unable to set boundaries around caregiving during the pandemic. Caregivers frequently found themselves on their own in providing care. This meant that caregivers had little time to themselves to rest and recharge. Whereas prior to the pandemic, caregivers may have had help with caregiving through services like respite and adult daycare, these services were no longer options. This reduced level of external support and care influenced caregivers' socioemotional wellbeing. Workplace policies, such as the ability to work remotely and working flexible hours, helped to ease caregiver burden. These findings have implications for both caregivers and workplaces during future crises and disasters.

### CARING FOR HOMEBOUND VETERANS DURING COVID-19 IN THE US DEPARTMENT OF VETERANS AFFAIRS MEDICAL FOSTER HOME PROGRAM

Chelsea Manheim[1], Maya Katz[1], and Leah Haverhals[2], *1. Rocky Mountain Regional VAMC, Aurora, Colorado, United States, 2. VA Eastern Colorado Health Care System, Rocky Mountain Regional VA Medical Center, Denver, Colorado, United States*

The COVID-19 pandemic made older, homebound adults with multiple chronic conditions increasingly vulnerable to contracting the virus. The United States (US) Department of Veterans Affairs (VA) Medical Foster Home (MFH) program cares for such Veterans residing in private homes of non-VA caregivers. In this qualitative study, we assessed adaptations to delivering safe and effective healthcare during the early stages of the pandemic for Veterans living in rural MFHs, interviewing (n=37) VA MFH care providers at 19 MFH programs across the US. We identified themes reflecting adaptations to care provision, including care providers increasing communication and education to caregivers who prioritized Veteran safety. Caregivers coordinated increasing telehealth visits, applied creative strategies to mitigate social isolation of Veterans and themselves, and Veterans were prioritized for in-home COVID-19 vaccinations. Study findings illustrate the importance of clear, regular communication and intentional care coordination to ensure high quality care for vulnerable, homebound populations during crises.

### SELF-DIRECTED CARE IN HOME-BASED LONG-TERM CARE DURING THE PANDEMIC: POLICY AND PRACTICE IMPLICATIONS

Carrie Wendel-Hummell[1], Tracey LaPierre[1], Darcy Sullivan[1], Jennifer Babitzke[1], Lora Swartzendruber[1], and Danielle Olds[2], *1. University of Kansas, Lawrence, Kansas, United States, 2. St. Lukes Hospital, Kansas City, Missouri, United States*

The COVID-19 pandemic highlighted strengths and challenges of the self-directed care model for home-based long-term care. We discuss policy and practice implications drawing on interviews with over 50 home-and-community-based-services consumers, caregivers, workers, and providers in Kansas. Low-pay, lack of benefits, rising wages in competing sectors, enhanced unemployment and COVID-19 concerns exacerbated workforce shortages that compromised consumer safety and well-being. The lack of budget authority for self-directed consumers in Kansas limited their ability to address these issues. Furthermore, the self-directed model was excluded from emergency funding sources that would have enhanced pay and benefits for workers, including sick pay for quarantine, pointing to the need for targeted funding. Emergency flexibility allowing paid family caregivers addressed care needs for some but is temporary and should be expanded. In the managed care model, MCOs still kept their capitated payment despite significant unfilled care hours, and thus pay-for-performance incentives need to be revisited.

### SESSION 2730 (SYMPOSIUM)

### WORKING WITH COMMUNITIES TO INCREASE DIVERSITY, EQUITY, AND INCLUSION IN GERONTOLOGICAL RESEARCH

Chair: Stephanie Chow, Co-Chair: Ronica Rooks

Gerontological researchers are encouraged to include older adults from underrepresented populations in their research and to think broadly about how they conceptualize diversity, equity, and inclusion (DEI) efforts in their work. This includes not only research on older adults from minority racial and ethnic communities, but also lesbian/gay/bisexual/trans*/queer+ (LGBTQ+) communities, as well as other cultural, religious, and socioeconomic groups. One of the most successful ways for reaching older adults in these populations is through community-based participatory research (CBPR). CBPR is a partnership approach to research that equitably involves community members, organizational representatives, researchers, and others in all aspects of the research process, with all partners in the process contributing expertise and sharing in the decision-making and ownership. Two papers in this symposium use CBPR to identify and include older adults from minority populations in their studies. A third paper reports on working with a Community Advisory Board to recruit bereaved dementia caregivers for research. The final paper walks us through steps to increase DEI efforts by meticulous research question development and thoughtful determinations regarding literature review terminology, recruitment efforts, data collection, and reporting and dissemination techniques.

### CONDUCTING DIVERSITY, EQUITY, AND INCLUSION-INFORMED RESEARCH WITH OLDER ADULTS

Ashley Taeckens-Seabaugh, *University of Denver, Denver, Colorado, United States*

Researchers are encouraged to think broadly and holistically about how they conceptualize diversity, equity, and inclusion (DEI) efforts in their work. Each element of a research project must be rigorously devised to identify or address existing gaps in the literature, which will continue to be increasingly important as the US population of older adults continues to expand and diversify. Intentionality throughout every stage of a project is bound to result in a better understanding of the focus populations' needs, and how to best address those needs through their perspective. Moreover, consider weighing the pros and cons of working to implement culturally competent versus multiculturally competent research and whether either approach is as culturally appropriate and responsive as possible. This work summarizes guidance to increase DEI efforts by meticulous research question development and thoughtful determinations regarding literature review terminology, recruitment efforts, data collection, and reporting and dissemination techniques.

### LGBTQ+ OLDER ADULTS AND COMMUNITY NEEDS IN METRO DETROIT: ADAPTING METHODS TO EVOLVING CONDITIONS

Angela Perone, *University of California - Berkeley, Berkeley, California, United States*

Gerontological researchers have long called for more research on diverse older adults, including diverse communities of lesbian/gay/bisexual/trans*/queer+ (LGBTQ+) older adults (e.g., race/ethnicity, economic status, religion). This paper identifies lessons learned from a community-based participatory action research (CBPR) project with LGBTQ+ older adults across four counties. Initiated by LGBTQ+ older adults of color and transgender older adults concerned about access to affordable and culturally responsive housing as they age, researchers worked with a coalition of 8 LGBTQ+ focused organizations to design, implement, and analyze a mostly online survey (n=270). Topics include: Capturing diverse perspectives in survey design (8 focus groups, N=35); addressing external threats to data integrity; implementing multiple types of outreach in phases to expand participant diversity; and working with the coalition and other partners through different stages of survey design, recruitment, data analysis, and identification/enactment of goals for change informed by data, while also navigating a pandemic.

### CBPR: AN EFFECTIVE APPROACH TO RECRUIT AND RETAIN MINORITY OLDER REFUGEE ADULTS IN COMMUNITY-BASED RESEARCH

Sudha Shreeniwas, *UNC Greensboro, Greensboro, North Carolina, United States*

Community based participatory research (CBPR) builds mutually beneficial partnerships between diverse communities and researchers, yielding improved communication among stakeholders, more interest in research participation among community older adults, and improved insights on health disparities. CBPR approaches have great utility for culturally appropriate research design, data collection and analyses, and dissemination with minority underserved communities. We describe outreach, research methods, and community capacity-building from three projects with refugee communities in Greensboro, NC: the Montagnard Hypertension Research Project; The Refugee Older Adults Needs Assessment Project; and the Elder Abuse and Neglect exploratory study. These projects were initiated by community leaders, with technical partnership provided by university researchers and students. We used community-centric methods, and collected a range of data including interviews, photographs, oral histories, surveys, and biological specimens. These efforts have advanced social justice and health equity agendas for both academic and community partners, working to advance health of older adults.

### RECRUITING THE ULTRA-INVISIBLE: COMMUNITY RECOMMENDATIONS FOR FORMER DEMENTIA CAREGIVER RECRUITMENT

Zachary Baker, *Arizona State University, Tempe, Arizona, United States*

Bereaved dementia caregivers often refer to themselves as "Ultra-Invisible" (contrasting with caregivers' "Invisibility"). We report on a series of open-ended discussions with the "Supporting Dementia Caregivers After Death" community advisory board (CAB) about how best to recruit bereaved dementia caregivers for research. The 12-member CAB includes current and former caregivers of

PLWD due to early- and normal-onset Alzheimer's, Lewy body, and Parkinson's, an Alzheimer's Association senior program manager, the Indian Health Board of Minneapolis Spiritual Health leader, the Engaging communities of Hispanics/Latinos for Aging Research Network leader, and a Federally Qualified Healthcare Center Equity Director. Strategies included: partnering with local religious institutions who may be primary source of bereavement services across cultures, multiple survey formats (e.g., recruiting Latino caregivers by phone; written surveys for Indians on rural reservations with limited phone/internet access). 3) Broadened age group considerations (e.g., one African American CAB-member's young children contribute meaningfully to caregiving).

### SESSION 2740 (SYMPOSIUM)

### INTERDISCIPLINARY PUBLIC POLICY DISCUSSION SESSION

Chair: George Taffet Discussant: Brian Lindberg

This interactive session is an interdisciplinary look at policy issues in aging with the speakers representing the four sections of GSA, ESPO, and AGHE. This session, organized by the GSA Public Policy Advisory Panel, will provide both GSA section leadership and attendees an opportunity to have an open dialogue on important public policy issues of significance in the field of aging.

### SESSION 2750 (SYMPOSIUM)

### HOW OLD DO YOU FEEL? CONSIDERING THE CONTEXTS, DYNAMICS, AND ASSESSMENT OF SUBJECTIVE AGE

Chair: Anna Kornadt Co-Chair: Jennifer Bellingtier

How old people feel is a highly effective predictor of later life health and well-being. Despite a wealth of research, the developmental dynamics of the construct as well as its antecedents and consequences are not well understood. Our symposium brings together research that models dynamic trajectories in subjective age over long- and short periods of time and links it to psychological constructs and objective indicators of health and functioning. First, Weiss and colleagues present longitudinal findings of subjective age trajectories in a lifespan sample that highlight the reciprocal dynamics between subjective age and social contexts. Bellingtier and colleagues link the age people feel on a daily basis to the age people want to feel and find that when people felt closer to the age they desired, their affect was more positive. Rupprecht and colleagues measured subjective age as well as affect, stress and physical activity on 21 consecutive days. Data attest to the relevance of daily experiences for subjective age. In a similar approach, Tingvold and colleagues show the relationship of momentary subjective age with subjective and physiological stress in late-midlife adults' daily lifes. Finally, Touron and Hughes found that momentary fluctuations in subjective age are associated with current task engagement and enjoyment. Together, the findings show that innovative perspectives and research designs are needed to understand how people respond to the question "How old do you feel" and why it predicts how well they actually age.

### I WISH I MAY, I WISH I MIGHT FEEL THE AGE I WISH TONIGHT

Jennifer Bellingtier[1], Fiona Rupprecht[2], Shevaun Neupert[3], and Frieder Lang[4], *1. Friedrich Schiller University Jena, Jena, Thuringen, Germany, 2. University of Vienna, Vienna, Wien, Austria, 3. North Carolina State University, Raleigh, North Carolina, United States, 4. Friedrich-Alexander-Universität Erlangen-Nürnberg, Nürnberg, Bayern, Germany*

Subjective age has traditionally been considered by comparing felt age to chronological age, with those who feel younger reporting more adaptive developmental outcomes. Here we consider a new approach: subjective age discordance, which compares felt ages to the ideal ages of participants. Across eight study days, 116 older and 107 younger adults reported their daily felt and ideal ages. On the majority of days, both older and younger adults idealized ages younger than they felt. The opposite pattern, idealized ages older than felt ages, was rare and primarily seen in younger adults. Days when felt ages were less discordant from ideal ages were characterized by higher levels of positive affect than days with greater subjective age discordance. These findings suggest that positive developmental outcomes can occur not only from feeling younger, but through a greater alignment of ideal and felt ages.

### THE DYNAMIC NATURE OF SUBJECTIVE AGE ACROSS THE LIFE SPAN

David Weiss, *Martin-Luther-University of Halle-Wittenberg, Halle, Sachsen-Anhalt, Germany*

A large body of research has confirmed that from childhood to old age most individuals feel significantly younger or older than their chronological age. Up to now, however, there is no clear theoretical understanding as to why younger adults tend to feel on average older and older adults tend to feel on average younger. We adopt a motivated social-cognition perspective on subjective age and examine age-differential antecedents and correlates of subjective age across the adult life span. Results from a cross-sectional study (N = 1652, 18-84 years) and a 9-month longitudinal study (N = 814; 18-84 years) highlight the dynamic link between subjective age bias and individual (motivation and emotion) as well as social factors (social comparison, meta stereotypes). We discuss the role of reciprocal dynamics between individuals and social contexts in explaining why individuals adopt a younger or older subjective age.

### FEELING YOUNG TODAY, FEELING GOOD TOMORROW? MICROLONGITUDINAL DYNAMICS IN SUBJECTIVE AGE

Fiona Rupprecht[1], Laura Schmidt[2], Monika Sieverding[2], Jana Nikitin[1], and Hans-Werner Wahl[3], *1. University of Vienna, Vienna, Wien, Austria, 2. Heidelberg University, Heidelberg, Baden-Wurttemberg, Germany, 3. Universität Heidelberg, Heidelberg, Baden-Wurttemberg, Germany*

Insights into the short-term dynamics and micro-longitudinal consequences of subjective age can drive our understanding of its long-term mechanisms across adulthood. Using data from 80 newly retired individuals (aged 59 to 76 years; 59% women) collected on 21 days, we made use of a recent methodological advance—multilevel dynamic structural equation modeling. As possible same-day correlates and micro-longitudinal consequences of subjective age, we investigated physical activity, step number, sleep quality, affect, and stress, which were either assessed via wearables (FitBit Charge HR) or daily diaries. Analyses suggest a weak autoregression of subjective age, indicating that how old one feels is determined via daily rather than lasting experiences. Indeed, there were significant same-day relations to all suggested correlates. The one effect lasting across several days was from an older subjective age on subsequent negative affect—a potential short-term mechanism contributing to the detrimental long-term influence of an older subjective age.

### MOMENTARY VARIATION IN SUBJECTIVE AGE, PERCEIVED STRESS, AND PHYSIOLOGICAL REACTIVITY IN LATE MIDLIFE

Maiken Tingvold, Lisa Borgmann, and Anna Kornadt, *University of Luxembourg, Esch-sur-Alzette, Diekirch, Luxembourg*

The age people feel, their subjective age, can vary on both daily and momentary levels. Previous longitudinal and daily-diary studies have shown that hormonal and self-reported measures of stress covary with subjective age. Our study aims at exploring momentary fluctuations in subjective age and their relation to objective and subjective measures of stress assessed in people's daily lives. 54 participants aged 50 – 62 years (Mage = 56.1 yrs, 75% female) wore sensors recording their physiological reactivity and reported on perceived stress and subjective age six times per day for one week. We found that a lower subjective age was related to a greater heart rate variability and less perceived stress on a momentary level. Our findings confirm and expand studies showing the association of stress and subjective age and their importance for aging processes in late midlife.

### THE EXPERIENCE OF SUBJECTIVE AGE DURING EVERYDAY LIFE

Dayna Touron, and Matthew Hughes, *University of North Carolina at Greensboro, Greensboro, North Carolina, United States*

Empirical work has shown that subjective age is susceptible to momentary fluctuation throughout the day, and in certain contexts like challenging cognitive evaluations. We propose and test a contextual model that describes how momentary experiences impact subjective age, which in turn impacts daily activities and well-being. Using an experience sampling approach, 200 participants were asked to complete 6 surveys per day for a week. Questions asked about the task they were engaged in, including mental, physical and social engagement, challenge, motivation, confidence, and enjoyment. Participants also completed a manipulation of mental challenge and reported their momentary subjective age. Preliminary multilevel models show that certain momentary factors, such as whether participants were enjoying a task or found the task engaging, predict fluctuations in subjective age. We will also discuss moderators, subjective age domains, time of day effects, and the cross-lagged influence of cognitive stressors over time.

### SESSION 2760 (AWARD LECTURE)

### IRVING S. WRIGHT AWARD, VINCENT CRISTOFALO AWARD, AND TERRIE FOX WETLE AWARD PRESENTATIONS AND LECTURES

Chair: James Kirkland

The Irving S. Wright Award of Distinction Lecture will feature an address by the 2022 recipient Thomas M. Gill, MD of Yale University. The Vincent Cristofalo Rising Star Award in Aging Research lecture will feature an address by the 2022 recipient Jamie Nicole Justice, PhD, of Wake Forest University. The Terrie Fox Wetle Award lecture will feature an address by the 2022 recipient Benjamin H. Han, MD, MPH, of the University of California San Diego. These awards are given by the American Federation for Aging Research, Inc.

### 2022 IRVING S. WRIGHT AWARD

Thomas Gill, *Yale School of Medicine, New Haven, Connecticut, United States*

Dr. Gill is a leading international authority on the epidemiology and prevention of disability among older persons. His nomination for the Wright Award lauded his groundbreaking research on the mechanisms underlying and interventions targeting functional decline and disability among community-living older persons., He collaborates with investigators throughout the country, is a leader of multisite clinical trials, and is a devoted mentor. He has published more than 350 original reports and has been continuously funded by the National Institutes of Health (NIH) and multiple foundations since 1997. Dr. Gill is the Humana Foundation Professor of Medicine (Geriatrics) and Professor of Epidemiology (Chronic Diseases) and of Investigative Medicine; Director, Yale Program on Aging; Director, Claude D. Pepper Older Americans Independence Center; Director, Yale Center for Disability and Disabling Disorders; and Director, Yale Training Program in Geriatric Clinical Epidemiology and Aging-Related Research.

### BREAKING GROUND IN TRANSLATIONAL GEROSCIENCE: FROM BIOMARKERS TO CLINICAL TRIALS

Jamie Justice, *Wake Forest School of Medicine, Winston-Salem, North Carolina, United States*

The geroscience hypothesis posits that common biological mechanisms of aging drive susceptibility of aged individuals to functional decline, multi-morbidity, and death. The promise of geroscience is that some of these mechanisms may be intervenable, thereby preventing or delaying declines, and providing new therapeutic opportunities for hard-to-treat chronic diseases. This is supported by specific examples of translational research models, and interventions that are at the point of entering human clinical trials. This award presentation will review how we are reimagining existing resources and creating new translational frameworks to test the geroscience hypothesis in humans.

### OPIOID USE DISORDER AMONG OLDER ADULTS: ADDRESSING MULTIMORBIDITY AND GERIATRIC CONDITIONS

Benjamin Han, *UC San Diego, La Jolla, California, United States*

Dr. Han will present multidisciplinary research focused on understanding the healthcare needs of older adults with opioid use disorder. Nationally, there is a sharp increase in older adults with opioid use disorder (OUD) and the number of older adults dying of opioid-related overdoses. This lecture will first describe the prevalence of geriatric conditions and comorbidities among older adults who receive care in opioid treatment programs. Then, Dr. Han will present patterns of acute healthcare utilization among older adults with opioid use disorder. Qualitative data from patient experiences of aging with opioid use disorder and the challenges of managing chronic diseases will then be presented. The lecture will conclude with a discussion about opportunities for developing multidisciplinary models of care to best deliver geriatric-based interventions for this population.

### SESSION 3000 (PAPER)

### ASSESSMENT AND MEASUREMENT

### CONSUMERS AND PROVIDERS ON THE NEEDS OF LONG-TERM SURVIVORS AND PEOPLE AGING WITH HIV IN NEW YORK STATE

Maria Brown[1], Eugenia Siegler[2], Marz Albarran[3], John Wikiera[4], Angie Partap[5], Courtney Ahmed[6], Sheriden Beard[7], and Thomas Heslop[8], *1. Syracuse University, Syracuse, New York, United States, 2. Weill Cornell Medical College, New York, New York, United States, 3. HIVStopsWithMe.Org, Renssaelaer, New York, United States, 4. NY Statewide Peer Network, Rome, New York, United States, 5. Stony Brook Medicine, Patchogue, New York, United States, 6. NYSDOH AIDS Institute, Brooklyn, New York, United States, 7. NYSDOH AIDS Institute, New York, New York, United States, 8. Weill Cornell Medical College, Washgton, District of Columbia, United States*

Objectives: To document the practical needs, and develop quality initiatives to address those needs, of the growing population of long-term survivors (LTS) and older people with HIV (OPH) in New York State.

Methods: The HIV+ Aging/LTS/Perinatally Diagnosed Subcommittee of the NYS Quality of Care Consumer Advisory Council used community based participatory research methods to design a statewide survey based on categories identified in August 2020 virtual town halls with consumers and providers across New York. Syracuse University launched the survey, open to consumers aged 18 and over who were LTS or OPH, clinicians, and social services providers, in June 2021 using Qualtrics™. Participants chose the three most important barriers and recommendations for each category. Responses were characterized using basic descriptive statistics.

Results: Participants included 124 consumers from 26 counties, 20 clinicians, and 24 social service providers. Two thirds of participants were cisgender men (67%), 27% were African American, 80% identified as both LTS and OPH. On average, consumers were 58 years old, had been living with their HIV+ diagnosis for 27 years, and reported 4 additional conditions, most commonly depression (30%). LTS and OPH were concerned about clinical and financial needs, particularly coordination of clinical care, unmet housing needs, cultural representation in mental health services, and financial support of LTS and OPH. Implications: Community based participatory research can inform and stimulate changes in clinical care for LTS and OPH. Survey results are informing a plan for functional screening of OPH and LTS that can be performed by certified peer workers.

### MODE EFFECTS ON COGNITIVE FUNCTIONING ASSESSMENTS IN THE HEALTH AND RETIREMENT STUDY

Jessica Faul[1], Ben Domingue[2], Ben Stenhaug[3], Brady West[1], Kenneth Langa[1], and David Weir[1], *1. University of Michigan, Ann Arbor, Michigan, United States, 2. Stanford, Stanford, California, United States, 3. Stanford, Sanford, California, United States*

As the population of the US ages, there is interest in assessing health conditions associated with age and longevity, such as age-related decline in cognitive functioning. As a result, there is an increased focus on measuring cognitive functioning in surveys of older populations. One challenge relates to conducting comparable measurement across survey modes (e.g., phone vs. web). Compounding this is that mode of survey administration is often not assigned randomly making inter-group comparison more difficult. This paper addresses these issues using a novel experiment embedded within the Health and Retirement Study (HRS). The HRS, a US-based cohort of people over 50, has measured cognition since its inception using both in-person and telephone modes. In 2018, a sample of approximately 3700 respondents was identified as web-eligible based on a prior report of internet access along with other selection criteria. Of these, 60% were randomly selected for the web sample with the remainder serving as controls, assigned to telephone mode for comparison purposes. We deploy techniques from item response theory (IRT) and differential item functioning (DIF) to estimate the difference in cognitive functioning between web and phone respondents in 2018 based on longitudinal cognition data collected prior to 2018. Second, we estimate the overall effect of taking the survey via the web as compared to the phone. Third, we examine item-level variation in the magnitude of the mode effect and suggest possible methods for adjustment to support longitudinal consistency. These results are important in guiding future research that utilizes web-based cognitive measures.

### PERCEIVED AND OBJECTIVE EXECUTIVE DYSFUNCTION JOINTLY PREDICT BRAKING REACTION TIME IN OLDER DRIVERS

Caitlin Pope[1], Tyler Bell[2], Benjamin McManus[3], and Despina Stavrinos[3], *1. University of Kentucky, Lexington, Kentucky, United States, 2. University of California, San Diego, La Jolla, California, United States, 3. University of Alabama at Birmingham, Birmingham, Alabama, United States*

Motor vehicle crashes are a leading cause of injury and death for older adults in the United States. Braking reaction time (BRT), how quickly a driver responds to the situational demands of driving, is a known predictor of driver fitness and hypothesized as sensitive to difficulties with executive functioning. Unclear is how BRT may vary across different levels of executive functioning, including objective test performance and perceived executive dysfunction, and if awareness of executive dysfunction in the presence of objective difficulties jointly predicts BRT. Using data from a simulated driving study, 50 adults aged 65-94 years old (49.1% female) completed computerized EF tests (inhibition [Stroop], working memory [Automated Operation Span]), the Behavior Rating Inventory of Executive Function, the Big Five Inventory, and a simulated drive. Multilevel modeling analyses controlling for covariates of empirical and design relevance (simulated road segment, age, gender, and neuroticism) were performed. Our results show a significant interaction between perceived executive dysfunction and objective test performance on BRT. Specifically, associations with BRT were stronger for individuals who perceived more executive dysfunction in the presence of worse inhibition ($b = 0.79$, 95%CI = 0.27, 1.30) and worse working memory ($b = 0.94$, 95%CI = 0.26, 1.62). Findings provide further justification for the role of executive functioning in monitoring driver fitness in older age. Future directions and implications are discussed.

### QUALITY OF LIFE IN ADULTS LIVING WITH COGNITIVE IMPAIRMENT: DEVELOPMENT OF A UNIVERSAL MEASURE

Keith Anderson[1], Lisa Peters-Beumer[2], Megan Westmore[3], and Rebecca Logsdon[4], *1. University of Mississippi, Oxford, Mississippi, United States, 2. Concordia University Chicago, River Forest, Illinois, United States, 3. University of Texas at Arlington, Arlington, Texas, United States, 4. University of Washington, Seattle, Washington, United States*

Quality of life is one of the most important and holistic measures of perceived overall well-being across the lifespan. For older adults living with cognitive impairment, quality of life has been conceptualized as consisting of an array of intertwined domains, such as relationship quality, physical health and ability, and opportunities for roles and activities. A subset of measures have been developed to gauge quality of life in older adults living with cognitive impairment, yet no “gold standard” in measurement has emerged. Quality of Life – Alzheimer’s Disease (QOL-AD) is one of the most commonly used and long-standing measures, yet it was developed in 2002 and has yet to be updated to reflect the evolution of our understanding of quality of life in adults living with cognitive impairment. Drawing from the literature on adults living with intellectual and developmental disabilities (IDD), the researchers identified several domains and items to update and expand the applicability of the QOL-AD measure. The proposed revised measure, Quality of Life – Alzheimer’s Disease/Intellectual and Developmental Disabilities (QOL-AD/IDD), is intended for adults living with cognitive impairment regardless of age or cause of impairment (e.g., dementia, IDD). The QOL-AD/IDD retains the format of the original measure, most notably the useful prompts that accompany each item and the supplemental proxy measure for caregivers. In this presentation, the researchers will discuss the development of the QOL-AD/IDD and ongoing (e.g., Delphi review) and planned steps to evaluate the reliability and validity of this promising measure.

### GUIDELINES FOR DEVELOPING EVIDENCE-BASED RISKY DRIVING COUNTERMEASURES THAT INCLUDE OLDER DRIVERS

Jennifer Zakrajsek[1], Lisa Molnar[1], David Eby[1], Lidia Kostyniuk[1], Nicole Zanier[1], David J. LeBlanc[1], and Tina B. Sayer[2], *1. University of Michigan Transportation Research Institute, Ann Arbor, Michigan, United States, 2. Toyota Motor North America Research & Development, Ann Arbor, Michigan, United States*

Driver behavior will continue to play a critical role in driving safety for the foreseeable future. Utilizing behavior change theory appropriately presents opportunities to improve the effectiveness of risky driving countermeasures that have been under-utilized to date. Older drivers should not be excluded from consideration of risky behaviors. Forty-six drivers (33% age 65+) completed surveys, then drove for three weeks with data collection during all trips. The Theory of Planned Behavior guided a two-phased regression analysis approach: 1) behavioral intentions were predicted using attitudes about behaviors and demographics; 2) observed risky behavior was predicted using behavioral intentions, theory constructs, personality/psychosocial characteristics, demographics, and driving exposure. Results were synthesized and the emergent themes were used to formulate guidelines for developing theory-based education and communication risky driving countermeasures. Guidelines focused on four risky driving behaviors observed in a large proportion of participants (72% - 96%): holding/using a cellphone; eating/drinking; speeding; and tailgating. Twenty-six guidelines were developed across four categories: 1) relationships among risky behaviors; 2) characteristics or underlying dimensions of risky driving (e.g., time, location, emotion); 3) behavior change theory constructs; 4) audience and message factors. While older drivers self-reported low frequencies of risky behaviors, low intentions for future risky behaviors, and less favorable attitudes toward risky behaviors than younger drivers they were regularly observed engaging in risky behaviors: distracted behaviors in 79% of trips and 2.1 speeding events per trip. Risky driving countermeasures are appropriate for older drivers and the emergent guidelines will be presented with recommended variations for older drivers.

### SESSION 3001 (BIOLOGICAL SCIENCES INVITED SYMPOSIUM)

### SEX DIMORPHISM IN AGING

Chair: Bérénice Benayoun

In this symposium, the speakers will discuss how sex can influence aging trajectories using complementary perspectives and approaches across multiple biological scales and systems. Indeed, accumulating evidence across species has revealed that aging is a highly sex-dimorphic process. On the one hand, women outlive men consistently across populations, and most supercentanarians are women. On the other hand, especially after the onset of menopause, women are at increased risk for most age-related diseases (e.g. Alzheimer's disease, osteoporosis, etc.). However, the molecular pathways underlying such sex-differences in aging and longevity are still largely unexplored and poorly understood. Dr. Austad will discuss how compatibility between the mitochondrial and nuclear genomes during aging is influenced as a function of sex. Dr. Dubal will discuss the impact of sex chromosome complement on cognition with aging in mouse models, and how sex chromosomes may underlie sex-differences in brain aging. Dr. Ucar will discuss work from her lab revealing that the genomic signatures of immune aging in humans are specific to sex. Finally, Dr. Benayoun will discuss how aging of innate immune cells (e.g. macrophages or neutrophils) is regulated by the female vs. male milieu, with sex-specific age-related trajectories remodeling the immune compartment in mice.

### SEX-DIMORPHIC REGULATION OF INNATE IMMUNITY DURING AGING

Bérénice Benayoun, *University of Southern California, Los Angeles, California, United States*

Aging is accompanied by striking changes in chromatin and gene expression across cell types and species. Yet, how chromatin landscapes change with age and regulate transcription, and how epigenomic changes in turn influence aging in response to external or internal cues, is largely unknown. In addition, accumulating evidence indicates that sex hormones play a key role in driving aspects of cellular and molecular sex-dimorphism. In parallel to sex hormones, karyotypic sex (i.e. XX vs. XY) is also likely to have important impact outside of gonadal sex determination. A key compartment whose activity can be actively modulated by sex-dimorphic mechanisms throughout life is the immune system, whose function declines sharply with aging and may be actively modulated by sex. Indeed, aspects of the immune responses differ between sexes, with a more robust immune response in females and an increased susceptibility to infection in males. Thus, our main cell models of study are key components of the innate immune system and the inflammatory response: macrophages, which accomplish key tasks such as phagocytosis, antigen presentation and cytokine production, and neutrophils, the most abundant leukocyte type serving as a "first line of defense" against infection. Excitingly, we and others have observed strong sex-related differences in the transcriptional and functional phenotypes of these cells and have observed sex-dimorphic "omic" trajectories for these cells with aging. Based on our data and published literature, it is likely that mechanisms involving both gonadal hormones and sex chromosomes may fine-tune different aspects of immunity and, thus, overall health and lifespan.

### THE ROLE OF MITOCHONDRIAL-NUCLEAR INTERACTIONS IN SEX DIFFERENCES IN AGING

Steven Austad, *University of Alabama at Birmingham, Birmingham, Alabama, United States*

Sex differences in longevity and the aging phenotype are rampant if not ubiquitous. They are also highly variable and content dependent. That is, although there appears to be an overall female longevity bias in many species, that pattern has many exceptions. In virtually all of the best-studied species, with humans being a notable exception, longevity sex bias is context-dependent. To try to understand sex differences in aging, it is useful to consider the impact of mitochondria, which are exclusively inherited through the female lineage. Mitochondria are endosymbiotic bacteria that have given away most of their genes to the nucleus. Therefore, mitochondrial function depends on compatibility and coordination of the mitochondrial genome with the nuclear genome. The evolutionary success of mitochondrial genomes, specifically, will depend on how effectively they interact with female nuclear genomes, male nuclear genomes being evolutionary dead-ends for them. This talk will employ this logic to examine observations of sex differences in the aging phenotype.

### X CHROMOSOME-DERIVED MECHANISMS OF SEX DIFFERENCES IN LIFESPAN AND BRAIN AGING

Dena Dubal, *University of California San Francisco, San Francisco, California, United States*

Women live longer than men worldwide – and also show cognitive resilience in many aging populations. One major source of biologic difference between the sexes is that females have two X chromosomes and males have one. This difference in sex chromosome complement causes unique X-derived mechanisms that are sex-specific. In mammalian development, one X randomly inactivates in XX cells. One X-derived sex difference is that females are mosaics with the active X chromosome in each cell being either maternally-derived (Xm) or paternally-derived (Xp), whereas males harbor only a maternally-derived X (Xm) in all cells. Interestingly, some females show considerable or complete skew toward Xm or Xp. We utilized several genetic models of sex biology to understand mechanisms of sex difference in aging. We found that the X chromosome contributes to longevity and better cognition in male and female mice. In aging, a genetic manipulation in females to express only the maternally-derived X (Xm), like males, accelerated cognitive decline and epigenetic brain aging. This suggests that Xm is harmful and that female mosaicism (Xm+Xp) provides a buffer to deleterious processes in aging. To assess if Xm alters transcription, we used mice with nuclear localized genetic reporters and sorted Xm from Xp neurons from young and aging XX hippocampi. We found that Xm imprinted several genes within aging hippocampal neurons, suggesting silenced cognitive loci. Our data suggests that Xm – the maternal X – accelerates brain aging and causes cognitive deficits. Understanding how Xm impairs brain function could increase understanding of female heterogeneity and of sex differences in cognitive heath – and unlock new X-derived pathways against cognitive deficits and brain aging of males, females, or both.

### SEXUAL-DIMORPHISM IN HUMAN IMMUNE SYSTEM AGING

Duygu Ucar[1], George Kuchel[2], Jacques Banchereau[3], Sathyabaarathi Ravichandran[1], Onur Karakaslar[4], Fernando Erra Diaz[5], Eladio Marquez[6], and Radu Marches[1], *1. The Jackson Laboratory for Genomic Medicine, Farmington, Connecticut, United States, 2. University of Connecticut, Farmington, Connecticut, United States, 3. Immunai - NY, New York City, New*

*York, United States, 4. Leiden University Medical Center, Leiden, Zuid-Holland, Netherlands, 5. CONICET, Buenos Aires, Ciudad Autonoma de Buenos Aires, Argentina, 6. Dragonfly Tx, Waltham, Massachusetts, United States*

Differences in immune function and responses contribute to health- and life-span disparities between sexes. However, the role of sex in immune system aging is not well understood. Here, we characterize peripheral blood mononuclear cells from 172 healthy adults 22–93 years of age using ATAC-seq, RNA-seq and flow cytometry. These data reveal a shared epigenomic signature of aging including declining naïve T cells and increasing monocyte and cytotoxic cell functions. These changes are greater in magnitude in men and accompanied by a male-specific decline in B-cell-specific loci. Interestingly, genomic differences between sexes increase after age 65, with men having higher innate and pro-inflammatory activity and lower adaptive activity. In a separate cohort (n=39) we studied how older adults respond to two available pneumococcal vaccines: T-dependent Prevnar and T-independent Pneumovax. How older adults respond to these vaccines at the molecular and cellular level, and whether there are pre-vaccination predictors for vaccine responsiveness, is poorly understood. To address this, we characterized serum antibody responses and peripheral blood leukocyte composition using flow cytometry and transcriptional profiles using RNA-seq, in healthy older adults before and after vaccination. The vaccines induced comparable serological responses and increases in the number of ICOS+ T follicular helper cells. A shared plasmablast transcriptional signature (IGHG2, IGHA1, IGHA2) was induced by both vaccines ten days after vaccination, which is distinct from influenza vaccine responses. Importantly, the pre-vaccination (baseline) ratio of Th1/Th17 cells predicted responsiveness to Prevnar but not to Pneumovax. Interestingly, women had higher levels of Th1/Th17; and responded stronger to Prevnar compared to men. This study uncovered how older adults respond to different pneumococcal vaccines and demonstrated the significance of considering biological sex and the immune cell composition for precision vaccinology.

### SESSION 3010 (PAPER)

### CAREGIVING AND CAREGIVER WELL-BEING: CULTURAL AND SOCIAL DETERMINANTS OF HEALTH EXAMPLES

### DOES DISTANCE CARE MAKE A DIFFERENCE IN THE STATUS OF CARE RECIPIENTS OVER AN 18-MONTH PERIOD?

Tomoko Wakui[1], Ichiro Kai[2], and shuichi Awata[1], *1. Tokyo Metropolitan Institute of Gerontology, Tokyo, Tokyo, Japan, 2. The University of Tokyo, Tokyo, Tokyo, Japan*

Aging in place is a priority for policy makers and individuals, and the continuity of care provided at home is one of the major concerns. Co-resident caregiving has been a dominant caregiving arrangement in Japan; however, the solitary residence of older care recipients has become more frequent. This study thus examined the impact of distance care on the status of care recipients over an 18-month period. Longitudinal data were collected from family caregivers of older adults in 17 municipalities of north-central Japan beginning in 2010. The status of care recipients after 18 months was categorized as receiving care at home, being institutionalized, deceased, hospitalized, moved out from the community, recovered, or untraceable. Care recipients' age, gender, activities of daily living, dementia symptoms, and caregivers' age, gender, health status, economic status, and caregiving burden were measured. We analyzed data of 2280 care recipients–caregiver dyads. Care recipients' mean age was 84.7 (SD=7.3), and 66.7% were women. Of those, 65.0% had some dementia symptoms. Multivariate logistic regression models predicting the status of care recipients after 18 months showed that distance care had significant effects on care recipients' status. Those who received distance care were more likely to be moved out, institutionalized, and deceased after 18 months, even after controlling for their care needs and caregiving situations. There were no significant effects on being hospitalized. This study suggests that the more difficult situation pertaining to staying at home is experienced by care recipients who received distance care, and support is required.

### DYADIC EFFECTS ON DEPRESSIVE SYMPTOMS OF SPOUSE CAREGIVERS AND THEIR CARE RECIPIENTS: EVIDENCE FROM CHINA

Jinyu Liu[1], Yifan Lou[1], Lydia Li[2], Hongwei Xu[3], and Zhenmei Zhang[4], *1. Columbia University, New York, New York, United States, 2. University of Michigan, Ann Arbor, Michigan, United States, 3. Queens College in the City University of New York, Queens, New York, United States, 4. Michigan State University, East Lansing, Michigan, United States*

Objectives: The likelihood of providing care to a spouse in middle and older ages has increased as life expectancy increases, but knowledge about how the caregiver and care recipient influence each other's mental health is limited. This study examined whether a partner's physical, cognitive, and mental health in a spousal caregiving dyad are associated with the other partner's depressive symptoms in China and whether the dyadic effects vary by gender.

Methods: This study used data from Wave 3 (2015) and Wave 4 (2018) follow-up surveys of the China Health and Retirement Longitudinal Study (CHARLS). The analytic sample featured 1,245 dyads of care recipients aged 45 or older and their spouse caregivers. The Actor-Partner Interdependence Model was used to test the dyadic effects among all couples in the analytic sample, couples with wife caregivers and couples with husband caregivers, respectively.

Results: We found that caregiver's depressive symptoms at Wave 3 were significantly associated with care recipient's depressive symptoms at Wave 4 in the full sample. Regardless of caregiver or care recipient roles, wives' mental health was impacted by their husbands' depressive symptoms, but not vice versa. Wife recipient's cognitive impairment was associated with husband caregiver's lower depressive symptoms. Discussion: This study sheds light on the mental health of couples in the context of caregiving in China. The findings indicate that interventions to support couples in a caregiving dyad need to consider the influence they have on each other, and the gender and health conditions of each in the dyad.

### INCREASING RISK FOR ELDER MISTREATMENT? A QUALITATIVE STUDY OF CAREGIVER EXPERIENCES DURING COVID-19

Kelly Marnfeldt[1], Lilly H. Estenson[2], Julia Marinez[3], Zach Gassoumis[4], and Kathleen Wilber[3], *1. University of Southern California, Santa Monica, California, United States, 2. University of Southern California, Pasadena, California, United States, 3. University of Southern California, Los Angeles, California, United States, 4. Keck School of Medicine, USC, Los Angeles, California, United States*

Family caregivers (CGs) of community-dwelling older adults faced unprecedented psychosocial challenges in the face of the COVID-19 pandemic. Social isolation, lack of social support, truncated care networks, financial hardship and other factors put many older adults at risk for elder mistreatment (EM). Even in normal times, incidents of EM are only identified and formally reported in about one out of every 24 cases. Social distancing restrictions in the caregiving context, including decreased access to healthcare providers, rendered older adults with higher dependency needs inordinately vulnerable to abuse and neglect. We collected qualitative data on the experiences of 24 CGs receiving telephonic support sessions between March 2020 and February 2021. Data from 100 distinct post-session case notes were analyzed using thematic content analysis to generate codes and themes. Caregivers reported rapid deterioration of care receiver's (CRs) physical health, resulting in higher levels of dependency. Declines in mental health and cognitive impairment, including behavioral issues, were an indicator of stress for both CR and CG. CGs reported lack of access to services and social supports, fractured care networks, and increased feelings of isolation. Preliminary content analysis indicates that serious risk factors for EM were present across all 13 months of the study, and those risk factors continued or worsened over time. Findings suggest that the unintended consequences of the public health response to COVID-19 may have amplified the risk for EM in vulnerable community-dwelling older adults.

### THE IMPACT OF CHILDHOOD ADVERSITY ON CAREGIVERS' HEALTH

Elizabeth Avent[1], Jeanine Yonashiro-Cho[2], and Zach Gassoumis[3], *1. University of Southern California, Los Angeles, California, United States, 2. Keck School of Medicine of USC, Alhambra, California, United States, 3. Keck School of Medicine, USC, Los Angeles, California, United States*

Caregivers tend to report fair or poor health compared to non-caregivers. Adverse childhood experiences (ACEs), which are traumatic events experienced before age 18, have been associated with several health conditions and overall poor health in adulthood. The additive effect of early-life stressors and caregiving stressors may have a compounded impact on the health of caregivers, contributing additional stress and burden to their caregiving situation. Data from the 2019 and 2020 Behavioral Risk Factor Surveillance System (BRFSS) were used to analyze self-rated health (SRH) in the context of caregiving and ACEs, based on responses from Florida, Georgia, Tennessee, Virginia, and Utah (N= 41,334). Of the 8,368 caregivers, nearly 23% reported 4 or more ACEs, compared with 13% of non-caregivers. Nested regression models showed that caregivers have lower SRH (b=-0.04, p=0.0002); however, after including the combination of ACEs and being a caregiver, there was no significant impact of being a caregiver alone on SRH. The effect of ACEs alone on SRH persisted, with the strongest effect for individuals with 4 or more ACEs (b=-0.41, p< 0001). The finding that caregivers' childhood adversity accounts for SRH moreso than their caregiver status highlights the importance of a lifespan approach when considering caregiver health and, potentially, burden. This has implications for practice, suggesting that screening caregivers on their history of ACEs may be a valuable tool to identify caregivers at higher risk for poor health outcomes who may benefit from additional resources to support their health and well-being.

### TOOLS TO SUPPORT NEEDS ASSESSMENTS ON DEMENTIA, COGNITIVE HEALTH, AND CAREGIVING

John Shean[1], Talyah Sands[2], Priya Shah[2], and Kelsey Donnellan[3], *1. Alzheimer's Association, Chicago, Illinois, United States, 2. Association of State and Territorial Health Officials, Arlington, Virginia, United States, 3. Assoc. of State and Territorial Health Officials (ASTHO), Arlington, Virginia, United States*

Dementia, cognitive health, and caregiving are not always included in community health needs assessments, leading to their absence in public health improvement and strategic plans, and subsequent lack of action and funding to address these needs. In response, the Alzheimer's Association and the Association of State and Territorial Health Officials published the Needs Assessment Toolkit for Dementia, Cognitive Health, and Caregiving. Designed for state, local, and territorial public health and aging officials, this toolkit helps communities identify unmet needs of older adults, scale up existing community strengths, and promote healthy aging. The toolkit embeds health equity as a cornerstone to assessing community health. It offers approaches to operationalize equity in the assessment process by including wide representation of people from different racial and ethnic backgrounds, geographic locations, and levels of socioeconomic status and educational attainment. The toolkit's five steps are modular in design and allow jurisdictions to enter the process at any stage. It considers alignment with jurisdiction-wide plans including Alzheimer's plans, health improvement plans, aging plans, and the Healthy Brain Initiative Road Map, helping public health agencies advance attention to Alzheimer's through their networks. Developed through an iterative process, expert input from public health and aging practitioners guided the development and organization. Over 1,600 comments were reconciled to ensure usability among stakeholders, elevate equity to an essential component throughout the assessment process, and add value to allied strategic plans. This presentation complements Learn, Plan, Do: Public Health Planning to Address Dementia and Caregiving, abstract ID 1229181.

### SESSION 3020 (SYMPOSIUM)

### CAREGIVING AND COGNITIVE IMPAIRMENT IN MEXICO AND THE US

Chair: Phillip Cantu Co-Chair: Sunshine Rote Discussant: Joseph Gaugler

Dementia is one of the most common causes of disability and dependence in the world. The growing dementia population in Mexico is exemplary of many low- and middle-income countries (LMICs). While the U.S. has a highly developed formal long-term care system, the use of institutional care among Latinos has been low. Mexico lacks a publicly financed long-term care system, and it does not have a national-level mandatory registry of institutions, compulsory standards of care, nor a regulatory body to oversee management, quality of care standards for services, or the accreditation and evaluation of service providers. There are no policies, public programs, or services to provide dependency care, including support for people living with dementia and their family caregivers. As in other LMIC's, there is limited public support for the aging population in Mexico, leaving families with the main responsibility of providing care and economic security for older adults. "Informal" and family caregivers for older adults with cognitive impairment are critical components of how older adults with cognitive impairment are able to remain in the community. This symposium will reflect the broad spectrum of caregivers and care context in the U.S. and Mexico to shed light on sources of care for older adults living with cognitive impairment.

### RACIAL AND ETHNIC DIFFERENCES IN HEALTH OF SPOUSES OF COGNITIVELY IMPAIRED OLDER ADULTS

Phillip Cantu[1], Rafael Samper-Ternent[2], and Lu Chen[3], *1. The University of Texas Medical Branch, Galveston, Texas, United States, 2. University of Texas Medical Branch, Galveston, Texas, United States, 3. UTMB, Galveston, Texas, United States*

Introduction: A large body of research in the U.S. shows that non-Hispanic black (black) and Hispanic older adults have higher rates of cognitive impairment and dementia compared to Whites. Little research has examined how cognitive life courses differentially affect spouses of cognitively impaired older adults.

Objectives: This paper will describe the different life courses of spouses of cognitively impaired older adults by race and ethnicity in the U.S.

Methods: Using data from the 2010 Health and Retirement Survey, we examine differences in the health of spousal dyads where one partner becomes cognitively impaired over follow up, by 2016. Spousal dyads are limited to Hispanic (n=464 dyads), i black (n=345 dyads), or white (n=2,409 dyads) couples who were both cognitively normal at baseline and had cognition information at follow up.

Results: Hispanic and Black dyads were more likely to have at least one partner become cognitively impaired over the follow up period despite being younger than White dyads. A total of 23% of Hispanic dyads, 26% of Black dyads and 16% of White dyads were N/CI at follow up. Despite fewer chronic conditions at baseline, a higher proportion of Hispanic dyads with no chronic conditions had at least one member become cognitively impaired (20%) compared to Whites (6.28%).

Conclusion: Our results show that health of couples changes differently by race and ethnicity. A higher proportion of Hispanic and Black dyads have partners with cognitive impairment and the cognitive impairment happens earlier in life.

### MEXICAN AMERICAN CAREGIVER HEALTH AND TURNOVER: THE ROLE OF STRESSORS, RESOURCES, AND BACKGROUND FACTORS

Sunshine Rote[1], Jacqueline Angel[2], and Kyriakos Markids[3], *1. University of Louisville, Louisville, Kentucky, United States, 2. The University of Texas at Austin, Austin, Texas, United States, 3. UTMB, Galveston, Texas, United States*

The purpose of this study is to explore the role of caregiver background, stressors, and resources for Mexican American caregiver turnover and depressive symptoms. Using two waves of the Hispanic Established Epidemiologic Study of the Elderly Caregiver Supplement (H-EPESE CG, 2010/2011-2016 N=333) and informed by the sociocultural caregiver stress process model, we estimate logistic and OLS regressions of caregiver turnover and caregiver depressive symptoms over five years. Neuropsychiatric expressions of dementia were significantly associated with caregiver turnover and relative to caregivers who were adult children of the care recipient, grandchildren caregivers and other kin and non-kin caregivers were more likely to experience caregiver turnover. While depressive symptoms were relatively low at both waves, there was a greater increase in depressive symptoms for caregivers who completed the interview in Spanish, which was partially explained by lower caregiver resources (e.g., support from others). The findings demonstrate the need for dementia care supports for Mexican American caregivers, improving support systems for Spanish-speaking caregivers, and supporting Mexican American caregivers who may be entering the role unexpectedly (e.g., grandchildren or non-kin).

### PATHWAYS OF CARE: THE EXPERIENCE AND COSTS OF CARING FOR FAMILY MEMBERS LIVING WITH DEMENTIA IN MEXICO

Mariana López-Ortega, *NATIONAL INSTITUTE OF GERIATRICS, NATIONAL INSTITUTES OF HEALTH, Mexico CIty, Distrito Federal, Mexico*

Introduction: Formal health and social security institutions are not unequipped to respond to significant dementia prevalence, and currently, no publicly funded national level dementia care policies are in place. However, little is known about the economic, social and health consequences for those providing care.

Methods: This paper presents families' health and social dementia care seeking trajectories and the structural factors that shape them. It is based on results of almost 2 years of highly inductive fieldwork, carried out before and during the ongoing COVID-19 pandemic, and includes 50 in-depth interviews with 23 unpaid family caregivers.

Results: Lack of awareness and knowledge about dementia and normalization of cognitive impairment as part of aging result in long delays in seeking care. When there is a diagnosis, health professionals concentrate on clinical aspects of illness, but seldom explain possible changes, behaviours, symptoms, or provide care strategies. For most carers, high opportunity costs are reported with high impact as they stop or change to more flexible employment, change living arrangements, in addition to the large economic impact of paying for most care inputs such as medicines or home adaptations.

Conclusions: In Mexico, these unpaid family carers struggle to navigate complicated care pathways, with little or no government or public support. Health system institutions should increase awareness and knowledge about dementia and prioritize developing and coordinating programs that provide timely diagnosis within primary care services and support strategies for people living with dementia and their carers, using person centred care.

### LONGITUDINAL CHANGE IN LIVING ARRANGEMENTS AND COGNITIVE IMPAIRMENT

Jiwon Kim[1], Phillip Cantu[2], and Jacqueline Angel[3], *1. University of Texas at Austin, Austin, Texas, United States, 2. The University of Texas Medical Branch, Galveston, Texas, United States, 3. The University of Texas at Austin, Austin, Texas, United States*

Objective: To examine changes in living arrangements and cognition in the U.S. and Mexico.

Methods: Latent class analysis to document the joint progression of cognitive impairment and changes in living arrangements using the Hispanic -EPESE and the Mexican Health and Aging Study (MHAS)

Results: In the H-EPESE, vital status analyses reveal that those who died were more likely than survivors to report likely dementia and co-morbid physical conditions and one third of older adults lived alone. Almost one-fifth of the H-EPESE sample suffered a decline in cognition over time. Despite declines in cognitive function, there is a great deal of stability in living alone across waves. We expect even greater stability and change in living alone in the event of cognitive decline in the MHAS given cultural traditions and fewer options in care and a lower propensity to live alone in Mexico.

### SESSION 3030 (AWARD LECTURE)

### CLARK TIBBITTS AWARD AND HIRAM J. FRIEDSAM AWARD PRESENTATIONS AND LECTURES

Chair: Kara Dassel

The Clark Tibbitts Award Lecture will feature an address by the 2022 award recipient Laura M.K. Donorfio, PhD, FAGHE, of The University of Connecticut. AGHE's Clark Tibbitts Award was established in 1980 and named for an architect of the field of gerontological education. The award is given each year to an individual or organization that has made an outstanding contribution to the advancement of gerontology and geriatrics education. The Hiram J. Friedsam Award Lecture will feature an address by the 2022 award recipient Rona J. Karasik, PhD, FGSA, FAGHE, of Saint Cloud State University. Hiram J. Friedsam was the professor, co-founder, and director of the Center for Studies in Aging and dean of the School of Community Service at the University of Northern Texas. Dr. Friedsam was an outstanding teacher, researcher, colleague, and mentor to students, faculty, and administrators, as well as a past president of AGHE. The purpose of this award is to recognize those who emulate Dr. Friedsam's excellence in mentorship.

### PEDAGOGY OF AGING: A JOURNEY TOWARD AUTHENTICITY AND WHOLENESS

Laura Donorfio, *University of Connecticut, Waterbury, Connecticut, United States*

Innovation in gerontological education has been a driving catalyst since the beginning of my career over 30 years ago. While sometimes seen as a curse, this catalyst has shaped my teaching pedagogy in unimaginable ways. Examples include field trips with 40+ students, developing an aging toolbox for students to make the material more concrete, creating a new interactive television teaching platform over multiple university campuses, developing experiential curriculum and an undergraduate minor in aging, and proudly, developing AGHE's first teaching institute now in its 11th year. Unexpectedly, at this stage of my career, I have become very aware that my teaching about aging has intersected with my personal aging, shaping my personal pedagogy in profound ways--increased empathy, sensitivity, and social awareness. I am living the subject matter I thought I intimately knew. I am the other. Has my teaching been inauthentic? Hypocritical? Ageist? Please join me for a healthy discussion on how this insight can be leveraged to teach the next generation of aging scholars.

### FULL CIRCLE: MENTORING IN GERONTOLOGY AND GERIATRICS EDUCATION

Rona Karasik, *St. Cloud State University, St. Cloud, Minnesota, United States*

More than just a buzzword in business and education, mentoring is a complex interactional process that, at its best, fosters personal and professional development for all involved. In other words, a good mentoring relationship can be both transformative and reciprocal. This raises the question of what is (and is not) a good mentoring relationship? Moreover, how does one enter into and capitalize on the benefits of mentorship? While some mentoring relationships are intentionally created, others seem to evolve so organically that participants are not immediately aware of mentoring's presence. The current presentation looks at the nature of mentoring relationships within the context of gerontology and geriatrics education from both the mentor and mentee perspectives.

### SESSION 3040 (SYMPOSIUM)

### COGNITIVE AGING IN LOW- AND MIDDLE-INCOME COUNTRIES

Chair: Yuan Zhang Co-Chair: Joseph Saenz

Cognitive aging is a highly complex, dynamic, and context-dependent process; and is influenced by individual and community characteristics and experiences across the life course. Low- and middle-income countries (LMICs) are critical components of the international dementia research community because the burden of dementia is rapidly rising in LMICs, and low-resource settings may have unique social and economic determinants of cognitive health. However, little population-based research focuses on these regions. The papers in this symposium use high-quality population survey data from Mexico, Brazil, South Africa, and China to study critical social determinants of cognitive function in countries across the globe. These papers cover a range of social and economic factors, including food insecurity, education, household material resources, and urban-rural settings and policy. First, Saenz will present findings on how food insecurity, throughout life, relates with cognitive function among older Mexican adults. Next, Farina will present evidence on both indirect and direct pathways from education to cognitive function among older Brazilians. Third, Kobayashi will present longitudinal data evaluating level and change in household resources in relation to subsequent cognitive function in South Africa. Finally, Zhang will present evidence on the interplay of rural/urban residence and household registration system across the life-course in creating cumulative advantage/disadvantage in cognitive aging using longitudinal data from China. The papers in this symposium provide new insights into social determinants of cognitive health in diverse contexts and have significant policy implications for improving cognitive health in LMICs across the globe.

### FOOD INSECURITY ACROSS THE LIFE COURSE AND COGNITIVE FUNCTION AMONG OLDER MEXICAN ADULTS

Joseph Saenz[1], Jamie Kessler[2], and Ehlana Nelson[1], *1. University of Southern California, Los Angeles, California, United States, 2. University Of Southern California, Los Angeles, California, United States*

Food insecurity is a global public health problem and is related with poorer cognition among older adults. Few have examined how food insecurity, throughout life, relates with cognition among older Mexican adults. Data came from the 2015 Mexican Health and Aging Study (n=11,507, aged 50+). Early- and late-life food insecurity were self-reported. We evaluated how food insecurity related with cognition (Verbal Learning, Verbal Recall, Visual Scanning, and Verbal Fluency), controlling for health and sociodemographic confounders. In descriptive analyses, respondents who experienced food insecurity in early- or late-life performed worse on all cognitive tasks compared to the food secure. When adjusting for health and sociodemographic confounders, early-life food insecurity predicted worse Verbal Learning performance and late-life food insecurity related with poorer Visual Scanning. Food insecurity negatively related with cognition among older Mexican adults. The significance of effects depended on cognitive task and when in life food insecurity was experienced

### DIRECT AND INDIRECT PATHWAYS FROM EDUCATION TO COGNITIVE FUNCTIONING AMONG OLDER BRAZILIANS

Mateo Farina, *University of Southern California, Los Angeles, California, United States*

Cognitive impairment in Brazil is a growing population health concern. Studies suggest that Brazil may have one of the highest age-standardized prevalence of Alzheimer's disease and related dementias (ADRD) in the world. However, improving education attainment among older adults may lead to a decline in cognitive impairment across the population in the coming years. The purpose of this study is to examine the pathways that link educational attainment to cognitive functioning in Brazil, a highly unequal, resource rich society. I use the 2016 Brazilian Longitudinal Study of Aging, a nationally representative study of Brazilian older adults 50+. I use linear regression models to predict overall cognitive functioning as well as specific domains of cognition (memory vs. non-memory). Preliminary findings provide evidence of both indirect and direct pathways from education to cognitive functioning. The indirect pathways, however, were only found for adults with 8+ years of education.

### TRENDS IN HOUSEHOLD MATERIAL RESOURCES AND COGNITIVE HEALTH IN A LONGITUDINAL COHORT STUDY OF AGING IN SOUTH AFRICA

Lindsay Kobayashi[1], Chodziwadziwa Kabudula[2], Mohammed Kabeto[1], Xuexin Yu[1], Stephen Tollman[2], Kathleen Kahn[2], Lisa Berkman[3], and Molly Rosenberg[4], *1. University of Michigan, Ann Arbor, Michigan, United States, 2. MRC/Wits Rural Public Health and Health Transitions Research Unit (Agincourt), University of the Witwatersrand, Johannesburg, Gauteng, South Africa, 3. Harvard Center for Population and Development Studies, Harvard T.H. Chan School of Public Health, Cambridge, Massachusetts, United States, 4. Department of Epidemiology and Biostatistics, Indiana University School of Public Health-Bloomington, Bloomington, Indiana, United States*

Material resources that affect daily living conditions may be salient for cognitive aging in low-income settings, but evidence is limited on this topic. We investigated relationships between long-term trends in household material resources and subsequent cognitive function among 4,580 adults aged ≥40 in a population-representative cohort in Agincourt sub-district, South Africa, from 2001-2015. Household material resources (dwelling materials, water, sanitation, sources of power, modern amenities, and livestock) were assessed biennially from 2001-2013. We evaluated mean resources, volatility in resources, and change in resources over this period in relation to cognitive function in 2014/2015. Higher mean household resources and larger improvements over time in resources were positively associated with subsequent cognitive function, independent of confounders. Findings were largely driven by modern amenities for food preparation, transportation, and communication outside of the household. Access to these amenities may support cognitive aging through boosting nutrition and cognitive reserve and should be investigated further.

### CUMULATIVE (DIS)ADVANTAGE IN COGNITIVE HEALTH: RURAL/URBAN RESIDENCY, HUKOU, AND COGNITIVE AGING IN OLDER CHINESE

Yuan Zhang[1], Qian Song[2], and Jen-Hao Chen[3], *1. University of North Carolina at Chapel Hill, Chapel Hill, North Carolina, United States, 2. Universiy of Massachusetts Boston, Boston, Massachusetts, United States, 3. National Chengchi University, Taipei City, Taipei, Taiwan (Republic of China)*

Urban residency benefits cognition in later life. In China, the household registration system (hukou) creates another place-based advantage/disadvantage for cognitive aging. We exam the interplay of rural/urban residence and hukou across the life-course in creating cumulative advantage/disadvantage in cognitive aging trajectories using longitudinal data from the 2011-2018 China Health and Retirement Longitudinal Study and its Life History Survey. Results from linear mixed effect models show that always urban dwellers with urban hukou exhibited the highest level of cognitive function and the slowest decline, and the always rural dwellers with rural hukou had the worst cognitive function and experienced a faster decline. Rural-to-urban shifts had better cognitive health than always rural dwellers, but the advantages depend on urban hukou attainment and specific mechanisms. This research suggests cumulative advantage/disadvantage in cognitive aging by residence and hukou throughout life and demonstrates that institutional discrimination and exclusionary policy may create inequality in aging processes.

### SESSION 3050 (SYMPOSIUM)

### DEMENTIA CARE REIMAGINED: THE MULTIMODAL PREVENTING LOSS OF INDEPENDENCE THROUGH EXERCISE (PLIÉ) PROGRAM

Chair: Deborah Barnes Co-Chair: Francesca Nicosia
Discussant: Theresa Allison

Over 6 million people in the United States and 50 million people worldwide are living with some form of dementia. Current pharmacotherapies for people living with dementia (PLWD) can provide some symptomatic relief but do not alter the disease course, have numerous side effects, and do not improve quality of life. Non-pharmacologic and behavioral interventions are increasingly recognized as inexpensive and effective ways to improve a wide range of outcomes for PLWD. Yet few interventions target multiple domains related to dementia quality of life. This symposium overviews the Preventing Loss of Independence through Exercise (PLIÉ) program, an evidence-based, integrative mind-body group movement program that targets key domains associated with better quality of life in PLWD: physical function, cognitive function, well-being, social connection, and self-esteem. This symposium provides an overview of the broad impact of this novel, non-pharmacologic, multimodal intervention on quality-of-life outcomes for PLWD, including expansion to include care partners and people with mild cognitive impairment and implementation in varied settings including adult day programs, nursing homes, and online delivery. It includes a participatory component for attendees to experience PLIÉ in action. Discussion focuses on the need for programs that support dementia quality of life through engagement in mind-body movement and social connection.

### OVERVIEW AND EVIDENCE-BASE FOR PREVENTING LOSS OF INDEPENDENCE THROUGH EXERCISE (PLIÉ)

Deborah Barnes, Margaret Chesney, Linda Chao, and Wolf Mehling, *University of California, San Francisco, San Francisco, California, United States*

PLIÉ was initially developed and tested in the adult day setting. Caregivers reported qualitative improvements in PLWD in psychological, physical, cognitive, and social domains that were supported by class video-recordings. Paired PLIÉ was adapted for the community setting and expanded to include dyads of PLWD and care partners (CPs) who participated in group classes together. PLWD reported significant improvements in self-rated quality of life, and CPs reported psychological, physical, cognitive, and social benefits for PLWD as well as themselves. We then adapted PLIÉ for people with mild cognitive impairment (MCI) and found that it significantly increased default mode network connectivity on resting-state functional MRI scans, while improving quantitative measures of cognitive function, social isolation, physical performance and self-regulation. We also implemented PLIÉ in a VA skilled nursing facility, where a post-implementation evaluation determined that residents, staff and family members all reported psychological, physical, cognitive and social benefits.

### DEVELOPMENT AND TESTING AN INTERPROFESSIONAL PLIÉ TRAINING PROGRAM FOR VA NURSING HOME STAFF

Jennifer Lee[1], Francesca Nicosia[2], Catherine Pham[3], Nikita Shirsat[4], Matthew Miller[2], Margaret Chesney[2], and Deborah Barnes[2], *1. San Francisco VA Health Care System / Together Senior Health, San Francisco, California, United States, 2. University of California, San Francisco, San Francisco, California, United States, 3. San Francisco VA Health Care System, San Francisco, California, United States, 4. UC Irvine School of Medicine, Irvine, California, United States*

In this study, we collected formative data from key stakeholders (nursing home staff and leadership, dementia care experts), developed, pilot tested, and refined a 10-week remote training program at 2 VA nursing homes with 15 interprofessional staff (e.g., recreation therapists, social workers, registered nurses). Training components included weekly livestreaming didactic sessions for staff that demonstrated PLIÉ guiding principles and themes. Weekly experiential sessions, facilitated by the lead PLIÉ instructor, included staff and residents with dementia participating together online. All training sessions were video recorded and available for review along with a training manual. Staff completed weekly surveys, post-training focus groups, and expressed high confidence using 6 of 8 PLIÉ principles; 100% rated their overall training satisfaction as "very good" or "excellent." The remote PLIÉ training was an efficient model for staff training that leveraged interprofessional knowledge and skills. This presentation includes an experiential demonstration of PLIÉ in action.

### MOVING TOGETHER ONLINE FOR PEOPLE LIVING WITH MILD COGNITIVE IMPAIRMENT/DEMENTIA AND CARE PARTNERS

Francesca Nicosia[1], Amanda Lee[2], Margaret Chesney[1], Jennifer Lee[2], Cynthia Benjamin[2], Wolf Mehling[1], Rebecca Sudore[1], and Deborah Barnes[1], *1. University of California, San Francisco, San Francisco, California, United States, 2. Together Senior Health, San Francisco, California, United States*

This study used Human Centered Design methods to develop a digital platform tailored for PLWD and test an online version of PLIÉ, called Moving Together. We assessed feasibility and satisfaction of a 12-week online program using completion rates, attendance, and evaluation surveys. 39 people (18 PLWD, 7 MCI, 14 CP) attended 75% classes; 96% rated classes as excellent or good; and 88% were highly likely to recommend Moving Together. Participants reported benefits in social connection ("camaraderie," "like a family"), emotional well-being ("happier," "less stressed"), physical function ("stronger," "more range-of-motion"), cognitive function ("more focus," "learning new things"), and present-centered body awareness ("more conscious of breathing patterns"). PLWD/MCI experienced improved self-concept ("less immediate shame and more confidence"); and Care Partners reported improved caregiving efficacy ("greater purpose," "more comfortable"). Online delivery of Moving Together is feasible for people with dementia or mild cognitive impairment and care partners.

### SESSION 3060 (SYMPOSIUM)

### DESIGN AND DEVELOPMENT OF MHEALTH FOR DEMENTIA: CURRENT AND FUTURE DIRECTIONS

Chair: Tina Sadarangani Discussant: Richard Holden

Alzheimer's disease and related dementias (ADRD) represent an increasingly prevalent group of neurodegenerative chronic diseases for which there is no cure. Progressive functional and cognitive decline in individuals with ADRD places considerable demands on those involved in their care, including clinicians and care partners. Mobile health technology has the potential to support people living with dementia and their care team members, as well as potentially prevent the onset of dementia symptoms. In this symposium we first examine best practices in mhealth application development for dementia care based on applications developed for other chronic illnesses. We then examine mhealth applications being developed in three different NIH-funded studies that address the needs of clinicians, care partners, adult day centers involved in the care of people with dementia, as well as an application for people with mild cognitive impairment. The Aliviado Dementia Care app is a resource that enables clinicians to access best practices for managing behavioral and psychological symptoms of dementia at the point of service. CareMOBI streamlines communication between care partners, adult day centers, and primary care providers, allowing care team members to track and exchange information relevant to dementia care and identify emerging clinical problems. Finally, Spanish-language information and culturally relevant motivational messages to promote physical activity and delay progression to ADRD among those with mild cognitive impairment within the Tiempos Juntos intervention are presented. Our discussion summarizes the current and future direction of mhealth application development in ADRD, and key learnings from presenters' work.

### CAREMOBI: AN APP CONNECTING CAREGIVERS, ADULT DAY CENTERS, AND HEALTHCARE PROVIDERS CARING FOR PEOPLE WITH DEMENTIA

Tina Sadarangani[1], Jonelle Boafo[2], and Jie Zhong[1], *1. New York University, New York, New York, United States, 2. Rory Meyers College of Nursing, New York, New York, United States*

Fragmented communication between care partners, health care providers, and adult day centers around the needs of persons living with dementia (PLWD) contributes to avoidable health care utilization. CareMOBI, is a user-centered mobile application that streamlines information exchange around datapoints relevant to the care of PLWD. CareMOBI facilitates team-based communication around emerging clinical problems by uniquely integrating the knowledge and day-to-day observations of adult day center staff and care partners. We present a stakeholder engaged approach to iteratively designing and validating an initial prototype. Through interviews and focus groups with adult day center staff (n=31), primary care providers (n=22), and family caregivers (n=13) we identified barriers to communication across settings. We then visually mapped the domains of a future app which we subsequently validated and refined with end-users (n=25). We present our current prototype which synthesizes these findings and addresses key barriers to information exchange across community settings serving PLWD.

### DEVELOPING AND RAPIDLY SCALING AN MHEALTH APP IN A 25-SITE PRAGMATIC TRIAL OF ALIVIADO DEMENTIA CARE IN HOSPICE

Abraham Brody[1], Aditi Durga[2], Ariel Ford[3], and Shih-Yin Lin[2], *1. HIGN at the NYU Rory Meyers College of Nursing, New York, New York, United States, 2. New York University, New York, New York, United States, 3. New York University Rory Meyers College of Nursing, New York, New York, United States*

Following stakeholder engagement in our pilot phase of a large-scale 2-phase NIA funded pragmatic clinical trial in hospice (HAS-QOL Trial) of Aliviado Dementia Care, we utilized a co-design process to develop over a 6-month period and rapidly scale an mHealth application to assist clinicians in performing best practices in care for persons living with dementia receiving hospice. Throughout the 36 months of intervention period, the app has been utilized by at least 6,580 hospice team members, both to complete training, receive mobile push notification nudges, and: 1) perform 6,682 clinical assessment such as the PAINAD for pain and NPI-Q for behavioral symptoms; 2) develop 2,204 symptom specific care plans; and 3) Review specially developed education materials to 15,128 caregivers and send them to 671 directly. This session will also discuss the process of implementation and scaling, including the technological hurdles in working with a workforce with sometimes limited technical skills.

### OPPORTUNITIES FOR MHEALTH IN DEMENTIA CARE/CARE MANAGEMENT: LESSONS LEARNED FROM OTHER CLINICAL DISCIPLINES

Hayley Belli, *New York University School of Medicine, New York, New York, United States*

The use of mHealth applications in dementia care/care management has recently become of increasing interest to patient stakeholders, clinicians, and researchers working in the field. However, there exists a rich literature of successful examples of the development, implementation, and efficacy of mHealth applications across a variety of health care disciplines. Thus there is high potential to take these lessons learned from other clinical fields and apply such successes to dementia care/care management. We will walk through several examples of mHealth tools used for managing hypertension, diabetes, migraines, and weight loss, while highlighting specific opportunities for dementia care/care management. Points of discussion will include optimizing the user-centered design process during the development phase of mHealth applications, increasing patient engagement with mHealth applications – especially among underrepresented individuals in research, promoting adherence to mHealth interventions via principles from behavioral economics, and scaling up mHealth interventions to test for efficacy at a population-level.

### MHEALTH DESIGN AND IMPLEMENTATION IMPLICATIONS TO PROMOTE PHYSICAL ACTIVITY IN OLDER HISPANICS/LATINOS WITH MCI

G. Adriana Perez, *University of Pennsylvania, Philadelphia, Pennsylvania, United States*

Addressing physical inactivity for older Hispanics/Latinos with mild cognitive impairment (MCI) is a public health priority, since MCI increases the risk of developing Alzheimer's Disease and related dementias (ADRD). Compared to non-Latino Whites, Hispanics/Latinos are 2X more likely to develop ADRD. One promising approach to promoting physical activity includes use of mobile health (mHealth) strategies that may deliver Spanish-language information and culturally relevant motivational messages to enhance intrapersonal/interpersonal factors for health behavior change. The purpose of this study is to test mHealth strategies as a mechanism to deliver booster sessions for reinforcing physical activity goals/progress among older Latinos with MCI who complete the Tiempos Juntos intervention treatment. Our hypothesis is that culturally adapting mHealth strategies may improve efficacy and maintenance of physical activity effect; as well as cognitive health outcomes (6 months post-intervention). Results among the first wave of participants will be discussed, including challenges and opportunities for future research. While the use of mHealth is not new, these approaches commonly exclude individuals with limited-English proficiency and thus, designing interventions that center the needs of older Hispanics/Latinos with MCI/ADRD and other historically excluded communities, is an important first step in promoting physical activity and advancing cognitive health equity.

### SESSION 3070 (SYMPOSIUM)

### DIVERSITY MATTERS: INTERSECTIONAL INEQUITIES IN HEALTH AND WELL-BEING THROUGH AGING

Chair: Rachel Koffer Co-Chair: Johanna Drewelies
Discussant: Deborah Carr

Different positions within social hierarchies receive unequal access to resources, leading to health disparities in later life (Agenor, 2020). Research addressing inequities must increasingly account for the many social categorizations (e.g., race, gender, socioeconomic status) that affect individuals' lived experiences. This symposium examines the role of intersecting social contexts on health and well-being across the life course. Surachman and colleagues use the U.S.-based National Growth and Health Study to examine the intersectionality between early life socioeconomic context and race on women's metabolic syndrome severity. Their findings have implications for societal factors leading to accelerated aging across young adulthood and early midlife. Koffer and colleagues use the U.S.-based Study of Women's Health Across the Nation to demonstrate differential midlife exposure to types and number of major life events across race/ethnicity and education. They subsequently find that major life events increase risk of cardiovascular disease events, indicating the importance of studying the life experiences of diverse women across midlife. Drewelies and colleagues use the Germany-based Berlin Aging Study II to look at the associations among multidomain identity and social background factors on physical, cognitive, and psychological aging. Implications from their work demonstrate the interplay of diversity on health and well-being in older adulthood. Conjointly, findings indicate that intersectional identities play an important role in shaping key outcomes of human functioning across adulthood and aging. Dr. Deborah Carr will critically discuss the three contributions from a life course perspective and provide considerations for future research and policy promoting equity for diverse older adults.

### LIFE EVENTS AND CARDIOVASCULAR DISEASE EVENTS: THE STUDY OF WOMEN'S HEALTH ACROSS THE NATION

Rachel Koffer[1], Rebecca Thurston[2], and Karen Matthews[2], *1. Arizona State University, Phoenix, Arizona, United States, 2. University of Pittsburgh, Pittsburgh, Pennsylvania, United States*

Cardiovascular disease (CVD) is the number one cause of death for women, and major life events across midlife may contribute to CVD risk. The present study aimed to test whether greater exposure to major life events across nearly two decades of longitudinal follow-up would be associated with higher risk of clinical cardiovascular disease events. 3,222 middle-aged women from the multi-ethnic Study of Women's Health Across the Nation reported and provided up to 15 years of major life events, non-fatal incident CVD events, traditional biobehavioral and sociodemographic factors, and death certificates. Cox proportional hazards models were used to test the association between average annual life events and incident fatal and nonfatal CVD events. Each additional major life event was associated with a 1.16-fold (95% CI: 1.08-1.23) increase in CVD events. CVD risk will be discussed considering evidence of racial/ethnic disparities in exposure to major life events.

### THE ROLE OF MULTIDOMAIN IDENTITY AND SOCIAL BACKGROUND FACTORS FOR AGING OUTCOMES

Johanna Drewelies[1], Sandra Duezel[2], Ilja Demuth[3], Elisabeth Steinhagen-Thiessen[4], Ulman Lindenberger[5], Jan Goebel[6], Gertraud Stadler[7], and Denis Gerstorf[8], *1. Humboldt University Berlin, Berlin, Berlin, Germany, 2. Max-Planck-Insitute for human developement, Berlin, Berlin, Germany, 3. Charité Universitätsmedizin Berlin,*

*Berlin, Berlin, Germany, 4. Charité - Universitätsmedizin Berlin, Berlin, Berlin, Germany, 5. Max Planck Institute for Human Development, Berlin, Berlin, Germany, 6. German Institute for Economic Research, Berlin, Berlin, Germany, 7. Charité – Universitätsmedizin Berlin, Berlin, Berlin, Germany, 8. Humboldt Universität zu Berlin, Berlin, Berlin, Germany*

Domains of social privilege are predictive of a number of key aging outcomes, such as physical and cognitive functioning. However, less is known about the dynamic interplay of - related but distinct- domains of privilege and how they are associated with cognitive and physical functioning. In the following project, we aim to close this gap by examining the role of multidomain identity and social background factors for initial levels and subsequent changes in psychological, cognitive and physical functioning. Specifically, we identified predefined social privileges within the Berlin Aging Study II (BASE-II) sample (e.g., gender identity, gender conformity, sexuality, education, socioeconomic status, religion) and related these categories, interactively, to markers of physical health (multimorbidity), perceptual speed (digit symbol), and psychosocial functioning (loneliness). Results highlight the dynamic interplay (i.e., intersectionalism) of multidomain identity and social background factors with aging related outcomes across a number of key domains.

### CHILDHOOD SES IS ASSOCIATED WITH FASTER WORSENING METABOLIC SYNDROME SEVERITY FOR BLACK RELATIVE TO WHITE WOMEN

Agus Surachman[1], Nancy Adler[1], Barbara Laraia[2], and Elissa Epel[1], *1. University of California, San Francisco, San Francisco, California, United States, 2. University of California Berkeley, Berkeley, California, United States*

This analysis examined whether early life SES was associated with longitudinal changes in metabolic syndrome (MetS) severity among Black and white women. Data were from 531 women (non-Hispanic Black=263; non-Hispanic white=268, Mage=39) in the National Growth and Health Study (NGHS). Information about parental education was collected during the baseline survey when participants were 9. Information regarding MetS severity were collected during year-7 (Mage=16), year-10 (Mage=19), and year-30 (Mage = 39) follow-up studies. Controlling for baseline body mass index, smoking status, and marital status, Black participants showed a faster increase in MetS severity across two decades. Early life SES ($b=.03$, $SE=.01$, $p<.05$), independent of current SES, was associated with faster worsening MetS severity among Black relative to white women. The socioeconomic context of early rearing is an important factor for racial disparities in accelerated aging among these early midlife Black and white women.

### SESSION 3080 (SYMPOSIUM)

### DIVERSITY, DISCOVERY, AND AGING REIMAGINED: A CHAT WITH NIA SENIOR LEADERSHIP

Chair: Amy Kelley Discussant: Amy Kelley

The National Institute on Aging (NIA) at the National Institutes of Health, Department of Health and Human Services, is the federally designated lead agency on aging research and supports research to understand the nature of aging and to extend the healthy, active years of life. In the last six years, NIA has experienced a tripling of its budget. In keeping with NIA's scientific mission, funding includes significant investments in Alzheimer's disease (AD) and AD-related dementias research, as well as in non-AD research. This symposium will provide a forum for exploration of the implications of the NIA budget for the general research community. NIA's senior staff will discuss research priorities and programs supported by the Institute. A question-and-answer session will follow these remarks on current funding and future priorities and research directions of NIA.

### OVERVIEW OF NIA BUDGET

Richard Hodes, *National Institutes of Health, Bethesda, Maryland, United States*

Dr. Hodes will discuss budget and overall research priorities for the National Institute on Aging.

### NIA DIVISION OF GERIATRICS AND CLINICAL GERONTOLOGY

Basil Eldadah, *National Institute on Aging, Bethesda, Maryland, United States*

Dr. Eldadah will discuss research priorities for the Division of Geriatrics and Clinical Gerontology. Dr. Eldadah will also be available for small group discussion.

### NIA DIVISION OF AGING BIOLOGY

Ronald Kohanski, *National Institute on Aging, Bethesda, Maryland, United States*

Dr. Kohanski will discuss research priorities for the Division of Aging Biology. Dr. Kohanski will also be available for small group discussion.

### NIA DIVISION OF NEUROSCIENCE

Molly Wagster, *National Institute on Aging, Bethesda, Maryland, United States*

Dr. Wagster will discuss research priorities for the Division of Neuroscience. Dr. Wagster will also be available for small group discussion.

### NIA DIVISION OF BEHAVIORAL AND SOCIAL RESEARCH

Dana Plude, *National Institute on Aging, Bethesda, Maryland, United States*

Dr. Plude will discuss research priorities for the Division of Behavioral and Social Research. Dr. Plude will also be available for small group discussion.

### NIA OFFICE OF SPECIAL POPULATIONS

Patricia Jones, *Natioal Institute on Aging, Bethesda, Maryland, United States*

Dr. Jones will discuss the work of the NIA Office of Special Populations. Dr. Jones will also be available for small group discussion.

### NIA DIVISION OF EXTRAMURAL ACTIVITIES

Kenneth Santora, *National Institute on Aging, Bethesda, Maryland, United States*

Dr. Santora will discuss the work of the NIA Division of Extramural Activities. Dr. Santora will also be available for small group discussion.

### SESSION 3090 (SYMPOSIUM)

### EXPLORATORY STRUCTURAL PROCEDURES IN HEALTH SERVICES RESEARCH

Chair: Martina Roes Discussant: Barbara Resnick

When analysing data where little knowledge of relationships is available, exploratory structuring techniques, e.g., clustering and scaling techniques, are applied, using an inductive approach to construct analogies and thus suggest how to organize the data. These procedures in Health Service Research mainly focus on latent variables and are particularly used in the development of instruments or theories. Our symposium includes three presentations on research studies in which exploratory structural procedures were used for data analysis, that were considered appropriate for Health Service Research. Dr. Johannes Bergmann will present a concept mapping technique of the German Preferences for Everyday Living Inventory (PELI-D), for structuring the questionnaire from the perspective of professional nurses. This is a mixed methods participatory approach that combines interpretation and decisions by the researchers with a sequence of multivariate statistical analyses (multidimensional scaling, cluster analysis).. Jan Dreyer will present a Multiple Correspondence Analysis and Hierarchical Cluster Analysis as exploratory data reduction procedures. His aim was to identify types of home-based care arrangements for people living with dementia. To analyse the relationships between care arrangement variables, he performed Multiple Correspondence Analysis. To cluster the care arrangements, he performed Hierarchical Cluster Analysis. Anna Louisa Hoffmann will present an alternative, modified procedure of Multiple Correspondence analysis. Since Multiple Correspondence Analysis underestimates the true quality of data representation, she used Adjusted Multiple Correspondence Analysis to explore construct validity of the Dementia Policy Questionnaire (DemPol-Q). This procedure was considered appropriate to assign categorical variables to latent variables.

### USING ADJUSTED MULTIPLE CORRESPONDENCE ANALYSIS TO EXPLORE LATENT VARIABLES FOR THE DEMPOL-Q

Anna-Louisa Hoffmann[1], Johannes-Michael Bergmann[2], Bernhard Holle[3], Martina Roes[1], and Rebecca Palm[4], *1. German Center for Neurodegenerative Diseases (DZNE), Witten, Nordrhein-Westfalen, Germany, 2. German Center for Neurodegenerative Diseases (DZNE), Göttingen, Nordrhein-Westfalen, Germany, 3. German Center For Neurodegenerative Diseases (DZNE), Witten, Nordrhein-Westfalen, Germany, 4. University of Witten/Herdecke, Witten, Nordrhein-Westfalen, Germany*

Nursing home administrators are responsible to operationalize dementia-specific person-centred care into their care practice, e.g. by internal policies. To assess the degree of existence of internal policies on person-centred care for dementia-specific care in German nursing homes, we developed the Dementia Policy Questionnaire (DemPol-Q). After pretesting, the instrument consists of 19 dichotomous items. We aimed to explore the construct validity of the DemPol-Q. We used a secondary data set of a national survey with a representative sample of 134 nursing homes. For data analysis we conducted an Adjusted Multiple Correspondence Analysis. Results show that nine items of the DemPol-Q were significantly assigned to two latent variables. Since the items per latent variable vary in their content, they cannot be explicit denominated with a specific sub-dimension of dementia-specific person-centred care. Nonetheless, Adjusted Multiple Correspondence Analysis is an uncommon but appropriate exploratory data reduction procedure for nursing science.

### USING MULTIDIMENSIONAL SCALING IN COMBINATION WITH CLUSTER ANALYSIS TO STRUCTURE THE PELI-D QUESTIONNAIRE

Johannes-Michael Bergmann[1], Tobias Stacke[2], Daniel Purwins[3], Christina Manietta[3], Mike Rommerskirch-Manietta[4], Armin Stroebel[5], and Martina Roes[6], *1. German Center for Neurodegenerative Diseases (DZNE), Göttingen, Nordrhein-Westfalen, Germany, 2. Community Bergkamen, Bergkamen, Nordrhein-Westfalen, Germany, 3. DZNE, Witten, Nordrhein-Westfalen, Germany, 4. German Center For Neurodegenerative Diseases (DZNE), Witten, Nordrhein-Westfalen, Germany, 5. Center for Clinical Studies, Erlangen, Bayern, Germany, 6. German Center for Neurodegenerative Diseases (DZNE), Witten, Nordrhein-Westfalen, Germany*

Background: One way to contribute person-centered care is the use of a preference-based approach. In our project PELI-D the original PELI USA-version for Nursing Homes was (1) translated into German (2) and structured to explore construct validity of the instrument. Objective and Method: The PELI-D instrument (72-items) is used to illustrate how structuring procedures can be used for concept analysis. The approach allows to analyze the instrument from the perspective of the professional caregivers and to identify theoretical relations in further steps, which can be finally validated. For this purpose, the data of 58 professional nurses are analyzed.

Results: We computed a three-dimensional concept map of preferences enabled us in a second step to identify an appropriate cluster solution. The five-cluster solution, which explained 79 percent of the total variance, seemed to offer the best balance between statistical analysis and detailed categorisation through qualitative interpretation.

### USING MULTICORRESPONDENCE ANALYSES AND CLUSTER ANALYSIS TO CONSTRUCT TYPES OF CARE ARRANGEMENTS

Jan Dreyer[1], Johannes-Michael Bergmann[1], Kerstin Koehler[2], Iris Hochgraeber[2], Christiane Pinkert[2], Martina Roes[3], and Bernhard Holle[4], *1. German Center for Neurodegenerative Diseases (DZNE), Göttingen, Nordrhein-Westfalen, Germany, 2. German Center For Nuerodegenerative Diseases (DZNE), Witten, Nordrhein-Westfalen, Germany, 3. German Center for Neurodegenerative Diseases (DZNE), Witten, Nordrhein-Westfalen, Germany, 4. German Center For*

*Neurodegenerative Diseases (DZNE), Witten, Nordrhein-Westfalen, Germany*

Most persons with dementia live at home and are cared for by family carers and professional carers. Together, they form care arrangements to address the needs of persons with dementia. The aim of this study is to (1) uncover the underlying structures of home-based care arrangements for persons living with dementia, (2) construct types of these care arrangements, and (3) compare these types with regard to their stability. In this secondary analysis, data from 320 care arrangements for persons with dementia are analysed using multiple correspondence analysis and hierarchical cluster analysis. The multiple correspondence analysis identified 27 axis that explained the entire variance between all care arrangements. The subsequent cluster analysis identified four types of care arrangements. Two types included spouse-centred care arrangements, and two types included child-centred care arrangements at different phases of the dementia and care trajectory. The types differ with regard to their stability.

### SESSION 3100 (SYMPOSIUM)

### EXPLORING STRATEGIES FOR IMPROVED COORDINATION AND INNOVATION IN TRANSPORTATION FOR OLDER PEOPLE

Chair: Nina Silverstein Co-Chair: Taylor Jansen Discussant: Alycia Bayne

Communities across the nation have outgrown their capacities to meet the transportation needs of older people. While a struggle in the past, for many communities it now is a crisis given an increase in older people with impairments in critical driving skills that lead to driving cessation and/or limitations that preclude them from successfully navigating public transit. It is time for new strategies and innovations to meet these growing challenges. Jansen will describe a pilot study of a regional transit authority's use of customized software with four Councils on Aging to increase riders' trips and destinations, improve efficiencies in reporting and dispatching, and increase shared rides across the communities. Gleason will present results of a qualitative study of paratransit managers across the U.S. and their attempts to innovate and adapt to evolving market expectations spurred on by the emergence of transportation network companies (TNC) like Uber and Lyft. Schwartz describes a mixed-methods study of Project TRIP, a rural transportation program designed to increase access to healthcare and other locations for low income and vulnerable populations in rural eastern North Carolina. Lynott introduces AARPs RideSheet, an open-source ride scheduling software application designed for small, demand-responsive transportation providers that incorporates a transactional data specification (TDS) enabling two or more providers to interoperate more efficiently, improving service for their clients. Bayne concludes as discussant bringing in her own work with the CDC on barriers and facilitators to ride sharing and reflecting on the themes presented. Transportation and Aging and Qualitative Research IGs collaboration.

### BRIDGING SILOS: EVALUATION OF AN INNOVATIVE REGIONAL SENIOR TRANSPORTATION SERVICE

Taylor Jansen, Nina Silverstein, Beth Dugan, Chae Man Lee, Shu Xu, and YanJhu Su, *University of Massachusetts Boston, Boston, Massachusetts, United States*

A pilot study was conducted for a regional transit authority (RTA) to evaluate the implementation of a customized software to increase ridership, trips, destinations and coordination of rides among four Councils on Aging (COA) using RTA vans to serve rural communities in north central Massachusetts. Baseline and follow up ride data were collected from each COA from Fall 2019 (N= 178 riders; N= 4,230 trips) and Fall 2021 (N= 131 riders; N= 2,020 trips) and from 59 stakeholder interviews with riders, drivers, dispatchers, and staff from the COAs, RTA, and software company. The evaluation found after 6 months that while the goals of the pilot were not yet achieved, due in part to external factors such as the COVID-19 pandemic and driver shortage, valuable lessons were learned including the importance of balancing provider goals of efficiency and reporting with rider needs of driver hands-on assistance and friendly scheduling support.

### THE FUTURE OF PARATRANSIT: A STUDY OF PROVIDER EXPERIENCES AND HOPES FOR CHANGE

Shayna Gleason, and Nina Silverstein, *University of Massachusetts Boston, Boston, Massachusetts, United States*

The present study examined paratransit managers' perceptions of a changing transportation market, and what resources or supports they might need to adapt to evolving market expectations. As transportation network companies (e.g., Uber, Lyft) have emerged, the landscape of demand-responsive transportation has changed. However, the experiences of those closest to the operations of paratransit programs have been largely neglected in research. In-depth, semi-structured interviews were conducted with 16 managers of paratransit services. The resulting transcripts were coded iteratively using NVivo software, using both inductive and deductive approaches. We found that participants were already innovating and often wanted to be even more creative with their services, but were hampered by inadequate funding, driver shortages, regulatory or policy challenges, and other barriers. This study's findings advance the literature toward greater understanding of how policymakers can leverage existing paratransit infrastructure to better serve the transportation needs of older adults and other transportation disadvantaged groups.

### A MIXED METHODS EVALUATION OF PROJECT TRIP: KEY FINDINGS AND EXPANSION

Abby Schwartz[1], Alice Richman[2], Mallary Scott[2], Haiyong Liu[2], Weyling White[3], and Caroline Doherty[3], *1. East Carolina University, School of Social Work, Greenville, North Carolina, United States, 2. East Carolina University, Greenville, North Carolina, United States, 3. Roanoke Chowan Community Health Center, Ahoskie, North Carolina, United States*

Project TRIP is an innovative individualized transportation program for low-income adults in rural eastern North Carolina (eNC), that aims to increase access to healthcare and reduce health disparities. This paper presents results from a 3-year grant which sought to evaluate TRIP and expand it to a neighboring eNC county. The concurrent mixed methods findings from the process/implementation, outcome/effectiveness, and economic evaluation, and qualitative interview data with TRIP stakeholders (N=40) will be presented. Findings include relationships between pre and post TRIP enrollment health data indicating improvements in health outcomes and decreases in health care utilization, including estimated costs savings of Project TRIP. Qualitative interview data will be discussed in substantiating the quantitative evaluation data. Interviews with stakeholders in the TRIP replication and expansion county site will also be presented including pilot test data in that county. The presentation will conclude with implications for sustainability and further expansion of TRIP.

### SESSION 3110 (SYMPOSIUM)

### GERONTOLOGIC BIOSTATISTICS: MERGING WITH DATA SCIENCE AND TOWARD PERSONALIZED MEDICINE

Chair: Michelle Shardell Co-Chair: Terrence Murphy Discussant: Heather Allore

The sub-discipline of gerontologic biostatistics (GBS) was introduced in 2010 to emphasize the special challenges encountered in the design and analysis of research studies of older persons. These challenges center on the multifactorial nature of human aging, characterized by the parallel and progressive deterioration of diverse organ and cellular systems that eventually results in death. Ten years after the introduction of GBS, which initially focused on important aspects of design and analysis that ensure their statistical validity, we update how GBS has been enriched by evolving practices. We present individual sessions on three seminal developments in the practice of GBS: integration of data science and multiple streams of data, including those automated and or multidisciplinary in nature; enhanced methods of addressing the heterogeneity of treatment effects from health-related interventions for older patients; and how interactive visualization can help specific patients locate themselves along the continuum of individualized treatment effects. We conclude our presentation with a session that reviews three prominent trends in the validation of the heterogeneity inherent to the assessment of health among older adults. Reflecting this era of big gerontological data, we discuss several established modeling approaches for validation, the proliferation of signal intensive behavior phenotypes, and the deep characterization of phenotypes through OMICS studies and multimodal approaches. All talks discuss pitfalls and areas of future development and draw from published studies. We are submitting as an interest group collaborative panel submission between two interest groups: Epidemiology of Aging and Measurement, Statistics and Design.

### GERONTOLOGICAL BIOSTATISTICS AS THE FOUNDATION OF INTERDISCIPLINARY DATA SCIENCE IN AGING

Thomas Travison, *Harvard Medical School, Boston, Massachusetts, United States*

Explosive growth in computing power has increased by orders of magnitude the complexity of data structures and the number of analyses that may be performed per unit time. Contemporary gerontology utilizes diverse data ranging from continuous longitudinal assessments (e.g. motion capture) to complex single-timepoint assessments (e.g. bioimages) to systems-level administrative descriptions of the healthcare delivery environment. Paradoxically, this abundance of resources presents a considerable challenge, as the availability of information threatens to overwhelm mechanistic models of aging supportive of intervention development. To inform precision medicine, gerontological biostatistics therefore embraces the opportunity of collaborating with allied quantitative disciplines to bolster the coherence, reproducibility, and generalizability of findings. This presentation will demonstrate the salient advantages of such interdisciplinary collaborations using the example of design and analysis of an intensely longitudinal study of wearable and environmental sensors, conducted with teams in exercise science and architectural design.

### TIME TO EMBRACE HETEROGENEITY IN INTERVENTIONAL STUDIES OF OLDER ADULTS

Qian-Li Xue, *Johns Hopkins University, Baltimore, Maryland, United States*

Adopting evidence-based medicine in clinical care of older patients is challenging because the “best” evidence available may not be directly applicable due to exclusion or underrepresentation of older adults in clinical trials. Interventions shown to be beneficial in trial populations therefore often exhibit heterogeneous treatment effects (HTEs) in older adults, particularly among the most vulnerable. This talk will review the concept, causes, and estimation of HTEs. We distinguish clinical heterogeneity from methodologic heterogeneity, defined respectively as the variation in biological mechanisms leading to similar aging phenotypes and variation in study design, measurement, and analysis. We use examples drawn from geriatric medicine to introduce novel study designs and data analytics used to study HTEs. This talk highlights the importance of moving beyond post-hoc subgroup analysis to an approach that integrates theories, observational and experimental data, and data science in the study of HTEs.

### FINDING YOURSELF: PERSONALIZED MEDICINE, DATA SCIENCE, AND INTERACTIVE VISUALIZATIONS

Michael Griswold[1], James Henegan[2], Chad Blackshear[2], Beverly Gwen Windham[3], and Thomas Mosley[3], *1. The MIND Center at UMMC, Jackson, Mississippi, United States, 2. UMMC MIND Center, Jackson, Mississippi, United States, 3. UMMC-The MIND Center, Jackson, Mississippi, United States*

Personalized medicine is care that is tailored to an individual patient. In contrast, randomized trials are designed to report evidence of benefits and harms "on average". The average trial participant though, is often healthier than older patients seen in clinical practice. A variety of methods have been proposed which offer improvements to traditional (but questionable) practices of one-subgroup-at-a-time examinations of treatment effect heterogeneity, but translating evidence from these advancements has received less attention. Data Science initiatives have allowed broader data sharing, data harmonization and data synthesizing approaches, as well as an enormous maturing of interactive visualization techniques. Motivated by the SPRINT study and using advanced approaches of marginalized standardization and the Predictive Approaches to Treatment effect Heterogeneity (PATH) statement, we show how researchers, regulators, clinicians and patients can "find themselves" on the treatment effect continuum and be better informed of potential individualized evidence through interactive visualizations.

### SIGNAL DETECTION AND VALIDATION IN AN ERA OF BIG GERONTOLOGICAL DATA

Karen Bandeen-Roche, *Johns Hopkins Bloomberg School of Public Health, Baltimore, Maryland, United States*

Older adult health assessment long has posed measurement challenges—multidimensionality of sentinel outcomes like functioning and frailty, for example. This presentation discusses three developments creating opportunities for gerontologic biostatistics (GBS) over the past 10 years. Firstly, modeling to internally validate measurements or to quantify systematic heterogeneity in assessing older adult health has become considerably more widespread. Confirmatory latent variable modeling, harmonization, and mixture models will be addressed. Secondly, signal intensive behavioral phenotypes are proliferating, e.g. accelerometry, sleep actigraphy, and ecological momentary assessment. Functional data analysis is described as a data analytic technique to extract signal capturing main behavioral features or most relevant for health outcomes. Thirdly, "deep" characterization is under hot pursuit—whether by single- or multi-'omics studies, or by multimodal phenotyping as is increasingly common in the study of cognition. Techniques to accomplish this replicably are discussed. Throughout, potential pitfalls and implications for gerontologic data science development are identified.

### SESSION 3115 (PAPER)

### MENTAL HEALTH (PAPERS)

### CHRONIC PHYSICAL HEALTH CONDITIONS, DEPRESSIVE SYMPTOMS, AND SELF-RATED HEALTH IN GRANDMOTHERS

Christina Henrich[1], Carol Musil[1], Jaclene Zausniewski[1], Christopher Burant[2], Elizabeth Tracy[1], and Alexandra Jeanblanc[1], *1. Case Western Reserve University, Cleveland, Ohio, United States, 2. Case Western Reserve University, Parma, Ohio, United States*

Grandmothers often participate in caregiving to grandchildren, ranging from informal caregiving to having responsibility for raising grandchildren, but health problems may affect their ability to do so. To better understand potential health problems in grandmother caregivers, this secondary analysis examined 1) longitudinal relationships between chronic physical health conditions, depressive symptoms, and self-rated health with race, age, & marital status, and 2) causal ordering of depressive symptoms and self-rated health, controlling for baseline race, age, marital status, and chronic conditions of 328 Ohio grandmothers across 3 time points over 2 years. The study was guided using Lenz's Theory of Unpleasant Symptoms. Measures included chronic physical health conditions (modified Charlson), depressive symptoms (CES-D), self-rated health (SF-36), and demographic variables of race (white/nonwhite), age, and marital status (married-partnered/not married). Analyses included correlations and structural equation modeling. Pearson product moment correlations indicated significant relationships (ranging from 0.26 to 0.56) between chronic conditions, depressive symptoms and self-rated health at all three time points. Next, a bivariate autoregressive model was tested using structural equation modeling. The data fit the model well. The goodness of fit results included: Chi square=55.39, TLI=0.94, CFI=0.97, and RMSEA=0.07. Results indicated consistency of depressive symptoms and self-rated health over time, and these were affected by chronic conditions; the causal order indicated that self-rated health impacts depressive symptoms. This study contributes new knowledge about the relationships and causal sequence of self-rated health and depressive symptoms. Implications on practice and research are discussed.

### DEPRESSIVE SYMPTOMS AND MULTIMORBIDITY IN CONTEXT: INTERPERSONAL, SOCIOCULTURAL, AND TEMPORAL EFFECTS

Irina Mindlis[1], and Tracey Revenson[2], *1. The Graduate Center, City University of New York, New York City, New York, United States, 2. The Graduate Center, City University of New York, New York, New York, United States*

Among older adults with multimorbidity (MM), disease-related stressors (e.g., pain) are associated with greater depressive symptoms. However, the contextual factors influencing this relationship remain understudied. We explored the moderating effects of interpersonal, sociocultural, and temporal factors as buffers of this relationship. Adults ≥ 62 years with MM (n=366) recruited through a national health volunteer registry and an online panel platform completed validated scales assessing diagnoses, disease-related stressors (pain intensity, subjective cognitive function, physical function, somatic symptoms), depressive symptoms. Potential moderators: age, expectations regarding aging, perceived social support, and difficulty affording medications (proxy for SES). Data were analyzed with structural equation modeling. Participants were 62-88 years old and living with many illnesses (M = 3.5; range: 2-9); 15% reported moderate-to-severe depressive symptoms. Among those with low social support, the effect of disease-related factors on depressive symptoms was greater (B = .70, SE = .06, p <.001) than for those with high social support (B = .46, SE = .06, p < .001)]. The negative effect of disease-related factors on depressive symptoms was stronger for those with worse expectations of aging (B = .68, SE = .07, p <.001), compared to those with more positive expectations (B = .47, SE = .06, p < .001). Age and difficulties affording medications were not significant moderators. Among older adults with MM, garnering social support and addressing low expectations for old age may be key to preventing the detrimental effect of MM on mental health.

### JOINT TRAJECTORIES OF SOCIAL ISOLATION AND LONELINESS AND THEIR EFFECTS ON ACCELERATED BIOLOGICAL AGING

Xiang Qi[1], Daniel Belsky[2], Yang Yang[3], Ted Ng[4], and Bei Wu[5], *1. New York University, New York City, New York, United States, 2. Mailman School of Public Health, Columbia University, New York City, New York, United States, 3. University of North Carolina at Chapel Hill, Chapel Hill, North Carolina, United States, 4. Arizona State University, Phoenix, Arizona, United States, 5. New York University, New York, New York, United States*

Social isolation and loneliness (SI/L) are associated with a variety of physical and mental health conditions. Biological aging is a proposed mechanism through which psychosocial factors drive health disparities. However, it is not known how SI/L separately and jointly affect biological aging. Using longitudinal data (N=1,965 adults, aged 50+) in Health and Retirement Study (2006 to 2016), we tested the joint trajectories of SI/L and their differential effects on biological aging as quantified by five DNA Methylation clocks (Horvath, Hannum, PhenoAge, GrimAge, and DunedinPoAm). A group-based mixture modeling approach was used to fit the joint trajectories of SI/L. Biological aging was measured using residuals from regressing each clock on chronological age. Linear regressions were used to test associations of joint trajectories of SI/L with biological aging. Four SI/L trajectories were identified, including Rarely isolated and rarely lonely (47.8%), Moderately isolated and rarely lonely (15.5%), Rarely isolated and severely lonely (20.2%), and Persistently isolated and lonely (16.5%). Of all joint trajectory groups, the Rarely isolated and rarely lonely group had the youngest biological age as estimated by the Hannum, PhenoAge, and GrimAge clocks. Compared with the Rarely isolated and severely lonely group, the Moderately isolated and rarely lonely group had higher age acceleration for PhenoAge ($\beta$=0.10; 95% CI=0.01, 0.19), and GrimAge ($\beta$=0.20; 95% CI=0.06,0.33), and DunedinPoAm ($\beta$=0.21; 95% CI=0.09,0.33). Different trajectories of SI/L convey differential risks to biological aging. Future research is needed to investigate the differences and similarities of SI/L trajectories and identify intervention targets for ameliorating aging.

### PREDICTORS OF DEPRESSION AMONG MIDDLE-AGED AND OLDER MEN AND WOMEN IN EUROPE: A MACHINE LEARNING APPROACH

Stephen Aichele[1], Elizabeth Handing[2], Carolin Strobl[3], Yuqin Jiao[4], and Leilani Feliciano[5], *1. Colorado State University, Fort Collins, Colorado, United States, 2. Colorado State University, Golden, Colorado, United States, 3. University of Zurich, Zurich, Zurich, Switzerland, 4. Colorado State University at Fort Collins, Fort Collins, Colorado, United States, 5. University of Colorado Colorado Springs, Colorado Springs, Colorado, United States*

The high prevalence of depression in a growing aging population represents a critical public health issue. It is unclear how social, health, cognitive, and functional variables rank as risk/protective factors for depression among older adults and whether there are conspicuous differences among men and women. We utilized random forest analysis (RFA), a machine learning method, to compare 56 risk/protective factors for depression in a large representative sample of European older adults (N = 67,603; ages 45-105y; 56.1% women; 18 countries represented) from the Survey of Health, Ageing and Retirement in Europe (SHARE Wave 6). Self-rated social isolation and self-rated poor health were the most salient risk factors, jointly accounting for 22.0% (in men) and 22.3% (in women) of variability in depression. Difficulties in mobility (in both sexes), difficulties in instrumental activities of daily living (in men), and higher self-rated family burden (in women) accounted for an additional but small percentage of variance in depression risk (2.2% in men, 1.5% in women). To our knowledge, this is the first large, multi-country study to use machine learning to compare a broad range of social, health, functional, and cognitive variables as concurrent risk/protective factors for depression in middle-aged and older adults—and with analyses conducted independently for women and men. The results point to the importance of screening for depression risk within this age demographic during routine medical visits (i.e., when assessing general health status) and further indicate that such screening may be improved by inclusion of measures of perceived social isolation.

### SESSION 3120 (SYMPOSIUM)

### HEALTHY AGING: POLICIES AND RESOURCES TO SUPPORT AN AGE-FRIENDLY ECOSYSTEM

Chair: Patricia D'Antonio Discussant: Brian Lindberg

Our public health system is advancing activities to prioritize the health and well-being of older adults and recognizes the importance of policies to support us all as we age. Faculty will discuss national efforts underway to inform and support an age-friendly ecosystem.

### HEALTHY PEOPLE 2030 AND HEALTHY AGING

Yen Lin, *Office of Disease Prevention and Health Promotion, Rockville, Maryland, United States*

The US Department of Health and Human Services, Office of Disease Prevention and Health Promotion (ODPHP) sets science-based health promotion and disease prevention objectives for the nation with 10-year targets through the Healthy People Initiative. In addition to objectives specific to older adults, many of our population-based objectives in other condition-specific topics also contain data for older adults. Healthy People also developed the Department of Health and Human Service's Social Determinants of Health model. This presentation will highlight ODPHP's policy initiatives and campaigns specifically for older adults, including the Dietary Guidelines for Americans 2020-2025, Move Your Way © for Older Adults and provide an update on the Physical Activity Guidelines Midcourse Report on Older Adults.

### BUILDING OUR LARGEST DEMENTIA INFRASTRUCTURE FOR ALZHEIMER'S ACT: POLICIES ADVANCING HEALTHY AGING

Lisa McGuire, *CDC, Atlanta, Georgia, United States*

While public health experts cannot yet say how to prevent Alzheimer's disease and related dementias (ADRD), emerging science indicates that ADRD may be slowed through risk reduction strategies, early diagnosis, and better education and training of front-line health care professionals. Recognizing these scientific advances, the National Plan to Address Alzheimer's Disease: 2021 Update added a new goal, Goal 6, to the 10-year-old plan emphasizing risk reduction and healthy aging: Accelerate Action to Promote Healthy Aging and Reduce Risk Factors for Alzheimer's Disease and Related Dementias. CDC is authorized through Building Our Largest Dementia (BOLD) Infrastructure for Alzheimer's Act (P.L. 115-406) to create a uniform national public health infrastructure through BOLD Program and Public Health Centers of Excellence. This presentation will highlight CDC activities that not only build a uniform national public health infrastructure but also promote healthy aging consistent with Goal 6 of the 2021 National Plan.

### ELEVATING HEALTHY AGING: HOW PUBLIC HEALTH IS SUPPORTING OLDER ADULTS

Karon Phillips[1], and Megan Wolfe[2], *1. Trust for America's Health, Silver Spring, Maryland, United States, 2. Trust for America's Health, Washington, District of Columbia, United States*

Trust for America's Health (TFAH) leads the national Age-Friendly Public Health Systems initiative which focuses on expanding the public health role in healthy aging. The roles of public health include creating and leading policy, systems, and environmental changes; connecting and convening multi-sector stakeholders to improve older adult health and well-being; and collecting and translating data on older adult health to inform interventions in communities and states. TFAH will share examples of how public health departments are elevating healthy aging as a core function.

### SESSION 3130 (PAPER)

### HOSPITAL TRANSITIONS, HEALTH CARE UTILIZATION, AND OUTCOMES OF CARE

### ASSOCIATIONS BETWEEN SOCIAL ISOLATION AND HOSPITAL STAYS, NURSING HOME ENTRY, AND MORTALITY OVER TIME

Mary Louise Pomeroy[1], Gilbert Gimm[2], Alison Cuellar[3], Emily Ihara[3], and Thomas Cudjoe[4], *1. Johns Hopkins University, Fairfax, Virginia, United States, 2. George Mason University, Vienna, Virginia, United States, 3. George Mason University, Fairfax, Virginia, United States, 4. Johns Hopkins School of Medicine, Baltimore, Maryland, United States*

This study examined social isolation as a risk factor for hospitalization, nursing home stays, and mortality among a longitudinal sample of 12,860 community-dwelling older adults ages 65+ between 2006-2018. Using seven waves of Health and Retirement Study (HRS) data, we examined associations between social isolation and hospitalization, nursing home stays, and mortality. Social isolation scores ranged from 0-6 using an established typology with six objective measures of social interactions: 1) marital status; 2) living arrangement; 3) monthly communication with children; 4) family; 5) and friends; and, 6) monthly participation in groups, clubs, organizations, or religious services. Covariates included fixed demographics and time-variant characteristics including loneliness, depression, Medicaid enrollment, lifestyle behaviors, activities of daily living, number of chronic conditions, and Alzheimer's disease or related dementias. Logistic regression analyses were conducted with panel data and random effects to examine associations between social isolation and each outcome over time. About 15% of the sample was socially isolated, with an average isolation score of 1.29. Social isolation significantly increased the odds of having a nursing home stay ($OR = 1.22$, $p < 0.001$) and mortality ($OR = 1.14$, $p < 0.001$). However, it was not significantly associated with overnight hospitalizations ($OR = 0.97$, $p = 0.09$). Social isolation may put older adults at greater risk for nursing home stays and mortality over time, as compared to social integration. Policies and practices to reduce social isolation can support aging at home or the community, delay nursing home entry, and reduce the risk of mortality.

### PREDICTIVE MODEL OF 30-DAY HOSPITAL READMISSION FOR PATIENTS WITH ALZHEIMER'S DISEASE

Elham Mahmoudi, Cyrus Najarian, Wenbo Wu, James Aikens, and Julie Bynum, *University of Michigan, Ann Arbor, Michigan, United States*

Hospitals are insufficiently equipped for patients with Alzheimer's disease and related dementia (ADRD). Thus, 30-day hospital readmission is higher and costlier among ADRD patients than the general population of older adults. Our objective was to develop a risk-assessment tool for hospitalized patients with ADRD. We used 2016-2019 electronic medical record (EMR) data from the University of Michigan health system and applied machine learning techniques to develop a readmission risk-assessment tool. We identified 2,899 individuals with ADRD who had at least one index hospital admission. All data features available in EMR – demographics, lab results, prior counts of healthcare use, and characteristics of index hospitalization – were included in our predictive models. Additionally, we geocoded the street address of patients using the National Neighborhood Data Archive using the U.S. Census tract-level information to include two composite measures of socioeconomic status: disadvantage and affluence. The readmission rate for ADRD patients was 22% versus 17% for the general population. The best predictive model was the Random Forest (area under the receiver operating characteristic curve=0.66; sensitivity=0.64; specificity=0.61). The accuracy of our model (0.61) was 42% higher than the LACE score (0.43), which is currently used by the hospital for all patients. The top 5 predictors of 30-day readmission among people with ADRD included length of hospital stay, frailty index, living in a disadvantaged neighborhood, and total prior-year healthcare charges. Our risk-assessment tool identifies ADRD patients at high risk of readmission and why they are at higher risk. The tool enables better decision-making before discharge.

### SOCIAL DETERMINANTS OF HOSPITAL READMISSION FOR OLDER VETERANS

Portia Cornell[1], Christopher Halladay[2], Gina Chmelka[3], Caitlin Celardo[3], Robert Burke[4], Jennifer Silva[3], James Rudolph[5], and Kali Thomas[6], *1. Providence VA Medical Center / Brown University, Providence, Rhode Island, United States, 2. Providence VA Medical Center, Providence, Rhode Island, United States, 3. Veterans Health Administration, Washington, District of Columbia, United States, 4. University of Pennsylvania Perelman School of Medicine, Philadelphia, Pennsylvania, United States, 5. Veterans Health Administration, Providence, Rhode Island, United States, 6. Brown University, Providence, Rhode Island, United States*

The objective of this study was to estimate the effect of social risk on the likelihood of hospital readmission. Our study sample included 156,690 hospitalizations from 2016 - 2019 at one of 36 VA medical centers that participated in a national social-work staffing program. Using information from outpatient screenings, social workers' assessments, and diagnosis codes, We identified Veterans' social risks categorized into nine specific categories: intimate partner violence, financial need, housing instability, legal problems, social isolation, mental health, transportation, food insecurity, and functional need; and two general categories: nonspecific psychosocial and neighborhood deprivation. We estimated linear probability models of unplanned hospital readmission to a VA or a community hospital within 30 days of discharge, adjusted for demographics, clinical characteristics known to predict readmission (length of stay, primary diagnosis, admission from emergency department, chronic comorbidities, previous hospitalizations), and year and hospital fixed effects. 15.3 percent of hospital stays were followed by an unplanned readmission within 30 days. The prevalence of specific social risks ranged from 1.2% (food insecurity) to 13.9% (financial need). Social risk factors are important predictors of unplanned hospital readmission among Veterans after adjusting for medical risk. The risk categories with the strongest adjusted association with 30-day readmission were legal need, risk difference .033 (p=.015); interpersonal violence (r.d.=.022, p<.001); mental health (r.d.=.022, p=.002); social isolation (r.d.=.010, p<.001); and nonspecific psychosocial (r.d.=.017, p<.001). These social risk indicators could be used to target care-transition intervention and follow-up by a social worker to address social needs and avert unplanned hospital readmission.

### THE INCREMENTAL EFFECTS OF DEMENTIA ON HEALTHCARE USE AND ECONOMIC STATUS IN THE US: A QUASI-EXPERIMENTAL APPROACH

HwaJung Choi[1], Kenneth Langa[1], Edward Norton[1], Tsai-Chin Cho[2], and Cathleen Connell[1], *1. University of Michigan, Ann Arbor, Michigan, United States, 2. University of Michigan School of Public Health, Ann Arbor, Michigan, United States*

This research is to assess the impact of dementia on healthcare use and economic status over the course of dementia. We used a nationally representative, longitudinal study of older adults, specifying two propensity-score matched samples: 1) 3,476 adults aged 55+ at baseline who would have dementia in two years (hereafter "dementia group"), and 2) 3,476 adults who had never transitioned to dementia but had similar baseline sociodemographic and health status (hereafter "control group"). In two-year follow-up, the total care hours from unpaid helpers (44 hours per month vs. 20) and other in-home medical care (24% vs. 15%) were substantially higher with vs. without dementia. In the dementia group, the percentage of using nursing homes increased from 4% at the baseline to 27% at 8-year follow-up, compared to the increase from 3% to 10% in the control group. There was little change in OOP costs in the control group but a substantial increase in the dementia group over time (from $4,443 at baseline to $11,806 in an 8-year follow-up). The percent having Medicaid increased from 17% at baseline to 33% in 8-year follow-up in the dementia group. By using a quasi-experimental approach with longitudinal data, the study clarifies the incremental effects of dementia on health care and economic status. The effects of dementia on both home-based and facility-based care are substantial, with major changes occurring at the onset. Dementia exacerbated financial resources with increasing OOP medical costs, which may have profound impacts on families and society.

### THE LOOMING POST-ACUTE REORGANIZATION: IMPLICATIONS FOR OLDER ADULTS TRANSITIONING FROM THE HOSPITAL

Taylor Bucy[1], John McHugh[2], and Dori Cross[1], *1. University of Minnesota School of Public Health, Minneapolis, Minnesota, United States, 2. Columbia University Mailman School of Public Health, New York, New York, United States*

Post-acute volume plays an important role in the care of millions of older adults transitioning from a hospital stay. To help reduce the total cost of care, many hospitals have been shifting more volume from institutional skilled nursing care to less expensive home health care. In fact, in 2017, home health volume eclipsed skilled nursing facility volume as the preferred post-acute destination for the first time. The COVID-19 pandemic further exacerbated this trend with many older adults preferring to avoid institutional care. Using 2016-2019 MedPAR data, we explored changes over time in hospital discharges to skilled nursing facilities versus to home health. We regress the ratio of home health to skilled nursing facility volume on a month-year variable. We find that the ratio of discharges to home health versus to skilled nursing facility increases by .07 percentage points per month (coefficient = .00073, 95% CI [.00063 to .00084]). This translates to a nearly one percentage point change in the ratio of home health to skilled nursing volume per year. These trends vary by patient characteristics (e.g. hospital diagnosis), organizational characteristics (e.g. hospital market relationships), and indicators of market capacity of post-acute services. This growing trend has implications on vulnerable older adults; health system leaders need policy guidance and incentives to invest in value-oriented care practices across this changing post-acute landscape.

### SESSION 3140 (SYMPOSIUM)

### INNOVATIONS IN AGING: CREATING A TRANSDISCIPLINARY RESEARCH COLLABORATIVE GRADUATE FELLOWSHIP PROGRAM

Chair: Diane Martin Co-Chair: Nia Latimer Discussant: Katherine Bowers

Social isolation impacts nearly one-quarter of community dwelling adults aged 65+ in the United States. It is associated with poor physical and mental health, cognitive decline, higher healthcare costs, and early mortality. Older adults are at greater risk because they are more likely to live alone, experience a shrinking social network from loss of family and friends, and encounter limitations with driving and mobility resulting from chronic health conditions and sensory impairments. Social isolation in later life is not new; however, the COVID-19 pandemic brought to light the increased risk for negative outcomes. Reducing social isolation is a priority area for our state's Department of Aging (DoA), and the updated State Plan on Aging highlights the need for innovative approaches to develop and strengthen initiatives addressing social isolation among older adults. The DoA is advancing multiple projects, including a partnership with our public university to create a transdisciplinary graduate fellowship program. The program brought together professional students from medicine, nursing, pharmacy, and social work and resulted in a community of practice in which fellows engaged with DoA and university faculty to share best practices and receive training in transdisciplinary research. In this session, the first presentation will focus on development of the fellowship program and highlight the success of the inaugural year; the second session will focus on the future goals of the fellowship program, and the third presentation will share how we plan to expand the partnership between our public university and our state's Department of Aging.

### YEAR ONE AND BEYOND: SUSTAINING A COLLABORATIVE AGING FELLOWSHIP PROGRAM

Diane Martin, *University of Maryland, Baltimore Graduate School, Baltimore, Maryland, United States*

A graduate aging fellowship experience can provide participants with unique opportunities for professional development in interdisciplinary and transdisciplinary research, community engagement, and team science. Program success is contingent on multiple factors beyond participant recruitment and customizable curriculum to meet the educational goals of individual participants. Stakeholder buy-in, strategic planning, and identification of funding streams are all necessary to support an internal program beyond its first year. In this session, we will offer guidance in planning, building, and sustaining a fellowship program for current graduate students with a professional interest in supporting older adults.

### LEVELING THE PLAYING FIELD: ONBOARDING AGING FELLOWS REPRESENTING DIVERSE DISCIPLINES

Catherine Kim[1], Nia Latimer[2], Katherine Bowers[3], Loretta Anderson[4], and Diane Martin[5], *1. Veterans Affairs Maryland Health Care System, Baltimore, Maryland, United States, 2. University of Maryland, Baltimore, Baltimore, Maryland, United States, 3. University of Maryland, Baltimore, Maryland, United States, 4. University of Maryland, Baltimore, Reisterstown, Maryland, United States, 5. University of Maryland, Baltimore Graduate School, Baltimore, Maryland, United States*

The transdisciplinary Innovations in Aging Graduate Fellowship program brought together graduate students from medicine, nursing, pharmacy, and social work. As a result, fellows entered the program with a range of exposure as to how aging impacts the body, social interactions, health needs, and society. Therefore, onboarding modules were utilized to provide an integrated perspective on aging. Topics for these onboarding modules included ageism, dimensions of wellness, and health and well-being in later life. Through these modules, fellows were able to customize their learning to meet their unique educational needs and goals related to their involvement in the fellowship program. In this session, we will share development of the fellowship program and highlight factors, such as the onboarding modules, that led to the program's success.

### SESSION 3150 (SYMPOSIUM)

### INSIGHTS INTO HEALTH-RELEVANT INTERPERSONAL DYNAMICS IN AGING COUPLES FROM THE LAB AND DAILY LIFE

Chair: Theresa Pauly Co-Chair: Karolina Kolodziejczak Discussant: Christina Röcke

Emerging research demonstrates that long-term and daily health indicators are closely linked in aging partners. Yet, not much is known about exactly how partners get under each other's skin. This symposium investigates different positive and negative interpersonal contexts, including physical intimacy, positive and negative emotional experiences, and couple conversations (e.g., conflict, recounting distressing memories, discussing enjoyable topics), and their link with neuroendocrine and cardiovascular markers. The four talks feature a variety of study designs ranging from laboratory research to ambulatory assessment methods which recruited couples in midlife and old age. Kolodziejczak et al. use ambulatory assessment data to examine links between (experienced and wished for) physical intimacy, affect, and cortisol in older couples. Pauly et al. pool data from three ambulatory assessment aging studies to analyze how own and partner positive and negative affective states are intertwined with everyday cortisol secretion in old age. Wilson et al. invited middle-aged to older couples to engage in two different conversations (recounting a difficult memory, conflict) and investigated concurrent changes in affect and cardiovascular activity. Meier and colleagues make use of automated language analysis to examine how positive and negative emotion word use during a positive and negative conversation in the laboratory relates to cardiovascular reactivity in middle-aged couples. The discussion by Christina Röcke will delineate insights gained from these four papers, discuss their strengths and limitations, and outline directions for future inquiry.

### PHYSICAL INTIMACY IN DAILY LIVES OF OLDER ROMANTIC COUPLES: LINKS WITH MOMENTARY AFFECT AND DAILY CORTISOL LEVELS

Karolina Kolodziejczak[1], Johanna Drewelies[2], Theresa Pauly[3], Nilam Ram[4], Christiane Hoppmann[5], and Denis Gerstorf[6], *1. Medical School Berlin, Berlin, Berlin, Germany, 2. Humboldt University Berlin, Berlin, Berlin, Germany, 3. University of Zurich, Zurich, Zurich, Switzerland, 4. Stanford University, Stanford, California, United States, 5. University of British Columbia, Vancouver, British Columbia, Canada, 6. Humboldt Universität zu Berlin, Berlin, Berlin, Germany*

Physical intimacy is assumed to benefit well-being through stress-buffering and mood-improving processes. Although partnered older adults often report wishing for and experiencing physical intimacy, inquiries about how intimacy is linked to affect and stress in older couples' daily lives remain scarce. We examined self-report and salivary cortisol data from 120 German couples (Mage= 71.6, SDage= 5.94) obtained up to seven times per day over seven consecutive days. In moments when participants experienced more physical intimacy, women reported less negative affect, and men more positive affect. Experiencing more overall physical intimacy was associated with more positive affect and less negative affect in women, and lower daily cortisol in men. More overall intimacy wished was related to more negative affect in women and men, and to higher daily cortisol in men. We conclude that physical intimacy relates to indicators of well-being in older couples' daily lives and consider routes for future inquiry.

### "WHAT'S YOURS IS MINE": A DYADIC STUDY OF EVERYDAY EMOTIONAL EXPERIENCES AND CORTISOL IN OLDER COUPLES

Theresa Pauly[1], Karolina Kolodziejczak[2], Johanna Drewelies[3], Maureen Ashe[4], Kenneth Madden[5], Nilam Ram[6], Denis Gerstorf[7], and Christiane Hoppmann[5], *1. University of Zurich, Zurich, Zurich, Switzerland, 2. Medical School Berlin, Berlin, Berlin, Germany, 3. Humboldt University Berlin, Berlin, Berlin, Germany, 4. The University of British Columbia, Vancouver, British Columbia, Canada, 5. University of British Columbia, Vancouver, British Columbia, Canada, 6. Stanford University, Stanford, California, United States, 7. Humboldt Universität zu Berlin, Berlin, Berlin, Germany*

Older partners' health is linked; individuals' physiological arousal can be shaped by their own and their partner's emotional experiences. This study examines those associations using repeated daily life assessments (7 days) of physiological arousal (cortisol), positive affect (PA), and negative affect (NA) obtained from 321 older couples. Results from multi-level models revealed, at the between-person level, that individuals with higher average cortisol had partners with higher overall NA; and, at the within-person (momentary) level, that cortisol was lower in moments when the partner's PA was higher than usual and when one's own PA was higher and NA was lower than usual. On a day level, cortisol output was lower on days with lower own NA and with higher partner PA, particularly when relationship satisfaction was high, but unrelated to own PA. The findings emphasize the importance of considering how members of a dyad influence each other's health in old age.

### FEELING BLUE WITH YOU: AGING COUPLES' REACTIVITY TO UPSETTING PERSONAL MEMORIES AND MARITAL CONFLICT

Stephanie Wilson[1], M. Rosie Shrout[2], and Janice K. Kiecolt-Glaser[3], *1. Southern Methodist University, DALLAS, Texas, United States, 2. Purdue University, Lafayette, Indiana, United States, 3. Ohio State University, Columbus, Ohio, United States*

Reactivity to marital conflict has long been considered a critical mechanism linking marriage to health and well-being. Yet, developmental theories suggest conflict may subside with age. To compare mood and cardiovascular responses to two novel contexts—both partners' upsetting personal experiences—with marital conflict reactivity, 107 couples ages 40-87 recounted a difficult personal memory to the partner, discussed a marital problem, and provided baseline and post-task mood, blood pressure (BP), and heart rate (HR). Although older adults relived their upsetting personal memories with less emotional intensity, negative mood and systolic BP increased most after this task. Compared to disagreement, the spouse's upsetting memory triggered larger increases in negative mood, HR, and among older adults, SBP. Results suggest that both partners' upsetting personal experiences are as relevant for mood and cardiovascular activity as marital conflict in aging couples, and that spousal distress may grow increasingly important with older age.

### EMOTION WORD USE AND CARDIOVASCULAR REACTIVITY DURING MARITAL INTERACTIONS

Tabea Meier, Jacquelyn Stephens, and Claudia Haase, *Northwestern University, Evanston, Illinois, United States*

Cardiovascular reactivity in midlife may predict health problems in later life, but few studies have examined cardiovascular reactivity during marital interactions, as an important interpersonal context, and potentially modifiable linguistic correlates. This laboratory-based study examined emotion word use (i.e., positive and negative emotion words derived using automated language analysis) and cardiovascular reactivity (i.e., heart rate changes from baseline) across two marital interaction contexts (i.e., positive and conflict conversation) in 46 married couples (92 individuals; age: M = 42.6, SD = 8.5). Results showed that (1) spouses who used more negative emotion words during conflict showed higher cardiovascular reactivity. Moreover, (2) when husbands used a more diverse negative emotion word vocabulary during positive conversations, their wives showed higher cardiovascular reactivity and (3) when wives used a more diverse positive emotion vocabulary, their husbands showed lower cardiovascular reactivity. Findings highlight the relevance of couples' emotion word use for cardiovascular reactivity in midlife.

### SESSION 3160 (PAPER)

### PREDICTORS OF HELP-SEEKING AND SERVICE AND HEALTH CARE UTILIZATION

### HEALTHCARE-SEEKING BEHAVIORS AMONG OLDER AMERICANS WITH SUBJECTIVE COGNITIVE DECLINE

Seoyoun Kim[1], Hyun Woo Yoon[2], and Yuri Jang[3], *1. Texas State University, San Marcos, Texas, United States, 2. Kongju University, Gongju-si, Ch'ungch'ong-namdo,*

*Republic of Korea, 3. University of Southern California, Los Angeles, California, United States*

Subjective cognitive decline (SCD) in older adults is increasingly recognized as a harbinger of dementia. However, since SCD is, by definition, based on self-reports, it is unclear whether those who experience memory loss discuss these issues with physicians. Access to regular healthcare can be a catalyst for early detection of SCD because asymptomatic cognitive changes may not be apparent unless directly addressed with primary care physicians (PCP). Further, given the widely-known racial/ethnic disparities in healthcare access, minority adults might be further disadvantaged in healthcare seeking for SCD. Using a large national dataset, Behavioral Risk Factor Surveillance System (BRFSS) in 2019, the goals of the study are to a) examine the link between having a PCP and discussing memory issues with physicians and b) examine how race and ethnicity and the availability of PCPs are associated with discussion of SCD. The sample included 7,900 individuals 50 and older who reported SCD in the past year. We conducted a logistic regression adjusting for BRFSS survey weights, missing data, state-level clustering, and a set of covariates. Among those with SCD, those with a PCP were almost twice as likely to discuss their cognitive decline (OR=1.98, p<.001). There was also a significant interaction between Hispanic ethnicity and having a PCP (OR=0.25, p<.05, F=4.65, p=.03). Surprisingly, Hispanics with a PCP were less likely to discuss memory issues compared to Hispanics without one. The findings highlight the significance of routine healthcare service utilization for adults with SCD while providing a nuanced understanding of ethnic differences.

### MEALS ON WHEELS UTILIZATION AND THE RISK OF HOSPITALIZATION AMONG MEDICARE ENROLLEES OVER TIME

France Weaver[1], Sarah Walsh[2], and Jennifer Chubinski[3], *1. West Virginia University, Morgantown, West Virginia, United States, 2. Eastern Michigan University, Ypsilanti, Michigan, United States, 3. Xavier University, Cincinnati, Ohio, United States*

Meals on Wheels (MOW) services provide nutritional and social support for clients and may influence acute care use as a result. This study estimates the associations between MOW utilization and the risk of hospitalization for Medicare enrollees aged 65 and over, according to the degree of home-boundness (an eligibility criterion for MOW access). Attention is paid to the heterogenous role of MOW according to gender, race, and ethnicity. The analysis uses the 2014-2020 National Health and Aging Trends Study (NHATS) and includes 17,345 person-year observations. The study uses longitudinal complex survey Probit models of the likelihood of hospitalization, including interaction terms between lagged MOW usage and the extent of homeboundness. Control variables include sociodemographic indicators, living arrangements, health status, region and time fixed effects. Over the study period, the mean probability of hospitalization for Medicare enrollees aged 65+ is 20.5 percent, and the proportion of MOW users is 5.2 percent. Some 11.4 percent report being partially homebound and 5.6 percent mostly/fully homebound. Results reveal that MOW utilization is associated with a lower risk of hospitalization for those who are partly homebound (p= 0.058). This negative association is the largest and statistically significant for males (p=0.034) and whites (p=0.035) only. Further investigations will assess whether these associations are explained by differences between MOW and non-MOW users. The findings suggest that MOW services may delay or limit, to some extent, hospitalizations among partly homebound Medicare enrollees.

### OLDER ADULT PREFERENCES FOR THE FORMAT AND CONTENT OF PRESCRIPTION MEDICATION LABELS

Rahul Malhotra[1], Sumithra Suppiah[1], Yi Wen Tan[1], Semra Ozdemir Van Dyk[1], Pildoo Sung[2], Sarah Siew Cheng Tay[3], Ngiap Chuan Tan[3], and PROMISE Study Group PROMISE Study Group[1], *1. Duke-NUS Medical School, Singapore, Singapore, 2. Centre for Ageing Research and Education, Duke-NUS Medical School, Singapore, Singapore, 3. SingHealth Polyclinics, Singapore, Singapore*

The risk of misinterpreting prescription medicine labels (PMLs) is higher among older adults. Given the limited space on PMLs, their format and content should cater to older adults' medication information and legibility needs. We established the ranking of seven medication-related content variables (indication, precautions, interaction or paired medicines, food instructions, side effects, expiry date, and missed dose action; provision of these content is currently not legally mandated) to be prioritised on PMLs using best-worst scaling (BWS), and determined the relative utilities of five format attributes (presentation of dose-frequency instructions, font size, presentation of dose, presentation and colour of precautions) when incorporated onto PMLs using discrete choice experiment (DCE), from the perspective of older Singaporeans. The variables and attributes were informed by our prior qualitative study and best practices guidelines for PMLs. We recruited 280 older adults (mean age: 68.8±5.4 years). A BWS object case was used to evaluate the relative importance of medication-related content variables (range: 0 to 1). A mixed logit model estimated the relative utilities of attribute levels, which enabled calculation of importance scores for ranking the format attributes (range: 0% to 100%). The three most-preferred content variables were indication (1.000), precautions (0.853) and interaction or paired medicines (0.845). The top three format preferences were tabular presentation of dose-frequency instructions (43.7%), large font size (22.3%) and precautions in red colour (13.6%). The findings inform an actionable way to improve the content and format of PMLs in the real-world, which is aligned with the stated priorities of older adults.

### REASONS OLDER ADULTS START ATTENDING CONGREGATE MEAL PROGRAMS AND WHO ATTENDS FOR SOCIALIZATION

Kristen Robinson[1], Lauren Palmer[2], Angela Gasdaska[3], Michael Hayes[4], and Sara Nderitu[5], *1. Social & Scientific Systems, Inc., a DLH Holdings Corp (SSS), Silver Spring, Maryland, United States, 2. RTI International, Waltham, Massachusetts, United States, 3. RTI International, Research Triangle Park, North Carolina, United States, 4. RTI International, Research Triangle, North Carolina,*

*United States, 5. Accenture, Charlotte, North Carolina, United States*

The Older Americans Act Nutrition Services Program's congregate meals support food security and nutrition, promote socialization, and improve quality of life. Understanding what drives people to first attend a congregate meal program may support efforts to increase involvement. This study used the 2019 National Survey of Older Americans Act Participants to analyze the open-ended survey responses of congregate meal participants (N=1,072) on why they started attending the program. The top three reasons were that they sought socialization (36.3%), had a medical or age-related need (18.7%), or accompanied or were referred by a friend or relative (12.3%). We conducted descriptive analyses on differences in the demographic, socioeconomic, and health characteristics of those attending for socialization compared to others. Results indicated a relationship between attending congregate meals for socialization and several characteristics: these individuals are more often non-Hispanic white ($p< 0.05$), widowed ($p< 0.05$), aged 75 to 84 ($p< 0.05$), and have at least a high school education ($p< 0.01$). People who attend for socialization are also less likely to be lower income ($p< 0.01$), living in cities ($p< 0.001$), have food insecurity ($p< 0.01$), and living with three or more ADLs ($p< 0.01$). Findings suggest two subpopulations of congregate meal attendees: those who have the choice to attend and do so for socialization, and those who attend because of unmet needs (e.g., food insecurity or disability). Identification of different categories of participants and what drives them to attend congregate meal sites has implications for improvements to advertising congregate meal services, targeting of certain populations, and ultimately to increasing participation.

### SIMULATING WELL-BEING AND LITERACY INTERVENTIONS TO REDUCE ELDER SCAM SUSCEPTIBILITY

Marguerite DeLiema[1], and Aparajita Sur[2], *1. University of Minnesota, Twin Cities, Minneapolis, Minnesota, United States, 2. University of Minnesota, Minneapolis, Minnesota, United States*

Finanical fraud targeting older adults is on the rise, with annual losses in the billions of dollars. There is little longitudinal research on the causal relationships between known risk factors and scam susceptibility, including poor psychological well-being and poor health and financial literacy. Interventions designed to enhance well-being and/or literacy may reduce scam susceptibly among older adults. In this study, we use repeated measures from the Rush Memory and Aging Project to simulate how different trajectories in well-being and literacy might impact scam susceptibility among older adults alive over a seven year period. We simulated the effects of interventions of varying degrees -- 10%, 50%, and 100% increase in well-being/literacy from baseline scores. Simulations were performed for all participants as well as by education and income subgroups. Simulation models show that intervening on well-being causes a greater reduction in average scam susceptibility over time compared to intervening on total literacy. Even a 10% increase in baseline well-being significantly reduces scam susceptibility over time, regardless of participants' baseline income or educational attainment. Both interventions caused slightly greater reductions in susceptibility for those who are not college educated and those with an annual household income of less than $30,000. This study suggests that interventions that target self-efficacy and sense of purpose may help reduce older adults' scam susceptibility even more than interventions that improve health and financial literacy, but that both are promising targets for intervention.

### SESSION 3170 (SYMPOSIUM)

### PRESIDENTIAL SYMPOSIUM: REIMAGINING AGING AND LONG-TERM CARE FOR HEALTH EQUITY DURING AND POST-COVID-19: A HEALTH SCIENCES FOCUS

Chair: Kirsten Corazzini Discussant: Deb Bakerjian

In line with the theme of the conference, this symposium highlights exemplars of health sciences scholars who are identifying key issues and opportunities to re-imagine aging and long-term care, through a health equity lens, as highlighted by COVID-19. This lens is inclusive of the wide range of long-term care stakeholders whose well-being and health outcomes have been affected throughout the COVID-19 pandemic, including older adults, family caregivers, and the healthcare workforce. We focus in particular on vulnerable aging populations, such as older adults who are living with neurocognitive disorders, racial/ethnic minority older adults, and older adults receiving palliative care. Finally, we consider these issues at both individual and systems-levels. Our first presenter examines person and family engagement in assisted living for older adults living with ADRD and the impact of COVID-19. The second presenter provides insights into palliative care needs in nursing homes, and the implications for transition to an endemic. The third presenter examines long-term care needs in the community, with a focus on the consequences of unmet needs for racial/ethnic minority older adults. The fourth presenter highlights the role and capacity of the nurse practitioner in nursing homes during COVID. Our final presenters provide a systems-level look at the COVID-19 response, focusing on self-organizing community coalitions to support nursing homes. Presentations reveal how re-imagining aging and long-term care in health sciences, requires consideration of health inequities experienced throughout COVID-19, whether newly emerging inequities, or long-standing challenges and inequities that have been exacerbated by COVID-19.

### IMPACT OF COVID ON PERSON AND FAMILY ENGAGEMENT IN ASSISTED LIVING

Anna Beeber, *Johns Hopkins University, Baltimore, Maryland, United States*

Advancing person-centered care in assisted living (AL), while minimizing safety risks (e.g., injury, elopement, or medication errors), requires effective partnerships among residents, family members, and staff. The COVID-19

pandemic adversely affected capacity across and within AL settings to establish and nurture these relationships, ultimately affecting and changing person and family engagement in care. Using data from qualitative interviews, we will report findings about person and family engagement in the safety of AL from the perspectives of 104 residents, families, and staff. Particular attention will be given to examining the effect of COVID-19 on person and family engagement for residents at increased risk for disparities in assisted living, including residents who are living with mild cognitive impairment or dementia. The presentation highlights both challenges and promising practices that emerged from the COVID-19 pandemic. Implications for AL are presented to support the transition from pandemic to endemic.

### PALLIATIVE CARE FOR NURSING HOME RESIDENTS: APPLYING LESSONS LEARNED FROM COVID-19

Kathleen Unroe, *Indiana University Center for Aging Research, Regenstrief Institute, Inc., Indianapolis, Indiana, United States*

Many people receive care near or at the end of life in nursing homes, including 70% of people with Alzheimer's Disease and Related Dementias (ADRD). Studies have documented unmet needs for symptom management and frequent transitions of care for nursing home residents. Despite this, access to palliative care for nursing home residents is inconsistent. The COVID-19 pandemic both highlighted and exacerbated inequities in access to care, including in US-based nursing homes, as well as globally. COVID-19 specific guidance for nursing homes at state and federal levels, while designed to protect residents, contributed to increased social isolation and functional decline. Drawing upon data from an ongoing study to advance palliative care for residents living with ADRD, this presentation will highlight promising practices and opportunities to deliver palliative care in this setting.

### ADDRESSING UNMET LONG-TERM SERVICES AND SUPPORTS NEEDS FOR RACIAL/ETHNIC MINORITY OLDER ADULTS

Jasmine Travers, *NYU, New York City, New York, United States*

The COVID-19 pandemic magnified several long-standing problems with the delivery of long-term services and supports, including access to care in the community setting. A disproportionate rise in nursing home use among Black and Latino older adults reflects the inadequacy of existing programs and policies to support aging in place for these most at-risk populations. Enabling aging in the community and preventing avoidable nursing home placements is widely considered a priority by federal, state, and local entities along with families and older adults. Yet, it is unclear what is needed to support Black and Latino older adults to remain in the community. In this presentation, Dr. Travers will discuss unmet long-term services and supports needs among the Black and Latino population, issues particularly faced by these populations during COVID-19, and opportunities to move forward as we transition to an 'endemic' COVID-19 landscape.

### THE LONG-TERM CARE STAFFING CRISIS AND COVID-19: ROLE OF THE NURSE PRACTITIONER

Katherine McGilton, *KITE Research Institute: Toronto Rehabilitation Institute-UHN, Toronto, Ontario, Canada*

The residential long-term care sector has historically suffered from seemingly intractable staffing challenges in terms of ensuring adequate clinical expertise and a supportive work environment to address the complex health care needs of residents. Considerable evidence has demonstrated the devastating effect of COVID-19 on this fragile residential long-term care staffing structure, resulting in adverse outcomes among staff and residents alike, with the potential for permanent devastation without directed intervention. Drawing upon data from an Ontario-based study of nurse practitioner deployment during COVID-19, this talk will share an emergent approach to re-shaping expertise and capacity in Ontario, Canada through embedding nurse practitioners in residential long-term care homes. Results of this work helped to inform health policy action in the province to scale-up the use of nurse practitioners in long-term care homes, in order to enhance staff expertise and tackle the significant inequities of access to care among nursing home residents.

### A LOOK INSIDE THE MISSOURI NURSING HOME COVID-19 EXPERIENCE

Amy Vogelsmeier, and Lori Popejoy, *University of Missouri, Columbia, Missouri, United States*

The COVID-19 pandemic exposed the vulnerabilities of US nursing homes to manage widespread viral outbreaks including an ill prepared/under-resourced workforce, a physical environment not conducive to infection prevention or management, and isolation from community emergency response planning. In this session, we will share real-life, real-time experiences of diverse Missouri nursing homes as they responded to the COVID-19 pandemic. We will also report on emerging data about the impact of nursing homes' pandemic response on resident outcomes. Strategies such as community-based efforts to respond to resource scarcity, and creative workforce solutions to address staffing needs, will be shared. Critical next steps should focus on the implementation of community coalitions to create sustainable healthcare partnerships at the local and state level and enhanced workforce solutions that include registered nurses and advanced practice registered nurses working within nursing homes to guide clinical care and infection prevention and management strategies.

### SESSION 3180 (SYMPOSIUM)

### READY, FIRE, AIM: DOES INTEGRATING ACUTE AND LONG-TERM SERVICES WORK?

Chair: Robert Applebaum Discussant: Richard Browdie

Due to the increasing costs of Medicaid and Medicare and concerns about how these two programs fail to work together to deliver quality care, there has been a growing enthusiasm for integrated care programs. The Financial Alignment Initiatives (FAI), implemented by the Centers for Medicare and Medicaid Services (CMS) in 2011 and tested in 13 states were designed to test the impacts of a program that offers Medicare and Medicaid services under one organization to individuals who are dually eligible for both programs. Previous studies of the expansion of managed long-term services have generated considerable interest over the last two decades however, research results have been mixed. There is also limited information about the implementation of these efforts, as demonstrations have served varying target populations with very different intervention strategies. The lack of conclusive results means that states, now faced with decisions about continued implementation of these initiatives do not have good information to make sound policy decisions. The national evaluation of the FAI states did not include Medicaid costs. Our study is designed to gain a better understanding of Ohio's FAI MyCare Demonstration. This symposium provides data from a comprehensive impact analysis that examined both Medicaid and Medicare claims data using a difference- in- differences treatment and comparison analysis (n= 390,000) and an in-depth process evaluation (using interviews with 487 participants) to gain an understanding of program effects. After reviewing results the symposium will discuss the future of these and other reform efforts to integrate Medicaid and Medicare services.

### WHEN YOU GET TO A FORK IN THE ROAD, TAKE IT: SHOULD STATES FOLLOW YOGI'S ADVICE ON MANAGED LONG-TERM SERVICES?

Robert Applebaum, *Miami University, Oxford, Ohio, United States*

Today's Medicaid challenges, coupled with the baby boom demographics, have every state in the nation recognizing the need to do something different in their Medicaid programs. Although achieving a better balance between institutional and home and community-based services has been an important reform in many states, it does not appear to be enough to create a working system. Medicaid managed long-term care and efforts to integrate Medicare and Medicaid is a growing option. Designed to control the acute and long-term care costs of older people and individuals with disability, the approach also is directed at linking the two disparate systems. A review of the array of studies examining this area shows mixed results, despite the popularity of this option at the state level. This paper introduces the evaluation of Ohio's MyCare integrated care demonstration, raising questions about the important elements of these initiatives for policy makers, providers, and consumers.

### EVALUATING EXPENDITURES AND UTILIZATION OF OHIO'S INTEGRATED MEDICARE AND MEDICAID PROGRAM

John Bowblis, Robert Applebaum, and Matt Nelson, *Miami University, Oxford, Ohio, United States*

In 2014, 29 of 88 Ohio counties implemented MyCare, which integrated Medicare and Medicaid for dually eligible Ohioans. Using an intent-to-treat, difference-in-difference framework we examined medical expenditures and utilization associated with the implementation of MyCare. Specifically, we compared dually eligible Ohioans in MyCare counties to those in non-MyCare counties from 2012 to 2018. Overall medical expenditures were lower in the MyCare counties post implementation compared to non-Mycare counties, with most of the difference attributed to Medicaid. The effects were larger for individuals in the community compared to long-term services and supports (LTSS) users. The implementation of MyCare is associated with a decrease in the use of nursing homes, a large increase in hospice, and among LTSS users not in a nursing home decreases in the utilization of home and community-based services. Interestingly, the proportion of individuals in MyCare counties classified as an LTSS user increased after the implementation of MyCare.

### PROGRAM THEORY VERSUS IMPLEMENTATION: THE IMPORTANCE OF PROCESS IN UNDERSTANDING PROGRAM IMPACT

Jennifer Heston-Mullins[1], Athena Koumoutzis[2], Katherine Abbott[2], Dayna Bennett[2], and Karen Williams[2], *1. Scripps Gerontology Center, Miami University, Oxford, Ohio, United States, 2. Miami University, Oxford, Ohio, United States*

An extensive MyCare Ohio process evaluation was conducted to understand factors affecting everyday implementation. This involved a review of MyCare membership enrollment data and qualitative interviews and focus groups with state-level stakeholders (n=29), regional stakeholders comprised of Area Agency on Aging and MyCare Ohio Plan personnel and HCBS service providers (n=418), and MyCare members (n=40); which were audio-recorded, transcribed, and checked for accuracy prior to coding in Dedoose. Results show that while MyCare was originally envisioned as a program to coordinate Medicare and Medicaid services for dual-eligible, physically-disabled older adults, many MyCare members are under age 65 (47%), have opted out of the Medicare Advantage portion of MyCare (42%), and live with behavioral health diagnoses. This presentation will discuss how process evaluation is instrumental to understanding program impact and how younger members, members with behavioral health concerns, and opted-out members have shaped the implementation of MyCare Ohio.

### THE BLACK BOX OF CARE MANAGEMENT IN MANAGED LONG-TERM SERVICES AND SUPPORTS

Katherine Abbott[1], Jennifer Heston-Mullins[2], Athena Koumoutzis[1], Dayna Bennett[1], Karen Williams[1], and Robert Applebaum[1], *1. Miami University, Oxford, Ohio, United States, 2. Scripps Gerontology Center, Miami University, Oxford, Ohio, United States*

Within Ohio's MyCare demonstration, two distinct care management models were selected by the participating MyCare Ohio health plans (MCOPs): fully-delegated waiver care management and waiver service coordination. The purpose of this presentation is to describe the components of care management operating in MyCare Ohio. Qualitative interviews with n=91 Area Agency on Aging (AAA) and n=131 MCOP care management personnel were audio-recorded, transcribed, and checked for accuracy prior to thematic coding in Dedoose. Results indicate that comprehensive care management is the core element of MyCare Ohio. Fully-delegated care management models were viewed by participants as beneficial to reducing confusion for members however 'scope creep' challenged the already strained AAAs. Effective teamwork was identified for waiver service coordination models but the division of labor and communication needed between the AAA and MCOP care management personnel created tensions. The discussion will focus on practice recommendations for training, caseloads, and support staff.

### SESSION 3190 (SYMPOSIUM)

### REVISITING THE ROLE OF PERCEIVED TIME HORIZONS IN SOCIOEMOTIONAL SELECTIVITY THEORY

Chair: Li Chu Discussant: Laura Carstensen

This symposium will consider the ways that time horizons may influence motivation and emotional well-being. Socioemotional selectivity theory postulates that goals and motivation shift from ones about learning and exploration to ones about emotional meaning as time horizons grow constrained as people age. This theory maintains that a focus on emotional goals explains why older adults tend to show better emotional well-being compared to younger adults. Many studies use the Future Time Perspective scale developed by Carstensen and Lang (1996) to measure perceived time left in life. However, several studies find more limited future time perspective does not contribute to better emotional well-being nor more positivity bias in cognitive processing and in some cases predicts poorer emotional well-being. The first presentation will focus on the future time perspective scale and its underlying structure. The second presentation will discuss the association between future time perspective and psychosocial well-being during the COVID-19 pandemic. The third presentation will review different measures of time perception and examine the relationship between future time perspective on age-related positivity effect. The last presentation will introduce a new concept and measurement, time savoring. Laura Carstensen will synthesize findings and offer insights regarding future life-span research on motivation and well-being.

### THE DIMENSIONALITY OF FUTURE TIME PERSPECTIVE

Daniel Grühn, and Rebekah Knight, *NC State University, Raleigh, North Carolina, United States*

There have been suggestions that the measure of future time perspective shows a two-factor structure. However, the two-factor structure coincides with positively- and negatively-framed items potentially indicating a method factor rather than a content factor. By using reversed-scored items in an adult sample (N = 1421, aged 19 to 79, M = 39.1, SD = 11.1), we found evidence that the two-factor structure is mainly due to the framing of the items representing method factors rather than representing separate content factors. Item framing might be more important in aging-related research than expected.

### LIMITED FUTURE TIME PERSPECTIVE IS ASSOCIATED WITH LOWER EMOTIONAL WELL-BEING DURING THE COVID-19 PANDEMIC

Yochai Shavit[1], Jessica Barnes[2], and Laura Carstensen[2], *1. Stanford Center on Longevity, Stanford University, Stanford, California, United States, 2. Stanford University, Stanford, California, United States*

Socioemotional selectivity theory postulates that limited future time perspective (FTP) motivates older adults to prioritize emotionally meaningful goals, explaining documented age advantages in emotional well-being. During the early months of the COVID-19 pandemic, we collected data from 945 community dwelling adults and 156 assisted living facilities residents living in the United States (N= 1101, age-range: 18-98). Participants reported their FTP using the scale developed by Carstensen and Lang (1996), as well as the frequency and intensity of sixteen positive and thirteen negative emotions. Age association with limited FTP was comparable to past studies. Contrary to our hypotheses, limited FTP was associated with lower emotional well-being across ages and suppressed (rather than mediated) a general trend towards higher emotional well-being in older ages. Findings suggest that there may be conditions under which perceptions of limited time horizons have negative implications. Theoretical implications are discussed.

### EXAMINING THE RELATIONSHIP BETWEEN FUTURE TIME PERSPECTIVE AND POSITIVITY EFFECTS

Sarah Barber[1], Hyunji Kim[1], and Noelle Lopez[2], *1. Georgia State University, Atlanta, Georgia, United States, 2. San Francisco State University, San Francisco, California, United States*

When compared to younger adults, older adults favor positive over negative information in attention and memory. This positivity effect is typically interpreted through the lens of socioemotional selectivity theory. According to this view, older adults often have a limited future time perspective (FTP), which leads them to prioritize emotional well-being goals. However, data from our lab suggest that a limited FTP is actually associated with increased negativity, rather than with increased positivity. More specifically, we found that older adults with a limited FTP (self-reported on the Carstensen and Lang FTP scale) rated their autobiographical memories more negatively. We also found that among older adults with high executive function abilities, a limited FTP was associated with reduced positivity in an episodic memory task. In discussing why these counterintuitive relationships occurred, we will also present data showing that a limited FTP is associated with negative attitudes about the future and the present.

### TIME HORIZONS VERSUS TIME SAVORING: WHICH BEST PREDICTS AGE-RELATED IMPROVEMENTS IN EMOTIONAL WELL-BEING?

Tyler Matteson[1], Li Chu[2], and Laura Carstensen[1], *1. Stanford University, Stanford, California, United States, 2. Stanford University, Menlo Park, California, United States*

Previous research has shown that time horizons, as measured by the future time perspective (FTP) scale, yields mixed findings about the relationship between perceived time and emotional well-being. Expansive time horizons often predict better well-being than limited time horizons, raising questions about a core postulate of socioemotional selectivity theory (SST). The present study introduces a more nuanced construct about perceived time and introduces a new scale, time savoring, which captures the heightened value of time as time becomes more limited. Based on 1,384 participants (Mage = 54.55, age range = 18-96), time savoring and age are positively correlated (r(1,382) = .23, p < .001). Time savoring partially mediates age and well-being (i.e., greater happiness, lower depressive symptoms, and higher life satisfaction). Parallel mediation with multiple mediators demonstrated the measure's discriminant validity from FTP and personality measures (e.g., agreeableness and conscientiousness), offering a promising way to measure heightened value of time.

### SESSION 3200 (PAPER)

### UNDERSTANDING PEOPLE'S PERCEPTIONS OF PALLIATIVE CARE AND PRACTICES IN ASSISTED LIVING

### ANTIPSYCHOTIC MEDICATION USE IN ASSISTED LIVING/RESIDENTIAL CARE: DO ORGANIZATIONAL CHARACTERISTICS MATTER?

Sarah Dys[1], Ozcan Tunalilar[2], and Paula Carder[2], *1. Vital Research, LLC, Los Angeles, California, United States, 2. Portland State University, Portland, Oregon, United States*

This study investigates how assisted living/residential care (AL/RC) and memory care (MC) contexts are associated with the prevalence of antipsychotic medication use (APU). Primary data were collected from a statewide representative sample of AL/RC settings through the Oregon Community-Based Care study from 2017-2019 and combined with publicly available administrative data. Framed by Donabedian's model of care quality, we examine associations among 90-day prevalence of APU, organizational, care process, and AL/RC resident population characteristics using random intercepts regression models. Every licensed AL/RC setting in Oregon receives an annual mailed survey to provide aggregate resident demographics, health acuity, health service use, payment type and organizational policies. Organizational measures (e.g., profit status, license type, geographic designation) were collected from state websites. The average 90-day prevalence of APU among all Oregon AL/RC settings is 30.7%, though rates differ by MC endorsement (23.9% in AL/RC and 42.7% in MC). Compared to care processes and resident population characteristics, organizational characteristics were associated with larger magnitudes of difference in rates of APU. Nonprofit settings were associated with lower rates of APU in both AL/RC (β= -4.4 percentage points, [95% CI: -8.4, -0.4]) and MC (β= -12.4, [95% CI: -21.2, -3.6]. Compared to low-Medicaid settings, settings with very high proportions of Medicaid residents were associated with higher prevalence of APU, +8.9 in AL/RC (95% CI: 1.7, 16.1) and +11.0 in MC (95% CI: 2.3, 19.8). Policymakers might consider how organizational resource capacity influences APU in AL/RC settings, especially if APU prevalence is treated as a quality indicator.

### CAUSAL ANALYSES OF PALLIATIVE CARE OUTCOMES USING OBSERVATIONAL DATA: A REVIEW OF CURRENT LITERATURE

Narae Kim[1], Jingjing Jiang[2], Melissa Garrido[3], Mireille Jacobson[1], David Mockler[4], and Peter May[2], *1. University of Southern California, Los Angeles, California, United States, 2. Trinity College Dublin, Dublin, Dublin, Ireland, 3. Boston University School of Public Health, Boston, Massachusetts, United States, 4. University of Dublin, Dublin, Dublin, Ireland*

People with serious medical illnesses disproportionately account for health care spending, and the last year of life is typically the most costly. Economic evidence to inform improvement efforts in care for this population is long recognized as thin relative to policy importance. Palliative care studies rely heavily on routinely collected data since conducting randomized controlled trials with subjects nearing the end of life is particularly challenging; however, observational studies face high risk of bias. We conducted a systematic review of the peer-reviewed and grey literature to identify quasi-experimental studies evaluating palliative care's effect on costs and health care utilization. Eligible study designs were those that controlled for unobserved confounding using causal inference methods (e.g. difference-in-differences). Eligible outcomes were costs, health care use, and quality-of-life. Among 806 search results, we included 17 studies: seven used difference-in-differences methods, five used interrupted time series analysis, and five used instrumental variables. Reporting quality was variable. Studies reported a general pattern of improved outcomes associated with palliative care. However, the incidence of studies finding a significant difference was lower than cohort studies that don't attempt to control for unobserved confounding. Studies that don't control for unobserved confounding may be overestimating true effects due to bias. Given the large volume of routine data collection in end-of-life care, there exists clear potential to increase the application of the causal inference methods to palliative care research. Such studies would strengthen the evidence base on an understudied topic and potentially inform other types of economic analysis in palliative care.

### PROFILE OF DECEDENTS FROM A NATIONAL COHORT OF ASSISTED LIVING RESIDENTS

Emma Belanger[1], Nicole Rosendaal[1], Xiao (Joyce) Wang[1], Joan Teno[2], Pedro Gozalo[1], David Dosa[1], and Kali Thomas[1], *1. Brown University, Providence, Rhode Island, United States, 2. Oregon Health & Science University, Portland, Oregon, United States*

An increasing number of older adults reside in assisted living (AL) toward the end of life, and it remains unclear if this trend represents an additional place of care and end-of-life transition before eventual nursing home admission. Our objective was to examine the characteristics and healthcare utilization of AL residents who died during a two-year follow-up. We conducted a prospective cohort study of Medicare beneficiaries residing in large AL communities (25+ beds) in January 2017, and followed them until the end of 2018 using a variety of administrative healthcare claims data. The national population of Medicare beneficiaries in AL included 273,722 fee-for-service (FFS) beneficiaries, and 143,258 Medicare Advantage beneficiaries. From 2017 to the end of 2018, 23.7% of residents died. Of the 66,605 FFS Medicare beneficiaries who died during follow-up, 77.0% were 85 years old or older, 72.2% were diagnosed with Alzheimer's disease and related dementia (ADRD) and 80.8% were diagnosed with heart failure or chronic obstructive pulmonary disease. Most FFS decedents (97.3%) resided in AL during their last 12 months of life, with 23.0% leaving AL before the last month of life. Half of FFS decedents died in AL, while another 24.1% died in a nursing home. AL communities represent an increasingly common place of end-of-life care for dying Medicare beneficiaries. These findings point to the need for state and federal policies to protect a growing population of frail and vulnerable AL residents.

### REGULATORY PROFILES AND POTENTIALLY BURDENSOME TRANSITIONS AMONG ASSISTED LIVING DECEDENTS

Xiao (Joyce) Wang, Lindsey Smith, Joan Teno, Nicole Rosendaal, David Dosa, Pedro Gozalo, Kali Thomas, and Emma Belanger, *Brown University, Providence, Rhode Island, United States*

Potentially burdensome transitions at the end of life (i.e. repeated hospitalizations or transitions in the last 3 days of life) are common among assisted living (AL) residents, and are associated with lower care satisfaction by family members. AL regulations vary widely within and between states. This study aimed to describe the rate of burdensome transitions by AL regulatory profile, using a retrospective cohort study combining state AL regulations and multiple administrative claims data. The sample included 4,911 ALs serving 67,319 residents, who 1) died between 2017 and 2019; 2) resided in AL 120 days before death; 3) were continuously enrolled as fee-for-service one-year before death; and 4) resided in ALs with 25+ beds. The independent variable was a categorical variable indicating AL regulatory profile (i.e. housing, hybrid, hospitality, healthcare, rebalancing, hybrid healthcare), identified with a previously published methodology. These profiles differ in the allowance of third-party services, skilled nursing, medication administration, and requirements for medication review and licensed nursing staff. We first ran regression models to estimate the rate of burdensome transitions, accounting for AL-level resident demographic, socioeconomic, case-mix, and market characteristics. Results showed 'hybrid' ALs (i.e. low prevalence of skilled nursing allowed) had the highest rate of burdensome transitions (24.9%), whereas 'healthcare' ALs (i.e. high specificity for all types of services) had the lowest rate (22.2%). The rate of potentially burdensome transitions did not differ much by AL regulatory types, requiring more work to determine the drivers for burdensome transitions beyond AL regulations.

### UNDERSTANDING THE PALLIATIVE CARE INFORMATION CIRCULATING ON TWITTER DURING THE CORONAVIRUS PANDEMIC

Megumi Inoue, Mahdi Hashemi, Meng-Hao Li, Rajendra Kulkarni, and Naoru Koizumi, *George Mason University, Fairfax, Virginia, United States*

Palliative care is a growing medical specialty. While its value has become particularly evident during the pandemic, it faces various challenges including misconceptions among the general public, a lack of awareness of its benefits, and limited/sporadic access and coverage by Medicare. Given the increasing number of people sharing their health information and seeking healthcare information on social media where misinformation is widely spread, this study examined types of information on palliative care circulated on Twitter. A total of 26,495 English Tweets were collected 01/01/2020 – 12/30/2021 using the keywords "Palliative" and "Covid" or "Corona". We manually developed a framework for coding/classifying the first 6,000 Tweets. Of those, 5,308 were unrelated Tweets (e.g., advertising palliative care seminars/conferences). Among the remaining Tweets, persistent myths were observed (e.g., palliative care is only for dying people) and were labeled accordingly. In addition, while some people mentioned negative impact of the pandemic on palliative care (e.g., shortage of beds), others found value in palliative care and reported positive changes due to the pandemic (e.g., telehealth in palliative care). Consequently, the following categories were defined for Tweets: i) Recognized benefits (203 Tweets); ii) Positive impact of the pandemic on palliative care (120 Tweets); iii) Negative impact of the pandemic on palliative care (333 Tweets); iv) Myth (63 Tweets). We will use these manually classified Tweets to train machine-learning algorithms to automatically classify the remaining tweets, aiming to obtain a comprehensive understanding of palliative care information circulating on Twitter and seek ways to promote palliative care use.

### SESSION 3210 (SYMPOSIUM)

### THE EXPERIENCES OF OLDER PEOPLE IN ENGLAND DURING THE COVID-19 PANDEMIC: EVIDENCE FROM ELSA

Chair: Paola Zaninotto

In this session we will present results from the English Longitudinal Study of Ageing (ELSA) Covid-19 substudy. Data were collected during the pandemic at two separate occasions to capture the experiences of older people and to compare them to pre-covid periods. The session will include results among people with cognitive impairment, people who contracted infections and explore the consequences on their mental health, well-being and health in general

### MENTAL HEALTH AND FINANCIAL HARDSHIP AMONG OLDER ADULTS WITH PROBABLE COVID-19 INFECTION

Paola Zaninotto[1], Eleonora Iob[1], and Andrew Steptoe[2], *1. UCL, London, England, United Kingdom, 2. University College London, London, England, United Kingdom*

We investigated the immediate and longer-term impact of probable COVID-19 infection on mental health, wellbeing, and financial hardship among older people living in England. Data were analysed from 5146 older adults participating in the English Longitudinal Study of Ageing who provided data before the pandemic (2018-19) and at two COVID-19 assessments in 2020 (June-July and November-December). The associations of probable COVID-19 infection (first COVID-19 assessment) with depression, anxiety, poor quality of life (QoL), loneliness, and financial hardship at the first and second COVID-19 assessments were tested using linear/logistic regression and were adjusted for pre-pandemic outcome measures. Participants with probable infection had higher levels of depression and anxiety, poorer QoL, and greater loneliness scores compared with those without probable infection at both the first (ORdepression=1·62 [95%CI:1·16,2·26];ORanxiety=1·59[95%CI:1·00,2·51]; bpoorQoL=1·34[95%CI:0·66,2·02]; bloneliness=0 ·49[95%CI:0·25,0·74]) and second (ORdepression =1·56[95%CI:1·17,2·09];ORanxiety=1·55[95%CI:1·02 ,2·37];bpoorQoL=1·38[95%CI:0·74,2·03]; bloneliness= 0·31[95%CI:0·04;0·58]) assessments. Participants with probable infection also experienced greater financial difficulties than those without infection at the first assessment (OR=1·50[95%CI:1·10,2·05]).

### THE EXPERIENCES OF PEOPLE WITH COGNITIVE IMPAIRMENT DURING THE COVID-19 PANDEMIC IN ENGLAND

Brian Beach[1], and Paola Zaninotto[2], *1. UCL (University College London), London, England, United Kingdom, 2. UCL, London, England, United Kingdom*

The COVID-19 pandemic presented challenges that may have impacted people with cognitive impairment in disproportionate ways. Using the ELSA COVID-19 sub-study collected in 2020, we examined the experiences of people across three cognitive function groups (no impairment, mild impairment, and dementia) with respect to a range of social and health outcomes, including: shielding and self-isolation; access to health and care services; changes in lifestyle behaviours during the pandemic, and the impacts on mental health, wellbeing, and other psychosocial measures. Differences among cognitive function groups varied according to both outcomes and time. For example, people with dementia were around 2.4 times more likely to be shielding in June/July than those with no impairment, but no difference was found for November/December. On many measures, people with dementia fared similarly to those with no impairment once controlling for other factors.

### ASSOCIATION OF HEALTH BEHAVIORS WITH WEIGHT AND OBESITY DURING THE COVID-19 PANDEMIC AMONG THE UK OLDER POPULATION

Jingmin Zhu, Giorgio Di Gessa, and Paola Zaninotto, *UCL, London, England, United Kingdom*

Using a sample of 4,182 UK adults aged 50 and above, this study explored the association of changes in health behaviours with weight and obesity during UK lockdown in Jun/Jul and Nov/Dec 2020. Over 30% adults reported more sitting, more TV watching or less exercise. Around 20% adults were engaged in eating more or sleeping less. More alcohol drinking happened in 12.3% adults. Results suggested that more sedentariness, more TV watching, less exercise, more eating and more alcohol drinking were associated with a significant increase in weight. Meanwhile, less sedentariness or less eating significantly reduced weight in Nov/Dec 2020. A higher risk of obesity was found in adults sitting, eating, or sleeping more than usual. Considering potential health risks associated with obesity in older population, weight management is necessary nationwide.

### THE CORONAVIRUS JOB RETENTION SCHEME AND MENTAL HEALTH AMONG OLDER WORKERS: EVIDENCE FROM ENGLAND

Giorgio Di Gessa, and Paola Zaninotto, *UCL, London, England, United Kingdom*

The COVID-19 pandemic has led to major economic disruptions. In March 2020, the UK implemented the Coronavirus Job Retention Scheme –known as furlough –to minimize the impact of job losses. So far, little is known on the mental health impact of this scheme on older workers, and on whether this varies by job characteristics. Exploiting longitudinal data from Wave 9 (2018/19) and two COVID-19 sub-studies (June/July 2020; November/December 2020) of the English Longitudinal Study of Ageing we use logistic and linear regression models to investigate associations between changes of employment and mental health during the pandemic. About 10% of respondents aged 52-67 were furloughed in the initial phase of the pandemic. Overall, employment disruption was associated with changes in mental health, although results suggest differences by pre-pandemic job characteristics (i.e. hours worked, physical effort, social class, and stress measured by the effort-reward imbalance model).

### SESSION 3220 (SYMPOSIUM)

### UNDERSTANDING SOCIAL AND CULTURAL DIVERSITIES AND AGING FOR HEALTH AND WELL-BEING IN KOREAN AND KOREAN AMERICANS

Chair: Seunghye Hong Co-Chair: Michin Hong Discussant: Kathryn Braun

Guided by the socio-ecological model and the cultural diversity perspective, this symposium aims to enhance the understanding of critical issues in health and well-being among Koreans and Korean Americans with three primary focuses: aging, social-ecological and multi-level factors, and identifying social and cultural contexts.

Five studies examined multilevel factors—individual, relational/interpersonal, community, and societal—that are associated with health and well-being, conducted in Korea as well as in the United States. Study 1 examined psychological well-being among older Koreans, specifically its association with intergenerational relationships and social support using longitudinal multilevel modeling to estimate depression trajectories. Study 2 examined childhood experiences and midlife cultural engagement associations among middle-aged Korean couples, considering the influences of their spouses' experiences and cultural resources. Study 3 explored the experiences of the nature-based virtual reality program among older Korean Americans, using in-depth interviews and providing an innovative approach using technology as a therapeutic tool. Study 4 examined social determinants of health associated with Korean American immigrants' willingness for end-of-life discussions and the factors affecting willingness (awareness of hospice, communication with family/doctors, and social isolation). Study 5 examined health insurance coverage and its association with immigration-related factors (English proficiency, generational status, and age at immigration) among Korean Americans using national data. The various health, mental health, and well-being issues in Koreans and Korean Americans will be discussed from contextually responsive approaches. This symposium will provide implications for practices, education, research, and policy to promote health, mental health, and well-being in the Korean and Korean American populations.

### HEALTH INSURANCE COVERAGE, AGE, AND IMMIGRATION-RELATED FACTORS AMONG KOREAN AMERICANS

Seunghye Hong[1], Ki Tae Park[2], Yeonjung Lee[3], Mei Linn Park[4], and Nan Sook Park[5], *1. University of Hawaii at Manoa, Thompson School of Social Work & Public Health, Honolulu, Hawaii, United States, 2. Regional Skills Council, Seoul, Seoul, Seoul-t'ukpyolsi, Republic of Korea, 3. University of Hawai'i at Mānoa, Honolulu, Hawaii, United States, 4. University of Hawai'i at Mānoa, Thompson School of Social Work & Public Health, Honolulu, Hawaii, United States, 5. University of South Florida, Tampa, Florida, United States*

The purpose of this study was to examine health insurance coverage and its association with demographic and immigration-related factors (English proficiency, generational status, and age at immigration) among Korean Americans (KAs, n=11,462). Data were drawn from the 2017 American Community Survey. KA older adults aged 65+ had significantly higher odds of having any health insurance compared to those aged 41 to 64. KAs who speak English well or only speak English were more likely to have any health insurance compared to those with limited English proficiency (i.e., don't speak English well or at all). First-generation KAs had lower odds of having any health insurance compared to those born in the U.S. Those who immigrated after 41 years of age were less likely to have health insurance compared to those born in the U.S. These findings underscore the need to consider immigration-related factors in efforts to increase healthcare accessibility for KAs.

### CHILDHOOD AND MIDLIFE CULTURAL ENGAGEMENT AMONG KOREAN MARRIED COUPLES

Kyungmin Kim[1], Jeffrey Burr[2], Gyounghae Han[1], and Bon Kim[3], *1. Seoul National University, Seoul, Seoul-t'ukpyolsi, Republic of Korea, 2. University of Massachusetts Boston, Boston, Massachusetts, United States, 3. Jeju National University, Seoul, Gyeonggi, Republic of Korea*

Cultural reproduction theory posits that cultural resources transmit to the next generation, suggesting a lingering effect of parental influences on cultural experiences in adulthood. Further, middle-aged adults' cultural engagement may not only be influenced by their own childhood experiences but also their spouses' experiences. This study extends our understanding of childhood and midlife cultural engagement of married couples, using a sample of 1,271 couples (age 49–66) from the 2012 Korean Baby Boomer Panel Study and Korean Forgotten Generation Study. Results from Actor Partner Interdependence Models showed that beyond one's own childhood cultural engagement, spouse's childhood cultural engagement was associated with levels of perceived cultural engagement at midlife (for both husbands and wives) and number of arts and cultural activities at midlife (only for husbands). Given the cross-spousal associations in cultural engagement among Korean middle-aged couples, both spouses' cultural resources need to be considered for policy implications.

### MOBILE-BASED MULTIDOMAIN LIFESTYLE INTERVENTION AND HEALTH PROMOTION IN OLDER KOREAN AMERICANS

Junhyoung Kim, *Indiana University, Bloomington, Indiana, United States*

Prior studies have suggested that the nature-based virtual reality (VR) program serves as a therapeutic tool to increase health and wellbeing among older adults. While much research on virtual reality indicates their health benefits among older adults, there are research gaps in the current literature on the nature-based VR experiences of older Korean Americans. The objective of this study was to gather preliminary information on the health outcomes of the program as well as challenges to participation. Based on in-depth interviews with five participants, three main salient themes as positive outcomes emerged: (a) enjoyment, (b) stress reduction, and (c) cognitive function. On the other hand, there were three main challenges that participants experienced: (a) challenges in the immersion experience itself, (b) difficulties with the controllers, and (c) cybersickness. This finding suggests that the nature-based virtual reality program can serve older immigrant population as a therapeutic tool to increase their mental and cognitive health.

### SOCIAL DETERMINANTS OF HEALTH AND WILLINGNESS FOR END-OF-LIFE DISCUSSION AMONG KOREAN AMERICAN IMMIGRANTS

Hyunjin Noh[1], Eunyoung Choi[2], Lewis Lee[3], and Hee Lee[4], *1. The University of Alabama School of Social Work , Tuscaloosa, Alabama, United States, 2. School of Global Public Health, New York University, Los Angeles, California, United States, 3. School of Social Work, The University of Alabama, Tuscalossa, Alabama, United States, 4. University of Alabama, Birmingham, Alabama, United States*

This study aimed to examine social determinants of health (SDH) associated with Korean American (KA) immigrants' willingness for end-of-life discussions with family and doctors. A self-administered, cross-sectional survey was conducted with 259 KAs in Alabama. Demographic, health, acculturation, and SDH information were collected. Binary logistic regression analyses were conducted to examine associations between SDH and willingness for end-of-life discussion. Most participants were willing to discuss with family (94%) and doctors (82%). Those aware of hospice care were more likely to have willingness for discussion with family and doctors. Those who could not see a doctor because of cost and who had higher threats to interpersonal safety were less likely to have willingness for discussion with family. Those with more chronic conditions and higher social isolation were less likely to have willingness for discussion with doctors. Interventions aimed to promote KAs' end-of-life discussions should consider the SDH identified in this study.

### SESSION 3230 (SYMPOSIUM)

### USING REAL-WORLD EVIDENCE TO SUPPORT THE FDA REGULATORY APPROVAL: METHODOLOGICAL CONSIDERATIONS

Chair: Kevin Lu Discussant: Patricia Slattum

Evidence from clinical trials has traditionally been used to support regulatory drug approval, whereas real-world evidence (RWE) has been used for post-marketing surveillance studies. With the enaction of the 21st Century Cures Act, the Food and Drug Administration (FDA) is evaluating the potential use of RWE to support new indications for an approved product or to satisfy post-approval study requirements. Yet the substantial evidence standard remains unchanged for FDA approval. To date, the FDA has issued guidelines or frameworks for using RWE for regulatory decisions. However, many methodological challenges remain unanswered when using RWE in gerontology research. This Pharmaceutical Care and Outcomes Research Interest Group-sponsored symposium consists of leading experts in the country to address these problems. The four papers in this symposium will address methodological challenges that are unique in gerontology research. Specifically, The first paper will introduce the FDA drug evaluation process, focusing on the key considerations for evaluating RWE in regulatory decision making; the second paper will discuss possible challenges related to data sources from which RWE are generated in aging research; the third paper will provide a case example of some of the challenges in using RWD sources to generate RWE in the Alzheimer's Disease setting; and the fourth paper will provide recommendations for improving gerontology research based on RWE going forward.

### CHALLENGES RELATED TO DATA SOURCES FOR RWE IN AGING RESEARCH

Kevin Lu, *University of South Carolina, Columbia, South Carolina, United States*

Numerous challenges remain in research to generate RWE for FDA approvals and other health care decision-makers or stakeholders. This is particularly true for research to evaluate the effectiveness of pharmaceutical care and patient outcomes in older populations. For example, data sources available for RWE in the older populations are limited and data access can be daunting; identifying appropriate data sources for FDAapprovals remain challenging. Most of the FDA approvals are based on RWE rely on multiple data sources instead of a single database. In addition, pharmacoepidemiologic studies in older adults using RWD are often limited in capturing important clinical information and prognostic factors that are useful for decision-makers. Polypharmacy, frailty, and patient outcomes measures are often inconsistent depending on specific databases and thus produce controversial results. We will discuss different data sources appropriate for pharmacoepidemiology research and make comparisons and contrasts of different research approaches using different databases.

### KEY CONSIDERATIONS FOR EVALUATING REAL-WORLD EVIDENCE IN SUPPORT OF FDA REGULATORY DECISION-MAKING

Jie (Jenni) Li, *FDA CDER Office of Surveillance and Epidemiology, Silver Spring, Maryland, United States*

For decades, FDA has been using RWE to monitor and evaluate the safety of drug products in the post-marketing setting. Under the FDA RWE Program, FDA has accepted RWE in support of effectiveness claims. For example, the approval of tacrolimus (Prograf) for the prophylaxis of organ rejection after lung transplant was based on RWD from a national transplant registry. The key considerations for evaluating RWE for safety and effectiveness assessments include: (1) whether the RWE are fit for use (e.g., data relevance and reliability); (2) whether the study design can provide adequate scientific evidence to answer the regulatory question of interest (e.g., whether the lack of blinding impacts the reporting of the outcome events); and (3) whether the study conduct meets FDA regulatory requirements (e.g., study monitoring and standardization of data collection). FDA has issued draft guidances on EHR/Claims data, registries, data standards, and regulatory considerations, respectively, to detail these key considerations. Carefully designed and analyzed RWE studies can be used to support safety and effectiveness claims.

### USE OF RWD TO GENERATE RWE IN THE ALZHEIMER'S DISEASE SETTING: CHALLENGES AND FUTURE DIRECTIONS

Julia DiBello, *Merck & Co., Inc., West Point, Pennsylvania, United States*

There is considerable interest by many stakeholder groups in developing new therapies in Alzheimer's disease (AD). Increasing interest in the treatment of pre-clinical disease and measurement of associated long term outcomes using RWD is being explored as the use of RCTs in this context can be limited. Many challenges exist in generating RWE to support regulatory submissions in this disease population including lack of important clinical variables such as disease severity and progression in existing data sources. We will discuss the AD RWD landscape with a focus on challenges and future directions for the use of RWD to generate RWE supportive of regulatory submissions for pharmaceutical products.

### USE RWE TO IMPROVE DRUG INFORMATION FOR GERIATRIC PATIENTS

Minghui Li, and Chelsea Watts, *University of Tennessee Health Science Center, Memphis, Tennessee, United States*

Older adults are the main users of medications. However, they are usually excluded from clinical trials due to comorbidity and polypharmacy. Older adults, especially those 75 years and older, are underrepresented in clinical trials. This creates a unique opportunity to use RWE to improve the quantity and quality of drug information for geriatric patients. The quality of RWD is critical to have information on patient population, comparator, measurement, and endpoints. Data linkage is encouraged if key variables are not included in one data source. In addition, external controls based on RWE can be used when clinical trials are unethical or impractical. They can be applied for older adults, rare diseases, and under-represented minorities. For conditions common in older adults (e.g. Alzheimer's disease, lung cancer, and osteoporosis), RWE is greatly needed for all geriatric age groups. The entire spectrum of geriatric populations could identify age differences in efficacy and safety outcomes.

### SESSION 3240 (SYMPOSIUM)

### CAREGIVING NETWORKS OF DIVERSE FAMILIES: CONTEXT AND CULTURE INFLUENCES ON DEMENTIA CARE

Chair: Karen Roberto Co-Chair: Jyoti Savla

Older adults living with dementia and their primary caregivers are embedded within larger family and formal care networks that are shaped by cultural and contextual influences. Yet information about diverse caregiving networks is sparse. This symposium focuses on the composition, structure, responsibilities, and influences of caregiving networks in supporting persons living with dementia and their primary family caregivers. Savla and colleagues identify six distinct care networks among dementia family caregivers living in economically diverse areas of rural Appalachia. Networks varied by caregivers' emotional connectivity with family/friends, caregiver strain, role overload, loneliness, and service attitudes and utilization. Ashida and colleagues focus on changes in rural and urban dementia family caregiving networks during the COVID-19 pandemic. Network members engaging in negative social interactions increased while those engaging in uplifting interactions decreased over time, especially among urban caregivers. Bonds Johnson and colleagues examine shared decision making between African American caregivers and their relative living with dementia. Dyadic analysis revealed that while care recipients were involved in the decision-making processes, their care network's level of the involvement varied. Angel and colleagues explore the role of caregiver characteristics, stressors, and resources for Mexican American caregivers. Depressive symptoms were significantly associated with dementia caregiver turnover, with adult children and grandchildren caregivers more likely to experience turnover than spouses. Collectively, the findings presented extend prior research on community-based dementia caregiving, broadening the focus from a single caregiver to a network of caregivers and capturing the complexities of place-based context and culture variations in care provision.

### CARE NETWORKS OF RURAL APPALACHIAN FAMILY CAREGIVERS AND PERSONS LIVING WITH DEMENTIA

Jyoti Savla[1], Karen Roberto[2], and Rosemary Blieszner[1], *1. Virginia Tech, Blacksburg, Virginia, United States, 2. Virginia Polytechnic Institute & State University, Blacksburg, Virginia, United States*

Dementia care research typically focuses on primary family caregivers with limited consideration of their engagement with a broader care network and the geographic area in which they reside. Using a mixed-methods approach, we analyzed care networks of 163 primary caregivers and their relative living with dementia in rural Virginia. Six distinct care network types emerged based on the primary caregiver's gender, relationship to the person living with dementia, and presence of other informal caregivers. Networks differed by the caregiver's emotional connectivity with family/friends and feelings of caregiver strain, role overload, and loneliness. Caregivers' service use attitudes and support service utilization varied across network types and across divergent economic resources of the rural counties in which families resided. Findings establish a framework for understanding the types and influences of care networks and tailoring services to support dementia family caregivers in diverse rural areas.

### CHANGES IN RURAL AND URBAN FAMILY CAREGIVING NETWORKS IN THE MIDWEST DURING THE COVID-19 PANDEMIC

Sato Ashida[1], Hyunkeun Cho[2], Lena Thompson[2], Kristine Williams[3], Laura Koehly[4], Haley Schneider[1], Maria Donohoe[1], and Lubna Hossain[1], *1. University of Iowa College of Public Health, Iowa City, Iowa, United States, 2. University of Iowa, Iowa City, Iowa, United States, 3. University of Kansas Medical Center, Lawrence, Kansas, United States, 4. National Institutes of Health, Bethesda, Maryland, United States*

This study elucidates the changes in family caregiving networks during the COVID-19 pandemic and its implications on caregiver well-being. Eighty-two caregivers of individuals diagnosed with dementia within the past 2 years participated in this study to test a post-diagnosis intervention that provides a community care planning service that connects caregivers directly to community-based services. Caregivers completed telephone surveys at baseline and 3- and 6-month follow-up. The number of network members engaging in malfeasant (negative) social interactions increased by 0.798 every 3 months (p=0.002). Members engaging in uplifting interactions decreased, especially among intervention participants, by 1.93 every 3 months (p=0.047); urban caregivers reported greater decrease than rural (p=0.006). Participants in intervention group showed a trend for greater decrease in COVID-19 related distress (10-point scale) over time compared to control group (p=0.059); those with more members engaging in uplifting interactions reported lower distress (p=0.017) regardless of intervention status, network size, and rurality.

### CONTEXT MATTERS IN DECISION-MAKING: CAREGIVING NETWORKS OF AFRICAN AMERICAN DEMENTIA DYADS

Kalisha Bonds Johnson[1], Fayron Epps[2], MinKyoung Song[3], Karen Lyons[4], Kenneth Hepburn[1], and Martha Driessnack[3], *1. Emory University, Atlanta, Georgia, United States, 2. Emory University, Fairburn, Georgia, United States, 3. Oregon Health & Science University, Portland, Oregon, United States, 4. Boston College, Chestnut Hill, Massachusetts, United States*

Limited research explores the contextual and cultural nuances within African American dementia dyads within the United States and how these factors influence decision-making processes. Through a secondary data analysis of semi-structured interviews, we examined decision-making processes in five African American dementia dyads related to how they navigated decisions for the person living with Alzheimer's disease and related dementias (ADRD) across five unique contexts (e.g., mother with multiple daughters, mother with son and daughter where the son was the "primary" caregiver). Analysis revealed that within dyads, persons living with ADRD were involved in the decision-making processes, but the level of the involvement in decision making by the caregiving networks varied across dyads. Understanding the context in which decisions are made (i.e., within the dyad, across multiple family members in a caregiving network) has important implications in clinical practice and research. Interventions should be tailored to reflect these contextual and cultural nuances.

### TURNOVER AND DEPRESSIVE SYMPTOMS AMONG MEXICAN AMERICAN CAREGIVERS OF PERSONS LIVING WITH DEMENTIA

Jacqueline Angel[1], Sunshine Rote[2], and Kyriakos Markides[3], *1. The University of Texas at Austin, Austin, Texas, United States, 2. University of Louisville, Louisville, Kentucky, United States, 3. UTMB, Galveston, Texas, United States*

This study explored the role of caregiver background, stressors, and resources for Mexican American caregiver turnover and depressive symptoms. Using two waves of the Hispanic Established Epidemiologic Study of the Elderly (H-EPESE, 2010/2011-2016 N=333) Caregiver Supplement and informed by the sociocultural caregiver stress process model, we estimate logistic and OLS regressions of change in dementia and change in caregiver over five years. Neuropsychiatric expressions were significantly associated with caregiver turnover. Adult children and grandchildren caregivers were more likely to experience caregiver turnover than spouses. While depressive symptoms were relatively low at both waves, there was a greater increase in depressive symptoms occurred for caregivers who completed the interview in Spanish rather than English, which was partially explained by greater perceived stress at baseline. Findings demonstrate the need to provide dementia care supports for Mexican American caregivers, reduce stress for Spanish-speaking caregivers, and support Mexican American grandchildren who unexpectedly become caregivers.

### SESSION 3241 (BIOLOGICAL SCIENCES INVITED SYMPOSIUM)

### SENESCENCE AND INFLAMMATION

Chair: Marissa Schafer

Cell senescence and inflammation are interconnected mediators of aging and age-related disease. Recent advances in molecular and cellular profiling methods and research models are aiding in our ability to decipher mechanisms through which senescent cells drive inflammatory dysfunction, and inversely, to discover mechanisms through which aging immune cells may drive senescence, inflammation, and pathology. This symposium will feature exciting advances that span the emerging conceptual framework of how senescence and inflammation influence mammalian aging. Dr. Birgit Schilling will discuss the use of advanced mass spectrometric methods for profiling the senescent cell proteome, which reveal new insights into protein pathways and mechanisms of aging and disease. Dr. Matt Yousefzadeh will share how endogenous DNA damage can invoke cellular senescence, which enhances inflammation in both a cell autonomous and non-autonomous manner to drive tissue dysfunction and impact health. Dr. Daniel Tyrrell will discuss discovery of a novel population of age-associated CD8 T-cells that enhance local tissue senescence and inflammation and promote atherosclerosis. Dr. Xu Zhang will share a characterization of senescent cells in skeletal muscle using single-cell RNA-sequencing and the potential recruitment of immune cells by senescent fibroadipogenic progenitors. Dr. Marissa Schafer will discuss cell senescence as a mediator of age-related brain inflammatory cell composition and senescent cell targeting as a strategy to prevent cognitive decline. Importantly, discoveries discussed in this symposium may reveal new avenues for therapeutic development, to ultimately improve human healthspan.

### CELL SENESCENCE IS A FEATURE AND MODULATOR OF THE AGED INFLAMMATORY BRAIN CELL LANDSCAPE

Marissa Schafer, Xu Zhang, Chase Carver, Vesselina Pearsall, Elizabeth Atkinson, Benjamin Clarkson,

Ethan Grund, and Nathan LeBrasseur, *Mayo Clinic, Rochester, Minnesota, United States*

New strategies to preserve cognitive function could broadly benefit the aging population. Cellular senescence is a hypothesized mediator of inflammation-related tissue dysfunction in aging. Using single-cell RNA-sequencing, we discovered an age-related brain myeloid population exhibiting overlapping senescent and disease-associated activation signatures, including upregulation of chemoattractant factors. We confirmed senescent brain myeloid cells promote peripheral immune cell chemotaxis in vitro. Through mass cytometry, we demonstrate age- and sex-dependent increases in activated resident and infiltrating brain immune cells are reduced through systemic targeting of p16-positive senescent cells, which is associated with improvements in executive function and spatial learning tasks. Our high-dimensional results reveal dynamic remodeling of the age-dependent brain immune cell landscape and implicate senescent cell targeting as a strategy to counter inflammatory changes and cognitive decline.

### SENESCENCE-DERIVED PROTEIN BIOMARKERS DURING AGING AND OSTEOARTHRITIS

Birgit Schilling[1], Sandip Patel[2], Jacob Rose[2], Judith Campisi[2], Joanna Bons[2], Christina King[2], and Charles Schurman[2], *1. Buck Institute for Research on Aging, Novato, California, United States, 2. Buck Institute, Novato, California, United States*

Aging is a complex biological process associated with progressive loss of physiological function and susceptibility to several diseases, such as cancer and neurodegeneration. As senescence burden increases with aging and becomes a risk factor for many age-related diseases we are specifically interested in senescence-derived aging signatures. We use cutting-edge proteomic workflows to investigate both the senescence-associated secretory phenotype (SASP) in tissue cultures. We are subsequently examining exosome proteins as well as lipid cargo in plasma from human young (20–26 yrs) and old cohorts and old (60–66 yrs) individuals. We will also present current work assessing senescence markers in cartilage and bone targeting underlying mechanisms and options for intervention for osteoarthritis. Overall, our focus is specifically directed towards senescence-derived biomarkers of aging.

### ENDOGENOUS DNA DAMAGE AS A DRIVER OF AGING AND TISSUE DYSFUNCTION

Matt Yousefzadeh, *University of Minnesota, Minneapolis, Minnesota, United States*

Aging is a complex multifactorial process that enhances stress or impairs the ability to cope with it, whereby increasing the risk of morbidity and mortality. Many factors can contribute to aging, such as macromolecular damage (including DNA damage) that accumulates in a time-dependent manner. DNA damage is also known to induce cellular senescence, a cell fate that is known to play a causal role in aging. To investigate the effects of DNA damage and cellular senescence on aging, animal models lacking Ercc1, an important DNA repair gene, were utilized. Ercc1-deficient mice age faster than wild-type littermates and exhibit the senescent cell burdens that are comparable to that of a naturally aged mouse. To investigate cell autonomous and non-autonomous effects of DNA repair deficiency on senescence and aging, Ercc1 was deleted in mice in a tissue-specific manner. Increased senescent cell burden and dysfunction were present in tissues specifically targeted for Ercc1 deletion. However, enhanced senescence was also present in some non-targeted tissues, suggesting that these occur through cell non-autonomous mechanisms.

### AGE-ASSOCIATED GRANZYME K-EXPRESSING CD8+ T-CELLS ENHANCE ATHEROSCLEROSIS IN MICE

Daniel Tyrrell[1], and Daniel Goldstein[2], *1. University of Alabama at Birmingham, Birmingham, Alabama, United States, 2. University of Michigan, Ann Arbor, Michigan, United States*

A novel population of age-associated Granzyme K (GZMK)-expressing CD8 T cells was found in mice and humans. These cells enhance local tissue senescence and inflammation and are distinct from central memory and conventional Granzyme B- or Interferon γ-producing effector memory CD8 T cells. It is unknown whether these cells drive chronic disease; thus, we induced atherosclerosis in young (3-mo) and aged (18-mo) wild-type mice via the PCSK9-AAV model and used scRNAseq to demonstrate that this GZMK-CD8 T cell population homes to atherosclerotic lesions exclusively in aged mice. Neutralizing CD8 T cells demonstrates that GZMK-CD8 cells drive age-enhance atherosclerosis. Finally, we transferred GZMK-CD8 cells from different aged donors into young CD8-/- hosts and demonstrate that GZMK-CD8 cells from aged but not young donors drive atherosclerosis. In conclusion, we identified a novel role for this recently described population of aging-specific GZMK-expressing CD8+ T cells as a critical driver of chronic disease.

### IDENTIFICATION AND INVESTIGATION OF SENESCENT CELLS IN SKELETAL MUSCLE AGING

Xu Zhang, Leena Habiballa, Zaira Aversa, Yan Er Ng, Joao Passos, and Nathan LeBrasseur, *Mayo Clinic, Rochester, Minnesota, United States*

Skeletal muscle aging is marked by the loss and atrophy of resident fibers, and the accumulation of functionally diverse cell types including fibroblasts, adipocytes, and immune cells. Senescent cells amass in multiple tissues with advancing age where they contribute to aging, chronic disease, and physical decline. The role of senescence in mediating muscle aging has become a popular and sometimes contentious topic. However, to date, this concept has not been methodically tested. In this study, we characterized the changes in cell abundance and, importantly, cell-specific transcriptional profiles with skeletal muscle aging using scRNAseq. Interestingly, we identified a small population of p16 positive fibro-adipogenic progenitors (FAPs) which, upon further investigation using immunohistochemical methods, were found to express other senescence markers. This subpopulation of FAPs did not exhibit elevation in p21 levels with age. Instead, terminally differentiated myofibers were the source of the p21 increase. Myofibers with high p21 expression exhibit a strong inflammatory phenotype, which includes activated p53 signaling pathways together with strong cytokine-cytokine receptor interactions. We further identified large amounts of cross-talk between different cell types, suggesting that senescent FAPs and myofibers could contribute to skeletal muscle aging in a paracrine manner. Importantly, these observations in mice were confirmed in human samples, suggesting the strong translational power of these findings.

### SESSION 3250 (PAPER)

### COGNITION

### AN AGE-RELATED POSITIVITY EFFECT IN HUMOR: AN ASIAN CONTEXT

Zoe Ziyi Ng[1], and W. Quin Yow[2], *1. Raffles Institution, Singapore, Singapore, 2. Singapore University of Technology & Design, Singapore, Singapore*

Humor plays a pivotal role in our interaction with people. According to the age-related positivity effect, older adults (OA) demonstrate more positive emotions and are better able to modulate negative emotions than younger adults (YA), suggesting an increase in humor appreciation with age. However, given the heterogeneity and complexity of humor, it is unknown if this effect holds across various types of humor in a culture with ambivalent attitudes toward humor (Yue, 2016). We explored age-related effects in the perception of four types of humor with Asian adults: incongruity-resolution/benign humor, aggressive humor, local-slang humor, and self-vs.-other-deprecating humor. Fifty-six Singaporean-Chinese OA (n=26;M=64.33) and YA (n=30;M=22.48) watched four short videos depicting the four types of humor in a randomized order. Participants rated how funny each video was (from a scale of 1-not funny-at-all to 5-very-funny), ranked them in the order of most-to-least-entertaining and answered questions on Future-Time-Perspective-scale and self-rated humor.Repeated-measures ANOVA found significant main effects of age, $F(1,59)=6.60$, $p=.013$, where OA gave higher ratings than YA (M=3.00 vs. 2.47), and humor-type, $F(3,177)=26.87$, $p<.001$. There was a significant linear contrast in humor-type where aggressive and benign humor had the lowest ratings while self-vs.-others-deprecating humor had the highest ratings. Ranking analyses revealed that OA preferred local-slang and others-deprecating humor while YA preferred other-deprecating humor significantly above chance. Interestingly, there was no relationship between self-rated humor and future time perspective with age. Overall, OA displayed a greater appreciation of humor than YA, supporting an age-related positivity effect in an Asian context, despite preference differences. We would like to thank Prof Suzanne Flynn, Department of Linguistics and Philosophy, MIT, for her guidance, and the MOE Tier 2 Academic Research Fund Tier 2 (T2MOE2005) and SUTD Growth Plan Grant for Healthcare (SGPHCRS1902) awarded to the last author.

### COGNITIVE RESERVE AND RISK OF MOBILITY DISABILITY IN OLDER ADULTS

Roee Holtzer[1], Xiaonan Zhu[2], Andrea Rosso[2], and Caterina Rosano[2], *1. Yeshiva University and Albert Einstein College of Medicine, New York, New York, United States, 2. University of Pittsburgh, Pittsburgh, Pennsylvania, United States*

Background: Cognitive Reserve (CR) protects against cognitive decline and dementia but its relation to mobility disability has not been established. To address this important gap in the literature, we conducted a longitudinal investigation to test the hypothesis that higher baseline CR was associated with a lower risk of developing mobility disability in older adults.

Methods: Participants were dementia-free older adults who received a brain magnetic resonance imaging and had gait speed assessments during follow-up. Using the residuals approach, CR was derived from the modified-Mini-Mental Status Examination (3MS) total score by removing variance accounted for by measures of structural brain integrity, education and race. Mobility disability was defined using a validated cutoff score in gait speed of 0.8 m/s. Logistic regression models using General Estimating Equations (GEE) were utilized to examine longitudinal associations between baseline CR and the risk of developing of mobility disability across repeated assessments.

Results: Of the participants (n=237; mean age=82ys; %female=56%) who were free of mobility disability at baseline, 103 developed mobility disability during follow-up (mean=3.1ys). Higher CR at baseline was associated with lower risk of developing incident mobility/disability [odds ratio (OR)=.819, 0.67 to 0.98, p=.038 (unadjusted); OR=.815, 0.67 to 0.99, p=.04 (adjusted for socio-demographic variables and depression); OR=.819, 0.68 to 0.88, p=.035 (adjusted for illness history); OR=.824, 0.68 to 0.99, p=.045 (adjusted for white matter hyperintensities); OR= .795, 0.65 to 0.95, p=.016 (adjusted falls history)].

Conclusion: We provided first evidence that higher CR was associated with lower risk of developing mobilitydisability in older adults.

### CONCURRENT CHANGES IN COGNITIVE FUNCTION AND FUNCTIONAL LIMITATIONS: A BIVARIATE LATENT TRAJECTORY ANALYSIS

Elizabeth Handing[1], and Stephen Aichele[2], *1. Colorado State University, Golden, Colorado, United States, 2. Colorado State University, Fort Collins, Colorado, United States*

With a rapidly aging global population, declines in cognition and functional abilities are an increasingly salient challenge and fear among many older adults. Researchers and clinicians often study changes in cognition and changes in functional limitations as separate trajectories although they frequently co-occur. In this study, we used two-stage structural equation modeling (SEM) to estimate longitudinal trajectories of cognitive and functional limitations using data from the Health and Retirement Study (2010-2016) (N = 12,431, aged 50–91). First, individuals' longitudinal factor scores (at three measurement occasions, spanning 6 years) were estimated for cognition (indicated by verbal fluency, numeracy, and recall memory) and functional limitations (indicated by difficulties performing daily living activities, instrumental activities, and mobility). A bivariate latent trajectory model was then fit to these scores. Model fit was acceptable (CFI = .91; RMSEA = .036). Parameter estimates showed that, on average, cognition declined slightly (-.08SD/decade) and functional limitations increased gradually (0.38SD/decade). Importantly, individual cognitive declines were significantly correlated with increases in functional limitation ($r = -.19$, $p < .001$). This study fills an important research gap by using advanced statistical modeling (bivariate latent growth models) to examine the joint trajectory of cognition and functional decline which underscores the need to consider the interplay of these two abilities. Methods that quantify both individual and group trajectories can aid in better identification of aging patterns and lead to new ways of thinking about interventions to reduce the burden of concurrent decline.

### CUEING EFFECT ON DUAL TASK COORDINATION AMONG YOUNGER AND OLDER ADULTS

Yue Hu, and Helene Fung, *The Chinese University of Hong Kong, Hong Kong, Hong Kong*

Dual task coordination, which refers to the ability to coordinate the cognitive processes involved in performing two tasks with a temporal overlap, is evident in many if not all situations in the daily life of the older adults. The dual-task performance of older adults has been shown to be associated with driving performance, risk of falls, risk of car accidents and the absence of mild cognitive impairment. However, some researchers found that healthy older adults had worse dual-task performance than their younger counterparts, whereas other researchers found no age difference in dual-task performance. To address the mixed findings, the present work examined the effect of cue words on dual task coordination based on the selection, optimization, and compensation model. A total of 65 younger adults (24 females, mean age 21.80±1.99) and 91 older adults (36 females, mean age 65.38±5.61) were recruited via the platform Prolific. All participants judged the orientation of two arrows displayed with a shorter or longer temporal overlap. Before the onset of the first arrow, “difficult”, “easy” or a blank screen occurred on the computer screen. A significant cue effect was detected among younger adults regardless of the cue word. Yet, older adults had better dual-task performance under the condition of “difficult” cue word as compared with the other conditions. The findings highlight the potentially crucial role of higher expected task difficulty in preparing older adults to compensate for their age-related decline in dual-task coordination.

### STATE AND TRAIT INFLUENCES OF REPETITIVE NEGATIVE THINKING ON WORKING MEMORY PERFORMANCE

Karra Harrington[1], Riki Slayday[1], Stacey Scott[2], and Martin Sliwinski[1], *1. The Pennsylvania State University, University Park, Pennsylvania, United States, 2. Stony Brook University, Stony Brook, New York, United States*

Repetitive negative thinking (RNT) has been associated with faster cognitive decline and accumulation of Alzheimer’s disease pathology in otherwise healthy older adults. According to the cognitive debt hypothesis, RNT functions as a psychological mechanism that depletes cognitive resources and increases vulnerability for dementia. The aim of this project was to examine trait and state RNT influences on working memory performance in daily life. Data for this project were drawn from the ESCAPE study, a systematic probability sample recruited from residents of the Bronx, NY. Participants (n = 265, aged 25-65, 64.9% female) completed brief ecological momentary assessments on a smartphone five times per day for 14 days, including ratings of current thought valence (pleasant-unpleasant) and a brief working memory task. Multi-level models were used to disaggregate trait (between-person) and state (within-person) effects of thought valence on working memory performance, while controlling for demographic and contextual covariates. The models also included linear and quadratic functions of time and session number to account for time of day and retest effects on working memory performance across the assessment period. At moments when individuals were experiencing more unpleasant thoughts than usual (state RNT), they made significantly more errors on the working memory assessment ($\beta$= 0.013, SE = 0.004, p = .002). However, there was no significant relationship between trait RNT and working memory performance. The results of this study provide support for the potential for RNT to deplete cognitive resources in the moment, thus contributing to poorer cognitive performance.

### SESSION 3251 (SYMPOSIUM)

### APA GUIDELINES FOR THE EVALUATION OF DEMENTIA AND AGE-RELATED COGNITIVE CHANGE: OVERVIEW AND CLINICAL APPLICATION

Chair: Benjamin Mast Discussant: Peter Lichtenberg

Dementia is a leading cause of cognitive and functional decline and the number of people living with dementia or involved in caring for another person with dementia continues to rise. There is a growing need for health professionals who are well trained in dementia assessment and intervention. In 2021, the American Psychological Association and its Committee on Aging (CONA) published a revision of the APA Guidelines for the Evaluation of Dementia and Age-Related Cognitive Change with the goal of promoting proficiency and expertise in this growing practice area. This symposium presents an overview of the APA Guidelines and their clinical application in various practice situations and settings. The first presentation (Mast) provides an overview of the guidelines and highlights areas of significant revision, including greater attention to multicultural competence, person-centered principles, and assessment of family caregivers. The second presentation (Steffen) presents the application of the APA Guidelines to working with older adults before formal assessment and diagnosis has been initiated. The third presentation (Allen) describes use of the APA Guidelines in the post-evaluation period and integrates GSA’s revised KAER Toolkit for Primary Care Teams. The final presentation (Bush) presents ethical considerations before, during, and after dementia assessment.

### APA GUIDELINES FOR THE EVALUATION OF DEMENTIA: OVERVIEW AND KEY UPDATES

Benjamin Mast[1], and Shellie-Anne Levy[2], *1. University of Louisville, Louisville, Kentucky, United States, 2. University of Florida, Gainesville, Florida, United States*

This presentation will provide a historical background and a broad overview of the APA Guidelines for the Evaluation of Dementia and Age-Related Cognitive Change. This will begin with an overview of general and procedural guidelines pertaining to dementia assessment and will be followed by an in-depth discussion of key updates in the 2021 revision. The newly revised guidelines recognize racial and cultural disparities in dementia outcomes, and emphasize the importance of multicultural competence in all aspects of dementia assessment. The revised guidelines also integrate person-centered principles into the assessment process and place greater emphasis on understanding the person living with dementia. New guidelines were included to address (1) the assessment of behavioral and psychological changes in the context of dementia, and (2) the importance of assessing the health and well-being of family caregivers.

### BEFORE A NEUROCOGNITIVE EVALUATION: ROLES AND COMPETENCIES OF BEHAVIORAL HEALTH PROVIDERS

Ann Steffen[1], and Rebecca Allen[2], *1. University of Missouri-St. Louis, St. Louis, Missouri, United States, 2. The University of Alabama, Tuscaloosa, Alabama, United States*

Neurocognitive disorders are among the most stigmatized medical conditions in the USA and across the globe. Cultural beliefs about cognitive aging also impact how individuals, families and healthcare teams respond to concerns about cognitive impairment. GSA's revised KAER Toolkit for Primary Care Teams (Fall, 2020) provides useful information for the early stages of this process, including kickstarting the conversation about brain health concerns and conducting screenings. This presentation outlines additional competencies for behavioral health clinicians involved in patient, family, and health care team discussions regarding cognitive health concerns. Culturally responsive strategies and resources are suggested to help clinicians explore the decision for a formal evaluation and shape the nature of the referral question(s).

### AFTER A NEUROCOGNITIVE EVALUATION: ROLES AND COMPETENCIES OF BEHAVIORAL HEALTH PROVIDERS

Rebecca Allen[1], and Ann Steffen[2], *1. The University of Alabama, TUSCALOOSA, Alabama, United States, 2. University of Missouri-St. Louis, St. Louis, Missouri, United States*

GSA's revised KAER Toolkit for Primary Care Teams (Fall, 2020) provides useful information for referring individuals and families to community resources following a neurocognitive disorder diagnosis. Mental health practitioners must develop competencies and professional identities within integrated care teams to facilitate this process. This presentation outlines post-assessment suggestions for clinicians across disciplines (psychology, social work, nursing, medicine, pharmacy, +) in building such teams. There may be needs for serial assessment of specific individual capacities, treatment plans to facilitate shared decision-making between individuals and family care partners, and evaluations of the family care partner's capacity to provide care. Behavioral health providers assist patients, families, and health care teams across a range of residential settings in understanding and implementing treatment recommendations. Emerging science indicates the value of initiating planning discussions with lifestyle modifications to enhance functioning within dyads. Culturally responsive strategies will be suggested for engagement in planning for future care.

### SESSION 3260 (PAPER)

### COVID-19 II

### AGE AS ONE OF THE DETERMINANTS OF RESILIENCE TO THE COVID-19 PANDEMIC AND ITS IMPACTS ON SEX LIFE

Liza Berdychevsky, *University of Illinois at Urbana-Champaign, Champaign, Illinois, United States*

Recent research started exploring the impacts of the COVID-19 pandemic on sex life. However, there is a dearth of research on the roles of age in the overall adjustment to the pandemic and its impacts on sex life. The purpose of this study was to address this gap. Data collection was based on the online survey methodology (N=675, age range:18-76yo). Data analysis included one-way ANOVA and multivariate multiple regression. Age differences were found in the participants' overall ability to adjust to the pandemic-related lockdown conditions, with older adults (51+yo group) adjusting better than the rest of the sample and young adults (18-30yo group) adjusting significantly worse. Resilience to the impacts of COVID-19 on sex life also varied by age. Sexual desire deteriorated the worst among middle-aged participants (31-50yo group); likely, due to increased childcare responsibilities. However, the issues with physical sexual difficulties have worsened the most among older adults. Regression results also showed that age had negative effects on the frequencies of intimate and sexual behaviors (sexual touching and caressing, sexual intercourse, and oral sex) and sexual desire and satisfaction with sex life during the pandemic. While older adults' overall adjustment capacity to the pandemic's conditions was the highest in the sample, the pandemic's impacts on their sex lives and sexual health were the worst. The pandemic has exacerbated existing vulnerabilities and further jeopardized older adults' sexual wellbeing. This study provides age-specific insights for sexual counseling and age-targeted sexual health education for older adults during the pandemic and its aftermath.

### EXAMINING AGE, RACE, AND COMMUNITY ON PREVENTIVE HEALTH AND COVID-19 VACCINATION AMONG ADULTS 65+

Taylor Patskanick[1], Sophia Ashebir[2], Alexa Balmuth[2], Sophia Lee[2], Joie Le[2], Lisa D'Ambrosio[2], and Joseph Coughlin[2], *1. Massachusetts Institute of Technology, Somerville, Massachusetts, United States, 2. Massachusetts Institute of Technology, Cambridge, Massachusetts, United States*

During the ongoing spread of COVID-19 and its variants, older adults remain an age group particularly at-risk for poorer health outcomes, not only related to infection with COVID-19, but also due to disruptions in access to preventive health services, including routine vaccination. In the U.S., older adults have generally had high uptake of the COVID-19 vaccines, but differences persist regionally and between older adults from minority racial backgrounds. The purpose of the following study was to better understand how groups of Black and white-identifying adults ages 65+ described the impact of the COVID-19 pandemic on their preventive health behavior and healthcare use, including what contributed to their decision to receive or not receive a primary COVID-19 vaccination series. Seventy-five participants were purposively sampled and stratified into virtual focus groups based on their age, racial identity, vaccination status, and relationship to a local community. Findings leverage data from a pre-group questionnaire and focus groups conducted in November 2021. Analyses revealed differences among sub-groups about how the pandemic has impacted their relationship to their local community. Participants described the extent of the pandemic's disruption to their healthcare access, including modifications to in-person care, use of telehealth, and engagement in new health behaviors. Decision-making related to the COVID-19 vaccine differed among the vaccinated and unvaccinated and white and Black-identifying groups, including factors related to interpersonal and systemic trust, independent research, and bodily autonomy. Implications of this research for public health and practitioners working with older adults will be discussed.

### RACIAL AND ETHNIC DISPARITIES IN COVID-19 DISRUPTIONS TO SOCIAL ACTIVITIES AMONG OLDER ADULTS IN THE UNITED STATES

Felicia Wheaton[1], Farhana Islam[1], Matilda Johnson[2], and Sai Raj Kappari[3], *1. Xavier University of Louisiana, New Orleans, Louisiana, United States, 2. Bethune Cookman University, Daytona Beach, Florida, United States, 3. HEMA Inc, Harahan, Louisiana, United States*

Racial disparities in COVID-19 exposure, illness, hospitalization and mortality have been well-documented, however, less is known about potential disparities in the pandemic's effect on social activities. Data from the early release of the 2020 Health and Retirement Study (HRS) were used to examine the relationship between race (White/African American/other) and ethnicity (Hispanic/Non-Hispanic) and COVID-19 disruptions to social activities (N=4,500). Participants were asked, "due to the coronavirus pandemic, did you experience any of these changes in activities: 1) unable to visit a family member in a care facility, nursing home or group home, 2) family celebrations canceled/restricted, 3) unable to visit a close family member in the hospital, 4) unable to attend in-person funeral or religious service for a family member or friend, 5) unable to visit family after birth of a baby (1=yes, 0=no/not relevant) and, "overall, how stressful have changes in contacts with family and friends been for you?" (1=not at all stressful, 5=extremely stressful). Results from logistic regression controlling for age and gender showed that relative to Whites, African Americans had significantly higher odds of all disruptions except for family celebrations and relative to non-Hispanics, Hispanics had significantly higher odds of three types of disruptions. OLS regression results showed that only Hispanics had higher average stress due to these changes. These findings indicate that in addition to the previously documented disparities in COVID-19, older African Americans and Hispanics experienced greater disruptions to social activities. These impacts could further influence both short and long-term physical and mental health.

### SHIFT IN ACTIVITY PATTERNS AMONG OLDER ADULTS BEFORE AND AFTER THE COVID-19 PANDEMIC: A SEQUENCE ANALYSIS

Wenxuan Huang[1], and Jiao Yu[2], *1. Johns Hopkins University, Baltimore, Maryland, United States, 2. University of Minnesota, Minneapolis, Minnesota, United States*

Background: Previous research has discovered significant heterogeneity in older adults' activity patterns, as well as its associations with well-being. Studying activity pattern during the COVID-19 pandemic sheds new light on infection risks and coping strategies stratified by socioeconomic status. This study answers two research questions: (1) what are the typical activity patterns among older adults in the U.S.? and (2) how do different sociodemographic groups react to the COVID-19 pandemic by alternating activity pattern? Data: This study used data from the American Time Use Survey (2019 and 2020) to create an analytic sample of 5,068 older adults aged 65 and older. Method: First, we classified daily activity sequences into five typical activity patterns using cluster analysis. Second, we conducted multinomial logistic regression by including survey year and key covariates interactions to examine shifts between sociodemographic characteristics and activity pattern. Findings: We identified five distinct activity patterns including versatile (involves diverse types of activities), housework (daytime occupied by housework activities), work-centered (daytime dominated by paid work), leisure-intensive (long time spent in social and leisure activities), and inactive (intensive TV watching). Our findings show that SES does not affect predicted membership of the versatile group in pre-pandemic time, while an educational gradient becomes salient in 2020. Highly educated older adults are more likely to maintain the diversity of daily activities. Furthermore, the racial difference is more pronounced in the inactive group during the COVID-19 pandemic.

Conclusion: Our findings suggest a buffering effect of social advantages on the impact of public health crisis.

### TRENDS IN RISK FACTORS FOR DISABILITY AND REDUCED PHYSICAL FUNCTIONING AMONG US ADULTS DURING THE COVID-19 PANDEMIC

Theresa Andrasfay[1], and Eileen Crimmins[2], *1. University of Southern California, Leonard Davis School of Gerontology, Los Angeles, California, United States, 2. University of Southern California, Los Angeles, California, United States*

The COVID-19 pandemic has caused widespread societal disruptions that may have impacted risk factors for disability and functional limitations. Current research suggests that physical inactivity, BMI, and smoking increased during the early phase of the pandemic, but less is known about whether these risk factors remained elevated as the pandemic progressed and mitigation measures were less disruptive. In this study, we assess trends in exercise frequency, BMI, and smoking among American adults aged 45 and over during the first year of the COVID-19 pandemic. We use data from the Understanding America Study (UAS) Coronavirus in America study, a nationally representative online survey that followed American adults from March 2020 to July 2021. We find that exercise frequency was highly seasonal throughout the pandemic, increasing during warmer months and decreasing during the colder winter months. Throughout this period, exercise frequency was highest among those with more education and those who were retired. Average BMI remained similar throughout this period, but less educated individuals and those working away from home and those who were unemployed experienced increases in BMI. Consistent with media reports suggesting increased interest in quitting smoking during the pandemic, we find decreases in smoking prevalence and frequency of smoking over this period. Decreases in smoking prevalence were concentrated among less educated individuals, who had higher prevalence of smoking to begin with. In sum, changes in risk factors for disability and reduced physical functioning during the pandemic were mixed and varied by education and employment status.

### SESSION 3270 (SYMPOSIUM)

### DETERMINANTS AND CONSEQUENCES OF LEISURE ACTIVITY ENGAGEMENT AND CONSTRAINTS IN OLDER ADULT POPULATIONS

Chair: Angie Sardina Co-Chair: Alyssa Gamaldo
Discussant: Alyssa Gamaldo

Older adults engage in approximately 7.1 hours/day in leisure activities; however, much of the leisure activity engagement comprises of passive activity engagement (e.g., watching TV). An increasing amount of literature suggests that regular engagement in cognitively and physically stimulating activities, rather than strictly passive activity engagement, is associated with better physical and mental health as well as maintenance of social networks. As the aging population continues to increase and levels and types of activity engagement are shifting across our more diverse older adult populations, it is imperative to understand levels and types of activities older adults are participating in, as well as the psychosocial and contextual factors related to leisure activity engagement. This symposium will include presentations from studies that explore the following: (1) leisure activity interests, engagement, and constraints; and (2) determinants and/or consequences of leisure activity engagement. Specifically, Sardina and colleagues examined daily variability between affect and leisure engagement, and explored potential sociodemographic moderators for these associations. Tian and colleagues explored the association between leisure activities and modes of transportation. Tian and colleagues explored leisure activity engagement with prospective daily diary methods, and examined associations between leisure activities and physical health. Janke and colleagues explored associations between facilitators, constraints, and constraint negotiation and self-reported physical activity levels for older adults with arthritis.

### DAY-TO-DAY VARIABILITY BETWEEN AFFECT AND LEISURE ACTIVITY ENGAGEMENT IN BLACK ADULTS

Angie Sardina[1], Alyssa Gamaldo[2], Jason Allaire[3], and Keith Whitfield[4], *1. UNC Wilmington, Wilmington, North Carolina, United States, 2. Pennsylvania State University, State College, Pennsylvania, United States, 3. North Carolina State University, Raleigh, North Carolina, United States, 4. University of Nevada, Las Vegas, Las Vegas, Nevada, United States*

This study examined daily variability in affect and leisure engagement, within community-dwelling Black adults (age range=50-80 years). Associations between affect and leisure engagement, and moderating sociodemographic factors, were explored. Fifty adults (78% women; M education=11.62) reported affect and leisure engagement across 8-occasions over 2-3 weeks. Participants averaged 3-leisure activities/day with more engagement in watching TV, walking, reading, and visiting others. Significant within-person variation across daily affect and domains of leisure engagement were observed. Greater negative affect was significantly associated with lower social and total leisure engagement, particularly for adults with lower levels of education (p's<.05). Results demonstrate within-person changes in the type of leisure engagement among Black adults, with potential factors related to interconnections between affect and sociodemographics (e.g., education). This study reveals promising directions for future research to implement models estimating both between- and within-person effects of daily leisure engagement, within minority populations and across racial groups.

### RELATIONSHIPS BETWEEN LEISURE CONSTRAINTS AND MODES OF TRANSPORTATION AMONG OLDER ADULTS IN SUBSIDIZED HOUSING

Junyan Tian[1], Shyuan Ching Tan[2], Angie Sardina[3], Alyssa Gamaldo[4], and Lesley Ross[5], *1. Penn State University, State College, Pennsylvania, United States, 2. California State University San Marcos, San Marcos, California, United States, 3. UNC Wilmington, Wilmington, North Carolina, United States, 4. Pennsylvania State University, State College, Pennsylvania, United States, 5. Clemson University, Clemson, South Carolina, United States*

Transportation needs among lower-income older adults is understudied, particularly regarding how transportation utilization is related to leisure constraints. This preliminary study included 39 adults residing in subsidized housing in North Carolina and Pennsylvania (M=68.03, SD=10.26, female=74.4%) and assessed reported modes of transportation for daily activities and perceived leisure activity constraints. Much of the sample reported driving (57.9%) or relying on others to drive (70.3%) with a significant sample reporting use of public transportation (48.6%). Transportation utilization was differentially correlated with leisure constraints. Perceived difficulties getting to/from activities in the community was associated with greater utilization of having 'others drive you' (r=0.43, p=.011), but lower utilization of 'driving oneself' (r=-0.40, p=.019). Walking as a mode of transportation was associated with lower (r=-0.41, p=.014) perceived difficulties getting to/from activities in the housing complex. These results indicate the importance of further exploring the association between transportation and leisure needs of these lower-income older adults.

### CHARACTERISTICS OF OLDER ADULTS' LEISURE ACTIVITIES: A DAILY DIARY INVESTIGATION

Junyan Tian[1], Abigail Stephan[2], and Lesley Ross[2], *1. Penn State University, State College, Pennsylvania, United States, 2. Clemson University, Clemson, South Carolina, United States*

Leisure activity engagement in older adults' daily lives is well-documented, however, relatively few studies include participant-reported activities using prospective daily diary methods. The goal of the study was to examine the proportion of three types of reported leisure activities and how physical health moderated such activities. Two weeks of self-reported leisure activities examined among healthy sedentary community-dwelling adults from the Cognitive and Physical Exercise Study (N=33, 65-81 years, 442 unique data points). Each day, participants listed leisure activities performed, estimated time spent on each activity, and labeled the activities as cognitive, physical, and/or social. Physical health was assessed by the SF-12 at baseline. Across 14 days, 25.14% of reported activities were labeled as cognitive activities, 26.61% were labeled as social activities, and 22.97% were labeled as physical activities. Multilevel models indicated participants' engagement in different domains of activities were not influenced by physical health and did not change over time.

### FACILITATORS AND CONSTRAINTS TO SPORT ACTIVITY AMONG ADULTS WITH ARTHRITIS

Jill Juris[1], Julie Son[2], Jen Wong[3], Guangzhou Chen[4], Toni Liechty[5], Stephanie West[6], and Megan Janke[7], *1. Appalachian State University, Boone, North Carolina, United States, 2. University of Idaho, Moscow, Idaho, United States, 3. The Ohio State University, Columbus, Ohio, United States, 4. University of Illinois at Urbana Champaign, Champaign, Illinois, United States, 5. University of Illinois Urbana-Champaign, Champaign, Illinois, United States, 6. James Madison University, Harrisonburg, Virginia, United States, 7. Berry College, Mount Berry, Georgia, United States*

This study examines types of facilitators, constraints, and constraint negotiation strategies and their associations with self-reported physical activity levels for older adults with arthritis. A national sample of U.S. adults (N=288; age range =50-85, M=64.8) who participated in a larger study of sport participation completed an online questionnaire on their involvement in leisure activities. The sample was predominantly White (91.3%), female (65.2%), and unmarried (55.6%). As expected, individuals reporting more constraints engaged in significantly less physical activity ($\beta$=-.19, p=.01) while those using greater constraint negotiation strategies reported significantly more activity engagement ($\beta$ =.18, p=.03). Facilitators were examined (intrapersonal, interpersonal, and structural), but only interpersonal facilitators significantly predicted greater levels of physical activity ($\beta$ =-.07, p=.03). Adults reporting sport engagement during the past year were also more active ($\beta$ =.24, p<.001). The discussion will focus on the implications of findings and how barriers to activity in this population can be addressed.

### SESSION 3280 (SYMPOSIUM)

### DIVERSITY, DISCOVERY, AND AGING REIMAGINED: NIA SESSION FOR EARLY-CAREER RESEARCHERS

Chair: Melinda Kelley Discussant: Melinda Kelley

The National Institute on Aging (NIA) at the National Institutes of Health, Department of Health and Human Services, supports biomedical and behavioral research with a lifespan focus. NIA research seeks to understand the basic processes of aging, improve prevention and treatment of diseases in later life, and improve the health of older persons, in addition to a focus on Alzheimer's disease and related dementias. NIA also supports the training and career development of scientists focusing on aging research and the development of research resources. This symposium, meant for junior faculty and emerging scholars, will provide an update on the latest research findings from NIA followed by a segment on funding mechanisms and strategies. An opportunity will be provided to meet and consult with NIA extramural staff.

### OVERVIEW OF NIA MISSION AND RESEARCH

Richard Hodes, *National Institutes of Health, Bethesda, Maryland, United States*

Dr. Hodes will provide an overview of NIA's structure and mission, in addition to discussing research foci from across the Institute's scientific divisions.

### HOW TO GET AN NIA GRANT

Kenneth Santora, *National Institute on Aging, Bethesda, Maryland, United States*

Dr. Santora will provide an overview of the NIA application process and will share information on relevant policy changes.

### SESSION 3290 (PAPER)

### ENVIRONMENT, HEALTH, EQUITY, DIVERSITY, AND INCLUSION

### A COMPARISON OF SUBJECTIVE COGNITIVE DECLINE AND RELATED LIMITATIONS AMONG TRANSGENDER COMMUNITIES IN THE US

Ethan Cicero[1], Michael Goodman[1], Lisa Barnes[2], Molly Perkins[3], Jason Flatt[4], and Vin Tangpricha[1], *1. Emory University, Atlanta, Georgia, United States, 2. Rush Alzheimer's Disease Center, Rush University Medical Center, Chicago, Illinois, United States, 3. Emory University School of Medicine, Atlanta, Georgia, United States, 4. University of Nevada, Las Vegas, School of Public Health, Las Vegas, Nevada, United States*

Background: The transgender population is composed of subgroups that are diverse in gender identity (e.g., transgender women[TW], transgender men[TM], nonbinary[NB] individuals). Compared to cisgender adults, transgender adults are more likely to report subjective cognitive decline (SCD). It remains unclear if SCD prevalence and related limitations vary by transgender subgroups.

Methods: 2015-2020 Behavioral Risk Factor Surveillance System data, representing 38 U.S. states that assessed SCD (confusion/memory loss happening more often/getting work over previous 12months) and gender identity were used to examine differences in SCD prevalence and SCD-related limitations by transgender subgroups, TW(n=442), TM(n=298), and NB(n=183). Age-adjusted odds ratios (OR) along with 95% confidence intervals (CI) were calculated to investigate group differences in SCD prevalence. Separate analyses compared SCD-related limitations, demographics, and health across groups among participants reporting SCD.

Results: SCD prevalence was highest among NB(21.3%), followed by TW(16.3%) and TM(14.1%). After accounting for age, subgroup differences remained; odds of SCD were 1.6x higher among TW compared to TM (CI:1.1–2.4, p=0.012). Among those with SCD, TW were less likely to receive help they needed with day-to-day activities when compared to TM (OR=7.9; CI:0.1–0.2, p< 0.001) and NB (OR=5.0; CI:0.1–0.4, p=0.001); and TW were more likely to be deaf (OR=4.2; CI:1.7–10.1, p=0.002) and have asthma (OR=2.8; CI:1.4–5.7, p=0.005) when compared to NB adults. No other differences were found.

Conclusion: Health and social inequities are not uniformly experienced across transgender subgroups, and it is important to understand how these factors impact the brain health of TW, TM, and NB adults.

### BLACK PLACEMAKING: THE BODY, HOME, AND PUBLIC SPACE THROUGH THE LENS OF OLDER WOMEN

Laurent Reyes[1], Jarmin Yeh[2], and H. Shellae Versey[3], *1. UC Berkeley, Berkeley, California, United States, 2.*

University of California, San Francisco, San Francisco, California, United States, 3. Fordham University, Bronx, New York, United States

African American communities are frequently depicted as victims of urban conditions. However, a rich culture of grassroots community development and organizing, often led and stewarded by Black women, exists. Many of these efforts involve enhancing economic, political, and educational opportunities and centering ethics of care and caregiving. This is the notion of Black placemaking, which is explicitly community-focused, shaping the social fabric of everyday life and allowing for the development of Black vernacular spaces that became vital to African-American culture. This paper examines how Black older women engage in placemaking by presenting three select case studies. Using a narrative inquiry approach, we conducted secondary data analysis of interviews drawn from larger qualitative studies about aging in communities that took place in San Francisco and New York City. Black feminist spatial imagination, embodiment, and intersectionality theory were our guiding frameworks. Our analysis revealed how the aging Black body is a site that is subjected to socio-political regulation and violence and illuminates how Black women are agents of community resilience, creativity, and transformation. Creating and holding space (i.e., placemaking) with bodies and physical structures that center the Black community is an act of care, self-determination, and resistance to white supremacy. These embodied processes of placemaking have wide-ranging implications for the ways Black neighborhoods are framed and discussed in popular media, empirical research, and policy. Furthermore, they invite a shift in our current approach to placemaking in later life, one that centers the strengths, history, and traditions of the Black community.

### COGNITIVE DIFFICULTIES AMONG ASIAN AMERICAN OLDER ADULTS: RESULTS FROM THE AMERICAN COMMUNITY SURVEY

Duy Nguyen[1], Rui Liu[2], and Yookyong Lee[3], *1. Sacred Heart University, Teaneck, New Jersey, United States, 2. Sacred Heart University, Fairfield, Connecticut, United States, 3. University of Alabama at Birmingham, Birmingham, Alabama, United States*

While Asians are the fastest growing racial group in the United States, limited research exists on their health needs, especially among older adults. Cognitive difficulties increase disease and caregiving burdens, but little is known about patterns of cognitive health among Asian American older adults. This study fills the knowledge gap by using data from the 2015-2019 American Community Surveys to examine the relationship between Asian ethnicity and gender on cognitive health. This analysis focuses on respondents aged 65 and over from the six most populous Asian American groups: Chinese, Filipino, Indian, Japanese, Korean, and Vietnamese (n=100,538). Weighted, adjusted logistic regression analyses tested for the effects of Asian ethnicity and gender on cognitive difficulties. Multivariate analyses showed Filipino, Indian, and Vietnamese older adults were more likely than Chinese to report cognitive difficulties. Additionally, Asian American women were more likely to report cognitive difficulties compared to Asian American men. Joint effect analyses were statistically significant, thus we examined separate regression analyses by Asian ethnicity. Results showed that Vietnamese women were more likely than Vietnamese men to report cognitive difficulties. Across Asian ethnic groups, family relationships and economic factors play a major role in having a cognitive difficulty. Overall, the results suggest that ethnic-specific analysis of the Asian American reveals a fuller picture of health in older adulthood. Future research needs to dive more deeply into the sociocultural and economic dimensions of health. Further, culturally appropriate policy and practices are needed to promote successful aging among older Asian Americans.

### HOW DO INTERGENERATIONAL RELATIONSHIPS IMPACT THE HEALTH OF ASIAN AMERICAN OLDER ADULTS?

Duy Nguyen[1], Yookyong Lee[2], and Rui Liu[3], *1. Sacred Heart University, Teaneck, New Jersey, United States, 2. University of Alabama at Birmingham, Birmingham, Alabama, United States, 3. Sacred Heart University, Fairfield, Connecticut, United States*

Intergenerational relationships and filial piety are important values common to many Asian-ethnic groups. While a limited literature exists examining the health outcomes of older adults living with grandchildren in Asian countries, Asians in America have received less attention from researchers and policymakers. This study fills the knowledge gap by using data from the 2015-2019 American Community Surveys to examine the relationship between Asian ethnicity and living with grandchildren on health outcomes. This analysis focuses on respondents aged 65 and over from the six most populous Asian American groups: Chinese, Filipino, Indian, Japanese, Korean, and Vietnamese (n=100,538). Roughly half the sample lived with grandchildren. Weighted, adjusted logistic regression analyses tested for the effects of Asian ethnicity and living with grandchildren on 4 health outcomes: ambulatory, independent living, hearing, and vision difficulties. Multivariate analyses showed Filipino and Vietnamese older adults were more likely than Chinese to report difficulties across health outcomes. Additionally, individuals living with grandchildren were less likely to report ambulatory, independent living, and hearing difficulties. Joint effect analyses revealed Indian, Filipino, and Japanese Americans living with grandchildren reported more difficulties compared to the reference group. Overall, the results suggest that living with grandchildren can be a protective factor for the health outcomes of older Asian Americans, while having different impacts depending on ethnic origin. Future research needs to differentiate the impact of living with grandchildren across Asian ethnic groups. Further, culturally appropriate policy and practices are needed to promote successful aging among older Asian Americans living with grandchildren.

### KEEPING COMMUNITY DURING A PANDEMIC: LGBTQ+ OLDER ADULTS AND THE VIRTUAL SENIOR CENTER

Suzanne Marmo[1], Manoj Pardasani[2], and David Vincent[3], *1. Sacred Heart University, Fairfield, Connecticut, United States, 2. Adelphi University, Garden City, New York, United States, 3. SAGE Advocacy and Services for LGBTQ+ Elders, New York, New York, United States*

During the initial stages of the pandemic, 96% of all senior centers ceased in-person programming, leaving many older adults without resources for meals, socialization, and critical services (NCOA, 2020). As a result of this shutdown, risk factors such as being a member of the underserved LGBTQ+ community, identifying as part of a racial or ethnic minoritized group, and/or experiencing poverty contributed to an increased likelihood of experiencing difficulties in meeting basic needs, reduced immunity to COVID and experiencing isolation (Berg-Weger and Morley, 2020; Kuehn, 2021). Despite the closure of many senior centers, some organizations were well positioned to strategically utilize pre-existing resources to help the community (Pendergrast, 2021). One organization, SAGE Advocacy and Services for LGBTQ+ Elders, the first publicly funded senior advocacy organization for LGBTQ+ older adults in the US, was one of the first to effectively transition to becoming a virtual senior center within days after the start of the pandemic (NYC DOA, 2020). Having a group of front-line workers who were highly embedded in their community, helped facilitate effective organizational adaptation and transition to a virtual senior center. This presentation seeks to describe how staff and program facilitators became vital resources for maintaining connection to the community of LGBTQ+ older adults. Focus groups with SAGE senior center employees and program facilitators were conducted in summer of 2021. Data identified resiliencies and barriers for maintaining community, providing vital services, and mitigating isolation with LGBTQ+ elders. Lessons learned and implications for organizations facing crises will be shared.

### SESSION 3300 (SYMPOSIUM)

### GSA'S CONGRESSIONAL UPDATE

Chair: Brian Lindberg

This popular annual session will provide cutting-edge information on what the 117th Congress has and has not accomplished to date, and what may be left for the lame duck session to address. Speakers will discuss key issues such as Social Security, Medicare, Medicaid, Older Americans Act, Build Back Better Act, social isolation, serious illness care, and funding. Predictions for the 118th Congress may be provided.

### SESSION 3310 (SYMPOSIUM)

### IMPLEMENTATION SCIENCE IN DEMENTIA CARE: METHODOLOGICAL, CULTURAL, AND SOCIODEMOGRAPHIC CONSIDERATIONS

Chair: Joseph Gaugler Co-Chair: Laura Gitlin Discussant: Beth Prusaczyk

The gap between aging science and practice persists. Getting the best possible evidence into the real world is a particular challenge in gerontology because of the complex needs of older persons, the family caregiving experience, the workforce shortage, and underlying structural concerns with how services for older people are regulated, paid for, and valued. The purpose of this symposium is to consider contextual dimensions to facilitate implementation, methodological considerations, strategies to inform cultural adaptation of evidence-based innovations, and how creativity and innovation in low- and middle-income countries can better inform implementation practices in resource rich countries and vice-versa. Specifically, Dr. Marie Boltz and colleagues will examine the conceptual and theoretical underpinnings of organizational readiness for implementation and the operationalization of the construct. Dr. Joseph Gaugler and co-authors will provide case examples of three "hybrid effectiveness" trials in dementia care in different care settings including community-based and residential long-term care settings, each of which incorporate implementation methods alongside traditional evaluations of effectiveness. Dr. Lauren Parker and her team will demonstrate how cultural adaptation strategies were applied to facilitate the implementation of a community-based, evidence-based dementia care intervention. Dr. Baker and colleagues consider implementation issues and innovations in low- and middle-income countries. Dr. Beth Prusaczyk, an implementation expert, will serve as Discussant and highlight future implementation issues to advance best practices that facilitate and expedite the translation of aging science.

### USING HYBRID EFFECTIVENESS DESIGNS TO FACILITATE IMPLEMENTATION: THREE CASE STUDIES IN DEMENTIA CARE RESEARCH

Joseph Gaugler[1], Rosa Baier[2], Richard Fortinsky[3], Allison Gustavson[4], Nancy Hodgson[5], Miranda McPhillips[5], Justine Sefcik[6], and Laura Gitlin[6], *1. University of Minnesota, Minneapolis, Minnesota, United States, 2. School of Public Health, Brown University, Providence, Rhode Island, United States, 3. University of Connecticut Center on Aging, Farmington, Connecticut, United States, 4. Minneapolis VA Health Care System, Minneapolis, Minnesota, United States, 5. University of Pennsylvania, Philadelphia, Pennsylvania, United States, 6. Drexel University, Philadelphia, Pennsylvania, United States*

This individual symposium presentation will provide case examples of three "hybrid effectiveness" trials in dementia care in different care settings including community-based and residential long-term care settings, each of which incorporate implementation methods alongside traditional evaluations of effectiveness. We offer considerations related to conceptualization, study design, sampling, data collection, and analysis that may guide gerontologists who aspire to adopt hybrid effectiveness designs in their own work. We conclude with methodological recommendations to incorporate an implementation science lens throughout the lifecycle of gerontological (and/or dementia care) intervention development. By applying key principles from implementation science throughout the intervention development process, a truncated and more efficient implementation pipeline may be achievable in gerontological research. Moreover, incorporation of implementation science methods into standard intervention development and testing methodologies will result in older persons, their families, healthcare providers, and communities having the best evidence available at their disposal.

### A CONCEPTUAL MODEL OF ORGANIZATIONAL READINESS FOR IMPLEMENTATION OF EMBEDDED PRAGMATIC DEMENTIA RESEARCH

Marie Boltz[1], Kimberly Van Haitsma[2], Rosa Baier[3], Justine Sefcik[4], Nancy Hodgson[5], and Ann Kolanowski[6], *1. Penn State, Pennsylvania State University, Pennsylvania, United States, 2. Pennsylvania State University, University Park, Pennsylvania, United States, 3. School of Public*

Health, Brown University, Providence, Rhode Island, United States, 4. Drexel University, Philadelphia, Pennsylvania, United States, 5. University of Pennsylvania, Philadelphia, Pennsylvania, United States, 6. Penn State University, Penn State University, Pennsylvania, United States

Organizational readiness for implementation refers to the collective willingness and efficacy of people, processes, and internal and external contexts within study sites to implement an intervention; it is a salient consideration when planning and conducting embedded pragmatic trials. This paper examines the conceptual and theoretical underpinnings of organizational readiness for implementation and the operationalization of the construct. We synthesize the literature to offer a conceptual model for explicating and measuring organizational readiness for implementation and describe the unique characteristics and demands of implementing evidence-based interventions targeting persons with dementia and/or their care partners. Our model was derived from the Consolidated Framework for Implementation Research (CFIR), and Weiner's Determinants and Outcomes of Organizational Readiness for Change. We discuss how it can serve as a guide when planning and conducting embedded pragmatic implementation trials in settings that care for persons with dementia.

### CULTURAL ADAPTATION OF THE ADULT DAY SERVICE PLUS PROGRAM FOR HISPANIC/LATINO DEMENTIA CAREGIVERS

Lauren Parker[1], Katherine Marx[2], manka Knimbeng[3], Elma Johnson[4], Sokha Koeuth[5], Joseph Gaugler[3], and Laura Gitlin[5], *1. Johns Hopkins Bloomberg School of Public Health, Baltimore, Maryland, United States, 2. Johns Hopkins School of Nursing, Baltimore, Maryland, United States, 3. University of Minnesota, Minneapolis, Minnesota, United States, 4. University of Minnesota School of Public Health, Minneapolis, Minnesota, United States, 5. Drexel University, Philadelphia, Pennsylvania, United States*

Although Hispanic/Latinos are at disproportionate and increased risk for Alzheimer's disease and related dementias, few evidence-based supportive care interventions have been specifically developed for or adapted for this population. Adapting a supportive care intervention requires more than Spanish language translation and necessitates an understanding of cultural nuances and care preferences of Hispanic/Latino families and staff who implement the intervention. This paper reports on the cultural adaptation of the Adult Day Service Plus (ADS Plus) intervention for delivery by staff to Hispanic/Latino caregivers which was guided by the Cultural Adaptation Process Model. Also, using the Framework for Reporting Adaptations and Modifications-Enhanced (FRAME),we discuss: 1) when modifications were made, 2) who determined modifications needed, 3) what aspects of the intervention were modified, 4) the relationship to fidelity and how fidelity was maintained, and 5) reasons for modifications. Modifications to the delivery and content were changed to reflect values and norms of both the Hispanic/Latino staff and the caregivers they serve. As supportive interventions for dementia caregivers are developed and implemented into real world settings, inclusion of cultural elements may enhance research participation from Hispanic/Latino provider sites and caregivers. We suggest in this paper that cultural adaptation is an essential consideration in developing an intervention as well as adapting evidence-based previously tested interventions, and in implementation science. Cultural adaptation offers an important lens by which to identify contextual factors impacting intervention adoption interventions and needed adaptations to assure equity in the reach of evidence-based programs.

### DESIGNING INTERVENTIONS FOR OLDER ADULTS WITH LOW- AND MIDDLE-INCOME COUNTRIES SETTINGS IN MIND

Zachary Baker[1], Manka Nkimbeng[2], Pearl G. Cuevas[3], Ana Quiñones[4], Karmeet Kang[5], Joseph Gaugler[2], Ladson Hinton[6], and Tetyana Shippee[2], *1. Arizona State University, Tempe, Arizona, United States, 2. University of Minnesota, Minneapolis, Minnesota, United States, 3. Centro Escolar University, Manilla, Sta Rosa City, Laguna, Philippines, 4. Oregon Health and Science University, Portland, Oregon, United States, 5. Chitkara University, Rajpura, Punjab, India, 6. University of California Davis, Sacramento, California, United States*

Most people living with dementia (PLWD) live in low- and middle-income countries (LMIC) and the proportion in LMICs is poised to continue growing. But 99.9% of dementia funding is awarded to researchers in high-income countries (HIC). Our team of scientists from India, Cameroon, the Philippines, the USA, Ukraine, and Germany draw on our involvement in interventions in 12 countries to suggest one way to help meet the needs of PLWD living in LMICs. We suggest that researchers in HICs who are developing new interventions might consider the needs of LMICs during intervention development. By thinking through implementation scenarios in different settings or countries where barriers and facilitators to implementation vary in type, or in importance, it might speed future adaptation of those interventions to LMICs. We outline anticipated challenges, case studies from our own work, benefits for individual researchers, benefits for public health, and recommendations for employing this strategy.

### SESSION 3320 (SYMPOSIUM)

### INTEGRATING HOUSING, HEALTH, AND SUPPORTIVE CARE IN AFFORDABLE SENIOR HOUSING: EVALUATION OF THE R3 PROGRAM

Chair: Edward Miller Co-Chair: Marc Cohen

This symposium reports evaluation findings from the Right Care, Right Place, Right Time (R3) program. The initiative is designed to integrate housing, health, and supportive care to residents of affordable senior housing using a wellness team (nurse and social worker). The embedded team works directly with residents to address health-related, educational, and informational needs and access to services - focusing on proactive outreach and prevention, coordination with providers, constant contact with residents, and targeting high-risk residents. The initiative aims to create a replicable, scalable, and sustainable model of housing with supportive services that enables independent living while reducing health care costs. Two wellness teams served approximately 400 participants at seven Boston-area buildings. The R3 program was implemented in two phases. The 18-month pre-intervention period was January 2016-March

2017, while the 18-month Phase 1 intervention period was July 2017-December 2018. The 21-month Phase 2 implementation period (known R32), which introduced a targeting strategy to identify high risk residents, was January 2019-September 2020. Evaluation activities included quantitative and qualitative components. Program participants and non-participants at intervention and comparison sites were surveyed on program-related experiences. Program, housing, and community partners were interviewed. Key performance indicators were tracked. Medicare claims data were analyzed using comparison groups. Focus groups were completed with payers, housing providers, and community stakeholders. The purpose of this symposium is to identify the experiences of program participants and key stakeholders with the R3 program, and to assess program impact. Edward Miller and Marc Cohen will serve as chair and co-chair, respectively.

### THE EXPERIENCES OF PROGRAM PARTICIPANTS WITH THE R3 PROGRAM

Elizabeth Simpson, Edward Miller, Yan Lin, Pamela Nadash, and Marc Cohen, *University of Massachusetts Boston, Boston, Massachusetts, United States*

The voluntary nature of the R3 program means that the experiences of residents who enroll in the program are critical to program success. Surveys were distributed to participants and non-participants in the intervention and control buildings during R32. Findings indicate that most enrollees are interacting with R32 staff; they trust them and feel that they provide important information; and they feel safer, less alone, and healthier because of their participation in the program. Roughly nine-in-ten are satisfied with the program and three in five would recommend it to a friend. Fully one-third believe that the program is helping them avoid medical emergencies necessitating a trip to the emergency room. When faced with a serious medical concern, residents in R32 buildings are far less likely to view calling 911 or going to the emergency room for treatment as a way to address their issue compared to individuals living in control buildings.

### HOUSING AND COMMUNITY PARTNER VIEWS ON THE BENEFITS OF THE R3 PROGRAM

Edward Miller, Elizabeth Simpson, Pamela Nadash, Natalie Shellito, Yan Lin, Taylor Jansen, and Marc Cohen, *University of Massachusetts Boston, Boston, Massachusetts, United States*

Housing with services programs have potential to benefit residents and operators of affordable senior housing communities. This study aimed to understand housing and community partner views on the benefits of the R3 program. Data derived from 31 interviews with R3 program managers and wellness team members, executives and direct service staff at the intervention sites, and community partners. Overwhelmingly positive overall assessments of the R3 program were reported. Concrete benefits to residents included receiving additional attention and support; connecting with family and health care resources; enhancing group programming; preventing ambulance transports; helping with transitions; and improving health and quality of life. Psychosocial benefits to residents included: empowering program participants; providing socio-emotional support; and alleviating psychosocial distress. Benefits to housing sites included augmenting staffing levels, skills, and capabilities; proactively tracking and responding to resident needs; lowering resident turnover; and learning from and adopting procedures and processes underlying the R3 philosophy.

### TARGETING PARTICIPANTS AT HIGH RISK FOR ADVERSE HEALTH OUTCOMES IN THE R3 PROGRAM

Marc Cohen, Edward Miller, and Pamela Nadash, *University of Massachusetts Boston, Boston, Massachusetts, United States*

The R32 program seeks to ensure that individuals at high risk for adverse health outcomes are identified, engaged, and linked to needed services. The program pays particular attention to identifying individuals with risks related to mental health, memory, nutrition, food insecurity, and emergency department or inpatient hospitalizations. Key performance indicators were tracked, including the number and proportion of participants whose needs were addressed by R32. Results indicate that the program has succeeded in engaging the vast majority (>90%) of individuals with specific risk factors and connecting them with needed services. Viewed in the context of managed care plans, this level of performance is noteworthy and would earn the program a 5 Star rating – the highest rating available. Findings underscore the strong advantage offered by having a wellness nurse and wellness coordinator embedded on site in senior housing and using this platform to manage prevention and care services to residents.

### THE IMPACT OF THE R3 PROGRAM ON HEALTH SERVICES UTILIZATION AND COSTS

Jane Tavares, Elizabeth Simpson, Edward Miller, Pamela Nadash, and Marc Cohen, *University of Massachusetts Boston, Boston, Massachusetts, United States*

Relatively few housing with services evaluations use rigorous designs when assessing program impacts on health services utilization. This study thus employed a pre/post difference-in-difference quasi-experimental design, including building-level comparisons using Medicare fee-for-service claims data across two stages of the R3 intervention. Intervention sites included seven buildings with roughly 1,200 individuals. Key outcomes included hospital admissions, 30-day hospital readmission, and emergency department admissions. Results indicate that adding the R3 intervention to low-income housing sites led to meaningful reductions in service utilization and costs when compared to buildings where the program was not operating. The introduction of risk-targeting in the second stage of the intervention further strengthened this effect. Findings demonstrate that residents of affordable senior housing communities, who tend to be in poorer health than their counterparts in the community, benefit from the R3 program and that additional investment in this type of initiative would benefit the health care system.

### ACHIEVING SUSTAINABILITY IN HOUSING WITH SERVICES: INSIGHTS FROM R3 PROGRAM

Pamela Nadash, Edward Miller, Elizabeth Simpson, Molly Wylie, Natalie Shellito, Yan Lin, Taylor Jansen, and Marc Cohen, *University of Massachusetts Boston, Boston, Massachusetts, United States*

There has been a long-standing interest in developing housing models that integrate the supportive services that older people need to remain at home as long as possible. This approach has boomed for better-off people who can afford the cost of private-pay independent living and assisted living environments. For those with fewer resources, however, options are limited, given that they are largely dependent on public financing. This study focuses on how to develop sustainable models of housing with services for low-income older people. Using data from 31 key informant interviews and three focus groups, it reports and analyzes expert perspectives on how programs such as R3 can achieve financial sustainability. Four major themes emerged: (1) funding as the key to sustaining housing with services; (2) funding housing with services through participating health plans; (3) other potential funding sources for housing with services; and (4) gaining buy-in for housing with services.

### SESSION 3330 (SYMPOSIUM)

### INTEGRATION OF DIGITAL TECHNOLOGY IN RESEARCH INVOLVING OLDER ADULTS: LESSONS FROM COMMUNITY-BASED TRIALS

Chair: Hae-Ra Han

Working with study participants in community-based clinical trials during COVID-19 pandemic has created diverse challenges to study teams. Globally, COVID-related restrictions were implemented including country-wide lockdowns and social distancing, and study teams had to quickly adjust their study protocols to work in a virtual environment. Meeting virtually for recruitment activities or intervention delivery may be particularly challenging when the target group is older adults—one of the vulnerable populations to experience the digital divide due to limited digital access and limited digital literacyThis symposium covers the lessons learned related to use of digital technology in participant recruitment and intervention delivery across a range of populations, including community-dwelling Korean American older adults to African American older women living with pain and low mood, caregivers of persons living with heart failure, and low-income cancer survivors with multiple chronic conditions. The discussion will include 1) findings from screening over 1,000 older Korean Americans to enroll them into a multi-site community-based trial, 2) lessons in diversifying intervention delivery methods to African American older women, 3) the integration of virtual modality into a self-care and social support intervention for caregivers of persons with heart failure, and 4) the deployment of mHealth to deliver a home-based exercise program to ethnically diverse low-income cancer survivors with co-morbid conditions in the setting of the COVID-19 pandemic. This symposium seeks to build the evidence related to recruitment and intervention delivery targeting diverse groups of older adults in community settings using technology by sharing common challenges, experiences, and opportunities.

### WEB-ENABLED RECRUITMENT STRATEGIES FOR COMMUNITY-DWELLING OLDER ADULTS

Deborah Min[1], Ja'Lynn Gray[2], Chad Parslow[2], Anushka Jajodia[3], Ji-Young Yun[2], and Hae-Ra Han[4], *1. NYU Grossman School of Medicine, New York, New York, United States, 2. Johns Hopkins School of Nursing, Baltimore, Maryland, United States, 3. MICA Center for Social Design, Baltimore, Maryland, United States, 4. Johns Hopkins University, Baltimore, Maryland, United States*

Older adults are vulnerable to experiencing the digital divide. Identifying effective web-enabled recruitment strategies to target older adults is an important research focus. Web-enabled recruitment strategies have become increasingly popular amidst virtual working environments due to unprecedented challenges of the COVID-19 pandemic. Yet, it is unclear which web-enabled recruitment strategies have been successful among older adults in community-based intervention trials. We describe lessons learned in using web-enabled strategies to recruit Korean American older adults with probable dementia and their caregivers in a community-based intervention trial and compare our findings with the web-enabled recruitment strategies targeting older adults reported in relevant published studies. Data sources included: study team meeting minutes, community consultant interviews, and a PubMed search. Five themes emerged: unfamiliarity with technology, differences in internet access across older age groups, providing technological support to promote recruitment, successful and unsuccessful recruitment using social media, and other diverse online methods of recruitment.

### DEPRESSION AND PAIN PERSEVERANCE THROUGH EMPOWERED RECOVERY (DAPPER) PILOT PROGRAM: A CASE STUDY ON TECHNOLOGY

Catherine Clair[1], Tonisha Melvin[2], Sarah Szanton[3], Martha Abshire Saylor[3], Roland J. Thorpe, Jr.[4], and Janiece Taylor[4], *1. Johns Hopkins Bloomberg School of Public Health, Baltimore, Maryland, United States, 2. Johns Hopkins School of Nursing, Baltimore, Maryland, United States, 3. Johns Hopkins University School of Nursing, Baltimore, Maryland, United States, 4. Johns Hopkins University, Baltimore, Maryland, United States*

There is a paucity of work focusing on the engagement of older African American women with technology. The aim of the pilot study was to test the Depression and Pain Perseverance through Empowered Recovery (DAPPER) program in a sample of older African American women living with pain and low mood (N=19). The research team hypothesized that most of the women would prefer virtual visits, thus both in-person and virtual options were available for program delivery. So far, of 11 participants, five women have opted to do in-person visits. When asked why, one participant said that having the nurse come to her house was “rewarding, [especially] when you live alone.” Another participant commented that she did not have a computer in her home. These findings demonstrate that older adults exist on a spectrum of comfort with and access to technology.

### WHEN AND WHERE: LESSONS LEARNED ABOUT TECHNOLOGY FROM AN INTERVENTION FOR CAREGIVERS OF PERSONS WITH HEART FAILURE

Martha Abshire Saylor[1], Catherine Clair[2], Noelle Pavlovic[1], Katie Nelson[3], Lyndsay DeGroot[1], Janiece Taylor[3], and Sarah Szanton[1], *1. Johns Hopkins University School of Nursing, Baltimore, Maryland, United States, 2. Johns Hopkins Bloomberg School of Public Health, Baltimore, Maryland, United States, 3. Johns Hopkins University, Baltimore, Maryland, United States*

Use of technology in older adult populations is growing, therefore it is important to understand opportunities for healthcare initiatives that support older adults using technology. The aim of the pilot study was to test Caregiver Support, a self-care and social support intervention, for caregivers of persons living with heart failure (N=24). Originally, the protocol was designed with in-person visits. We expected this option to reduce participant burden: the caregiver would not have to travel, and the interventionist would gain more insight about the home context to aid with intervention delivery. However, due to the COVID-19 pandemic, it became necessary to conduct the visits virtually. All participants completed the 5-component intervention via virtual meeting and there were no dropouts related to technology use. When asked about the virtual modality, participants emphasized the flexibility of virtual meetings. In summary, the intervention visits conducted virtually were perceived as a caregiver-centered approach.

### CHALLENGES AND SOLUTIONS FOR MOBILE HEALTH INTERVENTION RESEARCH IN OLDER ADULTS WITH MULTIPLE CHRONIC CONDITIONS

Nada Lukkahatai, and Junxin LI, *Johns Hopkins University, Baltimore, Maryland, United States*

Advances in smart phone technologies resulted in exponential growth in the use of mobile technology in health care. The mobile health or mHealth became a tool in engaging patients in self-care, monitor symptoms and provider-patients communication. Older adults living with Multiple Chronic Conditions (MCCs) are required to self-manage their complex conditions. These health demands are greater in low-income older adults. The mHealth can be beneficial in supporting health and increase access to care for this population. Our study examined a feasibility of combination of the mHealth and tailored exercise program for low SES older adults. We found that age-related physical and cognitive decline, acceptance of technology, complexity of user interface, and financial burden hinder the benefit and accessibility of the mHealth. The research team purpose solutions include improve user interface, create training session and technical support, provide incentives and technology and involve caregivers for these challenges.

### SESSION 3340 (SYMPOSIUM)

### INTRODUCTION TO THE STUDY OF MUSCLE, MOBILITY, AND AGING (SOMMA) AND PRELIMINARY RESULTS

Chair: Steven Cummings Co-Chair: Anne Newman

SOMMA is a new cohort study conducted at Wake Forest and University of Pittsburgh, coordinated by the San Francisco Coordinating Center, with management of biological specimens by Adventist Health Research Institute. We aim to discover the biological basis of mobility disability and have collected tissues and measurements for future studies of the biology of human aging. SOMMA has enrolled ~875 older men and women (aged □70 years), and will follow-up participants by phone and in-person every six months for up to three years. An additional cohort of younger adults (aged 30-69) is being enrolled. In this symposium, we will describe the underlying conceptual construct for the study; the baseline characteristics of the cohort; recruitment techniques to enroll participants. We will present preliminary data from ~425 participants, including the association between mitochondrial energetics and muscle function (strength and power); the interrelationship between physical and cognitive measures; and the association of D3Cr muscle mass with strength and walking speed. Finally, we will describe how to obtain data and specimens for analyses and ancillary studies.

### INTRODUCTION TO SOMMA: CONCEPTUAL CONSTRUCT AND PARTICIPANT CHARACTERISTICS

Steven Cummings[1], Peggy Cawthon[2], Theresa Mau[3], Stephen Kritchevsky[4], Russell T. Hepple[5], Paul Coen[6], Bret Goodpaster[6], and Anne Newman[7], *1. San Francisco Coordinating Center, San Francisco, California, United States, 2. California Pacific Medical Center Research Institute, San Francisco, California, United States, 3. California Pacific Medical Center, San Francisco, California, United States, 4. Wake Forest School of Medicine, Winston-Salem, North Carolina, United States, 5. University of Florida, Gainesville, Florida, United States, 6. AdventHealth, Orlando, Florida, United States, 7. University of Pittsburgh, Pittsburgh, Pennsylvania, United States*

SOMMA aims to characterize key muscle characteristics and their longitudinal association with declines in mobility. To assess mitochondrial energetics in muscle, respiration assays were performed on biopsies of the vastus lateralis and the capacity to generate ATP (ATPmax) was determined by 31P MR spectroscopy. Other properties examined in biopsies include denervation, fiber type and size, capillary density and oxidative damage. We archive ~150mg for ancillary science. In a subset, we obtained biopsies of subcutaneous adipose for measurements including cell senescence and for archiving. To assess body composition, SOMMA used whole body MR for quadriceps volume and D3 Creatine dilution for total skeletal muscle mass. We measured fitness by cardiopulmonary exercise testing. Participants also had many other assessments of physical and cognitive performance including chair stands, balance, gait speed, leg power, stair climbing, Trails B, MOCA, and patterns of physical activity by actigraphy.

### RECRUITMENT OF SOMMA PARTICIPANTS: TECHNIQUES TO ENROLL OLDER ADULTS IN A COMPLEX STUDY

Anne Newman[1], Peggy Cawthon[2], Theresa Mau[3], Stephen Kritchevsky[4], Russell T. Hepple[5], Paul Coen[6], Bret Goodpaster[6], and Steven Cummings[7], *1. University of Pittsburgh, Pittsburgh, Pennsylvania, United States, 2. California Pacific Medical Center Research Institute, San Francisco, California, United States, 3. California Pacific Medical Center, San Francisco, California, United States, 4. Wake Forest School of Medicine, Winston-Salem, North Carolina, United States, 5. University of Florida, Gainesville, Florida, United States, 6. AdventHealth, Orlando, Florida, United States, 7. San Francisco Coordinating Center, San Francisco, California, United States*

We screened approximately 4200 individuals to enroll 881 including 41% men and 59% women with a mean age =73.4 ± 5SD years; 13% were Black, 85% White and 2% of other race and ethnic groups. We will add 80 individuals aged 30-69 years. Exclusions included unstable medical conditions and inability to walk 400 meters(m). The median baseline 400m gait speed at usual pace was 1.05 ± 0.18SD m/s. Techniques to recruit and enroll a diverse population for this complex project included the use of in-person and video-conference information sessions to explain the complicated study to interested adults and to screen potential participants as needed The COVID pandemic created numerous challenges; while SOMMA was able to meet its recruitment goals, the enrollment period lasted 33 months (instead of the originally planned 18 months). Attendance at follow-up in-person and phone-based assessments is excellent.

### PRELIMINARY SOMMA DATA: MITOCHONDRIAL ENERGETICS, LEG POWER, AND CARDIORESPIRATORY FITNESS

Theresa Mau[1], Paul Coen[2], Stephen Kritchevsky[3], Anne Newman[4], Giovanna Distefano[5], Russell T. Hepple[6], Bret Goodpaster[2], and Steven Cummings[7], *1. California Pacific Medical Center, San Francisco, California, United States, 2. AdventHealth, Orlando, Florida, United States, 3. Wake Forest School of Medicine, Winston-Salem, North Carolina, United States, 4. University of Pittsburgh, Pittsburgh, Pennsylvania, United States, 5. Advent Health Translational Research Institute, Orlando, Florida, United States, 6. University of Florida, Gainesville, Florida, United States, 7. San Francisco Coordinating Center, San Francisco, California, United States*

To assess mitochondrial function, max OXPHOS (maximal complex I and II supported state 3 respiration) was determined in vitro by high-resolution respirometry of permeabilized muscle fibers from thigh muscle (vastus lateralis) biopsies, and ATPmax was determined in vivo by 31P magnetic resonance spectroscopy (n=468, mean=76.9yr). Max OXPHOS was strongly associated with leg power (β= 10.7 Watts per 1 SD increment, p<0.001) and peak VO2 (β= 83.7 mL/min, p<0.001). ATPmax had weak associations with leg power (β= 4.2 Watts per SD increment, p=0.13). In contrast to its weaker relationship with leg power, ATPmax was strongly associated with VO2 peak (β= 70.1 mL/min per SD increment, p<0.001). Skeletal muscle mitochondrial energetics were significant determinants of leg power and VO2 peak in older adults.

### PRELIMINARY SOMMA DATA: COGNITIVE AND PHYSICAL FUNCTION RELATIONSHIPS IN OLDER ADULTS

Stephen Kritchevsky[1], Anne Newman[2], Peggy Cawthon[3], Theresa Mau[4], Russell T. Hepple[5], Paul Coen[6], Bret Goodpaster[6], and Steven Cummings[7], *1. Wake Forest School of Medicine, Winston-Salem, North Carolina, United States, 2. University of Pittsburgh, Pittsburgh, Pennsylvania, United States, 3. California Pacific Medical Center Research Institute, San Francisco, California, United States, 4. California Pacific Medical Center, San Francisco, California, United States, 5. University of Florida, Gainesville, Florida, United States, 6. AdventHealth, Orlando, Florida, United States, 7. San Francisco Coordinating Center, San Francisco, California, United States*

We used canonical correlation (n=424) to examine the relationships between a set of physical function measures (400m usual pace, chair stands/sec, 4m walk pace, standing balance times, VO2 peak, muscle power, four-square step test (FSST) time, stair climb time and stair climb power) and a set of cognitive measures (Trail Making Test B (sec), Digit Symbol Substitution Test (DSST), the Montreal Cognitive Assessment, and California Verbal Learning Test). Canonical correlation derives synthetic variables comprised of linear combinations within each set of variables (physical and cognitive) that maximize the correlations between synthetic variables. The FSST was most strongly correlated with the cognitive synthetic variable (- 0.42). The DSST score was the cognitive measure most strongly correlated with the physical synthetic variable (0.48). It is notable that only the timed cognitive and physical tests are inter-associated

### PRELIMINARY SOMMA DATA: D3CR MUSCLE MASS AND STRENGTH AND WALKING SPEED IN OLDER MEN AND WOMEN

Peggy Cawthon[1], Stephen Kritchevsky[2], Anne Newman[3], Theresa Mau[4], Russell T. Hepple[5], Paul Coen[6], Bret Goodpaster[6], and Steven Cummings[7], *1. California Pacific Medical Center Research Institute, San Francisco, California, United States, 2. Wake Forest School of Medicine, Winston-Salem, North Carolina, United States, 3. University of Pittsburgh, Pittsburgh, Pennsylvania, United States, 4. California Pacific Medical Center, San Francisco, California, United States, 5. University of Florida, Gainesville, Florida, United States, 6. AdventHealth, Orlando, Florida, United States, 7. San Francisco Coordinating Center, San Francisco, California, United States*

D3Cr muscle mass, grip strength, leg extension 1-rm max (Keiser), and walking speed (400m usual walk) were collected with standardized protocols. We calculated sex-stratified unadjusted Pearson correlation coefficients, and partial Pearson correlation coefficient adjusted for body size (height, weight). D3Cr muscle mass was positively correlated with grip strength (men: unadjusted r=0.28, p<.001; women: unadjusted r=0.16, p=0.02) and leg extension strength (men: unadjusted r=0.44, p<.001; women: unadjusted r=0.31, p<.001). D3Cr muscle mass was correlated with 400-m walk speed only after adjustment for body size among both men (partial r=0.29, p<.001) and women (partial r=0.16, p=0.02). Associations of total thigh muscle volume by MRI with strength and walking speed were of similar magnitude to the association between D3Cr muscle mass with strength and walking speed.

### SESSION 3350 (AWARD LECTURE)

### JOSEPH T. FREEMAN AWARD AND EXCELLENCE IN REHABILITATION OF AGING PERSONS AWARD PRESENTATIONS AND LECTURES

Chair: Cynthia Brown

The Joseph T. Freeman Award lecture will feature an address by the 2022 Freeman Award recipient Neil M. Resnick, MD, FGSA, of the University of Pittsburgh. The Joseph T. Freeman Award is a lectureship in geriatrics awarded to a prominent clinician in the field of aging, both in research and practice. The award was established in 1977 through a bequest from a patient's estate as a tribute to Dr. Joseph T. Freeman. The Excellence in Rehabilitation of Aging Persons Award lecture will feature an address by the 2022 Excellence in Rehabilitation Award recipient Patricia C. Heyn, PhD, FGSA, FACRM, of Marymount University. The Excellence in Rehabilitation of Aging Persons Award is designed to acknowledge outstanding contributions in the field of the rehabilitation of aging individuals.

### INTO THE VOID: A CAREER IN INCONTINENCE... AND BEYOND

Neil Resnick, *University of Pittsburgh, Pittsburgh, Pennsylvania, United States*

Long-believed inevitable with age, urinary incontinence (UI) --like dementia – has proved neither ineluctable nor untreatable. Moreover, older adults remain continent despite abnormal lower urinary tract (LUT) function! The key is that the LUT is just one risk factor, and incontinence results from contributors at every level: from drugs and diseases beyond the LUT, to problems of brain and physical function, to multiple issues within the LUT itself. Such insights have provided the rationale for a nontraditional diagnostic and therapeutic strategy, one that has not only improved outcomes but also enabled creation of previously-believed heretical approaches that address the LUT last and only if necessary. In addition, this approach enabled development of Medicare's Minimum Data Set for frail elderly and, more importantly, may facilitate development of scalable ways to raise the floor of care for other geriatric syndromes as well.

### TWO DECADES EVALUATING EXERCISE TREATMENTS FOR ALZHEIMER'S DISEASE: THE ANECDOTES OF AN EVIDENCE-BASED PATH

Patricia Heyn, *Center for Optimal Aging, Marymount University, Fairfax Station, Virginia, United States*

The last 20 years have produced accumulative evidence supporting the benefits of regular exercise to improve cognition in older adults with cognitive impairments (OAwCIs). Thus, multiple systematic reviews, including meta-analyses, have contributed to the current literature. Although the field advanced significantly our understanding of the role of exercise on AD, still there is limited information on the overall prescription effectiveness. Thus, a novel overview review to evaluate the available meta-analysis studies from the randomized exercise trials for OAwCI will help in synthesizing the best evidence to generate prescription precision. Dr. Heyn will discuss her path, her studies, and the future of AD research and exercise prescription.

### SESSION 3360 (SYMPOSIUM)

### MEET THE END-OF-LIFE CARE EXPERTS: INSIGHTS FROM FORMER CAREGIVERS OF OLDER ADULTS LIVING WITH SERIOUS ILLNESS

Chair: Emily Mroz Co-Chair: Emika Miller Discussant: Karen Moss

High-quality end-of-life care relies on the collective efforts of professional care providers and family caregivers; however, care practice development is rarely guided by experiential perspectives of former family caregivers (i.e., those whose care recipient has died). Former family caregivers typically carry rich, untapped insight into what works well, and what still does not, in end-of-life care. While current caregivers sometimes describe being over-burdened and unable to engage meaningfully in research, former caregivers express the desire to share insights and lessons learned to improve the experiences of future caregivers. Grounded in research methodologies which leverage insights from participants (e.g., qualitative analysis, community-based participatory research), this symposium draws on perspectives of former caregivers of seriously ill older adults to enhance end-of-life care practices, improve intervention designs, and enrich research agendas. First, Dr. Emily Mroz will describe the pathways by which recent caregiving experiences for parents living with dementia shape former caregivers' perspectives on, and engagement in, their own end-of-life care planning. Next, Emika Miller will characterize the advance care planning perspectives of former family caregivers of African Americans who lived with dementia, highlighting areas for improvement in culturally-tailored interventions. Lastly, Jennifer Dickman Portz will present on caregivers' use of patient portals near their care recipient's end-of-life. Our discussant, Karen Moss, will lead us in conversation by outlining the unique value added to end-of-life care research when incorporating perspectives of former caregivers from diverse backgrounds. She will also describe future directions in caregiver-informed end-of-life interventions, practices, and policies to improve end-of-life care outcomes.

### REFLECTION AND INTENTION: END-OF-LIFE PLANNING IN FORMER CAREGIVERS OF PARENTS LIVING WITH DEMENTIA

Emily Mroz[1], Tara Matta[2], Talha Ali[3], Amanda Piechota[4], Anissa Abboud[5], Shubam Sharma[6], Joan Monin[4], and Terri Fried[3], *1. Yale University, New Haven, Connecticut, United States, 2. University of South Florida, Tampa, Florida, United States, 3. Yale School of Medicine, New Haven, Connecticut, United States, 4. Yale School of Public Health, New Haven, Connecticut, United States, 5. Yale School of Public health, New Haven, Connecticut, United States, 6. Kennesaw State University, Kennesaw, Georgia, United States*

Family caregivers for parents living with Alzheimer's Disease or Related Dementias (ADRD) provide support through health decline, care planning, and death. Guided by the Consensual Qualitative Research method and grounded in a life story framework, this study examines recalled caregiving experiences and descriptions of personal end-of-life planning in 32 midlife former caregivers of parents with ADRD (range: 40-65; 44% male). Former caregivers often expressed appreciation for end-of-life planning but varied in their engagement in planning since their loss. Descriptions of hesitation with planning were rooted in salient challenges from caregiving experiences and reflected relational concerns (e.g., about burdening others with care needs), planning complexity (e.g., perceiving too many factors to account for), or mortality denial (e.g., aversion to thinking more about death). These caregiving experiences may clarify the value of end-of-life planning while, in some cases, impeding decision-making, leading to gaps between former caregivers' planning intentions and engagement.

### REIMAGINING ADVANCE CARE PLANNING: FORMER CAREGIVERS OF AFRICAN AMERICANS WITH DEMENTIA PERSPECTIVES

Emika Miller[1], Karen Moss[2], Kathy Wright[3], Kimberly Wilson-Lawson[4], Mary Beth Happ[2], Karen Rose[2], Todd Monroe[5], and Celia Wills[5], *1. Ohio State University, Columbus, Ohio, United States, 2. The Ohio State University, Columbus, Ohio, United States, 3. The Ohio State University College of Nursing, Columbus, Ohio, United States, 4. African American Alzheimer's and Wellness Association, Columbus, Ohio, United States, 5. Ohio State University College of Nursing, Columbus, Ohio, United States*

Advance care planning disparities persist among lower socioeconomic status African American older adults living with dementia despite multiple decades of research. The perspectives of former family caregivers are rarely used in developing effective interventions. This study aims to characterize the advance care planning and other health-related decision-making perspectives of former family caregivers of African Americans who lived with dementia. This qualitative study is part of an ongoing mixed-methods study to co-create a culturally tailored advance care planning intervention. Former caregivers (n=11) participated in semi-structured interviews and identified the following needs: Caregiver education, caregiver recognition by healthcare providers, and removal of caregiving obstacles. Participants also acknowledged their need to restore identity following the death of their care recipient and a desire to assist other caregivers through research participation. This study fills a critical gap by including former caregivers' perspectives in co-creating a culturally tailored advance care planning intervention.

### HOW CAN PATIENT PORTALS SUPPORT CAREGIVERS PROVIDING END-OF-LIFE CARE?: A MIXED-METHODS STUDY

Jennifer Dickman Portz[1], John David Powers[2], Megan Baldwin[2], Kathy Gleason[2], Rebecca Boxer[2], Ted Palen[2], and Elizabeth Bayliss[2], *1. University of Colorado Anschutz, Aurora, Colorado, United States, 2. Kaiser Permanente Colorado, Denver, Colorado, United States*

Patient portal design is rarely centered around patient caregivers—family members, friends and other designated persons who may access these digital tools for the patient's health needs. This study examines: (a) portal features used by caregivers during their loved-one's end-of-life; and (b) caregiver perceptions regarding the use of patient portals for caregiving near the end-of-life.Using sequential mixed-methods, we conducted a retrospective cohort analysis of caregiver proxy (N=137) use from 2016-2019 during their loved-ones' (N=5,284) last 12 months of life and interviews with (N=31) 16-former and 15-current caregiver proxies. Caregiver proxy portal use in this population was lower than expected; however, caregivers who used the portal as proxies found value in tools for caregiving near the end-of-life. To leverage the patient portal to better support caregivers, future strategies should target caregiver portal adoption, awareness of beneficial portal tools, and the addition of caregiver specific resources and education.

### SESSION 3380 (SYMPOSIUM)

### ON THE THRESHOLD: CREATIVITY IN MID-LIFE

Chair: Carolyn Adams-Price Co-Chair: Desmond O'Neill

While late-life creativity has attracted significant scholarly inquiry, it is not clear that the same focus has applied to creativity in midlife and its relationship to creativity across the lifespan. Allied to a proposed symphonic concert by the Indianapolis Symphony of works from late midlife creativity with gerontologically-themed pre-concert talks occurring alongside GSA 2022, an activity curated by the Humanities, Arts and Cultural Gerontology Advisory Panel of GSA, this symposium will provide insights into a relatively under-explored aspect of creativity and aging, that of midlife creativity and its relationship to creativity at other stages of life. For many artists the most significant breakthroughs in style and critical reception are found in the works of midlife, and the symposium will explore the phenomenology and underlying themes of midlife creativity. This will include a review of the literature, a focus on midlife breakthrough and development in the creativity of Brahms and Janacek, interaction with the program of the parallel symphony concert, and a focus on arts and crafts creativity in people newly undertaking arts and crafts in mid-life. This will be complemented by a review of creativity and well-being from the age of 50 onwards.

### MIDLIFE CREATIVITY AMONG COMPOSERS AND NOVELISTS

Desmond O'Neill, *Trinity College Dublin, Dublin, Dublin, Ireland*

Although the study of midlife has increased in recent years, it still lags behind study of life at the extremes of age. As a key threshold stage in the lifespan, our understanding can be enhanced by exploration of novelists and composers whose most substantive work began to be produced in midlife. This presentation will draw off the works of midlife works of composers who also composed into later life - Giussepe Verdi, Richard Strauss and Johannes Brahms - from the concert proposed by the Indianapolis Symphony during GSA 2022, as well as reflecting on the works of Anita Brookner as representative of other novelists and poets whose work and careers came to prominence in midlife.

### WE'RE ALL MAKERS NOW: ARTS AND CRAFT PARTICIPATION IN MIDLIFE

Carolyn Adams-Price, *Mississippi State University, STARKVILLE, Mississippi, United States*

Recent research has focused on the benefits of participation in creative activities for older adults, even those who experiencing cognitive deficits. However, many people begin participating in creative activities in midlife, and continue their participation into later life. Research in my lab suggests that middle aged people turn to crafts that involve making things to reduce stress and increase feelings of productivity. Glaveanu's sociocultural theory will be introduced to explain the benefits middle aged people get from creating crafts, and how those benefits are accrued from the beginner stage through the development of expertise.

### ARTS AND CREATIVITY AND THEIR IMPACT ON HEALTH AND WELL-BEING: A SYSTEMATIC REVIEW OF THE EVIDENCE

Roger O'Sullivan[1], and Laura McQuade[2], *1. Institute of Public Health in Ireland, Belfast/Dublin, Not Applicable, Ireland, 2. Institute of Public Health, Belfast, Northern Ireland, United Kingdom*

Aim: The aim of this mixed method systematic review was to develop a better understanding of the evidence on the impact of arts and creativity on the physical health and well-being of people aged 50+. Method: 73 studies were eligible for inclusion within the review and were appraised using the Mixed Methods Appraisal Tool.

Results: Dance was associated with improved balance, lower body physical strength and flexibility, aerobic fitness. Promising evidence showed that music and singing were associated with improved cognitive function, quality of life, affective states, and a sense of well-being. Preliminary evidence showed that visual and creative arts were associated with reduced feelings of loneliness, improved sense of community and social connectedness. Initial evidence showed that theatre and drama were associated with emotional well-being.

Conclusion: These findings highlight the value of participation in the arts and have implications for both public health and the arts and creativity agenda.

### SESSION 3390 (PAPER)

### PALLIATIVE CARE MODELS AND PROGRAMS

### AN INNOVATIVE MEDICAL LEGAL PARTNERSHIP TO SERVE COMMUNITY-DWELLING OLDER ADULTS

Rachel Lessem, and Tamara Kushnir Groman, *CJE SeniorLife, Chicago, Illinois, United States*

According to the Legal Services Corporation, each year, 56% of older adults have at least one civil legal problem yet 87% receive inadequate or no professional legal help for their civil legal problems. Unmet legal needs can have cascading catastrophic effects on the health and well-being of older adults. To address this, we took the innovative approach of creating a Medical-Legal Partnership (MLP) within a community based social service agency on aging. MLPs have been successful by addressing the social determinates of health. However, previous studies have not investigated MLPs aimed at older adults. We used a single arm, pretest-posttest design to investigate the effects and feasibility of providing legal services to community dwelling older adults. The MLP served over 88 clients referred both internally from the agency as well as external partners. The participants had a mean age of 73, (age range 45-98, n=88), were 60% female. African Americans represent 9% of the clients, 7% are Hispanic/Latino and 74% Caucasian. Additionally, 5% were veterans. 52% of clients were at or below the Federal Poverty line at baseline. 50% of clients reported greater financial stability as a result of legal services. 75% reported improved health of between 1 and 3 points on a 5 point likert scale (pre-test=2.75; post-test 3.9 p<.01). This pilot project demonstrates a successful intervention to work across professional silos coordinating lawyers, medical providers and social workers to better meet the legal needs of older adults by creating MLPs in social service agencies to support their well-being.

### BEST PRACTICES IN COMMUNICATING ABOUT PALLIATIVE CARE TO PATIENTS AND PHYSICIANS

Alexis Coulourides Kogan[1], Anna Rahman[2], Sindy Lomeli[2], and Susan Enguidanos[2], *1. Keck School of Medicine of USC, Alhambra, California, United States, 2. University of Southern California, Los Angeles, California, United States*

Home-based palliative care (HBPC) programs are proliferating across the U.S, yet face significant, documented challenges in promoting uptake of services and sustaining sufficient patient referrals. There is a tremendous need to understand effective methods for engaging physicians, patients, and caregivers in palliative care. Thus, the purpose of this study was to elicit best practices in how to communicate about HBPC to both healthcare providers and patients/caregivers. Focus groups with nine California-based HBPC organizations were conducted between January and April 2020. Discussions lasted approximately 54 minutes, were guided by a semi-structured protocol, audio recorded, and transcribed verbatim. Thematic analysis was used to identify themes and codes from the data. Twenty-five interdisciplinary HBPC staff members participated in a focus group. Most identified as white (76%), female (76%), and working in their current position for five years or less (56%). Three themes were identified from the data: (1) value of relationships; (2) communication do's and don'ts; and (3) need for education. Participants discussed actionable recommendations for each theme. Study findings highlight several best practices for HBPC programs to communicate- and foster relationships with healthcare professionals and patients/families about palliative care, with education at the crux. Lessons learned about key words and phrases to say and to avoid are particularly valuable for budding HBPC programs. Our results suggest that HBPC providers exert enormous efforts to increase patient referrals and enrollment through strategic, continuous outreach and education to physicians, patients, and their caregivers; however, palliative care educational interventions are needed.

### DOES RACE MATTER IN HEALTH MESSAGING? IMPACT OF RACE CONCORDANCE ON PALLIATIVE CARE KNOWLEDGE SCORE

Deborah Hoe[1], Mutian Zhang[2], and Susan Enguidanos[1], *1. University of Southern California, Los Angeles, California, United States, 2. University of Southern California, Los Angele, California, United States*

Palliative care (PC) programs have expanded rapidly across the United States in the last decade. However, there remains a wide disparity in access to PC among ethnic minorities. Therefore, it is necessary and critical to develop specific health-related educational tools targeted for the vastly diverse, community-based older adult population. This study seeks to investigate how race concordance in health-related messaging might affect changes in PC knowledge scores among Black and White, English-speaking, community-based adults aged 50 and older. We recruited 406 Black and White adults online and from community-based sites. Sixty-four percent were shown race concordant patient stories, the remainder were shown videos of opposite race patients. Pretest-posttest study design was employed and participants completed a 20-question survey about PC knowledge, intent to seek PC, and perceptions about the videos. Regression analysis was conducted to test if race concordance improved knowledge after viewing patient stories.PC knowledge score (max=13) improved from an average of 5.70 to 11.23 (t=23.2, p< 0.001). Participants that watched same-race videos for both Black and White registered greater significant score increases (White:1.26±0.36 points, p=0.001; Black:0.95±0.38 points, p< 0.05) compared with those who watched different-race videos. Further, intent to enroll oneself in PC revealed statistically significant relationships in all groups except among White participants who viewed different-race videos (p=0.157, Fisher's exact test, two-tailed). This study suggests that while PC role model stories improved PC knowledge across the board, the improvement is greater in race concordant groups, which also has implications for changes in intent to enroll in PC.

### EVALUATING IMPLEMENTATION COSTS FOR A TRANSITIONAL PALLIATIVE CARE INTERVENTION TO SUPPORT RURAL CAREGIVERS

Brystana Kaufman[1], Diane Holland[2], Catherine Vanderboom[2], Cory Ingram[2], Alice Chun[1], Erica Langan[1], Henry Baer-Benson[2], and Joan Griffin[2], *1. Duke University, Durham, North Carolina, United States, 2. Mayo Clinic, Rochester, Minnesota, United States*

Compared to urban caregivers, rural caregivers experience greater burdens accessing coordinated care for their loved ones during and after hospital discharge. The impact of technology-enhanced transitional palliative care (TPC) on patient and caregiver outcomes is currently being evaluated in a randomized control trial. This study evaluates resource use and health system costs of this caregiver-focused TPC intervention. Rural caregivers of hospitalized patients in Minnesota, Wisconsin, and Iowa were enrolled in an 8-week intervention consisting of video visits, conducted by a registered nurse, supplemented with phone calls and texts (n=207). Labor costs were estimated using the Bureau for Labor Statistics median hourly rate for a registered nurse and compared to a scenario analysis using a nurse practitioner or social worker wages. Hours spent conducting the visits and charting were calculated using study data. A one-way sensitivity analysis estimated resource use over a range of visits per caregiver and time per visit. Caregivers received 8.8 visits on average over the study period at 45 minutes per visit. In the base case, TPC cost $330 per caregiver facilitated by a registered nurse, compared to $281 and $489 if facilitated by a social worker or nurse practitioner, respectively. The number of visits had the greatest influence on total costs of the intervention (low of $198, high of $463). TPC is a feasible, low cost strategy to enhance caregiver support in rural areas. These results pose an opportunity to consider reimbursement mechanisms to evaluate the sustainability of transitional palliative care interventions to support caregivers.

### IMPACT OF SUBSIDY ON THE USE OF PERSONALIZED MEDICINE IN BREAST CANCER

Cynthia Chen[1], Jue Tao Lim[2], Swee Ho Lim[3], Ern Yu Tan[4], Veronique Kiak Mien Tan[5], Su Ming Tan[6], Tira Tan[7], and Mikael Hartman[8], *1. National University of Singapore, Singapore, Singapore, 2. National University of Singapore, Singapore, Singapore, 3. KK Women's and Children's Hospital, Singapore, Singapore, 4. Tan Tock Seng Hospital, Singapore, Singapore, 5. Singapore General Hospital, Singapore, Singapore, 6. Changi General Hospital, Singapore, Singapore, 7. National Cancer Centre Singapore, Singapore, Singapore, 8. NUS Yong Loo Lin School of Medicine, Singapore, Singapore*

Advances in adjuvant therapy have led to increased survival rates after cancer prognosis. Herceptin, a targeted therapy, had first been introduced to Singapore in 2006. We aimed to assess whether subsidies for Herceptin from 2012 will lead to changes in uptake among Human Epidermal Growth Factor Receptor 2 (HER2) positive patients by socio-economic groups. Two-level random-intercept logistic regression was used to model diagnostic test and Herceptin uptake using the Singapore Breast Cancer Cohort from 2006 to 2018, adjusting for covariates such as education, housing type and marital status before and after subsidies. Interrupted time series (ITS) analysis was used to evaluate the impact of Herceptin subsidy on treatment uptake. The concentration index was also computed to measure inequality in uptake by ethnicity and education. We found that the odds of diagnostic testing were not associated with socioeconomic factors. However, before subsidies, the highest education attained (OR = 4.57, 95% CI= (1.90, 11.02), P< 0.01) significantly increased the odds of Herceptin uptake. These odds were levelled after the introduction of subsidies to Herceptin treatment in 2012. After subsidy, we also found that Herceptin uptake increased significantly by 11.4% (95% CI= (3.47%, 19.4%), P=0.016). Also, inequality of Herceptin use decreased especially amongst the Indians, where at least 40% were used in the higher educated group prior to the subsidy. Subsidies have lowered the barriers to Herceptin uptake for marginalized individuals. Having targeted subsidies for socio-economically disadvantaged groups may work more efficiently in providing ease of access than a blanket subsidy in Herceptin.

### SESSION 3400 (PAPER)

### PHYSIOLOGICAL MECHANISMS AND AGING

### AGING-RELATED HUMAN MONOCYTE TRANSCRIPTOMIC PATHWAYS PREDICT WORSENING MULTIMORBIDITY

Jingzhong Ding[1], Kurt Lohman[2], Alain Bertoni[3], Steven Shea[4], Wendy Post[5], James Pankow[6], Stephen Kritchevsky[3], and yongmei Liu[2], *1. Wake Forest University, Winston Salem, North Carolina, United States, 2. Duke University School of Medicine, Durham, North Carolina, United States, 3. Wake Forest School of Medicine, Winston-Salem, North Carolina, United States, 4. Columbia University, New York, New York, United States, 5. Johns Hopkins University, Baltimore, Maryland, United States, 6. University of Minnesota, Minneapolis, Minnesota, United States*

In a cross-sectional analysis, we identified aging-associated monocyte transcriptional modules associated with the multimorbidity in the Multi-Ethnic Study of Atherosclerosis (MESA) study. Here we examine whether these modules predict worsening multimorbidity over time. Transcriptomic profiles were determined in circulating monocytes from 1,264 MESA participants aged 55-94 (51% female, 53% minority). The multimorbidity index was defined as the number of prevalent diseases: cardiovascular disease, type-2 diabetes, hypertension, cancer, dementia, chronic kidney disease, chronic obstructive pulmonary disease, and hip fracture. The mean index at baseline was 1.0 (33%, 45%, and 22% with 0, 1, and 2-6 diseases respectively). At baseline, the index was associated with 5 co-expressed transcriptional modules (FDR< 0.05). During a 6-year follow-up, 449 individuals developed new morbidities. In Cox proportional hazards regression models, 4 (p< 0.05) of these 5 modules including those enriched for downregulation of apoptosis (RH=1.11 per SD increment, p=0.03) and upregulation of complement subcomponent C1q (RH=1.14, p=0.005) predicted incident morbid diseases after adjusting for age, sex, race/ethnicity, study site, and the baseline multimorbidity index. Persons having above the median expression of both modules developed an average of 0.63 new diseases, while those with both below the median developed an average of 0.40 new diseases. These two modules predicted increasing morbidities independently of one another and IL6 levels (p< 0.05). In conclusion, transcriptomic analysis of human immune cells provided evidence that decreased apoptosis and increased immune response predict the onset of a variety of age-related diseases. Whether targeting these pathways will change morbidity risk remains to be demonstrated.

### EIGHT-YEAR CHANGES IN DEFICIT ACCUMULATION FRAILTY: RELATION TO GLYCEMIC CONTROL AND DIABETES MEDICATION USE

Felicia Simpson[1], Edward Boyko[2], Chloe Ferris[3], Jamie Justice[4], Stephen Kritchevsky[4], Jeanne Mccaffery[5], Scott Pilla[6], and Mark Espeland[4], *1. Winston-Salem State University, Winston-Salem, North Carolina, United States, 2. University of Washington School of Medicine, Seattle, Washington, United States, 3. Washington University School of Medicine, St. Louis, Missouri, United States, 4. Wake Forest School of Medicine, Winston-Salem, North Carolina, United States, 5. University of Connecticut, Storrs, Connecticut, United States, 6. The Johns Hopkins University School of Medicine, Baltimore, Maryland, United States*

Type 2 diabetes mellitus (T2DM) has been linked to accelerated biological aging and the accumulation of health deficits. It is unknown whether glycemic control can slow the progression of aging, as expressed by deficit accumulation frailty indices (FIs). We examined the cross-sectional and longitudinal associations that glycemic control, diabetes medication use, and weight change (predictors) had with a FI calculated as the percent of 36 deficits in behavioral, functional, and clinical characteristics (outcome). We drew data from 4177 participants across 8 years of follow-up in the Look AHEAD clinical trial of a multidomain intensive lifestyle intervention in individuals aged 45-76 years with T2DM and overweight or obesity. At baseline, the means(SD) FI(as a percent) for participants grouped as HbA1c< 7%, 7-7.9%, and >8% were: 19.93(6.45), 20.82(6.89), and 21.53(7.27), p< 0.001. Compared with HbA1c>8%, HbA1c< 7% at baseline was associated with 23% less mean 8-year FI progression: 2.42(7.35) vs 3.14(8.02), p< 0.001). Maintaining average HbA1c< 7% vs >8% during follow-up was associated with 47% less 8-year FI progression: 2.05(7.35) vs 3.14(8.02), p< 0.001. With adjustment for HbA1c, sustained weight loss >5% compared with weight gain >5% was associated with an 81% reduction in 8-year FI progression: 1.18(7.25) vs 3.89(8.09), p< 0.001. Use of metformin across >50% of annual visits was associated with a 30% reduction in 8-year progression of FI as compared with less use or no use: 2.25(7.27) vs 3.22(7.90), p=0.002. We conclude that better control of HbA1c, and sustained weight loss (>5%) and metformin use, may slow the accelerated aging associated with T2DM.

### EPIGENETIC AGING IN OLDER BREAST CANCER SURVIVORS: THE THINKING AND LIVING WITH CANCER (TLC) STUDY

Kelly Rentscher[1], Traci Bethea[2], Wanting Zhai[2], Harvey Cohen[3], Brent Small[4], Tim Ahles[5], Jeanne Mandelblatt[2], and Judith Carroll[6], *1. Medical College of Wisconsin, Milwaukee, Wisconsin, United States, 2. Georgetown University, Washington, District of Columbia, United States, 3. Duke University, Durham, North Carolina, United States, 4. University of South Florida, Tampa, Florida, United States, 5. Memorial Sloan Kettering Cancer Center, New York, New York, United States, 6. University of California, Los Angeles, Los Angeles, California, United States*

Cancer and its treatments increase risk for age-related disease, and biological aging may be a key mechanism; however, no research to date has examined epigenetic markers of aging in long-term breast cancer survivors. We used data from a national, prospective cohort to test whether older breast cancer survivors had accelerated epigenetic aging compared to non-cancer controls. Non-metastatic breast cancer survivors ages 62–84 years who received chemotherapy with or without hormonal treatment (n=29) or hormonal treatment alone (n=51), and controls frequency-matched on age, race, education, and time between blood draws (n=101), provided two blood samples between 24- and 60-months post-diagnosis (time between samples average=1.8 years). DNA methylation profiling (Illumina Infinium EPIC array) derived epigenetic aging measures: extrinsic, intrinsic, phenotypic, Grim, and Dunedin Pace of Aging (PoAm). Mixed-effects models tested effects of treatment group and change over time on epigenetic aging, adjusting for chronologic age and comorbidities. Survivors who received chemotherapy +/- hormonal treatment had a biological age 1.9–2.6 years older than controls based on extrinsic, intrinsic, and Grim estimates (p=.045, .045, and .001, respectively). Survivors who received hormonal treatment alone had an extrinsic biological age 1.6 years older than controls (p=.032) and a faster Dunedin PoAm (p=.040). Survivors who received chemotherapy +/- hormonal treatment had a trend for accelerated extrinsic aging over time compared to controls (p=.087). Older breast cancer survivors, especially those receiving chemotherapy, showed an accelerated epigenetic aging profile compared to matched women without cancer. Future research is needed to examine associations with age-related survivorship outcomes.

### GENOME-WIDE ASSOCIATION META-ANALYSIS OF PHYSICAL PERFORMANCE IN OLDER ADULTS

Adam Santanasto[1], Douglas Kiel[2], David Karasik[3], Ryan Cvejkus[4], Mary Biggs[5], Kathryn Lunetta[6], Joanne Murabito[7], and Joseph Zmuda[4], *1. School of Public Health, University of Pittsburgh, Oakmont,*

*Pennsylvania, United States, 2. Harvard Medical School, Boston, Massachusetts, United States, 3. Hebrew SeniorLife, Roslindale, Massachusetts, United States, 4. University of Pittsburgh, Pittsburgh, Pennsylvania, United States, 5. University of Washington, Seattle, Washington, United States, 6. Boston University School of Public Health, Boston, Massachusetts, United States, 7. Boston University, Boston, Massachusetts, United States*

Although measures of physical function such as walking speed and time to complete chair-rises are highly heritable, the genetic architecture underlying these phenotypes remains poorly defined. To identify potentially novel genes and pathways underlying physical performance in older adults, we conducted a genome-wide association meta-analysis of the short physical performance battery (SPPB) (Score 0-12) and one of its components, chair-rise time (seconds) in 24,033 Caucasian adults aged 60+ from 13 cohorts (mean cohort age 66.2 ± 5.3 to 84.3 ± 4.1 years; 56.5% women). Cohorts had a genome wide scan imputed to either the Haplotype Reference Consortium or Trans-Omics for Precision Medicine imputation panels. Single nucleotide polymorphism (SNPs) with a minor allele frequency ≥0.1% and imputation quality score ≥0.7 were included (range 7.5-10.5 million per cohort). Analyses were adjusted for age, sex, height, and population substructure. Meta-analysis was performed using a fixed-effects model. Although no genome-wide significant loci were identified, 67 and 60 suggestive loci ($p< 5*10^{-5}$) were detected for SPPB score and chair-rises time, respectively. Pathway-based analyses indicated significant enrichment of genes affecting negative regulation of calcium channel activity (Bonferroni corrected p-value < 0.05). Sex-stratified gene-based analyses identified clathrin vesicle-associated sec14 protein 1 (CLVS1), significantly associated with chair-rise time in women ($p= -1.5*10^{-7}$). CLVS1 is highly expressed in the cerebellum, which is involved in postural and motor function control. A larger sample size is needed to confirm and extend our findings, but our results potentially implicate a novel pathway and locus for physical performance in older women.

### PLASMA PROTEOMIC SIGNATURE OF DECLINE IN GAIT SPEED AND GRIP STRENGTH

Xiaojuan Liu[1], Stephanie Pan[2], Vanessa Xanthakis[2], Vasan Ramachandran[2], Anne Newman[3], Jason Sanders[4], Thomas Austin[5], and Michelle Odden[1], *1. Stanford University, Stanford, California, United States, 2. Boston University School of Public Health, Boston, Massachusetts, United States, 3. University of Pittsburgh, Pittsburgh, Pennsylvania, United States, 4. Vertex Pharmaceuticals, Boston, Massachusetts, United States, 5. University of Washington, Seattle, Washington, United States*

Physical function predicts health-related quality of life. The biological mechanisms underlying declines in physical function with age remain unclear. We examined the plasma proteomic profile associated with longitudinal changes of physical functions measured by gait speed and grip strength in community-dwelling adults. We applied aptamer-based platform to assay 1,161 plasma proteins on 2,871 participants (60% women, aged 76 years) in Cardiovascular Health Study (CHS) in 1992/1993 and 1,550 participants (55% women, aged 54 years) in Framingham Offspring Study (FOS) in 1991-1995. Gait speed and grip strength were measured annually for 6 years in CHS and at cycles 7 (1998-2001) and 8 (2005-2008) in FOS. The associations of individual protein levels (log-transformed and standardized) with longitudinal changes of gait speed and grip strength in two populations were examined separately by linear mixed effect models. Meta-analyses were implemented using random effect models with a Bonferroni correction for multiple testing. We found that plasma levels of 18 and 12 proteins were associated with changes in gait speed and grip strength, respectively (Bonferroni-corrected $p < .05$). The proteins most strongly associated with gait speed decline were growth/differentiation factor 15 (GDF-15) (uncorrected Meta-analytic p = 1.60E-15), pleiotrophin (PTN) (1.29E-08), and metalloproteinase inhibitor 1 (TIMP-1) (2.02E-08). For grip strength decline, the strongest associations were for GDF-15 (1.39E-07), carbonic anhydrase III (6.60E-07), and TIMP-1 (3.21E-06). Several statistically significant proteins are involved in the alternative complement pathway, extracellular matrix remodeling or immune function. These novel proteomic biomarkers may inform our understanding of the pathophysiology of functional decline.

### SESSION 3410 (SYMPOSIUM)

### PREPARING FOR THE FUTURE OF TECHNOLOGY TO SUPPORT OLDER ADULTS: PERSPECTIVES FROM THE CREATE CENTER

Chair: Patricia Heyn

The rapid advancement of technology promises new opportunities to help older adults maintain health, wellbeing, community and productive engagement, and purpose in life. However, the potential of technological innovation will not be met unless technology solutions account for the needs, preferences, and abilities of older users and involve older adults in all stages of the design process. This has been the primary focus of the Center for Research and Education on Aging and Technology Enhancement (CREATE). This symposium will discuss threats to the promise of these solutions and approaches to overcome these barriers. This session will start with N. Charness presenting an overview of digital inequity and the current state of the age-related "digital divide." J. Sharit will then present an experimental study examining older adults' willingness to adopt new technologies and attitudinal barriers to adoption. W. Boot will discuss CREATE research that has focused on the potential of virtual reality to improve the lives of older adults and potential facilitators and barriers to successful virtual reality experiences among older adults. W. Rogers will discuss the potential of voice interfaces for emerging technologies, challenges related to the success of this approach, and research gaps. Finally, S. Czaja will conclude with a broad discussion of future applications of technology to support older adults, including how developments in artificial intelligence, sensing technologies, and robotics that can be used to foster everyday activities, cognitive, physical, and emotional health.

potential barriers older adults may face to obtaining these benefits. In particular, this presentation will focus on the construct of presence, the sense of "being there" in a virtual environment, which has been described as the defining characteristic of successful VR experiences, usability challenges, and challenges related to cybersickness.

### ACHIEVING DIGITAL EQUITY FOR OLDER PERSONS WITH EMERGING TECHNOLOGY: THE CASE OF NORTH AMERICA

Neil Charness, *Florida State University, Tallahassee, Florida, United States*

The United Nations' theme for the International Day of Older Persons 2021 was "Digital Equity for All Ages". I define digital equity, then compare progress in North America, focusing on Canada, the USA, and Mexico for internet, smartphone, and broadband access. An age-related digital divide for internet and computer use, first identified in the USA in 1999, persists. Where year-to-year comparisons are available, I also discuss the impact of the pandemic on technology adoption. National data sets measuring internet use show that there are similarities and differences in the USA and Canada in factors influencing adoption. For smartphone ownership, Mexico showed gender differences favoring men, unlike the case in the USA and Canada. For broadband access in the USA inequity was seen as a function of age, race/ethnicity, education and income, and urban/rural residence. I discuss potential reasons for inequitable access and potential approaches to achieve greater equity.

### OLDER ADULTS' WILLINGNESS TO ADOPT TECHNOLOGIES: AN EXPERIMENTAL STUDY

Joseph Sharit[1], Jerad Moxley[2], and Sara Czaja[3], *1. University of Miami, Davie, Florida, United States, 2. Weill Cornell Medicine, New York, New York, United States, 3. Weill Cornell Medicine, New York City, New York, United States*

Understanding why older adults are willing to consider using technology is critical for ensuring that that the advantages of technology for improving the quality of their lives can be realized. Prior emphasis in this area has primarily been on questionnaire studies directed at capturing relatively general attitudes older adults have toward technology. We report on an empirical study that included 187 male and female adults 65-92 years of age who were given presentations on each of five technologies spanning domains that included transportation, leisure, health, and new learning. Each presentation was followed by a series of ratings on measures hypothesized to influence participants' willingness to adopt the technology. We will overview the Technology Assessment Procedure, the methodology developed to evaluate willingness to adopt a technology, and measures that were collected. We will also present a model used to predict willingness to adopt the technology and provide some initial findings.

### POTENTIAL AND PITFALLS IN THE USE OF EXTENDED REALITY TO SUPPORT OLDER ADULTS

Walter Boot, and Andrew Dilanchian, *Florida State University, Tallahassee, Florida, United States*

Extended Reality (XR), including Virtual Reality (VR) and Augmented Reality (AR), holds tremendous potential with respect to being able to support older adults' performance of Instrumental Activities of Daily Living (IADLs), activities crucial for independence, and Enhanced Activities of Daily Living (EADLs), activities that promote enjoyment, wellbeing, and quality of life. This presentation will draw from multiple empirical studies conducted within the CREATE Center, and other studies from the literature, on

### EXPLORING THE POTENTIAL OF VOICE INTERFACES FOR EMERGING TECHNOLOGIES: OPPORTUNITIES AND CHALLENGES

Wendy Rogers[1], George Mois[2], Kenneth Blocker[3], and Raksha Mudar[1], *1. University of Illinois Urbana Champaign, Champaign, Illinois, United States, 2. University of Illinois Urbana-Champaign, Urbana Champaign, Illinois, United States, 3. University of Illinois Urbana-Champaign, Champaign, Illinois, United States*

Successful human-technology interactions require that people be able to communicate their intentions to the technology. Emergent technologies such as virtual assistants, digital home assistant, smart phones, robots, and smart appliances are enabling voice activation and communication. This is potentially a positive trend as people often express a preference for using familiar methods of communicating with other people in their interactions with technologies. Presumably they expect these methods to be easier to learn and more effective. However, current voice technology interfaces are often dependent on specific language, cadence, and terminology that pose challenges for older adults to master. Moreover, age-related changes in voice characteristics, speech patterns, and the variability between individuals may lead to comprehension failures on the part of the technology. We have utilized research findings to develop implementation guidelines for use of voice interfaces with older adults. We also identify research gaps to provide a roadmap for future efforts.

### TECHNOLOGY TO SUPPORT AGING ADULTS: FUTURE APPLICATIONS

Sara Czaja[1], Walter Boot[2], Neil Charness[2], Wendy Rogers[3], and Joseph Sharit[4], *1. Weill Cornell Medicine, New York City, New York, United States, 2. Florida State University, Tallahassee, Florida, United States, 3. University of Illinois Urbana Champaign, Champaign, Illinois, United States, 4. University of Miami, Davie, Florida, United States*

The aging of the population is projected to continue for the upcoming decades. Thus, innovative strategies are needed to support current and future cohorts of older adults. In this regard, there are exciting developments in technology that have the potential to meet the needs of population aging. This summary presentation will discuss emerging technology applications such as developments in artificial intelligence, sensing technologies, and robotics that can be used to foster everyday activities, cognitive, physical, and emotional health. A framework will be provided to characterize current and emerging technology applications for older adults within life domains. Current research examining the efficacy, feasibility, and acceptability of these technologies with older adult populations will be also summarized. Recommendations for needed future research in the aging and technology domain will also be provided to ensure that the emerging technology applications are designed to meet the needs, abilities, and preferences of aging adults.

### SESSION 3420 (SYMPOSIUM)

### PRESIDENTIAL SYMPOSIUM: EMBRACING OUR DIVERSITY IN GERONTOLOGY EDUCATION: PERSPECTIVES ON STRATEGIES AND LEARNERS

Chair: Joann Montepare Co-Chair: Dana Bradley
Discussant: Tamara Baker

Our populations are not only becoming older, but also becoming more diverse on several fronts. Our new social structures call for new strategies for embracing our diversity in how we design and deliver education about aging in and beyond the classroom. In this AGHE Presidential Symposium, educators will discuss innovative and needed ways we can integrate diversity in gerontological pedagogical practices. To begin, Rona Karasik (St. Cloud State University) will discuss the call for integrating anti-racist strategies in classroom practices, and offer examples of several such strategies. Next, Brian Carpenter (Washington University in St. Louis) will discuss shrinking geropsychology pipelines, especially for students from racial and ethnic groups, and will share recommendations for expanding these pipelines. Aaron Guest (Arizona State University) will then discuss the need to extend diversity and education efforts to include LGBTQ individuals and issues. Karen Lincoln (University of Southern California) will discuss the need to move educational efforts beyond our classrooms to diverse learners in our communities. To this end, she will describe a dynamic academic–community partnership that provides aging-focused education to African-American older adults. Dana Burr-Bradley (University of Maryland, Baltimore County) will give the final presentation with a discussion about the need for an international lens that appreciates the global diversity of the aging experience. Tamara Baker (University of North Carolina, Chapel Hill) will serve as the discussant and offer her perspective on how the need to embrace our diversity in gerontology education cuts across all GSA units and connects them with AGHE's educational mission.

### STRATEGIES FOR INTEGRATING ANTIRACIST PEDAGOGY INTO GERONTOLOGICAL EDUCATION

Rona Karasik, and Kyoko Kishimoto, *St. Cloud State University, St. Cloud, Minnesota, United States*

Talking about diversity in gerontology is not new. Documenting racial disparities among older adults is not new. So what is different about applying antiracist pedagogy to teaching about aging and why should gerontology educators embrace it? Anti-racist pedagogy seeks to move beyond simply incorporating racial content into gerontology coursework. It involves acknowledging structural racism's existence and relation to aging, uncovering its root causes in historical and political context, and studying its impact not just over the life course, but from generation to generation. Anti-racist pedagogy challenges students to learn how racial disparities among older adults emerged, why they persist, and what can be done about them. Applying anti-racist pedagogy can also challenge gerontological educators, who must find/prepare appropriate materials, as well as engage students in work that is potentially unfamiliar and/or uncomfortable. This presentation offers some strategies, examples, and pitfalls to avoid when incorporating anti-racist pedagogy into the gerontological curriculum.

### DIVERSIFYING THE PATHWAY: A MULTIFACETED APPROACH WITHIN GEROPSYCHOLOGY

Brian Carpenter[1], Flora Ma[2], and Kimberly Hiroto[3], *1. Washington University in St. Louis, St. Louis, Missouri, United States, 2. Stanford HealthCare Psychology, Palo Alto, California, United States, 3. VA Palo Alto Health Care System, Palo Alto, California, United States*

Like other health services disciplines, geropsychology is facing a paucity of diverse intersectional identities within a larger shortage of interest in aging across all levels of training. We need an incisive and comprehensive plan to enhance the representation of historically marginalized groups within geropsychology and address systemic factors limiting entrance into the field. In this presentation, we review the projected need for mental health services attuned to equity, diversity, and inclusion and current trends in the geropsychology pathway. Then we describe several initiatives to expand attention to cultural humility and antiracism. These include the development of foundational competencies that include attention to diverse representations, which can be disseminated to pre- and post-licensure generalists and specialists; diversity-infused offerings within recent conferences and webinars; and efforts to intensify the focus on diversity within psychology's aging-related professional organizations. Finally, we conclude with additional suggestions to expand and sustain attention to diversity.

### ENSURING COMPETENCE: ADDRESSING THE NEED FOR LGBTQ+ AGING EDUCATION

Aaron Guest, *Arizona State University, Phoenix, Arizona, United States*

The continued demographic shifts in population aging are coupled with an increase in self-identified, LGBTQ+ individuals entering older age. While they share some common age-related experiences with non-LGBTQ+ individuals, they also face unique challenges as they age, including additional intersectional pressures beyond their sexual or gender identity. These challenges are exacerbated by pre-existing health inequities and heteronormative social support systems. Of particular concern is the ability to locate affirming and accessible social and health services. Gerontologists have a critical role in assisting older LGBTQ+ individuals in navigating complex social systems. To do so requires they have the appropriate education to develop the cultural humility needed to understand and assist these individuals. It necessitates the engagement of gerontology and geriatric education programs in educating future gerontologist professionals about LGBTQ+ older individuals. Critically, it requires the identification of appropriate instructors who are equipped to educate on LGBTQ+ aging topics.

### IF THEY BUILD IT, THEY WILL COME: ENGAGING OLDER AFRICAN AMERICANS IN EDUCATION AND RESEARCH

Karen Lincoln, *University of Southern California, Los Angeles, California, United States*

Engaging African Americans in health education and research is a mechanism for addressing health disparities and achieving health equity. The science of engagement and retention requires strong community partnerships, authentic leadership, and educational opportunities for students and the broader community. In this presentation, Karen Lincoln will discuss how findings from her research on health and mental health disparities laid the foundation for her community-based health education program and public scholarship. She will highlight Advocates for African American Elders (AAAE), the health education program that she founded and directs. AAAE provides culturally competent health education for older African Americans throughout Los Angeles County and has become a hub for her research, training and mentoring.

### EXPLORING DEI IN A GLOBAL PERSPECTIVE: AGHE INSIGHTS INTO OUR WORK SUPPORTING GERONTOLOGY AND GERIATRIC EDUCATORS

Dana Bradley[1], and Judith Howe[2], *1. University of Maryland, Baltimore County, Baltimore, Maryland, United States, 2. Ichan School of Medicine at Mount Sinai, Bronx, New York, United States*

Gerontologists across the globe make a sincere effort to create a learning, teaching, and research space that promotes the dignity of older persons. Indeed, this impetus imbues our work where all are treated with dignity and respect. The authors (AGHE chair and past chair) reached out to international educators to better understand how AGHE is supporting their efforts and how we may do so in the future. Our starting point was the GSA policy on Diversity, Equity, and Inclusion (2021). These policies laid out a groundwork that embraces inclusive membership cultivates practices that build a culturally inclusive workforce, foster research practices, and ensure that people from disproportionately affected and marginalized communities thrive in GSA. This paper offers insight into how our international community is thinking about DEI and incorporating it into teaching, research and engagement work.

### SESSION 3430 (SYMPOSIUM)

### REIMAGINING ADRD IN CONTEXT

Chair: Kristine Ajrouch Co-Chair: Toni Antonucci
Discussant: Damali Martin

This symposium embraces diversity and discovery to address contextual issues in aging, specifically issues of race and ethnicity in the study of cognitive health and Alzheimer's Disease and Related Dementia (ADRD). Rooks and colleagues compare dementia risk among African American and White men and women in the context of work using the longitudinal Health, Aging, and Body Composition data. They consider the effects of productive activities on dementia risk in gender stratified models, adjusting for socio-demographic and genetic factors. Siddiq and colleagues consider the contexts of migration. Using a multi-method approach, they establish priorities for interventions addressing ADRD risk among older adult immigrants and refugees from Afghanistan and the Middle-East and North Africa (MENA) in California. Sayed also investigates the context of migration, and uses qualitative data (N=31) to identify the psychosocial impact of COVID-19 on cognitive aging in Middle Eastern/Arab Americans immigrants and refugees in Michigan. Finally, Meier and colleagues consider contexts of metal exposure for cognitive decline among Latinos aged 65 and older using the Sacramento Area Latino Study on Aging. In total, this symposium highlights the benefits of reimagining contextual factors that influence ADRD to improve our understanding and the potential to reduce health disparities research in underrepresented racial and ethnic populations.

### ALZHEIMER'S DISEASE AND RELATED DEMENTIAS RISK AND PSYCHOSOCIAL HEALTH NEEDS OF OLDER ADULT IMMIGRANTS AND REFUGEES

Hafifa Siddiq[1], Nahla Kayali[2], and Kristine Ajrouch[3], *1. Charles R. Drew University, Garden Grove, California, United States, 2. Access California Services, Anaheim, California, United States, 3. Eastern Michigan University, Ypsilnati, Michigan, United States*

To establish priorities for an intervention addressing Alzheimer's Disease and Related Dementias (ADRD) risk among older adult immigrants and refugees from Afghanistan and the Middle-East and North Africa (MENA). A multi-methods Community-Based Collaborative Action Research design was employed to gather qualitative and quantitative data. Twenty community leaders and stakeholders participated in interviews and community needs survey responses of 124 ethnically diverse adults over age 55 were examined. We analyzed community needs survey data using descriptive statistics. A step-wise Thematic Analysis was used to analyze interview transcripts. A major theme and standout priority was identified: needing culturally-sensitive services and resources addressing social isolation among the elderly. Other psychosocial priorities included: accessibility needs, caregiving support, safety and end-of-life resources, digital literacy, and English language services. This study exemplifies a priority-setting partnership between clinician-researchers and refugee service providers. The priorities highlighted ADRD risk factors and guide the collaborative development of a community-based intervention.

### RACE, GENDER, AND LATE-LIFE PRODUCTIVE ACTIVITIES AND DEMENTIA IN THE HEALTH ABC STUDY

Ronica Rooks[1], Wassim Tarraf[2], Lindsay Ryan[3], and Toni Antonucci[4], *1. University of Colorado Denver, Denver, Colorado, United States, 2. Wayne State University, Detroit, Michigan, United States, 3. The University of Michigan, Ann Arbor, Michigan, United States, 4. University of Michigan, Ann Arbor, Michigan, United States*

We hypothesized productive activities, e.g., working and volunteering, would reduce dementia risk and mitigate racial disparities using longitudinal Health, Aging, and Body Composition data (n=3,069). We ran gender-stratified logistic regression models for dementia with socio-demographic and APOE adjustments. Among older men, no significant relationships existed between dementia with work, volunteering, or race in any models. Among older women, in unadjusted models, working vs. not (Yr1) was significantly related to 30% lower odds of dementia ($OR=0.70, p\leq 0.05$), and volunteering vs. not (Yr1) was significantly related to 24% lower odds of dementia ($OR=0.76, p\leq 0.05$). But, these activities were not significant in other models. Black vs. White women had 35% ($OR=1.35, p\leq 0.05$) and 32% ($OR=1.32, p=0.052$) higher odds of dementia in working and volunteering models with socio-demographic adjustments, respectively, but race was not significant after APOE adjustment. Productive activities are possible contextual interventions to reduce racial disparities in incident dementia among older women.

### METALS EXPOSURE AND COGNITIVE DECLINE IN LATINO ELDERS: RESULTS FROM THE SACRAMENTO AREA LATINO STUDY ON AGING

Helen Meier[1], Kelly Bakulski[2], and Allison Aiello[3], *1. University of Michigan, Ann Arbor, Michigan, United States, 2. University of Michigan School of Public Health, Ann Arbor, Michigan, United States, 3. Carolina Population Center, University of North Carolina at Chapel Hill, Chapel Hill, North Carolina, United States*

Latinos have a higher risk of Alzheimer's disease and related dementias (ADRD) and clinical presentation could take place 7 years earlier, on average, than non-Hispanic whites. This health disparity will likely intensify as the US Latino population over 65 years is predicted to grow from 4 million in 2016 to 19.9 million in 2060. Environmental exposures, such as metals, are of interest as contextual factors contributing to ADRD due to their known neurotoxic effects, particularly in early life. Using data from the Sacramento Area Latino Study on Aging, we examined the association between blood concentrations of five metals and cognitive outcomes. We hypothesized that Latino elders with higher concentrations of non-essential metals will have greater cognitive decline than those with lower concentrations of non-essential metals. Initial results suggest non-essential blood metal levels are not sociodemographically patterned in SALSA participants. Higher lead and mercury concentrations was associated with cognitive decline.

### THE PSYCHOSOCIAL IMPACT OF COVID-19 ON COGNITIVE AGING ARAB AMERICAN IMMIGRANTS IN MICHIGAN

Linda Sayed, *Michigan State University, East Lansing, Michigan, United States*

This study examines the psychosocial impact of COVID-19 on cognitive aging Middle Eastern/Arab Americans immigrants and refugees in Michigan. Sociopolitical experiences as an immigrant and/or refugee may have unique effects on late-life cognitive health. Given that social engagement reduces the risk of ADRD, this study sought to examine the consequences of COVID-19 socialization restrictions on familial and communal support systems for aging Middle Eastern/Arab immigrants and refugees. Three focus groups discussions with 8-10 participants each (N=31) conducted at local Arab organizations and religious institutions were conducted followed by inductive analysis. Preliminary results indicate two prominent themes: 1) Social and Familial Hardships; and 2) Isolation. Narratives illustrate the prevalence of psychosocial ADRD risk from COVID-19. Findings are discussed within a stress framework to advance universal and unique elements of Arab immigrant and refugee cognitive aging.

### SESSION 3440 (PAPER)

### SOCIAL DETERMINANTS OF HEALTH (PAPERS)

### COHORT EFFECTS IN OLD-AGE COGNITIVE AGING: A STUDY OF SOCIAL CONTEXT IN CHILDHOOD IN 140,030 OLDER ADULTS

Stacey Scott[1], Zared Shawver[1], Sean Clouston[1], Yun Zhang[1], Stacey Voll[2], Patrick O'Keefe[3], Joeseph Rodgers[4], and Scott M. Hofer[2], *1. Stony Brook University, Stony Brook, New York, United States, 2. University of Victoria, Victoria, British Columbia, Canada, 3. Oregon Health and Science University, Portland, Oregon, United States, 4. Vanderbilt University, Nashville, Tennessee, United States*

Researchers have reported that later-born cohorts often have higher scores on cognitive tests, potentially indicating that some of the differences normally attributed to cognitive aging may reflect developmental differences. The present study examined the hypotheses that social factors at birth and in early adolescence might partially explain birth cohort effects. This secondary analysis of data collected prospectively as part of four internationally comparable and nationally representative studies of individuals aged 50 and older residing in 17 European countries and the United States (N=140,030). Cognition was prospectively measured longitudinally 360,150 observations spanning, on average, 4.27 (SD=5.12) years allowing us to leverage age/cohort variability. Multilevel longitudinal modeling was used incorporated random intercepts and slopes at the country and individual levels to model lifetime cognition while adjusting for contextual factors. Birth cohort was associated with height (B=0.095, SE=0.002, P< 1E-06), episodic memory (B=0.105, SE=0.001, P< 1E-06), and verbal fluency (B=0.217, SE=0.002, P< 1E-06). Approximately 7.72% (95% C.I.=[7.60-7.86]) of participants were exposed to at least two years of famine in childhood, and were born into countries with moderate levels of income inequality. Multivariable adjustment accounted for 15.10-24.96% of birth cohort effects in episodic memory and verbal fluency respectively. Longitudinal modeling revealed that after adjusting for famine, war, income inequality, educational attainment, and height, these factors explained 81.58-63.65% of the birth cohort effect in episodic memory and verbal fluency respectively. Global research has reported that early life factors can have an impact on development, so this study extends this to suggest these factors have a lifelong impact.

### GRANDPARENTS' EDUCATION IS ASSOCIATED WITH GRANDCHILDREN'S EPIGENETIC AGE IN THE NGHS STUDY

Agus Surachman[1], Elissa Hamlat[1], Anthony Zannas[2], Barbara Laraia[3], and Elissa Epel[1], *1. University of California, San Francisco, San Francisco, California, United States, 2. University of North Carolina, Chapel Hill, North Carolina, United States, 3. University of California Berkeley, Berkeley, California, United States*

We leveraged information solicited from three generations (grandparents, mothers, and grandchildren) to examine the association between mothers' childhood SES (based on grandparents' educational attainment) and their children's epigenetic age and whether the association was mediated by mothers' life course socioeconomic and health-related factors. Mothers were recruited to the NHLBI Growth and Health Study when they were 9 or 10 and followed for ten consecutive years (1987-1998). Grandparents reported their highest education during the baseline interviews. Mothers were then re-contacted three decades later (ages 37-42) to participate in the National Growth and Health Study (NGHS), and health information of their youngest children (i.e., grandchildren; N = 241, ages 2-17) were collected, including their saliva samples to calculate epigenetic age. Two epigenetic ages were estimated (Horvath and Hannum), and DNA methylation age accelerations (DNAmAAs) were calculated using residuals from regressing chronologic age on each epigenetic age metrics. Mothers' life course socioeconomic and health-related mediators included childhood BMI trajectories (from age 9 to 19), highest education level, adult health behavior, and adult c-reactive protein (CRP). Adjusted for age and sex, grandchildren with college degree grandparents showed significantly slower Horvath's DNAmAA than those with no college degree. The association between grandparent's education level and grandchildren's DNAmAA was partially mediated by mothers' life course socioeconomic and health-related factors, especially mothers' education, health behavior, and CRP. Grandparents' educational attainment is a critical socioeconomic context of mothers' early rearing, and it might have a long-lasting impact on their grandchildren's epigenetic marker.

### IMPACT OF OBJECTIVE AND SUBJECTIVE SEP ON AGING TRAJECTORIES OF FUNCTIONAL CAPACITY

Deborah Finkel[1], Charlotta Nilsen[2], Shireen Sindi[3], and Ingemar Kåreholt[2], *1. Indiana University Southeast, New Albany, Indiana, United States, 2. Jönköping University, Jönköping, Jonkopings Lan, Sweden, 3. Karolinska Institutet, Solna, Stockholms Lan, Sweden*

Long-term stress is associated with adverse health outcomes in aging. It is important to identify not only factors that influence functioning in late adulthood, such as accumulated stress, but also the timing of such factors. The aim of the current analysis was to examine how socioeconomic stressors throughout the life course are associated with aging in functional capacity. Data were available from 740 adults ranging in age from 40 to 83 at intake (mean = 62.4, SD = 8.2) who participated in up to 8 waves of data collection (mean = 3.9, SD = 2.4). A Functional Aging Index (FAI) was created by combining measures of sensory, pulmonary, gait, and grip functioning. Both childhood and adulthood measures of objective socioeconomic position (SEP) and perceived SEP (financial strain) were available. Latent growth curve models (corrected for twinness) were used to estimate the trajectory of change in FAI over age and the impact of child and adult SEP measures on the trajectories. Results indicated that both childhood and adult objective SEP independently influenced rates of change in FAI in adulthood: higher SEP was associated with higher mean functioning and slower rates of decline. In combination, model fitting indicated that if SEP is above the median in adulthood, then childhood SEP has no impact on FAI trajectories; however, if SEP is below the median in adulthood, then childhood SEP can play a role. In addition, results indicated possible long-term effects of childhood financial strain on rates of change in FAI in adulthood.

### IN THE EYES OF THE BEHOLDER: RACE, PLACE, AND HEALTH

Alfredo Velasquez[1], Jason Douglas[1], Fangqi Guo[2], and Jennifer Robinette[2], *1. Chapman University, Orange, California, United States, 2. Chapman University, Chapman/Orange, California, United States*

Racial and ethnic health disparities are fundamentally connected to neighborhood quality. For example, racial and ethnic minorities are more likely to live in neighborhoods with signs of physical disorder (e.g., graffiti, vandalism), and physically disordered environments have been noted to associate with increased risk for chronic illness. Given that older adults may spend more time in their neighborhoods than younger adults as they transition out of the workforce, examining associations between neighborhood physical disorder and health among older minorities is of critical importance. Using 2016-2018 Health and Retirement Study (HRS) data, a representative sample of US adults aged 51 years and older (n= 9,080, mean age 68 years), we conducted a series of weighted linear regressions to examine links between neighborhood disorder as rated by third parties and both participant-perceived neighborhood safety and self-rated health. Study results indicated that higher neighborhood physical disorder was significantly related to more neighborhood safety concerns among non-Hispanic White and Hispanic residents, but not among non-Hispanic Blacks. On the other hand, neighborhood physical disorder was significantly associated with poorer health among all racial/ethnic groups. These patterns persisted after adjusting for education, sex, age, and census tract concentrated disadvantaged, population density, and racial/ethnic diversity. Our results indicate that community level interventions targeting neighborhood physical disorder may improve community health and minimize racial/ethnic health disparities.

### SOCIAL STRESSORS ASSOCIATED WITH AGE-RELATED T LYMPHOCYTE PERCENTAGES IN OLDER US ADULTS: EVIDENCE FROM THE HRS

Eric Klopack[1], Eileen Crimmins[2], Steve Cole[3], Teresa Seeman[3], and Judith Carroll[4], *1. University of Southern California, Leonard Davis School of Gerontology, Los Angeles, California, United States, 2. University of Southern California, Los Angeles, California, United States, 3. University of California Los Angeles, Los Angeles, California, United States, 4. University of California, Los Angeles, Los Angeles, California, United States*

Exposure to stress is a risk factor for poor health and accelerated aging. Immune aging, including declines in naïve and increases in late memory and terminally differentiated T cells, plays a role in immune health and tissue specific aging, and may contribute to elevated risk for poor health among those who experience high psychosocial stress. Past data have been limited in estimating the contribution of life stress to the development of accelerated immune aging and investigating mediators such as lifestyle and CMV infection. This study utilizes a national sample of 5744 US adults over age 50 to assess the relationship of social stress (viz., everyday discrimination, stressful life events, lifetime discrimination, life trauma, and chronic stress) with flow cytometric estimates of immune aging, including naïve and terminally differentiated T cell percentages and the ratio of CD4+ to CD8+ cells. Experiencing life trauma and chronic stress was related to a lower percentage of CD4+ naïve cells. Discrimination and chronic stress were each associated with a greater percentage of terminally differentiated CD4+ cells. Stressful life events, high lifetime discrimination, and life trauma were related to a lower percentage of CD8+ naïve cells. Stressful life events, high lifetime discrimination and chronic stress were associated with a higher percentage terminally differentiated CD8+

cells. High lifetime discrimination and chronic stress was related to a lower CD4+:CD8+ ratio. Lifestyle factors and CMV seropositivity partially reduced these effects. Results identify psychosocial stress as a contributor to accelerating immune aging by decreasing naïve and increasing senescent T cells.

### CORRELATES OF ELEVATED C-REACTIVE PROTEIN AMONG BLACK OLDER ADULTS: EVIDENCE FROM THE HEALTH AND RETIREMENT STUDY

Heather Farmer[1], Courtney Sinclair Tobin[2], and Roland J. Thorpe, Jr.[3], *1. University of Delaware, Newark, Delaware, United States, 2. Fielding School of Public Health, University of California Los Angeles, Los Angeles, California, United States, 3. Johns Hopkins University, Baltimore, Maryland, United States*

Substantial evidence documents gender and racial disparities in C-reactive protein (CRP), a measure of systemic inflammation, among older adults. Yet, the comparative approaches of these studies may obscure distinct risk and protective factors associated with elevated CRP among older Black Americans. To pinpoint opportunities for intervention, this study utilizes a "within-group approach" to identify the sociodemographic, psychosocial, behavioral, and health-related determinants of elevated CRP among older Black women and men. The sample consisted of 2,420 Black respondents aged 51+ in the Health and Retirement Study (2006-2016). Gender-stratified, random effects logistic regression models were used to examine determinants of elevated CRP (>3.0 mg/L). More than 50% of Black women had elevated CRP, and we found that younger age, Medicaid, lower mastery, religiosity, overweight/obesity, physical inactivity, and ADLs contributed to elevated CRP among this group. In contrast, elevated CRP was reported among only 37.25% of Black men, for whom financial distress was associated with lower odds of elevated CRP; religiosity, less neighborhood cohesion, current smoking, overweight/obesity, ADLs, and more chronic conditions were associated with greater odds of elevated CRP among this group. Sociodemographic factors had a limited association with elevated CRP among older Black Americans. Rather, a range of psychosocial, behavioral, and health-related factors were more influential determinants of elevated CRP among older Black Americans. Most notably, findings demonstrate distinct determinants of CRP among Black women and men, underscoring the critical need to further evaluate the risk and protective mechanisms undergirding disparities among this aging population.

### SESSION 3450 (PAPER)

### SUCCESSFUL AGING (PAPERS)

### COMMUNITY DISADVANTAGE AND WORKFORCE PARTICIPATION AMONG LATE MIDDLE-AGED MEDICAID BENEFICIARIES

Rodlescia Sneed, *Wayne State University, Detroit, Michigan, United States*

There have been numerous efforts to increase workforce participation among Medicaid beneficiaries in an effort to reduce program costs. We know little, however, about whether Medicaid beneficiaries with low workforce participation live in communities that support employment. The purpose of the current study was to determine if community disadvantage was associated with workforce participation among late middle-aged Medicaid beneficiaries, an age group that commonly faces difficulty entering the workforce. Participants were 1418 Medicaid beneficiaries ages 51-64 who participated in the 2016 wave of the Health and Retirement Study, a population-based study of community-dwelling adults aged >50. Community disadvantage was measured using the Social Vulnerability Index (SVI). We evaluated the association between community social vulnerability and reduced workforce participation (defined as working < 20 hours per week). We performed additional analyses to determine if the association between community disadvantage and reduced workforce participation was driven by any one of four subthemes of the SVI:(1) community socioeconomic status, (2) household composition/disability, (3) minority status & language and (4) housing type & transportation. Community disadvantages was associated with reduced workforce participation (OR: 2.82; 95% CI: 1.05-7.56). Analyses by SVI theme showed that the association between community disadvantage and reduced workforce participation was largely driven by community socioeconomic status (SES). Those living in low SES communities were more likely to report reduced workforce participation than those living in high SES communities: (OR: 5.48; 95% CI: 3.28-8.18). Policy interventions that focus on disadvantaged communities are likely needed to improve employment rates among late middle-aged Medicaid beneficiaries.

### FRAILTY-FOCUSED COMMUNICATION (FCOM) FOR OLDER ADULTS

Cathy Maxwell[1], Sally Miller[2], and Deborah Lee[3], *1. Vanderbilt University, Hermitage, Tennessee, United States, 2. Vanderbilt University, Nashville, Tennessee, United States, 3. Middle Tennessee State University, Murfreesboro, Tennessee, United States*

Frailty-focused communication (FCOM) equips older adults with a better understanding of factors and biological mechanisms related to decline, frailty, and eventual end-of-life, while also empowering them to engage in proactive measures for healthy aging in 8 domains (physical activity, nutrition, safety, mind/body health, relationships/community, sleep/rest, finances, health care decision-making). We aimed to develop and test: 1) an educational tool targeted to older adults, and 2) a FCOM workshop to train health care clinicians on how to explain the concept of frailty and facilitate goal-setting for proactive aging. We tested the tool and workshop in 2 prospective observational studies among community-dwelling older adults (N= 126) and clinicians (N=29) who provide care to older adults. Testing of the FCOM tool revealed that content on bioenergetics of aging increased motivation to make a plan for proactive aging (N=60, 68%), particularly among adults age 55-64 as compared to other age groups (p=.024). Testing of the training workshop demonstrated increased knowledge of frailty (pre score: 5.8, post score: 7.6; d= .55, p=.006) and increased self-reported competency with patient/clinician interaction (pre score: 33.2, post score: 42.0; d=.63, p=.001). Post course evaluations regarding understanding of best practice empathic skills was rated 3.6 (range: 1-4). FCOM promotes awareness of the importance of measures to mitigate development of chronic conditions and frailty, and provides a resource for clinicians in multiple settings to engage in conversations with older adults while physiologic compensatory mechanisms are still intact. FCOM discussions can facilitate a shift from disease-oriented discussion to patient-centered, holistic care.

### LESSONS ON LEARNING IN LATER LIFE WITH THE MIT AGELAB 85+ LIFESTYLE LEADERS

Taylor Patskanick[1], Julie Miller[2], Lucas Yoquinto[2], Lisa D'Ambrosio[2], and Joseph Coughlin[2], *1. Massachusetts Institute of Technology, Somerville, Massachusetts, United States, 2. Massachusetts Institute of Technology, Cambridge, Massachusetts, United States*

Previous research has established the role of lifelong learning in promoting psychological wellbeing and active aging. Population aging necessitates an understanding of the unique opportunities and challenges around formal and informal learning in later life. This paper will share findings from a mixed methods study with the MIT AgeLab 85+ Lifestyle Leaders, a panel comprised of octogenarians and nonagenarians from across the United States. Drawing on an online survey and virtual focus groups with 29 Lifestyle Leaders from January 2021, findings suggest the Lifestyle Leaders most often learned new things from talking with others (46%) and reading print (54%) or online (54%) sources. The majority were familiar with attending in-person lectures or classes (89.7%) and were now using videoconferencing to do these (78.6%). A majority (56.7%) had or are currently participating in a lifelong learning program. Most consider themselves lifelong learners and described this around remaining curious and engaged with life, choices around what one learns, and greater enjoyment of learning. In the survey, a plurality of Lifestyle Leaders indicated the top two challenges affecting their ability to learn were sensory burdens (e.g., hearing loss, declining eyesight) (35%) and their energy level (32.4%); focus group data revealed that recall also is a barrier. Focus group data further highlighted generational experiences around early life learning and career paths, specifically how gender roles, diagnoses of learning disabilities, and evolving digital technology have affected these and changed over the course of their lifetimes.

### METHODOLOGICAL AND ETHICAL REFLECTIONS FROM RESEARCH ON NEIGHBOURHOOD-BUILT ENVIRONMENT AND DEMENTIA

Kishore Seetharaman[1], Habib Chaudhury[1], Lillian Hung[2], Shannon Freeman[3], Mark Groulx[3], and Cari Randa[1], *1. Simon Fraser University, Vancouver, British Columbia, Canada, 2. University of British Columbia, Vancouver, British Columbia, Canada, 3. University of Northern British Columbia, Prince George, British Columbia, Canada*

The neighbourhood is widely regarded as a setting that affords emotional and practical support and opportunities to maintain community-based activities and social participation for people living with dementia (PLWD). Creating a supportive neighbourhood built environment that facilitates outdoor mobility, wayfinding, and access to community destinations is key to making our communities dementia-inclusive. Research on the built environment and dementia-inclusive planning is relatively sparse in the broader research domain of neighbourhoods and dementia. Further, how PLWD perceive, interpret, and interact with the neighbourhood built environment is not adequately understood. Although it is acknowledged that PLWD should be more meaningfully included and engaged in research in this area, there is a lack of guidance on methodological and ethical considerations necessary to explore people-place relations in the neighbourhood built environment through the lens of the lived experience of PLWD. To address this gap, our paper draws from the Dementia-inclusive Streets and Community Access, Participation, and Engagement (DemSCAPE) study to highlight reflections on conducting walk-along interviews, embodied videography, photo documentation, semi-structured sit-down interviews, and visual elicitation to explore the influence of the neighbourhood built environment on the outdoor walking experience of PLWD. We discuss 1) methodological strengths, including the triangulated strategy of capturing experiential data in-situ in real time and in retrospect, and flexibly working around memory and communication-related challenges experienced by PLWD, 2) ethical challenges and measures for mitigation, 3) logistical difficulties in undertaking complex fieldwork with PLWD, limitations of the research methods, and potential alternative methods to explore in future research.

### OLDER PEOPLE'S ACTIVITY PARTICIPATION (OPAP): FACTOR STRUCTURE AND PSYCHOMETRIC PROPERTIES OF A SCALE

Daniel Rong Yao Gan[1], Yuxin Cao[2], Wen Liu[3], John Chye Fung[2], and Tze Pin Ng[2], *1. Simon Fraser University, Vancouver, British Columbia, Canada, 2. National University of Singapore, Singapore, Singapore, 3. University of Iowa, Iowa City, Iowa, United States*

Older people's participation in activities is critical to their health and well-being. Active lifestyle in old age reduces the risk of mortality, prevents chronic diseases, promotes physical and mental wellbeing, and is conducive to active and healthy aging. While various measures of participation have been proposed, a holistic measure that includes both social and individual activity participation is currently unavailable. To enable lifestyle medicine recommendations, we developed the Older People's Activity Participation (OPAP) scale to understand the constituent factors of older people's everyday activities. This study examined OPAP's internal consistency using Cronbach's alpha, convergent validity using regression analysis, as well as factor structure using exploratory and confirmatory factor analyses in a dense urban setting. Preliminary items assessed engagement in 27 health-related, fitness, recreational, social, productive, and cognitive activities, and were administered to 270 community-dwelling adults aged 50 and older in Singapore. The 17-item OPAP showed acceptable internal consistency (Cronbach's alpha= .69), and demonstrated convergence with a validated measure of social cohesion (B=.27, p<.001) and Instrumental Activities of Daily Living (B=.32, p<.001). It has a 5-factor structure, namely socializing (alpha=.63), physical training

(alpha=.56), listening (alpha=.69), home-making (alpha=.59), and outing (alpha=.58). Acceptable model fit was obtained (RMSEA=0.48, SRMR=0.06, CFI=.90). The OPAP scale is valid and reliable to assess activity participation in Asian older adults. It can be a useful tool to understand older people's everyday life and lifestyles, relationships between activity participation and health outcomes, and further guide the development and use of effective interventions to promote active and healthy aging.

### SESSION 3460 (SYMPOSIUM)

### THE POWER OF RURAL PARTNERSHIP: MEETING NEEDS AND FOSTERING INNOVATION THROUGH THE WYOMING GWEP

Chair: Christine McKibbin Co-Chair: Catherine Carrico

The challenges of supporting the health and social needs of older adults and caregivers in rural and frontier areas are well-documented. It is common for rural older adults to experience barriers in accessing geriatric specialists, care coordination services, and caregiver support and education programs. The Wyoming Geriatric Workforce Enhancement Program (WyGWEP) is an innovative partnership comprising an academic geriatrics program, primary care delivery sites, single-unit on aging representing community-based organizations, and a tribal health program. This partnership, funded by the Health Resources and Services Administration, provides the infrastructure to assess needs, provide education and training, create programs to address gaps in care, implement practice innovation, and advocate for needs of rural older adults. This symposium includes five presentations detailing the unique projects of the WyGWEP partnership and the impact of this collaborative work on a variety of stakeholders. The results of a mixed-methods evaluation of the WyGWEP partnership will describe the benefits to the partners and areas for growth. A collaborative effort to assess the needs of older adults informs recommendations to support rural aging in place. Schenck et al. will describe the adaptation of the widespread ECHO model for use with dementia caregivers in rural and remote locations. Representatives from Wyoming's only Program for All-Inclusive Care of the Elderly (PACE) will explain the cost of a recent decision to de-fund and close this important program. Finally, the impact of a novel Chronic Care Management implementation program will be discussed, including sustainable billing revenue produced by rural primary care clinics.

### EVALUATING THE WYOMING GWEP PARTNERSHIP

Catherine Carrico[1], Irene Lujan[2], Trena Anastasia[3], and Christine McKibbin[1], *1. University of Wyoming, Laramie, Wyoming, United States, 2. Eastern Shoshone Tribal Health, Ft. Washakie, Wyoming, United States, 3. Thrive Alive, Masonville, Wyoming, United States*

The goal of the Wyoming Geriatrics Workforce Enhancement Program (WyGWEP) is to utilize partnerships to improve health outcomes for older adults by developing a healthcare workforce trained to address unmet health and social determinant needs of Wyoming's older residents. The purpose of this presentation is to report results from the novel mixed-methods evaluation of the WyGWEP partnership. Data were gathered from WyGWEP stakeholders through the Partnership's Self-Assessment Tool (n=17; Center for the Advancement of Collaborative Strategies in Health, 2002), which assesses domains of Synergy, Leadership, Resource Sharing — Non-financial and Financial, Benefits and Drawbacks, Decision Making, and Administration/Management and Satisfaction. Semi-structured interviews (n=12) with partnership participants provided additional information and context. Evaluation results indicate that WyGWEP partners are highly satisfied across all domains of the Partnership's Self-Assessment Tool. WyGWEP partners from Eastern Shoshone Tribal Health will share what has contributed to a successful collaborative. Additional results related to how this type of partnership has influenced participants' ability to complete objectives and activities will be reviewed.

### ADAPTING THE ECHO MODEL TO MEET THE NEEDS OF RURAL DEMENTIA CAREGIVERS

Sabine Schenck, Catherine Carrico, and Stacy Carling, *University of Wyoming, Laramie, Wyoming, United States*

Caring for an individual with dementia may result in caregiver stress and burden (Cassie & Sanders, 2008), which can lead to detrimental health outcomes if not managed with effective coping skills and support (Shulz & Martire, 2004). Access to support and psychoeducation is limited in rural and frontier communities, and solutions are needed to reach caregivers across sparsely populated regions. Project ECHO (Extension for Community Health Outcomes) is an evidence-based model designed to improve patient outcomes through healthcare provider education. The Wyoming Dementia Together Caregiver Network is the first of its kind to adapt the ECHO model for family caregivers of persons with dementia. This presentation details evaluation results of 24 session across 52 weeks (n=162). Results suggest that adaptation of Project ECHO for family caregivers is feasible and palatable. In addition, the adapted program shows promise for improving caregiver outcomes such as depressive symptoms and caregiver burden.

### CONNECTING THE DOTS: CHRONIC CARE MANAGEMENT PROGRAM IMPLEMENTATION IN RURAL PRACTICES

Faith Jones[1], Kevin Franke[2], Tonja Woods[2], Emma Bjore[3], and Elizabeth Punke[4], *1. HealthTech, Brentwood, Tennessee, United States, 2. University of Wyoming, Laramie, Wyoming, United States, 3. Ivinson Medical Group, Laramie, Wyoming, United States, 4. University Of Wyoming, Laramie, Wyoming, United States*

As the focus of healthcare changes from a "sick" care model to a population health model, primary care and specialty clinic practices have new opportunities supported through Medicare reimbursement. The incorporation of team-based care coordination programs into clinic practices is an important step towards value-based care and achieving the Triple Aim: better health for the population, better care for individuals, and lower costs through improvements. Since 2019, six rural Wyoming primary care practices have completed training to implement and expand care coordination programs. HealthTechS3 provides participating clinics with team-based training in the implementation of the Chronic Care Management Program, Behavioral Health Integration, and other billable care coordination services. To date, 301 patients have enrolled in care coordination services. These practices have generated $350,000 in revenue. Using a consolidated implementation framework as a guide, critical components of successful rural care coordination program implementation are discussed.

### COLLABORATIVE NEEDS ASSESSMENT OF RURAL OLDER ADULTS AND PRIORITIES FOR AGING IN PLACE

Bernard Steinman[1], Jennifer Tabler[1], Jeff Clark[2], and Lisa Osvold[3], *1. University of Wyoming, Laramie, Wyoming, United States, 2. Wyoming Department of Health Aging Division, Cheyenne, Wyoming, United States, 3. Wyoming Department of Health - Aging Division, Cheyenne, Wyoming, United States*

To assess aging-related needs of state residents, the Wyoming GWEP collaborated with the state's Department of Health--Aging Division to conduct a state-wide survey as the basis for Wyoming's 4-year state plan on aging. Survey items addressed concerns related to community living and caregiving, as well as general concerns related to aging in Wyoming's rural setting. Results suggest older residents are committed to aging at home; and when that is not feasible, wish to remain in their communities when possible. Concerns were identified regarding attributes of the state that place older residents at risk for poorer physical/mental health, dependence, financial jeopardy, social isolation, and negative secondary outcomes that are comparatively more costly to address in institutional settings. We discuss the collaborative needs-assessment process, as well as priorities for future service delivery that promote good social support, transportation options, and home modifications to increase independence and safety.

### THE END OF PACE: OPPORTUNITY FOR ADVOCACY AND EDUCATION

Gregory O'Barr[1], Lindsay Bonazinga[2], Rebecca Carey[2], Tracy Brosius[3], Anne Alexander[4], and Barbara Dabrowski[4], *1. Cheyenne Regional Medical Center, Windsor, Colorado, United States, 2. Cheyenne Regional Medical Center, Cheyenne, Wyoming, United States, 3. Wyoming Healthworks, Cheyenne, Wyoming, United States, 4. University of Wyoming, Laramie, Wyoming, United States*

The Program for All-Inclusive Care for the Elderly (PACE) is a Medicare/Medicaid Managed Care benefit for frail older adults age 55 years or older who, although certified by the state as long-term care (LTC) eligible, choose to live independently in the community. PACE features comprehensive medical and social services coordinated by an interdisciplinary team whose goal is to promote independence. Cheyenne Regional Medical Center has served as Wyoming's only PACE site since 2007. However, an effort to reduce Medicaid expenditures resulted in elimination of funding and closure of the program by the state. The purpose of this study was to examine the impacts of PACE closure on transition to LTC, concomitant Medicaid expenditures, as well as impacts on participant well-being. Results showed, as expected, that nursing home placement exceeded that projected, thereby increasing Medicaid expenditures in Wyoming. Additional impacts on participant well-being and advocacy benefits of GWEP partnership are discussed.

### SESSION 3480 (SYMPOSIUM)

### WELL-BEING DURING THE COVID-19 PANDEMIC: THE ROLES OF DEMOGRAPHICS, PERSONALITY, AND SOCIAL TIES

Chair: Lindsay Ryan Co-Chair: Heather Fuller Discussant: Aurora Sherman

The COVID-19 pandemic continues to exert widespread impacts on individuals, particularly older adults (Tyrrell & Williams, 2020). This symposium capitalizes on a variety of data sources to advance our understandings of the psychosocial impact of the pandemic on older adults. The first two papers consider the importance of personality characteristics in understanding the effects of social distancing. Fiori et al. highlight the potential for sociability to act as a liability during times of social distancing, finding that sociability exacerbated the effects of social distancing on mental health outcomes in a sample of community-dwelling older adults. Ryan's paper focuses on the Big Five Personality traits, age, and population density as key characteristics explaining differences in subjective well-being during the pandemic. Next, Van Vleet et al. apply a mixed-methods approach to investigate when older adults expect life to go back to normal, finding that expectations about the future became more positive with the passage of time. The final two papers consider the importance of adults' home social context during the pandemic. Newton examines relationships between living alone and well-being outcomes among older Canadian women, finding that perceived COVID-19 impact was significant only at T1 and living alone was linked to poorer well-being by T2. Birditt et al. examine how individuals' and partner's COVID-19 stress and couples' racial composition are related to affective experiences measured via ecological momentary assessments, finding that husbands' stress impacted both partners' well-being, and that associations differed by race. Sherman will lead a discussion to synthesize these new findings.

### WELL-BEING DURING THE COVID-19 PANDEMIC: A FUNCTION OF PERSONALITY, AGE, AND URBANICITY

Lindsay Ryan, *The University of Michigan, Ann Arbor, Michigan, United States*

The COVID-19 pandemic has differentially impacted population sub-groups over the last two years. For example, engagement in social isolation may have been particularly difficult for extroverted adults. The virus spread widely in densely populated regions and is more risky to health with increasing age. This paper explores the ways in which personality, age, and urbanicity are associated with subjective well-being during the pandemic. Longitudinal data from the Health and Retirement Study (N = 4316; M age = 69.0, Range 31 - 99) investigates Big Five personality characteristics from 2016 and interactions with age on life satisfaction and loneliness during the pandemic. Models are then stratified by Beale Rural-Urban Continuum codes denoting urban, suburban, and ex-urban residence. Results indicate the benefit of high conscientiousness on life satisfaction is weaker among older adults (p<.05) and associations of extroversion and age on loneliness are driven by individuals living in urban areas (p<.05).

### OLDER CANADIAN WOMEN'S LIVING ARRANGEMENTS AND WELL-BEING DURING COVID-19

Nicky Newton, *Wilfrid Laurier University, Waterloo, Ontario, Canada*

Living alone has been associated with lower COVID-related well-being over the course of the pandemic, although the results for women – particularly older Canadian women – depend on myriad factors. This study examines relationships between COVID-19 impact, living arrangement, and two types of well-being – psychological well-being and meaning in life – among Canadian women (N = 106) with Mage = 70.11 in two waves of data (July-September 2020 and March-May 2021). There were no differences in levels of well-being between T1 and T2, although regression analyses showed that correlates differed for type of well-being measured. Surprisingly, COVID-19 impact was only significant for psychological well-being at T1. Additionally, while levels of well-being did not differ by living arrangement at T1, by T2 both were lower for those living alone. Findings suggest adaptation to COVID-19 constraints and highlight changes in factors associated with well-being as well as the necessity of measuring well-being in multiple ways.

### PANDEMIC-RELATED STRESS AND DAILY WELL-BEING AMONG MIDDLE-AGED AND OLDER COUPLES: RACE AND GENDER DIFFERENCES

Kira Birditt, Angela Turkelson, and Akari Oya, *University of Michigan, Ann Arbor, Michigan, United States*

Pandemic-related stress may have important implications for well-being among middle-aged and older couples and these effects may vary by race. Participants included 30 married/cohabiting couples (10 Black, 23 White, 2 Mixed race) ages 44 to 84 who completed baseline interviews and 5 days of ecological momentary assessment (EMAs) 6 times a day. Every three hours individuals reported how stressed they felt about COVID-19 and negative affect. Actor-partner interdependence models revealed that greater pandemic stress among Black husbands was associated with their own and their wives greater negative affect ($b = 0.22$, $SE = 0.08$, $p < .01$; $b = 0.16$, $SE = 0.08$, $p < .05$). Greater pandemic related stress among White husbands was associated with wives' lower negative affect ($b = -0.15$, $SE = 0.07$, $p < .05$). Findings are consistent with structural racism theory indicating that Black individuals may be more negatively affected by pandemic-related stress than White individuals.

### THE ROLE OF SOCIABILITY IN OLDER ADULTS' ADAPTATION TO COVID-19

Katherine Fiori[1], Amy Rauer[2], Christina Marini[1], Christine So[1], and Amna Khan[1], *1. Adelphi University, Garden City, New York, United States, 2. University of Tennessee Knoxville, Knoxville, Tennessee, United States*

Some older adults may be particularly sensitive to the negative effects of social disruptions due to COVID-19 (Tyrrell & Williams, 2020). For example, the unique circumstances of the pandemic may have made greater sociability a liability (Wijngaards et al., 2020). The current study used a community sample of 136 older adults (M age = 67.77, range 50-91; 69.3% females; 93% White) to explore whether sociability moderated links between disruptions to contact with friends and family and indicators of mental health. Using a series of hierarchical linear regressions, we found that sociability moderated the association between disruptions in family interactions and depressive symptoms. For more sociable individuals, disruptions in family interactions were more strongly positively associated with depressive symptoms. Given the potential for future social disruptions due to COVID-19, our findings point to the importance of considering the role of sociability in developing interventions targeting older adults.

### "IT'S GOING TO BE A NEW NORMAL": OLDER ADULTS' FUTURE PERCEPTIONS DURING COVID-19

Bryce Van Vleet, Heather Fuller, Brittany Hofmann, and Andrea Huseth-Zosel, *North Dakota State University, Fargo, North Dakota, United States*

This study explores older adults' perceptions of the future related to COVID-19. Participants (N=76) of a larger study aged 70-97 were asked four times throughout the first year of the pandemic when they thought life would return to normal. Their open-ended responses were coded, and themes were identified at each timepoint. A resilient future perspective was identified at each timepoint; themes of a negative or unstable view of the future emerged over time. Additionally, responses were quantified into a 5-point scale for perceived timescale of a return to normal (5 = very long time) and attitude towards the future (5 = very positive). Repeated measures ANOVA revealed that perceived time to normal increased between the beginning and six months into the pandemic, then decreased by 12 months. Attitudes towards the future became more positive over time. These findings indicated that older adults were largely resilient, if uncertain, about the future.

### SESSION 3490 (SYMPOSIUM)

### "COUNT YOUR AGE BY FRIENDS, NOT BY YEARS": AN INTERPERSONAL PERSPECTIVE ON SUBJECTIVE VIEWS OF AGING

Chair: Amit Shrira Co-Chair: Yoav Bergman Discussant: Anna Kornadt

Humans are social beings, and as we age, meaningful and close relationships become increasingly important. However, the literature on older adults' subjective views of aging (VoA) tends to focus on the link between such views and health outcomes, overlooking important interpersonal correlates. This symposium consists of four presentations that focus on various concepts, study designs, and populations, and offers novel insights regarding how VoA operate in the social domain. The first presentation (Bergman et al.) establishes, using a diary study, the mediating role of understanding others' emotional states (i.e., Theory of Mind) in the connection between VoA and positive social relationships. The second presentation (Mejía & Hooker) examines, through daily assessment, how VoA differentiate the processes by which older adults construct interactions with their closest social partners to progress toward health goals in daily life. The third presentation (Neupert & Can) employs a diary study and demonstrates that daily fluctuations in both subjective age and ageist attitudes are important determinants for older adults' well-being. The fourth presentation (Shrira), a clinical study, focuses on how orthopedic patients' rehabilitation is affected by their own and their healthcare professionals' VoA. Together, the findings highlight the relevance of VoA to interpersonal functioning and health, and offer valuable information for both researchers and clinicians regarding the importance of this direction in future explorations of older adults' VoA and physical/psychological well-being.

### SUBJECTIVE VIEWS OF AGING AND SOCIAL RELATIONSHIPS AMONG OLDER ADULTS: THEORY OF MIND AS A MEDIATOR

Yoav Bergman[1], Amit Shrira[2], and Orel Swisa[3], *1. Ashkelon Academic College, Ashkelon, HaDarom, Israel, 2. Bar-Ilan University, Ramat Gan, HaMerkaz, Israel, 3. Bar-Ilan University, Ramat-Gan, HaMerkaz, Israel*

Research has demonstrated the importance of subjective views of aging (VoA) for older adults' well-being. However, little is known regarding how understanding others' emotional states may affect this association. Accordingly, we examined whether Theory of Mind (ToM), or the ability to comprehend others' emotions, may mediate the effect of VoA on older adults' social functioning. Thirty community-dwelling older adults (mean age= 71.23, SD= 8.57, 50% women) filled out daily questionnaires concerning ageist attitudes, attitudes toward aging, and satisfaction with social relationships for two weeks. Positive VoA (i.e., low ageist attitudes and positive attitudes toward aging) were associated with increased ToM and higher satisfaction with social relationships. Moreover, ToM was a significant mediator for the VoA-social relationships link, as positive VoA on a given day were indicative of higher ToM levels, which were associated with increased positive social relationships. Results are discussed from the perspective of Socioemotional Selectivity Theory.

### REHABILITATION OUTCOMES AS A FUNCTION OF PATIENTS' AND HEALTHCARE PERSONNEL'S VIEWS OF AGING

Amit Shrira, *Bar-Ilan University, Ramat Gan, HaMerkaz, Israel*

The effect of subjective views of aging (VoA) on health outcomes is well established, yet this effect was rarely examined among older adults undergoing hospitalization or rehabilitation. Moreover, the additive effect of healthcare personnel's ageist attitudes on treatment outcomes is unknown. Accordingly, these effects were examined within older adults hospitalized for osteoporotic fractures – a frequent late-life condition with cardinal functional implications. Study 1 (N=147 patients, mean age=79.3) found that feeling younger at admission predicted better rehabilitation outcomes. The reverse effect of functional independence at admission on subjective age at discharge was non-significant. Study 2 (N=52 patients, mean age=81.8, and N=55 hospital staff members, mean age=32.3) replicated these findings with additional VoA indices, and further demonstrated that rehabilitation outcomes were better when occupational and physical therapists reported low levels of ageist attitudes. Findings suggest that successful rehabilitation may be promoted by reducing negative VoA among patients as well as healthcare personnel.

### SELF-PERCEPTIONS OF AGING, SUPPORT FROM CLOSE SOCIAL TIES, AND HEALTH GOAL PROGRESS IN EVERYDAY LIFE

Shannon Mejía[1], and Karen Hooker[2], *1. University of Illinois at Urbana-Champaign, Champaign, Illinois, United States, 2. Oregon State University, Corvallis, Oregon, United States*

Subjective aging is interpersonal, it embodies processes of thinking about the self in relationship to others. We utilize data from the 100-day microlongitudinal Personal Understanding of Life and Social Experiences Project (N = 99; observations = 7,049; Mage = 63, range = 52-88) to explore how self-perceptions of aging differentiate the processes by which older adults shape interactions with close social ties to support progress toward a meaningful health goal in everyday life. Those with more positive self-perceptions of aging identified more friends among their closest social ties and reported a higher levels of goal progress and support toward that goal during the study period. Further, multilevel random coefficient models showed that goal progress on a given day was more sensitive to received support on that day among those with more positive self-perceptions of aging. The implications for adult development and shared experiences of aging within friendship networks is discussed.

### DAILY EMOTIONAL AND PHYSICAL WELL-BEING PREDICTED BY PERSONAL AND OTHER VIEWS OF AGING

Shevaun Neupert, and Reyyan Can, *North Carolina State University, Raleigh, North Carolina, United States*

Individual differences in personal views of aging and how older adults perceive others contribute to health. We examined these relationships as they unfold within persons over time. A 14-day daily diary study of 428 participants aged 50-85 (M = 63.51) assessed subjective age (how old one feels), ageism, negative affect, and physical health each day. Increases in daily subjective age corresponded to increases in negative affect, but there was no effect of daily ageism. In contrast, increases in daily subjective age and daily ageism were each uniquely associated with increases in daily physical health problems. Further, these views of aging interacted; daily increases in ageism were especially detrimental to physical health when accompanied by increases in subjective age. These results suggest that daily fluctuations in personal views of aging are important for emotional and physical well-being, whereas personal views as well as perceptions of others work together for physical well-being.

### SESSION 3495 (SYMPOSIUM)

### HEALTHY AGING DURING THE PANDEMIC AND BEYOND: LESSONS FROM FIVE YEARS OF THE NATIONAL POLL ON HEALTHY AGING AND AARP

Chair: Erica Solway Co-Chair: Teresa Keenan Discussant: Brian Lindberg

The past five years have brought enormous changes in the lives of older adults and their families. During this time the University of Michigan National Poll on Healthy Aging (NPHA), co-sponsored by AARP and Michigan Medicine, has gathered the experiences and perspectives from nationally representative samples of US adults age 50-80 on a range of topics related to healthy aging. These polls have been used to elevate the voices of the older adults in the development of policies and practices to improve the health of people as they age. During the past two years, poll reports have captured how changes in lifestyle and behaviors during the COVID-19 pandemic have impacted the health and well-being of older adults. Members of the NPHA team from the University of Michigan and AARP will begin with an overview of the poll including its goals and methods. The session will then highlight key findings from the first five years of the NPHA that have important implications during and beyond the COVID-19 pandemic, with special attention to poll findings related to joys and stresses and aging in place. The presenters will also discuss how to access publicly available NPHA data. The session will wrap up with a discussion of how the perspectives and experiences of older adults gathered through the NPHA and AARP polling can be used to inform policy and advocacy to support healthy aging.

### LESSONS FROM FIVE YEARS OF POLLING THROUGH THE NATIONAL POLL ON HEALTHY AGING

Erica Solway[1], Teresa Keenan[2], Cheryl Lampkin[2], Matthias Kirch[1], Dianne Singer[1], Jeffrey Kullgren[1], and Preeti Malani[1], *1. University of Michigan, Ann Arbor, Michigan, United States, 2. AARP, Washington, District of Columbia, United States*

Since 2017, the National Poll on Healthy Aging (NPHA) has surveyed older adults on a vast array of topics related to health, health care and health policy. With robust nationally representative samples of 2,000 adults age 50-80, the poll's format allows for the fielding of timely questions with important public health and policy implications. For example, polls examining delayed care during the COVID-19 pandemic and experiences of physical deconditioning and falls helped to demonstrate the ways in which the pandemic may have long-lasting effects if strategies are not implemented to address them. In this presentation we will discuss how the NPHA has increased awareness of the facilitators and barriers to healthy aging and where gaps exist in supporting older adults to live with optimal health and well-being. This presentation will also describe how researchers can access publicly available NPHA data to inspire their own future research.

### REIMAGINING AGING: LESSONS ON HEALTHY AGING FROM AARP

Teresa Keenan, and Cheryl Lampkin, *AARP, Washington, District of Columbia, United States*

This presentation will include insights on healthy aging based on data obtained from multiple recent AARP surveys fielded among nationally representative samples of midlife and older adults. Samples were weighted to reflect population estimates (i.e., gender, ethnicity, etc.). Studies also included oversamples of Black/African American and Hispanic/Latino respondents when possible, enabling enhanced analyses. This multi-analysis presentation will provide valuable insights on healthy aging in general, the impact the COVID-19 pandemic has had on the older populations' ability to maintain healthy habits, and how our "new normal" suggests the need to reimagine healthy aging. This presentation will showcase exciting new findings from AARP Research on diet, exercise, resiliency, and mental health, among others. Augmented with qualitative data, this presentation will also allow participants to hear the voices of older adults on these issues. Additionally, new data collected in late fall 2022 will provide a preview of up-to-the-minute insights.

### OLDER ADULTS' PREPAREDNESS TO AGE IN PLACE: FINDINGS FROM THE NATIONAL POLL ON HEALTHY AGING

Sheria Robinson-Lane[1], Erica Solway[2], Dianne Singer[2], Matthias Kirch[2], Jeffrey Kullgren[2], and Preeti Malani[2], *1. University of Michigan School of Nursing, Ann Arbor, Michigan, United States, 2. University of Michigan, Ann Arbor, Michigan, United States*

Though aging in place is a stated goal for many older adults, complications of advanced disease or disability can derail these plans. Little is known about the current preparedness of older adults to age in place. The January 2022 National Poll on Healthy Aging surveyed adults age 50-80 (n=2,277) about their home and available supports. Findings indicate that while 80% of adults believe they have the necessary features in their home to support aging in place, less than one in five had homes that were wheelchair accessible and fewer than half had other common safety and accessibility features. Older adults were most confident they could obtain help with household chores (51%) and were least confident about getting assistance with personal care (33%). Persons with disabilities (n=683) and those living alone (n=639) reported the most concerns. Several opportunities exist to better prepare older adults to successfully age in place.

### JOY AND STRESS DURING THE COVID-19 PANDEMIC: FINDINGS FROM THE NATIONAL POLL ON HEALTHY AGING

Jessica Finlay, Lindsay Kobayashi, Erica Solway, Dianne Singer, Matthias Kirch, Jeffrey Kullgren, and Preeti Malani, *University of Michigan, Ann Arbor, Michigan, United States*

This poll aimed to better understand the complex impacts of the COVID-19 pandemic on the emotional and mental health of older adults. In August 2021, the National Poll on Healthy Aging surveyed a national sample of adults age 50–80 about joys, stresses, and resilience during the pandemic. Most reported feeling a lot (30%) or some (53%) joy these days, while 17% reported feeling very little or no joy. Reports of joy and stress differed substantially by age, sex, physical health, mental health, and household income. For example, people age 50–64 were more likely to report feeling a lot of stress compared with those age 65–80 (25% vs 13%), as were women compared with men (24% vs 15%). The findings may inform public health and policy efforts to support older adults at-risk for poor emotional and mental health, and help cultivate resilience during and after the pandemic.

### SESSION 3500 (PAPER)

### CAREGIVING AND FAMILY ISSUES

### COMPETENCY DEVELOPMENT FOR A PROGRAM TO SUPPORT CAREGIVERS OF OLDER ADULTS WITH DEMENTIA: A MODIFIED E-DELPHI METHOD

Madison Huggins, Gloria Puurveen, and Barb Pesut, *UBC, Kelowna, British Columbia, Canada*

Nav-CARE is a program that utilizes volunteer navigators to support older adults with life-limiting illnesses who are living in the community. Currently, Nav-CARE is being adapted and expanded to support family and/or friend caregivers of older adults with dementia. In order to begin the process of adapting this program a modified e-Delphi method was utilized. This method consisted of presenting three sequential questionnaires to an expert panel (n=50) of individuals with knowledge of and/or experience in caregiving, dementia, volunteerism and/or navigation. Consensus was established regarding the importance of various caregivers' needs and the competencies volunteer navigators require in order to meet these needs. This process resulted in a final list of 46 caregivers' needs and 41 volunteer navigator competencies. Two key findings suggest that there is a crucial need for increased access to respite, which influences the caregivers' ability to benefit from additional supports, and that dementia stigma is prominent, which negatively impacts the caregiving experience. Additional findings highlight the need for increased knowledge of dementia among providers, caregivers, and community members, the importance of establishing strong volunteer navigator-caregiver relationships, and the need to balance the agency of older adults living with dementia and their caregivers. Findings from this study informed the development of training curriculum for volunteer navigators, and will be used to guide the ongoing development of the adapted Nav-CARE program.

### DEPRESSIVE SYMPTOM TRAJECTORIES AND COGNITIVE FUNCTION AMONG OLDER COUPLES: A DYADIC PERSPECTIVE

Dexia Kong[1], Peiyi Lu[2], Jean Woo[3], and Mack Shelley[4], *1. The Chinese University of Hong Kong, Hong Kong, Hong Kong, 2. Columbia University, New York, New York, United States, 3. Faculty of Medicine, Hong Kong, Hong Kong, 4. Iowa State University, Ames, Iowa, United States*

Despite the well-documented health interdependence in the spousal context, empirical evidence on how psychological wellbeing of one's partner might affect one's cognitive function remains limited. Using dyadic data, the objective of this study is to examine trajectories of depressive symptoms and associated cognitive function outcomes among U.S. older married couples. Longitudinal Health and Retirement Study data (2004-2016) were used (N=6,289 heterosexual couples). Latent class growth analysis characterized depressive symptom trajectories for wives and husbands, separately. Structural equation models examined the actor and partner effects of depressive symptom trajectories on cognitive function in 2016 after adjusting for covariates. Four distinct depressive symptom trajectories were identified, including persistently low (wives: 73.61%; husbands: 79.59%), increasing (wives: 8.60%; husbands: 8.27%), decreasing (wives: 12.80%; husbands: 8.32%), and persistently high (wives: 4.99%; husbands: 3.81%). Compared to the low trajectory, increasing and high depressive symptom trajectories were associated with poorer cognitive function for wives and husbands (β_(wife,increasing,actor)=-0.92,95%CI=-1.30,-0.54; β_(wife,high,actor)=-0.71,95%CI=-1.19,-0.23; β_(husband,increasing,actor)=-0.81,95%CI=-1.16,-0.45; β_(husband,high,actor)=-1.20,95%CI=-1.78,-0.63). Notable gender discrepancies in partner effects were observed. Specifically, wife's depressive symptom trajectories were not associated with husband's cognitive function (P>0.05). However, husband's decreasing depressive symptom trajectory was linked to wife's better cognitive function. One's own depressive symptom trajectories predicts his/her own cognitive function. Specifically, older adults with increasing and persistently high depressive symptoms over time may experience poorer cognitive function, and thereby warrant additional policy and clinical attention. Psychosocial interventions targeting depressive symptoms among older men may be beneficial to their spouses' cognitive function. Future studies need to validate such gender differences.

### PERCEPTIONS OF DISCHARGE READINESS AND ENGAGEMENT IN DISCHARGE PLANNING FOR SPOUSAL VERSUS NONSPOUSAL CAREGIVERS

Catherine Elmore, Alycia Bristol, Lisa Barry, Eli Iacob, Erin Johnson, and Andrea Wallace, *University of Utah, Salt Lake City, Utah, United States*

Informal caregivers are frequently excluded during hospital discharge planning, potentially impacting their ability to effectively care for older adults at home. Few studies have examined experiences of spousal versus non-spousal caregivers during hospital discharge planning. In a secondary analysis of a mixed-method study, we quantitatively examined how spousal relationships impact caregivers' (n=266; 51.8% identified as a spouse or partner) scores of patient discharge readiness using the Readiness for Hospital Discharge Scale (RHDS-CG). We then conducted semi-structured interviews with a participant subset (n=23), and analyzed transcribed interviews using content analysis. First, comparing scores on the RHDS-CG, spouses/partners (88.4%) were more likely than non-spouses (75%) to report RHDS scores of 7+ corresponding with moderate to high readiness (X2 (1) = 8.070, p=.005). Among those interviewed, spouses/partners (65.2%) described their role as long-term, and shared strategies they had learned over time regarding how to seek involvement with healthcare professionals (HCPs). In contrast, non-spousal caregivers (34.8%) viewed their role as short term and struggled with how to communicate with HCPs, citing patient privacy rules and patient autonomy as perceived barriers. Overall, spousal caregivers had more experience with the healthcare system and felt better prepared to assume post-discharge care duties. Exploring the experiences of non-spousal caregivers, which make up more than one-third of our sample, is important since caregiving roles shift away from spouses to adult children and others as people age. Further consideration is necessary regarding how to support non-spousal caregivers in navigating the healthcare system.

### RESILIENCE IN FAMILY CAREGIVERS OF ASIAN OLDER ADULTS WITH DEMENTIA: AN INTEGRATIVE REVIEW.

Thitinan Duangjina[1], and Valerie Gruss[2], *1. university of illinois Chicago, Chicago, Illinois, United States, 2. College of Nursing, University of Illinois Chicago, Chicago, Illinois, United States*

Resilience is the ability to rebound from adversity and the amount of resilience varies depending on the sociocultural contexts. Little is known about resilience in Asian family caregivers of older adults with dementia, although Asian countries show the fastest growth of dementia. An integrative review focused on resilience in Asian family caregivers was guided by Whittemore and Knafl (2005). A systematic search of five databases (CINAHL, EMBASE, PsycINFO, MEDLINE, PubMed) was conducted and limited to English-language empirical studies published between 2016 and 2021. A constant comparison approach was used for the data analysis. A total of 15 studies conducted in nine Asian countries were selected for review. Results revealed Asian family caregivers were most commonly adult children, female, and providing home care. Resilience was impacted by multiple interrelated factors. Two influences emerged from this review including risk factors (burden, stigma, family stress, social stress) and protective factors (positive aspect of caregiving [PAC], religiosity/spirituality, social support). Filial piety, derived from religiosity/spirituality, played a role in both risk and protective factors. Asian caregivers with a deep-rooted belief in filial piety exhibited more depression than US caregivers (d=.21, p <.05), as Asian caregivers restricted their other activities and dedicated themselves to caregiving. Other Asian caregivers who balanced a belief of filial piety experienced a PAC and were resilient. Future interventional research should focus on minimizing risk factors (burden) and maximizing protective factors (PAC and religiosity/spirituality) to promote resilience among Asian family caregivers. A culturally aligned family-centered approach to care should be considered.

### TYPOLOGIES OF PARENT-CHILD RELATIONSHIPS AND ASSOCIATED HEALTH OUTCOMES: A CROSS-CULTURAL COMPARISON

Dexia Kong[1], Peiyi Lu[2], and Merril Silverstein[3], *1. The Chinese University of Hong Kong, Hong Kong, Hong Kong, 2. Columbia University, New York, New York, United States, 3. Syracuse University, Syracuse, New York, United States*

Background: This study investigates types of parent-child relationships and associated health outcomes among older adults of U.S. and China. Method: Cross-sectional data from Health and Retirement Study in the U.S. and Health and Retirement Longitudinal Study in China were used (N_(U.S.)=3918, N_China=4058). Relationship indicators included co-residence, living nearby, having weekly contacts, receiving assistance with daily activities, providing grandchild care, and having financial transfer from/to children. Latent class and regression analyses were conducted.

Results: Four classes were identified for older Americans, including (1) distant and uninvolved (6.58%); (2) geographically proximate with frequent contacts and downward support (47.04%); (3) co-resident with frequent contacts and upward support (13.1%); and (4) geographically proximate with frequent contacts (33.28%). By contrast, three classes were identified among older Chinese, including (1) co-resident with frequent contacts and upward support (37.46%); (2) coresident/interdependent (25.65%); (3) geographically proximate with frequent contacts and upward financial support (36.89%). For both countries, providing downward support was associated with fewer functional limitations and better cognitive function. Receiving instrumental support from children was associated with more depressive symptoms and functional limitations, and poorer cognitive function among Chinese older adults only.

Conclusions: Cultural contrasts were evident in parent-adult child relationship types and their associations with health outcomes. Overall, child-parent relationships in China tend to be more tight-knit than that of the U.S. Receiving financial support from children and co-residence are unique features of child-parent relationships in China. Cultural differences in child-parent relationships call for culturally-relevant strategies to address needs of older adults from various cultures.

### SESSION 3510 (PAPER)

### COGNITIVE ASSESSMENTS AND FUNCTION

### ADAPTATION OF FACE-TO-FACE COGNITIVE ASSESSMENTS FOR TELEPHONE ADMINISTRATION: A POTENTIAL THREAT TO VALIDITY

Jason Smith[1], Laura Gibbons[2], Dan Mungas[3], Maria Glymour[4], Laura Zahodne[5], Elizabeth Mayeda[6], Richard Jones[7], and Alden Gross[1], *1. Johns Hopkins Bloomberg School of Public Health, Baltimore, Maryland, United States, 2. University of Washington, Seattle, Seattle, Washington, United States, 3. University of California, Davis, Sacramento, California, United States, 4. University of California, San Francisco, San Francisco, California, United States, 5. University of Michigan, Ann Arbor, Michigan, United States, 6. University of California, Los Angeles Fielding School of Public Health, Los Angeles, California, United States, 7. Warren Alpert Medical School, Brown University, Providence, Rhode Island, United States*

Telephone-administered cognitive assessments are a cost-effective, feasible, and sometimes necessary alternative to in-person assessments. However, there is a scarcity of information in large cohort studies concerning mode effects, or differences in cognitive performance attributable to assessment method instead of underlying cognition, as a potential measurement threat. We evaluated mode effects on individual cognitive items and overall cognitive score using a population-based sample of community-living older adults aged 65-79 in the US in the 2014 Health and Retirement Study for whom interview mode was randomized (n=6825). We assessed mode differences in test means and reliability, whether mode modifies associations of cognition with criterion variables, and formal measurement invariance testing by mode. Relative to those assessed face-to-face, people assessed by telephone tended to have higher scores for memory and calculation items (0.06 to 0.013 standard deviations (SD)) and lower scores for non-memory items (-0.09 to

-0.01 SD). We also found evidence that estimated cognition was significantly differentially related to IADL score depending on mode of assessment, observing a stronger association among participants completing telephone interviews. Measurement invariance testing identified the largest mode differences in memory and attention items: immediate noun recall, delayed noun recall, and serial 7s scores were higher when administered by telephone. Differences by telephone vs face-to-face mode of administration are apparent in cognitive measurement in older adults, and most pronounced for tests of memory and attention that can be easier to answer via telephone. Future investigations are warranted to further evaluate methods to correct for such differences.

### AFFECTIVE TRAJECTORIES: RISK OF DEMENTIA AND UNDERLYING STRUCTURAL BRAIN VARIABLES IN OLDER WOMEN

Andrew Petkus[1], Xinhui Wang[2], Susan Resnick[3], Daniel Beavers[4], Mark Espeland[5], Joshua Millstein[2], Margaret Gatz[2], and Jiu-Chiuan Chen[2], *1. University of Southern California, LOS ANGELES, California, United States, 2. University of Southern California, Los Angeles, California, United States, 3. National Institute on Aging, Baltimore, Maryland, United States, 4. Wake Forest School of Medicine, Winston-Salem, North Carolina, United States, 5. Wake Forest School of Medicine, Winston Salem, North Carolina, United States*

Understanding how trajectories of positive and negative affect relate to dementia risk and underlying structural brain variables is important for dementia prevention. We examined associations between annually assessed Positive and Negative Affect Scale subscales and dementia risk (2000-18) among cognitively-intact community-dwelling women (N=948; aged 72.9±3.7) from the Women's Health Initiative Study of Cognitive Aging (years 2000-2010) and Magnetic Resonance Imaging Study (2005-2006). Joint latent class mixture models were constructed to identify latent classes of women with similar trajectories of affect and dementia risk over time. Multinomial and logistic regressions examined whether structural MRI measures predicted latent class membership (adjusted for sociodemographic, lifestyle, clinical characteristics, and intracranial volume). Two latent classes of positive affect (high stable:88% and decreasing:12%) and four classes of negative affect (Minimal stable:75%; high stable:4%; emerging:12%; moderate decreasing:9%) were identified. With the high stable trajectory as referent, women with decreasing positive affect were more likely to develop dementia (HR=4.33;p<.001). The odds of being classified as this high-dementia risk group were increased among women with more (per SD) global small vessel ischemic disease (SVID;OR=1.42;p<.001), deep white matter SVID (OR=1.93;p<.001), and smaller parahippocampal volumes (OR=1.41;p=.016). For negative affect, with minimal stable negative affect as referent, women with smaller hippocampal volumes were more likely to be classified as having moderate decreasing negative affect (OR=1.45;p=.024) while emerging negative affect was associated with higher dementia risk (HR=2.00;p=.014). These findings highlight the importance of changes in affect in later-life with dementia risk and potential underlying role of cerebrovascular disease and medial temporal lobe structures.

### COGNITIVE FUNCTION CONTRIBUTES TO THE NONLINEAR RELATIONSHIP BETWEEN AGE AND DUAL-TASK GAIT IN MID-AGED POPULATION

Junhong Zhou[1], Gabriele Cattaneo[2], Wanting Yu[3], Natalia Gouskova[3], Lewis Lipsitz[4], Alvaro Pascual-Leone[3], David Bartres-Faz[5], and Brad Manor[6], *1. Harvard Medical School/Hebrew SeniorLife, Roslindale, Massachusetts, United States, 2. Universitat Autònoma de Barcelona, Barcelona, Catalonia, Spain, 3. Hebrew SeniorLife, Roslindale, Massachusetts, United States, 4. Hebrew SeniorLife, Brookline, Massachusetts, United States, 5. Institut Universitari de Neurorehabilitació adscrit a la UAB, Barcelona, Catalonia, Spain, 6. Hebrew SeniorLife/Harvard Medical School, Roslindale, Massachusetts, United States*

The capacity to maintain safe walking is critical to functional independence in older adults. However, the timing/stage when such capacity starts to diminish, and its potential contributors have not been well characterized. To explore that, we here conducted analysis based upon the data of 651 participants of age between 40 and 65 years from Barcelona Brain Health Initiative Study. Each participants completed: 1) one 45-second trial of walking normally (single-task) and while performing a serial-subtraction-by-three task (dual-task), of which gait was measured using a smartphone-based gait-assessment application; and 2) a battery of cognitive tests. The dual-task cost (DTC) (i.e., percent changes from single- to dual-task condition) to mean stride time (ST) and stride time variability (STV) and the score of global cognitive function were obtained. The LOESS analyses demonstrated nonlinear relationships between age and DTCs with a turning point at age of 54 years (R2>3%). Regression models showed significantly greater associations (p=0.01~0.03) between age and DTCs (i.e., older age, worse gait) (β=0.22~0.28, p< 0.006), as well as between global cognitive function and DTCs (β=-0.28~-0.18, p< 0.002), in older group (i.e., age≥54 years) compared to younger group. The structural-equation-modeling suggested that in older group, cognitive function mediated the relationship between age and dual-task gait (p< 0.02) with a contribution of 43~47% to such relationship. The observations here revealed that as early at age of 54 years, dual-task gait starts to significantly diminish, and its dependence on cognitive function dramatically increases, providing critical knowledge for the management of mobility and cognitive aging in mid-age population.

### RACIAL/ETHNIC DIFFERENCES IN SUBJECTIVE COGNITIVE DECLINE AMONG US ADULTS AGED ≥45 YEARS, 2015–2020

Benjamin Olivari[1], Karen Wooten[1], Eva Jackson[2], Lisa McGuire[1], and Janet Croft[1], *1. CDC, Atlanta, Georgia, United States, 2. Alzheimer's Association, Chicago, Illinois, United States*

Recent studies report that older minorities have a higher prevalence of subjective cognitive decline (SCD), the self-reported experience of worsening or more frequent memory loss or confusion, than older non-Hispanic Whites. This study describes the prevalence of SCD by race/ethnicity and select demographic and social characteristics among U.S. adults aged ≥45 years and whether those reporting symptoms of SCD talked with a health care professional about them. Data collected from 215,406 respondents by the Behavioral Risk

Factor Surveillance System (BRFSS) in 2015-2020 were used in the analyses. Statistical comparisons were made using chi-square tests and p value of < 0.05. SCD was reported by nearly 10% of the study population. Asian/Pacific Islanders (5.0%) were least likely to report experiences of SCD and American Indian/Alaska Natives (16.7%) were most likely to report the experience compared to other racial/ethnic groups. Among adults with SCD, Asian/Pacific Islander (34.5%) and Hispanic (40.5%) adults were less likely to talk with a health care professional about their SCD symptoms compared to non-Hispanic White (48.5), non-Hispanic Black (49.5%), and American Indian/Alaska Native (50.5%) adults. Early detection of SCD symptoms can be important to identify early signs of dementia or other potentially treatable conditions and establish a care plan to help people remain as healthy and independent for as long as possible. Health care professionals, especially those working with groups with increased prevalence of SCD, could consider initiating discussions with adults as young as 45 years of age to identify early signs of dementia-like symptoms.

### THE FLYNN EFFECT IN SHORT-TERM COGNITIVE DECLINES OF AMERICANS AGED 65 YEARS AND OLDER: SMARTER AND MAYBE SLOWER

Yun Zhang[1], Joeseph Rodgers[2], Patrick O'Keefe[3], Graciela Muniz Terrera[4], Stacey Voll[5], Frank D. Mann[6], Scott Hofer[5], and Sean Clousten[1], *1. Stony Brook University, Stony Brook, New York, United States, 2. Vanderbilt University, Nashville, Tennessee, United States, 3. Oregon Health and Science University, Portland, Oregon, United States, 4. Ohio University, Athens, Ohio, United States, 5. University of Victoria, Victoria, British Columbia, Canada, 6. Stony Brook University, New York, New York, United States*

To contribute to our understanding of cohort differences and the Flynn Effect in cognitive declines, this study aims to: 1) describe and compare cognitive decline trends of two nationally representative American older cohorts; 2) investigate significant determinants of cognitive declines and the cohort differences. The analysis used data from the National Health and Aging Trends Study (NHATS, 2011-2019), including one nationally representative cohort of older Americans in 2011 and another from 2015. We used mixed-effect models adjusted for cohort, wave, baseline age, sex, education, race, familiarity, and follow-up years, as well as survey designs, to describe and compare the intercepts and slopes in cognitive functions of the two NHATS cohorts. We included Cohort 1 (N=7,325) respondents (2011-2015), and Cohort 2 (N=7,330) respondents (2015-2019). Compared to Cohort 1, Cohort 2 has a significantly higher intercept and a slower decline for episodic memory, and a significantly higher intercept but a significantly faster decline for global cognition, orientation function, and executive function. Consistently, older age, poorer educational attainment, and minority races/ethnicity are associated with worse cognitive performances. Our results provide a comprehensive image of cohort declines for Americans aged 65 years and older. Our findings are consistent with the Flynn Effect in that the general levels of cognition of later cohorts improved. Furthermore, we found support for the Flynn Effect in a short term. We also found significant effects of older age, poorer educational attainment, and minority race/ethnicity on cognitive function.

### SESSION 3520 (SYMPOSIUM)

### DELIVERING PERSON-CENTERED CARE IN TIMES OF CRISIS

Chair: Liza Behrens Discussant: Tonya Roberts

Preference-based person-centered care (PCC) is the gold standard for delivering high quality care in nursing homes (NH). A public health crisis makes it difficult for NH leaders to make decisions that honor residents' preferences for care and activities while balancing the safety of others in the NH community. Based on the Operational Framework to Guide Decision-making During the COVID-19 Pandemic, the purpose of this symposium is to discuss several projects that are growing the evidence-base for person-centered risk management during a public health crisis. Data presented can guide NHs in balancing safety and risk during a public health crisis. Our first presentation speaks to the adoption of preferences as an important measure of care quality prior to a crisis, the next two presentations speak to how risk perceptions of NH staff and residents can support PCC delivery during a crisis, and the final presentation provides a potential evidence-based quality improvement strategy for integrating PCC in the pandemic recovery phases. First Dr. Abbott will present on the impact of using the preferences for everyday living inventory as a quality and safety indicator in NHs, then Dr. Carpenter will present on direct-care staff members risk perceptions while during the COVID crisis, followed by Dr. Behrens who will discuss residents' risk perceptions around having their preferences honored during the COVID crisis, and finally Ms. Heilbrunn will discuss an ongoing clinical trial evaluating the impact of using the ECHO platform to improve infection control and care quality during the COVID crisis.

### "I'M NOT A RISK-TAKER": RISK PERCEPTIONS OF NURSING HOME RESIDENTS WITH DEMENTIA

Liza Behrens[1], Hannah Anderson[2], Kalei Kowalchik[1], Jacqueline Mogle[3], Kimberly Van Haitsma[1], and Marie Boltz[4], *1. Pennsylvania State University, University Park, Pennsylvania, United States, 2. Children's Hospital of Philadelphia, Philadelphia, Pennsylvania, United States, 3. The Pennsylvania State University, University Park, Pennsylvania, United States, 4. Penn State, Pennsylvania State University, Pennsylvania, United States*

Persons living with dementia (PLWD) in nursing homes (NH) are often left out of care conversations about their health and safety. These omissions impinge on their personhood and rights to have care preferences heard and honored. PLWD maintain the ability to communicate values and preferences long after their decision-making abilities are affected by cognitive changes. This study explored risk perceptions of PLWD associated with their care preferences. As part of a larger focused ethnography conducted during the COVID-19 pandemic, in-depth interviews explored risk perceptions of residents (N=7) with dementia (BIMS M=9.29). Using a risk propensity survey, residents self-identified as risk avoiders (M=3.2) and content analysis of interviews revealed that PLWD perceive physical and psychosocial harms (e.g., high blood sugar, falls, choking) and benefits (e.g., feeling good, social interactions, reminiscing) related to care preferences. Results suggest it is possible for PLWD with varying levels of cognitive decline to participate in discussions about their health and safety.

### THE IMPACT OF IMPLEMENTING THE PREFERENCES FOR EVERYDAY LIVING INVENTORY (PELI) ON SURVEY DEFICIENCY OUTCOMES

Katherine Abbott[1], John Bowblis[1], Jane Straker[2], and Kimberly Van Haitsma[3], *1. Miami University, Oxford, Ohio, United States, 2. Scripps Gerontology Center at Miami University, Oxford, Ohio, United States, 3. Pennsylvania State University, University Park, Pennsylvania, United States*

The Ohio Department of Medicaid added the PELI as one of five quality incentive points used to determine Medicaid per diem reimbursement rates with the goal of improving person-centered care among Ohio's nursing homes (NHs). The purpose of this study was to explore if the degree of PELI implementation had an impact on quality as defined by deficiencies and star rating. Multinomial logistic regression of 1,300 NH-year observations was used to understand the relationships between survey star ratings/deficiency scores on PELI implementation controlling for organizational characteristics of the NH. We find complete PELI implementation statistically significantly increased the probability of being a 4- or 5-star survey-rated facility. Complete PELI implementation is associated with small improvements each year that add up. Organizational change takes commitment and persistent effort over time to see changes in quality as reflected by star ratings and deficiencies. Recommendations for practice and policy will be discussed.

### PROJECT ECHO FOR NURSING HOMES: A PATIENT-CENTERED, RCT TO IMPLEMENT INFECTION CONTROL BEST PRACTICES IN NHS

Emily Heilbrunn[1], William Calo[2], Erica Francis[3], Lan Kong[2], Ellie Hogentogler[3], Abbey Fisher[3], Nancy Hood[4], and Jennifer Kraschnewski[5], *1. Project ECHO, Penn State University College of Medicine, Hershey, Pennsylvania, United States, 2. Penn State College of Medicine, Department of Public Health Sciences, Hershey, Pennsylvania, United States, 3. Penn State College of Medicine, Department of Medicine, Project ECHO, Hershey, Pennsylvania, United States, 4. University of New Mexico, Project ECHO, Albuquerque, New Mexico, United States, 5. Penn State College of Medicine, Department of Medicine, Hershey, Pennsylvania, United States*

Nursing homes (NHs) have been devastated by COVID-19. Only 3% of designated infection preventionists in NHs have taken a basic infection control course. Little is known about the implementation of effective infection control practices in NHs. This study utilizes Project ECHO (Extension for Community Health Outcomes), an evidence-based tele-mentoring model, to connect subject matter experts with NH staff to proactively support evidence-based infection control guideline implementation. This study will determine how guidelines can be implemented effectively in NHs, including reducing COVID-19 diagnoses and improving other key patient-centered outcomes (e.g., quality of life) NHs (N=136) were recruited and assigned to ECHO or ECHO Plus using a randomized design. A multipronged approach to improving infection control and emergency preparedness in NHs is important. The ECHO model has significant strengths allowing for remote learning delivered by a multi-disciplinary team and utilizes case discussions that match the context and capacity of NHs.

### NURSING HOME STAFF RISK PERCEPTIONS DURING THE COVID-19 PANDEMIC

Joan Carpenter[1], Nia Kooiman[2], Kalei Kowalchik[3], Jacqueline Mogle[4], Jasmine Newman[2], and Liza Behrens[3], *1. University of Maryland School of Nursing, Baltimore, Maryland, United States, 2. University of Maryland, Baltimore, Maryland, United States, 3. Pennsylvania State University, University Park, Pennsylvania, United States, 4. The Pennsylvania State University, University Park, Pennsylvania, United States*

Nursing home (NH) staffs' risk perceptions related to NH residents' health and safety are a barrier to honoring residents' preferences for care and activities; this was exacerbated during the COVID-19 pandemic. This study intended to describe NH staffs' self-perceived risk perceptions during the COVID-19 crisis as measured by the validated Risk Propensity Scale. Participants (N=27) included licensed and unlicensed nursing staff (e.g., RN, LPN, CNA), social workers, and activities directors, were mostly female (85%), White (74%), non-Hispanic (93%), and had more than three years of experience working in NHs (78%). Survey results indicated that males considered themselves risk seekers (M=7.5 out of 9) and females risk avoiders (M=4.0). Despite identifying as a risk seeker or risk avoider, the majority of participants agreed with "safety first", preferred to avoid risk, and viewed risks as "a challenge." Findings will be discussed considering implications for person-centered risk management during a public health crisis.

### SESSION 3530 (SYMPOSIUM)

### DEMENTIA IN INCARCERATED SETTINGS: MAKING A DIFFERENCE IN OHIO'S PRISON SYSTEM

Chair: Margaret Sanders

The number of individuals living in incarcerated settings with dementia will double by 2030 and triple by 2050. Older adults are the fastest growing demographic in incarcerated settings and age is the number one risk factor for dementia. The HRSA-sponsored Geriatric Workforce Enhancement Program, Ohio Council for Cognitive Health, Benjamin Rose Institute on Aging and the Ohio Department of Rehabilitation and Correction (ODRC) worked together to develop Dementia Friends for Incarcerated Settings to impact the lives of those living with dementia within the correctional setting. This pilot furthered the development of this Dementia Friends programming and measured ODRC staff knowledge/attitudes about dementia, as well as uncovered the experiences and needs of staff within the ODRC. Online Dementia Friends sessions were conducted in all 27 Ohio prisons with voluntary, anonymous pre- and post-session surveys based on the Brief Tool for Dementia-Friendly Education and Training Sessions developed to measure the impact of dementia-friendly education efforts on knowledge, attitudes/perceptions, and interacting with people living with dementia and their caregivers. The results of this pilot underscored the need, usefulness and applicability of dementia education throughout the ODRC system, indicating teaching effective communication techniques within a correctional facility are helpful for those working within such settings. The results indicate benefits on both a personal and professional level. These sessions provide a starting point for potential future educational sessions to serve all members of the correctional sector. Future proposed projects will focus on people living in incarcerated settings and those transitioning back into the community.

### DEVELOPMENT OF DEMENTIA FRIENDS FOR INCARCERATED SETTINGS CURRICULUM

Bonnie Burman[1], and Elizabeth Kinzig[2], *1. Ohio Council for Cognitive Health, New Albnay, Ohio, United States, 2. Ohio Council for Cognitive Health, New Albany, Ohio, United States*

This presentation describes the working relationships and steps that were taken to develop Dementia Friends for Incarcerated Settings. This included identifying population needs, approval processes, curriculum development specific to this population, learner coordination, implementation, measurement and reporting. Dementia Friends, a one-hour session to help everyone in a community understand five key messages about dementia, was used as the framework for an educational program targeting staff at correctional facilities. Dementia Friends is a standardized program that can be modified depending on the target audience or sector. Our sector-specific program was piloted in all 27 Ohio prisons to ensure that all staff are knowledgeable about ways to make a difference in the lives of those living with dementia.

### THE DEVELOPMENT AND IMPLEMENTATION OF PILOT PROGRAM EVALUATION MEASURES

Denise Kropp, *Northeast Ohio Medical University, Rootstown, Ohio, United States*

This presentation describes measurement tools and techniques for the pilot. Anonymous pre- and post-surveys were based on the Brief Tool for Dementia-Friendly Education and Training Sessions, modified for the ODRC population. The Brief Tool is a 7-item scale measuring the impact of dementia education sessions on knowledge, attitudes/perceptions, and interacting with people living with dementia. Based on ODRC feedback, we added an item on recognizing dementia. The pre-session survey asked about experiences with dementia prior to the session. The post-session survey included items on satisfaction, including the usefulness of the session in their work within the ODRC, whether they learned new information, and whether they would recommend the training. Open-ended items asked about dementia-related topics participants wanted to learn more about. All four online pilot sessions were led by the Ohio Council of Cognitive Health. Recordings of the sessions were made available for people unable to attend a scheduled session.

### PILOT PROGRAM EVALUATION RESULTS OF DEMENTIA FRIENDS FOR INCARCERATED SETTINGS

Jessica Bibbo, and Sarah Nicolay, *Benjamin Rose Institute on Aging, Cleveland, Ohio, United States*

This presentation will present the findings from the pilot sessions. 266 people completed both pre- and post-session surveys (Mage=46.10, SDage=11.47; Female=62.03%; Full time=97.36%). Sixty-five percent knew a person living with dementia (PWD) and 28.57% said they worked with PWD. Four out of the five attitude items (e.g., I am confident interacting with PWD; I am able to recognize behaviors that may be due to dementia) significantly improved ($ps<0.001$). There was no change in knowledge items ($ps \geq 0.18$); Ninety-five percent agreed they would use the information in their work and 95.82% would recommend the session to the people they worked with. Topics participants wanted to learn more about focused on communication with and activities for PWD in an incarcerated setting, learning more about dementia, adjusting the physical environment for PWD, and ensuring proper diet. Further, participants felt the session should be required for all ODRC staff.

### IMMEDIATE AND FUTURE IMPLICATIONS

Martha Williman, *Ohio Council for Cognitive Health, Findlay, Ohio, United States*

ODRC leadership was engaged in developing and promoting this first-of-its-kind initiative. Success was enhanced by making the training mandatory and providing CEs for employees in ODRC's medical, behavioral health, mental health, dental and dietary departments. Our goals are to expand to all the ODRC's over 12,000 employees and to increase dementia education in correctional settings across the U.S. In addition, we propose further developing the program to include people living in incarcerated settings and those being released into the community. With 95% of staff agreeing they would be able to use what they learned in their workplace, and over 63% of attendees stating they know someone who lives/lived with dementia, this training has implications for not only professional development, but personal support as well and provides a starting point for future education sessions to serve both staff and those living in incarcerated settings within and beyond Ohio.

### SESSION 3540 (PAPER)

### A MOBILE APPLICATION FOR MUSIC EXPOSURE MEASUREMENT IN DEMENTIA RESEARCH: A PROOF-OF-CONCEPT STUDY

### INNOVATIONS IN TECHNOLOGY FOR SOCIAL ENGAGEMENT AMONG OLDER ADULTS AND TO IMPROVE CAREGIVER COMMUNICATION AND TRAINING

Hongdao Meng[1], Sean Barbeau[1], Mingyang Li[1], Cassidy Doyle[1], Kelly Wiechart[2], Robert Hammond[1], and William Kearns[1], *1. University of South Florida, Tampa, Florida, United States, 2. KellyW Consulting, Tampa, Florida, United States*

Music-based interventions (MBIs) have been shown to benefit persons with dementia. To develop new MBIs and build the evidence base for motor, cognitive, emotional, and behavioral outcomes, accurate measures of music exposure (e.g., content, dose, and duration) are critically important. However, most commercial music services require internet access and/or subscription fees without providing convenient ways for researchers to access music exposure data. Our research team developed the open-source MUsic to Support Engagement and Resilience (MUSER) Android application with an associated data processing engine. Caregivers can use MUSER to deliver individualized music selections via smartphones/tablets, with music exposure data being generated for research use. We used the Kotlin programming language for application development and Java/Python/Microsoft BI for data processing and analytics instrumentation. The MUSER application was tested for music exposure measurement feasibility with 16 users (ten research team members and six family caregivers of persons with dementia) for two weeks between July and November of 2021. Semi-structured interviews were conducted pre-post application testing to gather qualitative and quantitative data to enrich the understanding of user experiences. Family caregivers had a mean age of 57.8 (SD=26.2; range 20-77). The average number of listening days was 6 (SD=2.5), and the average music listening duration was 203 minutes (SD=123.2). Caregivers played an average of 52 (SD=36.9) songs from 10 (SD=4.2) albums involving 34 (SD=36.3) artists. Given the ease of deployment and integrated data collection and reporting capabilities, this application supports collaborative software development among researchers to advance MBI research.

### INTEGRATING HEALTH AND HOME CARE THROUGH TECHNOLOGY: ACTIONABLE SOLUTIONS FOR PROGRESS

Lauren Dunning, Caroline Servat, and Nora Super, *Milken Institute Center for the Future of Aging, Santa Monica, California, United States*

Shifts toward virtual care driven by the pandemic have continued to blur the previously sharp lines between health care and home care. A new care ecosystem is emerging, bringing with it the opportunity to integrate health and home care through technology. Over the course of the pandemic, Medicare telehealth visits increased 63-fold between 2019 and 2020, innovative programs enabled acute hospital care at home using digital tools, and people in need of care could reach their providers through a broad range of devices and modalities. But many of the policy changes underlying these advancements and others were temporary, and permanent actions are needed to sustain momentum. To characterize the emerging landscape, the Milken Institute conducted informational interviews with over 40 experts representing health, technology, government and policy, research and academia, philanthropy, advocacy, and community-based organizations. From these interviews, themes emerged on barriers, innovations, and opportunities. Findings were further refined through a roundtable workshop and survey to develop guiding principles and consensus-built recommendations. Three sets of recommendations were then developed on: 1) pandemic-related and larger-scale policy and program design to support tech-enabled care, 2) practices and policies to develop a systems approach that integrates health and home care and bolsters equity, and 3) collaboration and coordination to accelerate efforts. This paper focuses on the interview themes and progress made to advance solutions in partnership with stakeholders.

### PREDICTORS OF PARTICIPATION IN A TELEPHONE-BASED SOCIAL CONNECTEDNESS INTERVENTION FOR OLDER ADULTS

Omolola Adepoju[1], Ben King[1], Jiangtao Luo[2], LeChauncy Woodard[1], Jessica Dobbins[3], Jason Pierett[3], and William Glasheen[3], *1. University of Houston College of Medicine, Houston, Texas, United States, 2. University of Houston, Houston, Texas, United States, 3. Humana, Louisville, Kentucky, United States*

Background: Social isolation is a well-documented contributor to poor mental and physical health, and interventions promoting social connectedness have been shown to be protective. This study examined predictors of participating in a telephone-based social connectedness intervention for socially isolated older adults.

Methods: Data was obtained from a social connectedness intervention that paired students with older adults. Eligible participants included Houston area older adults, 65 years or older, enrolled in Medicare Advantage plans. Eligible participants were contacted telephonically and asked to complete the 3-item UCLA Loneliness Scale. Those who screened positive for loneliness were invited to participate in the social connectedness intervention. Logistic regression models, that accounted for sociodemographic, clinical and functional indices, were used to identify predictors of participation.

Results: Females (OR: 1.23, 95% CI: 1.04-1.45), and racial/ethnic minorities (African American OR: 1.39, 95%CI: 1.16-1.68; Hispanic OR: 1.43, 95%CI: 1.04-1.99) were more likely to participate in a telephone-based social connectedness intervention. Older adults with a disability (OR: 2.37, 95%CI: 1.96-2.87), higher CMS Hierarchical Condition Category risk scores (OR: 1.15, 95%CI: 1.06-1.25) and those who reported having 1+ social needs (OR: 1.38, 95%CI: 1.14-1.66) were more likely to participate. Of all Charlson Comorbidity Index flags examined, diabetes was the single strongest predictor of participation (OR=2.49, p=0.02). Older adults who reported functional comorbidities for anxiety and depression were also more likely to participate.

Conclusion: Telephone-based social connectedness interventions can reach vulnerable older adults with clinical and social needs, and can be useful in addressing racial/ethnic health equity gaps in socially isolated older adults.

### PUTTING THE 4MS INTO PRACTICE: IMPLICATIONS FOR TRAINING AND TECHNOLOGY

Jennifer Crittenden[1], Rachel Coleman[2], Jennifer Jain[2], David Wihry[3], Lenard Kaye[2], Susan Wehry[4], and Judith Metcalf[4], *1. UMaine Center on Aging, Bangor, Maine, United States, 2. University of Maine, Bangor, Maine, United States, 3. University of Maine, Orono, Maine, United States, 4. University of New England, Biddeford, Maine, United States*

The AgingME Geriatrics Workforce Enhancement Program (AgingME GWEP), a statewide collaboration led by the University of New England and the University of Maine, is guided by an annual statewide survey of community and professional stakeholders. The aim of this research is to identify training and resource gaps related to age-friendly healthcare and topics of interest. Of the 245 survey respondents, 15% indicated existing knowledge of the 4Ms framework. The top sources of 4M's framework exposure included trainings or webinars (30%) and web-based resources (19%). Of those with knowledge of the 4M's, 33% of providers and 29% of older adults/community members reported employing the 4Ms in their professional practices and personal lives, respectively. Respondents also noted the need for more training on how to use technology to locate healthcare information (33%), using technology to reduce isolation and loneliness among older adults (29%), and keeping providers connected with older patients (26%).

Additional write-in responses (11%) suggest a need for general technology training and improving access to technology overall. Respondents' (N = 157) top five categories for needed aging-related training topics were community resources for older adults (15%), aging-in-place (14%), exercise and nutrition (11%), improving provider/patient communication (9%), and 4M's of age-friendly healthcare overview (8%). Responses also identified themes related to improving patient/provider communications, availability of resources, and ageism that could be addressed through upcoming GWEP activities. Results indicate a need to facilitate the translation of 4Ms knowledge into practice and increasing technology training and access.

### SUPPORTING RESIDENT ENGAGEMENT VIA WEB-BASED ASSESSMENT: EXPERIENCE OF ACTIVITY PROFESSIONALS IN LONG-TERM CARE

xiaoli li[1], and Cheng Yin[2], *1. University of North Texas, Frisco, Texas, United States, 2. University of North Texas, Corinth, Texas, United States*

Background: Resident engagement and activities have positive relationships with emotional wellbeing and physical health. However, regularly offered activities may not be suitable for all individuals and may be unsustainable when there are changes in residents' health. Loneliness, depression, and social isolation are observed among residents in long-term care facilities. A web-based assessment system, "Elder Engagement Performance Improvement (EEPI)," has the potential to support resident engagement. Activity professionals are crucial in utilizing EEPI and engaging residents in meaningful activities, especially regarding their role in helping enhance resident engagement and wellbeing. This study aims explores activity professionals' experiences in implementing the EEPI assessment. Method: Semi-structured interviews with 23 long-term care activity professionals (e.g., certified activities professionals, therapeutic recreation specialists, and activity assistants) were conducted and analyzed using qualitative content analysis.

Results: Activity professionals` experiences showed the EEPI was a means in improving residents' engagement quality and making engagement a more targeted outcome. Four main themes emerged: (a) Lack of assessment tools, (b) Acceptance of a web-based assessment system, (c) Barriers to using the EEPI (time arrangements, staffing issues, and being on the same page), and (d) Areas for job improvement (person-center care and measurable practices). Conclusions: The EEPI may serve as a valuable tool in long-term care facilities. The web-based assessment system facilitated activity professionals' daily work and involved the residents' input in their care, which may enhance resident engagement and reduce the risk of social isolation in long-term care facilities.

### SESSION 3550 (SYMPOSIUM)

### INTEGRATING DIVERSE CANCER PERSPECTIVES: FROM DEVELOPMENT TO IMPLEMENTATION

Chair: Sean Halpin

Cancer among older adults is pervasive, putting excessive strain at the individual, caregivers, and wider society levels. In our symposium, we bring together researchers from varied disciplines—with a focus on easing the strain of cancer on older adults by identifying important gaps in care and developing and implementing innovative methods for improving health services. First, Carrion will discuss the multifactorial experience of fears and beliefs about cancer and cancer prevention in 57 older Latino men. Krok-Schoen will discuss the longitudinal association between religiosity and cognitive functioning among older adults with hematological cancers. Next, Seaman will discuss engaging hard-to-reach patients and those who underutilize the health system. Blackberry will then describe an implementation and impact framework of a five-year research program to improve care in older people with cancer. Lastly, Halpin will discuss the 36-month implementation of a video-based patient education program for patients with multiple myeloma who are preparing for autologous stem cell transplant. Understanding the development and implementation of programs aimed at improving health services among older adults with cancer will help improve understanding potential methods for identifying and addressing health services challenges in these populations.

### IMPLEMENTATION OF AN EDUCATION INTERVENTION FOR MULTIPLE MYELOMA PATIENTS RECEIVING STEM CELL TRANSPLANT

Sean Halpin[1], and Michael Konomos[2], *1. Evidera, Decatur, Georgia, United States, 2. Emory University, Atlanta, Georgia, United States*

Older adults have an increased risk for multiple myeloma (MM), yet they are often unable to collect sufficient healthy stem cells for autologous stem cell transplant (ASCT). We sought to assess the implementation of educational intervention videos, termed Ready for Transplant (R4T), for MM patients both during the 18-month study and at 12 and 18-months post-study. Guided by the Reach, Effectiveness, Adoption, Implementation and Maintenance (RE-AIM) framework, we observed 152 clinical encounters for 70 patients who received the intervention. Patients who reported viewing the R4T videos (56%) tended to be older and male and were more likely to admit for transplant than those who did not report viewing the videos (risk ratio, 2.3; 95% CI, 0.61, 3.88). At 12- and 18-months-post, nurses reported still using the R4T videos and found them particularly useful during times when patients were unable to attend the hospital for in-person visits due to the pandemic.

### IMPLEMENTING AND MEASURING IMPACT OF A FIVE-YEAR TRANSLATIONAL GERIATRIC ONCOLOGY RESEARCH PROGRAM

Irene Blackberry[1], Christopher Steer[2], Tshepo Rasekaba[1], Stacey Rich[3], Nicole Webb[2], and Kim Young[1], *1. La Trobe University, Wodonga, Victoria, Australia, 2. Border Medical Oncology and Haematology, Albury, New South Wales, Australia, 3. La Trobe University, John Richards Centre, Wodonga, Victoria, Australia*

The Geriatric Oncology Regional Victorian Trials Alliance, Linkages, Special populations, Equity program (GO ReViTALISE) is part of a five-year state-wide grant to increase access to cancer clinical trials in regional Victoria, Australia. The initiative covers engagement with key stakeholders and trial sites, co-design of research programs with consumers, implementation of multiple interventional and non-interventional research, and capacity building.

Employing a framework prospectively is critical to guide and scaffold implementation of complex interventions and capture the process of how several activities interact. Likewise, there is a need to show research impact beyond successful implementation and clinical outcomes. Using the GO ReViTALISE as a case study, we will demonstrate how an implementation framework and the Framework to Assess the Impact from Translational health research can deliver a cohesive, impactful and measurable program of research to increase older adults' participation in cancer clinical trials, trials availability and build capacity in regional cancer sites.

### NOT HAVING THE FAMILY, INSURANCE, AND MONEY AND EXPERIENCING LOTS OF PAIN: FEARS ABOUT CANCER AMONG OLDER LATINO MEN

Iraida Carrion[1], and Malinee Neelamegam[2], *1. University of South Florida, Tampa, Florida, United States, 2. University of North Texas Health Science Center, Fort Worth, Texas, United States*

Cancer is the second leading cause of death among Latino men. A total of 44.1% of Latino males 60-years or older have a lifetime probability of developing invasive cancer with lower survival rates, even when accounting for age and stage distribution. Given the population growth is essential to examine older Latinos' understanding of a cancer diagnosis. The study consisted of 200 surveys that included a qualitative component consisting of open-ended questions in Spanish. We separately analyzed the responses of the 57 immigrant Latino men to understand their perspective regarding cancer, prevention, diagnosis, and treatment. The median age is 68 years and the length of stay in the US is 27 years. Utilizing a grounded theory approach and thematic analysis, codes were developed based on five priori interview themes: leaving people behind, worry and stress about payment, emotions of sadness and fear of uncertainty, struggle with acceptance, and faith as comfort.

### THE ROOM WHERE IT HAPPENS: ENGAGING EQUITABLY TO OPTIMIZE CANCER SURVIVORSHIP CARE

AARON SEAMAN, and Nitin Pagedar, *University of Iowa, Iowa City, Iowa, United States*

Cancer survivorship care programs, services, and research would benefit from including people living with a cancer history who have ceased their survivorship care in their engagement efforts. Our prior research with head and neck cancer survivors has shown that a considerable number of patients discontinue their post-treatment survivorship care despite still having care needs. Those patients who continue their care are also the ones most often engaged in research and community partnership; as the ones who are "in the room where it happens," they consequently have the most influential voices in shaping survivorship care. In this paper, we underscore the necessity of including those who discontinue survivorship care in research and community engagement efforts. We use our work with head and neck cancer survivors as a model for strategizing ways to inclusively and respectfully do so.

### THE ASSOCIATION BETWEEN RELIGIOSITY AND COGNITIVE FUNCTIONING AMONG OLDER ADULTS WITH HEMATOLOGICAL CANCERS

Jessica Krok-Schoen[1], Ashley Rosko[2], Ying Huang[3], Allesia Funderburg[2], Katy Dvorak[2], and Erin Stevens[2], *1. The Ohio State University, Columbus, Ohio, United States, 2. The Ohio State University Comprehensive Cancer Center, Columbus, Ohio, United States, 3. The Ohio State University, Division of Hematology, Columbus, Ohio, United States*

Religiosity is positively associated physical health outcomes, yet longitudinal research on this association among older adults with cancer is limited. Participants were enrolled in a longitudinal study assessing chemotherapy's impact on the health of older adults with hematological cancers. Religiosity was assessed by the Duke University Religion Index (DUREL), with higher scores indicating higher religiosity. Cognitive functioning was assessed by the Blessed Dementia Scale, with higher scores indicating more impairment. Participants (n=97) had a median age of 70 years (range:60-88) and were 58% male and 97% white. DUREL scores were unchanged with a mean score of 22 (range:5-27). Cognitive functioning significantly improved with a median score of 4 (range:0-22) at baseline to 0 (range:0-20) at end-of-treatment. Religiosity was not significantly correlated with cognitive functioning over time. Future research should explore the trajectory of cognitive functioning and other sources of coping (social support, palliative/supportive care) among older adults with hematological cancers.

### SESSION 3555 (SYMPOSIUM)

### DYING WELL WITH DEMENTIA: SUPPORT NEEDS AND DIVERSITY ACROSS CULTURES AND SETTINGS

Chair: Jenny van der Steen

Dementia is increasingly recognized as a terminal illness but the end of life may comprise an extended period with death difficult to predict. Progressive cognitive impairment negatively affects autonomy. Family caregivers of people with dementia often fulfil important roles as spokespersons, informants, partners in care and proxy decision-makers. However, across cultures and individuals, values around autonomy, being in control, and family involvement are diverse. Therefore, perspectives on end of life with dementia and what is good care during the last phase of life may vary as well. Understanding and measuring good end-of-life care in dementia can help improve person and family centered caregiving at the end of life. This symposium will give insights into what is good end-of-life care in dementia and will show cultural and contextual differences drawing on studies in various countries. The contributions are based on diverse innovative and classical qualitative and quantitative methods including those engaging patient and public. Further work addresses how to support good end-of-life care during the COVID-19 pandemic, given the limitations of family restrictions and the need to provide care virtually. We present research on decision aids, care planning tools and care programs with a focus on ensuring that the perspectives of the person with dementia and family are at the forefront, and address individual differences in perceptions of a good end of life with dementia.

### A GOOD END OF LIFE WITH DEMENTIA ACROSS CULTURES

Jenny van der Steen, *Leiden University Medical Center, Leiden, Zuid-Holland, Netherlands*

Conceptualizations of a good end of life with dementia vary across cultures and understanding differences and similarities can help develop care planning tools and help understand validity of measuring it in the context of cross-cultural comparisons. We have analysed (2019-2020) 14 cross-cultural studies covering qualitative data of 121 people with dementia and 292 family caregivers. Nine cross-culturally common themes emerged, ranging from basic care needs addressed to satisfaction with life and spiritual well-being. Good relationships were essential in all themes, but an emphasis on "care for caregivers" differed the most across cultures. Based on this work, we expanded an existing dementia care goals model by refining palliative care goals beyond comfort and functioning, and in an ongoing Delphi study (2021-2022) it was evaluated by experts of over 30 countries. This session will report on these studies, focusing on relevant cross-cultural differences, and connecting it to available measures of comfort and end-of-life care.

### SUPPORTING RESIDENTS WITH DEMENTIA LIVING AND DYING IN LONG-TERM CARE AND THEIR FAMILIES DURING COVID-19

Sharon Kaasalainen[1], Abigail Wickson-Griffiths[2], and Rose McCloskey[3], *1. McMaster University, Hamilton, Ontario, Canada, 2. University Of Regina, Regina, Saskatchewan, Canada, 3. University of New Brunswick, Fredericton, New Brunswick, Canada*

TThis presentation will share research findings about experiences during COVID-19 about implementing a virtual palliative toolkit in long-term care in Canada. The toolkit includes tools and practices to: (a) engage residents and families with dementia within a palliative approach to care, (b) develop workforce capacity through online education modules, (c) reduce stress and improve psychological health of residents, families, and staff, and (d) develop organizational structures and processes to promote a palliative approach to care. Individual interviews were conducted with residents, family members, and staff before implementing a palliative toolkit and after using it. Findings highlighted the negative impacts of COVID-19 on resident health due to isolation within home, preventing family from being at the bedside and cancelling stimulatory activities especially at end of life that were exacerbated by the lack of resources and government supports. Families appreciated the virtual supports and stated that they helped prepare them for their loved ones' death while feeling more empowered, engaged, and supported in their journey. Although feedback from families was mostly positive, stating the virtual toolkit improved accessibility to information and supports, it was clear that some misunderstood terms, particularly what a palliative approach to care means; and others had challenges navigating the virtual platform to use the toolkit. Future work is needed to make the virtual tools more user-friendly so that they can be scaled up more widely.

### DEATH AND DYING IN GREEN CARE FARMS

Judith Meijers[1], Kirsten Smit[2], Bram de Boer[1], Hilde Verbeek[1], and Sascha Bolt[3], *1. Maastricht University, Maastricht, Limburg, Netherlands, 2. University Utrecht, Utrecht, Utrecht, Netherlands, 3. Tilburg University, Tilburg, Noord-Brabant, Netherlands*

Green care farms form an alternative to traditional nursing homes for people with dementia and combine agriculture production with health-related, social and educational services. Twenty-four-hour care green care farms offer end-of-life care. The aim of the study was to explore the experiences of healthcare workers and family caregivers with end-of-life care for people with dementia who died on a green care farm in the Netherlands. Semi-structured, in-depth interviews were conducted to explore their experiences with end-of-life care, including topics such as advance care planning, bereavement support and the influence of COVID-19. The study showed that most experiences were characterized by personal attention for the resident and family caregivers, and tailored holistic care. The duration of the dying phase was typically short, and most residents remained active until their final days. Despite the COVID-19 measures, healthcare workers and family caregivers still experienced adequate end-of-life care.

### SUPPORTING DECISIONS ABOUT END-OF-LIFE CARE IN DEMENTIA: FEASIBILITY OF A DECISION AID

Nathan Davies, Narin Aker, Victoria Vickerstaff, Elizabeth Sampson, and Greta Rait, *UCL, London, England, United Kingdom*

Decisions about end-of-life care are often left to family caregivers to make with professionals. Caregivers find these decisions difficult. A decision aid is one option to support family caregivers. We aimed to test the acceptability and feasibility of a co-produced decision aid for family carers of people with severe dementia or those towards the end of life in the UK. We aimed to recruit 30 family caregivers for a 6 month pre-post test feasibility study. Primary outcome was the feasibility of the study. We included quantitative measures at baseline, 3 month and 6 months, including: Decisional Conflict scale (DCS) and Kessler Psychological Distress Scale (K10). We recruited 28 carers (93% target), 26 completed baseline and 20 completed 6 month follow up. All outcomes changed indicating improvement at 6 months. Qualitative interviews reported the decision aid was acceptable. We met our success criteria, the study is feasible and the decision aid acceptable to caregivers.

### SESSION 3560 (SYMPOSIUM)

### INTERSECTIONALITY OF SENSORY LOSS AND RACE: IMPLICATIONS FOR BRAIN AND MENTAL HEALTH IN OLDER ADULTS

Chair: Jennifer Deal Co-Chair: Willa Brenowitz Discussant: Roland J. Thorpe, Jr.

Despite recent evidence that sensory loss may increase risk of dementia, mood disorders, and poor physical health in older adults, nearly all research to-date has been conducted in populations that are predominantly White and from high-income countries. This session will investigate the intersectionality of race with sensory loss with a focus on health equity and inclusion of traditionally underrepresented populations. It will evaluate sensory loss across multiple senses, how patterns vary by race, and whether socioeconomic status can explain these differences. In the US, prevalence of impairment in vision, touch, and smell is lower in Whites compared to Black or Hispanics. Interestingly, hearing loss prevalence is lower in Blacks than Whites, but dementia incidence is higher. This session will describe incident dementia risk associated with hearing loss in Black compared to White Americans. It will also address the relationship between dual (hearing and vision) loss and depressive symptoms in India to explore the generalizability of findings to lower middle-income countries. Sensory impairments may intersect with racial discrimination to exacerbate social and health disparities. This session will also describe racial disparities in food insecurity among Americans with vision loss. Finally, we will present results of a randomized controlled trial of an innovative, community-delivered approach to affordable, accessible hearing care. The study represents the largest U.S.-based trial cohort to date of low-income older adults and older African American adults with hearing loss.

### PREVALENCE OF SENSORY IMPAIRMENTS VARY BY RACE IN OLDER US ADULTS

Faaizah Arshad[1], Jayant Pinto[2], Kristine Yaffe[1], and Willa Brenowitz[1], *1. University of California, San Francisco, Los Angeles, California, United States, 2. University of Chicago, Chicago, Illinois, United States*

Few studies have examined racial/ethnic disparities in sensory function. We studied 3,005 US adults (aged 57-85, mean 69.3 years); 10% Hispanic, 17% Black, 71% White; National Social Life, Health, and Aging Project). Impairment was defined by established criteria for objectively measured vision, smell, taste, touch and interviewer-rated hearing. Vision (22%), hearing (19%), smell (23%), taste (15%), and touch (19%) loss were common. Hispanic and Blacks showed the highest prevalence of vision, smell, and touch dysfunction. Findings persisted after adjustment for age, sex, education, and cardiometabolic conditions. Blacks had higher odds of impaired vision (adjusted Odds Ratio [aOR]:1.61; 95%CI:1.12, 2.32), smell (aOR:2.64; 95%CI:1.81, 3.84)) and touch (aOR:1.81; 95%CI:1.23, 2.64)) compared to Whites. Hispanics had higher odds of impaired smell than Whites (aOR:2.33; 95%CI:1.47, 3.67). Racial/ethnic minorities face marked disparities in function of the classical senses. Understanding how these differences arise, including potential systemic/social mechanisms, may catalyze interventions that promote health equity.

### HEARING LOSS AND INCIDENT DEMENTIA OVER 7 YEARS IN BLACK AND WHITE OLDER ADULTS IN ARIC-NCS

John Shin[1], Kening Jiang[2], Nicholas Reed[1], David Knopman[3], Thomas Mosley[4], Richey Sharrett[5], Frank Lin[2], and Jennifer Deal[6], *1. Johns Hopkins Bloomberg School of Public Health, Baltimore, Maryland, United States, 2. Johns Hopkins Cochlear Center for Hearing and Public Health, Baltimore, Maryland, United States, 3. Department of Neurology, Mayo Clinic, Rochester, Minnesota, United States, 4. UMMC-The MIND Center, Jackson, Mississippi, United States, 5. Department of Epidemiology, Johns Hopkins Bloomberg School of Public Health, Baltimore, Maryland, United States, 6. Johns Hopkins University, Baltimore, Maryland, United States*

Hearing loss is a risk factor for dementia; whether this association differs by race is unknown. Although hearing loss is less prevalent in Blacks than Whites, we hypothesized the hearing loss-dementia relationship is stronger in Blacks. All-cause dementia and mild cognitive impairment (MCI) were adjudicated using longitudinal cognitive data. Hearing was measured using pure tone better-ear thresholds (0.5-4 kHz). Cox proportional hazards models adjusted for demographic and clinical covariates and included an interaction term between hearing and race. In 3,605 participants from a population-based cohort (68-89 years, 23% Black, 59% female), estimates for 7-year dementia risk per 10 decibels increase in hearing loss were stronger in Blacks (hazard ratio [HR]:1.24, 95% Confidence Interval (CI):1.07,1.43) than Whites (HR:1.07; 95%CI:0.96,1.19). Hearing loss is a risk factor for dementia in Black Americans. These findings emphasize the need to address existing racial disparities in hearing healthcare.

### DUAL SENSORY LOSS AND DEPRESSIVE SYMPTOMS: FINDINGS FROM THE LONGITUDINAL AGING STUDY IN INDIA (LASI)

Ethan Wang[1], Emmanuel Garcia Morales[1], Frank Lin[2], Nicholas Reed[3], and Jennifer Deal[4], *1. Cochlear Center for Hearing and Public Health, Baltimore, Maryland, United States, 2. Johns Hopkins Cochlear Center for Hearing and Public Health, Baltimore, Maryland, United States, 3. Johns Hopkins Bloomberg School of Public Health, Baltimore, Maryland, United States, 4. Johns Hopkins University, Baltimore, Maryland, United States*

Prior work suggests that vision and hearing loss may be risk factors for depressive symptoms in older adults. However, few studies have focused on the relationship of both vision and hearing loss (dual sensory loss, DSL) and depressive symptoms. Using the first nationally representative population study from LASI in India conducted from 2017-2019, Poisson models tested the association of DSL with depressive symptoms. Self-reported vision and hearing loss were associated with increased depressive symptoms (PR 1.29; 95%CI, 1.25-1.33 and 1.15; 95%CI, 1.09-1.22) after adjusting for age, sex, education, economic status, marital status, urbanicity, region, diabetes, heart disease, hypertension, stroke, and smoking. DSL had a 46% higher risk (95% CI, 1.37-1.56) of depressive symptoms. Poor vision and/or hearing loss may be a risk factor for depressive symptoms in adults in India aged ≥45 years. The risk of depressive symptoms in those with sensory loss should be investigated in further studies.

### RACIAL DISPARITIES IN THE ASSOCIATION BETWEEN VISION IMPAIRMENT AND FOOD INSECURITY IN THE US

Varshini Varadaraj[1], Priyanka Kumar[2], Jessica Brinson[3], Jiangxia Wang[4], Laura Samuel[1], Adrienne Scott[5], and

Bonnielin Swenor[1], *1. Johns Hopkins University, Baltimore, Maryland, United States, 2. Johns Hopkins University School of Medicine, Baltimore, Maryland, United States, 3. Howard University College of Medicine, Washington, District of Columbia, United States, 4. Johns Hopkins Bloomberg School of Public Health, Baltimore, Maryland, United States, 5. The Johns Hopkins Wilmer Eye Institute, Baltimore, Maryland, United States*

We examined racial disparities in household food insecurity among low-income Americans with and without vision impairment in the National Health Interview Survey, years 2011-2018. Among 257,620 U.S. adults below a threshold of 150% poverty, 15% of White, 16% of Black, and 8% of Asian Americans, and 18% of American Indians and Inuits reported vision impairment. In analyses adjusted for sociodemographic variables, vision impairment was associated with >100% greater odds (95% CI=2.01-2.31) of 30-day food insecurity, as compared to no vision impairment. Further, odds of household food insecurity were higher among Black Americans (OR=1.37, 95% CI=1.29-1.47), and American Indians and Inuits (OR=1.38, 95% CI=1.15-1.66) than White Americans, while Asians had lower odds (OR=0.45, 95 %CI=0.36-0.57). These findings highlight that low-income adults with vision impairment and racial minorities experience food disparities and dietary inadequacy, an area of disadvantage that can influence overall health, in a nationally representative sample in the U.S.

### ADVANCING HEARING HEALTH EQUITY FOR OLDER ADULTS: FINDINGS FROM THE HEARS RCT

Carrie Nieman[1], Joshua Betz[2], Emmanuel Garcia Morales[3], Jonathan Suen[4], Nicole Marrone[5], Hae-Ra Han[4], Sarah Szanton[6], and Frank Lin[7], *1. Johns Hopkins University School of Medicine, Baltimore, Maryland, United States, 2. Cochlear Center for Hearing & Public Health, Baltimore, Maryland, United States, 3. Cochlear Center for Hearing and Public Health, Baltimore, Maryland, United States, 4. Johns Hopkins University, Baltimore, Maryland, United States, 5. University of Arizona Department of Speech, Language, and Hearing Sciences, Tucson, Arizona, United States, 6. Johns Hopkins University School of Nursing, Baltimore, Maryland, United States, 7. Johns Hopkins Cochlear Center for Hearing and Public Health, Baltimore, Maryland, United States*

Hearing loss is highly prevalent but disparities exist in hearing care. Delivering care in partnership with community health workers (CHWs) is an established approach to addressing disparities but has not been robustly studied in hearing care. We recruited older adults with hearing loss from community sites in Baltimore, MD. The 2-hour intervention consists of fitting a low-cost amplification device and counseling.151 participants were randomized. The primary outcome was change in communication function (Hearing Handicap Inventory for the Elderly-Screening [HHIE-S]; range 0-40; higher scores indicate poorer function). Communication function significantly improved in the intervention group, with an intention to treat estimated average treatment effect of a -12.98 point change (95% CI: -15.52, -10.42). In the first randomized control trial of a CHW-delivered hearing care intervention for older adults using low-cost amplification devices, participants receiving the intervention demonstrated a treatment effect comparable to prior studies of conventional hearing aids fit by audiologists.

### SESSION 3570 (SYMPOSIUM)

### IT'S ABOUT WHO YOU KNOW: RELATIONSHIPS BETWEEN SOCIAL NETWORKS, COGNITIVE FUNCTION, AND BRAIN HEALTH IN LATER LIFE

Chair: Lucas Hamilton Discussant: Sara Moorman

Evidence has been steadily increasing about the benefit of social connectedness for healthy aging. Yet, thanks to the complexity of human social interactions, it remains difficult to pinpoint specific benefits and mechanisms by which such benefits emerge. We offer this symposium to address this issue by presenting new findings from ongoing work at Indiana University in collaboration with the Indiana Alzheimer's Disease Research Center. First, an overview of recent literature will be presented alongside a brief introduction to egocentric network analysis and its utility in probing social determinants of health. Next, we turn our attention to characteristics of social networks and their importance for cognitive health in later life. Different types of social enrichment will be evaluated in terms of their respective influence on cognitive reserve (i.e., neuropsychological evaluations, brain health). Additional attention will be paid to the comparison of emotionally supportive (i.e., social bonding) and more informationally supportive (i.e., social bridging) ties as promoters of brain health. Then, the final talks will describe potential avenues for increasing social connectedness in older adulthood. Individual differences in social cognitive function, namely Theory of Mind (i.e., reading another person's thoughts, feelings, and intentions), has been implicated as mechanism by which older adults can maintain larger networks with more weak ties, in turn, promoting cognitive function. Separately, the presence of ambivalent ties (i.e., connections that beget positivity and negativity) may serve as a window of opportunity whereby older adults can leverage strengths in emotion regulation to maintain these ties without negative health consequences.

### AGING AND THE SOCIAL BRAIN: THE ROLE OF SOCIAL NETWORKS IN ALZHEIMER'S DISEASE AND RELATED DEMENTIAS

Brea Perry, *Indiana University, Bloomington, Indiana, United States*

Research suggests social connectedness reduces dementia risk and helps older adults with neuropathology maintain cognitive functionality and quality of life. However, little is known about the specific underlying social and biological mechanisms. This presentation provides an overview of three promising pathways through social bridging (i.e., cognitive enrichment through expansive social networks), social bonding (i.e., neuroendocrine benefits of integration in cohesive social networks), and social stress (i.e., HPA axis dysregulation resulting from social losses, role exits, and dysfunction or strain in relationships). It discusses how personal social network methodology combined with tests of general and social cognitive function and/or biomarkers can identify specific etiological mechanisms. These insights can be leveraged to develop policies and programs that support brain health and cognitive function in older adults. This presentation sets the stage for the remainder of the symposium, which presents empirical findings examining these mechanisms from a social network perspective.

### NEUROLOGICAL CORRELATES OF SOCIAL BONDING AND BRIDGING

Mohit Manchella[1], Paige Logan[2], Brea Perry[3], Siyun Peng[3], Lucas Hamilton[3], Shannon Risacher[2], Andrew Saykin[2], and Liana Apostolova[2], *1. University of Southern Indiana, Carmel, Indiana, United States, 2. Indiana University School of Medicine, Indianapolis, Indiana, United States, 3. Indiana University, Bloomington, Indiana, United States*

Social connectedness has been linked to decreased rates of cognitive decline in later life. However, recent work suggests that particular social network characteristics (i.e., bonding and bridging) may buffer against age-related degeneration. The present study analyzes social network and structural MRI data of 176 older adults from the Social Networks and Alzheimer's Disease (SNAD) study. Results indicate that increased social bridging is associated with greater grey matter (GM) volume in several limbic structures. Increased social bonding is associated with greater GM volumes in several cerebral cortex structures as well as greater volumes in some components of the limbic system. Most notably, the effects of bridging are primarily lateralized in the left hemisphere while the effects of bonding are observed mostly in the right hemisphere. These results suggest that the neurocognitive benefits of social connectedness depend on the preponderance of bridging and/or bonding ties in older adults' social networks.

### LINKS BETWEEN SOCIAL CONNECTEDNESS AND COGNITIVE HEALTH OPERATE THROUGH SOCIAL STIMULATION AND COGNITIVE RESERVE

Siyun Peng[1], Anmoldeep Singh[2], Mohit Manchella[3], and Brea Perry[1], *1. Indiana University, Bloomington, Indiana, United States, 2. Indiana University, Newburgh, Indiana, United States, 3. University of Southern Indiana, Carmel, Indiana, United States*

The link between social connectedness and dementia risk and resilience has been examined using a diverse set of measures. Though different measures of connectedness reflect distinct social processes and underlying mechanisms (e.g., stress buffering, cognitive stimulation), few studies have compared them. Using data from two social network studies of older adults (N=283), we compare associations between 29 measures of social connectedness and general cognitive function (MoCA), and non-verbal (Rey) and episodic memory (Craft). Measures of social participation (e.g., volunteering, working, attending church) and social support were unassociated with cognitive outcomes, net of controls. Quality of friendships ($p<.05$), family relationships ($p<.01$), and marriage ($p<.05$) were sporadically associated. Measures indicating large, diverse, and expansive networks were strongly and consistently related to all cognitive outcomes (e.g., number of phone contacts [$p<.001$], network size [$p<.001$], density [$p<.001$], racial homophily [$p<.05$], age heterogeneity [$p<0.01$], and diversity [$p<.001$]). We discuss implications for theories of cognitive reserve.

### SOCIAL COGNITIVE DEFICITS RELATE TO KEY ASPECTS OF OLDER ADULTS' SOCIAL NETWORKS

Anne Krendl, and Brea Perry, *Indiana University, Bloomington, Indiana, United States*

Social connectedness confers benefits to older adults' cognition, including slowing the progression of Alzheimer's disease (AD). Social connectedness is facilitated by social cognitive function – how people understand, store, and apply information about others – which declines over the lifespan. We examined whether two core social cognitive skills – face memory and theory of mind (the ability to infer others' mental states) – predicted older adults' social network structure and composition. Cognitively normal older adults (OA; N=119) and OA with mild cognitive impairment (MCI) or AD (N=96) completed a social network interview, a face memory task, and a theory of mind measure. Social cognitive deficits were highest among OA with MCI and AD. Face memory predicted network size, whereas theory of mind predicted network composition. Neuroimaging results describing OA's social cognitive deficits are also discussed. Social cognitive function may be an important intervention target for preserving older adults' social connectedness.

### TO LOVE AND LOATHE: EXAMINING THE COSTS AND BENEFITS OF AMBIVALENT TIES IN OLDER ADULTHOOD

Lucas Hamilton, Siyun Peng, Anne Krendl, and Brea Perry, *Indiana University, Bloomington, Indiana, United States*

Ambivalent ties are relationships that offer support but beget stress, which generally has a detrimental impact on health. Existing theory suggests that older adults gradually remove such ties over time; however, it is not uncommon for ambivalence to exist in older adults' close relationships (i.e., partners, children). Social network data was used from 286 older adults with about half having mild cognitive impairment. Roughly two-thirds of the sample reported at least one ambivalent tie, most commonly partners, children, and friends. Logistic regressions revealed distinct characteristics of these ties. Participants who reported at least one ambivalent tie (most notably, partners and friends) had social networks with structures known to confer cognitive benefits. Importantly, these effects dissipate with diminished cognitive status. Altogether, ambivalent ties may confer benefits when resources are available to manage such relationships. When resources are taxed, however, ambivalent ties may contribute to cascading health declines.

### SESSION 3580 (SYMPOSIUM)

### KUAKINI HHP CENTER FOR TRANSLATIONAL RESEARCH ON AGING: LATEST FINDINGS FROM MICE TO HUMANS

Chair: BRADLEY WILLCOX Co-Chair: Richard Allsopp
Discussant: Peter Martin

Kuakini Medical Center (Kuakini) was funded by NIH in late 2019 to create an interdisciplinary Hawai'i-based, Center of Biomedical Research Excellence (COBRE), for translational research on aging. This Center is building upon Kuakini's five-decades of prior NIH-funded research. These resources include clinical data from the 57-year ongoing Kuakini Honolulu Heart Program (HHP), Honolulu-Asia Aging Study, HHP Offspring Study and a large biorepository. The Center's overarching aim is to increase infrastructure for collaborative aging research in Hawaii. The first step is to grow the Center's faculty by hiring and mentoring research project leaders (RPLs) from diverse disciplines to become independent, R01-funded, investigators on aging. Our first RPL has graduated after obtaining R01-funded status. His project utilizes novel CRISPR methods to i) improve the safety and efficacy of delivering potentially therapeutic genes (such as FOXO3) to the mouse genome, and ii) test whether temporal enhancement of FOXO3 expression improves healthy aging in this mouse model - both key steps for potential translation to human clinical therapies. This work will be highlighted in the Program Overview session followed by current RPL findings. The first of these current RPL talks presents data on a potential relation between the FOXO3 gene and vascular dementia in elderly Japanese-American males. The second talk reports on how strong social networks mitigate risk for dementia in elderly Japanese-American males. The third talk will report a relation between FOXO3-associated resilience to hypertension and lower intracerebral hemorrhagic stroke risk in elderly Japanese-American males. Supported by NIGMS 5P20GM125526 and NIA R01AG027060.

### FOXO3-MEDIATED PROTECTION AGAINST VASCULAR DEMENTIA RISK IN A CASE-CONTROL STUDY OF ASIAN AMERICAN MEN

Philip Davy[1], Kalpana Kallianpur[2], Randi Chen[1], Timothy Donlon[1], Brian Morris[1], Richard Allsopp[3], Bradley Willcox[1], and Kamal Masaki[1], *1. Kuakini Medical Center, Honolulu, Hawaii, United States, 2. Kuakini Medical Center, Honolulu, Hawaii, Honolulu, Hawaii, United States, 3. University of Hawaii, Honolulu, Hawaii, United States*

The longevity-associated allele of FOXO3 is associated with a significant reduction in cardiovascular disease (CVD) risk in older adults. We hypothesized that dementia, which shares several risk factors with CVD, might also be related to FOXO3 genotype, particularly vascular dementia (VaD). Therefore, we utilized the Kuakini Honolulu Heart Program/Honolulu Asia Aging Study dataset to assess the relation of FOXO3 genotype to dementia risk. Preliminary analyses suggested that VaD had the strongest relation to FOXO3 genotype. Therefore, we performed larger, more detailed study of FOXO3 genotype and VaD risk in a 9-year nest case-control study. Chi-Square test was used to assess the association of dementia with FOXO3 genotype in a sample of 1504 Japanese-American male study participants, aged 71-93 years, at the baseline exam. General Linear Model was utilized to compare VaD risk factors between VaD cases and controls. Multivariate Logistic Regression was used to assess the association of VaD with FOXO3 genotype adjusting for birth year, education, APOE4 and other vascular risk factors. We found a significant protective association for carriers of the principal FOXO3 longevity-associated allele (SNP rs2802292 G allele-carriers) against VaD risk (OR = 0.66, 95% CI = 0.44-0.98, p = 0.0388). The protective association retained significance when controlling for common risk factors for VaD in a multivariate model. These data indicate a potential neuroprotective role for the FOXO3 longevity-associated genotype against vascular dementia. This finding merits validation studies in other cohort studies.

### LATE-LIFE SOCIAL NETWORKS AND INCIDENT ALZHEIMER'S DISEASE: THE KUAKINI HONOLULU-ASIA AGING STUDY

Kalpana Kallianpur[1], Kamal Masaki[2], Randi Chen[2], Bradley Willcox[2], Richard Allsopp[3], Philip Davy[2], and Hiroko Dodge[4], *1. Kuakini Medical Center, Honolulu, Hawaii, Honolulu, Hawaii, United States, 2. Kuakini Medical Center, Honolulu, Hawaii, United States, 3. University of Hawaii, Honolulu, Hawaii, United States, 4. Oregon Health & Science University, Portland, Oregon, United States*

We assessed longitudinal associations between social networks and incidence of all-cause dementia, Alzheimer's disease (AD) and vascular dementia in Kuakini Honolulu-Asia Aging Study participants over a 10-year follow-up period. Median split of Lubben Social Network Scale (LSNS) scores defined weak/strong social networks among 2636 men who were dementia-free at baseline (median age 77 years) and during the first 3 years. Kaplan-Meier curves showed that those with strong networks at baseline were less likely to develop all-cause dementia ($p<0.0001$) and AD ($p=0.0006$); probability of dementia-free survival at 10 years for strong and weak social network groups was 93.8% and 89.0%, respectively. Cox regression models adjusting for age and other baseline factors revealed associations of weak networks with increased risk of all-cause dementia (HR=1.52, 95%CI=1.11-2.08, p=0.009) and AD (HR=1.67, 95%CI=1.11-2.51, p=0.014). As strong social networks may protect against incident dementia and AD, and are associated with other health benefits, prevention of social isolation of older adults should be considered a priority.

### FOXO3 LONGEVITY GENOTYPE MITIGATES THE IMPACT OF HYPERTENSION ON RISK OF INTRACEREBRAL HEMORRHAGE

Kazuma Nakagawa[1], Randi Chen[1], Steven Greenberg[2], Webster Ross[3], BRADLEY WILLCOX[1], Timothy Donlon[1], Brian Morris[1], and Kamal Masaki[1], *1. Kuakini Medical Center, Honolulu, Hawaii, United States, 2. Massachusetts General Hospital, Boston, Massachusetts, United States, 3. Pacific Health Research and Education Institute, Honolulu, Hawaii, United States*

The longevity-associated FOXO3 G allele of SNP rs2802292 is associated with protection against mortality, however the impact of FOXO3 genotype on the association between hypertension and risk of intracerebral hemorrhage (ICH) has not been assessed. Therefore, we utilized the Kuakini Honolulu Heart Program (KHHP), a prospective population-based cohort of Japanese American men living in Hawaii that began in 1965 to study this relation. After excluding baseline prevalent stroke and those missing FOXO3 data, 6,469 men were included in the analysis. Age-adjusted prevalence of ICH by hypertension was assessed for the whole cohort after stratifying by FOXO3 genotype. Cox regression models, adjusted for age, cardiovascular risk factors, FOXO3 and APOE genotypes, were utilized to assess relative risk of hypertension on ICH. All models were created for the whole cohort and stratified by FOXO3 genotype, namely G allele carriers versus TT genotype. Overall, 183

subjects developed ICH over the 34-year follow-up period. Age-adjusted ICH prevalence was 0.90 versus 1.32 per 1,000 person-years follow-up in those without and with hypertension, respectively (p=0.002). After stratifying by FOXO3 genotype, this association was no longer significant in G allele carriers. In the whole cohort, hypertension was an independent predictor of ICH (RR=1.70, 95%CI 1.25, 2.32; p=0.0007). In stratified analyses, hypertension remained an independent predictor of ICH among the FOXO3 TT allele group (RR=2.02, 95% CI 1.33, 3.07; p=0.001), but not in FOXO3 G allele carriers (RR=1.39, 95% CI 0.88, 2.19; p=0.15). Thus, the longevity/resilience-associated FOXO3 G allele may mitigate the impact of hypertension on ICH risk in this population. Further study is needed in other populations.

### SESSION 3585 (SYMPOSIUM)

### NARRATIVE AS A BOUNDARY CONCEPT IN GERONTOLOGY: THE STORY BEHIND THE STORIES

Chair: Kate de Medeiros Co-Chair: Desmond O'Neill

Ever since the “narrative turn” was taken up by the humanities and social sciences in the 1980s and early 1990s, there has been an increased interest in narrative as a medium for gaining knowledge in gerontology. Narrative gerontology in particular assumes that the "life lived is inseparable from the life told" (Bruner 1987). Central to this notion is the metaphor of life as story or, as Kenyon and Randall put it, "[W]e not only have stories, we are stories" (1999). As a boundary concept, “narrative” inhabits several disciplinary worlds. Its analysis can focus on individual identification strategies, on the storytelling process and its context, or on aesthetic dimensions, for instance when analyzing cultural representations including film and fiction. Often loosely defined yet broadly applied, narrative work can range from personal life stories to master narratives that convey cultural and/or political values. The purpose of the Humanities and Arts symposium is to take a critical look at "narrative" within gerontology to include mapping the uses of narrative as actions, objects, ways of knowing and acts of resistance. The first paper presents a conceptual structure of narrative based on findings from a guided review of narrative definitions and approaches in gerontology. The second argues for the normative importance in studying cultural narratives of dementia. The third paper examines narrative in the context of the medical humanities and narrative medicine, pointing to ways in which narrative competence translates into medical practice. The final paper considers the narrative of aging through music.

### HOW HAS NARRATIVE BEEN USED IN GERONTOLOGY?: FINDINGS FROM A QUALITATIVE EVIDENCE SYNTHESIS

Kate de Medeiros, *Miami University, Oxford, Ohio, United States*

Narrative gerontology emerged as a concept in the 1990s to describe the ways that people age biographical as well as biologically. Since then, narratives and narrative approaches have gained popularity as methods and ways of knowing. This paper presents findings from a qualitative evidence synthesis (QES) of narrative within the gerontological literature. ). QES describes methods to systematically review qualitative evidence (e.g., extracted text). The purpose is to gain a deeper understanding of a concept rather than evaluate study designs or findings. An initial search returned 1,480 papers. Upon further analysis, the following three large categories with regards to narrative were identified: definitions, accounts, processes, and possibilities. Overall, findings provide an comprehensive overview of uses of narrative within gerontology.

### EXPLORING THE NARRATIVE OF AGING THROUGH MUSIC

Desmond O'Neill, *Trinity College Dublin, Dublin, Dublin, Ireland*

With increasing interest in exploring narratives of aging through literary/oral sources, a less prominent area of inquiry has been possibilities afforded by narratives inherent in music across the life-span and into later-life. Two possible areas of interest are the arc of development and change over the life-course, and inherent content of the music. The latter element is less prominent in narrative analysis, providing a potentially rich field of discovery. While popular song parallels these possibilities, further opportunities arise from instrumental, orchestral and operatic music, allied to strengths in musicological analysis. Later compositions whose elements provide insights into life course review (Shostakovich Viola Sonata and Metamorphosen, Richard Strauss) as well as romantic love in later life across generations (The Cunning Little Vixen, Leos Janáček). The analysis will seek to delineate to possible avenues of interpretation of musicological analysis in understanding the phenomenology and opportunities arising from aging.

### UNDERSTANDING CULTURAL NARRATIVES OF DEMENTIA: TASKS AND TOOLS FOR HUMANITIES SCHOLARSHIP

Nancy Berlinger, *The Hastings Center, Garrison, New York, United States*

Humanities scholarship on dementia has long focused on the depiction of dementia in literature, film, and other genres. Recent research on neurodiversity includes humanistic scholarship on creativity within dementia. It is time for interdisciplinary humanities scholarship to focus on narratives of dementia that circulate within aging societies, are embedded in policy, and shape experiences of typical people living with dementia or providing dementia care. This paper argues for the normative importance of studying values-laden cultural narratives, recognizing competing or evolving narratives within a society, and demonstrating how to reframe flawed narratives beyond necessary attention to ageist and ableist language. It presents examples of approaches to social narrative analysis; describes tools and training that could be integrated into humanities scholarship on dementia and aging, and considers the potential role of social narrative analysis in articulating and launching policy ideas for aging societies.

### NARRATIVE MEDICINE AS INTERDISCIPLINARY PRACTICE

Ulla Kriebernegg, *University of Graz, Graz, Steiermark, Austria*

When dealing with patients who talk about their illnesses, medical doctors need to interpret the stories they hear. Also, they need to make sense of their own experiences regarding their medical encounters. Narrative and its analysis plays a central role in such processes that require empathy and self-reflection. The interdisciplinary practice of teaching medical students concepts and theories from literary studies as well as discussing literary texts with them can expand their scientific medical understanding. This paper explores the concept of narrative in the context of geriatric medical humanities and narrative medicine, looking at how narrative competence translates into medical practice. Presenting findings from a seminar on Narrative medicine at the Medical University of Graz, Austria, it also addresses some methodological and didactic challenges of this interdisciplinary approach.

### SESSION 3590 (SYMPOSIUM)

### NEAR: NEW OPPORTUNITIES FOR AGING RESEARCH

Chair: Debora Rizzuto Co-Chair: Scott Hofer

The global increase in life expectancy is one of the greatest achievements of the last half century. However, the demographic developments towards an older population also challenge many parts of the society, especially the health care. Promoting healthy ageing is therefore one of the most important commitments of the 21st century and to succeed, scientifically based knowledge of older individuals' health- and care requirements are needed. To better understand the individual and population aging process, the National E-infrastructure for Aging Research (NEAR) was founded in 2018 to build and run a national infrastructure by integrating existing databases from the 15 major longitudinal studies on aging and health in Sweden. To show the added value of NEAR, this symposium will present results from four ongoing NEAR projects: 1) Developing a metric of global brain integrity in multiple Swedish studies with different scanners; 2) Functional aging trajectories and drug interactions; 3) Long-term prediction of dementia using machine learning algorithms; 4) The new aging – how different aspect of ageing has changed over half a century. The creation of national infrastructures is needed to achieve broad, multidisciplinary research perspectives that cannot be achieved by individual databases. Moreover, to address the increased health demands of an older population and enhance new opportunities for aging research, a critical mass of data is needed to increase sample sizes, variations, representativeness, and generalizability. Ultimately, this can lead to the identification of sustainable intervention strategies for better health and care for older persons during the coming decades.

### FUNCTIONAL AGING TRAJECTORIES AND DRUG INTERACTIONS

Thais Lopes Deliveira[1], and Sara Hägg[2], *1. Karolinska Institutet, Stockholm, Sodermanlands Lan, Sweden, 2. Karolinska Institutet, Stockholm, Stockholms Lan, Sweden*

Markers of functional aging can be used to track biological aging longitudinally and how it changes in response to the environment, e.g., drugs. We aimed to investigate how different drug classes may alter functional aging trajectories using data from the National E-infrastructure of Aging Research (NEAR) in Sweden. Data were harmonized across several longitudinal cohorts of aging for general cognitive performance, grip strength, walking speed, sensory ability (visual and hearing), lung function and assessment of frailty using the accumulation deficit model known as the frailty index. Selected drug classes were lipid lowering, glucose lowering and blood pressure lowering medications that are commonly used in old adults. Preliminary analysis using data from one longitudinal cohort shows that using glucose lowering drugs was associated with lower frailty. Additional analyses are ongoing to increase sample sizes. We anticipate that several drug classes may be important for changing functional aging trajectories in late life.

### THE NEW AGING: HOW DIFFERENT ASPECTS OF AGING HAVE CHANGED OVER HALF A CENTURY. THE H70 STUDIES WITHIN NEAR

Ingmar Skoog[1], and Therese Rydberg Sterner[2], *1. University of Gothenburg, Mölndal, Vastra Gotaland, Sweden, 2. Karolinska Institutet, Stockholm, Stockholms Lan, Sweden*

The number and proportions of older people are increasing world-wide. The Gothenburg H70 Birth Cohort Studies started in 1971 and are still on-going. The studies include representative birth cohorts born 1901-02, 1906-07, 1911-12, 1922, 1923-24, 1930, and 1944 followed longitudinally from age 70 until death. The studies include psychiatric, somatic, audiological, opthalmological, psychological, social, genetic, dietary, functional, and psychometric examinations, personality, collection of blood, plasma, serum, and cerebrospinal fluid, and examinations with MRI. During the study period, cognitive and physical functions, and hearing, has improved considerably, the prevalence and incidence of dementia has decreased, the prevalence of depression and psychotic disorders has decreased, and sexual activity and alcohol consumption has increased considerably. Even personality has changed. Present older people are less neurotic and more extrovert. Our study shows that aging is a changing concept, and that previous knowledge needs to be continuously updated, as new generations reach old age.

### LONG-TERM PREDICTION OF DEMENTIA USING MACHINE LEARNING ALGORITHMS

Johan Sanmartin Berglund[1], and Ashir Javeed[2], *1. Blekinge Institute of Technology, Karlskrona, Blekinge Lan, Sweden, 2. Karolinska Institutet, Stockholm, Stockholms Lan, Sweden*

The core interest of this project is the development of predictive estimates and the identification of modifiable risk factors for the development of neurocognitive disorders based on multifactorial data from multiple health databases. We are conducting epidemiological research on predicting neurocognitive diseases of older adults included in the National E-Infrastructure on Aging Research (NEAR) using deep learning and other AI methods. Exploring the impact of lifestyle and environment on dementia development in the subjects together with biomarkers and images and predicting dementia using machine learning techniques would give insights into the long-term development. In addition, the possibility of screening a large number of persons and consequent early prediction of dementia based on optimized machine learning techniques could be of great importance for early treatment.

### SESSION 3600 (SYMPOSIUM)

### NOVEL INTERVENTIONS AND TARGETS FOR THE PREVENTION OF DEMENTIA AND FRAILTY: 2021 EDITOR'S CHOICE ARTICLES FROM JGMS

Chair: Lewis Lipsitz Discussant: Beverly Gwen Windham

This symposium will present four 2021 "Editor's Choice" articles from the Journal of Gerontology Medical Sciences that focus on novel interventions and targets for the prevention of dementia and frailty. Jeffrey Scherrer and colleagues, in their article "Lower Risk for Dementia Following Adult Tetanus, Diphtheria, and Pertussis (Tdap) Vaccination," examine the links between adult vaccinations and decreased dementia risk. Katharine Brewster, Justin Golub (presenter), and colleagues, in "Age-Related Hearing Loss, Late-Life Depression, and Risk for Incident Dementia in Older Adults," investigate hearing loss and depression as independent risk factors for eventual conversion to dementia. By understanding these potentially reversible mechanisms for dementia, targets for early interventions could be identified. "Association Between Long-Term Aspirin Use and Frailty in Men: The Physicians' Health Study," written by Ariela Orkaby and colleagues, examines the association between long-term aspirin use and frailty, furthering our understanding of the benefits of anti-inflammatory medications even in older adults. Kristen George and coauthors, in their article, "Impact of Cardiovascular Risk Factors in Adolescence, Young Adulthood, and Midlife on Late-Life Cognition: Study of Healthy Aging in African Americans," examine how cardiovascular risk factors early in life may affect late life cognition in Black Americans. Beverly Gwen Windham, the discussant, will highlight commonalities and lessons learned from these studies.

### ADULT TETANUS, DIPHTHERIA, AND PERTUSSIS VACCINATION AND INCIDENT DEMENTIA

Jeffrey Scherrer[1], Joanne Salas[2], Timothy Wiemken[2], Christine Jacobs[2], John Morley[2], and Daniel Hoft[2], *1. Saint Louis University School of Medicine, St. Louis, Missouri, United States,*
*2. Saint Louis University, St. Louis, Missouri, United States*

Adult vaccinations may reduce risk for dementia. We determined whether tetanus, diphtheria, pertussis (Tdap) vaccination is associated with lower risk for dementia. Analysis conducted with Veterans Health Affairs (VHA) administrative medical record data and replicated in private sector medical claims data. Patients were ≥65 years of age and free of dementia for 2 years prior to index date. Tdap vaccination in 2011 or 2012. Follow-up through 2018. Controls had no Tdap vaccination for the duration of follow-up. After controlling for confounding, patients with, compared to without Tdap vaccination, had a significantly lower risk for dementia in both cohorts (VHA: HR=0.58; 95%CI:0.54 - 0.63 and MarketScan: HR=0.58; 95%CI:0.48 - 0.70). Results were replicated in two cohorts with different clinical and sociodemographic characteristics. Several vaccine types are linked to decreased dementia risk, suggesting that these associations are due to nonspecific effects on inflammation rather than vaccine-induced pathogen-specific protective effects.

### IMPACT OF CARDIOVASCULAR RISK FACTORS IN ADOLESCENCE, YOUNG ADULTHOOD, AND MID-LIFE ON LATE-LIFE COGNITION

Kristen George[1], Paola Gilsanz[2], Rachel Peterson[3], Lisa Barnes[4], Charles DeCarli[3], Elizabeth Mayeda[5], Dan Mungas[6], and Rachel Whitmer[7], *1. University of California Davis School of Medicine, Daivs, California, United States, 2. Kaiser Permanente Division of Research, Oakland, California, United States, 3. University of California Davis School of Medicine, Sacramento, California, United States, 4. Rush Alzheimer's Disease Center, Rush University Medical Center, Chicago, Illinois, United States, 5. University of California, Los Angeles Fielding School of Public Health, Los Angeles, California, United States, 6. University of California, Davis, Sacramento, California, United States, 7. University of California Davis School of Medicine, Davis, California, United States*

Using the Study of Healthy Aging in African Americans (STAR), we assessed cardiovascular risk factors (CVRFs) in adolescence, young adulthood, and midlife with late-life cognition. Among 755 participants, body mass index, blood pressure, glucose, and total cholesterol were collected during Multiphasic Health Checkups (1964-1985). At STAR baseline (2018-2019; mean age=69(SD=9)), executive function (EF), verbal episodic memory (VEM), and semantic memory (SM) were measured using the Spanish and English Neuropsychological Assessment Scales. Linear regression models associated CVRFs with cognition. Hypertension was associated with worse late-life EF (β[95% CI]:-0.14[-0.28, -0.00]) and VEM (β[95% CI]:-0.22[-0.37, -0.07]). Diabetes was associated with worse EF (β[95% CI]: -0.43[-0.83, -0.03]). In age-stratified analyses, adolescent hypertension was associated with lower late-life EF (β[95% CI]:-0.39[-0.67, -0.11]). Young adulthood hypertension (β[95% CI]:-0.29[-0.49, -0.09]) and midlife hyperlipidemia (β[95% CI]:-0.386[-0.70, -0.02]) were associated with lower VEM. These results emphasize the importance of lifecourse cardiovascular health on the aging brain among Black Americans.

### ASSOCIATION BETWEEN LONG-TERM ASPIRIN USE AND FRAILTY IN MEN: THE PHYSICIANS' HEALTH STUDY

Ariela Orkaby[1], Laiji Yang[2], Alyssa Dufour[3], Thomas Travison[4], Howard Sesso[5], Jane Driver[6], Luc DJousse[5], and J Gaziano[1], *1. VA Boston, Boston, Massachusetts, United States, 2. Hebrew Senior Life, Boston, Massachusetts, United States, 3. Hebrew SeniorLife, Boston, Massachusetts, United States, 4. Harvard Medical School, Boston, Massachusetts, United States, 5. Brigham & Women's Hospital, Boston, Massachusetts, United States, 6. VA Boston Healthcare System, Brockton, Massachusetts, United States*

The role of anti-inflammatory medications such as aspirin to prevent frailty is unknown. We studied 12,101 men ≥60 years who participated in the Physicians' Health Study I, a completed aspirin trial. Annual questionnaires collected data on aspirin use, lifestyle, and clinical variables. Average aspirin use was dichotomized: ≤60 vs >60 days/year. Frailty was assessed using a 33-item index. Propensity score methods addressed confounding by indication. Logistic regression models estimated odds of frailty as a function of categories of average aspirin use. Mean age was 71 years. Following an average of 11 years of follow-up, 15% reported aspirin use ≤60 days/year; 20% were frail. The OR (95%CI) for frailty was 0.85 (0.76-0.96) for average aspirin use >60 days/year vs ≤60 days/year. Results were similar using an alternate definition of frailty. Long-term regular aspirin use is inversely associated with frailty among older men, even after consideration of multi-morbidity and health behaviors.

### AGE-RELATED HEARING LOSS, LATE-LIFE DEPRESSION, AND RISK FOR INCIDENT DEMENTIA IN OLDER ADULTS

Justin Golub[1], Katharine Brewster[2], Mei-Chen Hu[3], Sigal Zilcha-Mano[3], Patrick Brown[4], Melanie Wall[3], Steven Roose[5], and Bret Rutherford[5], *1. Columbia University, New York, New York, United States, 2. New York State Psychiatric Institute, New York, New York, United States, 3. New York State Psychiatric Institute, Columbia University College of Physicians and Surgeons, New York, New York, United States, 4. New York State Psychiatric Institute, Columbia University College of Physicians and Surgeons, New York., New York, New York, United States, 5. New York State Psychiatric Institute, Columbia University College of Physicians and Surgeons,, New York, New York, United States*

The interrelationships between hearing loss (HL), depression, and dementia remain poorly understood. 8529 participants ≥60 years without cognitive impairment were analyzed from the National Alzheimer's Coordinating Center uniform dataset. Participants had no HL, untreated HL, or treated HL. Outcomes included depression (15-item GDS≥5) and dementia conversion. A longitudinal logistic model examined the association between HL and changes in depressive symptoms. Cox proportional hazards models examined HL and incident dementia. Treated HL (vs no HL) had increased risk for depression (OR=1.26, 95%CI=1.04-1.54) and dementia conversion (HR=1.29; 1.03-1.62). Baseline depression was a strong independent predictor for dementia conversion (HR=2.32, 1.77-3.05). Development/persistence of depression was also associated with dementia (HR=1.89, 1.47-2.42), but only accounted for 6% of the direct hearing-dementia relationship suggesting no significant mediation of depression. In conclusion, both HL and depression are independent risk factors for dementia conversion. Understanding the mechanisms linking these later-life disorders may identify targets for early interventions.

### SESSION 3610 (SYMPOSIUM)

### OPPORTUNITIES AND CHALLENGES OF INNOVATIVE QUALITATIVE RESEARCH METHODS

Chair: Melinda Heinz Discussant: Keith Kleszynski

Using new qualitative methods can be exciting and add value to the field. However, Institutional Review Boards (IRB) approval can be a challenge if the methods are relatively new. This symposium provides an overview of 1) how several new methodologies have been used 2) IRB approval challenges and resolutions and 3) how varied disciplines can work together to understand phenomena. The first presentation explains how older adults used photographs to document meaning/purpose in their lives. IRB expressed concerns over identifying information in photographs. Discussion of how interdisciplinary collaborations create opportunities for diverse data dissemination will be included. The second presentation focuses on intersectionality and the benefits/challenges to understanding socially constructed identities. Discussion explains how to implement this methodology, including how category complexity creates additional possibilities for data analysis but also difficulties with narrowing down approaches. In the third presentation, examples of how to conduct co-research, such as photovoice with older adults will be reviewed, including best practices. Smartphones offer extensive possibilities for co-research; but developing IRB protocols to address concerns are needed. The fourth presentation outlines digital storytelling between older adults and university students. An explanation of how digital storytelling is connected to the narrative method and critical gerontology framework will be addressed. Advantages and pitfalls of digital storytelling with also be discussed. The last presentation focuses on using reminiscence with older migrant workers who have dementia, using a decolonial framework. The project seeks to improve participatory action research, with increased focus on "hearing" participants.

### PHOTO DOCUMENTATION: USING NEW METHODS IN COLLABORATION WITH ART

Melinda Heinz, Laura Gleissner, and Nathan Benton, *Upper Iowa University, Fayette, Iowa, United States*

Narrative analysis has been used extensively in qualitative research, but photo analysis is relatively new. In the present study, older adults took photographs and wrote narratives for 15 days about something that brought meaning and purpose to their lives. This presentation explains how both methods were paired together and the challenges of seeking Institutional Review Board (IRB) when new and "unproven" approaches are used. In our case, IRB reviewers were particularly concerned with identifying information in photographs and recommended that personal information (e.g., medication bottle) should be blurred when sharing photographs. Discussion of how using diverse qualitative methods can create opportunities for new dissemination methods and partnerships with other disciplines will also be noted. In our study, several pop-up art exhibits of participant photos and narratives were hung in public community spaces to promote greater discussion and reflection on purpose and meaning in later life.

### INTERSECTIONALITY AND QUALITATIVE HEALTH RESEARCH: POTENTIALS TO IMPACT QUALITATIVE RESEARCH

Martina Roes[1], Franziska Laporte-Uribe[1], Jem Bhatt[2], Carolien Smits[3], Aud Johanessen[4], Viktoria Peters-Nehrenheim[5], and Huerrem Tezcan-Guentekin[6], *1. German Center for Neurodegenerative Diseases (DZNE), Witten, Nordrhein-Westfalen, Germany, 2. UCL, Unit for Stigma Research, London, England, United Kingdom, 3. Pharos, Utrecht, Utrecht, Netherlands, 4. University of South-Eastern Norway, Kongsberg, Vestfold, Norway, 5. DZNE, Göttingen, Nordrhein-Westfalen, Germany, 6. ASH, Berlin, Berlin, Germany*

With a growing interest in intersectionality, critical qualitative researchers acknowledge the benefit from incorporating an intersectional lens. Incorporated intersectionality can be beneficial since it allows researchers from different disciplines to consider and account for the participant identities as multidimensional, fluid and, interdependent at each stage of the research process. In this presentation we will offer insights how qualitative research can be enhanced by incorporating an intersectional lens when analyzing the interconnectedness of numerous socially constructed identities (such as sex/gender/ethnicity/transnational families). Furthermore, we will address challenges how to conduct intersectional qualitative research (e.g. intersectional oriented interviews) and analysis (e.g. anticategorial, intracategorial and intercategorial complexity). This approach challenges on many levels, but since qualitative and interdisciplinary research is about utilizing meaning of phenomena, incorporating an intersectional lens will not only expand the methodological horizon of researchers but also stimulate ethical and critical inquiry throughout the research process.

### PLACE AND MEANING OF HOME: RESEARCH IN A VIRTUAL, PHOTOVOICE LENS

Joyce Weil, *University of Northern Colorado, Greeley, Colorado, United States*

Photovoice is built upon the principle of empowerment. With the increased use of Smartphones, photovoice provides a more readily available qualitative option for co-research with older adults. This presentation provides examples from the current study illustrating the process of creating a project that uses photovoice to see how older adults construct the meaning of home and place. While the benefits of this design are many, some issues do arise during implementation, such as: creating study protocols to address Institutional Review Board (IRB) concerns; working with organizations to ensure that the design is truly co-created; and navigating and conducting photovoice in a completely virtual arena. Examples will also be drawn from technical training received as part of LeadingAge LTSS Center @UMass Boston and Collective Insight's Aging Centered Outcomes Research Learning Collaborative, as well as the Healthier Black Elders Center, which is affiliated with the University of Michigan and Wayne State University.

### DIGITAL STORYTELLING AND INTERGENERATIONAL COLLABORATIONS: OLDER ADULTS AND COLLEGE STUDENTS

Angela Lavery, *West Chester University, West Chester, Pennsylvania, United States*

The use of digital storytelling can be a helpful tool within community work, health and social work research and policy. Digital storytelling refers to life-story telling that can be done in a variety of ways and used to encourage social change and transformation. This presentation will include experience on how this method was used in a study and an intergenerational project between older adults and university graduate and undergraduate students. This group of older adults specifically shared their experiences with equine interactions and activities, while the university students worked with the older adults to create a digital story. For this study and project, recruitment included students enrolled in different disciplines. Discussion on digital storytelling's connection to the narrative method and critical gerontology framework will be noted. Challenges and barriers, including Institutional Review Board and ethical considerations while preparing for this method will also be discussed.

### HEARING THE VOICE OF OLDER MIGRANTS WITH DEMENTIA: A DECOLONIAL APPROACH OF PARTICIPATIVE ACTION RESEARCH

Saloua Berdai Chaouni, *University College Karel de Grote Antwerp, Antwerp, Antwerpen, Belgium*

Gerontological research has been proven not always to succeed in engaging older migrants and their families. Various attempts are made to give voice to this under-researched population. Qualitative methods like participative action research (PAR) have been put forward as a way to engage this population. However, this approach does not always succeed to achieve this goal. Drawing on insights from decolonial frameworks, we present a learning process in engaging older migrants with and without dementia and their family members in developing a migration-sensitive reminiscence approach as a psycho-social intervention for older migrants with dementia. The emphasis of decolonial perspectives on seeing this population as the "Knower", deep reflection on own coloniality of mind as a researcher while critically looking at exclusive aspects of epistemology offers a supporting gaze to reshape PAR as an approach where this population is not only given voice but also heard.

### SESSION 3620 (PAPER)

### PHYSICAL EXERCISE AND FRAILTY

### A LIVE ONLINE EXERCISE PROGRAM FOR OLDER ADULTS' IMPROVED DEPRESSIVE SYMPTOMS: A PILOT RCT

Giulia Coletta, Angelica McQuarrie, Julia Delvecchio, Paula Bochnak, Meridith Griffin, Ada Tang, and Stuart Phillips, *McMaster University, Hamilton, Ontario, Canada*

Exercise improves mental health and effectively alleviates cognitive and physical declines. Unfortunately, engagement in physical activity decreases as individuals age and this was likely exacerbated by the COVID-19 pandemic. New technologies to deliver live online home-based group exercise classes may help mitigate mental and physical health declines in older adults while maintaining social connectivity. We evaluated the feasibility of an age-appropriate and ability-modified at-home exercise program via live video stream. The impact on loneliness, anxiety, and depression in older adults were exploratory outcomes. In this two-arm pilot RCT, we randomly assigned sedentary community-dwelling adults (65-80 years) to a waitlist control (CON) or an active group (ACTIVE) of thrice-weekly, 8-wk online live exercise program delivered via Zoom by trained exercise professionals. Attendance was recorded, and participant satisfaction to ACTIVE was assessed. Pre- and post-intervention loneliness, anxiety, and depression were collected using the revised

UCLA Loneliness Scale (R-UCLA), the Geriatric Anxiety Inventory (GAI), and the Geriatric Depression Scale (GDS). 32 participants were randomized (ACTIVE: n=16, mean age 70 ± 4, 69% women, 30 ± 5 kg/m2; CON: n=16, mean age 71 ± 5; 88% women; 29 ± 5 kg/m2). Attendance to online classes was >80% and all ACTIVE participants reported being satisfied with the exercise sessions. There was no intervention effect compared to CON on loneliness and anxiety. An effect of the intervention was observed for depression (ACTIVE: -1.94; CON: -0.07; p=0.015). We demonstrated good feasibility, satisfaction, and preliminary efficacy of a live online exercise program on older adults' mental health.

### ACCEPTABILITY OF A VIDEOCONFERENCE-BASED MULTICOMPONENT EXERCISE PROGRAM (VIVIFRAIL) FOR THE OLDEST-OLD

Ana Tiecker[1], and Angelo Jose Bos[2], *1. PUCRS, Porto Alegre, Rio Grande do Sul, Brazil,*
*2. Pontifical Catholic University of Rio Grande do Sul, PORTO ALEGRE, Rio Grande do Sul, Brazil*

Introduction: Previous studies have shown the effectiveness of supervised multicomponent exercise programs on counteract age-related changes in functional capacity and quality of life in the oldest-old. During the quarantine imposed by COVID-19 pandemic, social isolation has been a barrier to implement face-to face exercise programs. Objective: To study the acceptance and adequacy of an exercise protocol in oldest-old. Methodology: Quasi-experimental study with initial, intermediate and post-intervention evaluation. The functional capacity assessment was carried out by the Vivifrail test (Short Physical Performance Battery and 4 falls risk assessment tests), by videoconference at the three moments of the project. For twelve weeks, the older adults performed multicomponent ViviFrail exercises with monitoring and acceptance assessment (with a maximum score of 24 points) by weekly contact by videoconference.

Results: This study concluded 14 oldest-old (89.07 ± 6.30 years). In the 12 weeks, the participants showed an average increase of 4.2 points in acceptance, with a significant correlation (p < 0.001). It was possible to observe an improvement in the functional capacity of the oldest old, although not significant, with a decrease in the time to perform the Time Up and Go tests, sit and stand, and in walking time, which is the most evident change.

Conclusions: This study demonstrated that a home training program with weekly monitoring by videoconference was well accepted and suitable for oldest-old people in a period of social isolation imposed by COVID-19. In addition, it proved to be an effective intervention for maintaining and improving the functional capacity of oldest-old people.

### IMPACT OF FRAILTY ON THE DIGNITY OF COMMUNITY-DWELLING OLDER PEOPLE

Ali Darvishpoor Kakhki, Fereshteh Moradoghli, and Roghayeh Esmaeili, *Shahid Beheshti University of Medical Sciences, Tehran, Tehran, Iran*

The population of people aged 60 and older is rapidly increasing in developing countries such as Iran due to declining birth rates and increased life expectancy. Old age is associated with increased risk for frailty and reduced dignity. This study investigated the impact of frailty on the dignity of older people in Tehran, Iran. This cross-sectional study was conducted on 200 individuals aged 60 years and older. Data collection relied on the Demographic Questionnaire, Frailty Index for Elders (FIFE) and the Patient Dignity Inventory (PDI). Data were analyzed with SPSS 25. The mean age of the participants was 68 (±5.05) years; 62% of the participants were at risk for frailty, and 69% had few dignity-related problems. The multiple regression results showed that frailty was significantly associated with dignity (ß = -0.571, p < 0.001). The association was significant across all the dimensions of dignity measured by the PDI. The highest predictors of frailty included dependency (ß = -0.584, p < 0.001), followed by existential distress (ß = -0.560, p < 0.001), symptom distress (ß = -0.400, p = 0.400), social support (ß = -0.391, p < 0.001), and peace of mind (ß = -0.338, p < 0.001) in dignity. The results show that higher levels of frailty in older people are associated with decreases in their dignity, and frailty was the leading predictor of dignity. Providers should develop programs to prevent and reduce frailty in those at risk and to enhance the dignity of the already frail.

### PHYSICAL ACTIVITY TRENDS IN A NATIONWIDE MHEALTH PROGRAM: A POPULATION-BASED COHORT STUDY

Gregory Ang[1], Sarah Edney[1], Chuen Seng Tan[1], Nicole Lim[2], Jeremy Tan[2], Falk Riemenschneider[1], and Cynthia Chen[3], *1. National University of Singapore, Singapore, Singapore, 2. Health Promotion Board, Singapore, Singapore, 3. National University of Singapore, Singapore, Singapore*

Physical inactivity is a global public health challenge, leading to an increase in chronic diseases. Effective large-scale physical activity interventions are needed. We examined the effectiveness of a nationwide mHealth intervention in Singapore, National Steps Challenge Season 3 (NSC3). NSC3 included gain-framed financial incentives for reaching pre-defined daily step targets, nudging via reminders and real-time feedback on physical activity levels. Our study includes 411,528 participants, with 53,371 (13.0%) participants aged 59 and above. Regression discontinuity design examined changes to daily step counts prior to and during NSC3. NSC3 was associated with an overall mean increase of 1437 steps per day (95% CI: 1408 to 1467). Participants in older age groups were associated with larger mean step increases. Females aged between 59 to 68 and 69 and above were associated with average increases of 1640 steps per day (95% CI: 1510 to 1770) and 2300 steps per day (95% CI: 2050 to 2550), respectively. Males in the same age groups were associated with average increases of 1360 steps per day (95% CI: 1200 to 1520) and 1640 steps per day (95% CI: 1370 to 1900), respectively. We provide real-world evidence that suggests NSC3 improved participants' step counts, with a larger increase among older adults. As older adults have a higher risk of chronic diseases, lower physical inactivity has potential health benefits such as reducing the incidence of hypertension and improving cardiovascular health biomarkers. While results are promising, further investigations are necessary to ensure sustained engagement among older adults.

### SESSION 3630 (PAPER)

### PHYSICAL, SOCIAL, AND PLEASANT ACTIVITIES AND HEALTH

### COMPARING EXERCISE DETERMINANTS BETWEEN BLACK AND WHITE OLDER ADULTS WITH HEART FAILURE

Navin Kaushal[1], Donya Nemati[1], Dylan Mann-Krzisnik[2], and Adrian Noriega de la Colina[3], *1. Indiana University, Indianapolis, Indiana, United States, 2. McGill University, Montreal, Quebec, Canada, 3. McGill University, Westmount, Quebec, Canada*

Heart Failure (HF) is a leading cause of mortality among older-adults (OA); however, engaging in moderate-to-vigorous intensity of physical activity (MVPA) improves survival rates. Limited research has applied behavior theory to understand determinants of MVPA among OA with HF and compared the effects between races. This study aimed to use the Health Belief Model (HBM) to compare MVPA determinants between Black and White OA diagnosed with HF.

Methods: The HF-ACTION Trial is a multi-site trial that helped facilitate physical activity among individuals with HF. The present study used structural equation modeling to test the change in HBM determinants and MVPA across 12 months among OA.

Results: Participants were OA 72.28 (SD= 5.41) years old, (Black: n= 230; White, n= 441). The model found MVPA to be facilitated by perceived benefits (Black: $\beta$=.27, p=.001; White: $\beta$=.28; p<.001), self-efficacy (Black: $\beta$=.20, p=.003; White: $\beta$=.24; p<.001) and deterred by perceived barriers (Black: $\beta$=-.25, p=.001; White $\beta$=-.24; p<.001). Perceived threats deterred Black OA ($\beta$=-.23; p=.002) but not White OA ($\beta$=-.07; p=.278) from MVPA. Experimental arm predicted the development of self-efficacy (Black: $\beta$=.21. p=.001; White: $\beta$=.23; p=.001), and perceived benefits (Black: $\beta$=.21. p=.001; White: $\beta$=.23; p=.001) but not barriers (p>.05) for both races.

Conclusions: Perceived barriers was a strong determinant to MVPA, and it was not resolved in the experimental arm for either race. The experimental arm did not resolve perceived threat, which was a deterrent for Black OA. Individual characteristics need to be considered when designing programs for HF patients to reduce health disparity.

### SOCIAL PARTICIPATION AND DEPRESSIVE SYMPTOMS AMONG SPOUSAL AND ADULT CHILD CAREGIVERS

Elliane Irani, and Fei Wang, *Case Western Reserve University, Cleveland, Ohio, United States*

Participating in social activities through formal (e.g., social or religious organizations) and informal (e.g., gatherings with family members or friends) avenues is known to positively contribute to mental wellbeing. Family caregivers are at risk for limited social participation and increased depressive symptoms. However, little is known about which social activities are associated with depressive symptoms among spousal and adult child caregivers. The purpose of this study was to examine the effects of participating in various social activities on depressive symptoms among spousal (n=422) and adult child (n=1,112) caregivers using data from the 2017 wave of the National Study of Caregiving. Caregivers reported on participating in six social activities in the past month (e.g., visiting family and friends, attending religious services, doing volunteer work, working for pay) and completed the 2-item Patient Health Questionnaire to assess depressive symptoms. Data were analyzed using multiple linear regression, with separated models for spousal and adult child caregivers. Sociodemographic characteristics, self-rated health, and dementia caregiving status were included as covariates. For spousal caregivers, visiting friends or family and attending religious services were associated with lower depressive symptoms (b=-.55, p=.010 and b=-.33, p=.036, respectively). For adult child caregivers, going out for enjoyment was associated with lower depressive symptoms (b=-.80, p<.001). Findings suggest that spousal and adult child caregivers may benefit from participating in different types of social activities. Interventions targeting social participation to reduce depressive symptoms need to be tailored to the needs and preferences of spousal and adult child caregivers to be most effective.

### THE MULTIDIMENSIONAL SUCCESSFUL AGING SCALE: DEVELOPMENT AND VALIDATION

Edwin K. H. Chung, and Dannii Yeung, *City University of Hong Kong, Kowloon, Hong Kong*

Subjective successful aging involves a self-appraisal of individual aging process over multiple dimensions of later life (Feng et al., 2015). However, studies often ignore its multidimensional nature and simply utilize a single item as the measurement (e.g., Stewart et al., 2019). Two studies were hence conducted to develop and validate the Multidimensional Successful Aging Scale (MSAS). In Study 1, MSAS was administered among 414 community-dwelling older Chinese adults (Mage=64.50 years, SD=4.01; range = 60 – 82; 55.3% females). Repeated exploratory factor analysis revealed a nine-factor solution of 25 items, accounting for 64% of the variance. The nine factors include harmonious family, active engagement, supportive friendship, adaptive coping, social contribution, living independently, civic awareness, positive attitudes, and perceived constraints. The nine factors exhibit similar strength of associations with most of the well-being measures, but certain factors show stronger correlation with depressive symptoms, suggesting the uniqueness of each factor. In Study 2, the nine-factor model of MSAS was replicated in a sample of 558 older adults (Mage=69.11 years, SD=6.68; range = 60 – 97; 59.2% females) and the results of CFA indicated a good model fit ($\chi 2$ = 527.86, df = 239; CFI =.97; TLI =.96; RMSEA =.05; SRMR = .04). The strict and metric forms of measurement invariance were shown across age groups and gender, respectively. Overall, the MSAS demonstrates satisfactory psychometric properties. These findings disclose that the older adults' perceptions of successful aging cover broader dimensions than those identified in the Rowe and Kahn's (1997) model.

### SESSION 3640 (SYMPOSIUM)

### POLICY ATTENTION NEEDED TO IMPROVE DIRECT CARE WORKER WAGES AND OTHER CHALLENGES

Chair: Denise Tyler Discussant: Robyn Stone

Direct care workers (DCWs), including nursing assistants, home health aides, and personal care assistants, play an essential role in the health and well-being of over 20 million

Americans who receive long-term services and supports (LTSS) at home, in nursing facilities, and other settings. In 2020, nearly 4 million DCWs supported older adults and people with disabilities in completing self-care and other daily tasks. Their efforts require considerable technical and interpersonal skills, but these essential workers receive low pay and rarely receive benefits. The COVID-19 pandemic has increased the attention paid to DCWs in the media and among policymakers. This symposium presents the results of three studies conducted by RTI International and the Office of the Assistant Secretary for Planning and Evaluation (ASPE) focused on DCWs. First, we present the results of a study examining how the wages of DCWs compare to the wages of other entry-level workers across all 50 states and report the effect of state policies aimed at improving DCW wages. Second, we report findings from a study exploring the experiences of home care aides during the pandemic as well as the federal and state policies implemented to assist these DCWs during the pandemic. Finally, we present the results of a study that assessed the effect of the COVID-19 pandemic on DCW staffing in nursing facilities. Together these studies suggest that more policy attention is needed to improve direct care work and attract the millions of additional DCWs that are expected to be needed in coming years.

### EFFECT OF STATE WAGE POLICIES ON DIRECT CARE WORKER WAGES

Denise Tyler[1], Olga Khavjou[2], Melissa Hunter[3], Marie Squillace[4], Judith Dey[5], and Iara Oliveira[5], *1. RTI International, Waltham, Massachusetts, United States, 2. RTI International, Research Triangle Park, North Carolina, United States, 3. RTI International, Durham, North Carolina, United States, 4. ASPE, Washington, District of Columbia, United States, 5. Office of the Assistant Secretary for Planning and Evaluation, Washington, District of Columbia, United States*

Direct care workers (DCWs) have low wages and some states have tried to improve their wages through policies such as Medicaid wage pass-throughs and wage floors specific to DCWs. The purpose of this study was to examine the wages of DCWs in comparison to those of other entry level workers and assess the effect of state wage policies on changes in DCW wages. We analyzed state-level hourly wages using Bureau of Labor Statistics (BLS) data for two categories of DCWs separately—(1) home health and personal care aides and (2) nursing assistants and compared these to wages for other entry-level workers. Results show that many states that implemented policies to improve the wages of DCWs reduced the gap between these workers' wages and the wages of other entry-level workers, but the gap was still substantial in many states. Additional efforts will be needed to increase DCW wages.

### COVID-19 INTENSIFIED HOME CARE WORKFORCE CHALLENGES

Melissa Hunter[1], Denise Tyler[2], Kristie Porter[1], Judith Dey[3], Marie Squillace[4], William Haltermann[4], and Iara Oliveira[3], *1. RTI International, Durham, North Carolina, United States, 2. RTI International, Waltham, Massachusetts, United States, 3. Office of the Assistant Secretary for Planning and Evaluation, Washington, District of Columbia, United States, 4. ASPE, Washington, District of Columbia, United States*

The COVID-19 pandemic affected home care workers in several important ways. The purpose of this study was to detail the challenges faced and the state and federal policy responses implemented to address those challenges. We conducted scans of federal and state policies enacted during the pandemic, home care agency practices described in media reports and the literature, and interviews with 25 stakeholders. Some challenges encountered were entirely new and resulted directly from the pandemic, such as limited access to personal protective equipment due to workers not being recognized as "essential." In other cases, the pandemic worsened long-standing challenges, such as recruitment and retention. State policy responses included Medicaid rate increases, hazard pay, and changes to staff training requirements. Federal policy responses included disaster relief funding as well as allowing non-physician practitioners to order services. Stakeholders suggested additional policy changes will be needed to address ongoing challenges experienced by this workforce.

### COVID-19 PANDEMIC INCREASED NURSING FACILITIES' RELIANCE ON CONTRACT STAFF TO ADDRESS STAFFING SHORTAGES

Kristie Porter[1], Micah Segelman[2], Angela Gasdaska[3], Marie Squillace[4], Judy Dey[5], Iara Oliveira[5], and Denise Tyler[6], *1. RTI International, Durham, North Carolina, United States, 2. Research Triangle Institute, Washington, District of Columbia, United States, 3. RTI International, Research Triangle Park, North Carolina, United States, 4. ASPE, Washington, District of Columbia, United States, 5. Office of the Assistant Secretary for Planning and Evaluation, Washington, District of Columbia, United States, 6. RTI International, Waltham, Massachusetts, United States*

Nursing facilities (NFs) used a variety of strategies to mitigate worsening nursing staff shortages during the COVID-19 pandemic. We conducted analysis of payroll based journal PBJ data and interviewed 9 experts to understand COVID's impact on NF staffing, especially changes in contract staffing. In 2020, NFs increased their use of contract staff hours per resident day (HPRD) by 24%. Use of contract staff during the first year of the pandemic (comparing 2020 to 2019) varied by some facility characteristics (e.g., profit status). NF providers, and industry and academic experts confirmed facilities used multiple strategies beyond using contract staffing, such as temporary nurse aides, hazard pay and flexible schedules to maintain staffing throughout the pandemic. Competition for nursing staff from other healthcare settings and non-healthcare industries has grown throughout the pandemic, continuing to threaten the stability of the NF workforce.

### SESSION 3650 (SYMPOSIUM)

### POLYPHARMACY IN ASSISTED LIVING: WHERE ARE WE NOW AND EFFECTIVE APPROACHES TO IMPROVE MEDICATION MANAGEMENT

Chair: Barbara Resnick

There are many definitions for polypharmacy used within clinical practice and research and many factors contributing to this problem. Definitions vary from being based on the number of medications the older adult is taking to a more qualitative evaluation of the appropriateness of medication based on the benefit of the drug for a specific problem. For research purposes the definition is more commonly conceptualized as being equivalent to taking five or more medicines. Polypharmacy is noted to be presented in about 40% of older adults living in the community. Limited research has focused specifically on polypharmacy in assisted living settings. In addition to concerns about polypharmacy in assisted living there has also been a focus on the use of psychotropic medication and opioids in these settings as prevalence ranges from 53% to 68%. Although there are not regulations related to decreasing polypharmacy via deprescribing or to decrease use of psychotropics or opioids in assisted living, there are currently major initiatives in geriatrics to focus on these areas. This symposium will provide current data on medication use and polypharmacy among a large sample of 781 assisted living residents from 85 communities across three states and address the impact of a Function Focused Care approach on decreasing polypharmacy and use of psychotropics and opioids. Lastly data will be provided on the value of Deprescribing Networks to help decrease polypharmacy within these settings. The findings from this symposium will provide recommendations for future research as well as guidance for clinical practice.

### THE IMPACT OF FFC-AC-EIT ON DECREASING USE OF PSYCHOTROPIC MEDICATIONS AND OPIOIDS IN ASSISTED LIVING

Barbara Resnick, *University of Maryland, Baltimore, Maryland, United States*

The secondary aim of the study testing the impact of Function Focused Care for Assisted Living Using the Evidence Integration Triangle (FFC-AL-EIT) was to decrease psychotropic medications and opioids among assisted living residents. Function Focused Care is a philosophy of care in which direct care workers are taught how to evaluate older adults' underlying function and physical activity and optimize their participation in all activities. This randomized controlled trial included 85 communities and 781 residents across three states. A total of 501 out of 794 participants (63%) received at least one psychotropic medication or opioid. Except for opioid use at four months, there was no significant difference in the intervention groups with regard to medication use at any time point. The findings suggest that encouraging participation in physical activity during all care interactions does not result in an increase in the need for and prescribing of psychotropic medications or opioids.

### POLYPHARMACY IN ASSISTED LIVING

Elizabeth Galik, *University of Maryland, Baltimore, Baltimore, Maryland, United States*

The purpose of this study was to describe polypharmacy in assisted living settings, evaluate the factors that influence polypharmacy and the impact of polypharmacy on clinical outcomes. Baseline data from the study entitled, Dissemination and Implementation of Function Focused Care for Assisted Living Using the Evidence Integration Triangle (FFC-AL-EIT) was used. Total number of drugs taken daily among the 781 participants was 5.16 (SD=2.40) and over half (N=484, 62%) were exposed to polypharmacy. None of the predicted variables (age, gender, race, setting, diagnoses, and cognition) were associated with polypharmacy (Wald = .207, p=.65). Similarly, controlling for age, gender, race, setting, diagnoses, cognition, function, and physical activity, polypharmacy was not associated with falls, emergency room visits or hospitalizations. Factors not included in the data contributing to the high rate of polypharmacy in assisted living settings will be discussed and recommendations for further research and practice implications reviewed.

### DEPRESCRIBING IN ASSISTED LIVING

Barbara Zarowitz[1], and Nicole Brandt[2], *1. University of Maryland School of Pharmacy, Las Vegas, Nevada, United States, 2. University of Maryland, Baltimore, Baltimore, Maryland, United States*

Deprescribing is defined as the thoughtful process of "tapering, stopping, discontinuing, or withdrawing drugs, with the goal of managing polypharmacy and improving outcomes." There are multiple clinical issues that deprescribing can address such as: antibiotic resistance caused by inappropriate and excessive use, the ongoing opioid epidemic, as well as over treatment particularly at the end of life. Networks have been established to address deprescribing across settings including assisted living nationally and internationally. Fourteen key informants from these networks were interviewed including different disciplines. From the interviews, six major themes across two domains were identified. The two domains included regional resources and knowledge gaps and the six themes included: (a) network structure, (b) public perception, (c) policy implications, (d) implementation, (e) challenges, and (f) recommendations. Overall, the importance of collaboration among interprofessional team members will be critical to the success of deprescribing as this clinical issue moves ahead.

### SESSION 3660 (PAPER)

### RESEARCH TO ADDRESS THE NEEDS OF OLDER ADULTS USING LONG-TERM SERVICES AND SUPPORTS

### CONTENDING WITH UNCERTAINTY: IMPLEMENTING THE CMS ACUTE HOSPITAL CARE AT HOME WAIVER PROGRAM IN THE UNITED STATES

Ksenia Gorbenko[1], Emily Franzosa[1], Abigail Baim-Lance[1], Gabrielle Schiller[2], Heather Wurtz[1], Sybil Masse[1], David Levine[3], and Albert Siu[1], *1. Icahn School of Medicine at Mount Sinai, New York, New York, United States, 2. CUNY School of Public Health, Astoria, New York, United States, 3. Mass General Brigham, Boston, Massachusetts, United States*

As Congress considers renewing the Acute Hospital Care At Home (AHCaH) waiver, which provides a full hospital payment for Hospital at Home (HaH) care, evaluating uncertainty around the future of HaH payment is critical. Our qualitative study explored HaH leaders' experiences with implementing HaH (N=18, clinical/medical directors, operational and program managers) from 14 new and pre-existing programs across the U.S. We conducted semi-structured interviews with HaH programs diverse by size, urbanicity, and geography. We analyzed transcripts using a thematic approach. Participants across settings and regions wanted greater clarity about the waiver's future. Lack of clarity affected staffing (nurses reluctant to take temporary jobs) and investment in establishing programs (building EMR components, changing workflows, creating inpatient processes in an outpatient setting). Programs adapted to uncertainty in multiple ways: 1) operating parallel waiver and non-waiver programs; 2) seeking to determine/ calculate the HaH value for their institution; 3) determining which patients would benefit most from HaH; and 4) seeking additional health system financing options beyond the CMS reimbursement (new programs) or relying on existing contracts with payers (existing programs). Implementing HaH is a complex and resource intensive process. Greater clarity from CMS regarding the waiver's future state will encourage programs to invest the resources that they need to establish their programs long-term. Waiver extension/ permanence would also enable programs to develop and test measures of value, making rigorous evaluations possible to optimize different HaH components.

### EXPLORING QUALITY AND COVID MEASURES OF NEW YORK STATE LONG-TERM CARE FACILITIES INVOLVED IN THE SAFE STAFFING LAWSUIT

Jason Semprini[1], and Brian Kaskie[2], *1. University of Iowa College of Public Health, Iowa City, Iowa, United States, 2. University of Iowa, Iowa City, Iowa, United States*

In April 2021, New York's "Safe Staffing" law capped Long-Term Care Facility (LTCF) profits. LTCFs with "excess profits" are now challenging the law in court. This study examined how LTCFs involved in the lawsuit differed from other NY state LTCFs before and during the COVID-19 pandemic. LTCF "Safe Staffing" lawsuit data were obtained from Long Term Care Community Coalition, then linked with Centers for Medicare and Medicaid Services COVID-19 and Small Business Association Paycheck Protection Program (PPP) data. First, we tested for differences across quality measures. We found that, compared to LTCFs not involved in the lawsuit, LTCFs in the lawsuit were more likely to be located outside of a hospital, report more certified beds and higher occupancy rates, and have higher overall quality scores. LTCFs in the lawsuit also reported lower staff ratings and staffing hours, which have previously been identified as a determinant of higher mortality in LTCFs. To create valid comparisons given these systematic differences, we specified "Doubly Robust" Augmented Inverse Probability Weighted regression models and tested if lawsuit involvement was associated with COVID-19 outcomes. Despite finding higher rates of admitting patients infected with COVID-19 in "excess profit" LTCFs, we did not find that COVID-19 deaths differed by lawsuit involvement. Finally, lawsuit involvement was associated with a higher probability of receiving a PPP loan. Before and during the pandemic, LTCFs with "excess profits" appeared fundamentally different than other NY LTCFs. How these differences impact the health of older adults receiving long-term care beyond the pandemic remains unknown.

### FACTORS ASSOCIATED WITH THE AVAILABILITY OF SPECIALIZED RESOURCES TO TACKLE OBESITY IN HIGH MEDICAID NURSING HOMES

Gregory Orewa[1], Ganisher Davlyatov[2], Rohit Pradhan[3], Justin Lord[4], and Robert Weech-Maldonado[1], *1. University of Alabama at Birmingham, Birmingham, Alabama, United States, 2. University of Oklahoma, Oklahoma City, Oklahoma, United States, 3. West Liberty University, West Liberty, West Virginia, United States, 4. Louisiana State University at Shreveport, Bossier City, Louisiana, United States*

The purpose of this research is to explore the factors associated with the availability of specialized resources required to care for obese residents in high Medicaid nursing homes (NHs) (85% or higher). Due to the vagaries of payment models--Medicaid payments lag other modes of NH reimbursements—high Medicaid NHs typically report poorer quality and financial performance. Operating in a financial perilous environment, and with obesity among the elderly on the rise, high Medicaid NH may particularly struggle to obtain the appropriate resources essential to cater to obese residents' needs. Utilizing the resource-dependent theory, we hypothesized that occupancy rate, acuity index, and payer mix may be positively associated with the availability of obesity related specialized equipment in high Medicaid NHs. The study was conducted by merging survey and secondary data sources for the year 2017-2018. Obesity related data was collected via mail surveys sent to Directors of Nursing in high Medicaid NHs, The survey data was merged with the following secondary data sources: Brown University's LTC Focus, Area Health Resource File, and the Medicare cost reports. The dependent variable was the summative obesity score that ranged from 0-19 with the larger number indicating greater availability of obesity-related equipment/services. An ordinary least square regression with propensity score weights (to adjust for potential non-response bias), and appropriate organizational/market level control variables were used for our analysis. Results suggest that payer mix (>Medicare residents) and acuity index were positively associated with the summative obesity score ($p < 0.05$). Policy and managerial implications are discussed.

### INFORMATION SHARING TO SUPPORT CARE TRANSITIONS FOR PATIENTS WITH COMPLEX MENTAL AND BEHAVIORAL HEALTH NEEDS

Taylor Bucy, and Dori Cross, *University of Minnesota School of Public Health, Minneapolis, Minnesota, United States*

Information sharing practices between hospitals and skilled nursing facilities (SNFs) are insufficient to effectively support patient handoffs. Information needs are even greater for SNFs that admit patients with complex behavioral needs. It is unclear whether these needs have prompted hospital investment in enhanced information sharing with these SNFs, and what strategies these facilities are using to meet informational needs. We use data from a 2019 nationally representative SNF survey (N=265, response rate 53%) designed to gather information on information sharing practices with hospital partners. 122 SNFs (57% of respondents) report accepting at least two of the following complex conditions: serious mental illness, substance use disorder, or medication assisted treatment. Using logistic regression models that adjust for facility ownership and rurality, SNFs that accept complex patients are significantly more likely to receive information on behavioral, mental, and functional status compared to facilities who accept none or only one type of complex patient (odds ratio=2.42; p=0.023). Unadjusted models indicate that facilities that accept complex patients lag in IT-facilitated access to hospital information, and report more difficulty securing timely access to information. The significance of these findings do not persist after adjustment, suggesting structural differences in the types of SNFs that hospitals are partnering with to improve information sharing. We conclude that while SNFs that accept complex patients are mostly keeping pace or even doing slightly better in terms of access to hospital information that supports transitional care, further investment is needed to improve hospital information sharing behaviors.

### REBALANCING LONG-TERM CARE: THE ROLE OF MEDICAID-MANAGED LONG-TERM SERVICES AND SUPPORTS

Howard Degenholtz, *University of Pittsburgh, Pittsburgh, Pennsylvania, United States*

From 2018 to 2020, Pennsylvania conducted a phased implementation of a mandatory Medicaid managed long-term services and supports program called Community HealthChoices (CHC). The new program covers people receiving Medicaid financed long-term services and supports (LTSS) in nursing homes and home and community-based settings. The three participating MCOs are incentivized to serve people in the community. This study took advantage of the phased implementation to generate causal estimates of the effect of the new program on both the extensive margin (the proportion of people receiving HCBS vs. nursing homes), and the intensive margin (the type and amount of HCBS services). Medicaid claims data for the years 2013 to 2020 were analyzed to examine pre-program trends and program effects. There was a long-term pre-program trend away from nursing homes. The implementation of managed care did not appear to accelerate this trend, however, analysis of data from 2020 is complicated by the COVID-19 pandemic. However, MLTSS did appear to control to growth in hours of personal care per person both in the aggregate and longitudinally within the same individuals over time. There were decreases in the use of adult day services and home delivered meals. However, the decline in home delivered meals was more than offset by an increase in uptake of the supplementary nutritional assistance program (SNAP). This is the first causal analysis of the effects of managed care on use of long-term services and supports. The findings have implications for other states that are considering implementing similar policies.

### SESSION 3670 (SYMPOSIUM)

### TECHNOLOGY DESIGN AND IMPLEMENTATION TO PROMOTE EQUITY, INCLUSION, AND DIVERSITY

Chair: Walter Boot

The design of technology and technology-based solutions for the challenges many older adults face must consider principles of equity, inclusion, and diversity, or there is a risk of exacerbating digital divides in our society. This session focuses on the design, evaluation, and implementation of technologies to support older adults considering the diversity of the older adult population with respect to factors such as race and ethnicity, income level, health conditions, and cognitive status. M. Harris will discuss the facilitators and barriers identified by Black older adults related to the use of wearable devices for health monitoring. J. Chung will present on the development of a smart speaker application to support wellness among low-income senior housing residents. C. Berridge and E. Sanders will discuss designing technology solutions related to diversity with respect to cognitive status, with Berridge discussing preferences of older adults with mild Alzheimer's disease for a technology-delivered planning tool, and Sanders presenting technology-based solutions to support the prospective memory of older adults with diverse cognitive impairments. Finally, Schiaffino will present a study examining the acceptability of community-based telehealth programs among vulnerable older adults as a function off health, language, and ethnicity. General themes of inclusive design to ensure that all older adults can benefit from existing and emerging technology-based solutions will be highlighted.

### EXPLORING THE POTENTIAL OF WEARABLE DEVICES FOR HEALTH MONITORING: A FOCUS ON BLACK OLDER ADULTS

Maurita Harris[1], Shaunik Kapoor[2], and Wendy Rogers[3], *1. University of Illinois Urbana-Champaign, Champaign, Illinois, United States, 2. skapoo21@illinois.edu, Champaign, Illinois, United States, 3. University of Illinois Urbana Champaign, Champaign, Illinois, United States*

Chronic diseases are some of the top conditions leading to death in the United States. Management of these chronic diseases could benefit from monitoring risk factors, such as physical activity. Wearable devices (e.g., Fitbit) have the capability to track and give information to allow the individual to take control of their health. However, wearables are typically advertised to young, physically active, and individuals who belong to racial groups with the highest population. As such, the purpose of this study was to understand Black adults' opinions and attitudes towards wearable design to support usage, focusing on both users and non-users. Participants were interviewed to explore their wants and needs towards wearables to support usage (e.g., aesthetics) and to overcome barriers (e.g., usability concerns). The findings from the thematic analysis will be presented to illustrate these facilitators and barriers identified by Black adults. These insights can guide future inclusive design.

### ENGAGING LOW-INCOME SENIORS IN PARTICIPATORY DESIGN OF SMART SPEAKER APPLICATIONS FOR WELLNESS

Jane Chung[1], Jodi Winship[1], Tracey Gendron[2], Rachel Wood[3], Natalie Mansion[2], and George Demiris[4], *1. Virginia Commonwealth University, Richmond, Virginia, United States, 2. Virginia Commonwealth University College of Health Professions, Richmond, Virginia, United States, 3. Bon Secours Mercy Health System, Cincinnati, Ohio, United States, 4. University of Pennsylvania, Philadelphia, Pennsylvania, United States*

Low-income senior housing (LISH) residents are at a high risk of unmanaged health conditions, loneliness, and limited healthcare access. Smart speakers have the potential to improve wellness in LISH settings. We conducted a user-centered process with primarily African American, LISH residents (N=25) to develop prototypes of smart speaker applications for wellness and social connections. Five focus groups were conducted to elicit feedback about challenges with maintaining wellness and attitudes towards smart speakers. Participants expressed their desires for using the technology for safety and health. Through design workshops, they identified several smart speaker functionalities perceived as necessary for improving wellness and social connectedness. Then, seven low-fidelity prototypes and scenarios were developed in the following categories: wellness check-ins, befriending the virtual agent, community involvement, and mood detection. We demonstrate how smart speakers can provide a tool for their wellness and increase access to applications that provide a virtual space for social engagement.

### PILOT STUDY OF A SELF-ADMINISTERED ADVANCE PLANNING TOOL FOR TECHNOLOGY USE WITH DEMENTIA CARE DYADS

Clara Berridge, Natalie Turner, and Liu Liu, *University of Washington, Seattle, Washington, United States*

We present feasibility and preliminary efficacy findings from a novel web-app intervention to educate and facilitate dyadic communication about four categories of technologies used in dementia care and to document preferences of people living with mild AD regarding these technologies. A pre/post-test design was conducted. Eighty-eight percent of the 66 enrolled participants completed the study for a total of 29 dyads. Participants gave favorable ratings of satisfaction, helpfulness, clarity, and other usability measures. While improvements on multiple measures were of greater magnitude for care partners than for people living with dementia (PLWD), paired t-tests showed statistically significant improvement with medium and large effect sizes in PLWD (p<.001) and care partners' ( p<.001) self-reported understanding of the technologies, as well as care partners' perceptions of the PLWD's understanding (p<.01). After completing the web-app together, care partners felt significantly more prepared to make decisions about these technologies (p<.01).

### UNDERSTANDING THE UNIQUE NEEDS OF VULNERABLE OLDER ADULTS IN A COMMUNITY-BASED TELEHEALTH PROGRAM

Melody Schiaffino[1], Zhan Zhang[2], Pratik Chaudhari[2], and Jina Huh-Yoo[3], *1. San Diego State University, San Diego, California, United States, 2. CSIS, New York, New York, United States, 3. College of Computing and Informatiics, Philadelphia, Pennsylvania, United States*

Vulnerable older adults benefit from community-based telehealth programs (CTP) that facilitate remote health monitoring with support from trained personnel. This study assessed acceptability with such technology as a self-reported measure of comfort among participants in an on-going CTP, the Telehealth Intervention Program for Seniors (TIPS). We analyzed data from participants across 20 sites (N=2279), 38% responded to their comfort with technology (n=866). We modeled self-reported factors to explore the association with technology acceptability. There was more comfort with technology than not (53.5% vs 46.5%). Participants under age 65, those reporting better vs poorer health (p<0.0001) and a happier mood state (p<0.0001) were more likely to be comfortable. Older adults and much older adults reported greater odds of comfort compared with those under 65. Better health status was associated with 84.5% greater odds of acceptability compared to those with poor (AOR 1.85; 95CI 1.28-2.65). Happier participants reported 56% greater odds of comfort compared with those reporting unhappiness. Though only marginally significant, non-English speaking participants reported greater odds of comfort compared to English proficient. While ethnicity was not associated, our marginal significance for language suggests a need to continue exploring. Our work demonstrates the need to address the unique needs of older adults.

### ACCOUNTING FOR DIVERSITY IN COGNITIVE STATUS IN THE DESIGN AND EVALUATION OF INCLUSIVE PROSPECTIVE MEMORY SOLUTIONS

Edie Sanders[1], Walter Boot[2], and Robin Stuart[2], *1. Florida State University, TALLAHASSEE, Florida, United States, 2. Florida State University, Tallahassee, Florida, United States*

Technology holds tremendous promise for supporting older adults' performance of important everyday activities. However, truly inclusive design of technology-based solutions must account for diversity with respect to cognitive status. This talk will focus on empirical studies conducted under the umbrella of the Enhancing Neurocognitive Health, Abilities, Networks, & Community Engagement (ENHANCE) Center with an emphasis on designing inclusive prospective memory solutions for older adults with cognitive impairments. Initial usability studies will be discussed examining the usability and efficacy of novel technology solutions, including the use of smartwatches and digital assistants, to support prospective memory, the ability to remember and carry out an intention in the future, which is crucial for maintaining health, independence, and social connections.

### SESSION 3680 (SYMPOSIUM)

### THE GEROPSYCHIATRIC NURSING COLLABORATIVE: ADDRESSING BEHAVIORAL NEEDS OF PERSONS WITH DEMENTIA

Chair: Lauren Massimo Co-Chair: Lauren Hunt Discussant: Kathleen Buckwalter

Over the last decade, the Geropsychiatric Nursing Collaborative (GPNC) has sought to improve care of older adults with mental health needs such as those with dementia. Geropsychiatric nurses are well-poised to deliver person-centered care to address the psychosocial needs of persons with dementia, which may include behavioral expressions. In this interdisciplinary symposium, we will highlight approaches members of the GPNC are taking to address mood and behavior in persons with dementia. The first session will describe how persons with mild-cognitive impairment draw upon their inner strength after they receive a diagnosis. The second session will discuss how common behavioral expressions such as anxiety, depression and apathy contribute to difficulty with everyday functional activities in persons with Alzheimer's disease. The third session will describe an interprofessional communication tool to improve communication of behavioral expression in the nursing home setting. The last session will share findings from a nurse practitioner-led team care management model to address cognitive vulnerability in older adults with dementia, depression, and/or delirium. Together, these presentations describe how geropsychiatric nurses are investigating mood and behavioral symptoms in persons with dementia and addressing their mental health needs with innovative person-centered interventions.

### EXPERIENCES OF INNER STRENGTH AT THE TIME OF MILD COGNITIVE IMPAIRMENT DIAGNOSIS

Brianna Morgan[1], Lauren Massimo[1], Sharon Ravitch[1], Jason Karlawish[2], and Nancy Hodgson[1], *1. University of*

*Pennsylvania, Philadelphia, Pennsylvania, United States, 2. University of Pennsylvania Perelman School of Medicine, Philadelphia, Pennsylvania, United States*

Inner strength is a person's internal process of moving through challenging circumstances, such as receiving a diagnosis of Mild Cognitive Impairment (MCI). This study describes experiences of inner strength using qualitative methodologies to identify themes within semi-structured dyadic and individual interviews with persons diagnosed with MCI within 12 months at a Memory Center and their care partners. We analyzed data in NVivo using reflexive thematic analysis methods. Trustworthiness was maintained through vetted interview guides, verbatim transcription, field notes, peer group analysis, and audit trails. One overarching theme and three subthemes explained inner strength. An overarching theme, Finding Ways to Live with It, described how participants live within the circumstances of MCI. Three subthemes were Defining Strength by Recalling the Past, Seeking Relief and Dwelling in It, and Finding Purpose & Meaning. Implications include supporting inner strength at the time of MCI diagnosis through reminiscence therapy and meaning making interventions.

### APATHY AND ANXIETY ARE RELATED TO POOR FUNCTION IN PERSONS WITH EARLY-ONSET ALZHEIMER'S DISEASE

Adele Crouch, and Lauren Massimo, *University of Pennsylvania, Philadelphia, Pennsylvania, United States*

Neuropsychiatric symptoms are prevalent in persons with early-onset Alzheimer's disease (EOAD) and may contribute to the inability to perform instrumental activities of daily living. We examined associations between frequently observed symptoms in persons with EOAD: apathy, anxiety, depression, and patient function. Caregivers of 94 persons with EOAD completed questionnaires including the Neuropsychiatric Inventory and the Functional Activities Questionnaire. Regression analyses were performed for each neuropsychiatric symptom as a predictor with covariates (age, sex disease duration) and our outcome was patient function. We then performed multivariate analysis with the significant predictors. We observed that apathy explained 20.51% [$F(4,68)=5.65$, adjusted $R2=0.2051$; $p<0.001$], anxiety explained 6.63% [$F(4,70)=2.31$, adjusted $R2=0.0663$ $p<0.05$], and depression was not a significant predictor of patient function. In a multivariate model, apathy and anxiety explained 21.03% [$F(5,67)=4.83$, adjusted $R2=0.2103$; $p<0.001$] of the variance in patient function. These results suggest apathy and anxiety contribute to diminished ability to complete functional activities.

### DEMENTIA CARE IN NURSE PRACTITIONER-LED CARE MANAGEMENT FOR COGNITIVELY VULNERABLE OLDER ADULTS

Richard Fortinsky[1], and Shawn Ladda[2], *1. University of Connecticut Center on Aging, Farmington, Connecticut, United States, 2. UConn Center on Aging, University of Connecticut School of Medicine, Farmington, Connecticut, United States*

Care management approaches are being widely tested in the Medicare-eligible population to manage chronic conditions, but few have focused on cognitive vulnerability as the pathway to optimizing independence in the community-dwelling older population. Cognitive vulnerability refers to living with dementia, depression, and/or a history of delirium. This presentation features a nurse practitioner-led team care management model (3D Team) to address cognitive vulnerability, tested in an ongoing clinical trial with older adults in a Medicare Advantage population. For older adults with dementia and their families served by the 3D Team, the nurse practitioner works closely with occupational therapists (OTs) delivering a nonpharmacological dementia care intervention. Preliminary results presented will include: characteristics of dyads that have received the dementia care intervention (N=70 dyads to date), how the nurse practitioner and OTs communicate, how the nurse practitioner reinforces dementia care skill-building strategies introduced by OTs, and process evaluation results to date.

### SESSION 3690 (SYMPOSIUM)

### WHAT MAKES A BEAUTIFUL DAY IN THE NEIGHBORHOOD? PLACE AS A CONTRIBUTOR TO FUNCTION IN LATER LIFE

Chair: Andrea Rosso

Neighborhood environments are increasingly recognized as an important determinant of health and function in older adults. Environmental supports such as density of intersections and available community resources can promote activity and participation which in turn promotes physiological health. In contrast, barriers such as disorder and high traffic can limit activity and participation, particularly for those at high risk for mobility limitations and falls. Here, we present five papers exploring these relations. First, Kate Duchowny presents work assessing relations of the built and social environment with muscle strength in the Health and Retirement Study. Two papers utilizing walkability assessments using Google Street View in a physical activity intervention trial are presented; Kyle Moored demonstrates relations of neighborhood walkability with Global Positioning System (GPS)-derived time out of home and Anisha Suri assesses how the relation between actigraphy-derived gait quality and daily step counts differs by walkability. Next, Philippa Clarke presents data on the association of neighborhood environment with diabetes risk in those with low visual function in an administrative claims database. Finally, Pam Dunlap describes results of a systematic review of outdoor environmental risk factors for falls and fear of falling. Together, these papers will demonstrate the breadth of ways in which neighborhood environments and function relate to determine health outcomes for older adults.

### WHICH NEIGHBORHOOD FEATURES MATTER MOST FOR MUSCLE STRENGTH? FINDINGS FROM THE HEALTH AND RETIREMENT STUDY

Kate Duchowny[1], L. Grisell Diaz-Ramirez[1], W. John Boscardin[1], Peggy Cawthon[2], Maria Glymour[1], and Scarlett Lin Gomez[1], *1. University of California, San Francisco, San Francisco, California, United States, 2. California Pacific Medical Center Research Institute, San Francisco, California, United States*

Linking data from the National Neighborhood Data Archive (NaNDA) to the 2006-2018 Health and Retirement

Study (N=22,245), we fit linear mixed models to assess which of 22 built and social neighborhood environment variables predicted grip strength, a measure of total-body muscle strength. Among 22,245 respondents (mean age=63 years, SD=9.2) with up to 4 grip strength measures, neighborhood physical disorder (B= -0.25 kg, 95% CI= -0.37,-0.13), number of parks (B= 0.05 kg, 95% CI= 0.01, 0.10), number of gyms/fitness centers (B=-0.44 kg, 95% CI= -0.82, -0.07), proportion of highly developed land (B=-2.06 kg, 95% CI=-4.06,-0.07), and % urban (B=-0.66 kg, 95% CI=-1.27, -0.05) were associated with grip strength level after adjustment. No social neighborhood variables were associated with grip strength. Although preliminary, findings suggest that highly developed urbanized land may be a barrier to maintaining muscle strength in later life, but resources such as parks are associated with better outcomes.

### NEIGHBORHOOD WALKABILITY IS ASSOCIATED WITH GPS-DERIVED LIFE-SPACE MOBILITY OF OLDER ADULTS

Kyle Moored[1], Breanna Crane[1], Pamela Dunlap[2], Michelle Carlson[1], and Andrea Rosso[2], *1. Johns Hopkins Bloomberg School of Public Health, Baltimore, Maryland, United States, 2. University of Pittsburgh, Pittsburgh, Pennsylvania, United States*

For community-dwelling older adults, living in a more walkable neighborhood may encourage greater travel outdoors (i.e., life-space mobility). We assessed associations between neighborhood walkability and objective, GPS-derived life-space mobility. Participants were 149 adults (Age: M=77.1±6.5, 67% women) from a randomized trial to improve walking in older individuals. Participants carried a GPS at baseline that passively collected real-time location data for 5-7 days. Life-space mobility was quantified using median percentage time spent outside of home (pTOH). Neighborhood walkability was assessed using a modified Active Neighborhood Checklist and Google Street View, and a composite factor score was derived (M=-0.13±0.90, greater score=less slopes, more mixed residential/commercial land use). Each 1-point higher walkability score was associated with 2.5% greater pTOH (95%CI: 0.21-4.69, β=.23, p=.032), after adjusting for age, sex, race, device, season, and clustering on Census tract. Future work will examine how neighborhood walkability and functional status may interact to influence life-space mobility.

### NEIGHBORHOOD WALKABILITY INFLUENCES ASSOCIATIONS BETWEEN PHYSICAL ACTIVITY AND GAIT MEASURES IN OLDER ADULTS

Anisha Suri[1], Xiaonan Zhu[2], Geeta Acharya[2], Alyson Harding[3], Jessie VanSwearingen[4], Mark Redfern[1], Ervin Sejdic[5], and Andrea Rosso[2], *1. Swanson School of Engineering, University of Pittsburgh, Pittsburgh, Pennsylvania, United States, 2. University of Pittsburgh, Pittsburgh, Pennsylvania, United States, 3. University of Minnesota, Minneapolis, Minnesota, United States, 4. School of Health and Rehabilitation Sciences, Pittsburgh, Pennsylvania, United States, 5. University of Toronto, Toronto, Ontario, Canada*

Neighborhood walkability can influence physical activity and of older adults. Residential neighborhoods of participants (N=186, 77±6 years, 70% females) were audited for walkability using Google Street View. Factor analysis categorized neighborhood walkability as high, medium, and low. Gait quality was derived from a 4-m instrumented walkway (pace, variability, walk-ratio) and accelerometry signals at the lower back during a 6-minute walk test (adaptability, similarity, and smoothness). Activity was step-count from seven-day actigraphy. We studied associations between gait variables and step-count across high, medium, and low walkability neighborhoods using linear regression (age and sex as covariates). Pace(m/s) [High(β=0.46, p<.05), Medium(β=0.43, p<.05), Low(β=0.25, p>.05)], adaptability(m/s2) [High(β=0.47, p<.05), Medium(β=0.24, p<.05), Low(β=0.37, p<.05)], and similarity [High(β=0.39, p<.05), Medium(β=0.28, p<.05), and Low(β=0.18, p>.05)] were associated with step-count, stronger associations for high walkability neighborhoods (p for interactions <0.01). No associations with variability, walk-ratio, and smoothness were found. Associations between gait and activity differed by neighborhood walkability.

### RISK OF DIABETES AMONG ADULTS AGING WITH VISION IMPAIRMENT: THE ROLE OF THE NEIGHBORHOOD ENVIRONMENT

Philippa Clarke[1], Anam Khan[1], Kenzie Mintus[2], and Joshua Ehrlich[1], *1. University of Michigan, Ann Arbor, Michigan, United States, 2. Indiana University Purdue University Indianapolis, Indianapolis, Indiana, United States*

Experiencing vision loss as a result of the aging process may be different from aging with vision impairment (VI) acquired earlier in life. Adults aging with VI may be at risk for Type-2 Diabetes (T2DM) due to community barriers in accessing health care, healthy food, and recreational resources. We examined the relationship between neighborhood characteristics and incident diabetes in 22,719 adults aging with VI (without prevalent T2DM) in a private medical claims database (2008-2019). The primary outcome was time to incident T2DM diagnosis over 3+ years of enrollment. Cox models estimated hazard ratios (HRs) for incident diabetes (adjusted for age, sex, and comorbidities). Residence in neighborhoods with greater intersection density (HR=1.26) and traffic (HR=1.22) increased risk of T2DM, while broadband internet access (HR=0.67), optical stores (HR=0.62), supermarkets (HR=0.78), and gyms/fitness centers (HR=0.63) were associated with reduced risk. Results emphasize the importance of neighborhood context for aging well with VI.

### OUTDOOR ENVIRONMENTAL RISK FACTORS FOR FALLS AND FEAR OF FALLING: A SYSTEMATIC REVIEW

Pamela Dunlap[1], Zachary Hubbard[2], Erica Fan[1], Elsa Strotmeyer[3], and Andrea Rosso[1], *1. University of Pittsburgh, Pittsburgh, Pennsylvania, United States, 2. University of pittsburgh Geriatric Psychiatry Neuroimaging Program, Pittsburgh, Pennsylvania, United States, 3. School of Public Health, University of Pittsburgh, Pittsburgh, Pennsylvania, United States*

The purpose of this systematic review was to identify outdoor environmental risk factors associated with falls and/or fear of falling among older adults. PubMed, EMBASE, and CINAHL were searched through February 19, 2021. Studies were included if they measured outdoor environment, falls or fear of falling as an outcome, and included adults aged ≥ 45 years. Excluded studies were not published in English and/or did not include original results. Two study-team members completed abstract screening. For full text reviews, a third reviewer resolved conflicts. A total of 5,727 abstracts were screened and 462 full texts were reviewed. After full-text review, approximately 25 studies will be assessed for risk of bias and data extracted by two independent reviewers. Based on the initial review, uneven outdoor surfaces, busy traffic areas, and neighborhood disorder were associated with falls/fear of falling among older adults. Modifiable outdoor environmental factors may be targets for fall prevention.

### SESSION 3700 (SYMPOSIUM)

### WHAT PANDEMIC RECOVERY WILL LOOK LIKE FOR OLDER, MULTICULTURAL WORKERS

Chair: Cassandra Burton Co-Chair: Katherine Bridges
Discussant: Aisha Cozad

It is no surprise to many that the impact of Covid-19 on older adults, particularly LGBTQ and people of color has been detrimental, not just physically, but mentally, socially, and economically. Many African American/Black adults have suffered disproportionately during the pandemic. In terms of Social Security more than nine in 10 (93%) older Black Americans report that having adequate Social Security benefits is important, but for the many who were forced to retire early due to the pandemic, they will be at a disadvantage. LGBTQ older adults experience persistent discrimination due to sexual orientation or gender identity. When the LGBTQ individual was African American/Black, experiences of discrimination were higher and often occurred in the workplace. These forms of discrimination are directly impacts ones earning potential both during active work years and in retirement savings. AARP has a long history of being an advocate for marginalized and vulnerable adults. AARP staff will discuss policy needs and what the post pandemic workplaces needs to ensure that older LGBTQ people can thrive in the workplace with dignity and respect. The 2021 AARP's Vital Voices research will be used to showcase the economic impact the pandemic has had on older adults, African American communities, Hispanic/Latino communities, Asian Pacific Islander communities and LGBTQ communities. AARP staff will discuss strategies and tactics needed to ensure that opportunities for economic recovery for older adults. The survey gathers information to gauge opinions on a range of topics as well as breaking and current issues.

### WHAT PANDEMIC RECOVERY WILL LOOK LIKE FOR OLDER, MULTICULTURAL WORKERS

Cassandra Burton[1], Aisha Cozad[2], and Katherine Bridges[3], *1. AARP, Atlanta, Georgia, United States, 2. AARP, Washington, District of Columbia, United States, 3. AARP, Deer Isle, Maine, United States*

AARP has a long history of being an advocate for marginalized and vulnerable adults. AARP staff will discuss policy needs and what the post pandemic workplaces needs to ensure that older LGBTQ people can thrive in the workplace with dignity and respect. The 2021 AARP's Vital Voices research will be used to showcase the economic impact the pandemic has had on older adults, African American communities, Hispanic/Latino communities, Asian Pacific Islander communities and LGBTQ communities. AARP staff will discuss strategies and tactics needed to ensure that opportunities for economic recovery for older adults. The survey gathers information to gauge opinions on a range of topics as well as breaking and current issues. Among other issues, it explores concerns about racism and experience with age discrimination and employment experience among older adults.

### SESSION 3710 (AWARD LECTURE)

### DISTINGUISHED FACULTY AWARD AND RISING STAR EARLY CAREER FACULTY AWARD PRESENTATIONS AND LECTURES

Chair: Kara Dassel

The Distinguished Faculty Award Lecture will feature an address by the 2022 recipient Christine A. Fruhauf, PhD, FGSA, FAGHE, of Colorado State University. The Distinguished Faculty Award recognizes persons whose teaching stands out as exemplary, innovative, of impact, or any combination thereof. The Rising Star Early-Career Faculty Award Lecture will feature an address by 2022 recipient M. Aaron Guest, PhD, MPH, MSW, of Arizona State University. The Rising Star Early-Career Faculty Award acknowledges new faculty whose teaching and leadership stand out as influential and innovative.

### STARS AND WISHES: INSIGHTS AND SUGGESTIONS FOR ACADEMICS IN GERONTOLOGY AND GERIATRICS EDUCATION

Christine Fruhauf, *Colorado State University, Fort Collins, Colorado, United States*

In 2002, I presented a student paper at the annual meeting and educational leadership conference of the Association for Gerontology in Higher Education (AGHE) about co-teaching with my mentor an undergraduate adult development and aging course. Little did I know then, presenting about collaborative teaching methods and proven student assessment of learning such as, 'Stars and Wishes', that the AGHE presentation would lead me on a rewarding career path resulting in receiving the 2022 AGHE Distinguished Faculty Award. The purpose of this presentation is to discuss seven 'Stars' that I believe were pivotal points in my career whereby gerontological education and students studying gerontology benefited, and my 'Wishes' for gerontology and geriatrics education to inspire future academics as they navigate the next 20 years.

### DIRECTIONS UNCLEAR: DEMYSTIFYING AGING AND FINDING OUR FUTURE GERONTOLOGISTS

Aaron Guest, *Arizona State University, Phoenix, Arizona, United States*

When did you first learn about gerontology? Becoming a gerontologist is rarely at the top of any student's potential career list. How many of us only found the prospect of a gerontological career in graduate school – or after entering the workforce. We often hear that too few students show interest in jobs working with older persons. In turn, dedicated homes for gerontological degrees are threatened with closure and consolidation. Rather than students being uninterested, I believe the perceived ambiguity of careers and lack of exposure to aging content prevent many from seeking out these careers. In this lecture, I will share my own experience in navigating the ambiguity of a career in aging and how I now use it as a strength to recruit students. I aim to emphasize the unique role of gerontological education and the transdisciplinary approaches and interprofessional strengths that it provides for our aging society.

### SESSION 3720 (PAPER)

### COMPLEX CHRONIC AND END-OF-LIFE CARE ISSUES

### ASSOCIATION OF SOCIAL SUPPORT AND LONELINESS WITH FRAILTY AND FUNCTIONAL STATUS

Kay Thwe Kyaw[1], Thomas Flagiello[2], Alec Levine[2], and Joel Salinas[2], *1. NYU Grossman School of Medicine, Hartsdale, New York, United States, 2. NYU Grossman School of Medicine, New York, New York, United States*

Observational studies suggest psychosocial factors such as social support and loneliness are associated with vulnerability for frailty in older adults, but less is known about the generalizability of these putative psychosocial mechanisms in underrepresented groups. Thus, we evaluated whether better telecommunication social support and lower levels of loneliness were associated with decreased frailty and increased functional ability using a unique longitudinal cohort of rural South African older adults. We conducted generalized estimating equation and robust regression analyses using cross-sectional data from 347 participants in the HAALSI (Health and Aging in Africa: A Longitudinal Study of an INDEPTH Community in South Africa) Dementia Cohort. Social support via telecommunication and self-reported loneliness were measured using standard assessments and modeled as exposure variables. Outcomes were frailty (measured using Fried's frailty phenotype) and functional status (measured by instrumental activities of daily living, IADLs). Lower level of telecommunication social support was associated with higher impairment in ability to perform IADLs. This association persisted after additional adjustments for depression (beta -0.007, 95 % CI -0.016, 0.001) and vascular risk factors (beta -0.012, 95 % CI -0.020, 0.003). No associations were observed in relating telecommunication social support and loneliness with frailty. Among rural South African Black older adults, lower telecommunication-based social support was associated with greater risk of impairment in functional ability. Although further validation is required for possible reverse causality, our findings suggest that future intervention studies focused on promoting telecommunication-based social support to preserve independent functioning may be merited.

### CHRONIC CONDITIONS DURING THE COVID PANDEMIC: MORTALITY PATTERNS IN THE UNITED STATES

Julia Kravchenko[1], Masudul Hoque[2], and Igor Akushevich[2], *1. Duke University School of Medicine, Durham, North Carolina, United States, 2. Duke University, Durham, North Carolina, United States*

Patients with chronic diseases, especially the older adults, are at increased risk of death during the COVID pandemic. We analyzed monthly patterns of mortality rates for patients with diabetes, arterial hypertension, cerebrovascular disease, heart failure, and kidney disease using the provisional Multiple Cause of Death data (2018-2022), for age-, gender, and race/ethnicity-specific population groups. Since population is available at annual basis, we used interpolation of population at risk to have the estimates of population at monthly basis. For all studied diseases, there were substantial increases in mortality among patients that peaked in late-Spring 2020 and Winter 2020/2021. Increases of COVID-related deaths were greater in older (aged 65+) than in younger (55-64) patients. For majority of diseases, Black patients predominantly had their maximum/peaks of COVID-related mortality in late-Spring 2020, while White, American Indian, and Hispanic patients had their max in Winter 2020/2021, and Asian patients had both peaks. Additionally to increased COVID-related deaths, higher mortality rates were also observed among patients with above diseases who did not have COVID in their death records. These increases were more pronounced in younger (55-64) than in older (65+) age groups, and they varied by the studied disease. Increased mortality not directly related to COVID could be due to the relocations of medical resources, lower access to medical care (e.g., limited use of telemedicine), non-COVID related complications caused by the earlier COVID infection, undiagnosed and/or unregistered COVID cases in patients with chronic disease, or other causes. Disease-specific differences in mortality patterns were analyzed and discussed.

### DELIRIUM SUPERIMPOSED ON DEMENTIA IN POST-ACUTE CARE: NURSE DOCUMENTATION OF SYMPTOMS AND INTERVENTIONS

Andrea Sillner[1], Diane Berish[2], Tanya Mailhot[3], Logan Sweeder[4], Donna Fick[5], and Ann Kolanowski[6], *1. Pennsylvania State University Ross and Carol Nese College of Nursing, University Park, Pennsylvania, United States, 2. Pennsylvania State University, University Park, Pennsylvania, United States, 3. Université de Montréal, Montreal, Quebec, Canada, 4. The Pennsylvania State University, State College, Pennsylvania, United States, 5. Penn State University, University Park, Pennsylvania, United States, 6. Penn State University, Penn State University, Pennsylvania, United States*

Delirium is common in older adults and across settings of care, including post-acute care (PAC). Nurses have an important role in identifying, preventing and managing delirium. Even though best practice guidelines highlight the need to accurately document delirium and to deliver non-pharmacological, nurse-driven interventions, it is unclear how this is done in PAC. The aim of this research was two-fold: 1) to describe how nurses document DSD symptoms in PAC nursing notes and 2) to determine if appropriate non-pharmacological nursing interventions are included in their documentation when DSD is present. The sample (N=281) was drawn from a large, single-blinded randomized controlled trial (Recreational Stimulation For Elders As A Vehicle To Resolve DSD (Reserve-DSD) across 8 facilities. Participants tended to be white, female, and had a high-school education. A total of 115 participants (40.6%) had full delirium per the CAM upon admission to PAC, while the remainder 168 (59.4%) had subsyndromal delirium. All had a baseline of dementia. Symptoms of 'Confusion or Acute Confusion' were reported for more than 50% of patients. Approximately 90% of had the symptom 'Confusion or Acute Confusion' documented and this was also the most commonly documented symptom for which a nursing-driven intervention was provided. Overall delirium symptoms and interventions were poorly documented by nurses. Implications for future research and practice include understanding how the pandemic and subsequent resource deprivations impacted delirium documentation and intervention in this setting. Also there is a need for expanded nurse and other healthcare provider education.

### END-OF-LIFE HEALTH LITERACY: VALIDATION STUDY OF A NEW INSTRUMENT, THE END-OF-LIFE HEALTH LITERACY SCALE (EOL-HLS)

Clément Meier[1], Sarah Vilpert[1], Gian Domenico Borasio2, Ralf J. Jox[3], and Jürgen Maurer[1], *1. University of Lausanne, Lausanne, Vaud, Switzerland, 2, Lausanne University Hospital and University of Lausanne, Lausanne, Vaud, Switzerland, 3. Lausanne University Hospital and University of Lausanne, Lausanne, Vaud, Switzerland*

Measuring health literacy allows to assess individuals' competencies to deal with health issues; it influences how individuals perceive their health problems, communicate with healthcare providers, or make medical decisions. The end of life is commonly characterized by one or several diseases, healthcare services' uses and requires individuals to make complex medical decisions. Although the end-of-life concerns everyone, the level of competencies of individuals to get through this stage of life has been little explored. This study aims to fill this gap by validating a new instrument, the End-of-life Health Literacy Scale (EOL-HLS), in a representative sample of older adults aged 58+ living in Switzerland. We use the Swiss wave 8 (2019/2020) of SHARE. Based on the seminal work of Nutbeam (2000), end-of-life health literacy skills are measured using questions on the difficulty in understanding medical interventions, finding information, communicating, deciding in advance, and choosing end-of-life care options. In addition, we compare the findings to the European Health Literacy Survey questionnaire (HLS-EU-Q16). The results confirmed the suitability for performing factor analysis (KMO = 0.924, Bartlett's test of sphericity statistically significant), a three-factor model was established and showed good fit properties (CFI = 0.964, TLI = 0.958, RMSEA = 0.047, SRMR = 0.067) and good reliability ($\alpha = 0.93$). The associations found between individuals' sociodemographic characteristics and the HLS-EU-Q16 were also present in our instrument, but higher EOL-HLS scores were associated with more positive end-of-life outcomes. The EOL-HLS is a reliable and valid instrument to target individuals with low end-of-life health literacy.

### SESSION 3730 (SYMPOSIUM)

### DAILY CONTEXTS AND HEALTH OUTCOMES ACROSS ADULTHOOD

Chair: Joanna Hong Co-Chair: Meng Huo Discussant: David Almeida

Different daily contexts (i.e., social, behavioral, emotional) influence psychological and physical health. In particular, daily contexts change across adulthood and identifying salutary daily contexts is vital for improving the health of middle-aged and older adults. This symposium adds to a burgeoning literature and presents studies that examine how different daily contexts (social - friend interactions, behavioral - electronic usage, emotional - affective response) impact health outcomes across adulthood. Ng et al. found that friend interactions were associated with poorer heart rate variability throughout the day among Black, but not White adults. Kim et al. showed that evening computer use, a health-impairing behavior, is associated with greater sleep disturbances on the following nights among older adults. Rush et al. found that low and high levels of negative affect reactivity to stressors (increased negative affect in response to stress) were associated with poorer health. Yet, moderate levels of stress reactivity predicted better health. Similarly, Hong et al. found that less positive affect reactivity to stressors (decreased positive affect in response to stress) was protective against elevated systolic blood pressure across stressful days. Lastly, Huo et al. identified an association between older adults' empathy and daily rumination (i.e., repetitive thoughts) over distress, but observed that the link was attenuated on days when older adults maintained more stable levels of negative affect. Together, findings may inform future interventions aimed at increasing health across adulthood. Dr. Almeida will serve as the Discussant and summarize these studies with regard to their theoretical and methodological contributions.

### WHAT SAVES A STRESSFUL DAY: PROTECTIVE ROLE OF POSITIVE AFFECTS AGAINST DAILY STRESS

Joanna Hong[1], Jody Greaney[2], Jacqueline Mogle[3], and David Almeida[3], *1. The Pennsylvania State University, State College, Pennsylvania, United States, 2. The University of Texas of Arlington, Arlington, Texas, United States, 3. The Pennsylvania State University, University Park, Pennsylvania, United States*

Experiencing a greater number of stressful days is associated with a heightened risk of cardiovascular disease. This study examined whether positive affect reactivity, the trait-like change in positive affect in response to daily stressors, moderates the association between the number of stressful days and blood pressure. Participants were 664 adults from the National Study of Daily Experiences II (Midlife in the United States sub-study). Participants reported stressors and positive affect across 8 days and provided resting blood pressure measures during a separate clinic visit. A greater number of stressful days was associated with increased systolic blood pressure, but only among individuals with higher positive affect reactivity (B = 15.11, SE = 6.36, p = 0.02). Results suggests that individuals who maintain positive affect when experiencing stressors may have lower risk of heightened systolic blood pressure, contributing to the growing evidence that positive affective reactivity may be protective against daily stress.

### DO MORE EMPATHIC OLDER ADULTS RUMINATE MORE? EMOTION REGULATION MATTERS

Meng Huo[1], Kate Leger[2], Kira Birditt[3], and Karen Fingerman[4], *1. University of California, Davis, DAVIS, California, United States, 2. The University of Kentucky, Lexington, Kentucky, United States, 3. University of Michigan, Ann Arbor, Michigan, United States, 4. The University of Texas at Austin, Austin, Texas, United States*

Older adults differ in their responses to distress, and those who tend to ruminate report poor health. We sought to examine whether trait empathy, the tendency to share and understand others' distress, underlies rumination, and whether this association varies by emotion regulation. Participants included 289 adults aged 65+ in the Daily Experiences and Well-being Study. They reported demographics, empathy, general preferences for emotion regulation strategies, and affect throughout the day as well as daily rumination. Empathy was associated with greater rumination, which was particularly evident in older adults who preferred avoidant strategies. We also found that the link between empathy and rumination was attenuated on days when older adults had lower negative affect variability. This study identifies empathy as a key factor that underlies individual differences in rumination, a key precursor of psychopathologies, and also suggests emotion regulation as a promising target of interventions that can promote older adults' health.

### DAILY EVENING ELECTRONIC MEDIA USE, SEDENTARY BEHAVIORS, AND SLEEP IN LATER LIFE

Yijung Kim[1], Nicole Richards[2], and Karen Fingerman[1], *1. The University of Texas at Austin, Austin, Texas, United States, 2. The University of Texas, Austin, Austin, Texas, United States*

Sleep complaints and disorders are two of the most common disturbances to health and well-being in later life. This study examined how evening electronic media use and daytime sedentary behaviors affect subsequent sleep hours and perceived sleep quality, and whether consistent sleep hours (i.e., sleep regularity) moderate these associations. Data were drawn from 241 older adults (Mage = 74.02) from the Daily Experiences and Well-being Study who completed ecological momentary assessments and wore an accelerometer for four days on average. A series of conditional fixed-effects models indicated that older adults reported more sleep disturbances on nights following the evening computer use. Sedentary behaviors and evening television viewing were not associated with sleep quantity and quality. Older adults with more consistent hours of bedtime reported better sleep quality regardless of their evening electronic media use and daytime sedentary behaviors, thereby highlighting the importance of sleep regularity in later life.

### DO FRIENDS GET UNDER THE SKIN? DAILY INTERACTIONS AND CARDIOVASCULAR FUNCTIONING AMONG BLACK AND WHITE AMERICANS

Yee To Ng[1], Sae Hwang Han[2], Karen Fingerman[3], and Kira Birditt[4], *1. UT Austin, Ann Arbor, Michigan, United States, 2. University of Texas at Austin, Austin, Texas, United States, 3. The University of Texas at Austin, Austin, Texas, United States, 4. University of Michigan, Ann Arbor, Michigan, United States*

Scarce research has examined racial differences in cardiovascular health in the context of social interactions. This study investigated whether (a) friend interactions were associated with better cardiovascular functioning, and (b) such associations vary among Black and White adults. This study employed dual assessment techniques—ecological momentary assessments (EMA) and ambulatory physiological assessments—to examine the co-occurrence of social interactions and cardiovascular functioning. Multilevel models revealed no racial differences in ambulatory HRV and frequency of friend interactions throughout the day. Findings revealed a between-person link of friend interactions and better HRV in the overall sample. Race-stratified models found a within-person link between friendship interaction and worse HRV and a between-person link between friendship interaction and better HRV for Blacks but not for Whites. Findings suggest friend interactions are more salient for Blacks' cardiovascular health and may serve as a modifiable factor for preventing cardiovascular diseases.

### NONLINEAR ASSOCIATIONS OF DAILY STRESS REACTIVITY WITH HEALTH AND WELL-BEING

Jonathan Rush[1], Anthony Ong[2], Jennifer Piazza[3], Susan Charles[4], and David Almeida[5], *1. University of Victoria, Victoria, British Columbia, Canada, 2. Cornell University, Ithaca, New York, United States, 3. California State University, Fullerton, Fullerton, California, United States, 4. University of California, Irvine, Irvine, California, United States, 5. The Pennsylvania State University, University Park, Pennsylvania, United States*

Research has repeatedly demonstrated that greater affective reactivity to daily stressors is associated with detrimental health outcomes (e.g. inflammation, mortality). However, most research has only considered linear effects, which precludes an examination of whether moderate levels of stress reactivity may be beneficial. Using daily diary data from the National Study of Daily Experiences (N=2,018) we fit multilevel SEMs to simultaneously model daily within-person associations between stress and negative affect (i.e., stress reactivity), and individual differences in the linear and quadratic associations between stress reactivity and life satisfaction, psychological distress, and chronic conditions. Significant quadratic effects were found for each of the three outcomes (estimates=-20.23; 11.49; 20.81, ps<.001, respectively), indicating a U-shaped pattern where both low and high levels of stress reactivity were associated with poorer health, whereas moderate levels of daily stress reactivity predicted better health outcomes. The results suggest that some affective response to daily stressors can be beneficial.

### SESSION 3740 (SYMPOSIUM)

### EMBRACING THE DIVERSITY OF GRANDFAMILIES: UNDERSTANDING THE INFLUENCE OF CULTURE AND RACISM

Chair: Nancy Mendoza Discussant: Loriena Yancura

In line with this year's conference theme, we examine diversity in different forms, such as racial, cultural, and familial diversity. We discuss the importance of diversity's influence on the experiences of grandfamilies and the impact it has on their members. Findings from a study with Latinx grandparent caregivers suggest that those raising grandchildren often do not view themselves as "raising," but instead "helping." Such findings highlight the importance of understanding cultural norms to appropriately tailor services and resources. In an exploration of the experiences of Korean custodial grandparents, findings demonstrated the influence of patrilineality and stigma surrounding divorce for Korean grandparent-headed families, indicating the importance of considering a grandparents' position in the family when providing services. In a study with custodial grandparents during the COVID-19 pandemic, researchers examined the role of racial discrimination on grandparents' depressive symptoms and access to health services. Results indicated a higher level of perceived racial discrimination was associated with more depressive symptoms. These results imply the need to address racial/ethnic disparities experienced by these caregivers. Similarly, in a study of emerging adults raised by grandparents, race moderated the influence of attachment on symptoms of racial discrimination. Findings support the use of interventions addressing attachment and ethnic identity to decrease symptoms of racial trauma in grandfamilies. Together these four studies reiterate the diversity of grandfamilies, enrich our understanding of these families, and encourage us to reimagine how to best serve them.

### LATINX GRANDPARENTS RAISING GRANDCHILDREN: THE INFLUENCE OF CULTURAL NORMS

Nancy Mendoza, and Cherrie Park, *The Ohio State University, Columbus, Ohio, United States*

Few studies have focused on the experiences of Latinx grandparents. Using a grounded theory approach, nine Latinx grandparent caregivers were interviewed in an effort to understand how the Latinx culture influences their experiences. Major themes that emerged during data analysis were related to reasons for caring. These included cultural norms of caring and reasons for the non-usage of services. Findings suggest Latinx grandparents tend to care for their grandchildren because they view it as part of their role as a grandparent. Thus, the biggest "barrier" to acquiring services could be that they do not view themselves as primary caregivers. These findings emphasize the need to understand the experiences of these grandparents and the importance of tailoring education and resources to this subgroup. This study provides professionals with a new way of looking at services for Latinx grandparents and opens the door to a new set of implications for practice.

### CUSTODIAL GRANDPARENTS' DEPRESSION AND ACCESS TO HEALTH CARE DURING COVID-19: THE ROLE OF RACIAL DISCRIMINATION

Yanfeng Xu[1], and Theresa Harrison[2], *1. University of South Carolina, Columbia, South Carolina, United States, 2. University of South carolina, Columbia, South Carolina, United States*

The COVID-19 pandemic has exacerbated racism against racial minorities and widened racial/ethnic disparities in health outcomes and access to health care services. This study analyzed cross-sectional data (N=219) collected from custodial grandparents via Qualtrics Panels in February 2022 to understand the role of race and perceived racial discrimination in contributing to custodial grandparents' depressive symptoms and access to health care services. Results indicated that a higher level of perceived racial discrimination was positively associated with grandparents' more depressive symptoms, but it was also associated with lower odds of custodial grandparents' access to health care services. Furthermore, racial/ethnic disparities in depressive symptoms and access to telemental health services among custodial grandparents were identified. Results imply the importance of addressing racial/ethnic disparities in depressive symptoms and access to health care services among custodial grandparents.

### EXPERIENCES OF KOREAN GRANDFAMILIES: DIFFERENCES BETWEEN MATERNAL AND PATERNAL GRANDPARENTS

Youjung Lee, *Binghamton University, Binghamton, New York, United States*

Grandparent-headed families in South Korea have been growing prominent in the country's cultural landscape. Approximately 153,000 Korean grandparent-headed households existed in 2015; this number is expected to double by 2035. This qualitative study explored Korean custodial grandparents' experiences of raising grandchildren and the cultural significance of multigenerational caregiving in Korea. Using a phenomenological approach, semistructured interviews with 22 custodial grandparents were conducted. Significant functions of patrilineality and stigma surrounding divorce for Korean grandparent-headed families were found. Considering the complicated cultural factors, social/family service programs must pay attention to the unique needs of grandparent-headed families and consider the circumstances related to grandparents' positions in the family (i.e., paternal vs. maternal grandparent caregivers). Korean government programs and policies could better help marginalized grandparent-headed families with an empowerment approach to help marginalized grandparent-headed families gain positive attitudes toward their caregiving situation.

### RACIAL TRAUMA IN EMERGING ADULTS RAISED BY GRANDPARENTS: PROTECTING AGAINST DISCRIMINATION

Acacia Lopez[1], Rachel Scott[1], Marin Olson[2], and Danielle Nadorff[2], *1. Mississippi State University, Starkville, Mississippi, United States, 2. Mississippi State University, Mississippi State, Mississippi, United States*

Experiences of racial trauma are linked with psychopathology, but a strong ethnic identity may serve as a protective factor. Grandparents primarily influence the development of ethnic identity, and BIPOC children are increasingly being raised by grandparents. Secure attachments influence stronger ethnic identities, yet custodial grandchildren are at higher risk of disrupted attachments. The current study investigated whether ethnic identity would mediate the relation between attachment and racial trauma symptoms in emerging adults previously raised by their grandparents and their peers (N = 370; 33% raised by grandparents; 25.6% non-white), with race as a moderator. Across all races, there were group differences in symptoms of racial trauma, with those not raised by grandparents experiencing a direct effect of race on ethnic identity. Attachment was a significant predictor of trauma symptoms of discrimination, moderated by race. Implications may provide support for clinical interventions addressing attachment and ethnic identity to decrease trauma symptoms.

### SESSION 3750 (SYMPOSIUM)

### ESPO AND HEALTH SCIENCES SECTION SYMPOSIUM: INCLUDING DIVERSE STAKEHOLDERS IN HEALTH INTERVENTION DESIGN: LESSONS LEARNED FOR ADVANCING HEALTH EQUITY

Chair: An Nguyen Co-Chair: Kyle Moored Discussant: Kyle Moored

The COVID-19 pandemic exposed persistent disparities in the effectiveness of healthcare services and long-term care among the older adult population. For example, significant gaps disproportionately affected older adults living in rural areas, racial and ethnic minorities, individuals with cognitive impairments, and those from low socioeconomic backgrounds. To improve health equity, research is needed to design, implement, and disseminate health interventions that address the heterogeneity of older adults and caregivers. Reflecting on lessons learned from including underrepresented key stakeholder groups in intervention research may reveal strategies to design health interventions that are more accessible and effective for diverse populations of older adults and their caregivers. This symposium highlights five studies that included diverse populations of older adults and/or caregivers in the design of health interventions. Dr. Schiaffino will share how disparities in co-morbid cancer and dementia across racial, ethnic, and age groups will inform improvements to care delivery processes for this population. Ms. Crane will discuss barriers to designing exergame interventions that are accessible to older adults with multiple chronic conditions and functional limitations. Dr. Díaz-Santos will describe results of piloting and refining a social connection intervention to reduce loneliness among Latinx older adults at risk for dementia. Dr. del Pino will highlight strategies to improve implementation of a screening intervention for at-risk drinking among African American older adults living with HIV. Mr. Cotton will present results of tailoring a crisis intervention to improve cultural fit for African American caregivers of people with dementia.

### ENGAGING A COMMUNITY ADVISORY BOARD TO VALIDATE A CULTURALLY TAILORED INTERVENTION MODEL

Quinton Cotton[1], Gina Green-Harris[1], Juliet Chang[2], Laura Block[3], and Andrea Gilmore-Bykovskyi[2], *1. University of Wisconsin School of Medicine and Public Health, Madison, Wisconsin, United States, 2. University of Wisconsin-Madison, Madison, Wisconsin, United States, 3. University of Wisconsin-Madison School of Nursing, Madison, Wisconsin, United States*

National dementia research priorities call for interventional studies addressing the needs of vulnerable populations experiencing disparities and for greater inclusion of dementia care recipients and their family caregivers in research. While studies document disparities, high needs and experiences of African American dementia caregivers, there is limited translation of this knowledge into the development, implementation, and dissemination of dementia-specific culturally tailored interventions. Research methods that facilitate engagement capable of strengthening data collection and culturally congruent interpretation of caregiving perspectives are needed to inform intervention development. Drawing on a qualitative study utilizing Grounded Dimensional Analysis, we employed a longitudinal engagement method with a Community Advisory Board (CAB) to validate a culturally tailored crisis intervention model for African American dementia caregivers. We identified lessons learned through a review of transcripts, memos and research team discussions. CAB members identified language, family structure, cultural history, knowledge, and respect as important features for cultural tailoring.

### INCLUDING FEEDBACK FROM OLDER ADULTS WITH VARYING HEALTH CONDITIONS TO INFORM EXERGAME DESIGNS

Breanna Crane[1], Brittany Drazich[2], Janiece Taylor[3], Kyle Moored[1], Omar Ahmad[3], John Krakauer[3], and Michelle Carlson[1], *1. Johns Hopkins Bloomberg School of Public Health, Baltimore, Maryland, United States, 2. University of Maryland, Baltimore, Maryland, United States, 3. Johns Hopkins University, Baltimore, Maryland, United States*

Exergames are emerging technologies that combine “exercise” and “video games” to promote enjoyable cognitive and physical activities. Exergame studies often exclude individuals at greatest risk for adverse health outcomes (e.g., oldest-old, living with chronic conditions, comorbidities, or functional limitations). Thus, it is important to 1) understand motivators and barriers for joining and adhering to exergame studies and 2) capture perspectives from individuals commonly excluded from these studies. We conducted three focus groups among 14 older adults (mean age=79±9 years) with varying health conditions who participated in a novel three-dimensional exergame feasibility study. Data were analyzed using the “Sort and Sift, Think and Shift” approach. Motivators for joining were generativity, peer referrals, self-improvement, and curiosity. Motivators for retention were accomplishment, enjoyability, and exercise. Barriers to participation included frustration and pain/fatigue. We also discuss how participants' feedback influenced future exergame design. Findings will aid in promoting scalable, enjoyable, and accessible exergame interventions for all.

### TALKING ABOUT AT-RISK DRINKING WITH OLDER ADULTS AGING WITH HIV

Homero del Pino, *Charles R. Drew University of Medicine and Science, Los Angeles, California, United States*

Older adults living with HIV (OALWH) may have health conditions that amplify the negative health effects of alcohol use. Traditional alcohol screening tools do not consider comorbidities or age. We adapted the Comorbidity Alcohol Risk Evaluation Tool screening tool for at-risk drinking to reflect HIV-related conditions and medications. We tested a brief intervention with Black OALWH and collected usability data. Participants, n=27, 53.2 average age in years, 21 average years living with HIV. Three themes: (1) Expectations of doctors ("Doctors better ask me questions about my alcohol use..."); (2) It's how you say it ("[Doctors] talk down to me like I don't know my freaking disease …"); and (3) Effects of surviving HIV ("I am fully aware of the effects of alcohol…. I'm probably better informed than most people…"). We identified strategies to address at-risk drinking with OALWH.

### A SOCIAL CONNECTION HEALTH INTERVENTION FOR ISOLATED LATINX OLDER ADULTS AT RISK OF DEMENTIA

Mirella Díaz-Santos[1], Sandy Alcantar[2], Maribel Magana[3], Sujey Martinez[3], and Taya Varteresian[3], *1. University of California, Los Angeles, Los Angeles, California, United States, 2. Los Angeles County Department of Mental Health, Long Beach, California, United States, 3. Los Angeles County Department of Mental Health, Los Angeles, California, United States*

COVID-19 exacerbated the "epidemic of loneliness." Although loneliness and social isolation are social determinants of health and Alzheimer's dementia (AD), culturally and linguistically responsive social connection interventions tailored to Spanish-Speaking community-dwelling immigrant older adults at higher risk of AD are lacking. UCLA's Hispanic Neuropsychiatric Center of Excellence (HNCE) collaborated with Los Angeles County Department of Mental Health (LACDMH) to develop and implement a community-partnered social connection group intervention via Telehealth. All clients are homebound due to physical, psychiatric, and/or medical conditions. The main objective was to reduce feelings of loneliness by combining brain health education with skill-based exercises promoting self-efficacy, trust, belonging, and resilience by effectively communicating their needs in a psychologically safe group space. The effectiveness of the pilot intervention, however, required multiple refinements accounting for structural-social barriers, culture, language, and histories of trauma in country of origin and post-immigration to the United States.

### RACIAL, ETHNIC, AND AGE-RELATED DISPARITIES IN COMORBID CANCER AND DEMENTIA

Melody Schiaffino[1], James Murphy[2], Vinit Nalawade[3], Jessica Schumacher[4], William Dale[5], and Alison Moore[3], *1. San Diego State University, San Diego, California, United States, 2. UC San Diego, La Jolla, California, United States, 3. University of California, San Diego, La Jolla, California, United States, 4. University of Wisconsin, Madison, Wisconsin, United States, 5. City of Hope Comprehensive Cancer Center, Duarte, California, United States*

Older adults make up over 60% of new cancer cases and will be 73% of survivors by 2040. Dementia, which is cognitive impairments that includes Alzheimer's, affects 10.3% of this age group with a greater burden on Black and Latino/a older adults. It is essential to accurately characterize the prevalence of comorbid dementia and cancer (ADRD+Ca) to ensure guideline concordant care. The goal of our study was to characterize pre-existing ADRD+Ca across cancer sites (cervical, breast, prostate, colorectal, head and neck, and lung). Using a validated CMS algorithm, we found 5.6% ADRD+Ca in our sample. Female patients were more likely to report ADRD+CA vs men (62.5% vs 37.5%, $p<0.0001$); similarly, age differences were observed with the biggest difference among 85+ ages 33.8% vs 11.7% respectively. The proportion of Black patients with ADRD+Ca was nearly double compared to Ca only groups. Patients with additional multiple comorbidities, unstaged site, and single/other marital status were also more likely to report ADRD+Cancer ($p<0.0001$). Among cancer sites, we observed the highest prevalence in colorectal and cervical cancer both over 8%, the lowest was in prostate cancer (2.64%). Our findings show inequities in social factors and cancer subtypes among fee-for-service Medicare beneficiaries that warrants investigation.

### SESSION 3760 (SYMPOSIUM)

### FACTORS THAT IMPACT SUCCESSFUL AGING AMONG OLDER ADULTS LIVING WITH HIV/AIDS

Chair: Erin Robinson Co-Chair: Molly Perkins Discussant: Alexis Bender

The past two decades have brought significant medical advances in antiretroviral therapies (ART) for people living with HIV/AIDS (PLWHA). This has enabled PLWHA to live longer and healthier lives than ever before. In fact, nearly half of all PLWHA in the United States are age 50+ and HIV is treated as a chronic disease, instead of a terminal illness. Despite these advancements older adults living with HIV/AIDS (OALWHA) are still a highly stigmatized population who faces challenges with their health and overall well-being. This symposium will highlight recent research on factors that play a role in successful aging among OALWHA. Our first presentation leverages a quantitative dataset of OALWHA and examines the relationship between childhood sexual abuse and ART adherence, with a particular focus on substance abuse as a potential mediator. Our second presentation features a qualitative study with OALWHA that explores their conceptions of successful aging. Our third presentation includes a sample of medical case managers who serve OALWHA and examines the feasibility of using a cognitive screening tool with this population. Cognitive screening with OALWHA can help provide early indicators of cognitive decline and initiate early intervention. Finally, our fourth presentation includes a sample of older women living with HIV/AIDS and examines their support needs and resources, particularly to help understand their complex caregiving needs.

Findings from these four studies advance our understanding of OALWHA and their needs to ensure continued successful aging. This symposium informs further research and direct practice with this population.

### CHILDHOOD SEXUAL TRAUMA, SUBSTANCE USE, AND ART ADHERENCE AMONG OLDER ADULTS LIVING WITH HIV

Monique Brown, *University of South Carolina, Columbia, South Carolina, United States*

Childhood sexual abuse (CSA) is associated with lower ART adherence among people living with HIV and is linked to substance use (SU) in adulthood. However, studies examining the mediating role of SU in the association between CSA and ART adherence are lacking. Therefore, the aim of this study was to examine SU as a mediator in the association between CSA and ART adherence among older adults living with HIV (OALH). Data were obtained from 91 OALH. Adjusted path models were used to determine the mediating role of SU. After controlling for sociodemographic characteristics and depression, CSA had a direct association with ART adherence ($\beta$=-3.20; p<0.001) and with SU ($\beta$=-2.46; p=0.023). The association between SU and ART adherence was not statistically significant ($\beta$=0.071; p=0.444). Therefore, the indirect effect between CSA, SU, and ART adherence was not statistically significant ($\beta$=0.175; p=0.468). Future research should examine alternative pathways between CSA and ART adherence.

### CONCEPTIONS OF SUCCESSFUL AGING AMONG OLDER MEN AND WOMEN LIVING WITH HIV

Anna Rubtsova[1], Tonya Taylor[2], Marcia Holstad[3], Gina Wingood[4], Ighovwerha Ofotokun[5], Deborah Gustafson[6], Dilip Jeste[7], and David Moore[8], *1. Emory University, Atlanta, Georgia, United States, 2. SUNY Downstate Medical Center, Brooklyn, New York, United States, 3. Emory University Nell Hodgson Woodruff School of Nursing, Atlanta, Georgia, United States, 4. Columbia University Mailman School of Public Health, New York, New York, United States, 5. Emory University School of Medicine, Atlanta, Georgia, United States, 6. SUNY Downstate, Brooklyn, New York, United States, 7. University of California San Diego, La Jolla, California, United States, 8. University of California, San Diego, La Jolla, California, United States*

The aim of this qualitative study was to compare conceptions of successful aging (SA) among older men and women living with HIV. Participants were recruited through 1) Women's Interagency HIV Study (Atlanta and Brooklyn sites); and 2) HIV Neurobehavioral Research Program at the University of California, San Diego. Our sample included 31 participants: 17 women living with HIV (WLH) and 14 men living with HIV (MLH), age range 51 to 73, 58% Black. We conducted semi-structured interviews in 2018-2021, using the same interview guide for MLH and WLH. The interviews lasted ~90 minutes and were fully transcribed. The thematic and comparative analysis was conducted within the social constructivist paradigm, using MAXQDA software. Although there was some overlap in SA conceptions between MLH and WLH, we found important differences. Whereas maintaining function and general health was more emphasized by MLH, WLH stressed accepting and celebrating aging within the life course perspective.

### DETERMINING FEASIBILITY FOR USE OF GERIATRIC COGNITIVE SCREENINGS BY MEDICAL CASE MANAGERS SERVING OLDER (50+) PLWH

Monica Maly[1], and Erin Burk-Leaver[2], *1. Colorado School of Public Health, Aurora, Colorado, United States, 2. International Association of Gerontology and Geriatrics, Denver, Colorado, United States*

Cognitive impairment (CI) is a significant age-related risk exacerbated by HIV. However, AIDS Service Organizations (ASO) have not implemented effective screening tools for detection of CI in older adults living w/HIV (OALH). This study examined feasibility for use of those tools by medical case managers (MCM) serving OALH. MCMs from nine Colorado ASOs self-administered a questionnaire measuring baseline knowledge of warning signs and risk factors (WS/RF) of CI in OALH, evaluating familiarity with screening of/response to WS/RF of CI within this population. Data from 24 MCMs showed OALH comprised 47% of all clients, and 68% of respondents reported no related training. Similarly, 67% reported not feeling confident identifying WS/RF. 100% of respondents reported not screening for CI, and many endorsed limited awareness/availability of screening tools (87%), referral resources (87%), and training (83%). This identified a need and desire for use/implementation of cognitive screening tools to improve ASO care for OALH.

### SUPPORT NEEDS AND RESOURCES OF OLDER WOMAN LIVING WITH HIV

Jasmine Manalel[1], Jennifer Kaufman[2], and Mark Brennan-Ing[1], *1. Hunter College, New York, New York, United States, 2. Brookdale Center for Healthy Aging Hunter College, CUNY, New York, New York, United States*

People living with HIV are at increased risk for social isolation and sparse support networks, making it difficult to navigate complex caregiving needs as they age. This study aimed to identify the support needs and resources of older women living with HIV, an understudied population in this field, and their association with well-being. Participants included 217 heterosexual women aged 50 to 77 (Mage=59) from the ROAH 2.0 Study. Almost half of the sample (47.7%) had never required assistance because of HIV or other illness or disability, whereas the remainder required current or past assistance. Preliminary findings controlling for sociodemographics indicated that availability of emotional support, but not practical support (i.e., having someone to count on for activities of daily living), was associated with lower depressive symptomology. Identifying sources and types of support will help older women leverage existing social ties or form new ones to promote successful aging with HIV.

### SESSION 3770 (PAPER)

### FINANCIAL SECURITY, ECONOMICS, HEALTH, AND HOUSING

### "WE WANT TO PAY AND WE WANT TO STAY": OLDER ADULTS MANAGING PROPERTY TAX BURDEN IN A GROWING URBAN COUNTY

Anthony Traver[1], Katie White[1], Marisa Sheldon[2], Holly Dabelko-Schoeny[2], and Bethany Sanders[3], *1. The Ohio State University, Columbus, Ohio, United States, 2.*

*Ohio State University, Columbus, Ohio, United States, 3. Franklin County Auditor's Office, Columbus, Ohio, United States*

Aging in place is a goal for many older adults. As many older adults own their homes, strategies designed to promote aging in place must account for threats to the financial sustainability of ownership and occupancy later in life. One such threat is property taxes, which have risen substantially in many metropolitan areas over the last decade as home values soar. Property tax relief programs offered by state and local governments are designed to ease the housing cost burden of older adults. Yet, recent research indicates that such programs do little to ensure affordability for low-income homeowners. This study reviewed local property tax relief programs and interviewed local older adult homeowners and housing professionals to understand the circumstances of older adult homeowners in one growing U.S. County. Four major themes emerged from the interviews: housing market dynamics, personal finances, local housing resources, and wellbeing. Results indicate that unaffordability is a growing concern among older adult homeowners and services providers alike. Current property tax relief programs are thought to do little to reduce the cost burden posed by property taxes. Implications for social policy include expanding eligibility criteria and indexing the benefit to a local economic metric so that the relief remains relevant in areas with dynamic markets. Implications for practitioners include understanding the property tax relief programs in one's area and referring clients when appropriate.

### DISABILITY STATUS, FINANCIAL STRAIN, AND SUBJECTIVE HEALTH AND WELL-BEING FOR PEOPLE WITH LTSS NEEDS IN CALIFORNIA

Lei Chen[1], Kathryn Kietzman[2], and Fernando Torres-Gil[2], *1. University of California, Los Angeles, Los Angeles, California, United States, 2. University of California, Los Angeles (UCLA), Los Angeles, California, United States*

Many people with needs for Long-Term Services and Supports (LTSS) are vulnerable to financial strain, a chronic economic stressor that may negatively affect a person's well-being. This study examines the extent to which financial strain mediates the relationship between people's disability status and subjective health and well-being, controlling for select demographic characteristics. Disability status refers to the intensity of disabilities that people report, including cognitive impairments, and/or difficulties performing activities of daily living and/or instrumental activities of daily living. Financial strain measures the number of challenges that participants incurred during the last year in acquiring food, housing, health care, and other basic needs. We use the first cycle of data (2019-2020) from the California Long-Term Services and Supports (LTSS) survey, merged with select data from the California Health Interview Survey (CHIS) (N = 2,030). Drawing from Pearlin's Stress Process Model, we use Conditional Process Analysis (CPA) to examine the hypothesized mediation relationships. Findings show that the intensity of disability status has a direct association with self-rated health (c' = -.2054, $p < .0001$) and psychological distress (c' = .7247, $p < .0001$). Furthermore, financial strain experienced by people with LTSS needs mediates the relationship between their disability status and 1) self-rated health (ab = -.0178, BootCI= -.0285 to -.0082) and 2) psychological distress (ab = 0.19, BootCI= .1323 to .2648). These results have policy and practice implications for national and state programs, such as Medicaid, the Universal Basic Income (UBI) program, and the Master Plan for Aging in California.

### FOOD INSECURITY AMONG OLDER ADULTS WITH A HISTORY OF INCARCERATION: A MIXED METHODS STUDY

Rodlescia Sneed[1], and Tamara Jordan[2], *1. Wayne State University, Detroit, Michigan, United States, 2. Michigan State University College of Human Medicine, Flint, Michigan, United States*

The purpose of this study was to use a mixed methods approach to describe the association between history of incarceration (HOI) and food insecurity (FI) among older adults. Quantitative data were obtained from the Health and Retirement Study, a population-based study of community-dwelling adults (n=12,702) aged >50. Qualitative data were obtained via key informant interviews with formerly incarcerated older adults and the human service providers serving them (n=15). Multiple logistic regression was used to estimate the association between HOI and FI, adjusting for demographic variables. HOI was associated with increased odds of FI (OR 1.83; 95% CI 1.52-2.21). Race/ethnicity moderated the association between history of incarceration and food insecurity, with effects observed among Non-Hispanic Blacks (OR 1.78; 95% CI 1.29-2.46) and Whites (OR 2.27; 95% CI 1.74-2.97), but not Hispanics (OR 1.11; 95% CI 0.69-1.77) or those of other racial/ethnic groups (OR 1.79; 95% CI 0.71-4.52). Explanations for the association between HOI and FI obtained from qualitative interviews included ineligibility for food assistance programs due to felony conviction, lack of safe places to store healthy food, and difficulty using technologies needed to enroll in food assistance programs. The most common barrier associated with inaccessibility to healthful foods for this population, according to interviewees, is ineligibility for food assistance programs. FI is an important issue among older adults with a HOI. Re-examination of policies and procedures for accessing food assistance programs may be needed to reduce FI in this population.

### RECESSION EVENTS AND SLEEP PROBLEMS IN MIDLIFE AND AGING ADULTS

Aarti Bhat[1], Jose Diaz[2], and David Almeida[2], *1. The Pennsylvania State University, State College, Pennsylvania, United States, 2. The Pennsylvania State University, University Park, Pennsylvania, United States*

Adverse economic events can negatively impact aspects of health, including sleep quality. Poor sleep can increase risk of developing or exacerbating health conditions such as cardiovascular and metabolic disease, cancer, and suicidal ideation. It is critical to examine how economic hardships may amplify health disparities in midlife and aging, a rapidly growing demographic in the U.S. This study examines the effect of recession hardships on sleep issues in midlife and aging adults using waves 2 and 3 of the Midlife in the United States study (MIDUS; N = 2602; M age = 63.47;

56.99% women; 15.76% Black). Participants reported chronic sleep problems experienced in the past year, alongside frequency of experiencing sleep disruptions (trouble with onset, maintenance, feeling unrested). Participants also reported economic impacts (financial, housing, and job-related) experienced in the aftermath of the recession; with 75.56% reporting at least one adverse recession event. Regression indicated that recession events were significantly associated with higher odds of chronic sleep problems and high frequency sleep disruptions in wave 3, even when controlling for sleep problems in wave 2. When examining race and age as moderators, Black participants who experienced adverse events were more vulnerable to chronic sleep problems than white participants, whereas age showed no significant interactions. Results indicate that adverse economic events can impact sleep quality for midlife and aging adults, and that policy mitigating economic effects on sleep may especially benefit Black adults. Subsequent analyses will examine the effect of recession events on daily sleep patterns.

### THE EFFECTS OF HOUSING AND LIVING STANDARDS ON LONELINESS AND SOCIAL EXCLUSION AMONG OLDER PEOPLE

Charles Waldegrave[1], Chris Cunningham[2], Catherine Love[3], and Giang Nguyen[3], *1. Family Centre Social Policy Research Unit, Lower Hutt, Wellington, New Zealand, 2. Research Centre for Maori Health and Development, Wellington, Wellington, New Zealand, 3. Family Centre Social Policy Research Unit, Wellington, Wellington, New Zealand*

Introduction: Housing security is a likely indicator of loneliness (Gierveld et. al. 2015, Gonyea et. al. 2018) but there are few studies that focus specifically on the relationship between the two. This paper presents findings from a New Zealand Ageing Well National Science Challenge research programme that presents findings on the impacts of housing and living standards on loneliness and social exclusion. Method: This presentation will provide results from a study of 200+ Māori (indigenous New Zealanders) aged 50 years and over. Key questions around loneliness and social isolation were co-created with the participants and responses compared with standard international scales to help identify both universal aspects of loneliness and culturally specific aspects. Questions relating to housing security, affordability, living standards and neighbourhood safety were also asked. Regression analysis was used to test the statistical significance of the various relationships between differing aspects of housing and loneliness.

Results: The results demonstrate statistically significant relationships between housing quality, affordability, living standards and neighbourhood suitability with both the universal and culturally specific scales of loneliness. Overall greater housing security and quality was shown to be negatively associated with loneliness, which suggests it contributes to the reduction of loneliness.

Conclusion: Addressing the quality of social connections has often been seen as the key way to address loneliness. The results of this study suggest housing security and living standards play an important role in people's perception of loneliness and their experience of it, as well.

### SESSION 3780 (SYMPOSIUM)

### IMPLEMENTATION RESEARCH: INTERGENERATIONAL PROGRAMS ACROSS UNIVERSITY SETTINGS

Chair: Jill Juris Co-Chair: Skye Leedahl Discussant: Natalie Douglas

Implementing intergenerational programs within university settings has been associated with benefits for all generations involved, which often includes young children, university students, and older adults. However, from conceptualization to pilot testing to evaluation, challenges and opportunities present themselves. This symposium will highlight implementation realities for intergenerational programs within higher education settings. This symposium will specifically address dimensions of: geography (rural versus urban), modality (such as in-person, virtual, or a mix), community/university partnerships, and scholarship for faculty balancing instruction and research demands. Addressing stages of implementation, the papers reflect a continuum from pre-planning to pilot to more advanced stages of implementation. First, Lisa Borrero will highlight challenges and opportunities of conceptualizing fully online intergenerational programming, including the pre-implementation planning, execution, and evaluation stages. The second paper from Ladan Ghazi Saidi will describe pre-implementation tasks completed to establish interest in intergenerational programming in a rural setting, as well as challenges stemming from the pandemic. Third, Jill Juris and colleagues will highlight an online intergenerational technology program offered from a rural Western North Carolina university that began implementation during the pandemic.Fourth, Rachel Scrivano and colleagues will describe a community-based participatory research method that bridged the gap between expectations and reality of implementing a 5-year intergenerational program focused on healthy food access. The fifth paper from Skye Leedahl and colleagues will discuss the implementation experiences of an intergenerational, reverse mentoring, technology program that has evolved for seven years at a Rhode Island public university and utilizes community partners from across the state.

### OPTIMIZING ONLINE DELIVERY OF INTERGENERATIONAL STUDENT EXPERIENCES: LEVERAGING OPPORTUNITIES

Lisa Borrero, *University of Indianapolis, Indianapolis, Indiana, United States*

Conceptualizing intergenerational opportunities for students in fully online courses can initially appear daunting or impractical, given the absence of a common physical classroom and community environment. However, it could be argued that online learning serves as a facilitator, rather than a barrier to intergenerational programming. This session will focus on the connections between intergenerational programming and the principles of quality online teaching – emphasizing the ability of online modalities to increase connections with a global community, remove the limitations of geographic-based access to experiences, and elevate the level of student engagement typically expected within online courses. Leveraging opportunities to overcome real and perceived challenges to the planning, delivery, and evaluation of intergenerational student experiences in online courses will be discussed, as well as opportunities for future growth in the current era.

### INTEGRATING RESEARCH, TEACHING, AND SERVICE: AN INTERGENERATIONAL BONDING PROGRAM AT THE UNIVERSITY OF NEBRASKA

Ladan Ghazi Saidi, *Universoty of Nebraska at Kearney, Kearney, Nebraska, United States*

To establish an Intergenerational Bonding (IGB) program that pairs undergraduate students at the University of Nebraska at Kearney with older adults of this rural community, we first tested acceptability and feasibility of such a program. We surveyed both groups to identify their interest level in IGB and identify their common interests in activities. Older adults residing in independent dwellings, assisted living homes, and nursing homes and members of community groups were invited to participate in the survey. However, the response rate was low. Given the restrictions of the pandemic, accessing community older adults was challenging. Internet access was low, which made virtual and digital participation modalities impossible; recruitment from assisted living facilities was not successful; and, establishing collaboration with nursing homes was not easy despite initial interest. Building trust and working with community activity group facilitators was ultimately beneficial in recruiting older adults. Collaboration with community-based organizations facilitated the study.

### EVALUATING IMPLEMENTATION OF AN ONLINE INTERGENERATIONAL TECHNOLOGY PROGRAM

Jill Juris[1], Rachel Scrivano[2], Erin Bouldin[3], and Katherine Uva[4], *1. Appalachian State University, Boone, North Carolina, United States, 2. The Ohio State University, Columbus, Ohio, United States, 3. University of Utah, Salt Lake City, Utah, United States, 4. Appalachian State university, Boone, North Carolina, United States*

Social isolation and loneliness contributed to poor health outcomes among rural older adults prior to COVID-19. Physical distancing protocols during COVID-19 exacerbated social disconnect of rural older adults and paused in-person intergenerational programs. Pilot data from a Cyber-Seniors program that was adapted for virtual delivery and participation during the pandemic (2020-2021) indicates positive health outcomes for older adults (improved social isolation and loneliness). This presentation will address a gap between outcomes and the implementation process of the intergenerational technology program. Multi-method data on student experiences as technology mentors working at a distance suggest a need for an evaluation of program implementation. The type 1 effectiveness-implementation hybrid design includes quantitative measures of student outcomes (ageism, social isolation, and loneliness) and qualitative analysis of students' program implementation experiences. Students identified challenges accessing and working with the online platforms, scheduling meetings with older adults, and providing support with online systems by phone.

### THE IMPLEMENTATION PROCESS OF AN INTERGENERATIONAL COMMUNITY RESEARCH PROJECT: A NARRATIVE REVIEW

Rachel Scrivano[1], Jill Juris[2], and Shannon Jarrott[1], *1. The Ohio State University, Columbus, Ohio, United States, 2. Appalachian State University, Boone, North Carolina, United States*

Food for a Long Life (FFLL) was a five-year USDA Children, Youth and Families at Risk intergenerational community research project that sought to increase healthy food consumption, knowledge, and access among preschoolers and older adults living in food insecure communities of Ohio and Virginia. Using the community-based participatory action research approach, community stakeholders jointly participated in all stages of the project to co-create context-specific programming to address needs within their communities with the goal of promoting program sustainability beyond the grant funding period. This presentation will provide a narrative review to explore the implementation process of FFLL by comparing initial project expectations to reality using the community-based participatory research conceptual model (Wallerstein et al., 2008; Wallerstein et al., 2018). By exploring project barriers such as COVID-19, facilitators such as flexible partner relationships, and strategies including promoting early buy-in, we provide an in-depth discussion of project successes and lessons learned.

### SEVEN YEARS OF IMPLEMENTING AN INTERGENERATIONAL PROGRAM WITH UNIVERSITY/COMMUNITY PARTNERSHIPS

Skye Leedahl, Erica Estus, Kristin Fratoni Souza, and Alexandria Capolino, *University of Rhode Island, Kingston, Rhode Island, United States*

The University of Rhode Island Engaging Generations Cyber-Seniors Program was first implemented in the fall semester of 2015. URI students from 15+ majors have supported the digital competency of older adults in Rhode Island each semester and summer session since its inception. Gradually over time, we modified and expanded this in-person program to meet student, older adult, community partner, and faculty needs. The pandemic led to our program expanding exponentially due to new grant funding opportunities, new partnerships, and student interest. This presentation will address strengths and challenges of implementing this program state-wide in a small, mostly urban state with community partners, mostly involving senior centers. We will describe moving from in-person to phone/virtual and now to a hybrid model. Last, we will explain the program's efforts to conduct formative and summative evaluation research to assess program output and examine outcomes for students and older adults in the program.

### SESSION 3790 (SYMPOSIUM)

### INDIGENOUS UNDERSTANDINGS AND APPROACHES TO SUCCESSFUL AGING

Chair: Jordan Lewis Co-Chair: Steffi Kim

Indigenous peoples worldwide face unique challenges growing old. Many of these challenges are founded in historical colonization practices, present oppressive systems, racism, and underrepresentation in research, service provision, health education, and successful aging theories. The focus of this symposium is to discuss specific barriers and challenges related to Indigenous aging in rural and urban communities, as well as community strategies supporting aging well. The first presentation by Zayla Asquith-Heinz and colleagues will share the results of what successful aging or "Eldership" means in the Norton Sound southern subregion of Alaska. Results indicate that family plays a central role within the Norton Sound model of successful aging. The second presenter, Steffi Kim and Jordan Lewis, are discussing the role of cultural influences and Elder identity on successful aging in the context of Alaska Native Elders migrating from rural traditional communities to a western urban community. The third and fourth presentations by Sarah Russell and Rachel Quigley will share the results on what aging well means within Torres Strait Islander people. The results suggest that the availability and accessibility to traditional practices, language and foods can facilitate aging well within these communities. They will also describe the development and implementation of a toolbox of culturally appropriate screening tools and interventions. Lastly, Jordan Lewis will explore the Indigenous concept of "doing" successful aging rather than having good health. He will outline differences and similarities with BIPOC studies on successful aging.

### ALASKA NATIVE SUCCESSFUL AGING IN NORTHWEST ALASKA: AGING IN A GOOD WAY STARTS WITH YOUR FAMILY

Zayla Asquith-Heinz[1], Jordan Lewis[2], and Steffi Kim[3], *1. University of Minnesota Medical School, Minneapolis, Minnesota, United States, 2. University of Minnesota Medical School, Duluth, Minnesota, United States, 3. University of Minnesota, Minneapolis, Minnesota, United States*

Alaska Natives (AN) view aging from a holistic perspective. One of the challenges of researching with cultural groups is the lack of data, or research, on culture and aging. This research explored successful aging from an AN perspective. A community-based participatory research (CBPR) model was used to engage participants at every stage of the research process. Semi-structured interviews were conducted with 16 AN men and 25 women. Kleinman's Explanatory Model of Illness was adapted to gain a sense of the beliefs about aging and to guide the data analysis to establish an AN understanding of successful aging or attaining "Eldership" in Northwest Alaska. The foundation of the Norton Sound southern sub-region Model of Successful Aging is family, which contributed to Elders' feelings of emotional well-being, ability to engage in their Native Way of life, maintain their physical health, and continue spiritual practices.

### AN EXPLORATION OF CULTURAL INFLUENCES ON SUBJECTIVE SUCCESSFUL AGING

Steffi Kim, *University of Minnesota, Minneapolis, Minnesota, United States*

Indigenous peoples worldwide face unique challenges growing old. Many of these challenges are remnants of previous colonization practices and current oppressive systems often leading to out-migration from rural to urban environments. Despite Anchorage having the highest population of Alaska Native Elders little is known about the experience of relocation. This study investigated the impact of culture on the experience of successful aging within the Alaska Native context. Twenty-five semi-structured qualitative interviews with rural (N=13) and urban Elders (N=12; ages 48-84) were conducted. The use of Gee's discourse analysis tools provided the framework for analyzing the discourse of Elders based on location and traditional or western influences on subjective successful aging. We explored the use of language within two identified discursive patterns: cultural discourse and Elder identity discourse. Social and contextual determinants of successful aging involve aspects of minority and majority culture and self-appraisal of successful aging based on cultural assumptions.

### ON THE "ACTION" OF DOING HEALTHY AGING AS OPPOSED TO "HAVING" GOOD HEALTH

Jordan Lewis, *University of Minnesota Medical School, Duluth, Minnesota, United States*

The field of successful aging continues to grow and expand as authors investigate this topic in BIPOC populations, broadening the scope of successful aging, including Indigenous communities in the Arctic. Successful aging in Alaska Native communities is still a young, but growing, field of research, and this field of study places Indigenous voices at the forefront. This presentation will present data from 11 years of interviews with 108 Alaska Native Elders from rural and urban communities across the State of Alaska and highlight how successful aging is more of an "action" of doing healthy aging as opposed to the Western notion of "having" good health. The findings of this presentation will discuss how Alaska Native Elders' understanding, or doing, successful aging can be applied to everyday life, similarities and differences with BIPOC studies on successful aging, and serve as an act of resistance against western notions of successful aging.

### SESSION 3800 (SYMPOSIUM)

### INTEGRATING AGE INCLUSIVITY WITH DEI EFFORTS ON AGE-FRIENDLY UNIVERSITY (AFU) CAMPUSES

Chair: Joann Montepare Co-Chair: Kimberly Farah
Discussant: Peter Lichtenberg

The pioneering Age-Friendly University (AFU) initiative, endorsed by GSA's Academy for Gerontology in Higher Education (AGHE), calls for institutions of higher education to respond to shifting demographics and the needs of age-diverse, older populations through more age-friendly programs, practices, and partnerships. Over 85 institutions in the United States, Canada, European countries, and beyond have joined the global network and endorsed the 10 AFU principles, with even more showing interest in becoming partners in the movement. One key foundational area identified by AFU research efforts and partners is integrating age inclusivity with ongoing diversity, equity, and inclusion (DEI) efforts on campuses. This symposium explores the need for this integration featuring AFU partners who will offer their observations and recommendations. Bowen and colleagues will open the session with data from their national study of age-friendliness in U.S. institutions to describe their insights regarding the state of age diversity on campuses and the experiences of students, faculty, and staff that call for greater age inclusivity. Morrow-Howell and colleagues will present data from interviews with DEI officers that identify institutional considerations for inclusion efforts. Andreoletti and colleagues will offer specific curricular and related strategies for connecting age-inclusivity efforts with DEI campus efforts. Gugliucci will discuss considerations regarding age-inclusive images and messages in health professions education including the inclusion of identifiable DEI objectives in syllabi. As discussant, GSA president Lichtenberg will comment on age-inclusivity efforts in higher education within GSA's broader commitment to diversity, equity, and inclusion.

### MAKING THE CASE FOR AGE INCLUSIVITY IN HIGHER EDUCATION

Lauren Bowen[1], Joann Montepare[2], Nina Silverstein[1], Susan Whitbourne[3], and Celeste Beaulieu[1], *1. University of Massachusetts Boston, Boston, Massachusetts, United States, 2. Lasell University, Newton, Massachusetts, United States, 3. University of Massachusetts Amherst, Amherst, Massachusetts, United States*

Often viewed as institutions primarily servings 18-to-24-year-old student populations, U.S. colleges and universities are age-diverse. In our recent national study of AFU institutions, 21 campuses maintaining age data reported that 12,718 faculty (39.24% of faculty) and 20,361 staff (42.31% of staff) were ages 50+. Additionally, 22 campuses reported 7,080 students (1.58%) ages 50+. Despite higher education's attention to diversity, equity, and inclusion (DEI), age is often overlooked; therefore, universities may need strategies for improving age inclusivity. Across 2,447 open-ended survey responses from our study, faculty, staff, and students describe experiences with age inclusivity (e.g., feeling valued) and exclusion (e.g., feeling unwelcome), and many call for greater sensitivity to aging in existing DEI efforts, such as more age-inclusive language in classrooms and attention to age bias in hiring and promotions. In addition, this presentation will examine responses that raise important considerations for integrating age inclusivity with other higher education DEI efforts.

### AGE AS A DIVERSITY FACTOR IN HIGHER EDUCATION: INSIGHTS FROM DEI OFFICERS

Nancy Morrow-Howell, Natalie Galucia, and Michele Dinman, *Washington University in St. Louis, St. Louis, Missouri, United States*

This study described how DEI officers across universities/colleges currently think about AGE as a diversity factor; and identified strategies used to increase age-inclusivity. Data were generated through review of university websites, focus groups and one-to-one interviews with DEI staff. Findings suggest that age is acknowledged as a diversity factor but there is less action toward strategies to increase age-inclusion. Examples of initiatives include: training human resource staff to be age-neutral in hiring; eliminating birthdates and other years from applications; workshops on multigenerational workplaces and classrooms; presentations on ageism; and specific programs to support non-traditionally aged students. Some of the motivation to address ageism stems from legal mandates rather than being mission-driven. There is the concern that focusing more on age may require moving attention and resources away from other diversity factors. It appears that there is interest in elevating age as an important factor in DEI efforts.

### STRATEGIES FOR CONNECTING AGE INCLUSIVITY TO DEI EFFORTS: A CAMPUS CASE STUDY

Carrie Andreoletti, and Andrea June, *Central Connecticut State University, New Britain, Connecticut, United States*

We've employed a multi-pronged approach to connecting our AFU initiatives, which promote age inclusivity, to our university's DEI efforts. First, we asked our DEI office if they would collaborate. They agreed to link our AFU webpage to the DEI webpage and supported Ageism First Aid training for faculty and staff. Second, we participated in equity, justice, and inclusion (EJI) efforts on campus by ensuring that gerontology courses qualified for the EJI designation. This helps expand aging education across the curriculum as all students must take one EJI designated course. We also volunteered to speak about ageism to the first cohort of John Lewis Institute Scholars. Third, we partnered with our Center for Teaching and Innovation to offer programming on age inclusivity and generational diversity in the classroom. Taken together, these efforts have helped us to expand our reach and ensure that age is part of DEI conversations on our campus.

### AGE-FRIENDLY MESSAGES AND IMAGES: A VIEW FROM HEALTH PROFESSIONS EDUCATION

Marilyn Gugliucci, *University of New England College of Osteopathic Medicine, Biddeford, Maine, United States*

The World Health Organization (WHO) report published March 2021 emphasizes that older adults are often subjected to a variety of negative stereotypes including helplessness, frailty, and child-like qualities. Ageism has both real mental and physical health consequences, including a decreased will to live, less desire to live a healthy lifestyle, an impaired recovery from illness, increased stress, and a shortened life span. Health professions education has a responsibility to prepare future providers in more than mindful physical care of older adults, it must address ageism that has proliferated negative personal biases that have triggered reduced overall health and quality of life. Preparation of lectures and learning materials in health professions education requires mindfulness of diversity as well as the implicit bias faculty may portray regarding age. This presentation will bring these nuances to light for consideration and as a reference to instill change.

### SESSION 3810 (BIOLOGICAL SCIENCES INVITED SYMPOSIUM)

### GENE REGULATION IN AGING

Chair: Alexander Mendenhall

The regulation of genes can both influence aging, and be influenced by the aging process itself. That is, the rate of aging can be altered by changing the gene expression program. And, the aging process itself causes changes in gene expression, including the ways in which genes are regulated. Understanding the consequences of differences in gene expression on aging rate, and the consequences of aging on gene regulation will continue to have profound impacts on our ability to manipulate the aging process. This symposium on gene regulation in aging will focus on how genes are regulated by the aging process and can be regulated differently to affect the aging process. We have an expert on the regulation of gene expression in the immortal germline and soma of the hydra, Dr. Celina Juliano. Dr. Roger Brent is an expert on the mechanisms of cell to cell variation in gene expression. Dr. Monica Driscoll is an expert on both the genetics of aging and gene expression changes with age. Finally, Dr. Alex Mendenhall's studies are focused on understanding intrinsic (epigenetic) variation in the regulation of gene expression as a cause and consequence of aging. Together these experts will present their research as it relates to gene regulation and the aging process.

### HOW THE SAME GENETIC PROGRAM RUNS DIFFERENTLY IN INDIVIDUAL ANIMALS TO AFFECT AGING AND DISEASE

Alexander Mendenhall, Bryan Sands, and Soo Yun, *University of Washington, Seattle, Washington, United States*

Monozygotic human twins will age at different rates. The same is true for isogenic laboratory animals. Some of these differences in the rates of aging are caused by differences in the expression of genes. And, some of the differences in gene expression between isogenic individuals are caused by seemingly non-heritable, stochastic epigenetic differences. Here we discuss how differences in chaperone expression can influence aging and a model of Ras-driven neoplasia risk and survival in the model nematode Caenorhabditis elegans. We review evidence suggesting differences in epigenetic silencing machinery contribute to differences in chaperone gene expression. We suggest models for germline and somatic epigenetic regulation of chaperones. We discuss potential means of targeted epigenome modification, and potential implications for human health during aging.

### MECHANISMS OF DEVELOPMENT AND REGENERATION IN HYDRA

Celina Juliano, Jack Cazet, and Abby Primack, *University of California, Davis, Davis, California, United States*

Hydra vulgaris is a small and simple aquatic animal capable of whole-body regeneration and has negligible senescence. The entire animal, including the nervous system, is composed of about 25 cell types, and can regenerate from a fragment of tissue as small as ~300 cells. In addition, all cell types are continually renewed in the uninjured adult as part of normal homeostasis; every differentiated cell type is replaced approximately every 20 days, which likely contributes to its lack of aging. The remarkable features of Hydra are enabled by three distinct populations of stem cells that support the three lineages that make up the adult Hydra – the ectodermal epithelial lineage, the endodermal epithelial lineage, and the interstitial lineage (includes the neurons). A major goal of our laboratory is to understand the gene regulatory networks that control the specification of all Hydra cell types in the uninjured (homeostatic) state and then understand how injury triggers these differentiation pathways at unexpected locations during regeneration. Using high throughput genomics approaches such as scRNA-seq, ATAC-seq, and Cut&Tag, we have transcriptionally defined every cell type in Hydra and identified putative transcriptional regulators for each cell type. This includes the 11 neuronal subtypes that comprise the nerve net that spans the entire length of the Hydra body. We are currently leveraging these data to conduct functional testing of key putative regulators and to identify injury inputs into cell specification events during regeneration.

### MISEXPRESSION OF GENES LACKING CPG ISLANDS IS A SHARED TRAIT OF MAMMALIAN AGING

Samuel Beck, *Boston University School of Medicine, Boston, Massachusetts, United States*

Changes in the 3-D architecture of chromatin are observed in various diseases and are also a hallmark of aging. Disruption of the nuclear lamina and associated heterochromatin are commonly observed in various aging contexts, including premature aging diseases, cellular senescence, and normative aging. Although these conserved structural changes have been reported for over two decades, their impacts on transcription and contribution to age-related degenerative changes remain unknown. By performing a large-scale computational analysis and experimental validation, here we show that genes lacking CpG islands (CGI- genes), which form heterochromatin when transcriptionally silent, are globally misexpressed in aged nuclei with disrupted chromatin architectures. We demonstrate that CGI- gene misexpression is a common feature of mammalian aging and explains the molecular basis of various age-associated defects, ranging from loss of cellular identity and increased transcriptional noise to age-associated chronic inflammation. Our findings reveal that CGI- gene misexpression is directly associated with age-related physiological deterioration, thus providing a novel biomarker of aging.

### A SIMPLE ANIMAL MODEL OF EXERCISE REVEALS A MOLECULAR DETERMINANT OF LONG TERM HEALTH MAINTENANCE

Monica Driscoll, *Rutgers, The State University of New Jersey, New Brunswick, New Jersey, United States*

At all stages of life, both dynamic gene expression changes and events that "lock in" particular programs that promote health and maintenance are critical factors in aging trajectories. We have a long-term interest in the fundamental biology of healthy maintenance, a topic that has led us to consider multiple facets of healthspan. A powerful whole-organism intervention with maintenance-promoting, anti-disease, anti-aging impact is exercise. The molecular and cellular mechanisms that mediate long-term system-wide exercise benefits, however, remain poorly understood, especially as applies to "off target" tissues that do not participate directly in training activity. We are investigating the basic biology of exercise benefits using the simple 959-celled model C. elegans. We found that multiple daily swim sessions are essential for exercise adaptation, leading to enhanced expression of muscle structural genes and improved locomotory performance. Importantly, swim exercise training enhances whole-animal health parameters such as mitochondrial respiration and mid-life survival, increases functional healthspan of pharynx and intestine, and enhances nervous system health by increasing learning ability of adults and protecting against neurodegeneration in models of tauopathy, Alzheimer's disease, and Huntington's disease. Remarkably, swim training only during early adulthood induces long-lasting systemic benefits that in several cases are still detectable well into mid-life. Our investigation of the molecular requirements for long-term maintenance ("legacy effects") revealed that deletion of the sole C. elegans extracellular superoxide dismutase gene SOD-4 changes this pattern. sod-4 mutants are able to swim train to gain measurable benefits, but do not maintain the healthspan improvements that wild type animals do, defining extracellular SOD-4 as a powerful mid-life maintenance factor associated with exercise experience. Our talk will discuss transcriptomic analysis of exercise and SOD-4, as well as our current understanding of the molecular mechanisms operative. Notably, mammalian extracellular SOD ecSOD has been shown to promote exercise associated health and protection against oxidative stress insults (PMID: 32220789 Yan, Spaulding 2020), implicating ecSOD in a conserved role for health maintenance and underscoring potential for therapeutic translation.

### SESSION 3820 (PAPER)

### LABOR FORCE PARTICIPATION: A GLOBAL PERSPECTIVE

### CHANGES IN SUBJECTIVE WELL-BEING DURING RETIREMENT TRANSITION: A 10-YEAR COHORT STUDY OF AGING ADULTS IN CHINA

Shuai Zhou, *The Hong Kong Polytechnic University, Hung Hom, Kowloon, Hong Kong*

The literature on retirement adjustment remains inconclusive whether retirement transition is a stressor or a relief. This study examined the effects of retirement on subjective well-being of Chinese ageing adults in various phases of retirement. Drawing on a representative sample of "baby boomers" born in 1945-1965 (N = 3,328) from the China Family Panel Studies (2010-2018), the author examined the within-individual changes in subjective well-being, measured by life satisfaction and confidence in the future, using time-distributed fixed-effects regression technique. After adjusting for a wealth of potential confounders such as financial standing and health, the data reveal that the two outcomes of interest started increasing at two years before retiring. In addition, the improvements persisted in four or more postretirement years. Urban adults experienced greater well-being boost than rural residents during the immediate pre- and post-retirement period, but not in two or more postretirement years. Furthermore, the subjective well-being of urban females and rural males increased upon retirement and fluctuated significantly, whereas rural females and urban males experienced a stable increase in subjective well-being since the first preretirement year. Results suggest that the transition into retirement generally represents a relief for urban ageing adults in China, but the well-being improvements are stratified by residency and gender. Therefore, retirement policies should ensure subjective well-being of disadvantaged pre-retirees and attend to the needs of retired urban women and rural men.

### DETERMINANTS OF RETIREMENT-AGE WOMEN'S LABOR FORCE PARTICIPATION: A RUSSIA AND THE US COMPARISON

Oksana Dikhtyar, *Miami University, Oxford, Ohio, United States*

Factors such as one's health, education, marital status, and family caregiving were found to be associated with people's retirement timing. Few studies looked at those factors separately for men and women and few cross-cultural studies were conducted on the topic. Women tend to accumulate less income and retirement savings throughout their working careers compared to men due to having more intermittent careers and lower paying jobs. Women, in general, live longer than men and are more likely to be divorced or widowed at older ages. Thus, they must spend fewer financial resources over a longer time in retirement. One possible solution is to continue employment after reaching pensionable age. Therefore, it is important to know factors that affect women's labor force participation at or after pensionable age.This quantitative study examines the relationship between women's personal and family factors and their labor force participation after reaching pensionable age in Russia and the U.S. Using data from the Background Questionnaire of the Program for the International Assessment of Adult Competencies (PIAAC) survey, we analyzed a sample of Russian women ages 55 and older and American women ages 66 and older.For retirement-age Russian women, having highest level of education, living in a larger household, and having spouse or partner in the labor force were positively associated with women's labor force participation. Likewise, American women of pensionable age with more education and a working spouse were more likely to be in the labor force. Implications for policy and future research are discussed.

### RE-EMPLOYMENT MANDATE IN SINGAPORE: EMPLOYMENT AND HEALTH OUTCOMES

Ngee Choon Chia[1], Cynthia Chen[2], Jemima Koh[1], Kia Yee Lim[1], and Yuet Yan Tsoi[1], *1. National University of Singapore, Singapore, Singapore, 2. National University of Singapore, Singapore, Singapore*

Older workers can be a crucial resource to meet manpower needs for aging society. This is especially so for countries where older adults are expected to live longer and healthier. In 2012, Singapore implemented the Retirement and Re-employment Act (RRA) which obliged employers to offer re-employment to eligible workers. With RRA, mature workers have the flexibility to work beyond retirement age. Using the Retirement and Health Study (RHS) data and regression discontinuity design, we find that, after adjusting for education, marital status, housing asset and expenditure, the re-employment mandate helps delay retirement in the sample by 8.7%, with a larger impact of 9.1% among the males, as compared to the females of 7.3%. More non-retirees reported that they have very good or excellent health compared to retirees. Retirement leads to increase in healthcare utilization. Does the work environment affect how one responds to retirement option? We observe a correlation between post-retirement wellbeing and stress at work. Our study also suggests that mandatory re-employment offers are less effective in encouraging those in physically demanding jobs to continue working. Re-employment mandate helped raise the employment rate for workers in high-routine, high-manual jobs by 6.1 per cent. These would include craft workers and trade workers. It raised the employment rate for workers in highly routine and low-manual occupations by 26.9 per cent. These include workers in the hospitality, retail and services sectors. In sum, the mandate together with other inclusive work policies have been useful to mitigate impacts of societal ageing in Singapore.

### SESSION 3830 (PAPER)

### MEALTIMES AND NUTRITION

### BREADTH OF ADVERSE OUTCOMES ASSOCIATED WITH ANOREXIA/APPETITE LOSS IN OLDER POPULATIONS

Roger Fielding[1], Francesco Landi[2], Karen Smoyer[3], Thomas McRae[4], Lisa Tarasenko[4], and John Groarke[4], *1. Jean Mayer USDA Human Nutrition Research Center on Aging, Tufts University, Boston, Massachusetts, United States, 2. Fondazione Policlinico Universitario Agostino Gemelli IRCSS, Rome, Lazio, Italy, 3. Envision Pharma Group, Philadelphia, Pennsylvania, United States, 4. Pfizer Inc, New York, New York, United States*

The scope of the clinical problem of anorexia or appetite loss (AL) in older populations is potentially underappreciated. A systematic literature review (SLR) was conducted following PRISMA guidelines to summarize associations of anorexia/AL in older adults with a range of clinical outcomes. Searches were run January/1/2011–July/31/2021 in PubMed, Embase®, and Cochrane databases to identify English-language studies of adults (aged ≥65 years) with anorexia/AL. Two independent reviewers screened titles/abstracts and full text against pre-defined inclusion/exclusion criteria. In all, 146 studies underwent full-text review; 58 met eligibility criteria. Most were from Europe (34/58 [58.6%]) or Asia (16/58 [27.6%]); only 3 (5.2%) were from the United States (US). Although mortality (n=18) and malnutrition (n=15) were the most reported outcomes, no US study reported on these outcomes. Other commonly reported outcomes included sarcopenia (n=7), functional status (n=6), need for increased care (n=6), and hospitalization (n=4). Significant associations of anorexia/AL were most consistently observed with mortality (17/18 studies), malnutrition (15/15 studies), and functional status (5/6 studies). Although the overall number of studies is small, associations have emerged with falls (2/3 studies), health related quality of life (2/3 studies), depression (2/2 studies), and cognition (2/2 studies). However, associations with hospitalization (1/4 studies), sarcopenia as assessed by muscle strength (2/4 studies) or by muscle mass (1/3 studies), and need for increased care (3/6 studies) were less consistent. Together, these studies highlight the breadth of adverse outcomes associated with anorexia/AL in older populations and a need for more US-based research into this common clinical problem.

### DYSPHAGIA FOLLOWING HOSPITALIZATION IN NURSING HOME RESIDENTS WITH ALZHEIMER'S DISEASE AND RELATED DEMENTIAS

Nicole Rogus-Pulia[1], Raele Robison[1], Sweta Patel[2], Jennifer Bunker[3], James Rudolph[2], and Joan Teno[2], *1. University of Wisconsin-Madison, Madison, Wisconsin, United States, 2. Brown University, Providence, Rhode Island, United States, 3. Oregon Health and Science University, Portland, Oregon, United States*

Dysphagia frequently occurs in nursing home (NH) residents with Alzheimer's Disease and Related Dementias (ADRD), often leading to serious health outcomes (e.g., pneumonia, malnutrition, reduced quality of life). While it is known that hospitalized NH residents with ADRD experience high rates of iatrogenic complications, dysphagia following discharge has not been examined. A retrospective cohort of all NH residents in the US (older adults aged ≥66) with advanced ADRD (Cognitive Function Scale ≥2), hospitalized between 2013-2017, and without a feeding tube or reported dysphagia on a Minimum Data Set (MDS) 3.0 assessment within 120 days prior to hospitalization was constructed. Treatment with intermittent mandatory ventilation (IMV) or non-invasive ventilation (NIV) during hospitalization and dysphagia status on the first post-hospitalization MDS was recorded. Data were analyzed using descriptive statistics and random effects multivariate logistic models that adjusted for age, gender, race/ethnicity, CFS score, ADL score, and comorbidities. Among the 805,199 residents with ADRD who survived the hospitalization and returned to the NH, new onset dysphagia occurred in 53,807 (6.7%; 95% CI 6.6-6.7) of residents. After adjustment, invasive mechanical ventilation (IMV) use was associated with increased risk of new onset of dysphagia (AOR 1.5; 95% CI 1.4-1.6) and non-invasive mechanical ventilation (NIMV) only slightly increased the risk (AOR 1.3; 95% 1.2-1.3). NH residents with ADRD are at risk for dysphagia following hospitalization. These findings emphasize the importance of swallowing evaluation and dysphagia treatment during hospitalization for ADRD patients, especially those treated with IMV or NIV, to prevent further negative health outcomes.

### FACTORS ASSOCIATED WITH EATING PERFORMANCE IN NURSING HOME RESIDENTS WITH DEMENTIA AND MULTIPLE COMORBIDITIES

Wen Liu[1], Elizabeth Galik[2], and Barbara Resnick[3], *1. University of Iowa, Iowa City, Iowa, United States, 2. University of Maryland, Baltimore, Baltimore, Maryland, United States, 3. University of Maryland, Baltimore, Maryland, United States*

Eating performance is the functional ability to get food and drink into the mouth. Nursing home residents with dementia commonly experience compromised eating performance and subsequent nutritional consequences. Resident characteristics including cognition, physical capacity, and functional ability are associated with eating performance. This study aimed to test the association between functional ability, behavioral symptoms, psychotropic medication use, and comorbidities and eating performance. This was a secondary analysis using baseline data from two randomized controlled trials testing the impact of Function Focused Care on function and behavioral symptoms between 2014-2020. A total of 889 residents with moderate-to-severe dementia (mean age 86.58 years, 72% female, 70% white, 69.5% severe dementia) from 67 nursing homes in two states were recruited. Eating performance (dependent variable) was measured using the single self-feeding item on the

Barthel Index. Independent variables included functional ability (Barthel Index total score excluding self-feeding item score), behavioral symptoms (agitation, depression, resistiveness to care), and psychotropic medication use (anti-depression, sedative, anti-psychotics, anti-seizure, anti-anxiety), and comorbidities. Residents had on average 5 documented comorbidities (SD=3.06, range=0-12) and were on approximately 1 psychotropic medication (range=0-5, SD=1.24). Thirty-eight percent of residents were dependent in eating performance. Functional ability (OR=1.052, 95% CI=1.043,1.061, p<.001), depression(OR=.931, 95% CI=.887,.978, p=.004), and anti-anxiety medication use(OR=.632, 95% CI=.409, .978, p=.039) were associated with eating performance. Findings supported better functional ability, lower depression, and less anti-anxiety medication use were associated with better eating performance. Targeted efforts including maintaining functional ability, minimizing anti-anxiety medication use, and managing depression are encouraged to support eating performance.

### FACTORS ASSOCIATED WITH MEALTIME LANGUAGE CHARACTERISTICS IN NURSING HOME STAFF AND RESIDENTS WITH DEMENTIA

Wen Liu[1], Ying-Ling Jao[2], and Si On Yoon[3], *1. University of Iowa, Iowa City, Iowa, United States, 2. Pennsylvania State University, University Park, Pennsylvania, United States, 3. The University of Iowa, Iowa City, Iowa, United States*

Interactive staff-resident communication is crucial for nursing home residents with dementia requiring mealtime assistance. Effective communication may facilitate food intake and promote function and nutrition. However, understanding of staff and resident language characteristics in mealtime interactions is limited. This study examined language characteristics and associated factors in mealtime interactions. This was a secondary analysis using data from videotaped mealtime observations (N=160) involving 36 staff and 27 residents with moderate-to-severe dementia (53 staff-resident dyads) in 9 nursing homes. The dependent measures were 1) the number of words produced in each utterance (expression length), and 2) whether staff named the resident in each utterance (naming the resident). Mixed-effects models examined the effect of utterance quality (positive vs. negative utterances), intervention (pre- vs. post-communication training), and subject speaking (staff vs. resident), adjusting resident comorbidities and dementia stage. Staff (mean=4.30, SD=2.98) produced significantly longer utterances than residents (mean=2.64, SD=2.27). Expression length was modulated by utterance quality and intervention. Staff's negative utterances were shorter than their positive utterances, while residents' negative utterances were longer than their positive utterances. Staff's negative utterances became longer after the intervention while the length of positive utterances remained similar pre- and post-intervention. Staff named the resident in 16.72% of their utterances and was more likely to name residents with severe dementia. Findings emphasize the potential benefit of communication training on mealtime interactions. Findings also highlight the need to further examine the impact of language characteristics on food intake, which may guide intervention development to promote nutrition for residents with dementia.

### FIFTEEN-YEAR TRENDS IN THE PATTERNS OF MEAL TIMING AND EATING FREQUENCY AMONG US ADULTS

Samaneh Farsijani, Ziling Mao, and Anne Newman, *University of Pittsburgh, Pittsburgh, Pennsylvania, United States*

Understanding patterns of eating behavior may provide insights into contributors to obesity and metabolic diseases. To characterize trends in meal timing and frequency in US population age >19 years, we performed a serial cross-sectional analysis of 8 National Health and Nutrition Examination Survey (NHANES) cycles (2003-2018) on 34,470 adults (52.5% women and 21% black). Time of food and beverage intake was extracted from two 24-hour food recalls. The following meal timing measures were defined: 1) The time of the last and first calorie intake either from food or drink, 2) Eating window: The time elapsed between the first and last food intake, 3) Calorie midpoint time: The time when half of the calories for the day were consumed, 4) Late night eating: Consumption of ≥33% of total daily energy between 5:00pm and midnight, and 5) Eating frequency: Frequency of food or beverage consumption >0 kcal which were >15 minutes apart. From 2003 to 2018, survey-weighted mean (±SE) of eating window decreased from 12.21±0.06 to 12.02±0.05 hours/d (Ptrend=0.002). Time of the last calorie intake significantly decreased from 20.31±0.04 to 20.09±0.03 hours/d, while the time of first calorie intake and eating frequency remained unchanged. More than two-third of participants consumed ≥33% of total daily energy after 5:00pm. Over the 15-year span, composition of diet has also changed, including a decline in total calorie intake and percentage of energy from carbohydrate intake, while percentage of energy from dietary fats increased, Ptrend< 0.05. Patterns of eating behavior have changed in American adults over time.

### SESSION 3840 (SYMPOSIUM)

### MITIGATING LONG-TERM CONSEQUENCES OF COVID-19 ON OLDER ADULTS IN HONG KONG, SINGAPORE, THAILAND, AND THE US

Chair: Ladda Thiamwong Discussant: Vivian Wei Qun Lou

The impact of COVID-19 on older adults goes beyond a higher risk for serious infection to long-term consequences such as decreased well-being and disability. In 2020, we formed an international and interdisciplinary research group and adopted a biopsychosocial model as a conceptual framework to guide our collaboration. Our ultimate goal is to mitigate the long-term health consequences of the pandemic and utilize affordable technology-based interventions to enhance the quality of life for older adults in Hong Kong, Singapore, Thailand, and the US. Five empirical studies will be presented with topics ranging from quality of life, fear of falling, attitudes toward technology, and the utilization of technology-based interventions. A path analysis from the first study indicated that physical frailty was the strongest direct power on quality of life followed by depressive symptoms, and life-space mobility. The second study found that fear of falling was predicted by falls risk, resistance, COVID fear, and health conditions. The third study revealed a strong connection between the COVID severity and positive attitudes toward technology, and social support facilitates the adoption of technology. The fourth study reported the benefits of using a technology-based body-mind intervention to alter a mismatch between fear of falling and actual fall risk and to increase the accessibility of the fall intervention. The final study indicated that utilizing an alternative online exercise program during the pandemic increased exercise regularity and enhanced motivation. Together, all five studies contribute to strategic implications on global mitigating long-term health consequences and essential issues for further research.

### A TECHNOLOGY-BASED BODY-MIND INTERVENTION FOR LOW-INCOME AMERICAN OLDER ADULTS.

Ladda Thiamwong[1], Rui Xie[1], Joon-Hyuk Park[1], Nichole Lighthall[1], Victoria Loerzel[2], and Jeffrey Stout[1], *1. University of Central Florida, Orlando, Florida, United States, 2. College of Nursing, University of Central Florida, Orlando, Florida, United States*

Research is limited on the use of technology to help individuals who have a mismatch between physiological fall risk (Body) and perceived fall risk (Mind) and are unable to access traditional fall interventions. We examined the feasibility and acceptability of a technology-based body-mind intervention in low-income older adults during the COVID-19 pandemic and explored barriers to access and adopting the technology. Data were collected using a survey, balance test, accelerometer-based physical activity (PA), and semi-structured interviews with twenty participants who engaged in an 8-week intervention at a low-income setting in Florida. We found that: 1) the technology-based intervention is feasible, 2) participants tend to accept technology to alter their perceptions of fall risk and balance capacity, 3) tailored activities to each component are not a one-size-fits-all approach. There were no statistically significant changes in sedentary time, light PA, and moderate to vigorous PA between pre and post-intervention.

### A MULTISITE INVESTIGATION OF FACTORS INFLUENCING QUALITY OF LIFE IN THAI OLD ADULTS DURING THE COVID-19 PANDEMIC

Inthira Roopsawang[1], suparb Aree-Ue[1], Teepatad Chintapanyakun[2], Yuwadee Saraboon[3], and Sirirat Intharakasem[3], *1. Mahidol University, Bangkok, Krung Thep, Thailand, 2. Department of Nursing, Bangkok, Krung Thep, Thailand, 3. Mahidol University Amnatcharoen Campus, Amnatcharoen, Amnat Charoen, Thailand*

Coronavirus-19 outbreak situation is a significant problem affecting the older population globally. The aim of this study was to investigate the association among physical activity, physical frailty, social frailty, life-space mobility, and depressive symptoms on quality of life in Thai older persons with non-communicable diseases prior to the COVID-19 pandemic. A path analysis was used for data analysis with the Mplus program. A total of 2,000 participants aged rang 60 – 89 years with a mean age of 65.13 years (SD = 4.62 years) participated in the study. Path analysis indicated that physical frailty was the strongest direct power on quality of life followed by depressive symptoms, and life-space mobility, respectively at the time of COVID-19's first outbreak. Monitoring and managing changes in physical frailty, depressive symptoms, and life-space mobility are suggested to promote active aging and maintain a good quality of life in this population, specifically age-friendly living areas.

### COVID-19 AND THE FEAR OF FALLING AMONG OLDER ADULTS IN SINGAPORE: AN ELECTRONIC SURVEY

Wayne Chong[1], Tharshini Lokanathan[2], and W. Quin Yow[2], *1. Nanyang Technological University, Singapore, Singapore, Singapore, 2. Singapore University of Technology & Design, Singapore, Singapore*

We investigated the predictors of fear of falling in Singapore during the COVID-19 pandemic. Older adults aged 60 to 85 (N=144) participated in an electronic survey that assessed their attitudes toward technology, perceived severity and fear of COVID, frailty, social engagement and falls risk. Hierarchical linear regressions revealed that the fear of falling was first predicted by falls risk, beta = .26, p = .001, F(1, 142) = 10.50, then by resistance, beta = .18, p = .03, F(2, 141) = 7.96. When COVID-19 fear, beta = .14, p < .001, and health conditions, beta = -.22, p = .005, were added together, falls risk became non-significant, beta = .14, p = .083, F(4, 139) = 10.50. The interaction between COVID-19 fear and health conditions was found to add significant variance in the final model, beta = -.31, p < .001, R square = .37, R square change = .09.

### HOW HAS COVID-19 SHAPED SINGAPORE OLDER ADULTS' ATTITUDES TOWARD TECHNOLOGY?

W. Quin Yow[1], Tharshini Lokanathan[1], and Wayne Chong[2], *1. Singapore University of Technology & Design, Singapore, Singapore, 2. Nanyang Technological University, Singapore, Singapore, Singapore*

This study investigated Singapore older adults' attitudes toward technology during the COVID-19 pandemic when in-person socializations have been negatively impacted. One hundred and forty-four older adults (range = 60-85 years old) participated in a large-scale survey that included questions such as their attitudes toward technology, severity and fear of COVID, as well as frequency of social support and contact with relatives and friends. Results revealed that the more severe the COVID situation in their area was, the more they believe that technology is useful and could enhance their effectiveness, and the more they like the idea of using technology in their daily activities, Fs>6.60, Bs>1.71, ps<.01. Furthermore, social support could facilitate the adoption of technology in older adults, F=6.31, B=.89, p=.013. Paradoxically, the fear of COVID could collaterally increase the hesitation of using technology in older adults for fear of making mistakes they cannot correct, F=8.50, B=1.30, p=.004.

### ONLINE EXERCISE CLASS EXPERIENCE AMONG OLDER ADULTS' MEMBERS OF COMMUNITY CENTERS IN HONG KONG DURING COVID-19

Janet Lok Chun Lee[1], Vivian Wei Qun Lou[2], and Rick Kwan[3], *1. The Hong Kong Polytechnic University, Hong Kong, Hong Kong, 2. The University of Hong Kong, Hong Kong, Hong Kong, Hong Kong, 3. Tung Wah College, Hong Kong, Hong Kong*

Due to the social distancing measures, community-centres in Hong Kong has been converting exercise classes to online delivery mode through video-conferencing software since the outbreak of COVID-19. The phenomenon is new, and no research has been done to investigate older adults' experience on it. This study adopted a descriptive qualitative methodologically orientation to understand the phenomenon. Twenty-three older adults (aged 55-89 years), with experience of participating online exercise class since COVID-19 participated in the study. Six major themes regarding their experiences emerged. Regarding positive experiences, older adults experienced convenience, increased exercise regularity, technical transformation and motivation in this new form of home-based exercise. At the same time, they experienced specific technical barriers and compensated supervision quality from the instructor. The findings of the study gave important future research direction and implications to the development of smart aging in community centres in Hong Kong.

### SESSION 3850 (SYMPOSIUM)

### MULTIDISCIPLINARY PERSPECTIVES ON LATER-LIFE DEPRESSION: THE ROLE OF BRAIN HEALTH

Chair: Ann Steffen Discussant: Jennifer Moye

Bi-directional associations between depression and cognitive functioning are magnified among aging individuals, challenging behavioral health providers who treat older adults experiencing clinical and subsyndromal depression. This symposium contributes to the science and practice of assessing and treating later-life depression while also attending to issues in professional training. The first paper presents pre-treatment data from the multi-site Optimum Study of older adults experiencing treatment-resistant depression (n = 529). The relevance of positive psychological constructs is supported with analyses showing important relationships among cognitive functioning, social participation, and psychological well-being. The second paper describes the development of an updated measure to assess behavioral health providers' knowledge of later life depression, including brain health concerns. Psychometric data for the measure were generated from a random pool of licensed social workers (N=900) who were mailed the survey packet. The third presentation features an experimental study demonstrating that foundational information about aging, including debunking misconceptions about cognitive aging, influenced continuing education preferences of generalist Licensed Professional Counselors (LPCs). Among the randomly generated pool of LPCs (N = 120), participants who received aging-specific information were more likely to later choose an aging-specific continuing education option. The fourth paper highlights recommendations for mental health practitioners working in primary care and general medical settings with older adults who have co-existing depressive symptoms and cognitive concerns. The fifth and final presentation describes cognitive behavioral clinical tools to address brain health concerns in the context of later-life depression, using the new Brain Health module of a published client workbook.

### PSYCHOLOGICAL WELL-BEING IN OLDER ADULTS WITH TREATMENT-RESISTANT DEPRESSION

Selmi Kallmi[1], Ann Steffen[1], and Eric Lenze[2], *1. University of Missouri-St. Louis, St. Louis, Missouri, United States, 2. Washington University School of Medicine in St. Louis, St. Louis, Missouri, United States*

Geriatric psychiatry research has documented the importance of psychological well-being to older adults diagnosed with depression (Lenze et al., 2016). This presentation utilizes pre-treatment data from the Optimum Study of treatment-resistant depression in older adults recruited from five USA and Canadian metropolitan areas (N = 529). Social participation was measured with the PROMIS scale: Ability to Participate in Social Roles and Activities (Hahn et al., 2014). Positive affect and life satisfaction were assessed using scales from the Psychological Well-Being subdomain (Salsman et al., 2013) of the NIH Toolbox for Assessment of Neurological and Behavioral Function - Emotion Battery (NIHTB-EB). Along with associations between social participation and positive affect ($r = .38$, $p < .01$) and between social participation and life satisfaction ($r = .26$, $p < .01$), path analyses explored social participation as a mediator of the relationship between cognitive functioning (NIHTB-CB; Weintraub et al., 2013) and psychological well-being.

### MEASURING PROFESSIONAL KNOWLEDGE OF LATER LIFE DEPRESSION

Abby Laine, and Ann Steffen, *University of Missouri-St. Louis, St. Louis, Missouri, United States*

Research linking behavioral health providers' knowledge of later life depression to their clinical practices and patient outcomes is restricted by outdated measures. A multi-stage study was conducted to ameliorate this gap. Qualitative interviews with geropsychology content experts (N = 5) generated 49 true/false items capturing relevant concepts in later life depression, including brain health concerns. Additional content experts (N = 10) completed the questionnaire and a card sort task placing items into categories (Psychopathology, Assessment/Diagnosis, Treatment, Other) to understand measurement structure. This resulted in retention of 42 items. A random pool of licensed social workers (N=900) were mailed the survey packet with option to complete via return mail or online. This presentation will review scale psychometrics, reliability, and construct validity among MSWs from diverse clinical backgrounds. Associations between participant characteristics and knowledge of cognitive and other depressive symptoms will also be highlighted.

### SHAPING PREFERENCES FOR AGING-SPECIFIC CONTINUING EDUCATION BY LICENSED PROFESSIONAL COUNSELORS

Nicholas Schmidt[1], and Ann Steffen[2], *1. Boston VA Healthcare System, Boston, Massachusetts, United States, 2. University of Missouri-St. Louis, St. Louis, Missouri, United States*

Although the prevalence of depression, like many other mental health disorders, declines with age, older adults who seek behavioral health services often present with depressive symptoms mixed with cognitive complaints. This creates challenges for generalist practitioners, who are responsible for the majority of mental health care of aging individuals. Ageism and misconceptions about older adults are theorized barriers to clinicians seeking aging-specific specialty training. This completed experimental study showed that assignment to receiving foundational information about aging, including debunking common misconceptions about cognitive aging, influenced choice of continuing education (CE) preferences of generalist Licensed Professional Counselors (LPCs). Among the randomly generated pool of LPCs (N=120), participants who received aging-specific information were more likely to choose an aging-specific CE option, F(4, 107) = 5.35, p<.001. Demographic variables, perceived competence for working with older adults, knowledge of aging, and ageist beliefs were also collected; analyses including these variables will also be presented.

### MANAGEMENT OF DEPRESSION IN OLDER ADULTS RECEIVING CARE IN MEDICAL SETTINGS

Cecilia Poon, *Nebraska Medicine, Omaha, Nebraska, United States*

Although rates of major depressive disorder are lower among older adults, depressive symptoms are a common presentation for aging individuals in medical settings. Unique challenges arise when treating depressive symptoms co-occurring with brain health concerns in older adults with a complex medical history. This presentation reviews how cognitive behavioral interventions for later-life depression are relevant for mental health practitioners who work in primary care and general medical settings. Specific clinical and multicultural considerations will be highlighted to support clinicians and interdisciplinary teams to work effectively with older adults who have co-existing depressive symptoms and cognitive concerns.

### CLINICAL TOOLS TO ADDRESS BRAIN HEALTH CONCERNS IN THE CONTEXT OF LATER-LIFE DEPRESSION

Kelly Bergstrom, *University of Missouri-St. Louis, St. Louis, Missouri, United States*

GSA's revised KAER Toolkit for Primary Care Teams (Fall, 2021) is an important resource, yet the complexities of depressive and cognitive symptoms in aging individuals create particular challenges for generalist behavioral health providers. Mental health practitioners and their patients can benefit from evidence-based clinical intervention materials that address the intersection of depression and brain health concerns. This presentation highlights treatment strategies and clinical tools from the new Brain Health module of the revised 2021 client workbook Treating Later-Life Depression: A Cognitive Behavioral Therapy Approach from Oxford University Press. Examples will be presented of large print within-session "Learn pages" that inform both providers and patients about normative cognitive aging and ways in which cognitive functioning can be affected by depression. Between-session "Practice forms" will also be demonstrated that address lifestyle factors to promote brain health, consideration of whether to complete a cognitive evaluation, and strategies to manage brain health changes.

### SESSION 3860 (SYMPOSIUM)

### NEIGHBORHOOD CHARACTERISTICS INFLUENCE OLDER ADULTS' HEALTH AND MENTAL HEALTH OUTCOMES

Chair: Weidi Qin Discussant: Weidi Qin

Both social and physical aspects of neighborhood characteristics are related to a wide range of health and mental health outcomes. There has been increasing evidence pointing to the link between neighborhood-level factors and health among older adults. Specifically, older adults living in disadvantaged neighborhoods with under-resourced infrastructure may experience more daily activity limitations, mental health symptoms, and increased morbidity and mortality. Positive aspects of the neighborhood, such as social cohesion, may serve as a social capital resource and protect against adverse health outcomes. On the contrary, negative aspects of the neighborhood, such as physical disorder, can be a substantial stressor leading to poor health. The neighborhood environment also disproportionally affects racial and ethnic minorities in the US. This symposium session will present four studies exploring important topics related to neighborhood factors of health among older adults. Collectively, the findings will inform neighborhood-level interventions to promote health and well-being among community-dwelling older adults. This session will start with a talk by Dr. Chan on the link between neighborhood and disability across six ethnic groups of older Asian Americans residing in New York City. Dr. Perry will present a qualitative study to explore the environmental and infrastructure challenges in the neighborhood from the perspective of older adults in Detroit. This will be followed by Dr. Jiang's talk on the relationship between neighborhood cohesion and mortality among a sample of older Chinese in Chicago. Finally, the session will conclude with Dr. Qin's presentation on how neighborhood characteristics affect older adults' mental health trajectories.

### ETHNIC AND NEIGHBORHOOD DIFFERENCES IN POVERTY AND DISABILITY AMONG OLDER ASIAN AMERICANS IN NEW YORK CITY

Keith Chan[1], and Christina Marsack-Topolewski[2], *1. Hunter College, City University of New York, New York, New York, United States, 2. Eastern Michigan University, Ypsilanti, Michigan, United States*

Asian Americans are the fastest growing and aging U.S. population, and occupy both extremes of socioeconomic and health indices. Using the 2016 NYC.gov dataset, multi-level logistic regression analyses were conducted to examine the relationship of poverty, acculturation and neighborhood-level variables with disability for different ethnic groups of Asian older adults (Chinese, South Asian, Filipino, Japanese, Korean and Vietnamese) in New York City. Findings indicated that South Asian older adults had higher odds for disability compared to other ethnic groups. Living in a neighborhood with higher percentages of persons of the same ethnicity was protective for Chinese older adults only. There is an important opportunity for interprofessional collaborations through education, awareness, screening and intervening to enhance systems of care for Asian older adults. Social workers can play a pivotal role in providing key linkages to form interprofessional solutions and shared efforts to address the needs of this understudied and under-resourced population.

### UNDEREXPLORED SOLUTIONS IN DETROIT'S NEIGHBORHOODS: THE IMPORTANCE OF LEGACY TO ADDRESS ENVIRONMENTAL CONCERNS

Tam Perry[1], Evan Villeneuve[2], Brenda Butler[3], Fatima Hazimeh[4], and Ventra Asana[5], *1. Wayne State University, Detroit, Michigan, United States, 2. ClearCORPS Detroit, Detroit, Michigan, United States, 3. Detroit's Eastside Community Network/Marlowe Stoudamire Wellness Hub, Detroit, Michigan, United States, 4. Wayne State University, Dearborn, Michigan, United States, 5. Independent Scholar, Detroit, Michigan, United States*

Neighborhood infrastructure challenges and changes are possibly understood and experienced in unique ways across the lifespan. This study presents findings on neighborhood conditions and underexplored solutions from the perspective of older adults in Detroit, Michigan. This project obtained multiple perspectives on these issues from older Detroiters through interviews (n-19) and professionals working on climate concerns in the region (n=5). The research was designed using community-based research approaches including having older adults as members of the research team from instrument design to dissemination. One emergent theme, the importance of legacy as motivation in addressing environmental concerns, will be highlighted. This presentation will conclude with a discussion of next steps for this work.

### NEIGHBORHOOD COHESION, LIVING ALONE, AND ALL-CAUSE MORTALITY IN COMMUNITY-DWELLING OLDER CHINESE AMERICANS

Yanping Jiang, Mengting Li, and Tammy Chung, *Rutgers, The State University of New Jersey, New Brunswick, New Jersey, United States*

This study aimed to examine whether neighborhood cohesion would mitigate the adverse effect of living alone on all-cause mortality in community-dwelling older Chinese Americans. Data were drawn from the Population Study of Chinese Elderly (PINE, N = 3,157, 59-105 years, 58% female), a longitudinal study started in 2011. Mortality was tracked through December 2021 (N = 642 deceased). Cox regression indicated that neighborhood cohesion moderated the association between living alone and all-cause mortality (HR = 0.74, 95%CI [0.57, 0.97]), showing that among participants living alone (N = 678), those with high neighborhood cohesion had a 41% lower mortality risk than their counterparts with low neighorhood cohesion. In contrast, among participants living with others, those with high and low neighborhood cohesion had a similar mortality risk. These findings highlight that strong neighborhood cohesion may protect against the increased risk of premature mortality associated with living alone in older Chinese Americans.

### NEIGHBORHOOD CHARACTERISTICS AND TRAJECTORIES OF DEPRESSIVE SYMPTOMS AND ANXIETY

Weidi Qin, *University of Michigan, Ann Arbor, Michigan, United States*

This study aims to identify the trajectories of mental health among older adults and to examine the association between neighborhood characteristics (i.e. social cohesion and physical disorder) and the identified trajectories. Data came from nine waves of the National Health and Aging Trend Study (N=6,951). Group-based trajectory modeling was used to identify the trajectories of depressive symptoms and anxiety respectively. Multinomial logistic regressions were used to examine the relationship between neighborhood and trajectories. Four trajectories were identified, namely "constantly low" "increasing" "decreasing" and "constantly high". Results show that higher levels of social cohesion and the absence of physical disorder in the neighborhood demonstrated beneficial and protective effects on older adults' mental health trajectories. The findings suggest that social cohesion may be an important social capital to cope with negative mental health experiences. On the contrary, negative physical features may be a stressor that adversely affect older adults' mental health trajectories.

### SESSION 3870 (SYMPOSIUM)

### ORAL HEALTH AND DENTAL CARE AMONG OLDER ADULTS IN DIVERSE POPULATIONS: ANALYSES OF LARGE COHORT DATA

Chair: Bei Wu Co-Chair: Stephen Shuman Discussant: Michele Saunders

Using large cohort surveys, this symposium includes five studies examining the linkages between oral health and systemic conditions, and social and behaviors factors affecting oral health and dental care utilization in older adults. The first study used the English Longitudinal Study of Ageing (ELSA) and analyzed the effect of changes in self-rated dental conditions on memory among adults age 51+. This study also examined the mediation effect of stroke on this relationship. Using the same data (ELSA), the second study examined the longitudinal relationships between cognitive function and changes in diabetes and oral health status. Results showed that participants with co-occurrence of diabetes and poor oral health had an accelerated decline in cognitive function over the study period. The third study was conducted among 8,744 adults age 51+ using data from the Health and Retirement Study over a 10-year period. The authors found that social isolation had a significant effect on dementia onset and that both dental visits and tooth status had a mediating effect on the association. The fourth study analyzed the Medical Expenditure Panel Survey from 2009-2016 and found that individuals with cognitive impairment had a significantly lower probability of annual dental visits. Using data from the Population Study of Chinese Elderly in Chicago collected between 2017-2019, the fifth study found that spousal support was significantly associated with a lower likelihood of having any dental visit. Findings illustrate the importance of understanding how different aspects of social relationships might play a role in dental care use.

### THE IMPACTS OF CHANGES IN DIABETES AND ORAL HEALTH CONDITIONS ON COGNITIVE TRAJECTORIES

Bei Wu[1], Chenxin Tan[1], Huabin Luo[2], and Xiang Qi[3], *1. New York University, New York, New York, United States, 2. East Carolina University, Greenville, North Carolina, United States, 3. New York University, New York City, New York, United States*

Despite the emerging research studying the relationship between diabetes mellitus (DM), oral health problems, and cognitive function, little is known about how changes in DM and oral health status affected the trajectories of cognitive decline. Using data of 12,802 participants aged 51+ from the 2006-2018 English Longitudinal Study of Ageing, this study examined the longitudinal relationships of cognitive functions with time-varying DM and oral health status – measured by edentulism, self-rated dental condition, and oral impact on daily performance (OIDP) scale at five time-points from 2006 to 2018. Results showed that participants had DM and edentulism throughout the study period had an accelerated decline in verbal fluency (mean=20.9; b=-0.17,

95% CI=-0.28, -0.07); and those had both DM and OIDP-problem had an accelerated decline in memory function (mean=10.4; b=-0.09, 95% CI=-0.15, -0.03). Prevention and treatment of multiple chronic conditions are essential for cognitive health in later life.

### SELF-REPORTED DENTAL CONDITIONS AND MEMORY DECLINE: THE MEDIATION ROLE OF STROKE

Chenxin Tan[1], Huabin Luo[2], Xiang Qi[3], and Bei Wu[1], *1. New York University, New York, New York, United States, 2. East Carolina University, Greenville, North Carolina, United States, 3. New York University, New York City, New York, United States*

The relationship between oral health and cognitive function has been studied extensively; however, little research has examined the underlying pathways. Using a cohort of 6,403 adults aged 51+ from the 2006-2010 English Longitudinal Study of Ageing, we analyzed the effect of changes in self-rated dental conditions on memory function (ranges 0-20) and the mediation effect of stroke using a causal mediation analysis framework. Controlling for socio-demographics, lifestyle, and health status, we found that compared with participants whose dental conditions remained the same, those who reported deterioration of dental conditions in 2006-2010 (N=1,755) experienced a steeper decline in memory function, with an average total effect of -0.22 (95% CI=-0.36, -0.07). The stroke incident had an average mediation effect of -0.007 (95% CI=-0.014, -0.001), representing 3% of the average total effect. The findings accentuate the importance of dental care access for older adults with exacerbated oral health problems.

### SOCIAL ISOLATION AND DEMENTIA ONSET: THE ROLE OF DENTAL VISITS AND TOOTH STATUS

Xiang Qi[1], and Bei Wu[2], *1. New York University, New York City, New York, United States, 2. New York University, New York, New York, United States*

Using data from the Health and Retirement Study, this study investigated the mediating effects of dental visits and tooth status (measured by edentulism) on the association between social isolation and dementia onset. Social isolation (exposure) and covariates in 2008, mediators (dental visits and edentulism) in 2012, and the onset of dementia between 2012 and 2018 were obtained. Dementia was identified through self- or proxy-reported physician diagnosis. We included 8,744 participants, and 576 (6.6%) had dementia during follow-up. There was a significant effect of social isolation on the onset of dementia (Hazard Ratio [HR], 1.14; 95% CI, 1.01-1.28). Controlling for mediators, the effect of social isolation was reduced to 1.10 (95% CI, 0.98-1.25), leaving an indirect effect of 1.03 (95% CI, 1.02-1.04). The proportions mediated by dental visits and edentulism were 4.4% and 7.5%, respectively. Our findings highlight the importance of improving oral health and dental care for older adults.

### DISPARITIES IN ACCESS TO DENTAL CARE IN COMMUNITY-DWELLING OLDER ADULTS WITH COGNITIVE DISABILITY

Preeti Zanwar[1], Elizabeth Wood[2], and Gilbert Gimm[3], *1. Jefferson College of Population Health, Philadelphia, Pennsylvania, United States, 2. Washington State University, Spokane, Washington, United States, 3. George Mason University, Vienna, Virginia, United States*

Older adults with cognitive disability are worse off with being up-to-date with preventive care and routine dental care is not considered a clinical preventive visit covered by insurance for this population. I examine disparities in access to dental and clinical preventive services (e.g. dental visit, blood pressure visit, flu shot) in the past year in 28,068 older adults with cognitive disability using the nationally representative Medical Expenditure Panel Survey from 2009-2016. I conduct multivariate logistic regressions and find older adults with cognitive disability vs. no disability have higher odds of receiving annual blood pressure check (AOR, 1.97, 95% CI 1.34-2.88) but lower odds for having an annual dentist visit (AOR, 0.61, 95% CI 0.53-0.71) with only one-tenth of those with cognitive disability report having dental insurance. These findings have implications for integrated community-and-clinical care partnerships for closing the gap for routine dental care services among older adults with cognitive disability.

### SOCIAL SUPPORT, SOCIAL STRAIN, AND DENTAL CARE UTILIZATION AMONG OLDER CHINESE AMERICANS

Weiyu Mao[1], Bei Wu[2], Iris Chi[3], Wei Yang[4], and XinQi Dong[5], *1. University of Nevada, Reno, Reno, Nevada, United States, 2. New York University, New York, New York, United States, 3. University of Southern California, Los Angeles, California, United States, 4. University of Nevada Reno, Reno, Nevada, United States, 5. Rutgers, New Brunswick, New Jersey, United States*

Regular dental care utilization is instrumental to good oral health. This study aimed to examine how positive and negative aspects of social relationships jointly exert influences towards dental care use among foreign-born older Chinese Americans. Data came from the Population Study of Chinese Elderly in Chicago collected between 2017 and 2019 (n = 3,000). Dental care utilization was dichotomized into "no dental visit" versus "any dental visit" in the past two years (including dental visit overseas). Social support and strain were measured by the Health and Retirement Study's scale from spouse, other family members, and friends (1= having any support/no strain). In stepwise logistic regression, accounting for chronic conditions, oral health problems, and sociodemographics, spousal support remained to be significantly associated with a lower likelihood of having any dental visit. Findings illustrate the importance of understanding how different aspects of social relationships might play a role in dental care use.

### SESSION 3880 (SYMPOSIUM)

### OUTDOOR FALLS PREVENTION: PROMOTING SAFETY IN URBAN NEIGHBORHOODS

Chair: Tracy Chippendale

Outdoor falls present a significant challenge to the well-being of community dwelling older adults. There are a number of existing evidence-based programs that address fall risk, including multifactorial and exercise-based programs. However, despite the difference in risk factors for indoor and outdoor falls, no existing program specifically targets outdoor falls. To fill this gap, the Stroll Safe program was developed and refined based on a prior feasibility study. The 7-week group-based manualized program is focused on promoting safe behavioral strategies to reduce the risk of outdoor falls. In addition to presentations and community mobility coaching by the group leader, an occupational therapist, the program includes group discussion and problem solving, capitalizing on the life experiences of participants. Action planning facilitates implementation of the strategies discussed. Given that the neighborhood environment impacts both risk and fear of falling, a walkability audit (i.e. the SWAN) focused on environmental hazards is included and is used to promote awareness of hazards and as a tool for self-advocacy. Data science and machine learning facilitate the creation of resources for route planning. Vision Zero resources help to identify hotspots for pedestrian injuries, and a map of shadow is used to create a user friendly map of potential hotspots for black ice. In this symposium, we will present 1) Findings from the efficacy trial for Stroll Safe, and 2) Describe data science research that can be used to inform outdoor falls prevention programs.

### EFFICACY OF THE STROLL SAFE OUTDOOR FALLS PREVENTION PROGRAM

Tracy Chippendale[1], and Steven Albert[2], *1. New York University, New York, New York, United States, 2. University of Pittsburgh, Pittsburgh, Pennsylvania, United States*

Informed by the Ecological and Health Belief models, the manualized Stroll Safe program addresses the unmet need of a targeted outdoors falls prevention program. We examined the efficacy of the program in partnership with eight Naturally Occurring Retirement Community program sites in one U.S city (N=86). Participants age 60 and older with a history of falls or a fear of falling were recruited. Community sites were randomly assigned to the treatment or wait list control group. Results of independent samples t-tests reveal a statistically significant improvement in knowledge of outdoor fall risks and safe community mobility strategy use for the treatment group as compared to the wait list control group on the Outdoor Falls Questionnaire (OFQ) and Falls Behavioral Scale for the Older Person (FaB) (ps<.001, Cohen's d=1.9 & 1.2). Improvements were retained at 6-week follow up. Implementation strategies are being developed to facilitate program adoption by other occupational therapists.

### MAPPING SIDEWALK FALL RISKS USING BIG DATA AND MACHINE LEARNING

Fabio Miranda[1], and Maryam Hosseini[2], *1. University of Illinois at Chicago, Chicago, Illinois, United States, 2. New York University, Brooklyn, New York, United States*

Outdoor falls are a leading cause of fatal and non-fatal injuries in the US. These falls are more likely to occur due to environmental hazards, inclement weather, unsafe construction zones, or poor sidewalk condition. Fall prevention programs targeted at older adults must therefore be informed by the interplay between weather condition, urban development, and built environment. Current practices, however, are limited by the lack of comprehensive data describing the condition of pedestrian facilities at fine, human scale, limiting the effectiveness of these programs. To address these shortcomings, we propose a multi-pronged approach leveraging urban data and machine learning techniques to create city-scale inventories describing sidewalk features that can inform safe strategies and programs for community mobility. We will cover the creation of multiple data sets, including computing shadow / shade from building geometries, detection of sidewalk surface material from street-level images, and creation of sidewalk networks from satellite images.

### SESSION 3890 (SYMPOSIUM)

### PRESIDENTIAL SYMPOSIUM: THE INTERSECTION OF AGE AND RACE: IMPACT ON REIMAGINING AGING

Chair: Shannon Jarrott Co-Chair: Karen Lincoln
Discussant: Becca Levy

In this BSS Presidential Symposium, we address the conference theme of "Embracing our diversity; enriching our discipline; reimagining aging" by attending to the intersection of age and race. In our panel, speakers will address the intersection of age and race as it relates to experience of ageism and its effects on individuals' opportunities to live meaningful lives. Researchers who apply an intersectional lens to their study of aging, ageism, and productive aging among diverse populations will share relevant theoretical concepts, methodological approaches, and associated findings. Links between racism, ageism, and health hold practice implications across health care, work, leisure, and other community settings. Researchers link life course influences, including discrimination, and cognitive health (Muñoz) and productive aging (Gonzales); Robinson-Lane presents a health care model of Culturally and Linguistically Appropriate Services Standards designed to reduce racism and improve care; and, Muramatsu offers practice and policy recommendations to reduce structural racism experienced by older persons and those paid to care for them. Presenters will address the following as we discuss how to reimagine aging from an anti-ageist, anti-racist perspective: 1. Evidence of the intersection of age and race in the experience of ageism. 2. Research models that attend to the intersection of age and race.3. Efforts to reduce ageism as it intersects with race in order to reimagine aging through practice and policy.

### PLACE, TIME, AND STRUCTURAL RACISM: GLOBAL PERSPECTIVES FOR REIMAGINING AGING

Naoko Muramatsu, *University of Illinois Chicago, Chicago, Illinois, United States*

Racism is embedded throughout society, producing enduring health inequities. Structural racism is pervasive across racial or ethnic groups and at the intersection of racism, ageism, and sexism. However, only the tip of the iceberg is visible in the literature or media. This presentation highlights aging and long-term care experiences of racial and ethnic groups that are often invisible in structural racism discussions (e.g., Asians). It also features direct care workers, many of whom are minorities or immigrants. I present a conceptual model of how structural racism (institutionalized, personally mediated, internalized) impacts well-being (mental, physical, cognitive, social). The model integrates the concepts of "place" (where we live, work, play, learn), "time" (effects of history, cohort, age), and invisibility. Global perspectives of structural racism will help engage gerontologists, policy makers, practitioners, and the public to maximize everyone's opportunities to live meaningful lives.

### ENDING AGEISM ISN'T ENOUGH: INFUSING AN INTERSECTIONAL LENS TO ADVANCE PRODUCTIVE AGING RESEARCH AND HEALTH

Ernest Gonzales, *New York University, New York, New York, United States*

This presentation integrates anti-racism, anti-ageism, and health equity lenses into the productive aging scholarship. Ageism and racism undermine population health and compromises choices to work and volunteer. These isms, among others, intersect and disproportionately impact populations of color and older adults; nonetheless, these oppressive systems create a culture of intergenerational conflict within the workplace and in general society. I will review key theoretical concepts and values in productive aging scholarship and how intersectionality as a framework has informed the development of new and important research questions for the field. This presentation will also analyze a variety of methodological approaches to examine productive aging and health inequities by race, ethnicity, gender, and age. A discussion on the implications for research, policy, and practice will conclude the presentation.

### PAIN, PLACE, AND POSITIONALITY: SYSTEMIC APPROACHES TO IMPROVING EQUITABLE HEALTH OUTCOMES FOR OLDER ADULTS

Sheria Robinson-Lane, *University of Michigan School of Nursing, Ann Arbor, Michigan, United States*

Racism and other "isms" are systemic problems that operate across multiple domains and affect access to care and services, available treatments, and long-term health outcomes for older adults. As such, changing these systems requires diverse methodological approaches that can identify and examine the contextual factors that influence these outcomes, along with continued strong advocacy that will result in related policy change. This presentation will highlight challenges in care delivery and outcomes for Black older adults in pain management, COVID care, and long term care placement and examine how mixed methodological approaches and implementation of the Culturally and Linguistically Appropriate Services (CLAS) Standards as outlined by the U.S. Department of Health and Human Services can make a difference.

### NO SILVER BULLET: ADDRESSING COGNITIVE HEALTH IN RACIALLY AND ETHNICALLY DIVERSE OLDER ADULTS

Elizabeth Muñoz, *The University of Texas at Austin, Austin, Texas, United States*

The growing racial and ethnic diversity of the older adult population, along with continued inequities in brain health outcomes highlight the need to examine risk-factors for reduced cognitive health in racial-ethnic minoritized adults. Stress is an important risk factor for reduced cognitive health, but the conditions under which stress operates among minoritized older adults is poorly understood. This presentation will discuss how studying sources of stress across multiple levels of analyses, including neighborhoods and interpersonal interactions, and the timing and duration of these experiences is needed. Further, addressing within group heterogeneity and shifting from between group comparison will enrich our understanding of risk-factors. Theoretical and empirical support for these propositions will be discussed, particularly as they relate to how neighborhood contexts and experiences with discrimination are associated with reduced cognitive health across multiple timescales.

### SESSION 3900 (PAPER)

### ADVANCING THE IMPLEMENTATION SCIENCE OF HOME MODIFICATION FOR OLDER ADULTS: AN UMBRELLA REVIEW

### PROGRAMS, POLICIES, AND SERVICES TO FACILITATE AGING IN PLACE

Mengzhao Yan, Emily Nabors, and Jon Pynoos, *University of Southern California, Los Angeles, California, United States*

Rooted in the person-environment-fit theory, home modification has been tested as an intervention to support older adults to age safely at home by improving functioning and reducing fall risks. Home modification is often a complex process, involving multiple steps and various stakeholders. As such, proper implementation is important to its effectiveness. However, limited research has focused on how to implement home modification from a comprehensive perspective. To advance the implementation science of home modification for older adults and achieve a better understanding of promising practices, we conducted a scoping analysis of review articles, referred to as umbrella review, to identify strategies that have been found effective in implementing home modification. Guided by the Preferred Reporting Items for Systematic Reviews and Meta-Analysis Protocols (PRISMA-P), after performing a thorough literature search from six electronic databases, including MEDLINE, PubMed, Embase, CINAHL, Web of Science, and Cochrane Review, we identified 16 highly relevant review articles from 1,310 articles retrieved. From these, we derived a set of practical strategies for service delivery professionals and developed an implementation matrix with the strategies clustered under two dimensions: (1) types of home modification based on demands of effort and level of evidence, including evidence-based practices, best practices, emerging practices; and (2) the critical stages of home modification, including preparation, home assessment, installation, follow-up visit. Our findings highlight the importance of maintaining cohesion between different stages of home modification and sustaining consensus among service delivery professionals and residents when implementing home modification for older adults.

### CORRELATES OF SOCIAL COHESION AMONG COMMUNITY-DWELLING OLDER PERSONS IN SOUTH-WESTERN NIGERIA

Eniola Cadmus[1], Lawrence Adebusoye[2], and Eme Owoaje[3], *1. College of Medicine/ University of Ibadan, Ibadan, Oyo, Nigeria, 2. Chief Tony Anenih Geriatric Centre, University*

*College Hospital, Ibadan, Oyo, Nigeria, 3. University of Ibadan, Ibadan, Oyo, Nigeria*

Existing research shows that older persons prefer to 'Age in Place' in their homes or community for as long as possible and are attached to both the physical space as well as the social environment. Despite growing attention to the relationship between social cohesion and health outcomes, few studies have focused on its correlates, especially in low- and middle-income countries, including Nigeria. This study identified the factors related to neighbourhood social cohesion among older adults aged >60years dwelling in a selected rural and urban community in Oyo State, southwestern Nigeria. Data were collected using a semi-structured, interviewer-administered questionnaire and analysed using Stata version 15. Overall, 1180 (588 rural and 592 urban) respondents were interviewed (mean age 74.2 ± 9.5 years). Over half (55.3%) of the respondents had an adequate level of social cohesion. In the rural environment, positive predictors of social cohesion were male gender [OR: 1.73 (95%CI: 1.10-2.73)], formal education [OR:2.70 (95%CI: 1.52-4.81)] being employed [OR:3.68 (95%CI: 2.50-5.42)], homeownership [OR: 1.82 (95%CI: 1.22-2.71)] and good self-rated health (SRH) [OR:2.73 (95%CI: 1.78-4.20)]. Among the urban respondents, factors predictive of good social cohesion were male sex [OR:1.68 (95%CI: 1.06-2.68)], being employed [OR:2.38 (95%CI: 1.67-3.41) and reporting good SRH [OR:2.13 (95%CI: 1.45-3.11)]. Older respondents were less likely to have good social cohesion compared with the younger respondents in the rural [OR:0.54 (95%CI: 0.35-0.84)] and urban setting [OR:0.68 (95%CI: 0.47-1.00)]. The study findings underscore the importance of socio-economic status and homeownership on older adults' social cohesion and areas for targeted policy and intervention

### HOUSEHOLD COMPOSITION AND THE RESILIENCE OF OLDER ADULTS AGING IN COMMUNITY DURING COVID-19

Samara Scheckler, Jennifer Molinsky, and Christopher Herbert, *Harvard University, Cambridge, Massachusetts, United States*

Household composition impacts older adults' financial needs, earnings capacity, and benefits eligibility. It is also related to formal and informal LTSS access and may be associated with disparities between racial and ethnic groups since Black and Hispanic older adults are more likely than white older adults to live in multigenerational homes. The relationship between household composition and community-based aging is highly salient as multigenerational households and older people living alone are expected to increase in both number and share in coming years. This research framed the pandemic period as a stress-test to detect differences in resilience, defined as financial and LTSS stability and number of hardships, associated with older adults living alone, with partner, or with family or unrelated coresidents. Using the state-identified Health and Retirement Study (HRS), researchers developed a pre-pandemic profile of financial resources and public benefit utilization, informal and professional LTSS, and a vector of wellbeing measures by household composition type. Researchers then conducted two analyses to identify different pandemic experiences by composition type. First, wave over wave variation was regressed by household composition, noting any increased rates of instability in the 2020 wave. Researchers then used the HRS COVID-19 supplemental survey to describe pandemic hardships by household composition. About a third of HRS respondents lived in each household type. Findings, which offer a profile of resources by housing composition type and two analyses of resilience associated with composition, suggest complex relationships between household composition and resilience, but overall, residents living alone appear more vulnerable to instability.

### UNDERSTANDING THE USE OF FLEXIBLE WORK ARRANGEMENTS AMONG OLDER INFORMAL CAREGIVERS

Shanika Koreshi, and Fiona Alpass, *Massey University, Palmerston North, Manawatu-Wanganui, New Zealand*

The increasing provision of informal caregiving and the extension of working lives will result in many older workers combining paid work and informal caregiving responsibilities. Specific flexible work arrangement policies have been enacted in many countries to support working caregivers. Flexibility in the workplace has been suggested to promote prolonged employment among older workers. This study primarily focuses on the question of whether use of flexible work arrangements differs between caregivers and non-caregivers and how potential differences can be explained. Participants were 296 carers and 1611 non-carers (aged 55–70 years) who completed wave 8 of the New Zealand Health, Work and Retirement survey. The use of flexible work arrangements was analyzed based on five categories; Flexibility in number of work hours, flexible schedule, flexible place, options for time off, and other options. Hierarchical regressions were used to investigate caregiving as an independent predictor of use of flexible work arrangements after controlling for demographic and work characteristics. Results indicate that the studied informal caregivers on average used more workplace flexible arrangements than non-caregivers, both in flexible work hours, flexible schedules, and time off. The caregiver status difference in use of the three significant categories of flexible work arrangements can be explained by differences in socio-demographic and work characteristics. This difference in use of FWAs among older caregivers and non-caregivers warrants attention in discussions about prolonged employment and reconciliation of care and paid work.

### SESSION 3910 (SYMPOSIUM)

### REIMAGINING THE FUTURE OF ASSISTED LIVING TO PROMOTE QUALITY

Chair: Sheryl Zimmerman

Assisted living (AL) has existed in the U.S. for decades, evolving in response to older adults' need for supportive care and distaste for nursing homes and older models of congregate care. Today, AL is the largest provider of residential long-term care in the country, with more than 996,000 AL beds in almost 29,000 AL communities. Unfortunately, that growth has spawned notable concern, with a recent report concluding that the initial key constructs of AL have become mired under key tensions such that "the current model of AL

has been taken as far as it can go." This symposium includes five presentations that respond to some of those tensions. The first two presentations respond to the increased acuity of AL residents: one addresses challenges and recommendations for residents' palliative care needs based on numerous studies, and the other provides expert consensus panel recommendations for pragmatic medical and mental health care provision in AL. The next two presentations respond to concerns related to quality in AL, one focusing on frameworks, measures, and implementation of quality metrics, the other envisioning use of administrative data to study and promote quality. The final presentation speaks to the complexity of regulation, and how related research can be improved to better examine consequential differences between models of AL care. The discussant, a nationally recognized and highly regarded leader from the American Health Care Association/National Center for AL, will discuss how the presentations can be used to reimagine the future of AL to promote quality.

### EXPERT CONSENSUS PANEL RECOMMENDATIONS FOR MEDICAL AND MENTAL HEALTH CARE IN ASSISTED LIVING

Sheryl Zimmerman[1], Philip Sloane[2], Christopher Wretman[3], Kevin Cao[4], Johanna Silbersack[1], Paula Carder[5], Kali Thomas[6], and Suzanne Meeks[7], *1. University of North Carolina, Chapel Hill, North Carolina, United States, 2. University of North Carolina-Chapel Hill, Chapel Hill, North Carolina, United States, 3. University of North Carolina at Chapel Hill School, Chapel Hill, North Carolina, United States, 4. University of Illinois, Chicago, Illinois, United States, 5. Portland State University, Portland, Oregon, United States, 6. Brown University, Providence, Rhode Island, United States, 7. University of Louisville, Louisville, Kentucky, United States*

Assisted living (AL) is now the largest residential provider of long-term care in the U.S., and the acuity of AL residents has been rising. However, AL is not a health care setting, and concern has grown about the medical and mental health care needs of AL residents. Because there is no guidance to inform this care, a Delphi consensus panel of 19 experts was convened, and their recommendations were compared to actual practice. Panelists rated 200 discrete items, 43 of which met standard recommendation cut points (rated as important by at least 75% of panelists) and reflected four critical components of AL: the tenets of AL, the role AL plays in providing long-term care for persons with dementia, the need for pragmatism in response to the diversity of AL, and workforce needs. Next steps regarding implementation of the recommendations will be discussed.

### CHALLENGES AND RECOMMENDATIONS TO MEETING THE PALLIATIVE CARE NEEDS OF RESIDENTS IN ASSISTED LIVING

Debra Dobbs[1], Hongdao Meng[1], William Haley[1], Carlyn Vogel[1], and Harleah Buck[2], *1. University of South Florida, Tampa, Florida, United States, 2. Csomay Center for Gerontological Excellence, Iowa City, Iowa, United States*

Of the more than 800,000 assisted living (AL) residents in the U.S. approximately 25% remain until the end of life and can benefit from palliative care (PC) and hospice care. Although the AL population is the fasting-growing segment of hospice care, increasing by 40% in the past five years, ALs still have special challenges in providing quality end-of-life care. ALs have a lack of nurse staffing and adequate training about how to care for those with serious illnesses. We present findings from studies over the last decade in Florida ALs (N=100) related to PC and hospice care practices. Some of the findings reveal gaps in care related to PC staff education, documentation of pain and advance directives and recognizing the need for more family involvement in decisions, especially for those with dementia. Recommendations on how to improve processes of care for residents who can benefit from PC will be discussed.

### QUALITY METRICS IN ALFS: FRAMEWORK, MEASURES, AND IMPLEMENTATION

Tetyana Shippee[1], Taylor Bucy[2], Odichinma Akosionu[1], and Tricia Skarphol[1], *1. University of Minnesota, Minneapolis, Minnesota, United States, 2. University of Minnesota School of Public Health, Minneapolis, Minnesota, United States*

Concerns have surfaced regarding the quality of assisted living (AL) with calls for quality metrics reporting as essential for consumer choice and organizational accountability. This study presents a framework for quality metrics in ALs using a) results from literature review and environmental scan of existing domains and indicators used to assess quality in AL and 2) a survey of results from MN stakeholders (n=822) on their priorities regarding these quality measures. Our findings showed that consumer-reported measures (resident quality of life and family satisfaction) were rated as top priority, followed by staff-based measures (e.g., job satisfaction, turnover). Other domains included residents' safety, resident health outcomes, care services and integration, and the environment of the ALs. These results have implications for states looking to develop and implement quality measures in ALs, especially in the context of rising AL resident acuity and the continued trend away from institutional models of long-term care.

### A VISION FOR THE FUTURE OF ASSISTED LIVING RESEARCH: LEVERAGING ADMINISTRATIVE DATA TO STUDY QUALITY

Kali Thomas[1], Paula Carder[2], Lindsey Smith[1], and Sheryl Zimmerman[3], *1. Brown University, Providence, Rhode Island, United States, 2. Portland State University, Portland, Oregon, United States, 3. University of North Carolina, Chapel Hill, North Carolina, United States*

Because the assisted living industry is neither federally regulated nor federally licensed, methodologies to study the quality of long-term care used in other settings (e.g., annual inspection reports, resident assessment data) do not exist for the large and growing number of assisted living communities and their residents. The objective of this presentation is to present a vision for the future of assisted living research to inform quality using large, national datasets. To do this, the presenter will discuss existing approaches to identify and characterize assisted living communities and their residents using administrative data. A specific focus will be to highlight the opportunities and challenges associated with each approach. The presenter will offer suggestions for possible enhancements in existing approaches and next steps in the study of assisted living aimed at understanding and improving the quality of care delivered in these settings.

### REIMAGINING POLICY-INFORMED RESEARCH FOR THE FUTURE OF ASSISTED LIVING

Paula Carder[1], Kali Thomas[2], Lindsey Smith[2], and Sheryl Zimmerman[3], *1. Portland State University, Portland, Oregon, United States, 2. Brown University, Providence, Rhode Island, United States, 3. University of North Carolina, Chapel Hill, North Carolina, United States*

Researchers have long known that states are responsible for licensing assisted living (AL). Recent analyses indicate that the regulatory landscape is more complex, as states have numerous license classifications that can be combined in multiple ways through various subtypes and designations applied to settings under the AL umbrella. Other sources of complexity include within-state policy variation in terms of affordability and access, person-directed care, emergency preparedness, workforce, and diversity and inclusion. This presentation describes a vision and framework for future research informed by this complex policy landscape. We describe how researchers can better conceptualize types of ALs, and provide guidance for how and why AL researchers might shift from the current practice of analyzing AL settings by the “state” versus by license type, among other categorizations.

### SESSION 3920 (SYMPOSIUM)

### THE FACES OF ELDER ABUSE: A CROSS-CULTURAL PERSPECTIVE

Chair: Pamela B. Teaster Teaster Discussant: Georgia Anetzberger

This presentation gives a real world and compelling picture of the abuse of older adults, with authors representing three different World Health Regions (i.e., Regional Office for Africa, Regional Office for Southeast Asia, and the Regional Office for the Western Pacific) each locating the problem within an area’s historic and present societal treatment of older persons. An actual and emblematic case study of the abuse of an older adult will frame each presentation. Presenters will synthesize empirical data and research on the problem, explaining its usefulness and limitations as well as guiding frameworks utilized to address the problem, highlighting efforts of leading figures in each area or region who are addressing the problem and explaining existing policies and future initiatives to address the abuse of older adults. Dr. Eniola Cadmus will present perspectives on elder abuse in Nigeria. Drs. Noriko Tskudada and Asako Katsumata and will present perspectives from Japan. Drs. Farida Ejaz and Mala Shankardass will present perspectives from India. Dr. Georgia Anezberger will tie together the differences and commonalities within each region drawing upon a holistic and person-centered picture of the issue and problem of elder abuse.

### THE FACE OF ELDER ABUSE IN AFRICA: THE NIGERIAN PERSPECTIVE

Eniola Cadmus, *College of Medicine/ University of Ibadan, Ibadan, Oyo, Nigeria*

Elder abuse is increasingly recognized as a serious problem in many African societies, including Nigeria. Contributing factors include a high level of poverty, unemployment, diminished availability, willingness, and ability of primary caregivers to provide for their older persons. Traditionally, elder abuse is considered taboo, and high secrecy surrounding its occurrence further contributes to challenges reporting and addressing the problem. Additionally, the patriarchal nature of the Nigerian society accounts for the gender-based differences in forms of abuse experienced with increased vulnerability among older women. This paper presents case studies that typify the experience of abuse among male and female older persons. The prevalence rates of abuse are drawn from community surveys across the six geopolitical zones. Although the methodologies vary, the total prevalence of any form of abuse experienced ranged from 30-81.1%. However, most studies relied on self-reports with no attempts at verification or data and records for triangulation of information.

### WHAT CAN WE LEARN FROM THE THREE MURDER CASE IN A FEE-BASED NURSING HOME IN JAPAN?

Noriko Tsukada[1], and Asako Katsumata[2], *1. Nihon University, Tokyo, Tokyo, Japan, 2. Rehabilitation Institution, Chiba, Chiba, Japan*

Japan enacted “The Act on the Prevention of Elder Abuse, Support for Caregivers of Elderly Persons and Other Related Matters” on 2006 April 1. Although the Act enabled the implementation of new programs and procedures to address abuse, elder abuse remains a cause of concern, particularly in institutional settings. This paper presents an elder abuse case that occurred in 2014 in a fee-based nursing home where a staff member murdered three residents. Although extreme, we chose to present this case because there is minimal research focused on fee-based nursing homes. Moreover, this case provides an opportunity to look broadly at factors that need to be addressed to reduce the likelihood of elder abuse in institutional settings. This paper first introduces facts about this case and discusses what can be learned to help increase an understanding about preventing future elder abuse cases in institutional settings.

### ELDER MALTREATMENT IN INDIA: EXAMINING THE SENIOR CITIZENS ACT OF 2007 AND ITS IMPLEMENTATION

Farida Ejaz, *Benjamin Rose Institute on Aging, Cleveland, Ohio, United States*

Although cultural traditions place a high regard for older adults, elder maltreatment is on the rise in India. Prevalence rates vary widely. A recent national survey demonstrated a prevalence rate as low as 5% (LASI, 2020) while another report (HelpAge, 2015) found that 50% of older adults felt emotionally abused by family members. This presentation focuses on an important legislation, ‘The Maintenance and Welfare of Parents and Senior Citizens Act’ of 2007, amended in 2013 and 2018. It makes it obligatory for adult children and heirs to care for their parents/stepparents, address their needs, provide a monthly allowance and protect their property. As part of the implementation of this Act, tribunals have been set up to prosecute perpetrators. Police stations also have a helpline for Senior Citizens to address complaints amicably. Through two case studies, we will discuss how the Act is implemented in India, bringing critical nuances to light.

### SESSION 3930 (SYMPOSIUM)

### THE MANY VOICES OF CARE PARTNERS ACROSS THE CONTINUUM OF CARE

Chair: Aimee Fox Co-Chair: Abigail Latimer Discussant: Deborah Waldrop

Caregiving and care-sharing is a dynamic, stressful, and physically demanding responsibility, often leading to adverse psychological and physical outcomes. Caregiving for chronic illness and disease involves increasing complexity and scope of responsibilities and expectations. Utilizing strengths-based approaches and a variety of qualitative methods, this symposium highlights the many voices of care partners across the continuum of care; pre-, during and post-caregiving. First, Latimer and colleagues will present a case study of an older adult with multiple chronic illnesses, offering insight into anticipating care needs and coping with daily stressors of multi-morbidity in late life. Second, Morgan and colleagues will present findings from interviews with care partners on supporting the inner strength of those recently diagnosed with mild cognitive impairment. Third, Fox and colleagues share results from a dyadic, multi-modal intervention for pain management. Care partners who both experience persistent pain discuss changes in their relationship as a result of participating in the intervention together. Fourth, Wladkowski discusses caregiver's perspectives on live discharge and re-enrollment into hospice care. Her findings demonstrate how the anticipated "end" isn't always the end for ADRD caregivers. Fifth, Buck and colleagues will share caregivers' responses to a psychotherapy intervention for the treatment of complicated grief post-death of the care partner. Discussant Deborah Waldrop will contextualize these findings and offer suggestions for future research and interventions to enhance care partners' well-being across the continuum of chronic illness and care.

### MULTIMORBIDITY IN LATE LIFE: APPRAISAL, COPING, AND ANTICIPATING CARE NEEDS

Abigail Latimer, Jia-Rong Wu, and Patricia McGuire, *University of Kentucky, Lexington, Kentucky, United States*

Older adults diagnosed with multiple chronic and serious illnesses are confronted with complex, at times unpredictable, and burdensome medical realities. We present a case study of a 76-year-old woman with heart failure, kidney disease, and arthritis early in her illness course. We use the Theory of Stress and Coping to guide our discussion of her coping appraisals and strategies that promote her psychological and emotional well-being. Although she dismissed her heart failure symptoms as "old age," she identified pain and disability associated with her arthritis as more of an immediate and serious threat to her autonomy, control, and personal goals. As she is functioning independently and still working, we share her perspective on anticipated caregiving needs and expectations for the last decades of her life. Insights from this case study can inform future research directions and have implications for education and practice.

### THE ROLE OF CARE PARTNERS IN SUPPORTING INNER STRENGTH OF PERSONS NEWLY DIAGNOSED WITH MILD COGNITIVE IMPAIRMENT

Brianna Morgan[1], Lauren Massimo[1], Sharon Ravitch[1], Jason Karlawish[2], and Nancy Hodgson[1], *1. University of Pennsylvania, Philadelphia, Pennsylvania, United States, 2. University of Pennsylvania Perelman School of Medicine, Philadelphia, Pennsylvania, United States*

Inner strength is one's internal process of facing challenging circumstances, like receiving a mild cognitive impairment (MCI) diagnosis. However, the role of care partners in this process is unknown. This study explores the role of care partners in supporting inner strength at the time of diagnosis using qualitative methodologies. We interviewed persons diagnosed with MCI (N=5) at a Memory Center within 12 months and their care partners (N=5). We analyzed data in NVivo using reflexive thematic analysis methods. Trustworthiness was maintained through vetted semi-structured interview guides, verbatim transcription, field notes, peer analysis, and audit trails. Care partners supplant cognitive losses (e.g., redistribution of self, organizing and coordinating) to allow the person with MCI to thrive, which is built on a foundation of the care partner's personal resources and the nature of their relationship with the person with MCI. Implications include incorporating care partners into diagnostic processes and tailoring caregiving supports.

### EXPERIENCING PAIN TOGETHER: CARE PARTNERS REFLECT ON A DYADIC INTERVENTION FOR PAIN SELF-MANAGEMENT

Aimee Fox[1], Arlene Schmid[2], Jennifer Dickman Portz[3], Marieke Van Puymbroeck[4], Heather Leach[2], Julia Sharp[2], and Christine Fruhauf[2], *1. Kansas State University, Manhattan, Kansas, United States, 2. Colorado State University, Fort Collins, Colorado, United States, 3. University of Colorado Anschutz, Aurora, Colorado, United States, 4. Clemson University, Clemson, South Carolina, United States*

When caregivers and care receivers (caregiving dyad) both experience persistent pain, there is increased risk for shared adverse health outcomes, including social isolation and decreased relationship satisfaction. Yet, there are few non-pharmacological pain interventions for the caregiving dyad. The purpose of this study was to understand changes in the caregiving dyad after participating in a dyadic, multi-modal intervention for pain self-management. Fifteen caregiving dyads with pain (N=30) participated in the Merging Yoga and self-management to develop Skills (MY-Skills) intervention. Open-ended questions were included in the post-intervention evaluation tool to discuss changes in the dyadic relationship. Qualitative methods were used to analyze data, develop a coding scheme, and identify themes. Findings suggest the intervention strengthened relationships by improving communication, enhancing emotional connection, and increasing physical activity. This study demonstrates the importance of dyadic approaches to interventions for care partners with pain.

### UNDERSTANDING CAREGIVER PERSPECTIVES ON LIVE DISCHARGE AND RE-ENROLLMENT ONTO HOSPICE CARE

Stephanie P. Wladkowski, *Bowling Green State University, Bowling Green, Ohio, United States*

Hospice care improves end-of-life outcomes for adults with Alzheimer's Disease and related dementias (ADRD), yet with eligibility limited to a six-month prognosis, the patient-caregiver dyad can experience a live discharge from hospice. In 2019, nearly 350,000 patients with an ADRD diagnosis received hospice services in the US, and 6.5% of hospice patients were discharged due to being 'no longer terminally ill.' Caregivers of adults with ADRD who experienced a live discharge (n=24) were interviewed and thematic analysis was conducted. More than half (58%) noted specific support in their caregiving roles while 46% cited feeling relief. Eleven participants were enrolled for one hospice episode, and six re-enrolled at least one time. While participants would consider re-enrolling, they are waiting for a health crisis (n=7) for eligibility, while others question the meaning of hospice for ADRD patients (n=10). Implications for policy, practice, and research to support the patient-caregiver dyad are discussed.

### CAREGIVERS' EXPECTATIONS OF A MIND-BODY THERAPY FOR COMPLICATED GRIEF

Harleah Buck, *Csomay Center for Gerontological Excellence, Iowa City, Iowa, United States*

Accelerated Resolution Therapy (ART) is a psychotherapy for the treatment of complicated grief, defined as unusually prolonged, functionally impairing grief. The purpose of this study was to qualitatively examine caregiver's expectations of ART. The sample included 29 primarily female, older (67.4 + 7.1 years) former informal caregivers; a little over half (n=18) had been married to their care recipient. Thematic analysis resulted in three themes and six sub-themes arising: The role of knowledge in expectations (sub-themes uncertainty, prior knowledge); The role of personality in expectations (sub-themes openness, positive affect); and Expecting a process (sub-themes cognitive processes, affective processes) which described the interaction of person and process in shaping expectations of our intervention. An across theme analysis of the specificity of the participants' expectations uncovered that knowledge and personality inform expectations of ART and that individuals who verbalize a process for recovery tend to be very specific in their expectations.

### SESSION 3940 (SYMPOSIUM)

### THE RAISE FAMILY CAREGIVER ADVISORY COUNCIL: STRATEGIES TO BOLSTER CAREGIVERS' FINANCIAL SECURITY

Chair: Pamela Nadash

The RAISE Family Caregiving Advisory Council, created under the Recognize, Assist, Include, Support, and Engage (RAISE) Family Caregivers Act (2018) has been tasked to support the Secretary of Health and Human Services in developing a national family caregiving strategy. The Council began by (in 2021) identifying five key Goals critical to supporting family caregivers, which were reported to Congress in the Council's Initial Report; the next step (in 2022) was to identify how these Goals are to be operationalized via specific actions, as well as the stakeholders that needed to be involved. This symposium discusses Goal 4, which states that "Family caregivers' lifetime financial and employment security is protected and enhanced," a goal incorporating diverse components, including federal legislation (expanding FMLA, for example), enhancing workplace security for working caregivers, and ways to pay family caregivers for providing supportive services. The first paper, by Salom Teshale, PhD, will provide an overview of the Council's work and the strategies that have been chosen to support the overall national strategy. The second paper, by Eileen J. Tell, MPH, will describe strategies to improve the ability of caregivers to remain and thrive in the workplace. Pamela Nadash, PhD, will report on the research that identified the expansion of self-directed programs to incorporate payment for family caregivers as key, and the fourth paper by Rani Snyder will conclude by identifying the research needed to move these efforts forward. Greg Link of the Administration for Community Living will act as discussant.

### STRATEGIES TO CREATE CAREGIVER-FRIENDLY WORKPLACES

Eileen Tell[1], Pamela Nadash[2], and Siena Ruggeri[3], *1. ET Consulting, LLC, Belmont, Massachusetts, United States, 2. University of Massachusetts Boston, Boston, Massachusetts, United States, 3. Community Catalyst, Boston, Massachusetts, United States*

An estimated one in six employees is juggling work and being a family caregiver. Research suggests working caregivers neglect their own health and experience higher levels of stress and poor mental health. Their employers also experience negative outcomes, most typically in terms of increased absenteeism, lost productivity, difficulty recruiting and retaining workers, and higher health care claim costs. A wide range of strategies have emerged for employers to better support working caregivers and, hopefully, thereby reducing the financial impact both for the company and the caregiver. In this session, we discuss recommendations that emerged from research with both public and private sector stakeholders to identify best practice models and action steps for a national strategy supporting working family caregivers. Recommendations range from employer education, voluntary recognition programs, tax credits for employer-paid caregiver support programs, expanded PFMLA, and even federal LTSS financing reform.

### A RESEARCH AGENDA TO ADVANCE FAMILY CAREGIVERS' FINANCIAL SECURITY

Rani Snyder[1], and Eileen Tell[2], *1. The John A. Hartford Foundation, New York, New York, United States, 2. ET Consulting, LLC, Belmont, Massachusetts, United States*

The research supporting the RAISE Family Caregiver Advisory Council engaged a broad range of stakeholders who were committed to supporting actions that make a difference to family caregivers' financial security, with many focusing on actions that sustain continued employment; 103 different organizations participated, including employer representatives. Participants strongly agreed on the need for more data. On a broad level, there was agreement on federal-level coordination regarding data collection on elements specific to family caregivers across federal agencies. In addition, they called for better information on the return on investment of varying strategies for workplace supports for employers, the added value of caregiver employees, and evidence regarding best practices in supporting family caregivers, with a goal of making a business case for family caregiver workplace supports.

### THE RAISE COUNCIL'S WORK IN DEVELOPING A NATIONAL FAMILY CAREGIVING STRATEGY

Salom Teshale, *National Academy for State Health Policy, Washington, District of Columbia, United States*

Since 2019, the RAISE Family Caregiver Advisory Council (FCAC) has met regularly to carry out its work of developing a national family caregiving strategy. This strategy incorporates five goals to support family caregivers, and key actions that a range of stakeholders can carry out centered around these goals. This overview will describe the RAISE FCAC's work in developing the national family caregiving strategy, and highlight the development of recommendations and key actions to support the fourth goal, "Family caregivers' lifetime financial and employment security is protected and enhanced." This goal's recommendations include supporting caregivers through flexible workplace policies; supporting affordable long-term services and supports; supporting financial education and planning; and reducing overall negative financial impacts of caregiving short and long-term.

### WHAT DO FAMILY CAREGIVERS WANT? PAYMENT FOR PROVIDING CARE

Pamela Nadash, *University of Massachusetts Boston, Boston, Massachusetts, United States*

Although the primary goal of self-directed programs providing long term services and supports (LTSS) is to maximize choice and control for service recipients, such programs may also benefit family caregivers by compensating them for providing supportive services. This study draws on qualitative data from research supporting the RAISE Family Caregiver Advisory Council, finding that family caregivers themselves see the expansion of self-directed programs as a policy priority due to their need for financial security. The request for compensation was the strongest finding, with respondents highlighting the incompatibility of work with caregiving and their inability to rely on the existing paid workforce due to supply and quality issues; the consequences of this loss of earned income were reported as severe. Ultimately, respondents saw payment for providing care as an issue of fairness. This evidence supports the policy case for expanding access to self-directed programs that permit the employment of family caregivers.

### SESSION 3950 (PAPER)

### WORKFORCE ISSUES ACROSS THE LONG-TERM CARE CONTINUUM

### DIRECTORS OF SOCIAL SERVICES: OVERLOOKED LEADERSHIP NEEDS AT SKILLED NURSING FACILITIES (SNFS)

John Paul Abenojar, *Vinson Hall Retirement Community, Arlington, Virginia, United States*

Skilled Nursing Facilities (SNFs) provide ongoing care to the elderly and chronically ill. To maximize the quality of this care, SNF staff must be trained to respond to patient care crises and communicate across departments. Although researchers have studied the leadership styles, strategies, and interactions of facility administrators and nursing directors, little was known about the leadership styles and strategies employed by the directors of social services (DSSs). The aim of this phenomenological study was to explore how DSSs influenced leadership policies, prepared subordinates for crisis intervention and management, perceived that social workers influenced patient care decisions, and believed that communication amongst SNF staff about patient care could be improved. The conceptual framework was based on the multilevel leadership model construct, the situational leadership model construct, and the complex adaptive leadership model construct. Participants included a purposive sample of 10 DSSs working in a large, corporate SNFs in Virginia. Data were collected via in-person, semi structured interviews consisting of open-ended questions. Data were analyzed via Hycner's phenomenological approach. Findings from this investigation helped clarify roles and responsibilities of DSSs, thereby improving the leadership they provide to subordinate social workers. Findings may be used to improve communication across professionals within SNFs and their roles in patient care decisions.

### DOES HIGHER WORKER RETENTION BUFFER AGAINST CONSUMER COMPLAINTS? EVIDENCE FROM OHIO NURSING HOMES

Katherine Kennedy[1], and John Bowblis[2], *1. Providence VA Medical Center, Providence, Rhode Island, United States, 2. Miami University, Oxford, Ohio, United States*

This study examined the relationship between nursing home (NH) quality using consumer complaints and certified nursing assistant (CNA) annual retention rates among Ohio freestanding NHs with complete data (n = 691). Core variables came from the 2017 Ohio Biennial Survey of Long-term Care Facilities and CMS Automated Survey Processing Environment Complaints/Incidents Tracking System. Four quartiles were created to compare NHs on their CNA retention rates: low (0-48%), medium (49-60%), high (61-72%), and very high retention (73-100%). Negative binomial regressions were estimated on total, substantiated, and unsubstantiated counts of allegations and complaints. All regressions controlled for facility and county-level factors and clustered facilities by county. Controlling for other factors, NHs in the top 50% (high and very high) of retention received 1.92 fewer allegations than those in the bottom 50%, representing a 19% difference; this trend was significant and negative across all outcomes. Using quartiles revealed a non-linear pattern: high retention NHs received the fewest number of allegations and complaints. The difference between high and low retention on total allegations, substantiated allegations, and unsubstantied allegations were 33% (3.73 fewer), 34% (0.51 fewer), and 32% (3.12 fewer) respectively. Unexpectedly, very high retention NHs received more unsubstantiated allegations than high retention NHs controlling for other variables. Research, policy, and practice efforts are critical to increase CNA retention for better consumer experiences by substantively improving CNA job quality through career advancement, high wages, and benefits. Given some turnover is desirable, effective strategies for recruitment are also needed.

### EFFECT OF ACA MEDICAID EXPANSION ON THE LABOR SUPPLY OF DIRECT CARE WORKERS

Lili Xu, and Hari Sharma, *University of Iowa, Iowa City, Iowa, United States*

Direct care workers (DCWs) such as personal care aides, home health aides, and nursing assistants provide critical care to patients and residents in different settings including at home, nursing homes, and hospitals but DCWs earn low wages with limited benefits. The Affordable Care Act Medicaid expansion increased health insurance access among low-income individuals but there are concerns that public insurance may disincentivize labor supply. In this study, we examine whether Medicaid expansion affected the labor supply of low-educated DCWs at both extensive and intensive margin overall, and by different healthcare settings. Using annual American Community Survey data from 2010 to 2019 retrieved via Integrated Public Use Microdata Series, we identify 100,676 adult DCWs (age: 19-64) with a high school or less degree from 50 states and DC. We examine the potentially causal effect of Medicaid expansion on labor supply of DCWs using difference-in-differences and event-study regressions We find that Medicaid expansion is associated with a 2.9 percentage-point ($p< 0.01$) increase in full-time employment (>=35 hours) and a 1.9 percentage point ($p< 0.05$) decrease in part-time employment (20-34 hours). We also find that unemployment decreased by 0.8 percentage points ($p< 0.1$) among DCWs mainly driven by those working in the long-term care industry. Our study suggests that Medicaid expansion does not have a negative impact on labor supply among low-educated DCWs. States that have not expanded Medicaid can consider policies to increase insurance coverage for DCWs as a strategy to strengthen this workforce.

### HOME HEALTHCARE WORKFORCE AND NEIGHBORHOOD CHARACTERISTICS

Chenjuan Ma, *New York University, New York, New York, United States*

Despite the rapid increase in the needs for home- and community-based services (HCBS), including home health care which is the most commonly used HCBS, workforce shortage has become a critical challenge to home health agencies in providing quality care to meet the needs of millions of homebound Americans. This study aimed to examine the availability of home health care workforce and its variations by neighborhood characteristics. We linked several national datasets from 2019 and included information from 11,005 HHC agencies in 1,849 counties. The unit for analysis is county. We found that on average county had fulltime equivalent (FTE) 83 (SD=351) home health care nurses, 120 (SD=411) FTE skilled home health providers (e.g., nurses, physical/occupational therapists) and 37 (SD=411) FTE aides. For every 1,000 persons, on average counties had 0.7 (SD=4.6) FTE nurses, 0.9 (SD=4.7) FTE skilled providers, and 0.2 (SD=0.8)) FTE aides. For every 1,000 older adults (>=65), on average counties had 3.6 (SD=23.9)) FTE nurses, 4.8 (SD=24.6) FTE skilled providers and 1.2 (SD=4.4) FTE aides. We also found that counties with moderate (2nd tertile) proportion of Black and Hispanic Americans; counties with highest (3rd title) proportion of Black and Hispanic Americans had the lowest number of FTE home health care aides per every 1,000 persons. Our findings highlight the staff shortage facing home health care and suggest the existence of disparities in availability of home health care workforce.

### THE RELATIONSHIP BETWEEN ADULT DAY HEALTH CENTER OWNERSHIP, STAFFING, AND PARTICIPANT OUTCOMES

Joanne Spetz[1], and Jason Flatt[2], *1. University of California, San Francisco, San Francisco, California, United States, 2. University of Nevada, Las Vegas, School of Public Health, Las Vegas, Nevada, United States*

Growing demands for specialized care for older adults living with disabilities, including those with Alzheimer's Disease and related dementia (ADRD), and for caregiver respite have resulted in rising use of adult day health centers (ADHCs). ADHCs vary in size, ownership, participant demographics, and services offered, with a many operated by multi-site chain organizations and with for-profit ownership. This study examines whether ADHC ownership is associated with their scope of services, staffing models, and outcomes. We used facility-level data from the restricted-use 2014 National Post-Acute and Long-Term Care Study (NPALs) Adult Day Services Center module, which collects primary data on ADHCs through a nationally representative survey. Key outcome variables were measures of ADHC staffing, and rates of participants' emergency department visits, hospitalizations, and falls. The first part of the analysis was descriptive, focused on participant and staffing patterns. We then estimated ordinary least squares multivariate regressions to learn whether staffing differences exist holding other ADHC characteristics constant, such as size, region, and other services offered. We also estimated Poisson regression models to learn whether there are differences in rates of emergency room visits, hospitalizations, and falls. We found little difference in staffing or participant outcomes between for-profit vs. not-for-profit ADHCs. We found that chain-affiliated ADHCs had different participant populations and sources of revenue. They also had lower levels of staffing in general and for licensed nurses, activity staff, and social workers. Rates of falls and emergency department visits were higher in chain-affiliated versus independent ADHCs when controlling for other characteristics.

### SESSION 3960 (SYMPOSIUM)

### PANDEMIC POLICIES MOVING FORWARD: WHAT HAVE WE LEARNED

Chair: Brian Lindberg

Leading aging and health policy advocates will present their findings and viewpoints regarding pandemic and post-pandemic policy and programmatic changes and innovations. Issues will include elder justice, home and community-based services, Medicaid, nursing home care, and social isolation. The group will discuss what has changed and how will programs and services be different in the future.

### SESSION 4001 (BIOLOGICAL SCIENCES INVITED SYMPOSIUM)

### MITOCHONDRIAL COMMUNICATION AND AGING

Chair: Changhan Lee

Mitochondria evidently originate from endosymbiotic bacteria that presumably provided several advantages for eukaryotic life. For the past 1–2 billion years, mitochondria co-evolved with the ancestral cell to coordinate various cellular functions. Coordination requires communication and mitochondrial signaling has been shown to be vital to cellular fitness and aging. In this symposium, the speakers will discuss the role of mitochondria as a signaling organelle and their impact on key cellular functions in the context of aging. Consistent with emerging evidence of the complexity and sophistication of mitochondrial communication mechanisms, some of the mitochondrial-to-nuclear communication modes, including nuclear transcriptional programs and cellular signaling networks, that regulate molecular and cellular processes to promote fitness will be discussed. Dr. Chen will discuss how a mitochondrial metabolic checkpoint can regulate stem cell quiescence and maintenance that is important to stem cell aging. Dr. Picard will discuss the impact of mitochondrial stress on increased energetic cost of living (i.e. hypermetabolism) and cellular lifespan. Finally, Dr. Lee will discuss how signaling peptides that are encoded withing the mitochondrial genome regulate cellular homeostasis, increase physiological resilience, and promote healthy aging.

### MITOCHONDRIA: POWERHOUSE, SLAUGHTERHOUSE, AND SPEAKER OF THE HOUSE

Changhan Lee, *University of Southern California, Los Angeles, California, United States*

The nuclear and mitochondrial genomes have co-evolved since the union forged 1-2 billion years ago between our ancestral cell and free-living bacteria. The bi-genomic system is coordinated by close communication between the two genome-possessing organelles. More recently, peptides that are encoded in the mitochondrial genome have been identified and shown to communicate mitochondrial messages. In this symposium, I will discuss MOTS-c as a mitochondrial-encoded communication factor in the context of aging. MOTS-c can translocate to the nucleus under metabolic stress to directly regulate adaptive nuclear gene expression. In humans, MOTS-c levels increase in skeletal muscle and in circulation upon exercise. In mice, MOTS-c treatment significantly reversed physical decline in aged mice (22 mo.), including doubling in treadmill running time. Studies from our lab and others point to a novel class of mitochondrial-encoded longevity genes that coordinate cellular homeostasis.

### MITOCHONDRIAL METABOLIC CHECKPOINT, STEM CELL AGING AND REJUVENATION

Danica Chen, *University of California - Berkeley, Berkeley, California, United States*

Cell cycle checkpoints are surveillance mechanisms in eukaryotic cells that monitor the condition of the cell, repair cellular damages, and allow the cell to progress through the various phases of the cell cycle when conditions become favorable. Recent advances in stem cell biology highlight a mitochondrial metabolic checkpoint that is essential for stem cells to return to the quiescent state. As quiescent stem cells enter the cell cycle, mitochondrial biogenesis is induced and mitochondrial stress is increased. Mitochondrial unfolded protein response and mitochondrial oxidative stress response are activated to alleviate stresses and allow stem cells to exit the cell cycle and return to quiescence. These processes are critically regulated by several sirtuin family members, which are NAD+-dependent deacetylases. Because loss of stem cell quiescence results in the depletion of the stem cell pool and compromised tissue regeneration, deciphering the molecular mechanisms that regulate the mitochondrial metabolic checkpoint in stem cells will increase our understanding of tissue homeostasis and how it becomes dysregulated under pathological conditions and during aging. More broadly, this knowledge is instrumental for understanding the maintenance of cells that convert between quiescence and proliferation to support their physiological functions.

### CROSSTALK BETWEEN UBIQUITINATION AND ADP-RIBOSYLATION IN THE MAINTENANCE OF MITOCHONDRIAL HOMEOSTASIS

Valentina Perissi, *Boston University School of Medicine, Boston, Massachusetts, United States*

Oxidative stress and increased ROS production, as observed in aging and obesity, commonly lead to the accumulation of mitochondrial dysfunctions. This is met by the activation of a robust mitochondria-to-nucleus stress response promoting the rewiring of nuclear gene expression to limit cellular and tissue damage and promote organelle adaptation. Here we will review previous work uncovering the transcriptional cofactor G-Protein Pathway Suppressor 2 (GPS2) as a mediator of mitochondria retrograde signaling and a key nuclear regulator of nuclear-encoded mitochondrial genes, including mitochondrial chaperones/proteases, mitokines, and other protective enzymes such as the ADP-ribosyltransferase NEURL4. We will also discuss unpublished work showing that shuttling of GPS2 between organelles plays a role in coordinating the transcriptional and translational regulation of antioxidant factors and pro-apoptotic genes by promoting the ubiquitination of mitochondria-associated translation factors.

### DOES THE ENERGETIC COST OF STRESS ACCELERATE BIOLOGICAL AGING AND SHORTEN LIFESPAN?

Martin Picard, *Columbia University, New York, New York, United States*

In humans, chronic activation of cellular stress responses predict functional decline, accelerate aging, and increase mortality, but the cellular basis for the stress-aging cascade remains unclear. Here we induced chronic stress in primary human fibroblasts from multiple donors with constant i) ATP-synthase inhibition (Oligomycin-1nM) or i) glucocorticoid stimulation (Dexamethasone-100nM) cultured for up to 10 months. Stressors triggered mtDNA instability and activated integrated stress responses resulting in both transcriptional activation and secretion of cytokines and metabokines. In parallel, chronic stress increased cellular energy expenditure or the “cost of living” by 62–108% ($Ps<0.001$). Thus, chronically stressed cells considerably expend more energy to undergo each cell division. This severe state of hypermetabolism led to faster rates of telomere shortening and of genome-wide DNA methylation-based epigenetic aging monitored across the cellular lifespan, reflecting mito-nuclear signaling. This accelerated aging phenotype culminated in 20–40% fewer maximal cell divisions (i.e., Hayflick limit). Based on findings that hypermetabolism and increased energy flux through mitochondria may shorten lifespan, we pharmacologically inhibited carbon entry (glutamine, pyruvate, long-chain fatty acids) into the Krebs cycle across the entire cellular lifespan. While this manipulation successfully decreased OxPhos activity, it increased glycolysis-derived ATP synthesis and total energy expenditure, exacerbating the accelerated aging phenotype. Combined, our longitudinal bioenergetic and multi-omic profiling of primary human cells show that chronic heterotypic stressors converge on an acceleration of metabolism (i.e., hypermetabolism), and commensurately accelerate the progression of multiple aging hallmarks. These findings also implicate long-term mito-nuclear signaling in the stress-aging cascade in a human model.

### SESSION 4010 (PAPER)

### COVID-19 CAREGIVING

### ALL HANDS ON DECK: CONCEPTUALIZING AND OPERATIONALIZING COLLABORATION WITHIN CARE NETWORKS

Athena Koumoutzis[1], Katrina Ellis[2], Jordan Lewis[3], Zhiyong Lin[4], Yuanjin Zhou[5], William Chopik[6], and Richard Gonzalez[2], *1. Miami University, Oxford, Ohio, United States, 2. University of Michigan, Ann Arbor, Michigan, United States, 3. University of Minnesota Medical School, Duluth, Minnesota, United States, 4. University of Texas at San Antonio, San Antonio, Texas, United States, 5. University of Texas at Austin, Austin, Texas, United States, 6. Michigan State University, East Lansing, Michigan, United States*

Care recipients often report multiple caregivers that provide assistance. Yet, consideration of conceptual and methodological issues of caregiving networks has yet to be fully explored. This paper proposes a care collaboration index for each care network that predicts variation in collaboration among multiple networks. The association between network size, race/ethnicity, and dementia status with collaboration was also examined. Data came from the 2015 waves of NHATS and NSOC. Operationalization of collaboration was derived from 1,298 caregivers within 552 care networks. Care recipients were older adults (Mage = 83.69, SD = 7.73), most were women (71.6%), 47.9% had possible/probable dementia, and 38.9% identified as persons of color. The collaboration index considered shared care tasks and scope of assistance while controlling for the size of the immediate and broader care networks. This measure also considered task overlap among care networks as a collaboration process. A series of regression models were run to analyze whether care collaboration differed for older adults by key predictors and if the association between care collaboration and predictors varied across care tasks. Care networks with more caregivers garnered greater collaboration overall, both in general and across most types of tasks. Greater collaboration was observed among Black, Hispanic, and Other (non-White) care recipients and those with possible/probable dementia. This index provides a way to examine care network behaviors and highlights the importance of collaboration-informed approaches. Implications regarding the relationship between care collaboration and outcomes for caregivers and recipients will be discussed.

### CHALLENGES AND SUPPORTS FOR VETERAN CAREGIVERS DURING THE COVID-19 PANDEMIC: A MIXED-METHODS STUDY

Lauren Penney[1], Polly Noel[1], Karla Hernandez-Swift[2], Luci Leykum[1], Ranak Trivedi[3], Stuti Dang[4], Andrea Kalvesmaki[5], and Jorie Butler[6], *1. South Texas Veterans Health Care System, San Antonio, Texas, United States, 2. Texas State University, San Antonio, Texas, United States, 3. Stanford University, Palo Alto, California, United States, 4. Miami Veterans Affairs Healthcare System, Miami, Florida, United States, 5. Informatics, Decision-Enhancement and Analytic Sciences Center, Salt Lake City, Utah, United States, 6. Salt Lake City Veterans Health Care System, Salt Lake City, Utah, United States*

Informal caregivers face challenges in supporting older or medically-complex Veterans, which could be exacerbated by the COVID-19 pandemic. Our mixed methods observational study explored Veteran caregivers' supports, challenges, and self-identified impacts during the pandemic. Caregivers whose veterans needed help with at least one activity of daily living for the last year and received care at one of five Veterans Health Administration (VA) study sites were eligible. Survey participants (n=46) were 96% female, 32-83 years old (median 59); most (83%) cared for a spouse. A majority (67%, n=31) reported increased stress since the start of the pandemic. Top sources of increased stress included worry about COVID-19 infection, increased caregiving responsibility, delayed access to care, concerns about vaccine safety, and employment or financial concerns. Caregiver interviews (n=26) qualitatively analyzed using a rapid, templated approach identified the following themes: (1) the benefits and challenges of VA COVID precautions to Veteran care access (e.g. telehealth, getting care for new problems), (2) supports afforded by and limits of the expansion of the VA Caregiver Support Program, (3) declines in Veteran physical and cognitive functioning, (4) increased caregiver role in Veterans' support and care, (5) changes in work and living situations to address increased caregiving needs and/or reduce risk of exposure, and (6) loss of and then return to more usual routines and social outlets amid ongoing COVID-related uncertainties. Recommendations include targeted, personalized outreach to engage caregivers in existing supports, removing barriers and streamlining processes for obtaining services, and creating durable caregiver-to-caregiver, peer support opportunities.

### EVALUATION OF A CAREGIVER FINANCIAL PREPAREDNESS PROGRAM: SIGNIFICANT SHORT- AND LONG-TERM IMPACTS

Katherine Judge[1], Claire Grant[2], Monica Moreno[3], Kerry Lanigan[3], Lauren Stratton[3], and Sam Fazio[3], *1. Cleveland State University, Cleveland, Ohio, United States, 2. Cleveland State University, Cleveland Heights, Ohio, United States, 3. Alzheimer's Association, Chicago, Illinois, United States*

Over 50 million informal caregivers provide care to an older adult age 50 or older. Negative financial impacts include significant out-of-pocket expenses, decreased income and future earnings. The program, Managing Money: A Caregiver's Guide to Finances, was developed to address the lack of evidence-based programs and evaluated using a randomized control trial across 5 time points: Time 1 (baseline); Time 2 (after condition completion); Time 3 (30 days after Time 2); Time 4 (60 days after Time 2); and Time 5 (90 days after Time 2). The mean age of participants was

55.45 (SD=14.85); 80% female; 72% White and 69% married. Forty-six percent provided care to their parent/in-law, 36% to a spouse/partner, and 40% were employed full-time. Participants were randomly assigned to the program (N=76) or waitlist condition (N=98). No significant differences on demographic or care context variables were found between conditions. A 2 x 5 repeated measures analyses examined significant change across time based on condition. For significant outcomes, post-hoc analyses examined whether change was short-term (T2,T3); long-term (T4,T5); or both. Results found: 1) significant short- and long-term improvements for Unmet Needs (F(1,4)=8.34,p=.01); Self-efficacy (F(1,4)=4.27,p=.01); and Behavioral Intentions (F(1,4)=3.63,p=.01) and 2) significant long-term improvements for Unmet Needs Distress (F(1,4)=5.82,p=.01) and Behavioral Actions (F(1,4)=4.55,p=.01). Results indicated the program was efficacious in positively impacting key financial preparedness measures. Discussion highlights key study elements including program accessibility and scalability; generalizability of findings and limitations; and contextualizing results within the larger literature.

### IT WAS TERRIBLE, I DIDN'T SLEEP FOR TWO YEARS: A MIXED-METHODS EXPLORATION OF SLEEP AMONG HOSPICE FAMILY CAREGIVERS

Lauren Starr[1], Karla Washington[2], Miranda McPhillips[1], George Demiris[1], and Debra Parker Oliver[2], *1. University of Pennsylvania, Philadelphia, Pennsylvania, United States, 2. Washington University in St. Louis, St. Louis, Missouri, United States*

Due to overnight caregiving demands and inadequate social support, over 1.2 million hospice family caregivers in the U.S. and millions more worldwide are at risk of poor sleep and resulting negative effects on health. Sleep problems are a modifiable source of global health inequities. The purpose of this study was to describe hospice family caregiver sleep experiences, efforts to improve sleep, and effects of sleep. Developed using the Symptom Management Model, this mixed methods study featured a concurrent nested design prioritizing qualitative reflexive thematic analysis. In 2021, 47 family caregivers of hospice care-recipients from two randomized clinical trials in the United States (NCT03712410, NCT02929108) were interviewed. Sleep-related questions from PHQ-9 and GAD-7, and self-rated health and energy were administered at baseline. Three themes emerged: quality of sleep, factors influencing sleep, and effects of sleep. Hospice family caregivers commonly experienced “interrupted” sleep with frequent night-waking due to “on-call” “vigilance” and anxiety overnight and, sometimes, in bereavement. Negative effects included exhaustion, mental and physical health decline, and reduced performance. 72.5% described sleep quality as “fair” or “poor.” At baseline, 35.5% of caregivers were bothered by trouble falling asleep, staying asleep, or sleeping too much at least “more than half the days” in a week. Caregivers were reluctant to take sleep medications. Over half quit jobs or reduced work hours to provide care; few reported adequate support. Hospice family caregivers commonly experience disordered sleep with negative effects. Clinicians must assess sleep, offer tailored sleep interventions, and provide more supports to hospice family caregivers.

### SESSION 4030 (PAPER)

### FAMILIES AND CAREGIVING

### ALTRUISTIC AGING: ON THE MORALITY OF “AGING WELL”

Anne Barrett[1], and Katia Vecchione[2], *1. Florida State University, Tallahassee, Florida, United States, 2. University of Trento, Trento, Trentino-Alto Adige, Italy*

A dominant aging narrative emphasizes what individuals should do to age well, namely remain active and productive. Underlying these promotional messages, however, are others about what should be avoided. At their core is a proscription against becoming dependent, thus “burdensome.” To critically examine this aging narrative, we develop the concept of “altruistic aging,” which captures a cultural expectation that older adults adopt a selfless concern for the well-being of others. We use data from interviews with 28 Italians aged 65 and older to illustrate how the goal of altruistic aging shapes older adults’ behaviors in the present and their framing of care options in the future. It motivates physical and social activity, as well as healthy eating – all aimed, in part, at extending the duration of one’s self-sufficient years. Altruistic aging also involves expunging the idea of ever living with one’s children and emphasizing the benefits of nursing homes. This observation suggests that altruistic aging may be constructed as a familial duty, particularly in nations like Italy with family-centered models of care. Our analyses reveal that taken-for-granted, and culturally celebrated, orientations toward aging may mask an internalization of ageism that devalues the self to the point of selflessness.

### DETERMINING EVIDENCE FOR EFFECTIVE FAMILY CAREGIVER COMMUNICATION: STRATEGIES LEADING TO BREAKDOWN AND REPAIR

Carissa Coleman[1], Jinxiang Hu[2], and Kristine Williams[3], *1. University of Kansas, Kansas City, Kansas, United States, 2. University of Kansas Medical Center, Kansas City, Kansas, United States, 3. University of Kansas Medical Center, Lawrence, Kansas, United States*

Communication is fundamental for dementia care and identifying evidence for strategies that facilitate or impede communication is needed. We analyzed 221 videos from a randomized controlled trial of a family caregiver telehealth intervention (FamTechCare) using Noldus second-by-second behavioral coding of communication behaviors and breakdowns in interactions between 53 caregiver-person with dementia dyads. Coded data from 3,642 30-second observations were first analyzed using penalized regression for feature selection (least absolute shrinkage and selection operator; LASSO) to identify strategies most important for predicting prevention and successful repair of communication breakdown. Bayesian mixed modeling was then used to identify communication strategies associated with successful versus unsuccessful prevention and repair of communication breakdown taking into account of the dyadic structure of our data. Results showed that given our data, communication breakdown was associated with caregivers changing topic (Mdn=11.23, 95% CrI [4.39, 24.43]), ignoring (Mdn=11.47, 95% CrI [4.75, 24.17]), making commands (Mdn=10.55,

95% CrI [3.41, 23.22]) and taking over the task (Mdn=4.04, 95% CrI [1.69, 7.23]). Successful repair of breakdown was associated with caregivers verbalizing understanding (Mdn=-0.47, 95% CrI [-0.88, -0.09]), tag questions, (Mdn=-2.44, 95% CrI [-5.35, -0.33), and silence (Mdn=-0.78, 95% Crl [-1.18, -0.40) while ignoring and changing topic were associated with unsuccessful repair (Mdn=3.63, 95% Crl [-2.56, 4.78; and 2.51 [1.39, 3.74]). These results provide evidence for development and testing of evidence-based communication strategy training for family caregivers of persons with dementia. Future analyses will identify effects of dementia stage, diagnosis, and dyad characteristics on associations.

### EMPLOYING DESIGN THINKING METHODS TO CREATE BEHAVIORAL INTERVENTIONS THAT SUPPORT AFRICAN AMERICAN CAREGIVERS

Leila Aflatoony[1], Kenneth Hepburn[2], Molly Perkins[3], Drenna Waldrop[4], and Karah Alexander[2], *1. Georgia Institute of Technology, Atlanta, Georgia, United States, 2. Emory University, Atlanta, Georgia, United States, 3. Emory University School of Medicine, Atlanta, Georgia, United States, 4. Emory, Atlanta, Georgia, United States*

Design Thinking provides an empathy-based creative problem-solving approach to intervention and/or patient-centered care design that seeks to articulate the right problem space and propose appropriate solutions to challenges encountered by individuals in specific contexts, such as healthcare. This abstract illustrates the application of the Design Thinking approach in the context of dementia caregiving. Emory's Roybal Center for Caregiving Mastery conducted a three-part design studio workshop with African American dementia family caregivers to understand and identify prioritized solutions to their caregiving challenges. The workshops followed the Double-Diamond design process model that is divided into four phases (Discover, Define, Develop, and Deliver) and that emphasizes divergent and convergent modes of thinking. The process first encourages divergent thinking to explore a problem space and then employs convergent thinking to identify the salient problem and develop the right solution. The workshop's Discover stage provided a broad and deep contextual understanding of the range of unmet needs African American caregivers experience in their interactions with healthcare personnel and systems. The Define phase enabled the convergent review and prioritization of the list of needs to be addressed. The Develop phase generated 17 intervention ideas. Finally, the Deliver phase focused on converging and validating the proposed interventions. The workshops concluded that having access to caregiver-focused resources supporting mental/physical self-care, such as support groups and peer programs, can be beneficial interventions to potentially improve knowledge, awareness, and caregiving mastery among African Americans American caregivers.

### HOW DO OLDER AFRICAN AMERICAN COUPLES TAKE CARE OF EACH OTHER? A DYADIC, QUALITATIVE ANALYSIS

Amy Rauer[1], Wendy McClean Cooke[2], and Lyndsey Hornbuckle[2], *1. University of Tennessee Knoxville, Knoxville, Tennessee, United States, 2. University of Tennessee, Knoxville, Tennessee, United States*

Guided by Lyons and Lee's (2018) Dyadic Theory of Illness Management, the current study explored covariation in dyadic health management behaviors within a sample of seventeen older African American married couples (Mage=61.68 years; Mmaritalduration=32.29 years). We also examined if spouses agreed on how they took care of each other's health and whether these patterns changed after couples participated in a health-focused intervention together. Prior to beginning a 12-week walking plus resistance training exercise intervention, spouses completed questionnaires asking them to list the common things they did to help take care of their partner's health as well as what their partner did for them. Thirteen couples completed these questionnaires post-intervention. Data from both waves were analyzed using thematic analysis (Braun & Clarke, 2006). Five health management behaviors domains were identified (diet, exercise, self-care, medical compliance, relationship maintenance). Although both partners reported encouraging healthier diets and exercise, wives also reported promoting other health management behaviors. Couples had greater congruence in their appraisals of wives' health management behaviors compared to husbands', as wives recognized many things husbands did to take care of them that husbands did not report themselves. Patterns appeared stable over time. Findings suggest the incongruence in couples' health management behaviors represented complementary efforts to support each other and revealed that husbands may be underestimating how much care they are providing to their wives. A promising method for addressing health disparities in this population may involve capitalizing on this clear investment that older African American couples have in each other's health.

### SESSION 4040 (PAPER)

### FRAILTY, RESILIENCE, AND FUNCTION

### A SIMPLIFIED APPROACH FOR CLASSIFYING PHYSICAL RESILIENCE AMONG OLDER ADULTS: THE HEALTH ABC STUDY

Chenkai Wu, and Tianze Lin, *Duke Kunshan University, Kunshan, Jiangsu, China (People's Republic)*

Physical resilience is an emerging concept within the context of aging and geriatric medicine, and we previously developed and validated one such measure based on the mismatch between persons' frailty level and comorbidity burden. We sought to develop a simplified version for classifying physical resilience. We also examined the agreement between the simplified version and the original approach and evaluated its predictive validity. We included 2,457 older adults from the Health, Aging, and Body Composition Study. We constructed a simplified version for quantifying physical resilience based on the comorbidity burden and level of frailty (score: 0-10). Participants were grouped by the number of diseases and classified into three groups—adapters, expected agers, and premature frailers—based on the mean and SD of frailty score (less than, within, or above one standard deviation of the mean). The Cohen's kappa between the novel resilience classification and the original approach was 0.70, and the percentage of absolute agreement was 85.4%. We observed a steep increase in years of healthy and able life from premature frailers to adapters in the simplified resilience classifications. We developed a simplified version for quantifying physical resilience in a cohort of initially well-functioning older Black and White adults. The agreement between the simplified version and the original approach is high. Adapters had a longer healthy lifespan than expected agers and premature frailers. This user-friendly measure could help assess patients' physical resilience in clinical settings.

### CHANGES IN FUNCTIONAL STATUS AMONG US ADULTS IN LATE MID-LIFE FROM THE HEALTH AND RETIREMENT STUDY 2004–2016

Adit Doza[1], Elham Mahmoudi[1], and Wassim Tarraf[2], *1. University of Michigan, Ann Arbor, Michigan, United States, 2. Wayne State University, Detroit, Michigan, United States*

Reducing racial/ethnic disparities in health and functioning among older adults are salient key goals in the U.S. health policy. This study examined whether and how the functioning have progressed between White and minority populations in late midlife in recent years. We analyzed the Health and Retirement Study (HRS) among adults ages 45-64 years across two time periods (2004-2010 and 2010-2016). Using generalized linear regression, we modeled changes in activities of daily living (ADL) and changes in instrumental activities of daily living (IADL) as a function of race/ethnicity and sequentially adjusted for period specific risk factors including sociodemographic factors, health insurance, health behaviors and social networks. Oaxaca-Blinder Regression Decomposition (OBRD) techniques are used to assess the contributions of these factors to the observed trends in ADL/IADL. We find changes in ADL is significantly higher for Blacks (Odds Ratios of 1.23, CI 1.01-1.48) and US-born Hispanics (Odd Ratios of 1.54, CI 1.12-2.12) after adjusting for all the risk factors in the second period. About two-thirds of the disparity in functional status of adults is explained in our OBRD model and more than 70% of these explained differences among minorities is related to socio-demographic factors. This probably occurred due to the differences in income and net wealth which began to widen during the time of 2008 financial crisis. Although recently there is a decline in disability trends for older adults in U.S., our findings point to an increase in racial/ethnic disparities in functional status for adults in late midlife.

### PROXIES OF FRAILTY AND INFLAMMATION PREDICT ALTERED WHITE MATTER MICROSTRUCTURE IN OLDER ADULTS

Andrew Bender[1], Sandra Duezel[2], Ulman Lindenberger[3], Ilja Demuth[4], Elisabeth Steinhagen-Thiessen[5], Thad Polk[6], and Simone Kühn[3], *1. Michigan State University, East Lansing, Michigan, United States, 2. Max-Planck-Insitute for human developement, Berlin, Berlin, Germany, 3. Max Planck Institute for Human Development, Berlin, Berlin, Germany, 4. Charité Universitätsmedizin Berlin, Berlin, Berlin, Germany, 5. Charité - Universitätsmedizin Berlin, Berlin, Berlin, Germany, 6. University of Michigan, Ann Arbor, Michigan, United States*

Age-related elevations in inflammation are associated with both neurodegeneration and increased frailty in older adults. Here we used state-of-the-art diffusion-MRI (dMRI) methods to examine how specific markers of white matter (WM), including fiber density [FD], fiber cross-section [FC], and extracellular cerebral spinal fluid [CSF] are linked to inflammation and frailty in a population-based aging cohort. We hypothesized that increased inflammation and frailty would be associated with reduced FD and FC and increased CSF. Participants included 255 older adults (Mage=70.46; SD=3.79 years; 42% women) recruited from the Berlin Aging Study-II (BASE-II) for MRI scanning. Measures of blood serum ferritin and grip strength (corrected for sex and body mass) served as proxies of inflammation and frailty, respectively. Processing of dMRI data (b=1000 s/mm2; 60-directions) followed the MRtrix3 fixel-based analysis (FBA) pipeline, extended for voxelwise estimation of FD, FC, and CSF, with data transformed to a sample-specific template. The voxelwise estimates of FD, FC, and CSF were then regressed on the measures of blood serum ferritin and grip strength, correcting for multiple comparisons. Greater inflammation (ferritin) and frailty (grip strength) predicted lower FD in the internal capsule and anterior commissure. Higher ferritin also predicted lower FC in dorsal cingulum and forceps minor. Finally, frailty, but not inflammation, predicted increased CSF in multiple regions. These findings demonstrate common and specific associations of inflammation and frailty with WM in older adults and highlight the utility of newer methods for characterizing WM alterations.

### THE PHYSIOLOGIC COMPLEXITY OF BEAT-TO-BEAT BLOOD PRESSURE FLUCTUATION IS ASSOCIATED WITH FRAILTY IN OLDER ADULTS

Junhong Zhou[1], Yurun Cai[2], Yi Guo[3], and Xin Jiang[3], *1. Harvard Medical School/Hebrew SeniorLife, Roslindale, Massachusetts, United States, 2. University of Pittsburgh, Pittsburgh, Pennsylvania, United States, 3. Shenzhen People's Hospital, Shenzhen, Guangdong, China (People's Republic)*

Background: Beat-to-beat blood pressure (BP) is an important cardiovascular output and regulated by neurophysiologic elements over multiple temporal scales. The multiscale dynamics of beat-to-beat BP fluctuation has thus been characterized using "BP complexity" and has been linked to age-related adverse health outcomes. We here aimed to examine the relationship of BP complexity to frailty, and if BP complexity mediates the association between arterial stiffness and frailty. Method: A total of 350 older adults completed assessments for frailty (i.e., Fried frailty phenotype criteria), arterial stiffness (i.e., average brachial-ankle pulse wave velocity), and beat-to-beat finger BP. The complexity of beat-to-beat systolic (SBP) and diastolic (DBP) BP series were measured using multiscale entropy. The relationships between frailty, BP complexity and arterial stiffness were examined using ANOVA and linear regression models. The effects of BP complexity on the association between arterial stiffness and frailty were examined using mediation analyses.

Results: Compared to non-frail, pre-frail and frail groups had significantly elevated lower SBP and DBP complexity ($F>11$, $p< 0.001$), and greater arterial stiffness ($F=16$, $p< 0.001$). Greater arterial stiffness was associated with lower BP complexity ($\beta<-0.42$, $p< 0.001$). SBP and DBP complexity mediated the association between arterial stiffness and frailty (indirect effects>0.28), account for at least 47% of its total effects on frailty (Mediated proportion: SBP: 50%, DBP: 47%).

Conclusion: This study demonstrates that BP complexity is associated with frailty in older adults, and mediates the association between arterial stiffness and frailty, suggesting that this metric would serve as a marker to characterize important functions in older adults.

### SESSION 4050 (SYMPOSIUM)

### HEARING, COGNITION, AND PHYSICAL FUNCTION: LEVERAGING LARGE-SCALE EPIDEMIOLOGIC STUDIES TOWARD INTERVENTION

Chair: Carrie Nieman Co-Chair: Jennifer Deal Discussant: Margaret Wallhagen

Hearing loss and aging represent the intersection of two major public health challenges. Over 1.5 billion individuals or almost 20% of the global population have hearing loss, where the largest burden is among older adults secondary to age-related hearing loss. By 2030, the end of the UN Decade of Healthy Aging, almost 2 billion individuals are expected to be living with hearing loss. Evidence continues to grow related to the negative consequences of hearing loss in the context of aging, both on cognitive and physical function. This symposium will share the latest findings on hearing loss and its relationship to a range of negative consequences from diverse large-scale cohorts. Specifically, the session will cover hearing loss and its relationship to cognitive health, including subjective cognitive decline and neuropsychiatric symptoms among persons living with dementia, as well as physical health, including frailty and physical function. Each presentation draws upon datasets from a large-scale epidemiologic study with varying types of data on hearing status and measures of cognitive and physical health. The covered datasets include nationally representative cohorts, such as the National Health and Aging Trends Study (NHATS), to longitudinal cohorts, such as the Atherosclerosis Risk in Communities Study (ARIC). The session will serve as a primer on available datasets that include data on hearing status and hearing care, including strengths, limitations, and analysis considerations. This symposium will provide tangible ways to leverage existing data in order to move toward intervention and build capacity around hearing health within gerontology.

### HEARING LOSS, HEARING AID USE, AND NEUROPSYCHIATRIC SYMPTOMS IN DEMENTIA: FINDINGS FROM NACC

Carrie Nieman[1], Emmanuel Garcia Morales[2], Jeannie-Marie Leoutsakos[1], Constantine Lyketsos[3], and Esther Oh[4], *1. Johns Hopkins University School of Medicine, Baltimore, Maryland, United States, 2. Cochlear Center for Hearing and Public Health, Baltimore, Maryland, United States, 3. Johns Hopkins Bayview, School of Medicine, Baltimore, Maryland, United States, 4. Division of Geriatric Medicine, Johns Hopkins University School of Medicine, Baltimore, Maryland, United States*

Hearing loss is highly prevalent among persons living with dementia (PWD) and associated with an increased risk of neuropsychiatric symptoms (NPS), while hearing aid use may be protective. We analyzed data from the National Alzheimer's Coordinating Center's (NACC) uniform data set. We estimated the association between hearing loss and number or severity of NPS in a negative binomial regression adjusting for demographic and clinical characteristics. In the subsample with hearing loss, we estimated the association between hearing aid use and number or severity of NPS.10,054 participants were included with a mean age of 75 years. 2,416 (24%) self-reported hearing loss with 71% reporting 2+ NPS. In PWD and hearing loss, 1,325 (54.8%) reported hearing aid use. After a well-balanced matching, in adjusted models, hearing aid use was associated with fewer (IRR=0.82; 95%CI=0.77,0.87) and less severe NPS (IRR=0.74; 95%CI=0.69,0.80). Hearing aid use may represent an underutilized, non-pharmacological intervention to address NPS.

### HEARING LOSS AND SUBJECTIVE COGNITIVE DECLINE IN THE ARIC-NEUROCOGNITIVE STUDY

Jennifer Deal[1], Natalie Lenzen[2], marjan majid[3], Kening Jiang[4], and Nicholas Reed[5], *1. Johns Hopkins University, Baltimore, Maryland, United States, 2. Boys Town National Research Hospital, Omaha, Nebraska, United States, 3. Rice University, Houston, Texas, United States, 4. Johns Hopkins Cochlear Center for Hearing and Public Health, Baltimore, Maryland, United States, 5. Johns Hopkins Bloomberg School of Public Health, Baltimore, Maryland, United States*

Subjective cognitive decline (SCD) impacts quality-of-life, healthcare utilization and predicts dementia. If hearing loss is associated with SCD is unknown. We estimated the cross-sectional association between hearing loss and SCD in 2,536 participants (79.3±4.4 years, 60% female, 20% Black) in the Atherosclerosis Risk in Communities Neurocognitive Study. SCD was defined as self-reported persistent decline in memory (yes/no) among adults with normal cognition. Hearing was measured using pure tone better-ear thresholds (0.5-4 kHz), speech-in-noise, and self-report. Using Poisson models with robust standard errors, after adjustment for demographic and clinical covariates, self-reported moderate or greater trouble hearing (vs. excellent/good) was associated with a 30% increase in the prevalence of SCD (95%CI:1.12,1.51). Audiometric hearing and speech-in-noise performance were not associated. Use of self-report to approximate audiometric hearing warrants caution, particularly when the outcome is also self-reported; our findings suggest the association between self-reported hearing and SCD may be due to correlated measurement error.

### HEARING LOSS AND FRAILTY: LONGITUDINAL ASSOCIATIONS

Nicholas Reed[1], and Kening Jiang[2], *1. Johns Hopkins Bloomberg School of Public Health, Baltimore, Maryland, United States, 2. Johns Hopkins Cochlear Center for Hearing and Public Health, Baltimore, Maryland, United States*

Hearing Loss (HL) is common among older adults and is associated with factors (e.g., walking speed and social isolation) that may mediate an association with frailty. In the National Health and Aging Trends Study (NHATS) 2011-2018 data, frailty was defined by the physical frailty phenotype composite (exhaustion, low physical activity, weakness, slowness, and shrinking) while HL was self-report. Among, 6897 baseline participants in 2011, 1132 (16%) were frail. In a cox proportional hazard model adjusted for demographic, socioeconomic, and clinical risk factors, HL (n=1607; Hazard Ratio[HR]=1.15; 95% Confidence Interval[CI]=1.00-1.32) was associated with higher risk of incident frailty relative to those with no HL (n=5290). In a generalized estimating equation longitudinal model (2011-2018), HL was associated with higher odds of frailty at baseline (Odds Ratio[OR]=1.52; 95%CI=1.49-1.96) and a 1.06 (95%CI=1.04-1.09) annual increase in odds of frailty. Future research should focus on mechanisms underlying association and determine the impact of HL treatment.

### MIDLIFE PHYSICAL ACTIVITY AND HEARING IMPAIRMENT IN LATE LIFE: ATHEROSCLEROSIS RISK IN COMMUNITIES (ARIC) STUDY

Pablo Martinez-Amezcua[1], Erin Dooley[2], Emmanuel Garcia Morales[3], Nicholas Reed[1], Frank Lin[4], Jennifer Schrack[1], Jennifer Deal[5], and Priya Palta[6], *1. Johns Hopkins Bloomberg School of Public Health, Baltimore, Maryland, United States, 2. Epidemiology, Birmingham, Alabama, United States, 3. Cochlear Center for Hearing and Public Health, Baltimore, Maryland, United States, 4. Johns Hopkins Cochlear Center for Hearing and Public Health, Baltimore, Maryland, United States, 5. Johns Hopkins University, Baltimore, Maryland, United States, 6. General Medicine, New York City, New York, United States*

Background: Cardiovascular risk factors are associated with worse hearing, but the role of mid-life physical activity (PA) on hearing loss at older ages is yet to be investigated.

Methods: Among 3,198 participants of the Atherosclerosis Risk in Communities study, we investigated the association between self-reported mid-life PA (meets PA recommendations [≥150 minutes of moderate-to-vigorous PA/week] vs. not) and hearing loss (audiometric battery [pure-tone and speech-in-noise]) at older ages. We estimated differences in hearing between those who met and did not meet PA recommendations at mid-life and at late life adjusting for demographics, medical conditions, and noise exposure.

Results: 43.3% participants met PA recommendations at mid-life. These participants, compared to those who did not meet recommendations, had lower better hearing by 1.51 (0.46, 2.55) decibels, and 0.37 (0.01, 0.74) more words identified in the speech-in-noise test.

Conclusions: Meeting PA recommendations in mid-life was associated with better hearing at older ages.

### SESSION 4060 (PAPER)

### INTERVENTIONS (BSS)

### A MIXED-METHODS, RANDOMIZED, CONTROLLED EVALUATION OF REMOTE ACTIVITY MONITORING FOR PERSONS LIVING WITH DEMENTIA

Joseph Gaugler[1], Christina Rosebush[1], Rachel Zmora[2], and Elizabeth Albers[3], *1. University of Minnesota, Minneapolis, Minnesota, United States, 2. Boston University, Boston, Massachusetts, United States, 3. University of Minnesota School of Public Health, Minneapolis, Minnesota, United States*

Remote activity monitoring (RAM) technology has the potential to allow at-home caregivers to track the behaviors and activities of persons with dementia in real-time, thus facilitating more proactive symptom management. The aim of the present study was to assess whether RAM technology was associated with reductions in negative health transitions and service utilization for persons with Alzheimer's disease or related dementias (ADRD). An embedded experimental mixed methods design was used that included 179 caregivers who were followed over a 1.5 year study period. Participants were randomly assigned to receive the RAM technology system or usual care. Follow-up surveys were administered on a bi-annual basis over an 18-month period that collected information on dementia caregiver and care recipient characteristics and outcomes. We developed multilevel mixed effects models to estimate odds ratios for binary outcomes (falls, wandering, health care admissions) and categorical outcomes (frequency of falls and wandering). In adjusted models, RAM technology use was statistically and significantly associated with lower odds of emergency department visits (p<.05) and less frequent falls (p=.05) for people living with dementia over the 1.5 year study period. Technologies that prevent or delay the onset of negative health events via improved care management and monitoring may enhance the care of persons with dementia. As dementia continues to pose an array of challenges for someone living with ADRD, RAM or similar technologies may offer a solution to the conundrum of dementia care.

### EFFECTIVENESS OF SMOKING CESSATION INTERVENTIONS WITH LDCT LUNG CANCER SCREENING AMONG AFRICAN AMERICAN SMOKERS

Tung-Sung Tseng, Yu Hsiang Kao, and Mirandy Li, *LSUHSC, New Orleans, Louisiana, United States*

Low dose computed tomography (LDCT) screening can detect lung cancer early and decrease lung cancer-specific mortality for current smokers but remains under-utilized among these populations. Although African-American smokers tend to smoke less and have lower smoking pack-year histories, they have lower quit rates, higher rates of mortality from lung cancer than other racial/ethnic groups. This study examined the effectiveness of a smoking cessation intervention integrating LDCT screening among African-American smokers. This study recruited 60 African-American daily smokers over the age of 55 who qualified for LDCT screening. Participants were randomly and equally assigned into two groups (intervention and control). Overall, the mean age was 61.0 years old (standard deviation, 5.5), 61.7% of the participants were female and, 91.7% had lower incomes (<$20,000). Descriptive statistics were used to summarize demographics, smoking status, knowledge, attitudes, and stage of change for smoking cessation. The findings showed that participants in the intervention group had a lower number of daily cigarettes smoked (9.5 vs. 11.0) and a higher reduction in the number of daily cigarettes smoked (-2.3 vs. -0.9) than those in the control group. Participants in the intervention group were more likely to be in the preparation stage of the stages of change model (50.0% vs. 40.0%), progress in the stage of change (36.7% vs. 16.7%), or report already having had quit smoking (10.0% vs. 3.3%) than those in the control group. LDCT screening represents a potential "teachable moment" for African-American smokers, which may encourage them to consider these strategies for smoking cessation.

### HOLISTIC WELLNESS COACHING INTERVENTION FOR OLDER ADULTS IN RESIDENTIAL COMMUNITIES

Philip Clarke[1], Matthew Fullen[2], and Jennifer Smith[3], *1. Wake Forest University, Winston-Salem, North Carolina, United States, 2. Virginia Tech, Blacksburg, Virginia, United States, 3. Mather Institute, Evanston, Illinois, United States*

Many wellness programs for older adults focus on physical health or specific conditions, such as heart disease prevention or diabetes management. To supplement these efforts, there is a need for holistic wellness programming to enhance well-being in later life. We developed and piloted a novel wellness coaching program for residents of senior living communities to address this need. The theoretical framework of the program is based on a holistic, multidimensional wellness model and self-determination theory. Staff members from eight residential communities were trained on wellness coaching techniques, such as motivational interviewing, active listening, and group facilitation skills, before they led the 9-week resident wellness coaching program. A total of 79 residents, ages 71 to 97 (M = 84.3, SD = 6.5), completed the pilot program. The resident wellness coaching program included a mixture of individual and group coaching sessions. The coaching sessions provided wellness education, social support, and space to share progress on wellness goals. Participants completed surveys at three time points: pre-program, post-program, and 1-month follow-up. Residents reported high satisfaction with the overall program and its components. Repeated measures ANOVAs were conducted to examine changes in wellness over time. Comparison of pre- and post-test scores revealed significant improvements in health satisfaction, physical and psychological quality of life, purpose, loneliness, relatedness, and competence. Some of these changes, such as increases in psychological quality of life and decreases in loneliness, persisted at the one-month follow-up. These findings have implications for the development and implementation of wellness coaching programs with older adults.

### IMPACT OF AN ONLINE SOCIAL INTELLIGENCE TRAINING PROGRAM ON CUSTODIAL GRANDMOTHERS' WELL-BEING

Megan Dolbin-MacNab[1], Gregory Smith[2], Frank Infurna[3], Britney Webster[4], Luxin Hu[4], Saul Castro[3], Max Crowley[5], and Carol Musil[6], *1. Virginia Tech, Blacksburg, Virginia, United States, 2. Kent State University, Stow, Ohio, United States, 3. Arizona State University, Phoenix, Arizona, United States, 4. Kent State University, Kent, Ohio, United States, 5. Pennsylvania State University, University Park, Pennsylvania, United States, 6. Case Western Reserve University, Cleveland, Ohio, United States*

Despite widespread evidence that the circumstances leading to care, combined with the stress of full-time parenting, have profound effects on psychological, physical, and social functioning of custodial grandmothers (CGM) and their adolescent grandchildren (GC), evidence-based interventions for these families are scarce. To address this gap, we conducted a randomized clinical trial (RCT) with 349 nationally recruited CGMs which compared an online social intelligence training intervention (SIT; n=185) to an attention-control (AC; n=164) condition. The SIT focused on enhancing CGMs' capacity to develop and sustain positive social ties; an important goal since working models of attachment and caregiving are challenged and re-shaped by the off-time and demanding nature of parenting a GC. To investigate initial impact of SIT, we conducted multi-domain latent difference score models (Mplus 8) on a full intent-to-treat basis comparing the two RCT conditions on changes across key outcomes from baseline to immediate post-intervention. In comparison to AC, SIT yielded statistically significant improvement in CGMs' depressed affect, self-esteem, relationship quality with the GC, and attachment avoidance and attachment anxiety with GC. Contrary to expectations, no significant differences were found between the two conditions on outcomes indicative of social competence (e.g., perspective taking, social awareness, social information processing, social self-monitoring). We conclude that CGMs may have applied information obtained from the SIT primarily to their relationship with GC. Our findings point to the potential benefits of the self-guided SIT, given that it can be delivered online and therefore has widespread reach to a vulnerable population. [Funded by R01AG054571]

### SESSION 4070 (SYMPOSIUM)

### JOURNAL OF GERONTOLOGY SOCIAL SCIENCES: FEATURED 2021 EDITOR'S CHOICE ARTICLES

Chair: Jessica Kelley

Social science inquiry on age, aging, and the life course spans many topics and methodologies. This symposium highlights papers that were selected as Editor's Choice articles in the Journal of Gerontology Social Sciences in 2021. These papers highlight methodological innovations, important advancements in our state of knowledge in an area, or emerging issues in the study of aging and older adults. Nguyen et al. discuss a qualitative study of the causes and consequences of financial exploitation of older adults. Newmyer et al. present a 31-country comparative study of measures of loneliness with their reliability and validity. Rurka et al. present a mixed-methods study of sibling dynamics and tensions when caring for an older parent. Dennison and Lee provide a novel method for studying intergenerational selection effects of education and older adult health. Lin and Brown demonstrate gender differences in the economic consequences of later-life divorce and potential impact of repartnering versus staying single.

### PERCEIVED TYPES, CAUSES, AND CONSEQUENCES OF FINANCIAL EXPLOITATION: NARRATIVES FROM OLDER ADULTS

Annie Nguyen, and Duke Han, *University of Southern California, Alhambra, California, United States*

We investigate the perception of financial exploitation and its causes and consequences by older adults who have firsthand experience of being exploited. Thirty-one cognitively healthy older adult participants aged 50 or older were drawn from the Finance, Cognition, and Health in Elders Study. In-depth, one-on-one interviews were conducted. Interview transcripts were analyzed using an iterative, data-driven, thematic coding scheme and emergent themes were summarized. Categories of financial exploitation included (a) investment fraud, (b) wage theft/money owed, (c) consumer fraud, (d) imposter schemes, and (e) manipulation by a trusted person. Themes emerged around perceived causes: (a) element of trust, (b) promise of financial security, (c) lack of experience or awareness, (d) decision-making, and (e) interpersonal dynamics. Perceived consequences included negative and positive impacts around (a) finances, (b) financial/consumer berhaviors, (c) relationships and trust, (d) emotional impact, and (e) future outlook. These narratives provide important insights into perceived financial exploitation and experiences.

### THE CAREGIVER IDENTITY IN CONTEXT: CONSEQUENCES OF IDENTITY THREAT FROM SIBLINGS

Marissa Rurka[1], J Jill Suitor[2], and Megan Gilligan[3], *1. University of Michigan - Center for Health and Research Transformation, Superior Charter Township, Michigan, United States, 2. Purdue University, West Lafayette, Indiana, United States, 3. Iowa State University, Ames, Iowa, United States*

We draw from theories of identity and stress to examine the impact that siblings have on caregivers' psychological well-being. Using data collected from 404 caregivers nested in 231 families as part of the Within-Family Differences Study, we conduct mediation analyses to examine whether perceived sibling criticisms are associated with caregivers' depressive symptoms (a) directly and/or (b) indirectly through sibling tension. Qualitative data from the same caregivers give insight into the processes underlying statistical associations. We found an indirect relationship whereby perceived sibling criticisms were associated with greater sibling tension, which in turn was associated with higher depressive symptoms. Qualitative interviews show that efforts to mitigate the negative impact of sibling criticisms can lead to caregiver strategies that fuel sibling tension. These findings demonstrate how identity processes, as well as the family networks in which caregiving takes place, shape the experiences and consequences of parent care.

### ADULT CHILDREN'S EDUCATIONAL ATTAINMENT AND PARENT HEALTH IN MID- AND LATER LIFE

Christopher Dennison, and Kristen Schultz Lee, *University at Buffalo, SUNY, Buffalo, New York, United States*

While intergenerational models of adult health contend that children's educational attainments influence the health of their parents, background characteristics that predict both can confound the results. Data from the National Longitudinal Study of Adolescent to Adult Health Parent Study are used to examine how having no children who completed college influences parents' self-rated health and depressive symptoms. We use propensity scores to assess this relationship net of potential confounders and test for heterogeneity in the consequences associated with having no children who completed college. Having no children who completed college is negatively associated with parents' self-rated health and positively associated with depressive symptoms. Among parents with the highest propensity for having no children who complete college, the consequences on depressive symptoms are greatest. These findings are important given the call for investments in children's educational opportunities as a vehicle for promoting health among adults and their older parents.

### THE ECONOMIC CONSEQUENCES OF GRAY DIVORCE FOR WOMEN AND MEN

I-Fen Lin, and Susan Brown, *Bowling Green State University, Bowling Green, Ohio, United States*

Surprisingly little is known about the consequences of gray divorce (after age 50) and how women and men fare economically during the aftermath. Using longitudinal data from the 2004-2014 Health and Retirement Study, we estimated hybrid fixed/random effects models comparing women's and men's economic well-being prior to, during, and following gray divorce and subsequent repartnering. Women experienced a 45% decline in their standard of living, whereas men's dropped by just 21%. These declines persisted over time for men, and only reversed for women following repartnering, which essentially offset women's losses associated with gray divorce. Both men and women experienced roughly a 50% drop in wealth. Although repartnering seems to reverse most of the economic costs of gray divorce for women, few form new co-residential unions after divorce. This study offers insight about the financial aftermath of gray divorce, which is likely to contribute to growing economic disadvantage among older adults.

### MEASURING OLDER ADULT LONELINESS ACROSS COUNTRIES

Lauren Newmyer[1], Ashton Verdery[1], Rachel Margolis[2], and Lea Pessin[1], *1. The Pennsylvania State University, University Park, Pennsylvania, United States, 2. Western University, London, Ontario, Canada*

The topic of older adult loneliness commands increasing media and policy attention around the world. Are surveys of aging equipped to measure it? We assess the measurement of loneliness in large-scale aging studies in 31 countries. In each country, we document available loneliness measures, examine correlations between different measures, and assess how these correlations differ by gender and age group. There is substantial heterogeneity in available measures of loneliness across countries. Within countries with multiple measures, the correlations between measures are high (range .38-.78). Differences by age and gender group are relatively small. Correlations between loneliness measures and living alone and being without a spouse are positive and similar in magnitude across countries, supporting construct validity. We establish that even single-item measures of loneliness contribute meaningful information in diverse contexts, with reliable and consistent measurement properties within many countries.

### SESSION 4080 (SYMPOSIUM)

### MULTIDIMENSIONAL CONSTRUCTS OF SLEEP IN OLDER ADULTS

Chair: Christopher Kaufmann Discussant: Katie Stone

Sleep disturbances are common in older adults and are associated with a variety of adverse mental and physical health outcomes. While most studies have characterized sleep based on a single dimension of sleep (typically sleep duration or self-reported sleep quality), there is a growing paradigm shift to characterize “Sleep Health” as multidimensional. More research is needed to identify the clinical utility of a multidimensional approach to characterizing sleep specifically in older adults. In this symposium, we will present five studies that explore how multidimensional Sleep Health relates to age-related health outcomes (e.g., fall risk). Our first presentation will identify associations between subjective/objective sleep quality and physical activity, and examine the moderating role of chronotype. Second, we will examine the extent to which a multidimensional index of Sleep Health and its individual dimensions relate to fall risk. Third, we will ascertain the impact of mindfulness on various Sleep Health dimensions. Fourth, we will identify the extent to which discordance in subjective and objective sleep metrics are related to cognitive functioning. Finally, we will explore how the accumulation of disturbances in Sleep Health may increase risk for early mortality. Findings from this symposium will highlight the clinical relevance of a multidimensional approach to Sleep Health in older adults, and identify the ways in which sleep interventions can be tailored to specific sleep dimensions to promote health in later life.

### THE ASSOCIATION OF SLEEP AND PHYSICAL ACTIVITY AMONG OLDER ADULTS AND THE MODERATION OF CHRONOTYPE

Jing Huang[1], Mengchi Li[2], Miranda McPhillips[3], Nada Lukkahatai[1], and Junxin LI[1], *1. Johns Hopkins University, Baltimore, Maryland, United States, 2. Johns Hopkins, University, Baltimore, Maryland, United States, 3. University of Pennsylvania, Philadelphia, Pennsylvania, United States*

This study aimed to examine the associations of both subjectively and objectively measured sleep with self-reported level of physical activity among older adults, and explore the possible moderative role of chronotype in these associations. Baseline data of 116 community-dwelling older adults without dementia from three prior studies were included. Pittsburgh Sleep Quality Index and Actigraphy were used as subjective and objective sleep measures respectively. The Morningness-Eveningness Questionnaire was used to measure chronotype, which was further dichotomized into morning type and non-morning type. Physical activity was assessed by the Physical Activity Scale for Elderly. Multiple linear regressions were performed to examine the associations, controlling for demographic and health characteristics. We found better subjective sleep quality, shorter actigraphy sleep duration, and higher sleep efficiency were independently associated with greater physical activity. Being morning type might alleviate the association between poor subjective sleep quality and physical activity among older adults.

### MULTIDIMENSIONAL SLEEP HEALTH AND RECURRENT FALLS: THE OSTEOPOROTIC FRACTURES IN MEN STUDY (MROS)

Jane Cauley[1], Stephanie Harrison[2], Peggy Cawthon[3], Andrew Kubala[4], Kristine Ensrud[5], Carolyn Crandall[6], Daniel Buysse[7], and Katie Stone[8], *1. University of Pittsburgh, Pittsburgh, Pennsylvania, United States, 2. California Pacific Medical Center, Research Institute, San Francisco, California, United States, 3. California Pacific Medical Center Research Institute, San Francisco, California, United States, 4. Sleep, Tactical Efficiency, and Endurance Lab (STEEL) Research Group, San Diego, California, United States, 5. University of Minnesota Medical School and Minneapolis VA Health Care System, Minneapolis, Minnesota, United States, 6. University of California, Los Angeles, Los Angeles, California, United States, 7. UPMC, Pittsburgh, Pennsylvania, United States, 8. University of California, San Francisco, Castro Valley, California, United States*

Prospective studies have reported associations between individual dimensions of sleep and subsequent falls. Considering sleep as a multidimensional construct may provide a more comprehensive assessment. Using data from the MrOS Sleep study, we calculated a multidimensional index of sleep, operationalized as the number of disturbances in 5 dimensions of sleep (self-reported “poor”: satisfaction, duration, daytime sleepiness, latency and sleep midpoint) and categorized as: 0, 1-2, >=3. A total of 3111 men, mean age 76.4 years, were studied. Logistic regression generalized estimating equations were used to model repeated measures of annually self-reported recurrent falls (≥2 falls) over 4 years of follow-up. Compared to men with no poor sleep dimensions, the Odds Ratio (OR) of recurrent falls in men with scores of 1 or 2 was 1.13 (95% Confidence Intervals, 0.96, 1.34), and in men with scores >3, the OR was 1.46(1.15, 1.84). Worse multidimensional sleep health was associated with recurrent falls.

### FOCUS ON PRESENT MOMENT FOR YOUR SLEEP: MINDFULNESS PROMOTES MID-LIFE ADULTS' SLEEP HEALTH

Claire Smith, Christina Mu, Angelina Venetto, Arooj Khan, and Soomi Lee, *University of South Florida, Tampa, Florida, United States*

Sleep health during midlife sets the stage for health over the lifespan. Mindfulness, or present-moment awareness and attention, is shown to benefit sleep, yet mechanisms explaining these benefits are missing. Applying self-regulation theory, we test affective and cognitivemechanisms linking mindfulness to quantitative and qualitative sleep health. Across two independent samples of nurses (N1=60; N2=84), ecological momentary assessment (measuring mindfulness, sleep quality and sufficiency, positive and negative affect, and rumination) and actigraphy (sleep duration and wake-after-sleep-onset [WASO]) were collected over fourteen days. Multilevel mediation revealed that, between people, mindfulness generally associated with better sleep quality and sufficiency, mediated by lower rumination and negative affect. Within people, daily mindfulness associated with better sleep sufficiency, mediated by higher positive affect. Our findings position mindfulness as an emotional and cognitive self-regulation strategy that promotes midlife adults’ qualitative – but not quantitative -- sleep health. Results may inform health interventions during this pivotal life stage.

### CON/DISCORDANCE BETWEEN SUBJECTIVE AND OBJECTIVE SLEEP PARAMETERS AND THEIR ASSOCIATION WITH COGNITIVE FUNCTION

Kristin Calfee, and Soomi Lee, *University of South Florida, Tampa, Florida, United States*

Studies report that subjective and objective sleep parameters often do not agree with each other. This study examined if discordance between subjective and objective sleep measures are associated with cognition. Participants from the Midlife in the United States study (n=627) provided subjective (self-report) and objective (actigraphy) sleep duration and sleep quality. Discordance was assessed by absolute difference between subjective and objective z-scores in each sleep variable. Cognitive function was measured by the Brief Test of Adult Cognition by Telephone (BTACT). The correlations between subjective and objective sleep measures were weak-to-moderate (r=.43, .18 for duration and quality, respectively). Discordance scores in sleep duration and quality were each significantly associated with BTACT, such that greater discrepancy was associated with poorer cognitive function. These associations remained persistent after adjusting for sociodemographic differences. Results suggest that discordance between subjective and objective sleep parameters may relate to cognitive abilities needed to accurately monitor sleep characteristics.

### A LONGITUDINAL INCREASE IN SLEEP HEALTH PROBLEMS IN MIDDLE ADULTHOOD PREDICTS EARLY MORTALITY

Soomi Lee[1], Christina Mu[1], Meredith Wallace[2], Ross Andel[1], David Almeida[3], Orfeu Buxton[3], and Sanjay Patel[4], *1. University of South Florida, Tampa, Florida, United States, 2. University of Pittsburgh, Pittsburg, Pennsylvania, United States, 3. The Pennsylvania State University, University Park, Pennsylvania, United States, 4. University of Pittsburgh, Pittsburgh, Pennsylvania, United States*

Negative consequences of sleep health problems are common in middle-age but poorly understood. This study investigated multidimensional sleep health in middle adulthood and mortality risk. Participants from the Midlife in the United States Study reported sleep characteristics in 2004-2006 (T1; n=9,640, Mage=52.72) and again in 2013-2016 (T2; n=4,334). Deaths since each survey were logged. Multidimensional sleep health composite captured Regularity, Satisfaction, Alertness, Efficiency, and Duration. Cox regression adjusted for sociodemographics and known risk factors (BMI, smoking, depression/anxiety, diabetes, and hypertension) indicated that each unit higher sleep health problems at T1 was associated with 245% and 324% increase in hazard rates for all-cause (Hazard Ratio; HR=3.45, p<.001) and heart disease (HR=4.24, p<.001) mortality, respectively. Those with an increase in sleep health problems at T2 compared to T1 had a 182% increase in all-cause mortality risk (HR=2.82, p<.05), but not heart disease mortality risk. Improving sleep health may reduce early mortality risk.

### SESSION 4090 (PAPER)

### OLDER ADULT PROTECTION AND ABUSE

### A SCOPING REVIEW OF OUTCOMES IN ELDER ABUSE INTERVENTION RESEARCH: THE CURRENT LANDSCAPE AND WHERE TO GO NEXT

David Burnes[1], Andie MacNeil[1], Aliya Nowaczynski[1], Christine Sheppard[2], Erica Nekolaichuk[3], Mark Lachs[4], and Karl Pillemer[5], *1. University of Toronto, Toronto, Ontario, Canada, 2. Wellesley Institute, Toronto, Ontario, Canada, 3. University of Toronto Libraries, Toronto, Ontario, Canada, 4. Weill Cornell Medicine, New York, New York, United States, 5. Cornell University, Ithaca, New York, United States*

Researchers, practitioners, and policy-makers worldwide recognize elder abuse (EA) as a major threat to the health and well-being of older adults, but rigorous intervention research has greatly lagged behind this interest. A major weakness is the lack of cohesive understanding of appropriate program outcomes to be measured. To address this knowledge gap, we conducted a scoping review of the EA intervention research literature to understand the range of outcomes considered to date and to provide guidance for future research. We searched Ovid MEDLINE, Ovid Embase, Ovid PsycInfo, Ovid Social Work Abstracts, Ebsco AgeLine, Ebsco CINAHL, Wiley Cochrane Central, and Proquest Sociological Abstracts for studies evaluating community-based EA response programs. Two independent reviewers completed record search, screening, and data extraction procedures. We identified 52 eligible studies (1986-2019) that employed a total of 184 outcomes (range: 1–16, mean = 3.5). This study revealed that a large range of outcomes has been employed in EA intervention studies to date, mostly attached to victims or the intervention process itself, with inconsistent operational definitions and measurement procedures. Several key recommendations for future EA intervention research are: 1) implementing intervention outcomes that reflect multiple levels of eco-systemic influence, 2) heightening the analysis of intervention process outcomes beyond description toward identifying factors that mediate or moderate successful case outcomes, 3) conducting qualitative research with EA victims and other relevant stakeholders to understand meaningful intervention outcomes from their perspectives, and 4) establishing common EA outcome measures for implementation across studies to facilitate greater data pooling and synthesis.

### FINANCIAL FRAUD EXPERIENCES AT AGE 85+: AN EXPLORATORY STUDY WITH THE MIT AGELAB 85+ LIFESTYLE LEADERS PANEL

Taylor Patskanick[1], Sophia Ashebir[2], Julie Miller[2], Lisa D'Ambrosio[2], and Joseph Coughlin[2], *1. Massachusetts Institute of Technology, Somerville, Massachusetts, United States, 2. Massachusetts Institute of Technology, Cambridge, Massachusetts, United States*

Financial exploitation of older adults has emerged as one of the most common and costly forms of elder abuse. While prior research has examined the risk and protective factors that contribute to older adults' experiences of being defrauded, less has specifically explored the prevalence of these factors among the 85+ age demographic. This not only includes hearing from the 85+ directly about the factors that have contributed to successful financial exploitation, but also the factors that contributed to their experiences of unsuccessful and 'near miss' exploitation. This paper shares findings from a mixed methods study with the MIT AgeLab 85+ Lifestyle Leaders, a U.S.-based panel of octogenarians and nonagenarians. Leveraging an online survey (n=32) and virtual focus groups (n=18) conducted in September 2021, findings distinguish among three categories of experiences with financial fraud – those had been, almost were, or have never been a victim of financial fraud within the past five years.

In particular, 'almost victims' frequently scored in between 'have been' and 'have never been' victims with regard to several questionnaire variables. Six perceived risk factors for financial fraud victimization emerged from both data sources, including being older, cognitive decline, misplaced trust and a lack of knowledge, loneliness, technology use, and stereotypes of older adults. The majority of Lifestyle Leaders identified protective factors including, knowledge about protecting oneself from financial scams, a lifetime of experience and one's social network. These findings will be discussed in further detail along with their implications for multidisciplinary professionals working with older adults.

### FINANCIAL SCAMS TARGETING OLDER ADULTS DURING COVID-19

Scott Beach[1], Sara Czaja[2], Richard Schulz[3], David Loewenstein[4], and Peter Lichtenberg[5], *1. University Of Pittsburgh, Pittsburgh, Pennsylvania, United States, 2. Weill Cornell Medicine, New York City, New York, United States, 3. University of Pittsburgh, Pittsburgh, Pennsylvania, United States, 4. University of Miami, Miami, Florida, United States, 5. Wayne State University, Detroit, Michigan, United States*

The COVID pandemic afforded financial scammers with new opportunities to target older adults. This paper presents data from a telephone survey conducted June–September, 2020 with 380 participants from a larger National Institute of Aging study examining financial exploitation among older adults. The survey assessed COVID-related scams in three areas: (1) products, testing, treatments; (2) financial assistance (e.g., stimulus checks); and (3) charities. Questions focused on scam exposure / attempts, mode of contact, responses, and whether the older adult reported it to someone. The sample (284 Pittsburgh; 96 New York City) was 64% female; mean age = 73.6; and 47% White, 41% African American, 12% Hispanic. Across all scam types, 18.4% reported scam attempts / exposure; 24% of those exposed engaged / responded without getting scammed (11%); or were actually scammed (13%); and 40% told someone about it. The most frequent modes of contact were: telephone (54%), internet / email (40%), or mail (29%). Controlling for sociodemographics, participants from NYC were more likely to be exposed (OR = 1.91; p=.036); as were those reporting more loneliness (8-item UCLA scale; OR = 1.06; p=.042); and those reporting that COVID had worsened their emotional well-being to a greater extent (OR = 1.57; p=.032). Older adults who were more socially isolated / lonely were also more likely to have been scammed and less like to tell anyone about it. Psychosocial factors play an important role in exposure and response to scams during pandemics. Implications for policy, intervention, and general scam susceptibility are discussed.

### RESIDENT-TO-RESIDENT AGGRESSION: PREVALENCE AND RISK FACTORS

Elsie Yan[1], Daniel WL Lai[2], Vivian Wei Qun Lou[3], Habib Chaudhury[4], Karl Pillemer[5], and Mark Lachs[6], *1. The Hong Kong Polytechnic University, Hong Kong, Hong Kong, 2. Hong Kong Baptist University, Hong Kong, Hong Kong, 3. The University of Hong Kong, Hong Kong, Hong Kong, 4. Simon Fraser University, Vancouver, British Columbia, Canada, 5. Cornell University, Ithaca, New York, United States, 6. Weill Cornell Medicine, New York, New York, United States*

This study examines the rates and risk factors of resident-to-resident aggression in long term care facilities in Hong Kong. A total of 800 personal care worker participated. Participants averaged 42.03 years of age (SD=7.63), were mostly female (92.7%), married (79.1%) and reported an average of 6.28 years of experience in long term care. 96.9 percent of the participants provided care to residents with dementia but 58.9% considered the training they received insufficient. Resident-to-resident aggression was common: All participants reported having witnessed verbal aggression (100%), 18% disruptive behaviors, 11.8% physical violence, and 3.1% sexual aggression.Resident-to-staff aggression was commonly reported with verbal aggression being the most common (97.6%) following by other disruptive behaviors (13.7%), physical violence (10.7%), and sexual aggression (8.5%). Logistic regression analysis indicated that disruptive behaviors and physical violence were associated with perpetrator male gender, dementia, and neuropsychiatry symptoms, as well as staff prior and current experience of taking care of persons with dementia, not having received training in dementia care, and perceived insufficiency of training. Sexual aggression was associated with perpetrator male gender and staff female gender. There is an urgent need to provide supportive services to prevent and intervene resident-to-resident aggression in long term care facilities. Improving management of behavioral and psychological symptoms of dementia through sufficient staff training and adequate staffing ration may be helpful in this aspect.

### USE OF MOTIVATIONAL INTERVIEWING IN THE CONTEXT OF ELDER ABUSE INTERVENTION: THE RISE PROJECT

Andie MacNeil[1], David Burnes[1], Marie-Therese Connolly[2], Erin Salvo[3], Patricia Kimball[4], Geoff Rogers[5], and Stuart Lewis[6], *1. University of Toronto, Toronto, Ontario, Canada, 2. University of Southern California, Los Angeles, California, United States, 3. Department of Health and Human Services, Augusta, Maine, United States, 4. Elder Abuse Institute of Maine, Brunswick, Maine, United States, 5. Silberman School for Social Work of Hunter College, New York, New York, United States, 6. Geisel School of Medicine, Hanover, New Hampshire, United States*

Despite the increasing number of elder abuse (EA) cases, many EA victims are reluctant to engage with formal support services, such as Adult Protective Services (APS). For EA interventions to be effective, it is important to overcome this client reluctance. This study examined the use of motivational interviewing (MI) by elder advocates, as a component of a larger EA intervention model, RISE (Repair Harm, Inspire Change, Support Connection, Empower Choice), developed in partnership with Maine APS and the Elder Abuse Institute of Maine. The advocate role was developed, in part, to increase service acceptance/utilization among EA victims. Advocates are trained in MI, a collaborative, client-centered approach designed to help individuals explore and resolve ambivalence around making a change. This study conducted qualitative interviews and a focus group interview with all advocates (n = 4) working within the RISE model to understand how MI is applied in the context of an EA intervention. Three domains were identified: (1) therapeutic relationship, which describes the importance of foundational relationship building to support EA victims; (2) techniques, which refers to the MI strategies that advocates apply and adapt in the context of EA intervention; and (3) implementation challenges, which discusses the difficulties that advocates encounter when using MI with victims of EA. Overall, the experiences of advocates suggest MI is a beneficial and amenable approach to help EA victims navigate feelings of ambivalence and explore their motivation for change. This study represents the first in-depth exploration of MI in the context of EA intervention.

### SESSION 4100 (PAPER)

### OUTPATIENT CARE AND ER VISITS

### CHARACTERIZING AVOIDABILITY OF NURSING HOME RESIDENTS: COMPARING THE CLAIMS-BASED ALGORITHM AND NURSE ASSESSMENT

Justin Blackburn[1], Jennifer Carnahan[2], Susan Hickman[3], Greg Sachs[4], and Kathleen Unroe[2], *1. IU Richard M. Fairbanks School of Public Health at IUPUI, Indianapolis, Indiana, United States, 2. Indiana University Center for Aging Research, Regenstrief Institute, Inc., Indianapolis, Indiana, United States, 3. Indiana University School of Nursing, Indianapolis, Indiana, United States, 4. Indiana University (IU) Center for Aging Research, Regenstrief Institute, Inc., Indianapolis, Indiana, United States*

The elevated risks associated with transferring nursing home residents to the hospital are problematic, but identifying which transfers can be avoided is complex. Using billing claims to determine "avoidability" based on hospital discharge diagnostic codes ignores resource constraints, clinical comorbidities, and asymmetrical information between nursing home staff making the transfer decision at the onset of clinical changes and hospital billing departments following treatment and diagnostic procedures. Conversely, relying on clinical staff assessments at the time of transfer may be an impractical and resource-intensive strategy to drive payment reform and improve quality. Using Medicare claims data representing emergency department and hospitalization transfers from 38 nursing facilities in Indiana from 2016-2020, we compared classification of transfers using a claims-based algorithm and trained nurse assessments of avoidability. Among 960 transfers, nurses judged 48.4% were potentially avoidable while 30.8% were classified as such using claims data. Of concordant assessments, 15.3% were avoidable and 36.0% as not avoidable. Of discordant assessments, 33.1% were judged avoidable by nurses only and 15.5% via the claims-based algorithm (Kappa=0.0153). Discordance was most frequent among transfers with heart failure (64%, n=42), psychosis (74.5%, n=34), acute renal disease (50%, n=28); and lowest among urinary tract infections (31.3%, n=64). No resident demographic or clinical characteristics were associated with discordance (age, race, sex, cognitive function scale, activities of daily living, or CHESS scale). High discordance in determining avoidability may be driven by presentation of symptoms or other condition-specific factors. Policies to reduce avoidable hospitalizations must not rely on overly simplistic approaches for identification.

### HEALTH RISKS AMONG OLDER ADULTS ACCORDING TO PRIMARY LANGUAGE: EVIDENCE FROM MEDICARE ANNUAL WELLNESS VISITS

Amjed Alsulaily[1], Kristen Berg[2], Lorella Luezas-Shamakian[2], Douglas Einstadter[2], and Adam Perzynski[3], *1. Case Western Reserve University, Qatif, Ash Sharqiyah, Saudi Arabia, 2. The MetroHealth System, Cleveland, Ohio, United States, 3. MetroHealth and Case Western Reserve University, Cleveland, Ohio, United States*

Emerging evidence suggests that older adults benefit from the Medicare Annual Wellness Visit (AWV). Lind and colleagues found that between 2011 and 2016, utilization of the AWV increased from 8.1% to 23.0%. However, compared with non-Hispanic whites, AWV utilization is 11.6% points lower for Hispanic/Latinos and 10.2% lower for non-Hispanic Blacks. AWV differences by primary language have not been previously reported. We examined the rate of AWV utilization for older adults with English vs. Limited English Proficiency (LEP) among patients seen at a large urban health system in northeastern Ohio. Using Bonferroni-corrected chi-square and t-tests, we also evaluated the association between LEP and health risks (e.g., depression, falls, activities of daily living, cognitive status) assessed during the AWV. Using the electronic health record, we identified 41,262 Medicare patients aged 65+ who were eligible for the AWV. Of those identified, 42.8% completed an AWV between 2019 to 2021. Persons who were white (41.7%), Hispanic (37%), male (39.9%), Spanish-speaking (37.2%) or other LEP (41.2%) had lower utilization of AWVs (p<.001). Pain and fatigue ratings, depressive symptoms, and oral health problems and were similar between language groups, while cognitive impairment (p<.001), functional independence (p<.001), and self-rated health (p<.001) were substantially worse among non-English speakers. LEP is associated with a lower rate of AWV utilization and worse self-rated health. A clearer understanding of how speaking a different language from that in which the AWV is conducted is important for clarifying discrepancies and disparities in minority population health.

### IMPACT OF NON-NATIVE ENGLISH SPEAKING ON MEDICATION PROBLEMS AND ER VISITS AMONG COMMUNITY-DWELLING OLDER ADULTS

YUJUN ZHU[1], and Susan Enguidanos[2], *1. University of Southern California, SOUTH PASADENA, California, United States, 2. University of Southern California, Los Angeles, California, United States*

Older adults have higher risk of experiencing medication related problems (MRPs), leading to increased morbidity, healthcare utilization, and mortality. Few studies have examined the relationship between non-native English speaking (NNES) and MRPs and their association with health service use. This study aimed to explore language disparities in MRPs and their relationship to emergency room (ER) visits among community-dwelling older adults. We used secondary enrollment data from a community medication program for older adults (N=180). We conducted linear regression to examine the relationship between NNES and MRPs, and logistic regression to explore the association between MRPs and ER visits. Generalized structural equation modeling (GSEM) with bootstrapping was used to test the indirect effect between NNES through MRPs to ER visits. The sample was diverse with 40% White, 30% Hispanic, 22% African American, and 8% of other races; 18% were non-native English speakers. Analysis showed NNES was associated with having 2.7 more MRPs than native English-speaking participants, while race was not significant, after controlling for covariates. Each additional MRP was associated with a 9% increased probability of having an ER visit. The GSEM results found a significant indirect effect between NNES through MRPs to ER visits (β, 0.27; 95% CI, 0.07 to 0.61). Although NNES was not directly related to ER visits, it may have inhibited the ability of Hispanics to read important medication instructions, contributing to their elevated risk of experiencing MRPs, thus indirectly increasing potential risks leading to ER visits. Further research is needed to better understand this relationship.

### TRENDS IN FALL-RELATED EMERGENCY DEPARTMENT VISITS INVOLVING ALCOHOL AMONG OLDER ADULTS: 2011–2020

Yara Haddad[1], Gwen Bergen[1], Iju Shakya[2], Keming Yuan[1], Royal Law[1], Briana Moreland[3], and Tadesse Haileyesus[1], *1. Centers for Disease Control and Prevention, Atlanta, Georgia, United States, 2. Oak Ridge Institute for Science and Education, Oak Ridge, Tennessee, United States, 3. Cherokee Nation Operational Solutions, Atlanta, Georgia, United States*

Falls disproportionately affect older adults (≥65) resulting in three million emergency department (ED) visits annually. The relationship between alcohol and falls is unclear, but side effects of alcohol consumption include gait and balance impairments, which are risk factors for falls. This study examined trends of older adult fall-related ED visits involving alcohol from 2011-2020. Alcohol data are not routinely collected during ED visits. Alcohol involvement was identified from National Electronic Injury Surveillance System-All Injury Program free-text narratives using natural language processing and text analytic techniques. Joinpoint regression was used and cross-validated with SAS to determine significant older adult fall changes from 2011-2020 by sex and to compare older adult trends to adults < 65 (20-34, 35-54, 55-64). From 2011-2020, 2.2% of all ED visits for older adult falls involved alcohol. Alcohol-involved fall ED visit rates for older adults increased annually by 7.2% (95%CI:6.1%-8.4%). These older adult ED visit rates increased by 6.8% (95%CI:5.4%-8.2%) for males and 7.4% (95%CI:6.0%-8.9%) for females annually. For adults aged 20-34 and 35-54, alcohol-involved fall ED visit rates increased annually by 1.3% (95%CI:0.1%-2.6%) and 3.8% (95%CI:2.7%-5.0%), respectively, until 2017 and then decreased by 4.4% (95%CI:1.2%-7.5%) and 4.0% (95%CI:0.3%-7.5%). For adults aged 55-64, ED visit rates increased annually by 8.5% (95%CI:6.8%-10.2%) until 2018 and then stabilized. Documenting alcohol use when treating falls is important for monitoring trends. Assessing alcohol use in conjunction with assessments for other fall risk factors can inform healthcare providers on effective strategies to recommend to reduce fall risk.

### SESSION 4110 (SYMPOSIUM)

### PANDEMIC VULNERABILITY AND RESILIENCE: QUANTITATIVE AND QUALITATIVE FINDINGS FROM THE COVID-19 COPING STUDY

Chair: Jessica Finlay Co-Chair: Lindsay Kobayashi

The COVID-19 Coping Study aims to investigate social, behavioral, health, and economic impacts of the pandemic on the health and wellbeing of aging Americans. A total of 6,938 adults aged ≥55 were recruited from all 50 US states, the District of Columbia, and Puerto Rico from April-May, 2020, using a multi-frame online recruitment strategy. Participants completed a 20-minute baseline survey and a subset were recruited to complete monthly follow-up surveys for one year. A random subsample of 57 participants completed semi-structured interviews in May-July, 2021. This symposium will share novel mixed-methods insights into the ongoing experiences, perspectives, significant life events, and mental health changes among aging US adults during the COVID-19 pandemic. First, Kobayashi will describe the dynamic, longitudinal relationships between loneliness, anxiety, and COVID-19 worry with cognitive function and abilities. Second, Abrams investigates long-term employment outcomes and mental health trajectories among a particularly vulnerable group of older adults: those who experienced work disruptions and job loss early in the pandemic. Third, Meltzer will share how particular groups of older adults were more likely to report greater closures of key places to socialize in their neighborhoods since the pandemic onset. Fourth, Finlay will present a qualitative thematic analysis of ongoing pandemic sources of risk and vulnerability among marginalized and underserved aging adults. The symposium highlights diverse strengths and resiliencies to cope with adversities of the pandemic. Findings may inform individual-strategies and community-level policies to support aging adults during times of collective stress and trauma.

### ACUTE RELATIONSHIPS BETWEEN MENTAL HEALTH AND COGNITIVE FUNCTION AMONG AGING ADULTS DURING THE COVID-19 PANDEMIC

Lindsay Kobayashi[1], Brendan O'Shea[1], Carly Joseph[2], and Jessica Finlay[1], *1. University of Michigan, Ann Arbor, Michigan, United States, 2. Central Michigan University, Ann Arbor, Michigan, United States*

The acute impacts of COVID-19-related mental health concerns on cognitive function among aging adults are unknown. We investigated whether between-person (BP) differences and within-person (WP) changes in loneliness, anxiety, and COVID-19 worry were related to cognitive function and abilities in the COVID-19 Coping Study over nine months from August/September 2020 through April/May 2021 (n=2,262). Marginal structural models accounting for depression as a time-varying confounder and incorporating attrition and sampling weights estimated the BP and WP relationships of loneliness, anxiety, and COVID-19 worry with PROMIS cognitive function and abilities scores over time. In any given month, experiencing a loneliness or anxiety score higher than the sample mean (BP difference) as well higher than one's personal mean across the nine-month period (WP change) were negatively associated with cognitive function and abilities in that month. Future research should explore long-term impacts of loneliness and anxiety experienced during the pandemic for population cognitive aging.

### OLDER ADULTS' WORK DISRUPTIONS IN APRIL/MAY 2020: IMPLICATIONS FOR WORK STATUS AND MENTAL HEALTH OVER 6 MONTHS

Leah Abrams[1], Jessica Finlay[2], and Lindsay Kobayashi[2], *1. Tufts University, Department of Community Health, Medford, Massachusetts, United States, 2. University of Michigan, Ann Arbor, Michigan, United States*

Using the COVID-19 Coping Study, we sought to determine how work disruptions for older adults in April/May 2020 related to labor force status in September/October 2020 and mental health throughout those six months (N=2,367). One-third of respondents who lost their job in April/May were unemployed at the end of follow-up, while 15% were unemployed after furloughs and 9% after reduced hours/income. One-quarter of those furloughed in April/May were out of the labor force at follow-up – evidence of a potential pathway from furloughs into retirement. Being employed at follow-up was most common after work-from-home in April/May (90%). Multi-level models revealed differences in mental health trajectories over six months according to baseline work disruptions, including persistently high anxiety following job loss and delayed upticks in anxiety and depressive symptoms when working from home. This research provides insights into longer-term economic and mental health ramifications of pandemic-related work disruptions among older workers.

### SOCIAL PLACES: SHIFTING NEIGHBORHOOD PERCEPTIONS AMONG AGING AMERICANS DURING COVID-19

Gabriella Meltzer[1], Brendan O'Shea[2], Michael Esposito[3], Lindsay Kobayashi[2], Ashly Westrick[2], and Jessica Finlay[2], *1. Columbia University, New York, New York, United States, 2. University of Michigan, Ann Arbor, Michigan, United States, 3. Washington University in St. Louis, St. Louis, Missouri, United States*

This study examines individual and community factors related to older adults' perceived losses in places to socialize with people of similar and different ages in their neighborhoods during COVID-19. In the 11-month wave of the COVID-19 Coping Study from March-April 2021, responses to perceived availability were "Less," "About the Same," or "More." Most respondents reported less availability in places to socialize with those of similar (68.0%) or different (68.4%) ages. Ordinal logistic regressions showed respondents who lived alone perceived less availability in places to socialize with those of similar or different ages than those living with others (ORs 0.67, 95% CI 0.47, 0.97). Those living in metropolitan compared to non-metropolitan areas also perceived less availability in places to socialize with those of similar ages (OR 0.62, 95% CI 0.39, 0.98). These findings enhance our understanding of COVID-19-related losses in community resources that facilitate healthy aging in place.

### STRUGGLES AND STRENGTH: A QUALITATIVE STUDY OF DIVERSE VOICES FROM THE COVID-19 COPING STUDY

Jessica Finlay, Marisa Eastman, and Lindsay Kobayashi, *University of Michigan, Ann Arbor, Michigan, United States*

This paper aims to explore complex realities and nuanced lived experiences in how diverse older adults are making sense of and dealing with the COVID-19 pandemic. We conducted semi-structured video and phone interviews with 57 COVID-19 Coping Study participants (average age 70.7 years, 44% female, 49% white) from May-July 2021. Qualitative thematic analysis identified physical, mental, social, and economic struggles. These included heightened COVID-19 risk given comorbidities, difficulties accessing healthcare, distressing political events, diminished sense of safety, distance to family and support networks, inability to collectively mourn, and exacerbated financial instability. Coping strategies included exercise, hobbies, spirituality, online activities, engaging with family and friends, and self-care practices. Community-level sources of resilience included vaccines, telemedicine, stimulus checks, and community groups/services. These results highlight profound resiliency and strength to cope with adversities of the pandemic and may inform strategies to support underrepresented and underserved older adults during and after the pandemic.

### SESSION 4120 (PAPER)

### PHYSICAL FUNCTION AMONG OLDER ADULTS AND CAREGIVERSARTHRITIS AMONG CAREGIVERS—BRFSS 2017, 2019

Eva Jackson[1], Michael Boring[2], Lisa McGuire[2], John Omura[2], Benjamin Olivari[2], Erica Odom[2], and Janet Croft[2], *1. Alzheimer's Association, Chicago, Illinois, United States, 2. CDC, Atlanta, Georgia, United States*

Unpaid caregivers are people who provide support to a family member or friend with a health condition or disability. While there are benefits to caregiving, it can negatively affect caregivers' physical and mental health. Arthritis and caregiving affect 58.5 million and 53 million U.S. adults, respectively, and these numbers are expected to increase with increasing numbers of older adults. Little is known about the experience of caregivers with arthritis. Data from the 2017 and 2019 BRFSS were combined for all states that administered the optional Caregiving and the core Arthritis modules (17 states; n=91,224). Analyses accounted for the complex sampling design using SUDAAN (version 11.0; RTI International). Statistical significance was determined at $\alpha$=0.05.Overall, 20.6% of respondents were caregivers, among whom 35.1% had arthritis compared with 24.5% of non-caregivers ($p < 0.001$). Arthritis among caregivers was higher in all 17 states compared to non-caregivers. Among adults with arthritis, caregivers were more likely to be younger, female, White, and more physically active than non-caregivers. Caregivers were also more likely to experience arthritis-related activity and work limitations than non-caregivers. Arthritis-related limitation outcomes varied at the individual state levels.Among caregivers, those with arthritis provided more care in weekly hours and duration (years) and were more likely to report difficulty walking or climbing stairs, dressing or bathing, and doing errands alone than caregivers without arthritis. Given the disparities between caregivers with and without arthritis, it is crucial for public health to collect data on the experience of caregivers with arthritis.

### ASSOCIATIONS OF GAIT CHARACTERISTICS AND VARIABILITY WITH WALKING EFFICIENCY IN OLDER ADULTS

Fangyu Liu[1], Yurun Cai[2], Laura Skow[1], Amal Wanigatunga[1], Qu Tian[3], Eleanor Simonsick[4], and Jennifer Schrack[1], *1. Johns Hopkins Bloomberg School of Public Health, Baltimore, Maryland, United States, 2. University of Pittsburgh, Pittsburgh, Pennsylvania, United States, 3. National Institutes on Aging, Baltimore, Maryland, United States, 4. National Institute on Aging, Baltimore, Maryland, United States*

Higher energetic cost of walking per unit distance has been linked to many adverse health outcomes in older adults. Aging-related changes in gait characteristics have been postulated to contribute to energetic inefficiency, but previous studies focused on younger adults, and/or examined only a few gait characteristics. In the Baltimore Longitudinal Study of Aging, 507 older adults ≥50 years (72.3±9.8 years, 48.3% women, 48.3% black) without stroke and Parkinson disease had concurrent measurements of usual-paced gait characteristics using 3D motion analysis and energetic cost of walking using indirect calorimetry during a 2.5-min usual-paced overground walk. We tested the associations of the mean and the coefficient of variance (CV) of cadence (steps/min), swing time (ms), double support time (ms), stance time (ms), step time (ms), step length (cm), and step width (cm) with energetic cost of walking using linear regression models, adjusting for demographics, body composition, comorbidities, and gait speed. We found that a 5-cm shorter step length was associated with 0.40 ml/kg/100m higher cost of walking ($p < 0.001$). A 1% higher CV in swing time and 1% lower CV in step width was associated with 0.152 ml/kg/100m higher ($p=0.044$) and 0.023 ml/kg/100m lower ($p=0.022$) cost of walking, respectively. Our results suggest that mean step length and variability in swing time and step width could potentially contribute to the rising cost of walking in older adults. Future longitudinal studies are needed to understand whether changes in gait variability can predict increased energetic cost of walking and can be intervened to preserve energy efficiency.

### STAIR CLIMB TIME AND FUNCTIONAL POWER ASSOCIATIONS TO MUSCLE FUNCTION AND PHYSICAL PERFORMANCE

Elsa Strotmeyer[1], Li-Yung Lui[2], Nancy W. Glynn[1], Adam Santanasto[3], Stephen Kritchevsky[4], Peggy Cawthon[5], Anne Newman[6], and Jane Cauley[6], *1. School of Public Health, University of Pittsburgh, Pittsburgh, Pennsylvania, United States, 2. University of California at San Francisco, San Francisco, California, United States, 3. School of Public Health, University of Pittsburgh, Oakmont, Pennsylvania, United States, 4. Wake Forest School of Medicine, Winston-Salem, North Carolina, United States, 5. California Pacific Medical Center Research Institute, San Francisco, California, United States, 6. University of Pittsburgh, Pittsburgh, Pennsylvania, United States*

Repeated stair climbing assesses sustained performance and neuromuscular components of movement, including functional muscle power (force*velocity). However, repeated stair climb associations to standard muscle and physical function measures are not established in older adults. We hypothesized that stair climb time (sec), and ascend power (peak and average; Watts=W) over 3 stair climb laps were associated with standard muscle function, physical function and risk factors in the Study of Muscle, Mobility and Aging (SOMMA; preliminary baseline N=455; 76.9+/-5.3 years; 58.0% women; 85.7% White). Adjusting for age, sex, race, and BMI using multivariate linear regression, stair climb time, peak power and average power were significantly associated with all standard muscle (Keiser leg press 1-RM strength and power; grip strength) and physical function (400m walk speed, SPPB and components) measures. Women had worse stair climb performance vs. men (all $p < 0.01$) including: longer total time (29.6+/-8.1 vs. 27.4+/-6.7 sec), lower peak power (121.9+/-34.5 vs. 161.4+/-39.0 W), and lower average power (94.9+/-24.3 vs. 124.1+/-28.4 W). Adjusting for age, sex, race, BMI, CHAMPS total physical activity/week, CES-D depressive symptoms, and comorbidity count using multivariate linear regression, older age was related to slower stair climb time and lower peak/average power. Other known risk factors were also associated with worse stair climb performance: non-White race (average power only), lower physical activity (peak/average power only), BMI, depressive symptoms, and higher comorbidity (time only). Repeated stair climb time and power may capture unique aspects of functional decline with aging and are associated with standard muscle and physical function measures.

### TILT TEST TOLERANCE AFTER TWO-WEEKS BED REST IN OLDER ADULTS IS NOT PROTECTED BY DAILY AEROBIC AND RESISTANCE EXERCISE

Andrew Robertson[1], Richard Hughson[2], Eric Hedge[2], Carmelo Mastrandrea[2], and Federico Granados Unger[2], *1. University of Waterloo, Waterloo, Ontario, Canada, 2. Schlegel-UW Research Institute for Aging, Waterloo, Ontario, Canada*

Bed rest is associated with increased risk of orthostatic intolerance on return to upright posture and, at least in young adults, regular exercise during bed rest is proposed as protective. 22 older adults (55-65 years, half women) participated in a randomized trial with 14-days of continuous 6-degree head-down bed rest. Half completed 60 min/day of aerobic and high-intensity interval cycling plus resistance exercises (Ex) while the other half were control (Con). A passive 80-degree head-up tilt (HUT) to a maximum of 15-min assessed orthostatic tolerance while cardio- and cerebrovascular responses were monitored to a pre-syncopal level of systolic blood pressure < 90 mmHg. In pre-bed rest baseline, all but 3 women (Ex) completed 15-min HUT. After 14-days bed rest, only 3 men (1 Ex) and 1 woman (Ex) completed 15-min with no differences in tolerance between men and women or Ex and Con. Vasovagal syncope presented in 5 participants (2 M-Con, 1 M-Ex, 1 F-Con, 1 F-Ex), most other non-finishers had progressive reduction in stroke volume without adequate vasoconstriction. Reductions in cerebral blood velocity measured during HUT by Doppler ultrasound revealed strong linear relationships to both end-tidal PCO2 and mean arterial pressure across all participants. These data indicate that neither older men nor older women were protected from orthostatic intolerance by 60-min per day exercise including high-intensity interval training. These data contrast with previous findings in younger adults in controlled trials of bed rest pointing to the need for greater understanding of mechanisms regulating blood pressure and brain blood flow.

### SESSION 4130 (SYMPOSIUM)

### PRESIDENTIAL SYMPOSIUM: RACIAL AND ETHNIC HEALTH DISPARITIES ACROSS THE LONG-TERM SERVICES AND SUPPORTS CONTINUUM: NEED FOR POLICY AND ACTION

Chair: Debra Dobbs Discussant: Larry Polivka

The pandemic further exacerbated racial and ethnic health disparities with BIPOC (Black, Indigenous and People of Color) communities experiencing significantly higher infection rates and higher hospitalization rates compared to White populations. Underlying medical conditions that disproportionately affect BIPOC populations (e.g., cardiovascular disease, diabetes, COPD) and social determinants of health (income, community and environmental factors, access to health care and job security) can account in large part for increased rates of morbidity and mortality. Within the long term services and supports (LTSS) continuum, health disparities exist in both who receives and provides care. The four papers in this Social Research Policy and Practice Presidential Symposium will highlight factors that result in health disparities across the LTSS continuum and propose possible policy and practice solutions to address the disparities. The first paper presented will be an overview of existing nursing home workforce issues that continue to impact disparities in the quality of resident care. The second paper presented examines nursing home resident quality of life disparities from a measurement perspective. The third paper will highlight issues on elder abuse and mistreatment of rural elders, American Indian and Alaskan Natives. The fourth paper will present recommendations from key stakeholders of the RAISE Family Caregiving Advisory Council on how to meet the needs of underrepresented groups (AAPI, BIPOC, Indigenous peoples) of family caregivers. Policy and practice solutions to reduce disparities will be discussed.

### REBUILDING THE NURSING HOME WORKFORCE: A TIME FOR CHANGE

Louisa Holaday[1], and Jasmine Travers[2], *1. Icahn School of Medicine at Mount Sinai, New York, New York, United States, 2. New York University, New York, New York, United States*

The COVID-19 pandemic magnified several long-standing problems in nursing homes, including issues within the nursing home workforce. Issues include staffing shortages, high turnover, low pay, inadequate training, poor treatment and limited access to resources. Research suggests that workforce issues of these kinds have the potential to significantly threaten and further increase disparities in the quality of care for nursing home residents. Thus, solutions are needed that ensures the nursing home workforce receives adequate investment so that these critical personnel are able to more effectively do the work that they do. In this presentation, workforce issues salient within the nursing home setting will be elaborated and potential solutions to mitigating these issues will be discussed.

### MECHANISMS TO ADDRESS RACIAL/ETHNIC DISPARITIES IN NURSING HOME QUALITY OF LIFE

Tetyana Shippee, *University of Minnesota, Minneapolis, Minnesota, United States*

Evidence documents racial/ethnic disparities in access, quality of care, and quality of life (QoL) among nursing home (NH) residents who are Black, Indigenous and persons of color. Yet, little is known about mechanisms for these disparities. This presentation examines the mechanisms for racial/ethnic disparities in QoL in high-proportion BIPOC facilities while highlighting variability in QoL disparities across these facilities. The presentation uses data from a 5 year mixed-methods project involving 96 resident interviews; 61 staff interviews; and 614 hours of observations in high proportion BIPOC facilities in MN, coupled with resident clinical Minimum Dataset assessments linked with survey data on residents' QoL. The findings show significant racial/ethnic disparities in QoL with need for system level changes. Given the increasing racial/ethnic diversity of NHs, ensuring equity in QoL for BIPOC residents is an urgent priority for NHs to remain relevant in the future.

### ELDER ABUSE IN SPECIAL POPULATIONS IN THE US: AMERICAN INDIANS AND ALASKA NATIVES AND THOSE LIVING IN THE RURAL SOUTH

Pamela B. Teaster Teaster, *Virginia Polytechnic Institute & State University, BLACKSBURG, Virginia, United States*

The number of older adults who experience abuse, neglect, and exploitation as well as the complexities of the cases continue to increase, despite advances in prevention and intervention of the problem. At once a multifaceted problem that involves intertwining individual, relational, community, and societal contexts, issues related to elder abuse become even more complicated among special populations, which require a nuanced understanding and approach to prevention, detection, and intervention. This presentation is a compilation of the findings from studies of the mistreatment of older American Indians and Alaska Natives (AIANs) and adults living in the rural south, all populations that are understudied. Both research and practical policy and practice solutions that have the potential to resolve disparities in these populations will be discussed.

### SUPPORTING DIVERSE FAMILY CAREGIVERS

Pamela Nadash, *University of Massachusetts Boston, Boston, Massachusetts, United States*

The RAISE Family Caregiving Advisory Council, created under the Recognize, Assist, Include, Support, and Engage (RAISE) Family Caregivers Act (2018) has been tasked to support the Secretary of Health and Human Services in developing a national family caregiving strategy. This presentation reports on research commissioned to support the activities of the Council, which aimed to engage a broad range of stakeholders in identifying concrete strategies to carry out the Goals identified as critical to supporting family caregivers. One priority was to engage with organizations representing the diversity of family caregivers, including groups working with Blacks, Indigenous people, Asian-Americans, Pacific Islanders, and other people of color, along with groups such as faith organizations that work with under-resourced communities. Respondents had much to say regarding mechanisms for ensuring access to services and supports among diverse family caregivers, most notably identifying support for "community ambassadors" as key, as well as targeted awareness campaigns.

### SESSION 4140 (SYMPOSIUM)

### REACHING THE LIMIT: CENTENARIANS IN THE HEALTH AND RETIREMENT STUDY

Chair: Peter Martin Discussant: Jennifer Ailshire

Although life expectancy has increased significantly over the last century, it is still unlikely that individuals reach the century mark of their lives. As a result, it is difficult to study a large enough sample of centenarian survivors; it is even more difficult to follow developmental trajectories of those who survive into very late life. The AHEAD sample of the Health and Retirement Study (HRS) contains longitudinal data of older adults who first participated in 1993. More than 500 HRS participants survived to at least 98 years of age. In this symposium, we present three uses of the data: first, we compare centenarians to older adults who did not survive into their nineties. Second, we compare different cohorts of centenarians with regard to health and psychosocial behavior. Third, we follow participants from their eighties to 100 years of age. The first presentation provides an overview of the HRS subsample. The second presentation highlights the personality profiles of centenarians. The third presentation traces health and psychological well-being among centenarians in the HRS. Finally, we discuss trajectories of cognition and functional limitations for three cohorts of centenarians. The results provide important information for policies and practical implications for families and service providers to older adults, highlighting available resources and health and well-being changes in very late life.

### INTRODUCING THE CENTENARIANS IN THE HEALTH AND RETIREMENT STUDY

Peter Martin[1], Gina Lee[1], Rotem Arieli[2], and Yeon Ji Ryou[1], *1. Iowa State University, Ames, Iowa, United States, 2. Iowa State University, Bellevue, Washington, United States*

The Health and Retirement Study (HRS) is a nationally representative sample of older adults, and data are available for 15 waves. This presentation presents an overview of the oldest cohort of the HRS (the AHEAD sample) as they become centenarians. Basic characteristics of these centenarians and near centenarians (98 years and older, N = 516) indicate that they are primarily female (77 percent), White (82 percent) with on average 10.6 years of education. The average age was 84.2 years at baseline and 98.3 years at wave 14. We analyze data by comparing centenarian survivors to nonsurvivors, by following them longitudinally, and by comparing different cohorts of centenarians over historical time. Results indicate that centenarians continue to enjoy life, and they have special survivorship characteristics. Newer cohorts, however, show a more compromised quality of life. The results are relevant to family members of oldest-old adults and to community service providers.

### PERSONALITY PROFILES OF CENTENARIANS AND NEAR-CENTENARIANS IN THE HEALTH AND RETIREMENT STUDY

Yeon Ji Ryou, *Iowa State University, Ames, Iowa, United States*

Centenarians are typically considered exceptional survivors who maintain their general health. The current study aims to track the change of personality over time and examine the differences between centenarians (defined as 98+ years; n = 516) and a group of non-survivors (died before 98; n = 6,771) using data from the Health and Retirement Study. Findings demonstrated that extraversion, agreeableness, conscientiousness, and openness decreased in 2012 and climbed again in 2014. Conversely, however, neuroticism increased in 2012 and then decreased in 2014. Interestingly, a pattern similar to that of 2012 appeared in 2020. Significant differences between centenarians and non-survivals were found in 2006 and 2010. Centenarians were more extraverted, agreeable, conscientious, and open-minded than non-survivors in 2006. Similarly, centenarians were more extraverted, agreeable, and open-minded than their counterpart in 2010. These findings provide a cornerstone for future research investigating the change of personality and its role in longevity among centenarians.

### EXAMINING PHYSICAL HEALTH AMONG COHORTS OF CENTENARIAN SURVIVORS IN THE HEALTH AND RETIREMENT STUDY

Rotem Arieli, *Iowa State University, Bellevue, Washington, United States*

This research highlights cohort differences in physical health among centenarians/near-centenarians in the Health and Retirement Study. Across 14 waves, participants aged 98 or older (n=494) were compared by three cohorts (i.e., 1890-1900, 1901-1910, and 1911-1920). Cohorts were examined at respective waves where participants were 96, 98, and 100 years of age. One-way ANOVA results presented significant differences in functional health at age 96 ($F[2,324]=5.01$, $p<.01$), subjective health at age 98 ($F[2,212]=7.94$, $p<.001$), and health conditions at ages 98 ($F[2,213]=12.52$, $p<.001$) and 100 ($F[2,115]=3.45$, $p<.05$). The oldest cohort had significantly better functional health than the youngest cohort (age 96), better subjective health than the two younger cohorts (age 98), and fewer health conditions than the youngest cohort (ages 98 and 100). Consistently, the oldest cohort performed better on subjective and objective health markers, providing implications for health care, disease prevention, and policy related to the shrinking "healthspan" among exceptional agers.

### THE TRAJECTORIES OF COGNITION AND FUNCTIONAL LIMITATIONS OF CENTENARIAN SURVIVORS

Gina Lee, *Iowa State University, Ames, Iowa, United States*

The purpose of this study is to explore cohort differences in the trajectories of cognition and functional limitations among oldest-old adults. Using 1992-2018 data from the Health and Retirement Study, participants included those who survived to at least 98 years (N=494). The study included three cohorts (i.e., 1890-1900, 1901-1910, and 1911-1920) predicting intercept and slope of cognition and functional limitations. Two separate latent growth curve models were computed, and models of cognition, $\chi 2(5)=6.935$, $p=.226$ and functional limitation, $\chi 2(7)=7.743$, $p=.35$ fit the data well. There was a significant decline in cognition levels and a significant increase in functional limitation over time. When adding cohort, results indicated that older cohorts were more likely to have a decrease in cognition and increase in functional limitations compared with the younger cohort. Future research should investigate trajectories of other health variables for a better understanding of health changes of centenarians.

### SESSION 4150 (SYMPOSIUM)

### RECRUITMENT FOR A MULTISITE PRAGMATIC TRIAL OF DEMENTIA CARE STRATEGIES: BARRIERS AND SUCCESS DURING COVID-19

Chair: David Reuben Discussant: Katie Maslow

With 2176 participants recruited, D-CARE is the largest pragmatic clinical trial of dementia care strategies, to date. At four clinical trial sites (CTS), D-CARE will compare the effectiveness of three dementia care strategies over 18 months: 1) by nurse practitioners or physician assistants within a health care system, 2) by social workers or nurses at community-based service organizations (CBO), or 3) usual care. Primary outcomes include person with dementia (PWD) behavioral symptoms and caregiver strain. Other outcomes include the PWD quality of life and ability to reach personal goals, and caregiver self-efficacy, distress, and depressive symptoms. Recruitment began in June 2019 with a basic protocol in which participating providers reviewed lists generated from the electronic health records (EHR) of patients who had a diagnosis of dementia, allowing the removal of patients who should not be contacted and giving an opportunity to provide information about the family caregiver. Some practices gave "blanket" referral allowing research staff to recruit participants directly. Other practices provided direct referrals via EHR communications to the research team. Self-referrals triggered by public postings in clinics and CBOs, social media, and media coverage were also accepted if a dementia diagnosis was confirmed in the EHR. By March 16, 2020, all in-person recruitment visits were suspended due to COVID-19. In response, informed consent was switched to telephone with verbal consent as permitted by State and Institutional regulations. This symposium describes the creative approaches employed by CTS' to respond to these challenges and reach the recruitment goal in January 2022.

### ELECTRONIC HEALTH RECORD SCREENING FOR PERSONS LIVING WITH DEMENTIA IN D-CARE

Mia Yang[1], Kristin Lenoir[2], Darcy McCurry[3], Katherine Currie[3], Nicholas Pajewski[3], and Jeff Williamson[1], *1. Wake Forest School of Medicine, Winston-Salem, North Carolina, United States, 2. Center for Healthcare Innovation, Wake Forest School of Medicine, Winston Salem, North Carolina, United States, 3. Department of Internal Medicine-Section on Gerontology & Geriatric Medicine, Wake Forest School of Medicine, Winston Salem, North Carolina, United States*

Pragmatically identifying persons living with dementia (PLWD) for research is a persistent challenge, reflecting limitations in data elements. As part of the D-CARE study, the recruitment coordinator and analyst collaborated to create a screening algorithm to identify potentially eligible patients. A combination SAS/R script was applied on a monthly basis (Oct 2020-Dec 2021) to a clinical data warehouse, identifying living patients 50 years or older with ICD-10 codes for dementia or related conditions within the past two years, and an encounter within the past year. This process identified 6,478 unique patients, leading to the recruitment of 837 PLWD into the study (22% Black or Hispanic). Algorithmic components that enhanced recruitment efforts included the incorporation of data elements necessary to contact and enroll patients, formatting data consistent with reporting requirements, and tracking information on primary care provider, diagnosis dates with the set of providers that agreed to recruit for the trial.

### FOCUSING ON DIVERSITY: UTMB RECRUITMENT STRATEGY FOR THE D-CARE STUDY

Rafael Samper-Ternent[1], Alice Williams[2], Roxana Hirst[2], Rebecca Galloway[2], and Elena Volpi[1], *1. University of Texas Medical Branch, Galveston, Texas, United States, 2. Sealy Center on Aging, University Of Texas Medical Branch, Galveston, Texas, United States*

The University of Texas Medical Branch (UTMB) is the main healthcare system in Galveston County where about 15% of older adults identify as Hispanic. Our recruitment efforts for the Dementia (D-CARE) study included adapting and translating in Spanish the screening, recruitment, and intervention materials. The study is being conducted by a bilingual team, and actively engages a highly diverse Local Patient and Stakeholder Council. After obtaining a partial HIPAA waiver from the Institutional Review Board, we created a dementia registry in the UTMB Epic (TM) electronic health record which captured patients diagnosed with ICD-9/10 codes of dementia. Referral letters from UTMB primary care and neurology providers authorized us to contact eligible patients. We utilized outpatient clinic appointment schedules, direct referrals, and community outreach to meet our enrollment goal. This recruitment strategy resulted in inclusion of 478 patient-caregiver dyads with 27.4% of participants identifying as Hispanic and 17% as Black.

### ENROLLING PARTICIPANTS WITH DEMENTIA IN A RURAL SETTING: GEISINGER RECRUITMENT STRATEGIES FOR D-CARE

Maya Lichtenstein[1], Pamela Borek[2], and Ilene Ladd[3], *1. Geisinger Health System, Wilkes Barre, Pennsylvania, United States, 2. Office of Clinical Research Operations, Geisinger Wyoming Valley, Wilkes Barre, Pennsylvania, United States, 3. Genomic Medicine Institute, Geisinger Health System, Danville, Pennsylvania, United States*

Geisinger Health System serves a large, geographically stable population in central and northeast Pennsylvania and was chosen to participate in D-CARE due to its rural population, which is often under-represented. To reach potential participants, we used a multi-pronged recruitment approach including local radio, television, and newspaper advertisement; publication in a health system newsletter targeted to 55+; outreach to senior-care managers and primary care providers to encourage referrals; and leveraged an extensive electronic health record (EHR) database to identify a pool of almost 9,000 potential participants. Initial barriers to enrollment stemmed from geographic challenges and the dyad's inability and/or unwillingness to travel long distances to lengthy in-clinic appointments. A post-pandemic transition to virtual visits helped ease the travel burden and increase study enrollment. Despite varied recruitment strategies, by far the most effective recruitment method remained direct referrals of patients and caregivers with whom the provider had discussed the D-CARE study.

### CLINIC-BASED RECRUITMENT STRATEGIES WITHIN AN INTEGRATED HEALTH CARE SYSTEM FOR THE DEMENTIA CARE STUDY (D-CARE)

Alan Stevens, Aval-Na'Ree Green, Sitara Abraham, and Jordan Reese, *Baylor Scott and White Health, Temple, Texas, United States*

At Baylor Scott and White Health (BSWH), 4,564 patients with a diagnosis of dementia were identified via search in the EHR, direct referrals from BSWH Providers and self-referrals. Of 2,296 potential participants (dyads of person with Dementia [PWD] and their family caregivers) screened, 2,034 were deemed eligible, and 626 dyads were enrolled. The BSWH D-CARE leadership team provided a 20-minute presentation to Medical Directors and Providers of 32 BSWH primary care clinics followed by a request to review Provider-specific patient lists. After Provider review, research team members sent letters and/or made phone calls to dyads. Providers were also invited to make direct referrals using an EHR referral link and the research team pinned appointment notes to upcoming PWD clinic appointments to remind Providers of the project. Recruitment strategies shared during monthly meetings of the Recruitment & Retention Committee also proved helpful in the identification of successful outreach and recruitment activities.

### SESSION 4160 (SYMPOSIUM)

### REIMAGINING AGING WITH DISABILITY THROUGH TECHNOLOGY

Chair: Elena Remillard Discussant: Margaret Campbell

Individuals aging with long-term disabilities are likely to require support for managing challenges with everyday activities. To effectively promote independence and aging-in-place for this population, we must consider innovative technology solutions that go beyond traditional direct care services. This symposium will highlight technology research and development efforts from the Rehabilitation Engineering Research Center on Technologies to Support Aging-in-Place for People with Long-Term Disabilities (RERC TechSAge). TechSAge is an interdisciplinary collaboration between Georgia Tech, the University of Illinois Urbana-Champaign, and Georgia State University, dedicated to understanding the needs of, and developing supportive technologies for people aging with long-term vision, hearing, and mobility disabilities. First, Remillard et al., will present interview findings from individuals aging with long-term vision loss on challenges performing instrumental activities of daily living. Next, Mitzner et al., will describe TechSAge research-driven guidelines for designing and delivering wellness classes via video-conferencing to older adults with and without disabilities. Blocker et al., will highlight a field trial evaluating how digital home assistants (e.g., Amazon Echo devices) and smart home devices, along with user-friendly support guides, can support older adults with mobility disabilities at home. Finally, Jones et al., will describe the latest feature of the SmartBathroom laboratory – a SmartBathing Transfer Testbed prototype that will enable us to study of how different bathing environment configurations can impact transfer performance. Margaret Campbell (Retired Senior Scientist for Planning and Policy Support, National Institute on Disability, Independent Living, and Rehabilitation Research) will serve as the discussant for the symposium.

### AGING WITH VISION LOSS: EXPLORING EVERYDAY ACTIVITY CHALLENGES AND OPPORTUNITIES FOR TECHNOLOGY INNOVATION

Elena Remillard[1], Lyndsie Koon[2], Tracy Mitzner[3], and Wendy Rogers[4], *1. Georgia Institute of Technology, Atlanta, Georgia, United States, 2. University of Kansas, Lawrence, Kansas, United States, 3. Georgia Institute of technology, Atlanta, Georgia, United States, 4. University of Illinois Urbana Champaign, Champaign, Illinois, United States*

Vision loss can impact the health of older adults on multiple domains, including physically (e.g., mobility challenges), psychologically (e.g., depression), and socially (e.g., isolation). Older adults with vision loss, and particularly those with long-term vision loss, are likely to experience challenges in performing Instrumental Activities of Daily Living (IADLs), as these activities often require abilities like reading, fine motor movements, and navigating. We conducted structured interviews with 60 older adults aged 60-79 with vision loss for at least 10 years about their challenges with select IADLs (e.g., household tasks, transportation, shopping, finances, and managing health). An analysis of in-depth interview data revealed specific challenge themes associated with engaging in different IADLS, such as challenges with accessibility, physical limitations, assistance from others, communication, and finances. Findings highlight opportunities for technology solutions to support IADL participation and independence for people aging with vision loss.

### TECHSAGE TELEWELLNESS TOOL: GUIDELINES FOR DELIVERING INCLUSIVE WELLNESS CLASSES VIA VIDEO-CONFERENCING

Tracy Mitzner[1], Elena Remillard[2], Kara Mumma[2], and Lelah Cochran[2], *1. Georgia Institute of technology, Atlanta, Georgia, United States, 2. Georgia Institute of Technology, Atlanta, Georgia, United States*

Many older adults, particularly those with mobility disabilities, experience barriers to participation in exercise classes, including lack of accessibility and transportation. Tele-technologies (e.g., Zoom) provide an opportunity to facilitate both physical and social wellness through remote group classes. We developed the TechSAge Telewellness tool to provide guidelines for designing wellness classes delivered via video-conferencing for older adults with and without disabilities. The protocols and guidelines presented within it have been tested for usability and acceptance with older adults with long-term mobility disabilities. Throughout the tool, we present examples from the TechSAge Tele Tai Chi study as a case study to provide helpful insights on developing a Telewellness program. The tool includes guidance on selecting a software, safety considerations, class organization and logistics, social time, and tech support. The contents of the tool provide insights for research and design regarding telehealth technology, as well as health and wellness interventions.

### ASSESSING THE POTENTIAL OF DIGITAL HOME ASSISTANTS TO SUPPORT OLDER ADULTS WITH MOBILITY DISABILITY

Kenneth Blocker[1], Ki Lim[1], Husna Hussaini[1], Kyreon Williams[2], Ramavarapu S. Sreenivas[1], and Wendy Rogers[3], *1. University of Illinois Urbana-Champaign, Champaign, Illinois, United States, 2. university of Illinois Urbana-Champaign, Champaign, Illinois, United States, 3. University of Illinois Urbana Champaign, Champaign, Illinois, United States*

Digital home assistants (e.g., Amazon Echo devices) hold great potential for supporting older adults in completing a wide range of daily living activities and improving their overall quality of life. As these devices can wirelessly connect with other smart technologies to increase their capabilities (e.g., environmental control), digital assistance technologies may benefit older adults who need support within their home such as those living with mobility disabilities. However, research is needed to better understand the facilitators and barriers of using these technologies by these individuals, as well as the resources that best support their onboarding and continued use. To assess the potential efficacy of integrating smart and connected technologies into the homes of older adults with mobility disability, we are conducting a field trial to understand how best to introduce these devices and support their use. Design considerations and initial findings are discussed.

### SMARTBATHROOM 102: STUDYING BATHING TRANSFER PERFORMANCE

Brian Jones[1], Jon Sanford[2], Peter Presti[1], Susan Lee[2], Sutanuka Bhattacharjya[2], and Kyle Murphy[3], *1. Georgia Institute of Technology, Atlanta, Georgia, United States, 2. Georgia State University, Atlanta, Georgia, United States, 3. Georgia Institute Of Technology, Atlanta, Georgia, United States*

Age-related changes in functional ability, particularly among people aging with long-term disabilities, can impact safe performance of toilet and bathtub transfers and severely limit their ability to successfully age-in-place. The SmartBathroom is a state-of-the-art bathroom laboratory that features mechanically adjustable features and an array of sensors that can measure walking performance, location of feet and hands, and forces applied to the bathroom surfaces and fixtures. To complement the SmartToilet system, TechSAge recently developed the SmartBathing Transfer Testbed prototype that will enable us to study how different bathing environment configurations can impact transfer performance. The testbed consists of a height adjustable tub wall and three-wall, height and angle adjustable grab bars with integrated sensors to measure hand location and grip and load forces. This session will describe the SmartBathing Testbed and present results of studies with individuals both with and without ambulatory impairments performing simulated transfers within the testbed.

### SESSION 4170 (PAPER)

SOCIAL SUPPORT

### OLDER INDONESIAN WOMEN'S EXPERIENCE TO MAINTAIN INDEPENDENCE IN FOOD-RELATED ACTIVITIES

Widya Ramadhani[1], and Lynne Dearborn[2], *1. University of Illinois Urbana-Champaign, Urbana, Illinois, United States, 2. University of Illinois Urbana-Champaign, Champaign, Illinois, United States*

The ability to conduct food-related activities such as cooking, eating, and cleaning are central to older adults' health. Such ability is especially critical for older women who are more likely to live alone or become caregivers for their spouses and other family members. We investigated the experience of older Indonesian women, an understudied population in aging research, when engaging in food-related activities. Using the grounded theory approach, we examined the challenges and adaptive behavior of twelve community-dwelling older women in Indonesia (60+) when conducting food-related activities at home. We employed two data collection strategies: photo and video elicitation, followed by an interview. Through photo and video elicitation, participants took photos and videos of the space where they conducted food-related activities to provide physical environmental data. In the interview, participants explained their routine, activity challenges, and adaptation strategies in food-related activities. We discovered that participants' view of their role in cooking food for the family is central to their sense of identity. When faced with age-related challenges, participants accepted assistance from others for activities less connected to food production, such as sweeping, mopping, or cleaning the kitchen. However, they are less likely to accept assistance for cooking activities. Instead, they adapted by modifying the physical environment, simplifying the tasks, and adjusting the method to remain engaged in cooking. Older Indonesian women's cultural identity influenced their strategy to face age-related challenges and maintain independence. This finding highlights the importance of a culturally sensitive approach when planning support for older adults.

### TRANSITIONS IN SOCIAL SUPPORT EXCHANGE PROFILES OVER TIME AMONG OLDER ADULTS

Pildoo Sung[1], Rahul Malhotra[2], Grand Cheng[3], and Angelique Chan[2], *1. Centre for Ageing Research and Education, Duke-NUS Medical School, Singapore, Singapore, 2. Duke-NUS Medical School, Singapore, Singapore, 3. National University of Singapore, Singapore, Singapore*

Despite increasing interest in social support exchanges among older adults, little is known about the interplay between giving and receiving social support, how social support exchanges change over time, and factors associated with such change. Using data on 1,305 older Singaporeans participating in two waves of a national, longitudinal survey conducted in 2016-2017 and 2019, we investigated (1) distinct social support exchange profiles that comprise different types of giving and receiving social support, (2) transitions in social support exchange profiles over time, and (3) association of sociodemographic characteristics and health status with such transitions. Gender-stratified random intercept latent transition analysis (RI-LTA) produced three main findings. First, we identified four social support exchange profiles—multi-exchange, provider, receiver, and low exchange—for both males and females at both waves, although the distribution of profiles varied by gender and waves. Second, males were more likely to transition from the multi-exchange profile to other types, whereas females were relatively more likely to transition into the multi-exchange profile over time. Third, among males, those older, of ethnic minority, unmarried, employed, and with depressive symptoms were more likely to transition into the receiver profile from other types. Females who were younger, of ethnic majority, married, and less educated were more likely to transition into the multi-exchange type from low or receiver profiles. The findings capture the temporal dynamics in social support exchange profiles and their gendered characteristics. Policy interventions should focus on older adults who lack social support exchanges and those who lose social support exchanges over time.

### WHEN LOVE HURTS: AMBIVALENT TIES INFLUENCE THE LINK BETWEEN FUNCTIONAL LIMITATIONS AND WELL-BEING

Misha Haghighat[1], and Meng Huo[2], *1. University of California, Davis, Davis, California, United States, 2. University of California, Davis, DAVIS, California, United States*

Functional limitations may co-occur with worse emotional well-being in older adulthood. Research has found that this association likely varies by social factors, with social support buffering well-being from physical declines, and strain exacerbating it. Yet, older adults' functional limitations may contribute to more ambivalence in their social relationships, which involves both support and strain. Such ambivalence may exacerbate older adults' emotional well-being in the face of physical limitations, but we know little about whether and how this occurs. The current study innovatively examined how older adults' overall ambivalence moderates the association between functional limitations and depressive symptoms, and captured emotional mood among older adults with functional limitations when they had encounters with ambivalent partners throughout the day. Study participants (N = 313; ages 65+) were from the Daily Experiences and Well-being Study. Participants reported on functional limitations, depressive symptoms, and relationship quality with each social partner in a 2-hour in person interview, followed by reports of social encounters every 3-hours across 5-6 days. We observed a significant moderating effect of overall ambivalence on the association between functional limitations and depressive symptoms, such that this association was more salient among older adults with more ambivalent social networks. Further, only unpleasant encounters with ambivalent social partners, but not those with non-ambivalent social partners, compromised mood among older adults with functional limitations. These findings may advance our understanding of older adults' ambivalent ties in the context of physical declines, and help identify therapies that may enhance well-being for older adults with health limitations.

### WITHIN-FAMILY DIFFERENCES IN INTERGENERATIONAL CONTACT: THE ROLE OF GENDER NORMS IN A PATRILINEAL SOCIETY

Jeremy Lim-Soh[1], Dahye Kim[2], and Kyungmin Kim[3], *1. Duke-NUS Medical School, National University of Singapore, Singapore, Singapore, 2. National University of Singapore, Singapore, Singapore, 3. Seoul National University, Seoul, Seoul-t'ukpyolsi, Republic of Korea*

Intergenerational contact is important for older adults' quality of life. However, few studies have examined older adults' patterns of intergenerational contact across multiple children and whether these patterns are gendered within families, which has been a distinct characteristic in countries with patrilineal traditions. A sample of older adults aged 65 and above with mixed-gender children (N = 3,228) from the 2006 Korean Longitudinal Study of Ageing reported the frequency of meeting and communication with each of their children (N = 14,110). To identify "within-family" patterns of contact, we applied latent profile analysis to three family-level indicators of contact across children: (a) family means, (b) family standard deviations, and (c) gender differences between sons and daughters. Multinomial logit regression was estimated to examine differences in parents' characteristics between the derived patterns. Nearly half of parents exhibited gender-balanced patterns of meeting across children: "equally high contact" (14%) and "equally low contact" (34%). We also found two gendered patterns of contact: "more frequent contact with daughters" (13%) and "more frequent contact with sons" (39%). Communication also exhibited similar four patterns across children with a higher proportion of "equally high contact" (77%). The derived patterns of contact differed by parents' age, education, marital status, employment situation, and depressive symptoms. The results highlighted substantial within-family differences in older parents' contact with their children, revealing more frequent contact with sons. This points to the enduring nature of patrilineal traditions as well as the lack of frequent physical contact in some Korean families.

### SESSION 4180 (SYMPOSIUM)

### STORIES FROM THE INSIDE OF DEMENTIA: CONSTRUCTING IDENTITIES AND RELATIONSHIPS THROUGH THE DISCOURSE OF EXPERIENCE

Chair: Kate de Medeiros Co-Chair: Pamela Saunders

Dementia is often characterized as a time of decline and loss. The grand narrative (i.e., the biomedical narrative) of dementia describes it as a time of meaninglessness given the inevitable loss of memory and language, fraught with difficulties recognizing familiar faces, recalling autobiographical events, and orienting to time and place. This unfortunate depiction negatively positions people living with dementia as living a 'social death' and overlooks the potential of forming meaningful relationships and experiencing a sense of community. Elements often overlooked can be found in the careful reading and analysis of individual interactions that make up constructions of identity, relationships, and community. In this symposium, we draw data from the "Friendship Study," a 6-month ethnographic study on the social environments of people living with dementia in long-term care to consider meaning-making in dementia through three perspectives: phenomenological, gerontological, and discursive. The first paper considers the phenomenology of friendship, moving away from linear narratives to look at micro-constructions of identity. The second considers the co-construction of friendship through discursive elements found in conversational interactions. The third challenges staff's construction of residents, highlighting how negative assumptions about resident capabilities can affect personal relationships and the social environment.

### EXPERIENCE BEYOND NARRATIVE: A PHENOMENOLOGICAL ACCOUNT OF MEANING-MAKING WHILE LIVING IN THE WORLD WITH DEMENTIA

MaryKatherine Gaurke, *Georgetown University, Washington, District of Columbia, United States*

Narrative-based approaches in dementia care emphasize the importance of understanding humans as beings who make sense of themselves and others through constructing life narratives, taking this process to be morally significant for constituting or characterizing identities (and for holding others into their identities) (Kitwood, 1997). Yet, placing too much emphasis on the formation of a cohesive life narrative risks dismissing or otherwise failing to acknowledge the activities of meaning-making and narrative-sharing/constructing that those with dementia engage outside of, or even counter to, a unified narrative. This paper offers a Heideggerian-inspired phenomenological analysis of interactions detailed in the Friendship Study (de Medeiros et al. 2011) and from supporting published accounts and personal observation in clinical settings. This account reveals significant meaning-making possibilities engaged and shared among those living with dementia in long-term care settings that may be left unexamined in traditional narrative-based approaches suggesting that micro-narrative construction needs further attention.

### FRIENDSHIP IS IN THE DETAILS: CO-CONSTRUCTION OF RELATIONSHIPS AMONG PERSONS WITH DEMENTIA IN LONG-TERM CARE

Pamela Saunders, *Georgetown University, Washington, District of Columbia, United States*

Friendships have been linked to psychological and emotional wellbeing and better physical functioning in older adults. Conversely, negative consequences (e.g., depression) are associated with losing friendships and shrinking social networks. While cognitive decline might be a limiting factor for persons with dementia (PWD) to establish friendships, this has not been proven in the literature. This paper reports on 20 interactions between PWD collected during the Friendship Study (de Medeiros et al. 2011), an ethnographic study of friendship in long-term care (LTC). Participants are male and female residents in an LTC community. Diagnoses range from mild to severe Alzheimer's disease. Conversational interactions were transcribed and coded for linguistic and discursive devices signaling friendly interactions. Findings reveal that friendships are co-constructed by PWD using 4 primary linguistic discursive devices, including topic (meals, religion, medication, furniture, directions, baking), co-constructed narrative, repetition, and alignment. Implications for future research on friendship among PWD are discussed.

### NONE OF THEM ARE REALLY FRIENDS: STAFF PERCEPTIONS OF THE SOCIAL WORLD OF PEOPLE LIVING WITH DEMENTIA IN LONG-TERM CARE

Kate de Medeiros, *Miami University, Oxford, Ohio, United States*

Nursing homes are charged with the care and protection of residents. Consequently, staff exercise tremendous control over residents' day-to-day lives, including their social worlds. This is especially true for people living with dementia who may have challenges with communication. This paper examined how staff viewed friendships among dementia residents. Eleven staff members were asked to describe the friendship among the 20 residents in their charge to include who was friends with whom and what were the key features of the friendships. Results revealed that nearly all staff viewed residents as incapable of forming friendships among other residents. One exception was that many staff viewed felts that the male residents enjoyed a level of friendship that female residents did not. Overall, the findings point to the need to help staff move beyond a limited view of residents' capabilities and discover ways to help foster a rich social environment

### SESSION 4190 (SYMPOSIUM)

### TECHNOLOGY TO SUPPORT OLDER ADULTS' HEALTH, SAFETY, AND WELLBEING

Chair: Walter Boot

Technology has revolutionized the ability to deliver interventions to support older adults' health, safety, and wellbeing, and technology-based solutions have become increasingly important in the delivery of remote care during the COVID-19 pandemic. However, these interventions and novel approaches require a careful understanding of older adults' needs, preferences, and abilities, and need to undergo tests of feasibility, acceptability, and efficacy. This session will present a sampling of research examining the development, testing, and implementation of technology-based solutions to support older adults. R. Azevedo will present on the development of a novel digital therapeutic for the self-management of hypertension medication adherence among older adults. S. Kwon will present on the initial efficacy of an app-based mindfulness-meditation intervention to alleviate stress and depressive symptoms among bereaved older adults. Y. Du will discuss the use of a commercial fitness tracker to facilitate activity self-monitoring among overweight diabetic older adults with and without kidney disease. S. Dimmick will discuss the development of a novel augmented reality (AR) safety checklist to reduce fall risk among older adults. Finally, F. Jain will present on the development of a new mobile application platform to meet the needs of family dementia caregivers informed by focus groups and an inductive and deductive mixed method analytic approach. Themes of the necessity of a user-centered design approach in the development of technology-solutions will be emphasized.

### A TRANSDISCIPLINARY TEAM APPROACH TO TECHNOLOGY DEVELOPMENT TO SUPPORT HEALTH AND WELL-BEING

Renato Ferreira Leitao Azevedo[1], Jennifer Nicholas[2], Jeannie Lee[3], Kathleen Insel[3], and Wendy Rogers[4], *1. University of Illinois Urbana-Champaign (UIUC), Urbana Champaign, Illinois, United States, 2. The University of Arizona, Tucson, Arizona, United States, 3. University of Arizona, Tucson, Arizona, United States, 4. University of Illinois Urbana Champaign, Champaign, Illinois, United States*

Diverse disciplinary and experiential knowledge is required to understand the challenges and develop workable solutions to support the health and wellbeing of a rapidly aging population. We are developing a theory-based, digital therapeutic for self-management of hypertension medication adherence for older adults; namely, the Medication Education, Decision Support, Reminding, and Monitoring (MEDSReM) system. This development process has necessitated a transdisciplinary approach with members co-learning and working collaboratively to advance the MEDSReM system. Our group includes complementary expertise in pharmacy, nursing, human factors, cognitive aging, gerontology, and technology development. We describe the challenges of transdisciplinary teamwork, our collaborative processes, and strategies that led to successful integration of expertise in advancing MEDSReM. Lastly, we discuss useful approaches to technology conceptualization, and digital therapeutic development and implementation, as well as lessons learned in effective communication and coordination among the diverse team members to ensure that the MEDSReM goals are achieved.

### INITIAL EFFICACY OF AN APP-BASED MINDFULNESS MEDITATION FOR BEREAVED KOREAN OLDER ADULTS DURING THE PANDEMIC

Soonhyung Kwon[1], and Jaesung An[2], *1. University of Illinois Urbana Champaign, Urbana Champaign, Illinois, United States, 2. California State University East Bay, Cupertino, California, United States*

Given the onset of COVID-19, older adults who recently lost their significant others feel more stressed. There yet exist a study utilizing smartphones for web-based delivery of mindfulness intervention among bereaved older adults. Therefore, this study aimed to test the initial efficacy of an app-based mindfulness-meditation (AMM) to alleviate stress and depressive symptoms and improve stress resistance, social support, and self-esteem in Korean older adults experiencing bereavement. Participants included 22 Korean older adults who had been bereaved within the preceding year. AMM involved sound therapy, breathing exercises, and narrated meditation sessions, and the program was conducted over eight weeks. The linear regression results showed that stress level among participants was significantly lower after the intervention, with decreased scores from the baseline. By confirming that AMM is an effective way of reducing stress, more active usage of devices like smartphones should be promoted to develop mental health interventions for older adults.

### SELF-MONITORING OF LIFESTYLES FOR DISEASE MANAGEMENT IN OVERWEIGHT DIABETIC SENIORS WITH/WITHOUT KIDNEY DISEASE

Yan Du[1], Sharma Kumar[1], Valerie Ramirez[2], Dennis Brittany[2], Shiyu Li[2], Chengdong Li[3], and Jing Wang[3], *1. UT Health San Antonio, San Antonio, Texas, United States, 2. Center On Smart And Connected Health Technology, School Of Nursing, UT Health San Antonio, San Antonio, Texas, United States, 3. College of nursing, Florida State University, Tallahassee, Florida, United States*

Overweight/obesity, diabetes, and chronic kidney disease (CKD) are common multiple chronic conditions (MCCs) in seniors. Fitbit fitness trackers were used to track physical activity and food intake over 12-week in 15 overweight/obese diabetic seniors (age=70.5±4.8) with/without CKD. The associations of percentage of days with tracked steps (PDWTS), percentage of days with food logs (PDWFL) and 7-day mean step count with body mass index, fasting glucose, HbA1c and renal function were examined using Pearson Correlation analysis. Qualitative data assessing perceived factors related to self-monitoring and lifestyle behaviors was collected and analyzed using content analysis. At week 12, PDWTS and PDWFL was positively and negatively associated with renal function ($P<0.05$) and HbA1c ($P<0.05$), respectively. Motivation and perceived benefits of self-monitoring were frequently cited facilitators for self-monitoring. Self-monitoring might help seniors manage MCCs. Motivating self-monitoring and providing health education on the benefits of self-monitoring in lifestyle interventions to manage MCCs are warranted.

### MOBILE INTERVENTION FOR FAMILY DEMENTIA CAREGIVERS: FROM FOCUS GROUPS TO SMARTPHONE PLATFORM

Felipe Jain[1], Saira Madarasmi[2], DJ Ursal[2], Paulina Gutierrez-Ramirez[2], Abu Sikder[3], Sreya Banerjee[2], and Liliana Ramirez Gomez[4], *1. Depression Clinical Research Program / Massachusetts General Hospital / Harvard Medical School, Boston, Massachusetts, United States, 2. Massachusetts General Hospital, Boston, Massachusetts, United States, 3. Innovation Studio, Children's Hospital, Los Angeles, Los Angeles, California, United States, 4. Massachusetts General Hospital, Harvard Medical School, Boston, Massachusetts, United States*

There is growing recognition that in-person delivery of caregiver interventions limits scalability due to distance from locations where interventions are available, and costs associated with locating substitute care for the person living with dementia. Internet-based interventions are often not optimized for smartphones, which are more accessible than desktops to minoritized populations and those of lower socioeconomic status. In this study, focus groups were conducted with 17 English language and 12 Spanish language family dementia caregivers regarding needs for smartphone-based technological intervention. We employed an inductive and deductive driven mixed method analytic approach. Identified needs included psychoeducation regarding dementia, provision of caregiver skills information, activities for the person living with dementia, and relaxation techniques. Similarities and differences among preferences between the two populations were identified. Family dementia caregivers endorsed several needs for mobile intervention. The development of a new mobile application platform to meet these needs will be described.

### SESSION 4200 (SYMPOSIUM)

### THE BALTIMORE LONGITUDINAL STUDY OF AGING: READY TO SHARE

Chair: Eleanor Simonsick Discussant: Luigi Ferrucci

Beginning January 2023, the new National Institutes of Health (NIH) Data Sharing Policy for NIH-funded and conducted research is scheduled to go into effect. In anticipation of this requirement, scientists and staff of the Baltimore Longitudinal Study of Aging (BLSA) launched a comprehensive effort to create a user friendly well-documented accessible database scheduled for a "soft" release just in time for GSA. The BLSA, established in 1958 to study normative aging, continues to advance knowledge and understanding of aging mechanisms and their impact on and response to disease processes. During a 3-day clinic visit, the 1200+ BLSA participants undergo comprehensive evaluation covering the full range of physical functional and cognitive performance capacity as well as extensive imaging studies, biological sample collection and clinical examination. In this symposium, Dr. Griswold will introduce the data sharing platform and demonstrate its facility. Dr. Simonsick will elaborate on development of a fitness percentile-based approach to define exceptional aging and the factors associated with maintaining exceptional status. Dr. Moore will provide findings on life-course physical activity and CT derived muscle parameters and homeostatic regulation in later life. Dr. Tian will share her ongoing work aimed at identifying metabolomic signatures of brain atrophy and Alzheimer's disease risk. Dr. Tanaka will report on her work analyzing the metabolomic profile of different dietary patterns and their association with frailty. Our Discussant, Dr. Ferrucci will briefly share future directions for the BLSA and then invite attendees to comment on the BLSA data sharing platform and scientific priorities.

### FAIR SHARING OF THE BLSA DATA ECOSYSTEM

Michael Griswold[1], James Henegan[2], Chad Blackshear[2], Ann Moore[3], Thomas Smith[4], Deric McGowan[4], Eleanor Simonsick[5], and Luigi Ferrucci[5], *1. The MIND Center at UMMC, Jackson, Mississippi, United States, 2. UMMC MIND Center, Jackson, Mississippi, United States, 3. TGB NIA NIH, Baltimore, Maryland, United States, 4. NIA, Baltimore, Maryland, United States, 5. National Institute on Aging, Baltimore, Maryland, United States*

The BLSA is an invaluable resource for the study of human aging with uniquely rich measures taken longitudinally on a continuous replenishment cohort since 1958. Tremendous interest has been expressed by the United States and international research communities in expanded access to and use of BLSA data to address emerging scientific questions. Here, we will describe recent study leadership initiatives into: (1) making the BLSA data more FAIR (Findable, Accessible, Interoperable, and Reusable), (2) growing a BLSA data ecosystem that enables scalable sharing, and (3) leveraging and developing platforms for accessing the ecosystem. Lastly, we will demonstrate current platform functions that empower researchers to find and access BLSA data, metadata, protocols, code and supporting materials to make new discoveries.

### CORRELATES OF PHYSICAL ACTIVITY HISTORY IN PARTICIPANTS OF THE BALTIMORE LONGITUDINAL STUDY OF AGING

Ann Moore[1], Eleanor Simonsick[2], Bennett Landman[3], and Luigi Ferrucci[2], *1. TGB NIA NIH, Baltimore, Maryland, United States, 2. National Institute on Aging, Baltimore, Maryland, United States, 3. Vanderbilt University, Nashville, Tennessee, United States*

Physical activity across the life course contributes to physical function and health in later life. Here we characterize a measure of physical activity history, newly implemented in participants of the Baltimore Longitudinal Study of Aging (n = 690). Participants selected one of four levels to describe activity during each decade of life from age ten to the present. Recalled levels of physical activity are positively associated with activity assessed in current and prior decade study visits, suggesting that the recalled estimates are consistent with historic activity. A summary measure based on ranking activity patterns was associated with measures of physical performance, muscle and fat areas quantified from computed tomography images as well as some indicators of homeostatic dysregulation ($p < .05$). The observed associations suggest that an estimate of physical activity across decades provides complementary information to estimates of current activity and reemphasizes the importance of consistently engaging in physical activity.

### METABOLOMIC SIGNATURES OF BRAIN ATROPHY PATTERNS IN AGING AND ALZHEIMER'S DISEASE

Qu Tian[1], Brendan Mitchell[2], Guray Erus[3], Christos Davatzikos[3], Susan Resnick[2], and Luigi Ferrucci[2], *1. National Institutes on Aging, Baltimore, Maryland, United States, 2. National Institute on Aging, Baltimore, Maryland, United States, 3. University of Pennsylvania, Philadelphia, Pennsylvania, United States*

Can plasma metabolomics reveal mechanisms of brain aging? We investigated metabolomic signatures of brain atrophy patterns related to cognitive decline and Alzheimer's disease(AD) risk. Relationships between metabolomics(Biocrates-p500) and annual rates of change in two neuroimaging-based brain atrophy patterns(SPARE-BA indexing brain aging, SPARE-AD indexing AD-related atrophy) were examined using multivariable linear regression in 477 Baltimore Longitudinal Study of Aging participants aged 60+, adjusted for demographic variables and BMI. Higher concentrations of sarcosine, triglycerides, diglycerides, and ceramides and lower concentrations from phosphatidylcholines and cholesteryl ester were associated with faster rates of SPARE-BA increase longitudinally. Higher concentrations of diglyceride and alpha-amino-butyric acid and lower concentrations of tryptophan, hippuric acid, cholesteryl ester, phosphatidylcholine and sphingomyelin were associated with faster rates of SPARE-AD. Metabolites have differential associations with age-related and AD-related brain atrophy patterns, which may provide new insights into preventive and therapeutic interventions.

Future studies should examine whether metabolite changes precede brain atrophy patterns.

### METABOLOMIC PROFILE OF DIFFERENT DIETARY PATTERNS AND THEIR ASSOCIATION WITH FRAILTY

Toshiko Tanaka[1], Sameera Talegawkar[2], Yichen Jin[3], Qu Tian[4], Eleanor Simonsick[1], and Luigi Ferrucci[1], *1. National Institute on Aging, Baltimore, Maryland, United States, 2. Department of Exercise and Nutrition Sciences, Milken Institute School of Public Health, The George Washington University, Washington, District of Columbia, United States, 3. Nutrition Sciences, Milken Institute School of Public Health, The George Washington University, Washington, District of Columbia, United States, 4. National Institutes on Aging, Baltimore, Maryland, United States*

Diet quality is an important behavioral determinant of healthy aging, however the mechanisms underlying the protective effect of diet are not fully understood. To address this question, we explored whether plasma metabolites mediate the relationship between diet and frailty index (FI) in the Baltimore Longitudinal Study of Aging. Adherence to different dietary patterns was evaluated using the Mediterranean diet score (MDS), Mediterranean-DASH Diet Intervention for Neurodegenerative Delay (MIND), and Alternate Healthy Eating Index 2010 (AHEI). Higher adherence to the dietary patterns was associated with lower FI. There were 127 plasma metabolites that were associated with all three dietary patterns including lipids, fatty acids, ceramides, amino acids, and bile acids. Metabolomic signatures of diet mediated 42%, 70%, and 61% of the association between MDS, MIND, and AHEI with FI, respectively. Overall, we found common metabolomic biomarkers of dietary patterns that explain part of the relationship between diet and frailty.

### DEVELOPING A FITNESS AGE-NORMALIZED PERCENTILE-BASED APPROACH TO DEFINE EXCEPTIONAL AGING

Eleanor Simonsick[1], Ann Moore[2], Toshiko Tanaka[1], Qu Tian[3], and Luigi Ferrucci[1], *1. National Institute on Aging, Baltimore, Maryland, United States, 2. TGB NIA NIH, Baltimore, Maryland, United States, 3. National Institutes on Aging, Baltimore, Maryland, United States*

An operational definition of 'well-aged' is integral to research identifying biological, psychological and behavioral parameters that predict and/or support healthy aging but remarkably few measures exist. A major challenge is incorporating age-related physiologic decline – a normal feature of healthy aging. We defined well-aged using fast 400m walk performance, a measure of cardiovascular fitness and hallmark of healthy aging. We determined the sex-specific, age-normalized 75th percentile using 4000+ observations collected over 15 years from BLSA participants aged 50-96y (51% women). In 1257 BLSA participants mean age 68.9y (52% women) we compared well-aged to average-aged (25th-75th percentile) adjusting for age, sex and race, and found more favorable WBC, triglycerides, CRP, depressive symptoms, waist/height ratio, eGFR and FEV1 z-score distinguished concurrent well-aged status (all p<.003). Waist/height ratio and pulse-wave velocity (both p<.001) predicted retained well-aged status 2-plus years later. Future work aims to identify physiologic and behavioral facilitators of healthy aging.

### SESSION 4210 (SYMPOSIUM)

### THE INFLUENCE OF NURSING HOME CONTEXT ON IMPLEMENTATION: EARLY FINDINGS

Chair: Carole Estabrooks Co-Chair: Anna Beeber
Discussant: Amy Vogelsmeier

Evidence suggests that organizational context, including context of care in nursing homes (NHs) is associated with implementation and improvement success and that optimized context contributes to improved resident and staff outcomes. However, evidence on mechanisms of these influences is scarce. The longitudinal Translating Research in Elder Care program (2007 to present) is our data source. This symposium reports findings of a secondary analysis of longitudinal organizational structural and contextual, staff worklife, and resident care quality data collected in nursing homes from 2007-2021, in a cohort of 94 Canadian nursing homes. Our data include continuously collected administrative data (RAI-MDS 2.0), 5 waves of primary surveys from multiple care providers in NHs (15,000 cases to date), extensive qualitative case study data, and data from two large scale pragmatic trials. The TREC program was framed originally and continues to be, using the Promoting Action on Research Implementation in Health Services (PARiHS) framework. Each of our 5 papers will report on a critical aspect of the findings. The symposium will conclude with a discussion of implications of these findings of phase one and a description of phase two and how it is shaped by our interactions with expert panels (context and implementation experts, policymakers, NH managers, paid care providers, and residents and families). Early implications of this work for future research in nursing homes and for practice and policy will be mentioned.

### DEVELOPMENTAL ADVANCES OF THE PARIHS FRAMEWORK OVER THE LAST DECADE: A CRITICAL INTERPRETIVE SYNTHESIS

Whitney Berta[1], Yinfei Duan[2], Alba Iaconi[2], Jing Wang[3], Janelle Perez[4], Yuting Song[5], Stephanie Chamberlain[2], and Carole Estabrooks[2], *1. University of Toronto, Toronto, Ontario, Canada, 2. University of Alberta, Edmonton, Alberta, Canada, 3. University of North Carolina, Chapel Hill, North Carolina, United States, 4. University of North Carolina Chapel Hill, Chapel Hill, North Carolina, United States, 5. Qingdao University, Qingdao, Shandong, China (People's Republic)*

The original Promoting Action on Research Implementation in Health Services (PARiHS) and i-PARiHS frameworks contend that the successful implementation of evidence-based practices is a function of the core elements evidence, context, facilitation, and the capacity of intended recipients to apply research to practice. While applied widely, a number of theoretical and practical challenges associated with the framework's application have been identified. Our critical interpretive synthesis examines how the last decade of research has advanced understanding of the conceptualizations of, relationships between, and dynamics amongst, PARiHS core elements. We find that work over the past decade affords more nuanced conceptualizations of context and facilitation; reveals myriad conceptualizations of implementation success, suggesting the need for a typology; demonstrates contradictory effects of context on facilitation that warrants more study; leads us to question the contextual primacy of leadership; and generally under-examines the interactions and dynamics amongst PARiHS core elements and their sub-elements.

### UNDERSTANDING LEADERSHIP IN NURSING HOME CONTEXT THROUGH ADAPTIVE LEADERSHIP FRAMEWORK FOR CHRONIC ILLNESS

Jing Wang[1], Janelle Perez[2], Anna Beeber[3], Ruth Anderson[4], Whitney Berta[5], Holly Lanham[6], and Carole Estabrooks[7], *1. University of North Carolina at Chapel Hill, Chapel Hill, North Carolina, United States, 2. University of North Carolina Chapel Hill, Chapel Hill, North Carolina, United States, 3. Johns Hopkins University, Baltimore, Maryland, United States, 4. UNC Chapel Hill, Chapel Hill, North Carolina, United States, 5. University of Toronto, Toronto, Ontario, Canada, 6. University of Texas Health San Antonio, San Antonio, Texas, United States, 7. University of Alberta, Edmonton, Alberta, Canada*

The case studies were conducted as an early component of a pan-Canada project entitled Translating Research in Elder Care (TREC). This sub-project provided insights into the challenges and leadership of facilitating care model changes, care quality improvement, and quality of work enhancement of three long-term care (LTC) facilities in Canada. Through the lens of Adaptive Leadership Framework for Chronic Illness (ALFCI), leadership emerged as a critical element of their organizational context. Staff reported that contextual factors of high intensity of work and inadequate staff were barriers that added to the complexity of challenges facing them. Data collectors observed that frontline staff exhibited leadership behaviors in knowledge transmission, information sharing, teamwork, and person-centered strategies to address challenges. However, top-down facilitation can lead to misunderstanding and a lack of motivation from the frontline staff to follow the facilitation. The findings also suggested tailored facilitation about including frontline staff in formal interactions.

### CONTEXT AND TRUST: DIRECT CARE WORKER RELATIONSHIPS, CARE PRACTICES, FACILITATION, AND IMPLEMENTATION OF INNOVATIONS

Janelle Perez[1], Jing Wang[2], Anna Beeber[3], Ruth Anderson[4], Whitney Berta[5], Holly Lanham[6], and Carole Estabrooks[7], *1. University of North Carolina Chapel Hill, Chapel Hill, North Carolina, United States, 2. University of North Carolina, Chapel Hill, North Carolina, United States, 3. Johns Hopkins University, Baltimore, Maryland, United States, 4. UNC Chapel Hill, Chapel Hill, North Carolina, United States, 5. University of Toronto, Toronto, Ontario, Canada, 6. University of Texas Health San Antonio, San Antonio, Texas, United States, 7. University of Alberta, Edmonton, Alberta, Canada*

This paper examines the relationships between Direct Care Workers (DCW) and other nursing home (NH) staff. Specifically, we examine how trust, and the context in which trust exists, influences care practices and implementation of innovations in NHs. This secondary analysis of interviews and field observations from three separate NHs in different Canadian provinces shows differing levels of trust that between HCAs and other staff. We also analyze the organizational context can mediate trust in these relationships. Specifically, we analyzed trust DCWs have at the interpersonal, institutional, organizational levels and what ways they act as facilitators of programs aimed at improving care practices health outcomes of residents in NHs. Using the Alberta Context Tool's dimensions of organizational context as a framework, this research shows how trust in the relationships between DCWs and other LTC staff influences caregiving practices and the uptake of new innovations.

### ORGANIZATIONAL CONTEXT AND QUALITY INDICATORS IN NURSING HOMES: A MICROSYSTEM LOOK

Yinfei Duan[1], Alba Iaconi[1], Yuting Song[2], Matthias Hoben[1], Leslie Hayduk[1], Peter Norton[3], and Carole Estabrooks[1], *1. University of Alberta, Edmonton, Alberta, Canada, 2. Qingdao University, Qingdao, Shandong, China (People's Republic), 3. University of Calgary, Calgary, Alberta, Canada*

This cross-sectional quantitative sub-project assessed the association of organizational context (modifiable elements of work environments) with quality indicators (QIs) at the clinical microsystem (care unit) level. We used TREC data collected 09/2019-03/2020. The sample included 285 care units within 91 Western Canadian nursing homes. Outcomes included thirteen practice-sensitive QIs derived from the Minimum Data Set 2.0. Results from random-intercept logistic regression for each dichotomized QI showed that higher unit-aggregated scores on contextual elements as identified by the Alberta Context Tool, specifically care aide participation in decision-making (OR=3.7-8.4, $p<.05$), care aide perceived staffing (OR=2.6, $p<.05$) and time for completing tasks (OR=5.1-7.0, $p<.05$), and care aide rated unit-level leadership (OR=20.1, $p<.05$), were associated with a better unit-level performance on delirium symptoms, indwelling catheter use, behavioral symptoms, pain, and late-loss physical function. The findings suggest that targeting modifiable contextual elements is an important avenue for quality improvement interventions in nursing homes.

### ORGANIZATIONAL CONTEXT'S EFFECT ON CARE AIDES' PSYCHOLOGICAL EMPOWERMENT IN WESTERN CANADA

Alba Iaconi[1], Yinfei Duan[1], Yuting Song[2], Matthias Hoben[1], Leslie Hayduk[1], Peter Norton[3], and Carole Estabrooks[1], *1. University of Alberta, Edmonton, Alberta, Canada, 2. Qingdao University, Qingdao, Shandong, China (People's Republic), 3. University of Calgary, Calgary, Alberta, Canada*

This quantitative cross-sectional sub project investigated the effects of organizational context and individual characteristics on psychological empowerment of care aides working in nursing homes. We analyzed data collected from 3765 care aides from 91 nursing homes across Western Canada between 09/2019 and 03/2020. From the random-intercept mixed effects regression models we identified significant predictors at different levels for each component of psychological empowerment. At the organizational outer context level: region and home ownership model. At the inner context (care unit) level: formal interactions (β=-0.07, p=0.03; competence), evaluation (β=0.20, p<0.02; self-determination), culture (β=0.20, p<0.02; self-determination), communication (β=0.16, p<0.001; self-determination), and social capital (β=0.22, p=0.01; impact). At the individual level: care aides' sex, language and job satisfaction.These findings suggest important ways in which contextual elements may influence staff quality of work life characteristics and underscore the need to consider context operating at different levels, as well as consider individual and contextual interaction.

### SESSION 4220 (PAPER)

### TRANSPORTATION AND COMMUNITIES

### ASSOCIATIONS BETWEEN LIFE SPACE MOBILITY AND ASPECTS OF HOUSING AND SERVICE INFRASTRUCTURE

Bjorn Slaug, Magnus Zingmark, and Susanne Iwarsson, *Lund University, Lund, Skane Lan, Sweden*

To achieve the widespread policy goal of active aging, it is central that older adults can participate and be independent in activities both in and outside the home. Life-space mobility refers to the extent of the area where a person performs different activities, including frequency and level of independence, and is considered an indicator of active aging. There is, however, a lack of studies on the relationship between life-space mobility and aspects of the home and neighborhood environment. The aim of this study was to explore associations between life-space mobility and (a) perceived usability of the home and (b) satisfaction with commercial and societal services in the surrounding neighborhood. We utilized the baseline survey of the Swedish RELOC-AGE project (N=1,964; mean age=69; 45% men), comprising data from adults aged 55+ with an interest for relocation. We found weak but significant correlations between life-space mobility and usability of the home (r=0.10; p< 0.001), and between life-space mobility and satisfaction with service infrastructure (r=0.09; p< 0.001). The correlations were stronger for men and for adults aged 65-74 years, compared to younger and older age groups. Those with their independent life space limited to the home and the close exterior surroundings (balcony, garden etc) assessed the usability of the home significantly lower (p=0.04) than those with more extended independent life space. There was no difference with regard to infrastructure satisfaction. These results suggest that improvement of both the home environment and service infrastructure may be important in supporting extended life space mobility among older adults.

### FIT2DRIVE: DEVELOPMENT AND TESTING OF A PREDICTOR OF DRIVING CAPACITY OF OLDER ADULTS WITH COGNITIVE CONCERNS

Ruth Tappen[1], David Newman[1], Monica Rosselli[2], and Joshua Coniff[2], *1. Florida Atlantic University, Boca Raton, Florida, United States, 2. Florida Atlantic University, Davie, Florida, United States*

The decision to stop driving has been reported by caregivers of persons with dementia as one of the most difficult ones they confront. Additionally, primary care providers to whom they often turn for guidance report being unprepared to provide them with evidence-based information. Our aim was to develop and test a predictive model employing 2 or 3 brief, easily administered cognitive tests to predict the individual's likelihood of passing an on road driving test. Participants were licensed drivers recruited from our Memory Center's driver evaluation program and the community to obtain a broadly representative sample of older drivers. A total of 357 drivers age 60 to 97 (mean 81) completed an established on-road driving test and battery of short cognitive tests. Two-thirds of the sample were white, non-Hispanic, one third were Black, African American, 59% male and 41% female, mean MMSE score of 24. Employing Receiver Operating curve analysis, the best set of predictors included participant age, MMSE utilizing world spelled backwards (a better predictor than serial 7's), Trails B time in seconds and participant age yielding 95% AUC (area under the curve). The model was invariant across gender, education and ethnic group. A website with an interactive calculator in which this data is entered and likelihood of passing an on-road driving test prediction is presently under construction and will be available first to providers and later to individuals with a concern about continuing capacity to drive. Funded by Florida Department of Health Ed and Ethel Moore Alzheimer's Disease Initiative.

### IMPACT OF COVID-19 MOVEMENT CONTROL ON OLDER ADULTS' HEALTHCARE UTILIZATION

Vanessa Tan[1], Gregory Ang[1], Kim Huat Goh[2], Adrian Yeow[3], Gamaliel Tan[4], and Cynthia Chen[5], *1. National University of Singapore, Singapore, Singapore, 2. Nanyang Technological University, Singapore, Singapore, 3. Singapore University of Social Sciences, Singapore, Singapore, 4. Ng Teng Fong General Hospital, Singapore, Singapore, 5. National University of Singapore, Singapore, Singapore*

Singapore was one of the first countries affected by COVID-19. Measures to contain the spread of COVID-19 include raising the Disease Outbreak Response System Condition (DORSCON) risk assessment to Orange and instituting a movement control order, termed as the Circuit Breaker. These measures have caused significant disruption in primary care and chronic disease management. As the first point of contact in testing suspected cases, primary care providers shifted their focus from non-COVID-19 services. Using an interrupted time series analysis, we examine the associations of DORSON Orange and Circuit Breaker on acute and chronic primary care visits among older adults aged above 50. We found significant reductions in both acute and chronic primary care visits immediately following DORSCON Orange and Circuit Breaker. DORSCON Orange was associated with a drop of 231 mean acute and chronic daily visits (95% CI -356 to -106). Circuit Breaker was associated with a further drop of 268 mean daily visits (95% CI -426 to -111). These reductions were also observed for acute and chronic visits separately. Routine chronic care appointments were deferred or cancelled to reduce the risk of transmission as patients with underlying medical conditions were at higher risk of developing severe complications. Delayed access to primary care can have profound health implications, especially for older adults. Ensuring accessibility to primary care is a key priority in maintaining population health. Understanding the impact of COVID-19 tightening measures on older adults' primary care utilisation will be useful for future public health planning.

### PROFILE OF OLDER PUBLIC TRANSPORTATION USERS IN THE UNITED STATES: IMPLICATIONS FOR AGE-FRIENDLY COMMUNITIES

Afnan Gimie[1], Andrea Melgar Castillo[2], C. Daniel Mullins[2], and Jason Falvey[3], *1. University of Maryland School of Medicine, Baltimore, Maryland, United States, 2. University of Maryland School of Pharmacy, Baltimore, Maryland, United States, 3. University of Maryland Baltimore, Baltimore, Maryland, United States*

Public buses, trains, and trams are a growing mode of transportation for older adults in the United States, yet many environmental and health related barriers to use have been reported. Characterizing the population of older public transit users is essential for developing age-friendly communities. We used data from 5696 urban, community dwelling older adults in round 5 of the National Aging and Trends Study (NHATS), an annual nationally representative survey of late-life disability. Using SAS (version 9.4), weighted frequencies were calculated and compared between public transit and non-transit users using procedures that account for the complex design of the NHATS survey. Compared to non-transit users, those who reported using transit within the last month (n=555, 9.8%; weighted n=3,122,583) were significantly more likely to identify their race/ethnicity as Black or Hispanic (50% vs 28%) and reported difficulty meeting financial needs for housing, utility, and food (12% vs 7%), and to speak a language other than English (14% vs 8%). Transit users were significantly less likely to use a walker (9% vs 14%) or wheelchair/scooter (4% vs 9%). Additionally, 15% of transit users did not have a working cell phone and 42% did not have a working computer. Over 20% of transit users (weighted n=658,850) rely on these services to get to their doctor. These findings highlight the clinical, social, and financial barriers that disproportionately affect over 3 million older adult transit users in the United States, and inform initiatives oriented towards improving community access for older adults.

### REFINING CARFREEME, A DRIVING RETIREMENT PROGRAM FOR PERSONS WITH DEMENTIA AND THEIR CARE PARTNERS

Colleen Peterson[1], Katie Louwagie[2], Robyn Birkeland[2], Stephanie N. Ingvalson[2], Lauren Mitchell[3], and Joseph Gaugler[2], *1. University of Michigan Transportation Research Institute, Ann Arbor, Michigan, United States, 2. University of Minnesota, Minneapolis, Minnesota, United States, 3. Emmanuel College, Boston, Massachusetts, United States*

Persons living with dementia (PLWD) are at increased risk for roadway crashes and subsequent injury or death. Navigating driving retirement while respecting the PLWD's autonomy and supporting continued independence can be challenging. CarFreeMe™, originally developed in Australia, is a driving retirement intervention providing tailored psychoeducational telecoaching modules to PLWD and/or their care partners. Session topics include living with dementia, balancing independence and safety, adjusting to loss and change, exploring others' experiences with driving retirement, planning for alternative transportation, lifestyle planning, advocacy and support, and problem solving. Phase I enrolled 16 care partners and 11 PLWD. Mixed methods data from Phase I's 1- and 3-month follow-up surveys and post-intervention interviews demonstrated feasibility and acceptance of CarFreeMe™ with a U.S. audience. Phase I participants found the program valuable and would recommend it to others (96% care partners, 100% PLWD). Care partners and PLWD reported improved Readiness of Mobility Transition scores at the 3-month survey. Several felt the program may be most useful early in the decision making process. The program offered strategies and education that facilitated conversations both during and outside of the intervention sessions to support the PLWD's agency and acceptance of driving retirement. Participant feedback and lessons learned from Phase I informed Phase II development and deployment. Phase II is enrolling 50 care partners, PLWD, or dyads and includes 3- and 6-month follow-up surveys. Preliminary CarFreeMe™ Phase II utility, acceptance, and driving related outcomes will be discussed as well as next steps for evaluation.

### SESSION 4230 (AWARD LECTURE)

### DONALD P. KENT AND ROBERT W. KLEEMEIER AWARD LECTURES

Chair: Peter Lichtenberg

The Donald P. Kent Award lecture will feature an address by the 2021 Kent Award recipient Luigi Ferrucci, MD, PhD, FGSA, of the National Institute on Aging. The Kent Award is given annually to a member of The Gerontological Society of America who best exemplifies the highest standards of professional leadership in gerontology through teaching, service, and interpretation of gerontology to the larger society. The Robert W. Kleemeier Award lecture will feature an address by the 2021 Kleemeier Award recipient Kenneth F. Ferraro, PhD, FGSA, of Purdue University. The Kleemeier Award is given annually to a member of The Gerontological Society of America in recognition for outstanding research in the field of gerontology.

### DUAL FUNCTIONALITY IN LATER LIFE

Kenneth Ferraro, *Purdue University, West Lafayette, Indiana, United States*

Longevity and quality of life are core interests in gerontology, but debate has ensued as scholars have sought to integrate the two. I propose the concept of dual functionality to examine how humans reach advanced ages while maintaining both physical and cognitive function. Using a large national sample, my colleagues and I operationalize dual functionality and identify life course factors that predict it. Analyses of 33,310 respondents 50 years or older from the Health and Retirement Study show an estimated median age of 74 for loss of dual functionality. Lifetime stress exposure leads to earlier loss of dual functionality, even after adjustment for socioeconomic status and lifestyle factors.

Estimates of dual-function life expectancy, moreover, reveal greater racial-ethnic disparities than those for life expectancy per se. Dual functionality may be useful for assessing the quality of longevity across societies and social categories and for identifying exceptional longevity.

### A JOURNEY FROM GERIATRIC MEDICINE TO GEROSCIENCE

Luigi Ferrucci, *National Institute on Aging, Baltimore, Maryland, United States*

In 1984 LZ Rubenstein group demonstrated that geriatric assessment improved function and QoL in frail in frail, older patients. I heartly joined the international sparkle of enthusiasm generated by these results although later work did not match our expectations. Understanding the complexity of older person is an extraordinary tool for geriatricians, but coding the nuances of making the "best choice" in a randomized trial remains difficult. Frailty is difficult to reverse because it occurs when resilience is exhausted. Geroscience postulates that chronic diseases and frailty stem from the biological mechanism of aging and that interventions that slow down aging will successfully improve resilience. This approach have shown great potential but whether it will lead to prevention or improvement of frailty is unknown. While we continue to provide optimal care to frail older patients, we need to push forward the translation arm of geroscience both in area of prevention and care of older patients.

### SESSION 4225 (BSS FLASH POSTERS)

### BSS FLASH POSTER SESSION 1: SOCIAL DETERMINANTS OF LATE LIFE HEALTH: A LIFE COURSE PERSPECTIVE

### ADVERSE CHILDHOOD EXPERIENCES AND DEPRESSIVE SYMPTOMS AMONG RACIALLY/ETHNICALLY DIVERSE OLDER ADULTS IN THE US

David Camacho[1], Julia Vazquez[2], Laura Vargas[3], Charles Henderson[4], and Brenda J Jones-Harden[2], *1. University of Maryland, Baltimore, Norwalk, California, United States, 2. University of Maryland, Baltimore, Baltimore, Maryland, United States, 3. University of Colorado School of Medicine, Department of Psychiatry, Aurora, Colorado, United States, 4. Cornell University, Ithaca, New York, United States*

Adverse childhood experiences (ACEs) and depression are major public health concerns. However, few studies have examined the relationship between ACEs and mid- and late-life depression among racially/ethnically diverse groups. We explore this relationship among U.S. racially/ethnically diverse community-dwelling midlife and older adults (≥50 years of age). Guided by ACEs and Minority Stress Frameworks, we used general linear models to examine this relationship with data from Wave 3 of the National Social Life, Health, and Aging Project. We created an ACEs composite ranging from 0 to 7 (e.g., violence, health, poverty) and assessed the role of individual ACEs on depressive symptoms (CES-D). Final adjusted models (n:1424) included key demographic, health (e.g., chronic disease), social (living alone, social isolation, loneliness), and minority stress factors (e.g., limited access to healthcare and treatment, perceived discrimination). Results indicated that higher composite score ACEs (particularly childhood violence and poor health) were positively associated with higher levels of depressive symptoms. We found no interactions between race/ethnicity and ACEs. Our results suggest that ACEs contribute to the presence and severity of depressive symptoms into mid- and late-life adulthood. Consistent with Minority Stress Framework, common life-course stressors for minoritized groups may explain a lack of significant interactions in our models. Future research should explore the association of ACEs and other important health outcomes in diverse midlife and older adults. Finally, research is needed to examine if and how culturally appropriate depression interventions can be adapted to address the role of ACEs in later life health.

### CHILDHOOD PHYSICAL ABUSE INCREASES THE RISK OF SUBJECTIVE MEMORY IMPAIRMENT

Lauren Roach[1], Tyler Bell[2], and Caitlin Pope[3], *1. University of Kentucky, Holland, Michigan, United States, 2. University of California, San Diego, La Jolla, California, United States, 3. University of Kentucky, Lexington, Kentucky, United States*

Subjective memory impairment, defined as self-reported difficulties in recall and learning, doubles the risk of Alzheimer's Disease and related dementia, despite being weakly related to objective memory decline. Because of its strong stability over time, it may be possible that subjective memory impairment reflects earlier life risk factors for dementia such as adverse childhood experiences. It is reported that over a fifth of older adults worldwide experienced physical abuse during childhood. Previous cross-sectional studies suggest physical abuse is associated with later cognitive impairment. Still unclear, are the longitudinal associations between childhood abuse and subjective memory impairment in later life. Using a sample of adults drawn from the Health and Retirement Study (n = 19,185, Mage = 67.05, SD = 11.33) we assessed associations between reported physical abuse by a parent before the age of 18 and subjective memory impairment (current memory problems and perceived memory decline) over periods of up to 18 years. Generalized linear mixed models examined longitudinal associations between childhood physical abuse and subjective memory impairment while controlling for depressive symptoms and other empirically relevant covariates. Experiencing childhood physical abuse was associated with increased likelihood of reporting more current memory problems (OR = 1.17, 95% CI 1.04, 1.33) and perceived memory decline in later life (OR = 1.27, 95% CI 1.13, 1.43). Findings suggest childhood physical abuse is associated with subjective memory impairment, a strong predictor of dementia. Understanding early life conditions, including adverse childhood experiences may help explain associations between subjective memory impairment and dementia risk.

### SEQUENCING OF PLANNED AND UNPLANNED BIRTHS AND IMPLICATIONS FOR MID- AND LATER-LIFE HEALTH AMONG NLSY79 WOMEN

Mieke Thomeer[1], Clifford Ross[1], Rin Reczek[2], and Christina Bijou[2], *1. University of Alabama at Birmingham, Birmingham, Alabama, United States, 2. Ohio State University, Columbus, Ohio, United States*

Existing studies demonstrate that unplanned births (e.g., unwanted, mistimed) are associated with worse health for mothers in the short-term and—according to some preliminary evidence—in mid- and later-life. Yet as life course and reproductive career frameworks highlight, childbearing experiences often unfold over a number of years, with a considerable amount of diversity in pregnancy and birth experiences even for the same individual. For example, a person may have an unplanned birth in late adolescence followed by only planned births in early adulthood. In order to provide a more holistic understanding of how birthing experiences births are associated with midlife health, we use Sequence Analysis (SA) on the 1979 National Longitudinal Survey of Youth (NLSY79; N=3,992) to examine how patterning of planned and unplanned births is associated with physical and mental health at ages 50 and 60 (SF-12). Preliminary analysis indicates that compared to respondents with only planned births, respondents with unplanned birth(s) followed by planned birth(s) have worse physical and mental health at midlife, but there is no difference in health for respondents with only planned births, only unplanned births, and planned birth(s) followed by unplanned birth(s). Future analysis with SA will consider how more detailed sequences (e.g., timing, number and type, ordering, spacing) are associated with these mid- and later-life health outcomes, taking into account selection factors such as childhood SES and educational attainment. This project demonstrates the need for life course perspectives on the long-term health implications of unplanned births, recognizing diversity within and between individuals.

### CHILDHOOD MALTREATMENT AND PROSOCIAL BEHAVIOR: A QUALITATIVE COMPARATIVE STUDY OF IRISH OLDER ADULT SURVIVORS

Shauna Rohner[1], Aileen Salas[2], Alan Carr[3], and Myriam Thoma[4], *1. University of Zürich, Zürich, Zurich, Switzerland, 2. University of Zürich, Zurich, Zurich, Switzerland, 3. University College Dublin, Dublin, Dublin, Ireland, 4. University of Zurich, Zurich, Zurich, Switzerland*

Although childhood maltreatment can have lasting effects into later life, positive outcomes have also been observed, including an increased tendency towards prosocial behavior. However, little is known about the link between childhood maltreatment and later life prosocial behavior. Therefore, this study aimed to explore older adult's experiences of childhood maltreatment and identify mechanisms linked to prosocial behavior in later life. The individual level, but also broader cultural and contextual mechanisms, were considered by comparing two adversity contexts and applying conceptual frameworks (socio-interpersonal framework model of trauma and recovery, motivational process model of altruism born of suffering). Semi-structured interviews (60-120 minutes) were conducted with 29 Irish (older) adult survivors of childhood maltreatment: 17 institutional (welfare care) abuse survivors (mean age: 61 years, range: 50-77), 12 familial abuse survivors (mean age: 58 years, range: 51-72). Interviews were analyzed using Framework Analysis. In both groups at the individual level, enhanced empathy, amelioration, and identity-related mechanisms were linked to prosocial behavior, with connections to caring roles and coping strategies from childhood. On a social contexts level, the limited resources or opportunities for help in childhood, and the social norms and beliefs of that time, influenced participants' motivation to help others in later life. Group-specific mechanisms were also observed, such as compassion fatigue in the familial sample; and denouncing detrimental societal values in the institutional sample. The identification of individual, adversity-context, and culture-specific mechanisms linked to later-life prosocial behavior can promote a greater understanding of resilience and adaptability in older adult survivors of childhood maltreatment.

### THE NEIGHBORHOOD CONTEXT AND ALL-CAUSE MORTALITY AMONG OLDER ADULTS IN PUERTO RICO

Catherine Garcia[1], Mary McEniry[2], and Michael Crowe[3], *1. Syracuse University, Syracuse, New York, United States, 2. University of Wisconsin-Madison, Madison, Wisconsin, United States, 3. University of Alabama-Birmingham, Birmingham, Alabama, United States*

The neighborhood contexts in which older adults live are increasingly being recognized for their role in influencing disease processes and risk of death among the U.S. population. However, few studies have focused on neighborhood impacts among older populations residing in Puerto Rico– a U.S. territory –who are especially vulnerable to the effects of the environment as they "age in place" in the context of a budget crisis, the great recession, the debt crisis, and Hurricanes Irma and María. The combination of these events can obstruct access to neighborhood resources, services, and contexts considered necessary for promoting healthy aging. Thus, it is warranted to understand the effects of place on mortality in Puerto Rico, whose social and economic contexts differ from the U.S. and are more similar to that of other Latin American and Hispanic-Caribbean countries. We used 2000 U.S. Census data at the block-group level linked to the 2002 Puerto Rican Elderly Health Conditions Project with mortality follow-up to 2021 to examine neighborhood characteristics that are conceptualized as influencing mortality (e.g., residents without a high school degree; households receiving public assistance income; residents living below the poverty level; unemployed residents; residential stability; age structure). Multilevel mixed-effects parametric survival models with a Weibull distribution were estimated. Overall, results show that neighborhood socioeconomic disadvantage is associated with an increased risk of mortality among older Puerto Ricans. This suggests that older Puerto Ricans clustered in disadvantaged communities are more likely to experience a cumulative burden of social disadvantages that adversely impacts their longevity.

### IS THE HEALTH OF OLDER AMERICANS WITH A GED EQUIVALENT TO THEIR PEERS WITH A HIGH SCHOOL DIPLOMA?

Esme Fuller-Thomson, Robin Grossman, and Andie MacNeil, *University of Toronto, Toronto, Ontario, Canada*

Previous research has found higher levels of educational attainment to be strongly associated with better health outcomes in later life, such as better cognitive functioning and fewer functional and sensory impairments. However, most studies have grouped General Educational Development (GED) recipients with high school graduates, neglecting potential differences in socioeconomic status, health behaviours, and health outcomes among these two groups. The aim of the current study is to identify differences in the age-sex-race-poverty adjusted prevalence and odds of cognitive impairment, hearing impairment, vision impairment, limitations in activities of daily living (ADLs), and ambulation limitations among three groups of older American adults: high school dropouts, GED recipients, and high school graduates with no post-secondary education. The present study uses secondary analysis of the 2017 American Community Survey, a nationally representative survey of community-dwelling and institutionalized older adults aged 65 years and older, of whom 20,489 were GED recipients, 154,892 had a high school diploma and 49,912 had finished grade 8 but had not completed high school. Our findings indicate that there is a gradient in health outcomes among Americans aged 65-84, with the highest prevalence and odds of cognitive impairment, hearing impairment, vision impairment, ADL limitations, and ambulation limitations occurring among high school dropouts, followed by GED recipients, and the lowest prevalence among high school graduates. These findings suggestion that although GED recipients have better health outcomes than high school dropouts, there is still a significant disparity in health status between GED recipients and high school graduates.

### COHORT DIFFERENCES IN EARLY-LIFE SOCIOECONOMIC STATUS AND LATE-LIFE COGNITIVE IMPAIRMENT IN MEXICO

Brian Downer[1], Mariela Gutierrez[1], Silvia Mejia Arango[2], and Rebeca Wong[1], *1. University of Texas Medical Branch, Galveston, Texas, United States, 2. Colegio de la Frontera Norte, Tijuana, Baja California, Mexico*

Socioeconomic characteristics over the life course are associated with late-life cognitive impairment. However, evidence is lacking from countries like Mexico where population aging is occurring in the context of rapidly changing socioeconomic conditions. We used the Mexican Health and Aging Study to investigate differences between participants aged 60-76 in 2001 (n=5085) and 2018 (n=5947) in childhood (home with indoor toilet, parents' education) and midlife (education, longest held occupation) socioeconomic characteristics and late life cognitive impairment. Cognitive impairment was defined as a low score on >2 out of five assessments. Most participants in the 2018 cohort lived in a home with an indoor toilet as a child (58.1%) and 36.9% had parents who both completed at least some education compared to 41.9% and 28.7% of participants in the 2001 cohort, respectively. Men and women in 2018 had on average 2.34 and 1.83 more years of education than men and women in 2001, respectively. The percentage of women with no main job and men who worked in agriculture were lower in 2018 than 2001 (women: 27.0% vs. 34.6%; men: 23.3% vs. 30.4%). The 2018 cohort had lower odds for cognitive impairment when adjusting for age, sex, marital status, and living in a rural/urban community (OR=0.67 95% CI=0.56-0.81). This difference was reduced after adjusting for childhood socioeconomic measures (OR=0.76 95% CI=0.67-0.86) and was no longer statistically significant after adding midlife socioeconomic measures (OR=0.98 95% CI=0.86-1.12). These findings suggest that improved early-life socioeconomic conditions in Mexico contribute to birth-cohort differences in late-life cognitive impairment.

### SESSION 4240 (PAPER)

### COVID-19 AND MENTAL HEALTH AND WELL-BEING OF OLDER ADULTS

### AN ACTION PLAN FOR ORGANIZATIONS SERVING OLDER ADULTS AND THEIR CAREGIVERS DURING PUBLIC HEALTH EMERGENCIES

Alycia Bayne[1], Lauren Isaacs[2], Jessica Fox[1], Rachel Singer[1], Roy Ahn[3], Desirae Leaphart[2], Felicia Cerbone[4], and Madeleine Liotta[1], *1. NORC at the University of Chicago, Bethesda, Maryland, United States, 2. NORC at the University of Chicago, Chicago, Illinois, United States, 3. NORC, Chicago, Illinois, United States, 4. NORC, Bethesda, Maryland, United States*

During public health emergencies, it is critical to maintain the continuity of services and resources essential to health and safety. Public health emergencies can disproportionately affect older adults and their caregivers. Organizations, including community-based, faith-based, rural, and tribal organizations, can play a vital role during a public health emergency response given their familiarity with the community's unique needs and resources. With support from the CDC Foundation and technical assistance from the Centers for Disease Control and Prevention, NORC at the University of Chicago conducted a study to identify public health interventions to meet the needs of older adults and their caregivers during public health emergencies. Methods included an extensive search of peer-reviewed and grey literature in Spanish and English to identify interventions on six topics: deconditioning; deferral of medical care; elder abuse and neglect; management of chronic conditions; social isolation; and caregiving. NORC identified 300 public health interventions to support older adults and their caregivers during public health emergencies with a focus on underserved populations, including programs to support racial and ethnic minority populations, people with disabilities, and rural and tribal communities. NORC developed Search. Find. Help., a virtual resource library, and an Action Plan to support organizations in using these interventions. Search. Find. Help., which houses the Action Plan, has had 34,000 unique users. This session focuses on how organizations that serve older adults and caregivers can use the Action Plan's four phases to select, adapt, implement, and evaluate public health interventions before or during a public health emergency.

### OLDER ADULT PERSPECTIVES ON AGEISM DURING COVID-19: A QUALITATIVE STUDY

Carson De Fries[1], Pilar Ingle[2], Matthew Schilz[1], Andrew Steward[3], Emma Baker[1], and Leslie Hasche[1], *1. University of Denver, Denver, Colorado, United States, 2. University of Denver, Lakewood, Colorado, United States, 3. University of Wisconsin-Milwaukee, Milwaukee, Wisconsin, United States*

Since the COVID-19 pandemic began, there has been a reported surge of ageism toward older adults. Research demonstrates that events perpetuating negative attitudes towards older adults can increase ageism and associated negative outcomes. The purpose of this phenomenological qualitative study was to explore how older adults navigated experiences of ageism and their social relationships during the COVID-19 pandemic. Semi-structured interviews with adults ages 60 and older were conducted between February and April of 2021

over Zoom. Data were coded using an iterative, inductive approach and thematic analysis was performed to draw themes from the data. A total of 24 participants ages 61-80 (mean = 70.6) were interviewed. Most participants identified as white (n = 19) female (n = 14), retired (n = 21) and had at least a bachelor's degree (n = 22). Findings showed that participants experienced ongoing ageism but did not report ageist experiences associated specifically with COVID-19. Ageist experiences, unrelated to COVID-19, as shared by participants included assumptions about older adults' (in)ability to use technology, ageism in professional settings, and feelings that ageism is an inevitable part of growing older. Future research should examine the impact of intersectionality on this topic within more diverse populations and explore potential differences that may have occurred throughout different stages of the pandemic.

### PANDEMIC CAREGIVING: A LONGITUDINAL ASSESSMENT OF THE TRAJECTORIES AND CORRELATES OF STRESS

Kristin Litzelman, Christina Kim, and Margaret Kerr, *University of Wisconsin-Madison, Madison, Wisconsin, United States*

In public health emergencies, caregivers are a crucial but often overlooked human resource. The purpose of this longitudinal study was to assess the well-being of caregivers and non-caregivers over the first 18 months of the COVID-19 pandemic. We used three waves of data from the Survey of the Health of Wisconsin's COVID-19 Community Impact Survey (Wave 1: May-June 2020; Wave 2: January-February 2021; Wave 3: June 2021; n=2,434 observations of 1,653 unique respondents). Caregivers were identified as those providing care for an adult with an illness or disability. Perceived stress (Global Stress Scale; mean=5.08, SD=4.81) was regressed on caregiver status and covariates in mixed models accounting for repeated measures. On average, caregivers had higher stress than non-caregivers (beta=2.10, $p< 0.0001$). Across the sample, stress increased between summer 2020 and winter 2021 (mean of 4.8 versus 5.8, $p<.01$), and lowered somewhat by summer 2021 (mean=5.0, $p<.05$); this trajectory was similar on average for caregivers and non-caregivers. Respondents who transitioned into a caregiving role during the pandemic had the highest stress (beta=2.55, $p< 0.01$ compared to non-caregivers). Other factors associated with higher stress ($p<.01$) include marginalized racial/ethnic identity (beta=1.74), being employed (beta=1.47) or female (beta=0.66), or caregiver having more health conditions (beta=0.22 per condition). Public benefits use and higher self-efficacy were associated with lower stress (betas=-1.18 and -0.30, respectively, $p<.01$). The findings emphasize the adverse outcomes experienced by caregivers and non-caregivers over the course of the pandemic and highlight potential factors that can inform risk stratification and interventions to support well-being in future crises.

### REOPENING UNDER COVID-19: THE IMPACT OF REOPENING SOCIETY ON OLDER ASIAN AMERICAN'S DEPRESSIVE SYMPTOMS

Yifan Lou[1], Ethan Siu Leung Cheung[1], Jinyu Liu[2], and Bei Wu[3], *1. Columbia University, New York, New York, United States, 2. Columbia University, New York City, New York, United States, 3. New York University, New York, New York, United States*

Background:

The lockdown due to COVID-19 has influenced individuals' lives in many aspects. Yet, the impact of reopening under an ongoing pandemic is understudied. This study aims to investigate the impact of reopening policy on older Asian Americans' depressive symptoms and whether the impact varies by their sociodemographic characteristics.Method: We used interview data collected from 519 Chinese and Korean aged 60 and older in New York City between 5/23/2021 to 7/30/2021. Interrupted time series model was used to test whether there are significant level and slope changes in depressive symptoms (PHQ-9 scale) before and after the reopening on 7/1/2021 in NYC. We then ran the models in stratified sample by gender, education, income, self-reported health, and social connectedness through living arrangements, use of technology, and social interactions.

Results:

Older Asians' depression increased immediately following the reopening (ß=1.52, $p< 0.05$), and then slowly decreased then after (ß=-0.12, $p< 0.001$). A decrease in depression following reopening was significantly associated with the male gender, good health, higher income, living alone, having received or provided social support, daily texting, and no engagement in the discussions related to COVID-19 in social media. Discussions: While reopening may have long-term benefits on mental health, older Asians were anxious about their safety at the beginning of reopening under an ongoing pandemic. Older adults with worse health, lower SES, and limited social connectedness struggled to adjust to "back-to-normal" life. We discussed research, policy, and practice implications to support these disadvantaged older adults after reopening.

### THE COMBINED EFFECTS OF SOCIAL ISOLATION AND LONELINESS ON PSYCHOLOGICAL WELL-BEING DURING THE COVID-19 PANDEMIC

Ke Li, and Fengyan Tang, *University of Pittsburgh, Pittsburgh, Pennsylvania, United States*

Social isolation and loneliness are related but distinct constructs. A number of studies have examined these two constructs separately; however, the combined and interactive effects of social isolation and loneliness on health outcomes have rarely been studied. Using the most recent data of the Health and Retirement Study (HRS 2020) collected during the pandemic, this study aimed to explore the latent classes of social isolation and loneliness among adults aged 60 and older (N=3,486) and to examine the associated psychological well-being. Social isolation was measured by five indicators, including living alone, no membership in any organizations, and less than once a month contact with children, relatives, and friends. Loneliness was measured by the 3-item UCLA scale. Four classes were identified by the Latent Class Analysis (LCA): neither isolated nor lonely (class 1, 35%), living alone and lonely (class 2, 25%), no social participation and lonely (class 3, 18%), and highly isolated and lonely (class 4, 23%). The results of multivariate regression indicated that compared to respondents who were neither isolated nor lonely, those who were in the class of living alone and lonely and the class of highly isolated and lonely had more depressive symptoms, stress, anxiety, worry, loneliness during the pandemic. The latent class of no social participation and lonely was associated with more depressive symptoms and covid-related stress. This study emphasizes the importance of specialized intervention strategies targeting the unique needs of older adults with different experiences of social isolation and loneliness.

### SESSION 4241 (BIOLOGICAL SCIENCES INVITED SYMPOSIUM)

### AGING-RELATED ADIPOSE REMODELING AND DYSFUNCTION

Chair: Qiong (Annabel) Wang

Aging is associated with insulin resistance, cardiovascular dysfunction, and many other chronic metabolic disorders, significantly shortening healthspan and lifespan. Fat (adipose) tissue, as the major site for energy storage, maintains whole-body energy homeostasis and insulin sensitivity. Adipose tissue has extraordinary plasticity, and it was not until recently that fat tissue remodeling during aging is considered to play an essential role in aging-associated metabolic disorders. Benefiting from recent technology advances, especially the single-cell technology and comprehensive genetic mouse models, we are beginning to unmask how adipose tissue remodels during aging cellularly and molecularly. This symposium features internationally-renowned aging research scientists whose work focuses on how aging remodels adipose tissues and how adipose tissue is vital for healthy aging and longevity. We will hear from Philipp Scherer from The University of Texas Southwestern Medical Center, who will present his research on the impact of adipocyte-derived factors on Healthspan and Lifespan; Hei Sook SUL from the University of California Berkeley will discuss "Aging dependent changes in adipose precursors"; Annabel Wang from the City of Hope will introduce her recent discovery of a new type of adipocyte progenitor cell that promotes aging-related visceral adiposity; and lastly, Gina Wade from the University of Wisconsin-Madison who will talk about "Regulation of aging energy expenditure by plasma lipid signaling". Attendees will learn about the latest breakthroughs in adipose tissue aging, and the role of adipose tissue in maintaining and restoring metabolic health in aged individuals.

### A UNIQUE ADIPOCYTE PROGENITOR POPULATION PROMOTES AGE-RELATED ADIPOSITY

Qiong (Annabel) Wang, *City of Hope, City of Hope, California, United States*

The average fat mass in adults increases dramatically with age, and older people often suffer from visceral obesity and related adverse metabolic disorders. Unfortunately, how aging leads to fat accumulation is poorly understood. It is known that fat cell (adipocyte) turnover is very low in young mice, similar to that in young humans. Here, we find that mice mimic age-related fat expansion in humans. In vivo lineage tracing shows that massive adipogenesis (the generation of new adipocytes), especially in the visceral fat, is triggered during aging. Thus, in contrast to most types of adult stem cells that exhibit a reduced ability to proliferate and differentiate, the adipogenic potential of adipocyte progenitor cells (APCs) is unlocked by aging. In vivo transplantation and 3D imaging of transplants show that APCs in aged mice cell-autonomously gain high adipogenic capacity. Single-cell RNA sequencing analyses reveal that aging globally remodels APCs. Herein, we identify a novel committed preadipocyte population that is age-specific (CP-A), existing both in mice and humans, with a global activation of proliferation and adipogenesis pathways. CP-A cells display high proliferation and adipogenesis activity, both in vivo and in vitro. LIFR serves as a functional maker of CP-A, which promotes CP-A proliferation. Together, these findings define a new fundamental mechanism involved in fat tissue aging and offer prospects for preventing and treating age-related metabolic disorders.

### ADIPOCYTE-DERIVED FACTORS: IMPACT ON HEALTHSPAN AND LIFESPAN

Philipp Scherer, *The University of Texas Southwestern Medical Center, Dallas, Dallas, Texas, United States*

Adipocytes secrete numerous lipid and protein factors with profound effects on systemic energy homeostasis. One such adipokine that we first identified in the early 1990's, adiponectin, has garnered significant attention as a potent mediator of insulin sensitivity and cell survival. FGF21 is another factor that is secreted by a number of cell types (including adipocytes), which has a beneficial effect on metabolism. Our group generated a novel mouse model that overexpresses, in an inducible fashion, physiological levels of FGF21 from adipocytes in the adult mouse. While comparable levels of constitutive overexpression of FGF21 from the liver do not have an impact on aging, adipocyte-derived FGF21 exerts a profound beneficial impact on health- and lifespan. This demonstrates that selective manipulation of adipose tissue per se has the potential to significantly reduce mortality and extend lifespan. The adipocyte-specific FGF21 transgenic animals have increased energy expenditure, weigh considerably less and exhibit an improvement in all systemic metabolic parameters examined to date. The mice further display unique immune-metabolic profiles of their adipose tissue depots, which defy the conventional changes associated with aging. Importantly, all these phenotypic alterations are achieved without a significant impact on adipose tissue beiging/browning. Moreover, at least some beneficial aspects of FGF21 appear to be mediated through a lowering of leptin, which leads to central leptin sensitization. Combined, these efforts shed additional light on the physiological effects of FGF21.

### AGING-DEPENDENT CHANGES IN ADIPOSE PRECURSORS

Hei Sook Sul, and Frances Lin, *University of California, Berkeley, Berkeley, California, United States*

Adipose tissue mass and adiposity change throughout the lifespan. During aging, while visceral adipose tissue (VAT) tends to increase, peripheral subcutaneous adipose tissue (SAT) decreases significantly. Unlike VAT, which is linked to metabolic diseases, including type 2 diabetes, SAT has beneficial effects. However, the molecular details behind the aging-associated loss of SAT remain unclear. Here, by comparing scRNA-seq of total stromal vascular cells of SAT from young and aging mice, we identify an aging-dependent regulatory cell (ARC) population that emerges only in SAT of aged mice and humans. ARCs express adipose progenitor markers but lack adipogenic capacity; they secrete high levels of pro-inflammatory chemokines, including Ccl6, to inhibit proliferation and differentiation of neighboring adipose precursors. We also found Pu.1 to be a driving factor for ARC development. We identify an ARC population and its capacity to inhibit differentiation of neighboring adipose precursors, correlating with aging-associated loss of SAT.

### HDL-LOCALIZED PLASMA CERAMIDES SUPPORT BODY TEMPERATURE REGULATION

Gina Wade, Judith Simcox, *University of Wisconsin - Madison, Madison, Wisconsin, United States*

As organisms age, the balance of energy expenditure is disrupted. One of the major ways in which this disruption is observed is in an inability to maintain body temperature. Our lab is interested in understanding the signals that regulate this energy balance, and we focus on lipids as energy substrates and signaling molecules to regulate this process. During cold exposure, plasma lipids produced by peripheral tissues are required to fuel and activate heat production in the brown adipose tissue. One of the lipid classes that increases with cold exposure is plasma ceramides. Beyond cold exposure, ceramide lipids are also elevated in aged individuals and are associated with increased cardiovascular disease risk and age-related diseases such as Alzheimer's disease where they are thought to signal inflammation. However, the functions of ceramides in non-disease states are unknown. To address this gap in knowledge, we have shown that ceramide production in 12-week-old C57Bl6/J mice is required for body temperature maintenance in the cold. Moreover, plasma ceramide levels in 2-year-old mice are unchanged in the cold, and these mice are unable to maintain their body temperature. In disease states, ceramides are transported through the plasma in LDL, but ceramides are enriched in the HDL plasma fraction in the cold. This differential plasma lipid transport suggests dynamic modes of lipid uptake and tissue targeting to regulate energy expenditure. This work will identify molecular mechanisms governing ceramide function in the mammalian response to cold and better our understanding of the systemic lipid metabolism dysregulated in disease.

### SESSION 4250 (PAPER)

### DISTRUPTING AGEISM AND STIGMA THROUGH EDUCATION

In this session, innovative and unique ways to combat ageism through educational efforts in varied classes are described.

### "HOW OLD IS OLD?" ADDRESSING AGEISM AND UNCONSCIOUS BIAS AMONG MEDICAL STUDENTS DURING GERIATRICS CLERKSHIP

Ravishankar Ramaswamy, Stephanie Chow, Noelle Marie Javier, Rosanne Leipzig, Gregory Hinrichsen, and Amy Kelley, *Icahn School of Medicine at Mount Sinai, New York, New York, United States*

Ageism (stereotyping, prejudice and discrimination based on age) has deleterious consequences on older adults' health. Medical students have variable attitudes and biases toward older people. We hypothesized that an embedded ageism curriculum within the Ambulatory Care-Geriatrics clerkship would increase ageism awareness and commitment to reduce ageism in the clinical environment for third year medical students. The 2021 curriculum included assigned pre-reading, videos, short didactics, expert-facilitated small group discussion of clinical vignettes, reflective journaling, and posting of personal commitments on a virtual messaging board. We surveyed students at the start and end of the clerkship to evaluate baseline awareness, change in UCLA Geriatrics Attitudes Scale, and satisfaction with curricular components. Of the 95 students who thusfar participated in the curriculum, we received 92 pre- and 48 post-curriculum survey responses. Pre-curriculum students reported the median age for "old" was 65 years (range 35-90) and 42% of students expressed preference for younger patients (33% neutral). Proportion of students with self-assessed ability to identify ageist remarks/actions increased from 52%(pre) to 92%(post), and ability to minimize own ageist biases increased from 23%(pre) to 83%(post). 86% of students found the curriculum useful; discussion with experts and viewing an ageism TED Talk were the most favorably scored components. Integrating an ageism curriculum with pre-work, didactic, guided discussion and reflection components in the Geriatrics clerkship increased medical student awareness and confidence in addressing ageism-related behaviors. This curriculum complements students' clinical interactions with older adults and has the potential to reduce the future impact of ageism in medicine.

### IMPACT OF AN EDUCATIONAL INTERVENTION ON KNOWLEDGE AND ATTITUDES ABOUT MEMORY LOSS AND CARE ISSUES

Colleen Galambos[1], and Julie Ellis[2], *1. University of Wisconsin-Milwaukee, Milwaukee, Wisconsin, United States, 2. University of Wisconsin Milwaukee, Milwaukee, Wisconsin, United States*

While previous studies have evaluated the impact of education interventions on knowledge and attitudes about Alzheimer's disease and other dementias (Kaf, Barboa, Fisher, & Snavely, 2011; Kimzey, Mastel-Smith, & Alfred, 2016; Yamashita, Kinney, & Lokon, 2011), there is little research about the impact on knowledge and attitudes about issues related to caregivers of those with dementia. This paper reports on the results of an education intervention to increase college students' knowledge, and positive attitudes on memory loss, Alzheimer's disease and other dementias, and related caregiver issues. The intervention consisted of a series of lectures and readings, including a caregiver memoir. The intervention was conducted in social work and nursing classes over two semesters. A questionnaire, using established scales that measured knowledge and attitudes about dementia and caregiving issues, was administered to the students using a pretest-posttest design. The educational intervention tested successfully increased knowledge and positive attitudes about Alzheimer's disease, dementia, memory loss, and caregiving (p=.0001). This finding held across courses, grade levels, previous experience with older adults, knowledge of someone with memory loss, and relationships with caregivers and persons with memory loss. A memoir as a storytelling informational narrative, and structured lectures and readings served as effective learning tools that influenced both knowledge and attitude levels about Alzheimer's disease, dementia, memory loss, and caregiving. Gains made in knowledge and attitude changes were true for both face- to- face and online teaching venues. Implications for training, classroom teaching, and workforce development will be discussed.

### WHAT AND HOW DID IT WORK? A SYSTEMATIC REVIEW OF THE INTERVENTION STUDIES OF CHANGING PERCEPTION TOWARD OLDER ADULTS

Yeonjung Lee[1], Lun Li[2], and Laxman Shrestha[3], *1. Chung-Ang University, Seoul, Seoul-t'ukpyolsi, Republic of Korea, 2. MacEwan University, Edmonton, Alberta, Canada, 3. University of Calgary, Calgary, Alberta, Canada*

Social workers are one of the major professionals serving for older adults. However, social work students often rank gerontology at the bottom of their future professional practice. Studies show that students have negative attitudes about, and perceptions of older adults and aging. Such stereotypes result in social work students considering practice areas other than gerontological social work. The objective of this study is to conduct a systematic review and identify any types of the intervention studies of changing perception and attitudes toward older adults and aging among social work students. This systematic review identified empirical research studies written in English that were published in a peer-reviewed journal before November 2021. Systematic search was carried out within four electronic databases (AgeLine, Education Research Complete, Social Work Abstracts, SocINDEX with full text) with the key words such as "social work student" and "aging or ageing or elderly or older adults or seniors or geriatrics or gerontology". A total of 470 abstracts were identified, and after careful review of 143 full articles yielded from the databases, 43 journal articles were included in this study. Analysis of the selected literature provides that major interventions have been tried include service learning, lecture, curriculum development, simulation, training program, and intergenerational program. This systematic review highlights the importance of the experiential learning, partnership development with community, and interprofessional collaboration to provide the best model of practice for changing perception toward aging and older adults among social work students.

### SESSION 4260 (SYMPOSIUM)

### EMBRACING OUR DIVERSITY: ENHANCING THE GEROFIT PROGRAM FOR PATIENT EXPERIENCE

Chair: Katherine Hall Co-Chair: Monica Serra

The Veterans Health Administration has long been at the forefront of telehealth development and advances. Gerofit to Home (GTH) is a novel, telehealth-based adjunct to a widely successful Gerofit facility-based program for Veterans ages 65 and older with multi-morbidities and functional limitations. Of note, participants are allowed to remain in the program as long as they wish, all of which enhances social engagement and camaraderie. Now disseminated to nearly 30 VA medical centers across the country, all of our sites transitioned within 6 weeks of the COVID-19 pandemic to group-based virtual classes, aptly called GTH. Because of the rapid and continuously evolving changes in telehealth, we had near daily Veteran feedback on what was working and what was not working. We cast a wide net and used multiple methods to obtain Veteran feedback. These were quite successful and allowed us to make ongoing improvements to our classes. The first presentation describes results from a telephone survey deployed to all patients who transitioned from facility-based Gerofit to GTH. The second presentation describes the development of a Gerofit Veterans Advisory Panel and the perspectives they provided in terms of scheduling, patient-requested content, feasibility and access. The third presentation explores patient satisfaction and retention in GTH participants, with an emphasis on rural vs non-rural settings. The fourth paper details an innovative approach to integrating student learners in to the Gerofit program, and explores the impact of these intergenerational interactions.

### GEROFIT TELEHEALTH-DELIVERED GROUP EXERCISE IMPACTS SOCIAL SUPPORT AND HEALTH AMONG OLDER VETERANS

Lauren Abbate[1], Rebekah Harris[2], Sarah Jordan[3], Kathryn Nearing[4], Janet Prvu Bettger[5], Megan Pearson[6], Miriam Morey[7], and Katherine Hall[7], *1. Eastern Colorado Health Care System, Aurora, Colorado, United States, 2. VA Boston, Boston, Massachusetts, United States, 3. University of Colorado, school of Medicine, Aurora, Colorado, United States, 4. VA Eastern Colorado Geriatric Research Education and Clinical Center, Denver, Colorado, United States, 5. Duke University, Durham, North Carolina, United States, 6. Durham VA Healthcare System, Durham, North Carolina, United States, 7. Durham VA Health Care System, Durham, North Carolina, United States*

Group exercise provides social support, which is especially important among older adults. Gerofit conducted a telephone survey with participants (n=258) from 15 sites to learn about Veterans' experiences with transition to Gerofit-specific telehealth-delivered group exercise to guide continuous improvement of the program. One, open-ended question was asked at the end, inviting respondents to reflect on any other thoughts they had about the telehealth-delivered exercise sessions. This yielded robust qualitative responses which constitute the data analyzed in this qualitative descriptive study. Key themes that emerged from participant responses included: 1) telehealth-delivered group exercise provides structure, motivation, and accountability; and 2) transitioning from in-person to telehealth-delivered exercise has many beneficial impacts on health such as retaining strength and ability to recover from injury, weight management, and positive impacts on mental health and quality of life. Telehealth-delivered group exercise sessions provide social support and are associated with positive health impacts.

### GEROFIT TO HOME: REDUCING BARRIERS TO GROUP EXERCISE TO RURAL AND NONRURAL OLDER ADULTS IN A SUCCESSFUL FASHION

Neil Gregor[1], Eva-Marie Christman[2], David Crivello[1], and Jamie Schukle[1], *1. Veteran Affairs, Martinez, California, United States, 2. Veteran Affairs, Redding, California, United States*

Transportation, scheduling and travel distance can present as barriers to participation in facility based exercise programs. Gerofit to Home(GTH) eliminates aforementioned burdens commonly associated with in-person programs. Understanding participant satisfaction and retention in rural(R) and non-rural(NR) groups assists in planning to better serve these populations with group exercise. This study aims to explore GTH participant satisfaction and retention in over 100 participants, through 3 months, for veterans over 65yo in rural and non-rural areas. Current results illustrate "Highly Satisfied" in majority of both R and NR groups. Retention at 3 months, in both groups, and factors associated with 3-month retention will be discussed in this presentation. Further research into the reasoning for participant stoppage prior to 3 months will aide in understanding how to better cater group exercise to these groups. Satisfaction scores in this study endorse further efforts for virtual group exercise in older adults.

### GEROFIT STUDENT VOLUNTEERS: INTERGENERATIONAL APPROACH TO MOBILITY

Cathy Lee[1], Steven Castle[1], Katherine Hall[2], and Miriam Morey[2], *1. VA Greater Los Angeles, Los Angeles, California, United States, 2. Durham VA Health Care System, Durham, North Carolina, United States*

The Institute of Medicine (IOM) has reported that innovative models of care will need to be used to increase the workforce to care for an aging population and to encourage older adults "to be active partners in their own care" (IOM, 2008). Use of student volunteers in an intergenerational approach could be used to supplement the workforce to motivate older adults for self-care in clinical practice. Gerofit@UCLA is a student organization created to support Gerofit at the Greater Los Angeles VA. These students completed an Expectation Regarding Aging (ERA-12) Survey. ERA-12 is a 12-item survey that measures expectations regarding aging in physical health, mental health and cognitive function with scores ranging from 0-100, higher scores indicating higher expectations with aging. After volunteering in Gerofit, students indicated highest expectations for mental health (78.33 ± 5.9) compared to cognitive health (53.34 ± 5.3) and physical health (39.17 ± 3.7). Although these students recognize the physical changes with aging of this 80-102 year old group, they appreciate that group physical activity in a program like Gerofit has positive effects on mental health and cognition, for the patients and for themselves.

### VETERANS VOICES: ACCEPTABILITY AND ENRICHING THE GEROFIT TO HOME EXPERIENCE FOR OLDER VETERANS

Cheryl Parham[1], and Janet Prvu Bettger[2], *1. Baltimore Gerofit Program, Baltimore, Maryland, United States, 2. Duke University, Durham, North Carolina, United States*

The pandemic helped us overcome many of the barriers we had struggled with for group based exercise. Within six weeks, all of our sites had transitioned to group-based virtual classes, aptly called Gerofit to Home (GTH). Because of the rapid and continuously evolving changes in telehealth, we had near daily Veteran feedback on what was working and what was not working. Part of this planning included retaining a smaller group of Veterans on a regular basis who would serve as an ongoing Gerofit Veterans Advisory Panel (VAP). The VAP is a racially and geographically diverse group of 12 men and women Veterans from 7 different sites, with varying levels and duration of participation in Gerofit (some exclusively remote, some in-person then transition to remote with the pandemic). This presentation will feature a moderated discussion with VAP representatives, summarizing the perspectives provided on Veteran preferences, needs, and motivators to engage in GTH.

### SESSION 4270 (SYMPOSIUM)

### ENRICHING OUR DISCOVERY BY TRAINING FOR ENGAGEMENT: METHODS FOR PATIENT/PERSON ENGAGEMENT IN RESEARCH

Chair: Rachel Lessem Co-Chair: Martina Roes Discussant: Katherine Abbott

There has been a recent movement towards greater involvement of the patient/person voice in aging research. However, many researchers embark on engaged research without adequate training or knowledge and without the skills or experience to train those they hope to engage. We define engaged research as research topics, designs, methods, analyses and/or dissemination strategies that have been informed through collaboration with or input from those who have a direct stake in research. This symposium explores education as a method of engaged research and is premised on the notion that engaged research enriches our discovery and ultimately leads to better outcomes in interventions as well as policy. There is an identified need for training of researchers as well as stakeholders in order to do successful engaged research. This symposium details educational methods for training those who utilize engaged research: student, researcher, and patient/person stakeholder, culminating in an examination of supporting patients in being trainers themselves. The first presentation investigates an overarching educational strategy to change the research paradigm. The second presentation explores a unique method for training researchers to engage older adults as advisory board members. The third presentation details a strategy for training a specific group of older adults to be active in engaged research. The symposium concludes by highlighting a method by which older adults engage in a research intervention as trainers. Attendees will be able to identify and evaluate the outcomes of the different educational methods and translate these methods to new settings.

### TRAINING RESEARCHERS TO CREATE RESEARCH ADVISORY BOARDS WITH OLDER ADULTS

Rachel Lessem[1], and Rebecca Berman[2], *1. CJE SeniorLife, Chicago, Illinois, United States, 2. Leonard Schanfield Research Institute, Chicago, Illinois, United States*

The Sage Resource Project's goal was to prepare researchers to use the Sage Resources to create Sage Model boards in order to continue to expand and amplify the voice of older adults in aging PCOR/CER research. Training researchers is essential to amplify the voice of older adults in research. This project culminated in the creation of a training manual as well as critical lessons learned for the advancement of engaged research with older adults receiving Long Term Services and Supports (LTSS). After developing a series of webinars for national dissemination, 5 research centers were identified as potential sites for intensive and tailored training on The Sage Model and, ultimately, two research centers completed the training with quite different approaches and results. This session will review the training that was developed as well as the lessons learned from training researchers on creating research advisory boards with older adults.

### BUILDING CAPACITY FOR PATIENT-CENTERED OUTCOMES RESEARCH IN AGING RESEARCH: TRAINING ACROSS THE CONTINUUM

Erin McGaffigan[1], and Marc Cohen[2], *1. UMASS Boston Gerontology Institute, North Reading, Massachusetts, United States, 2. University of Massachusetts Boston, Boston, Massachusetts, United States*

Patient-centered outcomes research (PCOR) models are increasingly used to ensure stakeholders inform evidence-based systems of care. Unfortunately, older adults are often left out of PCOR, leading to continued health and service disparities. The Aging PCOR Learning Collaborative was funded by the Patient-Centered Outcomes Research Institute in 2020 to conduct a series of training activities to change how older adults are viewed and engaged in research. Training was extended to Gerontology students, researchers new to engagement, established researchers, and to older adults to advance the strategies needed to infuse PCOR within aging research design and implementation. Project staff analyzed data relevant to these activities, which demonstrated the project's reach to over 300 older adults, researchers, funders, and academic leaders. This session will review the steps required to implement training, our project reach, outputs, and mid-term outcomes, providing one of the first glimpses into how to measure this shift in our research paradigm.

### TRAINING LONG-TERM SURVIVORS OF HIV TO ENGAGE IN RESEARCH TO IDENTIFY PRIORITIES FOR AGING WITH HIV

Andy Rapoport[1], Rachel Lessem[2], and Margaret Danilovich[3], *1. Leonard Schanfield Research Institute, CJE SeniorLife, Chicago, Illinois, United States, 2. CJE SeniorLife, Chicago, Illinois, United States, 3. Leonard Schanfield Research Institute, Chicago, Illinois, United States*

With effective antiretroviral therapies, HIV has become a chronic disease. Currently, >50% of people living with HIV are 50+ years old, and they face dual challenges of aging and HIV management. Overall, <15% of published studies in HIV engage stakeholders in research. These rates are even lower for older adults with HIV. Thus, there is a critical need to engage long-term survivors in developing meaningful research questions for aging with HIV. To address this gap, we created the SHARE (Survivors of HIV Advocating for Research Engagement) board. Presenters will discuss the process for building the board's research capacity and results of a community needs assessment that board members designed and conducted to ascertain priorities for HIV-aging research. Presenters will discuss evidence-based educational strategies utilized to build member research knowledge, and pre-post training changes in knowledge, confidence, and understanding. Presenters will focus on novel training approaches implemented in the remote environment.

### RECRUITING, FACILITATING, AND SUPPORTING PERSONS LIVING WITH DEMENTIA AS TRAINERS IN RESEARCH

Jen Hirsch[1], Fei Sun[1], Dan Cohen[2], Ha-Neul Kim[1], Steven Barbieri[3], and Brian LeBlanc[3], *1. Michigan State University, East Lansing, Michigan, United States, 2. Right to Music, Mineola, New York, United States, 3. National Council of Dementia Minds, Alma, Michigan, United States*

Research demonstrates that dementia education programs are effective in improving knowledge of dementia, but it takes more effort to change attitudes or skill sets. Empathy toward people living with dementia (PLWD) is low among college students due to lack of experience interacting with PLWD. Exposure to PLWD is recommended in training aimed to improve dementia literacy. We engaged PLWD from an active advocacy group, The National Council of Dementia Minds, to be co-trainers in an intervention with college students in health and social care professions to provide quality care for PLWD. Presenters will discuss the process for recruiting, engaging, and training PLWD successfully, including explanation of informed consent, preparation for training sessions, and structural support around communication and information processing needs. Emphasis will be on lessons learned directly from our co-trainers for successful support mechanisms, particularly in an online environment as all interaction occurred primarily over zoom and email.

### SESSION 4280 (SYMPOSIUM)

### EXCURSIONS INTO HEALTHCARE SYSTEMS TO HELP INFORMAL CAREGIVERS MANAGE DEMENTIA

Chair: Richard Fortinsky Discussant: Christopher Callahan

With the establishment in 2019 of the National Institute on Aging-funded IMPACT Collaboratory, the era of designing and implementing pragmatic clinical trials in partnership with health care systems to improve care and health-related outcomes for people living with dementia (PLWD) is well underway. An important focus for Collaboratory-funded work involves targeting and engaging family and other informal caregivers of PLWD across health care system settings. In this Symposium, we feature three Collaboratory-funded investigators who are partnering with health care systems to determine how to engage informal caregivers most pragmatically, with the goals of improving caregivers' capacity to manage dementia and provide sustained input into the clinical care of PLWD. Symposium presenters Quincy Samus and Hillary Lum are the first two Collaboratory-funded Health Care System Scholars and Richard Fortinsky is a pilot study awardee. Dr. Samus will present on her experiences and lessons learned in her multiple efforts to adapt and embed the MIND at Home family-focused care management model into health care systems, including her current work with a large managed care organization. Dr. Lum will explain ongoing capacity-building stakeholder engagement activities at a large academic health system in preparation for pragmatic clinical trials for PLWD and their caregivers. Dr. Fortinsky will present on experiences and lessons learned from ongoing efforts to identify caregivers of PLWD to join caregiver support programs, and store caregiver data electronically, in two health system outpatient care settings. Dr. Christopher Callahan, Leader of the Training Core for the Collaboratory, will serve as Symposium Discussant.

### PILOT STUDY TO PRAGMATICALLY EMBED CAREGIVER DEMENTIA EDUCATION AND SUPPORT IN HEALTH CARE SYSTEMS

Richard Fortinsky, *University of Connecticut Center on Aging, Farmington, Connecticut, United States*

Evidence-based interventions offering meaningful benefits to informal caregivers of people living with dementia (PLWD) would be attractive to office-based practitioners if pragmatic linkages could be made between these interventions and outpatient care settings. This presentation will explain experiences and lessons learned in an ongoing pilot study in which we are: pragmatically identifying and inviting caregivers of PLWD to join online dementia education and support programs; collecting and storing caregiver outcomes data into electronic health records where these data are accessible to clinicians. Participating outpatient health care settings are a geriatrics practice at UConn Health and a memory care clinic at Emory Healthcare. Caregivers recruited at both sites participate in either Tele-Savvy or Caregiving During Crisis programs. Outcomes data will inform effects of program participation on caregivers' competence and stress, and help clinicians gain insights into caregivers' capacity to manage PLWD. Implementation evaluation strategies and results also will be discussed.

### BUILDING CAPACITY FOR DEMENTIA CAREGIVER TOOLS AND PRAGMATIC TRIALS THROUGH STAKEHOLDER ENGAGEMENT

Hillary Lum, *University of Colorado Anschutz Medical Campus, Aurora, Colorado, United States*

Caregivers face challenges communicating and coordinating health-related needs on behalf of persons living with dementia (PLWD). This presentation will highlight capacity building activities at UCHealth, a large academic health system in Colorado and Wyoming, to prepare for pragmatic clinical trials to test integrated health informatics tools to improve the care of PLWD and their family caregivers. We will describe foundational activities of: 1) convening diverse patient and family caregivers to identify communication priorities and refine health informatics tools, 2) engaging health system leaders to develop a system-based value proposition, and 3) partnering with health informatics teams to adapt tools (i.e., dementia registry, refined patient portal tools) and implement reports of pragmatic outcomes. Through support as an NIA IMPACT Health Care System Scholar, we will discuss lessons learned related to highlighting the preferences of diverse caregivers, especially related to use of health informatics tools in the context of dementia care.

### FLEXIBILITY MEETS COMPLEXITY: LESSONS FROM EMBEDDING A DEMENTIA CARE COORDINATION MODEL IN PRACTICE

Quincy Samus, *Johns Hopkins School of Medicine, Baltimore, Maryland, United States*

People living with dementia (PLWD) are among the highest-need and highest-cost individuals because of the complexity, duration, and range of medical, behavioral, environmental, and social needs. There is a growing evidence base showing that family-centered active management approaches that include activation and empowerment of care partners are well suited to improve care quality and health-related outcomes, and have potential to curb high ADRD-related healthcare costs. This presentation will outline key experiences and lessons learned after a decade of work developing, adapting and embedding a comprehensive family-focused care management model called MIND at Home into practice. The work, supported in part by the IMPACT Collaboratory Health Care System Scholars Award to partner with Centene Corporation, a large managed care organization, illustrates two overriding principles: (1) the necessity of "meeting people and health systems where they are" (literally and figuratively), and (2) the importance of effectively matching intervention to outcome and context.

### SESSION 4290 (PAPER)

### FRAILTY AND PHYSICAL AGING

### ABNORMALITIES IN THE CITRATE CYCLE METABOLISM ARE ASSOCIATED WITH DECLINED INTRINSIC CAPACITY IN OLDER ADULTS

Yiming Pan, Pan Liu, Yun Li, and Lina Ma, *Xuanwu Hospital Capital Medical University, Beijing, Beijing, China (People's Republic)*

Background:

Aging is accompanied by a decline in physical and mental functions, manifested as the declines in intrinsic capacity. However, there is still a lack of understanding about the metabolic mechanisms underlying the declining intrinsic capacity. Objective: To explore the metabolic characteristics and pathways of the declines in intrinsic capacity with the assistance of metabolomics methods.

Methods: Our study recruited 38 participants in total. The Short Physical Performance Battery, Mini-Mental State Exam, 30-item Geriatric Depression Scale, self-reported hearing/visual impairment and Mini Nutritional Assessment were used to assess the five domains of intrinsic capacity respectively. The untargeted liquid chromatography-mass spectrometry-based metabolomics was performed on the serum of participants. Multivariate statistical analysis and pathway analysis were then implemented.

Results: Among the 38 participants aged 66.50±15.20 years, 22 were with the declines in at least one domain of intrinsic capacity. In the 349 identified metabolites, 35 were candidate biomarkers for declining intrinsic capacity with variable importance in the projection > 1.5 and $p < 0.05$. Citrate cycle, tryptophan metabolism, phenylalanine and tyrosine metabolism, and Arginine biosynthesis were significant pathways associated with the declines in intrinsic capacity.

Conclusions: Decreased intrinsic capacity is an age-related change with characteristic metabolomics features. Many pathways, especially the dysregulation of the citrate cycle metabolism, may be involved in the pathogenesis of intrinsic capacity decline.

### ARE INTRINSIC CAPACITY AND MULTIMORBIDITY ASSOCIATED TO FRIED'S FRAILTY REVERSIBILITY?

Emmanuel Gonzalez Bautista[1], Aarón Salinas Rodríguez[2], Ana Rivera Almaraz[2], and Betty Soledad Manrique Espinoza[2], *1. Institute of Aging. Toulouse University Hospital (CHU Toulouse)., Toulouse, Midi-Pyrenees, France, 2. National Institute of Public Health, Cuernavaca, Morelos, Mexico*

Our objective was to characterize older adults with reversible frailty in their baseline intrinsic capacity and multimorbidity status. MethodsWe used data from the most recent waves of the SAGE Mexico study (3 and 4), representative of older adults at a national level. Study n=749. We objectively measured gait speed and grip strength based on Fried's frailty criteria. Weight loss exhaustion was self-reported and physical activity (using the IPAQ). Reversible frailty was defined as going from frailty to pre-frail or robust and from pre-frail to robust. Worsening and stable frailty were coded similarly. Intrinsic capacity was measured using a summary index (0-100) based on five domains: cognition, locomotion, sensory, nutrition, and psychological. Multimorbidity was coded yes/no if the participant self-reported two or more chronic conditions. We compared the odds of being in the reverse group versus not in it according to baseline intrinsic capacity score and multimorbidity status, adjusting for age, sex, rural/urban, and wealth. ResultsReversible frailty=33% of those pre-frail or robust at baseline. Intrinsic capacity was higher in the reverse group than in those with worsening frailty but not significantly higher than those with stable frailty.Having multimorbidity significantly decreased the chances of frailty reversibility, adjusting for covariates. ConclusionsAs an expression of lifecourse damage to the physiological reserve, multimorbidity limits the chances of reversible frailty in Mexican older adults. High levels of intrinsic capacity did not characterize frailty reversibility in our study. Yet, low intrinsic capacity levels are a marker for older adults prone to frailty worsening.

### PATTERNS OF AGING CHANGES IN BODY WEIGHT AND BMI MAY PREDICT CHANCES OF ALZHEIMER'S DISEASE AND LONGEVITY

Svetlana Ukraintseva, Konstantin Arbeev, Hongzhe Duan, Rachel Holmes, Igor Akushevich, Arseniy Yashkin, Heather Whitson, and Anatoliy Yashin, *Duke University, Durham, North Carolina, United States*

Background: Lower bodyweight/BMI was previously linked to AD and frailty; however, the role of long-term changes in the bodyweight/BMI in both AD and longevity is not well understood, as is the role of APOE polymorphism in such changes.

Methods: Using longitudinal data from the Framingham Heart Study (FHS) and the Health and Retirement Study (HRS), we estimated trajectories of the weight and BMI at ages 40 to 75, and compared them between individuals who did and who did not develop AD at ages 75+. We also evaluated associations between APOE4 carrier status and key characteristics of the age-trajectories of weight/BMI, including the age at peak value of the bodyweight/BMI (AgeMax), and slope of the decline in bodyweight/BMI after reaching the maximum.

Results: Women with late-onset-AD had lower bodyweight/BMI values up to three decades before AD diagnosis. They reached the peak of bodyweight in their 50s, about 10 years earlier than AD-free women. Younger AgeMax was associated with lower survival chances after age 80 in women. APOE4 carriers showed earlier/faster declines in weight and BMI than non-carriers; however, relevance of this to AD was unclear.

Conclusion: Younger age at peak value of the bodyweight/BMI indicates higher chances of late-onset-AD, while older age can predict better survival later in life and may favor longevity in women. The earlier start of the decline in bodyweight/BMI values could be sign of accelerated aging, which may contribute to AD. Relevance of APOE4 effects on age-trajectories of weight/BMI to AD warrants further investigation.

### THE ROLE OF SYSTEMIC INFLAMMATION IN HEALTH NOW, LATER, AND CHANGE OVER TIME: EVIDENCE FROM MIDUS

Julie Ober Allen, *University of Oklahoma, Norman, Oklahoma, United States*

Systemic inflammation is theorized to be a biological pathway through which chronic stressors (e.g., discrimination, social marginalization) contribute to adverse health outcomes and disparities among older adults. Cross sectional research documents associations between inflammation and a broad array of health outcomes. Less is known about whether and how inflammation may influence health longitudinally. This study investigated whether a composite measure of inflammatory burden predicted physical health: a) concurrently, b) approximately 7 years later, and c) changes during that time. Data derive the National Survey of Midlife in the United States (MIDUS) participants who provided inflammatory biomarker data (2004-2009 Biomarker study) and health data in MIDUS 2 (2004-2006) and MIDUS 3 (2013-2014) (N=931, Mage 57, 45% male, 91% White). Inflammatory burden reflected the number of inflammatory biomarkers in the highest risk quartile, out of five: C-reactive protein, interleukin-6, fibrinogen, E-selectin, and intracellular adhesion molecule. Three general indicators of physical health were examined: number of commonplace chronic physical health conditions, out of 7; functional limitations; and self-rated physical health. Multivariate regression analyses indicated that inflammatory burden predicted all three concurrent physical health outcomes and the same health outcomes seven years later (p-values <.02). Inflammatory burden was unrelated to changes in these outcomes over time. This study adds to the literature on stress-related biological mechanisms of health and identifies a potential clinical indicator of enduring health problems. It also suggests that while inflammatory burden may demonstrate consistent relationships with physical health over time, other mechanisms may account for health changes and deterioration.

### SESSION 4300 (SYMPOSIUM)

### INNOVATIVE CARE ENVIRONMENTS FOR PEOPLE WITH DEMENTIA

Chair: Hilde Verbeek Co-Chair: Bram de Boer Discussant: Marie Boltz

As part of the ongoing culture movement within long-term care several innovative care concepts for people with dementia are developing. These concepts radically change their physical, social and/or organizational environment in order to align care services with needs and demands of people with dementia, and providing meaningful activities. The current symposium will discuss the effects and possible working mechanisms of innovative caring environments for people with dementia across three European countries and the US. The symposium will start with a presentation describing the Homestead care model in the Netherlands, which is a care model that was developed following a co-creation study focused on translating scientific knowledge on nursing home care environments into practice. This is followed by three presentations about the effects of three types of innovative care environments for people with dementia: (1) farm based day care in Norway, (2) shared housing arrangements in Germany, (3) green care farms providing 24-hour nursing care in the Netherlands. The three studies on the effects of innovative care environments present a variety of designs (case study, cluster randomized controlled multi-center intervention study, and a qualitative study with semi-structured interviews) to study the effects on various outcomes (activities, physical effort, social interaction, mood, the number of hospital admissions for people with dementia, quality of life, challenging behavior, risk of falls, stabilization of cognitive abilities, daily life). The symposium will conclude with a reflection on these innovative care concepts from a US perspective.

### TRANSLATING SCIENCE INTO PRACTICE TO DEVELOP AN INNOVATIVE DEMENTIA CARE MODEL: THE HOMESTEAD CARE MODEL

Bram de Boer, *Maastricht University, Maastricht, Limburg, Netherlands*

This study reports on a participatory research approach to translate a scientific conceptual framework on innovative dementia care into practice. This led to the development of the Homestead care model. Results indicate that achieving positive outcomes for people with dementia, (in)formal caregivers, and the community is dependent on how well the physical, social and organizational environment are congruently designed. Physical aspects are related to interior design, outdoor areas, architecture and sensory elements. Social aspects include interactions with others in the environment, such as residents, staff, family, and the wider community (local entrepreneurs, societies, schools). Organizational aspects are related to the organizational culture and leadership of a care facility. These theoretical underpinnings of the conceptual model have been translated into three main pillars of the Homestead care model: activation, freedom, and relationships. The Homestead care model has a unique physical, social and organizational environment, which can affect daily life of residents.

### TURNING THE ORDINARY INTO THE EXTRAORDINARY: EXPERIENCES OF PROVIDING FARM-BASED DAYCARE FOR PEOPLE WITH DEMENTIA

Ingeborg Pedersen, *Norwegian University of Life Sciences, Ås, Akershus, Norway*

This study gives a deeper understanding of how service providers think and work when offering farm-based days care (FDC) services for people with dementia. Seven semi-structured interviews with service providers, analyzed using text condensation, resulted in the five main themes; "On fertile ground"; "The foundation"; "Activities with rippling effects", "Positive social and emotional environment" and, "Making room". The theme "On fertile ground", reflects the ordinary aspect of the farm context; closeness to nature and the home-like atmosphere. "The foundation" describes the extraordinary aspect of FDC; the service providers relentless effort to tailor all aspects of the service to the attendees. These two themes lay the foundation for the activities, the social setting, and possibilities for having a personal focus, reflected in the three remaining themes. The study gives an enhanced understanding of the key role of the service provider and how they offer person-center care in the farm context.

### RISK REDUCTION OF HOSPITAL ADMISSIONS FOR PEOPLE WITH DEMENTIA IN SHARED-HOUSING ARRANGEMENTS: THE DEMWG STUDY

Karin Wolf-Ostermann[1], Susanne Stiefler[2], Annika Schmidt[2], Anna-Carina Friedrich[2], Elmar Gräßel[3], Carolin Donath[3], Jennifer Scheel[3], and André Kratzer[3], *1. University of Bremen, Bremen, Bremen, Germany, 2. Institute of Public Health and Nursing Research, University of Bremen, Germany, Bremen, Bremen, Germany, 3. Universitätsklinikum Erlangen, Department of Psychiatry and Psychotherapy, Center for Health Services Research, Erlangen, Bayern, Germany*

People living with dementia (PlwD) have an increased risk of hospital admission which may result in severe consequences. The DemWG-study therefore aims to investigate whether a complex intervention in shared housing arrangements (SHA) for PlwD reduces the number of hospital admissions. Furthermore, quality of life, risk of falls, and stabilization of cognitive abilities are assessed. The intervention includes motor and cognitive training for PlwD and education of caregivers and general practitioners. The study is conducted in 88 SHA in Germany with a total of 330 PlwD and a 6 months intervention. Data are collected up to 18 months after baseline, including quantitative and qualitative data. Results from caregivers' perspectives on the intervention designate a perceived improved social interaction, which in turn has a positive effect on living together in the SHA. Based also on the results of the ongoing quantitative assessments recommendations for better care and support will be derived.

### HOW DO GREEN CARE FARMS FOR PEOPLE LIVING WITH DEMENTIA SUCCESSFULLY INTEGRATE OUTDOOR SPACES?

Katharina Rosteius, Bram de Boer, and Hilde Verbeek, *Maastricht University, Maastricht, Limburg, Netherlands*

Green Care Farms (GCFs) are an innovative care environment for vulnerable groups. This study explores the design, and the successful integration of the natural environments of GCFs. By actively integrating outdoor spaces into daily care, they may encourage residents to participate in meaningful activities. A case study was conducted on a Dutch GCF using mixed ethnographic methods. 129 hours of observations of daily life were combined with 24 semi-structured interviews with residents, family, volunteers, staff and management, and a focus group with staff. The physical environment was assessed quantitatively. Data was analyzed thematically and triangulated. Results indicate that some factors supported the integration of the outdoor spaces into daily care. First, the physical environment was specifically designed to stimulate residents. Second, the outdoors was easily and openly accessible for residents. Third, residents' security was supported in several ways. Lastly, staff members creatively motivated residents to use the outside spaces.

### SESSION 4310 (SYMPOSIUM)

### LESSONS FROM CAPABLE: DRIVERS, IMPLEMENTATION EXPERIENCE, AND MARKET READINESS CONSIDERATIONS

Chair: Deborah Paone Co-Chair: Sarah Szanton

External, organizational, and individual factors which drive or restrain adoption, implementation, and scaling of CAPABLE provide lessons for other programs to move from research to practice and promote sustainability. Lessons are offered for policymakers, payers, consumers, and communities to support evidence-based programs that improve function and reduce disability for older adults. This symposium presents results from a 3-year study of implementation of CAPABLE by 40 organizations, an analysis of the drivers and restrainers of CAPABLE dissemination, and a 1-year pilot study on engaging unpaid care partners in CAPABLE. We will explore the lessons from each--finding synergy and unique insights. CAPABLE is a case example to guide efforts at the organizational, system, and societal levels.

### CAPABLE IMPLEMENTATION: PROGRESS AND BARRIERS

Deborah Paone[1], and Sarah Szanton[2], *1. Johns Hopkins University, Baltimore, Maryland, United States, 2. Johns Hopkins University School of Nursing, Baltimore, Maryland, United States*

CAPABLE is an evidence-based 4-to-6-month program that improves daily function and the home environment based on the older adult's goals. Study aims were to: (1) understand context and readiness factors in implementation, (2) identify barriers and facilitators, and (3) examine the utility of two frameworks. The unit of analysis was the organization. We employed the CFIR and RE-AIM frameworks for this qualitative study, using multiple data sources and traced the implementation of 40 organizations over 3 years. Leadership support, perceived value of the program, initial funding, strategic importance, and program champion were common supportive factors. Common challenges included difficulty with recruitment, staffing and infrastructure readiness and sustainability of funding. This study indicates readiness, technical support, and resources needed for implementation and sustainability of CAPABLE and suggests external environmental supports needed. It offers a practical way to monitor, evaluate, and report on ongoing implementation of evidence-based programs.

### DRIVERS AND RESTRAINERS OF CAPABLE DISSEMINATION

Sarah Szanton[1], and Deborah Paone[2], *1. Johns Hopkins University School of Nursing, Baltimore, Maryland, United States, 2. Johns Hopkins University, Baltimore, Maryland, United States*

Using the technique of Force Field Analysis, the co-developer of CAPABLE examined drivers and restrainers of dissemination for the CAPABLE program. This generated 19 distinct drivers including robust research findings, demonstrated clinical and economic utility, and high value to older adults. The environmental shift toward value-based approaches in Medicare and Medicaid also increased the perceived value of CAPABLE. In addition, there were 8 restrainers, including component complexity, siloed health and housing sectors, and the operational lift needed to build the program. Lessons are offered from this case study to move from research to embedded practice.

### UNDERSTANDING POTENTIAL INVOLVEMENT OF CARE PARTNERS IN CAPABLE

Beth Fields[1], and Pamela Toto[2], *1. University of Wisconsin-Madison, Madison, Wisconsin, United States, 2. University of Pittsburgh, Pittsburgh, Pennsylvania, United States*

This qualitative study focused on understanding how to involve unpaid care partners ('family members or friends') of older adults who are participating in CAPABLE. Using field notes, focus groups, and key informant interviews, the study revealed considerations for involving care partners, including providing choices, defining roles, sharing information in a collaborative manner, and reinforcing knowledge and skills training. These findings can help guide organizations interested in involving care partners in their older adult programs.

### SESSION 4320 (PAPER)

### MEASURES AND PROFILES OF FRAILTY AND FUNCTION

### ASSOCIATION BETWEEN COGNITIVE FRAILTY AND ORAL HEALTH IN COMMUNITY-DWELLING OLDER ADULTS IN KOREA

Yoonjung Ji[1], Jane Chung[2], and TaeWha Lee[1], *1. Yonsei University, Seoul, Seoul-t'ukpyolsi, Republic of Korea, 2. Virginia Commonwealth University, Richmond, Virginia, United States*

Cognitive frailty(CF) is a condition where physical frailty and mild cognitive impairment (MCI) co-exist without dementia. CF is related to adverse health outcomes, such as functional disability, hospitalization, depression, and mortality. Studies suggest that poor oral health is also associated with a greater risk of frailty, a significant healthcare challenge in aging populations. However, no previous studies have investigated the association between oral health and CF. This study aimed to assess the prevalence and its association with oral health. A secondary analysis was conducted using the 7th wave dataset from the Korean Longitudinal Study of Aging collected in 2018. Individuals aged ≥65 years, without dementia and pre-frailty were included (N=750, mean age 73.2 years). Participants were classified into either robust (n=635) or CF (n=115) based on the presence or absence of CF. Oral health was assessed using the Geriatric Oral Health Assessment Index (GOHAI). Poor oral health was prevalent in both robust and CF groups (58.6% and 95.7%, respectively). Logistic regression analyses were conducted to investigate the associations of CF with oral health. CF was significantly associated with GOHAI scores (B = −0.153, 95% CI: 0.833-0.884, p < 0.001). The association remained significant after adjusting for age, sex, educational level, smoking, drinking, comorbidities, and body mass index (B = −0.140, 95% CI: 0.836-0.904, p < 0.001). The presence of oral health problems was associated with greater risks of developing CF in older age. The identification and management of poor oral health is critical to preventing cognitive frailty in older adults.

### CREATION AND VALIDATION OF A POLYSOCIAL SCORE FOR PHYSICAL FRAILTY AMONG COMMUNITY-DWELLING OLDER ADULTS IN THE US

Chenkai Wu, and Junhan Tang, *Duke Kunshan University, Kunshan, Jiangsu, China (People's Republic)*

The interrelatedness between social determinants of health impedes researchers from identifying important social factors for health investment. A new approach is needed to quantify the aggregate effect of social factors and develop person-centered social interventions. Data are from the Health and Retirement Study; 6,075 older adults initially not frail (non-frail or prefrail) were included. Frailty was assessed by slowness, weakness, exhaustion, inactivity, and shrinking. Persons were classified as “nonfrail” (0 criteria), “prefrail” (1–2 criteria), or “frail” (3–5 criteria). We included 24 social factors from five categories (economic stability, neighborhood environment, education, community/social context, and healthcare system) and used forward stepwise regression to screen for important ones. Polysocial score was created using 15 social factors and was classified as low (0-29), intermediate (30-42), and high (43+). We used the Poisson regression to estimate the risk of incident frailty by three polysocial score categories. We found that 444 (34.5%), 651 (17.9%), and 108 (9.4%) cases of incident frailty at the 4-year follow-up among participants with a low, intermediate, and high polysocial score, respectively. In the multivariable-adjusted Poisson model, the risk of frailty among participants in the intermediate and high polysocial score categories was 35% and 59% lower than those in the low polysocial score category, respectively. We found a universal association between polysocial scores and frailty across race/ethnicity and sex subgroups. The polysocial approach may offer possible solutions to monitor social environments and suggestions for older people to improve their social status for specific health outcomes.

### DISTINCT PROFILES OF SEDENTARY BEHAVIORS IN OLDEST OLD ADULTS

Katelyn Webster[1], and Weijiao Zhou[2], *1. Indiana University, Indianapolis, Indiana, United States, 2. University of Michigan, Ann Arbor, Michigan, United States*

Sedentary behavior (SB) is a significant health risk. Emerging research suggests that mentally active SB (such as computer use and reading) were associated with better health than mentally passive SB (such as watching TV). However, this has not been examined among the oldest old (age ≥80). The aims of this study were to (1) identify distinct profiles of oldest old adults based on six domains of SB (watching TV, using a computer/tablet, talking to friends or family members, doing hobby or other activities, transportation, and resting/napping); and (2) compare health-related outcomes across identified profiles, using the National Health and Aging Trends Study (NHATS) dataset. Latent profile analysis was used to identify distinct profiles of SB. Design-based linear and logistic regressions were used to examine associations between different profiles and health outcomes, accounting for sociodemographic characteristics. We identified four profiles and named them based on total sedentary time (ST) and passive/active pattern (n=852, “Low ST”, “High ST-passive”, “Medium ST-TV”, “High total ST-mentally active”). Compared to the “High ST-passive” group, “Low ST” group was associated with fewer difficulties with activities of daily living, fewer problems limiting activities and higher cognitive function; “High ST-mentally active” group was associated with the above outcomes, as well as lower anxiety and depression. This study, with a national representative sample of the oldest old population, suggests that both total ST and SB pattern matter when evaluating health outcomes of being sedentary. Interventions should encourage oldest old adults to reduce ST and especially target mentally passive ST.

### FRAILTY AND CARDIOVASCULAR DISEASE EVENTS IN COMMUNITY-DWELLING HEALTHY OLDER ADULTS

A R M Saifuddin Ekram[1], Sara Espinoza[2], Joanne Ryan[3], Michael Ernst[4], Andrew Tonkin[3], Christopher Reid[5], John McNeil[3], and Robyn Woods[3], *1. Monash University, Nunawading, Victoria, Australia, 2. Sam and Ann Barshop Institute, UT Health San Antonio, San Antonio, Texas, United States, 3. Monash University, Melbourne, Victoria,*

*Australia, 4. The University of Iowa, Iowa City, Iowa, United States, 5. Curtin University, Perth, Western Australia, Australia*

BACKGROUND: Frailty is associated with adverse outcomes, but whether it independently increases cardiovascular disease (CVD) risk requires clarification.

METHODS: This study examined the association between frailty in a cohort with no previous CVD events and subsequent CVD outcomes in 19,114 community-dwelling older people from the ASPREE trial. Frailty was assessed using the modified Fried phenotype, comprising weakness, exhaustion, low body mass index (BMI), slowness and low physical activity, and a deficit accumulation frailty index (FI) of 66 items. CVD event was defined as a composite of CVD death, non-fatal myocardial infarction, non-fatal stroke and hospitalization for heart failure.

Results: Over a median 4.7-years of follow-up (interquartile range: 3.6 to 5.7 years), pre-frail/frail participants were more likely to develop CVD events (Hazard ratio (HR): 1.33; 95% Confidence Interval (CI): 1.16, 1.53 for pre-frail and HR: 1.68; 95% CI: 1.19, 2.38 for frail participants) according to Fried phenotype. Subtypes of CVD (fatal/non-fatal myocardial infarction and heart failure hospitalization) similarly increased HRs except fatal or non-fatal stroke. These effect sizes were more prominent when frailty was assessed using the FI than that assessed by Fried phenotype.

CONCLUSION: Pre-frail and frail participants were at significantly increased risk of developing CVD and its subtypes (particularly fatal/non-fatal myocardial infarction and hospitalization for heart failure). Addressing pre-frailty and frailty in older people could contribute to CVD prevention strategies.

### TRANSLATION OF A MEDICARE CLAIMS-BASED FRAILTY ALGORITHM FROM ICD-9-CM TO ICD-10-CM

Keturah Faurot[1], Emilie Duchesneau[1], Shahar Shmuel[2], Jihye Park[1], Til Stürmer[2], Michele L. Jonsson-Funk[1], and Jennifer L lund[1], *1. University of North Carolina at Chapel Hill, Chapel Hill, North Carolina, United States, 2. Gillings School of Global Public Health, Chapel Hill, North Carolina, United States*

Clinical trials often have insufficient power to estimate drug effects in older adult populations. Medicare claims data are a valuable resource for studying healthcare delivery for older adults. The Faurot frailty index (FFI) is a validated algorithm that uses demographic, enrollment, and ICD-9-CM-based claims information to predict ADL dependency as a proxy for frailty. The original FFI consists of 20 constructs, including those related to geriatric syndromes, of which 16 are based on ICD-9-CM diagnosis codes (e.g., arthritis, dementia). In October 2015, the US healthcare system transitioned to ICD-10-CM. In this study, we updated and validated the FFI for the ICD-10-CM era . We used General Equivalence Mapping (GEM) to translate the ICD-9-CM codes to ICD-10-CM. For each construct, we manually reviewed the ICD-10-CM codes after GEM, assessed the suitability, and plotted the monthly prevalence before and after the transition (2013-2017). Code lists for all but 1 construct required editing after translation using GEM (adding and/or removing codes). We observed increasing monthly prevalence for several constructs, although trend lines were consistent across the ICD-9-CM and ICD-10-CM eras: bladder dysfunction (1.8-2.5%), weakness (2.7-3.9%), and psychiatric illnesses (6.9-9.1%). Rehabilitation services did not translate well using diagnosis codes, however, adding CPT codes improved capture. The updated FFI was strongly associated with frailty-related geriatric outcomes (1-year mortality, hospitalization, SNF admission). Studies of drug effects in older adults should use validated indices, such as the FFI, to reduce bias. Thorough evaluation of claims-based algorithms is essential to support healthcare services research using these measures.

### SESSION 4330 (SYMPOSIUM)

### MOVING, THINKING, AND SLEEPING: NOVEL INSIGHTS INTO PHYSICAL AND COGNITIVE HEALTH FROM ACCELEROMETRY DATA

Chair: Jennifer Schrack Discussant: Amal Wanigatunga

Physical activity and sleep are well-established predictors of health and longevity with aging. Wrist accelerometers, that produce high-frequency time series data, capture multiple aspects of daily physical activity and sleep 24-hours/day. Historically, the majority of accelerometry-based activity research has employed summary metrics to understand the associations of total daily physical activity and sleep with physical and cognitive health. Although these measures are important for understanding conformity with physical activity and sleep recommendations, they underutilize the potential of these data. Further, the summary metrics may differ by accelerometer type/brand, making it difficult to translate results across device types and studies. This symposium will examine the associations between accelerometry-derived physical activity and various aging-related health outcomes, and compare the measurement properties of two commonly used accelerometers for measuring sleep. Ms. Marino will discuss the association of physical activity volume and fragmentation with the presence of the Apolipoprotein-ε4 genotype in the Baltimore Longitudinal Study of Aging (BLSA), overall and by time of day. Dr. Wanigatunga will present evidence on the association of physical activity patterns with beta amyloid plaques in the BLSA. Dr. Schrack will present the association of physical activity fragmentation and diurnal patterns with peripheral artery disease in the Hispanic Community Health Study/Study of Latinos (HCHS/SOL). Finally, Ms. Liu will compare measurement of sleep variables derived from two commonly used accelerometers. Collectively, these presentations highlight ways to utilize the richness of accelerometry data to illuminate more sensitive associations between movement and health outcomes to advance prevention science and promote health aging.

### ANKLE BRACHIAL INDEX (ABI) AND ACCELEROMETER PATTERNS OF DAILY PHYSICAL ACTIVITY IN HCHS/SOL

Jennifer Schrack[1], Venus Chiu[2], Amal Wanigatunga[1], Daniela Sotres-Alvarez[3], Shoshana Ballew[2], Matthew Allison[4], Robert Kaplan[5], and Kunihiro Matsushita[2], *1. Johns Hopkins Bloomberg School of Public Health, Baltimore, Maryland, United States, 2.*

*Johns Hopkins University, Baltimore, Maryland, United States, 3. University of North Carolina, Chapel Hill, North Carolina, United States, 4. University of California San Diego, La Jolla, California, United States, 5. Albert Einstein College of Medicine, Bronx, New York, United States*

The benefits of routine engagement in physical activity (PA) on vascular health are well established; lesser known is whether vascular health associates with accelerometry-based daily patterns of PA in mid-to-late life. Using linear regression models adjusted for demographics and comorbidities, we modeled the cross-sectional associations between ankle brachial index (ABI) and PA patterns (diurnal activity and activity fragmentation) in 7,848 HCHS/SOL participants (45.5% men, 56.4±0.2 years). Compared to those with normal ABI (1.0-1.4), those with low ABI (≤0.9) had more fragmented daily activity (B=1.0%, SE=0.4, p=.04); primarily between 12 and 8 PM (B=1.66%, SE=0.55, p<.01). Similarly, those with low ABI had lower daily activity throughout the day (B=-0.09 log-counts, SE=0.05, p<.05) also mainly between 12 and 8 PM (B=-0.16 log-counts, SE=0.55, p<.01). Results suggest a diminished, less favorable PA profile patterns in those with low ABI. Future longitudinal studies should consider whether patterns of PA assist in determining PAD risk.

### OBJECTIVELY MEASURED PHYSICAL ACTIVITY PATTERNS OVER 24-HOURS BY APOE-E4 CARRIER STATUS

Amal Wanigatunga[1], Francesca Marino[2], Fangyu Liu[1], Eleanor Simonsick[3], Qu Tian[4], Jennifer Deal[5], Susan Resnick[3], and Jennifer Schrack[1], *1. Johns Hopkins Bloomberg School of Public Health, Baltimore, Maryland, United States, 2. Johns Hopkins Bloomberg School of Public Health, Washington, District of Columbia, United States, 3. National Institute on Aging, Baltimore, Maryland, United States, 4. National Institutes on Aging, Baltimore, Maryland, United States, 5. Johns Hopkins University, Baltimore, Maryland, United States*

The Apolipoprotein-ε4 (APOE-ε4) genotype is associated with earlier onset and greater risk of β-amyloid deposition, but whether APOE-ε4 status is associated with physical activity amount or fragmentation remains unclear. In 563 BLSA participants aged 55-97 years with APOE-ε4 and ≥3 days of wrist accelerometry data, multivariable linear regression models were fitted to estimate differences in activity amount (daily or by 6-hour time blocks) or fragmentation by APOE-ε4 status and to test whether age modifies these effects. Overall, daily activity amount and fragmentation did not differ by APOE-ε4 status (p=0.28 and p=0.93, respectively). Yet, age significantly modified the APOE-ε4 effect. Per each additional year of age, APOE-ε4 carriers had less activity during early morning (12:00-6:00AM) than non-carriers (p=0.035). This association was non-significant among the oldest-old (p=0.697 at age 80 years, p=0.347 at age 90 years), suggesting possible decreases in the predictive power of APOE-ε4 with increasing age.

### DIFFERENCES IN PHYSICAL ACTIVITY AMOUNT AND FRAGMENTATION OVER 24 HOURS BY Β-AMYLOID STATUS AND AGE

Francesca Marino[1], Fangyu Liu[2], Eleanor Simonsick[3], Qu Tian[4], Jennifer Deal[5], Amal Wanigatunga[2], Susan Resnick[3], and Jennifer Schrack[2], *1. Johns Hopkins Bloomberg School of Public Health, Washington, District of Columbia, United States, 2. Johns Hopkins Bloomberg School of Public Health, Baltimore, Maryland, United States, 3. National Institute on Aging, Baltimore, Maryland, United States, 4. National Institutes on Aging, Baltimore, Maryland, United States, 5. Johns Hopkins University, Baltimore, Maryland, United States*

β-amyloid is a hallmark of Alzheimer's disease and can be measured in vivo by PET imaging. Whether PiB+ (elevated β-amyloid) individuals exhibit distinct physical activity patterns throughout the day (i.e., activity amount or fragmentation) is unknown. Using data from 125 BLSA participants (56-95 years) with PET imaging and ≥3 days of wrist accelerometry, we examined associations between PiB status and activity patterns daily and during 6-hour time blocks using multivariable linear regression models. Modification by age was also examined. Daily total activity counts and activity fragmentation did not differ by PiB status (p=0.34 and p=0.70, respectively). Yet, per each additional year of age, PiB+ individuals were more active during early morning (p=0.04) and evening (p=0.029) but trended towards less active in mid-morning (p=0.069) than PiB- individuals. Though replication of findings is needed, older PiB+ participants appeared more active in the late evening/early morning, possibly reflecting altered sleep-time, agitation, or restlessness.

### COMPARISON OF SLEEP PARAMETERS FROM WRIST-WORN ACCELEROMETERS IN OLDER ADULTS

Fangyu Liu[1], Jennifer Schrack[1], Sarah Wanigatunga[2], Jill Rabinowitz[1], Linchen He[1], Amal Wanigatunga[1], Eleanor Simonsick[3], and Adam Spira[4], *1. Johns Hopkins Bloomberg School of Public Health, Baltimore, Maryland, United States, 2. Johns Hopkins, University, Baltimore, Maryland, United States, 3. National Institute on Aging, Baltimore, Maryland, United States, 4. Department of Mental Health, Johns Hopkins Bloomberg School of Public Health, Baltimore, Maryland, United States*

Researchers often prefer different accelerometers for measurement of sleep and physical activity (PA) in older adults, but wearing multiple accelerometers increases costs and participant burden. We tested the hypothesis that the Actiwatch-2, commonly used to measure sleep, and the ActiGraph GT9X Link, commonly used to measure PA, yield comparable sleep parameters among 331 participants (aged 71+/-14 years, 50.5% women) who wore them simultaneously for 7+/-1.6 nights in the BLSA. Compared to the Actiwatch-2, the ActiGraph estimated 50% greater wake after sleep onset, 89% longer wake bout length, 18% lower sleep fragmentation index (SFI), 5% lower total sleep time (TST) and 5% higher sleep efficiency. Yet, despite yielding different absolute values of TST and SFI, the devices generated similar between-person (relative) differences in TST and SFI. These devices may only be interchangeable for measurement of some relative sleep parameters in older adults. Studies with both devices and gold-standard polysomnography are needed.

### NOVEL INSIGHTS INTO GAIT, MOTOR CONTROL, AND THE BRAIN: IMPLICATIONS FOR COGNITIVE AGING

Andrea Rosso[1], Sarah Fraser[2], Roee Holtzer[3], Talia Salzman[2], Caterina Rosano[1], Emma Baillargeon[1],

Briana Sprague[1], and Qu Tian[4], *1. University of Pittsburgh, Pittsburgh, Pennsylvania, United States, 2. University of Ottawa, Ottawa, Ontario, Canada, 3. Yeshiva University and Albert Einstein College of Medicine, New York, New York, United States, 4. National Institutes on Aging, Baltimore, Maryland, United States*

What can muscles, gait, and brain activity tell us about cognitive decline? Are there certain markers we can track that are early predictors of cognitive status years later? In the current symposia, our goal is to address these questions with recent pilot, longitudinal and cross-sectional studies that measure potential markers of cognitive decline in different older adult cohorts. The first speaker will present findings on changes in skeletal muscle adiposity and 10-year change in global cognition from the Health Aging and Body Composition (Health ABC) study. The second speaker explores cross-national comparisons of gait speed and its association with cognitive function from the Cohort Studies of Memory in an International Consortium (COSMIC). The third speaker investigates changes in brain activity with functional near infra-red spectroscopy (fNIRS) from single to dual-task walking and its relation to changes in several gait quality parameters. The fourth speaker presents pilot work that examines dual-task gait and tapping with fNIRS and compares the dual-task performance and brain activity of older adults who report experiencing subjective cognitive decline to those that do not. The final speaker presents findings from the Baltimore Longitudinal Study on Aging (BLSA) that demonstrate that early markers of slow gait and metabolic dysfunction could identify those at risk of progression to dementia 7 years prior to onset. Taken together, the findings from this symposium present novel markers of changes in cognitive function in older adults and ultimately targets for prevention or slowing of cognitive declines in older adults at risk for dementia.

### WHAT CAN DUAL-TASK WALKING AND TAPPING TELL US ABOUT SUBJECTIVE COGNITIVE DECLINE? AN FNIRS STUDY

Talia Salzman[1], Hannah Perreault[1], Farah Farhat[1], Diana Tobon Vallejo[2], and Sarah Fraser[1], *1. University of Ottawa, Ottawa, Ontario, Canada, 2. Universidad de Medellin, Medellin, Antioquia, Colombia*

Older adults who pass standard cognitive tests but report subjective cognitive decline (SCD) may be identifying early changes in cognition at a stage when intervening can prevent further declines. Changes may be subtle highlighting the need for novel approaches, such as divided attention tasks, to distinguish between those with and without SCD. This pilot study examined 15 older women (9 SCD, 6 non-SCD) completing dual-task walking and tapping. Brain (cerebral oxygenation) and behavioural (gait and tap speed, accuracy, and vocal response) measures were assessed during single and dual-tasks. Older adults with SCD were marginally less accurate during dual-task tapping ($p < .06$) and had greater cerebral oxygenation than the non-SCD group ($p = .01$). SCD did not moderate gait speed from single to dual-task while non-SCD did ($p = .02$). Findings suggest that challenging dual-task paradigms may help identify different behavioural and brain activity markers of SCD and intervention targets.

### INTERMUSCULAR ADIPOSITY: A NOVEL RISK FACTOR FOR COGNITIVE DECLINE IN A BIRACIAL COHORT OF OLDER ADULTS

Caterina Rosano[1], Anne Newman[1], Xiaonan Zhu[1], Adam Santanasto[2], Bret Goodpaster[3], and Iva Miljkovic[1], *1. University of Pittsburgh, Pittsburgh, Pennsylvania, United States, 2. School of Public Health, University of Pittsburgh, Oakmont, Pennsylvania, United States, 3. AdventHealth, Orlando, Florida, United States*

Skeletal muscle and brain health both decline with age. Poorer skeletal muscle characteristics may be early markers of cognitive decline. The Health ABC study obtained repeated measures of thigh intermuscular adiposity via CT (IMAT, Years 1 and 6) and Mini-Mental State Exam (MMSE, Years 1 through 10), in 1634 adults (69-79 years, 48% women, 35% black). Linear mixed effects models accounted for change (Years 1 and 6) in weight, muscle (strength, area), and adiposity characteristics (abdominal subcutaneous, visceral, total fat mass) and for dementia risk factors (education, APOE4, diabetes, hypertension, physical activity). IMAT increased by 0.97 cm2/year (SD:1.16), and MMSE declined by 0.4 points/year (0.02). Each SD of IMAT corresponded to a MMSE decline of 0.22 points/year ($p<0.0001$), similar in adjusted models and stratified by race or gender. Aging-related IMAT increase may be a novel predictor of cognitive decline beyond traditional risk factors, with potential implications for muscle-brain cross talk.

### DUAL-TASK CHANGES IN PREFRONTAL ACTIVATION AND GAIT QUALITY IN OLDER ADULTS

Emma Baillargeon[1], Anisha Suri[2], Theodore Huppert[1], Ervin Sejdic[3], and Andrea Rosso[1], *1. University of Pittsburgh, Pittsburgh, Pennsylvania, United States, 2. Swanson School of Engineering, University of Pittsburgh, Pittsburgh, Pennsylvania, United States, 3. University of Toronto, Toronto, Ontario, Canada*

We compared the impact of performing dual-task walking on gait quality and prefrontal cortical activation assessed by functional near-infrared spectroscopy (fNIRS). We hypothesized a greater increase in fNIRS averaged over the left prefrontal cortex during dual-task walking would be associated with a greater decrease in gait quality (increased step-time variability; decreased gait speed, cadence, smoothness, and adaptability). In older adults ($n=60$, $75\pm5.8$ years, 57% female), we quantified the change in fNIRS and gait metrics from single-task walking (even surface) to walking with attentional (reciting every-other letter of the alphabet) and physical (uneven surface) dual-task challenges using four 15m repetitions of each task. Gait metrics were computed from a tri-axial accelerometer at the lower-back. Changes in fNIRS from single to dual-task walking were not associated with changes in gait quality for both attentional and physical challenges (Spearman correlations, all $p>0.08$). Variability in response across individuals may contribute to our findings.

### COGNITIVE CORRELATES OF GAIT SPEED AMONG OLDER ADULTS FROM SIX COUNTRIES: FINDINGS FROM THE COSMIC COLLABORATION

Briana Sprague[1], Xiaonan Zhu[1], Andrea Rosso[1], Joe Verghese[2], Kim Delbaere[3], Darren Lipnicki[4],

Perminder Sachdev[4], and Caterina Rosano[1], *1. University of Pittsburgh, Pittsburgh, Pennsylvania, United States, 2. Albert Einstein College of Medicine, Bronx, New York, United States, 3. Neuroscience Research Australia, Randwick, New South Wales, Australia, 4. Centre for Healthy Brain Ageing (CHeBA), Sydney, New South Wales, Australia*

Using data from population-based cohorts of older adults 65+ from six countries across three continents (N=6472), we aimed to (1) describe and (2) identify predictors of usual and rapid gait speed from studies participating in the Cohort Studies of Memory in an International Consortium (COSMIC) collaboration. We investigated whether clinical (BMI, hypertension, stroke, APOE status), psychological (cognition, mood, general health) and behavioral factors (smoking, drinking, physical activity) predicted gait speed similarly across cohorts; we used regression models controlling for demographics. Unadjusted usual gait speed ranged from 0.52-1.09 m/s and rapid gait speed ranged from 1.20-1.68 m/s. Lower BMI and better cognitive function consistently predicted faster usual and rapid gait speed in all cohorts. These patterns were not attenuated by demographics. Other psychological and behavioral factors were less consistently associated with gait. Taken together, gait speed is variable across ethnogeographic regions, but the influence of cognitive on gait is remarkably consistent.

### EARLY CHARACTERISTICS AND BIOMARKERS OF COGNITIVE IMPAIRMENT AND DEMENTIA WITH PRIOR MOTOR IMPAIRMENT

Qu Tian[1], Susan Resnick[2], and Luigi Ferrucci[2], *1. National Institutes on Aging, Baltimore, Maryland, United States, 2. National Institute on Aging, Baltimore, Maryland, United States*

Motor impairment is one early feature of preclinical Alzheimer's disease and strongly predicts future dementia. Whether older persons with dementia who had prior motor impairment present specific characteristics is less clear. In the Baltimore Longitudinal Study of Aging, we compared characteristics of participants with cognitive impairment or dementia who had slow gait on average 6 years before symptom onset (<1.0m/sec)(n=65,81±7 years,35%women,25%black) to those who did not (n=93,77±7 years,61%women,16%black). Early cognition and key biomarkers on average 7 years before onset were compared between groups using multivariable linear regression, adjusted for demographics. Those with prior slow gait had early unfavorable metabolic panel (higher fasting glucose,higher 2hr-OGTT insulin,lower HDL,higher triglyceride,higher BMI and waist,higher blood pressure), lower manual dexterity and processing speed, and lower lysophosphatidylcholine 18:1 and 18:2, critical in the synthesis of cardiolipin-an essential component of mitochondrial membranes. Strategies to prevent metabolic dysfunction at an early stage may slow down dementia progression.

### SESSION 4350 (SYMPOSIUM)

### NURSING FACILITY QUALITY, CLOSURES, AND STAFFING

Chair: Denise Tyler

The quality of care provided in nursing facilities has long been a societal concern and an important focus of policy at the federal and state level. The COVID-19 pandemic has drawn increased attention to nursing facilities due to high infection and mortality rates as well as increased media attention on staffing shortages that have occurred in the wake of the pandemic. The Office of the Assistant Secretary for Planning and Evaluation (ASPE) and RTI International have recently completed a series of studies focused on nursing facility quality and staffing. This symposium will present the results of four of those studies. First, we will report the results of a study exploring states' use of value-based payment (VBP) programs as part of their nursing facility Medicaid payment systems. Second, we will present the results of a study examining inappropriate discharges from nursing facilities. Third, we will report findings from a study that examined the factors associated with nursing facility closures. Finally, we will present findings from a study that explored the effect of the pandemic on nursing facility staffing. Together these studies provide important information about the state of nursing facilities in the wake of the COVID-19 pandemic and suggest key areas of focus for policymakers.

### STATE USE OF VALUE-BASED PAYMENT IN NURSING FACILITIES

Denise Tyler[1], Kristie Porter[2], Melissa Hunter[2], Iara Oliveira[3], Marie Squillace[4], and Judith Dey[3], *1. RTI International, Waltham, Massachusetts, United States, 2. RTI International, Durham, North Carolina, United States, 3. Office of the Assistant Secretary for Planning and Evaluation, Washington, District of Columbia, United States, 4. ASPE, Washington, District of Columbia, United States*

Some states use value-based payment (VBP) programs as part of their nursing facility Medicaid payment systems. The purpose of this study was to determine which states use these programs, their goals, the measures used and other elements of these programs. An environmental scan was conducted to identify states that have VBP programs for nursing facilities, the key design elements of those programs, and the goals that states are aiming to achieve. Interviews with seven stakeholders were also conducted. We found that 20 states and the District of Columbia use VBP for nursing facilities with the goals of improving quality and increasing efficiency. However, due to lack of evaluation, it is not known if these programs are achieving their goals. Additional research is needed to determine which state programs are most successful. The results of such research would help in the development of future VBP programs.

### ASSESSING THE FACTORS ASSOCIATED WITH NURSING HOME CLOSURES

Kelly Hughes[1], Qinghua Li[2], Zhanlian Feng[3], Iara Oliveira[4], and Judith Dey[4], *1. RTI International, Research Triangle, North Carolina, United States, 2. RTI International, Waltham, Massachusetts, United States, 3. RTI International, Research Triangle Park, North Carolina, United States, 4. Office of the Assistant Secretary for Planning and Evaluation, Washington, District of Columbia, United States*

The media has reported recent increases in nursing home closures. This study examined closures from 2011-2019, identified facility and market characteristics associated with closures, and assessed the impact of closures on quality and access. We identified closures using termination dates and gaps in certification surveys and conducted descriptive and multivariate analysis. We identified 1,220 closures, with large increases in closure rates in 2018 and 2019 and geographic clusters. Chain facilities, urban facilities and smaller facilities were more likely to close, as were facilities with higher percentages of non-white and Medicaid residents. Staffing and quality five-star ratings had a nonlinear relationship with closure, which suggests Medicaid funding may impact closures rates. We found both the number of beds per 1,000 elderly and occupancy rates decreased, including in high-quality facilities. Closures should be examined further in the context of the COVID-19 pandemic.

### THE COVID-19 PANDEMIC MAY HAVE EXACERBATED NURSE STAFFING DISPARITIES IN NURSING FACILITIES

Kristie Porter[1], Angela Gasdaska[2], Marie Squillace[3], Judith Dey[4], Iara Oliviera[4], Zhanlian Feng[2], and Micah Segelman[5], *1. RTI International, Durham, North Carolina, United States, 2. RTI International, Research Triangle Park, North Carolina, United States, 3. ASPE, Washington, District of Columbia, United States, 4. Office of the Assistant Secretary for Planning and Evaluation, Washington, District of Columbia, United States, 5. Research Triangle Institute, Washington, District of Columbia, United States*

Nursing facilities (NFs) have historically struggled to maintain adequate nurse staffing. We used PBJ data linked with other publicly available sources and conducted stakeholder interviews to understand nurse staffing between 2019 and 2020. We found large declines in the population of NF residents and in staffing hours. Measured in hours per resident day (HPRD) to account for the size of the NF resident population, there were slight increases in staffing. Staffing was nonetheless a major challenge for NFs because they required increased staffing due to the impact of the pandemic. NFs in higher quartiles of percentage of minority residents lost nurse staffing HPRD relative to NFs in the lowest quartile of minority residents. Stakeholders explained that NFs with more minority residents were likely to employ staff who live in more vulnerable communities with a greater concentration of minorities, who were more impacted by COVID.

### FACTORS ASSOCIATED WITH HIGH-RISK DISCHARGES FROM NURSING FACILITIES

Kelly Hughes[1], Zhanlian Feng[2], and Iara Oliveira[3], *1. RTI International, Research Triangle, North Carolina, United States, 2. RTI International, Research Triangle Park, North Carolina, United States, 3. Office of the Assistant Secretary for Planning and Evaluation, Washington, District of Columbia, United States*

Although some nursing facility discharges are appropriate or an individual's choice, others may be facility-initiated discharges (FIDs) that are potentially inappropriate. Since FIDs cannot be observed directly in available data, this study focused on live discharges of residents with risk factors for FIDs from 2011-2017. We defined risk factors as characteristics identified in prior literature that were more prevalent among residents discharged live than residents not discharged in our analysis. We found the following risk factors: severe behavioral symptoms; impairments requiring more staff time; and transition to Medicaid eligibility. Psychiatric and mood disorders were not identified in prior literature but also associated with live discharge. Risk factors were consistent across years but varied by state and facility type. For-profit, chain, and government facilities were most likely to discharge residents with risk factors. Furthermore, the rates of post-discharge acute care use were highest among residents with risk factors.

### SESSION 4360 (SYMPOSIUM)

### PROGRAMS TO IMPROVE QUALITY OF LIFE AND HEALTH: WHAT DO OLDER ADULTS AND PRACTITIONERS THINK?

Chair: Ashley Ritter Co-Chair: Allen Glicksman Discussant: Robyn Stone

Numerous programs and services are designed to help older adults improve their health and quality of life. Impact evaluations typically focus on health outcomes. Often, less attention is paid to the opinions of professionals tasked with administering these efforts or the older persons and caregivers about the need for the program or the way the program is administered. This panel addresses the question with four presentations, each of which uses data collected from practitioners and/or older adults to gain their perspectives on programs to help maintain quality of life and health. Sweeny examines the experience of older LTBTQ adults as they navigate the care and social service systems. Dictus and colleagues will describe a community level effort to develop a master plan for aging in one county. The method for including the views of the older adults in the process can be a model for other communities. Corpora and colleagues report on efforts to ensure that staff are aware of important preferences of nursing home residents, with a focus on the predictors of successful implementation. Glicksman and colleagues examine the impact of migration on the attitudes and perceptions of older Spanish speaking migrants toward the formal long-term care system. Finally, Stone will address the question of how these types of data can be used to help shape policy and practice, using research to help older adults and professionals at all levels to support older persons in community and facility care.

### COMING OUT AND GOING BACK IN: AGING AND STRATEGIC IDENTITY DISCLOSURE AMONG LGBTQ ADULTS

Brian Sweeny, *Long Island University, Brookville, New York, United States*

In addition to facing social, economic, and health disparities, LGBTQ older adults face prejudice and discrimination across various social domains, leading some to re-enter the closet. When might older adults openly identify as LGBTQ, and when might they conceal this identity? Based on participant observation and interview data collected in two naturally occurring retirement communities in New York City, this paper examines LGBTQ identity and coming out in relation to aging processes, especially changing care networks and family structures, decreasing autonomy, and increasing engagement with health and social services. The older LGBTQ adults in the study feel the benefits of broad social changes and increasing cultural competencies regarding LGBTQ issues, yet they also speak of unsafe spaces, experiences of exclusion, and uncertainties. I analyze three specific identity disclosure strategies older LGBTQ adults use as they navigate prejudice and discrimination and work to affirm identities as aging gender and sexual minorities.

### ENGAGING OLDER ADULTS AND STAKEHOLDERS TO DEVELOP A COMMUNITY MASTER AGING PLAN: A CASE EXAMPLE

Cassandra Dictus[1], Sallie Allgood[2], Ryan Lavalley[3], Janice Tyler[4], and Cherie Rosemond[5], *1. University of North Carolina - Chapel Hill, Chapel Hill, North Carolina, United States, 2. Center Of Innovation To Accelerate Discovery And Practice Transformation (ADAPT), Health Services Research And Development, Durham VA Health Care System, Durham, North Carolina, United States, 3. Occupational Science And Therapy, University of North Carolina - Chapel Hill, Chapel Hill, North Carolina, United States, 4. Orange County Department on Aging, Chapel Hill, North Carolina, United States, 5. Partnerships in Aging Program, University of North Carolina - Chapel Hill, Chapel Hill, North Carolina, United States*

As our population ages, communities are creating policies and programs to support older adults. Yet, there is a lack of input from older adults about which policies and programs meet their needs. This case example describes how one community in North Carolina (NC) engaged community stakeholders (older adults and organizational leaders) in all phases of their Master Aging Plan (MAP). Community stakeholders participated in a community assessment through surveys (n=860), focus groups (n=13, 63 participants), key stakeholder interviews (n=34), and two community-wide listening sessions. Organized by the WHO's age-friendly framework domains, community stakeholders formed workgroups to create, implement, and track the MAP. An evaluation was completed using the tracking documents, ripple effect mapping, and workgroup leader interviews. Orange County, NC's experience with collaborative community engagement can serve as a guide for other communities seeking to involve community stakeholders in the development and evaluation of their own MA

### THE INFLUENCE OF NURSING HOME CHAMPIONS ON THE IMPLEMENTATION OF A PERSON-CENTERED QUALITY IMPROVEMENT PROJECT

Miranda Kunkel[1], Kamryn Kasler[1], Alexandra Heppner[1], Kimberly VanHaitsma[2], and Katherine Abbott[1], *1. Miami University, Oxford, Ohio, United States, 2. The Pennsylvania State University, University Park, Pennsylvania, United States*

Preferences for Activity and Leisure (PAL) Cards are a communication tool designed to alert nursing home staff to important resident preferences. This study explored the role of nursing home provider champions implementing (PAL) Cards in their community. Champions (n=35) created PAL Cards for 15-20 residents. A total of 88 monthly interviews were audio-recorded, transcribed, checked for accuracy, and coded using the domain "Characteristics of the Individual" from the Consolidated Framework for Implementation Research. Each construct was assigned a general valence rating based on consensus. Four constructs received the highest positive valence ratings and were identified as important facilitators to implementation success, and one construct received a neutral valence. Champions who anticipated the benefits of PAL Cards for their community and recruited a supportive team exhibited high implementation success. Additionally, the champion's perception of help from staff seemed to increase their own self-efficacy to achieve their goals.

### PAST AS PRESENT: HOW LIVED EXPERIENCE INFLUENCES INTERACTION WITH LTSS AMONG OLDER SPANISH SPEAKERS

Allen Glicksman[1], Misha Rodriguez[2], Lauren Ring[3], and Michael Liebman[4], *1. NewCourtland, Haverford, Pennsylvania, United States, 2. APM, Philadelphia, Pennsylvania, United States, 3. Private, Philadelphia, Pennsylvania, United States, 4. IPQ Analytics Inc, Kennett Square, Pennsylvania, United States*

Older persons who migrated to the mainland United States bring with them their experiences and culture from their homelands and the process of migration and settlement in their new communities. The attitudes, values and assumptions that emerge will contribute to the interaction of the individual with the formal Long Term Care Services and Support (LTSS) system. Our research focused on better understanding of that process among older Spanish speakers. Data from four focus groups (# of participants) conducted in Spanish about the participants experience with LTSS was used in this analysis. Findings demonstrate the importance of understanding the ways in which the worldview of these persons shape their expectations and use of the formal LTSS system. The findings will facilitate the development of a Social Interaction Model to enable planners, policy makers and practitioners in LTSS to more effectively work with older migrants.

### SESSION 4370 (PAPER)

### SOCIAL DETERMINANT OF HEALTH, UNMET NEEDS, AND DISCRIMINATION

### A MIXED-METHOD SOCIAL NETWORK ANALYSIS OF LOW-INCOME DIVERSE OLDER VOLUNTEERS

Katy (Qiuchang) Cao[1], Holly Dabelko-Schoeny[2], Keith Warren[3], and Mo-Yee Lee[3], *1. The Pepper Institute on Aging and Public Policy and The Claude Pepper Center, Florida State University, Tallahassee, Florida, United States, 2. Ohio State University, Columbus, Ohio, United States, 3. The Ohio State University, Columbus, Ohio, United States*

Although the health benefits of volunteering among older adults are well established in gerontology, older migrants' abilities and interests in social participation are hardly recognized. To address the gap, we collected focus groups and survey information in Russian, Khmer, Somali, Nepali, and English to understand the volunteering experiences, social networks, and feelings of loneliness among low-income diverse volunteers in the Senior Companions Program (SCP) in Columbus, Ohio (N=41). The grounded theory approach informed the qualitative analysis. Exponential Random Graph Modeling (ERGM) was utilized to identify statistically significant structural features in the volunteers' network. Five major themes emerged from the focus groups: (1) Expanding and strengthening social networks through volunteering; (2) Experiencing and coping with loneliness; (3) Experiencing and managing the social impact of COVID; (4) Exploring and loving the program; (5) Social connections outside of the program. Graphs and preliminary ERGM results demonstrate that participants tend to form homophily-based relationships with other volunteers of the same gender (β=2.45, p< 0.001) and from the same country (β=4.86, p< 0.001). However, participants tend to form friendships with people from different racial (β= -1.12, p< 0.001) and different educational backgrounds (β = -0.88, p< 0.001). The tendency to reciprocate (β= 0.96, p< 0.001) and to form triads (β= 9.90, p< 0.001) are both positively significant in the networks. Findings imply that practitioners should attend to within- and cross-cultural relationships in programs for diverse older adults. Addressing language barriers and other sources of homophily may facilitate cross-cultural friendships.

### DIFFERENCES IN HEALTH AMONG LOW-INCOME OLDER IMMIGRANTS: DOES ETHNICALLY HOMOGENEOUS ENVIRONMENT MATTER?

BoRin Kim[1], ByeongJu Ryu[2], Jihye Baek[3], Sojung Park[3], Chung Hyeon Jeong[1], and Jina Park[3], *1. University of New Hampshire, Durham, New Hampshire, United States, 2. Chuncheon Hyoja Social Welfare Center, Chuncheon-si, Kangwon-do, Republic of Korea,*
*3. Washington University in St. Louis, St. Louis, Missouri, United States*

Older immigrants comprised 14% of the US older population, which is predicted to continue to rise. Despite the importance of ethnic attachment in older immigrants' well-being, little is known how ethnically homogeneous housing environment affect older adults' health. This study aims to fill the gaps in knowledge on low-income older immigrants living in subsidized senior housing and to explore the associations between immigrant status, senior housing environments, and self-rated health among this population. Our survey data was collected from 18 subsidized senior housing communities in IL, MO, and NH (N=459). Bilingual surveyors fluent in Chinese, Russian, and Korean were also available for the participants with limited English proficiency as the sampled housings were the multi-ethnicity properties. Hierarchical Linear Modeling was employed for our analysis to address our participants were clustered within senior housing.More than a half (56%) of our sample lived in ethnically homogeneous senior housing (either all immigrants or all non-immigrants). Immigrants consist of 53% of our sample, and 48% of them lived in ethnically homogeneous housing. Our finding showed that immigrants were more likely to report low level of self-rated health (OR=0.885, p< 0.01). However, ethnically homogeneous senior housing environments positively influence the association between immigrant status and self-rated health (OR=1.219, p<.05).This study highlights the importance of ethnically homogeneous living environment for low-income older immigrants in senior housing. Although ethnic diversity may be beneficial in broader society, concentration of ethnic minority older immigrants could play an important role in terms of social support and service delivery.

### MENTAL HEALTH IMPACT OF DISCRIMINATION: THE PROTECTIVE FUNCTION OF SOCIAL CAPITAL IN DIVERSE GROUPS OF OLDER ADULTS

Mengzhao Yan, Yuri Jang, and Kathleen Wilber, *University of Southern California, Los Angeles, California, United States*

Discrimination occurs in complex social contexts leading to various levels and types of outcomes. Although the negative health impact of discrimination is well-documented, there is a need to investigate patterns among discrimination, social factors, and health outcomes in diverse racial/ethnic groups of older adults to inform interventions. For example, social capital, such as social cohesion, social ties, and safety, is anticipated to be directly associated with mental health and also to modify the impact of discrimination. In the present study, we examined (1) racial/ethnic differences in perceived discrimination, social capital, and depressive symptoms and (2) the direct effect of perceived discrimination and social capital, as well as their interactions, on depressive symptoms among different racial/ethnic groups. Data were drawn from the National Social Life, Health, and Aging Project (NSHAP) Round 3 (2,988 non-Hispanic Whites, 719 non-Hispanic Blacks, and 499 Hispanics; 68 mean age). Compared to non-Hispanic Whites, non-Hispanic Blacks had a significantly higher level of perceived discrimination, lower social capital, and more depressive symptoms. Findings from multivariate linear regression models demonstrated that, in all racial/ethnic groups, frequent experiences of discrimination and low levels of social capital were associated with increased symptoms of depression. A significant interaction between discrimination and social cohesion was observed in non-Hispanic Whites and Hispanics. In both groups, the negative impact of discrimination was lower among those with higher levels of social cohesion. Our findings support efforts such as improving well-being for older adults by promoting age-friendly communities to build greater social cohesion.

### NEIGHBORHOOD CHARACTERISTICS AND QUALITY PERFORMANCE OF HOME HEALTH AGENCIES: A COHORT STUDY

Chenjuan Ma, *New York University, New York, New York, United States*

Home health care (HHC) is a core source of home- and community-based services to older adults "aging in place'. This study examined variation in HHC quality by neighborhood characteristics in the US by using 5 years (2014-2019) of national Home Health Compare data linked to Providers of Services (POS) files and Area Health Resources Files (AHRF). HHC quality was measured by agency rates of timely initiation of care, hospitalization, and ED visits. We examined 7,487 HHC agencies (37,435 agency-years) in 1,760 counties. At baseline, the mean rates of timely initiation of care, hospitalization, and ED visit were 91.5%, 15.8%, and 12.5%. On average, county percentage of Blacks, Hispanic, individuals with college degree, unemployment, and being in poverty were 12.9% (range: 0.1% – 76.0%), 20.6% (0.4% - 95.8%), 30.4% (7.3% - 77.6%), 5.4% (2.1% - 24.0%), and 14.4% (3.1% - 38.0%) respectively in 2015. Estimates from multivariate regressions showed that neighborhoods with higher proportion of Blacks (β= -0.04, p=0.000) and Hispanics (β= -0.02, p=0.000), higher rate of unemployment (β= -0.07, p=0.035) were less likely to have HHC agencies providing timely initiated care; neighborhoods with higher proportions of Blacks (β= 0.01, p=0.000) and higher poverty rates (β= 0.02, p=0.027) were more likely to have HHC agencies with higher hospitalization rates; and neighborhoods with higher poverty rates (β= 0.04, p=0.000) were more likely to have HHC agencies with higher ED visit rates. Our findings indicate that racial/ethnic minorities in neighborhoods with low socioeconomic status were less highly to access high quality HHC.

### UNMET NEEDS AMONG ADULTS AND OLDER ADULTS IN BRAZIL, ELSI-2019

Flavia Andrade[1], and Xiayu Chen[2], *1. University of Illinois--Urbana-Champaign, Urbana, Illinois, United States, 2. University of Illinois at Urbana-Champaign, Urbana, Illinois, United States*

Many older adults in Brazil struggle to receive adequate assistance in later life. To better understand this issue, this study examines the prevalence and characteristics of unmet needs of adults ages 50 and older with difficulties performing activities of daily living (ADL), instrumental activities of daily living (IADL), the caregiving patterns, and the consequences of unmet needs in Brazil. We use data from the second wave of the Brazilian Longitudinal Study of Aging (ELSI-Brazil) study conducted in 2019-2021 (N=9,949). Results indicate that the prevalence rate of ADL was 8.9% (95% CI 7.6 – 10.5), and the prevalence of IADL was 15.6% (95% CI 13.1 – 18.5). Among those with ADL difficulties, 11.5% (95% CI 8.2 – 16.0) reported having unmet needs. Among those with IADL difficulties, 8.8% (95% CI 6.1 – 12.4) reported having at least one activity with unmet needs. Unmet needs were higher among younger adults, those living alone, Black or mixed race, or the Northeast region. Most of the care (87%) was provided by family members, primarily daughters, and wives. About 20% of caregivers had to stop working or studying to provide the needed care. Those with ADL unmet needs were less likely to visit friends or family, whereas IADL unmet needs were associated with lower odds of visiting a doctor. Our study provides the first glimpse of the characteristics of those with currently unmet needs among adults in Brazil and of caregivers. Unmet needs are associated with social isolation and worse healthcare use.

### SESSION 4380 (SYMPOSIUM)

### HOUSING, AGING, AND HEALTH: NEW FINDINGS AND FRAMEWORKS FROM HOUSING-FOCUSED RESEARCH IN THE CONTEXT OF COVID-19

Chair: Nancy Berlinger

Older adults have been the most vulnerable population to severe illness, hospitalization, or death from COVID-19. During the pandemic, housing became a site of health and safety for some, while reinforced inequities for others due to underlying problems of affordability, accessibility, safety, and service access. This symposium showcases five housing-focused studies reflecting the pandemic context, including research by early-career investigators. The first speaker will present findings from a study of materials preserving promising practices and policy ideas generated by housing-focused pandemic responses to middle-income and low-income community-dwelling older adults. The second speaker will discuss narrative lessons from "avoidance hotels" – an infection-reduction strategy that transferred older adults from shelters to hotel rooms – with potential to guide planning for the needs of older adults facing homelessness and serious illness. The third speaker will analyze findings from multi-city research on the pandemic housing experiences of lower-income Black women, who faced severe intersectional threats to housing security. The fourth speaker will share data from a qualitative study of a Medicare-financed home care program for residents of HUD housing, including recommendations for improving experiences of participants and providers. The fifth speaker will describe the evolution of the pandemic-response roles of HUD service coordinators, based on findings from surveys in 2020 and 2021. We will conclude with audience discussion about ways for age-focused researchers to collaborate in crafting policy solutions and effective public narratives about housing equity in America's aging society.

### SEEING THROUGH A HOUSING LENS: STUDYING HOUSING-FOCUSED RESPONSES TO THE NEEDS OF OLDER ADULTS DURING THE PANDEMIC

Nancy Berlinger, *The Hastings Center, Garrison, New York, United States*

This paper draws on a study of unindexed literature produced by local and state policymakers, Area Agencies on Aging, nonprofit organizations, for-profit companies, age-friendly initiatives, affordable housing providers, and other groups responding to the needs of older adults at home during the COVID-19 pandemic. By collecting and analyzing descriptions of how service providers pivoted or improvised to meet urgent challenges, we sought to identify promising practices and policy ideas preserved in reports, newsletters, websites, and other communications. This paper reviews study methods and recommendations, and explores a concept that emerged from this research: recognizing services and support for older adults at home as "housing-focused" care that relies on the affordability, accessibility, and safety of a person's home. The applications of the "housing lens" to future research on housing, aging, and health and to making progress on better integrating housing policy and health policy in aging societies will be discussed.

### TEMPORARY ADDITIONS TO TEMPORARY HOUSING: LESSONS FROM HEALTH EXPERIENCES OF COVID AVOIDANCE HOTEL RESIDENTS

Ian Johnson[1], Michael Light[2], Tam Perry[3], and Terri Lewinson[4], *1. University of Tennessee, Knoxville, Tennessee, United States, 2. University of Washington Palliative Care Training Center, Seattle, Washington, United States, 3. Wayne State University, Detroit, Michigan, United States, 4. The Dartmouth Institute for Health Policy and Clinical Practice, Lebanon, New Hampshire, United States*

COVID-19 amplified system burdens and health risks within the housing care continuum, in which older adults with chronic serious illness are disproportionately represented. We present retrospective chart review data about the health experiences of older adults with serious illness living in and moving through temporary avoidance hotels during the COVID-19 pandemic. Through narratives of fourteen residents, we illustrate trends across two nine-month phases. Trends illustrate how avoidance hotels created opportunities for continuity of care, connection to services, and health-affirming relationships with place. We also identified challenges in catering to diverse medical, behavioral, and psychosocial-spiritual needs of older and seriously ill residents, as well as negative consequences to the geographic dispersion caused by de-congregating homeless shelters. Avoidance hotels present important lessons in considering future housing and healthcare intervention and implementation for older people facing homelessness while seriously ill.

### BRINGING HOME CARE TO HUD HOUSING: AN EVALUATION OF NEW JERSEY'S ASSISTED LIVING PROGRAM

Karen Zurlo, *Rutgers, The State University of New Jersey, New Brunswick, New Jersey, United States*

The Assisted Living Program (ALP) in New Jersey provides home care services, financed by Medicaid and self-pay, to older adults living in low-income HUD housing. Because the program had attracted few providers and had low uptake, we designed a qualitative study aimed at understanding experiences of ALP participants compared to non-participants. After pandemic-related postponements, the study began in June 2021, collecting data from 63 respondents. Findings revealed differences in the demographics and needs of older New Jerseyans as well as differences between participants and non-participants in ALP. This paper will review the study and discuss investigators' conclusions about how to strengthen this highly viable model of home-based care and better serve low-income older adults in New Jersey. Opportunities for improvement include modifications to regulations and financing; marketing of services, and collaboration among ALPs and housing providers.

### BRIDGING THE GAP: SERVICE COORDINATION IN HUD HOUSING DURING COVID-19

Samara Scheckler, and Jennifer Molinsky, *Harvard University, Cambridge, Massachusetts, United States*

Older HUD residents were vulnerable to COVID-19 due to their age, health, income, race, ethnicity, and resources. In combination, these factors increased risk to health and housing stability. While many HUD properties employ a service coordinator, this role is not universally adopted and impacts of these services are not well understood. This research surveyed service coordinators in mid-2020 and late-2021 to characterize older adult experiences living in public housing during the pandemic and ways service coordinators helped manage disruptions. Surveys were disseminated to 3,500 service coordinators. Findings identified needs in transportation, personal care, sociality, mental and physical healthcare, and food. Interventions included resource procurement, benefits management, technology access improvement, and linking residents to services. Tools included information dissemination, needs assessments, and partnership development. This work offers insight into the role of a service coordinator to mitigate health and housing risk. It becomes increasingly salient as HUD residents trend demographically older.

### SESSION 4390 (SYMPOSIUM)

### THE IMPLEMENTATION OF GERIATRIC EMERGENCY DEPARTMENT ACCREDITATION (GEDA) IN RURAL CRITICAL ACCESS HOSPITALS

Chair: Ellen Flaherty Discussant: Kevin Biese

The largely rural setting of Northern New England offers unique challenges to implementing improved acute care for the growing geriatric population. Northern New England is one of the United States' most rapidly aging regions, with Vermont and New Hampshire being the second and third oldest US states respectively by median age (U.S. Census 2017). There is a need to expand innovations in geriatric emergency medicine to reach older adults in rural areas such as Northern New England. Dartmouth-Hitchcock Medical Center and the West Health are collaborating on a project leveraging telehealth to extend the reach of a GED to rural hospitals, as well as investigate the opportunities for scaling and sustaining this concept to other rural facilities across Northern New England and throughout the country. This symposium will focus on our experience implementing a hub and spoke model to achieve our goal of improving the care of older adults in rural emergency departments.

### LEVERAGING THE STRENGTH OF UNIQUE PARTNERS TO IMPROVE GERIATRIC EMERGENCY CARE IN RURAL CRITICAL ACCESS HOSPITALS

Scott Rodi, *Dartmouth-Hitchcock Medical Center, Lebanon, New Hampshire, United States*

Developing a level 1 accredited geriatric emergency department during the pandemic was challenging. The greater challenge has been to support rural critical access hospitals on their journey of becoming level 2 accredited geriatric emergency departments. This has been accomplished through a novel collaborative approach between the level 1 accredited emergency department, a newly established Geriatric Center of Excellence at Dartmouth-Hitchcock Health and a HRSA funded Geriatric Workforce Enhancement Program. This talk will focus on bringing together system partners and funders who have a history of success through: (a) development, testing and dissemination of tool kits and implementation guides; (b) boot camp training that includes interprofessional team training, QI and specific geriatric content training; (c) implementation support and coaching using data and QI methodology through learning collaboratives; (d) scaling successful models of evidence based geriatric care; (e) expertise in implementation science; (f) expertise in evaluation and (g) disseminating findings to achieve our goals.

### ALIGNING GERIATRIC EMERGENCY CARE WITH THE 4MS OF THE AGE-FRIENDLY HEALTH SYSTEM FRAMEWORK

Jennifer Raymond, *Dartmouth-Hitchcock Medical Center, Lebanon, New Hampshire, United States*

In 2019 Dartmouth-Hitchcock Medical Center (DHMC) was recognized by the Institute for Healthcare Improvement as a level 2 Age Friendly Health System (AFHS). Over the past two years DHMC leveraged the strengths of the AFHS to become a level 1 American College of Emergency Physicians (ACEP) accredited geriatric emergency department. Leveraging the paradigm of 4M care including: What Matter's Most, Mobility, Medication and Mentation, DHMC achieved alignment between the geriatric emergency department accreditation and the AFHS framework. Pathways focusing on the 4Ms have been identified as a model to improve transitions of care post ED. This talk will focus on lessons learned and outcome data specific to the creation of the 4M pathways and transitions of care,

### SAFE TRANSITIONS: POST-ED DISCHARGE FROM A RURAL CRITICAL ACCESS HOSPITAL

Kristie Foster, *Alice Peck Day Memorial Hospital, Lebanon, New Hampshire, United States*

This talk will focus on leveraging the strengths of small rural communities to improve transitions of care post ED discharge. We moved beyond post discharge telephone calls, and partnered with already existing community based organizations. These collaborative partnerships enabled the use of standardized bundles of interventions targeting specific risks such as falls, depression, polypharmacy and the lack of documentation around goals of care.

### ENHANCED COMMUNICATION BETWEEN A RURAL CRITICAL ACCESS HOSPITAL, A RESIDENTIAL CARE FACILITY, AND PRIMARY CARE

Lee Morissette, *Mount Ascutney Hospital, Windsor, Vermont, United States*

A need to improve the transitions of care between a small rural emergency department, a residential care facility and a primary care practice emerged early on in our work to become a level 2 accredited emergency department. One challenge is bringing together 3 partners all with distinct types of health records, unique foci of care and a variety of disciplines and workforce. This paper will focus on our journey of creating an individualized plan of care to help mitigate problems of poor communication with a focus on the 4Ms framework including: What Matters Most, Mobility, Medication and Mentation.

### SESSION 4400 (SYMPOSIUM)

### TRAINING THE NEXT GENERATION OF GERONTOLOGICAL LEADERSHIP: WHO AND HOW?

Chair: Neil Charness Co-Chair: Patricia Heyn Discussant: James Appleby

Knowing how to train the next generation of gerontological leaders involves understanding where we are now and where we want to be in the coming decades. We outline the results of a survey of the membership of Directors of Aging Centers. The Directors of Aging Centers interest group in GSA has representation from the USA, Canada, and Europe. A survey was sent to the membership in late December with reminders in January and had 31 respondents. We discuss the results of the survey, highlighting the demographics of the current leadership (Neil Charness), perceived need for training by current leaders (Peter Lichtenberg), and consensus content of leadership training programs (Patricia Heyn). Patricia D'Antonio provides a perspective on GSA's approach to professional development programs and avenues for soliciting funding for leadership training. Our discussant (James Appleby, CEO of GSA) will reflect on the need for training in the context of building the next generation of gerontological leadership. We plan to devote significant symposium time to solicit audience input on next steps for supporting the effort to improve the quantity, quality, and diversity of the gerontological leadership workforce.

### CHARACTERISTICS OF LEADERS OF AGING CENTERS

Neil Charness, *Florida State University, Tallahassee, Florida, United States*

In December of 2021 and January of 2022, current leaders of Centers on Aging belonging to the GSA Age Directors interest group were invited to fill out a survey inquiring about their backgrounds, training for leadership, and perceived needs for training via an online Qualtrics survey. Thirty-one responses were received though a few responses were missing for most questions. The sample mostly resided in the USA (86%), and identified mainly as female (63% female, 37% male). Most were married (90%). The average age of a respondent was 58 years (SD=9; range 37-74). Respondents were not diverse: 97% White. Most had doctoral level education (1 Master's level). The results indicate that there is a need to enhance and develop leadership skills in diverse mid-career gerontologists in order to provide replacements for an aging cohort of directors. Results are also consistent with prior National Academies' recommendations for training the gerontological workforce.

### GSA R13 DIVERSITY WORKSHOP: THE CAREER JOURNEY OF A THOUSAND EXPERIENCES BEGINS WITH ONE STEP

Patricia Heyn[1], and Keith Whitfield[2], *1. Center for Optimal Aging, Marymount University, Fairfax Station, Virginia, United States, 2. University of Nevada, Las Vegas, Las Vegas, Nevada, United States*

After 23 years, since the NIH recognized the need to increase the contribution of underrepresented minority (URM) scientists in the biomedical sciences, modest gains have been made to address the significant under-representation in biomedical sciences. By the time URM scholars have gained the necessary research skills to succeed as scientists, they have overcome many social and professional barriers, yet they still experience the social burdens of disadvantage and discrimination. In an effort to increase representation, the GSA has been successfully delivering Diversity Mentoring and Career Development Technical Assistance Workshops (GSA DMCDTAW) since 2018 with support from grants from the NIH/NIA. Many trainees and faculty from diverse backgrounds participated in DMCDTAW. The program promoted peer mentoring opportunities, professional training, and networking engagements and underwent a series of evaluations and focus group discussions. The program framework, curriculum, and evaluation will be presented. Recommendations for future mentoring development and evaluation will be discussed.

### WHAT LEADERSHIP SKILLS ARE NEEDED TO DIRECT GERONTOLOGY PROGRAM: SURVEY RESULTS FROM LEADERS ACROSS THE COUNTRY

Peter Lichtenberg, *Wayne State University, Detroit, Michigan, United States*

Gerontology programs across the country, including Centers, Institutes and Departments play a key role in promoting the field and the careers of the scientists they connect with. A recent survey of 30 Gerontology leaders from the GSA interest group found that 60% of them received no leadership training before becoming a Director or Chair in their program. Only 50% of those who received some generic leadership training found that it prepared them for their current role. This presentation will highlight some of the potential areas of focus for leadership training more specific to Gerontology programs. Among the topics most highly valued by the Gerontology Program leaders include budgets, managing people, negotiating difficult situations, understanding different leadership styles, teamwork, ethics and diversity, equity and inclusion. This session will frame some of the key aspects of leadership training including a deeper understanding of universities as organizations; and negotiating for resources.

### GSA ACTIVITIES TO SUPPORT CAREER DEVELOPMENT: RELEVANCE TO DIRECTORS OF AGING CENTERS

Patricia D'Antonio, *The Gerontological Society of America, Washington, District of Columbia, United States*

GSA continues to support our members through career development activities. These activities are designed to empower members to develop the necessary skills to advance their careers as leaders in the field of aging. Through activities like the GSA NIA R13 Diversity, Mentoring, and Career Development Technical Assistance Workshop, we strive to support, promote, and advance the training of diverse students in aging. This presentation will discuss how the experiences with this R13 project and other career development efforts inform additional activities of the Society through a well-framed aging lens. We also discuss the process of soliciting funding to support leadership training and the role that GSA can play in the application process.

### SESSION 4410 (SYMPOSIUM)

### UPDATE IN GEROSCIENCE FOR NONBIOLOGISTS

Chair: George Kuchel Co-Chair: Steven Austad

Geroscience began as a result of discoveries made by biologists in aging cells, simple organisms and animal models. Such advances, the discovery of strategies for modifying rate of aging, and appreciation of aging as a shared risk factor for chronic diseases led to the Geroscience Hypothesis. It states that interventions modifying aging biology can slow its progression, resulting in the prevention or delay of onset of multiple diseases. However, for these discoveries to truly impact human health, nonbiologists – scientists in other disciplines and clinicians, must also engage in geroscience. An obstacle to a wider engagement by disciplines and GSA members other than basic biologists in geroscience has been the fact that varied fields engaged in aging issues tend to use language (jargon), methods and scientific approach that can make their work less accessible and understandable to other disciplines. To that end, speakers have been selected not only for their scientific contributions, but also for their ability to overcome such barriers. Steve Austad (UAB) will discuss the range of animal models and experimental approaches that led to the Geroscience Hypothesis. Anne Newman (U Pittsburgh) will review evidence that these findings are relevant to and might be translatable to humans. John Newman (UCSF/Buck) will seek to frame these considerations in terms of broader societal discussions regarding aging while providing a framework by which nonbiologists may critically evaluate discoveries and actual products claiming benefits in terms of aging. (Sponsored by Nathan Shock Coordinating Center U24AG056053; Geroscience Education and Training Network R25AG073119).

### ANIMAL MODELS AND THE EMERGENCE OF GEROSCIENCE

Steven Austad, *University of Alabama at Birmingham, Birmingham, Alabama, United States*

Aging, meaning the generalized deterioration in physical and mental function with time, influences nearly all living species. Moreover, many underlying biological mechanisms of aging appear to be conserved. At the same time, there appear to be great differences in the rate at which different species age. Research using both traditional and nontraditional animal species, employing both laboratory and field studies has offered important insights into the discovery of underlying causes of aging. Also, a variety of studies using well-established animal models of aging have now identified both repurposed medications and new compounds and other approaches that appear to slow biological aging with potential improvements in lifespan and healthspan. It is these findings, when framed in the context of the emergence of multiple chronic diseases with aging, that have led to the Geroscience Hypothesis.

### STUDYING BIOLOGICAL AGING AND GEROSCIENCE IN HUMANS

Anne Newman, *University of Pittsburgh, Pittsburgh, Pennsylvania, United States*

For geroscience to impact human health, its scientific underpinnings observed in animal models must also be relevant to humans, and therapies grounded in geroscience principles must be translatable to humans. Given the heterogeneity of human aging, chronological age alone is not a sufficient indicator of susceptibility to specific diseases, disabilities, or death, nor does it provide insights into the likelihood of benefiting from specific geroscience-inspired approaches. Bringing these metrics to large epidemiological studies and clinical trials has provided a number of important insights. For example, indices developed from aggregates of biomarker, physiological and clinical data have been shown to perform well in terms of predicting healthy aging, specific aging-related traits and mortality. Functional or composite outcomes have been favored for capturing health span in translational trials.

### ASSESSING PURPORTED AGING THERAPIES IN THE CLINIC

John Newman, *Buck Institute and UCSF, Novato, California, United States*

Aging is a universal human experience that looms large in popular and commercial culture. It can be difficult for patients and providers alike to sort fact from unproven plausibility and from potentially harmful fiction. Geroscience is an emerging field, and while some interventions involve repurposed or over-the-counter drugs with early clinical data, it is important to note that none yet have a specific aging-related indication and none have a consensus recommendation for clinical use in an aging context. Even highly plausible links to mechanisms of aging do not ensure that an intervention will be effective or risk-free, and not all purported therapies align with preclinical science. Finally, some purported therapies fall into potentially problematic regulatory gaps. With all of these considerations in mind, attendees will gain insights into applying their existing skills to evaluate this field, and new tools for critically evaluating geroscience literature to assist in patient-centered decision making.

### SESSION 4420 (SYMPOSIUM)

### US RACIAL AND ETHNIC DISPARITIES IN DEMENTIA LIFE EXPECTANCY, RISK/PROTECTIVE FACTORS, AND CAREGIVING STRESSORS

Chair: Maria Aranda

The health, emotional, and economic impacts of dementia are far-reaching and impossible to fully calculate. Late-life dementia is not equitably distributed across population subgroups including older people and racial/ethnic minorities. Clear evidence underscores disparities in social/personal resources, environmental exposures, racism/discrimination, increased burden of caregiving, and uneven access to quality care especially for Blacks and Hispanics and caregivers. Each paper in this symposium addresses high-priority, disparity research areas highlighted in the recent NASEM Decadal Survey of Behavioral and Social Science Research on ADRD: characteristics of population groups that influence disease ascertainment and dementia life expectancy; quantification of mid- and late-life risk/protective factors; pesticide exposure and cognition, and caregiving stressors among racial/ethnic minorities. All presentations utilize nationally representative data on older people in the US. Garcia et al. utilize HRS data to create dementia life expectancy estimates using four competing algorithmic classifications of dementia status by race/ethnicity (Blacks, Whites), nativity status (US-, non-US-born Latinos), and gender. Applying the Lancet Commission Dementia Prevention framework to HRS data, Ferdows and Aranda examine risk/protective factors and quantify the relative contribution of individual variables in explaining racial/ethnic dementia disparities. Chanti-Ketterl et al. elucidate the association between organochlorines pesticides (total scores, specific type) and cognition based on NHANES data to ascertain racial/ethnic differences. Based on NHATS, Moon et al. explore differences in primary stressors by dementia care and race (Blacks, Whites) during COVID-19. Findings provide evidence of potential multi-level targets for interventions and public policies. All primary authors are early-career scholars dedicated to mitigating ADRD health disparities.

### DEMENTIA LIFE EXPECTANCIES: NEW KNOWLEDGE AND CONSIDERATIONS FROM THE HEALTH AND RETIREMENT STUDY

Marc Garcia[1], Wassim Tarraf[2], Chi-Tsun Chiu[3], Joseph Saenz[4], and Adriana Reyes[5], *1. Syracuse University, Syracuse, New York, United States, 2. Wayne State University, Detroit, Michigan, United States, 3. Institute of European and American Studies, Taipei City, New Taipei, Taiwan (Republic of China),*
*4. University of Southern California, Los Angeles, California, United States, 5. Cornell University, Ithaca, New York, United States*

Alzheimer's disease and related dementias (ADRD) are a growing public health crisis. Estimates on the prevalence and incidence of ADRD across and within population-based studies have varied in part due to competing measures to assess dementia status. Disentangling these inconsistencies is crucial for understanding dementia disparities among racial/ethnic, and nativity groups among older adults. Based on the Health and Retirement Study we examined across (Whites, Blacks) and within-group differences (US- and non-US-born Latinos) in estimates of dementia life expectancy, using four competing algorithmic techniques (i.e., the Langa-Weir, Expert, Hurd, and Lasso) for the classification of dementia ascertainment. Estimates of dementia life expectancy across algorithms largely point to dementia disparities in the prevalence of the disease across racial/ethnic, and nativity groups, regardless of the algorithmic technique utilized. Elucidating algorithms that can be utilized with different racial/ethnic groups may reduce bias in dementia assessment in the future.

### RACIAL/ETHNIC DISPARITIES IN RISK AND PROTECTIVE FACTORS OF DEMENTIA: A LIFESPAN APPROACH

Nasim B. Ferdows[1], and Maria Aranda[2], *1. Northeastern University, Boston, Massachusetts, United States, 2. University of Southern California, Los Angeles, California, United States*

We examined risk and protective factors associated with dementia and decomposed racial/ethnic disparities in factors associated with dementia. We performed a retrospective analysis of 3,495 individuals 65+ who participated in the 2016 Harmonized Cognitive Assessment Protocol, linked to Health and Retirement Study. Using the MMSE score, individuals were classified as having dementia vs cognitively normal. Risk factors included cardiovascular factors and hearing loss in midlife, current smoking, depression, and physical inactivity. Protective factors included educational attainment and wealth. Using Oaxaca-Blinder decomposition we quantified the relative contribution of individual variables in explaining racial/ethnic disparities. We found that Blacks and Hispanics have higher dementia risk compared to Whites. Accounting for demographics, mid-and later-life risk and protective factors explained one-third of the difference in Blacks and Whites' cognitive functioning, and two-thirds of the difference in Hispanics and Whites. The findings highlight disparate dementia rates, and the need to elucidate other factors beyond race/ethnicity.

### ORGANOCHLORINE EXPOSURES AND COGNITIVE FUNCTION IN OLDER LIFE: THE ROLE OF RACE/ETHNICITY

Marianne Chanti-Ketterl[1], Maria Aranda[2], and Brenda Plassman[1], *1. Duke University, Durham, North Carolina, United States, 2. University of Southern California, Los Angeles, California, United States*

Organochlorines (OCs) are persistent pesticides that remain in the environment and body for extended periods after exposure. Little is known about their long-term impact on cognition in later life. Some studies, but not all, have shown that higher levels of selected OCs are associated with lower cognitive function (CF). Using weighted linear generalized estimating equations, we explored whether the association between lipid-adjusted levels of 8 OCs and global CF is modified by race/ethnicity in 985 adults age 60+ from the National Health and Nutrition Examination Surveys (2011-2014). Adjusting for covariates, results indicated that higher levels of grouped-OCs are not associated with lower CF (p=0.121), but higher levels of specific OCs were associated with lower CF: Mirex[p=0.029]; Oxychlordane[p=0.036]; p,p'-DDE[p=0.009]; p,p'-DDT[p=0.003]; and trans-Nonachlor[p=0.023]. These associations did not differ by race/ethnicity. Disentangling the complex impact of OCs on late-life cognition—whether in combination or singularly—may lead to more equitable environmental health policies.

### IMPACT OF CAREGIVING DURING THE COVID-19 PANDEMIC: COMPARISON BETWEEN DEMENTIA AND NONDEMENTIA CAREGIVERS BY RACE

Heehyul Moon[1], Sunshine Rote[1], Jeanelle Sears[2], and Stacey Schepens Niemiec[3], *1. University of Louisville, Louisville, Kentucky, United States, 2. Bowling Green State University, Bowling Green,, Ohio, United States, 3. University of Southern California, Los Angeles, California, United States*

Despite evidence of increased caregiving intensity, stress, and burden during the COVID-19 crisis and clear racial disparities in COVID-19, there is a lack of research on the impact of dementia (vs. non-dementia) care provision on caregivers' (CGs) quality of life by race. The current study used the National Health and Aging Trends Study COVID-19 Family Members and Friends data (N=1,462, 15% Black and 85% Non-Hispanic White CGs) to explore differences in primary stressors (changes in the provision of help with ADL and IADL tasks and emotional support) by dementia care and race during COVID-19. Most respondents were adult children. Dementia CGs in particular reported increases in stressors (ADL and IADL provision) during the pandemic. Black dementia CGs reported significantly more provision of help with ADL tasks before and after the COVID-19 onset and a significantly higher level of emotional support to their care recipients compared with the other three groups (Black non-dementia, White dementia, and White non-dementia CGs). Black dementia CGs also reported the highest care burden and worst psychological well-being and self-rated health during COVID-19. Findings revealed significant differences in the magnitude and nature of the changes in CG help provision before and amidst the pandemic, by race and dementia care status. Future studies should examine the longitudinal impact of the pandemic on the well-being of CGs from other racial and ethnic minority groups.

### SESSION 4430 (PAPER)

### USING QUALITATIVE METHODS TO GAIN A DEEPER UNDERSTANDING INTO AGEISM, HOMELESSNESS, AND DEATH EXPERIENCES

### AGING IN PLACE: THE REALITIES OF OLDER HOMELESS WOMEN NAVIGATING URBAN STREETS AND SHELTERS

Judith Gonyea[1], and Arden O'Donnell[2], *1. Boston University, Boston, Massachusetts, United States, 2. Boston University, Medfield, Massachusetts, United States*

Older homeless women have often been an invisible population; however, this is changing as their numbers grow. Today, women age 50 and older represent 30% of the shelter population. In this qualitative study, we explored the question, What does "aging in place" mean to women in their fifties who are navigating the urban streets and shelter system?" Our study's purpose was to gain a deeper understanding of the impacts of daily displacement and migration and, through the women's accounts, engage in a critical analysis of the shelter system's practices and policies. Fifteen older chronically homeless women were recruited via a flyer posted at a city's women's day shelter to participate in an onsite interview about their homelessness experiences. Using a phenomenological approach, in depth semi-structured interviews were conducted, recorded and transcribed verbatim. Our analysis process was inductive and iterative with the culminating phases being theme generation and interpretation. Participants identified challenges centered on six themes: 1) loss of a sense of control, self-determination, and independence, 2) lack of physical security, 3) worsening physical and emotional health, 4) lack of comfort, familiarity, and routine, 5) weakening or loss of social connections, and 6) a diminished sense of self-worth. The findings highlight the links between place, sense of belonging, and identity. The data underscored that shelters were dehumanizing places that further diminished women's sense of self. We discuss reforms needed to disrupt shelter and housing systems' practices that further disempower and stigmatize the women and fail to support pathways out of homelessness.

### DEEP AGING: THE CONNECTION THAT SURVIVES DEATH AND ITS ROLE IN INCREASING HUMAN–NONHUMAN RELATIONSHIPS

Hong-Jae Park, *Western Sydney University, Penrith, New South Wales, Australia*

Ageing is part of life, and so is death. Although death will involve all of us over time, it is often regarded as a taboo topic, and bonds with the dead are seldom acknowledged in contemporary times. The aim of this paper is to present selected insights on the connection that survives death and its role in increasing human/non-human relationships, learned from two indigenous knowledges—whakapapa (genealogical connections in Maori) and filial piety (respect/care for parents and ancestors). Data were collected from semi-structured interviews with 49 key informants (Maori=25; Korean=24) between 2019 and 2021 in New Zealand and South Korea. A modified thematic analysis method was used to analyse the data obtained in a bilingual research context. The research findings show that the connectedness with ancestors or deceased loved ones is a significant part of the participants' mental and social lives, emphasised in both whakapapa and filial piety/ancestor veneration traditions. Maori participants were likely to consider their natural environment (for example, land and water) as a common good for all generations, while Korean counterparts viewed it (for example, a mountain) as the place where ancestors were remembered and venerated. Participants' awareness of the post-mortem relationships was associated with their connection with nature and spiritual practices. Overall, this study suggests that there are several possible ways that older people could do 'something' prior to death for their remaining families and friends, ranging from activities concerning death talk, end-of-life preparation, legacy building and after-life planning, to connectedness with nature and non-human beings.

### EXPLORING THE LIVED EXPERIENCE OF RACIALIZED AGEISM AMONG OLDER ADULTS: A PHENOMENOLOGICAL STUDY

Andrew Steward[1], Carson De Fries[2], Annie Zean Dunbar[2], Miguel Trujillo[2], Yating Zhu[3], Nicole Nicotera[2], and Leslie Hasche[2], *1. University of Wisconsin-Milwaukee, Milwaukee, Wisconsin, United States, 2. University of Denver, Denver, Colorado, United States, 3. University of Denver, Dener, Colorado, United States*

Ageism is a prevalent, insidious social justice issue which has harmful effects on the health of older adults. Preliminary literature explores the intersectionality of ageism with sexism, ableism, classism, and ageism experienced among LGBTQ+ older adults. To our knowledge, the intersection of ageism with racism, or racialized ageism, is largely absent from the literature. This qualitative study explored the following research question from a phenomenological perspective: “What is the lived experience of racialized ageism among diverse older adults?” Twenty participants 60+ years of age (M=69) in the U.S. Mountain West identifying as Black, Latino(a), Asian-American/Pacific Islander, Indigenous, or White engaged in a one-hour interview between February and July 2021. A three-cycle coding process used constant comparison methods. Five coders independently coded interviews, engaging in critical discussion to resolve disagreements. An audit trail, member checking, and peer debriefing enhanced credibility. Six umbrella themes and 20 sub-themes were identified. In this paper, the following six sub-themes are highlighted with direct implications for practice:1) compounding oppression, 2) being avoided or categorized due to others’ discomfort, 3) increased disrespect, 4) microaggressions, 5) acts of hate, and 6) cultural values/respect for elders as a protective factor. Implications include informing how practitioners may enhance support for older adults in preventing and coping with experiences of racialized ageism. Enhanced understanding of racialized ageism should also inform existing anti-ageism initiatives at the community level. Future research should focus on the experience of racialized ageist microaggressions and the relationship between racialized ageism and specific health outcomes.

### MID- AND LATER-LIFE COMMUNITY COLLEGE STUDENTS: OFF-TIME EDUCATION AND THE SIGNIFICANCE OF INTRACOHORT DIVERSITY

Kathryn McGrew[1], Phyllis Cummins[2], and Oksana Dikhtyar[3], *1. Miami University Scripps Gerontology Center, Oxford, Ohio, United States, 2. Miami University, Oxford, Ohio, United States, 3. Miami University, Oxford, Ohio, United States*

Workers ages 45 and older make up about 45% of the U.S. labor force. To meet labor market demands in the U.S., employers will need to rely on mid- and later-life (MLL) (age 40 and older) workers. Through a gerontological lens, using both qualitative and quantitative data, we investigated the “off-time” enrollment of MLL community college students to explore how their enrollment decisions and academic goals are situated in the timing and intersection of life events and transitions, such as the responsibilities of work and family. We conducted 12 focus group interviews with MLL community college students (n=68) in Ohio and conducted a quantitative analysis of the distribution of academic goals by age group. We conclude that the foremost characteristic of MLL students as a group is its within-group heterogeneity and the greatest barrier to serving MLL students is in how we have been thinking—or failing to think—about them. We take a first slice at disaggregating “adult” students and turn away from deficit narratives to identify opportunities and assets among MLLs. Finally, we offer a theory to explain how especially wide within-group diversity of MLL academic goals is produced by cohort divergence of life events, transitions and trajectories; diverse enrollment precipitants; and individual point-of-time appraisals of lifetime elapsed and lifetime remaining. We suggest that community colleges, already leaders in adult education, are in a unique position to effectively serve MLL students by recognizing and accommodating their diversity. We present implications and strategies for colleges, policymakers and researchers.

### SESSION 4440 (SYMPOSIUM)

### WHAT NEEDS TO BE DONE TO PREVENT ELDER ABUSE IN JAPAN?

Chair: TsuAnn Kuo Discussant: Georgia Anetzberger

This symposium first introduces the Japanese Act on the Prevention of Elder Abuse, including its elder abuse definitions and trends in elder abuse in both institutional and domestic settings. It then describes a recent amendment to operational standards made by ministerial ordinance, which aims to reduce elder abuse. Second, it presents analysis of elder abuse data (N=497) from Matsudo-City in Chiba prefecture, which found that only about 65% of reported elder abuse cases were deemed to meet the legal definition of elder abuse and the rest were excluded due to how the law defines abusers. Findings suggest the definitions in the Act be broadened to encompass a wider set of abuse situations. Third, it presents analysis using the same data, of characteristics of abusers. Findings indicate that about 24% of abusers were caregivers with disabilities, suggesting the importance of supporting caregiver health and welfare to help prevent abuse. The symposium concludes by presenting findings from a longitudinal survey of older adults aged 75+ (N=769) indicating that physical restraints use is associated with “having been diagnosed as intractable neurological diseases,” “unstable general condition or at the terminal stage,” “higher levels of care-need,” “having medical treatment,” and “family members’ long-term care burdens.”

### A NEW STEP TO PREVENT ELDER ABUSE IN JAPAN

Noriko Tsukada[1], Naoki Ikeda[2], and Asako Katsumata[3], *1. Nihon University, Tokyo, Tokyo, Japan, 2. Kamihonnmachi sougou houritsu jimusyo, Tennouji-ku, Osaka, Japan, 3. Rehabilitation Institution, Chiba, Chiba, Japan*

This paper first introduces major aspects of the Japanese Act on the Prevention of Elder Abuse, including trends in substantiated elder abuse cases in both institutional and domestic settings, definitions of elder abuse, and abusers and reporting systems. It then introduces the recent amendment of operational standards in institutional settings made by ministerial ordinance. The amendment includes a requirement to develop a committee to prevent occurrence and recurrence of elder abuse and to appoint a designated individual to establish guidelines and implement training. It is hoped to further prevent elder abuse in Japan.

### DETECTION OF ELDER ABUSE IN JAPAN NOT COVERED BY THE ELDER ABUSE PREVENTION LAW IN COMPARISON WITH WHO DEFINITION

Tadashi Wada[1], Hitoshi Suda[2], and Kana Satoh[3], *1. Irahara Primary Care Hospital, Matsudo City, Chiba, Japan, 2. SEITOKU University, Matsudo city, Chiba, Japan, 3. Teikyo University of Science, Adachi, Tokyo, Japan*

The Elder Abuse Prevention Law mainly defines "elder abuse" inflicted by caregivers. Elder abuse by non-caregivers is therefore rarely covered by the law. We investigated domestic elder abuse reported in Matsudo City, Chiba, Japan, to determine the number of domestic elder abuse cases excluded from legal protection but constituting elder abuse defined by WHO. From April 2017 to March 2020, the municipal elder protection services agency received 525 reports of domestic elder abuse. We studied the effective records of 497 of these cases. 299 cases were confirmed elder abuse cases defined by the law, with 198 excluded. However, 176 out of the 198 excluded cases constitute elder abuse defined by WHO. In these 176 cases, abuse was perpetrated by non-caregivers. Due to the current formation of the law, this abuse went undetected and unprotected. We think amendment of the law is required to protect these excluded cases.

### NEED FOR SUPPORT FOR PERSONS WITH DISABILITIES IN ELDER ABUSE

Hitoshi Suda[1], Tadashi Wada[2], and Kana Satoh[3], *1. SEITOKU University, Matsudo city, Chiba, Japan, 2. Irahara Primary Care Hospital, Matsudo City, Chiba, Japan, 3. Teikyo University of Science, Adachi, Tokyo, Japan*

Among the elder abuse cases reported to Matsudo City, Chiba Prefecture (population 487,091 as of April 1, 2017) from fiscal year (FY) 2017 to FY 2019. Of the 525 reported cases, 497 for which records could be judged to be accurate were included in the study. There were 119 cases (23.9%) in which the abusers were presumed to have a disability. Out of these 119 cases, the abusers of 86 (72.3%) cases were not involved with health and welfare policies for people with disabilities or such involvement had been suspended. If the abuser had received support in the field of health and welfare for the disabled or as a person in need at an early stage, the elder abuse might not have occurred. Support for people with disabilities and support for persons in financial need are required before they become abusers.

### PHYSICAL RESTRAINTS AT HOME AND THEIR FACTORS: A ONE-YEAR LONGITUDINAL SURVEY AMONG HOME CARE SERVICE USERS IN JAPAN

Noriko Yamamoto-Mitani, Hanako Numata, and Chie Fukui, *The University of Tokyo, Tokyo, Tokyo, Japan*

We conducted a one-year survey of homecare service users aged 75 years and older. Data from 769 clients were examined. Altogether 47 (6.1%) received physical restraint at least once among the five survey points conducted over a one-year period. Among them, 27 (57.4%) received restraint only at one point, and only 2 received restraint in all five points. In the bivariate analyses, the factors associated with the use of physical restraint were: diagnosis of neurological disease, unstable general condition or terminal stage, high level of care required, medical treatment, and family burden. When those who were released from the restraint at 12 months (n=30) were compared with those who were not (n=17), not having stroke, not receiving suctioning, and not having home-visit medical care were significant factors. Physical restraints at home were not common but we must be careful when we serve clients with significant factors of prolonged restraints.

### SESSION 4450 (SYMPOSIUM)

### EAST MEETS WEST: MODELS OF "AGING IN COMMUNITIES": DEMENTIA, CAREGIVING, AND ACTIVE AGING BETWEEN THE US AND TAIWAN

Chair: TsuAnn Kuo Discussant: Fernando Torres-Gil

As countries around the world are entering the super aged societies, aging in place will need to be planned not only as an individual but also as a multi-disciplinary and age-friendly community. Many models are built based on the concept of "Aging in communities" in order to consider the multi-levels of needs and interests that older adults face when living in the same area. With Asia being the next fastest aging continent, innovative models and programs are also developed in Taiwan as it tries to promote healthy aging, combating dementia and supporting family caregivers. This symposium will include 4 papers by introducing a national program for frailty prevention, a community friendly model for people with dementia and a collaborative network to prevent family caregivers from being burned out. In addition, the village model developed in the U. S. will be compared to show how east meets west to demonstrate communities meeting the needs of older adults.

### FOSTERING MULTIPARTNERSHIP IN DEVELOPING A DEMENTIA-FRIENDLY COMMUNITY WITH AN OPEN DEMENTIA DAY CENTER IN TAIWAN

Ling-Hui Chang[1], Men-An Pan[2], Li-Xue Wu[2], Chi-pang Lu[3], Liwen Sung[4], and Pai--Chuan Huang[5], *1. National Cheng Kung University, Tainan City, Tainan, Taiwan (Republic of China), 2. Pingtung County Government, Pingtung City, Pingtung, Taiwan (Republic of China), 3. Research Center For Humanities And Social Sciences, Tainan City, Tainan, Taiwan (Republic of China), 4. Department of Architecture, Tainan City, Tainan, Taiwan (Republic of China), 5. Occupational Therapy, College of Medicine, Tainan City, Tainan, Taiwan (Republic of China)*

Introduction. Supporting persons with dementia without increasing the demands of formal care is an issue that many countries face today. Pingtung County proposed an innovative practice of an open dementia day center situated in a dementia-friendly community (DFC), where people with dementia in the center can freely go outside and enjoy community living. This presentation examines the experience of an university academic team in fostering a municipality-academic-community partnership as we tried to "flip" the care of a traditional dementia day center to adopt an "open" care model and concurrently to make the nearby community dementia-friendly. Method. A pragmatic research paradigm was adopted to address the socially situated situation in which we encountered when building a dementia-friendly community and the strategies we adopted to respond to the problem. Results/Discussion. Three themes: (1) Partnering with local government, e.g. to reduce the cognitive and physical barriers for outdoors activities and to increase the competence of local officials in DFC. (2) Partnering with local community and business: e.g. to increase the DFC awareness of community volunteers and to initiate a DFC business project, (3) building an academic team: e.g. to engage faculties who were motivated to pioneer an innovative practice, to fulfill their social responsibility, bridge the gap between theory, research, and practice. Conclusion. Significant changes were made in the community and the day center towards DFC. Community stigma towards dementia and community inertia was major obstacles. The allocation of city resources, personal drive towards DFC, and regular communication were key, despite the process was fraught with compromise, power dynamics, and conflicts of interests.

### AGING IN COMMUNITY MODELS IN THE US: FRAMEWORKS FOR COMMUNITY SUPPORTIVE NETWORK DIALOGS BETWEEN WEST AND EAST

Su-I Hou, *University of Central Florida, Orlando, Florida, United States*

Aging in Community (AIC) is the preferred way to age. This session shares promising AIC models in the U.S. and analyzed model characteristics. Four models were identified with the potential to achieve person-environment (P-E) fit, including village, naturally occurring retirement community (NORC), cohousing, and university-based retirement community (UBRC). Empirical studies show these AIC models enhance social support and improving older adult's well-being, remain independence at homes, and community social and civic participation. Each model has a unique way to help community-dwelling older adults with their aging needs. This session aims to stimulate dialogs on opportunities and challenges of forming AIC supportive networks considering Eastern versus Western cultural and societal differences. Promising U.S. AIC models shared will serve as conceptual frameworks to facilitate discussions on various model's strengths and weakness and how they might be adapted in Taiwan's society considering eastern cultural values, societal norms, and healthcare system. Examples of Taiwan versions of these similar promising U.S. AIC models will be shared. Continued research is needed to address the challenges of engaging older adults with lower socio-economic status, meeting older adults' diverse and dynamic needs, and conducting comparative studies to share lessons learned across the globe.

### THE ESTABLISHMENT OF A COMMUNITY NETWORK TO SUPPORT FAMILY CAREGIVERS

Tsuann Kuo, *Chung Shan Medical University, South District, Taichung, Taiwan (Republic of China)*

In 2021, 114 Caregiver Resource Sites were established in Taiwan to provide case management and services for family caregivers who may experience caregiving burnout due to long-term care. Each Caregiver Resource Site is responsible for serving 60-100 caregivers. An effort was made to recruit volunteers and gather resources so caregivers can be surrounded in the care-friendly community network, including care counseling, support groups, caregiving skill classes, relaxation activities, caregiver café, and in-home coaching for hard-to-care moments. By forming this community network to support caregivers, it has reduced caregiver burnout and avoided possibly negative tragedies due to caregiving.

### SESSION 4460 (SYMPOSIUM)

### BEREAVEMENT ACROSS THE ADULT LIFE COURSE: VARIATIONS IN EFFECTS ON HEALTH AND WELL-BEING

Chair: J Jill Suitor Co-Chair: Megan Gilligan

This symposium considers the consequences of deaths of family members across the life course on psychological well-being, physical health, and mortality in the middle and later years. The first two papers focus on intergenerational losses, using data from the HRS. Donnelly, Lin and Umberson examine the ways in which the impact of parental death on adult children's health in later life are shaped by children's ages at loss, social isolation, and race/ethnicity. Next, Huo and Kim investigate the extent to which the effect of child loss on parents' well-being in mid- and later-life is moderated by volunteering. The third and fourth papers focus on the role of spousal loss in health and well-being. Rodin and colleagues use HRS data to explore whether the association between widowhood and mortality differs depending on the cognitive health of the surviving spouse. Next, Vedder and colleagues provide a systematic review of variations in patterns and predictors of loneliness by marital status in middle and later life. Finally, Miles and Olsen examine the association between recent loss and binge drinking among spouses and adult children who had served as caregivers to the deceased, using data from the Georgia Behavioral Risk Factor Surveillance Survey. This diverse set of studies reveals how the impact of the death of family members on survivors' health and well-being is shaped by a complex set of structural and socioemotional factors, shedding new and important light on health disparities by sex/gender and race/ethnicity in the middle and later years.

### RACIAL/ETHNIC DISADVANTAGE IN PARENTAL DEATH ACROSS THE LIFE COURSE, SOCIAL ISOLATION, AND HEALTH IN LATER LIFE

Rachel Donnelly[1], Zhiyong Lin[2], and Debra Umberson[3], *1. Vanderbilt University, Nashville, Tennessee, United States, 2. University of Texas at San Antonio, San Antonio, Texas, United States, 3. The University of Texas at Austin, Austin, Texas, United States*

Prior research has not considered how exposure to parental death across the life course may contribute to lasting social isolation and, in turn, poor health among aging adults. The present study uses longitudinal data from the Health and Retirement Study (HRS; 1998-2016) to consider linkages of parental death, social isolation, and health (self-rated health, functional limitations) for Black, Hispanic, and White older adults. Findings suggest that exposure to parental death before later life is associated with higher levels of isolation, greater odds of fair/poor self-rated health, and more functional limitations in later life. Moreover, social isolation partially explains associations between parental bereavement and later-life health. Racial disparities in bereavement are central to disadvantage: Black and Hispanic adults are more likely to experience parent death early in the life course and this differential exposure to bereavement has implications for racial and ethnic disparities in social isolation and health throughout life.

### LONG-TERM PSYCHOLOGICAL CONSEQUENCES OF PARENTAL BEREAVEMENT PRIOR TO MID-LIFE: VOLUNTEERING HELPS

Meng Huo[1], and Kyungmin Kim[2], *1. University of California, Davis, DAVIS, California, United States, 2. Seoul National University, Seoul, Seoul-t'ukpyolsi, Republic of Korea*

Losing a child prior to midlife may be a uniquely traumatic event that continues to compromise parents' well-being in later life. This study compared psychological well-being between bereaved and non-bereaved parents, and examined whether volunteering protects bereaved parents. We analyzed a pooled sample of parents aged 50+ (N = 12,023) from the Health and Retirement Study, including parents who lost a child prior to 50 and those who never lost a child. Bereaved parents reported more depressive symptoms and lower life satisfaction than non-bereaved parents, which was more evident among parents with fewer children alive. Among bereaved parents, volunteering, particularly volunteering 100+ hours/year, was associated with better well-being at baseline; yet, volunteering 1–99 hours/year led to a larger increase in life satisfaction over time. This study adds to our understanding of lasting effects of parental bereavement and suggests volunteering as a potential intervention aimed at helping bereaved older parents.

### THE WIDOWHOOD EFFECT IN COMPLEX SERIOUS ILLNESS: THE IMPACT OF SPOUSAL DEATH ON MORTALITY IN DEMENTIA

Rebecca Rodin[1], Alex Smith[2], Edie Espejo[2], W. John Boscardin[2], Siqi Gan[2], Lauren Hunt[2], Katherine Ornstein[3], and Sean Morrison[1], *1. Icahn School of Medicine at Mount Sinai, New York City, New York, United States, 2. University of California, San Francisco, San Francisco, California, United States, 3. Johns Hopkins, University, Baltimore, Maryland, United States*

Numerous studies suggest that there is an association between widowhood and mortality. This "widowhood effect" may be heightened in patients with dementia, who have high support needs and for whom spouses typically provide extensive caregiving support. Yet there are limited data on widowhood and mortality that account for dementia status. To determine the relative mortality risk of widowhood among those with and without dementia, we conducted a retrospective cohort study among community-dwelling, married/partnered persons, ≥65 years, enrolled in the Health and Retirement Study, 2000-2018. Among the 12,308 persons (n=390 with dementia), widowhood was not associated with increased mortality, after adjusting for age and dementia status, in men or women (adjusted HR 1.04; 95%C.I.(0.95-1.13); HR 0.96; 95%C.I.(0.87-1.95), respectively). These findings suggest that dementia, age, or other unmeasured confounding variables may account for the previous finding of increased mortality following spousal death. Further research is needed to confirm these findings in diverse populations.

### THE LONG-TERM LONELINESS OF WIDOWHOOD: A SYSTEMATIC REVIEW OF MARITAL STATUS DIFFERENCES

Anneke Vedder[1], Jeffrey Stokes[2], Kathrin Boerner[2], Henk Schut[3], Paul Boelen[3], Bibi Schut[3], and Margaret Stroebe[4], *1. Faculty of Social & Behavioural Sciences, Utrecht, Utrecht, Netherlands, 2. University of Massachusetts Boston, Boston, Massachusetts, United States, 3. Utrecht University, Departement of Psychology, clinical psychology, Utrecht, Utrecht, Netherlands, 4. University of Groningen, Experimental Psychopathology, Department Clinical Psych. & Exp. Psychopathology, Groningen, Groningen, Netherlands*

Loneliness can be prominent in bereavement, possibly leading to compromised mental and physical health. We systematically reviewed the extent of loneliness across marital status groups, examining the prevalence, intensity, risk factors, and correlates of loneliness in widowhood, compared to other marital statuses. Studies that met predefined criteria as well as investigated marital status (comparisons) were included in the review. For reporting, we followed the PRISMA statement. Thirty-eight studies were included. Widowhood was associated with a greater likelihood and intensity of loneliness when compared to other marital statuses, and especially the divorced. Widowers were on average lonelier than widows. Findings suggest that , widowed persons are uniquely vulnerable to loneliness, and that, in the long-term, loneliness may be more pronounced among the widowed than the divorced. However, methodological shortcomings (e.g., heterogenous samples, different measures of loneliness) of available studies must be considered, and future research should aim to overcome these limitations.

### NEW BEREAVEMENT IS A RISK FACTOR FOR BINGE DRINKING, SMOKING, AND POOR MENTAL HEALTH (2019 GEORGIA BRFSS)

Toni Miles, *Rosalynn Carter Institute, Athens, Georgia, United States*

This presentation advances studies of population health by estimating the association between new bereavement and binge drinking rates among adults aged 50 years and older. In other reports, bereavement is associated with significantly higher rates of binge drinking among older adults. In 2019, the state of Georgia used an optional module in the Behavioral Risk Factor Surveillance Survey (BRFSS) to measure the prevalence of new bereavement. This is the first effort to capture population-level data on bereavement in Georgia. 45% of adults (4 million persons) aged 18 and older were newly bereaved in the 24 months prior to survey. Highest bereavement rates: adults aged 55 to 64 years (51%), unemployed (49%), and Black respondents (60%). Binge drinking rate for bereaved was 31 % (versus 23.6% not bereaved). Combined bereavement and binge drinking increased risk for poor mental health (OR = 2.83) and smoking (OR = 4.54).

### SESSION 4461 (BIOLOGICAL SCIENCES INVITED SYMPOSIUM)

### NEUROBIOLOGY OF AGING

Chair: Jasmin Herz

### MENINGES HARBOR IMMUNE MEMORIES OF LIFE EXPERIENCES

Jasmin Herz[1], Khalil Alves de Lima[2], Andrea Francesca Salvador[2], Mackenzie Lemieux[2], Taitea Dykstra[2], Igor Smirnov[2], and Jonathan Kipnis[1], *1. Washington University in St. Louis, School of Medicine, St. Louis, Missouri, United States, 2. Washington University in St. Louis, St. Louis, Missouri, United States*

The adaptive immune system relies on formation of the memory of past microbial challenges to accelerate protective immune responses in the event of reinfection. This memory is accomplished in part by the retention of antigen-specific B and T cells within barrier tissues. As the healthy central nervous system parenchyma is virtually devoid of adaptive immune cells, the meningeal spaces carry out the vital function of coping with environmental threats during aging. We conducted an extensive molecular and functional analysis of meningeal T cells to test the hypothesis that the meninges in the brain sense and respond to internal and external cues throughout life, and that alterations in the meningeal T cell repertoire alter brain function. We found presumably self-reactive tissue-resident T cells in the meninges of naive mice. Using models of pathogen exposure, we describe a neuroimmune axis in which antigen experienced resident Tcell subsets dynamically record immune perturbations, which resulted in behavioral abnormalities that were exacerbated with aging. Our findings elucidate molecular properties of T cells that survey the brain borders under both homeostatic and pathological conditions and provide insights linking CNS immune privilege with memory.

### REGULATION OF NEURONAL MITOCHONDRIAL BIOENERGETICS BY LIPID METABOLITES UPREGULATED IN DEMENTIA

Stephanie Heimler,[1] K. Allison Amick,[2], Jaclyn Bergstrom,[1] Mohit Jain[1], Anthony J. Molina[1], *1. UC San Diego School of Medicine, San Diego, California, United States*
*2. Wake Forest School of Medicine, Winston Salem, North Carolina, United States*

Mitochondrial dysfunction occurs early in Alzheimer's disease (AD) progression and is evident in the Central Nervous System (CNS) and peripheral circulating cells. While there is evidence indicating that bioenergetic decline can drive the early pathogenesis of AD, little is known about the extracellular factors that alter neuronal bioenergetic capacity. We hypothesized that circulating metabolites contribute to neuronal bioenergetic decline associated with AD. Using a cohort comprised of participants with normal cognition, mild cognitive impairment, and dementia, we show that human serum harbors circulating, non-cellular factors capable of mediating neuronal bioenergetic differences according to the cognitive status of the serum donor. We developed a novel screening-based approach to identify candidate "mito-active" lipid metabolites in human serum that could mediate differences in bioenergetic capacity. Among these, Nervonic Acid and 15-epi-PGA1 were predicted to be mitochondrial inhibitors upregulated in participants with dementia. Neurons exposed to physiologically-relevant ranges of these molecules in-vitro exhibited a dose-dependent reduction in maximal mitochondrial respiration. We found that 500ug/mL of Nervonic Acid and 9ug/mL of 15-epi-PGA1 reduced maximal mitochondrial respiration by 62.3% and 63.3%, respectively, thereby validating our screening and prediction approach. Future experiments may be directed towards investigating if and how these and other mito-active metabolites of interest cross the blood-brain barrier to meaningfully affect AD. Furthermore, identified mito-active molecules can be investigated in other clinical cohorts to examine their role/s in multiple age-related or neurological conditions. This work expands our mechanistic understanding of how extrinsic factors associated with age and cognitive status contribute to neuronal bioenergetic decline.

### R1P DISPERSION IN WHITE MATTER CORRELATES WITH COGNITIVE IMPAIRMENT IN OLDER ADULTS

Fatemeh Adelnia, Taylor Davis, Lealani Mae Acosta, Amanda Puckett, Feng Wang, Zhongliang Zu, Kevin Harkins, John Gore, *Vanderbilt University Medical Center, Nashville, Tennessee, United States*

Alzheimer's disease is the most frequent form of dementia in older adults and has a total estimated worldwide cost that rises to $2 trillion by 2030. Much previous neuroimaging research in AD has focused on the roles of amyloid and tau proteins using PET, but there have also been several studies that have implicated microvascular changes as an early indicator of damage related to later dementia. Here, we developed and refined a non-invasive 3D-R1ρ dispersion imaging technique using different locking fields to quantify microvasculature changes within brain tissues in persons with mild cognitive impairment (MCI) compared to healthy controls. The fractional difference in R1ρ comparing different locking fields provides a unique way of characterizing changes in the geometry and structure of microvasculature. After providing informed consent, 40 adults aged 62 to 82 years (n=17MCI) underwent cognitive assessments and MRI scan at 3T. We found the fractional change in R1ρ of the whole brain white matter is significantly greater in persons with MCI, and the correlation remained significant (ß=-0.4, p-value=0.01) after introducing age (ß=0.2, p-value=0.2) and sex (ß=-0.1, p-value=0.5) as covariates. The white matter hypertonicity lesion volume measured from conventional MRI was also correlated with the health status ($p$-value $< 0.05$); however, the size of the regression coefficient was substantially smaller (53% lower), and it was no longer significant (p-value=0.14) after adjusting for age and sex. This work establishes a new non-invasive method that can potentially characterize changes in microvasculature anatomy with the progression of cognitive impairment regardless of an age effect.

### SESSION 4465 (SYMPOSIUM)

### REIMAGINING DISASTER AND EMERGENCY PREPAREDNESS IN LONG-TERM CARE: EXAMPLES FROM FLORIDA AND TEXAS

Chair: Lindsay Peterson Co-Chair: Ross Andel Discussant: Sue Anne Bell

A thread common to all disasters is the effect on human health that results when the health care infrastructure and delivery of health services are disrupted. This view has reinforced the importance of an all "all-hazards approach" to preparedness, whereby disaster response planning incorporates principles common to all events and can be adapted to specific contingencies. Preparing for and responding to a disaster in long-term care (LTC) requires a broad view of multiple events that can disrupt daily life and needed services for LTC residents (e.g. hurricanes, pandemics). This symposium will examine the effects of varied emergency events on older adults residing in nursing homes (NHs) and assisted living communities (ALCs) using quantitative and qualitative methodologies. The first presentation will discuss morbidity and mortality of NH residents exposed to Hurricane Harvey (Texas). The second is an investigation of NH direct care staffing during the recent Winter Storm Uri (Texas). The third presentation qualitatively explores the challenges of providing care to residents living with dementia during the COVID-19 pandemic, based on interviews with Florida NH and ALC administrators. The fourth quantitatively and qualitatively explores issues related to resident-to-resident contact restrictions in Florida ALCs. Finally, we will discuss the application of the 4Ms Age Friendly framework (What Matters, Medication, Mentation, and Mobility) to disaster preparedness in LTC. This symposium will provide information that can be used to develop or revise public policies to improve preparedness for and response to a range of emergency events in NHs and ALCs.

### ASSOCIATION BETWEEN EXPOSURE TO HURRICANE HARVEY AND MORTALITY AMONG NURSING HOME RESIDENTS

Cassandra Hua[1], Kali Thomas[2], Sweta Patel[2], David Dosa[2], Dylan Jester[3], and Ross Andel[4], *1. Brown University School of Public Health, Providence, Rhode Island, United States, 2. Brown University, Providence, Rhode Island, United States, 3. University of California San Diego, La Jolla, California, United States, 4. University of South Florida, Tampa, Florida, United States*

Hurricane Harvey made landfall in Southeast Texas in August 2017, causing catastrophic flooding. We examined the association between exposure to Hurricane Harvey and rates of 30-and 90-day mortality among Texas nursing home residents. Using Medicare data, we compared a cohort of residents exposed to the storm in 2017 (n= 18,693) to a cohort of residents not exposed to the storm in 2015 (n=19,688). We fit generalized estimating equations with a sandwich estimator, adjusting for resident demographic and clinical characteristics. Exposure to Hurricane Harvey was not associated with 30-day rates of mortality. However, it was associated with a 15% increase in the odds of dying at 90 days holding resident characteristics constant (adjusted odds ratio: 1.15 [95% Confidence interval: 1.06, 1.25). Health effects due to severe flooding, such as those related to decreased ability to manage chronic conditions, may not be apparent until several weeks after the storm.

### TEXAS NURSING HOMES INCREASED DIRECT-CARE STAFFING LEVELS DURING WINTER STORM URI

Dylan Jester[1], Lindsay Peterson[2], Ross Andel[2], and David Dosa[3], *1. University of California San Diego, La Jolla, California, United States, 2. University of South Florida, Tampa, Florida, United States, 3. Brown University, Providence, Rhode Island, United States*

Prior work suggests that nursing homes (NHs) increase their registered nurse (RN), licensed practical nurse (LPN), and certified nursing assistant (CNA) staffing levels in anticipation of a major hurricane. However, less is known about NHs' ability to increase staffing levels during a winter storm. We examined RN, LPN, and CNA staffing levels in 1,170 Texas NHs during Winter Storm Uri from February 13-20, 2021. This storm was characterized by cold temperatures and snow/ice accretion that affected the Texas power grid. Linear mixed effects models were adjusted for profit status, continuing-care retirement community status, resident census, overall star rating, a weighted deficiency score, number of citations for infection control, number of substantiated complaints, and county fixed-effects. After adjustment, Texas NHs decreased RN ($\beta$=-0.005;b=-0.002;p=.004) but increased LPN ($\beta$=0.022;b=0.009;p<.001) and CNA ($\beta$=0.044;b=0.026;p<.001) staffing levels. NHs face unprecedented challenges during winter storms, including maintaining adequate staffing levels to meet the needs of their residents.

### WE DID MORE DAMAGE: HOW COVID-19 COLLAPSED THE CONVOYS OF CARE FOR RESIDENTS LIVING WITH DEMENTIA

Sara Hackett, Lindsay Peterson, Carlyn Vogel, and Debra Dobbs, *University of South Florida, Tampa, Florida, United States*

COVID-19 has posed a multitude of challenges for nursing homes (NHs) and assisted living communities (ALCs). However, little information is known about how the pandemic impacted residents living with dementia. Using the convoys of care framework, we conducted a qualitative descriptive study to gain insight regarding administrators' perceptions of how care for residents living with dementia was altered. Forty-two participants, representing 16 NHs and 43 ALCs in Florida, participated in one 60-minute semi-structured interview. Thematic analysis revealed that the convoys of care for those living with dementia were strained by the competing demands associated with maintaining residents' social and medical care. Explicitly, participants emphasized how diminished family involvement, the increasing responsibilities of staff, and the climate of the industry, contributed to disrupted convoys of care. Consequently, this study may help inform policy and practice as it details which care strategies worked, which did not, and considerations for moving forward.

### RESTRICTIONS ON RESIDENT CONTACT IN ASSISTED LIVING DURING COVID-19: A MIXED-METHODS STUDY

Lindsay Peterson, Sara Hackett, Kallol Kumar Bhattacharyya, and Debra Dobbs, *University of South Florida, Tampa, Florida, United States*

Efforts to protect assisted living residents from COVID-19 by limiting contact warrant attention. Assisted living was developed as a social model where care is provided in a home-like environment. Given the social dimensions of assisted living, we sought to better understand the effects of COVID-19-based restrictions in assisted living. We surveyed (online) assisted living community (ALC) administrators (N=130) between October 2020 and March 2021 as part of a larger project on COVID-19 in Florida. We then interviewed a subset of participants (N=26). Administrators of chain-affiliated ALCs (compared to non-chain) were 2.7 times more likely to report resident-contact limitations had disrupted care (p=0.02). Larger ALCs (25 or more beds) were marginally more likely to report care disruptions (p<0.10). Three main themes emerged from our qualitative interviews

– varying interpretation of COVID-19 guidelines, effect of precautions on residents, assisted living as a home. Policy implications of these findings will be discussed.

### USING THE INSTITUTE OF HEALTHCARE IMPROVEMENT'S 4M FRAMEWORK TO IMPROVE DISASTER PREPAREDNESS FOR NH POPULATIONS

David Dosa[1], Dylan Jester[2], Lindsay Peterson[3], Debra Dobbs[3], Lisa Brown[4], and Kathy Black[5], *1. Brown University, Providence, Rhode Island, United States, 2. University of California San Diego, La Jolla, California, United States, 3. University of South Florida, Tampa, Florida, United States, 4. Palo Alto University, Palo Alto, California, United States, 5. University of South Florida, Sarasota-Manatee, Florida, United States*

Prior studies have repeatedly demonstrated that exposure to natural disasters such as hurricanes, earthquakes, and flooding events have profound effects on nursing home (NH) residents. Previous efforts to improve disaster preparedness have often been predicated on the decision to evacuate or shelter in place from a disaster. Unfortunately, evacuation of NH residents is often not possible and nursing homes are often required to make difficult decisions to protect the lives of their residents without data. Recognizing that healthcare systems increasingly provide care for complex individuals; the Institute of Healthcare Improvement (IHI) and the John H. Hartford Foundation introduced a framework for evaluating age-friendly healthcare systems based on 4 evidence-based core elements that matter to older adults. We believe that this 4 M's paradigm: What Matters, Medication, Mentation, and Mobility, provides a step off point for defining a practical approach to disaster preparedness for nursing homes exposed to future disasters.

### SESSION 4470 (PAPER)

### COMMUNICATION AND LANGUAGE

Paper

### AGING COUPLES' SATISFYING RELATIONSHIPS AND WE-TALK PROMOTE CARDIOVASCULAR HEALTH DURING CONFLICT

M. Rosie Shrout[1], Stephanie Wilson[2], Megan E. Renna[3], Annelise A. Madison[4], and Janice K. Kiecolt-Glaser[4], *1. Purdue University, Lafayette, Indiana, United States, 2. Southern Methodist University, DALLAS, Texas, United States, 3. University of Southern Mississippi, Hattiesburg, Mississippi, United States, 4. Ohio State University, Columbus, Ohio, United States*

Marital conflict poses health risks that intensify as couples grow older. Dyadic stress theories suggest spouses' marital satisfaction and communication patterns alter cardiovascular function, a key pathway from troubled relationships to poor health. Despite these risks, older spouses are more likely to have a strong couple identity where they think and talk in relational terms. This communication pattern, termed we-talk, is shown when spouses use words like "we" rather than "you" or "me," reflecting that they are thinking about resolving conflict as a couple rather than as two separate individuals. We examined how both spouses' relationship satisfaction and we-talk reduced conflict's cardiovascular toll, and if the health benefits were greatest when both spouses were satisfied and used we-talk. Married couples (n=107) ages 40-87 engaged in a 20-minute conflict discussion while wearing heart rate monitors to assess heart rate variability (HRV), a measure of cardiac flexibility. Couples' conflicts were transcribed to measure we-talk, or the proportion of first-person plural pronouns, such as we, us, and our. Results showed a person's HRV was higher and thus healthier when both spouses were satisfied and their partner used we-talk more often. In contrast, HRV was lower and less healthy when neither or only one spouse was satisfied and their partner used we-talk less often. Thus, a couple's mutually satisfying relationship along with a partner's we-talk provided a health advantage during conflict. Talking in relational terms may help reduce conflict's biological toll in aging couples, particularly when their relationships are satisfying.

### LANGUAGE-BASED DISCRIMINATION RELATES TO MEMORY COMPLAINTS AMONG LATINX/HISPANIC OLDER ADULTS

Elizabeth Soto[1], Silvia Chapman[2], Indira Turney[3], Laura Zahodne[4], Stephanie Cosentino[3], Adam Brickman[5], Jet Vonk[6], and Jennifer Manly[6], *1. Columbia Medical Center, New York City, New York, United States, 2. Columbia University Irving Medical Center, New York City, New York, United States, 3. Columbia University Medical Center, New York, New York, United States, 4. University of Michigan, Ann Arbor, Michigan, United States, 5. Columbia Medical Center, New York, New York, United States, 6. Columbia University, New York, New York, United States*

Memory Complaints (MCs) are a risk factor for dementia, but research in this area has largely been limited to non-Latinx White adults. Previous studies have shown that Latinx/Hispanic individuals are at higher risk for Alzheimer's Disease and Related Disorders (ADRD). The mechanisms underlying this disparity in ADRD are multidimensional and can include negative social stressors such as discrimination. Language discrimination can be a source of stress in this population and might be an important contributor to MCs. We examined if MCs varies as a function of language-based discrimination in a total of 495 Latinx/Hispanic older adults without dementia from the community-based Washington Heights Inwood Columbia Aging Project. Language-based discrimination was measured with a dichotomous item that inquired if individuals had been discriminated against because they do not speak English well (yes or no). A linear regression was conducted to examine the cross-sectional association between language-based discrimination and MCs adjusted for age, education, sex/gender, socioeconomic status (income), and depressive symptoms. The experience of language discrimination was independently associated with MCs (B= 0.83, 95% CI=0.13, 1.54, SE= 0.36, p= .021). Results demonstrate a relationship between negative stressors and the expression of MCs. Future studies should comprehensively examine other discriminatory stressors which may also impact the expression of MCs and the risk for progression to ADRD. Identifying language-based and other discriminatory stressors that negatively affect Latinx/Hispanic communities will allow us to develop models that aim to assess and prevent discrimination, as a protective mechanism for the development of MCs and ADRDs.

### LINGUISTIC STRATEGIES FOR AGE-INCLUSIVITY: ANALYSIS OF HAIKU-BASED INTERGENERATIONAL INTERACTION

Harumi Maeda[1], Emily Wan[2], and Yoshiko Matsumoto[2], *1. Stanford University , Stanford, California, United States, 2. Stanford University, Stanford, California, United States*

Many studies have examined communicative and linguistic strategies helpful to the interactional inclusion of persons living with dementia (PLwD) and the promotion of an age-inclusive society (e.g., Wray, 2020; Stickle, 2020). Building on this existing body of research, our study examines question-answer sequences in conversations (Schegloff & Sacks, 1973) among younger adults and PLwD using discourse analysis methodology to illuminate effective communicative practices for facilitating more inclusive and meaningful interactions between these individuals. Findings from this methodology are complemented by analysis of post-interaction interviews. The data set for this study consists of audio-video recordings of (1) intergenerational conversations around haiku poetry in English between older adults with mild cognitive impairments (MCI) and college students, and (2) post-interaction interviews that assess participants' feelings about the experience. The data was collected as part of a larger study designed to promote social inclusion of individuals of all ages and cognitive conditions, particularly during the pandemic, via an online intergenerational haiku-making activity. The audio-video recordings were transcribed for qualitative analysis. Findings from the analysis demonstrate the effectiveness of open and emotion-focused questions (Matsumoto, Liao, Xue, 2020; Worley, 2005) in encouraging persons with MCI to express themselves on their own terms and promoting their interactional participation. By presenting the benefits of this type of question and related linguistic strategies, this study hopes to provide a basis for developing more inclusive communicative environments for people of all ages and PLwD to help improve quality of life and well-being across all stages of life.

### MAPPING BARRIERS AND FACILITATORS OF ACCESS TO HEARING HEALTHCARE RESOURCES TO THE COM-B FRAMEWORK

Debra Schutte[1], Thomas Templin[1], and Brian Schutte[2], *1. Wayne State University, Detroit, Michigan, United States, 2. Michigan State University, East Lansing, Michigan, United States*

Age-related hearing impairment (ARHI) affects 20-40% of older adults. However, many people with hearing impairment fail to seek professional evaluation and treatment, delay seeking treatment, or abandon treatment. The purpose of this study was to identify barriers and facilitators of access to hearing healthcare resources in a rural setting and to characterize these barriers within the COM-B (capability, motivation, and opportunity) framework. Survey packets were handed out following services at a rural community church; 72 completed surveys were returned. The mean age of participants was 54.4 years. Thirty-one percent of respondents reported moderate to severe hearing impairment; of these individuals, 7 (31%) were currently using a hearing aid. Rates of hearing protection use fell below the Healthy People 2020 target of 53% across occupational and leisure noise exposure. Forty-eight percent (N=34) of participants had never had their hearing tested as an adult. Overall perceived barriers were significantly correlated with age (r= .260, P = .034) and perceived hearing loss (r=-.277, P= .025). Barriers were identified across all domains and subdomains of the COM-B framework. Barriers clustered in the reflective motivation domain, in which participants undervalue the significance of hearing loss. Barriers also clustered within the social and physical opportunity domains, suggesting a lack of access to providers and an undervaluation of hearing loss by others (family and health care providers). These findings provide evidence regarding the accessibility of hearing healthcare services in rural settings and will serve as a foundation for the design of a community-tailored hearing health intervention.

### SESSION 4480 (PAPER)

### DEATH, DYING, AND END OF LIFE

### DEATH FEARS AND ANXIETY ACROSS THE LIFE COURSE: IMPLICATIONS FOR DEATH REFORM

Amber Thompson[1], and Rebecca Utz[2], *1. University of Utah, Sandy, Utah, United States, 2. University of Utah, Salt Lake City, Utah, United States*

Western culture is experiencing a movement for death reform which includes aspects such as death acceptance and supporting individual choice concerning how one wishes to experience end-of-life (EOL). The underlying goal of this reform is to foster natural curiosity about death and dying in order to soothe death fears and anxiety. Over the past few decades in the U.S. there has been an increase in public support for the EOL option of medical-aid-in-dying (MAID), or legally prescribed pharmaceuticals intended to cause death. There have been mixed findings regarding the effects of age on attitudes and perspectives towards death and dying. The aim of this study was to determine the relationship between age, death fears and anxiety, values and perspectives, demographic characteristics, and approval of MAID at EOL in a U.S. nationally representative sample (N = 1994), using a partially latent structural equation model (SEM). Results show that age has a curvilinear effect on death fears and anxiety, and that as death fears and anxiety increase support for MAID decrease. This suggests that the ability to provide person-and-family-centered care at EOL must address the biopsychosocial aspect of death fears and anxiety across the life course. These findings demonstrate the need for more research documenting changes in attitude and perspectives towards EOL for different populations (i.e., ages). The potential benefits of this approach provide a basis for expanding education and awareness of both death and dying in general, and EOL needs and wants for older adult populations.

### FAMILY DECISION-MAKING IN SERIOUS ILLNESS SCENARIOS: ACCURACY AND PERCEIVED ACCURACY OF ADULT CHILD SURROGATES

Jessica Hahne, and Brian Carpenter, *Washington University in St. Louis, St. Louis, Missouri, United States*

Adult children often help older adults make important decisions in serious illness situations, but previous research suggests adult children are not uniformly accurate in predicting parents' preferences. In this project we examined the accuracy with which adult children (N= 68; M age= 45; 69.1% female) were able to predict medical preferences of their parents (N= 34; M age= 75; 94.1% female). Parents expressed their preferences in four serious illness scenarios, and children predicted parents' preferences. Parents and children also estimated the accuracy of children's predictions and the frequency of communication about medical care. On average across all scenarios, children predicted parents' preferences accurately 42.3% of the time. Parents were willing to take more risk with treatment for more severe disease, as their children predicted, although children generally overestimated parents' willingness to live with increasing disability (M difference = 9.77%). Overall, children were poor at predicting their own accuracy (Rs = -.017, p= .893). Parents were slightly more insightful about their children's accuracy (Rs= .261, p=.034), but there was no association between accuracy and parent or child estimate of frequency of conversations about medical preferences (Parent Rs= .067, p=.586 ; Child Rs= .164, p= .182). These findings suggest that neither are adult children highly accurate at predicting parents' medical preferences, nor do they or their parents have a precise sense of their accuracy. Results from this study may inform interventions to help families communicate about advance care planning.

### PERSONALITY TRAITS AND END-OF-LIFE ATTITUDES AND BEHAVIORS IN OLDER ADULTS: EVIDENCE FROM SHARE

Valerie-Anne Ryser[1], Sarah Vilpert[2], Gian Domenico Borasio3, and Jürgen Maurer[2], *1. Swiss Centre of Expertise in the Social Sciences, Lausanne, Vaud, Switzerland, 2. University of Lausanne, Lausanne, Vaud, Switzerland, 3, Lausanne University Hospital and University of Lausanne, Lausanne, Vaud, Switzerland*

Personality traits (PTs) - Neuroticism, Extraversion, Openness to experience, Agreeableness, Conscientiousness - are related to decision-making styles, as well as to specific end-of-life (EOL) healthcare preferences. Building on this knowledge, our study aims to explore the association of PTs with attitudes and behaviours towards the EOL in general and EOL care planning in particular. Our analyses are based on a paper-and-pencil self-completion questionnaire (n=1'524) administered as part of wave 6 and 7 of SHARE in Switzerland. PTs are measured with the Big-Five inventory ten (BFI-10). Attitudes and behaviours related to EOL are: outlook –avoid thinking about death, think about and discuss EOL preferences–, administrative arrangements –testament, power of attorney–, approval and completion of advance care planning. Results show that older adults with higher scores on openness are more likely to think, plan and discuss EOL, as well as to have made arrangement in advance such as a testament, and a power of attorney. Higher scores on extraversion are linked with having a testament. Individuals who express higher scores on neuroticism more often think about their wishes for EOL, whereas those scoring higher on agreeableness are less likely to be in favour of advance care planning. To conclude, older adults in Switzerland remain unfamiliar with EOL topics, since only individuals with higher openness to experience are more prone to prepare for the EOL. PTs should be taken into account when educating and encouraging people to plan for their EOL.

### UNEQUAL LOSS: DISPARITIES IN RELATIONAL PROXIMITY TO A COVID-19 DEATH AMONG US OLDER ADULTS

Alicia Riley, *University of California, Santa Cruz, Santa Cruz, California, United States*

The COVID-19 pandemic has devastated communities of color in the U.S. at disproportionate rates. Racial-ethnic and language disparities in COVID grief may be even more extreme than those in individual mortality. We drew on the National Social Health and Aging Project (NSHAP) COVID Study, a supplement to the nationally-representative longitudinal survey. The analytic sample consisted of 2,554 community-dwelling older adults in the U.S. interviewed between September 2020 and January 2021. We used descriptive analysis to evaluate disparities by race/ethnicity/language subgroups in relational proximity to a COVID-19 death (aquaintaince < friend/family member < household member < spouse) and multiple logistic regression to evaluate disparities in experiencing at least one COVID-19 death in one's social network. English-speaking, Non-Hispanic Black and Latino older adults were over-represented in every category of proximity to a COVID-19 death, but overrepresentation in proximity to a COVID-19 death was greatest among Spanish-speakers of any race. Although Spanish-speaking respondents were only 4.6% of the full sample, half of the respondents who lost a spouse to COVID-19 were Spanish-speakers. Disparities by language and race/ethnicity persisted even with the inclusion of controls for age, sex, marital status, education, and nativity. The most extreme disparities in closeness to COVID-19 death were experienced by Spanish-speakers of any race/ethnicity. It is unclear if this disparity is driven by language alone or the intersection of language and nativity and associated systemic vulnerabilities. Observed disparities could also reflect variation by language and immigrant identity in network size and structure, including connections to relatives in other countries.

### WORKING MEMORY MODERATES DEPRESSIVE SYMPTOMS AFTER PARTNER MORTALITY: HEALTH AND RETIREMENT STUDY

David Brush[1], Daniel Paulson[2], and Robert Dvorak[3], *1. University of Michigan, Ann Arbor, Michigan, United States, 2. University of Central Florida, Maitland, Florida, United States, 3. University of Central Florida, Orlando, Florida, United States*

Grief is conceptualized as a process after which the individual becomes better adapted to changed living conditions after a loss. The Selection, Optimization, and Compensation with Emotion Regulation (SOC-ER) model posits that working memory may be necessary for mitigation and resolution of grief. This study examined the hypothesis that working memory facilitates successful grieving following the loss of an intimate partner. Participants include 3,599 respondents of the longitudinal Health and Retirement Study (HRS) who experienced spousal mortality between 1994 and 2014. Working memory was measured assessed using Serial 7's, and depressive symptoms were assessed using the 8-question CES-D. Latent-growth models were used to estimate rate of change in depressive symptoms up to loss-of-spouse (baseline event), and then subsequent to that loss. Missing data were handled using full-information maximum likelihood. Sample participants had an average age 78.04 (SD = 7.32) at the time of their spouse's death and were disproportionately female (69.10%), White/Caucasian (82.30%), non-Hispanic (92.37%), and completed an average of 11.61 (SD = 3.42) years of education. The hypothesized level 2 model fit the data very well: $\chi 2(56)=61.323$, p=.29 RMSEA=0.005 [0.000 0.012]; CFI=0.998, SRMR=0.028. Initial depressive symptom endorsement was significantly related to working memory ability. Working memory also moderated the relationship between depressive symptom endorsement and time, where individuals with better working memory tended to report lower depressive symptoms and demonstrated a lesser increase in depressive symptoms. In conclusion, working memory may be one determinant of successful bereavement. Findings support application of the SOC-ER model to the study of grief.

### SESSION 4490 (PAPER)

### DIVERSE GERIATRIC SYNDROMES ISSUES

### ASSOCIATION OF LIVER STIFFNESS BY TRANSIENT ELASTOGRAPHY WITH MOBILITY DIFFICULTY IN OLDER ADULTS WITH NO CIRRHOSIS

Paulo H Chaves, *Florida International University, Miami, Florida, United States*

Growing experience with non-invasive ultrasound transient elastography (TE) offers now a major opportunity to improve understanding about the functional impact of non-advanced, asymptomatic liver dysfunction. We assessed the relationship of liver stiffness measurement (LSM) and controlled attenuation parameter (CAP) obtained by TE, surrogates for liver fibrosis and steatosis, respectively, with mobility difficulty, an early stage of the disablement process. Cross-sectional study utilizing National Health and Nutrition Examination Survey 2017-2018 data. Analytic sample (n=1,203) included participants aged 60 years and older without known cirrhosis with available LSM and CAP. Logistic regression assessed the odds of mobility difficulty (walking ¼ mile or climbing stairs) according to LSM and/or CAP, with adjustment for demographics, diseases, metabolic syndrome, body mass index, aminotransferases, gamma-glutamyl transferases, albumin, and platelets. LSM and CAP were linearly, positively associated with the probability of mobility difficulty. In the fully adjusted model, LSM, though not CAP, remained strongly associated with mobility difficulty. Those with LSM in the top (>7 kilopascals [kPa]) and intermediate quintiles (4-7 KPa) had higher odds of mobility difficulty; i.e., odds ratio (OR): 1.81; 95% confidence interval (CI): 1.15-2.86; p=0.01) and OR:1.45; 95%CI: 1.01-2.08; p=.046), respectively. LSM was independently associated with early mobility disability in older adults without known cirrhosis. A direct effect through sarcopenia promotion pathways might be speculated. Whether TE screening could be useful to identify adults with non-advanced liver fibrosis who might benefit from preventive interventions – e.g., diet and exercise for non-alcoholic fatty liver disease patients vis-à-vis mobility disability prevention remains to be tested.

### DEMOGRAPHIC DIFFERENCES IN THE LONGITUDINAL EFFECTS OF SYMPTOMS ON FALLS AND FALL-RELATED OUTCOMES

Wenting Peng[1], Christina Miyawaki[2], Cen Mo[1], Yuqian Luo[1], Siyuan Tang[1], and Minhui Liu[1], *1. Central South University, Changsha, Hunan, China (People's Republic), 2. University of Houston, Houston, Texas, United States*

Research has shown an association between symptoms and falls and fall-related outcomes among older adults. However, this association was primarily drawn from cross-sectional studies or studies with a single symptom. Using the 2011-2018 waves of the National Health and Aging Trends Study, we examined whether 1) prior-wave common co-occurring symptoms predicted later-wave fall-related outcomes and 2) demographics moderated the longitudinal effects of symptoms on fall-related outcomes among community-dwelling older adults. Falls and fall-related outcomes were self-reported falls, multiple falls, fear of falling (FOF), and FOF limiting activity. The number of symptoms (from 0 to 6) was calculated based on the presence of pain, insomnia, breathing difficulty, depressive symptoms, anxiety, and fatigue. Binomial logistic regression was used for data analyses. Our sample consisted of 9,060 participants who contributed 34,327 observations. These observations were aged between 65 and 79 years old (57.7%), female (58.4%), and non-Hispanic White (70.5%). Each additional symptom was associated with an increased risk of falls (Adjusted Odds Ratio [AOR]: 1.13, 95% CI: 1.11-1.15), multiple falls (AOR: 1.15, 95% CI: 1.12-1.18), FOF (AOR: 1.21, 95% CI: 1.18-1.24) and FOF limiting activity (AOR: 1.25, 95% CI: 1.21-1.29). Age, race/ethnicity, education, and living arrangement significantly moderated the relationships between symptoms and falls and fall-related outcomes. However, gender did not moderate the effects of symptoms on any outcomes. These findings suggest that symptoms longitudinally predict falls and fall-related outcomes. Common symptoms assessment and individual demographics should be incorporated into fall risk assessments and interventions.

### HOME-BASED TRANSCRANIAL DIRECT CURRENT STIMULATION AND CLINICAL PAIN IN OLDER ADULTS: A RANDOMIZED CLINICAL STUDY

Hyochol Ahn[1], Geraldine Martorella[1], Kenneth Mathis[2], and Hongyu Miao[1], *1. Florida State University, Tallahassee, Florida, United States, 2. University of Texas Health Science Center at Houston, Houston, Florida, United States*

Knee osteoarthritis (OA) is one of the leading causes of pain in older adults. Previous studies indicated clinic-based transcranial direct current stimulation (tDCS) was effective to reduce pain in various populations, but no published studies have reported the efficacy of home-based self-administered tDCS in older adults with knee OA using randomized clinical study. Thus, the purpose of this study was to evaluate the efficacy and safety of tDCS on clinical pain intensity in adults with knee OA pain. One hundred twenty participants aged 50–85 years with knee OA pain were randomly assigned to receive fifteen daily sessions of 2 mA tDCS for 20 min (n = 60) or sham tDCS (n = 60) over 3 weeks with remote supervision via telehealth. Clinical pain intensity was measured by asking participants to rate their knee pain using a numeric rating scale from 0 (no pain) to 100 (worst pain imaginable). Participants (68% female) had a mean age of 66 years and the mean body mass index in the sample was 32.59 kg/m2. There have been no adverse events. Active tDCS significantly reduced pain intensity compared to sham tDCS after completion of the fifteen daily sessions (t = 6.57, df = 110, p < .001). We demonstrated that home-based self-administered tDCS was safe and reduced clinical pain intensity in older adults with knee OA, which can increase its accessibility. Future studies with multi-site randomized controlled trials with various populations are needed to validate our findings.

### THE ASSOCIATION BETWEEN MEDITERRANEAN DIET ADHERENCE AND ALLOSTATIC LOAD IN OLDER ADULTS

Alexandra Fiocco[1], Anik Obomsawin[2], and Danielle D'Amico[3], *1. Toronto Metropolitan University, Toronto, Ontario, Canada, 2. X (Ryerson) University, Toronto, Ontario, Canada, 3. Toronto Metropolitan University, York, Ontario, Canada*

Allostatic load (AL) is a multisystemic index of biological wear and tear which is associated with poor health outcomes. In recent years, researchers have examined the association between dietary pattern intake and AL; however, no studies to date have examined the relationship between AL and consumption of a Mediterranean diet. Blood and urine samples were collected from 201 community-dwelling older adults who completed a Food Frequency Questionnaire (FFQ). A Mediterranean Diet Score (MDS) was calculated based on previous recommendations and a sex-based AL index was calculated using a count-based approach for 16 biomarkers associated with neuroendocrine, immune, cardiovascular, or metabolic function. It was hypothesized that a higher MDS would associate with lower AL, and that this association would be particularly robust for the immune and metabolic subcomponents of the AL index. In support of the study hypotheses, generalized linear models revealed a significant inverse relationship between MDS and AL (ß = -0.03, P = 0.037). Furthermore, higher MDS was significantly associated with lower immune (ß = -0.06, P = 0.38) and metabolic (ß = -0.05, P = 0.039) subsystem scores, but was not associated with cardiovascular or neuroendocrine subsystem scores. Exploratory analyses further showed that the association was more robust in male than female participants. The current findings are interpreted with caution given the study design and sample characteristics. However, these findings contribute to the literature supporting the Mediterranean diet as an important lifestyle behavior that may support healthy aging.

### SESSION 4500 (SYMPOSIUM)

### RACIAL AND ETHNIC VARIATIONS IN DEMENTIA DIAGNOSIS, SURVIVAL, AND END-OF-LIFE CARE QUALITY

Chair: Olga Jarrín Co-Chair: Zahra Rahemi Discussant: Michael Gusmano

In the United States most adults have a preference to die at home and is an indicator of good end-of-life care. In the context of dementia, family members and caregivers are decision makers and part of good and equitable care involves understanding cultural variation in attitudes and social norms related to dementia, death and dying, and the meaning of a good death. This symposium explores racial and ethnic variation in lifetime dementia diagnosis and end-of-life care quality indicators. The first presentation examines racial, ethnic, and geographic variation in the rarely discussed lifetime prevalence of dementia and survival time from dementia diagnosis to death using national Medicare data. The second presentation describes the relationship between end-of-life care planning and satisfaction with end-of-life care using data from the Health and Retirement Study. The third presentation describes variation in place of death, a key indicator of end-of-life care quality, by dementia diagnosis and race/ethnicity using national Medicare data. The fourth presentation examines variation in hospice use, another indicator of end-of-life-care quality, and place of death by dementia diagnosis, race, and ethnicity using national Medicare data. The symposium concludes with a presentation examining the relationship between place of death and satisfaction with care received using data from the Health and Retirement Study. The Institute for Healthcare Improvement's Triple Aim (improving the experience of care, improving the health of populations, and reducing per capita costs of health care) serves as a lens for discussing policy and practice implications of the major findings from each presentation.

### SURVIVAL AFTER DEMENTIA DIAGNOSIS: VARIATION BY RACE/ETHNICITY AND GEOGRAPHIC FACTORS

Haiqun Lin[1], Olga Jarrín[2], Bei Wu[3], Paul Duberstein[4], Anum Zafar[2], and Maria Lopez[2], *1. Rutgers University, Newark, New Jersey, United States, 2. Rutgers, The State University of New Jersey, New Brunswick, New Jersey, United States, 3. New York University, New York, New York, United States, 4. Rutgers School of Public Health, Piscataway, New Jersey, United States*

This study describes racial and ethnic variation in dementia diagnosis and survival time between diagnosis and death for Medicare beneficiaries aged 50 years and older who died in 2018 (n=1,998,282). The prevalence of diagnosed dementia was higher among non-Hispanic white (45.7%), Black (45.5%), and American Indian/Alaska Native (44.1%) decedents compared to Asian American/Pacific Islander (42.7%), and Hispanic (38.5%) decedents. Median survival time in years was shorter for American Indian/Alaska Native (2.51, IQR 0.7-6.0) and white (2.85, IQR 0.8-6.3) beneficiaries, and longer for Asian American/Pacific Islander (3.36, IQR 1.0-7.3), Black (3.38, IQR 0.9-7.4), and Hispanic (3.83, IQR 1-8.2) beneficiaries. Median survival time also varied by geography and was shortest for Hispanic decedents in Arizona (2.37, IQR 0.6-6.0) and New Mexico (2.92, IQR 0.8-7.0) compared to New Jersey (4.85, IQR 1.5-8.9) and Puerto Rico (5.45, IQR 1.1-12.8). Among Black decedents, survival was longest in New Jersey (3.80, 1.1-7.9) and Arkansas (4.22, 1.0-8.5).

### END-OF-LIFE CARE PLANNING AND SATISFACTION AMONG THE HEALTH AND RETIREMENT STUDY DECEDENTS

Zahra Rahemi[1], Ayse Malatyali[2], Tom Cidav[3], Olga Jarrín[4], Cheryl Dye[5], and Christopher McMahan[5], *1. Clemson University, Greenville, South Carolina, United States, 2. University of Central Florida, Orlando, Florida, United States, 3. Johns Hopkins University, Baltimore, Maryland, United States, 4. Rutgers, The State University of New Jersey, New Brunswick, New Jersey, United States, 5. Clemson University, Clemson, South Carolina, United States*

The frequency and timing of advance care planning among individuals living with cognitive impairments vary by race/ethnicity and other sociodemographic factors. This study examined relationships between advance care planning and end-of-life care satisfaction among participants in the Health and Retirement Study (Exit files 2002-2018). Among decedents with cognitive impairment (n=3,102), Black and Hispanic participants were less likely to have a living will (OR=0.22, 0.19) and less likely to discuss end-of-life care (OR=0.422, 0.544) compared to White and non-Hispanic participants, respectively. Black and Hispanic participants were more likely to prefer all possible end-of-life care (OR=3.29, 3.34) and less likely to refuse extensive-care measures (OR=0.34, 0.48) compared to White and non-Hispanic participants. Participants dissatisfied with end-of-life care were 48% less likely to have a living will. End-of-life care planning disparities among racial/ethnic groups can inform interventional and educational programs to improve equity in end-of-life care.

### PLACE OF DEATH AMONG MEDICARE BENEFICIARIES: VARIATIONS BY DEMENTIA DIAGNOSIS AND RACE/ETHNICITY

Olga Jarrín[1], Haiqun Lin[2], Bei Wu[3], Anum Zafar[1], Maria Lopez[1], and Paul Duberstein[4], *1. Rutgers, The State University of New Jersey, New Brunswick, New Jersey, United States, 2. Rutgers University, Newark, New Jersey, United States, 3. New York University, New York, New York, United States, 4. Rutgers School of Public Health, Piscataway, New Jersey, United States*

Death at home is preferred by most people and has become a key indicator of the end-of-life care quality; however cultural attitudes about death and dying may vary by race and ethnicity. This study describes racial and ethnic variation in place of death among Medicare beneficiaries aged 50 years and older who died in 2018 with a dementia diagnosis (n=902,208) and without a dementia diagnosis (n=1,096,074). Among those with dementia, 60% died at home, with the highest percentage for non-Hispanic white (62%) beneficiaries, followed by Black/African American (55%), Hispanic (61%), Asian American/Pacific Islander (58%), and American Indian/Alaska Native (58%) beneficiaries. Among those without dementia 61% died at home, with the highest percentage for non-Hispanic white (60%) beneficiaries, followed by Black (57%), Hispanic (65%), Asian American (65%), and American Indian (60%) beneficiaries. Among those who died at home, about half without dementia received hospice care compared to the majority with dementia.

### HOSPICE UTILIZATION AND PLACE OF DEATH BY DEMENTIA DIAGNOSIS AND RACE/ETHNICITY

Olga Jarrín[1], Haiqun Lin[2], Bei Wu[3], Paul Duberstein[4], Maria Lopez[1], and Anum Zafar[1], *1. Rutgers, The State University of New Jersey, New Brunswick, New Jersey, United States, 2. Rutgers University, Newark, New Jersey, United States, 3. New York University, New York, New York, United States, 4. Rutgers School of Public Health, Piscataway, New Jersey, United States*

Timing of hospice referral is critical to patient's and family's quality of life and satisfaction with hospice care. This study examined racial/ethnic variation in hospice use and factors associated with death at home among Medicare beneficiaries aged 50 years and older who died in 2018 (n=1,998,282). Hospice use was most frequent among non-Hispanic white beneficiaries (55.8%) followed by Hispanic (46.2%), Asian American (44.5%), Black (42.7%), and American Indian (42.1%) beneficiaries. Among decedents diagnosed with dementia, initiation of hospice prior to the last day of life was significantly associated with death at home among all racial/ethnic groups: non-Hispanic white (OR=2.62), Black (OR=2.04), Hispanic (OR=2.17), Asian American (OR=2.54), and American Indian (OR=2.76). Among decedents not diagnosed with dementia, initiation of hospice prior to the last day of life was less strongly associated with death at home: white (OR=1.33), Black (OR=0.83), Hispanic (OR=1.32), Asian American (OR=1.69), and American Indian (OR=1.56).

### PLACE OF DEATH AND CARE SATISFACTION AMONG THE HEALTH AND RETIREMENT STUDY DECEDENTS

Ayse Malatyali[1], Zahra Rahemi[2], Tom Cidav[3], Cheryl Dye[4], Olga Jarrín[5], and Christopher McMahan[4], *1. University of Central Florida, Orlando, Florida, United States, 2. Clemson University, Greenville, South Carolina, United States, 3. Johns Hopkins University, Baltimore, Maryland, United States, 4. Clemson University, Clemson, South Carolina, United States, 5. Rutgers, The State University of New Jersey, New Brunswick, New Jersey, United States*

The place of care at the time of death can influence the satisfaction, type, and cost of end-of-life care. This study investigated factors associated with place of death among older adults in the Health and Retirement Study (Exit files 2002-2018) with cognitive impairment (n=3,102). Black and Hispanic participants were more likely to die in the hospital (OR=1.80, 1.47) and less likely to die in a nursing home (OR=0.54, 0.37) than white and non-Hispanic participants. Hispanic participants were also 50% more likely to die at home than non-Hispanics. Compared to other places, participants who died at home were 43% more satisfied, and participants who died at nursing homes were 32% less satisfied with the care they received. There was no significant effect of ethnicity on the relationship between place of death and satisfaction with care. Investigating the moderating role of other demographic factors can shed more light on this relationship.

### SESSION 4510 (PAPER)

### ECONOMIC AND EMOTIONAL COSTS OF FAMILY CAREGIVING

### FINANCIAL DISTRESS OF ADULT CHILDREN TAKING CARE OF AGING PARENTS

Mengya Wang, and Suzanne Bartholomae, *Iowa State University, Ames, Iowa, United States*

Providing care to parents is often a rewarding experience for adult children, however, one trade-off is the toll it takes on the caregiver's own financial security. Using the framework of life course theory, the current research examines the sources of support (family, community and the government) that may reduce parental caregivers' financial distress. In this study, we examine whether intensive parental caregivers are experiencing financial distress and whether the association between caregiving and financial distress is ameliorated by the presence of having siblings, community support, and government policy supportive of caregiving. This study utilizes data provided by the fifth wave of the survey of health and retirement in Europe (SHARE). Preliminary analyses are consistent with previous studies, that providing intensive care to parents is associated with an increased risk of experiencing healthcare hardship. Moreover, the findings align with life course theory as having support from family members and the government is associated with lower levels of financial distress. However, the effect of community support is not significant. This study suggests that adult children, especially adult daughters, sacrifice their own financial security for the benefit of caring for their parents. In addition, having a broad support network, such as caregiving support groups and caregiving educational programs, can reduce adult children's financial distress, and enable them to provide care longer, which avoids or delays the need for costly institutional care. Ongoing analyses examine the potential long-term consequences of caregiving.

### LATINO FAMILY CAREGIVERS' EXPERIENCES COVERING OUT-OF-POCKET COSTS WHEN CARING FOR SOMEONE LIVING WITH DEMENTIA

Susanna Mage[1], Donna Benton[2], Alexander Gonzalez[3], Gabriela Zaragoza[4], Kathleen Wilber[2], Reginald Tucker-Seeley[5], and Kylie Meyer[6], *1. University of Southern California, Leonard Davis School of Gerontology, Los Angeles, California, United States, 2. University of Southern California, Los Angeles, California, United States, 3. University of Southern California, LOS ANGELES, California, United States, 4. UTHSCSA, San Antonio, Texas, United States, 5. University of Southern California, Los Angele, California, United States, 6. Case Western Reserve University, Cleveland, Ohio, United States*

Family caregivers to persons living with dementia spend an average of $11,293 annually out-of-pocket on caregiving costs. Costs include home modifications, medications, and other daily expenses. Financial strain from caregiving affects caregiver mental and physical health and is unequally distributed across race/ethnicity groups. While other caregivers spend approximately one-quarter of their income on caregiving, the cost for Latinos family caregivers comprises nearly half of annual income. To gain insight into this disparity, eleven 1-hour in-depth qualitative interviews were conducted with Latino caregivers of persons living with dementia living in California and Texas. Interview transcripts were thematically coded, guided by the Tucker-Seeley Material–Psychosocial–Behavioral Model of financial strain. We identified four themes: 1) caregivers were concerned with meeting the daily needs of care recipients and would incur additional costs, such as credit card interest, to address immediate needs; 2) high levels of psychological distress were expressed regarding caregivers paying for the care of loved ones and themselves in the future; 3) caregivers wanted to use community resources that help older adults and their families but described stressful barriers accessing support. Further, Latino culture affected spending. For example, caregivers incurred shared living costs given reluctance to place loved ones in a nursing facility, exemplifying the concept of familismo. By understanding the factors contributing to high caregiving costs for Latino caregivers and the challenges met when addressing these costs, we can build approaches at both the individual- and policy-levels and develop culturally relevant interventions to help Latino families to lower care costs.

### RESOURCE UTILIZATION AMONG FAMILY CAREGIVERS: INDIVIDUAL AND NEIGHBORHOOD CHARACTERISTICS

Hyojin Choi[1], Kristin Litzelman[1], Autumn Harnish[1], and Maija Reblin[2], *1. University of Wisconsin-Madison, Madison, Wisconsin, United States, 2. University of Vermont, Burlington, Vermont, United States*

Resources in the community play a pivotal role in increasing well-being of both care recipients and caregivers and ensuring equitable access to needed resources is a crucial priority for policy and practice. Drawing on the Andersen Behavioral Model, this study explored the longitudinal relationship between individual and neighborhood factors and social resource utilization (i.e., paid caregivers, respite care, support group, training, financial help, or transportation) among family caregivers. Unpaid family caregivers for community-dwelling older adults were identified using data from the National Study of Caregiving and National Health and Aging Trends Study (Time 1: 2015, Time 2: 2017; n=616). Neighborhood characteristics were indicators of the Social Vulnerability Index and provider density at the census-tract level. Poisson regression was used to assess predictors of greater resource use (interpreted as incident rate ratios [IRR] with 95% confidence intervals [CI]). Nearly two-thirds of the sample reported using one or more services at each timepoint. Enabling factors were key predictors of resource use at Time 2, including income above 400% federal poverty level (IRR[CI]=1.53 [1.11,2.09]), better self-rated health (IRR[CI]=1.11 [1.01,1.22]), and resource use at Time 1 (IRR[CI]=1.64 [1.29,2.08]). Need-based predictors included greater frequency of personal care (IRR[CI]=1.13 [1.05,1.22] and longer care duration (IRR[CI]=0.99 [0.98,1.00]). Neighborhood factors were not associated with resource use in this analysis, nor were other need factors including caregiving burden. The findings highlight potential disparities in resource use by income, health status, or experience with systems navigation, with implications for policy and outreach.

### THE MASON CARES RANDOMIZED STUDY: ASSESSING CAREGIVER BURDEN FOLLOWING A STRESS MANAGEMENT PROGRAM

Gilbert Gimm[1], Harveen Pantleay[2], Emily Ihara[2], Megumi Inoue[2], Shannon Layman[2], and Catherine Tompkins[2], *1. George Mason University, Vienna, Virginia, United States, 2. George Mason University, Fairfax, Virginia, United States*

OBJECTIVE: The Mason CARES study examines the impact of a virtual stress management program and personalized music intervention on the stress of family caregivers of older adults with dementia. This study presents early findings on 31 participants (Cohort 1) who enrolled in January 2022.

METHODS: Using primary data, we analyzed caregiver stress levels among 31 participants in Weeks 1 and 5 of a stress management program. Caregiver stress was measured using the Zarit Burden Interview (ZBI) score with a range from 0 to 48 (Bédard, 2001). Higher values (ZBI >=17.0) are associated with higher levels of stress.

RESULTS: Among the 31 caregivers, 51.6% were spouses and 48.4% were non-spouses (e.g., adult children, nephew, etc.). While mean ZBI scores in Week 1 were similar for spouses and non-spouses (24.2 vs. 24.9), caregiver stress was higher when older adults had advanced versus moderate dementia

(26.2 vs. 23.2). Between Weeks 1 and 5, spouses reported a greater decrease in stress (-2.6 vs. -2.2) compared to non-spouses. However, caregivers of older adults with advanced dementia experienced less of an improvement (-1.5 vs. -3.0).

CONCLUSION: Findings show that all caregivers had high stress levels in Week 1 (ZBI score of 17 or more) with no difference between spouses and non-spouses. Between Week 1 and 5, spouses reported greater stress improvement than non-spouses. However, caregivers of older adults with advanced dementia had less improvement in stress. Future research will examine caregiver stress levels after random assignment to a Phase 2 music intervention group and control group.

### TIME-USE AND WELL-BEING IN FAMILY AND OTHER UNPAID CAREGIVERS OF OLDER ADULTS

Sol Baik[1], Amanda Lehning[2], Paul Sacco[2], John Cagle[2], and Nancy Kusmaul[3], *1. University of Virginia Weldon Cooper Center for Public Service, Charlottesville, Virginia, United States, 2. University of Maryland, Baltimore, Baltimore, Maryland, United States, 3. University of Maryland Baltimore County, Baltimore, Maryland, United States*

Due to the intensive time commitment for caregiving, caregivers report limited freedom to engage with others, participate in physical activities, pursue leisure activities, and have adequate time for sleep. Few studies have focused on caregivers' time-use across different activities, particularly how different patterns of time-use are associated with well-being. This study aimed to: (1) identify time-use profiles of family caregivers of older adults and (2) examine associations between identified time-use profiles and caregiver well-being. We analyzed 1,640 family caregivers of community-dwelling older adults by combining secondary data from Round 7 (2017) of the National Study of Caregiving and the National Health and Aging Trends Study. We conducted latent profile analysis to estimate time-use profiles including covariates and outcomes. Three classes of caregivers emerged based on time-use patterns. The High Committed class (20%) spent the longest time in non-eldercare related committed activities, such as household activities and paid work. The High Discretionary class (49%) spent the highest amount of discretionary time, including social activities, physical activities, and other free-time activities. They also spent the least amount of non-eldercare committed time compared to the other two caregiver types. Lastly, the Balanced class (31%) allocated time relatively evenly in all activities. When comparing well-being outcomes between time-use profiles, caregivers in the High Discretionary class had worse self-rated health but lower levels of anxiety than the Balanced class. Research on time-use and caregiver well-being may help identify at-risk caregiver groups based on lifestyle profiles and develop targeted policies to promote better caregiver well-being.

### SESSION 4520 (SYMPOSIUM)

### EVOLVING AGE-FRIENDLY UNIVERSITIES: ENHANCING STUDENT SUPPORT SERVICES AND PROMOTING SKILL DEVELOPMENT

Chair: Allyson Graf Co-Chair: Katherina Terhune
Discussant: Heidi Ewen

Students in higher education face numerous challenges, many of which have been exacerbated by the pandemic. Globally, universities have capitalized on age-friendly programs and practices to respond to rapid changes in age demographics, build more age-inclusive and intergenerational programming, and create new forms of support campus-wide. This symposium features campus leaders representing universities that have drawn on the Age-Friendly University (AFU) principles to generate new and creative forms of support and skill-building for students of all ages. These initiatives represent the diverse ways that AFU-based programs and practices can be utilized to respond to opportunities and challenges in higher education. Graf et al. will discuss how age and age-related bias impact adult learners' experiences in the classroom, and ways to inform training programs for students, faculty, and staff. Felsted and Eaton will describe a GSA/AARP-funded grant initiative aimed at increasing access, inclusion, and support for older adult learning at the university, resulting in a partnership with Emeritus faculty to pursue the AFU designation. Hancock and Kutcher will describe how service-learning and intergenerational learning opportunities provide a meaningful space for students to develop realistic notions of their own aging experiences. Newsham et al. will discuss a virtual intergenerational mentoring project designed to improve social connectedness and well-being, to improve expectations regarding aging, and to decrease ageism. Terhune et al. will discuss campus-wide data used to assess the prevalence and impact of unpaid caregiving on students and will explore community and campus connections to better support students in caregiving roles.

### HOW AGE-FRIENDLY UNIVERSITIES CAN COMBAT AGEISM

Allyson Graf[1], Heather Han[2], Will Phillips[2], and Jessica Wiley[2], *1. Northern Kentucky University, Florence, Kentucky, United States, 2. Northern Kentucky University, Highland Heights, Kentucky, United States*

At a time of growing enrollment among adult learners, student bodies are increasingly age diverse. Identifying and reducing age-related bias on campus may be multipurpose in supporting those who experience ageism while promoting professional skills to combat ageism in students' future careers. The extant literature has primarily focused on ageism in the workplace and ageism experienced by faculty and staff in higher education while less has focused on ageism experienced by adult students and the possible disruption this creates to feeling included as part of the campus community. We present data from a mixed methods study of adult learners assessing the extent to which they experience and are impacted by age-related bias on campus. Data are used to justify faculty and student training to improve intergenerational contact in the classroom and beyond under the broader umbrella of DEI initiatives.

### UNDERSTANDING AND RESPONDING TO THE NEEDS OF STUDENT CAREGIVERS THROUGH THE AGE-FRIENDLY UNIVERSITY LENS

Katherina Terhune[1], Allyson Graf[2], Kate Wade[3], and Faith Greer[3], *1. Northern Kentucky University, Lexington, Kentucky, United States, 2. Northern Kentucky University, Florence, Kentucky, United States, 3. Northern Kentucky University, Highland Heights, Kentucky, United States*

Caregiving has steadily increased over the years, with approximately 44 million individuals in the U.S. providing over 37 billion hours of unpaid, informal care for adult family members and friends with chronic illnesses or conditions that impact their functionality and daily activities. While much caregiving research has been completed over the years, research on student caregivers is scant, and less is known about the experiences and the needs of students in caregiving roles. This presentation examines data from a campus-wide survey to assess the prevalence and impact of unpaid caregiving on students. Utilizing the Age-Friendly University framework, this study aims to identify sources of unmet caregiving need, as well as community and campus connections to better support students in caregiving roles. Recommendations for assessing student caregiver needs and ways to generate and enhance caregiver support through the AFU lens will be highlighted.

### INTERGENERATIONAL CONVERSATIONS AS GOOD AGE-FRIENDLY UNIVERSITY PRACTICE

Cynthia Hancock, and Katie Kutcher, *UNC Charlotte, Charlotte, North Carolina, United States*

Service-learning is an established practice in gerontological education. At UNC Charlotte, our service-learning is embedded in our foundational gerontology course which is open to students across the University. Students engage in 10 hours of conversations utilizing topic prompts that align with course content to engage in comfortable conversations with an older adult they have never before met. Students then complete five reflective writings to process the experience. Our model aligns with the AFU principles of promoting "intergenerational learning" and increasing "the understanding of students of the longevity dividend and the increasing complexity and richness that aging brings to our society." Through a qualitative analysis of student reflective writings, the authors will share how this service-learning model leads to increased student gerontological literacy and in particular a better understanding of their future aging selves.

### IMPLEMENTING A VIRTUAL, AGE-FRIENDLY INTERGENERATIONAL PROGRAM

Tina Newsham[1], Elizabeth Fugate-Whitlock[2], Natalie Quinn[2], and Sky Bergman[3], *1. UNC Wilmington, Wilmington, North Carolina, United States, 2. University of North Carolina Wilmington, Wilmington, North Carolina, United States, 3. Cal Poly, San Luis Obispo, San Luis Obispo, California, United States*

As the University of North Carolina Wilmington (UNCW) seeks to become more age-friendly and stakeholders pursue endorsement of the Age-Friendly University principles, a virtual intergenerational mentoring project was implemented. The aim was to connect the community's older adults, including retired faculty, with current students. Older adults and students were paired and asked to engage in a series of conversations, watch a documentary, and attend a Q&A with the filmmaker. The goals were to improve social connectedness and well-being, improve expectations regarding aging for members of both groups, and to decrease ageism. Impacts of the project were assessed through the Expectations Regarding Aging scale prior to any interactions between partners and again at the end of the project along with an open-ended questionnaire about their experiences. Expectations regarding aging improved on 75% of the items, and participants indicated qualitatively that their experiences with the intergenerational interactions were positive.

### PAVING THE WAY FOR AGE-FRIENDLY UNIVERSITY DESIGNATION: ACCESS AND SUPPORT FOR OLDER ADULT LEARNING INITIATIVES

Katarina Friberg Felsted[1], and Jacqueline Eaton[2], *1. University of Utah, Salt Lake City, Utah, United States, 2. University of Utah College of Nursing, Salt Lake City, Utah, United States*

Through a GSA/AARP funded grant, the University of Utah implemented an initiative to promote awareness of a state bill which provides reduced tuition for residents 62 and older. The initiative provided tuition waivers to older adult students, which enhanced enrollment and increased engagement with key age-friendly stakeholders. Implemented over four semesters, student use of tuition waivers increased 875%. This initiative was a crucial step in seeking Age Friendly University (AFU) designation, as it led to mentorship from members of the AFU global network and a partnership with our university's Emeritus Faculty Board. Strategic planning guided the preparation of a four-step plan towards designation: 1) complete a cross-campus AFU inventory and inform campus stakeholders, 2) report progress to the Council of Deans, 3) present to Academic Vice Presidents, and 4) obtain AFU endorsement from the University President. During this presentation we will present lessons learned as we progress towards designation.

### SESSION 4530 (SYMPOSIUM)

### EXISTING DATA SETS FOR FAMILY CAREGIVING AND BEREAVEMENT RESEARCH: HIGH-VALUE APPLICATIONS AND KEY CONSIDERATIONS

Chair: Djin Tay Discussant: Rebecca Goodwin

Aging and the end of life is marked by greater reliance on others to address increasingly complex care needs. At the end of life, nine in 10 caregivers are family members. Reliance on family caregivers will continue to increase as the home outpaces hospitals and nursing facilities as place of death. As the population ages and dependence on family caregivers increases, it is critical to examine the impact of family on aging and end-of-life experiences and conversely, the impact of end-of-life caregiving and bereavement on families' health and well-being. Population-based data, such as registries that link administrative, health and vital records or nationally representative datasets, minimize the challenges of selection bias, low response rates, and underrepresentation of racial and ethnic minority groups that are common in prospective clinical research. However, few population-based datasets exist that can facilitate research on family caregiving. This symposium includes five presentations focused on the use of population-based data for family caregiving research-- the first examines the social and caregiving network of adults who age solo using the National Health and Aging Trends Study data. The next two presentations examine how family characteristics are associated with place of death and end of life treatment utilization among nursing home decedents using linked statewide data. The final two examine how family caregivers are impacted by different death and dying experiences in Utah and Denmark. Leveraging population-based data to understand the end-of-life dynamics within a family system has the potential to advance family caregiving research, clinical practice, and policy.

### INFORMAL CARE NETWORKS AMONG ADULTS AGING SOLO

Jane Lowers[1], Duzhi Zhao[2], Evan Bollens-Lund[2], Dio Kavalieratos[1], and Katherine Ornstein[3], *1. Emory University, Atlanta, Georgia, United States, 2. Icahn School of Medicine at Mount Sinai, New York, New York, United States, 3. Johns Hopkins, University, Baltimore, Maryland, United States*

More than 20% of older adults lacks proximal family caregivers. Yet is it unclear who provides support for this growing population. Using 2015 National Health and Aging Trends Study data for 2,998 community-dwelling Medicare beneficiaries who received help with at least one task (e.g., bathing, shopping, insurance help) in the past month, we identified individuals aging solo (no spouse/partner or children residing in the same state) and determined social and caregiving network size and composition. Compared to married peers with nearby children, adults aging solo (7.6% of sample) had social networks of similar size but greater diversity (i.e., more friends, neighbors). Adults aging solo were significantly more likely to rely on paid help and a wider network of informal caregivers, including distal family, and friends. Social networks can anchor interventions to help adults aging solo prepare for future care needs and inform policies to support informal caregiving.

### FAMILIAL AND SOCIODEMOGRAPHIC DETERMINANTS OF PLACE OF DEATH: A RETROSPECTIVE STUDY OF THE UTAH POPULATION DATABASE

Brenna Kelly[1], Heidi Hanson[1], Huong Meeks[1], Michael Hollingshaus[1], Djin Tay[1], Lee Ellington[2], Caroline Stephens[1], and Katherine Ornstein[3], *1. University of Utah, Salt Lake City, Utah, United States, 2. University of Utah and Huntsman Cancer Institute, Salt Lake City, Utah, United States, 3. Johns Hopkins, University, Baltimore, Maryland, United States*

Consistent with preferences, home deaths in the US increasing — yet most Americans still die in hospitals or other healthcare facilities. Although declining health has been considered the primary factor influencing place of death, few studies have examined how family support and sociodemographic factors influence place of death. This study examined a population-based cohort of 205,932 decedents aged 50+ who died in Utah between 1998 and 2016. Using multivariate logistic regression models, we found that having a living spouse or child was associated with decreased odds of a healthcare facility death (spouse: AOR= 0.62, CI 0.65-0.59; child: AOR = 0.80, CI 0.79-0.82). Additionally, educational attainment (graduate degree: AOR = 0.95, CI 0.91-0.99) and non-Hispanic/Latinx ethnicity (AOR = 0.81, CI 0.79-0.85) were associated with decreased odds of a home death. Our findings highlight the importance of families in place of death and suggest that sociodemographic and economic disparities persist even in death.

### UTILIZING POPULATION DATA TO CHARACTERIZE NURSING HOME DECEDENTS AND THEIR FAMILIES AT THE END OF LIFE

Caroline Stephens[1], Michael Hollingshaus[1], Brenna Kelly[1], Djin Tay[1], Katherine Ornstein[2], Ken Smith[1], and Rebecca Utz[1], *1. University of Utah, Salt Lake City, Utah, United States, 2. Johns Hopkins, University, Baltimore, Maryland, United States*

Over 2/3 of nursing home (NH) residents are eligible for palliative care (PC), but do not receive it. Utilizing the Utah Population Database, we examined EOL characteristics for 39,672 NH residents who died in Utah between 1998-2016. Dementia was the leading cause of death (36.6%), followed by cardiovascular disease (23.9%) and COPD (16.7%). Women were more likely than men to die in a NH (21.0 vs 15%) and more likely to die in a NH with heart disease (20.0% vs. 11.7%) or cerebrovascular disease (25.9% vs. 21.0%), compared to men. Use of PC, hospice, and life-sustaining treatments was low within this NH sample, particularly among persons with dementia. First-degree family characteristics varied at EOL with presence of a spouse exerting the greatest influence on EOL care utilization. Understanding population-based NH resident EOL life characteristics can help inform the development of resident- and caregiver-centered PC interventions and health policies.

### HEALTH AND WELL-BEING OF FAMILY CAREGIVERS FOLLOWING HOSPICE USE

Rebecca Utz[1], Michael Hollingshaus[1], Katherine Ornstein[2], Djin Tay[1], Caroline Stephens[1], and Ken Smith[1], *1. University of Utah, Salt Lake City, Utah, United States, 2. Johns Hopkins, University, Baltimore, Maryland, United States*

Some patterns of hospice use are potentially disruptive and stressful for both patient and family. Using a decedent sample of 17,320 hospice users from the Utah Population Database, linked to the health records of spouse and first-degree relatives (child, parent, sibling, Mean=3.36 per decedent). Stroke decedents had higher odds of delayed admission (i.e., <7 days of hospice), while those with dementia and COPD had higher odds of extended use (i.e., >6 months of hospice), compared to cancer decedents. Family members, especially spouses, who experienced delayed admission or extended use had higher odds of hospitalization and increased use of antidepressants, both prior to and following the death, compared to those with more normative hospice use (6 months or less, typical of cancer patients). The hospice experience has both immediate and lasting consequences on the family; eligibility criteria for the hospice benefit may need to be modified by admitting diagnosis.

### SERIOUS MENTAL ILLNESS EXACERBATION POST-BEREAVEMENT: A POPULATION-BASED STUDY OF PARTNERS AND ADULT CHILDREN

Djin Tay[1], Lau Thygesen[2], and Katherine Ornstein[3], *1. University of Utah, Salt Lake City, Utah, United States, 2. University of Southern Denmark, Odense, Syddanmark, Denmark, 3. Johns Hopkins, University, Baltimore, Maryland, United States*

The death of a family member may trigger exacerbations among individuals with serious mental illness (SMI). We hypothesized that bereavement would be associated with SMI exacerbations among bereaved partners and adult children diagnosed with SMI. Using linked population-based registries in Denmark, we identified partners and adult children diagnosed with schizophrenia, schizoaffective disorder, bipolar disorder, and major depression in the five years preceding the family member's death. Generalized estimating equations were used to estimate the odds of SMI exacerbation two years after decedent death. Partners had increased odds of SMI exacerbation at 3 months into bereavement compared to 9-12 months prior to partners' death (AOR=1.43, [1.13-1.81]). Children with a history of SMI had lower odds of SMI exacerbation in the second year of bereavement. Sociodemographic characteristics and co-occurring alcohol and substance abuse disorders were associated with higher odds of SMI exacerbations. These findings have implications for targeted bereavement support.

### SESSION 4540 (SYMPOSIUM)

### FOSTERING SOCIAL CONNECTIVITY AND RESILIENCE IN SEXUAL AND GENDER MINORITY OLDER ADULTS

Chair: Hyun-Jun Kim

While social connectedness and engagement are key aspects of well-being over the life course, sexual and gender minority (SGM) older adults are found to experience elevated risks of social isolation and limited social connectivity, which have been linked to loneliness. Social isolation and loneliness have been significant health concerns, increasing risk of premature death, cognitive decline, and poor health and well-being, yet SGM older adults are resilient populations. To address the strengths and challenges found in evidence-based intervention development, it is critical to understand how they have managed to build and maintain their social resources and reduce social isolation and loneliness. To this end, this symposium will analyze and incorporate the results using data from both Aging with Pride: National Health, Aging and Sexuality/Gender Study (NHAS), the first longitudinal study of SGM older adults in the United States and Innovations in Empowerment and Action (IDEA) Café, the first clinical trial pilot study addressing social isolation among SGM older adults living with dementia. Dr. Kim and colleagues with present a factor analysis suggesting a multi-dimensional construct of social resources unique to SGM older adults. Dr. Fredriksen Goldsen and colleagues will identify sexual and gender diverse older adults at heightened risk of loneliness and examine risk and protective factors. Dr. Emlet and colleagues will present findings from a pilot study, IDEA Café, designed to enhance social connectedness, physical functioning, and quality of life among socially isolated SGM older adults with cognitive impairment.

### UNDERSTANDING THE DIMENSIONS OF SOCIAL RESOURCES AMONG SEXUAL AND GENDER MINORITY OLDER ADULTS

Hyun-Jun Kim, Karen Fredriksen Goldsen, and Meghan Romanelli, *University of Washington, Seattle, Washington, United States*

Despite the growing number of recent studies on the positive role of social resources on health and well-being among SGM older adults, the multi-dimensional construct of social resources has not been examined due to a lack of adequate measures and data. This study describes the rationale behind the measurements of social resources used in the National Health, Aging, and Sexuality/Gender Study (NHAS). We analyze NHAS data collected in 2014 (N = 2,450) to assess the reliability of these social resource measurements. Out of 28 indicators of social resources ($\alpha = .82$), three distinct factor structures emerged: relational social connectedness ($\alpha = .71$), collective social connectedness ($\alpha = .82$), and perceived social connectedness ($\alpha = .79$). All item-rest correlations for each scale were moderate to strong. The scales could be used to understand various aspects of social resources and its role in enhancing health and well-being in the historically marginalized populations.

### LINKING LIVES: DISRUPTING THE CYCLE OF SOCIAL ISOLATION

Karen Fredriksen Goldsen[1], Hyun-Jun Kim[1], Charles Hoy-Ellis[2], and Christi Nelson[1], *1. University of Washington, Seattle, Washington, United States, 2. University of Utah, Salt Lake City, Utah, United States*

Loneliness has been found to be associated with increased risk for early mortality and dementia, with sexual and gender diverse older adults at elevated risk of both social isolation and loneliness. Based on the Health Equity Promotion Model and Iridescent Life Course, we examine factors associated with increased risk of loneliness over time, utilizing 2014 to 2016 data from the Aging with Pride: National Health, Aging, Sexuality/Gender Study, a longitudinal national study of LGBTQ+ midlife and older adults. The findings illustrate that sexual and gender diverse older adults had nearly double rates of loneliness compared to the general population, with those living alone and having cognitive decline at increased risk. We found that higher mastery, LGBTQ+ community engagement, larger network size, and being partnered/married were associated with less loneliness over time. Loneliness is ripe for the development of interventions; additional longitudinal data is needed to further assess trajectories in loneliness.

### REACHING THE SOCIALLY ISOLATED OLDER ADULTS IN DEMENTIA RESEARCH

Charles Emlet[1], Karen Fredriksen Goldsen[2], Hyun-Jun Kim[2], Linda Teri[2], Hailey Jung[2], and Brittany Jones[2], *1. University of Washington, Tacoma, Washington, United States, 2. University of Washington, Seattle, Washington, United States*

Sexual and gender minority (SGM) older adults are at greater risk for dementia and social isolation compared to their heterosexual peers. Lack of an available caregiver is of major concern. Yet, most dementia interventions to date have been designed primarily for caregiver-care recipient dyads or solely caregivers. A pilot study evaluated a 9-session virtual program designed to address social isolation, physical functioning and quality of life among SGM older adults living with dementia without a caregiver. Participants in the experimental group (n = 14) showed high attendance (75%) and retention rates. Social support was associated with intervention adherence. Compared to controls, the experimental group showed a significant increase in physical functioning and quality of life and decrease in depressive symptomatology and perceived stress. To meet the needs of this population without adequate social resources, research must address the different configurations of resources to better serve socially isolated SGM older adults.

### SESSION 4550 (SYMPOSIUM)

### IMPROVING DEMENTIA CARE IN CO-CREATION BETWEEN SCIENCE, PRACTICE, AND EDUCATION

Chair: Simone de Bruin Discussant: Simone de Bruin

Long-term care is transitioning from a medical-somatic model of care to a psychosocial model of care. This transition requires new ways of working, and thus different competences of health and social care professionals. Innovative learning environments of multiple stakeholders in long-term care are increasingly being created in the Netherlands to foster co-creation between science, practice (including persons with dementia) and education of current and future health and social care professionals. In this symposium, we will share experiences from the Netherlands. The first presentation describes the challenges current and future health and social care professionals are facing with regard to innovation in dementia care. We will further explain how these experiences were used to set up a learning community 'living well with dementia'. The second presentation is dedicated to a participatory action research to develop new collaboration practices to address the often under-addressed theme of meaning in life among community dwelling older adults. The third presentation will focus on a co-creation project dedicated to a care innovation for migrants with dementia, being a guideline for managing challenging behaviour at home. In the plenary discussion after the three presentations, participants are invited to share experiences from their own countries and discuss the lessons learned.

### WORKING TOWARD A LEARNING COMMUNITY ENABLING LIVING WELL WITH DEMENTIA

Simone de Bruin, and Franka Bakker, *Windesheim University of Applied Sciences, Zwolle, Overijssel, Netherlands*

Health and social care professionals play an important role in enabling to live well with dementia. Supporting living well from a psychosocial approach to care requires new ways of working and different competences compared to a medical-somatic approach to care. Interviews and group meetings with future (i.e. students) and current health and social care professionals organized in 2021, however, revealed that training to develop such competences and adopt these new ways of working receive little attention in the curricula of existing training programs. They further experience that their organisations insufficiently facilitate them (e.g. in terms of time, creating a positive learning climate) to actually use these competences and innovate their ways of working. Therefore, there is a need for creating innovative learning environments in the form of learning communities. These collaborations of stakeholders from science, practice, and education aim to accelerate innovation in dementia care based on lifelong learning and development.

### MULTISTAKEHOLDER COLLABORATION TO BETTER ADDRESS MEANING IN LIFE: PARTICIPATORY ACTION RESEARCH

Franka Bakker[1], and Theo van Leeuwen[2], *1. Windesheim University of Applied Sciences, Zwolle, Overijssel, Netherlands, 2. Department of Theology and Philosophies of Life, Windesheim University of Applied Sciences, Zwolle, Overijssel, Netherlands*

Meaning in life of older adults is related to a good life. Health and social care professionals are expected to address tensions in finding meaning in life among their clients. From 2020 to 2022, we conducted a 24-month participatory action research to discover how professionals in spiritual care, social workers, and volunteers can collaborate to better recognize, address and support questions regarding meaning in life. Four groups of co-researchers were composed, each consisting of a social worker, professionals in spiritual care, older adult, volunteer, students and researcher. They had monthly meetings to discuss actions and findings. In-between, collaboration activities were shaped and qualitative data were gathered about e.g. experiences. Results show that collaboration can be shaped by activities like providing workshops, expert consultation, coaching-on-the-job, jointly shaped interventions for clients, dialogue sessions, and multidisciplinary meetings. Thereby, for realising sustainable collaboration and ongoing mutual learning, structural and person-independent encounters should be realised.

### CO-CREATING A GUIDELINE FOR MANAGING CHALLENGING BEHAVIOR OF MIGRANTS WITH DEMENTIA AT HOME

Leontine Groen-van de Ven[1], Serena van der Kaaij[2], Geraldine Visser[1], Judith Huis in het Veld[2], Robbert Gobbens[2], and Simone de Bruin[1], *1. Windesheim University of Applied Sciences, Zwolle, Overijssel, Netherlands, 2. InHolland University of Applied Sciences, Amsterdam, Noord-Holland, Netherlands*

Challenging behaviour is common in people with dementia at home. Nurses guide people with dementia and their informal caregivers managing challenging behaviour. However, common nursing guidelines for challenging behaviour in home care are unsuitable to the situation of migrants with dementia. We started a co-creation project with home care nurses, dementia case managers, informal caregivers of migrant background and student nurses as co-researchers that aims to develop an appropriate guideline. Semi-structured interviews at the start explored the current experiences with challenging behaviour. Interview results informed several working sessions where we discussed the results and used creative working methods such as role play to develop a first prototype of the guideline. The prototype was implemented in the daily practice of six home care teams and followed up during reflection meetings with our co-researchers. Our guideline puts special attention to getting familiar with each other before taking steps in managing challenging behaviour.

### SESSION 4555 (BSS FLASH POSTERS)

### BSS FLASH POSTER SESSION 2: THE POWER OF PURPOSE AND PERSONAL TIES IN LATE LIFE

### PURPOSE IN LIFE, STRESS REACTIVITY, AND COGNITIVE AGING: A LONGITUDINAL INVESTIGATION

Niccole Nelson, and Cindy Bergeman, *University of Notre Dame, Notre Dame, Indiana, United States*

This study examined (1) the time-varying relationship between purpose in life and perceived stress reactivity, (2)

the trajectory of perceived stress reactivity as it relates to both within- and between-person purpose in life, as well as (3) the predictive utility of perceived stress reactivity and its rate of change on cognitive ability and allostatic load. The sample comprised 933 participants from the Notre Dame Study of Health & Well-being, a 10-year study of annual questionnaire packets and biennial daily diary bursts. Analyses included three-level multilevel models from which random effects were extracted and used to predict allostatic load and cognitive ability. Results indicated that individuals were affectively reactive to perceived stress, and that perceived stress reactivity declined over time. Considering the effects of purpose in life on these processes, there were two cross-level interaction effects indicating (1) more purposeful individuals were less stress reactive than less purposeful individuals, and (2) more purposeful individuals declined less in negative affect over time than less purposeful individuals. There was also preliminary evidence for a within-person interaction effect between yearly purpose in life and daily affective reactivity such that when individuals felt particularly purposeful, they also tended to be less stress reactive. Finally, higher perceived stress reactivity, as well as less decline in this construct, was predictive of better cognitive ability. These findings indicate purpose in life buffers against environmental and maturational effects on negative affect, and that perceived stress reactivity may indicate a different, more adaptive process than affective reactivity to experienced stressors.

### OLDER ADULTS SHARE CHALLENGES IN THEIR LIFE STORY: SENSE OF PURPOSE AS A RESOURCE FOR RESILIENCE

Semper Habib[1], Ameesha Narine[1], Susan Bluck[2], and Shubam Sharma[1], *1. Kennesaw State University, Kennesaw, Georgia, United States, 2. University of Florida, Gainesville, Florida, United States*

Maintaining a sense of purpose promotes mental and physical well-being in older adults (Musich et al., 2018). Drawing on one's sense of purpose is thus important for late life resilience. How older adults' sense of purpose manifests in their everyday lives remains understudied. This study used qualitative methods to amplify older adults' voices regarding purpose and resilience through analysis of their life stories. This study 1) explored what factors contribute to maintaining purpose in older adulthood, and 2) identified how older adults draw on their purpose during major challenges, particularly in the context of the COVID-19 pandemic. Eighteen older men and women (Mage = 79.1; including the young-old, old-old, and oldest-old) participated in semi-structured life story interviews that asked about participants' individual interpretations of purpose in their lives and their experiences navigating the COVID-19 pandemic. Thematic analysis was conducted using established methods (Braun & Clarke, 2012). To address the first research question, analyses revealed that older individuals largely maintain their purpose through engaging in acts of service to others, fostering connections with close others, and actively setting and achieving goals. Regarding the second question, older adults described how drawing on purpose through acts of service and connections with others fostered resilience through the COVID-19 pandemic. Overall, older adults' own expressions of their life stories illuminated how they are guided by purpose. Findings demonstrate the functionality of purpose in late life and how purpose can be practically fostered, specifically within the context of universally challenging experiences such as the COVID-19 pandemic.

### BLACK GREEK LETTER ORGANIZATIONS: FACILITATING HEALTH PROMOTION FOR AFRICAN AMERICANS ACROSS THE LIFECOURSE

Chivon Mingo, *Georgia State University, Atlanta, Georgia, United States*

African Americans remain underrepresented in accessing and utilizing evidenced-based health promotion interventions (EBIs). Challenges with dissemination and implementation of EBIs further corroborate existing racial/ethnic health/healthcare disparities. Therefore, there is a need to identify effective ways to increase the widespread adoption of health promotion behaviors among African Americans across the life course. It is plausible that engaging in non-traditional partnerships (i.e., community groups or organizations valued in the community with the capacity and infrastructure) could result in greater adoption and improved utilization of EBIs among African Americans. Although frequently overlooked as a study variable in empirically sound public health research, Black Greek Letter Organizations (BGLO) could be an innovative and practical approach to advancing health in the African American community. It is necessary to gain preliminary evidence of feasibility (e.g., motivation, target population reach, acceptability, ). Therefore, the purpose of this study was to conduct a content analysis to identify the intentions and communication trends of BGLOs as it pertains to public health and the African American community and assess population reach and perceptions by evaluating responses to communication specific to health promotion.We assessed health promotion patterns of four BGLOs in a ten-county metropolitan area. Coded content included communication via the organization's webpage, Facebook, Twitter, YouTube, Instagram, and LinkedIn from a five-year time period. Findings confirm that BLGOs are invested in the health and well-being of the community, place emphasis on mitigating health inequities, and are uniquely positioned to serve as stakeholders for the translation of EBIs to end-users.

### THE ROLE OF EDUCATION AND LITERACY SKILLS ON MIDDLE-AGED AND OLDER VOLUNTEERS BY RACE AND ETHNICITY IN THE US

Donnette Narine[1], Takashi Yamashita[2], Wonmai Punksungka[3], Abigail Helsinger[4], Jenna Kramer[5], Rita Karam[6], and Phyllis Cummins[7], *1. University of Maryland, Baltimore, Baltimore, Maryland, United States, 2. University of Maryland, Baltimore County, Baltimore, Maryland, United States, 3. University of Maryland - Baltimore County, Baltimore, Maryland, United States, 4. Miami University, Oxford, Ohio, United States, 5. RAND Corporation, Arlington, Virginia, United States, 6. RAND*

*Corporation, Santa Monica, California, United States, 7. Miami University, Oxford, Ohio, United States*

Volunteer participation is a form of civic engagement that benefits both the individual and society over the life course. Although education, basic skills (e.g., literacy), and race/ethnicity are individually associated with volunteering, detailed interrelations are yet to be explored. Guided by the integrated theory of volunteer work and the notion of productive aging, the goal of this study was to examine the roles of education and adult literacy in the context of volunteering in later life across racial and ethnic groups (Whites, Blacks, Hispanics) in the U.S. Using the nationally representative sample of middle-aged and older adults (age 45+; n = 3,770) from the 2012/2014/2017 Program for International Assessment of Adult Competencies (PIAAC), structural equation modeling was constructed to evaluate mediation relationships among education, literacy, and volunteering by racial and ethnic groups. Results show no statistically significant mediation (a.k.a., indirect) effect of education on volunteering through literacy, nor was there statistically significant difference in the mediation effect across racial and ethnic groups. However, there were statistically significant differences in the direct effect of education on volunteering between Black adults and White adults [b(Black) = 0.44 versus b(White = 0.24), $p < 0.05$], as well as Black adults and Hispanic adults [b(Black) = 0.44 versus b(Hispanic) = 0.08, $p < 0.05$]. These findings indicate that higher education was more strongly associated with volunteering among older Black adults, compared to White and Hispanic counterparts. Suggested policy implications include support for the promotion of volunteer participation through culturally and socioeconomically sensitive approaches.

### COMPASSIONATE LOVE AND LONELINESS: LATER LIFE MENTAL HEALTH IN THE UNITED STATES

Nirmala Lekhak[1], Tirth Bhatta[2], Eva Kahana[3], and Joel Synder[1], *1. University of Nevada, Las Vegas, Nevada, United States, 2. University of Nevada Las Vegas, Las Vegas, Nevada, United States, 3. Case Western Reserve University, Cleveland, Ohio, United States*

Loneliness is a serious public health problem that affects over 25% of older adults and is associated with an increased risk of depression, cognitive decline, and premature death. Previous research on social support mechanisms that contribute to loneliness has consistently illustrated the role of emotional support in reducing loneliness. However, the importance of compassionate love in reducing loneliness and, as a consequence, improving psychological well-being in later life has received little attention. Neurobiology indicates that the brain regions associated with loneliness and compassion overlap, suggesting that increasing compassion-related emotions may help alleviate loneliness. Using data from a nationwide web-based survey (n=1,861), we examined the influence of compassionate love on loneliness and assessed whether loneliness mediates the relationship between compassionate love and mental health outcomes. Even after controlling for emotional support, estimates from an ordinary least squares regression (OLS) model suggest that older adults who felt loved had significantly lower levels of loneliness (b=-0.84, p< 0.001). Feeling of love also contributed to significantly fewer depressive symptoms (b=-2.03, p< 0.001) and anxiety (b=-1.07, p< 0.001). Loneliness completely mediated the effect of compassionate love on anxiety (b=-0.82, p< 0.001) and significantly mediated its influence on depressive symptoms (b=-1.18, p< 0.001). Our findings underscore the need to design interventions that increase compassionate love to reduce loneliness and improve psychological wellbeing among older adults.

### SOCIAL SUPPORT AND LONELINESS AS DETERMINANTS OF THE ONSET OF DISABILITY AMONG PUERTO RICAN OLDER ADULTS

Amilcar Matos-Moreno[1], Neil Mehta[2], Eduardo Villamor[3], Lu Wang[3], and Carlos Mendes de Leon[4], *1. The Pennsylvania State University, Bayamon, Pennsylvania, Puerto Rico, 2. UTMB, Galveston, Texas, United States, 3. University of Michigan, Ann Arbor, Michigan, United States, 4. University of Michigan, Ann arbor, Michigan, United States*

Background: The effect of loneliness and social support on health is poorly understood among older Puerto Ricans. As family size continues to decrease in Puerto Rico due to out-migration, a higher number of older adults have fewer family members to rely on, which may lead to detrimental health outcomes.Method: Using both waves (2002-03 and 2006-07) of the Puerto Rican Elderly: Health Conditions database, we examined the association between social support, living alone, and incident disability among a sample of older adults over 60 years of age residing in Puerto Rico. Disability was defined as the occurrence of difficulties with Activities of Daily Living (ADLs).

Results: 13.4% of older adults in our sample developed some form of disability. Older adults who developed a disability indicated receiving higher levels of social support (2.04 vs. 1.64) and loneliness (30.7% vs. 22.8%). Using multivariate logistic regression, we found that receiving social support increased the odds of developing a disability by 17% (OR: 1.17; CI: 1.02 – 1.35). Older adults who live alone had 58% higher odds of developing a disability (OR: 1.58; CI: 1.01 – 2.46).

Conclusion: The presence of social support and loneliness was correlated with a population prone to developing disabilities. Our findings concur with the well-established literature on psychosocial determinants in late life. However, this study represents the first attempt to understand psychosocial measures and disability in Puerto Rico. Public health organizations and healthcare systems must develop new societal mechanisms of support for older adults at risk of developing disabilities.

### EXAMINING THE CUMULATIVE EFFECTS OF SOCIAL ISOLATION AND LONELINESS IN OLDER ADULTS WITH HIV

Moka Yoo-Jeong, *Northeastern University, Melrose, Massachusetts, United States*

Social isolation exists when one has limited or lacks social contact with others and is distinct from loneliness, an affective state on the perception of isolation. Social isolation and loneliness are recognized as risks to well-being among older adults. Less in known about the cumulative effects of social isolation and loneliness in older persons with HIV (OPWH). Using cross-sectional data on OPWH (age ≥50) recruited from an outpatient HIV clinic in Atlanta, GA (N=146), we aimed to 1) describe the overlap between social isolation and loneliness and 2) examine the combined effects of social isolation and loneliness on quality-of-life (QoL), HIV-related stigma, depressive symptoms, and comorbidity burden. Loneliness and social isolation were assessed using the PROMIS-Social Isolation Scale and Social Network Index, respectively. Participants were grouped into four categories into 'lonely only,' 'isolated only,' 'both lonely and isolated,' or 'neither.' Bivariate and adjusted associations were conducted. Among participants (mean age=56.53), 26.7% (n=39) were considered 'lonely' only, 12.3% (n=18) 'isolated' only, 15.1% (n=22) 'both lonely and isolated,' and 45.9% (n=67) 'neither.' In bivariate analyses, individuals categorized as 'both lonely and isolated' were likely to have past homelessness and higher depressive symptoms, stigma, and comorbidity burden, and lower QoL. In adjusted models, 'both isolated and lonely' significantly predicted QoL, stigma, and depressive symptoms. Findings highlight the critical emphasis on targeting OPWH who are both isolated and lonely.

### A NEW NORMAL: EXAMINING THE LINKAGES BETWEEN SOCIAL TECHNOLOGY AND LONELINESS

Yeon Ji Ryou[1], Natasha Peterson[1], Mengya Wang[1], and Joseph Svec[2], *1. Iowa State University, Ames, Iowa, United States, 2. Iowa state university, Ames, Iowa, United States*

As the COVID-19 pandemic continues to shape social landscapes, social isolation and loneliness are major issues with mental and physical ramifications. In recent years, individuals of all ages are turning to social technology (ST) to communicate with loved ones. However, the viability of ST as a substitute to in-person interaction remains hotly debated. Moreover, few studies examined how psychosocial factors interact with ST in mental health outcomes. Using the 2020 survey data (N = 1,969) from Health and Retirement Study (HRS), this research examines whether and to what extent ST ameliorates loneliness among 65+ individuals. We identify which personality dimensions moderate the relationship between ST and loneliness using the conceptual framework of the five-factor and the unified theory of acceptance and use of technology model. Linear regression analyses are conducted to determine direct and interaction effects. Results indicate that greater ST use corresponds negatively with loneliness. We also observe that particular personality traits (e.g. extraversion) are negatively associated with loneliness, while neuroticism corresponds positively with loneliness. In the interaction between neuroticism and ST, neuroticism mitigates the association between social technology and loneliness. These findings indicate that ST can be a positive source of social connectivity but the extent may be conditioned on personality profiles. Plausibly, neurotic individuals may exacerbate behavioral propensities by technology use. As evidenced by the robust independent effects of ST and conditional nature of technology connections with neurotic profiles. These findings imply that future intervention should consider individual differences when developing mental health programs using ST.

### SESSION 4560 (SYMPOSIUM)

### INTERACTIONS BETWEEN SLEEP AND BIOLOGICAL MARKERS OF AGING

Chair: Christopher Kaufmann Discussant: Katie Stone

There is well documented evidence that sleep architecture and circadian patterns change as people age in part due to accumulation of comorbidities, polypharmacy, and other factors inherent to the aging process. While a number of studies have examined sleep in the context of these factors, recent advances in the assessment of biological aging (including epigenetic age, metabolomics, and measurement of inflammatory biomarkers) have made it possible to identify potential mechanisms by which sleep impacts the aging course. In this symposium, we will highlight research that investigates the link between sleep disturbances and biological factors in order to identify whether sleep could be a modifiable risk factor for accelerated aging. Our first presentation will examine the association between insomnia symptoms and short sleep duration with epigenetic age acceleration using data from the Health and Retirement Study. Second, we will describe whether objectively measured sleep characteristics are associated with a number of metabolomic markers of aging. The third presentation will center around the extent to which social disparities in presence of inflammatory biomarkers may be mediated by sleep quality. Finally, we will examine how daytime sleepiness may be associated with longitudinal BMI trajectories. These presentations will, as a whole, highlight the importance of sleep as a contributor to healthy aging and longevity, and inform the development of interventional approaches targeting biological mechanisms to promote successful aging more broadly.

### SHORT SLEEP AND INSOMNIA ARE ASSOCIATED WITH ACCELERATED EPIGENETIC AGE

Cynthia Kusters[1], Eric Klopack[2], Eileen Crimmins[3], Teresa Seeman[4], Steve Cole[4], and Judith Carroll[1], *1. University of California, Los Angeles, Los Angeles, California, United States, 2. University of Southern California, Leonard Davis School of Gerontology, Los Angeles, California, United States, 3. University of Southern California, Los Angeles, California, United States, 4. University of California Los Angeles, Los Angeles, California, United States*

Short sleep (<6 hours) and insomnia are independently associated with greater risk for age-related disease suggesting that insufficient sleep may accelerate biological aging. Epigenetic age acceleration is an estimate of biological aging that predicts morbidity and mortality. We tested whether insomnia symptoms and short sleep duration relates to epigenetic age in 2783 participants in the Health and Retirement Study. Insomnia and short sleep were associated with an 0.70(95%CI:0.23-1.17;P: 0.005) and 1.45(95%CI:0.67-2.24;P:0.001) years acceleration of GrimAge, respectively, as well as a faster pace of aging (DunedinPoAm; 0.015(95%CI: 0.005-0.024; P:0.006); 0.021(95%CI: 0.006-0.037; P:0.009)). Compared to healthy sleepers, Individuals with the combination of short sleep and insomnia had an accelerated GrimAge (1.34;95%CI: 0.49-02.19; P:0.003) and a greater DunedinPoAm (0.025; 95%CI: 0.009-0.041; P:0.004). Our findings indicate short sleep and insomnia are linked to epigenetic age acceleration, suggesting that these individuals have an older biological age that may contribute to risk for comorbidity and mortality.

### ASSOCIATIONS BETWEEN SLEEP AND REST: ACTIVITY CHARACTERISTICS AND METABOLOMIC PROFILES IN OLDER MEN

Qian Xiao[1], Joshua Sampson[2], Aladdin Shadyab[3], Andrea LaCroix[3], Kristine Yaffe4, and Katie Stone4, *1. The University of Texas Health Science Center at Houston,*

Houston, Texas, United States, 2. FDA, Silver Spring, Maryland, United States, 3. University of California, San Diego, La Jolla, California, United States, 4 University of California, San Francisco, San Francisco, California, United States

The association of sleep and rest-activity rhythms (RAR) with metabolic health is not fully understood. Previous studies have identified multiple metabolite markers in amino acid and lipid pathways associated with various characteristics of sleep. However, most of the studies used self-reported sleep, and limited studies have examined 24-hour RAR profiles, a more complete picture of activity. We studied 950 older men and measured metabolomics from fasting blood samples. We identified numerous metabolomic markers that were cross-sectionally associated with actigraphy-based measures of sleep (total sleep time, sleep efficiency, sleep timing) and RAR (amplitude, acrophase, mesor and overall rhythmicity). The majority of the associated metabolites were amino acids and lipids from a wide range of pathways, including metabolism pathways of branched chain amino acid metabolism, fatty acids, and gamma-glutamyl amino acids. Our preliminary findings suggest that sleep and RAR are widely involved in human metabolism.

### SOCIAL DISPARITIES IN INFLAMMATORY BIOMARKERS MEDIATED BY POOR SLEEP QUALITY

Joseph Obiagwu, Christina Mu, and Soomi Lee, *University of South Florida, Tampa, Florida, United States*

This study investigated whether sleep quality mediates the relationship between race/SES and biomarkers (CRP, IL6, IL10, TNF-α). Participants in the Midlife in the United States Study (n=1,689; Mage=53.02) completed the Pittsburgh Sleep Quality Index and provided information on eight life-course indicators to measure SES. Black individuals and those with lower SES had poorer sleep quality and higher inflammation compared to their counterparts. Poorer sleep quality mediated the relationship between being Black and higher CRP (β=0.02, 95%CI [0.002, 0.04]), IL6 (β=0.008, 95%CI [0.0002, 0.02]), IL10 (β=0.008, 95%CI [0.0004, 0.02]), and TNF-α (β=0.004, 95%CI [0.0002, 0.01]). Poorer sleep quality also mediated the relationship between lower SES and higher CRP (β=-0.01, 95%CI [-0.01, -0.001]), IL6 (β=-0.003, 95%CI [-0.007, -0.00]), IL10 (β=-0.003, 95%CI [-0.01, - 0.0003]), and TNF-α (β=-0.002, 95%CI [-0.004, -0.0002]). Improving sleep quality may help reduce the risk of inflammation in at-risk groups and subsequently reduce health disparities.

### DAYTIME SLEEPINESS AND WEIGHT CHANGE AMONG ADULTS: FINDINGS FROM THE WISCONSIN SLEEP COHORT STUDY

Yin Liu[1], Jodi Barnet[2], Erika Hagen[2], Paul Peppard[2], Eric Reither[1], Emmanuel Mignot[3], and David Plante[4], *1. Utah State University, Logan, Utah, United States, 2. University of Wisconsin Madison, Madison, Wisconsin, United States, 3. Stanford University, Palo Alto, California, United States, 4. University of Wisconsin-Madison, Madison, Wisconsin, United States*

BMI trajectories are associated with nighttime sleep, but it is less clear how they relate to daytime sleepiness. We examined the association between levels and changes in daytime sleepiness and BMI among men and women using growth curve models among 1047 participants in the Wisconsin Sleep Cohort Study (mean [sd] age = 51.1 [8.0] years at baseline). The outcome variable was BMI (kg/m2). Key predictors were self-reported sleepiness measured by the Epworth Sleepiness Scale (ESS), and the objective Multiple Sleep Latency Test (MSLT) scores at each data collection wave. Men, but not women, who were sleepier had higher BMI levels. Age moderated the association between changes in ESS and MSLT sleepiness and BMI trajectories. The association was weaker for older men, but stronger for younger men; such effect was the opposite for women. The MSLT models further suggested that women who were sleepier had steeper increases in BMI over time.

### SESSION 4570 (SYMPOSIUM)

### NEW SPINS ON CLASSIC IDEAS ABOUT CONTEXT IN ADULT EMOTIONAL DEVELOPMENT

Chair: Tabea Springstein Co-Chair: Tammy English

Individuals often experience improvements in emotional well-being into old age. Understanding mechanisms contributing to these emotional outcomes in daily contexts can inform ways to support healthy aging. Development is embedded within various contexts that shape individuals' experiences. Novel perspectives are emerging on how to conceptualize context and the way it can contribute to emotional development during the aging process. This symposium illustrates four innovative ways to consider contextual contributions to emotional well-being across adulthood. The first talk will use experience sampling to illustrate age differences in how daily situations contribute to emotion regulation related processes, showing that older adults can more easily distinguish between emotions when in familiar situations. The second talk will take a fresh perspective on psychosocial contexts by distinguishing between types of social interactions in couples, highlighting the important role of affection for well-being in adulthood. The third talk will introduce the idea that the body itself provides context for emotional processes, showcasing that the way this context affects emotional experience changes as individuals age. The fourth talk will center on how renewing our classical developmental models of context in modern ways can help to overcome shortcomings of previous research and provide insight into how engagement with environmental features contributes to well-being across the lifespan. In sum, this symposium features innovative perspectives on how context can be leveraged to gain a deeper understanding of psychosocial development into old adulthood and illustrates specific ways individuals can navigate their social world to preserve or improve mental health across adulthood.

### FAMILIAR CONTEXTS, FAMILIAR EMOTIONS? A NEW PERSPECTIVE ON CONTEXT-SPECIFIC EMOTION PROCESSES IN OLDER ADULTHOOD

Tabea Springstein, and Tammy English, *Washington University in St. Louis, St. Louis, Missouri, United States*

As people age, their emotional well-being tends to be maintained or improves. Theories of adult development suggest that features of the context (e.g., more familiar environments) and successful management of emotions contribute to this effect. This talk focuses on how familiarity may promote emotional differentiation (e.g., knowing whether one feels angry or sad) in daily life, an important predecessor to emotion regulation success. A sample (N=290) of community participants between the ages of 25 and 85 years old completed an experience sampling study (6x/10days). When people were more familiar with their current situation, they differentiated more between emotions. The relationship between familiarity and differentiation was stronger for older adults than younger adults. These results support the perspective that emotional well-being benefits from wisdom accrued over the lifespan, such that older adults are at more of an advantage when their daily contexts afford for them to draw on their prior experiences.

### ALL YOU NEED IS LOVE? SPOUSES' AFFECTION ACROSS MARITAL INTERACTION CONTEXTS

Claudia Haase, Jacquelyn Stephens, and Tabea Meier, *Northwestern University, Evanston, Illinois, United States*

Affection is a positive emotion that plays an important role in intimate relationships. In a laboratory-based dyadic study of 52 middle-aged married couples (104 individuals) from highly diverse socioeconomic backgrounds, we examined spouses' subjective experience of affection across different marital interaction contexts, correlates, and specificity. Results showed that (1) wives and husbands experienced lower affection in a conflict conversation compared to a positive conversation. Moreover, (2) for wives, higher affection in the conflict conversation was associated with lower physiological reactivity (i.e., less heart rate acceleration from baseline to conflict discussion). For wives, higher affection was also associated with higher marital satisfaction for themselves and their husbands. These findings were specific to affection and did not robustly emerge for other positive emotions. These findings support views of affection as a positive, context-sensitive emotion that may become particularly important in intimate relationship conflict. Directions for future research are discussed.

### AGING BODY AS CONTEXT: THE ROLE OF INTEROCEPTIVE AGING IN MID- AND LATE-LIFE EMOTION

Jennifer MacCormack, *University of Virginia, Charlottesville, Virginia, United States*

Interoceptive sensations (e.g., racing heart, clenched gut) are often closely tied to emotion. Older adults tend to exhibit reduced sensitivity and awareness of their interoceptive sensations relative to younger adults (e.g., Khalsa et al., 2009; Mikkelsen et al., 2019; Murphy et al., 2019), yet the emotional implications of such interoceptive differences remain unclear. Herein, I present two behavioral cross-sectional studies (N=350) documenting age differences in how adults (18-75 yrs) link their interoceptive sensations to emotions (Study 1: cognitive behavioral task; Study 2: experience sampling). Results reveal that from midlife into late life, coherence between interoceptive sensations and emotions increasingly weakens, both behaviorally and in self-reports. Age effects are most prominent for high arousal sensations and states. These findings provide converging evidence with recent neuroimaging results (MacCormack et al., 2020) showing the importance of interoceptive aging and related shifts in physiological arousal as potential pathways by which emotions transform across adulthood.

### INFLUENCE OF EARTH, WIND, AND FIRE ON WELL-BEING: THE CLASSIC CONCENTRIC CIRCLES MODEL OF CONTEXT IS ALSO FROM THE 1970S

Michelle Ng[1], Angelina Garron[1], Denis Gerstorf[2], and Nilam Ram[3], *1. Stanford University, Palo Alto, California, United States, 2. Humboldt Universität zu Berlin, Berlin, Berlin, Germany, 3. Stanford University, Stanford, California, United States*

Classic bioecological models of human development (Bronfenbrenner, 1979) highlight the influence that five layers of context have on individuals' function and growth. Modern versions specifically highlight five specific aspects of context (socio-economic, social, physical, care/service, and technology) that influence both how older adults negotiate their daily lives and how they age. Using a collection of empirical findings obtained from our analyses of longitudinal panel data from the German SOEP, experience sampling data from iSAHIB, and screenome data from the Stanford HSP, we illustrate how differences in individuals' engagement with green spaces (earth), ambient air pollution (wind), and smartphones (fire) may contribute to differences in daily emotional well-being and developmental changes in life satisfaction. We then use these examples to elucidate shortcomings of the hierarchical models used to frame investigations of context and development for the past 40 years – and suggest the classic models be renewed rather than continually recycled.

### SESSION 4580 (PAPER)

### OPIOID USE AND PRESCRIBING PATTERNS

### CHARACTERISTICS OF OLDER ADULTS WHO DIED FROM OPIOIDS AS A CAUSE OF DEATH DURING THE COVID-19 PANDEMIC

Armiel Suriaga, and Ruth Tappen, *Florida Atlantic University, Boca Raton, Florida, United States*

There was a 1,886% increase in opioid overdose deaths among people 55 years and above, from 518 in 1999 to 10,292 in 2019 in the U.S. The National Institute on Drug Abuse reported a 30% increase in drug overdose deaths in 2020, where it claimed nearly 100000 American lives. However, few studies focused on the individual opioids that caused more deaths in the aging population and their death circumstances. This retrospective study aims to describe the sociodemographic characteristics of older adults who died from opioids as a cause of death (COD) in Florida during the COVID-19 pandemic in 2020, using descriptive statistics in SPSS 28. De-identified data from the Florida Department of Law Enforcement in 2020 were used in this study. A total of 1656 cases of decedents (ages > 65 years) were analyzed; 348 out of 1656 cases had opioids as a COD. Age ranged from 65 to 103, mean age of 73.31 (SD= 7.690). Majority of decedents were males (67.3%), non-Hispanic whites (86.5%). Nearly 80% died from accidents, suicide at 18.1%; 99.1% of cases happened in urban counties. Fentanyl caused more deaths (n=135), then morphine (n=48), oxycodone (n=42), and hydrocodone (n=29).The study results have direct clinical practice implications to patient safety, particularly the opioid deaths that resulted from accidents. Opioid harm reduction interventions should be tailored to older adults' specific needs, specifically in urban counties. Healthcare providers should pay extra attention to opioids that cause more deaths, i.e., fentanyl, morphine, oxycodone, and hydrocodone to safeguard public safety.

### CONTINUED OPIOID USE REDUCES PERCEIVED CHANCE OF LIVING TO AGE 75 AMONG YOUNG-OLD INDIVIDUALS WITH CHRONIC PAIN

Gillian Fennell, *University of Southern California, Los Angeles, California, United States*

Pain limits older adults' predictions of their own life expectancies. Due to their analgesic properties and simultaneous linkages to depression, poor subjective health, and functional disability, it is uncertain if the use of opioids augments or contributes to limitations in subjective life expectancy (SLE). This study uses 2016 and 2018 data from the Health and Retirement study to assess the predictive value of prescription opioid use transitions on perceived chances of living to age 75 (SLE). Our sample includes 50 to 62 year-olds who reported pain in both waves (N=1,977). The OLS model controls for pain intensity and activity interference, socio-demographic factors, six chronic conditions, depression, and the 2016 baseline outcome measure. Relative to non-opioid users, respondents who transitioned off of opioids after 2016 reported significantly higher SLEs in 2018 (b=5.56, p=.05). Respondents who used opioids in both waves reported significantly lower SLEs relative to non-opioid users (b=-4.17, p=.02). Individuals who began using opioids following the 2016 wave were no different on SLE than non-opioid users (p=.11). Higher 2016 SLE predictions and being female were associated with higher SLEs. Being never married and ever having diabetes were negatively associated with SLE. Continued opioid use among individuals with chronic pain reduced anticipated chances of living to 75 whereas transitioning off of such medications had the opposite effect. Despite their analgesic properties, prescribed opioid use has negative effects on future outlook independent of pain, health status, and depression. As a consequence, continued opioid users may poor health and/or financial planning decisions.

### DISCHARGE LOCATIONS AFTER HOSPITALIZATIONS INVOLVING OPIOID USE DISORDER AMONG MEDICARE ENROLLEES

Patience Moyo[1], Kimberly Goodyear[1], Eric Jutkowitz[2], Kali Thomas[2], and Andrew Zullo[1], *1. Brown University School of Public Health, Providence, Rhode Island, United States, 2. Brown University, Providence, Rhode Island, United States*

Hospitalizations involving opioid use disorder (OUD) are increasing in the Medicare population. Rising OUD-related acute care use emphasizes the need to understand where post-acute care is provided and opportunities to facilitate OUD treatment in those settings. We conducted a retrospective cohort study using 2016-2018 Medicare Provider Analysis and Review (MedPAR) files linked to enrollment data and the Residential History File (RHF) for 100% of Medicare fee-for-service beneficiaries aged ≥18 years. We used diagnostic codes for opioid dependence or "abuse" to identify OUD from MedPAR data. The RHF identified hospital discharge locations. Our analysis included 459,763 OUD-related hospitalizations. Overall, individuals < 65 years (60.3%), female (53.7%), or Medicare-Medicaid dually enrolled (59.1%) were the majority. The mean (standard deviation) length of stay was 5.5 (6.6) days and 31.3% of OUD-related hospitalizations required intensive care use. Seventy percent of patients with OUD-related hospitalizations were discharged home, 15.8% to skilled nursing facilities (SNFs), 9.6% to non-SNF institutional facilities, 2.5% home with home health services, and 1.8% died in-hospital. Within 30 days of hospital discharge, the range for 30-day all-cause readmissions was 27.7% (home) to 47.5% (non-SNF institutional setting). The 30-day all-cause mortality ranged from 3.1% (home without health) to 6.4% (non-SNF institutional settings). Given that over one-quarter of OUD-related hospitalizations resulted in discharge to locations other than home and that many institutional post-acute care settings cannot obtain OUD medications, modifying Drug Enforcement Administration regulations and expanding access to OUD medications for aging adults in post-acute and long-term care settings must be a near-term policy focus.

### MEDICATIONS ASSOCIATED WITH GERIATRIC SYNDROMES AND HOSPITALIZATION RISK IN HOME HEALTHCARE PATIENTS

Jinjiao Wang[1], Jenny Shen[2], Fang Yu[3], Yeates Conwell[2], Yue Li[4], Thomas Caprio[4], Avantika Shah[5], and Sandra Simmons[6], *1. University of Rochester School of Nursing, Rochester, New York, United States, 2. University of Rochester Medical Center, Rochester, New York, United States, 3. Arizona State University, Phoenix, Arizona, United States, 4. University of Rochester, Rochester, New York, United States, 5. Johns Hopkins University, Baltimore, Maryland, United States, 6. Vanderbilt University Medical Center, Nashville, Tennessee, United States*

Polypharmacy is common in the home health care (HHC) setting. This study examined the prevalence, predictors, and impact of medications associated with geriatric syndromes (MAGS) use on subsequent hospitalization in HHC patients. Data from the HHC electronic medical records, the Outcome and Assessment Information Set (OASIS), and Medicare HHC claims of 6,882 adults ≥ 65 years old receiving HHC from a large, non-profit HH agency in New York State in CY 2019 were used. MAGS use was identified from active medications reconciled during HHC visits and defined as total count and in quartiles. Hospitalization during the HHC episode was operationalized as a time-to-event variable. Regression analyses were conducted to identify predictors of MAGS use, and survival analyses were conducted to examine the association between MAGS use and hospitalization. Nearly all (98%) of the HHC patients used at least one MAGS and 41% of all active medications were MAGS. A higher MAGS count was found among HHC patients who were non-white, community-referred, with more comorbidities, depressive symptoms, and functional limitations. A higher MAGS count was also related to increased risk for hospitalization. Moreover, higher quartiles of MAGS

use combined with having ≥10 diagnoses predicted a 2.5-fold increase in hospitalization risk, relative to the lowest quartile of MAGS use and having < 10 diagnoses. In conclusion, over 40% of medications taken by HHC patients are MAGS, which, along with multimorbidity, increased hospitalization risk. HHC clinicians should identify MAGS use to inform deprescribing discussion with patients and their prescribers.

### SESSION 4590 (SYMPOSIUM)

### OPPORTUNITIES AND OUTCOMES OF CENTERING OLDER ADULTS IN PATIENT-ORIENTED AND COMMUNITY-ENGAGED RESEARCH

Chair: Carrie Leach Co-Chair: Carol Geary Discussant: Discussant should be Carol Geary also

Community engagement is critical for achieving context-sensitive and culturally appropriate communication, implementation and research approaches that can lead to better public and patient health outcomes. Though patient and community-engaged approaches are increasingly utilized in social research, the inclusion of older adults remains limited, as are methodologies that will help inform adult-centric programs and interventions. To advance the science of community engagement and implementation, this symposium brings together methodological innovations to help researchers shift from involving older adults as participants in research, toward collaboratively engaging and partnering with older adults in the design and conduct of research. The first presentation centers on place-based health disparities by starting with a statewide survey to identify communities that researchers could engage. The second highlights how the involvement of older adults led to the development of tactics for connecting with the hardly-reached, people over the age of 75, with health enhancing services and support. The third discusses a model for greater involvement of older adults to draw out lessons for implementation science in long-term care. The final presentation demonstrates an innovative approach to pilot new solutions with multidisciplinary and community partners in clinical settings. This CEnR and PPER interest group collaborative symposium concludes by imagining ways to integrate the diverse perspectives to advance the science of engaged research and implementation in community and clinical settings. This symposium represents diverse perspectives on involving community stakeholders meaningfully and equitably with research, to bend the later life health equity arc toward greater inclusion and justice.

### COMMUNITY PERSPECTIVES ON COMMUNICATION DISPARITIES IN LATE OLD AGE AND TACTICS TO CONNECT WITH THE HARDLY REACHED

Carrie Leach, and Thomas Jankowski, *Wayne State University, Detroit, Michigan, United States*

Individuals who face the greatest health burdens often have the fewest communication resources to draw from to help them cope. Communication infrastructure theory provides a framework for understanding how communication resources and context constrain and enable connections to health enhancing information. Data collected from 1,609 older adults through in-depth interviews and a random sample survey in one midwestern county in Michigan as part of a multi-year, multi-level community engaged study and a statewide random digit dial telephone survey showed that age influenced integration in a communicative structure. Findings suggest that the dynamics of living into late old age result in communication disparities through diminishing interpersonal resources, activity, and opportunities to congregate. Actionable knowledge and strategies provided to community leaders on how to connect with the hardly reached, individuals age of 75 and older, are presented.

### MULTISTAGE SAMPLING FOR TRANSLATIONAL COMMUNITY RESEARCH: ZOOMING OUT TO HONE IN ON PLACE-BASED HEALTH DISPARITIES

Daniel Rong Yao Gan, *Simon Fraser University, Vancouver, British Columbia, Canada*

Whereas researchers strive for generalizability, community-engaged research (CEnR) typically involves only a few specific communities. Drawing on Weberian ideal type, I outline the use of an innovative blended-methods approach to sample the communities in which CEnR practitioners would collect in-depth data. To complement typical practices of entering a community without preconceived ideas, understanding how communities in the sampling frame relate to one another is important for equigenic (place-based health equity) implementations. The selection of neighborhood communities from quadrants in 2x2 matrices allows pertinent concepts to emerge and relevant solutions to be drawn from thriving communities to aid program co-creation and implementation in other communities. For example, this has led to the identification of communities in British Columbia with differing socioeconomic status, social capital, and coping during COVID-19. This methodological innovation is congruent with asset-based community development (ABCD) to minimize arbitrariness in sampling decisions and advance health equity in our cities.

### A SUCCESSFUL MODEL FOR AMPLIFYING THE VOICES OF OLDER ADULTS IN LONG-TERM CARE AND SERVICES AND SUPPORT RESEARCH

Rachel Lessem[1], Rebecca Berman[2], and Amy Eisenstein[3], *1. CJE SeniorLife, Chicago, Illinois, United States, 2. Leonard Schanfield Research Institute, Chicago, Illinois, United States, 3. RRF Foundation for Aging, Chicago, Illinois, United States*

The experiential voice of older adults receiving long term services and supports (LTSS) is largely absent from health research hampering the development of effective interventions. While many have the capacity to participate in the design, development, and delivery of research, researchers traditionally do not recognize such capacity, may be unsure how to seek input, or may not appreciate the extent to which such input can improve the research enterprise. But the participation of these older adults can ensure that patient-centered research meaningfully addresses their care preferences and desired health outcomes, and can improve the effectiveness of care and patients' quality of life. Through three PCORI-funded projects, we developed a successful model for addressing barriers to amplify these voices. We outline barriers to the dissemination and implementation of this model, and suggest next steps to test strategies to amplify the voices of older adults in long-term care.

### USING COLLABORATIVE ACTION RESEARCH TO PILOT TELEPRESENCE ROBOTS IN LONG-TERM CARE

Lillian Hung, *University of British Columbia, Vancouver, British Columbia, Canada*

The COVID-19 pandemic has exposed the fragile state of patient involvement in research and presented new opportunities. The telepresence robot project aims to tackle social isolation and loneliness in older people with dementia in Long-Term Care (LTC) homes. My team took a Collaborative Action Research (CAR) approach to work with stakeholders, emphasising meaningful involvement of patient partners (people with dementia), family partners, frontline healthcare workers throughout all phases of the research process. In this paper presentation, I will discuss how we apply the core principles of CAR to engage stakeholders to carry out the study during the COVID-19 pandemic – a challenging time. It is precisely at times like this that we need to work with patient partners and frontline workers to uncover lessons learned for meaningful solutions to address the most pressing social problems. I will share stories about challenges and creative strategies used. Finally, practical lessons learned will be discussed.

### SESSION 4600 (SYMPOSIUM)

### PERCEIVED AND PERFORMANCE FATIGABILITY: NOVEL FINDINGS ON CORRELATES, CONSEQUENCES, AND CANDIDATE GENES

Chair: Nancy W. Glynn Co-Chair: Jennifer Schrack
Discussant: Eleanor Simonsick

Characterizing fatigability enables quantification of an individual's susceptibility to fatigue in the context of standardized physical task(s). This approach eliminates self-pacing, and is a less-biased, more sensitive approach to assess the presence and severity of fatigue, a critical prodromal symptom of chronic and acute health conditions. Collectively, we have established the Pittsburgh Fatigability Scale (PFS) and Borg Rating of Perceived Exertion at the end of a standardized 5-minute treadmill walk (RPE Fatigability) as prognostic indicators of aging-related outcomes. Recently, we extended our methodological work in developing a novel accelerometry-based performance fatigability measure of slowing down during in-lab walking (Pittsburgh Performance Fatigability Index, PPFI). This symposium presents findings from the Study of Muscle, Mobility, and Aging (SOMMA), Long Life Family Study (LLFS), and Baltimore Longitudinal Study of Aging (BLSA). Specifically, using SOMMA, Ms. Qiao will share development and validation of the PPFI using a usual-paced 400m walk, Ms. Moffit will report the association of fitness (i.e., VO2peak) with PFS physical and mental fatigability, and extending the SOMMA findings, Dr. Moored will detail the dual roles of fitness and fatigability on life-space mobility. Using LLFS data, Ms. Gay will report associations of variants (SNPs) in 272 candidate autosomal genes involved in energy metabolism (previously associated with mitochondrial dysfunction disease) with PFS physical fatigability. Lastly, using BLSA data, Dr. Wanigatunga will compare the discriminatory power of RPE Fatigability versus general fatigue symptoms for predicting mortality. Our Discussant, Dr. Simonsick, will provide critical review in the context of new directions for fatigability research.

### VALIDATION OF THE PITTSBURGH PERFORMANCE FATIGABILITY INDEX FROM USUAL-PACED 400 M WALK

Yujia (Susanna) Qiao[1], Jaroslaw Harzelak[2], Pamela Toto[3], Kyle Moored[4], Bret Goodpaster[5], Adam Santanasto[6], barbara Nicklas[7], and Nancy W. Glynn[8], *1. University of Pittsburgh, Schoold of Public Health, Pittsburgh, Pennsylvania, United States, 2. Indiana University, School of Public Health in Bloomington, Bloomington, Indiana, United States, 3. University of Pittsburgh, Pittsburgh, Pennsylvania, United States, 4. Johns Hopkins Bloomberg School of Public Health, Baltimore, Maryland, United States, 5. AdventHealth, Orlando, Florida, United States, 6. School of Public Health, University of Pittsburgh, Oakmont, Pennsylvania, United States, 7. Wake Forest School of Medicine, Winston-Salem, North Carolina, United States, 8. School of Public Health, University of Pittsburgh, Pittsburgh, Pennsylvania, United States*

The Pittsburgh Performance Fatigability Index (PPFI, higher=greater fatigability), a novel accelerometer-based performance fatigability measure, was recently developed for adults aged ≥60 years. We validated PPFI during a usual-paced 400m walk in 429 individuals enrolled in the Study of Muscle, Mobility and Aging (age=76.9±5.3years, 57.6% women, gait speed=1.0±0.2 m/s from 4-meter walk). PPFI quantifies percent of performance decrement (i.e., slowing down) during 400m walk by comparing area under the observed cadence trajectories to the hypothetical area that reflects sustaining maximum cadence for the entire walk. PPFI scores (mean=2.1%±2.5%, range: 0-21.7%) demonstrated convergent validity with Short Physical Performance Battery (SPPB, Pearson correlation (r)=-0.32) and mobility (time to walk 400m, r=0.47). PPFI scores discriminated higher versus lower physical function (SPPB: 1.6% (≥10) vs. 2.9% (<10), p<.001), adjusted for age, sex, height, and weight. PPFI is the first validated accelerometer-based objective risk assessment tool for measuring performance fatigability, an established marker of functional decline.

### ASSOCIATION BETWEEN AEROBIC CAPACITY AND PERCEIVED FATIGABILITY: THE STUDY OF MUSCLE, MOBILITY, AND AGING (SOMMA)

Reagan Moffit[1], Daniel Forman[1], Paul Coen[2], barbara Nicklas[3], Kyle Moored[4], Yujia (Susanna) Qiao[5], Peggy Cawthon[6], and Nancy W. Glynn[7], *1. University of Pittsburgh, Pittsburgh, Pennsylvania, United States, 2. AdventHealth, Orlando, Florida, United States, 3. Wake Forest School of Medicine, Winston-Salem, North Carolina, United States, 4. Johns Hopkins Bloomberg School of Public Health, Baltimore, Maryland, United States, 5. University of Pittsburgh, Schoold of Public Health, Pittsburgh, Pennsylvania, United States, 6. California Pacific Medical Center Research Institute, San Francisco, California, United States, 7. School of Public Health, University of Pittsburgh, Pittsburgh, Pennsylvania, United States*

Peak aerobic capacity declines with age concomitant with greater fatigability and slower gait speed. We explored these relationships with cross-sectional SOMMA data (N=422, age=76.7±5.1, 57.4% women, gait speed= 0.97±0.18 m/s from a 4m walk). Participants completed a treadmill peak oxygen consumption test (VO2peak) and the self-administered Pittsburgh Fatigability Scale (PFS, range: 0-50; higher=greater fatigability). Mean VO2peak was 19.4±4.2 ml/kg/min, PFS Physical score was 16.9±8.5 points, and PFS Mental score was 8.1±8.2 points. Pearson correlations between VO2peak and PFS Physical and Mental scores were r=-0.36 and r=-0.23, respectively. For every one standard deviation higher VO2peak, PFS Physical and Mental scores were lower by 2.07 points (CI: -3.00, -1.13) and 1.10 points (CI: -2.07, -0.17), respectively, when adjusted for clinic site, age, race, sex, and self-reported physical activity. Future SOMMA analyses will evaluate a likely bidirectional association between VO2peak and fatigability, as well as examine their potential mediating effects on physical function.

### DUAL ROLES OF FITNESS AND FATIGABILITY IN THE LIFE-SPACE MOBILITY OF OLDER ADULTS

Kyle Moored[1], Yujia (Susanna) Qiao[2], Andrea Rosso[3], Frederico Toledo[3], Steven R. Cummings[4], Bret Goodpaster[5], Stephen Kritchevsky[6], and Nancy W. Glynn[7], *1. Johns Hopkins Bloomberg School of Public Health, Baltimore, Maryland, United States, 2. University of Pittsburgh, Schoold of Public Health, Pittsburgh, Pennsylvania, United States, 3. University of Pittsburgh, Pittsburgh, Pennsylvania, United States, 4. San Francisco Coordinating Center, San Francisco, California, United States, 5. AdventHealth, Orlando, Florida, United States, 6. Wake Forest School of Medicine, Winston-Salem, North Carolina, United States, 7. School of Public Health, University of Pittsburgh, Pittsburgh, Pennsylvania, United States*

Objective fitness and perceived fatigability may interact to limit mobility within the larger environment (life-space mobility). We assessed this cross-sectionally in SOMMA (N=371, mean age=76.4±5.0). Life Space Assessment scores (range: 0-120, 5-point difference=clinically relevant, e.g., going outside additional 1-3 days/week) incorporated level, frequency, and assistance used for life-space mobility (Mean=82.7±18.8). Fitness was measured as VO2peak (Mean=19.5±4.2 mL/kg/min) from a symptom-limited treadmill test. Higher fatigability was defined as a Borg Rating of Perceived Exertion (RPE, range: 6-20) ≥10 after a five-minute steady-state treadmill test. Each 1-SD higher VO2peak was associated with a 2.2-point higher life-space score (95% CI: 0.25-4.07, p=.027). After adjustment for demographic, lifestyle, and health confounders, the association between fitness and life-space mobility was significant only for those with higher fatigability (RPE≥10: B=5.70, 95% CI: 0.79-10.60, p-interaction=.008). Older adults with both lower fitness (objective capacity) and higher fatigability (perceived capacity) may be at greatest risk of reduced real-world mobility.

### ENERGY METABOLISM RELATED CANDIDATE GENE ASSOCIATION STUDY OF PERCEIVED PHYSICAL FATIGABILITY

Emma Gay[1], Adam Santanasto[2], Ryan Cvejkus[3], Mary Wojczynski[4], Mary Feitosa[5], and Nancy W. Glynn[6], *1. University of Pittsburgh School of Public Health, Pittsburgh, Pennsylvania, United States, 2. School of Public Health, University of Pittsburgh, Oakmont, Pennsylvania, United States, 3. University of Pittsburgh, Pittsburgh, Pennsylvania, United States, 4. Washington University, Saint Louis, Missouri, United States, 5. Washington University, St Louis, Missouri, United States, 6. School of Public Health, University of Pittsburgh, Pittsburgh, Pennsylvania, United States*

Mitochondrial energy production decreases while fatigability increases with age. Genes associated with energy metabolism may contribute to fatigability. Using Long Life Family Study (LLFS), we initially assessed variants (SNPs) in 272 candidate autosomal genes involved in energy metabolism (previously associated with mitochondrial dysfunction disease) with perceived physical fatigability. Two generations of LLFS enrollees (N=2342, mean age=73.7, range 60-108 years) completed the Pittsburgh Fatigability Scale (PFS, 0-50, higher=greater fatigability) at Visit 2 (2014-2017). Physical fatigability prevalence was 42.1% (PFS≥15). Generalized linear mixed models assessed the association of each SNP with continuous PFS (GENESIS R package) adjusted for age, sex, field center, and family relatedness. We found no associations with perceived physical fatigability, all p>2.5E-7 (Bonferroni multiple comparison corrected p-value). Next steps will examine variants in the mitochondrial genome and BTBD3, another promising candidate gene recently discovered. Genetic biomarker(s) may identify individuals susceptible to greater fatigability to target for early intervention.

### PERCEIVED FATIGABILITY AND MORTALITY IN MID-TO-LATE LIFE IN THE BALTIMORE LONGITUDINAL STUDY OF AGING

Amal Wanigatunga[1], Xiaomeng Chen[2], Francesca Marino[3], Eleanor Simonsick[4], Luigi Ferrucci[4], and Jennifer Schrack[1], *1. Johns Hopkins Bloomberg School of Public Health, Baltimore, Maryland, United States, 2. Johns Hopkins School of Medicine, Baltimore, Maryland, United States, 3. Johns Hopkins Bloomberg School of Public Health, Washington, District of Columbia, United States, 4. National Institute on Aging, Baltimore, Maryland, United States*

This study hypothesized that perceived fatigability, fatigue standardized to walking, would better discriminate mortality risk than general fatigue symptoms. We leveraged perceived fatigability, self-reported tiredness, self-reported low energy and mortality data from 1,112 participants aged ≥50 years. Hazard ratios (HRs) of all-cause mortality (198 [17.8%] events) by either z-scored fatigability, tiredness, and energy level were estimated using Cox proportional hazards models. Model discrimination was compared using Harrell's C-statistics (C). After full covariate adjustment, 1 SD higher perceived fatigability, tiredness and low energy level were associated with higher mortality risk (HR=1.20, 95% confidence interval [CI]:1.05-1.37; HR=1.28, 95% CI:1.11-1.48; HR=1.26, 95% CI:1.10-1.45, respectively). The model for perceived fatigability (C=0.8039) had comparable discrimination to tiredness (C=0.8085,p=0.26) and low energy level (C=0.8067,p=0.41). While perceived fatigability was associated with higher mortality risk, it did not exhibit superior ability to discriminate mortality risk than self-reported fatigue symptoms among persons in mid-to-late life.

### SESSION 4610 (SYMPOSIUM)

### PRESIDENTIAL SYMPOSIUM: BUILDING AND LEVERAGING COMMUNITY PARTNERSHIPS THAT EMBRACE DIVERSITY, ENRICH DISCOVERY, AND REIMAGINE AGING

Chair: Kalisha Bonds Johnson Co-Chair: Brianna Morgan Discussant: Katherine Abbott

Are you an ESPO member or early careerist who is interested in creating meaningful and engaging partnerships with different stakeholders? Want to learn how to leverage partnerships across diverse research settings and make impactful change? Or are you unsure of all the “hype” surrounding engaging communities and key stakeholders? If you answered “yes” to any of these questions, this symposium is for you. The ESPO Presidential Symposium will highlight three speakers who will share their career stories and a discussant with expertise in building and keeping important collaborations that influence aging research. Speakers include Drs. Christopher G. Engeland, Andrea Gilmore-Bykovskyi, and Martina Roes. Dr. Katherine Abbott will serve as the discussant. Starting at the basic science end of the research spectrum, Dr. Engeland will focus on communicating and collaborating at the intersections between research settings to carry science from the bench to the bedside. Next, moving to research that focuses on aging older adults in clinical research, Dr. Gilmore-Bykovskyi will discuss how she has established, maintained, and evolved her career in Alzheimer’s disease and related dementias across diverse research paradigms. Lastly, Dr. Roes will provide and international perspective and shares how she builds trust with the co-creation of research with persons living with dementia and their significant others.

### PROS AND CONS OF PERFORMING RESEARCH AT AN INTERSECTION OF MULTIPLE DISCIPLINES

Christopher Engeland, *Pennsylvania State University, University Park, Pennsylvania, United States*

In the last few decades, team science has become increasingly common and the need for interdisciplinary researchers has likewise increased. There is a stronger need than ever for individuals who can speak across disciplines, serving as a hub (and sometimes a translator) to unite researchers in different fields. Researchers with diverse methods (from those using animal models and bench-side science to biobehavioral observations and clinical approaches) all have a strong need for understanding each other’s work and to express it clearly to others. The ability to function and communicate at multiple intersections is needed to optimize the interpretation and understanding of scientific findings, along with their translation to clinical practice, implementation to intervention, and dissemination to the public. Performing research at these complex intersections of fields and roles is both challenging and rewarding. The pros and cons of a research career situated at these intersections will be discussed.

### ENGAGED RESEARCH: HUMILITY, LISTENING, BETTER QUESTIONS, BETTER ANSWERS

Andrea Gilmore-Bykovskyi, *University of Wisconsin-Madison, Madison, Wisconsin, United States*

The expansive field of aging science comprises diverse disciplines, perspectives, and approaches to address questions that extend from strengthening our understanding of biological aging to promoting wellbeing among diverse aging populations. Engaged research practices represent a cross-cutting series of diverse epistemological beliefs and pragmatic practices in the conduct of research with broad relevance to areas of inquiry across the translational aging research continuum. This presentation will describe experiences with the establishment, maintenance, and continual evolution of engaged research practices in an interdisciplinary program of health services research focused on Alzheimer’s disease and related dementias (AD/ADRD). Specifically, this presentation will discuss: a) conceptualizations of engagement and its relationship to equitable research, b) engagement as praxis for the conduct of equitable research, c) personal reflections on experiences with and the practice of humility, listening, and valuing stakeholders’ distinctive expertise, and d) practical recommendations for early career scholars approaching engaged research.

### ENGAGING AND INVOLVING PEOPLE LIVING WITH DEMENTIA IN RESEARCH: WHAT TO (NOT) EXPECT

Martina Roes, *German Center for Neurodegenerative Diseases (DZNE), Witten, Nordrhein-Westfalen, Germany*

Collaborating across disciplines, professions, and countries as well as engaging a variety of stakeholders has been a goal for researchers for a long time. Interestingly, when people living with dementia and their significant others are actively involved and become a co-researcher or a member of a patient advisory board, the perspective of what collaboration and/or engagement and involvement means, becomes blurry. The intersection between the individual reality of people living with dementia and researchers can be understood as (a) a door that opens up and leads to new (multiple) perspectives and understandings or (b) a disaster because misconceptions of dementia hinder mutual trust building. This presentation will provide examples on how to build trust and how sustain a relationship over the course of time (and progression of the disease). Since living with dementia is highly stigmatized this presentation will also reflect on the impact of misconceptions of dementia on engagement.

### SESSION 4620 (SYMPOSIUM)

### PSYCHOSOCIAL AND BEHAVIORAL PREDICTORS OF HEALTH AND AGING

Chair: Eileen Graham Co-Chair: Nicholas Turiano

Crucial to understanding the aging process is to identify modifiable characteristics that can be targeted in hope of optimizing health across adulthood. This symposium brings together a diverse set of talks about the biopsychosocial and behavioral processes that influence aging outcomes. Turiano used a national sample to demonstrate that individuals with higher levels of neuroticism often use food to cope with stressors which ultimately results in a greater central adiposity and elevated glycated hemoglobin. Zavala used an epidemiological sample of long-banked frozen blood samples to successfully extract DNA and methylation biomarkers from 140 adults and found that stress measures are associated with accelerated biological aging. Luo used coordinated analysis to explore how correlations between personality traits and four health outcomes fluctuate over time, and found the associations between personality (level/change) and health increased in strength through middle adulthood and early stage of late adulthood but weakened in very old age. Jackson found that individuals who experienced high and increasing loneliness in older adulthood had worse cognitive function and steeper cognitive decline than expected given their observed post-mortem neuropathology (i.e., worse cognitive resilience). Lastly, Pfund investigated how personality and social support change together and found within and between person associations between personality and social support, and evidence that retirement was associated with an increase in social support. In sum, this symposium presents novel evidence for the associations among personality, stress, social support, and lonliness are associated with physical and cognitive health in old age.

### PERSONALITY TRAITS PREDICT DIABETES RISK IN ADULTHOOD: THE MEDIATING EFFECTS OF USING FOOD TO COPE WITH STRESS

Nicholas Turiano[1], Jacob Alderson[1], Meredith Willard[1], Sina King[1], and Páraic Ó Súilleabháin[2], *1. West Virginia University, Morgantown, West Virginia, United States, 2. University of Limerick, Limerick, Limerick, Ireland*

Identifying the individual characteristics that predict which adults will develop obesity and diabetes is crucial. This study included national data from 902 participants (aged 25-75) in the Midlife Development in the U.S. (MIDUS) study. Participants completed the Big-5 personality trait measure in 1995-1996, and behavior/health variables between 2004-2009. We tested whether levels of certain personality traits would predict an elevated risk of diabetes via hemoglobin A1c (HbA1c) levels through eating behaviors. A structural equation modeling framework demonstrated good fit when testing indirect effects (CFI = 0.95; RMSEA = 0.05). Indirect effects revealed that higher levels of neuroticism predicted greater waist circumference and higher HbA1c levels due to an increased use of food to cope with problems (IE =0.10; $p<0.05$). Moreover, indirect effects were found for conscientiousness, albeit in a protective direction. Our findings suggest that personality traits may be an early predictor of behavior and thus long-term adverse health outcomes.

### APPLYING NEW METHODS TO EXISTING DATASETS TO TEST STRESS EFFECTS ON BIOLOGICAL AGING

Daisy Zavala[1], Natalie Dzikowski[2], Shyamie Gopalan[3], Kerry Reid[4], Jennifer Graham-Engeland[5], Christopher Engeland[6], Krishna Veeramah[4], and Stacey Scott[2], *1. Stony Brook University, Queens, New York, United States, 2. Stony Brook University, Stony Brook, New York, United States, 3. Duke University, Durham, North Carolina, United States, 4. Stony brook University, Stony Brook, New York, United States, 5. Pennsylvania State University, Pennsylvania State University, Pennsylvania, United States, 6. Pennsylvania State University, University Park, Pennsylvania, United States*

Novel opportunities to examine how life experiences connect to health and mortality through biological aging have increased due to the affordability of genomic analysis. Although epidemiological studies suggest associations between stress and accelerated biological aging, most if not all measures in the past have been global, data cross-sectional, and from primarily white samples. As part of a completed measurement study, a diverse sample (n=140, 25-65 years, Mage=47, 65% Female) provided detailed stress measures, including EMA and lifetime stress assessments, and consented to future analysis of their blood samples. We successfully used decade-old blood samples to estimate methylation-based epigenetic clocks and examined them within the context of various stress measures as a preliminary phase of a future longitudinal study. Additional whole genome sequencing will also allow for future work that incorporate continuous measures of genomic ancestry when examining lifetime discrimination-related stress, rather than simply testing for demographic group differences.

### DOES PERSONALITY ALWAYS MATTER? EXAMINING THE MODERATING EFFECT OF AGE ON THE PERSONALITY-HEALTH LINK

Jing Luo[1], Bo Zhang[2], Eileen Graham[1], and Daniel Mroczek[1], *1. Northwestern University, Chicago, Illinois, United States, 2. Texas A&M University, College Station, Texas, United States*

The current research examined how the associations between the level and changes in the Big Five personality traits and different types of health outcomes (self-rated, physical, cognitive, and physiological health outcomes) differ across ages over the lifespan (Sample 1, age range: 15-100) and during the aging process (Sample 2, age range: 50-109) in particular. Using data from the two large longitudinal studies, we observed three important patterns based on the results. First, levels and changes in personality traits demonstrated substantial effects on health across different life phases, with the effects observed even in very old ages. Second, overall, the prospective relations between personality traits/changes in personality traits and health outcomes increased in strength in mid adulthood and/or early stages of late adulthood; however, the strength of their connections diminished in very old ages. Finally, there were some trait-specific and health outcome-specific patterns in the age-differential associations between personality and health.

### ASSOCIATIONS BETWEEN LONELINESS AND COGNITIVE RESILIENCE TO NEUROPATHY IN OLDER ADULTS

Kathryn Jackson[1], Emily Wilroth[1], Jing Luo[1], Bryan James[2], Anthony Ong[3], David Bennett[2], Daniel Mroczek[1], and Eileen Graham[1], *1. Northwestern University, Chicago, Illinois, United States, 2. Rush University, Chicago, Illinois, United States, 3. Cornell University, Ithaca, New York, United States*

Loneliness in the aging population is a concern, as increased loneliness is associated with decreased cognitive function and increased neuropathology. Less is understood about the relationship between loneliness and cognitive resilience. Cognitive resilience is defined as the discordance between a person's actual and expected cognition given their neuropathology and can be estimated by extracting residuals from a model regressing cognition on neuropathology. Using data from two longitudinal aging cohorts (MAP/MARS), we estimated cognitive resilience proximate to death and cognitive resilience over time to use as the key outcomes. We then regressed these two cognitive resilience indicators onto loneliness level and slope. Higher baseline loneliness and increasing loneliness over time were both associated with lower cognitive resilience. Our results suggest that loneliness should be included into resilience-based prevention models, and interventions aimed at optimizing cognitive function across older adulthood should include loneliness reduction as a potential area of focus.

### CHANGES IN PERSONALITY TRAITS AND SOCIAL SUPPORT BEFORE AND AFTER RETIREMENT

Gabrielle Pfund[1], Patrick Hill[2], Mathias Allemand[3], Marie Kivi[4], Anne Ingeborg Berg[4], Valgeier Thorvaldsson[4], and Isabelle Hansson[4], *1. Northwestern University, Chicago, Illinois, United States, 2. Washington University in St. Louis, St. Louis, Missouri, United States, 3. University of Zurich, Zurich, Zurich, Switzerland, 4. University of Gothenburg, Gothenburg, Vastra Gotaland, Sweden*

Aging is tied to transitions in occupational, social, and personal contexts, which can have implications for changes in who one is and how connected they feel to others. The current study uses data from 5,844 older adults (Ages: 60-66) with six annual reports on the Big Five personality traits and three distinct social support types (family, friends, relationships) to investigate how personality and social support change together, and the role retirement plays in these changes. Random intercept-cross lagged panel models were conducted to evaluate the associated changes in each trait and social support type while accounting for the time-varying covariate of retirement status. Higher social support across all relationship types was associated with higher agreeableness, extraversion, and conscientiousness and lower neuroticism both between- and within-person. Furthermore, entering retirement predicted an increase in social support with friends and relationship partners, but no consistent change in any of the personality traits.

### SESSION 4630 (PAPER)

### SUICIDE

### ASSESSING THE PSYCHOMETRIC PROPERTIES OF THE GERIATRIC SUICIDE IDEATION SCALE (GSIS) IN MIDDLE-AGED AND OLDER MEN

Marnin Heisel[1], and Gordon Flett[2], *1. The University of Western Ontario, London, Ontario, Canada, 2. York University, Toronto, Ontario, Canada*

Middle-aged and older men have high rates of suicide, necessitating focused risk detection. We developed the Geriatric Suicide Ideation Scale (GSIS; Heisel & Flett, 2006) as an age-specific, multidimensional suicide risk assessment tool. The GSIS has shown strong psychometric properties in clinical, community, and residential samples (see Heisel & Flett, 2016), yet research has lagged investigating its utility with middle-aged and older men. The purpose of the present study was thus to assess the psychometric properties of the GSIS administered to 82 men, 55 years and older (M=63.3, SD=4.6 years), who participated in a meaning-centered psychological intervention group for those concerned about or struggling with the transition to retirement (Heisel et al., 2020). Psychometric analyses included investigation of participant response characteristics, internal consistency, and construct validity. Findings demonstrated acceptable internal consistency for GSIS totals ($\alpha$ =.88) and for its Suicide Ideation, Death Ideation, Loss of Personal and Social Worth, and Perceived Meaning in Life subscales ($\alpha$ =.62-.81). Positive associations between the GSIS and negative psychological factors (depression, anxiety, hopelessness, loneliness, perceived lack of mattering to others, and history of suicidal behavior; r =.30 to .51) and negative associations with positive factors (life satisfaction, psychological well-being, perceived support, and meaning in life; r = -.21 to -.51) supported its construct validity. These and other findings will be discussed in the broader context of upstream population level approaches to suicide risk detection and prevention.

### IS NARCISSISTIC PERSONALITY ASSOCIATED WITH SUICIDE IDEATION IN MIDDLE-AGED AND OLDER MEN?

Marnin Heisel[1], Gordon Flett[2], and Paul Links[3], *1. The University of Western Ontario, London, Ontario, Canada, 2. York University, Toronto, Ontario, Canada, 3. McMaster University, Hamilton, Ontario, Canada*

Older adults have high rates of suicide, necessitating theory and research on factors that contribute to suicide risk and may be targets for intervention. Clark (1993) theorized that age-related losses and transitions can trigger narcissistic injury in older men, and lead to mood decline, substance misuse, loss of insight, and suicide ideation and behavior. We tested elements of Clark's model in a secondary analysis of a geriatric depression clinic database (n=574), and reported a positive association between clinician-diagnosed Narcissistic Personality (NP) and suicide ideation and behavior, controlling for depression severity (Heisel et al., 2007). We sought to replicate and extend these findings in 82 community-residing men, 55 years and older (M=63.3, SD=4.6), who participated in a trial of Meaning-Centered Men's Group (MCMG; Heisel et al., 2020) for those concerned about or struggling with the transition to retirement. Participants completed the Pathological Narcissism Inventory (Pincus et al., 2009), Geriatric Suicide Ideation Scale (Heisel & Flett, 2006), and Geriatric Depression Scale (Yesavage et al., 1983). Linear regression analyses indicated a significant association between PNI-Contingent Self-Esteem and GSIS totals (t=2.41, p=.019) and GSIS Loss of Personal Worth (t=2.10, p=.040) and Perceived Meaning in Life Subscales (t=2.80, p=.007), controlling for depressive symptom severity (Geriatric Depression Scale; Yesavage et al., 1983), suggesting an association between suicide ideation and self-esteem issues. These and other findings will be discussed in a broader thematic context regarding masculinity, loss, life transitions, and suicide prevention.

### RETIREMENT TRANSITIONS AND COMPLETED SUICIDE DURING RECOVERY FROM THE GREAT RECESSION: EVIDENCE FROM THE NVDRS

Briana Mezuk[1], Aparna Ananthasubramaniam[2], Linh Dang[2], and David Jurgens[2], *1. University of Michigan, School*

*of Public Health, Ann Arbor, Michigan, United States, 2. University of Michigan, Ann Arbor, Michigan, United States*

One million older Americans retire annually. While these transitions are not generally associated with poor mental health, the broader macro-economic context in which retirement transitions take place may shape how they relate to mental health. The objective of this study was to use state-of-the-art natural language processing (NLP) to develop a model to identify retirement transitions from textual data in the National Violent Death Reporting System (NVDRS), and to use that model to examine how the number of suicides related to retirement transitions changed during the recovery from the Great Recession. Data come from the NVDRS (2003 - 2018, n=62,165), a state-based registry of suicide deaths. We used RoBERTa to train a NLP model to identify retirement transitions (e.g., recent retirement, anticipated retirement, unable to retire despite wanting to) from 1,291 annotated sentences from NVDRS text narratives of suicide decedents aged ≥55 (model performance: F1=0.92). Applying this model, 19.35 of every 1,000 suicides among decedents aged ≥55 years mentioned a retirement transition. Decedent characteristics associated with retirement transitions were younger age (< 75 years), having a college education and experiencing financial problems. The probability that a narrative referenced a retirement transition increased 1.495-fold during the Great Recession (2007 - 2009) and declined during recovery (2009-2016) before beginning to increase again. Findings illustrate the utility of NLP methods to identify workforce transitions from NVDRS narratives, and further understanding the impact of macro contextual events like the Great Recession on population mental health.

### SESSION 4640 (PAPER)

### TECHNOLOGY APPLICATIONS

### FACTORS ASSOCIATED WITH ELECTRONIC WEARABLE DEVICE USE AMONG US OLDER ADULTS

Mengchi Li[1], Qiwei Li[2], Chakra Budhathoki[3], Sarah Szanton[4], and Junxin LI[3], *1. Johns Hopkins, University, Baltimore, Maryland, United States, 2. Johns Hopkins School of Nursing, Baltimore, Maryland, United States, 3. Johns Hopkins University, Baltimore, Maryland, United States, 4. Johns Hopkins University School of Nursing, Baltimore, Maryland, United States*

Electronic wearable devices(EWD) have been increasingly used in the adult population. Although health benefits of using EWDs may be appealing through health self-monitoring(e.g., physical activity, heart rate, and sleep), safety alerts(e.g., fall risk detection), or stress reduction(breathing technique), there is a lack of understanding in EWD use among older adults. This cross-sectional study used self-reported data of 3370 older adults aged 65 to 104 years from the Health Information National Trends Survey 2019 and 2020. We examined the prevalence of EWD use and used Logistic Regression Models to identify social factors (gender, race/ethnicity, education, and income) associated with the use of EWDs in older Americans. We found that EWD users increased from 13.3% in 2019 to 17.4% in 2020 in older Americans. After adjusting for age, insurance, smoking, marital-status, chronic conditions, region, and all other social factors of interest, we found that older men(Odds Ratio[OR]= 0.58, 95%Confidence Interval[CI]:0.45, 0.73), Black/African American older adults(OR=1.50, 95%CI: 0.41, 0.68), older adults who did not attend college(OR=0.62, 95%CI: 0.46, 0.83), and those with household income lower than $35,000(OR=0.32, 95%CI: 0.24, 0.46) were less likely to use EWDs. Interestingly, before adjustment, EWD use in Black/African American older adults(OR: 1.02, 95%CI:0.74, 1.34) did not differ from that in White/Caucasian older adults. Our findings suggest inequality of EWDs use in older Americans: those with lower education and income had less access to EWDs and fewer potential health benefits from EWDs. This study highlights the needs of equal access to EWDs and addressing social inequalities among older Americans.

### IS ONLINE ACCESS AND ACTIVITY ASSOCIATED WITH BETTER MOOD IN OLDER ADULTS LIVING ALONE DURING THE COVID-19 PANDEMIC?

Yael Koren[1], and Suzanne Leveille[2], *1. University of Massachusetts Boston, Brookline, Massachusetts, United States, 2. University of Massachusetts Boston, Boston, Massachusetts, United States*

Older adults living alone during the COVID-19 pandemic face risks for social isolation and mental health problems. The goal of this study was to investigate the relationship between access to internet-capable devices or utilizing email/texting, social networking [SN], or health-related internet use [HIU] and psychological outcomes (depressive and anxiety symptoms) among older adults living in the community during the early months of the pandemic, March, 2020-May, 2020. A cross-sectional analysis of the National Health and Aging Trends Study (NHATS, Round 10) was conducted, using multivariable logistic regression. Adults aged ≥85y were more likely to live alone than those aged 65-84y (34.3% and 24.9% respectively, p< 0.001). Additionally, 39.6% of women lived alone compared with 21.2% of men (p< 0.001). Fewer people living alone had access to an internet-enabled device than those living with others (10.4% and 5.4%, respectively). Among older adults living alone, utilizing SN and HIU was associated with lower likelihood of depression or anxiety (Depression: adj.OR 0.36, CI 0.17-0.77; Anxiety: adj.OR 0.31 CI 0.13-0.76). Alternatively, among those living with others, using email/ texting was associated with lower likelihood of depression and anxiety (Depression: adj.OR 0.23, CI 0.13-0.46, Anxiety: 0.33 CI, 0.17-0.67). In summary, internet activity was associated with reduced risk for depression and anxiety among older adults who lived alone or with others early in the pandemic. Further research is needed to better understand the potentially mitigating role of internet activities among older adults at risk for isolation and associated mood disturbances.

### PATIENT PERCEPTIONS OF USING VOICE-BASED DIETARY ASSESSMENT TOOLS AMONG OLDER ADULTS

Tiffany Driesse[1], Xiaohui Liang[2], Michael Fowler[3], Jing Yuan[2], Hillary Spangler[3], David Lynch[4], and John Batsis[3], *1. UNC School of Medicine, University of North Carolina at Chapel Hill, Chapel Hill, North Carolina, United States, 2. University of Massachusetts*

*Boston, Boston, Massachusetts, United States, 3. University of North Carolina at Chapel Hill, Chapel Hill, North Carolina, United States, 4. UNC, Chapel Hill, North Carolina, United States*

Poor diet among older adults is a risk factor for developing multiple chronic diseases. Dietary recall comprises an important component in intervention research and clinical care. Commonly used tools include the web-based automated self-administered 24-hour assessment (ASA-24). Yet voice assistant (VAS) systems (i.e., Amazon Alexa) have not been developed for this purpose. Hence, we evaluated patient perceptions on performing a VAS-based dietary assessment among older adults. Community-dwelling adults (age 65+ years) participated in two virtual sessions who reported their past 24-hour intake, first using ASA-24, and then using a VAS. All completed a Likert questionnaire (binary, % strongly agree/strongly disagree reported) regarding the simplicity of using both systems, completion time, and user satisfaction. Semi-structured interviews allowed us to ask about technology use. Of the 40 participants (100% enrolled), mean age was 69±1.0 years (85% female, 100% white, 5% Latinx). Only 40% owned a VAS; 60% reported having VAS experience prior to the study. After completing both sessions, 80% preferred a VAS over the ASA-24. Participants reported that web-based recalls were unnecessarily complex (60%), time-consuming (50%), and 60% did not wish to use them. Comparatively, VAS recalls were intuitive (75%), easily reportable (85%), and there was willingness to report food while preparing meals (85%). In 16 participants, we evaluated themes of VAS use including easier navigation, less time, and ability to have a natural conversation. A VAS provides a more convenient, conversational, and computerless interaction to report meals over web-based solutions suggesting they hold promise for dietary recall in older adults.

### PATIENT PORTAL USE WITHIN OLDER ADULTS' FAMILY CAREGIVING NETWORKS

Julia Burgdorf[1], Chanee Fabius[2], and Jennifer Wolff[2], *1. Center for Home Care Policy & Research, VNS Health, New York, New York, United States, 2. Johns Hopkins Bloomberg School of Public Health, Baltimore, Maryland, United States*

The patient portal (hereafter, "portal") is an online platform allowing patients to view health information, perform health management tasks, and directly message clinicians. Millions of older adults co-manage their health care with family caregivers, yet little is known about the extent to which caregivers use the portal alongside or on behalf of patients. We examined linked data from the 2017 National Health and Aging Trends Study (NHATS), National Study of Caregiving (NSOC), and American Community Survey for 1,675 (weighted n=8,511,568) older adults with disability. We measure older adult and caregivers' respective use of the older adult's portal from NHATS and NSOC. We use weighted, multivariable, ordered logistic regression to model transition from older adult using the portal alone, to co-using the portal with caregivers, to a caregiver using the portal on the older adult's behalf. We find that 9% of older adults use the portal on their own, 2% co-use the portal with a caregiver, and 15% have a caregiver who uses the portal on their behalf (74% have no portal use). In adjusted models, older adults had higher odds of a caregiver using the portal on their behalf (compared to the older adult using the portal alone or in tandem with a caregiver) if they were older (aOR: 1.12; p< 0.001), female (aOR: 2.08; p< 0.01), had probable dementia (aOR: 7.04; p< 0.001), were homebound (aOR: 3.78; p< 0.01), attended doctors' visits with a caregiver (aOR: 3.10; p< 0.01), or lived in a census tract with low broadband accessibility (aOR: 5.76; p< 0.01).

### SESSION 4650 (SYMPOSIUM)

### THE LANDSCAPE OF ALZHEIMER'S DISEASE AND RELATED DEMENTIAS CAREGIVER COMMUNICATION RESEARCH

Chair: Debra Dobbs Co-Chair: Jessica Yauk Discussant: Harleah Buck

Alzheimer's Disease and Related Dementias (ADRD) affect one in nine adults aged 65 and over. Past research has demonstrated the need for services that improve quality of care for those with ADRD and their caregivers. Those with ADRD are at a greater risk for negative health outcomes. Therefore, it is crucial that those with ADRD receive care consistent with their preferences. However, more research is needed to shed light on effective tools available and patterns of advance care planning (ACP) when it comes to those with ADRD and their caregivers. This symposium highlights the landscape of ACP communication among those with ADRD and their caregivers. The session begins with a study that explored experiences of apathy and preferences for dyadic-communication via mobile health (mHealth) among adults with mild cognitive impairment and their care-partners. The second presentation demonstrates the feasibility and effectiveness of a palliative care education in assisted living program for nurses and administrators on increasing ACP discussions with family caregivers of residents with dementia. The third presentation investigates ACP among African Americans with ADRD and their caregivers and the critical role the community can play in facilitating communication. The final presentation explores how ACP differs by race, ethnicity and ADRD status using data from the 2018 Wave of the Health and Retirement Study. Future directions for implementation of these innovations for improving ACP communication among caregivers and persons with ADRD will be discussed.

### EFFECTIVENESS OF A PC EDUCATION PROGRAM IN ASSISTED LIVING IN IMPROVING DISCUSSIONS WITH DEMENTIA FAMILY CAREGIVERS

Debra Dobbs[1], Jessica Yauk[1], Carlyn Vogel[1], Dany Fanfan[2], Harleah Buck[3], William Haley[1], and Hongdao Meng[1], *1. University of South Florida, Tampa, Florida, United States, 2. University of Florida, Gainesville, Florida, United States, 3. Csomay Center for Gerontological Excellence, Iowa City, Iowa, United States*

There is a need for improved communication in assisted living communities (ALCs) for staff and family caregivers of persons with dementia. This study is a cluster randomized trial in 10 ALCs (N=23 nurses and administrators; N= 122 residents with dementia) of a PC education (PCEAL)

program for dementia care providers facilitated by hospice nurse educators. Baseline, 3- and 6-month data was collected for documentation of advance care planning (ACP) discussions. The PCEAL program had a moderate effect on ACP discussions at the 3-month follow in the treatment (N=17) compared to the control group (N= 6) (Cohen's d = 0.72, 95% CI [0.25, 1.19]). The findings indicate that the PCEAL program potentially improves ACP discussions with family members of persons with dementia. We will discuss recommendations for tailoring the PCEAL program to include other direct and administrative staff and to elicit the perspective of families about quality of communication.

### DIFFERENCES IN ADVANCE CARE PLANNING AMONG RACIAL AND ETHNIC MINORITIES AND PERSONS WITH ADRD

Jessica Yauk, Hongdao Meng, and Debra Dobbs, *University of South Florida, Tampa, Florida, United States*

Advance care planning (ACP) is an important process to enable individuals to make their preferences known in the event of dementia onset. Racial and ethnic minorities have an increased risk of developing dementia. Understanding more about ACP differences by race and dementia status will help improve ACP communication among minority populations. For the current study the 2018 wave of the Health and Retirement Study (N=8,663) to test the relationship between dementia status and ACP and if there were differences by race. Findings indicated that both African Americans and Hispanics are less likely to have completed ACP than non-Hispanic Whites. In unadjusted models, having a dementia diagnosis indicated a lower likelihood of completing ACP; dementia was not significant in the final model when controlling for predisposing and enabling factors. More research on the predisposing and enabling factors that influence ACP among minority and dementia populations is needed.

### EXPERIENCES WITH APATHY AND MHEALTH PREFERENCES AMONG ADULTS WITH MILD COGNITIVE IMPAIRMENT

Pilar Ingle[1], Evan Plys[2], Danielle Kline[2], Emma Stark[2], and Jennifer Dickman Portz[3], *1. University of Denver, Lakewood, Colorado, United States, 2. University of Colorado School of Medicine, Aurora, Colorado, United States, 3. University of Colorado Anschutz, Aurora, Colorado, United States*

Apathy is common early in dementia, often increases in severity as illness progresses, and is one of the most pervasive neuropsychiatric symptoms associated with Alzheimer's Disease and related dementias (ADRDs). This study explored experiences of apathy and preferences for dyadic-communication via mobile health (mHealth) among adults with mild cognitive impairment (MCI) and their care-partners. Semi-structured interviews were conducted with 10 ADRD-related health providers (5 behavioral neurologists, 2 nurse practitioners, 2 geriatricians, 1 nurse, 1 social worker), 9 patients with MCI (5 male, mean age = 73), and 6 of their care-partners. Participants expressed interest in the development of mHealth dyadic communication tools targeting apathy symptom monitoring, dyadic understanding of apathy, spiritual and religious coping, sharing ADRD health information, and resources for addressing burdensome behavioral symptoms of MCI. This research provides preliminary insight to the development of apathy specific mHealth communication strategies that may improve patient and care-partner quality of life.

### EMBRACING COMMUNITY: ADVANCE CARE PLANNING FOR AFRICAN AMERICAN OLDER ADULTS LIVING WITH DEMENTIA

Karen Moss[1], Kathy Wright[2], Emika Miller[3], Kimberly Lawson[4], Karen Rose[1], Todd Monroe[5], and Celia Wills[5], *1. The Ohio State University, Columbus, Ohio, United States, 2. The Ohio State University College of Nursing, Columbus, Ohio, United States, 3. Ohio State University, Columbus, Ohio, United States, 4. African American Alzheimer's and Wellness Association, Westerville, Ohio, United States, 5. Ohio State University College of Nursing, Columbus, Ohio, United States*

Lower socioeconomic status African American older adults living with dementia and their family caregivers face unique advance care planning challenges, including achieving appropriate preference-consistent healthcare near the end of life. The purpose of this project within a larger multi-stakeholder study is to assess community stakeholder perspectives on the needs of African American older adults living with dementia and their family caregivers. Diverse community-based organization leaders (n=9) were interviewed and identified the following thematic needs: Reframing of Advance Care Planning, Clarification of Misinformation, and Expansion of Available Resources. Community leaders expressed strong desires to assist families with advance care planning. The use of creative, culturally-tailored, non-traditional approaches to this process offers innovative ways to promote advance care planning through community engagement. This will change the narrative on advance care planning from a cultural perspective while embracing diversity, enriching discovery, and reimaging aging in the community

### SESSION 4660 (SYMPOSIUM)

### THE LEGACY OF HELEN KIVNICK: THE IMPACT OF VITAL INVOLVEMENT AND CREATIVITIES ON WELL-BEING IN LATE LIFE

Chair: Carolyn Adams-Price

Helen Kivnick was an extraordinary woman whose body of work strongly influenced and continues to influence the field of gerontology. Her classic work on vital involvement in late life, some of which was conducted with Erik and Joan Erikson, could be considered the foundation of current perspectives on positive gerontology, and creativity and aging. The current symposium will include both personal and theoretical takes on the legacy of Kivnick's work, especially her interests in vital involvement and creativity. Kivnick emphasized the possibility for older adults to adapt to new situations, even in very late life, and while experiencing physical and cognitive decline. Two of the speakers will speak on their personal experiences with Kivnick, while two others will emphasize research and theory derived from Kivnick's work. Dr. Adams-Price will introduce the symposium by addressing the significance of vital involvement as a concept in aging, as well as the ways creativity can improve well-being in late life. Dr. Wyatt-Brown will describe her long friendship with Dr. Kivnick, including Kivnick's comments in their book with Ruth Karpen and Margaret Gullette. The Big Move, which describe Wyatt-Brown's life with her husband in continuing care. Dr. Chee will discuss her research on patients with dementia who participated in a story-telling intervention, which increased their meaningful engagement in their environment. Finally, Ms. Linda Davis will recall her long partnership with Kivnick and the results of the national study they conducted on the benefits of creativity interventions for at risk- older adults in HUD housing.

### VITAL INVOLVEMENT IN LATE LIFE: HELEN KIVNICK'S VITAL CONCEPT FOR GERONTOLOGY+

Carolyn Adams-Price, *Mississippi State University, Mississippi State, Mississippi, United States*

Carolyn Adams-Price will lead off the symposium with a discussion of Kivnick's impact on gerontology and the arts. Her various arts interests will be discussed, including her interests in choral and African music. Much of the talk will focus on the concept of vital involvement, why it was revolutionary, and how it connects to current research on positive gerontology, creativity, and adaptation in late life. Adams-Price will address in detail the benefits that participation in creative activities can impart to older adults, based on her own research on creative benefits, and will provide a framework for studying those benefits in the future.

### MY LONG CLOSE FRIENDSHIP WITH HELEN KIVNICK

Anne Wyatt-Brown, *University of Florida, Gainesville, Florida, United States*

Anne Wyatt-Brown will discuss meeting Dr. Kivnick at a conference at Case Western Reserve in the 1980s, organized by David Van Tassel, who was introducing academics to the field of Gerontology. Kivnick was invited to discuss her work with Erik Erikson. Wyatt-Brown will discuss her long friendship with Kivnick, which continued until Kivnick's death at age 70. Wyatt-Brown will also discuss Kivnick's chapter in their book The Big Move with Ruth Karpen and Margaret Gullette, where Kivnick comments with compassion and insight about the steps Wyatt-Brown took to become vitally involved in the eldercare community, where she lived with her seriously ill husband.

### THE ART, WELLNESS, AND VITAL INVOLVEMENT IN AGING PROGRAM: MY WORK WITH HELEN KIVNICK

Linda Davis, *Aging, Wellness and the Arts, INDIANAPOLIS, Indiana, United States*

Linda Davis will discuss her partnership with Helen Kivnick on The Art, Wellness and Vital Involvement in Aging (AWVIA) Program. AWVIA began in 2009 as an innovative approach to health programming in HUD Affordable Senior Housing, employing engaging creative activities to attempt to address the despair and loneliness often found in older adults who have low social engagement, due to health, mobility, or economic circumstances. In 2013, Davis teamed up with Helen Kivnick to develop the Vital Involvement Model, which employs creative activities to increase meaningful engagement with the world. Over 6 years, AWVIA was employed by 84 HUD Senior Housing communities across the U.S. Kivnick and Davis received funding from NEA for a quasi-experimental study on the participants. Information from more than 2,000 qualitative narratives suggests the program decreased isolation, increased self-reliance, generativity, and a sense of mastery in participants. Examples of the narratives will be shared.

### IT COULD BE ANYTHING YOU WANT!: VITAL INVOLVEMENT OF PERSONS WITH DEMENTIA IN CREATIVE GROUP STORYTELLING

Kyong Hee Chee, Seoyoun Kim, and Olga Gerhart, *Texas State University, San Marcos, Texas, United States*

The benefit of vital involvement (VI) in dementia care is largely unknown. The purpose of this study is to demonstrate VI elements in a creative group storytelling program for persons living with dementia (TimeSlips). After offering 6 weekly storytelling sessions for 4 small groups in a memory care community, we interviewed participants (n = 21), family members (n = 2), and care associates (n = 6) to obtain feedback on the program. Themes from narratives in the audiorecorded and transcribed interviews suggest that participants enjoyed the program, enacting their personal values, strengths, and interests, and incorporating their past and current experiences, as supported by their sociospatial environment (e.g., facilitator, co-participants, researchers, shared table). When paired with the VI practice, creative group storytelling has potential to magnify favorable outcomes for participants with dementia, who may express more fully their meaningful engagement with their inner (psychological) and external (social and physical) world.

### SESSION 4670 (SYMPOSIUM)

### USING ANOCRITICISM AS AN ANALYTICAL FRAMEWORK: PERSPECTIVES FROM THE HUMANITIES

Chair: Kate de Medeiros Co-Chair: Roberta Maierhofer

To date, Gender Studies and Age Studies (predominantly humanities-based approaches to studying old age) have mostly remained two separate areas of research despite the ontological and epistemological similarities of both fields. As Maierhofer points out, "[a]ge/[a]geing studies would not have been established as a field without the theoretical and methodological approaches established through feminist theory." Age studies research has largely focused on cultural representations (e.g., novels, films) and social manifestations of either age or gender but not both. Considering this limitation in research, this interdisciplinary panel discusses cultural and social intersections of age and gender from a theoretical as well as an empirical perspective. Taking Maierhofer's analytical approach of 'anocriticism' as a starting point, the contributions of this panel explore intersections of age and gender in society, culture, and cultural representations. The first paper traces the historical development of anocriticism, from its feminist origins to its current application in Age Studies scholarship. The second paper presents research from two studies which used anocriticsm as an analytical framework. The third reports findings from an analysis of two novels. Here, an anocritical lens was used to deconstruct heteronormative assumptions about gender and age. The final paper presents an anocritical, qualitative analysis of narratives from five older men with regarding their performance of masculinities and power. As the papers demonstrated, anocriticism as theoretical framework, allows for a multi-dimensional yet nuanced analysis of cultural and social intersections of age and gender.

### ANOCRITICISM: THEORIZING AND DISCOVERING INTERSECTIONS OF AGE AND GENDER

Roberta Maierhofer, *University of Graz / Center for Inter-American Studies , Graz, Steiermark, Austria*

This paper presents historical research on the theoretical foundations and application of 'anocriticism' as a method to trace portrayals of female aging in American literary texts to understand what it means to be "aged by culture" (Gullette 2004). Originally developed in the 1990s in context of American Studies as an analytical framework for the analysis of cultural representations of intersections of age and gender in literature, anocriticism brings together the feminist tradition of Elaine Showalter's (1985) "gynocriticism" – a study of women writers and of the history, styles, themes, genres and structures of writing by women – through analysis of narratives and Germaine Greer's (1992) use of "anophobia" (from Latin "anus" for "old woman") to describe the fear of old women. The paper presents examples of anocriticism in practice in age studies and gerontology and highlights its potential for future multidisciplinary and interdisciplinary application.

### ANOCRITICAL READINGS OF THE INTERSECTIONS OF AGE AND GENDER IN AMERICAN LITERATURE: A FEMINIST CRITIQUE

Nicole Haring, *University of Graz, Graz, Steiermark, Austria*

Intergenerational relationships have been present features in texts from the American literary canon that often problematizes the intersections of age and gender. This paper investigates how representations of intergenerationality provide the opportunity to explore the concepts of relationality and intersectionality through a feminist lens. Maierhofer's concept of anocriticism was used in in the analysis of Julia Alvarez' In the Time of the Butterflies (1994) and Yaa Gyasi's novel Homegoing (2016) to validate individual experiences of gendered ageing. This paper presents ways in which this particular intersection can be viewed as a potential site of resistance towards what it means to grow old as a woman. Placing literature in a cultural, social, and political context, traditional paradigms can be reconstructed and heteronormative assumptions of age and gender can be deconstructed by focusing on the individual narratives and their potential for resistance.

### ANOCRITICISM AND THE PERFORMANCE OF MASCULINITY IN OLDER MEN

Kate de Medeiros, *Miami University, Oxford, Ohio, United States*

While anocriticism is a feminist framework often applied in the context of literary gerontology, this paper uses an anocriticism approach to consider how hegemonic masculinities and old age reveal a separation between cultural stereotypes of age and chronological age. Narratives from 5 men aged 65 and over, who participated in a qualitative study on everyday attributions of depression, were analyzed using an anocriticism framework. Results revealed that participants both claimed and contested their chronological age as an identity marker. Of particular note were claims related to the stereotype of loss of vigor in later life, with each man making a clear point to affirm his virility and sexual prowess independent of other interview questions. Overall, findings point to an important way to read interview data through an anocritical lens, paying particular attention to ways that power are questions and performed in relation to age.

### SESSION 4680 (SYMPOSIUM)

### USING PHOTOVOICE TO EXPLORE THE STRUCTURED ENVIRONMENT AND ADVANCE POLICY IN MULTIPLE CONTEXTS

Chair: Melissa Hladek Discussant: Hae-Ra Han

Contextual factors, including social determinants of health, have gained recognition for greatly influencing aging trajectories and chronic disease progression. Although traditional qualitative research gives a rich understanding of the internal psychological experience, it is unable to assess socio-cultural and built environment aspects of life apart from the verbal descriptions from participants. This symposium examines the use of Photovoice as a tool to assess the socio-cultural and structural context surrounding the participant. This symposium will present a narrative review on the use of current Photovoice methodologies in dementia care; results from three studies using photovoice, one with participants with mild cognitive impairment and their caregivers to explore their perceptions of aging in their home, the second exploring the lived experience and structural barriers among older adults with pre-frailty or frailty awaiting kidney transplant, and the third exploring the older adult peer mentors' experience providing community-delivered hearing care. The talks will also address practical aspects of using photovoice such as institutional review board tips, logistical challenges, interview preparation and coding approaches. The fifth talk will describe Photovoice result dissemination strategies, including photo exhibits and stakeholder meetings, used to engage the community and advance policy to improve disparities. Photovoice as a research tool originally aimed to reinforce empowerment for those marginalized in society to describe their lived environment. This important aim is augmented in this symposium by improving researchers' understanding of context to design more impactful research.

### NARRATIVE LITERATURE REVIEW OF PHOTOVOICE STUDIES TO EXPLORE METHODOLOGY, ANALYSIS, AND THE DEMENTIA EXPERIENCE

Emerald Jenkins[1], Phatt Thaitrong[2], Janiece Taylor[1], jenni Seale Reiff[1], Grace Huynh[1], Melissa Hladek[1], and Sarah Szanton[3], *1. Johns Hopkins University, Baltimore, Maryland, United States, 2. University of Alabama, Tuscaloosa, Alabama, United States, 3. Johns Hopkins University School of Nursing, Baltimore, Maryland, United States*

Dementia is a serious public health concern requiring effective interventions to address older adults' unmet needs. Photovoice, an underutilized and effective communication method for persons with waning abilities, provides insight into lived experiences and diverse needs. The purpose of this study is to understand current methodologies, analysis, and participant insights from photovoice literature in persons with dementia. A narrative review was conducted through PubMed, CINAHL Plus, and PsycINFO. From eleven eligible studies, insights were categorized via the 5-level Hierarchy Model of Needs in Dementia incorporating the 3-level Blackfoot Native American belief. Synthesized findings suggest providing dyadic opportunities to: (1) spend time in the natural world, (2) link activities for healthy aging with a need for hard work, and (3) recognize the importance of social determinants of health/disease when focusing on brain health. This review fills the knowledge gap regarding the lived experience of persons with dementia using relevant photovoice methodology.

### USING PHOTOVOICE TO UNDERSTAND THE LIVED EXPERIENCE AND ENVIRONMENT OF OLDER ADULTS AWAITING KIDNEY TRANSPLANT

Melissa Hladek[1], Deborah Wilson[2], Meera Shanbhag[1], Katie Krasnansky[1], Jeremy Walston[1], Qian-Li Xue[1], Mara McAdams-DeMarco[3], and Sarah Szanton[2], *1. Johns Hopkins University, Baltimore, Maryland, United States, 2. Johns Hopkins University School of Nursing, Baltimore, Maryland, United States, 3. NYU Grossman School of Medicine and NYU Langone Health, Baltimore, Maryland, United States*

End-stage renal disease (ESRD) causes age-accelerating disease and disproportionally affects older and minority patients; the preferred treatment is kidney transplantation (KT). Rates of KT have risen 5-fold for older adults since 1990 but there is great heterogeneity in KT outcomes by frailty and waitlist status. The person-environment fit theory posits that improving a person's lived environment will facilitate optimal individual functioning. We recruited a balanced sample of 20 participants (mean age 60, range 35-82) with respect to their frail/ pre-frail and inactive/ active waitlist status to explore personal experience and the lived environment through the use of photovoice, gaining insight into the person-environment fit of those older adults awaiting KT. We will discuss IRB issues, recruitment and logistical tips with using Photovoice. This work will help to inform what vulnerable and marginalized older adults awaiting KT must be prepared for psychologically and physically, including modifications needed to their lived environments.

### PHOTOVOICE STUDY TO EXPLORE THE PHYSICAL DISABILITY EXPERIENCE IN OLDER ADULTS WITH MCI/EARLY DEMENTIA

Emerald Jenkins[1], Janiece Taylor[1], Allyson Evelyn-Gustave[1], Erika Hornstein[1], Jennifer Wolff[2], George Rebok[2], and Sarah Szanton[3], *1. Johns Hopkins University, Baltimore, Maryland, United States, 2. Johns Hopkins Bloomberg School of Public Health, Baltimore, Maryland, United States, 3. Johns Hopkins University School of Nursing, Baltimore, Maryland, United States*

Adults aging with mild cognitive impairment (MCI)/early dementia often experience physical disability, yet most research is focused on cognitive or physical disability rather than both. Understanding the experiences of this population and their care partners will help identify unmet needs and potential solutions. The purpose of this study is to explore perceptions of daily experiences relevant to aging in place in this population. We used human-centered design, critical ethnography, photos, written narratives, and semi-structured interviews with community-dwelling care partners (mean age: 63 years (SD= 7.48)) and older adults with MCI/early dementia and physical disability (mean age: 84.5 years (SD =2.60)) (total n= 9). Example participant insights included: preferred nonpharmacological remedies for pain, environmental constraints resulting in further isolation, systems of reminders relevant to the older adults' preferences, and the importance of shared dyadic activities. This study demonstrates participants' lived experiences and challenges faced and informs tailoring of an evidenced-based intervention.

### CAPTURING OLDER ADULT PEER MENTORS' EXPERIENCE WITH COMMUNITY-DELIVERED HEARING CARE THROUGH PHOTOVOICE

Carrie Nieman[1], Audrey Mossman[2], Julie Yi[3], Jonathan Suen[3], Frank Lin[4], Sarah Szanton[5], and Hae-Ra Han[3], *1. Johns Hopkins University School of Medicine, Baltimore, Maryland, United States, 2. University of Washington School of Medicine, Seattle, Washington, United States, 3. Johns Hopkins University, Baltimore, Maryland, United States, 4. Johns Hopkins Cochlear Center for Hearing and Public Health, Baltimore, Maryland, United States, 5. Johns Hopkins University School of Nursing, Baltimore, Maryland, United States*

While age-related hearing loss is common, disparities in care exist by race, ethnicity, and socioeconomic position. HEARS is a hearing care intervention that incorporates over-the-counter hearing technology, partnering older adults with peer mentors via a community health worker (CHW) model to address disparities. Through a randomized controlled trial, 8 older adult peer mentors delivered the HEARS intervention. We aimed to understand the CHWs' perspectives on their role through photovoice. Participants took photos illustrating the intervention, their role, and hearing health. Semi-structured interviews (n=4) and a focus group were conducted. CHWs responded positively to the intervention and were satisfied with their role. A notable theme was the value of serving as a peer mentor. Participants valued the opportunity for generativity, learning as older adults, and associated social benefits. Our findings demonstrate an opportunity to engage older adults in the evaluation process, expanding access to hearing care, and in peer mentorship.

### "WE WILL SURVIVE … BUT I JUST WANT MORE FOR US": DISSEMINATING PHOTOVOICE FINDINGS TO ADVANCE FOOD POLICY AND EQUITY

Anika Hines[1], Rebecca Brody[2], Zehui Zhou[3], Kennedy McDaniel[4], Chiazam Omenyi[3], Edgar Miller, III[5], Lisa Cooper[5], and Deidra Crews[6], *1. Virginia Commonwealth University School of Medicine, Richmond, Virginia, United States, 2. Williams College, Williamstown, Massachusetts, United States, 3. Johns Hopkins Center for Health Equity, Baltimore, Maryland, United States, 4. Johns Hopkins School of Nursing, Baltimore, Maryland, United States, 5. Johns Hopkins School of Medicine, Baltimore, Maryland, United States, 6. Johns Hopkins University, Baltimore, Maryland, United States*

Poor food environments and disproportionate food insecurity among African Americans (AAs) represent determinants of chronic disease disparities by race/ethnicity. We used PhotoVoice to explore the food environment in Baltimore, MD, through the perspectives of Black residents with hypertension. Findings illuminated structural racism and classism as root causes of "food desert" conditions. Respondents provided critical analysis of existing food access barriers, including problematic policies, and offered asset-based solutions. Using an exhibition of participants' photographs to set the tone of the discussion, we will convene a stakeholder meeting including study participants, community advisory board members, local and state government agencies, civic organizations, community farmers, agricultural organizations, non-profit food providers, churches, local restauranteurs, convenience store owners, insurance payers, and transportation providers. Discussion will center on three areas of action informed by findings: systems level issues; individual activation, agency, and advocacy; and community-based solutions. Meeting proceedings will be disseminated through academic and lay publications.

### SESSION 4690 (SYMPOSIUM)

### AGING IN CROSS-NATIONAL CONTEXTS

Chair: Jennifer Ailshire Discussant: Eunyoung Choi

Cross-national comparative research is a useful tool for identifying common aspects of, and risk factors for, healthy and unhealthy aging across populations and sociocultural contexts. The papers in this symposium use harmonized data from the Gateway to Global Aging to examine a range of topics in aging and provide new insights into long-standing and emerging questions in aging research. Using data on 31 countries, Ehrlich estimates the population attributable fraction of dementia due to vision impairment, a treatable and thus potentially viable target for interventions to slow progression to dementia. Seligman et al., use a frailty index validated in multiple countries to provide new evidence for which aspects of socioeconomic status (SES) – education, income, rural residence – are most strongly linked to frailty in Brazil, China, and India, three highly populated and rapidly aging countries. Using recent harmonized data on stress, Chen et al. examine the association between cumulative social stressors and cognitive function trajectories in the US and UK, finding interesting patterns in the relationship with status and change over time. Finally, several harmonized aging studies collected data throughout the COVID-19 pandemic, and Mair et al., take advantage of this to gain insights into how family structure impacted experiences of loneliness among older adults during the pandemic in the U.S. and across Europe. The papers in this symposium demonstrate the tremendous potential for using cross-national comparisons to deepen our understanding of health and well-being among older adults.

### VISION IMPAIRMENT IN A GLOBAL LIFE COURSE MODEL OF POTENTIALLY MODIFIABLE DEMENTIA RISK FACTORS

Joshua Ehrlich[1], Jenna Goldstein[1], Bonnielin Swenor[2], Heather Whitson[3], Kenneth Langa[1], and Phillip Veliz[1], *1. University of Michigan, Ann Arbor, Michigan, United States, 2. Johns Hopkins University, Baltimore, Maryland, United States, 3. Duke University, Durham, North Carolina, United States*

Vision impairment (VI) is a risk factor for accelerated cognitive decline and incident dementia. An estimated 90% of VI is preventable. Nonetheless, VI has not been included in the dominant life-course models of dementia risk factors. We sought to strengthen existing models of potentially modifiable dementia risk factors through the inclusion of VI using cross-sectional survey data from the Gateway to Global Aging project (G2Aging) from 31 countries. Prevalence rates and communalities from the G2Aging will be used to estimate the population attributable fraction (PAF) of dementia due to VI. It is expected that the PAF for VI will range from 1.0%-2.0%, suggesting that >10,000,000 prevalent dementia cases globally may potentially have been prevented through healthy vision. Since a large majority of VI can be treated with cost-effective but underutilized interventions, this may represent a viable target for future interventional research that aims to slow cognitive decline and prevent dementia.

### FRAILTY AND SOCIOECONOMIC STATUS IN BRAZIL, CHINA, AND INDIA

Benjamin Seligman[1], Arunika Agarwal[2], and David Bloom[2], *1. David Geffen School of Medicine at UCLA, Los Angeles, California, United States, 2. Department of Global Health and Population, Harvard T.H. Chan School of Public Health, Boston, Massachusetts, United States*

Frailty, the vulnerability to poor health outcomes following physiologic stress, acts as a measure of physiological age that can be used to compare the health of older populations. Using a frailty index validated in multiple countries, we looked at the relationship between four measures of socio-economic status (SES) and frailty in Brazil, China, and India. For SES measures, we considered within-country income and wealth deciles, education level, and rural residence. Analysis used beta regression, first within each country, then with combined data with country fixed effects and country-by-SES interactions. We found evidence of strong, statistically significant negative associations between education level and frailty in all three countries. Associations between frailty and income or wealth decile or rural residence were small and inconsistently statistically significant. This demonstrates the key role of education for healthy aging and the value of harmonized frailty measures for comparative studies of older populations.

### ASSOCIATIONS BETWEEN CUMULATIVE STRESS AND COGNITIVE FUNCTION IN HRS AND ELSA

Ruijia Chen[1], Harold Lee[2], Sakurako Okuzono[3], and Laura Kubzansky[3], *1. University of California, San Francisco, San Francisco, California, United States, 2. University Of North Carolina At Chapel Hill, Chapel Hill, North Carolina, United States, 3. Department of Social and Behavioral Sciences, Harvard T.H. Chan School of Public Health, Boston, Massachusetts, United States*

Despite growing research linking stressors with poorer cognition, less research explicitly considers stress from several sources, which often co-occurs and accumulate to influence health. We used the Global Gateway Harmonized data (HRS: n=8,888; mean age: 74 years; ELSA: n=6,715; mean age: 65 years) to examine whether higher cumulative stress is associated with lower levels of and faster decline in cognitive function. We fit linear mixed effect models to assess the stress-cognition associations. After adjusting for all covariates, baseline cumulative stress was associated with lower baseline cognitive function in both HRS (β=-0.02, 95%CI -0.04, -0.01) and ELSA (β=-0.03, 95%CI -0.05, -0.01). Unexpectedly, higher baseline cumulative stress was associated with slower cognitive decline in HRS (βtime*stress=0.001, 95%CI 0.002,0.01) but not in ELSA. The stress-cognition associations may differ among adults in the US and the UK. Future research should investigate how cumulative stress may operate differently to influence cognitive function across different populations.

### UNPARTNERED AND CHILDLESS OLDER ADULTS' RISK OF LONELINESS DURING COVID-19 BY COUNTRY CONTEXT

Christine Mair[1], Bruno Arpino[2], Nekehia Quashie[3], and Radoslaw Antczak[4], *1. University of Maryland, Baltimore County (UMBC), Baltimore, Maryland, United States, 2. Department of Statistics, Computer Science, Applications, Firenze, Toscana, Italy, 3. University of Rhode Island, Kingston, Rhode Island, United States, 4. Institute of Statistics and Demography, Warszawa, Warminsko-Mazurskie, Poland*

Older adults with non-traditional family structures (unpartnered and childless) may be at higher risk for loneliness. Yet, experiences of loneliness during COVID-19 can vary depending on a country's context (e.g., culture, demography, COVID-19 mitigation policies, severity of the pandemic). We explore associations between older Europeans' family structure and loneliness to assess risks for those with non-traditional family structures (e.g., unpartnered vs partnered, childless vs parents) during the pandemic across a variety of country contexts. We analyze data from the Survey of Health, Ageing and Retirement in Europe (SHARE) collected before (2019/2020) and twice during (2020, 2021) the pandemic to examine if unpartnered and childless older adults are at higher risk of loneliness, and compare results by multiple indicators of country context (fertility and partnership rates, stringency of COVID mitigation policies, population age composition, and COVID fatality rates). Results of this study can potentially inform current and future pandemic mitigation strategies.

### SESSION 4691 (SYMPOSIUM)

### PUBLIC HEALTH POLICY: EXPERIENCE OF 2022 GSA SUMMER POLICY INTERNS

### CHRONIC PAIN'S PAINFUL IMPACT ON COGNITIVE FUNCTIONING

Lilian Azer,[1] Hanamori Skoblow,[2] Eileen Flores,[3] Danielle Llaneza,[4] Weiwei Zhang,[1] and Patricia D'Antonio[5], *1. University of California, Riverside, Riverside, California, United States, 2. University of Missouri, Columbia, Missouri, United States, 3. Penn State, State College, Pennsylvania, United States, 4. Rutgers University, Piscataway, New Jersey, United States, 5. The Gerontological Society of America, Washington, District of Columbia, United States*

One in five Americans adults report experiencing chronic pain and stated it has negatively impacted their lives by limiting their engagement in activities. The magnitude of pain experienced is more severe in older adults, which may be due to age-related differences in pain perception pathways leaving older adults more vulnerable to the negative impact of pain. The most common type of chronic pain experienced by older adults involves musculoskeletal disorders, which is consistent with age-related declines muscle strength and mass. Declines in handgrip strength is associated with chronic pain perception and poor cognitive functioning. Older adults who experience severe chronic pain have worse working memory and attention. The experience of chronic pain is attentionally demanding on cognitive processes making it difficult for older adults to draw their attention away from pain perception during cognitively demanding tasks. Chronic pain is also associated with accelerated decline in cognition, dementia, and Alzheimer's Disease pathogenesis. As a GSA policy intern, I represented GSA in the Protecting Access to Pain Relief Coalition to explore policy initiatives and recommendations in relation to chronic pain experienced by older adults. Research on chronic pains' impact on cognitive and physical function is often conducted in isolation. However, they seldom occur in isolation during real-world tasks. Therefore, research on tasks that involve simultaneous physical and cognitive functioning in relation to chronic pain is imperative. In addition, handgrip strength should be considered as a useful cost-effective screening tool as a measure for cognitive decline in older adults with chronic pain.

### THE INTERSECTIONS OF AGING BELIEFS AND POLICY

Hanamori Skoblow,[1] Lilian Azer,[2] Danielle Llaneza,[3] Eileen Flores,[4] and Patricia D'Antonio[5], *1. University of Missouri, Columbia, Missouri, United States, 2. University of California, Riverside, Riverside, California, United States, 3. Rutgers University, Piscataway, New Jersey, United States, 4. Penn State, State College, Pennsylvania, United States, 5. The Gerontological Society of America, Washington, District of Columbia, United States*

Ample evidence and stereotype embodiment theory indicate that older adults' internalized beliefs about aging are related to their health and well-being over time. Aging beliefs are constructed and reinforced socioculturally, interpersonally, and structurally, the latter of which includes federal, state, and local policies. For instance, policies affect resource allocation for Older Americans Act programs, job security and safeguarding from workplace age discrimination, access to medical services or treatments, and financial well-being via tax and retirement law. Heterogeneity in community and organization-level public health policies during the first year of the COVID-19 pandemic also shaped and were shaped by beliefs about aging. Further, policies that acknowledge and address ageism not only promote positive health, social, and financial outcomes for older adults, but also allow all of the public to benefit from older adults' participation in society. As a GSA policy intern, I tracked and analyzed legislation that is relevant for optimal aging outcomes, identified implicit age bias in policy, and framed policy recommendations that promote positive aging beliefs. Effective policies and collective action that reduce ageism, facilitate intergenerational contact, and ensure access to necessary services have the potential to benefit all of us as we age, not only directly, but also indirectly through beliefs about aging.

### PUBLIC HEALTH POLICY IN AN AGING POPULATION

Eileen Flores,[1] Danielle Llaneza,[2] Hanamori Skoblow,[3] Lilian Azer,[4] and Patricia D'Antonio[5], *1. Penn State, State College, Pennsylvania, United States, 2. Rutgers University, Piscataway, New Jersey, United States, 3. University of Missouri, Columbia, Missouri, United States, 4. University of California, Riverside, Riverside, California, United States, 5. The Gerontological Society of America, Washington, District of Columbia, United States*

In the U.S., cancer and its related issues have an impact on public health, such as increased death rates and economic burdens due to lost productivity and associated treatment costs. Along the cancer control continuum, there are ample opportunities to enact policies that can improve cancer prevention, detection, and survivorship. As the US population ages, health policies that address barriers to cancer prevention and optimal survivorship care for older adults will be key. The intersection of policy and public health has gained momentum in the last decade. Changes resulting from the Affordable Care Act and President Biden's recently set goals (i.e. reduce age-adjusted death rates from cancer by at least 50% and improving the experience of people and their families living with and surviving cancer) for the Cancer Moonshot program have ignited the role of public health policy and its implications among the US population. As a policy intern, I had the opportunity to advocate on behalf of GSA, the nation's largest aging organization, for research initiatives (e.g. ARPA-H) that amplify opportunities to address policies in public health. Advocating for initiatives that accelerate the government's biomedical and health research to embrace a geroscience approach will continue to spearhead much needed opportunities to directly impact policies surrounding aging and cancer.

### POLICY IMPLICATIONS ON CLINICAL PRACTICE: AN EXPLORATION

Danielle Llaneza,[1] Eileen Flores,[2] Lilian Azer,[3] Hanamori Skoblow,[4] and Patricia D'Antonio[5], *1. Rutgers University, Piscataway, New Jersey, United States, 2. Penn State, State College, Pennsylvania, United States, 3. University of California, Riverside, Riverside, California, United States, 4. University of Missouri, Columbia, Missouri, United States, 5. The Gerontological Society of America, Washington, District of Columbia, United States*

Clinical practice is direct and indirect work with and for patients to improve their wellbeing. Typically, clinical practice is specific to the discipline and institutional body in which providers work. Additionally, caring for older adults requires a review of the biological, psychological, and social aspects of their health to address their needs and diseases. Therefore, policy has significant albeit gradual effects on clinical practice. Health policies and legislation change clinical guidelines, options, and barriers. Federal and state-level policies influence clinical practice factors such as billing, provider knowledge, student training, treatment access, and availability of interventions. Understanding the needs of both patients and providers is imperative to creating clinical practices in line with policy. The collaboration between researchers, providers, and policymakers promotes inclusive and equitable recommendations for health care practices. Yet, often health policy at the federal level is a formidable dynamic that requires expertise and cooperation for all stakeholders that is often not readily accessible. A critical component of this work is reaching providers and patients where they are most often located with information dissemination and outreach efforts. As a GSA policy intern, I explored policy initiatives that directly and indirectly influenced clinical practice across disciplines from the Department of Health of Human Services, National Institute of Aging at NIH, Centers for Medicare & Medicaid Services, and other agencies and reviewed methods that promoted health equity in health care. We need better policy to improve our clinical practice to increase treatment options, expand access, and tailor delivery for our patients.

### SESSION 4700 (PAPER)

### COGNITION AND DEMENTIA

### BEHAVIORAL AND PSYCHOLOGICAL SYMPTOMS OF DEMENTIA (BPSD) IN HOSPITALIZED OLDER ADULTS WITH AD/ADRD

Allison Marziliano, Alex Makhnevich, Edith Burns, Michael Diefenbach, and Liron Sinvani, *Northwell Health, Manhasset, New York, United States*

The epidemiology of Behavioral and Psychological Symptoms of Dementia (BPSD) in hospitalized older adults with Alzheimer's Disease (AD) and AD-related dementias (ADRD) has not been well-characterized. The purpose of this abstract is to examine the prevalence, patient-level factors and hospital outcomes associated with BPSD in hospitalized older adults with AD/ADRD. Data was extracted from the electronic health record (EHR) of older adults (aged 65+ years) with AD/ADRD, admitted to one of 7 hospitals in the greater New York metropolitan area during 2019. Three independent coders reviewed the EHR and recorded the presence or absence of the 11 domains of the Neuropsychiatric Inventory (NPI), a validated measure of BPSD. Of N = 1,865 hospitalized older adults with AD/ADRD, 1,564 had BPSD (prevalence = 83.9%). The most commonly reported BPSD were agitation (72.3%) and anxiety (17.7%). Older age (M = 84.6, SD = 7.6 versus M = 82.4, SD = 7.8, p = .000) and antipsychotic medication use prior to hospital admission (91% of the older adults who used home antipsychotics had BPSD, p = 000) was associated with BPSD. With regard to hospital outcomes, BPSD was associated with: increased mortality (of those who died, 90.6% had BPSD, p = .006), and increased likelihood of readmission to the hospital within 30 days of discharge (of those readmitted, 89.3% had BPSD, p = .007). Given its high prevalence and associated poor outcomes, recognizing and managing BPSD in hospitalized patients with AD/ADRD is critical to improving quality of care for this vulnerable population.

### COGNITIVE FUNCTION AND HELP-SEEKING AMONG US CHINESE OLDER ADULTS WITH ELDER MISTREATMENT

Ying-Yu Chao[1], An-Yun Yeh[2], Peijia Zha[1], and XinQi Dong[3], *1. Rutgers, the State University of New Jersey, Newark, New Jersey, United States, 2. College of Staten Island CUNY, New York, New York, United States, 3. Rutgers, New Brunswick, New Jersey, United States*

Purpose: Elder mistreatment (EM) in immigrants with cognitive decline is an understudied public health problem. Cognitive function plays an important role in individuals' ability to seek help. This study aimed to examine the associations between cognitive function and help-seeking among U.S. Chinese older adults reported EM.

Methods: Data were from the Population Study of Chinese Elderly in Chicago (PINE). Five instruments were used to measure cognitive function, including the Mini-Mental State Examination, East Boston Memory Test Immediate Recall and Delayed Recall, Digit Span Backwards, and Symbol Digit Modalities Test. Informal/formal help-seeking intentions and behaviors were measured. Descriptive statistics, linear regression, and logistic regression were performed.

Results: A total of 450 participants reported EM. Most victims sought help from informal sources (53.48%), followed by any sources (11.42%) and formal sources (3.34%). About one-third of participants did not seek any help. Higher episodic memory was associated with an increase in help-seeking intentions among Chinese older adults with financial mistreatment (p < .05) and poly-victimization (p < .01). Lower working memory was associated with an increase in help-seeking intentions among those with caregiver neglect (p < .001). Compared to not seeking help, higher executive function was associated with a higher likelihood to seek help from any sources among Chinese older adults with psychological mistreatment (p < .05). Conclusion/implication: This study highlights the associations between cognitive function and help-seeking among this underserved population. Culturally tailored interventions are suggested to promote help-seeking for different types of cognitive impairment among Chinese older adults with EM.

### EXPLORING THE USE OF MACHINE LEARNING TO IMPROVE DEMENTIA DETECTION: AUTOMATING CODING OF THE CLOCK-DRAWING TEST

Mengyao Hu[1], Yi Lu Murphey[2], Song Wang[2], Tian Qin[2], Zixuan Zhao[2], Richard Gonzalez[1], Vicki Freedman[1], and Laura Zahodne[1], *1. University of Michigan, Ann Arbor, Michigan, United States, 2. University of Michigan-Dearborn, Dearborn, Michigan, United States*

Alzheimer's disease and related dementias, a leading cause of disability among older adults, has become a critical public health concern. The clock-drawing test (CDT), which asks subjects to draw a clock, typically with hands showing 11:10, has been widely used as a screening tool to detect dementia in clinical research and surveys. The clock-drawings are often coded into binary (e.g., normal vs. abnormal) or ordinal scores. A limitation in large-scale studies is that the manual-coding of CDT could result in biases if coders interpret and implement coding rules differently. Several small-scale studies have explored the use of machine learning methods to automate CDT coding. Such studies either have had limited success with ordinal coding or have used methods that are not designed specifically for complex images. This study aims to create and evaluate an intelligent CDT-scoring system that automatically codes ordinal CDT scores using deep learning neural networks (DLNN) methods including resnet101, EfficientNet-B3, and Visual Transformer. We used a large repository of CDT images from the 2011-2019 National Health and Aging Trends Study, a panel study of Medicare beneficiaries ages 65 and older. Results show that 1) DLNN has achieved a high scoring accuracy; 2) DLNN generates more consistent and accurate scores than trained lay coders, compared to expert coders with a neuropsychology background; 3) DLNN-coded CDT scores are highly correlated with self-reported dementia diagnosis and performance-based assessments of memory. This study offers a model for automating coding of other drawing tests used to evaluate a variety of cognitive functions.

### INFLUENCE OF POSTTRAUMATIC STRESS AND ABNORMAL SPIROMETRY ON COGNITIVE PERFORMANCE IN 9/11 WTC RESPONDERS

Andrea Zammit[1], Charles Hall[2], Sean Clousten[3], and Benjamin Luft[3], *1. Rush Alzheimer's Disease Center, Chicago, Illinois, United States, 2. Albert Einstein College of Medicine, Bronx, New York, United States, 3. Stony Brook University, Stony Brook, New York, United States*

Post-traumatic stress disorder (PTSD) and abnormal spirometry are highly prevalent mental and health conditions in World Trade Center (WTC) responders. We tested the hypothesis that PTSD symptomatology and abnormal spirometry are conjointly and synergistically associated with poorer cognitive performance. A total of 1,326 responders (mean age = 53.1, SD = 8.1, 92% males) from the WTC Health Program took part in the study. PTSD symptomatology was assessed using the PCL-IV, and we calculated the FEV1/FVC ratio to measure pulmonary function. Cogstate assessments measured cognitive performance. Linear regressions were employed to evaluate PTSD and pulmonary function on cognitive performance while adjusting for age, sex, education, smoking status, and comorbidity. Higher PTSD symptomatology and lower pulmonary function were independently and conjointly negatively associated with cognitive performance. Further, a significant synergistic effect was present in that higher severity of PTSD symptomatology in the presence of lower pulmonary function was associated with poorer cognitive performance (estimate = -0.096, SE = 0.03, p <0.001). Results suggested that chronic stress and lung damage might share underlying biological mechanisms, including inflammatory and oxidative stress pathways, which may also be affecting the brain. Early intervention efforts to mitigate preventable cognitive decline in high-risk populations should be studied.

### PSYCHOMETRIC PROPERTIES OF THE NEUROPSYCHIATRIC INVENTORY QUESTIONNAIRE FOR THAI OLDER ADULTS WITH DEMENTIA

Kaipeng Wang[1], Ladson Hinton[2], Sue Levkoff[3], Siranee Sihapark[4], Komatra Chuengsatiansup[5], and Hongtu Chen[6], *1. University of Denver, Denver, Colorado, United States, 2. University of California Davis, Sacramento, California, United States, 3. University of South Carolina, Columbia, South Carolina, United States, 4. Boromarajonani College of Nursing, Khon Kaen, Khon Kaen, Thailand, 5. Society and Health Foundation & Sirindhorn Anthropology Center, Nonthaburi, Nonthaburi, Thailand, 6. Harvard University, Boston, Massachusetts, United States*

Psychiatric and behavioral symptoms are highly prevalent among persons living with dementia. Clinical evaluation of those symptoms is critical for assessment of dementia progression and development of effective treatment. The Neuropsychiatric Inventory Questionnaire (NPI-Q) is a brief informant-based instrument to assess the severity of 12 types of neuropsychiatric symptoms among persons with dementia and associated caregiver distress. Although Thailand is experiencing a rapid increase in the prevalence with dementia, there is no validated instrument to assess neuropsychiatric symptoms in Thailand. This study aims to examine the psychometric properties of the NPI-Q instrument among older adults with dementia in Thailand. Data were collected from 353 participants aged 60 and over with probable dementia, at least one neuropsychiatric symptom, and an adult family caregiver willing to participate in the study. We examined the internal consistency, factorial validity, and criterion validity of the NPI-Q instrument. NPI-Q has acceptable internal consistency (Cronbach's $\alpha$=0.77). Principal component analysis yielded a five-factor solution, which accounted for 63% of the variance. The five factors included (1) agitation, anxiety, and irritability, (2) delusions, hallucinations, apathy, and disinhibition, (3) euphoria, motor disturbance, and night disturbance, (4) depression, and (5) abnormal appetite. We found that higher NPI-Q severity score was significantly associated with higher geriatric depression, associated caregiver distress, caregiver burden, and caregiver depression and lower quality of life among older adults and their caregivers, which confirmed the criterion validity of the instrument. Findings provide support for use of the NPI-Q among older adults with dementia in Thailand.

### TOXIC STRESS, RESILIENCY RESOURCES, AND TIME TO DEMENTIA DIAGNOSIS AMONG AMERICANS AGED ≥50 YEARS IN THE HRS 2006–2016

Allan Nkwata[1], Ming Zhang[2], Xiao Song[2], Bruno Giordani[1], and Amara Ezeamama[3], *1. University of Michigan, Ann Arbor, Michigan, United States, 2. University of Georgia, Athens, Georgia, United States, 3. Michigan State University, East Lansing, Mississippi, United States*

Toxic stress (TS), resiliency-promoting factors (RPFs) and their interactions were investigated in relationship to incident dementia in a sample (n = 6516) of US adults ≥50 years old enrolled in the HRS between 2006-2016. TS included experiences of everyday discrimination and RPF included personal mastery. Race/ethnicity was self-reported as African American (AA), Caucasian, or Other. Multivariable Cox proportional hazards regression models estimated TS-, RPF- and race-associated hazard ratios (HR) for dementia diagnosis and 95% confidence intervals (CIs) with adjustment for comorbidity and socio-demographic factors. Discrimination-associated risk of dementia diagnosis on average increased with education level [discrimination x education, p = 0.032; HR = 1.75 (95% CI: 1.01–3.03) if < high school, HR = 5.67 (95% CI: 2.94–10.94) if high school completed and HR = 2.48 (95% CI: 1.53–4.00) if ≥some college education]. Likewise, AA vs. Caucasian race disparity in new-onset dementia was evident (HR = 2.12, 95% CI: 1.42–3.17) among adults with high-mastery while absent (HR = 1.35, 95% CI: 0.75–2.41) among adults with low mastery (Mastery x Race, p = 0.01). TS is a contextual driver of incident dementia that seemingly operates in a race and RPF-dependent manner. Heterogeneity pattern suggests that TS may overwhelm the cognitive reserve benefit of RPF particularly in status-inconsistent contexts including persons subjected to discrimination despite high education and in AA individuals despite high mastery. Policies that reduce discrimination and promote equitable treatment by race/ethnicity may support cognitive resiliency and reduce the risk of dementia diagnosis in adult Americans.

### SESSION 4710 (SYMPOSIUM)

### COMMUNITY ENGAGEMENT: INDIGENOUS AND NONINDIGENOUS RESEARCHERS

Chair: Jordan Lewis Co-Chair: Steffi Kim

In response to populations that have been chronically underserved and underrepresented within the social sciences, scholars have outlined the need for community-based engagement strategies and research methodologies that are able to represent the voices of those communities to promote culturally informed, appropriate, and driven health initiatives. This symposium introduces and discusses Indigenous research methodologies that are empowering and emphasize the knowledge and wisdom of Indigenous Elders, shares urban Elder engagement strategies, explores engagement strategies for Indigenous and non-Indigenous researchers, and describes the impacts of exclusion of Indigenous voices from research. The first presentation by Maria Crouch and her colleagues will introduce an Elder-centered research methodology as exemplified by two research studies. The second presentation by Steffi Kim focuses on outreach and research engagement strategies to reach Alaska Native Elders in the urban environment. The third presenter, Jordan Lewis, shares and discusses community-based research as an insider and outsider in your own community, outlining research practices, positionality, and accuracy in research. Finally, Lyn Holley will discuss themes emerging from her review of the literature that explored the impacts of the application of “top-down” Eurocentric approaches to inquiry on American Native people and the implications of these findings.

### ELDER-CENTERED RESEARCH METHODOLOGY: RESEARCH THAT DECOLONIZES AND INDIGENIZES

Maria Crouch[1], Steffi Kim[2], Zayla Asquith-Heinz[3], Elyse Decker[4], Nyche Andrew[5], Rosellen Rosich[6], and Jordan Lewis[7], *1. Yale School of Medicine, New Haven, Connecticut, United States, 2. University of Minnesota, Minneapolis, Minnesota, United States, 3. University of Minnesota Medical School, Minneapolis, Minnesota, United States, 4. Swarthmore College, Swarthmore, Pennsylvania, United States, 5. Yale University, New Haven, Connecticut, United States, 6. University of Alaska Anchorage, Anchorage, Alaska, United States, 7. University of Minnesota Medical School, Duluth, Minnesota, United States*

Alaska Native (AN) Elders have historically been underrepresented in research. Innovative AN research posits that practice-based evidence is fundamental to culturally grounded, multifaceted methods. Semi-structured interviews were conducted with 34 AN Elders and 12 AN and non-Native caregivers in two studies exploring cultural understandings of memory and successful aging. The design and implementation of these studies employed Elders at every level of the research, ensuring cultural relevancy, outcomes, and dissemination activities. Findings reflect the benefits of engaging AN Elders in research and reveal methods for best practices: 1) creating Advisory councils; 2) identifying stakeholders 3) weaving together Elder and Western knowledge systems; and 4) the reciprocal nature of Elder engagement and wellbeing. This complimentary research builds on Elder-centric principles and lays the foundation for an Elder-centered research methodology that can be adapted and applied in other studies to encourage engagement of older adults in meaningful, restorative, and enculturated ways.

### URBAN ENGAGEMENT STRATEGIES FOR ALASKA NATIVE ELDER PARTICIPATION IN RESEARCH

Steffi Kim, *University of Minnesota, Minneapolis, Minnesota, United States*

Given the current threats to health for older Indigenous people, it is important to understand the health needs and perceptions of older Indigenous people who play a pivotal role in the health and wellbeing of their communities. The underrepresentation of Indigenous voices has led to research, health promotion efforts, and community engagement that has largely been unsuccessful in facilitating culturally grounded and effective health promotion. Engaging Indigenous Elders in research has often been a challenge for researchers – Indigenous and non-Indigenous. Reaching Indigenous Elders within the urban environment can even be more challenging at times based on dispersed communities due to challenging historical contexts, experiences of racism, and mistrust. This presentation is based on the employed engagement strategies of urban-based Alaska Native Elders. Developing policies and programs to promote healthy aging in Indigenous communities requires the voices of those living within their communities.

### CONDUCTING COMMUNITY-BASED RESEARCH AS AN INSIDER AND OUTSIDER IN YOUR OWN COMMUNITY

Jordan Lewis, *University of Minnesota Medical School, Duluth, Minnesota, United States*

As an Alaska Native from the Bristol Bay region, this presentation will discuss being an insider and outsider in your own community and how it impacts research studies. Using case studies from my dissertation research, this presentation will discuss positionality and how to collect objective data, the lack of objectivity in getting, analyzing, and reporting data, and how research methods influenced my study. I will also discuss challenges and benefits of being an insider, including enhancing the validity of the research process, data collection, and analysis, understanding the group’s culture. The results include trusting relationships with communities, the Elders’ voices guiding the findings, an appreciation for the challenge of conducting research as an insider and outsider. This presentation will conclude with a discussion on how to obtain objective data and ensure the accuracy of findings through community-engagement principles and the benefits of working in your own community.

### EXAMINING EXCLUSION FOR GUIDANCE TO INCLUSION: A GOOD IDEA?

Lyn Holley, *University of Nebraska at Omaha, Omaha, Nebraska, United States*

This paper is based on a literature review that informed writing of a chapter for a new edited book, Moving beyond the rhetoric - research with older service users – why ethics and integrity matter. (Emerald, in review). This literature review was undertaken to ground guidance for the chapter in the book that addresses ethics in research with American Indians of all tribes on lands currently part of the USA. A theme that emerged uninvited was the many ways in which inquiry that was unintentionally or deliberately unethical also operated to reduce inclusion of American Indians in research – often the very research needed to discern or demonstrate value of various clinical, social, or other types of interventions needed to improve policies or practices. This paper explores that theme and its implications for research with American Indians.

### SESSION 4720 (SYMPOSIUM)

### CONTINUOUS WORK-BASED LEARNING IN THE NURSING HOME SETTING

Chair: Judith Meijers

Nursing homes face challenges caused by increasing numbers of older adults with frailty and multimorbidity, and complexity of care in combination with decades of under resourcing and severe workforce challenges. In most countries the vast majority of direct care in LTC homes is provided by a large unregulated workforce that is largely unregulated, female, racialized and poorly remunerated. The results are severe difficulties in recruiting and retaining staff at all levels and a tendency to substitute less qualified workers for more highly qualified and more highly remunerated. Less well reported are the significant pressures that critical LTC home managers face. However, they are equally under duress especially in the wake of a global pandemic. Developing a culture of continuous learning and integrating this into daily nursing practice is one promising way to attenuate these challenges, ensure a high quality of care and a better equipped nursing staff. However, insight into strategies and interventions that contribute to such a continuous work-based learning culture in nursing homes is still relatively limited. In this symposium we will focus on different approaches to increasing workplace learning in long term care.

### ELEMENTS OF AN INTERPROFESSIONAL LEARNING CULTURE IN NURSING HOMES

Judith Meijers[1], Merel (E.A.) Van Lierop[1], Frank Verbeek[2], Miranda Laurant[2], Erik van Rossum[3], Sandra Zwakhalen[1], and Anneke Van Vught[2], *1. Maastricht University, Maastricht, Limburg, Netherlands, 2. HAN University, Nijmegen, Gelderland, Netherlands, 3. Zuyd University of Applied Sciences, Maastricht, Limburg, Netherlands*

Healthcare professionals in nursing homes need to be able to keep up with changes at work, deal with a growing complexity in healthcare demands and have to keep developing themselves within a team to be able to plan and deliver high quality of care. To keep up with these changes and demands, an interprofessional learning culture must be stimulated. Insight about developing such a culture in nursing homes is lacking. A scoping review was performed. Articles were examined on actions that positively influence the development of an interprofessional learning culture. 51 Actions were identified. The influencing factors were divided in eight main themes i.e. (1) communication, (2) shared goals, (3) tasks and responsibilities, (4) learning and knowledge, (5) work approaches, (6) attitude, (7) leadership, and (8) safety, respect and transparency. These actions form a basis for an interprofessional learning culture but should be operationalized and tailored to practice use.

### LINC TOOL: USING QUALITY DATA, COACHING, AND AN IMPROVEMENT CYCLE FOR LEARNING AND IMPROVEMENT IN LONG-TERM CARE

Merel (E.A.) Van Lierop[1], Judith Meijers[1], Erik van Rossum[2], and Sandra Zwakhalen[1], *1. Maastricht University, Maastricht, Limburg, Netherlands, 2. Zuyd University of Applied Sciences, Maastricht, Limburg, Netherlands*

Providing high quality of care can be challenging for nursing staff because of the continuous changes in healthcare. Nursing staff needs to learn continuously to remain employable and deal with these challenges. Therefore, availability of a continuous learning and improvement tool (LINC tool) using nursing quality data and an improvement cycle to maintain high quality of care in long-term care is important. The development of this tool took place in co-creation with researchers, experts, educational staff and nursing staff working in long-term care. Taskforces with researchers and experts on quality data and education were established to develop the main steps of the tool. Nursing staff was involved in the development and testing of the tool. The LINC tool consists of 1) Assessment of the current learning climate and preferred quality data for nursing staff to work with, 2) Discussing findings from step 1 with the nursing staff team and formulate learning topics, 3) Start improvement cycle (PDCA cycle) using one learning climate topic and one quality data topic as a case to improve quality of care and improve the learning climate. During this cycle, nurse coaches are available to help nursing staff use the LINC tool materials. The LINC tool can be used to improve long-term care and has proven to be feasible. However, the LINC tool is currently tested in more nursing staff teams to increase its usability and effectiveness. Further development of the LINC tool is therefore a constant cycle of developing, implementing, testing, and evaluating, all in cooperation with stakeholders.

### SUPPORTING NURSING HOME MANAGERS TO ACT ON PERFORMANCE FEEDBACK DATA: A PROVINCEWIDE IMPLEMENTATION

Carole Estabrooks[1], Seyedehtanaz Saeidzadeh[1], Stirling Bryan[2], Peter Norton[3], Liane Ginsburg[4], Matthias Hoben[1], and Don McLeod[5], *1. University of Alberta, Edmonton, Alberta, Canada, 2. Michael Smith Health Research, Vancouver, British Columbia, Canada, 3. University of Calgary, Calgary, Alberta, Canada, 4. York University, Toronto, Ontario, Canada, 5. Translating Research in Elder Care, Edmonton, Alberta, Canada*

INFORM (Improving Nursing home care through Feedback on perfoRMance data) is a tested research intervention targeting care managers that demonstrated positive two year follow up results and has subsequently been shaped into an operationally acceptable "implementation package". This package or innovation is being scaled up in one Canadian province's total Long-Term-Care (LTC) home population with in depth process evaluation during the first cohort of LTC homes. This evaluation will, among other things, assess sector needs for adaptation (vs fidelity). At its core INFORM is designed to address managers' learning needs with respect to using data to make positive change in a continuous learning loop. We will discuss the transformation of a research intervention to a sector innovation and report on interim process evaluation results.

### SESSION 4730 (PAPER)

### DISCRIMINATION

### CORRELATES OF RACIAL DISCRIMINATION TRAJECTORIES AMONG BLACK MIDDLE-AGED AND OLDER ADULTS

Kellee White[1], Shuo Huang[2], and Bethany Bell[3], *1. University of Maryland College Park School of Public Health, College Park, Maryland, United States, 2. University of Maryland, College Park, Maryland, United States, 3. University of Virginia, Charlottesville, Virginia, United States*

Single cross-sectional measures of racial discrimination may mask the dynamic nature and cumulative impact of repeated exposures to discrimination. Characterizing racial discrimination trajectories, patterns of repeated exposure, may provide insight on age-related patterns of health risk and resilience. However, there is a dearth of research exploring racial discrimination trajectories. This study characterizes racial discrimination trajectories and identifies trajectory correlates among a nationally representative sample of middle-aged and older black adults who participated in the Health and Retirement Study (2006-2018). Repeated measures latent profile analysis was employed to identify racial discrimination trajectories. Logistic regression models, estimating odds ratios (ORs) and 95% confidence intervals (CIs), were conducted to investigate sociodemographic and neighborhood-level predictors of racial discrimination trajectories. Two racial discrimination trajectories were identified: low to moderate (82%) and persistently high (18%). Individuals 65+ (OR: 0.45; 95% CI: 0.32, 0.64) and female sex (OR: 0.59; 95% CI: 0.45, 0.76) were associated with a lower odds of belonging to the persistently high racial discrimination trajectory class. Individuals reporting higher levels of major lifetime experiences of discrimination (OR: 1.47; 95% CI: 1.33, 1.63) and greater neighborhood social cohesion (OR: 1.30; 95% CI: 1.1, 1.50) predicted membership in the persistently high racial discrimination trajectory class. These findings suggest heterogeneity in the cumulative patterning of discrimination among black middle-aged and older adults. Discrimination trajectories may enable greater precision in estimating the health consequences of cumulative exposure to discrimination. Future studies are warranted to determine whether membership in specific trajectory classes confers differential risk to age-related conditions.

### PERCEIVED DISCRIMINATION, DEPRESSIVE SYMPTOMS, AND COGNITIVE CONTROL AMONG MEXICAN-ORIGIN WOMEN

Elizabeth Muñoz[1], Hin Wing (Florence) Tse[2], Jinjin Yan[3], and Su Yeong Kim[1], *1. The University of Texas at Austin, Austin, Texas, United States, 2. Department of Human Development and Family Sciences, The University of Texas at Austin, Austin, Texas, United States, 3. University of Texas at Austin, Austin, Texas, United States*

Mexican-origin adults comprise over 60% of the Latino population in the U.S., and are at greater risk for cognitive impairment compared to non-Latino Whites. Older Mexican-origin women are also disproportionally affected by cognitive impairment compared to their non-Latino White counterparts. Identifying early-life precursors for reduced cognitive function can inform prevention efforts. Perceived discrimination is a ubiquitous stressor with emergent evidence linking it to cognitive function and no studies testing mechanisms of this association among Mexican-origin women. Using data from a three-wave longitudinal study of Mexican-origin women spanning 8 years (n = 595, mean baseline age = 38.89 (5.74)) living in central Texas, we tested the longitudinal associations between baseline daily and ethnic discrimination on a task of cognitive control at Wave 3 and the mediating effects of Wave 2 depressive symptoms. We also tested the moderating effects of age, education, and financial difficulties on these links. Structural equation model results indicated that baseline daily discrimination was associated with slower reaction times at Wave 3 and that depressive symptoms at Wave 2 mediated this association; models controlled for age, education, and baseline depressive symptoms. Ethnic discrimination was positively associated with Wave 2 depressive symptoms, but not with cognitive control. Greater daily discrimination was associated with faster reaction times among those with lower financial difficulties. Findings show that greater daily discrimination is subsequently associated with lower cognitive control through increased depressive symptoms and highlight the need for future research to consider the socioeconomic context under which these links operate among Mexican-origin women.

### RISK AND RESILIENCE: HIGHLIGHTING MINORITY STRESS IN A LIFESPAN SAMPLE OF SEXUAL MINORITIES

Michael Vale[1], and Toni Bisconti[2], *1. Sacred Heart University, Fairfield, Connecticut, United States, 2. University of Akron, Akron, Ohio, United States*

Older sexual minorities (OSM), or those older adults sexually attracted to the same sex, are at risk for poorer well-being, which is rooted in the stresses of being marginalized. There is limited knowledge on how older age contextualizes the minority stress process. OSM have witnessed more stigmatized sociopolitical climates, yet report less minority stress compared with younger adults, which may be due to having accrued lifetime practice with stigma. The current study explores age differences in the daily experiences of minority stress in a sample of sexual minorities stratified across the lifespan. Data from a baseline sample of 355 sexual minorities (ages 18-90) confirmed previous research that OSM experienced lower minority stress compared with their younger counterparts (p<.05). Additionally, an age-stratified subset of this sample (N=112; ages 19-79) answered daily surveys for 3 weeks (N=1,923). Using multilevel modeling, we found that older age moderated the daily stress-distress relationships between outness disclosure and positive affect (B=-.005, p<.05); internalized homonegativity and positive affect (B=.004, p <.05) and negative affect (B=-.005, p<.05), and experienced microaggressions and negative affect (B=.007, p<.05). OSM had weaker relationships among four different daily minority stressors and worse daily well-being. There also was support that the associations between age and minority stress were partially mediated by lifelong exposure with marginalization. This project highlights the intersection of sexuality and aging and emphasizes the unique resilience of OSM. The results of this study inform both theory and practice and contribute to a better understanding of how to address OSM' health disparities.

### SEXUAL AND MARITAL SATISFACTION IN OLDER ADULTHOOD: EFFECTS OF CHILDHOOD AND ADULTHOOD VIOLENCE EXPOSURE

Jooyoung Kong[1], Scott Easton[2], and Yan Zhang[1], *1. University of Wisconsin-Madison, Madison, Wisconsin, United States, 2. Boston College, Boston, Massachusetts, United States*

Objectives: Guided by Finkelhor and Browne's traumagenic dynamics model, the current study examines: 1) the long-term effects of childhood sexual abuse (CSA) and other adverse childhood experiences (ACEs) on older adults' sexual and marital satisfaction, 2) the mediating role of intimate partner violence (IPV) victimization in the association between childhood adversities and sexual and marital satisfaction, and 3) whether gender moderates the mediational association. Research Design and

Methods: Using the Wisconsin Longitudinal Study, we analyzed data from 4,239 older adults between the 2004/2005 (Wave 1, Mean age = 65 years) and 2011 (Wave 2, Mean age = 72 years) data collections. The structural modeling approach was employed where the independent variables were CSA and ACEs, the dependent variables were sexual and marital satisfaction in Wave 2, and the mediators were IPV victimization and sexual and marital satisfaction in Wave 1.

Results: Both CSA and ACEs were associated with lower levels of sexual satisfaction and marital satisfaction in late adulthood. IPV victimization mediated the association between ACEs and marital satisfaction. The hypothesized moderated mediation by gender was not supported. Discussion and Implications: Using a population-based sample, this is one of the first studies demonstrating that distal life events such as CSA and ACEs can undermine older adults' sexual and marital health, and revictimization in adulthood may serve as a mechanism for the association. Timely intervention is needed to prevent persistent negative effects of childhood violence.

### THE ROLE OF COPING IN THE ASSOCIATION BETWEEN DISCRIMINATION AND HEALTH OVER TIME IN SEXUAL MINORITY OLDER ADULTS

Christi Nelson, *University of Washington, Edmonds, Washington, United States*

While research on lesbian, gay, and bisexual (i.e., sexual minority) middle-aged and older adults has increased over the past decade, there is still a critical need for more research on the health and resilience in this marginalized group, especially longitudinal research. This study examined the differences in the associations between perceived daily discrimination and the physical and mental health of sexual minority and heterosexual adults over time and assessed the moderating effects of problem-focused and emotion-focused coping on these associations. The sample for this study consisted of sexual minority (n=162) and propensity matched heterosexual (n=324) middle-aged and older adults (mean age at baseline=43 years, SD=12.5) with data from three waves of the Midlife in the United States (MIDUS) study, spanning approximately 20 years. Results found that, for sexual minority participants, reporting higher perceived discrimination was associated with a greater number of chronic conditions at baseline, but was also associated with a significant decrease in the number of chronic conditions over time. For the heterosexual participants, both high and low perceived daily discrimination was associated with an increase in the number of chronic conditions over time. The results of this study also found significant moderating effects of coping on the number of chronic conditions and self-rated mental health over time. Problem-focused coping may protect against the negative effects of discrimination on health. These results are suggestive of a "steeling" effect or a resistance to stress achieved through exposures to adversity for the sexual minority middle-aged and older adults in this study.

### SESSION 4750 (PAPER)

### EXPERIENTIAL SERVICE LEARNING IN GERONTOLOGY

In this session, gerontology educators describe how different experiential strategies can be used to enhance students' aging education across a range of classrooms.

### AN INNOVATIVE MULTIDISCIPLINARY PAID APPRENTICESHIP PROGRAM TO DEVELOP A NEW WORKFORCE IN GERONTOLOGY

Anita Sharma, *University of Louisiana Monroe, Monroe, Louisiana, United States*

There is an acute need to develop new workforce talent in the Gerontology industry due to (a) a rapidly increasing older population; (b) new and evolving needs of older adults due to increased life spans and the long-term impact of COVID-19, and (c) high attrition rates of the current workforce due to death and retirement. One practical strategy to increase new talent is to develop paid apprenticeship opportunities for undergraduate students from different disciplines. The author developed multiple hybrid apprenticeship opportunities to create an "earn and learn" program through funds received from the Louisiana Board of Regents. The program was open to students from all majors because Gerontology is a multi-disciplinary field and there is a need to provide gerontological training to professionals from different disciplines to prepare them to serve older adults in different types of settings. As a pilot project, ten students were selected after an application process. Students in this project were required to complete two three-credit hour fully online courses in Gerontology with a grade of “B” or better. They were also required to complete an eight-hour orientation to help them develop soft skills to work with older adults during their apprenticeship period. They were then required to complete a 150-hour paid summer-apprenticeship in an organization that serves older adults and/or people with disabilities. The program was successful with a 100% completion rate. The students enrolled in this program completed the final step by presenting their apprenticeship experiences at a conference organized by the author.

### DEATH AND GRIEF IN INTERGENERATIONAL SERVICE LEARNING: EXPLORATIONS FROM THE FIELD

Sara Bartlett[1], and Phyllis Solomon[2], *1. Cal Poly, San Luis Obispo, San Luis Obispo, California, United States, 2. University of Pennsylvania, Philadelphia, Pennsylvania, United States*

Intergenerational service-learning has been established as an effective pedagogical strategy for undergraduate gerontology education but can challenge students and instructors alike if an older adult participant dies during such a program. Currently, there is a lack of literature to provide guidance to instructors about how to address such an event. Examination of a case study of such an occurrence during the Lives Well Lived intergenerational service-learning program seeks to advance the field by describing an example of such an event and how an instructor might seek to address it. Grief literature, including theories such as Kübler-Ross' stage model, Worden's TEAR model, and Wortman and Silver's approach, as well as Doka's concept of disenfranchised grief are examined and applied to this challenging situation. Applying these theories as a framework, recommendations are given for addressing death and grief in service-learning including: (1) assessment of the course, instructor, student, and situation; (2) creating opportunities for grief processing and (3) resources for on and off campus grief and instructor support. Providing education on death, grief and grieving as integral course content and allowing student choice in how to proceed with the project should a death occur are key recommendations to consider. Implications call for more research related to this topic.

### QUALITATIVE COMPARISON OF TWO INTERGENERATIONAL SERVICE-LEARNING PROGRAMS

Sara Bartlett[1], Sky Bergman[1], Phyllis Solomon[2], and Zvi Gellis[2], *1. Cal Poly, San Luis Obispo, San Luis Obispo, California, United States, 2. University of Pennsylvania, Philadelphia, Pennsylvania, United States*

Intergenerational service-learning programs are an effective and frequently used training modality in undergraduate education and are often examined using qualitative methods. It is less common to qualitatively examine two such programs to compare their outcomes. This study reports qualitative findings from a mixed-methods study comparing two intergenerational service-learning programs in an undergraduate Psychology of Aging class. The longer, more relational intervention, the Lives Well Lived program, matched students and older adults exemplifying “successful aging” in a mutual interviewing, life review project utilizing documentary film, photography, and memoir creation. The comparison intervention, the Meals That Connect/Lunch Bunch program, also exposed students to older adults exemplifying successful aging, but in a shorter, less relational way. A convenience sample of 128 students (65 in the intervention group and 63 in the comparison group) answered post-intervention open-ended questions about what they liked/disliked about the program in which they participated, as well as any viewpoints about aging they felt changed or were reinforced by the project. Thematic analysis revealed students in both groups experienced decreased ageism and improved attitudes about aging. However, those participating in the Lives Well Lived program had closer relationships with the older adults participating in the project, expressed more positivity about their own aging process, and indicated more willingness to engage in future intergenerational relationships. Use of a comparison project in qualitative examination of intergenerational service-learning adds greater insight into such programs' outcomes, enhancing quantitative effectiveness examinations.

### TEACHING GERONTOLOGY TO SOCIAL WORK STUDENTS: APPLYING THE EXPERIENTIAL LEARNING USING ETHNOGRAPHIC INTERVIEW

Lun Li[1], and Yeonjung Lee[2], *1. MacEwan University, Edmonton, Alberta, Canada, 2. Chung-Ang University, Seoul, Seoul-t'ukpyolsi, Republic of Korea*

There is an increasing need for well-trained social workers to support the growing aging population in Canada. Still, concerns arise regarding social work students' insufficient knowledge and understanding of aging and aging-related issues. This study aims to examine social work students' experience when experiential learning through ethnographic interview with older adults is applied as a pedagogical approach. This study was conducted based on two cohorts of social work undergraduate students who enrolled in a gerontology course in a Canadian university between 2020 and 2021. Students conducted an ethnographic interview with older adults aged 70 years and older and wrote a reflection paper as an assignment. We did a thematic analysis of eight reflection papers in which consent was obtained from students. We find that students connect aging-related theories/models to various topics discussed during their ethnographic interview, reflect on their personal experiences with aging family members, and show a positive perception of aging and attitude towards working with aging. The findings also suggest the benefit of adopting an approach of experiential learning through the ethnographic interview with older adults to teach gerontology to social work students. We offer recommendations for educators to create opportunities for students, especially from social work or other helping professions who traditionally have shown a lack of interest in working with older adults, to meet and interact with older adults, and to further enhance students' competencies and interests in the fields of senior care.

### TRANSFORMING AN INTERGENERATIONAL SERVICE-LEARNING PROJECT DURING THE COVID-19 PANDEMIC

Renee' Zucchero, and Annaliet Delgado-Rodriguez, *Xavier University, Cincinnati, Ohio, United States*

The COVID-19 pandemic has required transformation in the delivery of higher education and pedagogy that is used. The Co-Mentoring Project links undergraduate Psychology of Aging students with older adult volunteers for an intergenerational service-learning experience. Prior to the pandemic, the Project was delivered via an in-person format. During the pandemic, the Project transitioned to a virtual format. Self-reported postproject evaluations from undergraduate students (n= 30) and older adults (n= 27) during the two academic years prior to the pandemic were compared to evaluations from students (n= 26) and older adults (n= 28) during two years of the pandemic. Mann-Whitney U Tests revealed no significant differences in older adult and student postproject evaluation outcomes between in-person and virtual formats. For example, there were no differences in older adult level of enjoyment between the in-person (Md= 5, n= 27) and virtual formats (Md= 5, n= 28), U = 405.00, z = .84, p = .40. Likewise, there no differences in student level of comfort interacting with older adults between in-person (Md= 5, n= 30) and virtual formats (Md= 5, n= 26), U = 389.00, z = -.02, p = .99. Qualitative information from the postproject evaluations indicated participants were glad to have had the opportunity to meet virtually during the pandemic, however they preferred an in-person format. These results support the conclusion that intergenerational service-learning can be successfully implemented virtually. This paper will describe the transformation of the Project from an in-person to virtual format, and advantages and disadvantages of both formats.

### SESSION 4760 (SYMPOSIUM)

### FUNCTION FOCUSED CARE FOR ACUTE CARE: OVERCOMING RECRUITMENT, MEASUREMENT, AND IMPLEMENTATION POST-COVID-19

Chair: Barbara Resnick

To help patients spend more time engaged in physical activities and avoid the complications that occur Function Focused Care for Acute Care was developed (FFC-AC-EIT). FFC-AC-EIT includes the implementation of four steps: (1) Environment and policy assessments; (2) Education of staff; (3) Establishing patient goals; and (4) Mentoring and motivating of staff, patients, and families. A total of 600 patients from 12 hospitals will be included. Eligibility of patients is based on being 55 years of age and older, admitted for a medical reasons excluding COVID-19, and demonstrating evidence of dementia. Outcome measures are obtained at baseline, discharge, 1, 6 and 12 months post discharge and include physical function, physical activity, pain and pain management, psychological and behavioral symptoms associated with dementia, delirium and adverse events (falls, rehospitalizations, nursing home admissions). Due to COVID-19 innovative approaches were implemented to be able to initiate and continue with the study. These included: identifying potential participants that were COVID-19 free off site; transitioning some intervention activities with staff to online; completing verbal consent with proxies versus face to face; adjusting follow up MotionWatch 8 deliveries and placements to be done without face to face interaction; and adjusting recruitment time periods and intervention activities to fit with intermittently high periods of COVID-19. This symposium will describe intervention challenges, solutions and lessons learned, describe an effective process and measurement model for identification of participants with dementia; and provide optimal ways to measure pain and physical activity among older adults with dementia.

### OVERCOMING RECRUITMENT APPROACHES TO IDENTIFY PARTICIPANTS WITH DEMENTIA

Elizabeth Galik, *University of Maryland, Baltimore, Baltimore, Maryland, United States*

Clinically the work up for dementia often includes a history and physical, neuropsychiatric screening measures and neuroimaging. These assessments are neither practical nor realistic when identifying participants for research studies. To confirm a diagnosis of dementia for study participants in the FFC-AC-EIT study we developed a measurement model. The model included four measures: the AD8, the Functional Activities Questionnaire, the Clinical Dementia Rating Scale, and the Saint Louis University Mental Status Examination. In the first 346 patients consented, 176 were enrolled and 158 were ineligible. The mean age of the participants was 80.70 (SD=9.60) and the majority were female (64%) and white (66%). There was evidence of reliability based on internal consistency and construct validity based on model fit using Rasch analysis and Structural Equation Modeling. All four measures are recommended as a pragmatic way in which to comprehensively determine evidence of dementia for research studies.

### OVERCOMING CHALLENGES TO PAIN ASSESSMENT AND FACTORS THAT INFLUENCE PAIN IN PATIENTS WITH DEMENTIA

Ashley Kuzmik, *Penn State University, University Park, Pennsylvania, United States*

The purpose of this study was to describe the optimal way to measure pain among older hospitalized patients with dementia and evaluate the factors that influence pain. The PAINAD is described as a reliable and valid observation measure of pain in this population based on Rasch analysis and was invariant to gender or racial biases. Using this measure and a protocol for observation of pain, pain and associated factors were obtained on the first 112 participants from 6 hospitals in the FFC-AC-EIT study. For descriptive purposes and to guide interventions, factors that were associated with pain were tested. The following factors explained 61% of the variance: functional focused care activities performed, delirium, and quality of care interactions. Those that performed more functional activities, had less delirium, and had higher (more positive) quality of care interactions were less likely to have pain. The findings also supported the use of the PAINAD.

### MEASUREMENT CHALLENGES AND SOLUTIONS WHEN EVALUATING PHYSICAL ACTIVITY AMONG HOSPITALIZED PATIENTS WITH DEMENTIA

Brittany Drazich, *University of Maryland, Baltimore, Maryland, United States*

Obtaining subjective reports of physical activity from individuals is known to be biased. With increased creation, dissemination, and use of technology, approaches for objective measurement have improved. Although actigraphy is increasingly used, challenges include a lack of clearly established cut off levels for vigorous, moderate, and low level activity in older individuals, decreased arm movement with walking, and the impact of comorbidities (e.g., Parkinson's Disease) on actigraphy readings. The advantages of the MotionWatch 8 over other devices will be discussed and our protocol for use shared. The findings from the first 200 participants will be described and challenges to interpretation addressed. The majority of the study participants were willing to wear the MotionWatch 8 during hospitalization. Using innovative approaches to avoid direct patient contact due to concerns related to COVID-19, follow up activity was obtained at 1 month post discharge. Lastly, recommendations for interpretation of findings will be provided.

### IMPLEMENTING FUNCTION-FOCUSED CARE IN THE HOSPITAL: LESSONS LEARNED

Marie Boltz, *Penn State, Pennsylvania State University, Pennsylvania, United States*

The mentorship provided by the nurse interventionist is an important implementation strategy to support Function-focused care adoption in hospitalized older adults with dementia. The nurse interventionist works with unit champions and interdisciplinary stakeholder teams over a 12 month period. The first interaction includes using a brainstorming approach to develop unit-specific goals to support the integration of function-focused care into routine care delivery. Implementation strategies at the unit level include flexible staff training, involving staff in evaluations of care interactions, targeting pragmatic measures, and a feedback loop. Based on content analysis of field notes we will describe stakeholder goals established, action plans, and barriers and facilitators to meet goals in four hospital units. Key findings include the influence of leadership support, communication strategies, engagement of direct care staff, interdisciplinary collaboration, access to human and material resources, and contextual factors within the hospital setting (including pandemic-related challenges).

### SESSION 4770 (PAPER)

### HEALTH AND SOCIAL SERVICES: FROM COMMUNITY AND POST-ACUTE CARE INTERVENTIONS TO TRAUMA-INFORMED CARE SURVEY RESEARCH

### A CITATION REVIEW OF DISSEMINATION AND IMPLEMENTATION MODELS UTILIZED IN AGING RESEARCH WITHIN THE UNITED STATES

Jennifer Sullivan[1], Anna Rae Montano[2], Heather Davila[3], Marlena Shin[4], Chelsea Hawley[5], Jaime Hughes[6], Kelly O'Malley[4], and Camilla Pimentel[5], *1. VA LTSS COIN / Brown University, Providence, Rhode Island, United States, 2. VA Providence Healthcare System, Providence, Rhode Island, United States, 3. VA Iowa, Iowa City, Iowa, United States, 4. VA Boston Healthcare System, Boston, Massachusetts, United States, 5. VA Bedford Healthcare System, Bedford, Massachusetts, United States, 6. Wake Forest, Wake Forest, North Carolina, United States*

The application of implementation science in aging research has been growing. To our knowledge, there has been no study detailing the Dissemination and Implementation (D&I) Models utilized in aging research. The goal of this citation review is to further understand D&I models frequency and nature of their use in aging research. We identified 111 Dissemination and Implementation (D&I) Models compiled on the dissemination-implementation.org website. We then conducted a citation analysis on them, searching Web of Science and PubMed databases. We extracted key data from identified articles up to January 28, 2022. Search terms were broad and included aging, older, elderly and geriatric. To be included, articles had to be in peer-reviewed journals, in English, and occur in the United States. We identified 297 articles meeting our eligibility criteria. The nature of the way D&I models used to advance evidence-based practice in aging research and practice varied as did the number of citations over time. Of the D&I models included in this review, only one (4E Framework) was developed within the aging research field. The top five models included: CFIR, RE-AIM 1.0, Behavior Change Wheel, Greenhalgh Diffusion of innovation in Service Organization and CBPR. Citations were distributed across many frameworks and yet only totaled less than 1% of all D&I Model citations suggesting there are many ways the field can grow in the future.

### COMPLEXITY IN PSYCHOSOCIAL INTERVENTIONS: CASE STUDIES FROM A STROKE TRANSITIONS TRIAL

Emmanuel Chima[1], Amanda Woodward[2], Anne Hughes[1], Michele Fritz[1], Paul Freddolino[1], Sarah Swierenga[1], Constantinos Coursaris[3], and Mathew Reeves[1], *1. Michigan State University, East Lansing, Michigan, United States, 2. Michigan State University, Ann Arbor, Michigan, United States, 3. HEC Montreal, Montreal, Quebec, Canada*

The Michigan Stroke Transitions Trial (MISTT) tested whether in-home social work case management (SWCM) or SWCM combined with access to a website providing stoke-related information improved outcomes relative to usual care for patients discharged home post-stroke and their caregivers. The aims of this secondary analysis are 1) to describe the actual support social work case managers (SWCM) provided to MISTT participants and 2) use select case studies to illustrate the relationship between SWCM and quantitative patient and caregiver outcomes. Data for the study were derived from SWCM case notes on 157

patients and their caregivers who received the MISTT intervention. Case notes were coded in two steps with a subset of cases coded by two researchers and reviewed for interrater reliability in each step. The first round of coding was guided by primary SWCM intervention goals. The second round of coding identified SWCM sub-themes within each primary goal. Key themes indicate SWCMs aided with understanding the post-hospitalization period, helped patients navigate a range of systems and services, identified needs and supported patient goals, provided psychosocial support, and centered support on stroke recovery and prevention. Case studies illustrate ways in which SWCM were key supports during the transition period, but that support does not cleanly align with quantitative findings from patient-reported outcomes. This study aligns with a growing body of work documenting the complexity of transitions of care and has implications for how we support patients and caregivers as they move from inpatient to outpatient care and measure outcomes.

### HOME-DELIVERED MEAL DELIVERIES AND FEELINGS OF SAFETY FOR OLDER AMERICANS ACT PARTICIPANTS

Claire Pendergrast[1], and Heather Menne[2], *1. Syracuse University, Syracuse, New York, United States, 2. RTI International, Washington, District of Columbia, United States*

Home-delivered meals supported by the Older Americans Act (OAA) serve a dual purpose of improving nutritional intake and providing regular social contact for older adults. This regular contact can increase feelings of safety experienced by meal recipients. The benefits of home-delivered meal services may vary between meal recipients based on sociodemographic characteristics. Variation in home-delivered meal clients' reports of feeling safer at home because of regular meal delivery visits was examined to support ongoing efforts to increase social engagement and equity through the delivery of OAA services. Using data from the 2019 National Survey of Older Americans Act Participants (NSOAAP) home-delivered meal module, descriptive statistics and logistic regression were conducted to identify the characteristics associated with feeling safer at home because of meal delivery visits. The majority (85%) of meal recipients report feeling safer because of meal delivery visits, and rates were especially high for rural recipients (92%), those with a high school education or less (89%),racial/ethnic minorities (94%), and those with three or more ADL limitations (90%). Logistic regression found that rural residence (OR=3.3), lower educational attainment (OR=2.0), racial/ethnic minority status (OR=4.7), living alone (OR=1.6), and having 3+ ADLs (OR=1.9) were significantly associated with higher odds of feeling safer at home because of meal delivery visits; however, age, gender, and suburban residence were not significant. Findings suggest that benefits of home-delivered meal programs are supporting the needs of traditionally disadvantaged groups and broadly increasing meal recipients' sense of safety in their homes.

### LINKING DISADVANTAGED HEALTHCARE PATIENTS TO HOME- AND COMMUNITY-BASED SERVICES: CAN NEEDS BE MET?

Farida Ejaz, Miriam Rose, and Courtney Reynolds, *Benjamin Rose Institute on Aging, Cleveland, Ohio, United States*

As part of a larger study examining the social determinants of health, the current analysis focuses on 254 older and disabled, Medicare Advantage patients from 19 primary care clinics in Texas. The patients faced challenges such as depression, limitations in activities of daily living (ADL) and dementia. They received a home-based, social work intervention to examine their needs, an individualized care plan was created, they were offered home and community-based services, and followed over a four-month period. The median age of the sample was 69 years, 71% were Hispanic/Latino, 80% had a high school education or less, and 76% had a monthly income of less than $1,361. A total of 823 needs were identified in these patients, and 1,126 service recommendations were made. Some needs required more than one service recommendation or vice versa. The most frequently identified needs involved food assistance (136 patients received 220 nutritional service recommendations), home modifications/housing (118 patients offered 159 services), and ADLs (115 patients, 147 services). During the four-month period, social workers reported that services related to food assistance met patient needs 61% of the time; 52% for home modifications/housing; and 75% for ADLs. Reasons for unmet needs included service applications still in process/waiting lists; services being unavailable (e.g., lack of mental health providers); and refusals by patients, and family/friends. Practice and policy implications include the possibility that four months is not enough time to fully address needs, and some patients may need more intensive assistance and motivation to apply for and access services

### SESSION 4780 (SYMPOSIUM)

### HOME HEALTHCARE ACCESS AND UTILIZATION AMONG VULNERABLE POPULATIONS IN THE US AND PUERTO RICO

Chair: Maricruz Rivera-Hernandez

Home health (HH) care utilization among Medicare beneficiaries has exploded, serving over 3.3 million users (The Medicare Payment Advisory Commission, 2021). With the aging of the population and the preference for Home- and Community-Based Services and/or person-centered care delivered in the home, HH care use is expected to continue increasing. Prior research has highlighted inequitable access and use of HH rooted in structural and social determinants of health (Fashaw-Walters et al., 2022). However, more research is needed about disparities in timely access to quality of care and outcomes among racial and ethnic and rural populations. Furthermore, the literature regarding post-acute care has often excluded Puerto Rico HH beneficiaries (Rivera-Hernandez et al., 2020). Given the need to ensure equity of care post-pandemic, there is a pressing need to understand disparities in care and outcomes, specifically using representative data. This symposium will feature four presentations that provide novel insight regarding HH care utilization and outcomes among vulnerable populations. Individual presentations will describe 1) Home health care services utilization among Medicare beneficiaries in Puerto Rico; 2) Public reporting role in exacerbating disparities in access; 3) Differences in home healthcare latency following hospitalization for ADRD patients by race/ethnicity and rural and urban locations; 4) Racial disparity in the start of home healthcare in high-risk ADRD patients by the quality of home health and impact on rehospitalization. In addition, studies in this panel will discuss policy and clinical implications, as well as directions for future research regarding equitable access and health outcomes among HH users.

### THE IMPACT OF PUBLIC REPORTING ON INEQUITIES IN HIGH-QUALITY HOME HEALTH UTILIZATION

Shekinah Fashaw-Walters[1], Momotazur Rahman[2], Gilbert Gee[3], Vincent Mor[2], and Kali Thomas[2], *1. University of Minnesota School of Public Health, Minneapolis, Minnesota, United States, 2. Brown University, Providence, Rhode Island, United States, 3. UCLA, Los Angeles, California, United States*

Literature suggests that public reporting of quality may exacerbate disparities in access to high-quality post-acute and long-term care for older adults. The objective of this study was to evaluate the impact of the home health (HH) 5-star ratings on changes in high-quality HH agency (HHA) use, by race, ethnicity, income level, and place-based factors. Using a difference-in-differences framework, we found that after introduction of 5-star ratings adjusted rates of high-quality HHA use increased for all HH users, except for Latinx, Asian American/Pacific Islander, and low-income HH users, and disparities in access to high-quality HHAs were exacerbated for each of these groups. Last, we found that users within predominantly Latinx and lower income neighborhoods had a significantly greater decrease in their use of high-quality HHAs. Policymakers should be aware of the potential unintended consequences of public reporting and should consider adding measures of equity to the publicly reported information.

### RACE AND RURAL-URBAN DIFFERENCES IN HOME HEALTHCARE LATENCY IN PATIENTS WITH DEMENTIA

Amit Kumar, *Northern Arizona University, Flagstaff, Arizona, United States*

Given the need to ensure equity of timely access to post-acute care among minority groups in rural areas, there is a pressing need to understand disparities in the start of home healthcare among minorities with Alzheimer's disease and Related Dementia (ADRD). This study investigates differences in the timing of initiation of home healthcare services following acute hospitalization for ADRD patients by race/ethnicity and rural and urban locations. A secondary analysis was conducted among older adults with ADRD using Medicare data, discharged to home following an episode of acute hospitalization in 2016-2017. The study outcome was a delay in the start of home healthcare after two days of hospital discharge, defined as a home health latency. Compared to non-Hispanic Whites residing in urban areas, Blacks living in urban areas and Hispanics living in rural areas have a significantly higher odd of home health latency 1.15 (95%CI;1.11-1.18) and 1.06 (95%CI;1.02-1.10), respectively.

### RACIAL DISPARITY IN THE START OF HOME HEALTHCARE IN HIGH-RISK ADRD PATIENTS BY THE QUALITY OF HOME HEALTH

Indrakshi Roy, *Northern Arizona University, FLAGSTAFF, Arizona, United States*

Improving the quality and timely access of home health care is a new quality measure and particularly crucial in high-risk ADRD adults following hospitalization. However, a significant portion of older patients waits longer than 2 days to receive home healthcare. In this study, we examine how the quality of home health agency and race are associated with the delay in care among ADRD patients receiving home healthcare and how this delay mitigates the risk of rehospitalization. We find that Black patients in low rated home health agencies have 28% higher odds of delay in care compared to White patients in high rated home health agencies (Odds ratio (95% CI) =1.28 (1.21 - 1.36)). Timely initiation of home health care also reduces the risk of rehospitalization in minority older adults with ADRD.

### SESSION 4790 (SYMPOSIUM)

### INNOVATIONS IN REMOTE SUPPORT FOR DEMENTIA FAMILY CAREGIVERS

Chair: Kylie Meyer Co-Chair: Lyndsey Miller Discussant: Jeffrey Kaye

Remote delivery of dementia caregiver interventions can decrease delivery costs, and make it more feasible to provide evidence-based interventions to caregivers across the country. As the science behind remote delivery develops, new technologies and their applications can ensure preservation of important intervention components and principles, as well as novel forms of data collection. In this symposium, investigators will present on studies that demonstrate how technology can be used to improve delivery and assessment of remote caregiver interventions. Walter Dawson, D.Phil, will share findings from the Support via Technology: Living and Learning with Advancing Alzheimer's disease and related dementia (STELLA) intervention. Using secondary data collected via weekly survey, he examined the association between costs of care and behavioral symptoms of dementia. Next, Allison Gibson, PhD, MSW, will present results from focus groups about caregivers' experiences of the Harmony at HOME (H@H), a telehealth intervention to improve person-environment fit and limit behavioral symptoms of dementia. Kylie Meyer, PhD, will present results from the Learning Skills Together intervention, which uses teleconferencing to teach family caregivers how to provide complex care tasks while adhering to self-efficacy theory. Lastly, Shirin Hiatt, MPH, MS, RN, will present findings from the REmote Assessment and Dynamic Response (READyR) study, which tests the application of remote monitoring technology to assess adherence to value-based care (e.g., autonomy) among spousal family care partners. Each study was supported by the Emory University Roybal Center for Dementia Caregiving Mastery or Oregon Roybal Center for Care Support Translational Research Advantaged by Integrating Technology.

### STICK TO THE THEORY: FINDINGS FROM A COMPLEX CARE INTERVENTION FOLLOWING TRANSITION TO DIGITAL DELIVERY

Kylie Meyer[1], and Carole White[2], *1. Case Western Reserve University, Cleveland, Ohio, United States, 2. UT Health San Antonio, San Antonio, Texas, United States*

Two thirds of family caregivers to persons living with dementia provide complex care tasks, including medical/nursing tasks, and nearly half worry about making a mistake. Learning Skills Together (LST) was designed to prepare caregivers to provide complex care through hands-on instruction (e.g., practice using a gait belt). Consistent with self-efficacy theory, the in-person intervention integrated behavioral modeling, strengths-based feedback, and knowledge-building. COVID-19 prompted a transition to digital delivery of LST over Zoom. Intervention content was modified to accommodate a digital approach while continuing to adhere to self-efficacy theory. Results from a pre- and post-test pilot study (N=35) indicate improvement in self-efficacy on (mean difference (MD)=1.0, SD= 1.6, p-value=0.004). Caregiver comments during qualitative interviews affirm intervention objectives were met. For example, caregivers described the importance of peer learning (modeling) during discussion. Results indicate that complex care intervention can be digitally delivered to family caregivers to improve self-efficacy surrounding complex care.

### THE READYR PROGRAM'S INTERIM FEASIBILITY, ACCEPTABILITY, AND EFFECTS ON VALUES-BASED CARE AND RELATIONSHIP QUALITY

Shirin Hiatt[1], Joel Steele[2], Chao-Yi Wu[1], and Lyndsey Miller[1], *1. Oregon Health & Science University, Portland, Oregon, United States, 2. Portland State University, Portland, Oregon, United States*

This study evaluated preliminary feasibility, acceptability and efficacy of the READyR program, a remote intervention for early stage dementia dyads designed to assess and address values-based care and relationship quality. Measures of program acceptability, preparation for future care, and relationship quality were examined pre- and post-intervention. Feasibility was high among the first 10 enrolled dyads who completed phase 1: 39 out of 40 sessions were completed with 100% retention. Acceptability was also high with nearly 95% reporting that participation was easy and that they would recommend it to others. Insufficient time was the only critique reported by 17%. Medium/large effects were found in improvements to positive interactions (d=-0.71 95%CI [-1.34-.00]), relationship strain (d=0.53 95%CI [.-.15-1.18]), and awareness (d=-0.91 95%CI [-1.64- -0.15]) and avoidance (d=-0.53 95%CI [-1.18-.15]) of preparation for future care needs. The READyR program shows preliminary feasibility, acceptability, and efficacy in improving values-based care and relationship quality remotely.

### INCREASING DEMENTIA CARE MASTERY FOR RURAL CAREGIVERS: LESSONS FROM ADAPTING THE HARMONY @ HOME INTERVENTION

Allison Gibson[1], Laura Henley[2], Vickie Fairchild[3], David Goss[3], Carolyn Baum[4], Gregory Jicha[1], and Elizabeth Rhodus[1], *1. University of Kentucky, Lexington, Kentucky, United States, 2. HealthPRO Heritage, Louisville, Kentucky, United States, 3. Northeast AHEC, St. Clair Healthcare, Morehead, Kentucky, United States, 4. Washington University School of Medicine in St. Louis, St. Louis, Missouri, United States*

Providing care support to rural Alzheimer's disease and related dementia (ADRD) caregivers has always been challenging, but particularly with the COVID-19 pandemic, new barriers have emerged for families in accessing care support. Delivered via telehealth, Harmony at HOME (H@H) provides dementia care mastery for caregivers of persons with ADRD in the skills of assessing and modifying the home environment to promote "person-environment fit," whereby increasing functional activity engagement and minimizing maladaptive behaviors. To enhance care supports specific to the needs of rural communities, this study reports the findings of two focus groups with rural caregivers who participated in the H@H intervention. Focus groups were aimed to identify the needs of caregivers in rural communities to guide adaptation approaches for future implementation of the H@H intervention in these underserved areas. Additional studies to assess real-world efficacy of the adaptations to H@H are needed as a next step toward implementing this intervention in clinical, home care settings.

### WHAT DO 'BEHAVIORS" COST? UNDERSTANDING COSTS IN DEMENTIA CARE PARTNER-BASED RESEARCH

Walter Dawson[1], Sarah Gothard[1], Nora Mattek[2], Jeffrey Kaye[1], and Allison Lindauer[1], *1. Oregon Health & Science University, Portland, Oregon, United States, 2. Oregon Health and Science University, Portland, Oregon, United States*

The out-of-pocket costs to care for individuals living with dementia are high, and is exacerbated by the expense of managing behavioral and psychological symptoms of dementia (BPSD) (e.g., depression, irritability). STELLA (Support via TEchnology: Living and Learning with Advancing AD) is a telehealth-based intervention that provides a personalized approach to teach care partners (CPs) strategies to manage BPSD. To understand the relationship between BPSDs and costs, weekly surveys were administered to CPs (n=12). Surveys asked about out-of-pocket costs incurred from: hospitalizations and emergency department (ED) utilization, primary care visits, use of paid in-home care, prescription drugs, and over-the-counter medications. The most frequent cost reported by CPs was prescription drug related (11 CPs), while costs associated with hospitalizations and ED were the least frequently reported (4 CPs). A longitudinal weekly survey-based approach to quantify CP costs is a novel approach examine an intervention outcome (cost) that matters to families and policymakers.

### SESSION 4800 (SYMPOSIUM)

### NOVEL BEHAVIORAL INTERVENTIONS TARGETING COGNITIVE-MOTOR NEUROPLASTICITY IN OLDER ADULTS

Chair: Tanvi Bhatt Co-Chair: Susan Hughes Discussant: Susan Hughes

Comparatively little is known about the ability of physical activity to improve or maintain cognitive function in older adults. Our Midwest Roybal Center uses social/behavioral theory to promote engagement in physical activity in order to improve this outcome. In 2019, we received funding to establish a Multimodal Connectomics Core that enabled investigators to examine concurrent changes in cognitive function and brain connectivity by providing access to fMRI imaging. The Connectomics Core enabled investigators for the first time to examine cognitive function and changes in brain connectivity simultaneously by providing access to fMRI imaging as well as MRI data storage and data analysis. It enabled measuring of standardized participant outcomes selected from the NIH toolbox. This Symposium describes findings from the first three completed pilots and discusses their implications for the field. The first pilot by Bronas examined effect of a 6-month home-based walking program in people chronic kidney disease on cognitive function and intervention induced neuroplasticity compared to usual care. The second pilot by Marquez adapted the Fit & Strong! program to test the combined impact of physical activity and interactive health education for managing mood on cognitive function among older adults with depression. The third pilot by Bhatt examined the effectiveness of 4 weeks of exergaming combined explicit cognitive training on behavioral measures of fall-risk (physical and cognitive) and their associations with brain integrity measures. The discussant will initiate discussion based on outcomes of these pilots and future directions for developing them into larger studies or clinical translation.

### DUAL-TASK TRAINING FOR IMPROVING COGNITIVE-MOTOR INTERFERENCE

Tanvi bhatt[1], and Lakshmi Kannan[2], *1. University of Illinois at Chicago, Chicago,, Illinois, United States, 2. University of Illinois at Chicago, Chicago, Illinois, United States*

Dr. Bhatt will discuss the differences in types of cognitive motor interference patterns experienced for different tasks (gaitand volitional versus reactive balance) in cognitively intact versus people with mild cognitive impairment. She will discuss the effectiveness of dual-task training and exergaming on gait, volitional and reactive balance control in older adults with mild cognitive impairment. After 4 weeks of such training, the results showed beneficial effects on improving volitional based performance when performed with a cognitive task (i.e., spatial memory and executive function) and had significant improvement in NIH toolbox (cognitive- increased working memory, episodic memory and executive function, and motor-increased gait speed). However, its positive effects on dual task reactive balance control were limited. Additionally, the speaker will go on to discuss the associations of balance control deficits in mild cognitive impairment with neural correlates (structural and functional brain integrity) to understand the attributing factors to increased fall risk in people with mild cognitive impairment.

### ENHANCING WELL-BEING THROUGH EXERCISE DURING OLDER AGE

David Marquez, *University of Illinois Chicago, Chicago, Illinois, United States*

Dr. Marquez's Roybal pilot Enhancing Well-Being Through Exercise During Older Age piloted an adaptation of a group exercise evidence-based program for people with arthritis symptoms, Fit & Strong!, that included education on emotional wellbeing and mental health. The pilot used a waitlist design to test the 8-week program among Black residents reporting depressive symptoms living in a senior housing facility. A battery of physical, emotional, and cognitive outcomes were collected pre and post intervention and a subset of participants (n=11) received MRIs pre and post. The sample included 28 participants (n=13 intervention, n=15 waitlist) who had a mean age of 69.8, were 89% female, reported a mean of 3.6 chronic conditions, and an average WOMAC pain score of 5.7 (moderate pain). Though under-powered, analyses show greater improvements in key physical activity and cognitive domains among the intervention group compared to the waitlist group.

### EFFECT OF A 6-MONTH HOME-BASED WALKING PROGRAM ON COGNITIVE FITNESS IN OLDER ADULTS WITH CHRONIC KIDNEY DISEASE

Ulf Bronas, *University of Illinois Chicago, Chicago, Illinois, United States*

We have previously demonstrated that cognitive status and white matter integrity are highly associated with physical fitness in older adult patients with chronic kidney disease (CKD). We present data from a two group RCT determining effect of a 6-month home-based walking intervention on physical and cognitive fitness compared to usual care. The intervention included use of a wearable activity monitor, weekly didactic phone meetings, interactive tools and monthly coach-delivered feedback. The intervention group had a 78% compliance rate to the 6-month exercise program (159.9 (149.2) minutes/week). Executive function composite score and global cognitive for intervention group > than control group. Additionally, global white matter integrity and functional connectivity improved in the intervention group and declined in the usual care group. We conclude that a home-based physical activity intervention can have significant improvement on cognitive health and neuroplasticity in older adults with CKD and lowering their risk of developing AD/ADRD

### SESSION 4810 (SYMPOSIUM)

### NOVEL GENOMIC AND EXCEPTIONAL LONGEVITY FINDINGS FROM THE LONG LIFE FAMILY STUDY

Chair: Mary Wojczynski Co-Chair: Nancy W. Glynn
Discussant: Evan Hadley

The Long Life Family Study (LLFS), funded by the National Institute on Aging, is an international collaborative study of the genetics and familial components of exceptional longevity and healthy aging. We phenotyped 4,953 individuals from 539 two-generational families (1,727 proband; 3,226 offspring) at baseline (2006-2009). A second visit (2014-2017) was conducted for 2,904 (478 proband; 2,426 offspring) participants. The longitudinal, comprehensive in-person visits measured domains of healthy aging, including physical performance, cognition, and blood markers. Extensive genetic analyses were performed using the baseline blood draw, including genotyping with the Illumina 2.5M Human Omni array, linkage analyses with the families, whole genome sequencing using the TopMED protocol, and metabolomic assays. Collectively, this symposium will present novel findings that examined differences in end of life events and cause of death between non-exceptional and exceptional long-lived women, elucidate potential rare variants associated with exceptional longevity, and examine new potential metabolomics pathways involved in gait speed and cardiovascular disease. Specifically, Dr. Galvin will share results from three Danish nationwide studies (including LLFS) on different end of life events in long-lived female siblings. Then, Dr. Gurinovich will share findings on new uncommon variants associated with extreme longevity. Next, Dr. Kuipers will discuss associations between lipid metabolomics and vascular health. Lastly, Dr. Santanasto will discuss lipid metabolomics associated with lower odds of slow gait speed. Dr. Evan Hadley, NIA, will be the discussant and will share insights and propose future directions for LLFS.

### END-OF-LIFE EVENTS AND CAUSES OF DEATH IN DANISH LONG-LIVED FEMALE SIBLINGS

Angeline Galvin[1], Svetlana Ukraintseva[2], Konstantin Arbeev[2], Mary Feitosa[3], Anne Newman[4], and Kaare Christensen[5], *1. University of Southern Denmark, Odense, Syddanmark, Denmark, 2. Duke University, Durham, North Carolina, United States, 3. Washington University, St Louis, Missouri, United States, 4. University of Pittsburgh, Pittsburgh, Pennsylvania, United States, 5. Southern Denmark University, Odense, Syddanmark, Denmark*

Long-lived siblings have better health and survival compared to “sporadic” long-lived individuals, but it is unknown whether they also differ in end-of-life events and causes of death. Deceased Danish long-lived female siblings (n=833, mean age at death=95.6) were identified through national health registers compared to controls matched on sex, year-of-birth, and year-of-death. End-of-life events (hospitalizations, emergency room visits, medication within the five years before death) and causes of death were analyzed using linear models with fixed effects and multinomial logistic models, respectively. End-of-life events and causes of death were not statistically significantly different between long-lived female siblings and “sporadic” long-lived individuals. However, long-lived female siblings presented non-significant higher risk of ischemic heart disease and cancer – and lower risk of mental diseases and accidents. The analyses will be extended to include men, a longer follow-up, and focus on dementia in the last years of life.

### GENOME-WIDE ASSOCIATION STUDY OF EXTREME LONGEVITY USING WHOLE-GENOME SEQUENCING DATA

Anastasia Gurinovich[1], Harold Bae[2], Zeyuan Song[3], Anastasia Leshchyk[3], Mengze Li[3], Stacy Andersen[4], Thomas Perls[3], and Paola Sebastiani[1], *1. Tufts Medical Center, Boston, Massachusetts, United States, 2. Oregon State University, Corvallis, Oregon, United States, 3. Boston University, Boston, Massachusetts, United States, 4. Boston University School of Medicine, Boston, Massachusetts, United States*

Extreme longevity (EL) runs in families which supports the hypothesis that this is a genetically regulated trait. However, with the exception of APOE, genome-wide association studies (GWAS) of EL have not identified many genetic variants that replicate in independent studies. The majority of GWAS of EL have used imputed genotype data. Recently, the Long Life Family Study has generated the largest whole-genome sequencing data of centenarians and matched controls. We perform single-variant and gene-based tests of EL in these data using the nf-gwas-pipeline with the saddle point approximation adjustment of the p-values. These analyses suggest that, in addition to the APOE/TOMM40 region, some uncommon variants of GRM7 (chr3), AUTS2 (chr7), KIF13B (chr8), SLC2A14 (chr12), and ADCY9 (chr16) genes, and other intergenic SNPs in chromosomes 5, 10, and 20 may be implicated with EL.

### ASSOCIATION OF PLASMA LIPID METABOLITES WITH ANKLE-BRACHIAL INDEX IN THE LONG LIFE FAMILY STUDY

Allison Kuipers[1], Ryan Cvejkus[1], Bharat Thyagarajan[2], Gary Patti[3], Mary Feitosa[4], Jonas Mengel-From[5], Emma Barinas-Mitchell[1], and Joseph Zmuda[1], *1. University of Pittsburgh, Pittsburgh, Pennsylvania, United States, 2. University of Minnesota Twin Cities, MINNEAPOLIS, Minnesota, United States, 3. Washington University, Department of Chemistry, Genetics, and Medicine, St. Louis, Missouri, United States,*

*4. Washington University, St Louis, Missouri, United States, 5. University of Southern Denmark, Odense, Syddanmark, Denmark*

We tested the association of lipid metabolites with ankle-brachial index (ABI) in the LLFS. Minimum ABI from doppler was used. Participants underwent phlebotomy after >8 hours fasting. Plasma metabolites were isolated via solid phase extraction. Lipid metabolites (N=193) were measured using RPLC-MS and corrected for batch effects in 948 participants. We used linear mixed models adjusted for age, sex, site, fasting, and relatedness. Twenty metabolites were associated with ABI (FDR P<0.05), of which 17/20 were triacyclglycerol (TAG) species, plus cholesterol ester (22:6), phosphatidylcholine (35:7), and phosphatidylethanolamine (38:7). After additional adjustment for traditional ABI risk factors, only two TAGs (50:1, 42:0) were significantly associated after multiple testing correction. While not significant in the base model, the metabolite lysophosphatidylcholine (18:3) demonstrated a marginally significant protective association after full adjustment (P=0.014). While triglycerides are known correlates of atherosclerotic deposition, these findings may identify novel metabolic correlates of peripheral vascular health in long-lived individuals.

### LIPIDOMIC ANALYSIS OF GAIT SPEED IN THE LONG LIFE FAMILY STUDY

Adam Santanasto[1], Mary Fiestosa[2], Michael Lustgarten[3], Kaare Christensen[4], Gary Patti[5], Joseph Zmuda[1], Ryan Cvejkus[1], and Mary Wojczynski[5], *1. School of Public Health, University of Pittsburgh, Oakmont, Pennsylvania, United States, 2. Washington University School of Medicine in St. Louis, St. Louis, Missouri, United States, 3. Tufts University, Boston, Massachusetts, United States, 4. Southern Denmark University, Odense, Syddanmark, Denmark, 5. Washington University in St. Louis, St. Louis, Missouri, United States*

Gait speed is a predictor of overall health and mortality in older adults. Metabolomics may provide insights into biological mechanisms underlying gait speed. Herein, we examined the association between 193 lipid metabolites with gait speed in 1,717 adults (52.1% women) aged 82.0 ± 14.5. Lipidomic analysis was performed using liquid chromatography-mass spectrometry. Gait speed was measured over 4-meters and slowness was defined as <0.8m/s. Logistic regression, adjusted for age, sex, field center, height, fasting-duration and familial-relatedness, were used to examine the association between log-transformed metabolites with slowness. A false discovery rate (FDR) of p<0.05 was employed to account for multiple comparisons. Gait speed was 0.83 ± 0.32 and 53.4% had slowness. Three lipid metabolites were significantly associated with lower odds of slowness: an acylcarnitine, sphingomyelin and a ceramide non-hydroxy fatty acid-sphingosine. Our results potentially link lipids involved with mitochondrial beta-oxidation and nerve signal transduction to gait speed in older adults.

### SESSION 4820 (PAPER)

### NURSING HOME RESOURCES, PATTERNS OF CARE DURING THE PANDEMIC, AND QUALITY

### DOES FIVE-STAR QUALITY RATING MATTER FOR NURSING HOME QUALITY DURING THE COVID-19 PANDEMIC?

Lei Yu, Xiao Qiu, and Na Sun, *Miami University, Oxford, Ohio, United States*

Objectives Nursing homes (NH) confronted tremendous difficulties considering confirmed residents Covid-19 cases and deaths in the U.S. The Centers for Medicare & Medicaid Services (CMS) applies the Five-Star Quality Rating (FSQR) to indicate the quality of care in nursing homes based on health inspection surveys, staffing as well as care process and resident outcomes during the COVID-19 pandemic. This study aims to examine whether FSQR was related to the total number of NH resident Covid-19 cases and deaths.Design This study analyzed 6,978 nursing homes across the country with data from CMS Nursing Home Compare, CMS COVID-19 Nursing Home Public File, Long-term Care Focus, Payroll Based Journal, Rural-Urban Commuting Area. Negative binomial regressions were used to investigate associations between FSQR and NH COIVD-19 outcomes controlling for state fixed effects and clustering of nursing homes within counties. The characteristics of facility, residents, payer-mix, nursing staff, and geographic location were also controlled. ResultsComparing to NH with 1-star in Health Inspection, Staffing, or Overall ratings, NH with better performance have lower risk of having increased number of COVID cases and deaths among residents. Further, nursing home Quality Measures rating is not significantly associated with residents' COVID-19 deaths. ConclusionOverall, the FSQR is a useful measure of quality in part when investigating NH's performance during the COVID-19 pandemic. Future policymakers should pay special attention to providers performing poorly in FSQR when improving the quality of nursing homes, particularly regarding infection control.

### HOSPICE UTILIZATION AMONG LONG-STAY NURSING HOME RESIDENTS DURING THE COVID-19 PANDEMIC

Xiao (Joyce) Wang[1], Emma Belanger[1], Kali Thomas[1], Debra Dobbs[2], and David Dosa[1], *1. Brown University, Providence, Rhode Island, United States, 2. University of South Florida, Tampa, Florida, United States*

Background: The COVID-19 pandemic has had significant impacts on nursing home residents. This study aims to examine how hospice utilization changed among long-stay nursing home residents between January and September in 2020, as compared with the same period in 2019, nationally. Design: A retrospective cohort study of residents present in US nursing homes as long-stay as of January 1st, 2019 and 2020, respectively. A subgroup of residents who died from January to September in each year was also examined. We utilized the Minimum Data and multiple administrative claims data. We compared hospice utilization rate between 2019 and 2020 nationally and by state. Outcomes: This study examined: 1) any hospice utilization among long-stay residents from January to September in 2019 and 2020 respectively, and 2) hospice utilization in the last 30 days of life among the decedent subgroup, which we also tracked as a factor of percent change in mortality rate at the state level.

Results: The hospice utilization rate among long-stay residents was 19.4% in 2019 and 19.7% in 2020. The rate was 27.5% in 2019 and 24.2% in 2020 among the decedent subgroup ($\chi2=553.1$, $p< 0.001$), although the absolute number of decedents using hospice in the last 30 days of life was higher in 2020 than 2019. Substantial state variation in hospice utilization was observed, mostly following patterns in community-level infections.

Conclusions: Hospice managed to continue service delivery despite many challenges. The pandemic highlights the importance of integrating hospice and palliative care into emergency preparedness planning.

### NORTH CAROLINA NURSING HOME AND PUBLIC HEALTH RESILIENCE DURING 2021 OF THE COVID-19 PANDEMIC

Sandi Lane, Trent Spaulding, Adam Hege, Maggie Sugg, and Lakshmi Iyer, *Appalachian State University, Boone, North Carolina, United States*

Since the onset of the COVID-19 pandemic many healthcare organizations have operated in a climate of uncertainty. Building resilience is one method used to survive and thrive during periods of uncertainty. Measuring an organization's resilience (ability to prepare for, respond and adapt during time of change) allows the organization to identify its vulnerabilities and set priorities to avert negative outcomes during an untoward event. Through collaboration with four long-term care professional associations, the Benchmark Resilience Tool (BRT-13) resilience survey was disseminated to North Carolina long-term care leaders in April 2021. The BRT-13 survey was also sent to North Carolina public health officials via email during the same timeframe. The BRT-13 contains 13 resilience (RES) items divided into two factors of adaptive capacity (AC) and planning (PL) on a five-point Likert scale of strongly disagree (1) to strongly agree (5). Organizational factors surveyed included type of facility, rural-urban classification area designation, ownership type, level of debt, level of profitability, and employee satisfaction. A total of 142 completed surveys were received, 101 (71%) from long-term care leaders and 41 (28.9%) from Public Health officials. Overall average resilience scores ranged from 3.96 for public health respondents to 4.46 for continuing care retirement communities (CCRC) respondents. Analysis of Variance (ANOVA) was employed to compare the three factors (AC, PL, and RES) to the organizational factors. Resilience was significantly associated with one factor, employee satisfaction. Our findings indicate that organizations can build resilience through processes that contribute to staff satisfaction.

### REFRAME, REFORM, AND TRANSFORM: POLICY APPROACHES TO IMPROVE NURSING HOME QUALITY IN THE UNITED STATES

Angela Perone, *University of California - Berkeley, Berkeley, California, United States*

Nursing homes (NHs) are one of the most heavily regulated industries in the United States. However, concerns about quality of care remain, especially for more vulnerable residents, including those with dementia. Concerns only increased during recent natural disasters and the COVID-19 pandemic. Community and industry advocates have suggested diverse reforms that prompted new federal policies. This study poses the following research questions: (1) How do federal policies compare with proposals by community (staff and patient advocates) and industry (NH) advocates to improve quality of care in NHs before and during the pandemic; and (2) How do federal policies address micro, mezzo, and macro level issues? To address these questions, this study employs a multi-level comparison case study design. Primary data includes systematically collected and analyzed federal bills, CMS regulations, and community and industry reports, press releases, and website data from 2018 through 2022. Findings revealed that policy proposals fell into three categories: reframe, reform, and transform. Reframe approaches included minimal transformation at the micro-level. Reform approaches involved more mezzo and macro-level changes. Transform approaches proposed significant structural changes at the macro-level. Community advocates presented far more transformative changes than industry advocates and federal policymakers. Federal policies focused on reframe and reform solutions at the micro level and rarely proposed transforming macro-level structures (e.g., workforce structural inequities). This study has important implications for research, policy, and practice by exposing limitations and strengths of proposed solutions for addressing resident care and concludes with suggestions for better aligning with community advocates.

### THE IMPACT OF SOCIAL DEPRIVATION ON COVID-19 INFECTIONS IN NURSING HOMES

Robert Weech-Maldonado[1], Justin Lord[2], Ganisher Davlyatov[3], Akbar Ghiasi[4], and Gregory Orewa[1], *1. University of Alabama at Birmingham, Birmingham, Alabama, United States, 2. Louisiana State University at Shreveport, Bossier City, Louisiana, United States, 3. University of Oklahoma, Oklahoma City, Oklahoma, United States, 4. University of the Incarnate Word, San Antonio, Texas, United States*

Health inequities vary along social and economic gradients. The COVID-19 pandemic and nursing home infections have highlighted this fact. Using the Centers for Medicare and Medicaid Services' Nursing Home COVID-19 Public File, Brown University's LTCFocus, Robert Graham Center's Social Deprivation Index, and CMS Nursing Home Payroll Based Journal Staffing Data. We examined the relationship between community resource scarcity, as conceptualized by the Social Deprivation Index (SD) and COVID-19 incidence rates in nursing homes. After controlling for interstate differences, organizational enabling factors, as well as, facility-level resident and community-level characteristics, nursing homes located in communities with medium levels of social deprivation had 4.4% more COVID-19 infection rates (Incidence Rate Ratio [IRR] = 1.04; $p < 0.05$) and communities with high levels of social deprivation had 7.5% higher COVID-19 infection rates (Incidence Rate Ratio [IRR] = 1.07; $p < 0.01$) as compared to nursing facilities located in areas of low social deprivation. From a policy perspective, nursing homes, that are located in socially deprived communities, may need additional resources, such as, funding for staffing and personal protective equipment in the face of the pandemic. The COVID-19 pandemic has sharpened the focus on healthcare disparities and societal inequalities in the delivery of long-term care.

### SESSION 4830 (SYMPOSIUM)

### NURSING HOME SOCIAL SERVICES DIRECTORS' ROLE AND RESPONSIBILITIES

Chair: Mercedes Bern-Klug Discussant: Colleen Galambos

While most of the nursing home literature focuses on residents and nurses, there is a growing literature on social services staff and family members of nursing home residents. Social services staff include social workers as well as non-social workers with responsibility for assessing and care planning for resident psychosocial issues, as well as a host of other responsibilities including working with families. This symposium presents findings from the National Nursing Home Social Services Directors Survey (NNHSSDS), a cross-sectional (2019) nationally representative study (n=924) that collected data on social services staff characteristics, departmental role responsibilities, and training needs. In this session three papers developed from these data are presented to document the extent to which social services staff are involved with family members, their interest in receiving training on how they can support other staff in understanding resident abuse and exploitation (at the hands of family, fellow residents, or staff), and neglect. As demanding as the social services role is, many holding the position of social services director report they are thriving (not just surviving) in the role. A final paper describes which variables help to distinguish those who report they are thriving versus those who do not. Implications to be discussed include what administrators could do to make the social services director's job more attractive. The RRF Foundation for Aging provided data collection support for the NNHSSDS.

### THRIVING OR SURVIVING? FACTORS ASSOCIATED WITH NURSING HOME SOCIAL SERVICE DIRECTORS REPORTING THRIVING AT WORK

Nancy Kusmaul[1], Mercedes Bern-Klug[2], Kevin Smith[3], and Dana Cheek[3], *1. University of Maryland Baltimore County, Baltimore, Maryland, United States, 2. University of Iowa, IOWA CITY, Iowa, United States, 3. University of Iowa, Iowa City, Iowa, United States*

Nursing homes (NHs) provide care to medically complex residents. Social services (SS) are primarily responsible for addressing residents' psychosocial needs. High staff turnover in nursing homes is a significant problem. When SS staff leave, training and experience goes with them. Much research has explored nursing staff turnover, far less about SS staff turnover. Social service workers who are thriving are more focused, innovative, and engaged. This study explored characteristics contributing to NH SS directors reporting thriving at work. We surveyed 924 NH SS directors from randomly selected nursing homes. Guided by Spreitzer's model, a hierarchical logistic regression was conducted utilizing unit contextual factors and individual agentic behaviors to examine likelihood of thriving. Unit contextual factors, i.e. being valued, feeling influence, and the NH providing quality care had the greatest influence on SS directors reporting thriving at work. These results suggest great potential for NHs to foster thriving in SS directors.

### ELDER ABUSE TRAINING NEEDS AMONG NURSING HOME SOCIAL SERVICES DIRECTORS

Amy Roberts[1], Georgia Anetzberger[2], Kevin Smith[3], and Mercedes Bern-Klug[4], *1. Miami University, Oxford, Ohio, United States, 2. Case Western Reserve University, Cleveland, Ohio, United States, 3. University of Iowa, Iowa City, Iowa, United States, 4. University of Iowa, IOWA CITY, Iowa, United States*

This study aims to identify elder abuse training needs among social services directors. Using nationally representative data from the 2019 National Nursing Home Social Services Directors Survey, we found that approximately three-quarters of directors reported either a moderate or strong interest in receiving training in the following areas: (1) the types of abuse, neglect, and/or exploitation by staff, residents, family and/or other visitors…; (2) establishing trust with a resident and/or family member who feels mistreated by the nursing home or a staff member…; and (3) interacting with visitors whose behaviors are perceived as threatening to residents or staff. Hierarchical logistic regressions found significant associations between strong interest in each training topic and facility, department, and director characteristics. Additional training may enhance directors' skills in explaining basic information about elder abuse and supporting other staff with strategies to address elder abuse and difficult interpersonal dynamics among staff, residents, families, and visitors.

### A DEGREE IN SOCIAL WORK INCREASES FAMILY ENGAGEMENT WITH THE SOCIAL SERVICES DEPARTMENT

Amy Roberts[1], Nancy Kusmaul[2], Amber Alaniz[3], and Mercedes Bern-Klug[4], *1. Miami University, Oxford, Ohio, United States, 2. University of Maryland Baltimore County, Baltimore, Maryland, United States, 3. University of Iowa, Iowa City, Iowa, United States, 4. University of Iowa, IOWA CITY, Iowa, United States*

This mixed-methods presentation will examine the role of social services directors in supporting the family members of nursing home residents. A logistic, hierarchical regression (n= 924) found a positive association between the social services director having a degree in social work and strong agreement with "family members regularly engage the expertise of the social services department." A qualitative analysis of written-in comments to the question, "What do you like about your job?" identified 234 comments that described directors' view of the importance of working with and supporting families. A content analysis revealed that two types of support are provided to families, instrumental and emotional. Instrumental support includes connection to resources for adjustment, financial needs, and discharge planning. Emotional support includes activities such as helping people through transitions, and coping with grief and loss. Appropriately trained social services directors can offer different types of support and be valued resources for families.

### SESSION 4840 (SYMPOSIUM)

### POINT-OF-CARE TESTING FOR THE FRAILTY PHENOTYPE PREDICTS POSTSURGICAL OUTCOMES

Chair: Mamoun Mardini Co-Chair: Christopher Kaufmann

Frailty is a cornerstone in geriatric medicine and is known to be strongly associated with poor postsurgical outcomes. Fried and colleagues defined frailty as a syndrome with five interlinked components, including weakness, low energy, slow walking speed, decreased physical activity, and unintentional weight loss. Consequently, frailty could serve as a useful measure for the purposes of pre-probability risk assessment for clinical outcomes after a surgery. Electronic health records (EHR) are a rich source of information, and when harnessed appropriately, could provide a valid presurgical frailty screening metric and be used towards anticipating adverse postsurgical outcomes. The growing adoption of EHR in clinical settings can help in identifying patients in need of enhanced intervention to promote recovery following a surgery. However, to date, few studies have fully characterized how individual frailty components, as assessed in the clinic, could be a predictor of postsurgical outcomes. This symposium will examine how presence of frailty and its individual components predict three postsurgical clinical outcomes: length of stay, 30-day mortality, and discharge disposition. We will present data from a unique dataset of approximately 14,000 patients who underwent frailty assessment at the University of Florida-Health (UFHealth) presurgical clinic as part of their standard clinical evaluation. Our presentations will examine each frailty syndromic components and overall frailty as predictors of the outcomes of interest. Findings will demonstrate the utility of electronic health records data for the allocation of at-risk patients for poor postsurgical outcomes. Funding: UF Claude D. Pepper Older Americans Independence Center P30AG028740 and R01 AG055337.

### IDENTIFYING THE FRAILTY COMPONENTS THAT ARE MOST IMPORTANT IN PREDICTING POSTSURGICAL OUTCOMES

Mamoun Mardini, Emily Smail, Patrick Tighe, Catherine Price, Christopher Kaufmann, and Todd Manini, *University of Florida, Gainesville, Florida, United States*

Presurgical frailty among patients is strongly associated with poor postsurgical outcomes. In the prior presentations of this symposium, we assessed the associations between each individual frailty syndromic component and three postsurgical outcomes. However, it remains unclear whether individual relationships remain after accounting for presence of the other components. In regression models that included all frailty components, demographic information, and medical history, results showed that weight loss and slow walking speed were significantly associated with length of stay and 30-day mortality, respectively. Of note, the odds of mortality in patients with slow walking speed was over three times higher than patients with normal walking speed. Lastly, weaker grip strength was strongly associated with discharge to more intensive care (vs. to home). These findings suggest that frailty screening should be universally applied before surgical procedures for better risk stratification.

### IMPACT OF UNINTENTIONAL WEIGHT LOSS AND EXHAUSTION ON CLINICAL OUTCOMES FOLLOWING SURGERY

Christopher Kaufmann, Mamoun Mardini, Emily Smail, Patrick Tighe, Catherine Price, and Todd Manini, *University of Florida, Gainesville, Florida, United States*

For patients undergoing surgery, unintentional weight loss and exhaustion, two hallmarks of frailty, may be salient markers for identifying patients at risk for adverse postsurgical outcomes. This study examined how these frailty components impact postsurgical outcomes including length of stay, 30-day mortality, and discharge disposition. Patients with weight loss and exhaustion pre-surgery experienced greater length of stay, and higher odds of 30-day mortality as well as lower likelihood of being discharged home. After adjustment for confounders including age, sex, race, and disease history, the above results for the most part remained significant, although statistical significance was lost for 30-day mortality and some discharge dispositions. Results highlight importance of assessing frailty components to enhance surgical care in older adults.

### LOW PHYSICAL ACTIVITY AND SLOW GAIT AS PREDICTORS OF POSTSURGICAL CLINICAL OUTCOMES

Emily Smail, Mamoun Mardini, Patrick Tighe, Catherine Price, Christopher Kaufmann, and Todd Manini, *University of Florida, Gainesville, Florida, United States*

Low physical activity and slow gait are independently associated with adverse health outcomes of aging (e.g., hospital readmission and mortality). The objective of this study was to build on current knowledge using presurgical indicators of frailty to predict postsurgical outcomes (i.e., length of stay,

30-day mortality, and discharge disposition). In a sample of over 8,900 patients, low physical activity and slow gait were both associated with a greater length of stay, higher odds of mortality, and lower odds of being discharged to home (vs. more intensive care). Associations remained significant for 30-day mortality and discharge disposition in fully adjusted models (adjusting for age, sex, race, and disease history). These results suggest that measuring movement-based presurgical markers of physical activity and slow gait may be useful for identifying patients at risk for poor postsurgical outcomes.

### PRESURGICAL GRIP STRENGTH PREDICTS POSTSURGICAL OUTCOMES IN OLDER ADULTS

Todd Manini, Mamoun Mardini, Emily Smail, Patrick Tighe, Catherine Price, and Christopher Kaufmann, *University of Florida, Gainesville, Florida, United States*

Muscle weakness is a strong predictor of a myriad of health outcomes among older adults. However, measures of muscle strength are not incorporated in healthcare settings which is partly due to a lack of established clinical applicability. Maximal grip strength was measured in 11,668 older patients (73.2+/-6.0 yrs, 50.3% female) during a standard presurgical consultation. Each kilogram of maximal grip strength was associated with approximately 1-hour shorter length of stay. Patients categorized as having weak muscle strength according to sex-based norms had a higher 30-day post-surgery mortality than their non-weak counterparts (Odds Ratio: 2.5, p=0.002 after statistical adjustment of age, sex, race and disease history). Additionally, patients with weak muscle strength were less likely to be discharged home than patients without weakness (68.8% vs. 83.9% returning home, p<0.01). Grip strength is a consistent predictor of post-surgical outcomes that might be used to improve care management.

### SESSION 4850 (SYMPOSIUM)

### POSITIVE AFFECT IN DEMENTIA CARE DYADS

Chair: Joan Monin Discussant: Claudia Haase

Positive affect is important for physical health, well-being, and relationships. Yet, most studies of dementia care dyads have focused on negative outcomes, such as depression and caregiver burden. We present findings from studies that address this important research gap. The first two speakers present findings from actor partner interdependence models using secondary data from a randomized controlled trial of persons with early-stage dementia (PWD) and their spouses. Specifically, Dr. Monin will show that actor quality of life is significantly related to positive affect cross-sectionally, controlling for functional status of each partner and PWD behavioral symptoms. Ms. Piechota will present longitudinal findings demonstrating that when spouses were high in difficulty regulating emotions, the PWD's positive affect decreased over three months, controlling for intervention arm and covariates. The second two speakers will present findings from two different studies of persons living with behavioral-variant frontotemporal dementia (bvFTD), Alzheimer's disease (AD), and controls that engaged in laboratory conflict discussions. Dr. Brown will show that caregivers of individuals with bvFTD reported greater decreases in positive emotion across the discussion relative to caregivers of individuals with AD and language variants. She will also discuss how caregivers who reported greater decreases in positive emotion had higher levels of depressive symptoms, controlling for their partner's diagnosis and level of cognitive impairment. Finally, Dr. Chen will provide evidence that bvFTD caregivers had lower emotional well-being according to the SF-36 than AD caregivers and controls, and this effect was fully mediated by bvFTD caregivers' lower positive emotional connections.

### ASSOCIATIONS BETWEEN HEALTH AND POSITIVE AFFECT IN SPOUSAL DEMENTIA CARE DYADS

Joan Monin[1], and Kalisha Bonds Johnson[2], *1. Yale School of Public Health, New Haven, Connecticut, United States, 2. Emory University, Atlanta, Georgia, United States*

Little is known about the conditions that foster greater positive affect in the daily lives of spousal dementia care dyads in the early stages of dementia. This study aimed to examine the extent to which multiple indicators of health, including activities of daily living needs, quality of life, and the person with dementia's behavioral symptoms were associated with each partner's positive affect in daily life. Using secondary baseline data from a randomized controlled trial testing a stress reduction intervention in 63 couples (N=126), we examined whether individuals' multiple health indicators were associated with their own positive affect (actor effects) and their partner's positive affect (partner effects). Actor partner interdependence model results showed that for both persons with dementia and spouses, actor quality of life was the greatest predictor of positive affect, controlling for all other actor and partner health indicators ($\beta$=.04, SE=.01, t(67.1)=3.36, p=.001).

### EMOTION REGULATION AND POSITIVE AFFECT IN SPOUSAL DEMENTIA CARE DYADS

Amanda Piechota[1], Joshua Novak[2], Thi Vu[3], and Joan Monin[1], *1. Yale School of Public Health, New Haven, Connecticut, United States, 2. Auburn University, Auburn, Alabama, United States, 3. Yale University, New Haven, Connecticut, United States*

The early stages of dementia can be a time of stress and uncertainty for spouses, yet little attention is paid to positive experiences. It is important to understand whether there are individual differences in emotion regulation that impact the positive affect of the individual and the partner. Drawing from interdependence theory, we hypothesized that one spouse's greater difficulty regulating emotions would be associated with their spouses lower positive emotions over time. We used self-report data from an intervention study with three assessment points (baseline, two weeks, and three months) of 45 older adult married couples (N=90) where one spouse has early-stage dementia. Both partners completed the Difficulties in Emotion Regulation Scale and the Positive and Negative Affect Scale. Results from the longitudinal actor partner interdependence model showed that when care partners were high in difficulty regulating emotions, their partner's positive affect decreased ($\beta$ = -0.343, p <.01), controlling for intervention arm and covariates.

### INFORMAL DEMENTIA CAREGIVERS' EMOTIONAL EXPERIENCES DURING CONFLICT AND DEPRESSIVE SYMPTOMS

Casey Brown[1], Yuxuan Chen[2], and Robert Levenson[3], *1. Georgetown University, Washington, District of Columbia, United States, 2. UC Berkeley, Berkeley, California, United States, 3. University of California, Berkeley, Berkeley, California, United States*

Emotional interactions may change as a result of dementia and negatively impact caregivers' mental health. We examined 62 dementia caregivers' emotional experiences during conflict with their partners, including 22 individuals with behavioral variant frontotemporal dementia (bvFTD), 20 with Alzheimer's disease (AD), and 20 with a language variant of frontotemporal dementia (language variant). Dyads engaged in a 10-minute conversation involving conflict. Caregivers watched recordings of their conversation while rating their emotional valence using a rating dial. Caregivers of individuals with bvFTD reported greater decreases in positive emotion across the conversation relative to caregivers of individuals with AD and language variants. Caregivers who reported greater decreases in positive emotion across the conversation had higher levels of depressive symptoms, even after accounting for their partner's diagnosis and level of cognitive impairment. Findings suggest bvFTD caregivers experience the greatest declines in positive emotion during conflict, with potential implications for depression.

### POSITIVE EMOTIONAL CONNECTION AND CAREGIVER WELL-BEING IN BEHAVIORAL-VARIANT FRONTOTEMPORAL DEMENTIA

Kuan-Hua Chen[1], Claire Yee[1], Casey Brown[2], Anna Sapozhnikova[3], Peter Pressman[4], Jennifer Merrilees[5], Barbara Fredrickson[6], and Robert Levenson[1], *1. University of California, Berkeley, Berkeley, California, United States, 2. Georgetown University, Washington, District of Columbia, United States, 3. Pacific Anxiety Group, San Francisco, California, United States, 4. University of Colorado School of Medicine, Aurora, Colorado, United States, 5. University of California, San Francisco, San Francisco, California, United States, 6. University of North Carolina at Chapel Hill, Chapel Hill, North Carolina, United States*

Behavioral-variant frontotemporal dementia (bvFTD) is characterized by impairment in socioemotional functioning. Spouses caring for individuals with bvFTD often experience profound health/well-being declines, compared to Alzheimer's disease (AD) caregivers and non-caregiving older adults. We hypothesized that disrupted positive emotional connections between spousal caregivers and individuals with bvFTD contribute to caregivers' lower emotional well-being. 23 bvFTD-caregiver, 23 AD-caregiver, and 17 control dyads had a 10-minute conflict conversation in the laboratory. Positive emotional connections were measured as the covariation of partners' positive emotional behaviors during the conversation. Caregiver emotional well-being was assessed via questionnaire (SF-36). We found that bvFTD caregivers had lower emotional well-being than AD caregivers and controls (who did not differ from each other, $t=.80$, $p=.43$), $c=-.70$, $p<.01$. Importantly, this effect was fully mediated by bvFTD caregivers' lower positive emotional connections, $c'=-.38$, n.s. We speculate that lower positive emotional connections can cause social isolation and contribute to bvFTD caregivers' health/well-being declines.

### SESSION 4860 (SYMPOSIUM)

### PRECLINICAL DEMENTIA CARE AND PREVENTION IN CONTEXT: FROM INSIGHTS TO ACTION

Chair: Daniel Rong Yao Gan Co-Chair: Patricia Heyn Discussant: Emily Greenfield

Dementia affects people with lower socioeconomic statuses disproportionately. People with undiagnosed Alzheimer's disease may or may not express dementia symptoms depending on their brain reserve, cognitive reserve, and their ability to compensate for brain pathology. Much of these depend on their childhood education, but late-life social engagement may also play a role. As lifespan increases globally, preclinical dementia care and prevention in community settings will be increasingly important. The characteristics of everyday environments that are kind to various changes amid declining cognition may enhance the quality of life of older adults with and without dementia. Such environments may be available or unavailable in diverse communities of varying socioeconomic statuses. This collaborative symposium between the ADRD and CEnR interest groups brings together multidisciplinary scholars to imagine psychosocial interventions that could mitigate place-based disparities in cognitive health. The first presentation frames the importance of relational perspectives in a biopsychosocial-ecological model of care amid cognitive decline. The second presentation shows the cognitive impact of networks among kinless older adults across Europe. The third presentation explores community perspectives on preventive brain health programs in various neighborhoods. The fourth presentation centers the voice and action of people with dementia to improve their neighborhood social environments. The final presentation identifies policy implications for community-based care and prevention from network perspectives. Discussions will distil the significance of community for persons living with and without dementia diagnosis to generate place-based interventions that may better support care and prevention in diverse settings, and identify structural barriers to these efforts.

### A BIOPSYCHOSOCIAL-ECOLOGICAL, FAMILY-FRAMED APPROACH TO DEMENTIA CARE

Carol Podgorski, *University of Rochester, Rochester, New York, United States*

An individual's experience of cognitive impairment is shaped by biopsychosocial factors, including their own perceptions of illness as well as interactions with family members, healthcare providers, and the communities in which they live. With advancing illness an individual's dementia care requires the involvement and commitment of others, usually family. Hence, the quality of a person's illness experience is shaped largely by relationships with family members and others throughout their respective communities. Current models of dementia care recognize family members as an important part of the care team, but fail to consider a patient's family system and relationships as social determinants that affect care outcomes. This presentation will introduce a biopsychosocial-ecological, family-framed approach to dementia care that addresses factors that influence care considerations at both the individual and relational levels of the social ecological networks that the patient and their family members occupy.

### KINLESSNESS AND COGNITIVE FUNCTION AMONG EUROPEAN OLDER ADULTS

Christine Mair[1], and Katherine Ornstein[2], *1. University of Maryland, Baltimore County (UMBC), Baltimore, Maryland, United States, 2. Johns Hopkins, University, Baltimore, Maryland, United States*

So-called "kinless" older adults (unpartnered and childless) are a growing population across the globe who may be at higher risk for social isolation and cognitive decline. Yet, this group of older adults is understudied in dementia-related research and their risk of cognitive decline likely varies by country context. We analyze data from the Survey of Health, Ageing & Retirement in Europe (SHARE) to explore cognitive decline by family structure and country. We assess cognitive decline along a gradient of "kinlessness" by comparing older adults with a) partner and child, b) partner and no child, c) no partner and child, and d) no partner and no child across 20 European countries. Results of this study will outline the dementia risks faced by the growing population of older adults with non-traditional family structures and highlight characteristics of country context that might enhance or reduce risk of cognitive decline for "kinless" older adults.

### "PEOPLE DON'T LIVE IN A VACUUM": CO-DEVELOPING A BRAIN HEALTH PILOT PROGRAM IN THE COMMUNITY THROUGH CITIZEN SCIENCE

Claire Wang[1], Daniel Rong Yao Gan[2], Eireann O'Dea[3], and Brain Health in the Community (BHIC) Steering Committee[2], *1. Johns Hopkins University , Baltimore City, Maryland, United States, 2. Simon Fraser University, Vancouver, British Columbia, Canada, 3. Simon Fraser University, North Vancouver, British Columbia, Canada*

The Psychosocial Model of Everyday Cognitive Resilience identifies social identity as an important determinant of older adults' wellbeing as they experience cognitive decline in community settings. We engaged community-dwelling older adults to assess the model and co-develop programs that address existing gaps through twelve focus groups. N=55 older adults were recruited from various community organizations. Two 1-hour sessions discussed (1) variables that were important to older adults, namely neighbourhood friendship and social experiences, and (2) how these mediated the effects of self-expression, time outdoors, and communal provisions on mental wellbeing. Many participants highlighted the importance of strong friendship for deeper needs such as grief support, whereas others pointed out the relevance of meaningful activities or volunteering opportunities for a sense of purpose. Overall, a speed-friending program with an emphasis on listening was desirable for connecting and contributing socioemotionally to develop "happy medium" friendships, while piloting evidence-based interventions for brain health.

### FLIPPING STIGMA ON ITS EAR: A TOOLKIT FROM PARTICIPATORY ACTION RESEARCH

Mariko Sakamoto[1], Jim Mann[1], Deborah O'Connor[2], and Alison Phinney[1], *1. University of British Columbia, Vancouver, British Columbia, Canada, 2. School of Social Work - University of British Columbia, Vancouver, British Columbia, Canada*

People living with dementia face persistent stigma, discrimination and social exclusion, with significant emotional, physical and social consequences. Addressing this requires changing attitudes and fostering actions for communities to include people with dementia as citizens with agency and self-determination. This presentation highlights the work of an Action Group (AG) of people living with dementia. As part of a four-year Participatory Action Research study aimed at addressing the stigma, discrimination and social exclusion that is so common to the dementia experience, members of the AG in partnership with the research team developed the Flipping Stigma on its Ear Toolkit. Focus will be on the action-oriented nature of this research project, an overview of the toolkit, and exploration of the communal space that was created by AG members in the process of working together, learning from one another, and making a collective contribution towards addressing stigma, discrimination and social exclusion.

### SOCIAL NETWORKS AND COGNITIVE FUNCTION: POLICY IMPLICATIONS FROM RECENT RESEARCH

William McConnell[1], Katherine Haggar[1], Mohit Manchella[2], Adam Roth[3], and Brea Perry[3], *1. Florida Atlantic University, Boca Raton, Florida, United States, 2. University of Southern Indiana, Carmel, Indiana, United States, 3. Indiana University, Bloomington, Indiana, United States*

This presentation will describe a series of research projects using Social Network Analysis (SNA) to measure the personal social networks of community-dwelling older adults at-risk for or currently experiencing dementia. The first part of the presentation will provide a brief overview of SNA methods, including advantages of SNA compared to more traditional social activity scales. The second part of the presentation will present evidence from multiple studies to identify distinct pathways linking upstream social network characteristics to downstream pathophysiological processes in the aging brain. In particular, we will distinguish between cognitive stimulation experienced in expansive social networks and neuroendocrine benefits derived from cohesive social networks. The third part of the presentation will present implications for psychosocial interventions to reduce health disparities in dementia care and prevention. We recommend leveraging the multidimensional functionality of social networks across the lifecourse to influence multiple cognitive health pathways simultaneously.

### SESSION 4870 (SYMPOSIUM)

### REIMAGINING ALZHEIMER'S RESEARCH: ON THE ROAD TO NEW DISCOVERIES

Chair: Stacy Andersen Co-Chair: Lenora Smith Discussant: Allison Gibson

Organized by the Alzheimer's disease and related dementias (ADRD) Interest Group, this symposium aims to highlight unique approaches to address 1) ADRD risk reductions through social and biological factors, and 2) quality of life of those with dementia. The first two presentations introduce remote communication technologies used for behavioral interventions, one for preventing social isolation and the resultant cognitive decline using videochats by recruiting socially isolated older adults with mild cognitive impairment (MCI). The results of the recently completed randomized controlled trial provide evidence of a positive effect on cognition suggesting enhanced cognitive reserve. Another is for delivering a behavioral symptom intervention for community-dwelling persons with ADRD using telehealth. Researchers will highlight the feasibility, opportunities, and challenges of remote behavioral interventions. The third presentation focuses on micro-level communication between persons living with dementia and their family caregivers. By examining video-recorded home care observations, findings suggest caregiver education on positive interactions could facilitate more meaningful interaction with persons with ADRD. The fourth presentation explores pain among those with ADRD, a multifaceted phenomenon with health and functional consequences. Using the NHATS dataset, researchers show a continued need to detect and address pain in clinical settings particularly among persons with ADRD. The symposium concludes with a presentation using the CHARLS study to investigate correlations between inflammation or kidney-related metabolic biomarkers and cognition. Results provide longitudinal evidence of an association of kidney function and inflammation with cognitive test scores supporting the need to broaden dementia etiology research beyond the amyloid-cascade theory.

### I-CONECT RESULTS: ENHANCING SOCIAL INTERACTIONS IMPROVES COGNITIVE FUNCTION IN SOCIALLY ISOLATED PARTICIPANTS

Hiroko Dodge[1], Chao-Yi Wu[1], Kexin Yu[2], Peter Lichtenberg[3], Laura Struble[4], Kathleen Potempa[4], and Lisa Silbert[1], *1. Oregon Health & Science University, Portland, Oregon, United States, 2. Oregon Health & Science University, Palo Alto, California, United States, 3. Wayne State University, Detroit, Michigan, United States, 4. University of Michigan, Ann Arbor, Michigan, United States*

Epidemiological studies suggest that social isolation is a risk factor of dementia although the underlying mechanisms are not well known. A core component of social isolation is a lack of conversational interactions. We recently completed a NIH-funded multi-center randomized controlled trial called Internet-Based Conversational Engagement Clinical Trial (I-CONECT, ClinicalTrials.gov: NCT02871921), which aimed to examine whether social engagement, specifically conversational interactions through webcam and internet, could improve cognitive function in socially isolated older adults with mild cognitive impairment (MCI) or normal cognition. The data was un-blinded in August, 2021. We found strong evidence of efficacy in the primary (global cognitive function, Cohen's $d = 0.73$, $p=0.03$) and secondary (memory function, Cohen's $d=0.67$, $p=0.03$) outcomes at Month 6 (high-dose post-trial endpoint) and Month 12 (maintenance-dose post-trial-endpoint), respectively, using the mixed model for repeated measures. We will present the background, COVID-19 pandemic related protocol modifications and the primary results of this intervention project.

### USE OF TECHNOLOGY TO DELIVER AND MONITOR NONPHARMACOLOGICAL INTERVENTION FOR ADRD WITH BEHAVIORAL SYMPTOMS

Elizabeth Rhodus, Rosmy George, Richard Kryscio, Justin Barber, Allison Gibson, Donna Wilcock, and Gregory Jicha, *University of Kentucky, Lexington, Kentucky, United States*

Onset of the COVID-19 pandemic created numerous barriers to providing care supports and clinical research for persons living with Alzheimer's disease and related dementias (ADRD). Technology offered one avenue for continued care support and clinical data collection. This study reports on the use of technology to deliver a 6-week, non-pharmacological care intervention directed toward caregivers of persons with ADRD and remote data collection including cognitive assessment, biometric data, and survey data for community-residing persons with ADRD and behavioral symptoms (N=28). Benefits and challenges of such technology use for intervention delivery and data collection will be discussed. Benefits include increased geographical outreach, no travel time, and greater scheduling flexibility. Challenges include access to technology (equipment and/or internet), internet connection quality, ease of use, and equipment return at study completion. These findings offer specific aspects to consider while designing and implementing remote care programming and clinical research for community-residing persons with ADRD.

### MICROLEVEL COMMUNICATION BETWEEN FAMILY CAREGIVERS AND PERSONS LIVING WITH DEMENTIA

Sohyun Kim[1], and Wen Liu[2], *1. University of Massachusetts, Amherst, Massachusetts, United States, 2. University of Iowa, Iowa City, Iowa, United States*

Little is known about the relationship between family caregivers and persons living with dementia communication at micro-level (smallest verbal and nonverbal behavior unit). Micro-level communication was assessed second-by-second using 75 in-home video recordings from 19 caregiver-care recipient dyads. Each care recipient (N = 38) and caregiver (N = 42) behavior unit was paired and compared using Spearman's partial correlation. Two-hundred twenty care recipient-caregiver behavior unit pairs were correlated (n = 148 verbal, n = 72 nonverbal pairs, rs range = .456 - .990, all $p < .05$). Ninety-one caregiver facilitative-care recipient engaging behavior unit pairs (e.g., comfort/empathetic touch, using humor) were positively correlated (rs range = .460 - .990, all $p < .05$). Eighteen caregiver disabling-care recipient challenging behavior unit pairs (e.g., withdrawing) were positively correlated (rs range = .456 - .697, all $p < .05$). Individualized caregiver education for matching and adjusting communication is needed to facilitate meaningful interaction.

### IMPLICATION OF PAIN ON MOBILITY, FALLS, AND PSYCHOLOGICAL OUTCOMES FOR PEOPLE LIVING WITH DEMENTIA

Annalisa Na[1], Leslie McClure[1], Rose Ann DiMaria-Ghalili[2], and Laura Gitlin[1], *1. Drexel University, Philadelphia, Pennsylvania, United States, 2. Drexel University, College of Nursing and Health Professions, Philadelphia, Pennsylvania, United States*

Dementia and pain can challenge mobility, increase fall risk, and result in psychological consequences; however, the extent of these relationships among community-dwelling people living with dementia (PLWD) is unclear. Using the National Health and Aging Trends Study, we examined 9,974 community-dwelling older adults (female=57%, age>85yrs=18%) for pain prevalence, mobility (i.e., Short Physical Performance Battery [SPPB]), falls, and psychological outcomes (i.e., depression and anxiety) using four groups with and without dementia (+PLWD/-PLWD) and pain (+Pain/-Pain). Groups were compared at baseline with ANOVA and chi-square and over time with repeated-measures ANOVA models. Pain (+PLWD= 56.8%; -PLWD=49.8%) most commonly affected the low-back and knee. Group differences were significant for baseline mobility, falls, and psychosocial outcomes, p<0.01, but not SPPB over time. Therefore, pain is common, especially at low back and knee, and associated with health and function among PLWD, indicating a need for early and effective interventions that can preserve quality of life.

### THE ASSOCIATION BETWEEN COGNITION AND METABOLIC BIOMARKERS AMONG PARTICIPANTS FROM THE CHARLS LONGITUDINAL COHORT

Diefei Chen, *Johns Hopkins Bloomberg School of Medicine, Baltimore, Maryland, United States*

Inflammatory or kidney-related metabolic biomarkers have been correlated with cognition and risk for developing dementia among older adults. Using the China Health And Retirement Longitudinal Study, we investigated the cross-sectional correlations between metabolic biomarkers and cognitive outcomes that were ascertained in the same wave, as well as measures lagged by data collection waves. After excluding participants with diagnoses of dementia, we analyzed the association of each biomarker (e.g., White Blood Cell count, Creatinine, Blood Urea Nitrogen, Uric Acid, Cystatin C, C-Reactive protein) with scores from cognitive tests. We then identified biomarkers that showed consistent associations across waves. Having elevated CRP (1-3 mg/L) was positively associated with cognitive test scores; whereas abnormal levels of Creatinine (Low: <0.74 for male and <0.59 for female; High:>1.35 for male and >1.04 for female) were associated with poorer outcomes. These results provide biomarker evidence of the importance of kidney function and inflammation in dementia research.

### SESSION 4880 (SYMPOSIUM)

### THE BLUES AND OLDER MINORITY MUSICIANS: MORE THAN JUST MUSIC XXIX

Chair: John Migliaccio Co-Chair: Michael Marcus

The “ ’Bo Diddley’ Track”; GSA, 2022 Indianapolis, INLIndianapolis has been described as “The Crossroads of America,” with a long history of fostering the music and entrepreneurial spirit of Black Americans. Starting in 1915 with Mrs. C.J. Walker, America’s first female millionaire and her cosmetic enterprise, and the Starr Piano Company and its Gennett Records Studio – identified as “The Cradle of Recorded Jazz” in the 1920s and 30s – both were instrumental in national distribution of the works of the earliest blues, jazz, country, and gospel artists including Louis Armstrong, King Oliver, Alberta Hunter, and Charlie Patton among others.Indianapolis continues to host a thriving Blues music community, and will host the 29th consecutive year of GSA’s “Bo Diddley Track” with local older minority musicians, one of GSA’s most popular and fun events. This year will feature a lecture, interview, and mini-performance with leading local blues musicians exploring their life, influences, music and resilience, followed by a typically raucous live blues performance that evening at a local blues hot-spot, with prizes for the first 50 GSA attendees. This has been going on for 29 years for a reason, folks---don’t miss it!

### THE BLUES AND OLDER MINORITY MUSICIANS: MORE THAN JUST MUSIC XXIX

John Migliaccio, *Maturity Mark Services Co., LLC, White Plains, New York, United States*

The Blues and Older Minority Musicians: More Than Just Music XXIXThe “ ’Bo Diddley’ Track”; GSA, 2022 Indianapolis, INLecture/Interview/ Performance: TBAIndianapolis has been described as “The Crossroads of America,” with a long history of fostering the music and entrepreneurial spirit of Black Americans. Starting in 1915 with Mrs. C.J. Walker, America’s first female millionaire and her cosmetic enterprise, and the Starr Piano Company and its Gennett Records studio – identified as “The Cradle of Recorded Jazz” in the 1920s and 30s – both were instrumental in national distribution of the works of the earliest blues, jazz, country, and gospel artists including Louis Armstrong, King Oliver, Alberta Hunter, and Charlie Patton among others.Indianapolis continues to host a thriving Blues music community, and will host the 29th consecutive year of GSA’s “Bo Diddley Track” with local older minority musicians, one of GSA’s most popular and fun events. This year will feature a lecture, interview, and mini-performance with leading local blues musicians, followed by a typically raucous live blues performance that evening at a local blues hotspot, with prizes for the first 50 GSA attendees. This has been going on for 29 years for a reason folks---don’t miss it!

### SESSION 4890 (SYMPOSIUM)

### THE ELDER MISTREATMENT EMERGENCY DEPARTMENT TOOLKIT: ADDRESSING ELDER MISTREATMENT DURING COVID-19 AND BEYOND

Chair: Rebecca Stoeckle Co-Chair: Kristin Lees Haggerty
Discussant: Terry Fulmer

Elder Mistreatment (EM) is a prevalent problem that too often goes undetected. Hospital emergency departments (ED) offer a unique opportunity to screen for elder mistreatment and connect patients at risk with needed services but most do not have training and protocols in place to help staff screen for and respond to EM. In 2020, the National Collaboratory to Address Elder Mistreatment partnered with five hospitals to test the feasibility of implementing a streamlined set of tools and training known as the Elder Mistreatment Emergency Department Toolkit, and conducted two qualitative studies to better understand related barriers and facilitators to addressing EM. The presentations in this symposium share results from case studies at two feasibility test sites: Heywood Hospital, a small community hospital in central Massachusetts, and Lyndon B. Johnson Hospital, the busiest Level III Trauma Center in Texas, each presented by the respective site's local clinical champion. Following presentations by the clinical champions, National Collaboratory expert core faculty will report results from two related qualitative studies. The first explores barriers and facilitators to communication between EDs and Adult Protective Services (APS) and the second explores older adult survivors' own perspectives on the screening and response tools used in the feasibility study. The results of these studies highlight the need for streamlined and easy-to-use tools for identifying and responding to EM in busy EDs, the need for a personalized and trauma-informed approach to screening, and the importance of a personalized approach to connecting the ED and APS.

### IMPLEMENTATION OF THE ELDER MISTREATMENT EMERGENCY DEPARTMENT TOOLKIT AT LYNDON B. JOHNSON HOSPITAL

Sherry Plummer[1], Jason Burnett[2], Ruthann Froberg[3], Randi Campetti[4], and Kim Dash[4], *1. Lyndon B. Johnson General Hospital, Houston, Texas, United States, 2. The University of Texas McGovern Medical School at Houston, Houston, Texas, United States, 3. Education Development Center, Inc., Waltham, Massachusetts, United States, 4. Education Development Center, Waltham, Massachusetts, United States*

Lyndon B. Johnson Hospital (LBJ) has the busiest Level III trauma and emergency department (ED) in Texas. Located in the busiest Houston zip code for Adult Protective Services reports, LBJ staff routinely assess older adults for mistreatment with no formal screening and response protocol. An ED-wide staff assessment revealed formal training needs for elder mistreatment (EM) detection, management, and reporting. Between October and December 2019, 55% of ED bedside nurses were trained along with 75% of charge nurses, and 12 clinical and nurse case managers, resulting in improved knowledge regarding EM screening and response best practices. In January 2021, LBJ staff implemented the Elder Mistreatment Screening and Response Tool (EM-SART). This resulted in 1,218 complete screens, 23 cases of suspected EM (2%), and 4 confirmed EM cases. Despite the pandemic and other challenges, LBJ staff demonstrated resilience and dedication, and reported EM training, screening, and response protocol efficacy.

### IMPLEMENTATION OF THE ELDER MISTREATMENT EMERGENCY DEPARTMENT TOOLKIT AT HEYWOOD HOSPITAL

Carol-Lynne Papa[1], Randi Campetti[2], Ruthann Froberg[3], Kristin Lees Haggerty[3], and Kim Dash[2], *1. Heywood Hospital, Gardner, Massachusetts, United States, 2. Education Development Center, Waltham, Massachusetts, United States, 3. Education Development Center, Inc., Waltham, Massachusetts, United States*

Beginning in 2016, Heywood Hospital, a 134-bed community hospital located in Central Massachusetts, partnered with the National Collaboratory to Address Elder Mistreatment to design and test a care model for identifying and responding to elder mistreatment (EM) in hospital emergency departments (ED). In February 2020, Heywood Hospital began implementing the care model known as the Elder Mistreatment Emergency Department (EMED) Toolkit as part of a five-site feasibility study. This presentation shares local results from the feasibility study and strategies for implementing the EMED Toolkit. 95% of Heywood staff participated in an online training program and, over 9 months, 4,588 (84%) of all older ED patients were screened for EM. Of these, 19 screened positive and 11 were reported to Adult Protective Services, a more than 7-fold (annualized) increase over the prior year. Most of the remaining positive cases were connected with community-based resources through Heywood's Social Services Program.

### EMERGENCY DEPARTMENT: ADULT PROTECTIVE SERVICES RELATIONSHIP IN RESPONSE TO ELDER MISTREATMENT

Theresa Sivers-Teixeira[1], Erin Thayer[2], Carmen Van Den Heever[1], and Bonnie Olsen[1], *1. University of Southern California, Alhambra, California, United States, 2. University of Southern California - Keck School of Medicine, Alhambra, California, United States*

Universal screening of older adults in emergency departments (ED) effectively improves identification of elder mistreatment (EM). To realize improved response, collaboration between ED providers and staff and local Adult Protective Services (APS) is essential. Qualitative data was derived from transcribed and coded key informant interviews with 10 APS workers and 5 ED clinical providers from 6 states. Results indicate each entity approaches EM from a different global perspective which informs key strategies to build effective response. ED providers focus on acute medical needs while APS focuses on long-term psychosocial needs. Assignment of specific individuals in both entities to be liaisons between ED and APS would support improved communication and collaboration. Education should focus on 1) capabilities and limitations in EM response and 2) clarity regarding bilateral sharing of information. Both entities report participation in multidisciplinary teams is valuable to foster collaboration on effective interventions in EM cases.

### PERSPECTIVES OF THE EM-SART: INTERVIEWS WITH ADULT PROTECTIVE SERVICES CLIENTS

Jason Burnett[1], Kristin Lees Haggerty[2], Randi Campetti[3], and Rebecca Stoeckle[2], *1. The University of Texas McGovern Medical School at Houston, Houston, Texas, United States, 2. Education Development Center, Inc., Waltham, Massachusetts, United States, 3. Education Development Center, Waltham, Massachusetts, United States*

Older adults who experience mistreatment are more likely to visit emergency departments (ED), yet screening and response protocols in these health care settings are sorely lacking. Protocols designed with the mistreated older adult's perspective in mind are needed to maximize efficacy and effectiveness. In this study, 18 mistreated older adults completed semi-structured perspective interviews regarding the ED Elder Mistreatment Screening and Response Tool (EM-SART). The findings highlight the importance of training healthcare staff to ask elder mistreatment (EM) questions in a preset context and to ask EM questions with empathy, concern, privacy, and clarity. Participants also stressed the desire to be reported to Adult Protective Services, but to also be included in the safety planning. These findings have direct implications for training health care workers to screen and respond to EM in the ED.

### SESSION 4900 (PAPER)

### STATE- AND SYSTEMS-LEVEL RESEARCH INTO ELDER ABUSE AND NEGLECT

### A POLICY ANALYSIS OF ELDER ABUSE PREVENTION LEGISLATION: TRANSLATION OF NATIONAL INITIATIVES TO THE STATE LEVEL

Autumn Decker[1], Kathryn Bruzios[1], Raven Weaver[1], and Cory Bolkan[2], *1. Washington State University, Pullman, Washington, United States, 2. Washington State University Vancouver, Vancouver, Washington, United States*

Elder abuse (EA) is an under-recognized global public health threat in need of enhanced research and policy attention, with 1 in 6 older adults experiencing at least one form of abuse/neglect. Public policy changes, such as the federal 2017 Elder Abuse Prevention and Prosecution Act (EAPPA), aim to address EA, but little is known regarding translation and implementation of the initiatives to state-levels. Applying a thematic analytic approach, we identified four major themes from the EAPPA: criminal investigation support; abuse reporting; financial exploitation; and training/technical assistance. Then, we conducted a comparative policy analysis to evaluate each state's EA statutes enacted since 2017 to determine whether statutes were updated to reflect the EAPPA's goals. Preliminary findings from 10 states (i.e., states with the greatest proportion of older adults in each Health & Human Services region) showed that eight states had updated statutes relevant to an identified theme(s). Eight states updated statutes related to financial exploitation, highlighting the growing concern over this specific type of abuse. Four states updated statutes related to reporting, reflecting problems with identifying and detecting elder abuse. Two states updated statutes regarding investigation, demonstrating minimal emphasis on elder abuse investigation. No states updated statutes relevant to training/technical assistance. These findings indicate significant state-level variability in translation and implementation of recent legislation. More recognition, research, and policy attention to EA, in the U.S. and globally, is needed. We conclude with recommendations for innovation and prevention policy efforts coupled with multidisciplinary collaborations to strengthen implementation against EA at the state-level.

### ADULT MALTREATMENT OUTCOMES OF A TARGETED CASE MANAGEMENT INTERVENTION FOR SELF-NEGLECT

Courtney Reynolds, Farida Ejaz, and Miriam Rose, *Benjamin Rose Institute on Aging, Cleveland, Ohio, United States*

A longitudinal study to prevent self-neglect among at-risk, older (age 60+) or disabled (age 18+) healthcare patients was conducted collaboratively by a gerontological research institute, a health care system, and Texas Adult Protective Services (APS). Selected patients (n = 285) from 19 primary care clinics received targeted case management services for four months by a trained social worker. Their median age was 70, 72% were Hispanic/Latino, 79% had a high school education or less, and 73% had monthly income less than $1,361. A screening tool was used to identify adult maltreatment during baseline and posttest interviews. Consistent with national prevalence rates, 86% of participants at baseline, and 90% at posttest, reported they had not recently experienced abuse. In this sample, we found a significant decrease in mean scores on the screening tool from baseline ($M = 0.24$, $SD = 0.63$) to posttest ($M = 0.14$, $SD = 0.48$); $t(241)$, $p < 0.05$. Six of the seven participants reported by project social workers to APS had at least one allegation of self-neglect. APS administrative data were also examined and included another 10 study participants reported to APS during the study. In total, 21 reports were made, with 26 allegations of maltreatment, of which 35% were validated, all for self-neglect; 70% of self-neglect allegations reported by project social workers were validated. Results suggest that targeted case management services could reduce the incidence of self-neglect. Similar targeted interventions may help to identify, report, and address other forms of adult maltreatment.

### APS PERSPECTIVES ON CHALLENGES AND STRATEGIES SERVING CLIENTS DURING THE COVID-19 PANDEMIC SURGE

Alyssa Elman[1], Elaine Gottesman[2], Lena Makaroun[3], E-Shien Chang[4], Sunday Clark[1], and Tony Rosen[5], *1. Weill Cornell Medical College / NewYork-Presbyterian Hospital, New York, New York, United States, 2. New York-Presbyterian Hospital/Weill Cornell Medical Center, New York, New York, United States, 3. VA Pittsburgh Healthcare System, Pittsburgh, Pennsylvania, United States, 4. Weill Cornell Medical College, New York, New York, United States, 5. Weill Cornell Medical College / NewYork-Presbyterian Hospital, PELHAM, New York, United States*

The initial surge of the COVID-19 pandemic and public health measures in response dramatically impacted Adult Protective Services' (APS) ability to conduct investigations and provide services, requiring agencies to quickly adapt. Our goal was to describe challenges for APS and strategies they developed to respond. We conducted 6 focus groups and 7 interviews during March-April 2021 used a semi-structured topic guide, with 31 participants from APS leadership, supervisors, and case workers in New York City, a community hard hit by the initial COVID surge. Focus groups and interviews were recorded and transcribed, with data analyzed to identify themes. Participants identified 9 major challenges, including: clients using concern about COVID-19 to refuse APS workers' access to their home, necessity to perform home visits/wellness checks on behalf of other agencies who had suspended home visits, and dependence on in-office activities including receiving paper mail. Participants reported 30 formal and informal strategies adopted to address challenges. These included 13 focusing on maximizing client engagement while maintaining client and APS worker safety, such as: adding a pre-investigation before a home visit to assess COVID-19 risk/exposure, offering clients masks and hand sanitizer to build trust, and close collaboration with other agencies/programs including joint visits. Also, 17 strategies were reported to allow for remote work and support staff, including: modifying processes to replace paper mail with electronic communication/processing, offering counseling services, formally recognizing excellent performance, and leadership reaching out personally to check on staff members. These findings may inform APS planning for future large-scale societal disruptions.

### RISK FACTORS AND IMPACT OF FINANCIAL FRAUD ON OLDER ADULTS AND DISADVANTAGED COMMUNITIES

Marguerite DeLiema[1], and Paul Witt[2], *1. University of Minnesota, Twin Cities, Minneapolis, Minnesota, United States, 2. Federal Trade Commission, Washington, District of Columbia, United States*

In 2021, Americans age 80 and older reported median fraud losses of $1,300 per incident, substantially higher than younger age groups (Federal Trade Commission, 2022). Using data from the Federal Trade Commission's Sentinel Fraud Complaint Database and the Area Deprivation Index, this study assesses the demographic and contextual correlates of consumer fraud victimization in the United States. Models show a positive association between living in a disadvantaged community and reporting victimization (versus reporting no losses after being targeted). Black and Hispanic consumers are significantly more likely report victimization, although non-Hispanic White victims report higher average fraud losses. Results also show that older adults are significantly less likely to report fraud victimization relative to people age 30 and younger, but older victims lose hundreds of dollars more, on average. Applying a sentiment analysis to the consumer complaint narratives, we find that compared to emotionally neutral complaints, emotionally positive and emotionally negative complaints are positively associated with reporting victimization versus reporting no losses. Gift cards are the most common method of payment demanded by fraud criminals overall, but victims who pay using cash, wire transfer, or cryptocurrency lose significantly more money, on average. To limit the impact of fraud on the aging population, consumer protection agencies need to focus their efforts on educating older adults and members of socially and economically disadvantaged communities about scams, as well as enforce greater controls on cryptocurrency exchanges and retail gift card sales.

### SELF-NEGLECT OF OLDER ADULTS: HOW CAN ADULT PROTECTIVE SERVICES HELP?

Pi-Ju Liu[1], Zach Hass[1], Sara Stratton[2], Karen Conrad[3], and Ken Conrad[3], *1. Purdue University, West Lafayette, Indiana, United States, 2. San Francisco Adult Protective Services, San Francisco, California, United States, 3. University of Illinois Chicago, Chicago, Illinois, United States*

Adult Protective Services (APS) is the only government agency dedicated to investigating elder mistreatment. This frontline agency also provides and coordinates needed services to its clients, but their service effectiveness has not been extensively investigated. Over half of all APS cases investigated are self-neglect. Although no universal definition of self-neglect exists in the literature, self-neglecting older adults fail to perform self-care tasks, such as getting adequate nutrition, maintaining personal hygiene, obtaining medical care, protecting oneself from danger, managing one's finances– all of which may threaten their health or safety. In partnership with San Francisco and Napa APS, levels of self-neglect were measured using the Elder Self-Neglect Assessment Short-Form during case investigation (before APS interventions) and at case closure (after APS interventions). Services accepted and received by clients at case closure were also documented. A total of 634 older adults' (on average 77 years old; 50% females) were found to be self-neglecting during the six-month pilot demonstration. Overall, levels of self-neglect decreased after APS interventions ($t=-16.97$, $p<.001$). Most common services included care/case management (7%) and mental health services (12%). Services associated with decreased levels of self-neglect included in-home assistance services ($b=-1.14$, $p<.05$) and care/case management ($b=-0.79$, $p<.10$). Using standardized measures before and after implementing the services needed by older adults allows identification of services that decrease mistreatment. In this study, standardized assessment by APS impacted both practice and research to help advance the understanding of this elder mistreatment type.

### SESSION 5000 (PAPER)

### ATTITUDES ABOUT AGING

### AGE GROUP DIFFERENCES IN VIEWS ON ONE'S OWN OLDER ADULTHOOD

Shelbie Turner[1], Shannon Jarrott[2], and Karen Hooker[3], *1. Weill Cornell Medical College, Pitman, New Jersey, United States, 2. The Ohio State University, Columbus, Ohio, United States, 3. Oregon State University, Corvallis, Oregon, United States*

Most of the current scholarship on views on one's aging focuses on middle and older adulthood, prompting lifespan theorists to call for studies about views on one's aging throughout the entire lifespan (Kornadt et al., 2020). In this study, we established a factor structure to Kornadt et al.'s (2020) Domain Specific Future Selves Scale, which prompted respondents to rate their thoughts on their self in old age across 9 different items. We then utilized measurement invariance testing to analyze how the factor structure differed between 433 young (18-39), 485 middle-aged (40-64), and 286 older adults (65 and older). Via Exploratory and Confirmatory Factor Analyses, three latent factors emerged, constituting future older selves in the domain of social lives ($a=0.78$), work and finances ($a=0.62$), and physical health ($a=0.76$). The model maintained configural ($RMSEA = 0.06$, $CFI = .96$; $TLI = .95$), metric ($RMSEA = 0.06$, $CFI = .97$; $TLI = .96$), and scalar ($RMSEA = 0.06$; $CFI = .96$; $TLI = .95$) invariance across all three age groups. Latent mean testing revealed that older adults thought more positively about their future social lives ($p=0.005$), work and finances ($p=0.05$), and health ($p=0.02$) than did younger adults, and more positively about their social lives (p=0.001) and health (p=0.05) than middle-aged adults. Results from this study offer additional psychometric validation of a measure of future selves that researchers can use across the lifespan, and deeper theoretical understanding of how domain-specific views on one's own older adulthood might differ between age groups.

### ASSOCIATIONS BETWEEN MEDIA CONSUMPTION AND AGEIST ATTITUDES: A CROSS-NATIONAL ANALYSIS

Erfei Zhao[1], Eileen Crimmins[2], Elizabeth Zelinski[2], and Eunyoung Choi[3], *1. University of Southern California, Leonard Davis School of Gerontology, Los Angeles, California, United States, 2. University of Southern California, Los Angeles, California, United States, 3. School of Global Public Health, New York University, Los Angeles, California, United States*

The mass media has been thought to be associated with public opinion, often creating and sustaining stereotypes. However, little is known about the role of media exposure in people's ageist attitudes, particularly at a cross-national level. This study examines whether the daily use of different media types is associated with personal attitudes towards older people. We analyzed data from 59,103 adults across 54 countries, using the World Value Survey wave 6 (2010-2014). Personal ageist attitudes were assessed by whether participants agree that older people are a burden on society. We used logistic regression, controlling for individual- and country-level factors. Our findings suggest that people's exposure to media is significantly associated with their attitudes towards older adults, but differently by the platform and respondents' age. Those who used newspaper (OR:1.66, CI:1.38-1.98), magazines (OR:1.67, CI:1.27-2.20), and radio (OR:1.42, CI:1.23-1.65) were more likely to have negative attitudes toward older people, whereas those who used TV (OR:0.62, CI:0.53-0.72) and internet (OR:0.76, CI:0.65-0.89) were less likely to. Further, the effects of newspaper and radio consumption on people's attitudes were moderated by respondents' age. Younger adults' ageist attitudes had a stronger negative relationship with these media types compared to those that are older. For the older age group, in contrast, more consumption of newspapers and radio are associated with less ageist attitudes. Future studies may focus on the content of each platform and assess their effect on people's ageist attitudes by age groups in order to understand how to foster a more age-friendly media environment.

### DEMENTIA-RELATED STIGMA IS ALIVE AND NOT WELL AMONG UNREGULATED HOME CARE WORKERS

Marie Savundranayagam, Shalane Basque, and Audrey Gruneberg, *Western University, London, Ontario, Canada*

Dementia-related stigma is pervasive despite the growing rates of dementia globally. The social cognitive model of stigma contends that stigma is created and maintained through negative stereotypes, prejudice, and discrimination towards people who belong to a group. Research on dementia-related stigma highlights the prevalence of stigmatizing views among formal care providers. However, little is known about how stigma is created and maintained by the ways in which frontline care providers talk about persons living with dementia. Accordingly, this study aimed to identify ways in which stigmatizing language is used by home care workers when describing routine care interactions with clients living with dementia. Semi-structured interviews were conducted with 30 unregulated home care workers, who shared their experiences caring for clients with dementia. We used conventional content analysis to identify themes related to dementia-related stereotypes, prejudice, and discrimination. Under stereotypes, persons with dementia were portrayed as objects, infants, not engaging, and cognitively and behaviorally unstable. Under prejudice, persons with dementia invoked pity, fear, disdain, and emotional fatigue. Under discrimination, participants shared experiences of excluding/ignoring, controlling, and using patronizing communication with persons living with dementia. Uncovering common examples of stigmatizing language offers opportunities to train home care workers to use person-centered communication. It is noteworthy that stigmatizing language emerged, when not asked about directly. Our findings underscore the persistence of dementia-related stigma and the need for training to eliminate stigma.

### DO BELIEFS THAT OLDER ADULTS ARE INFLEXIBLE SERVE AS A BARRIER TO RACIAL EQUITY?

Kimberly Chaney[1], and Alison Chasteen[2], *1. University of Connecticut, Storrs, Connecticut, United States, 2. University of Toronto, Toronto, Ontario, Canada*

Past research has demonstrated that older adults are stereotyped as less malleable than younger adults, such that older adults are perceived to be less capable of changing their beliefs and learning new things. Moreover, beliefs that people are less malleable are associated with lower confrontations of prejudice, as perpetrators are seen as less capable of changing their (prejudiced) behavior. The aim of the present research was to integrate these lines of research to demonstrate that endorsement of ageist beliefs that older adults are less malleable will lead to lower confrontation of anti-Black prejudice espoused by older adults. Across four experimental studies (N = 1,310), people were less likely to confront anti-Black prejudice espoused by an 82 year-old compared to a 62, 42, or 20 year-old, due, in part, to beliefs that older adults are less malleable. Further exploration demonstrated that malleability beliefs about older adults were held across young, middle-aged, and older adult samples, though older participants were the least likely to confront prejudice, regardless of perpetrator age. Alternative mechanisms are explored, including respect, perceived social influence, and perceived awareness of egalitarian norms. These findings demonstrate how stereotypes about older adults can impede racial equality and highlight that interventions geared towards reducing ageism could, in turn, lead to greater racial equality.

### NEIGHBORHOOD ADVERSITY AND COGNITIVE HEALTH: THE MODIFYING ROLE OF SELF-PERCEPTIONS OF AGING

Eunyoung Choi[1], Elizabeth Zelinski[2], Jennifer Ailshire[2], and Yuri Jang[2], *1. School of Global Public Health, New York University, Los Angeles, California, United States, 2. University of Southern California, Los Angeles, California, United States*

Research has documented the increased risk of cognitive impairment among older adults living in socioeconomically disadvantaged neighborhoods. Much less is known about the factors that moderate this risk. We conceptualized self-perception of aging (SPA) as a potential moderator because it reflects core beliefs about the self at older ages but is also closely linked to late-life health. Guided by the diathesis-stress model that postulates the interactive roles of cognitive styles and stressors in shaping health outcomes, we hypothesized that more positive SPA would buffer the effects of neighborhood adversity on cognitive function. Using data from the Health and Retirement Study (2008–2016), the analytic sample consisted of adults aged 54 and older (N=5,902). Cognitive function was assessed by the Telephone Interview for Cognitive Status. The neighborhood indicators included 1) poverty rates at the census tract level, 2) perceived neighborhood social cohesion, and 3) perceived neighborhood disorder. Three-level growth curve models were separately estimated for each neighborhood indicator's effect as well as its interaction with SPA on the 8-year cognitive function trajectories. Findings showed that higher poverty rates, more disorder, and less cohesion were associated with lower initial levels of cognitive function but slower rates of cognitive decline. SPA partially moderated the linkage between neighborhood adversity and the level of cognitive function. More positive SPA was associated with reduced negative effects of living in neighborhoods with higher poverty rates and more physical disorder. These findings highlight the intersection of an individual-level psychological factor and a contextual-level factor in shaping late-life cognition.

### SESSION 5001 (BIOLOGICAL SCIENCES INVITED SYMPOSIUM)

### BS GSA AND AMERICAN AGING ASSOCIATION JOINT SYMPOSIUM

Chair: Blanka Rogina

### AGING-RELATED VEGF IMPAIRS MUSCLE REGENERATION

Yori Endo[1], Charles Hwang[2], Yuteng Zhang[2], Ronald Neppl[2], Shailesh Argawal[2], and Indranil Sinah[2], *1. Harvard Medical School, Boston, Massachusetts, United States, 2. Brigham and Women's Hospital, Boston, Massachusetts, United States*

Purpose: Aging is associated with frailty, a parameter that correlates with mortality and loss of muscle mass. The molecular mechanisms behind aging-associated impairment of muscle regeneration remain incompletely understood. We hypothesized VEGF-A with known role in angiogenesis and muscle progenitor differentiation to regulate regeneration in aged skeletal muscle.

Methods: Young C57BL/6 (10 weeks old) and old C57BL/6 mice (24 months old) were subjected to muscle cryoinjury to induce regeneration. Quantifications of cross-sectional area (CSA) of regenerating myofibers were performed. Tibialis anterior muscle lysates was used for quantifying VEGF-A. To evaluate the role of VEGF in muscle regeneration, a similar experiment was performed on VEGFlo mice with a 75% decrease in VEGF-A activity and littermate controls. ML228, a hypoxia signaling activator that increases VEGF-A levels, was injected into young and old mice as well as VEGFlo and littermate controls.

Results: Old mice exhibited marked reduction in the VEGF-A protein levels and regenerating myofiber CSA on DPI 10 (1250 vs. 833µm2, p<.001). Similarly, VEGFlo mice exhibited significantly smaller regenerating fiber CSA as compared to littermate controls on DPI 10 (541 vs. 238µm2, p=.0011). Pharmacological augmentation of VEGFA using ML228 increased muscle VEGF levels by 2 folds and skeletal muscle regeneration in both old mice (25% increase in regenerating fiber CSA, p<.01) and VEGFlo (20% increase in regenerating fiber CSA, p<.01) mice, but not young or littermate controls.

Conclusions: Muscle regeneration declines with aging in correlation with loss of VEGFA levels within skeletal muscle. Supplementation of VEGFA represents a therapeutic target for sarcopenia.

### BODY WEIGHT INFLUENCES MUSCULOSKELETAL ADAPTATION TO LONG-TERM VOLUNTARY WHEEL RUNNING DURING AGING

Yukiko Kitase[1], Vallejo Julian[2], Yixia Xie[3], Mark Dallas[3], Sarah Dallas[3], Mark Johnson[3], Michael Wacker[4], and Lynda Bonewald[1], *1. Indiana University School of Medicine, Indianapolis, Indiana, United States, 2. University of Missouri-Kansas City, School of Dentistry and Medicine, Kansas City, Missouri, United States, 3. University of Missouri-Kansas City, School of Dentistry, Kansas City, Missouri, United States, 4. University of Missouri-Kansas City, School of Medicine, Kansas City, Missouri, United States*

Frailty is a key hallmark of aging and exercise has been shown to delay aging effects. This study was initiated based on the hypothesis that voluntary wheel running (VWR) starting at 12 mo until 18 or 22 mo of age would benefit the female murine musculoskeletal system. Based on the final body weight, the mice were separated into high (HBW) and low body weight (LBW) subgroups. Beneficial effects of VWR were observed on soleus muscle mass and contractile force at both ages, although HBW led to greater increases at 22 mo. VWR increased fiber cross-sectional area by 20%, leading to more type I and fewer IIA fibers in soleus. HBW mice were resistant to age-related decline in Extensor digitorum longus (EDL) mass and contractile force. EDL in 18 mo HBW also showed 15% higher contractile force following VWR while muscle from 18 & 22 mo LBW responded to VWR with greater osteocyte protective factor secretion. Skeletal adaptation to VWR was also dependent on body weight, with HBW showing higher femoral cortical thickness and area under sedentary conditions. VWR maintained osteocyte dendrite number in HBW. VWR increased periosteal and endosteal circumferences in HBW, suggesting compensation for loss of material strength. Consistent with this, VWR maintained higher bone mechanical properties in 18mo LBW. In summary, VWR alters musculoskeletal parameters depending on body weight with HBW contributing to more muscle mass and strength to prevent sarcopenia while bone retains better mechanical properties in LBW but HBW contributes structural modification to prevent osteopenia.

### LIPID REGULATION BY DGA1, CUT6, AND SRE1 CONTRIBUTE TO THE GENOTOXIC STRESS RESPONSE IN FISSION YEAST

Wilber Escorcia, Mauricio Dominguez, Maryam Khzyr, Sam Burek, Jack Pluth, Meghan Graber, Hayley Barta, and Alex Rausenberger, *Xavier University, Cincinnati, Ohio, United States*

Lipids are essential for cell growth and maintenance, intracellular signaling, and cellular energetics. When exposed to environmental insults, cells activate lipid metabolism programs that facilitate the response, recovery, and exit from stressful conditions. Failure to regulate lipid storage and utilization is associated with accelerated aging, which compounds genomic instability. In this study, we test the physiological consequences of disruptions to three lipid regulator genes frequently found to be mutated in human cancers. Since the products of these genes show functional homology in fission yeast, we examine how lipid deregulation by two enzymes (Dga1DGAT2 and Cut6ACC) and one transcription factor (Sre1SREBF1) influence the response to genotoxic stress in this organism. Using microscopy quantification of lipid staining, we observe abnormal homeostatic control of lipid levels in cells lacking Dga1, Cut6, and Sre1. These phenotypes are enhanced in sub-lethal doses of DNA damage (UV-C light) and are associated with altered cell fitness and proliferation. Furthermore, in response to genotoxicity, these lipid regulator mutants exhibit disrupted nuclear segregation, abnormal cell dimensions, and altered lifespan. These data suggest functional mechanisms that may contribute to the deregulated metabolic and physiological environments of prematurely aged cells and of tumors in human cancers.

### THE ROLE OF PPARδ-DRIVEN β-OXIDATION IN BONE HEALTH DURING AGING

Matt Prideaux[1], Tom O'Connell[1], and Yukiko Kitase[2], *1. Indiana University, Indianapolis, Indiana, United States, 2. Indiana University School of Medicine, Indianapolis, Indiana, United States*

Musculoskeletal disorders are a significant complication of aging, leading to increased morbidity and mortality. However, current understanding of the mechanisms by which aging affects skeletal health is limited. Osteocytes are the most numerous and long-lived bone cells and play key roles in maintaining bone mass by responding to anabolic signals such as mechanical loading. Energy metabolism is dysregulated in many cells with aging, however regulation of energy metabolism in osteocytes and how this is affected during aging and by mechanical loading remains undefined. To investigate this, we first used IDG-SW3 osteocyte cells to determine the effects of mechanical loading on osteocytes in vitro by applying fluid flow shear stress (FFSS). FFSS increased Pparδ and Cpt1 expression, key promoters of fatty acid β-oxidation (FAO). Pharmacological antagonism of PPARδ or CPT1 resulted in dysregulated expression of key bone remodeling genes and impaired ATP release in response to FFSS. In vivo, mechanical loading significantly increased FAO in tibia cortical bone. However, FAO was impaired in the bones from aged mice. To further elucidate the role of osteocyte FAO, we deleted PPARδ specifically in osteocytes (PPARδ cKO), which resulted in decreased FAO and bone volume in female PPARδ cKO mice. Lastly, treatment of aging mice with the PPARδ activator GW0742 resulted in significantly increased bone mineral content, density and trabecular bone volume. These findings suggest important functions of osteocyte energy metabolism during aging and with mechanical loading on bone and identify PPARδ-driven FAO as a novel therapeutic target for improving skeletal health with aging.

### CLARIFYING THE ROLE OF RESILIENCE IN NONHUMAN PRIMATE AGING USING A BABOON CELL MODEL

Daniel Adekunbi[1], Cun Li[1], Peter Nathanielsz[1], and Adam Salmon[2], *1. University of Wyoming, Laramie, Wyoming, United States, 2. The University of Texas Health Science Center at San Antonio, San Antonio, Texas, United States*

Aging is associated with defects in homeostatic maintenance. Delineating the dynamics of the homeostatic network is challenging in vivo and often limited by endpoint measurements. However, we have developed carefully controlled translatable cellular models to bridge some of these challenges using primary cells obtained from male and female baboons across the life course (4 to 21 years). We recently published findings showing that donor age corresponds to loss of cellular resilience to oxidative stress in males but not in females. Responses to thapsigargin-induced endoplasmic reticulum (ER) stress were also age and sex dependent, though with different pattern than that of oxidative challenge. Young females alone were vulnerable to ER stress, while young males and old females were not affected. These data highlight that primary cells retain donor characteristics regarding molecular mechanisms associated with aging including those dependent on sex. To further investigate these mechanisms, we investigated whether the proteostatic machinery contributes to cellular resilience outcome following ER stress. In female donors, we find significant decrease in 20 S proteasome activity and expression of its catalytic subunit (PSMB 8) in female-derived fibroblasts under basal or ER stress conditions when compared to males. ER oxidoreductin 1 (ERO1) as well as autophagy marker, LC3-II/I ratio, were significantly higher in young females compared to young males during ER stress. These data highlight the propagation of sexual dimorphisms in cellular resilience at the molecular level and suggest that sex differences in maintenance of proteostasis contributes to the vulnerability of young females to ER stress.

### SESSION 5010 (PAPER)

### CARE MODELS AND DEMENTIA

### AN INNOVATIVE MODEL OF CARE FOR SUBJECTS WITH DEMENTIA IN ITALY—IL PAESE RITROVATO—THROUGH THE SARS-COV-2 PANDEMIC

Paolo Mazzola[1], Gaia Ferraguzzi[2], Mariella Zanetti[3], and Giuseppe Bellelli[4], *1. Università degli Studi di Milano-Bicocca, Monza, Lombardia, Italy, 2. Università degli Studi di Milano, Milano, Lombardia, Italy, 3. Cooperativa La Meridiana, Monza, Lombardia, Italy, 4. University of Milano-Bicocca, Monza, Lombardia, Italy*

"Il Paese Ritrovato" is an innovative long-term facility dedicated to subjects with mild-to-moderate Alzheimer's disease or other dementias, located in Monza (Lombardia, Italy).By living in a village that resembles a community neighborhood, it aims at people's wellbeing and represents a shift in the paradigm of traditional nursing home care, allowing the guests to visit the hair stylist, the supermarket, and various activities according to their personal habits, always supported by trained professionals.We performed a retrospective cohort study on 60 subjects, comparing their characteristics before and during the SARS-CoV-2 pandemic, from June 2018 to December 2020. We collected demographic information, cognition, comorbidity, functional status, engagement in the activities through Popularity Index (PI) and Engagement Social Index (ISE), and use of psychoactive medications.The follow-up is performed every 6 months from admission: T0, T6, T12, and T18 are the time-points. The assessment performed during the pandemic is called Tcovid.Compared to a relative stability in cognition before the pandemic, the assessment at Tcovid showed an accelerated worsening of MMSE and CDR scales. We observed an increase in the prescription of antipsychotics (+5%), antidepressants (+11.7%), and benzodiazepines (+1.7%). As expected, engagement in activities dropped (lower PI and ISE). Functional status gradually worsened during the follow-up, according to the natural progression of dementias, but Tcovid slightly accelerated this process and overall worsened the balance performances.Although we are currently unable to quantify the negative effect of this reorganization, future studies need to address the real impact of pandemic on the guests' performances.

### CHANGING TALK (CHAT) ONLINE COMMUNICATION EDUCATION FOR ADULT DAY CARE STAFF: EVALUATION AND FUTURE DIRECTIONS

Kristine Williams[1], Carissa Coleman[2], Maria Hein[3], Clarissa Shaw[4], Yelena Perkhounkova[4], and Tim Beachy[5], *1. University of Kansas Medical Center, Lawrence, Kansas, United States, 2. University of Kansas, Kansas City, Kansas, United States, 3. University of Iowa College of Nursing, Iowa City, Iowa, United States, 4. University of Iowa, Iowa City, Iowa, United States, 5. University of Iowa, Iowa City, Kansas, United States*

Communication education has been effective in reducing elderspeak use for nursing home staff and subsequently reduces resistiveness to care and inappropriate antipsychotic medication use in residents living with dementia. Communication is fundamental for dementia care and educating staff in other long-term service and support settings such as Adult Day Care (ADC) is needed. Tailoring education to the specific setting is indicated to optimize acceptability and effectiveness. ADC staff (N=22) from 3 ADC centers participated in the Changing Talk: Online Training (CHATO). Results for the 19 staff who completed the education program were analyzed. A focus group was conducted with a subset of participants to evaluate feasibility, applicability, and directions for tailoring for ADC settings. Knowledge gain was demonstrated by score increases 15.0 percentage points (p<.001) from pre- to post-education on the Changing Talk Scale (CHATS). Confidence in Dementia Care (CODE) scores significantly increased after CHATO (p=.05), indicating an increase in confidence in caring for clients with dementia. The mean Modified Diffusion of Innovation scale score was 2.2 (1=intend to use skills in practice to 5=don't intend to use), indicating intent to use with clients. Program evaluation ratings were positive indicating satisfaction. Focus group participants reported that the CHATO was valuable and recommended ADC specific scenarios. The results support the value of CHATO for ADC staff as well as specific directions to better tailor the education to increase applicability in this LTSS setting.

### IDENTIFYING ASSISTED LIVING SAFETY PRIORITIES: A DELPHI PANEL WITH RESIDENT, FAMILY, AND PROFESSIONAL STAKEHOLDERS

Cassandra Dictus[1], Youngmin Cho[1], Victoria Bartoldus[1], Matthias Hoben[2], Stephanie Chamberlain[2], and Anna Beeber[3], *1. University of North Carolina - Chapel Hill, Chapel Hill, North Carolina, United States, 2. University of Alberta, Edmonton, Alberta, Canada, 3. Johns Hopkins University, Baltimore, Maryland, United States*

While assisted living (AL) communities emphasize resident safety, one barrier to resident safety is a lack of information about AL stakeholders' safety priorities. As part of a larger research project to create a toolkit to foster resident and family engagement in safety in AL, we created a stakeholder panel that includes 13 AL residents, family members, and professionals (i.e., direct care workers, administrators, researchers, and policymakers). This paper describes a web-based Delphi process to create a ranked safety priority list with stakeholders. After three rounds involving online surveys and Zoom group discussions, the stakeholder group came to a consensus on a final list of 14 ranked safety priorities. Using verbatim transcripts of the Zoom discussions and chat, we conducted content analysis to highlight the rationales for the 14 ranked safety priorities on the final list. Reasons to prioritize safety concerns included the seriousness of the impact on AL residents and system-level root causes. These findings will be used to guide the development of a toolkit to improve resident and family engagement in the safety of AL. This list can also help AL communities and researchers at large to better understand what safety priorities are most important to a broad range of AL stakeholders and why.

### THE SHORT- AND LONG-TERM IMPACTS OF DEMENTIA ON PREVENTIVE CARE UTILIZATION AND HEALTH BEHAVIORS

Zhuoer Lin, and Xi Chen, *Yale University, New Haven, Connecticut, United States*

Identifying early and consistent signals of dementia risk may offer people more time to prepare for the future, helping to delay the onset or slow the progression of dementia. Using the 1995-2018 waves of Health and Retirement Study, we offered novel evidence on the impacts of dementia and its precursors on a rich set of preventive care utilization and health behaviors in a longitudinal sample of Americans aged 50 and older. Leveraging both within- and between-individual variations in an event study, we characterized short- and long-term dynamic changes in preventive care and health behaviors relative to the incidence of dementia, suggesting early behavioral indicators of the disorder. 3,488 persons with incidence of dementia were identified and 22,619 consecutive person-waves around the incidence of dementia were included to estimate short-term effect. Individuals who never developed dementia were further included as the comparison group for long-term estimation. Results from statistical models showed that incidental dementia was associated with significant reductions in preventive care utilization, including cholesterol test, flu shot, mammogram, prostate test, and dental visit. Significant declines were also found in physical activities and body weights. Moreover, growing declines were observed in preventive care utilization, physical activities, and body weight 4-6 years prior to the incidence of dementia, with escalating impact over time. These findings highlight vulnerability and costs dementia risk may accrue to disrupt preventive care utilization and health behaviors. Targeted interventions are needed to detect signals associated with dementia risk to shield individuals from adverse behavioral and health consequences.

### EVALUATION OF COGNITION IN PRIMARY CARE (CPC): A PILOT STUDY TO INCREASE EARLY DETECTION AND MANAGEMENT OF DEMENTIA

Annette Fitzpatrick[1], Barak Gaster[1], Jaqueline Raetz[1], Basia Belza[1], Monica Zigman Suchsland[1], Karen Tracy[2], Benjamin Olivari[3], and Lisa McGuire[3], *1. University of Washington, Seattle, Washington, United States, 2. GSA, Washington, District of Columbia, United States, 3. CDC, Atlanta, Georgia, United States*

Despite evidence that early detection of mild cognitive impairment and dementia leads to improved patient care, these conditions remain under-diagnosed in primary care. To address this gap at University of Washington Medicine, we have developed and pilot-tested a program to increase cognitive evaluations and improve dementia care by primary care providers (PCPs). The Cognition in Primary Care (CPC) program was designed utilizing stakeholder-selected components of the GSA KAER (Kickstart-Assess-Evaluate and Refer) Model and Toolkit (2020 Edition), developed by the Gerontological Society of America (GSA). In this presentation we will describe evaluation of the CPC Program including (a) education training; (b) use of assessment tools integrated within the electronic health record (e.g. potentially harmful medications, depressive symptoms, alcohol use, sleep apnea, etc.); (c) practice recommendations such as dedicated evaluation appointments, diagnoses and referrals, and (d) PCP use of community resources for post-diagnosis support. A total of 66 PCPs participated in the series of 3 CME educational seminars. Over 93% of participants reported the content to be highly relevant and expected to incorporate it into their practice; commitments to use specific tools were made. Analysis is underway to determine impact of the program by comparing the above metrics before and after the training as well as between trained and untrained PCPs. Results from this evaluation will inform modifications to increase utilization of the CPC Model within UW Medicine, will be used to package the program to share with other primary care systems, and will guide future enhancements to the GSA KAER Toolkit.

### SESSION 5020 (SYMPOSIUM)

### CARING FOR OLDER ADULTS ACROSS THE CARE CONTINUUM: EXPERIENCES OF OLDER ADULTS AND FORMAL CAREGIVERS

Chair: Rose Ann DiMaria-Ghalili Discussant: Rose Ann DiMaria-Ghalili

As individuals age, they are at risk of experiencing health challenges that can contribute to poorer health outcomes and increase health care costs. During acute care crises and through the continuum of dealing with chronic diseases, older adults rely on formal caregivers to provide care and support. Formal caregivers' delivery of care can be challenged during difficult patient cases (e.g., providing wound care to a person exhibiting behavioral and psychological symptoms of dementia) and during high stress times, such as working through a pandemic. However, during difficult times, resilience among formal caregivers can have a positive affect on care delivery. This symposium identifies some of the health challenges experienced by aging adults, challenging experiences of nurses, levels of resilience among formal caregivers, and discusses the implications for nursing care and future research. Ms. Coates examines trends in adverse drug admissions among older adults in the United States using the 2018 Healthcare Cost and Utilization Project's National Inpatient Sample. Dr. Hwang explores the relationship between nutrition, inflammation, and physical and mental health in aging adults with chronic leg wounds. Dr. Sefcik discusses nurses' experiences caring for community-dwelling persons living with dementia and chronic wounds. Mr. Hathaway examines factors that contribute to resilience in staff of a continuing care retirement community during the late stage of the COVID-19 pandemic. Dr. DiMaria-Ghalili, discussant, will synthesize these findings and offer insights on designing interventions to address health challenges and recommendations for supporting formal caregivers.

### TRENDS IN ADVERSE DRUG EVENT ADMISSIONS AMONG OLDER ADULTS IN THE UNITED STATES

Martha Coates[1], Patricia Shewokis[1], and Rose Ann DiMaria-Ghalili[2], *1. Drexel University, Philadelphia, Pennsylvania, United States, 2. Drexel University, College of Nursing and Health Professions, Philadelphia, Pennsylvania, United States*

Older adults experience adverse drug events (ADEs) putting them at risk for increased morbidity and mortality. Utilizing the 2018 Healthcare Cost and Utilization Project's National Inpatient Sample, we identified the prevalence of ADE admissions among adults 65 and older and examined the differences in characteristics and discharge outcomes in those with and without a primary diagnosis of an ADE (n=2,647,673). ADEs accounted for 7.4% of discharges and had higher odds of needing post-discharge care including transfer to a skilled nursing facility (OR=1.08, 95% CI [1.07, 1.09]) and home-health care (OR=1.1, 95% CI [1.09,1.1]). In the ADE group, hospital charges were higher ($39,609 vs. $38,649, p< .01) and length of stay (6+ days) longer (OR=1.53, 95% CI [1.52,1.55]). Opiates, diabetic agents, benzodiazepines and narcotics were frequently associated with ADEs. Older adults discharged after an

ADE have increased healthcare utilization. Education on medication self-management is needed to prevent ADEs in older adults.

### NUTRITION, INFLAMMATION, AND PHYSICAL AND MENTAL HEALTH IN AGING ADULTS WITH CHRONIC WOUNDS

Yeji Hwang[1], Zachary Hathaway[1], Isabella Stoll[1], Shelby Hufnal[1], Peter Lewin[1], and Rose Ann DiMaria-Ghalili[2], *1. Drexel University, Philadelphia, Pennsylvania, United States, 2. Drexel University, College of Nursing and Health Professions, Philadelphia, Pennsylvania, United States*

Nutritional status can impact the healing of chronic leg wounds (CLW). This secondary data analysis examined characteristics associated with nutritional status among aging adults (N=62, M= 57.5, SD 12.1 years of age) living with a CLW who were enrolled in a randomized control trial to test a novel ultrasound intervention on wound healing. Nutritional status (Mini-Nutrition Assessment, [MNA]), physical and mental health (36-Item Short-Form Health Status Survey), and inflammation (high-sensitivity C-Reactive Protein [CRP]) were measured at baseline. Majority of the participants were male (54.8%), non-Hispanic Black (67.7%) and either malnourished or at risk of malnutrition (64.5%). Compared to individuals with normal nutrition, individuals with malnutrition or risk for malnutrition had on average poorer physical health (38.7 vs 33.4, p <0.01), mental health (52.4 vs 45.8, p=0.03) and greater inflammation (CRP=13.4 vs 24.0, p=0.05). Tailored interventions targeting nutritional status, inflammation, physical and mental health are needed in aging adults with CLW.

### NURSES' EXPERIENCES CARING FOR PERSONS LIVING WITH DEMENTIA AND CHRONIC LEG WOUNDS IN THE COMMUNITY

Justine Sefcik[1], Olivia Hernandez[1], Isabella Stoll[1], Zachary Hathaway[1], Ellen Bass[1], and Rose Ann DiMaria-Ghalili[2], *1. Drexel University, Philadelphia, Pennsylvania, United States, 2. Drexel University, College of Nursing and Health Professions, Philadelphia, Pennsylvania, United States*

A gap exists with understanding the care required for community-dwelling persons living with dementia (PLWD) who have chronic wounds. As one step to address this gap we explored the experiences of nurses who care for community-dwelling PLWD that have chronic diabetic foot and venous leg ulcers. A qualitative descriptive approach with a conventional content analysis was undertaken. We conducted 5 focus groups with Home Health Nurses (n = 13) and nurses holding specialty certifications (e.g., wound care) (n = 3); 87.5% female, 69% White, non-Hispanic, mean age = 52 (range 32-67), mean years of experience as a nurse = 23.8. The main themes identified were 1) the challenges with dementia (e.g., confusion, taking dressings off), 2) concerns about the person (e.g., medical issues, financial considerations), and 3) adjusting interventions (for wounds and behavioral symptoms). Study findings can inform the develop of future novel interventions for PLWD and chronic wounds.

### COVID-19 PANDEMIC AND RESILIENCE IN STAFF OF A CONTINUING CARE RETIREMENT COMMUNITY

Zachary Hathaway[1], Kathleen Fisher[1], Martha Coates[1], Shelby Hufnal[1], Isabella Stoll[1], Justine Sefcik[1], and Rose Ann DiMaria-Ghalili[2], *1. Drexel University, Philadelphia, Pennsylvania, United States, 2. Drexel University, College of Nursing and Health Professions, Philadelphia, Pennsylvania, United States*

Residents and staff of continuing care retirement communities (CCRC) experienced many challenges during the COVID-19 pandemic including loss, social isolation, and staff turnover. This study examined factors that contribute to resilience in staff during the late stage of the pandemic using the Connor-Davidson Resilience Scale. Resilience scores ranged from 0 (low) to 100 (high). A total of 96 staff (76% female) were enrolled, and average age was 48.41 years (SD = 16.16). Average resilience in staff was 75.16 (SD = 11.81). Those under 35 years of age reported lower resilience scores (M = 67.38) compared to those 35-49 years of age (M = 76.65), 50-64 (M = 75.83), and 65 years and older (M = 82.71), p <.05. Staff who were married scored higher than those who were not (M = 76.63 vs 69.05), p < .05. Findings can inform professional development programs aimed at increasing coping skills in staff.

### SESSION 5030 (SYMPOSIUM)

### DEMENTIA CARE THROUGHOUT THE TRAJECTORY AND ACROSS SETTINGS: RESULTS FROM THE DEVELOP AD RESEARCH STUDY

Chair: Lauren Hunt Co-Chair: Katherine Ornstein
Discussant: Lauren Massimo

About 1 in 8 older adults in the United States has dementia, making it one of the biggest public health issues facing society today. The impact of dementia ranges across health and social systems, yet consideration of these contexts--which are of great relevance to patients and families--has been underexplored in prior research. In this symposium, we will present findings from the Deploying High-Value Longitudinal Population-Based data in Dementia Research (DEVELOP AD Research) study. This multicenter collaboration leverages existing population-based data sources to examine the health and social impacts of dementia throughout the trajectory of decline and across acute, long-term, and community-based settings. Session one will describe how acute care utilization increases in the years before the onset of incident dementia. Session two will examine the association between dementia and increased mortality risk after disruptive medical events, including hip fracture and pneumonia. Session three will report on the prevalence and patterns of medication overuse and misuse in community-dwelling people with dementia. Session 4 will estimate how many community-dwelling individuals, including those with dementia, could benefit from home-based medical care. Session 5 will focus on hospice use patterns and hospice care quality rating in people admitted to hospice for dementia versus other conditions. Together, these presentations provide a wide lens on the health and social experiences and needs of people with dementia at the national level, leading to a better understanding of how society can better address the challenges of caring for this growing population.

### EMERGENCY DEPARTMENT AND INPATIENT HOSPITAL HEALTHCARE UTILIZATION IN THE YEARS PRECEDING INCIDENT DEMENTIA

Raj Kumar[1], Katherine Ornstein[2], Evan Bollens-Lund[1], Jing Li[3], Ken Covinsky[4], and Amy Kelley[1], *1. Icahn School of Medicine at Mount Sinai, New York, New York, United States, 2. Johns Hopkins, University, Baltimore, Maryland, United States, 3. Cornell University, Ithaca, New York, United States, 4. University of California, San Francisco, San Francisco, California, United States*

There is evidence health utilization increases after incident dementia, particularly toward the end of life. However, less is known about utilization in the years before dementia. Our study objectives were to compare outpatient emergency department (ED) and inpatient hospital utilization in the six years preceding incident dementia compared to a reference group without dementia. We obtained data on n=5,547 Beneficiaries from the Health and Retirement Study-Medicare linked sample, and defined dementia using a validated algorithm. Those with (n=1,241) and without (n=4,306) dementia were balanced on confounders using inverse probability weighting applied to longitudinal Generalized Estimating Equation models. We found persons with dementia had greater odds of ED (OR=1.46, 95% CI: 1.21, 1.77) and inpatient hospital (OR=1.35, 95% CI: 1.12, 1.63) usage in the years preceding dementia compared to those without dementia across a comparable timespan. This study provides evidence to suggest greater healthcare burden may exist before manifestation of dementia.

### THE EFFECT OF DISRUPTIVE MEDICAL EVENTS ON MORTALITY IN PEOPLE WITH AND WITHOUT DEMENTIA

Lauren Hunt[1], Sean Morrison[2], Siqi Gan[1], Edie Espejo[1], W. John Boscardin[1], Rebecca Rodin[2], Katherine Ornstein[3], and Alexander Smith[1], *1. University of California, San Francisco, San Francisco, California, United States, 2. Icahn School of Medicine at Mount Sinai, New York, New York, United States, 3. Johns Hopkins University, Baltimore, Maryland, United States*

Disruptive medical events such as pneumonia and hip fracture occur more frequently among older adults with dementia than those without dementia. It is not well-understood whether these events increase the risk of mortality to a greater extent for people with dementia (PWD) compared to people without dementia (PWoD). Using data from the Health and Retirement Study linked to Medicare claims, we estimated the impact of hip fracture and pneumonia on risk of mortality among 700 PWD and 12,438 PWoD using a Cox proportional hazards model. PWD had a higher risk of mortality both in the case of hip fracture (HR 1.64, 95% CI 1.31, 1.96) and pneumonia (HR 1.21 95% CI 1.09, 1.34) compared to PWoD who experienced those events. This study provides evidence that dementia may increase mortality after a disruptive medical event and suggests that the clinical course of dementia may not always be slow and gradual.

### UNNECESSARY AND HARMFUL MEDICATION USE IN COMMUNITY DWELLING PERSONS WITH DEMENTIA

W. James Deardorff[1], Bocheng Jing[1], Matthew Growdon[1], Kristine Yaffe[1], Kenneth Boockvar[2], and Michael Steinman[1], *1. University of California, San Francisco, San Francisco, California, United States, 2. Icahn School of Medicine at Mount Sinai, New York, New York, United States*

Persons with dementia (PWD) often have multiple comorbidities which results in extensive medication use despite potentially limited benefit and increased risk of adverse events. Compared to the nursing home, little is known about medication overuse and misuse among the ~70% of PWD in the community. Therefore, we examined medication use from Medicare Part D prescriptions among 1,289 community-dwelling PWD aged ≥66 from the Health and Retirement Study. We classified medication overuse as over-aggressive treatment of chronic conditions (e.g., insulin/sulfonylurea use with hemoglobin A1c<7.5%) and medications inappropriate near the end of life. We classified medication misuse as medications that negatively affect cognition (strongly anticholinergics/sedative-hypnotics) and problematic medications (using Beers and STOPP criteria). We describe the prevalence and patterns of different types of medication overuse/misuse. Frequently problematic medications included antipsychotics (9%), benzodiazepines (12%), and gabapentinoids (13%). Our findings highlight the burden of unnecessary/harmful medications among PWD and inform future deprescribing interventions.

### WHO CAN BENEFIT FROM HOME-BASED MEDICAL CARE?

Katherine Ornstein[1], Bruce Leff[1], Jennifer Reckrey[2], Evan Bollens-Lund[2], Margaret Salinger[3], Yihan Wang[2], and Christine Ritchie[4], *1. Johns Hopkins University School of Medicine, Baltimore, Maryland, United States, 2. Icahn School of Medicine at Mount Sinai, New York, New York, United States, 3. Harvard Medical School/Massachusetts General Hospital, Boston, Massachusetts, United States, 4. Massachusetts General Hospital, Boston, Massachusetts, United States*

Leaving the home to access medical care may result in undue burden for patients with dementia and other serious illnesses and their caregivers. While home-based medical care (HBMC) may be beneficial for many older adults, it is not clear how to best identify individuals who could benefit from such services. Using the 2015 NHATS linked to Medicare claims we estimated prevalence across multiple overlapping subtypes: Individuals who have moderate/severe dementia; are homebound; have serious illness; are frail; rely on assistive devices; have high caregiving needs; those with minimal primary care and high ED use; and those who met previously established criteria for Independence at home. Using these criteria, more than half of community-dwelling older adults could benefit from HBMC and more than 25% meet multiple criteria. Medicare and other payers can benefit from targeted identification of patients who could benefit from HBMC.

### SPECTRUM OF DEMENTIA IN OLDER HOSPICE RECIPIENTS

Lauren Hunt[1], Irena Cenzer[1], Alexander Smith[1], Amy Kelley[2], Melissa Aldridge[2], Kenneth Covinsky[1], and Krista Harrison[1], *1. University of California, San Francisco, San Francisco, California, United States, 2. Icahn School of Medicine at Mount Sinai, New York, New York, United States*

We know little about differences between hospice enrollees with dementia co-existing with another terminal illness (like cancer), those dying from dementia (as principal hospice diagnosis), and those dying with no dementia. We used the National Health Aging and Trends Study linked to Medicare claims to compare characteristics, hospice use patterns, and care quality ratings. Among 1,105 decedent hospice-enrollees age 70+, we found 39% were dying with coexisting dementia, 17% from dementia, and 44% without dementia. In adjusted analyses, those dying with dementia had similarly high rates of functional impairment, higher rates of clinical needs, and worse measures of care quality compared to elders dying from dementia. Hospice use patterns were different for elders dying with dementia compared to elders without dementia. In summary, 56% of older hospice enrollees have dementia, mostly in addition to another terminal illness. Their differing hospice experience implies changes are needed to hospice care and policy.

### SESSION 5050 (SYMPOSIUM)

### ENRICHING THE LIVES OF OLDER VETERANS: THE GEROFIT WAY

Chair: Katherine Hall Co-Chair: Adam Gepner

Gerofit is a clinical exercise program for Veterans ages 65 years and older with multi-morbidities and functional limitations that place them at elevated risk for institutionalization. It was declared a VHA "Best Practice" in 2017 and was selected for widespread dissemination and implementation, with over 30 Gerofit programs spanning the country. The original Gerofit program consists of supervised, facility-based exercise, offered 3 days per week in a group setting. Each exercise prescription incorporates aerobic training, progressive resistance training, and specialized exercises for balance and physical function. Everything changed in March 2020 when facilities began receiving orders to shut down face to face encounters and social distancing measures were put in place. Within six weeks, all of our sites had transitioned to group-based virtual classes, aptly called Gerofit to Home (GTH). This symposium includes 2 presentations that explore the effect of GTH on physical functioning and health-related quality of life outcomes in veteran participants during the pandemic: one focusing on resilience among GTH adopters vs. non-adopters in patients formerly participating in facility-based Gerofit; and the second focusing on patients enrolled directly in to GTH (no facility-based experience). The third presentation describes differences in physical function and health gains among Gerofit participants with and without peripheral artery disease (PAD). The fourth and fifth presentations describe augmented models of care to enrich the Gerofit experience for a) participants with supplemental nutrition programming and b) advanced physical therapy trainees through a Gerofit resident training model.

### GEROFIT TO HOME (GTH) AS A NEW MODEL OF CARE

Rebekah Harris[1], Miriam Morey[2], Elisa Ogawa[1], Alan Wesley[3], Jonathan Bean[1], and Katherine Hall[2], *1. VA Boston, Boston, Massachusetts, United States, 2. Durham VA Health Care System, Durham, North Carolina, United States, 3. Puget Sound VA, Seattle, Washington, United States*

Gerofit, a facility-based program transitioned to GTH, a completely virtual structured exercise program, at the onset of the pandemic and previously demonstrated that Veterans already engaged sustained performance with this transition (In-Person vs GTH, n=46; arm curls 19.7 vs 19.0 reps; 30-second chair stand 14.3 vs 15.9 reps). This study investigated whether gains in performance are achieved and sustained in Veterans who participated in GTH only, as it is unknown if virtual programs are as robust as facility-based programs. Measures of performance (3-month mean change, n=45, Arm Curls +3.4 reps; 30-second chair stand +1.5 reps) and self-reported function (SF-36 + 1.8 points) will be assessed from baseline to 3,6, and 12 months. Pooled results from 14 sites have been accumulated and change scores, age and gender-based percentile changes and clinically meaningful thresholds will be presented. This knowledge can have implications to support programs for older adults aging in place.

### CAN VIRTUAL EXERCISE PROMOTE PHYSICAL RESILIENCE DURING THE COVID-19 PANDEMIC AMONG ACTIVE OLDER ADULTS?

Kenneth Manning[1], Stephen Jennings[1], Megan Pearson[1], Richard Sloane[2], Katherine Hall[3], Kyle Bourassa[1], and Miriam Morey[3], *1. Durham VA Healthcare System, Durham, North Carolina, United States, 2. Duke University Medical Center, Durham, North Carolina, United States, 3. Durham VA Health Care System, Durham, North Carolina, United States*

Background: Gerofit is a facility-based exercise and health promotion program for older Veterans that transitioned to virtual delivery in March 2020. Little is known about how virtual exercise would promote resilience in the physical function of individuals previously participating in-person.

Methods: Preliminary data from 1 of 14 sites was gathered 72 Veterans returning to facility-based exercise after COVID mandated shutdowns. 39 individuals chose not to participate virtually, and 33 actively participated virtually for over 1 year. Re-entry data were then compared to the patients' most recent test. Assessment means were compared within groups.

Results: Change scores from T1 to T2 were: -1.19 versus +2.4 repetitions for 30-second arm curls; +1.57 repetitions for 30-second chair stands; and -113.87 versus -77.3 yards for six-minute walk distance for non-virtual versus virtual groups. Implications: Participation in virtual exercise interventions may promote resilience and resistance to functional decline in previously active individuals during enforced isolation.

### VIRTUAL NUTRITION EDUCATION AND PRODUCE DELIVERY INCREASES DIETARY QUALITY AMONG OLDER VETERANS

Elizabeth Parker[1], Sarah Cassatt[2], Jamie Giffuni[3], Leslie Katzel[4], Heidi Ortmeyer[5], and Odessa Addison[6], *1. University of Maryland School of Medicine, Baltimore, Maryland, United States, 2. University of Maryland,*

*Baltimore and Baltimore County, Baltimore, Maryland, United States, 3. Geriatric Research Education & Clinical Center, Baltimore, Maryland, United States, 4. VAMHCS/UMB, Baltimore, Maryland, United States, 5. Baltimore VA Medical Center, Baltimore, Maryland, United States, 6. Department of Physical Therapy and Rehabilitation Science, Baltimore, Maryland, United States*

This project assessed the impact of a 14-week virtual group nutrition education plus produce delivery on diet quality of Baltimore GEROFIT participants. RD led classes focused on healthy aging content. Participants could receive an optional bag of local produce. Dietary intake assessed via a 24-hour recall was used to calculate healthy eating index (HEI-2015; higher scores=higher diet quality). Twelve participants enrolled in the program. Two participants discontinued produce delivery due to physical/environmental limitations prohibiting preparation; seven completed recalls at pre/post and received produce (86% male; 71% Caucasian; age=73 years; BMI=33 kg/m2). There were no changes in overall diet. However, among participants who received >80% of produce (n=4), HEI scores increased (+5.4 points). Daily F&V increased by 1.9 servings. Daily protein intake decreased (-25g). This study highlights areas for program improvement including a stronger emphasis on protein intake and incorporating physical/environmental adaptations that may increase participation among older Veterans with disabilities.

### UTILIZING PHYSICAL THERAPY RESIDENTS' AS HEALTH PROMOTION EXPERTS TO IMPROVE QOL FOR OLDER VETERANS

Susan Patel, *Cincinnati VA Medical Center, Cincinnati, Ohio, United States*

Gerofit, an evidence-based supervised exercise and health promotion program for older Veterans, has demonstrated success with its best practice model through the improved health, physical function, and well-being of participants. In 2017, Cincinnati VA Medical Center (CVAMC) launched an innovative approach to address the staffing needs of this wellness program by utilizing a Physical Therapy (PT) resident model for Gerofit group supervision/functional testing. As licensed PTs, residents engage in year-long advanced training in geriatrics across a variety of settings. They encounter adults who have unhealthy behaviors (lack of physical activity, smoking, poor nutrition, inadequate sleep, and stress), and demonstrate the knowledge/skills to reduce risk factors, prevent/treat chronic disease, prescribe physical activity/exercise, perform interventions consistent with the biopsychosocial paradigm, design/deliver individualized exercise prescriptions, and lead telehealth group exercises classes for Veterans. This presentation will explore how PT residents are in an ideal position to promote health/wellness in the Gerofit Program.

### FUNCTIONAL IMPROVEMENTS AMONG OLDER VETERANS WITH PAD FOLLOWING 12 MONTHS OF EXERCISE TRAINING

J Antonio Gutierrez[1], Megan Pearson[2], Lin Gu[1], Katherine Hall[3], Sunil Rao[4], and Miriam Morey[3], *1. Durham VA Medical Center, Durham, North Carolina, United States, 2. Durham VA Healthcare System, Durham, North Carolina, United States, 3. Durham VA Health Care System, Durham, North Carolina, United States, 4. DURHAM VA MEDICAL CENTER, DURHAM, North Carolina, United States*

The prevalence of peripheral artery disease (PAD) increases exponentially with age and is associated with heightened risk of functional impairment. We assessed baseline function and compared changes in mobility among older Veterans with PAD participating in Gerofit, a clinical exercise program. A total of 545 Veterans (mean age: 79 years) completed baseline, 3-, 6-, and 12-month mobility assessments and were divided into the following cohorts: PAD (n=99) and no PAD (n=446). Assessments included 10-m walk speed, 6-min walk distance, 30-s chair stands, and 8-ft up-and-go time. Veterans with PAD performed worse on all measures than non-PAD Veterans at baseline. Veterans with PAD demonstrated significant improvements ($p < 0.05$) in all measures from baseline at 3, 6 and 12 months. There were no significant differences in change scores between PAD and non PAD across all time points, controlling for age, except for 8-ft up-and go. PAD Veterans benefit from function-based exercise.

### SESSION 5060 (PAPER)

### INFLAMMATION

### AFFECTIVE REACTIVITY TO DAILY STRESSORS AND IMMUNE GENE EXPRESSION IN THE MIDUS STUDY

Abner Apsley[1], Sun Ah Lee[2], Aarti Bhat[3], David Almeida[2], and Idan Shalev[2], *1. Pennsylvania State University, University Park, Pennsylvania, United States, 2. The Pennsylvania State University, University Park, Pennsylvania, United States, 3. The Pennsylvania State University, State College, Pennsylvania, United States*

Past research indicates that an individual's affective reactivity to daily stressors is associated with elevated levels of inflammatory biomarkers such as interleukin-6 and C-reactive protein. Here, we assessed individuals' positive and negative affective reactivity to daily stressors, and their association with expression levels of genes in the conserved transcriptional response to adversity (CTRA; proinflammatory and type-1 interferon antiviral genes). We hypothesize that gene expression levels of CTRA genes are a possible explanatory mechanistic link between higher levels of affective reactivity and inflammatory markers. Our study included 199 individuals from the Midlife in the United States Refresher study (ages 26-71, 52.8% female, and 17.9% non-white) who participated in the Gene Expression and Daily Diary Projects. Individuals provided whole blood for gene expression analysis and completed an 8-day telephone interview to assess daily experiences. Positive and negative affective reactivity scores were used to indicate magnitude of an individual's changes in daily affect on stressor-days versus non-stressor-days, and were computed using multilevel modeling. Three CTRA gene expression scores were created for each individual, representing the average expression of proinflammatory, type-1 interferon, and a combination of both. Preliminary findings suggest a negative association between the CTRA composite score and negative affective reactivity to daily stressors (β = -0.963, p = 0.034), controlling for covariates. Further analyses will use affective reactivity to predict individual CTRA genes, correcting for multiple testing. This study will be the first to examine the relationship between daily assessments of emotions and gene expression levels in a representative U.S. cohort.

### RACIAL AND ETHNIC DIFFERENCES IN THE LIFE COURSE INFLUENCES OF SOCIOECONOMIC STATUS ON ADULT SYSTEMIC INFLAMMATION

AMBER KELLY[1], Patricia Morton[2], Fatima Eid[3], and Kenneth Ferraro[4], *1. Wayne State University, Ferndale, Michigan, United States, 2. Wayne State University, Detroit, Michigan, United States, 3. Wayne State University, Dearborn, Michigan, United States, 4. Purdue University, West Lafayette, Indiana, United States*

Two fundamental causes of health disparities in the U.S. are race and socioeconomic status (SES). Although these disparities emerge early in the life course, there is limited research on how race/ethnicity intersects with SES over the life course to influence later-life systemic inflammation—a key pathogenic process of health for older adults. Moreover, life course studies on systemic inflammation often focus on Black/White differences. Analyzing a sample of Black, White, and Hispanic Americans, this study examined the intersecting influences of childhood SES, race/ethnicity, and adult education on three time points of later-life inflammation levels (measured using C-reactive protein [CRP]). Longitudinal mixed-effects models were estimated using a sample of U.S. men (n=4,137) and women (n=6,187) over age 50 from the Health and Retirement Study during a decade of observation (2006-2016). Black men who experienced low childhood SES had higher levels of CRP than White men with low childhood SES; education did not moderate these associations. Education had a stronger protective effect against systemic inflammation for Hispanic men who experienced childhood socioeconomic disadvantage compared to their White counterparts. For women, the protective effect of education on systemic inflammation was stronger for White women compared to Black and Hispanic women. There were no significant childhood SES interactions for women. Findings indicate that the effects of SES on later-life systemic inflammation are contingent upon race/ethnicity, suggesting that intersectionality operates over the life course to produce health disparities in later-life. However, adult education acts as a leveler of life course inequality for Hispanic men.

### SOCIAL DIFFERENCES IN ALLOSTATIC LOAD TRAJECTORIES ACROSS ADULTHOOD

Christine Walsh[1], Yang Yang[2], Daniel Belsky[3], Sarah Mills[2], Marissa Hall[2], and Shelley Golden[4], *1. Carolina Population Center, University of North Carolina, Chapel Hill, North Carolina, United States, 2. University of North Carolina at Chapel Hill, Chapel Hill, North Carolina, United States, 3. Mailman School of Public Health, Columbia University, New York City, New York, United States, 4. University of North Carolina at Chapel Hill, Chapel HIll, North Carolina, United States*

Chronic psychosocial stress has been indicated as a potentially important driver of U.S. health disparities. Allostatic load (AL) is an indicator of cumulative biological risk or "wear and tear" on the body resulting from repeated adaptations to life stressors. A population-level examination of AL may be particularly useful in understanding the intermediate physiological dysregulation that underlies social inequalities in health. No study has yet documented changes in allostatic load over a wide range of ages and intersecting sociodemographic groups. To address this gap, we pooled biomarker data from three U.S. longitudinal cohort studies (National Longitudinal Study of Adolescent to Adult Health, Midlife Development in the U.S., and the Health and Retirement Survey) to create an integrated dataset covering individuals ages 24 to over 95 from several birth cohorts. We conducted growth curve models to examine associations of AL trajectories with cohort and multiple dimensions of social identity. We found more recently born cohorts were associated with higher mean levels in AL as compared to earlier-born cohorts. Net of cohort, education, and other covariates, Black men and women and Hispanic men had significantly higher AL than their White counterparts. These differences were present in early adulthood and persisted at all ages. Hispanic women experienced the largest increases in AL with age, surpassing nearly all other groups at older ages. These findings contribute new knowledge about the temporal dynamics and social patterning of AL as individuals age, particularly intersecting stress exposures as an underlying and enduring source of health inequity.

### VIGILANCE, STRESS COPING, AND DISPARITIES IN METABOLIC HEALTH OVER THE LIFE COURSE

Viktoryia Kalesnikava[1], Michele Evans[2], Alan Zonderman[3], and Briana Mezuk[1], *1. University of Michigan, School of Public Health, Ann Arbor, Michigan, United States, 2. National Institute on Aging, Bethesda, Maryland, United States, 3. National Institute on Aging, National Institute of Health, Baltimore, Maryland, United States*

Recent investigations of the mechanisms through which stress may impact cardiometabolic health point to the potential importance of cognitive tendencies (e.g., vigilance, coping strategies) in this relationship. This study examined the associations between vigilance and stress coping with metabolic risk, and whether these associations varied by race and life course socioeconomic status (SES). Data come from the Healthy Aging in Neighborhoods of Diversity across the Life Span (HANDLS) study, a cohort of urban adults (n=3720, baseline age 30-64 years; balanced by race, sex, age and household income). Vigilance was measured by the MacArthur Reactive Responding subscale; stress coping was measured by the Brief Cope scale. Metabolic risk was operationalized by metabolic syndrome z-scores (MetS-Z). Linear mixed models were used to examine longitudinal associations between cognitive tendencies and MetS-Z; interaction terms were used to examine effect modification by race and life course SES. Black participants reported higher adaptive coping; participants experiencing persistent poverty or downward mobility reported higher vigilance and avoidant coping and lower adaptive coping. Overall, vigilance was not associated with metabolic risk. Avoidant and adaptive coping were inversely associated with baseline MetS-Z and further varied by sociodemographic factors. Higher adaptive coping was associated with lower MetS-Z among White adults, while higher avoidant coping was associated with lower MetS-Z among Black adults. Lower stress coping were associated with higher MetS-Z among White participants with low lifecourse SES or downward socioeconomic mobility. In sum, stress coping may modulate the stress-health relationship, but these associations must be interpreted within the intersection of contextual factors.

### DEPRESSION, INFLAMMATION, AND THE MODERATING ROLE OF METFORMIN: RESULTS FROM THE MIDUS STUDY

Sumaiyah Syed[1], Iris Yang[1], and Stephanie Wilson[2], *1. Southern Methodist University, Dallas, Texas, United States, 2. Southern Methodist University, DALLAS, Texas, United States*

There is a well-established link between depression and aging-related inflammation, where depression can spur inflammation and affect physical health. Previous animal trials have shown compelling evidence that the use of metformin, the first line of treatment for diabetes, can offset inflammation. These findings have not yet been extended to human samples. This study examined the association between depression and inflammation, specifically the moderating role of metformin usage in middle-aged and older adults. We predicted that metformin would attenuate the link between depression and inflammation. Participants in Project 4 of the Midlife in the United States (MIDUS) Study (n=1255, Mage = 57) provided data on medication use, depressive symptoms, and inflammatory cytokines interleukin (IL)-6, tumor necrosis factor (TNF)-α, and C-reactive protein (CRP). Controlling for age, sex, body mass index (BMI), and comorbidity burden, metformin use moderated the association of depressive symptoms with IL-6 and CRP, but not TNF-α. Higher levels of depressive symptoms were significantly associated with higher interleukin 6 (IL-6) ($p < .001$) and C-reactive protein (CRP) ($p = .033$) among those not using metformin. However, this effect was not significant among those using metformin, suggesting that metformin usage may attenuate the relationship between depression and inflammation. These findings support the potential of metformin in mitigating the link between depression, a well-known behavioral risk factor, and inflammation, a key source of biological aging. Metformin, an accessible and commonly prescribed drug, may have a hidden anti-aging role that may protect against the inflammatory effects of depression.

### SESSION 5070 (SYMPOSIUM)

### LATE-LIFE CARE FOR OLDER ADULTS: FINDINGS FROM NHATS AND NSOC

Chair: Mengyao Hu Co-Chair: Sarah Patterson

With the aging of the U.S. population, caregiving has become an emerging public health issue that affects the health and quality of life for millions of care recipients and their caregivers. An increasing number of older adults rely on their family caregiver networks and home-based clinical services for physical, social and emotional support. Caregiving plays an important role in health and wellbeing of older adults both in daily life and in the context of health care, e.g., in post-acute caregiving after hospital discharge. There is a growing interest in identifying caregivers in greatest need of support and developing programs and interventions to help these caregivers. This symposium describes caregiver network and examines the roles of the caregiver network and family caregiving support on care recipients' and caregivers' quality-of-life outcomes using the National Health and Aging Trends Study (NHATS) and linked National Study on Caregiving (NSOC). This symposium will 1) evaluate typologies of the structures and compositions of caregiver network and examine their effects on care recipients' well-being; 2) describe findings on associations between caregiving network and caregiver supports with unmet needs among older adults; 3) describe the role of unpaid caregivers after hospital discharge; 4) evaluate effects of family caregiving support in facilitating the use of home-based clinical services by older adults; 5) examine the effects of family disagreement on caregivers' emotional difficulty and overload in dementia caregiving. Together, these presentations suggest important public health implications for research, policy and practice for improving late-life caregiving.

### TYPOLOGIES OF CAREGIVING NETWORKS AND OLDER ADULTS' QUALITY OF LIFE

Mengyao Hu, Vicki Freedman, and Sarah Patterson, *University of Michigan, Ann Arbor, Michigan, United States*

This study identifies typologies of caregiving networks of older adults and their effects on older adults' well-being and unmet needs in late life using the National Health and Aging Trends Study (NHATS). NHATS identifies the entire care network of older adults, cataloguing all activities each respondent assists with and the amount of time they spend helping, providing a unique opportunity to evaluate caregiving networks. Using network analysis and cluster analysis, we find that older adults have different types of caregiving networks including large and small networks with a primary caregiver and networks where caregivers share caregiving responsibilities more evenly. Size and structure of the networks are significantly associated with older adults' well-being and unmet needs. These findings highlight the importance of considering the full care network in developing policies and programs to support family caregivers.

### THE ROLE OF FAMILY CAREGIVING CONFIGURATIONS AND SUPPORTS IN OLDER ADULT OUTCOMES

Natalie Chong, *Brandeis University, Waltham, Massachusetts, United States*

This study uses data from the 2017-2018 National Health and Aging Trends Study and the 2017 National Study of Caregiving to examine whether caregiving network configuration factors (e.g., if and how multiple caregivers of the same care recipient distribute tasks, responsibility and disagreement among caregivers, involvement of paid caregivers) and caregiver supports (e.g., emotional support, skills training, respite services) are associated with consequences of unmet needs among older adults. Findings from this study will provide greater understanding of how caregiving contexts shape the quality of care and health of older adults who rely on help from family caregivers.

### A PROFILE OF POST-ACUTE CAREGIVING

Claire Ankuda, Amy Kelley, and Subashini Rajagopalan, *Icahn School of Medicine at Mount Sinai, New York, New York, United States*

Post-acute care is increasingly shifting from institutional settings into the home, but the impact on family caregivers is unknown. Using NHATS, we identified a cohort of 2,124 older adults enrolled in fee-for-service Medicare with a survey interview conducted within 90 days of discharge from discharge. We first examined the hospitalization characteristics associated with both receipt of family and friend assistance in the 90 days post-discharge and average hours of assistance per week. We then used linked NSOC data to a subgroup of 365 individuals to compare the caregiver reports for caregivers of older adults in the post-acute period vs. not. We found that both the receipt of post-acute family/friend assistance and hours of assistance varied by hospitalization length of stay and discharge services. We also found that post-acute caregivers differed from non-post-acute caregivers in their caregiving tasks and difficulties related to providing care.

### THE ROLE OF FAMILIES IN FACILITATING USE OF HOME-BASED SERVICES

Jennifer Reckrey[1], Duzhi Zhao[1], Christine Ritche[2], and Katherine Ornstein[3], *1. Icahn School of Medicine, The Mount Sinai Hospital, New York, New York, United States, 2. Massachusetts General Hospital, Boston, Massachusetts, United States, 3. Johns Hopkins University, Baltimore, Maryland, United States*

Homebound older adults rely on home-based clinical services (e.g., home based medical care) and long-term services and supports (e.g., caregiving support, environmental modifications) to remain living at home, however patterns of service use are unknown. Using data from the 2015 National Health and Aging Trends Study linked to Medicare Claims Data, we identified a population of homebound older adults enrolled in fee-for-service Medicare (n=1066). Latent class analysis identified 3 distinct patterns of home-based service use: low (36%), medium (51%), and high (13%) service use. High family caregiving support was particularly prevalent in the medium service group (35% with over 40 hours of family care per week), but receipt of home-based medical care was minimal in this group. Additional support to connect family caregivers with other home-based services may improve care and outcomes for older adults living at home.

### WHEN FAMILIES DISAGREE: CORRELATES AND STRESS PROCESS OUTCOMES OF FAMILY CAREGIVER DISAGREEMENT

Amanda Leggett[1], Hyun Jung Koo[2], Hannah Lee[2], and Elaina Baker[2], *1. Wayne State University, Ypsilanti, Michigan, United States, 2. University of Michigan, Ann Arbor, Michigan, United States*

Dementia caregiving is a family affair, yet prior research suggests that families often disagree about care provision. We explore correlates of such disagreements and how disagreements relate to stress process outcomes of care. The current study examines 552 family caregivers for Medicare-eligible older adults with dementia from the 2017 National Study of Caregiving. We consider demographic and care characteristics as predictors of family disagreement in a multinomial logistic model, and family disagreement as the key predictor of caregiving overload, emotional difficulty, and gains in linear regressions. Females had greater odds of disagreement, while those with friends and family to talk to and providing assistance with household care activities had lower odds of disagreement. Family disagreement was significantly associated with overload ($B=0.95$, $p<.001$) and emotional difficulty ($B=0.45$, $p<.05$), but not gains. Interventions to help families manage care disagreements and coordinate care may have great impact on caregiver well-being.

### SESSION 5080 (SYMPOSIUM)

### OLDER ADULT ENGAGEMENT IN COMMUNITY GERONTOLOGY

Chair: Natalie Pope

Community gerontology integrates scholarship on aging and communities through the lens of mesolevel environments, the diverse continua of settings linking micro and macro contexts of aging. This interdisciplinary symposium brings together research exploring the interconnections of older adults' engagement across and within the socio-ecological levels of their lived experiences, place-based communities, and policy contexts. Yeh et al.'s paper elucidates the promises of visual methods in critical qualitative research with older adults to subvert power dynamics and advance social justice in gerontology. Latham-Mintus' paper explores how "weak ties" created by the interaction of social infrastructure within geospatial places and spaces can facilitate longer, healthier lives. Reyes' paper uses an intersectional life course perspective to explore placemaking as civic participation among Latinx immigrants and African American older adults across time and social environments. Plasencia's paper focuses on aging in community among diverse older adults using a photovoice to assess their perceptions and needs within the lived urban environment. Pope and colleagues explore how a multi-state sample of older adults perceive their roles in age-friendly initiatives at individual, organizational, and community levels. Taken together, the papers discuss and contextualize the multidirectional ways older adults engage their place-based communities within larger macro sociopolitical structures – an analysis that highlights the embeddedness of mesolevel community environments both within and between micro and macro spheres. This session further speaks to the importance of centering older adults' experiences in community gerontology by illuminating the ways older adults not only age in community but through layers of community.

### OLDER ADULT ENGAGEMENT IN AGE-FRIENDLY COMMUNITY WORK

Natalie Pope[1], Katy (Qiuchang) Cao[2], and Emily Greenfield[1], *1. Rutgers, The State University of New Jersey, New Brunswick, New Jersey, United States, 2. The Pepper Institute on Aging and Public Policy and The Claude Pepper Center, Florida State University, Tallahassee, Florida, United States*

Drawing on 22 interviews with older adults across four states, this qualitative study explores how older adults perceive their age-friendly community work and the personal meaning they derive from participation. Based on iterative coding, findings indicate ways in which geographic differences in the structure and framing of local age-friendly community initiatives set the boundaries, scope, and potential customization of older adult engagement. Across geographies, motivations for starting and sustaining engagement included: perceived personal growth; social connectivity; outlets for advancing skills and passions; and satisfaction from making a difference in the lives of older adults and their broader community. These findings highlight the importance of conceptualizing older adult engagement in age-friendly community work as a dynamic process embedded within intrapersonal, interpersonal, organizational, and community contexts. Demonstrating multiple ways older adults contribute to placemaking, the findings have implications for older adult engagement at various levels and stages of age-friendly community initiatives.

### EMANCIPATORY VISIONS: USING VISUAL METHODS TO CO-CONSTRUCT KNOWLEDGE WITH OLDER ADULTS

Jarmin Yeh[1], Laurent Reyes[2], and H. Shellae Versey[3], *1. University of California, San Francisco, San Francisco, California, United States, 2. UC Berkeley, Berkeley, California, United States, 3. Fordham University, Bronx, New York, United States*

Research is a political activity. Researchers are responsible for theories and methods used to explore, explain, or ignore injustices. A need exists for developing new tools and pathways of knowledge based on experiences, language, and intellect of older adults from Black, Indigenous, and People of Color communities. This presentation argues for the utility of visual methods in critical qualitative research as a medium that allows researchers and participants to co-construct knowledge. Lessons learned about implementing projects using visual methods from the intersectional standpoint of the authors – three younger women of color – will be discussed. Promises and complexities of navigating interpersonal dynamics, decolonizing knowledge-production, and scaling visual methods on multiple levels will be elucidated. Collectively, we argue that visual methods are rigorous for subverting power dynamics rooted in extractive research practices, and provide a vehicle for community-engaged participatory action research that has potential to advance social justice in gerontology.

### UNDERSTANDING THE ROLE OF SOCIAL INFRASTRUCTURE FOR OLDER ADULT ENGAGEMENT ACROSS GEOGRAPHIC CONTEXTS

Kenzie Latham-Mintus, Lucas Montgomery, and Jeffrey Wilson, *IUPUI, Indianapolis, Indiana, United States*

Using semi-structured interviews of older adults, living alone or with a partner in the community, this research explores themes related to aging in place, social infrastructure, and community engagement across geographic contexts in Indiana. In particular, we are interested in understanding how older adults experience and use the existing social infrastructure in their communities and how these experiences vary across the rural-suburban-urban continuum. Additionally, we examine how social interactions supported by social infrastructure influence the maintenance of social relationships including weak social ties. Because COVID-19 has significantly changed patterns of community engagement among older adults, respondents were asked to discuss their behavior pre- and post-COVID-19. Applying a thematic analysis approach to the data, we explore the interconnections among social infrastructure, community engagement, social relationships, and geographic settings with an emphasis on older adults' experiences and perceptions.

### PLACEMAKING AS CIVIC PARTICIPATION: A STORY OF AGENCY AND RESISTANCE AMONG BLACK AND LATINX IMMIGRANT OLDER ADULTS

Laurent Reyes, *UC Berkeley, Berkeley, California, United States*

Placemaking is a collective process that happens every day. However, little has been written about the ways older African Americans and Latinx immigrants create place in their everyday lives. Less has been said about the ways these efforts are examples of civic participation in the context of systemic inequality and oppression. Results from this phenomenological study that applies an intersectional life course perspective shows how older Black and Latinx immigrant adults maintain, preserve, and build their place in a society that continuously threatens their erasure. These results shine light on the ways Black and Latinx communities work to maintain the physical space (i.e., community safety and sanitation), preserve their culture and history, and build structures to ensure access to resources that will empower their communities. Their efforts across the micro, meso, and macro social environments and across time, demonstrates a concerted effort towards creating a dignified and just place in our society to nourish and sustain future generations.

### SESSION 5081 (SYMPOSIUM)

### DUALS AND ASSISTED LIVING: EXAMINING ACCESS AND OUTCOMES

Chair: Kali Thomas Discussant: Hyunjee Kim

Assisted living, a popular long-term care option for older adults needing personal care assistance, is increasingly serving a vulnerable population of low-income older adults dually-enrolled in Medicare and Medicaid (duals). However, we know very little about the needs of this population, their access to assisted living, the quality of care duals are receiving, and how this varies across states and assisted living providers. This symposium will include five presentations using national data and focused on access to assisted living for duals and the quality of care that duals in assisted living settings receive. The first two presentations take different approaches to investigate access to assisted living and how it relates to Medicaid policy: one examines Medicaid policies and their relationship to geographic access, and the other presents results from an analysis examining the association between Medicaid financing and segregation of duals. The next three presentations highlight important findings related to the quality of care for duals in assisted living: one focuses on concentration of duals in assisted living and its relationship with hospitalization and nursing home placement, one examines injury-related emergency department visits among duals, and the final presentation discusses the ability of duals in assisted living to age in place toward the end of life. The discussant, an expert in Medicaid policy, will conclude with a discussion of states' different approaches for covering services in assisted living through their Medicaid programs, highlighting the opportunities for increasing equitable access and ensuring high quality care is delivered to this vulnerable population.

### MEDICAID POLICY AND ASSISTED LIVING ACCESS

Lindsey Smith, *Brown University, Providence, Rhode Island, United States*

Funding for home and community-based services (HCBS), including assisted living (AL), using Medicaid is limited to either Medicaid program waivers or state plan amendments. The Affordable Care Act (ACA) created a new option for the state plan, the Community First Choice 1915(K), that mandates all care is self-directed and inclusive of families in decision making while disallowing waiting lists. This presentation will provide an overview of the various combinations of Medicaid waivers and amendments used by states to cover services in AL. We will then share the results of a study that used coincidence analysis, a configurational comparative method, to compare states' approaches to HCBS Medicaid coverage and a measure of AL geographic access. We found that both Medicaid waivers and 1915(K) amendments are associated with increased geographic access to AL.

### MEDICAID FINANCING AND SEGREGATION OF DUALS IN ASSISTED LIVING

Kali Thomas[1], Portia Cornell[2], Cassandra Hua[3], and Momotazur Rahman[1], *1. Brown University, Providence, Rhode Island, United States, 2. Providence VA Medical Center / Brown University, Providence, Rhode Island, United States, 3. Brown University School of Public Health, Providence, Rhode Island, United States*

We used 2018 Medicare enrollment data, a national directory of licensed ALs, and Medicaid state policies. We identified a cohort of 474,661 AL residents and a comparison cohort of 58,911,266 community-residing individuals. We compared the distribution of duals across ALs to the distribution of community-dwelling duals across ZIP codes by taking the ratio of AL Gini index for each state over the community Gini index for each state (the "Gini ratio" or GR). On average, states with both waivers and state plans covering services in AL had the lowest Gini ratio (less segregated than community; GR=0.87;) States with no Medicaid financing for AL had the highest Gini ratio (more segregated than community, GR=1.16). Medicaid coverage for home and community-based services in AL is associated with increased access to AL for duals.

### STRUCTURAL INEQUITIES IN OUTCOMES FOR DUAL-ELIGIBLE RESIDENTS IN ASSISTED LIVING

Portia Cornell[1], Cassandra Hua[2], Momotazur Rahman[3], Gauri Gadkari[2], and Kali Thomas[3], *1. Providence VA Medical Center / Brown University, Providence, Rhode Island, United States, 2. Brown University School of Public Health, Providence, Rhode Island, United States, 3. Brown University, Providence, Rhode Island, United States*

We examined the association of AL residents' dual-eligibility and the concentration of dually eligible residents in AL communities with residents' risk of hospitalization and long-term nursing home admission. The exposure was dual status interacted with AL concentration: no-duals, minority-duals [<=50%] (reference group), and majority-duals [>50%]. We found that duals in AL have higher risk of hospitalization and nursing home admission than non-duals. For both duals and non-duals, moving to an AL with a high concentration of duals conferred excess risk of hospitalization. Among duals, however, lower concentration of duals in ALs increases risk of long-term nursing home admission for duals, whereas it is protective for non-duals. The association of higher hospitalization with concentration of duals suggests that quality may be a concern in communities that specialize in care for duals. However, majority-duals ALs may be better equipped to provide more comprehensive care as an alternative to nursing homes.

### DUAL ELIGIBILITY AND INJURY-RELATED EMERGENCY DEPARTMENT VISITS AMONG ASSISTED LIVING RESIDENTS

Cassandra Hua[1], Portia Cornell[2], Elizabeth White[3], Katherine Kennedy[4], Ian Nelson[5], and Kali Thomas[3], *1. Brown University School of Public Health, Providence, Rhode Island, United States, 2. Providence VA Medical Center / Brown University, Providence, Rhode Island, United States, 3. Brown University, Providence, Rhode Island, United States, 4. Providence VA Medical Center, Providence, Rhode Island, United States, 5. Miami University, Oxford, Ohio, United States*

Using 2018 Medicare data, we examined the relationship between dual eligibility and injury-related emergency department use among a cohort of assisted living residents (n=116,754). We fit multilevel models with random intercepts at the assisted living community and license type levels. The baseline rate of injury-related emergency department emergency department use was 0.17. After controlling for resident characteristics (i.e., age, sex, race, and chronic conditions), license type characteristics (i.e., dementia care licensure, staffing regulations), and assisted living community characteristics (i.e., size and percentage of residents with dementia), being dually eligible for Medicare and Medicaid was associated with a 12% increase in the probability of having an injury-related emergency department visit (b=.02; p<.001). Assisted living communities that serve duals may have fewer resources and staff to provide personal care, potentially leading to increased rates of injuries.

### RETENTION OF DUALLY ELIGIBLE BENEFICIARIES IN ASSISTED LIVING AT THE END OF LIFE

Susan Hayes[1], Nicole Rosendaal[2], Xiao (Joyce) Wang[2], Kali Thomas[2], and Emma Belanger[2], *1. Brown University School of Public Health, Providence, Rhode Island, United States, 2. Brown University, Providence, Rhode Island, United States*

To examine to what extent dually eligible beneficiaries (duals) residing in assisted living remain there toward the end of life, we conducted a prospective cohort study of 98,944 Medicare beneficiaries present at validated AL ZIP codes in January 2017, and who died during a two-year follow-up. The outcome was AL residence in the last 30 days of life. We compared decedents who were not duals (80,156 decedents), with those newly dually eligible in 2017-2018 (3,722 decedents), and those already dually eligible in 2016 (15,066 decedents). Only 36.7% of new dual decedents resided in

AL in the last 30 days of life, compared to 66.2% among those dually eligible in 2016, and 84.5% of those without Medicaid. While 29 states retained over half of all decedents in AL until death, only 8 states retained a majority of dually eligible decedents.

### SESSION 5090 (SYMPOSIUM)

### RESEARCH AT THE INTERSECTION OF TECHNOLOGY AND AGING

Chair: Jodi McDaniel

New and emerging technologies offer opportunities for the delivery of wide-ranging, adaptable interventions to improve quality of life of older adults. Novel technologies can also improve the quality of measurement methods used in aging research. In this symposium five presentations will describe advanced technologies to promote health and function in older adults. The first will discuss the Urban Aging Residents Coalition (UARC), an organization founded in partnership with an African American older adult community leader to address engaging urban older adults with technology. A primary goal of UARC is to prevent social isolation and promote mental wellness through education and computer literacy. The second presentation will describe a project testing socially assistive robots (SARs) to complement personnel resources in older adults with cognitive impairment in long term care facilities. Study participants were successfully engaged in a participatory design involving repeated sessions with SARs that resulted in prototype refinement. The third presentation will describe current applications of patient communication technologies in acute-critical care settings with a focus on the user experience among older adult patients. The fourth will report on the implementation of an ongoing, in-home Smarthealth technology intervention for two older adult family caregivers of persons with dementia. Study findings showed the intervention improved self-awareness of emotional care and reactions to care recipients. The final presentation will explain an advanced wound measurement system using artificial intelligence to track healing progress in clinical research. The presentations will highlight technologies to support healthy aging and discuss implication for practice, policy, and research.

### INTERRATER RELIABILITY OF AN ADVANCED NONCONTACT WOUND MEASUREMENT SYSTEM USING ARTIFICIAL INTELLIGENCE

Jodi McDaniel[1], Alai Tan[2], and Narasimham Parinandi[3], *1. Ohio State University College of Nursing, Columbus, Ohio, United States, 2. The Ohio State University College of Nursing, Columbus, Ohio, United States, 3. The Ohio State University College of Medicine, Columbus, Ohio, United States*

Venous leg ulcers (VLU) are recurring, disabling wounds that develop primarily in older adults with comorbidities. In an ongoing clinical trial testing a nutritional intervention researchers use advanced technology (Swift Skin and Wound) to measure VLU healing over time. This noncontact wound measurement system uses artificial intelligence to automatically calculate wound measurements and track healing progress. However, a subjective component of the Swift system involves delineating wound perimeters on photographs captured with a tablet or smartphone. To evaluate interrater reliability of the system in the current study, measurements of 11 wounds by two independent raters were assessed using an intraclass correlation coefficient (ICC). ICC estimates and their 95% confidence intervals were calculated using SPSS statistical package version 27 based on a mean-rating ($k = 2$), consistency, 2-way mixed-effects model. For area measures, ICC = 0.99 with 95% confidence interval = 0.998-1.0, indicating the Swift measurement system has excellent interrater reliability.

### EXPERIENCES USING A SMARTHEALTH SYSTEM: TWO CASES OF DEMENTIA FAMILY CAREGIVERS

Karen Rose[1], Eunjung Ko[1], Sooyoung Kim[2], Kristina Gordon[3], Hongning Wang[4], and John Stankovic[4], *1. The Ohio State University, Columbus, Ohio, United States, 2. Ohio State University, Columbus, Ohio, United States, 3. The University of Tennessee Department Of Psychology, Knoxville, Tennessee, United States, 4. University of Virginia, Charlottesville, Virginia, United States*

Family caregivers for persons with dementia often times experience stress and burden while caregiving. The COVID-19 pandemic has worsened this situation with increased physical and social isolation, further resulting in health risks in this population. Technology-based interventions have become more commonplace in today's world to address ongoing caregiver needs although gaps in technological literacy and usage still exist among many users. This case study aims to report on the implementation of an ongoing Smarthealth technology intervention for two older adult family caregivers of persons with dementia and to explore their experiences with this system. Data were collected through acoustic monitoring, survey administration, and semi-structured interviews. Intervention effects on changes in emotional states will be discussed. Study findings showed the intervention improved self-awareness of emotional care and reactions to care recipients. Findings in this study highlight the importance and challenges of real-time technology-based intervention implementation in older adult caregiving populations.

### URBAN AGING RESIDENTS COALITION (UARC): A HYBRID MODEL TO BRIDGE THE TECHNOLOGY GAP

Kathy Wright[1], Karen Moss[2], Judith Tate[3], Karen Rose[2], and Pamela Shields[4], *1. The Ohio State University College of Nursing, Columbus, Ohio, United States, 2. The Ohio State University, Columbus, Ohio, United States, 3. Ohio State University, College of Nursing, Columbus, Ohio, United States, 4. Urban Aging Residents Coalition, Columbus, Ohio, United States*

The COVID-19 pandemic spotlighted technology barriers for urban Black/African American older adults that lead to chronic stress, social isolation, and inability to fully engage with the world. In partnership with an African American older adult community leader, the Urban Aging Residents Coalition was founded in May 2020 to address engaging older adults with technology. A primary goal of UARC is to prevent social isolation and promote mental wellness through education. Using a hybrid model of videoconferencing and socially-distanced meetings, volunteer health professionals and community members (e.g., Information Technology Technician, Psychologist, and Nurse) delivered education to older adults. Participants also met for computer training to build confidence by sharing technology accomplishments with others (e.g., online bill paying). UARC has 75 active members and conducted over 12 videoconferences and in-person programming. The UARC project serves as a model for community/academic partnerships to support mental wellness and technology use in urban older adults.

### PARTICIPATORY DESIGN: AN ESSENTIAL PROCESS FOR SOCIALLY ASSISTIVE ROBOTIC ACTIVITIES IN LONG-TERM CARE SETTINGS

Mion Lorraine[1], Emily Latshaw[1], Yi Chun Lin[1], Miroslava Migovich[2], Ritam Ghosh[2], Nibraas Khan[2], Nilanjan Sarkar[2], and Judith Tate[3], *1. The Ohio State University, Columbus, Ohio, United States, 2. Vanderbilt University, Nashville, Tennessee, United States, 3. Ohio State University, College of Nursing, Columbus, Ohio, United States*

We conducted participatory design research for long term care (LTC) socially assistive robotic activities comprised of social, cognitive and physical components and enhanced human-robot (HRI) and human-human interactions (HHI). Repeated sessions were conducted with 10 geriatric experts (physicians, activity directors, nurses, occupational therapist) and 12 LTC residents (ages 70–92). Two robots, animal and humanoid, were used in combination with virtual reality. Four collaborative activities for paired older adults were designed and evaluated: playing drums to music, completing paintings, a fishing game, and training a dog with simple commands. Within each activity, three levels of difficulty were designed. Stakeholder feedback was obtained through observations and interviews. Numerous modifications were made following each session that addressed hardware, software and activity issues. Modifications were necessary both for the HRI and HHI aspects of the activity. Our experience demonstrates the necessity for participatory design in the deployment of technology for LTC settings.

### CURRENT APPLICATIONS OF PATIENT COMMUNICATION TECHNOLOGIES IN GERIATRIC CRITICAL CARE

Mary Beth Happ[1], Emika Miller[2], Judith Tate[3], and JiWon Shin[4], *1. The Ohio State University, Columbus, Ohio, United States, 2. Ohio State University, Columbus, Ohio, United States, 3. Ohio State University, College of Nursing, Columbus, Ohio, United States, 4. University of California, Davis, Sacramento, California, United States*

The availability and utility of patient-centered communication technologies in acute-critical care settings have evolved slowly over the past 30 years with wide variability, little standardization, and few randomized controlled clinical trials (RCT). The COVID-19 pandemic forced rapid expansion and use of communication technologies, particularly between patients and remote family caregivers. To capture changes responsive to the pandemic, this paper reviews current literature (< 5 years) on communication technologies in acute-critical care settings focusing on the user experience among older adult patients. We supplement these findings with case-based evidence from a pilot RCT of an electronic tablet communication application provisioned to mechanically ventilated ICU patients, and efforts toward hospital-wide implementation. Recent literature on patient communication technology consists primarily of qualitative, descriptive accounts of video communication (i.e., ICU visits) or provision of augmentative and alternative communication. Recommendations for required skills, standardization, and research regarding patient communication technology are provided.

### SESSION 5091 (BIOLOGICAL SCIENCES INVITED SYMPOSIUM)

### PROTEOSTASIS: NOVEL INSIGHTS AND TECHNOLOGIES

Chair: Andrew Pickering

### GENETIC AND PHARMACOLOGIC PROTEASOME AUGMENTATION AMELIORATES ALZHEIMER'S-LIKE PATHOLOGY IN MICE AND FLIES

Andrew Pickering, *University of Alabama at Birmingham, Birmingham, Alabama, United States*

The proteasome has key roles in neuronal proteostasis, including removal of misfolded and oxidized proteins, presynaptic protein turnover, as well as synaptic efficacy and plasticity. Proteasome dysfunction is a prominent feature of Alzheimer's disease (AD) (1–3). Artificial impairment of proteasome function can mimic many neurodegenerative phenotypes (4, 5). We report impaired proteasome function to represent an early-stage marker of AD preceding many other markers of the disease. Significantly, we show that prevention of proteasome dysfunction by genetic manipulation in fly and cell culture models of AD delays mortality, cell death, and cognitive deficits. We developed a transgenic mouse with neuronal-specific proteasome overexpression which, when crossed with a mouse model of AD showed reduced mortality and cognitive deficits. To establish translational relevance, we developed a set of novel TAT-based proteasome-activating peptidomimetics. These agonists stably penetrate the blood-brain-barrier and enhance 20S as well as 26S proteasome activity. We show that treatment with these agonists protects against cell death in a cell culture model of AD as well as both cognitive decline and mortality in fly and mouse models of AD. The protective effects observed from proteasome overexpression in our models appear to be driven at least in part by increased turnover of the amyloid precursor protein (APP) by the proteasome. We conclude that the proteasome plays an important role in AD progression. Furthermore, augmentation of proteasome function is protective against AD-like pathogenesis in diverse models of the disease, representing a new therapeutic target for treatment of AD.

### PROTEOSTASIS: NOVEL INSIGHTS AND TECHNOLOGIES

Constanza Cortes, *University of Alabama at Birmingham, Birmingham, Alabama, United States*

Skeletal muscle has recently arisen as a novel regulators of Central Nervous System (CNS) function and aging, secreting bioactive molecules known as myokines with proteostasis and metabolism-modifying functions in targeted tissues, including the CNS. Myokine secretion is heavily modified by exercise, suggesting that myokine signaling in the periphery may underlie the well document geroprotective benefits of exercise on the brain. The following studies address muscle proteostasis, a pathway highly activated during exercise, as a potential new regulator of the neurocognitive benefits of exercise. We have recently generated a novel transgenic mouse with enhanced muscle proteostasis via moderate overexpression of Transcription Factor E-B (TFEB), a powerful master regulator of cellular clearance and proteostasis. We have discovered that the resulting enhanced skeletal muscle proteostasis function can significantly ameliorate proteotoxicity and reduce neuroinflammation in the aging CNS. We derived cTFEB;HSA-Cre transgenic mice in the P301S MAPT background and we detected a significant reduction in hyperphosphorylated tau[AT8 phospho-tau antibody] in whole hippocampal lysates and in the dentate gyrus of cTFEB;HSACre;P301S mice compared to their single transgenic P301S littermate controls. Nanostring nCounter®AD panel analysis reveals displayed reductions in microglia activation modules in P301S MAPT/cTFEB:HSACre hippocampi, suggesting reduced neuroinflammation. We also determined that these CNS benefit sin P301S MAPT/cTFEB:HSACre mice were accompanied by activation of exercise-associated neurotrophic signaling and reduced markers of advancing tau-associated pathologies in the hippocampus. These provocative results suggest that enhanced skeletal muscle proteostasis modifies the accumulation of pathogenic tau isoforms and reduces neuroinflammation in the CNS of P301S MAPT mice via activation of exercise-associated signaling in the CNS.

### INTERPLAY BETWEEN LOSS OF PROTEOSTASIS AND CELLULAR SENESCENCE: A SPOTLIGHT ON MISFOLDED PROTEIN QUALITY CONTROL

Rahul Samant, *Babraham Institute, Cambridge, England, United Kingdom*

Loss of protein homeostasis ('proteostasis') and onset of cellular senescence are two conserved hallmarks of ageing. Healthy proteostasis relies on tightly-regulated intracellular quality control circuits that co-ordinate clearance of potentially toxic misfolded proteins arising from various internal or external stresses throughout an organism's lifespan. Proteostasis imbalances are mechanistically linked to a broad range of ageing-associated diseases, and are also characteristic of cellular senescence—a permanent cell cycle arrest that prevents uncontrolled proliferation during development, injury repair, and tumorigenesis, but drives ageing-associated frailty, degeneration, and therapy resistance. Across a range of replicative and stress-induced senescence models in primary human cells, we have discovered differences in how misfolded proteins are triaged when compared with proliferating, quiescent, or immortalised cells—especially at the level of ubiquitin-mediated protein clearance systems. Given recent findings that proteostasis modulators act as senolytics with geroprotective properties, our work highlights the need for an improved fundamental understanding of how different ageing hallmarks are inter-connected in order to drive advances in human healthspan.

### PARALLEL MEASUREMENTS OF PROTEIN AND CELL TURNOVER REVEAL HOW TISSUE CONTEXT AND AGING SHAPE PROTEIN LIFETIMES

Abigail Buchwalter[1], Kevin Welle[2], Jennifer Hryhorenko[3], Sina Ghaemmaghami[4], and John Hasper[5], *1. University of California, San Francisco, San Francisco, California, United States, 2. Mass Spectrometry Resource Lab, University of Rochester, Rochester, New York, United States, 3. Mass Spectrometry Resource Laboratory, University of Rochester, Rochester, California, United States, 4. Department of Biology, University of Rochester, Rochester, New York, United States, 5. Cardiovascular Research Institute, University of California, San Francisco, San Francisco, California, United States*

The lifespans of proteins can range from moments to years within mammalian tissues. Protein lifespan is relevant to organismal aging, as long-lived proteins can accrue damage over time. It is unclear how protein lifetime is shaped by tissue context, where both cell division and proteolytic degradation contribute to protein turnover. We have developed turnover and replication analysis by 15N isotope labeling (TRAIL) for parallel quantification of protein and cell lifetimes. We have deployed TRAIL over 32 days in 4 mouse tissues to date to quantify cell proliferation with high precision and no toxicity and determine that protein lifespan varies independently of cell lifespan. Variation in protein lifetime is non-random: multiprotein complexes such as the ribosome have consistent lifetimes across tissues, while mitochondria, peroxisomes, and lipid droplets have variable lifetimes across tissues. To model the effects of aging on tissue homeostasis, we apply TRAIL to a mouse model of Hutchinson-Gilford progeria syndrome and uncover fat-specific alterations in cell lifetime and proteome composition, as well as a broad decrease in protein turnover flux. These data indicate that environmental factors influence protein turnover in vivo and provide a framework to understand proteome aging in tissue context.

### PHOSPHORYLATION OF ULK1 AT S555 IS REQUIRED FOR METABOLIC ADAPTATIONS TO CALORIC RESTRICTION

Joshua Drake, Anna Nichenko, Orion Wiloughby, Matt Brisendine, Garrett Hays, Grace DiGirolamo, Zach Weingrad, and Ryan McMillan, *Virginia Polytechnic Institute and State University, Blacksburg, Virginia, United States*

Unc-51 Like Autophagy Activating Kinase 1 (Ulk1) is responsible for initiating selective degradation of damaged/dysfunctional mitochondria (mitophagy) once phosphorylated at S555 in response to energetic stress. Mitophagy is integral for mitochondrial health and Ulk1 has been implicated to be important for metabolic adaptation to exercise. Caloric restriction (CR), which extends lifespan and healthspan, has profound metabolic benefits, including improved mitochondrial health. However, the contribution of Ulk1 in adaptation to CR is unknown. To decipher a functional role of Ulk1(S555) in adaptations to CR we used CRISPR-Cas9 generated, loss-of-function Ulk1(S555A) mice, in which Ulk1 cannot be phosphorylated at S555. 6-month-old, male and female homozygous Ulk1(S555A) mice and C57BL6/J (wild type, WT) mice were placed on a 40% CR diet for 8 weeks. Body mass in both male and female Ulk1(S555A)

and WT mice was reduced with CR (p < 0.001), however female Ulk1(S555A) were heavier than their WT counterparts (p=0.02). Via nuclear magnetic resonance (NMR), male and female Ulk1(S555A) mice did not lose fat mass during CR. In addition, periovarian (female) and epididymal (male) fat mass was greater in Ulk1(S555A) compared to WT mice post-CR (p < 0.001). Furthermore, fasting blood glucose increased in male and female Ulk1(S555A) post-CR (p < 0.0001), suggesting altered substrate metabolism. In support of this notion, glucose oxidation in both quadriceps muscle and liver of male mice increased in WT following CR but not in Ulk1(S555A) mice (interaction effect p< 0.002). In sum, these data suggest that phosphorylation of Ulk1 at S555 is required for metabolic adaptations to CR.

### SESSION 5100 (PAPER)

### ADULT PROTECTION AND ELDER ABUSE

### A DIGITAL ELDER ABUSE INTERVENTION FOR THE EMERGENCY DEPARTMENT

Fuad Abujarad[1], Esther Choo[2], James Dziura[3], Chelsea Edwards[3], Michael Pantalon[3], Karen Jubanyik[3], Gail D'Onofrio[3], and Thomas Gill[4], *1. Yale University, Orange, Connecticut, United States, 2. Oregon Health & Science University, Portland, Oregon, United States, 3. Yale University, New Haven, Connecticut, United States, 4. Yale School of Medicine, New Haven, Connecticut, United States*

Elder abuse continues to grow as a national issue with significant gaps in standards for identifying victims. The emergency department provides an opportune setting for affected older adults to report abuse privately, but traditional screening methods may miss certain types of elder abuse when there are no visible signs of abuse. Community resources and reporting methods may not be known or can be stigmatized, therefore creating a challenging scenario for victims of abuse to navigate without fear. The VOICES Elder Abuse Intervention (EAI) is a unique self-administrated tablet-based tool with an automated digital coach that combines major components of screening, educational content, and brief psychoeducational interviewing to empower the older adults and encourage self-identification and self-reporting of abuse. The digital format of the tool allows this EAI to be integrated within an existing elder abuse workflow protocol in the emergency department, rather than replacing it entirely. We will discuss our completed study that included (N=1,002) participants over the age of 60, which suggests that patients find the EAI user-friendly and easy to use. For example, 93% of participants agreed that VOICES was appropriate for learning about abuse, and 94% agreed that using VOICES was very easy to use. Informed by these findings, we plan to adapt and evaluate VOICES EAI for use in other older adult populations and healthcare settings.

### BARRIERS TO REDUCING AND PREVENTING INVOLUNTARY TREATMENT: THE PERSPECTIVE OF PROFESSIONAL CAREGIVERS

Michel Bleijlevens, Jules Willems, Valeria Lima Passos, and Jan Hamers, *Maastricht University, Maastricht, Limburg, Netherlands*

Measures to which a person resits and/or does not provide consent for are defined as involuntary treatment. The use of involuntary treatment violates the autonomy of (older) persons and causes more harm than benefit. Moreover, it contradicts the values of person-centred care. Nevertheless, its use among Persons Living with Dementia (PLWD) is still common practice and remains difficult to prevent and/or reduce. The aim of this study was to gain insights into the barriers towards the prevention and/or reduction of involuntary treatment in long-term geriatric care. We conducted a cross-sectional, mixed-methods study, including an online survey for professional caregivers, and a semi-structured focus group interview with professional caregivers. A total of 218 participants completed the questionnaire. The percentage of participants that experienced barriers in one of the twenty-two survey items ranged from 15% to 42%. Lack of time; the experienced need to use involuntary treatment; uncertainty about responsibilities of stakeholders; and a lack of knowledge on methods to prevent and/or reduce its use were most seen as barriers. Nursing staff experienced a lack of time more often than other professional caregivers. Working in home care and having no former experience with involuntary treatment usage increased perceived barriers. Participants of the focus group interview confirmed these findings. One out of four professional caregivers experience barriers hindering prevention and/or reduction of involuntary treatment. More research is needed to gain better understanding on how professional caregivers can be supported aiming to, remove barriers and consequently prevent and/or reduce the use of involuntary treatment.

### ELDER MISTREATMENT, MORTALITY, AND HOSPITAL READMISSION AMONG MEDICARE BENEFICIARIES, 2015–2018

Monique Pappadis[1], Leila Wood[2], Allen Haas[2], Yong-Fang Kuo[2], and Charles Mouton[2], *1. University of Texas Medical Branch, Galveston, Texas, United States, 2. University of Texas Medical Branch at Galveston, Galveston, Texas, United States*

Elder mistreatment (EM) is a growing public health and safety crisis, with long-term consequences for individuals, families, and communities. We explored whether older adults hospitalized with a primary diagnosis of EM was associated with an increased risk of mortality and unplanned hospital readmission compared to those with a secondary EM diagnosis. We further examined whether EM type and hospital setting was associated with risk of mortality and unplanned hospital readmission. Using 100% of 2015-2018 Medicare files of hospitalized Medicare Fee-for-Service beneficiaries aged 66 and over, we used Kaplan-Meier and Cox proportional hazard models to estimate mortality and unplanned readmission rates by primary versus secondary EM diagnosis, EM type, and facility type. 11,023 patients were hospitalized with an EM diagnosis. The majority were female (64.1%) and Non-Hispanic/Latinx White (74.3%). Neglect was the most common EM type. The three-year mortality rate was 56.7% and one-year readmission rate was 53.8%. Compared to other EM types, patients diagnosed with neglect had a 2.20 (95% Confidence Interval [CI]=1.88-2.56) and 3.21 (95% CI=2.32-4.43) times greater risk for mortality within and after 50-days from discharge, respectively. Patients discharged from a skilled nursing facility (SNF) were at an increased risk of mortality and unplanned readmission compared to those discharged from an acute hospital. Hospitalized patients with a primary EM diagnosis were associated with an increased risk of mortality and readmission compared to those with a secondary diagnosis. Future work should explore care patterns before and after EM diagnosis to identify potential time points for medical and social intervention.

### MENTAL HEALTH RESILIENCE IN SWISS OLDER ADULT SURVIVORS OF CHILD WELFARE–RELATED MALTREATMENT

Myriam Thoma[1], Florence Bernays[2], Andreas Maercker[1], and Shauna L. Rohner[1], *1. University of Zurich, Zurich, Zurich, Switzerland, 2. University of Zurich, Zürich, Zurich, Switzerland*

Minors affected by child welfare practices in Switzerland during the last century had a high risk for exposure to childhood trauma and maltreatment. Several studies with this cohort demonstrated substantially higher levels of clinically-relevant psychopathology in older adult survivors in comparison to non-affected control individuals. However, these studies also revealed that not all affected individuals developed mental health disorders over their lifespan. To date, this mental health resilience in survivors of an advanced age is still insufficiently understood. Therefore, this study aimed to assess and compare the resilience profiles of older adults who were formerly affected by child welfare-related trauma and maltreatment (risk group, RG; n = 132; Mage = 71 years) and non-affected, age-matched controls (control group, CG; n = 125). Within the RG, approximately one-third of the individuals had no current or lifetime DSM-5 mental health disorders. In comparison to the survivors with a history of mental ill-health, these individuals were older, had a higher income, and expressed a higher subjective satisfaction with their socio-economic status. Furthermore, they reported less early-life physical abuse, and had lower levels of neuroticism, as well as empathy-related characteristics. In addition, they showed higher levels of self-esteem and trait resilience. Group differences between the RG and CG highlight the importance of considering past adversity in the understanding of mental health resilience in later life.

### SELF-ADMINISTRATED ELDER ABUSE INTERVENTION FOR OLDER ADULTS WITH COGNITIVE IMPAIRMENT

Fuad Abujarad[1], Chelsea Edwards[2], Brent Vander Wyk[2], Laura Mosqueda[3], Ula Hwang[2], Judith Neugroschl[4], and Richard Marottoli[2], *1. Yale University, Orange, Connecticut, United States, 2. Yale University, New Haven, Connecticut, United States, 3. University of Southern California, Alhambra, California, United States, 4. Mount Sinai, New York, New York, United States*

Many elder abuse interventions and tools designed to screen for abuse exclude older adults with cognitive impairments (CI) due to the challenges associated with screening and whether the older adult with CI can reliably report elder abuse. However, it has been shown that older adults with CI are among the most vulnerable to experiencing elder abuse. VOICES is an innovative, automated tablet-based elder abuse screening and prevention intervention that is self-administered by the older adult in the provider's waiting room or office. The VOICES Elder Abuse Intervention (EAI) provides screening, educational modules, and brief psychoeducational intervention to enhance and improve identification of elder abuse when there are no visible signs of abuse. The VOICES EAI was already proven successful in terms of feasibility and acceptability in cognitively intact older adults in a busy emergency department setting with (N=1,002). In this study we tested the VOICES EAI with (N=30) participants 60 and above with cognitive impairment at a geriatric center using the Montreal Cognitive Assessment (MoCA) to determine cognitive capacity. Experts in the field of geriatrics and cognitive impairment assisted in grouping participants within three cognitive categories: Mild cognitive impairment (MoCA 23-25), mild dementia (MoCA 16-22) or moderate dementia (MoCA 8-15). Of the (N=30) participants, 29 were able to successfully use the VOICES EAI independently, and most participants were satisfied with the tool. We will discuss the findings of this preliminary study and the implications for future research with older adults with CI.

### SESSION 5110 (SYMPOSIUM)

### ADULTS' VIEWS OF AGING AS AN UNDERESTIMATED RISK FACTOR FOR HEALTH, WELL-BEING, AND LONGEVITY

Chair: Manfred Diehl Co-Chair: Susanne Wurm Discussant: Becca Levy

Worldwide population aging has greatly increased the diversity of the "aging enterprise." Research has established a solid portfolio of evidence showing that positive and negative views of aging represent independent resilience or risk factors for health, well-being, and longevity. Indeed, the effects of views of aging remain significant beyond the effects of other risk factors for health and mortality. This raises the following questions: What do we currently know about the effect of self-perceptions of aging (SPA) and subjective age (SA) on health, well-being, and longevity? What are recent advancements and perspectives? Which research questions should be addressed to stimulate further, sustainable developments in research and practice? This symposium addresses these questions with a diverse set of presentations and from different perspectives. Wahl and colleagues will discuss the role of SPA in the clinical context, namely in a sample of older adults with terminal cancer comparing them to older adults without a terminal illness. Based on a population-based sample, Wurm and Schaefer will report findings on the impact of different gain- and loss-related SPA and SA on mortality over a 23-year period. Building on an earlier meta-analysis, Westerberg and colleagues evaluated data from over 100 studies and will present the findings of a systematic review on the role of SPA and SA for health and longevity. Finally, Nehrkorn-Bailey et al. will present findings from a clinical trial that addressed views of aging as a mechanism to promote physical activity. Dr. Becca Levy will serve as the discussant.

### LIFETIME CLOSE TO THE END: EFFECTS ON PERCEIVED TIME AND AGING IN OLDER ADULTS WITH ADVANCED CANCER

Hans-Werner Wahl[1], Katsiaryna Laryionava[2], Anton Schönstein[2], Pia Heussner[3], Wolfgang Hiddemann[4], and Eva Winkler[2], *1. Universität Heidelberg, Heidelberg,*

*Baden-Wurttemberg, Germany, 2. Heidelberg University, Heidelberg, Baden-Wurttemberg, Germany, 3. Klinikum Garmisch-Partenkirchen GmbH, Garmisch-Partenkirchen, Bayern, Germany, 4. Ludwig-Maximilians University, Munich, Baden-Wurttemberg, Germany*

This study addressed two questions: (1) Does advanced cancer in later life affect a person's awareness of time and their subjective age? (2) Are awareness of time and subjective age associated with distress, perceived quality of life, and depression? We assessed patients with terminal cancer (OAC, n = 91) and older adults with no life-threatening disease (OA, n = 89). All participants were age 50 or older. OAC perceived time as being a more finite resource and felt significantly older than OA controls. Feeling younger was significantly related with better quality of life and lower levels of distress. In the OA group, feeling younger was also associated with reduced depression. Perceiving time as a finite resource was related to higher quality of life in the OA group. Indicators of an older person's awareness of time and subjective aging differ between those with advanced cancer versus controls without a terminal disease.

### GAIN- BUT NOT LOSS-RELATED VIEWS ON AGING PREDICT MORTALITY OVER A PERIOD OF 23 YEARS

Susanne Wurm[1], and Sarah Schaefer[2], *1. University Medicine Greifswald, Greifswald, Mecklenburg-Vorpommern, Germany, 2. Leibniz Institute for Resilience Research, Mainz, Rheinland-Pfalz, Germany*

Some 2 decades ago, Levy et al. (2002) published their seminal study on the impact of SPA on mortality over a period of 23 years. Our study aimed at replicating and extending these findings. Based on a large German population-based sample of individuals aged 40+ (N = 2,400), for whom mortality was also documented over 23 years (1996–2019), we investigated the impact of gain- and loss-related SPA and SA on mortality. Data were analyzed with hierarchical Cox proportional hazard regressions. For individuals who perceived aging as ongoing development risk of death was half that of individuals with less gain-related SPA. Viewing aging as associated with physical or social losses could not predict mortality after controlling for covariates (age, gender, education, health-related variables, and psychological variables). Neither could SA predict mortality. The results suggest that mainly gain-related SPA explain differences in mortality and should thus be addressed in intervention studies.

### THE EFFECT OF SELF-PERCEPTIONS OF AGING ON PHYSICAL ACTIVITY: RESULTS FROM THE AGINGPLUS STUDY

Abigail Nehrkorn-Bailey[1], Han-Yun (Heidi) Tseng[2], Diana Rodriguez[2], Kaigang Li[2], George Rebok[3], David Roth[4], Shang-En Chung[4], and Manfred Diehl[2], *1. University of Wisconsin-Green Bay, Green Bay, Wisconsin, United States, 2. Colorado State University, Fort Collins, Colorado, United States, 3. Johns Hopkins Bloomberg School of Public Health, Baltimore, Maryland, United States, 4. Johns Hopkins University, Baltimore, Maryland, United States*

The AgingPLUS program targets negative self-perceptions of aging (SPA) as one mechanism to increase physical activity (PA) in adults. This study utilized a mediation model to examine the effect of AgingPLUS on subsequent PA with SPA included as a mediator. Data came from 184 participants (Mage = 59.91 years; SDage = 8.14 years) from the ongoing trial. Although the direct effect from condition to Week 8 PA was not significant, the pathway from condition to Week 4 SPA was significant. Additionally, the pathway from Week 4 SPA to Week 8 PA was marginally significant ($\beta = .11$, $p = .07$). However, the indirect effect was not significant. Given that (1) the AgingPLUS program resulted in significantly more positive SPA and (2) more positive SPA marginally predicted more minutes of subsequent PA, these results provide preliminary support for the efficacy of the ongoing program.

### THE EFFECT OF SUBJECTIVE AGING ON HEALTH AND SURVIVAL: A SYSTEMATIC REVIEW OF LONGITUDINAL DATA

Gerben Westerhof[1], Abigail Nehrkorn-Bailey[2], Allyson Brothers[3], Jelena Siebert[4], Han-Yun (Heidi) Tseng[3], Manfred Diehl[3], Hans-Werner Wahl[5], and Susanne Wurm[6], *1. University of Twente, Enschede, Overijssel, Netherlands, 2. University of Wisconsin-Green Bay, Green Bay, Wisconsin, United States, 3. Colorado State University, Fort Collins, Colorado, United States, 4. Heidelberg University, Heidelberg, Baden-Wurttemberg, Germany, 5. Universität Heidelberg, Heidelberg, Baden-Wurttemberg, Germany, 6. University Medicine Greifswald, Greifswald, Mecklenburg-Vorpommern, Germany*

Longitudinal effects of subjective age on adult health and survival are well-documented (Westerhof et al., 2014). This systematic review provides an updated and expanded evaluation of the state of the field. A systematic search in PsycInfo, Web of Science, Scopus, and PubMed resulted in 103 articles: 19 articles from the 2014 meta-analysis and 84 newer articles. Sixty-five articles focused on physical health (e.g., subjective health, objective health, and health behaviors), 41 studied mental health outcomes (e.g., mental health disorders, well-being, and quality of life), and 18 articles used mortality as the outcome. Important developments during the past years include new instruments, particularly multidimensional assessment of subjective aging, and a more fine-grained representation of health outcomes, including bio indicators. A major outcome is that most studies confirmed the longitudinal effects of subjective aging on health as reported previously, but on a much broader study portfolio.

### SESSION 5120 (SYMPOSIUM)

### AGING RESEARCH IN CRIMINAL LEGAL SYSTEMS: IMPLICATIONS FOR POLICY AND PRACTICE

Chair: Jennifer James Discussant: Brie Williams

ADD

### INSIDE-OUTSIDE COMMUNITY ADVISORY BOARDS: ONE MODEL FOR ENGAGING OLDER BIPOC AND WOMEN IN CLINICAL RESEARCH

Jennifer James, *University of California, San Francisco, San Francisco, California, United States*

Historical legacies of unethical research performed on people in prison, coupled with stringent policies intended to protect those who are incarcerated from exploitation, has meant that much clinical research about the health of people who are incarcerated is conducted at a distance, without directly engaging those who are incarcerated in the formulation of studies. As a result, research conducted about correctional healthcare and the healthcare needs of incarcerated persons may not reflect specific stakeholder values or priorities. In this session, we will describe barriers in engaging incarnated older women and BIPOC patients, their healthcare providers, and their loved ones in the research process and new models for the development of research questions that center stakeholder perspectives. We will describe identified priority areas for future research, insight on the ethics of research consent and participation with this population, and methodological considerations for clinical, social, and behavioral research with this vulnerable population.

### THE ROLE OF PEER HEALTH WORKS IN ENGAGING INCARCERATED PATIENTS IN RESEARCH

Fernando Murillo, *University of California, San Francisco, San Francisco, California, United States*

Incarcerated peers play a critical role in the provision of care for incarcerated older adults. Peer counsel is part of the culture in carceral settings and official and unofficial caregiving is a necessity for visually, mobility, and cognitively impaired residents of these institutions. Due to their experiences of exploitation by the prison system and research institutions, most older incarcerated people are reluctant to trust community partners or correctional staff members who conduct research in correctional settings. Many older residents who are offered participation in research do not believe any real systemic changes will occur with the publication of findings. This presentation will draw on autoethnographic experiences of a peer health worker in the palliative care setting. We will describe the role that peer health workers play in guiding patients in decision-making around research and interrogate a model of partnering with peer health workers to engage incarcerated older adults in research.

### ASSESSING PHYSICAL FUNCTION AND MENTAL HEALTH AMONG OLDER PERSONS IN PRISON

Lisa Barry, *University of Connecticut Center on Aging, Farmington, Connecticut, United States*

As the number of older incarcerated persons grows, evaluating changes in their physical and mental health over time may be important for appropriate planning and needs assessment. Based on findings from the Aging Inmates' Suicidal Ideation and Depression Study (Aging INSIDE), we will provide recommendations for assessing both objective and subjective physical function in older incarcerated persons. We will also discuss our experiences with assessing depression and suicidal ideation in this population and describe how the processes for collecting data needed to be modified during the COVID-19 pandemic. Those attending this session will learn about the pros and cons of using face-to-face assessments and mailed surveys to assess physical function and mental health among older persons in the prison setting and will learn how these outcomes may differ between those who have a life sentence versus those expecting to be released from incarceration in late life.

### STRATEGIES FOR PARTNERING WITH CARCERAL SETTING STAFF AND LEADERSHIP

Stephanie Grace Prost, *University of Louisville, Louisville, Kentucky, United States*

The mission of carceral agencies--the pursuit of public safety andsecurity--often conflicts with the primary aim of social justice-oriented scholarship, most notably research objectives related to health promotion in jails and prisons. However, such research is essential to awareness building, policy reformation, and revisions today-to-day practices that increase the health and well-being of historically marginalized populations. As a result, the conduct of aging research within carceral settings requires specialized knowledge and skills including frequent, targeted communication,transparency, humility, and flexibility. This presentation includes discussion of strategies for building and maintaining successful partnerships with local and state-level agencies with examples drawn from four distinct aging research projects, specifically. These partnerships resulted in primary data collection with over 800 carceral constituents including prison hospice program representatives, persons who are incarcerated, and experts in correctional health and art therapies. Lessons learned and opportunities for future partnerships are also described.

### SESSION 5130 (PAPER)

### ASSESSMENT OF PERSONS WITH ADRD IN COMMUNITY AND ACUTE CARE SETTINGS

### ASSESSING DEMENTIA CLASSIFICATION IN THE NATIONAL CORE INDICATORS AGING AND DISABILITY SURVEY

John Mulcahy[1], Taylor Bucy[2], Tetyana Shippee[1], and Eric Jutkowitz[3], *1. University of Minnesota, Minneapolis, Minnesota, United States, 2. University of Minnesota School of Public Health, Minneapolis, Minnesota, United States, 3. Brown University, Providence, Rhode Island, United States*

Improving the quality of long-term care services for older adults is a national priority. The National Core Indicators Aging and Disability (NCI-AD) collects data from multiple states to evaluate long-term care recipients' service satisfaction and needs. Many analysts are interested in exploring the service environments of people living with dementia. However, dementia measurement and reporting varies in NCI-AD between states. Dementia status may be obtained from administrative records or self-reported during the survey. We explored the measurement of dementia in NCI-AD and the implications of relying on administrative or self-reported dementia status. We analyzed NCI-AD data from 2015-2018 representing 24,569 respondents age 65+, of which 5,502 (22.4%) were identified as having dementia. 42.9% of respondents had dementia status determined administratively and 57.1% had it determined during the survey. To assess dementia accuracy by data source, we fit separate LASSO models for both the administrative and survey subpopulations predicting dementia status using demographic and functional predictors. We then used each model to predict dementia status in the subpopulation with discordant dementia data source. Using the administrative model to predict survey reported status resulted in a higher sensitivity than using the survey model to predict administrative status (44.6% vs 32.2%). The diminished predictive accuracy of the survey model suggests administrative records may capture cases of dementia diagnosis that would be missed by self-report. These findings highlight analytical caveats for researchers interested study long-term care quality for people with dementia using NCI-AD, and emphasize the importance of moving towards more standardized dementia reporting.

### DEVELOPMENT OF A FRAILTY INDEX FOR PEOPLE WITH DEMENTIA IN THE HEALTH AND RETIREMENT STUDY

Rachel Wilkie, and Jennifer Ailshire, *University of Southern California, Los Angeles, California, United States*

Frailty indices (FI) have been found to predict adverse outcomes, such as, mortality, hospitalization, and institutionalization in older adults. However, traditional FIs often exclude people with dementia (PWD), who may not be able to consent to or complete all of the standard frailty items. While frailty is a known risk factor for onset of dementia and PWD have higher rates of frailty, little is known regarding how frailty predicts outcomes among PWD. Our study aims to develop an FI for PWD and to examine how this index relates to mortality, hospitalization, and nursing home stays. We used data from the Health and Retirement Study to create a 52-item FI for community-dwelling adults aged 50 years and over classified as having dementia (n = 1,107) in 2014. The index includes deficits in four domains: chronic health conditions, functional status, sensory problems, and overall health and wellbeing. A standardized FI score between 0 and 1 was calculated for each respondent. We used logistic regression to examine associations with FI and 2-year mortality, hospitalization, and nursing home stay, adjusting for age and gender. We found that a 0.1 unit increase in FI was significantly associated with higher odds of 2-year mortality (OR 1.39, $p< 0.001$), hospitalization (OR 1.45, $p< 0.001$), and nursing home stay (OR 1.38, $p< 0.001$) for people with dementia. This study developed an FI which is predictive for adverse outcomes among PWD. Future work should explore how socioeconomic and neighborhood factors contribute to the relationship between frailty and adverse outcomes among PWD.

### EVALUATING THE FEASIBILITY AND ACCEPTABILITY OF THE WITS WELLNESS COGNITIVE HEALTH PROGRAM

Julie Bobitt[1], Laura Payne[2], Neha Gothe[3], Chelsey Byers[4], and Molly Hofer[5], *1. University of Illinois at Chicago, Chicago, Illinois, United States, 2. University of Illinois Urbana-Champaign, Champaign, Illinois, United States, 3. University of Illinois at Urbana Champaign, Urbana, Illinois, United States, 4. University of Illinois Extension, Champaign, Illinois, United States, 5. University of Illinois Extension, Chicago, Illinois, United States*

About 11% of U.S. older adults are at risk for or have subjective cognitive decline. Although some factors that affect brain health cannot be changed, research indicates lifestyle changes (i.e., physical activity, social engagement, heart-healthy diet) can delay or reduce cognitive decline. Drawing from existing research, UI Extension developed Wits Wellness, a 12-week program designed to enhance brain health among people ages 50 and older. Wits Wellness addresses multiple factors that affect cognitive health (e.g., physical activity, stress, sleep, social isolation, diet). Using a two-arm randomized control trial, 285 participants (mean age = 66.6 years, 24 males) were randomly assigned to the Wits Wellness intervention (in person or virtual) or a waitlist control group. Summative program evaluation surveys indicated adherence to the intervention was high, with 68% of participants attending 9 or more of the 12 sessions. Program acceptability was high, with 82.7% evaluating the program as great to excellent, and 90.8% reporting great to excellent interaction with program leaders. Approximately 63.6% of participant write-in comments included positive statements about the program challenging their brains, and 59.1% indicated the program positively affected participants' socialization. For program feasibility, 90.4% rated the 60-minute weekly sessions 'just right' for program length, while 66.3% rated the 12-week program duration 'just right' and 27.7% rated it 'too long'. Thus, preliminary results suggest that Wits Wellness delivered both in-person and virtually was well attended and highly acceptable in the community setting. Future studies will examine the program's efficacy in improving physical and mental health related outcomes.

### FINDING THE MINDS OF OUR ELDERS: TESTING THE MINORITY STRESS AND COGNITION MODEL WITH INDIGENOUS OLDER ADULTS

Cliff Whetung, *NYU Silver School of Social Work, New York, New York, United States*

This study used data from the Health and Retirement Study (HRS) data to investigate how an understudied group of Indigenous Older Adults (IOAs) in the United States fared over a 14-year period (2006-2020) in the domain of global cognitive function. The number of IOAs, defined here as Native American and Alaska Natives, will more than double in the next 30 years. Concurrently, the number of IOAs living with cognitive impairments will also increase. Guided by the Minority Stress and Cognition Model, we tested the hypothesis that discriminatory stress increases the risk of cognitive impairment in later life. Using a robust set of psychosocial (e.g. educational quality, perceived everyday discrimination experiences), behavioral (e.g. substance use, exercise), and physiological (e.g. diabetes, hypertension, obesity) risk factors, we modeled the cognition trajectories 186 IOAs using mixed growth curves. We found that one third of these IOAs reported experiencing everyday discrimination at least once per month, the highest of any ethnic group. They also reported high rates of other risk factors for cognitive impairment like low education, SES, and physical activity, and high rates of depression and chronic health conditions. Our analysis found that everyday discrimination was negatively associated with total cognition among IOAs but that this relationship was mediated by allostatic loads. On average, the total cognition scores of IOAs declined significantly faster than those of Whites. This study has important implications for the integration of stress as a mechanism for cognitive decline and the health equity of Indigenous older adults.

### RACIAL/ETHNIC DISPARITIES IN THE EFFECTS OF RECEIVING A DIAGNOSIS OF DEMENTIA ON SOCIAL RELATIONSHIPS

Takashi Amano[1], Addam Reynolds[2], Clara Scher[3], and Yuane Jia[2], *1. Rutgers University-Newark, Newark, New Jersey, United States, 2. Rutgers University, New Brunswick, New Jersey, United States, 3. Rutgers, The State University of New Jersey, New Brunswick, New Jersey, United States*

Although early diagnosis has been recognized as a key strategy to improve outcomes for those with dementia, receiving a diagnosis may negatively influence social relationships. Absent from the literature are considerations of how race/ethnicity may alter these associations. This study examines racial/ethnic variation in the effects of receiving a diagnosis of dementia on social relationships of older adults. Data from the three waves of the Health and Retirement Study were utilized as part of this study. This study examined whether receiving a new diagnosis of dementia changed subsequent social relationships (social networks, social engagement, social support). Regression analyses with inverse probability weighting were performed to estimate the impact of receiving a dementia diagnosis on changes in social relationships. Receiving a new diagnosis of dementia reduced both informal (b = -0.476, p = 0.011, 95% CI = [-0.842, -0.110]) and formal (IRR = 0.688, p = 0.034, 95% CI = [0.487, 0.971]) social engagements. The negative impact of receiving a diagnosis on formal social engagement was stronger among non-Hispanic Blacks (IRR = 0.047, p = 0.007, 95% CI = [0.005, 0.432]). We found no statistically significant impacts of receiving a diagnosis of dementia on social networks and social support. Results suggest that receiving a new diagnosis of dementia may have unintended negative consequences on social engagement and may be more salient for racial/ethnic minorities, such as non-Hispanic Blacks. Practitioners and policymakers should be aware of these consequences and should identify strategies to alleviate the negative implications of receiving a diagnosis.

### SESSION 5140 (PAPER)

### DEPRESSION

### COVID-19 ERA EFFECTS ON OLDER ADULTS' COGNITIVE COMPLAINTS, DEPRESSIVE SYMPTOMS, AND STRESSFUL EVENTS

Suzanne Segerstrom[1], Paris Crosby[1], Dakota Witzel[2], Maria Kurth[3], Soyoung Choun[3], and Carolyn Aldwin[3], *1. University of Kentucky, Lexington, Kentucky, United States, 2. Penn State University, State College, Pennsylvania, United States, 3. Oregon State University, Corvallis, Oregon, United States*

Older adults have adjusted better to the COVID-19 pandemic in terms of their psychological well-being than younger adults. We investigated individual differences in vulnerability within older adulthood as pandemic severity changed, providing a more refined prediction of older adults' adjustment to COVID-19. Participants from this longitudinal study were included if they had at least one semiannual assessment before and one during the COVID-19 era (N = 111, 65% women, age range = 62-96 at onset of COVID-19 era in the US). There were 1,098 pre-COVID-19 assessments (M=9.9, 1/5/2018-1/22/2020) and 265 post-COVID-19 (M=2.4, 1/23/2020-10/31/2021). At each assessment, participants reported on six cognitive complaints (MOS), five depressive symptoms (Geriatric Depression Scale), and six domains of undesirability-weighted stressful life events (Louisville Older Persons Event Scale). Daily national, state, and regional COVID-19 case and death rates were obtained from the Centers for Disease Control and summed for the week preceding each assessment. In multilevel ZIP models, the COVID-19 era significantly increased depressive symptoms (0.68 to 1.18, p < .0001) and stressful events (30.9 to 48.5, p < .0001), but did not significantly affect severity of cognitive complaints. Older age was associated with greater impact of COVID-19 on depressive symptoms and stressful events; women reported more stressful events when pandemic severity was high, but men reported more stressful events when pandemic severity was low. Although older adults in general have adjusted better to the pandemic than younger adults, the old-old had greater vulnerability to this unavoidable event than the young-old.

### EFFECTS OF DEPRESSIVE SYMPTOMS ON COGNITIVE BEHAVIORAL INTERVENTIONS WITH CUSTODIAL GRANDMOTHERS

Carol Musil[1], Alexandra Jeanblanc[1], Christopher Burant[2], and Jaclene Zausniewski[1], *1. Case Western Reserve University, Cleveland, Ohio, United States, 2. Case Western Reserve University, Parma, Ohio, United States*

Over 25% of custodial grandmothers report elevated depressive symptoms, which may affect situational appraisals and learning/retaining new information, and thus the effectiveness of cognitive-behavioral interventions. We examined the effect of depressive symptoms on self-appraised stress and reward associated with caregiving to co-residential grandchildren in a sample of 342 grandmothers who participated in a nationwide behavioral RCT testing two methods of stress reduction. Participants completed a baseline questionnaire, one of 2 cognitive-behavioral interventions (guided journaling or journaling with Resourcefulness Skills Training ©), and 3 subsequent questionnaires over 6 months. We analyzed self-appraised stress and reward with RM-ANOVAs to evaluate whether level of depressive symptoms (CES-D below-16, 16-29, 30+) affected the effectiveness of each arm with regard to self-appraised stress and reward. We found differences by depressive symptom category and interaction effects of depressive symptoms by arm in self-appraised stress. The below-16 group had relatively constant appraised stress over time, but the 16-29 and 30+ groups had significant decreases in stress over time. The Resourcefulness Skills Training © group had an average decrease in stress of .68 over time compared to .2 points for the journal-only group; there were no intervention effects in the below-16 group. The highest symptom group showed a stronger effect for the journal-only intervention, perhaps reflecting the challenges in learning and applying new skills. Self-appraised reward increased over time across groups. Participants' level of depressive symptoms have significant effects on the effectiveness of interventions and should be taken into consideration when designing studies and analyzing effectiveness.

### GENETIC RISK FOR ADHD ON LATER LIFE EMOTIONAL AND COGNITIVE HEALTH: TESTING MECHANISMS OF EFFECT

Thalida Arpawong, and Jimi Huh, *University of Southern California, Los Angeles, California, United States*

Attention Deficit Hyperactivity Disorder (ADHD) is associated with emotional regulation and cognitive processing challenges throughout life. Not well-understood is how ADHD genetic risk (ADHD-GR) affects depressive symptoms (DepSx) and cognitive functioning in older age. Furthermore, less is known about the mechanisms through which some individuals show better outcomes despite higher ADHD-GR. We evaluated potential mechanisms using the Health and Retirement Study with data from 7,871 European Americans (EAs) and 1,226 African Americans (AAs), ages 50-93, and ADHD-GR calculated from a mixed ancestry genomewide scan. Mediators included closeness of parental relationships during childhood, sense of purpose in life in adulthood, and educational attainment. Outcomes included validated scales for DepSx and cognitive functioning. Structural equation models were stratified by race/ethnicity due to potential differences in genetic effects, adjusted for age, gender, ancestry, and health conditions with DepSx and genetic risk for dementia with cognitive functioning. Among EAs, ADHD-GR significantly predicted lower sense of purpose ($p<.05$) and worse parental relationships ($p<.001$), with higher levels of both, in turn, predicting less DepSx ($p's< 0.01$). ADHD-GR also showed significant direct effects on DepSx ($p<.01$), controlling for mediators. Additionally, ADHD-GR predicted less education ($p<.001$), but more education predicted better cognitive functioning ($p< 0.001$). No relationships with ADHD-GR were significant among AAs. Findings imply that among EAs, mediators could be targets to mitigate negative effects on psychological and cognitive health, two hallmark challenges for individuals with ADHD. More work is needed to characterize ADHD-GR on outcomes among older AAs.

### HEARING LOSS AND ITS CONSEQUENCES FOR SPOUSAL MENTAL HEALTH: EVIDENCE FROM THE HEALTH AND RETIREMENT STUDY

Jessica West, Sherri Smith, and Matthew Dupre, *Duke University, Durham, North Carolina, United States*

Hearing loss (HL) is an increasingly prevalent chronic stressor among older adults and is associated with numerous adverse health outcomes. The life course perspective and stress process framework highlight that an individual's stressors may have a short and/or long-term impact on the health of others. However, little is known about how HL influences the proliferation of stress within married couples. Drawing on nationally-representative data from 11 waves (1998-2018) of the Health and Retirement Study (n=9,000 individuals, 4,500 couples), we use age-based mixed models to examine how one's own HL, spouse's HL, or both spouses have HL shape the level and changes in depressive symptoms. For men, we find that their wives' HL, their own HL, and both spouses having HL are each associated with an increase in depressive symptoms—and that the associations persist as spouses age. For women, we find that their own HL and both spouses having HL is associated with an increase in depressive symptoms. Furthermore, we find that the differences in women's depressive symptoms between spouses who both have HL and those who do not have HL significantly declines with age. We also find no evidence to suggest that husbands' HL is associated with wives' depressive symptoms. Together, these findings suggest that the connections between spouses' HL and their depressive symptoms are a dynamic process that unfolds differently by gender over time. Interventions that recognize the proliferation of stress associated with HL may help both individuals with HL and their spouses reduce their depressive symptoms.

### LONG-TERM GENDERED PATHWAYS OF RELIGIOUS INVOLVEMENT POST WIDOWHOOD

Frances Hawes[1], and Jane Tavares[2], *1. University of Wisconsin-Eau Claire, Eau Claire, Wisconsin, United States, 2. University of Massachusetts Boston, Boston, Massachusetts, United States*

Widowhood is associated with decreased emotional well-being, particularly increased depression. Prior research suggests that religiosity may help improve mental health among widowed individuals. However, longitudinal studies exploring the role of religiosity on emotional well-being among widowed older adults is lacking, as are studies which examine different dimensions of religiosity. This longitudinal study analyzed data from the 2006-2018 waves of the nationally representative Health and Retirement Study (HRS). Ordinary least squares (OLS) regression analysis was used to examine the relationship between widowhood and depression as well as the role of religiosity as a moderator of this association. Analysis was stratified by gender to further explore these interactions. Results show that men and women show similar levels of depression at widowhood, but men are far less likely to be depressed prior to widowhood. Women also show a better recovery pattern over time post-widowhood. Furthermore, religiosity (particularly attending church) is an effective way of coping with widowhood and mitigating depression for both genders. However, men are significantly less religious than women. This study highlights the long-term effects of widowhood on depressive symptomology among older adults. Practical implications of this study include intervention development around increased screening and treatment for depression for widowed older adults (in particular, for widowers) as well as connecting this vulnerable population with resources. These findings may also inform program outreach (such as hospice bereavement services) that aim to facilitate healthy grieving among widowed older adults.

### SESSION 5150 (PAPER)

### LONELINESS

### FACTORS THAT PREDICT LONELINESS FOR MIDDLE-AGED AND OLDER ADULTS DURING THE COVID-19 PANDEMIC

Angela Curl, and Katie Wolf, *Miami University, Oxford, Ohio, United States*

The Protection Motivation Theory (Eberhardt & Ling, 2021; Rogers, 1975) describes factors that influence intention and engagement in preventive health behaviors, including knowledge/experience, threat appraisal, and coping/efficacy appraisal. Public health responses to the covid-19 pandemic were designed to increase knowledge, emphasize potential severity of being infected, and promote preventive health behaviors – all with the goal of reducing infection transmission. However, these efforts may have inadvertently increased loneliness, particularly for older adults. This study used 2020 Health and Retirement Study data (N=1,687 adults over age 50) to examine predictors of loneliness based on the PMT framework, controlling for demographic factors and 2016 loneliness scores. Structural Equation Model results indicate that being at higher risk for covid-19 complications and death was associated with lower feelings of control over health (B=-.09), greater likelihood of knowing someone who died from covid-19 (B = .20), and higher overall concerns about covid-19 (B=.12), all p<.01. Higher concerns about covid-19 and knowing someone who died from covid-19 were associated with more protective behaviors (B=.41, p<.01; B=.05, p<.05; respectively). Not knowing someone who died of COVID-19, and lower perceived control over health and social life were all significantly associated with higher loneliness scores. These results suggest that interventions that promote perceived control over one's health and social life may be effective in reducing feelings of loneliness in this population, and that feelings of control over health also increases preventive health behaviors that reduce the risk of covid-19 infection.

### LONELINESS IN COMMUNITY-DWELLING US OLDER LATINX ADULTS

David Camacho[1], Kelly Pacheco[2], Todd Becker[3], Cary Reid[4], and Elaine Wethington[5], *1. University of Maryland, Baltimore, Norwalk, California, United States, 2. New York University, New York City, New York, United States, 3. University of Maryland, Baltimore, Baltimore, Maryland, United States, 4. Weill Cornell Medicine, New York City, New York, United States, 5. Cornell, Ithaca, New York, United States*

Loneliness is a critical public health problem yet little is known about older Latinx's experiences with loneliness. We conducted narrative interviews (Cultural Formulation Interview), with 17 Spanish-speaking Latinx (60 years+) living with loneliness (based on 3-item Loneliness scale) in New York or Los Angeles. Interviewers were bilingual (English/Spanish) gerontological social workers with training in qualitative methods, culturally-sensitive clinical research and elicitation of "sensitive data". Our thematic analysis was informed by Biopsychosocial and Minority Stress Models, and our gerontological clinical experience with Latinx groups. Most participants immigrated from Mexico, Dominican Republic, Ecuador or Uruguay and lived in the US for more than 20 years. Interviews ranged from 60 to 120 minutes. Most participants described comorbid depression or pain. A substantial majority (82%) had never discussed feelings of loneliness with family or friends. No participant reported seeking professional help to address loneliness. Other identified themes demonstrated how cultural and contextual factors shape loneliness: 1) Descriptions and interpretations rooted in familism and views of aging ("it seems I no longer matter to my children"); 2) perceived causes e.g. discrimination, family conflict, bereavements, immigrant stress, violence; 3) religious and cognitive coping to mitigate loneliness ("I pray that God gives me strength, "I befriend loneliness"); and 4) psychosocial functioning impact ( "there is no reason to live"). Our findings support development of culturally congruent loneliness prevention and treatment practices for older Latinx adults. Future quantitative research should explore the association of cultural and minority stress factors related to loneliness in diverse samples of older Latinx.

### NETWORK SIZE, SOCIAL ACCOMPANIMENT, AND GENDER: PROTECTIVE OR RISK FACTOR FOR REAL-TIME LONELINESS, AND FOR WHOM?

Ellen Compernolle[1], and Alyssa Goldman[2], *1. NORC at the University of Chicago, Chicago, Illinois, United States, 2. Boston College, Chestnut Hill, Massachusetts, United States*

Large personal networks are a key marker of social integration that protect against loneliness. Yet how such social integration shapes loneliness on a momentary basis remains unclear. This study examines (1) the role of personal network size in shaping older adults' momentary loneliness, (2) how momentary social accompaniment modifies this association (protective or risk factor), and (3) whether this moderation varies by gender. We use three waves of ecological momentary assessments (n = 12,251) and personal network data collected from 333 older adults as part of the Chicago Health and Activity in Real-Time (CHART). Findings from multilevel regression models suggest that older adults with large social networks experience greater intensity loneliness when momentarily alone compared to those with smaller social networks. This association is also more pronounced among men compared to women. Results support our deviation-from-baseline hypothesis, which is further supported by additional analyses that find a similar association between more frequent interaction with network ties and heightened loneliness when alone. Additional analyses provide insight into observed gender differences as well: women's networks and momentary social company tend to be familial ties characterized by caregiving, compared to men's ties, which are characterized as peers or companions. Here, moments of momentary social isolation may be a welcome reprieve from otherwise demanding social relationships ("tethering"). We conclude that extending the well-documented links between global measures of social integration and loneliness into a real-time framework offers novel insight into existing, high-level trends in loneliness among older adults.

### POST-TRAUMATIC STRESS AND LONELINESS AMONG OLDER PUERTO RICANS: HOW DOES DISCRIMINATION MATTER?

Kaipeng Wang[1], Daniel Vélez Ortiz[2], Lisa Colón[3], and Fei Sun[2], *1. University of Denver, Denver, Colorado, United States, 2. Michigan State University, East Lansing, Michigan, United States, 3. Washington State University, Pullman, Washington, United States*

Loneliness is associated with worse mental health conditions, more functional limitations, and higher mortality among older adults. Almost one third older Puerto Ricans have experienced loneliness. Research has shown that post-traumatic stress has been associated with higher risk of loneliness among older adults; however, such an association has not been examined among Puerto Ricans despite their high prevalence of post-traumatic stress disorder. In addition, perceived discrimination, a critical risk factor of social isolation, is likely to reduce help-seeking behaviors and aggregate loneliness among individuals who have experienced trauma. Nonetheless, few studies have focused on the moderating role of perceived discrimination on the association between post-traumatic stress and loneliness. This study examines the association between post-traumatic stress and loneliness and whether that association differs by everyday perceived discrimination among older Puerto Ricans. Data were collected from 304 Puerto Ricans aged 60 and above living in the Greater Boston area who responded to questionnaires. We used ordinary least squares regression to examine the association between post-traumatic stress, perceived everyday discrimination, and loneliness. We found that post-traumatic stress was significantly associated with higher levels of loneliness; however, the association differed by perceived everyday discrimination. The association between post-traumatic stress and loneliness was stronger for those that perceived a higher level of everyday discrimination. Findings underscored the role that perceived discrimination plays in exacerbating loneliness among older Puerto Ricans experiencing post-traumatic stress and have clinical and public health implications for service delivery and mental health promotion among older Puerto Ricans.

### SOCIAL ISOLATION AND LONELINESS AMONG OLDER ADULTS IN SUBSIDIZED SENIOR HOUSING: DOES IMMIGRATION STATUS MATTER?

Chung Hyeon Jeong[1], Jihye Baek[2], ByeongJu Ryu[3], Jina Park[2], BoRin Kim[1], and Sojung Park[2], *1. University of New Hampshire, Durham, New Hampshire, United States, 2. Washington University in St. Louis, St. Louis, Missouri, United States,*
*3. Chuncheon Hyoja Social Welfare Center, Chuncheon-si, Kangwon-do, Republic of Korea*

Older immigrants are at increased risk for social isolation and loneliness due to cultural and linguistic barrier to forming social networks. The vulnerability to social isolation could be exacerbated by the gaps between older immigrants' cultural expectations on social relations and actual social connectivity. Guided by the cognitive discrepancy model of loneliness, this study examined how social isolation influences loneliness among low-income older residents living in subsidized senior housing and if the relation varies by immigration status. Survey data were collected in 2019 and 2020 from 231 residents in subsidized senior housing communities located in St. Louis and Chicago. Social isolation was measured by frequency of social contacts, accounting for the types of relationships (family vs. friends) and the modes of contacts (in-person vs. remote). Loneliness was measured by the Revised University of California Los Angeles loneliness scale. Perceived quality of friendship was also included in the analytic models. The results of multiple linear regression showed that having positive friendship ($b=-1.00$, $p<.001$) and more frequent in-person contacts with friends ($b=-1.29$, $p<.001$) were negatively associated with loneliness, respectively. A significant moderating effect was found that older immigrants felt less lonely than non-immigrant counterparts when they had more in-person family contacts ($b=-1.09$, $p<.05$). The results suggest that the impacts of social isolation on loneliness among senior housing residents could differ depending on immigration status. The findings of this study could contribute to developing culturally sensitive interventions to enhance social connectedness and reduce loneliness among older immigrants in subsidized senior housing.

### SESSION 5160 (SYMPOSIUM)

### METAMORPHOSES: THE CONTINUING EVOLUTION AND ADAPTATION OF THE SAVVY CAREGIVER PROGRAM

Chair: Kenneth Hepburn Discussant: Joseph Gaugler

This symposium traces the nearly 30 years of development projects, trials, and transformations that continue to occur in efforts to make an evidence-based psychoeducation program (Savvy Caregiver) available to family members and friends who provide care to community-dwelling persons living with dementia illnesses. Savvy seeks to enhance caregivers' knowledge and skills for providing care and to enhance their competence and confidence (sense of mastery) in being able to guide their persons through days that are as safe, calm, and pleasant as possible. Over this period of time, Savvy has clarified its target participant group (focusing on primary caregivers rather than larger family groups), moved from having concurrent sessions for caregivers and persons living with dementia, continued to develop strategies that enable broad implementation while maintaining program fidelity, demonstrated the effectiveness of a synchronous/asynchronous online version that extends program reach (Tele-Savvy), and is currently exploring in an ongoing pilot how best to deliver a fully asynchronous version of the program that employs continuing education principles (Tele-Savvy@Home). The individual presentations will describe the main developmental milestones (investigator led to protocol-trained to online trained interventionist; in-person to synchronous/asynchronous to fully asynchronous) and the structural adaptations that enabled them. Particular emphasis will be placed on design decisions linked to maintaining a psychoeducational orientation and to program emphasis on developing or strengthening caregivers' self-efficacy for providing effective day-to-day care. Savvy's experience may help investigators in designing new interventions, particularly online interventions or in considering augmentation or delivery platform change of established interventions.

### HISTORY: MINNESOTA FAMILY WORKSHOP TO SAVVY CAREGIVER: CENTRALITY OF SELF-EFFICACY

Kenneth Hepburn, *Emory University, Atlanta, Georgia, United States*

This presentation will trace the development of the Savvy Caregiver program from its beginnings as the Center for Nursing Research-supported Minnesota Family Workshop (1993; PI: Sharon Ostwald) through a further test as the Partners in Caregiving Program (1997; PIs: Hepburn 1RO1NR04517-01) to the Alzheimer's Association-supported Savvy program (1997; PIs Hepburn & Lewis). Three main developments occurred over this period: the program solidified its identity as a caregiver training program and its mechanism of action as that of self-efficacy development through an active learning approach; it sharpened its focus by concentrating on the principal family caregiver (moving away from a broader family approach and eliminating concurrent care recipient programming); and it developed interventionist training materials and programs to enable broader reach. Since 2002, Savvy has enjoyed wide dissemination as an evidence-based in-person psychoeducation program, fostered by support to sponsoring organizations by the Administration on Community Living.

### TELE-SAVVY: CHALLENGES AND OPPORTUNITIES OF A SYNCHRONOUS/ASYNCHRONOUS PROGRAM

Mariya Kovaleva, *University of Nebraska Medical Center, Omaha, Nebraska, United States*

This presentation will trace the development of the Savvy Caregiver program from its beginnings as the Center for Nursing Research-supported Minnesota Family Workshop (1993; PI: Sharon Ostwald) through a further test as the Partners in Caregiving Program (1997; PIs: Hepburn 1RO1NR04517-01) to the Alzheimer's Association-supported Savvy program (1997; PIs Hepburn & Lewis). Three main developments occurred over this period: the program solidified its identity as a caregiver training program and its mechanism of action as that of self-efficacy development through an active learning approach; it sharpened its focus by concentrating on the principal family caregiver (moving away from a broader family approach and eliminating concurrent care recipient programming); and it developed interventionist training materials and programs to enable broader reach. Since 2002, Savvy has enjoyed wide dissemination as an evidence-based in-person psychoeducation program, fostered by support to sponsoring organizations by the Administration on Community Living.

### TELE-SAVVY@HOME: CONVERTING TO A COMPLETELY ASYNCHRONOUS DELIVERY APPROACH

Fayron Epps, *Emory University, Fairburn, Georgia, United States*

It is well established that family caregiving is taxing and stressful. Group-based psychoeducational programs such as the Tele-Savvy program demonstrated that the acquisition of skills, knowledge, and caregiving mastery can ameliorate caregiving stress – and enhance PLWD quality of life. Many factors, however, preclude caregivers' attendance in the synchronous portions of Tele-Savvy limiting the program's scalability. To make Tele-Savvy more accessible and available to a growing number of dementia family caregivers, we designed a fully asynchronous version of the Tele-Savvy program, Tele-Savvy@Home. To accomplish this, we consulted facilitators and caregivers who participated in Tele-Savvy. We also reviewed the existing curriculum, re-wrote portions, reordered modules, and created new videos to cover synchronous content. In addition, we partnered with education design specialists to create interactive and reflection exercises to maintain a psychoeducation orientation and strengthen caregivers' self-efficacy. This strategy may help investigators in their augmentation of the delivery strategies of established interventions.

### BRINGING INTERACTIVITY TO FULLY ASYNCHRONOUS PROGRAMS

Carolyn Clevenger, *Emory University, Atlanta, Georgia, United States*

Asynchronous online learning brings both new opportunities as well as challenges to an asynchronous psychoeducation program like Tele-Savvy@Home. While asynchronous learning offers engagement flexibility for time-strapped family caregivers, a lack of scheduled meetings represents a lack of contact and interactivity, threatens accountability, and produces a high incompletion rate. To engage participants in the next generation of this asynchronous, self-paced course, we have implemented a small cohort structure (10 participants each) and created a combination of automated prompts and optional touchpoints. The cohort structure and touchpoints mimic important shared and active learning components of the facilitated sessions. Prompts include weekly messages that nudge learners with reminders of their ideal progress point and offer value propositions for staying on-time. A pacing guide allows caregivers to build the course into their schedule in advance of the course's start. Optional touchpoints include unit-by-unit post-tests, a cohort-based discussion board, and free text reflective exercises.

### SESSION 5170 (SYMPOSIUM)

### SOCIAL HEALTH IN THE CASE OF DEMENTIA: AN INTERNATIONAL PERSPECTIVE

Chair: Karin Wolf-Ostermann Co-Chair:
Myrra Vernooij-Dassen Discussant: Rene Melis

Dementia is one of the major age-related societal challenges and causes enormous demands for persons living with dementia (PlwD) and their families. We do not understand the origins of this multifactorial syndrome and there is still no cure for dementia. New thinking by the exploration of paradigms has scope to improve knowledge about this complex condition. Social health can be understood as a driver for stimulating the use of cognitive reserve through active facilitation and utilization of the individual's capacities. It allows to slow cognitive impairment or to maintain cognitive functioning in old age and therefore seems to be a promising approach to a better understanding of the developmental mechanism of dementia.In this international symposium we therefore aim to explore the role of social health in the onset and progress of dementia. The first presentation will present a new framework on understanding social health in dementia. The second presentation will describe convoys of care in a family based culturally sensitive ADRD caregiving intervention reducing care burden and family conflicts. The third presentation reports on the role of immune system and neurodegeneration markers in the association between social health and cognitive brain aging in older adults. The final presentation presents newly derived results from a mixed research synthesis on underlying mechanisms of the interrelation of social health and cognitive functioning, elaborated by the international SHARED-consortium. Our discussant will synthesize the research findings and lead a discussion of future directions for research and practice to successfully fight challenges in dementia care.

### CONCEPTUAL ADVANCEMENT FOR SOCIAL HEALTH IN DEMENTIA RESEARCH

Myrra Vernooij-Dassen[1], Eline Verspoor[1], Marieke Perry[1], and Karin Wolf-Ostermann[2], *1. Radboud University, Nijmegen, Gelderland, Netherlands, 2. University of Bremen, Bremen, Bremen, Germany*

The lack of conceptual clarity on social health in dementia research hinders its articulation. We aim to apply concept advancement for social health to provide conceptual clarity by building from a conceptual meaning to domains. The procedure is underpinned by theoretical models and epidemiological evidence on the relation between social heath and cognitive functioning.This led to considering social health as a reciprocal relational concept that refers to the influence that an individual has on others (social environment), and vice versa. We distinguished three domains defining the individual level, representing the social competences of the individual, and three domains defining the social environmental level (structure , function and appraisal of the relationship. We hypothesize that social health acts as a driver for stimulating the use of cognitive reserve. This conceptual advancement promotes developments that integrate neurobiological and social sciences and new interventions to support older people with and without dementia.

### CONVOYS OF CARE

Toni Antonucci[1], and Kristine Ajrouch[2], *1. University of Michigan, Ann Arbor, Michigan, United States, 2. Eastern Michigan University, Ypsilnati, Michigan, United States*

Many existing Alzheimer's disease (AD) caregiving interventions focus narrowly on thechallenges and needs of a primary caregiver rather than the family systems in which they areembedded. We advance a family systems framework by invoking convoys of caregiving to adaptan existing AD caregiver intervention to Middle Eastern/Arab American families in metroDetroit (N=56). The composition of caregiving networks is described, followed by assessment ofcare burden, depressive symptoms, care satisfaction and family conflict. Results show thatsiblings and children are the predominate support network members who accompanied theprimary caregiver to the program. Paired t-tests show that care burden and family conflictdecreased while caregiving satisfaction increased following program participation. Depressivesymptoms did not change. Findings illuminate how convoys of care may serve as valuablesupport resources, yet may also be the source of stress and conflict.

### SOCIAL HEALTH AND IMMUNE SYSTEM IMBALANCE: SEX-SPECIFIC ASSOCIATIONS AND CAUSAL LINKS TO BRAIN AGING

Isabelle van der Velpen[1], Amber Yaqub[1], Meike Vernooij[1], Marieke Perry[2], Myrra Vernooij-Dassen[2], Mohsen Ghanbari[1], Arfan Ikram[1], and Rene Melis[3], *1. Erasmus MC, Rotterdam, Zuid-Holland, Netherlands, 2. Radboud University, Nijmegen, Gelderland, Netherlands, 3. Radboud university medical center, NIjmegen, Gelderland, Netherlands*

Background: We explored whether the balance between innate and adaptive immune system links social health to cognitive brain aging in community-dwelling older adults.

Methods: Social health markers (social support, marital status, loneliness) were measured in the Rotterdam Study in 2002-2008. Balance of the immune system was assessed using white blood-cell-based indices (neutrophil-to-lymphocyte ratio (NLR), platelet-to-lymphocyte ratio (PLR), systemic immune-inflammation index (SII)) during the same visit. Cognitive function and total brain volume were measured at the 2009-2014 follow-up visit.

Results: In 8375 adults (mean age 65.7, 57% female), never married participants had higher NLR, PLR and SII compared to married peers, indicating imbalance towards innate immunity. Widowed/divorced males, but not females, had higher NLR, PLR and SII. Immune system balance did not mediate associations between social health and cognitive brain aging. Discussion: Social health is sex-differentially associated with immune system balance, but does not link to cognitive brain aging through mediation.

### MAPPING THE INTERPLAY OF SOCIAL HEALTH AND COGNITIVE FUNCTIONING: A MIXED RESEARCH KNOWLEDGE SYNTHESIS

Henrik Wiegelmann[1], Imke Seifert[1], Marta Lenart-Bugla[2], Mateusz Łuc[2], Ansgar Gerhardus[1], Dorota Szcześniak[2], Joanna Rymaszewska[2], and Karin Wolf-Ostermann[1], *1. University of Bremen, Bremen, Bremen, Germany, 2. Wroclaw Medical University, Wroclaw, Dolnoslaskie, Poland*

Introduction: Dementia is a syndrome with complex underlying bio-psycho-social mechanisms relevant for prevention and intervention. This work presents a mixed research knowledge synthesis, mapping the multidimensional interplay of social health and cognitive functioning in dementia.

Methods: Data integration from 1) systematic review, 2) group model building workshops, (3) iterative integration of multi-national cohort studies (4) ongoing revisions of the social health concept via expert discussions.

Results: The map comprises more than 50 markers, clustered in six domains (social health, psychological pathways, physiological pathways, health behavior pathways, brain/cognitive reserve, cognitive functioning). The social health domain is structured in six sub-domains representing a novel conceptual understanding. Three pathways (physiological, psychological, health behavior) reflect principal mechanisms connecting social health with brain/cognitive reserve and cognitive functioning.

Conclusion: The map depicts dynamic relationships between social health and cognitive functioning that can serve as a basis for recommendations, both for prevention and for improved dementia care.

### SESSION 6000 (POSTER)

### AGHE POSTER SESSION I

Explore innovative age-friendly, intergenerational, dementia-inclusive, and other approaches to gerontology education.

### CONNECTING GENERATIONS AND PRESERVING LIVED EXPERIENCES: AN ILLINOIS AGE-FRIENDLY INITIATIVE

Alyssa Stanfield[1], Matthew Splitstone[2], George Mois[3], Chelsey Byers[4], and Wendy Rogers[5], *1. University of Illinois--Urbana-Champaign, Champaign, Illinois, United States, 2. University of Illinois--Urbana-Champaign, Urbana Champaign, Illinois, United States, 3. University of Illinois Urbana-Champaign, Urbana Champaign,*

*Illinois, United States, 4. University of Illinois Extension, Champaign, Illinois, United States, 5. University of Illinois Urbana Champaign, Champaign, Illinois, United States*

The University of Illinois Urbana-Champaign was recently designated an age-friendly university (AFU).Our AFU program coordinated by CHART (Collaborations in Health, Aging, Research, and Technology) collaborates with our campus University of Illinois Extension office. We are partnering with a student organization called Sage to engage undergraduate students in AFU principles (i.e., promote intergenerational learning and increase the understanding of students of longevity divide). Sage is a social venture run by the Illinois chapter of the global nonprofit organization called Enactus, a social entrepreneurship organization. Sage was established in the spring of 2019 by students who noticed discomfort and lack of understanding among their fellow students of the older generations. By fostering communication, Sage aims to combat ageist social processes, reduce social isolation, and increase mutual understanding and access to the often undervalued wisdom of older adults. Sage helps facilitate life review and develops intergenerational relationships by pairing older adults with college students who listen to and capture their stories through writing to boost the morale and self-confidence of participants. The stories and wisdom they share are then preserved through the production of a cherished legacy booklet or digital media. CHART and campus Extension services support the Sage efforts by assisting with materials development and recruiting older adults from the community to participate. We will share our lessons learned to encourage the development of similar programs at other universities.

### DISCUSSION OF AGE-FRIENDLY UNIVERSITY PRINCIPLES WITH OLDER LEARNERS

Emiko Takagi, and Madeleine Marroquin-Serrano, *San Francisco State University, San Francisco, California, United States*

The purpose of this project was to study how older learners think a university campus currently meets the 10 Age-Friendly University principles and what they see as potential steps to create an age-friendly campus community. Online focus group interviews were conducted with 17 members of Osher Lifelong Learning Institute at San Francisco State University in 2020. The participants were 60 years or older, and the majority were female and non-Hispanic white. The study participants received information about the 10 Age-Friendly University principles presented by the Age-Friendly University Global Network and were asked to discuss their thoughts about how the university satisfies these principles. The interview recordings were transcribed for the thematic analysis of qualitative data. The analysis yielded three themes. The first theme described the diversity of older adults' learning needs and desires that the university must recognize and accommodate. The second theme represented older adults' sense of optimism and anxiety about intergenerational learning. The third theme highlighted the challenges older adults tend to experience in accessing information, educational programs, and/or university facilities. The interviews with older learners were found valuable and indispensable in the work of Age-Friendly University assessments. The presentation is focused on the discussion regarding how older adults' voices can be incorporated in the assessments and ways in which higher education institutions should combat ageism on and off campus as part of work to address the issues identified in this study.

### "DEMENTIA-FRIENDLY": WHAT DOES IT MEAN TO UNDERGRADUATE STUDENTS?

Nasreen Sadeq, *University of South Florida, Tampa, Florida, United States*

Experiential learning assignments may be a potential method of educating undergraduate students about relevant issues in gerontology. Experiential learning involves applying knowledge learned in the classroom to real-world situations and requires students to engage in reflection. The current study explores undergraduate students' perspectives on what it means to be "dementia-friendly" through an activity completed outside of the classroom. Students enrolled in an Alzheimer's Disease Management course completed a module about quality of life, which included information about dementia-friendly communities. Then, students visited a location of their choice that is often frequented by older adults. Based on what they learned in class, they explored the location and documented observations about its dementia-friendly characteristics, or lack thereof. Following their visit, students wrote a reflection on their experience and suggested ways to improve dementia-friendliness. Consensual qualitative methods were used to explore themes in student responses. Among students (N=56), the majority visited grocery or big-box stores, followed by retail shops and restaurants. The three most common themes that emerged about essential dementia-friendly characteristics were 1) availability of helpful people, 2) easy to read signs, and 3) easy to navigate with few fall hazards. Themes from students' suggestions for improving dementia-friendliness included 1) items with larger font sizes, 2) providing staff with dementia training, and 3) more amenities (e.g., store maps, personal shoppers). Student reflections suggested that the assignment also increased awareness and empathy about challenges faced by older adults who wish to age in place.

### DISMANTLING AGEISM: EXPLORING PERSONAL AND SOCIETAL ATTITUDES ABOUT AGING IN UNDERGRADUATE CLASS ACTIVITIES

Erica Srinivasan, *University of Wisconsin, La Crosse, La Crosse, Wisconsin, United States*

Ageism and fear of aging are influenced by both personal and societal attitudes about aging. Ageist beliefs reflected in society or in individuals can impact both our own aging process and how we treat others (Cooney et al, 2020). Thus, it is imperative to explore personal and societal attitudes about aging. In this poster, themes will be presented from a study on two class activities that explore ageism and attitudes about aging, as well as directions for these activities. Students (n=34) in an undergraduate college class were asked to describe their thoughts on aging in 6 words. They were next prompted, with no word limit, to describe fears and what they look forward to about aging. Content analysis of responses revealed themes including the complexity of aging, fears about dementia, physical changes, and the unknown nature of life, and positive interest in career and family goals. In a second activity, based off the AARP social experiment in which millennials were asked "what do you consider to be old?," students asked peers "what image comes to mind when I say "old person?" and "what three words come to mind when I say old person?" Content analysis of responses included themes relating primarily to negative stereotypes, such as "confused and nursing home," with some positive associations, such as "grandparents or wisdom." Both activities prompted rich discussion about the connection between fears of aging and ageism, the importance of addressing ageism and of exploring both positive and challenging aspects of personal aging.

### LET'S PLAY TOGETHER: INTERGENERATIONAL EDUCATION BY CO-DESIGN ESCAPE GAME WITH OLDER ADULTS

Li-Chuan Liu, *National Taitung University, Taitung, Taitung, Taiwan (Republic of China)*

People always think that the older adults are only suitable for participating in activities and games suitable for older adults, and cannot complete games with young people, such as escape game. However, if we can understand the characteristics and needs of the elderly, we find that the older adults still have mobility and vitality to participate in games with young people. Through the intergenerational learning of university's students and the older adults, we use design thinking and PDCA as class frameworks, and let two groups can jointly together to design escape game for the older adults. During the planning stage, the survey and interview of older adults, game design training; the practical operation in the implementation stage; the team discussion and correction in the reflection stage; and the sharing with older adults in the re-action stage (celebration). As a result, these processes allow students and older adults to communicate and share life experiences and wisdom. In one hand, students have a better understanding and concerns about older adults, and they find that the older adults also have vitality and enthusiasm; on the other hand, older adults also more understand the life of students, and feel the creativity and investment of students. In other words, both groups have changed their cognition to each other, and discovered the possibility of mutual cooperation and communication.

### BREATHING LIFE INTO CONVERSATIONS ON DEATH: USING A DEATH CAFÉ MODEL IN A SERVICE-LEARNING PROJECT

Marjorie Getz, *Methodist College, Peoria, Illinois, United States*

This poster presentation describes a project from an upper-level course on death and dying (part of a gerontology program at a small Midwest college). The course derives from the work of Phillipe Ariès's book, The Hour of Our Death. Ariès traced five patterns across time and showed why death had become an uncomfortable truth for those of us living now. In essence, death has been banished from our daily lives. Other research suggests that younger healthcare professionals are particularly uncomfortable interacting with dying clients and may withdraw from them at this important life juncture. Finally, frustration with textbook development of cultural approaches to dying and death (often varied cultural groups, e.g., Asian Americans, are lumped together as if all Asian-Americans share death related practices) led to design of this unique service-learning project. Students, and community members who are experts on various faith/cultural practices take part in a modified death café. Students from an OLLI program are invited to participate, too. We report on qualitative data taken from student reflections. Content analyses of reflective essays identified these themes: (a) new insights/questions about death encounters/attitudes/practices in America (b) willingness to assimilate and accommodate various aspects of other cultural approaches to death into one's own philosophy; (c) feelings of accomplishment/awareness of new skills in providing community-based support to people isolated during the pandemic; (d) understandings related to the importance/value of community service and civic engagement; and (e) new found comfort in openly discussing death concepts in this supportive environment.

### IMPROVING MEDICAL STUDENT RECOGNITION AND UNDERSTANDING OF MORAL INJURY IN MILITARY VETERANS

Maribel Rodriguez-Gonzalez[1], Michael Alan Silverman[2], Maura Miller[3], Sandra DiScala[4], Mythili Bharadwaj[4], and Anthony Beazley[4], *1. Florida Atlantic University, West Palm Beach, Florida, United States, 2. FAU-West Palm Beach VA Medical Center, West Palm Beach, Florida, United States, 3. West Palm Beach VA Medical Center, Jupiter, Florida, United States, 4. West Palm Beach VA Medical Center, West Palm Beach, Florida, United States*

Developing a curriculum for medical students is essential in teaching specific skill sets to better care for patients. There are 18,000,000 living military Veterans in America with unique needs not usually included in medical school curriculum. Military personnel are trained to kill or neutralize the enemy, a doctrine that goes against the moral, religious, and societal principle of thou shalt not kill. Combat Veterans are frequently placed in circumstances where they are forced to commit or participate in acts that go against their moral beliefs leading to a dimensional problem called Moral Injury (an affliction of the soul) with accompanying psychological, behavioral, social, and symptoms such as lack of trust, spiritual distress, fatalism, regret, depression, self-loathing, and apathy, etc. and can be a significant problem encountered in older Veterans precipitated by the normative changes of aging. Discussion with more than one hundred second- and third-year medical students over a 3-year period showed that the students were unfamiliar with Moral Injury. The Geriatrics Department at the West Palm Beach VA Medical Center has created an educational module for medical students that focuses on 1) recognizing the symptoms of Mora Injury, 2) how to approach and converse with Veterans including specific questions to ask and what not to say, 3) the importance of a team approach including a chaplain, 4) interventions such as providing forgiveness and life review, and 5) being cognizant of the trust issues of older LGBTQ Veterans. The response of the medical students has been both positive and enthusiastic.

### SKILL BUILDING BLOCKS: ASYNCHRONOUS ONLINE LEARNING THROUGH INTERACTIVE THEMATIC INSTRUCTION

Harvey Sterns[1], and Vicki de Klerk-Rubin[2], *1. The University of Akron, Akron, Ohio, United States, 2. Validation Training Institute, Pleasant Hill, Oregon, United States*

Whether professional or as a family carer, interactions with an older adult living with or without cognitive decline should be person-centered. Learning behavioral skills requires more than watching a demonstration or cognitive learning; it requires practice, feedback and more practice. Online learning has become the best way to reach carers of older adults, partially due to the COVID pandemic and partially due to time, money and energy resources being stretched to their limits. The Validation Training Institute has developed 6 self-directed, online Skill Building Blocks that uses thematic instruction strategy, interactive exercises, and videos demonstrating why each skill is important and how to best utilize the skill in real life situations. Learners have completed pre and post-questionnaires using the Likert scale and the IMMS (Instructional Material Motivation Survey) which measures attention, relevance, confidence, and satisfaction. An online focus group of participants concluded the data collection process. Techniques taught through Skill Building Blocks proved to be relevant to the learners' life experience. There was effective retention and transfer of skills, and the training proved to be motivating for continued application in the real world. Learners expressed confidence and satisfaction with the user-friendly, thematic design of each block.

### AFTER THE STORM: SOCIAL WORK STUDENTS ASSISTING WITH FEMA DISASTER RELIEF FOR OLDER ADULTS

Joy Ernst, *Wayne State University, Detroit, Michigan, United States*

This poster describes a partnership during Fall 2021 between the Wayne State School of Social Work and the Federal Emergency Management Agency (FEMA) to assist older adults whose homes sustained damage during flooding in June 2021. This short-term project helped people access resources to repair and restore damaged homes, repair, or replace mechanical and electrical systems, deal with mold and other health hazards, and replace their personal belongings. Social work students trained by FEMA made over 700 outreach calls to homeowners whose applications for relief were rejected. The students worked with the homeowners to facilitate access to help that either resulted in the approval of their FEMA application (due to assistance with technical issues such as missing documentation or errors in the application) or connected them with alternative sources of help and support. A focus group with students provided insights on their motivations, training experiences, issues affecting the applicants, and skills developed. While FEMA-required training offered little relevant assistance, SSW staff and faculty overseeing the program provided ongoing support that students considered vital as they worked to assist applicants. Some homes were extensively damaged and resources available from FEMA were insufficient. Some homeowners suspected the students were scammers; students also learned of exploitation by contractors. Students honed empathy, reflection, and supportive listening skills as they heard stressors associated with maintaining their homes amidst losses due to covid and their knowledge of community resources expanded. Lessons learned to aid in future efforts to assist in disaster relief are described.

### AGE-FRIENDLY CAMPUS PRACTICES IN HIGHER EDUCATION: A HEAT MAP VIEW

Janelle Fassi[1], Celeste Beaulieu[2], Lauren Bowen[2], Joann Montepare[3], Susan Whitbourne[4], and Nina Silverstein[2], *1. University of Massachusetts Boston, Goffstown, New Hampshire, United States, 2. University of Massachusetts Boston, Boston, Massachusetts, United States, 3. Lasell University, Newton, Massachusetts, United States, 4. University of Massachusetts Amherst, Amherst, Massachusetts, United States*

The Age-Friendly University (AFU) initiative aims to increase the participation of age-diverse older adults in higher education communities. The present study investigated age-friendly practices across 23 institutions in the United States. The ICCS Inventory (Silverstein et al., 2022), which identifies 192 potential age-friendly campus practices was completed by administrators representing major campus units. A heat map was used to graphically represent age-friendly practices and identify where universities differed in the presence of those practices. Heat map findings indicated campuses are low in some auxiliary services that assist retired faculty and staff. However, campuses consistently gave retired faculty and staff access to university library services. Campuses also had limited age-friendly teaching and learning services. None of the campuses reported having resources to help faculty deliver teaching materials in formats specifically geared toward older learners. In addition, none of the campuses reported having teaching and learning staff visiting campus departments to provide resources for older learners, and very few campuses offered courses that focused on aging and age diversity issues. Common age-friendly practices were seen with respect to providing instructional technology support for faculty/staff/students and community partnerships for intergenerational activities. Physical environment and personnel evidenced the most frequent age-friendly practices likely because they are mandated by the ADA (e.g., clear signage, handicapped parking close to buildings, spaces free of obstacles, training in ageism as a form of discrimination). Overall, the present study highlighted the areas where college campuses are most age inclusive, while also revealing areas for improvement in age inclusive practices.

### EVALUATING THE EFFECTIVENESS OF AN ONLINE TRAINING COURSE FOR MEETING NEEDS ASSOCIATED WITH CAREGIVING BURDEN

Shera Hosseini, Lorraine Carter, Donna Thomson, and Michelle Howard, *McMaster University, Hamilton, Ontario, Canada*

Informal caregivers make up a critical part of long-term support in the communities due to their important role in caring for older adults living at home. Modern-day caregivers are facing greater responsibility and burden for managing their care recipients. The present study aimed to explore the effect of a caregiving training program in alleviating caregiver burden. This program included four standalone online modules each with specific foci. Completion of activities across the modules allowed for the creation of a Caregiver Action Plan which offered a personal and practical resource to the informal caregivers. This evaluation study was qualitative and a used thematic analysis method of data analysis. Data stemmed from semi-structured interviews with the caregivers and their reflections on the program's discussion board. Most caregivers provided care for persons with dementia. Interviews with the family caregivers were conducted, transcribed, and thematically analyzed. Themes were identified through constant comparison and in an iterative process. The family caregivers demonstrated consensus on the efficacy of the program in raising competence and confidence and contributing to ameliorating burden levels. Important themes were identified in association with areas for which the caregivers needed support: Early dementia education, planning for future care, learning about navigating healthcare systems, peer support, enhancing self-care, and coping with emotional burden and self-blame. The findings will be informative in shaping the program based on the caregiver's identified needs through addressing those areas that they would need support. These findings may offer recommendations to other programs designed to support the family caregivers.

### COMBATING STIGMA: AN EDUCATIONAL RESOURCE ON SUBSTANCE USE DISORDER IN OLDER ADULTS

Marissa Mackiewicz, Patricia Slattum, and Leland Waters, *Virginia Commonwealth University, Richmond, Virginia, United States*

Substance Use Disorder (SUD) affects people from all walks of life and age groups. Special considerations for older adults with SUD should be made secondary to the unique pathophysiological and socio-economic complexities of aging populations. Successful SUD treatment and recovery depends on access to essential support systems comprised of interprofessional teams as well as family, friends, and caregivers. Education on SUD for healthcare providers, caregivers and the community is a key component for combating the national opioid epidemic and the stigma that can be a barrier to recovery. A ten-minute video and an accompanying discussion guide were developed to present to clinical faculty and students participating in an interprofessional care coordination wellness clinic servicing low-income older adults living independently in the community setting. The video was designed to highlight stigma and ageism facing older adults with SUD and serve as the basis for discussion among learners. The process for creating and evaluating the video and discussion guide will be shared including the role of older adults with lived experience of SUD in the process. Information will be provided on SUD as a chronic disease and the importance of interprofessional and individualized patient care in recovery. Participants will be able to identify the significance of SUD related stigma in providing and seeking care, asses the role of mental/behavioral health in SUD, and learn about treatment options for SUD. This will allow for better identification of characteristics of older adult populations that place them at greater harm from the consequences SUD.

### TUTORIAL-STYLE EDUCATION FOR UNDERGRADUATES IN AGING

Karley Deason, Nasreen Sadeq, and Brianne Stanback, *University of South Florida, Tampa, Florida, United States*

At almost every university, there is an option for undergraduates to enroll in directed readings or research, independent studies, or some other form of tutorial-style education, although not much is known about them or best practices associated with them for undergraduate students interested in aging. This project reviews available literature about tutorial-style education and describes the multidimensions involved in these learning experiences. Search phrases such as "history of Oxford tutorials", "tutorial classes", and "student traits in tutorials" were used in Google Scholar to find literature, returning 142 articles. Articles were included if the literature was published in a periodical, findings were not redundant, and measures connected to the objectives of this project, and, by these criteria, ten articles comprised the final sample for review. Student characteristics, instructors, and other factors, like the time intensity of the tutorial course and the student's connection to the subject area, have been found to impact tutorial-style education. The review is the first of its kind to use its findings to propose a set of best practices to enhance the experience for students interested in aging, including the fit of student and instructor, skillsets of successful students, and modalities, and advance a research agenda to better understand and communicate practices in tutorial-style education for undergraduates in aging.

### CREATING AN AGE-FRIENDLY UNIVERSITY: THE CASE OF ST. CLOUD STATE UNIVERSITY (SCSU)

Phyllis Greenberg[1], Rona Karasik[2], Jessica VanderWerf[2], Claudia Burgos Zuniga[2], and Maria Kroeber[2], *1. St. Cloud State University, St Cloud, Minnesota, United States, 2. St. Cloud State University, St. Cloud, Minnesota, United States*

As universities seek ways to address decreasing enrollments and enhance Diversity, Equity, and Inclusion (DEI) initiatives, the concept of Age-Friendly Universities (AFU) has gained momentum. The AFU Global Movement is a way to situate a university to explore means to increase student enrollments, and expand DEI Initiatives to include age. This poster details the steps taken by St. Cloud State University, a comprehensive Minnesota State university, to become designated as an AFU, as well as the unique challenges encountered. Actions taken both before and after being designated as an Age Friendly University (December, 2021) are discussed. Pre-designation steps included evaluating the campus' current age friendliness, gathering university and community support at all levels, participating as a "champion" in the UMass-Boston & LaSalle University's Age-Friendly University Inventory and Campus Climate survey, and piggy-backing on Minnesota's Age-Friendly State and the local community's Age-Friendly initiatives. The SCSU Gerontology program was awarded a grant from the MinnState Innovation Fund to explore ways of "Creating an Age-Friendly SCSU." The grantors saw this as a potential template for other universities in the system who were seeking to expand their student base. Post benefits, challenges, strategies and next steps working towards meeting the 10 Principles of an Age-Friendly University are also discussed.

### USING CONNECTED LEARNING THEORY GUIDED ADOPTION IN SUCCESSFUL IMPLEMENTATION OF 4MS AGE-FRIENDLY CARE

Leah Tobey[1], Danette Scherer[2], and Robin McAtee[3], *1. University of Arkansas for Medical Sciences/AR Geriatric Education Collaborative, Little Rock, Arkansas, United States, 2. UAMS: AR Geriatric Education Collaborative, Little Rock, Arkansas, United States, 3. University of Arkansas for Medical Sciences, Little Rock, Arkansas, United States*

As one of the HRSA funded Geriatric Workforce Enhancement recipients, the Arkansas Geriatric Education Collaborative worked with the Reynolds Institute on Aging Thomas and Lyons Longevity Clinic to implement Age

Friendly care. An adult learning theory was used to develop trainings on the 4M's of Age-Friendly Healthcare and to pursue Age-Friendly Certification from The Institute for Healthcare Improvement. The Connected Learning Theory is a proven method to reliably help a trainee's adoption of new principles and ideas by connecting familiar relationships which are easily recognized. This theory supports various learner pathways and proposes that students learn best when they form familiar connections to the materials. In an effort to provide enhanced training to healthcare professionals who were already familiar with much of the content, associations were made between M&M's candy with each of the 4Ms, What Matters, Mobility, Mind and Medications of Age-Friendly Care. Baseline and 6-months post training data on two Merit-Based Incentive Payment Systems (MIPS) measures, depression and falls risk screening, were used to evaluate outcomes. Staff were satisfied with the 4Ms Age-Friendly Framework and found it easily applicable to their older adult patient workflow. MIPS evaluation of data showed significant changes in depression screens with a 23% improvement and a 3% improvement in screenings for future fall risk 6 months after training. This clinic achieved Level I Age-Friendly Certification in June 2021. Although the staff were already specialists in geriatrics, the training increased application of the 4 M's, improved staff acceptance, and improved knowledge via M&M connections.

### THE EFFECT OF COMMUNICATION SKILLS TRAINING FOR NURSING STUDENTS BY AUGMENTED REALITY SIMULATION SYSTEM

Masaki Kobayashi[1], Miyuki Iwamoto[2], Saki Une[3], Ryo Kurazume[4], Atsushi Nakazawa[2], and Miwako Honda[3], *1. Rochester Regional Health System, Rochester, New York, United States, 2. Kyoto University, Kyoto, Kyoto, Japan, 3. National Hospital Organization Tokyo Medical Center, Tokyo, Tokyo, Japan, 4. Kyushu University, Fukuoka, Fukuoka, Japan*

Background: Educational methods for improving communication with dementia patients are lacking. Our study aimed to evaluate the efficacy of simulated communication skills training for nursing students using augmented reality (AR).

Methods: A randomized controlled study. 25 nursing students enrolled and learned standardized multimodal communication skills using textbooks. Participants were randomly assigned to AR training or conventional mannequin training group and each had one-hour training of basic nursing care. The mannequin of AR group was superimposed a computer graphic of an elderly woman's face, which reacts to participants' communication. Further, participants' gaze and voice were evaluated by artificial intelligence (AI) and the participants received real-time feedback to their head-mounted display. The conventional group had the self-training with nursing mannequins. Participants performed basic nursing care to simulated patients before and after training, which were video-recorded by eye-tracking cameras and fixed cameras, then the videos were analyzed by AI. The primary outcome was the proportion of eye contact, verbal, and multimodal communication time during the care to simulated patients. Additionally, participants' empathy was evaluated by the Jefferson Scale.

Results: The interactive communication in AR group significantly increased than conventional group (eye contact 13.6% versus 4.4%, verbal communication 27.7% versus 20.1%, multimodal communication 9.6% versus 3.8%, P< 0.05, respectively). The empathy score significantly increased in AR group, whereas it decreased in conventional group [Mean (SD): 9.1 (6.6) versus -1.3 (3.8), P< 0.01].

Conclusions: The simulated communication skills training for nursing students using AR was associated with increased interactive communication skills and empathy to patients.

### MOVING FROM ENDORSEMENT TO ACTION: ALIGNING AFU EFFORTS WITH UNIVERSITY STRATEGIC PLANS AND GOALS

Sarah Walsh[1], Andrea Zakrajsek[1], Cassandra Barragan[1], and Stephanie P. Wladkowski[2], *1. Eastern Michigan University, Ypsilanti, Michigan, United States, 2. Bowling Green State University, Bowling Green, Ohio, United States*

The Age-Friendly University global initiative has the opportunity to unite nations around the world towards making higher education spaces inclusive of older adults. Once a university joins the AFU Global Network and endorses the 10 guiding principles, the real work of living up to the principles begins. Each institution has unique geographical, political, social, and financial characteristics that require tailored approaches to this work. At our regional comprehensive university, we completed three separate analyses (external, internal, future directions) as part of our Environmental Scan. The goal of the Environmental Scan was to understand the initial strengths, weaknesses, and opportunities to age-friendliness and inclusiveness for older learners within our campus community. We utilized a multi-pronged data collection approach, including individual stakeholder interviews, online surveys of older leaners, policy analysis, and internal and external audits. Many aspects of data collection for the environmental scan occurred concurrently with data analysis. Data from each of the eight data collection methods were used to generate key findings and recommendations and will be presented. Furthermore, we found that aligning our recommendations with the University's marketing and enrollment efforts as well as the University's goals relating to diversity, equity and inclusion allowed for greater administrative support for strategies to increase age-friendliness on campus. with implications for similar work at other institutions. The Age-Friendly University global initiative has the opportunity to better support the inclusion of older adults in higher education. Our Environmental Scan of age-friendliness, from inception to completion will apply to institutions across the globe.

### SESSION 6010 (POSTER)

### BIOLOGY OF AGING (BS)

### BDNF VAL66MET MODERATES THE EFFECTS OF HYPERTENSION ON EXECUTIVE FUNCTIONING IN OLDER ADULTS DIAGNOSED WITH AMCI

Peter Louras[1], Lisa Brown[2], Rowena Gomez[2], Stacie Warren[2], and Kaci Fairchild[3], *1. VA Palo Alto Health Care System, Palo Alto, California, United States, 2. Palo Alto University, Palo Alto, California, United States, 3. VA*

*Palo Alto Healthcare System, Palo Alto, California, United States*

Brain-derived neurotrophic factor (BDNF) has a demonstrated role in promoting memory functions and neuronal survival. A common variant in the BDNF gene, the Val66Met polymorphism, has been found to reduce BDNF expression and increase vulnerability to neurocognitive impairments and age-related cognitive declines. Research suggests that BDNF Val66Met may be associated with hypertension, a well-established risk factor for health and brain functioning across the lifespan. While BDNF is most studied for its effect on learning and memory, its role in moderating other cognitive domains is not as well understood, especially those most affected by hypertension. Furthermore, no study to date has investigated these relationships exclusively with older adults and those at increased risk for cognitive decline. Therefore, the aim of this study was to investigate the effect of BDNF Val66Met and hypertension on the executive function ability of older adults (mean age 71.3±9.2 years) diagnosed with amnestic Mild Cognitive Impairment (aMCI) (N = 108). Results showed that BDNF Val66Met moderated the relationship between hypertension and executive functioning, such that hypertensive carriers of the BDNF Met allele performed significantly worse compared to Val-Val homozygotes, and independent to the effects of aging. These results indicate that genetic and vascular risk interactions can predispose older adults with aMCI to impairments in multiple cognitive domains, and potentially increase susceptibility to further declines or conversion to dementia. These findings have implications for future research on dementia disease pathology, and highlight the importance of strategies that target fixed and modifiable risk factors to promote cognitive resilience.

### EXOME-WIDE STUDY IDENTIFIED 12 PLEIOTROPIC LOCI ASSOCIATED WITH ALZHEIMER'S AND CARDIOVASCULAR DISEASE RISKS

Yury Loika, Elena Loiko, Konstantin Arbeev, Eric Stallard, Anatoliy Yashin, Irina Culminskaya, and Alexander Kulminski, *Duke University, Durham, North Carolina, United States*

Health of brain, heart and blood vessels are closely connected, which means that Alzheimer's (AD) and cardiovascular (CVD) diseases may have overlapping etiologies including genetic component. Most of previous genetic association studies considered different traits (AD, CVD, and their risk factors) separately. The analysis of pleiotropic predisposition to these traits may shed light on the trait-specific mechanisms in protection against AD. We carried out pair-wise pleiotropic exome-wide association study (~250K common genetic variants) in predisposition to AD and each of 17 traits in a sample of 118K individuals from UK biobank. The analysis included seven qualitative traits (CVD, coronary-heart disease, type 2 diabetes, stroke, myocardial infarction, heart failure and hypertension), as well as their 10 risk factors (blood glucose, body-mass index, height, weight, 4 lipid traits, systolic and diastolic blood pressure). Fisher's method and omnibus test were used in pleiotropic analyses. In addition to the APOE-TOMM40 locus, the analysis identified 12 genetic loci in which genetic polymorphisms demonstrated significant associations with AD at $p \leq 5 \times 10\text{-}4$ and pleiotropic associations at genome-wide level, $p \leq 5 \times 10\text{-}8$. The identified genes are involved in processes of phosphorylation; regulation of cell growth, differentiation, and brain development; signal transduction and neurotransmission; mitochondrial and cytoskeleton organization; regulation of gene expression, apoptotic processes, and adaptive immune response. Our results provide novel evidence supporting the hypothesis of complex pleiotropic mechanisms contributing to the development and progression of AD and provide more insights into understanding of underlying biological functions and regulatory mechanisms behind these effects.

### REGULATION OF NEURONAL MITOCHONDRIAL BIOENERGETICS BY LIPID METABOLITES UPREGULATED IN DEMENTIA

Stephanie Heimler[1], K. Allison Amick[2], Jaclyn Bergstrom[3], Mohit Jain[3], and Anthony Molina[4], *1. UC San Diego School of Medicine - Division of Geriatrics, Gerontology, and Palliative Care, LA JOLLA, California, United States, 2. Wake Forest School of Medicine, Winston-Salem, North Carolina, United States, 3. UC San Diego School of Medicine, La Jolla, California, United States, 4. UC San Diego School of Medicine - Division of Geriatrics, Gerontology, and Palliative Care, La Jolla, California, United States*

Mitochondrial dysfunction occurs early in Alzheimer's disease (AD) progression and is evident in the Central Nervous System (CNS) and peripheral circulating cells. While there is evidence indicating that bioenergetic decline can drive the early pathogenesis of AD, little is known about the extracellular factors that alter neuronal bioenergetic capacity. We hypothesized that circulating metabolites contribute to neuronal bioenergetic decline associated with AD. Using a cohort comprised of participants with normal cognition, mild cognitive impairment, and dementia, we show that human serum harbors circulating, non-cellular factors capable of mediating neuronal bioenergetic differences according to the cognitive status of the serum donor. We developed a novel screening-based approach to identify candidate "mito-active" lipid metabolites in human serum that could mediate differences in bioenergetic capacity. Among these, Nervonic Acid and 15-epi-PGA1 were predicted to be mitochondrial inhibitors upregulated in participants with dementia. Neurons exposed to physiologically-relevant ranges of these molecules in-vitro exhibited a dose-dependent reduction in maximal mitochondrial respiration. We found that 500ug/mL of Nervonic Acid and 9ug/mL of 15-epi-PGA1 reduced maximal mitochondrial respiration by 62.3% and 63.3%, respectively, thereby validating our screening and prediction approach. Future experiments may be directed towards investigating if and how these and other mito-active metabolites of interest cross the blood-brain barrier to meaningfully affect AD. Furthermore, identified mito-active molecules can be investigated in other clinical cohorts to examine their role/s in multiple age-related or neurological conditions. This work expands our mechanistic understanding of how extrinsic factors associated with age and cognitive status contribute to neuronal bioenergetic decline.

### AN OPTIMIZED MOUSE PARABIOSIS PROTOCOL FOR INVESTIGATION OF AGING AND REJUVENATIVE MECHANISMS

Sonia Rodriguez, Chase Carver, Katayoun Ayasoufi, Felicia Duke Boynton, and Marissa Schafer, *Mayo Clinic, Rochester, Minnesota, United States*

Surgical parabiosis is widely used to study the mechanistic influence of the circulating milieu on aging and regeneration.

This powerful model presents diverse complications based on age, strain, sex, and other experimental parameters. In young (Y) and old (O) male and female C57BL6 mice, we optimized heterochronic (n=12) and isochronic (n=10 Y-Y, n=7 O-O) parabiosis. Throughout protocol development, we identified several complications including variable responses to anesthesia, external and internal dehiscence, dehydration, and weight loss. We identified and implemented solutions during surgical and post-surgical periods, including titrated anesthesia, reinforced internal sutures, topical agents to promote wound healing, and increased supplementation. By consistently adopting protocol changes we were able to significantly increase survival. Separately, we confirmed the time course of chimerism in heterochronic pairs of C57BL6 and Tg(act-EGFP)Y01Osb (eGFP) mice. Baseline and longitudinal blood samples were collected via tail vein. Flow cytometry was used to visualize GFP-positive cells from the parabiont blood sample. Through blood analysis we found that chimerism occurs as early as 2 days post-operatively. Exploitation of our optimized protocol may enable others to efficiently adopt the surgical parabiosis model to dynamically study mechanisms of aging and regeneration.

### EPIGENETIC AGING OF THE DEMOGRAPHICALLY NONAGING NAKED MOLE RAT

Csaba Kerepesi, *Institute for Computer Science and Control (SZTAKI), Budapest, Budapest, Hungary*

The naked mole-rat (NMR) is an exceptionally long-lived rodent that shows no increase of mortality with age, defining it as a demographically non-aging mammal. Here, we perform bisulfite sequencing of the blood of>100 NMRs, assessing>3 million common CpG sites. Unsupervised clustering based on sites whose methylation correlates with age reveals an age-related methylome remodeling, and we also observe a methylome information loss, suggesting that NMRs age. We develop an epigenetic aging clock that accurately predicts the NMR age. We show that these animals age much slower than mice and much faster than humans, consistent with their known maximum lifespans. Interestingly, patterns of age-related changes of clock sites in Tert and Prpf19 differ between NMRs and mice, but there are also sites conserved between the two species. Together, the data indicate that NMRs, like other mammals, epigenetically age even in the absence of demographic aging of this species.

### IMPACT OF COMBINATIONS OF THE APOE Ɛ4 ALLELE AND TOMM40-APOC1 VARIANTS ON SURVIVAL TO OLDER AGES AND ALZHEIMER'S RISK

Alexander Kulminski[1], Ethan Jain-Washburn[2], Ian Philipp[1], Konstantin Arbeev[1], Eric Stallard[1], Mary Feitosa[3], Nicole Schupf[4], and Kaare Christensen[5], *1. Duke University, Durham, North Carolina, United States, 2. DUKE UNIVERSITY, Durham, North Carolina, United States, 3. Washington University, St Louis, Missouri, United States, 4. Columbia University, New York, New York, United States, 5. Southern Denmark University, Odense, Syddanmark, Denmark*

Multifactorial diseases, health-related traits, and lifespan are polygenic phenotypes with complex genetic architectures. Polygenicity implies that multiple variants can impact the risks of these phenotypes independently or jointly. Recently, we showed that carriers of minor alleles of rs429358 (APOE ε4 encoding polymorphism), rs2075650 (TOMM40), and rs12721046 (APOC1) polymorphisms have up to 4.4 times higher risk of Alzheimer's disease (AD) than APOE ε4 carriers without the minor alleles of rs2075650 and rs12721046. Here, we examined the chances of living to older ages—85 years and above—for carriers of compound genotypes combining these three polymorphisms using data from the Long Life Family Study, Framingham Heart Study, Cardiovascular Health Study, and UK Biobank. Consistent with their higher AD risk, carriers of the APOE ε4 allele with one or two minor alleles of rs2075650 and rs12721046 had 24.3% lower log odds of living to 85+ years (β=-0.243, p=2.22×10-2) than APOE ε4 carriers without either minor allele. Counterintuitively, AD did not mediate this risk. With AD-affected subjects excluded from the analysis, the effect size for the log odds of living to 85+ years (β=-0.352, p=2.35×10-3) was 1.45 times larger for APOE ε4 carriers with one or two minor alleles of rs2075650 and rs12721046. The chances of survival could be associated with lipid- and immunity-related mechanisms, whereas the risk of AD may be associated with amyloid-β-related mechanisms, among others. Targeting heterogeneous polygenic profiles of individuals at higher risks of multifactorial phenotypes may be a promising strategy for translating genetic discoveries to health care.

### CONVERGENT CELL NONAUTONOMOUS PATHWAYS REWIRE METABOLISM TO SLOW AGING

Scott Leiser, Shijiao Huang, Hillary Miller, Safa Beydoun, Elizabeth Dean, Hyo Choi, Ajay Bhat, and Marshall Howington, *University of Michigan, Ann Arbor, Michigan, United States*

An organism's ability to perceive and respond to changes in its environment is crucial for its health and survival. Our approach to identify molecular mechanisms of aging is to focus on common mechanisms downstream of multiple pathways. This approach led to our discovery of a gene, flavin-containing monooxygenase (fmo)-2, that is both necessary and sufficient to increase lifespan and healthspan downstream of several longevity interventions, including dietary restriction and hypoxia. Surprisingly, we also find that in both hypoxia and dietary restriction models, fmo-2 is induced by cell non-autonomous signaling pathways, consistent with the worms' perceiving the stress (e.g. low oxygen, lack of food) and changing physiology as a result. Our current work focuses on 1) the signaling networks that regulate stress perception and integrate multiple signals to change physiology, and 2) the mechanism of FMO-2-mediated longevity. Our new data suggest that these cell non autonomous networks pathways utilize both overlapping and distinct signaling mechanisms to converge on upregulation of the same gene. They also suggest that these pathways can be manipulated by small molecule drugs to increase lifespan by "tricking" the organism into activating stress response networks. We further find that FMO enzyme expression has a drastic effect on endogenous metabolism, primarily through tryptophan and one carbon metabolism. Ultimately, we aim to leverage our results in a translational framework to identify key signals, genes, and mechanisms where organisms respond to the perception of environmental stress to improve health and slow aging.

### INFLAMM-AGING IS ASSOCIATED WITH PRO-INFLAMMATORY PROGRAMMING OF INNATE IMMUNE CELLS IN THE COLON

Ji Yeon Noh[1], Hongying Wang[1], Yuhua Farnell[2], Xiao-Di Tan[3], Gus Wright[4], Andrew Hillhouse[4], and Yuxiang Sun[1], *1. Texas A&M University, College Station, Texas, United States, 2. Texas A&M AgriLife Research, Texas A&M University, College Station, Texas, United States, 3. Ann & Robert H. Lurie Children's Hospital of Chicago, Feinberg School of Medicine, Northwestern University, Chicago, Illinois, United States, 4. College of Veterinary Medicine & Biomedical Sciences, Texas A&M University, College Station, Texas, United States*

Chronic low-grade inflammation is prevalent in aging, which is called inflamm-aging. Immune cells are important mediator of inflammatory state of host and the innate immunity is the first responder to various insults. Gastrointestinal track, especially colon, is the site where immune cells are abundant. Aging is associated with increased gut dysbiosis and functional decline. We hypothesize that colonic innate immune cells contribute to the age-associated inflammation in the colon. We found that macrophages in colon mucosa are elevated in aging, and it is accompanied by pro-inflammatory cytokine expression and increased gut permeability. Specifically, we used flow cytometry to assess colonic innate immune cells collected from 5-, 7-, 14-, 19-, 24-, and 28-month-old mice. Aging significantly increased the populations of pro-inflammatory cytokine producing innate immune cells in the colon, including neutrophils, dendritic cells, monocytes and macrophages. Interestingly, the infiltrating Ly6Chi macrophages and pro-inflammatory CD11c+ macrophages were much higher, while anti-inflammatory CD206+ macrophages were decreased in colon of the aged mice. In line with the immune profile, aged mice showed increased gut permeability tested by fluorescein isothiocyanate-dextran, and the gene expressions of gap junction proteins in the colon were decreased, supporting increased gut permeability in the aged mice. Collectively, our results suggest that innate immune cells play an importance role in age-associated gut inflammation; targeting the innate immune cells in the colon may present a novel therapeutic strategy for prevention and treatment of aging leaky gut.

### CELLULAR RESPIRATION AND RESILIENCE AS A POTENTIAL BIOLOGICAL MECHANISM DRIVING SEX DIFFERENCES IN AGING

Adam Salmon[1], and Daniel Adekunbi[2], *1. The University of Texas Health Science Center at San Antonio, San Antonio, Texas, United States, 2. University of Wyoming, Laramie, Wyoming, United States*

There are substantial differences in the progression of aging between males and females including in progression and prevalence of disease and longevity. Not all can be explained solely by sex-specific endocrine regulation and growing evidence suggests there are basic biological and genetic differences in sex that drive disparity in physiological function. In this study, we describe metabolic differences at the cellular level that both define some of these biological differences as well as provide a potential mechanism for delineating relevant molecular mechanisms of aging. Using HET3 mice, a genetically heterogeneous model with a consistent female advantage in longevity, we show that primary fibroblast lines retain functional metabolic characteristics, including mitochondrial response and stress resilience, which are representative of the sex of the donor animal. These differences persist even after several rounds of passage using standard culturing techniques suggesting these differences are not driven by direct impact of circulating sex hormones. Moreover, we find that differences in these cellular responses have some predictive power to determine both sex- and individual-specific responses to physiological challenge including obesity and longevity. In addition, we are able to use this model to delineate how donor sex affects cellular responses within defined pillars of aging including proteostasis, metabolic function, and adaption to stress. Overall, our model then provides valuable in defining the cellular responses that contribute to the mammalian aging process.

### THE FOXO3 LONGEVITY GENOTYPE PROTECTS AGAINST MECHANISMS OF CELLULAR AGING IN OKINAWANS

Trevor Torioge[1], D. Craig Willcox[2], Michio Shimabukuro[3], Timothy Donlon[2], Randi Chen[2], Richard Allsopp[4], and Bradley Willcox[2], *1. John A. Burns School of Medicine, University of Hawaii, Manoa, Honolulu, Hawaii, United States, 2. Kuakini Medical Center, Honolulu, Hawaii, United States, 3. Fukushima Medical University, Fukushima City, Fukushima, Japan, 4. University of Hawaii, Honolulu, Hawaii, United States*

The genetic association of FOXO3 genotypes with human longevity is well established, although the mechanism is not fully understood. We now report on the relationship of the FOXO3 longevity variant rs2802292 with telomere length, telomerase activity, FOXO3 expression and inflammatory cytokine levels in men and women. In agreement with earlier work, the FOXO3 longevity variant conferred protection against telomere shortening of peripheral blood mononuclear cells from adults aged 55 and older. This was accompanied by higher levels of telomerase activity in carriers of the longevity-associated FOXO3 G-allele ($P=0.015$). FOXO3 mRNA expression increased slightly with age in both young ($P=0.02$) and old ($P=0.08$) G-allele carriers. Older female G-allele carriers displayed a modest decline in levels of pro-inflammatory cytokine IL-6 with age ($P=0.07$). In contrast, older male G-allele carriers displayed an age-dependent increase in levels of anti-inflammatory cytokine IL-10 with age ($P=0.04$). Thus, FOXO3 may act through several different pro-longevity mechanisms, which may differ by age and sex.

### LATE-LIFE MORTALITY GWAS IN FLIES IDENTIFIES DIABETES AND OBESITY REGULATED TO REGULATE MORTALITY AND RESILIENCE

Tyler Hilsabeck[1], Sudipta Bar[2], Kenneth Wilson[2], Jennifer Beck[3], Christopher Nelson[3], Rachel Brem[4], and Pankaj Kapahi[2], *1. Buck Institute, Santa Rosa, California, United States, 2. Buck Institute for Research on Aging, Novato, California, United States, 3. Buck Institute, Novato, California, United States, 4. University of California at Berkeley, Novato, California, United States*

Variations in rate of aging in geneticaly heterogenious populations supports the hypothesis that aging is at least partially genetically regulated. However, genetically identical individuals also vary in their time of death. We have observed in D. melanogaster that this variation is genotype-dependent, as specific genotypes have characteristic survival curve shapes that are largely reproducible. Typical aging studies reduce a strain's lifespan down to a population-level value, i.e. mean lifespan. While these metrics can represent the trends in a population, they are unable to encapsulate the variation in the aging of individuals from the same distinct population. Instead, we used two values that characterize the logistic fit of a strain's mortality late in life: the risk of initial mortality (α) and the rate of aging (β). To identify regulators of the rate of aging, we performed a Genome-Wide Association Study (GWAS) of β for 160 different fly strains from the DGRP collection on two different diets late in life. This approach identified the candidate gene Diabetes and Obesity-Regulated (DOR), which has known roles in stress response, autophagy, and senescence, as having a role in the late-life mortality. DOR inhibition leads to a significant increase in late-life mortality that is preceded by a reduction in healthspan-related traits. Further, germline-specific inhibition is sufficient to increase senescence-related factors and shorten lifespan. We conclude that a decrease in DOR, a conserved gene, compromises an organism's resilience through increased inflammation, senescence, and increased mortality, providing a potential target for bolstering the decline seen in human aging.

### VITAMIN D DEFICIENCY AND FASTER EPIGENETIC AGING: RESULTS FROM THE BERLIN AGING STUDY II (BASE-II)

Valentin Vetter[1], Yasmine Sommerer[2], Christian Kalies[3], Dominik Spira[1], Lars Bertram[2], and Ilja Demuth[4], *1. Charité - Universitätsmedizin Berlin, Berlin, Berlin, Germany, 2. University of Lübeck, Lübeck, Schleswig-Holstein, Germany, 3. Charité – Universitätsmedizin Berlin, Berlin, Berlin, Germany, 4. Charité Universitätsmedizin Berlin, Berlin, Berlin, Germany*

Particularly older people are at risk for deficient vitamin D levels, as their capability for cutaneous synthetization is lower and they are often less exposed to sunlight. However, the resulting impact on one individual's health is not fully understood. To examine potential consequences of low vitamin D levels for health of older people, we examined its relationship with a DNA related biomarker of aging, DNA methylation age acceleration (DNAmAA). Several epigenetic clocks, that are used to estimate this parameter from methylation fractions of specific cytosine-phosphate-guanine sites, are available and differ in aspects of aging they represent best. Five clocks, 7-CpG clock, Horvath's clock, Hannum's clock, PhenoAge, and GrimAge, were available in the longitudinal BASE-II cohort (n=1,100) at follow-up examination. DNAmAA estimated from 7-CpG clock and GrimAge was associated with vitamin D levels in covariate adjusted regression analysis. Additionally, a quasi-interventional study design was employed and showed 2.6 year lower 7-CpG DNAmAA (p=0.011) and 1.3-year lower Horvath DNAmAA (p=0.042) in vitamin D deficient participants that chose to start vitamin D supplementation during the follow-up period of 7.4 ± 1.5 years compared to a matched control-group of participants with untreated vitamin D deficiency. No statistically significant difference was found between sufficiently treated participants with vitamin D deficiency and a matched group of participants that reached sufficient vitamin D level without supplementation. Although validation of our results in a randomized controlled trial is needed, our findings suggest, that vitamin D deficiency associated higher rates of epigenetic aging can potentially be reversed through supplementation.

### SHORT TELOMERES ASSOCIATE WITH HYPERPHOSPHORYLATED TAU BURDEN IN PRIMARY AGE-RELATED TAUOPATHY

Corey McMillan[1], Bandita Adhikari[1], Kurt Farrell[2], David Wolk[1], PART Working Group[2], Edward Lee[1], John Crary[2], and F. Bradley Johnson[1], *1. University of Pennsylvania, Philadelphia, Pennsylvania, United States, 2. Icahn School of Medicine at Mount Sinai, New York City, New York, United States*

Aging is a major risk factor for hyperphosphorylated tau burden (p-tau). Telomeres lengths (TL) shorten with age and various sources of DNA damage, thus provide a measure of biological age. Additionally, DNA methylation (DNAm) changes over time and may contribute to changes in TL. We hypothesize that shorter TL will be associated with increased risk of p-tau burden and that this process may be mediated by DNAm. We extracted DNA from frontal cortex of 113 individuals (Age=87.3 + 9.3; 37% Female) that met neuropathological criteria for primary age-related tauopathy (PART), characterized by p-tau in the absence of amyloid pathology. We measured mean TL using qPCR to determine the copy number of telomere repeat DNA in comparison to a single copy gene. We also measured DNA methylation using the Illumina MethylationEPIC Kit for ~850K CpGs. P-tau was measured in medial temporal cortex using an Aperio Digital Pathology Slide Scanner. Linear regression revealed that shorter TL was associated with increased p-tau burden (B=-0.28; p=-.003), including adjustment for age (B=0.003; p=0.003). eWAS identified six CpGs associated with TL (all q< 0.05). Causal mediation analyses identified that two of these CpGs mediate the TL and p-tau association: proportion mediated by cg08701686 (UNC5D) and cg24533059 (near IFNGR1 and OLIG3) was 32.5% and 48.6%, respectively. Shorter TL is associated with increased p-tau pathological burden in PART and may be mediated in part by DNAm at particular loci. These findings support the concept that biological aging, as measured with TL and DNAm, may contribute to tauopathy beyond chronological aging.

### SPERMIDINE TOXICITY IN MITOCHONDRIAL DNA-DEFICIENT SACCHAROMYCES CEREVISIAE

Wei-Hsuan Su, Omar Ocegueda, Catherine Choi, Jessica Smith, Kelsey Lee, Yihan Wan, Jacqueline Yao, and Sam Schriner, *University of California, Irvine, Irvine, California, United States*

Mitochondrial dysfunction is thought to play a significant role in aging and in manyhuman diseases. Over the last 20 years or so, a number of drugs have been found toextend lifespan in model organisms. Using ethidium bromide to deplete the yeastSaccharomyces cerevisiae of its mitochondrial DNA (mtDNA), we evaluated thedependence on functional mitochondrial in the action of five of these lifespan-extending compounds; dinitrophenol, metformin, rapamycin, resveratrol, andspermidine. None of them extended lifespan in mtDNA-deficient cells, demonstratinga requirement for functional mitochondria in their action. However, we found thatspermidine significantly shortened lifespan in these cells, decreasing the medianlifespan from 6 days to 4 days. Despite this, spermidine, nor any of the othercompounds tested, had any effect of growth rates in mtDNA-deficient cells. Spermidine is thought to extend lifespan through the induction of autophagy. Wepredict that spermidine shortened lifespan in mtDNA-deficient cells through anincreased need for ATP, for which these cells were not able to provide. Given thatmitochondrial dysfunction might be a common feature of aging and disease, ourresults suggest that if spermidine were used as an anti-aging treatment in humans, itmay be harmful.

### ETHNICITY-SPECIFIC EXTREME LONGEVITY VARIANTS IN A CONSORTIUM OF FOUR CENTENARIAN STUDIES

Harold Bae[1], Anastasia Gurinovich[2], Zeyuan Song[3], Cristina Giuliani[4], Marianne Nygaard[5], Nir Barzilai[6], Annibale Puca[7], and Paola Sebastiani[2], *1. Oregon State University, Corvallis, Oregon, United States, 2. Tufts Medical Center, Boston, Massachusetts, United States, 3. Boston University, Boston, Massachusetts, United States, 4. University of Bologna, Bologna, Emilia-Romagna, Italy, 5. University of Southern Denmark, Odense, Syddanmark, Denmark, 6. Albert Einstein College of Medicine, Bronz, New York, United States, 7. IRCCS Multimedica, Milan, Lombardia, Italy*

It has been shown that some longevity variants including the APOE variants have ethnicity-specific effects on EL within European ethnicities. The goal of the present study is to identify genetic variants whose effects vary by ethnicity by conducting a genome-wide association study of extreme longevity (EL: defined as living past the age at which less than 1% individuals from the 1900 - 1920 birth year cohorts survived) that includes the SNP-by-ethnicity interaction terms using a consortium of four centenarian studies: the New England Centenarian Study, the Long Life Family Study, the Southern Italian Centenarian Study, and the Longevity Gene Project. We used the Uniform Manifold Approximation and Projection (UMAP), a non-linear dimension reduction technique, to identify distinct ethnic clusters. The UMAP analysis revealed four distinct ethnic groups (Danish, Italian, Ashkenazi Jewish, and middle European) in the aggregated data set with 2223 cases and 5673 controls. Using a mixed effects logistic model with SNP-by-ethnicity interaction terms, we found 29 loci, in which the test of any interaction effect produced p< 10-5. The results showed that some variants had ethnicity-specific effects on EL. We sought for replication in two independent studies of longevity: the Danish Longevity Study and the Italian Longevity Study. In the Italian Longevity Study, rs7907949 (PFKP), rs11667516 (intergenic: RAB11B;MARCHF2), rs13245505 (intergenic: ZC3HC1;KLHDC10) replicated with a nominal significance level of 0.05. In the Danish Longevity Study, rs79853795 (PLCB1) and rs4072601 (SLC39A11) replicated. Future drug development should account for ethnic-specific differences in the genetic effects for higher efficacy for more diverse populations.

### EVALUATING T-CELL AGE-RELATED IMMUNE PHENOTYPES IN THE CONTEXT OF BIOLOGICAL AGING IN HEALTH AND RETIREMENT STUDY

Ramya Ramasubramanian[1], Helen Meier[2], Sithara Vivek[3], Eric Klopack[4], Eileen Crimmins[5], Jessica Faul[2], Janko Nikolich-Žugich[6], and Bharat Thyagarajan[3], *1. University of Minnesota Twin Cities, Minneapolis, Minnesota, United States, 2. University of Michigan, Ann Arbor, Michigan, United States, 3. University of Minnesota Twin Cities, MINNEAPOLIS, Minnesota, United States, 4. University of Southern California, Leonard Davis School of Gerontology, Los Angeles, California, United States, 5. University of Southern California, Los Angeles, California, United States, 6. University of Arizona, Tucson, Arizona, United States*

Cellular changes in the adaptive immune system accompanies the aging process and contributes to an age-related immune phenotype (ARIP) characterized by decrease in naïve T (TN) cells and increase in memory T (TM) cells. However, a population level marker of ARIP associated with biological aging and age-related chronic conditions has not been evaluated previously. We developed two ARIP measures based on well understood age related changes in T cell distribution: TN/ (TCM (Central Memory) + TEM (Effector Memory) + TEFF (Effector)) or TN/ TM in CD4+ and CD8+ T cells. We compared them with more commonly used ARIP measures such as CD4/CD8 ratio and CD8+ TN cells by evaluating associations with chronological age and phenotypic age using linear regression and association with multimorbidity using multinomial logistic regression. CD8+ TN and TN/TM had the strongest inverse association with chronological age (beta estimates: -3.41, -3.61; p-value< 0.0001). CD4+ TN/TM had the strongest inverse association with phenotypic age (beta estimate = -0.74; p-value < 0.0001) after adjustment for age, sex, race and CMV serostatus. CD4+ TN/TM was inversely associated with co-occurring chronic conditions (odds ratio for 2 conditions and 3 conditions vs. 0 conditions: 0.74 (95%CI: 0.63-0.86) and 0.75 (95% CI: 0.63-0.90), respectively) after adjustment for age, sex, race, CMV serostatus, smoking, and BMI. CD4+ TN/TM had a stronger association with phenotypic age and age-related morbidity compared to other ARIP measures. Future longitudinal studies can help us evaluate if CD4+ TN/TM can predict risk of aging related outcomes.

### MEGA-ANALYSIS OF A CONSORTIUM OF FOUR CENTENARIAN STUDIES IDENTIFIES NOVEL EXTREME LONGEVITY VARIANTS

Harold Bae[1], Anastasia Gurinovich[2], Zeyuan Song[3], Anastasia Leshchyk[3], Mengze Li[3], Nir Barzilai[4], Thomas Perls[3], and Paola Sebastiani[2], *1. Oregon State University, Corvallis, Oregon, United States, 2. Tufts Medical Center, Boston, Massachusetts, United States, 3. Boston University, Boston, Massachusetts, United States, 4. Albert Einstein College of Medicine, Bronz, New York, United States*

The meta-analysis of extreme longevity (EL) conducted in 2017 using a consortium of four longevity studies—the New England Centenarian Study (NECS), the Long Life Family Study (LLFS), the Southern Italian Centenarian Study (SICS), and the Longevity Gene Project (LGP)—confirmed previous associations with APOE alleles and identified additional candidate genes. The current study builds upon this work. Using an aggregated set of the four studies (mega-analysis), we sought to identify additional longevity variants. The current study includes 234 additional cases with a total of 2304 cases (median age=103, age range=[96, 119]), defined as living past the age at which less than 1% individuals from the 1900 - 1920 birth year cohorts survived and approximately 6,000 controls. Approximately 10 million genotyped and imputed

SNPs that passed imputation quality score threshold of 0.7 and that had a minor allele count of 3 or greater were analyzed. We ran a mixed effects logistic model for EL adjusting for sex, top principal components, indicator for living in Denmark, indicator for living in Italy, and the genetic relationship matrix. The analysis identified 21 novel loci that are genome-wide significant, both common and rare, and these loci were not previously published. The genome-wide significant loci include genes such as ADGRL2 (p=6.46-15), HLA-DPA1 (p=1.06-8), GRK5 (p=2.01-8), TECTB (p=3.38-8), KCNQ1 (p=7.53-10), TEAD2 (p=9.53-10), and several intergenic regions. We are currently in the process of seeking replication of the top results in independent cohorts.

### MEDICAL MORBIDITY RISK IN A SAMPLE OF OLDER WOMEN WITH BINGE EATING

Savannah Hooper[1], Victoria Marshall[1], Preston Bridges[2], Sara Espinoza[3], Pamela Keel[4], Andrea LaCroix[5], Nicolas Musi[1], and Lisa Kilpela[1], *1. UT Health Science Center San Antonio, San Antonio, Texas, United States, 2. The University of Texas Health Science Center San Antonio, San Antonio, Texas, United States, 3. Sam and Ann Barshop Institute, UT Health San Antonio, San Antonio, Texas, United States, 4. Florida State University, Tallahassee, Florida, United States, 5. University of California, San Diego, La Jolla, California, United States*

Binge eating (BE; consuming an abnormally large amount of food in one sitting while feeling out of control) is the most common form of disordered eating among older women, and aging may confer greater risk for medical morbidities. While the clinical phenotype of older women with BE is unknown, understanding health impacts of BE on the aging process is important. The objective of this study was to describe physiological characteristics of older women (aged 60+) with clinical levels of BE (i.e., ≥weekly episodes). Participants (N = 21; M age = 66.0±4.59) underwent a DEXA, indirect calorimetry, anthropometric measurements, and fasting blood labs. 76% of participants reported a BE age of onset in midlife or later (age 42+); the sample mean BMI was 35.08±8.64, with an average waist-to-hip ratio of 0.90±.047. DEXA scans indicated 90.48% were classified as overweight or obese. Using age- and gender-matched norms, mean fat percentile was 84.75±17.43; mean total body fat percentage was 47.0±4.5. Mean Resting Energy Expenditure (kcal/lean mass) was 34.2±3.71; average respiratory quotient was 0.81±0.06. HbA1c values indicated that 61.90% had pre-diabetes or diabetes; 47.61% had high fasting blood glucose and 76% had high LDL cholesterol. Mean bone mineral density (BMD) fell within a healthy range (1.11 g/cm2±.14); 33.33% had a BMD z-score of less than zero. Cardiovascular comorbidities were rare in this sample (9.5% had standard hypertension). Overall, metabolic disturbances were prevalent, while a minority had cardiovascular or skeletal morbidities. Findings suggest that BE may interfere with healthy aging predominantly through metabolic morbidities.

### THE TOLL OF BINGE EATING ON AGING: THE PHYSICAL FUNCTIONING OF OLDER WOMEN WITH WEEKLY BINGE EATING

Savannah Hooper[1], Victoria Marshall[1], Preston Bridges[2], Sara Espinoza[3], Pamela Keel[4], Andrea LaCroix[5], Nicolas Musi[1], and Lisa Kilpela[1], *1. UT Health Science Center San Antonio, San Antonio, Texas, United States, 2. The University of Texas Health Science Center San Antonio, San Antonio, Texas, United States, 3. Sam and Ann Barshop Institute, UT Health San Antonio, San Antonio, Texas, United States, 4. Florida State University, Tallahassee, Florida, United States, 5. University of California, San Diego, La Jolla, California, United States*

Binge eating (BE) is defined as eating an unusually large amount of food while feeling a loss of control; BE prevalence rates range from 12-26% in older adult women. BE is closely linked with obesity, depression, and poor micronutrient intake, as well as with cardiovascular issues and pain in younger populations. Among older adults, such morbidities can negatively affect quality of life and mobility. Yet, little is known about the toll of BE on the aging body in terms of physical function. Thus, the current investigation assessed the physical functioning of older women (60+ years) with clinical levels of BE (i.e., ≥weekly BE). Participants (N = 21) underwent several physical function tests including lower extremity functioning (Short Physical Performance Battery; SPPB), endurance (Six-Minute Walk Test), and grip strength. The majority of participants (76%) had a BE age of onset in midlife or later (age 42+). The average BMI was 35.08±8.64, and 90.48% met criteria for overweight or obesity. On the SPPB, 47.62% scored < 10, suggesting mobility limitations and predicting all-cause mortality. Most women (90.48%) scored >1SD below the age/gender matched norm on the Six-Minute Walk Test (*M*=355.96±67.04) while 61.9% scored >1SD below the norm for grip strength (*M*=24.91±6.40). Results indicate that older women struggling with BE also demonstrate diminished physical functioning, which confers risk for future health problems and poorer quality of life. Our findings suggest that intervention development for older populations struggling with BE may need to incorporate physical rehabilitation to promote healthy aging in this population.

### IDENTIFICATION AND INVESTIGATION OF SENESCENT CELLS IN SKELETAL MUSCLE AGING

Xu Zhang, Leena Habiballa, Zaira Aversa, Yan Er Ng, Joao Passos, and Nathan LeBrasseur, *Mayo Clinic, Rochester, Minnesota, United States*

Skeletal muscle aging is marked by the loss and atrophy of resident fibers, and the accumulation of functionally diverse cell types including fibroblasts, adipocytes, and immune cells. Senescent cells amass in multiple tissues with advancing age where they contribute to aging, chronic disease, and physical decline. The role of senescence in mediating muscle aging has become a popular and sometimes contentious topic. However, to date, this concept has not been methodically tested. In this study, we characterized the changes in cell abundance and, importantly, cell-specific transcriptional profiles with skeletal muscle aging using scRNAseq. Interestingly, we identified a small population of p16 positive fibro-adipogenic progenitors (FAPs) which, upon further investigation using immunohistochemical methods, were found to express other senescence markers. This subpopulation of FAPs did not exhibit elevation in p21 levels with age. Instead, terminally differentiated myofibers were the source of the p21 increase. Myofibers with high p21 expression exhibit a strong inflammatory phenotype, which includes activated p53 signaling pathways together with strong cytokine-cytokine receptor interactions. We further identified large amounts of cross-talk between different cell types, suggesting that senescent FAPs and myofibers could contribute to skeletal muscle aging in a paracrine manner. Importantly, these observations in mice were confirmed in human samples, suggesting the strong translational power of these findings.

### IDENTIFICATION AND INVESTIGATION OF SENESCENT CELLS IN SKELETAL MUSCLE AGING

Xu Zhang, Leena Habiballa, Zaira Aversa, Yan Er Ng, Joao Passos, and Nathan LeBrasseur, *Mayo Clinic, Rochester, Minnesota, United States*

Skeletal muscle aging is marked by the loss and atrophy of resident fibers, and the accumulation of functionally diverse cell types including fibroblasts, adipocytes, and immune cells. Senescent cells amass in multiple tissues with advancing age where they contribute to aging, chronic disease, and physical decline. The role of senescence in mediating muscle aging has become a popular and sometimes contentious topic. However, to date, this concept has not been methodically tested. In this study, we characterized the changes in cell abundance and, importantly, cell-specific transcriptional profiles with skeletal muscle aging using scRNAseq. Interestingly, we identified a small population of p16 positive fibro-adipogenic progenitors (FAPs) which, upon further investigation using immunohistochemical methods, were found to express other senescence markers. This subpopulation of FAPs did not exhibit elevation in p21 levels with age. Instead, terminally differentiated myofibers were the source of the p21 increase. Myofibers with high p21 expression exhibit a strong inflammatory phenotype, which includes activated p53 signaling pathways together with strong cytokine-cytokine receptor interactions. We further identified large amounts of cross-talk between different cell types, suggesting that senescent FAPs and myofibers could contribute to skeletal muscle aging in a paracrine manner. Importantly, these observations in mice were confirmed in human samples, suggesting the strong translational power of these findings.

### SESSION 6050 (POSTER)

### BIOLOGY OF AGING: BEHAVIORAL AND SOCIAL SCIENCES SECTION I

### C-REACTIVE PROTEIN AND BRAIN MORPHOLOGY

Rahul Pearce[1], Lisa Eyler[1], Tyler Bell[2], Jeremy Elman[1], Christine Fennema-Notestine[3], Donald Hagler[1], Carol Franz[1], and William Kremen[1], *1. University of California San Diego, La Jolla, California, United States, 2. University of California, San Diego, La Jolla, California, United States, 3. University of California San Diego, San Diego, California, United States*

The process by which aging leads to increased risk for Alzheimer's disease and dementia is not entirely understood, but one hypothesized contributor is the occurrence of low-grade inflammation in older age. In this study, we examined how peripheral levels of C-reactive protein (CRP), a measure of systemic inflammation, relate to brain structure and diffusion in a group of older adult men. After excluding twenty-seven participants for confounding medical conditions, we analyzed a final sample of 426 community-dwelling men from the Vietnam Era Twin Study of Aging, who were assessed at average age 68 for plasma levels of CRP and underwent structural and diffusion brain imaging. Linear mixed models adjusting for family relatedness, age, medical morbidity, and BMI examined associations of CRP with whole brain volume, whole gray and white matter volumes, global fractional anisotropy (FA), and global mean diffusivity (MD). Higher CRP was related to lower whole brain volume ($\beta$ = -.13, p = .006), including lower whole white matter volume ($\beta$ = -.22, p <.001) but not whole gray matter volume (p = .08). Higher CRP was related to lower global FA ($\beta$ = -.51, p = .012) but not global MD (p = .203). Regionally, a relationship of higher CRP to lower FA was found in the anterior thalamic radiation ($\beta$ = -.51, p = .010), which is implicated in a variety of higher order cognitive processes. These results suggest a link between peripheral inflammation and lower white matter integrity in older adult men, the implications of which for cognitive aging and dementia should be further explored.

### SOCIODEMOGRAPHIC DIFFERENCES IN IMMUNOSENESCENCE IN OLDER AGE: EVIDENCE FROM THE HEALTH AND RETIREMENT STUDY

Grace Noppert[1], Rebecca Stebbins[2], Jennifer Dowd[3], and Allison Aiello[4], *1. University of Michigan, Ann Arbor, Michigan, United States, 2. King's College London, London, England, United Kingdom, 3. Leverhulme Centre for Demographic Science, University of Oxford, Oxford, England, United Kingdom, 4. Carolina Population Center, University of North Carolina at Chapel Hill, Chapel Hill, North Carolina, United States*

Population patterns of immunosenescence are not well described. We characterized markers of immunosenescence and assessed sociodemographic differences in a population of individuals ages 56 years and older using newly released venous blood data from the nationally representative U.S. Health and Retirement Study (HRS) (n=8,400). Median values of the CD8+:CD4+, effector memory (em)RA:naïve CD4+ and emRA:naïve CD8+ T cell ratios were higher among older participants (more aged immune profile) and were lower among those with additional educational attainment (less aged immune profile). Racialized minority populations had immune markers suggestive of a more aged immune profile: Hispanics had a CD8+:CD4+ median value of 0.37 (95% CI: 0.35, 0.39) compared to Whites (0.30, 95% CI: 0.29, 0.31). Blacks had the highest median value of the emRA:naive CD4+ ratio (0.08; 95% CI: 0.07, 0.09) compared to Whites (0.03; 95% CI: 0.028, 0.033). Our regression analyses showed that race/ethnicity and education were associated with large differences in T-cell markers of aging, which were orders of magnitude greater than age. By standardizing regression coefficients to estimate years of immunological aging, we found that each additional level of education was associated with roughly an additional decade of immunological age, and racialized minorities had on average an immunological age two to four decades higher than Whites. As one of the first large-scale population-based investigations of immunosenescence, our study advances understanding of the immune mechanisms underlying age-related disease, with implications for risks such as vulnerability to novel pathogens (e.g., SARS-CoV-2).

### INVESTIGATING LINKS BETWEEN CHILDHOOD HEALTH AND FAMILY LIFE AND ACCELERATED BIOLOGICAL AGING

Marina Larkina, and Jacqui Smith, *University of Michigan, Ann Arbor, Michigan, United States*

An epigenetic clock measure, DNA methylation (DNAm) PhenoAge, differentiates individual aging trajectories and has been shown to predict mortality and morbidities. Despite suggestions that early-life experiences may shape epigenetic aging, little is known to date about specific risk and protective factors. We use data from Health and Retirement Study (HRS) to investigate the role of childhood factors, including health and family financial situation, in epigenetic age acceleration. The sample (N = 3952, M age = 64, range 50-100) included participants from the HRS 2016 Venous Blood Study and those, who reported about their childhood health and family. Using logistic regression, we predicted DNAm PhenoAge acceleration, calculated as the residuals resulting from regressing DNAm PhenoAge on chronological age and coded as 0 or 1 (1 = positive values, faster epigenetic aging rate). Participants with more years of education were less likely to have accelerated epigenetic aging (OR: 0.963, 95%CI[0.941-0.985], $p < .001$). However, self-reported chronic illnesses before age 16 (15 possible conditions), self-rated childhood health, and family financial situation before age 16 were not associated with accelerated aging. In addition, men had higher likelihood of accelerated aging than women (OR: 1.145, 95%CI[1.007-1.301], $p < .05$). Race/ethnicity and age cohort (e.g., being younger or older age 65) were not significant predictors. Our results highlight that investigation of relation between childhood disadvantages and DNAm PhenoAge acceleration might need to include other indicators (e.g., residential history). Future work is needed also to identify life course moderators of the clock efficacy.

### GENETIC ARCHITECTURE OF SUBJECTIVE HEALTH: RELATIONSHIP WITH PHYSICAL HEALTH, COGNITION, AND DEPRESSION.

Deborah Finkel[1], and Margaret Gatz[2], *1. Indiana University Southeast, New Albany, Indiana, United States, 2. University of Southern California, Los Angeles, California, United States*

The fact that self-rated health (SH) predicts mortality and a variety of other health outcomes independent of objective health measures generates questions about mechanisms and etiologies. SH can be considered an indicator of physical health, per se, resulting from active cognitive processing of explicit information about one's own health and intuitive knowledge of symptoms and physical sensations. The extent to which SH taps shared cultural ideas about health should be reflected in estimates of the shared environmental component of variance (C). SH has also been associated with emotional health measures, such as neuroticism and depression. Previous analyses have been limited by sex (only women), sample size, age (range = 63-76), and failure to include cognitive function. The current analysis used data from 8291 adults ranging in age from 22 to 102 from the international Interplay of Genes and Environment Across Multiple Studies (IGEMS) consortium to investigate the genetic architecture of SH. Genetic influences on self-rated health (SRH) were investigated in the context of CIRS (Cumulative Illness Rating Scale), MMSE (Mini-Mental Status Exam), and depression (CES-D or CAMDEX). Independent pathways modeling indicated that all genetic variance for SRH was shared with CIRS, MMSE, and depression. Comparison of groups older and younger than 74 indicated age differences in genetic architecture of SRH. Evidence suggests that the discordance between objective and subjective health increases in late adulthood, possibly as a result of greater emphasis on psychological rather than physical components of subjective health assessments by older adults.

### TOWARD A NEUROECOLOGICAL MODEL OF FINANCIAL CAPACITY AMONG OLDER ADULTS

Michael Barnett[1], Samuel Van Vleet[2], Kailee Prentice[1], Annika Wurm[1], Danica Bass[1], Yenifer Morales Mejia[1], Jeanné Dube[1], and Samuel Van Vleet[2], *1. The University of Texas at Tyler, Tyler, Texas, United States, 2. Miami University, Oxford, Ohio, United States*

In recent decades, technology has changed how individuals interact with their money and with each other. A combination of financial vulnerability and low technological literacy puts many older adults at risk for identity theft, fraud, and financial exploitation. We reviewed the literature on financial capacity, financial exploitation, and digital literacy. Extant models and measures of financial capacity among older adults emphasize numeracy and basic functional skills, such as writing checks and counting change; these may not reflect the digital nature of contemporary financial activity. We propose the neuroecological model of financial capacity among older adults. This function-led model contends that financial capacity consists of neurocognitive abilities to make sound financial decisions in a complex environment, to use technology to monitor and carry out financial activities, and the ability to protect personal information and guard against fraud. This points to a need for more ecologically valid measures of financial capacity and vulnerability to financial exploitation that addresses the role of technology in everyday financial activities.

### SESSION 6060 (POSTER)

### ALZHEIMER'S DISEASE AND RELATED DEMENTIAS (SRPP)

### PLACE OF DEATH FOR PEOPLE WITH DEMENTIA: EVIDENCE FROM THE CDC WONDER MORTALITY DATA

Takashi Amano, *Rutgers University-Newark, Newark, New Jersey, United States*

Previous studies have found that patients with dementia experience poorer end-of-life care compared to patients with cancer. Dying in the preferred place has become a common measure of the quality of end-of-life care, and it has been consistently reported that the majority of people prefer to die at home. Thus, this study examines whether dying from dementia is a significant determinant of the place of death in mortalities among older adults. The Mortality Data on Center for Disease Control and Prevention (CDC) Wide-ranging Online Data for Epidemiologic Research (WONDER) between 2010 and 2019 were utilized. This study examined whether dying from dementia was associated with place of death (hospital, home, hospice facility, nursing home/long-term care facility) among deaths at 65 years or older. A multinomial logistic regression analysis was performed to estimate the association adjusted for covariates. We analyzed a total of 15,855,034 death records of which 12.34% were dementia deaths. The percentage of deaths at nursing homes was higher in deaths from dementia (56.5%) than other deaths (21.85%), whereas percentages of death at home were similar (20.7% for dementia death and 29.1% for other deaths). Multinomial logistic regression revealed that dementia deaths were 2.74 times more likely to occur at nursing homes than at hospitals compared to other deaths. Results suggest that dementia deaths are more likely to occur at nursing homes/long-term care facilities than other deaths. Further research should investigate the ways to improve the quality of end-of-life care for people with dementia in nursing homes/long-term care facilities.

### GOOD ALZHEIMER'S CARE DEFINED BY FAMILY CAREGIVERS AND FORMAL CAREGIVERS IN KOREA

Gawon Chung, *Korean Women's Development Institute, Seoul, Seoul-t'ukpyolsi, Republic of Korea*

The purpose of this study is to explore the meaning of good Alzheimer's care and the policy needs from the perspective of family caregivers and formal caregivers(in long-term care settings) in Korea and to provide policy suggestions to mainstream "care" and "caregivers" into the Korean National Dementia Plan. By using mixed methods, the Korean Government's policy efforts under the National Dementia Plan to respond to the issue based on Alzheimer's patients' individual needs and care environments and to alleviate family's caregiving burden are analyzed. Also, the formal caregivers' working conditions, difficulties, and the level of knowledge/expertise are examined to find out whether they are well prepared for providing good Alzheimer's care. The findings reveal that both family caregivers and formal caregivers desire Alzheimer's care experts' consulting and honest communication among the care triads. Family caregivers want formal caregivers not to infantilize the patients, to provide more cognitive services based on their individual history and characteristics. Also, the vulnerabilities of patients' spouses are identified that in many cases there is no one other than the spouse who can provide care for the patient, their self-rated health status is poorer, and their tendency to seek professional help is relatively lower. Formal caregivers want the Alzheimer's patient-formal caregiver ratios to be lowered for better care and their Alzheimer's care expertise to be acknowledged and properly compensated. Thus, it is crucial to reflect the experiences of the care triads(patients, family caregivers, and formal caregivers) in the Korean National Dementia Plan more rigorously.

### LEARN, PLAN, DO: PUBLIC HEALTH PLANNING TO ADDRESS DEMENTIA AND CAREGIVING

Talyah Sands[1], Priya Shah[1], Kelsey Donnellan[2], and Heidi Holt[3], *1. Association of State and Territorial Health Officials, Arlington, Virginia, United States, 2. Assoc. of State and Territorial Health Officials (ASTHO), Arlington, Virginia, United States, 3. CDC, Atlanta, Georgia, United States*

To respond to growing rates of dementia, as well as emerging scientific evidence, there is an urgent need for states to leverage public health strategies to reduce the risk of dementia, promote early detection, and prevent avoidable health issues related to dementia as well as systematically support dementia caregiving. The BOLD Infrastructure for Alzheimer's Act passed into law on December 31, 2018. The law is designed to promote implementation of CDC's Healthy Brain Initiative State and Local Public Health Partnerships to Address Dementia: The 2018-2023 Road Map Series and the Healthy Brain Initiative Road Map for Indian Country. Its goal is to create jurisdiction infrastructure and promote planning and action to address dementia across the life-course. With support from CDC, the Association of State and Territorial Health Officials developed an e-learning module to guide public health departments on engaging partners in jurisdiction planning on dementia. The module aligns with the Healthy Brain Initiative Road Map and offers approaches to operationalize health equity throughout its resources. During this session, presenters will overview the module contents and detail the process developed to help jurisdictions assess their dementia and caregiving needs, prioritize actions from the Road Map, and create an action plan. They will also share examples of state planning efforts. Participants will learn how to get started with their own planning process using the tools offered in the module and examples from other states. This presentation complements Tools to Support Needs Assessments on Dementia, Cognitive Health, and Caregiving, abstract ID 1226183.

### LEVERAGING NATURAL LANGUAGE PROCESSING TO IDENTIFY CAREGIVER AVAILABILITY FOR PATIENTS WITH ALZHEIMER'S DISEASE

Elham Mahmoudi, Wenbo Wu, Cyrus Najarian, James Aikens, Julie Bynum, and Vinod Vydiswaran, *University of Michigan, Ann Arbor, Michigan, United States*

Identifying caregiver availability for patients with Alzheimer's disease and related dementia (ADRD) can inform healthcare systems and hospital administrations on appropriate discharge planning. However, caregiver availability data is not systematically collected in the healthcare system. Our objective was to use medical notes (from physicians, nurses, or social workers) to assess caregiver availability and types of caregivers for hospitalized patients with ADRD. We used 2016-2019 telephone-encounter notes from a single institution. Rule-based natural language processing (NLP) was used to develop an algorithm to identify whether the patient (1) resides at home; (2) resides at an institution; (3) has a formal caregiver; and (4) has an informal caregiver. We evaluated the data at the note level and used chart abstraction as the "gold standard." We examined the validity of the algorithm for both training (n=749) and test sets (n=227). Our results indicated a high level of accuracy and reliability for identifying an informal caregiver (F1=0.942; accuracy=0.947, sensitivity=0.970, and specificity=0.928). Identifying whether the patient lived at an institution was the least reliable measure (F1=0.638, accuracy=0.899, sensitivity=0.512, specificity=0.978). The most common causes of misclassifications were: (1) incomplete or misspelled names of the facilities; (2) past/uncertain/undecided situations; (3) lack of specificity; (4) use of uncommon abbreviations; and (5) irregular use of templates. Our NLP algorithm was able to identify whether the patient lived at home vs. an institution and whether the patient had a formal or informal caregiver. There is merit in continuing the NLP approach to identify more granular, caregiver-related information (e.g., hours available).

### THE SENIOR COMPANION PROGRAM PLUS: A PROMISING LAY PROVIDER MODEL FOR ADRD FAMILY CAREGIVING

Ling Xu[1], Noelle Fields[2], Ishan Williams[3], Joseph Gaugler[4], and Daisha Cipher[2], *1. University of Texas at Arlington, Arlington, Texas, United States, 2. The University of Texas at Arlington, Arlington, Texas, United States, 3. University of Virginia, School of Nursing, Charlottesville, Virginia, United States, 4. University of Minnesota, Minneapolis, Minnesota, United States*

Considerable disparities exist in the prevalence of Alzheimer's Disease and related dementia (ADRD) in older African American (AA) populations compared to older white populations. Given the need for caregiver interventions for family caregivers from racial and ethnic minority groups that hold potential for translation, implementation, and sustainability in their communities, peer-led, lay provider models may offer promise. We developed the Senior Companion Program Plus (SCP Plus), a peer-led psychoeducational program for AA family caregivers (CGs) provided by Senior Companions (SCs). Guided by sociocultural stress and coping model, the study was a randomized controlled trial that assessed the effects of SCP Plus on AA ADRD family caregivers' stress and burden, coping skills, and social support. Although COVID-19 ended the trial early, study participants included Senior Companion-Caregiver dyads (N=20). Friedman tests for non-normally distributed variables, one-way repeated measures ANOVA for normally distributed variables, and post hoc between group significance tests were conducted. CGs in SCP Plus group reported significantly decreased caregiving burden ($F(2,6) = 17.65$, $p = 0.003$) especially between pre- and follow up-tests, as well as increased coping skills, especially at post-test ($F(2,6) = 4.93$, $p = 0.05$), satisfaction with social support ($\chi 2(2) = 6.53$, $p = 0.04$), and positive aspect of caregiving ($\chi 2(2) = 6.53$, $p = 0.04$) (especially between pre- and post-tests as well as between pre- and follow up-tests). Implications for future research are offered as well as lessons learned for culturally congruent, lay provider interventions for ADRD family caregivers.

### EFFICACY OF A BRAIN HEALTH PROGRAM FOR OLDER MICHIGANDERS: SINGLE-GROUP DESIGN FINDINGS

Ha-Neul Kim, Fei Sun, and Paul Freddolino, *Michigan State University, East Lansing, Michigan, United States*

This study aims to examine the effectiveness of a brain health education intervention addressing four areas: dementia false beliefs, Alzheimer's disease knowledge, confidence in managing cognitive changes, and dementia worries among older adults. A quasi-experimental design was used with 6 groups of participants of older adults in Michigan who received the intervention over an 8-week period via Zoom. Participants (n=39) included male = 8, female = 31; Caucasian = 19, African American = 20 that were 60 or older (M = 71.74, SD = 6.80). Participants' MoCA score ranged from 18 to 30 (M = 26.36, SD = 2.43). Paired t-tests were used to compare pre-post differences on four main areas. At post-test, participants reported decreased false beliefs to dementia ($p<.001$); increased Alzheimer's disease knowledge ($p=.003$); and improved confidence in managing cognitive changes ($p=.018$) compared to their baseline scores. However, although the mean score of 'dementia worries' decreased on post-test (pre-test: 15.67, post-test: 14.53), it did not show statistical significance ($p=.33$). The 8-week brain health intervention showed a positive impact on lessening participants' false beliefs on dementia, and enhancing knowledge of Alzheimer's disease and confidence in managing their cognitive changes. The findings of this study laid the groundwork for future studies that use a larger sample size and randomized control trial design to test brain health education programs among older adults.

### QUALITY CARE AND CONFLICTING RIGHTS AMONG RESIDENTS WITH DEMENTIA, FAMILIES, AND STAFF IN LONG-TERM CARE FACILITIES

Angela Perone, *University of California - Berkeley, Berkeley, California, United States*

Conflicting rights between residents with dementia and staff pose unique problems for quality of care in long-term care (LTC) facilities. Residents relying on staff for care may be afraid to express concerns. A disproportionate female (and often women of color) staff may be exposed to discrimination that stems from a resident's loss of executive functioning and inability to control emotions. Family often find themselves somewhere in the middle. This study, thus, poses two research questions: (1) How do residents, families, and LTC staff understand their rights when conflicts arise between residents with dementia and staff?; (2) How can we leverage collective expertise of residents, families, and LTC staff to deliver a more holistic approach for addressing these conflicts? To answer these questions, I employed a multi-method qualitative design using semi-structured interviews (n=90) and participant observation (n=8 months). Building on legal consciousness theory—which explains how individuals invoke (or do not invoke) legal principles to define everyday experiences—findings revealed that staff rarely invoked legal framings to describe interactions whereas residents readily invoked rights rhetoric. Families mostly avoided rights rhetoric and instead focused on storytelling. All groups (residents, staff, and families) employed an array of emotional literacy and life experiences to navigate conflicts that coalesced around empathy. This paper concludes with research, policy, and practice suggestions for invoking principles of empathy to advance quality of care for residents with dementia by improving interactions among residents, staff, and family and workforce conditions for staff that flow from these interactions.

### EXPLORING RACIAL DISPARITIES IN AD/ADRD RISK: EVERYDAY DISCRIMINATION AS A SOCIAL STRESSOR

Katharine Gary, Masudul Hoque, and Igor Akushevich, *Duke University, Durham, North Carolina, United States*

Using evidence from the Health and Retirement Study (HRS), we explore racial disparities in Alzheimer's Disease and Related Dementia (AD/ADRD) risk. From a stress process perspective, there is substantial evidence in the literature that everyday discrimination is a chronic strain for Black individuals that acts as a social determinant of illness. However, few studies have examined specific relationships between this social stressor, race and AD/ADRD risk. Using generalized linear regression and Cox proportional hazard models, we explored racial differences in exposure and vulnerability to everyday discrimination. Results suggest that Black individuals experience more frequent exposure to everyday discrimination than their counterparts, and this chronic strain predicts AD/ADRD risk. However, contrary to the stress process model, White respondents were more vulnerable to the effect of everyday discrimination on AD/ADRD risk. Racial bias from medical professionals during the diagnostic process as well as mortality selection bias may explain these unexpected findings, especially given that racial minorities who experience high rates of everyday discrimination may not live long enough to reach the average age of diagnosis. These results provide further evidence that discrimination is a key factor in explaining racial disparities in late-life disorders, while also taking into consideration that many racial minorities with high rates of this type of stress may not survive to late-life.

### LOS ANGELES COUNTY BOLD INITIATIVE: LAYING THE GROUNDWORK FOR STRATEGIC PLANNING ON DEMENTIA

Tony Kuo[1], Noel Barragan[2], Mariana Reyes[2], Mateeha Zafar[3], Cinthie Lopez Paz[3], and Ariane Thomas[2], *1. University of California, Los Angeles, Los Angeles, California, United States, 2. Los Angeles County Department of Public Health, Los Angeles, California, United States, 3. Los Angeles County Department of Workforce Development, Aging and Community Services, Los Angeles, California, United States*

Funded by the Centers for Disease Control and Prevention's Building Our Largest Dementia Public Health Program through U.S. Congressional appropriations for the BOLD Infrastructure for Alzheimer's Act, the Los Angeles County BOLD initiative (LA BOLD) was established in 2021 with the goal of addressing the silent but burgeoning epidemic of Alzheimer's disease and related dementias (ADRD) in Los Angeles County (LAC). In 2019, approximately 166,857 LAC residents were living with Alzheimer's disease; this number is expected to increase 150% by 2040. The initiative brings together a diverse group of multi-sector stakeholders, including two Area Agencies on Aging, two major academic centers, the largest Medicaid health plan in the region, the county's safety net health agency (Public Health, Health Services, Mental Health), and other key community partners. The primary objectives of LA BOLD are: (i) establish a countywide coalition to address ADRD locally, and (ii) develop a strategic plan to guide the coalition's efforts. The present study describes the first year of this initiative, highlighting the formation of the coalition's steering group, identifying the three focus areas for strategic planning – risk reduction, early detection, advance care planning – and sharing lessons learned from navigating the contextual realities of ADRD care and prevention in LAC's diverse communities. Data and program accomplishments for this early phase of LA BOLD are discussed within the context of medical advancement, the financial realities of ADRD programming and services, and the geopolitical factors that are being considered as part of the strategic planning and implementation processes.

### UTILIZING BOOT CAMP TRANSLATION TO ENCOURAGE HISPANIC/LATINX PARTICIPATION IN ALZHEIMER'S RESEARCH

Stacey Moeller[1], Samantha John[1], and Luis Medina[2], *1. University of Nevada, Las Vegas, Las Vegas, Nevada, United States, 2. University of Houston, Department of Psychology, Houston, Texas, United States*

Hispanic/Latinx (H/L) individuals are 50% more likely to develop Alzheimer's disease (AD), relative to non-Hispanic whites. Despite higher prevalence rates and earlier age of onset, H/L individuals are underrepresented in aging and AD research. The Engaging Communities of Hispanics for Aging Research (ECHAR) Network is an interdisciplinary, multi-site project that aims to address these disparities. ECHAR uses Boot Camp Translation (BCT), a community-based participatory research (CBPR) approach that partners research faculty and medical experts with community stakeholders to translate medical jargon into action-based, locally-relevant messages. Researchers at University of Nevada, Las Vegas recruited 14 community members to participate in an AD-specific BCT. The process was completed between June 2021 through January 2022 to identify: 1) key messages, 2) target audience(s), and 3) dissemination strategies. BCT members identified three messages to reflect community concerns and highlight values: 1) "Amar es saber" ("To love is to know"), which acknowledges the importance of family and addresses cultural norms that may discourage disease conversations; 2) "The community cares. Let's talk."; and 3) "Podemos afrontar el Alzheimer juntos." ("We can face Alzheimer's disease together."). Target audiences include younger and middle-age adults who may be or become caregivers and local non-profit and community organizations. Members identified social media, community education events, community influencers, and educational products as dissemination strategies. BCT is an effective tool for engaging local communities and exploring Hispanic/Latinx beliefs and attitudes toward AD. It leverages community member expertise to create meaningful, tailored, and culturally-relevant messages that address health disparities and encourage action.

### RESULTS FROM A PLATFORM-BASED CLINICAL TRIAL FOR PERSONS DIAGNOSED WITH MILD COGNITIVE IMPAIRMENT

Allison Gibson, Caitlin Pope, Kelly Parsons, Elizabeth Rhodus, Mark Huffmyer, Julia Johnston, and Gregory Jicha, *University of Kentucky, Lexington, Kentucky, United States*

The process of receiving a diagnosis of mild cognitive impairment (MCI) is overwhelming. Individuals may experience adaptive or maladaptive responses to the diagnosis. Five specific areas of maladaptive response were previously identified, including 1) failure to plan for future decline, 2) decreased compliance and interaction with medical care providers, 3) decreased confidence and reduced social engagement, 4) increased physical limitations and mobility, and 5) decreased medication compliance. This pilot study reports on the delivery of the platform-based trial for persons diagnosed with MCI with survey data and qualitative focus groups data (n=38). The study consisted of a single-site platform trial examining the intervention group. Using this approach allowed the participants to explore different biopsychosocial arms of the intervention. Second, the platform design allowed researchers to determine the effects of the interventions on patient help-seeking and adherence behavior in real-world care. Feasibility, opportunities, and challenges will be discussed. Opportunities include the group's cohesion with the group-based intervention, which increased engagement for study group participation. Additionally, participants were most susceptive to intervention components that were novel (i.e., mindfulness) and administered by a professional (i.e., pharmacist, physical therapist) as opposed to self-facilitated activities. Challenges include frequency of study visits, the study partner requirement, and the in-person delivery of the intervention. These challenges were further compounded by the COVID-19 pandemic. Findings from this study offer considerations in implementing support programming and clinical research for persons diagnosed with MCI. The presentation will also include discussion on COVID-19 pandemic-related protocol modifications of this intervention study.

### BUILDING A COUNTYWIDE ALZHEIMER'S DISEASE AND RELATED DEMENTIA RESOURCE INVENTORY

Mariana Reyes[1], Noel Barragan[1], Tony Kuo[2], and Ariane Thomas[1], *1. Los Angeles County Department of Public Health, Los Angeles, California, United States, 2. University of California, Los Angeles, Los Angeles, California, United States*

As a BOLD Public Health Program grantee, the Los Angeles County (LAC) Department of Public Health (DPH) was funded to translate national ADRD priorities to the local level. Guided by the social ecological framework, DPH aims to address ADRD by engaging a multi-pronged approach that meaningfully considers the intersection of social, political, and environmental realities when developing and implementing key strategies. As part of this effort, DPH developed a resource inventory and simultaneously created a dedicated website to host and disseminate this information. The target audience for this resource inventory includes the general public; people living with ADRD and their caregivers and families; health professionals; and community-based and allied health professionals. The resource inventory encompasses a wide range of educational information, training materials, local programs, and services related to ADRD such as trainings for healthcare providers to strengthen their knowledge and ability in screening and diagnosing ADRD and legal/financial planning tips for caregivers. In developing the inventory's scope, DPH relied on multi-sector partners to understand the needs of LAC's diverse communities especially keeping in mind communities disproportionately impacted by ADRD (e.g., Black and Latinx communities). Stakeholders highlighted the need to ensure that resources addressed both ADRD topics and critical community needs related to the social determinants of health. The final inventory drew extensively from a wide range of existing national, state, and local resources. This presentation will outline DPH's resource inventory development process and share how results will be used to identify resource gaps and inform local ADRD-focused efforts.

### THE IMPACT OF A FACILITATED DISCUSSION SERIES IN IMPROVING DEMENTIA CAREGIVER STRESS, RESILIENCY, AND WELL-BEING

Robin Bonifas[1], and Wendy Cohen[2], *1. Indiana State University, Terre Haute, Indiana, United States, 2. Duet: Partners in Health & Aging, PHOENIX, Arizona, United States*

Emotional stress stemming from the complex grief associated with dementia care takes a greater toll on a family caregiver's health than the physical effort of providing care. Finding Meaning and Hope, a 10-week workshop for caregivers of persons with dementia designed to promote understanding of grief and teach self-care concepts that mitigate stress. Each session features a video of a dementia care expert sharing strategies, followed by a facilitator-led discussion. Home practice activities help caregivers incorporate focal self-care strategies into their lives. This evaluation research assessed the association between workshop participation and caregiver outcomes in stress and well-being. 208 caregivers, 83 percent age 60 and older, completed the workshops between 2019 and 2021. Two standardized instruments were administered before and after workshop participation: the 12-item Caregiver Self-Assessment Questionnaire (American Medical Association, 2015) and the 11-item Sense of Competence Questionnaire (Vernooij-Dassen, 2008). Multivariate Linear Regression was used to assess factors associated with participant outcomes. Participation in more than half the sessions had a positive impact on stress management, emotional well-being, and coping, but fewer gains were made by caregivers with health limitations and those who began the workshops with lower emotional well-being ( $F = 15.923$, $p < .001$). Given that family caregivers' mental and emotional health has been negatively impacted by the COVID-19 pandemic (Czeisler et al. 2020), strategies to manage stress and bolster well-being are vital in enabling them to continue care provision when isolation may exacerbate the challenges they encounter. Finding Meaning and Hope offers a promising approach.

### THE ROAD MAP STRATEGIST INITIATIVE: A NATIONWIDE EFFORT TO BUILD LOCAL PUBLIC HEALTH CAPACITY ON DEMENTIA

Meghan Fadel[1], Peter Holtgrave[2], Shelby Roberts[3], Eva Jackson[3], John Shean[3], Lisa Garbarino[4], and Lisa McGuire[5], *1. Alzheimer's Association, Albany, New York, United States, 2. National Association of County and City Health Officials (NACCHO), Washington, District of Columbia, United States, 3. Alzheimer's Association, Chicago, Illinois, United States, 4. Centers for Disease Control and Prevention, Atlanta, Georgia, United States, 5. CDC, Atlanta, Georgia, United States*

The Road Map Strategist Initiative is a nationwide effort to build local health department (LHD) capacity to address cognitive health and dementia. Through a collaboration between the Alzheimer's Association and the National Association of County and City Health Officials (NACCHO), the initiative aims to make policy, systems, and environmental change, with a focus on health equity. Participating health departments receive funding, training, and technical assistance to establish a part-time Healthy Brain Initiative (HBI) Road Map Strategist, a public health professional serving as a system change agent. With support and education from the program sponsors, Road Map Strategists conduct a community health needs assessment, train LHD staff and leadership, and collaborate with community partners to implement and accelerate public health actions on dementia. Actions taken by the Road Map Strategists are informed by the Healthy Brain Initiative's State and Local Public Health Partnerships to Address Dementia, The 2018-2023 Road Map. Presenters will share lessons learned from the first cohort of eight (8) LHDs during each stage of the initiative, with a focus on approaches used for Road Map implementation efforts in their communities. This session will share evaluation findings on successes and barriers for changing health department capacity to support local dementia efforts. Data from key indicators related to knowledge gain and capacity change will be shared, including the way LHD characteristics influenced success. The session will also cover effective approaches toward advancing health equity in dementia in local communities and plans for future iterations of the initiative.

### SESSION 6070 (POSTER)

### ALZHEIMER'S DISEASE, FAMILY CAREGIVING, AND SOCIAL RELATIONSHIPS

### CORRELATIONS OF GPS-BASED COMMUNITY MOBILITY METRICS BETWEEN PERSONS WITH DEMENTIA AND FAMILY CAREGIVERS

Jane Chung, Joseph Boyle, and David Wheeler, *Virginia Commonwealth University, Richmond, Virginia, United States*

Currently, the majority of dementia care is provided at home by informal caregivers. Most informal caregivers share a routine with their loved ones and change their activity patterns to adapt to a new routine of persons with dementia (PWDs). Given the dementia caregiving context, caregivers' mobility behaviors and PWDs' mobility may be positively associated. This study aimed to characterize patterns of GPS-derived community mobility in dementia dyads and examine relationships between PWDs' and caregivers' mobility patterns. Six dyads wore a GPS data logger inside and outside the home for 8-11 days. Twelve participants generated valid GPS track files (N=110). Four temporal and spatial mobility metrics were derived from GPS data (total distance, time use, median speed, and convex hull area). Then we calculated Pearson correlation coefficients between PWDs and their caregivers over all tracks. All dyads made active out-of-home trips, indicated by mean daily distance (range: 6,198 - 115,592m for PWDs; 5,125 - 108,857m for caregivers). Median speed of movement ranged from 0.09 to 1.29 km/hour for PWDs, and from 0.21 to 0.97 km/hour for caregivers. The mean size of convex hull over the monitoring period indicates a limited space usage level in both PWDs and caregivers, meaning restricted community mobility despite relatively large distance trips. The correlation coefficient was positive and significant for each metric ($r = 0.70$-$0.97$, $p < .001$). These results suggest substantial agreement in the mobility metrics between PWD and their caregivers, indicating a high level of dyadic effects of a partner's experience of community mobility.

### SOCIAL CONNECTIVITY IS ASSOCIATED WITH INCREASED PERFORMANCE ON MEDIAL TEMPORAL LOBE FUNCTION IN OLDER AFRICAN AMERICANS

Genesis Tan[1], Zuzanna Osiecka[1], Bernadette Fausto[1], Darlingtina Esiaka[2], and Mark Gluck[1], *1. Rutgers University - Newark, Newark, New Jersey, United States, 2. Robert Wood Johnson Medical School, Rutgers University, Newark, New Jersey, United States*

Research links social connectivity to cognitive function in older adults. However, many of the studies relating social connectivity to cognitive function focused on global cognition—orientation/attention, memory, verbal fluency, language, and visuospatial ability domains. In this study, we aimed to assess the association of social connectivity with generalization, a domain of cognition which relies on hippocampal function, in a population of older African Americans residing in the Greater Newark area. Specifically, we examined the impact of social connectivity on generalization using sensitive measures that tap into medial temporal lobe (MTL) function. Participants (N = 74; M = 73.84) from an ongoing study, Pathways to Healthy Aging in African Americans—a Rutgers University-Newark community partnership fostered over 16 years of community engagement, health education, and public service—responded to measures of cognitive function, social network, social engagement, mental health, and demographic details. Also, they completed a task-based functional Magnetic Resonance Imaging. Results showed that marital status was negatively associated with MTL function, with those that are either single, divorced, or separated outperforming those that are married. Similarly, depressive symptoms had a negative association with MTL function. Further, the linear combination of social network variables and covariates significantly predicted MTL function. Our findings illuminate the benefits of social connectivity and resources on cognitive skills, and amplify the need to study the brain in the social context.

### EXAMINING SUBJECTIVE BURDEN AMONG SPOUSAL CAREGIVERS OF PARTNERS WITH DEMENTIA: THE IMPACT ON MARITAL QUALITY

Jeremy Yorgason, and Avalon White, *Brigham Young University, Provo, Utah, United States*

Although sometimes caregiving is associated with closeness in marriage, at other times the stresses may affect marriage relationships negatively. In the current study we explore how caring for a partner with dementia, and subjective burden are associated with marital quality. We further explore how dementia care and subjective burden might interact (suggesting a pileup of stress) to affect marital quality. Using data from 1,066 spousal caregivers that participated in the NSOC study and their corresponding care recipients from the NHATS study, the current analysis explored cross-sectional associations between spousal caregiving (primary vs. Secondary caregiver, subjective burden, gender, age, education) and partner care recipient characteristics (dementia classification, household income) predicting positive and negative marital quality. Results suggested that dementia classification and subjective burden were associated with lower positive marital quality and higher negative marital quality. The relationship between subjective burden and positive marital quality was moderated by whether or not the care recipient had dementia. Specifically, when dementia was present, the negative association between subjective burden and positive marital quality was stronger. Spousal caregivers often carry substantial burden to help their partners with activities of daily living. Care provision can alter the marriage relationship in negative ways when perceived burden is high. The current study suggests that the negative association between burden and positive marital quality is even stronger when caring for a spouse with dementia. Gender differences need further exploration, as well as how these patterns play out in early vs. later stages of dementia and across time.

### A MOBILE MUSIC APPLICATION FOR CAREGIVERS OF PERSONS WITH DEMENTIA: A USABILITY TESTING STUDY

Cassidy Doyle[1], Debra Dobbs[1], Kelly Wiechart[2], Rohan Wadhwa[1], and Hongdao Meng[1], *1. University of South Florida, Tampa, Florida, United States, 2. KellyW Consulting, Tampa, Florida, United States*

More than 11 million Americans spend an average of 26.3 hours caring for a person with dementia (PWD). Managing behavioral and psychological symptoms of dementia (BPSD) is a significant source of caregiver stress. Music-based interventions (MBIs) have been shown to be a pleasant activity enjoyed by both PWD and their caregivers, with the potential to help alleviate BPSD. However, current commercial music streaming services are not designed for families coping with dementia due to subscription and/or network connection requirements. This mixed-methods usability study tested caregivers' experience with an open-source Android application (MUsic to Support Engagement and Resilience, or MUSER) as a potential platform for home-based MBI delivered by caregivers of PWD. In-lab and in-home testing sessions were conducted with 6 caregivers (2 former and 4 current caregivers, 1 male and 5 females). Semi-structured interviews were conducted before and after the in-home testing period between July and November 2021 to better understand User Experiences (UX). Family caregivers had a mean age of 57.8 (SD=26.2, range 20-77). The main themes identified from the interviews included ease of use, device and music preferences, and prior experience with technology. Overall, users reported high satisfaction and would recommend the application to other caregivers. Users reported that listening to music on the app elicited positive emotional effects (e.g., calmness and relaxation). The MUSER mobile music application can be used by caregivers to deliver MBI in the community. Future research should test whether a caregiver-delivered MBI would be efficacious in reducing BPSD among PWD.

### CARE TO PLAN: TAILORING RESOURCES FOR DEMENTIA CAREGIVERS ACROSS A HEALTH SYSTEM

Ashley Millenbah[1], Katie Louwagie[1], Zachary Baker[2], Christine Jensen[3], and Joseph Gaugler[1], *1. University of Minnesota, Minneapolis, Minnesota, United States, 2. Arizona State University, Tempe, Arizona, United States, 3. Riverside Center for Excellence in Aging and Lifelong Health, Williamsburg, Virginia, United States*

Each day, millions of persons with Alzheimer's disease or related dementias (ADRD) rely on family members for necessary care and support. While there are many well-documented interventions to support ADRD caregivers, they lack individualization for specific caregiver needs and contexts. Care to Plan (CtP) was designed to address this gap in tailoring recommendations to support caregivers. After a Phase I pilot, CtP was refined and implemented within a moderately sized health system in the eastern US. A mixed-methods randomized controlled trial aimed to provide insights into the efficacy, acceptability, and utility of CtP. Twenty-two caregivers were randomly assigned to the treatment group (to review the CtP tool with a senior care navigator) and 21 were assigned to receive usual care. After completion of usual care procedures, 7 participants joined a waitlist control group, following treatment procedures. Three- and six-month outcomes included caregiver self-efficacy and distress, caregiver use of recommended support, and service utilization by the person with memory loss. Most caregivers were white (81%), female (74%), and cared for a parent (56%) or spouse (35%). The majority of caregivers agreed that the tool was helpful (70%) and would recommend it to other caregivers (83%). While 48% of caregivers felt that resources provided by the tool were not new to them, the checklist scores and interviews indicate that the senior care navigators were valuable in discussing the recommendations. Phase II findings will guide future evaluation and dissemination, and inform future models of CtP for health system usage.

### MINDFULNESS AND CAREGIVING EXPERIENCE IN ADRD CAREGIVERS

Magdalena Tolea[1], Iris Cohen[1], Simone Camacho[2], and James Galvin[3], *1. University of Miami Miller School of Medicine, Boca Raton, Florida, United States, 2. University of Miami Miller School of Medicine, Boynton Beach, Florida, United States, 3. University of Miami School of Medicine, Miami, Florida, United States*

Mindfulness (being present in the moment without judgement) has been linked to greater caregiver emotional health. Recent mindfulness-based interventions report improved coping skills, mood, and reduced stress in dementia caregivers. In this cross-sectional study of 141 ADRD caregivers, we assessed whether the relationship between caregiver mindfulness and caregiver experience varies by caregiver gender, relationship to patient (spouse-vs-child), etiology (AD-vs-LBD), or stage (MCI-vs-dementia). A stratified univariate analytic approach was used. Four mindfulness parameters (AMPS scale) were used: global score (GS), decentering (F1), positive (F2), and negative emotional regulation (F3). Outcomes included positive and negative appraisals of caregiving (PANAC), preparedness, care confidence, and depression. GS was linked to positive outcomes in male (rPANAC+=0.32/p=0.005), spouse caregivers (rPANAC+)=0.32/p=0.006 ) of ADRD patients regardless of etiology (rPANAC+=0.31/p=0.013 for AD; rconfidence=0.31/p=0.036 for LBD) and stage (rPANAC+=0.33/p=0.010 and rpreparedness=0.38,/p=0.008 for MCI; rPANAC+=0.29/p=0.011 and rconfidence=0.31/p=0.007 for dementia). Inverse relationships were observed with negative outcomes in male (rPANAC-=-0.46/p=0.002 and rdepression=-0.41/p=0.005), spouse caregivers (rPANAC-=-0.25/p=0.035 and rdepression=-0.30/p=0.009) of AD patients (rPANAC-=-0.25/p=0.043 and rdepression=-0.33/p=0.009) in early stages (rdepression=-0.41/p=0.001). F2 contributed to most relationships, with F3 and F1 significant in some but not all caregiver groups. Specifically, male spouse caregivers of AD patients regardless of stage may benefit from full-scope (F1-F3) programs while those of LBD patients from programs focused on improving emotional regulation (F2-F3). Wives of AD and LBD patients may in turn benefit from programs to improve positive emotional regulation (F2). Findings suggest that tailoring mindfulness-based interventions to specific caregiver groups may be effective in improving caregiver experience and mood.

### POLICIES AND PERSPECTIVES AROUND SEXUAL ACTIVITIES AMONG RESIDENTS WITH COGNITIVE IMPAIRMENT OR DEMENTIA IN LTC

Mijin Jeong, and Sarah Jen, *University of Kansas, Lawrence, Kansas, United States*

Many older adults remain sexually interested and active in later life. However, little is known about how sexual policies and practices in skilled-nursing facilities (SNF) address sexual activities of residents with cognitive impairment and dementia. This study seeks to identify the current sexual policies and staff's perspectives related to residents with cognitive impairment or dementia in SNFs in Kansas. Online surveys and mailed surveys were distributed to administrators from all 364 SNFs in Kansas in June 2020. 60 long-term care facilities (16.5%) answered the survey. Of 60 survey respondents, 22 facilities (36.7%) have a policy addressing sexual expression and 19 of those policies (94.7%) address issues related to cognitive impairment, competency, or dementia. 77.4% had trained their staff on the impact on sexual expression for those with cognitive impairment or dementia once or more than once during the past year. 73.3% of administrators stated that their staff would respond differently to sexual expression among individuals with dementia or cognitive impairment compared to other residents, often noting issues related to consent and capacity. 55.2% reported any sexual expression among residents with dementia within the past year. Findings indicated that there is a lack of overall sexual policies, but those that exist are likely to address residents with cognitive impairment or dementia. Although there is evidence of training and attention to issues related to sexual expression in individuals with dementia or cognitive impairment, there is a need for further efforts to establish practice norms and policies around more complex or nuanced situations.

### SESSION 6080 (POSTER)

### BIOBEHAVIORAL HEALTH

### PSYCHOLOGICAL DISORDERS LINKED TO OSTEOPOROSIS IN A POPULATION-BASED COHORT STUDY

Kanya Godde Chrisco, Margaret Gough Courtney, Josephine Roberts, and Yadira Quintero, *University of La Verne, La Verne, California, United States*

Psychosocial disorders can stem from or have profound effects on one's health, having been linked to many negative health outcomes. In this study, we hypothesize psychological disorders are associated with a higher risk osteoporosis diagnosis. Self-reported information from years 2012-2016 of the public-use, longitudinal cohort-based Health and Retirement Study, was evaluated from 11,716 American respondents aged 50-90 years old. The odds of scores on the Center for Epidemiological Studies Depression (CESD) scale, and broader psychological disorders (emotional, nervous, psychiatric) on osteoporosis diagnosis (outcome), were estimated with a logistic regression using survey weights, while controlling for sex, logged age, education level, race/ethnicity, family structure during childhood (number of adults), having thyroid disease, allostatic load, and body weight. A McFadden's R2 (0.18) shows the model fits relatively well. The results demonstrate that as CESD score goes up, there is a 10% increase in odds (OR = 1.1, $P < 0.001$) of an osteoporosis diagnosis. Similarly, if a respondent reported a doctor told them they had other psychological disorders, the odds of an osteoporosis diagnosis increased by 52% (OR = 1.52, $P < 0.001$). It is unknown whether the components of broader psychological disorders are caused by decreased quality of life and/ or other limitations from osteoporosis or if they contribute to bone health changes in this sample, or both. However, as CESD is a short-term measure (reflecting on the week prior) it is deduced to be as a result of a decreased quality of life associated with some cases of osteoporosis.

### SYMPTOMS CONTRIBUTING TO SLEEP PROBLEMS IN OLDER ADULTS WITH TYPE 2 DIABETES

Min Jung Kim[1], Cynthia Fritschi[2], and Eunhee Cho[1], *1. College of Nursing, Yonsei University, Seoul, Seoul-t'ukpyolsi, Republic of Korea, 2. University of Illinois Chicago, Chicago, Illinois, United States*

Sleep problems are common in older adults. Those with diabetes are more vulnerable to sleep disorders since diabetes-specific symptoms can interfere with sleep quality. Yet little is known which diabetes symptoms most strongly affect sleep in older adults. This study aimed to examine the associations between diabetes symptoms and sleep and to identity the symptoms that most strongly disrupt sleep in older adults in the United States. Diabetes symptoms were assessed using the Diabetes Symptom Checklist-Revised. Sleep impairment and sleep disturbance were self-reported using The Patient-Reported Outcomes Measurement Information System. Demographic (age, sex, race/ethnicity) and other variables (body mass index, depressive symptoms, diabetes duration, glycemic control) were also assessed. Multivariate regression analyses were used with standardized coefficients. A total of 82 adults aged ≥ 60 years were included (mean age = 68.32 ± 5.29 years, White 76.83%, female 56.1%). After controlling for demographic and other variables, increased hypoglycemia ($\beta = .35$), hyperglycemia ($\beta = .38$), fatigue ($\beta = .65$), cognitive ($\beta = .48$), and ophthalmologic ($\beta = .25$) symptoms and neurological pain ($\beta = .42$) significantly increased sleep impairment. Of these, fatigue was the strongest contributor to sleep impairment. Similarly, increased hyperglycemia symptoms ($\beta = .30$), fatigue ($\beta = .34$), and neurological pain ($\beta = .37$) significantly increased sleep disturbance while neurological pain was the strongest contributor. To improve sleep quality of older adults with diabetes, their diabetes symptoms should be comprehensively assessed, and potential contributor to poor sleep such as increased fatigue and neurological pain should be addressed.

### EXERCISE BEHAVIOR IS DETERMINED BY PANDEMIC DISTRESS AND TASK BURDEN AMONG CAREGIVERS OF OLDER ADULTS

Donya Nemati, NiCole Keith, and Navin Kaushal, *Indiana University, Indianapolis, Indiana, United States*

Background: Caregivers who have dependents with dementia are at a much higher risk of heart disease and mental illnesses compared with non-dementia caregivers. Consequently, these outcomes have been exacerbated by societal barriers that resulted from the pandemic. Engaging in regular physical activity at a moderate-to-vigorous level (MVPA) is beneficial for caregivers has it has been shown to prevent several adverse health outcomes. However, pandemic-related (COVID-19) distress likely worsened caregiver burden which in turn compromised their MVPA levels. The purpose of this study was to understand how caregiving burden impacts MVPA when accounting for physical activity determinants from an augmented Theory of Planned Behavior (TPB) model.

Methods: Participants (n=127) were caregivers for older adults (65+) who have dementia. Participants completed measures of MVPA (behavior), TPB, pandemic-related distress (COVID Caregiver Risk Index) and burden scale for family caregivers. The study was investigated using a structural equation model.

Results: Participants were 45.5 (SD=3.4) years old, 76.4% female. Attitudes (β=.22, p=.012) and perceived behavioral control (β=.19, p<.001) predicted intention. Attitudes and perceived behavioral control mediated the relationship between past behavior and intention (β=.17, p=.02). Covid distress predicted caregiver burden (β=.35, p<.001), and caregiver burden mediated the effects between distress and behavior (β=-.12, p=.01).

Conclusions: Caregiver burden findings suggest that societal changes and demographic-specific burdens related to caregivers need to be considered for caregivers with dependents who have dementia. Taken together, exercise programs that focus on traditional behavioral determinants also need to include specific approaches to buffer caregiving burden experienced in this demographic.

### CHRONIC CONDITIONS AND MORTALITY: MODERATION BY SELF-RATED HEALTH

Lauren Voss[1], Elizabeth Teas[2], and Elliot Friedman[1], *1. Purdue University, West Lafayette, Indiana, United States, 2. Purdue University, Lafayette, Indiana, United States*

Chronic conditions become more common with age and greater numbers and severity of chronic conditions, in turn, increase the risk of mortality. However, individuals with similar disease burden often have different mortality rates. The purpose of the present study is to examine potential explanations for divergent mortality outcomes. Self-rated health (SRH), or perceptions of one's own health, consistently predicts mortality. Thus, we hypothesized that participants' SRH would modify the association between chronic conditions and mortality. Data were from the second wave of the Midlife in the US (MIDUS study, N=5,524). Mortality data were collected through 2018. Chronic conditions were measured in two different ways. To assess disease severity, each chronic condition was weighted by its propensity to cause disability; these were then summed. The second was a count of chronic conditions, a common measure in many studies. SRH was measured on a scale of 1-5 (1 = poor, 5 = excellent). Results from logistic regression models showed probability of mortality increased significantly with greater disease burden (measured both as counts and severity of conditions) and decreased with higher ratings of SRH. Importantly, compared to lower ratings, higher ratings on SRH were associated with lower probability of mortality at the same levels of disease severity. In fact, participants who rated their health as excellent showed no increase in probability of mortality with increasing number or severity of conditions. Overall, this study suggests that even in the context of chronic diseases, positive perceptions of health predict greater longevity.

### REMOTE ELECTRONIC DATA CAPTURE OF MEDICATION USE AND AGITATION SYMPTOMS IN OLDER ADULTS WITH MCI OR DEMENTIA

Wan-Tai Au-Yeung[1], Lyndsey Miller[2], Sarah Gothard[2], Allison Lindauer[2], and Jeffrey Kaye[2], *1. Oregon Health and Science University, Portland, Oregon, United States, 2. Oregon Health & Science University, Portland, Oregon, United States*

Timely and objective knowledge regarding behavioral and psychological symptoms (BPS) in older adults with cognitive impairment or dementia residing in the community setting is challenging to obtain. The Monitoring Dementia-Related Agitation using Technology Evaluation (MODERATE) Study aims to identify longitudinal changes of agitation and related symptoms for dyads living at home with dementia. To date, MODERATE has enrolled seven dyads (a person living with MCI or dementia and a spousal caregiver). The mean age of the participants is 75.7 (8.5) years, the age of the spousal caregivers is 71.0 (6.0) years, and 5 out of 7 of the participants with MCI or dementia are male. We created an online survey, which is sent weekly to the caregivers, to inquire of any changes in medication and frequency of symptoms of agitation experienced by the persons with MCI or dementia in the past week. As of 02/22/2022, 102 weekly surveys (mean per participant = 14.6) were sent out via email; 99 responses were returned (response rate of 97.1%). One caregiver used a personal computer (PC) only, 2 used smartphone only and 4 used both PC and smartphone to complete the surveys. The median time to complete the online survey was 3.83 minutes with IQR 1.72 – 9.14 minutes. "Complaining, negativism, refusal to follow directions" were the most commonly reported agitated behaviors reported (43% of surveys). Caregivers can provide regular detailed symptom profiles and medication reports online. This approach may be used for more timely and informative management of BPS in dementia.

### SESSION 6090 (POSTER)

### COVID-19 PANDEMIC

### THE IMPACT OF COVID-19 ON SERVICE USAGE IN A RURAL "VILLAGE"

Joonyoung Cho[1], Ruth Dunkle[2], and Julie Tarr[3], *1. University of Michigan-Ann Arbor, Ann Arbor, Michigan, United States, 2. University of Michigan, Ann Arbor, Michigan, United States, 3. ShareCare of Leelanau, Leland, Michigan, United States*

The Covid-19 pandemic disrupted service access and use for many older adults aging in place. This study focuses on understanding how a rural "Village", where services are primarily provided by volunteers to older adults, adjusted to Covid-19. The sample is drawn from service users of at least one or more services between January and October 2020 (N=233). Survey and qualitative data were gathered via telephone-interview (N=80). This study examined (1) the impact of Covid-19 on service use and ability to stay at home, 2) services offered, and 3) changes to day to day life. In the sample, 53.75% is 80+ in age, 70% female, and 43.75% lived alone. 15% of respondents reported reduction in service used, 10% an increase, 65% no change, and 6.25% used no services. Some services such as rides to medical care, and grocery delivery increased, other services (e.g., caregiving) were reduced. 26.24% respondents believed that the organization's services helped them stay at home during Covid-19. Other services were developed to better serve older adults such as phone reassurance and expansion of delivery of medication and food. Open ended responses identified how Covid-19 impacted day to day life. While many reported negative ways such as changing mental health perspectives and limiting medical care, others report life being more peaceful and providing time to enjoy nature. Findings provide an understanding that services while disrupted continued for one volunteer organization with some service users showing resilience in their day to day lives.

### ASSOCIATIONS BETWEEN COVID-19 VACCINE HESITANCY AND SOCIOSPATIAL FACTORS IN NYC TRANSIT WORKERS 50 YEARS AND OLDER

Gabriella Meltzer[1], Jordan Harris[2], Michelle Heffner[3], Paula Lanternier[4], Robyn Gershon[5], and Alexis Merdjanoff[5], *1. Columbia University, New York, New York, United States, 2. Purdue University, West Lafayette, Indiana, United States, 3. Colorado State University, Fort Collins, Colorado, United States, 4. University of Texas at Austin, Austin, Texas, United States, 5. New York University School of Global Public Health, New York, New York, United States*

This analysis aimed to investigate how age, race/ethnicity, and geographical location contributed to vaccine hesitancy in a sample of New York City (NYC) Metropolitan Transit Authority (MTA) workers. Transport Workers Union, Local 100 members completed online surveys in August 2020 about their COVID-19 history, workplace protections and policies, fear of COVID-19 exposure, vaccination attitudes, and sociodemographic and health characteristics. We conducted univariate and bivariate analyses, followed by multivariate logistic regression, to determine the association between respondent age (younger than 50 vs. 50+) and vaccine hesitancy (willing vs. unwilling/unsure). We also produced spatial visualizations to examine these factors by participants' zip codes. Of 645 respondents, 59% were 50 years or older, 53% were non-White, and 71% expressed vaccine hesitancy. MTA workers ages 50+ were 46% less likely to be vaccine hesitant than their younger counterparts (OR 0.64; 95% CI 0.42, 0.97). Compared to Whites, non-Whites (OR 3.95; 95% 2.44, 6.39) and those who did not report their race (OR 3.10; 95% CI 1.87, 5.12) were significantly more likely to be vaccine hesitant. Those who were not concerned about contracting COVID-19 in the community had 1.83 greater odds (95% CI 1.12, 2.98) of being vaccine hesitant than those who were concerned. Spatial visualizations revealed that the oldest respondents tended to reside in Queens. Zip codes with high vaccine hesitancy were clustered in Brooklyn, where non-White respondents tended to reside. The trends observed in COVID-19 vaccine hesitancy based on race and age persist in a population of high risk, non-healthcare essential workers.

### COVID-19 CASE AND MORTALITY RATES IN GREEN HOUSES AND TRADITIONAL NURSING HOMES IN NEW YORK STATE

Yuchi Young[1], Ashley Shayya[1], Thomas O'Grady[1], and Ya-Mei Chen[2], *1. SUNY at Albany School of Public Health, Rensselaer, New York, United States, 2. National Taiwan University, Taipei City, Taipei, Taiwan (Republic of China)*

Introduction. Green Houses (GHs) have features that distinguish them from traditional nursing homes (NHs) including small size, home-like settings, humane model of care, and a sense of community. Literature shows these features have contributed to lower staff turnover, higher resident satisfaction, and lower COVID-19 case and mortality rates. Few studies use longitudinal data to quantify the differences between GHs and NHs by examining COVID-19 case and mortality rates. Methods. Nursing Home COVID-19 Data from CMS were used to compare case and mortality rates between GHs (n=4) and NHs (n=614) from 5/2020 to 1/2022. Case and mortality rates were calculated for GHs and NHs. Incidence rate ratio (IRR) of case and mortality rates were provided. Results. The preliminary results indicate GHs have lower COVID-19 case (3.76 vs. 6.8 per 1,000 resident weeks) and mortality rates (0.35 vs 1.21 per 1,000 resident weeks) compared to NHs. The IRR for COVID-19 cases is significantly higher in NHs compared to GHs (IRR = 1.8; 95% CI 1.55, 2.11), likewise, for mortality (IRR = 3.45; 95% CI 2.09, 5.75). Conclusions. The findings illuminate key differences in COVID-19 case and mortality rates among GHs and NHs. Factors such as GH size and their unique care model may contribute to the differences observed in COVID-19 case and mortality rates when compared to NHs. Future studies may include facility or resident characteristics in the study design.

### POST-COVID-19 ASSESSMENT: HEALTH LITERACY AND PREVENTION PRACTICES IN LTC FACILITIES IN INDIA: PROVIDER PERSPECTIVE

Shantha Balaswamy[1], Raja Samuel[2], and Rufus Singh[2], *1. Ohio State University, Dublin, Ohio, United States, 2. Madras School of Social Work, Chennai, Tamil Nadu, India*

There were no governmental issued guidelines directed specifically towards Long-Term Care residential facilities (LTCF) for older adults in India during COVID-19 pandemic (Rajagopalan 2020) and no data has been collected on care for resident in NGO's and private facilities (WHO, 2021). Further, studies of level of knowledge of providers on health indicators of residents, health literacy and risks factors of contracting the virus is absent. In India, older adults who live in LTCF have higher rates of chronic diseases (hypertension, diabetes, respiratory, heart problems), live in crowded communal space, less access to medical care for underlying health conditions, and have untrained, multiple caregivers, thereby putting them at increased risk for exposure and contracting the COVID-19 virus, and significant risk of infection, illness and mortality. LTCF includes NGO's, government funded old age homes and private paid retirement homes and is considered a multi-residence housing facility. This exploratory study, using online survey of providers of approximately 125 LTC facilities, examines the providers general health literacy level, prevention practices at their facility interventions, demands and rewards of caring for residents during COVID. Preliminary results: less than 20% of providers tracked prevalence of illness, health symptoms, infection rate, hospitalization, mortality rate, or post-recovery. Majority reported difficulty in accessing vaccination, getting residents vaccinated, and accessing resources ( food/ medicine/sanitizers) and problem coordinating care due to staffing shortage. Only a small percent use and track health and psychological assessments of residents. Findings have implications for advocating for standardization care and support National and State LTCF policy.

### NURSING HOME ADMINISTRATOR EXPERIENCES DURING COVID-19: AN EXPLORATORY STUDY

Susanny Beltran[1], Jennifer Wagner[2], and Vivian Miller[2], *1. University of Central Florida, Orlando, Florida, United States, 2. Bowling Green State University, Bowling Green, Ohio, United States*

Nursing homes have been the epicenter for the COVID-19 pandemic; 149,107 residents and over 2,200 staff have died of COVID-19. In addition to the loss of lives, 99% of nursing homes report staffing shortages. Various exploratory studies have emerged gathering the experiences of frontline nursing home staff during COVID-19, however less is known about the experiences of Nursing Home Administrators (NHA) responsible for overseeing personnel and operating a facility in line with shifting state and federal mandates. Thus, this study explores the experiences of NHA during the pandemic. A cross-sectional online survey was conducted. In addition to demographic and facility-level questions, open-ended questions explored prior training on infection prevention, day-to-day operational challenges, needs, and considerations of leaving their role as administrator. The total sample (N=60) included 47 NHA of record and 13 assistant administrators/other; 53% worked in corporate NHs and 23% were part of continuing care retirement communities. Respondents report prior infection prevention training, but indicate it was not adequate preparation for COVID-19. Moreover, administrators describe challenges in recruiting and retaining staff, and in supporting staff mental health needs (e.g., burnout, PTSD). The majority of NHAs endorse a desire to step away from their role, but indicate a commitment to residents keeps them from resigning. Findings indicate that NHAs, like other members of the NH team, have experienced the effects of COVID-19, and point to specific training and support needs to equip NHAs for work in the context of this pandemic and future emergencies.

### RESEARCH, (UN)INTERRUPTED? EFFECTS OF THE PANDEMIC ON A 1 YEAR FOLLOW-UP TO AN ONLINE RCT

Alexandra Jeanblanc[1], Carol Musil[1], Christopher Burant[2], and Jaclene Zausniewski[1], *1. Case Western Reserve University, Cleveland, Ohio, United States, 2. Case Western Reserve University, Parma, Ohio, United States*

The pandemic profoundly disrupted research, largely due to the interruption of data collection. Our online-only behavioral RCT, evaluating two methods of stress-reduction for grandmothers with co-resident grandchildren, completed the originally planned 4 data collection points prior to the pandemic. When it began, we were in the process of collecting a 5th questionnaire (Q5) from a sample of 152. Our pre-existing work from home policies allowed us to continue data collection uninterrupted.Of 152 potential grandmothers, 51 (58 eligible) grandmothers completed Q5 prior to the pandemic and 62 (94 eligible) completed it after it began, (total N=113, 74.43% response rate. We compared Q5 responses pre/post pandemic, finding no significant differences on resilience, mindfulness/decentering, subjective support, instrumental support, or the friendship scale. Post-pandemic participants had significantly lower scores on the social resourcefulness subscale (t=-1.723, p=.044), but not personal or overall resourcefulness. They reported more depressive symptoms for the CES-D positive affect and depressed affect subscales (t=1.997, p=.048; t=1.673, p=.049). The post-pandemic sample reported worse self-rated health over the last year (t=2.753, p=.003) and worse overall health (t=1.781, t=.039).The higher levels of depressive symptoms, worse self-reported health, and lower social resourcefulness aligns with pandemic-related changes observed by other studies. We did not need to adapt our research procedures for the pandemic and, due to the lower post-pandemic response rate, our subsamples were of relatively equal size. Consequently, it is likely that the differences we observe here are real and reflect the negative effect of the pandemic on individual health and psychosocial well-being.

### WHAT DO LONG-TERM CARE LEADERS SAY ABOUT THEIR PREPAREDNESS FOR COVID-19? A QUALITATIVE STUDY

Darren Liu[1], Sandi Lane[2], Betty Burston[3], and Robert Rados[4], *1. West Virginia University, Morgantown, West Virginia, United States, 2. Appalachian State University, Boone, North Carolina, United States, 3. University of Nevada Las Vegas, Nevada, Las Vegas, Nevada, United States, 4. Southern Illinois University Carbondale, Carbondale, Illinois, United States*

The post-acute and long-term care (PALTC) sector has, in some respects, served as the epicenter in the U.S. during the COVID-19 pandemic. Many decisions were carried out by administrative and/or clinical leaders during this pandemic. The decisions were made based on their professional experiences, and recommendations by the Centers for Medicare & Medicaid Services (CMS). However, little research has reported on the perspective of those administrators who took the lead during this most difficult time. This study aims to understand how responses and decisions were formed during COVID-19 to ensure resources were available protect staff and residents. Accordingly, this study tried to answer two key questions: 1) What did the PALTC administrative and clinical leaderships learn? 2) What can we do better not if, but when COVID ever "…hits again?" We interviewed nursing home and/or assisted living administrators in two conveniently selected states: Pennsylvania and North Carolina. These interviews (each of which took about 30 minutes) were conducted over Zoom using structured and open-ended questions. The transcripts were entered and analyzed using NVivo – a qualitative data analysis software. The results revealed several themes including communications, relationship building, experience as an administrator, fears and resilience, as well as successful activities to support their staffs such as recognition, bonuses, and food bags prepared for their family. The findings highlight some important administrators' thoughts which recommend key future strategies. These include whether preparedness assets, knowledge, resources, and policies were adequate and where the future efforts should focus.

### APPLYING A COLLABORATIVE, INTERDISCIPLINARY APPROACH TO INCREASE COVID-19 VACCINE EQUITY, ACCESS, AND CONFIDENCE

Alice Prendergast[1], and Kristi Fuller[2], *1. Georgia Health Policy Center, Georgia State University, Atlanta, Georgia, United States, 2. Georgia State University, Atlanta, Georgia, United States*

The COVID-19 pandemic has impacted the health and well-being of older adults, people with disabilities, and caregivers in myriad ways. Subgroups of these populations, particularly people of color, experience higher rates of infection and mortality from COVID-19, and are also at increased risk for social disconnection, depression, economic hardship, and a host of other adverse outcomes brought on or exacerbated by the pandemic. COVID-19 vaccines are effective in preventing serious illness and play an important role in protecting vulnerable groups, yet uptake varies considerably across communities and health disparities are growing. Researchers from the Georgia Health Policy Center conducted a mixed-methods study involving a literature review, secondary data analysis, and focus groups to gather information about drivers of vaccine hesitancy and inequity. The team partnered with a multiagency collaborative, branding firm, and subject matter experts to synthesize and apply findings in the development of a statewide, multichannel vaccine communications campaign. In this session, researchers will provide an overview of the study, highlight key findings, and discuss lessons learned throughout this complex, collaborative project.

### COVID-19 CASES AND DEATHS IN NURSING HOMES: ROLE OF QUALITY IN MULTIPLE WAVES

Xiaochuan Wang, and Camilo Osorio Castaneda, *University of Central Florida, Orlando, Florida, United States*

The COVID-19 pandemic has disproportionally impacted nursing homes in the United States. As of February 2022, nursing homes have reported over 980,000 resident cases and nearly 150,000 resident deaths, accounting for a high share of total COVID-19 cases and deaths in the U.S. Earlier studies have identified various factors that relate to nursing homes' vulnerability to COVID-19 (e.g., ownership status, facility size). Quality has a critical role in affecting nursing home COVID-19 outcomes. However, research on this topic has been relatively limited and inconsistent. Using publicly available data from CMS COVID-19 Nursing Home Dataset, Nursing Home Compare, Long-Term Care Focus, and The New York Times, this study examines the role of nursing home quality (e.g., infection control deficiency, quality of care deficiency, and five-star quality rating) on COVID-19 resident cases and deaths during multiple waves. Of the over 15,000 nursing homes examined, approximately 64% have had at least one infection control deficiency and 82% have had at least one quality of care deficiency during the two years preceding the COVID-19 pandemic. Results from regression models indicate that the likelihood of having at least one case is more related to facility and resident characteristics (e.g., facility size, percentage of Medicaid residents) and environmental factors (e.g., county case rates) than quality indicators. Whereas the roles of quality on the odds of having resident deaths, and the magnitude of resident cases and deaths, are more consistent in the later waves of the pandemic. Implications for future research and practice will be discussed.

### COMMUNICATION DURING COVID-19: A COMPARISON BETWEEN INSTITUTIONALIZED AND COMMUNITY DWELLING OLDER ADULTS

Victoria Dube[1], Mollie George[1], Sarah Hubner[2], and Julie Blaskewicz Boron[3], *1. University of Nebraska at Omaha, Omaha, Nebraska, United States, 2. University of Nebraska Omaha, Omaha, Nebraska, United States, 3. University of Nebraska, Omaha, Omaha, Nebraska, United States*

COVID-19 has created communication and socialization challenges for many older adults (OAs) living in community or institutional settings (CECs, IECs respectively) and their essential contacts (ECs). An essential contact is someone who provides emotional, physical, and social support for an older adult. While social distancing measures have decreased the spread and infection rate, these measures have placed strain upon communication and socialization needed for wellbeing which may increase the risk of loneliness and subsequently depression, cognitive functioning, and mortality among OAs. This study compared how general, in-person, and distanced communication between CECs and IECs has changed due to COVID-19. Self-identified ECs to OAs (N=546) completed a Qualtrics questionnaire via Amazon Mechanical Turk. Respondents (Age Range: 19-77; Mean=44.3; SD=14.2) were generally female (54.8%), white (81.0%), and CECs (57.3%). Pearson chi-square was used to evaluate the association between EC and communication type. Phi and Cramer's V were used to measure effect size. IECs were significantly more likely than CECs to report much less general (41.6% vs. 10.7; medium effect) and in-person (60.0% vs. 22.4%; medium effect) social interaction due to COVID-19. IECs were significantly more likely to report much more distanced communication than CECs (42.8% vs 5.2%; small effect). Overall, this suggests community dwelling OAs' communication and socialization were less impacted by COVID-19 distancing than institutionalized OAs. Thus, institutionalized OAs may be at greater risk for negative effects of social distancing. Future efforts need to focus on prioritizing ways for institutionalized adults to communicate with their ECs if social distancing is needed.

### THE ASSOCIATION BETWEEN CHRONIC LUNG CONDITIONS AND COVID-19 MORTALITY AMONG 65+ IN CONNECTICUT AND RHODE ISLAND

Taylor Jansen, Chae Man Lee, Shu Xu, YanJhu Su, Nina Silverstein, and Beth Dugan, *University of Massachusetts Boston, Boston, Massachusetts, United States*

Older adults aged 65 and older have accounted for 81% of all deaths from COVID-19 in the US. Individuals with chronic lung diseases have higher risk for severe COVID-19 and death. The aim of this research is to examine the association between town-level rates of asthma and COPD and deaths from COVID-19 in 208 towns in Connecticut and Rhode Island. This study utilized data from multiple publicly available datasets: the public health departments of Connecticut and Rhode Island, the 2021 Connecticut and 2020 Rhode Island Healthy Aging Data Reports (www.healthyagingdatareports.org), and the Census Bureau's 2014-2018 American Community Survey (ACS). A multi-step analysis was conducted to examine the association between town-level chronic lung conditions and COVID-19 mortality. Bivariate correlations and mapping found strong, positive associations between town-level asthma prevalence and COVID-19 mortality (rs [206] = 0.15; P = .03) and

COPD prevalence and COVID-19 mortality (rs [206] = 0.15; P = .03). After controlling for town-level factors associated with chronic lung conditions and COVID-19 mortality, linear regression models did not find support for an association between chronic lung conditions and COVID-19 mortality. However, town-level factors like percentage of the 65+ African American population (β: 0.19, SE: 249.18, p= .02) and 65+ with low educational attainment (β: 0.45, SE: 278.98, p= .001) were significant predictors of COVID-19 mortality. This research adds to the current knowledge base that high rates of racial minorities and low education among the 65+ population are significant predictors of town level COVID-19 mortality.

### SENIOR CENTER RESPONSE TO COVID-19: INVOLVEMENT WITH VACCINE DISTRIBUTION

Ceara Somerville, Caitlin Coyle, and Jan Mutchler, *University of Massachusetts Boston, Boston, Massachusetts, United States*

In early 2021, access to a COVID-19 vaccine was prioritized for older adults and people with multiple co-morbidities. Between high demand, emerging supply, and new systems for booking a vaccine appointment, many people had challenges getting an appointment. Senior centers became a crucial resource for access to the vaccine and additional information about its efficacy and safety. This poster presents survey data collected from 282 senior centers in Massachusetts regarding their involvement with the COVID-19 vaccine distribution in 2021. Nearly all senior centers reported making vaccine appointments on behalf of residents and were responsible for over 166,000 appointments made. Nearly 100,000 hours were spent by senior center staff or volunteers to make appointments. About a third of senior centers participated in hosting nearly 2,000 vaccine clinics, which vaccinated over 175,000 adults. Respondents reported challenges faced during the booking process, including the length of time and the developing technologies to book an appointment. Almost two-thirds of senior centers reported assisting community members under age 60, operating beyond the traditional scope of their services. Other assistance provided by senior centers included providing information about eligibility and guidelines (84%), information about vaccine efficacy and safety (62%), offering transportation to appointments (70%), and providing physical assistance for appointments (31%). Evidence from this poster emphasizes the important role that senior centers play in the community, not just for older adults but also for the community at large.

### MEANING IN CHALLENGING TIMES: SENSE OF MEANING SUPPORTS COPING WITH PANDEMIC STRESSES

Katherine Kero[1], Anja Podlesek[2], and Voyko Kavcic[1], *1. Wayne State University, Detroit, Michigan, United States, 2. University of Ljubljana, Ljubljana, Ljubljana, Slovenia*

The COVID-19 pandemic created disruptions in daily life and trauma for many individuals. Traumatic experiences often trigger reflection on meaning in life (MIL), which may result in either resilience or despair. The purpose of this study was to evaluate the role of MIL in mediating the relationship between COVID-related stressors and well-being outcomes. Further, age-related differences in MIL were examined. Web-based surveys were completed by 831 Slovenian participants in April of 2020. Demographic data; perceptions of stressors related to lacking necessities, movement restrictions, and concerns at home; MIL; perceived overall health status; anxiety; emotional state; and perceived stress were measured. Overall, a moderately strong sense of MIL (M = 5.0, SD = 0.74, range 1-7) was reported by participants. Older age was a significant predictor of greater MIL, (B = 0.098, SE = .036, p < .01). Older adults also experienced less perceived stress, negative emotions, and home-related stressors compared to younger adults, but greater stress from lacking necessities (p < .05). MIL partially mediated the relationship between stressors and well-being outcomes, especially stressors related to lacking necessities and concerns at home (13-27% mediation) and outcomes of anxiety, perceived stress, and negative emotions. A strong sense of MIL was associated with improved well-being and a buffering of the effects of pandemic-related stressors. Older adults were less vulnerable to stress and had a greater sense of MIL than younger individuals. Public health initiatives and media may help improve resilience to pandemic trauma by emphasizing the collective meaning in challenging situations.

### UNDERSTANDING OLDER HEALTH CARE WORKERS' BURNOUT DURING COVID-19

Jihee Woo[1], and Rafael Engel[2], *1. University of Minnesota, St. Paul, Minnesota, United States, 2. University of Pittsburgh, Pittsburgh, Pennsylvania, United States*

The prolonged battle against the COVID-19 pandemic has left many health care workers physically, mentally, and emotionally exhausted, exacerbating burnout that was already endemic in the healthcare sector. Burnout places not only an individual's well-being but patient care at risk. In this study, we examine protective and risk factors contributing to burnout specifically among older health care workers during COVID-19. We address these questions using data collected from an online survey conducted on health care workers employed at hospitals in Pittsburgh, Pennsylvania in February 2022. Health care workers ages 50 and older (n=165) –a subset of surveyed health care workers– were included in the analytic sample. Participants were asked a series of questions about burnout, mental health, workplace stressors, intent to leave the job, and demographics. Participants were predominantly female (88%) and white (91%) with a mean age of 57.2 years. Almost 70% were registered nurses and the remaining were service, clerical, and technical workers; the mean years of work experience was 21.5 years. Most participants (77%) experienced moderate levels of burnout. Preliminary regression analysis suggests that perceived inadequate staffing (β=2.12, p<.001) and workplace discrimination (β=1.23, p=.001) were positively associated with burnout while job autonomy (β=-1.77, p<.05) and schedule flexibility (β=-.2.09, p=.001) were negatively associated with burnout. Burnout was in turn positively associated with depression, anxiety, and intent to leave the job. These findings demonstrate the need for workplace support to address older health care workers' burnout, better accommodate their needs, and keep them safe and healthy in their jobs.

### SESSION 6100 (POSTER)

### COVID-19, ACUTE, AND END-OF-LIFE CARE

### CLINICAL CHARACTERISTICS AND MORTALITY IN ADULTS 85 YEARS AND OLDER HOSPITALIZED WITH SARS-COV-2

Valerie Danesh[1], Jinmyoung Cho[2], Kristen Tecson[3], and Alan Stevens[4], *1. Baylor Scott & White Health, Dallas, Texas, United States, 2. Baylor Scott & White Health,*

*Temple, Texas, United States, 3. Baylor Scott & White Research Institute, Dallas, Texas, United States, 4. Baylor Scott and White Health, Temple, Texas, United States*

Severe acute respiratory syndrome coronavirus 2 (SARS-CoV-2) places older adults at increased risk of hospitalization and mortality due to coronavirus disease 2019 (COVID-19). To date, research and regulatory data describe adults 65 years and older, with few reporting on characteristics specific to older adults 85 and older ("oldest-old"). A consecutive 12-month case series including 1,510 oldest-old patients hospitalized with SARS-CoV-2 is presented to describe clinical characteristics, Intensive Care Unit (ICU) utilization and in-hospital mortality from 26 urban, suburban and rural-serving hospitals in an integrated health system in Texas. Known predictors of poor prognosis in COVID-19 include older age, male sex, metabolic risk factors (dyslipidemia, obesity, diabetes) and respiratory failure requiring oxygen support. Compared with hospitalized patients < 85 years, oldest-old patients more often had 4+ chronic conditions (31% vs 12%, p< 0.0001), but less often had metabolic risk factors associated with increased risk for mechanical ventilation and mortality. Specifically, diabetes prevalence was similar between age groups (27% oldest-old vs 30%, NS), while obesity prevalence was lower (18% oldest-old vs 55%, p< 0.0001). Oxygen support requirements were largely similar to the general population, with 4 of 5 hospitalized patients requiring oxygenation support. Of 1,510 oldest-old patients hospitalized for SARS-CoV-2, 269 (18%) required admission to an ICU, 1,206 (80%) required oxygen support, and 1,221 (81%) survived. Following hospitalization, 78% required supportive care, including hospice (15%). Hospitalization characteristics across the lifespan suggest an accelerated and compounded need for ICU recovery and post-hospitalization rehabilitation attributed to the COVID-19 pandemic.

### IMMUNE RESPONSES TO MODERNA COVID-19 MRNA VACCINE IN FRAIL NURSING HOME PATIENTS

Charles Semelka[1], John Sanders[1], and Martha Alexander-Miller[2], *1. Wake Forest School of Medicine, Winston Salem, North Carolina, United States, 2. Wake Forest School of Medicine, Winston-Salem, North Carolina, United States*

Prior COVID-19 mRNA vaccine trials included healthy older adults, but mRNA vaccine responses were not studied in frail older adults. We postulated that frailty was associated with immune responses of reduced quality and quantity following mRNA vaccination. A cohort of 15 older adults in a retirement facility were followed from the first Moderna mRNA-1273 vaccine dose in February 2021 with blood collections at baseline and weeks 4 (boost), 6, 18 and 28. Outcomes were IgG titers to SARS-CoV-2 Spike protein with secondary outcomes of T cell responses. Statistical analysis used log transformed geometric mean antibody titers in multivariable regression models with clinical predictors including, age, sex, prior infection status, and clinical frailty scale (CFS) score. Cellular immune response analysis used multivariable regression for function and phenotyping of T cell subsets. All participants with median (IQR) age: 90 years (84, 96) and CFS score: moderately frail 6 (5, 7) generated robust antibody responses with mean peak titer levels 10-fold higher than baseline. In the adjusted model, individuals with severely frail scores CFS=7 had lower antibody levels compared to mildly frail CFS=5, OR: 0.55 (0.35, 0.87) p=0.017. Both chronological age and sex had non-significant relationships with antibody titers. Spike peptide specific CD4 cells and T follicular helper cells were significantly decreased in more frail individuals (p=0.011 and p=0.008 respectively), though the relationship with antibody titers was non-significant. Frailty scores were a better predictor than age for serologic and cellular immune responses to COVID-19 mRNA vaccination in very old adults.

### DEPRESSION, ANXIETY, STRESS, AND HEADACHE IN BRAZILIAN OLDER PEOPLE IN THE CONTEXT OF THE COVID-19 INFODEMIC

Alexandre Bulgarelli[1], Camila Mello Dos Santos[1], Ricardo Cavalcante[2], and Karla Frichembruder[1], *1. University of Rio Grande do Sul, PORTO ALEGRE, Rio Grande do Sul, Brazil, 2. Federal University of Juiz de Fora, PORTO ALEGRE, Rio Grande do Sul, Brazil*

Objective: The aim of the present study was to analyze the prevalence and factors associated with headaches in Brazilian older people in the context of COVID-19 Infodemic.

Methods: This is a cross-sectional study carried out with 3,307 older Brazilians through a virtual questionnaire, self-completed using a cell phone, tablet, or computer with internet access. The questionnaire was composed of the Geriatric Depression Scale(GDS), Geriatric Anxiety Inventory(GAI), and the Perceived Stress Scale (PSS). Data collection was developed between June 2020 and January 2021. The analysis model consisted of variables distributed into four blocks: exogenous variables, primary determinants, health behaviors, and health conditions. It was used the Goodness-of-fit test to assess the quality of fit of the final model. Poisson regression with robust variance was used to estimate the associations.

Results: The prevalence of headache was 31.7% (CI 95%: 30 – 33). This outcome was associated with the use of psychotropic drugs (p < 0,001), concern with information about COVID-19(p < 0,001), symptoms of depression and anxiety(p < 0,001), and perception of stress(p < 0,001).

Conclusion: Anxiety, depression, and stress are thought to be associated with headaches in older adults who are exposed to excess information and fake news about COVID-19. It is considered that in the COVID-19 Infodemic scenario, headache in older people who have access to information is an important marker of mental health associated with suggestions of depression, anxiety, and stress.

### SHARED DECISION-MAKING WITH COST INFORMATION IN THE CONTEXT OF AGE-FRIENDLY CARE

Nicole Iny, *FAIR Health, New York, New York, United States*

The proposed poster session will explore shared decision making—the patient-clinician communication to decide on tests, treatment and care based on clinical evidence, balancing risks and outcomes with patient and caregiver preferences and values—and how evidence-based strategies, such as shared decision-making tools, or decision aids, can support older adults and their caregivers/care partners as they navigate the healthcare system. Notably, the session will share findings from FAIR Health's grant-funded initiatives to advance shared decision making through decision aids, which combine clinical and cost information for specific clinical scenarios, and are freely available for consumers on fairhealthconsumer.org and for healthcare providers on fairhealthprovider.org. As such, the session will offer diverse perspectives and a unique spotlight on the financial and patient- and caregiver-centered aspects of healthcare decision making. Through FAIR Health's needs assessment for a current project funded by The John A. Hartford Foundation, which has as its goal to advance shared decision making among older adults with serious illness and family caregivers, FAIR Health collected qualitative feedback from older patients, family caregivers and healthcare providers regarding perspectives on navigating the healthcare system. Through a national survey of older patients and family caregivers, FAIR Health found that: (1) though a significant proportion of older adults consider healthcare costs to be an important factor when making healthcare decisions, more than a third have difficulty getting such cost information; and (2) family caregivers/care partners expressed an appetite and need for healthcare information, resources and tools that facilitate better decisions about their care receiver's care.

### WHAT IS THIS COVID? COMMUNITY PERCEPTIONS OF COVID-19

Karen Kopera-Frye, and JoJo Delgado, *New Mexico State University, Las Cruces, New Mexico, United States*

The COVID pandemic was complicated by the varied amounts and sources of information available on what exactly COVID was, e.g., CDC website, social media; some fact-based and some not. The purpose of the project was to learn how people defined COVID. The surveyed sample consisted of 155 community dwellers (M age = 45.7 years), with above average education, primarily female (66%), and slightly over one-half identifying as Caucasian and approximately 40% Latinx. The sample were all residents of the Southern Border region, having very high rates of COVID infection in this area, and a statewide mask mandate both inside and outside. The open-ended question inquiring "What is COVID?" was answered by the respondents and thematic analysis focused on two dimensions: 1) was the response factual or not (incorrect in some manner); and 2) the definition of COVID in terms of a virus, flu, etc. Interestingly, despite the median education being around 15 years, 44.5% of the sample gave an incorrect definition of COVID. The three most predominant themes emerging from the definitions were (in descending order): 1) a virus with specific facts noted; 2) a disease/infection; and 3) an affective reaction such as annoying. The results highlight the diverse conceptualizations in a very high-risk area, especially focused on oftentimes an incorrect understanding of COVID.

### A DATA-BASED PROPOSITION FOR THE CREATION OF NEW ICD-10 DIAGNOSIS CODES FOR POST-ACUTE SEPSIS SURVIVORS

Sungho Oh[1], Melissa O'Connor[2], Michael Stawnychy[3], Yolanda Barrón[4], Patrik Garren[1], Elaine Sang[1], and Kathryn Bowles[3], *1. University of Pennsylvania, Philadelphia, Pennsylvania, United States, 2. Villanova University, M. Louise Fitzpatrick College of Nursing, Villanova, Pennsylvania, United States, 3. University of Pennsylvania School of Nursing, Philadelphia, Pennsylvania, United States, 4. Visiting Nurse Service of New York, New York, New York, United States*

Sepsis survivors have the second highest readmission rate among Medicare beneficiaries, next to heart failure. I-TRANSFER is an implementation science study to improve transitions and reduce readmissions among sepsis survivors transitioning from acute to home health care (HHC). A total of 63 semi-structured interviews were conducted with stakeholders among 12 hospitals and 5 affiliated home health agencies (HHAs). The purpose of this secondary analysis was to: 1) examine how the sepsis diagnosis is reflected in HHC documentation, 2) identify barriers in sepsis information transfer to HHC, 3) recommend a documentation strategy to enhance information transfer and HHC documentation. We analyzed the diagnosis coding within a national OASIS dataset and eight I-TRANSFER interviews with hospitalists, documentation specialists, and HHC coders. Findings include: a) sepsis diagnoses were nearly invisible in HHC records being documented for only 10% of 165,000 sepsis survivors transitioned to HHC, b) sepsis information in referral documentation can be unclear to HHC coders, c) the lack of sepsis diagnosis documentation might make HHC clinicians unaware of the patient's risk for readmission, d) HHC coders recommended improved language in the acute care discharge summary to link the need for HHC to sepsis, thereby supporting the use of ICD-10 sepsis 'A' codes in HHC. The use of terms, such as "History of Sepsis" or "Sepsis Resolved," leads to HHC using non-specific codes, thus is not recommended. We highlight the need for new ICD-10 codes for "Sepsis Aftercare" and "Post-Sepsis Syndrome" to clearly communicate and document the needs of sepsis survivors.

### CORRELATIONS BETWEEN OLDER PATIENTS' FIRST COVID-19 VACCINATION TIMING AND MEDICATION ADHERENCE

Thomas Marano[1], Renae Smith-ray[2], Clifford Jones[3], Balamurugan Sivakolunthuvel[3], Linda Schultz[3], Michael Taitel[4], and Kevin Leung[3], *1. AllazoHealth, Forest Hills, New York, United States, 2. Walgreen Co., Deerfield, Illinois, United States, 3. AllazoHealth, New York, New York, United States, 4. Walgreen Co, Deerfield, Illinois, United States*

Medication adherence and COVID-19 vaccinations are both essential to improving health outcomes and avoiding hospitalizations. When vaccines first became available in early 2021, the CDC recommended prompt vaccination for patients age 65-and-over. Vaccine hesitant older adults are among those at the highest risk for severe outcomes from a COVID-19 infection. A retrospective study was conducted using prescription and vaccine claims data from a pharmacy chain. Patients were included if they: 1) were age 65+ as of 1/1/21; 2) had at least two prescriptions of RAS antagonists, non-insulin anti-diabetics, or statins (in 2021); and 3) received at least two doses of mRNA COVID-19 vaccine between 1/1/21 and 9/21/21. Patients were then split into two evenly-distributed groups based on the number of days into 2021 when they received their first vaccine dose. The outcome was the proportion of optimally adherent patients (PDC ≥ 80%) by condition as of 12/31/21. Two-proportion z-tests were performed comparing the adherent proportions of the "earlier to vaccinate" group versus the "later to vaccinate" group as of 12/31/21. Age groups analyzed were "65-74" and "75+". The proportion of adherent patients as of 12/31/21 in the "Earlier to Vaccinate" group is higher than those in the "Later to Vaccinate" group for all three adherence metrics by 4.4% (65 to 74) and 2.2% (75+, p=0.00108*) for Diabetes, by 5.1% and 2.6% for Hypertension, and by 6.0% and 3.9% for Statins (*all other p< 0.00001). The timeliness of a patient's first COVID-19 vaccination dose was predictive of medication adherence in 2021.

### REAL-WORLD EFFECTIVENESS ENHANCED INFLUENZA VACCINES IN OLDER ADULTS DURING THREE CONSECUTIVE INFLUENZA SEASONS: 2017–2020

Ian McGovern, *Seqirus (A CSL Company), Summit, New Jersey, United States*

Unenhanced influenza vaccines often fail to elicit adequate immune responses in adults ≥65 years due to immunosenescence. Enhanced influenza vaccines, MF59-adjuvanted trivalent inactivated influenza vaccine (aTIV) and high-dose vaccine (HD-TIV), were specifically developed to overcome this problem. The relative vaccine effectiveness (rVE) of aTIV vs HD-TIV and standard, egg-derived quadrivalent influenza vaccines (QIVe) was estimated in 3 retrospective cohort studies during the 2017-2020 US influenza seasons. Influenza-related medical encounters (IRMEs) were identified using diagnostic codes specific to influenza disease (ICD J09*-J11*) from a dataset that integrated electronic primary care medical records with pharmacy and medical claims. rVE was estimated using propensity score methods adjusting for variables including age, sex, race, ethnicity, geographic location, week of vaccination, frailty and health status. Subgroup analyses included specific age groups and those with high-risk medical conditions. aTIV demonstrated a consistent benefit over QIVe and HD-TIV across all three seasons in the overall study populations, and greater rVE versus QIVe was consistently observed over all three seasons in all age subgroups as well (65-74, 75-84, 85+). aIIV3 was comparable to HD-TIV and more effective than QIVe in a subgroup with high-risk medical conditions. These findings lend further support to the use of aTIV to prevent influenza-related medical encounters in older individuals.

### REGIONAL VARIATION IN COVID-19 MITIGATION PRACTICES AMONG HOME- AND COMMUNITY-BASED LONG-TERM CARE PROVIDERS

Jessica Lendon, Christine Caffrey, Priyanka Singh, Zhaohui Lu, and Sengupta Manisha, *National Center for Health Statistics, Hyattsville, Maryland, United States*

Residential care communities (RCC) and adult day services centers (ADSC) were greatly impacted by COVID-19. As state-regulated home- and community-based care settings (HCBSs), they experienced changing regulations, outbreaks among care recipients and staff, and new challenges to safely provide services. Using a nationally representative sample of over 11,600 RCCs and census of nearly 5,500 ADSCs from the 2020 National Post-acute and Long-term Care Study, this study examined the prevalence and regional variation ($p<.05$) of practices to mitigate COVID-19. Preliminary data show that most RCCs (87%) and ADSCs (72%) screened for symptoms. Most RCCs limited communal activities (83%) and ADSCs reduced hours or temporarily closed (73%). More RCCs used video telemedicine than audio-only (40% vs 34%) to assess, diagnose, or treat users. However, more ADSCs used audio-only than video telemedicine (25% vs 17%). Over 80% of all providers always or sometimes imposed in-person restrictions on family, visitors, volunteers, and non-essential services providers. More ADSCs in the Midwest (82%) and South (80%) screened symptoms than Northeast (68%) and more ADSCs in the Midwest (85%) limited hours/temporarily closed than Northeast (72%). More RCCs in the Midwest (43%) and South (41%) used video telemedicine than Northeast (36%); however, a lower percentage of ADSCs in the Midwest (9%) and South (12%) used video telemedicine than Northeast (25%). Results show a variety of experiences in the first year of the COVID-19 pandemic among these two HCBSs. Practices to mitigate COVID-19 while continuing to provide needed services were common, with some differences across settings and US regions.

### ANXIETY IN COMMUNITY-DWELLING OLDER ADULTS AND ITS RELATED FACTORS DURING THE COVID-19 PANDEMIC

Yu Ma[1], Chieko Greiner[2], and Hirochika Ryuno[3], *1. Kobe University, Kobe, Hyogo, Japan, 2. Kobe University, Kobe-city, Hyogo, Japan, 3. Kobe University, Kobe city, Osaka, Japan*

The rate of infection and magnitude of the effects of the coronavirus disease 2019 (COVID-19) pandemic has far exceeded the realm of what most people had previously experienced in their lives. There is an urgent need to ensure that strategies are in place to prevent and reduce anxiety in older adults. The present study aimed to assess anxiety in community-dwelling older adults and the related factors during the COVID-19 pandemic in Japan. A community-based cross-sectional study was completed in July 2021. The Japanese version of the Geriatric Anxiety Inventory was used to screen for anxiety symptoms. Demographical data and data for the following five other factors were collected: loneliness, mental well-being, perceived social support, coping behaviors to COVID-19, and fear of COVID-19. The association between anxiety and potential predictors was analyzed using binomial logistic regression. A total of 385 participants aged 65 to 86 years were included in the present study. 17% developed anxiety symptoms. Factors associated with anxiety were age, living alone, loneliness, significant people support, fear of COVID-19, and health monitoring as a COVID-19 coping behavior. Older age, living alone, and significant people support were associated with lower anxiety, and loneliness, fear of COVID-19, and health monitoring as a COVID-19 behavior were associated with higher anxiety in community-dwelling older adults during the COVID-19 pandemic. These findings can help to develop effective measures for reducing anxiety and will support the development of a psychological intervention for tackling the mental health of community-dwelling older adults during the COVID-19 pandemic.

### PROFILE OF COMMUNITY-DWELLING OLDER ADULTS ONE YEAR INTO COVID-19

Helen Lach[1], Devita Stallings[1], Janice Palmer[2], and Rebecca Lorenz[3], *1. Saint Louis University, St. Louis, Missouri, United States, 2. Webster University, St. Louis, Missouri, United States, 3. University at Buffalo, Buffalo, New York, United States*

We surveyed community-dwelling older adults in 2020, primarily in the Midwest, to learn about their experiences during the initial months of the COVID-19 pandemic. In summer 2021, we collected quantitative and qualitative one-year follow-up data on 118 participants from the original cohort who agreed to be contacted later (n=246;

48% response rate). Respondents included mostly women (75.2%), White (95%) with a mean age of 76.4 years and 15 mean years of education; half were married (49.5), and 40.7% lived alone. Compared to 2020, participants had a higher mean score on the CESD10 (9.76 vs. 6.97); although self-rated health and quality of life scores were similar (94-95% good-excellent). Mean scores on the Brief Resilient Scale were similar. In this group, 99.3% had been vaccinated, and had a concern score of 3.7 out of 5 that there would be more COVID outbreaks. Themes from their reported adaptations during the pandemic included taking steps for physical and mental self-care, maintaining connections with others, and finding credible sources of information. Connecting with others online was the biggest technological change during this time, and the computer was the most valued technology. These educated, middle class older adults continued to adapt to the COVID-19 pandemic, but those who did not respond to the second survey may have had more health or mental health challenges. These findings suggest a need for health care providers to screen older adults for depression so that early interventions can take place.

### TRIPLE MALADY: OLDER PERSONS WITH MENTAL ILLNESSES AND COVID- 19: INSIGHTS FROM A PSYCHOGERIATRIC CLINIC IN NIGERIA

Olubukola Omobowale[1], Olufisayo Elugbadebo[2], and Oluwagbemiga Oyinlola[3], *1. University of Ibadan, Nigeria, Ibadan, Oyo, Nigeria, 2. College of Medicine, University of Ibadan, Ibadan, Oyo, Nigeria, 3. University College Hospital, Ibadan, Ibadan, Oyo, Nigeria*

Background Older persons with mental illnesses have been differentially affected by COVID-19 because of reduced access to routine health care, the adverse social impacts of the preventive strategies, and inadequate knowledge of the ongoing COVID -19 pandemic.Adequate knowledge is crucial to ensuring adherence to the right preventive practices among older persons.

Methods A hospital-based comparative study was conducted among older persons attending the Psychogeriatric and Healthy Ageing clinics of the Geriatric Centre, University College Hospital, Ibadan. Data were gathered with a semi-structured interviewer administered questionnaire and SPSS version 23 was used to analyze the data. The level of significance was set at 5%.

Results A total of 390 respondents aged 60 and above were sampled in the two groups: 195 with a psychiatric diagnosis (PD) and 195 with a non-psychiatric diagnosis (nPD). Their mean age was PD: 72.2 (±7.4) years and nPD: 71.0 (±8.0) years. About 14.9% had dementia and mild cognitive impairment; 14.6% psychotic disorders; 13.8% mood disorders and 6.6% anxiety and somatoform disorders. The majority were aware of the ongoing pandemic (PD: 95.9%; nPD: 96.4%). The use of facemask (PD: 89.7%; nPD: 86.7%) was the commonest preventive practice. Male gender (OR:2.09, CI; 1.14-3.86, p= 0.018) and education (OR: 5.10, CI; 1.15-22.67, p= 0.032) were predictors among PD and nPD respectively.

Conclusion: Older persons with psychiatric diagnoses have gaps in their knowledge of COVID-19 compared to those without symptoms. Health education about the ongoing pandemic and prevention programs targeting the older population with mental illnesses would be beneficial.

### SHOT ON THE SPOT VACCINATION COMMUNITY OUTREACH

Alissa McIntyre[1], Rebecca Allen[2], Brian Cox[3], Loretta Wilson[4], Pamela Payne-Foster[5], JoAnn S. Oliver[6], Yan Luo[7], and Hee Lee[8], *1. University of Alabama, Tuscaloosa, Alabama, United States, 2. The University of Alabama, TUSCALOOSA, Alabama, United States, 3. Alabama Research Institute of Aging, Tuscaloosa, Alabama, United States, 4. Rural Alabama Prevention Center, Eutaw, Alabama, United States, 5. Rural Health Institute/Community Rural Medicine, Tuscaloosa, Alabama, United States, 6. Capstone College of Nursing, Tuscaloosa, Alabama, United States, 7. The University of Alabama, Tuscaloosa, Alabama, United States, 8. University of Alabama, Birmingham, Alabama, United States*

Due to the COVID-19 pandemic disproportionately affecting Black communities, The University of Alabama (UA) partnered with the Rural Alabama Prevention Center (RAPC), a community-based healthcare organization to improve the vaccination rate from 34% vaccinated Alabama Black Belt residents to 70% over a year. Health literacy training is provided to community health workers and students who, along with the team members, volunteer at pop-up Shot on the Spot vaccination sites to administer surveys collecting demographics and vaccine hesitancy data. Team members provide health literacy information and answer questions non-vaccinated individuals have. This vaccination intervention has led to drastic rate increases, such as, Choctaw County having a 36.6% increase since the beginning of the project in August (30.7% to 67.30%). However, some counties have low vaccination rate changes, such as, Crenshaw County with a rate change of 15.4% (19.5% to 34.90%). Notably, the Alabama Black Belt currently stands at a higher vaccination rate compared to Tuscaloosa County (UA's location), only having a rate of 44.3%. Within one year, there have been a total of 44 administered first and second vaccine doses and 435 booster doses, resulting in 50.24% vaccinated Black Belt residents. As the virus evolved into different variants, team members were able to observe an increase in administered booster doses in congruence with the rise of a new coronavirus variant. The partnership formed between RAPC and UA scientists and students is an important step in improving vaccination rates and building community research on minority and diverse populations.

### OLDER ADULT'S SEPSIS CARE: CROSSING THE CONTINUUM: LESSONS LEARNED, OPPORTUNITIES PRESENTED

Teresa Cranston, Joseph Kontra, and Lanyce Roldan, *Penn Medicine Lancaster General Health, Lancaster, Pennsylvania, United States*

Sepsis is the body's overwhelming response to infection that can lead to tissue damage, organ failure, and death. Delays in identification and treatment are associated with increased morbidity and mortality especially for older adults. It is key that clinicians practice collaboration and communication when diagnosing & treating patients that may have a different presentation. The Continuous Monitoring Unit

(CMU) consists of RNs, who monitor the Sepsis Dashboards 24 hours/7 days a week. These nurses evaluate all BPA data points. They dismiss the irrelevant and align the significant while looking for possible notes of infection. They act upon the BPA when the patient meets criteria. They collaborates with the provider to initiate recommended sepsis care. Once the sepsis diagnosis is confirmed, the CMU RN communicate with inpatient nursing via EMR shift reporting and Care planning tools. Nursing utilizes these tools for daily rounds with the health care team communicating patient discharge needs. Identifying the Sepsis survivor on admission allows for early sepsis survivor education, discharge preparation and outpatient follow up referrals. Case Management utilizes an electronic application for outpatient referrals and includes a Sepsis Survivorship alert. Community resources, alert to a sepsis diagnosis will follow up within 24-48 hours of discharge to insure that the patient is having a smooth transition. Referred providers can escalate care to the PCP for early intervention therefore reducing worsening state or readmission. Specifically, our home care agency monitors the patient closely upon discharge due to the sepsis survivor's tenuous state and reinforces sepsis education.

### A NURSING HOME–BASED COVID-19-ONLY REHAB PROGRAM: VARIABLES RELATED TO SUCCESSFUL COMMUNITY DISCHARGE

Orah Burack[1], Joann Reinhardt[1], Wingyun Mak[2], and Ruth Spinner[1], *1. The New Jewish Home, New York, New York, United States, 2. The New Jewish Home Research Institute on Aging, New York, New York, United States*

Many COVID-19 patients continue to test positive for COVID-19 beyond the typical quarantine period. Persistent positive testing complicated hospital discharges to nursing home (NH) based rehabilitation facilities when (in May 2020) New York State (NYS) required a negative COVID result prior to NH admissions. To meet the needs of medically stable persistent positive patients, NYS approved 19 NHs as COVID-only facilities (COF). These facilities provided rehabilitation services and allowed hospitals to regain critical space for incoming acute care patients. In the present study we describe the (1) establishment of a 100 bed COF, (2) patients admitted to the COF and care provided, and (3) predictors of successful discharge to the community.Information on the establishment of the COF was obtained from interviews with NH leadership and clinical staff. Patient, treatment, and outcome data were obtained from the NH's electronic health record. Of 319 COF admissions over four months, 54% were female and the mean age was 80 years (SD=10.56). 51% had cognitive impairment and the mean number of comorbidities was 6.75 (SD=2.47). All patients received physical and occupational therapy while 37% received speech therapy. Medical treatment included: anticoagulants (52%), oxygen (37%), antibiotics (35%), inhaler/nebulizers (31%), and oral steroids (15%). 59% of COF stays resulted in successful community discharges. This outcome was more likely for patients who had fewer comorbidities, less speech therapy, and did not require antibiotics. Implications for COVID care as well as leveraging NH potential during crisis will be discussed.

### FILE OF LIFE: OLDER ADULT PERSPECTIVES AND SUGGESTIONS TO INCREASE FOL USAGE

Kaye Dandrea, Teresa Williams, and Marilyn Gugliucci, *University of New England College of Osteopathic Medicine, Biddeford, Maine, United States*

IntroductionIn a home emergency, an accurate patient medical history is vital for proper medical decision-making. One method to provide medical information in an emergency is through the FILE OF LIFE™ (FOL); a document distributed by local medical centers and public service organizations completed by community adults and placed on their refrigerator in a magnetic folder. To date, there was no known study, until this one, conducted with older adults to check the FOL design suitability for older adults; the goal of this project. MethodsOlder adults were recruited through the UNE COM Geriatrics Education Mentor (GEM) program for this pilot qualitative research. Twenty-one GEMs volunteered to participate in five 90-minute focus groups. Questions were specific for each section - Patient Identifiers, Emergency Contacts, Medical Data, Medical Conditions, Allergies, and Medical Insurance Information, and the usability of the FOL. Qualitative content analysis followed by NVivo 12+ analysis applied inter-rater reliability methods and changes for each FOL section were based on reaching data saturation. ResultsFILE OF LIFE™ comments (N=328) were coded to the following themes: Layout, Font Size, Spacing, and Section Ordering. Sections were edited accordingly with "Identifiers," Medical Data, Medical Conditions, Allergies, and Emergency Contacts requiring substantive changes. Other minor changes were made throughout the form. ConclusionFurther research will ensure efficient usability by older adults and emergency response personnel. The FILE OF LIFE™, although a trusted resource for older community dwelling residents, has a chance to increase usability by incorporating the suggested changes from this research.

### REIMAGINING END OF LIFE: ALTERNATIVE APPROACHES TO SERIOUS ILLNESS CARE CONVERSATIONS

Maresi Berry-Stoelzle[1], Cristina Ortiz[2], Jessica Trepanier[1], Sarah Murray[1], Melissa Steffen[1], Bruce Alexander[3], and Kenda Stewart Steffensmeier[1], *1. Iowa City VA HCS, Iowa City, Iowa, United States, 2. University of Iowa VA, Iowa City, Iowa, United States, 3. Iowa City VA Health Care System, Iowa City, Iowa, United States*

Serious illness care conversations are intended to document and protect patients' medical treatment wishes. In the outpatient management of chronic diseases, it has been difficult to pinpoint ideal timing for these conversations. The goal of this project is to analyze current practice within a VA health care system for identifying and referring veterans for palliative care services in the last years of life. To develop a targeted intervention, we relied on different forms of information to identify life events which should trigger a goals of care conversation, possibly even a formal palliative care referral. This study describes how we operationalized knowledge gained from: 1) Veteran community members via a project-dedicated engagement panel, 2) semi-structured interviews with providers, and 3) review of patients' clinic notes written by clinicians during the last 5 years of life. Tapping into the knowledge and experience of multiple parties provided nuance and complexity to our approach to intervention development. From the Veteran community member engagement panel, we learned that trusting relationships were essential. Providers emphasized that having enough time during appointments and timing of the conversation during a patient's disease trajectory were important. Finally, chart review indicated a need for broaching serious illness conversations early and throughout the duration of their care. The goal of an earlier intervention is to engage veterans when they are medically stable and prior to prolonged hospitalization. These findings structured our ongoing intervention that focuses on identifying and reaching out to Veterans with serious illnesses.

### FEASIBILITY OF ECOLOGICAL MOMENTARY ASSESSMENT OF SURROGATES FOR COGNITIVELY IMPAIRED ICU PATIENTS: A PILOT STUDY

Tracy Taylor[1], Emily Burke[2], James Slaven[3], Bruce Pfeffer[4], Shelley Varner Perez[4], Sarah Banks[5], and Alexia Torke[2], *1. Regenstrief Institute, Inc., North Vernon, Indiana, United States, 2. Indiana University (IU) Center for Aging Research, Regenstrief Institute, Inc., Indianapolis, Indiana, United States, 3. Indiana University, Indianapolis, Indiana, United States, 4. Indiana University Health, Indianapolis, Indiana, United States, 5. Indiana University School of Medicine, Indianapolis, Indiana, United States*

We explore the feasibility of ecological momentary assessment (EMA) with surrogate decision makers (SDMs) of critically ill hospitalized adults, to obtain in-the-moment participant-reported data. In this small pilot, 23 SDMs were approached. 82.6% were white, 53.9% were female, and 53.9% were 55 years and older. 25% were spouse or partner, 25% were adult children, 30% were parents, and 20% were other relations. Average ICU length of stay was 13.46 days (range 1.73-77.79). 13 SDMs enrolled (56.52%). Using Twilio, an automated text was sent to SDMs containing a secure link to the surveys. Up to five questions about anxiety, depression, distress, communication, and spiritual well-being were sent up to twice per day. Most participants (84.62%) completed EMA surveys for 5 or more days. Most SDMs stated that the surveys were not burdensome. Of those who declined participation (n= 10), accessibility to a smartphone, stress, and availability to complete texted surveys were noted as barriers to participation. The data suggest that EMA is a valuable tool to inform clinical interventionists and care providers interacting with SDMs. Future research will assess EMA with a larger group of SDMs, utilizing questions that help identify gaps in communication and opportunities to provide support to SDMs. Although surrogates are often under high stress in the ICU setting, they are willing to provide real-time EMA feedback on their experiences. EMA can provide a "just in time" option for research and clinical intervention that focuses on bridging gaps that may contribute to better outcomes for SDMs.

### SESSION 6110 (POSTER)

### DEMENTIA CARE

### THE RISK OF DEMENTIA AMONG INDIVIDUALS WITH DEPRESSION AFTER A TRAUMATIC BRAIN INJURY

Joan Ashby[1], Miriam Nuno[1], Beatrice Ugiliweneza[2], Maya Deshmukh[1], and Maxwell Boakye[3], *1. University of California, Davis/Davis, Davis, California, United States, 2. Univerisity of Louisville, Louisville, Kentucky, United States, 3. Univerisity of Louisville/Louisville, Louisville, Kentucky, United States*

Although traumatic brain injury (TBI) has been found to be associated with dementia and depression, large-scale studies of US non-Veteran populations are limited. This study assesses the role of depression on dementia risk after TBI. Data was analyzed from 80,423 individuals with TBI age 55+ in the IBM MarketScan Commercial Claims and Encounter Database and the Medicare Supplemental Database between January 1, 2000 and December 31, 2019. Dementia diagnosis was captured from inpatient or outpatient visits with a "wash out" period of one year. Depression was defined based on documented diagnosis and/or the prescription of antidepressants. The median age was 69 years, 51% female, with a majority covered by Medicaid (51%). 62% of TBIs were moderate/severe and 16% mild. 44,234 (55.1%) individuals were diagnosed with depression with a median of 2.1 months after TBI and 24.4 months prior to dementia. Depression rates differed by sex (female: 54%, male: 44%, $p < 0.0001$) and insurance (Commercial: 36%, Medicaid: 49%, $p < 0.0001$). The median time to dementia was 158 months. The median time to dementia was 128 months among those with depression, while patients without depression did not reach this estimate within 250 months of follow-up ($p < 0.0001$). The risk of dementia increased significantly over time after depression among patients with TBI. This study provides evidence to support that depression after TBI represents a symptom of TBI rather than a prodrome of dementia. Future research will investigate the role of depression in the risk of dementia by race/ethnicity, insurance status, and TBI severity.

### DOES THE MICROBIOME INFLUENCE THE DEVELOPMENT OF DEMENTIA IN OLDER ADULTS?

Naomi Tesema, Kristen Wroblewski, Jayant Pinto, and Martha McClintock, *University of Chicago, Chicago, Illinois, United States*

Some data implicate microbes in the pathogenesis of Alzheimer's Disease and Related Dementias (ADRD). Whether the use of antibiotics predisposes to ADRD remains unknown. To investigate the relationship between antibiotics and subsequent cognitive function, we analyzed data from a longitudinal, nationally representative sample of older US adults (N=2,906, the National Social Life, Health and Aging Project). The use of antibiotic medications was collected by home interview at baseline. Five years later, cognition was assessed using a survey adapted version of the Montreal Cognitive Assessment (MoCA). Additionally, ADRD status was measured by a report of a physician's diagnosis (self or proxy). The association between baseline antibiotic use and an interval dementia diagnosis/MoCA scores was tested using multivariate logistic regression, controlling for age, sex, race and ethnicity, education, co-morbidities (modified Charlson Comorbidity Index), and cognition at baseline (Short Portable Mental Status Questionnaire). Older US adults who used antibiotics had lower MoCA-SA scores at 5-year follow-up (OR=3.94, 95% CI= 1.79-8.66). However, the use of antibiotics did not predict a subsequent diagnosis of dementia (OR=1.48, 95% CI= 0.44-4.95). Thus, antibiotic use may cause deleterious effects on cognitive function, but does not appear to have a clinical impact in terms of diagnosis of dementia. Further study of the role of microbes and drugs that modulate them may be useful in understanding ADRD.

### TAI CHI OR CONVENTIONAL EXERCISE ON PHYSICAL PERFORMANCE, SLEEP, AND QUALITY OF LIFE IN OLDER PEOPLE WITH DEMENTIA

Ran Gao[1], Chieko Greiner[2], Hirochika Ryuno[3], and shuhong Gao[4], *1. Kobe University, Kobe, Hyogo, Japan, 2. Kobe University, Kobe-city, Hyogo, Japan, 3. Kobe University, Kobe city, Osaka, Japan, 4. Hainan nursing home, Haikou city, Hainan, China (People's Republic)*

Aims: This study was to compare the therapeutic efficacy of Tai chi and conventional exercise on physical performance, sleep efficiency and quality of life among older people with dementia.

Methods: Eighty-one elderly mild to moderate older people with dementia living in a nursing home in China were screened and randomized to three groups. The intervention groups received a 12-week Tai chi or conventional exercise intervention. Participants were assessed at baseline, week 6, and week 12 between December 2020 and July 2021. The primary outcome was short physical performance battery, secondary outcomes including the sleep efficiency and quality of life. Generalized estimating equations and one-way analysis of variance were the main statistical tests.

Results: The mean age was 80.7 (SD 7.7) years and 74.1% of participants were women. Analysis indicated that there was a significant difference in both intervention groups in short physical performance battery, sleep efficiency and quality of life relative to control from 0-12 weeks, and the Tai chi group had a substantial advantage over the conventional exercise group.

Conclusions: Tai chi and conventional exercise could improve physical performance, sleep efficiency and quality of life in older people with dementia. Furthermore, Tai chi could be a more effective approach to improve physical performance.

### VALIDITY AND RELIABILITY OF THE ROWLAND UNIVERSAL DEMENTIA ASSESSMENT SCALE: IMPLICATIONS OF AN INDONESIAN STUDY

Renata Komalasari, and Elias Mpofu, *University of North Texas, Denton, Texas, United States*

Standardized dementia screening self-report assessments from developing countries are increasingly adopted in developing countries. Yet the cross-cultural transportability of dementia screening tools cannot be assumed. The Southeast Asia region has one of the fast-growing geriatric populations but no locally developed or validated measures of cognitive decline among older adults. This study aimed to determine the validity and reliability of scores from the Rowland Universal Dementia Assessment Scale (RUDAS) for use in the multiethnic older adult population in Indonesia. Participants were 135 Indonesian older adults (females = 61.5%, males = 38.5%; age range = 60-82) who were patients of a geriatric nursing centre. They completed the Indonesian translation of the Rowland Universal Dementia Assessment Scale (RUDAS-Ina). Exploratory factor analysis and confirmatory factor analysis of the scores yielded two factors explaining a total of 55.13% (the first factor explained 38.18%; the second factor explained 16.96%) of the variance for the entire set of variables. The reliability of scores from the RUDAS-Ina was marginally satisfactory (Cronbach $\alpha$ = .61), suggesting a need for locally developed measures for use with the Indonesian geriatric population. Future studies should consider the feasibility of developing and calibrating an adapted RUDAS to the Indonesian setting, which also may be studied in other Southeast Asian countries.

### INCIDENCE AND ASSOCIATED FACTORS OF DEMENTIA IN PEOPLE WITH SCHIZOPHRENIA: A POPULATION-BASED COHORT STUDY

Yingyang Zhang, Pengcheng Wang, Jennifer Y.M. Tang, Gloria H.Y. Wong, and Hao Luo, *The University of Hong Kong, Hong Kong, Hong Kong*

Existing evidence suggests that people with schizophrenia may have an increased risk of dementia. This study aimed to quantify the incidence of dementia and examine putative risk factors associated with dementia in people with schizophrenia. This is a cohort study using population-based electronic health records of people who visited public hospitals in Hong Kong. We included participants (≥ 45 years) with schizophrenia diagnosis between 2009 to 2018 without dementia and followed them until March 2021. Incidence of dementia was calculated, and Cox proportional hazard regression was utilized to estimate hazard ratios of dementia, adjusting for covariates. A total of 20,901 individuals (mean [SD] age, 58.4 [10.3] years, 55.7% were women) were followed for 154,630 person-years (median [interquartile range] follow-up, 7.6 [4.7-10.3] years). The incidence of dementia was 5.1 [95% CI, 4.7-5.4] per 1000 person-years (those aged 45-64: 2.0 [1.8-2.3]; those 65 and above: 17.0 [15.6-18.5] per 1000 person-years). Factors independently associated with all-cause dementia were age (Hazard Ratio, 1.11 [95% CI, 1.10-1.11]), diabetes (1.62 [1.36-1.93]), bipolar disorders with at least five-year duration (2.12 [1.50-3.01]), and schizophrenia duration (1.02 [1.01-1.04]). Stratified analysis indicated that the association of these factors with dementia had differences in different sex and age groups. The incidence of dementia after schizophrenia diagnosis was high and two risk factors of dementia that are different from the well-established ones were identified.

### PEOPLE LIVING WITH DEMENTIA GUIDED THE WAY: ADAPTIVE STAKEHOLDER ENGAGEMENT TO DESIGN A PRAGMATIC CLINICAL TRIAL

Michael Lepore, *University of Maryland School of Nursing, Baltimore, Maryland, United States*

Research on non-pharmacologic interventions for people living with dementia (PLWD) has shown many benefits, but healthcare systems do not offer these interventions widely. To strengthen understanding of the real-world effectiveness of evidence-based non-pharmacologic interventions for PLWD requires a pragmatic approach to trialing interventions in the contexts of healthcare systems where they are standardly delivered. Designing pragmatic trials with the engagement of primary stakeholders can support trial feasibility and meaningful outcome measurement, but little evidence is available on approaches for engaging PLWD in designing pragmatic trials. This longitudinal case study introduces adaptive stakeholder engagement, a three-phase approach to engaging PLWD in planning a pragmatic trial. First, a multistakeholder workshop including PLWD was held where participants prioritized topics for research, including research on the impact of increasing social activity. Second, an evidence-based non-pharmacological intervention that addresses this priority area was piloted with PLWD and qualitative feedback was collected from PLWD over the course of intervention piloting to inform the trial design. Finally, PLWD were engaged in facilitated monthly meetings to provide input on the pragmatic trial design. In addition to informing intervention selection, input collected from PLWD informed the pragmatic trial in several ways, including approaches to PLWD recruitment, disclosure of research to PLWD, and intervention orientation processes. The adaptive stakeholder engagement model involved PLWD in different roles, as workshop participants, intervention participants, and trial advisors. This model enabled PLWD to be engaged in priority setting, infrastructure development, and trial protocol design, and may be useful for researchers planning future pragmatic trials.

### THE PRESCRIPTION DRUG LANDSCAPE OF ALZHEIMER'S DISEASE AND RELATED DEMENTIAS

Lauren Bangerter[1], Callahan Clark[2], Megan Jarvis[3], Emma Coates[4], Natalie Sheils[5], Alyssa Wong[4], and Ken Cohen[2], *1. United Health Group, Edina, Minnesota, United States, 2. United Health Group, Minnetonka, Minnesota, United States, 3. Optum Labs, Minnetonka, Minnesota, United States, 4. UnitedHealth Group, Minnetonka, Minnesota, United States, 5. United Health Group, Eden Prarie, Minnesota, United States*

Alzheimer's Disease and Related Dementias (ADRDs) are debilitating conditions that impact cognition and function of nearly 5 million adults in the U.S. This study describes the landscape of prescription drugs to treat cognitive and behavioral symptoms of ADRD among Medicare Advantage Part D enrollees with ADRD (Dementia, Alzheimer's Disease, Parkinson's, Frontotemporal Dementia, Lewy Body, Vascular Dementia, or Mild Cognitive Impairment). We assessed prevalence of prescription fills in the years pre- and post-ADRD diagnosis. Prescription fills to manage cognitive symptoms, were assessed by acetylcholinesterase inhibitors and NMDA receptor antagonists. Prescription fills to manage behavioral and psychological symptoms of dementia were assessed by antipsychotics, mood stabilizers and anticonvulsants, antidepressants, sedative-hypnotic z-drugs, and benzodiazepines. The final sample (N=161,368) consisted of 79.2% white, 62.2% female with median age 83 (IQR 77-89). Most drug fills increased from pre-diagnosis year to post diagnosis year including Acetylcholinesterase inhibitor (15.3% to 35.5%), NMDA Receptor Antagonists (5.8% to 16.6%), mood stabilizers and anticonvulsants (15.8% to 18.2%), and antidepressants (19.4% to 24.2%). Benzodiazepine use remained stable (13.0% to 13.6%) as did Sedative hypnotic z- drugs (1.9% 1.8%). Results reveal polypharmacy (median of 11 prescriptions post-ADRD diagnosis) and high healthcare utilization (median of 10 specialty types visited post-ADRD diagnosis). This study shows discrepancies between real-world pharmacologic ADRD management and evidence-based practices. This work highlights the need for innovative care models that simultaneously address polypharmacy and optimally coordinated care for people with ADRD.

### DELIRIUM-LIKE BEHAVIORAL SYMPTOMATOLOGY IN COMMUNITY-DWELLING OLDER ADULTS WITH DEMENTIA

Jasmine Vickers[1], Danny Wang[2], and Carolyn Pickering[2], *1. University of Alabama at Birmingham, Birmingham, Alabama, United States, 2. The University of Alabama at Birmingham, Birmingham, Alabama, United States*

Delirium, a sudden and acute confusional state, is known to be more prevalent and deleterious in older adults with dementia; yet the literature on estimates of delirium in community-dwelling older adults with dementia is scarce. The aim of this study was to determine the patterns and frequency of delirium-like symptoms in community-dwelling older adults with dementia, as reported by their caregivers (n=50) in a 21-day diary study (n=1389 diaries). Caregiver reports of severity scores of care-recipient behaviors that were one standard deviation above the mean were considered outliers, representing a sudden and acute change. Caregivers that reported outlier scores for two consecutive days were considered delirium-like symptoms. Descriptive statistics were used to determine the number of caregivers who reported delirium-like symptoms and the percentage of days with symptoms present. Additionally, chi-square tests and Independent samples T-test were used to determine the associations between delirium-like symptoms and participant characteristics. Caregivers were predominately White/Caucasian (89%), female (94%), and a son/daughter (60%) of the care-recipient. Caregivers had an average age of 52.6 years and were caregiving an average of 3.9 years. The care-recipients had dementia for an average of 4.3 years. Caregivers reported delirium-like symptoms for 12(24%) care-recipients and this rate was consistent with previous research findings. Caregivers reported delirium-like symptoms, on average, for 6.9 days (33%) of the 21-day diary period. More research is needed to understand the occurrence and patterns of delirium in community-dwelling older adults with dementia to improve home care services, outpatient services, and support for caregivers.

### UTILIZATION TRENDS OF GLUCOSE-LOWERING DRUGS FOR TYPE 2 DIABETES IN OLDER ADULTS WITH AND WITHOUT DEMENTIA

Chandrasekar Gopalakrishnan[1], Dae Kim[2], and Elisabetta Patorno[3], *1. Brigham and Women's Hosp./ Harvard Medical School, Boston, Massachusetts, United States, 2. Hebrew SeniorLife, Boston, Massachusetts, United States, 3. Brigham and Women's Hospital, Harvard Medical School, Boston, Massachusetts, United States*

Dementia is an important consideration in the therapeutic management of older adults with type 2 diabetes (T2D). Given the recent availability of numerous second-line therapeutic agents, there is limited evidence on uptake of these newer medications by dementia status. Using Medicare fee-for-service data from 2013-18, we identified a cohort of patients with T2D who initiated a glucose-lowering drug (N=3,355,725; mean [SD] age, 74.8 (6.9) years) and stratified our analysis based on the presence of a diagnosis for dementia in the year prior to treatment initiation. Amongst patients with dementia (N=511,835; mean [SD] age, 79.9 (7.7) years), metformin use remained stable from 25.8% to 24.8%, whereas sulfonylureas (20% to 17.5%) and insulin (31.8% to 26.2%) use declined. Amongst patients without dementia (N=2,843,890; mean [SD] age, 73.8 (6.3) years), metformin (31.7% to 24.7%), sulfonylurea (22.1% to 19.4%) and insulin use (18.7% to 14.6%) decreased. DPP-4i and glitazones use remained largely stable whereas the use of newer agents such as SGLT-2i and GLP-1 RA increased steadily in both patients with and without dementia though the uptake was much higher in patients without dementia. By the end of 2018, 19.6% of patients initiated either a SGLT-2i or a GLP-1 RA amongst those without dementia whereas only 11.1% did so amongst those with dementia. In conclusion, older medications such as metformin, sulfonylureas and insulin accounted for about two-thirds of initiated glucose-lowering medications and were more frequently used by patients with dementia, though their use declined steadily over time with the availability of newer agents.

### QUALITY OF LIFE PREDICTS END-OF-LIFE CARE TRANSITIONS AMONG PERSONS WITH DEMENTIA OVER NINE YEARS

Suzanne Sullivan, Chin-Shang Li, and Yu-Ping Chang, *University at Buffalo, Buffalo, New York, United States*

Physical, financial, and emotional burdens are profound and affect the quality-of-life of persons with dementia (PWD) and their caregivers, particularly as cognitive decline accelerates and death approaches, which leads to burdensome, non-beneficial transitions in care that can lead to further decline of PWD. Measures of quality-of-life for PWD and their caregivers include important physical, psychological/cognitive, existential, caregiving burden, and socio-demographic factors that may be useful for predicting and preventing these end-of-life transitions. This secondary data analysis of the National Health and Aging and Trends Study (NHATS) linked to the National Study of Caregiving (NSOC) investigates the relationship between quality-of-life factors and end-of-life care transition (overnight hospitalization, hospice use, and place of death) over nine years (2011-2019) among caregiver/PWD dyads using multivariable logistic regression for hospice and place of death and multivariable logistic regression with generalized estimating equations for longitudinal overnight hospitalizations. Overnight hospitalizations are predicted by overall health (p=0.048), pain (p=0.016), and having a regular doctor (p=0.012). Predictors of hospice include health prevents enjoying life (p=0.0002) and receiving food stamps (p=0.008). Place of death is predicted by PWD needing >30 mins to fall asleep (p=0.007), dementia status (p=0.015), health preventing enjoying life (p=0.0005), race (p=0.033), and census division (p=0.012). End-of-life care transitions can be predicted far in advance by quality-of-life and socio-demographic factors of PWD and their caregivers. With this knowledge it may be possible to develop upstream interventions to facilitate appropriate transitions and improve end-of-life quality-of-life far in advance of avoidable care transitions of PWD.

### ACCEPTABILITY OF A TAILORED MUSIC LISTENING INTERVENTION FOR SLEEP IN OLDER ADULTS WITH DEMENTIA

Sophia Geisser[1], Isabella Ciccone[1], Justine Sefcik[2], Nancy Hodgson[3], and Darina Petrovsky[1], *1. Rutgers, The State University of New Jersey, New Brunswick, New Jersey, United States, 2. Drexel University, Philadelphia, Pennsylvania, United States, 3. University of Pennsylvania, Philadelphia, Pennsylvania, United States*

There is a lack of non-pharmacological interventions aimed at improving sleep disturbances among persons living with dementia (PLWD). In this randomized clinical trial, we examined the acceptability of a tailored music listening intervention on sleep disturbances in PLWD. We recruited 33 PLWD age >60 and their care partners (‘dyad’); 30 dyads completed the study. Dyads were randomly assigned to a wait-list control or intervention group, which included listening to tailored sleep-inducing music 30 minutes every night for 4 weeks. Acceptability outcomes included a questionnaire of perceived benefits and satisfaction with study participation; and a single item examining the PLWD’s level of enjoyment while listening to music. In addition, we conducted semi-structured exit interviews. We used descriptive statistics and conventional content analysis to examine the qualitative data. Most PLWD and care partners identified themselves as female, Black and non-Hispanic. One third responded that the study benefited care partners (n=11, 36.7%) and helped improve PLWD quality of life (n=11, 37.9%). The majority (n=28, 93.3%) felt that this study was clearly explained to them, while a third felt that the study required too much work (n=10, 33.3%). Most dyads enjoyed listening to music that was selected for them (n=38, 93.3%) and reported that the intervention and music selection was acceptable. We identified four themes regarding the music selections: music was relaxing, enjoyable, promoted sleep, and brought back memories. Overall, this intervention shows promise for decreasing sleep disturbances among PLWD. Future studies involving music interventions for sleep should examine the mechanism of action.

### ASSOCIATION OF HBA1C WITH BEHAVIORAL SYMPTOMS OF DEMENTIA IN VA NURSING HOME RESIDENTS WITH DIABETES (2013–2019)

Alexandra Lee[1], Ying Shi[2], Matthew Growdon[2], Kristine Yaffe[2], Kenneth Covinsky[2], and Sei Lee[2], *1. University of California, San Francisco, San Francisco, California, United States, 2. UCSF, San Francisco, California, United States*

For people with dementia, behavioral symptoms of dementia are often signs of distress and may be caused by pain, confusion, agitation, or negative moods. In people with diabetes, pain, confusion, and agitation may result from aspects of diabetes management, including poor glycemic control, episodes of very low or high blood sugar (hypo- and hyperglycemia), insulin injections, or fingerstick pricks to check blood glucose. Few studies have examined how diabetes control contributes to behavioral symptoms of dementia. We conducted a retrospective study in VA nursing home (NH) residents aged 65+ with both dementia and diabetes, and a NH stay>45 days. We used the second assessment of the Minimum Dataset (MDS 3.0) to identify behavioral symptoms of verbal or physical outbursts during the prior 7 days. We conducted mixed-effects logistic regression, with clustering by NH facility. The 5060 residents were overwhelmingly male (99%), had an average age of 78, with mean HbA1c was 7.1 (standard deviation 1.6). The overall prevalence of any behavioral symptoms was 16.7%. Compared to residents with HbA1c < 6%, behavioral symptoms were slightly more common among residents with HbA1c 6-7%, 7-8%, and >9%, but not statistically significantly different. Behavioral symptoms were significantly more common in residents with HbA1c 8-9% (prevalence 19.6%) than in residents with HbA1c < 6% (prevalence 15.5%; odd ratios adjusted for NH facility: 1.35, 95% confidence interval: 1.05-1.75). Additional research is needed to determine which aspects of diabetes management may be contributing to excess risk of behavioral symptoms of dementia.

### COSTS OF DEMENTIA CARE AND EARLY-STAGE PREVENTIVE NURSING: A COMPARATIVE BIG DATA STUDY IN OSAKA

XUJING HU[1], Reiko Kanaya[2], Ryohei Yamamoto[1], Hiroshi Toki[3], Yasushi Takeya[1], and Miyae Yamakawa[1], *1. Osaka University, Suita, Osaka, Japan, 2. Osaka University, Osaka, Osaka, Japan, 3. Health and Counseling Center, Suita, Osaka, Japan*

Data show a substantial increase in the costs of dementia care nationally and globally. We assessed the factors increasing financial pressure, and found an association between the costs and the severity of dementia. We suggest that early diagnosis and preventive nursing care can reduce the deterioration associated with dementia, and therefore the overall costs of dementia care. The purpose of this study was to determine the relationship between the costs of dementia care and early-stage preventive nursing care in a sample of people with dementia aged 65 to 85 years, using the Medical Care Insurance and Osaka Prefecture Long-Term Care Insurance Database (N = 17973). We found that early care might have a significant effect on formal care costs in the early period of dementia but did not influence the medical care costs of dementia. During the first 5 years, the long-term care costs of people with dementia with an initial care level of Support 1/2 (one type of care level of Japanese long-term care insurance system, and the participants receive preventive services) were $4,389, while people with an initial care level of Care 1/2 (the other type of care level, and the participants receive nursing services) were $12,333. About 20% of people who received preventive care recovered, suggesting that preventive nursing care can restore the ability of people with early-stage dementia. This might be the reason for the reduction in long-term care costs. This research provides a basis for supporting preventive nursing care and extending healthy lifespans.

### PAIN IN PERSONS WITH DEMENTIA AND APATHY IN NURSING HOMES

Yo-Jen Liao[1], and Ying-Ling Jao[2], *1. Pennsylvania State University, Pennsylvania State University, Pennsylvania, United States, 2. Pennsylvania State University, University Park, Pennsylvania, United States*

Apathy and pain commonly occur in persons with dementia and significantly impact their quality of life. However, communication barriers in persons with dementia make pain assessment challenging. Apathy further complicates pain management in dementia due to decreased facial expression and verbal communication. This study aims to examine pain management in persons with dementia and apathy. This descriptive study included 13 residents with dementia and apathy from two nursing homes in Pennsylvania. Data on pain, pain-related diagnoses, and treatments were extracted from medical records. Participants' mean age was 90 years old, and their mean apathy level was 54.6. All 13 participants had pain-related diagnoses with an average of 3.1 pain-related diagnoses (range=1-7). Four participants (30.7%) had pain reported in their medical records with osteoarthritis being the most common diagnosis (38.5%). Eight participants (61.5%) had pain-related diagnoses but did not have regular pain medication administered, and 3 of them (37.5%) had pain reported. In addition, five participants had one or more acute pain-related diagnoses, including surgery, fractures, and falls, and only 2 (40%) of them had pain reported. The average number of prescribed pain medications was 0.4 and 1.1 for regularly administered and as-needed medications, respectively. Acetaminophen was the most common administered medication. Overall, the results pointed out the potential issue that pain may be underrecognized and undermanaged in this population. More research is needed to examine the pain assessment and treatment in this population to promote pain management in persons with dementia and apathy.

### REJECTION OF CARE AND AGGRESSION AMONG OLDER VETERANS WITH DEMENTIA WITH AND WITHOUT POSTTRAUMATIC STRESS DISORDER

Bada Kang[1], Wei Pan[2], Michele Karel[3], Kirsten Corazzini[4], and Eleanor McConnell[2], *1. Yonsei University, Seoul, Seoul-t'ukpyolsi, Republic of Korea, 2. Duke University, Durham, North Carolina, United States, 3. VA Central Office, Washington, Washington, United States, 4. University of New Hampshire, Durham, New Hampshire, United States*

Veterans with co-occurring dementia and posttraumatic stress disorder (PTSD) living in residential long-term care encounter a range of physical and social stimuli, which may trigger trauma-related distress that can be exacerbated and manifested with care rejection and aggression. Yet, it is largely unknown how PTSD influences manifestation of care rejection and aggression in older veterans with dementia. Guided by the need-driven dementia-compromised model, this study examined the moderation effect of PTSD on pathways from background factors, and interpersonal triggers to care rejection and aggression among veterans with dementia with and without co-occurring PTSD. In this secondary analysis study, a multi-group structural equation modeling was conducted using program evaluation data of 315 veterans with dementia from the STAR-VA behavioral intervention implemented in 76 Veterans Health Administration-operated nursing homes. Although no moderation effect of PTSD on the overall model was found, findings revealed distinct patterns of relationships among background factors, interpersonal triggers, and care rejection and aggression between veterans with dementia with and without PTSD. The magnitude of the direct effects of interpersonal triggers on care rejection was greater in veterans with PTSD. Findings on the indirect effect of depression via interpersonal triggers on care rejection and direct effect of functional status on aggression only in veterans with PTSD implies that different mechanisms may underlie distressed behavior depending upon whether or not a veteran has PTSD. This study also underscores the importance of an enhanced focus on trauma-informed care, and individualized multi-component symptom management approach for veterans with dementia and PTSD.

### USE OF HIGH COST CARE AMONG VETERANS WITH COMORBID MENTAL ILLNESS AND ALZHEIMER'S DISEASE AND RELATED DEMENTIAS

Katherine Miller[1], and Megan Shepherd-Banigan[2], *1. University of Pennsylvania, Durham, Pennsylvania, United States, 2. Durham VA Medical Center, Durham, North Carolina, United States*

As Vietnam-era Veterans age, VA faces a growing need to manage aging-related conditions, such as Alzheimer's disease and other dementia (ADRD), for this complex patient population. ADRD is of particular concern as military related factors, such as psychiatric illnesses, increase ADRD likelihood and complicate care management. In particular, decreasing use of low value care (e.g., emergency department (ED) care) is one approach to promote appropriate supportive care for Veterans with comorbid ADRD and psychiatric illness, but is understudied. We describe differences in potentially low value health care among older Veterans with ADRD 12 months after a new ADRD diagnosis. We compare Veterans with mental illness (MI) pre-ADRD diagnosis (major depressive disorder, post-traumatic stress disorder, or generalized anxiety disorder) to those without pre-existing MI. Potentially low value care includes ED use, hospitalization, and 30-day readmissions 12 months after a new ADRD diagnosis. Compared to Veterans with no pre-existing MI, Veterans with pre-existing MI are more likely to be younger (79 vs. 82 years old), male, married, White, Hispanic. In the year after new ADRD diagnosis, 16.9%, 21.6% and 2.6% of Veterans with MI had any ED visit, hospitalization, and 30-day readmission, respectively. In contrast, only 7.3%, 9.5%, and 0.9% of Veterans without pre-existing MI had any ED visit, hospitalization, and 30-day readmission, respectively. Our findings suggest that reducing low-value care may be an appropriate intervention target to improve care quality for Veterans with ADRD and mental illness.

### DIFFERENCES IN NEUROPSYCHIATRIC SYMPTOMS AMONG RACIAL AND ETHNIC GROUPS LIVING WITH DEMENTIA IN HOME HEALTHCARE

Rebecca Lassell[1], Kimberly Convery[1], Jason Fletcher[1], Tracy Chippendale[1], Shih-Yin Lin[1], Tessa Jones[1], Aditi Durga[1], and Abraham Brody[2], *1. New York University, New York, New York, United States, 2. HIGN at the NYU Rory Meyers College of Nursing, New York, New York, United States*

The relationship between dementia and neuropsychiatric symptoms is well documented. Yet, little is known about the prevalence of neuropsychiatric symptoms among diverse homebound persons living with dementia. Guided by an intersectionality framework we asked: 1) Is there an association between the presence of individual neuropsychiatric symptoms and racial and ethnic groups? 2) If so, do these symptoms differ by dementia stage among groups? We conducted a cross-sectional study of n=190 receiving skilled home healthcare in Utah, New York, and New Jersey and enrolled in the DSM-H trial. We prospectively measured symptom prevalence with the Neuropsychiatric Inventory Questionnaire and dementia stage using the Quick Dementia Rating System. We performed Chi-square tests to determine the association of individual symptom prevalence with race and ethnicity and cross tabs to descriptively stratify individual symptom prevalence by dementia stage among groups. Participants were 11.9% Hispanic, 28% non-Hispanic Black, 56.1% non-Hispanic white (white). The prevalence of delusions was significantly higher in Hispanic and non-Hispanic Black groups than whites (41.7% & 41.8% vs. 22.5%) and elation/euphoria in Hispanic and non-Hispanic Black groups than whites (20.8% &12.7% vs. 5.4%). Delusions were most prevalent in non-Hispanic Black groups with mild dementia (53.8%) and Hispanics with moderate dementia (50.1%), while elation/euphoria was most prevalent for Hispanics with severe dementia (33.3%). Other neuropsychiatric symptoms showed no significant differences. These findings expand our knowledge of differences in neuropsychiatric symptoms among racial and ethnic persons living with dementia, which can inform future studies and targeted interventions that address disparities and improve care.

### DEMENTIA CARE IN THE LAST YEAR OF LIFE: EXPERIENCES IN A COMMUNITY PRACTICE

Mairead Bartley[1], Jennifer Manggaard[2], Karen Fischer[2], Diane Holland[2], and Paul Takahashi[2], *1. Mayo Clinic Rochester, Rochester, Minnesota, United States, 2. Mayo Clinic, Rochester, Minnesota, United States*

Objectives: People with dementia often have high care needs at end of life. We compared care delivery in the last year of life for people living with dementia in the community (home/assisted living facilities, ALF) to those in skilled nursing facilities (SNF).

Methods: This was a retrospective study of people who died with a diagnosis of dementia in the community compared with SNF between 2013 and 2018. Primary outcomes of hospitalizations/ED visits in the last year of life were measured. Secondary outcomes included advance care planning (ACP) completion, hospice enrollment, provider visits and ICU admissions.

Results: Of 1203 people with dementia, 581 (48.3%) were living in a SNF and 622 (51.7%) in the home/ALF. At least one hospitalization was recorded for 70.7% in the home/ALF compared with 50.8% in the SNF ($p< 0.01$), similar to ED visits (80.2% versus 58% in home/ALF and SNF groups, respectively, $p< 0.01$). People in the SNF had more provider visits (median 9 (IQR 6, 12) compared with 5 (IQR 3,9) in the home/ALF group ($p< 0.01$). There was no ACP for

12% of those in the home/ALF, compared with 4.6% in the SNF(p< 0.01). Approximately half (56.5%) in the SNF were enrolled in hospice compared with 68.3% in the home/ALF group (p< 0.01), Median time in hospice was 26.5 days in the SNF versus 30 days in the home/ALF (p=0.67).

Conclusions: People living with dementia have frequent acute care utilization in the last year of life. Hospice care was more common in the home/ALF. Time in hospice was short.

### DEMENTIA AND THE ROLE OF EVIDENCE-BASED GUIDELINES IN PRIMARY CARE: RESULTS OF A CLUSTER RANDOMIZED CONTROLLED TRIAL

Sonia Lech[1], Julie L. O'Sullivan[2], Robert P. Spang[3], Jan-Niklas Voigt-Antons[4], Paul Gellert[1], and Johanna Nordheim[1], *1. Charité – Universitätsmedizin Berlin, Berlin, Berlin, Germany, 2. Charité-Universitätsmedizin Berlin, Berlin, Berlin, Germany, 3. Technische Universität Berlin, Berlin, Berlin, Germany, 4. University of Applied Sciences Hamm-Lippstadt, Hamm, Nordrhein-Westfalen, Germany*

General practitioners (GPs) play a key role in the care of people with dementia (PwD). However, the potentials of evidence-based dementia guidelines in primary care remain unclear. The main objective of the present study was to examine the role of guideline-based dementia care in general practices and to evaluate the effectiveness of a tablet-based intervention aiming at improving guideline-based care. A two arm, cluster-randomized controlled trial (cRCT) with an intervention group (tablet-based intervention) and a control group (treatment as usual) was conducted from April 15, 2018, to July 31, 2021, in Berlin and the surrounding area. Primary outcome was defined as adherence to dementia guideline recommendations after 9 months. Secondary outcomes included various health outcomes assessed in PwD (e.g., quality of life). Linear Mixed Models were used to analyse cross sectional baseline data. Primary and secondary outcomes were analysed by intention-to-treat analysis and using Mixed Models. Data of N = 28 GPs and N = 91 PwD was analysed. PwD were on average 80.5 years old (SD = 6.3), 59% were female. Self-reported overall guideline adherence of GPs was on average 71% (SD = 19.4, range: 25–100). Further, lower guideline adherence was found to be significantly associated with higher numbers of patients ($\gamma 10 = -5.58$, CI = – 10.97, – 0.19, p = .04). The effectiveness of the cRCT is still under evaluation. Final results will be presented and discussed at the GSA Annual Scientific Meeting. Further, implications and conclusions of the trial will be provided.

### FORECASTING PREVALENCE AND MORTALITY OF ALZHEIMER'S DISEASE AND RELATED DEMENTIAS USING PARTITIONING MODELS

Igor Akushevich[1], Arseniy Yashkin[1], Mikhail Kovtun[2], Julia Kravchenko[3], Konstantin Arbeev[1], and Anatoliy Yashin[1], *1. Duke University, Durham, North Carolina, United States, 2. Duke university, Durham, North Carolina, United States, 3. Duke University School of Medicine, Durham, North Carolina, United States*

A forecasting model for prevalence and mortality based on a trend partitioning approach which models trends in age-adjusted prevalence and incidence-based mortality in terms of changes in interpretable epidemiologic quantities such as disease incidence and survival, is developed and applied to generate forecasts of Alzheimer's disease (AD) and related dementia (ADRD) prevalence and mortality up to 2035 using health data drawn from a 5% sample of the total Medicare population. Forecasts are generated for entire AD population and for unique subgroups characterized by age groups and the presence of high-impact health conditions (stroke, traumatic brain injury, pneumonia, hypertension, diabetes) prior to AD diagnosis. Then methodology using B-splines in key time points allows is used to analyze scenarios of possible interventions focused on the prevention and treatment of AD/ADRD. Prevalence of AD/ADRD is predicted to be stable between 2017 and 2035 primarily due to a decline in prevalence of pre-AD/ADRD-diagnosis stroke. Mortality, on the other hand, is predicted to increase. In all cases the resulting patterns come from a trade-off of two disadvantageous processes: increased incidence and disimproving survival. The projections are constructed with the assumption that future trends represent a superposition of historic trends in different time periods taken with weights. Sensitivity to assumptions on choice of specific weights are studied, and the approach to choose an optimal combination of weights and therefore, to minimize uncertainties of future forecasts of AD/ADRD prevalence and mortality is suggested and discussed.

### SESSION 6120 (POSTER)

### DIVERSITY, EQUITY, INCLUSION, AND ACCESSIBILITY

### RETURN MIGRATION AND AGING OF JAPANESE INDIVIDUALS LIVING IN GREATER NEW YORK: A QUANTITATIVE ANALYSIS

Itsuko Toyama[1], and Taeko Nakashima[2], *1. St. Andrew's University, Izumi City, Osaka, Japan, 2. Nihon Fukushi University, Handa-city, Aichi, Japan*

Our previous quantitative research studies have revealed that large numbers of older Japanese individuals living in overseas communities either maintain hope of receiving culture-oriented care or harbor aspirations of returning to Japan to live out their final days. Presently, by analyzing a quantitative research study on older Japanese individuals living in Greater New York in 2018, we clarify the characteristics of those individuals who have decided to return to Japan. In the research study, 2,057 questionnaires were distributed, and the overall return rate was 29.7%. Respondents were divided into three groups based on their answers regarding their intentions for the location of their final abode: the United States, Japan, or indecisive. We performed a multinomial logistic regression analysis based on age, gender, immigration status, having children in the United States, and academic background. The following is a summary of the findings: (1) as participants aged, the ratio of selecting either "Japan" or "indecisive" as opposed to "the United States" decreased, (2) compared to those holding citizenship, individuals with permanent residence selected both "Japan" and "indecisive" at greater rates (by approximately 3 times and 8.7 times, respectively); (3) compared to those having children in the United States, individuals without children in the United States selected both

"Japan" and "indecisive" at greater rates (by approximately 1.8 times and 2.2 times, respectively); (4) individuals with a graduate career displayed a tendency to select "the United States," and (5) gender and language proficiency were not critical attributes in determining the likelihood of selecting "Japan."

### BUILDING CAPACITY FOR PATIENT-CENTERED OUTCOMES RESEARCH IN THE AGING RESEARCH COMMUNITY

Taylor Gray[1], Sophia Webber[2], and Erin McGaffigan[3], *1. UMass Boston, Waltham, Massachusetts, United States, 2. University of Wisconsin- Madison, Madison, Wisconsin, United States, 3. UMASS Boston Gerontology Institute, North Reading, Massachusetts, United States*

Patient-centered outcomes research (PCOR) models are increasingly used to ensure stakeholders inform evidence-based systems of care. Unfortunately, older adults, especially those of low income, frailty, and color, are often left out of PCOR, leading to continued health and service disparities. The Aging PCOR Learning Collaborative was funded by the Patient-Centered Outcomes Research Institute in 2020 to conduct a series of multi-media education, training, and mentoring activities to change how older adults are viewed and engaged in research. This project was strongly guided by multiple advisory structures, including two stakeholder advisory boards, two older adult paid advisors, and two student advisors, all of whom assumed an integral role in the design and implementation of project activities, dissemination of products, and analysis and reporting of evaluation methods. Project staff applied a multi-domain evaluation framework to analyze data relevant to these activities, which demonstrated the effective role of advisory structures and the project's reach to over 300 older adults, researchers, funders, and academic leaders. This session will review the steps required to implement these activities as well as our project reach, outputs, and mid-term outcomes, providing one of the first glimpses into how to measure this significant shift in research paradigm.

### THE RELATIONSHIP BETWEEN HEALTHCARE STEREOTYPE THREAT AND ADVANCE CARE PLANNING AMONG HISPANIC PATIENTS

Valeria Cardenas[1], Aaron Storms[2], Sindy Lomeli[3], Carin van Zyl[4], and Susan Enguidanos[3], *1. University of Southern California, San Diego , California, United States, 2. Keck School of Medicine of USC, Los Angeles, California, United States, 3. University of Southern California, Los Angeles, California, United States, 4. USC, Los Angeles, California, United States*

Experiencing ethnic/racial stereotype threat, the psychological experience of confronting stereotypes in the healthcare system, has been found to negatively impact healthcare outcomes (e.g. dissatisfaction with healthcare, poorer mental and physical health, higher physician distrust). Few studies have examined healthcare stereotype threat among Hispanics, and no study has tested how it might impact advance care planning outcomes among Hispanic hospitalized patients. This study aims to examine the relationship between healthcare stereotype threat and advance care planning activities among Hispanic hospitalized patients. We analyzed secondary data collected from a feasibility study of a palliative care educational intervention among 50 Spanish-speaking, Hispanic patients, over the age of 40, who were hospitalized in a large public hospital. Fifty percent had discussed future care if they were to become seriously ill, 18% had written instructions about their care (living will or advance directive), and 32% had a durable power of attorney (proxy) for healthcare. Results of logistic regressions revealed that greater healthcare stereotype threat scores were associated with lower odds of having discussed future care or medical treatment with others (OR=0.49, p=.03). However, healthcare stereotype threat scores were not associated with having provided written instructions about future medical care (OR=0.44; p=0.07), or with having a durable power of attorney for healthcare (OR=0.89; p=0.76). Future research should investigate ways to improve the cultural sensitivity in broaching discussions of future medical treatment among seriously ill patients. Additionally, more research and attention to policy implication are needed to reduce racial stereotype threat experienced in the healthcare system.

### EXPERIENCE OF UNFAIR TREATMENT IN HEALTHCARE SETTING AND RELATED STRESS AMONG OLDER AMERICANS: RACIAL/ETHNIC GAPS

Eun-Hye Yi[1], Michin Hong[2], and Cherish Bolton[1], *1. Indiana State University, Terre Haute, Indiana, United States, 2. Indiana University, Indianapolis, Indiana, United States*

Guided by the intersectionality framework, this study examined the experience of unfair treatment in healthcare settings over lifetime and related stress. A subsample drawn from the California Health and Interview Survey 2017, including residents age 55 or higher, was used (N=12,261). Significant differences existed in unfair treatment and corresponding stress among racial/ethnic groups, including Whites, African Americans (AA), Hispanics, and Asian Americans (AS). Using weighted chi-square tests, we found that most Whites (75.99%) never experienced unfair treatment, while around 60% of AA answered never. Whites tended to feel extreme stress more when mistreated (23.47%) than Hispanics (14.83%) and AS (15.69%). Weighted logistic regression analyses revealed that younger older adults with lower mental health were more likely to experience unfair treatment across all race/ethnic groups. Intersectional factors contributing to unfair treatment experience were identified for each race/ethnic group. Being a female, living in poverty, poor health, being a naturalized citizen, and living in an urban area were factors for Whites while having higher education was a factor for AA. Mental health was associated with extreme stress for the unfair treatment in all racial/ethnic groups. Different contributors to the stress were found by race/ethnicity. Gender, poverty, citizenship, and length of staying in the U.S. were significant for Whites. For AA, poverty, healthcare insurance, and obesity were significant, and for AS, physical health and obesity were. This study highlights the importance of culturally/ethnically sensitive approaches shaping interventions and policies to enhance awareness about unfair treatment and preventing discrimination toward diverse older adults.

### RACIAL DISCRIMINATION IN HEALTHCARE SETTINGS OF OLDER ADULTS: SUBJECTIVE REASONS AND CONTRIBUTORS

Eun-Hye Yi[1], Michin Hong[2], and Cherish Bolton[1], *1. Indiana State University, Terre Haute, Indiana, United States, 2. Indiana University, Indianapolis, Indiana, United States*

Racism is prevalent in the United States; however, literature exploring racial discrimination experienced by older adults is still limited. The current study examined subjective reasons for discrimination and compared race/ethnic groups. Then, we examined the contributors to racial discrimination in healthcare settings. An older adult sample aged 55 or higher was drawn from California Health and Interview Survey 2017 for analysis (N=12,261). African Americans were the highest (13.06%) among five racial-ethnic groups who reported racial discrimination experienced in a lifetime in getting medical care, while Whites were the lowest (1.57%). Perceived reasons for discrimination were significantly different by racial/ethnic group. Only 3.5% of Whites perceived they were discriminated against due to their race, whereas racial/ethnic minorities perceived the main reason for discrimination was their race/skin color (African American: 55.43%, Others: 24.06%, Asian Americans: 20.26%, Hispanics: 18.22%). The weighted logistic regression analyses revealed that being a racial/ethnic minority, economic status, mental health status, citizenship, the length of living in the United States, and age were significantly associated with the experience of racial discrimination of older people. Analyses by race/ethnic groups found different contributors. For example, poverty was the most prominent factor in racial discrimination for Whites, while education was for African Americans. This study identified an apparent gap in lifetime discrimination toward racial/ethnic minority older people. Also, we found racial discrimination experience combined with systematic barriers. The findings of this study support the need for interventions for race/ethnicity-based trauma of older people and anti-racism framework education for healthcare professionals and researchers.

### FORMAL AND INFORMAL VOLUNTEERING AMONG OLDER ADULTS: ASSOCIATIONS WITH HEALTH OUTCOMES ACROSS SEXUAL ORIENTATIONS

Joseph Frey[1], Huei-Wern Shen[1], and Yi Wang[2], *1. University of North Texas, Denton, Texas, United States, 2. University of Iowa, Iowa City, Iowa, United States*

Volunteering is associated with positive physical and mental health outcomes among older adults. However, little is known about the impact of volunteering on health among older lesbian, gay, and bisexual (LGB) Americans. Using nationally representative 2016 Health and Retirement Study data, the present study explored associations between volunteering and health outcomes (i.e., self-rated health, current memory rating) across sexual orientations among older Americans. Two types of volunteering were considered in the study—formal volunteering (doing unpaid work for religious, educational, health-related, or other charitable organizations) and informal volunteering (providing unpaid help to friends, neighbors, or relatives who do not co-reside). We included 235 (5.7%) respondents who self-identified as LGB or something else, and 3,894 (94.3%) heterosexual-identified respondents. Controlling for health-related variables (e.g., ADLs/IADLs), SES (e.g., income), contextual features (e.g., urban/rural), and sociodemographics (e.g., age, gender, race), results from eight stratified OLS regression models showed that for heterosexual-identified older adults, both formal and informal volunteering promoted self-rated health ($p<.001$, respectively) and memory rating ($p<.001$, respectively). However, for LGB-identified older adults, only informal volunteering was detected to increase memory rating ($p<.038$). Findings suggest that the well-documented health benefits of formal volunteering may not be supported in the LGB population. The current theoretical model and discourse about productive aging should be modified to be more inclusive with respect to sexual orientation. Further research identifying the pathways (e.g., identity-related stigmatization and discrimination) between various types of volunteering and health outcomes among older LGB adults is urged and possible directions will be discussed.

### BARRIERS TO VACCINE UPTAKE IDENTIFIED BY COMMUNITY-BASED ORGANIZATIONS FOR INSITUTIONALLY UNDERSERVED GROUPS

Tracy Wharton[1], Emily Costello[2], Vincent Lafronza[3], and Oscar Espinosa[3], *1. National Network of Public Health Institutes, Kensington, Maryland, United States, 2. National Network of Public Health Institutes, Atlanta, Georgia, United States, 3. National Network of Public Health Institutes, Washington, District of Columbia, United States*

Through a competitive proposal process for a recent funding opportunity, 38 community based organizations submitted proposals that addressed vaccine hesitancy among adults who are members of racial or ethnic minority communities. Proposals were required to include discussion of barriers identified in the community of focus and evidence to support all assertions. Submissions ranged from single employee projects to large collaborative networks, and were submitted from all regions of the US, including both tribal and territorial areas, including more than 45 ethnic and language groups. Barriers were coded by two reviewers and six (6) primary themes were identified: access related to transportation, distance, or time; lack of culturally responsive materials or sensitivity of providers; structural issues such as poor data collection, historical inequity, mistrust, or systemic racism; messages coming from untrusted sources; misinformation or no information available; and differing cultural perspectives. The most commonly identified issues were related to mistrust, historical structural issues or fear and racism (n=23), and lack of access due to transportation, distance, or time (n=17). This group of proposals for funding represent a small cross-section of communities who continue to have significant pockets of unvaccinated persons. While it is possible to see themes for barriers that are encountered in increasing vaccination rates among adults, communities demonstrate extremely nuanced realities, filtered through a range of culturally and paradigmatically different ways of knowing. Success in public health initiatives requires intensive focus on the variance across these different perspectives and careful attention to appropriately focus outreach and messaging.

### EMBRACING GENDER DIVERSITY: REIMAGINING HEALTH AND HEALTH EQUITY FOR TRANSGENDER OLDER ADULTS

Teri Kennedy[1], Courtney Marsh[2], Quinnlyn Walcott[1], Paddy Qiu[3], Angela Li[1], and Sally Maliski[1], *1. The University of Kansas Medical Center, Kansas City, Kansas, United States, 2. The University of Kansas Medical Center, Overland Park, Kansas, United States, 3. The University of Kansas, Lawrence, Kansas, United States*

This presentation will share results of in-depth focus groups exploring eight transgender older adults' (TOAs) experiences of health and healthcare and related themes, explain weathering and factors contributing to health and healthcare disparities, and apply the Sexual and Gender Minority Health Disparities Research Framework to explore strategies to address individual, interpersonal, community, and societal factors to foster health and health equity. TOAs represent an underserved population at elevated risk for lower socioeconomic status, higher rates of disability and chronic illness, and poorer overall physical and mental health compared to their non-transgender LGBQ and cisgender peers. Due to their unique life experiences including difficult decisions about "coming out" and transitioning, lack of acceptance of being transgender, and physiological impacts of hormonal and surgical treatments, transgender individuals experience long-term chronic stress and accumulate health-related risks as they age, contributing to significant health disparities. Higher levels of illness, disability, and premature death among historically disadvantaged groups can be explained by weathering, the accumulated impact of social disadvantages and socio-political inequalities over the life course producing premature biological aging. A 2019 systematic review of LGBTQ aging literature found that only 10% of studies included trans-identified participants and few addressed the needs of transgender older adults. Understanding the lived experiences of TOAs including healthcare access and receipt, and psychological, social, and financial experiences across the life course, is crucial to understanding current disparities in health and healthcare, developing strategies to improve care and reduce inequities, and fostering health and health equity of transgender older adults.

### EFFECTIVENESS OF A HUMAN RIGHTS–BASED APPROACH ONLINE PROGRAM FOR GERONTOLOGICAL SOCIAL WORKERS

Minhong Lee[1], An Sok[2], and Kyeongmo Kim[3], *1. Dong-eui University, Busan, Pusan-jikhalsi, Republic of Korea, 2. Daegu University, Gyeongsan City, Kyongsang-bukto, Republic of Korea, 3. Virginia Commonwealth University, Richmond, Virginia, United States*

The human rights perspective is emphasized through the social work code of ethics and practice principles. However, the efforts to improve the human rights perspective of social workers were insufficient in gerontological social work education in South Korea. Thus, the study aimed to prove the effects of a human rights-based approach intervention for gerontological social workers, which consisted of nine online sessions using Zoom. PI developed the program based on McPherson's integrated framework for Human Rights Practice in Social Work. A quasi-experimental design was used. The experimental group consisted of 36 social workers, and the control group was 31 social workers at senior centers. The pre-post tests for the experimental group and posttest for the control group were conducted. The socio-demographic characteristics of the two groups were equivalent. The key measure was the Human Rights Lens in Social Work (HRLSW) scale consisting of two sub-dimensions: social problems as rights violations and clients as experiencing rights violations.The effect sizes of the online program's effects on the social worker's rights-based perspective were large over .90. In addition, the degrees two dimensions of the HRLSW at the posttest were significantly higher than those of the control group (social problems as rights violations: $d = 1.04$, $p < .001$; clients as experiencing rights violations: $d=0.98$, $p<.001$). Based on the findings of this study, the implications of human rights education and training were discussed to improve the human rights-based perspective of gerontological social workers.

### SESSION 6130 (POSTER)

### EDUCATION AND TRAINING

### AGEISM, CONTACT, AND OLDER ADULT ADLS RELATE TO MENTAL HEALTH TRAINEES' INTEREST IN GEROPSYCHOLOGY

Grace I. L. Caskie[1], Abigail Voelkner[1], MaryAnn Sutton[2], Anastasia Canell[3], and Eve Root[1], *1. Lehigh University, Bethlehem, Pennsylvania, United States, 2. UPMC Psychological Associates, Harrisburg, Pennsylvania, United States, 3. Lehigh University, Mineola, New York, United States*

Despite growing demand, few mental healthcare professionals specialize in clinical work with older adults. A better understanding of factors related to geropsychology interest may increase the pipeline of future geropsychologists. Graduate-level trainees (N=460; 67.4% doctoral; age=21-64) completed the Fraboni Scale of Ageism, Contact with Older Adults Scale, six indicators of interest in work with older adults, and an imagined "typical" older adult's ability to complete activities of daily living (ADLs). 60% imagined full ADL independence. Between 14.1%-25.7% expressed strong interest in education/training related to treating older adults and having some older adult clients; only 6.7% planned to specialize in clinical work with older adults. In regression analyses (R-squared=16%-32%), more ageist attitudes, less contact, and being a master's trainee were related to less interest. ADL status was significant only for interest in specialization; imagining more ADL-dependence related to higher interest in specializing in older adults. ADL status significantly moderated the relation of contact to interest in learning about issues related to older adults; ADL-based differences in interest were non-significant at low and average contact, but at high contact, interest in learning about older adults was significantly higher when the older adult was imagined as ADL-dependent rather than ADL-independent. Findings may indicate benevolent ageism partially motivates trainees' interest in learning about/working with older adults whom they imagine need more help with basic tasks of daily living. Increasing contact, reducing ageist attitudes, and providing more clinical opportunities with older adults may facilitate trainees' readiness and interest in future clinical work with older adults.

### ACCEPTABILITY AND FEASIBILITY OF A CAREGIVER FINANCIAL PREPAREDNESS PROGRAM

Lauren Stratton[1], Claire Grant[2], Monica Moreno[1], Kerry Lanigan[1], Katherine Judge[3], and Sam Fazio[1], *1. Alzheimer's Association, Chicago, Illinois, United States, 2. Cleveland State University, Cleveland Heights, Ohio, United States, 3. Cleveland State University, Cleveland, Ohio, United States*

Caregivers are often financially impacted, through health care costs, long-term care costs, and decreased income. A financial literacy program, Managing Money: A Caregiver's Guide to Finances, was developed to provide information on financial impacts in caregiving. The virtual program was delivered synchronously with a live presenter through an online platform and was rated with high acceptability. The virtual program was then shifted to an online format. Changes included removing the live presenter and group format in favor of a voiceover and self-paced movement, the addition of videos, and online resources. The asynchronous, self-paced online delivery of the program was evaluated through a survey of 146 caregivers. Participants had a mean age of 45.85 (SD=5.65); 55% female; 75% White; 46% cared for their grandparent and 45% to their parent. A Likert scale was utilized to indicate satisfaction with the program (1=strongly disagree, 5=strongly agree). Overall, participants indicated that the program provided important information on managing money (M=3.97, SD=.83); helped to understand the content (M=3.89, SD=.91); was easy to read and understand (M=3.99, SD=.90); and would recommend the program (M=3.91, SD=.84). The explanation of content areas were rated on a Likert scale (1=poor, 4=excellent); participants rated areas including having conversations about finances (M=3.05, SD=.80); avoiding financial fraud and abuse (M=2.98, SD=.81); covering care costs (M=2.98, SD=.81); and organizing legal plans (M=3.09, SD=.80). Participants reported no difficulties that needed to be resolved when moving through the program. Overall, both the virtual and online formats were acceptable and feasible to caregiving populations.

### EXPLORING HEALTHCARE WORKERS' GERIATRIC EDUCATION AND SUBSEQUENT COMMUNICATION WITH OLDER ADULTS

Tina McQuaid, *University of Guelph, Mississauga, Ontario, Canada*

Research shows that older adults are living longer than ever, are the fastest growing population, and can have increasingly complex health-related issues. However, the health knowledge and literacy of older adults can be limited, and these adults may have difficulty understanding the terminology that healthcare workers use to communicate with them about their health. For impoverished older adults especially, this can contribute to poor health decisions and decreased care. Given this, educating healthcare practitioners to communicate effectively with older adults becomes essential to older patients' quality of care. Using predominantly North American studies of healthcare workers' practices, education, and their interactions with older adults (aged 65-85, primarily), this review paper finds that: i) older adults are responsible for their communication with healthcare workers, but practitioners, because of their implied authority, control the narrative, and therefore it is necessary for them to become more educated in communicating with older adults; ii) some current communication practices by healthcare workers (with older adults) are not reflective of sufficient care; and iii) new gerontology education can foster increased empathy and shared communication practices among healthcare workers, and this can aid patients to better control and have confidence in their healthcare decisions. Social and cultural factors that may explain the health literacy divide in older adults are discussed, as are recommendations and best practices for healthcare workers working with older adults.

### EDUCATIONAL PREPAREDNESS IN MEETING THE NEEDS OF AN AGING POPULATION

Donnette Narine[1], Abigail Helsinger[2], Phyllis Cummins[3], Rita Karam[4], Wonmai Punksungka[5], Jenna Kramer[6], and Takashi Yamashita[7], *1. University of Maryland, Baltimore, Baltimore, Maryland, United States, 2. Miami University, Oxford, Ohio, United States, 3. Miami University, Oxford, Ohio, United States, 4. RAND Corporation, Santa Monica, California, United States, 5. University of Maryland - Baltimore County, Baltimore, Maryland, United States, 6. RAND Corporation, Arlington, Virginia, United States, 7. University of Maryland, Baltimore County, Baltimore, Maryland, United States*

Community colleges play an important role in training adults in healthcare-related occupations, such as nurses and medical technicians, to meet the needs of an aging population in the U.S. In addition, community colleges with open access policies, have a long-standing record of serving a diverse population, including students of all ages. However, the relatively low program completion rates (e.g., about 30% in Ohio) are of concern in general, and among older students in particular. One of the reasons for the low completion rate is the lack of postsecondary education readiness or basic skills, such as literacy, numeracy, and digital skills. The COVID-19 pandemic has created additional challenges, including faculty shortages, lack of internet access, poor digital skills, and transitions to online classes. This study examined characteristics of health-related occupational programs at community colleges located in Washington, Indiana, and Ohio, focusing on adult student characteristics, and their administrators' and faculty's views on basic skills and program completion. In addition, potential employers were interviewed regarding the skill expectations of community college hires. Using the semi-structured qualitative interview, community college administrators, faculty, and potential employers reported that numeracy skills, excellent grammar, and critical thinking skills are important to succeed both in the classroom and in the workplace. Creating a supportive atmosphere in the classroom and providing tutoring, either by the instructor or at a tutoring center, are also important for student success. More detailed results as well as other policy implications are discussed in this study.

### EVALUATION OF FALL RISK PREVENTION EDUCATION THROUGH AGE-FRIENDLY PARTNERSHIPS

Solymar Rivera-Torres[1], and Jennifer Severance[2], *1. University of North Texas, Denton, Krum, Texas, United States, 2. UNTHSC-TCOM, Fort Worth, Texas, United States*

Falls in the home and community environments are the leading cause of injuries and long-term disabilities for the aging population. This study examines the outcomes of a partnership among an academic institution, government agency, community nonprofit, and emergency services organization to expand access to a fall prevention training program by targeting delivery in postal codes identified as underserved with high rates of falls emergencies (hot spots) and non-high rates (non-hot spots). A total of 354 adults aged 50 and older participated in a fall prevention education program, with 188 (53%) participants completing at least five sessions (completers), of which 35% resided in hot spot areas. Descriptive statistics for frequency, percentage, mean, and standard deviations values were calculated for demographic variables. A paired t-test analysis was conducted to compare initial and final scores for self-assessed general health and fall efficacy programs. The paired sample t-test statistics revealed significant improvements in fall efficacy for completers in hot spots ($t= -6.23$; $p < 0.001$) and non-hot spots areas ($t= - 11.17$; $p < 0.001$). No statistical differences ($p > 0.05$) were observed between the initial and final scores of the self-assessed general health for completers in hot spots and non-hot spot areas. Cross-sector collaboration to deliver targeted falls prevention training at various community locations can effectively reach underserved, at-risk older adults, although additional retention strategies must be considered. In conclusion, identifying at-risk older adults to mobilize partnerships, limited resources can be allocated towards improving retention and program outcomes of community-based fall prevention education.

### IMPROVING THE PERFORMANCE OF COMMUNITY HEALTH WORKERS IN A BEHAVIORAL LIFESTYLE INTERVENTION FOR OLDER ADULTS

Xinran Liu[1], Yuxin Lin[2], Sara Baumann[1], and Steven Albert[1], *1. University of Pittsburgh, Pittsburgh, Pennsylvania, United States, 2. Peking University Health Science Center, Beijing, Beijing, China (People's Republic)*

Community health workers (CHWs) play essential roles in implementing community-based behavioral lifestyle interventions. This qualitative analysis aimed to explore what program factors and CHW characteristics would help improve CHW performance in the Mobility and Vitality Lifestyle Program (MOVE UP), a community-based behavioral weight-management intervention for improving mobility among overweight or obese older adults. The MOVE UP program was implemented from January 2015 to June 2019 at 23 community-based sites in Pittsburgh, Pennsylvania. Trained and supported CHWs delivered 32 group sessions over 13 months. We collected data from semi-structured interviews with 21 CHWs and 14 community key informants (community site directors or coordinators); seven focus group discussions with participants from 9 sites, and 124 pieces of graduation advice provided by MOVE UP graduates for future participants; onsite CHW performance observation at 18 sites, and 124 meeting memos of support phone calls with CHWs. Interviews and focus groups were audio-recorded and transcribed verbatim. All data were thematically analyzed in a deductive approach, using the CHW generic logic model. Emerging themes were: Trained CHWs still require onsite support by experienced CHWs or training staff to deliver the first session; Participants need a Q&A session by specialists to address their questions beyond CHWs' knowledge; Desirable CHW characteristics could increase CHWs' relation and accessibility to participants, such as the same age group, similar weight loss experience, and living or working in the same community. These findings provide important insight as to recruiting and supporting CHWs in a community-based behavioral lifestyle intervention for older adults.

### WORKFORCE DEVELOPMENT: AGING NETWORK CARE MANAGERS AS BEHAVIORAL HEALTH PROVIDERS FOR OLDER ADULTS IN COMMUNITIES

Mbita Mbao[1], and Bronwyn Keefe[2], *1. Salem State University, Salem, Massachusetts, United States, 2. Boston University, Boston, Massachusetts, United States*

Background and Objectives: The population of older adults (65 and over) continues to increase with projections of one in five by 2030. Furthermore, one in four older adults have a behavioral health problem, and over 63% are not receiving behavioral health (BH) services. Many older adults living in the community depend on aging networks for home- and community-based services. However, most care managers' current education and training do not cover the skills and competencies to provide adequate care for older adults with BH needs. This study aimed to evaluate the effectiveness of a training intervention on the perceived self-efficacy of care managers working with older adults with BH issues in the aging network. Research Design and

Methods: The study used a quasi-experimental design with a pre-and post-tests approach. The study used convenience sampling (n=90).

Results: We found a significant difference in the mean self-efficacy scores related to working with clients with mental health problems between pre-test (M=62.31, SD=10.11) and post-test (M=65.88, SD=7.40). In addition, results indicated a significant difference in the mean self-efficacy score between the pre-test (M=59.81, SD=10.68) and post-test (M=65.60, SD=9.85) related to working with clients with substance use problems. Discussion and Implications: The study found that self-efficacy was higher at post-test than pre-test. In addition, there was no difference in the self-efficacy scores of those participants who had previously completed a mental health course or certification and those who had not.

### KNOWLEDGE DEFICITS ABOUT THE SAFE USE OF PRESCRIPTION OPIOIDS AND NALOXONE IN OLDER ADULTS IN ARKANSAS

Patricia Savary, Gohar Azhar, Naomi Amstrong, Regina Gibson, Elizabeth Eoff, Amanda Pangle, Robin McAtee, and Jeanne Wei, *University of Arkansas for Medical Sciences, Little Rock, Arkansas, United States*

Older adults might be more likely to experience adverse effects of opioid medications, or accidental overdose because of age-related multi-morbidities and polypharmacy. Arkansas has the second highest legal opioid prescription rate in the nation hence disseminating information to older individuals is key in fighting the opioid epidemic. As part of an Opioid Prevention for Aging and Longevity Program, we conducted a community-based, cross-sectional, anonymous survey of individuals >59 years about opioids, the common side effects and use of naloxone. The total N was 304, and the response per item varied from 236-298. Respondents' knowledge was strongest for preventative safety measures: to avoid drinking alcohol when using opioids 90.94% and to lock opioids in a secure location when children are nearby (89.49%). About three-quarters of respondents correctly identified medications classified as opioid (70.34%) or non-opioid (74.48%). Similarly, 75.36% recognized a non-opioid, ibuprofen, as the appropriate pain medication for back pain. The side effects of opioids not as well recognized. Sleepiness and trouble driving was the best understood side effect (72.18%). In contrast, the side-effect of constipation was correctly identified in just 34.17% of responses and trouble urinating in only 26.79%. In addition, a key emergency safety measure in case of opioid over-dose was far less known: Only 63.98% were able to correctly identify naloxone as an antidote for opioid overdose.Older adults exhibited gaps in knowledge about the common side effects of prescription opioids and were unaware of the life-saving role of naloxone in reversing the effects of opioid overdose.

### IMPACT OF AN ONLINE EMOTION-FOCUSED COMMUNICATION TRAINING ON STAFF KNOWLEDGE AND SELF-EFFICACY

Katherine Abbott[1], Allison Heid[2], Megan Kelley[1], Alexandra Heppner[1], and Kimberly Van Haitsma[3], *1. Miami University, Oxford, Ohio, United States, 2. Ardmore, Pennsylvania, United States, 3. Pennsylvania State University, University Park, Pennsylvania, United States*

The way we respond emotionally to others can impact how we provide care. Emotional intelligence is vital for care team members whose entire day involves interacting with other people. We have developed an interactive, online, self-paced course for people providing care to others with the specific goal of increasing awareness of emotions and helping to identify emotions in others. The training is supported by research indicating that individuals who can better manage their own emotions are better positioned to manage behaviors and emotions in others. The central concept of the training is to build relationships that enhance person-centered care through increasing care providers' emotional intelligence. These skills allow care providers to better manage their own behavior and emotions, which results in improved quality of their care work. The purpose of this study was to assess the knowledge, self-efficacy, acceptability, and appropriateness of the online training. The data for this study came from n = 130 individuals (19% direct care worker, 16% activities, 13% health care provider, and 12% case manager) who completed assessments pre-and post-training. Knowledge of emotion-focused communication strategies and self-efficacy in using emotion-focused communication strategies both increased significantly with training ($t(129) = -5.40$ $p < .001$ and $t(124) = -6.42$ $p < .001$). In addition, high levels of acceptability, appropriateness, and satisfaction were reported. Findings indicate the benefits of online training for emotion-focused communication for caregivers. The discussion will focus on recommendations for practice, policy, and research.

### SESSION 6140 (POSTER)

### HEALTH INEQUITIES, DISPARITIES, AND PERSON-CENTERED CARE

### DOES AGEISM ACCELERATE BIOLOGICAL AGING

Mingxin Liu, Alan Cohen, Tamas Fulop, Véronique Legault, Carine Betrisey, and Melanie Levasseur, *Université de Sherbrooke, Sherbrooke, Quebec, Canada*

Defined as, "stereotype, prejudice, and discrimination directly towards people because of their age", ageism may contribute to adverse health outcomes, accelerate aging process, and increase the burden on health and social services. Little is known about the ageism impact on biological aging. Secondary analysis of the American Health and Retirement Study (2012 and 2016 waves) was carried out. Participants were asked: the self-perception of aging (SPA), the causes of receiving discrimination, including ageism as one of the causes, and the frequency of receiving such discrimination. The aging rate was measured using two distinct measurements: homeostatic dysregulation (using Mahalanobis distance on 44 biomarkers, n= 9934, 2016 wave) and epigenetic aging clocks (n=4018, 2016 wave). The influence of perceived ageism (current or previous waves) on the aging rate was modelled with linear models using biological aging (aka. homeostatic dysregulation and epigenetic age) as the dependent variable (outcome), ageism as the exposure, with considering confounders: sex, depressive symptom. The results show that more negative SPA, either from the previous (2012) or the same wave (2016), is associated with elevated homeostatic dysregulation (e.g. the slope increases from 1.20 to 1.34, p< 0.001, previous wave) and increasing epigenetic age (e.g. DNAm PhenoAge, the slope increases from 53.81 to 61.14, p< 0.001, current wave). The association between the ageism receiving frequency and biological aging is similar but less significant. The results demonstrate that ageism is associated with accelerated biological aging. More interventions are called to combat ageism and foster the health and wellbeing of the older adults.

### ASSOCIATION BETWEEN PERCEIVED CONTROL AND COGNITIVE FUNCTION AMONG STROKE SURVIVORS IN CHINA: A LONGITUDINAL STUDY

Zhijian Liu[1], Huanzhi Zhu[1], Jing Wang[2], Bei Wu[3], Qin Wang[4], and Juan Li[5], *1. Naval Military Medical University, Shanghai, Shanghai, China (People's Republic), 2. University of North Carolina at Chapel Hill, Chapel Hill, North Carolina, United States, 3. New York University, New York, New York, United States, 4. Linyi People's Hospital, Linyi, Shandong, China (People's Republic), 5. Huashan Hospital affiliated to*

*Fudan University, Shanghai, Shanghai, China (People's Republic)*

This study explored the association between perceived control and cognitive function among stroke survivors in China. We conducted a longitudinal study and assessed perceived control (by Perceived Control in Health Care Questionnaire) and cognitive function (by Montreal Cognitive Assessment, MoCA) of 231 stroke survivors at the acute stage, 3, 6 months after onset from two stroke centers in Shanghai and Linyi from June to December 2020. General linear mixed model was used for analysis. Perceived control was at a moderate level, and the average score of MoCA showed cognitive impairment at 3 waves. Both perceived control and cognitive function improved with time. Perceived control was positively associated with cognitive function ($\beta$=0.08, $p < 0.001$). After controlling for stroke severity, risk factors of cognitive impairment, age, gender, and education, the association was still significant ($\beta$=0.04, $p < 0.001$). These findings suggest that perceived control may be a potential target in cognitive interventions for stroke survivors.

### DIFFERENCES IN COMMUNITY AND PROVIDER STAKEHOLDERS' PERCEPTIONS ON BRAIN HEALTH SERVICE GAPS AND SCREENING NEEDS

Annie Rhodes, Lana Sargent, and Faika Zanjani, *Virginia Commonwealth University, Richmond, Virginia*

Introduction: Alzheimer's Disease and Related Dementias (ADRD) management and prevention is a priority for providers and community members. Aligning perceptions regarding resources and screening supports person-centered care while addressing the increased dementia burden attributed to systemic inequity and health disparities. This project aimed to identify needs in lower-income diverse populations. Research Question: What are the differences in perceived service gaps and screening preferences for brain health and ADRD?

Methods: A convenience sample of 15 providers and 20 community stakeholders (55+) completed a 2021 survey about Richmond's service gaps and screening needs for brain health and ADRD.

Results: 40% of providers were ADRD focused, 65% of community members reported a memory concern. Overall, providers reported fewer service gaps: Administration on Aging Service Gaps 46.7%/75%, Caregiving gaps 80%/85%, Specialty Health gaps: 53.3%/80%. Clinical ADRD gaps 73.3%/80%, Memory Case Management gaps 86.7%/90%, Memory Social Services gaps 33.3%/90%, Memory Screening gaps 53.3%/85%. Provider and Community stakeholders were more aligned regarding screenings that should be included in a brain health/ADRD management program: Advance Care Planning screening: 80%/80%, Caregiving Status screening: 93.3%/90%, Memory Loss screening: Lifestyle Risk screening: 100%/95%, Cognitive-Comorbidity Risks screening: 100%/75%, Psychosocial Risks screening:100%/100%, Depression screening 100%/100%, Clinical Health screening 73.3%/90%, Substance Use Disorder screening 86%/75%, Sleep Problem screening 93%/75%

Conclusion: Although there were areas in which both providers and community members aligned, results show that providers underestimate brain health/ADRD service gaps experienced by the community. Both groups are amenable to comprehensive screening for services. However, providers are interested in more expansive ADRD screeners.

### CROSS-CULTURAL DIFFERENCES IN AGE ESTIMATION AND AGE BIAS

Hannah Benz, Hailey Scherer, Gustavo Rodriguez, and Jenessa Steele, *Radford University, Radford, Virginia*

Our society is aging rapidly with older adults composing a continuously growing proportion of the population. This expected shift in population age is likely going to carry societal consequences, such as an increase in age discrimination. Previous research has shown that ageism (the systematic stereotyping and categorizing of people based on their age) is the most experienced kind of prejudice across Europe, with individualistic, industrialized countries like the USA and Germany showing greater levels of age bias towards the elderly. The current study aimed to investigate cross-cultural differences in age estimation and attitudes towards older adults. Pilot measures included 102 participants (65 American, 37 German) who estimated the age of 12 male celebrities representing three different age groups (young, middle, and older adult) and completed the Fraboni Scale of Ageism (FSA), a survey measurement investigating ageism. Although the Fraboni scale has been validated in other countries, it has not yet been translated to German, nor tested on a primarily German-speaking population. Preliminary analyses showed that both the original FSA scale and the German translation were reliable ($\alpha$Original = 0.909, $\alpha$German = 0.703), however, t-test revealed significant differences between the FSA mean scores of the original scale (M = 1.78, SD = .34) and the translated version (M = 3.15, SD = .28), $t(100) = -20.90$, $p < .001$. The researchers are currently recruiting 400 additional participants to explore the effects of culture, race, and participant age on age estimation and further validate the German translation of the scale.

### INCLUSION OF ETHNICALLY DIVERSE POPULATIONS IN CLINICAL TRIALS OF HEALTHSPAN: IMPLICATIONS FROM THE SALSA STUDY

Sara Espinoza, Tiffany Cortes, and Helen Hazuda, *UT Health San Antonio, San Antonio, Texas, United States*

Background: Geroscience-guided clinical trials focused on healthspan may seek to enroll older adults initially free of chronic diseases and disability. Here we examine healthspan in the San Antonio Longitudinal Study of Aging (SALSA), a cohort of 749 community-dwelling older (65+ years) Mexican Americans (MA) and European Americans (EA), and describe prevalence and characteristics associated with poor healthspan.

Methods: Poor healthspan was defined at the SALSA baseline exam as presence of any one of: 1) chronic disease (diabetes, myocardial infarction, congestive heart failure, stroke, chronic obstructive pulmonary disease, or cancer);

2) dependence in basic or instrumental activities of daily living; or 3) mini mental state exam score < 18. Frailty was defined by Fried phenotype criteria. The association of poor healthspan with age, sex, ethnic group, socioeconomic status (SES), and frailty was assessed using chi-square or t-tests.

Results: 544 (72.6%) participants met criteria for poor healthspan, which was associated with older age (69.6 ±3.4 vs. 69.3 ±3.4, p< 0.05), male sex (77.3% vs. 69.2%, p< 0.05), MA ethnicity (77.4% vs. 67.3%, p< 0.05), lower income (11.3 ±3.1 vs. 12.2 ±2.9, p< 0.001 and education (10.4 ±4.6 vs. 12.7 ±3.2, < 0.0001), and frailty (95.5% vs. 4.6%, p< 0.001).

Conclusion: Poor healthspan was highly prevalent (>70%) in SALSA and associated with MA ethnicity, low SES, and frailty. Geroscience-guided clinical trials of potential interventions to improve healthspan by preventing chronic diseases and disability may under-represent individuals of ethnic minority background and lower SES and, thereby, jeopardize generalizability of the findings to the broader population.

### LIVING WITH MULTIMORBIDITY: SHARED EXPERIENCES OF PATIENTS, FAMILY CAREGIVERS, AND HEALTHCARE PROFESSIONALS

Anna Peeler[1], Katie Nelson[2], Sarah Badawi[2], Lara Street[3], David Hager[3], Patricia Davidson[4], and Binu Koirala[2], *1. Johns Hopkins University School of Nursing, Baltimore, Maryland, United States, 2. Johns Hopkins University, Baltimore, Maryland, United States, 3. Johns Hopkins Hospital, Baltimore, Maryland, United States, 4. University of Wollongong, Wollongong, New South Wales, Australia*

Over 50% of patients in intermediate care units (IMCU) present with multimorbidity, two or more chronic conditions. Balancing the effects of multimorbidity and their treatments with quality-of-life can be a challenge. This experience-based co-design project aimed to elicit experiences of patients, family caregivers, and healthcare professionals in IMCU, in the context of challenges and intricacies of multimorbidity management, to inform the development of a symptom management toolkit. Patients aged 55 years and older were recruited and interviewed in person. Healthcare professionals working in IMCU (i.e., physicians, nurses, respiratory therapists, social workers, etc.) were recruited and interviewed virtually. Participants were asked questions about their role in recognizing and treating symptoms, factors affecting quality of life, symptom burden and trajectory over time, and symptom management strategies that have and have not worked. An inductive thematic analysis approach was used for data analysis. Twenty-three interviews were conducted: 9 patients, 2 family-caregivers, and 12 healthcare professionals. Patients' mean age was 67.5 (± 6.5) years, over half (n=5) were Black or Hispanic, and average number of multimorbidity was 3.67. Five major themes emerged: 1) importance of patient-provider relationship; 2) open and honest communication; 3) accessibility of resources during hospitalization and at discharge; 4) caregiver support, training, and education; and 5) care-coordination and follow-up care. Patients, caregivers, and healthcare professionals often have different priorities for multimorbidity management, treatment, and education. However, given the growing population of patients experiencing multimorbidity, it is imperative to identify shared priorities and target holistic interventions considering their experiences to enhance outcomes.

### PATIENT PARTICIPATION IN HEALTHCARE ACTIVITIES: NURSES' AND PATIENTS' PERSPECTIVES IN TAIWAN

Hsueh-Fen Kao[1], Chang-Chiao Hung[2], Bih-O Lee[3], Su-Ling Tsai[2], and Oscar Moreno[4], *1. The University of Texas at El Paso, El Paso, Texas, United States, 2. Chung-Gung University of Science and Technology, Puzi City, Chiayi, Taiwan (Republic of China), 3. Kaohsiung Medical University, Kaohsiung, Kaohsiung, Taiwan (Republic of China), 4. M B Care Home Health, LLC, El Paso, Texas, United States*

Patient participation in healthcare activities is key to producing successful patient-centered care. However, little is known about both nurses' and patients' perspectives regarding patient participation in East Asia. This paper compared and contrasted perspectives of patient participation in healthcare activities between nurses and patients (age=61.53 ±8.75), using a qualitative study with a purposive sample of 39 nurses and 15 patients. Inclusion criteria for patient participants were: (1) speaking Mandarin Chinese or Taiwanese dialect, (2) aged 20 or older, (3) hospitalized at the unit for at least 3 days, (4) able to provide written informed consent, and (5) well enough to endure 40–50 minutes of individual interview. A semi-structured interview was applied to focus groups for nurses, and to face-to-face interviews for patients to prevent nosocomial infection. Content analysis was utilized to analyze the data, common themes and subthemes were identified showing three similarities in perspectives between nurses and patients—authoritative culture, participation behaviors, and obstacles to participation, as well as two differences—sources of acquiring patient-related health information, and responsible party. Nurses and patients did not entirely view participation in healthcare activities congruently. Relevant clinical practices are also suggested, including respecting patients' autonomy, nurses' using layman's language for explanations, patients' understanding the meaning behind their participation behaviors, recognizing obstacles faced in enhancing patient participation with adjusted nursing workload, actively providing needed health information, and leading patients to realize that they will be responsible for their health behaviors after discharge.

### PLACE-BASED HEALTH DISPARITIES: FUNCTIONAL DISABILITY IN APPALACHIAN WEST VIRGINIA

Carly Pullen, Kareem Ibrahim-Bacha, and Julie Hicks Patrick, *West Virginia University, Morgantown, West Virginia, United States*

Place-based health disparities contribute to disability across the lifespan. Additional examinations of contributors to morbidity and disability at mid-and late-life are needed to inform policies and programs. Using data from the 2020 Centers for Disease Control and Prevention's (CDC) Behavioral Risk Factor Surveillance System (BRFSS), we examine some of the social determinants of health (e.g., age, gender, education, income) as predictors of access to health care and predictors of functional ability. The study included 5,880 adults living in

West Virginia all between the ages of 18 to 65+ years. Access to healthcare was indexed by three variables, including whether one had medical insurance, saw personal physicians, and avoided medical treatment due to cost. Functional ability was indexed by reports of difficulty in three ADLs and the number of chronic health conditions. Although most paths were significant, our initial model fit the data poorly, X2 (DF = 39, N =5880) = 2503.6, p < .001, CFI = .70, RMSEA = 1.0. We re-ran the model, using age as a moderator. In this set of analyses, the model fit well for middle-aged and older adults, but a different set of predictors characterized the relations for younger adults. Our results suggest that policies and programs that increase medical access for current middle-aged and older adults might decrease functional disability. Moreover, as younger adults age into midlife, they enter with lower economic and educational resources, further exacerbating their lack of access to health care and increasing disability.

### A LONGITUDINAL INQUIRY OF PATIENT-PROVIDER RELATIONSHIPS AMONG AN UNDERSTUDIED GERIATRIC POPULATION

Rosana Bravo[1], and Angela Gutierrez[2], *1. Western University of Health Sciences, Pomona, California, United States, 2. Ohio University, Heritage College of Osteopathic Medicine, Athens, Ohio, United States*

Patient-provider relationships have a direct impact on patient outcomes. This study explored patient-provider relationships among an understudied geriatric population of color— foreign-born Latinos¬ participating in an all-inclusive specialized program aimed at controlling patients' costs and enhancing access to care. Thirteen older adult Latinos with multimorbidities from nine Program of All-Inclusive Care for the Elderly (PACE) centers in Southern California were recruited. Researchers conducted three in-depth interviews in Spanish with each participant (39 interviews total) over 13 months. The first interviews were conducted face-to-face and lasted one hour on average. Subsequent interviews were conducted over the phone (Range: 60-90 minutes). Data were analyzed using codes, identifying categories and themes. The concepts of time and trust were used to analyze the process of relationship development and to capture changes over time. The patient-provider relationship developed on a continuum across time and trust, establishing three stages to the patient-provider relationship. In the first stage emerged the concept of el buen doctor (the good doctor). In the second stage, trust was perceived to have been established, and was only strengthened as the doctor continued to demonstrate trustworthy characteristics over time. The third stage embodied all that a person of trust was plus an additional advocacy dimension. The longitudinal and specialized geriatric program design illuminated the nature of quality of care and patients' perceptions on relationship development over time. Controlling patient/provider costs and enhancing access to care in an all-inclusive program are beneficial in enhancing patient-provider relationships.

### ADVANCE CARE PLANNING FOR SPANISH-LANGUAGE SPEAKERS: PATIENT, FAMILY, AND INTERPRETER PERSPECTIVES

Geraldine Puerto[1], Germán Chiriboga[1], Susan DeSanto-Madeya[2], Vennesa Duodu[1], Dulce Cruz-Oliver[3], and Jennifer Tjia[1], *1. University of Massachusetts Chan Medical School, Worcester, Massachusetts, United States, 2. University of Rhode Island, Providence, Rhode Island, United States, 3. Johns Hopkins Medical School, Baltimore, Maryland, United States*

Language access is a challenge to advance care planning (ACP). Spanish-language speakers are the largest non-English speaking population in the US. While ACP tools have been translated into Spanish, it is unclear how heterogeneity in country of origin may affect the generalizability of translations across diverse US Spanish-speaking populations. The study objective is to describe challenges and facilitators to ACP for diverse populations of Spanish-language speakers. We conducted 3 focus groups with a total of 29 participants from members of Spanish-language speaking communities whose countries of origin were predominantly from the Caribbean, Central, and South America. Eligibility included being 18 years or older, being a native Spanish-speaker, and having direct experience with ACP as a patient, caregiver, or medical interpreter. We conducted thematic analysis with axial coding. Themes include: 1. Linguistic Challenges with Current ACP Translations; 2. Effect of Country of Origin and Culture on ACP Understanding; 3. Impact of Local Healthcare System Cultural on ACP; and 4. Need for ACP to be Normalized into the Local Community. ACP is both a cultural practice and a clinical practice. Recommendations for improving ACP completion for non-English speakers extend far beyond translation, since simply translating ACP tools without a cultural context is neither equitable nor inclusive. A key step is normalizing ACP into the local community. Understanding the intersection of local healthcare systems of ACP practice with the patient's and family's cultures of origin will facilitate introducing ACP in a culturally sensitive manner.

### COLORECTAL CANCER SCREENING (CRC) DISPARITIES: A ZIP CODE–LEVEL ANALYSIS

Roshni Singh, Carla Parraga, Rachel Lin, Leonardo Tamariz, and Ana Palacio, *University of Miami Miller School of Medicine, Miami, Florida, United States*

CRC is the third leading cause of cancer-related deaths among older adults in the US. CRC screening can prevent disease by early identification, yet there are disparities in CRC screening. This study aimed to determine the impact of race, social determinants, and geographic location at zip-codes level on CRC screening.We conducted a retrospective cross-sectional study of CRC screening among different races, evaluating the relationship with the social deprivation index (SDI) and annual income as health determinant factors using the public available data of 2016-2019 CDC 500 cities project and PLACES project 2020 database combined with 2019 American Community Survey for zip code-based analysis. We conducted a multivariate analysis and a confirmatory factor analysis among race, income, lack of health insurance, access to check-up visits and SDI. Increasing SDI tertile increased the likelihood of being Black and Hispanic and having lower median household income (p< 0.01). Lack of health insurance and lower regular checkup visits were less common in the third tertile of SDI (p< 0.01). The multivariate analysis showed that being black, Hispanic, having a lower income, not having health insurance, not having regular check-ups and SDI were related to decreased screening. In the confirmatory factor analysis, the variables most associated with decreased screening are SDI and access to health insurance.Race, SDI, insurance status, socioeconomic status, all impact CRC screenings, but the two most important factors are SDI and access to healthcare. These data may help implement interventions that specifically target these barriers to promote CRC screenings within disadvantaged communities.

### ETHNORACIAL DIFFERENCES IN HOME HEALTHCARE USE: FINDINGS FROM THE NATIONAL HEALTH AND RETIREMENT STUDY

Adam Simning[1], Jinjiao Wang[2], Thomas Caprio[1], Brian McGarry[1], and Yue Li[1], *1. University of Rochester, Rochester, New York, United States, 2. University of Rochester School of Nursing, Rochester, New York, United States*

This study seeks to identify ethnoracial differences in self-reported home health care (HHC) use among older adults. To do so, we examined 8,817 people aged 65 and older from the 2016 wave of the Health and Retirement Study (HRS), a nationally representative survey of older adults in the U.S. The dependent variable was whether HRS participants reported any HHC service use in the past two years. The primary independent variable was ethnoracial grouping, which included non-Hispanic White, non-Hispanic Black, and Hispanic groups. Multivariable logistic regressions stratified by ethnoracial grouping identified correlates of HHC use. We found that HHC use was more prevalent among non-Hispanic Blacks (14.9%) than in non-Hispanic Whites (10.1%) or Hispanics (10.7%). For all ethnoracial groups, increasing age, dementia, activities of daily living impairment, medical comorbidity (except for Hispanics), and hospitalization (in the past two years) were associated with an increased likelihood of HHC use. In addition, we identified ethnoracial differences in the correlates of HHC use. Among non-Hispanic Whites, more formal education and Medicaid insurance were associated with a higher likelihood of using HHC. For non-Hispanic Blacks, residing in rural areas was associated with a decreased likelihood of HHC use, whereas being single and living alone were associated with an increased likelihood of HHC use. This study thereby identified notable ethnoracial differences in the correlates of HHC use among older adults.

### PERSON-CENTERED CARE: WHY TAKING INDIVIDUALS' CARE PREFERENCES INTO ACCOUNT MATTERS

Frances Hawes[1], Jane Tavares[2], Marc Cohen[2], and Ann Hwang[3], *1. University of Wisconsin-Eau Claire, Eau Claire, Wisconsin, United States, 2. University of Massachusetts Boston, Boston, Massachusetts, United States, 3. Harvard, Cambridge, Massachusetts, United States*

Person-centered care has been recognized as an integral part of a high-quality health care system. Utilizing the 2014 to 2018 waves of the Health and Retirement Study, we explore trends in person-centered care among those 50 and older by examining the extent to which they feel their care preferences are being taken into account, that is, that they are being heard by providers. We analyze the impact of not receiving person-centered care on health care utilization, health outcomes, and preventative care utilization. One-third of respondents reported that their care preferences were "sometimes" or "never" considered. Findings show that wealth and racial disparities in person-centered care are worsening over time. From 2014 to 2018, the percentage of non-Hispanic White respondents who reported that their care preferences were never taken into account decreased while the percentage for Hispanic and non-Hispanic Black individuals increased. Similar trends were seen for low-income individuals. Having a usual source of care was associated with a greater likelihood of having care preferences considered as well as significantly better control of chronic condition and greater use of preventive care. When care preferences are not being taken into account, there is less utilization of health care services, less preventive care usage, poorer control of chronic conditions, and increased risk for higher health care costs. These findings highlight the importance of assuring that people feel listened to by health care providers and emphasize a need for strategies to advance person-centered care for people of color and low-income populations.

### SYSTEMATIC EXCLUSION AT STUDY COMMENCEMENT MASKS EARLIER MENOPAUSE FOR BLACK WOMEN IN THE STUDY OF WOMEN'S HEALTH ACROSS THE NATION (SWAN)

Alexis Reeves[1], Michael Elliott[2], Carrie Karvonen-Gutierriez[2], and Sioban Harlow[2], *1. Stanford University, School of Medicine, Berkeley, California, United States, 2. University of Michigan, Ann Arbor, Michigan, United States*

Assumptions regarding "normative" aging, including average age at onset of disease, are often based on White populations. However, evidence suggests that Black and Hispanic populations may experience "weathering" or accelerated health declines compared to Whites due to the cumulative impact of social and economic marginalization. If "weathering" leads to a differential probability of inclusion into a cohort study, it will likely misinform understanding of aging in minoritized populations. Using SWAN, a longitudinal multi-ethnic cohort of midlife women, and its cross-sectional screening study, we quantified the extent of potential selection bias at study commencement and re-estimated racial/ethnic differences in age at menopause, the main outcome of SWAN, with adjustment for various forms of potential selection bias. Left truncation was corrected for using inverse probability weighting and right censoring using multiple imputation. Two selection mechanisms were identified, eligibility and participation. Black and Hispanic women had the lowest probability of eligibility stemming from a high prevalence of surgical menopause. Their eligibility rates decreased with increasing age faster than in White women. Correcting for selection biases showed that uncorrected analyses overestimated of the median age of menopause in Black and Hispanic women, thereby underestimating racial/ethnic disparities. After adjustment, Black women had earlier natural and surgical menopause (average 1.2 years) versus White women. Overall, this study found that failure to account for different forms of selection can lead to mis-estimation of racial/ethnic disparities in health and aging. Selection bias is particularly acute at study commencement, and particularly affects minoritized populations.

### VOICES OF OUR ELDERS: ATTITUDES, BELIEFS, AND PERSPECTIVES ABOUT RESEARCH IN MINORITY OLDER ADULTS

Trudy Gaillard[1], Donna Shambley-Ebron[2], Giovanni Garcia[1], Ryan Romero[1], Donna Neff[3], Phildra Swagger[3], Dawn Gardier[1], and Fern Webb[4], *1. Florida International University, Miami, Florida, United States, 2. University of Cincinnati, Cincinnati, Ohio, United States, 3. University of Central Florida, Orlando, Florida, United States, 4. University of Florida (UF) College of Medicine, Jacksonville, Jacksonville,, Florida, United States*

The U.S. Census Bureau projects that the numbers of adults 65 and older will double from 46 million in 2020 to 90 million by 2050, thus representing the fastest growing segment of the population. However, older adults, especially those from minority groups, remain underrepresented in clinical research. It is imperative to understand what older adults believe about research and research participation to enhance recruitment efforts. The aim of this presentation is to present preliminary findings from our qualitative study which explored the attitudes, beliefs, and perspectives of older minority adults regarding research and research participation. We conducted 12 focus groups via Zoom, in South Florida with minority adults (African American, Caribbean, Hispanic) over the age of 65 (N=49). An interview guide was used to query the participants about their attitudes, beliefs, and perspectives of research and research participation. Focus groups were video-recorded and transcribed. NVivo software was used for data management and analysis. We found that participants: 1) thought research was necessary to expand understanding and knowledge of health conditions; 2) stated research should be conducted by trusted scientific institutions; 3) relied heavily on their adult children for advice regarding research participation; 4) expressed reluctancy regarding invasive procedures; and 5) were influenced by personal experiences when considering research participation. Our preliminary findings suggest that older minority adults believe in the value of research, however, may be hesitant about participating. We propose continued strategies aimed at increasing engagement of minority older adults into health research.

### RURAL LIVING AND DISABILITY IN OLDER ADULTS: THE ROLE OF ALTERNATIVE SUPPORT RESOURCES AS MEDIATORS

Bernard Steinman[1], and Daniel Barbakoff[2], *1. University of Wyoming, Laramie, Wyoming, United States, 2. Weill Cornell Medical College, New York, New York, United States*

Rural communities are often characterized by sparse service environments offering limited care, services, and conveniences that help with daily activities. In lieu of community services that target older adults to assist with aging-in-place, alternative supportive features, including environmental modification and informal social networks may be especially important in rural settings to preserve functional independence. The purpose of this study was to assess the role of alternative support resources as potential mediators between service environments and Activities of Daily Living (ADL) functioning of older adults living in rural settings. Data from the National Health and Aging Trends Study (NHATS) were analyzed. Guided by the International Classification of Functioning, Disability and Health, regression models included covariates for sociodemographics, chronic conditions, mobility functioning, and participation. Service environments were quantified using a measure of the number of services (e.g., help with bathing) available in communities. Two potentially important support features were tested as mediators. Environmental modification was operationalized using indicators of whether homes had been modified (e.g., with features such as grab bars). Size and quality of individuals' social networks were calculated using indicators of whom participants spoke to about important things in their life. Measures of ADLs served as key dependent variables. Results suggest a negative statistical relationship between service environments and disability that is explained in part by the availability of alternative support resources. Implications are that older adults who live in rural communities may often benefit by employing home modifications and relying on informal care options to meet their needs.

### DEPRESSION IS ASSOCIATED WITH POORER CLINICAL FUNCTIONING AMONG HISPANIC OLDER ADULTS

Andres Duarte[1], Dilianna Padron[1], Joanna Gonzalez[2], Patricia Garcia[3], Lisandra Mendoza[4], Ranjan Duara[2], and Miriam Rodriguez[1], *1. Albizu University, Miami, Florida, United States, 2. Mt. Sinai Medical Center, Miami Beach, Florida, United States, 3. Rehabilitation Hospital of Indiana, Indianapolis, Indiana, United States, 4. Department of Veterans Affairs Bay Pines, Cape Coral, Florida, United States*

Objective: The aim of the current study was to examine associations between depression and clinical functioning among a multi-ethnic sample.

Methods: 35 cognitively normal and Mild Cognitive Impairment (MCI) participants were included and self-identified as Hispanic or white non-Hispanic (WNH). The Hispanic group (n=18), had a mean age of 70.83 (SD=7.66) and 15.59 mean years of education (SD= 3.43). The WNH group (n=17) had a mean age of 71.76 (SD=6.9) and 16.81 mean years of education (SD=2.59). Subjects were given the Alzheimer's disease Cooperative Study (ADCS) ADL inventory, the modified Clinical Dementia Rating scale (mCDR), and the Geriatric Depression Scale (GDS). Linear regressions were conducted to analyze the predictive associations between GDS scores and ADL functioning while controlling for the effect of diagnosis and age.

Results: Among Hispanics, the overall regression was significant (R2 = .622, F(2,17)= 12.32, p<.001). Higher GDS scores was found to significantly predict worse mCDR scores ($\beta$ = .676, p<.001) when controlling for the effects other factors. When examining ADCS-ADL scores, the overall model was also found to be significant (R2= .413, F(2,17)=5.28, p<.05). Higher GDS scores significantly predicted worse ADCS-ADL scores ($\beta$ = =.652, p<.01) when controlling for the effects of other factors. Diagnosis and age did not significant predict ADL scores. Among the WNH group, the regression model was not significant and depression was not a significant predictor of ADL functioning.

Conclusions: The results suggest Hispanics are more vulnerable to the effects of depression on ADL function which has important implications for AD diagnosis.

### ADDRESSING ALZHEIMER'S DISPARITIES AMONG BLACK POPULATIONS WITH BRAINGUIDE BY USAGAINSTALZHEIMER'S

Brooks Kenny, *UsAgainstAlzheimer's, Kensington, Maryland, United States*

Older, Black Americans are disproportionately impacted by Alzheimer's disease (AD), accounting for 15% (~1 million) of all individuals aged 65+ living with AD (5.8 million). Stigma, fear, and gaps in education contribute to 60% of undetected AD cases. In Georgia, AD remains the 6th leading cause of death. By 2029, cases are projected to spike by 46%, from 130,000 to 190,000. Given these alarming statistics and in response to AD health disparities in this population, UsAgainstAlzheimer's, in partnership with community leaders and organizations, launched a pilot outreach program to promote AD prevention and brain health awareness in Atlanta. Program goals included: increasing knowledge about brain health, emphasizing the importance of early detection and diagnosis, raise awareness of BrainGuide™ by UsAgainstAlzheimer's and other brain health resources, and develop a network of organizations for ongoing collaboration, awareness, and education. Program strategies included accessing highly saturated, faith-based spaces like mega churches, circulating key messaging through paid and earned media, and hosting widely received community webinars. UsAgainstAlzheimer's collected participant feedback and examined BrainGuide website traffic to evaluate the effectiveness of community engagement on increasing brain health awareness and addressing AD stigma in Atlanta. Preliminary findings indicate a 96% increase in BrainGuide traffic from Atlanta and 70% increased engagement with BrainGuide resources, compared to the national average. UsAgainstAlzheimer's' pilot program suggests that brain health promotion, grounded in community engagement from trusted influencers, has potential to raise brain health awareness and empower people to take action. Further research and learnings are required to determine program scalability.

### SOCIAL EPIGENETICS OF RACIAL DISPARITIES IN AGING

Isabel Yannatos[1], Sharon Xie[1], Rebecca Brown[2], and Corey McMillan[1], *1. University of Pennsylvania, Philadelphia, Pennsylvania, United States, 2. University of Pennsylvania School of Medicine, Philadelphia, Pennsylvania, United States*

Racial disparities in many aging-related health outcomes are persistent and pervasive among older Americans. There are well-documented inequities in social and physical environmental exposures which may contribute to these disparities, but we lack understanding of the biological intermediates by which environmental exposures affect disparate health outcomes. DNA methylation (DNAm) aging captures the residual between biological age, robustly measured by GrimAge and Dunedin Pace of Aging methylation (DPoAm), and chronological age. We hypothesize that neighborhood social environment and air pollution exposures contribute to racial disparities in DNAm aging. We performed retrospective cross-sectional analyses among non-Hispanic participants (N=2611 White, N=639 Black) in the Health and Retirement Study whose 2016 DNAm age is linked to survey responses and geographic data. We observed Black individuals have significantly accelerated DNAm aging on average compared to White individuals according to GrimAge (599%) and DPoAm (498%). We implemented linear regression models and Kittagawa-Blinder-Oaxaca decomposition to identify exposures that contribute to this disparity. Exposure measures include census-tract-level Social Deprivation Index, perceived social stress, particulate matter (PM2.5), nitrogen dioxide, and ozone. Individual-level determinants include socioeconomic status, healthcare access, health status, and health behaviors. Results suggest these individual-level factors account for ~43% of the disparity in GrimAge and ~34% in DPoAm. Higher neighborhood socioeconomic deprivation for Black participants significantly contributes to the disparity in GrimAge, while greater vulnerability to PM2.5 contributes to the disparity in DPoAm. DNAm aging may play a role in the environment "getting under the skin" and contributing to age-related health disparities between Black and White Americans.

### SESSION 6150 (POSTER)

### IMPROVING ADRD CARE THROUGH MEASUREMENT, ASSESSMENT, AND METHODS

### SACCADES TO SCREEN AND ASSESS COGNITIVE IMPAIRMENT IN OLDER ADULTS: A SYSTEMATIC REVIEW AND META-ANALYSIS

Qiaoqin Wan, Shifang Zhang, xiuxiu huang, and Xiaoyan Zhao, *Peking University, Beijing, Beijing, China (People's Republic)*

Objective: To systematically summarize the evidence of saccade as a screening and assessing for patients with mild cognitive impairment (MCI) and dementia.

Methods: English databases including PubMed, EMBASE, the Cochrane Library, Web of science, and PsycINFO and Chinese databases including CNKI, Wanfang and VIP were searched. Studies that analyzed the metrics of saccade in people with health cognition, MCI, or dementia were included. The quality of the included studies was evaluated with Cross-sectional/ Prevalence Study Quality from Agency for Healthcare Research and Quality (AHRQ). Study characteristics, participants' characteristics, sample size, saccade procedure, and metrics were extracted from the included studies.

Results: Twenty-two studies involving 1595 participants were included. Meta-analysis showed that peak velocity (SMD= -0.27°/s, 95% CI (-0.44, -0.11), latency (SMD=-0.36ms , 95%CI (-0.51,-0.20), and accuracy rate (SMD=0.42%, 95%CI (0.17,0.68) of prosaccade between older adults with and without cognitive impairment had significant difference. The performance in latency (SMD=-0.56ms, 95%CI(-0.72,-0.39), accuracy rate (SMD=1.32%, 95%CI(1.07,1.56), and corrected errors (SMD=1.23%, 95%CI(0.98,1.47) of antisaccade in people with health cognition was better than that in older adults with cognitive impairment. The results of subgroup analysis revealed that the accuracy rate of prosaccade, latency and accuracy rate of antisaccade demonstrated crucial difference between health older adults and people with MCI, while only accuracy rate of antisaccade showed significant difference between people with MCI and dementia.

Conclusions: The metrics of saccade, especially antisaccade, can be a potential screening and assessing tool for MCI and dementia in elderly persons.

### AUTOMATIC CLASSIFICATION OF ADRD CAREGIVERS' ONLINE INFORMATION WANTS: A MACHINE LEARNING APPROACH

Yuelyu Ji[1], Ning Zou[1], Bo Xie[2], Daqing He[1], and Zhendong Wang[1], *1. University of Pittsburgh, Pittsburgh, Pennsylvania, United States, 2. The University of Texas at Austin, Austin, Texas, United States*

Social media platforms are used by caregivers for persons with Alzheimer's Disease and its Related Dementias (ADRD) to obtain care information. Understanding what types of information caregivers want is critical to developing interventions to provide tailored information. Existing research on caregivers' information wants has relied primarily on manual data analysis, unable to handle the vast amount of data available on social media. In our prior work, we adapted the validated Health Information Wants (HIW) framework to the ADRD caregiving context, forming the HIW-ADRD framework that includes 7 types of information commonly wanted by caregivers. Our longer-term goal is to develop machine algorithms that use the HIW-ADRD framework to automatically classify caregivers' information wants from vast social media posts. Towards this end, we first scrapped posts from ADRD-related subgroups on Reddit, a popular social media platform. We then used few-shot learning, a machine learning method, to extract HIW from the posts. Using questions with question marks as a primary indicator for HIW, we filtered out those sentences and their corresponding background information. Next, we combined the questions and their background information as summaries of the posts. Finally, we sent the summaries to a classification model that classified these summaries based on HIW categories. We used 200 annotated posts to train the model, then tested it on 16779 posts. The evaluation results showed that our model achieved 62.35% in accuracy. These findings provide preliminary evidence for both the deep learning process and the algorithms. This study has implications for future research.

### HEALTH SURVEILLANCE OF PERSONS LIVING WITH DEMENTIA AND CAREGIVER DYADS

Annie Robitaille[1], Neil Drummond[2], Linda Garcia[1], Himasara Marasinghe[3], and David Barber[4], *1. University of Ottawa, Ottawa, Ontario, Canada, 2. University of Alberta, Edmonton, Alberta, Canada, 3. University of Calgary, Calgary, Alberta, Canada, 4. Queen's University, Kingston, Ontario, Canada*

The relationship between individuals living with dementia and their caregivers is important and can impact their dementia journey. However, limited national longitudinal data exists about the caregiver-person living with dementia dyad. Availability of such data would provide important information about joint trajectories and help to better identify the needs of caregivers and persons living with dementia across the dementia journey. The objective of this study was to develop a linked national longitudinal database of persons living with dementia and their caregivers. The Canadian Primary Care Sentinel Surveillance Network (CPCSSN) extracts and de-identifies clinical data from electronic medical records (EMR) from approximately 2 million patients across Canada. CPCSSN data is used to identify persons living with dementia and caregivers willing to participate in the study. CPCSSN data from participating dyads are linked (e.g., chronic and mental health conditions, diagnoses, laboratory test results) and additional information about the experiences of persons living with dementia and their caregivers (e.g., ethnicity, amount and type of care provided, burden, availability of support) are collected yearly using surveys. The growing database contains linked, de-identified, comprehensive information about persons living with dementia and their caregivers that will become a rich source of data for researchers, clinicians, and policymakers. Specifics around how the database was developed, and lessons learned will be discussed as these findings can be used as a template to develop similar linked health surveillance databases.

### NORMALIZING COGNITIVE EVALUATIONS IN ADULTS: GETTING PEOPLE IN THE SCREENING PIPELINE EARLY

Laura Mehegan, and Chuck Raineville, *AARP, Washington, District of Columbia, United States*

To understand how adults and healthcare providers perceive a dementia diagnosis and cognitive evaluations, AARP fielded a nationally representative survey of adults aged 40 and older (N=3,022) along with a companion survey of healthcare providers in a position to diagnose dementia (N=500). Like healthcare providers, Americans aged 40 and older understand the benefits of diagnosing dementia at an early stage, 81% agree that early detection would motivate them to engage in healthier behaviors to slow the progression of the disease. Adults look to healthcare providers as authorities to determine when a cognitive evaluation should be triggered with 63% saying they would get an evaluation if their doctor recommended it and 49% of healthcare providers saying evaluations should be done if the doctor feels it is necessary and 39% saying it should be done yearly beginning at age 65. The research also showed that most adults (76%) want to know if they have dementia. Additionally, majorities of adults would engage in healthy behaviors if they knew it's good for their brain health. Given the desire to prevent and improve outcomes, routine screening for cognitive function should be as commonplace as getting a screening colonoscopy. Unlike a colonoscopy, routine screening for cognitive function is not a normalized process yet. Data from both populations suggest an opportunity for routine evaluation due to a shared understanding of the long and short-term benefits of screening.

### INVOLVING PERSONS LIVING WITH DEMENTIA AS KEY STAKEHOLDERS: RESEARCH "WITH" RATHER THAN RESEARCH "ON"

Ashlee Cordell[1], Christa Wilk[1], Silvia Orsulic-Jeras[1], Sara Powers[2], Farida Ejaz[1], and Beth Sanders[3], *1. Benjamin Rose Institute on Aging, Cleveland, Ohio, United States, 2.*

*The Benjamin Rose Institute on Aging, Cleveland, Ohio, United States, 3. LifeBio, MARYSVILLE, Ohio, United States*

Involving persons living with dementia (PLWD) as members of the research team is an important step in establishing an environment of conducting research “with” as opposed to research “on”. Emerging research is also capturing PLWDs' lived experiences to inform product development. This presentation highlights the importance of conducting product development research with PLWD as key stakeholders and the valuable insight they provide to researchers and developers. Five community-dwelling PLWD with varying diagnoses (60% mild cognitive impairment; 20% Alzheimer’s Disease; 20% mixed dementia) and tMMSE scores ranging from 22-26 (Mean=25) participated in two rounds (9 months apart) of 60-minute-long virtual focus groups via Zoom to test multiple versions of LifeBio MemoryTM: an app-based product designed for PLWD which utilizes advanced technology to improve an existing life story intervention. In Time 1, PLWD provided feedback on prototypes of the app and associated training that was used to inform the final product. In Time 2, participants reviewed the final product. A thematic analysis was conducted at each time point to inform further development of the app. Findings determined the following themes: 1) life stories can help with providing better person-centered care, 2) potentially sensitive topics require more training, 3) simple and clear instructions are vital for success. Topics further discussed in this presentation will include PLWDs': 1) thoughts on the concept of LifeBio and its adaptation, 2) comfort with answering life story interview questions, 3) opinions on dementia-friendly materials, and 4) reactions to the use of app-based technology.

### ADJUSTING FOR COUNTRY-LEVEL VARIATION IN DEMENTIA PREVALENCE WITH CLASSIFICATION ALGORITHMS IN SHARE

Matthias Klee[1], Kenneth Langa[2], and Anja Leist[3], *1. University of Luxembourg, Esch-sur-Alzette, Not Applicable, Luxembourg, 2. University of Michigan, Ann Arbor, Michigan, United States, 3. University of Luxembourg, Esch-sur-Alzette, Grevenmacher, Luxembourg*

Background. Population-level dementia prevalence depends on societal factors and individual-level risk and protective factors. To improve our understanding of how these factors interact, we can use cross-national surveys such as the Survey of Health, Ageing and Retirement in Europe (SHARE). However, in absence of validated cognitive assessments, adjusting for underdiagnosis of dementia is needed. The present study sought to explore the usefulness of the Langa-Weir and alternative algorithms to detect probable dementia while accounting for country-level variation in estimated prevalence and underdiagnosis of dementia.Method. Data from 57,880 respondents aged 60 years and older to wave 7 of SHARE (2017) with non-missing data on variables related to sociodemographics and cognition were used. Adaptations of the Langa-Weir classification algorithm were compared to a weighted logistic regression model and an XGBoost classifier applying the synthetic minority oversampling technique. Different specifications of algorithms were tested globally and for individual countries and compared with the World Alzheimer’s Report (2015)’s country-level projections of dementia prevalence for 2018. Results. All algorithms accurately classified self-reported diagnosis of dementia (accuracy = 0.90-0.96), with the Langa-Weir classification based on recall and a cutoff reflecting country-specific prevalence outperforming other algorithms regarding compensation of underdiagnosis. Algorithmically detected probable dementia is associated with newly self-reported dementia diagnosis, drop-out and death two years later. Discussion. Identifying probable dementia through classification algorithms can increase statistical power and improve validity in cross-national investigations. Further research is needed to replicate the findings in validated cognitive assessments and to identify causes of cross-national variation in dementia underdiagnosis.

### SESSION 6160 (POSTER)

### KNOWLEDGE AND COMMUNICATION AS ADRD INTERVENTIONS

### NONVERBAL STRATEGIES TO ENHANCE PERSON-CENTERED COMMUNICATION WITH PEOPLE LIVING WITH DEMENTIA

Emma Bender, Marie Savundranayagam, Laura Murray, and Joseph Orange, *Western University, London, Ontario, Canada*

Many people living with dementia experience difficulties comprehending language, and benefit from nonverbal communication (NVC). Yet, little published empirical evidence exists for care partners regarding NVC strategies that support person-centered communication with perons living with dementia. This study aimed to determine whether NVC strategies used by personal support workers accompany verbal communication demonstrating person-centered communication indicators (facilitation, negotiation, recognition, validation). Secondary data analysis of video-recorded interactions (n=40) between personal support workers and simulated persons living with dementia was conducted. The recordings were transcribed according to communication-units, which were coded for person-centered communication and NVC, using a novel coding system consisting of ten NVC strategies. The overlap between NVC strategies and verbal person-centered communication was examined. Findings revealed that personal support workers frequently accompany verbal person-centered communication with NVC strategies. Out of 1848 communication-units in which person-centered verbal communication was used, 69% overlapped with NVC strategies. Gaze overlapped with all person-centered communication indicators frequently, both individually (40% – 49% of overlapping communication-units) and when combined with touch (13-24%). Gestures using objects (with and without gaze) frequently accompanied facilitation (17%) and negotiation (21%), while positive facial expressions (with and without gaze) were commonly found in recognition (16%) and validation (16%). The use of NVC strategies which support person-centered communication may lead to communication enhancement, in turn improving interactions and relationships between persons living with dementia and their care partners.

### FEASIBILITY OF THE ACTPLAN PROGRAM FOR AFRICAN-AMERICAN DEMENTIA CAREGIVERS: A SELF-DIRECTED MULTIMEDIA DELIVERY

Yashika Watkins[1], Rita Tharpe[2], Neelum Aggarwal[3], and Gloria Bonner[4], *1. Chicago State University, Cliffside Park, New Jersey, United States, 2. ACTPlan Study, Chicago, Illinois, United States, 3. Rush University, Chicago, Illinois, United States, 4. University of Illinois at Chicago College of Nursing, Chicago, Illinois, United States*

African Americans (AA) are less likely than White Americans to complete advance care plans or end-of-life treatment documents. They face significantly greater risk of Alzheimer's Disease, a silent epidemic for this population, and other dementias. The healthcare system's lack of dementia support for AAs contributes to disparate care. A four-session caregiver group education program was conducted on advance care planning for AA dementia family providers. The program was based on Kolb's Experiential Learning Model and initially found effective in an R01 study using in-person delivery by a professional. The present pilot assessed feasibility of delivering the program in a self-directed multimedia format without professional facilitation, using Session 1 on tube feeding decisions as the test session. Twenty-six AA dementia caregivers completed the session in groups of 5 to 8 at a church equipped with a large TV screen. On-screen prompts guided navigation through the program which included recorded lecture, slides, short videos on decision-making, and group discussions. Using quantitative and qualitative methods, pre-and post-survey instruments were administered and interviews conducted. Usability ratings averaged 84%. Knowledge and self-efficacy gains exceeded those of the R01, with a 35% increase in correct responses on knowledge items, versus 18% for the R01 subjects; and increase in perceived decisional self-efficacy of 31% versus 30% for the R01 subjects. Qualitative feedback was universally positive. These findings confirm the feasibility of the self-guided multimedia approach to delivery of the program. A large RCT is planned which, if successful, will support wide dissemination to AA caregivers in need.

### DOES KNOWLEDGE MATTER? AN INVESTIGATION INTO KNOWLEDGE OF ALZHEIMER'S DISEASE AND CAREGIVER BURDEN

Kaitlin Grelle, Kristen Hardin-Sigler, and Rebecca Deason, *Texas State University, San Marcos, Texas, United States*

A caregiver for someone with Alzheimer's disease often experiences burden, which can negatively impact their own physical and mental health, social participation and network, and financial stability (Chaio et al.; 2015, George & Gwyther, 1986). There is much interest in determining how to mitigate burden and studies suggest knowledge of the disease and recognizing positive aspects of caregiving may help reduce burden (Abdollahpour et al., 2018; Graham et al., 1997). The purpose of this study was to better understand how certain caregiver characteristics may impact burden. Participants (N=112) completed questionnaires regarding burden, disease knowledge, specific characteristics, and their caregiver experience. Pearson's correlations were conducted to examine the relationship between knowledge, burden, and caregiver characteristics. There was no significant correlation between burden and knowledge ($p = .457$), but burden was significantly correlated with positive aspects of caregiving ($p < .001$) and life satisfaction ($p < .001$). To better understand how well these two variables predicted burden, a multiple regression was conducted, $R^2 = .352$, $F(2, 90) = 24.48$, $p < .001$. Our findings provide further evidence that a focus on the positive aspects of caregiving and higher levels of life satisfaction are predictors of reduced caregiver burden. Some prior studies suggested knowledge of Alzheimer's disease led to decreased burden, whereas other studies found more knowledge increased anxiety, thus increased burden (Zawadzki et al., 2011). Our failure to find a correlation between knowledge and burden levels suggests this relationship may not be straightforward and future research needs to explore more specific aspects of this question.

### IDENTIFYING GAPS AND MISCONCEPTIONS AND EXAMINING PREDICTORS OF ALZHEIMER'S DISEASE KNOWLEDGE IN KOREAN AMERICANS

Michin Hong[1], and Sang Lee[2], *1. Indiana University, Indianapolis, Indiana, United States, 2. San Jose State University, San Jose, California, United States*

This study examined Alzheimer's disease (AD) knowledge and its predictors among Korean Americans (KAs). A total of 268 KAs in the Greater Washington metropolitan area participated in the study and completed a cross-sectional survey. Using the Alzheimer's Disease Knowledge Scale (ADKS), overall and domain knowledge was assessed. Multiple regression analyses were conducted with predictors including exposure to AD, social engagement, sources and frequency of health-related information, stigmatic beliefs (pity, antipathy, and social distance), English proficiency, and education. KAs reported 59% accuracy in overall AD knowledge. They were most knowledgeable about assessment and diagnosis domain and least knowledgeable about caregiving domain. Regression analyses showed that having more education is associated with greater overall and certain domain knowledge. Having more pity stigmatic beliefs is related to greater knowledge in both life impact and caregiving domains while having less pity stigmatic beliefs is associated with more risk factor knowledge; having less social distance stigmatic beliefs is associated with greater life impact knowledge; and having less antipathy stigmatic beliefs is related to better caregiving knowledge. Our findings revealed areas of misconceptions and knowledge gaps in KAs which need to be addressed in educational interventions. Different knowledge status across the domains demonstrates a multi-dimensional nature of AD knowledge. Multivariate findings confirmed the robust role of education in AD knowledge. Effect of different AD stigmatic beliefs on certain AD knowledge domains suggests ways of how stigma change can be efficient for the purpose of increasing AD domain knowledge in KAs.

### TARGETING KNOWLEDGE, BARRIERS, AND FACILITATORS TO PARTICIPATION IN DEMENTIA RESEARCH (THE POWER PROJECT)

Martha Sajatovic[1], Lynn Herrmann[2], Clare Knebusch[3], Kaylee Sarna[4], Alan Lerner[1], Edna Fuentes-Casiano[5], and Christopher Burant[6], *1. Case Western Reserve University School of Medicine, Cleveland, Ohio, United States, 2. Northern Illinois University, DeKalb, Illinois, United States, 3. University Hospitals Cleveland Medical Center,*

*Cleveland, Ohio, United States, 4. Case Western Reserve University, Cleveland, Ohio, United States, 5. Case Western Reserve University School of Medicine, Cleveland, Ohio, United States, 6. Case Western Reserve University, Parma, Ohio, United States*

Racial and ethnic minorities are under-represented in dementia research. We tested a conceptually-driven health communication approach targeting evidence-based barriers and facilitators to research participation vs. dementia awareness training. Input from the minority advisory board (MAB) of the Cleveland Alzheimer's Disease Research Center (CADRC) informed development of 2 brief health communication videos which differentially focused on research barriers and facilitators (POWER) versus an education control (EDU). A randomized controlled on-line survey compared POWER vs. EDU among 200 individuals ≥ 50 years. We examined pre/post video change in research motivation measured by the transtheoretical model, dementia knowledge and cumulative barriers and facilitators. Interim analysis included 129 individuals who completed the pre-video survey, mean age 58.8 (SD 14.9), 101 (78.3 %) women, 54 (41.9 %) non-white. 100 (77.5 %) completed the post-video survey. Whole sample dementia knowledge improved from pre (mean 4.6, SD 1.3) vs. post (5.8, SD 0.7) video (p <.01), as did facilitators to research from pre (22.4, SD 2.7) vs. post (22.8, SD 3.0) video (p=.02), while there was no significant change in barriers. Research motivation was increased pre (10.8, SD 2.0) vs. post (22.8, SD 3.0) video (p=.02). Overall, dementia knowledge improved more with EDU vs. POWER (p=.04). African-American participants trended towards greater improvement in dementia knowledge with EDU vs. POWER (p=.07), while non-African-Americans had greater facilitator improvement with POWER vs. EDU (p=.03). Targeted communication approaches that address knowledge gaps and both barriers and facilitators to participation may improve engagement of diverse groups in dementia research.

### OLDER ADULTS' ONLINE INFORMATION SEEKING FOR ALZHEIMER'S DISEASE

Matthew Picchiello, Payton Rule, and Brian Carpenter, *Washington University in St. Louis, St. Louis, Missouri, United States*

Many older adults use the internet to gather health information. Yet past research shows that older adults display less understanding of internet concepts and score lower on information-retrieving tasks than younger adults, making them more vulnerable to internet misinformation. To address this, a variety of guidelines (e.g., HONCode) have been developed to help consumers identify credible sources. This is especially relevant for Alzheimer disease (AD), as research has shown that online AD information is mixed in its quality. The purpose of this study was to examine how older adults gather online AD information and evaluate their ability to find credible sources to improve knowledge. We recruited 83 older adults (Mage = 70.0, 60.2% female; 85.5% White) who completed the Alzheimer Disease Knowledge Scale (ADKS; Carpenter et al., 2009) before and after engaging in six AD-related search tasks (e.g., identify age of onset, risk factors, treatment, etc.). Older adults utilized a variety of search strategies, visiting both reputable (i.e., not-for-profit or university-based) and less scrutinized (i.e., commercially sponsored) websites, and a vast majority (95.0%) were unaware of HONCode guidelines for evaluating website quality. While most participants could locate some AD-related information (97.6% identified risk factors), other information was more elusive (29.3% could not identify age of onset). Despite this, participants showed improved ADKS scores, $t(74) = 5.55$, $p < 0.001$. Our research shows that many older adults can gather some accurate online information about AD but may benefit from additional resources on using effective search strategies and identifying credible sources.

### SESSION 6170 (POSTER)

### MENTAL HEALTH (POSTERS)

### DEVELOPMENT AND VALIDATION OF THE LEISURE EUSTRESS-DISTRESS SCALE

Jaesung An[1], Laura Payne[2], and Toni Liechty[2], *1. California State University East Bay, Cupertino, California, United States, 2. University of Illinois Urbana-Champaign, Champaign, Illinois, United States*

Encountering stressors is a part of everyday life and can even occur when people engage in leisure activities. This is more prevalent among older adults due to the onset of chronic condition(s), lack of resources, and social isolation. Therefore, leisure related stressors could provoke complex emotions derived by positive and/or negative responses to stressors before, during, and after older adults engage in leisure activities. Different from the concept of leisure constraints, which emphasizes causes of stress that prevents leisure engagement in the first place, the concepts of leisure eustress and distress are associated with the emotional experience of stress in the context of leisure. Despite the presence of leisure-based eustress and/or distress among older adults, there is no measurement scale that assesses this phenomenon. Therefore, the purpose of this study was to develop and validate a leisure eustress-distress scale (LEDS). Phase one of this study included in-depth interviews, expert panelists review, and a pilot study to develop and refine items. In the second phase, a target study was conducted to examine the factor structure using exploratory and confirmatory factor analysis. For validity, LEDS showed partial support for construct and criterion validity. Strategies to strengthen the scale's validity will be discussed as well as future recommendations and practical implications of the LEDS.

### BA FOR DEPRESSION AND DIABETES MANAGEMENT: EXAMINED VIA SINGLE-CASE DESIGN

Anna Robertson[1], and Leilani Feliciano[2], *1. University of Colorado, Colorado Springs, Colorado Springs, Colorado, United States, 2. University of Colorado Colorado Springs, Colorado Springs, Colorado, United States*

Type 2 Diabetes Mellitus (T2DM) is a pervasive, chronic disease whose prevalence increases with age. The most common treatment options for T2DM include medication, dietary changes, and engagement in physical activity, which require considerable lifestyle changes. Further, individuals with T2DM are highly likely to develop physical and mental health comorbidities that can complicate effective T2DM management. In fact, individuals with T2DM and other comorbidities are frequently excluded from behavioral and other interventions in randomized controlled trials due to their comorbidities. Individualized treatment is necessary to address these comorbid diseases and to effectively navigate lifestyle changes. As part of a larger study evaluating an at-home diabetes management program with patients at a Federally Qualified Health Care Facility, the current study utilized a single-case experimental design approach using changing criterion design to evaluate behavioral activation to treat depression and diabetes in a 68 year-old Hispanic woman with cognitive impairment. Across her intake, five treatment sessions, and two follow-up appointments, we collaborated with her to set and achieve behavioral goals while effectively managing her diabetes. By end of treatment, she reported significant improvements in depression and overall well-being, increased engagement in exercises, increased blood glucose tracking, and rated improvement in her overall efficacy in life that maintained through follow-up. Results revealed the effectiveness of behavioral activation in treating comorbidities in a low-income, diverse individual. Attendees will learn about a promising treatment avenue for older individuals with complex medical and psychiatric conditions and the utility of single case design.

### UNDERSTANDING THE EXPERIENCE OF EUSTRESS AMONG OLDER ADULTS WITH CHRONIC CONDITIONS

Jaesung An[1], Laura Payne[2], Toni Liechty[2], and Yoshitaka Iwasaki[3], *1. California State University East Bay, Cupertino, California, United States, 2. University of Illinois Urbana-Champaign, Champaign, Illinois, United States, 3. San Jose State University, San Jose, California, United States*

In their longitudinal study of stress and health among adults, McGonigal and colleagues (2013; 2016) suggested that how person perceives stress is a key factor affecting their actual physiological health. This is related to the concept of eustress (i.e., positive response to a given stressor) which has received very little attention in the literature. Besides, older adults with chronic conditions deal with symptoms that are difficult to manage and can contribute to chronic stress, which can then affect their health status and quality of life. Thus, helping older adults to shift their perceptions and interpretations of stressors from distress to eustress, rather than trying to avoid stress seems to be a reasonable approach to help them experience healthy aging. The purpose of this study was to explore the experience of eustress among older adults with chronic conditions in the context of leisure. Sixteen participants were recruited from three settings in a Midwestern city: a retirement community, an assisted living center, and a community senior program. Findings showed that participants' health conditions were one of their greatest stressors, however, they modified their leisure engagement based on their conditions to enable their engagement in valued leisure activities. In the context of leisure, five themes emerged as facilitators that helped older adults to experience eustress: leisure satisfaction, sense of control, high levels of perseverance, positive attitude, and spiritual belief. Direct quotes and further discussion on how the themes are represented in the stress, leisure, and gerontology literature will be presented.

### AGE AND COGNITIVE ABILITY AS PREDICTORS OF EMOTION REGULATION STRATEGY USE

Claire Growney, and Tammy English, *Washington University in St. Louis, St. Louis, Missouri, United States*

Emotion regulation (ER) is viewed as a cognitively demanding process and involves selecting and implementing specific strategies in support of one's emotional goals. Older adults are theorized to maintain emotional wellbeing by selecting ER strategies that are consistent with their available resources, which may involve engaging more with lower-arousal stimuli and less with higher-arousal stimuli, especially when cognitive resources are limited. The aim of this study was to examine relationships between age, cognitive ability, and use of different types of ER strategies. Participants (N = 287) aged 25-85 (M = 54.33, SD = 17.19) completed assessments of cognitive ability from the NIH Toolbox Cognitive Battery. In a laboratory task, participants viewed a series of film clips eliciting low-arousal emotions (sadness, contentment) and high-arousal emotions (disgust, amusement) under instructions to regulate pro-hedonically using any available strategy. They reported their use of disengagement (e.g., distraction, suppression), engagement (e.g., perspective-taking, awareness), and positive-focus strategies (e.g., savoring, positive reappraisal). Results from multilevel models revealed that with low-arousal stimuli, age was associated with higher engagement and lower disengagement, regardless of fluid cognitive ability. With high-arousal stimuli, a fluid cognition X age interaction indicated that there was an age-related increase in engagement among individuals with higher fluid cognitive ability, but not lower fluid cognitive ability. Findings support the idea that older adults tend to engage with emotional stimuli, which may aid with processing and support emotional memory for future regulation attempts. Fluid cognitive ability may be an important resource supporting older adults' engagement with high-arousal stimuli.

### COMMUNITY MOBILITY PATTERNS OF OLDER ADULTS DURING THE COVID-19 PANDEMIC

Arne Stinchcombe[1], Nadia Mullen[2], John Timinski[1], Shawn Marshall[1], Gary Naglie[3], Mark Rapoport[4], and Michel Bédard[2], *1. University of Ottawa, Ottawa, Ontario, Canada, 2. Lakehead University, Thunder Bay, Ontario, Canada, 3. Baycrest Health Sciences, Toronto, Ontario, Canada, 4. University of Toronto, Toronto, Ontario, Canada*

The COVID-19 pandemic has had appreciative impacts on the lives of older adults. At various times during the pandemic, Canadian provinces put in place public health measures to prevent the spread of the virus, including closures of non-essential businesses and services. The Candrive Driving Cessation sub-study is a 12-year, mixed methods, longitudinal study. During the COVID-19 pandemic, participants from four sites (n=124, mean age=85.5 years) were asked to report how their community mobility patterns during the pandemic compared with before the pandemic. They also completed the COVID-19 Anxiety Syndrome Scale and the Assessment of Readiness for Mobility Transition (ARMT) tool. The results indicated that during the pandemic, participants were less likely to drive ($\chi2=7.11$, $p=.004$) or ride a public bus ($\chi2=20.05$, $p<.001$) when leaving their home. During the pandemic, they reported fewer trips to the supermarket ($\chi2$=99.00, p<.001) and fewer visits with family ($\chi2$=68.00, p=.001). We observed a statistically significant relationship between COVID-19 Anxiety Syndrome Scale scores and ARMT (Anticipatory Anxiety subscale; r= .810, p<.001), such that higher COVID-19-related anxiety was associated with greater anxiety related to anticipating changes in mobility. These results emphasize the impacts of the COVID-19 pandemic on the mobility patterns of older adults. Promoting community mobility for older adults alongside preventative public health measures is essential.

### FACTOR ANALYSIS OF CORNELL SCALE FOR DEPRESSION IN DEMENTIA AMONG ASSISTED LIVING RESIDENTS

Rachel McPherson[1], and Barbara Resnick[2], *1. University of Maryland Baltimore and Baltimore County, Catonsvile, Maryland, United States, 2. University of Maryland, Baltimore, Maryland, United States*

The Cornell Scale for Depression in Dementia (CSDD) was developed to measure depressive symptoms among older adults with dementia. The psychometric qualities of the CSDD have been inconsistent regarding the factor structure, with some studies showing a four-factor model and others a five-factor model. The purpose of this study was to test the factor structure of the CSDD as a measure of depression among a sample of assisted living residents. It was hypothesized that a four-factor version of the CSDD would provide a better fit than a five-factor version of the CSDD. The present study used baseline data from the Function-Focused Care for Assisted Living Using the Evidence Integration Triangle (FFC-AL-EIT) intervention study. A total of 511 residents from 85 assisted living facilities were included in the analyses. Confirmatory factor analyses were conducted to examine the factor structure and a chi-square difference test was conducted to compare model fit. Three items were removed from both models due to small factor loadings. The chi-square difference test indicated that the five-factor model fit the data significantly better ($\chi2$ = 796.08, $\Delta\chi2$= 22.86, $\Delta$df = 4, p < .001) than the four-factor model, although both the five-factor and four-factor models produced very poor model fits. These findings may be due to the fact that the CSDD relies on information from caregivers, and the measure might benefit from including observational signs of depression. Future work should examine other factors or items that belong in a depression measure among assisted living residents.

### FEAR OF FALLING IS RELATED TO ANXIETY

Hyeon Jung Kim[1], Jennifer Yentes[2], Dawn Venema[3], and Julie Blaskewicz Boron[4], *1. University of Nebraska Omaha, Omaha, Nebraska, United States, 2. Texas A&M University, College Station, Texas, United States, 3. University of Nebraska Medical Center, Omaha, Nebraska, United States, 4. University of Nebraska, Omaha, Omaha, Nebraska, United States*

Fear of falling (FOF), defined as a psychological symptom, is related to increased fall risk, anxiety, and depressive symptoms. As physical function changes with age, FOF can appear beginning in midlife. FOF may result in avoiding physical activities, resulting in weakness of postural balance. Neuroticism is associated with anxiety and depression; thus, those high on neuroticism may have an increased FOF. This study aimed to address the relationships between FOF, neuroticism, and number of falls from external perturbations while standing. Participants included 45 participants (female: n=24; age range: 42-77yrs; age=62.44±9.25yrs). Five standing trials were completed on a force platform with perturbations. Hierarchical regression was used to investigate the impact of neuroticism (Big Five Inventory) and number of falls on FOF (The Falls Efficacy Scale International), accounting for age, anxiety (Geriatric Anxiety Scale), and depressive symptoms (Center for Epidemiological Studies Depression Scale). Anxiety was significantly associated with FOF (model 1; age, anxiety, depression), B=0.31, $\beta$=.55, t(41)=3.08, R2=.30, p=.004, 95% CI [0.11, 0.51]. Neuroticism was not significantly related to FOF (model 2; $\Delta$R2=.05, $\Delta$F=2.94, p=.094), nor was number of falls (model 3; $\Delta$R2=.00, $\Delta$F=0.01, p=.974). Results revealed that anxiety levels had the strongest relationship with FOF. This suggests that strategies to reduce daily anxiety may decrease FOF. Future research should examine how anxiety is related to history of falls, and how changes in physical functions (e.g., mobility, vision, proprioception) may impact FOF.

### MARITAL DISSOLUTION AND DEPRESSIVE SYMPTOMS IN MIDDLE AND LATE LIFE: AN ANALYSIS OF THE HEALTH AND RETIREMENT STUDY

Julia Tucker[1], and Sae Hwang Han[2], *1. The University of Texas at Austin, New Braunfels, Texas, United States, 2. University of Texas at Austin, Austin, Texas, United States*

Literature to date has long indicated that marital relationships serve as one of the most important sources of health and well-being across adulthood. Despite the well-documented link between marital status and health, however, studies that have examined strict within-person linkages between martial transitions and subsequent health are limited . Drawing from the strength and vulnerability integration theory and the life course perspective, we investigated within-person mental health consequences of two forms of marital dissolution (i.e., divorce and widowhood), and whether the mental health consequences vary depending on the timing of marital dissolution (i.e., midlife vs late adulthood). Data for this study came from 11 waves of longitudinal data from the Health and Retirement Study (1998-2018). Study sample included 22,911 individuals 50 and older (11,429 women, 11,482 men) who were selected at the first wave at which they were identified as married during the observation period and subsequently followed up for up to 14 years (person-wave observations N=127,081). Approximately 4% and 17% of the study sample experienced a divorce and widowhood, respectively, during the observation. Mental health was assessed with an 8-item version of the Center for Epidemiologic Studies Depression scale. Study findings based on multilevel models indicated a robust within-person association between transition to widowhood and higher levels of depressive symptoms, but such association was not found regarding divorce. For women only, the mental health consequences of widowhood experienced during midlife were more detrimental compared to late-life widowhood. The findings are discussed in the context of intervention strategies.

### SUICIDAL IDEATION AMONG OLDER ADULTS LIVING WITH CHRONIC ILLNESS

Abigail Poe, and Frank Puga, *The University of Alabama at Birmingham, Birmingham, Alabama, United States*

Older adults living with chronic illnesses, such as diabetes, cancer and heart disease, have an increased risk of poor mental health outcomes. While a link between living with chronic illness and depression have been examined in previous studies, relatively little is known about factors that increase and decrease the risk of suicidal ideation among older adults living with chronic illness. Using data from the third wave of the Midlife in the United States (MIDUS) database, we examined the relationship between living with a chronic illness were more likely to endorse thinking about death in the previous two weeks ( OR=3.17, CI: 1.16 – 8.65, p=0.24). The analysis also revealed that the likelihood of suicidal ideation in the previous two weeks increased with the number of chronic conditions reported by participants (OR=1.128, CI: 1.04 – 1.23, p=0.005). The results of this study are consistent with previous studies suggesting a relationship between older adults living with a chronic condition are at a higher risk of suicidal ideation. Findings from this study can help inform intervention development to support mental health of older adults living with chronic illness. Future studies are needed to examine additional psychosocial factors that may mediate the relationship between living with a chronic illness and suicidal ideation among older adults.

### THE IMPACT OF SLEEP ON MINDFULNESS, WELL-BEING, AND DEPRESSIVE SYMPTOMATOLOGY IN MIDDLE TO OLDER AFRICAN AMERICANS

Folasade Ilori[1], Sanaz Dabiri[1], Mara Ramirez-Ruiz[1], George Daniel[1], Robert Head[1], Regina Wright[2], and Denee Mwendwa[1], *1. Howard University, Washington, District of Columbia, United States, 2. University of Delaware, Newark, Delaware, United States*

The decline in physical and mental well-being is common with age. The negative impact of poor quality of sleep and depressive symptomatology contributes to the decline in well-being among middle to older adults. African Americans are more likely to report a decrease in well-being as they age. They are also disproportionately affected by poorer sleep quality and may be at greater risk for depressive symptomatology. Dispositional mindfulness is linked to improved well-being. Research has shown dispositional mindfulness is related to adaptive health behaviors, improved sleep quality, and reduced negative affect. Mindfulness allows engagement of early regulation of intense emotional responses because of nonjudgmental acceptance of thoughts and emotions. Therefore, this study aims to examine the impact of mindfulness on physical/mental well-being and depressive symptomatology through sleep. 131 African American participants were recruited for the study, with an average age of 58 years. The results showed a significant effect of mindfulness on well-being and depressive symptoms. Additionally, the impact of mindfulness on mental and physical well-being was mediated by sleep quality (B = .7920, CI [.1482, 1.6702]) and sleep disturbance (B = 2.874, CI [.1582, 6.335]). The results also showed an indirect effect of mindfulness on depressive symptoms through sleep quality (B = –6.139 CI [-12.871, -.5298]). This study demonstrates the positive impact of mindfulness on well-being and depressive symptomatology. Sleep quality also plays an important role in the relationship between dispositional mindfulness and physical/mental well-being and depressive symptomatology in middle to older African Americans.

### GRANDPARENTING AND DEPRESSIVE SYMPTOMS IN URBAN CHINA: LIVING ARRANGEMENTS AND WORK STATUS AS MODERATING EFFECTS

Luyan Ma[1], and Zhaowen Cheng[2], *1. Peking University, Beijing, Beijing, China (People's Republic), 2. China Development Research Foundation, Beijing, Beijing, China (People's Republic)*

Scholars worldwide are divided as to whether grandparenting can benefit to older adults' well-being. Evidence from rural China supports that custodial grandparenting worsens grandparents' psychological well-being. However, given the great urban-rural gap, the situation in urban China might be different. To clarify this, we use CHARLS 2011-2013 (900 respondents) and examine the association between length and intensity of grandchild care and depressive symptoms among older adults living in urban China. We also explore the moderating effect of grandparents' living arrangements and work status. Multiple linear regression with interaction analysis is applied in our analysis. We find that moderate levels of grandchild care, without distinguishing between living arrangements and work status, are associated with reduced depressive symptoms ($\beta = -1.146$ , $p < .05$). Grandparents are likely to have more depressive symptoms when working full-time and providing care at the same time. Comparing with not living with grandchildren, living with grandchildren is beneficial to grandparents' mental health. Findings suggest that psychological counseling services and economic support policies should be launched to relieve the work pressure of grandparents in financial difficulties.

### CHANGE IN SOCIAL INTERACTION AND MENTAL HEALTH AMONG OLDER AMERICANS DURING COVID-19 PANDEMIC

Krystyna Keller[1], Jielu Lin[1], Melissa Zajdel[1], Fiona Gilpin Macfoy[1], Philip Shaw[1], Brenda Curtis[2], Lyle Ungar[3], and Laura Koehly[1], *1. National Institutes of Health, Bethesda, Maryland, United States, 2. National Institutes of Health, Baltimore, Maryland, United States, 3. University of Pennsylvania, Philadelphia, Pennsylvania, United States*

Recent research has shown the mental health consequence of social distancing during the COVID-19 pandemic, but longitudinal data are relatively scarce. It is unclear whether the pattern of isolation and elevated stress seen at the beginning of the pandemic persists over time. This study evaluates change in social interaction over six months and its mental health impact among older adults. We drew data from a panel study with six repeated assessments of social interaction and mental health conducted monthly May through October 2020. The sample included a total of 380 White, Black and Hispanic participants aged 50 and over, of whom 33% had low income, who residing in fourteen U.S. states with active stay-at-home orders in May 2020. The analysis examined how change in living arrangement, in-person interaction outside the household, quality of relationship with family and friends, and perceived social support affected trajectories of isolation stress, COVID worry and sadness. While their living arrangements and relationship quality remained stable, older adults experienced fluctuations in perceived social support and increases in in-person conversations outside the household. Living with a spouse/partner stabilized isolation stress and COVID worry over time. Individuals with better relationship quality with friends became happier over time. Changes in social support were associated with greater fluctuations in isolation stress and COVID worry. During the pandemic, social interactions are protective and lack of stability in feeling supported makes older adults vulnerable to stress. Efforts should focus on (re)building and maintaining companionship and support to mitigate the pandemic's negative impact.

### AT THE INTERSECTION OF CULTURE AND MENTAL HEALTH

Daniel Jimenez, *University of Miami Miller School of Medicine, Miami, Florida, United States*

Although mental health disorders affect people across the lifespan, older adults face unique issues associated with accessing mental health treatment. These structural and psychosocial challenges are further exacerbated among older racial and ethnic minorities. Often compared to their white counterparts, racial and ethnic minority older adults face specific cultural factors and other systemic barriers that create stark disparities in diagnosis, treatment, and access to care. Increasing research on identifying barriers to treatment for older racial and ethnic minority adults has been recognized as an integral component in enhancing treatment access to improve behavioral health outcomes among these marginalized groups. Therefore, this review article aims to investigate the intersection of mental health and culture through the lens of three racial and ethnic minority groups in the United States – Blacks/African-Americans, Asian Americans, and Latinos. Authors provide unique insights on the differing needs of these under-researched communities by exploring psychiatric comorbidity, experiences with seeking, accessing, and engaging in treatment, and the unique cultural and psychosocial factors that affect treatment outcomes for these diverse groups. Future directions and recommendations to provide appropriate mental healthcare to Black/African-American, Asian American, and Latino communities are discussed with special attention placed on cultural adaptations, models of care, prevention, and practical strategies that can be implemented to reduce disparities and increase health equity.

### EXPLORING THE PHYSICAL FUNCTION PROFILES OF OLDER ADULTS WITH SERIOUS MENTAL HEALTH CONDITIONS

Julia Browne[1], Miriam Morey[1], Eric Elbogen[1], Kim Mueser[2], and Katherine Hall[1], *1. Durham VA Health Care System, Durham, North Carolina, United States, 2. Boston University, Boston, Massachusetts, United States*

Older adults with serious mental health conditions experience high rates of comorbid chronic medical diseases, which affect the physical function and long-term aging trajectory of this population. Existing physical function research in this population has focused almost exclusively on endurance with little to no attention paid to other important domains such as balance, mobility, and strength, which are critical to promoting independence and improved quality of life. The primary aim of this study was to explore the multiple dimensions of function that are important to maintaining independence and health in older adults with serious mental health conditions. This study examined publicly available data from the 2014 and 2016 waves of the Health and Retirement Study, a national longitudinal panel study of older adults. Analyses were limited to participants that self-reported lifetime diagnoses of serious mental health conditions (schizophrenia, bipolar disorder, depression, posttraumatic stress disorder). The sample comprised 428 older adults over age 50 (M=67.1, SD=10.1; 68% female) who completed standardized assessments of grip strength, single-leg standing balance, and usual walking speed. Scores on these three measures were compared to established age-and/or gender-based norms for each assessment to characterize performance. Overall, 65% of the sample demonstrated deficits in strength and 31% of the sample performed below established norms for both balance and walking speed. These findings highlight that physical function in this population is compromised across multiple domains and suggest a need for multicomponent interventions.

### DIFFERENT LEVELS OF LEISURE-TIME PHYSICAL ACTIVITY AND MENTAL HEALTH FOR OLDER ADULTS WITH DIABETES IN THE PANDEMIC

Jungjoo Lee[1], Junhyoung Kim[2], and Sua Han[2], *1. Indiana University, Blooimgton, Indiana, United States, 2. Indiana University, Bloomington, Indiana, United States*

This study is an initial investigation of the association of different levels of LTPA engagement with mental health (e.g., loneliness, happiness, and positive and negative affect) among older adults with diabetes during the COVID-19 pandemic. The COVID-19 has led public health researchers to improve mental health among older adults with diabetes. Leisure-time physical activity (LTPA) has been emerged to cope with mental health difficulties in the pandemic. Total 301 respondents were extracted from the Health and Retirement Study (HRS) based on the following two criteria: over 50 years old and the onset of diabetes. Multiple questionnaire items were used to assess mental health (i.e., loneliness, happiness, and positive and negative affect) for older adults with diabetes. Multivariate Analysis of Variance was utilized to investigate the relationships between the fixed variable (i.e., LTPA) and outcome variables (i.e., mental health). We categorized LTPA participation into three groups (i.e., low, mid, high) and examined mental health following a different level of LTPA participation. LTPA participation showed a significant group mean differences for loneliness, happiness, and positive affect, but not for negative affect. High-LTPA respondents presented lower loneliness) and higher happiness than Low-LTPA respondents. High-LTPA and Mid-LTPA respondents indicated higher positive affect than Low-LTPA respondents. This study provides evidence of the benefits of LTPA on mental health for older adults with diabetes. Our study supports the evidence that high-LPTA involvement can be effective in promoting mental health among older adults with diabetes in the COVID-19 era.

### AGE DIFFERENCES IN EXPOSURE TO NOVEL SITUATIONS IN DAILY LIFE AND ASSOCIATED EMOTIONAL EXPERIENCE

Li Chu[1], Yochai Shavit[2], Nilam Ram[3], and Laura Carstensen[3], *1. Stanford University, Menlo Park, California, United States, 2. Stanford Center on Longevity, Stanford University, Stanford, California, United States, 3. Stanford University, Stanford, California, United States*

Exposures to novelties are cognitively beneficial in later adulthood, but their impact on emotional well-being is still unknown. Novel situations may bring excitement as well as anxiety, and this may be different across ages According to socioemotional selectivity theory, older adults prioritize familiar and positive experiences that likely contribute to better emotional well-being over novel and negative ones, suggesting that older adults may be motivated to avoid novel situations, especially if these experiences are associated with negative emotions. This study examined age differences in novelty experienced in daily life and the associated emotions. We utilized experience sampling data collected five times a day for one week from 375 participants (age range=18-94). Contrary to the hypothesis, older age exhibited a quadratic association with novel daily experiences such that situation novelty was lowest in middle adulthood. Results from multilevel models suggested that people with higher overall exposure to novel situations had higher overall levels of negative emotions, and that when in more novel situations the prototypical individual experienced more negative emotions in general. However, consistent with SST postulates, one of these associations were moderated by age; older adults experienced lower positive emotions during novel situations than younger individuals. In contrast, the association between situation novelty and negative emotions did not differ with age. Together, these findings suggest that older adults do often find themselves in novel situations but may experience them less positively than younger adults. Implications on learning-related programs and interventions designed to expose older adults to novelties will be discussed.

### COMPASSIONATE LOVE AND LONELINESS: LATER LIFE MENTAL HEALTH IN THE UNITED STATES

Nirmala Lekhak[1], Tirth Bhatta[2], Eva Kahana[3], and Joel Synder[1], *1. University of Nevada, Las Vegas, Nevada, United States, 2. University of Nevada Las Vegas, Las Vegas, Nevada, United States, 3. Case Western Reserve University, Cleveland, Ohio, United States*

Loneliness is a serious public health problem that affects over 25% of older adults and is associated with an increased risk of depression, cognitive decline, and premature death. Previous research on social support mechanisms that contribute to loneliness has consistently illustrated the role of emotional support in reducing loneliness. However, the importance of compassionate love in reducing loneliness and, as a consequence, improving psychological well-being in later life has received little attention. Neurobiology indicates that the brain regions associated with loneliness and compassion overlap, suggesting that increasing compassion-related emotions may help alleviate loneliness. Using data from a nationwide web-based survey (n=1,861), we examined the influence of compassionate love on loneliness and assessed whether loneliness mediates the relationship between compassionate love and mental health outcomes. Even after controlling for emotional support, estimates from an ordinary least squares regression (OLS) model suggest that older adults who felt loved had significantly lower levels of loneliness ($b=-0.84$, $p< 0.001$). Feeling of love also contributed to significantly fewer depressive symptoms ($b=-2.03$, $p< 0.001$) and anxiety ($b=-1.07$, $p< 0.001$). Loneliness completely mediated the effect of compassionate love on anxiety ($b=-0.82$, $p< 0.001$) and significantly mediated its influence on depressive symptoms ($b=-1.18$, $p< 0.001$). Our findings underscore the need to design interventions that increase compassionate love to reduce loneliness and improve psychological wellbeing among older adults.

### DAILY STRESS, ANTICIPATORY ANXIETY, RUMINATION, AND NEGATIVE AFFECT IN LATE LIFE

Zexi Zhou, and Karen Fingerman, *The University of Texas at Austin, Austin, Texas, United States*

Chronic and daily stress are risk factors to older adults' health and well-being. However, most studies of stress have focused on the reactivity and recovery process following the onset of stress. Relatively little research has investigated the worries that precede stress (e.g., anticipatory anxiety) and how such worries may contribute to older adults' subsequent emotional experiences and rumination, especially in daily settings. This study investigated the joint associations of daily stress and anticipatory anxiety on rumination and negative affect in older adults' everyday life. We leveraged the ecological momentary assessment (EMA) data over 5 to 6 days from the Daily Experiences and Well-being Study ($N = 267$, Mage = 73.72). Anticipatory anxiety was moderately correlated with stresses experienced that day, $r = .23$, $p < .001$. We found significant joint associations between daily stress and anticipatory anxiety with rumination and negative affect. Higher daily anticipatory anxiety (M+1SD) combined with higher stress generated the highest rumination and negative affect, whereas lower daily anticipatory anxiety (M-1SD) paired with lower stress generated the lowest rumination and negative affect. Higher daily anticipatory anxiety paired with lower stress, or lower anticipatory anxiety paired with higher stress, led to a moderate level of rumination and negative affect. These results suggest that anticipatory anxiety toward potential stress has distinct negative effects on older adults' daily experiences, and these effects may contribute to stressors and to heightened rumination. The findings highlight the role that proactive emotional expectations may play in older adults' everyday life.

### GENERATIVITY AND OLDER ADULTS' COVID-19 ADJUSTMENT AND MENTAL HEALTH

Christine So[1], Katherine Fiori[1], Amy Rauer[2], and Christina Marini[1], *1. Adelphi University, Garden City, New York, United States, 2. University of Tennessee Knoxville, Knoxville, Tennessee, United States*

Generativity, the capacity to be productive, caring, and concerned with the well-being of the next generation, has been linked to positive mental health outcomes and posttraumatic growth (Bellizzi, 2004). Generativity may be particularly important nowadays as older adults adjust to the pandemic and its aftermath. For example, after months of social distancing, the availability of vaccines has enabled many older adults to begin resuming social activities. Considering the meaning-making function of generativity, generativity may be associated with more positive outcomes, including better mental health and views on quality of life and family relationships during this post-pandemic adjustment period. The current study used a community sample of 136 older adults (M age = 67.77, range 50-91; 69.3% females; 93% White) to explore whether generativity predicted older adults' anxiety and depressive symptoms, and attitudes about how the pandemic affected their quality of life and family relationships. Using hierarchical linear regressions controlling for age and gender, we found that generativity was negatively linked to anxiety and depressive symptoms. Furthermore, those with greater generativity were more likely to report that their family relationships improved because of the pandemic. In contrast, generativity was not associated with positive growth in the personal domain or with perceptions that the pandemic had harmed either personal or family domains. Our findings are consistent with Erikson's theory on the important role that generativity plays in shaping well-being and psychological health in older adults, and our findings suggest these effects may be especially pronounced during this post-pandemic adjustment period.

### THE ASSOCIATION OF INCREASING RESILIENCE WITH POSITIVE HEALTH OUTCOMES AND QUALITY OF LIFE AMONG OLDER ADULTS

Shirley Musich[1], Shaohung Wang[2], James Schaeffer[2], Sandra Kraemer[3], Ellen Wicker[4], and Charlotte Yeh[4], *1. Optum Labs, Bloomfield Hills, Michigan, United States, 2. Optum Labs, Minnetonka, Minnesota, United States, 3. UnitedHealthcare, Minnetonka, Minnesota, United States, 4. ASI, Inc., Washington, District of Columbia, United States*

Background: Resilience has been recognized as a concept central to successful aging. Higher resilience has been associated with positive mental health while a direct impact on physical health outcomes has been less consistent. Objective: To investigate three levels of resilience (low, medium, and high), identify characteristics associated with medium and high resilience and measure the impact of increasing resilience on selected health outcomes: quality-of-life (QOL), and healthcare utilization and expenditures.

Methods: The study sample was identified from adults age ≥65, covered by an AARP® Medicare Supplement Insurance Plan from UnitedHealthcare, who had completed a health survey during May-June 2019 (N=3,573). Resilience was categorized to three levels: low, medium, and high. Other positive resources, including purpose-in-life, locus of control, social connections, and optimism, were dichotomized as high/low and counted with equal weighting as a continuous variable. Quality of life was measured from the health survey; healthcare utilization and expenditures from administrative databases.

Results: Among weighted survey respondents, the prevalence of low, medium, and high resilience levels were 27%, 29%, and 43%, respectively. The strongest predictors of medium and high resilience included increasing number of other positive resources, lower stress, and no depression. Individuals with medium and high resilience had significantly higher QOL, lower healthcare utilization and reduced healthcare expenditures.

Conclusions: Interventions promoting the maintenance and/or increase of resilience should include a focus on other positive resources and stress/depression management. Resilience strategies integrated into healthy aging programming could be associated with improved health outcomes.

### POSITIVE AND NEGATIVE RELATIONSHIPS, OUTLOOK, AND DEPRESSION PREDICT LONELINESS IN OLDER ADULTS DURING COVID-19

Benjamin Hougaard[1], Vennisia Mo[2], Anahita Eskandari[2], and Rowena Gomez[2], *1. Palo Alto University, Mountain View, California, United States, 2. Palo Alto University, Palo Alto, California, United States*

The COVID-19 pandemic has changed the way many older adults live and function in the United States. Because of the isolation from stay-at-home mandates and higher risk of death from infection, many older adults are experiencing depressive symptoms, anxiety, and increased loneliness (National Council on Aging [NCOA], 2021; Center for Disease Control [CDC], 2021). One factor might be whether one has a spouse, children, or friends and if those relationships are positive or negative (Vanderhorst & McLaren, 2005). Besides the quality and nature of the relationship, another factor might be the amount of virtual contact while in isolation during COVID-19 (Regis College Online, 2020.In this study, we examined the relationships between outlook (optimism, pessimism, hopelessness), depression, loneliness, and social relationships in older adults during the COVID-19 pandemic in a sample of 808 adults 60 years of age or older. The data was gathered from self-reported questionnaires from the psychosocial and lifestyle questionnaire and health questionnaire from the Health and Retirement Study (HRS) 2020 version (HRS, 2021).We found that loneliness scores were significantly predicted by self-reported measures of positive and negative relationships, along with optimism, pessimism, hopelessness, depressive symptom scores, and COVID-19 life changes (Adjusted R2 = .447, $F(12, 795) = 55.26$, $p < .001$). COVID-19 has changed the way many older adults live and function. Finding ways to increase the level of communication older adults are able to have with their close relationships is crucial to combating this epidemic of loneliness.

### SESSION 6180 (POSTER)

### MINORITY AND DIVERSITY POPULATIONS

### HEALTHCARE DISPARITY IN THE ASSESSMENT OF DEMENTIA IN THE PRIMARY CARE SETTING

Regina Gibson, Maneetha Kodali, Priya Priyambada, Priya Mendiratta, Danish Hasan, Melodee Harris, Jeanne Wei, and Gohar Azhar, *University of Arkansas for Medical Sciences, Little Rock, Arkansas, United States*

Literature suggests racial implicit bias can significantly affect quality of care. Data demonstrates that older adult Blacks are twice as likely to have dementia as their White counterpart. Inappropriate cognitive screening by providers or diagnosing dementia just based on clinical impression could easily result in an over or under-estimation of the dementia. We reviewed the rate and method of cognitive screening in randomly selected patient records (n=75) at a primary care university clinic. Our results indicated that the cognitive screening rate for Black patients was lower compared to their White counterpart (43.4% Blacks vs 68.5% Whites, p < 0.05). We designed a quality improvement project to identify any contributory causes and challenges involved in screening for dementia with the goal to reduce racial disparity in dementia diagnosis. We identified knowledge deficits in providers in their approach towards patients with dementia and a lack of experience in their use of appropriate instruments for cognitive screening. A multipronged educational program, with videos, case conferences, presentations and one-to-one training by dementia experts and a neuropsychologist was employed to reduce the bias and train the providers in appropriate screening methods. Post-educational intervention, screening rates greatly improved in n=75 randomly selected patients from both races. In Whites, the screening rate increased by 20.9% to 89.4% and in Blacks by 38.9% to 82.3% (p < 0.05). Overall, the quality improvement driven educational intervention improved the self-efficacy of providers and improved the standardized dementia screening rates in Black patients to levels comparable to those of White patients.

### RACIAL DIFFERENCES IN THE ASSOCIATION BETWEEN LONELINESS AND COGNITIVE STATUS AMONG BLACK AND WHITE MEN

Marino Bruce[1], Bettina Beech[2], Gillian Marshall[3], and Roland J. Thorpe, Jr.[4], *1. University of Houston Tilman J. Fertitta Family College of Medicine, Houston, Texas, United States, 2. University of Houston, Houston, Texas, United States, 3. University of Washington, Seattle, Washington, United States, 4. Johns Hopkins University, Baltimore, Maryland, United States*

Background: Loneliness is a stressor that has been found to increase the likelihood of poor health and dementia. Few studies have focused on this association among men and even fewer studies have examined racial disparities in loneliness and cognitive functioning among this group. The purpose of this study was to examine racial differences in the association between loneliness and cognitive functioning among national sample of men aged 50 years and older.

Methods: Data were drawn from Black and White men in the 2016 Health and Retirement Study who completed the Leave Behind Questionnaire (n=2226). Cognitive function was the primary outcome and was measured by a dichotomous variable derived from a modified version of the Telephone Interview for Cognitive Status. Loneliness was the primary independent variable and was derived from the 3-item UCLA Loneliness Scale.

Results: Black men made up 18.5% of the study sample; however, the proportion of this group with scores indicating cognitive impairment or dementia (35.9%) doubled the corresponding percent of white men (17.6%). Findings from race-stratified modified Poisson regression models indicated that loneliness was associated with a higher prevalence of cognitive impairment or dementia for White men (PR=1.24, CI:1.05-1.47), but Black men (PR=0.92, CI:0.73-1.16).

Conclusions: Results from this study raise important questions about the salience of pooled analyses and suggest a need for tailored approaches to mitigate cognitive decline. Additional studies focusing on Black men are needed to develop effective interventions preserving cognitive functioning among this population.

### ADJUSTMENT TO AGING AMONG LBGT+ OLDER ADULTS

Sofia von Humboldt, Francis Carneiro, and Isabel Leal, *ISPA - Instituto Universitário, Lisbon, Lisboa, Portugal*

Intervention programs that highlight predictors of adjustment to aging (AtA)(1) for minority older lesbian, gay and bisexual (LGB) populations are scarce(2).The aim of this preliminary study is to build a structural model to explore whether socio-demographic, health and lifestyle-related variables, are correlates of AtA in a group of LGB older adults(3).The sample comprised 287 LGB older adults aged 75 years old and older.Convenience sampling was used to gather questionnaire data.Measures encompassed the Adjustment to Aging Scale, the Satisfaction with Life Scale, demographics and lifestyle and health-related characteristics.Structural equation modeling was used to explore a structural model of the self-reported AtA, comprising all the above variables.The structural model indicated the following significant correlates: perceived health($\beta$=0.456; p< 0.001), leisure($\beta$=0.378; p< 0.001), income($\beta$=0.302; p< 0.001), education($\beta$=0.299; p=0.009), spirituality($\beta$=0.189; p< 0.001), sex($\beta$=0.156; p< 0.001), physical activity($\beta$=0.142; p< 0 .001), satisfaction with life($\beta$=0.126; p< 0.001), and marital status($\beta$=0.114; p=0.008).The variables explain respectively 76.4% of the variability of AtA. These outcomes suggest that policy making and community interventions with LGB older adults may benefit of including variables, such as, perceived health, leisure and income, as these were pointed out as significant for this group of older adults for promoting adjustment to aging in late adulthood. 1.von Humboldt S et al. How do older adults experience intergenerational relationships? Different cultures, ambivalent feelings. Educ. Gerontol.2018;44(8):501-513. 2.von Humboldt S et al. Analyzing adjustment to aging and subjective age from Angolan and Portuguese community-dwelling older adults' perspectives. Int.J.Gerontol.2013;7(4):209-215. 3.von Humboldt S et al. What influences the subjective wellbeing of older adults?: A systematic review of the literature. Rev. Argent.Clín.Psicol.2014;23(3):219-230.

### PHYSICAL ACTIVITY AND EPISODIC MEMORY IN AFRICAN AMERICAN OLDER ADULTS

Rio Tate, and Brent Small, *University of South Florida, Tampa, Florida, United States*

African Americans are more likely to have Alzheimer's Disease (AD) yet less likely to be included in AD research. Additionally, memory complaints may signal the clinical genesis of AD. Interventions such as physical activity that can help individuals maintain their cognitive functioning as they age have merit in research. The objective of this study is to examine the influence of physical activity of various intensities on episodic memory in African American older adults at the interindividual and intraindividual levels over time. We used data from the Health and Retirement Study over 12 years. Our analyses included an indicator of episodic memory and we examined physical activity of three intensities as predictors while controlling for relevant demographic and health variables. Preliminary data indicates that African American older adults who engaged in more frequent physical activity (regardless of intensity) had better episodic memory. Individuals who engaged more frequently in physical activity had a slightly greater rate of decline than those who engaged less frequently in physical activity. Physical activity may act as a buffer against cognitive decline for older adults. Intensity need not be vigorous to observe changes.

### UNDERSTANDING THE NEEDS AND FUTURE OF OLDER BLACK AND HISPANIC WORKERS IMPACTED BY COVID-19

Kendra Jason, *University of North Carolina at Charlotte, Charlotte, North Carolina, United States*

COVID-19 infection and associated deaths are unequally distributed, as 78.5% of decedents are adults over 65 years of age; and Hispanics accounted for 24.2% of deaths. Black people account for 30% of infections and 18.7% of deaths, although they represent only 12.5% of the population. Older Black and Hispanic adults are in "Double Jeopardy" with their experiences shaped by racism and ageism, thus, putting them at higher risk for exposure to SARS-CoV-2, the virus that causes COVID-19, and poor health outcomes. Centering the intersections of race, age, and socioeconomic status and utilizing a scoping review (N=55), this study identifies the four primary risks faced by Black and Hispanic adults that help explain disparate COVID-19 work outcomes: (1) being an essential worker, (2) type of work performed, (3) workplace risks; and (4) community and geographic risk factors. This study also (1) explores the impacts of COVID-19 influence work participation, and (2) identifies processes linking ageism, racism, health, and employment situations in shaping the health and work-ability of older working adults. This research centers populations in which COVID-19 has had the most devastating financial impact: Black and Hispanic workers, Black women, and low-wage workers. This study increases our understanding of older Black and Hispanic adults lived experiences of managing COVID-19 – information that is critical for planning intervention and support services to ameliorate impact of the disease on Older Black and Hispanic adults; and informs policy and practice for economic recovery from the pandemic for other marginalized populations.

### ADVERSE CHILDHOOD EXPERIENCES AND DEPRESSIVE SYMPTOMS AMONG RACIALLY/ETHNICALLY DIVERSE OLDER ADULTS IN THE US

David Camacho[1], Julia Vazquez[2], Laura Vargas[3], Charles Henderson[4], and Brenda J Jones-Harden[2], *1. University of Maryland, Baltimore, Norwalk, California, United States, 2. University of Maryland, Baltimore, Baltimore, Maryland, United States, 3. University of Colorado School of Medicine, Department of Psychiatry, Aurora, Colorado, United States, 4. Cornell University, Ithaca, New York, United States*

Adverse childhood experiences (ACEs) and depression are major public health concerns. However, few studies have examined the relationship between ACEs and mid- and late-life depression among racially/ethnically diverse groups. We explore this relationship among U.S. racially/ethnically diverse community-dwelling midlife and older adults (≥50 years of age). Guided by ACEs and Minority Stress Frameworks, we used general linear models to examine this relationship with data from Wave 3 of the National Social Life, Health, and Aging Project. We created an ACEs composite ranging from 0 to 7 (e.g., violence, health, poverty) and assessed the role of individual ACEs on depressive symptoms (CES-D). Final adjusted models (n:1424) included key demographic, health (e.g., chronic disease), social (living alone, social isolation, loneliness), and minority stress factors (e.g., limited access to healthcare and treatment, perceived discrimination). Results indicated that higher composite score ACEs (particularly childhood violence and poor health) were positively associated with higher levels of depressive symptoms. We found no interactions between race/ethnicity and ACEs. Our results suggest that ACEs contribute to the presence and severity of depressive symptoms into mid- and late-life adulthood. Consistent with Minority Stress Framework, common life-course stressors for minoritized groups may explain a lack of significant interactions in our models. Future research should explore the association of ACEs and other important health outcomes in diverse midlife and older adults. Finally, research is needed to examine if and how culturally appropriate depression interventions can be adapted to address the role of ACEs in later life health.

### BLACK GREEK LETTER ORGANIZATIONS: FACILITATING HEALTH PROMOTION FOR AFRICAN AMERICANS ACROSS THE LIFECOURSE

Chivon Mingo, *Georgia State University, Atlanta, Georgia, United States*

African Americans remain underrepresented in accessing and utilizing evidenced-based health promotion interventions (EBIs). Challenges with dissemination and implementation of EBIs further corroborate existing racial/ethnic health/healthcare disparities. Therefore, there is a need to identify effective ways to increase the widespread adoption of health promotion behaviors among African Americans across the life course. It is plausible that engaging in non-traditional partnerships (i.e., community groups or organizations valued in the community with the capacity and infrastructure) could result in greater adoption and improved utilization of EBIs among African Americans. Although frequently overlooked as a study variable in empirically sound public health research, Black Greek Letter Organizations (BGLO) could be an innovative and practical approach to advancing health in the African American community. It is necessary to gain preliminary evidence of feasibility (e.g., motivation, target population reach, acceptability, ). Therefore, the purpose of this study was to conduct a content analysis to identify the intentions and communication trends of BGLOs as it pertains to public health and the African American community and assess population reach and perceptions by evaluating responses to communication specific to health promotion.

We assessed health promotion patterns of four BGLOs in a ten-county metropolitan area. Coded content included communication via the organization's webpage, Facebook, Twitter, YouTube, Instagram, and LinkedIn from a five-year time period. Findings confirm that BLGOs are invested in the health and well-being of the community, place emphasis on mitigating health inequities, and are uniquely positioned to serve as stakeholders for the translation of EBIs to end-users.

### EXPLORING AGE AND RACE DIFFERENCES IN THE REPORT OF ADVERSE CHILDHOOD EXPERIENCES AMONG WOMEN AGING IN CUSTODY

Alex Bishop, and Claire Stolfa, *Oklahoma State University, Stillwater, Oklahoma, United States*

Survey data for this study was collected from N = 544 women, aged 18 and older (M = 39.03; SD = 10.32), under correctional custody in Oklahoma. Participants reported an average of M = 4.94; SD = 2.94 childhood adversities. Chi-square analyses revealed that age was associated with the proportion of women who experienced parental divorce ($\chi$(2, N = 525) = 8.03, p < .05., lived with a substance abuser ($\chi$(1, N = 528) = 7.14, p < .05), lived with a mentally ill household member ($\chi$(2, N = 524) = 15.04, p < .05) and lived with household member who went to prison ($\chi$ = (2, N = 528) = 18.99, p < .001). Race was also associated with the proportion of participants who reported experiencing a household member go to prison ($\chi$(1, N = 538, = 15.92, p < .001). Furthermore, race was associated with the proportion of women who reported not feeling loved by family ($\chi$(1, N = 525), = 4.87, p < .05), being physical hurt or injured by a parent ($\chi$(2, 525) = 8.01 p < .05), and being verbally insulted or humiliated ($\chi$ = 7.38 (1, N = 537, p < .01). Results suggest that age and race are associated with the self-disclosure of past childhood adversities among women in custody. This has implications relative to how clinicians such as social workers, counselors, and psychiatrists provide rehabilitative services and programs to women-of-color aging under correctional control.

### ACCULTURATION ON PSYCHOLOGICAL DISTRESS AMONG OLDER KOREAN AMERICANS: DOES ETHNIC COMMUNITY SOCIAL CAPITAL MATTER?

Juyoung Park, Maria Aranda, Yeon Jin Choi, and Yuri Jang, *University of Southern California, Los Angeles, California, United States*

Responding to the rapid growth of the older immigrant population and building upon the literature on the critical role of acculturation in older immigrants' health and well-being, we focused on the role of ethnic community social capital (social cohesion, social engagement, safety, and negative interactions in ethnic communities) in older Korean Americans. Guided by social capital and stress-buffering theories, we examined the direct effect of acculturation and ethnic community social capital on psychological distress, as well as their interactions. We hypothesized that the negative impact of low acculturation on mental health would be lowered by positive perceptions of and experiences in ethnic communities. Using data from 2,150 participants in the Study of Older Korean Americans (Age range = 60-99, M [SD] = 73.4 [7.97]), the direct and interactive effect models were examined. Results showed that low acculturation posed a significant risk to mental health and all four types of ethnic community social capital had a significant direct effect. Furthermore, significance was observed in the interaction of acculturation with social cohesion (B [SE] = .01 [.01]. p < .05) and with negative interaction (B [SE] = -.01 [.01]. p < .01). The negative impact of low acculturation was attenuated among those with a high sense of ethnic community social cohesion but intensified among those with frequent experiences of negative interactions with ethnic community members. Our findings highlight the importance of social capital that forms within ethnic communities and provide implications for programs and services to promote older immigrants' mental well-being.

### CULTURAL AND SOCIAL ACTIVITIES AND MENTAL HEALTH AMONG INDIGENOUS OLDER ADULTS IN THE UNITED STATES

Yeonjung Lee[1], Kathryn Braun[2], Yanyan Wu[3], Collette Adamsen[4], and Shelly Davis[5], *1. University of Hawai'i at Mānoa, Honolulu, Hawaii, United States, 2. University of Hawai'i at Mānoa, Thompson School of Social Work & Public Health, Honolulu, Hawaii, United States, 3. University of Hawai'i at Mānoa, Honolulu, Hawaii, United States, 4. University of North Dakota, Grand Forks, North Dakota, United States, 5. University of North Dakota School of Medicine & Health Sciences, Grand Forks, North Dakota, United States*

Although the number and percentage of indigenous older adults are growing, little is known about the risk and protective factors associated with mental health among this population. We estimated regression models for mental health using data from the 2017-2020 needs assessment of indigenous elders (aged 55+) administered by the University of North Dakota, which included 19,143 indigenous elders, including 17,184 American Indians, 1,521 Alaska Natives, and 438 Native Hawaiians. The Mental Health Index (MHI) was used, with five questions: during the past month, how much of the time (1) were you a happy person, (2) have you felt calm and peaceful, (3) have you been a very nervous person, (4) have you felt downhearted and blue, and (5) have you felt so down in the dumps that nothing could cheer you up? The answers for MHI-5 were standardized on a 0-100 scale, where higher scores indicate optimal mental health. Having ADL and/or IADL difficulties was associated with worse mental health. Frequent engagement in social activities was positively associated with mental health. Data also suggest that those engaged in cultural practice all the time compared to people who engaged in cultural practice less frequently had higher levels of MHI-5, controlling for other variables. These findings underscore the importance of cultural activities, as well as social activities and physical health, in the management of mental health.

### RACIAL DIFFERENCES IN COPING STRATEGIES AND MENTAL HEALTH OUTCOMES

Jordan McWilliams[1], Meghan Custis[2], Natasha Peterson[3], and Jeong Eun Lee[3], *1. Iowa State University, Ankeny, Iowa, United States, 2. Iowa State University, Muscatine, Iowa, United States, 3. Iowa State University, Ames, Iowa, United States*

Previous literature has examined the consequences of loneliness coping methods; however, scarce research has examined racial/ethnic disparities and the differential association between coping activities and psychological outcomes. This study aimed to fill this gap in the literature by examining racial/ethnic differences in loneliness coping strategies and their impact on anxiety and depression among middle-aged and older adults. Data were from the 2018 Loneliness and Social Connections survey (N=3,233) conducted by AARP. Findings revealed minorities reported higher levels of loneliness and coped with loneliness by socializing with friends in person, socializing with friends using technology, and individualized activity significantly more compared to white counterparts. Logistic regressions revealed that minorities who engage in risky behaviors are 1.66 times more likely to be depressed (95% CI [1.07, 2.58]) and 1.98 times more likely to be anxious (95% CI [1.51, 2.59]). Moreover, the odds of being anxious increased by 58% if minorities coped with loneliness by socializing with friends via technology (95% CI [1.12, 2.21]). However, minorities who socialize with friends in person are 48% less likely to be anxious (95% CI [.35,.79]). In general, socializing methods have more implications for mental health among minorities. Findings suggest that differential coping strategies may have differential outcomes for minorities. The results of this study point to the need for further longitudinal research examining factors contributing to coping strategies and the direction of causality between coping strategies and psychological outcomes.

### PREPARING THE GROUND: DEVELOPING COMMUNITY-BASED STRATEGIES TO ENGAGE OLDER BLACK MEN IN HEALTH RESEARCH

Jamie Mitchell[1], Kent Key[2], Elena Flores[1], Sean Knurek[3], Tam Perry[4], Vanessa Rorai[4], Joan Ilardo[5], and Alexandra Sass[1], *1. University of Michigan, Ann Arbor, Michigan, United States, 2. Michigan State University, Flint, Michigan, United States, 3. Michigan State University, Corunna, Michigan, United States, 4. Wayne State University, Detroit, Michigan, United States, 5. Michigan State University, East Lansing, Michigan, United States*

Black men across the lifespan are overburdened by poor health and underrepresented as participants in health research. The Flint Healthier Black Elders program seeks to engage more older Black men in research that could contribute to health discoveries by developing and testing strategies to recruit Black men into a community participant research pool (PRP). The PRP recruitment strategies account for the influence of gender role norms, mistrust derived from current and historical research and medical abuses, and other factors that affect older Black men's willingness to participate in safe and ethical research. This initiative also focuses on building trust in research engagement by foregrounding the voices of local older men as community stakeholders and research gatekeepers, and tailoring multimedia recruitment materials to represent older Black men more fully and positively. Videos and print materials developed as recruitment tools specifically tailored to older Black men were pilot tested for messaging and impact, and the results of this community-driven process can serve as an innovative model for equitable and trustworthy research recruitment in Black communities. We would like to acknowledge the contributions of the Flint Healthier Black Elders Community Advisory Board: Yaushica Aubert, Rev. Dr. Sarah Bailey, E. Hill DeLoney, Luther Evans, Ella Greene-Moton, Cynthia Howell, Bishop Bernadel Jefferson, Beverly Lewis, Geraldine Redmond, Sharon Saddler, Arlene Sparks, Erica Thrash-Sall.

### PSYCHOSOCIAL AND BEHAVIORAL INDICATORS OF CHRONIC PAIN AMONG MIDDLE-AGED AND OLDER BLACK MEN

Anna Wakita[1], Melicia Whitt-Glover[2], Amber Brooks[3], Jaylyn Clark[4], Katrice Perry[5], Knashawn Morales[6], and Tamara Baker[7], *1. Gillings School of Global Public Health, Durham, North Carolina, United States, 2. Gramercy Research Group, Winston Salem, North Carolina, United States, 3. Wake Forest School of Medicine, Winston-Salem, North Carolina, United States, 4. Atrium Health Wake Forest Baptist, Winston-Salem, North Carolina, United States, 5. Cecil G. Sheps Center For Health Services Research, Chapel Hill, North Carolina, United States, 6. Perelman School of Medicine at the University of Pennsylvania, Philadelphia, Pennsylvania, United States, 7. University of North Carolina, Chapel Hill, Cary, North Carolina, United States*

There is a lack of data describing the pain experience of Black men. This may be the result of social messaging that men are to project strength and avoid any expression of emotion or vulnerability. This avoidant behavior(s) however, often comes too late when illness/symptoms are more aggressive and/or diagnosed at a later stage. This begins to address two larger issues – the willingness to acknowledge pain and to seek medical attention when experiencing pain. To address this lack of data, this study aimed to determine the influence psychosocial indicators have in the pain experience of Black men 40+ years old. Participants from the Active & Healthy Brotherhood study were categorized based on the pain question from the Health-Related Quality of Life Questionnaire. Demographics, general health, and psychosocial measures were compared across pain groups. A multivariable logistic regression model was used to determine predictors of pain, while adjusting for other factors. Results showed that the men (n=303) were mostly married (54%) and employed (53%). Those reporting arthritis (OR=4.5; 95% CI 2.0, 10.0), good health (2.8; 1.2, 6.6), higher somatization (3.2; 1.2, 8.8) compared to excellent/very good self-rated health, were more likely to self-report pain in the past 30 days. Findings from this study show that continued efforts are needed to identify the unique pain experiences of Black men, while recognizing the impact it has on their identity as a man, a person of color, and as a person living with pain.

### IS ETHNIC HERITAGE MEANINGFUL IN ADULTHOOD? ETHNIC IDENTITY AND WELL-BEING IN MEXICAN-ORIGIN FEMALE ADULTS

Hin Wing (Florence) Tse[1], Jiaxiu Song[2], Elizabeth Muñoz[2], and Su Yeong Kim[2], *1. Department of Human Development and Family Sciences, The University of Texas at Austin, Austin, Texas, United States, 2. The University of Texas at Austin, Austin, Texas, United States*

Being the largest immigrant group in the U.S., the process of exploring, forming, and retaining ethnic identity is a critical component to Mexican immigrants' successful adaptation and wellbeing. Despite recognizing the dynamic nature of ethnic identity development, extant research has predominantly focused on adolescence, overlooking the development and impact of ethnic identity in adulthood, when individuals' experiences are continuously shaped by critical life events (e.g., moving to a new country). Additionally, there is a lack of longitudinal research on mid-late adulthood. Filling these gaps, this study utilized a three-wave longitudinal dataset of 595 Mexican-origin female adults (Mage.wave1 = 38.39) to examine their initial levels and trajectories of ethnic identity development (i.e., exploration, centrality, and resolution) and understand how these individuals' initial levels and trajectories of ethnic identity are associated with their wellbeing (i.e., life meaning, resilience, and depressive symptoms) at Wave 3. Using latent growth curve modeling, unconditional models revealed that initial levels of ethnic identity in Mexican-origin female adults were moderately high, and that their centrality and resolution of ethnic identity remained stable while their exploration of heritage identity increased over time. The conditional model also showed that Mexican-origin female adults' higher initial levels of centrality and resolution were associated with a greater sense of life meaning (and resiliency, only true for resolution levels) but not with depressive symptoms. These findings suggest that ethnic heritage is associated with more positive perception in life and may inform interventions on developing a positive ethnic identity that is related to better wellbeing.

### LIMITED ENGLISH PROFICIENCY AND HEALTH LITERACY IN KOREAN OLDER ADULTS: MEDIATING EFFECT OF ACCULTURATION

Hae Sagong, and Pao-Feng Tsai, *Auburn University, Auburn, Alabama, United States*

Language proficiency and comprehension in the culture and health systems of the host country are imperative factors enabling appropriate health literacy (HL) for non-English speaking immigrants. Older immigrants with limited English proficiency have fewer opportunities and limited abilities to improve their English skills. Increasing individuals' acculturation levels can be an effective strategy for older immigrants than solely educating English language skills. The purpose of this study is to investigate the mediating effect of acculturation between English proficiency and HL in older Korean immigrants. From June to October of 2020, a total of 244 older Korean immigrants aged 50 years or older residing in the Southern US were recruited. English proficiency was categorized into limited (very limited to fair) and fluent (fluent to very fluent). HL and acculturation were measured using the Health Literacy Survey-12 Questionnaires (HLS-Q12), and the East Asian Acculturation Measure (EAAM), respectively. Covariates were gender, age, monthly income, education, and length of residency in the U.S. The statistically significant differences were shown in age, gender, education, perceived health, and length of residency in the US by the level of English proficiency The limited English proficiency was negatively associated with HL ($\beta$= -.192, p=.002) and acculturation had a mediating effect between English proficiency and HL ($\beta$= -.133, p=.001). To alleviate the language barrier that causes low HL in older Korean immigrants, enhancing their understanding of the host country's culture as well as improving organizational HL should be considered.

### CORRELATES OF HEALTH LITERACY IN THE BLACK BELT AREAS OF ALABAMA: IMPORTANCE OF INTERNET ACCESS

Avani Shah[1], Yan Luo[2], LeAnna Roberts[1], and Hee Lee[3], *1. University of Alabama, Tuscaloosa, Alabama, United States, 2. The University of Alabama, Tuscaloosa, Alabama, United States, 3. University of Alabama, Birmingham, Alabama, United States*

Health literacy, which is defined as being able to understand and utilize information related to one's health, is an essential part of the health care process as it is related to health outcomes. However, little is known about health literacy in Black Belt communities in Alabama although this rural area has shown very high rates of health concerns such as diabetes, cardiovascular disease, stroke, and cancer (CDC Interactive Atlas, 2022). Study participants were recruited from the Black Belt areas of Alabama. A total of 180 African-American participants with a mean age of 57.5 completed a survey. A regression analysis was conducted to understand if sociodemographic and other relevant factors would predict health literacy. Lower education, gender, age, perceived racism, and perception that race impacts health care quality predicted health literacy. Interestingly, those with less internet access had significantly lower health literacy. Access to internet and smart phones was a reported concern for over a third of the participants. Only a quarter of participants reported use of the internet to assist with lifestyle modifications while almost half reported internet use to obtain health information; suggesting this as a potential means to improve health literacy and even potential lifestyle modification in health behaviors. Recommendations are made for interventions to improve health literacy in minority populations of this underserved region.

### HOW IS MENTAL HEALTH IN LATE LIFE SHAPED BY STRUCTURAL RACISM?

Reed DeAngelis[1], Patricia Homan[2], and Tyson Brown[3], *1. University of North Carolina at Chapel Hill, Chapel Hill, North Carolina, United States, 2. Florida State University, Tallahassee, Florida, United States, 3. Duke University, Durham, North Carolina, United States*

Less than one percent of studies on the link between race and health have focused on structural racism. Empirical research on how structural racism affects health in later life is especially rare. Moreover, the conceptualization of structural racism in the race theory literature has often differed from the measurement strategies used in aging and health research. This study advances the field by 1) utilizing a novel, theory-informed latent measure of structural racism in states across multiple domains, including political participation, education, economics, housing, and the judicial system, 2) mapping structural racism across states, and 3) quantifying the association between structural racism and mental health outcomes (depressive symptoms and frequency of poor mental health days) among Black and White older adults. We use administrative data measuring state-level racial stratification linked to geocoded individual-level demographic and health data from the HRS (N=9,126) and the

BRFSS (N=308,029). Results show that, whereas structural racism is consistently associated with worse mental health for Black people, it is either unrelated to health or predictive of better health among Whites. Findings highlight the utility of rigorously conceptualizing and measuring structural racism and its impact on health among older adults.

### PROMOTING POSITIVE AGING IN COMMUNITY-DWELLING SOUTH ASIAN AMERICANS: AN EXPLORATORY QUALITATIVE STUDY

Mushira Khan[1], Sheetal Shah[2], and Ajla Basic[1], *1. Mather Institute, Evanston, Illinois, United States, 2. University of California (Davis), Sacramento, California, United States*

Between 2010 and 2017, South Asians were the fastest-growing major ethnic minority group in the US, growing at a rate of 40% over the previous decade (SAALT, 2019). This exponential growth, along with a rapidly aging US population, implies that a significant proportion of the South Asian American population will be 65 years or older in the coming years; yet research on the lived experiences/needs of older South Asian Americans is limited. To address this gap, this qualitative study explored barriers and facilitators to healthy or positive aging in a sample of community-dwelling South Asian Americans 50 years and older. In-depth, semi-structured interviews were conducted with 32 South Asian American older adults (18 women and 14 men). Thematic analysis of the interview data showed that level of acculturation, proficiency in English, cultural beliefs/practices, awareness about available health and social services, degree of religiosity, and the density of social networks were key determinants of healthy aging. Compared to those who were US-born or had immigrated earlier in life, participants who had immigrated later in life (post-retirement) appeared more financially and/or emotionally dependent on their adult children and expressed ambivalence vis-à-vis future caregiving arrangements and intergenerational co-residence. Nearly all participants shared that helping their adult children with childcare, cooking, or other household chores gave them a sense of purpose and made them feel valued. Study findings suggest that along with culturally appropriate programs and policies to support healthy aging, increased volunteering opportunities may enhance subjective well-being in South Asian American older adults.

### SESSION 6190 (POSTER)

### SEXUAL HEALTH IN LATER LIFE

### HETERONORMATIVE DEFINITIONS OF SEX: IMPLICATIONS FOR LGBT+ WOMEN'S PREVENTIVE HEALTHCARE

Jessica Noblitt, and Anne Barrett, *Florida State University, Tallahassee, Florida, United States*

The dominant cultural definitions of sex, which is heteronormative, has implications for preventive health screening among LGB+ women. Medical recommendations for women's screening exclude some same-sex behaviors from this definition, and they center on reproduction – both of which can discourage LGB+ women's preventive health screening. Qualitative studies have found that sexual minority women, as well as their doctors, are less likely to see sexual health exams as important for sexual minority women's healthcare because many are not engaging in penile-vaginal intercourse. However, we are aware of no study that has used a large, nationally representative dataset to examine potential differences in health screening by sexual identity. We used data from the National Health Interview Survey (2018; n=1394) to examine differences by sexual identity in having Pap tests and mammograms. We found that sexual minority women were about 40% less likely than heterosexual women to have ever had a Pap test. Moreover, among sexual minority women, lesbian women were about 50% less likely than bisexual women to have ever had one. Sexual minority women also were 22% less likely than heterosexual women to have had a Pap test in the last 12 months. Differences by sexual identity in receiving mammograms were less striking. We found, however, that bisexuals were 25% less likely than lesbians to have ever had a mammogram. In addition, these differences in health screening were more pronounced in younger than older women.

### SEXUAL ACTIVITY OF OLDER ADULTS: WE'RE ASKING THE WRONG QUESTIONS

Janie Steckenrider, *Loyola Marymount University, Los Angeles, California, United States*

Sexual activity of older adults is an under researched area as most surveys on sexual behavior end at age 60 reflecting the myth that older adults are not sexually active. Only recently has survey data asked specifically about sexual activity of those ages 60 to 95. Their consensus is over half of males and a third of females over 70 are sexually active. Most striking is the current body of research almost exclusively defines sexual activity measured as partnered sexual behaviors of intercourse, fondling, kissing, touching. Given the reality for many older adults lacking an active sex partner due to death, sexual dysfunction, or serious illness, the aim of this study was to determine if the right survey questions are being asked for older adults. Seven major surveys, underlying most current research, were analyzed regarding solitary sex (masturbation) compared to partnered sex. Results of this study found extensive questioning about aspects of partnered sex including pleasure, satisfaction, pain, relationship status, sexual functioning, anxiety, individual sexual acts, etc. compared to only two questions about masturbation, both only about frequency. The psychological and physiological benefits of sexual activity, both partnered and solo sex, are well documented and correlate with higher life satisfaction for older adults. Sexual activity needs to be redefined to also include solitary sex and this begins by asking the right questions. This study has implications for the need to bring a broader perspective in promoting a healthy sex life among older adults, defined both as partnered and solitary sex.

### CONTEXT-DEPENDENT SEXUAL CHANGES DURING WOMEN'S MIDLIFE TRANSITIONS

Amber Watts, and Sarah Jen, *University of Kansas, Lawrence, Kansas, United States*

For women, midlife represents an important stage of transition, including shifts in physiological, social, and sexual experiences. Prior research demonstrates that women's sexuality is more dynamic and context-dependent than men's. Most research focused on women's sexuality in mid- to later-life emphasizes physiological changes, while largely ignoring changes stemming from social, psychological, and relational contexts. The present study examined midlife women's diverse sexual experiences within the context of their lives. We conducted semi-structured interviews with 27 women, ages 39-57 and used interpretive phenomenological analysis to investigate perceptions and interpretations of midlife sexual experiences and changes. Themes included changes in sexual engagement, unwanted sexual experiences, body image, and sexual healthcare. Participants reported changes in frequency of sex and sexual desire within the context of their diverse social roles and identities, prior intimate relationships, and sexual health. Women contrasted perceptions of their own bodies with societal perceptions of sexiness. Frequently reported negative experiences with sexual healthcare informed a distrust of healthcare systems. The diverse and changing nature of participants' experiences supports prior evidence of sexual fluidity and context-dependence. By questioning societal expectations around sexuality and body image, participants illustrated the potential of counternarratives to combat dominant beliefs and stereotypes about midlife women's sexuality. To improve sexual health and education, psychoeducational interventions and improved training for healthcare professionals are needed.

### SEXUAL MOTIVES, SEXUAL FREQUENCY, AND SEXUAL SATISFACTION AMONG MIDDLE-AGED DIFFERENT-SEX AND SAME-SEX COUPLES

Hye Won Chai, Sara Mernitz, and Debra Umberson, *The University of Texas at Austin, Austin, Texas, United States*

Reasons for having sex and frequency of sex are significant correlates of sexual satisfaction. However, the possible interplay between sexual motives and sexual frequency remains unexplored. Also, prior studies on sexual satisfaction largely focused on heterosexual couples and less is known about the experiences of same-sex couples. Using dyadic survey data collected from 838 middle-aged spouses in 419 gay, lesbian, and heterosexual marriages, this study examined whether the associations between sexual motives and sexual satisfaction differed by sexual frequency and whether these dynamics varied across gay, lesbian, and heterosexual couples. Results showed that intrinsic sexual motives (e.g., for enjoyment and pleasure) were associated with higher sexual satisfaction only in the context of more frequent sex, and this association did not differ for same- and different-sex couples. On the other hand, extrinsic sexual motives (e.g., to please spouse) were associated with lower levels of sexual satisfaction in the context of high-frequency sex only among men married to men and women married to men, wherein the association was stronger for heterosexual couples compared to same-sex couples. These associations were not significant with less frequent sex. Results suggest that while frequent engagement in sex with intrinsic sexual motives benefits middle-aged adults' sexual satisfaction regardless of relationship type, the sexual satisfaction of individuals married to men is vulnerable to the negative consequences of engaging in sex due to external pressure. These findings highlight the importance of considering how sexual experiences of men and women in midlife same-sex marriages compare to those of different-sex marriages.

### SESSION 6200 (POSTER)

### SOCIAL DETERMINANTS OF HEALTH (POSTERS)

### CAN SOCIAL ENVIRONMENT OFFSET THE GENETIC RISK OF MYOCARDIAL INFARCTION AMONG OLDER ADULTS?

Chenkai Wu[1], and Chen Sheng[2], *1. Duke Kunshan University, Kunshan, Jiangsu, China (People's Republic), 2. Fudan University, Shanghai, Shanghai, China (People's Republic)*

The interrelatedness between social determinants of health impedes researchers to identify important social factors for cardiovascular health. Additionally, it remains largely unknown whether a derivable social environment could offset the genetic risk for cardiovascular events. We developed a polysocial score approach to quantify the aggregate effect of social factors on myocardial infarction (MI). We also examined the association of polysocial score and polygenic risk scores (PGS), and their interaction, on MI. Data are from the Health and Retirement Study, a longitudinal cohort of a nationally representative sample of Black and White Americans with pre-calculated PGS for MI (N=6,036). We included 24 social factors from five categories (economic stability, neighborhood environment, education, community, and social context, and healthcare system) and used forward stepwise regression to screen for important ones. Polysocial score was created using 14 social factors and was classified as low (< 28), intermediate (29-39), and high (40+). The incidence of MI was 4.5, 8.5, 10.5 per 1000 person-years among Whites with a low, intermediate, and high PGS, respectively; no graded association was found among Blacks. Polysocial score stratified the rate of MI in each tertile of PGS among Whites. We found a significant additive interaction between PGS and polysocial score. The difference in MI rate was 10.3 per 1000 person-years among individuals with a high genetic risk, while the difference significantly reduced to 3.5 per 1000 person-years among those with a low genetic risk. Desirable social environment could possibly offset the increased risk of MI associated with genetics among Whites.

### ASSOCIATION BETWEEN VIOLENT CRIME INCIDENT PROXIMITY AND COGNITIVE FUNCTION IN OLDER AFRICAN AMERICANS

Genesis Tan[1], Alejandro Gimenez-Santana[1], Zuzanna Osiecka[1], Darlingtina Esiaka[2], Bernadette Fausto[1], and Mark Gluck[1], *1. Rutgers University - Newark, Newark, New Jersey, United States, 2. Robert Wood Johnson Medical School, Rutgers University, Newark, New Jersey, United States*

Research on Area Deprivation Index (ADI) suggests that the built environment and neighborhood stressors (e.g., violent crime incidents) play a role in later-life cognitive function. However, most of the research linking ADI and cognitive function was conducted on majority White American samples. Further, while ADI is useful in facilitating efficient integration of social determinants of health (SDOH) into models of cognitive aging, it does not account for the impact of micro-level measures of neighborhood stressors on cognitive function. Therefore, the purpose of the current study was to determine whether violent crime incident proximity (VCIP) contributes to later-life cognitive function above and beyond ADI in older African Americans. Participants (N=147; M= 68.34) from an ongoing study, Pathways to Healthy Aging in African Americans—a Rutgers University-Newark community partnership fostered over 16 years of community engagement, health education, and public service—responded to measures of cognitive ability, SDOH, and demographic details. The results show that VCIP is a trending predictor of cognitive performance, when adjusting for age, gender, education, depression, and ADI. The result aligns with our hypothesis that individuals living in areas with greater VCIP will have poorer performance on cognitive tasks. Our findings suggest that for African Americans in an urban setting, hyper-local VCIP appears to be more useful at capturing the impact of neighborhood disadvantage on cognitive decline and Alzheimer's disease risk. Therefore, for later-life cognitive health in African Americans, it is important to consider micro-level measures of neighborhood stressors such as VCIP.

### CHILDHOOD PHYSICAL ABUSE INCREASES THE RISK OF SUBJECTIVE MEMORY IMPAIRMENT

Lauren Roach[1], Tyler Bell[2], and Caitlin Pope[3], *1. University of Kentucky, Holland, Michigan, United States, 2. University of California, San Diego, La Jolla, California, United States, 3. University of Kentucky, Lexington, Kentucky, United States*

Subjective memory impairment, defined as self-reported difficulties in recall and learning, doubles the risk of Alzheimer's Disease and related dementia, despite being weakly related to objective memory decline. Because of its strong stability over time, it may be possible that subjective memory impairment reflects earlier life risk factors for dementia such as adverse childhood experiences. It is reported that over a fifth of older adults worldwide experienced physical abuse during childhood. Previous cross-sectional studies suggest physical abuse is associated with later cognitive impairment. Still unclear, are the longitudinal associations between childhood abuse and subjective memory impairment in later life. Using a sample of adults drawn from the Health and Retirement Study (n = 19,185, Mage = 67.05, SD = 11.33) we assessed associations between reported physical abuse by a parent before the age of 18 and subjective memory impairment (current memory problems and perceived memory decline) over periods of up to 18 years. Generalized linear mixed models examined longitudinal associations between childhood physical abuse and subjective memory impairment while controlling for depressive symptoms and other empirically relevant covariates. Experiencing childhood physical abuse was associated with increased likelihood of reporting more current memory problems (OR = 1.17, 95% CI 1.04, 1.33) and perceived memory decline in later life (OR = 1.27, 95% CI 1.13, 1.43). Findings suggest childhood physical abuse is associated with subjective memory impairment, a strong predictor of dementia. Understanding early life conditions, including adverse childhood experiences may help explain associations between subjective memory impairment and dementia risk.

### IS THE HEALTH OF OLDER AMERICANS WITH A GED EQUIVALENT TO THEIR PEERS WITH A HIGH SCHOOL DIPLOMA?

Esme Fuller-Thomson, Robin Grossman, and Andie MacNeil, *University of Toronto, Toronto, Ontario, Canada*

Previous research has found higher levels of educational attainment to be strongly associated with better health outcomes in later life, such as better cognitive functioning and fewer functional and sensory impairments. However, most studies have grouped General Educational Development (GED) recipients with high school graduates, neglecting potential differences in socioeconomic status, health behaviours, and health outcomes among these two groups. The aim of the current study is to identify differences in the age-sex-race-poverty adjusted prevalence and odds of cognitive impairment, hearing impairment, vision impairment, limitations in activities of daily living (ADLs), and ambulation limitations among three groups of older American adults: high school dropouts, GED recipients, and high school graduates with no post-secondary education. The present study uses secondary analysis of the 2017 American Community Survey, a nationally representative survey of community-dwelling and institutionalized older adults aged 65 years and older, of whom 20,489 were GED recipients, 154,892 had a high school diploma and 49,912 had finished grade 8 but had not completed high school. Our findings indicate that there is a gradient in health outcomes among Americans aged 65-84, with the highest prevalence and odds of cognitive impairment, hearing impairment, vision impairment, ADL limitations, and ambulation limitations occurring among high school dropouts, followed by GED recipients, and the lowest prevalence among high school graduates. These findings suggestion that although GED recipients have better health outcomes than high school dropouts, there is still a significant disparity in health status between GED recipients and high school graduates.

### HISTORY OF JOB STRAIN AND RISK OF LATE-LIFE DEPENDENCY: A NATIONWIDE SWEDISH REGISTER-BASED STUDY

Ingemar Kåreholt[1], Charlotta Nilsen[1], Janne Agerholm[2], Susanne Kelfve[3], Jonas Wastesson[2], Kirsten Nabe-Nielsen[4], and Bettina Meinow[5], *1. Jönköping University, Jönköping, Jonkopings Lan, Sweden, 2. Karolinska Institutet, Stockholm, Stockholms Lan, Sweden, 3. Linköping University, Linköping, Ostergotlands Lan, Sweden, 4. University of Copenhagen, Copenhagen, Hovedstaden, Denmark, 5. Stockholm Gerontology Research Center, Stockholm, Stockholms Lan, Sweden*

There is substantial evidence that work plays a significant role in post-retirement health. Yet little is known about its role in when late-life dependency may occur. We examined associations between job strain and the risk of entering late-life dependency. Individually linked nationwide Swedish registers were used to identify people 70+ alive in January 2014, and who did not experience the outcome (late-life dependency) during two months prior to the start of the follow-up. Late-life dependency was operationalized as use of long-term care. Information about job strain was obtained via a job exposure matrice and matched with job titles. Cox regression models with age as time-scale (adjusted for living situation, educational attainment, country of birth, and sex) were conducted to estimate hazard ratios (HR) for entering late-life dependency during the 24 months of follow-up (n=993,595). Having an initial high starting point of job strain followed by an increasing trajectory throughout working life implied a 23% higher risk of entering late-life dependency at a younger age, compared with the reference group (low starting point with a decreasing trajectory). High initial starting point followed by a stable trajectory implied a 12% higher risk of entering late-life dependency at a younger age. High initial starting point followed by a decreasing trajectory implied a 10% risk reduction, and a low starting point with a stable trajectory implied a 22% risk reduction, of entering late-life dependency at a younger age. Reducing stressful jobs across working life may contribute to postponing late-life dependency.

### SEQUENCING OF PLANNED AND UNPLANNED BIRTHS AND IMPLICATIONS FOR MID- AND LATER-LIFE HEALTH AMONG NLSY79 WOMEN

Mieke Thomeer[1], Clifford Ross[1], Rin Reczek[2], and Christina Bijou[2], *1. University of Alabama at Birmingham, Birmingham, Alabama, United States, 2. Ohio State University, Columbus, Ohio, United States*

Existing studies demonstrate that unplanned births (e.g., unwanted, mistimed) are associated with worse health for mothers in the short-term and—according to some preliminary evidence—in mid- and later-life. Yet as life course and reproductive career frameworks highlight, childbearing experiences often unfold over a number of years, with a considerable amount of diversity in pregnancy and birth experiences even for the same individual. For example, a person may have an unplanned birth in late adolescence followed by only planned births in early adulthood. In order to provide a more holistic understanding of how birthing experiences births are associated with midlife health, we use Sequence Analysis (SA) on the 1979 National Longitudinal Survey of Youth (NLSY79; N=3,992) to examine how patterning of planned and unplanned births is associated with physical and mental health at ages 50 and 60 (SF-12). Preliminary analysis indicates that compared to respondents with only planned births, respondents with unplanned birth(s) followed by planned birth(s) have worse physical and mental health at midlife, but there is no difference in health for respondents with only planned births, only unplanned births, and planned birth(s) followed by unplanned birth(s). Future analysis with SA will consider how more detailed sequences (e.g., timing, number and type, ordering, spacing) are associated with these mid- and later-life health outcomes, taking into account selection factors such as childhood SES and educational attainment. This project demonstrates the need for life course perspectives on the long-term health implications of unplanned births, recognizing diversity within and between individuals.

### COGNITIVE AGING IN THE SHADOW OF THE CLOSET: THE EFFECTS OF IDENTITY CONCEALMENT AND DISCRIMINATION

Douglas Hanes, and Sean Clousten, *Stony Brook University, Stony Brook, New York, United States*

Lesbian, gay, and bisexual (LGB) people tend to experience poorer cognitive health as they age; discrimination and identity concealment are common LGB experiences that carry emotional, health, and cognitive tolls. Previous research utilized data on research participants in same-sex relationships (SSR) to successfully identify a subset of LGB people and analyze their aging experiences. The present study relied on one situation in which identity concealment was legally mandated: military service. Until 2011, LGB people were banned from participating in military service in the U.S., leading to either concealment or institutionalized discrimination (i.e., discharge) for most LGB servicepeople. Using Health and Retirement Study (HRS; 1998–2016) data, this project analyzed the combined effects of veteran status and SSR on cognitive performance. Using multilevel longitudinal modelling, we found that while non-SSR veterans had higher mean scores than non-SSR civilian participants (the reference group), SSR civilians and veterans both had mean scores lower than the reference (SSR: $\beta$=-0.514, p=0.031; SSR+veteran ($\beta$=-1.065 p=0.081). These results may be limited due to the low number of SSR+veteran participants (n = 28). Nevertheless, these results suggest the potential for using veteran status among LGB people to study the health effects of identity concealment and discrimination on aging.

### EARLY-LIFE EXPOSURE TO THE CHINESE FAMINE OF 1959–1961 AND LATER-LIFE HEALTH: EARLY LIFE AS A CRITICAL PERIOD

Mengling Cheng, Nicolas Sommet, Daniela Jopp, and Dario Spini, *University of Lausanne, Lausanne, Vaud, Switzerland*

Barker's fetal origins hypothesis and the critical period theory suggest that early life events have long-term health effects. However, evidence of the famine exposure in early life and its effects on health in later life is scarce and inconsistent. To explore the effects of early-life exposure to the Chinese famine of 1959-1961 on later-life multimorbidity, we performed Poisson growth curve models using CHARLS Life History 2014 and CHARLS 2011-2018 (42,775 observations from 12,060 respondents). Our analyses revealed two findings. First, there was an overall detrimental effect of the early-life famine exposure on multimorbidity, although there was no effect of severity of famine exposure. Second, there was no overall interaction between famine exposure and life stages, although a more parsimonious model suggested that the detrimental effect of famine exposure was more pronounced in earlier life stages than in later life stages. Findings suggest that early life is a critical period in the life course and provides developmental origins of health and disease in later life.

### LIFE-COURSE SOCIOECONOMIC STATUS AND MORBIDITY AND MORTALITY IN THE MIDUS NATIONAL SAMPLE

Yanping Jiang[1], Li Chen[2], and Samuele Zilioli[3], *1. Rutgers, The State University of New Jersey, New Brunswick, New Jersey, United States, 2. Augusta University, Augusta, Georgia, United States, 3. Wayne State University, Detroit, Michigan, United States*

Socioeconomic status (SES) is a well-established social determinant of health shaping the distribution of the burden of morbidity and mortality. In this area, a less understood topic is how life-course SES affects physical health. This study aimed to examine the effects of childhood and adulthood SES and social mobility on morbidity and all-cause mortality. Data were drawn from the Midlife in the United States study (MIDUS, N = 7,108, ages 25-74 years, 88.4% of White, 51.6% of female). Childhood and adulthood SES were assessed at baseline (i.e., 1995-1996). Mortality was tracked through June of 2018 (N = 1,425 deceased). The number of comorbidities was assessed at baseline and the third wave of MIDUS in 2013-2014 (N = 2,786). Results showed that lower childhood SES was associated with a larger increase in the number of comorbidities at the third wave of MIDUS and higher mortality risk (ps < .05). However, when including adulthood SES in the model, adulthood SES, but not childhood SES, was associated with morbidity and mortality (ps < .01). Childhood SES was indirectly associated with morbidity and mortality through adulthood SES (ps < .01). Participants achieving upward mobility (i.e., low childhood SES, high adulthood SES) had a smaller increase in the number of comorbidities and lower mortality risk (ps < .01) than those reporting persistent low SES in childhood and adulthood. These findings indicate the unique effect of adulthood SES on physical health and highlight the potential health benefits of upward mobility in middle-aged and older US adults.

### LONGING FOR LOVE: FINANCIAL STRAIN AND LATER LIFE MENTAL HEALTH IN THE US

Tirth Bhatta[1], Jeffrey Albert[2], Stacy Torres[3], Eva Kahana[2], Nirmala Lekhak[4], and Timothy Goler[5], *1. University of Nevada Las Vegas, Las Vegas, Nevada, United States, 2. Case Western Reserve University, Cleveland, Ohio, United States, 3. University of California, San Francisco, San Francisco, California, United States, 4. University of Nevada, Las Vegas, Nevada, United States, 5. Norfolk State University, Norfolk, Virginia, United States*

Objectives: Emotional support has been consistently identified as a mechanism through which socioeconomic resources influence mental health outcomes. Despite emerging evidence that compassionate love has a beneficial effect on mental health, its distribution across levels of financial strain and subsequent role in mediating the effect of financial strain on later life mental health have yet to be examined.

Methods: Based on our nationwide web-based survey of adults aged 50 years and older (n=1751), we conducted a mediation analysis to estimate the direct and indirect effects (via two mediators, feeling loved and emotional support) of financial strain on depressive symptoms and anxiety.

Results: We documented a statistically significant overall effect of financial strain on depressive symptoms (b=0.14, p-value< 0.001) and anxiety (b=0.196, p-value< 0.001). We found a statistically significant path-specific effect (from financial hardship) through compassionate love alone on depressive symptoms (b=0.018, p-value=0.003) and anxiety (b=0.016, p-value=0.005), but did not find effects for paths through emotional support. There was also a significant direct effect of financial strain on both depressive symptoms and anxiety. Discussion: Our study advances a new line of research by looking at the role of compassionate love in transmitting the effects of financial strain on mental health in later life. Findings suggest that the detrimental effect of financial hardship on mental health operates through its negative effect on the receipt of compassionate love, rather than by its impact on emotional support.

### SUBJECTIVE SOCIAL STATUS MODERATES BACK PAIN AND MENTAL HEALTH: A LONGITUDINAL ANALYSIS OF OLDER MEN

Christina Mu[1], Dylan Jester[2], Peggy Cawthon[3], Katie Stone[4], Nancy Lane[5], and Soomi Lee[1], *1. University of South Florida, Tampa, Florida, United States, 2. University of California San Diego, La Jolla, California, United States, 3. California Pacific Medical Center Research Institute, San Francisco, California, United States, 4. University of California, San Francisco, Castro Valley, California, United States, 5. University of California, Davis Health, Davis, California, United States*

Objectives. This study tested the longitudinal relationship between back pain and mental health and examined the moderating role of subjective social status (SSS). Method. Community-dwelling older men from the MrOS Study provided four study visits of data collected between 2000-2016 (15,975 observations nested within 5,979 participants). Back pain frequency and severity were assessed at visits 1-4. General mental health was measured at each visit by the 12-item Short Form Survey Mental Component Score (SF-12 MCS; higher scores representing better mental health). National and community SSS were assessed at visits 1 and 3 with the MacArthur Scale. Growth curve models tested longitudinal within-person change associations after accounting for the repeated measures within each person. Age was used as the primary time variable. Results. At baseline, those with higher back pain-frequency/severity reported lower SF-12 MCS. After accounting for this between-person difference, there were bidirectional within-person associations between back pain frequency/severity and SF-12 MCS. On follow-up visits when back pain frequency/severity increased from baseline, participants reported lower SF-12 MCS (p<.001). On follow-up visits when SF-12 MCS decreased from baseline, participants also reported higher back pain frequency/severity (p<.001). Higher national and community SSS at baseline and having increases or consistently higher SSS over time attenuated the negative relationships between back pain frequency/severity and SF-12 MCS. Results were consistent after controlling for an extensive list of baseline health covariates and pain medications. Discussion. These findings highlight how self-perceived social status may buffer the relationship between greater back pain frequency/severity and lower mental health.

### MULTIMORBIDITY AND ITS ASSOCIATION OF WIDOWHOOD, RACE, AND ETHNICITY IN OLDER ADULTS

Maki Karakida[1], Qian Song[2], Jeffrey Stokes[3], Suzanne Leveille[3], and Beth Dugan[3], *1. UMass Boston, Boston, Massachusetts, United States, 2. Universiy of Massachusetts Boston, Boston, Massachusetts, United States, 3. University of Massachusetts Boston, Boston, Massachusetts, United States*

Living with chronic disease is common in later life and over 60% of adults 65+ have two or more conditions. Spousal loss, also common in late adulthood, is linked to chronic diseases, like heart attacks, cardiovascular disease, and strokes. This study examines the associations between widowhood, race/ethnicity, and onset of multiple chronic conditions (i.e., cancer, diabetes, heart disease, stroke) among 4,187 Americans aged 60+. Using the RAND HRS Longitudinal File 2018, we analyzed how married/partnered adults without major chronic conditions in 2010 experienced widowhood and developed cancer, diabetes, and/or cardiovascular disease during the subsequent eight years. We used ordinal logistic regressions to examine the associations between marital status, race/ethnicity, and multimorbidity onset, controlling for age, gender, birthplace, living conditions, income, education, smoking habits, physical activities, self-reported BMI, and Medicaid enrollment. Among participants, 69.41% were non-Hispanic White, 13.04% were non-Hispanic Black, 14.19% were Hispanic (any race), and 3.37% were non-Hispanic other. The mean age was 70.33 years, more than half (54.79%) were female, and 83.85% were married or partnered in 2018. Older widowed group were more likely to be non-Hispanic White women without smoking habits nor multimorbidity. The onset of multimorbidity at follow-up was elevated among divorced or separated non-White/Hispanic participants (OR = 1.83, 95%, CI 0.896, 3.744) unlike widowed non-White individuals (OR = 0.71, 95%, CI 0.433, 1.152). Age, previous smoking behavior, BMI, and birth in the South were all significantly associated with increased multimorbidity risk ($p<0.017$). The association of widowhood and health outcomes may vary by race and ethnicity.

### RACIAL/ETHNIC DISPARITIES IN THE RISK OF DEMENTIA: THE ROLE OF STRESS EXPOSURE IN LATE LIFE

Wen-Hua Lai, *Michigan State University, East Lansing, Michigan, United States*

The number of individuals with Alzheimer's disease and related dementias is expected to triple from 5 million to 13.7 million by 2050 in the US. Current studies suggest that older Black and Latinx adults experience poorer cognitive health; their dementia prevalence is 1.5 to 2 times higher than their white counterparts. Excessive stress exposure (e.g., traumatic experiences and discrimination) may serve as contributors to the cognitive health inequalities faced by racial/ethnic minorities. However, extant studies on cognitive health in diverse aging populations remained limited. In particular, little is known about how the level and effect of stress exposure account for cognitive health disparities across racial/ethnic groups. This study examines whether racial/ethnic differences in stress exposure (traumatic events, stressful life events, perceived everyday discrimination, lifetime discrimination, and financial strain) shape cognitive health disparities by race/ethnicity. Data from the 2006-2012 Health and Retirement Study are used to address the research question (N=9,251). Preliminary results based on ordinal logit regression models and moderation analyses suggest that all racial/ethnic minority older adults, especially Blacks, were more exposed to stressors and experienced poorer cognitive health than their White peers. However, even though some stressors are associated with adverse cognitive health, greater stress exposure among minority older adults explains little cognitive disparities faced by them. Moreover, the magnitude of the stressor effects on cognitive health is smaller for Blacks and Latinxs than for Whites. This study will discuss the implication of these unexpected findings and areas of promising future research.

### THE NEIGHBORHOOD CONTEXT AND ALL-CAUSE MORTALITY AMONG OLDER ADULTS IN PUERTO RICO

Catherine Garcia[1], Mary McEniry[2], and Michael Crowe[3], *1. Syracuse University, Syracuse, New York, United States, 2. University of Wisconsin-Madison, Madison, Wisconsin, United States, 3. University of Alabama-Birmingham, Birmingham, Alabama, United States*

The neighborhood contexts in which older adults live are increasingly being recognized for their role in influencing disease processes and risk of death among the U.S. population. However, few studies have focused on neighborhood impacts among older populations residing in Puerto Rico– a U.S. territory –who are especially vulnerable to the effects of the environment as they "age in place" in the context of a budget crisis, the great recession, the debt crisis, and Hurricanes Irma and María. The combination of these events can obstruct access to neighborhood resources, services, and contexts considered necessary for promoting healthy aging. Thus, it is warranted to understand the effects of place on mortality in Puerto Rico, whose social and economic contexts differ from the U.S. and are more similar to that of other Latin American and Hispanic-Caribbean countries. We used 2000 U.S. Census data at the block-group level linked to the 2002 Puerto Rican Elderly Health Conditions Project with mortality follow-up to 2021 to examine neighborhood characteristics that are conceptualized as influencing mortality (e.g., residents without a high school degree; households receiving public assistance income; residents living below the poverty level; unemployed residents; residential stability; age structure). Multilevel mixed-effects parametric survival models with a Weibull distribution were estimated. Overall, results show that neighborhood socioeconomic disadvantage is associated with an increased risk of mortality among older Puerto Ricans. This suggests that older Puerto Ricans clustered in disadvantaged communities are more likely to experience a cumulative burden of social disadvantages that adversely impacts their longevity.

### ARE PERCEIVED NEIGHBORHOOD CHARACTERISTICS ASSOCIATED WITH CHRONIC PAIN IN MIDDLE-AGED AND OLDER ADULTS?

Yulin Yang, *University of California, San Francisco, San Francisco, California, United States*

Neighborhoods predict many health outcomes including functional limitations and disability; however, how neighborhoods are associated with chronic pain remains poorly understood. This study uses examined the association between four perceived neighborhood characteristics (social cohesion, physical disorder, safety, and social ties) and chronic pain in a nationally representative sample of adults aged 51+ in the US. Among 14,069 participants (58.65% women; mean age 68.81) in the Health and Retirement Study (2006-2008), we investigate whether neighborhood social cohesion, neighborhood physical disorder, neighborhood safety, and neighborhood social ties were associated with odds of having moderate-severe and limiting pain using adjusted logistic regression. After adjusting socio-demographic factors (age, gender, race/ethnicity, education, wealth, marital status), residence in neighborhoods with high perceived social cohesion, safety, and more social ties were associated with lower odds of having moderate-severe and limiting pain. Perceived neighborhood physical disorder did not predict whether respondents have moderate-severe and limiting pain or not.

### RURAL ADULTS' BEHAVIOR CHANGES DURING COVID-19: A LATENT CLASS ANALYSIS

Tim Killian, and Betsy Garrison, *University of Arkansas, Fayetteville, Arkansas, United States*

The advent of the SARS-CoV-2 virus necessitated behavior changes for most older adults. Changes were largely associated with voluntary or enforced compliance with social distancing policies. Whereas the value of social distancing for mitigating the spread of the dangerous SARS-CoV-2 virus is not the subject of serious debate among public health advocates, policies that encouraged or enforced social distancing have been met with skepticism in the general public. Understanding patterns in behavior changes may be helpful to public health professionals and policymakers who develop health communication and revised guidelines in the efforts to contain the spread of the SARS-CoV-2 and future public health infections. Our study examined patterns in behavior changes. This study had two aims. First, using survey responses from 661 adults living in rural areas about changes they made to 20 different behaviors, we identified four classes of behavior changes: (1) non-social distancers (10.7%), (2) social distancers (54.0%), (3) social distancers making civic exceptions (24.0%), and (4) social distancers making religious exceptions (11.3%). Second, polytomous logistic regression was used to predict the probability of membership in those latent patterns of behavior change. Predictor variables included demographic variables (i.e., age, sex, and race) and variables theoretically predicted to covary with patterns of behavior change (i.e., social trust, self-assessment of health, experience of pain, and depressive symptoms). This study provides insight into behavior changes during the outbreak of the SARS-CoV-2 virus that may be used by practitioners to understand variations in how people responded to the outbreak.

### SOCIAL DETERMINANTS OF HEALTH AND QUALITY OF LIFE AMONG OLDER ADULTS NEW TO LONG-TERM SERVICES AND SUPPORT

Onome Osokpo[1], Karen Hirschman[1], Liming Huang[1], and Mary Naylor[2], *1. University of Pennsylvania, Philadelphia, Pennsylvania, United States, 2. University of Pennsylvania - School of Nursing, Philadelphia, Pennsylvania, United States*

The number of older adults requiring long-term services and support (LTSS) is increasing. Social determinants of health (SDoH)—underlying economic, social, and structural factors—fundamentally shape people's health including quality of life (QoL). However, the influences of SDoH factors on QoL are relatively unexplored among older adults new to LTSS. The purpose of this study was to examine the relationship between SDoH factors and overall QoL ratings among older adults new to LTSS using data from a prospective NIA funded longitudinal study of change in health related QoL in this population. QoL was measured using a single item: "How would you rate your overall quality of life at the present time?" (1- Poor to 5- Excellent). SDoH variables included years of education, marital status, food security, spirituality, social isolation, financial resources, and social support measures. A full multivariable regression analysis was performed adjusting for age, sex, cognitive status, functional status, physical and emotional health, and LTSS type. Among the 470 participants, 71% were female, 51% were white, 20% were married, and the average age was 81 years (+/- 8 years). Having adequate financial resources to pay for health care needs ($p < .0001$) was positively associated with QoL. No other SDoH factors were significant. Access to resources to meet health care needs is critically important in promoting the quality of life of older adults especially at the start of LTSS. Implications for future research and policy will be discussed.

### SOCIAL DETERMINANTS OF HEALTH UNDERLIE RACIAL/ETHNIC DISPARITIES IN PSYCHOLOGICAL HEALTH AND WELL-BEING

Dylan Jester[1], Jordan Kohn[1], Lize Tibirica[2], Michael Thomas[3], Lauren Brown[4], James Murphy[1], and Dilip Jeste[1], *1. University of California San Diego, La Jolla, California, United States, 2. University of California, San Diego, San Diego, California, United States, 3. Colorado State University, Fort Collins, Colorado, United States, 4. San Diego State University School of Public Health, San Diego, California, United States*

This study sought to determine the impact of selected social determinants of health on psychological health and well-being (defined as depression, cognition, self-rated health) among Black and Hispanic/Latinx adults relative to White adults aged 51 to 89. We measured disparities in depression, cognition, and self-rated health among 2,306 Non-Hispanic/Latinx Black, 1,593 Hispanic/Latinx, and 7,244 Non-Hispanic/Latinx White adults from the Health and Retirement Study (n=11,143). Blinder-Oaxaca decomposition was used to examine whether differences in selected social determinants of health explained a larger share of the disparities than age, sex, measures of health, health behaviors, and healthcare utilization. Selected social determinants included education, parents' education, number of years worked, marital status, veteran status, geographic residence, nativity status, income, and insurance coverage. Black and Hispanic/Latinx adults reported worse depression, cognition, and self-rated health compared to White adults. Selected social determinants of health explained a larger proportion of the Black-White disparities in depression (51%), cognition (39%), and self-rated health (37%) than did age, sex, measures of health, health behaviors, and healthcare utilization. Social determinants of health explained a larger proportion of the Hispanic/Latinx-White disparity in cognition (76%) and self-rated health (75%), but age and functional status primarily drove the disparity in depression (28%). Education, parents' education, years worked, income, and insurance parity were social determinants associated with the disparities.In conclusion, differences in social determinants of health underlie racial/ethnic disparities in depression, cognition, and self-rated health among older adults. Education, income, number of years worked, and insurance parity are key social determinants of health.

### SOCIOECONOMIC CHARACTERISTICS AND HEALTH OF OLDER ADULTS IN RURAL CAMBODIA: ROLES OF SOCIAL AND WELFARE SUPPORT

Kakada Kuy, *WASHINGTON UNIVERSITY ST LOUIS, UNIVERSITY CY, Missouri, United States*

Considering differences in support systems and socioeconomic characteristics, findings from studies of high-income nations could be biased if generalized to population of low- and middle-income countries. Insights into roles of social support in socioeconomic characteristics and health relationships are valuable for health interventions targeting older adults in low-resource communities, such as rural Cambodia, but such knowledge remains unexplored. This study examined mediating roles of social and welfare support in associations between socioeconomic characteristics and health among older Cambodians. Data came from a 2019 survey of older Cambodians age 60 and older (N=220) living in three rural provinces. Dependent variables included health complaints and psychological well-being index. Independent variables were wealth index derived from household items and living conditions, and subjective socioeconomic characteristic derived from three self-perceived economic circumstances. Social support and welfare support were operationalized using Social Support and Social Network scale and status of welfare assistance, respectively. Mediation analysis using Structural Equation Modeling substantiated a full mediation effect of social support on the relationship between subjective socioeconomic characteristic and psychological health. A partial mediation of social support was also found in the subjective characteristic and physical health relationship. However, no mediation was found in relationships between wealth index and health. Welfare support did not mediate any relationship between socioeconomic characteristics and both health outcomes. Healthy aging interventions in low-resource settings could benefit from improving existing support system of family and friends, which may buffer the impact of economically negative self-image on one's health and well-being in the community.

### SOCIAL DETERMINANTS OF COGNITIVE HEALTH

Amber Rusch, Janella Camp, and Julie Hicks Patrick, *West Virginia University, Morgantown, West Virginia, United States*

Social determinants of health (SDOH), such as age, race, sex/gender, socioeconomic status, and access to healthcare, influence wellbeing, and shape day-to-day life. To what degree SDOH influence cognitive decline is less well understood. Thus, we applied frameworks of SDOH to understand cognitive health better. We used data from the CDC's 2020 Behavioral Risk Factor Surveillance System, in which 61,770 adults (mean age = 64.7, SD = 10.4, range 24-80+ yrs.) supplied demographic information and answered questions about cognitive decline. We conducted a logistic regression and interpreted the Odds Ratios (ORs). A significant equation emerged, X2 (DF = 8) = 438.214, $p < 0.001$. Each indicator of age, sex, race, and education uniquely contributed to the equation. Older adults, those with less education, and those identified as American Indian (OR = 1.25) were more likely to express cognitive decline. Using this equation, we correctly classified 0%, so we added a second step that included SDOH beyond demographic predictors: number of health conditions, access to health care, urban/rural status, and depression. The model retained its significance (X2 (DF=12) = 3400, $p < .001$. The OR showed adults with depression were 3.8 times more likely to report cognitive decline. Thus, examining a range of SDOH beyond demographics increases our understanding of the context in which cognitive decline occurs.

### SESSION 6210 (POSTER)

### SOCIAL DIMENSIONS OF COVID-19: HEALTH AND CAREGIVING DURING THE PANDEMIC

### "MISERY LOVES COMPANY": THE OTHER SIDE OF COVID-19 FOR SPOUSAL CAREGIVERS

Jing Geng[1], Toni Calasanti[2], and Sadie Giles[2], *1. Virginia Tech, BLACKSBURG, Virginia, United States, 2. Virginia Tech, Blacksburg, Virginia, United States*

Gerontologists have voiced much concern about the potential for the pandemic to create social isolation risks for older people, especially older caregivers, for whom the lockdown cut off many sources of support. This presentation is based on a national study of older same-sex and heterosexual people who care for their spouses with Alzheimer's disease and related dementias. The qualitative, interview-based research began before the pandemic, and we were able to ask caregivers about their experiences during this public health crisis. Drawing on a total of 48 interviews, this presentation documents the surprising finding that, although caregivers discussed some challenges, they found much to say about the positive aspects of the lockdown and continued restrictions, including not having to rush for appointments or social engagements and being less frustrated with their care receivers as a result. They also described instances of greater social connection rather than less through the use of technology. In addition, some caregivers found that being with their care receiver all day gave them greater insights into disease progression and how to manage it, as well as greater intimacy. Finally, the phrase "misery loves company" was used to convey that the pandemic provided a situation wherein other people also were restricted in ways that some caregivers had been prior to the pandemic; as a result they felt others had more of an appreciation of what their lives had been like.

### PROTEIN CONSUMPTION SINCE COVID-19 PANDEMIC AMONG DIFFERENT RACES

Lillie Monroe-Lord[1], Azam Ardakani[2], and Lily Spechler[1], *1. University of the District of Columbia, Washington, District of Columbia, United States, 2. Howard University, Beltsville, Maryland, United States*

Covid-19 has changed individuals eating behavior. This study examines how protein consumption changed since Covid-19 pandemic. This is a cross-sectional study design with a total of 10,035 adults aged 40-100 years old surveyed through Qualtrics by dietary screening tool. The protein consumption was questioned by "How often do you eat chicken or turkey?" and "How often do you eat not-fried fish or seafood?". Pre and since pandemic responses were compared by Wilcoxon signed-rank tests. Participants were 57% female,

43% male, 75% White, 14% African American, %7 Asian, and 4% Hispanic. According to the analysis, chicken, or turkey (p <.001) and fish or seafood (p <.001) consumption significantly reduced since Covid-19 pandemic. Chicken or turkey consumption reduced significantly among African American (p < .01), White (p < .01), and Hispanic (p < .05) participants while this reduction was not significant among Asians. Moreover, Fish or seafood consumption also significantly reduced among African American (p < .05) and White participants (p < .01), while did not change among Asians and Hispanics. In conclusion, since Covid-19 pandemic, protein consumption significantly reduced among some races (i.e., African American and White) while changed at a lower grade (i.e., Hispanic), or remained unchanged (i.e., Asian) among other races. It could be justified by considering that foods with high amount of protein and especially fish or seafood is part of the eating culture of some races. Reduction in protein consumption resulted from changes in the daily routine of people and financial instability resulting from the Covid-19 pandemic.

### TRAJECTORIES OF ANXIETY SYMPTOMS DURING THE COVID-19 PANDEMIC AMONG OLDER ADULTS

Soyoung Choun[1], Dylan Lee[2], Maria Kurth[1], Hye Soo Lee[1], Heidi Igarashi[1], and Carolyn Aldwin[1], *1. Oregon State University, Corvallis, Oregon, United States, 2. Touro University Nevada, Henderson, Nevada, United States*

Older adults face particular challenges during the COVID-19 pandemic, including increased risk for morbidity and mortality (CDC, 2021). Social distancing and lockdown to prevent contagion may create social isolation and loneliness, adversely affecting mental and physical health. We examined anxiety symptom trajectories of older adults and identified risk and protective factors during the early months of the COVID-19 pandemic. We also examined how anxiety symptoms were associated with both between- and within-person variations in loneliness, social contacts, and physical problems. We sampled 247 older adults (Mage = 71.1, SD = 7.3, range = 51 - 95), who participated in eight weekly longitudinal online surveys from April 28 to June 23, 2020. Multilevel modeling analysis controlling for age, gender, marital status, and education showed that anxiety symptoms significantly decreased in the first few weeks, but then increased around week 6 of the study period. At the between-person level, individuals with higher levels of both loneliness and physical problems were at risk for experiencing higher levels of anxiety, but social contacts were not significant. Middle-aged participants reported higher anxiety symptoms than older participants. Women experienced higher anxiety symptoms than men. As a protective factor, individuals who were high in resilience had significantly lower anxiety symptoms over time, compared to those with low in resilience. In the within-person level, anxiety symptoms were positively coupled with both loneliness and physical symptoms over time. We conclude that lonely individuals and those in poor health were at greater risk of poor mental health during the pandemic.

### EFFECTS OF CHANGES IN THE FREQUENCY OF SOCIAL OUTINGS ON DEPRESSION AND WELL-BEING DURING THE COVID-19 PANDEMIC

Koji Abe, *Kagoshima University, Kagoshima, Kagoshima, Japan*

In Japan, in order to reduce the risk of infection with COVID-19, a state of emergency was declared in areas the infection had spread to, and priority measures were issued in equivalent areas. As a result, many senior citizens who were not good at using the Internet lost the opportunity to communicate with others through interpersonal interaction. Previous studies have shown that reduced interpersonal interaction negatively affects the mental health of senior citizens. However, little research has been done on the effects of socially created restrictions on interpersonal interaction. The purpose of this study was to focus on the changes in the frequency of going out among senior citizens and to examine the effect on mental health. An internet survey of a nationwide sample of individuals was conducted in October 2021. In total, there were 1600 respondents (males, 47.8%; females, 52.2%; mean age, 70.39(SD=4.67) years). During the pandemic, 47.1% participants had decreased the frequency of going out, and 52.9% had either no change or a slight increase in the frequency of going out compared to the period before the pandemic. Multiple regression analysis showed that social capital and decreasing frequency of going out had a significant effect on depression. However, a decrease in the frequency of going out had no effect on subjective well-being, and only social capital had a significant effect. The results showed that a decrease in frequency of going out increased the depressive mood, but social capital had an even greater impact on depression and subjective well-being.

### CAREGIVING FOR PEOPLE WITH DEMENTIA DURING THE PANDEMIC: A SCOPING REVIEW

Phillip Cantu[1], Joanna Chyu[2], Neil Mehta[2], and Markides Kyriakos[2], *1. The University of Texas Medical Branch, Galveston, Texas, United States, 2. UTMB, Galveston, Texas, United States*

Background: The COVID-19 pandemic has had a large impact on the roles and responsibilities of caregivers for older adults with dementia. An increasing number of studies have examined the unique challenges faced by caregivers during this time, including extended work hours, anxiety around contracting COVID-19, and adhering to public health guidelines. Informal caregivers may also face greater strain on their personal lives during lockdown. Objective: To conduct a scoping review to examine factors impacting well-being of caregivers of older adults with dementia during the first year of the COVID-19 pandemic. Method: We conducted a PubMed search using the terms "COVID-19," "older adults," and "caregiving" or "caregiver." Sixty-seven papers were identified published between June 2020 and December 2021.

Results: All papers identified were cross-sectional and conducted after the pandemic began and prior to the availability of COVID-19 vaccines. Articles highlighted increasing burdens such as financial and physical stress, as well as worsened psychological wellbeing through increased anxiety and depression among caregivers during the pandemic. Additionally, themes of protective factors on wellbeing in the form of social connection and telehealth interventions emerged. Discussion: Limitations to our review include lack of longitudinal information on caregiver experience to better identify broad impacts on caregiver well-being. Studies not only identified new, pandemic-related risk factors for caregiver burden, but also a heightened effect of pre-existing risk factors (e.g. income, living situation, gender) on burden. Caregiver psychiatric outcomes reflect the overall population's increase in mental illness since the start of the pandemic.

### MAKING "NONESSENTIAL" FAMILY/VOLUNTEER CAREGIVING ESSENTIAL IN LONG-TERM CARE HOMES

Annie Robitaille, Linda Garcia, and Michaela Adams, *University of Ottawa, Ottawa, Ontario, Canada*

For individuals living in long-term care (LTC), loneliness is often a concern. With the COVID-19 pandemic, this is only exacerbated as strict restrictions are put in place on visits between residents and their loved ones and on volunteer presence. Understanding how these changes affect residents, family caregivers, and volunteers is paramount to best implement changes with regards to how family/volunteer caregiving presence is managed during pandemics. The objective of this study was to gain a better understanding of the response to COVID-19 that pertains to the family caregiver and volunteer presence in LTC and increase the evidence about the impact of reduced levels of family caregivers and volunteers on residents, caregivers, and volunteers. A total of 64 semi-structured interviews were conducted with caregivers and volunteers. Of these interviews, 49 were one-time interviews and 15 were weekly interviews over a 5-month period to examine the impact of the ever-changing pandemic restrictions on caregivers, volunteers, and residents. Thematic analysis was used to analyze the interviews and two independent researchers coded each interview. Results highlight the importance of connections in LTC, the feeling that human rights were neglected, the importance of flexibility amongst staff, the role of caregivers as advocates for residents, increased caregiver guilt, and resident decline in physical and emotional well-being. The role of family caregivers and volunteers as essential in LTC homes will be discussed and recommendations to revisit policies on the family caregiver and volunteer presence to improve the preparedness for future pandemics and outbreaks will be presented.

### OPTIMAL AGING AND SATISFACTION WITH SOCIAL SUPPORT DURING THE COVID-19 PANDEMIC

Hye Soo Lee[1], Soyoung Choun[1], Maria Kurth[1], Heidi Igarashi[1], Dylan Lee[2], and Carolyn Aldwin[1], *1. Oregon State University, Corvallis, Oregon, United States, 2. Touro University Nevada, Henderson, Nevada, United States*

Social support is important for optimal aging, especially during the COVID-19 pandemic when older adults are at risk of social isolation and its attendant health problems. Providing support may be especially protective of health outcomes. We examined whether actual received, provided, and satisfaction with support were related to optimal aging early in the pandemic (April-May, 2020). Survey participants (N=238) were on average 71.2 years old (SD=7.3), 73% female, 92.6% White, and highly educated (48% with post-graduate degrees). Optimal aging (Aldwin & Igarashi, 2016) was indicated by a latent variable of health outcomes, including depressive symptoms, cognitive lapses, and physical symptoms. Nearly all older adults (90+%) reported receiving or providing actual support from or to at least one family member or friend. We investigated the associations between age, summed social support, satisfaction with support, and health outcomes, controlling for chronic illnesses. Two SEM models were estimated for received support and provided support, respectively. After trimming non-significant paths, both models had acceptable fits (CFI > .90, RMSEA < .08, SRMR < .08). Age and chronic illnesses had negative associations with health outcomes, but neither received nor provided social support was significant. However, satisfaction with both received and provided support were significant and independently associated with optimal aging in both models. Thus, isolation levels in this sample were surprisingly low, as indicated by high levels of social support received and provided. However, only the quality of (or satisfaction with) support was important for optimal aging during this unique and shared stressful experience.

### HEALTHCARE ACCESSIBILITY AND UTILIZATION AMONG LGBTQ+ OLDER ADULTS

Jessika Dayrit[1], Ana Maria Pasatiempo[2], Shirley Salom-Bail[3], and Cesz Islaya[3], *1. Washington University in St. Louis, Menlo Park, California, United States, 2. U.S. Department of Veterans Affairs, Anchorage, Alaska, United States, 3. US Department of Veterans Affairs, Menlo Park, California, United States*

Background: Studies show disparities in healthcare have exacerbated during the COVID-19 pandemic, especially among Lesbian, Gay, Bisexual, Transgender, and Queer + (LGBTQ+) older adults. The aim of this paper is to understand health care utilization and accessibility among LGBTQ+ older adults and to examine if social assets, such as income, education, and employment are associated with health care utilization and accessibility.

Methods: Data from BRFSS 2020 was used. Study focused on LGBTQ+ and non-LGBTQ+, age 65 and older, comparing health care utilization and accessibility. Data was weighted for complex sampling design. Logistic regression was used to examine the odds of health care accessibility and health care utilization while controlling for socioeconomic status.

Results: Total sample size of study was 14,453. 6.14% of participants identified as LGBTQ+, which 11% of them were unemployed (CI=0.08-0.17), 21% earned less than $15,000 annually (CI=0.15-0.28), and 35% did not graduate from high school (CI=0.27-0.44). Our analyses indicated LGBTQ+ are less likely to have primary health care provider than non-LGBTQ+ (OR=0.64; CI=0.48-0.84); they are less likely to have health care insurance than non-LGBTQ+ (OR=0.60; CI=0.39-0.93). We did not find association if LGBTQ+ are less likely to seek medical help, when needed, due to cost (OR=0.72; CI=0.53-1.02). Our findings suggest disparities in socioeconomic status, such as income, employment, and education have significant association with health care accessibility and health care utilization. Limitation of study includes recall bias due to self-report. Longitudinal and qualitative research regarding healthcare utilization and accessibility among older LGBTQ+ population need to be explored.

### AGE DIFFERENCES IN SELF-CONTINUITY REMAIN ROBUST IN RESPONSE TO THE COVID-19 PANDEMIC

Yi Lu, and Corinna Löckenhoff, *Cornell University, Ithaca, New York, United States*

Self-continuity, the perceived connectedness to one's past and future selves, predicts well-being and tends to be higher among older adults (Rutt & Löckenhoff, 2016). Self-continuity is also susceptible to major life changes (Sani, 2008). The pandemic brought dramatic changes in multiple life domains, especially for older adults who experienced higher rates of COVID-19 morbidity and mortality and may have been more likely to restrict their behavior as a result. This study examined whether age differences in self-continuity remained robust in response to the pandemic using representative U.S. survey data. Pre-pandemic data (Fall 2016, N = 230, aged 18 – 87, M = 50.85, SD = 16.40, 47.83% female, 67.83% white) and mid-pandemic data (Summer 2020, N = 230, aged 18 – 88, M = 50.20, SD = 19.49, 48.27% female, 64.78% white) were demographically matched using propensity scores. Participants rated their self-continuity one, five, and ten years into the past and future. Multi-level modeling examined the effects of age, temporal distance (in years), temporal direction (past vs. future), and assessment time (pre- vs. mid-pandemic) on self-continuity. Consistent with prior research, self-continuity was lower for more distant intervals, especially for the past. An interaction between temporal direction and assessment time indicated that future self-continuity was lower mid-pandemic than pre-pandemic. Across samples, however, advanced age was associated with higher self-continuity and the size of this effect did not vary by assessment time. Overall, findings suggest that even though future self-continuity decreased during the pandemic, existing age effects remained robust to this disruption.

### COPING AND UNCERTAINTY IN THE CONTEXT OF COVID-19: DIFFERENCES BETWEEN PARTNERED AND UNPARTNERED OLDER ADULTS

Ashley Tate[1], Christina Marini[2], Suyoung Nah[3], and Lynn Martire[1], *1. Pennsylvania State University, University Park, Pennsylvania, United States, 2. Adelphi University, Garden City, New York, United States, 3. Pennsylvania State University, State College, Pennsylvania, United States*

The initial conditions of the COVID-19 pandemic made it such that individuals—especially older adults—experienced uncertainty about their own health/well-being, as well as that of their loved ones and communities. The current study examined how older adults' social context shaped their well-being (i.e., anxiety, depressive symptoms, and sleep quality) early in the pandemic. Specifically, we tested whether there were differences in uncertainty, communal coping, and well-being among partnered individuals who indicated that their partner was their primary source of emotional support during the pandemic versus un-partnered individuals who indicated another social tie as their primary source of emotional support. Data were collected between May and August 2020 from 101 older adults (Mage = 80.63, SD = 8.96). Correlational analyses showed that uncertainty about one's own health/well-being was positively associated with anxiety ($p < .05$) and communal coping (i.e., viewing the pandemic as a stressful period that will be worked through together) was negatively associated with depressive symptoms ($p = .001$) and anxiety ($p < .01$). Results from independent samples t-tests showed that, on average, partnered older adults reported fewer depressive symptoms ($p < .05$), less uncertainty about their own health/well-being ($p < .05$), and higher communal coping ($p < .001$) compared to un-partnered older adults. Unexpectedly, no group differences were found for anxiety or sleep quality. Results suggest the unique benefit of having a partner as an emotional support confidant in the context of the pandemic, perhaps because older adults were largely confined to their homes during lockdown.

### HOW COVID-19 NEWS AFFECTS OLDER ADULTS' MENTAL HEALTH

Grace Li[1], Zoe Ziyi Ng[2], and W. Quin Yow[3], *1. Palo Alto High School, Palo Alto, California, United States, 2. Raffles Institution, Singapore, Singapore, 3. Singapore University of Technology & Design, Singapore, Singapore*

Media affects the trajectory of many individuals' mental health. With the rise of COVID-19 cases, the media not only provides informative updates on the pandemic, it also fuels the hysteria and paranoia of citizens across the world. Studies show that older adults (OA) who consume media frequently were at a higher risk for declining mental health (Negarestani et al., 2021) and that individuals experience negative bias more than positive bias (Vaish et al., 2013). However, no studies have directly investigated how much positive and negative COVID-19 news affect OA's mental health and emotions. Sixty-nine OA (aged 55-95) answered questions about their weekly media consumption, how closely they followed COVID-19 news, and the General Health Questionnaire. They were then randomly assigned to read either positive or negative COVID-19 news (n=35 and 34 respectively), and asked if the news made them feel happy/fearful and if they wanted to read more about/ignore the news. Analysis revealed that the more OA consumed media and closely followed COVID-19 news, the more they felt unhappy and depressed, rs>.25, ps<.05. There was a significant condition effect, where OA who read positive news reported more positive emotions than those who read negative news, $t(67)=5.21$, $p<.001$. Chance analyses revealed that positive news evoked positive emotions significantly above chance, $t(34)=8.99$, $p<.001$, but negative news evoked negative emotions only marginally above chance, $t(33)=1.92$, $p=.064$. These findings suggest that media consumption of COVID-19 news does negatively impact OA's mental wellbeing, and OA appear to have a strong positivity bias for COVID-19 news.

### OLDER ADULTS DURING THE COVID-19 PANDEMIC: ROLE OF HEALTH CONDITIONS IN RESILIENCY AND PERCEIVED STRESS

Allyson Sloan, *Johns Hopkins University School of Medicine, Baltimore, Maryland, United States*

The COVID-19 pandemic has necessitated protracted lockdowns, exacerbating the challenges associated with social isolation. Older adults, who often suffer from social isolation, may have a harder time coping with the added isolation imposed by COVID-19. In this study, we investigated the resiliency and coping skills of older adults amidst the COVID-19 pandemic, and whether they were associated with different co-morbidities. We conducted a 45-minute telephone survey of 107 participants to assess their experiences during the COVID-19 pandemic. Participants were recruited from existing Johns Hopkins studies. The survey included the Brief Resilient Coping Scale, the Perceived Stress Scale, and questions about current health conditions (arthritis, osteoporosis, high blood pressure, heart disease, and depression/anxiety) and whether these conditions worsened, improved, or remained unchanged since the beginning of the pandemic. We regressed scores for resiliency and coping scores on a series of indicators of whether co-morbidities had worsened during the pandemic. On average, participants were 76 years old and 63% were female. There was no association between resiliency scores and any of the co-morbidities (r2=2.8%). With respect to perceived stress, participants who reported their depression/anxiety worsened during the pandemic also reported greater levels of perceived stress (B=0.83, 95% confidence interval: 0.30, 1.36). We were surprised that additional co-morbidities did not affect resiliency or stress. In order to better serve geriatric populations, health professionals should closely monitor those patients who have depression and anxiety during times of social distancing, epidemics, or pandemics.

### OLDER AGE IS (STILL) ASSOCIATED WITH MENTAL HEALTH BENEFITS IN JUNE–JULY 2021 OF THE COVID-19 PANDEMIC

Amanda Chappell[1], Kelly Smith[2], and JoNell Strough[2], *1. West Virginia University, Morgantown, West Virginia, United States, 2. WVU, Morgantown, West Virginia, United States*

In the early months of the COVID-19 pandemic, older age was associated with less anxiety and depression (Bruin de Bruin, 2021). Similar results were found for data collected during the June-July 2020 spike in cases (Smith et al., 2021). Theorists have suggested that benefits of age for well-being may be reduced when stressors are prolonged and unavoidable (Charles, 2010). Here, we investigated whether older age continued to be protective in June-July 2021, when vaccines had become widely available, but the pandemic persisted. Secondary data analysis was conducted from the Understanding America Study, based on n=5,535 (M=52.69 yrs., SD=16.04) participants who responded to online self-report surveys. Participants reported symptoms of anxiety and depression (assessed by the Patient Health Questionnaire, PHQ-4), engagement in protective behaviors (e.g., wearing a mask), and coping strategies (e.g., getting extra exercise). Multiple regression analyses predicted anxiety and depression from age, coping strategies, and protective behaviors, controlling for marital status, gender, and income. Coping through exercise and calling family/friends were significantly associated with less anxiety and depression, whereas coping by using social media and engaging in protective behaviors was significantly associated with more anxiety and depression. The harmful effects of protective behaviors may reflect the people engaging in these strategies most often are also those most worried about COVID-19. Even after accounting for coping strategies and protective behaviors, older age was still associated with fewer symptoms of anxiety and depression. Implications of older adults' resilience in the face of a prolonged stressor for promoting mental health are discussed.

### "ADAPTING TO A NEW REALITY": OLDER ADULTS' EXPERIENCES DURING THE COVID-19 PANDEMIC

Jodie Oshana[1], Mariana Guzzardo[2], and Irina Todorova[3], *1. University of Connecticut, Storrs, Connecticut, United States, 2. California State University, Hayward, California, United States, 3. Northeastern University, Boston, Massachusetts, United States*

Older adults were uniquely affected during the COVID-19 pandemic, given that they were most at risk of serious complications and death if infected. Numerous preventative measures were abruptly mandated, including stay at home orders and social distancing that led to social isolation. Given this context, the aim of this qualitative study is to explore the lived experiences, perceived challenges, and mechanisms for resilience of older adults during the first wave of the pandemic. Our sample (n=50), derived from a larger international study with data from 15 countries, includes individuals 60 or older in Puerto Rico and mainland United States. Following thematic analysis of responses from an online survey, three themes were identified: resilience through reflection and adaptation, resilience through critique of systemic problems and injustices, and resilience through reaffirmation of values. Findings support various recommendations for improving support to older adults in future crises.

### ARE OLDER ADULTS ABLE TO DRAW UPON PRIOR EXPERIENCES WHEN COPING WITH THE NOVEL COVID-19 STRESSOR?

Maria Kurth, Heidi Igarashi, Hye Soo Lee, Soyoung Choun, and Carolyn Aldwin, *Oregon State University, Corvallis, Oregon, United States*

Despite higher physiological vulnerability to stress, older adults may accumulate resources through prior experiences that can promote resilience (Aldwin & Igarashi, 2016). During the COVID-19 pandemic, older adults drew on prior experiences and resources to cope (McKinlay et al., 2021; Herron et al., 2021), although these events were typically not specified. Some found vulnerability due to prior trauma (Galica et al., 2021). We examined whether older adults drew upon specific experiences or more general resilience resources in coping with this novel stressor. Data were collected using an online survey from April 28-May 4, 2020 from 235 older adults in Oregon (Mage = 71.35, SD = 7.39; 74% female; 92% White). We examined open-ended responses from a question that asked whether prior experiences influenced how they were dealing with the COVID-19 situation. Nearly 2/3 provided valid responses (n=144). After inductive open coding, preliminary consolidation resulted in three broad categories: past experiences (74%), resources (19%), and both (8%). The most common prior experiences were illness (n = 20) and work (n = 19). Some (n=10) reported specific coping strategies learned during prior stressful experiences. Resources include personal characteristics (e.g., being "introverted" or "resilient"), financial ("financially secure") and social resources ("loving spouse"). Five reported experiences that made COVID-19 more difficult ("PTSD/anxiety prior to COVID-19 makes this even worse"). Although 1/3 of the sample could not draw upon a prior experience in coping with this novel stressor, many older participants could utilize their lived experience when coping with problems during the COVID-19 pandemic.

### CHANGES IN LIFE SATISFACTION DURING THE FIRST YEAR OF THE COVID-19 PANDEMIC: A LONGITUDINAL STUDY OF JAPANESE ADULTS

Takeshi Nakagawa, Taiji Noguchi, Ayane Komatsu, Sayaka Okahashi, and Tami Saito, *National Center for Geriatrics and Gerontology, Obu, Aichi, Japan*

The outbreak of the novel coronavirus (COVID-19) has exerted a major impact on daily life. Despite older adults being at a higher risk of severe illness from COVID-19, research has indicated that older adults experienced the same or even higher levels of well-being during the early phase of the pandemic than younger adults. To investigate adverse long-term effects, we examined how individuals' well-being changed during the first year of the pandemic. A total of 5,281 Japanese adults aged 15—89 years participated in an online survey in February 2020 (before the first state of emergency) and were followed up in March 2021 (during the second state of emergency; follow-up rate 54.7%). Well-being was assessed as a one-item indicator of life satisfaction, with a range of 0 - 10. Counterintuitively, results of a latent change score model demonstrated that life satisfaction increased over time (unstandardized coefficient of 1.17, SE = 0.16, standardized coefficient of 0.58). Individuals aged 64 years and below, with low levels of education, living alone, and perceiving worse health and economic status showed less increase in life satisfaction. In an additional analysis, those more concerned about the pandemic at follow-up displayed less increase. The findings suggest that most people did not exhibit a decrease in life satisfaction during the first year of the COVID-19 pandemic. However, certain individuals could be vulnerable to the outbreak due to the lack of resources. Researchers should better understand the factors that drive the resilience of older adults in the face of adversity.

### COGNITIVE COPING AND PERSPECTIVE-TAKING AMONG OLDER ADULTS DURING THE COVID-19 PANDEMIC: AN ECOLOGICAL INVESTIGATION

Kristin Heideman[1], Eric Griffith[2], Daniel O'Connor[3], Irina Orlovsky[3], Elizabeth Alwan[3], and Bruna Martins-Klein[4], *1. Yale Child Study Center, New Haven, Connecticut, United States, 2. Duke University, Durham, North Carolina, United States, 3. University of Massachusetts Amherst, Amherst, Massachusetts, United States, 4. University of Southern California, Los Angeles, California, United States*

In response to the COVID-19 pandemic, older adults report engaging in coping to manage sources of pandemic stress (e.g., loneliness, reduced healthcare access, ageism). Variability in cognitive coping strategies, however, has not been explored through an ecological model lens. In this study, we interviewed older adults online in the northeastern US (N=33, age 63-92) regarding stress and coping strategies during the pandemic, using the taxonomy of cognitive strategies from the Cognitive Emotion Regulation Questionnaire. Consistent with Bronfenbrenner's ecological model, stress and cognitive coping were qualitatively coded using NVivo in reference to individual, immediate environment (microsystem), connections between environments (mesosystem), and societal (macrosystem) factors. Microsystem-level stressors were prevalent and endorsed in all interviews, but stress was reported at multiple ecological levels. Common microsystem stressors included changes to work/personal life, travel restrictions, and loss of face-to-face socialization. Many participants reported engaging in adaptive cognitive coping (87%), with perspective-taking being one of the most common strategies (78%). Perspective-taking was endorsed across all levels of the ecological model (e.g., comparing the pandemic to other personal experiences, considering family-level resilience, and reflecting on impact to community). The multi-level structure of pandemic stress and coping highlights that older adults subjectively experience stress simultaneously at multiple ecological levels. In turn, they confront stressors using individualized patterns of cognitive coping that extend beyond intrapersonal experience/insight to help fully contextualize the COVID-19 experience. Future studies should explore the use of perspective-taking at intra- and interpersonal levels as integrated coping approaches for managing naturalistic stressors in daily life.

### FUTURE TIME PERSPECTIVE MODULATES AFFECTIVE REACTIVITY TO SOCIAL PROBLEMS DURING THE PANDEMIC

Elizabeth Zambrano Garza[1], Yoonseok Choi[1], Theresa Pauly[2], Denis Gerstorf[3], and Christiane Hoppmann[1], *1. University of British Columbia, Vancouver, British Columbia, Canada, 2. University of Zurich, Zurich, Zurich, Switzerland, 3. Humboldt Universität zu Berlin, Berlin, Berlin, Germany*

Socioemotional Selectivity Theory posits that individuals with a limited future time perspective (FTP), prioritize emotionally meaningful, positive social interactions. Due to the high value placed on positive social interactions, individuals with a limited FTP might be particularly vulnerable to experiencing elevated negative affect when problems that involve other people do occur as compared to an experience of non-social problems. This project examined the role of FTP in modulating social problem–negative affect links during the pandemic, a time when people were particularly aware of their mortality, and thus their remaining time in life. This study used data from 150 Canadian adults (Mage =43 years, SD= 19, range 18-83, 78% women) who participated in two measurement bursts (one in 2020 and one in 2021). Participants provided FTP information at baseline and then repeatedly reported their negative affect and everyday problem characteristics in 10 consecutive daily diaries across both bursts. Results from multi-level models reveal a differentiated picture. The within-person association between social problem occurrence and elevated negative affect was significant among individuals with a more limited FTP. In contrast, at the between-person level, experiencing more social problems was associated with higher overall negative affect among those with a more extended FTP. To better understand these differential associations, follow-up analyses will explore if type of person involved in the problem and how the problem was handled matter for how they respond to social problems. Findings will be discussed in the context of the Strength and Vulnerability Integration model.

### LONG-TERM POSITIVE AND NEGATIVE CONSEQUENCES OF THE COVID-19 PANDEMIC AMONG OLDER ADULTS

Carolyn Aldwin[1], Soyoung Choun[1], Maria Kurth[1], Hye Soo Lee[1], Dylan Lee[2], and Heidi Igarashi[1], *1. Oregon*

*State University, Corvallis, Oregon, United States, 2. Touro University Nevada, Henderson, Nevada, United States*

Despite their greater physiological vulnerability, community-residing older adults have shown surprising psychological resilience, at least at the beginning of the COVID-19 pandemic. However, a handful of reports suggest that older adults' well-being has decreased after a few months, although others have suggested a recovery after a year (Schlomann et al., 2021). The purpose of this study was to examine change in change from baseline (April-May, 2020) to a 13- month follow-up (June, 2021). We analyzed data from 162 older adults with complete data at both time points. Mean age at baseline was 72, SD = 7.6, range = 51-96; 71% were female, 13% were minorities; 74% were married, 71% retired, and most (85%) had at least a BA. Linear modelling showed that there were only marginal increases in the number of problems across time (B = .25, p = .08), but their severity did not increase. There were no significant changes in depression, anxiety, loneliness or physical symptoms over this time period. The modest increase in problems may have been offset by an end to being in lockdown and an increase in social contacts which doubled over this time period, B = 1.65, p <.001). More troubling was that self-reported resilience decreased, B = -.92, p < .01), as did the ability to perceive positives in this situation, B = -2.46, p < .01), and self-reported cognitive problems increased, B=.67, p < .01). Thus, the results showed decidedly mixed effects, suggesting individual differences in long-term adaptation to COVID.

### SOCIAL CONNECTION IN OLDER ARKANSANS DURING COVID-19: CHANGES AND SATISFACTION

Robin McAtee[1], Jennifer Peszka[2], Anne Goldberg[2], Laura Spradley[1], Noura Musallam[2], Lauren Allen[2], Sophie Vogle[2], and Luke Simpson[2], *1. University of Arkansas for Medical Sciences, Little Rock, Arkansas, United States, 2. Hendrix College, Conway, Arkansas, United States*

Social support is important to the health and well-being of older adults. Changes in social support (both frequency and satisfaction with) and barriers to social support during the pandemic remain unknown. During Spring 2021 an automated phone survey of adults 65 and older in Arkansas was completed. Calls were placed to 27,296 households where 8,745 individuals answered, and 867 (N=723 White, non-Hispanic) older Arkansans completed the 18 question self-report survey. Results indicated that: 41% had curtailed their in-person interactions significantly; 61% continued to engage in in-person social interactions outside of their home once a week or more; and social group activities decreased from 52% to 41%. Additionally, it was reported that 88% used technology for social interactions once a week or more and 60% reported technology interactions made them feel socially connected. Despite this, only 67% reported being satisfied with their social connection during the pandemic compared to 93% prior to the pandemic. While many participants continued in-person social interactions, social activity and satisfaction decreased during the pandemic. Social technology alternatives were used by many and for some, social connection was reported to be satisfactory. The value of what was learned from this survey has application outside pandemic times. Understanding and acknowledging that social isolation exists for older adults in normal times and improving technological access to social activities has great value. This knowledge can be used to substantiate the expansion and improvement of older adult friendly virtual platforms therefore contributing to reducing social isolation.

### HOW CAREGIVERS AND OLDER US VETERANS MANAGED SOCIAL ISOLATION DURING THE COVID-19 PANDEMIC

Leah Haverhals[1], Roman Ayele[2], and Hillary Lum[3], *1. VA Eastern Colorado Health Care System, Rocky Mountain Regional VA Medical Center, Denver, Colorado, United States, 2. VA Eastern Colorado Health Care System, Aurora, Colorado, United States, 3. University of Colorado Anschutz Medical Campus, Aurora, Colorado, United States*

To protect themselves from contracting the SARS-CoV-2 virus, many older adults managing multiple medical conditions experienced increased social isolation. The objective of our qualitative research study was to describe how older Veterans receiving care from the United States (US) Department of Veterans Affairs (VA) healthcare system, and their caregivers, managed increased social isolation during the pandemic. We recruited Veterans and their caregivers residing in rural and urban areas who received care from either a tele-palliative care or a tele-geriatrics clinic connected to one VA Medical Center, inviting them to participate in phone interviews. From May-September 2021, we interviewed N=23 participants (n=9 Veterans and n=14) caregivers. We applied a deductive and inductive approach to thematic analysis to analyze interview data. Findings revealed that while caregivers experienced increased anxiety, which they attributed to pandemic-related changes, they also expressed solidarity in that others were experiencing similar stressors. Many caregivers and Veterans shared experiences of increased loneliness, which some found difficult to manage as communication with their social networks was sparse. At the same time, the pandemic made them value relationships with others more than before. Some Veterans noted they kept busy with hobbies and did not feel much loneliness despite increased isolation. Caregivers caring for Veterans with dementia stated they experienced confusion about their narrower social networks because they could not remember reasons why they were not regularly spending time with them. Findings demonstrate the need to identify strategies and policies to better support caregivers and older Veterans during times of crisis.

### FACTORS ASSOCIATED WITH NURSING HOME DIRECT CARE PROFESSIONALS' TURNOVER INTENT DURING COVID-19

Verena Cimarolli[1], Natasha Bryant[1], Francesca Falzarano[2], and Robyn Stone[1], *1. LeadingAge, Washington, District of Columbia, United States, 2. Weill Cornell Medicine, Douglaston, New York, United States*

The negative effects of the COVID-19 pandemic on the well-being of direct care professionals (DCPs; nursing assistants and aides) in nursing homes (NHs) has led to high rates of DCPs' turnover and staff shortages - both issues that were already prevalent before the pandemic. More optimal staffing levels and less turnover are essential for optimal NH quality of care, but little is known about factors associated with turnover in DCPs in NHs during the pandemic. Hence, the purpose of this study was to examine the mediating roles of 1) quality of employer communication related to COVID-19; 2) DCPs' perceived preparedness to care for residents with COVID-19; and 3) DCPs' job satisfaction in the relationship between overall COVID-19-related work stress and intent to remain in one's job (an indicator of turnover). Path analyses (N=809) demonstrate a significant, indirect effect between COVID-19-related stress and intent to remain in one's position through the variables of communication, preparedness, and job satisfaction. Higher levels of COVID-19 related stress were associated with poorer communication quality, lower levels of preparedness, and lower job satisfaction, which was subsequently associated with a reduced likelihood of intent to remain in one's job. However, direct effects show that better communication quality was associated with better preparedness correlating with higher job satisfaction which increased the likelihood of intent to remain in one's job. Findings underscore the importance of employer supports in DCPs' job satisfaction and turnover in NHs and, thus have implications for how to improve quality of care in NHs.

### SESSION 6220 (POSTER)

### AGHE POSTER SESSION II

Explore how education, evaluation, and related efforts in gerontology can inform community connections and practices.

### COLLABORATIVE PARTNERSHIP BETWEEN STATE AGENCIES AND ACADEMIA ADDRESSING LONG-TERM CARE WORKFORCE NEEDS

Katie Ehlman, Karla Diekemper, and Kevin Valadares, *University of Southern Indiana, Evansville, Indiana, United States*

The rural areas of southwestern Indiana are challenged with insufficient healthcare workers in the long-term care industry. The University of Southern Indiana (USI) Geriatric Workforce Enhancement Program (GWEP) is collaborating with state agencies to address shortages in licensed administrators, activity professionals and social service designees. These roles, vital in the nursing facility, hold educational requirements through the Indiana State Department of Health (ISDH) and the Indiana Professional Licensing Agency (IPLA). USI gerontology faculty addressed workforce challenges in five phases: 1) Evaluation of state educational requirement and comparison to current USI gerontology courses; 2) Development of new coursework to support state requirements; 3) Expansion of preceptor pool; 4) Proposal to state agencies; 5) USI curriculum modifications. Faculty evaluated the educational and training requirements for these roles and compared the requirements with current courses. Preceptors were needed for students' field experiences; a preceptor pool was developed through funded training. The requirements and course alignment were included in the program proposal to the state agencies. Upon ISDH, IPLA and University New Program Development Committee approval, additional instructors were recruited. Results of this initiative include a: 1) Fully approved and launched Administrator-in-Training (AIT) residency embedded in curriculum at undergraduate and graduate level; 2) Fully approved Activity Professional Certificate Program; 3) Pool of 10 certified preceptors; and 4) Onset of the social service designee certificate program. This collaborative partnership developed between USI, ISDH and IPLA ensure state agency requirements are met academically for students entering the long-term care workforce.

### DEMENTIA CARE COORDINATION FOR CAREGIVERS OF VETERANS DIAGNOSED WITH DEMENTIA: AN INNOVATIVE VA INITIATIVE

Judith Howe[1], Eve Gottesman[2], and Nicholas Koufacos[2], *1. Ichan School of Medicine at Mount Sinai, Bronx, New York, United States, 2. James J. Peters VA Medical Center, Bronx, New York, United States*

There are expected to be 335,425 Veterans with dementia by 2033. Dementia has a significant impact on families, especially caregivers. Caregivers often report high levels of stress and burden, which can cause them to develop physical and mental illness, impacting the care they provide to family members with dementia. In 2021, under a Veterans Health Administration (VHA) directive, the Bronx VA Geriatric Research, Education and Clinical Center developed a Dementia Care Coordination program, funded by the VHA Office of Rural Health sponsored Geriatric Scholars Program. Led by a Dementia Care Coordinator (DCC), who is also a clinical social worker, the program identified caregivers of Veterans with dementia who reported experiencing stress and burnout while caring for Veterans with dementia. The DCC provided support via telephone and VVC and also shared resources focused on stress reduction, relaxation techniques, and coping with grief. During 2021, 23 caregivers of diverse racial and ethnic backgrounds were enrolled in the program and 17 were found to have significant caregiver burden. Over 100 support calls were made on the topics of stress reduction, problem solving and community resources. Common problems reported by caregivers included sundowning, sleep problems, grief, and increased social isolation due to the COVID-19 pandemic. Caregivers showed a willingness to participate in the program, indicating that the information, resources and support provided by the DCC helped reduce their stress levels and allowed them to provide ongoing care. Recent program developments, findings from an IRB approved survey of participants, and expansion plans will be reported.

### TRAINING PHYSICIAN-SCIENTISTS IN SOCIAL AND BEHAVIORAL SCIENCE: INDIANA ADRD MEDICAL SCIENTIST TRAINING PROGRAM

Nicole Fowler[1], Brittney-Shea Herbert[1], Christopher Callahan[2], Siyun Peng[3], Brea Perry[3], Karmen Yoder[1], Gary Landreth[1], and William Truitt[1], *1. Indiana University School of Medicine, Indianapolis, Indiana, United States, 2. Indiana University School of Medicine, IU Center for Aging Research, Indianapolis, Indiana, United States, 3. Indiana University, Bloomington, Indiana, United States*

There is a critical need to grow and strengthen the pipeline of physician scientists who have expertise in sociomedical and behavioral research and are dedicated to addressing the nation's challenges posed by Alzheimer's disease and related dementias (ADRD). In 2021 The Indiana ADRD Medical Scientist Training Program (IADRD MSTP) was designed to meet this need and is built on the infrastructure of a robust portfolio of ADRD research, graduate training programs in medical neurosciences and sociology, and our existing MD-PhD program at Indiana University School of Medicine. The Aims of the IADRD MSTP are: 1) To recruit and train a competitive pool of diverse students who have an interest and commitment to social and behavioral research and patient care focused on ADRD; 2) To engage MD-PhD students early in mentored sociomedical and behavioral research that integrates IUs systems-based medical training curriculum with our cutting edge ADRD research that reinforces commitment and minimizes attrition of physician-scientists ADRD; and 3) To graduate students with dual MD-PhD degrees with strong methodological training in social and behavioral science and experts in ADRD who will be successful independent investigators at the best academic medical centers nationwide. The program includes rigorous didactic training in social, behavioral, and clinical research methods, with flexibility to allow students to focus their effort on one methodological area of interest; early initiation of ADRD research experiences with multidisciplinary teams of mentors and advisors; and the provision of educational experiences that enhance students' abilities to become independent researchers.

### ASSOCIATION BETWEEN EDUCATION LEVEL AND DEPRESSIVE SYMPTOMS AMONG COMMUNITY-DWELLING OLDER ADULTS IN ICELAND

Milan Chang Gudjonsson[1], Chiharu Nishizuka[2], and Sigurveig Sigurdardottir[2], *1. Norwegian Institute of Public Health, Oslo, Norway, 2. University of Iceland, Reykjavik, Iceland*

Objectives. Previous research studies have suggested that a higher education level has a positive impact on one's health throughout life. The aim of this study was to examine the association between education level and depressive symptoms among community-dwelling older adults aged 65 years and older in Iceland. Method. This is a cross-sectional study using data from the Age, Gene/Environment Susceptibility–Reykjavik Study (AGES–RS) cohort (n = 3,989, mean age = 76.2, 56.3% women). Education was classified into four levels: primary (n = 895), secondary (n = 1,980), college (n = 630), and university (n = 484). For the analysis, education was categorized into two groups: primary education versus higher than primary education (secondary, college, and university). Depressive symptoms were assessed by using the 15-item Geriatric Depression Scale. High depressive symptoms (HDEPS) were defined if the GDS score was 6 or higher. Results. Education was highly significantly associated with the 15-GDS score ($p < .001$). Compared to people with primary education, those with higher than primary education level were significantly less likely to have HDEPS after adjusting for confounders including age, gender, body mass index, physical activity, coronary diseases, and smoking status. Discussion. Older adults with higher than primary education were less likely to have HDEPS compared to those with primary education in the current cross-sectional analysis. The findings indicate that level of education is highly associated with depressive symptoms of community-dwelling older adults in Iceland.

### UTILIZING COMMUNITY ANALYSIS TO INCREASE AWARENESS OF HEALTH DISPARITIES IN OLDER OKLAHOMANS

Donna Sylvester, *Langston University, Drumright, Oklahoma, United States*

Where we live affects us. The state of Oklahoma consistently ranks in the bottom ten percent for health outcomes, health policies, and health behaviors of older adults. Physical therapy students in Oklahoma must recognize the impact that social determinants of health have on older adults in order to fully meet the needs of their future patients. Prior to final clinical experiences, third-year Doctor of Physical Therapy students explore these concepts through individual community analysis projects in urban and rural communities across the state. The students visit their chosen community to assess access to transportation services, food, housing, and healthcare-specific to older adults. Additionally, students explore opportunities for exercise classes targeted toward older adults and the walkability of the community. Students come away with a better understanding of the wide array of social determinants of health experienced by older adults in Oklahoma and learn of opportunities to advocate for future patients in their communities.

### AGING AGENCIES' AND PROVIDERS' REASONS FOR HOSTING OR NOT HOSTING GERONTOLOGY INTERNS

Rona Karasik[1], Phyllis Greenberg[2], Maria Kroeber[1], Jessica VanderWerf[1], and Claudia Burgos Zuniga[1], *1. St. Cloud State University, St. Cloud, Minnesota, United States, 2. St. Cloud State University, St Cloud, Minnesota, United States*

Hands-on experience is essential to gerontological education and associated with numerous student benefits. Much less is known about the community agency perspective, including why they do/don't host gerontology interns. In this national study (n=281), non-profit, for-profit, and government aging-service providers (e.g., Social Services, Health Care, Housing) were surveyed regarding their perceptions of internship benefits, challenges, and areas to improve. Unique to this study is the inclusion of both hosting (n=129) and non-hosting (n=142) agencies. Hosts' reasons for accepting interns included: share experience/knowledge (n=81, 87%); future university relationships (n=71, 76%); intergenerational interaction for students (n=69, 74%) and clients (n=60, 65%); attract future employees (n=68, 73%); and fills unmet need at agency (n=47, 50%). Non-host reasons for abstaining included: lack of applicants (n=58, 41%); never considered it (n=40, 28%); insufficient staffing (n= 37, 26%); unsure where to start (n=34, 24%) or find/attract interns (n=34, 24%), and too time-consuming (n=25,18%). Additional reasons for nonparticipation included: lack of space; insufficient intern-suitable work; risk/liability; and agency policy. Both hosting and non-hosting agencies anticipated/realized student-related challenges (e.g., limited motivation, inconsistent availability, inadequate preparation) and agency-based factors (e.g., insufficient time, staffing). Given the ongoing need for suitable gerontology internship sites and the finding that almost half of the non-hosting agencies indicated possible future interest in accepting gerontology interns (n=80, 49%), attention to agency recommendations (e.g., increased faculty communication/feedback, stronger student commitment/preparation, recognition of agency contributions) is warranted. Strategies for addressing identified challenges and implementing requested changes to enhance the community agency experience with gerontology interns are discussed.

### CRAFTING AN UNDERGRADUATE CONCENTRATION IN AGING ACROSS THE UNIVERSITY AND ASSESSING COMPETENCIES

Kristine Mulhorn, *Drexel University, Wallingford, Pennsylvania, United States*

In the last few years, our Health Administration Department has grown its undergraduate offerings in aging and now is promoting those courses as a concentration available for any major. A recent collaborative effort between our college and the honors college, our university has created a university-wide set of courses across all majors related to topics in aging. In our university, students can pursue a concentration with as few as three courses that are relevant to their majors. Usually, the courses in a concentration are taught by those in the major. However, the global issue of aging can be taught as a specialty area for many majors. To ensure students meet the learning objectives, the following assessment plan was developed for three key courses offered by the Health Administration Department—Healthy Aging, Aging and the Law and either Aging in a Global Context or Aging Services Management. First, a curricular map of the areas of competency within our program are outlined and then the content and areas of assessment are considered based on whether the competencies are "Introduced", "Reinforced," and "Mastered." Next, the key assignments in each course within the concentration were aligned with the Program Objectives for the Health Administration bachelor's program to assess student achievement of key competencies. In the process of offering the concentration in Aging across multiple majors, it is important for various departments to understand how courses in aging add valuable competencies regardless of their major.

### FLINT INTERGENERATIONAL LIVING EXPERIENCE

Jennifer Blackwood, and Elizabeth Yost, *University of Michigan-Flint, Flint, Michigan, United States*

Previous studies indicate that students who have a formative experience with an older adult, in a personal or professional setting, are more likely to choose to work in geriatrics. With a growing population of older adults, the need for empathetic health professionals who desire to work in geriatrics is needed. In Fall, 2021 four graduate students (2 physical therapy, 2 occupational therapy) moved into a senior housing community in Flint, Michigan. From that moment, the Flint Intergenerational Living Experience (FILE) began. The purpose of FILE is to mold these students into empathetic future health professionals who choose to work in geriatrics within their careers. These four twenty-something year-olds live, learn, study, eat, and share life with their senior neighbors with the hope of gaining a better understanding of aging issues of older adults who chose to remain in Flint. In exchange for completing service hours, the FILE students live in an apartment complex rent-free. This presentation will describe the activities performed, outcomes achieved, and perceptions of the FILE program after one-year of being in the program. Based on data gathered within the first year of the program, activities that impacted the personal growth of the students to understand the impact of aging in an urban setting will be described. The influence of loss (e.g. death, physical abilities) and the framework of rehabilitation needs of the urban dwelling older adults will be discussed. Lastly, strengths and weaknesses of this lived experience will be presented.

### LONGITUDINAL ASSOCIATION BETWEEN HOSPITALIZATION AND TRANSFER TO RESIDENTIAL CARE FACILITIES AMONG OLDER ADULTS

Reza Amini[1], and Amat Sidhu[2], *1. University of Michigan-Flint, Ann Arbor, Michigan, United States, 2. University of Michigan-Flint, Flint, Michigan, United States*

Dementia increases the risk of post-hospitalization transfer to residential care facilities (RCF), increasing mortality risk. The risk of falling is significantly higher among people with dementia, leading to more hospital admissions (HA). This study examines the association between HA and transferring to RCF controlling for dementia and falling. This secondary data analysis used nine years of data from the National Health and Aging Trends Study (2011-2019) with 2,548 participants (each wave). Transfer to RCF was measured based on the residency status, HA and history of falling by survey questions, and dementia (probable, possible, no dementia) by combining Clock Drawing Test, word recall test, and previous dementia diagnosis. Falling increased the risk of transition to RCF by 31%; however, this association was not statistically significant. Dementia was a significant predictor for transition to RCF (Odds Ratio (OR)=3.889). Transferring to RCF was significantly associated with HA (OR=1.835); after including falling, OR increased to 2.067, showing falling moderates this association. Including dementia into the model dropped HA's OR to 1.620. The interaction between falling and dementia showed that frequent HA could increase the risk of transition to RCF by 61%. Probable dementia plus a history of falling can increase the risk of transition to RCF by 22 times and 14 times for those cases of probable dementia without a history of falling. These results may reflect the necessity of screening for the risk of falling and dementia among community-dwelling older adults to prevent falling, hence, frequent HA and ultimately transition to RCF.

### ADDRESSING COVID-19 HEALTH DISPARITIES THROUGH VIRTUAL EDUCATION AND COLLABORATION

Kelly Boyd, Jacob Walker, Jenil Benett, Jeffrey Graupner, and Katherine Thompson, *University of Chicago, Chicago, Illinois, United States*

The South Side of Chicago, made up of predominantly Black and Latinx neighborhoods, has higher rates of COVID-19 infection and death rates and lower COVID-19 vaccination rates than other areas of Chicago. People aged 65 and older comprise nearly 15% of the region's population. The SHARE Network at the University of Chicago has a long history of providing healthy aging education to organizations serving older adults on Chicago's South Side. Using this pre-existing curricular structure, investigators created a monthly virtual workshop for community members to address COVID-19 related health disparities for older adults. Topics address aging concerns (i.e. dementia, mobility) and pandemic concerns (i.e. health disparities, vaccine hesitancy). At the end of the sessions, participants can ask questions to experts and share resources. Attendance has ranged from 15-20 participants, predominantly formal and informal caregivers. In post-attendance surveys, 80% of respondents identified as Black or African-American. Of the 20 respondents, 95% felt more knowledgeable about the topics discussed and all felt more knowledgeable about local health resources. Three-quarters felt more confident in the caregiving role, and 95% planned to make healthy lifestyle changes to benefit themselves or a loved one (i.e. more exercise, seeking respite care). These results demonstrate the positive impact of a timely community-informed virtual workshop. Providing virtual education on COVID-19 and healthy aging directly to older adult-focused community organizations and their clients is an early step health systems can take to address health disparities.

### DEVELOPMENT OF PODCAST IN SERVICE OF THE NEXT GENERATION OF GEROPSYCHOLOGISTS

Branden Schaff[1], Rachel Weiskittle[1], and Lindsey Jacobs Dodson[2], *1. University of Colorado Colorado Springs, Colorado Springs, Colorado, United States, 2. University of Alabama Tuscaloosa, Tuscaloosa, Alabama, United States*

The current geropsychology workforce falls woefully short of meeting the mental healthcare needs of older adults, with only 3% of licensed psychologists identifying Geropsychology as their primary or secondary specialty. Formal training in geriatric mental health is extremely limited. Out of the over 400 APA-accredited doctorate-level psychology programs in the U.S., only five provide specialty training in Geropsychology. Consequently, the vast majority of psychologists who treat older adults have never received formal education or training in geriatrics. It is imperative that evidence-based geropsychology education is disseminated to a wide audience of mental health providers across all levels of training and career development. The Geropsychology Podcast was developed to address this gap as a novel pedagogical resource commensurate with educational competencies established by the 'Pikes Peak Model for Professional Geropsychology.' Topics include geriatric assessment methods, intervention, consultation, and foundational knowledge on adult development and aging. The feasibility and acceptability of the podcast was evaluated with a two-year pilot study from 2019 to 2021. During this time, eleven episodes were published across multiple podcast platforms. Preliminary results found the episodes were downloaded by over 1,500 unique listeners across 34 countries. These results indicate that The Geropsychology Podcast is scalable, acceptable, and potentially fulfilling an educational need that people are actively seeking. This poster describes the podcast's model of curriculum development and ongoing quality metrics and impact analysis. Future work holds that this podcast will continue to be a vital resource for the field.

### DOES THE NUMBER OF DISEASES OR DISEASE SEVERITY AFFECT THE AGING ATTITUDES IN HEALTH RETIREMENT STUDY?

Da Jung Chang, *Miami University, Oxford, Ohio, United States*

The number of older adults is increasing worldwide, coupled with an increase in chronic disease. Evidence has shown a relationship between health conditions and changes in attitudes toward own aging (ATOA) and thus may contribute to a negative ATOA among older adults with more health conditions. Additionally, studies also show that ATOA can affect the health of older people and become a vicious circle. To promote a healthier life for older adults, it is important to assist them to maintain a more positive ATOA. Therefore, the purpose of this study wants to determine the relationship between the diseases number and ATOA for older adults (N = 6303). Although the relationship between the number of the diseases has been discussed in previous studies, this study will use 2012 and 2016 waves Health and Retirement Study data to update the relationship by using generalized estimating equation. To understand whether it is the disease severity that affects ATOA, this study uses Charlson Comorbidities Index to measure the burden of diseases and use structural equation models to understand the relationship between variables and do the model pathway analysis. The initial results show that an individual with a greater number of diseases ($p < .01$, $r=-.039$), and a higher score of CCI have more negative ATOA ($p < .01$, $r=-.04$). These findings suggest both the number and severity of diseases contribute to ATOA in older adults. Furthermore, the CCI provides a method to identify the disease severity and can be used in the national databases.

### OPTIMIZING OLDER ADULT BLOOD PRESSURE SCREENING IN A COMMUNITY SETTING BY INTERPROFESSIONAL STUDENTS

Erin Miller[1], Rima Charara[2], Ali Turfah[3], and Jennifer Mendez[4], *1. Wayne State University School of Medicine, Detroit, Michigan, United States, 2. Wayne State University Eugene Applebaum College of Pharmacy and Health Sciences, Dearborn, Michigan, United States, 3. University of Michigan School of Public Health, Ann Arbor, Michigan, United States, 4. Wayne State University, Toronto, Ontario, Canada*

A novel program was implemented in an effort to optimize the blood pressure taking skills of medical and pharmacy students, in collaboration with older individuals in Detroit. Under the supervision of Interprofessional faculty, pharmacy and medical students provided blood pressure screenings at the popular Riverwalkers program, a community exercise program designed for older Detroiters. Prior to volunteering at the program, students were required to complete the AMA blood pressure modules to develop proficiency in blood measurement best practices. Over the course of two months,

72 students provided blood pressure screenings for 79 older individuals during 266 distinct encounters. We believe this program can serve as a model for other professional schools, as it efficiently accomplishes several distinct

### PAEAN: PAIN IN AGING, EDUCATIONAL ASSESSMENT OF NEED: OPIOID DIAGNOSES AND IMPACTS ON PAIN DIAGNOSIS IN OLDER ADULTS

Beth Hogans[1], Bernadette Siaton[2], Julie Witter[2], Leslie Katzel[3], Lana Brown[4], Laura Frey-Law[5], and John Sorkin[6], *1. VAMHCS/Johns Hopkins School of Medicine, Baltimore, Maryland, United States, 2. UMB, Baltimore, Maryland, United States, 3. VAMHCS/UMB, Baltimore, Maryland, United States, 4. Central Arkansas Veterans Healthcare System, North Little Rock, Arkansas, United States, 5. Carver School of Medicine, University of Iowa, Iowa City, Iowa, United States, 6. UMB/VAMHCS, Baltimore, Maryland, United States*

Pain is prevalent in older adults of both genders but more so in women. The extent to which this is impacted by opioids is not well established. We sought to evaluate common pain diagnoses among older adults noting whether the pattern of female preponderance holds across all conditions and whether opioids might impact this. Utilizing the 2017 Medicare 5% standard analytical claim sample we examined the rate of pain condition diagnoses in older females and males with or without opioid-related diagnoses. We have previously reported that low back pain and opioid diagnosis coding rates interact in older adults, we here expand this finding. We established a condition definition file for opioid use, not including overdose-related codes. We segmented the older adult Medicare part B claims file into those with and those without opioid diagnoses, separated by CMS-coded gender. Diagnostic rates of the 100 most common pain diagnoses in each of the subpopulations showed low back pain was the most prevalent pain diagnosis regardless of gender or opioid diagnosis status. Chest pain unspecified, neck pain, and abdominal pain unspecified, were also highly prevalent. All pain conditions were diagnosed at higher rates in the opioid subpopulation. Few diagnoses were more common in males, these included sex-specific conditions, diabetic neuropathy, and vascular claudication. Although pain conditions are common in older adults, diagnosis rates are elevated in those with opioid diagnoses. This impacts the educational needs of geriatricians, and other healthcare providers, and may indicate needs for more research in pain and aging.

### IMPLEMENTING A HEALTHY BRAIN INITIATIVE IN THE NATION'S LARGEST COUNTY JURISDICTION

Tony Kuo[1], Noel Barragan[2], Mariana Reyes[2], and Ariane Thomas[2], *1. University of California, Los Angeles, Los Angeles, California, United States, 2. Los Angeles County Department of Public Health, Los Angeles, California, United States*

In 2019, there were an estimated 166,857 Los Angeles County (LAC) residents living with Alzheimer's disease. By 2040, this number is expected to rise by 150% to 405,382, with 152,980 and 35,341 of them, respectively, being Black and Latinx. Locally, there is special interest in reducing dementia risk among the two communities, as modifiable risk factors such as untreated hypertension, diabetes, and smoking are disproportionately higher in these groups (e.g., hypertension – Black, 35.9% vs. White, 27.3%; diabetes – Black and Latinx, 14.4% and 13.6%, respectively, vs. White, 8.8%). In LAC, considerable investments have been made to help create and implement the local arm of the California Healthy Brain Initiative (Healthy Brain Initiative: Los Angeles or HBI-LA). Launched in 2019, HBI-LA utilized a multi-level, multi-sector approach to expand the breadth and quality of tools available to the Black and Latinx communities and key stakeholders who work with these communities. The initiative set out to complete the following State-approved objectives: (i) educate the public and health professionals about dementia care and prevention; (ii) develop policies that improve access to health, aging, and prevention services for those with cognitive decline; and (iii) disseminate reliable, culturally sensitive information and tools to promote best practices on brain health. The present investigation examines the stepwise process that LAC took to achieve these objectives, including the development of two culturally tailored educational workshops (one for community members, one for healthcare and social services professionals) and how a U.S. Congressperson and County Supervisor supported dissemination efforts.

### ADAPTING PRIMARY CARE WORKFLOWS TO PROMOTE ADVANCE CARE PLANNING WITH AN EMPHASIS ON A DEDICATED ACP FACILITATOR

Kevin Valadares[1], Suzanne Leahy[1], Katie Ehlman[1], and Robin Arnold[2], *1. University of Southern Indiana, Evansville, Indiana, United States, 2. Deaconess Clinic, Evansville, Indiana, United States*

The COVID-19 pandemic has sharpened attention regarding the need to proactively plan for a future medical crisis. Advance Care Planning (ACP) has emerged in the last 30 years as a potential way to improve individuals' end-of-life care by ensuring that patients explore and communicate personal values, goals, and preferences regarding future medical care to surrogate decision makers and medical providers. Elevating ACP as a core function of direct patient care, the University of Southern Indiana's (USI) Geriatric Workforce Enhancement Program (GWEP) embedded a multi-modal ACP initiative in a primary care clinic, anchored by the Medicare Annual Wellness Visit (AWV). Within this initiative, the role of a dedicated ACP Facilitator embedded in the practice is highlighted to promote and bill (Medicare CPT codes 99494, 99498) for ACP conversations. The addition of an ACP Facilitator (who is a licensed clinical social worker) as part of the primary care team complements the efforts of the providers to focus on What Matters most to patients. This is particularly important to patients with serious illness. This presentation will summarize the re-alignment efforts of the primary care clinic to prioritize ACP conversations for older adults, amounting to 10,000 visits per year.

### ADVANCING LATE-LIFE TRAUMA-INFORMED CARE EDUCATION: DEVELOPMENT AND EVALUATION OF AN EDUCATIONAL PODCAST

Rachel Weiskittle[1], Lola Baird[2], Hannah Bashian[3], Anica Pless Kaiser[4], Kelly O'Malley[5], Anna Etchin[4], and Jennifer Moye[5], *1. University of Colorado Colorado Springs, Colorado Springs, Colorado, United States, 2.*

*Boston VA Medical Center, Jamaica Plain, Massachusetts, United States, 3. VA Boston HCS, Boston, Massachusetts, United States, 4. VA Boston Medical Center, Jamaica Plain, Massachusetts, United States, 5. VA Boston Healthcare System, Boston, Massachusetts, United States*

Posttraumatic stress disorder (PTSD) may emerge or reemerge in the context of life-threatening illness, retirement, and life review, leading to complications in disease management and end-of-life care. Symptoms of PTSD in later life can be misattributed or overlooked by healthcare professionals, caregivers, and the older adults experiencing PTSD themselves. The "Talking Later" podcast was developed as an accessible educational product to improve recognition and trauma-informed responses to late-life PTSD. Each of the ten episodes had distinct goals and content objectives, which were identified through problem identification and needs assessment by a multidisciplinary team of geriatric clinicians. In the first three months of its publication in November 2021, the podcast has been downloaded by 1,473 unique listeners across 19 countries. The podcast was evaluated via feedback survey (N=39). Approximately 97% of respondents reported the episodes as engaging and informational. 87% stated that no more than general knowledge of PTSD was required to enjoy the podcast. Qualitative analysis of open-ended feedback items found that participants were interested in learning about additional comorbidities and diversity issues related to late-life trauma reengagement. The present poster will additionally describe ongoing efforts to address these and other feedback responses with the ongoing development of a second season of the podcast.

### ASSESSING TELEHEALTH USE AMONG OLDER ADULTS WITH LIMITED RESOURCES DURING THE COVID-19 PANDEMIC

Marie Dezine[1], Naushira Pandya[2], Sashah Damier[2], and Sweta Tewary[2], *1. Nova Southeastern University - - Ft Lauderdale, FL, North Miami, Florida, United States, 2. Nova Southeastern University - - Ft Lauderdale, FL, Fort Lauderdale, Florida, United States*

The COVID-19 pandemic created new barriers to accessing primary care services, particularly among older adults who has faced barriers related to access to care, transportation, health literacy, and social isolation. Nova Southeastern University South Florida Geriatric Workforce Enhancement Program (NSU SFGWEP) partnered with primary care clinics and a local community partner to conduct wellness calls to older adult patients identified through clinic EHR. This project aimed to provide educational and telehealth support to vulnerable adults with limited resources in the Tri-County region of Florida. Wellness calls were made to determine educational and technical support needs of the older adults designated as underprivileged. We identified 44 participants to receive telehealth devices. Samsung tablets were mailed with educational resources, developed by NSU SFGWEP related to COVID-19 pandemic. The information included health, vaccine education, and instructions to access telehealth services. They had the tablets for six months. We conducted bi-weekly calls to offer peer training to access the educational materials. The participants were asked a series of questions to assess the effectiveness of the peer training support. Among participants, 36% (n=16) found the education materials impactful. Most participants, 89% (n=39), used the tablet, and 23% (n=10) reported using it daily. 11% (n=5) used it for telehealth, 7% (n=3) to connect with friends and family, and 7% (n=3) to connect with faith. This pilot project suggests that tablets were beneficial in assisting the participants in accessing education materials and resources encouraging the use of telehealth appointments and eliminating some of the social isolation.

### SESSION 6230 (POSTER)

### AGING IN PLACE

### MOTIVATIONS TO VOLUNTEER: SURVEY OF A SERVICE AGENCY IN RURAL MICHIGAN

Rita Hu[1], Ruth Dunkle[1], and Julie Tarr[2], *1. University of Michigan, Ann Arbor, Michigan, United States, 2. ShareCare of Leelanau, Leland, Michigan, United States*

While volunteers are the foundation of service agencies in rural communities, they are difficult to recruit. This study aims to understand the characteristics of volunteers in a rural community that could aid in recruitment. All volunteers (N=127) for an organization providing services to keep older adults in their homes in rural Michigan were approached to participate in the study. Data were gathered via telephone-interview survey (N=76) and in-person focus groups (N=14) in the summer of 2021 to understand demographic factors as well as benefits and community factors contributing to volunteer participation. Most volunteers were female (61%), married (71%), over age 70 (M=71), and lived with others (72%). In the past year, 49% volunteered 1-5 hours per week and with multiple organizations (70%). They identified the primary benefits from volunteering as getting to know new people (66%). Volunteers agreed that they feel they contribute to the community (97%), have a strong attachment to the community (91%), and have the ability to make a difference in the community (95%). Volunteers were less aware of what could be done to meet the needs in the community (54%) and understand the needs and problems facing the community (59%). Focus group data suggest that volunteers desire information about community needs and aging in place. Due to the lack of centralized locations, social and educational hot spots such as coffee shops could attract new volunteers. Having a sense of community is an important component of volunteering and should be fostered when recruiting new volunteers.

### SELF-RATED ORAL HEALTH SATISFACTION AMONG OLDER ADULTS RECEIVING ONE HOME AND COMMUNITY-BASED SERVICE

Steffany Chamut[1], and Shahdokht Boroumand[2], *1. Harvard School od Dental Medicine, Boston, Massachusetts, United States, 2. National Institutes of Health (NIH), Bethesda, Maryland, United States*

Objective: To assess self-rated oral health (OH) satisfaction among recipients of the home and community-based services (HCBS) administered by the Administration on Aging.

Methods: This cross-sectional study is representative of the national population of the 2014 National Survey of Older

Americans Act Participants (NSOAAP), and analyzed baseline OH data from community-dwelling adults (n= 3,995) age 60 and older covering case-management, congregate-meals, home-delivered-meals, homemaker-services, and transportation.

Results: Among the 3,995 participants, 35% received only one HCBS, the majority being age 75-84 (42%), females (67%), Non-Hispanic Whites (72%), and living in urban areas (53%). About 54% reported having a dental visit in the last twelve months, 30% were not satisfied with their OH, and 45% reported having 4-6 medical conditions. Congregate-meals (67%), home-delivered-meals (23%), and transportation (6%) were the most services provided (n=1,130). Being not satisfied with general health (OR=4.44, CI95=2.81-7.00) p<.0001, experiencing difficulties with three or more activities of daily living (ADLs) (OR=2.06, CI95=1.15-3.70) p= 0.0149, and not having a dental visit in the last twelve months (OR=1.66, CI95=1.06-2.59) p= 0.0248 were the strongest indicators negatively associated for not being satisfied with OH among HCBS participants.

Conclusion: Oral diseases and oral microbiota are known to be precursors of dementia, Alzheimer's, multiple systemic diseases (i.e. diabetes), and mortality. Understanding OH issues among HCBS recipients could refine policies that focus on improving functional status with person-centered services, and help identify opportunities to revamp older adults' oral and systemic health, promote healthy aging and longevity allowing them to continue living independently at home.

### UPDATING ESTIMATES OF DRIVING LIFE EXPECTANCY

Jonathon Vivoda, *Miami University, Oxford, Ohio, United States*

Information is limited about driving life expectancy, and the amount of time between loss of driving ability and death. The most often cited study (by Foley and colleagues, 2002) analyzed data collected in the mid-1990s and used life expectancy estimates to determine survival probabilities. Although that study was well designed, longitudinal data from the Health and Retirement Study (HRS) are now available to use participants' actual date of death to assess issues related to driving life expectancy. HRS data from 1996-2018 were assessed; only participants who had answered the driving ability question for at least one wave, and had a reported death date were included. The percentages of participants were determined who never reported the ability to drive, always reported the ability to drive, and those who transitioned from driving to non-driving. Time between loss of driving ability and death was calculated among former drivers by subtracting the interview date when respondents reported an inability to drive (after having previously reported being able to drive) from the participants' date of death. Only about 3% reported never driving, with a nearly even split between those who stopped driving (49%) and those who always drove (48%). Women were more likely to report being a never/former driver (65%) compared to men (36%). Among former drivers, the average time between inability to drive and death was 1312 days (3.59 years), and was significantly longer for women (437 days). Among former drivers, the average age for reporting inability to drive was about 83 years old.

### PRIORITIZING FUTURE RESEARCH ON NONDRUG DEMENTIA CARE INTERVENTIONS

Eric Jutkowitz[1], Peter Shewmaker[1], and Fernando Alarid-Escudero[2], *1. Brown University, Providence, Rhode Island, United States, 2. Center for Research and Teaching in Economics (CIDE), Mexico City, Aguascalientes, Mexico*

We previously used an evidence-based mathematical model to evaluate the cost-effectiveness of psychosocial interventions that reduce the risk of a nursing home admission for people with dementia from a healthcare payer perspective. We found the incremental cost-effectiveness of MIND, an in-home intervention for people with mild-moderate dementia, compared to usual care was $271,456 per quality-adjusted life-year (QALY). The incremental cost-effectiveness of NYU Caregiver Intervention (NYUCI), which is for people with moderate dementia, compared to usual care was $3,964/QALY. Here we quantify the uncertainty around our cost-effectiveness estimates. First, we calculated the expected value of partial perfect information (EVPPI), which is the value of eliminating uncertainty around the treatment effect (i.e., risk of entering a nursing home) of MIND and NYUCI, and represents the maximum willingness-to-pay for a study to inform these estimates. Given a willingness-to-pay of $110,000/QALY, population EVPPI for MIND and the NYUCI were $81,000,000 and $395,000,000, respectively. Second, we calculated the expected value of sample information (EVSI), the expected net benefit of sampling (ENBS) and the optimal sample size (OSS). EVSI is the amount of uncertainty reduced from a pragmatic trial evaluating the risk of entering a nursing home for people in the intervention compared to usual care. ENBS is the return of a pragmatic trial with a fixed ($1,050,000) and per person ($2,000) cost to conduct the study. The OSS is the sample size that maximizes ENBS and was 3,571 for MIND and 5,357 for NYUCI. There is value in conducting pragmatic trials on MIND and NYUCI.

### RACIAL AND ETHNIC DISPARITIES IN COGNITIVE DIFFICULTY AMONG OLDER ADULTS: EVIDENCE FROM NEW YORK CITY

Ethan Siu Leung Cheung[1], and Jinyu Liu[2], *1. Columbia University, New York, New York, United States, 2. Columbia University, New York City, New York, United States*

This study examined racial and ethnic disparities in cognitive difficulty among older adults in New York City (NYC). Also, we tested whether physical health, family structure, individual socioeconomic status (SES), and neighborhood SES explained the disparities. Based on community districts, individual-level data from the 2019 American Community Survey were merged with neighborhood data from NYC Community District Profile. A sample of 5,622 NYC residents aged 60 or older was included across 55 community districts. The outcome variable, cognitive difficulty, was measured by a binary variable in which respondents' self-reported challenges with cognitive health (1=having challenge, 0=no). Racial and ethnic groups included Whites, Blacks, Latinos/Hispanics, and Asians. We used multilevel logistic regressions for analysis. Results show that Latinos/Hispanics had the highest odds of reporting cognitive difficulty across groups.

Physical health, marital status, individual SES, and access to parks were significantly associated with cognitive difficulty. Physical health, family structure, and multilevel SES partially explained or influenced the racial and ethnic disparity in cognitive difficulty. However, such influence varied by race and ethnicity. Physical health and individual SES contributed to the disparities for Latinos/Hispanics and Blacks, compared to Whites. Neighborhood SES attenuated the disparity in cognitive difficulty between Latinos/Hispanics and Whites. Also, family structure uniquely explained the disparity for Blacks. No significant disparity was identified between Asians and Whites. This study shed light on the important roles of multilevel factors in predicting racial and ethnic disparities in cognitive difficulty. Findings provide direction for interventions to reduce racial and ethnic disparities in cognitive difficulty.

### AGING IN COMMUNITY: LESSONS LEARNED IN THE FIRST YEAR OF DEVELOPING A GRASSROOTS, RURAL PROGRAM

Emily Kinkade, Melisa Hajdar, and Heather Fuller, *North Dakota State University, Fargo, North Dakota, United States*

The North Dakota Aging in Community project has an overarching goal of creating programs that improve the quality of life of rural older adults by increasing community-level support to help them age in place in two rural communities. The progress of program development and implementation was assessed across the project's first year through program data and steering committee surveys. Program data, including steering committee meeting minutes and monthly activity reports completed by program staff, were qualitatively analyzed for successes, challenges, and lessons learned by finding common themes within these data. Successes included the use of an apprenticeship/mentorship model utilizing consultants from similar grassroots, rural organizations, recruitment of an optimistic, enthusiastic, and collaborative steering committee, and employing patience to develop community-led efforts based on unique community needs. Challenges included hiring qualified staff in rural communities, limitations in infrastructure opportunities, and need for creative marketing strategies. Survey results aligned with these conclusions, yet demonstrated the strengths and challenges related to incorporating the ideas and needs of diverse stakeholders. These findings highlight the unique challenges in developing programs to support rural aging-in-place, yet also highlight unique strategies that leverage rural strengths. Based on the findings of this first-year evaluation, future directions include conducting needs assessments to identify new avenues for program development, ensuring local program ownership through continued development of local steering committees, and continuing the mentorship/apprenticeship model while creating strategies to successfully foster independence. By sharing lessons learned, this program may serve as a replicable model to foster rural aging-in-place.

### FOOD INSECURITY AMONG OLDER ADULTS IN NEW YORK CITY: DOES LOCATION MATTER?

Ethan Siu Leung Cheung, *Columbia University, New York, New York, United States*

Having access to adequate and appropriate food sources is essential to addressing food insecurity among older adults. However, the role of locational characteristics in explaining food insecurity remains unclear, especially in urban areas. This study investigated the association of distance to grocery stores, neighborhood disadvantage, and social cohesion with food insecurity among older adults in New York City. Individual-level data were drawn from a 2-year Poverty Tracker Study. The sample included New York City residents aged 65 or older (baseline N = 710). Based on the respondents' residential address and neighborhood ZIP codes, the individual-level data were merged with two spatial datasets: American Community Survey and ReferenceUSA. ArcGIS 10 (near analysis) was used to manage spatial data and calculate the distance to grocery stores. Hierarchical logistic regression models were employed for analyses. Descriptive results show that more older adults in neighborhoods with economic disadvantage and lower level of social cohesion reported more food insecurity. Logistic regressions suggested that after controlling for individual-level characteristics (e.g., age, gender, race and ethnicity, and education), living farther (0.26–0.50 miles and 0.51–0.70 miles) from the nearest grocery store was positively associated with food insecurity. Residing in economically disadvantaged neighborhoods also increased the odds of food insecurity. Community social cohesion was a marginally significant protective factor against food insecurity. Findings suggest that locational characteristics play a significant role in predicting food insecurity in New York City, suggesting that community outreach and grocery delivery programs are needed to mitigate the risk.

### CONNECTING RURAL OLDER ADULTS WITH DISABILITIES TO HOME MODIFICATIONS WITH A REMOTE HOME ASSESSMENT

Emily Nabors[1], Catia Garell[2], and Jon Pynoos[1], *1. University of Southern California, Los Angeles, California, United States, 2. Thrive for Life LLC, Honolulu, Hawaii, United States*

Older adults living in rural areas have particular challenges to accessing critical supportive services such as home modifications to promote functioning and safety. Conducting remote home assessments through telehealth has the potential to reduce time spent and overall cost that occur in conducting in-person assessments. During the pandemic, providers turned to telehealth to preserve continuity of assessment services with few research-based practices to guide them. With support from a NIDLRR SBIR grant, Thrive for Life LLC in partnership with the USC Leonard Davis School of Gerontology conducted research to develop a remote home assessment that aims to connect health and home modification providers with rural older adults (65+) with disabilities, a population that may not receive home modifications otherwise. Research included a literature review, key informant interviews with five experts in the field, and individual phone interviews with 30 rural older adults who have disabilities. The literature was analyzed and used to inform the interview questions. Key informant interview responses were analyzed for models, potential challenges, lessons learned, and opportunities to impact priority needs. Consumer interview responses were analyzed for needs, preferences, concerns, and challenges related to technology use. Findings demonstrate common barriers such as lack of access to broadband and smart technology; circumstances in which remote assessments are, and are not, likely to be successful; and the potential value of conducting remote home assessments in rural areas to ensure equity of access to home modifications for older adults with disabilities during the pandemic and beyond.

### OPENING THE DOOR TO AGING IN PLACE: FINDINGS FROM THE INTERGENERATIONAL TORONTO HOMESHARE PROGRAM

Jessica Hsieh[1], Raza Mirza[2], Jackie Tanner[3], Amanda Bull[2], Andrea Gardiola[4], Carley Moore[2], Deirdre Kelly-Adams[2], and Christopher Klinger[1], *1. University of Toronto, Toronto, Ontario, Canada, 2. National Initiative for the Care of the Elderly, Toronto, Ontario, Canada, 3. Natioinal Initiative for the Care of the Elderly, Toronto, Ontario, Canada, 4. National Initative for the Care of the Elderly, Toronto, Ontario, Canada*

Older adults prefer to live in their own homes for as long as possible — to 'age in place' — but for myriad reasons, may be unable to do so. To address this, a number of housing alternatives have been explored, including homesharing, or homeshare, an exchange-based shared housing approach with the potential to empower older adults to age in place by enabling them to obtain additional income, companionship, and assistance with completing household tasks in exchange for renting out a room in their home.An intergenerational homesharing pilot program in Toronto matched older adults (55+) with postsecondary students. With limited research in the area, a mixed methods research study was embedded within the pilot project with the goals of: 1) conducting a scoping review to map and synthesize the literature related to outcomes of homeshare participation for this population, 2) conducting in-depth interviews with homeshare participants (N=22) to learn about their experiences, and 3) conduct a full evaluation and exit survey to better understand the implications of the project. Results were organized around the following themes: (1) benefits and challenges of participating in homeshare for older adults; (2) intergenerational engagement as social exchange; and (3) the key role of agency facilitation as a determinant of the experience of homesharing for older adults. Results spoke to the unique benefits and challenges of participating in homeshare for this population. Findings were used to derive implications for policy and practice, as well as highlight areas for future research.

### INTEGRATED CARE POLICY AND PRACTICE IN THE US: THE SCENARIO OF AGING IN CHINATOWN

Yuanyuan Hu, and Qingwen Xu, *New York University, New York, New York, United States*

Background: Older Chinese adults, the fastest-growing population among older immigrants, experience multiple barriers to access quality physical and behavioral health care, including low English proficiency, low health literacy, and segregation between health care and social care sectors (Tsoh et al., 2016). While integrated care attempts to address these issues, there is still a lack of culturally sensitive integrated care practices to address the needs of older Chinese immigrants.

Methods: This article reviews the definition and history of integrated care policies in the U.S., and compares four integrated care models on the service user and community levels, including the Chronic Care Model (CCM), Program of All-Inclusive Care for the Elderly (PACE), Patient Navigation Model, and Delivery System Reform Incentive Payment (DSRIP) Program.

Results: Taking the community-dwelling older Chinese immigrants as the context, this article discusses factors that are essential to this group of older adults and proposes a framework to integrate social determinants of health in the development of integrated care practice with the infusion of cultural values and norms.

Conclusion: Integrated care for older immigrants asks for a complicated mass reconstruction of current care systems. We propose an innovative framework that fully takes advantage of CBO's capacity in providing culturally appropriate services is proactive and preventive in nature by addressing social determinants of health directly, recognizes the role of family and community in older immigrants' life and aging process, and provide equal attention to the older adults' needs in health, mental health, and elderly care.

### SESSION 6240 (POSTER)

### AGING IN PLACE: SOCIAL DETERMINANTS OF HEALTH

### AGING IN PLACE: TURNING TO THE VOICES OF EXTENSION EDUCATORS

Susan Koerner[1], Brianna Anderson[2], and Sehyun Ju[1], *1. University of Illinois - Urbana-Champaign, Urbana, Illinois, United States, 2. Saint Louis University, St. Louis, Missouri, United States*

Most adults report a preference for aging-in-place (AIP) – remaining safely in their own home and community as they age, even as they become more dependent on others. When attempting to determine options and feasibility for AIP, older adults and/or their families – especially those living in non-metropolitan rural areas and small towns, may turn to Extension educators for information and guidance. For the current study we interviewed seven family-focused Extension educators responsible for 25 counties throughout a Midwestern state to explore the challenges, supports, patterns of experience, and service/policy recommendations that these professionals find relevant to AIP in their regions. The principal investigator (PI) conducted each semi-structured interview by phone; each audio-recorded interview lasted approximately 60 minutes. Two trained research assistants and the PI applied combined deductive-inductive thematic analyses to the transcribed interview data following Braun and Clarke (2012), utilizing MAXQDA, and ensuring trustworthiness during the coding process. Five major categories with sub-themes emerged: Challenges to AIP (e.g., transportation), Supports to AIP (e.g., churches), Most-Challenged Populations (e.g., middle-income families who neither can afford in-home assistance nor are eligible for government aid), Attitudes Toward AIP (e.g., caution against social isolation), and Recommendations for Services/Policies to Facilitate AIP (e.g., government funding, in-home technology assistance). Some variation across counties was apparent with, for example, one county making concerted efforts to retain young adults in its communities (i.e., reducing out-migration), thus enhancing family presence making AIP more feasible, non-isolating. Our findings will be especially noteworthy to social service providers and aging-focused public policymakers.

### SPIRITUAL RESILIENCE? THE ROLE OF RELIGIOSITY IN BUFFERING THE EFFECT OF NEIGHBORHOOD DISORDER ON COGNITIVE DECLINE

Haena Lee[1], Yeon Jin Choi[2], and Jong Hyun Jung[3], *1. USC, Los Angeles, California, United States, 2. University of Southern California, Los Angeles, California, United States, 3. Sungkyunkwan University, Seoul, Seoul-t'ukpyolsi, Republic of Korea*

Living in neighborhoods with high levels of disorder and danger can induce psychological distress and compromise cognitive function. However, not all individuals who live in difficult life circumstances have poor health outcomes. Research on resilience shows that some older adults maintain healthy profiles despite adversity, but this has not been tested with respect to cognitive aging. In this paper, we focus on religiosity–religious belief and attendance–as a source of resilience and how it can reverse or reduce cognitive risks in later life that result from long-term exposure to neighborhood disorder. We used 2006-2016 Health and Retirement Study (HRS) to investigate how religiosity moderates the relationship between neighborhood disorder and cognitive decline. We assessed trajectories of cognitive functioning using the Telephone Inventory for Cognitive Status. We measured neighborhood disorder and neighborhood unsafety using the 2006/2008 HRS interviewer observation data and Housing data. We found that individuals living with higher levels of neighborhood disorder had lower cognitive functioning at baseline. The disorder effect was mitigated by religious belief—for instance, poor neighborhood conditions were negatively associated with cognitive function only for those with lower religious belief. The protective effect of religious belief was more pronounced among older Black women. This is consistent with prior literature that spirituality serves as a protective factor in the African American community, especially among women, for triumphing over adversity and lack of secular resources over the life course.

### GEOGRAPHIC PROXIMITY TO NEIGHBORHOOD RESOURCES AND DEPRESSIVE SYMPTOMS AMONG KOREAN OLDER ADULTS

JuHyeong Lee, Donghee Shin, Yeoju Kim, and Giyeon Kim, *Chung-Ang University, Seoul, Seoul-t'ukpyolsi, Republic of Korea*

Given the importance of geographic proximity to neighborhood resources especially during the COVID-19 pandemic, this study examine whether the relationship between geographic proximity to neighborhood resources (e.g. hospitals, public transportation, etc.) and depressive symptoms varied by geographic location (i.e., rural vs. urban areas) among older adults in South Korea and whether this relationship was mediated by participation in social activities (e.g. education, club, community, etc.). The nationally representative samples, Korean older adults aged 65 or older, were drawn from the 2020 Survey of Living Conditions and Welfare Needs of Korean Older Persons (N=9,732, Urban=6,975, Rural=2,757). Hierarchical regression models, Baron and Kenny's steps, and Sobel Test for the mediation effect were conducted. Results showed that geographic proximity was negatively associated with depressive symptoms in urban areas (B=-.041, $p<.001$), while positively associated in rural areas (B=.034, $p<.01$). Participation in social activities partially mediated the relationship in urban areas (Z=-2.162, $p<.05$), while there was no significant mediation effect in rural areas. Additionally, geographic proximity to hospitals or public transportation was significantly associated with depressive symptoms in rural areas. The findings suggest that geographic proximity to neighborhood resources helps older adults reduce social isolation, which may improve mental health of older adults living in urban areas during the pandemic. However, geographic proximity to neighborhood resources could make older adults living in rural areas become depressed, emphasizing that the characteristics of the urban and rural areas need to be considered to create an aged-friendly environment.

### NEIGHBORHOOD PREDICTORS OF MENTAL HEALTH OF OLDER AMERICANS: EVIDENCE FROM A 5-YEAR LONGITUDINAL STUDY

AFOLABI OREKOYA[1], Henry Egbuchiem[2], Olawale Olanisa[3], Abimbola Arisoyin[4], Ujunwa Ebili[5], Adeleye Adaralegbe[6], and Khuzeman Abbasi7, *1. Wellspring Medical Center, Riverdale, Georgia, United States, 2. The Everest Foundation, Los Angeles, California, United States, 3. IQVIA Biotech, Morrisville, North Carolina, United States, 4. TCA health, Lagos, Lagos, Nigeria, 5. Emel Specialists and Pediatric Center, Festac Town, Lagos, Nigeria, 6. University of Connecticut, Storrs, Connecticut, United States, 7 Vanderbilt University Medical Center, Glen Burnie, Maryland, United States*

With increasing dependence on other people in old age, environmental resources become an important asset for older adults to experience healthy aging. Data on the longitudinal relationship between neighborhood and mental health in late life is scanty. This study utilized hierarchical multiple regression model analysis to investigate whether and which neighborhood factors predicted depression and anxiety among older Americans followed up for over five years within the same neighborhood. Two waves of data containing a cohort of 1,731 older adults from the NSHAP project were used. Outcome measures were depression and anxiety. Predictors were four neighborhood factors: Social cohesion (NSC), social ties (NSC), neighborhood problems (NP), and perceived neighborhood danger (PND). We adjusted for demographic and physical health characteristics. The mean age of the respondents was 71.4 ± 6.5 years and were mostly females (55.5%). Lower NSC and a higher PND significantly predicted depression. However, the model only explained 2.8% of the variance in depression. In the covariate-adjusted model, none of the neighborhood factors predicted depression, but the model significantly improved to 32.5%. NP was the only significant predictor of anxiety in the final model and explained 27.8% of the variance in anxiety. Covariates, which are primary determinants of mental health disparity, have a much larger role to play. This study sheds some light on the complexity of the relationship between neighborhood and mental health in older adults. Future policy development and interventions should target improving both physical and social environments to enhancing the mental wellbeing of older adults.

### CHARACTERISTICS OF LONG-TERM HOME CARE PATIENTS WITH DEMENTIA

Takashi Yamanaka[1], Maiko Mizuki[1], Kiwami Kidana[1], Ryonosuke Yamaga[2], and Masahiro Akishita[1], *1. Graduate School of Medicine, The University of Tokyo, Bunkyo-ku, Tokyo, Japan, 2. Tsubasa Home Care Clinic, Funabashi-city, Chiba, Japan*

This study aimed to clarify the characteristics of older adults with dementia receiving physician home visits. We prospectively registered 179 patients aged ≥65 years, estimated to receive physician home visits for over 6 months, in a clinic in Chiba, Japan, in 2020–2021. The patients' mean (±SD) age was 85.1±7.4 years and ranged from 68 to 102 years. Out of the total patients, 57.5% were men. We collected clinical information; employed the Dementia Assessment Sheet in Community-based Integrated Care System 21 items (DASC-21) and EuroQOL five dimensions 5-level (EQ-5D-5L) every 6 months; and calculated the incidence of death. Eighty-two patients (45.8%) were diagnosed with dementia at the commencement of physician home visits. The characteristics of older adults diagnosed with dementia (D+ group) were compared with the characteristics of those who were not (D- group). The D+ group was older than the D- group (86.4±6.6 years vs. 84.1±8.0 years, p=0.04118). Total cholesterol levels and DASC-21 scores were higher in the D+ group compared to the D- group (180.2±41.7 vs. 163.7±51.2, p=0.04091; 62.3±14.5 vs. 50.2±16.2, p=0.00004, respectively). The EQ-5D-5L was not significantly different between the groups (0.439±0.255 vs. 0.397±0.267, p=0.32409). The proportion of those living in assisted living facilities or fee-based homes for the elderly was higher in the D+ group ($\chi2$=8.5177, df=2, p=0.01414, V=0.2258). The mortality rate after 6 months was 20.7% in the D+ group and 16.5% in the D- group ($\chi2$=0.5305, df=1, p=0.46641). In conclusion, the characteristics of patients with dementia should be further elucidated to provide better care.

### PARTICIPATORY RESEARCH APPROACH FOR EVALUATING HOME ENVIRONMENT FOR OLDER ADULTS WITH DISABILITIES

Juliana Tissot[1], Widya Ramadhani[2], Lizandra Vergara[1], and Wendy Rogers[3], *1. Federal University of Santa Catarina, Florianópolis, Santa Catarina, Brazil, 2. University of Illinois Urbana-Champaign, Urbana, Illinois, United States, 3. University of Illinois Urbana Champaign, Champaign, Illinois, United States*

Regardless of where they live, older adults' ability to retain their independence depends on their health and capacity. Considering that older adults are more likely to experience disabilities, there are opportunities for built environmental intervention to help them conduct everyday activities to achieve successful aging in place. We conducted participatory research with designers, architects, and aging specialists to identify aspects and characteristics of the physical space that can support or limit older adults' ability to do everyday activities. The research was conducted at the McKechnie Family LIFE Home, a home simulation facility. Participants were asked to walk through the home spaces and identify potential challenges and solutions for older adults with different disabilities (i.e., mobility, cognitive, sensory, socio-emotional), followed by a group discussion. Preliminary findings showed that the bathroom and kitchen had the most barriers. Recommendations to improve bathroom safety include installing high friction flooring, grab bars on both toilet sides, adequate wheelchair maneuvering space, and color contrast. Cooking was mentioned as a difficult activity and solutions included appliances at waist level, layout in "C" or a "U", lights under cabinets, and in the foot space. Also, the cabinets doors should be translucent to allow them to check what is inside. The house floor plan should be designed with considerations of users' everyday activities and the relationship between these activities, such as having the bathroom nearby bedroom, laundry nearby the kitchen to improve users 'mobility. We have developed a protocol for designers to follow when evaluating home spaces.

### SENIOR HOUSINGS' ROLE FOR AGING IN PLACE: FOCUSING ON THE NATIONAL PROFILE OF INDEPENDENT LIVING FACILITIES IN KOREA

Sojung Park[1], and ByeongJu Ryu[2], *1. Washington University in St. Louis, St. Louis, Missouri, United States, 2. Chuncheon Hyoja Social Welfare Center, Chuncheon-si, Kangwon-do, Republic of Korea*

Understanding the changing preference for alternative housing arrangements is important due to the confluence of a global population aging, increasing cost concern for long-term care, and the desire of older people for aging in place (AIP). To date, little is known about to what extent the current housing models meets the needs of AIP for older people. Guided by the Person-Environment fit perspective, we examined the South Korean case using the first national data on independent living facilities (ILF) for older adults. Employing a convergent mixed-method design, we conducted a quantitative analysis of 277 ILFs and qualitative interviews with executive directors and managers from 10 facilities. We found the majority of the housings serve the low-income, and the residents' health status varies widely (i.e., ranging from fully independent to those homebound). However, the characteristics and nature of services available and staff composition are not adequate to meet the needs of the residents. Notably, privacy issues (i.e., a shared restroom and bedroom) in congregate settings stood out, decreasing the occupancy rate. Our findings suggest that the housing model needs to be diversified corresponding to the resident's needs for health and social care and economic affordability and should respect their privacy needs. We discussed ways to reform the current policy governing the ILFs, including strengthening service coordination programs to alleviate gaps between the residents' needs and the service availability. Our findings provide implications for improving and reforming the existing housing model to age in place in Korea and potentially other aging societies.

### SOCIAL INTERACTIONS OF COMMUNITY SPACE ATTENDEES AND ANTICIPATED FUNCTION OF THE SPACE AMID THE COVID-19 PANDEMIC

Mai Takase[1], Ryogo Ogino[2], Ryoichi Nitanai[1], Riko Nakayama[1], Hongjik Kim[1], Neo Kazembe[1], Jun Goto[3], and Katsuya Iijima[1], *1. The University of Tokyo, Tokyo, Tokyo, Japan, 2. Saga University, Saga, Saga, Japan, 3. Tokai University, Hiratsuka, Tokyo, Japan,*

Introduction: A regional community space in Japan, Chiiki-Katsudokan, was founded to facilitate the social interactions of older adults through activities. Meanwhile, the COVID-19 pandemic occasionally forced this space to close or limited the volume of its participants. In this research, the operation of the space was reviewed by monitoring the changes in the social interactions of the attendees, and by investigating the anticipated function from the attendees' needs. Method: A semi-structured interview targeting the attendees of Chiiki-Katsudokan was conducted in December 2021 (N=19, main age:80s). The level of social interaction before and during the pandemic and the newly anticipated function of the space were examined.

Results: First, deep and light interactions were observed. Those with deep interactions initially had wide social connections and used Chiiki-Katsudokan to interact with friends. Meanwhile, those with light interactions only talked to other attendees while attending the space. During the quarantine, those with deep interactions stayed connected with others and met privately, while those with light interactions faced a higher risk of social isolation. Second, the most popular newly anticipated functions of the space were "Place to gather with friends (n=14)" and "Interaction with younger generation (n=14)." "Opportunity to learn about the new pandemic-lifestyle (n=11)" was also rated high, while need for "online events" was rated the lowest (n=7).

Conclusion: The pandemic re-emphasized older adults' need for direct interaction. Low interest in technology-based countermeasures suggested that community spaces should expand their operation method while considering offline methods (e.g., pen-pal system) that could enhance social interaction.

### TYPES OF ATTENDEES IN COMMUNITY SPACE IN JAPAN: TOWARD DESIGNING AN ENVIRONMENT FOR SOCIAL PARTICIPATION

Mai Takase[1], Ryogo Ogino[2], Ryoichi Nitanai[1], Riko Nakayama[1], Hongjik Kim[1], Neo Kazembe[1], Jun Goto[3], and Katsuya Iijima[1], *1. The University of Tokyo, Tokyo, Tokyo, Japan, 2. Saga University, Saga, Saga, Japan, 3. Tokai University, Hiratsuka, Tokyo, Japan*

Introduction: Designing community space for social participation of older adults is essential for healthy aging. The behavioral characteristics of the attendees have much to offer as they relate to social settings designed in the space. This study aims to elucidate the basic typology of the attendees considering their motivation for attendance and state of social connection.

Methods: Semi-structured interviews targeting attendees of Chiiki-Katsudokan, a space designed for social participation in a larger aging estate in Japan, were conducted in December 2021 (N=16, 16% male). Attendees were asked about their reasons for attending events at Chiiki-Katsudokan, interaction levels with other attendees, and levels of social participation during the COVID-19 pandemic.

Results: Based on the interviews, attendees were classified into three types: Seeker, Hobbyist, and Socializer. First, Seekers (n=3) used Chiiki-Katsudokan as the primary means of preventing social isolation. Amongst the three types, the social interaction level of Seekers was the lightest, and the opportunity decreased when Chiiki-Katsudokan closed during the quarantine. Second, the main motivation for Hobbyists (n=5) was the event contents which matched their interests. Hobbyists were also likely to engage in hobby networks, which continued privately during the pandemic. Finally, Socializers (n=8) attended Chiiki-Katsudokan to communicate with friends. Many were initially socially active and engaged in social interaction outside Chiiki-Katsudokan, e.g., teatime with friends, during the quarantine.

Conclusion: This study suggested that designing community space for social participation requires defining the varying levels of engagement and expecting relationships outside the space based on the behavioral characteristics of the attendees.

### MEANINGFUL ACTIVITY ENGAGEMENT AND WELL-BEING AMONG DISABLED OLDER ADULTS: THE MODERATED ROLE OF ENVIRONMENT

Yi-Hsuan Tung[1], Iris Chi[1], Shinyi Wu[1], and Shiau-Fang Chao[2], *1. University of Southern California, Los Angeles, California, United States, 2. National Taiwan University, Taipei, Taipei, Taiwan (Republic of China)*

Engaging in meaningful activities has been seen as an important way to sustain the well-being of older adults with disabilities and to achieve person-centered care. Yet, it is still unclear whether and to what extent meaningful activity engagement promotes well-being for community-dwelling older adults with disabilities, and how the environmental factors could affect these relationships. This study aims to investigate the relationship between meaningful activity engagement and psychological well-being, and to explore the moderated role of environmental factors (physical, attitudinal, service/support, policy). Survey data conducted in Taiwan between April and July of 2018 were analyzed by using multiple regression (N=1,244). Three types of meaningful activities (instrumental, social, and leisure) were identified based on a self-rated activity meaningfulness measure. Findings showed that higher levels of engagement in three types of meaningful activities were associated with better quality of life (QOL), but only engaging in meaningful leisure was associated with less depressive symptoms while adjusting functional status. Perceived better policy-related environment (e.g., long-term care services) could reinforce the positive effects of three types of meaningful activity engagement on QOL and depressive symptoms. Findings also indicated perceived attitudinal environment moderated the association between meaningful leisure activity engagement and QOL. These results established the influences of meaningful activity engagement on the well-being of community-dwelling older adults with disabilities and highlight the importance of age-friendly environment in supporting meaningful activity engagement and older adults' well-being.

### SESSION 6250 (POSTER)

### ATTITUDES ABOUT AGING (POSTERS)

### OLDER ADULTS' INDIVIDUAL TRAJECTORIES IN SOCIAL STATUS AND AGING ANXIETY

Tim Kuball, and Georg Jahn, *Chemnitz University of Technology, Chemnitz, Sachsen, Germany*

A high social standing in comparison to others is associated with positive psychological and health outcomes. Highest social standing is assigned to the group of middle-aged adults, hence, on average, older adults face a loss in status relative to younger age groups and relative to their former selves. Experienced and expected age-related changes in subjective social status and their association with aging anxiety have not yet received much attention in aging research. Using a new methodological approach, respondents indicated their perceived and expected social status for five points in time: 10 years ago, 5 years ago, now, in 5 years, in 10 years, which allowed for inter- and intrapersonal comparisons. They did the same for the average status of members of their age group. Early and later in old age (N = 191; range 65 – 88; MW = 73.5 years), participants expected higher losses in status than they have experienced in the past. However, low personal status in relation to others showed higher associations with aging anxiety ($R2 = .16$) than disadvantageous age-related intrapersonal changes ($R2 = .14$). Perception of a stable subjective status trajectory as well as distancing oneself from the group older adults, as in perceiving one's personal status above the groups' status, was related to reduced anxiety of aging. Taken together, analysis of individual status trajectories can help to gain new insights on attitudes toward aging. Implications for creating a more positive perception of aging are discussed.

### QUALITY OF LIFE AND ATTITUDES TOWARD AGING IN OLDER ADULTS DURING THE COVID-19 PANDEMIC

Morgan Inman[1], Maria MacLean[1], Olive Bryanton[2], William Montelpare[1], and Jessica Strong[1], *1. University of Prince Edward Island, Charlottetown, Prince Edward Island, Canada, 2. Prince Edward Island, Charlottetown, Prince Edward Island, Canada*

Research has shown that positive or negative views of aging are associated with quality of life. Prior research has found that community dwelling older adults aged 65 and older with more negative views of aging have lower scores on quality of life scales, whereas those with higher views of aging have higher scores on quality of life scales. A group of 264 community dwelling older adults (Mean age = 72.4 years, 62-92 years old) living in Prince Edward Island, Canada, completed a survey measuring attitudes towards aging and quality of life during the COVID-19 pandemic. The sample consisted of a majority of retired (n=206) older adults, living in an urban area (n=151), and approximately 55% receiving a household income of $26,000 to $75,000 per year. Regression analysis found that attitudes towards aging significantly predicted quality of life ($F(1,127)=24.9$, $p< 0.01$), with positive attitudes predicting higher quality of life scores and negative attitudes predicting lower quality of life scores. The model showed that the predictor, attitudes towards aging, explained 16.5% of the variance in quality of life ($B=-2.7$, $t=-4.9$, $p< 0.01$). This suggests that attitudes towards aging play a role in predicting quality of life in older adults during the COVID-19 pandemic.

### INTERACTIONS WITH PEOPLE WITH DEMENTIA, LEARNING EXPERIENCES, AND PUBLIC STIGMA AGAINST DEMENTIA

Taiji Noguchi[1], Takeshi Nakagawa[1], Ayane Komatsu[1], Erhua Shang[2], Chiyoe Murata[3], and Tami Saito[1], *1. National Center for Geriatrics and Gerontology, Obu, Aichi, Japan, 2. Aichi Toho University, Nagoya, Aichi, Japan, 3. Tokai Gakuen University, Nagoya, Aichi, Japan*

Overcoming stigma around dementia is a global challenge. This cross-sectional study examined the association of experiences of interacting with people with dementia (PwD) and learning about dementia, with the public-stigma against dementia. We recruited—via an internet survey—710 Japanese adults (mean age = 46.3 years; 49.3% females) without any medical or welfare license, or dementia-related work experience. Public-stigma against dementia was assessed using the Japanese version of Phillipson et al.'s scale (2012) exploring dementia-related attitudes in the context of "personal avoidance," "person centeredness," "fear of labeling," and "fear of discrimination." Multivariable linear regression analysis was employed to examine the association of interacting with PwD and learning regarding dementia as explanatory variables with dementia stigma score, adjusting for sociodemographic variables. Regarding interactions with PwD, talking or activities with PwD were associated with low "personal avoidance" ($\beta = -1.47$, $p = 0.002$), "fear of labeling" ($\beta = -0.96$, $p = 0.020$), and "fear of discrimination" ($\beta = -0.396$, $p = 0.043$). Experiences of living with PwD were associated with low "personal avoidance" ($\beta = -2.35$, $p = 0.002$) and "fear of discrimination" ($\beta = -0.789$, $p = 0.013$). Learning experiences at school regarding dementia were associated with low "personal avoidance" ($\beta = -4.01$, $p < 0.001$), and self-learning experiences were associated with low "personal avoidance" ($\beta = -1.73$, $p = 0.049$) and high "person centeredness" ($\beta = 1.27$, $p = 0.037$). However, workplace learning was not associated with any area. Interacting with PwD and learning about dementia might reduce associated public-stigma.

### AWARENESS OF AGE-RELATED GAINS AND LOSSES AND THEIR ASSOCIATIONS WITH PHYSICAL AND COGNITIVE FUNCTION

Sarah Barber[1], Vanessa Martinez Lenis[1], and Kate Hamel[2], *1. Georgia State University, Atlanta, Georgia, United States, 2. San Francisco State University, San Francisco, California, United States*

Awareness of age-related change (AARC) refers to people's recognition of how aging has affected their performance, behavior, and ways of experiencing life. Sometimes these age-related changes are perceived as losses (AARC-Losses), such as when people notice declines in their health. However, other times these age-related changes are perceived as gains (AARC-Gains), such as when people notice they have developed a better sense of what is important to them. Past research has shown that higher AARC-Losses (and to a lesser extent lower AARC-Gains) are associated with poorer self-rated health. However, no research has yet examined whether AARC also relates to an objective performance-based measure of health. To address this, we examined the cross-sectional relationships between AARC-Losses and AARC-Gains with gait speed (i.e., a measure of physical function) in 164 community-dwelling older adults. Participants in this study also completed health-related questionnaires and the NIH Toolbox Cognition Battery. Results showed that AARC-Losses were most strongly predicted by depression levels, but higher AARC-Losses were also predicted by slower gait speeds. A different pattern emerged for AARC-Gains. After controlling for demographic factors, depression, and other self-reported measures of health, we found that higher AARC-Gains were predicted by poorer cognition and slower gait speed. The counterintuitive relationship between AARC-Gains and objective cognition has previously been reported in the literature. However, this study is the first to document that AARC-Gains are also associated with poorer performance on an objective measure of physical function. We discuss features of the AARC questionnaire that may lead to these paradoxical effects.

### FUTURE SELF, PERCEIVED CONTROL, AND AGING PREPARATION BEFORE AND AFTER THE PANDEMIC: MODERATED MEDIATION EFFECT

Yue Hu, Zhixuan Lin, and Helene Fung, *The Chinese University of Hong Kong, Hong Kong, Hong Kong*

Aging preparation, which can be seen as an adaptation to age-related challenges, is always oriented toward the future. The present study aims to explore how thinking about the future self and aging preparation are related. Domain-specific aging preparation was assessed in a longitudinal sample including 359 Hong Kong adults aged from 20 to 98 yrs. In both the pre- (2018) and during pandemic (2020) waves, perceived control fully mediated the positive relationship between thinking clarity on the near future self and aging preparation in the financial domain. However, the mediation effect of perceived control on aging preparation was moderated by age in the pre-pandemic wave, with a higher effect for older adults than for younger adults. Yet, these age differences were no longer significant during the pandemic. The present findings suggested the critical role of perceived control in preparation for the age-related changes and the age-equating effect of COVID-19 pandemic outbreak.

### PHYSICAL FUNCTION, SELF-PERCEPTIONS OF AGING, AND DEPRESSIVE SYMPTOMS AMONG OLDER ADULTS

Suyoung Nah[1], Lynn Martire[2], and Ashley Tate[2], *1. Pennsylvania State University, State College, Pennsylvania, United States, 2. Pennsylvania State University, University Park, Pennsylvania, United States*

Poor physical function has been linked to greater depressive symptoms among older adults. On the other hand, older adults' perceptions of positive and negative age-related changes provide personal strength and vulnerability to stressful events, respectively. We therefore expected that positive self-perceptions of aging (SPA) would be associated with fewer depressive symptoms, while negative SPA would be related to greater depressive symptoms, beyond the effect of physical function. We further tested the hypotheses that positive SPA would buffer the association between physical function and greater depressive symptoms, whereas negative SPA would exacerbate this association. This study used data from 108 older adults (mean age = 81.09) in independent-living or retirement communities. Results from a linear regression revealed that more positive SPA ($B = -0.21$, $p = .02$) and less negative SPA ($B = 0.21$, $p = .06$) were associated with fewer depressive symptoms, even after controlling for physical function, both types of SPA, and other covariates. In contrast, physical function was no longer significantly associated with depressive symptoms ($B = -0.15$, $p = .19$), after controlling for both types of SPA. There were no significant moderating effects of positive and negative SPA. Findings suggest that how positively and negatively older adults perceive their own aging may be important for their mental health while experiencing less physical function in late life.

### ADVANCE DIRECTIVES: SOLO AGERS RISK OF BECOMING "UNBEFRIENDED"

Rupal Parekh[1], Kathryn Betts Adams[1], and Donna Schuman[2], *1. University of Connecticut, West Hartford, Connecticut, United States, 2. University of Texas at Arlington, Arlington, Texas, United States*

Solo agers are an at-risk population of almost 14 million older U.S. adults currently living alone (Administration for Community Living, 2018). Having been independent for most of their lives they often enter older age unprepared for declining health. Even though the numbers of solo agers are rapidly increasing with the aging of the Baby Boomer generation, research remains scant on this population (Colby & Ortman, 2014). A mounting concern facing solo agers is finding themselves "unbefriended" (i.e., having no one to act as health care proxy in the event of incapacitation due to a medical crisis). The risk of unbefriended status emphasizes the critical importance of advance care planning; however, many solo agers have no advance directives and factors influencing advance care planning in this group are unclear. In this mixed-methods study, we examined factors influencing advance care planning among solo agers. Survey data were collected from 467 members of a Facebook group for self-identified "elder orphans," six of whom subsequently participated in qualitative interviews. Among these solo agers, 55% indicated they had advance directives. Hierarchical logistic regression results indicated financial and overall wellbeing predicted having advance directives; however, perceived health risk did not. An interpretive phenomenological analysis of data from six in-depth interviews revealed emergent themes of fears of the future and reluctance to plan for end of life despite acknowledging health risks. Findings can inform policies to meet the growing needs of solo agers who may be at elevated risk of becoming unbefriended.

### ATTITUDES TOWARD AGING AND WELLNESS ENGAGEMENT IN LIFE PLAN COMMUNITIES

Jennifer Smith[1], Matthew Fullen[2], and Philip Clarke[3], *1. Mather Institute, Evanston, Illinois, United States, 2. Virginia Tech, Blacksburg, Virginia, United States, 3. Wake Forest University, Winston-Salem, North Carolina, United States*

Life Plan Communities, also known as Continuing Care Retirement Communities, typically offer a wide range of wellness programs and services, including fitness classes, educational lectures, volunteer opportunities, and social events. Despite the convenience of onsite wellness resources, some residents choose not to participate in wellness offerings available at the community. One factor that may influence engagement in wellness behaviors is older adults' attitudes toward aging, which has been associated with differences in health and well-being over time. The purpose of this research was to examine the relationship between attitudes toward aging and wellness engagement among residents of Life Plan Communities. A total of 447 residents (ages 59 to 97; $M = 81.82$, $SD = 6.55$; 65% female) of 10 communities completed surveys that measured their attitudes toward aging, self-reported wellness, interest in improving wellness, participation in wellness programs, and barriers to wellness participation. Multiple regression analyses revealed that more positive attitudes toward aging were associated with better wellness, higher interest, more frequent participation, and fewer barriers, controlling for age, gender health, education, and marital status. Follow-up analyses revealed that these effects could be accounted for by specific types of aging attitudes (i.e., psychological growth, psychosocial loss, and physical change). For example, greater psychological growth was associated with greater wellness, interest in improving wellness, and wellness participation, whereas greater psychosocial losses was associated with greater perceived barriers and lower wellness. These findings have implications for the development, implementation, and promotion of wellness programs for older adults.

### WHO PLANS FOR LATER LIFE? AN EXAMINATION OF MIDDLE-AGED AND OLDER FLORIDIANS

Anne Barrett, Hope Mimbs, Cherish Michael, and Jessica Noblitt, *Florida State University, Tallahassee, Florida, United States*

The extension of life expectancy highlights the importance of understanding how people conceptualize – and plan for – their later years. We address this issue using data from an online survey of over 3,400 Floridians aged 50 and older that was conducted between December 2020 and March 2021 and funded by the Florida Department of Transportation. We examine five types of planning: for health care needs, financial well-being, living arrangements, driving retirement, and end-of-life care. We find that the likelihood of planning varies considerably across these types. Only 23 percent of respondents reported planning "some" or "a lot" for driving retirement, compared with 74 percent for health care needs, 76 percent for end-of-life care, 77 percent for living arrangements, and 83 percent for financial well-being. Likelihood of planning varied by age, gender, socioeconomic status, health, and race or ethnicity. Across all types of planning, older adults and those with at least a college degree and higher income were more likely to have planned. Women were more likely than men to plan for their financial future, living arrangements, driving retirement, and end-of-life care. Those in better health were more likely to plan for their financial future and end-of-life care. The effects of race or ethnicity were less consistent across the types of planning. White respondents were more likely than other race or ethnic groups to report planning for their living arrangements and end-of-life care, while Hispanic respondents were more likely than other groups to plan for driving retirement.

### SESSION 6260 (POSTER)

### BRAIN HEALTH, HEALTH BEHAVIORS, AND SOCIAL RELATIONSHIPS

### AGITATION IN ALZHEIMER'S DISEASE: A DECISION TREE FOR HEALTHCARE PROVIDERS

George Grossberg[1], Angela Sanford[2], Susan Scanland[3], Karen Tracy[4], and Richard G Stefanacci[5], *1. St Louis University School of Medicine, St Louis, Missouri, United States, 2. St Louis University, St Louis, Missouri, United States, 3. U of Pittsburgh, Pittsburgh, Pennsylvania, United States, 4. GSA, Washington, District of Columbia, United States, 5. Thomas Jefferson University, Philadelphia, Pennsylvania, United States*

Agitation in Alzheimer's Disease (AAD) impacts nearly 80% of persons with Alzheimer's Disease and is a cause of significant distress for patients and family/professional caregivers.Clinicians need guidance relative to assessment as well as non-pharmacologic and pharmacologic treatment approaches to AAD. To this end, the Gerontological Society of America (GSA) convened a group of experts representing Geriatric Psychiatry, Gerontological Nursing, Geriatric Medicine, and Long-Term Care to develop a "Decision Tree for Healthcare Providers" in the evaluation and management of AAD. The "Decision Tree" is a practical, user-friendly template for clinicians which provides a step-by-step approach to differential diagnosis of AAD, including conditions such as delirium, pain/discomfort, environmental and psycho-social stressors. The "Decision Tree" also walks the clinician through a range of non-pharmacologic treatment options such as music therapy; activities therapy, environmental modification and de-prescribing. The "Decision Tree" highlights rational use of pharmacotherapies as well as their hazards in this vulnerable population.

### ASSOCIATIONS BETWEEN DAILY DRINKING AND DAILY MEMORY LAPSES AMONG MIDDLE-AGED AND OLDER ADULTS IN THE MIDUS STUDY

Sara Miller[1], Jacqueline Mogle[2], Ashley Linden-Carmichael[3], and David Almeida[2], *1. Pennsylvania State University, University Park, Pennsylvania, United States, 2. The Pennsylvania State University, University Park, Pennsylvania, United States, 3. Pennsylvania State University, Pennsylvania State University, Pennsylvania, United States*

Alcohol use predicts short- and long-term memory impairment. However, it is unclear how alcohol influences subjective memory, which refers to perceptions of one's own memory functioning. This study examined associations between daily alcohol use and memory lapses (i.e., subjective memory) and evaluated perceived impact of memory lapses on daily life. Participants (n=953; Mage=55.2) were non-abstaining adults from the Midlife in the United States (MIDUS) study who participated in the 8-day daily diary project. Survey items included number of drinks and 9 memory lapse items (1=yes). After each reported memory lapse, participants indicated memory lapse irritation (range=1-10) and interference (range=1-10). Covariates included age, gender, race, education, and cognitive functioning. Multilevel logistic regression analysis revealed that within-person alcohol use (OR=1.07; $p< 0.01$), but not between-person alcohol use ($p>0.05$), was associated with greater odds of reporting any memory lapses. When assessing retrospective and prospective lapses separately, only the prediction of prospective lapses from between-person alcohol use was significant (OR=1.11; $p=0.01$). Finally, multilevel linear regression analysis indicated that neither within-person nor between-person alcohol use was predictive of reported irritation or interference from memory lapses ($p>0.05$). Study findings revealed between- and within-person associations between alcohol use and subjective memory. At the daily-level, individuals were more likely to notice memory lapses on days they drank more than usual. At the person-level, people who drank more relative to others were more likely to report prospective memory lapses. Future studies should assess how associations between alcohol use and subjective memory relate to objective cognitive functioning outcomes.

### INVESTIGATION INTO THE PREDICTORS OF HELP-SEEKING BEHAVIOR IN OLDER ADULTS

Michael Kann[1], Silvia Chapman[2], Martina Azar[3], Jillian Joyce[4], Leah Waltrip[5], Shaina Shagalow[4], Sandra Rizer[5], and Stephanie Cosentino[5], *1. Columbia University, Pittsburgh, Pennsylvania, United States, 2. Columbia University Irving Medical Center, New York City, New York, United States, 3. VA Boston Healthcare System, Boston, Massachusetts, United States, 4. Columbia University, New York City, New York, United States, 5. Columbia University Medical Center, New York, New York, United States*

Early help seeking (HS) among patients with emerging Alzheimer's disease and related dementias (AD/ADRD) can have considerable implications for treatment course, access to clinical trials, lifestyle, and future quality of life. Previous studies in older adults suggest cognitive impairment itself does not lead people to seek help; rather, HS appears to be driven by Subjective Cognitive Decline (SCD), personality, and mood. It is possible, however, that tests used to measure objective cognition were not sensitive to detect subtle cognitive impairments that may influence HS behavior. In this study of 142 cognitively healthy older adults, we examined if utilizing cognitive tasks sensitive to preclinical AD (i.e., short term memory binding, associative memory, and susceptibility to semantic interference) revealed an independent association between objective cognition and HS, or if SCD continued to be a primary driver. Participants were assessed for HS, SCD severity, personality traits (conscientiousness and neuroticism), depressive symptoms, and demographics (age, gender, education). Partially and fully adjusted regression models were conducted to examine the association between cognitive tests and HS whilst adjusting for demographics, personality, depressive symptoms, and SCD. Associative memory was the only cognitive marker significantly worse in those who help seek (B=- 0.07, SE=0.03, p=.031); however, it did not withstand adjustment for SCD. Only increased SCD (B=0.06, SE=0.02, p=.005) and educational attainment (B=0.42, SE=0.15, p=.005) had independent associations with HS. Ongoing work is establishing possible moderators of these associations to unravel the multifaceted influences on HS and to guide strategies to increase HS in older adults with SCD.

### PATTERNS OF HEAVY ALCOHOL USE AMONG OLDER MARRIED AND COHABITING COUPLES

Angela Curl, Jennifer Bulanda, and Amy Roberts, *Miami University, Oxford, Ohio, United States*

Heavy drinking has increased among older adults in recent years. Research suggests romantic partners influence each other's alcohol use, but this influence may vary by partnership type and relationship quality. Heavy drinkers are more likely to form cohabiting versus marital unions and, relative to marriage, cohabitation is linked with a higher risk of heavy episodic drinking. Although some studies have explored older married couples' alcohol consumption, we lack research on older cohabitors despite the substantial growth in this form of partnership during later life. We use data from the 2014-2018 waves of the Health and Retirement Study to examine drinking behavior among a sample of older married and cohabiting couples (n=1,876) using Actor-Partner Interdependence Models (APIM). Heavy drinking is measured as consuming more than 3 drinks on a given day or 7 drinks per week for women, or 4 drinks on a given day or 14 per week for men (NIAAA, 2021). Compared to first marriages, being in a cohabiting union is associated with significantly higher odds of one (OR=5.22) or both (OR=3.82) partners becoming heavy drinkers over the observation period, but there are no significant differences between those in first marriages and remarriages. Relationship quality is also a significant correlate of heavy drinking risk, with male partners' negative relationship quality and female partners' positive relationship quality associated with a higher risk of heavy drinking over time. Results suggest the importance of accounting for partnership type and relationship quality in understanding partners' drinking behavior.

### DEVELOPMENT OF A BEHAVIORAL FRAMEWORK FOR DEMENTIA CARE PARTNERS' FALL RISK MANAGEMENT

Yuanjin Zhou[1], Clara Berridge[2], Nancy Hooyman[2], Tatiana Sadak[2], Tracy Mroz[2], and Elizabeth Phelan[2], *1. University of Texas at Austin, Austin, Texas, United States, 2. University of Washington, Seattle, Washington, United States*

Although older adults living with dementia (OLWD) are at high risk for falls, few strategies that effectively reduce falls among OLWD have been identified. Dementia care partners may have a critical role in fall risk management (FRM). However, little is known in what ways care partners behave that may be relevant to FRM and how to effectively engage them in FRM. Semi-structured, in-depth interviews were conducted with 14 primary care partners (Age: 48-87; 79% women; 50% spouses/partners; 64% college-educated; 21% people of color) of community-dwelling OLWD to ascertain their own behaviors and those of care partners who were secondary in the caring role. The analysis of interview data suggested a novel behavioral framework consisting of eight domains of FRM behaviors adopted across four stages. The domains were 1. functional mobility assistance, 2. assessing and addressing health conditions, 3. health promotion support, 4. safety supervision, 5. modification of the physical environment, 6. receiving, seeking, and coordinating care, 7. learning, and 8. self-adjustment. Four stages of FRM included 1. supporting before dementia onset, 2. preventing falls, 3. preparing to respond to falls, and 4. responding to falls. FRM behaviors varied by the care partners' caring role. Primary care partners engaged in behaviors from all eight domains. Secondary care partners were reported to assist in health promotion support, safety supervision, modification of the physical environment, and receiving, seeking, and coordinating care. This multi-domain and multi-stage framework will inform intervention development to engage care partners in managing fall risk for community-dwelling OLWD.

### LONGITUDINAL ASSOCIATIONS OF SOCIAL CONNECTEDNESS, SOCIAL CONTRIBUTION, SOCIAL ENGAGEMENT, AND LATE-LIFE DRUG USE

Atami De Main[1], Daniel Powers[2], and Bo Xie[2], *1. Weill Cornell University, New York City, New York, United States, 2. The University of Texas at Austin, Austin, Texas, United States*

Substance misuse among older adults is a growing and complex problem with implications for society, aging adults, and their families. It is understudied in research and clinical practice. Limited and mixed evidence exists that suggests relationships between changes in older adults' social environment and their drug use over time. This study addressed this gap by examining potential associations between social environment and drug use among community-dwelling older adults. Data were drawn from 3-waves of the national longitudinal survey of Midlife Development in the United States (MIDUS) (N= 2,020; age range=55-94 years; Mean=63.10, SD=5.66 at Wave 1). We estimated multilevel logistic growth models to assess the relationships between social environment indicators (social connectedness, social contribution, social engagement) and drug use, controlling for age, marital status, race/ethnicity, education level, household income, employment status, and number of chronic conditions. Our findings showed significant odds of drug use over the 20-year period of study, which were increased among older adults with multiple chronic conditions with a 59.5% risk of drug-related problems. Social contribution (feelings of being valued by society) negatively predicted older adults' drug use whereas social connectedness and social engagement were not significant predictors over time. The models also indicated a moderating effect of social contribution on drug use, showing low changes in drug use over time with low social contribution. Our findings illuminate the differential roles of social environment indicators in drug use. Future research, policy, and practice may particularly focus on the role of social contribution in late-life drug use.

### DEMENTIA CAREGIVERS: THE IMPACT OF SOCIAL DISTANCING ON RELIGIOUS AND SPIRITUAL PRACTICE AND BEHAVIORAL SYMPTOMS

Katherine Britt[1], Kavita Radhakrishnan[2], Liam Fry[2], and Kathy Richards[2], *1. University of Pennsylvania, Austin, Texas, United States, 2. University of Texas at Austin, The, Austin, Texas, United States*

Prevalent among older adults with Alzheimer's disease related dementia (ADRD), behavioral symptoms can cause adverse consequences for those with ADRD and their caregivers. Prompting earlier institutionalization and increased rapid cognitive decline, behavioral disturbances are difficult to manage. Social isolation prompted by the COVID-19 pandemic limited social and religious activities utilized by caregivers and older adults with ADRD for coping and may have affected behavioral symptoms and overall well-being. This qualitative study aimed to explore the impact of the pandemic on behavioral symptoms, religious and spiritual (R/S) utilization for coping, and well-being in caregivers and older adults with ADRD. A purposive sample of 7 home family caregivers and 7 nursing home caregivers (4 indirect family caregivers, 3 nurse practitioners) actively caring for an older adult with ADRD participated in semi-structured telephone interviews. Directed content analysis was used to analyze data. While behavioral symptoms increased overall for older adults with ADRD and social R/S practice abruptly declined, they still utilized individual R/S resources for coping such as prayers, reading religious texts, and holding a prayer book which provided a calming effect, decreased their anxiety, and prompted memories. Caregivers participated in R/S virtual groups which calmed them, connected them with others, helped them transition to sleep, and prompted mindfulness. Resources and interventions utilizing social and individual R/S for coping are warranted as they have the potential to promote cognitive stimulation, connection with others, decrease neuropsychiatric symptoms, and decrease stress.

### SESSION 6270 (POSTER)

### CARE INTERVENTIONS AND INNOVATIONS

### TOILETING AND MOBILITY ASSISTANCE PREFERENCES OF PATIENTS ON AN ACUTE CARE FOR ELDERS (ACE) UNIT

Lucy Wilson[1], Emily Hollingsworth[1], Avantika Shah[2], and Sandra Simmons[1], *1. Vanderbilt University Medical Center, Nashville, Tennessee, United States, 2. Johns Hopkins University, Baltimore, Maryland, United States*

While the Agency for Healthcare Research and Quality (AHRQ) fall prevention guidelines include recommendations for providing consistent toileting and mobility assistance during hospitalization, little is known about hospitalized older adults' preferences for receiving such assistance. The aim of this study was to identify older adults' perceived need and preferences for toileting and mobility assistance during their hospital stay. We interviewed 150 patients aged 50 or older and asked about their perceived need for toileting and mobility assistance and how frequently hospital staff provided it. A total of 75 patients (50%) reported a need for toileting assistance; and, of those who reported a need for assistance, 95% reported that staff provided assistance at a frequency that met their needs. A total of 72 patients (48%) reported a need for mobility assistance; however, an additional 41 patients (27%) had not yet attempted to ambulate at the time of the interview. Across all patients, 100 (67%) reported either no attempt or having ambulated only once during their hospital stay. Most patients who required toileting or mobility assistance stated it was "very" or "extremely important" to receive assistance (82% and 76%, respectively). In summary, approximately one-half of older patients on an ACE unit require toileting and/or mobility assistance. Efforts to provide consistent toileting and mobility assistance during hospitalization could require significant staff time based on the number of patients who need it. Further research is needed to determine how to comprehensively implement AHRQ fall prevention guidelines in the hospital setting for older patients.

### 4MS AGE-FRIENDLY HEALTHCARE INITIATIVE IMPLEMENTATION ON AN ACUTE CARE FOR ELDERS UNIT

Lucy Wilson, Chelsea Rick, Sarah Welch, Sarah Nelson, Olivia Lawson, Hannah Boren, and Mariu Duggan, *Vanderbilt University Medical Center, Nashville, Tennessee, United States*

Although evidence supports 4Ms [Medication, Mentation, What Matters Most (WMM), Mobility] care for older adults, successful implementation at an academic hospital is not well described. The aim of this study was to describe the implementation methods and process measures for 4Ms delivery on the Acute Care for Elders (ACE) unit at an academic hospital. We used the Institute for Healthcare Improvement's Model for Improvement to guide efforts. We set SMART goals for each M: best possible medication reconciliation, identification of potentially inappropriate medications, and recommendations to deprescribe (medication), delirium screen documented 2x/day and delirium nonpharmacologic protocol in place (mentation), documentation of WMM and care alignment (WMM), Johns Hopkins-Highest Level of Mobility screen assessed during the patient's hospitalization, mobilization of patient 3x/day offered and documented, and restraints avoided (mobility). We mapped current and ideal workflows. We sought community grant funding to expand the implementation team, supporting a nurse educator to train the unit and data analyst to extract real-time data from the electronic medical record to inform improvement processes. Multiple Plan-Do-Study-Act cycles were run iteratively and discussed at weekly team meetings. We included patients >65 years old, admitted for >48 hours, and excluded patients admitted on hospice. Of 519 eligible patients admitted from 04/2021-01/2022, goals were met by 454 (87%) for medication, 187 (36%) for mentation, 130 (25%) for WMM, and 6 (1%) for mobility. We found implementing 4M care processes at an academic hospital to be feasible. Further exploration of barriers to meeting the mobility target is warranted.

### ADDRESSING GAPS IN PRIMARY CARE DIAGNOSIS OF COGNITIVE IMPAIRMENT VIA A NURSE CONSULTATION

So Young Shin[1], Nhat Minh Bui[2], Rachel Kiekhofer[2], Collette Goode[2], Sarah Dulaney[2], and Katherine Possin[2], *1. Inje University, Busan, Pusan-jikhalsi, Republic of Korea, 2. Memory and Aging Center University of California, San Francisco, San Francisco, California, United States*

Purpose: This study aimed to identify the concerns and unmet needs of patients and care partners after incident primary care diagnosis of cognitive impairment.

Methods: Primary care providers referred older adults who had newly diagnosed with cognitive impairment for a telephone encounter, the 'Brain Health Consultation' (BHC), with a dementia expert nurse. The nurse assessed for questions or concerns regarding immediate needs, cognitive, neuropsychiatric, functional, or other symptoms; cognitive assessment results and the diagnosis; care planning including safety, prognosis, treatments, advance care planning, and community services.

Results: Patients (N=37) and care partners (N=30) completed the BHC. The patients were racially/ethnically diverse; 51% Asian, 18% Non-Hispanic White, 10% Hispanic, 10% Black, 11% other). Most patients (70%) and caregivers (70%) endorsed cognitive concerns, and many patients endorsed mood (65%), sleep or fatigue (49%), and pain (10%) concerns. All patients and care partners had questions about the assessment results and diagnosis, and some patients (11%) and caregivers (13%) expressed concerns about disease progression. Few patients and caregivers expressed care planning needs.

Conclusion: Following incident cognitive impairment diagnosis in primary care, patients and families have unmet needs around understanding their assessment and diagnosis. Care planning may be reserved for a follow-up consultation after the patient and family have had time to understand and accept the diagnosis. While we used a dementia expert nurse to perform the BHC, given the types of concerns identified, a supervised, trained, unlicensed health professional (e.g., a care team navigator) may be appropriate to perform the BHC.

### A VIRTUAL STAFF TRAINING TO IMPROVE COMMUNICATION FOR OLDER ADULTS DURING MASK WEARING

Sara Mamo, Katherine Garrity, and Kara Wheeler, *University of Massachusetts Amherst, Amherst, Massachusetts, United States*

The need for masking protocols in care centers for older adults exacerbated communication challenges in an already difficult environment. The purpose of this study was to alleviate the burden of communicating while wearing masks by providing a virtual staff training that introduced a headset amplifier and communication tips. The training was delivered via Zoom to small groups of staff at three Programs for All-inclusive Care for the Elderly (PACE®) organizations. The training included education about the impact of age-related hearing loss, instructions for using a headset amplifier, and communication tips. Staff were encouraged to use the amplifier with as many participants as possible to ease communication while wearing masks—rather than targeting participants based on hearing status. A pre/post quasi-experimental approach was undertaken. Fifty-one staff members completed the training and immediate pre/post questionnaires to measure knowledge gain. Follow-up questionnaires (including open-ended responses) were collected at 2- (n = 29), 4- (n = 23), and 6-months (n = 23) post-training. In addition, we completed one focus group (n = 5) and one in-depth interview regarding the feasibility of participation in the research project and brainstorming to increase use of the amplifiers. By integrating quantitative and qualitative findings, we highlight communication improvements when using the amplifiers and tips for integrating amplifiers into group care programs. The findings from this study will contribute to the development of a large-scale intervention to address hearing loss and support communication for older adults in group care settings.

### SIGNIFICANCE OF SECOND YEAR MEDICAL STUDENTS PARTICIPATING IN THE 48-HOUR HOSPICE HOME IMMERSION PROJECT, 2017–2018

Samuel Hanlon, Benjamin Packard, and Marilyn Gugliucci, *University of New England College of Osteopathic Medicine, Biddeford, Maine, United States*

Medical education on palliative medicine and end-of-life care is generally lacking in the medical curricula. The University of New England College of Osteopathic Medicine (UNECOM) Learning by Living: 48 Hour Hospice Home Immersion Project is an immersion-based learning model whereby UNECOM 2nd year students live in an18-bed acute care hospice house to care for dying patients, provide family support, and conduct post-mortem care. This project determined if and in what ways immersion experiences were valuable in augmenting student medical end-of-life care education during AY 2017-2018.Retrospective ethnographic/

autobiographic data were analyzed from the eight randomly selected student hospice immersion journals (approx. 200 pages) who participated during academic year 2017-18. Prefieldwork, fieldwork, post-fieldwork journals were reviewed and analyzed using manual content analysis followed by NVivo 12+ analysis. Thematic coding resulted in representative quotes, key words, and native concepts. Inter-rater reliability was established with the use of a codebook and agreed upon thematic definitions. Four key themes included: Subversion of End of life (EOL) Expectations; Character Development/Introspection; Exposure to Diverse Cultural/Spiritual Perspectives; and Skills to Bring into Future Practice. Proximity to death/dying resulted in reflections on values and priorities, and a renewed sense for compassionate patient care. Students developed skills for future practice, including competency in EOL and post-mortem care, navigating difficult, emotionally laden family dynamics, and contributing to an interprofessional staff team even in uncomfortable situations. This immersion positively affected student perspectives about death and end-of-life care; creating life-altering experiences in patient-centered-care. Students stated significant impacts to employ as a physician.

### AN INNOVATIVE APPROACH TO ENHANCING COPD CARE AND MANAGEMENT IN A RURAL NORTHERN COMMUNITY

Shannon Freeman[1], Anthon Meyer[2], Kelly Skinner[3], and Laura Peach[3], *1. University of Northern British Columbia, Prince George, British Columbia, Canada, 2. Northern Health Authority, Prince George, British Columbia, Canada, 3. University of Waterloo, Waterloo, Ontario, Canada*

Background: COPD is the third leading cause of death worldwide. Rural communities often face challenges to provide high quality chronic disease care for aging populations. Despite these longstanding challenges, there was an intention to improve the care setting by developing and fostering a shared vision for quality care, as evidenced by enhancing COPD screening and care. To ensure consistent and longitudinal patient access to high quality of care as well and ongoing physician recruitment and retention a new rural program was developed. Objective-In this presentation we will describe a new rural community based COPD program from conceptualization and development through to current functioning highlighting areas of innovation. Methods-A process evaluation guided by Moore et al.'s framework to assess program implementation, mechanisms of impact, and context was conducted. Qualitative thematic analysis was undertaken of stakeholder interviews conducted in 2021 (n=11) and document review (n=60;~500 pages) of key clinic documents dated back to pre-program development.

Results: We describe five phases of program development: Survive; Reorganize and Stabilize; Assess and Respond; Build and Refine; and Sustain and Share. Outreach and localizing resources improved access to the program. Acquiring secured physician compensation, capturing quality data, and improving patient and provider self-efficacy built the capacity of the system and stakeholders within it. Finally, relationships were forged through building an integrated facility, collaborative networking, and patient engagement. The key elements of program implementation were the resources required to ensure its operation, categorized as hardware, software, organizational, and human.

### EVALUATING IMPLEMENTATION FIDELITY TO A NURSE-LED CARE MODEL IN NURSING HOMES: A MIXED-METHODS STUDY

Raphaelle Ashley Guerbaai[1], Sabina De Geest[2], Michael Simon[1], Lori Popejoy[3], Nathalie Wellens[4], Kris Denhaerynck[1], and Franziska Zúñiga[5], *1. The Institute of Nursing Science, Basel, Basel-Stadt, Switzerland, 2. Institute of Nursing Science, Basel, Basel-Stadt, Switzerland, 3. University of Missouri, Columbia, Missouri, United States, 4. Haute Ecole La Source, Lausanne, Vaud, Switzerland, 5. University of Basel, Basel, Basel-Stadt, Switzerland*

Implementation fidelity assesses the degree to which an intervention is delivered as intended. Little is known about how it acts as a moderator between an intervention and its intended outcome(s) and which factors affect the fidelity trajectory over time. We exemplify implementation fidelity in INTERCARE, a nurse-led care model implemented in eleven Swiss nursing homes (NH) successfully decreasing unplanned hospital transfers. A mixed-methods design was used, guided by the Conceptual Framework for Implementation Fidelity. Fidelity to INTERCARE's core components was measured with 44 self-developed items at 4-time points (baseline, 6, 12 months after intervention start, 9 months post-intervention; fidelity scores were calculated for each component and overall. Structured notes from NH meetings were used to identify moderators affecting the fidelity trajectory over time. Generalized linear mixed models were computed to analyze the quantitative data. Deductive thematic analysis was used for the qualitative analysis. The quantitative and qualitative findings were integrated using triangulation. A higher overall fidelity score showed a decreasing rate of unplanned hospital transfers (OR: 0.65 (CI=0.43-0.99), $p=0.047$). Higher fidelity score to advance care planning was associated with lower unplanned transfers (OR= 0.24 (CI 0.13-0.44), p= < 0.001) and a lower fidelity score for communication tools (e.g., ISBAR) to higher rates in unplanned transfers (OR= 1.69 (CI 1.30-2.19), p= < 0.003). High implementation fidelity to INTERCARE was necessary to achieve a reduction in unplanned transfers. In-house physicians with a collaborative approach and staff's perceived need for nurses working in extended roles were important factors for high fidelity.

### AN INTERPROFESSIONAL APPROACH TO DEPRESCRIBING: A CURRICULAR FRAMEWORK

Winnie Sun[1], Cheryl Sadowski[2], and Barb Farrell[3], *1. Ontario Tech University, Lindsay, Ontario, Canada, 2. University of Alberta, Edmonton, Alberta, Canada, 3. Bruyere Continuing Care, Ottawa, Ontario, Canada*

Deprescribing is an important approach for managing polypharmacy and reducing harm from potentially inappropriate medications. Healthcare professionals identify barriers to deprescribing, including lack of knowledge and skill. This is not surprising as pre-licensure education does not consistently incorporate components of deprescribing into curricula. As such, there is a clear need to consider how to promote deprescribing competencies, teach related knowledge and skills and assess learning outcomes. The Canadian Deprescribing Network (CaDeN) Health Care Professional Committee undertook a consensus process to develop a proposed competency framework that describes essential knowledge, teaching strategies, and assessment protocols to promote deprescribing skills and advocate for consistent education about deprescribing principles and practices. The framework is informed by the deprescribing process, which includes gathering and interpreting patients' medication history and clinical information within their context, using tools that help identify potentially inappropriate medications, weighing potential benefit and harm of continuing or deprescribing medications, using shared decision-making to make decisions about deprescribing, communicating deprescribing and monitoring plans, and monitoring progress and outcomes. The competency framework considers interprofessional learning and how to involve patients and care partners in deprescribing decisions. Integrating deprescribing competencies in healthcare curricula requires an intentional and structured approach across all years of the program, focusing on interprofessional collaboration. Learning activities should be active and practical, progressing from early to advanced learner skills and include integration of deprescribing during experiential education. This framework includes a review of the competencies, learning outcomes, and assessment strategies, with a discussion of strategies to incorporate interprofessional learning activities.

### CONSIDERATIONS OF AGE-FRIENDLY 4M PRINCIPLES IN DEPRESCRIBING INTERVENTIONS: A SCOPING REVIEW

Jinjiao Wang[1], Jenny Shen[2], Yeates Conwell[2], Kobi Nathan[3], Fang Yu[4], Erika Ramsdale[2], Donna Fick[5], and Sandra Simmons[6], *1. University of Rochester School of Nursing, Rochester, New York, United States, 2. University of Rochester Medical Center, Rochester, New York, United States, 3. St. John Fisher College, Rochester, New York, United States, 4. Arizona State University, Phoenix, Arizona, United States, 5. Penn State University, University Park, Pennsylvania, United States, 6. Vanderbilt University Medical Center, Nashville, Tennessee, United States*

The 4Ms – Medication, Mentation, Mobility, and What Matters - represent the key components in the Age-Friendly Health Systems initiative and provide a conceptual framework for research in the older adult population. Over 40% of older adults experience polypharmacy, which can be addressed by deprescribing unnecessary medications. This review aimed to assess the degree to which the 4Ms were considered in intervention design, sample selection, and outcome assessment in deprescribing trials by keyword search in six databases and snowballing. Thirty-seven of the 564 trials identified met the review eligibility criteria. Imbalanced consideration of 4Ms in the deprescribing trials was observed. Intervention design: "Medication" was considered in all trials; "Mentation" was considered in 8 trials; "Mobility" (n=2) and "What Matters" (n=6) was less often considered. By targeting providers, most of the trials lacked consideration of patient-centeredness - aligning what matters most to older adults and their families with deprescribing decision making and implementation. Sample selection: "Medication" was considered in 15 trials (e.g., samples including patients taking ≥ 5 medications or specific types of medications), whereas "Mentation" (n=6), "Mobility" (n=6) and "What Matters" (n=0) were less often considered. Outcome assessment: "Medication" was the most commonly assessed outcomes (n=33), followed by "Mobility" (n=13) and "Mentation" (n=10) outcomes, with no study examining "What Matters" outcomes. 4Ms were not purposefully considered in the intervention design, sample selection, and outcome measurement of existing deprescribing trials. Future deprescribing trials need a more balanced and complete consideration of the 4Ms in the trial design and implementation.

### HIGH-RISK ELDERLY SCREENING IN MEDICAL INTENSIVE CARE UNIT

Gowrishankar Gnanasekaran[1], and Narendrakumar Alappan[2], *1. Cleveland Clinic Foundation, Cleveland, Ohio, United States, 2. Fairview Hospital, Cleveland Clinic, Cleveland, Ohio, United States*

Geriatrics care in medical intensive care units (MICU) establishes a unique opportunity in early screening of High Risk Elderly (HRE) patients admitted for critical care. Many MICUs do not have a standard protocol to screen for HRE patients as part of their daily huddle. Our program is a quality improvement initiative to improve early identification of HRE patients in the MICU. HRE patients were identified based on nursing specific screening triggers at one of the Regional Hospitals of a large teaching hospital in Northeast Ohio.The program was designed as a part of geriatrics care expansion at regional hospital sites. Identified patients were discussed in daily huddles to determine unique geriatric needs in caring for these patients. A geriatrics co-management team was engaged in comprehensive geriatric assessments and care transition when it was needed. Geriatrics care in MICU demonstrates a unique opportunity in early identification of HRE patients. This helps to support a patient -centered approach in caring for critically ill elderly patients. The program would lay foundations in early screening for risk factors and optimizing elderly care in MICU.

### RESULTS OF A PILOT IMPLEMENTATION OF AN ADL GUIDELINE FOR NURSING PROFESSIONALS IN THE NETHERLANDS

Svenja Cremer[1], Michel Bleijlevens[1], Silke Metzelthin[1], Janneke de Man-van Ginkel[2], and Sandra Zwakhalen[1], *1. Maastricht University, Maastricht, Limburg, Netherlands, 2. Julius Center for Health Sciences and Primary Care, Utrecht, Utrecht, Netherlands*

Nursing care in activities of daily living (ADL), such as washing, dressing, or eating is frequently provided while being poorly informed by scientific evidence. To address nursing professionals' need for guidance we developed a practice guideline on ADL-care. This guideline comprises eleven core recommendations on involving care receivers and informal caregivers in ADL-care, identifying ADL-care needs, and effective ADL-interventions. Since the success of this guideline hinges on its actual use by nursing professionals, we assessed the use and determined influencing factors to guide targeted strategies for future implementation in different nursing care settings. In a mixed-method study, nursing professionals documented the number of core recommendations applied over three weeks using recording forms and a self-administered questionnaire to identify barriers and facilitators. In addition, we conducted focus groups to capture team experiences in applying the 11 core recommendations and clarifying survey results. Seven nursing care teams participated from various settings: hospital (n=1), rehabilitation (n=2), home-care (n=1), and long-term care (n=3). Preliminary results reveal that participants consider the core recommendations compatible with and adaptable to current practices, and their care settings. Participants experienced advantages over existing practices. However, core-recommendation usage and perception of influencing factors appeared to be highly context-dependent, especially regarding the involvement of informal caregivers in ADL-care. Limited knowledge on the application of interventions to improve ADL limited the use of some recommendations. Our results underline the necessity of careful selection of targeted strategies for each setting tailoring the core recommendations to the different qualifications and roles of nursing professionals.

### DEVELOPMENT OF A HOPE-BASED HEALTH PROMOTION PROGRAM TO IMPROVE HEALTH BEHAVIORS AMONG DIVERSE OLDER ADULTS

Britteny Howell[1], Amber Worthington[2], and Leslie Redmond[3], *1. University of Alaska Anchorage, Anchorage, Alaska, United States, 2. UNIVERSITY OF ALASKA ANCHORAGE, Anchorage, Alaska, United States, 3. University of Manitoba, Anchorage, Alaska, United States*

Studies have shown that health education can improve dietary intake, exercise, and energy balance, which improves health outcomes and quality of life. However, diverse low-income older adults may have low self-efficacy coupled with potentially negative opinions regarding the aging process. Emerging research suggests that asset-based approaches utilizing persuasive and hopeful messages may be particularly effective with diverse populations of older adults who may be struggling with negative perceptions of self-efficacy. Research also shows that health behavior change interventions are most likely to be successful among diverse populations when they incorporate messages of resilience and facilitate information sharing and diffusion among participants. This presentation outlines the program curriculum development process of a student-led health promotion program by an interdisciplinary team of university researchers. Public health, dietetics, and communication faculty utilized Persuasive Hope Theory (PHT) and Self-Efficacy Theory to create a 15-week program to improve fruit and vegetable intake and physical activity among low-income adults aged 55+ living in Anchorage, Alaska. We will also detail the curricular components for this NIA-funded program which will be delivered in Fall 2022.

### A DEDICATED APPROACH TO PALLIATIVE DEMENTIA CARE

Judith Meijers[1], Sascha Bolt[2], Sandra Zwakhalen[1], Chandni Khemai[1], Jos Schols[1], Daisy Janssen[1], Jenny van der Steen[3], and Jesper Biesmans[4], *1. Maastricht University, Maastricht, Limburg, Netherlands, 2. Tilburg University, Tilburg, Noord-Brabant, Netherlands, 3. Leiden University Medical Center, Leiden, Zuid-Holland, Netherlands, 4. Zuyderland, Zuyderland, Limburg, Netherlands*

Nursing staff play a central role in the palliative care for people with dementia. Development of their palliative care competences may support timely recognition and addressing of individual needs of persons with dementia and their family caregivers in long term care. In the DEDICATED (Desired Dementia Care Towards End of Life) project, we aim to develop materials to support nursing staff in providing palliative dementia care. The first step of the project concerned a needs assessment, mapping the perspectives of nursing staff, family caregivers and people with dementia (scoping review, surveys and semi-structured interviews). Using these studies' results as a starting point, an intervention (the DEDICATED approach) was built, using an iterative co-creation approach involving nursing staff and educators (N=12). These 'ambassadors' were also trained to disseminate the approach within their care teams and nursing curricula. First reactions from the pilot study regarding the DEDICATED-materials are positive and nurses and nurse educators are eager to use the materials (www.dedicatedwerkwijze.nl). Currently the DEDICATED-approach is being evaluated using a mixed methods pretest-posttest controlled design comparing three groups: a design group (12 wards), a test group (28 wards, 28 new-trained ambassadors) and a control group (12 wards). The main outcomes of study are e.g. self-efficacy, empowerment and engagement in providing palliative care for people with dementia. A secondary outcome involves longitudinal trends of the quality of dying of persons with dementia within the group.

### ASSESSING INTERPROFESSIONAL ORAL HEALTH EDUCATION AND PRACTICE FACILITATION OUTCOMES WITHIN LONG-TERM CARE

Jennifer Crittenden[1], Rachel Coleman[2], David Wihry[3], Labrini Nelligan[4], and Denise O'Connell[4], *1. UMaine Center on Aging, Bangor, Maine, United States, 2. University of Maine, Bangor, Maine, United States, 3. University of Maine, Orono, Maine, United States, 4. Lunder-Dineen Health Education Alliance of Maine, Bangor, Maine, United States*

Older adults residing in long-term care (LTC) settings are at an increased risk of poor oral health due to comorbidities and limited ability to provide self-care. Routine oral care is essential for maintaining overall health. Coupled with resident vulnerability is a traditional lack of training and focus on oral health care in LTC. MOTIVATE (Maine's Oral Team-Based Initiative: Vital Access to Education) is a pilot (N = 8 sites) interprofessional education program focused on daily oral health care within LTC, providing education and technical assistance to advance staff knowledge, skills, and attitudes about oral health. An evaluation was carried out using a pre/post survey design with instruments administered immediately before and after learning module completion along with a survey administered one month after implementation. Knowledge, attitudes, and oral health practices were assessed. A statistical comparison between baseline (N = 491) and post-launch (N = 215) scores revealed a statistically significant improvement ($p < 0.001$) across all knowledge and attitudes measures including the perceived importance of oral health, understanding of interprofessional roles among the care team, the role of oral health in supporting resident dignity and quality of life, and confidence in providing oral health care. Factors facilitating the transfer of knowledge to practice (N = 478) included personal interest in the topic (46.4%), knowing where to obtain information when needed (47.6%), and knowing how to apply learning to LTC daily care responsibilities (62.4%). Findings underscore the importance of oral health training and implications for practice transformation in an interprofessional context.

### EFFECTS OF SPECIAL NURSING UNITS IN NURSING HOMES: FOCUSING ON HEALTH OUTCOMES AND SATISFACTION

Eunhee Cho, YeaSeul Yoon, Seonhwa Choi, and Eunkyo Kim, *Yonsei University, Seoul, Seoul-t'ukpyolsi, Republic of Korea*

This quasi-experimental study aimed to evaluate the effectiveness of special nursing units in nursing homes by comparing health outcomes and satisfaction between the general nursing units and special nursing units across nursing homes in South Korea. Surveys—paper, phone, and online—were conducted five times (March, August, and November 2019; June and October 2020) for health outcomes (health pattern changes and the number of residents with new health problems) and four times (March and August–November 2019; June and October 2020) for satisfaction. Descriptive analysis, $\chi^2$-test, paired t-test, and McNemar test were performed using the SPSS 25.0 program. The results showed an improvement in the health outcomes of residents in the special nursing units. Regarding health pattern changes, there was a decrease in the number of residents facing problems related to consciousness, cardiovascular function, urination, defecation, and pain and of those with new health problems such as aches, falls, pressure sores, and urinary incontinence. Furthermore, caregivers' satisfaction in the special nursing units was higher than that of their counterparts in the general nursing units. The results of this study will be used as evidence to expand this special nursing unit's services in the future.

### HEALTHCARE AIDE-FOCUSED INTERVENTIONS TO IMPROVE PAIN MANAGEMENT IN LONG-TERM CARE

Jennifer Knopp-Sihota[1], Megan Nuspl[2], Tara MacGregor[2], Jennifer Reeves[2], and Ahsan Saleem[2], *1. Athabasca University, Edmonton, Alberta, Canada, 2. University of Alberta, Edmonton, Alberta, Canada*

Pain is endemic for residents of long-term care homes, with many residents experiencing pain daily. Given that healthcare aides provide most daily care for residents, they are ideally situated to deliver timely assessment and non-drug interventions for managing resident pain. In this Cochrane-style systematic review, we searched 7 databases to identify intervention studies that included long-term care residents aged ≥60 years who received interventions to reduce chronic pain. Interventions were either delivered by healthcare aides at the resident level or were directed at healthcare aides to improve their pain management practices. We screened 400 titles/abstracts and 152 full-text articles. Nine studies met inclusion criteria and were included in a narrative review. Due to the limited number of studies and variety of study designs, data were insufficient to perform meta-analyses or thematic analysis. Three studies described pain interventions delivered by healthcare aides at the resident level reporting significant improvement of pain. Six studies described pain interventions delivered to healthcare aides. Results of these interventions were inconsistent; 2 reported significant improvements in pain-related outcomes (e.g., resident pain, monitoring of pain), 3 reported insignificant changes, and 1 reported a positive correlation between measured pain and pain medication use. We concluded that despite the paucity of research in this area, this systematic review provides preliminary support for pain interventions by healthcare aides for long-term care residents. Future research exploring interventions for healthcare aides to take greater roles in pain management could unlock further improvements in resident care.

### DELIVERING FOOD RESOURCES AND KITCHEN SKILLS TO OLDER ADULTS WITH FOOD INSECURITY AND HYPERTENSION

Rebecca Rivera[1], Richard Holden[2], Mariah Adams[3], Emily Dawkins[3], Wanzhu Tu[1], and Daniel Clark[1], *1. Indiana University School of Medicine, Indianapolis, Indiana, United States, 2. Indiana University School of Public Health, Indianapolis, Indiana, United States, 3. Eskenazi Health, Indianapolis, Indiana, United States*

Non-Hispanic Black adults experience higher rates of food insecurity, poor diet quality, and hypertension compared with their non-Hispanic White counterparts. Delivering Food Resources and Kitchen Skills (FoRKS) is an integrated intervention that concurrently addresses food insecurity, nutrition literacy, and chronic disease self-management among older patients at a Federally Qualified Health Center. Two clinical dietitians led virtual hypertension self-management, cooking skills, and nutrition education classes twice per week for 16 weeks. Participants registered for classes, ordered free meal ingredients, and received encouragement from the dietitians through the FoRKS mobile technology application. This study presents diet quality descriptive results from the FoRKS pilot intervention conducted at Eskenazi Health from September 2021 to January 2022 in Indianapolis, Indiana. Participants (n=13) with hypertension (systolic blood pressure ≥120 mm Hg) and who identified as food insecure per the 18-item US Household Food Security Survey Module completed the 2018-version of the National Cancer Institute's Automated Self-Administered 24-hour (ASA24) Dietary Assessment Tool at baseline and post-intervention assessments. Healthy Eating Index (HEI)-2015 scores range from 0-100 and were calculated from ASA24 data. Participants were non-Hispanic Black (n=12) or non-Hispanic White (n=1) with mean age 58 years (range 53-65 years). Mean±SD HEI-2015 scores improved from 51.5±11.9 at baseline to 55.2±12.5 after the intervention. This preliminary data supports further investigation into the efficacy of integrated lifestyle interventions, i.e., FoRKS, using a randomized controlled study design. Addressing food and culinary skills can lead to improvements in diet quality that may translate to improved disease management among diverse older adults.

### PATIENTS' SPIRITUAL CONCERNS AND NEEDS DURING GOALS OF CARE CONVERSATIONS: HEALTHCARE CHAPLAINS' PERSPECTIVES

Jung Kwak[1], Shilpa Rajagopal[2], Neha Dronamraju[1], George Handzo[3], and Brian Hughes[3], *1. The University of Texas at Austin, Austin, Texas, United States, 2. Project*

*Horseshoe Farm, Pomona, California, United States, 3. HealthCare Chaplaincy Network, New York, New York, United States*

Healthcare chaplains are critical members of interdisciplinary healthcare teams and routinely facilitate advance care planning (ACP) and goals of care conversations with patients with serious illnesses. These conversations involve discussion of one's deeply held personal values and beliefs as well as uncertainty, fears, and hopes for current and future health care needs. However, there is limited empirical data on how patients' spiritual concerns and needs are addressed during these conversations. An online survey of 563 board-certified chaplains was conducted from March to July 2020 to identify chaplains' roles in facilitating goals of care conversations and included three open-ended questions about patients' hopes and fears and how chaplains addressed them. Quantitative data and written qualitative responses from 244 chaplains were analyzed using descriptive analysis and content analysis, respectively. Majority of participants were white (83.6%), female (59%), Protestant (63.1%), employed full-time (82%), and worked in community hospital settings (45.5%) or academic medical centers (32.8%). Six major themes emerged from chaplains' qualitative responses: (1) the utility of ACP; (2) focus on the present moment; (3) achieving a personally defined "good death"; (4) spiritual and religious matters and beliefs; (5) caring for and connection with family; and (6) how to address hopes and fears during ACP. ACP and goals of care conversations reflect profound human desires and needs; they require deeper listening and engagement by interdisciplinary team members to provide adequate spiritual and psychosocial support in addition to providing medical information to fulfill the intended meaning and purpose of ACP.

### HEALTH SYSTEM IMPLEMENTATION, SCALING, AND IMPACT OF THE 4MS FRAMEWORK TO ADVANCE AGE-FRIENDLY CARE

Julia Adler-Milstein, Courtney Lyles, and Stephanie Rogers, *University of California, San Francisco, San Francisco, California, United State*

There is a critical need to redesign the healthcare system to provide more effective and tailored care to older adults. The 4Ms Framework (What Matters, Medication, Mentation and Mobility) offers a blueprint to guide health system efforts to deliver more age-friendly care. We sought to characterize and assess real-world implementation experiences with the 4Ms in three health systems (University of California, San Francisco, University of Utah, & Anne Arundel Medical Center). Specifically, we conducted semi-structured interviews (N=29) with diverse stakeholders from each site to characterize the approach to operationalize the framework and assess the implementation experience. Via cross-site content analysis of interview transcripts, we identified four themes. First, the 4Ms offer a compelling conceptual framework around which to organize health system efforts to advance age-friendly care, but each "M" required distinct, complex implementation work that resulted in a fragmented implementation experience. Second and relatedly, inpatient and ambulatory efforts to implement the 4Ms became disconnected, with missed opportunities for synergies and gave little attention to the 4Ms during care transitions. Third, non-physician leadership was key to initiating 4Ms implementation efforts, but obtaining physician buy-in, which often came later, was essential to full implementation. Fourth, sustained 4Ms implementation efforts required both top-down vision and communication from leadership as well as bottom-up culture change that engaged and motivated frontline workers. As efforts to spread the 4Ms Framework advance, these results offer important implementation guidance and also suggest domains in which the Framework may need to be adapted to accommodate real-world implementation experiences.

### NURSE PRACTITIONER–LED IMPLEMENTATION OF HUDDLES TO SUPPORT STAFF IN LONG-TERM CARE HOMES

Katherine McGilton[1], Alexandra Krassikova[2], Aria Wills[3], Margaret Keatings[3], Jennifer Bethell[3], Veronique Boscart[4], and Souraya Sidani[5], *1. KITE Research Institute: Toronto Rehabilitation Institute-UHN, Toronto, Ontario, Canada, 2. Toronto Rehabilitation Institute, University Health Network, Thornhill, Ontario, Canada, 3. Toronto Rehabilitation Institute, Toronto, Ontario, Canada, 4. Conestoga, Kitchener, Ontario, Canada, 5. Ryerson University, Toronto, Ontario, Canada*

Staff working in long-term care (LTC) homes frequently report experiencing moral distress related to lack of autonomy and not being able to provide quality care. Huddles have been used as a communication tool for many years in acute care settings to improve collaboration and safety culture. In LTC homes, huddles are implemented less often, despite evidence of their benefits in improving support and teamwork. In this pre-test post-test implementation study, huddles led by a nurse practitioner (NP) were introduced in a privately-owned not-for-profit LTC home with < 150 beds, located in a medium urban centre in Ontario, Canada. Objectives of the study were to 1) examine fidelity of huddle implementation; 2) examine the extent to which the huddles improved staff' outcomes of moral distress, job satisfaction, and support provided by the NP estimated with Bayesian proportional odds model. A total of 48 huddles were carried out by the NP over 15 weeks. Huddles were most commonly attended by personal support workers (98%) and registered practical nurses (96%), with an average of 7 individuals per huddle. Topics most often addressed at huddles were related to resident care (46%) and staff concerns (34%). Strong statistical evidence of a reduction in overall moral distress was evident for staff attending the huddles, when compared to staff who did not (posterior probability =.9933). No changes in job satisfaction and support provided by the NP were observed. Introducing huddles in LTC homes may be effective at reducing moral distress experienced by staff.

### SESSION 6280 (POSTER)

### CAREGIVING AND SOCIAL SUPPORTS

### WHAT MATTERS TO OLDER SURGICAL PATIENTS AND THEIR CARERS DURING THEIR HOSPITALIZATION?

Janani Thillainadesan[1], Helen Box[2], Leanne Kearney[2], Vasi Naganathan[2], Michelle Cunich[3], Sarah Aitken[2], and Sue Monaro[2], *1. Department of Geriatric Medicine and*

*Centre for Education and Research on Ageing (CERA), Concord Hospital, Concord, New South Wales, Australia, 2. Concord Hospital, Concord, New South Wales, Australia, 3. University of Sydney, Sydney, New South Wales, Australia*

Older adults account for a growing proportion of the surgical population. We investigated patient- and carer-reported experiences of hospitalization in this group. A mixed-methods study using telephone and postal surveys of recently hospitalized vascular surgical patients aged ≥ 65 years at an acute care academic hospital. Qualitative data were thematically analyzed using a framework approach. In total 46 patients (mean age 77 years, 78% male) and nine carers were surveyed. Nine (20%) patients were frail (Clinical Frailty Scale score >4). The majority of patients reported overall they felt their views were listened to (n=42, 89%), were kept informed (n=39, 83%) and were asked about their pain level (n=37, 79%). Among the nine carers, seven reported overall they felt their views were listened to (n=42, 89%) and were kept informed (n=39, 83%). Thematic analysis of responses to open-ended survey questions about their experience of hospitalization revealed four key themes: receiving fundamental care including hygiene and nutrition, importance of the hospital environment such as sleep and meals, being informed and involved in health care decision-making, and treating pain and deconditioning to help recovery. Findings from this study identified that older adults admitted to hospital for surgical care, and their carers, highly value care that meets fundamental needs and involves them in decision-making and recovery. These priorities can be addressed through Age-Friendly Health System initiatives such as the implementation of the "4Ms" of geriatrics care: what matters, medication, mobility and mentation.

### A SUCCESSFUL EDUCATIONAL INTERVENTION LINKING DEMENTIA PATIENTS AND FAMILIES WITH COMMUNITY RESOURCES

Stacy Barnes[1], Edmund Duthie[2], Kathryn Denson[2], Jennifer McAlister[3], Wendy Betley[3], Amanda Szymkowski[2], Michael Malone[4], and Deborah Simpson[4], *1. Marquette University, Milwaukee, Wisconsin, United States, 2. Medical College of Wisconsin, Milwaukee, Wisconsin, United States, 3. Alzheimer's Association Wisconsin, Milwaukee, Wisconsin, United States, 4. Advocate Aurora Health, Milwaukee, Wisconsin, United States*

Unpaid caregivers provided approximately 83% of the care for the 6.2 million Americans aged 65+ who were living with Alzheimer's dementia in 2021. Physicians and other health professionals are not well informed about dementia-related community resources and rarely refer patients/families despite strong evidence which supports incorporating community services as part of an ongoing treatment plan. To bridge this gap, partnership with community agencies is essential. The Alzheimer's Association's "Direct Connect" referral program is provider-initiated and connects patients/families with disease information, care consultations, support groups, a 24/7 Helpline, and other resources. As part of the Wisconsin Geriatric Workforce Enhancement Program award from HRSA (U1QHP28712) 1,023 health professionals working in Wisconsin hospitals and primary care clinics were trained to use Direct Connect. Our educational intervention re-framed dementia from a hopeless disease to a chronic condition requiring supportive services as a necessary component of the disease management plan. Results of a paired-samples t-test (n=675) indicated our educational intervention had a significant impact on trainees' self-reported pre/post-test knowledge in both hospitals [$t(444) = -34.67$, $p < 0.001$, $d = 1.64$] and primary care [$t(229) = -24.63$, $p < 0.001$, $d = 1.62$]. By the end of the 5-year project period, Direct Connect referrals totaled 1,352 and the program grew from less than 50 referrals to 290 annually. While these results are promising, Direct Connect remains underutilized and initiatives to further increase referrals from providers must be expanded.

### STRONGER MEMORY: EXPERIENCES OF OLDER ADULTS AND CAREGIVERS IN AN INTERVENTION PROGRAM FOR COGNITIVE PERFORMANCE

Hyun Kang[1], Catherine Tompkins[1], Kendall Barrett[1], Francesca Keesee[1], Catherine Magee[1], Mckenzie Lauber[1], Emily Ihara[1], and Jessica Fredericksen[2], *1. George Mason University, Fairfax, Virginia, United States, 2. Goodwin House Incorporated, Alexandria, Virginia, United States*

Subjective cognitive decline (SCD), defined as self-reported memory issues that have worsened over the past year, can be one of the first indications of Alzheimer's disease and related dementias. SCD affects nearly 9% of Virginians aged 45 or older, and 31% of these individuals live alone. A practice-based intervention is essential to addressing the challenges associated with cognitive impairment. This study examined the effect of StrongerMemory program, which involves a series of cognitive exercises. The StrongerMemory program provides a 12-week curriculum designed to stimulate the brain's prefrontal cortex, which governs the ability to retrieve memories. Participants are encouraged to spend 20 to 30 minutes a day reading aloud, writing or journaling and completing simple math problems. The Mason Gerontology Research Team investigated the pre- and post-cognitive outcomes among 43 participants who participated in the StrongerMemory program to determine the effectiveness of the program. The cognitive outcomes were evaluated using the Mini Montreal Cognitive Assessment 2.1 (Mini MoCA). The mean (SD) scores in Mini MoCA evaluated in the participants were 12.60 ± 2.1 (pre-test) and 13.53 ± 1.9 (post-test). The mean difference was statistically significant. Our results indicate that the StrongerMemory program is beneficial for cognition. The premise around the StrongerMemory program is that prior to a diagnosis or even with a diagnosis of mild cognitive impairment, a decline can be slowed and possibly prevented by participating in a program like StrongerMemory. The Mason Gerontology Research Team continues to evaluate the StrongerMemory program to examine its impact on SCD.

### PSYCHONEUROLOGICAL SYMPTOMS IN CANCER SURVIVORS AND THEIR CAREGIVERS: A LONGITUDINAL PILOT STUDY

Kristin Litzelman, Jen Stevens, Ashley Berghoff, Afnan Balubaid, and Kristine Kwekkeboom, *University of Wisconsin-Madison, Madison, Wisconsin, United States*

In cancer patients, stress is associated with a psychoneurologic symptom cluster of depressed mood, anxiety, pain, fatigue, and sleep disturbance. The stress of caregiving may trigger similar symptoms among caregivers and warrants investigation. The purpose of this analysis was to characterize correlates of psychoneurologic symptoms in cancer caregivers. Cancer survivor-caregiver dyads (n=29) provided eight weekly symptom reports using a web-based assessment system. Symptom burden was calculated as the sum of symptom severity (mean=7.5, SD=8.1, range=0-41). Primary and secondary stressors of caregiving were also assessed. Mixed modeling was used, accounting for repeated measures. Models controlled for person-mean of time varying covariates, as well as sociodemographic, clinical, and care-related covariates. A secondary analysis assessed interdependence in survivor and caregiver symptoms using Actor-Partner Interdependence Models. In descriptive analyses, caregivers most frequently reported feeling anxious (44% on average across timepoints), sleep problems (31%), fatigue (25%), depressed mood (24%), and feeling irritable/angry (24%). Within caregivers, greater hours of care (beta=0.11, p<.01) and more patient symptoms (beta=0.10, p<.01) were associated with greater symptom burden. Between caregivers, higher baseline perceived stress was associated with greater symptom burden (beta=0.38, p=.04) and coresidence with the survivor was associated with lower symptom burden (beta=-12.8, p<.01). Caregivers experienced higher symptoms when patients specifically reported anxiety, lack of appetite, or pain (beta=1.4, 2,4, and 3.0, p<.05). Cross-lagged Actor-Partner Interdependence Models indicated interdependence among survivor and caregiver symptom burden. The findings provide preliminary evidence of the interrelationship of psychoneurologic symptoms in survivors and patients, with implications for symptom management and supportive care practice.

### DEVELOPMENT OF A BRIEF MEASURE OF COMMUNICATION QUALITY: THE 5- AND 10-ITEM FAMILY INPATIENT COMMUNICATION SURVEYS

Emily Burke[1], James Slaven[2], Tracy Taylor[3], Patrick Monahan[2], Greg Sachs[1], and Alexia Torke[1], *1. Indiana University (IU) Center for Aging Research, Regenstrief Institute, Inc., Indianapolis, Indiana, United States, 2. Indiana University, Indianapolis, Indiana, United States, 3. Regenstrief Institute, Inc., North Vernon, Indiana, United States*

Communication quality between clinical care teams and families impacts important outcomes like satisfaction, depression, and anxiety. Assessment tools must be efficient and reliable to be useful in the clinical environment. We report the development of 5 and 10-item versions of the validated 30-item Family Inpatient Communication Survey (FICS). Data were from 364 surrogate decision makers (SDMs) for incapacitated older adults in the ICU. Most SDMs were adult children (66.8%). SDMs were 70.9% female, 68.9% white, with a mean age of 58.3. Exploratory factor analysis revealed high internal reliability for the single-factor FICS5 (α= .88) and two-factor FICS10 (α= .93). The FICS10 reliably measures two subscales: information (α= .91) and emotional support (α=.81). Good discriminant and predictive validity were demonstrated when comparing total scores to outcomes at 6-8 weeks after hospital discharge, including anxiety (correlation coefficient (ρ)= -.13; p=.0234 ), depression (ρ =-.15; p=.0076), decision regret (ρ =--.15; p=.0066), and satisfaction (ρ =-..48; p<.0001). Repeating analysis with a new sample (n=188) revealed similar results with Cronbach's alpha ranging from .81 to .93. The FICS5 revealed significant associations (p<.05) at 6-8 weeks after discharge with distress (-.22), while the FICS10 demonstrated significant associations with distress (-.28), anxiety (-.20), depression (-.19) and decision regret (-.27). Confirmatory factor analysis indicated adequate fit (CFI: FICS5= .994, FICS10= .994; RMSEA: FICS5= .093, FICS10= .103). The FICS provides clinicians and interventionists with a reliable, low burden tool to evaluate communication quality and respond quickly, which could impact satisfaction and other important outcomes for patients and families.

### THE VALIDITY AND RELIABILITY OF THE DISTRESS THERMOMETER IN FAMILY SURROGATES OF ICU PATIENTS

Emma O'Brien[1], Emily Burke[2], James Slaven[3], Tracy Taylor[4], and Alexia Torke[2], *1. Regenstrief Institute, Inc., Indianapolis, Indiana, United States, 2. Indiana University (IU) Center for Aging Research, Regenstrief Institute, Inc., Indianapolis, Indiana, United States, 3. Indiana University, Indianapolis, Indiana, United States, 4. Regenstrief Institute, Inc., North Vernon, Indiana, United States*

Brief, reliable assessment tools are highly valued in both research and clinical settings. The single-item Distress Thermometer (DT) asks participants to rank their overall level of distress from zero to ten. Similar measures of distress perform well in oncology populations, but the validity of the DT has not been well tested with other populations. To determine its validity and reliability, we analyzed data from family surrogates (n=188) of critically ill ICU patients. Surrogates were asked to rate their distress during the first four days of the patient's ICU stay and 6-8 weeks after discharge (n=127). Data were analyzed using Spearman non-parametric correlation due to the distributions of the data. DT scores at both baseline and follow-up were significantly correlated with anxiety (GAD-7: correlation coefficient (ρ)=.527, p<.0001; ρ=.543, p<.0001, respectively), depression (PHQ-9: ρ=.480, p<.0001; ρ=.399, p=.0002), distress (Kessler-6: ρ=.477, p<.0001; ρ=.528, p<.0001), and negative religious coping (ρ=.149, p=.0426; ρ=.238, p=.0074). Results also indicated that spiritual well-being at baseline and follow-up (FACIT: ρ=-.391, p<.0001, ρ=-.443, p<.0001) and positive religious coping at baseline (RCOPE: ρ=-.164, p=.0253) have an inverse relationship with overall distress. At baseline, surrogates with better positive religious coping and/or more involvement in organizational religious activity (ρ=-.189, p=.0106) were more likely to report lower distress. The DT could be an efficient, single item predictor of outcomes that impact patient and family care. Future research could confirm its validity as a measure of distress, in a variety of clinical populations and environments that could inform clinical care for patients and families.

### CHARACTERIZING GENERATIONAL CAREGIVING USING DATA FROM THE BRFSS

Benjamin Olivari[1], Nia Reed[1], Erin Bouldin[2], Eva Jackson[3], John Omura[1], Lisa McGuire[1], and Janet Croft[1], *1. CDC, Atlanta, Georgia, United States, 2.*

*University of Utah, Salt Lake City, Utah, United States, 3. Alzheimer's Association, Chicago, Illinois, United States*

As the proportion of family and friends in the U.S. providing informal care increases, it is important to understand how this may impact certain demographic groups. Millennials and older adults are two generational segments of caregivers of interest given the complexities associated with these groups. Millennials can be sandwiched between a growing older population of parents or grandparents while also raising young children, often providing care for both. Similarly, older adults frequently balance caring for spouses, themselves, and grandchildren. We characterized these two groups of caregivers using 2015-2019 Behavioral Risk Factor Surveillance System (BRFSS) data from the Caregiver Optional Module administered in 44 states, D.C., and Puerto Rico. Among 246,223 Module respondents, 21.3% reported providing care for a family member or friend. Among caregivers, 12.4% were identified as Millennials (aged 18-38 years), and 19.7% were older adults (aged≥65 years). About one-in-three Millennial caregivers reported providing care to a parent and half to another relative, such as a child or grandparent. While less than 4% of Millennial caregivers reported providing care for a spouse, nearly one-in-five older adult caregivers did. Although their care recipients may differ, both groups reported high intensity of care, with 30% of caregivers in each group providing 20 hours of care or more weekly. Caregiving among older adults can be further complicated by their own health difficulties, with over half of older adult caregivers reporting having two or more chronic conditions and one-third reporting a disability. BRFSS data may inform planning efforts pertaining to these caregiving groups.

### PERCEIVED NEEDS, FELT CONCERNS, AND QUALITY OF LIFE OF OLDER ADULTS IN MISSISSIPPI: AN INTERGENERATIONAL APPROACH

Muhammad Riaz[1], Laura Downey[2], Carolyn Adams-Price[3], Donna Peterson[2], Lori Staton[2], and Alisha Hardman[2], *1. Mississippi State University, Starkville, Mississippi, United States, 2. Mississippi State University, Mississippi State, Mississippi, United States, 3. Mississippi State University, STARKVILLE, Mississippi, United States*

The purpose of the study was to identify felt needs and concerns of older adults living in their own homes in Mississippi using intergenerational perspectives. This mixed-methods study used snowball sampling to collect data through semi-structured interviews and structured questionnaires that asked about the quality of life and current and future problems among aging adults. Three generations of Mississippians participated in the study, including grandparents (N = 22), adult children (N = 23), and grandchildren (N = 19). Quantitative data were analyzed using SPSS, while qualitative data were managed with MaxQDA. Respondents identified concerns with physical health, difficulty living independently, and mental health problems affecting life satisfaction and quality of life. Incongruently with the intergenerational stake hypothesis, adult children were more invested in their children than their parents. Older adults who live closer to significant others and family were more resilient despite having physical and mental issues. Physical issues related to mobility, access to daily need services, and help with basic tasks like food preparation and mowing lawns, and issues related to mental health seemed less pronounced. Consistent with other research studies, older adults showed a positive sense of self. Similarly, parents showed resilience without being intrusive and overbearing while considering themselves as support for their children. Results could inform the development of programs or initiatives for grandparents, adult children, grandchildren, and others involved in caregiving activities and planning for older adults in Mississippi.

### ASSOCIATION OF CARDIOVASCULAR DISEASE WITH RACE/ETHNICITY IN FAMILY CAREGIVERS OF PERSONS LIVING WITH DEMENTIA

Dawon Baik[1], Bryan McNair[2], and Sophia Centi[3], *1. University of Colorado, Aurora, Colorado, United States, 2. Colorado School of Public Health, University of Colorado Anschutz Medical Campus, Aurora, Colorado, United States, 3. CU Anschutz Medical Campus College of Nursing, Aurora, Colorado, United States*

Family caregivers of persons with dementia (dementia FCGs) experience high caregiving burden, and caregiving-related stressors have been linked to cardiovascular disease (CVD). Racially and ethnically diverse FCGs face additional challenges in managing their own health; however, racial/ethnic disparities in caregiving-associated cardiovascular health are understudied and poorly understood. To fill the gap, we evaluated racial/ethnic differences in CVD risk and CVD conditions among dementia FCGs in the U.S. Using 2015-2020 Behavioral Risk Factor Surveillance System data (N = 6,132), we compared CVD risk and CVD conditions in racial/ethnic dementia FCG minorities to non-Hispanic White dementia FCGs. Logistic regression models were used to estimate unadjusted (OR) and adjusted odds ratios (AOR) with 95% confidence intervals (CI) for association of race/ethnicity with CVD risk and CVD conditions, adjusting for potential confounders. Compared to non-Hispanic White FCGs, non-Hispanic Black FCGs (AOR 0.33, 95% CI 0.2-0.53) and non-Hispanic Asian FCGs (AOR 0.16, 95% CI 0.05-0.51) were less likely to have depressive symptoms. Hispanic FCGs (OR 0.69, 95% CI 0.5-0.95), non-Hispanic Black FCGs (AOR 0.6, 95% CI 0.41-0.86), and non-Hispanic Asian FCGs (AOR 0.51, 95% CI 0.28-0.94) were less likely to smoke. Non-Hispanic Black FCGs were less likely to exercise (OR 0.69, 95% CI 0.52-0.91) and more likely to be obese (AOR 1.64, 95% CI 1.14-2.36). The findings confirm racial/ethnic disparities in psychological, behavioral, and metabolic risk factors for CVD among dementia FCGs. Future studies should investigate how dementia FCGs' caregiving-related conditions affect CVD and how racial/ethnic considerations can inform culturally appropriate prevention-care strategies.

### CARING FOR INDIVIDUALS WITH DEMENTIA AND COGNITIVE IMPAIRMENT IN LOS ANGELES: A CAREGIVER PROFILE

Ariane Thomas[1], Noel Barragan[1], Mariana Reyes[1], and Tony Kuo[2], *1. Los Angeles County Department of Public Health, Los Angeles, California, United States, 2. University of California, Los Angeles, Los Angeles, California, United States*

In 2020, the Los Angeles County Department of Public Health launched Healthy Brain LA (HBLA). HBLA is a multi-pronged project designed to promote cognitive health, reduce the risk of dementia, and help develop a dementia-focused strategic plan for Los Angeles County (LAC) that prioritizes the needs of those impacted by dementia and cognitive impairment and their caregivers. To support this effort, the HBLA team analyzed weighted data from the 2019 and 2020 California Health Interview Survey (CHIS) to better understand the profile of adult caregivers of individuals with dementia and cognitive impairment. CHIS is a publicly available, population-based, web and telephone survey that asks California residents about a wide range of health topics, yielding representative data on all 58 counties in the state. In 2019-2020, an estimated 1.6 million adults provided care to individuals aged 18 years or older in LAC. About 322,880 of those caregivers provided care for individuals with dementia and cognitive impairment, representing 2% of LAC's adult population. Many of these caregivers were female (59%), Latino/Hispanic (44%), and between 50-64 years of age (32%). Most of them experienced challenges, such as financial stress (61%), physical/mental health problems (18%), or a change in job status (27%), due to caregiving. These data provide the first local estimate of dementia and cognitive impairment caregiving in nearly 15 years. Results offer critical information about this population that will be used by the HBLA team and its partners to guide efforts to effectively meet the needs of caregivers in LAC.

### HEALTH COACHING FOR DEMENTIA CAREGIVERS: LESSONS LEARNED FROM THE ICARE4ME STUDY

Lauren Fisher, Lauren Massimo, and Karen Hirschman, *University of Pennsylvania, Philadelphia, Pennsylvania, United States*

Frontotemporal dementia (FTD) is a common cause of young-onset neurodegenerative disease that causes progressive changes in behavior and personality. FTD is often diagnosed around age 60, creating complex care needs that result in caregivers of persons with FTD experiencing high rates of depression, burden, and poor self-care. The iCare4Me for FTD study (NCT04686266) randomized 15 caregivers to receive a virtual health coaching intervention over 6 months (10 sessions) and 15 caregivers to the control group. To better understand the caregivers' experience with the health coach intervention, two focus groups with intervention group caregivers (n=5) were held. Focus groups were recorded, transcribed and coded using content analysis. Caregivers reported the most valuable aspect was the relationship that was developed with their health coach. Caregivers particularly valued having someone to talk to who was outside their immediate social and support networks. It was noted the structured self-care curriculum served as a good backbone for discussions, but more specific coping conversations related to loss of patient empathy, prognostic uncertainty, and anticipatory grief are needed. One caregiver described being, "… awash in grief and it's affected my memory" while another described "grief is a big issue and I don't really find too many people understand it, because my husband is alive, but so many parts of him are gone". These findings will be used to inform future studies utilizing health coaches for caregivers of persons with FTD. Implications for evidence-based virtual health coaching interventions with caregivers of persons living with FTD will be described.

### QUALITY OF LIFE AND SOCIAL DETERMINANTS PREDICT HOSPICE IN DEMENTIA CAREGIVING DYADS: A MACHINE LEARNING APPROACH

Suzanne Sullivan, Wei Bo, Chin-Shang Li, Wenyao Xu, and Yu-Ping Chang, *University at Buffalo, Buffalo, New York, United States*

Hospice care is available to assist people with serious illness and their caregivers who wish to age in place, avoid unnecessary hospitalizations, and remain at home through the end-of-life. However, hospice care is under-utilized nationally despite the disproportionate prevalence of end-of-life dementia caregiving burdens among disadvantaged groups. The reasons are unclear, but emerging research suggests that systemic barriers may contribute to underutilization. Commonly used quality-of-life frameworks have long included social determinant of health (SDH) factors such as social, environmental, financial, and healthcare access needs. Investigating the link between quality-of-life and SDH concerns of persons with dementia (PWD) and their caregivers may help identify when a PWD might benefit from hospice care. This study uses machine learning techniques to longitudinally analyze caregiver/care-recipient dyads in the National Health and Aging and Trends Study (NHATS) linked to the National Study of Caregiving (NSOC) (2015-2018) to identify quality-of-life and SDH predictors of hospice use among 117 PWD and their primary caregivers. Results indicate that distinguishing features selected by Information Gain Ratio [IGR] predict that memory rating, receiving food stamps, whether health prevents enjoying life, having trouble chewing or swallowing, diabetes, a regular doctor, and nobody to talk to can predict hospice use well (accuracy=0.6848; sensitivity=0.8244; specificity=0.5371; AUC=0.7425). Quality-of-life/SDH factors are important longitudinal predictors of hospice that can be detected up to three years prior to death. Our study uses inductive, machine learning approaches to provide testable hypotheses for future research to improve the quality of end-of-life care through hospice for PWD and their caregivers.

### SELF-CARE AND QUALITY OF LIFE IN PEOPLE WITH PARKINSON'S DISEASE AND THEIR CAREGIVERS

EUNYOUNG KIM[1], JuHee Lee[1], Misook Chung[2], and Subin Yoo[1], *1. Yonsei University, Seoul, Seoul-t'ukpyolsi, Republic of Korea, 2. University of Kentucky, Lexington, Kentucky, United States*

Objective: To examine associations of self-care and the quality of life (QOL) in patients with Parkinson's disease (PD) and their caregivers dyads.

Background: Patients with PD engage in self-care with motor and non-motor symptom experiences. There is insufficient knowledge about associations of self-care and QOL in patients with PD and their caregivers though PD patients and family caregivers are interdependent. Method: A total of 73 PD patients and primary family caregivers from the Korean Parkinson's Disease Association or who visited outpatient clinics of the tertiary hospitals in Korea participated in this study. Dyad members completed the survey of the self-care (the Self-Care of Chronic Illness Inventory and Caregiver Contribution to Self-Care of Chronic Illness Inventory) and QOL (Parkinson's Disease Quality of Life Questionnaire and The Parkinson's Disease Questionnaire-Care). The comparisons of self-care and quality of life within two dyad members were conducted via paired t-test and Pearson's correlation using SPSS 26.0 Result: PD patients reported significantly higher levels of self-care maintenance (78.34 vs. 70.21, patients vs. caregiver respectively, p<.001) and management (65.9 vs. 60.49, p=.029) than caregivers. Patients' self-efficacy was significantly correlated with their QOL (r=.270, p=.021). Caregiver contribution to self-care management was significantly correlated with their own QOL (r=.365, p=.001) and patients' QOL (r=.234, p=.047).

Conclusion: The self-care efficacy of patients and the contribution of caregivers to self-care management can affect the QOL of both patients and caregivers. A dyadic approach for the intervention of self-care management is crucial to improve the QOL of patients with PD and caregivers.

### UNDERSTANDING THE CHARACTERISTICS AND WELL-BEING OF AMERICAN INDIAN AND ALASKA NATIVE GRANDPARENT CAREGIVERS

Ramona Danielson[1], Shelly Davis[2], and Collette Adamsen[3], *1. North Dakota State University, Fargo, North Dakota, United States, 2. University of North Dakota School of Medicine & Health Sciences, Grand Forks, North Dakota, United States, 3. University of North Dakota, Grand Forks, North Dakota, United States*

Background: American Indian/Alaska Native (AI/AN) grandparents have always had an important role in their grandchildren's lives. Grandparents being the primary caregiver of their grandchildren has become a more pronounced occurrence in AI/AN populations in recent years and warrants review.

Methods: Data come from the National Resource Center on Native American Aging's 2017-2020 needs assessment of AI/AN adults ages 55+. Analysis explored demographic and well-being indicators by caregiving status (N=19,855): not a caregiver of a grandchild (non-CG; 71%); part-time caregiver (PT-CG; 18%); and primary caregiver (PR-CG; 11%).

Results: Caregivers were more likely to be younger, be married, have higher education and incomes, and live with family in a single-family residence than non-CG. PR-CG were more likely to be female, employed full-time, and live with family on reservation/trust land than PT-CG and non-CG. Regarding indicators of well-being, caregivers were more likely to participate in cultural practices than non-CG. PT-CG were more likely to socialize and have recently participated in vigorous exercise, were less likely to have fair/poor health, and had fewer daily self-care restrictions than PR-CG and non-CG. PR-CG were more likely to be obese and daily smokers and had a higher measure of mental health concern, but were less likely to have recently binge drank or mainly eat alone than PT-CG and non-CG.

Conclusions: The demographics reviewed were all significantly different. PT-CG had better outcomes, pointing to a potential protective benefit, while PR-CG had more mental health strain. Further research is needed to better understand the interconnectedness of the indicators analyzed.

### CAREGIVER SINGING VERSUS MUSIC ACTIVITIES IN DEMENTIA CARE: DIFFERENT BENEFITS IN DIFFERENT OCCASIONS

Lena Hammar[1], Annica Lövenmark[1], and Anna Swall[2], *1. Mälardalen University, Västerås, Vastmanlands Lan, Sweden, 2. Dalarna University, Falun, Dalarnas Lan, Sweden*

Music has been used in the care of persons with dementia (PWDs) in decades, and research show that music activities such as background music, music listening, sing-along activities and playing instruments have positive effects on mood, emotions and interaction. However, Caregiver Singing (CS)- when caregivers are singing for or together with PWDs during care activities, are commonly in literature mixed up with other music activities. CS is a care intervention aiming to facilitate care situations and has shown to be useful to increase interaction and cooperation in care activities. This study aimed to describe caregivers' experiences of the differences between using CS compared to music in the care of persons with dementia.Data was three focus group interviews with 12 professional caregivers and residential facilities for PWDs. Data was analyzed with qualitative content analysis. Results revealed that CS are a successful tool to communicate, cooperate and bring out the PWDs hidden resources during care activities. The PWDs were told to be more adequate even thou the caregivers sings instead of speaking. Music activities were described to increase moods and socialization and are important part to increase a nice atmosphere. However, it does not increase cooperation in the way CS does. As interventions to facilitate caring situations with PWDs are needed, and CS has shown to be effective and should therefore be seen as an intervention not mixed up with traditional music activities. It is an important contribution in the education for staff in dementia care to include training in CS.

### FORMAL CAREGIVER BURDEN IN NURSING ASSISTANTS IN NURSING HOMES: A FEASIBILITY STUDY

Rachel Kunkle[1], Claudia Chaperon[2], and Ann Berger[2], *1. University of Nebraska Medical Center, Elkhorn, Nebraska, United States, 2. University of Nebraska Medical Center, Omaha, Nebraska, United States*

Formal caregivers are the direct care workforce that aid residents in nursing homes. Providing care to residents is hazardous and physically demanding. Formal caregiver burden encompasses five attributes – perceived stress, caring for another, dependency of the older adult, responsibility, and competence. Exploring the five attributes of formal caregiver burden using a mixed-methods approach will determine if the attributes are present and how the nursing home setting contributes to formal caregiver burden. The purpose of this feasibility study is to describe formal caregiver burden of nursing assistants who provide direct care to residents in a nursing home setting in the Midwest United States. Study site one was only able to enroll three participants; therefore, results were limited. However, study site two was able to recruit and enroll the desired sample size (N=9). Interviews and self-report measures (Background/COVID-19 Questionnaire, Perceived Stress Scale, Caring Behaviors Inventory, and Nursing Home Staff Competency Assessment) were completed, and the PI compared integrated mixed methods results. Results suggest all the attributes of formal caregiver burden were present, and no additional attributes were identified. The feasibility of virtual recruitment, enrollment, and data collection procedures were confirmed. Multiple challenges played a role in the unsuccessful recruitment of this feasibility study at site one; however, virtual recruitment was successful at site two. Further exploration will inform identification and measurement of formal caregiver burden to ensure support for nursing assistants, continue the vitality of the nursing assistant workforce in nursing homes/long-term care, and improve the lives of nursing assistants.

### ASSOCIATION BETWEEN INCREASED CAREGIVER BURDEN AND MENTAL HEALTH DURING THE COVID-19 PANDEMIC IN JAPAN

Isuzu Nakamoto[1], Hiroshi Murayama[2], Mai Takase[3], Yoko Muto[2], Tami Saito[4], and Takahiro Tabuchi[5], *1. Tohoku University, Tokyo, Tokyo, Japan, 2. Tokyo Metropolitan Institute of Gerontology, Tokyo, Tokyo, Japan, 3. The University of Tokyo, Tokyo, Tokyo, Japan, 4. National Center for Geriatrics and Gerontology, Obu, Aichi, Japan, 5. Osaka International Cancer Institute, Osaka, Osaka, Japan*

During the COVID-19 pandemic, informal caregivers' mental health was more deteriorated than non-caregivers. The objective of this study was to examine the association between increased caregiver burden and severe psychological distress (SPD) during the pandemic. We used cross-sectional data from a nationwide internet survey conducted between August and September 2020 in Japan. Of 25,482 participants aged 15–79 years, 1,920 informal caregivers were analyzed (mean age, 52.3 ± 15.9 years; men, 48.8%). SPD was defined as the Kessler 6 Scale (K6) ≥ 13. The self-rated change in caregiver burden was measured with the single question item. A binary logistic regression analysis was used to examine the association between SPD and increased caregiver burden, adjusted for demographic, socioeconomic, health, and caregiving variables. To examine the differential association between increased caregiver burden and SPD, interaction terms were added and binary logistic regression was separately conducted in all variables. Of total caregivers, 56.7% reported the increased caregiver burden and 19.3% were SPD. Increased caregiver burden was significantly associated with SPD, with an adjusted odds ratio 1.90 (95% confidence interval 1.37–2.66). The association between increased caregiver burden and SPD was stronger among married caregivers, with disease treatment, and with their care-receiver's care need level 1–2 (Japanese long term care service levels). This study demonstrated that increased caregiver burden was associated with SPD during the pandemic. Therefore, measures against mental health for the caregivers with increased caregiver burden need to be implemented immediately.

### POSITIVE CAREGIVING AND CAREGIVING RELATIONSHIP ASSOCIATION WITH MENTAL HEALTH AND PERCEIVED GENERAL HEALTH

Cristina de Rosa, Ashleigh Holmes, Weijun Wang, and Yu-Ping Chang, *University at Buffalo, Buffalo, New York, United States*

Caregiver burden is well understood as an important contributor to caregiver health. However, little is known about how positive aspects of caregiving (i.e., personal growth, gratitude, finding meaning) and the quality of caregivers' relationships with care recipients might play a role in caregiver health. The study aimed to examine whether positive caregiving and caregivers' relationship with care recipients were associated with caregiver mental health (depression and anxiety) and perceived general health. The sample consisted of 2,652 family caregivers in the National Study of Caregiving (NSOC) III (2017) providing care to older adults. A series of multiple regression models with covariate adjustments (i.e., caregiver's age, sex, and race/ethnicity) were performed to examine the associations. Results indicated that positive aspects of caregiving predicted caregiver mental health but did not predict perceived general health. Caregivers' relationship with care recipients and caregiver burden significantly predicted caregiver mental health ($b = 0.285$ [S.E. = 0.045], $p < .001$) and perceived general health ($b = 0.096$ [0.016], $p < .001$). After controlling for caregiver burden, only caregivers' relationship with care recipients remained a significant predictor of caregiver mental health ($b = 0.182$ [0.041], $p < .001$) and perceived general health ($b = 0.077$ [0.018], $p < .001$). Our results suggest that positive caregiving perceptions and quality of relationships between caregivers and care recipients are linked to better caregiver mental health. Interventions to reduce caregiver burden, including strategies to help caregivers maintain positive attitudes and positive relationships with care recipients, might be beneficial to improving caregiver health.

### ENGAGING AFRICAN AMERICAN FAMILY CAREGIVERS THROUGH ART AND CREATIVE ACTIVITIES: A CASE STUDY

Afeez Hazzan[1], Luvon Sheppard[2], and Joyce Hazzan[3], *1. State University of New York at Brockport, Hilton, New York, United States, 2. Rochester Institute of Technology, Rochester, New York, United States, 3. Liberty University, Lynchburg, Virginia, United States*

Studies have shown that engaging family caregivers of people with dementia in creative activities can provide therapeutic benefits by relieving stress and promoting well-being. However, there is a dearth of studies focusing on the involvement of racial minority family caregivers of people with dementia in research involving art and creative activities. The purpose of this study is to present the results of a case study interview with an artist-educator-community collaborator who acted as a key facilitator for the ACTION ARTS study. The purpose of the ACTION ARTS study was to utilize mobile technology applications for facilitating creative activities to enhance health and well-being of African-American family caregivers and their loved ones in the middle-to-late stages of dementia. The study included activities such as memory stimulation, art viewing, art making, and other forms of creative expressions. The research question for the current case study was: What are the best practices for engaging African-American family caregivers of older adults in the middle-to-late stages of dementia in research involving art and creative activities? Thematic analysis of the qualitative data from the case study interview yielded the following recommendations/results: (1) Design the program of art and/or creative activities in an easy way to facilitate interaction among participants; (2) Make the program as accessible and relevant as possible so everyone is able to relate to it; (3) Emphasize areas of commonality that could help in giving both the facilitators and participants food for thought.

### IMPACTS OF SOCIAL RELATIONSHIPS ON CO-OCCURRING SYMPTOMS AND FUNCTIONING IN OLDER ADULTS WITH COGNITIVE IMPAIRMENT

Bada Kang1, Sijia Wei[2], Eleanor McConnell[2], and Kirsten Corazzini[3], *1 Yonsei University, Seoul, Seoul-t'ukpyolsi, Republic of Korea, 2. Duke University, Durham, North Carolina, United States, 3. University of New Hampshire, Durham, New Hampshire, United States*

Social relationships are crucial for well-being of older adults with cognitive impairment (CI), however, evidence is lacking on how social relationships may influence symptom experience and functioning among those living with CI. This study aimed to identify subgroups of older adults with CI with distinct symptom and functioning profiles and to examine the association between latent class membership and social relationships. The sample included 927 older adults who were screened as having moderate or severe CI from wave 2 of the National Social Life, Health and Aging Project. Symptom (i.e., pain, fatigue, sleep disturbance, depression, anxiety, and stress) and functioning (i.e., instrumental activities of daily living, activities of daily living, and urinary incontinence) variables were used to identify subgroups. Latent profile analyses identified five distinct groups: most (51.7%) belonged to the low-symptom-high-functioning; few (7%) belonged to the high-symptom-low-functioning; while two groups had average symptom burden, one group (25%) had frequent urinary incontinence and normal daily functioning (poor-urinary-functioning), and the other group (5%) had normal urinary functioning and the worst daily functioning (worst-daily-functioning); interestingly, 13% belonged to high-symptom-normal-functioning group. Multinomial logistic regression modeling showed that, among social networks, support, strain, and engagement, members in groups with worse symptom burden and daily functioning were significantly more likely to have social strain, after adjusting for covariates (p-values < 0.01). Only severe CI was associated with worst-daily-functioning (OR = 3.24, p-value = 0.002). Interventions that ameliorate social strain may benefit symptom management and promote independent daily living among older adults with CI.

### SESSION 6290 (POSTER)

### COGNITION, AGING, AND THE SOCIAL ENVIRONMENT

### OBJECTIVE HEARING, SUBJECTIVE HEARING, AND PHONE-BASED MEMORY TESTING

Taylor Atkinson, and Ross Andel, *University of South Florida, Tampa, Florida, United States*

Hearing impairment may make responding to phone-based memory tests difficult. Words with fricative consonants may pose particular difficulty, as telephones exclude high-frequency sounds necessary for their intelligibility. We hypothesized that recall would be impacted by hearing ability and word type (fricative vs. non-fricative) in a phone-based word list task.Participants (N=1,352, mean age=69.1) in the 2016 and 2018 waves of the Health and Retirement Study completed a phone-based word list recall task evenly split (5 each) between fricative and nonfricative words. Hearing was measured objectively (OH) and subjectively (SH), and each were dichotomized into 'good' and 'poor' hearing. Covariates included age, sex, race, education, depression, a subjective health rating, and functional limitations. Separate mixed-design ANCOVAs were used for OH and SH, where word type recall was within-subject, and hearing ability was between-subject. For OH, there was a main effect of word type (F(1,1341)=5.67, p=.02) and hearing ability (F(1, 1341)=87.76, p<.001) but no interaction (p>.05). Participants recalled 0.35 fewer fricative words than nonfricative words, and participants with poor OH recalled 0.12 fewer words on average (ps<.001). For SH, there was still a main effect of word type (F(1,1341)=5.39, p =.02), but no effect of hearing ability (p>.05). OH and SH classifications had low agreement (Kappa=.22, p<.001). Hearing ability and word acoustic properties can affect word recall in phone-based tests. Objective hearing tests are important, as subjective ratings do not necessarily agree. Researchers should be careful when constructing telephone-based cognitive tests in order to avoid memory impairment misestimation.

### SHORT-TERM COUPLED ASSOCIATION BETWEEN BLOOD PRESSURE AND COGNITIVE FUNCTIONING

Tomiko Yoneda[1], Andrea Piccinin[2], Jonathan Rush[2], Nathan Lewis[2], Jamie Knight[2], Rebecca Vendittelli[2], and Scott Hofer[2], *1. Northwestern University, Evanston, Illinois, United States, 2. University of Victoria, Victoria, British Columbia, Canada*

An extensive body of research has investigated the association between cognition and blood pressure (BP). Limited research, however, has examined the association at the daily within-person level. No study has yet applied an intensive measurement design utilizing the convenience and objectivity of mobile devices for cognitive assessments and automated BP monitors. To address this gap in the literature, we recruited community-residing healthy adults (N=64; Mage=70.6, SD=3.5; 76.6% female), who recorded their BP and completed a battery of brief cognitive tasks twice daily for 14 days. Multi-level models estimated associations between systolic/diastolic BP (sBP/dBP) and cognitive functioning, adjusting for time-varying self-reported effort, stressors, and physical activity, as well as time-invariant variables (age, sex, education, and anti-hypertensive medication). At the interindividual level, results suggest that individuals with higher overall sBP and dBP relative to others performed worse on delayed-reproduction and visual short-term memory tasks. At the intraindividual level, on occasions when individuals had higher sBP compared to their personal average, they performed relatively worse on delayed-reproduction tasks. Mean sBP was also significant within these models, suggesting that this coupled relationship is stronger for individuals with higher sBP on average. Analyses did not indicate significant associations for working memory, reaction time, or cognitive interference assessments, which may have been due to relatively limited power for between-person analyses. Use of such digital health technology is critical for detecting the complex nature of and interplay between physiological and cognitive processes. Further, research based on intraindividual associations may contribute to strategies aiming to promote lifestyle modifications.

### CROSS-VALIDATION OF THE LICHTENBERG FINANCIAL DECISION RATING SCALE

Emily Flores, and Peter Lichtenberg, *Wayne State University, Detroit, Michigan, United States*

This study examines the cross-validation of the long form of the Lichtenberg Financial Decision Rating Scale in relation to decision making abilities and suspected financial exploitation.Ninety-five older adult community participants underwent an assessment session which included the LFDRS, cognitive tests and the Independent Living Scale financial management subtest. The LFDRS has four subscales including an intellectual factor that measures choice, understanding, appreciation, and the rationale of decisions. Demographic information was also collected.The concurrent validity of the subscales and LFDRS were examined by Pearson correlations in order to be able to examine the relationship between cognitive test scores, ILS money management scores and LFDRS risk scores. Hierarchical regression analysis was conducted to determine whether cognitive and financial management tests were contributed to the prediction or risk scores greater than demographic variables. LFDRS total was significantly related to executive functioning and female gender. The Financial Situational Awareness subscale showed significant correlations with Trailmaking B but was unrelated to demographics. The relationship of executive functioning to vulnerability to exploitation was largely driven by the Financial Situational Awareness subscale. This finding is consistent with previous studies of the LFDRS and adds to the evidence supporting the concurrent validity of the LFDRS with cognitive functioning.

### THE INTERPLAY BETWEEN MEMORY SPECIFICITY AND COGNITIVE INHIBITION

Jessie Chien, and Teal Eich, *University of Southern California, Los Angeles, California, United States*

Pattern separation studies suggest age-related declines in discriminating between a newly and a previously encountered overlapping mental representations. However, when similar experiences occur contemporaneously, shared features may serve as intrinsic factors that interfere with the ability to encode and retrieve events as distinct memories. If so, successful inhibition of competitors might also be necessary to maintain memory specificity. Here, we investigated the interplay between memory specificity and inhibitory control in younger (20-35;n=34) and older (65+;n=37) adults by testing whether and how presenting a target item with a lure item (varied in its similarity to the target) influenced recognition accuracy, probed using a 3-item (target, lure, and a non-presented control) forced-choice recognition test. Surprisingly, we found no age-related differences, including in target accuracy, which was high in both groups (young=80.25%; older=77.86%). While not age-specific, we did find that the pattern of errors varied as a function of the level of similarity between the items at encoding. When target and lure were highly similar, a greater proportion of control items were falsely-recognized relative to lure items. It is possible that inhibition of the lure spread to the target, leaving both inaccessible at retrieval (e.g., a retrieval-induced forgetting effect). When the target and lure were less similar, participants false alarmed more to lure than control, potentially because the lure provided less competition and thus did not necessitate inhibitory control. These results shed light on how inhibitory processes might be differentially involved, but age-invariant, depending on the similarity of encoded items.

### THE ROLE OF CAREGIVING IN COGNITIVE FUNCTION AND CHANGE: A REGARDS STUDY

Joanne Elayoubi, *USF, Land o' Lakes, Florida, United States*

Caregiving is often associated with elevated levels of stress and depressed mood. There is some evidence that higher caregiving strain is associated with worse cognitive functioning, however, findings are mixed. The current study examined the relationship between caregiving and cognitive functioning, and the potential mediating effects of depressive symptoms and high-sensitivity C-reactive protein (hsCRP). We identified participants in the Reasons for Geographic And Racial Differences in Stroke (REGARDS) study who were family caregivers at baseline assessment (n=3,118), and then used propensity matching on 14 sociodemographic and health variables to identify 3,118 non-caregivers. Participants completed repeated assessments of global cognitive functioning, learning and memory, and executive functioning and processing speed. Our results showed caregivers and non-caregivers did not differ on cognitive functioning at baseline or in rate of change in cognition over time. Caregivers had higher baseline depressive symptoms than non-caregivers but depressive symptoms and hsCRP did not mediate the relationship between caregiving strain and cognition. The present study, using several methodological advances not found in most previous papers on caregiving and cognition (large sample size, propensity matching of caregivers and non-caregivers, longitudinal methods) found no evidence that caregiving or caregiving strain significantly influenced cognitive performance. Results are consistent with recent research demonstrating that, while caregiving can be highly stressful, many caregivers are resilient and do not show marked declines in cognition and health, or higher mortality. A more balanced narrative of caregiving, emphasizing resilience and both the problems and benefits of caregiving, is indicated.

### AGE-RELATED CHANGES IN CUED TASK SWITCHING

Elizabeth Lydon[1], and Raksha Mudar[2], *1. University of Illinois Urbana-Champaign, Champaign, Illinois, United States, 2. University of Illinois Urbana Champaign, Champaign, Illinois, United States*

The task switching paradigm is widely used to examine cognitive switching, a critical subcomponent of cognitive control. Studies on aging suggest that switching is particularly vulnerable to age-related changes in cognition. However, the effects of manipulating the stimulus dimension on task switching performance is relatively understudied. In this study, 13 younger adults (YA; 11F; Mean Age= 22.31) and 13 older adults (OA; 8F; Mean Age= 65.85) completed a novel cued task-switching paradigm requiring speeded same-different judgments based on a perceptual (color) or conceptual (animal) dimension of the stimuli. Task switching performance was measured using switch cost, which is the difference in accuracy between switch and repeat trials. Overall both YA and OA exhibited switch costs, indicating increased cognitive demand when switching between judgments compared to repetition of the same judgement. In regard to group differences, YA and OA performed similarly when performance was collapsed across the stimulus dimensions; however, when examined separately, OA exhibited worse performance than YA when making conceptual judgements. These results highlight the importance of examining carefully manipulated stimulus-related factors in task switching paradigms to advance our understanding of cognitive control and aging.

### EXPLORING LINGUISTIC PATTERNS ON A STORY RECALL TASK IN PEOPLE WITH MILD COGNITIVE IMPAIRMENT

Angela Boland, Adelaide Jensen, Patrick Davidson, and Vanessa Taler, *University of Ottawa, Ottawa, Ontario, Canada*

Mild cognitive impairment (MCI) involves declines in language and episodic memory. Episodic memory is often assessed using language tasks. To prevent linguistic factors from confounding recall scores, memory and language should be jointly examined. We explored linguistic patterns on a story recall task among cognitively healthy adults aged 65+ (n=18) and people with amnestic MCI (n=18). Participants completed immediate and delayed (20-30min) recall on a set of novel story recall materials (6 pairs, i.e., 12 stories total). Stories were coded using a propositional coding scheme (where a proposition refers to the smallest unit of meaning), as well as a unit scoring scheme (i.e., individual words). Responses were coded as veridical (word-for-word), gist (general idea), and distortion (error). Linguistic features of the output were coded using the Linguistic Inquiry and Word Count (LIWC) program. Overall, people with MCI produced more verbs, fewer time-related words, and fewer total words than control participants. In the MCI group, delayed unit- and proposition-based veridical and gist recall scores were positively correlated with certainty and causation words, indicating that higher certainty about events and their causal links is associated with better memory. Total words were positively correlated with all immediate and delayed recall scores, indicating that amount of linguistic output is strongly linked to memory in MCI. Time-related words were positively correlated with immediate unit-based veridical recall, suggesting that, in MCI, more words denoting time signal better immediate recall of story details. Examining linguistic features of verbal output in memory tasks could improve detection of MCI.

### FACTORS ASSOCIATED WITH COGNITIVE FUNCTION AMONG OLDER ADULTS IN VIETNAM

Yasuhiko Saito[1], Choy Lye Chei[1], Nguyen Vu[2], and Linh Dong[3], *1. Nihon University, Chiyoda-ku, Tokyo, Japan, 2. Institute of Population, Health and Development, Hanoi, Ha Noi, Vietnam, 3. Institute of Population, Health and Development, Ha Noi, Ha Noi, Vietnam*

While Viet Nam is aging rapidly, there are few studies on cognitive function of older adults. We aimed to study status of cognitive function among older adults in Viet Nam and examine factors associated with cognitive function. The baseline data of the Longitudinal study of Ageing and Health in Viet Nam conducted in 2018/2019 was used for the study. LSAHV was a nationally representative survey of those aged 60 and above and computer-assisted personal interview (CAPI) using a tablet was conducted for data collection. The number of older person included in the study was 5,530. Cognitive function was measured by Short Portable Mental Status Questionnaire (SPMSQ). Factors such as demographic, socioeconomic, health behavior and daily activities such as reading newspaper were examined for their association with cognitive function by linear regression analyses. We found that better cognitive function was associated with engaging in physical activity in both male and female, listening to radio and receiving material support from children in male, and reading in female. Poorer cognitive function was associated with IADL limitation and financial support in both male and female, obesity in male, and hypertension and hearing loss in female. Our study provides an understanding of the associated factors with cognitive function of older people in Viet Nam. Some of the factors examined are modifiable and can be improved to prevent cognitive decline. It is also important to consider sex differences in planning and implementation of interventions and programs to prevent cognitive decline in Viet Nam.

### PSYCHOLOGICAL WELL-BEING AND PHYSICAL HEALTH ON SUBJECTIVE AND OBJECTIVE MEMORY IN OLDER ADULTS

Zhong-xu Liu[1], Brenda Whitehead[2], and Anda Botoseneanu[1], *1. University of Michigan Dearborn, Dearborn, Michigan, United States, 2. Grace College, Winona Lake, Indiana, United States*

Self-report measures of memory, often used in survey studies of older adults, are heavily influenced by stress, depression, and life satisfaction; this makes it difficult to tease memory performance apart from psychological well-being, and highlights the value of shifting to remotely-administered objective memory tasks when feasible. This study investigates how indicators of psychological well-being, psychological distress, and physical health differentially influence the subjective and objective memory measures in order to compare the extent to which they are explained by participants' psychological and physical health profile. 404 adults aged 55 and older without diagnosed cognitive impairment participated in an online survey which involved measures of physical health (PHY; multimorbidity, BMI), psychological well-being (PWB; life satisfaction, positive and negative affect), psychological distress (PDS; perceived stress, anxiety, depression) and subjective memory complaints (SM), along with remotely-administered objective memory tasks (OM). Regression analyses found all three health/well-being composite variables (PHY, PWB, PDS) maintained significant effects on SM ($p < .01$); PWB and PDS had no significant effects on OM, whereas PHY maintained significance on OM throughout ($p = .02$).So, SM measures are highly influenced by the psychological profile of the participant, highlighting the importance of controlling for these factors when relying on subjective memory measures. That physical health was the only significant predictor of the OM tasks in this study not only reveals remotely-administered OM tasks to be more immune to participants' psychological profile, but also supports previously-established links between physical health and brain function.

### METACOGNITION AND EMOTIONS IN OLDER AFRICAN AMERICAN ADULTS

Katherine Kero[1], Colt M. Halter[1], John L. Woodard[1], Bruno Giordani[2], Ana Daugherty[1], and Voyko Kavcic[1], *1. Wayne State University, Detroit, Michigan, United States, 2. University of Michigan, Ann Arbor, Michigan, United States*

Older adults in the earliest stages of cognitive decline often present with subjective cognitive complaints which may not be fully reflected in objective measures of cognition. Previous research suggests that a relationship exists between negative emotions, stress and metacognition, but these relationships have not yet been examined in the context of COVID-19. The purpose of this study was to examine the role of stress and emotions in perceived cognition in the context of the COVID-19 pandemic. Telephone screenings were administered to 206 older African Americans (aged 64–94 years). Objective cognition (Telephone Interview for Cognitive Status [TICS]), subjective cognition (Cognitive Change Questionnaire [CCQ]), perceived stress scale 4 (PSS-4), and survey questions about affective responses to COVID-19 experiences were measured. Objective TICS scores predicted subjective CCQ executive function scores ($F(1, 197)=4.37$, $p=.038$, $R2=.022$). Discrepancy scores were calculated as the standardized residual variance between objective and subjective measures. Survey items describing emotional states were summarized with emodiversity scores following Quoidbach and colleagues' (2014) formula. Discrepancy scores were correlated with perceived stress, as well as global and negative emodiversity (Spearman $r=.294$, $.279$, $.318$, $p<.001$). In conclusion, we have shown that objective and subjective measures of cognition are related, but discrepancies exist between objectively-measured and self-perceived cognition. Increased stress and greater negative emotions are associated with greater overestimation of cognitive difficulties relative to one's objective level of cognition. As stress and negative emotions have increased for many during the pandemic, individuals may also have depreciated their self-appraisal of cognitive abilities in the present climate.

### WHO NEEDS THEIR NEIGHBORS? EXPLORING NEIGHBORHOOD DISPARITIES IN COGNITIVE FUNCTION THROUGH PATH ANALYSIS

Daniel Rong Yao Gan, and John Best, *Simon Fraser University, Vancouver, British Columbia, Canada*

Neighborhoods are diverse and may or may not present opportunities for stress reduction or social engagement depending on their qualities. Based on the stress connectome (Dum et al., 2019), psychosocial stress may accelerate cognitive aging, which explains place-based disparities in cognitive function. This study examines two attributes of the neighbourhood environment, and their potential to influence cognitive function through semantic fluency.Using aggregated baseline (cross-sectional) data from N=1,010 neighborhoods with 5 or more respondents in the Canadian Longitudinal Study on Aging, we examined the effects of neighborhood greenness and cohesion on aggregates of age-, sex-, and education-adjusted cognitive test scores. Participants were community-dwelling adults aged 44 and above. Semantic fluency was assessed using the Animal Fluency Test (AFT). Delayed recall was assessed using Rey's Auditory Verbal Learning Test (RAVLT), whereas executive function was assessed using Mental Alternation Test (MAT). Neighborhood qualities were found to affect delayed recall ($B=.34$, $p<.001$) and executive function ($B=.42$, $p<.001$) through semantic fluency ($B=.08$, $.10$, $p<.01$). Semantic fluency fully mediated the effects of neighborhood attributes on cognitive function. Further stratifying these neighborhoods by socioeconomic status showed that cohesion has stronger effects in poorer neighborhoods (B indirect=.104) than richer neighborhoods (B indirect=.066). The effect of greenness was no longer significant upon stratification. Neighborhoods offer an important social arena for adults in mid- and late-life to practice conversing, especially in poorer neighborhoods, which improves cognitive function. Creating opportunities for socialization by improving cohesion and neighborhood parks may reduce place-based disparities in cognitive health. Causality remains to be ascertained.

### COGNITIVE PROFILES OF SPOUSAL AND PARENTAL CAREGIVERS: AN HRS STUDY

Katelyn Singer[1], and Benjamin Katz[2], *1. Virginia Tech Graduate School, Blacksburg, Virginia, United States, 2. Virginia Tech, Blacksburg, Virginia, United States*

Previous work analyzing the health and wellbeing correlates of caregiving has found increased rates of depression (Caputo, Pavalko, & Hardy, 2016), anxiety (Joling et al., 2015), and elevated risks of health problems, specifically cardiovascular-related incidents like hypertension (Capistrant, Moon, & Glymour, 2012), cardiovascular disease (Capistrant et al., 2012), and strokes (Haley et al., 2010). Research on specific cognitive effects that this responsibility may elicit has been less of an area of focus, especially when examining domains related to executive functioning. The present research investigated whether executive functioning mechanisms (such as processing speed, recall, and attention) are impacted in caregivers and if their function is mediated by interpersonal factors like birth year, gender, education, and depressive symptomology. Using the Health and Retirement Study, we compared spousal and parental caregivers of persons with medical conditions ($n = 143$) and non-caregivers ($n = 975$) who completed the 2015 Consumer and Activities Mail Survey (CAMS). Additional demographic and cognitive performance information was paired from the corresponding RAND 2016 data and the 2016 Harmonized Cognitive Assessment Protocol (HCAP) data. Preliminary ANCOVA results indicated, surprisingly, that spousal/parental caregivers were more likely to exhibit better executive function performance ($p < .05$) compared to non-caregivers, while delayed recall measures were not linked to caregiving status. These results indicate that intact executive function might be linked to one's ability to provide caregiving for parents and/or spouses; while the HCAP was only administered at a single time point, future research should examine the longitudinal effects of caregiving on cognitive function.

### THE EFFECT OF MOOD ON SELF-REPORTED COGNITION FOR INDIVIDUALS WITH HIV

Emilee Ertle[1], Darby Simon[2], Jax-Patrick Vogt[1], Benjamin Mast[1], and Shirish Barve[1], *1. University of Louisville, Louisville, Kentucky, United States, 2. University of louisville, Louisville, Kentucky, United States*

Older adults with HIV may experience cognitive changes. The Patient Assessment of Own Functioning Inventory (PAOFI) is a self-report measure of cognitive abilities with memory, language, and cognitive/intellectual functions domains. Self-reported cognition can be influenced by many factors. This study examined the relationship between demographics, performance-based cognitive tests, mood, and self-reported cognition in a sample of 23 HIV-positive individuals (mean age=56.7). The PAOFI memory domain positively correlated with depression (PHQ-9; r=.64, p=.001) and anxiety (GAD-7; r=.48, p=.019), but not tests of cognition. The PAOFI cognitive/intellectual functions domain also positively correlated with the PHQ-9 (r=.65, p=.001) and the GAD-7 (r=.66, p=.001), but not cognitive tests. Regression analysis found similar models for both domains. Both models included education, race, anxiety, and delayed recall. Depression was the only unique variable in the memory model, F(5, 17)=4.09, p=.013, R2=.55, and word reading was unique to the cognitive/intellectual functions model, F(5, 15) = 6.91, p = .002, R2 = .70. In contrast, the language domain correlated negatively with four cognitive measures: word reading (r=-.46, p=.038), learning of a word list (r=-.47, p=-.024), delayed recall of a word list (r=-.51, p=.012), and verbal fluency (r=-.49, p=.045). Regression analysis found age, gender, depression, word reading, and delayed recall predict PAOFI language scores, F(5, 15)=6.27, p=.002, R2=.68. These findings suggest mood, demographics, and performance on cognitive tests are related to self-reported cognition. Mood is likely a better predictor of self-reported memory and higher-level cognitive abilities, and cognitive tests may better predict self-reported language abilities.

### EVERYDAY PROSPECTIVE MEMORY LAPSES AND INFLAMMATION IN OLDER ADULTS

Erin Harrington[1], Jennifer Graham-Engeland[2], Martin Sliwinski[3], Mindy Katz[4], Karina Van Bogart[2], Jacqueline Mogle[3], Richard Lipton[4], and Christopher Engeland[1], *1. Pennsylvania State University, University Park, Pennsylvania, United States, 2. Pennsylvania State University, Pennsylvania State University, Pennsylvania, United States, 3. The Pennsylvania State University, University Park, Pennsylvania, United States, 4. Albert Einstein College of Medicine, New York, New York, United States*

Prospective memory (PM) refers to our memory for future intentions, such as attending an appointment or taking medication. Research suggests that PM deficits can distinguish healthy older adults from those in early stages of dementia. However, limited work has examined PM and biological markers associated with pathological memory decline. The current study examined older adults' everyday PM lapses and inflammation. Older dementia-free adults (n =237, Mage=76.86 years), enrolled in the ongoing Einstein Aging Study, completed a two-week ecological momentary assessment (EMA) as part of the first wave of data collection. Participants provided two blood samples (pre/post EMA) and self-reported daily PM lapses during nightly surveys. Inflammatory levels quantified from blood were averaged and included in regression analyses predicting total number of PM lapses (covarying for: age, education, race, health, BMI, depressive symptoms). Reporting more PM lapses was associated with higher circulating levels of interleukin [IL]-8 (p=.007); no significant associations emerged with C-reactive protein or other circulating or stimulated (ex-vivo) cytokines (IL-1β, IL-4, IL-6, IL-8, IL-10, TNF-a). Gender moderated the observed link between IL-8 and PM lapses (p=.015); specifically, higher levels of IL-8 were associated with more PM lapses among men (95%CI=[0.54, 4.72]) but not women (95%CI=[-1.56, 1.25]). Other researchers that found poor cognitive performance in association with elevated IL-8 have suggested that this relation may be indicative of neurodegeneration and future pathology. Future studies should continue to examine daily PM lapses and inflammation across genders to identify mechanisms through which these constructs may relate to neurodegeneration and dementia risk.

### DAILY STRESS, DYADIC COPING, AND COGNITIVE FUNCTIONS IN MIDLIFE SAME- AND DIFFERENT-SEX MARRIAGES

Yiwen Wang, *University of Texas at Austin, Austin, Texas, United States*

Little is known about same-sex couples' stress experience and cognitive performance at older ages as compared to different-sex couples. Sexual minority older adults may be at greater risk of cognitive impairment and dementia than their non-LGB counterparts due to life-long exposure to the discrimination and stigma associated with sexual minority status. In this study, I use daily dyadic longitudinal data from the Health and Relationships Project (HARP) to explore the patterns of daily cognitive functioning (memory failures and unconstructive repetitive thinking) among spouses in same-sex and different-sex marriages within the United States (N = 836 individuals, 419 couples). The primary focus of this study is to assess how daily stress—as reported by each spouse—is associated with cognitive function, and how these associations vary for women and men in same- and different-sex marriages. To examine how dyadic coping (the process through which couples manage stress together) matters, I also test whether collaborative dyadic coping buffers the health impact of daily stressors on cognitive function. Results suggest that same-sex couples experience more daily stress and have worse cognition compared to different-sex couples. While both respondent- and spouse-reported daily stress are negatively associated with cognitive function, the association between spouse-reported stress and cognition is stronger for women in same-sex marriages compared to men in different-sex marriages. Both men and women in same-sex marriages are more likely to cope with stress collaboratively than their counterparts in different-sex marriages, which helps buffer the negative consequences of daily stress on cognition.

### ASSOCIATIONS BETWEEN NEIGHBORHOOD CHARACTERISTICS AND COGNITIVE FUNCTIONING TRAJECTORIES BY RACE/ETHNICITY

Amy Thierry[1], and Heather Farmer[2], *1. Xavier University of Louisiana, New Orleans, Louisiana, United States, 2. University of Delaware, Newark, Delaware, United States*

Older adults living in neighborhoods with high physical disorder and low social cohesion may be especially vulnerable to experiencing declining health. However, less is known about how neighborhoods may influence age-related patterns of cognitive functioning for different racial/ethnic groups. Therefore, we examine whether perceived neighborhood characteristics (safety, cleanliness, and social cohesion) are associated with trajectories of cognitive functioning across race/ethnicity. Using data from the 2006-2016 waves of the Health and Retirement Study, our study includes 11,870 non-Hispanic White, non-Hispanic Black, and Mexican adults 65 and older. We conducted linear mixed models stratified by race/ethnicity using an age-accelerated design to understand how neighborhood characteristics are related to trajectories of cognitive functioning (measured by the Telephone Interview for Cognitive Status; range: 0-35). We also explore if sociodemographic, behavioral, and health characteristics account for this association. Analyses revealed that cognitive functioning showed a quadratic change with age. Negative neighborhood perceptions were associated with declines in cognitive functioning with increasing age for each racial/ethnic group. Among White adults, all neighborhood characteristics were negatively associated with cognitive functioning in fully adjusted models. For Black adults, perceived uncleanliness was significantly associated with trajectories of cognitive functioning. In Mexican adults, perceptions of uncleanliness and lower cohesion were significantly associated with worse cognitive functioning, but not in fully adjusted models. These findings suggest that the perceived neighborhood context is an important social determinant of cognitive health for older adults, and distinct aspects of the neighborhood may be more correlated with cognitive functioning for certain racial/ethnic groups than others.

### THE INTERACTIVE EFFECTS OF EDUCATION AND SOCIAL SUPPORT ON COGNITION IN AFRICAN AMERICANS

DeAnnah Byrd[1], Yanping Jiang[2], Samuele Zilioli[3], Peter Lichtenberg[3], Roland J. Thorpe, Jr.[4], and Keith Whitfield[5], *1. Arizona State University, Phoenix, Arizona, United States, 2. Rutgers, The State University of New Jersey, New Brunswick, New Jersey, United States, 3. Wayne State University, Detroit, Michigan, United States, 4. Johns Hopkins University, Baltimore, Maryland, United States, 5. University of Nevada, Las Vegas, Las Vegas, Nevada, United States*

This study examines whether the effects of receiving or providing social support on cognition differ by education. Data from 602 African American adults (48-95 years) enrolled in the Baltimore Study of Black Aging—Patterns of Cognitive Aging were analyzed using multiple linear regression. We found no main effects of receiving or providing social support on global cognition. However, a significant moderation effect was observed for memory, such that received social support was more strongly associated with higher working memory among less-educated individuals than those with high levels of education, adjusting for age, sex, marital status, chronic health conditions, and depressive symptoms. Study findings demonstrate that social support and education have joint effects on memory outcomes, highlighting the importance of considering psychosocial protective factors that might alleviate, reduce, or even eliminate cognitive health disparities in African Americans.

### ARE CAREGIVERS HEALTHIER?: ASSESSING CAREGIVERS' EPISODIC MEMORY IN A MATCHED AND UNMATCHED SAMPLE

Britney Veal, Jessica Yauk, and Hongdao Meng, *University of South Florida, Tampa, Florida, United States*

Recent findings using an advanced methodological technique of propensity matching have found that caregivers may have better cognitive health compared to non-caregivers. However, there are limited studies assessing how personality and other psychosocial variables may affect the relationship between caregiver status and cognition. Utilizing the healthy caregiver hypothesis (HCH), the current study examined the association between caregiving and episodic memory in a matched (N= 1,246) and unmatched (N=3,112) sample of caregivers from the 2016 wave of the Health and Retirement Study. The interaction between caregiving status and personality was also examined. Unadjusted models showed no difference between caregiver status and episodic memory in the samples; however, depression was significantly (p=<.0001) related to cognition in the unmatched sample. In adjusted models for the unmatched sample, conscientiousness (p=0.043), pessimism (p=0.006), and feeling constrained (p=0.028) were found to be significantly associated with episodic memory. In the matched adjusted models, conscientiousness was no longer a significant predictor, but number of chronic conditions was significantly related to episodic memory (p=0.001). The interaction between caregiving and extraversion also approached significance (p=0.076). Findings suggest extraverted caregivers may have better episodic memory performance. These findings highlight the importance of implementing propensity matching in caregiving research. Future research is needed to examine the relationship between coping style and personality specific domains in relation to the HCH.

### LONGITUDINAL ASSOCIATIONS BETWEEN PERSONAL GROWTH AND COGNITIVE FUNCTIONING IN ADULTHOOD

Masahiro Toyama, *Marshall University, Barboursville, West Virginia, United States*

While personal growth has been found to be associated with multiple aspects of health in adulthood, its associations with cognitive functioning have not been fully understood. The present study aimed to assess both directions of such longitudinal associations. Using data from the second wave (T1) and third wave (T2) of the Midlife in the United States (MIDUS) study (N = 4,206; mean age = 56.0 [SD = 12.3]), a longitudinal measurement model containing latent variables of episodic memory and executive function was first constructed. Built on the measurement model, a structural equation model was analyzed to assess cross-lagged relationships between personal growth and the two areas of cognitive functioning, in which T1 personal growth predicted residualized changes in episodic memory and executive function, and T1 episodic memory and executive function predicted change in personal growth, controlling for covariates. The results indicated that T1 personal growth significantly predicted smaller decreases in episodic memory, whereas it did not predict change in executive function. T1 episodic memory, but not T1 executive function, significantly predicted smaller decreases in personal growth. The present findings were unique, particularly implying potential longitudinal reciprocity between personal growth and episodic memory. These findings and implications can inform future research aimed at exploring approaches to promoting personal growth and cognitive functioning among aging adults.

### PREDICTED DIFFERENCES OF EPIGENETIC AGE AND DEMENTIA RISK AMONG DIFFERENT TRAJECTORIES OF LONGITUDINAL LONELINESS

Morgan Lynch, and Christopher Beam, *University of Southern California, Los Angeles, California, United States*

Different trajectories of loneliness in late adulthood may explain differences in the effects of aging on dementia risk. We tested whether greater or increasing loneliness across three time points demonstrated stronger associations between aging and dementia risk in a sample of 1,814 Health and Retirement Study participants. Dementia risk was quantified using modified Telephone Interview for Cognitive Status scores (TICSm), age was quantified using the epigenetic clock DNAm PhenoAge, and loneliness was measured with the UCLA Loneliness short-form scale. Growth mixture modeling was used to identify loneliness latent classes that best represented trajectories according to model fit statistics. Five groups were identified: low loneliness, high declining, low increasing, moderate and stable, and a moderate declining group. Multivariate analysis of variance was used to test whether class membership differentially predicted TICSm scores, PhenoAge, and the correlation between TICSm and PhenoAge.TICSm scores were statistically significantly lower (worse) for the high-declining compared to the low (-1.08 95% CI [-1.75, -0.42]) and low-increasing groups (-1.63 95% CI [-2.28, -0.98]), and the moderate group was lower than the low-increasing group (-1.11 95% CI [-1.66, -0.57] and the low group (-0.57 [-1.13, -0.002]) (p's < 0.05). No significant differences in PhenoAge or the correlation between PhenoAge and TICSm were found between groups. Analyses statistically adjusted for demographic characteristics, objective social isolation, depression, BMI, smoking, self-rated health, and polygenic risk score for cognition. Results suggest that epigenome-wide ages are unlikely to mediate the relationship between loneliness and dementia risk.

### AGE-RELATED DIFFERENCES IN THEORY OF MIND: A TIME-VARYING FUNCTIONAL CONNECTIVITY APPROACH

Samuel Naranjo Rincon[1], Richard Betzel[2], and Anne Krendl[2], *1. Indiana University at Bloomington, Bloomington, Indiana, United States, 2. Indiana University, Bloomington, Indiana, United States*

Theory of Mind (ToM) refers to the act of inferring someone's inner state, such as their emotions and thoughts. Previous work also demonstrated that ToM performance declines across age, but the mechanisms underlying this are not well-understood. The brain regions underlying ToM are generally in the default mode network (DMN) – a group of brain regions particularly vulnerable to pathological aging. Recent work suggests that declines in functional connectivity (FC; correlations between brain regions) during resting state (a period of undirected thought) predict age deficits in ToM. However, these findings come from aggregated FC, ignoring potentially informative details of dynamic changes during resting state. Researching these details may provide more specificity related to social cognitive aging. To test this, 35 older adults (OA; M = 75.61; 22 female) and 40 young adults (YA; M = 21.58; 25 female) underwent resting state and task-based fMRI. During the task, they completed a standard ToM task. We conducted time-varying functional connectivity analyses in the DMN to identify dynamic changes over time. Overall, OA had more variability than YA, but variability benefitted YAs, and not OAs. This occurred specifically in a DMN subnetwork associated with contextual information in memory. These results suggest that variability is useful, but only to an extent. With respect to ToM, variability may promote how YA integrate contextual information to memory, but is ineffective for OA.

### DOES PERCEIVED EFFICACY OF COGNITIVE TRAINING BIAS OUTCOMES?

Shenghao Zhang, Nicholas Gray, Neil Charness, and Walter Boot, *Florida State University, Tallahassee, Florida, United States*

Cognitive training has the potential to help older adults maintain or improve their cognitive abilities, but there has been debate regarding how susceptible cognitive outcomes in these studies are to placebo effects. Some previous research has found that expectations can shape performance on study outcomes, although this is not always a consistent finding. The degree to which perceived intervention efficacy can lead to changes in the performance of objective outcome measures typically assessed in these studies is unclear. The aim of the current study is to explore the effects of perceived effectiveness of cognitive training on actual improvements in a large sample of older adults. The current study utilized data from the Intervention Comparative Effectiveness for Adult Cognitive Training (ICE-ACT, NCT03141281) Trial. Two hundred and thirty older adults (Mean age = 72) were randomized into one of four training conditions: (1) a web-based brain game suite, Brain HQ, (2) a strategy video game, Rise of Nations, (3) Instrumental Activities of Daily Living (IADL) training, or (4) an active control condition of puzzle solving. Reasoning, memory, processing speed, and IADL performance were measured before and after the intervention. Perceived efficacy of training in those outcome domains were also measured. Bayesian ANOVA analysis showed strong evidence of group differences in perceived efficacy within all outcome domains across the training conditions whereas Bayesian regression showed moderate to strong evidence against influences of perceived efficacy on changes in performance. Discussion will focus on implications of those results on behavioral and cognitive intervention designs.

### SLEEP DURATION MEDIATES THE RELATIONSHIP BETWEEN LETTER FLUENCY AND COGNITION

Hyeon Jung Kim[1], Subhranshu Banerjee[1], and Julie Blaskewicz Boron[2], *1. University of Nebraska Omaha, Omaha, Nebraska, United States, 2. University of Nebraska, Omaha, Omaha, Nebraska, United States*

Changes in cognition are a normative part of aging. Verbal fluency is widely used for assessing cognitive function, including predicting mild cognitive impairment. As people age, sleep duration and quality decrease. This has been associated with cognitive decline; individuals with low-quality sleep (fewer hours and difficulties with initiation or maintenance of sleep) are more likely to complain of poorer cognition. Lack of sleep is detrimental to verbal fluency (word production). Longer sleep has been linked to memory deficits and is a potential indicator of mild cognitive impairment. This study aimed to address the relationship between verbal fluency and cognitive function as mediated by sleep duration. Participants included 31 middle-aged adults (female, n=19;age=53.55±7.18yrs) and 24 older adults (female=14; age=70.33±3.69yrs). The MoCA assessed cognitive functioning. Verbal fluency (90 seconds each) was measured via category word (animals; semantic verbal fluency) and letter word (phonemic verbal fluency; F,A,S). Mediation models used Baron and Kenny's (1986) approach for estimating mediation effects, controlling for age and education. Category results were non-significant. However, sleep duration partially mediated the relationship between letter fluency for each letter and cognitive function, respectively (F:F(2,52)=7.31, p=.002, R2=.22; A: F(2,52)=3.80, p=.029, R2=.18; S: F(2,52)=5.47, p=.007, R2=.17). Sleep hours were negatively associated with letter fluency, meaning fewer hours of sleep improved fluency performance, which in turn was positively associated with MOCA scores (F: b=-.378, 95%CI [-0.64, 0.02]; A: b=-.172, 95%CI [-0.42, 0.01]; S: b=-.24, 95%CI [-0.64, 0.02]. This coincides with the abovementioned literature and helps further understand how sleep and cognitive performance are related.

### RELIGION AND TRAJECTORIES OF COGNITIVE FUNCTION AMONG WHITE, BLACK, AND HISPANIC OLDER ADULTS

Madison Sauerteig-Rolston[1], Lisa Barnes[2], Patricia Thomas[3], and Kenneth Ferraro[1], *1. Purdue University, West Lafayette, Indiana, United States, 2. Rush Alzheimer's Disease Center, Rush University Medical Center, Chicago, Illinois, United States, 3. Purdue University--West Lafayette, West Lafayette, Indiana, United States*

Most prior research on the relationship between religious involvement and cognition among older adults is based on cross-sectional data and yields inconsistent results. We use longitudinal data from 14,161 older adults in the Health and Retirement study (HRS) to investigate whether religious involvement, measured by attendance, integration, and religiosity (i.e., beliefs, meanings, and values) is associated with trajectories of cognitive function from 2006 to 2016 among a diverse sample of respondents. We find that religiosity is associated with lower levels of cognition at baseline among White adults (b=-0.12, p < 0.001), but higher levels of cognition among Black adults (b=0.18, p < 0.05). In addition, growth curve analysis reveals that religious attendance is associated with higher cognition over time for Hispanic respondents (b=0.07, p < 0.001). Religious involvement is associated with later-life cognition, but this relationship differs for White, Black, and Hispanic older adults.

### COGNITIVE AGING AND SUCCESSFUL AGING: THE ROLE OF MEMORY

Neyda Ma. Mendoza Ruvalcaba[1], Elva Dolores Arias-Merino[2], Melina Rodríguez Díaz[2], and Maria Elena Flores-Villavicencio[2], *1. UNIVERSITY OF GUADALAJARA, Guadalajara, Jalisco, Mexico, 2. Universidad de Guadalajara, CUCS, Maestría en Gerontología, Guadalajara, Jalisco, Mexico*

The cognitive functioning as a general measure, is a criterion commonly used to define and operationalize successful aging(SA). This study aims to explore the specific contributions of the memory and meta-memory to understanding successful aging (SA).(Project-Conacyt-256589)Population based, random sample included n=656 community-dwelling older adults 60-years and older (mean age=72.8, SD=7.6 years, 58% women). Memory was measured with the Digit span test backward (WAIS-IV). and meta-memory through self-report. Objective SA was operationalized as no important disease, no disability, physical functioning, cognitive functioning, and being actively engaged. Subjective SA was an appreciation if they considered themselves as successful agers. Sociodemographic and health data were also asked. Pearson´s correlation test and MANOVAs were performed.In total 11.2% met the criteria for SA, although 76% considered themselves as successful agers. 54.8 has a bad perception of their own memory, while 41.9% have objective decline. Results of the multiple regression analysis emerged on a significant model using the entered method:F=1.13,p<.01, explaining 14.9% of the variance of SA. Specifically, memory and meta-memory explained 19.5% of the variance of objective and 14.4% od subjective SA, 18.4% of the variance in the criterion of life engagement. Knowledge generated by this study reveals the specific role of the memory and metamemory on the SA, both objective and subjective. Besides, sets a scenario to promote successful and healthy aging, through alternatives centered in the improvement of memory and the judgment that is made about the own memory in older adults.

### SESSION 6300 (POSTER)

### COGNITIVE IMPAIRMENT: SOCIAL DETERMINANTS WITHIN AGE GROUPS AND CULTURAL CONTEXTS

### AGING, COGNITION, AND COMMUNITY EXPERIENCE: CARE CONTINUA AND RISK RATIOS OF DAILY LIFE WITH MCI

Kendra Heatwole Shank, *Towson University, Towson, Maryland, United States*

Mild cognitive impairment (MCI) affects an estimated 15-30% of the population (Sachdev et al., 2015; Ward et al., 2012). Cognitive changes directly impact participation in instrumental activities of daily living that support the ability to age in place (Thoma-Lurken et al., 2018; Riley et al., 2014). To date, little is known about these initial challenges beyond self- and caregiver report. The purpose of this research was to understand the nature of out-of-home participation for individuals with cognitive change. Community-dwelling older adults with MCI and their care partners (n=10) were recruited in a suburban region. Data were collected utilizing the "go-along" interview method (Carpiano, 2009) in combination with geospatial mapping and photography. Results included three factors that influenced ongoing community participation related to cognitive changes, including the importance of familiarity of spaces developed over time, strategies related to roadways and parking, and the use of routine to create structure and to increase the support offered by others. In addition, the go-along methodology revealed additional insights about the experiential continuum of providing and receiving care, and how enacting a 'relative' risk and risk perceptions impacted participation. Results of this research offer a unique view into daily life of people with MCI and suggest ways that individuals and caregivers can be advised, and their wellbeing enhanced through continued out-of-home participation. There are also multiple applications for increasing the accessibility of community spaces for cognition inclusion. Theoretical implications are also addressed, via re-framing the selection, optimization, and compensation model for considering cognitive change.

### NEUROPSYCHIATRIC SYMPTOMS AND MORTALITY FOR COGNITIVELY NORMAL OLDER MEXICAN AMERICANS

Phillip Cantu[1], Soham Al Snih[2], and Kyriakos Markides[2], *1. The University of Texas Medical Branch, Galveston, Texas, United States, 2. UTMB, Galveston, Texas, United States*

Objectives: Neuropsychiatric symptoms (NPS) present in older adults with Alzheimer's disease (AD) and other dementias are related to mortality. Research on the relationship between NPS and mortality in a non-dementia population has not been conducted. This study examines NPS as a predictor of six-year mortality among community dwelling Mexican Americans aged 80 years and older

Methods: Data included 466 cognitively normal participants from wave 7 of the Hispanic Established Population for the Epidemiologic Study of Elderly. NPS were measured using the Neuropsychiatric inventory (NPI). Cox proportional hazard modes were used to estimate the hazard ratio (HR) of mortality.

Results: The hazard ratio death at six years was 1.02 (95 % CI, 1.00-1.04) as a function of having any NPI score and 1.09 (95% CI 1.02-1.17) for number of NPI conditions, controlling for demographic and health characteristics. Apathy, irritability, and aberrant motor behavior were all independently predictors of mortality.

Conclusions: NPS may be modifiable risk factors to increase survival time or may by indicative of underlying healthy problems. NPS may be related to underlying health conditions among older adults with normal cognitive functioning.

### FACTORS ASSOCIATED WITH SUBJECTIVE COGNITIVE FUNCTION IN OLDER ADULTS WITH TYPE 2 DIABETES

Min Jung Kim[1], Cynthia Fritschi[2], and Eunhee Cho[1], *1. Yonsei University, Seoul, Seoul-t'ukpyolsi, Republic of Korea, 2. University of Illinois Chicago, Chicago, Illinois, United States*

Subjective cognitive decline (SCD) could be an indicator of future cognitive impairment in older adults. Diabetes is a well-known risk factor of cognitive impairment, but little is known whether sleep and psychological problems common in older adults with diabetes can contribute to SCD. The study aimed to investigate whether self-reported sleep (sleep impairment and disturbance) and psychological problems (depressive symptoms and diabetes distress) were associated with subjective cognitive function in adults aged ≥ 60 years with type 2 diabetes. Sleep, depressive symptoms, and subjective cognitive function were self-reported using The Patient-Reported Outcomes Measurement Information System. Diabetes distress was assessed using the Diabetes Distress Scale. Covariates (age, sex, race/ethnicity, body mass index, and diabetes duration) were self-reported, and glycemic control (A1c) was measured using a fingerstick test kit. A total of 82 older adults were included (mean age = 68.32 ± 5.29 years, White 76.83%, female 56.1%). Multivariate regression analyses revealed that, after controlling for covariates and A1c, increased sleep impairment was associated with increased concerns on cognitive function reported by older adults ($r = -.47$, $\beta = -.56$). Increased depressive symptoms were also associated with decreased cognitive function perceived by older adults ($r = -.44$, $\beta = -.47$). Sleep disturbance and diabetes distress were not associated with subjective cognitive function. Sleep impairment and depressive symptoms were the two strongest predictors affecting subjective cognitive function. Improving sleep quality and addressing depressive symptoms could be an effective strategy to prevent SCD and potentially delay severe cognitive impairment in older adults with diabetes.

### IMPROVEMENT OF CAREGIVER MASTERY IS RELATED TO DECREASED ANXIETY AMONG PEOPLE LIVING WITH COGNITIVE IMPAIRMENT

Yeji Hwang[1], Miranda McPhillips[2], Liming Huang[2], G. Adriana Perez[2], and Nancy Hodgson[2], *1. Drexel University, Philadelphia, Pennsylvania, United States, 2. University of Pennsylvania, Philadelphia, Pennsylvania, United States*

Anxiety is common and distressing in people living with cognitive impairment. High caregiver mastery, a positive view of one's ability to provide care, is known to be protective against anxiety in people whom they are caring for. However, the longitudinal relationship between caregiver mastery and anxiety is unknown. Our objective was to examine whether improvement in caregiver mastery was related to decrease in anxiety in people living with cognitive impairment. This was a secondary data analyses using Healthy Patterns Clinical Trial (NCT03682185), an RCT of a home-based activity intervention designed to improve circadian rhythm disorders in people living with cognitive impairment. A total of 158 participants with cognitive impairment who provided data at both pretest (T1) and posttest (T2) were analyzed. Measures included Caregiver Mastery Scale and Neuropsychiatric Inventory. We used linear regression analyses to examine the relationship between changes in caregiver mastery and changes in anxiety. The sample was primarily female (66.7%), Black (63.1%), with mean age 73.3±8.4. The mean change of anxiety frequency (T2-T1) was -0.2±1.0; anxiety frequency decreased over time. The mean change of caregiver mastery (T2-T1) was 0.4± 3.0; caregiver mastery improved over time. After controlling for age, cognition, changes in sleep impairments, changes in depression, and intervention group assignment, improvement of caregiver mastery over time ($B=-0.087$, $SE=0.035$, $t=-2.51$, $p=0.013$) was related to decreased anxiety frequency over time ($R2= 0.112$, $F=2.31$, $p= 0.039$). Interventions to improve caregiver mastery may alleviate anxiety symptoms in people living with cognitive impairment. Additional research and practice implications will be discussed.

### WEALTH INEQUALITIES IN PHYSICAL AND COGNITIVE IMPAIRMENTS AMONG OLDER ADULTS ACROSS EUROPE AND JAPAN

Nekehia Quashie[1], Dung D. Le[2], Yoko Ibuka[2], and Martina Brandt[3], *1. University of Rhode Island, Kingston, Rhode Island, United States, 2. Keio University, Tokyo, Tokyo, Japan, 3. Technische Universität Dortmund, Dortmund, Nordrhein-Westfalen, Germany*

Although prior research has provided insights on the association between country-level factors and health inequalities, key research gaps remain. First, most previous studies examine subjective rather than objective health measures. Second, the wealth dimension in health inequalities is understudied. Third, a handful of studies explicitly focus on older adults. To bridge these research gaps, this study aims to: i) measure wealth-related health inequalities in physical and cognitive impairments; and ii) examines the extent to which welfare states moderate wealth inequalities in physical and cognitive impairments among older people across Japan and Europe. We utilized harmonized data of non-institutionalized individuals aged 50-75 for physical and cognitive impairments from the Japanese Study of Aging and Retirement (JSTAR) and the Survey of Health, Ageing and Retirement in Europe (SHARE), for (n= 31,969 and 31,348, respectively). We applied a concentration index to quantify the degree of wealth inequalities in impairments. Our multilevel linear regression analyses examined whether national public health spending and healthcare access resources explained cross-country differences in wealth inequalities in physical and cognitive impairments. Findings indicated that inequalities in both impairment outcomes favored wealthier individuals in all countries, but the magnitude of inequality varied across countries under examination. Furthermore, a higher share of public health spending, lower out-of-pocket expenditures, and higher investment in healthcare resources were associated with lower wealth inequalities especially for physical impairments. Our findings suggests that different health interventions and policies may be needed to mitigate specific impairment inequalities.

### COHORT DIFFERENCES IN EARLY-LIFE SOCIOECONOMIC STATUS AND LATE-LIFE COGNITIVE IMPAIRMENT IN MEXICO

Brian Downer[1], Mariela Gutierrez[1], Silvia Mejia Arango[2], and Rebeca Wong[1], *1. University of Texas Medical Branch, Galveston, Texas, United States, 2. Colegio de la Frontera Norte, Tijuana, Baja California, Mexico*

Socioeconomic characteristics over the life course are associated with late-life cognitive impairment. However, evidence is lacking from countries like Mexico where population aging is occurring in the context of rapidly changing socioeconomic conditions. We used the Mexican Health and Aging Study to investigate differences between participants aged 60-76 in 2001 (n=5085) and 2018 (n=5947) in childhood (home with indoor toilet, parents' education) and midlife (education, longest held occupation) socioeconomic characteristics and late life cognitive impairment. Cognitive impairment was defined as a low score on >2 out of five assessments. Most participants in the 2018 cohort lived in a home with an indoor toilet as a child (58.1%) and 36.9% had parents who both completed at least some education compared to 41.9% and 28.7% of participants in the 2001 cohort, respectively. Men and women in 2018 had on average 2.34 and 1.83 more years of education than men and women in 2001, respectively. The percentage of women with no main job and men who worked in agriculture were lower in 2018 than 2001 (women: 27.0% vs. 34.6%; men: 23.3% vs. 30.4%). The 2018 cohort had lower odds for cognitive impairment when adjusting for age, sex, marital status, and living in a rural/urban community (OR=0.67 95% CI=0.56-0.81). This difference was reduced after adjusting for childhood socioeconomic measures (OR=0.76 95% CI=0.67-0.86) and was no longer statistically significant after adding midlife socioeconomic measures (OR=0.98 95% CI=0.86-1.12). These findings suggest that improved early-life socioeconomic conditions in Mexico contribute to birth-cohort differences in late-life cognitive impairment.

### PSYCHIATRIC HISTORY AND LATER-LIFE COGNITIVE CHANGE: EFFECT MODIFICATION BY SEX, RACE, AND ETHNICITY

Maria Brown, and Miriam Mutambudzi, *Syracuse University, Syracuse, New York, United States*

Objective: To better understand life course influences affecting cognitive function and decline in later life, we explored sex and race/ethnicity differentials in the relationship between a history of psychiatric, emotional, or nervous problems and cognitive functioning in later life, while accounting for early life disadvantage and relevant covariates.

Methods: Multi-level growth curve models examined associations between psychiatric history and cognitive functioning, and differences by sex and race/ethnicity (SRE), in 20,155 Health and Retirement Study (1995-2014) participants aged 65 or older, by estimating cognition scores and plotting trajectories of change with age by SRE.

Results: A history of psychiatric, emotional, or nervous problems was significantly related to cognition scores and rates of decline. Hispanic and Black participants had significantly lower cognition scores at age 75 and steeper rates of decline than White females, and Black race and the Hispanic race-sex interaction erased the protective effects of being female.

Conclusions: Our findings indicate that members of minority groups with a history of psychiatric problems evidence lower cognitive function in later life, and as a result, have a greater need for community-based long-term care than their peers without this history. Future research should include longitudinal analyses of different components of cognitive function, specific psychiatric diagnoses, and life history data that capture socioeconomic and psychosocial experiences throughout the life course. Population level findings as reported here, along with aggregate findings from similar studies, can inform interventions and policies regarding support for populations that are vulnerable to mental illness and to subsequent cognitive decline.

### HEALTH ACROSS BORDERS: A CROSS-NATIONAL COMPARISON OF IMMIGRANT HEALTH IN EUROPE

Mara Sheftel[1], Rachel Margolis[2], and Ashton Verdery[3], *1. Penn State University, Brooklyn, New York, United States, 2. Western University, London, Ontario, Canada, 3. The Pennsylvania State University, State College, Pennsylvania, United States*

Although older immigrants are a growing share of the total population in many countries, evidence regarding health differentials by nativity in older adulthood remains underdeveloped. We examine whether foreign-born adults 50 and older in Europe are disadvantaged in terms of multiple health domains, what drives the potential immigrant health disadvantage, and whether such differences are contextually dependent or a general feature of the immigrant experience in Europe. We use the Survey of Health, Aging and Retirement in Europe (SHARE) to estimate physical, mental, and social health of middle age and older adults by nativity in 19 countries. We examine whether nativity-based health disparities can be attributed to demographic composition, socioeconomic factors, family and social support, and life course timing of migration. Last, we examine regional differences in nativity-based health disparities. We find that immigrants aged 50 and above in Europe are more likely to report fair/poor physical health, score worse on EURO-D depression scale, and are more likely to be lonely than the native-born. Socioeconomic status and age at migration partially explain these health differences, although immigrant health disparities remain after accounting for these and other factors. We document some contextual variation within Europe. Immigrants in Eastern, Western and Northern Europe are disadvantaged compared to native-born adults in those regions, while immigrants in Southern Europe are in comparable health to their native-born peers. This article offers new insights into the ways that aging immigrant populations will reshape older adult health profiles in a diverse array of countries.

### VARIATIONS IN COGNITIVE STATUS IN OLDER ADULTS WITH MEMORY DIFFICULTIES: THE ROLE OF PERSONALITY AND RESILIENCE

Elizabeth Barbour[1], Kodi van Antwerp[2], Majse Lind[2], Amanda Morgan[2], and Susan Bluck[2], *1. University of Florida, Royal Oak, Michigan, United States, 2. University of Florida, Gainesville, Florida, United States*

By approximately 70-years-old, two out of three Americans experience some cognitive impairment (Hale et al., 2020). Cognitive abilities that often decline with age include working and short-term memory (Cohen, 2019), both important for encoding and retaining information (Alloway & Copello, 2013). Depending on severity, affected individuals may face difficulties performing daily tasks. Beyond biological mechanisms, Self-Life Acceptance (Resilience; Wagnild & Young, 1993) and personality (i.e., Neuroticism, Openness; BFI-2-XS; John & Soto, 2017) may relate to variations in cognitive status. We collected measures of Self-Life Acceptance, Neuroticism, and Openness to investigate their relations to older adults' cognitive status (i.e., working and short-term memory; TICS; Brandt et al., 1988). The sample was comprised of older adults clearly experiencing memory difficulties (N = 49, Mage = 76.12). In a hierarchical regression, the interaction between Self-Life Acceptance and Neuroticism predicted higher cognitive status. Deconstructing this effect, for older people with low-to-moderate Neuroticism, having worse cognitive status was related to greater feelings of Self-Life Acceptance. These individuals show resilience; when cognitive status is worse, acceptance of oneself and life appears to 'kick in' allowing individuals to maintain well-being in the face of memory difficulties. Self-Life Acceptance, however, is not present for those high in Neuroticism. In a second regression, less Self-Life Acceptance and higher Openness were also related to better cognitive status. Our findings show psychosocial factors can predict variations in cognitive status. This work provides a window into how older individuals with different personality traits and varying capacity for resilience cope with memory loss.

### UNDERSTANDING CHALLENGES OF PERSONS WITH COGNITIVE IMPAIRMENTS THROUGH THEIR LIVED EXPERIENCES

Maurita Harris[1], Elizabeth Lydon[1], Widya Ramadhani[2], Raksha Mudar[3], and Wendy Rogers[3], *1. University of Illinois Urbana-Champaign, Champaign, Illinois, United States, 2. University of Illinois Urbana-Champaign, Urbana, Illinois, United States, 3. University of Illinois Urbana Champaign, Champaign, Illinois, United States*

Older adults aging with cognitive impairments face a variety of challenges related to their memory, thinking, and concentration in their everyday activities. Understanding their lived experiences s critical to inform the development of technology and supports that can help everyday activities and improve quality of life. We have designed an in-depth interview study to explore the everyday challenges of older adults with cognitive impairments and their response strategies. We will present two case studies to illustrate the richness of the data and its value for guiding intervention design: (1) one older adult with a post-stroke cognitive impairment (PSCI) and (2) one older adult with mild cognitive impairment (MCI). As expected, these individuals reported challenges in different functional activities and described varying solution strategies. The older adult with PSCI noted challenges with completing steps and remembering things when engaging with technology-mediated social activities. This individual reported responding to these challenges by having their own method, such as writing things down or receiving assistance from others. Challenges the older adult with MCI experienced when engaging with technology-mediated social activities, long-distance travel, and caregiving were planning, completing steps, remembering things, and experiencing emotions. This individual responded to their challenges by developing visualizations, methods, routine, and receiving assistance from others. These initial insights about the range of challenges with everyday activities and response strategies highlight the value of qualitative needs assessments in understanding the needs of those aging with cognitive impairment to guide future technology and support.

### EXPLORING CULTURALLY RESPONSIVE PHYSICAL ACTIVITY AS A RECRUITMENT AND RETENTION TOOL FOR AFRICAN AMERICAN WOMEN

Joyce Ogunrinde, *University of Houston, Houston, Texas, United States*

Physical activity (PA) is associated with lower cognitive decline and incident dementia for older adults. Yet, PA data (interventions) on older African American (AA) women, a population disproportionately affected by premature aging, are lacking. This limitation reduces the efficacy of PA to reduce cognitive decline, particularly for people of color and more so women of color whose race and gender create unique spaces for PA engagement. Although AA women desire to engage in PA, they face social, structural, and behavioral barriers to PA, challenges that parallel those faced in preventing premature aging (Li et al., 2018). Extant literature on AA women's PA investigates social determinants of health (SDoH) and calls for more attention as to how these factors intertwine to shape these women's PA over time (Fleury & Lee, 2006). Culturally responsive physical activity programs (CRPA) offer a framework for addressing these factors synergistically to promote PA in a way that is desirable to AA women. Specifically, CRPA provides a strength– based approach to explicate the ways PA can redress social, structural, and behavioral causes of cognitive decline and barriers to PA (cf., Joseph et al., 2020). The purpose of this poster is to explore the benefits of CRPA interventions on preventing cognitive decline. Implications include refining current models of PA as premature aging prevention measures by increasing our knowledge of the sociocultural factors shaping AA women's aging and PA behavior and providing greater insight into the mechanisms for recruiting and retaining AA women into PA-based cognitive decline interventions.

### SESSION 6310 (POSTER)

### FAMILY CAREGIVING I

### DEMENTIA CAREGIVER PERCEPTIONS OF TELE-DEMENTIA CARE FOR VETERANS DURING THE COVID-19 PANDEMIC

Sowmya Iyer[1], Victoria Ngo[2], Marika Humber[3], Marisa Brodrick[4], Christine Gould[5], and Ranak Trivedi[6], *1. VA Palo Alto Healthcare System, Palo Alto, California, United States, 2. VA Palo Alto Health Care System, Menlo Park, California, United States, 3. VA Palo Alto Health Care System, Palo Alto, California, United States, 4. VA Palo Alto Healthcare Syste, Palo Alto, California, United States, 5. VA Palo Alto, Palo Alto, California, United States, 6. Stanford University, Palo Alto, California, United States*

The estimated 5 million persons living with dementia in the United States have been greatly impacted by the medical and psychosocial impacts of the COVID-19 pandemic, respite program closures, social isolation, and Veterans seen within the Veterans Health Administration system are particularly vulnerable. Telemedicine provides needed specialty dementia care to these patients with complex needs in their homes, and its uptake has increased during the pandemic. This qualitative, observational study explored informal caregivers' perceptions of tele-dementia care for Veterans seen at 2 sites, Palo Alto and Cleveland, via semi-structured interviews. Twenty-five caregivers (Mean age = 67y, SD=12y, 88% women) were interviewed over telephone following a tele-dementia visit. Themes that emerged from the interviews were that tele-dementia visits: (1) saved caregivers 2.6h±1.5h (Range: 0.5 to 6h) of travel time, (2) required limited preparation compared to in-person visits, (3) mitigated COVID-19 risk and avoided needs for masking and social distancing, (4) avoided behavioral challenges during appointments, and (5) allowed participation from home with minimal disruption of routine. Caregivers described significant physical challenges that made leaving the home for appointments difficult including balance issues, incontinence, and difficulties getting into vehicle. Caregivers plan to continue using tele-dementia services beyond the pandemic due to the convenience. Taken together, these findings indicate that caregivers find tele-dementia care convenient, comfortable, helpful, and timesaving and highly satisfactory. A combination of both in-person and virtual visits would be an ideal future state. This study illustrates how caregivers experience virtual visits for dementia care and will shape future intervention design.

### IMPROVING CAREGIVER ASSESSMENT AND COMMUNICATION ABOUT PAIN IN RELATIVES WITH DEMENTIA

Catherine Riffin[1], Lilla Brody[2], Priya Mukhi[3], Cary Reid[4], Keela Herr[5], and Karl Pillemer[3], *1. Weill Cornell Medical College, New York, New York, United States, 2. Weill Cornell Medicine, New York, New York, United States, 3. Cornell University, Ithaca, New York, United States, 4. Weill Cornell Medicine, New York City, New York, United States, 5. University of Iowa, Iowa City, Iowa, United States*

Pain is under-detected and poorly managed in persons with dementia (PWD). Family caregivers are well situated to detect and facilitate management of pain in PWD, but they receive little guidance and training in these tasks. Our group developed the Pain Identification and Communication Toolkit (PICT), a manualized, multicomponent intervention to help caregivers recognize pain in their care recipients and communicate their observations to healthcare providers. PICT includes a) training in administering an observational pain assessment tool, b) coaching in effective pain communication, and c) building caregivers' skills through practice. To evaluate PICT's acceptability, feasibility, and preliminary efficacy, we conducted a pilot randomized controlled trial of N=34 caregivers (n=18 randomized to PICT; n=16 randomized to a control condition). Participants were from diverse racial and ethnic backgrounds (14% Black; 15% Hispanic; 8% Asian; 8% multiracial). Of the caregivers enrolled in the intervention group, 66.7% reported that PICT improved their confidence in identifying pain symptoms, and 83.3% reported that PICT improved their confidence in communicating pain-related concerns to providers. Retention was excellent: 100% of caregivers in the PICT group completed all intervention sessions (4 total); only 5% prematurely terminated the study (did not complete the 12-week post-assessment questionnaire). Notably, caregivers in the PICT group showed a significant improvement in confidence communicating with healthcare providers from baseline to 12-week follow-up (M=3.9 vs. M=4.4; p<.01). Collectively, these findings suggest PICT's potential as an intervention to help caregivers recognize and communicate about pain in PWD and that a full-scale efficacy trial with larger sample is warranted.

### INFORMAL CAREGIVING NETWORKS FOR PERSONS WITH DEMENTIA SUPERIMPOSED ON COMPLEX MULTIMORBIDITY

Mi-Kyung Song[1], Sudeshna Paul[1], and Mary Beth Happ[2], *1. Emory University, Atlanta, Georgia, United States, 2. The Ohio State University, Columbus, Ohio, United States*

Informal caregiving research has focused on the primary caregiver and caregiver-patient dyad. Thus, we know little about caregiving beyond the dyadic relationship. This study was to gain a comprehensive understanding of informal caregiving networks for individuals with dementia superimposed on complex multimorbidity. We used egocentric social network analysis to obtain caregiving information of 46 patients with moderate to severe cognitive impairment, 5 chronic conditions on average, and undergoing hemodialysis (4.3 mean years). Most patients (n=35, 77.8%) were Black, 22 (47.8%) male, and mean age of 73.9 years. Starting with the primary family caregiver (FCG), up to 2 additional FCGs were recruited for each patient, totaling 76 FCGs (46 primary, 30 non-primary). Most were a child of the patient (n=39, 51.3%), female (n=57, 75%), and 54.2 years of age. Of the 46 networks, 16 (35%) included only one FCG (singletons). Multimember networks (n=30, 65%) provided longer caregiving than singletons (7.7 vs 3.8 years, p=0.008). Average network size was 2.8, and 26 (54.5%) networks had at least one male caregiver. Among the 30 multimember networks, average size was 3.8, density (proportion of possible ties) was 0.9, and mean degree and maximum degree (number of ties per member to other network members) were 2.5 and 2.8, respectively. Higher mean and maximum degrees were associated with fewer 12-month patient hospitalizations (r=-0.47, p=0.01; r=-0.43, p=0.02, respectively). Including additional caregiver informants significantly increased network size, ties and maximum degree centrality compared to those based on primary caregiver only, allowing for fuller network description.

### RESOURCES AND EXPERIENCES AMONG DIVERSE DEMENTIA CAREGIVERS BY GEOGRAPHIC CONTEXT

Aya Yoshikawa[1], Erin Bouldin[2], Mónika López-Anuarbe[3], Tiffany Kindratt[4], Dominique Sylvers[5], and Noah Webster[5], *1. Texas Woman's University, Denton, Texas, United States, 2. University of Utah, Salt Lake City, Utah, United States, 3. Connecticut College, New London, Connecticut, United States, 4. The University of Texas at Arlington, Arlington, Texas, United States, 5. University of Michigan, Ann Arbor, Michigan, United States*

Rural caregivers are often underserved by caregiving services, yet little is known about how the intersectionality of geographic context and race/ethnicity relates to caregiving resources among dementia caregivers. We examined whether 1) caregiving resources and experiences differ across metro and non-metro areas; and 2) the use of caregiving resources is associated with geographic context by race/ethnicity, controlling for age, gender, and education. We analyzed a sample of caregivers of care recipients aged 65 years or older with 'probable' dementia (n= 808) in the 2017 National Health and Aging Trends Study (NHATS) and the associated National Study of Caregiving (NSOC). We defined geographic context by the recipient's residence in metro (urban) or non-metro (rural) counties and grouped formal (respite care, support groups, caregiving training) and informal (family or friend help) resources. Among minority caregivers, 47% of those living in metro and 36% in non-metro areas used a formal service, and 83% and 72%, respectively, used informal resources. Among White caregivers, estimates were 44%, 48%, 76%, and 66%, respectively. Multivariate regression analyses revealed that non-metro White dementia caregivers had 2.04 times higher odds (95% CI=1.10-3.78) of using formal resources than metro White dementia caregivers. This pattern was not observed among minority dementia caregivers. The use of informal resources did not differ across geographic contexts by race/ethnicity. Findings suggest the influence of geographic context on the use of formal caregiving resources varies by race/ethnicity. With higher rates of dementia in non-metro areas, formal caregiving resources among non-metro minority dementia caregivers need more attention.

### THE ROLE OF SUPPORT IN THE LIVED EXPERIENCE OF DEMENTIA CAREGIVERS: A SYSTEMATIC REVIEW AND THEMATIC SYNTHESIS

Paria Seyyedmirza, Clark Benson, Diane Ploch Emily, Anna Linden, and Andrea Gilmore-Bykovskyi, *University of Wisconsin-Madison, Madison, Wisconsin, United States*

People living with dementia have complex care needs, which are primarily met by unpaid family caregivers. Family caregivers are often underprepared and under-supported in these roles and often experience negative health impacts associated with their caregiving responsibilities. Research suggests caregiving experiences and associated outcomes can be improved through the use of supportive resources which vary widely in design, access, and implementation. Yet how dementia caregivers perceive, identify, and experience supports in the context of their lived experience is less well understood. Understanding caregivers' un-proscribed conceptualizations of "support" holds important implications for the optimal design of supportive interventions, which are often under-utilized. The objective of this qualitative evidence synthesis was to systematically identify, appraise, and synthesize evidence regarding dementia caregivers' conceptualization of support through qualitative studies focused broadly on eliciting caregivers' reports of lived experience. Forty-one qualitative studies were analyzed and synthesized according to methods suggested by Sandelowski (2007) and Graneheim & Lundman (2004). Six themes were identified and synthesized across included studies which include a range of domains from accessibility, awareness, usability, and match of informal and formal support for caregivers' needs and the needs of their care recipient. Caregivers conceptualized support broadly, extending beyond traditional resources to address aspects of their caregiving role. Findings demonstrate that caregivers readily distinguish between formal and informal support, but do not necessarily evaluate them uniformly and are perhaps focused on the fit of support that extends beyond the caregiving role and is more aligned with how caregivers view support in their daily lives.

### THEORETICAL IMPLICATIONS AND IMPACT OF SELF-COMPASSION IN CAREGIVERS OF PEOPLE LIVING WITH DEMENTIA

Claire Grant[1], and Katherine Judge[2], *1. Cleveland State University, Cleveland Heights, Ohio, United States, 2. Cleveland State University, Cleveland, Ohio, United States*

Caregivers of people living with dementia often report greater symptoms of depression, anxiety, and burden. Guided by the Stress Process Model, the study examined the impact of self-compassion on these key psychosocial well-being outcomes in a sample of dementia caregivers (N=99). Participants were 66.7% female, 67.7% White, and 74% were children/in law or grandchildren/in law, with a mean age of 38.61. Findings indicated self-compassion was a significant and unique predictor of symptoms of depression ($\beta$ = -.25, p = .03), anxiety ($\beta$ = -.36, p = .01), and burden ($\beta$ = -.25, p = .02). Additional analyses found support for dysfunctional coping meditating the relationship between self-compassion and caregiver burden ((B = -.20, SE = .06, 95%CI[-.3242, -.0924] dysfunctional coping mediating the relationship between self-compassion and anxiety (B = -.16, SE = .05, 95%CI[-.2257, -.0302] and emotion-focused coping meditating the relationship between self-compassion and depression (B = -.06, SE = .03, 95%CI[-.1388, -.0045]. Results demonstrate the important role of self-compassion along with the impact of coping style on psychosocial well-being outcomes. Discussion will highlight the theoretical implications of these findings along with how these results can be used to develop efficacious interventions for caregivers of persons with dementia. Specifically, tailoring an intervention for dementia caregivers grounded in self-compassion theory as well as Compassion Focused Therapy and Mindful Self-Compassion Therapy may assist caregivers in developing a self-compassionate mindset and skills to more effectively cope with caregiving challenges.

### UNDERSTANDING THE IMPACT OF CARE LITERACY ON PREVENTATIVE CARE: EVIDENCE FROM FAMILY CARERS IN JAPAN

Hiroko Costantini, *University of Tokyo, Tokyo, Tokyo, Japan*

To address the importance of family carers' understanding of care, encompassing their own care as well as the broader care and health social systems, this paper leverages the notion of 'care literacy'. The aim of this study is to understand the variation in care literacy and the impact of care literacy on preventative care. The empirical focus is on working family carers for older relatives in Japan, through a cross-sectional online survey that includes a novel operationalization of care literacy, established measure of health literacy, assessment of information used to understand care, and measures of preventative care. The participants' (n = 292) mean age was 53, with 44% women, and an average of 8.3 hours per week caring for their parent(s). The measure of care literacy is shown to be correlated, as expected, but distinct to health literacy (correlation 0.60). Based on regression analysis of care literacy, significant explanatory variables are health literacy (p< 0.001), gender (p=0.044), number of sources of information on care (p=0.029), and care hours (p< 0.001). In contrast, proximity in living arrangements of carer and care receiver, and severity of care needs were not significant predictors. Turning to the impact of care literacy, care literacy is a significant explanatory variable for use of preventative measures (p=0.002), in particular related to nutrition (p< 0.001), frailty (p=0.028), dementia (p=0.090) and general home renovations (p=0.018). The pattern of results from this cross-sectional analysis indicates the importance of understanding the potential for improved care literacy as an enabler of better care.

### DEMENTIA FAMILY CAREGIVERS EXPERIENCES USING COMMUNITY-BASED AND HEALTHCARE SERVICES DURING COVID-19

Amanda Leggett[1], Elaina Baker[2], Hannah Lee[2], Anna Webber[2], Sarah Wallace[2], Tyson Fang[2], and Florence Johnson[2], *1. Wayne State University, Ypsilanti, Michigan, United States, 2. University of Michigan, Ann Arbor, Michigan, United States*

Family caregivers are key medical decision makers for persons living with dementia (PLwD) and play a critical role in interfacing with community-based services and the health care system. The COVID-19 pandemic, however, had dramatic impact on service utilization, with many suspended, delayed, or moved to telehealth. Emerging data suggests the pandemic posed challenges to service use for family caregivers, yet how this impacted caregiver's care practice is unknown. An in-depth qualitative interview was conducted with 100 primary family caregivers for PLwD in 2021 on care management during the COVID-19 pandemic. Watkins' rigorous and accelerated data reduction technique was used to analyze qualitative data through open and focused coding and identify themes specific to family caregiver's access to and use of community-based and health care services. Themes emerged regarding challenges and benefits of service use during the pandemic. Caregivers struggled with not being allowed to attend medical appointments with the PLwD or to speak confidentially with the physician over a PLwD's telehealth appointment. Caregivers felt physicians couldn't always read the care situation over telehealth, but appreciated not having to leave the house. Losing the consistency of in-home caregivers or respite programs put additional strain on caregivers, but many found virtual programming engaging. Grocery delivery/pickup was a new service used by many caregivers and found to be supportive of in-home care. As the pandemic evolves, finding ways to support caregivers through continued involvement in health care visits and as they access community services is critical to caregiver and PLwD health and well-being.

### MENTAL HEALTH AND WELL-BEING OF OLDER CARERS DURING THE COVID-19 PANDEMIC: EVIDENCE FROM ENGLAND

Giorgio Di Gessa[1], and Debora Price[2], *1. UCL, London, England, United Kingdom, 2. The University of Manchester, Manchester, England, United Kingdom*

Older people caring at home or in the community play a vital role in supporting population health and wellbeing and in protecting health and care systems, often at cost to their own health. Yet there has been very little research or policy attention given to this group of carers during the pandemic. Exploiting longitudinal data from Wave 9 (2018/19) and the first two COVID-19 sub-studies (June/July 2020; November/December 2020) of the English Longitudinal Study of Ageing, we use logistic and linear regression models to investigate associations between changes in provision of informal care and mental health during the pandemic, controlling for socio-demographic characteristics, pre-pandemic physical and mental health, and social isolation measures.

During the first months of the pandemic, about a quarter of older people provided informal care (with ~10% caring for members living in the same household). Those caring in the household experience worse mental health during the pandemic. Even controlling for prior characteristics and lack of social interactions, those caring for family members in the household had higher odds of reporting elevated depressive symptoms (OR=1.67, 95%CI=1.07;2.62), poor self-rated health (OR=1.73, 95%CI=1.09;2.73), anxiety (OR=2.21, 95%CI=1.20;4.06) as well as lower quality of life (B=-0.85, 95%CI=-1.66;-0.05) and life satisfaction (B=-0.43; 95%CI=-0.78;-0.09) than those who were caring for friends and family outside the household. As we aim to build back society and restore the wellbeing of our populations, policies and services should be better directed to support those people who during the pandemic struggled to cope while caring for their family members.

### LEFT OUT TO DRY: UNMET NEEDS AND RISK OF DEPRESSION AMONG OLDER ADULTS

Haowei Wang[1], Ashton Verdery[2], Rachel Margolis[3], and Sarah Patterson[4], *1. Tufts University, Quincy, Massachusetts, United States, 2. The Pennsylvania State University, State College, Pennsylvania, United States, 3. Western University, London, Ontario, Canada, 4. University of Michigan, Ann Arbor, Michigan, United States*

Pre-pandemic research has shown adverse consequences of having unmet care needs for older adults' mental health. Due to the broad psychological distress and increased caregiving challenges during COVID-19, older adults' vulnerabilities to unmet needs may be amplified by the pandemic, especially for those with functional limitations and intense care needs. This study aims to examine (1) the associations between unmet needs and depression among older adults before and during the COVID-19 and (2) whether the excess mental health consequences from unmet needs and COVID-19 vary by older adults' dementia status. We pool data from the 2018, 2019, and 2020 rounds of National Health and Aging Trends Study, a nationally representative sample of U.S. Medicare beneficiaries. We analyze N=6,273 older adults aged 70 years and older who had limitations with self-care, household activities, or mobility. Results show that older adults with functional limitations experienced increased risk of depression over time. Before and during the pandemic, older adults with unmet needs and older adults with probable dementia had higher risks of depression compared to their counterparts, respectively. The risk of depression was highest among older adults who had probable dementia and could not have their care needs met. For older adults without dementia, their risks of depression increased significantly from pre-pandemic to COVID-19 if they had unmet care needs. Findings demonstrate the disproportionate impacts of COVID-19 on mental health among older adults. Older adults who have cognitive impairments and unmet needs are in particular need of mental health support.

### THE LONGITUDINAL EFFECT OF FINANCIAL DIFFICULTY ON CAREGIVER DEPRESSION

Susanna Mage[1], Kathleen Wilber[2], and Donna Benton[2], *1. University of Southern California, Leonard Davis School of Gerontology, Los Angeles, California, United States, 2. University of Southern California, Los Angeles, California, United States*

Informal caregivers are the backbone of the long-term healthcare system in the United States and crucial in supporting the rapidly increasing aging population. Previous studies have illustrated the negative effects that the caregiving role can have on caregiver mental health, but few studies have examined the singular role of experiencing financial difficulty on caregiver mental health. The aim of this study was twofold: to measure how financial difficulty in 2015 correlates with caregiver depression in 2015, and to examine whether financial difficulty in 2015 can predict caregiver depression in 2017. We used two-wave panel data from the National Study on Caregiving (N=1,125) to conduct both regression and lagged dependent variable (LDV) regression analyses to investigate the cross-sectional and longitudinal effect of financial difficulty on caregiver depression, respectively. Caregiver depression was measured using the 2-item version of the Patient Health Questionnaire-9 (PHQ-2), and financial difficulty was self-reported. Results show that caregivers who did not experience financial difficulty in 2015 had 0.46 times the odds of not being depressed (CI 0.30-0.70, p-value= 0.00) in 2015, compared to those who noted that they did experience financial difficulty. In performing a LDV analysis, outcomes showed that financial difficulty in 2015 was significantly associated with caregiver depression in 2017 (p-value< 0.05), implying that current reported financial difficulty may influence caregiver depression two years later. Conclusions from this work provide support for the development of financial interventions for caregivers experiencing financial difficulty that could play a role in alleviating their depressive symptoms.

### WORSENING OF DEPRESSIVE SYMPTOMATOLOGY SINCE BEFORE COVID-19 AMONG CAREGIVERS OF PERSONS LIVING WITH DEMENTIA

Matthew Smith[1], Chung Lin Kew[1], Gang Han[1], Lucas Wilson[1], Shinduk Lee[2], John Fitch[1], and Marcia Ory[1], *1. Texas A&M University, College Station, Texas, United States, 2. University of Utah, Salt Lake City, Utah, United States*

This study examines factors associated with self-reported worsening of depressive symptomatology since before the COVID-19 pandemic among caregivers of persons living with dementia (PLWD). Cross-sectional baseline survey data were analyzed from 76 paid and unpaid caregivers of PLWD participating in a larger NIH-funded study assessing the feasibility of using a novel in-situ sensor system. Using the PHQ-2, caregivers were asked to report their depressive symptomatology twice: (1) retrospectively before the COVID-19 pandemic started; and (2) since the COVID-19 pandemic. A change score was calculated, and whether or not caregivers' depressive symptomatology worsened since before the pandemic served as the dependent variable. A logistic regression model was fitted, controlling for contextual items related to the caregiver, care recipient, and caregiving logistics. On average, caregivers were age 58.09(±16.92) and the majority was female (84.2%). Thirty percent of caregivers reported worse depressive symptomatology since the COVID-19 pandemic than before the COVID-19 pandemic started. Caregivers who were female (OR=25.86, P=0.028), not paid for caregiving (OR=14.59, P=0.047), had lower annual household incomes (OR=0.50, P=0.032), and whose care recipients had higher Mini-Mental State Exam scores (OR=1.78, P=0.005) were more likely to report worse depressive symptomatology since the COVID-19 pandemic. Results suggest that the COVID-19 pandemic intensified depressive symptomatology for many caregivers, and unpaid, female caregivers with fewer financial resources have been especially impacted. As the pandemic continues, support for caregivers of PLWD should be tailored to specifically meet their needs and circumstances.

### SPOUSAL ASSOCIATIONS BETWEEN GRANDPARENT CAREGIVING AND WELL-BEING: FINDINGS FROM THE HEALTH AND RETIREMENT STUDY

Athena Chung Yin Chan[1], and Rodlescia Sneed[2], *1. University of Minnesota--Twin Cities, Minneapolis, Minnesota, United States, 2. Wayne State University, Detroit, Michigan, United States*

Numerous studies document the impact of grandparent caregiving on the health and well-being of grandparents; however, there has been little dyadic research on how the caregiving and health-related outcomes of one grandparent influence partner couples The purpose of this study was to determine the interdependence of grandparents' intensity of caregiving and well-being (i.e., depressive symptoms and self-rated health) over time. Participants were 7,133 dyads of American grandparents aged ≥ 50 who participated in the Health and Retirement Study, a population-based study of community-dwelling adults, in 2010 and 2012. Data on hours of grandparent caregiving in the past two years, depressive symptoms, and self-rated health were obtained via self-report. Two longitudinal, dyadic path analyses were conducted using the Actor-Partner Interdependence Model. Within individuals (actor effects), greater depressive symptoms and better self-rated health at baseline, predicted greater depressive symptoms and better self-rated health two years later. Between spouses (partner effects), an individuals' greater depressive symptoms predicted the spouses' greater depressive symptoms. However, grandfathers' better self-rated health predicted subsequent better grandmothers' self-rated health, but not vice versa. Further, greater depressive symptoms among grandmothers predicted lower subsequent caregiving intensity among both grandmothers and grandfathers. Additionally, better self-rated health among grandfathers predicted better self-rated health and lower subsequent self and spousal grandparenting caregiving intensity. Our findings demonstrate that depressive symptoms, self-rated health, and caregiving intensity are interrelated among grandparent couples. Interventions for improving well-being and caregiving outcomes that focus on couples may be more effective than those that focus on individuals.

### CONVERSATIONS ON THE COSTS OF CAREGIVING: COMPARING CAREGIVERS' AND FINANCIAL ADVISORS' PERCEPTIONS

Alexa Balmuth, Julie Miller, Lauren Cerino, Adam Felts, Lisa D'Ambrosio, and Joseph Coughlin, *Massachusetts Institute of Technology, Cambridge, Massachusetts, United States*

Costs of caregiving go beyond physical, mental, and emotional; the financial costs are astronomical. On average, family caregivers spend 26% of their income on caregiving (AARP 2021). As the need for family caregivers grows due to longer lifespans (AARP 2020), incorporating the topic of caregiving into financial planning for longevity conversations becomes increasingly necessary. Drawing on data from the MIT AgeLab Caregiver Panel, a research panel of over 1400 family caregivers, and from the MIT AgeLab Preparing for Longevity Advisory Network, a research panel of over 900 financial professionals, this presentation will describe mixed methods research findings that highlight caregivers' and financial professionals' attitudes and perceptions toward caregiving as a topic within client-advisor conversations. Results demonstrate that many caregivers wish they had financially planned for the costs of caregiving more than they did. However, caregivers rarely turn to their financial advisors for support; many perceive their value to be strictly financial and are unaware of their advisors' ability to support them. Despite caregivers' doubts, most financial professionals feel equipped and willing to have caregiving-related conversations with clients. Additionally, while over three-quarters of financial professionals reported making referrals to outside resources for caregiving-related support, some critical avenues – including social workers and therapists, support groups, and respite care providers – were underutilized. Implications of these findings for caregivers and professionals of various industries will also be discussed.

### DEVELOPING A RESOURCE FOR LEVERAGING POPULATION DATA SETS TO ADVANCE CAREGIVING SCIENCE

Rebecca Goodwin[1], Katherine Ornstein[2], Catherine Elmore[1], Rebecca Utz[1], Djin Tay[1], Lee Ellington[3], Ken Smith[1], and Caroline Stephens[1], *1. University of Utah, Salt Lake City, Utah, United States, 2. Johns Hopkins, University, Baltimore, Maryland, United States, 3. University of Utah and Huntsman Cancer Institute, Salt Lake City, Utah, United States*

Older adults often rely on other people to help care for them as they age. Caregiving has predominantly been studied through a primary caregiver lens with few studies focusing on the wider caregiving network. However, it is challenging to collect new data about family caregivers, particularly in contexts of serious illness and end of life. Selection and attrition effects, underrepresentation of marginalized or minoritized racial and ethnic groups, and low response rates can bias findings in prospective clinical research. Analyzing existing datasets, particularly population-based data (e.g., registries with linked administrative, health, and vital records) may hold promise to advance our understanding of the role families play throughout the caregiving and bereavement continuum to optimize the health and well-being of older adults. Little is known, however, about existing population-based datasets available for research on family caregiving. We conducted a scoping review of published literature and data repositories to identify datasets relevant for family caregiving research, explore the research reuse of these datasets, and describe potential validity and reliability concerns. Synthesized findings reveal: 1) methodological approaches for identifying the presence and type of caregivers and their level of engagement; 2) inclusion and measurement of key outcome variables; and 3) sampling and study design issues. We describe and compare a selection of high-value existing datasets relevant to family caregiving research. This new research information resource will advance research use of population datasets to improve care for older adults and their family caregivers across the lifespan.

### THE ROLE OF THIRD PLACES IN REDUCING LONELINESS AMONG CAREGIVING SPOUSES

Yeon Jin Choi, and Jennifer Ailshire, *University of Southern California, Los Angeles, California, United States*

Loneliness is a public health concern that is associated with poor mental and physical health. Caregiving spouses of community-dwelling older adults often have high levels of caregiving burden, which make them more vulnerable to social isolation and loneliness. There has been a growing interest in third places as mediums for social interaction. Research on third places shows a positive association of greater access to third places with social networks and social health. However, this has not been tested in the context of caregiving. In this paper, we examined the role of various types of third places in reducing loneliness among caregiving spouses. We used the 2006-2016 Health and Retirement Study and the National Neighborhood Data Archive to examine the relationship between the availability of third places per square mile and loneliness. Third places include food outlets (e.g. grocery stores), eating and drinking places (e.g., restaurants, coffee shops), commercial establishments (e.g., department stores), entertainment organizations (e.g., museum), exercise facilities (e.g., fitness), religious organizations (e.g., churches), civic and social organizations (e.g., social clubs), personal care services (e.g., barbershops, beauty salons), and social services for older adults (e.g., senior centers). We found that caregiving spouses living in neighborhoods with greater availability of third places had lower levels of loneliness. We also found gender differences in the association. For instance, greater availability of eating and drinking places was associated with loneliness only among females. Increasing access to places that provide opportunities for social interactions may prevent social isolation and reduce loneliness among caregiving spouses.

### CONTEXTUAL FACTORS RELATED TO CAREGIVER IDENTITY DISCREPANCY IN EMERGING ADULT CAREGIVERS

Anastasia Canell, and Grace I. L. Caskie, *Lehigh University, Bethlehem, Pennsylvania, United States*

Approximately 12-18% of unpaid family caregivers to older adults in the U.S. are 18-25 years old, yet minimal research focuses on this subgroup of caregivers (Levine, 2005). Because caregiver identity theory postulates that the extent to which informal caregivers integrate their caregiving roles and duties into their identity may relate to caregiver distress (Montgomery & Kosloski, 2012), contextual factors related to higher reported caregiver identity discrepancy should be assessed. In a sample of 135 emerging adult informal caregivers, the current study compared caregiver identity discrepancy across two factors: level of caregiving responsibility and caregiver gender. Caregiving responsibility had four groups: (1) primary with minimal help (n=54), primary but receiving some help (n=51), secondary (n=18), or shared (n=12). Caregiver identity discrepancy was measured using the five subscales of the Family Caregiver Identity Scale (Eifert et al., 2019). Caregiver identity discrepancy differed significantly by caregiving responsibility (p<.001) and gender (p=.046). Emerging adult caregivers identifying as women reported statistically more caregiver identity discrepancy in family obligation (p=.003) and master identity (p=.036) domains than men. Caregiving responsibility showed statistically significant differences in role engulfment (p<.001), loss of shared identity (p<.001), family obligation (p=.013), and master identity (p=.010) domains of identity discrepancy. Emerging adult caregivers identifying as the primary caregiver with minimal help from others reported significantly more identity discrepancy than all other groups across subscales. These results demonstrate that young caregivers who have little support in task responsibilities and identify as a woman may be at particular risk for experiencing caregiver identity discrepancy.

### SESSION 6320 (POSTER)

### FRAILTY (HEALTH SCIENCES POSTERS)

### A PHENOMENOLOGICAL STUDY ON THE PERCEPTION OF FRAIL ELDERLY IN SUBJECTIVE HEALTH AWARENESS AND SUCCESSFUL AGING

Jisu Seo[1], Rhayun Song[2], Moonkyoung Park[2], and Ahyun Ryu[2], *1. Nursing, Daejeon, Taejon-jikhalsi, Republic of Korea, 2. Chungnam National University, Daejeon, Taejon-jikhalsi, Republic of Korea*

BackgroundFrailty is a multi-dimensional concept including physical, functional, and psychological aspects. The frail elderly have been assessed by various instruments, yet the classification of being frail is not often consistent with the perception of individuals. PurposeThis study aimed to explore the perception of frail older adults in subjective health awareness and successful aging. Methods9 participants who met the following inclusion criteria: older than 70 years, defined as frail status screened by frailty index, and signed the consent form. Data was collected through in-depth interview with recording, and evaluated by phenomenological approach of Colazzi. Results The content analysis on the perception of 9 participants on subjective health awareness yielded 7 theme-clusters and 3 categories: (a)Recognize frailty, (b) Vague and endless emotions, (c)Reflecting on the past and thinking about death. The participants described ‘recognizing frailty through senility’ as a subjective health awareness. The perception of participants on successful aging was structured 6 theme-clusters and 4 categories: (a)Sustainable health, (b)The goal of successful goodbye, (c)Responsibility for parenting, (d) The best wisdom that aging gives life. The participants described ‘living in the present and successfully parting with it’ as a successful aging. ConclusionThe participants who were categorized as frail status did not perceive themselves as frail. Many participants perceive being frail as natural process of aging, and their successful aging as sustainable health and successful goodbye. It is pertinent to understand the meaning of successful aging and subjective health awareness of frail older adults to provide effective intervention to help them.

### SCREENING OF US VETERANS AT HIGH RISK FOR OUTCOMES FOLLOWING COVID-19 BY FRAILTY STATUS

Catherine Park[1], Aanand Naik[1], Molly Horstman[1], Benjamin Seligman[2], Ariela Orkaby[3], Orna Intrator[4], Kristine Lynch[5], and Javad Razjouyan[1], *1. Veterans Affairs, Houston, Texas, United States, 2. David Geffen School of Medicine at UCLA, Los Angeles, California, United States, 3. VA Boston, Boston, Massachusetts, United States, 4. Canandaigua VA Medical Center, Canandaigua, New York, United States, 5. University of Utah, Salt Lake City, Utah, United States*

Frailty increases risk of adverse outcomes in the presence of stressors such as COVID-19 infection. We examined the association between different levels of frailty and outcomes following COVID-19 infection. This is a retrospective cohort study of US Veterans aged ≥50 years, active Veterans Health Administration (VHA) care users, and tested positive for COVID-19 between 02/15/2020 and 09/30/2021. VHA frailty index (VA-FI) was calculated from one year prior to the COVID-19 testing and divided into three groups: robust (≤0.1), prefrail (0.1-0.2), and frail (>0.2). The risk of hospitalization, ICU admission, ventilator use, and in-hospital mortality were calculated using logistic regression adjusted by age, sex, body-mass-index, and race. The performance of VA-FI in predicting outcomes was compared to age or Charlson comorbidity index (CCI) using area under the curve (AUC). Of 204,426 COVID-19 positive Veterans, 32,965 were hospitalized (age: 71.4±10.4 years, BMI: 29.5±7.1 kg/m2). We observed higher odds of hospitalization (frail, adjusted odds ratio (aOR)=8.64; prefrail, aOR=2.57), ICU admission (frail, aOR=1.58; prefrail, aOR=1.32), ventilator use (frail, aOR=1.97; prefrail, aOR=1.57), and mortality (frail aOR=2.15; prefrail, aOR=1.55) in frail and prefrail compared to robust Veterans. We observed that VA-FI had higher AUC in predicting hospitalization (AUC 0.75) independent of age (0.59) and CCI (0.63). Veterans with COVID-19 who were frail and prefrail had a higher risk of hospitalization, ICU admission, ventilator use, mortality compared to robust. VA-FI may be a useful tool at the time of COVID-19 diagnosis to triage patients at risk for adverse outcomes.

### ASSOCIATION OF LOWER URINARY TRACT SYMPTOMS WITH INCIDENT FRAILTY AND MORTALITY AMONG OLDER COMMUNITY-DWELLING MEN

Peggy Cawthon[1], Scott Bauer[2], Charles E. McCulloch[3], Kristine Ensrud[4], Kaiwei Lu[5], Rebecca Scherzer[5], Kenneth Covinsky[3], and Lynn M. Marshall[6], *1. California Pacific Medical Center Research Institute, San Francisco, California, United States, 2. UCSF and San Francisco VA, San Francisco, California, United States, 3. UCSF, San Francisco, California, United States, 4. University of Minnesota Medical School and Minneapolis VA Health Care System, Minneapolis, Minnesota, United States, 5. San Francisco VA, San Francisco, California, United States, 6. OHSU-PSU School of Public Health, Portland, Oregon, United States*

Lower urinary tract symptoms (LUTS) are associated with increased risk of new mobility and functional limitations among older men. Our objective was to evaluate the longitudinal relationship between baseline LUTS severity and incident frailty or all-cause mortality among 3667 community-dwelling non-frail men age >70 years from the Osteoporotic Fractures in Men (MrOS) study. LUTS severity at analytic baseline was defined using the American Urologic Association Symptom Index (AUASI). Phenotypic frailty was defined at baseline and 2-year follow-up visits using modified Fried criteria and classified as robust (0), intermediate stage (1-2), or frail (3-5); men classified as frail at analytic baseline were excluded. Vital status was assessed every 4 months. Since the proportional odds assumption was not met, we used multivariable multinomial logistic regression to estimate odds ratios (OR) for the association between baseline LUTS severity and incident frailty or death at follow-up compared to robust. OR were adjusted for demographics, health-behaviors, comorbidities, and cognition. After a mean follow-up of 2.3 years, 37% of men were categorized as robust, 46% were intermediate stage, 9.2% developed incident frailty, and 7.9% had died. Per 4 point higher AUASI, the adjusted odds incident frailty versus robust was 1.23 (95% CI 1.12, 1.34). Odds of death versus robust was not statistically significant (OR=1.05, 95% CI 0.94, 1.18). In conclusion, non-frail men with greater LUTS severity at baseline have slightly greater odds of incident frailty within 2 years. Clinicians should be aware that LUTS severity is a prognostic marker for developing frailty in older men.

### ALIGN-CARE: IMPLEMENTATION AND FEASIBILITY OF NEW GERIATRIC SURGERY CO-MANAGEMENT MODEL FOR PHYSICAL AND SOCIAL FRAILTY

Fred Ko[1], Stephanie Chow[1], Abigail Baim-Lance[1], Amy Park[1], Gabrielle Schiller[2], Minji Kim[1], Kavya Sreevalsan[1], and William Hung[1], *1. Icahn School of Medicine at Mount Sinai, New York, New York, United States, 2. CUNY School of Public Health, Astoria, New York, United States*

Pre-operative geriatric evaluation in frail older patients that addresses both medical and psychosocial needs is often not completed prior to surgery, leaving frail older adults ill prepared for surgery. The ALIGN-CARE interprofessional co-management model uniquely serves to standardize care and coordination amongst the surgeon, geriatrician and social worker in ambulatory settings to allow a greater window of time to intervene for surgical optimization. Program elements include assessment of preoperative medications, functional status, cognition, nutrition, What Matters Most, advance care planning (ACP), and social determinants of health. Recommendations and action items from the geriatric-social work team are presented to surgeon and primary care physicians for follow-up and optimization. Program and process elements recorded in a REDCap database facilitate longitudinal evaluation of ALIGN-CARE model implementation feasibility. Initial data of 9 frail surgical oncology patients indicate increases in meaningful ACP discussions, patient-oriented decisions regarding surgery candidacy, and surgeon referrals for inpatient geriatric consultation post-operatively as a result of ALIGN-CARE. Current analyses to assess feasibility and quality of the program include thematic coding of program documents, semi-structured interviews of key stakeholders (surgeons, geriatricians, social workers, patients), and quantitative analysis of program outcomes, including uptake of program elements and process measures. Planned areas of analytic focus include workflow, cross-specialty coordination, and patient and primary care engagement and activation. We anticipate identifying specific barriers and facilitators of ALIGN-CARE implementation in our evaluation to aid the dissemination and upscaling of interprofessional co-management programs aimed to improve outcomes and quality of life for frail, older surgical patients.

### CLINICAL TRIAL OF METFORMIN FOR FRAILTY PREVENTION IN COMMUNITY-DWELLING OLDER ADULTS WITH PRE-DIABETES

Sara Espinoza, Chen-pin Wang, Joel Michalek, Daniel MacCarthy, Tiffany Cortes, Becky Powers, and Nicolas Musi, *University of Texas Health Science Center at San Antonio, San Antonio, Texas, United States*

Background: Metformin may reduce frailty through improving insulin resistance and inflammation, aging mechanisms known to increase frailty risk. We describe a randomized clinical trial of metformin for frailty prevention in community-dwelling older adults with pre-diabetes and provide baseline characteristics of randomized participants.

Methods: Older adults (65+ years) are studied in this randomized, double-blind, placebo-controlled trial of metformin (max 2,000 mg/day). Pre-diabetes, required for inclusion, is assessed by 2-hour oral glucose tolerance test (OGTT). Individuals with glomerular filtration rate < 45 mL/min and frail individuals (Fried criteria) are excluded. The primary outcome, frailty, is assessed by both Fried criteria and frailty index. Secondary outcomes are physical function (short physical performance battery), lower extremity strength (Biodex), 6-minute walk, inflammation (systemic and skeletal muscle tissue), muscle insulin signaling, insulin sensitivity (insulin clamp), glucose tolerance (OGTT), and body composition (dual-energy x-ray absorptiometry). Subjects are followed for 2 years with safety assessments every 3 months and frailty assessment and OGTT every 6 months.

Results: 145 participants (49% female, 35% Hispanic) are randomized. Mean age is 71.8 ± 5.4 years (range: 65-88), body mass index is 30.8 ±6 kg/m2, and Hemoglobin A1c is 5.6 ±0.4%. Using Fried criteria, 63.4% have frailty score of 0, 30.3% a score of 1, and 6.2% a score of 2.

Conclusion: Metformin is being examined as a potential therapeutic agent to prevent frailty in older adults with pre-diabetes. Findings from this trial may have future implications for screening and treatment of pre-diabetes in older adults for the prevention of frailty.

### DEPRESSIVE SYMPTOMS MEDIATE THE ASSOCIATION OF FRAILTY PHENOTYPE SYMPTOMS AND COGNITION FOR FEMALES BUT NOT MALES

Nicholas Resciniti[1], Anwar Merchant[2], and Matthew Lohman[2], *1. University of Southern California, Los Angeles, California, United States, 2. University of South Carolina, Columbia, South Carolina, United States*

We aimed to evaluate whether depressive symptoms mediated the relationship between frailty phenotype and cognitive function by sex. Frailty phenotype symptoms, cognitive function scores, and depressive symptoms were measured in 3,705 community-dwelling US adults from the Health and Retirement Study (HRS) from 20012-2016. Fried's frailty phenotype criteria (weakness, slowness, physical inactivity, low weight, and exhaustion) were used as a continuous score for frailty symptoms (0-5). Global cognition was measured by the Telephone Interview for Cognitive Status (range: 0-35). The Centers for Epidemiologic Studies-Depression Scale (CES-D) was used as a continuous measure to assess depressive symptoms (0-8). We used mediation analysis to estimate the direct and indirect effects of frailty symptoms on cognitive scores to evaluate the role of depressive symptoms as a potential mediator. Males had a larger total effect (β= -0.43; 95% CI: -0.66, -0.02; p< 0.001) for lower cognitive score for each increase in frailty symptom compared to females (β= -0.28; 95% CI: -0.47, -0.08; p=0.02), suggesting all five frailty symptoms was associated with 2.15 lower cognitive scores for males and 1.50 for females. A significant indirect effect from frailty phenotype to cognition was found through depressive symptoms for females (β= -0.03; 95% CI: -0.06, -0.00; p=0.02) but not males (β= -0.04; 95% CI: -0.08, 0.00; p=0.07). These results highlight the importance of identifying individuals with frailty and depressive symptoms to monitor and provide interventions to preserve cognitive function

### FRAILTY AND OSTEOARTHRITIS IN OLDER CHILEANS

Cecilia Albala[1], Carlos marquez[2], Barbara Angel[3], Rodrigo Saguez[3], and Lydia lera[3], *1. INTA, Universidad de Chile, Santiago de Chile, Region Metropolitana, Chile, 2. INTA, Universidad de chile, Macul, Region Metropolitana, Chile, 3. INTA, Universidad de chile, Santiago de Chile, Region Metropolitana, Chile*

The adverse consequences on health of frailty, make it early diagnostic very important. The coexistence of frailty with osteoarthritis, a frequent condition especially in older women, has been less studied Objective. to study the association between frailty and self-reported osteoarthritis

Methods: Cross-sectional study in994 people65 years and older (72.1%women, mean age72y±6.7) from the Alexandros cohort, designed to study disability associated with obesity in community-dwelling people60y and older living in Santiago/Chile. The frailty phenotype was defined as having ≥3 from the5 following criteria: weak handgrip dynamometry, unintentional weight loss, fatigue/exhaustion, five chair-stands/slow walking speed and low physical activity. Self-reported osteoarthritis was registered.We found a prevalence of osteoarthritis was much higher in women than in men (49.4% vs22.9%, p<0.01) Osteoarthritis was present in46.5% of frail people and30% of the robust ones (p=0.01). Frailty was present in53.3% of women with OA in and11.8% of men with OA. The crude OR for the association of frailty with Osteoarthritis was significant only for women (OR=1.43;95% CI:1.06-1.93). After sex and age adjusted logistic regression for frailty in Ostroarthritis, the OR for frailty was OR=1,39;95%IC (1,04-1,85), p=0,025, but in women with osteoarthritis the adjusted OR for frailty increase to OR=11.98 (5.637-13.23)Considering the severe consequences of frailty over health, the high burden of Osteoarthritis, its high frequency in women, and the strength of the association between both conditions, the screening for frailty is highly recommended In older women with Osteoarthritis

### INTERVENTIONS TO PREVENT THE FRAILTY IN OLDER WOMEN WITH FRAILTY: A SYSTEMATIC REVIEW AND META-ANALYSIS

Ha Na Jeong, Sun Ju Chang, Joo Ri Kim, and Gi Won Choi, *Seoul National University, Seoul, Seoul-t'ukpyolsi, Republic of Korea*

Background: Frailty is a health challenge related to adverse health outcomes in older adults. Older women are more likely to be frail than older men. However, few studies have reviewed the effectiveness of interventions for older women with frailty is scant. Objective: This systematic review aimed to explore the properties of interventions and to investigate their effectiveness in preventing the progression of frailty in older women with pre-frailty or frailty.

Methods: Narrative synthesis was conducted to identify the contents, outcome variables, and findings of the interventions. Then, a meta-analysis was performed to evaluate the effectiveness of exercise interventions on grip strength, sit-and-reach, sit-to-stand, and timed up and go tests.

Results: Twenty-six studies were selected, including 14 randomized controlled trials and 12 quasi-experimental studies. These studies implemented exercise (96.2%), nutrition (15.4%), hormone replacement (7.7%), toileting strategies (3.8%), and laughter interventions (3.8%). The selected studies assessed physical, psychological (11.5%), and cognitive health (11.5%), as well as quality of life (19.2%). The meta-analysis found significant effects of aerobic and resistance exercise interventions on the sit-to-stand (SMD = 1.30, 95% CI [0.70, 1.90], $p < 0.001$) and timed up and go scores (SMD = -0.56, 95% CI [-0.93, -0.19], $p = 0.003$).

Conclusion: Exercise interventions are essential to improve physical health, in particular mobility, in older women with pre-frailty or frailty. Future studies should consider theoretical frameworks and evaluate psychological and cognitive health as well as quality of life to develop and provide effective interventions to prevent the progression of frailty in older women.

### BLOOD-BASED BIOMARKER CHANGES IN A PHASE 2B TRIAL ASSESSING LOMECEL-B IN OLDER ADULTS WITH FRAILTY

Kevin Ramdas[1], Danial Mehranfard[1], Eric Naioti[2], Geoff Green[1], Lisa McClain-Moss[1], Dan Gincel[1], Joshua Hare[1], and Anthony Oliva[1], *1. Longeveron Inc., Miami, Florida, United States, 2. Longeveron, Inc., Miami, Florida, United States*

Aging frailty (AF) is a multidimensional geriatric syndrome that is characterized by physical and cognitive symptoms, increasing the vulnerability of affected older adults to adverse health outcomes. Mechanistically, a low-grade chronic inflammatory state (inflammaging), endothelial dysfunction, and decreased regenerative capacity are thought to be major contributors to AF pathophysiology. Lomecel-B is an allogenic medicinal signaling cell(MSC) formulation that can potentially ameliorate AF through pleiotropic mechanisms, including anti-inflammatory, pro-vascular, and pro-regenerative activities. We completed a Phase 2b randomized, double-blinded, placebo-controlled trial designed to assess Lomecel-B benefits for AF via change versus placebo in the six-minute walk test(6MWT), to assess the dose-response relationship, and to evaluate bioactivity via changes in blood-based biomarkers. Enrolled subjects were aged 70–85 years with mild-to-moderate AF, a reduced 6MWT of 200-400m, and Tumor Necrosis Factor-α of ≥ 2.5pg/mL indicative of inflammaging. In total, 143 subjects received a single intravenous infusion of Lomecel-B at doses of 2.5 x 107 cells (25M, N=35), 5.0 x 107 cells (50M, N=30), 1.0 x 108 cells (100M, N=33), or 2.0 x 108 cells (200M, N=16), or placebo (N=29). Safety and efficacy assessments were performed at 1, 3, 6, and 9 months post-infusion. Increases in 6MWT and decreases in serum levels of the blood-based biomarker Soluble-Tie-2 were observed at 9 months in the Lomecel-B groups versus placebo. Notably, both observations were seen in a dose dependent fashion with 200M showing the highest effect. Based on the findings, a next-phase trial is being developed to advance this clinical program and will be presented.

### POLYPHARMACY, FRAILTY, AND DISABILITY-FREE SURVIVAL IN COMMUNITY-DWELLING HEALTHY OLDER INDIVIDUALS

A R M Saifuddin Ekram[1], Robyn Woods[2], Joanne Ryan[2], Sara Espinoza[3], Julia Gilmartin-Thomas[4], Raj Shah[5], Mark Nelson[6], and Michael Ernst[7], *1. Monash University, Nunawading, Victoria, Australia, 2. Monash University, Melbourne, Victoria, Australia, 3. Sam and Ann Barshop Institute, UT Health San Antonio, San Antonio, Texas, United States, 4. Victoria University, Melbourne, Victoria, Australia, 5. Rush University, Chicago, Illinois, United States, 6. Menzies Research Institute, University of Tasmania, Hobart, Tasmania, Australia, 7. The University of Iowa, Iowa City, Iowa, United States*

BACKGROUND: Polypharmacy and frailty are two common geriatric syndromes. We examined the association between polypharmacy and frailty and if, together, they predicted disability-free survival (DFS), defined as time to the first event of dementia, persistent physical disability or death.

METHODS: We included 19,114 participants from the "ASPirin in Reducing Events in the Elderly" (ASPREE) clinical trial. Polypharmacy was defined as regular, concomitant use of five or more prescription medications. Frailty was assessed using a modified Fried phenotype and a deficit accumulation frailty index (FI) of 66 items. The association between polypharmacy and frailty was assessed by multinomial logistic regression. In addition, Cox regression was used to determine the association between polypharmacy-exposed frailty and DFS.

RESULTS: Individuals with polypharmacy (vs. < 5 medications) were 55% more likely to be pre-frail (Relative Risk Ratio or RRR: 1.55; 95%Confidence Interval or CI:1.44, 1.68) and three times more likely to be frail (RRR: 3.34; 95%CI: 2.64, 4.22) according to Fried phenotype. Frail individuals had a two-fold reduction in their survival free of dementia/disability (Hazard ratio or HR: 2.16; 95%CI: 1.56, 2.99), whereas frail individuals with polypharmacy had a four-fold reduction (HR: 4.24; 95%CI: 3.28, 5.47). Effect sizes were more prominent when frailty was assessed using the FI than when assessed by Fried phenotype.

CONCLUSION:

Polypharmacy was significantly associated with pre-frailty/frailty. Polypharmacy-exposed pre-frailty/frailty increased the risk of death, dementia or physical disability among older adults. Addressing polypharmacy in older people could ameliorate the impact of frailty on individuals' functional status, cognition and survival.

### DEVELOPMENT OF A TRANSITIONAL CARE PROGRAM FOR FRAIL OLDER ADULTS BETWEEN HOSPITAL AND HOME

Ji Yeon Lee, Eunhee Cho, Sue Kim, Gwang Suk Kim, Kyung Hee Lee, and Chang Oh Kim, *Yonsei University, Seoul, Seoul-t'ukpyolsi, Republic of Korea*

Frail older adults particularly need transitional care between hospital and home due to physical function decline and psychological instability after discharge. This study aimed to develop a transitional care for frail older adults in Korea who are discharged home following hospitalization. The Returning Home (Rehome©) program was established through the three phases according to the Medical Research Council's 2013 guidelines. 1) Identifying the evidence base phase included a systematic review of literature and needs assessments from interviews with frail older adults. The core intervention components (e.g., geriatric assessment, transitional care planning, home visits, phone follow-up, community service liaison, and family engagement) were determined. 2) At the phase of identifying theory, the transition theory was selected and modified to fit the target population in the context of the Korean healthcare system. 3) Phase three was for the modeling process and outcomes. Based on the result from phases 1 and 2, the Rehome program was developed considering clinically applicable strategies. The final Rehome program consisted of a comprehensive geriatric assessment at admission; structured discharge/transitional care planning (e.g., medication review, education for chronic disease management, emergencies, and geriatric syndromes, and community resource) at discharge; a home visit and six phone follow-up calls up to 12 weeks after discharge; and emotional support and engagement of the family during the entire period. The Rehome program showed good content validity. The Rehome as a frailty-focused transitional care program could improve the transition through implementing a tailored intervention that meets the care needs of these vulnerable populations.

### RISK OF FRAILTY ASSOCIATED WITH COGNITIVE DECLINE, SENSORY IMPAIRMENTS, AND FUNCTIONAL LIMITATION IN OLDER ADULTS

Jinhee Shin, Gwang Suk Kim, and Agani Afaya, *Yonsei University, Seoul, Seoul-t'ukpyolsi, Republic of Korea*

The purpose of this study was to examine the effects and risk factors of cognitive decline, sensory impairment, and functional limitation associated with frailty among older adults. We conducted secondary data analysis using data from the Korean Longitudinal Study of Aging (2006–2018). The participants of this study consisted of 1,789 older adults over the age of 65 in South Korea who had never experienced frailty at the beginning of the survey (2006). Participants were classified into two groups, frailty and non-frailty, based on frailty events over time. We used Kaplan–Meier analysis to determine the effects of frailty-related group differences on cognitive decline (K-MMSE), sensory impairment (visual and hearing), and functional limitation (K-ADL) from 2006 to 2018. To determine the risk factors associated with frailty, the Cox regression analysis was performed. This study established that over time, approximately 71.2% of the older adults (n = 1,274) developed frailty. The study also revealed that over time, both physical (sensory impairment and functional limitation) and psychological factors (cognitive decline) could lead to frailty. We also identified that demographic (age, female), physical (BMI, chronic disease, hearing, vision, and K-ADL), and psychological (K-MMSE) factors were associated with frailty. This study provides an opportunity for healthcare professionals and policymakers to implement intervention programs tailored to ensure regular monitoring prevention and among of these risk factors.

### TRAJECTORIES OF SKELETAL MUSCLE MASS AND FAT MASS AND THEIR IMPACTS ON MORTALITY IN OLDER JAPANESE ADULTS

Satoshi Seino[1], Yu Taniguchi[2], Miki Narita[1], Takumi Abe[1], Yu Nofuji[1], Yuri Yokoyama[1], Shoji Shinkai[3], and Yoshinori Fujiwara[4], *1. Tokyo Metropolitan Institute of Gerontology, Itabashi, Tokyo, Japan, 2. National Institute for Environmental Studies, Tsukuba, Ibaraki, Japan, 3. Kagawa Nutrition University, Sakado, Saitama, Japan, 4. Tokyo Metropolitan Institute of Gerontology, Tokyo, Tokyo, Japan*

Skeletal muscle mass and fat mass have differential impacts on mortality between men and women. We aimed to determine age-related trajectories of skeletal muscle mass index (SMI) and fat mass index (FMI) among men and women and to examined their impacts on mortality risks. This prospective study included 1,770 (863 men and 907 women) aged ≥65 years who participated in health checkups; the total number of observations was 6,110. SMI and FMI were determined using segmental multi-frequency bioelectrical impedance analysis, and their age-related trajectories from age 65–90 years were examined using group-based semiparametric mixture models. SMI and FMI age-related trajectories for all-cause mortality were determined by multivariate-adjusted hazard ratios (HRs) and 95% confidence intervals (CIs). SMI and FMI trajectories were classified into three trajectories in both sexes: the low- (28.6% and 34.0%), middle- (56.0% and 47.5%), and high-trajectory (15.4% and 18.5%) groups in men and the low- (27.6% and 40.9%), middle- (51.6% and 48.1%), and high-trajectory (20.8% and 11.0%) groups in women. The median follow-up was 5.3 years; 101 (11.7%) men and 56 (6.2%) women died. Compared with the low-trajectory groups, male multivariate-adjusted HRs for mortality in the middle- and high-trajectory groups were 0.89 (95% CI: 0.57–1.39) and 0.34 (0.13–0.93) for SMI, and 0.76 (0.47–1.23) and 1.13 (0.60–2.14) for FMI, respectively. Corresponding female multivariate-adjusted HRs were 1.00 (0.50–2.02) and 1.64 (0.62–4.36) for SMI, and 0.74 (0.38–1.43) and 0.37 (0.12–1.14) for FMI. Maintaining high skeletal muscle mass is important for prolonging life expectancy, especially in men.

### VULNERABILITY TO ANTICHOLINERGIC MEDICATION ASSOCIATED FRAILTY IN LOW-INCOME MINORITY OLDER ADULTS

Lana Sargent[1], Matthew Barrett[2], Huma Nawaz[2], Marissa Mackiewicz[2], Youssef Roman[2], Patricia Slattum[2], Sarah Hobgood[2], and Elvin Price[2], *1. VCU School of Nursing, Ashland, Virginia, United States, 2. Virginia Commonwealth University, Richmond, Virginia, United States*

Low-income minority older adults are highly susceptible to drug adverse effects of medications due to aging, comorbidities, and polypharmacy. Several studies have demonstrated anticholinergic medication is associated with frailty, supporting the hypothesis for mechanistic peripheral nervous system effects. The goal of this cohort study is to determine peripheral nervous system effects of anticholinergic medication exposure with frailty by conducting a sensitivity analysis using multiple anticholinergic tools. Spearman correlation matrix and intraclass correlation coefficients (ICC) are used to determine the function of five clinical Anticholinergic Burden Scales (ACBS): Anticholinergic Burden Scale (ACB), Anticholinergic Drug Scale (ADS), total standardized daily doses (TSDD), and Cumulative Anticholinergic Burden scale (CAB). Ordinal logistic regression and area under the curve (AUC) are used to evaluate anticholinergic burden-associated frailty models. The cohort included 80 individuals (mean age = 69 years; 55.7% female, 71% African American). Among individuals prescribed anticholinergics, 33% are robust, 44% pre-frail, and 23% frail. All scales are highly correlated with each other ($p < 0.001$), ICC3 = 0.66 ($p < 0.001$, CI 95% 0.53-0.73). All five of the scales predicted pre-frail and frail status ($p < 0.05$) with low misclassification rates for frail individuals (AUC = 0.70 – 0.80). Considering ACBS are highly correlated and all predict frailty; all of the scales can be used in future frailty research; however, the CAB and TSDD consider both potency and dose. Additional research is necessary to understand the peripheral nervous system effects of anticholinergic drug exposure and if deprescribing can improve frailty status.

### EXPLAINABLE MACHINE-LEARNING FOR PREDICTING PREOPERATIVE FRAILTY PHENOTYPE USING ELECTRONIC HEALTH RECORDS

Mamoun Mardini, Catherine Price, Patrick Tighe, and Todd Manini, *University of Florida, Gainesville, Florida, United States*

Pre-operative frailty among patients is strongly associated with poor post-operative outcomes. Operationalizing frailty in clinical practice is challenging due to the lack of resources and pragmatic complexities. Feasible tools are needed to cover the scarcity in this area. Harnessing electronic health records (EHR) to screen pre-operative frailty and its related post-operative outcomes would be important for decision making and planning care management. This study aimed to validate an EHR-based machine learning model for pre-operative frailty ascertainment. Measures of the frailty phenotype (slowness, weight loss, exhaustion, low physical activity, and grip strength) were collected on approximately 14,000 patients (aged 65-100 years) by nurses housed in the UF Health pre-surgical center. Patients with at least 3 out of 5 syndromic components were considered frail. We utilized an explainable machine learning algorithm, eXtreme Gradient Boosting (XGBoost), to build our models to predict pre-operative frailty phenotype. We extracted the important predictors that contributed to predicting the outcome and evaluated their relationship with the outcome. The machine learning model achieved an area under the curve (AUC) of 0.71 in recognizing pre-operative frailty across all surgical specialties. The top five predictors for frailty phenotype were hemoglobin level, sex, education level, history of COPD, and diabetes. Using explainable machine learning approaches on EHR data provides a moderate mapping of the frailty phenotype in pre-operative settings. Funding: UF Claude D. Pepper Older Americans Independence Center P30AG028740 and R01 AG055337.

### SESSION 6330 (POSTER)

### GLOBAL AGING AND HEALTH

### RISK FACTORS OF DISABILITY AMONG COMMUNITY-DWELLING OLDER ADULTS WITH HYPERTENSION IN THAILAND: THE HART STUDY

Utoomporn Wongsin, and Tuo-Yu Chen, *Taipei Medical University, Taipei City, Taipei, Taiwan (Republic of China)*

About 60% of the older adults in Thailand experience hypertension, a condition that increases the risks of disability. However, little to no study has investigated modifiable risk factors to prevent disability in this segment of population in Thailand. Therefore, this study aims to investigate factors associated with disability among community-dwelling older adults aged 60 years or above with hypertension in Thailand. Longitudinal data (wave-1 [2015] and wave-2 [2017]) were from the Health, Aging, and Retirement in Thailand (HART) survey. Descriptive analysis and logistic regression analysis were employed to analyze the data. Outcome variables were difficulty with activity of daily living, hearing-related disability, vision-related disability, and pain-related disability in wave-2. Sociodemographic information, health behaviors/health status, mental illness, chronic diseases, and disability at baseline were independent variables. The results showed that older age significantly predicted difficulty with activity of daily living and hearing-related disability among community-dwelling older adults with hypertension in Thailand. Drinking alcohol significantly predicted vision-related disability. No factors were found to be a significant factor for pain-related disability. Our analysis provided useful information for the prevention of disability among older adults with hypertension in Thailand.

### GLOBAL DIFFERENCES IN ADAPTATION TO SOCIETAL AGING: A CROSS-SECTIONAL COMPARISON OF 143 COUNTRIES

Cynthia Chen[1], Kenwin Maung[2], Jemima Koh[3], and John W. Rowe[4], *1. National University of Singapore, Singapore, Singapore, 2. University of Rochester, Rochester, New York, United States, 3. National University of Singapore, Singapore, Singapore, 4. Columbia University, New York City, New York, United States*

Societal ageing is our success story of advancing public health and medicine in reducing the risk of premature death, the control and prevention of diseases, and improvement in social and economic development. Almost all countries are experiencing growth in size and proportion of older persons. In 2019, 703 million older persons were aged 65 and above, projected to double to 1.5 billion by 2050. We construct a multidimensional ageing Index that extends the Ageing Society Index, to account for global differences in societal ageing from 2015 to 2019 for 18 OECD countries. The Index is a weighted sum of scores for five domains that are important for societal ageing: well-being, productivity and engagement, equity, cohesion, and security. We compare the overall index and domain scores between countries. High-income countries dominated the top rankings. The overall Ageing Society Index ranged between 35.0 for Gabon in Africa to 74.6 for Switzerland in Europe. Top-performing countries were high-income countries from the European and Oceania regions, while the worst-performing countries were countries from the African region. Domain scores ranged from 19 (India) to 91.6 (Singapore) for well-being, 12.8 (Mozambique) to 88.5 (Finland) for equity, 32.8 (Gabon) to 88.8 (Uzbekistan) for cohesion, and 27.8 (Madagascar) to 88.8 (Switzerland) for security. Our multidimensional index helps identify specific societal gaps for policymakers to address. Furthermore, the cross-country comparison can be instructive for policymakers to adapt the experiences of successful countries to domestic policies.

### THE IMPACT OF EARLY CONDITIONS ON THE PREVALENCE OF DIABETES ACROSS THREE COHORTS OF OLDER ADULTS IN MEXICO

Rafael Samper-Ternent[1], Jesús-Daniel Zazueta-Borboa[2], Neil Mehta[3], cesar González[4], and Rebeca Wong[1], *1. University of Texas Medical Branch, Galveston, Texas, United States, 2. Netherlands Interdisciplinary Demographic Institute, 's-Gravenhage, Zuid-Holland, Netherlands, 3. UTMB, Galveston, Texas, United States, 4. University of COlima, Colima, Colima, Mexico*

In low-and-middle-income countries like Mexico the impact of disadvantaged early childhood conditions (ECC) on later-life health in not well known and may be changing across birth cohorts. Our objective is to examine the impact of ECC on the propensity to have diabetes in three different cohorts of older adults aged 60-69 (born in 1930-1939, 1940-1949, 1950-1959). We examine to what extent differences in ECC, sociodemographic characteristics, and lifetime obesity explain the differences in the prevalence of diabetes across the cohorts. We use five waves of data from the Mexican Health and Aging Study (n=10,313). We estimate a series of logistic regression models and use counterfactual analyses to identify the roles of ECC and lifetime obesity on cohort-specific diabetes prevalence and on differences in diabetes prevalence across cohorts. Our results suggest that improvements in ECC would decrease prevalence of diabetes by 12.5% in the 1930-1939 cohort and 10% in the 1950-1959 cohort. However, the rise of obesity at age 50 counters this effect and leads to higher prevalence of diabetes in both cohorts. Our models show that if everyone had a normal weight at age 50, the prevalence of diabetes would decrease by 31.3% for the 1930-1939 cohort, 27.3% for the 1940-1949, and 26.7% for the 1950-1959 cohort. Despite improvements in socio-economic conditions during childhood in Mexico, the increase in lifetime obesity appear to offset the benefits derived from better ECC resulting in higher rates of diabetes.

### EVERYTHING HAS CHANGED FOR THE WORSE: EXPERIENCES OF ELDERS DISPLACED BY ARMED CONFLICT IN ETHIOPIA

Getachew Gebeyaw[1], Shambel Dessale[1], Eyayu Kasseye[1], and Margaret Adamek[2], *1. University of Gondar, Gondar, Oromiya, Ethiopia, 2. Indiana University, Indianapolis, Indiana, United States*

In November 2020 an outbreak of ethnically and politically motivated armed conflict started in Tigray, Ethiopia and expanded to the Amhara and Afar regions, bringing a devastating impact upon civilians and disadvantaged groups. Persons living in those areas, including elders, were forced to flee and seek refuge at internal displacement centers. The purpose of this study was to investigate the challenges faced by older individuals fleeing the war zone and settling in internal displacement centers during Ethiopia's armed conflict. A qualitative descriptive case study was used in this cross-sectional investigation. Purposive sampling was used to identify 13 displaced older adults. Data from the in-depth interviews with elders were supplemented with key informant interviews and observations. Narrative data was analyzed using thematic analysis. Findings revealed that the older adults faced a variety of challenges in the war zone while escaping their homes and in the displacement center, all of which negatively impacted their physical and psychosocial well-being. Elders fled on foot and walked for three days without food to reach the internal displacement center. In addition to food shortage, other themes included loss of significant others, family disintegration, and lack of care and support. The findings call attention to the need for practical access to social and economic integration of elders in the aftermath of war as well as ongoing psychosocial intervention. In Ethiopia and in other war-affected areas, displaced older people need tailored support.

### ETHIOPIAN CENTENARIANS: HEALTH CONDITIONS AND SENSORY/COGNITIVE FUNCTIONALITY

Samson Chane[1], and Margaret Adamek[2], *1. Bahir Dar University, Bahir Dar, Oromiya, Ethiopia, 2. Indiana University, Indianapolis, Indiana, United States*

Globally, the centenarian population is expected to increase in the coming three decades reaching 3 million by 2050. In Ethiopia, like other Sub-Saharan African countries, information about the health condition, sensory and cognitive functionality of centenarians is scanty. This study examined the health conditions and sensory/cognitive functionality of Ethiopian centenarians. A qualitative case study design was employed. Nine centenarians (1 woman, 8 men) between age 100 and 108 were identified using snowball sampling. Data were generated through in-depth interviews and analyzed using descriptive analysis. All 9 were Orthodox Christians and lived in rural areas either with their wives (3) or other relatives. Four centenarians could read and write, two attended grade eight and nine, and three were non-literate. Before retirement the centenarians engaged in different occupations as farmers (4), shoemaker, government employee, school teacher, priest, “bounty hunter” and mechanic. Most perceived that their sensory and cognitive functionality was normal until their later 90s except for minor complaints. Centenarians faced walking difficulty (5), joint tightness (4), back pain (4), fatigue, dental issues, stuttering, hypertension (3), insomnia, diabetes, anorexia, severe headache, constipation and anxiety. Two centenarians identified themselves as escapers. Centenarians faced hearing defect (4), sight problems, and a decrease in taste and smell. Two reported proper functioning of their sensory organs. Despite various health problems, the Ethiopian centenarians aspired to live longer resiliently. Findings call for further study and the need for social workers, caregivers, and health care practitioners to consider the health conditions, sensory and cognitive functionality of centenarians.

### LIFE ON THE STREETS IS HORRIBLE: OLDER RURAL-URBAN MIGRANTS COPE WITH HOMELESSNESS IN ETHIOPIA

Getachew Gebeyaw[1], Messay Kotecho[2], and Margaret Adamek[3], *1. University of Gondar, Gondar, Oromiya, Ethiopia, 2. Addis Ababa University, Addis Ababa, Adis Abeba, Ethiopia, 3. Indiana University, Indianapolis, Indiana, United States*

The prevalence of homelessness among older adults in Ethiopia is growing. While prior studies examined the push factors and challenges of homeless elders, little is known about how older people in Sub Saharan Africa cope with homelessness. This study explored the coping strategies of homeless older people in Kobo Town, Ethiopia. Purposive sampling was used to identify 10 homeless older people and four key informants. Study participants were homeless for a year or longer. Thematic analysis was used to analyze the data collected through in-depth interviews. To cope with the challenges faced on the street, homeless older people used various strategies including begging, holy water, drying leftover food, using river water for hygiene and sanitation, sleeping in church compounds, and creating their own social networks. Despite their efforts, the coping strategies used by elders were not sufficient. In the absence of family and government support, study participants relied heavily on begging to meet their survival needs. The findings call attention to the need for a national income support program and other supportive services for older adults. Homelessness is the product of a failing support system. Despite Ethiopia having a Plan of Action for Older Persons and Social Protection Policy for Vulnerable Groups, these policies have not been effectively implemented leaving older adults with no safety net. This study calls for the development of new policies to empower older people in Ethiopia and prevent them from turning to begging as their only recourse.

### SESSION 6340 (POSTER)

### GRIEF AND THE LIFE SPACE

### MEASURING A GOOD DEATH: AN ASSESSMENT ACROSS FOUR COUNTRIES

Sarah Clem, *University of Maryland, Baltimore, Baltimore, Maryland, United States*

A good death is often viewed as a common goal in end-of-life care, yet our understanding of what qualifies as a good death is still under development. Previous studies have attempted to conceptualize our understanding of a good death and have highlighted the need to improve our measurement of this construct. The present study examines a measure of a good death across four countries. Data for this study were derived from the Four-Country Survey on Aging and End-of-Life Medical Care (2017), in which participants were sampled using a random digit dial method. The study relies on a sample of 4,239 participants from the US, Italy, Japan, and Brazil. Confirmatory Factor Analysis (CFA) was employed to fit a measurement model to the full sample for a measure of the importance of a good death. Once model fit was deemed sufficient, a multi-group CFA was used to assess invariance across the four countries. Model fit was adequate at the configural and metric levels, highlighting that across the four countries importance of a good death was measured and understood the same. However, the model failed at the scalar level of invariance, implying that indicators for the importance of a good death are valued differently across the four countries. Further developing our understanding of a good death and how it is measured is essential for improving quality end-of-life care. This study identifies a quality measure of good death while simultaneously highlighting the limitations in using such a measure in a transnational context.

### THINGS LEFT UNDONE: RELATION OF GRIEF TO SENSE OF PURPOSE IN BEREAVED OLDER ADULTS

Shubam Sharma[1], Emily Mroz[2], and Susan Bluck[3], *1. Kennesaw State University, Kennesaw, Georgia, United States, 2. Yale University, New Haven, Connecticut, United States, 3. University of Florida, Gainesville, Florida, United States*

Sense of purpose is grounded in life experiences (Ko et al., 2019) and can be threatened by the loss of a loved one. The death of a spouse in late life can be particularly disruptive when the bereaved struggle with perceptions of unfinished business with the deceased. This mentality may also derail their sense of life purpose. This study examined the association between persistent grief symptomatology (Prigerson et al.,1995) and sense of purpose (Ryff, 1989) in conjugally-bereaved older adults ($N = 53$; Mage = 81.59). Unfinished business constructs (i.e., unfulfilled wishes, unresolved conflict; Holland et al., 2018) were tested as mediating grief-purpose relations. Older adults who reported greater grief symptomology also experienced lower sense of purpose ($r = -.27$, $p < 0.05$). Extent of unfulfilled wishes fully mediated the relation between grief symptomology and sense of purpose ($t = -2.26$, $p < 0.05$); unresolved conflict did not act as a mediator. Findings indicate that the association between experiencing pervasive grief symptomology and a disrupted sense of purpose may be due, in part, to ongoing rumination about unfulfilled wishes: missed opportunities to meaningfully engage with one's spouse before death. Results are poignant given recent COVID-19 physical distancing requirements that separated older couples during spouses' dying days, potentially prompting unfulfilled wishes across bereavement. Findings highlight the need for palliative care practices that facilitate optimization of relationships at end of life and grief support practices that re-ignite purpose after difficult spousal loss.

### SOCIAL SUPPORT AND STRAIN AS PREDICTORS OF MULTIMORBIDITY FOLLOWING A MARITAL TRANSITION

Emily Denning[1], Jason Newsom[1], Anda Botoseneanu[2], Heather Allore[3], Corey Nagel[4], David Dorr[5], and Ana Quiñones[6], *1. Portland State University, Portland, Oregon, United States, 2. University of Michigan Dearborn, Dearborn, Michigan, United States, 3. Yale University, Guilfrod, Connecticut, United States, 4. University of Arkansas for Medical Sciences, Little Rock, Arkansas, United States, 5. Oregon Health & Science University, Portland, Oregon, United States, 6. Oregon Health and Science University, Portland, Oregon, United States*

Marital transitions (MTs; widowhood or divorce) are stressful events that impact the health of older adults. This study examined the impact of social support and social strain on multimorbidity trajectories using data from the Health and Retirement Study (HRS). Participants were 377 adults age 50+ with a single MT between years 2006 and 2016. We used piecewise growth curve modeling to investigate whether social support and strain from one's spouse, children, family, or friends, measured prior to transition, predicted trajectories of chronic conditions (count of 8 conditions: hypertension, diabetes, cancer, lung disease, heart disease, stroke, arthritis, and cognitive impairment) following MT. Covariates included sex, age, education, race/ethnicity, and wealth. On average, chronic conditions were increasing before MT, B = .172, SE = .021, $p < .001$, and after MT, B = .211, SE = .031, $p < .001$. Participants had an average of 2.2 chronic conditions at MT. Spousal support prior to MT was associated with fewer chronic conditions at MT, B = -.863, SE = .427, p = .043, whereas support and social strain from friends were each associated with more chronic conditions at MT (support: B = .772, SE = .354, p = .025; strain: B = 1.288, SE = .387, p = .001). Support from children was positively associated with more chronic conditions following MT, B = .212, SE = .084, p = .011, which may reflect adult children providing support in response to parental health decline.

### CHANGES IN LIFE-SPACE WITH LOSS OF RELATIVES AND FRIENDS AMONG OLDER ADULTS: RESULTS FROM THE UAB STUDY OF AGING

Amelia Driggers[1], Richard Kennedy[2], and David Buys[1], *1. Mississippi State University, Mississippi State, Mississippi, United States, 2. University of Alabama at Birmingham, Birmingham, Alabama, United States*

Increasing age brings greater risk of the death of friends and family (hereafter referred to as loss). Loss may impact individuals' life-space mobility (LSM); however, no research has explored relationships between loss and LSM trajectories. Using the UAB Study of Aging, we examined differences in LSM trajectories of 1000 community-dwelling older Alabamians (65+years) with and without loss during the study period. We assessed LSM using the UAB Life-Space Assessment (LSA), a validated instrument measuring individuals' ability to move through zones ranging from their bedroom to out of town. We assessed loss every 6 months using a standard bereavement questionnaire capturing spousal, other relative, or friend loss. We used piecewise linear mixed effects models to compare LSA trajectories. Those who do not experience loss had a baseline mean LSA score of 49.5 and show a decline of 0.08 points per year ($p < 0.001$). Those that do experience loss had a baseline LSA score of 60 and decline by 1.0 point per year before the loss ($p < 0.001$), accelerating to 1.8 points per year after the loss ($p < 0.001$). Those with loss do not experience acute decline post loss but do have an acceleration of the pre-existing decline. More research is needed on this topic to better understand the impact of loss on LSM trajectories; but this finding suggests that more public health and clinical interventions may be needed for those who experience loss. Specifically, bereaved individuals may benefit from social, mental, or healthcare services for loss-related challenges.

### THE RELATION BETWEEN DEATH PREPARATION AND LIFE SATISFACTION AMONG KOREAN OLDER ADULTS

Suha You, and Giyeon Kim, *Chung-Ang University, Seoul, Seoul-t'ukpyolsi, Republic of Korea*

Objectives: There is a growing interest in death preparation as an important process to achieve ego integrity at the last stage of life. However, in South Korea, conversations about death remain taboo especially with older adults. Thus, this study aims to examine the association between preparation for one's death and life satisfaction among Korean older adults.

Methods: Data for this study were drawn from the 2020 National Survey of Older Koreans conducted by the Korean Ministry of Health and Welfare. The sample was limited to adults aged 65 and older (N=10,097). The survey measured preparation for death, life satisfaction, and various covariates (e.g., sociodemographic characteristics, physical conditions, psychological conditions). Hierarchical multiple regression analysis was used to examine whether death preparation influences life satisfaction among Korean older adults.

Results: Results from hierarchical multiple regression showed that after adjusting for covariates, those who reported more preparations for death mentally (B=.054, $p<.001$) and materially (B=.035, $p<.001$) were more likely to have greater satisfaction in life than their counterparts.

Conclusion: Findings suggest that it is necessary to generate proper information and recognition about death at the government level and activate discourses on death preparation in later life.

### SESSION 6350 (POSTER)

### HEALTH, MENTAL HEALTH, AND SOCIAL SERVICE INTERVENTIONS

### POETRY AND UNCOVERING THE MYSTERY OF SOCIAL PROBLEMS: AN ARTS-BASED INQUIRY TO AGING CARE

Heng Wu, and Peter Szto, *The University of Nebraska at Omaha, Omaha, Nebraska, United States*

This research focuses on a current social welfare problem: unemployment and the loss of employer-based health insurance among non-elderly adults aged 18 to 64. The literature covers this social problem in terms of health status, access to medical care, employed versus unemployed working-age adults, mitigating the risks of unemployment, and loss of health insurance via the Consolidated Omnibus Budget Reconciliation Act of 1985 (COBRA) and the Health Insurance Portability and Accountability Act of 1996 (HIPAA). As good as the findings are, they are methodologically limited by looking at only half the story. This research asks a different question: how might the humanities and arts-based perspective address the social problem? It aims at answering why the employment status is related to health and health insurance. Chinese poetry has a long rich tradition of expressing insights about the inner life since antiquity to the present. These reflections include practice wisdom and keen observations on aging. This poster presents case findings on the use of Chinese poetics to inform aging care in the West. Each case provides discussions on why social welfare system comes up short to solve the problem and how the social welfare system can be effectively changed. Researchers use survey data to compliment Chinese poetic insights about employment status and insurance status to illustrate the correlation between respondents' health status with versus without employment. Findings are hoped to interpret the role of family as a social unit where it consists in social welfare along with the speculative inquiry of poetry.

### INTERGENERATIONAL TRANSMISSION OF "SEVA": ELDERS MODEL SELFLESS SERVICE IN SOUTH ASIAN HOMES FOR CHILDREN

Connie Corley, Laura Sherwood, Dibendu Ghosh, and David Willis, *Fielding Graduate University, Santa Barbara, California, United States*

Life course theory is a framework for examining the role of elders in modeling altruistic behavior or "seva" (selfless service) in two homes for children: Unatti (Bhaktapur, Nepal) and Ramana's Garden (Rishikesh, India). Two American women, compelled to impact the plight of children subject to poverty, trafficking and/or caste discrimination (Dalits, the former "untouchables"), each founded homes 20+ years ago and modeled selfless service exemplified by children, some now in "emerging adulthood," who are giving back to their communities. In this intergenerational/intercultural multiple case study, narratives of the program founders now in mid- to late adulthood are presented along with narratives of six young adults who continue to provide nurturance to younger children and engage in projects to educate and feed residents of the homes and nearby communities. The life course principle of time and place situates the children in areas of their countries with relatively low literacy rates. The founders saw the value of education to empower young people, especially girls, to expand their opportunities and serve as role models for children coming into the homes after them. The linked lives principle is evident through shared relationships with the founders as these young people navigate political crises, disasters like the Nepal earthquake, and health crises like the Covid-19 pandemic. Some become leaders themselves as they have been mentored over their life course. A sense of agency is experienced in their young adulthood as more choices for their lives become available as an alternative to subsistence existence and early marriage/childbearing.

### DOES DEPRESSION IMPROVE DURING RETIREMENT AMONG BINGE DRINKERS?

Antonia Diaz-Valdes[1], Christina Sellers[2], and Julian Ponce[3], *1. Universidad Mayor, Las Condes, Region Metropolitana, Chile, 2. Simmons University, Boston, Massachusetts, United States, 3. University of California, Berkeley, California, United States*

Data from the Health and Retirement Study (1992-2016) was used. All non-institutionalized respondents aged 50+ were included in our sample (n=12,618). Mixed models were conducted to study the association between transitioning to retirement and depressive symptoms (CESD) among retirees, and to test the mediation effect of alcohol use.

Results: Those retired for at least 6 years had increased probability of binge drinking. Additionally, binge drinking mediated the association between the retirement transition and depressive symptoms, making the effect stronger for those retired for 3 to 5 years ($p < 0.05$), and weaker and no significant for those retired for 6+ years ($p > 0.05$). Thus, the decrease on depressive symptoms was higher for those retired between 3 to 5 years when being binge drinkers. Discussion and Implications: Addressing depressive symptoms and binge drinking among older adults is sorely needed. Binge drinking was associated with decreased depressive symptoms, consistent with the self-medication hypothesis. Treatment and screening for depression as well as binge drinking are especially important as both are associated with increased mortality. Treating depression would reduce the risk of increased alcohol use and its detrimental effects on health.

### MERGING AN AGE FRIENDLY UNIVERSITY WITH AN AGE FRIENDLY HEALTH CARE SYSTEM: CASE STUDY FINDINGS

John Schumacher[1], Nicole Brandt[2], Barbara Resnick[3], Anissa Nahabedian[2], Mojdeh Heavner[4], Giora Netzer[3], Lara Wilson[5], and Raya E Kheirbek[6], *1. University of Maryland, Baltimore County, Baltimore, Maryland, United States, 2. University of Maryland, Baltimore, Baltimore, Maryland, United States, 3. University of Maryland, Baltimore, Maryland, United States, 4. University of Maryland School of Pharmacy, Baltimore, Maryland, United States, 5. University of Maryland Shore Regional Health, Chestertown, Maryland, United States, 6. University of Maryland School of Medicine, Baltimore, Maryland, United States*

Age Friendly Health Systems (AFHSs) and Age Friendly Universities (AFUs) are distinct entities in the "Age Friendly" ecosystem. While Age Friendly entities function independently, they typically exist in close proximity (e.g., universities and community hospitals); yet they remain isolated in their Age Friendly efforts. We report on a collaboration between a mid-Atlantic Age Friendly University and a new AFHS using case study methodology. Our goal is to inform and inspire key stakeholders responsible for creating innovative healthy aging communities. The collaboration began with a shared stakeholder team who articulated focus areas and overlapping goals. A charter document was developed articulating commitments and responsibilities. Using a Quality Improvement (QI) approach, projects targeted the hospital's older patient needs that linked to the AFHS 4 M's of Matters, Medications, Mobility, and Mentation. University graduate students and faculty volunteered to teach and mentor hospital staff on the QI projects: 1) Get to Know Me Boards filled by staff caring for hospitalized older adults (Matters); 2) Medical Intensive Care Unit discharge opioid medication deprescribing (Medication); 3) UMove Mobility Screening addressing functional status (Mobility); 4) UB-2 Delirium Screening (Mentation). Data collection across projects demonstrated proof-of-concept and identified implementation challenges around communication, screening, data entry, and data extraction from electronic medical records. During Covid-19 pandemic, the collaboration allowed QI projects to conduct multiple Plan-Do-Check-Act cycles while contributing to the Age Friendly goals of both organizations. Partnerships between academic institutions and hospitals foster development of evidence- based healthy aging communities and provide opportunities for continuing education and research.

### HELP-SEEKING FOR SUICIDE RISK AMONG MIDDLE- TO OLD-AGE ADULTS

Xiaochuan Wang, Susanny Beltran, and Kim Gryglewicz, *University of Central Florida, Orlando, Florida, United States*

Suicide is a major public health concern. Suicide rates have increased steadily in the U.S. and are highest among middle-aged (ages 45-64) and older (age 75+) adults. Help-seeking represents an important coping behavior that can mitigate suicide risk. However, research on this topic is sparse, especially among older adults. To address this gap, a systematic review of the existing literature describing help-seeking for suicide risk among middle- to old-age adults was conducted. Using PRISMA guidelines, we searched electronic databases (e.g., ProQuest, EBSCOhost, PsycINFO, PubMed, Medline) and key journals with suicide and/or gerontology focuses for peer-reviewed publications in English between 2010-2020. The search yielded 4,732 unduplicated publications. After screening articles for relevance based on titles and abstracts, 52 articles were reviewed in full text. A total of 24 articles met inclusion criteria and were included in this review. The articles reviewed included a range of topics, including prevalence of service utilization, service use prior to suicide-related behaviors, and correlates of help-seeking. Overall, prevalence of service utilization was generally low and varied by suicidal history (e.g., greater prevalence among those with a history of suicide attempt, as compared to those with suicide ideation but no attempts). The systematic review also identified key service use facilitators (e.g., higher suicide literacy, previous or current suicidality) and barriers (e.g., stigma). Results of this systematic review highlight the need for future research and tailored services to improve suicide prevention and intervention strategies for middle-aged and older adults.

### ACHIEVING UNIVERSAL HEALTH COVERAGE FOR OLDER ADULTS: A REVIEW OF GHANA'S NATIONAL HEALTH INSURANCE SCHEME

Samuel Asante[1], and Grace Karikari[2], *1. Northeastern State University, Broken Arrow, Oklahoma, United States, 2. University of North Dakota, Grand Forks, North Dakota, United States*

Universal Health Coverage (UHC) is defined by the World Health Organization (WHO) as ensuring that all people receive quality health services they need without financial hardship. Predicated on the idea of providing universal healthcare to all Ghanaians, Ghana's National Health Insurance Scheme (NHIS), established in 2003, has done considerably well among some age categories than others. While most studies on this healthcare program have focused on the health-seeking behaviors among users, its implementation, financial sustainability, and the role of national politics in its administration, little has been done to understand the program's effectiveness in achieving a universal health coverage for and its impact on older Ghanaians. Consistent with WHO's objective of achieving UHC, as enshrined in the Global Strategy on Aging and Health, this review examines Ghana's progress toward achieving UHC for all. The paper specifically provides a narrative review of the National Health Insurance Scheme in advancing the interest and welfare of older Ghanaians. Findings revealed that not all individuals considered as older adults, either conventionally or legally, are beneficiaries of the programs; that WHO's objective of obtaining needed care without financial hardship may be far from reach for most older Ghanaians; and the program only offers basic protection to current beneficiaries. We address these areas of concern to achieving UHC. We also make recommendations for a path forward where all stakeholders—older adults, families, healthcare providers, and policymakers—involved can play a role in ensuring all eligible older adults get the quality of health care they deserve.

### SUBTYPES OF FRAILTY AND NONFRAIL HEALTH IMPROVEMENT OF DISABILITY PREVENTION PROGRAM: LATENT CLASS ANALYSIS

Ya-Mei Chen[1], Wan-Yu Chiu[1], Hui-Fen Mao[1], Ling-Hui Chang[2], and Shwu-Chong Wu[3], *1. National Taiwan University, Taipei City, Taipei, Taiwan (Republic of China), 2. National Cheng Kung University, Tainan City, Tainan, Taiwan (Republic of China), 3. College of Public Health, Taipei City, Taipei, Taiwan (Republic of China)*

Frailty has become a public health priority in many advanced countries. However, the effects of national implemented intervention remained unclear. Taiwan implemented a disability prevention program nationwide targeting on people aged 65 and over in 2019. We aimed to identify the subtypes of function outcomes of the national disability prevention programs and compare the different outcome subtypes between frail and non-frail older adults. The current study analyzed participants' outcomes before and after the program. Two years of data (2019-2020) from the national disability program were used for analysis. Among the participants, 622 were frail (3.8%), and 13084 are non-frail (81.3%). Outcomes were nine domains of Kihon checklist assessed, including nutrition, depressive mood, oral function, fall risk, cognitive function, socialization, independent function, mobility and health knowledge. Latent Class Analysis is used to identify the subtypes of classes in frailty and non-frail older adults. Four classes of outcomes subtypes for frail and non-frail older adults were identified, and both of them have groups of multi-aspect improvement and no improvement groups. The other two classes for frail people were cognitive and independent function improvement group and social and fall risk improvement group, whereas non-frail group had independent function improvement group, and independent and mobility function improvement group. The similar groups identified indicated both frail and non-frail can benefited and not benefited from the program. The two different subtypes identified benefited from the program. Further study investigating the characteristic of people in different outcome subtypes may shed some light for future tailored-made disability prevention.

### PUBLIC MENTAL HEALTH SERVICE USE AMONG US ADULTS AGE 50+ COMPARED TO YOUNGER AGE GROUPS

Namkee Choi, and Diana DiNitto, *University of Texas at Austin, Austin, Texas, United States*

Despite increasing numbers of older-adult mental health service users, few studies have examined their use of public mental health services. Using the 2018 and 2019 Mental Health-Client Level data (N=4,291,737 in 2018 and N=4,513,946 in 2019 for those age 18+), we examined age group differences in the types of mental disorders diagnosed in outpatient-only, both outpatient and inpatient, and inpatient-only service settings. Of all users, 25.3% were age 50-64 and 6.7% were age 65+. Multivariable logistic regression results, controlling for gender, race/ethnicity, census region, and alcohol/substance use disorder, showed that compared to the 30-49 age group, the 50-64 and 65+ age groups had higher odds of having depressive disorder in outpatient-only settings (aOR=1.28 [95% CI=1.28-1.29] and aOR=1.08 [95% CI=1.08-1.09] for the 50-64 and 65+ age groups, respectively). Both older groups also had higher odds of delirium/dementia disorder in all three service settings. In addition, they had consistently higher odds of a diagnosis of schizophrenia or other psychotic disorder in all three service settings (aOR=1.88 [95% CI=1.86-1.89], aOR=1.70 [95% CI=1.65-1.74], and aOR=1.44 [95% CI=1.39-1.49] in outpatient, both outpatient and inpatient, and inpatient-only settings, respectively). Community mental health centers (CMHC) are on the frontlines in serving vulnerable communities and received increased federal funding during the COVID-19 pandemic. Our findings indicate that CMHCs also need programs dedicated to and tailored for older adults. More research is also needed on older adults who receive public mental health services and unmet mental health needs among low-income older adults with serious mental illness.

### PARTNERING TO DEVELOP COUNTY-LEVEL DATA PROFILES

Karon Phillips[1], and Megan Wolfe[2], *1. Trust for America's Health, Silver Spring, Maryland, United States, 2. Trust for America's Health, Washington, District of Columbia, United States*

Building robust data systems that include information on the health of older adults is crucial to developing programs and services that meet their health and social needs. Organizations and agencies can use this information to target resources and identify community partners, as well as using data to support grant applications. But there are challenges to building such systems, even within the public health system, due to lack of expertise, funding, and access to underlying data sources. Through its Age-Friendly Public Health Systems initiative, Trust for America's Health's has worked directly with several state public health departments to explore opportunities to develop and expand their county-level data profiles on older adults. This session will highlight the unique collaborations and partnerships between departments of health and other institutions, including academia, to develop data profiles. Through these innovative partnerships, departments of health have been able to develop dynamic data profiles that they share with aging services providers to discuss strategies to support their older adults in their communities. Best practices and lessons learned through the profile development process will also be discussed.

### OLDER ADULT'S PERCEPTION OF PRIMARY HEALTH CARE REFORM IN A LOW-MIDDLE-INCOME COUNTRY (LMIC): IMPACT ON GERIATRIC CARE

Theresa Abah, *California State University, Sacramento, Sacramento, California, United States*

Health reforms are intended to provide equitable and beneficial care across population groups, it also transforms service delivery. We sought to identify current or potential implementation gaps that divert improvement efforts in service delivery, and practices that prevent holistic approaches in caring for older adults, especially in developing nations. This study examined a major health reform initiative in a LMIC aimed at integrating defragmented health organizations under one management, it also looked at extent to which reform agendas align with primary health care (PHC) principles. Using purposeful random sampling design, this quantitative study obtained participants' views of care received in 12 sites and 2 states in Nigeria. A total of 218 participants aged 50 years and older in both rural and urban areas. Partial correlations were carried out after accounting for other variables of interest. Findings show there was no significant differences in attributes of primary care received by geography, gender or by state. Although Ondo state scored higher than Federal Capital Territory in previous performance evaluations (66% and 43% in 2015), respondents from both states received only two out of the seven primary care attributes recommended by the World Health Organization. First contact (access to care) and Care continuity, p <.01 and p, 0.5, (d = 0.202 and 0.179) were significant. Overall, our findings suggest that performance improvement at management level dominated reform efforts over service delivery. It is unclear how this aligns with providing older adults with the health care that they need.

### EFFECTS OF A SPECIFIC NARRATIVE GROUP PRACTICE ON HEALTH OUTCOMES OF CHINESE OLDER ADULTS: AN RCT STUDY

Esther Chow, and Sai-fu Fung, *City University of Hong Kong, Kowloon, Hong Kong*

In view of the rising chronicity during older adulthood, a specific narrative group practice is developed to improve well-being. Narrative therapy (NT), views people as experts in their own lives, and have accrued their life wisdom through their lifespan. To test its effects, a randomized controlled trial design was used to assess and compare outcomes between the intervention and waitlist-controlled group. A total of 157 older adults were recruited, 82 of which was randomly assigned to 9 intervention groups to receive four 2-hour NT sessions, recording difficult life circumstances, through narrative conversations, which made them stronger. Quantitative assessment of well-being, and depression were conducted at baseline (T0), at the end of treatment (T1), and at two (T2) and 8 months after treatment (T3). By employing the SEM parallel-process latent growth curve model, the results from the 4 waves of longitudinal data suggesting that the NT intervention have positive impact the participants, in particular the improved well-being of the participants contributed to the reduction of depressive symptoms with standardised estimate -0.502, p = 0.007. The estimated model fulfilled the criteria of mediocre fit, with TLI = 0.943; CFI = 0.969; RMSEA = 0.053. No adverse reaction was recorded in any of the cases mentioned at all study-sites. These findings have significant theoretical and practice implications for both health and social care professions. NT offers an effective practice to develop a strength- and meaning-based group practice and ground a new theory for to develop a constructive perspective to late life development.

### MOTIVATIONAL FACTORS AFFECTING THE INTENTION TO VOLUNTEER IN ELDER CARE AGENCIES: A PILOT STUDY

Rong Fu[1], and Qi Xu[2], *1. Siena College, Loudonville, New York, United States, 2. China Agricultural University, Beijing, Beijing, China (People's Republic)*

The rapid rise of the aging population and the “4-2-1” family structure in China calls for speeding up the construction of the elderly care service system. To facilitate this process, it is desirable to encourage voluntary participation in elder care agencies. This study aims to identify motivational factors that may affect the intention to volunteer in elder care agencies in China. Data were collected in a sample of 199 residents of Beijing, China. Our results revealed gender differences in the demographic and socioeconomic factors that affect the volunteer intention. For men, earning more than 12,000 Yuan (about $1900) significantly increased the intention to participate in voluntary services (p = .043). For women, those younger than 60 years of age were more willing to participate in voluntary services than their older counterparts (p = .014). Participants identified three key motivational factors for providing volunteer services in elder care agencies: (1) to show love and kindness to older adults; (2) to fulfill oneself and take social responsibilities; and (3) to gain more life experience. We also found that most participants prefer to provide spiritual comfort services (e.g., chatting, shopping) and life care services (e.g., cooking, house cleaning) than other types of services. Our study suggested that effective interventions should be designed and implemented to match volunteering resources to the needs of elder care agencies in China.

### A META-ANALYSIS OF MUSIC THERAPY AND SLEEP QUALITY: TRENDS AND IMPLICATIONS FOR OLDER ADULTS

Noell Rowan[1], Angie Sardina[2], Tina Newsham[2], Richard Pond[2], Lenis Chen-Edinboro[1], Christine Leist[3], Daniel Johnson[2], and Michele Erich[4], *1. University of North Carolina Wilmington, Wilmington, North Carolina, United States, 2. UNC Wilmington, Wilmington, North Carolina, United States, 3. Appalachian State University, Boone, North Carolina, United States, 4. Novant Health New Hanover Regional Medical Center, Wilmington, North Carolina, United States*

Music therapy (MT) has been an established healthcare profession in the United States for over seven decades. MT interventions are non-pharmacological approaches, administered by board-certified music therapists, designed to promote physical, emotional, cognitive, and spiritual well-being. One aspect of health where MT may be of use to older adults is in improving poor sleep quality, which is associated with increased risk for morbidity and mortality. Many pharmacological treatments for insomnia and other conditions carry adverse effects. Investigating the impact of evidence-based non-pharmacological interventions (e.g., MT) on sleep could be beneficial, particularly among older adults who are at greater risk for polypharmacy-related challenges. The purpose of this study was to investigate the average effect of MT on sleep quality via a meta-analysis across 46 studies (N=2912). This study included a review of the effects of MT on sleep, the characteristics of strongest and weakest MT interventions, the role age plays on the efficacy of MT, and differences in effects between adults with and without dementia. Results revealed robust improvements in sleep quality, as a function of MT. These improvements were observed among teams that included a professional music therapist. Clinical and practical implications will be discussed, particularly as they apply to older adults and interprofessional practice.

### IN THE WEEDS: UNDERSTANDING CANNABIS USE AMONG OLDER ADULTS

Isabelle Sheck, and H. Shellae Versey, *Fordham University, Bronx, New York, United States*

The existing literature from the past decade shows an increase in the prevalence of cannabis use among older adults (OA) aged 50-65. Most commonly, studies have reported OA using cannabis to manage symptoms related to chronic pain, arthritis, insomnia or disordered sleep, and anxiety. Recent recreational cannabis legislation in several states has also contributed to increasing rates of cannabis use among older adults. Despite an increase in recreational and medicinal cannabis consumption, few studies have focused on cannabis attitudes and behaviors among older adults. Within the past ten years, studies reporting cannabis behavior have been limited to samples of younger adults. This paper provides a comprehensive review of qualitative and quantitative studies of cannabis use among older adults (OA). Additional research detailing the characteristics of cannabis use among OA is needed, including frequency, motivation, type of cannabis (medicinal/recreational), route of administration, suppliers, and social networks associated with cannabis use. This systematic review adds to our knowledge about aging and substance use addressing a gap in the literature regarding drivers of cannabis use among OA. Implications of this research extend to gerontological, public health, and community research as cannabis continues to become more easily accessible in various states.

### SESSION 6360 (POSTER)

### POLICY, FINANCING, AND DELIVERY SYSTEMS

### PROTECTING RETAIL CUSTOMERS FROM GIFT CARD PAYMENT SCAMS

Dionne Bailey[1], and Marti DeLiema[2], *1. University of Minnesota, Minneapolis, Minnesota, United States, 2. University of Minnesota - Twin Cities, Saint Paul, Minnesota, United States*

Older adults are frequently targeted by scams and fraud, a persistent and growing problem in the U.S. According to the Federal Trade Commission, gift cards were the most common method of payment requested by fraud criminals in 2021. The purpose of this AARP-sponsored study is to investigate gift card payment scams by analyzing the retail sector for current interventions and opportunities to protect customers from fraud. Through secret shopping at 23 retail locations in a large U.S. city, we documented the existing status of merchant controls designed to prevent suspicious gift card purchases, as well as how these measures work in practice. At each location, the "secret shopper" attempted to purchase between $400-$1000 in gift cards from a sales clerk, while another researcher stood in line nearby to observe the interaction. Nearly 80% of retail stores had signs warning customers about gift card frauds. Fewer than 50% imposed limits on the purchase amount; more than 40% required manager approval, and five of the transactions resulted in manager involvement. Current retail measures would only limit purchases in around 22% of gift card frauds and would completely halt about 9% of transactions. Results indicate that despite warnings, retailers are not doing enough to protect customers from fraud. New policies are needed to advocate for reduced gift card purchase limits, increase public awareness, educate retail employees on the red flags, and enforce anti-money laundering rules to limit fraud.

### BUDGET IMPACT ANALYSIS OF NONPHARMACOLOGICAL INTERVENTIONS FOR COMMUNITY-DWELLING PEOPLE LIVING WITH DEMENTIA

Eric Jutkowitz, Mauricio Lopez Mendez, and Peter Shewmaker, *Brown University, Providence, Rhode Island, United States*

Non-pharmacologic dementia care interventions significantly reduce the risk of a nursing home admission for people living with dementia. We used an evidence-based mathematical model to evaluate the budget impact for a healthcare payer that independently implemented four non-pharmacologic dementia care interventions that reduce the risk of transitioning to a nursing home for people living with dementia: 1) MIND, an at-home care coordination intervention; 2) NYU Caregiver (NYUCI), provides caregivers with counseling and ad-hoc support; 3) Alzheimer's and Dementia Care (ADC) program, a clinic based care coordination intervention; and 4) Adult Day Services Plus (ADS-Plus), an adult day based care coordination intervention. Healthcare payer costs included Medicare and Medicaid expenditures. We simulated a cohort of 302,630 community-dwelling people with dementia, which is the number of people who were diagnosed with dementia in 2018. We applied each intervention's inclusion criteria to determine the proportion of the cohort that would receive the interventions. Some people may die or enter a nursing home before receiving the interventions. MIND, NYUCI, ADC, and ADS-Plus reduced annual payer expenditures (relative to $25,000, which is the average amount Medicare-Medicaid pay per person with dementia) on average by 0.67%, 0.23%, 0.13%, and 0.58%, respectively over 5 years. Cost savings for the interventions varied by demographics. African American females between ages 95-100 who received NYUCI had the largest cost savings ($2,750.57). White females between ages 65-70 who received ADC had an increase in payer costs ($2,397.07). On average, non-pharmacologic dementia care intervention do not increase a healthcare payer's budget.

### SUPPLEMENTAL PAYMENTS AND NURSING HOME STAFFING: A CASE STUDY OF INDIANA

Hari Sharma, and Lili Xu, *University of Iowa, Iowa City, Iowa, United States*

Adequate reimbursements are key to improving quality in nursing homes (NHs), but Medicaid payments remain low. Indiana implemented Medicaid supplemental payment program to non-state government (NSGOs) owned NHs to boost Medicaid payment rates. Under the program, NSGOs contribute the state-share of the supplemental payments through intergovernmental transfers allowing the state to claim federal matching funds. The supplemental payments are distributed between NSGOs and NHs but little is known about whether NHs invested the supplemental payments to improve staffing levels. In this study, we examine whether NHs receiving supplemental payments, proxied by NSGO-ownership, have higher staffing hours per resident day (HPRD) for registered nurse (RN), licensed practical nurse (LPN), and certified nurse aide (CNA) compared with NHs without supplemental payments. We use facility-year data on NSGO ownership (State of Indiana), staffing levels (Nursing Home Compare), and facility and resident characteristics (LTCfocus.org) on 500 NHs from 2009 to 2017. We use difference-in-difference (DD) regressions with year and facility fixed effects to estimate the potentially causal effect of NSGO ownership on NH staffing controlling for facility and resident characteristics. DD estimates suggest that NSGO-ownership is associated with decreased staffing level across all three types of staffing. More specifically, RN staffing decreased by 0.019 HPRD ($p< 0.05$), LPN staffing decreased by 0.041 HPRD ($p< 0,05$), and CNA staffing decreased by 0.089 HPRD ($p< 0.001$). Policymakers should increase the transparency of supplemental payment programs and audit the expenditures to ensure that supplemental payments are tied to quality improvement.

### VARIATION AND CONSISTENCY IN ALLOCATION OF HCBS: MASSACHUSETTS AGING SERVICES ACCESS POINTS

Signe Flieger[1], Gabrielle Katz[2], and Christine Bishop[2], *1. Tufts University School of Medicine, Boston, Massachusetts, United States, 2. Brandeis University, Waltham, Massachusetts, United States*

Massachusetts' 27 Aging Services Access Points (ASAPs) are nonprofit organizations responsible for guiding clients and families in their geographic service areas through a maze of financial and functional eligibility criteria to receive needed home and community-based services (HCBS). These services aim to sustain function and promote engagement for older adults and persons with disabilities, and to maintain individuals in the community rather than in residential settings. Payment for HCBS flows from numerous state and Federal funding programs, including but not limited to state Medicaid waivers and Older American Act programs. We conducted semi-structured interviews with 10 ASAP directors and gathered information from documentary sources to delineate the pathways to funded HCBS that these agencies open for clients. We identified variation in the 27 ASAPs' contractual relationships with service providers. State data revealed variation in budgetary resources and area population characteristics. These nonprofit corporations appear to flexibly adapt to local conditions and needs. Understanding variation across ASAPs will support analysis of client data to examine whether ASAP processes result in different service outcomes for similar clients. Alternate payment models for health services (managed care, managed long-term services and supports, accountable care organizations) are broadening their reach, encouraging health systems to recognize the importance of functional supports and services. Some health systems are charging their own dedicated units with assigning HCBS to enrollees, avoiding the ASAPs. Understanding and modeling the allocation of services by the ASAPs will allow investigation of trends in services for persons with varying needs under various payment methods.

### EVALUATING THE STAFFING METHODOLOGY MODEL IN THE VETERANS HEALTH ADMINISTRATION'S COMMUNITY LIVING CENTERS

Lana Brown[1], Shelly Lensing[2], Sheila Cox Sullivan[3], Teresa Odom[3], Jade Moore[3], Pamela Billings[4], and Lisa Minor[3], *1. Central Arkansas Veterans Healthcare System, North Little Rock, Arkansas, United States, 2. Central Arkansas for Veterans Healthcare System, North Little Rock, Arkansas, United States, 3. Veterans Health Administration, Washington, District of Columbia, United States, 4. Veterans Health Administration, Nashville, Tennessee, United States*

The project purpose was to describe nursing hours per patient day (NHPPD) within the Veterans Health Administration (VHA) and to evaluate Staffing Methodology in the VHA Community Living Centers (CLCs) by comparing Staffing Methodology targeted NHPPD with actual NHPPD. Data were compiled retrospectively for each CLC unit over a one-year timeframe for calendar year 2019. VHA had 134 CLCs including 390 nursing units. Nursing units without 6 months of actual NHPPD data were excluded from the data set. For descriptive analyses, actual NHPPD were averaged across months for each unit. The percent of target was calculated as this average divided by the NHPPD target times 100. The percentage of months each unit met the targeted NHPPD was calculated. After data exclusions, the final data set included 322 nursing units from 133 CLCs. The mean for actual hours as a percent of target was 121.6% (95% CI, 118.5 to 124.7%) indicating that the units' average hours across 2019 were 21.6% significantly higher than target. Also, half of units met their targets 91.7% of months as reflected by the median. The actual NHPPD significantly differed across months ($p< 0.001$) with the 2019 months of January and October having the highest NHPPD. Similar results were seen for NHPPD as a percent of target. Veteran safety is a VHA priority and appropriate nurse staffing is key to providing care that improves Veteran outcomes and reduces adverse events. Further exploration is needed on how nurse staffing in the CLCs impacts Veteran outcomes, safety, and satisfaction.

### MINNESOTA'S 2021 LGBTQ AGING NEEDS ASSESSMENT

Rajean Moone, *University of Minnesota, St Paul, Minnesota, United States*

Every decade Minnesota embarked on a needs assessment of lesbian, gay, bisexual, transgender and queer (LGBTQ) older adults which has provided important insights into aging within these communities. In 2021, a research team replicated these past monumental studies into this Minnesota 2021 LGBTQ Aging Needs Assessment. 485 individuals responded to the survey, either online or by paper. Of those, 354 met the inclusion criteria and are included in the results. Like the 2012 study, LGBTQ older adults who participated in the study were more likely to be a caregiver than compared to the general population. At the same time they were less likely to have a caregiver and less likely to have children. Half of the participants experienced some form of discrimination and the vast majority more knew someone who experienced discrimination due to their sexual orientation or gender identity. Positively, study participants were more likely to have completed a health care directive and more likely to volunteer than the general population. Possibly one of the most striking changes over the last two decades is that 85% of respondents were confident they would receive sensitive services. In 2012 only 18% and 2002 only 9% expressed the same confidence. In congruence with the past studies, the majority of respondents continue to desire LGBTQ welcoming senior services rather than LGBTQ segregated services.

### SESSION 6370 (POSTER)

### REMINISCENCE AND LIFE REVIEW

### FROM PILOT TO PRODUCT: EVOLUTION OF A REMINISCENCE THERAPY INTERVENTION FOR PERSONS LIVING WITH DEMENTIA

Silvia Orsulic-Jeras[1], Farida Ejaz[1], Miriam Rose[1], Sara Powers[2], Ashlee Cordell[1], Christa Wilk[1], and Lisbeth Sanders[3], *1. Benjamin Rose Institute on Aging, Cleveland, Ohio, United States, 2. The Benjamin Rose Institute on Aging, Cleveland, Ohio, United States, 3. LifeBio Inc., Marysville, Ohio, United States*

Reminiscence therapy (RT) and life story work are effective techniques for eliciting engagement in persons living with dementia (PLWD) and helping inform direct care staff about residents' lives and preferences to facilitate quality person-centered care. However, there are challenges in bringing such programs to scale in a feasible and cost-effective way. Two studies were conducted to test development and evolution of an RT program, LifeBio Memory. A pilot study of the original LifeBio protocol was conducted with 238 residents in 16 Ohio nursing homes. Most residents (88%) indicated at posttest that they enjoyed telling their life stories, and 87% would recommend the program to other residents. However, manually gathering and processing life story data led to challenges with feasibility. Subsequently, novel software was developed to streamline the process using machine learning and artificial intelligence via a tablet application. Acceptability and feasibility were examined in two rounds, 9 months apart, of seven focus groups (n=35) conducted with PLWD, family care partners, and residential care staff. Audiotapes of the groups were transcribed; thematic data analysis was used to generate a list of recommended changes and showed high levels of acceptability and feasibility. Based on these results, it was determined that LifeBio Memory was preferred for obtaining life story information over the previous manual method (e.g., handwriting life story notes). Potential barriers to implementing the new platform in residential care settings were also identified. Implications include the importance of maintaining person-centered care practices when creating technological solutions to address PLWD needs.

### MULTIPLE SCALES OF SOCIAL RELATIONS IN REMINISCENCE ACTIVITIES FOR OLDER ADULTS

Jessica Robbins-Panko, *Wayne State University, Detroit, Michigan, United States*

Memory, especially autobiographical memory, is central to personhood in contemporary North America and Europe (Herskovits 1995). Cultivated practices of reminiscence, in which older adults are prompted to remember events and experiences from the past, can be pleasurable, meaningful, and therapeutic activities that shape personhood and contribute to wellbeing (Coleman, 1999; Sherman, 1987). Although reminiscence practices include both individual (e.g., Butler, 1963; Woods et al., 2018) and social (Hash et al., 2021; Sass et al., 2021) elements of remembering, less attention has focused on the myriad scales of social relations that are part of reminiscence activities. Moreover, the mechanisms through which reminiscence creates positive transformations remain unclear (Menn et al., 2020). Anthropological theories of personhood show that social relations at multiple spatiotemporal scales play key roles in transforming personhood (Carsten, 1995; Robbins, 2021), thus suggesting a nuanced framework within which to understand how social relations shape personhood and contribute to wellbeing for older adults. Drawing on a systematic review of interdisciplinary literature on reminiscence activities for older adults, this paper documents the multiple scales of social relations (interpersonal, institutional, regional, [trans]national) that are part of reminiscence activities, and interpretively analyzes their significance for personhood and wellbeing. Key findings of this study are: 1) collective memory plays an important role in reminiscence activities; and 2) fostering reminiscence activities that focus on meaningful elements of the shared past can promote wellbeing for older adults. These findings have implications for 1) refining gerontological theories of reminiscence, and 2) improving reminiscence-based practices for older adults.

### THE TELLEGACY PROGRAM

Jeremy Holloway, *University of North Dakota, Fargo, North Dakota, United States*

Emerging research displays how social isolation and loneliness (SIAL) before and during the Coronavirus pandemic are linked to health issues caused by SIAL. These issues are estimated to increase the risk of early death by 26 percent, the equivalency of smoking 15 cigarettes a day. The program started soon after the pandemic as an intergenerational program created to combat SIAL among older adults. University students, known as Legacy Builders, connect through virtual or via phone conversations to communicate meaningfully with the older adult. Students are prepared with a curriculum on mindfulness, SMART goal-setting, growth mindset, visualization goal-setting exercises, and reminiscence therapy as well as the skill and art of listening. At the end of the encounters, a legacy book is given to the older adult with a description of the older adult's life shared during their conversations with the Legacy Builder. The UCLA Loneliness Scale was utilized along with older adult self-reports. Sixteen residents in a memory care facility, ages 58 to 86, with multiple levels of dementia participated in the program of Summer 2021. The UCLA Loneliness Scale revealed that 13 of the 16 residents originally reported that they feel lonely or isolated. After 5 weeks of participation 7 out of the 16 residents report that they felt less lonely. Qualitative analysis showed improved perceptions of aging and an interest in Geriatrics among the student participants. These results also displayed positive older adult perceptions of the younger generation, and similar responses of the younger generation towards older adults.

### SESSION 6380 (POSTER)

### SUCCESSFUL AGING (POSTERS)

### TOPICS ADDRESSED IN CENTENARIAN RESEARCH: A SCOPING REVIEW OF PUBLICATIONS SINCE 2000

Daniela Jopp[1], Charikleia Lampraki[1], Kim Uittenhove[1], Flavien Delhaes[2], Daniele Zaccaria[3], Carla Gomes Da Rocha[4], Garnelle Ziade[1], and Melanie Becker[5], *1. University of Lausanne, Lausanne, Vaud, Switzerland, 2. University of Geneva, Geneva, Geneve, Switzerland, 3. University of Applied Sciences and Arts of Southern Switzerland, Manno, Ticino, Switzerland, 4. HES-SO Valais-Wallis, Sierre, Valais, Switzerland, 5. University of Zurich, Zurich, Zurich, Switzerland*

Over the past decades, centenarians have increasingly attracted the attention of the research community. This development reflects the constant rise of the numbers of very old individuals, but also the need to better understand longevity mechanisms and what characterizes life at age 100. This scoping review provided an overview of trends in centenarian research from all disciplines since 2000, and identified the most frequently mentioned and neglected topics. Scientific articles meeting the following eligibility criteria were included: (1) publication between 2000 and 2021; (2) in English, French or German language; (3) study population of at least 95 years of age on average, or, for studies with larger age ranges, provision of specific insights for the

95+ group. Following the standard procedures for scoping reviews, we identified a total of N = 3955 articles. After removal of duplicates and exclusion due to unmet criteria, we used content coding to identify research themes of N = 1117 articles. Data confirmed that research articles offering findings on centenarians have increased substantially over the past two decades. Content coding led to 37 main topics: Most frequently, studies addressed higher-order topics such as physical, biological, and mental (e.g., cognitive), which were mostly investigated within disciplinary boundaries. Few multidisciplinary articles examined content domains in conjunction. The least investigated topics in centenarian research included the sub-domains of pain, stress, anxiety, and psychiatric disorders. In sum, findings inform the research community about the existing centenarian research, suggest that multidisciplinary publications are infrequent, and offer guidance for future studies.

### IMPACT OF COGNITIVE RESERVE IN OBJECTIVE AND SUBJECTIVE SUCCESSFUL AGING

Neyda Ma. Mendoza Ruvalcaba,
Elva Dolores Arias-Merino,
Maria Elena Flores-Villavicencio, and
Melina Rodríguez Díaz, *University of Guadalajara, Guadalajara, Jalisco, Mexico*

Cognitive reserve (CR) refers to the capacity of the brain to withstand changes due to age or disease-related pathology, showing how flexibly and efficiently the individual makes use of available brain resources. This study aims to explore the contributions of the CR to understanding successful aging (SA).(Project-Conacyt-256589)Population based, random sample included n=656 community-dwelling older adults 60-years and older (mean age=72.8, SD=7.6 years, 58% women). CR was measured by their main indicators: education, life-long learning, being bilingual, participation, use of information and communications technology. Objective SA was operationalized as no important disease, no disability, physical functioning, cognitive functioning, and being actively engaged. Subjective was an appreciation if they considered themselves as successful agers. Sociodemographic and health data were also asked. Pearson´s correlation test and MANOVAs were performed.In total 11.2% met the criteria for SA, although 76% considered themselves as successful agers. CR was significantly related to subjective and objective criteria of SA(p<.000), except to no-important diseases. CR explains in general 20% of the variance in objective SA, specifically explains 28% of variance in the criteria of high cognitive function, 18% of the variance in disability, 11.3% of life engagement, 8% of physical functioning, and 2% of disease-free criteria. Also, CR explains 10% of the variance in subjective SA. This study has shown that CR the is related to SA, this set possible targets for cognitive interventions to promote resiliency of the brain not only for preventing cognitive pathologies, but also for encouraging successful and healthy aging in older adults.

### OLDER ADULTS SHARE CHALLENGES IN THEIR LIFE STORY: SENSE OF PURPOSE AS A RESOURCE FOR RESILIENCE

Semper Habib[1], Ameesha Narine[1], Susan Bluck[2], and Shubam Sharma[1], *1. Kennesaw State University, Kennesaw, Georgia, United States, 2. University of Florida, Gainesville, Florida, United States*

Maintaining a sense of purpose promotes mental and physical well-being in older adults (Musich et al., 2018). Drawing on one's sense of purpose is thus important for late life resilience. How older adults' sense of purpose manifests in their everyday lives remains understudied. This study used qualitative methods to amplify older adults' voices regarding purpose and resilience through analysis of their life stories. This study 1) explored what factors contribute to maintaining purpose in older adulthood, and 2) identified how older adults draw on their purpose during major challenges, particularly in the context of the COVID-19 pandemic. Eighteen older men and women (Mage = 79.1; including the young-old, old-old, and oldest-old) participated in semi-structured life story interviews that asked about participants' individual interpretations of purpose in their lives and their experiences navigating the COVID-19 pandemic. Thematic analysis was conducted using established methods (Braun & Clarke, 2012). To address the first research question, analyses revealed that older individuals largely maintain their purpose through engaging in acts of service to others, fostering connections with close others, and actively setting and achieving goals. Regarding the second question, older adults described how drawing on purpose through acts of service and connections with others fostered resilience through the COVID-19 pandemic. Overall, older adults' own expressions of their life stories illuminated how they are guided by purpose. Findings demonstrate the functionality of purpose in late life and how purpose can be practically fostered, specifically within the context of universally challenging experiences such as the COVID-19 pandemic.

### BENEFITS OF VOLUNTEERING ON RESILIENCE WITH AGING: A CASE STUDY

Sanghee Lee[1], Jinmoo Heo[2], and Sanghee Chun[3], *1. Korea University, Seoul, Seoul-t'ukpyolsi, Republic of Korea, 2. Yonsei University, Seoul, Seoul-t'ukpyolsi, Republic of Korea, 3. Brock University, St. Catharines, Ontario, Canada*

As the study of volunteering among older adults continues to evolve, questions of the benefits of volunteering are of growing interest to many researchers. Volunteering may develop resilience in older adults as it can serve as a coping strategy as they recover from adverse events or other life challenges. We explored the perceived benefits of volunteering on resilience in later life among older adults who perform Korean traditional dance on voluntary basis. We used a qualitative design with a case study method. In this study, older adults' volunteer dance performing was taken as a case. A case design enables researchers to understand social and cultural phenomenon in depth using a wide range of data collection and analysis methods. Thirteen volunteer performers of Korean traditional dance whose ages ranged between 61-74 years were recruited for in-depth interviews (11 females and 2 males). The analysis of the transcripts generated five themes related to the benefits of volunteering: (1) finding a sense of self-worth through serving others, (2) finding a sense of purpose, (3) experiencing gratitude, (4) renewing a younger self, and (5) building companionship. The findings of this study provided an empirical support showing how volunteering experience benefited resilience among the volunteers in the face of challenges associated with aging by maximizing positive physical, social, and psychological outcomes through involvement in voluntary dance performance. The findings also provide guidance for researchers and practitioners in positions to better serve older adults and thereby suggest volunteering as a resilience strategy in later life.

### SWISS CENTENARIANS: PRELIMINARY FINDINGS FROM THE SWISS100 PHONE STUDY

Daniela Jopp[1], Stefano Cavalli[2], Armin von Gunten[3], Francois Herrmann[4], Mike Martin[5], and Kim Uittenhove[1], *1. University of Lausanne, Lausanne, Vaud, Switzerland, 2. University of Applied Sciences and Arts of Southern Switzerland, Manno, Ticino, Switzerland, 3. Lausanne University Hospital, Prilly, Vaud, Switzerland, 4. Geneva University Hospitals, Thônex, Geneve, Switzerland, 5. University of Zurich, Zurich, Zurich, Switzerland*

Increase in very old individuals is observed in all developed countries around the world. The number of centenarians has also been rising, requiring the investigation of the characteristics of these exceptionally long-lived individuals as well as their experience of life at age 100. In the present study, we present findings from the first nation-wide Swiss centenarian study SWISS100. Given the ongoing COVID-19 pandemic, we conducted a telephone study with centenarians and a family member as proxy informant, using a mixed-methods approach to investigate specific characteristics, their life circumstances and their experience during the pandemic. Recruitment was conducted with the help of the national address registry. A total of 64 centenarians and 62 family members participated, leading to data for 119 centenarians. Centenarians were on average 102 years old, with a range of 100 to 108 years. In line with higher survival rates in females, 76% were women and 24% were men. Most centenarians had received basic education and had completed an apprenticeship. Concerning their residence, 43% lived in private homes and 57% lived in institutions. Of those living in private, half lived alone, one fourth lived with their spouse and one fourth lived with a child. The majority was widowed. Over 80% had children. Although over 70% of the centenarians reported health restrictions, 60% reported good to excellent subjective health. Over 90% of the sample were aware of COVID-19. Despite substantial COVID-19-related restrictions, life-satisfaction was high. Overall, Swiss centenarians show health-related vulnerability but also psychological resilience.

### VOLUNTEERING AND RISK OF HEART ATTACK IN LATER LIFE: THE MODERATING ROLE OF PURPOSE IN LIFE?

Mallory Bell[1], and Kenneth Ferraro[2], *1. Purdue University, Center on Aging and the Life Course, Lafayette, Indiana, United States, 2. Purdue University, West Lafayette, Indiana, United States*

Background and Objectives: Although research on the health benefits of volunteering has proliferated in recent decades, most studies are cross-sectional, and none prospectively examine the relationship between volunteering engagement, purpose in life, and heart attack. Research design and methods: This study uses seven waves of data (2006-2018) from the Health and Retirement study--a nationally representative survey of adults over age 50 (N=5,093). Event history analysis using Cox proportional hazards were used to examine if volunteering engagement in later life reduced the risk of first heart attack and if the effects of volunteering vary by level of purpose in life.

Results: Results reveal that volunteering, and doing so at a low time commitment, reduces the risk of first heart attack in later life. Additionally, the effect of volunteering varies by sense of purpose in life, such that volunteers with a strong sense of purpose in life had the lowest risk of heart attack. Implications: The findings of this study will inform public health and policy interventions dedicated to extending and improving later life health.

### PURPOSE IN LIFE, STRESS REACTIVITY, AND COGNITIVE AGING: A LONGITUDINAL INVESTIGATION

Niccole Nelson, and Cindy Bergeman, *University of Notre Dame, Notre Dame, Indiana, United States*

This study examined (1) the time-varying relationship between purpose in life and perceived stress reactivity, (2) the trajectory of perceived stress reactivity as it relates to both within- and between-person purpose in life, as well as (3) the predictive utility of perceived stress reactivity and its rate of change on cognitive ability and allostatic load. The sample comprised 933 participants from the Notre Dame Study of Health & Well-being, a 10-year study of annual questionnaire packets and biennial daily diary bursts. Analyses included three-level multilevel models from which random effects were extracted and used to predict allostatic load and cognitive ability. Results indicated that individuals were affectively reactive to perceived stress, and that perceived stress reactivity declined over time. Considering the effects of purpose in life on these processes, there were two cross-level interaction effects indicating (1) more purposeful individuals were less stress reactive than less purposeful individuals, and (2) more purposeful individuals declined less in negative affect over time than less purposeful individuals. There was also preliminary evidence for a within-person interaction effect between yearly purpose in life and daily affective reactivity such that when individuals felt particularly purposeful, they also tended to be less stress reactive. Finally, higher perceived stress reactivity, as well as less decline in this construct, was predictive of better cognitive ability. These findings indicate purpose in life buffers against environmental and maturational effects on negative affect, and that perceived stress reactivity may indicate a different, more adaptive process than affective reactivity to experienced stressors.

### SOURCES OF INTRINSIC MOTIVATION IN LATER ADULTHOOD: A QUALITATIVE ANALYSIS

Melinda Heinz, Nathan Benton, and Laura Gleissner, *Upper Iowa University, Fayette, Iowa, United States*

A distinct sense of purpose and motivation is increasingly recognized as a contributing factor in successful aging and prolonged health in older adults. While prior studies have established the relationship between intrinsic motivation, defined purpose, and health outcomes, it is essential for care providers and researchers to understand the underlying sources of purpose and motivation for older adults. This study examined qualitative data collected over a two-week period from a sample of older adults (N = 15) in the Midwest. Participants documented daily, through written journal entries and photography, aspects of their lives that they felt illustrated sources of purpose and motivation. The journal entries were then transcribed, aggregated, coded, and analyzed for potential themes. Two researchers coded the data; and a codebook was used to increase coding consistency. Using latent thematic analysis, three distinct themes developed from the dataset. First, engagement in behaviors and activities that incorporated aspects of mindful practices, particularly sensory awareness and grounding, proved to be motivating for many of the participants. The second theme discovered that participants found purpose in active social participation, where a subtheme regarding the impact of technology on maintaining relationships emerged. In the third theme, a goal-oriented mindset proved to be a key aspect of ongoing motivation, with a particular focus on cognitive and physical development, lifelong learning, and planning for the future. Overall, the data pointed to three distinct themes that can be used to foster ongoing opportunities for greater meaning in the lives of older adults.

### LAY PERSPECTIVES ON SUCCESSFUL AGING IN ROMANIA

Angélique Roquet[1], Mariana Barbut[2], Cornelia Pocnet[3], and Daniela Jopp[1], *1. University of Lausanne, Lausanne, Vaud, Switzerland, 2. Lausanne University Hospital, Lausanne, Vaud, Switzerland, 3. lausanne university, Lausanne, Vaud, Switzerland*

Successful aging is a topic of world-wide interest, yet much work focuses on specific and highly industrialized countries, such as the USA. The present study investigated lay perspectives of the concept of successful aging in young, middle-aged, and older adults from Romania. Ninety-three participants aged between 20 and 84 years were asked about definitions and determinants of successful aging. Based on a previous study, codes were developed to capture common themes among the answers, resulting in 14 categories. Participants mentioned four themes on average to describe successful aging, which varied by sociodemographic, health and psychological variables: individuals of younger age, with higher levels of education, in better health and those more satisfied with their aging mentioned more themes. Regarding specific topics, Social resources and Health were mentioned most frequently, followed by Quality of life, Financial situation, and Attitudes/Mechanisms. Young adults were more likely to mention Social resources, Success/Respect, and Aging themes, while older adults mentioned more often Meaning of life themes. Finally, we found that the themes mentioned were related to self-perception of aging, particularly among older adults: Older adults more satisfied and with positive views own aging were more likely to mention Social resources, Independence, and Attitudes/Mechanisms themes, while those with negative views on aging were more likely to mention Micro-environment themes. In sum, findings support the multidimensionality of lay perspectives of successful aging and offer insights on the understanding of successful aging in Eastern Europe, including topics and links to general and personal aging experiences.

### MODELS OF OLDER ADULT GROUP ENGAGEMENT TO IMPROVE HEALTH MANAGEMENT

Mary Hynes[1], Nicole Anderson[2], Monika Kastner[3], and Arlene Astell[1], *1. University of Toronto, Toronto, Ontario, Canada, 2. University of Toronto, North York, Ontario, Canada, 3. North York General Hospital, Toronto, Ontario, Canada*

We are studying the use of peer-to-peer group intervention as a means of promoting older adult health self-efficacy and self-management. To explore how older adults have worked together to improve health behaviors, a scoping review was conducted of older adult peer coaching in health maintenance or health improvement groups. Seventeen studies met all search criteria, including interventions examining the value of peer support in self-management of diabetes, a peer led program for fear of falling, and the effect of self-help groups on quality of life. Two models of peer engagement were identified: peer support and mutually supportive environments. Ten studies trained older adults to be peer mentors or leaders with training periods varying from two days to 30 weeks, although many did not include details of the training. The other seven studies examined mutually supportive environments for peer engagement such as a clinician-led with peer-support model, an app-based program with a social support component, and a prevention focused mutual support group. These studies included research comparing self-care and quality of life results after self-help group therapy and a study that analyzed the impact and role of volunteering at a seniors' centre on maximizing member self-efficacy. While all studies reported on peer self-health engagement, there were many different goals ranging from evaluating health improvement programs to comparing peer and professional health group leadership. One consistent theme was improved perceived self-efficacy though peer group engagement.

### SESSION 6390 (POSTER)

### TECHNOLOGY

### THE IMPACT OF BEHAVIORAL LIFESTYLE INTERVENTION ON INFLAMMATORY CYTOKINES IN OLDER ADULTS WITH TYPE 2 DIABETES

Rozmin Jiwani[1], Monica Serra[2], Sara Espinoza[3], and Darpan Patel[2], *1. UTHSCSA, San Antonio, Texas, United States, 2. UT Health San Antonio, San Antonio, Texas, United States, 3. Sam and Ann Barshop Institute, UT Health San Antonio, San Antonio, Texas, United States*

Chronic inflammation, a hallmark of type 2 diabetes (T2D) and adiposity, increases the risk for age-related co-morbidities, including physical frailty. This study aimed to examine the effects of a mobile technology-enhanced behavioral lifestyle educational intervention on frailty and associated clinical, inflammatory, and laboratory outcomes in overweight older adults with T2D. Twenty participants (≥65 years, BMI ≥25 kg/m2) with T2D were recruited to complete a single-arm 6-month lifestyle intervention modified from the Action for Health in Diabetes (Look AHEAD) study, enhanced with technology for self-monitoring of diet and physical activity. Clinical assessments were collected at baseline and end of the study and analyzed with paired t-tests. Inflammatory cytokines (interleukin [IL]2, IL4, IL6, IL8, IL10, granulocyte-macrophage colony-stimulating-factor [GM-CSF], interferon [IFNγ], tumor necrosis factor [TNFα]) were quantified using a commercially available multiplex kit (Millipore Sigma, Burlington, MA, USA). Clinical lab analysis was performed by Quest Diagnostics (Dallas, TX, USA). Eighteen participants completed the study (71.5±5.3 years; 56% female; 50% Hispanic). At baseline, 13 participants were pre-frail, 4 were frail based on Fried frailty criteria. Inflammatory cytokines and liver enzymes values were within normal limits. At follow-up, the following outcomes significantly improved: frailty score -44% (p=0.01), BMI -3% (P< 0.00), alanine transaminase -18% (p=0.03), aspartate aminotransferase -13% (p< 0.00), IL2 -33% (p=0.01), IL4 -31% (p=0.05), IFNγ -36% (p=0.03), GM-CSF -43% (p=0.03). No other significant differences were observed. The results suggest the efficacy of a technology-enhanced lifestyle intervention on frailty and associated clinical, inflammatory, and laboratory outcomes in overweight older adults with T2D.

### TIME-VARYING ASSOCIATIONS BETWEEN SOCIAL INTERACTION MODALITY AND POSITIVE AFFECT DURING TIMES OF CHALLENGE

Tiana Broen[1], Yoonseok Choi[1], Theresa Pauly[2], Elizabeth Zambrano Garza[1], Denis Gerstorf[3], and Christiane Hoppmann[1], *1. University of British Columbia, Vancouver, British Columbia, Canada, 2. University of Zurich, Zurich, Zurich, Switzerland, 3. Humboldt Universität zu Berlin, Berlin, Berlin, Germany*

The COVID-19 pandemic compromised psychological wellbeing, especially given public health guidance that restricted social contact. Engaging in daily social interactions is especially important for maintaining wellbeing, and virtual interactions may be a surrogate for face-to-face interactions when in-person contact is not possible. Embracing the idea that virtual communication may facilitate social connectedness, this project seeks to better understand the potential of virtual social interactions for supporting wellbeing. Using repeated-daily-life-assessments from an adult lifespan sample (N=164, Mage=42.53 years), we examined how number of virtual and face-to-face social interactions were associated with positive affect in individuals' daily lives. We expected that face-to-face interactions would have a stronger relationship with positive affect than virtual social interactions, but that engaging in virtual interactions would be positively related to affect during these unprecedented times. In line with our hypotheses, number of daily in-person (r(812)=0.14, p<.001) and total number of social interactions (r(812)=0.14, p<.001) were associated with higher positive affect at the bivariate level; number of daily virtual social interactions showed a positive trend with affect (r(812)=0.06, p=.07). Multilevel models including quantity of face-to-face and virtual interactions indicate that only face-to-face interactions were associated with concurrent elevations in positive affect (b=0.58, p=.03) and overall higher positive affect (b=0.72, p<.001). Future analyses will examine whether social interactions involving close ties are more powerful drivers of positive affect than distal ties, whether everyday closeness to others mediates the relationship between social interaction modality and positive affect, and we will unpack age as a moderator in these relationships.

### FACTORS RELATED TO MOBILE INTERNET USE AMONG MIDDLE-AGED AND OLDER ADULTS: A MODERATING EFFECT OF DISABILITY

Eunjin Yang, and Kyung Hee Lee, *Yonsei University, Seoul, Seoul-t'ukpyolsi, Republic of Korea*

Although mobile devices are preferred as internet access devices, and the number of older adults with and without disabilities is rapidly increasing, little is known about the disability moderating effect on mobile internet use among older adults. This study examined the moderating effect of disability on older adults' mobile internet usage. This study was a secondary data analysis using 2020 Digital Divide Survey in South Korea. A total of 3,629 participants aged 55 and above were included. With the complex sample design in mind, multiple regression analysis was conducted to identify factors related to mobile internet use and test the moderating effects of disability on mobile internet use. Older adults with disabilities used mobile internet less than older adults without disabilities. However, disability status had a moderating effect on the relationships between mobile internet use and (1) operational skills of mobile devices (B=0.31, P=.004), (2) skills of internet use (B=1.46, P<.001), (3) motivation to use digital devices (B=0.46, P=.01), and (4) attitude towards new technology (B=0.50, P=.002). The results revealed that these positive relationships were stronger among older adults with disabilities than those without disabilities. Although older adults and people with disabilities are considered vulnerable populations for technology adoption, disability creates a stronger positive association between several factors and actual mobile internet use. Therefore, policymakers and practitioners should deliver tailored information and technological education for older adults with disabilities. Older adults with disabilities could be the primary beneficiaries of various mobile services.

### STAKEHOLDER ENGAGEMENT IN THE DESIGN OF A FALL RISK PREDICTION SYSTEM FOR SOCIALLY VULNERABLE OLDER ADULTS

George Demiris[1], Nancy Hodgson[1], Therese Richmond[1], Marjorie Skubic[2], Sean Harrison[1], Justine Sefcik[3], Sungho Oh[1], and HanNah Cho[1], *1. University of Pennsylvania, Philadelphia, Pennsylvania, United States, 2. University of Missouri, Columbia, Missouri, United States, 3. Drexel University, Philadelphia, Pennsylvania, United States*

Falls and fall-related injuries are significant public health issues for adults 65 years of age and older. Fall-related injuries are among the most expensive medical conditions. Mild cognitive impairment (MCI) and housing conditions are each independent risk factors for multiple falls among older adults and there is increasing evidence of the adverse effect of MCI on balance, stride, gait speed and increasing fall risk. We developed an innovative technology-supported intervention called Sense4Safety to 1) identify escalating risk for falls through real-time in-home passive sensor monitoring (using depth sensors); 2) employ machine learning to inform individualized alerts for fall risk; and 3) link 'at risk' socially vulnerable older adults with a tele-coach who guides them in implementing evidence-based individualized plans to reduce fall-risk. The purpose of this study was a) to examine preferences and attitudes of low-income older adults towards an in-home sensor-based system to inform fall prevention strategies; and b) solicit feedback from and validate the intervention protocols by clinical experts in fall prevention, fall risk assessment and geriatrics. We conducted one hour interview sessions with a total of 10 older adults and 10 clinical experts. Sessions were transcribed and analyzed. Findings included perceived benefits and challenges, recommendations for refinement of the intervention (including educational components and different visualization approaches for sensor data) and the role of family members or trusted others in addressing fall risk. We highlight clinical and ethical implications resulting from the use of passive monitoring for socially vulnerable older adults with MCI.

### DIGITAL TECHNOLOGY USE AND INTERGENERATIONAL INTEGRATION: THE MEDIATING ROLE OF DIGITAL LITERACY

Hayoung Park, Bomi Choi, Susanna Joo, Yoon-Myung Kim, and Hyoun K. Kim, *Yonsei University, Seoul, Seoul-t'ukpyolsi, Republic of Korea*

This study aimed to analyze the mediating effect of digital literacy on the association between digital technology use and intergenerational integration in later life. The sample included 315 older Korean adults aged 65 and above (M=68.83, SD=4.19, range=65-84) who participated in an online survey in December 2021. The participants' use of digital technology was measured by the sum of thirteen questions about how often they used each digital technology in their daily lives. Intergenerational integration was measured by the sum of thirty questions concerning awareness of understanding and empathizing with characteristics and cultures between generations to live harmoniously in the times through communication and cooperation. The mediating variable, digital literacy, was measured by the sum of thirty-five questions from the New Media Literacy Scale, asking individuals' ability to find, evaluate, and communicate information in a digital society. Age, gender, education level, household income, marital status, and living conditions were controlled for to reduce confounding effects. According to the mediation path analysis using SPSS PROCESS macro and bootstrapping, digital literacy significantly mediated the effects of digital technology use on intergenerational integration in later life; the higher the level of digital technology use, the higher the level of digital literacy, and this had a positive effect on the level of awareness of intergenerational integration among older adults. This study is significant in that it confirms the mechanism underlying the link between digital technology use and intergenerational integration and provides a new empirical model to promote intergenerational integration in terms of digital aging.

### PREDICTORS OF MHEALTH MINDFULNESS USE IN OLDER ADULT CAREGIVER POPULATION

Danielle Llaneza[1], and Elissa Kozlov[2], *1. Rutgers University, Piscataway, New Jersey, United States, 2. Rutgers School of Public Health, Piscataway, New Jersey, United States*

Caregivers of older adults with MCI have multiple competing demands that limit their ability to seek mental health care, despite being at high risk for stress, anxiety, burnout, and depression. MHealth mindfulness interventions have preliminary efficacy to reduce distress for caregivers, but it is unknown which caregivers are most likely to use and benefit from mHealth mental health interventions. Understanding older adult caregiver mHealth use is an important step in providing personalized mental health care. This study investigated caregiver characteristics that predicted mHealth mindfulness mobile application use in a sample of n=58 caregivers of older adults with MCI. Using linear regression models, it was determined that years spent caregiving, having someone to help with caregiving tasks and education level were all significant predictors of the total amount of minutes spent using the mindfulness application. Our results contribute to the growing body of literature on mHealth strategies to address caregiver distress. Future work should address the expansion of mHealth innovations for older adults and their care persons.

### TECHNOLOGY USE AMONG OLDER CHINESE ADULTS: BENEFITS ON SUBJECTIVE WELL-BEING

Dannii Yeung, Natalie Chow, and Edwin Chung, *City University of Hong Kong, Kowloon, Hong Kong*

The growth of Information and Communication Technology (ICT) in recent years has led policy-makers and researchers to look into how these developments affect older adults' daily lives. The educational attainment of older people in Hong Kong is relatively low, with more than 60% of them only completing primary education or having no formal education. Therefore, their utility of ICT remains low as compared with that of their Western counterparts. One of the objectives of this study is to understand the usage of various forms of ICT among older Chinese adults, including technology use in social, financial, medical, and leisure domains. This study also investigates whether social technology use (such as social media, instant messages, video call) could contribute to better subjective well-being through enhancing their social support. A total of 452 older adults joined this study (M=68.7 years, SD=6.27, Range=60-89; 66.7% female). Among the four domains of ICT, higher usage was observed in the social and leisure domains, particularly among the young old. However, among the participants aged 75 and above, more than 70% of them did not use any form of ICT due to a lack of ICT knowledge. The mediation analyses reveal significant indirect effects of social technology use on life satisfaction (B=0.19, SE= 0.08, p=.010) and positive emotions (B=0.02, SE=0.02, p=.014) through social support, thereby suggesting that social technology use could increase older adults' perceived support from friends, which subsequently improves their life satisfaction and positive emotions. Practical implications of these findings will be discussed.

### IMPLEMENTING A HYBRID TELEHEALTH HOME-BASED TRANSITIONAL CARE PROGRAM: PRE-IMPLEMENTATION CHALLENGES

Lauren Penney[1], Teresa Damush[2], Dawn Bravata[2], Cathy Schubert[3], Alaina Preddie[2], Sean Baird[2], and Ashley Schwartzkopf[4], *1. South Texas Veterans Health Care System, San Antonio, Texas, United States, 2. Richard L. Roudebush VA Medical Center, Indianapolis, Indiana, United States, 3. Richard L. Roudebush VA Medical Center in Indianapolis, Indianapolis, Indiana, United States, 4. Richard L Roudebush VA Medical Center, Indianapolis, Indiana, United States*

The reach of home-based clinical programs for medically complex, older adults can be limited by geographic scope. Implementing telehealth versions can expand reach but create constraints for comprehensive assessments and technological barriers for users. We describe challenges and lessons learned during the pre-implementation period for the randomized trial of a hybrid video -modality of the Geriatrics Resources for Assessment and Care for Elders (GRACE) Program at the Indianapolis Veterans Health Care System. In TeleGRACE, a health technician makes home visits to facilitate clinical activities (e.g., medication reconciliation) using telehealth technology for a clinical team (social worker, nurse practitioner) who conduct the visits remotely. Data used in this one-year pre-implementation evaluation included: periodic reflections with the clinical and evaluation staff, planning and interdisciplinary team meeting fieldnotes, and interviews with clinical team members. Data were summarized by selected constructs from the Consolidated Framework for Implementation Research; implementation challenges and problem solving were identified. Pre-implementation challenges occurred in: assuring assessment devices worked correctly for and were trusted by staff (e.g., connectivity, virtual stethoscope), technician fit with existing GRACE team, ensuring technician welfare (e.g., COVID-19 exposure, guns in the home), caseload balance amid staffing shortages, travel logistics, and sampling to adequately power the trial. Building on an existing strong team dynamic and a culture of feedback for quality improvement, challenges were addressed through pilot-testing, monitoring for barriers and impacts, and group reflecting conversations. Adaptations to the initial plan resulted in a more focused and targeted implementation effort to test the model and its effectiveness.

### IMPLEMENTATION OF LIFE STORY TECHNOLOGY IN RESIDENTIAL CARE SETTINGS: BENEFITS AND BARRIERS

Christa Wilk[1], Ashlee Cordell[1], Silvia Orsulic-Jeras[1], Sara Powers[2], Farida Ejaz[1], and Beth Sanders[3], *1. Benjamin Rose Institute on Aging, Cleveland, Ohio, United States, 2. The Benjamin Rose Institute on Aging, Cleveland, Ohio, United States, 3. LifeBio, MARYSVILLE, Ohio, United States*

Life story work is a promising intervention in residential care settings to engage persons with dementia (PWD) in meaningful conversation and promote person-centered care. Utilizing technology to streamline the life story collection process brought gerontologists and tech developers together to create LifeBio Memory™: an app which utilizes machine-learning and artificial-intelligence to collect life story data more efficiently than collection by hand. This presentation highlights focus group findings related to the benefits and barriers of this adaptation. Two waves (Time 1 and Time 2) of 60–90-minute (mean=80 SD=14.31) focus groups were conducted via Zoom nine months apart to evaluate the acceptability and feasibility of LifeBio Memory™. In Time 1 (n=35), community-dwelling PWD, residential care staff, and current/former LifeBio program users were asked to provide feedback on 1) their experience with the original version of LifeBio and 2) a prototype of the app. A thematic analysis yielded a list of recommended changes utilized to develop the app. In Time 2 (n=28), participants were asked to provide feedback on 1) training, 2) experience using the app, 3) implementation feasibility, 4) impact on person-centered care. A second thematic analysis was conducted. Across time 1 and 2, participants reported high levels of acceptability and feasibility for the LifeBio Memory™ concept and provided valuable feedback to the tech developers related to future implementation barriers of such technology. This presentation will discuss focus group findings related to 1) benefits of the adaptation, 2) barriers to using the tablet app, 3) implementation of the adaptation.

### PATTERNS OF SOCIAL COMMUNICATION IN MINORITY ADULTS: ASSISTIVE AND INTERACTIVE TECHNOLOGY USE DURING COVID-19

Akankshya Chataut[1], Sarah Hubner[1], and Julie Blaskewicz Boron[2], *1. University of Nebraska Omaha, Omaha, Nebraska, United States, 2. University of Nebraska, Omaha, Omaha, Nebraska, United States*

Literature suggests aging adults are more likely to be socially isolated, particularly given the COVID-19 pandemic. This has been associated with diminished well-being and increased morbidity and mortality. Assistive and interactive technologies (AITs) may help reduce isolation by bolstering communication. However, rapid advancement of AITs can create new barriers, especially for marginalized/underserved communities. The purpose of this study was to explore isolation and AITs in a majority-non-white sample. Data collected were part of a larger survey. Participants (N=80: Non-White=92.5%, Black=36.5%, Hispanic=36.5%) completed a Qualtrics survey via Amazon Mechanical Turk. Respondents included younger (YA: N=34, ages:19-34, M=27.7±4.6), middle-aged (MA: N=27, ages:35-49, M=41.9±4.1), and older (OA: N=20, ages:50-70, M=57.6±6.5) adults. Surveys consisted of likert-style and free-response questions. Content analysis with inductive coding was performed independently by two reviewers. Qualitative results suggested most adults used AITs regularly. Phone-based functions (texting/calling, smartphone-use) were favored for communication between 2020-2021. These functions were the largest group reported for all ages, although YA reported them more frequently (65%) compared to MA (48%) and OA (59%). Data also suggested that people had more in-person interaction at the time of survey completion (majority September 2021), compared to one-year previous (46.4%). Increased distanced socialization negatively correlated with overall socialization (r(78)=-.231, p=.05). Results suggest that although AITs are widely used, they may not adequately reduce isolation, particularly for those relying on them for primary contact. This may be especially true for underrepresented adults. Future research should investigate if this trend is universal and if existing AITs are sub-optimal in specific populations.

### A WHOLE NEW WORLD: EXPLORING VIRTUAL REALITY USE AND PERCEPTIONS WITH COMMUNITY-DWELLING OLDER ADULTS

Mary Milnamow, Emily Quinones-Visot, Lillian Agyemang, and Louanne Bakk, *State University of New York, University at Buffalo, Buffalo, New York, United States*

Virtual reality (VR) is an emerging technology that can facilitate and support active engagement and social interactions through shared immersive experiences. While limited evidence suggests VR can improve the health and well-being of older adults in residential and clinical care settings, research has not examined the potential benefits of a shared VR experience with community-dwelling older individuals despite their increased risk for social isolation. This exploratory, longitudinal study used a mixed-methods approach to assess the impact of VR programming among persons 65+ years of age with limited mobility attending a Program of All-Inclusive Care for the Elderly (PACE) and participating in a group-level VR intervention (n=9). Quantitative measures pertaining to health, well-being, social isolation, and comfort with technology were collected at baseline, six weeks, and twelve weeks. Qualitative, semi-structured focus groups were also conducted to assess participants' perceptions of the VR intervention. Paired sample t-test results indicated improvements over time in participants' pain level, well-being, and comfort in relation to using VR technology ($p <.05$). Qualitative data illustrated that participants were extremely receptive to using VR and found the experience pleasurable, realistic, and exciting. The most common themes suggested VR programming was regarded as a highly immersive, stimulating experience that can bolster camaraderie and positive feelings. Moreover, participants felt empowered by the experience. These findings demonstrate that utilizing VR programming with community-dwelling older adults can provide interactive and engaging experiences. The potential benefits of this intervention provide a basis for developing programming that can improve well-being and connectedness.

### DIGITAL PHENOTYPING TO UNDERSTAND HEALTH-RELATED OUTCOMES: A SCOPING REVIEW

Kyungmi Lee[1], Sidharth Kumar[2], Frank Puga[2], Andres Azuero[2], Arif Kamal[3], Carolyn Pickering[2], and J. Nicholas Dionne-Odom[2], *1. The University of Alabama at Birmingham, Hoover, Alabama, United States, 2. The University of Alabama at Birmingham, Birmingham, Alabama, United States, 3. Duke University, Durham, North Carolina, United States*

Modeling passive data collected by smartphones, or digital phenotyping, may help detect the early signs of declines in daily health and lead to steps to prevent declines. Health-related outcomes have been typically acquired through self-report measures, however this broad approach has several limitations, such as recall and social desirability bias. A potential solution is digital phenotyping. Guided by the Arksey and O'Malley methodological framework, the aim of this scoping review was to identify studies that modeled passive smartphone-sensor data into behavioral markers that correlate or predict health-related outcomes. A literature search of peer-reviewed or conference studies in PubMed, Scopus, Compendex, and HTA yielded 3,170 articles. 40 studies met the inclusion criteria. Studies were organized and assessed for their: 1) data collection approaches (e.g., smartphone passive sensor data and questionnaire), 2) feature extraction (e.g., converting bedtime or wake time into sleep), 3) data analytics (e.g., identifying accelerometer data into sleep patterns using machine learning), 4) behavioral markers, which is observing the activity pattern for an extended period of time and then modeling the activities into appropriate behaviors, and 5) health-related outcomes. The findings of this study demonstrate how to transform passive smartphone-sensor data into behavioral markers that correlate or predict health-related outcomes, which could provide information for more grounded predictive use and successful implementation of digital phenotyping in future research.

### TAILORED INTERNET-BASED SEXUAL EDUCATION FOR OLDER ADULTS: NEEDS ASSESSMENT AND ALGORITHM DEVELOPMENT

Liza Berdychevsky[1], Galit Nimrod[2], and Wendy Rogers[3], *1. University of Illinois at Urbana-Champaign, Champaign, Illinois, United States, 2. Ben-Gurion University of the Negev, Beer Sheva, HaDarom, Israel, 3. University of Illinois Urbana Champaign, Champaign, Illinois, United States*

Many older adults are sexually active, but ageism fuels the neglect of older adults' sexuality in research and sexual education. The purpose of this study was to (1) assess older adults' willingness to adopt technology tools to receive innovative, tailored sexual health education via the internet, (2) investigate their sexual health needs, and (3) contribute to development of an assessment tool and tailoring algorithm. Tailoring is a precision approach that matches educational content to users' needs instead of offering the same suboptimal one-size-fits-all content to everyone. We conducted an online survey using Qualtrics research panels of older adults ($N = 836$, with equal representation by gender and balanced representation in each 5-year cohort from age 60 to 80+). We found that 30% reported they were likely to try tailored internet-based education for older adults; an additional 34% were unsure. The results establish priority areas in sexual health needs, including behavioral patterns, knowledge gaps, health-related constraints, and attitudinal and communicational issues. These components provide the basis for the tailoring assessment questionnaire. Respondents also rated the personal relevance of 28 educational modules covering sex in later life (e.g., sexual ageism, sex as quality-of-life issue, sex-related communication, body image, health conditions, gender-specific issues, sexual attitudes, broadening sexual definitions and repertoires, sexual adaptations, inhibitions, sex aids/toys/robotics, medications/treatments, orgasm, self-stimulation/masturbation, dating, risk-taking, celibacy, LGBTQIA+, sexual rights, abuse). Statistical relationships between sexual health needs and personally relevant modules contribute to the development of a tailoring algorithm for creating educational bundles matched to older adults' unique needs.

### DIGITAL TECHNOLOGY USE AND SOCIAL CAPITAL: THE MODERATING EFFECT OF SOCIAL CLASS

Bomi Choi, Hayoung Park, Susanna Joo, Yoon-Myung Kim, and Hyoun K. Kim, *Yonsei University, Seoul, Seoul-t'ukpyolsi, Republic of Korea*

This study aims to elucidate the heterogeneous associations between digital technology use and social capital by social class. The sample comprises 315 Korean older people who are 65 years old or older and participated in an online survey. Digital technology use was measured by the frequency of independent use in four areas of digital technology: primary, cultural, economic, and public areas. Social capital was measured with ten items asking the perceived support availability from both online and offline relationships. Social class was measured with education, household income, and subjective social position to reflect both objective and subjective aspects of social class. Using SPSS 25 PROCESS Macro 3.5, linear regression with moderation analyses was performed. A simple slope and the region of significance were tested for a significant interaction. Results showed that subjective social position significantly moderated the relationship between digital technology use and social capital. The positive association between digital technology use and social capital was strengthened when the level of subjective social position was higher. Education and household income did not moderate the relationship between digital technology use and social capital. The results of this study indicate that the effects of digital technology use vary depending on perceived aspects of social class. This study also demonstrates that people with higher social classes enjoy more benefits from digitalization, supporting digital inequality among the older population.

### BOLSTERING SOCIAL CONNECTEDNESS DURING COVID-19: COMMUNICATION BETWEEN ESSENTIAL CONTACTS AND OLDER ADULTS

Joanna George[1], Sarah Hubner[2], and Julie Blaskewicz Boron[3], *1. Oklahoma State University College of Osteopathic Medicine, Tulsa, Oklahoma, United States, 2. University of Nebraska Omaha, Omaha, Nebraska, United States, 3. University of Nebraska, Omaha, Omaha, Nebraska, United States*

Social isolation may increase morbidity and mortality, particularly for aging adults. Research suggests that COVID-19 has significantly disrupted social networks, exacerbating isolation and risk. However, the extent of disruption and its implications for older adults and their essential contacts (ECs) is unknown. ECs, those who provide support/engagement to older adults, play a significant role in networks and help shape communication patterns. Understanding the effects of COVID-19 on social connectedness between ECs and older adults is vital to promoting their well-being. The purpose of this study was to investigate ECs' patterns of communication with adults aged 60+ during the COVID-19 pandemic , considering preferences, needs, and barriers. Self-identified ECs (N=546, Aged 19+, MAge=44.3±14.2) completed a Qualtrics survey via Amazon Mechanical Turk. Participants were ECs for community-dwelling (CDECs=57.3%) and institutionalized adults (IECs=42.7%). In addition to likert-style questions, the survey included free-response sections, examining communication quality, frequency, and method. Content analysis was conducted independently by two reviewers, using inductive coding. Qualitative results revealed distanced communication was widely utilized. Audiovisual/video communication was more frequently noted as desirable by IECs (32.8%) than CDECs (11.85%). Further, preference for audiovisual communication negatively correlated to expression of COVID-19-related barriers (IECs=12.5%, CDECs=7.7%, (r(546)= -.128, p=.01). Comparisons suggested that IECs reported absence of in-person contact with older adults more frequently than CDECs (recent contact=Never: 51% and 13.4% respectively). This may support the utility of video-communication to meaningfully supplement connectedness in the absence of in-person contact. The experiences described can reveal avenues for loneliness interventions and may guide future technology innovations.

### COMMUNICATION BETWEEN OLDER ADULTS AND ESSENTIAL CONTACTS: ISOLATION AND ASSISTIVE AND INTERACTIVE TECHNOLOGIES

Dinesh Karki[1], Sarah Hubner[1], and Julie Blaskewicz Boron[2], *1. University of Nebraska Omaha, Omaha, Nebraska, United States, 2. University of Nebraska, Omaha, Omaha, Nebraska, United States*

Essential contacts (ECs), people providing support/engagement to older adults, are vital to their physical and socio-emotional well-being. Research suggests that COVID-19 has significantly affected the connectedness of aging populations and their ECs, limiting interaction and potentially weakening support networks. Assistive and interactive technologies (AITs) may be useful for mitigating disruptions by facilitating communication. The goal of this study was to explore AIT preferences and needs of ECs for connecting with adults aged 60+. Participants completed a Qualtrics survey via Amazon Mechanical Turk. An initial sample (N=580,MAge=44.3±14.3,White=81%) was collected (December 2020-February 2021). A minority sub-sample (N=79,MAge=38.9±11.6,Non-White=87.3%) was subsequently accrued (September-December 2021). Participants were initial/minority-ECs for community-dwelling (initial-CDECs=57%,minority-CDECs=89%) and institutionalized adults (initial-IECs=43%,minority-IECs=11%). Minority respondents overwhelmingly identified as CDECs. Results revealed that recent ,"daily/often" phone-based, video-based, and in-person communication were all, more-frequently reported in minority-ECs compared to the initial-EC sample. Phone-based communication was most-frequent in minority-IECs (61%,versus initial-IECs=49%), but was relatively ubiquitous among all (minority-CDECs=49%,initial-CDECs=43%). Initial-CDECs had the least video-based contact (17%); the remaining groups were relatively similar (minority-CDECs=36%,minority-IECs=33%,initial-IECs=32%). CDECs reported the most in-person communication (minority-CDECs=70%,versus initial-CDECs=49%); IECs reported the least (minority-IECs=44%, versus initial-IECs=14%,). Results suggest that this minority sub-set may communicate more, respectively. This may be influenced by population and data-collection-time differences. Still, CDECs may have maintained better access to in-person communication and relied less-heavily on distanced networks through the pandemic. Ultimately, individual characteristics (i.e., ethnicity) and experiences (i.e., COVID-19) may drive communication patterns and end-user needs; this could inform innovation. Future investigation into these topics is warranted.

### LEVERAGING VIDEO CONFERENCING TECHNOLOGY TO FACILITATE SOCIAL ENGAGEMENT IN OLDER ADULTS

George Mois[1], Elizabeth Lydon[2], Shraddha Shende[3], Madina Khamzina[3], Dillon Myers[4], Raksha Mudar[3], and Wendy Rogers[3], *1. University of Illinois Urbana-Champaign, Urbana Champaign, Illinois, United States, 2. University of Illinois Urbana-Champaign, Champaign, Illinois, United States, 3. University of Illinois Urbana Champaign, Champaign, Illinois, United States, 4. OneClick, Philadephia, Pennsylvania, United States*

Social engagement is critical for maintaining well-being and quality of life in older adults. However, typical age-related changes across bio-psychosocial dimensions as well as age-related conditions such as mild cognitive impairment (MCI), pose many challenges for older adults to remain socially connected. The development of technologies, specifically video conferencing, can be leveraged as a tool to facilitate the delivery of social engagement opportunities, both enhancing access and minimizing social isolation. For successful adoption of these tools, the needs and preferences of older adult users must be considered. We employed an iterative research process for designing and developing a social-engagement intervention using a platform called OneClick.chat. We provide community-dwelling older adults with opportunities to engage in casual conversations and reminisce with others on topics of shared interests (e.g., nature, food, hobbies). Our iterative design process involved an interdisciplinary team of engineers, human factors specialists, gerontologists, and neuropsychologists. We also evaluated the system with eight participants (aged 50-64) who provided their insights pertaining to the content, delivery, perceived ease of use, usefulness, and potential adoption of this video-conferencing platform. Based on the feedback received, we have optimized the intervention in preparation for a randomized controlled trial. Furthermore, we provide key insights related to the implementation of social engagement through video technologies with the aim of facilitating social connectivity. Our learnings can be used to guide future work involving video-technology-based interventions to facilitate social connectivity for older adults with varying cognitive abilities.

### THE ASSOCIATION BETWEEN SUBJECTIVE AGE AND TECHNOLOGY USE AMONG OLDER ADULTS

Tomoko Ikeuchi[1], Tomoko Wakui[1], Sakiko Itoh[2], Hiroyasu Miwa[3], and Kentaro Watanabe[3], *1. Tokyo Metropolitan Institute of Gerontology, Tokyo, Tokyo, Japan, 2. Osaka University, Suita, Osaka, Japan, 3. National Institute of Advanced Industrial Science and Technology, Kashiwa, Chiba, Japan*

Background: Subjective age (SA) (i.e., felt age) has been found to be a biopsychosocial marker of aging. This study examined the associations between SA and frequency of technology usage of older adults.

Methods: Data were collected via an online survey conducted in 2020. The study analyzed participants aged 65 to 89 (M = 71.9, SD = 3.91) years resided in Japan (N = 1855, 54.3% women). SA was indexed by asking participants to specify in years how old they felt. Proportional discrepancy scores (PDS) ((SA - chronological age) / chronological age) were calculated to indicate younger or older SAs and used as an independent variable. Participants were asked about the frequency of computer, smartphone, flip phone, and SNS use.

Results: Nearly 90% reported using computers for more than 2-3 days a week, 64.3% smartphones, 22.9% flip phones, and 36.6% SNS. Logistic regression analyses revealed that lower PDS (i.e., feeling younger) was associated with a significantly higher frequency of smartphone use (OR: 0.75; 95% CI: 0.59, 0.96) after adjusting for age, gender, education, and subjective health. No such association was found for computer, flip phone, and SNS use. Implications: Older adults who use smartphones daily may feel younger than those who do not. Since the present study was administered during the COVID-19 pandemic, the daily use of smartphones may have helped older adults stay in touch with friends and family members and obtain information they need. The use of smartphones possibly contributed to better mental health outcomes while practicing social distancing.

### REMEMBER STUFF: A PILOT FEASIBILITY TRIAL OF DYADIC-FOCUSED TECHNOLOGY TO SUPPORT PEOPLE WITH ADRD

Sneha Manoharan[1], Matthew Schroeder[2], James Slaven[3], Anna Higbie[4], Mary Ellen Mullholand[5], and Nicole Fowler[6], *1. Regenstrief Institute, Inc, Indianapolis, Indiana, United States, 2. Regenstrief Institute, Inc., Indianapolis, Indiana, United States, 3. Indiana University, Indianapolis, Indiana, United States, 4. Regenstrief Institute, Indianapolis, Indiana, United States, 5. Eperture, LLC, Fishers, Indiana, United States, 6. Indiana University School of Medicine, Indianapolis, Indiana, United States*

Technology to support caregivers of people with Alzheimer's disease or related dementias (ADRD) with tasks may be the next frontier for caregiving research. This single-arm 90-day pilot trial tested the usability, feasibility, and acceptability of a software system called RememberStuff® (R/S) by Eperture. We also tested R/S's impact on caregiver burden. R/S includes a web-based portal where caregivers add information to a touch screen used by patients. R/S is organized around four main features– Calendar, Messaging, Activities, and Remember, a personalized task list. We collected data from dyads at baseline, 30-, 60-, and 90-days. Measures included the Healthy Aging Brain Care (HABC) monitor, System Usability Scale (SUS), and satisfaction scales indicating caregiver's willingness to use and behavioral intention. We approached caregivers of patients with ADRD seen in primary care clinics. Of the 469 participants reached, 278 (59.28%) refused, 156 (33.26%) were ineligible due to nursing home placement and 35 dyads were enrolled (7.46%). Among enrolled participants, 65.7% completed data up to 90 days. 73.5% of the caregivers lived with the patients. Mean caregiver age was 59.1 years; 67.7% are female; 88.2% are white. Willingness to use R/S was consistent across time from 30 days (M=3.9, SD=0.7) through 90 days (M=3.8, SD=1.1), while usability decreased (30 days M=57.7, SD=7.5 to 90 days M=54.9, SD 8.7). Caregiver's overall HABC monitor also decreased from baseline (M=29.0,

SD=13.1) to 90 days (M=27.2, SD=12.2) indicating less burden at 90 days. These findings support the feasibility of R/S technology to support caregivers of people with ADRD.

### SERIAL MEDIATION BY DIGITAL ASSISTANCE, INTERNET SELF-EFFICACY, DIGITAL CITIZENSHIP, AND UNMET NEEDS

Susanna Joo, Bomi Choi, Hayoung Park, Changmin Lee, Yoon-Myung Kim, and Hyoun K. Kim, *Yonsei University, Seoul, Seoul-t'ukpyolsi, Republic of Korea*

Although many social services are getting digitalized with the rapidly growing digital technology, there is little information available on the specific process of meeting personal needs in digital aging contexts. The present study examined the serial mediation model from digital assistant to unmet needs via internet self-efficacy and digital citizenship. An online survey was used to collect data in December 2021, and the sample included 223 older Korean adults aged 65 and above (M=68.79, SD=4.18, range=65-84) who owned at least one digital device. Unmet needs, the dependent variable, was the number of problems (e.g., housing, physical functioning) that participants responded to as 'unmet.' The independent variable was digital assistance, meaning getting help from friends or others when having problems with the digital device. The first mediator was internet self-efficacy, and the second mediator was five subtypes of digital citizenship (internet political activism, technical skills, local/global awareness, critical perspective, and networking agency). Covariates were gender, age, education, and income. SPSS Process Macro was utilized for serial mediation analysis. The result showed that only a serial mediation path from digital assistance to unmet needs via internet self-efficacy and internet political activism was significant. Additionally, a simple mediation path from digital assistance to unmet needs via internet political activism was significant. Technical skills, local/global awareness, critical perspective, and networking agency did not reveal significant mediational paths. Findings imply providing digital assistance system may be useful to reduce unmet needs among older adults by enhancing self-efficacy and political participation in the current digitalized world.

### A COMPARISON OF TRADITIONAL REMOTE MONITORING (RPM) DEVICES VERSUS A NOVEL, TOILET SEAT–BASED MONITORING DEVICE

Carla VandeWeerd, Erica Sappington, Lydia Poon, Chidi Okeahialam-Iheanyi, and Mitchell Roberts, *UFHealth Precision Health Research Center-The Villages, The Villages, Florida, United States*

As the aging population increases, older adults are disproportionately susceptible to cardiovascular disease(CVD). Routinely monitoring vital signs can alleviate CVD's burden. Current telemonitoring technologies targeting vital signs can be complex, generating inconsistent outputs and suboptimal adherence. In response, Casana developed the Heart Seat(THS), a passive toilet seat-based remote patient monitoring (RPM) device. This study aimed to compare older adults' (1) adherence between traditional telemonitoring(TT) devices and THS and (2) satisfaction across devices. A randomized control study (UFIRB202100869) was conducted in an active lifestyle retirement community-The Villages, Florida. Using a crossover design, participants (N=49) with CVD were followed for 12 weeks (8/12/2021-12/21/2021), beginning in TT or THS Arm and crossing over arms at 6 weeks. Adherence was calculated daily as ≥1 use for ≥90 seconds for THS, and ≥1 use of the blood pressure cuff and scale for TT. Descriptive statistics and t-tests were used to evaluate adherence rates and participant's experience and perception of RPM devices. Participants mean age was 73.4 years (SD=7.1,51% female). The majority (94%) of participants had previous experience using telemonitoring devices (e.g. scale, blood-pressure cuff, smartwatch) and 87.7% strongly agreed to feeling comfortable with at-home health monitoring. Overall, participants demonstrated greater adherence to THS than TT (t(48)=4.86, p<.001). These findings suggest older adults are becoming increasingly experienced and comfortable with at-home monitoring of health parameters. Overall, adherence was greatest in THS, suggesting that passive, easy-to-use telemonitoring devices may increase adherence, promote real-time health monitoring, and support adverse event prevention in older adults.

### INTERNET LITERACY FROM GRANDCHILDREN OR YOUNGER GENERATION: A NEW DIRECTION FOR GERONTECHNOLOGY

Ananya Sarker[1], Jeffrey Kahana[2], and Eva Kahana[1], *1. Case Western Reserve University, Cleveland, Ohio, United States, 2. Mount Saint Mary College, Newburgh, New York, United States*

Internet literacy offers a modern set of tools that can help older adults achieve the three pillars of successful aging, especially in the post-pandemic world. In the United States, older adults have shown significant growth in the usage of the internet and online services. However, information and social connectivity for older adults with lower incomes and education levels still lag. The current pandemic raises the public question of whether internet access and education should be deemed as a basic human right. Internet access is even more critical for older adults who need additional technology access and training, tailored to fit their varying experience, disability, and cognitive ability levels. The pandemic has demanded numerous technological skills for daily life: videoconferencing to carry on work, ordering groceries, taking exercise classes online, talking to family members and healthcare providers, and now registering for vaccination. Thus older adults need help with technology use more than ever. We need awareness of the "digital divide" and collaboration from designers, developers, services, products, and programs or interventions from various public and private sources. Identifying social and logistical barriers to technology literacy, we propose a conceptual model of using intergenerational training strategies to ameliorate the digital divide experienced by older adults. In this paper, we will review successful intergenerational programs like Students to Seniors, and Zoomers to Boomers, and introduce a similar model for internet literacy. The intergenerational mentoring model is advocated due to its wide-ranging potential benefits for both the older generation and the younger generation.

### UNDERSTANDING THE ADOPTION OF A MOBILE APPLICATION TO SUPPORT WORKFLOW OF HEALTHCARE AIDES

Christine Daum[1], Antonio Miguel-Cruz[2], Hector Perez[1], Emily Rutledge[1], Sharla King[2], and Lili Liu[1], *1. University of Waterloo, Waterloo, Ontario, Canada, 2. University of Alberta, Edmonton, Alberta, Canada*

Healthcare aides are unlicensed support personnel who provide direct care, personal assistance and support to persons living with health conditions. Workflow issues have a negative impact on health care aides' job satisfaction and quality of care. The implementation of information communication technologies could improve workflow. In collaboration with an industry partner, we developed a mobile application intended to support the workflow of health care aides who provide services to long-term care residents living with dementia. The purpose of this study was to investigate the technology acceptance and usability of a mobile application in a real-world environment when used by health care aides of a care facility. We used a sequential explanatory mixed methods approach. Our study included pre and post paper-based questionnaires with no control group (n=60). This was followed by two focus groups with a subsample of health care aides informed by qualitative description (n=12). We found: (a) acceptance of the mobile application was high; (b) usefulness was the strongest predictor of intention to use the mobile application, and (c) intention to use the mobile application predicted usage behaviour. Focus group findings supported the quantitative findings and highlighted participants' strong belief that the mobile application was useful, portable, and reliable. An area for improvement was user interface adjustments. Overall, these results support the assertion that our mobile application assisted health care aides in addressing their workflow issues and thus, has potential to improve the quality of care provided.

### SESSION 6400 (POSTER)

### TECHNOLOGY AND AGING

### EXPLORING COMMUNICATION DISPARITIES IN LATE OLD AGE THROUGH THE LENS OF COMMUNICATION INFRASTRUCTURE THEORY

Carrie Leach, and Thomas Jankowski, *Wayne State University, Detroit, Michigan, United States*

Health disparities are driven, in part, by communication disparities. Those who face the greatest health burdens often have the least communication resources to draw on to help them to manage and cope. As part of a multi-year, multi-level community engaged study to understand how to reduce the communication disjuncture between community-based service providers and older adults age 75 and older, we draw on data collected from 1,609 older adults through in-depth interviews (n=20) and a random sample survey (n=989) in one county in Michigan, along with a random digit dial telephone survey (n=600) conducted statewide. Findings point to key channels for disseminating information to hard-to-reach older adults being healthcare professionals and home delivered mail. Despite optimistic reports that technology usage and adoption rates are increasing for older users, findings suggest that old adults continue to face digital exclusion. Challenges with connecting through interpersonal networks are discussed. Limited resources for connecting with others through technology combined with increased social distancing due to the pandemic may exacerbate older adults' diminishing social networks and dwindling communication resources to draw upon for managing their health in later old age. Understanding how to connect with the hard-to-reach is pressing particularly as connecting via tele-health normalizes. This information may be employed to inform strategies for disseminating information to older adults, including those ages 75 and older who are hardest-to-reach, and likewise, outreach and recruitment by researchers to engage them in studies that aim to reduce health disparities.

### DOES TRUST IN SOCIAL SECURITY INHIBIT ACCEPTANCE OF ASSISTIVE TECHNOLOGY FOR ADL HELP?

Tomoko Wakui[1], Sakiko Itoh[2], Hiroyasu Miwa[3], Tomoko Ikeuchi[1], and Kentaro Watanabe[3], *1. Tokyo Metropolitan Institute of Gerontology, Tokyo, Tokyo, Japan, 2. Osaka University, Suita, Osaka, Japan, 3. National Institute of Advanced Industrial Science and Technology, Kashiwa, Chiba, Japan*

In 2000, Japan introduced a mandatory long-term care insurance program to facilitate aging-in-place of older care recipients; there has been a great demand for assistive technologies such as AI (artificial intelligence) and robots in care settings to reduce the burden of caregivers and long-term care costs in society. This study examined the relationship between the trust in social security and acceptance of assistive technology for ADL help and discussed the challenges in introducing technology in a well-developed social security system. An online survey was conducted in August 2020 among community-dwelling individuals aged between 40 and 89 across Japan to find out their acceptance of help provided via AI or robotics technology in five dimensions of ADL. In addition, a 5-point Likert scale was used to assess the trust in social security.A total of 4,047 respondents were analyzed in this study. The respondents' mean age was 60.6 (SD=11.3), and 53.2% of them were female. Of those, 13.2% preferred help from humans in ADL, while for 86.8%, the use of some assistive technology was acceptable. Logistic regression revealed that the female and younger respondents and those who had better health and had completed higher education were more likely to accept AI or robotics technology in all/some ADL if they needed assistance; those with higher trust in social security, however, were less likely to accept technology (OR=.894; p=.011).The challenges in introducing assistive technology under a well-developed social security system will be discussed.

### EXAMINATION OF THE DIGIAGE CYBER-SENIORS PILOT STUDY FOR OLDER ADULTS FROM LOWER INCOME COMMUNITIES

Skye Leedahl, Alexandria Capolino, Melanie Brasher, Kristin Fratoni-Souza, and Erica Estus, *University of Rhode Island, Kingston, Rhode Island, United States*

The University of Rhode Island (URI) team implemented a pilot program designed to bridge the digital divide between older adults and younger generations. Through this project, the goal was for older Rhode Islanders to become digitally literate by engaging them in a formal program that provides digital devices (i.e., Apple iPads), connectivity (i.e., internet connection through HotSpots), and training by supervised and trained university student mentors. This project specifically worked to promote social and economic equity by recruiting participants from lower-income communities and areas hit hardest by the COVID-19 pandemic, including those who speak English and Spanish. The final sample (N=124) for the study was primarily female (79%) and low income (82%). They were 41% Non-White and 23% Spanish speaking with 23% having a college degree or more. We conducted preliminary analysis on the first 38 participants who completed both a pre- and post-survey. Based on these analyses, the program participants have shown statistically significant improvements in frequency of tablet use (p<.05), digital competency across all questions (p<.05), quality of life (p<.05), and social isolation (p<.05). In addition, the loneliness and depression questions/variables are trending in the expected direction, so it is possible that with a more robust sample size we may see significant improvement in these areas as well. This presentation will describe the key elements of the pilot intervention and explain the findings from the full sample. The presentation will also discuss the plan for state-wide implementation of the program.

### THE EXPLORATION OF CORRELATES OF LONELINESS AMONG COMMUNITY-DWELLING OLDER PEOPLE'S TECHNOLOGY LITERACY

Suk-Young Kang[1], Jeungkun Kim[2], and Antonina Poplawski[3], *1. Binghamton University, State University of New York, Binghamton, New York, United States, 2. Kangnam University, Youngin, Kyonggi-do, Republic of Korea, 3. Binghamton University, Binghamton, New York, United States*

Social isolation is a major public health risk among older adults, even considered to be as harmful as smoking. The aim of this study was to explore the benefits of digital literacy and the correlates related to levels of loneliness on older people.Mail-survey data were collected from community-dwelling adults over the age of 65. There were 192 total participants, with information from 180 people being included in the analysis.The six-item De Jong Loneliness Scale was used to measure levels of loneliness. Among the 180 participants, the mean was 2.02 (SD = 1.68).Regression analysis revealed that the equation explained 48.2% of variance (adjusted R-squared = .446) in levels of loneliness (F (11,162) = 13.682, p <.000). Variance inflation factor (VIF) values were smaller than 10, indicating that multicollinearity among the correlates was not an issue. Correlates of levels of loneliness included aging anxiety, depression, social network, and self-evaluated technology literacy with computer software programs compared to peers. Results confirmed the correlates related to loneliness among older people identified in previous studies. This shows quality of life related to mental health may be improved by a positive attitude towards technology use.This study further found that digital literacy affects loneliness when controlling for income, depression, level of network, and physical health.Participants who could increase their digital literacy are interested in more opportunities on how to use new technology. Policy changes that allow for lifelong learning options could support this, while also decreasing levels of social isolation and loneliness.

### EXISTING KNOWLEDGE ASSOCIATED WITH SMART HOME HEALTH TECHNOLOGIES IN THE CARE OF OLDER PERSONS: A SYSTEMATIC REVIEW

Yi Jiao (Angelina) Tian[1], Nadine Andrea Felber[2], Félix Pageau[3], Delphine Roulet Schwab[4], and Tenzin Wangmo[2], *1. Institute for Biomedical Ethics, University of Basel, Basel, Basel-Stadt, Switzerland, 2. University of Basel, Basel, Basel-Stadt, Switzerland, 3. University of Laval, Quebec City, Quebec, Canada, 4. School of Nursing Sciences, University of Applied Sciences and Arts Western Switzerland, Lausanne, Vaud, Switzerland*

As a greater part of the global population reaches the golden age, smart home technologies are said to allow older persons to remain independent at a place of residence, or "home", of their own choice. Though their development has been making their way to the market, there has not been a systematic review of the empirical literature on the knowledge associated with their use for persons who are 65 years or older. Hence, we conducted a systematic review of empirical peer-reviewed English, German, and French articles in ten electronic databases. Data was textually described, separated into key characteristics, logged into a customized data extraction document, and analysed using narrative synthesis. The search across ten databases revealed 144 empirical papers that were admissible to our inclusion criteria. Of which, we discovered 5 first-order categories of benefits and 5 of barriers of smart home health technologies with further sub-themes that together form the concurrent array of existing knowledge. These categories included, for example, allows older persons to live independently at home, reminds older persons to promote self-care, and alternatively, concerns about usability, cost, and social acceptance. These systematically-derived categories of benefits and barriers could be a starting point for researchers interested in caregiving for older persons to conduct further empirical and reflective research. Furthermore, having this understanding of existing challenges and opportunities associated with smart home health technologies then allows the research and technical communities to collaborate upon a joint foundation to inform policy and improve caregiving for the global aging population.

### IMPACT OF OLDER ADULTS' INTERNET USE ON THE ECONOMIC BURDEN OF INFORMAL CAREGIVING

Jeehoon Kim[1], Heesoo Joo[2], and Sally Hageman[1], *1. Idaho State University, Pocatello, Idaho, United States, 2. University at Albany, Albany, New York, United States*

Objectives: Digital technology enables older adults to live independently by handling health-related tasks or shopping/banking online, thus can reduce care burden. This study examines the impact of older adults' Internet use on formal and informal care hours and its influence on estimating cost savings with active and potential Internet users.

Methods: Medicare beneficiaries ages 65 and above with functional difficulties were sampled from the 2015 National Health and Aging Trends Study (N=1,806). Care is measured by total hours for help older adults received in the past month, separated by formal and informal care. Internet use for health-related tasks and shopping/banking were measured. A survey weighted two-part model with gamma distribution was estimated. The cost savings of informal caregiving was estimated by a replacement approach.

Results: Older adults' health-related Internet use substantially reduced informal care hours by 27.7% (19.8 hours per month, $p < .05$), but not for formal care hours. A monthly estimated cost reduction of informal caregiving was $3,094 per an older adult with functional difficulties by applying 2020 median wage of home health aide workers. The annual savings in the cost of informal caregiving associated with older adults' health-related Internet use is estimated to be $8.1 billion with the active users and additionally $20.1 billion with the potential health-related Internet users in the US. Discussion: Promoting health-related Internet use among older adults with functional difficulties to relieve economic burden of informal caregiving will be discussed.

### DIGITAL LITERACY AND ASSOCIATED FACTORS IN COMMUNITY-DWELLING OLDER ADULTS IN SOUTH KOREA: A QUALITATIVE STUDY

Jiwon Baek[1], JiYeon Choi[2], Heejung Kim[1], Soyun Hong[1], Yesol Kim[1], Seongmi Choi[1], and Eunkyung Kim[1], *1. Yonsei University, Seoul, Seoul-t'ukpyolsi, Republic of Korea, 2. Yonsei University College of Nursing, Mo-Im Kim Nursing Research Institute, Seoul, Seoul-t'ukpyolsi, Republic of Korea*

Background: Digital literacy has gained growing importance in the health agenda. However, the level of digital information use and access among Korean older adults is still low at 54%. It is important to understand digital literacy among older adults to provide healthcare and social connectivity, especially during the COVID-19 pandemic. Objective: This study aims to explore the digital literacy and identify barriers to learning and using digital devices among community-dwelling older adults in urban South Korea.

Methods: A qualitative study was conducted using a semi-structured interview guide according to the DigComp 2.0 framework which emphasizes the competencies for full digital participation in five categories: information, communication, content creation, safety, and problem solving. Our sample consisted of 14 older adults (age 68 – 79, 12 women).

Results: Participants reported varying competency of using digital devices for search, communication, and self-management of lifestyle and health. They actively sought help from family and community members to troubleshoot issues related to installation, maintenance, setup software or applications. However, they were passive or evasive in the use of digital devices because of concerns about invasion of privacy or personal information. They struggled to use digital devices owing to physical and cognitive changes associated with aging.

Conclusion: Our findings depict current state and barriers of digital literacy in urban older adults in Korea.

### SESSION 6410 (POSTER)

### THE COST OF CARING: RISK AND REWARD

### CAREGIVER DEPRESSION AND PROGRESS TOWARD VALUES: AN AUTOREGRESSIVE CROSS-LAGGED PANEL MODEL

Jacob Gossner[1], Elizabeth Braungart Fauth[1], Joshua Novak[2], Michael Levin[1], and Spencer Bradshaw[1], *1. Utah State University, Logan, Utah, United States, 2. Auburn University, Auburn, Alabama, United States*

Acceptance and Commitment Therapy (ACT) is an established in-person therapy but can be provided self-guided online. Recently, online ACT has been adapted to support dementia family caregivers. A primary goal of ACT is to connect individuals with their self-identified values, with the assumption that increased progress towards values will lead to improved well-being. While there are established negative associations between depression and progress towards values, the lagged influence of progress toward values at one time point on another time point within intervention is less understood. Our sample was 51 dementia caregivers who completed an online self-guided ACT intervention. Participants were 80.3% female, 53% spouse, with a mean age of 65.98 (SD =11.66). We created an auto-regressive cross-lagged panel model to examine longitudinal associations between progress towards values and depressive symptoms at three time points. Controlling for time 1 (pre-intervention) depressive symptoms, progress towards values at time 1 significantly predicted depression at time 2 ($\beta = -.427$, $p < 0.01$) and progress towards values at time 2 ($\beta = .716$, $p < 0.001$). Depression at time 2 significantly predicted progress towards values at time 3 ($\beta = -.515$, $p < 0.001$) and depressive symptoms at time 3 ($\beta = .580$, $p < 0.001$). The R^2 range from .30-.43. Our analyses suggest that participants' initial progress toward values strongly negatively predicted their subsequent depressive symptoms, which then predicted later progress towards values. These findings demonstrate the potential reciprocal relationship between progress towards values and depressive symptoms within ACT intervention.

### CAREGIVING STRESSORS AND MENTAL HEALTH AMONG OLDER DEMENTIA CAREGIVERS: THE MEDIATING ROLE OF LIFE DISRUPTION

Fei Wang, and Elliane Irani, *Case Western Reserve University, Cleveland, Ohio, United States*

Providing care to a loved one with dementia can take a mental toll on older adult caregivers who struggle to balance their caregiving duties and self-care. However, few caregiving studies focus on older adults as caregivers. Further, the extant research mainly investigates stressors directly related to the demands of caregiving without accounting for the disruptions in different areas of life triggered by dementia caregiving. Guided by the Stress Process Model, we examined how life disruption mediates the association between caregiving stressors and caregiver mental health. A total of 360 older adult caregivers (age 65 or above) of individuals with dementia (age 65 or above) in the U.S. were selected from the 2017 National Study of Caregiving. An index of life disruption was created based on seven types of life challenges due to caregiving, such as constriction of social life and job-caregiving conflict. Results from a path analysis show that both objective and subjective caregiving stressors (i.e., care assistance and role overload) were positively associated with life disruption, which in turn, were positively associated with depressive symptoms and anxiety. Caregiver socio-demographics and self-rated health were included in the analysis as covariates. These findings inform a novel clinical assessment framework for practitioners to identify the unique challenges faced by older dementia caregivers in the U.S. Moreover, this study elucidated life disruption as a modifiable factor to mitigate mental health problems in this population. Future research should compare the effects of different types of life disruption on mental health among older dementia caregivers.

### UNDERSTANDING THE PREDICTIVE FACTORS THAT INFLUENCE THE PERCEIVED BURDEN OF DEMENTIA CAREGIVERS

Kaitlyn Lucas[1], and Katherine Judge[2], *1. Cleveland State University, Westlake, Ohio, United States, 2. Cleveland State University, Cleveland, Ohio, United States*

Burden is a significant negative outcome for caregivers of individuals with dementia. Guided by the Stress Process Model, the following study examined the impact of several novel constructs on burden including: compassion for others, family functioning, coping styles, and positive and negative affect. Data were gathered through Amazon Mechanical Turk from 102 dementia caregivers. Participants were 62% female, 72% Caucasian with a mean age of 39.5 years old. Burden was significantly related to positive affect ($r = -.31$, $p = .01$), negative affect ($r = .56$, $p = < .01$), family functioning ($r = .43$, $p < .01$), and dysfunctional coping ($r = .37$, $p < .01$). Compassion for others was not correlated with burden. Multiple regression analysis results found the set of predictors accounted for 65.0% of the total variance in burden ($F(5,96) = 14.17$, $p < .01$, $R2 = .65$), with positive affect ($\beta = -.26$, $p = .01$), negative affect ($\beta = .19$, $p = .05$), and dysfunctional coping ($\beta = .31$, $p = .01$) predicting significant and unique variance. Results from mediation analyses indicated dysfunctional coping mediated the relationship between positive affect and burden (effect= -.14 95% bootstrap CI= -.26, -.04) as well as the relationship between negative affect and burden (effect= .24, 95% bootstrap CI= .07, .48). A mediating relationship between dysfunctional coping and family functioning and burden was not supported. Discussion will highlight the implications of the study findings in developing innovative caregiver programs aimed at reducing dysfunctional coping and the broader theoretical implications of the role of affect in family caregivers of individuals with dementia.

### SUFFERING BUT SATISFIED: OLDER ADULTS' LIFE SATISFACTION WITHSTANDS DEPRESSION AND DEMENTIA

Samuel Van Vleet[1], Danica Bass[2], Yenifer Morales Mejia[2], Jeanné Dube[2], Brandee McKinney[2], Kailee Prentice[2], Logan Guillory[2], and Michael Barnett[2], *1. Miami University, Oxford, Ohio, United States, 2. The University of Texas at Tyler, Tyler, Texas, United States*

Among older adults with neurocognitive disorders, lower life satisfaction has been linked with poorer aspects of physical and mental health that affect quality of life, including depression. However, these associations between life satisfaction and mental health problems have not been found universally. In this study, we further investigated life satisfaction and depression within clinical and healthy older adult populations. Older adults ($N = 98$), ages 60 to 90 years either with ($n = 25$) or without ($n = 73$) a neurocognitive diagnosis, were administered self-report questionnaires measuring depression and life satisfaction. Results showed that although depression was negatively correlated with life satisfaction among both samples, older adults with dementia had higher depression scores but equivalent life satisfaction scores as older adults without dementia. In fact, both the clinical and healthy samples of older adults averaged on the positive or satisfied half of the life satisfaction scale. Older adults with neurocognitive disorders may generally experience higher depressive symptoms, yet despite challenges to quality of life, they seem to find satisfaction in their daily lives. This may stem from older adults viewing current depressive symptoms as a relatively short-term problem while evaluating life satisfaction across their whole lives. The best protective factors for older adults suffering from dementia and depression may therefore be found both in their present and in their past.

### SESSION 6411 (POSTER)

### COGNITION AND ALZHEIMER'S DISEASE

### ATIP LEVELS ARE ASSOCIATED WITH LESS AMYLOID-B BURDEN IN POSTMORTEM BRAINS OF OLDER ADULTS WITH ALZHEIMER'S DEMENTIA

Caglar Cosarderelioglu[1], Alma Plaza-Rodriguez[2], Pablo Iglesias[1], C. Conover Talbot[1], D. Brian Foster[1], Robert N. Cole[1], Jeremy Walston[1], and Peter M. Abadir[1], *1. Johns Hopkins University, Baltimore, Maryland, United States, 2. Krieger School of Arts and Sciences, Baltimore, Maryland, United States*

Angiotensin system, aging, and Alzheimer's disease are tightly linked. Of the brain angiotensin receptors, the subtype 2 receptor (AT2R) is relatively less studied. Canonically, the AT2R functions through nitric oxide release, and its activation has been linked to vasodilatation and neurite outgrowth as well as anti-inflammation. How AT2R signals is not known, however, an AT2R-binding protein (ATIP) has been recently described. Controversies exist on the link between ATIP and AT2R functions. Here, we describe the development of the first non-antibody based ultra-sensitive and specific quantitative mass spectrometry assay for ATIP. Using a technique that permits targeted analysis of multiple peptides across multiple samples in a single mass spectrometry run, known as TOMAHAQ, we have identified specific human tryptic peptides that permit quantification of ATIP abundance. We have used this method to quantify ATIP in postmortem frontal cortex samples of older adults (n= 60) with Alzheimer's dementia (AD). We correlated levels of ATIP to brain RAS receptors, and biomarkers of AD pathogenesis including oxidative stress, inflammation, mitochondrial dysfunction as well as amyloid-β, and tau burden. Our results show that ATIP expression (ANLKNPQIMYLEQELESLK sequence of ATIP) is positively correlated with neuronal nitric oxide synthase (nNOS) ($p = 0.009$, $r = 0.337$). Furthermore, expression of ATIP is negatively correlated with amyloid-β load in several brain regions including hippocampus ($p= 0.014$, $r= -0.317$), entorhinal cortex ($p= 0.010$, $r= -0.331$), frontal cortex ($p= 0.023$, $r= -0.294$), and overall ($p= 0.004$, $r= -0.365$). These results highlight a potential protective role for ATIP in Alzheimer's disease.

### IS CEREBRAL AMYLOID ANGIOPATHY A PRECIPITATING EVENT IN THE ETIOLOGY OF ALZHEIMER'S DISEASE

Steven Edland[1], Chris Zarow[1], Benjamin Shifflett[2], Margaret Flanagan[3], and Lon White[4], *1. University of California, San Diego, San Diego, California, United States, 2. University of California, San Diego, La Jolla, California, United States, 3. Northwestern University, Evanston, Illinois, United States, 4. Pacific Health Research and Education Institute, Honolulu, Hawaii, United States*

Amyloid plaque and cerebral amyloid angiopathy (CAA) are early neuropathologic changes observed in the setting of clinical Alzheimer's disease (AD), typically preceding biomarker evidence of intraneuronal tau neurofibrillary tangle (NFT) development and associated cognitive decline. Here we apply structural equation models to explore the potential causal associations between amyloid-beta lesions (i.e. plaques and CAA) and NFTs as they affect cognitive performance in the elderly.Brain autopsy data used in this study are from the Honolulu Asia Aging Study (HAAS) of Japanese American men from the state of Hawaii (n=600), and the Nuns Study (NS) of Roman Catholic Sisters largely from the upper midwestern United States (n=378). Cognitive performance within three years of death was assessed by multi-domain dementia rating scales.We found that neocortical neuritic plaque and CAA effects on cognitive performance were mediated by neocortical NFTs for both the HAAS ($p < 0.002$) and NS ($p < 0.043$). There were no direct effects of neuritic plaque and CAA after controlling for the mediating effect of NFTs ($p > 0.54$).These data are consistent with the hypothesis that the association between neuritic plaque and CAA on cognition is mediated by NFT load. This may inform our understanding of the etiology of NFT lesion pathology in aging and AD. The disruption of arterial smooth muscle function by CAA has predictable effects on glymphatic processes and on the regulation of capillary blood flow. These effects are plausibly relevant to the AD neurodegenerative process, and these pathways deserves continued attention by the AD research community.

### DETECTION OF PRECLINICAL AND PRODROMAL ALZHEIMER'S DISEASE USING A MULTIDISEASE DIAGNOSTIC PLATFORM

Cassandra DeMarshall[1], Jeffrey Viviano[1], Sheina Emrani[2], George Godsey[1], Abhirup Sarkar[1], Benjamin Belinka[1], David Libon[2], and Robert Nagele[2], *1. Durin Technologies Inc., Mullica Hill, New Jersey, United States, 2. Durin Technologies Inc., Mullica Hill, New Jersey, United States, 2. Rowan University School of Osteopathic Medicine, Stratford, New Jersey, United States*

AD-related pathological changes begin in the brain long before symptoms emerge. In the present study, we demonstrate the utility of a panel of AD-related autoantibodies capable of detecting AD at the earliest points along the AD continuum, including preclinical AD, years before the onset of symptoms, and prodromal AD (mild cognitive impairment, MCI). Using a customized panel of AD-specific autoantibody biomarkers and Luminex xMAP® technology, sera from ADNI subjects with preclinical AD or MCI were screened to demonstrate preclinical and prodromal AD detection. A panel of eight autoantibodies with increased titer in MCI and preclinical AD relative to controls was evaluated using Random Forest and Receiver Characteristic Operating curves for their ability to distinguish diseased subjects from age- and sex-matched controls, as well as from individuals with other neurodegenerative and non-neurodegenerative diseases. Results showed that this panel of biomarkers was capable of differentiating patients with MCI from age- and sex-matched controls with high overall accuracy, sensitivity, and specificity. These biomarkers also identified cognitively normal subjects who later converted to MCI and AD. Furthermore, this autoantibody biomarker panel distinguished MCI and preclinical AD subjects from Parkinson's disease and breast cancer subjects, demonstrating excellent disease specificity. Results demonstrate the utility of our blood-based autoantibody biomarker panel as an accurate, non-invasive, and inexpensive diagnostic screener, not only for the detection of prodromal AD, but also the earlier, preclinical stages of AD pathology. This multi-disease diagnostic platform has been demonstrated to be useful for multiple neurodegenerative diseases including AD, Parkinson's disease and Multiple Sclerosis.

### INTEGRATIVE MULTI-OMICS ANALYSIS REVEALS THE CRITICAL ROLE OF PBXIP1 GENE IN AGING-RELATED ALZHEIMER'S DISEASE

Zuyun Liu, Jingyun Zhang, and Xiaoyi Sun, *Zhejiang University School of Medicine, Hangzhou, Zhejiang, China (People's Republic)*

Alzheimer's disease (AD) is a neurodegenerative disorder, and its strongest risk factor is aging. A few studies have explored the relationship between aging and AD, while the underlying mechanism remains unclear. We assembled data cross multi-omics (i.e., epigenetics, transcriptomics, and proteomics, based on frozen tissues from the dorsolateral prefrontal cortex) and neuropathological and clinical traits from the Religious Orders Study and Rush Memory and Aging Project (ROSMAP). Aging was assessed using six epigenetic clocks (including Horvath clock, Hannum clock, Levine clock, Horvathskin clock, Lin clock, and Cortical clock) that capture mortality risk in literature. After accounting for age, we first identified a gene module (including 263 genes) that was related to most epigenetic clocks (e.g., $P=3.61\times10{-}5$ for Levine clock) and three neuropathological traits of AD (i.e., β-amyloid, Tau tangles, and tangle density). Interestingly, among 20 key genes with top intramodular connectivity of the module, PBXIP1 was the only one that was significantly associated with all three neuropathological traits of AD at the protein level after Bonferroni correction. Furthermore, PBXIP1 was associated with clinical diagnosis of AD in both ROSMAP and two independent datasets. The results suggest the critical role of PBXIP1 in aging-related AD and support the potential and feasibility of using multi-omics data to investigate mechanisms of complex diseases. However, more validations in different populations and experiments in vitro and in vivo are required in the future.

### LOCI RESPONSIBLE FOR RACIAL DISPARITY BETWEEN WHITE AND BLACK AMERICANS IN ALZHEIMER'S DISEASE

Stanislav Kolpakov Nikitin, and Igor Akushevich, *Duke University, Durham, North Carolina, United States*

We tested the following genotype-related mechanisms generating race-dependent disparity in Alzheimer's Disease (AD) risk: i) the frequencies of SNPs with genotypes associated with AD are higher in the Black subpopulation; ii) the effects on AD risk of genotypes associated with increased AD risk is higher in the Black subpopulation; iii) there is a small group of SNPs with a large difference in the effects on AD race-dependent risk generating disparities; alternatively, the disparities are generated by a collective effect of multiple SNPs with minor or moderate effects. We modified the GWAS algorithm to use it with the Cox regression multivariable model and the outcome accounting for race-related differences in AD risk. Using the modified GWAS we have identified loci in charge for the disparity between Whites and Blacks and used the results along with SNPs of a special interest which were identified from the literature in the Cox multivariable regression model and generalized Oaxaca-Blinder approach. The following genes had the strongest effects on racial disparities: i) NYAP1 neuronal tyrosine-phosphorylated phosphoinositide-3-kinase adaptor; ii) RPA3-UMAD1 loci RPA3 replicates protein A, interacting with gastric tumor and hepatocellular carcinoma; iii) TOMM40 associated with an increased risk of developing late-onset AD; iv) ACE - responsible for making the enzyme which converts angiotensin which regulates blood pressure; v) PSME3IP1 - proteasome activator subunit 3 interacting protein 1; vi) MARCHF1 regulator of glucose-tolerance and lipid storage and vii) LINC01146 which belongs to coagulation cascade pathway and linked to hepatocellular carcinoma.

### A NOVEL FRAMEWORK LINKING MNEMONIC AND HIPPOCAMPAL INTEGRATION TO LATE-LIFE REAPPRAISAL EFFICACY

Bruna Martins-Klein[1], Irina Orlovsky[2], and Kristin Heideman[3], *1. University of Southern California, Los Angeles, California, United States, 2. University of Massachusetts Amherst, Amherst, Massachusetts, United States, 3. Yale Child Study Center, New Haven, Connecticut, United States*

Socioemotional theories suggest that surviving challenging experiences enhances emotional resilience with age, yet the role of memories is overlooked in most models of emotion regulation. In parallel, cognitive accounts focus on age-related memory deficits associated with overlapping hippocampal neural representations across unique memories (neural dedifferentiation). We propose a novel framework supporting enhanced late-life reappraisal via hippocampal dedifferentiation of memory representations across current and past experiences. We review classic studies supporting mood benefits from integrated positive narratives following adverse autobiographical events. We also discuss multivariate neuroimaging evidence supporting overlapping hippocampal representations and ventromedial prefrontal cortex (vmPFC) involvement in meaning-making processes. We posit that greater hippocampal dedifferentiation across life memories may facilitate associative binding of current and past stressors as well as reappraisals in vmPFC. This process may provide avenues for generalizing past reappraisals to novel contexts and reducing cognitive demands of reappraisal. In addition, we discuss the possible age-related facilitation of this process, as a greater number of life experiences may become increasingly integrated with one another over the lifespan. These integrated neural associations may serve to make reappraisals from the past more readily accessible and applicable in new contexts over time, increasing routes to positive narratives following stress. We discuss future directions for testing components of the proposed model using multivariate neuroimaging methods. We conclude by briefly reviewing the possible clinical impact of mnemonic emotion regulation in promoting emotional well-being among older adults, using a strengths-based approach that leverages wisdom from experience and neural processes facilitated with age.

### SPATIALLY RESOLVED MAPPING OF CELL-SPECIFIC SENESCENT PROFILES IN AGING MOUSE BRAIN

Chase Carver, and Marissa Schafer, *Mayo Clinic, Rochester, Minnesota, United States*

Cellular senescence is a conserved mechanism of aging characterized by cell cycle arrest and secretion of a senescence-associated secretory phenotype comprised of proinflammatory cytokines, chemokines, proteases, and growth factors that disrupt cellular homeostasis by promoting sterile inflammation and aberrant tissue remodeling. Tissue microenvironment, induction mechanism, and duration since senescence onset contribute to senescent cell heterogeneity, making identification challenging. Senescent cell contribution to age-related cognitive decline suggests that the hippocampus may be a key brain region for uncovering functional identities of senescence. Therefore, our goal is to contextually and spatially map senescent cells in comparison of cell-type specific morphological, transcriptional, and functional features that distinguish them among highly-ordered brain microenvironments. GeoMx Digital Spatial profiling was used to investigate cell identity and senescence markers in the aging brain to develop transcriptomic profiles from 22-24-month old mice. We also adapted Imaging Mass Cytometry with a curated panel of antibody-labeled proteins in fixed brain sections. We observed that senescent-like phenotypes of cells from myeloid and non-myeloid origin accumulate in the hippocampus and cortex, whereas CD45+ immune cells primarily accumulate in the white matter regions, in close proximity to microglia and GAL3+ cells. We profiled differences in ramified and ameboid microglia, in comparison of young and old mice, with emphasis on the chemotactic microenvironment involving activation of inflammatory signals. These advanced, multiplex imaging modalities enable in-depth characterization of senescent cell features in the aging brain. Beyond morphological profiling, we demonstrate enhanced resolution and detection of individualized senescence in the aging brain.

### HOW INTERPLAY BETWEEN INFLAMMASOME AND MIRNA IS ASSOCIATED WITH ALZHEIMER'S DISEASE

Anatoliy Yashin[1], Deqing Wu[1], Konstantin Arbeev[1], Alexander Kulminski[1], Eric Stallard[1], Jonas Mengel-From[2], Olivia Bagley[1], and Svetlana Ukraintseva[1], *1. Duke University, Durham, North Carolina, United States, 2. University of Southern Denmark, Odense, Syddanmark, Denmark*

The results of recent analyses showed that neuroinflammation—a key contributor to Alzheimer's disease (AD)—is activated by protein complexes known as inflammasomes. The NLRP3 inflammasome protein complex is mostly studied in the central nervous system; it includes a protein encoded by the NLRP3 gene. Connections between inflammasomes, neuroinflammation, and AD may be regulated by small non-coding RNAs called microRNAs. These molecules have multiple ways to influence AD. For example, microRNA miR-107 may influence AD through regulation of the BACE1 gene expression involved in Aβ production. It remains unclear whether interplay of miR-107 and NRLP3 is associated with AD and whether mechanisms of such interplay differ among participants of LLFS and other studies. To address these questions, we evaluated associations with AD of interactions between genetic variants (SNPs) located in DNA encoding miR-107 and NLRP3 in participants of the LLFS and Framingham Heart Study using a logistic regression model with an interaction term. The facts that both datasets deal with data on related individuals and that many SNPs from either miR-107 or NLRP3 are in linkage disequilibrium were accounted for in these studies. The analysis of the Framingham data showed that the association with AD of the interaction between SNPs from miR-107 and the NLRP3 gene was statistically significant(p=6.71E-03; Bonferroni correction=7.14E-03). The association of the same interaction with AD was replicated in the analysis of LLFS data. These results indicate that in addition to regulating BACE1 expression and Aβ production, miR-107 may influence AD in interaction with the NLRP3 gene.

### SLEEP IS REQUIRED FOR ODOR EXPOSURE TO CONSOLIDATE MEMORY AND REMODEL OLFACTORY SYNAPSES

Rashmi Chandra[1], Fatima Farah[2], Fernando Muñoz-Lobato[1], Anirudh Bokka[2], Kelli Benedetti[1], Fatema Saifuddin[1], Miri VanHoven[2], and Noelle L'Etoile[1], *1. University of California, San Francisco, San Francisco, California, United States, 2. San Jose State University, San Jose, California, United States*

Olfactory dysfunction precedes dementia in several neurodegenerative disorders such as Alzheimer's disease (AD) or Parkinson's Disease (PD), and AD/PD are associated with progressive sleep abnormalities. However, how sleep affects cognitive performance remains unclear, perhaps due to the complexities of the human nervous system. Here we demonstrate that the transparent model organism C. elegans which has well defined neural connection sleeps after repeated odor trainings. This provides us with a platform to dissect how sleep affects memory at a synaptic resolution. We identified that sleep after training is required for the animal to retain a long-term memory of the odor. We found that if animals do not sleep in the first two hours after training, memory is not consolidated. After identifying the neurons that are required for the memory, we show that the sensory-interneuron connections within the circuit are downscaled after sleep. Therefore, we found a time-specific requirement of sleep that modulates synaptic downscaling to preserve memory. Conversely, lack of sleep post-training erases the long-term memory and destabilizes the synaptic downscaling, indicating that modulating the amount of sleep is sufficient to modulate memory. These results make C. elegans an excellent tool to ask what molecular mechanisms, cell biological processes and circuit level reorganizations are engaged during sleep to promote memory. This understanding will provide insights into the functions of sleep that affects cognitive performance in neurodegenerative diseases.

### SLEEP, COGNITION, AND BRAIN FUNCTION IN OLDER ADULTS WITH CHRONIC KIDNEY DISEASE AND MILD COGNITIVE IMPAIRMENT

Thanakrit Jeamjitvibool, Mary Hannan, and Ulf Bronas, *University of Illinois Chicago, Chicago, Illinois, United States*

Older adults with chronic kidney disease (CKD) are at risk for cognitive impairment and commonly report sleep disturbances. The association between sleep and brain function remains unclear. This cross-sectional study investigated the relationship between sleep, cognition, and brain function in older adults with CKD and mild cognitive impairment (MCI). Older adults with CKD and MCI (n=37, age=68 + 4.93, eGFR=43.7 + 10.98, 73%=Black, 70%=Female), were evaluated via a comprehensive cognitive function battery and brain MR-imaging. Seven-day actigraphy was scored utilizing the Cole-Kripke algorithm to evaluate sleep quality and duration. Ordinary least square regression controlling for age and education quality indicated that >9 hours of sleep, as compared to < 7 hours of sleep, was associated with worse executive function (unstandardized beta=-6.86, 95% CI:-13.19, -0.53, p=.04). Less sleep (< 8 hours) was associated with better learning/memory (unstandardized beta=2.61, 95% CI: 0.84, 4.38, p=.01). Better sleep efficiency was associated with increased global cerebral blood flow (unstandardized beta=3.30, 95% CI: 0.65, 5.95, p=.02). Longer sleep time (>9 hours), as compared to shorter sleep time (< 7 hours), was associated with worse mean diffusivity (unstandardized beta=0.12, 95% CI: 0.03, 0.20, p=.01). Shorter sleep time (< 8 hours, compared to >=8 hours) was associated with better fractional anisotropy of the cingulum (unstandardized beta=0.03, 95% CI: 0.01, 0.05, p=<.001). Sleep duration and quality may be related to cognition and brain function in older adults with CKD and MCI providing a possible avenue for interventions to improve cognitive outcomes in this population.

### NEUROCORRELATES BETWEEN THEORY-OF-MIND AND BILINGUALISM IN GRAY MATTER VOLUME OF YOUNG AND OLDER ADULTS

W. Quin Yow[1], Xiaoqian Li[1], Kwun Kei Ng[2], Janice Jue Xin Koi[2], and Juan Helen Zhou[2], *1. Singapore University of Technology & Design, Singapore, Singapore, 2. National University of Singapore, Singapore, Singapore*

The experience of being bilingual may accrue cognitive reserve against age-related declines in older adults. Early bilingualism, e.g., second language age-of-acquisition (L2AoA), has been shown to be associated with better theory-of-mind (ToM) performance in older adults (Yow et al., 2021). Here, we aim to understand the brain structural correlates associated with bilingualism and ToM performance in normal aging. Forty-six young (YA, aged 19-30, M=21.87) and 51 older adult bilinguals (OA, aged 54-77, M=63.61) completed 1) ToM assessments, where they viewed vignettes and answered questions about the protagonists' mental states, 2) an anatomical MRI scan, 3) a demographic questionnaire including L2AoA and years of education, and 4) a general cognitive ability assessment by the Montreal Cognitive Assessment (MoCA) test. As expected, ANCOVA on ToM composite scores revealed a significant main effect of age – YA showed better ToM performance than OA, F(1,93)=9.48, p=.003, controlling for education and MoCA scores. Importantly, MRI data were preprocessed to obtain gray matter volume (GMV), proxy of neuronal density, of 430 brain regions. Partial least square correlation analysis identified one significant multivariate pattern linking individual differences in GMV with ToM score and L2AoA (48.7% covariance explained, p=.014). Regardless of age, larger GMV in several regions including prefrontal, frontal, medial temporal, and superior temporal cortices were associated with earlier L2AoA (p=.003) and higher ToM score (p=.004), indicating shared variance between ToM and L2AoA in brain morphology. Findings suggest that earlier bilingual acquisition might promote brain maturation that would preserve ToM ability well into later stages of life.

### SESSION 6420 (POSTER)

### TELEHEALTH, TECHNOLOGY, AND DEVICES

### DEVELOPMENT OF A MOBILE INTERVENTION TO SUPPORT HEALTHY EATING IN PERSON WITH CO-OCCURRING FRAILTY AND DEMENTIA

Oleg Zaslavsky, Kuan-Ching Wu, Shao-Yun Chien, and Kimiko Domoto-Reilly, *University of Washington, Seattle, Washington, United States*

New behavioral solutions are needed to improve health in persons with co-occurring frailty and dementia. Using dementia-specific principles of human-centered design, we developed a mobile intervention that includes a patient-facing app and clinician interface to promote a Mediterranean-style eating plan in this population. Our design processes were as follows. We first solicited iterative input from experts in dementia, accessible design, and technology concerning language, cognitive load, and overall accessibility needs in persons with early dementia. Next, we recruited seven people with mild dementia and their partners for interviews, experience sampling, and usability studies. Our primary findings were: 1) participants have basic knowledge of healthy eating tenets and want to learn more; 2) participants are unaware of anything specific to improve in their diet; 3) participants value simple meals with few ingredients; 4) participants strongly rely on physical cookbooks. Based on the results, we developed a high-fidelity prototype of the patient-facing mobile app, divided into tasks: 1) First time onboarding; 2) Setting the first dietary goal; 3) Finding a recipe; 4) Filter/sort for recipes; 5) Food tracking tool; 6) Mastering a dietary goal and starting a new one; 7) Reviewing progress. The resultant product was iterated in usability studies and informed a final prototype for further testing. In parallel, a web-based clinician interface was developed through individual interviews, a survey of potential end-users (n=24), and two rounds of usability studies (n=3 in each round). The online clinician product features these interactive modules: dashboard, tracking manager, patient portal, and individual patient page

### ELECTIVE TELEMEDICINE: ARE OLDER WOMEN IN THE POSTPRIMARY TREATMENT PHASE OF EARLY BREAST CANCER INTERESTED?

Caroline Buse, Kirsten Nyrop, Erin O'Hare Kelly, and Hyman Muss, *UNC Chapel Hill, Chapel Hill, North Carolina, United States*

Explosive growth in the use of telemedicine occurred during the COVID-19 pandemic. Telemedicine has the potential to lessen healthcare burden for older adults with frequent appointments, physical and cognitive disabilities, and reliance on caretakers. To benefit from telemedicine, patients must have the capacity to engage with technology, for which inexperience and access may pose barriers. This study aimed to better understand the perspectives of older women with non-metastatic breast cancer on telemedicine, in regards to visit convenience, completeness, and interpersonal satisfaction. In this qualitative study, semi-structured interviews were conducted in a convenience sample of women age 65+, post-primary treatment for Stage I-III breast cancer, who received in-person outpatient care at NC Cancer Hospital before transitioning to telemedicine after March 2020. Patients were interviewed about their perceptions of telemedicine (telephone, video) as compared to in-person visits. Audio files of interviews were transcribed and reviewed to identify themes established a priori in the interview protocol. 15 patients (telephone=5, video=10) were consented and interviewed (July-October 2021), mean age=74. 13/15 participants reported that they preferred a hybrid care model that included telemedicine care over in-person care alone. COVID-19, physical disability, and transportation burden were associated with telemedicine preference. Comfort with familiar patient-provider interaction and lack of physical exam were associated with in-person appointment preference. Patient-clinician conversations and clinic protocols guiding use of telemedicine should take into account newness of diagnosis, patient comfort and familiarity with the care team, travel burden, disability, and whether the physical exam is or is not essential.

### ARE PAIN APPS USABLE? MHEALTH LITERACY FRAMEWORK

Mary Milidonis[1], and Carolyn Menges[2], *1. Cleveland State University, HUDSON, Ohio, United States, 2. Cleveland State University, Cleveland, Ohio, United States*

Older adults are at risk for low health literacy and not adhering to self-care. Health professionals are shifting their practices to emphasize wellbeing through health promotion. Mobile pain applications may be a tool to improve health communication and individualize care. Mobile health apps do not always consider the needs of the older adult who may be less comfortable and confident with technology. The purpose of this qualitative review is to explore mobile pain applications for adults with a health literacy framework, and identify facilitators and barriers to usability. Databases that reviewed apps included Google, Google Scholar, ScienceDirect, PubMed, CINAHL plus full text, APTA EBP database, MEDline, and SportDiscus. Fifty-three apps found. Excluded criteria: if there was a cost, targeted for children, and without pain diary. Six apps identified to be specifically relevant to pain intensity, location quality, and impact on life. Mobile Application Rating System (MARS) tool assessed engagement, functionality, aesthetics, information quality, and subjective quality. All apps were engaging, allowed for tracking symptoms and life impact over time. Some apps were difficult to navigate, did not offer education support. Two of the apps individualized pain. Two apps are on only accessible on one app platform. One app allowed for feedback about the app design. Pain mobile health applications can improve tracking, managing and understanding pain for improved mobility and social engagement for older adults. The use of health literacy frameworks with mobile health applications may increase accessibility to care.

### ENHANCING TELEHEALTH CLINICAL SERVICES AND CHRONIC DISEASE SELF-MANAGEMENT FOR OLDER ADULTS DURING COVID-19

Nicolette Castagna[1], and Lisa Granville[2], *1. Florida State University College of Medicine , Tallahassee, Florida, United States, 2. Florida State University College of Medicine, Tallahassee, Florida, United States*

During the pandemic, providers shifted to telemedicine to ensure patient safety. Under a federally funded cooperative agreement, the Florida State University College of Medicine's Department of Geriatrics partnered with a Federally Qualified Health Center to assess rural older adults' perceptions about telehealth and identify potential barriers. The quality improvement study used a convenience sample to survey patients aged ≥ 65 (n=62) at one rural clinic. 54.8% of respondents indicated they would not use telehealth for any care or preferred to receive most or all care in person. Conversely, 32.8% indicated a willingness to receive some care via telehealth. The survey also assessed the need for self-management devices, including blood pressure cuffs. There was not a consistent positive relationship between persons requesting devices and those who were telehealth willing. Devices were distributed to enhance telehealth visits and at-home monitoring. Clinic staff provided patients hands on training for device use and connection to EMR. Informal qualitative reports suggest patients are more empowered to utilize telehealth services and self-manage conditions. COVID-19 also limited access to community blood pressure screenings. The Tallahassee Senior Center and FSU Physician Assistant program distributed devices to community-dwelling older adults. PA students provided hands on training to aid in device familiarity and digital connectivity. Almost all older adult participants aged >60 strongly agreed "after the presentation I feel ready to use the blood pressure cuff at home." Clinical and community-based activities can help older adults who lack digital fluency feel more confident in using telehealth services and self-management devices.

### MULTIMODAL AFFECTIVE ANALYSIS OF FACIAL AND VOCAL EXPRESSIVITY USING SMARTPHONE AND DEEP LEARNING ANALYSIS

Heejung Kim[1], Youngshin Cho[1], Sunghee Lee[2], and CHAEHYEON Kang[1], *1. Yonsei University, Seoul, Seoul-t'ukpyolsi, Republic of Korea, 2. BRFrame, Seoul, Seoul-t'ukpyolsi, Republic of Korea*

Limited expressivity of emotion is one of the most common symptoms of major depression, particularly in older adults. Although assessing facial and vocal expressivity is very important for accurate clinical evaluation of geriatric depression, research has rarely examined older adults via telehealth technology. This study aims to quantify facial and vocal expressivity via a multimodal affective system with deep learning. A total of 19 Korean adults aged over 65 years with severe depressive symptoms participated in this research. Using smartphone video recording, 1,429 facial and vocal data were collected between July and December 2020. Recorded videos were transmitted automatically to the cloud system. Basic facial movements were extracted using combined video frames and mel spectrogram images. Compared to the AI hub of Korean images from big data, mood status was classified into seven categories (anger, disgust, fear, happiness, neutrality, sadness, and surprise). Frequencies of each mood were coded into continuous variables for each participant in each recording. When comparing video and text prediction to determine "true labels," the overall accuracy was 0.69, with F1 scores ranging from

0.57 to 0.79. In addition, the most common emotions were angry, happy, neutral, sad, and surprised. This study suggests that smartphone-recorded video could function as a useful tool for quantifying mood expressivity. This study established a preliminary method of affective assessment for older adults for telecare use based on socially assistive technology at a distance from the clinic.

### ACCEPTABILITY OF ANIMATRONIC PETS AMONG COMMUNITY-DWELLING VETERANS LIVING WITH DEMENTIA AND THEIR CAREGIVERS

Melissa Harris[1], Edwin Alcorn[2], and Judith Davagnino[2], *1. Duke University, Durham, North Carolina, United States, 2. Durham VA Health Care System, Durham, North Carolina, United States*

Animatronic pets can improve psychosocial outcomes among older adults, but little is known about in-home use by Veterans living with dementia and the perceptions of informal caregivers regarding these tools. This quality improvement project examined the usability and acceptability of animatronic pets by community dwelling Veterans living with dementia from the perspective of informal caregivers. Usability and acceptability were examined through a structured telephone interview with 17 caregivers. Usability was operationalized as frequency of: pet use by the Veteran (1=Not at all, 4=Daily or all of the time), and reminders provided by caregivers (1=Not at all, 4=Daily or all of the time). Acceptability was defined as perceived benefit (measured using an acceptability tool; 0= Not at all, 2=A great deal beneficial) and satisfaction (measured using acceptability tool with close and open-ended response items) as reported by caregivers. Results indicated high usability (71% of Veterans used the pet daily with reminders provided by 30% of caregivers) and acceptability (Benefit: (M=1.5/2, SD=0.7); Satisfaction: 94% of caregivers would continue to encourage use of the animatronic pet by the Veteran and recommend one to others). Open-ended responses suggest that they may be less satisfying to those who lost a real pet or did not have an affinity for pets earlier in life. Animatronic pets can be used with minimal assistance by Veterans with dementia and are acceptable to informal caregivers. As VA initiatives support provision of these pets, additional research is needed to examine their broader impact on Veterans living with dementia specifically.

### COMMUNICATION WITH HEALTHCARE PROVIDERS DURING THE PANDEMIC: EXPERIENCES OF OLDER ADULTS

Logan Sweeder[1], Andrea Sillner[2], Marie Boltz[3], and Kimberly Van Haitsma[4], *1. The Pennsylvania State University, State College, Pennsylvania, United States, 2. Pennsylvania State University Ross and Carol Nese College of Nursing, University Park, Pennsylvania, United States, 3. Penn State, Pennsylvania State University, Pennsylvania, United States, 4. Pennsylvania State University, University Park, Pennsylvania, United States*

The COVID-19 pandemic introduced many societal changes, including the need to use technology to communicate with healthcare providers. For many this was a new form of communication. This study examines (1) who older adults identified as healthcare providers and (2) reflected on how communications with formal healthcare providers changed during the pandemic. This data is a subset of a cross-sectional descriptive survey that was distributed electronically to a sample of community-dwelling older adults aged 65 years and older (N=323). Qualitative data was analyzed using thematic analysis. Quantitative data was analyzed using descriptive statistics. Participants averaged 72.7 years of age and 74.5% were retired. Just over ¼ of the sample identified nurses as part of their formal healthcare team; however, participants who identified nurses as formal healthcare providers had fewer negative themes regarding telehealth communication with healthcare providers compared to those who did not. They were also more likely to support in-person and technology driven communication models in the future. Telehealth remains an evolving form of primary healthcare that was utilized throughout the COVID-19 pandemic. Many older adults in our study considered it a resource that requires further optimization while others were staunch opponents to its continued employment in healthcare settings. Nurses are a vital part of our healthcare system and frequently interact with patients and families to coordinate care. Further research should examine how different scopes of nursing practice can support the implementation of telehealth to improve care delivery among community-dwelling older adults.

### TURNING REAL-WORLD DATA INTO ACTIONABLE EVIDENCE FOR HEALTHCARE INTERVENTIONS IN NEURODEGENERATION

Rachel Poulsen, Jun Ha Chang, Danish Bhatti, Matthew Rizzo, and Jennifer Merickel, *University of Nebraska Medical Center, Omaha, Nebraska, United States*

Background: Age-related neurodegenerative disorders, including Alzheimer's disease and Parkinson's disease (PD), progressively reduce mobility and quality of life (QoL). Real-world mobility from actigraphy predicts PD disease severity. This pilot analysis assessed utility of actigraphy to screen early physical function-related QoL decline in PD. Method: Mobility was monitored for 4 weeks using wrist-worn ActiGraph recordings in 27 participants with idiopathic PD (age = 67.78 ± 5.64, 19 males). Days with >600 mins of wear time during non-sleep times were analyzed ($\mu$ = 29.78 days ± 3.78). Typical activity was quantified as average steps per hour. Participants completed demographic and health assessments. Disease severity and physical function QoL were measured using the clinically-validated Unified PD Rating Scale (MDS-UPDRS) and Short Form-36 (SF-36), respectively. Disease severity, QoL, and typical activity were compared using Spearman correlations.

Results: Lower typical activity from actigraphy was associated with more severe motor symptoms (MDS-UPDRS; $r = -0.40$, $p = 0.04$) and with increased impairment in physical function QoL ($r = 0.50$, $p < 0.01$). Daily activity from actigraphy did not predict symptom severity of non-motor and motor-related complications. Discussion: Pilot results show utility of actigraphic metrics for indexing real-world mobility and QoL declines in neurodegenerative disorders, in line with broader efforts to turn real-world data into actionable evidence for healthcare interventions. Ongoing discovery in larger populations should yield robust, clinically-relevant indices of daily activity in aging and neurodegenerative impairment.

### IMPACT OF EHR IMPLEMENTATION IN NURSING HOMES ON FINANCIAL PERFORMANCE

Neeraj Dayama[1], Holly Felix[2], Rohit Pradhan[3], Michael Morris[4], and Saleema Karim[5], *1. Texas Tech University Health Sciences Center, Lubbock, Texas, United States, 2. University of Arkansas for Medical Sciences, Little Rock, Arkansas, United States, 3. West Liberty University, West Liberty, West Virginia, United States, 4. The University of Texas Health Science Center at Tyler, Tyler, Texas, United States, 5. Virginia Commonwealth University College of Health Professions, Richmond, Virginia, United States*

Nursing homes (NHs) are a critical part of the US healthcare system providing care to the sick and fragile elderly, and research suggests that they can benefit from the implementation of health information technology (HIT). However, for multiple reasons—exclusion from HITECH incentives; limited financial wherewithal; hazy return on investments (ROI), they have lagged in HIT implementation compared to other significant components of the US healthcare system. Utilizing the resource-based view (RBV) of the firm, the purpose of this study was to examine the longitudinal impact of HIT on NH financial performance potentially building a sustainable economic argument for its implementation. To examine this relationship, we merged data from five different sources between the years 2009-2013-: Health Information and Management Systems Society, LTCFocus, Online Survey Certification and Reporting/Certification and Survey Provider Enhanced Reporting, Area Health Resource Files, and Medicare Cost Reports. The outcome variable was financial performance which was measured in four ways: operating cost and revenue, operating margin, and total margin. The independent variable of interest was HIT implementation and was measured using a novel HIT Adoption Score (HAS) reflecting NH implementation of five different HIT applications. Four separate multivariate linear regression models were estimated, and we included a series of organizational and market characteristics in the analyses. We report a positive association between NH implementation and improved profitability (operating margin) ($p \leq 0.01$), and lower operating cost ($p \leq 0.05$). Our results, suggesting a potential business case for EHR implementation within NHs, have important policy and managerial implications that are discussed.

### RACIAL/ETHNIC DISPARITY IN 4M-BASED PRIMARY CARE DELIVERY VIA TELEHEALTH IN PROVIDER SHORTAGE AREA

Rain Lee[1], Yi Hyun Kim[2], Laurie Kim[3], ji won Yoo[3], Andrea Maxi[1], and Chelin Hong[4], *1. Yeshiva University, New York, New York, United States, 2. University of California, Berkeley, Berkeley, California, United States, 3. University of Nevada, Las Vegas, Las Vegas, Nevada, United States, 4. Monash University, San Diego, California, United States*

Background: Telehealth is a promising alternative to primary care delivery in provider shortage areas. The purpose of this study was to evaluate the implementation of the 4Ms (i.e., Medication, Mentation, Mobility, What Matters) framework in telehealth-based primary care in provider shortage areas by ethnic status.

Methods: This study was a retrospective analysis of 184 older adults (60+) representing 5% of the total sample at urban primary care in ethnically and racially diverse populations. Data were retrieved from July 2020 to September 2021. 14 trained primary care providers participated in this study and provided the 4Ms as following: 1) Medication (e.g., deprescribe or reduce high-risk Medication); 2) Mentation (e.g., depression and cognition assessment with brief counseling); 3) Mobility (e.g., mobility and home safety assessments); 4) What matters (e.g., advance care planning). The current study measured components of the 4Ms per telehealth visit by ethnic/race status (white vs. non-white).

Results: Overall, advance care planning (i.e., what matters) was the most discussed via telehealth (79%), followed by mobility (46.2%), Medication (16.8%), and Mentation (14.7%). To examine the disproportion of accessing telehealth by patients' racial background, the independence test of chi-square showed that non-white populations were less likely to have access to telehealth than white patients ($p = .02$).

Conclusion: There was an ethnic and racial disparity in the 4M framework application via telehealth in an urban primary care clinic. To sustain telehealth for patients in a healthcare shortage, ethnically and culturally specific training is needed, and linguistically diverse curricula are recommended.

### THE EFFECTIVENESS OF INTERNET-BASED INTERVENTIONS IN REDUCING STRESS EXPERIENCED BY OLDER INFORMAL CAREGIVERS

Abdulmajeed Altukhys, and Richard Beaulaurier, *Florida International University, Miami, Florida, United States*

Purpose: This systematic review assesses and provides a critical review of the effectiveness of internet-based interventions to decrease informal caregiver stress. Method: A systematic review of the following databases: PsycINFO, Social Service Abstract, Social Work Abstract, PubMed, and Cochrane Library was conducted using terms related to informal caregivers and internet-based interventions from January 2010-February 2022. Only data conducting interventions in the U.S. included. This systematic review reviewed the quality of articles on the methods of randomized and non-randomized trials using the approaches recommended by the Cochrane Handbook for systematic review for intervention. The review was reported using a PRISMA chart.

Results: Eight studies met the review criteria. Most of the studies showed positive benefits in reducing caregiver stress. There were no clear patterns as to the variables such as study duration and complexity of intervention associated with better outcomes, although earlier studies typically had more negative outcomes.

Conclusions: Internet-based interventions can develop solutions to decrease the physical and psychological consequences resulting from caregiving and can help empower older caregivers of people with chronic diseases. Internet-based interventions were mainly effective in reducing aspects of caregiver stress and improving their well-being. Further studies can assess outcomes for older informal caregivers and their recipients' health, different technology delivery methods, and the cost of such interventions are needed to examine the psychological impacts of COVID-19 on older informal caregivers.

### IMPACT OF VIBRATION ON TREMOR IN OLDER ADULTS WITH PARKINSON'S DISEASE

Ingrid Pretzer-Aboff[1], R.K. Elswick[1], Amanda Watson[2], Minglong Sun[3], Woosub Jung[3], Ken Koltermann[3], Gang Zhou[4], and Leslie Cloud[5], *1. Virginia Commonwealth University, Richmond, Virginia, United States, 2. University of Pennsylvania, Philadelphia, Pennsylvania, United States, 3. College of William & Mary, Williamsburg, Virginia, United States, 4. William & Mary, Williamsburg, Virginia, United States, 5. Virginia Commonwealth University School of Medicine, Henrico, Virginia, United States*

This was a randomized trial to explore the safety and efficacy of the RMBand (Resonate Forward LLC, Delaware) device to alleviate Parkinson's Disease (PD) tremors. The RMBand applies vibration to the proximal arm and was worn on the side of tremor dominance. Thirty subjects with PD and associated tremors were randomized to receive high vs. low-frequency vibration. Tremor assessments occurred for 20 minutes before, during 20 minutes of continuous vibration, and for 20 minutes immediately post-vibration in a single 60-minute session. Assessments (MDS-UPDRS part 3 and FTM tremor scale) were performed during each of the 3 phases by blinded raters. A quantitative assessment of tremor was performed throughout the session using wearable sensors on the distal arms. Linear mixed models were used for group comparisons. No significant difference was observed between the high and low dose groups in the MDS-UPDRS part 3 or FTM tremor scale (p=0.83 and 0.48 respectively), which may be due to the crude nature of these scales. Quantitative wearable sensor data were used to assess total time with tremors during the pre-vibration, vibration, and post-vibration periods. Time with tremor during the pre-vibration period was significantly greater than that during and post-vibration (p< 0.0001) for both dose groups, suggesting that vibration therapy applied to the proximal arm may suppress PD tremor. No significant adverse events related to vibration therapy occurred. In conclusion, the RMBand appears safe and possibly effective for suppressing PD tremor. Further study is warranted.

### IMPROVING HOME-CARE SERVICES FOR HIGH-RISK OLDER ADULTS USING PEER-LED VIDEO VISITS TO HOME

Shiv Lamba[1], Sandra Garcia[2], Elizabeth Bast[3], Lauren Penney[4], Ana Palacio[3], Sonia Madera[3], Orna Intrator[5], and Stuti Dang[6], *1. Washington University, St. Louis, Missouri, United States, 2. University of Miami, Miami, Florida, United States, 3. Miami VA Healthcare System, Miami, Florida, United States, 4. South Texas Veterans Health Care System, San Antonio, Texas, United States, 5. Canandaigua VA Medical Center, Canandaigua, New York, United States, 6. Miami Veterans Affairs Healthcare System, Miami, Florida, United States*

Older Veterans at high-risk for institutionalization often require home- and community-based services (HCBS). Yet, current HCBS delivery often fails to meet the needs of high-risk Veterans due to decreased veteran engagement in outpatient programs and limited HCBS capacity. A promising approach to address these gaps is the use of Veteran-Peers to make home-visits. Peer-2-PACT is a peer-led needs-assessment intervention for high-risk older veterans. Two trained peers conducted a checklist-guided virtual and/or in-person home-assessment to identify unmet needs and home-safety concerns. Veterans with access, acceptance and ability for video-capable technology were offered video-visits. We report on the feasibility of video home-visits in this high-risk group, and the experience of the video-visits using the visit-data and interviews with peers.Eight of 27 Peer-2-PACT Veterans successfully completed initial video-visit to home. The video-visit participants (n=8) were age 74±9; Non-Hispanic Black (50%); males (100%), compared to initial in-person home-visit participants (n=19), age 75.3±10.8; Non-Hispanic Black (47%); males (89%). The commonest needs identified during video-home-visits were home-safety devices 5(62.5%), housing assistance 4(50%), and medication refills 2(25%). Peers report that identifying veterans suitable for video-visits was challenging. During video-visits, depth-perception by peers is limited and sometimes needed in-person follow-up. Main advantages of video-visits was ability to identify unmet needs, engage veterans, provide care during COVID, and tele-present to remote clinicians. Preliminary data suggest that peer-conducted video home-visits is a feasible way to identify unmet needs in some high-risk older adults. This is particularly important improve care of Veterans who live at a distance from the facility.

### DOES AGE, RACE, AND/OR RURALITY DETERMINE OLDER VETERANS' ABILITY TO ACCESS VA VIDEO CONNECT

Linda Sawyer[1], Christine Cigolle[2], Kristin Phillips[3], Janette Dunlap[4], and Dennis Sullivan[5], *1. Central Arkansas VA Healthcare System, Sherwood, Arkansas, United States, 2. VA Ann Arbor, Ann Arbor, Michigan, United States, 3. Ann Arbor VA, Ann Arbor, Michigan, United States, 4. North Florida South Georgia VA, Jacksonville, Florida, United States, 5. Central Arkansas VA, North Little Rock, Arkansas, United States*

The purpose of this project was to determine whether ability to use VA Video Connect (VVC) by older Veterans (OV) differed by age, race, or rurality. A service to help older Veterans learn to use VVC was developed. As part of an ongoing QI project, demographics, willingness to attempt a VVC test call and outcomes of test calls were collected on all referrals. Descriptive statistics, Chi-square, and Fisher's exact test were used to examine differences in success rates by group. Of the 66 OV (age 60+) referred by their primary care providers, we were able to contact 63 by phone. Of those, 46 (73%) scheduled a VVC test call, 7 (11%) chose not to participate, and 10 (16%) were already using VVC for appointments. Of the 40 who continued the VVC test call, 31 (77.5%) were successful without issues, 7 (17.5%) were successful with help resolving issues, and 2 (5%) disconnected before finishing the call because it became too difficult. Of the 63 OV contacted, 38 (57.5%) had a successful VVC test call. However, those residing in rural (vs. urban) settings were less likely to have a successful test call (43% vs. 57%, p=0.04). There was no statistically significant difference in success rates for the test calls between whites vs. non-whites (52% vs. 48%, p= 0.2), or those aged 75 years or above vs. 60-74 years (53% vs. 46%, p=0.6). More work is needed to identify barriers to use of VVC, especially among OV living in rural settings.

### INTERGENERATIONAL VIRTUAL YOGA INTERVENTION INVOLVING OLDER ADULTS AND COLLEGE STUDENTS: A PILOT STUDY

Nancy Martens, PhD[1], Debra Schutte[2], Thomas Templin[2], and Morgan Cinader-Galvan[3], *1. Wayne State University, Birmingham, Michigan, United States, 2. Wayne State University, Detroit, Michigan, United States, 3. Michigan State University, Flint, Michigan, United States*

Social isolation is common in older adults and students and is associated with poor health outcomes. While evidence suggests active and yoga interventions improve health, interventions encouraging intergenerational social interaction are limited. The objective was to implement and evaluate a twice-weekly, 6-week, intergenerational yoga intervention delivered virtually. Specific aims included evaluation of feasibility and acceptability, examination of estimates of efficacy on health outcomes, and examination of age cohort effects on acceptability, feasibility, and efficacy. Older adults were recruited through a community activity center and randomized to the control group (N=10) (Mage= 70.05, SD 5.83) or intergenerational older adult treatment group (N=11) (Mage= 72.32, SD= 5.81) with students (N= 12) (Mage= 25.98, SD= 5.64). Older adult outcome measures included Center for Epidemiological Studies Depression Scale (CES-D), PROMIS Social Isolation Scale (SI), Meaning-in-Life Questionnaire, and Tilburg Frailty Index; outcomes for the students included the CES-D, SI, and Image of Aging Scale. Outcomes were assessed at week 0, 3, 6, and 12 (6-weeks post-intervention). Attrition was minimal; 81% of older adults and 80% of students received the full intervention. Using the Acceptability of Intervention Measure, 77.8% of older adults and 100% of college-age adults indicated their willingness to participate again. There were no significant intergenerational effects; however, the effects of yoga were in the expected direction with significant values in CES-D, $P < .01$ and Meaning-in Life, $P < .01$. While the acceptability and feasibility of the intervention were supported; additional research is needed to strengthen the intergenerational component of the intervention.

### FEASIBILITY AND ACCEPTABILITY OF A WEB-BASED COGNITIVE TRAINING PLATFORM FOR OLDER ADULTS: THE BREAKFAST TASK

Sharon Sanz Simon[1], Daniel Ben-Eliezer[2], Maria Pondikos[1], Yaakov Stern[1], and Daniel Gopher[2], *1. Columbia University, New York City, New York, United States, 2. Technion Israel Institute of Technology, Haifa, Hefa, Israel*

Background: Developing efficient cognitive training for the older population is a major public health goal due to its potential cognitive benefits. A promising cognitive training target is executive control, critical for multitasking in everyday life. The aim of this pilot study was to establish the feasibility and acceptability of the Breakfast Task in older adults, a new web-based cognitive training platform that simulates real-life multitasking demands. Research Design and

Methods: A community-based sample of 24 cognitively healthy participants aged between 60 and 75 (M = 69.12, SD = 3.83) underwent an online 5-session training protocol. Each session lasted 40 minutes and occurred twice a week at participant's homes. Game performance was recorded, and participants completed questionnaires at baseline and after the intervention.

Results: Feasibility metrics showed overall high recruitment (82.7%), adherence and retention rates (100%). Acceptability was considered good based on participant`s quantitative and qualitative responses. On average, participants rated the game as interesting, enjoyable and did not report difficulties in accessing the game online or in understanding the instructions. Moreover, participants showed a learning curve across sessions, improvement in most game outcomes and benefits from the emphasis change approach. Discussion and Implications: The findings provide preliminary support for the feasibility and acceptability of the Breakfast Task training platform with community-dwelling older adults and demonstrate potential cognitive benefits. Results suggest the value of further research investigating the Breakfast Task features and dose-response relationship, as well as its efficacy in older adults via larger randomized controlled trials.

### SMART HOME DATA VISUALIZATION FOR PROACTIVE HEALTH MONITORING OF COMMUNITY DWELLING OLDER ADULTS

Katherine Wuestney[1], Javier Ramirez[2], Diane Cook[2], and Roschelle Fritz[1], *1. Washington State University, Vancouver, Washington, United States, 2. Washington State University, Pullman, Washington, United States*

The role of Ambient Assistive Living and smart home technologies, which utilize unobtrusive sensors to detect changes in health, is becoming increasingly important in the delivery of healthcare services to older adults. However, these technologies must be designed to meaningfully incorporate into clinicians' decision making. Research has shown when clinicians are engaged in the design process of smart home systems, the accuracy and efficacy of the systems are improved. We present the process undertaken by a team of nurse researchers and computer science engineers to design clinically meaningful behavior markers derived from smart home sensor data that can be used by nurses to proactively identify changes in patient status. During the first phase of design, nurse researchers qualitatively analyzed time series from smart home sensors installed in the homes of community dwelling older adults and identified patterns in these data related to significant health changes. From this analysis, we assembled a candidate list of 15 sensor-based behavior metrics, such as percent time spent in each room or frequency of bathroom use. During the second phase of design, we will build on lessons we learned from participatory design to create behavior markers and visualizations that are inspired by clinical experience. These include visualizing behavior change over time, highlighting behavioral anomalies at multiple time scales, and calculating markers that are not directly observable such as time spent out of home.

Lessons learned from clinicians using the data visualizations to proactively screen for health changes in near real time will also be discussed.

### FINDINGS FROM HIGH USERS OF VIDEO TELEHEALTH TO DELIVER OCCUPATIONAL THERAPY SERVICES DURING COVID-19

Megan Gately[1], Dylan Waller[2], Emily Metcalf[1], and Lauren Moo[1], *1. VA Bedford Health Care System, Bedford, Massachusetts, United States, 2. VA Portland Health Care System, Portland, Oregon, United States*

Occupational therapy (OT) helps older adults improve their ability to perform day-to-day tasks. Veterans Health Administration (VHA) is the single largest employer of occupational therapy (OT) practitioners in the United States and a forerunner in telehealth. As a result of COVID, OT video visits increased by nearly 2000% from 2019 to 2020. To ascertain barriers and facilitators to this shift in care delivery, we conducted interviews between January and April 2021 with OT practitioners (N=27) who were high users of VA Video Connect (VVC), VHA's videoconferencing software. OT participants were from rural and urban settings, and had completed an average of 536 VVC appointments each in 2020. Participants used VVC to deliver a variety of OT services, including mental health groups and home safety interventions. Facilitators to VVC included, a) Patient characteristics, such as positive perceptions of VVC and technological skill, b) OT clinician characteristics, like flexibility, level of experience, and desire to increase patient access to care, and, c) VHA's telehealth infrastructure. Barriers included, a) Patients' lack of familiarity or skills with technology, particularly older patients, b) challenges translating traditionally hands-on care to video, and c) unreliable internet connectivity, particularly for rural patients. This study broadens our understanding of video telehealth service delivery for care which has historically been delivered in brick-and-mortar settings. Understanding challenges and enablers to video telehealth highlights opportunities to increase access to those who face barriers, such as older, rural patients.

### IMPROVING VIDEO DEVICE USAGE AMONG OLDER RURAL VETERANS WITH USER-CENTERED DESIGN

Hillary Lum[1], Lynette Kelley[2], Kathryn Nearing[3], Leah Walker[4], Gerhardt Johnson[4], and Steven Barczi[4], *1. University of Colorado Anschutz Medical Campus, Aurora, Colorado, United States, 2. Department of Veteran Affairs, Denver, Colorado, United States, 3. VA Eastern Colorado Geriatric Research Education and Clinical Center, Denver, Colorado, United States, 4. Department of Veterans Affairs, Madison, Wisconsin, United States*

The Veterans Administration (VA) established pathways to provide VA-issued tablets and increased access to internet for Veterans without these resources. Veterans aged above 65, experience barriers with telemedicine such as access and usability. We sought to improve the usability and experience of telemedicine for rural, older Veterans receiving a VA-issued tablet by modifying materials and qualitatively evaluating their experience with set-up and preparing for their first appointment, guided by user-centered design. We conducted a rapid exploratory evaluation, to understand Veteran and care partner experiences setting up VA-issued tablets and logging into their first appointment using standard 9-page instructions. We interviewed telehealth technicians, and providers to better understand patient barriers from their perspective. Using insights from interviews and evidence-informed guidelines on educational materials for older adults, we created a two-page guidance. A group of Veterans and care partners reviewed the materials and provided feedback. Received feedback provided 17 suggestions, 8 of which were utilized, including enlarging graphics, clarifying abbreviations (or wording), consolidating the instructions further and emphasizing pertinent information further. Modified materials reduced standard written instructions from nine pages to two. Feedback suggests that updated materials are helpful, aesthetically pleasing and preferred over current materials. Utilizing user-centered design methods, addressed barriers experienced by older, rural Veterans with initial telemedicine device and appointment set-up. Veterans, care partners, providers, and telehealth technicians perceived materials adapted for older adults as supportive of video device usability, helping to alleviate barriers that prevent Veterans from initiating telemedicine.

### REMOTE TELEASSESSMENT AND TELEREHABILITATION OF A COMPREHENSIVE EXERCISE TRAINING PROTOCOL FOR OLDER ADULTS

Savitha Subramaniam[1], Edwina Wilson[2], and Tanvi bhatt[1], *1. University of Illinois at Chicago, Chicago,, Illinois, United States, 2. University of Illinois at Chicago, Chicago, Illinois, United States*

Background: With the increased implementation of interactive technologies for assessment and rehabilitation, it would be optimal to exhibit the reliability of physical assessment measures via tele-assessment. Aim: To determine the test-retest and intra-rater reliability of physical function outcome measures routinely used in the balance and gait rehabilitation using real-time online tele-assessment.

Methods: Community-dwelling healthy older adults (N=30) participated in three experimental tele-assessment sessions. During each session, a real-time online tele-assessment was performed on five major domains that evaluate balance and gait function: lower limb strength and endurance (30-second chair stand test), aerobic endurance (2-minute step test), static balance (One-legged stand test), dynamic balance (4-step square test), and gait (Tinetti).

Results: Coefficient of determination (R2) was used to determine the test-retest (TRT) and intra-rater (IR) reliability for all outcome variables. Excellent reliability was shown by Tinetti (TRT-1.0; IR-1.0) and one-legged stand test (non-dominant leg TRT-0.92; IR-0.97 and dominant leg TRT-0.89; IR-0.92). Excellent to good reliability was shown by 4-step square test (TRT-0.89; IR-0.74), 2-min step in place test (TRT-0.85; IR-0.78), and 30-second chair stand test (TRT-0.86; IR-0.80).

Conclusion: The reliability of individual outcome measures ranged from good to excellent, suggesting that these outcomes have sufficient sensitivity for detecting change with telerehabilitation.

### SESSION 6430 (POSTER)

### CANCER

### FACTORS INFLUENCING CANCER SURVIVORSHIP AMONG NIGERIAN WOMEN

Darlingtina Esiaka[1], Candidus Nwakasi[2], Runcie Chidebe[3], Khadijat Banwo Fatai[4], and Gloria Orji[5], *1. Robert Wood Johnson Medical School, Rutgers University, Newark, New Jersey, United States, 2. Providence College, Providence, Rhode Island, United States, 3. Health and Psychological Trust Center, Abuja, Federal Capital Territory, Nigeria, 4. Project PinkBlue, Abuja, Federal Capital Territory, Nigeria, 5. Network of People Impacted by Cancer in Nigera, Abuja, Federal Capital Territory, Nigeria*

Breast cancer is the most common cancer globally and among Nigerian women. With technological and medical advancements, especially in the early detection of chronic illnesses, there is a reduction in cancer deaths, and more women are living as cancer survivors in Nigeria. Despite the increase in the number of cancer survivors, many survivors struggle with life after cancer diagnoses. In this study, we interviewed 30 Nigerian women (Mage = 42.00) who completed cancer therapy in the past 12 months and above, to explore how women who underwent cancer therapy perceive their healing, health, and quality of life. An example of one of the interview questions is “How did cancer diagnoses affect your normal way of life?” Each interview lasted an average of 1 hour. Participant responses were transcribed, and theme coded for analysis. We found that fear of reoccurrence, loss of sense of purpose, shame from physical mutilation related to mastectomy, poor psychological health, social isolation, and ill-treatment from other members of the community were recurring themes across all of the women’s responses. The presence of cancer survivors' support groups and religiosity were important for the experience of life after a cancer diagnosis. Our findings draw attention to the need for interventions targeting cancer survivors. The result further indicates that addressing community perceptions of breast cancer is imperative to ensure the health of breast cancer survivors, especially in low-resource countries.

### SOCIAL SUPPORT DYNAMICS FOR SOUTH ASIAN BREAST CANCER PATIENTS: AN ANALYSIS CONDUCTED USING ATLAS CAREMAPS

Ranak Trivedi[1], Ambri Pukhraj[2], Shreya Desai[3], Rishabh Shah[2], Rashmi Risbud[4], Lidia Schapira[2], Dolores Gallagher-Thompson[2], and Karl Lorenz[5], *1. Stanford University, Palo Alto, California, United States, 2. Stanford University, Stanford, California, United States, 3. Veterans Affairs Palo Alto Health Care System, Menlo Park, California, United States, 4. University of California, Davis, Davis, California, United States, 5. Veteran Affairs Palo Alto Health Care System, Stanford, California, United States*

With a rise in the prevalence and a drop in mortality rates of breast cancer among South Asians (people with heritage from India, Pakistan, Nepal, Bhutan, Sri Lanka, Maldives, and Bangladesh), globally and in the US, there is an increasing number of South Asians managing breast cancer. The South Asian Family Approaches to Disease (SAFAD) study aims to better understand how South Asian breast cancer survivors are supported while managing breast cancer. We conducted semi-structured interviews to complete an adapted version of Atlas CareMaps, a visual representation of survivors' care networks at the time of the interview. Thirteen South Asian breast cancer survivors were enrolled. Survivors were on average 47y (SD=9.1y) and reported being diagnosed with stage 0 (n=1), stage 1 (n=3), stage 2 (n=6), or stage 4 (n=3) breast cancer. Analyses of the Atlas CareMaps suggest 1) South Asian breast cancer survivors received support from 13.7±3.5 individuals, while providing care to 3.3 ± 2.2 individuals; 2) at more advanced stages of breast cancer, patients provide less support to others (Stage 1=3.8±2.2, Stage 4=1.7±1.5); 3) older survivors received more support from abroad (< 40y=2.0±2.2, patients 50+=5.3±3.3). Atlas CareMaps can provide useful insights into the rich care networks of South Asian breast cancer survivors which can be used to develop clinical programs.

### UNDERSTANDING THE CARE NETWORKS OF INFORMAL CAREGIVERS OF SOUTH ASIANS WITH BREAST CANCER USING ATLAS CAREMAPS

Ranak Trivedi[1], Ambri Pukhraj[2], Shreya Desai[3], Akanksha Jain[4], Rashmi Risbud[5], Lidia Schapira[2], Dolores Gallagher-Thompson[2], and Karl Lorenz[6], *1. Stanford University, Palo Alto, California, United States, 2. Stanford University, Stanford, California, United States, 3. Veterans Affairs Palo Alto Health Care System, Menlo Park, California, United States, 4. Veterans Affairs Palo alto Health Care System, Menlo Park, California, United States, 5. University of California, Davis, Davis, California, United States, 6. Veteran Affairs Palo Alto Health Care System, Stanford, California, United States*

Breast cancer rates are increasing among individuals with a South Asian heritage, i.e., from India, Pakistan, Nepal, Bhutan, Sri Lanka, Maldives, and Bangladesh. Informal caregiving is vastly understudied among this population, despite being influenced by cultural mores such as collectivism, cancer-related stigma, and gender roles. The South Asian Family Approaches to Disease (SAFAD) study took a mixed-methods, observational approach to describe the care networks of informal caregivers via an adapted version of Atlas CareMaps. Thirteen caregivers (43.9+/-14.8y, 30.8% female) were interviewed and included 6 husbands, 1 wife, 2 daughters, 1 son, 1 brother, and 1 friend of the breast cancer survivors. Semi-structured interviews were designed to develop an adapted Atlas CareMap, a visual representation of the caregivers’ care network at the time of the interview. Atlas CareMaps depicted the number of people supported by caregivers, and who provide support; their relationship; the frequency, intensity, and type of care; and modes of communication used. Immediate or extended family members were the most common people included. Results indicated that: 1) caregivers reported 8.8□3.5 individuals in their care network, provided care to 3.5+/-1.8 individuals and received care from 7.3+/-3.5 individuals; 2) caregivers primarily received emotional support from others; 3) their survivors’ care teams were often included as a source of support and medical knowledge for survivors, but only three noted that the care teams supported them directly. Describing these networks is a key step to developing culturally-concordant programs that can support South Asian caregivers, even as they care for breast cancer survivors.

### CANCER CARE IN ELDERLY: CHALLENGES AND REQUIREMENTS OF INFORMAL CAREGIVERS

Esha Chakravarty[1], and Indrani Chakravarty[2], *1. Calcutta Metropolitan Institute of Gerontology, London, London, United Kingdom, 2. Calcutta Metropolitan Institute of Gerontology, Kolkata, West Bengal, India*

The affect of Covid -19 and changes in healthcare has shifted most of cancer care of elderly to home settings. Calcutta Metropolitan Institute of Gerontology (CMIG), Regional Resource and Training Centre in Ageing studied the daily tasks, requirements and emotional burden of caregivers in West Bengal, India. Caregivers of lung cancer, liver cancer and colorectal cancer patients (n=500) completed questionnaires provided by CMIG. The mean age of cancer patients was 72.5 years. CMIG found that 47% of the caregivers were the spouses of elderly patients, 33% were their adult children and 20% were other family members. Forty six percent of the caregivers were found to care for patients with metastatic disease, 33% cared for co morbidities, 63% cared for those undergoing chemotherapy or radiotherapy. Ninety eight percent of the caregivers provided assistance with Activities of Daily Living, 75% in administering medicine, 47% helped in pain management, 39% helped in hospital visits or deciding to call doctor. Twenty one percent of the caregivers reported poor health and 16% were not confident of the quality of care they provided. Sixty six percent reported suffering from stress and perceived cancer as a family disease, 45% thought they lacked social support. Of the adult children who provided care, 78% resported they needed more training to provide quality cancer care and transitioning to formal care would improve results. This study will help influence State policies for allocation of resources for training for cancer caregivers, build support groups for stress management and create cancer care helplines.

### THE ROLE OF VIRTUAL PEER HEALTH SUPPORT IN ALTERNATIVE ILLNESS APPRAISALS AND PATIENT SELF-REAPPRAISAL AFTER CANCER

Gul Seckin, *University of North Texas, Hickory Creek, Texas, United States*

Technology is transforming how older adults find health-related support and get connected with others who cope with similar healthproblems. This study examined the relationship between virtual peer health support and alternative appraisals of illness experience in old age among people who use the Internet for cancer related support (N=157, Age Range: 50-79, Mean = 57). Regression models with interaction terms examined whether and to what extent virtual peer health support was associated with differential illness appraisal as well as and post-diagnosis self-reappraisal. Results demonstrated that a) perceived benefits of virtual peer health support is a significant predictor of self-reported self-reappraisal ($\beta = .24$, $p < .01$) b) appraisals of illness experience as a traumatic event and/or as a life challenge are both significantly associated with virtual peer health support ($\beta = .18$, $p < .05$ and $\beta = .23$, $p < .01$), and c) appraisal of illness experience as a personal growth growth opportunity is not significantly associated with virtual peer health support ($\beta = .10$, $p < .23$). These results suggest the statistical interactions of the virtual peer health support with illness appraisals are significant predictors of self-reappraisal post-diagnosis. Specifically, those who appraised cancer to have been a traumatic experience and/or life challenge for them perceived virtual peer support to be positively influential on their self-perception after cancer diagnosis, which is in contrast to those who perceived cancer experience as an opportunity for personal growth and thus possibly not deriving further significant additional benefits from virtual peer support.

### "HOW WILL I LIVE THIS LIFE I AM TRYING TO SAVE?": EXPLORING DUALITIES IN CANCER SURVIVORSHIP EXPERIENCES IN NIGERIA

Candidus Nwakasi[1], Darlingtina Esiaka[2], Runcie Chidebe[3], Khadijat Banwo Fatai[4], and Gloria Orji[5], *1. Providence College, Providence, Rhode Island, United States, 2. Robert Wood Johnson Medical School, Rutgers University, Newark, New Jersey, United States, 3. Health and Psychological Trust Center, Abuja, Federal Capital Territory, Nigeria, 4. Project PinkBlue, Abuja, Federal Capital Territory, Nigeria, 5. Network of People Impacted by Cancer in Nigera, Abuja, Federal Capital Territory, Nigeria*

Objectives. Cancer mortality rate is high in Nigeria with about 102,000 new cases and 72,000 deaths per year. Cancer incidence rate is projected to increase with its rapidly aging population and growth — emphasizing its growing cancer burden. Due to Nigeria's weak health system, limited cancer therapy and expert oncological services, lack of cancer education and awareness, and sub-optimal implementation of its national cancer control plan, there is an increase in the risk of poor outcomes from cancer. To further understand the mechanism behind the poor outcomes, we explored cancer survivorship experiences of breast cancer survivors in Nigeria. Methods. The study employed a qualitative descriptive method. Semi-structured interviews were conducted with a purposive sample of 30 female breast cancer survivors in Abuja, Nigeria (Mage = 42 years). Their responses were transcribed, coded, and analyzed for themes. Results. The four major themes identified were: 1) life after cancer diagnosis (hope vs despair), 2) faith and religion (coping with faith vs issues with religion), 3) accessing cancer therapy (alternative therapy vs orthodox therapy), and 4) relationship with medical providers (encouragement from medical providers vs hinderance from medical providers). Discussion. Cancer death rates may be high but there is also a growing number of survivors in Nigeria. It is crucial to improve the health and well-being of Nigerians from the time of diagnosis until end of life, thus, the focus of cancer survivorship. The findings point to the critical need for policies to help strengthen cancer survivorship in Nigeria.

### DEVELOPMENT AND EVALUATION OF A WEB-BASED DECISION AID FOR LUNG CANCER SCREENING FOR OLDER CHINESE AMERICANS

Chien-Ching Li[1], Alicia K. Matthews[2], Xiaojun Gao[3], and Krystal Cheung[3], *1. Rush University, Chicago, Illinois, United States, 2. University of Illnois at Chicago, Chicago, Illinois, United States, 3. University of Illinois at Chicago, Chicago, Illinois, United States*

Low dose computed tomography (LDCT) lung cancer screening is effective in reducing lung cancer mortality. In the United States, Chinese Americans are disproportionately impacted by lung cancer compared to other Asian subgroups. Our prior study showed that over 25% of older Chinese male smokers met eligibility for LDCT screening based on the CMS guidelines. However, in general, cancer screening rates among Chinese Americans are lower compared to other Americans. Therefore, we developed a culturally and logistically web-based decision aid (DA) for LDCT screening "Lung Decision Coaching Tool (LDC-T)" to improve the older Chinese American's knowledge of lung cancer and screening and to facilitate the decision making with the physician. Compared to traditional paper-based DA, the LDC-T DA has key elements including video, pack-year calculator, eligibility determination, and visual aids to support comprehension and shared decision-making. We conducted acceptability and usability tests among 22 older Chinese American smokers. The tool was highly received by participants for its usefulness and ease to use. Over 80% reported the way of information presented in LDC-T is "good or very good" and the length of the information is "just right". Overall, the majority were satisfied with the LDC-T DA (95%) and agreed that the tool includes sufficient information for people to make the screening decision (90%) and prepare shared decision making with their healthcare providers (81%). Our study results will inform strategies for reducing lung cancer disparities among Chinese Americans and other underserved smokers thus filling important gaps in the literature.

### THE HOPES AND FEARS OF OLDER ADULT, LONG-TERM CANCER SURVIVORS: RACIAL AND GENDER DIFFERENCES

Gary Deimling[1], Spencier Ciaralli[2], Dyanna Burnham[1], and Colleen Kavanagh[1], *1. Case Western Reserve University, Cleveland, Ohio, United States, 2. Augustana University, Sioux Falls, South Dakota, United States*

Understanding the impact of cancer on the hopes and fears of older adult (age 60+), long-term (5 years +) survivors is important for assessing their quality of life after cancer. Prior quantitative research has shown the important role that cancer plays in the health and psycho-social well-being of survivors in the years and even decades after diagnosis and treatment. This presentation extends that research by examining both quantitative data and survivors' narratives revealing important issues survivors face such as altered sense of self/identity, feelings of social isolation, key sources of social support as well as cancer and other health-related concerns. These are examined in the context of broader existential issues related to their hopes and fears for the future and differences by race, gender and cancer type are also presented. The data analyzed are from an NCI funded, 10-year longitudinal study based on in-person interviews with 321 survivors of breast, colorectal and prostate cancer to document how cancer-related factors have affected their aspirations for the future. The findings are discussed in the context of how the issues that survivors identified as affecting their hopes and fears may be integrated into the dialogue clinical staff can have with older survivors and their families as part of after-cancer care.

### ASSOCIATION OF PSYCHOSOCIAL FACTORS WITH MORTALITY IN OLDER FEMALE SURVIVORS OF CANCER

Jennifer King[1], Kimberly Lind[2], Kristin Morrill[2], and Cynthia Thomson[2], *1. University of Arizona, Marana, Arizona, United States, 2. University of Arizona, Tucson, Arizona, United States*

Understanding factors associated with survival after a cancer diagnosis among older adults is critical as the population ages and cancer survivorship increases. The purpose of this study was to: (1) identify clusters of postmenopausal cancer survivor characteristics by demographic and lifestyle factors; 2) describe the characteristics of each cluster; and 3) evaluate the association of cluster assignment with survival. Participants from the Women's Health Initiative (WHI) who reported either a prevalent cancer diagnosis at baseline (n=14294) or were diagnosed with a first primary incident cancer within the first 10 years of WHI (n=12934) were included. Latent class analysis was used to identify survivor clusters using psychosocial variables. Clusters were characterized using descriptive statistics. We tested for differences in cluster characteristics using ANOVA and Chi-square tests as appropriate. Cox proportional hazards regression was used to evaluate the association between cluster and mortality. Prevalent (n=7) and incident (n=9) cancer survivors clusters were identified. Among both (prevalent and incident) sets of clusters, age at WHI baseline, age at menopause, race, ethnicity, income, education, body mass index, diet quality, smoking, alcohol consumption and exercise all differed by cluster ($p<.0001$ for all). The most racially and ethnically diverse cluster had higher mortality rates compared to the largest most homogenous cluster; hazard ratio (95%CI) 1.30 (1.15, 1.48) and 1.33 (1.16, 1.53), respectively. Understanding how clusters of risk factors influence cancer survival in postmenopausal women will inform future interventions to improve outcomes and reduce health disparities for cancer survivors.

### CANCER SURVIVORSHIP, AGING, AND SEXUAL HEALTH: GENDERED EXPERIENCES OF SEXUAL BEHAVIOR AND BODY IMAGE

Spencier Ciaralli[1], Gary Deimling[2], Dyanna Burnham[2], and Colleen Kavanagh[2], *1. Augustana University, Sioux Falls, South Dakota, United States, 2. Case Western Reserve University, Cleveland, Ohio, United States*

Previous research on aging has studied sexual health as well as cancer. However, less is known about the intersections of cancer survivorship, aging, and sexual health, particularly as it pertains to the gendered experiences of body image. The select studies available that consider the role of sex and intimacy after cancer have provided wholly quantitative or qualitative analyses. This study provides a mixed-methods approach of cancer survivorship and aging as it pertains to sexual health, particularly examining prostate, colorectal, and breast cancer survivors. In their narratives as to how cancer has influenced their sexual behavior, female breast cancer survivors construct their experiences around body image satisfaction. Men who are prostate cancer survivors note struggles around "performance" and impotence. Common issues for both men and women with colorectal cancer survivorship identify body image and impotence as affecting their sexual behavior. An underlying theme throughout all aging cancer survivors' narratives is the role of relationships and the perceptions of their partner. Race, marital status, and education are also examined. The data analyzed are from an NCI funded, 10-year longitudinal study based on in-person interviews with 321 cancer survivors. The findings illustrate the importance of medical professionals providing wholistic after-care that is inclusive of aging survivors' sexual health, relationships, and perception of self.

### INFORMATION SEEKING IN OLDER ADULT RURAL CANCER SURVIVORS: UNMET NEEDS AND SEARCH EXPERIENCES

Susan LaValley, Christina Crabtree-Ide, Kathryn Glaser, Rodney Haring, Martin Mahoney, Elisa Rodriguez, Christy Widman, and Elizabeth Bouchard, *Roswell Park Comprehensive Cancer Center, Buffalo, New York, United States*

Cancer survivors over the age of sixty-five have specialized supportive care needs related to maintaining their health and quality of life after treatment. However, cancer survivors living in rural areas may experience barriers like limited digital infrastructure and/or low health literacy when attempting to access necessary informational resources. This project sought to identify older adult rural cancer survivors' domains of unmet informational support and experiences searching for supportive informational resources. Data from a survey administered to cancer survivors (N=292 with complete age data) in rural areas of Western New York (RUCA codes 7-10) were analyzed using descriptive statistics. Variables of interest included Internet access, information-seeking activities, cancer-related topics of interest, and information search experiences. Fifty-four percent of participants in the sample self-identified as over the age of 65. Among older adult cancer survivors, 62% reported using the Internet. The following topics were identified as unmet cancer survivorship information needs: (1) decreasing the risk of cancer recurrence, (2) new symptoms that warrant contacting one's doctor, and (3) medical advances in treatment. Most older adult respondents (70%) were confident in their ability to find needed health information but 40% were concerned about the quality of the information they found and 31% found their search experience frustrating. While rural cancer survivors have access to digital resources and report confidence in their ability to find survivorship information, our findings highlight the need for tailored information on cancer survivorship and interventions to support information appraisal and selecting high quality informational sources.

### SESSION 6440 (POSTER)

### COGNITION AND LIFECOURSE DEVELOPMENT

### POTENTIAL PREDICTORS OF HEALTH LITERACY IN OLDER ADULTS

Stephanie Rubinstein, Gillian Fennell, and Elizabeth Zelinski, *University of Southern California, Los Angeles, California, United States*

Objective: Being able to effectively communicate health care needs and understanding health-related information is particularly important as people grow older. In this study, we sought to identify the factors related to subjective health literacy among older adults.

Methods: We examined eight potential predictors of health literacy: subjective memory, cognition, objective health literacy, self-rated health, age, sex, race, education, depression. Our data was derived from a large sample (N=1,272) of participants aged 50 and older who took part in the Health and Retirement Study (HRS; 2008 wave and 2009 internet survey).

Results: Controlling for all other variables, subjective memory ($b = .15$, $p = .02$) was positively and self-rated health ($b = -.09$, $p < 0.001$) was negatively associated with subjective health literacy. Neither objective health literacy, cognition, nor age were significantly associated with subjective health literacy. Women reported better health literacy than did men ($b = .09$, $p = 0.01$). The predictors in this model explained 9% of variation in subjective health literacy. Discussion: These findings may be better understood as we take into consideration the interplay between health literacy, cognition, education, and subjective memory established in extant literature. We discuss the implications of our findings as they relate to healthcare decision-making as well as plans for future research.

### ADHD POLYGENIC RISK AND COGNITIVE PERFORMANCE IN LATER LIFE

Marrium Mansoor[1], and Benjamin Katz[2], *1. Virginia Tech Graduate School, Blacksburg, Virginia, United States, 2. Virginia Tech, Blacksburg, Virginia, United States*

Attention-Deficit/Hyperactivity Disorder (ADHD) affects approximately 4.4% of adults in the US and impacts multiple domains of daily life including education, workplace performance and interpersonal relationships. Although an increasing number of individuals with ADHD are now entering later life, there is very little research on how ADHD risk may impact cognitive function during aging. As such, there is value in understanding the association between polygenic risk for ADHD and cognition during different stages of later life. This study utilized data from the Health and Retirement Study which surveys 37,000 Americans biennially and aimed to determine if there was an influence of ADHD risk on cognitive performance when individuals were young-old (ages 65-74) or middle-old (ages 75-84). Only participants who responded in 2006, 2016, and to the Venous Blood Study were selected. The resulting sample size of 403 African-ancestry individuals (AA) and 2286 European-ancestry individuals (EA) was compared on executive function-focused measures as well as delayed recall measures. Results showed that there was no significant effect of ADHD risk on memory-related measures at both time-points for AA and EA individuals. However, there was a statistically significant association between ADHD risk and performance on the executive function measure for EA older adults who were middle-old ($p = 0.028$), but not when they were young-old; no such association was observed for AA adults. This finding suggests that ADHD risk may influence cognition among older adults and has significant implications for treatment and care of individuals with ADHD throughout the life course.

### LONGITUDINAL EFFECTS OF HYPERTENSION ON COGNITIVE PERFORMANCE IN OLDER ADULTS

Allison Moll, and John L. Woodard, *Wayne State University, Detroit, Michigan, United States*

Hypertension impacts many older adults, but it is still not clear whether it has a negative effect on cognitive performance in older adults. The purpose of this study was to assess the longitudinal effect of hypertension on cognition in older adults (Mage=75.6 years, SD=8.3). Participants came from the National Alzheimer's Coordinating Center database. The cognitive assessment included the MoCA, Digit Span, Trail-Making Test A and B, WAIS-R Digit Symbol, Category Fluency, and Letter Fluency. Linear mixed effects modeling examined the random and fixed effects of clinician-assessed hypertension, months since first study visit, sex, age, and the interaction between hypertension and time since first visit on cognitive performance across five annual study visits. Results showed that hypertension had a significant main effect on Category Fluency, Trails B, Letter Fluency, and Digit Span–Forward and Backward. However, effect sizes were quite small (ηp2 range: 3.93x10-4 – 1.73x10-3). Main effects of age and months since first visit were significant predictors of all cognitive measures, such that older age was associated with worse cognitive performance and more months since the first visit was associated with better cognitive performance. This positive association is perhaps suggestive of practice effects across study visits. A significant interaction between hypertension and months since first visit for Category Fluency and Trails B showed that hypertensives and non-hypertensives performed differently at the initial visit but similarly by the last visit. However, effect sizes were small (ηp2 range: 3.86–9.64x10-4). These results suggest hypertension effects on cognition in older adults are minimal.

### LIFE COURSE CONDITIONS AND COGNITION IN A NATIONALLY REPRESENTATIVE SURVEY OF OLDER ADULTS

James Iveniuk, Ellen Compernolle, Riddhi Gupta, Jen Hanis, and Louise Hawkley, *NORC at the University of Chicago, Chicago, Illinois, United States*

By the time people reach older adulthood, their cognitive function may be conserved, or in decline, in part due to their social experiences over their entire life course. Researchers have gained a greater understanding, over recent decades, of the importance of life-course events for cognition in later life. Nevertheless, our understanding of many of these factors, especially in childhood, remains limited. Drawing upon Round 2 of the National Social life, Health, and Aging Project (NSHAP; N=3,377), and data linked to the 2010 census, the 1940s census, and air pollution data, we undertake a whole-life-course approach to understanding the determinants of cognitive function in older adults. Building on the work of the Lancet Commission on risk factors for dementia, we considered health conditions, low education,incarceration, and brain injury (ever); poor health behaviors and low social contact (current); and air pollution (average over past five years). We also considered adverse childhood experiences,, and home conditions in 1940. Similar to other studies, we found that female gender, identifying as white, and being born in the US were significantly associated with better cognitive function. Higher depression and lower social contact were associated with worse cognition. There were no significant associations between cognition and early childhood factors - with the exception that growing up in an urban area was associated with better cognitive function. Experiencing jail time was also negatively associated with cognitive function. Findings point towards the need for a more expansive consideration of life course conditions, as they impact cognition in late life.

### COGNITIVE RESERVE, PHYSICAL HEALTH, AND COGNITIVE FUNCTIONING IN OLDER ADULTS

Alyssa MacLean, and Jessica Strong, *University of Prince Edward Island, Charlottetown, Prince Edward Island, Canada*

Prior research has shown positive relationships between cognitive reserve (CR), physical health, and cognition, meaning that higher levels of physical health and CR are associated with higher cognitive functioning and vice versa. A group of community-dwelling older adults (N = 45, mean age = 70.5 years) completed a measure of CR (Life Experiences Questionnaire; LEQ), as well as cognitive tests, with number of physician diagnosed health conditions and number of medications measuring physical health. Initially, we ran correlations with the intention of running a mediation model (physical health factor as the independent variable, LEQ as the mediator, and cognitive test scores as dependent variables). Significant correlations were found between physical health and CR (r = -0.44, p = .01) with a medium effect size, and between CR and some test scores. However, there were no correlations between physical health and cognitive scores. Therefore, using linear regression analyses, the LEQ significantly predicted scores on some tests of executive functioning (DKEFS: Colour-Word Interference Test; Trial 3: F(1,39) = 7.42, p = .010), and processing speed (DKEFS: combined colour naming/reading: F(1,32) = 4.32, p = .046). However, the LEQ did not significantly predict verbal fluency, any set-switching tests, or a set-switching and inhibition test. Additionally, when physical health was added to the model, there was no significant improvement. The results suggest that CR may predict some types of executive functioning test scores, but not other executive functioning tests. Additionally, physical health did not predict cognitive test scores in this sample.

### AGING AND LONGITUDINAL EFFECTS ON NUMERACY

Ryan Best[1], Katherine Carman[2], and Andrew Parker[3], *1. West Virginia University, Morgantown, West Virginia, United States, 2. RAND Corportation, Santa Monica, California, United States, 3. RAND Corporation, Pittsburgh, Pennsylvania, United States*

Numeracy, the ability to competently make use of numbers and numerical information, is a skill associated with diverse positive outcomes across the lifespan. Numeracy is related to increases in education attainment, economic success, and the quality of health and financial decisions (e.g., Chesney, Bjälkebring, & Peters, 2015; Reyna et al., 2009; Smith, McArdle, & Willis, 2010). More generally, numeracy correlates positively with measures of fluid and crystallized intelligence, but accounts for a unique portion of the variance in models predicting risk comprehension (Cokely et al., 2012) and performance in many decision-making tasks (e.g., Peters et al., 2006; Peters, 2012). Age effects on cognitive functioning are well established, generally describing declines in fluid abilities and increases or stability in crystallized abilities (for a review, see Salthouse 2010), but little is known about the longitudinal trajectory of numeracy into older age. The current study investigates longitudinal age effects on numeracy using a sample of 524 adults (2008 Agerange = 20-78) from the RAND American Life Panel. Participants completed a numeracy measure in both 2008 and 2019, a span of 11 years. Results show that numeracy scores generally decreased between measurement points ($\beta = -.24$, $t = -2.27$, $p = .03$) and that increased age was predictive of a larger decrease in numeracy scores ($\beta = -.02$, $t = -2.07$, $p = .04$). Results are discussed as they relate to cognitive aging and how the trajectory of numeracy compares to other cognitive constructs.

### COHORT SHIFTS IN POPULATION COGNITIVE AGING

Patrick O'Keefe[1], Scott Hofer[2], Stacey Voll[2], Graciela Muniz Terrera[3], Linda Wänström[4], Sean Clousten[5], and Joeseph Rodgers[6], *1. Oregon Health and Science University, Portland, Oregon, United States, 2. University of Victoria, Victoria, British Columbia, Canada, 3. Ohio University, Athens, Ohio, United States, 4. Linkoping University, Linkoping, Ostergotlands Lan, Sweden, 5. Stony Brook University, Stony Brook, New York, United States, 6. Vanderbilt University, Nashville, Tennessee, United States*

Declines in cognitive functioning with increased age, on average and individually, is well documented and demonstrated to be related to genetics and a variety of life course risk factors, many of which are modifiable. Related to population cognitive aging is the phenomenon of the Flynn effect, the finding of increasing cognitive test scores across successive cohorts of young adults (e.g., Flynn 1987). This cohort effect has been repeatedly observed, in a wide variety of contexts, for over 30 years, with evidence that it has been occurring for at least a century. Our research looks at the interaction of the population cognitive aging and the Flynn effect. Using data from the English Longitudinal Survey of Ageing (ELSA) we show that, indeed, later born cohorts show significant (and meaningful) differences from earlier born cohorts. Using nonlinear Bayesian modeling we find that, on certain measures, later born cohorts have higher initial ability. This higher ability leads to a persistent advantage for later born cohorts, even as they experience (or will experience) cognitive decline. Additionally, we find that the advantage for later born cohorts is not present for every measure. Later born cohorts show an advantage for verbal fluency and episodic memory, however there is no cohort advantage on orientation scores. The lack of measurable differences on orientation is likely due to ceiling effects on orientation, which suggests that any substantial decline on that measure is indicative of pathology. We discuss the potential factors underlying both population cognitive aging and recent birth cohort trends.

### EARLY LIFE ADVERSITY, ALLOSTATIC LOAD, AND COGNITIVE FUNCTION AMONG MIDDLE-AGED AND OLDER ADULTS

Danielle D'Amico[1], Maya Amestoy[2], and Alexandra Fiocco[3], *1. Toronto Metropolitan University, York, Ontario, Canada, 2. University of Toronto Scarborough, Thornhill, Ontario, Canada, 3. Toronto Metropolitan University, Toronto, Ontario, Canada*

Early life adversity (ELA) is consequential for poor cognitive health in mid to late life. ELA is associated with higher allostatic load (AL), a biological indicator of physiological dysregulation due to cumulative wear-and-tear from chronic stress. Higher AL is also associated with poorer cognitive function across the lifespan. To date, however, a paucity of research has investigated AL as a mechanism through which ELA impacts cognition. Using cross-sectional data from the Midlife in the United States (MIDUS) Study, the objective of this study was to investigate the mediating role of AL in the relationship between ELA and cognitive performance (global cognition, episodic memory, executive function) among middle-aged and older adults without cognitive impairment (n=1541, mean age=53±12, 53% female). ELA, including physical, emotional, and sexual experiences, was measured retrospectively using the Childhood Trauma Questionnaire. AL was composed of 20 biomarker proxies of neuroendocrine, metabolic, inflammatory, and cardiovascular systems, stratified by sex. Cognitive performance was evaluated using a battery of neuropsychological tests from the Brief Test of Adult Cognition by Telephone. Controlling for age, education, and ethnicity, AL significantly mediated the relationship between ELA and global cognition (ß=-0.01,95%CI[-0.02,-0.003]) and executive function (ß=-0.01,95%CI[-0.02,-0.003]) such that higher ELA was associated with higher AL, and higher AL was associated with poorer global cognition and executive function. No such effects were found for episodic memory. Consistent with the biopsychosocial lifespan model of cognitive aging, findings suggest that ELA may become biologically embedded over time to negatively impact cognitive function in later adulthood in a domain-specific manner.

### FAMILY FLYNN EFFECTS AND LINKS TO MIDDLE-AGE HEALTH OUTCOMES

Linda Wänström[1], Patrick O'Keefe[2], Sean Clousten[3], Frank D. Mann[4], Graciela Muniz Terrera[5], Stacey Voll[6], Scott Hofer[6], and Joeseph Rodgers[7], *1. Linkoping University, Linkoping, Ostergotlands Lan, Sweden, 2. Oregon Health and Science University, Portland, Oregon, United States, 3. Stony Brook University, Stony Brook, New York, United States, 4. Stony Brook University, New York, New York, United States, 5. Ohio University, Athens, Ohio, United States, 6. University of Victoria, Victoria, British Columbia, Canada, 7. Vanderbilt University, Nashville, Tennessee, United States*

The Flynn effect (Flynn, 1984; 1987) refers to increases in cognitive performance, for later-born cohorts. It has been documented globally, occurring for more than a century. In a meta-analysis, Pietschnig and Voracek (2015) noted that the effect may be even stronger in adults than in children, though little research has addressed this topic (or its implications) for aging adults. Similarly, overall life-time health has improved, and incidences of cognitive impairment have decreased during the last two decades (Clouston et al., 2021). Using multilevel growth curve models, we found family Flynn effects in the National Longitudinal Survey of Youth; children in families with later-born mothers, and later-born first children, had higher PIAT math scores, and steeper developmental slopes. Although the link from childhood and adolescent cognitive function to later life outcomes has been well studied, research that takes advantage of the Flynn effect to facilitate interpreting that link is lacking. Clouston et al. (2021) emphasized the value of the Flynn effect in investigating links between childhood cognitive functioning and later adult Alzheimer's disease and related dementia (ADRD) risks. We linked our family level results to middle-age maternal health outcomes (factors that are related to ADRD risks). Canonical correlation analyses showed that mothers (at ages 40+ and 50+) from families with higher score levels and slopes tended to have better mental and physical health. Our results, showing a Flynn effect in child and adolescence scores, at the family level, with links to adult health, persisted after controlling for a known selection bias.

### PHYSIOLOGICAL DYSREGULATION AS A PREDICTOR OF COGNITIVE DECLINE

Elizabeth Mahon[1], Sharon Stein Merkin[2], Margie Lachman[1], Arun Karlamangla[3], and Teresa Seeman[2], *1. Brandeis University, Waltham, Massachusetts, United States, 2. University of California Los Angeles, Los Angeles, California, United States, 3. University of California, Los Angeles, Los Angeles, California, United States*

Preclinical indicators of disease such as inflammation, cortisol and glucose dysregulation, and multisystem dysregulation (allostatic load) are related to individual differences in the level of cognitive functioning across adulthood. This study examined whether individual biological systems and allostatic load are related to differential patterns of change in cognitive functioning over 9 years. Data are from the Midlife in the United States (MIDUS) study second (biomarker and cognitive) and third waves (cognitive). The sample includes 863 men and women who ranged in age from 35 to 85 when the data were first collected. MIDUS biomarkers include a comprehensive range of biological and anthropometric measurements reflecting cardiovascular functioning, glucose metabolism, lipid metabolism, inflammation, HPA axis function, as well as sympathetic and parasympathetic nervous system function. Summary indices of dysregulation in each of these major systems as well as an overall index of multi-system dysregulation, or allostatic load were examined in relation to 9-year changes in episodic memory and executive functioning from the Brief Test of Adult Cognition by Telephone. Regression analyses, controlling for preexisting diseases and medications, showed that higher allostatic load was associated with decreased executive functioning over time for those who started out with higher cognitive performance at baseline, after adjusting for age, gender, race, English language, education, neurological conditions, medication use and smoking. Identifying biomarkers as antecedents of cognitive changes in midlife and old age, can potentially aid in the early detection of cognitive impairments and increase the possibilities for preventive interventions.

### SESSION 6450 (POSTER)

### COGNITIVE FUNCTION (HEALTH SCIENCES POSTERS)

### USEFULNESS OF REVISED SIMPLIFIED SHORT-TERM COGNITIVE SCREENING TEST (STMT-R) IN ACUTELY ILL GERIATRIC PATIENTS

Hiroshi Yamamoto[1], Kenichi Ogawa[1], Yasushi Hisamatsu[1], and Tatsuya Ishitake[2], *1. Yamamoto Memorial Hospital, Imari-city, Saga, Japan, 2. Kurume University School of Medicine, Kurume-city, Fukuoka, Japan*

Background: Dementia can be a major cause of mortality and morbidity in geriatric patients. So, it would be essential to assess their mental state.Aims: We aim to appraise the impact of cognitive dysfunction on the long-term prognosis on STMT-R as a quicker and sensitive cognitive identification in acutely ill geriatric patients.

Methods: The inclusion criteria were to measure geriatric patients by STMT-R at admission, age≧50yo and being non-critical ill. Between October 2014 and September 2015, 836 were enrolled (52.4% female, mean age: 78.9 years). STMT-R≦4 was considered as cognitive dysfunction. Following the collection of clinical data, survival was subsequently measured for 7-8 years until January 2022. Cox's proportional hazards regression models were used to evaluate the hazard of death according to the dementia severity, with adjustment for potential covariates. Survival was estimated using Kaplan-Meier method.

Results: Among enrolled subjects, 144 were unable to complete the test due to severe dementia (ITG). 433 had cognitive dysfunction (STMT-R≦4; CDG) and 259 didn't have cognitive dysfunction (STMT-R>4; NCDG). The survival curves for death among three groups were significantly decreased in the CDG and ITG compared with the NCDG. The risks for mortality in the ITG and CDG are 3.92 (hazard ratio; 95% confidence interval:2.74-5.61, p< 0.001) and 1.82(1.33-2.51, p< 0.001) compared with the NCDG as reference.

Conclusion: 1) It was suggested that severity of cognitive dysfunction at admission has independently an impact on survival rate in acutely ill geriatric patients.2) STMT-R may also be useful for the future bedside or remote cognitive assessment.

### ASSOCIATION BETWEEN SUBJECTIVE COGNITIVE DECLINE AND STRENGTH TRAINING IN US ADULTS AGED 45+ YEARS

Matthew Schroeder[1], Molly Waring[2], Nicole Fowler[3], and Sherry Pagoto[2], *1. Regenstrief Institute, Inc., Indianapolis, Indiana, United States, 2. University of Connecticut, Storrs, Connecticut, United States, 3. Indiana University School of Medicine, Indianapolis, Indiana, United States*

Subjective cognitive decline (SCD) can be an early marker for Alzheimer's disease and related dementias. Data supports physical activity to delay cognitive impairment and to improve cognitive functioning. We examined strength training engagement by middle-aged and older US adults with and without SCD. We used data from 121, 059 participated aged 45 years or older from the 2019 Behavioral Risk Factor Surveillance System (BRFSS) from 31 states and Washington, D.C. SCD was assessed by asking participants if they had experienced confusion or memory loss during the past 12 months (yes/no). Participants reported how often they engaged in strength training (e.g., using weight machine, free weights) in the past month. We dichotomized strength training engagement as meeting physical activity recommendations (2+ times weekly) or not (< 2 times weekly). An adjusted logistic regression model, controlling for confounding variables, estimated the likelihood of strength training in relation to SCD. Analyses were weighted; results are nationally representative. SCD was reported by 11.0% (SE: 0.2%) of middle-aged and older US adults. Three in 10 (29.1%; SE: 0.7%) of US middle-aged and older adults who reported SCD engaged in strength training 2+ times a week compared to 34.0% (SE: 0.3%) of US adults without SCD (aOR, 0.9; 95% CI: 0.9-1.0). While middle-aged and older US adults with SCD were less likely to strength train than those without SCD, only a third engaged in recommended strength training regardless of SCD status. Primary care providers should encourage strength training among middle-aged and older adults regardless of cognitive status.

### HIGHER PHYSICAL FITNESS CLUSTERS SHOWED GREATER GLOBAL COGNITIVE OUTCOMES IN MIDDLE- TO OLDER-AGED ADULTS

Joshua Gills[1], Megan Jones[1], Anthony Campitelli[1], Sally Paulson[2], Kelsey Birk[3], Jennifer Myers[4], Jordan Glenn[4], and Michelle Gray[1], *1. University of Arkansas, Fayetteville, Arkansas, United States, 2. St. Elizabeth Healthcare, Cincinnati, Ohio, United States, 3. Neurotrack Technologies, Inc., Redwoods City, California, United States, 4. Neurotrack Technologies, Redwood City, California, United States*

Approximately, 6 million individuals in the United States are living with Alzheimer's disease. A new diagnosis occurs every 67 seconds, which will triple the rates by 2050. Recently, physical dysfunction has been associated with cognitive decline; however, usually, this is examined in one dimension of physical fitness (PF). A more robust way including multiple domains of PF would be beneficial in examining the relationship between PF and cognition. Therefore, the purpose of this investigation was to examine cognition in clustered PF variables among middle to older-aged adults. Participants (n=216;73% female) enrolled and completed a DXA scan, RBANS, handgrip, sit-to-stand power with TENDO, dual-task (4-meter and 10-meter), and 6-minute walk distance test. A hierarchal cluster analysis was utilized to identify PF cluster for participants, a one-way ANOVA was used to assess differences in cognition between clusters. Cluster 1 (C1;n=29) was characterized with the highest physical fitness values, cluster 2 (C2;n=74) was in-between C1 and C3, cluster 3 (C3;n=113) had the lowest values among PF variables. C1 had significantly higher global cognitive and visuospatial scores compared to C3 ($p < 0.05$). C1 and C2 had significantly higher values on line orientation and figure recall than C3 ($p < 0.05$). Data showed high PF clusters had higher global cognitive values when compared to lower PF clusters. Moreover, higher PF showed greater visuospatial and delayed memory values compared to lower PF. Clustering PF tasks served as a practical tool evaluating cognition—this may be useful for future interpretation of cognitive decline where higher PF represents higher overall cognition.

### THE RELATIONSHIP BETWEEN SKIPPING BREAKFAST AND DEMENTIA: A RETROSPECTIVE COHORT STUDY IN OSAKA

Hitomi Chikama[1], Reiko Kanaya[2], Yasushi Takeya[3], Hiroshi Toki[4], Ryohei Yamamoto[3], and Miyae Yamakawa[3], *1. Osaka University, Suita City, Osaka, Japan, 2. Osaka University, Osaka, Osaka, Japan, 3. Osaka University, Suita, Osaka, Japan, 4. Health and Counseling Center, Suita, Osaka, Japan*

In Japan, annual medical checkups are carried out to prevent lifestyle diseases. Studies have found that skipping breakfast is a risk factor for diabetes, and potentially also dementia. This retrospective cohort study aimed to evaluate the relationship between skipping breakfast and dementia in people with diabetes. The eligible cohort was anyone on the National Health Insurance Database of Osaka who had a checkup in FY 2013 and had diabetes (N = 283,410). The cohort was divided into two groups, those who skipped breakfast more than three times a week (7.38%) and those who did not. The database does not allow individuals to be identified. People with diabetes were defined as those prescribed diabetes drugs in the year before the checkup or with HbA1c of 6.5% or higher at the checkup. People with dementia were defined as those prescribed anti-dementia drugs between April 2014 and December 2017. Data also included body mass index (BMI), smoking, sex , age, and prescription of hypolipidemic or blood pressure drugs. Logistic regression analysis was used, with odds ratio (OR) and 95% confidence interval . Being male, younger, smoking, having lower BMI or HbA1c levels, and drug prescriptions were associated with skipping breakfast. Skipping breakfast was associated with dementia (OR 1.26, 1.14–1.41), as was lower BMI and being older. For people with diabetes, skipping breakfast is a risk factor for obesity and dyslipidemia, which are associated with dementia. This study therefore provides evidence for a health behavior approach to eating breakfast in people with diabetes.

### ASSOCIATION OF SUBJECTIVE AND OBJECTIVE COGNITION WITH DISEASE AWARENESS: EVIDENCE FROM BLOOD BIOMARKERS DATA

Zhuoer Lin[1], Mingqi Fu[2], and Xi Chen[1], *1. Yale University, New Haven, Connecticut, United States, 2. Wuhan University, Wuhan, Hubei, China (People's Republic)*

Impaired cognitive ability and its misperception contribute to poor decision-making of older adults; yet their impact on health-related decisions is less known. We examined how self-perceived and actual cognition were associated with chronic disease awareness using a nationally representative sample of Chinese older adults. Blood biomarkers data were collected in 2015 to identify participants' dyslipidemia and diabetes status. Among participants with identified dyslipidemia or diabetes, disease awareness was defined as self-reported diagnosis of the conditions as of 2018. Objective and subjective cognition were respectively assessed using the Mini-Mental State Examination and self-rated memory. The associations of subjective and objective cognition with chronic disease awareness were determined by weighted logistic regressions. Among 4,578 adults aged 60 and older with complete measurements, 1,442 and 759 individuals were identified having dyslipidemia and diabetes, with proportions of disease awareness being 38.0% and 58.1%, respectively. Individuals with mild (Odds Ratio [OR]=0.63; 95%CI: 0.45-0.89) or severe cognitive impairment (OR=0.46; 95%CI: 0.29-0.72) had lower odds of dyslipidemia awareness; and those with severe cognitive impairment had lower odds of diabetes awareness (OR=0.43; 95%CI: 0.23-0.79) than cognitively intact counterparts. However, adjusting for objective cognition, older adults with better subjective cognition had lower odds of dyslipidemia (OR=0.80; 95%CI: 0.63-1.02) and diabetes (OR=0.71; 95%CI: 0.55-0.92) awareness. Such associations were stronger for individuals with rural status, lower education, or living without children. Our findings highlight the great challenges deteriorated cognitive function and misperception may pose to chronic disease awareness, as well as the importance of targeted supports particularly for those more disadvantaged.

### SENSORY IMPAIRMENT, COGNITIVE FUNCTION, AND DEPRESSION AMONG CHINESE OLDER ADULTS

Shu Xu, and YanJhu Su, *University of Massachusetts Boston, Boston, Massachusetts, United States*

Sensory impairment (SI) is a contributor to poor mental health and cognitive decline for older adults, and the likelihood of having sensory impairment increases with age. However, the association between sensory impairment and cognition is still under-investigated and the potential mechanisms for the SI-cognition link is still not clear. This study examines the relationships between sensory impairment, depression, and cognitive function among older adults in China. Using nationally representative data from the China Health and Retirement Longitudinal Study 2018, we conducted cross-sectional analysis on adults age 60 years and older (n=7,026). Sensory impairment is defined as having vision impairment (VI) only or hearing impairment (HI) only or dual sensory impairment (DSI). Cognitive function was measured by the Mini-Mental State Examination and depression were assessed by the 10-item Center for Epidemiologic Studies Depression scale. Descriptive analysis showed that 10.66% of older adults experienced sensory impairment. Linear regression analyses revealed that HI and DSI were associated with cognitive declines among older Chinese adults (HI: $\beta$=-0.75, p<.01; DSI: $\beta$=-1.45, p<.01). SEM results showed that depression partially mediate the relationship between SI and cognition. Sensory-impaired older adults were more likely to have depression (HI: $\beta$=1.71, p<.001; DSI: $\beta$=4.76, p<.001), which lead to worse cognitive function (HI: $\beta$=-1.09, p<.001; DSI: $\beta$=-2.80, p<.001). Models were controlled for age, gender, education, social activities, and other covariates. Findings suggest that Chinese older adults experiencing sensory loss are at greater risk of cognitive function declining and that depression play an important role in the relationship between SI and cognition.

### IMPLEMENTATION BARRIERS AND FACILITATORS OF COGNITIVE STIMULATION THERAPY ACROSS CARE SETTINGS

Milta Little[1], Kim Johnson[2], Francis Karia[2], and Eleanor McConnell[2], *1. Duke University, Hillsborough, North Carolina, United States, 2. Duke University, Durham, North Carolina, United States*

Cognitive Stimulation Therapy (CST) is an evidence-based treatment for people living with dementia that is not in widespread use in the United States. To better understand barriers and facilitators to implementation of CST, we conducted virtual focus groups of newly trained facilitators of Cognitive Stimulation Therapy (CST). Of 12 facilitators trained, representing two settings of care (The Program for All-inclusive Care of the Elderly and a Continuing Care Retirement Community), 4 facilitators, representing Social Work n = 2, Speech Therapy n = 1, and Recreation Therapy n = 1, participated. We analyzed interview transcriptions using framework analysis guided by constructs from the Consolidated Framework for Implementation Research (CFIR). Themes that emerged across constructs were 1) balancing competing personal, stakeholder, and organizational needs; 2) lack of mastery experiences and the need for opportunities for more practice, reflection, and feedback from trainers; 3) building a community of facilitators to provide peer support; 4) logistical concerns; and 5) identifying appropriate participants who would most benefit from CST. The barriers to implementation imposed by the ongoing COVID-19 pandemic were also a cross-cutting theme. Many of these barriers are readily managed, and professionals who implement CST in their sites should be aware of and take into consideration these barriers and facilitators.

### IMPROVING COGNITION IN OLDER CANCER SURVIVORS USING NONPHARMACOLOGIC INTERVENTIONS: A SYSTEMATIC REVIEW

Jennifer Blackwood[1], and Abigail Simone[2], *1. University of Michigan-Flint, Flint, Michigan, United States, 2. University of Michigan--Flint, Flint, Michigan, United States*

Cancer-related cognitive dysfunction (CRCD) is a side-effect of chemotherapy, particularly among young and adult populations. Results indicate CRCD is also prevalent in older adult populations. While various non-pharmacological interventions for cognitive impairments have been studied in young cancer and older adult populations, limited information is available regarding non-pharmacologic interventions for older adults with cancer. The purpose of this systematic review is to describe the current non-pharmacologic interventions for CRCD in the older adult cancer population. Databases searched included PubMed, MEDLINE, CINAHL, and EMBASE. Articles meeting inclusion criteria were appraised by 2 reviewers independently. The Cochrane Risk of Bias Assessment was used to assess study quality. The search located 3441 articles; 4 met inclusion criteria. Cognitive domains assessed by included studies comprised executive function (n=2), attention (n=1), learning/memory (n=2), perceptual-motor (n=1), and a general measure of global cognitive function (n=3). Two studies used exercise interventions and 2 employed cognitive training interventions. One exercise intervention improved executive function, while attention and learning/memory improved following cognitive training. However, a limited number of studies utilizing non-pharmacological approaches for treating cognitive impairment in this population showed high methodological heterogeneity. Non-pharmacologic interventions demonstrated positive outcomes for CRCD, however, methodological concerns in the included studies prevented definitive recommendations from being made. Findings may guide additional studies needed in this field in order to make more robust conclusions.

### INFLAMMATORY BIOMARKERS ASSOCIATED WITH COGNITIVE FUNCTION AND DEMENTIA

Jiachen Chen[1], Yuan Fang[1], Kathryn Lunetta[1], Joanne Murabito[2], and Margaret Doyle[3], *1. Boston University School of Public Health, Boston, Massachusetts,*

*United States, 2. Boston University, Boston, Massachusetts, United States, 3. University of Vermont, Colchester, Vermont, United States*

Neuroinflammation is an important contributor to the pathogenesis of dementia, but the role of circulating inflammatory markers is unclear. The aim of the study is to identify circulating inflammatory biomarkers associated with cognitive function in the Framingham Heart Study Offspring cohort. We used OLINK inflammation panel to measure 92 inflammation protein biomarkers using stored plasma samples at Offspring examination 7 (1998-2001) from 879 participants (52% female, mean 61 years range 40 to 88 years, 22% APOE e4 carrier). Cognitive function was assessed with seven tests derived from a neuropsychological (NP) testing battery representing four cognitive domains collected closest to the 7th examination. 68 proteins with at least 50% values above the limit of detection (LOD) were clustered with hierarchical clustering, and data below LOD were replaced with LOD/√2. We identified nine clusters of highly correlated proteins and created cluster composite scores on the first principal component of each. Adjusting for age, sex and education, five of the seven NP tests are nominally associated ($p < 0.05$) with at least one of the protein clusters, and five of the nine clusters are associated with at least one of the NP tests. The executive function test Trails B–Trails A is nominally associated with three clusters, and the Boston Naming Test 30 items version with two clusters, both sharing one common cluster which includes IFN-γ-inducible chemokines involved in inflammatory conditions. Future analyses will explore individual protein associations with cognitive test performance and incident dementia and will investigate the biological relevance of the identified clusters.

### PREFRONTAL BLOOD FLOW DURING DUAL-TASK WALKING IN ADULTS WITH AND WITHOUT MILD COGNITIVE IMPAIRMENT

Cristina Udina Argilaga[1], Stella Avtzi[2], Turgut Durduran[2], Andrea Rosso[3], Miriam Mota[4], Joan Ars[1], and Marco Inzitari[1], *1. Parc Sanitari Pere Virgili, Barcelona, Catalonia, Spain, 2. Institut de Ciències Fotòniques, The Barcelona Institute of Science and Technology, Barcelona, Catalonia, Spain, 3. University of Pittsburgh, Pittsburgh, Pennsylvania, United States, 4. Vall Hebron Research Institute (VHIR), Barcelona, Catalonia, Spain*

Background: Blood flow differences in the prefrontal cortex (PFC) during dual-task walking are thought to indicate various degrees of neural efficiency. Individuals with poorer neural resources might need higher activation to meet behavioral performance. We aim to assess PFC cerebral blood flow (CBF) among older adults with and without mild cognitive impairment (MCI) during dual-task using functional Diffuse Correlation Spectroscopy (fDCS).

Methods: We assessed PFC CBF with DCS during dual-task paradigm: 1)Normal Walk(NW); 2)Forward-count(FWC); 3)Backward-count(BWC); 4)Obstacle negotiation(WWO). We assessed demographics, clinical variables, physical and cognitive function in those with MCI vs normal cognition (NC). Linear mixed effects models assessed changes of CBF across the tests in the dual-task paradigm and differences between MCI and NC.

Results: 49 older adults (median age=78 years, 51% women, 34 MCI) were included. MCI were older, with higher frailty, polypharmacy and comorbidity. Compared to NC, MCI showed worse cognitive and physical performance scores and lower Gait Speed (GS) during NW and WWO but not during FWC and BWC. N=12 were unable to perform BWC. CBF change from NW to FWC was higher in MCI compared to NC (estimate=0.35, 95%CI [0.03, 0.67], p=0.03). CBF change from NW to BWC and WWO was not different between groups. There was no effect of age or clinical covariates.

Conclusions: Higher NW-FWC CBF change seems due to the cognitive load of FWC in MCI. Higher activation in MCI compared to healthier counterparts could be explained by compensatory mechanisms. Further research should focus on better understanding dual-task related neural mechanisms.

### THE EQUIVALENCE OF CANTAB AS A COGNITIVE FUNCTION MEASURE FOR CHINESE STROKE SURVIVORS: A PILOT STUDY

Fanfan Li[1], Huanzhi Zhu[1], Bei Wu[2], Hanzhang Xu[3], Jing Wang[4], and Juan Li[5], *1. Naval Military Medical University, Shanghai, Shanghai, China (People's Republic), 2. New York University, New York, New York, United States, 3. Duke University, Durham, North Carolina, United States, 4. University of North Carolina at Chapel Hill, Chapel Hill, North Carolina, United States, 5. Huashan Hospital affiliated to Fudan University, Shanghai, Shanghai, China (People's Republic)*

This study aims to investigate the equivalence between Cambridge Neuropsychological Test Automated Battery (CANTAB) and Montreal Cognitive Assessment, Changsha version (MoCA-CS) as cognition measures for Chinese stroke survivors. Sixteen stroke survivors were recruited from stroke center in Shanghai, China. Participants completed Paired Associates Learning (PAL) task in CANTAB, MoCA-CS and questionnaire about demo-social characteristics. Pearson correlation and hierarchical regression were used for equivalence analysis. CANTAB-PAL task performances ( PAL First Attempt Memory Score, PALFAMS, PAL Total Attempts 2 Patterns, PALTA2 ) were significantly associated with score of MoCA-CS and delayed recall (β=0.528, p=0.014; β=0.198, p=0.043; β= -3.885 , p=0.017; β= -1.600,p=0.026, respectively ) after controlling for age, gender and education. The results suggest CANTAB-PAL is a reliable tool for measuring Chinese stroke survivors' cognitive function. Future studies are needed to establish the equivalence of CANTAB in multi-tasks in larger sample of Chinese stroke survivors.

### DOES NEGATIVE AFFECT MODERATE THE RELATIONSHIP BETWEEN SUBJECTIVE COGNITIVE CONCERNS AND OBJECTIVE FUNCTIONING?

Bradley Dixon, Kylie Kadey, and John L. Woodard, *Wayne State University, Detroit, Michigan, United States*

The onset of Alzheimer's disease can be insidious, slowly affecting an individual's cognitive abilities. Previous research demonstrated that informant-reported cognitive decline was associated with significantly worse baseline and longitudinal cognitive performance than was a participant's subjective perceptions of decline. What remains unclear is how negative affect (i.e., depressive symptoms) could moderate the relation between objective cognitive performance and subjectively perceived cognitive concerns (participant vs. informant). Using the National Alzheimer's Coordinating Center (NACC) database, we performed moderated multiple regressions to test whether Geriatric Depression Scale (GDS) score showed different relationships with cognitive measures (animal/vegetable fluency, digit span, Boston Naming Test, digit-symbol coding (DSC), Wechsler Memory Scale Logical Memory, Trail-Making Test, and Mini-Mental State Examination) for participant- and informant-reported cognitive decline (yes/no). Participants were aged ≥65 years and were cognitively healthy at baseline. Informants lived with the participant or visited the participant weekly (N = 9,354). Participant-reported cognitive concern interacted significantly ($p<.05$) with negative affect only for animal fluency while informant-reported cognitive concern interacted significantly with DSC. Depressive symptoms were associated more strongly with cognitive performance for participants who did not report a subjective cognitive decline compared to those who did report a subjective cognitive decline. Participant age showed significant negative relationships with all measures while GDS score showed significant negative relationships with all measures except immediate Logical Memory recall, regardless of decline status. In conclusion, negative affect generally did not moderate the relationship between participant- or informant-reported cognitive concerns and objective cognitive functioning except for animal category fluency and DSC.

### PREVALENCE AND FACTORS ASSOCIATED WITH MOTORIC COGNITIVE RISK IN A COMMUNITY-DWELLING OLDER SCOTTISH POPULATION

Donncha Mullin[1], Lucy Stirland[1], Tom Russ[1], Michelle Luciano[1], and Graciela Muniz Terrera[2], *1. University of Edinburgh, Edinburgh, Scotland, United Kingdom, 2. Ohio University, Athens, Ohio, United States*

Motoric Cognitive Risk (MCR) syndrome combines slow walking and self-reported cognitive complaints. It is a quick and simple way of identifying individuals at high risk of developing dementia. MCR has not been described in a Scottish population to date. This study describes the prevalence and associated factors of MCR in a community-dwelling sample of older Scottish people. The MCR concept was derived in the Lothian Birth Cohort 1936 (LBC1936) - a highly phenotyped cohort of over 1000 people followed up every 3 years since 2004. Uniquely, participants in LBC1936 had their IQ measured at age 11 in 1936. Authors found MCR prevalence of approximately 5.4% at baseline. Using logistic and linear regression analysis, as appropriate, they found that participants' age, depressive symptoms and cognitive measures of executive function were significantly associated with an increased likelihood of have MCR, but that IQ aged 11 was not associated. This study found rates of MCR in Scotland are within the typical range for this age group, albeit on the lower end. Interestingly, IQ at age 11 was not significantly associated with MCR, which was unexpected given MCR's prognostic value for dementia. That tests of executive function were associated with MCR adds further credence to the hypothesis that walking speed and executive function are linked. This points to further important work to ascertain if increasing walking speed can improve executive function.

### SEX DIFFERENCES IN THE ASSOCIATION BETWEEN BODY COMPOSITION AND 2-YEAR CHANGE IN COGNITIVE FUNCTION

Daehyun Lee[1], Miji Kim[2], Hyung Eun Shin[2], Jae Young Jang[2], and Chang Won Won[2], *1. Kyung Hee Univerisity, Seoul, Seoul-t'ukpyolsi, Republic of Korea, 2. Kyung Hee University, Seoul, Seoul-t'ukpyolsi, Republic of Korea*

In the process of aging, the loss of lean mass and increase in fat mass are associated with cognitive decline. This study investigated sex differences in the association between body composition and changes in cognitive function in community-dwelling older adults in Korea. A total of 1,420 participants (aged 70–84 years, 54.2% men) of the Korean Frailty and Aging Cohort Study with data from baseline and 2-year follow-up surveys were included. Body composition was measured using dual-energy X-ray absorptiometry and cognitive function was assessed using the Korean version of the Consortium to Establish a Registry for Alzheimer's Disease Assessment Packet. The total fat mass was lower in men than in women ($p< 0.001$), whereas total lean mass was higher in men than in women ($p< 0.001$). Total body fat mass was positively associated with the time taken to finish the Trail Making Test-A only in women (standardized beta coefficient [ß]= -1.371, p=0.018), and a negative association was observed between trunk fat mass and digit span total only in men (ß= -0.092, p=0.039). Appendicular lean mass was significantly positively associated with word list recognition only in women (ß=0.087, p=0.010) and was significantly positively associated with digit span total (ß=0.108, p=0.027) and digit span forward (ß=0.081, p=0.025) only in men. The results of this study indicated that higher fat mass was associated with the protection of decline in cognitive function only in women, while lean mass was positively associated with a change in cognitive function in both sexes.

### COPING WITH COGNITIVE DECLINE AMONG OLDER ADULTS WITH MILD COGNITIVE IMPAIRMENT OR MILD DEMENTIA: A SCOPING REVIEW

Youngmin Cho[1], Donruedee Kamkhoad[2], and Anna Beeber[3], *1. University of North Carolina - Chapel Hill, Chapel Hill, North Carolina, United States, 2. The University of North Carolina at Chapel Hill School of Nursing, Chapel Hill, North Carolina, United States, 3. Johns Hopkins University, Baltimore, Maryland, United States*

As cognitive impairment progresses, Persons with Mild Cognitive Impairment or Mild Dementia (PwMCI or MD) have trouble in adapting to changes in their cognitive functioning and, as a result, develop challenges in daily activities. If these challenges are not appropriately addressed, PwMCI or MD can experience poor health outcomes and quality of life. Coping, behavioral and cognitive efforts to regulate the distress from a certain stressful situation, is widely regarded as a fundamental determinant of health outcomes. We conducted a scoping review to understand what coping strategies PwMCI or MD used, and facilitators and barriers of the use of these coping strategies. Using the PRISMA-ScR

guideline, we reviewed peer-reviewed empirical studies exploring coping experiences for cognitive impairment among community-dwelling PwMCI or MD. We used thematic synthesis to generate themes relevant to the coping strategies and the facilitators and barriers. Of 1267 studies identified, 12 qualitative studies were reviewed. Under three dimensions of coping strategy (i.e., problem-solving, emotional, and maladaptive coping strategies), six themes were identified: independent coping (e.g., use of reminder and practice of cognitive activities), collaborative/dependent coping (e.g., asking help as needed), reframing, expression of unpleasant feelings, comparing self to others, and avoidance. The themes for facilitators and barriers were social response to one's cognitive impairment, assistance from informal care partners, and support from professionals. These findings can provide not only a better understanding of how people cope with their cognitive impairment but can also provide rationale for developing interventions to maximize the use of coping strategies.

### MENTAL-SOMATIC MULTIMORBIDITY IN GROUP-BASED TRAJECTORIES OF COGNITIVE FUNCTION FOR MIDDLE-AGED AND OLDER ADULTS

Siting Chen[1], Corey Nagel[2], Anda Botoseneanu[3], Heather Allore[4], Jason Newsom[5], Stephen Thielke[6], Jeffrey Kaye[1], and Ana Quiñones[7], *1. Oregon Health & Science University, Portland, Oregon, United States, 2. University of Arkansas for Medical Sciences, Little Rock, Arkansas, United States, 3. University of Michigan Dearborn, Dearborn, Michigan, United States, 4. Yale University, Guilfrod, Connecticut, United States, 5. Portland State University, Portland, Oregon, United States, 6. University of Washington, Seattle, Washington, United States, 7. Oregon Health and Science University, Portland, Oregon, United States*

Somatic and mental multimorbidity contributes to cognitive decline. The study aims to identify distinct trajectories of cognitive performance among middle-aged and older adults, and to examine the contribution of specific somatic and mental multimorbidity combinations on subsequent risk of cognitive impairment. We used group-based trajectory modeling to identify trajectories of cognitive impairment risk among participants in the Health & Retirement Study during years 1998-2016 (N=20,372). We included time-invariant sociodemographic factors (sex, race/ethnicity, education) to quantify their association with trajectory class membership, and time-varying multimorbidity combinations to examine their relative impact on observed trajectories. Four somatic-mental multimorbidity combinations were analyzed: somatic multimorbidity (two or more of the following: heart disease, lung disease, hypertension, arthritis, diabetes, cancer), stroke-multimorbidity (any somatic including stroke); depressive-multimorbidity (any somatic including high depressive symptoms); and stroke and depressive multimorbidity (including both stroke and high depressive symptoms). We identified three trajectory classes of cognitive impairment: low baseline risk with gradual increase (55.1%); low baseline risk with rapid increase (32.8%); and high baseline risk with gradual increase (12.1%). In the group with low baseline risk with rapid increase, stroke-multimorbidity (OR: 2.40, 95%CI: 2.11, 2.74) and depressive-multimorbidity (OR: 1.65, 95%CI: 1.50,1.81) each had higher odds of cognitive impairment relative to somatic multimorbidity. Similarly, stroke and depressive multimorbidity (OR: 3.46, 95%CI: 2.85, 4.21) was associated with higher odds of cognitive impairment compared with somatic multimorbidity. This study highlights the importance of risk modification for somatic and mental multimorbidity combinations from mid-life to inform interventions to sustain cognitive performance.

### PSYCHOSOCIAL WORKING CONDITIONS IN MID-LIFE AND COGNITIVE AND PHYSICAL IMPAIRMENT IN OLDER AGE

Shireen Sindi[1], Shadi Kiasat[1], Ingemar Kåreholt[2], and Charlotta Nilsen[2], *1. Karolinska Institutet, Solna, Stockholms Lan, Sweden, 2. Jönköping University, Jönköping, Jonkopings Lan, Sweden*

Background: Psychosocial working conditions have been associated with cognitive and physical impairment among older adults. However, less is known on whether psychosocial working conditions are associated with a combination of cognitive and physical impairments. The aim of this study was to investigate the associations between midlife psychosocial working conditions and physical and cognitive impairment among older adults, and to assess whether there are sex differences in these associations.

Methods: The data were derived from two Swedish nationally representative surveys (n=839) with a follow-up time of 20-24 years. Multinomial and binary logistic regressions were used to assess the associations between work stressors according to the job demand-control model, and a combination of cognitive and physical impairment.

Results: Low control jobs were significantly associated with higher odds of both cognitive and physical impairment as well as a combination of cognitive and physical impairment. Passive jobs (low control, low demand) were associated with higher odds of cognitive impairment, and cognitive and physical impairment in combination. Active jobs (high control, high demand) were associated with lower odds of cognitive impairment. Sex-stratified analyses showed stronger associations among men than among women. Among men passive jobs were significantly associated with both cognitive and physical impairment. Low strain jobs were significantly associated with less physical impairment.

Conclusions: These results highlight the importance of midlife psychosocial working conditions for late-life physical and cognitive impairment, and especially among men. Jobs characterised by higher control, lower strain and active jobs may promote resilience and cognitive reserve among older populations.

### SKELETAL MUSCLE ADIPOSITY IS ASSOCIATED WITH LOWER COGNITION IN AFRICAN CARIBBEAN WOMEN

Adrianna Acevedo-Fontanez, Ryan Cvejkus, Allison Kuipers, Joseph Zmuda, Caterina Rosano, and Iva Miljkovic, *University of Pittsburgh, Pittsburgh, Pennsylvania, United States*

Objective: Skeletal muscle adiposity (SMA) increases with aging and is recognized as a major risk factor for cardiometabolic diseases, disability, and mortality among older adults. Obesity is related to dementia and cognitive decline yet the relationship between SMA and cognition remains ill defined. The objective of this study was to assess SMA and cognitive function among African Caribbean women.Design and

Methods: Cross-sectional analysis of 448 African Caribbean women in the Tobago Health Study (mean age, 55 years; range, 39-84 years). Cognition was assessed by the Digit Symbol Substitution Test (DSST), a test of information processing speed with a range of 0-90; higher scores suggest better cognitive function (faster information processing speed). Calf SMA (muscle density) was assessed with computed tomography (Stratec XCT-2000). Linear regression was used to assess the association of SMA with DSST adjusted for age, education, muscle area, waist circumference, alcohol intake, smoking, physical activity, diabetes, and hypertension.

Results: Participants had a BMI of 30.7 kg/m2. Mean (SD) DSST scores and SMA were 39.2 (13.1) and 71.7 (5.3) mg/cm3, respectively. After full adjustment, we found that one SD greater skeletal muscle adiposity was associated with a 1.40 lower DSST score (p-value=0.025).

Conclusions: Our findings suggest that in African Caribbean women, greater SMA is associated with slower information processing speed, an early indicator of future dementia risk. Future studies using an expanded battery of cognitive tests and longitudinal follow-up should further advance our understanding of the role of SMA and dementia risk among African ancestry populations.

### ASSOCIATIONS BETWEEN PRESCRIPTION OPIOID AVAILABILITY AND LONGITUDINAL CHANGES IN COGNITION IN OLDER ADULTS

Nafisseh Warner, Andrew Hanson, Phillip Schulte, Elizabeth Habermann, David Warner, and Michelle Mielke, *Mayo Clinic, Rochester, Minnesota, United States*

Opioids are frequently prescribed to alleviate pain symptoms in older adults, yet the relationships between prescription opioid availability and long-term cognitive function are incompletely defined. We evaluated the associations between prescription opioid availability and neuropsychological function and incident mild cognitive impairment (MCI) by performing a secondary analysis of the Mayo Clinic Study of Aging (MCSA), a longitudinal population-based observational cohort study with participants enrolled between 11/1/2004 and 4/1/2019 aged 65 years or older at the time of enrollment and without dementia. A total of 4,218 participants (51% male) were included with median age of 76 (interquartile range 72, 82) years. In multivariable analyses, each receipt of an opioid prescription, regardless of duration, resulted in an additional -0.007 (95% CI: -0.009, -0.005) change in global cognitive z-score (p< 0.001), with significant effects seen in the domains of memory (-0.005, 95% CI: -0.007, -0.003; p< 0.001), language (-0.002, 95% CI -0.003, 0.000; p=0.024) and attention (-0.004, 95% CI: -0.006, -0.002; p< 0.001) but not visual-spatial function (0.000, 95% CI: -0.001, 0.001; p=0.897). Among 2,963 cognitively unimpaired subjects with longitudinal follow-up, 769 (26%) developed incident MCI. Receipt of any opioid prescription during follow-up was associated with incident MCI in adjusted analysis (hazard ratio 1.21, 95% CI: 1.04, 1.42; p=0.014). In conclusion, prescription opioid availability was associated with cognitive decline across multiple domains and with incident MCI. These findings suggest that prescription opioid availability may have long-term effects on cognitive function.

### SESSION 6460 (POSTER)

### COMMUNICATION AND LANGUAGE ACROSS CARE SETTINGS

### THE ASSOCIATION BETWEEN SMARTPHONE USE AND HEALTH BEHAVIORS AMONG OLDER ADULTS WITH DISABILITIES

Eunjin Yang, *Yonsei University, Seoul, Seoul-t'ukpyolsi, Republic of Korea*

People actively use smartphones to maintain and improve their health in the digital era. With this, evidence exists of the positive effect of smartphone use on the well-being of people with disabilities. This study aimed to examine the preferred sources of health information among older adults with disabilities and the association between smartphone use and healthy behaviors. This study is based on data from the 2017 national survey on persons with disabilities carried out in South Korea. A total of 4,014 participants aged 60 and above were included. Logistic regression analysis was carried out to verify the association between smartphone use and health behaviors. As the study reports, 37.7% were smartphone users and 62.3% were non-smartphone users. Among health information resources, participants identified mass media, medical institutions, and acquaintances as the first, second, and third preferred sources, regardless of smartphone use. However, a higher proportion of non-smartphone users reported having no health information available to them compared to smartphone users (5.7% vs. 1.3%, respectively). Regarding health behaviors, older adults with disabilities who use smartphones are more likely to have regular health checkups (Odd Ratio [OR], 1.51), cancer screenings (OR 1.33), dental examinations (OR 1.26), and exercise (OR 1.47), and to consume a balanced diet (OR 1.23) compared to non-smartphone users. Although these results do not prove causal relationship, smartphone use is associated with healthy behaviors in this population. Considering that healthy lifestyles are crucial to achieving active aging, resolving the disability digital divide is needed for older adults with disabilities.

### DETECTING NARCISSISM FROM DAILY LANGUAGE USE: A MACHINE LEARNING APPROACH

Shiyang Zhang[1], Sibo Gao[2], and Karen Fingerman[3], *1. University of Texas at Austin, Austin, Texas, United States, 2. School of Human Ecology, Austin, Texas, United States, 3. The University of Texas at Austin, Austin, Texas, United States*

High-quality social contacts are important for older adult's emotional well-being whereas narcissism, characterized by self-centeredness, may undermine the quality of personal relationships. Narcissism may be associated with poorer quality relationships because narcissistic adults are more self-centered in their daily social interactions. The current study examined the associations between linguistic features of conversation throughout the day and narcissism using a machine learning approach. Older adults aged 65–89 (N = 281, Mage = 74.07) wore an unobtrusive electronically activated recorder (EAR) throughout the day for 4 to 6 days. The EAR was activated 30 seconds every 7 minutes to capture conversations occurring in a natural setting. The sound files that contained participant's speech (N = 28,243) were transcribed verbatim. We used Linguistic Inquiry and Word Count (LIWC) to extract linguistic features from the transcriptions (nfeature = 81; e.g., function words, affective processes). Linguistic features were analyzed using random forest algorithm which is a supervised machine learning method that can evaluate features' performances in predicting older adult's narcissism. The model reached an accuracy of 62% and results showed that total word count, more auxiliary verbs (e.g., will), more swear words (e.g., damn), and less assent (e.g., agree) were the most powerful predictors of narcissism. Drawing on sound files collected from real-life interactions, findings indicate higher usage of aggressive and disagreeable words among more narcissistic individuals, which probably leads to poorer quality relationships with social partners. Interventions aiming to improve contact quality and emotional well-being may consider individuals' word use in daily interactions.

### CAN YOU HEAR ME? A MEDICAL STUDENT-LED QUALITY IMPROVEMENT INITIATIVE FOR HOSPITALIZED PATIENTS WITH HEARING LOSS

Stephanie Rennke, Rachel Warren, Wynton Sims, Shreya Menon, Sasha Binford, Megan Rathfon, and Margaret Wallhagen, *University of California, San Francisco, San Francisco, California, United States*

In acute care settings hearing loss (HL) is associated with impaired patient-provider communication, increased length of stay, and increased mortality. COVID-19 has exacerbated this problem with the widespread use of masks and eye shields which muffle speech and prevent lip reading. Our 800-bed tertiary care medical center lacked a standardized approach to identify patients with HL and address communication barriers. Three first year medical students spearheaded the initiative as part of a health systems improvement curriculum with support from a faculty coach and a faculty researcher. From September 2020 to October 2021, the students met with stakeholders, leadership and champions and unit staff, identified the current state and completed a gap analysis through interviews, surveys, and direct observation. The pilot in May 2021 included two week-long PDSA cycles on one hospital unit to: 1) screen patients aged 65 and older using the validated 10-item HHIE-S questionnaire, 2) implement an education and awareness campaign with bedside signage, posters, and conferences and 3) provide a personal amplifier (purchased in bulk by the medical center) with verbal and written instructions. A total of 29 patients screened positive and were given personal amplifiers. Post-pilot interviews reported increased provider awareness and knowledge around best communication practices. Patients and staff reported limited amplifier use due to poor sound quality, small dials, poorly fitting ear buds and a short battery life. Based on these results the team recommended discontinuing the personal amplifiers and identifying other communication tools including higher quality personal amplifiers and speech to text applications.

### PERSON-CENTERED LANGUAGE-BASED STRATEGIES USED BY HOME CARE WORKERS WHO CARE FOR PERSONS LIVING WITH DEMENTIA

Reanne Mundadan, Marie Savundranayagam, Joseph Orange, and Laura Murray, *Western University, London, Ontario, Canada*

Several studies recommend language-based strategies for communication with persons living with dementia. Language-based strategies improve coherence, clarity, reciprocity, and continuity of interactions. Person-centered communication (PCC) strategies are the gold standard, including facilitation, recognition, validation, and negotiation. Only one study has examined the overlap between language-based strategies and PCC in long-term care. Little is known about which language-based strategies support PCC in home care. Accordingly, this study investigated the overlap between language-based strategies and PCC in home care interactions. Conversation analysis of 30 audio-recorded routine care interactions between home care workers and persons living with dementia was conducted. The overlap between communication-units coded for PCC and 33 language-based strategies was analyzed. Of 11,347 communication-units, 2,664 overlapped with PCC. For facilitation, 21% were yes/no questions and 15% were announcements of action/intent. For recognition, 25% were yes/no questions and 22% were affirmations. For validation, the majority (81%) of communication-units were affirmations and positive feedback. Finally, for negotiation, 60% of communication-units were yes/no questions. This is the first study examining naturalistic interactions between home care workers and persons living with dementia. The findings highlight the person-centeredness of language-based strategies. Yet only six of 33 language -based strategies occurred in the top 50% of overlapping communication-units. Home care workers in this study use a uniform set of person-centered language-based strategies, illustrated by the frequent use of yes/no questions overlapping with most PCC indicators. Our findings emphasize the need for training among home care workers in the use of diverse language-based strategies that are potentially person-centered.

### SESSION 6470 (POSTER)

### DRIVING

### PSYCHOMETRIC EVALUATION OF CARS: A BRIEF SCREENING INSTRUMENT FOR DRIVING SAFETY

Angela Curl, *Miami University, Oxford, Ohio, United States*

Driving is critical to mobility, autonomy, freedom of choice, and engagement for older adults, but many eventually stop driving. While poor health often eventually leads to driving cessation, some drivers do not realize that their driving is impaired. The 4-item CARS instrument of driving safety has been created to serve as a screening tool by health professionals during assessments and routine appointments (Curl, 2014). To assess the construct validity of CARS, data were collected in 2015-2016 by The Knight Alzheimer's Disease Research Center from cognitively impaired (N=42)

and cognitively healthy participants (N=229), as well as "collateral sources" who answered the CARS items about the respondents' driving. Vision problems and cognitive impairment, both known predictors for driving problems, were correlated with CARS scores. Results indicate that worse eyesight in the right eye is correlated with higher CARS scores (Spearman rho = -.17, p<.01), but not the left eye. Poor eyesight (i.e., less than 20/40 vision) was correlated with higher CARS scores (Spearman rho = .12, p<.05). However, cognitive impairment (very mild/mild dementia vs. healthy cognitive functioning) was not statistically significant. Total CARS scores were moderately correlated for respondents and their collateral sources, r = .32, p<.01. Results indicate that this instrument may be more useful as a tool for initiating difficult conversations about driving safety than for identifying older adults who are at higher risk for negative driving outcomes. Future research is needed to identify additional/alternative items with greater psychometric support.

### VISUAL PROCESSING SPEEDS DIFFERENTIATE OUTCOMES OF DRIVING EVALUATIONS OF CONTROLS AND MEDICALLY AT RISK DRIVERS

Anne Dickerson, and Victoria Penna, *East Carolina University, Greenville, North Carolina, United States*

Visual processing speed is considered a critical factor for determining driving capacity in older adults. The specific research questions were: 1) is there a statistically significant difference in performance time between the medically at-risk (n=35) and controls (N=242), 2) does the type of medical condition (e.g., neurological, cognition, complex medical conditions) differentiate performance, and 3) can visual processing speed differentiate between fit and unfit drivers. A cross sectional quasi-experimental design was used to compare the visual processing reaction times between at-risk adult drivers and healthy controls. Participants were medically-at-risk drivers referred for a comprehensive driving evaluation to determine their fitness to drive. The Vision Coach™ "Full-Field-60" task was used to collect reaction times which required participants to tap 60 randomly illuminated red dots. One practice trial was followed by three testing trials that were averaged together. At-risk participants were divided into three diagnostic categories. Fitness-to-drive outcomes were pass, fail, or restrictions. A propensity score method based on age and gender was used to account for the difference in sample sizes by weighting the participants from the two studies for a fair comparison between the two groups. Independent t-tests found a significant difference t(275)=-6.42, p=< 0.001 in trial times between healthy controls (M=53 + 10.82) and medically-at-risk adults (M=72 + 17.04). No significant difference was found between the diagnostic groups (p=0.141), but the Vision Coach™ differentiated between those who "passed" and those who "failed" a driving evaluation (F(2,32)=8.28, p=0.001.

### ACHES AND PAINS: HOW DO THEY AFFECT TRANSITIONS FROM DRIVING?

Anne Barrett, and Cherish Michael, *Florida State University, Tallahassee, Florida, United States*

Chronic pain, which affects more than 1 in 4 middle-aged and older adults, can have profound implications for everyday activities, like driving. Although research has revealed pain's effect on driving performance, less is known about driving-related behaviors and self-assessments that are part of the process of transitioning from driving. We address this issue using data from an online survey of 3,441 Floridians aged 50 and older that was conducted between December 2020 and March 2021 and funded by the Florida Department of Transportation. We examine the association between pain and four driving-related outcomes: self-rated driving ability, self-regulated driving, perceived nearness of driving retirement, and planning for driving retirement. Results of multivariate regression analyses indicate that experiencing greater pain is associated with worse self-rated driving ability, more frequent self-regulated driving, and greater perceived nearness of driving retirement. Pain is not, however, associated with greater planning for driving retirement. These findings indicate that although greater pain may hasten the transition from driving, it may not lead to more planning for it. Both patterns suggest that pain may increase people's risk of experiencing the social isolation that can follow driving retirement. By focusing on transitioning from driving, our study reveals a largely overlooked benefit of reducing pain – It could extend people's years behind the wheel.

### COGNITIVE AND PSYCHOSOCIAL OUTCOMES OF DRIVING DIFFICULTIES IN OLDER ADULTS: A 5-YEAR STUDY

Yeji Hwang, *Drexel University, Philadelphia, Pennsylvania, United States*

Many older adults find it difficult to drive a car as they age. However, there are lack of studies on the outcomes of driving difficulties among older adults. The aim of this study was to examine the cognitive and psychosocial outcomes of driving difficulties in older adults. This study was a secondary data analysis using National Social Life, Health, and Aging Project Wave 2 (2010-2011) and 3 (2015-2016). This study followed 1,638 older adults that were of the age 65 and older, who had no difficulties driving a car at Wave 2. Montreal Cognitive Assessment Scale, Center for Epidemiological Studies Depression Scale, Hospital Anxiety and Depression Scale, and Perceived Social Isolation Scale were used. For data analysis, chi square tests, t-tests, and regression analysis were used. After 5 years, 11.1% of people began to have difficulties in driving a car (n=180), and 88.9% of people maintained to have no difficulties driving a car (n=1,441). Compared to people who maintained no difficulties of driving a car over time, people who began to have difficulties had more severe cognitive decline (t=4.59, p< 0.001) and more depressive symptoms over time (t=3.253, p=0.001). Univariate regression analysis also indicated that having difficulties of driving resulted in more severe cognitive decline over time (b=0.137, p< 0.001) and more depressive symptoms over time (b=0.097, p< 0.001). Driving difficulties were not related to anxiety or social isolation. As difficulties in driving are related to poor cognitive and psychological outcomes, healthcare professionals should pay more attention to people who experience driving difficulties.

### "IS IT TIME TO STOP DRIVING?": A RANDOMIZED TRIAL OF AN ONLINE DECISION AID FOR OLDER DRIVERS

Faris Omeragic[1], Lauren Meador[2], Nicole Fowler[3], Rachel Johnson[4], Elizabeth Boland[5], Ryan Peterson[6], and

Marian Betz[1], *1. University of Colorado School of Medicine, Aurora, Colorado, United States, 2. Stanford University School of Medicine, Stanford, California, United States, 3. Indiana University School of Medicine, Indianapolis, Indiana, United States, 4. Colorado School of Public Health, Aurora, Colorado, United States, 5. University of Colorado Anschutz Medical Campus, Aurora, Colorado, United States, 6. University of Colorado, Aurora, Colorado, United States*

The decision to stop or continue driving can be challenging for older adults. In a prospective two-arm randomized trial, we sought to test whether an online driving decision aid (DDA) would improve decision quality. We recruited 301 English-speaking licensed drivers, age ≥70 years, without significant cognitive impairment but with ≥1 diagnosis associated with increased likelihood of driving cessation, from clinics associated with study sites in three states. They were randomized to view 1) the online Healthwise® DDA for older adults addressing "Is it time to stop driving?"; or 2) a control condition of web-based information. Our primary outcome was decision conflict as estimated by the Decisional Conflict Scale (DCS; lower scores indicate higher quality). Secondary outcomes were knowledge and decision self-efficacy about driving decisions. We examined differences in post-randomization outcomes by study arm using generalized linear mixed-effects models with adjustment for site and pre-randomization scores. Intervention participants had a lower mean DCS score (12.3 DDA vs 15.2 control; p=0.017) and a higher mean knowledge score (88.9 DDA vs 79.9 control; p=0.038); we found no difference between groups in self-efficacy scores. The DDA had high acceptability; 86.9% of those who viewed it said they would recommend it to others in similar situations.The online Healthwise® DDA decreased decision conflict and increased knowledge in this sample of English-speaking, older adults without significant cognitive impairment. Use of such resources in clinical or community settings may support older adults as they transition from driving to other forms of mobility.

### PREVALENCE AND USE OF ADVANCED DRIVER ASSISTANCE SYSTEMS IN THE OLDER DRIVER POPULATION

Renee St. Louis[1], David Eby[1], Lidia Kostyniuk[1], Lisa Molnar[1], Jennifer Zakrajsek[1], Nicole Zanier[1], Raymond Yung[2], and Linda Nyquist[2], *1. University of Michigan Transportation Research Institute, Ann Arbor, Michigan, United States, 2. University of Michigan, Ann Arbor, Michigan, United States*

Research on advanced driver assistance systems (ADAS) in the older driver population has suggested the potential for ADAS to improve safety and driving comfort by helping aging drivers overcome functional declines commonly experienced in later-life. However, attaining anticipated ADAS benefits is dependent upon drivers' awareness, understanding, and use of ADAS in their own vehicles. Questionnaire data from 2,374 older drivers enrolled in the AAA LongROAD study were analyzed to investigate changes in the prevalence and use of 15 ADAS and how participants learned to use these technologies. From baseline to Year 3, the prevalence of each ADAS significantly increased, with the greatest percentage point increase being for backup/parking assist technology (from 41.5% to 58.8%). The prevalence of one or more ADAS in participants' vehicles increased from 59.0% to 72.0%, and the average number of ADAS per vehicle increased from 2.0 to 3.3. At both baseline and Year 3, approximately one-third of participants reported always using the ADAS available in their vehicle, but nearly one-quarter reported never using their ADAS. The largest proportion of participants at both baseline and Year 3 reported learning to use ADAS by figuring it out by themselves (45.5% and 50.8%, respectively), yet approximately 12.0% of participants at both time points reported never learning to use ADAS. To achieve the expected benefits of ADAS for older drivers, research is needed to better understand why ADAS are not being use more frequently when available, and to develop acceptable and accessible programs for training older adults to use ADAS.

### SESSION 6480 (POSTER)

### ELDER ABUSE: DETERMINANTS, DISRUPTORS, AND CONSEQUENCES

### INVESTIGATING THE CONNECTION BETWEEN AGEISM AND ELDER ABUSE

David Burnes[1], Karl Pillemer[2], and Andie MacNeil[1], *1. University of Toronto, Toronto, Ontario, Canada, 2. Cornell University, Ithaca, New York, United States*

Elder abuse is recognized as a pervasive public health problem with detrimental consequences for older adults and society. Although considerable research has examined elder abuse risk factors at the individual level, there is a growing call for the field to move beyond proximal causes and consider broader socio-cultural and structural factors that influence elder abuse. Illustrating this shift, organizations, advocacy groups and researchers have proposed a connection between ageism and elder abuse. However, despite the assertion that ageism is a causal factor for elder abuse, there is a scarcity of research to demonstrate this relationship, and a coherent theoretical framework linking ageism to elder abuse remains to be articulated. The purpose of the current study was to examine the conceptual pathways and limited empirical research connecting ageism and elder abuse, and to develop a conceptual model that links ageism and elder abuse. We conducted a comprehensive review and synthesis of the ageism/elder abuse literature, as well as research from other domains of interpersonal/family violence. Based on this synthesis, the proposed model includes plausible mediators (social isolation, devaluation, depersonalization, infantilization, powerlessness, blame) and moderators (intersection with socio-cultural identities, internalized ageism, policy/social norms) that could be targeted as mechanisms of change in interventions designed to address the issue. As such, it provides a framework for hypothesis-testing and future research on the topic. This study informs a research agenda to bring conceptual clarity and empirical evidence to the study of the connection between ageism and elder abuse.

### THE ASSOCIATION BETWEEN FINANCIAL RESOURCES AND STRESSORS AND FINANCIAL EXPLOITATION

LaToya Hall, Juno Moray, Evan Gross, and Peter Lichtenberg, *Wayne State University, Detroit, Michigan, United States*

The prevalence of financial exploitation (FE) experienced by older adults is increasing. Population-based survey estimates of FE in the older adult population range from 5% to 11%. Given the growing prevalence of FE victimization in older adult populations, understanding the population's vulnerability to FE has increased in importance. This study investigates a conceptual framework in an attempt to understand how financial stressors and resources are associated with substantiated FE in a sample consisting largely of black older adults. The study uses a cross-sectional design to investigate group differences among a total sample of 142 community-dwelling older adult participants, 62 of whom sought services to address FE and 80 with no FE history FE. Older adults who sought services to address FE were more likely to be unmarried and had fewer years of education. Financial literacy had a protective effect on FE, whereas and perceived financial vulnerability increased FE risk. According to the findings sociodemographic and financial stress and resource measures have significant relationships with FE. These findings support the conceptual framework describing their relationship. This new conceptual framework provides a guiding factor in better understanding older adult's vulnerability to financial exploitation.

### A MOMENT'S NOTICE: RECOGNIZING THE STRESSFUL LIFE EVENTS, EMOTIONS, AND ACTIONS THAT MAKE US SUSCEPTIBLE TO SCAMS

Alicia Williams[1], Lona Choi-Allum[1], Doug Shadel[2], Karla Pak[2], and Chuck Raineville[1], *1. AARP, Washington, District of Columbia, United States, 2. AARP, Seattle, Washington, United States*

Scamming has become a booming, multibillion-dollar industry with millions of Americans targeted by scams each year. To better understand the rates of exposure to scams and factors that may increase a target's susceptibility to financial loss resulting from a scam, AARP commissioned a national survey that screened over 9,000 adults ages 18+ about their rates of encounters and financial loss from 26 popular scams. Among the total sample, 15% (an estimated 33 million people) reported losing money to a scam in the past year. Additionally, a subset of 3,280 respondents, including 1,085 respondents who lost money to a scam, were surveyed more extensively about their experiences. The study uncovered environmental and emotional factors in which targets are more susceptible to fraud. Specifically, fraud victims reported experiencing twice as many stressful life events (i.e., 4.0 vs. 1.9), 58% more fraud encounters, stronger emotional arousal during fraud encounters, and significantly less family support and closeness than non-victims. Additionally, only two of the eight protection tools recommended by fraud experts (i.e., using a passcode and installing protective software on one's computer) were used by most respondents; and less than half of respondents reported using call blocking tools or online monitoring of their accounts, which can dramatically reduce exposure to scam encounters. Together, these findings show the importance of building public awareness of the role of stress, emotional arousal, and feelings of isolation in fraud susceptibility, and highlight the benefits of using fraud protection tools to limit one's exposure to fraud.

### EMPOWERING SENIORS TO RECOGNIZE ELDER MISTREATMENT THROUGH PEER SUPPORT: RESULTS FROM A COMMUNITY OUTREACH PROJECT

Jessica Hsieh[1], Sharon Tan[1], Celeste Pang[2], Raza Mirza[3], and Christopher Klinger[1], *1. University of Toronto, Toronto, Ontario, Canada, 2. Egale, Toronto, Ontario, Canada, 3. National Initiative for the Care of the Elderly, Toronto, Ontario, Canada*

Elder mistreatment, often understood in the context of abuse and neglect, is a growing concern for the health and wellbeing of seniors and their families. A 2015 Canadian prevalence study by the National Initiative for the Care of the Elderly (NICE) found that seniors who are mistreated are more willing to talk to other seniors, rather than clinicians, police or family. However, a lack of knowledge, access to resources, and community stigma may limit seniors' abilities to address mistreatment.This study evaluated the impact on knowledge, attitudes, and behaviours of having seniors deliver workshops on mistreatment to other seniors. A Seniors' Advisory Committee developed content for these workshops, delivered by seniors in sixteen communities across Ontario, Canada. Participants completed pre/post-surveys assessing changes in knowledge, attitudes, and behaviours. Results indicate that workshops effectively increased awareness of mistreatment issues among participants, on average, by 37%. Participants experienced a 44% increase in their perceived preparation to provide information to another older adult asking about mistreatment. Barriers to help-seeking among seniors include finding trustworthy sources, fear of retaliation, and a lack of legal protection. Prior to the training, healthcare providers were the main sources of information for participants (58%); depending on the severity of the situation, 60% of participants indicated eventually reporting to police, who are not the preferred source of information. Senior-led workshops about mistreatment appear to be effective for increasing knowledge and encouraging disclosure and help-seeking behaviours. Results support prevention models that empower seniors to educate other seniors on issues around mistreatment.

### THE ROLE OF RELATIONSHIP FACTORS IN HARMFUL CAREGIVER BEHAVIORS: OLDER CHILDREN CARING FOR PARENTS WITH DEMENTIA

Elizabeth Gallagher[1], Kathrin Boerner[1], Yijung Kim[2], Kyungmin Kim[3], and Daniela Jopp[4], *1. University of Massachusetts Boston, Boston, Massachusetts, United States, 2. The University of Texas at Austin, Austin, Texas, United States, 3. Seoul National University, Seoul, Seoul-t'ukpyolsi, Republic of Korea, 4. University of Lausanne, Lausanne, Vaud, Switzerland*

Elder abuse by family caregivers is an often-overlooked phenomenon that affects many older adults. Especially, retirement-aged children caring for their oldest-old parents with dementia may be at greater risk of engaging in harmful or abusive behaviors, given their own age-related health issues and other competing caregiving demands. Most of the elder abuse literature has focused on general demographic predictors of elder abuse, regarding the caregiver, care recipient, and the care environment. Less attention has been paid towards relationship factors, which may play a large role among these parent-child dyads. This study examined how relationship factors are associated with potentially harmful caregiver behaviors (PHCB; e.g., screaming), which have been identified as "early warning signs" for elder abuse. Relationship factors of interest include positive and negative relationship quality measured by caregivers' mean scores on the support and conflict subscales on the Quality of Relationship Inventory (QRI). We conducted in-depth interviews with 88 caregivers (65+) who are caring for their parents with dementia (90+) as the part of the Boston Aging Together Study. Regression models revealed that relationship conflict was significantly associated with higher levels of PHCB, accounting for caregiver, care recipient, and care environment characteristics. The creation of screeners to identify "high conflict" care dyads could prove useful in the early detection and intervention of potential elder abuse cases, given that caregivers may be more willing to report negative aspects of their relationship (e.g., fighting) than more obviously harmful or abusive behaviors.

### SOCIAL NETWORKS AND HELP-SEEKING AMONG US CHINESE OLDER ADULTS REPORTED ELDER MISTREATMENT

Ying-Yu Chao[1], Jin Young Seo[2], and XinQi Dong[3], *1. Rutgers, The State University of New Jersey, Newark, New Jersey, United States, 2. Hunter College, CUNY, New York, New York, United States, 3. Rutgers, The State University of New Jersey, New Brunswick, New Jersey, United States*

Purpose: Older immigrant adults are reported to be more tolerant of abusive situations and less likely to seek help. This study aimed to examine the associations between social networks and help-seeking among U.S. Chinese older adults reported elder mistreatment (EM).

Methods: Data were from the Population Study of Chinese Elderly in Chicago (PINE). Social networks were assessed with network size, volume of contact, emotional closeness, proportion kin, proportion female, and proportion coresident. Informal/formal help-seeking (intentions and actual behaviors) were measured. Descriptive statistics and regression analyses were performed.

Results: A total of 450 participants reported EM. Participants had a mean age of 72.73 ± 8.03 years old (range 60-97). Participants had a mean of 3.29 (SD ± 1.31) network members, a mean of 3.24 (SD ± 0.67) emotional closeness, and average contacts of 6.62 (SD ± 1.10) times per year with network members. Smaller network size (p = .00) and less emotional closeness (p = .03) were associated with an increase in intentions of seeking help from formal sources. Compared to not seeking help, smaller network size (p = .04) and more emotional closes (p = .03) were associated with a higher likelihood to seek help from informal sources among U.S. Chinese older adults who reported any EM. Conclusion/implication: This study highlights the dynamic nature of social networks of help-seeking among this underserved population. Culturally tailored interventions are suggested to promote help-seeking through increasing strong ties and improving the quality of social networks for U.S. Chinese older immigrants with EM.

### THE ROLE OF FORMAL RESOURCES ON ELDER MISTREATMENT HELP-SEEKING BEHAVIORS AMONG ASIAN OLDER ADULTS

Eun Jeong Lee[1], Ga-Young Choi[2], and Eun Koh[3], *1. Asian American Resource and Information Network, Wood Ridge, New Jersey, United States, 2. California State University, Los Angeles, Los Angeles, California, United States, 3. The Catholic University of America, Washington, District of Columbia, United States*

Elder mistreatment is one of the most difficult topics to talk about among Asian older adult immigrants in the U.S. because they are unfamiliar and uncomfortable to disclose the issue due to their unique cultural values and limited English language proficiency. We recruited 494 community dwelling older adults from five cities who identified themselves of Asian ethnicity, and were 55 years old and older. Participants had various Asian ethnic and language backgrounds (e.g. Korean, Chinese, Filipino, and Vietnamese). Most of them were foreign-born (84.6%) with the mean age of 69.0 (SD = 8.47). Using the participant survey data, we focused on examining difference regarding elder mistreatment experience, perceptions, and help-seeking intentions between the Senior Community Service Employment Program (SCSEP) group and the non-SCSEP group that may or may not participate in paid workforce. The SCSEP is the federally paid workforce program for low-income older adults aged 55 or above aiming at fostering older adults' community service and economic self-sufficiency. There was a significant difference in emotional mistreatment experiences between SCSEP and non-SCSEP participants. Furthermore, the two groups were significantly different in their likelihood of seeking help from Adult Protective Services (APS). Those who ever participated in the SCSEP were more likely to seek help from APS (X2 = 1, N = 284) = 4.674, p ≤ .05. Implications for future research and gerontology services are discussed to enhance Asian older adults' awareness of elder mistreatment, and APS and other formal sources of help.

### PSYCHOMETRIC PROPERTIES OF A NOVEL MEASURE OF FINANCIAL ABUSE OF OLDER ADULTS

David Hancock[1], Karl Pillemer[2], David Burnes[3], Sara Czaja[1], and Mark Lachs[4], *1. Weill Cornell Medicine, New York City, New York, United States, 2. Cornell University, Ithaca, New York, United States, 3. University of Toronto, Toronto, Ontario, Canada, 4. Weill Cornell Medicine, New York, New York, United States*

Background and Objectives: Elder abuse, or mistreatment affects at least 1 in 10 older adults. Financial abuse, or exploitation, of older adults is among the most commonly reported forms of abuse. Few validated measures exist to measure this construct. We aim to present a new psychometrically validated measure of financial abuse of older adults. Research Design and

Methods: Classical test theory and item response theory methodologies were used to examine a five-item measure of financial abuse of older adults, administered as part of the New York State Elder Mistreatment Survey.

Results: Factor analysis revealed a single factor best fits the data, which we labeled as financial abuse. Moreover, IRT analyses revealed that these items discriminated well between abused and non-abuse persons and provided information at high levels of the latent trait θ, as is expected in cases of abuse. Discussion and Implications: The FIVE has acceptable psychometric properties and has been used successfully in large scale survey research. We recommend this measure as an indicator of financial abuse in elder abuse, or mistreatment prevalence research studies.

### ANALYSIS OF SOCIAL PARTICIPATION, ELDER ABUSE, AND CORRESPONDING BEHAVIOR OF THE ELDERLY IN CHINA

Guanggang Feng, and Shumin Jiang, *Anhui University of Finance and Economics, Bengbu, Anhui, China (People's Republic)*

Introduction: Ancient Chinese wisdom under the Confucian culture show that “Parents raise children who in turn support them in old age” and "Don't wash one's dirty linen in public". The elderly in China brings up children with the aim to guard against troubles in their late years, but many older people with sons also suffer from various forms of elder abuse, and often choose to submit to humiliation when facing abuse from family members. Data: The Survey on the living conditions of the elderly in urban and rural China ( SSAPUR ) , four waves: 2015, 2016, 2017 and 2018 followed samples(N=224360)

Methods: The hierarchical Logit model was used to analyze the influence of social participation on elder abuse in China, and the risk of the elderly's choice to submit to humiliation. Results and conclusion: The results showed that the annual incidence of elderly abuse (include physical abuse, emotional abuse, financial abuse, and neglect of care) among the elderly in China was 5.36%, and 35.77% of them chose to submit to humiliation. The more the elder participate in society involvement outside the home, the less likely they are to be abused from family members, the less likely they submit to humiliation. The more sons, the higher the risk of elder abuse. Therefore , the relevant government and the grass-roots social organizations should strengthen the construction of social organizations for the elderly.

### THE ASSOCIATION OF SUBJECTIVE AGE AND FINANCIAL EXPLOITATION RISK: SOCIAL SUPPORT AS A MODERATOR

Gali Weissberger[1], and Yoav Bergman[2], *1. Bar Ilan University, Ramat Gan, HaMerkaz, Israel, 2. Ashkelon Academic College, Ashkelon, HaDarom, Israel*

Financial exploitation of older adults bears detrimental physical and psychological consequences. However, little is known regarding factors which may contribute to financial exploitation vulnerability (FEV) among this age-group. In line with the growing awareness of the importance of subjective perceptions of the aging process for older adults’ functioning and well-being, the current work examined the connection between subjective age (i.e., feeling younger/older than one’s chronological age) and FEV, and the possible moderating role of social support on this connection. Data were collected from a convenience sample of 137 Israeli older adults (age range 60-89, M=69.90, SD=6.85, 40.1% male), who completed scales assessing FEV, subjective age, and social support, as well as relevant socio-demographic information. Associations were examined using a hierarchical linear regression model which adjusted for age, sex, education, marital status, and self-rated economic and health status. The main effect of subjective age was not significant (B=1.24, p=0.14), while the main effect of social support was found (B=-1.30, p=.03) indicating that low levels of support were associated with increased FEV, a finding consistent with the literature. The interaction of social support × subjective age was significant (B= -1.17, p= .025), accounting for an additional 4.0% of variance. Older subjective age was associated with increased financial exploitation vulnerability when social support was low, but not when social support was high. Results are interpreted with regard to Socio-Emotional Selectivity Theory and provide initial information pertaining to the relevance of subjective age perceptions to FEV in older adults.

### SESSION 6490 (POSTER)

### ENVIRONMENT AND AGING

### COGNITIVE EFFECTS OF PM2.5 EXPOSURE FROM MID-LIFE TO EARLY OLD AGE

Carol Franz[1], Xin Tu[1], Yongmei Qin[1], Jeremy Elman[1], Christine Fennema-Notestine[2], Jaden DeAnda[3], Tsung-Chin Wu[4], and William Kremen[1], *1. University of California San Diego, La Jolla, California, United States, 2. University of California San Diego, San Diego, California, United States, 3. San Diego State University, La Jolla, California, United States, 4. University of California, La Jolla, California, United States*

Air pollution exposure is a notable public health hazard with adverse effects on multiple health outcomes as well as with increased risk of developing cognitive impairment, Alzheimer’s disease, and related dementias. Few studies examine associations between air pollution exposure in midlife or the transition from midlife to old age. We examined associations between exposure in midlife and cognitive functioning in early old age in ~800 men from the Vietnam Era Twin Study of Aging. Measures included PM2.5 and NO2 exposure in the three years prior to the time 1 (mean age 56; range 51-61) assessment, and cognitive performance in 5 domains at time 1 and time 2 (mean age 68; range 65-72). Analyses adjusted for multiple health and lifestyle covariates. Cognitive performance in all domains was worse at age 68 than at age 56. There was a main effect of midlife PM2.5 on verbal fluency; greater PM2.5 exposure was associated with worse fluency. This association was at a trend level for NO2. In addition, we found significant PM2.5-by-APOE genotype interactions. Increased exposure to PM2.5 in midlife was related to lower executive function and working memory performance in APOE-ε4 carriers, but not non-carriers. Both early executive deficits and APOE-ε4 status have been associated with increased risk for progression to mild cognitive impairment and Alzheimer’s disease. The present results indicate that midlife PM2.5 exposure in men is an additional factor contributing to poorer frontal-executive function, and that APOEε4 carriers are more susceptible to the deleterious effects of PM2.5.

### FRAMING CLIMATE-CHANGE INFORMATION TO PROMOTE ACTION IN OLDER ADULTS

Philip Lemaster, *Concordia College, Moorhead, Minnesota, United States*

Climate change poses an existential global risk, and mitigation requires population-level behavioral change. Due to their shortened time horizon, older adults may not perceive climate change as a personal risk within their lifetime and may be less likely to take action to combat climate change. Socioemotional selectivity theory (SST; Carstensen, 2006) posits that, as people age and perceive a shorter time horizon ahead, they become more focused on maintaining emotional wellbeing. This study, rooted in SST, manipulated the emotional framing of climate-change information in a sample of U.S. older adults. Participants were given EPA climate-change information localized to their state of residence and were instructed that, by taking action, the impact of climate change could be reduced (instilling hope) or that, by not taking action, the impact of climate change could be exacerbated (instilling fear). Afterward, they completed measures assessing their willingness to engage in pro-environmental behaviors and support of macro-level legislation to combat climate change. Compared to older adults who received the "hope" frame, older adults who received the "fear" frame reported greater willingness to engage in pro-environmental behaviors ($p = 0.007$) and greater support of legislation to combat climate change ($p = 0.038$), perhaps an effect of older adults' motivation to reduce the negative affective state induced by the "fear" frame. Implications of these results for catalyzing older adults toward climate action are discussed.

### ROLE OF HOME ENVIRONMENT IN LTSS USE AMONG OLDER ADULTS IN THE US

Shalini Sahoo, *University of Maryland, Baltimore, Hanover, Maryland, United States*

Older adults have shown increasing preference towards having help and health care in the comfort of their own homes rather than in an institutional setting. Research suggests that about 1 in 5 older adults with limitations in activities of daily living report needing more help than they receive. There is insufficient research examining how home inaccessibility and disorder relates to LTSS use for community-dwelling older adults in the U.S. This study addresses a gap in the literature by directly examining the relationship between home environment quality and LTSS use for a national sample of older adults. Data was analyzed from round eight (2018) of the National Health and Aging Trends Study (NHATS), an epidemiological panel study of nationally representative Medicare beneficiaries ages 65 and older living in the communities ($n = 4,806$). The final analytic sample included respondents with valid data on all variables ($n = 4,265$). Community dwelling adults with greater home accessibility had lower odds of having two or more LTSS caregivers, compared to the counterpart with lower home accessibility ($OR = 0.246$, $SE = 0.094$, $p < 0.01$). However, poor housing conditions had lower odds of having two or more LTSS caregivers ($OR = 0.333$, $SE = 0.156$, $p < 0.05$). Community-dwelling older adults who have accessible and organized homes are more likely to be independent and are less likely to depend on LTSS caregiving. Policies to improve home environment quality could benefit both older adults and those who care for them.

### COMMUNITY TYPOLOGY IN OLDER KOREAN AMERICANS: IMPLICATIONS FOR MENTAL/COGNITIVE HEALTH

Nan Sook Park[1], Yuri Jang[2], Soondool Chung[3], and David Chiriboga[1], *1. University of South Florida, Tampa, Florida, United States, 2. University of Southern California, Los Angeles, California, United States, 3. Ewha Womans University, Seoul, Seoul-t'ukpyolsi, Republic of Korea*

The purposes of this study were to: (1) identify community typology in older Korean Americans; and (2) examine the associations of typology with loneliness, mental distress, and cognitive health. Guided by social capital conceptualization, we hypothesized that distinct community groups would be identified and that they would be differentially associated with sociodemographic, immigration-related, health, and social characteristics and mental/cognitive health. Data were drawn from a survey with older Korean Americans aged 60 and older, collected during 2017–2018 in diverse locations ($n=2,138$). To identify community typology, a series of latent profile analysis (LPA) were conducted using 15 community-related variables in three domains (neighborhood characteristics, social cohesion, ethnic attachment). After examining characteristics of the identified groups in relations with study variables, hierarchical multiple regression models of loneliness, mental distress, and self-rated cognitive health were estimated. Based on several model evaluation criteria, LPA model with five community groups was identified as best-fit (BIC=64,619, Entropy=.94). The five groups were identified as “a. high safety/cohesion/ethnic attachment” (10%), “b. high safety/low cohesion/ethnic attachment” (10%), “c. moderate neighborhood/low ethnic attachment/cohesion” (38%), “d. moderate neighborhood/high cohesion/ethnic attachment” (31%), and “e. low safety/moderate cohesion/ethnic attachment” (11%). In reference to the group with high on all three domains (a), group with low ethnic attachment/cohesion in moderate neighborhood (c) and group in unsafe environment with moderate cohesion/ethnic attachment (e) were consistently associated with elevated loneliness/mental distress and poor rating of cognitive health. The results suggest the need to understand profiles of community characteristics and their relationships with health/well-being among older immigrants.

### EXAMINING THE ROLE OF OBJECTIVE AND SUBJECTIVE NEIGHBORHOOD CHARACTERISTICS ON HEALTH AND WELL-BEING IN MID-LIFE

Omar Staben[1], Frank Infurna[2], and Kevin Grimm[2], *1. RAND Corporation, Tempe, Arizona, United States, 2. Arizona State University, Phoenix, Arizona, United States*

There is a long-standing literature that has documented how one’s neighborhood context has the potential to shape mental and physical health across the adult lifespan. An abundance of research documents how various components of the neighborhood, such as disorder, cohesion, in addition to pollution and sidewalk quality are linked to short- and long-term mental and physical health outcomes across adulthood. One key component that has been less studied within this literature is the extent to which objective or subjective neighborhood indicators exert a more potent impact on mental and physical health. Up to this point, much of the research has focused on subjective indicators of neighborhood context. This study explores whether and to what extent objective neighborhood factors of income inequality, residential stability, and greenness and subjective neighborhood factors of social ties, collective efficacy, and neighborhood disorder are predictive of mental and physical health in midlife. We use data from a sample of participants in midlife (n=800, aged 40-65) to analyze our research questions. Structural equation models found that both subjective and objective indicators of neighborhood were related to health and well-being when considered separately. When considered simultaneously, subjective neighborhood indicators (sense of community) mediated the association between objective constructs and health. Our findings demonstrate the independent associations between objective and subjective neighborhood context and highlight the particularly strong association between subjective context and health and well-being. Our discussion elaborates on how our findings can inform interventions and sparks future research aimed at exploring potential mechanisms underlying the associations found.

### IMPACT OF NEIGHBORHOOD GREEN INFRASTRUCTURE ON ACCESS TO SOCIAL CAPITAL ACROSS THE LIFE COURSE

Noah Webster[1], Natalie Sampson[2], and Joan Nassauer[1], *1. University of Michigan, Ann Arbor, Michigan, United States, 2. University of Michigan-Dearborn, Dearborn, Michigan, United States*

While the impact of neighborhood characteristics on access to social capital is well established, less is known about how neighborhood landscape design interventions play a role in shaping access to this resource and how this varies across the life course. In this study we examined the association between age and perceived impact of recently installed neighborhood block-scale green infrastructure (GI) on frequency of social interactions with neighbors. We also examined age variation in how alternative GI designs were perceived (e.g., how well cared for), and how these perceptions were associated with the anticipated impact on frequency of neighbor interactions. Data are from the Neighborhood, environment, and water research collaborations for green infrastructure (NEW-GI) project based in Detroit, MI. Four neighborhood GI interventions were installed in two Detroit neighborhoods in 2016. Surveys were conducted with residents living around the interventions in 2017-18 (n=171), and in nearby neighborhoods (n=145). Age was significantly associated with perceived impact of the landscape interventions on frequency of social interactions with neighbors. Specifically, older adults were significantly more likely to report that the landscape interventions that they were most familiar with resulted in increased frequency of interactions with their neighbors. Further, design alternatives perceived as more well cared for were anticipated to result in greater increases in the frequency of interactions with neighbors among older compared to younger adults. Results suggest neighborhood landscape interventions can improve access to social capital particularly among older adults, and perceptions of landscape care play a role in this process.

### SESSION 6500 (POSTER)

### ENVIRONMENT, DISASTERS, AND EMERGENCY PREPAREDNESS

### THE AGE-FRIENDLY COMMUNITY ENVIRONMENT FACTORS CONTRIBUTING TO LIFE SATISFACTION OF OLDER ADULTS LIVING ALONE

Mi Sun Choi[1], and Hyunmin Lee[2], *1. Silla University, Busan, Pusan-jikhalsi, Republic of Korea, 2. Seoul, Seoul, Seoul-t'ukpyolsi, Republic of Korea*

Age-friendliness studies in Korea have investigated a few subsets of the local community environments for older adults' well-being. Little is known about comprehensively understanding which aspects in community contexts can help older individuals living alone improve their life satisfaction. To address the knowledge gaps, this study utilized the raw data from the 2020 National Survey of Older Koreans, and responses of 3,112 older adult single-person households were investigated. From an ecological perspective, hierarchical regression analysis was performed by distinguishing the community environments into three categories of the World Health Organization's age-friendly environments: physical, social, and service environments. The results showed that the perception of the physical environment (housing, living facilities, and space) was positively associated with life satisfaction. Second, the social environment (social involvement, neighbor interaction, respect for older individuals, and political activity) was positively related to life satisfaction. Third, perceived service environment (difficulty in utilizing service) was negatively related to life satisfaction. Based on the findings, we proposed political and practical recommendations. Specifically, as a physical environment aspect, effective budget allocation and policy attention should be required for the autonomy and independence of older adults living alone in their daily lives by ensuring that housing and circumstances are suitable for aging-in-place. As a societal component, initiatives must be established to promote participation in decision-making processes that result in a more favorable social perception through social inclusion. Finally, for the service environment, we must advocate for increased accessibility to community supportive services (mobility, health care, food delivery, communication, and information).

### COMMUNAL SUPPORT PREDICTS BETTER MENTAL HEALTH: KNOWLEDGE TRANSLATION AMONG OLDER ADULTS DURING COVID-19

Eireann O'Dea[1], Daniel Rong Yao Gan[2], Habib Chaudhury[2], Ziying Zhang[2], Andrew Wister[2], Lisa Cohen Quay[3], Shelley Jorde[4], and Claire Wang[5], *1. Simon Fraser University, North Vancouver, British Columbia, Canada, 2. Simon Fraser University, Vancouver, British Columbia, Canada, 3. Jewish Community Centre of Greater Vancouver, Vancouver, British Columbia, Canada, 4. South Vancouver Seniors Hub, Vancouver, British Columbia, Canada, 5. Johns Hopkins University , Baltimore City, Maryland, United States*

The well-being of older adults has been linked to the quality of their neighbourhood environment. Given that COVID-19 affected poorer neighbourhoods disproportionately, we partnered with community organizations to identify meso-level psychosocial factors that may improve loneliness, depressive mood, and cognitive function. Five variables were identified through focus groups with older adults and community organizations. These variables were drawn from validated scales, including communal provisions, neighbourhood friendship, self-expression, social experiences, and time outdoors. This paper presents preliminary findings from surveys administered to 151 community-dwelling older adults across British Columbia and interviews in four neighbourhoods.Purposeful and snowball sampling were used to recruit older adults (age 55+) from community centres and neighbourhood houses. Online surveys measured the five meso-level psychosocial exposure variables. Outcome variables included an index of loneliness, depressive mood, self-rated memory, semantic fluency and delayed recall. Data was geocoded and aggregated by Forward Sortation Area. Regression and cross-level mediation analysis were conducted. Four neighbourhoods were selected from a 2x2 matrix of high and low neighbourhood deprivation (CANUE, 2016). Mental health was associated with better social experiences (B=.26, p=.003). Time outdoors (B=.35, p=.047) was associated with better delayed recall. Mental health was better in poorer neighbourhoods (B=.20, p=.015). This was partially mediated by communal provisions (B=.19, p=.032). Social experiences (B=.23, p=.009) fully mediated these effects on mental health. Participants described being of local community services and took on opportunities to volunteer. Social experiences and neighbourhood resources may help support mental health and well-being among older adults during the pandemic and beyond.

### AGING, DISPLACEMENT, AND COPING WITH COVID-19 IN PRECARIOUS SPACES IN NORTHEAST NIGERIA

Adewale Adeyombo[1], and Abiodun Oyebode[2], *1. Bowen University, Offa, Kwara, Nigeria, 2. Federal Polytechnic Offa, Offa, Kwara, Nigeria*

Nigeria is witnessing internal displacements due to terrorism and kidnapping across many communities with more of these evitable challenges in the North Eastern region. These challenges are impacting on older people amongst other social categories that have been displaced, killed or kidnapped across different communities. The outbreak of covid-19 and the poor social support for older persons and other vulnerable groups in Nigeria imply that survivors in such precarious spaces are likely to face challenges with implications on their well-being and meanings attached to life. This paper explores ageing in precarious spaces that have been ravaged by terrorism and banditry and the outbreak of covid-19. The narratives of the 15 older persons aged 57 to 82 years living in Internally Displaced Camps revealed some dimensions of hopelessness, despair and the acceptance of present challenges as fate and structural defects. Awareness and adherence to covid-19 protocol was poor. Moving away from communities of residence represent diverse forms of loss that transcends material things. Sharing of experiences, prayers and relief materials from non-governmental agencies were considered as instrumental in coping with their daily challenges. The hope of returning to their communities of residence was doubted and considered risky. The belief that their seeds would have a better future was expressed by all the participants, with the females affirming such expectation than the men. Integrating displaced older persons would require more than existing efforts being taken by Nigerian government across levels.

### GEOGRAPHICAL VARIATIONS IN THE PRODUCTIVE ENGAGEMENT OF OLDER ADULTS

Peter Sun, *Washington University in St. Louis, Clayton, Missouri, United States*

While having opportunities to be productively engaged has implications for long, healthy lives, little is known about the geographical distribution of older adults engaged in productive activities. Using the nationally representative Health and Retirement Study, a sample of community-dwelling older adults age 65 and above in the year 2018 (n = 8,728) was used to compare working, volunteering, and caregiving rates between rural and urban areas and Census regions and divisions. Urban and rural rates of individuals who were working were significantly different ($p < .05$) in the Midwest region and the East North Central and Pacific divisions (e.g., 20.9% in rural Midwest vs. 14.8% in urban Midwest). Urban and rural caregiving rates were significantly different in the East South Central division (35.1% rural vs. 26.2% urban caregivers). Urban and rural rates of individuals who engaged in one or more productive activities (working, volunteering, and caregiving) were significantly different in the West North Central (64.9% rural vs. 56.6% urban) and Middle Atlantic (47.6% rural vs. 58.6% urban) divisions. Urban and rural volunteering rates were significantly different in the West region (37.0% rural vs. 30.2% urban). These findings suggest that older adults' participation in working, caregiving, and volunteering roles may depend on where they live, and older adults are not a homogeneous group as commonly misunderstood. Future studies should explore the factors that influence these patterns of engagement.

### THE IMPACTS OF WILDFIRES ON OLDER ADULTS: A SCOPING REVIEW

Carson De Fries, Colleen Melton, Rebecca Smith, and Lisa Reyes Mason, *University of Denver, Denver, Colorado, United States*

Climate change is leading to worsening disasters that disproportionately impact certain populations, including older adults (Benevolenza & DeRigne, 2019). Older adults are more likely to encounter life-threatening challenges during disaster evacuation, less likely to receive disaster warnings, and more likely to experience greater financial losses following disasters (Acierno et al., 2006). While research has begun to measure these disparities, there is a gap in examining the effects of wildfire-specific disasters, which are increasing in intensity and severity (Hoover & Hanson, 2020). To examine this gap, scoping review methodology was used to analyze peer-reviewed studies of how wildfires affect older adults by looking at impacts and the disaster recovery cycle (i.e., preparedness, response, recovery, and mitigation). Authors screened 263 titles and abstracts, and 138 were reviewed in full. Eighty-one studies were included for data extraction and analysis. Preliminary findings illustrate that most literature focuses on the health impacts that older adults endure during and following wildfires, with a specific focus on the short and long-term effects of smoke and poor air quality on respiratory health. While previous literature has cited a need for community response strategies that incorporate the needs of older adults, few findings addressed firsthand experiences of older adults during a wildfire event. However, one unique finding was the incorporation of Aboriginal and Indigenous Elders' knowledge into fire management strategies. While recommendations for incorporating the needs of older adults into policy planning were briefly mentioned, most articles focused on problem scope rather than evaluating potential solutions.

### HOUSING CHARACTERISTICS, NEIGHBORHOOD ENVIRONMENTS, AND SELF-RATED MENTAL HEALTH AMONG OLDER CANADIANS

Ethan Siu Leung Cheung[1], and Ada Mui[2], *1. Columbia University, New York, New York, United States, 2. COLUMBIA UNIVERSITY, New York City, New York, United States*

Previous studies have established a clear association between the surrounding environment and mental health. Whereas most literature has focused on neighborhood environment, very few studies have examined the role of housing characteristics in self-rated mental health (SRMH). Using data from the 2018 Canadian Housing Survey, this study investigated the relationships between housing characteristics, neighborhood environment, and SRMH among older Canadians and whether the relationships varied by education and gender. Using a sample of 21,725 Canadians, SRMH was measured by older adults' self-evaluation of mental health on a 5-point scale. We categorized education into three groups: high school or less, some college, and university or beyond. Hierarchical linear regressions showed that men and women with high school education and women with some college educations were more likely to report worse SRMH when living in low-income housing. Reporting a home maintenance need was a unique risk factor of SRMH for men with a university education, whereas living in uninhabitable conditions uniquely predicted better SRMH for men with some college education. Regarding neighborhood environment, safer community was a protective factor of SRMH for women with university education only. Sense of belonging was positively associated with SRMH for all subgroups, except for men with a university education. Expressing a need for community service was significantly associated with lower SRMH for women who completed a high school education or some college. Findings of this study shed light on the diverse need for environmental improvement and maintenance programs to improve SRMH among older Canadians.

### FACTORS IMPACTING EMERGENCY PREPAREDNESS AMONG COMMUNITY-DWELLING OLDER ADULTS

Alisha Thompson, Kristina Little, and Youn Kyoung (Lily) Kim, *Louisiana State University, Baton Rouge, Louisiana, United States*

Natural and manmade emergencies have become more frequent over the past 20 years and will continue to pose serious risks to public health and safety. Older adults are more adversely affected by—and less prepared for—emergencies than younger adults. However, little is known about the risk factors impeding emergency preparedness among older adults. This study uses the ecological systems approach to explore factors associated with emergency preparedness and how those factors influence adults 60 and older. The study analyzed cross-sectional data taken from 690 community-dwelling older adults who participated in Wave 5 of the National Poll on Healthy Aging. The sample was broken down into the following two groups for comparison: individuals aged 60–69 ($n = 383$) and individuals 70 or above ($n = 307$). The self-reported measures of sociodemographic characteristics, physical health, mental health, and previous experience of a disaster were utilized via regression analysis to predict emergency preparedness. Emergency preparedness was assessed using X dichotomous questions (60-69: $M = X$; 70+: $M = X$). The results revealed that living alone and having a Hispanic background were negatively associated with emergency preparedness among those aged 60–69, while mental health status was positively associated with emergency preparedness among those aged 70+. Previous experience of a disaster positively impacted emergency preparedness among the sample. Implications for policy and practice focus on shifting the perspective of the disproportional risks for older adults around emergencies to one that values and supports older adults' strengths and insights.

### RECENT ADVERSITY AND LIFE SATISFACTION AMONG CHINESE OLDER ADULTS: ROLE OF GENDER AND NEIGHBORHOOD COHESION

Yi Wang[1], Ying Ma[2], Man Guo[1], and Li Wang[3], *1. University of Iowa, Iowa City, Iowa, United States, 2. University of Houston, Houston, Texas, United States, 3. Anhui Medical University, Hefei, Anhui, China (People's Republic)*

A growing body of literature suggests that recent adversity impedes psychological wellbeing. Little is known about whether living in a cohesive neighborhood could buffer the negative consequences of adverse experience and whether such associations vary by gender. This study aims to examine the association of recent adverse events and perceived life satisfaction among Chinese older adults, and to explore the potential moderating role of gender and neighborhood cohesion. Data were from a cross-sectional study conducted with community-dwelling older adults aged 60 years and above in Anhui Province, China in 2014 ($N=1,960$). Recent adversity was measured by six events in the past two years (health decline, economic difficulty, loss of intimate people, loss of an important item, major life event, conflict with family or close friends/neighbors). Multilevel ordered logit regression with interaction term of adversity and neighborhood cohesion was performed. Models were further conducted on stratified samples to compare gender differences. Individuals experiencing any recent adverse event are 84% less likely ($p=.008$) to report a higher level of life satisfaction. Living in neighborhoods with better cohesion increased the likelihood of reporting better life satisfaction by 6% ($p=.031$). Modeling on the stratified samples showed that the above-mentioned significant relationships hold for male sample only. Recent adversity may negatively affect life satisfaction among Chinese older adults. Living in neighborhood with higher levels of cohesion could help buffer such negative influences, particularly for older men. Findings highlight the critical role of neighborhoods in combating the negative psychological consequences of adversity among Chinese older adults.

### ADAPTING STAKEHOLDER WALKABILITY/WHEELABILITY AUDIT TOOL IN NEIGHBORHOOD FOR SENSORY AND COGNITIVE DISABILITIES

Kishore Seetharaman[1], Atiya Mahmood[1], Bridget Disini[1], Hailey-Thomas Jenkins[1], Mike Prescott[1], Farinaz Rikhtehgaran[1], and W Ben Mortensen[2], *1. Simon Fraser University, Vancouver, British Columbia, Canada, 2. University of British Columbia, Vancouver, British Columbia, Canada*

Neighbourhood accessibility influences health, social inclusion, and overall wellbeing of older adults. It is important to assess neighbourhood accessibility in relation to the diverse needs and challenges brought on by the intersection of aging and disability, particularly sensory and cognitive disabilities. Given the paucity of neighbourhood audit tools tailored for this population, The user-led Stakeholders' Walkability/Wheelability Audit in Neighbourhoods (SWAN) tool was originally created for people with mobility disabilities and is now being adapted for seniors with sensory and cognitive disabilities to evaluate functionality, safety, appearance, supportive features, and social aspects in their neighbourhoods. In this paper, we present highlights and key takeaways from the process of adapting the SWAN tool for three user groups: people living with 1) Blindness or low vision, 2) Deafness and hearing loss, and 3) Dementia. Key steps in the iterative tool adaptation process include 1) identifying access needs/challenges for the three user groups based on a literature review, 2) online consultation with stakeholders with lived and/or professional experience (N = 4) to prioritize key access needs/challenges that will be captured through the SWAN tool and review draft versions of the tool, and 3) in-person pilot testing of tools with persons with lived experience (N = 2) in two urban/suburban neighbourhoods in British Columbia, Canada. Reflections of team members and input from stakeholders and pilot participants revealed issues that were addressed in tool development, namely 1) length of audit and participant fatigue, 2) legibility of tool, and 3) tailoring audit to participants' context and needs.

### SESSION 6510 (POSTER)

### EPIDEMIOLOGY, BIOLOGY, CHRONIC DISEASES, AND FUNCTION I

### ANNUALIZED AND CUMULATIVE MEASURES OF ANTICHOLINERGIC EXPOSURE FOR RESEARCH AND CLINICAL APPLICATIONS

Hussein Khalil[1], Eva Stachler[1], Khalid Alamer[1], Jared Ludlow[2], and Noll Campbell[3], *1. Purdue University, West Lafayette, Indiana, United States, 2. Indiana University Purdue University Indianapolis, Indianapolis, Indiana, United States, 3. Purdue University, Indianapolis, Indiana, United States*

Medications with anticholinergic properties are commonly used by older adults despite being associated with dementia.The anticholinergic total standardized daily dose (TSDD) is a continuous measure of exposure that has been associated with an increased risk of dementia at values >1095 over ten years in epidemiologic studies. We sought to determine a cumulative (cTSDD) and annualized (aTSDD) in a sample of community-dwelling older adults enrolled in the ongoing Reducing Risk of Dementia Through Deprescribing (R2D2) trial (NCT04270474). Participants were 65 years or older without dementia, attended at least one primary care visit within 12 months prior to enrollment, and were current users of strong anticholinergics according to the 2012 Anticholinergic Cognitive Burden Scale. Prescribed and over-the-counter medication details were collected during the baseline visit through self-report and included strength, frequency, units/dose and duration. The aTSDD was calculated for each participant assuming continuous use patterns throughout the year. The cTSDD was calculated by summing the aTSDD across the number of years since initiation. Of 66 participants, the median cTSDD was 2425 (IQR 5131), and 48 (72%) exceeded the threshold of dementia risk (>1095). Additionally, the aTSDD had a median of 730 (IQR 547), with 60 (90%) exceeding the threshold of dementia risk (109.5, one-tenth of the ten-year risk) while 11 (17%) exceeded dementia risk threshold of 1095 considering 1 year of exposure. Both measures identified the majority of anticholinergic users exceeding dementia risk thresholds despite some disagreement between the two approaches. However, both methods have potential for research and clinical applications.

### DIURNAL CORTISOL SECRETION AND SELF-REPORTED AND CAREGIVER-REPORTED QUALITY OF LIFE IN PEOPLE LIVING WITH DEMENTIA

Fanghong Dong, Subhash Aryal, and Nancy Hodgson, *University of Pennsylvania, Philadelphia, Pennsylvania, United States*

IntroductionPeople living with dementia (PLwD) report lower quality of life (QoL), compared to healthy older adults. The poorer QoL is not fully accounted for by the severity of dementia. Dementia is associated with prominent neuroendocrine changes, however, there is a lack of research examining whether biological factors are related to QoL in PLwD. This study examined relationships between cortisol, symptom severity, and QoL in PLwD.

Methods: A total of 143 participants aged 55-94 years (65.7% women) in the Healthy Patterns Study (NCT03682185) provided three saliva samples at wake-up (AM1), 30 minutes (AM2) after waking, and bedtime (PM) on two consecutive days. We derived cortisol awakening response (CAR), wake to bedtime cortisol slope, and diurnal mean cortisol secretion. Sociodemographic and severity of dementia were assessed by interviews and questionnaires. Self-reported and caregiver-reported QoL was measured using the Quality of Life in Alzheimer's Disease (QoL-AD).

Results: Poorer QoL was associated with more severe dementia rating. Flattened cortisol slope was significantly correlated with overall poorer self-reported QoL ($\beta = 0.43$, p=0.017), but not caregiver-reported QoL (p=0.12), after controlling for severity of dementia and demographic variables. We did not find a significant relationship between CAR and diurnal mean cortisol with QoL.

Conclusions: This study provides novel evidence linking neuroendocrine mechanisms to QoL in PLwD. The findings indicate that dysregulation of the hypothalamic-pituitary-adrenal axis is linked to poorer QoL, independently of the severity of dementia. Biopsychosocial approaches to QoL for

PLwD may lead to a greater understanding of the underlying mechanisms.

### HEALTH-RELATED QUALITY OF LIFE OF OLDER PEOPLE WITH DIABETES DEPENDING ON WHETHER THEY LIVE IN FAMILIES OR NOT

Gi Won Choi, and Sun Ju Chang, *Seoul National University, Seoul, Seoul-t'ukpyolsi, Republic of Korea*

Diabetes requires self-management, such as the use of insulin, oral antidiabetic drugs, diet, and exercise, for maintaining blood glucose. To promote self-management of older people with diabetes, family support is considered an important factor. However, older people living alone lack family support and have a lower health-related quality of life (HRQOL) than those living in families. The purpose of this study was to assess HRQOL and the factors that affect it between older people with diabetes living alone and those living in families. We performed secondary data analysis using the data of Korea National Health and Nutrition Examination Survey (KNHANES) 2017–2019. In total, 973 people with diabetes aged 65 years or older answered all questions about HRQOL. Complex sample analysis was performed. Each dimension of HRQOL was significantly lower in people living alone than those living in families. Restricted activity, bedridden, number of comorbidities, and perceived health status significantly predicted HRQOL in patients living alone. Health insurance, age, economic activity, unmet medical service needs, restricted activity, bedridden, number of comorbidities, perceived health status, and suicidal ideation significantly predicted HRQOL in patients living in families. This study indicated how differences in HRQOL exist between older patients with diabetes living alone and those living in families. This data will be useful in developing educational programs in the future, and it will be helpful when dealing with people living alone who have diabetes.

### ASSOCIATION BETWEEN DUAL TASK FUNCTION AND NEUROPSYCHOLOGICAL TESTING IN OLDER ADULTS WITH COGNITIVE IMPAIRMENT

Kelsi Petrillo, and Nima Toosizadeh, *University of Arizona, Tucson, Arizona, United States*

Cognitive impairment is an increasingly relevant health concern, as demonstrated through data estimates that by 2040 the number of older adults with dementia will surpass nine million in the United States. Despite this high prevalence, more than half of patients with dementia never receive an evaluation. This indicates that a quick and objective routine test for screening cognitive decline in older adults is needed. Poor dual-task gait performance has been associated with decreased executive and neuropsychological function. However, gait tests are not always viable for clinics or older patients. The aim of this study was to assess the relationship between a novel upper-extremity function (UEF) dual-task performance and neuropsychological test results in older adults. We recruited older adults at three stages: cognitively normal (n=33), mild cognitively impaired (n=34), and Alzheimer's disease (n=22). For UEF tasks, participants performed a consistent elbow flexion, while counting backwards in by threes. Wearable motion sensors were attached to forearm and upper-arm to measure accuracy and speed of elbow flexion kinematics and the UEF cognitive score. The results demonstrate a strong correlation between UEF cognitive score and MMSE, Mini-Cog, Category fluency, Benson complex figure copy, Trail making test, and MOCA (r values between -0.2355 and -0.6037 and $p< 0.0288$). UEF dual-task was associated with executive function, orientation, repetition, abstraction, verbal recall, attention and calculation, language and visual construction. The results from this study convey a promising future for the use of dual task UEF as a safe and convenient cognitive impairment screening.

### ASSOCIATION BETWEEN UPPER EXTREMITY FUNCTION AND ADVERSE OUTCOMES IN OLDER ADULTS WITH COPD

Mehran Asghari[1], Martha Ruiz[2], Miguel Peña[3], Haley Johnson[2], Sairam Parthasarathy[4], and Nima Toosizadeh[2], *1. University of arizona, Tucson, Arizona, United States, 2. University of Arizona, Tucson, Arizona, United States, 3. university of arizona, Tucson, Arizona, United States, 4. University of Arizona, Tuscon, Arizona, United States*

Chronic obstructive pulmonary disease (COPD) is the third leading cause of death in the United States. COPD adverse outcomes are required to identify vulnerable patients and enhance bronchodilator therapies. Although the 6-minute walk distance test is commonly used to assess functional capacity in COPD and predict adverse outcomes, it is not feasible for older adults with mobility impairments. Previously, we demonstrated that a simple upper-extremity function (UEF) test could identify frailty and cognitive impairment. We developed two indexes to predict the physical frailty (20-s rapid arm test) and cognitive impairment (60-s normal speed dual-task arm test and counting) among older adults. UEF tests were performed by 76 eligible older adults (age=67.421±6.363). All participants were followed up for one month to record the adverse outcomes. The measured health outcomes included: in-hospital outcomes (death, complication, and excessive length of stay), and 30-day outcomes (30-day death or readmission). Based on the results, 54% and 30% of patients had in-hospital and longitudinal adverse outcomes, respectively. The frailty index was significantly associated with all measured outcomes ($p< 0.018$). However, cognitive score showed significant association only with 30-day longitudinal outcomes ($p< 0.021$), but not for in-hospital outcomes ($p>0.05$). Among all the associations the highest effect size was observed between frailty score and longitudinal outcomes ($p< 0.007$; effect size=0.94). The results of this study suggest that a 20-s UEF test is a practical quick measure for predicting adverse in-hospital and 30-day outcomes among COPD patients.

### ASSOCIATION BETWEEN VISUOSPATIAL ABILITY, GAIT, AND FUNCTION IN OLDER SURVIVORS OF BREAST CANCER

Jennifer Blackwood[1], and Abigail Simone[2], *1. University of Michigan-Flint, Flint, Michigan, United States, 2. University of Michigan--Flint, Flint, Michigan, United States*

Research has highlighted the relationship between impaired cognitive function and physical mobility measures in older adults. This relationship may also exist in older survivors of breast cancer, however, specific cognitive domains like executive function or visuospatial ability may influence physical mobility. The purpose of this study is to describe associations between cognitive function and mobility in a group of community-dwelling older females with a history of breast cancer. Cross-sectional data of performance on cognitive and physical mobility measures from 38 older adults with a history of breast cancer were analyzed. Pearson's correlation coefficient was used to examine relationships between measures. Significant inverse relationships were found between TMT-A and usual gait speed (r = -.662, p<.001), fast gait speed (r = -.409, p = .012), and dual-task gait speed (r = -.631, p<.001), suggesting that as visuospatial ability became more impaired, gait speed decreased. Significant relationships were found between TMT-A and performance on the TUG (r = .664, p<.001), TUG-cognitive (r = .635, p<.001), and TUG-manual (r = .653, p<.001). Results indicate that visuospatial ability, as measured with the TMT-A, is associated with physical mobility measures in older survivors of breast cancer. While previous research has reported impairments in executive function in this population, impaired visuospatial ability may also play a role, suggesting that clinicians should include a visuospatial task when working on improving gait in older survivors of breast cancer.

### UTILITY OF SIX-MONTH PROGNOSIS TO PREDICT SURVIVAL AMONG NEWLY ADMITTED NURSING HOME RESIDENTS WITH CANCER

Long Vu[1], David Warner[2], Nicholas Schiltz[1], Johnie Rose[3], Jennifer Cullen[3], Cynthia Owusu[3], Martha Sajatovic[3], and Sara Douglas[1], *1. Case Western Reserve University, Cleveland, Ohio, United States, 2. University of Alabama at Birmingham, Birmingham, Alabama, United States, 3. Case Western Reserve University School of Medicine, Cleveland, Ohio, United States*

A prognosis of less than 6 months life expectancy has potential to be useful in planning and evaluating end-of-life care; however, its agreement with actual survival data has not been assessed. Here, we measured the sensitivity, specificity, positive predictive value (PPV), and negative predictive value (NPV) of the nursing home (NH) Minimum Data Set (MDS) 6-month prognosis indicator among individuals diagnosed with incident breast (female), colorectal, lung, pancreatic, or prostate cancer during the years 2004-2017 and admitted to a NH between 2012-2017 (n=254,183). Cancer patients were identified from the linked Surveillance, Epidemiology, and End Results (SEER) Registry. The median age was 80 years, 52.6% were women, and 15.15% were non-White. Only 7,541 (2.97%) of patients were deemed by a physician to have a life expectancy of less than 6 months as recorded in their first comprehensive MDS assessment; of those, 83.0% died within 6 months, and 12.3% died after 6 months. Sensitivity and specificity were 7.8% and 99.3%, respectively, for the prognosis indicator predicting mortality within 6 months; PPV was 83.0% and NPV was 69.9%. In conclusion, although very few patients had received a physician designation of their 6-month prognosis, a high percentage actually died within 6 months. Conversely, among those without a 6-month prognosis, nearly one-third died within 6 months. A terminal diagnosis appears reasonably predictive of death — when made. However, its low sensitivity demonstrates that a vast majority of terminal patients are not properly captured, calling into question its greater application.

### GLAUCOMA, CATARACT, AND EMOTIONAL DISTRESS IN NEW ENGLAND: FINDINGS FROM THE HEALTHY AGING DATA REPORTS

Shu Xu, Chae Man Lee, YanJhu Su, Taylor Jansen, Nina Silverstein, and Beth Dugan, *University of Massachusetts Boston, Boston, Massachusetts, United States*

Glaucoma and Cataract are two common leading causes of vision loss and major sources of disability and mental issues for older adults, but few studies investigated glaucoma and cataract among older adults from a community-level perspective. This study describes the rates of glaucoma and cataract among older adults 65+ in New England and examined the association of glaucoma and cataract and emotional distress (depression and anxiety) of older adults at the community level across 794 towns/cities (MA (351), NH (235), RI (39), and CT (169)) in New England. Data sources used to calculate rates of communities were: the American Community Survey (2014-2018 RI and CT, 2012-2016 MA and NH) and the Medicare Current Beneficiary Summary File (2016-2017 RI, 2015 MA and NH, 2016-2017 CT). Small area estimation techniques were used to calculate age-sex adjusted community rates for more than 150 health indicators (https://healthyagingdatareports.org/). Results showed CT had the highest rates of glaucoma (28.3%), RI had the highest rates of cataract (67.5%), and NH had the lowest rates of glaucoma and cataract (22.9% and 61.2%, respectively). After adjusting for age, gender, race, marital status, education, income, chronic diseases, communities with higher rates of glaucoma or cataract were significantly associated with higher rates of emotional distress compared to those with lower prevalence (glaucoma: β=0.06, p< 0.01, for depression, β=0.09, p< 0.001 for anxiety; cataract: β=0.08, p< 0.01 for depression, β=0.11, p< 0.01 for anxiety). Findings help practitioners and policymakers to allocate emotional support services to areas of high need due to eye diseases.

### STEP EXECUTION TIME: EXAMINING ITS RELIABILITY AND ASSOCIATION WITH PARTICIPATION IN OLDER ADULTS

Chad Tiernan[1], Ida Kolodziejczyk[1], Susan Talley[2], and Allon Goldberg[1], *1. University of Michigan-Flint, Flint, Michigan, United States, 2. University of Michigan-Flint, Ferndale, Michigan, United States*

The projected rise in the number of older adults warrants increased attention to selecting reliable and meaningful clinical assessments. Compared to more frequently used balance measures, step execution time (SET) has not been studied extensively. The purpose of the current study was twofold. First, we investigated the association of several balance measures [SET, Activities-specific Balance Confidence Scale (ABC-6), Five-times-sit-to-stand (5TSTS), Four-square step test (4SST), and maximum step length (MSL)] with participation (LIFE-H). Second, reliability of SET was investigated. The study included 32 community-dwelling older adults between the ages of 65 and 83 years (88% White, 66% female). Results indicated that SET was the only balance measure associated with both participation accomplishment (r = -.54, p = .001) and participation satisfaction (r = -.55, p = .001); 5TSTS (r = -.64, p < .001) and 4SST (r = .37, p = .039) were also significantly correlated with participation accomplishment while MSL (r = .37, p = .040) showed a significant association with participation satisfaction. SET demonstrated excellent test-retest reliability (ICC = .92). Bland-Altman analysis determined the 95% Limits of Agreement to be -258.5 ms to +271.5 ms (mean difference = 6.5 ms; 95% CI of difference = -43.1 to 56.1), suggesting that bias was not a concern. SEM (100.5 ms) and MDC95 (278.5 ms) for SET represented 9.3% and 25.8% of the mean, respectively. Collectively, findings suggest that SET may have clinical utility as a reliable assessment in older adults that relates to meaningful constructs, such as participation.

### METABOLOMIC PROFILES FOR THE EXPLORATION OF BIOMARKERS IN SEVERE SARCOPENIA AMONG OLDER MEN

Hyung Eun Shin[1], Miji Kim[1], Daehyun Lee[2], Jae Young Jang[1], and Chang Won Won[1], *1. Kyung Hee University, Seoul, Seoul-t'ukpyolsi, Republic of Korea, 2. Kyung Hee Univerisity, Seoul, Seoul-t'ukpyolsi, Republic of Korea*

The pathophysiology of sarcopenia is complex and multifactorial; however, it has not been fully elucidated. This preliminary study explored novel biomarkers of severe sarcopenia through a metabolomic analysis of plasma metabolites in community-dwelling older men. Twenty older men (mean age: 81.9±2.8 years) were randomly selected from the Korean Frailty and Aging Cohort Study. Participants with severe sarcopenia were compared to healthy, age-, and body mass index-matched controls (n = 10 each). Severe sarcopenia was diagnosed using the Asian Working Group for Sarcopenia 2019 criteria. Non-targeted metabolomic profiling of plasma metabolites was performed using capillary electrophoresis time-of-flight mass spectrometry. Among the 191 plasma metabolic peaks, 10 metabolites differed significantly between healthy controls and participants with severe sarcopenia. The plasma concentrations of l-alanine, homocitrulline, n-acetylserine, gluconic acid, n-acetylalanine, proline, and sulfotyrosine were higher, while the concentrations of 4-methyl-2-oxovaleric acid, 3-methyl-2-oxovaleric acid, and tryptophan were lower in participants with severe sarcopenia than in healthy controls (all, p < 0.05). Of the 57 metabolites quantified in target metabolites, L-alanine (area under the receiver operating characteristic curve [AUC] = 0.760, p = 0.049), gluconic acid (AUC = 0.800, p = 0.023), proline (AUC = 0.785, p = 0.031), and tryptophan (AUC = 0.800, p = 0.023) predicted the presence of severe sarcopenia. In conclusion, plasma metabolomic analysis demonstrated significant changes in amino acid, arginine, proline, and pentose phosphate metabolism in participants with severe sarcopenia. The identified metabolites could be helpful in understanding the underlying pathophysiology of sarcopenia.

### EARLY PHYSICAL AND MENTAL HEALTH PREDICTORS OF NIGHTTIME DRIVING DIFFICULTY IN OLDER ADULTS

Colleen Peterson[1], and Toben F. Nelson[2], *1. University of Michigan Transportation Research Institute, Ann Arbor, Michigan, United States, 2. University of Minnesota, Minneapolis, Minnesota, United States*

Older drivers are expected to make up about 25% of licensed U.S. drivers by 2050. Describing early predictors of driving difficulty can identify interventions that might delay driving cessation or support successful transition to driving retirement. In this exploratory study, we analyzed nationally representative data from the longitudinal National Social Life, Health, and Aging Project (NSHAP) conducted in 2005–06, 2010-11, and 2015-16. Our purpose was to ascertain mental health and physical functioning correlates of increased nighttime driving difficulty, which often precedes daytime difficulties. The sample included 1,454 drivers with observations at all 3 waves aged 57 to 85 at baseline. By wave three, adjusting for non-response and sampling bias, 59% reported no driving difficulty, 23% reported some or much difficulty, and 17% reported no longer being able to drive at night. Results of mixed-effect ordinal logistic models, adjusting for demographics, showed increased inability to drive at night was associated most strongly with self-report of poor or fair physical health (OR=10.4, 95%CI=6.2,17.5; OR=2.4, 95%CI=1.8,3.2), considering oneself "not at all active" (OR=2.9, 95%CI=1.3, 6.4), and being unable to balance heel-to-toe for 10 seconds (unsuccessful attempt: OR=4.8,95%CI=1.5,15.5; less than 10 seconds: OR=2.3, 95%CI=1.1,4.9). Although generalized self-reported mental health was not associated with increased nighttime driving difficulty, each additional depressive symptom (CESD-11) increased the odds of having greater driving difficulty by 12% (95%CI=1.1, 1.5). This study found early predictors of increased nighttime driving difficulty and retirement, providing opportunity to inform interventions to reduce roadway risk to older adults and others.

### ASSOCIATIONS BETWEEN MEASURES OF SLEEP QUALITY, PHYSICAL ACTIVITY, ANXIETY, AND STRESS AMONG ADULTS

Sally Paulson[1], Michelle Gray[2], Anthony Campitelli[2], Joshua Gills[2], Megan Jones[2], Erica Madero[3], Jennifer Myers[3], and Jordan Glenn[3], *1. St. Elizabeth Healthcare, Cincinnati, Ohio, United States, 2. University of Arkansas, Fayetteville, Arkansas, United States, 3. Neurotrack Technologies, Redwood City, California, United States*

Good quality sleep is important for physical and mental well-being and plays a vital role in healthy aging. Additionally, sleep quality is influenced by multiple factors associated with aging, such as lifestyle, depression, and physical activity levels. Therefore, the purpose of this study was to explore the relationship between self-reported measures of physical activity levels, anxiety, stress, and sleep quality among adults aged 45-75 years. Adults (N = 204) completed the short form International Physical Activity Questionnaire (IPAQ), Generalized Anxiety Disorder 7-Item Scale (GAD-7), Perceived Stress Scale (PSS), and the Pittsburgh Sleep Quality Index (PSQI). Participants were classified as either good quality sleepers (GQS, n = 65, 67.7% female, M±SD: age: 61.7±8.9 yrs; mass: 82.8±16.7 kg, height: 169.6±10.2 cm, education: 17.3 yrs) or poor quality sleepers (PQS, n = 139, 77.7% female, M±SD: age: 61.9±8.0 yrs; mass: 83.1±16.2 kg, height: 166.4±8.5 cm, education: 18.0 yrs) using their PSQI

global score. A score of ≥5 denoted PQS. Data were analyzed using descriptive statistics, Spearman correlation, and Chi-square ($p \leq .05$). Sleep quality was negatively associated with the PSS ($r = -.17$, $p = .02$) and GAD-7 ($r = -.19$, $p < .01$). Adults reporting PQS presented with higher levels of perceived stress and generalized anxiety. The relation between sleep quality and IPAQ was significant ($X2 = 8.47$, $p = .01$). These results suggest there was a greater tendency for PQS to report lower levels of physical activity and higher levels of stress and generalized anxiety.

### CARDIOVASCULAR DISEASE AND PHYSICAL ACTIVITY PHENOTYPES WITH FALLS: BEHAVIORAL RISK FACTOR SURVEILLANCE SYSTEM

Minsuk Oh[1], and Kelly Ylitalo[2], *1. Baylor University, Woodway, Texas, United States, 2. Baylor University, Waco, Texas, United States*

Cardiovascular disease (CVD) is associated with a higher risk of falls and physical activity (PA) can prevent falls. However, the role of PA in the association between CVD and falls is unknown. We examined the associations of four CVD–PA phenotypes (no CVD–PA, no CVD–no PA, CVD–PA, CVD–no PA) with any, recurrent, and injurious falls. Middle-aged and older adults ≥ 45 years (n=295,282; N=130,103,093) from the 2018 Behavioral Risk Factor Surveillance System who self-reported CVD, leisure-time PA, and falls in the previous 12 months were examined. The weighted prevalence ratio (PR) of any (≥ 1 fall), recurrent (≥ 2 falls), and injurious (≥ 1 injurious fall) falls across CVD–PA phenotypes was estimated using Poisson regression models with adjustment for sociodemographics and major health characteristics. The likelihood of any, recurrent, and injurious falls was greater across the unhealthier CVD–PA phenotypes in fully adjusted models. In the total sample, no CVD–no PA (PR: 1.19; 95% confidence interval (CI): 1.14, 1.24), CVD– PA (PR: 1.22; 95% CI: 1.15, 1.29), and CVD–no PA (PR: 1.29; 95% CI: 1.23, 1.35) phenotypes were more likely to report injurious falls, than no CVD–PA phenotype. Engaging in leisure-time PA may be an important lifestyle strategy for fall prevention in midlife and older adults, particularly those with CVD. Our findings could be useful for primary and/or secondary fall prevention efforts by healthcare providers who treat midlife and older adults with CVD.

### THE IMPACT OF SOCIAL DETERMINANTS ON SLEEP HEALTH AMONG MIDDLE-AGED AND OLDER ADULTS

Rebecca Lorenz, Heather Orom, Samantha Auerbach, Chin-Shang Li, and Yu-Ping Chang, *University at Buffalo, Buffalo, New York, United States*

Poor sleep health, including short or long duration and/or irregular timing may lead to a variety of chronic health conditions including diabetes and heart disease. An estimated 50-70 million adults in the United States have poor sleep health and this burden is disproportionately felt among systematically disadvantaged groups. While social and behavioral determinants of sleep duration and quality have been examined, sleep health, a multidimensional concept, has been less explored. The study aims to examine the impact of social determinants on sleep health among middle-aged and older adults. Data from the 2014 wave of the Health and Retirement Study were weighted and restricted to respondents of "Leave-Behind" questionnaire (n=5334). Sleep Health score was derived from sleep variables (range 0-100). Structural equation modeling was conducted using the R package lavaan. Sample mean age was 68.2 years (SD=10.1). Majority were female (60%) and white (76%) with mean Sleep Health score of 50 (SD=5.2). Black ($p< 0.0001$) and Latinx respondents ($p< 0.0001$) had worse sleep health than white respondents. Depression, financial strain, and neighborhood characteristics of socioeconomic status, social cohesion, and physical disorder mediated the relationship between race and sleep health. Ongoing chronic stress and everyday discrimination also mediated the relationship between race and Sleep Health among Black vs. white respondents. These findings suggest multiple individual and neighborhood-level determinants may negatively influence sleep health among a nationally representative sample of middle-aged and older Black and Latinx adults. Neighborhood-level characteristics may be modifiable factors that can be targeted to improve sleep and related health outcomes.

### INSTABILITY OF ECOLOGICALLY DERIVED MOOD, PAIN, AND FATIGUE SYMPTOMS IN OLDER ADULTS: THE ROAMM STUDY

Erta Cenko, Emily Smail, Roger Fillingim, Mamoun Mardini, Sanjay Ranka, Yashaswi Karnati, Jordan Alpert, and Todd Manini, *University of Florida, Gainesville, Florida, United States*

Background: Accurate assessments of mood, pain, and fatigue, are fundamental for evaluating and optimizing well-being in later life. However, traditional methods often do not capture instability – a measure of variability that incorporates the temporal order of the experiences. Objectives: Using the Real-time Online Assessment and Mobility Monitoring (ROAMM), a novel smartwatch platform, we sought to compare the instability of ecologically derived ratings of mood, pain, and fatigue symptoms among community-dwelling older adults.

Methods: Thirty-one participants answered questions on a smartwatch for approximately 2 weeks. Questions were delivered in the morning, afternoon, and evening, three days a week. Participants rated their current mood, pain, and fatigue on an anchored scale ranging from 0 to 10. We calculated the mean squared successive difference (MSSD) between ratings to determine the instability of each symptom.

Results: On average, instability was highest for mood (MSSD = 4.44) and lowest for pain (MSSD=1.51). Mood instability was moderately correlated with pain ($r= 0.42$, $p = 0.02$) and fatigue ($r= 0.45$, $p = 0.01$) instability, while fatigue and pain instability were strongly correlated ($r=0.82$, $p< 0.01$).

Conclusions: Results suggest that instability in pain, mood, and fatigue ratings are inter-correlated. The strong association between pain and fatigue instability suggests a co-occurrence that will aid in understanding the origins of these complex states. Repeated ecological assessment of common gerontological symptoms might provide new knowledge about the psychosocial well-being of older adults.

### SESSION 6520 (POSTER)

### FAMILIES AND INTERGENERATIONAL RELATIONS

### "BECAUSE HE'S MY BABY": HOW MOTHERS EXPLAIN FAVORING THEIR FIRST- AND LAST-BORN CHILDREN IN LATER LIFE

Reilly Kincaid[1], J Jill Suitor[1], Megan Gilligan[2], and Destiny Ogle[1], *1. Purdue University, West Lafayette, Indiana, United States, 2. Iowa State University, Ames, Iowa, United States*

Past research suggests that adult children's birth order is an important predictor of mothers' favoritism in later life. Older mothers are especially likely to favor their last-born children for socioemotional relationship dimensions such as emotional closeness. However, researchers have not examined how children's birth order affects the reasons underlying these patterns. To address this question, we examine: (1) how older mothers' reasons for favoritism differ by favored children's birth order, and (2) how these differences help explain mothers' disproportionate likelihood of favoring their last-borns. Qualitative data were collected during in-home interviews with 156 older mothers as part of the Within-Family Differences Study-II. Ninety-two of the mothers chose their last-borns and 64 chose their first-borns as the children to whom they felt most emotionally close. Findings suggest that last-borns were most often favored because they were seen as understanding and empathetic or in greater need of mothers' attention and support. First-borns were often favored based on birth order and geographical proximity or contact frequency. Birth order itself was mentioned in 17.39% and 23.44% of cases of favoritism toward last-borns and first-borns, respectively. These findings enrich and extend past research on the role of birth order in shaping intergenerational relationships by shedding light on the reasons why last-borns are often mothers' emotionally closest children. Given that poor parent–child relationship quality is linked to worse physical and psychological health for both adult children and their older parents, findings have important implications for research on family well-being in later life.

### INTERGENERATIONAL DIGITAL SOLIDARITY IN AGING FAMILIES AND ITS ASSOCIATIONS WITH RELATIONAL OUTCOMES

Xiaoyu Fu, Woosang Hwang, and Merril Silverstein, *Syracuse University, Syracuse, New York, United States*

Since the coronavirus disease outbreak, older adults have been isolated from family, as in-person contact declined, and many turned to digital contact to stay in touch. This form of contact, consisting of texting, email, and social media, is labeled digital solidarity. A key advantage of digital communication over in-person contact is that it requires less investment of time and no geographic proximity. However, it is unclear whether digital solidarity represents a separate dimension of intergenerational solidarity, and whether it compensates for low in-person contact. In this paper, we examined traditional and digital types of intergenerational communication between older parents and adult children, and their associations with older adults' perceived quality of communication and closeness with children. We used the 2016 wave of the Longitudinal Study of Generations to generate a sample of 580 older parents who reported on relationships with 1,489 adult children. Adopting a three-step latent class approach, we identified four classes of intergenerational communication: all-type contact, no contact, digital contact, and traditional contact. Older adults in both no contact and digital contact classes were less likely to report being emotionally close with their adult children when compared to those in the traditional contact class. No difference in perceived quality of communication was found between contact classes. Our findings indicate that digital solidarity is a distinct dimension of intergenerational solidarity and can compensate for reduced in-person contact with children. Discussion centers on the implications of these results for pandemic times and a replication using recently collected data from 2021-22.

### CHANGES IN INTERGENERATIONAL RELATIONSHIPS DURING THE COVID-19 PANDEMIC: THE IMPACT ON WELL-BEING

Catherine Stepniak[1], J Jill Suitor[1], Megan Gilligan[2], Destiny Ogle[1], and Yifei Hou[3], *1. Purdue University, West Lafayette, Indiana, United States, 2. Iowa State University, Ames, Iowa, United States, 3. Renmin University, Beijing, Beijing, China (People's Republic)*

A burgeoning body of research has revealed the COVID-19 pandemic's dramatic impact on the lives of family members in the U.S., including increasing caregiving burden and reducing in-person contact. However, the preponderance of this work has focused on how COVID-19 has reshaped a single role, rather than the set of role relationships individuals hold. In the present paper, we used mixed-methods data collected from 543 adults in later midlife (mean age=60) collected as part of the Within-Family Differences Study-III to explore the impact of the pandemic on intergenerational and intragenerational family relationships. Using a combination of quantitative and qualitative analyses, we classified which relationships were affected (e.g., parents, adult children, grandchildren, siblings, etc.), identified social structural characteristics that predicted pandemic related relational changes (e.g., gender, race, age, SES), provided a detailed picture of how family members experienced these changes, and studied the impact of these changes on respondents' depressive symptoms. Findings suggested that respondents were most likely to report changes in their relationships with their parents and adult children, with reduced in-person contact and direct care to parents, and increased coresidence with adult children. White women were most likely to report changes in in-person contact and care to parents, and they were also more likely to develop higher depressive symptoms due to these changes. Black respondents reported fewer changes in contact with parents or children due to greater coresidence compared to White respondents, and no association was found between changes and depressive symptoms in this group.

### THE LINGERING EFFECTS OF LOSS: WIDOWHOOD AND SUBSTANCE USE IN THE LGBT POPULATION

Anne Barrett, and Jessica Noblitt, *Florida State University, Tallahassee, Florida, United States*

The immense stress of widowhood can lead to unhealthy coping strategies, including substance use. Relatively little is known, however, about widowhood's effects on substance use among sexual minorities. Of the few studies examining LGB widowhood, none employ large, nationally representative samples or consider whether effects depend on current partner status. We address these issues using a sample of 2,258 respondents aged 50 and older who participated in the 2010 Aging with Pride: National Health, Ageing, and Sexuality/Gender Study. Multivariate regression analyses reveal that widowhood is associated with higher odds of being a current smoker and of using drugs in the past year. These results are found for individuals who are currently partnered, as well as those who are not. This observation suggests that widowhood's negative effects on use of these substances are not attenuated by the potentially protective effect of current partnership. In contrast, the effects of widowhood on alcohol use do appear to be diminished by current partnership. Among those who have experienced widowhood, those with a current partner are less likely than those without a partner to report problematic drinking. Our results suggest that current partnership may protect against problematic use of more socially acceptable substances like alcohol, but it may not prevent less acceptable behaviors like smoking or drug use. In sum, our study suggests that current partnership may not completely counter the lingering effects of partner loss in the LGB population.

### INTERGENERATIONAL PERCEPTIONS OF FAMILY DYNAMICS AMONG OLDER PARENTS AND ADULT CHILDREN ON THE FACES IV AND SFI II

Jessica Hahne, Eleanor A. Skemp, and Brian Carpenter, *Washington University in St. Louis, St. Louis, Missouri, United States*

Standardized measures of family dynamics are rarely used with late-life families in either research or clinical contexts. The purpose of this study was to examine perceptions of family dynamics from the perspective of older parents and their adult children on two widely used family assessment measures. To our knowledge, no previous studies have reported data on these measures with late-life families. Within a larger project examining decision-making in late life, 34 older parents and two of their adult children (total N= 102) completed the Family Adaptability and Cohesion Evaluation Scale (FACES) IV and the Self-Report Family Inventory (SFI) II. Parents were mostly mothers (94.1%; M age= 75), but adult children included both daughters (67.6%; M age= 46) and sons (32.4%; M age= 46). For comparison with published norms, we report subscale scores on both family measures for parents and adult children. The mean intrafamily ICC (two-way, mixed effects, single rater, absolute agreement) for individual items for the FACES IV was .561 (SD= .164, Min= .128, Max= .827), and the mean intrafamily ICC on the SFI II was .422 (SD= .178, Min= .014, Max= .750). Overall, family consistency was in the poor to moderate range, but with a high degree of variability across families. Moreover, within families, parents and children had highly variable inconsistency in their perception of family dynamics. Findings from this study provide insight as to how established measures function in late-life families, and they highlight the importance of assessing multiple family members in order to understand family dynamics.

### COUPLES' FINANCIAL BEHAVIORS AND SATISFACTION AMONG KOREAN BABY BOOMERS

Youngwon Nam, Kyungmin Kim, and Gyounghae Han, *Seoul National University, Seoul, Seoul-t'ukpyolsi, Republic of Korea*

The life-cycle theory posits that midlife is a peak time of earning and productivity. However, middle-aged adults also face complex financial decisions (e.g., mortgage, child tuition cost, care expenses for older parents, and their own health care expenses), which may influence their current and future financial well-being. While research has focused on individuals' financial management behaviors at midlife, less is known about the interdependence of financial behaviors and well-being within a couple, which may be a more relevant unit of financial decisions. To address this gap, we examined middle-aged couples' financial behaviors and satisfaction, using a sample of 1,111 couples (age 51–59) from the 2014 Korean Baby Boomer Panel Study (KBBPS). Wives showed consistently higher levels of financial behaviors in (a) recognizing financial status, (b) planning and monitoring financial goals, and (c) building and maintaining household wealth. Korean Baby Boomer couples also showed substantial within-couple similarity in planning and monitoring financial goals (ICC = .51) and building and maintaining household wealth (ICC = .65)—whereas they showed less shared awareness of household financial status (ICC = .23) between spouses. Results from the Actor-Partner Interdependence Models (APIM) revealed that beyond one's own financial behaviors, their spouses' financial behaviors were associated with higher financial satisfaction for both husbands and wives (partner effect). Further, intra-couple differences in financial behaviors were associated with lower financial satisfaction only for wives. These findings highlight the importance of shared process of financial decision making for financial well-being among couples in middle and later life.

### PASSING ON THE SILVER SPOON: THE ROLE OF EARLY LIFE CIRCUMSTANCES ON DOWNWARD INTERGENERATIONAL FINANCIAL TRANSFERS

Kent Jason Cheng, *Syracuse University, Syracuse, New York, United States*

Childhood circumstances are known to etch an indelible influence on individual outcomes across the life course such as education, income, and health. Yet relatively little is known about how early life exposures influence family outcomes. This study aims to determine how childhood exposures are associated with inter vivos downward intergenerational financial transfers among older adults aged 51-85 with at least one surviving adult child. Using cumulative (dis)advantage theory, I hypothesized that experiences in childhood shape intergenerational transfers patterns – with early-life advantage being able to provide more transfers than their disadvantaged counterparts. I used data from the US Health and Retirement Study waves 1998 to 2018 (n=32,095 individuals, 169,316 person-years) and estimated random effects models. The index of childhood socioeconomic status was constructed by adding the following dichotomized indicators: poor to fair family socioeconomic status, mother / father having less than HS education, father working for service sector, and moved due to financial difficulty. Downward transfers were defined as whether the respondent gave money, helped pay bills, or covered certain costs to children or grandchildren worth ≥US$ 500. The unadjusted model revealed that the probability of providing downward transfers among those with 1 and 2+ childhood socioeconomic status disadvantage was 5.4% and 3.8% less than those without disadvantage. The inverse association of early life disadvantage and downward transfers were preserved when basic demographic controls and household income and wealth were controlled for, although the magnitude slightly declined (4.0% and 3.1% lower probability for those with 1 and 2+ misfortunes, respectively).

### DAILY ARGUMENTS WITH SPOUSES AND NEGATIVE AFFECT: AGE AND EMOTION WORK AS MODERATORS

Dakota Witzel[1], Madeline Nichols[2], Justin White[2], and Robert Stawski[2], *1. Penn State University, State College, Pennsylvania, United States, 2. Oregon State University, Corvallis, Oregon, United States*

Daily interpersonal stressors, such as arguments with spouses, are predictors for increased negative affect within the literature (Witzel & Stawski, 2021). Emotion work, or activities done to promote another's positive emotional state, has been noted to have mixed effects on health and well-being (Umberson et al., 2020) and emotion work is more prevalent in times of increased stress (Rao, 2017). Moreover, with old age, individuals experience fewer daily interpersonal stressors (Witzel & Stawski, 2021). Using the 2015 wave of the Health and Relationships Study (Ncouples=756, Mage=48.22, Rangeage=35-65, Mmaritalduration=8.52; Umberson, 2015), we explored 1.) how daily arguments with spouses within diverse marriages were related to negative affect across 10 days and 2.) whether age and emotion work moderate these associations. HARP is a daily diary study exploring marriage and health in same-sex and different-sex marriages. Preliminary results using multilevel models suggest that individuals' negative affect significantly increased on days they reported spousal arguments. The interaction between spousal arguments and emotion work was significant ($p<.05$); on days when individuals reported more emotion work, negative affective reactivity associated with spousal arguments was higher, compared to days when less emotion work was reported. No significant age differences were found. Discussion will focus on the import of spousal arguments and emotion work for shaping daily well-being, and their relevance throughout adulthood, midlife, and older age. Future work should explore how being the recipient of emotion work may buffer or exacerbate the associations between daily arguments and negative affect and potential gendered patterning therein.

### DYADIC APPRAISALS OF FAMILY DECISIONS AND HEALTH TASKS IN MIDDLE-AGED AND OLDER COUPLES

Karen Lyons[1], Sae Hwang Han[2], Kyungmin Kim[3], and Lyndsey Miller[4], *1. Boston College, Chestnut Hill, Massachusetts, United States, 2. University of Texas at Austin, Austin, Texas, United States, 3. Seoul National University, Seoul, Seoul-t'ukpyolsi, Republic of Korea, 4. Oregon Health & Science University, Portland, Oregon, United States*

Dyadic theories, including the Theory of Dyadic Illness Management, emphasize the important protective roles of shared appraisals and collaborative tasks within the couple for optimizing health and well-being. Within illness contexts, research has also focused on shared decision-making and collaborative illness management within couples. However, less is known about how concordant middle-aged and older couples are regarding how they make decisions (e.g., one partner has final say, both have equal say) and how they complete health-related tasks (e.g., one partner does it, they do it together), factors associated with being more concordant as a couple, and implications of concordance for health and well-being of both members. Data from 2,761 couples (Mage = 64.94 for wives and 68.04 for husbands) from the Health and Retirement Study (2014/16–2016/18) were examined. The majority of couples were congruent regarding who makes family decisions (69.7%) and who completes medical forms (64.4%) within the couple; further, 62% agreed they make family decisions collaboratively versus 25.5% completing medical forms collaboratively. Greater concordance in these appraisals was significantly associated with greater marital support and length of marriage. While concordance in appraisals was not significantly associated with depressive symptoms 2 years later, the link between congruence in making major family decisions and self-rated health differed by age and gender— suggesting that the intersection of age and gender may shape how decisions within couples lead to potential health benefits.

### ASSOCIATION BETWEEN OLDER PARENTS' INFORMATION SHARING AND THEIR INTERGENERATIONAL AMBIVALENCE

Noriko Toyokawa[1], Nancy Darling[2], and Teru Toyokawa[3], *1. Southern Oregon University, Ashland, Oregon, United States, 2. Oberlin College, Oberlin, Ohio, United States, 3. California State University San Marcos, San Marcos, California, United States*

The current study examined (1) the relation between older parents' information sharing of their life issues with their children as the primary helpers (helper children) and parents' intergenerational ambivalence and (2) the effect of parents' reasons for not sharing the information with the helper children on parents' intergenerational ambivalence. Older parents (N=268, Mage =76, SD=5.41, range 65-94, female=59%) participated in an online survey and responded to how much they openly shared information on 10 issues of their lives developed based on a focus group study (Toyokawa et al., 2021). A series of one-way ANOVAs, followed by Bonferroni posthoc analysis, revealed that compared to parents who openly shared information on life issues and did not perceive the issues were not applicable for their lives, parents who did not openly share information reported significantly lower levels of intergenerational ambivalence on the issues of pain management, $F(2, 265)=7.51$, $p=.0007$, social partners, $F(2,265)=9.50$. $p=.0001$,what they do with other children, $F(2,256)=7.49$, $p=.0007$, financial management, $F(2,265)=3.80$, $p=.0234$, and their plan for the end-of-life, $F(2,256)$, $p=.004$. A series of t-tests showed that parents who did not share the information with the helper children because of avoiding unwanted interventions reported significantly higher scores of intergenerational ambivalence than parents who did not share the information to prevent the helper children from being worried about them in eight issues. The findings suggest parents maintain their autonomy by limiting the helper children's monitoring ability. Possible reasons for older parents' managing issue-specific information sharing will be discussed.

### TO CARE OR NOT TO CARE: CONTENT ANALYSIS OF MEDIA COVERAGE ABOUT ADULT CHILDREN CARING FOR PARENTAL PERPETRATORS

Jaime Goldberg, and Jooyoung Kong, *University of Wisconsin-Madison, Madison, Wisconsin, United States*

Though research about adult child survivors of childhood parental maltreatment who serve as caregivers for their perpetrating parent remains in its infancy, this topic has received some media attention in recent years. These publications, mainly focused on what, if any, duty an adult child survivor of parental childhood maltreatment has to care for their aging/ill parent, have garnered hundreds of online responses from readers that reveal vastly different beliefs, attitudes, and opinions about such caregivers. Content analysis was conducted on 513 comments across 5 online publications including the New York Times New Old Age blog, AgingCare blog, Slate Magazine, and Quora discussion forum. Several themes emerged from the data: (1) feelings of obligation; (2) setting boundaries; (3) validation of experience; (4) judgment of choices. It is evident from this analysis that the shame and stigma of being harmed by a parent in childhood persists into adulthood and the isolation of caregiving is palpable; participants posted to this online forum seeking connection in shared experience. The decision to participate in caregiving is particularly fraught when there is a history of maltreatment in the family system. For those who decide to become caregivers, they are at higher risk for experiencing ongoing challenges to their health, mental health, and overall wellbeing; for those who do not, they may still experience emotional turmoil. Gerontological researchers and clinicians' heightened awareness of and knowledge about such caregivers' experiences and needs are crucial to provide effective, trauma-informed support.

### THE POWER OF DAUGHTERS: THE ROLE OF SIBSHIP GENDER COMPOSITION IN MOTHER-ADULT CHILD RELATIONS

Robert Frase[1], J Jill Suitor[1], Megan Gilligan[2], Reilly Kincaid[1], and Catherine Stepniak[1], *1. Purdue University, West Lafayette, Indiana, United States, 2. Iowa State University, Ames, Iowa, United States*

Prior scholarship demonstrates that older mothers receive more care from daughters, prefer daughters as caregivers, and have stronger emotional bonds with daughters. Despite these clear gendered differences in care, care preferences, and closeness, less is known about whether the presence of daughters in a family affects mother-adult son relationships or whether the presence of sons in a family affects mother-adult daughter relationships. Drawing from theories of gender socialization and social exchange, we propose that mothers would, given the choice between daughters and sons, prefer to receive care from and engage in emotional exchanges with daughters. Therefore, we predict sons' care to and emotional closeness with older mothers will be inversely related to the number of daughters in the family. We test our hypotheses with mixed-method data from 1,577 mother-adult child dyads nested within 420 families collected as part of the Within-Family Differences Study-II. Findings support our hypotheses. The larger the number of daughters, the less likely sons are to provide care to their mothers, whereas the likelihood of daughters providing care is unaffected by the number of sons. Similarly, the larger the number of daughters, the lower closeness mothers report with their sons, whereas mother-adult daughter closeness is unrelated to the number of sons. In sum, our findings show ways in which both an adult child's gender and the gender composition of their sibship affect mother-adult child relationships, as well as highlighting the applicability of theories of gender socialization and social exchange to the context of aging families.

### LATER-LIFE TRAJECTORIES OF MARITAL QUALITY FOR WOMEN AND MEN

Jennifer Bulanda[1], Takashi Yamashita[2], and J. Scott Brown[1], *1. Miami University, Oxford, Ohio, United States, 2. University of Maryland, Baltimore County, Baltimore, Maryland, United States*

It is essential to better understand older adults' marital dynamics, as marital quality is consequential for older adults' physical and mental health, relationships with other social network members, and risk of divorce. Although numerous studies examine marital quality during the early life course, much less is known about later life trajectories. Earlier work suggested marital quality followed a U-shaped curve, characterized by a later-life upswing, but more recent research finds patterns of stability or decline over time. Gender differences in marital quality are well established, but it is less clear whether older men and women experience different marital quality trajectories during later life. We use nationally-representative data from a sample of continuously-married adults over age 50 in the Health and Retirement Study (n=2,175) with measures of both positive and negative marital quality at three time points (2006, 2010, and 2014). Latent growth mixture models in Mplus estimate trajectories of marital quality over the eight-year time period. Results show small declines in both positive ($b = -0.016$, $p < 0.05$) and negative ($b = -0.028$, $p < 0.05$) marital quality over the observation period, but the declines in positive marital quality are limited to those in remarriages. Women have lower initial positive marital quality and higher initial negative marital quality than men, but there are no significant gender differences in change over time. Results support the stability and continuous-decline patterns of marital quality over the life course rather than a U-shaped curve, and suggest persistence of gender differences over time.

### EFFECTS OF TRANSMISSIONS OF OLDER MOTHERS' INTERPERSONAL RELATIONSHIP QUALITY ON ADULT CHILDREN'S WELL-BEING

Destiny Ogle[1], Yifei Hou[2], J Jill Suitor[1], and Megan Gilligan[3], *1. Purdue University, West Lafayette, Indiana, United States, 2. Renmin University, Beijing, Beijing, China (People's Republic), 3. Iowa State University, Ames, Iowa, United States*

Prior research has rarely considered how the effects of social relationships on well-being can extend across generations. Drawing from the life-course perspective, we tested intergenerational transmission of relationship quality (with mothers and friends) as a mechanism through which mothers' relationship quality affects adult children's psychological well-being. We proposed that adult children bear the consequence of mothers' relationship quality because they tend to develop interpersonal relationships similar to their mothers' through socialization and observational learning, which in turn affects their psychological well-being. We investigated how this mechanism varied by adult children's gender using mixed-method data collected from 693 adult children in 270 families as part of the Within-Family Difference Study. Multilevel mediation analyses revealed that transmission of mother-child closeness and tension affected adult sons' but not daughters' depressive symptoms. In contrast, transmission of friendship closeness decreased daughters' but not sons' depressive symptoms. Mother's friendship tension affected both sons' and daughters' depressive symptoms, but the mechanisms differed by gender. Sons' depressive symptoms were affected by intergenerational transmission of friendship tension. In contrast, intergenerational transmission was not the mechanism via which mothers' friendship tension raised daughters' depressive symptoms. Qualitative analyses revealed daughters' well-being was affected by more diverse pathways besides intergenerational transmission of relationship quality and that these differences could be explained by gender socialization and differing meanings of social relationships for sons and daughters. These findings highlight the role of parental socialization in the mechanisms by which linked lives affect adult children's well-being, even decades after these processes were set into motion.

### ETHNIC IDENTIFICATION AND GRANDPARENT-GRANDCHILD RELATIONSHIPS IN ASIAN AND ASIAN AMERICAN YOUNG ADULTS

Catherine Ju[1], and Brian Carpenter[2], *1. University of Minnesota/Minneapolis VA, Eden Prairie, Minnesota, United States, 2. Washington University in St. Louis, St. Louis, Missouri, United States*

Asians and Asian Americans experienced an increase in exposure to racial discrimination during the COVID-19 pandemic. Ethnic minorities may counter discrimination by actively strengthening their ethnic identity and engaging in behaviors designed to enhance ethnic and cultural identification, such as reaching out to people who personify their culture. Grandparents are one such resource to whom young adults may turn to learn about their cultural heritage. The current study examined the degree to which facets of Asian and Asian American grandparent-grandchild relationships were related to ethnic identity, particularly in response to exposure to discrimination. Asian and Asian American young adults ($N = 102$) completed survey questions related to their experiences with COVID-19-related racial discrimination, ethnic identification, and relational closeness and frequency of contact with grandparents. Overall, exposure to discrimination was not significantly associated with strength of ethnic identity. However, there was a significant positive association between strength of ethnic identity and frequency of synchronous contact with grandparents, $rs(100) = .329$, $p < .001$. Strength of ethnic identity and relational closeness with grandparents were also significantly positively correlated, $rs(100) = .383$, $p < .001$. In contrast to some previous research, results show that discrimination encountered during the pandemic may not be related to strength of ethnic identification. However, there is a strong relationship between Asian and Asian American young adults' strength of ethnic identification and the nature of their relationships with their grandparents. These findings enhance understanding of how intergenerational relationships are related to ethnic identity.

### DO OPPOSITES ATTRACT OR AGGRAVATE? THE IMPACT OF INTERGENERATIONAL SOLIDARITY ON WELL-BEING

Rachel Scott[1], Acacia Lopez[1], Marin Olson[2], and Danielle Nadorff[2], *1. Mississippi State University, Starkville, Mississippi, United States, 2. Mississippi State University, Mississippi State, Mississippi, United States*

Family Systems Theory states that values are transmitted between generations within families and, while many of these values are similar between immediate generations, there may be more differentiation in values between generation gaps. Ideological differences between generations may potentially cause subsequent tension and fluctuations in well-being. The current study sought to examine the moderating effect of ideological differences (religious and political) on the relation between how emotionally close grandparents perceive themselves being with younger generations and grandparental well-being. Participants included 419 grandparents (age: $M = 76$, $SD = 5.33$), 716 adult children (age: $M = 53$, $SD = 4.16$), and 638 adult grandchildren (age: $M = 29$, $SD = 5.52$) from the 8th wave of the Longitudinal Study of Generations (LSOG) data set. Well-being in grandparents was positively correlated with perceived emotional closeness among both of the intergenerational dyads. Religious ideological differences between grandparents and grandchildren were found to negatively influence grandparental well-being; this relation was also found to be significantly different from political ideological differences within the same intergenerational dyad. While moderation was not achieved for either dyad, the overall model fit was found to be excellent, suggesting its utility for further research into the relation between the study variables. These findings indicate that there is a complex relation between perceived emotional closeness, ideological beliefs, and the well-being of grandparents that warrant additional attention within the literature and may inform interventions related to intergenerational communication and well-being. Further implications for these findings will be discussed.

### WHAT CAUSES OLDER PARENT–ADULT CHILD ESTRANGEMENT?: A QUALITATIVE INTERVIEW STUDY

Julia Nolte, Marie Cope, and Karl Pillemer, *Cornell University, Ithaca, New York, United States*

The life-course perspective's emphasis on "linked lives" highlights the importance of positive parent-child relationships for adults' well-being in mid-life and beyond. However, one in ten Americans experiences parent-child estrangement, defined as ending all contact and communication for a period of time. Despite the prevalence, the reasons underlying family estrangements are poorly understood. In this study, we conducted in-depth, qualitative interviews with 43 middle-aged and older adults (age range = 46-81, M = 65.05 years) who are or were estranged from an adult child (estrangement range = 1-26, mean = 6.53 years). On the basis of a jointly developed codebook, independent coding from the first and second author revealed 22 estrangement reasons across eight categories, with parents citing reasons from across four categories on average. Estrangement was most often attributed to the behavior, issues, or traits of the child (79%), problems surrounding a difficult in-law/ partner (72%), or disagreements about beliefs and values (67%). To a lesser degree, estrangements were linked to separation or divorce (49%), financial problems and conflicts (42%), adverse childhood experiences of the estranged child (30%), and interference from someone other than a partner (16%). More than half of the parents (58%) also endorsed other reasons, including their own traits and behaviors, communication issues, a family history of estrangement, and passive loss of contact. Older parent–adult child estrangements are perceived as multi-causal, with four out of five parents describing the estranged child as difficult or troubled. Implications of the findings for professionals working with older adults are discussed.

### SESSION 6530 (POSTER)

### FAMILY CAREGIVING, SOCIAL NETWORKS, AND SOCIAL SUPPORT

### IMPACT OF THE COVID-19 PANDEMIC: HEALTH AND MENTAL HEALTH CAPACITY OF CAREGIVERS AND CARE RECIPIENTS

Christina Miyawaki[1], and Nina Tahija[2], *1. University of Houston, Houston, Texas, United States, 2. The Council on Recovery, Houston, Texas, United States*

We developed a depression intervention, Caregiver-Provided Life Review (C-PLR) for people living with dementia and their family caregivers in 2018 and pilot-tested (N=19 caregiver (CG)-care recipient (CR) dyads). Encouraged by the outcomes, in 2020 we expanded the study to long-term care facility (LTCF) residents. Due to the COVID-19 pandemic, we adapted the in-person mode to virtual and recruited CGs who provided care for >8 hours/ week and CRs who were community-dwellers or LTCF residents with mild depression and dementia. Between August 2020 and December 2021, we contacted 195 LTCFs, 29 senior/community centers, and 148 community-dwellers and recruited 12 dyads nationwide. CGs were on average 54 years old, working (67%), college-educated (83%), female (92%), and in good-excellent health (75%). CRs were 83 years old (mean), single (58%), female (92%), and in fair health (50%). CRs' depression score significantly improved (p=0.0004), however, CG's burden and positive aspects of caregiving scores worsened (p=0.02). Amid the pandemic, LTCF closures and staff turnover made recruitment challenging. Despite a small sample, we found CRs enjoy the LRs alleviating depressive symptoms. However, CGs experienced more burden, which could be due to COVID fatigue and the LR as an additional responsibility. These results reminded us of the COVID impact and adjusted the C-PLR model further. Instead of a weekly visit, each dyad can meet at its own pace. We developed the C-PLR to improve the health of the dyads. Thus, we must consider both CGs' and CRs' health capacity because CR's wellbeing is dependent on that of CGs.

### SUPPORTING DEMENTIA CAREGIVERS IN HEALTHCARE DELIVERY: RECOMMENDATIONS FROM A NATIONAL CONSENSUS CONFERENCE

Catherine Riffin[1], Joan Griffin[2], Lauren Bangerter[3], Lilla Brody[4], Francesca Falzarano[5], Karl Pillemer[6], Sara Czaja[7], and Ronald Adelman[7], *1. Weill Cornell Medical College, New York, New York, United States, 2. Mayo Clinic, Rochester, Minnesota, United States, 3. United Health Group, Edina, Minnesota, United States, 4. Weill Cornell Medicine, New York, New York, United States, 5. Weill Cornell Medicine, Douglaston, New York, United States, 6. Cornell University, Ithaca, New York, United States, 7. Weill Cornell Medicine, New York City, New York, United States*

More than 6 million adults in the United States are affected by Alzheimer's Disease and related dementias (ADRD), the majority of whom rely on assistance from an unpaid caregiver (family, friends). The goal of the 2021 Conference on Engaging Family and Other Unpaid Caregivers of Persons with Dementia in Healthcare Delivery, funded by the National Institute on Aging, was to establish a policy- and practice-aligned research agenda for enhancing ADRD caregiver engagement and support in healthcare settings. The Conference convened multidisciplinary thought leaders from across the United States to establish a set of actionable recommendations to advance the field. Recommendations centered on five key topics: 1) Identification and assessment of ADRD caregivers, 2) Reimbursement and financing for provider time spent on ADRD caregiver, identification, assessment, and support, 3) ADRD caregiver training and support, 4) Healthcare provider education, and 5) Technology. To support future work in each of the five priority areas, conference participants highlighted the importance of leveraging lessons from implementation science and models of equity and inclusion. Recommendations are intended to inform federal agencies and foundations about high-priority areas and motivate multidisciplinary collaborations to design care delivery systems that effectively engage and support ADRD caregivers.

### UNDERSTANDING CHALLENGES AND STRENGTHS EXPERIENCED BY CHINESE AMERICAN FAMILY CAREGIVERS OF PERSONS WITH DEMENTIA

Jessica Cassidy[1], and Kathy Lee[2], *1. University of Texas at Arlington, Arlington, Texas, United States, 2. University of Texas at Arlington, Coppell, Texas, United States*

Chinese American family caregivers of persons with dementia are often challenged by barriers to utilize caregiving support services due to language barriers, minimal understanding of dementia, limited numbers of culturally competent health care providers, as well as conflicting cultural norms, such as the disease related stigma. Subsequently, a delayed diagnosis and a hesitance to seek social support services and formal resources are prevalent among Chinese American families caring for persons with dementia. However, the resilience of these caregivers is often noted as a strength of this sub-population. We aimed to identify the challenges and strengths expressed by Chinese American family caregivers caring for persons with dementia by exploring their caregiving experiences. Using a qualitative study design, we conducted semi-structured, in-depth interviews with 26 Chinese American family caregivers of persons with dementia during the midst of COVID-19 pandemic. The data were analyzed using thematic analysis. Two themes emerged as challenges: lack of knowledge regarding early signs of dementia and family caregivers' social isolation and loneliness. Two additional themes emerged as demonstrated strengths: utilizing technology throughout the caregiving trajectory and family caregivers' helping behaviors. Our findings indicate potential to promote disease-related education and reduce feelings of social isolation and loneliness among Chinese family caregivers caring for persons with dementia by using technology and peer-led approaches. The examined coping strategies of Chinese American caregivers may be leveraged across disciplines to increase disease related knowledge, rates of early diagnosis, as well as the utilization of formal supports and resources.

### "HE JUST WANTS HIS SERVICES BACK": IMPACTS OF HOME CARE WORKER DISRUPTIONS ON PATIENTS AND CAREGIVERS DURING COVID-19

Emily Xu[1], Patricia Kim[1], Meng Zhang[1], Jennifer Reckrey[1], Katherine Ornstein[2], and Emily Franzosa[1], *1. Icahn School of Medicine at Mount Sinai, New York, New York, United States, 2. Johns Hopkins University, Baltimore, Maryland, United States*

Home care workers (HCWs) have played a critical role in keeping homebound older adults safely at home during COVID-19, yet their essential work is often understudied. This study characterized the roles of HCWs during COVID-19 and examined how HCW service disruptions impacted patients and their caregivers. We performed a thematic analysis of medical records from 53 patients with HCWs in a home-based primary care practice in New York City. We abstracted unstructured clinical notes into a priori and emergent categories and identified core themes via team discussion. The following themes emerged: 1) Shifts to remote medical care and changing patient needs led to task shifting and new tasks for HCWs (i.e. getting food for patients experiencing food insecurity), 2) The risks associated with HCW tasks, such as exposure from caring for patients with COVID-19, increased during the pandemic, 3) Patient and family refusal of HCW services to avoid COVID-19 exposure as well as abrupt loss of HCW services due to HCW precarity or COVID-19 exposure left family caregivers with additional caregiving responsibilities, 4) Regulations surrounding return to work following COVID-19 exposure created additional difficulties in reinstating HCWs and left patients without adequate care, putting them at risk of hospitalization. In conclusion, pandemic-related disruptions created barriers to adequate home care, putting both patients and caregivers at risk. This analysis suggests a need for more robust HCW training and established regulations to protect HCW safety as well as a need for policies to support caregivers and ensure continuity of care during emergencies.

### SOMEBODY ELSE TO THINK ABOUT BESIDES MYSELF: VOLUNTEER EXPERIENCES WITH THE CARING CALLERS PROGRAM DURING COVID-19

Noelle Fields[1], Kathy Lee[2], and Jessica Cassidy[3], *1. The University of Texas at Arlington, Arlington, Texas, United States, 2. University of Texas at Arlington, Coppell, Texas, United States, 3. University of Texas at Arlington, Arlington, Texas, United States*

Social distancing during COVID-19 had significant implications for older adults, a subpopulation already at greater risk of social isolation and loneliness prior to the pandemic. Emerging evidence showed promise for implementing peer- and volunteer-led, telephone-based support programs in collaboration with community agencies to reach older adults at-risk for loneliness. Given a need for research in this area, the Caring Callers Program was developed as a telephone-based intervention using Senior Companion volunteers for at-risk older adults in a large metropolitan area in Texas. Volunteers were provided training via zoom to prepare them to provide supportive, weekly calls as well as to share community resources with their Caring Callers clients. The purpose of this study was to qualitatively explore the experiences of Senior Companion volunteers (N = 20) in the Caring Caller Program through the lens of productive aging. Rapid and Rigorous Qualitative Date Analysis (RADaR) was used to analyze the data and yielded four themes: 1) reciprocity, reflecting how volunteers described the program mutually benefited their feelings of well-being as well as, those contacted.; 2) purposeful use of time, illustrating how the volunteers perceived their role as meaningful and fulfilled their value for civic duty; 3) learning new skills, such as active listening skills; and 4) gaining perspective, capturing how the volunteers gained new inspiration from their experiences in the program. Recommendations for future research and strategies for designing peer-led, telephone-based interventions to promote social connectedness among both older volunteers and socially isolated older adults are offered.

### TRANSFERS, DIFFICULTIES, AND CARE STRESS AMONG FAMILY CAREGIVERS OF OLDER ADULTS LIVING WITH DISABILITIES IN CHINA

Lirong Yue, Shuangshuang Wang, and Nengliang Yao, *Shandong University, Jinan, Shandong, China (People's Republic)*

Family caregivers are critical sources of support to older adults living with disabilities, but they also need to cope with caregiving stress and difficulties brought by the caregiving process. This study aimed to explore the associations between family transfers (i.e., time transfer and financial transfer), perceived difficulties of caregiving, and caregiving stress among family caregivers of older adults living with disabilities. Respondents were randomly selected from 6 urban districts and 6 rural towns in Shandong province, China. Data from 220 older adults living with disabilities and their family caregivers were analyzed. Caregiving stress were divided into three categories (mild stress, moderate stress and high stress). Difficulties were divided into four categories (insufficient care abilities, economic hardship, time conflicts, and inadequate nursing equipment). Family caregivers reported moderate to high levels of caregiving stress. Results from multivariate ordinal logistic regression shows that family caregivers providing nine or more hours of care per day reported higher levels of caregiving stress than those who provided 8 or less hours of care per day (9 to 12 hours: OR=2.92, p<.05; 13 to 16 hours: OR=2.66, p<.05). Caregivers who perceived insufficient care abilities, economic hardship, and time conflicts were more likely to report caregiving stress. Respites in the forms of caregiver support group and day care attendance for older adults living with disabilities may reduce caregiver stress. Educational program and professional knowledge training courses may be helpful in reducing caregiving stress. The long-term care insurance system should be promoted to provide social support for family caregivers.

### DYADIC COPING AND WELL-BEING AMONG COUPLES TAKING CARE OF EACH OTHER

Vivian Wei Qun Lou[1], Huiying Liu[2], and Wenmiao Wu[1], *1. The University of Hong Kong, Hong Kong, Hong Kong, 2. Central South University, Chang Sha, Hunan, China (People's Republic)*

Background: In the context of population aging, more and more couples would have the chance to taking care of each other when one or both of them experience functioning decline. Objective: Taking a dyadic perspective, the objective of this study is to examine collaborative dynamics in mastering daily stressors, and the relationship between couples' practice of dyadic coping and individual well-being. Method: A total of 77 dyads were recruited from community elderly centres located in four different geographic locations of Hong Kong. Potential participants who met the following inclusion criteria: (1) both partners aged 60 or above; (2) both partners living alone in the same household; (3) one or both partners reporting at least one limitation in performing ADLs and/or IADLs, were invited to complete a survey questionnaire interviewed by trained researchers. Standardized measures by Dyadic Coping Inventory (DCI), Centre for Epidemiological Studies-Depression Scale (CES-D), EQ-5D, and Relationship Assessment Scale (RAS), respectively. The Actor-Partner Interdependence Model (APIM) framework was used for data analysis. Findings: The most adopted collaborative coping strategies were non-negative dyadic coping of oneself, non-negative dyadic coping of the partner, and common dyadic coping. Supportive and delegated dyadic coping strategies were strongly associated with marital satisfaction and health-related quality of life.

Conclusion: Collaborative dynamics among couples when one or both experience functional impairment is associated with quality of relationship. The findings advocate a shift from individual-based stress process on caregiving to a dyadic-based process that address joint needs of both spouses in super-aging societies globally.Funding: RGC 17605119

### UNDERSTANDING STROKE SURVIVOR–CARER DYNAMICS USING THE ACTOR-PARTNER INTERDEPENDENCE MODEL: A SYSTEMATIC REVIEW

Esther Chow, and Sai-fu Fung, *City University of Hong Kong, Kowloon, Hong Kong*

Background: Family members often take on the caregiver roles to stroke survivors due to kinships and cultural responsibilities. Yet, little is known about the the impact of survivor-caregiver dynamics on stroke rehabilitation. To understand the dyadic relationships between the dyads, a systematic review was conducted to examine studies which have adopted Actor–Partner Interdependence Model (APIM) analysis.

Methods: A systematic review and meta-analysis were conducted using the following electronic databases: PubMed, SAGE Journals, MEDLINE, PsycINFO and Cochrane Review to identify eligible studies published from their inception to December 2021, following the PRISMA guidelines.

Results: Nine studies involving 1183 stroke survivors (male = 57%) and 1181 caregivers (male = 37%) had met the inclusion criteria and were identified in this review. The interaction among the following outcomes were self-esteem, optimism, stress, depression, emotional distress, quality of life, and life satisfaction. The review provided evidence that (1) APIM is valuable tool in analyzing dyadic interactions; (2) support from caregivers has a significant impact on the stroke survivor's recovery; (3) the mental health status of caregivers can influence that of stroke survivors and vice versa; (4) Due to the interconnected nature of dyadic interaction, providing dyadic intervention have positive impact on dyads.

Conclusion: These findings highlight the interdependence nature between survivor-caregiver dyads in the context of stroke rehabilitation. The APIM provides conclusive evidence on the effectiveness of survivor-caregiver dyadic interactions, with significant theoretical and practice implications for both health and social care professions. More research is needed to support dyadic strategies for stroke survivors.

### CARING FOR THOSE WHO CARE: EVALUATION OF A COMPREHENSIVE CULTURAL COMPETENCY TRAINING CURRICULUM

Sara Powers[1], Rachel Cannon[1], David Bass[1], Lauren Pongan[2], Ocean Le[2], and Nina Darby[2], *1. The Benjamin Rose Institute on Aging, Cleveland, Ohio, United States, 2. Diverse Elders Coalition, New York, New York, United States*

By 2030, nearly 3 in 10 older Americans will identify as a member of a diverse or underrepresented group. To better support diverse, aging communities and help professionals meet the growing needs of family and friend caregivers, the Diverse Elders Coalition developed a comprehensive cultural competency training curriculum that, in the first year of development, engaged over 2,500 healthcare and social service providers. To evaluate the short and long-term impact of the training curriculum, this paper focuses on two types of anonymous evaluations that were electronically distributed to training attendees: 1) Subjective knowledge post-tests (n=162), and 2) 3-month post-training follow-ups (n=232). Majority of participants identified as female, White/Caucasian, and earned at least a college degree. Upon completion of the trainings, participants reported improved subjective knowledge about diverse communities, more confidence and preparedness to meet diverse caregivers' needs, and also indicated that because of the training they would engage in a variety of diversity related actions (e.g., use more inclusive language, share training resources with colleagues, attend additional diversity trainings). At the 3-month follow-up, 91.8% of respondents reported they engaged in two or more diversity related actions since attending the training. Respondents (73.8%) also indicated that their organization engaged in one or more diversity-related actions since attending the training (e.g., provided staff additional diversity-related trainings, translated materials). Discussion will focus on ways to improve healthcare and social service providers' advocacy efforts and awareness surrounding the needs of older adults and caregivers from diverse communities.

### FACTORS PREDICTING FAMILY MEMBER PROVISION OF ASSISTANCE WITH ACTIVITIES OF DAILY LIVING FOR SENIORS

Joanne Spetz, and Leslie Ross, *University of California, San Francisco, San Francisco, California, United States*

The aging of the U.S. population is leading to greater demand for assistance with activities of daily living. Much of this support is provided by family members, but changing family sizes and locations have raised concern that those needing support will be increasingly reliant on non-family assistance. This study examined patterns of provision of family, non-family, and paid caregiving to those 65 years and older who need assistance with activities of daily living, focusing on the impact of geographic location. We used the National Health and Aging Trends Study Restricted Metro-Nonmetro round five data file. We identified individuals who needed assistance with activities of daily living and/or instrumental activities of daily living. We merged information about the primary survey respondent (subject person, or SP) with data on "other people" (OP) in their supportive network, excluding spouses living with the respondents because nearly all spouses provided support. The final analytic data file consisted of 5,234 unique SPs. We used weighted and unweighted bivariate analyses as well as weighted multivariate logistic regression models to examine the association of family or non-family support and geographical location. We found that SPs who never were unmarried were more likely to have a non-spouse family helper compared to those married or living with a partner. Family who lived near the SP had a greater likelihood of providing help. SPs who were Black, non-Hispanic, multi-racial, and other non-Hispanic had more than twice the likelihood of having a family member helping them compared to White non-Hispanic SPs.

### AN EXPLORATORY DESCRIPTIVE STUDY OF CARE PARTNERS' PREFERENCES AND SKILL TRAINING NEEDS DURING HOSPITAL CARE

Kaitlyn Vanias[1], Andrea Yahr[2], and Beth Fields, *1. University of Wisconsin-Madison, Madison, Wisconsin, United States, 2. Geriatric Health Services Research Lab, Madison, Wisconsin, United States*

Care partners play a critical role in caring for an increasingly complex, aging population in the United States after hospital discharge. However, care partners' preferences and needs are often not formally assessed during their loved one's hospitalization. The Care Partner Hospital Assessment Tool (CHAT) is a standardized decision-support tool that facilitates the inclusion and training of care partners during hospital care. The purpose of this exploratory descriptive study was to understand the preferences and needs of 12 care partners of older adult patients admitted to a large academic hospital utilizing the CHAT. The majority (8 of 12) of care partners surveyed were spouses while the remainder were adult children of the patients. All care partners provided comparable support including physical and social support as well as healthcare management. Despite providing similar support, there were differences in the care preferences and needs of spouses compared to adult children. A greater proportion of spouses preferred to be present for care in the hospital and requested information on adaptive equipment and community services. All adult children desired access to the electronic medical record while there was a mixed response among spouses. The most common needs across care partners included training on mobility assistance and medical devices. Findings from this study demonstrate that care partners have varying preferences and training needs during hospital care and suggest differences across care partner relationships. Further investigation is necessary to better understand these patterns and improve hospital care for patients and their care partners.

### ASIAN AMERICAN FAMILY CAREGIVING EXPERIENCES COMPARED TO CAREGIVERS OF OTHER RACIAL/ETHNIC GROUPS

Christina Miyawaki[1], and Erin Bouldin[2], *1. University of Houston, Houston, Texas, United States, 2. University of Utah, Salt Lake City, Utah, United States*

Asian Americans (AAs) are the fastest-growing immigrant group in the U.S. While otherwise highly heterogeneous, AAs value filial piety and eldercare. This study compared the prevalence, health, and caregiving experiences of AA caregivers (18+ years) to AA non-caregivers and caregivers in other racial/ethnic groups. Data from the 2015-2019 Behavioral Risk Factor Surveillance System were used (N=252,602). Caregivers were classified as those who provided care/assistance to a parent/parent-in-law/grandparent with a long-term illness/disability. Chi-square tests were used to compare weighted estimates. Among 4,564 AAs, 4.1% (n=321) were caregivers. They were single (55.6%), college-educated (84.2%), working (64.7%), young (< 45 years) (68.2%), male (60.0%), and in excellent/very good/good health (86.3%). Compared to non-caregivers, caregivers were more often unmarried with more chronic health conditions. They assisted a parent/parent-in-law (73.4%) with personal care (52.7%) and household tasks (77.9%). Although overall caregiving experiences were similar across racial/ethnic groups, the prevalence of caregivers was higher among other racial/ethnic groups than AAs (8.3%, Hispanic to 14.8%, American Indian/Alaska Native, $p < 0.001$), which was a surprise due to their caregiving culture. Because caregiving is expected, they may not identify themselves as caregivers. The data were collected in English and Spanish but 44% of AAs had limited English proficiency. Those limitations may have affected the sample size. Despite the limitations, this study was unique because of the comparison among different racial/ethnic groups of caregivers across the nation. However, to examine a full picture of AA caregivers, we need to administer surveys for each AA ethnic group in various languages.

### IMPLICATION OF SLEEPING DURATION ON THE OVERALL HEALTH STATE OF FAMILY CAREGIVERS

Babatope Ogunjesa[1], Andiara Schwingel[2], Tosun Ozgur[3], and Alice Oloo[2], *1. University of*

*Illinois--Urbana-Champaign, Champaign, Illinois, United States, 2. University of Illinois, Urbana Champaign, Urbana Champaign, Illinois, United States, 3. Near East University, Nicosia, Nicosia, Cyprus*

Family caregiving is integral to long-term care for older adults. The National Alliance for Caregiving and AARP Public Policy Institute in 2020 estimated there are 53 million Americans providing care for loved ones. Caregiving intensity often impacts the health and well-being of caregivers, including influencing their sleep duration. The Centers for Disease Control and Prevention (CDC) recommended an average of 7 or more sleeping hours for adults. Unfortunately, most caregivers' average hours of sleep per night do not meet this recommendation. Using secondary data from the Long-Term Caregiving study conducted by the AP-NORC Center for Public Affairs Research, the present study establishes the effect of sleep duration on the overall health of family caregivers while adjusting for socio-demographic factors. A binary logistic analysis was conducted to infer the effects of sleep duration, age, gender education, race, employment status, and household income relative to reporting high or low overall health wellbeing. The binary logistic model was statistically significant, $\chi2(16) = 77.5$, $p < .0001$, Nagelkerke R2=0.111. Family caregivers with a sleeping duration of 7 hours or more were twice as likely to report a high overall health status than those who slept less than 7 hours (OR=2.08, 95%CI [1.52 – 2.84]). Increasing household income and being employed were positively associated with high overall health status. We do not find a statistically significant effect of gender, education level, age group, and race on overall health status. We recommend that policies that support family caregivers at a micro and macro level be implemented.

### CULTURALLY CONCORDANT CARE: CLINICIAN PERSPECTIVES ON PROVIDING CANCER CARE TO SOUTH ASIANS

Nainwant Singh[1], Veena Manja[1], Ambri Pukhraj[2], Rashmi Risbud[3], Shreya Desai[4], Lidia Schapira[2], Karl Lorenz[5], and Ranak Trivedi[6], *1. VA Palo Alto, Palo Alto, California, United States, 2. Stanford University, Stanford, California, United States, 3. University of California, Davis, Davis, California, United States, 4. Veterans Affairs Palo Alto Health Care System, Menlo Park, California, United States, 5. Veteran Affairs Palo Alto Health Care System, Stanford, California, United States, 6. Stanford University, Palo Alto, California, United States*

In many South Asian (SA) cultures, cancer is stigmatized, and family members are expected to become primary caregivers. Clinicians need to be familiar with these SA needs and values to provide culturally concordant care. The South Asian Family Approaches to Disease (SAFAD) study aims to understand cultural needs of SAs managing breast cancer. We conducted semi-structured qualitative interviews with multidisciplinary clinicians at a major academic medical center about caregiver interactions, cultural dynamics affecting clinical practice, unmet patient needs, and perceptions of culturally concordant care. Participants included physicians(8), a nurse practitioner(1), a social worker(1), and a physician assistant(1) with experience in palliative care(5), hematology/oncology(4), breast cancer(3), critical care(2), and radiation oncology(1) with one to 42 years in practice. Participants identified as Caucasian/White(6), South Asian(3), African American/Black(1), and Chinese(1). Clinicians noted the following: 1)SAs have greater family involvement in care and may defer treatment decisions to family members; 2)SAs seek clinician support with cancer management and nutrition; 3)SA emotions and hesitation around sensitive topics may result in non-disclosure; 4)SAs have diverse caregiver roles; and 5)individual SA needs cannot be generalized within the diaspora. "There's still some stigma… with breast cancer… [it] may lead a patient to not want to share their diagnosis… then set up a patient for having less support… [We can] help them feel more comfortable opening up selectively." Understanding such cultural needs is essential to cultivating trust and providing person-centered care. Interventions and resources to promote culturally concordant cancer care can target education in these areas.

### THE APPLICATION OF TIME BANKING IN ELDERLY CARING: A MIXED-STUDIES SYSTEMATIC REVIEW

Hui Ding[1], and Hui-Ling Li[2], *1. Soochow University, Suzhou, Jiangsu, China (People's Republic), 2. School of Nursing, Suzhou Medical College, Soochow University, Suzhou, Jiangsu, China (People's Republic)*

Aging has become a universal concern of the world, and its process is accelerating, which leads to the shortage of the care resources and increased burden of social development. Based on this problem, The concept of time banking has been gradually applied to geriatric caring, forming a model of mutual support for older people, which has been practiced in different countries. However, there is no consensus about the effect of time banking for older person. Thus, the aim of this review was to integrate research on the application of time banking to summarize the benefits for older people and challenges faced in different countries. Moreover, the review put forward solutions to provide references for the localization of time banking in China. PubMed, Wiley Online Library, Web of Science, SAGE Journals Online, Science Direct, EbscoHost, SpringerLink and CNKI databases were searched to include 22 eligible studies. Qualities of included studies were assessed, evidences about the benefits for older person and challenges regarding time banking was synthesized. Although the overall study quality was medium, consistent evidence was existed to indicate that time banking has beneficial effects which containing social values and personal values on the older people. Furthermore, the comprehensive evidences also showed that time banking would be improved in the following four aspects: diversity of volunteers, improvement of service quality, variety of incentives and scientific management. Additional well-designed and high-quality studies are needed for us to better understand the opportunities and challenges of time banking.

### EXAMINATION OF THE HEALTHY CAREGIVER EFFECT AMONG OLDER ADULTS FINDING FROM THE CANADIAN LONGITUDINAL STUDY ON AGING

Lun Li[1], Andrew Wister[2], and Barbara Mitchell[2], *1. MacEwan University, Edmonton, Alberta, Canada, 2. Simon Fraser University, Vancouver, British Columbia, Canada*

Objective. The Healthy Caregiver Hypothesis (HCH) suggests that caregiving is associated with beneficial health impacts for family caregivers. However, mixed results have been reported, particularly when different levels of caregiving intensity were examined. This study analyzes the relationship between caregiving intensity and three health indicators (functional health, chronic illness, and self-rated general health) among Canadian older adults over three years. Method. We drew upon a sub-sample of 11,344 participants aged 65 and older from the Baseline and Follow-up 1 data of the Canadian Longitudinal Study on Aging and used linear mixed models to test the hypothesis based on different levels of caregiving intensity. Results. Older adults who provided low-intensity care recently or continuously reported better functional health and self-rated health than non-caregivers. In contrast, older adults with low-intensity caregiving responsibility developed more chronic conditions over time compared to non-caregivers, but this association was not found for high-intensity caregivers. Discussion. This study elucidates the HCH by incorporating caregiving intensity to understand patterns of better functional health and perceived health, but more chronic conditions. The findings yielded from different health indicators suggest the impact of caregiving on health may be domain specific.

### SESSION 6540 (POSTER)

### LONG TERM SERVICES AND SUPPORTS

### COMORBIDITIES AMONG RESIDENTS DIAGNOSED WITH DEMENTIA IN RESIDENTIAL CARE COMMUNITIES

Christine Caffrey, Jessica Lendon, and Sengupta Manisha, *National Center for Health Statistics, Hyattsville, Maryland, United States*

Residential care communities (RCCs) are an important source of care for older adults, particularly those with dementia. Nationally in 2018, 34% of RCC residents had been diagnosed with dementia. Other common chronic conditions among RCC residents were high blood pressure (55%), depression (27%), arthritis (20%), diabetes (20%), and heart disease (17%). RCC residents with dementia who have comorbidities could present greater challenges when managing their care. This report presents national estimates of comorbidities among RCC residents with dementia and compares these estimates by selected characteristics. Estimates are from the 2018 National Post-acute and Long-term Care Study conducted by the National Center for Health Statistics. Among RCC residents with dementia, 62% had also been diagnosed with high blood pressure, 28% with depression, 23% with arthritis, 16% with diabetes, and 16% with heart disease. Among RCC residents with dementia, 17% were diagnosed with only dementia, 38% were diagnosed with dementia and one other common condition, 31% were diagnosed with dementia and two other conditions, and 14% were diagnosed with dementia and three to five other conditions. A greater percentage of RCC residents with dementia and two or more common conditions compared to only dementia or dementia and one other condition were aged 85 years and older (69% vs 62%), needed help with three or more activities of daily living (82% vs 73%), were hospitalized (34% vs 28%), and were taking more than 10 medications (48% vs 32%). Results may benefit efforts focused on implementing long-term care practices to manage dementia and comorbidities.

### PERCEPTIONS OF DEMENTIA CARE AMONG AFRICAN IMMIGRANTS IN MINNESOTA: INSIGHTS FROM COMMUNITY CONVERSATIONS

Hawking Yam[1], Manka Nkimbeng[1], Christina Rosebush[1], Wynfred Russel[2], Tetyana Shippee[1], and Joseph Gaugler[1], *1. University of Minnesota, Minneapolis, Minnesota, United States, 2. African Career Education And Resources Inc. (ACER), Brooklyn Park, Minnesota, United States*

Immigrants and persons from diverse racial and ethnic backgrounds have a higher prevalence of dementia compared with their U.S.-born counterparts, yet the estimates of dementia prevalence, perceptions, and needs among African immigrants are not fully understood. This qualitative study examined African immigrants' perceptions of dementia and dementia care. Specifically, it explored the community's attitudes, care practices, and barriers toward dementia care. Community conversations (focus groups) were conducted with 24 African immigrants residing in the Northwest Minneapolis suburbs. Conversation transcripts were analyzed using thematic analysis in Nvivo. The majority of participants were women, 79% from Liberia, 43% were over the age of 65 years, and 65% held a bachelor's degree or higher. Three themes were identified: 1) Perceptions of and attitudes toward mental health and dementia, which indicated that cultural issues limit awareness of dementia, but 2) Family care of a person living with dementia, meaning care happens in the home, and receiving care in a facility is the last resort; 3) Facilitators and barriers to dementia care, including distrust of health systems, immigrant status, and limited finances. This project offers unique insights on the dementia care and caregiving beliefs and attitudes among African immigrants that can inform policy and intervention efforts aimed at decreasing dementia care barriers in the African immigrant community.

### THE DEVELOPMENT OF AN UNMET NEEDS APPRAISAL SCALE FOR LONG-TERM CARE SERVICE USERS

Ya-Mei Chen, Shih-Cyuan Wu, Zi-Ting Zhong, and Yu-Chien Chang, *National Taiwan University, Taipei City, Taipei, Taiwan (Republic of China)*

Background: Taiwan's Long-Term Care (LTC) Plans 1.0 and 2.0 aimed to provide accessible LTC services since 2007. Currently, >40% of the people needing LTC are covered by these plans. Past studies have indicated that high unmet needs among LTC users may lead to adverse outcomes. Thus, whether the LTC services meet the needs of both the care recipients and their family caregivers merits further study. The purpose of this study was to develop a suitable "unmet needs appraisal scale (UNAS) for long-term care service users" and pilot test this tool on older Taiwanese adults.

Methods: The tool was developed through a systematic literature review. The tool underwent two rounds of the Delphi method with 26 experts, and the tool reached a high consensus among the experts. The UNAS includes 11 items assessing activities that need more assistance, including daily activities, psychological health, and spiritual health. We pilot tested the tool in older Taiwanese adults and examined the tool properties, including reliability and validity. We included short-form 8 as a concurrent validity measure. Result: The survey was conducted in 6 counties, and 255 service user questionnaires were collected. The overall Cronbach's α value was 0.91, indicating good internal consistency. Our confirmatory factor analysis also showed good construct validity [$\chi 2 = 146.514$, $p < 0.001$, CFI = 0.98, RMSEA = 0.06, SRMR = 0.02]. The UNAS scores showed a moderate negative correlation with quality of life scores ($r = -0.452$, $p < 0.001$), demonstrating good concurrent validity of the unmet needs scale.

### LONG-TERM CARE RESIDENT PERSPECTIVES ON PERSON-CENTERED CARE: INTERSECTIONS OF PRACTICE AND THEORY

Mary Lou Ciolfi[1], Catherine Taylor[2], Tracy Ericson[3], David Wihry[4], Jennifer Crittenden[5], Angela Hunt[3], Lenard Kaye[2], and Paul Nebenzahl[6], *1. University of Maine Center on Aging, Bangor, Maine, United States, 2. University of Maine, Bangor, Maine, United States, 3. The Cedars, Portland, Maine, United States, 4. University of Maine, Orono, Maine, United States, 5. UMaine Center on Aging, Bangor, Maine, United States, 6. The Mayer-Rothschild Foundation, Chicago, Illinois, United States*

The Designation of Excellence in Person-Centered Long-Term Care is a multi-year study to redefine standards of long-term care delivery according to the preferences of residents, family, staff, and leaders. During 2021, as one component of the study, 247 residents from 23 communities across the country engaged in a participatory message board activity inquiring about their lived experiences of receiving care and services in their long-term care communities. Residents commented on their personal care, personal and shared spaces; family, friends, and community; and wishes and feelings. Using a phenomenological approach to data analysis to better understand the universal and the unique aspects of the experience of living in long-term care, key themes centered on the importance of staff (1) knowing, responding, and being attentive to the details of residents' life history, care needs and preferences; (2) treating residents respectfully and as mature adults; and (3) demonstrating care and connection in resident interactions. These key themes and their related sub-themes intersect with several social science theories that underscore those factors promoting adaptability to changed environments while trying to preserve identity, belief systems, and values. Applying a conceptual lens to resident comments suggests programmatic strategies that long-term care providers can prioritize for promoting and operationalizing person-centered care. The poster will present a graphic representation of the intersections of person-centered care and existing theoretical frameworks using resident remarks to highlight central tenets of continuity theory, person-environment fit theory, and social identity theory.

### A SCOPING REVIEW OF HOW NURSING HOME STAFFING LEVELS AND QUALITY OF CARE ARE MEASURED

Mutian Zhang, Shelby Brown, Weizhou Tang, and Reginald Tucker-Seeley, *University of Southern California, Los Angele, California, United States*

The Centers for Medicare & Medicaid Services (CMS) is the largest payer of nursing home care in the US. CMS defines staffing levels as hours per resident day (HPRD) and staff turnover rates; it defines quality of care as nurse staffing levels, hospitalization rates, resident outcomes, and deficiencies (violations of regulations). However, these definitions have been inconsistently applied in the research literature, which has led to equivocal results in research on the association between staffing levels and the quality of care in nursing homes. We conducted a scoping review of how nursing home staffing levels and quality of care have been defined and measured in the research literature, guided by the PRISMA (Preferred Reporting Items for Systematically reviews and Meta-Analyses) framework. Out of the N=423 initially identified studies through PubMed, N=67 articles were selected, based upon the following inclusion criteria: 1) published after January 1, 2010; 2) published in the US; 3) focused on determinants of nursing home staffing levels or determinants of quality of care. Two independent reviewers conducted abstract screening and data extraction. The findings from our review showed that approximately 50% of studies partially adopted the definitions from CMS. For example, HPRD and deficiencies were utilized to measure staffing levels and quality of care in N=37 and N=12 studies, but none of them included all suggested measures. Future studies should carefully consider the approriate definitions as specific measures can yield contradictory associations between staffing level and quality of care, easily leading to confusion for policymakers.

### REGULATORY COMPLIANCE AND RESIDENT OUTCOMES IN OREGON ASSISTED LIVING COMMUNITIES

Ozcan Tunalilar[1], Sunny Lin[2], and Paula Carder[1], *1. Portland State University, Portland, Oregon, United States, 2. Washington University School of Medicine in St. Louis, St. Louis, Missouri, United States*

The outsized negative impact of the COVID-19 pandemic among residents of assisted living (AL) communities has drawn attention to the existing challenges that licensing agencies face in providing oversight in this setting. While regulatory compliance inspections in AL may be a critical tool for promoting high-quality care, no published research has examined the association between deficiencies and resident outcomes in AL. Using novel data collected from 331 AL communities in Oregon (response rate=62%) merged with deficiency data from inspections, we examined whether the number of deficiencies is associated with the following resident outcomes as measured at the community level: share of residents who fell at least one time (fall rate), who were hospitalized overnight (hospitalization rate), and who were treated in the hospital emergency room (ER rate) in the last 90 days. Negative binomial regression models that controlled for a robust set of community (e.g., size, rurality, profit status, "memory care" units) and resident (e.g., age, Medicaid use, ADL needs) characteristics showed a weak, but statistically significant, association between deficiencies and hospitalization rate (IRR=1.05). However, there was no statistically significant association between deficiencies and falls or ER visits among residents. The time lag between inspections and the resident outcome data did not moderate these observed associations. With more states moving to make available such deficiency data on public-facing websites, these results have policy implications for the role and content of regulatory compliance inspections for ensuring quality in AL communities and informing the public.

### NOWHERE ELSE TO GO: EFFECTIVENESS OF THE PASRR PROGRAM TO MEET THE NEEDS OF RESIDENTS WITH SMI ADMITTED TO NURSING HOMES

Taylor Bucy[1], Tetyana Shippee[2], Mark Woodhouse[2], John Bowblis[3], Shekinah Fashaw-Walters[1], and Nathan Shippee[1], *1. University of Minnesota School of Public Health, Minneapolis, Minnesota, United States, 2. University of Minnesota, Minneapolis, Minnesota, United States, 3. Miami University, Oxford, Ohio, United States*

The number of adults with serious mental illness (SMI) who receive care in nursing homes (NHs) continues to rise. The Preadmission Screening and Resident Review (PASRR) program requires screening for SMI prior to NH placement, in order to avoid inappropriate admission and unnecessary institutional care. We interviewed staff responsible for the processing of PASRR documentation at four NHs in Minnesota (n=15), and obtained and analyzed all completed PASRR-II assessments in Minnesota from 2019 (N=532). PASRR assessments overwhelmingly recommended 24-hour NH care (94.7%) with 94% of assessments indicating a need for mental health services while at the NH. Most NH staff interviewed noted that PASRR is not used in the care planning process and described PASRR as a regulatory hoop. Staff shared that PASRR assessments could provide insight into an individual's mental health history, current and future needs, and can be helpful in assessing NH capacity to provide such services. Although mental health services provided while at the NH are supposed to be facilitated in partnership with the county, there is a lack of follow-up and NH staff are largely left to deal with SMI in isolation. PASRR assessments are supposed to be a tool for care coordination, but leave the NH as the sole responsible point of contact for residents with SMI. A more integrated PASRR program that better focuses on incorporating PASRR into care planning and mental health service delivery in NHs and the broader community is necessary to improve the lives of individuals with SMI.

### SERIOUS MENTAL ILLNESS IN MINNESOTA NURSING HOMES: THE ROLE OF RESIDENT AND FACILITY CHARACTERISTICS

Tetyana Shippee[1], Taylor Bucy[2], Weiwen Ng[2], Mark Woodhouse[1], Shekinah Fashaw-Walters[2], and John Bowblis[3], *1. University of Minnesota, Minneapolis, Minnesota, United States, 2. University of Minnesota School of Public Health, Minneapolis, Minnesota, United States, 3. Miami University, Oxford, Ohio, United States*

Multiple studies have shown an increasing prevalence of adults with serious mental illness (SMI) in nursing homes. As adults with SMI age, the reality of care needs that span physical, medical, and psychosocial services necessitates further consideration of the role of comprehensive, ancillary mental health services in nursing homes (NH). Yet, little work examines characteristics of those with SMI, their care needs & the role of facility structural factors. Using the 2011-2017 Minimum Dataset (MDS) assessment data for Minnesota, we examined resident-level demographic characteristics of NH residents with and without SMI, and facility-level characteristics including quality of life (QoL), quality of care (QoC), and state recertification survey scores. We defined SMI as a diagnosis of bipolar disorder, schizophrenia or schizoaffective disorder, or psychotic conditions other than schizophrenia present on the reference assessment. Individuals admitted with SMI were younger, had better physical health, were more likely to be racial/ethnic minorities, and more likely to be admitted to a facility with a higher proportion of racial/ethnic minority residents. SMI-only admissions were concentrated in larger, for-profit facilities with a high-reliance on Medicaid. Lastly, SMI-only admissions were more likely to occur in facilities with lower QoL, QoC, and inspection scores. There is a growing need for behavioral health services in NHs, yet access to services is inadequate and lacks equity based on geography, race/ethnicity and other system-level disparities.

### NURSING HOME PALLIATIVE CARE DURING THE PANDEMIC: DIRECTIONS FOR THE FUTURE

Kacy Ninteau[1], and Christine Bishop[2], *1. Dana Farber Cancer Institute, Boston, Massachusetts, United States, 2. Brandeis University, Waltham, Massachusetts, United States*

For this qualitative, pilot study, seven Massachusetts nursing home Directors of Nursing (DONs) were interviewed remotely about palliative care provision before and during the COVID-19 pandemic. Interview data were analyzed using thematic analysis. Palliative care addresses physical, emotional, psychological, and spiritual suffering that accompanies serious illness. Symptom management and goals of care is especially valuable for seriously ill nursing home residents. We investigated the solutions nursing home staff developed to provide palliative care during the COVID-19 pandemic despite restrictions on external resources. Before the pandemic, palliative care was delivered primarily by nursing home staff depending on formal and informal consultations from palliative care specialists affiliated with hospice providers. When COVID-19 lockdowns precluded these consultations, nursing staff did their best to provide palliative care, but were often overwhelmed by shortfalls in resources, resident decline brought on by isolation and COVID-19 itself, and a sense that their expertise was lacking. Advance care planning conversations focused on hospitalization status and options for care given COVID-19 resource constraints. Nevertheless, nursing staff discovered previously untapped capacity to provide palliative care on-site as part of standard care, building trust of residents and families. Nursing staff rose to the palliative care challenge during the COVID-19 pandemic, albeit with great effort nursing home payment and quality standards should support development of in-house staff capacity to deliver palliative care while expanding access to the formal consultations and family involvement that were restricted by the pandemic.

### ROLE OF ACTIVITY PROFESSIONALS IN APPLYING APIE MODEL TO ENHANCE RESIDENTS' ACTIVITY IN LONG-TERM CARE SETTINGS

xiaoli li, *University of North Texas, Frisco, Texas, United States*

Background: Residents' activity is a strong contributor to quality of life in long-term care facility. Hence, activity professionals (AP) have an essential role to play in enhancing resident activity and engagement. The service process of AP includes Assessment, Planning, Implementation, and Evaluation (APIE model). Although AP have made significant advances in demonstrating its value, few studies have focused on the APIE model for activity professionals. Method: A survey was conducted to help define the role of AP in using APIE in long-term care. Questionnaires were completed by 195 AP working as activity directors and activity staff in long-term care settings. The major factors studied were job background and certification, and performance of AP using the APIE model.

Results: Findings showed that 11.8% of AP only had a high school education, while 12.3% of participants had no certification and 9.2% of activity professionals had invalid certifications. When examining the APIE model, the activity professionals used person-centered approaches when they assessed residents` needs and wants, planned and implemented the activities, but lacked evaluation when measuring the outcomes, 79.5% of the participants were not tracking the number of minutes residents spent on activities, and 72.8% did not measure the impact of resident engagement on clinical outcomes such as falls, depression, cognition, and medication use.

Conclusion: Lack of professional training and meaningful evaluation could lead administrators to underestimate the value and role of activity professionals. The study advocates for applying APIE mode and increased evidence-based research on the benefits of residents` activity and engagement.

### COMPLAINT PATTERNS IN US NURSING HOMES: 2013–2017

Kallol Kumar Bhattacharyya, and Victor Molinari,
*University of South Florida, Tampa, Florida, United States*

Nursing home (NH) quality of care is often short of meeting residents' and family expectations to maintain optimum quality of life. Using complaints as a facility-level outcome (i.e., complaints per NH), this study updates earlier published findings by replicating prior analyses with more recent data, and by analyzing the number of complaints, complaint allegations, and deficiency citations separately. This will allow us to determine whether any major change has taken place in the consumer complaint pattern in recent years. The result reveals, in the entire study period (2013-2017), overall, 458,101 complaints (5.9/NH/year) were identified that contain 949,466 allegations (12.2/NH/year), which resulted in the issuance of 156,135 deficiency citations (2.0/NH/year) in about 15,600 NHs across the country. Regarding the number of complaints, substantiated complaints, and deficiency citations, the results show a steady increase compared to previous years. Furthermore, there are marked differences among the ten CMS survey regions on the prevalence of overall complaints, substantiated complaints, and deficiency citations. The current study found a lower number of NHs with zero complaints and a higher number of NHs with five/more complaints in later years suggesting a steady increase in the number of complaints over the years. However, the average rate of substantiation of complaint allegations is showing a decreasing trend in recent years. This may be because people are now complaining more due to higher care expectations. Alternatively, it may be simply be because of the easier complaint lodging process developed in recent years. Other policy and practice implications will be discussed.

### THE IMPACT OF DIFFERENT PATTERNS OF HOME- AND COMMUNITY-BASED SERVICES AND FACTORS ON MULTIDIMENSIONAL UNMET NEEDS

Ya-Mei Chen, Zi-Ting Zhong, and Shih-Cyuan Wu,
*National Taiwan University, Taipei City, Taipei, Taiwan (Republic of China)*

Background: Long-Term Care (LTC) in Taiwan has been implemented over the last 15 years, focusing on providing home- and community-based services (HCBS). Previous studies have shown that care recipients' level of unmet needs is associated with adverse health outcomes. This study aimed to identify HCBS use patterns among LTC care recipients and examine the impact of different patterns of HCBS on unmet needs.

Methods: Surveys of service users were conducted in eight counties. Latent class analysis was used to identify the underlying subgroups of LTC recipients. Multiple regression analysis was used to assess the impact of HCBS patterns and care recipients' predisposing, enabling, and need factors on the level of unmet needs.

Results: A total of 952 participants from northern, middle, and southern Taiwan completed the surveys. Three subgroups based on HCBS use were identified, including home-based personal care (HB-PC), home-based personal care and medical care (HB-PC/MC), and community care (CC). Care recipients in the HB-PC group ($\beta = -1.922$, $p = 0.028$) and the CC group ($\beta = -2.177$, $p = 0.019$) had lower unmet needs than those in the HB-PC/MC group. A lower disability level, living in a highly urbanized city, and lower levels of quality of life were associated with higher unmet needs ($p < 0.05$).

Conclusions: Our results demonstrating that care recipients with lower disability levels had higher unmet needs may indicate insufficient funding support from Taiwan's LTC plans. However, multiple service users had high unmet needs, which warrants further investigation.

### ACTUAL AND DESIRED SERVICE USE OF COMMUNITY-DWELLING CONSUMERS OF HOME- AND COMMUNITY-BASED SERVICES

Taylor Bucy[1], John Mulcahy[2], Eric Jutkowitz[3], and Tetyana Shippee[2], *1. University of Minnesota School of Public Health, Minneapolis, Minnesota, United States, 2. University of Minnesota, Minneapolis, Minnesota, United States, 3. Brown University, Providence, Rhode Island, United States*

Federal and state long-term care policies are focused on helping people age in their community rather than in nursing homes or other institutional settings. Through home- and community-based (HCBS) waiver programs, states have the flexibility to target specific populations, providing supportive services meant to promote residence in the community. Although consumer needs and goals are a key component of HCBS delivery and effectiveness, little is known about how actual service use differs from the services individuals desire to meet their needs. Using the National Core Indicators-Aging and Disability (NCI-AD™) survey responses from 2018-2019 (N=14,202), an effort by state agencies to measure and track performance outcomes using standardized measures, we examined differences in actual versus desired use of HCBS among people living in the community. Services were categorized as delivered in home-based setting, day services, transportation, support/modification services, health and therapeutic services, and respite. Among NCI-AD respondents in the community (n=9,860), 11% used one service area, 49% used two service areas, and 36% used three or more service areas. The most common service area was home-based services (57%). About a third of community-dwelling respondents desired at least one additional service area. Factors significantly associated with wanting more services included being in combined Medicaid-Medicare, managed LTSS, a woman, an adult aged >65, living alone, and having a physical disability. While the majority of community-dwelling HCBS recipients do not list unmet service needs, there is heterogeneity by program category and other key demographic and social characteristics.

### SESSION 6550 (POSTER)

### PERSONALITY

### PERSONALITY FUNCTIONING FEATURES OF CLINICAL ANXIETY AND COVID-19 ANXIETY AMONG OLDER ADULTS

Lisa Stone, Colleen Mock, and Daniel Segal, *University of Colorado Colorado Springs, Colorado Springs, Colorado, United States*

Introduction: Anxiety is a significant mental health problem among older adults and is commonly comorbid with personality disorders (PD). However, specific relationships between personality functioning (a proposed feature of PDs) and late-life anxiety remain unclear. This study examined relationships between two models of personality functioning with late life clinical anxiety and COVID-19 anxiety. Method: Older adults (n = 222) completed the Geriatric Anxiety Scale (GAS), Coronavirus Anxiety Scale (CAS), Levels of Personality Functioning Scale-Self-Report (LPFS-SR), and Severity Indices of Personality Problems-Short Form (SIPP-SF).

Results: The GAS and CAS were significantly correlated to all LPFS-SR and SIPP-SF domains with large effect sizes (> .78); higher clinical and COVID-19 anxiety was associated with increased personality dysfunction. In regressions, the LPFS-SR domains significantly accounted for 65% of variance in the GAS and 59% of variance in the CAS. Identity and Self-Direction were the strongest predictors of each anxiety scale, with Empathy also significantly related to coronavirus anxiety. The SIPP-SF domains significantly accounted for 65% of variance in the GAS and 58% of variance in the CAS. Responsibility and Social Concordance were the strongest predictors of each anxiety scale, with Self-Control also significantly related to clinical anxiety. Discussion: Results indicate theoretically-supported and meaningful overlap between clinical and COVID-19 anxiety with personality dysfunction according to two different models. This extensive overlap questions the extent to which personality functioning differentiates from affective distress. The two personality dysfunction models also differed somewhat in their relationships to anxiety, suggesting the need for further research especially among older adults.

### MEASURING PERSONALITY IN DAILY LIFE: EVIDENCE FROM AN AGE-HETEROGENEOUS ADULT SAMPLE

Giselle Ferguson[1], Giancarlo Pasquini[2], Martin Sliwinski[3], and Stacey Scott[1], *1. Stony Brook University, Stony Brook, New York, United States, 2. Stony Brook University, Port Jefferson Station, New York, United States, 3. The Pennsylvania State University, University Park, Pennsylvania, United States*

Research on personality has theorized that repeated short-term experiences can lead to changes in personality traits across years or decades. Whereas much research on these short-term experiences relevant to personality has been done in samples of college students, this study intended to measure personality-relevant short-term experiences among an age-heterogeneous sample of adults. As part the Effects of Stress on Cognitive Aging, Physiology, and Emotions study, 260 participants (Mage=46.49 years, range=25-65 years) completed a measure of Big Five personality traits before completing a 14-day ecological momentary assessment (EMA) period during which participants reported their momentary negative and positive thoughts, emotions, and social interactions up to six times per day on study-provided smartphones. We hypothesized that these EMA reports could be used as daily markers of trait extraversion and trait neuroticism such that these daily experiences could be interpreted as manifestations of personality traits in daily life. Results of parallel multilevel confirmatory factor analyses showed good model fit (extraversion: CFI=0.96; TLI=0.95; RMSEA=0.03; neuroticism: CFI=0.95; TLI=0.94; RMSEA=0.04). For both extraversion and neuroticism, the latent trait factor and the latent daily-marker factor were positively correlated (extraversion: r=0.36; SE=0.07; p<.001; neuroticism: r=0.45; SE=0.07; p<.001). Results suggest that among an age-heterogeneous adult sample, momentary thoughts, feelings, and behaviors across a two-week period represented expressions of extraversion and neuroticism in daily life. Measuring these short-term experiences is meaningful for understanding how personality changes across adulthood, and future work can use longitudinal data to test if daily markers of personality are sensitive to fluctuations and changes in personality.

### ADIPOSITY CHANGE IN ADULTS: THE IMPACT OF TRAIT NEUROTICISM

Jacob Alderson, Meredith Willard, and Nicholas Turiano, *West Virginia University, Morgantown, West Virginia, United States*

Increased body weight is a risk factor for poor health and shortened life expectancy. Thus, it is imperative to understand how body weight changes across adulthood and to identify factors that predict weight gain so effective prevention strategies can be implemented. It is well-known that eating habits and physical activity are two of the most important factors (along with genetic factors) contributing to weight gain. However, we seek to determine if personality levels predict weight gain because individual differences in personality are thought to be the root-cause of many behaviors related to weight gain. We utilized longitudinal data on over 6,000 adults (aged 20-75 at baseline) from the Midlife Development in the U.S. Study (MIDUS). The Big 5 personality traits, body weight, waist circumference, and body mass index were measured three times from 1995-2015. We estimated a growth curve model to determine whether each adiposity measure changed over 20 years, controlling for age, gender, and education. There was a significant increase in all adiposity measures over time. The rate of adiposity change over time varied among persons (random: weight b = 0.543; waist b = 0.008; BMI b = 0.009; p values < .05). Higher levels of neuroticism predicted this variability (fixed: weight b = 0.211; waist b = 0.027; BMI b = 0.029; p values < .05) such that those scoring higher in trait neuroticism had a steeper increase in all three adiposity measures. These findings suggest that personality traits are important in the progression of weight-gain in adults.

### HEALTH LITERACY ACROSS PERSONALITY TRAITS AMONG OLDER ADULTS: CROSS-SECTIONAL EVIDENCE FROM SHARE

Valerie-Anne Ryser[1], Clément Meier[2], Sarah Vilpert[2], and Jürgen Maurer[2], *1. Swiss Centre of Expertise in the Social Sciences, Lausanne, Vaud, Switzerland, 2. University of Lausanne, Lausanne, Vaud, Switzerland*

Introduction: Personality traits (PTs) - Neuroticism, Extraversion, Openness to experience, Agreeableness, Conscientiousness - are related to how older adults deal with health-related issues. However, little is known about the relationship between PTs and health literacy (HL). HL measures individuals' ability to find, understand, appraise, and use health information to deal with health-related outcomes. Objectives: This research tries to understand better differences in HL across PTs in a nationally representative sample of adults aged 58 years and older in Switzerland. Method: Multivariable probit regressions to explore how respondents' PTs are independently associated with HL after controlling individuals' social, regional and health characteristics are based on a paper-and-pencil self-completion questionnaire (N= 1'555) administered as part of wave 8 (2019/2020) of SHARE in Switzerland. HL is measured using the short version of the European Health Literacy Survey questionnaire (HLS-EU-Q16), whose scores of dichotomized responses is grouped into two categories: inadequate and adequate HL. PTs are measured with the Big-Five inventory ten (BFI-10).

Results: Results show that two out of five PTs are significantly associated with HL among older adults. Individuals who score higher on neuroticism and thus have a persistent tendency to experience negative emotions are more likely to have inadequate HL. More open individuals who are more prone to engage in self-examination are also more likely to have adequate HL.

Conclusion: These findings call for targeted interventions, such as using adjusted health or eHealth information tools that would consider individuals' PTs when designing health policies to improve HL in the population.

### NEIGHBORHOOD CONDITIONS AND PERSONALITY CHANGE AMONG OLDER ADULTS

James Iveniuk[1], and Kathleen Cagney[2], *1. NORC at the University of Chicago, Chicago, Illinois, United States, 2. University of Michigan, Ann Arbor, Michigan, United States*

Conscientiousness is a key aspect of personality, associated with overall better health and wellbeing across the life course, including among older adults. The past ten years have seen a growing interest in how social-environmental factors impact personality traits, but the impact of neighborhood conditions is rarely investigated. Broken windows theory argues that changes to residents' character can be brought about through signals of disinvestment in local norms (i.e. visible disorder), pointing to neighborhood disorder as a possible environmental condition that can shape personality. Drawing upon three rounds of data from the National Social Life Health and Aging Project (2005/2006, 2010/2011, 2015/2016; N=1554), we investigate longitudinal associations between traits and changing neighborhood conditions using a Big Five self-assessment, and interviewer ratings of local disorder. Using a combination of mediation and cross-lagged models, we find mixed support for broken windows perspectives – significant mediation processes, but no cross-lagged effects.

### SESSION 6560 (POSTER)

### PERSPECTIVES ON FORMAL CAREGIVING: PRIMARY CARE, NURSING, AND LONG-TERM CARE

### NEUROPSYCHIATRIC SYMPTOMS IN PRIMARY CARE PATIENTS WITH DEMENTIA

Constanca Paul[1], Laetitia Teixeira[1], and Susana Sousa[2], *1. University of Porto, Porto, Porto, Portugal, 2. ICBAS, University of Porto, Porto, Porto, Portugal*

Neuropsychiatric Symptoms of dementia are mostly valued within care and burden of formal and informal caregivers and less considered as diagnosis predictors. Diagnosis of dementia in Primary Care Service (PCS) is frequently late and based mostly in general clinic assessment, and patient/family subjective complaints. We study the association of Neuropsychiatric Symptoms (NPI-Q) and existing diagnosis of dementia in PCS. The objectives are to know 1) the prevalence of symptoms identified by familial caregivers in people with a diagnosis of dementia and 2) if there is an association of symptoms with the existence of a diagnosis.Method: we randomly select a community based sample of a pool (N=2734) of primary care users with mental health concerns referred by General Physicians, (N=154), mean age 76 years (sd 7.8), 57% women. Caregivers (N=39) were interviewed and fulfil NPI-Q. Results of descriptive and logistic regression analysis showed that 39% (60) had a formal diagnosis of dementia not differentiating men and women. The neuropsychological symptoms frequency varied between 3.1% (hallucinations) and 16.3% (apathy/indifference), and the symptoms' mean was 4.5(sd 2.1). The amount of symptoms was not associated with the diagnosis. The symptoms that predict the diagnosis were Apathy/ Indifference OR 5.24(1.25-22.0),p.024 and Motor Disturbance OR 5.70(1.17-27.6), p.031. Qualitative data from caregivers interview show that they are not very comfortable with the terminology of NPI-Q, which may limit the accuracy of assessment.

Conclusion: some neuropsychological symptoms identified by caregivers seems to be relevant as predictors of diagnosis of dementia in Primary Care.

### PERSPECTIVES ON THE RISK FACTORS ASSOCIATED WITH MISSING INCIDENTS IN PERSONS LIVING WITH DEMENTIA

Christine Daum[1], Hector Perez[1], Antonio Miguel-Cruz[2], Elyse Letts[1], Emily Rutledge[1], and Lili Liu[1], *1. University of Waterloo, Waterloo, Ontario, Canada, 2. University of Alberta, Edmonton, Alberta, Canada*

Persons living with dementia are at higher risk of getting lost and going missing. The adverse outcomes of missing incidents are stressful for persons living with dementia and those who care for them. This study aimed to identify and describe the perspectives of persons with dementia, caregivers and community support organizations on risk factors. Generic qualitative description informed our methods. We conducted 30 virtual interviews with persons who live with dementia, professional and family caregivers and community support organization representatives. We used a card sort to elicit and describe perspectives on the importance of 27 risk factors commonly associated with missing incidents in persons living with dementia. Interviews were digitally recorded, transcribed verbatim, and subjected to content analysis to determine the presence of relevant words, themes, and concepts. Participants reported multiple experiences of a person going missing, impressions, and suggested relationships between factors such as environmental contexts. The most critical risk factors associated with getting lost and going missing were cognitive impairment, unmet needs, and inadequate concentration of services and resources. In contrast, race, education, and gender were perceived as unimportant pertaining to risk factors related to missing incidents in persons living with dementia. An understanding of the perceived importance of risks associated with missing incidents enhances a person-centered approach to addressing unmet needs, services and resources that balances quality of life with maintaining safety.

### SOCIAL INTERACTIONS BETWEEN CERTIFIED NURSING ASSISTANTS AND CLIENTS WITH DEMENTIA: A QUALITATIVE STUDY

Elizabeth Braungart Fauth, Jacob Gossner, Malinda Dokos, and Rebecka Schultz, *Utah State University, Logan, Utah, United States*

Certified nursing assistants (CNAs) provide direct care for persons with dementia in formal settings and are therefore positioned to engage socially with clients during care. Our prior published observational study in memory care (7000 minutes of observation over 1 year) identified infrequent negative interactions of CNAs with clients, but many opportunities where CNAs did not socially engage a client, including ignoring attempts for engagement. This followup study seeks to understand CNAs experiences and attitudes towards social interaction with clients with dementia. Through semi-structured interviews with 11 CNAs at two care facilities (not included in the prior study), we examined the perceived role that CNAs have towards engaging socially with clients with dementia, barriers to interactions, and behaviors and attitudes that promote quality social interactions. Thematic analyses with multiple trained coders identified that all CNA participants reported feeling personally responsible for engaging with clients socially, having learned this through observation and trial and error, rather than formal training. Barriers to social interaction were lack of time, training, and managing symptoms of dementia. In describing quality social interactions, CNAs highlighted multiple verbal and nonverbal behaviors, including touch, eye contact, body posture, and volume and tone of voice. CNAs emphasized that quality social interactions were facilitated by internal attitudes of valuing personhood, getting on the client's level, and being a friend. Our findings highlight that CNAs identify the importance of social interactions in their work in memory care, and they identify needing training in effective interaction techniques, specifically with their clients with dementia.

### DEFICIENCY CITATIONS ON INAPPROPRIATE PSYCHOTROPIC MEDICATION USE WHEN CARING FOR BEHAVIORAL SYMPTOMS OF DEMENTIA

Jung Min Yoon[1], Alison Trinkoff[2], Elizabeth Galik[3], and Nicole Brandt[3], *1. Stony Brook University, Saint James, New York, United States, 2. University of Maryland Baltimore School of Nursing, Baltimore, Maryland, United States, 3. University of Maryland, Baltimore, Baltimore, Maryland, United States*

The National Partnership to Improve Dementia Care in Nursing Homes in 2012, initiated by the Centers for Medicare and Medicaid (CMS), helped decrease antipsychotics use. However, inappropriate psychotropics use to control behavioral symptoms associated with dementia remains. Nursing homes (NHs) can be flagged for inappropriate psychotropics use as a deficiency of care citation (F-758 tag). The purpose of this study was to explore the nature of inappropriate psychotropic medication use deficiency, F-758 citations, in caring for NH residents with dementia using a mixed-methods study design. During the first quarter of 2018 (January–March), 444 NHs received F-758 tags related to care of residents with dementia. Information on deficiencies were obtained from 2018 Certification and Survey Provider Enhanced Reporting (CASPER) data. Deficiency reports were obtained from CMS Nursing Home Compare and ProPublica. Quantitative analysis was done to examine the frequency of involved psychotropic medications, scope and severity of F-758 tags, and reasons for the citations. Qualitative data analysis was conducted using content analysis with an inductive coding approach to summarize the inspection reports. Antipsychotics were the most involved drug category for F-758 tag citations. The three most common reasons for F-758 citations included failure to: identify and/or monitor behavioral symptoms (178 NHs), attempt gradual drug reduction (131 NHs), and maintain 14-day limitations on PRN psychotropic orders (121 NHs). This study suggests areas for improvement that could potentially reduce inappropriate psychotropics use. Supporting quality dementia care workforce and improving cooperation within healthcare professionals are recommended to ensure proper non-pharmacological and pharmacological interventions.

### DISTRICT NURSES' ATTITUDES TOWARD INVOLUNTARY TREATMENT IN DEMENTIA CARE: A CROSS-SECTIONAL STUDY

Vincent Moermans[1], Michel Bleijlevens[1], Hilde Verbeek[1], Valeria Lima Passos[1], Koen Milisen[2], and Jan Hamers[1], *1.*

*Maastricht University, Maastricht, Limburg, Netherlands, 2. KU Leuven, Leuven, Vlaams-Brabant, Belgium*

Persons Living with Dementia (PLWD) prefer to live in their home environment, making their own choices and living a self-determined life for as long as possible. However, recent studies have shown that one in two PLWD receives involuntary treatment. Involuntary treatment is care provided that they resist to and/or have not given consent to, such as physical restraints, psychotropic medications. Although district nurses play a key role in the care of PLWD, it is unknown how their attitudes and opinions influence the use of involuntary treatment. This study aims to investigate the attitudes of district nurses towards the use of involuntary treatment in dementia care at home, determinants, and their opinion about its restrictiveness and discomfort. In a cross-sectional study, data were collected in a convenient sample of 296 Belgian district nurses using an online version of the Maastricht Attitude Questionnaire - Home Care. Results show that district nurses perceive involuntary treatment as a regular part of nursing care, having neither positive nor negative attitudes towards its appropriateness. They consider involuntary treatment usage as moderately restrictive to PLWD and feel moderately uncomfortable when using it. Nursing staff with more years of experience in dementia care, perceiving care for PLWD as burdensome, and having a lower educational background were more accepting towards involuntary treatment, rating it as a regular measure in the nursing care repertoire. These findings underscore the need to increase the awareness of district nurses regarding the negative consequences of involuntary treatment use to PLWD.

### EXPERIENCES OF DISTRICT NURSES WITH MAKING DECISIONS ABOUT THE USAGE OF INVOLUNTARY TREATMENT.

Vincent Moermans[1], Michel Bleijlevens[1], Hilde Verbeek[1], Bernadette Dierckx de Casterlé[2], Koen Milisen[2], and Jan Hamers[1], *1. Maastricht University, Maastricht, Limburg, Netherlands, 2. KU Leuven, Leuven, Vlaams-Brabant, Belgium*

District nurses play a key role in care of older Persons Living with Dementia (PLWD) who still live in their own home environment. Studies show that they often have to deal with decision-making about and the usage of involuntary treatment. Involuntary treatment is care provided that they resist to and/or have not given consent to, such physical restraints, psychotropic medication. This study aimed to describe district nurses' experiences regarding the decision-making processes about and the usage of involuntary treatment among PLWD. A qualitative study design with a grounded theory approach was used. During semi-structured interviews, 16 district nurses shared experiences in 31 different cases of involuntary treatment use among PLWD. Results show that district nurses were often not involved in the decision-making process. In most cases, family caregivers decided solely to use involuntary treatment, and district nurses were asked to do also. District nurses experienced these situations as difficult to deal with. They felt that they were torn between providing "good care", wishes of PLWD, and meeting the expectations of family caregivers. Their experiences were negatively affected if PLWD verbally rejected the care provided, they were unable to enter into a dialogue with the caregivers about the care provided, and if they did not have sufficient knowledge about how to deal with PLWD. These findings indicate the importance, of proactive case management in dementia care at home. District nurses can facilitate this by going into dialogue between PLWD and his caregivers, in order to provide person-centered dementia care at home.

### NJGWEP RESUSCITATING THE VA COGNITIVE CLINIC: REBUILDING COMMUNITY PARTNERSHIPS DURING A PANDEMIC

Lisa Bodenheimer[1], Kevin Overbeck[1], Elyse Perweiler[1], Jennifer DeGennaro[1], Anita Chopra[1], Lavona Toplyn3, Richard Cetkowski[2], and Renee Berna[2], *1. Rowan University School of Osteopathic Medicine, Stratford, New Jersey, United States, 2. VA Wilmington, Vineland, New Jersey, United States*

The New Jersey Geriatrics Workforce Enhancement Program (NJGWEP), in collaboration with the Wilmington Veteran's Administration Community-Based Outpatient Clinic (VA-CBOC) in Vineland, NJ, implemented a multidisciplinary comprehensive, evidence-based geriatric care program to assess cognitive health and geriatric syndromes as well as offer caregiver support, dementia disease education and referrals to VA and community resources. The "Cognitive Clinic" evaluated 15 veterans during a 3-month pilot phase before suspension due to COVID-19 in March 2020. To maintain momentum established prior to COVID-19, NJGWEP/VA clinical providers routinely met virtually to update each other on their organizations' status, policies, and progress providing clinical care during the pandemic. These meetings offered opportunities to retain rapport and brainstorm strategies to modify the cognitive clinic.NJGWEP/VA providers successfully pivoted to include a telehealth model of care when appropriate and feasible; also helping to reduce barriers that limited the number of in-person contacts allowed on any given day. Implementation of telehealth proved successful at capturing caregivers who otherwise cannot attend appointments. The VA suggested de-coupling the back-to-back social work/physician appointments to ease scheduling concerns for VA providers. The Cognitive Clinic resumed employing a hybrid model of in-person physician assessments with referrals to social work for caregiver support offered via telehealth. From 1/19/22-3/2/22, 10 veterans were evaluated by a physician; 5 were diagnosed with dementia, all of whom identified a caregiver and received education, resources and referrals. In the upcoming year, the plan is to evaluate 4 veterans per month and train a VA geriatric APN to support program sustainability.

### SESSION 6570 (POSTER)

### PHYSICAL ACTIVITY AND FUNCTION

### FEASIBILITY OF AN EXERGAMING-BASED DANCE TRAINING PARADIGM IN THE HOME SETTING AMONG PEOPLE WITH CHRONIC STROKE

Savitha Subramaniam, and Tanvi bhatt, *University of Illinois at Chicago, Chicago,, Illinois, United States*

Background: Dance-based exergaming (DBExG) from laboratory (phase 1 -p1) to a safe and feasible home-based exercise program (HEP- phase 2-p2) could enhance physical activity (PA) behavior, and can be used as a maintenance therapy in PwCS. However, traditionally structured HEP are interfaced with impediments for employing best practice for fall prevention safety approach at home settings in PwCS, thus, making it essential to evaluate training strategies that foster safety. Aim: To evaluate the feasibility, compliance, and safety of a safety harness augmented rehabilitation using DBExG training paradigm (SHARP).

Methods: Community-dwelling PwCS (n=7) participated in the study and received DBExG training using the commercially available Kinect dance gaming "Just Dance 3". The first 6 weeks training (20 sessions) was provided in the laboratory setting (health coach stand by assistance – SBA). Followed with 4 more weeks (12 sessions) of SHARP in the participant's house with health coach SBA.

Results: The primary focus was feasibility, addressed by acceptability, and retention. All the seven participants completed the laboratory, and home-based DBExG. All participants reported enjoying the sessions and felt they were beneficial. Study retention and session adherence was 90% and 98%, at p1, and p2 respectively. There were no falls, and adverse safety events reported in either phase of the study. The intervention was safe with no falls, and major adverse events.

Conclusion: SHARP appears feasible and safe, thus promising for home-based PA rehabilitation for PwCS. A larger randomized controlled trial is recommended to further investigate efficacy.

### PHYSICAL FITNESS MODERATES THE AGE-RELATED ASSOCIATION BETWEEN EXECUTIVE FUNCTIONING AND MOBILITY

Emma Dupuy[1], Florent Besnier[1], Christine Gagnon[2], Juliana Breton[2], Catia Lecchino[2], Mathieu Gayda[1], and Louis Bherer[3], *1. Université de Montréal, Montreal, Quebec, Canada, 2. Montreal Heart Institute, Montreal, Quebec, Canada, 3. University of Montreal, Montreal, Quebec, Canada*

In older adults, executive functions are important for daily-life function and mobility. Evidence suggests that the relationship between cognition and mobility is dynamic and could vary according to individual factors, but whether cardiorespiratory fitness reduces the age-related increase of interdependence between mobility and cognition remains unexplored. One hundred eighty-nine participants (aged 50-87) were divided into three groups according to their age: middle-aged (MA; < 65), young older adults (YOA; 65-74), and old older adults (OOA; ≥75). Participants performed Timed Up and Go and executive functioning assessments (Oral Trail Making Test and Phonologic verbal fluency) remotely by videoconference. Participants completed the Matthews questionnaire to estimate their cardiorespiratory fitness (VO2 max in ml/min/kg). A three-way moderation was used to address whether cardiorespiratory fitness interacts with age to moderate the relationship between cognition and mobility. Results showed that the cardiorespiratory fitness x age interaction moderated the association between executive functioning and mobility ($\beta = -.05$, $p = .047$) ($R2 = .18$, $p <.0001$). At lower levels of physical fitness (< 19.16 ml/min/kg), executive functioning significantly influenced YOA's mobility ($\beta = -.48$, $p = .004$) and to a greater extent OOA's mobility ($\beta = -.96$, $p = .002$). Our results support the idea of a dynamic relationship between mobility and executive functioning during aging and suggest that physical fitness could play a significant role in reducing their interdependency.

### THE EFFECT OF ACTIGRAPHY MEASURED PHYSICAL ACTIVITY ON EXECUTIVE FUNCTION IN OLDER ADULTS

Pilar Thangwaritorn[1], Hilary Hicks[2], Alex Laffer[3], Genna Losinski[2], Kayla Meyer[4], Elizabeth Tran[4], Keri Cox[4], and Amber Watts[2], *1. University of Kansas, LAWRENCE, Kansas, United States, 2. University of Kansas, Lawrence, Kansas, United States, 3. University of Kansas, Lawrencee, Kansas, United States, 4. University of Kansas Alzheimer's Disease Research Center, Kansas City, Kansas, United States*

Executive function (i.e., decision making, self-control, planning) is important for facilitating independent living in older adults. Physical activity may preserve executive function, but previous research has demonstrated sex differences in both physical activity and executive function among older adults. Few studies have investigated sex differences in the association between the two. We examined associations between objectively measured physical activity and executive function with attention to sex differences. We recruited N = 204 participants (Mage =71, SD=6.36; 57% women) with (n=47) and without (n=157) Alzheimer's disease from the University of Kansas Alzheimer's Disease Research Center. We used wrist-worn accelerometers (Actigraph GT9X) to measure physical activity 24 hours a day for 7 days in a free-living environment. We categorized physical activity as moderate to vigorous (MVPA) based on the Montoye (2020) Adult Vector Magnitude cut-points. We evaluated sex differences in the association between executive function and MVPA using multiple regression with an interaction term, adjusting for age, education, and dementia status. We used a composite score to combine tests of executive function (Digit Symbol Substitution, Stroop Interference, Trail making Part B, and Verbal Fluency). Results indicated, older age and lower education were associated with lower executive function scores ($\beta=-2.12$, $p < 0.001$; $B=2.13$, $p < .05$). In contrast to previous research, we did not find evidence for sex differences in the MVPA, executive function, nor the association between the two in our sample. Future research should investigate whether individualized exercise-based interventions and treatment between men and women may differentially benefit cognitive function.

### PHYSICAL ACTIVITY MEDIATES LATIN DANCE PARTICIPATION AND FITNESS OUTCOMES IN OLDER LATINOS

Navin Kaushal[1], Guilherme Balbim[2], Michelle Jaldin[3], and David Marquez[3], *1. Indiana University, Indianapolis, Indiana, United States, 2. University of British Columbia, Vancouver, British Columbia, Canada, 3. University of Illinois Chicago, Chicago, Illinois, United States*

Background: The benefits of engaging in physical activity (PA) for older adults (OA) are well documented; however, participation rates remain low, especially among OA Latinos. Latin dance expresses and promotes culture among Latinos, and can be an effective approach to promote PA. However, the physical function and cardiorespiratory fitness (CRF) benefits of OA engaging in Latin dance have not been investigated. The purpose of this study was to test if PA from an 8-month dance trial yielded and explained improvements in physical function and CRF.

Methods: The study analyzed physical function and CRF outcomes from the BAILA trial. Participants (n= 333) were Latinos (age 55+) who were randomized to a dance or control condition for an 8-month study. PA was assessed using the Community Healthy Activities Model Program for Seniors (CHAMPS), physical function was assessed with the short physical performance battery protocol (SPPB) and estimated CRF was assessed using the Jurca non–exercise test model. Results. ANCOVA models found significant change in SPPB total scores(F1, 331= 4.01, p=0.046) and estimated CRF (F1, 331= 7.66, p= 0.006) over eight months in favor of the dance group. Follow-up mediation models found MVPA to mediate between group and SBBP scores, ($\beta$= 0.05, 95% CI [0.0128, 0.1147]). MVPA also mediated between group and CRF, ($\beta$= 0.06, 95% CI [0.0164, 0.1197]). Conclusion. The study supports organized Latin dance programs to be effective for improving physical and cardiorespiratory benefits among older adults. The findings also encourage future investigations to promote PA in culturally relevant forms.

### IMPROVEMENTS IN HEALTH-RELATED QUALITY OF LIFE FOR BINGOCIZE CLINICAL TRIAL PARTICIPANTS

Emily Smith[1], Matthew Shake[2], Callie Hamm[1], and Jason Crandall[2], *1. Western Kentucky University, Louisville, Kentucky, United States, 2. Western Kentucky University, Bowling Green, Kentucky, United States*

With the number of older adults increasing rapidly, researchers have increasingly focused on designing interventions to improve health-related quality of life (HRQOL) in older adulthood. However, many interventions struggle with adherence because older adults often perceive them as unenjoyable, condescending, or painful. Here, we report results from a clinical trial of Bingocize®, a community-based “in vivo” exercise and health education intervention for older adults, to determine if participation impacts participants' HRQOL. One-hundred and forty-three older adults ages 60+ were randomly assigned to one of four conditions that contrasted exercise and health education to non-intervention control groups. All conditions were matched on social engagement and met in group sessions twice weekly for 12 weeks. The CDC HRQOL measure was administered before and after the intervention. Session adherence was >90% across all sessions. Results from 2 (Time: Pre/Post) x 4 (Condition: Bingo-only Control vs. Bingo+Health Education vs. Bingo+Exercise vs. Bingo+Health Education+Exercise) ANOVAs found that all participants reported better sleep quality, reduced pain, and increased energy after completing the program (p-values <.05). Results from 2 (Time: Pre/Post) x 2 (Exercise/No Exercise) ANOVAs revealed interactions showing that exercise participants experienced greater decreases in days with anxiety and physically unhealthy days as compared to non-exercise participants (p-values <.05). These findings suggest that elements of Bingocize can contribute to improvements in older adults' mental and physical quality of life. The current research can help researchers and professionals further elucidate which intervention mechanisms play a role in determining older adults' health-related quality of life.

### USING ACTIGRAPHY TO ASSESS CHRONOTYPE AND PHYSICAL ACTIVITY IN OLDER ADULTS

Hilary Hicks[1], Genna Losinski[1], Pilar Thangwaritorn[2], Alex Laffer[3], and Amber Watts[1], *1. University of Kansas, Lawrence, Kansas, United States, 2. University of Kansas, LAWRENCE, Kansas, United States, 3. University of Kansas, Lawrencee, Kansas, United States*

Chronotype refers to the time of day that people prefer to be active or to sleep and varies predictably across the lifespan. In younger samples, the morning-chronotype is related to greater levels of physical activity (PA) and improved health outcomes. It is unclear whether this pattern holds in older adults, a group that commonly exhibits an “early bird” preference. We investigated differences in PA patterns between chronotypes in 109 older adults (Mage = 70.45 years) using wrist-worn ActiGraphs in a free-living environment. ActiGraphs captured data about PA and sleep using a novel approach to measuring chronotype with the mid-point of the sleep interval. We categorized participants as morning-, intermediate-, or evening-chronotypes. We used ANCOVA to predict total and average peak PA from chronotype, adjusting for age, sex, education, and BMI. Total PA significantly differed between chronotypes such that evening-types engaged in less PA than both morning- and intermediate-types, F (2,102) = 4.377, p =.015. Average peak activity did not differ between chronotypes, p =.112. Consistent with findings in younger samples, our evening type participants engaged in less overall activity. A unique finding was that evening-types did not differ from their morning- and intermediate-chronotype peers in peak activity levels. This implies a key distinction between total activity and peak activity levels consistent with recent trends in PA research using a 24-hour-a-day framework instead of average or total activity levels. Future research should consider whether these differences in activity patterns translate into meaningful differences in health benefits in this age group.

### SESSION 6580 (POSTER)

### PSYCHOSOCIAL WELL-BEING

### SENSE OF PURPOSE IN LIFE AND ALLOSTATIC BURDEN IN TWO LONGITUDINAL COHORTS

Nathan Lewis[1], Scott Hofer[1], and Patrick Hill[2], *1. University of Victoria, Victoria, British Columbia, Canada, 2. Washington University in St. Louis, St. Louis, Missouri, United States*

Sense of purpose in life has been linked with better physical health, longevity, and reduced risk for disability and dementia, but the mechanisms linking purposefulness with diverse health outcomes is unclear. Chronic activation and dysregulation of neural, immune, and other bodily systems, known as allostatic load, may contribute to these underlying mechanisms. Specifically, sense of purpose may promote better physiological regulation in response to stressors and health challenges, leading to lower allostatic burden and disease risk over time. Data from the nationally representative US Health and Retirement Study (HRS) and English Longitudinal Study on Ageing (ELSA) (Total N=5846; Mean Age=67.24, SD=10.68, 59.08% female) were used to examine associations between sense of purpose and repeated assessments of allostatic load across 8 and 12 years of follow-up. Allostatic load scores were constructed from 12 blood-based and anthropometric biomarkers of cardiovascular, metabolic, immune, and lung function, with higher scores representing higher allostatic burden. Population-weighted multilevel models revealed that sense of purpose in life was associated with lower overall levels of allostatic load in HRS (b = -0.22, 95% CI: -0.27,-0.17) and in ELSA (b = -0.19, 95% CI: -0.35,-0.03). Sense of purpose in life did not predict rate of change in allostatic load in either sample. Associations with sense of purpose were highest among cardiovascular and immune biomarkers, suggesting that preservation of these bodily systems may underlie associations between purposefulness and reduced risk for chronic health conditions. Discussion will focus on biological and behavioral pathways connecting sense of purpose in life and health.

### COPING STRATEGIES AND THEIR INFLUENCE ON DEPRESSIVE SYMPTOMS DURING THE SECOND COVID-19 PANDEMIC WAVE IN SWITZERLAND

Charikleia Lampraki[1], Daniela Jopp[1], Adar Hoffman[1], Angélique Roquet[1], Kim Uittenhove[1], and Stefan Sieber[2], *1. University of Lausanne, Lausanne, Vaud, Switzerland, 2. LIVES Centre, University of Lausanne, Lausanne, Vaud, Switzerland*

Coping strategies help individuals face stressful events and adapt to them. During the second wave of the COVID19 pandemic, individuals were confronted with increased governmental restrictions that aimed in impeding the propagation of the virus, but affected, at the same time, the life as we knew it, with negative consequences for mental health. This study aims at identifying the coping strategies that individuals used during this period, whether they changed over time and how they affected depressive symptoms in a life span sample in Switzerland. Our sample consisted of 736 individuals with age ranging between 18 and 81 years. The study was conducted in three waves with one-month intervals during the second pandemic-wave (i.e., October, November, December 2020). We used multilevel modelling to identify within-subject change and between-subject differences in depressive symptoms, with coping strategies and sociodemographic variables included as predictors. Older age, male gender, cohabiting with others, and being employed protected from feeling depressed. Results also indicated that seeking functional support, seeking emotional support, positive reappraisal and acceptance decreased, while self-distraction and depressive symptoms increased. When positive reappraisal decreased or/and when self-distraction increased, depressive symptoms also increased. This protective effect of positive reappraisal on depression differed in magnitude for younger and older individuals: Reduction in positive reappraisal was more strongly related to increases in depression for younger individuals. In sum, to adapt to the pandemic stress individuals changed the frequency of coping strategy use, but only changes in positive reappraisal and in self-distracting had an influence on depressive symptoms.

### GOAL ADJUSTMENT CAPACITIES DURING COVID-19: CONTEXT-DEPENDENT BENEFITS FOR EMOTIONAL WELL-BEING

Jeremy Hamm[1], Meaghan Barlow[2], Jaron Tan[1], Odalis Garcia[1], and Katherine Duggan[1], *1. North Dakota State University, Fargo, North Dakota, United States, 2. Wilfrid Laurier University, Waterloo, Ontario, Canada*

Increased constraints and lost opportunities inherent in the COVID-19 pandemic can threaten important life goals and erode emotional well-being. Theories of lifespan development have identified goal adjustment capacities (goal disengagement and goal reengagement) as core self-regulatory resources that can buffer against declines in well-being. However, little is known about the pandemic-related contextual circumstances under which goal adjustment capacities may become more or less beneficial for well-being. Using longitudinal data from a nationally-representative sample of Americans across the adult lifespan (aged 18-80, n=286), we examined the consequences of goal adjustment capacities for emotional well-being under circumstances when individuals reported lower or higher constraints than normal in their lives. Specifically, multilevel models tested whether the influence of between-person differences in (Level 2) goal disengagement and goal reengagement on well-being were moderated by (Level 1) within-person fluctuations in perceived constraints. Analyses controlled for age, sex, education, and income. We observed cross-level Goal Reengagement x Perceived Constraints interactions for depressive symptoms, perceived stress, and positive affect (bs = -.11 to .07, ps < .05), but not negative affect. Results showed that the benefits of goal reengagement for depressive symptoms, perceived stress, and positive affect were pronounced on occasions when participants reported lower (vs. higher) than average perceived constraints in their lives. Findings point to the moderating role of pandemic-related contextual circumstances and suggest that goal reengagement may be most beneficial when individuals have fewer constraints than usual in their lives and may thus able to capitalize on opportunities to pursue new attainable goals.

### WELL-BEING BEFORE AND DURING THE COVID-19 PANDEMIC: ASSOCIATIONS WITH RESILIENCE AND CHRONIC CONDITIONS

Jay Kayser, and Jacqui Smith, *University of Michigan, Ann Arbor, Michigan, United States*

Adults over the age of 60 tend to have higher well-being than younger adults, and high well-being is associated with a range of favorable mental and physical health outcomes. Resilience is related to the maintenance of well-being in the presence of challenging circumstances and stressors. Chronic health conditions are common in older adults and represent a stressor. Using longitudinal data, we address two research questions: 1) Are chronic health conditions and resilience predictive of changes in well-being before and during Covid-19? 2) Does the number of chronic health conditions and resilience interact in predicting changes in well-being before and during Covid-19? To answer these questions, we analyzed data from participants in the Health and Retirement Study (HRS) who completed both the 2016 and 2020 waves

(N = 2276). On average, these participants were age 71.44 (SD = 7.35) and reported 2.40 chronic conditions. In unadjusted models, both the number of chronic conditions (β = -.21) and resilience (β = .09) were related to changes in well-being (R2 = .32). When covariates were added, these values were attenuated but remained statistically significant. The interaction between resilience and chronic health conditions was not statistically significant. Though the number of chronic health conditions and resilience play an important role in well-being for older adults, high resilience does not moderate the relationship between the number of chronic health conditions and changes in well-being. Further investigations using longitudinal data are needed to understand the relationship between resilience and well-being for those with chronic health conditions.

### ASSOCIATION BETWEEN QUALITY OF LIFE AND DEPRESSION IN DYADS OF OLDER PRIMARY CARE PATIENTS AND FAMILY MEMBERS

Nicole Fowler[1], Anthony Perkins[1], Seho Park[1], Matthew Schroeder[2], and Malaz Boustani[3], *1. Indiana University School of Medicine, Indianapolis, Indiana, United States, 2. Regenstrief Institute, Inc., Indianapolis, Indiana, United States, 3. Indiana University School of Medicine, IU Center for Aging Research, Indianapolis, Indiana, United States*

Familial dyads experience illness as an interdependent unit. We evaluate the association of quality of life (QOL), as measured by physical (PCS) and mental health component (MCS) scores, with depression in dyads of older primary care patients and a family member. This is a cross sectional, descriptive study where QOL and depression were measured concurrently in the dyad using baseline data from 1809 dyads enrolled in a trial testing the benefits and harms of Alzheimer's disease and related dementias (ADRD) screening. QOL was measured with the SF-36, depression was measured with the PHQ-9, and the association of depression with QOL was examined using an actor-partner interdependence model with distinguishable dyads. Patient mean (SD) age was 73.7 (5.7) years; 53.1% women; 85.1% white; 13.4% black. Family member mean (SD) age was 64.2 (13) years; 67.7% women; 13.4% black. A patient's spouse/partner were 64.8% of family members. After controlling for dyadic relationship and gender, significant actor effects of depression on PCS for patient (β= -1.39; p< 0.001) and family member (β =-0.954; p< 0.001), and significant partner effects of depression on PCS for patient (β=-0.15, p< 0.05) and family member (β =-0.18; p< 0.01). There were significant actor effects of depression on MCS for patient (β =-1.2; p< 0.001) and family member (β=-1.2; p< 0.001), but depression had a significant partner effect on MCS only for patient (β = -0.08; p< 0.05). Among dyads participating in an ADRD screening trial, dyads with higher depression had lower QOL. Family member depression was associated with decreased family member and patient QOL.

### INTERPERSONAL DYSFUNCTION PREDICTS SUBSEQUENT FINANCIAL EXPLOITATION VULNERABILITY IN OLDER ADULTS

Duke Han[1], Aaron Lim[1], Laura Mosqueda[1], Annie Nguyen[1], Tyler Mason[2], Gali Weissberger[3], Laura Fenton[1], and Peter Lichtenberg[4], *1. University of Southern California, Alhambra, California, United States, 2. University of Southern California, Los Angeles, California, United States, 3. Bar Ilan University, Ramat Gan, HaMerkaz, Israel, 4. Wayne State University, Detroit, Michigan, United States*

The goal of this study was to test whether interpersonal dysfunction, characterized by loneliness and/or dissatisfaction with relationships, is an imminent predictor of financial exploitation vulnerability (FEV) among older adults within a 6-month observation period. This study also tests whether FEV prospectively predicts interpersonal dysfunction. Twenty-six adults aged 50 or older completed a study involving baseline data collection and 13 follow-ups over 6 months. Linear mixed models were used for primary analyses. After adjustment for demographic, psychological, and cognitive covariates, there were between-person effects of FEV and interpersonal dysfunction across follow-ups, suggesting that those with generally higher interpersonal dysfunction compared to other participants also reported greater FEV (B(SE)=1.09(.33), p=.003). There was a within-person effect (B(SE)=.08(.03), p=.007) of elevated interpersonal dysfunction predicting greater FEV two weeks later across all follow-ups. Within-person effect of FEV was not predictive of interpersonal dysfunction (B(SE)=.25(.15), p=.10). Among older adults, individuals with higher interpersonal dysfunction relative to others in the study reported greater FEV throughout the 6-month observation period. Increased loneliness and social dissatisfaction, relative to one's average level, predicts subsequent increases in FEV, and may be an imminent risk factor for exploitation.

### GIVING BACK TO FEEL GOOD? SOCIAL RESPONSIBILITY, AGE, AND NEGATIVE AFFECT

Madeline Nichols[1], Dakota Witzel[2], and Robert Stawski[1], *1. Oregon State University, Corvallis, Oregon, United States, 2. Penn State University, State College, Pennsylvania, United States*

Developmental theories suggest that midlife and older adulthood are stages in which individuals may begin to focus their time on contributing to society (Erikson, 1969). During these stages, individuals may engage in socially responsible behaviors that protect against negative affect resulting from a lower sense of purpose in later life (Greenfield & Marks, 2004). Social responsibility includes both subjective measures of an individual's felt contribution to society (i.e., generativity) and objective measures reporting actual time volunteering in different settings (Rossi, 2001). We utilized data from the Midlife in the United States Refresher survey study and Biomarker Project (N=735, Mage=51.56, SD=13.59, 50.20% Male) to explore how self-perceptions of generativity and time spent volunteering predicted negative affect for individuals in midlife and older adulthood. Preliminary analyses indicate that higher generativity (p<.001) and older age (p<.001), but not average time spent volunteering, were associated with higher negative affect. Further, we considered age as a potential moderator for the associations between generativity, volunteering, and negative affect. Age significantly interacted with generativity (p<.01), such that the effects of generativity on reducing negative affect decrease with age. Age did not significantly interact with time spent volunteering. Discussion will focus on how actual engagement in socially responsible behaviors and perceived societal contributions might yield different outcomes regarding protection against negative affect in mid- and later-life. Future directions may include exploring daily indicators of time spent volunteering, generative beliefs, and affect.

### MODERATION OF CUSTODIAL GRANDPARENT'S WELLNESS AND GRANDCHILD'S BEHAVIOR BY RELATIONSHIP QUALITY

Marin Olson[1], Karly Pyles[1], Acacia Lopez[2], Rachel Scott[2], and Danielle Nadorff[1], *1. Mississippi State University, Mississippi State, Mississippi, United States, 2. Mississippi State University, Starkville, Mississippi, United States*

A growing number of grandparents are assuming care of their grandchildren, and both grandparents and children report higher levels of stress than their peers, although the source of this stress remains largely unexplored. In Conger & Donnellan's Family Stress Model, a parent's distress can impact the levels of distress experienced by their child, and previous research indicates that parent/child relationship quality impacts children's mental health. This study extended this to custodial grandfamilies, by investigating whether the relationship quality of custodial grandparents (GP) and grandchildren (GC) would moderate the relation between the health of GC and the wellness of their GP. Participants include 322 custodial grandparents within the United States recruited via Qualtrics Research Panel Survey (m age = 55.66 yr., 86.1% female). Measures included the Achenbach Child Behavior Checklist, Kivnick's Grandparent Meaning Scale, and grandparents' wellness. Moderation of relationship quality on the relation between the GP's wellness and internalizing behaviors of the GC was tested using SPSS' Process macro. A significant interaction was found ($t = 2.31$, $p = .02$) wherein GP's wellness was not predictive of changes in GC's behavior for the group with high relationship quality, but greater GP wellness was associated with less GC internalizing behaviors when relationship quality was low ($p < .001$). These findings indicate that when a GP feels that they have a more positive relationship with their GC, there is a greater likelihood that they will report fewer health concerns and fewer GC's internalizing behavior symptoms.

### OLDER ADULTS' RESILIENT IDENTITY APPRAISALS: WHO AM I COMPARED TO MYSELF AND OTHERS?

Lauren Bouchard[1], and Lydia Manning[2], *1. Portland State University , Portland , Oregon, United States, 2. Dele Health Tech, Naperville, Illinois, United States*

Resilience is the adaptation of bouncing back from adverse life events. Older adults provide rich and varied resilience narratives of personal growth across the life course, especially related to others. Many people describe resilience as part of their identity, formed in childhood and maintained throughout adulthood. As adults age, they continually integrate new information into their identity, and aging presents long-term adversity and new life course challenges that older people must navigate. This qualitative study explored how older adults describe their sense of resilient identity compared to their younger selves and other older adults in their lives. Our narrative findings indicate resilience narratives often include comparative appraisals to the strengths and weaknesses of younger selves (e.g., "I am more emotionally resilient, but not as physically strong as I used to be"). Additionally, our findings show older adults comparing their resilience to others in a similar age category (e.g., "I do not complain like my friends"). Comparisons to other older adults fit two themes: empathic and connected and judgmental and distanced. We conclude the participants who view other older adults in an empathic and connected way maintained solid social connections and a general sense of interconnectedness, while those in the judgmental and distanced category might be reacting to internalized ageism. The social implications of these findings will be discussed in detail. In addition to specific examples, our study also provides limitations and future directions in resilient identity and resilience appraisals.

### THE PROTECTIVE EFFECT OF RESILIENCE ON SHRINKAGE OF FRIENDSHIP IN LATER LIFE

Ikuko Sugawara[1], Midori Takayama[2], and Yoshiko Ishioka[3], *1. Bunri University of Hospitality, Suginami-ku, Tokyo, Japan, 2. Keio University, Yokohama, Kanagawa, Japan, 3. O.P. Jindal Global University, Sonipat, Haryana, India*

The purpose of this study was to examine the protective effect of trait resilience, a positive personality characteristic that enhances individual adaptation to life events and adversity, in the face of declining social relationships in later life. Social relations have a significant effect on our development and well-being throughout life. However, it is known that a decline in physical, cognitive, and mental functioning makes it difficult to maintain active participation in society and leads to shrinking social networks in later life. Therefore, it is important to identify protective factors that enable people to maintain positive social relations when functioning in daily life declines in old age. We analyzed longitudinal survey data from a representative sample of older Japanese adults aged 74 to 86 years (N=1064). The interaction effect of resilience in the relationship between functions (physical, cognitive, and mental) and relationships with friends (social support exchange and companionship) was examined using multi-group Structured Equation Modeling (SEM). Respondents were divided into upper quartile and lower quartile groups according to their Resilience Scale (RS-14) scores. The results showed significant group differences for the effect of cognitive function on friendship. The positive correlation between cognitive functioning and friendship was stronger among respondents with low resilience. For high resilience participants, cognitive functioning was not associated with friendship. The results suggest that resilient older adults cope well with poor cognitive health and maintain positive social relationships in very late life.

### INCREASING THE SENSE OF IDENTITY AND INTEGRITY: AN INTERVENTION STUDY OF ACTIVE AGING AND AUTOBIOGRAPHY WORKSHOPS

Tomoko Ikeuchi[1], Mayuko Ono[1], Sachiko Yamazaki[2], Cullen Hayashida[3], Michiyo Tomioka[3], Chiho Shimada[4], and Hisao Osada[5], *1. Tokyo Metropolitan Institute of Gerontology, Tokyo, Tokyo, Japan, 2. Bunkyo Gakuin University, Fujimino, Saitama, Japan, 3. University of Hawaii, Honolulu, Hawaii, United States, 4. Saku*

*University, Saku, Nagano, Japan, 5. J.F. Oberlin University, Machida, Tokyo, Japan*

Identity and integrity (i.e., a sense of coherence and wholeness) are important elements for psychosocial development in later stages of life as described in Erikson's life-span developmental theory. This study aimed to develop an intervention program to increase the sense of identity and integrity among older adults. The program included two types of workshops to examine the combined impact of searching for one's post-retirement identity and clarifying personal goals and purpose for later life. The first workshop, Introductory of Active Aging, focused on providing the knowledge and skills necessary to prepare for life after retirement. The second workshop, Creating Your Life History, focused on a life review and composing one's life history using the guided autobiography method developed by Birren. The study used a crossover design with a one-week break interval between the two workshops. Each workshop was conducted weekly with a two-hour session over four weeks. Thirty-eight participants aged 60 to 80 attended both workshops between October and December 2021 at a community center in Tokyo, Japan. The sense of identity and integrity were assessed using the Japanese version of the Erikson Psychosocial Inventory Scale (EPSI) at baseline and after completing both workshops (9th week). The scores of both identity and integrity increased significantly among participants who completed two workshops in sequence. This crossover study showed no statistical difference in the effects of each workshop. Further work is needed to monitor the long-term impact of these workshops in clarifying one's identity and achieving satisfaction with self in later life.

### HOW GOAL REPRESENTATIONS MODULATE ASSOCIATIONS BETWEEN EVERYDAY AFFECT AND GOAL PURSUIT AMONG OLDER ADULTS

Yoonseok Choi[1], Denis Gerstorf[2], Maureen Ashe[3], Kenneth Madden[1], and Christiane Hoppmann[1], *1. University of British Columbia, Vancouver, British Columbia, Canada, 2. Humboldt Universität zu Berlin, Berlin, Berlin, Germany, 3. The University of British Columbia, Vancouver, British Columbia, Canada*

Individuals differ in the extent to which they represent their goals as hoped-for versus feared states. We examined the role of such goal representations for how everyday affective experiences and goal pursuit are intertwined. When goals are represented as hoped-for states, we expected stronger associations between daily positive affect and goal pursuit. In contrast, when goals are represented as feared states, we expected stronger associations between daily negative affect (particularly fear) and goal pursuit. We used seven days of repeated daily life assessments from 238 older individuals (Age: M = 70.5 years, SD = 5.99, 59-87 years; N = 119 couples). At baseline, participants reported three goals they planned to pursue over the study period and the extent to which each goal referred to something they hoped-for or feared. During the daily life assessments, participants reported their current affective states and momentary goal pursuit (goal engagement and goal progress) three times per day (11 AM, 4 PM, 9 PM). Multilevel analyses regarding participants' most salient goal provide initial evidence supporting the expected interactions of goal representations on everyday affect–goal pursuit links. Specifically, individuals with a strong hope-focus in their goals engaged in more goal pursuit when positive affect was up than individuals whose goals were low in hope-focus. In contrast, those with feared goals engaged in more goal pursuit when reporting increased fear. Findings are discussed in the context of the possible selves literature. Future analyses will examine lead-lag effects to address the temporal order underlying affect-goal pursuit associations.

### THE MEDIATING EFFECT OF SELF-OBJECTIFICATION AND BODY ESTEEM ON AGE AND MOOD

Sydney Tran, and Aurora Sherman, *Oregon State University, Corvallis, Oregon, United States*

Objectification theory is a well-established framework that outlines the consequences of being sexually objectified (Fredrickson & Roberts, 1997). One of the major consequences of sexual objectification is self-objectification, or the tendency to internalize an observer's perspective on one's own body and the learned behavior to value one's physical appearance over one's body functionality. Self-objectification has been linked to poorer subjective well-being and poorer body image in college-aged women but has not yet been examined among older adult women. This study recruited a group of younger (N = 132 ; M age = 20.93, range = 18-26) and older (N = 86; M age = 67.83, range = 48-90) adult women to examine age differences in self-objectification and their relationships with body esteem and negative mood and anxiety. This study tested a serial mediation model using Model 6 of the Hayes PROCESS Macro in SPSS, in which self-objectification and body esteem were hypothesized to mediate the relationship between age and negative mood and anxiety. After controlling for sexual orientation, marital status, and education, results indicated that the standardized indirect effect was significant, B = -.09, SE = .03, 95% CI [-.16, -.04], suggesting that self-objectification and its related consequences are not unique to young adult women and that women of all ages are negatively impacted by self-objectification.

### IN THE SHADOW OF THE GREAT RECESSION: THE ASSOCIATION BETWEEN RECESSION EXPERIENCES AND DAILY INDICES OF WELL-BEING

Jonathan Rush[1], Aarti Bhat[2], Duncan Thomas[3], Arun Karlamangla[4], David Almeida[5], and Teresa Seeman[6], *1. University of Victoria, Victoria, British Columbia, Canada, 2. The Pennsylvania State University, State College, Pennsylvania, United States, 3. Duke University, Durham, North Carolina, United States, 4. University of California, Los Angeles, Los Angeles, California, United States, 5. The Pennsylvania State University, University Park, Pennsylvania, United States, 6. University of California Los Angeles, Los Angeles, California, United States*

The current study examined the associations of positive and negative experiences during the Great Recession (GR) with levels of daily well-being. In 2012, participants from the Midlife in the United States Refresher survey reported on their positive or negative GR experiences related to job, housing, or finances. A subsample, selected into the National Study of Daily Experiences (N=782), also reported on their daily levels of health and well-being across eight consecutive days and provided saliva samples, from which cortisol was assayed. The number of negative GR experiences reported related to poorer daily well-being (negative and positive affect, physical symptoms, stress severity, and cortisol daily peak-to-nadir ratio), whereas, the number of positive GR experiences was only related to lower severity of daily stressors (β=–0.03, p=.03). Examining specific GR experiences revealed that individuals who reported bad housing experience during the GR reported higher daily levels of negative affect (β=0.14, p<.001), physical symptoms (β=0.90, p<.001), and frequency of stressor days (β=0.01, p<.001), and lower daily levels of positive affect (β=–0.19, p=.02). Bad financial experience was related to more physical symptoms (β=0.62, p<.001) and greater severity of daily stressors (β=0.14, p=.03). Conversely, positive financial experiences were related to greater cortisol daily peak-to-nadir ratio (β=1.98, p=.03), but also greater frequency of stressor days (β=0.05, p=.01). Results highlight the potential influence of major economic strains on our ongoing daily experiences. This work has implications for policy and interventions around supporting midlife and older adults facing economic strains, in order to improve daily well-being.

### PROFILES OF PERSONAL DETERMINANTS OF HEALTH AND ASSOCIATED HEALTH OUTCOMES IN OLDER ADULTS: A LATENT CLASS ANALYSIS

Melanie Serrao Hill[1], Timothy Barnes[1], Lizi Wu[1], Rifky Tkatch[1], Sandra Kraemer[2], James Schaeffer[1], and Charlotte Yeh[3], *1. Optum Labs, Minnetonka, Minnesota, United States, 2. UnitedHealthcare, Minnetonka, Minnesota, United States, 3. ASI, Inc., Washington, District of Columbia, United States*

Personal Determinants of Health (PDOH) are considered personal resources that contribute to successful aging. Positive PDOH factors have been associated with better health outcomes in older adults; however, research in this area is still limited. Using survey and claims data among adults age 65+ (N=2,866), profiles were identified based on five key PDOH factors (resilience, purpose in life, optimism, self-perception of aging, and social connection via loneliness) utilizing Latent Class Analysis (LCA). Differences in socio-demographics and healthcare outcomes were then explored utilizing multivariate regression models. Outcomes included emergency room (ER) visits, inpatient (IP) admissions, total medical cost, and quality of life (physical and mental health component scores). LCA models yielded three main classes within our sample: high PDOH (56%, n=1,150), moderate PDOH (40%, n=1,611), and low PDOH (4%, n=125). Those within the high PDOH class were significantly younger (Mage=75.6) compared to those within the moderate (Mage=78.2) and low (Mage=78.9) PDOH classes. In addition, the high PDOH class was significantly less likely to have an ER visit (OR=1.81, 95% CI:1.23-2.66) and had 28% better physical health and 52% better mental health components scores as compared to the low PDOH class. Results demonstrate support for the PDOH model as a potential successful aging model. Combinations used to create classes using resilience, purpose in life, optimism, self-perception of aging, and social connection via loneliness indicates that those who are positive in all five aspects show the most promising health outcomes. Future implications include targeting interventions at improving these variables for at-risk older adults.

### RURAL-URBAN DISPARITIES IN COMORBIDITY BETWEEN OBESITY AND DIABETES AMONG OLDER ADULTS

Youngjoon Bae, *University of Massachusetts Amherst, Amherst, Massachusetts, United States*

Obesity and diabetes are common among older Americans. While obesity is a risk factor for diabetes, research on rural-urban health disparities among older adults has reported paradoxical results. That is, living in rural areas is a risk marker of obesity, whereas rural residency is inversely associated with diabetes. This study utilized multiple national data resources to diagnose the inconsistency, including the 2006-2016 RAND HRS Longitudinal data, biomarker data, the 2010 US Census, and the USDA food access information. HRS survey participants 65 to 97 years of age comprised the study sample (N=6,476). General generalized estimating equation models with survey weights were used to estimate associations with measured obesity and diabetes. Age, race, Hispanic status, foreign-born status, education, marital status, working status, wealth, physical activity, smoking, drinking, ADL, IADL, loneliness, depression, cholesterol, HDL, CRP, and Cystatin C were included as control variables. Findings suggest that rurality is associated with a higher likelihood of obesity and a lower likelihood of diabetes, particularly for older women. However, by adjusting for confounders, these relationships disappeared. Instead, social isolation indicated significant associations with a lower likelihood of obesity and a higher likelihood of diabetes among older women. Contrary to prior research, the food desert and food insecurity measures did not correlate with both outcome variables. Further investigation on food consumption and dietary behavior among socially isolated older women is needed.

### VISION IMPAIRMENT AND SOCIAL ISOLATION IN OLDER AFRICAN AMERICANS: THE IMPACT ON COGNITIVE DECLINE

Emily Hayes[1], Avalon White[2], Corinna Trujillo Tanner[2], and Jeremy Yorgason[2], *1. Brigham Young University--Provo, Provo, Utah, United States, 2. Brigham Young University, Provo, Utah, United States*

Evidence suggests a consistent correlation between vision impairments, social isolation, and cognitive decline. The National Eye Institute reports that African Americans have an increased risk of developing certain vision impairments such as cataracts, glaucoma, and diabetic retinopathy. At the same time, older African Americans often receive care from family members and this family care may act as a buffer against social isolation and resulting cognitive decline. Using data from 737 African Americans that participated in waves 5, 6, and 7 of the National Health and Aging Trends Study (NHATS), we explored associations between vision impairment, social isolation, and cognitive functioning. Results showed that vision impairment at round 5 was related to increased social isolation, and higher social isolation at round 5 was related to decreased delayed word recall scores at the same wave. No significant longitudinal associations were found between these constructs. Findings suggest that concurrent associations exist between sensory impairments, social isolation, and cognitive functioning, but that these relationships are not robust across time. Despite support provided by unpaid family caregivers, African American older adults with vision impairment are at an increased risk for concurrent social and cognitive challenges. It may be that family support of those with sensory impairments helps so that these impairments aren't related to social isolation or cognitive functioning across time. Researchers and clinicians could benefit older African Americans with sensory impairments by providing and encouraging support during early stages of vision loss.

### CHILDHOOD MALTREATMENT AND PROSOCIAL BEHAVIOR: A QUALITATIVE COMPARATIVE STUDY OF IRISH OLDER ADULT SURVIVORS

Shauna Rohner[1], Aileen Salas[2], Alan Carr[3], and Myriam Thoma[4], *1. University of Zürich, Zürich, Zurich, Switzerland, 2. University of Zürich, Zurich, Zurich, Switzerland, 3. University College Dublin, Dublin, Dublin, Ireland, 4. University of Zurich, Zurich, Zurich, Switzerland*

Although childhood maltreatment can have lasting effects into later life, positive outcomes have also been observed, including an increased tendency towards prosocial behavior. However, little is known about the link between childhood maltreatment and later life prosocial behavior. Therefore, this study aimed to explore older adult's experiences of childhood maltreatment and identify mechanisms linked to prosocial behavior in later life. The individual level, but also broader cultural and contextual mechanisms, were considered by comparing two adversity contexts and applying conceptual frameworks (socio-interpersonal framework model of trauma and recovery, motivational process model of altruism born of suffering). Semi-structured interviews (60-120 minutes) were conducted with 29 Irish (older) adult survivors of childhood maltreatment: 17 institutional (welfare care) abuse survivors (mean age: 61 years, range: 50-77), 12 familial abuse survivors (mean age: 58 years, range: 51-72). Interviews were analyzed using Framework Analysis. In both groups at the individual level, enhanced empathy, amelioration, and identity-related mechanisms were linked to prosocial behavior, with connections to caring roles and coping strategies from childhood. On a social contexts level, the limited resources or opportunities for help in childhood, and the social norms and beliefs of that time, influenced participants' motivation to help others in later life. Group-specific mechanisms were also observed, such as compassion fatigue in the familial sample; and denouncing detrimental societal values in the institutional sample. The identification of individual, adversity-context, and culture-specific mechanisms linked to later-life prosocial behavior can promote a greater understanding of resilience and adaptability in older adult survivors of childhood maltreatment.

### SESSION 6590 (POSTER)

### QUANTITATIVE RESEARCH METHODS

### USING AGILE METHODOLOGY AND NUDGE STRATEGIES TO IMPROVE ENROLLMENT IN CLINICAL TRIALS

Peggy Bylund[1], Jade Mehta[2], Nandini Mathavan[3], Kimberly Trowbridge[1], Britain Taylor[4], Husam El Sharu[5], and Noll Campbell[6], *1. Regenstrief Institute, Inc., Indianapolis, Indiana, United States, 2. Indiana University School of Medicine, Indianapolis, Indiana, United States, 3. Indiana University School of Medicine/Center for Health Innovation and Implementation Science, Indianapolis, Indiana, United States, 4. Indiana University Bloomington, Bloomington, Indiana, United States, 5. Indiana University, Indianapoilis, Indiana, United States, 6. Purdue University, Indianapolis, Indiana, United States*

The enrollment of human subjects is crucial for the success of clinical trials. In the ongoing "Reducing the Risk of Dementia through Deprescribing" trial, the initial approach for enrolling subjects did not meet expected goals in the first 6 months, creating the need for innovative nudge strategies. We used an Agile methodology as the framework to understand the problem, then find and implement a solution. Our study aimed to examine the effectiveness of utilizing a texting nudge to enhance post-agreement recruitment of subjects with cognitive impairments. Prior to enrollment, eligible potential participants were contacted using a texting nudge. Potential participants received a second contact call to remind subjects of the enrollment appointment, introduce the person and the phone number that would call them, and the option of confirming or rescheduling. During the 1-week text-message experiment, 8 out of 9 subjects who agreed to participate in the study and received the text message enrolled, yielding an 89% post-agreement enrollment rate compared to a baseline rate of 44% prior to introducing this nudge. After implementing into the standard operating procedures, the 6-month average rate of enrollment among those that agreed rose to 80%, nearly doubling the rate from the first 6 months of the study and quadrupling the number enrolled each month. Inadequate recruitment has necessitated the use of innovative recruitment methods. Using the Agile problem-solving mindset, the texting nudge was developed to leverage the behavioral influences of the messenger, social commitments, priming and affect to increase subject enrollment.

### DISSOCIATING RETEST EFFECTS FROM DEVELOPMENTAL CHANGE FOR PREDICTING COGNITIVE STATUS

Nicholas Tamburri[1], Cynthia McDowell[2], and Stuart MacDonald[2], *1. University of Victoria, Montreal, Quebec, Canada, 2. University of Victoria, Victoria, British Columbia, Canada*

In longitudinal designs, unadjusted retest effects can confound developmental change estimates. This study utilized a measurement burst design and three-level multilevel modeling to a) independently parameterize short-term retest and long-term developmental change and b) employ these estimates as predictors of cognitive status at long-term follow-ups. Using data from Project MIND , participants (N=304; aged 64-92 years) were assessed across biweekly sessions nested within annual bursts (spanning up to 17 total assessments over four years). Cognitive impairment no dementia (CIND) status was classified at Years 4 (the final burst assessment) and 8 (the study end date). Response time inconsistencies (RTI) were computed to index intraindividual variability across RT trials of a one-back response time (BRT) task. Three-level multilevel models simultaneously yet independently estimated BRT RTI

change across weeks and years, indexing short-term retest and long-term developmental change, respectively. Individual slope estimates were extracted and utilized in multinomial logistic regression models contrasting short- vs. long-term RTI change as predictors of long-term cognitive status. Results from the three-level models indicated that retest and developmental slopes yielded non-redundant sources of variance, providing unique estimates of change that would otherwise be confounded. Further, short- and long-term RTI differentially predicted cognitive status at Years 4 and 8; failing to benefit from retest effects on the BRT task was associated with increased likelihood of cognitive impairment. This innovative approach to parameterizing retest effects can reduce systematic bias in estimates of long-term developmental change, as well as highlight the utility of retest effects as predictors of cognitive health.

### STRATEGIES TO ENSURE AUTHENTIC PARTICIPANTS AND VALID DATA WITH ONLINE RECRUITMENT OF FAMILY CAREGIVERS

Henrietta Armah[1], Jasmine Vickers[1], Danny Wang[2], and Carolyn Pickering[2], *1. University of Alabama at Birmingham, Birmingham, Alabama, United States, 2. The University of Alabama at Birmingham, Birmingham, Alabama, United States*

Over the past years, recruitment of participants for behavioral and biomedical research through the internet has become more popular. Online has become an advantageous approach to recruitment, especially since the covid pandemic has posed a great challenge to most in-person research activities. Typically, internet-based recruitment strategies include website posting, emailing list of potential participants as well as using social media such as Facebook, Twitter, and Instagram to deliver recruitment information to groups who are often unrepresented in research. Although this mechanism has reduced barriers to participation, it has posed serious threats to data quality and validity. For example, some studies on data validity estimate that up to 90% of online survey responses are fraudulent when they rely on screening questions and CAPTCHA alone. Others have shown that vetted panel data such as Mechanical Turk (mTurk), has high rates of participant misrepresentation. Therefore, the aim of this paper is to highlight the challenges associated with internet-based recruitment of family caregivers and describe strategies for researchers to ensure data integrity. We discuss multi-faceted approaches to detect and prevent fraudulent and suspicious activities such as duplicate and automated enrollment by software applications known as bots as well by fraudulent human participants. We discuss data on several strategies that have proven effective in our previous and ongoing trails. We will also demonstrate the need to implement several strategies and a "fail-safe" to detect fraud after enrollment. It is imperative that researchers understand the need to address these challenges to preserve data integrity and replicability.

### HAIR CORTISOL FEASIBILITY AND DEMOGRAPHIC CORRELATES IN A SAMPLE OF OLDER ADULTS FROM PUERTO RICO

Erin Ballard[1], Bulent Turan[2], and Michael Crowe[3], *1. University of Alabama at Birmingham, Hoover, Alabama, United States, 2. Koc University, Sariyer, Istanbul, Turkey, 3. University of Alabama-Birmingham, Birmingham, Alabama, United States*

Hair cortisol is increasingly being used as a biomarker of chronic HPA-axis activation. Studies using older adults often exclude a substantial portion of participants due to insufficient hair or non-detectable cortisol levels, but do not provide details on correlates of these factors. We examined feasibility of hair measurement and cortisol detectability in an ongoing study of older adults in Puerto Rico. Among the first 537 participants in the current follow-up of the Puerto Rican Elder Health Conditions (PREHCO) study (now age 78-106 years old), approximately 11% of participants refused to give a hair sample and 20% did not have enough hair to sample. Women (13%) were significantly more likely than men (4%) to refuse hair collection. However, men (47.7%) were significantly more likely than women (4.8%) to not have enough hair. Of participants with enough hair to take a sample (n=372), 23% had non-detectable levels of cortisol. Black participants were the most likely to have non-detectable hair cortisol (43%), followed by multiracial mestizo (32%). The two most common racial categories in our sample, multiracial trigueño (23%), and white (17%) were the least likely to have non-detectable cortisol. In terms of hair products (including frequency of hair washing and use of conditioner, dye, or perm), only the use of chemical hair straighteners was associated with higher likelihood of non-detectable cortisol (38%). Findings underscore the importance of measuring hair products when examining hair cortisol in older adults and suggest that older black participants may be disproportionately excluded due to non-detectable cortisol levels.

### TIME IS OF THE ESSENCE: RELIABLE MEASUREMENT OF HEART RATE VARIABILITY IN OLDER ADULTS

Cristina Pinheiro, Suzanne Segerstrom, and Justin Karr, *University of Kentucky, Lexington, Kentucky, United States*

Heart rate variability (HRV) decreases with age and is an important correlate of psychosocial and physical health. Recommendations for the minimum duration of EKG to accurately derive HRV vary from 1 minute to several minutes. However, the definition of "accuracy" or reliability depends on study design, including whether the focus of the study is on stable or momentary between-person differences or within-person changes in HRV. In a sample of 216 older adults (Mage = 72.7, 62.5% women), ECG was measured at 1000 Hz for 10 minutes every 6 months for up to 6.5 years. HRV was high-frequency power (0.15–0.40). A generalizability study determined the variance due to minute, occasion, person, and their interactions. The most variance was due to idiosyncratic occasion differences (46%), followed by stable person variance (18%). A decision study determined how accuracy in HRV measurement could be achieved. Between-person differences in HRV at a specific occasion could be reliably measured with 6 minutes of ECG (.80); within-person changes in HRV between occasions could be reliably measured with 3 minutes of ECG (.83). For stable individual differences, 10 or more occasions of 1-minute duration produced a more reliable estimate (.73) than increasing the length of a single ECG recording to 10 minutes (.25). The necessary duration of ECG for measuring HRV reliably depends on the study design and research question. Ultra-short durations (1-2 minutes) are generally not recommended for older adults.

### OPPORTUNITIES, FEASIBILITY, AND CHALLENGES OF USING IMMERSIVE VIRTUAL REALITY IN AGING RESEARCH

Cornelia Wrzus, *Ruprecht Karls University Heidelberg, Heidelberg, Baden-Wurttemberg, Germany*

Immersive virtual reality (iVR), that is, 3D-scenarios presented in head-mounted displays, is rarely used in aging research, although it gained much popularity recently in medical, educational, consumer, and gaming contexts and offers advantages such as real-life scenarios under experimental control. Still, iVR might be less suited for older adults, if they do not experience realism (i.e., presence) or feel strong cyber-sickness (e.g., nausea, headaches). The current preregistered project (osf.io/ryz2c) examined the opportunities, feasibility, and challenges of immersive Virtual Reality for studying age differences in socio-emotional processes. Up to now, 50 younger (age M = 23.5) and 50 older adults (age M = 67.9) saw different socio-emotional situations in iVR using a HTCvive headset, which included eye tracking, and then rated experiences of presence and cyber-sickness. Results showed that feelings of being present in the virtual reality were moderate to high, while cyber-sickness was generally low, and did not differ significantly between younger and older adults. Further analyses explore associations with technology acceptance, health, and personality. The findings suggest that using iVR with older adults is feasible, and creates similar levels of realism and low cyber-sickness as among young adults. The discussion highlights the opportunities and challenges of using iVR for studying age differences in cognitive, emotional, or social processes in experimentally controlled, real-life scenarios.

### SESSION 6600 (POSTER)

### SLEEP

### THE EFFECTS OF SLEEP DEFICIENCY ON MULTIMORBIDITY AMONG OLDER ADULTS IN THE PHILIPPINES AND VIETNAM

Tuo-Yu Chen[1], Grace Cruz[2], Nguyen Vu[3], and Yasuhiko Saito[4], *1. Taipei Medical University, Taipei City, Taipei, Taiwan (Republic of China), 2. University of Philippines, Diliman, Quezon, Philippines, 3. Institute of Population, Health and Development, Hanoi, Ha Noi, Vietnam, 4. Nihon University, Chiyoda-ku, Tokyo, Japan*

Multimorbidity increases the risks of disability and death and are prevalent among older adults. Evidence shows that sleep might play an important role in multimorbidity. However, previous research studies sleep quality and quantity in isolated manners, where in reality sleep quality and quantity are likely to affect each other. Besides, no study has been done to understand such relationship among older adults in low- and middle- income counties where the prevalence of multimorbidity increases rapidly. This study investigated the relationship between sleep deficiency (i.e., poor and insufficient sleep) and multimorbidity among community-dwelling older adults in the Philippines and Vietnam. Cross-sectional data were obtained from the Longitudinal Study of Ageing and Health in the Philippines (N = 3,562) and the Longitudinal Study of Ageing and Health in Vietnam (N = 3,936). Multimorbidity was defined by having two or more of chronic conditions (i.e., heart disease, heart attack, cardiovascular disease, hypertension, diabetes, lung diseases, renal diseases, liver diseases, and arthritis). Sleep deficiency was conceptualized as self-reported short sleep duration (< 6 hours), having trouble with falling asleep or maintaining sleep, and/or experiencing non-restorative sleep. Logistic regression was used to analyze the data adjusting for demographics, body mass index, sleep medications, naps, mental health, and lifestyle. The results showed that having deficient sleep was significantly related to increased odds of experiencing multimorbidity by about 81% in the Philippines and Vietnam. Our findings revealed that treating sleep deficiency among older adults in the Philippines and Vietnam can potentially reduce the risks of multimorbidity.

### THE MEDIATING ROLE OF FATIGUE/SLEEPINESS BETWEEN STATE MINDFULNESS AND SUBJECTIVE COGNITION

Aziz Rehman, Christina Mu, and Soomi Lee, *University of South Florida, Tampa, Florida, United States*

Previous studies have established a connection between higher mindfulness and cognitive abilities; however, few studies have considered the mechanism underlying this relationship. The cognitive benefit of mindfulness may be through reduced fatigue and daytime sleepiness. This study examined if higher, naturally occurring mindfulness is associated with higher subjective cognition and whether lower fatigue or sleepiness mediate this relationship. Two independent samples of nurses (N1=60 inpatient (IP); N2=84 outpatient (OP)) completed 14 days of ecological momentary assessment (EMA). Fatigue/sleepiness, mindfulness, and subjective cognition (mental speed, processing sharpness, memory) were assessed using EMA. The 5-item Mindful Attention Awareness Scale assessed state mindfulness. Multilevel mediations were conducted in Mplus to account for the nested data. At the within-person level, daily subjective cognition was higher than average on days when mindfulness was higher in both OP and IP samples. This association was mediated by lower levels of fatigue (IP indirect effect: B=2.08, p<.001; OP indirect effect: B=2.57, p<.001) and lower sleepiness (IP indirect effect: B=1.72, p=.001; OP indirect effect: B=0.92, p=.027). The daily indirect pathways were found after controlling for between-person differences; those with higher mindfulness reported higher subjective cognition through lower fatigue, and this effect was only significant in OP nurses (indirect effect: B=11.61, p=.001). Results highlight the importance of monitoring momentary mindfulness and intervening on daily fatigue and sleepiness as these may influence one's subjective cognition and ultimately their objective performance. These findings may help identify modifiable factors to promote quality of care in nurses and their own well-being.

### WORKING AROUND THE CLOCK: THE EFFECT OF SHIFT WORK AND SLEEP ON DEPRESSIVE SYMPTOMS

Cleothia Frazier, *Duke University, Durham, North Carolina, United States*

Shift work is increasingly prevalent in a 24-hour society where there is increased demand for round the clock service. However, shift work can disrupt circadian rhythms, which can negatively impact sleep. In turn, diminished sleep is associated with poor mental health. To expand prior research that reveal the independent effects of shift work and sleep on mental health, this study focuses on the interconnection between shift work, sleep, and depressive symptoms. Guided by the Stress Process Model (SPM), I examine the association between shift work and depressive symptoms and investigate whether sleep duration, sleep quality (insomnia symptoms), and sleep latency (the time it takes to fall asleep) mediate this relationship. Data was drawn from the age 50 health module of the National Longitudinal Survey of Youth 1979 cohort. The sample consisted of noninstitutionalized adults aged 51-60 (N=5,386). Findings show that shift workers had increased odds of short sleep, insomnia symptoms, and increased sleep latency compared to non-shift workers. Moreover, shift work was associated with increased depressive symptoms. However, part of the effect of shift work on depressive symptoms was indirect, operating through sleep. Specifically, short sleep during the week and on the weekend as well as insomnia symptoms mediated the relationship between shift work and depressive symptoms. The findings suggest that while engaging in shift work can negatively affect mental health, improving sleep duration and sleep quality can be effective in reducing the harmful effects of engaging in shift work during late midlife.

### CAREGIVER OUTCOMES RELATED TO SLEEP DISTURBANCES IN PEOPLE LIVING WITH COGNITIVE IMPAIRMENT

Miranda McPhillips[1], Darina Petrovsky[2], Justine Sefcik[3], Junxin LI[4], Glenna Brewster[5], Nalaka Gooneratne[1], Subhash Aryal[1], and Nancy Hodgson[1], *1. University of Pennsylvania, Philadelphia, Pennsylvania, United States, 2. Rutgers, The State University of New Jersey, New Brunswick, New Jersey, United States, 3. Drexel University, Philadelphia, Pennsylvania, United States, 4. Johns Hopkins University, Baltimore, Maryland, United States, 5. Emory University, Atlanta, Georgia, United States*

Sleep disturbances in people living with cognitive impairment (CI) may impose a great burden on caregivers. We examined the relationship between objective and subjective sleep measures in people living with CI and caregiver depression and mastery via a secondary analysis of The Healthy Patterns Clinical Trial (NCT03682185) baseline data (n=170). Objective sleep variables included total sleep time and sleep efficiency derived from 3 nights of actigraphy. Subjective sleep measures included PROMIS Sleep Related Impairment, Pittsburgh Sleep Quality Index, and Epworth Sleepiness Scale. Caregiver measures included Center for Epidemiological Studies Depression Scale and Caregiver Mastery Scale. People living with CI were female (67%), Black (80%), with mean age 73.4 ± 8.7. Caregivers were female (81%), family caregivers (80%) with mean age 56.5 ± 14.7. We used multiple regression analysis, adjusting for cognition, and examined if there were differences by caregiver gender. Poorer subjective sleep quality was significantly associated with more caregiver depression (B=0.407, SE= 0.198, p = 0.042). There were no significant sleep predictors for caregiver mastery; however, there was a moderating effect of gender on the association between subjective sleep quality and caregiver mastery. Female caregivers had increased caregiver mastery compared to males when the person living with CI had better sleep quality (B=0.555, SE= 0.218, p = 0.012). This study found that people living with CI sleep characteristics differentially influence caregiver outcomes. Sleep should be assessed using a combination of objective and subjective sleep measures in people living with CI to inform providers when interventions are needed.

### GOLDILOCKS AT WORK: JUST THE RIGHT AMOUNT OF JOB DEMANDS MAY BE NEEDED FOR YOUR SLEEP HEALTH

Monica Nelson[1], Soomi Lee[1], Tammy Allen[1], Orfeu Buxton[2], David Almeida[2], and Ross Andel[1], *1. University of South Florida, Tampa, Florida, United States, 2. The Pennsylvania State University, University Park, Pennsylvania, United States*

Objectives: It has been reported that job demands affect sleep, but how different levels of job demands affect sleep remains unclear. We examined whether curvilinear relationships exist between job demands and multiple sleep health outcomes. Design: Cross-sectional analyses with linear and quadratic effects, using self-administered survey data. Setting: A national sample of U.S. adults. Participants: Workers from Midlife in the United States Study (MIDUS2; n=2,927). Measurements: The Job Content Questionnaire assessed overall and five specific aspects of job demands (intensity, role conflict, work overload, time pressure, and interruptions). Habitual sleep health patterns across five dimensions (regularity, satisfaction/quality, daytime alertness, efficiency, and duration) were assessed. Age, sex, race/ethnicity, marital/partnered status, education, job tenure, work hours, body mass index, smoking status, and study sample were covariates.

Results: There were significant linear and quadratic relationships between job demands and sleep outcomes. Specifically, the linear effects indicated that higher job demands were associated with degraded sleep health, such as shorter duration, greater irregularity, greater inefficiency, and more dissatisfaction. The quadratic effects, however, indicated that the rate of degrade was decelerated in terms of sleep regularity and efficiency, such that these sleep outcomes were best with moderate levels of job demands. These effects were found for overall job demands as well as specific aspects of job demands. Stratified analyses further revealed that these curvilinear associations were mainly driven by participants with low job control.

Conclusions: Moderate levels of job demands, especially if combined with adequate job control, are related to better sleep health.

### SESSION 6610 (POSTER)

### SOCIAL DETERMINANTS OF HEALTH AND AGING

### HOSPITALIZATION AT HOME MAY ADDRESS SOCIAL DETERMINANTS OF HEALTH: RESULTS FROM A QUALITATIVE STUDY

Gabrielle Schiller[1], Heather Wurtz[2], Sybil Masse[2], Abigail Baim-Lance[2], Alex Federman[2], Emily Franzosa[2], Ksenia Gorbenko[2], and Linda DeCherrie[3], *1. CUNY School of Public Health, Astoria, New York, United States, 2. Icahn School of Medicine at Mount Sinai, New York, New York, United States, 3. Medically Home, Boston, Massachusetts, United States*

This project evaluated implementation of the Acute Hospital Care at Home (AHCaH) waiver from the U.S. Center for Medicare & Medicaid Services (CMS), launched in November 2020 to ensure availability of acute care as COVID-19 cases overwhelmed hospitals. We interviewed 18 HaH program leaders of 14 programs, from both existing and newly launched HaH programs, about their experiences applying for and implementing HaH under the CMS waiver. A thematic analysis of the interviews was completed. Informants described HaH programs as an opportunity for healthcare professionals to have "eyes in the home," referring to ways in which the HaH team was able to observe and intervene in aspects of the patient's life not accessible from traditional in-patient care. Informants described various ways that HaH programs addressed Social Determinants of Health (SDoH) including assessing patients' ability to obtain healthy foods, reducing potential safety hazards in the home, and providing education and support to patients' caregivers. Informants also suggested that HaH may reduce anxiety associated with hospital stay and separation from loved ones, helping people already contending with social inequities. Addressing SDoH is important for ensuring quality care and long-term positive changes in the patient's lifestyle and environment. As CMS shifts their aims to further address the SDoH, our findings suggest that the interdisciplinary care approach of HaH programs may be a model of addressing both social and medical needs.

### HOME AWAY FROM HOME: SOCIODEMOGRAPHIC AND HUMAN CAPITAL PREDICTORS OF NURSING HOME USAGE

Cayley Ryan, and Liying Luo, *Pennsylvania State University, University Park, Pennsylvania, United States*

The U.S. is experiencing a demographic shift characterized by a significant segment of the population entering older age groups and beginning to require specialized eldercare, however little is known about the influence of demographic characteristics on individuals' involvement with long-term care institutions. I use data from the Health and Retirement Study and logistic regression models to examine how the identities and resources people bring to the later stages of their lives influence their use of nursing homes. I find that individuals' levels of human capital – measured through their educational attainment – have the strongest influence on their use of nursing homes, with individuals with college degrees having significant increases in the odds of reporting any nursing home stay and in the expected count of nursing home stays. I posit multiple pathways by which education could increase the likelihood of using institutionalized care, including through the social mobility of other family members as well as the ability to navigate healthcare resources and evaluate eldercare options. This detailed examination of the intersection between older ages, individual characteristics, the accumulation of different kinds of capital over the life course, and the involvement with formal care facilities provides valuable insight into the relationship between individuals and institutions across older ages and can inform policies attempting to prepare the landscape of formal care institutions for the influx of possible consumers.

### DO AGE-FRIENDLY COMMUNITIES PROMOTE OLDER ADULTS' HEALTH?

Kyeongmo Kim[1], Denise Burnette[1], Thomas Buckley[2], and Seon Kim[3], *1. Virginia Commonwealth University, Richmond, Virginia, United States, 2. University of Pittsburgh, Pittsburgh, Pennsylvania, United States, 3. Virginia commonwealth university, Richmond, Virginia, United States*

The WHO developed the concept of age-friendly environments that contribute to older adults' better health and well-being. However, research on the effects of age-friendly communities (AFCs) on their health is limited. As AFC initiatives grow, it will be essential to examine whether older adults who live in an AFC have better health than those in other environments. This study uses data from the 2017 AARP AFC Surveys and the AARP Livability Index to assess whether AFCs promote the health of older adults. We analyze data for 3,027 adults aged 65 and older who reside in 262 zip code areas. Following AARP guidelines, we allocated the sample into two groups: the AFC group (livability score of 51+; n=2,364) and the non-AFC group (score≤50, n=663). The outcome variable was self-rated health (M=3.47; SD=1.09; range: 1-5). Findings employing an inverse probability weighting approach showed that older adults who lived in an AFC had better self-rated health than those in a non-AFC (b=0.08, p=.027). Compared to non-Hispanic whites, Black and Hispanic older adults reported worse self-rated health. Oldest old individuals had better health status than young-old adults. Consistent with previous literature, higher education, and frequent social interaction were associated with better self-rated health. The study adds to the growing literature on the role of age-friendly environments in older adults' health and suggests directions for future research. Since living in an AFC can promote the well-being of older adults, policymakers and practitioners should continue to build high-quality, accessible built and social environments.

### A CLINICAL-COMMUNITY PARTNERSHIP TO ADDRESS OLDER ADULTS' SOCIAL DETERMINANTS OF HEALTH NEEDS

Cory Bolkan[1], Raven Weaver[2], Erik Geissal[3], and Breanne Wise-Swanson[4], *1. Washington State University Vancouver, Vancouver, Washington, United States, 2. Washington State University, Pullman, Washington, United States, 3. The Vancouver Clinic, Vancouver, Washington, United States, 4. Area Agency on Aging & Disabilities of Southwest Washington, Vancouver, Washington, United States*

Social determinants of health (SDH) are non-medical social needs key to reducing health disparities and improving health outcomes. Adequately identifying patients' unmet SDH needs in primary care (PC) is a critical first step in addressing them, yet many questions remain regarding feasibility and implementation of screenings and how to effectively meet patients' needs and improve their outcomes. With formative and process evaluation analyses, we report on the development and implementation of a community-based pilot study to proactively target high-risk, low-income, older patients with SDH needs. Over a six-month planning period, leadership from a PC clinic and a community based aging services organization (CBO) collaboratively created a shared infrastructure for in-office SDH screening by clinicians with direct referral to CBO for SDH support. The research team addressed challenges of workflow and barriers to sharing/accessing electronic health records. The pilot program will cover a 2-year period (12-month enrollment; 12-month follow-up) in which patients are screened at annual visits and followed-up in the community. In the first 6 months, 286 patients were screened, from which 34 (12%) CBO referrals were made, and nine patients were receptive to receiving more information, suggesting a need to explore patient barriers and receptiveness to services/supports. We report on lessons learned, adaptations to the pilot, efforts to increase identification of eligible patients, and strategies to enhance uptake of services beyond the traditional health care setting. Investment in health and aging services partnerships is a viable pathway to reducing health care use and spending, especially for older adult populations

### HOUSING CONDITIONS, SOCIAL CAPITAL, AND SUBJECTIVE WELL-BEING AMONG MIGRANT OLDER ADULTS IN INDIA

SRUTHI ANILKUMAR HEMALATHA, and
NAWAJ SARIF, *International Institute for Population Sciences, Mumbai, Maharashtra, India*

Aging and migration are complex multidimensional processes affected by various factors at different levels. According to the most recent Census of India (2011), more than half of the older adults in India are migrants. Migration at an older age is considered risky and vulnerable because it may lead to many problems. Changes in the environment and living place at an older age may disconnect their social network and affect their health. Therefore, this study examines the housing conditions, social networks, and subjective well-being of older adult migrants in India using data from Longitudinal Aging Study in India, Wave 1 (2017-18). The bivariate and multivariate analyses show significant socio-economic differences in living arrangements and access to better housing facilities among older adult migrants. Additionally, there exist state-wide differences in the social capital of older migrant adults. The likelihood of having poor social capital is higher among migrant older adults than non-migrant older adults. It also reveals that the subjective well-being of older adult migrants is strongly associated with their housing conditions, social networks, and other socio-economic factors. Thus, the paper finds environmental factors and social support essential for better health and well-being, especially for older adults, especially those in new surroundings. Therefore, extensive research is needed to understand the situation of migrant older adults in India to formulate appropriate policies for the same.

### NEIGHBORHOOD IMPACTS ON KOREAN OLDER ADULTS' DEPRESSION

Seon Kim, *Virginia commonwealth university, Richmond, Virginia, United States*

Older adults are more influenced by their neighborhood than young adults, however neighborhood impacts on older adults are little known. In response to the increasing depression of older adults in Korea, this study aims to identify the effect of perceived and objective neighborhood characteristics in depression and find differences between rural and urban areas. This study uses the Korean Elderly Survey conducted in 2020 on 10,097 older adults aged 65 and over residing in 17 states, and Korean administration data by connecting them. Multilevel modeling results show that after controlling individual characteristics, depression decreases when older adults perceive their housing condition (b=-.64, p<.001), interaction with neighbors (b=-.29, p<.001), overall neighborhood environment (b=-.26, p<.001) positively. Although objective neighborhood characteristics do not significantly reduce depression in older adults living in urban area, for older adults living in rural areas, the number of social worker (b=- .42, p <.001), the number of senior center (b=-6.70, p<.001), and home care services (b=-49.5, p <.001) are negatively associated with depression in older adults. This study discusses that neighborhood characteristics may play an important role in older adults' depression, and that their effects may differ between rural and urban areas. This study encourages policy makers to consider neighborhood characteristics to improve the mental health of Korean older adults.

### OBJECTIVE AND SUBJECTIVE POVERTY AMONG OLDER PEOPLE AND ITS ASSOCIATION WITH HEALTH, FUNCTION, AND MORTALITY

Jeremy Jacobs[1], and Jochanan Stessman[2], *1. Hadassah Medical Center, Mount Scopus, Jerusalem, Yerushalayim, Israel, 2. Hadassah Medical Center, Jerusalem, Yerushalayim, Israel*

We examined objective and subjective poverty between ages 70-95, comparing and contrasting frequency, associated subsequent medical, functional, psychosocial impairment, and mortality. Data collection by the Jerusalem Longitudinal Study, following a representative community dwelling sample (born 1920-1921), assessed at ages 70, 78, 85, 90, and 95. We defined "objective poverty" as sole income from "national old-age pension" necessitating "supplemental income benefit" from the National Institute for Social Security; "subjective poverty" as "financial difficulty getting through each month". Subjective and objective poverty differed: frequency of objective poverty remained 13% throughout follow-up period, while subjective poverty was more frequent at younger age, decreasing from 24% at ages 70-85, to 14% at ages 90-95. Subjective poverty was significantly associated with depression at all ages (from HR=3.2, CI 95%:1.73-5.93 at age 70, to HR=2.45, CI 95%:1.32-4.54 at age 95), and poor self-rated health (from HR=2.69, CI 95%:1.63-4.46 at age 70, to HR=1.96, CI 95%:1.11-3.43 at age 95), however no consistent associations with objective poverty was observed. Both objective and subjective poverty were associated with low levels of physical activity, impaired functional and cognitive status. Mortality was significantly increased among subjects with subjective poverty at ages 70, 85, and 90 (ranging from HR=1.33, CI 95%: 1.02-1.74 to HR=1.50, CI 95%:1.11-2.03), while objective poverty was significantly associated with increased mortality at ages 78 and 85 (HR=1.43, CI 95%:1.01-2.03, and HR=1.55, CI 95%:1.16-2.07 respectively). In conclusion, subjective and objective poverty differ in their association with health, functional and psychosocial status, and mortality between ages 70-95.

### SESSION 6620 (POSTER)

### SPIRITUALITY AND RELIGION

### LONGITUDINAL ASSOCIATIONS BETWEEN SPOUSAL LOSS, RELIGIOUS INVOLVEMENT, AND COGNITIVE HEALTH IN LATER LIFE

Xinfang Yu, and Hyo Jung Lee, *Nanyang Technological University, Singapore, Singapore*

Death of a spouse is one of the major stressors which is more frequently observed in one's late life. Previous literature showed a link between the death of a spouse and late-life cognitive health and suggested a benefit of having more religious involvements. Applying Stress Process Model, this study examines multiple dimensions of religious involvements and whether they buffer a negative impact of spousal loss on cognitive health in old age. Using data from the Health and Retirement Study (2006 – 2016 waves), we selected older adults who were married at baseline (N = 6658). We conducted a) regression analyses to examine the main effect of spousal loss on cognitive health as well as buffering effect of religious involvement and b) growth curve modeling to analyze the trajectories of cognitive functions. Preliminary analyses showed that spousal loss in late life was associated with poorer cognitive function among older adults at baseline and follow-ups. More frequent attendance at religious services and prayer in private setting were associated with better cognitive function, yet, no buffering effect was found. We observed a gradual decline in cognitive function over time among the widowed respondents. Findings confirm that widowed individuals are more likely to experience cognitive decline over time, compared to married counterparts. While religious involvement might not mitigate the negative impact of spousal loss on cognition, the study supports that each dimension of religious involvement may exert different outcomes in terms of older individual's cognitive health during widowhood.

### THE RELATIONSHIP BETWEEN RELIGIOSITY AND NURSING HOME SERVICE UTILIZATION: A LONGITUDINAL ANALYSIS

Corina Ronneberg[1], and Edward Miller[2], *1. University of Massachusetts Boston, Chicago, Illinois, United States, 2. University of Massachusetts Boston, Boston, Massachusetts, United States*

On any given day 3.9% to 4.2% of the US population receives nursing home (NH) care. Given the critical role NHs play in caring for older adults, research has identified various demographic, biological, psychological, and social factors associated with NH utilization. Few studies, however, have explored religiosity as a predictor; none, longitudinally. This study thus aims to examine how one's religiosity (organizational, non-organizational, intrinsic) affects NH use. A total of n=7,564 respondents (>51 years) from the 2006 wave of the Health and Retirement Study, with no prior NH use, were assessed every two years, until 2014. Hierarchical modeling techniques (i.e., Generalized Estimating Equations Multinomial Logistic Regression) were used to model the relationship between religiosity and subsequent NH use, while controlling for other factors. Results indicate that high levels of organizational religiosity (i.e., service attendance) is associated with higher rates of NH utilization (B= 0.13, $p < 0.001$). Conversely, they indicate that higher non-organizational religiosity (i.e., private prayer) is associated with a lower likelihood of NH care use (B= -0.03, $p < 0.05$). Sex and race served as moderators in these relationships. The association between higher levels of service attendance and higher rates of NH utilization may reflect the sharing of information about available resources such as long-term care through one's social support networks, including congregations. In contrast, individuals who were more likely to pray on their own, may lack the social support provided by congregations, and as a result, experienced more limited knowledge transfer about available resources such as NHs.

### TESTING A PORTION OF THE OKLAHOMA AGING INMATE FORGIVENESS MODEL

George Randall[1], Alex Bishop[2], and Sydney Bellah[2], *1. Sam Houston State University, Huntsville, Texas, United States, 2. Oklahoma State University, Stillwater, Oklahoma, United States*

The study assessed the viability of the published Oklahoma Aging Forgiveness Model on women in custody using cross-sectional data collected from females, violent and non-violent, in custody in Oklahoma. The theoretical model led to a hierarchical regression of a measure of positive mental health (Positive Evaluation of Life) on a block of control variables (age, education, and crime type), a block consisting of items from the Duke Religiosity measure, and a final block utilizing forgiveness of self, others, and situation (Heartland Forgiveness Scale). Results from the complete sample, N=447, explained 36% of the variance in the outcome. Significant individual predictors included in the final model were crime type, religiosity, and forgiveness of self and situations. We split the sample on crime type and found that for the violent offenders (N=228), 39% of the variance in the outcome was explained; in addition to religiosity, all three assessments of forgiveness were significant predictors. For the non-violent offenders (N=209), 35% of the outcome's variability was explained. Religiosity, forgiveness of self and situation were significant predictors for this sub-sample. Discussion will focus on the Oklahoma Aging Forgiveness Model and how it works similarly for men in custody (published) and for women in custody. Further, the discussion will focus on the significant role played both by religiosity and forgiveness for those in custody. Findings from this study and that of studies with men clearly demonstrate that religiosity and forgiveness are important aspects of a prisoner's life.

### FAITH, HOPE, AND LOVE: UNDERSTANDING RELIGIOSITY AND HOPE AMONG OLDER AFRICAN AMERICAN COUPLES

Antonius Skipper[1], and Andrew Rose[2], *1. Georgia State University, Atlanta, Georgia, United States, 2. Texas Tech University, Lubbock, Texas, United States*

Older African Americans frequently turn to their religion or spiritual faith as a source of coping and resilience. Disproportionately burdened by numerous disparities and stressors, many African American utilize religion as a source hope for the future. Existing studies suggest that higher levels of positive religious coping are associated with higher levels of hope, and more frequent experiences with negative religious coping are associated with lower levels of hope. However, the relationship between religious coping and hope is underexamined among one of the most religious dyads in the U.S., older African American couples. This study utilizes data from 194 older African American couples (146 married and 48 cohabiting), with each partner between the age of 50 and 86 years, to examine the dyadic relationship between religious coping and hope. Actor Partner Interdependence Models revealed that men's religious coping was associated with their own hope, and women's religious coping was associated with their own hope. One unexpected partner effect was identified and found that women's positive religious coping was negatively associated with men's hope. Given the dearth of research on older African American couples, along with the need to better understand religious significance in psychosocial dyadic outcomes, this study offers several implications for engaging African American couples in relational counseling and therapy across the life course.

### THE ROLE OF SPIRITUALITY IN LATER LIFE: A STUDY OF OLDER ADULT UNIVERSITY STUDENTS IN PORTUGAL

Giuliana Casanova, *University of Aveiro/ University of Porto , Leca do Balio , Porto, Portugal*

In Portugal, little has been done to investigate spirituality and its impact on later life. This study aims to discover whether spirituality contributes to successful aging in the lives of Portuguese older adults. This mixed-methods research study is based in Porto, Portugal. The sample size is 58 students from universities of the third age, ages 65 to 89 with no cognitive deficit. Data was collected by a demographic questionnaire, Spirituality Scale, Scale of Psychological Well-Being, and a mix method questionnaire. The data analysis was conducted by descriptive statistics and thematic analysis.

Results: Participants rated high in both the Spirituality Scale and the Psychological Well-Being Scale. The data also showed a significant and positive correlation between the dimension of hope/optimism from the Spirituality Scale and the dimension of autonomy from the PWB Scale; as well as between global spirituality and the dimension of autonomy. The mix-methods questionnaire showed 90% of the participants believe in God or a higher power, 81% considered themselves spiritual while 89.4% felt spirituality had always been a part of their lives. The three most significant themes that emerged were the connection to a higher power/divine, personal well-being, and the importance of spirituality throughout their lifetime. Discussion: Spirituality demonstrated to be correlated with the participant's ability and perception of autonomy and independence; having their spirituality as a coping mechanism to deal with life´s adversities while being able to look at the future with optimism. Spirituality can provide purpose, guidance, and meaning in the later years of life.

### SESSION 6630 (POSTER)

### ADULT PROTECTION, ELDER ABUSE, AND AGEISM

### ATTITUDES TOWARD AGING AMONG COLLEGE STUDENTS: RESULTS FROM THE INTERGENERATIONAL CONNECTIONS PROJECT

Ling Xu[1], Noelle Fields[2], Alan Adolfo Kunz Lomelin[1], Kathryn Daniel[1], Daisha Cipher[2], and Brooke Troutman[3], *1. University of Texas at Arlington, Arlington, Texas, United States, 2. The University of Texas at Arlington, Arlington, Texas, United States, 3. Air Force Academy, Air Force Academy, Colorado, United States*

To improve college students' attitudes toward aging, an intergenerational intervention was implemented to connect them with community-dwelling older adults with cognitive impairment. College students were trained to make weekly phone calls to Meals on Wheels clients (age 65+). This study was an investigation of changes in college students' attitudes toward aging over the course of study participation.College students aged 18 to 30 from a large public university in North Texas were recruited. Participants (n = 41) completed surveys through QuestionPro at baseline, midway, and at the end of the study. The Fraboni Scale of Ageism was used to measure attitudes toward aging. Friedman tests and one-way repeated measures ANOVAs were computed. Results indicated that college students' total ageism scores significantly improved over time ($F(2, 76)=4.491$, $p=.014$), as well as their antilocution ageism scores ($F(2, 76)=5.075$, $p=.007$), and their avoidance ageism scores ($F(2, 76)=3.844$, $p=.026$). In addition, scores on six specific items significantly improved after participating in the study: "Many old people are stingy and hoard their money and possessions", "Many old people are not interested in making new friends,", "Many old people just live in the past", "I personally would not want to spend much time with an old person", "Most old people should not be trusted to take care of infants", and "Most old people would be considered to have poor personal hygiene". Results suggest that weekly engagement with older adults offers promise for improving attitudes towards aging among college students. Implications for research and practice are discussed.

### EXPLORING AGEISM WITHIN THE FAMILY

Stacey Gordon, and Ernest Gonzales, *New York University, New York, New York, United States*

It is well documented that ageism negatively affects older people's opportunities for productive aging, and access to the workplace and healthcare. Ageism as it manifests within families, however, has not yet been well studied despite significant implications for the wellbeing of older adults and families. This study presents the problem of ageism in the family through the lens of larger social structural factors shaping meso- and micro-level behaviors. Negative implicit ageism and stereotype embodiment theory underscore the unconscious perpetuation of negative age stereotypes and attitudes in families. To illustrate this concept, we describe three evidence-based case examples involving interactions among family members observed by a geriatric care manager. The first describes how ageism expressed by an adult child undermines choice, opportunity, and power of the parent when an older adult enlists the help of her adult children in downsizing from the family home to a smaller apartment. The second explores ageism when adult children attempt to persuade their older father to leave his home, pointing out dangers living alone at his age. The third describes a relatively healthy newly widowed woman who is coerced by her adult children to move to an assisted living facility by threatening social and emotional abandonment. These examples demonstrate how macro and meso-level factors combine with the implicit ageism of both adult children and older adults converge, influencing pathways to create healthy, tolerable and toxic living conditions within the family.

### ELDER MISTREATMENT AND RELATIONSHIP QUALITY: SECONDARY DATA ANALYSIS USING DATA FROM THE NSHAP

Wei-Lin Xue[1], Zach Hass[2], and Pi Ju Liu[1], *1. Purdue University, Lafayette, Indiana, United States, 2. Purdue University, West Lafayette, Indiana, United States*

As social networks shrink with age, older adults value the importance of interpersonal relationships with close others, such as partners, family and friends. Previous studies focused on perpetrators' and victims' characteristics; however, few studies examined the relationships with close others and the incidence of elder mistreatment. This study used the National Social Life, Health, and Aging Project Wave 1 data (2005-06) to examine the correlations between relationship quality with close others and the occurrence of mistreatment among older adults (N = 3005). Based on spousal relationship literature using the NSHAP data, factor analysis was used to estimate factor scores conceptualizing two domains of relationship quality: relationship support (positive dimensions of relationship), and relationship strain (negative dimensions of the relationship). Logistic regression models were used to test the relationship between the relationship quality factor scores and the likelihood of each mistreatment type while controlling for gender, education, age, and race. Psychological abuse was more likely for older adults experiencing relationship strain with spouse (OR=1.82, p<.001), family (OR=1.75, p<.001), and/or friends (OR=1.67, p< 0.001). Financial abuse was more likely for those experiencing poor relationship support with family (OR=1.34, p<.05) and those experiencing relationship strain with friends (OR=1.47, p<.01). However, relationship quality was not correlated with likelihood of physical abuse. Interpersonal relationships with close others could provide stronger support and care, but relationship strain might contribute to mistreatment. When determining safeguarding gatekeepers to protect older adults from potential mistreatment, interventions should consider the quality and composition of interpersonal relationships with close others.

### IS ACTIVE AGING POSSIBLE IN ETHIOPIA? PERCEPTIONS OF RURAL OLDER ADULTS

Abraham Teshome[1], Messay Kotecho[2], and Margaret Adamek[1], *1. Indiana University, Indianapolis, Indiana, United States, 2. Addis Ababa University, Addis Ababa, Adis Abeba, Ethiopia*

Despite historical veneration of elders, older adults are increasingly viewed as a burden in many Sub Saharan African nations. Using a hermeneutic phenomenological approach, in-depth interviews were conducted to explore the aging experiences of 20 adults aged 70 and older in rural Ethiopia. Themes that emerged from the interviews were analyzed in light of the three pillars of the World Health Organization's Active Aging Framework: health, security, and participation. Despite facing multiple barriers to active aging including lack of health care, financial hardship, ageism, and social exclusion, study participants were determined not to withdraw from activities in an effort to retain their autonomy, independence, and sense of dignity. As one participant shared, "the government does not care about us because it considers us as a useless segment of the society." Three themes reflected their major struggles: dwindling health and lack of access to health care, financial hardship, and social exclusion tied to ageism. The fourth theme--willful and purposive engagement--reflects the older adults' response to these struggles. Contrary to myths about rural aging, study participants faced multifaceted challenges that kept them from realizing active ageing as they were not provided with sustained opportunities for health, security, and participation. Study findings point to the need for policymakers and other concerned bodies to develop supportive policies and programs to promote older adults' well-being. The study calls for a paradigm shift that involves adopting the WHO's Active Aging Framework, developing rights-based policies and programs, popularizing active aging, and revitalizing intergenerational solidarity.

### A STUDY COMPARING TRENDS IN NUMBER OF AND CONTENT IN ARTICLES ABOUT DOMESTIC VIOLENCE AND ELDER ABUSE IN JAPAN

Asako Katsumata[1], and Noriko Tsukada[2], *1. Rehabilitation Institution, Chiba, Chiba, Japan, 2. Nihon University, Tokyo, Tokyo, Japan*

Since enactment in 2000, Japan's Domestic Violence (DV) Law has been amended four times. No amendments have been made to Japan's Elder Abuse Prevention Law, despite awareness of its imperfections. This study examined trends in the number of journal articles, and titles by content of those articles, using a KJ Method, to determine how the research literature has informed DV law, and to identify gaps in knowledge and further research needs related to elder abuse that could inform policy change. Using the CiNii database, in 2005-2021, we found1020 elder abuse articles (average 60 articles/year), and in 2001-2021, 836 DV articles (average 40 articles/year). Preliminary analyses revealed the per year annual average has been decreasing for each. Although the most used term was similar in both DV and elder abuse articles, which was "responses of professional personnel," 15.4% for elder abuse and 13.4% for DV, title terms differed, with titles of elder abuse articles are more likely to be related to caregiving, such as "support for caregiver" (who are actually abusers) (4.7%) and "dementia" (3.6%), while terms found in DV but not in elder abuse articles included "supports for victims" (7.1%) and "responses to abusers" (5.3%). Our findings indicate that more research on supporting victims of elder abuse may be imperative to inform lder abuse law changes.

### SESSION 6640 (POSTER)

### AGEISM: EDUCATION, INTERVENTION, AND ADVOCACY

### ASSESSING STEREOTYPES OF OLDER ADULTS THROUGH DRAWING: IMPLICATIONS FOR PEDAGOGY IN UNDERGRADUATE AGING COURSES

Melannie Pate, and Elizabeth Fugate-Whitlock, *University of North Carolina Wilmington, Wilmington, North Carolina, United States*

Negative stereotypes and ageism toward older adults remain an insidious problem in the United States. These harmful views of older adults can lead to disparities in health care, social isolation, and loss of functional ability. In order to supplement current research on the perceptions of older adults, the researchers focus on the views of undergraduate students before and after taking an aging course. The students included in this study are enrolled in an undergraduate health and aging course or an undergraduate introduction to gerontology course. Before beginning any coursework, students were asked to draw an older adult, using any combination of colored markers available and to write one sentence describing an older adult. At the end of the course, students were again given the same directive to draw an older adult, using any combination of colored markers, and write one sentence describing an older adult. Content analysis was used to analyze the drawings and the phrases. Colors used and depictions of activity in the drawings changed from the first day of class to the end of the course, showing a progression of their views. Drawings were chosen specifically to elicit honest responses and perceptions of older adults. The results of this study can be used to inform pedagogical choices made when teaching courses on aging to undergraduate students.

### CENTENARIANS IN SWEDISH MASS MEDIA: IS IT REVERSE AGEISM?

Cristina Torgé, and Rosita Nyman, *Jönköping University, Jönköping, Jonkopings Lan, Sweden*

Studies on ageism often deal with negative stereotypes due to increasing age. Seldom studied is the increase in status that seems to occur at the turning point of 100 years. This study explores how centenarians are portrayed in Swedish media texts and relates this to mechanisms of ageism and age coding. We used Retriever database, which is the biggest Nordic database containing news media, to search for press articles concerning 100- to 105-year-olds. Our search resulted in 1468 articles, consisting of both news articles and feature articles. Excluding articles that did not deal with persons, articles with a paywall and redundant articles resulted in 235 articles. We analyzed (1) the contexts in which centenarians appeared, (2) the relevance of the persons' age to the event and (3) feelings and values evoked in the articles. On the one hand, news reports evoke how centenarians ought to be shielded from harm and emphasize the indignity of even small wrongdoings. On the other hand, feature articles often use superhuman adjectives to describe centenarians, and emphasize their "youthfulness". In birthday features, there was also an expectation for centenarians to publicize their celebrations and there was a recurrent trope was that centenarians held well-kept secrets about ageing well. Portrayals of centenarians show that they are an esteemed group. However, we also interpret that these portrayals can hide other forms of ageism, particularly compassionate ageism, and a stereotyping of centenarians that play down ill health and emphasize youthfulness at the extremes of old age.

### EXAMINING CHANGES IN STUDENTS' ATTITUDES TOWARD WORKING WITH OLDER ADULTS

Christopher O'Brien, *Chatham University, Pittsburgh, Pennsylvania, United States*

Curricular intervention studies have examined if instruction in aging and gerontology affects undergraduates' attitudes, knowledge, and perceptions towards older adults. However, less is known about curricular impact on undergraduates' intentions to work with older adults. By identifying factors that increase undergraduates' intentions to work with older adults we may elucidate meaningful points of intervention to enhance pursuit of careers in the geriatric workforce. The current study examined baseline data from a longitudinal study examining the impact of an upper-level adult development psychology course on student attitudes towards working with older adults. It was hypothesized that there would be positive associations between attitudes towards working with older adults, knowledge about aging, and positive attitudes towards older adults. Participants were 19 undergraduate students enrolled in upper-level undergraduate psychology courses. Participants completed validated, self-report questionnaires related to their attitudes towards working with older adults, ageism attitudes, and attitudes and knowledge about aging. Bivariate correlation analyses were used to examine cross-sectional associations among main outcome variables. More positive explicit attitudes towards older adults were significantly associated with more willingness to work with older adults ($r= .49$ , $p=.04$). Additionally, knowledge of aging was positively correlated with perceived social norms around working with older adults ($r= .49$, $p=.04$). These initial findings suggest that knowledge and positive attitudes about aging may positively impact attitudes towards working with older adults. Future work will assess curricular impact on undergraduates' intentions to work with older adults, as well as evaluate predictors of change in intentions.

### STANDING UP TO AGEISM: PERCEPTIONS OF SIXTY AND EIGHTY YEAR-OLD ADULTS WHO REJECT BENEVOLENT AGEISM

Alison Chasteen[1], Zainab Saleem[1], and Michelle Horhota[2], *1. University of Toronto, Toronto, Ontario, Canada, 2. Furman University, Greenville, South Carolina, United States*

Older people encounter ageism in a variety of daily contexts, yet the consequences of confronting ageist behavior by older targets are not well understood. The present study asked whether the age of the older target who accepts versus rejects an act of benevolent ageism results in different impressions of the older target. Young ($N = 368$) and older ($N = 388$) adults read a scenario in which either a 60 or 80 year-old individual accepted or rejected benevolent ageism in the form of unwanted help. Participants then evaluated the older target's warmth, competence, and overall impression of the target. Consistent with pre-registered hypotheses, competence ratings increased but warmth and overall impression ratings decreased when the older target rejected benevolent ageism compared with accepting it. Also consistent with predictions, older participants rated the older target more positively on all measures compared with younger participants. Surprisingly, the 80 year-old target was rated as warmer and made a more positive overall impression than did the 60 year-old target. Target age did not moderate reactions to accepting or rejecting unwanted help, suggesting that perceivers did not distinguish between young-old and old-old targets when evaluating an older target that confronts benevolent ageism. Taken together, these findings are consistent with research on confronting prejudice in general, whereby such confrontations can result in both positive and negative outcomes. Although older adults may reaffirm their competence through rejecting benevolent ageism they will also face the social cost of being viewed as less warm.

### THE IMPACT OF COGNITIVE BEHAVIORAL THERAPY ON RELIEVING NURSES' AGEISM TOWARD OLDER ADULTS

Mohammad Rababa, *Jordan University of Science and Technology, Irbid, Irbid, Jordan*

Background: Previous research has approved the effectiveness of cognitive-behavioural therapy (CBT) in managing some psychological problems. However, no previous has examined its impact on nurses’ death anxiety and ageism.

Methods: A randomized controlled trial was conducted on a total of 110 nurses who were randomly assigned to the study groups to test the effectiveness of CBT to reduce nurses’ death anxiety and ageism. The intervention was comprised of multiple training sessions delivered in five modules with the incorporation of several CBT exercises which are guided by the principles of Terror Management Theory.

Results: Overall, using CBT revealed a significant improvement in nurses’ level of death anxiety and ageism among the intervention group compared to the control group. CBT nurses had lower levels of ageism and death anxiety than those in the control group.

Conclusions: the use of CBT is very effective in relieving nurses’ death anxiety and ageism. The findings of the study have proved CBT is an innovative and creative psychological intervention that improves nurses’ psychological well-being and relieves stress and anxiety experiences associated with caring for older adults such as death anxiety which is the precursor of ageism. Future studies are recommended to examine the impact of CBT on other types of prejudices, including sexism and racism among nurses.Relevance to clinical practice: These findings could contribute to improved quality of life and healthcare provided to older adults. CBT could be a crucial component of the ongoing training program for gerontological nurses to target their ageism.

### THE RELATIONSHIP OF AGEISM, INTENTION TO WORK WITH OLDER ADULTS, AND SOCIAL DESIRABILITY

Maria MacLean, and Jessica Strong, *University of Prince Edward Island, Charlottetown, Prince Edward Island, Canada*

Previous research demonstrates: 1) men and younger adults have higher negative ageism scores than women and older adults 2) higher scores of negative ageism are associated with lower intention to work with older adults and 3) women and older adults have higher scores for social desirability. It remains unclear how these factors interact. University students (N=547) aged 16 - 59 (Mean = 20.6) completed a survey measuring positive and negative attitudes towards older adults, intention to work with older adults, and social desirability. ANOVAs found a significant effects in negative ageism based on age, $F(1, 3) = 6.69$, $p = 0.01$, $\omega2 = 0.01$, and gender, $F(1, 3) = 11.43$, $p = 0.001$, $\omega2 = 0.02$, with a small effect size, but no significant interaction between age and gender. Young adults (M = 22.3) and males (M = 21.5) demonstrated more negative ageism than middle aged adults (M = 23.1) and females (M = 22.7) (lower scores indicate negative attitudes). An ANOVA of gender x age x social desirability was also significant for negative ageism, $F(11) = 2.00$, $p = 0.03$. However, there were no significant effects or interactions for gender or age on positive ageism and intention to work with older adults, or when social desirability was added. Although there were differences between demographic and social desirability groups for negative ageism, this relationship was not found for positive ageism. We expected social desirability to play a role in ageism, but this was not the case in the current sample.

### PERCEIVED WORKPLACE AGEISM AND OLDER WORKERS DURING THE GREAT RECESSION

Duygu Basaran Sahin, *The Graduate Center & CUNY Institute for Demographic Research, Brooklyn, New York, United States*

Age discrimination claims filed with the U.S. Equal Employment Opportunity Commission increased sharply at the beginning of the Great Recession (GR) and stayed elevated. Moreover, despite having lower unemployment rates compared to younger workers, older workers' unemployment lasted longer. Previous studies suggest that age discrimination might have extended the unemployment duration for older workers. However, no study has examined the role of perceived workplace ageism in older workers’ employment outcomes during the GR. This paper uses longitudinal data from the Health and Retirement Study to answer the following question: Were older workers who reported to perceive workplace ageism more likely to be unemployed or quit the labor force during the Great Recession? The baseline sample consists of workers aged 51 or older in 2006 (N=4,176). Multinomial logistic regression results show that older workers who agreed that in decisions about promotion, their employer gives younger people preference over older people, were more likely (RRR 2.05, $p=0.01$) to lose employment in 2008 than being employed, controlling for age, gender, race, education, health, marital status and job characteristics. Older workers who agreed that their co-workers make older workers feel that they have to retire before age 65, were more likely (RRR 1.63, $p=0.002$) to quit the labor force in 2008 than being employed, controlling for the aforementioned variables. These findings shine light on the important role that perceived workplace ageism played in older workers' employment outcomes during the GR.

### DEVELOPMENT AND VALIDATION OF THE FAMILIAL AGEISM SCALE

Ruth Walker[1], Emily Kinkade[2], and Alexandra Zelin[1], *1. The University of Tennessee at Chattanooga, Chattanooga, Tennessee, United States, 2. North Dakota State University, Fargo, North Dakota, United States*

Researchers have found a tie between family relationships and the physical and mental health outcomes of older adults (Kelly et al., 2017; Silverstein & Giarrusso, 2010). Researchers have also established the negative impact of ageism on the physical and mental health of older adults (Chang et al., 2020; Lyons et al., 2018). However, studies studying the impact of ageism perpetuated by family members is relatively unexplored. This may be due to a lack of measures to assess ageism perpetuated by family members specifically. To address this gap, the goal of the current study was to design a tool to measure experiences with familial ageism. Scale items were created based on the review and analysis of qualitative data on this topic (Walker & Kinkade, in prep). Items were reviewed by scholars for content and clarity. Two studies were then conducted to develop and validate our measure to assess experiences with ageism within the family context. In the first study, 151 participants completed the familial ageism items. The factor structure, convergent and divergent validity of the items were examined. We found evidence of strong psychometric properties for five related, but unique, factors in the retained items: Benevolent Ageism, Technological Competency, Out of Touch, Aging Appearance, and General Competence. Construct, convergent, and divergent validity was supported with small to large correlations with scales measuring general ageism, depression, and ego integrity. A second study with 282 participants confirmed the five-factor structure. Implications and recommendations for scale utilization will be discussed.

### CULTURAL VARIATION OF AGE DIFFERENCES IN DEVELOPMENTAL TRANSITIONS AND ATTITUDES

William Chopik[1], Alejandro Carrillo[1], and Hannah Giasson[2], *1. Michigan State University, East Lansing, Michigan, United States, 2. Arizona State University, Tempe, Arizona, United States*

The age-group dissociation effect posits that, as negative age stereotypes become more salient, older adults will psychologically distance themselves from their own age group. This phenomenon is also seen in various attitudes about development and older adulthood, such as how relatively old or young individuals feel and the point at which older adulthood starts. Previous research has shown that people push older adulthood further into the future with every year of life—placing it perpetually on the horizon. However, most research on age attitudes and age-group dissociation have been conducted within one culture, and variation across settings is rarely examined. A sample of 1,007,956 participants ranging in age from 10 to 89 (M = 27.45, SD = 12.45; 67.1% women) from 13 different countries completed attitudinal measures about aging and developmental transitions. Across all countries and attitudinal measures, we replicated the age-group dissociation effect ($\beta s > .36$). However, culture moderated many of the effects. For example, relative to the U.S., Chinese and Korean participants reported a younger age at which older adulthood started but showed a more dramatic age-group dissociation effect. In contrast, other countries reached an asymptote where older adults did not push the transition into the future as dramatically as middle-aged adults. Relative to the U.S. (and controlling for age), most countries showed a younger subjective age, and countries varied according to cultural values. The current project sheds light on how age-related attitudes and perceptual processes vary (and don't) across cultural contexts.

### AGEISM IN ARTIFICIAL INTELLIGENCE: A REVIEW

Charlene Chu[1], Kathleen Leslie[2], Shehroz Khan[3], Rune Nyrup[4], and Amanda Grenier[1], *1. University of Toronto, Toronto, Ontario, Canada, 2. Athabasca University, Athabasca, Alberta, Canada, 3. KITE-Toronto Rehab, Toronto, Ontario, Canada, 4. University of Cambridge, Cambridge, England, United Kingdom*

Background: Artificial intelligence (AI) has emerged as a major driver of technological development in the 21st century, yet little attention has been paid to algorithmic biases towards older adults. "Digital ageism" is a new form of ageism that is embedded into technology and AI systems. Aim: This review aimed to explore how age-related bias is encoded in AI systems to better understand digital ageism.

Methods: The scoping review follows a six-stage methodology framework developed by Arksey and O'Malley. The search strategy has been established in six databases and we will investigate grey literature databases, targeted websites, popular search engines. An iterative search strategy was used. Studies meet the inclusion criteria if they are in English, peer-reviewed, available electronically in full-text, and included the concepts 'bias' and old age. At least two reviewers independently conducted title/abstract screening and full-text screening.

Results: Our database searches resulted in 7 595 manuscripts that underwent title and abstract screening. Of these 49 papers, were included in the study. The word "ageism" was explicitly mentioned only in about half of these papers. Approximately half the papers mentioned how age-related bias could be encoded into AI systems. The most commonly used AI applicaiton was computer vision.

Conclusions: Our preliminary findings contribute foundational knowledge about the age-related biases that were encoded or amplified in AI systems. This work advances how AI can be developed in a manner consistent with ethical values and human rights legislation, particularly as it relates to an older and aging population.

### SESSION 6641 (POSTER)

### DISEASES, AGING, AND GEROSCIENCE

### KNEE OA AS A SYSTEMIC MODEL OF AGING

Dennis Lox, *Clearwater Fl, Clearwater, Florida, United States*

Knee osteoarthritis (OA) and aging, may be viewed differently by various patients and health care providers. Aging is now considered as a systemic interplay of molecular, cellular, tissue, hormonal, and body dysfunctions. Important drivers of these factors lead to osteoarthritis, coronary artery disease, cancer and dementia. An understanding of each as its progresses can alter morbidity. The proposed theories of aging hold true for knee OA. A simple joint compartment model, such as the knee, may help this understanding. A degenerative knee OA joint is a progressive disorder, just like aging. Knee OA may be accelerated by trauma, aging, decreased autophagy, cellular changes, cytokine production, disruption of the matrix, and cellular senescence. The associated SASP leads to the progressive cascade of degenerative changes. Understanding how to mitigate these affects provides a framework for a longer healthspan. Obesity, and it's adipokines exacerbate the inflammation, and compound weigh bearing stress. Promotion of longevity pathways, stimulate repair via Sirt, AMPK, FOXO and decreased stimulation of MTor, and FOXO4- P53 coupling. The resulting changes can altering the nature of the accelerated nature of progressive knee OA. Joint conservation, unregulated IL-4, IL-10, IRAP, and down regulation of NF-kB, and the ensuing cascade of HMBG1, and DAMP. Hippocrates wrote walking is man's best medicine, however, with knee OA this may need amendment, to encourage proper medical guidance for appropriate exercise and diet. It is time to merge knee OA and aging models to work in harmony for prolonged health span.

### STABILIZING ROLE OF HCN CHANNELS ON POST-CAMP MECHANISMS OF DETRUSOR MYOCYTE CONTROL

Ramalakshmi Ramasamy[1], Alya AlObaidi[2], and Phillip Smith[1], *1. UConn Health, Farmington, Connecticut, United States, 2. U Hart, Hartford, Connecticut, United States*

Aging is associated with an increased incidence of co-morbidities, including detrusor underactivity (DU). DU is defined as the failure to create sufficient and durable expulsive force to adequately empty the urinary bladder during a normal voiding timespan. DU is prevalent in older adults, as evidenced by its prevalence in nearly two-thirds of nursing home residents. Current treatments are mostly palliative or come with many side effects. β-adrenoceptor-mediated relaxation is the primary mechanism of detrusor relaxation, and Hyperpolarization-activated Cyclic Nucleotide-gated (HCN) channels have previously been identified by us and others as a very important mediator of this relaxation, however the role of HCN in detrusor relaxation has not been elucidated. Hence, we seek to characterize its role in adrenergic relaxation mechanisms and spontaneous myocyte activity. Male and female 10–12-month-old C57Bl/6 mice were used for this study. Pharmacomyography studies were performed to assess the effect of different drugs that act at various steps along the adrenergic relaxation pathway, +/- CsCl, an HCN blockade at [5mM]. As expected, we saw that increasing HCN opening probability by isoproterenol or forskolin (adenylyl cyclase/cAMP-agonist) or lamotrigine (HCN-activator) resulted in decreases in tonic tension, but were diminished in the presence of CsCl. Mechanisms modulated by H89 (PKA-inhibitor) and NS1619 (BK-channel-agonist) show no change in tonic tension, however spontaneous phasic activity significantly increases. These data support increased cAMP, not hyperpolarization, as the key inductor of HCN in adrenergic relaxation.

### THE ROLE OF PLATELET ENERGY METABOLISM IN PAIN AFTER WALKING IN BLACK ADULTS WITH KNEE OSTEOARTHRITIS

Jennifer Klinedinst[1], Weiliang Huang[2], Maureen Kane[3], Gary Fiskum[4], Apruva Borcar[4], Parisa Rangghran[4], and Susan Dorsey[5], *1. University of Maryland School of Nursing, Baltimore, Maryland, United States, 2. School of Pharmacy, Baltimore, Maryland, United States, 3. University of Maryland Baltimore, Baltimore, Maryland, United States, 4. University of Maryland School of Medicine, Baltimore, Maryland, United States, 5. University Of Maryland School of Nursing, Baltimore, Maryland, United States*

Nearly 30% of adults aged 60 or older suffer from knee osteoarthritis (KOA) that causes significant pain and disability. Walking is considered a "gold standard" treatment option for reducing KOA pain and maintaining joint mobility. However, pragmatic trials have shown that walking increases pain for some and relieves pain for others. The mechanism by which walking is helpful for KOA pain is unclear. The purpose of this study was to gain a better understanding of the mechanisms underlying walking for knee pain. We conducted a pre-test/post-test study using quantitative sensory testing to measure pressure pain sensitivity at the knee and examined protein signatures and measured energy metabolism in platelets in six adults with KOA before and after six weeks of walking three days/week at 100 steps/minute. All participants identified as Black/African American, five were female, average age 57±5.8. Pressure pain sensitivity increased for three participants and decreased for three participants. Protein signatures among KOA participants indicated differences in immune and energy metabolism pathways. Proteins in the energy metabolism pathways were significantly downregulated after walking in participants whose pain increased compared to participants whose pain decreased. Platelet energy metabolism was also lower among participants whose pain increased as compared to participants whose pain decreased. One goal of developing individualized interventions for KOA pain is to elucidate the mechanisms by which self-management interventions impact pain. The addition of therapies that target cellular energy metabolism may lower pain with walking among Black adults with KOA.

### COMMON NONCARDIOVASCULAR MULTIMORBIDITY PATTERNS AND OUTCOMES IN OLDER ADULTS WITH MAJOR CARDIOVASCULAR DISEASE

Stephanie Denise Sison[1], Joshua K Lin[2], Mehdi Najafzadeh[2], Elisabetta Patorno[3], Lily G Bessette[4], Heidi Zakoul[2], and Dae Kim[5], *1. Beth Israel Deaconess Medical Center, Brookline, Massachusetts, United States, 2. Brigham and Women's Hospital, Boston, Massachusetts, United States, 3. Brigham and Women's Hospital, Harvard Medical School, Boston, Massachusetts, United States, 4. Department of Medicine, Boston, Massachusetts, United States, 5. Hebrew SeniorLife, Boston, Massachusetts, United States*

Non-cardiovascular multimorbidity often coexists in older adults with cardiovascular disease (CVD), but their clinical significance is uncertain. Using Medicare data, we examined common non-cardiovascular comorbidity patterns and their association with clinical outcomes in beneficiaries with congestive heart failure (CHF), acute myocardial infarction (AMI), or atrial fibrillation (AF). We created 3 CVD cohorts of beneficiaries diagnosed with CHF (n=57,285), AMI (n=24,808), and AF (n=36,277) before January 1, 2016. Within each cohort, we applied latent class analysis to classify beneficiaries based on 7 non-cardiovascular comorbidities, including dementia, cancer, chronic kidney disease (CKD), chronic lung disease, depression, diabetes, and musculoskeletal disease. Mortality, cardiovascular and non-cardiovascular hospitalization, and home time lost until December 31, 2016 were compared across non-cardiovascular multimorbidity classes. Similar non-cardiovascular multimorbidity classes emerged from 3 CVD cohorts: 1) minimal, 2) depression (or lung-depression in AMI cohort), 3) diabetes-CKD, and 4) multi-system class. Compared to minimal class, multi-system class had the highest risk of mortality (hazard ratio [HR], 3.0 to 3.7), cardiovascular hospitalization (HR, 1.6 to 3.4) and non-cardiovascular hospitalization (HR, 2.8 to 6.0), and home time lost (rate ratio, 2.8 to 4.8), followed by lung-depression/depression or diabetes-CKD classes. In CHF and AF cohorts, multimorbidity classes were associated with greater increase in non-cardiovascular hospitalizations than cardiovascular hospitalizations. Our findings emphasize that improving health of older adults with CVD requires attention to non-cardiovascular multimorbidity.

### ELDERLY MOUSE DETRUSOR MAINTAINS ITS PEAK FORCE OF CONTRACTION OVER TIME

Nadav Mortman[1], Ramalakshmi Ramasamy[2], and Phillip Smith[2], *1. University of Connecticut School of Medicine, FARMINGTON, Connecticut, United States, 2. UConn Health, Farmington, Connecticut, United States*

Aging is a risk factor for urinary dysfunction. Detrusor weakening has been thought to contribute to the old bladder phenotype. Animal detrusor strips have been used to study bladder function. Strength of contraction in detrusor strips depends on length. This is referred to as the "length-tension relationship." The aim of our study was to investigate this relationship in a mouse model by comparing the length-tension relationship in old and mature mice. We hypothesize that aging is associated with no change in active detrusor tension capability, however maximal tension will occur at longer lengths.We used two groups of male C57/Bl6 mice for this study, mature 11-12 month old mice and old 22-23 month old mice. Longitudinal intact bladder strips were harvested and placed in a vertical tissue bath between tension recording transducer hooks. Passive tensions and KCl induced contraction tensions for step-wise increments of stretch were observed.In the old group, normalized strip length at the point of maximum active tension was increased by ~13% on average as compared to the mature group with a statistically significant difference (P value 0.0171). Interestingly, the maximum active tension between groups did not differ by age. In conclusion, detrusor from old mice achieve similar maximum active tensions as that from mature mice, however at an increased length. This finding argues against a common belief that the bladder weakens with age. Instead, the aging bladder may adapt to increased filling volumes with an ability to operate at a similar strength of a younger bladder.

### A ROUTINE BLOOD PARAMETER BIOLOGICAL AGE COMPOSITE IS ASSOCIATED WITH FRAILTY IN THE BERLIN AGING STUDY II (BASE-II)

Ilja Demuth[1], Valentin Vetter[2], Johanna Drewelies[3], Sandra Duezel[4], Dominik Spira[2], Elisabeth Steinhagen-Thiessen[2], Lars Bertram[5], and Denis Gerstorf[6], *1. Charité Universitätsmedizin Berlin, Berlin, Berlin, Germany, 2. Charité - Universitätsmedizin Berlin, Berlin, Berlin, Germany, 3. Humboldt University Berlin, Berlin, Berlin, Germany, 4. Max-Planck-Insitute for human developement, Berlin, Berlin, Germany, 5. University of Lübeck, Lübeck, Schleswig-Holstein, Germany, 6. Humboldt Universität zu Berlin, Berlin, Berlin, Germany*

High-quality biomarkers are needed to predict age-related phenotypes and evaluate health-related interventions. Telomere length has been known as a biomarker of aging for some time, however, its potential for clinical use is limited. Epigenetic age estimators and biological age (bioage) composites have been developed and refined over the past decade and their evaluation is ongoing. Aim of the current study was to further evaluate a recently developed bioage composite based on 12 routine laboratory blood parameters which have been shown to individually predict mortality risk. To this end, we assessed the relationship between the bioage composite, frailty and a number of functional assessments. The bioage composite was calculated for participants of the Berlin Aging Study II (BASE-II) aged ≥ 60 years (N=1,278, mean age 68.6 ±3.7 years, 53.3% women). Frailty was operationalized by Fried's five-point frailty score. Functional capacity was assessed with a battery of tests including the Tinetti mobility test, finger-floor distance (FFD), Mini-Mental State Examination (MMSE), Center for Epidemiologic Studies Depression Scale (CES-D), activities of daily living (ADL) and instrumented ADL (IADL). In covariate adjusted regression analyses the deviation of the bioage composite from chronological age was significantly associated with frailty (beta = -0.014, p= 0.014), the CES-D and ADL in men, and with the FFD in women. These results will be discussed with respect to their clinical relevance and compared to previously reported BASE-II data on the relationship between frailty, functional assessments and the biomarkers of aging derived from epigenetic clock algorithms and telomere length.

### REDUCED LEVELS OF NAD IN SKELETAL MUSCLE IN PEOPLE AGING WITH HIV INFECTION ON TREATMENT

Monty Montano[1], Karol Pencina[1], Michael Schultz[1], Thanh Tran[2], Zhuoying Li[1], Catherine Ghattas[3], Jackson Lau[4], and David Sinclair[1], *1. Harvard Medical School, Boston, Massachusetts, United States, 2. Boston University, Boston, Massachusetts, United States, 3. Brigham and Women's Hospitial, Boston, Massachusetts, United States, 4. Brigham and Women's Hospital, Boston, Massachusetts, United States*

People living with HIV (PLWH) are disproportionately burdened with multimorbidity and decline in physiologic function compared to their uninfected counterparts, but biological mechanisms that differentially contribute to the decline in muscle function in PLWH compared to uninfected people remains understudied. The study site was Brigham and Women's Hospital, Harvard Medical School, Boston MA. We evaluated skeletal muscle tissue for levels of total NAD, NAD+ and NADH in middle-aged asymptomatic PLWH, coinfected with hepatitis C virus (HCV) and/or cytomegalovirus (CMV) and compared them to uninfected control participants. Of the 54 persons with muscle biopsy data, the mean age 57 years with 33% women. Total NAD levels declined in skeletal muscle in association with HIV infection, and was exacerbated by HCV and CMV coinfection, with lowest levels of total NAD, NAD+ and NADH among persons that were coinfected with all three viruses (P=0.015, P=0.014, P=0.076; respectively). Levels of total NAD, NAD+ and NADH in skeletal muscle were inversely associated with inflammation (P=0.014, P=0.013, P=0.055; respectively). Coinfections were also associated with measures of inflammation (CD4/CD8 ratio: P< 0.001 and sCD163: P< 0.001), immune activation (CD38 and HLA-DR expression on CD8 T cells: P< 0.001). Additionally, coinfection was associated with increased physiologic frailty, based on the VACS Index 1.0 assessment (P=0.001). Further research is warranted to determine the clinical relevance of preclinical deficits in NAD metabolites in skeletal muscle in association with viral coinfection and inflammation, as well as the observed association between viral coinfection and physiologic frailty.

### OLDER PEOPLE LIVING WITH DEGENERATIVE SPINE DISEASE WHO UNDERGO SPINE SURGERY—TRYING TO BE NORMAL

Andrea Strayer[1], and Barbara King[2], *1. University of Iowa College of Nursing, Iowa City, Iowa, United States, 2. University of Wisconsin-Madison School of Nursing, Madison, Wisconsin, United States*

Lumbar degenerative spine disease (DSD), a consequence of aging, occurs globally in 266 million persons annually. As the population ages, the number of spinal surgeries will increase. The purpose was to investigate older peoples' understandings of living with and having surgery for DSD and the process they engage in to return to normal. Grounded theory (GT) was used to guide this study. Fourteen older people (≥ 65yrs) were recruited for 2 in-depth interviews (audio-recorded/transcribed verbatim) at 2-time-points: T1 during hospitalization and T2, 1-3-months post-discharge. All 14 interviewed at T1; 10-T2 (1mo); 2-T2 (2mo); 2-T2 (3mo) post-discharge (N=28 interviews). Consistent with GT, purposive and theoretical sampling was used. Data analysis (interdisciplinary team) included open, axial, and selective coding. A conceptual model was developed illustrating the phases older persons with DSD go through in their trajectory of trying to return to normal. Three key categories were identified (1) Losing Me (2) Fixing Me and (3) Recovering Me. All described a prolonged process of losing functional independence and being able to socialize. Fixing Me was proving they needed surgery and preparing for surgery. Recovering Me involved monitoring and ongoing progress. Conditions, including setbacks and delays, slowed recovery. Throughout, participants had to continually adjust their expectations. The conceptual model details how older people engage in living with and undergoing DSD surgery. Our model can serve as the foundation for developing interventions to guide older patient education programs, improve care transitions and develop patient-centered approaches for treating older people with DSD and spine surgery.

### OBESITY MAY UNDERLIE SKELETAL MUSCLE AUTOIMMUNITY IN OLDER ADULTS

Tan Zhang[1], Xin Feng[1], Juan Dong[1], Kristen Beavers[2], Stephen Kritchevsky[1], and barbara Nicklas[1], *1. Wake Forest School of Medicine, Winston-Salem, North Carolina, United States, 2. Wake Forest University, Winston-Salem, North Carolina, United States*

A mechanistic understanding of the biological basis for sarcopenia could lead to novel biomarkers and intervention targets. We recently reported a novel role of autoimmunity in sarcopenia, which is potentially mediated through cardiac troponin T (cTnT) and its autoantibody. Given the known role of obesity in both autoimmunity and sarcopenia, we further explored the association between circulating cTnT or skeletal muscle IgG and obesity in older adults. We found that, in 37 older adults (NCT01049698, BMI: 30.8 ± 2.3 kg/m²; Age: 69 ± 3.5 yrs) without any cardiac abnormality, serum cTnT was negatively correlated with skeletal muscle volume ($r = -0.37$, $p = 0.02$) and leg extension strength ($r = -0.38$, $p = 0.02$), but positively correlated with body fat/muscle volume ratio ($r = 0.37$, $p = 0.03$). In two other studies including older adults (n = 11 & 12) from a subset of subjects (NCT01049698 & NCT01298817), skeletal muscle IgG1 (GAPDH normalized level by immunoblot) was found to be positively associated with BMI or dual-energy X-ray absorptiometry derived regional fat measurements, including fat mass, abdominal subcutaneous fat, abdominal fat, or thigh fat. We also found that subjects with higher skeletal muscle IgG1 protein levels have significantly higher BMI but lower knee extensor strength. Our finding suggests that obesity may underlie increased circulating cTnT and skeletal muscle IgG infiltration. It also warrants additional investigation into its role in skeletal muscle autoimmunity in older adults.

### SESSION 6650 (POSTER)

### CHRONIC DISEASE MANAGEMENT

### CHRONIC CONDITION DISCORDANCE AND ALCOHOL USE AMONG OLDER COUPLES: THE MODERATING EFFECTS OF MARITAL QUALITY

Courtney Polenick, Angela Turkelson, James Cranford, Frederic Blow, and Kira Birditt, *University of Michigan, Ann Arbor, Michigan, United States*

Chronic condition discordance (i.e., the extent that two or more conditions have non-overlapping self-management requirements that heighten self-management complexity) may be stressful and contribute to increased alcohol use over time among older adults, especially for those with greater marital stress. In this study, we examined how chronic condition discordance at the individual level (i.e., within individuals) and the couple level (i.e., between spouses) predicted alcohol use across an 8-year period, and whether these links varied by negative marital quality. The U.S. sample included 1,116 wives (M = 54 years) and husbands (M = 57 years) from five waves (2006-2014) of the Health and Retirement Study. Dyadic growth curve models controlled for age, minority status, education, and own and partner time-varying number of chronic health conditions and depressive symptoms. Wives with greater individual-level discordance reported fewer initial drinks per week. Marital quality moderated the links between chronic condition discordance and alcohol use over time. Notably, when husbands reported lower negative marital quality and had greater individual-level chronic condition discordance, they had faster decreases in their own drinks per week over time. By contrast, when husbands reported higher negative marital quality and had greater individual-level discordance, their wives had faster increases in drinks per week over time. Overall, these findings indicate that better quality marriages may protect against increased alcohol use for both wives and husbands in the context of greater chronic condition discordance among husbands.

### APPLYING STRESS AND COPING THEORY TO UNDERSTAND DIURETIC ADHERENCE EXPERIENCES IN THOSE WITH HEART FAILURE

Rebecca Meraz[1], Kathryn Osteen[2], Jocelyn McGee[3], Paul Noblitt[4], and Henry Viejo[5], *1. Baylor University, Dallas, Texas, United States, 2. Baylor University Louise Herrington School of Nursing, Dallas, Texas, United States, 3. Baylor University, Waco, Texas, United States, 4. Baylor Scott & White Medical Center at Irving, Irving, Texas, United States, 5. Baylor Scott & White Heart and Vascular Hospital-Fort Worth, Fort Worth, Texas, United States*

Heart failure (HF) is a chronic and costly condition, affecting at least 6 million Americans. The ventricular dysfunction associated with HF results in fluid volume overload, which is commonly managed with diuretics. Despite the benefits of diuretics, some individuals with HF report bothersome and stressful side effects and intentionally do not adhere. Positive intrinsic motivators, such as positive emotions, may benefit individuals in their adaptation to medication-related stress. However, there has been limited study of these potential motivators in older adults with HF. The purpose of this descriptive qualitative study was to understand intrinsic factors underlying diuretic adherence in adults with HF using the transaction theory of stress and coping. Using a descriptive qualitative approach, 82 adults who were hospitalized for HF and prescribed home diuretics were interviewed. The findings of the current study revealed the ways participants appraised diuretics and how they overcame the perceived medication effects so they could adhere to the medication. Data analysis revealed three themes: (1) diuretics are bothersome (2) staying positive in the midst of hardship, and (3) adapting to endure. Adherent participants stayed positive amid the perceived medication-related hardship, maintaining resilient and grateful attitudes. Adherent participants adapted to bothersome diuretic effects and utilized creative strategies for adherence. More research is needed to understand the relationships between resilience, adaptive coping, and adherence to diuretics.

### STRUCTURAL EQUATION MODELING OF HEALTH-RELATED QUALITY OF LIFE AMONG PEOPLE WITH VISUAL IMPAIRMENT

Eunjin Yang[1], and Sun Ju Chang[2], *1. Yonsei University, Seoul, Seoul-t'ukpyolsi, Republic of Korea, 2. Seoul National University, Seoul, Seoul-t'ukpyolsi, Republic of Korea*

Visual impairment can lead to limited accessibility and activities and a decreased quality of life. This study aimed to test a hypothetical model based on the International Classification of Functioning, Disability, and Health to predict the health-related quality of life (HRQOL) of people with visual impairment. Data from 202 people with visual impairment were collected from August 2020 to November 2020 via telephone or e-mail. The overall fitness indices of the model for physical HRQOL (Normed $\chi 2$=2.076, GFI=.878, TLI=.900, CFI=.928, RMSEA=.073) and the model for mental HRQOL (Normed $\chi 2$=2.232, GFI=.884, TLI=.904, CFI=.932, RMSEA=.078) complied with the recommended criteria of structural equation model fitness, indicating that the models were appropriate for predicting the HRQOL of people with visual impairment. The socioeconomic status, multimorbidity, perceived environmental barriers, and activity limitation/participation restriction directly affected the participants' physical HRQOL. Moreover, depression, disability identity, and activity limitation/participation restriction directly affected participants' mental HRQOL. Age, socioeconomic status, depression, social support, and perceived environmental barriers affected the participants' physical and mental HRQOL by means of activity limitation/participation restriction. The findings of the study suggest that the strategies aimed at increasing the activity/participation of people with visual impairment will be helpful in improving their HRQOL. Based on the results of this study, nursing intervention programs and services for people with visual impairment should be developed and implemented to provide more comprehensive care for this group and expand nursing science.

### CHRONIC BACK PAIN, LIMITATIONS ON USUAL ACTIVITIES, AND TREATMENT AMONG OLDER BRAZILIANS

Flavia Andrade[1], and Xiayu Chen[2], *1. University of Illinois--Urbana-Champaign, Urbana, Illinois, United States, 2. University of Illinois at Urbana-Champaign, Urbana, Illinois, United States*

Chronic back pain is prevalent among Brazilian older adults, leading to substantial social and healthcare costs. It also disproportionately affects low-income and less-healthy people. Using the latest Brazil's National Health Survey (PNS-2019), multivariate logistic regressions were conducted to examine how biopsychosocial factors correlate with chronic back pain, limitations on usual activities, and pain treatment (N=17,184). PNS-2019 data showed that 31.3% (95% CI 30.1-32.5) of older adults ages 65 to 112 reported back pain. Chronic back pain was positively associated with being female, having more chronic conditions, depressive symptoms, and lower education. Among those who reported having back pain, 19.2% reported that pain limited their usual activities. Higher odds of having limitations were found among older adults with three or more chronic conditions, obesity, and depressive symptoms. However, it was lower among those with higher levels of alcohol consumption. Among older adults with back pain, 69.2% received some treatment for their pain - 24.1% exercised regularly, 14.3% received physical therapy, 48.1% medications/injections, 6.1% alternative methods, and 30.7% regularly visited health professionals. Treatment was higher among women and those with higher education. The results show significant disparities in the prevalence, limitations, and treatment of chronic back pain among older adults in Brazil. The findings point to the need for prevention and treatment programs for older Brazilians with lower socioeconomic and worse health conditions.

### INDIVIDUAL FACTORS AFFECTING GLYCATED HEMOGLOBIN LEVELS AMONG OLDER ADULTS WITH DIABETES: A CROSS-SECTIONAL STUDY

Jungkyung Min[1], and Idethia Harvey[2], *1. Texas A&M University, College Station, Texas, United States, 2. University of Missouri, Columbia, Missouri, United States*

Introduction: Many older adults fail to maintain glycemic control even though reasonable glucose control can prevent the onset of macro- and microvascular complications. This present study aimed to determine the independent association of demographic characteristics, clinical factors, treatment modalities, and lifestyle behaviors with glycated hemoglobin levels (HbA1c) among Korean older adults, a group at a very high risk of diabetes.

Methods: We analyzed data from a representative sample of participants (N = 707) aged > 65 years in the Korea National Health and Nutrition Examination Survey (KNHANES), 2016-2018. Participants were classified into two groups according to their age [group 1 (ages 65 to 74) and group 2 (age ≥75)]. Data were analyzed using t-test, analysis of variance, Pearson's correlation, and hierarchical multiple regression.

Results: Age (p=0.003), place of residence (p=0.01), treatment modality (p< 0.001), sedentary behavior (p=0.04), and moderate activity (p=0.002) significantly affected HbA1c levels. In comparison to the factors in two age groups, duration of diabetes was the variable more strongly associated with HbA1c levels (p< 0.001, β=0.283) in group 1, whereas treatment modality was the one strongly associated with HbA1c levels (p< 0.001, β=0.249) in group 2.

Conclusion: It is essential to consider the age duration of diabetes, and biochemical factors to manage HbA1c levels in older adults. Furthermore, our findings suggest including physical activity in the educational program to provide information for diabetes management. In the clinical setting, more close attention should be taken among healthcare providers to control the glucose level for older adults with diabetes.

### CHRONIC CONDITION SELF-MANAGEMENT FOR OLDER AFRICAN AMERICANS: THE CHALLENGE OF COMPLEXITY

Heather Fritz, and Malcolm Cutchin, *Pacific Northwest University of Health Sciences, Yakima, Washington, United States*

Older African Americans have higher rates of comorbidity and face substantive challenges with chronic condition self-management (CCSM), including less effective and supportive CCSM care than their white counterparts. Such disparities in care stem, in part, from physicians' lack of understanding about challenges older African Americans face as they engage in CCSM. Yet little is known about the CCSM experiences of older African Americans with comorbidity. In this study, we aimed to determine the central dynamics of those experiences. As part of a larger study on African American CCSM and physician empathy, we conducted in-depth qualitative interviews with 30 older African Americans living in Detroit aged 65 years and older with comorbidity. We used grounded theory analysis to distill findings into a core conceptual category as well as component domains and dimensions. "Complexity" emerged as the core category to describe CCSM in our sample. Complexity resides often at the intersection of race, age, and social position making the older African American CCSM experience inherently difficult for those of different races, ages, and social positions to understand. Data illustrate that domains of complexity include "care time", "care roles", "social context", "logistics", "learning", and "pain management". Each domain is composed of 2 to 4 dimensions, further fleshing out the nature of CCSM complexity for older African Americans with comorbidity. Findings provide a basis to enhance understanding and empathy for older African Americans with comorbidity. We discuss how the model will be used to test understanding of older African American CCSM by medical students.

### PHYSICAL FUNCTIONING AND BODY SIZE IN MID-LIFE WOMEN: THE STUDY OF WOMEN'S HEALTH ACROSS THE NATION

Minsuk Oh[1], Kelly Ylitalo[2], Aleda Leis[3], Brittney Lange-Maia[4], Elsa Strotmeyer[5], Jane Cauley[6], and Carrie Karvonen-Gutierriez[3], *1. Baylor University, Woodway, Texas, United States, 2. Baylor University, Waco, Texas, United States, 3. University of Michigan, Ann Arbor, Michigan, United States, 4. Rush University, Chicago, Illinois, United States, 5. School of Public Health, University of Pittsburgh, Pittsburgh, Pennsylvania, United States, 6. University of Pittsburgh, Pittsburgh, Pennsylvania, United States*

To evaluate patterns of age-related changes in physical functioning (PF) and associations with body size, we utilized data from the longitudinal Study of Women's Health Across the Nation (SWAN). Participants (n=1,793) with self-reported SF-36 PF data at visit 4 (2000-01; mean age:50.0 years ±2.7), visit 15 (2016-17), plus one additional visit were included. Body weight and waist circumference were measured at each visit; change from visits 4 to 15 was calculated. Five PF trajectories were identified using latent class growth modeling (% of women): (1) persistently low (4.1%); (2) start moderate and improve slightly (5.4%); (3) start high and decline slightly (24.0%); (4) persistently high (59.5%); and (5) start high and decline substantially (7.0%). Participants with persistently low PF (Group 1) lost weight during follow-up (mean change: -3.2% body weight) whereas there was little change in all other groups (ANOVA p< 0.0001). Women with persistently low PF (Group 1) had < 2% increase in waist circumference over follow-up; all other groups had more than double that increase (range: 4.1%-6.1%; ANOVA p=0.002). No statistically significant differences in anthropometry changes across the PF groups were observed after adjustment for sociodemographic and time-variant health characteristics. PF trajectories may be associated with changes in body weight or waist circumference during midlife and transition to older adulthood; however, these associations may be explained by other major health-related variables. Future research is needed to understand the complex interplay between PF and body size, particularly for women at-risk for late-life disability that may benefit from preventive efforts in mid-life.

### THE FIT OF THE HAPA MODEL TO THE EXPERIENCE OF EXERCISING AFTER STROKE: A DEDUCTIVE CONTENT ANALYSIS

Amy Silva-Smith, *University of Colorado at Colorado Springs, Colorado Springs, Colorado, United States*

Sustained physical activity is recommended for secondary stroke prevention. Persons with stroke leave rehabilitation having learned exercises to reduce disability. However, once discharged, people may be on their own to navigate psychological, emotional, social, and physical challenges of maintaining those activities and starting new ones. The Health Action Process Approach (HAPA) provides a framework for understanding how self-efficacy differs depending on where a person is in the process of engaging in physical activity. The purpose of this study was to assess the qualitative fit of the HAPA model to the experience of exercise after discharge from formal stroke rehabilitation using a deductive (directed) content analysis approach. Interviews with 12 stroke survivors were analyzed deductively using the HAPA model concepts task self-efficacy, coping self-efficacy, and recovery self-efficacy to create the analysis matrix. In this sample, a period of psychological adjustment interfered with maintaining exercise and included anxiety, depression, embarrassment, and fear of falling that affected motivation and intention to exercise. Experiences with physical activity and exercise as a child and routines prior to the stroke were factors influencing task, coping, and recovery self-efficacy and ease of dealing with interruptions in exercise, including the discharge from formal rehabilitation. The findings support the qualitative fit of the HAPA model with the experience of exercise after having a stroke. A HAPA model framed intervention is being developed to support the transition from formal rehabilitation support to living in the community.

### COMPARING OLDER ADULTS' EXPOSURE TO AND SHARING OF HEALTH-RELATED MESSAGES ON FACEBOOK BY CHRONIC CONDITION STATUS

Matthew Schroeder[1], Carrie Miller[2], and Sherry Pagoto[3], *1. Regenstrief Institute, Inc., Indianapolis, Indiana, United States, 2. VCU Fuemmeler Lab, Richmond, Virginia, United States, 3. University of Connecticut, Storrs, Connecticut, United States*

We compared older adult Facebook users with and without a chronic health condition on their frequency of exposure to and posting health-related messages. Demographics, social media use, and chronic condition status were collected via survey. Regular Facebook users aged 50+ years were recruited via Qualtrics. Participants reported if they had seen, posted, or shared: health-related information; about others'/their own health behaviors (e.g., exercise); and about others'/their own chronic condition. Responses were dichotomized as "Rarely" or "At least once a month". Six logistic regression models, controlling for demographics and Facebook login frequency, assessed whether viewing and/or posting health-related messages differed by chronic condition status. Respondents (N=697; 77.9% female; 87.9% non-Hispanic White) were on average 61.2 years old (SD=7.9). One-half reported a chronic condition (n=351; 50.4%). In adjusted models, those with a chronic condition had a higher likelihood of seeing posts from others with health information (OR=1.37; 95% CI: 1.01, 1.86) and about others' health conditions (OR=1.64; 95% CI: 1.20, 2.23) ≥ monthly (vs no chronic conditions). Similarly, those with a chronic condition had a higher likelihood of posting or sharing health information (OR=1.52; 95% CI: 1.03, 2.24) and about their chronic condition (OR=1.93; 95% CI: 1.16, 3.21) ≥ monthly. People with and without chronic conditions did not differ in how often they saw or posted about health behaviors. Older adults with chronic conditions were more likely than those without chronic conditions to regularly see and share health information on Facebook. The content and accuracy of this health information should be explored.

### INTEGRATING TELEHEALTH AND COMMUNITY HEALTH WORKERS TO ENHANCE QUALITY CARE ACCESS: A NARRATIVE REVIEW

Ilana Engel[1], Alice Zhang[2], Robert Stiles[2], Eve-Lynn Nelson[2], and Amber Watts[1], *1. University of Kansas, Lawrence, Kansas, United States, 2. University of Kansas Medical Center, Kansas City, Kansas, United States*

Community Health Workers (CHWs) often share cultural, geographic, or other lived experiences with patients and provide health education and support. Use of CHWs and telehealth approaches are promising strategies for addressing the needs of patients with metabolic syndrome (MetS). This narrative review analyzed how these approaches were integrated into programs expanding care access for patients with MetS. Searching PubMed, PSYCInfo, Embase, Web of Science, and Google Scholar resulted in 1,630+ abstracts screened and 12 articles meeting inclusion criteria. These studies examined implementation of tele-mentoring approaches (n=4), patient group classes via videoconferencing (n=2), or individual telehealth consultations facilitated by CHWs (n=7), with some programs including multiple intervention types. This review included adults ranging from 37-79 years old. Most studies focused on late mid-life (ages 50-64). Because health behaviors in midlife have important implications for MetS and related health concerns in later life, it is important to consider midlife interventions. Using the RE-AIM framework, we evaluated studies on five dimensions: reach, effectiveness, adoption, implementation, and maintenance. Reach and implementation indicators suggest reducing barriers to engagement (e.g., home visits) allows for higher participation and program completion rates. Measures of MetS-related behavioral outcomes were heterogeneous across study designs, making overall effectiveness difficult to determine. Adjusting time spent with patients according to health literacy and clinical needs is a strategy CHW programs use to provide equitable, cost-effective care. Programmatic considerations for implementing programs that include both CHWs and telehealth are discussed, with special consideration for what works in late middle age and in older adulthood.

### SESSION 6670 (POSTER)

### CIVIC ENGAGEMENT

### EXPOSURE TO GENERATIVE MESSAGES BOOSTS COGNITIVE PERFORMANCE IN OLDER ADULTS

Tara Gruenewald, Natalie Standridge, and Clarissa Tadros, *Chapman University, Orange, California, United States*

Both self-perceived and primed generativity has been found to be linked to better cognitive and physical functioning, psychological well-being, and longevity in older adulthood. A prior experiment also found that priming older individuals with messages regarding the generative, or contributory value of their social group (older adults) boosted cognitive performance compared to exposure to a message about the societal burden of one's group (Hagood & Gruenewald, 2018) . A key limitation of this earlier work was the lack of a neutral prime condition to determine whether being exposed to messages regarding the generativity of older adults enhanced their cognitive performance or being exposed to negative messages regarding the social burdens of one's age group suppressed performance. This limitation was addressed in the current online experiment of 300 U.S. adults age 55 and older who were randomly exposed to either a social generativity, social burden, or neutral prime presented as a test of reading comprehension and recall. Participants completed other measures of cognitive ability before and after the priming task, including an assessment of verbal memory. An ANCOVA model including age as a covariate indicated a significant effect of priming condition ($F(2,296) = 3.30$, $p = .038$). Mean verbal memory performance did not vary between the neutral and social burden priming conditions, while performance was significantly higher in the generativity prime condition ($d=.29$). Experimental findings provide support for the hypothesis that exposure to generative messages about one's social group can boost cognitive performance in a national sample of older adults.

### THE EFFECT OF STRUCTURAL AND FUNCTIONAL SOCIAL SUPPORT OF OLDER KOREAN VOLUNTEERS ON LIFE SATISFACTION

Meeryoung Kim, *Daegu University, Daegu, Kyongsang-bukto, Republic of Korea*

As well as helping others, senior volunteers, like other seniors, need their own social support. However, unlike general older adults, older volunteers can build many social networks through volunteering. This study aims to examine how structural and functional social support of older volunteers affects their life satisfaction.This study used the sixth additional wave (2016) and the seventh wave (2017) of the Korean Retirement and Income Study. The subjects of this study were volunteers who are aged 60 and older, and the sample size was 241. Multiple regression was used for data analysis. Demographic variables were controlled. As for independent variables, structural and functional social support were used. For the dependent variable, life satisfaction was used. Structural social support was measured by the number of related subjects. For functional social support, Cohen and Syme (1985)'s social support scale, which includes instrumental, emotional, informational, and evaluative support, was used.According to the results of the study, structural support did not significantly affect the life satisfaction of older volunteers, but functional support had a significant effect on life satisfaction. Structural support was not significant because volunteers had many networks through volunteering. However, the functional social support of receiving practical help significantly increased the life satisfaction of the volunteers. These findings imply that having someone who can help them is an important factor in increasing life satisfaction.

### THE ROLE OF EDUCATION AND LITERACY SKILLS ON MIDDLE-AGED AND OLDER VOLUNTEERS BY RACE AND ETHNICITY IN THE US

Donnette Narine[1], Takashi Yamashita[2], Wonmai Punksungka[3], Abigail Helsinger[4], Jenna Kramer[5], Rita Karam[6], and Phyllis Cummins[7], *1. University of Maryland, Baltimore, Baltimore, Maryland, United States, 2. University of Maryland, Baltimore County, Baltimore, Maryland, United States, 3. University of Maryland - Baltimore County, Baltimore, Maryland, United States, 4. Miami University, Oxford, Ohio, United States, 5. RAND Corporation, Arlington, Virginia, United States, 6. RAND Corporation, Santa Monica, California, United States, 7. Miami University, Oxford, Ohio, United States*

Volunteer participation is a form of civic engagement that benefits both the individual and society over the life course. Although education, basic skills (e.g., literacy), and race/ethnicity are individually associated with volunteering, detailed interrelations are yet to be explored. Guided by the integrated theory of volunteer work and the notion of productive aging, the goal of this study was to examine the roles of education and adult literacy in the context of volunteering in later life across racial and ethnic groups (Whites, Blacks, Hispanics) in the U.S. Using the nationally representative sample of middle-aged and older adults (age 45+; $n = 3,770$) from the 2012/2014/2017 Program for International Assessment of Adult Competencies (PIAAC), structural equation modeling was constructed to evaluate mediation relationships among education, literacy, and volunteering by racial and ethnic groups. Results show no statistically significant mediation (a.k.a., indirect) effect of education on volunteering through literacy, nor was there statistically significant difference in the mediation effect across racial and ethnic groups. However, there were statistically significant differences in the direct effect of education on volunteering between Black adults and White adults [b(Black) = 0.44 versus b(White = 0.24), $p < 0.05$], as well as Black adults and Hispanic adults [b(Black) = 0.44 versus b(Hispanic) = 0.08, $p < 0.05$]. These findings indicate that higher education was more strongly associated with volunteering among older Black adults, compared to White and Hispanic counterparts. Suggested policy implications include support for the promotion of volunteer participation through culturally and socioeconomically sensitive approaches.

### TYPES OF CULTURAL TOURISM PARTICIPATION AMONG KOREAN BABY BOOMERS

Kyungmin Kim[1], Bon Kim[2], Hwasung Song[3], Jeffrey Burr[4], and Gyounghae Han[1], *1. Seoul National University, Seoul, Seoul-t'ukpyolsi, Republic of Korea, 2. Jeju National University, Seoul, Gyeonggi, Republic of Korea, 3. Suwon*

*Research Institute, Suwon-si, Kyonggi-do, Republic of Korea, 4. University of Massachusetts Boston, Boston, Massachusetts, United States*

Cultural tourism (e.g., attending festivals, visiting museums and heritage sites) is an important part of leisure activity in middle and later life. As they age, midlife adults may anticipate more time for these activities when their family and work demands are less intense. However, the leisure-as-career perspective suggests that preferences, knowledge and skills for leisure and tourism activities develop throughout the life course. This study examines how Korean middle-aged adults participated in cultural tourism activities and whether their willingness to be involved in active leisure in later life differed by their current patterns of cultural tourism activities. We utilized data from the 2014 Korean Baby Boomer Panel Study (N = 4,053; age 51–60). Latent class analyses were applied to five types of activities (i.e., local festivals and events, exhibitions, museums, heritage sites, and international travels), and three distinct patterns were identified. The majority of the Korean baby boomer sample (81%) belonged to "Inactive cultural visitors"—not participating in any type of activities in the past two years, which may reflect persistent time pressures at midlife. "Casual cultural visitors" (11%) mainly visited local festivals and heritage sites. "Serious cultural visitors" (7%) engaged in all types of activities; they appeared to have more time and financial resources. Further, "serious cultural visitors" showed higher willingness to engage in active later life leisure compared to other two patterns, supporting the continuity of leisure/tourism activities. Our findings highlight the importance of prior leisure and tourism behaviors for understanding future leisure activity expectations.

### SESSION 6680 (POSTER)

### COGNITION AND AGING

### RETURNED MISSING PERSONS WITH DEMENTIA: WHAT ROLE CAN FIRST RESPONDERS AND SERVICE PROVIDERS PLAY?

Christine Daum[1], Lauren McLennan[1], Elyse Letts[1], Cathy Conway[2], and Lili Liu[1], *1. University of Waterloo, Waterloo, Ontario, Canada, 2. University of Waterloo, Toronto, Ontario, Canada*

The number of people living with dementia that wander and go missing is increasing. First responders and service providers play a role in the return of a missing person living with dementia. In the United Kingdom (UK), "return home interviews" are discussions between police and returned missing persons that offer support to the returned missing person to prevent repeat incidents. This study aims to explore and understand the role of first responders and service providers who follow-up with returned missing persons living with dementia. Eight service providers (e.g., social workers) and seven first responders (e.g., police officers) from Canada and the UK participated in online semi-structured interviews. Data were concurrently collected and analyzed using conventional content analysis. In the UK, police conduct "return home interviews" within 72 hours of the missing person's return. Some charities conduct interviews with vulnerable populations to prevent repeat missing incidents by understanding the circumstances of the missing incident and connecting the person to community supports. In Canada, although follow-up with returned missing persons is not routine, some police units offer support to returned missing older adults. Government and community support organizations also offer supports to returned missing older adults such as referrals for in-home support, technologies, and vulnerable person registries. Service providers and first responders have an important role to play in the prevention of repeat missing incidents. Findings will contribute to the development of a Canadian practice guide for conducting interviews with returned missing persons living with dementia.

### FAMILY SUPPORT AS A MEDIATOR OF COGNITIVE FUNCTIONING AMONG HISPANIC OLDER ADULTS WITH SENSORY IMPAIRMENTS

Corinna Trujillo Tanner[1], Jeremy Yorgason[1], Avalon White[1], Joshua Ehrlich[2], and Antonia Cash[1], *1. Brigham Young University, Provo, Utah, United States, 2. University of Michigan, Ann Arbor, Michigan, United States*

Background: Understanding the intersection of age, ethnicity, and disability will become increasingly important as the U.S. population ages and becomes more diverse. By 2060, Hispanics, the largest ethnic minority, will comprise 28% of the population. Although a heterogeneous group, originating from a variety of countries, Hispanics may share common cultural values. These familistic values may act as a buffer against social isolation and cognitive decline, commonly associated with sensory disabilities including vision, hearing, and dual sensory disabilities.

Methods: Our sample consisted of 557 Hispanic older adults that participated in the National Health and Aging Trends Study. Longitudinal mediation models across a three-year span were estimated using Mplus with vision, hearing, and dual sensory disabilities predicting cognitive decline directly and indirectly through social isolation. Bootstrapping with 5,000 draws adjusted the standard errors of indirect effects. Results and Discussion: Results suggest that vision disability and dual sensory disability were associated with declines in various cognitive functioning scores. Social isolation was linked with declines in some concurrent and some longitudinal cognitive measures. Although dual sensory disability (i.e., both vision and hearing disability) were linked with social isolation, individual vision or individual hearing disability were not associated with social isolation in this sample. Historically, vision and hearing disabilities have been associated with social isolation, yet in Hispanic cultures, social connections between generations may provide a buffer to this common result of sensory disabilities. Findings from this research suggest that Hispanic older adults experiencing dual sensory disabilities may benefit from interventions that foster social support.

### COGNITIVE DECLINE PUBLIC HEALTH SURVEILLANCE: EXPERT-GUIDED COLLABORATION FOR BRFSS MODULE REVISION

John Shean[1], and Benjamin Olivari[2], *1. Alzheimer's Association, Chicago, Illinois, United States, 2. CDC, Atlanta, Georgia, United States*

The Behavioral Risk Factor Surveillance System (BRFSS) Cognitive Decline Module collects population level data on subjective cognitive decline (SCD) — self-reported difficulties in thinking or memory — which can be one of the earliest warning signs of dementia. Originally devised in 2009, the module undergoes periodic revision to ensure it remains current, the data collected are actionable, and to encourage uptake of the module by state health agencies. In 2021, the Alzheimer's Association and the Centers for Disease Control and Prevention's Alzheimer's Disease Program formed an expert workgroup to examine the ongoing value and relevance of the module and propose any improvements. Workgroup members included researchers, chronic disease directors, epidemiologists, survey methodologists, policy analysts, and BRFSS coordinators. Using a consensus-building process, the workgroup utilized pre-meeting worksheets to identify areas of agreement and disagreement which informed key debates during subsequent meetings. Among many factors, the workgroup assessed the existing module for accuracy and utility. Discussion centered on aligning language and concepts contained within the module's questions with current scientific research on SCD and how to accurately and adequately assess the associated burden SCD imposes. People living with cognitive impairment were consulted during the revision process to ensure phrasing reflected their lived experience. The result was a consensus set of suggested revisions to the Cognitive Decline Module, submitted to CDC for review and approval. The robust process, involving a variety of stakeholders and perspectives, can serve as an efficient and effective model for ensuring the longevity and usefulness of population health surveillance.

### THE EFFECTS OF LIFETIME EXPERIENCES OF HOMELESSNESS AND INCARCERATION ON COGNITIVE AGING

Douglas Hanes, and Sean Clousten, *Stony Brook University, Stony Brook, New York, United States*

Alzheimer's disease and related dementias (ADRD) remain a pressing health concern in the U.S., and this burden and access to care are unequally distributed across the population. The U.S. has one of the highest incarceration rates globally, which is also unequally distributed; and groups like veterans, formerly incarcerated people, and sexual and gender minorities (SGM) face high risk of homelessness. Homelessness and incarceration are potentially traumatic experiences in themselves. Both experiences are more likely among people with less formal education, less economic security, and racialized groups, even as they reduce educational and economic opportunities. Using data from the Health and Retirement Study (HRS; 1998–2016), we investigate whether self-reported lifetime experiences of homelessness and incarceration (including time spent incarcerated) are associated with cognitive functioning and risk of possible dementia. Multilevel modeling adjusted for age, education, and other demographic covariates revealed that lifetime experiences of homelessness and incarceration are associated with lower cognition (Homelessness: $\beta$=-1.231, p< .001; Incarceration: $\beta$=-0.929; p < .001), but slower aging-related declines (Homelessness-slopes: $\beta$=0.044, p<.001; Incarceration-slope $\beta$=0.041; p<.001), and homelessness moderated the impact of prior incarceration ($\beta$=-1.789; p<.001), but less-steep declines ($\beta$=0.092; p <.001). Homelessness and incarceration, independent of their other risk factors and associated harms, have associations with ADRD risk.

### CHILDHOOD SOCIOECONOMIC POSITIONING AND LATE-LIFE COGNITIVE FUNCTIONING: A CRITICAL REVIEW

Hanamori Skoblow, and Christine Proulx, *University of Missouri, Columbia, Missouri, United States*

The predictors of cognitive functioning are varied and complex. Gerontologists are increasingly interested in the long arm of childhood, suggesting that late-life cognitive functioning may be partly the result of influences across the life span. Research shows that childhood economic hardship is associated with disparities in cognitive functioning in older adulthood. Framed by the life course perspective, we reviewed 27 articles that examine associations between childhood socioeconomic positioning (SEP), commonly assessed via parents' educational attainment, and late-life cognitive functioning in 11 different US datasets. The influence of childhood SEP on cognitive functioning is stronger when cognitive functioning is assessed at a single time point rather than as change over time, suggesting that childhood SEP might not affect the rate at which cognition declines in later life, but does impact where decline begins. The majority of research supported the pathway hypothesis, suggesting that childhood SEP's influence on adult cognitive functioning works primarily through the mechanism of adults' own educational attainment and SEP. Several studies support the accumulation of (dis)advantage hypothesis and point to the compensatory potential of upwards social mobility. Support for the latency model, which posits that early-life economic hardship results in enduring outcomes that influence cognitive functioning in older adulthood, above and beyond one's adult SEP, is present, although weaker than the other hypotheses. Implications include strengthening policies that relieve economic strain and promote educational access among families with young children and also among young adults, as addressing the precursors of cognitive functioning in tomorrow's older adults is paramount.

### RELATIONSHIP BETWEEN PHYSICAL AND MENTAL HEALTH WITH SUBJECTIVE COGNITIVE DECLINE AMONG OLDER ADULTS IN PUERTO RICO

Thomas Buckley, *University of Pittsburgh, Pittsburgh, Pennsylvania, United States*

Worse physical and mental health are risk factors for cognitive decline in older adults. In Puerto Rico, existing healthcare services are lacking, further exacerbating this risk. This study examines how mental and physical health factors affect subjective cognitive decline for older adults in Puerto Rico. Data comes from the 2020 Behavioral Risk Factor Surveillance System, restricted to adults age 60+ residing in Puerto Rico (n = 1603). Subjective cognitive decline was measured with two dichotomous variables (no/yes): increases in confusion or memory loss and difficulty making decisions in the past year. Multivariate logistic regression models were run for each outcome variables. Predictor variables were number of days in past month with poor mental health, diagnosis of depression or mood disorder, self-rated health, and access to healthcare services, along with covariates. Higher number of days with poor mental health (B = 2.44, p < .001), diagnosis of depression or mood disorder (B = 3.76, p < .001), and cost barriers to accessing healthcare (B = 2.90, p = .002) were associated with increased odds of increased confusion or memory loss. Significant predictors of increased odds of decision-making difficulty included higher number of days of poor mental health (B = 1.88, p < .001), diagnosis of depression or mood disorder (B = 3.80, p < .001), and worse self-rated health (B = 2.13, p < .001). To promote better cognitive health, intervention efforts should focus on those with poor mental and physical health, including identify strategies to improve access to healthcare services.

### IMMIGRATION EXPERIENCES AND COGNITIVE TRAJECTORIES AMONG OLDER CHINESE IMMIGRANTS

Fengyan Tang[1], Ke Li[1], Mary Rauktis[1], and Iris Chi[2], *1. University of Pittsburgh, Pittsburgh, Pennsylvania, United States, 2. University of Southern California, Los Angeles, California, United States*

Some studies have documented cognitive health among older immigrants in the United States; however, little is known about how the life-course immigration experiences were associated with cognitive trajectories among older Chinese immigrants. This study filled the research gap by identifying the patterns of cognitive change trajectory among older Chinese immigrants and examining the associations of immigration experiences (that is, age at migration, reasons for migration, acculturation, perceived discrimination, and preferred dialects) with cognitive trajectories. The sample comprised 2,075 participants from the Population Study of Chinese Elderly (PINE), who completed a battery of cognitive tests at four time points (2011-2019). Latent class growth analysis and multinomial logistic regression were utilized. Three latent classes of cognitive trajectories were identified: the low functioning with the fastest decline (LCF, 12%), the moderate functioning with a medium decline rate (MCF, 39%), and the high functioning with the slowest decline (HCF, 48%). Perceiving more discrimination reduced, while speaking Taishanese increased the odds of being in the LCF and MCF. High acculturation only distinguished MCF from HCF after controlling for the known factors of cognition such as age, education, and social engagement. This study identifies a group of older Chinese immigrants who are especially vulnerable to cognitive impairment, and indicates that the immigration-related risks for cognitive decline, such as late-life migration and lower acculturation, could be buffered by education in early life and social engagement post immigration. Practice and policy efforts are needed to increase socioeconomic and cultural opportunities for social integration among older immigrants.

### EXPLORING THE EFFICACY OF MOCA SCORE CORRECTIONS IN REDUCING THE INFLUENCE OF RACE/ETHNICITY

Rachel McCray[1], Omonigho Bubu[2], Judite Blanc[3], Azizi Seixas[3], Girardin Jean-Louis[3], and Arlener Turner[3], *1. New York University, New York City, New York, United States, 2. NYU Grossman School of Medicine, New York, New York, United States, 3. University of Miami, Miami, Florida, United States*

As studies have highlighted significant differences in test score distributions between ethnicities, we chose to examine if the MoCA corrections for education curb racial differences. Therefore, we use data from the NIA Alzheimer's Disease Research Center (ADRC) program to explore the efficacy of score corrections in reducing the influence of race/ethnicity. This study utilized the NACC dataset to analyze data covering UDS visits from September 2005 to February 2021. Participants included in the analyses (n= 11987, 64.9% women, 12.3 % Black/African American, mean age 73□9.460; 16□4.98 years of education) were all cognitively normal. The analyses uses the Montreal Cognitive Assessment (MoCA) with and without correction for education (addition of one point for less than 12 years of education), via cut off score derived cognitive status categories. A 2x3 contingency table revealed a statistically significant association between participants' race (black vs white) and performance on the uncorrected MoCA, X22,n=5291=188.971, p<.001, and the corrected MoCA X22,n=5282=167.073, p<.001. Additionally, a One-way ANCOVA analysis comparing the correlation of education and uncorrected MoCA score for Black/African American (r=.425, p<.001) and White participants (r=.198, p<.001) shows a significant difference between the two groups F1,5288=167.992, p<.001. Specifically, in Black/African American participants, the correlation is much stronger suggesting that years of education is a greater determinant of cognitive status. These results demonstrate that regardless of controlling for education via adding buffer points significant racial disparities in global cognition scores were still present. Alternative corrections for race and education should be considered for future test adaptations.

### STRUCTURAL DISCRIMINATORY FACTORS RELATED TO COGNITIVE FUNCTIONING AMONG OLDER HISPANICS: RESULTS FROM HRS

Ernest Gonzales[1], and Cliff Whetung[2], *1. New York University, New York, New York, United States, 2. NYU Silver School of Social Work, New York, New York, United States*

A growing body of research reveals how interpersonal and structural discriminatory factors undermine cognitive functioning among older Black and White adults, yet few studies have focused on older Hispanics in the United States. We examined cognitive health trajectories among 1,513 Hispanic men and women in the Health and Retirement Study (2008-2020) with mixed-effects regression. The majority (71%) of older Hispanics reported less than a college education. This older Hispanic sample often attributed everyday and major discrimination to nativity, race, age, and gender. In multivariate analyses, education, marital status, total household assets, mental health (depression), physical activity (regular vigorous exercise), and multi-morbidity (a weighted index) were all associated with cognitive functioning and in the expected directions. Surprisingly, cognitive functioning did not vary by gender. Living in neighborhoods characterized by physical disorder and social cohesion by respondents, were associated with cognitive functioning at the bivariate level, but only neighborhood physical disorder held association at the multivariate level. Perceived everyday and major discriminatory experiences were not associated with total cognition at the bivariate or multi-variate levels. Results from this study suggest interventions that target access to higher education, as well as chronic health conditions and neighborhood factors, can optimize cognitive functioning. Stratified analyses and interaction effects are needed to tease out the diversity of older Hispanic adults by nativity, acculturation, interpersonal discrimination, and cognitive health.

### SESSION 6690 (POSTER)

### DISABILITY AND HEALTH CARE

### FOLLOWING PHYSICIAN'S ADVICE IN LATE LIFE: THE ROLES OF LOCUS OF CONTROL AND HEALTH STATUS

Poshan Dahal[1], and Eva Kahana[2], *1. Case Western Reserve University, Shaker Heights, Ohio, United States, 2. Case Western Reserve University, Cleveland, Ohio, United States*

Adherence to physicians' recommendations plays an important role in health maintenance in old age (Kravitz, Hays, Sherbourne & DiMatteo,1993). However insufficient attention has been paid to the influence of patients' mental and physical health and locus of control on adherence to physician's recommendations. This paper studied subjective health appraisals, chronic illnesses, functional limitations, and cognitive impairment along with internal or external locus of control as influences on adherence to physicians' recommendations. We explored these associations among 684 older adults (mean age = 80.71 years) living in a retirement community in Florida. Respondents participated in the Elderly Care Research Center successful aging study (Kahana, Kelley-Moore & Kahana 2012). Older patients and those with more functional limitations reported greater adherence to physicians' recommendations ($b=.15$, $p< 0.05$). Those respondents with greater cognitive impairment were less adherent ($b=-.1$, $p< 0.05$). Internal locus of control, reflected in beliefs that patients play a major role in health outcomes was associated with less adherence ($b=-0.1$, $p< 0.05$). The belief that health professionals influence health outcomes was associated with greater adherence ($b=.1$, $< 0.05$). Lower adherence was also associated with the stronger belief that health is a matter of good fortune ($b=-.06$, $p< 0.05$). Our findings offer useful insights about the role of internal vs. external influences on health outcome expectations in late life and health behaviors of compliance with medical regimens.

### CHANGES TO CARE PROVISIONS TO OLDER ADULTS DURING COVID-19: IMPLICATIONS FOR CAREGIVER WELL-BEING

Esther Friedman[1], Allison Kirkegaard[2], and David Kennedy[2], *1. University of Michigan, Irvine, California, United States, 2. RAND, Santa Monica, California, United States*

Among the many consequences of the COVID-19 pandemic is its impact on caregiving for older adults. Pandemic-related physical distancing policies, avoidance of infection risk, and changing obligations at home and work has caused shifts in care that may not have occurred otherwise. We use nationally representative data on 2,363 caregivers to older family members and friends collected at the height of the pandemic to explore the extent to which family caregivers changed the amount and types of care they provided to a loved one with a disabling condition, and the impact of such changes on caregiver burden and wellbeing. About 41.5% of caregivers who continued to care for someone they helped prior to the pandemic, increased the amount of care provided, while only 7% reduced their intensity of help. Caregiving disruption in the amount of help – both increasing and decreasing care – was significantly associated with increased anxiety, depression, and loneliness of caregivers, but not with self-rated health. These findings persist even after controlling for a variety of sociodemographic, caregiving, and contextual characteristics, and the characteristics of support networks. This work points to an underexplored consequence of the pandemic: the impact on caregiving for older adults and caregiver burden. It will be important to explore the extent to which these effects persist as the pandemic runs its course.

### UNDERSTANDING OLDER ADULTS' FLU VACCINE HESITANCY: THE ROLE OF MEDICAL MISINFORMATION

Jessica Hsieh[1], Raza Mirza[2], James Hull[2], Siyu Peng[1], Muhammad Ibrar Mustafa[2], Carley Moore[2], Deirdre Kelly-Adams[2], and Christopher Klinger[1], *1. University of Toronto, Toronto, Ontario, Canada, 2. National Initiative for the Care of the Elderly, Toronto, Ontario, Canada*

Influenza persists as a common communicable disease and remains a significant cause of disease burden across the world. Despite preventative therapies, such as influenza vaccination to reduce its spread and transmission, influenza continues to be a source of morbidity and mortality, even in developed countries. For the population over the age of 65, the effects of influenza virus may be more severe when they are compounded by pre-existing conditions and reduced natural immune function.In light of plateauing vaccination rates, a scoping review was conducted to map the literature and determine why seniors aged 65 and above refuse or fail to receive seasonal influenza vaccination. Nine peer-reviewed academic databases covering both social sciences and medical research were searched, along with the grey literature. A total of 6562 references were identified; after the screening process, 118 references were included in the final review. Thematic analysis focused on the broad areas that positively or negatively influence older adults' decision-making regarding influenza vaccination, and this resulted in five main themes: (1) barriers to obtaining vaccination; (2) social factors; (3) personal characteristics; (4) individual subjectivity; and (5) direct clinical interventions.This review aims to identify gaps in knowledge and synthesize currently available information to make recommendations for future research, policy development and clinical practice. Increasing the vaccination rate among Canadian older adults will contribute to ongoing efforts to reduce the spread of the influenza virus among the population, reducing influenza-associated hospital admissions and deaths.

### ADVERSE CHILDHOOD EXPERIENCES (ACES) AND PARTICIPATION IN ROUTINE HEALTH SCREENING AMONG OLDER INDIVIDUALS

Ganesh Baniya[1], and Vijaya Tamla Rai[2], *1. University of Texas at San Antonio, San Antonio, Texas, United States,*

2. *University of Wisconsin - Milwaukee, Milwaukee, Wisconsin, United States*

Adverse Childhood Experiences (ACEs) are a collective term used to define traumatic events before the age of 18. Individuals with ACEs are at risk for various negative health behaviors and chronic diseases. Routine screenings help in early detection of the disease and minimize the impact of the disease, disability, early death, and higher medical costs. Research on ACEs focusing exclusively on older population has received inadequate attention. Additionally, it is not clear whether different types of childhood adversities impact older individuals to participate in routine health screenings. This study included 3997 individuals above 65 years from the 2015 Texas Behavioral Risk Factor Surveillance System (BRFSS) to examine the prevalence of ACEs and their health screening practices. The dependent variable was participating in routine health checkup in the past year and 8 ACEs were the independent variables where gender, age, race, and having a personal doctor were controlled. Logistic regression models showed that prevalence of four ACEs (Physical, sexual, and emotional abuses and parental divorce/separation) were likely to reduce older individuals' participation in routine checkup in the past year. Similarly, older individuals with personal doctor were twice more likely to visit for routine checkup compared to those without personal doctor. Lastly, older Black individuals were less likely to participate in routine checkup than their White counterparts. This study provided preliminary results to consider the impact of specific ACEs in routine health screening behaviors among older individuals. Future research might benefit from longitudinal study examining causal relationship between ACEs and health behaviors.

### PHYSICAL FRAILTY AS A PREDICTOR OF INCIDENT DISABILITY IN SPECIFIC-IADL ITEMS AMONG OLDER ADULTS

Jane Lee[1], Miji Kim[2], and Chang Won Won[2], *1. Yonsei University, Seoul, Seoul-t'ukpyolsi, Republic of Korea, 2. Kyung Hee University, Seoul, Seoul-t'ukpyolsi, Republic of Korea*

This study investigated the 2-year impact of physical frailty on disability (activities of daily living [ADL], instrumental ADL [IADL], and mobility) and mortality among community-dwelling older adults in Korea. We used data from 2,905 older adults aged 70–84 years who participated in the Korean Frailty and Aging Cohort Study (KFACS) at baseline (2016–2017) and Wave 2 (2018–2019) with all five components of Fried's physical frailty phenotype. Of these,277 (7.8 %) were frail and 1,312 (45.2 %) were robust. In the 2-year follow-up, multivariate analysis showed significant differences in frailty status for all disabilities (ADL, IADL, mobility) and mortality incidence. Both pre-frail (odds ratio [OR]=1.48, 95% confidence interval [CI]= 1.06–2.05) and frail (OR = 5.11, 95% CI=2.78–9.39) statuses showed increased risks of mobility disability. The incidence of ADL disability was significant only in frail older adults (OR =10.26, 95% CI=3.16–33.31). Both pre-frailty (OR =1.55, 95% CI=1.03–2.32) and frailty (OR =4.11, 95% CI=2.35–7.18) status were significantly associated with the incidence of IADL limitations. Frailty status was associated with disability in mobility-related items of IADL (going out, using transportation, and shopping) among men, and was associated with most IADL items among women after 2 years. The results of this study emphasize the need for sex-specific policies and frailty prevention programs, with a focus on detecting frailty before it leads to irreversible disability or other negative health outcomes. These findings may provide a framework for frailty prevention in community-dwelling older adults.

### WHAT MOTIVATES PHYSICIANS TO ADDRESS CAREGIVER NEEDS? THE ROLE OF EXPERIENTIAL SIMILARITY

Taeyoung Park[1], Karl Pillemer[1], Corinna Löckenhoff[1], and Catherine Riffin[2], *1. Cornell University, Ithaca, New York, United States, 2. Weill Cornell Medical College, New York, New York, United States*

Despite the negative emotional and physical consequences of caregiving, caregivers' needs and risks are often overlooked in health care settings. This study used survey data from a national random sample of primary care physicians (N=106) to examine the factors associated with physicians' perceived responsibility to identify caregiver needs and risks, focusing on three theoretically implicated variables: 1) experiential similarity (i.e., physicians having personal experience with caregiving), 2) structural similarity (i.e., physicians being older and female), and 3) secondary exposure to caregivers (i.e., more time seeing older patients and a higher percentage of older adults in their patient panel). Physicians in our sample consisted of 42.5% women; most of whom were white (73.6%). The majority (76.5%) agreed or strongly agreed that they were responsible for identifying caregivers' needs and risks. Multivariable models controlling for physicians' age and gender revealed that physicians who had personal experience with caregiving were four times more likely than those without caregiving experience to feel responsible to identify caregivers' needs and risks (adjusted odds ratio [aOR] 3.90; 95% confidence interval [CI] 1.34-11.41) and to assess caregivers' mental health concerns (aOR 3.58; 95% CI 1.29-9.94). Structural similarity and secondary exposure did not play significant roles in motivating physicians. Findings highlight the role of experiential similarity in physicians' motivation to assess caregivers' needs and risks. Future work may benefit from designing intervention programs for physicians that incorporate experiential learning activities (e.g., conversations in which caregivers share their experiences) and evaluating whether such programs enhance physicians' sensitivity toward family caregivers.

### SESSION 6700 (POSTER)

### EMPLOYMENT, DIRECT CARE WORKFORCE, AND RETIREMENT

### EXPERIENTIAL ASSESSMENT OF AN INNOVATIVE DEMENTIA CARE TRAINING SEQUENCE FOR HEALTH AND HUMAN SERVICES WORKERS

Susan Wehry[1], and David Wihry[2], *1. University of New England, Biddeford, Maine, United States, 2. University of Maine, Orono, Maine, United States*

This poster presents results of an assessment of an interdisciplinary dementia care training titled Dementia

Reconsidered (DR). In contrast to disease and deficit-focused training curricula, DR incorporates and emphasizes the strengths and humanity of people living with dementia and focuses on person-directed support for, and care of, persons living with dementia. For example, DR makes the case for an approach to shared and supported decision-making that optimizes the individual's— and significant others'—abilities. The on-line lectures were chunked to accommodate cognitive load and active learning; 20-30 second stretch breaks were interspersed to facilitate attention. This may account for the higher retention and utilization rates of DR than are often found in one-off trainings. A retrospective pre-post survey administered at the conclusion of DR sessions measured participant learning outcomes. Participants in DR sessions were representative of the fields of social work (42%), nursing (21%), and a significant "other" category (28%), mostly long-term care administrators. Among post-training survey respondents (N=36), 77% improved their knowledge of the key principles of person-directed dementia care; 73% (N=37) showed improved comfort in supporting residents with dementia during the CoVID-19 pandemic; 83% (N=24) showed improvement in ability to distinguish capacity and competency; and 83% (N=23) reported increased comfort in supporting people with dementia in making decisions in the face of diminished capacity. At six month follow-up, 63% (N=33) had used a skill gained from their training (17% no, 20% not sure). Implications for training in dementia care practices will be discussed.

### ORGANIZATIONAL PRACTICES FOR THE AGING WORKFORCE: A CROSS-CULTURAL VALIDATION OF THE LATER-LIFE WORKPLACE INDEX

Juergen Deller[1], Julia Finsel[2], Anne Wöhrmann[3], Max Wilckens[2], Xiuzhu Gu[4], and Eduardo Oliveira[5], *1. Leuphana University of Lueneburg, Lueneburg, Niedersachsen, Germany, 2. Leuphana University Lueneburg, Lüneburg, Niedersachsen, Germany, 3. BAuA, Dortmund, Nordrhein-Westfalen, Germany, 4. Tokyo Institute of Technology, Tokyo, Tokyo, Japan, 5. University of Porto, Porto, Porto, Portugal*

Successful employment of experienced employees becomes more important for both, individuals and organizations. To identify organizational practices that foster the motivation, health, and performance of experienced employees in particular, a holistic assessment of relevant organizational factors is needed. The Later Life Workplace Index (LLWI) provides such a measure for organizational practices for older employees by differentiating nine domains, namely organizational climate, leadership, work design, health management, individual development, knowledge management, transition to retirement, continued employment after retirement, and health and retirement coverage. So far, a German-language and an English-language version of the LLWI have been validated in Germany and the U.S in a multi-study procedure. The psychometric properties and measurement invariance of the English-language version of the LLWI will be presented. Preliminary findings from Japan and Portugal show promising results regarding reliability and validity of the LLWI in the respective country. The findings suggest that the multidimensional measurement model developed in Germany and the U.S. could be applicable to other regulatory and cultural contexts as well. A focus group consisting of the original authors of the LLWI and international scholars, whose research expertise lies in the field of employment and older employees, is currently developing a short version of the LLWI. We aim to provide researchers and practitioners from different countries with a validated measurement to holistically assess organizational practices. Researchers can utilize the LLWI to gain a comprehensive understanding of organizational influences on later life work, while practitioners are able to assess their organizational readiness for an aging workforce.

### PREPARING OLDER ADULTS FOR REMOTE EMPLOYMENT: OPPORTUNITIES AND CHALLENGES

Setarreh Massihzadegan[1], Shayna Gleason[2], Jan Mutchler[2], and Caitlin Coyle[2], *1. UMASS Boston, Cambridge, Massachusetts, United States, 2. University of Massachusetts Boston, Boston, Massachusetts, United States*

As Americans live longer, many are finding they need or want to remain longer in the workforce. When the COVID-19 pandemic transitioned much of the U.S. workforce into temporary or permanent remote employment, many older job seekers were left behind, wanting to compete in the ever-more technology-based job market but often without the requisite skills to do so. The present study evaluated a workforce training program (funded by a Department of Labor demonstration grant) that trained low-income workers over the age of 55 for remote employment. Approximately 60 older adults were trained across three 20-week cohorts. Our data sources included biweekly participant surveys, typing speed and Microsoft Office skill assessments, exit interviews with program "drop-outs," focus groups, training observations, data from participant applications, and instructor assessments of each participant's level of "job readiness" at the end of the program. Results revealed that participants had acute financial need for employment, a keen interest in working remotely, and a wide range of employment experiences and past job stability. Many of their career trajectories and workplace needs were affected by COVID-19. Results also showed promising improvements in participants' technology skills and confidence in their ability to conduct a job search over the course of the program. The need for greater connectivity between participants and employers was identified as an area for improvement for the program. The results of this study contribute to the literature on workforce development by exploring how training programs might better prepare older adults for an increasingly remote job market.

### LONGITUDINAL INVESTIGATION OF FACTORS PREDICTING RETIREMENT ADJUSTMENT AMONG RETIREES IN TAIWAN

Shiau-Fang Chao[1], Wei Ye[2], and Wei-Cheng Liu[3], *1. National Taiwan University, Taipei, Taipei, Taiwan (Republic of China), 2. Nation Taiwan University, Taipei, Taipei, Taiwan (Republic of China), 3. Taiwan Social Welfare League, Taipei, Taipei, Taiwan (Republic of China)*

BackgroundRetirement can substantially affect one's lifestyle and self-identity. However, little research has focused on how pre-retirement conditions are associated with retirement adjustment.MethodThis study utilized data from 1989 to 2015 Taiwan Longitudinal Study on Aging. A total of 1,471 cases who experienced retirement between waves and completed the subsequent four year and eight year follow-up surveys after retirement were included for analyses. High life satisfaction and low depressive symptoms represented good retirement adjustment. Multiple regression analysis was applied to test the hypothesized relationships. ResultsThe findings were as follows: 1. Retirees experienced increased depressive symptoms and worsened family relationships when transiting from pre-retirement to post-retirement.2. High cognition functioning, ideal family relationships and sufficient financial resources before retirement were consistently associated with few depressive symptoms and high life satisfaction, both at four and eight year follow-ups. 3. Of the 10 different leisure activities investigated, physically active leisure activities such as walking and participating group exercise before retirement predicted few depressive symptoms and high life satisfaction four years after retirement, but not eight year follow-up. DiscussionCognitive functioning, financial status, and family relationship before retirement can significantly affect both short-term (four years) and long-term (eight years) retirement adjustment, whereas physically active leisure activities only link to short-term retirement adjustment. At policy level, pre-retirement financial security should be addressed and well-prepared. At practitioner level, maintaining ideal cognition level, positive family relationship, and engaging physically active leisure activities should be the central intervention target when serving employees who are approaching retirement.

### DOES SOCIAL ENGAGEMENT FOSTER GENERATIVITY AND GOOD CITIZENSHIP AMONG THE THIRD AGERS IN JAPAN?

Keiko Katagiri, *Kobe University, Kobe, Hyogo, Japan*

The labor force rate of those in their sixties have been increasing in Japan. Active Theory assumes that continuing activity is good for their well-being. Several researches also elucidate positive effects of work on older adults. Further, Japan has a mandatory retirement age of 60 years; though, persons can restart their work under contract labor. Katagiri proposed a social engagement model for third agers, which assumes that work for third agers cannot be the purpose in life and suggests three activities social participation, civic activities and work. This study examines the effect of the three activities on third agers. Random sampling survey was conducted in 2019 on people living in Tokyo and Hyogo prefecture. The response rate was 43.0% (N = 665, aged 60-74). Hierarchical multiple regressions were conducted to examine the effect of the three activities on generativity, sense of community, social contribution orientation, interest in politics by gender, and the robust positive effects of social and civic activities for male and civic activities for female. Though no effect of work existed, interaction effects of work and other activities were observed. Those working with social or civic activities had higher generativity. Among people with civic activity, working males showed higher sense of community while working females showed higher interest in politics. Males engaged only in work showed no such sense. The results suggest engaging only in work do not foster their development and engagement in social and civic activities are required to enrich their lives.

### JOB SEPARATION OF LONG-TERM CARE FRONTLINE STAFF IN ENGLAND

Florin Vadean[1], and Eirini-Christina Saloniki[2], *1. University of Kent, Canterbury, England, United Kingdom, 2. University College London, London, England, United Kingdom*

Staff turnover in the long-term care (LTC) sector in England is perceived to be relatively high. Most job leavers do not leave the sector, but rather move to other LTC employers. Nevertheless, there are concerns that the high 'churn' has a negative impact on continuity and quality of care, care providers' recruitment and training costs, and the remaining staff workload and motivation. This study aimed to provide missing quantitative evidence on the drivers of LTC staff retention in England, with a focus on job quality. We used yearly data (2016 to 2019) from a large, employer-employee dataset covering about half of England's LTC market: the Adult Social Care Workforce Dataset. After controlling for observed individual, organisational, and local market characteristics as well as unobserved worker and employer heterogeneity, we found that, everything else being equal, wages and employment conditions (i.e. full time contracts and contracts with guaranteed working hours) significantly reduce job separation. For example, a 10 per cent wage increase from the sample mean would have reduced the job separation rate by about 3 ppt. Our results also show that the wage effect was substantially downward biased (i.e. closer to zero) when not accounting for unobserved effects: the estimated reduction in job separation rate of a 10 per cent wage increase was only 1 to 1.5 ppt. Our findings show that improving pay and employment conditions for LTC staff can improve staff retention in LTC and stress the importance of accounting for unobserved variable bias.

### NURSING HOME STAFF'S PERCEPTIONS AND EXPERIENCES COLLABORATING WITH HOSPICE

Susanny Beltran, Norma Conner, Adam Reres, and Morris Beato, *University of Central Florida, Orlando, Florida, United States*

Hospice has been associated with improved outcomes for terminally-ill patients and families, including in pain management, care satisfaction, and rates of hospitalizations. In 2016, 1/3 of Medicare hospice beneficiaries died in nursing homes (NH). The responsibilities for meeting the needs of the NH resident receiving hospice are shared by the NH staff and hospice team, making good communication and coordination of services between providers critical to the successful delivery of services. This exploratory study surveyed NH direct care and administrative staff about their perceptions of hospice, and barriers to collaboration. A total of 66 NH staff completed the online survey. The sample was 62.1% direct care staff (e.g., social work, nursing) and 37.8% administrators, predominately female (75%), non-Hispanic-white (43.9%), and employed full-time (87.8%). Over half of the NHs were non-profit organizations (56.1%). Respondents had on average 33.8 hours of hospice education and held positive hospice attitudes, with 76% strongly agreeing that hospice should be an option regardless of setting, and 87.8% believing they could collaborate with hospice toward the goal of a "good death." Although participants cited barriers to hospice related to COVID-19, this was not associated with overall hospice attitudes. Respondents cited resistance from family as the primary barrier to hospice, indicating lack of knowledge and a need for family education. They also cited concerns surrounding duplication of roles. Family members in previous studies have identified care allocation as a challenge, based on expectations that hospice would bring additional contact/services instead of replacing NH services. Implications will be discussed.

### INTERNATIONALLY EDUCATED NURSES CARING FOR OLDER ADULTS: A SCOPING REVIEW

SHERIF OLANREWAJU[1], Susan Loeb[2], and Manuel Rosaldo[2], *1. Penn State University, BOALSBURG, Pennsylvania, United States, 2. The Pennsylvania State University, State College, Pennsylvania, United States*

Internationally Educated Nurses (IENs) are nurses who are born and obtained their licenses in their home country and relocate to work in a different country. IENs are increasing being recruited to work in Western countries to address nursing shortages. Estimates indicate that IENs account for 5-8 percent of registered nurses in the United States (US). The purpose of this scoping review was to identify and synthesize research evidence on IENs' experiences caring for older adults. A 5-step process for Scoping Reviews was applied, which includes: identifying the research question; identifying relevant studies; study selection; charting the data; and collating, summarizing, and reporting the results. A search was conducted in Web of Science, PubMed, CINAHL, PsycINFO, and Google Scholar. Keywords included but were not limited to IENs, older adults, and long-term care setting. Inclusion criteria were (1) empirical studies examining IENs providing direct care for older adults in any healthcare settings and (2) original research published in English. A total of 13 articles were selected for inclusion (nine quantitative and four qualitative studies). The studies were conducted in the US (n=10), the Netherlands (n=1), Australia (n=1), and New Zealand (n=1). Results revealed three primary themes: transitional challenges, IENs' experiences working with older adults, and factors affecting IEN capacity to deliver services. Study findings are relevant to nursing leaders and policymakers in developing culturally relevant programs to help IENs transition successfully into the nursing workforce. Additional qualitative research is required to explore lived experiences of IENs caring for older adults.

### DIRECT CARE WORKFORCE IN THE US BEFORE AND DURING COVID-19

Christopher Kelly, Jerome Deichert, and Lyn Holley, *University of Nebraska at Omaha, Omaha, Nebraska, United States*

Purpose: This study describes the changes in the direct care workforce between 2019 and 2020. Design and

Methods: Data were from the 1% Public Use Microdata Sample (PUMS) of the 2019 and 2020 American Community Survey (ACS). Logistic regression was used to compare demographic and employment characteristics of direct care workers (DCWs) in 2019 and 2020.

Results: While the total number of DCWs increased by 0.08% and the number employed in home care increased by 3.45%, the number of DCWs employed in hospitals decreased by 3.73% and the number employed in residential care facilities decreased by 4.01% from 2019 to 2020. Compared to 2019, DCWs in 2020 were more likely to be nonmedical aides, under 25, over 65, non-white, to purchase health insurance directly, and to receive health insurance from Medicaid. DCWs in 2020 were less likely to be widowed, to be recently married, to be non-English speaking, to work year-round and full-time, to have moved in the last year, and to be below poverty than they were in the previous year. Implications: By the end of 2020, the early effects of the COVID-19 pandemic on the direct care workforce could be seen. While the total number of DCWs increased slightly, this was due to the accelerated growth of DCWs in home care, as the decline in the number of DCWs in hospitals and residential care facilities also accelerated. The direct care workforce in 2020 was more nonmedical, part-time, entry-level or exit-level and non-white than in the previous year.

### THE EFFECT OF EMPLOYMENT SUPPORT PROGRAM PARTICIPATION ON MID-LIFE AND OLDER ADULTS IN A STATE

Hyesu Yeo[1], and Steven Wilson[2], *1. University of Georgia, Athens, Georgia, United States, 2. echnical College System of Georgia, Atlanta, Georgia, United States*

BackgroundQuestions have been raised about the effectiveness of these government's efforts as older people aged 50 or older are affected by restrictions to participate in the labor market. This study examined the impact of the total programs under the Workforce Innovation and Opportunity Act in a state on older participants. MethodsThe study used the Technical College System of Georgia data between 2016 and 2020, with 11,390 participants aged 50 or over. The outcome of the program participation was employment status. The participants' characteristics consist of gender, race, age, earnings, and types of training they received. Bivariate analyses were performed to examine the relationship between the program's outcome and individuals' characteristics. ResultsIn 2016-2020, most older participants were female (60%), and they were African American (64%) and White (24%). Among the total participants (N=11,390), about 60% of older people received job training, and about 70 % of them completed their training. Many older participants received training in 1) health professions and related clinical sciences and 2) business, management, marketing, and related support services. A total of 3,143 older people succeeded in finding their jobs. As age increases, the probability of getting a job and wage increase in re-employed jobs decreases. Conclusion The study found that most older people participated in service profession-related training. Also, racial minorities were the majority of the participants, while the state's racial majority was White. This suggests considering program development to enhance racial minorities' job skills for increasing wages through the state's job training programs.

### SESSION 6710 (POSTER)

### EMPLOYMENT, OLDER WORKERS, AND RETIREMENT

### THE RELATIONSHIP BETWEEN EMPLOYMENT AND ALL-CAUSE MORTALITY IN JAPANESE COMMUNITY-DWELLING OLDER ADULTS

Yoshinori Fujiwara[1], Satoshi Seino[2], Yu Nofuji[2], Yuri Yokoyama[2], Takumi Abe[2], Toshiki Hata[2], Shoji Shinkai[3], and Akihiko Kitamura[4], *1. Tokyo Metropolitan Institute of Gerontology, Tokyo, Tokyo, Japan, 2. Tokyo Metropolitan Institute of Gerontology, Itabashi, Tokyo, Japan, 3. Kagawa Nutrition University, Sakado, Saitama, Japan, 4. Yao City Health Center, Yao City, Osaka, Japan*

It is well known that social contribution such as working maintains the functional health and prevents frailty of older adults. However, the effect of employment in old age with frailty on health outcomes is not well-established.This 3.6-year prospective study examined the influence of working on all-cause mortality in urban community-dwelling Japanese older adults. Participants were 10,591 initially non-disabled residents (5,180 men; 5,411 women) aged 65–84 years of Ota City, Tokyo, Japan. We applied the Cox proportional hazard model by gender and with/without frailty, controlling for age, years of education, equivalent income, number of chronic diseases, body mass index, instrumental activities of daily living (IADLs), smoking and drinking status, exercise habits, social activities, and social interaction for evaluating the predictive value of working status (full-time worker, part time worker, occasional worker and non-worker) at baseline for all-cause mortality.During a follow-up of 3.6 years, 328 (3.1%) individuals died. Compared with non-workers, independent multivariate-hazard ratios (95% confidence intervals) of part-time workers for all-cause mortality in men with/without frailty were 0.35 (0.13–0.97) and 0.54 (0.28–1.01), respectively. Corresponding female multivariate-adjusted HRs with/without frailty were 1.21 (0.34–4.34) and 0.23 (0.05–0.99). Also, for both genders, full-time workers and occasional workers did not have significantly lower adjusted hazard ratios of all-cause mortality, compared to non-workers, regardless of frailty. In conclusion, moderate employment resulted in lower risk of all-cause mortality, even in frail older men.

### NUMERACY SKILL USE AMONG MIDDLE-AGED AND OLDER WORKERS IN THE US

Takashi Yamashita[1], Donnette Narine[2], Abigail Helsinger[3], Wonmai Punksungka[4], Phyllis Cummins[5], Jenna Kramer[6], Rita Karam[7], and Thomas Smith[8], *1. University of Maryland, Baltimore County, Baltimore, Maryland, United States, 2. University of Maryland, Baltimore, Baltimore, Maryland, United States, 3. Miami University, Oxford, Ohio, United States, 4. University of Maryland - Baltimore County, Baltimore, Maryland, United States, 5. Miami University, Oxford, Ohio, United States, 6. RAND Corporation, Arlington, Virginia, United States, 7. RAND Corporation, Santa Monica, California, United States, 8. Northern Illinois University, DeKalb, Illinois, United States*

In numeric information-rich societies, numeracy is essential both at work and in everyday life (e.g., calculating a budget). Numeracy skills generally decline with aging. Thus, among middle-aged and older workers, maintaining and improving numeracy skills is crucial to securing employment as well as managing everyday life. One of the counteracting strategies is to practice numeracy. However, little is known about how and what kinds of numeracy skills are used among older workers. We analyzed a nationally representative sample of U.S. workers aged 45 to 74 (n = 3,850) from the 2012/2014/2017 Program for International Assessment of Adult Competencies (PIAAC) restricted-use-file. Six dichotomous numeracy use indicators (e.g., calculating a budget, using advanced statistics) at work and in everyday life were considered. Survey weighted latent class analysis (LCA) identified three subgroups with distinctive numeracy skill use patterns, including ubiquitous (both at work and in everyday life) users, occupational users, and non-users. For example, calculating a budget was common both in non-users (40%) and ubiquitous users (84%), whereas uncommon among occupational users (25%). Also, only ubiquitous users practiced advanced math and statistics (14%), while others did not (nearly 0%). The subsequent regression analysis revealed that higher educational attainment, higher income, certain racial group (i.e., Whites), and better self-rated health were associated with greater numeracy skill use. The lack of numeracy skill use leads to lower skill levels and, in turn, social and economic disadvantages in later life. In addition to more detailed LCA results interpretations, possible policy and educational interventions are evaluated.

### AGING ON THE JOB? THE ASSOCIATION BETWEEN OCCUPATIONAL CHARACTERISTICS AND ACCELERATED BIOLOGICAL AGING

Theresa Andrasfay[1], Jung Ki Kim[2], Jennifer Ailshire[2], and Eileen Crimmins[2], *1. University of Southern California, Leonard Davis School of Gerontology, Los Angeles, California, United States, 2. University of Southern California, Los Angeles, California, United States*

There is a common saying that demanding jobs can make workers age faster, but there is little empirical evidence linking occupational characteristics to accelerated biological aging. We examine how occupational category (professional/managerial, sales/clerical, service, and manual) and occupational characteristics (e.g., psychosocial stressors, physical demands) are associated with a novel measure of physiologic aging, expanded biological age, that incorporates 22 biomarkers and captures physiologic dysregulation throughout several bodily systems. We assess how occupational characteristics for working individuals aged 51-60 in 2010 Health and Retirement Study (HRS) are associated with expanded biological age in the 2016 Venous Blood Study (VBS). We find that, compared to same-age individuals working in professional or managerial positions, those working in manual occupations appear 0.96-years older biologically while those in service jobs appear 2.8-years older biologically. Individuals whose jobs are high-stress, physically demanding, or require long working hours are nearly one year older biologically than their same-age peers without these adverse working conditions. These findings largely persisted after adjustment for educational attainment. Together these findings suggest that occupational characteristics may be an independent social determinant of accelerated aging.

### TRAJECTORIES OF INFORMAL AND FORMAL SOCIAL PARTICIPATION AFTER RETIREMENT

Jeremy Lim-Soh[1], Shannon Ang[2], and Rahul Malhotra[3], *1. Duke-NUS Medical School, National University of Singapore, Singapore, Singapore, 2. Nanyang Technological University, Singapore, Singapore, 3. Duke-NUS Medical School, Singapore, Singapore*

Literature suggests that social participation, a component of successful ageing, declines on average after retirement, but to whom does this experience apply? We sought to identify contrasting longitudinal trajectories of social participation after retirement, and their associated individual-level correlates. Seven waves of the Korean Longitudinal Study of Ageing provided data on the informal and formal social participation, measured by frequencies of meeting a friend and attending a group respectively, of individuals 45 years and older who left work. Group-based trajectory modeling captured heterogenous changes over time in social participation after retirement. Multinomial logit regressions estimated individual-level correlates of the trajectories, including whether the individual returns to work. While a sizeable minority of respondents did experience decreasing trajectories of informal (17%) and formal (23%) participation, a majority exhibited stable trajectories of either type of social participation, and some experienced increasing formal (9%) participation. Employment type, age, gender, education, marital status, region, health, and economic satisfaction were associated with the trajectories. Returning to work, versus stopping work for an extended period, was associated with moderate stable or increasing trajectories of social participation. The findings challenge the belief that decline in social participation is the norm after retirement. They underscore the presence of heterogenous experiences of social participation after retirement, and identify vulnerable sub-groups that do experience decline. Furthermore, as returning to work may be beneficial for social participation, future studies should examine ways in which bridge employment can support successful aging.

### PRIMARY OCCUPATION AND PAIN AMONG ADULTS AGED 50 AND OLDER IN MEXICO

Sadaf Milani, and Rebeca Wong, *University of Texas Medical Branch, Galveston, Texas, United States*

Working in physically demanding jobs may be associated with pain in middle and old age. This has not been widely studied in Mexico, which has a rapidly aging population. We evaluated the association between primary occupation and pain over 17 years of follow-up among Mexicans aged 50 and older using data from the Mexican Health and Aging Study (2001-2018). Primary occupation was categorized as professional, agriculture, domestic/service, production/industrial, or no main job. We examined frequent pain (yes vs. no) and pain interference (none/non-interfering vs. interfering), defined as pain that limits daily activities. Mixed effects logistic regression was used to model the association between primary occupation and pain. In 2001, participants (n=11,339) were 62.1 years old on average (standard deviation: 9.3) and 55.7% female. About 25.1% of participants worked as professionals, 17.2% worked in agriculture, 16.9% in domestic/service, 22.4% in production/industry and 18.4% had no main job. Almost all of those reporting no main job were women (98.7%). Compared to professionals, those in production/industry had higher odds of frequent pain at baseline [odds ratio (OR): 2.42; 95% confidence interval (CI): 1.25, 4.67]. Those in agriculture had higher odds of frequent (OR: 2.09, 95% CI: 1.03, 4.25) and interfering pain (OR: 2.50, 95% CI: 1.10, 5.70) at baseline. Pain did not change over time by lifetime occupation. The final analysis will include an estimation of gender differences. Health care providers should consider lifetime occupational risks, which may help address pain and minimize pain interference in old age.

### CORRELATES OF JOB SATISFACTION AMONG FULL-TIME EMPLOYED OLDER ADULTS WITH CHRONIC CONDITIONS

Matthew Smith[1], Chung Lin Kew[1], Caroline Bergeron[2], Martha Parker[1], Oluyomi Oloruntoba[1], Kaysey Aguilar[1], Mark Benden[1], and Ashley Merianos[3], *1. Texas A&M University, College Station, Texas, United States, 2. University of Ottawa, Ottawa, Ontario, Canada, 3. University of Cincinnati, Cincinnati, Ohio, United States*

With a rapidly aging workforce and the rising prevalence of chronic conditions, efforts are needed to better understand the needs of older adult workers and how to create supportive working environments for them. This study examines factors associated with job satisfaction among full-time employed adults ages 60+ years with 1+ chronic conditions. Data were collected with an internet-delivered survey in January 2022. Analyses included 337 older adult workers with chronic conditions. An ordinal regression model was fitted to assess factors associated with higher levels of job satisfaction. The model adjusted for sociodemographics, disease characteristics, social engagement, work logistics, and perceptions about the workplace. On average, participants were age 65.14(±4.56) years and self-reported 3.05(±2.09) chronic conditions. Twenty-eight percent reported being very satisfied with their current job and 48.4% worked remotely 1+ days per work week. Higher job satisfaction levels were positively associated with being Hispanic (β=0.68, P=0.030), exhibiting stronger organizational citizenship behavior (β=0.16, P< 0.001), and working more days remotely (β=0.12, P=0.026). Higher job satisfaction levels were negatively associated with job-related stress (β=-0.26, P< 0.001), feelings of social disconnectedness (β=-0.12, P=0.010), and greater intentions of leaving current jobs within the next three months (β=-0.70, P< 0.001). Findings suggest that job satisfaction among older adult workers is rooted in their compatibility with their organizations' work environment, management of job-related stressors, and opportunity to engage in meaningful and fulfilling interactions with others. Strategies such as remote working are encouraged to give older adults flexibility to promote work-life balance and self-manage their chronic conditions.

### EXPLORING ADJUSTMENT TO RETIREMENT: LEISURE IN THE LIVES OF FIRST-GENERATION KOREAN IMMIGRANT MEN

Wonock Chung[1], and Toni Liechty[2], *1. University of Illinois at Urbana-Champaign, Champaign, Illinois, United States, 2. University of Illinois Urbana-Champaign, Champaign, Illinois, United States*

Baby boomers, born between 1946 and 1964, comprise a significant portion of the United States' older adult population. Retirement is also a hallmark of their current life stage. While a body of literature points to the benefits of leisure activities in later life, the roles and relevance of leisure during the retirement transition among first-generation immigrant baby boomers are not well understood. The purpose of this study was to explore leisure throughout the lifespan among first-generation Korean immigrant men (N = 19) and how their cultural values and leisure involvement played out during the retirement transition. Guided by continuity theory of normal aging (Atchley, 1989) and leisure innovation theory (Nimrod, 2008), findings from interview data through interpretive phenomenological analysis (Smith et al., 1995) indicated that (1) perceptions and definition of leisure is shaped by their cultural backgrounds; (2) leisure in the working years mostly involved family leisure activities with an emphasis on providing their children with educational values; (3) leisure activities such as golf and fishing were a particular interest for this demographic, but meanings changed over time; (3) leisure provided continuity during the retirement; and (4) retirement was viewed as an opportunity for new leisure activities, but limited availability of sport and recreation programs was perceived as a barrier. These findings yield meaningful implications in that (a) leisure engagement can provide continuity in maintaining their social roles over the lifespan; and (b) more community-based sport and recreation programs targeting older adults would help them successfully transition to retirement.

### STRESSOR REACTIVITY DEPENDS ON THE CORTISOL AWAKENING RESPONSE AND REACTIVITY TO WORK OVERLOAD

MacKenzie Hughes[1], Christopher Hertzog[1], Shevaun Neupert[2], and Scott Moffat[1], *1. Georgia Institute of Technology, Atlanta, Georgia, United States, 2. North Carolina State University, Raleigh, North Carolina, United States*

Ecological momentary assessment (EMA) was used to understand the influence of individual differences in stress reactivity measured by the Perceived Stress Reactivity Scale (PSRS) and the cortisol awakening response (CAR) on emotional reactivity to stressors, operationalized as the within-person change in negative affect (NA) associated with stressor exposure. Five times per day for 10 days, 178 working adults ages 20-80 years old (M = 49.22, SD = 19.07) reported in EMAs their current NA and whether they had experienced a stressor since the previous survey. During the same period, participants provided seven salivary cortisol samples per day. Samples collected at awakening and 30-minutes post-awakening were used to calculate the CAR. Steeper CARs are hypothesized to have a role in preparing individuals to cope with upcoming daily demands. Before the EMA period, participants completed the PSRS, including its Work Overload Reactivity subscale. Multilevel models revealed a significant 3-way interaction between Stressor Exposure x CAR x Work Overload Reactivity predicting daily NA. Individuals with high Work Overload generally reported greater NA, regardless of stressor exposure or the magnitude of their CAR. Individuals with low Work Overload reported lower levels of NA on days they experienced more stressors than usual and had steeper CARs. Effects remained significant after controlling for neuroticism and the Perceived Stress Scale. Findings suggest the CAR's potential role of preparing individuals for upcoming demands is moderated by work-related stress reactivity. Steeper CARs on days with more stressor exposure may provide enhanced emotional benefits for individuals low in workload reactivity.

### MEMBER CHECKING GERONTOLOGY: THE CASE OF RETIREMENT

David Ekerdt, *University of Kansas, Lawrence, Kansas, United States*

Gerontology has long been a public-facing field with an applied focus. As such, the credibility of gerontology's conclusions and guidance about aging are crucial, our advice having relevance and impact in proportion to its popular resonance. In 2021 I authored an article for a large-circulation newspaper that generated over 500 reader replies, creating an opportunity for member checking of a kind. The article reported my personal experience of having retired—what I expected and what was a surprise. All of my observations about emotions and lifestyle, while my own, were nonetheless grounded in the research literature. Public comments on the article came from a readership that skews male and highly educated, i.e., people like myself. Many comments affirmed my observations (e.g., about time use, awareness of finitude) as experiences we had in common. Some comments disputed my authority, as an academic, to say anything valid about the "real world." Opinion split on the value of continued work: it gives life meaning, it invites corrosive stress. Likewise, some retirees endorsed surrender to leisure while others urged engagement. One research takeaway: with no standard way to be retired or regard it, the quality of retired life remains a measurement challenge. Another takeaway: Retirees with partners commonly describe experience in the first-personal plural (we, us), suggesting that dyads are often apt units of analysis for retirement studies. This is but one case study, but it indicates that we must continually assess whether gerontology's knowledge is valid and whether the public is grateful for it.

### OLDER ADULTS LEAVING THE WORKFORCE: SENSORY LOSS, RETIREMENT, AND DISABILITY

Emmanuel Garcia Morales[1], Lama Assi[2], and Nicholas Reed[3], *1. Cochlear Center for Hearing and Public Health, Baltimore, Maryland, United States, 2. Louisiana State University Health Center, New Orleans, Louisiana, United States, 3. Johns Hopkins Bloomberg School of Public Health, Baltimore, Maryland, United States*

Hearing loss (HL), vision loss (VL), and their combination (dual sensory loss, DSL) are common among older adults. Sensory loss impacts labor productivity which might result in departures from the workforce. Whether older adults leave due to retirement or a disability, and how these responses are associated with sensory loss remains unexplored. Using the 2004-2018 rounds of the Health and Retirement Study, self-reported sensory loss (No Impairment/HL/VL/DSL) at baseline, and reason for leaving the workforce (retirement or disability) were observed. Competing risk models models for departures from the workforce treating retirement or disability as a competing risk were estimated. Among 5,201

adults employed at baseline, ages 50-94, 3,436 reported retirement, and 4254 reported a disability as a reason for not working. In Fine-Gray models, treating retirement as a competing risk and adjusting for sociodemographic and clinical characteristics, we found that compared to older adults without impairments, DSL was associated with a 50% increase in the rate of departures from the labor force due to disability among adults in the in the risk group (SHR=1.51; 95% CI=1.09,2.11). In contrast, when treating disability as the competing risk, HL was associated with a 22% increase in the rate of departures labor force due to retirement (SHR=1.22; 95% CI=1.10,1.36) among adults in the risk group when compared to those without impairments. In sample of older adults, we provide evidence that the presence of sensory impairments is associated with departures from the workforce. Our results highlight differences in the type of departures by sensory loss.

### STICKING WITH THE UNION? LABOR UNION MEMBERSHIP, WORKING CONDITIONS, AND POSTRETIREMENT HEALTH IN THE MIDWEST

Michal Engelman, and Yue Qin, *University of Wisconsin-Madison, Madison, Wisconsin, United States*

American Employment experiences over the past five decades have been shaped by growing prevalence of bad jobs – those that are precarious and offer few pension or health insurance benefits – and a marked decline in unionization. Previous health research has highlighted the deleterious implications of bad jobs and yielded mixed or inconclusive findings about union membership. However, most of this research focused on working-age adults, and few studies have examined the long-term impacts of working conditions and union membership. We fill this gap via data from the Wisconsin Longitudinal Study – a sample of men and women who graduated from Wisconsin high schools in 1957 and have been followed through their working years, past retirement, and into oldest-old ages. We estimated regression models examining the impact of union participation in 1975 on subsequent self-rated health and depressive symptoms (measured in 1993, 2004, and 2011). Our findings suggest that union participation was associated with poorer self-rated health in 1993 (OR=0.67, 95% CI (0.48, 0.96)), with a stronger negative effect for more active union members (OR=0.58, 95% CI (0.36, 0.96)), even after controlling for socioeconomic status in childhood and adulthood. This effect dissipated by 2004, when most WLS participants were nearing retirement and further diminished by 2011, when participants were in their 70s. We found no significant effects of union activity on depressive symptoms. Job characteristics and the historical decline in the prevalence and power of unions over the cohort's lifetime provide important contexts for interpreting these results.

### TOWARD AGE-FRIENDLY WORKPLACES: IDENTIFYING FACTORS THAT SUPPORT COGNITIVE FUNCTION IN OLDER WORKERS

Ashley Price[1], and Soomi Lee[2], *1. University of South Florida-Tampa, Tampa, Florida, United States, 2. University of South Florida, Tampa, Florida, United States*

Older adults in the workforce face natural age-related decline that may impede their work performance. Sleep and cognitive function, both of which are degraded with age, may affect work performance in older workers. Workplace demands and support may also play roles in older workers' performance. Yet there remains a lack of effort in identifying modifiable factors that contribute to older workers' performance. This review compiled previous studies on modifiable factors across personal and workplace domains that support or impede older workers' performance at work. Databases utilized for this systematic review include Google Scholar, AgeLine, and APA PsycINFO. Inclusion criteria were empirical studies conducted in developed countries, published in 2000 or later, that focused on older adults (age >55) working full-time (≥ 35 hours/week). Keywords included: sleep, older adults, workforce, cognition, aging, work performance, aging workforce, workplace support, management strategies. Of the 32 studies initially identified, 13 qualified for this analysis. In 6 studies, poorer sleep (measured by actigraphy) was prevalent in older workers and was negatively associated with their cognitive performance at work. Across 7 studies, demanding and non-supportive workplace characteristics (i.e., greater job demands, lower supervisor support, and higher agism) were identified as common risk factors for poorer sleep, poorer cognitive function, and lower work performance in older workers. Bridging this information together may help identify specific factors that may be modifiable by workplace interventions to support optimal performance in workers and promote more age-friendly work environments.

### SESSION 6720 (POSTER)

### END OF LIFE, HOSPICE, AND PALLIATIVE CARE

### TO TELL OR NOT TO TELL: TYPOLOGIES OF OLDER ADULTS PREFERENCES ON DIAGNOSIS DISCLOSURE OF CRITICAL ILLNESS IN CHINA

Yifan Lou[1], and Jinyu Liu[2], *1. Columbia University, New York, New York, United States, 2. Columbia University, New York City, New York, United States*

Background: A priori of advance care planning, that older adults should know their diagnosis, is not guaranteed nor legally supported in China. Typically, doctors will inform the family members of the diagnosis and prognosis of critical illness and let family members decide whether to inform or not. This study aims to explore how older Chinese prefer diagnosis disclosure of their critical illness and the factors related to each typology of desired roles.

Methods: We surveyed 571 older adults in Shanghai from late-2021. We included 7 items measuring values of diagnosis disclosure on three levels: to self, to significant others, and regarding physician disclosure approach. We characterized preference types using latent class analysis. Multinomial regression models on class memberships were used with cultural, sociodemographic, and healthcare experiences predictors.

Results: Three latent classes were identified: 34% of respondents preferred control over own diagnosis and respected significant others' rights to know their own diagnosis ("transparent"). 50% of respondents has conflicted values.

They preferred to know own diagnosis and to discuss with physicians privately, but chose to conceal others' diagnoses if their significant others have serious illness ("contradictory"). 16% preferred to delegate control over diagnosis to others and assumes that their families hold the same value ("avoidant"). Increased familism is significantly related to contradictory type. People with experience of hospitalization and making medical decisions for family members were more likely to be transparent. Discussions: We discussed research, practice, and policy implications on culturally-appropriate approaches to honor patients' autonomy with serious illness in China.

### THE RELATIONSHIP BETWEEN ADVANCE CARE PLANNING AND COGNITIVE FUNCTION IN A MULTICULTURAL COHORT

Simone Camacho[1], Magdalena Tolea[2], Nicole Mendez[3], Joyce Jimenez Zambrano[3], and James Galvin[4], *1. University of Miami Miller School of Medicine, Boynton Beach, Florida, United States, 2. University of Miami Miller School of Medicine, Boca Raton, Florida, United States, 3. University of Miami Miller School of Medicine, Miami, Florida, United States, 4. University of Miami School of Medicine, Miami, Florida, United States*

Engagement in Advance care planning (ACP) – the process of communicating preferences for future medical decisions, has been linked to increasing age, greater decision-making capacity, and higher education, and is more prevalent in White older adults. To advance knowledge of ACP in multicultural populations, we examined variations in the relationship between sociodemographics, cognitive functionality and ACP. A total of 303 older (mean-age: 69.4±10.5; 69.9% females; 29.6% Non-Hispanic White, 21.2% African American, and 49.2% Hispanic) participants in a community-based dementia screening program were included. ACP measures included life insurance, disability insurance, long-term care (LTC) insurance, power of attorney (POA), living will, and having a health care proxy (HCP). Although most participants had health insurance (92.4%, no differences by race), low rates of ACP engagement were found in racial/ethnic minorities across multiple ACP measures (all ≤40%). Black older adults had the highest use of life insurance (p<.001). Higher ACP rates (i.e., LTC, HCP, living will) were associated with better global cognition (MoCA; p< 0.05). Higher POA rates were associated with lower AD8 scores (30.1% in AD8< 2 vs 15.4% in AD8≥2, p=0.003). Finally, ACP rates varied across racial-SES groups being highest in high SES White participants and lowest in minorities with low SES, regardless of subjective or objective cognitive performance. Findings link ACP to greater cognitive functionality and highlight racial and SES disparities in ACP engagement, particularly related to non-insurance-based planning. Understanding cultural differences in decision making can lead to targeted interventions to increase early ACP in cognitive aging and dementia for older adults.

### URBANIZATION, FINANCIAL SOURCE, AND PLACE OF DEATH AMONG CHINESE LONGEVITY OLDER ADULTS

Rui Zhou, Shuangshuang Wang, and Nengliang Yao, *Shandong University, Jinan, Shandong, China (People's Republic)*

The research on place of death among Chinese oldest adults is scare. This study explored the associations of urbanization and financial source on the places of death among Chinese longevity older adults. The sample consists of 21,920 decedents (female=60%, died at home=89%, mean age at death=95) from 2000-2018 Chinese Longitudinal Healthy Longevity Survey (CLHLS), conducted by Peking University. Places of death were divided into two categories: home and other sites (including hospitals, institutions and others). Urbanization was measured by residential areas: rural or city. Financial source consisted of family financial support and retirement wage. Chi-square test showed that older adults living in rural areas and getting family finance support were more likely to die at home. Results from binary regression models show that controlling for covariates, decedents living in cities were 6.2 times more likely to die in other places than at home and those received retirement wage were 3.1 times respectively. Notably, older adults who lived in cities and also had retirement wage were 17.7 times more likely to die in other places comparing to those living in rural areas with only family finance support. Findings suggest that more older adults will choose to die at hospitals or institutions as the process of urbanization and the development of social pension system in China, which will put enormous pressure to the hospital-centered healthcare system. Improving the quality of healthcare in grassroots areas might be a feasible attempt to relive the pressure of hospital-centered healthcare system in cities.

### MEDICAL AID IN DYING: DOES POLICY PREVENT SUFFERING AT THE END OF LIFE?

Todd Becker[1], Nancy Kusmaul[1], Allison Gibson[2], and Cara L. Wallace[3], *1. University of Maryland Baltimore County, Baltimore, Maryland, United States, 2. University of Kentucky, Lexington, Kentucky, United States, 3. Saint Louis University, Saint Louis, Missouri, United States*

Medical aid in dying (MAID) reflects the legal provision for qualifying, terminally ill individuals to receive a prescription from their medical provider for self-ingestion to hasten death. This policy analysis examines current MAID policy with a focus on MAID's intended relief of suffering. Using Wallace's (2015) policy model, we evaluated MAID on: policy description, historical context, development of the policy, efficiency, alternative programs, effectiveness, and unintended consequences. Policy description: Currently legal in 10 jurisdictions (nine states and Washington, DC), common goals include relieving suffering and promoting self-determination. Historical context: Following a failed attempt at federal legalization, the U.S. adopted an incremental, jurisdiction-based approach to legalization beginning in Oregon (1994). Development of the policy: Although all statutes imitate Oregon's model, subtle differences exist. Efficiency: Although each policy includes waiting periods that vary in sequential order and duration, revisions have targeted waiting periods to improve efficiency. Alternative programs: Frequently considered an alternative to MAID, existing statutes encourage providers to discuss palliative options. Effectiveness: Despite appearing effective in promoting self-determination, MAID-related complications (e.g., vomiting) may exacerbate suffering. Unintended consequences: MAID's requirement of clinician participation may place providers in situations that challenge their self-determination and provoke distress. In sum, many questions remain unanswered regarding MAID's effectiveness and efficiency. Thus, more data are needed. Ethical implications will continue to be debated, though policy implications (e.g., who uses MAID, the number of unique providers, the emergence of complications, those present during death) greatly impact implementation and overall choices to limit suffering at the end of life.

### PALLIATIVE AND END-OF-LIFE CARE INTERVENTIONS FOR INDIVIDUALS EXPERIENCING HOMELESSNESS: A SCOPING REVIEW

Pilar Ingle, *University of Denver, Lakewood, Colorado, United States*

Despite the benefits of palliative and end-of-life (PEOL) care for individuals with life-limiting illness, barriers to accessing this care persist among older adults experiencing homelessness. A number of studies have identified barriers to PEOL care from the perspectives of unhoused individuals and other relevant stakeholders; however, few empirically studied interventions exist to support evidence-based guidelines for PEOL care for this population. This scoping review aimed to identify PEOL interventions for adults experiencing homelessness. The search was conducted in May 2021 and yielded 480 results, from which 50 full-text articles were closely reviewed for inclusion. Sources were included if they reported findings of original, empirical research or quality improvement projects evaluating PEOL services tailored for individuals experiencing homelessness. A total of 13 articles representing 12 studies published between 2006-2021 were included in the review. Over half (n = 7) of the studies described interventions to promote advance care planning documentation, five described interventions embedding PEOL services within homeless shelters, and one described a community outreach program to increase access to PEOL services. Nearly all studies reported positive outcomes on participant perspectives, advanced directive completion rates, or cost analysis. None of the studies included clinical palliative care outcomes. Although most studies reported promising results, service and healthcare providers expressed concerns about continuing challenges and longevity of the programs. The body of literature encompassing PEOL interventions for people experiencing homelessness is limited but growing. Future research may benefit from the inclusion of clinical outcomes and efforts to reduce structural barriers to providing care.

### RECRUITING PATIENT-CAREGIVER DYADS IN A PANDEMIC: CHALLENGES AND OPPORTUNITIES IN HOSPICE AND PALLIATIVE RESEARCH

Cara L. Wallace[1], Karla Washington[2], Kathryn Coccia[1], Verna Ferguson[1], Anna Wingo[1], Leslie Hinyard[3], Marla Berg-Weger[1], and Stephanie P. Wladkowski[4], *1. Saint Louis University, St. Louis, Missouri, United States, 2. Washington University in St. Louis, St. Louis, Missouri, United States, 3. Advanced HEAlth Data (AHEAD) Institute. Saint Louis University, St. Louis, Missouri, United States, 4. Bowling Green State University, Bowling Green, Ohio, United States*

Hospice and palliative care (HAPC) research has shown participation in research benefits both patients and caregivers. However, recruiting participants for HAPC research can be difficult and requires flexible, adaptive, and creative strategies. This presentation will review ongoing efforts for recruitment and subsequent alterations for an active NIH study of patients and their caregivers who have been discharged alive from hospice. A live discharge occurs when a hospice patient medically stabilizes, no longer meeting policy's prognosis eligibility criteria. In 2019, hospices in the United States served more than 1.6 million people, and 17.4% were discharged alive from hospice care, with 6.5% due to being 'no longer terminally ill' (NHPCO, 2021). Currently, there is not an explicit discharge process available to guide practitioners in transitioning patients and caregivers out of hospice care, creating challenges and opportunities for collaborative research.Despite existing relationships with hospice networks, our initial recruitment strategy of recruiting patients and caregivers through direct referrals has been insufficient. Incorporating creative solutions to boost recruitment efforts appears to be effective. First, we developed partnerships with hospital/health systems for identification of eligible partnerships using existing information available in Electronic Medical Records (EMRs), yielding an initial 80% increase in referrals. Additional recruitment efforts include using brief informational videos on social media platforms, such as YouTube, Twitter, and TikTok. Promising recruitment strategies for hard-to-reach populations such as HAPC patients and their caregivers, including identification and outreach using existing EMRs, expanding eligibility to include retrospective data, and integrating efforts across social media platforms.

### WHO GETS HOSPICE VISITS AT THE END OF LIFE: A LOOK AT MEDICARE'S HOSPICE SERVICE INTENSITY ADD-ON POLICY

Michael Plotzke[1], and Thomas Christian[2], *1. Abt Associates, Saint Louis, Missouri, United States, 2. Abt Associates, Cambridge, Massachusetts, United States*

In 2016, the Centers for Medicare & Medicaid Services (CMS) implemented the Service Intensity Add-on (SIA) payment for the Medicare Hospice Benefit. The SIA incentivizes direct patient care by registered nurses and medical social workers during the last seven days of life through additional payments to hospices. We examined 100% of Fee-for-Service Medicare hospice claims in Fiscal Year 2020 to determine whether SIA utilization varies by patient and hospice characteristics. Using hospice level fixed-effects regression, we examine the associations between SIA utilization and various beneficiary and hospice characteristics. Relative to beneficiaries with cancer as a principal diagnosis, we find that other diagnoses are associated with only a slight reduction in SIA utilization (a reduction between 0.6 and 1.1 percentage points - where the average SIA utilization overall is equal to 85.3%). Those identifying as black had a reduction in SIA of 1.3 percentage points compared to those identifying as white. Compared to a patient dying on a Sunday, a patient's death on Tuesday through Friday was associated with a higher likelihood of SIA (between 9.7 and 11.6 percentage points more likely) and more SIA minutes (between 29.9 minutes and 36 minutes). All reported results were statistically significant at the 0.1% level. Based on these results, we find that SIA utilization varied by certain characteristics. As a result, CMS should continue to monitor rates of SIA utilization to better understand whether any patient groups appear to be underserved at the end of life.

### LIVE DISCHARGE FROM HOSPICE WITH DEMENTIA: CHALLENGES TO SUPPORTING THE PATIENT-CAREGIVER DYAD

Stephanie P. Wladkowski[1], Cara L. Wallace[2], and Karla Washington[3], *1. Bowling Green State University, Bowling Green, Ohio, United States, 2. Saint Louis University, Saint Louis, Missouri, United States, 3. Washington University in St. Louis, St. Louis, Missouri, United States*

A live discharge from hospice disrupts care continuity and results in burdensome transitions for individuals with a life-limiting illness and their caregivers. In 2019, hospices served more than 1.6 million people across the United States, with nearly 63% of Medicare decedents age 85 or older. Of these patients, nearly 350,000 (20.9%) had a principal diagnosis of Alzheimer's Disease/Dementia/Parkinson's Disease. Research demonstrates that hospice care improves end-of-life outcomes for adults with Alzheimer's Disease and related dementias (ADRD), yet with eligibility limited to a six-month prognosis, hospice is not structured to meet longer-term needs. The result is a live discharge from hospice. In 2019, 17.4% of hospice patients were discharged alive from hospice, with 6.5% discharged due to being 'no longer terminally ill.' The majority of live discharges are either hospice-initiated due to patient stabilization (extended prognosis resulting in a situation in which a patient no longer meets the life expectancy hospice eligibility criteria) or are patient-initiated (revocation) where a patient or proxy chooses to leave hospice care, typically to access disease-directed therapies or inpatient hospitalization. Both present unique challenges and opportunities for hospice providers. Informed by over 10 years of practice experience and research, this presentation will discuss the impact of live discharge from hospice and the unique impact for patients and primary caregivers of individuals with ADRD, the service gaps that exist for this population, and our recommendations for policy reform.

### BUILDING A PROTOCOL FOR LIVE DISCHARGE FROM HOSPICE: UNDERSTANDING THE APPROACHES, CHALLENGES, AND OPPORTUNITIES

Stephanie P. Wladkowski[1], Tracy Schroepfer[2], and Susan Enguidanos[3], *1. Bowling Green State University, Bowling Green, Ohio, United States, 2. University of Wisconsin-Madison, Madison, Wisconsin, United States, 3. University of Southern California, Los Angeles, California, United States*

A live discharge from hospice can occur when a patient stabilized under hospice care no longer meets the life expectancy hospice eligibility criteria. In 2019, 278,400 hospice patients across the United States were discharged alive from hospice care, with 18,096 (6.5%) discharged due to being 'no longer terminally ill.' For these individuals and their caregivers, the result is a disruption of care continuity and an often burdensome transition. Hospice care improves end-of-life outcomes for some patients, and a live discharge results in lost access to important supportive services and resources, while the patient remains 'terminal.' Further, an increased burden is placed on primary caregivers who may be unprepared for this transition. Currently, there is no explicit discharge process available within hospice to guide practitioners in transitioning patients and their caregivers out of hospice care. This study aimed to garner a deeper understanding of current approaches and accompanying challenges to inform the development of an explicit live discharge protocol. Focus group interviews with hospice social workers at four hospice agencies across the U.S. were conducted. Using thematic analysis, four key themes emerged, including the logistical (n=13) and psychosocial (n=9) approaches, the need for clear professional roles during a live discharge (n=12), and specific challenges (n=14), such as needed services and desired discharge timeline to best support the patient-caregiver dyad. Findings demonstrate the complexities of conducting a live discharge, the uniqueness of each hospice agency, and the need for more research to support a standardized and reimbursable discharge process.

### THE RELATIONSHIP BETWEEN PHYSICIAN VISITS AND ADVANCE CARE PLANNING

YuHsuan (Olivia) Wang[1], and Susan Enguidanos[2], *1. USC, Los Angeles, California, United States, 2. University of Southern California, Los Angeles, California, United States*

Some studies have shown that advance care planning (ACP), discussions about future care given a serious illness, are associated with improved quality of death and better end-of-life care. Studies also have found that physicians play an important role in ACP. In fact, in 2016 CMS began reimbursing physicians for ACP discussions. However, little is known about the relationship between the number of physician visits and engaging in ACP . This study investigated the association between outpatient physician visits and ACP engagement. Only respondents over 65 were included in our study. We conducted logistic regressions using the 2016 Health and Retirement Study. We used ACP engagement (n=9838), advance directive (AD; eg, living will) completion (n=9746), and healthcare power of attorney (e.g., proxy) assignment (n=9724) as outcome variables. In addition to the frequency of physician visits, we controlled for basic demographics (age, gender, marital status, race, and socioeconomic status), number of chronic conditions, and self-rated health in our models. For each additional physician visit, the probability of having an ACP conversation, ($p<.001$), AD completion ($p<.001$), and healthcare proxy assignment ($p<.001$) increased by 1.6%,1.4%, and 1.9% respectively after controlling for covariates. Number of chronic conditions also was independently and positively associated with ACP-related activities. Additionally, greater perceived health was associated with higher odds of AD completion (OR= 1.08, $p<.05$) and healthcare proxy assignment (OR= 1.07, $p<.05$). This study found that frequent encounters with physicians are associated with higher rates of ACP engagement even after controlling for health conditions.

### DO DIFFERENT ADVANCE DIRECTIVES CREATE CONFUSION ON A PATIENT'S WISHES? (MOLST VERSUS FIVE WISHES)

Taylor Perre[1], and Yuchi Young[2], *1. Home Care Association of New York State, Albany, New York, United States, 2.*

*SUNY at Albany School of Public Health, Rensselaer, New York, United States*

Introduction. The objective of this study is to compare perspectives of young adults toward advance directives (ADs) and their preferences for life-sustaining treatment and care options. Methods. Participants include graduate students (n=30) attending a New York State university. Data were collected using a structured survey questionnaire, the Medical Orders for Life-Sustaining Treatment (MOLST) form and the Five Wishes form. Summary statistics were performed to address the study aim. Results. Of the participants, the average age was 24 years (60% were female, 60% White, and 27% Black). In Five Wishes, participants who are close to death, 70% wanted all or some forms of life support; when in a coma (47%), or with permanent and severe brain damage (36.6%) chose similar options. In MOLST, without pulse and/or breathing, 87% want CPR; while with pulse and breathing, 96% want artificially administered fluids and nutrition, 90% want mechanical ventilation, 67% want to be hospitalized, 67% want antibiotics, and 53% want unlimited interventions. Conclusion. (1) The majority of participants had not previously completed an AD; however, they were capable of making decisions about their life-sustaining treatments. (2) The discrepancies in treatment preferences may be due to the language of advance directives. Further studies in this respect are warranted.

### EXPLORING FIVE WISHES AND END-OF-LIFE CARE PLANNING IN YOUNG ADULTS

Taylor Perre[1], Arianna Stone[1], and Yuchi Young[2], *1. Home Care Association of New York State, Albany, New York, United States, 2. SUNY at Albany School of Public Health, Rensselaer, New York, United States*

Introduction. Advance care planning (ACP) allows individuals to plan ahead and express their preferences for medical treatment and care options to health care providers, family, and loved ones before they are no longer able to make or voice decisions due to the event of a serious illness or injury. Advance directives (ADs) allow individuals to record their preferences. While unintentional injuries are the leading cause of death among young adults, limited studies focus on ACP, ADs, and end-of-life treatment and care. Our study aims to (1) examine the perspectives of young adults towards Five Wishes, and (2) measure their preferences related to personal, emotional, spiritual, and medical values in end-of-life care planning. Methods. Data were collected using Five Wishes and a one-time questionnaire. Participants include graduate students (n=30) at a New York State university. The average age was 24 years old (60% were female, 60% White, and 27% Black). Results. In the case of permanent and severe brain damage without expectation to wake up or recover, 63% do not want life-support treatment. In the event of coma without expectation to wake up or recover, 53% do not want life-support treatment. When close to death, 80% want to have religious or spiritual readings and well-loved - poems read aloud. Conclusions. Young adults are capable of making their decisions regarding appointing a health care proxy and giving specific instructions for personal, emotional, spiritual, and medical care. The present findings intend to make contributions in promoting population-based healthcare decision-making, education, and awareness.

### ACCESS TO HOSPITAL-BASED PALLIATIVE CARE SERVICES FOR OLDER ADULTS IN IOWA

Hyesu Yeo[1], and Elisa Childs[2], *1. University of Georgia, Athens, Georgia, United States, 2. The University of Georgia, Athens, Georgia, United States*

Background: U.S. Medicare covers many palliative services; however, Iowa's rurality and high rate of older adults (OAs) aged 65 or over make it unclear whether older Iowans have equitable service access. Hospital-based palliative care services (HBPCSs) include curative treatment, whereas other providers of palliative care may not. Thus, this study only examined OAs' geographic access to HBPCSs in Iowa.

Methods: This study used the American Hospital Association and U.S. Census Bureau estimate in 2017. The geographical distribution of hospitals and HBPCSs was examined using a county-level approach, considering rural/urban status (using the Office of Management and Budget's definitions) and OA population proportion.

Results: Of the 99 counties in Iowa, 89 were rural. Of the 116 hospitals statewide, one urban county and nine rural counties had no hospital. A total of 52 hospitals provided HBPCSs in 43 counties across nine urban and 34 rural counties. HBPCSs were primarily located in central Iowa, with northern and southern Iowa having the lowest access to HBPCSs. The OA population ratio was higher in counties without HBPCSs (19%) than HBPCSs (15.7%). All 54 counties with over a 20% OA population were concentrated in rural counties, while only 37% of these rural counties had HBPCSs.

Conclusion: This study suggests a regional imbalance of HBPCS providers for OAs in Iowa. Results showed that rural communities with a high ratio of OAs lack HBPCSs and suggest HBPCSs should be expanded at hospitals in northern and southern Iowa counties where many OAs live.

### STARTING THE CONVERSATION: FINDINGS FROM AN ADVANCE CARE DIRECTIVE WORKSHOP

Kelly Parsons, Justin Barber, Allison Gibson, Gregory Jicha, and Renee White, *University of Kentucky, Lexington, Kentucky, United States*

Every adult that enters a hospital is asked about an advance care directive (ACD) document. The completion rates of care directives started to grow in 2016, when Medicare provided a reimbursement benefit in the annual wellness exam. Previous data shows only 37% of older Americans have an ACD, of that 64% are white. Previous studies report data from medical visits, however, advance directive discussions conducted in a community setting has not yet been explored. This pilot study examines readiness to have discussions with your appointed decision-maker, primary care provider, and complete ACD document. This was a community workshop with a physician-led presentation regarding medical choices, followed by break-out groups with trained moderators reviewing ACD materials. Data from pre/posttest along with qualitative comments addressing information seeking (pre) and additional comments (post) are included. The workshop increased participants readiness to identify an individual to make medical decisions and put into writing the type of medical care they desire. It also showed resistance to having conversations with a person's primary care provider and chosen health care agent. Challenges included the pre/

posttest completion rate and the inability to compare a control group. Viewing advance directive completion within the self-determination theory assists in understanding the need for a novel setting to promote relatedness. Further research needs to be done on community setting ACD workshops to provide a space for participants to be better informed in the ACD process. Community workshops need to be examined to have a broader spectrum of inclusion within historically marginalized communities.

### SESSION 6730 (POSTER)

### END OF LIFE AND PALLIATIVE CARE

### ORAL HEALTH AND MORTALITY AMONG OLDER ADULTS: A DOUBLY ROBUST SURVIVAL ANALYSIS

Weidi Qin[1], Jiao Yu[2], Wenxuan Huang[3], and Kristi Thomas[1], *1. University of Michigan, Ann Arbor, Michigan, United States, 2. University of Minnesota, Minneapolis, Minnesota, United States, 3. Johns Hopkins University, Baltimore, Maryland, United States*

Evidence on the association between oral health and mortality is inconclusive, and few studies have accounted for non-random selection bias in estimating their relationship. The present study aims to investigate the link between oral health and mortality in community-dwelling older adults by adjusting confounding factors with a doubly robust survival estimation. Data came from the third National Health and Nutrition Examination Survey (NHANES III, 1988-1994) and linked the National Death Index (NDI) mortality data through December 2015. The analytic sample consisted of 4,880 adults aged 50 years and above. Oral health measures included subjective oral health evaluation (self-rated oral health) and objective clinical indicators (edentulism, periodontitis, and untreated dental caries). Cox proportional hazards regression models and inverse probability weighting with regression adjustment (IPWRA) were utilized to assess the relationship between oral health and mortality. Good self-rated oral health was significantly associated with lower mortality risk (hazard ratio: 0.83, 95% CI: 0.74-0.93). Moderate periodontitis (hazard ratio: 1.18, 95% CI: 1.05-1.33), severe periodontitis (hazard ratio: 1.50, 95% CI: 1.21-1.87), and edentulism (hazard ratio: 1.13, 95% CI: 1.01-1.26) were significantly associated with higher risks of all-cause mortality, adjusting for socio-demographic characteristics, medical conditions, and health behaviors. Untreated dental caries was related to mortality in Cox models, but became non-significant when IPWRA estimators were applied. Both subjective and objective oral health are risk factors for mortality among older adults. Maintaining good oral health plays a crucial role in longevity.

### AWARENESS OF PARTNER PREFERENCES FOR END-OF-LIFE ASPECTS AND TREATMENTS IN OLDER ADULT COUPLES

Sarah Vilpert[1], Clément Meier[1], Ralf J. Jox[2], Gian Domenico Borasio3, and Jürgen Maurer[1], *1. University of Lausanne, Lausanne, Vaud, Switzerland, 2. Lausanne University Hospital and University of Lausanne, Lausanne, Vaud, Switzerland, 3, Lausanne University Hospital and University of Lausanne, Lausanne, Vaud, Switzerland*

Surrogate medical decision-making at the end of life is common and the patient's partner is often the person who must make these critical decisions. The challenge of surrogate medical decision-making is to make decisions that best fit the patient's wishes. This study investigates how accurately older adult couples assess each other's preferences for eleven end-of-life aspects and three medical treatments in a nationally representative sample of adults aged 58 and over living in Switzerland (N=573). The contribution of factors including end-of-life (EOL) discussion and care planning, self-rated knowledge of partner preferences, and EOL preferences homogamy in scoring higher in accurately assessing partner's preferences for the end of life is examined. Weighted proportions, assessment accuracy scores and ordinary least squares regressions controlled for individuals' characteristics are calculated. Results show that 44% of respondents accurately assessed their partners' preferences for aspects related to medical, psychosocial, anticipatory and burden concerns at end of life, and 62% correctly predicted the preferences for cardiopulmonary resuscitation, life-prolonging measures, and palliative sedation. Respondents whose partner discussed their EOL preferences with them, completed an advance directive, designated them as their healthcare proxy, as well as respondents reporting very/rather good knowledge of their partners' EOL preferences were more likely to have a higher accuracy score for their partners' EOL preferences. Being a couple is not enough to assess one's partner's EOL preferences correctly. Communication about EOL wishes among the couple and individual EOL care planning should be encouraged, as both improve the assessment accuracy of partners' EOL preferences.

### EFFECTS OF PATIENT QUESTION PROMPT LIST USE ON PARTICIPATION DURING OUTPATIENT PALLIATIVE CARE APPOINTMENTS

Meghan McDarby[1], Hannah Silverstein[2], and Brian Carpenter[2], *1. Memorial Sloan Kettering Cancer Center, New York, New York, United States, 2. Washington University in St. Louis, St. Louis, Missouri, United States*

Question prompt lists (QPLs) support patients who are seeking information during medical appointments by providing preset questions a patient might ask their provider. Prior research suggests that QPLs promote communication, including question asking, during appointments. The current study evaluated use of a 25-question QPL during initial outpatient palliative care appointments. We applied tenets of Social Cognitive Theory to investigate the relation between question asking and self-efficacy. Participants were patients attending their first outpatient palliative care appointment at an academic hospital. Participants were randomly assigned to receive the QPL before their appointment (n = 29) or to receive usual care (n = 30). Audio recordings of appointments were transcribed and coded for total questions asked. Participants also responded to a single-item measure of self-efficacy in question asking at pre- and post-appointment. On average, participants in usual care appointments asked 9.37 questions (SD = 10.3, range= 0 – 49), and participants in QPL appointments asked 11.24 questions (SD = 10.1, range = 0 – 45), but with no significant difference between groups,

$F(1,56) = 0.14$, $p = .71$. All participants reported an increase in self-efficacy from pre- to post-assessment, $F(1,83) = 18.76$, $p < .00$, but there was no interaction effect of time and condition, $F(1,83) = .81$, $p = .37$. Despite their promise in previous studies, our results suggest QPLs may lack potency to shift question asking, at least in palliative care appointments, and that other mechanisms of Social Cognitive Theory may better characterize the relation between question asking and self-efficacy.

### UNDERSTANDING THE RELATIONSHIP BETWEEN COPING STYLES AND DEATH ANXIETY IN OLDER ADULTS

Juliamaria Coromac-Medrano[1], Amber Watts[1], Christian Sinclair[2], and Ilana Engel[1], *1. University of Kansas, Lawrence, Kansas, United States, 2. University of Kansas Medical Center, Kansas City, Kansas, United States*

Research suggests that death anxiety stems from fear of pain, worry about loved ones, and uncertainty about what comes after death. Understanding the relationship between coping styles and attitudes towards death in older adults may help identify individuals who need support with death anxiety. This study explored the relationships between coping styles (active, disengaged, social) and death anxiety (fear, avoidance). We used the Death Attitude Profile Revised and three subscales from the Brief Coping Orientation to Problems Experienced (COPE) Inventory. We conducted linear regressions to determine which coping styles were associated with fear of death and death avoidance. In post-hoc analyses, we investigated the role of spirituality-based coping as a two-item subscale from the active coping scale. All models controlled for age, sex, marital and educational status. The sample included 87 community-dwelling older adults (Mage=72.72 (SD=5.88); 56.32% female; 86.21% White). Higher levels of disengaged coping were significantly associated with greater fear of death and death avoidance ($p < .05$). Use of social support coping was significantly associated with less fear of death ($\beta = -.10$, $p < .05$). Spirituality-focused coping was associated with lower death avoidance ($p < .05$). Disengaged coping may indicate higher death anxiety, whereas spirituality and social support coping strategies may indicate lower death anxiety. Our findings have implications for identifying individuals in need of extra support during critical points in the healthcare process. They may also inform design and implementation of psychosocial interventions for communication about healthcare goals in the context of serious or terminal illness.

### A QUALITATIVE EVALUATION OF A POSITIVE REAPPRAISAL INTERVENTION FOR HOSPICE CAREGIVERS

HanNah Cho[1], George Demiris[1], Debra Parker Oliver[2], and Karla Washington[2], *1. University of Pennsylvania, Philadelphia, Pennsylvania, United States, 2. Washington University in St. Louis, St. Louis, Missouri, United States*

For older adults who receive hospice services in the United States, family members often become their primary caregivers playing an essential role in delivering care. Nearly 25% of hospice patients and families report the need for improved communication and additional emotional support. However, there are few interventions targeting specifically caregivers. Whereas the term caregiving implies a unidirectional flow of benefits from the caregiver to the care recipient, caregivers also experience emotional, cognitive, behavioral, or interpersonal rewards from caring for a loved one. Research has demonstrated that positive gains are common among caregivers, often manifested with negative experiences. Positive reappraisal, a form of emotion-focused coping, is especially relevant when dealing with intractable stressors. Positive reappraisal is supplemental to problem-solving therapy interventions, yet this component has never been tested in a hospice setting. In this study, we wanted to understand hospice caregivers' impressions of a problem-solving therapy intervention enhanced with positive reappraisal modules (called PISCESplus). We conducted a qualitative study examining hospice caregivers' perceptions of positive reappraisal. Participants received the intervention (as part of a larger clinical trial). Caregivers' exit interviews were audio-recorded and transcribed. Thirty hospice caregivers' interviews were analyzed. Many participants reported a paradigm shift in their thought processes. Positive reappraisal helped them reframe negative emotions to positive ones, declutter their minds, and acknowledge that caregiving required work and effort. Study findings demonstrate the potential of this intervention to improve the coping skills of caregivers in the hospice setting.

### INDIVIDUALS' KNOWLEDGE ABOUT A LOVED ONE'S END-OF-LIFE CARE WISHES IS ASSOCIATED WITH THEIR OWN ADVANCE CARE PLANNING

Peiyuan Zhang[1], and John Cagle[2], *1. University of Maryland Baltimore, Baltimore, Maryland, United States, 2. University of Maryland, Baltimore, Baltimore, Maryland, United States*

Despite evidence that ACP can improve quality of life for both patient and family, it remains underutilized. Only 37% US adults have a complete advance directive (Kuldeep et al., 2017). Existing studies primarily examined factors associated with patients' ACP engagement but very few explored ACP among patients' family. The goal of this study is to examine whether individuals' knowledge about a loved one's end-of-life (EOL) care preferences is associated with their ACP engagement. Data are from the US arm of the 2015 Four-Country Survey on Aging and End-of-Life Medical Care. The sample included N=609 adults who experienced the death of a family or close friend in the past 5 years.Three binary dependent variables were measured by different facets of ACP: having a serious conversation about EOL medical care wishes (1) with loved ones, (2) with doctors and (3) documenting those wishes. The primary independent variable was participants' knowledge about their deceased family's EOL treatment wishes.Three separate logistic regression models were used. Individuals who had a greater knowledge about their loved one's EOL treatment wishes were twice as likely to have a conversation with family about their own EOL wishes (OR=2.32, $P< 0.001$) and documented wishes than those who didn't(OR=2.03, $P< 0.05$). Results have direct implications for clinicians who work with families in EOL care settings. They may have opportunities to engage individuals – other than patients – in ACP. Focusing the experience of involvement in a loved one's EOL care may be an effective strategy to enhance ACP engagement.

### UCHINANCHU AMERICAN OLDER ADULTS' INFORMATION PREFERENCES AND BEHAVIORS IN END-OF-LIFE DECISION-MAKING

Kristina Shiroma[1], and Bo Xie[2], *1. University of Texas at Austin, Austin, Texas, United States, 2. The University of Texas at Austin, Austin, Texas, United States*

End-of-life (EOL) decisions are unique and sensitive, requiring nuanced health information. Careful examination of ethnic minority older adults' information preferences and behaviors in EOL decision making from a cultural perspective is increasingly necessary as the national and global population grows older and more diverse. Little is known about the information preferences and behaviors of Asian American older adults in EOL decision-making, especially for underrepresented ethnic minorities such as Uchinanchu American older adults. This pilot study examined Uchinanchu American older adult's information preferences and behaviors in EOL decision making. In November 2019, I interviewed 4 Uchinanchu American older adults (age range: 67-85 years; Mean: 76 years; SD: 8.04) recruited from personal contacts and through Uchinanchu cultural groups in Texas and Hawaii. The interviews took place in-person and via phone. Each interview lasted approximately 90 minutes. Interviews were transcribed verbatim and analyzed using inductive thematic analysis. I identified three themes from the data: (1) EOL decision-making information not coming from healthcare professionals; (2) Funeral planning easier to talk about than advance EOL medical decision making; and (3) Cultural traditions may exclude people (women; non-first born children) from EOL decision making. My findings suggest possible misalignments of EOL communication between medical professionals and Asian ethnic minority older adults. These findings may inform the development of culturally competent interventions to enhance EOL decision making.

### INTERPROFESSIONAL TEAM MEMBERS' PERCEPTIONS OF PALLIATIVE SOCIAL WORKERS' CONTRIBUTIONS TO PATIENT-CENTERED CARE

Arden O'Donnell[1], and Judith Gonyea[2], *1. Boston University, Medfield, Massachusetts, United States, 2. Boston University, Boston, Massachusetts, United States*

Interprofessional collaborative practice (IPCP) is fundamental to the delivery of high-quality patient-centered care. An IPCP core tenet is that efficient and effective teams are crucial for the delivery of care that achieves patient satisfaction as well as positive patient-level outcomes. Although a growing number of IPCP studies explore how IPCP professionals work collaboratively, the vast majority focus on the interactions of physicians and nurses, few consider other professions or simply lump them into an "other" category. This qualitative study addresses this knowledge gap through the exploration of how hospital palliative care teams utilize the skills of advanced hospice and palliative care certified (APHSW-C) social workers to increase teams' overall effectiveness. A two-step recruitment process was used to create matched samples of 17 palliative social workers and 24 interprofessional team members (i.e., physicians, nurses, physician assistants, chaplains). Using a constructivist grounded theory approach, semi-structured interviews were conducted, recorded, and transcribed. Analysis revealed six themes surrounding other profession's perceptions of social workers' contributions: 1) providing continuity during and across admissions, 2) assessing and promoting the patient's bio-psychosocial needs, 3) offering clinical expertise and support to patients with complex psychosocial needs, 4) allocating time to initiate, process and revisit difficult conversations with patients and families, 5) modulating the pace and intensity of emotionally laden conversations, and 6) supporting team well-being. Findings underscore that interprofessional team members view social workers as playing a significant role in developing, building, and sustaining a therapeutic alliance between the patient and the team.

### SESSION 6740 (POSTER)

### EPIDEMIOLOGY, BIOLOGY, CHRONIC DISEASES, AND FUNCTION II

### AGE-RELATED IMMUNE CELL PHENOTYPES IN THE FRAMINGHAM HEART STUDY PARTICIPANTS

Yuan Fang[1], Jiachen Chen[1], Kathryn Lunetta[1], Joanne Murabito[2], and Margaret Doyle[3], *1. Boston University School of Public Health, Boston, Massachusetts, United States, 2. Boston University, Boston, Massachusetts, United States, 3. University of Vermont, Colchester, Vermont, United States*

Mounting evidence supports a role for the immune system in the pathophysiology of dementia. Alterations in the composition of circulating immune cells is one of the key changes associated with age. However, the role of age and sex on immune cell phenotypes and their contribution to disease pathogenesis remains to be unraveled. We identified a study sample of 996 participants (mean age 62 years, range 40 to 88 years, 52% female, 22% APOE-ϵ4 carriers) from the community-based Framingham Heart Study Offspring cohort who were dementia and stroke free at the seventh examination (1998-2001), the first exam with existing stored peripheral blood mononuclear cells (PBMC). We profiled 132 circulating immune cell phenotypes from cryopreserved PBMCs by flow cytometry using standardized protocols. T cells, B cells, NK cells, and monocytes were reported as percentage of gated lymphocytes or monocytes. All other subsets were reported as percentage of the appropriate parent (e.g. CD4+ and CD8+). We summarized the distributions of each phenotype with age, sex, and APOE-ϵ4 genotype. We also evaluated the differences in percentages of T-cell subtypes including CD4+, and CD8+ T cells and their naïve, effector memory, and central memory subsets across age groups and sex. We observed that for both CD4 and CD8, naïve cells decrease with age, whereas effector/memory cells increase with age with no difference by sex. Future analyses will explore the association between immune cell phenotypes with cognitive test performance and risk of dementia in the context of exposure to cytomegalovirus infection.

### THE EFFECT OF SHORT DURATION BED REST AND TYPE 2 DIABETES MELLITUS ON ARTERIAL STRUCTURE AND FUNCTION IN OLDER ADULTS

Kseniya Masterova[1], Tatiana Moro[2], and Elena Volpi[3], *1. University of Texas Medical Branch at Galveston, Houston, Texas, United States, 2. University of Padua, Padova,*

*Veneto, Italy, 3. University of Texas Medical Branch, Galveston, Texas, United States*

Type 2 diabetes mellitus (T2DM) and inactivity individually accelerate changes related to vascular aging. These changes increase cardiovascular risk and contribute to morbidity and mortality in the elderly. It is unknown if T2DM and bed rest have an additive, deleterious effect on vascular structure and function in older adults. The objective of this study is to determine the magnitude of the effect of bed rest on vascular structure in older adults with T2DM compared to healthy controls and determine if resistance exercise is protective of this effect. So far, we have recruited T2DM (n=9) and healthy control (n=17) subjects (age: 67.0 ± 6.7 years) to undergo five days of bed rest. During bed rest, subjects were randomized to receive intensive bedside resistance exercise physical therapy or standard of care in-bed passive physical therapy. On bed rest days 1 and 5, popliteal artery diameter, area, blood velocity, and blood flow were measured using Doppler ultrasonography. Our preliminary data show that the T2DM, non-exercise group had significantly greater decreases in popliteal artery diameter (-0.59 mm ± 0.18) than the control, non-exercise group (-0.17 mm ± 0.11). Resistance exercise did not prevent artery size changes in either controls or T2DM. Resistance exercise showed a trend in preventing blood velocity and flow reduction in both T2DM and control groups. These preliminary data suggest that older adults with T2DM had greater structural popliteal artery changes compared to controls. Resistance exercise appears to help maintain blood flow by maintaining/increasing popliteal artery blood velocity, but not size.

### MULTISENSORY IMPAIRMENTS AND BRAIN VOLUMES IN MID-TO-LATE LIFE

Amal Wanigatunga[1], Chenxin Tian[1], Yang An[2], Yurun Cai[3], Alden Gross[1], Eleanor Simonsick[2], Jennifer Schrack[1], and Yuri Agrawal[4], *1. Johns Hopkins Bloomberg School of Public Health, Baltimore, Maryland, United States, 2. National Institute on Aging, Baltimore, Maryland, United States, 3. University of Pittsburgh, Pittsburgh, Pennsylvania, United States, 4. Johns Hopkins School of Medicine, Baltimore, Maryland, United States*

Age-related sensory impairment, such as loss in vision or hearing, have been linked to poor brain health. Yet, the relationship between co-occurring sensory impairments and brain volumes remains unclear. We used cross-sectional sensory and brain imaging data from 208 cognitively normal participants of the Baltimore Longitudinal Study of Aging (mean age 72 years; 59% women). Sensory impairments were separately identified with vision, hearing, smell, proprioception, and vestibular function testing. Brain imaging volumes were derived using an automated multi-atlas approach. Multiple linear regression models were used to estimate brain volumetric differences by number of sensory impairments (0-5) or by multisensory impairment status (MSI; ≥2 impairments). For every one additional sensory impairment, there was lower volume in the orbitofrontal gyrus (beta=0.35 cc [SE=0.17], p=0.04) but higher volumes in the caudate (0.14 cc [0.05], p=0.006) and putamen (0.13 cc [0.06], p=0.043). Participants with MSI (versus no MSI) had lower volumes in superior frontal gyrus (-1.01 cc [=0.34], p=0.003), lower orbitofrontal gyrus (-0.91 cc [0.38], p=0.018), superior parietal lobe (0.68 cc [0.27], p=0.011), and precuneus (-0.74 cc [0.35], p=0.038), but similar volumes in the caudate (p=0.12) and putamen (p=0.14). A negative relationship between MSI and brain volume across multiple regions was largely observed yet our exploratory findings raise the possibility that MSI is associated with compensatory maintenance of brain volume structures in the basal ganglia. Future directions include replication of these findings in other studies and longitudinal analyses to determine how MSI relates to brain atrophy.

### UNTREATED OBSTRUCTIVE SLEEP APNEA INCREASES HOSPITALIZATION RISK IN MEDICARE BENEFICIARIES 65+ WITH HEART DISEASE

Jennifer Kirk[1], Emerson Wickwire[2], Virend Somers[3], and Jennifer Albrecht[4], *1. University of Maryland, Baltimore, Annapolis, Maryland, United States, 2. University of Maryland, School of Medicine, Balrtimore, Maryland, United States, 3. Mayo Clinic, Rochester, Minnesota, United States, 4. University of Maryland, School of Medicine, Baltimore, Maryland, United States*

Cardiovascular disease (CVD) is a leading cause of death and disability in the United States and worldwide. Untreated obstructive sleep apnea (OSA) is common among older adults with CVD and may increase their risk of hospitalization. However, prior research has been limited by smaller samples of participants undergoing diagnostic sleep testing. Our objective was to estimate the effect of untreated OSA on the risk of hospitalization among older Medicare beneficiaries with CVD age 65+. Using a 5% sample of Medicare administrative claims data from 2006 to 2013, beneficiaries were identified as cases diagnosed with OSA or controls without any sleep-related diagnoses. Then, we restricted only to beneficiaries with a CVD diagnosis by the index date (i.e., OSA diagnosis date or matched date in controls). Next, we assessed the risk of hospitalization, counting only one per beneficiary, during the year before the index date. Because OSA is typically present long before it is diagnosed, the year before OSA diagnosis was assumed to represent a period of untreated OSA. Covariates were balanced between cases and controls using inverse probability of treatment weighting (IPTW). Then using logistic regression, we estimated the risk of hospitalizations the year prior to the index date. Of the 142,893 beneficiaries with CVD, only 19,390 were diagnosed with OSA. In the IPTW model, beneficiaries with untreated OSA were more likely to be hospitalized (odds ratio, 1.82; 95% CI 1.77–1.87). Results suggest screening for OSA among Medicare beneficiaries with CVD may identify older adults at increased risk of hospitalization.

### IMPACT OF SEX AND ANTIHYPERTENSIVE MEDICATION ON GLOBAL COGNITION IN PRIMARY CARE OLDER ADULTS

Adrian Noriega de la Colina[1], Helen-Maria Vasiliadis[2], Djamal Berbiche[3], Louis Bherer[4], helene Girouard[4], and Navin Kaushal[5], *1. McGill University, Westmount, Quebec, Canada, 2. Sherbrooke University, Longueuil, Quebec, Canada, 3. Sherbrooke, Sherbrooke, Quebec, Canada, 4. University of Montreal, Montreal, Quebec, Canada, 5. Indiana University, Indianapolis, Indiana, United States*

Hypertension is one of the strongest modifiable risk factors for the development of cognitive impairment and dementia. However, there are conflicting reports regarding which class of antihypertensive medication is the best for reducing the risk of cognitive decline. The objective of this study is to determine whether sex determines the pharmacological therapy that is the most effective in preserving cognitive outcomes. This study examined 1607 participants from the ESA Services Study, a longitudinal survey of older adults over 65 years old in Quebec-Canada. They were examined for the Mini-Mental State Examination(MMSE) at baseline (T1) and followed up three (T2) and four years after (T3). Hypertensive women had the highest mean MMSE score at each time point (T1 28.591 (SE .064); T2 28.282 (SE .118); T3 28.524 (SE.119)), while hypertensive men had the worst (T1 28.038(SE.070); T2 27.694(SE.125); 27.809(SE.128)). Women taking angiotensin II receptor antagonists (ARBs) showed the highest MMSE scores (p<.003), and men taking diuretics and other antihypertensives had the lowest MMSE scores(p<.001) after a 3-year follow-up. Combination therapy of two or three antihypertensives drugs was associated with higher scores in women at T1 and T2 (p<.001). In men, taking three antihypertensives showed a sharp decrease in MMSE scores from T1 to T3 (p<.001). Sex differences in global cognition outcomes in older adults are in part due to the heterogeneity in effects related to the type and number of antihypertensive drugs used. Effective antihypertensive treatment should consider the impact of sex to optimize the effect of pharmacological interventions on cognition.

### SENSORY PROCESSING CORRELATES WITH DEPRESSION AND PERCEIVED STRESS IN OLDER ADULTS

Megan Chang[1], Chiung-ju Liu[2], and Erna Blanche[3], *1. San Jose State University, San Jose, California, United States, 2. University of Florida, Gainesville, Florida, United States, 3. University of Southern California, Los Angeles, California, United States*

Sensory processing is defined as the ability to respond to sensory information from the environment and to act accordingly to the situational demands. Sensory processing is associated with anxiety in middle-aged adults, specifically in those with sensory over-responsiveness and under-responsiveness. It remains unclear how age-related change in sensory processing is correlated with mental health. The purpose of this study is to examine the correlations between sensory processing patterns, depression, and perceived-stress in older adults. Respondents were recruited from community networks serving older adults. They were asked to complete an electronic survey, including the Adult Sensory Processing Scale (ASPS), the Perceived Stress Scale, and the Center of Epidemiologic Depression Scale-Revised. ASPS has 11 factors related to over-responsive, under-responsive, and sensory seeking in visual, auditory, tactile, vestibular and proprioceptive input.Of 148 older adults (Mean age = 72 years) completed the survey, 30% perceived moderate to high levels of stress, and 18% had depressive symptoms. The total score of the ASPS Scale is positively correlated with perceived stress (r=0.26; p=.001) and depression (r=0.27; p=.001). Specifically, those who were over-responsive to auditory and vestibular input, and under-responsive to proprioception had higher stress levels and greater depression.Sensory decline or impairment in older adults may alter older adults' ability to process sensory information. As sensory processing has significant impact on anxiety and perceived stress in older adults, it should be considered in evaluation and intervention, particularly on audition, vestibular and proprioception. Including sensory-based approach may help better manage their mental health.

### FREQUENT CAUSES OF 30-DAY READMISSION FOLLOWING A FIRST EPISODE OF HEART FAILURE IN OLDER ADULTS

Ali Vaeli Zadeh[1], Andrew Crawford[2], Alan Wong[2], Elias Collado[1], and Joshua Larned[3], *1. Holy Cross Health-Jim Moran Cardiovascular Research Institute, Fort Lauderdale, Florida, United States, 2. Holy Cross Health-University of Miami, Fort Lauderdale, Florida, United States, 3. Holy Cross Health- University of Miami, Miami, Florida, United States*

Older adults with heart failure (HF) have a greater propensity to experience poor clinical outcomes, hospital readmission, and functional decline. It is important that healthcare workers are aware of the comorbidities that may lead to poor outcomes. A deeper understanding of potential causes of readmission will help in adequate prevention. Employing an all-payers claims database, we aimed to examine the diagnoses related to 30-day readmission in patients aged 65-75 with an Elixhauser-Comorbidity Index score < 4 upon the first episode of HF. Exclusion criteria included in-hospital death, readmissions for the same condition, and discharge against medical advice. Using ICD codes 9-10, a cohort of 323,678 patients (mean age: 70, SD: 3.22; male: 60%) with the first episode of HF between 2015-2019 were identified. We found 7,729 (2.4%) readmissions within 30 days and examined the frequency of 118 diagnoses as the primary cause. Cardiovascular etiologies including HF, hypertension, arrhythmias, and coronary artery disease were the most prevalent causes of readmission (40.64%), with HF being the most frequent (13.25%). Non-Cardiovascular causes included pulmonary (5.94%), sepsis (7%), renal (3.8%), and cerebrovascular (1.58%). Metabolic, liver, gastrointestinal diseases, and other infections accounted for 19.32%. Understanding the diagnoses related to readmissions in different populations is crucial for improving care, conducting research, and lowering costs. We focused on the first episode of HF in a relatively healthy older population to control for confounding factors. Further studies considering demographics, HF characteristics, and baseline comorbidities are needed.

### HEALTHCARE UTILIZATION IN OLDER ADULTS (≥70 YEARS) UNDERGOING CELLULAR THERAPY FOR HEMATOLOGIC MALIGNANCIES

Christina Snider, Hina Budhwani, Melissa Logue, Parul Goyal, Kelly Pugh, Harvey Murff, Adetola Kassim, and Reena Jayani, *Vanderbilt University Medical Center, Nashville, Tennessee, United States*

Increasingly, older adults are receiving hematopoietic cell transplant (HCT) and chimeric antigen receptor T-cell therapy (CAR-T), intensive therapies for treatment of hematologic cancers which typically require prolonged hospital admissions. Older adults are at high risk of increased healthcare utilization and complications of prolonged hospitalization [Mudge, J Am Geriatr Soc, 2019]. We identified patients age ≥70 years who received HCT or CAR-T in a primary outpatient transplant program at Vanderbilt University Medical Center between 1/1/19 and 12/31/20. Healthcare utilization, including all visits and admissions, was captured from the start of conditioning chemotherapy through early post-therapy. Thirty-eight patients met inclusion criteria; 26 (68%) received autologous HCT (autoHCT), 7 (18%) allogenic HCT (alloHCT), and 5 (13%) CAR-T. Twenty-four patients (63%) had high HCT-Comorbidity Index (HCT-CI). Eighteen (69%) autoHCT, 6 (86%) alloHCT, and no CAR-T patients had at least one unplanned admission. The median number of total hospital days (LOS) was 7.5 (2-14), 8 (4-62), and 9 (7-9) days, respectively. One-year mortality was 12% (3) in autoHCT, 43% (3) in alloHCT, and 0% in CAR-T. Low performance status and high HCT-CI did not correlate with LOS (p=0.58 and p=0.16, respectively) or number of outpatient visits (p=1, p=0.19). In conclusion, most patients who received auto- or alloHCT in a planned primary outpatient setting experienced at least one unplanned admission. LOS duration varied widely with shorter LOS among autoHCT patients. Further research is needed to identify factors among older adults (≥70 years) at risk of increased healthcare utilization during HCT or CAR-T.

### THE EFFECT OF AGE WHEN USING LLLT TO TREAT PAIN IN OLDER ADULTS

Alex Habegger[1], Danny Curtis[2], Dawn James[1], Alexis Kendrick[2], LaVona S. Traywick[2], Antonio Varela[2], Teressa Brown[2], and Reed Handlery[2], *1. Arkansas College of Health Education, Fort Smith, Arkansas, United States, 2. Arkansas Colleges of Health Education, Fort Smith, Arkansas, United States*

Low-level laser therapy (LLLT) is commonly used to treat pain; however, little is know about the effects of age on outcomes of the treatment. Participants included community dwelling adults (22 females, 9 males) with a mean age of 55 (n=31, range 29 to 77 years). Prior to participation, physical therapist examined each subject to determine appropriateness for treatment with LLLT. Following the examination, subjects received 12 sessions of LLLT using a Class 3B laser device. Intervention was administered by researchers trained in appropriate application of the intervention. The WALT guidelines and specific anatomical location of pain determined dosage of LLT. The most common site of pain or discomfort was hip and thigh pain (23%). Subjects completed the Patient-Specific functional Scale (PSFS) pre- and post-treatment and used the Numeric Pain Rating Scale (NPRS) before and after each intervention session. An analysis of the data showed that age was positively correlated (r=xx) and statistically significant ($p < 0.05$) with changes in both current and worst pain ratings and the first item on the PSFS. Age explained 13.4% of worst pain (p=0.024). Although not statistically significant, age explained 9.2% of the variability in current pain (p =0.054) and 3.9% of variability in patient identified changes in function (p = 0.147). Results suggest that older patients treated with LLLT may experience greater positive changes in pain than younger patients.

### EFFECTS OF ASSISTIVE TENNIS SHOES ON GROUND REACTION FORCE FOR PATIENTS WITH PERIPHERAL ARTERY DISEASE DURING WALKING

Nathaniel Evans[1], Hafizur Rahman[2], Iraklis Pipinos[3], Jason Johanning[3], Mahdi Hassan[2], and Sara Myers[2], *1. University of Nebraska Omaha, Omaha, Nebraska, United States, 2. University of Nebraska at Omaha, Omaha, Nebraska, United States, 3. University of Nebraska Medical Center, Omaha, Nebraska, United States*

Peripheral artery disease (PAD) is caused due to buildup of atherosclerotic plaques, typically in the leg arteries, preventing adequate blood circulation and ultimately claudication. A previous study showed that the vertical ground reaction force (VGRF) curve is significantly flatter in claudicating patients, resulting in a lower and less fluctuant center of mass when ambulating. Patients with PAD also demonstrate significantly decreased propulsion forces in the anterior– posterior direction. Assistive tennis shoes (carbon fiber: CF, and spring-loaded: SL) can potentially assist push-off by substituting for muscle forces using energy stored in a carbon fiber plate or metal spring within the shoe. This study aims to examine how the CF and SL shoes impact walking performance in patients with PAD. A total of ten patients with PAD performed a progressive treadmill test using a pressure-instrumented treadmill for each shoe type: i) standard shoes, ii) CF shoes, and iii) SL shoes. We calculated the peak VGRF for three subjects to date as an average of ten stance phases for the beginning of the walking condition (pain free condition). Preliminary results from three subjects showed that patients with PAD generated a greater peak VGRF wearing CF and SL shoes compared to normal shoes during the heel contact (normal: 0.97±0.10BW, CF: 1.03±0.08BW, and SL: 1.09±0.10BW) and push-off (normal: 0.97±0.06BW, CF: 0.99±0.04BW, and SL: 1.03±0.05BW). In future, we will calculate the VGRF for the remaining patients in pain free and pain conditions and conduct statistical analysis to identify significant differences among shoe types.

### OLDER DYADS USING A WEARABLE PERSONAL SLEEP MONITORING DEVICE FOR SLEEP SELF-MANAGEMENT

Cynthia Jacelon[1], Maral Torrisan[2], and Sarah Fiske[3], *1. University of Massachusetts Amherst College of Nursing, Amherst, Massachusetts, United States, 2. U. Massachusetts, Amherst, Amherst, Massachusetts, United States, 3. Elaine Maarieb College of Nursing, University of Massachusetts Amherst, Amherst, Massachusetts, United States*

Normally occurring changes in sleep patterns can affect behavior, safety, and function in older individuals. In addition, altered sleep of one partner can affect the functioning of the other. We tested a novel intervention using an off the shelf, wrist-worn actigraph as a personal sleep monitoring device (PSMD) with dyads comprised of individuals who were 70 years old or older, and slept in the same house. Aims included: 1) Establish the feasibility of sleep self-monitoring using PSMDs, as a self-management strategy. 2) Establish the feasibility of PSMD data sharing among members of the dyad to improve sleep self-management and improve sleep quality; and 3) Evaluate the usability of PSMDs and data sharing for dyads of older individuals. Over the course of a five-week trial, we used a mixed methods approach. Data were comprised of daily sleep diaries, data from the PSMD, weekly questionnaires regarding sleep patterns and function, and qualitative interviews focused on sleep, self-management within the dyad, and the usability of the PSMD. Data were analyzed using qualitative or statistical methods. The use of the PSMD increased awareness of sleep patterns at the individual and dyadic levels, but the limitations of the PSMD were frustrating. Participants valued having a graphic image of how their daily activities affected their sleep patterns. The dyadic approach was effective in improving sleep patterns for older individuals, and using over the counter activity monitoring devices provide a relatively inexpensive way to assist older adults to improve their sleep self-management.

### MULTIMORBIDITY PROGRESSION AMONG MEDICARE BENEFICIARIES IN THE HEALTH AND RETIREMENT STUDY (1991–2015)

Ana Quiñones[1], Sheila Markwardt[2], Michelle Bobo[2], Heather Allore[3], Jason Newsom[4], Corey Nagel[5], Anda Botoseneanu[6], and David Dorr[2], *1. Oregon Health and Science University, Portland, Oregon, United States, 2. Oregon Health & Science University, Portland, Oregon, United States, 3. Yale University, Guilfrod, Connecticut, United States, 4. Portland State University, Portland, Oregon, United States, 5. University of Arkansas for Medical Sciences, Little Rock, Arkansas, United States, 6. University of Michigan Dearborn, Dearborn, Michigan, United States*

Older adults are at greater risk for developing and accumulating multimorbidity (2+ chronic conditions). This study describes the characteristics of older adults with multimorbidity using Medicare claims over a 24-year period. The HRS-Medicare data (1991-2015, N=17,199, age 67+ years) were used in descriptive analyses. We identified 8,800 (51%) beneficiaries who did not have multimorbidity at first observation (no multimorbidity), 6,270 (36%) with 2-4 conditions at the first observation (low multimorbidity), and 2,129 (12%) with 5+ conditions at the first observation (high multimorbidity). Older respondents (80+ years) had lower levels of multimorbidity at baseline compared with younger respondents (< 70 years); this appeared to be related to differences by birth cohort. Higher proportions of minoritized groups had high multimorbidity (15% non-Hispanic Black; 20% Hispanic; 11% non-Hispanic White). Despite only being 22% of the entire sample, persons with obese body mass index (>30) represented 41% of the high multimorbidity group. Higher incidence rates of disease accumulation were noted for Black (89/1,000 person years [py]) and Hispanic (94/1,000 py) compared with White respondents (82/1,000 py) in the high multimorbidity group. Hospitalization and ED rates increased linearly between the low and high multimorbidity groups. Functional loss was more prevalent in the high multimorbidity group compared with the no multimorbidity group; no difference was observed between no and low multimorbidity. Results highlight sociodemographic differences between Medicare fee-for-service beneficiaries with differing levels of multimorbidity, allowing new insights to guide interventions that address burdensome changes in late life.

### EFFECT OF HYPERTENSION ON PREFRONTAL CORTEX ACTIVATION IN OLDER ADULTS DURING INSTRUMENTED TRAIL WALKING TASKS

Manuel Hernandez[1], and Alka Bishnoi[2], *1. University of Illinois at Urbana-Champaign, Urbana, Illinois, United States, 2. University of Illinois at Urbana-Champaign, Urbana Champaign, Illinois, United States*

Hypertension (HTN) can lead to non-amnestic cognitive impairment. However, it is unclear if this cognitive impairment affects dual task walking in older adults with HTN. The objective of this study was to evaluate the differences in prefrontal cortex (PFC) oxygenated hemoglobin (HbO2) levels during comfortable walking (CW) and an instrumented trail walking task (TWT) in older adults with HTN. We hypothesized that older adults with HTN would show decreased activation, or lower PFC HbO2 using functional near-infrared spectroscopy, during TWT in comparison to older adults without HTN. There were 15 adults in HOA group (11 females; 65±9 years old), and 9 adults in HTN group (3 females; 68±8 years old). The experimental paradigm consisted of three tasks (CW, TWT-A, TWT-B) in each of two blocks, with difficulty increasing from TWT-A to TWT-B through the inclusion of task-switching. A linear mixed effect model was conducted to investigate the effects of cohort, task, block and interaction between them on HbO2. We found that: 1) HTN adults exhibited lower HbO2 levels compared to HOA ($p < 0.001$), during TWTs but not CW; and 2) increased HbO2 levels were observed in TWTs relative to CW. During TWTs, HTN adults showed decreased activation compared to HOA, which shows that their ability to focus on dual task execution is limited. The hypertensive adults face difficulty in matching the attentional demands of the dual task, similar to cognitively impaired older adults, which may be partly explained by capacity sharing theory.

### INFLUENCE OF TOTAL KNEE REPLACEMENT ON LEAN MASS MEASUREMENTS USING DUAL-ENERGY X-RAY ABSORPTIOMETRY

Jae Young Jang[1], Miji Kim[1], Daehyun Lee[2], Hyung Eun Shin[1], and Chang Won Won[1], *1. Kyung Hee University, Seoul, Seoul-t'ukpyolsi, Republic of Korea, 2. Kyung Hee Univerisity, Seoul, Seoul-t'ukpyolsi, Republic of Korea*

Sarcopenia and osteoarthritis often occur together. Many sarcopenic patients with osteoarthritis are managed by total knee replacement (TKR). The prevalence of TKR is increasing in older adults; however, metal implants can lead to the overestimation of lean mass (LM) in dual-energy X-ray absorptiometry (DXA). However, studies considering metal implants in DXA measurement of LM are scarce. Comparisons without and with automatic metal detection (AMD) are important for accurately measuring LM. Therefore, this study examined the effects of TKR on LM. Twenty-four subjects (mean age: 76.4±4.0 years) who underwent TKR were selected from the Korean Frailty and Aging Cohort Study. A GE Lunar iDXA (GE Healthcare Lunar, Madison, WI, USA) system was applied twice (with and without AMD). Leg LM was significantly overestimated in the right and left legs with TKR. The LMs with and without AMD were 6017.1±199.3 g and 5493.7±171.3 g, respectively ($p < 0.001$), in the right leg, and 5657.1±220.1 g vs. 5173.7±201.8 g, respectively (p< 0.001) in the left leg. The appendicular lean mass index (ALMI) without and with AMD was 6.5±0.6 kg/m2, 6.1±0.6 kg/m2 (p< 0.001). In addition, only one subject was classified as having low muscle mass without AMD, which increased to four based on AMD according to the Asian Working Group of Sarcopenia 2019 guidelines. The overestimated leg LM in subjects after TKR decreased with AMD. Furthermore, LM with AMD increased the prevalence of low appendicular LM in the diagnosis of sarcopenia. Therefore, metal implants should be considered to accurately measure LM using DXA.

### SESSION 6750 (POSTER)

### FAMILY CAREGIVING II

### CARING FOR PARENTS AND IN-LAWS IN CHINA: IMPLICATIONS FOR COGNITIVE FUNCTIONING OF ADULT CHILD CAREGIVERS

Zhiyong Lin[1], and Haoshu Duan[2], *1. University of Texas at San Antonio, San Antonio, Texas, United States, 2. University of Maryland, Baltimore, Maryland, United States*

Considerable research and public discourse on family caregiving portrays it as a stressful and burdensome experience with serious negative health consequences. Yet, there is also recent evidence indicating better health status in caregivers compared to non-caregivers. Especially for cognitive health, caring for others involves cognitively stimulating activities that can help prevent cognitive decline. Although the negative consequences of eldercare on the mental and physical health of caregivers are well-documented, how it would affect their cognitive functioning is underexplored. Using three waves of nationally representative data in China, the China Health and Retirement Longitudinal Study (CHARLS, 2011, 2013, 2018), this study investigated the longitudinal association between parental caregiving and cognitive functioning among adult child caregivers, and further examined how this association was conditioned on the relationship type (parent versus in-law), caregiving frequency, and gender of adult children. Descriptive analysis indicated that women are more likely to be occasional caregivers for their own parents and regular caregivers for parents-in-law compared to men. Results from multilevel mixed-effects models showed that caring for own parents was beneficial for adult children's cognitive functioning whereas caring for parents-in-law was not significantly associated with their cognitive functioning. Both sons and daughters benefited from caring for their own parents although the beneficial effect was stronger for sons. Thus, we recommend to consider relationship types between caregivers and care-recipients when investigating informal caregiving and various health outcomes. Furthermore, we suggest more policies and programs that aim to help children-in-law who look after their parents-in-law.

### CHANGES IN PATTERNS OF FORMAL AND INFORMAL HELP AND UNMET NEEDS AMONG KOREAN OLDER ADULTS

Seoyeon Lee[1], Seung-Eun Cha[2], and Kyungmin Kim[3], *1. Chonnam National University, Gwangju, Kwangju-jikhalsi, Republic of Korea, 2. The University of Suwon, Hwaseong-si, Kyonggi-do, Republic of Korea, 3. Seoul National University, Seoul, Seoul-t'ukpyolsi, Republic of Korea*

Despite the consistent expansion of social long-term care insurance (LTCI), informal care by family members is still dominant in Korea. This study aims to examine how older adults in need utilized formal and informal sources of help and how patterns of help and unmet needs changed over time, since LTCI has been introduced in 2008. Community-dwelling older adults (65+) from the Korean Longitudinal Study of Ageing (N = 2,501) reported their I/ADL limitations and who helped them—if they are in need in 2010 and 2018. We grouped older adults' sources of care into (a) no help, (b) informal help only, and (c) any formal help, and examined the patterns by wave and gender. Among older adults with I/ADL limitations, formal help use increased between waves (1% to 12%), suggesting the impact of LTCI expansion. Changes in the patterns of help and unmet need differed by gender. The proportion of older men with no help dramatically decreased (41% to 27%); more men received informal or formal help in 2018. In contrast, the proportion of older women who did not receive any help remained similar (33% to 34%); thus, although more women received formal help, less women relied on informal help in 2018. Within the context of LTCI expansion, older men may mobilize support from their family further, which contributes to lowered unmet needs. However, older women's unmet need did not decrease, while reduced use of informal help. These findings highlight the importance of considering gendered long-term care resources in Korea.

### ADVERSE SOCIAL EXPERIENCES, SOCIAL SUPPORT, AND HEALTH OUTCOMES AMONG BLACK AND WHITE FAMILY CAREGIVERS

Wesley Browning[1], Ghislaine Atkins[2], Pamela Bowen[1], Michael Crowe[3], Laura Dreer[1], Nicole Ruggiano[4], and Olivio Clay[1], *1. University of Alabama at Birmingham, Birmingham, Alabama, United States, 2. University of Tampa, Tampa, Florida, United States, 3. University of Alabama-Birmingham, Birmingham, Alabama, United States, 4. University of Alabama, Tuscaloosa, Alabama, United States*

Family caregivers provide care for loved ones who have difficulty performing activities of daily living. Previous research has examined the influence of direct and indirect stressors on caregiver's health outcomes but has often neglected the role of tertiary stressors (stressors that are not caused by caregiving but influence caregivers' appraisals of stress). This study examined the impact of tertiary stressors called adverse social experiences (ASEs), including negative interaction (criticism from others, excessive demands by others, being taken advantage of, and others prying into caregivers' personal affairs), experiences of discrimination, and low physician trust on caregiver health outcomes including depressive symptoms along with mental and physical health quality of life (QoL). This study also investigated the effects of social support on health outcomes in caregivers. Linear regression models were used to analyze these relationships within a group of 56 Black and White adult caregivers (18+) recruited online via social media and listservs from

April to June 2021. Caregivers provided at least 5 hours of care weekly for recipients aged at least 45 years old. Negative interaction predicted more depressive symptoms, whereas emotional support and satisfaction with support were predictors of less depressive symptoms and better mental health QoL (p's < .05). Experiences of discrimination and physician trust did not predict health outcomes. These findings suggest researchers should further investigate the impact of ASEs, particularly negative interaction, on caregiver health. Healthcare providers of caregivers should consider how ASEs and social support may affect caregivers' health and ability to care for their relatives.

### INTERSECTIONS OF SEXUAL ORIENTATION AND GENDER IN SPOUSAL CAREGIVING: A FOCUS ON COGNITIVE LABOR

Sadie Giles[1], Toni Calasanti[1], Jing Geng[2], and Brian de Vries[3], *1. Virginia Tech, Blacksburg, Virginia, United States, 2. Virginia Tech, BLACKSBURG, Virginia, United States, 3. San Francisco State University, Palm Springs, California, United States*

Our presentation adopts a lens of intersecting inequalities based on gender and sexual orientation in exploring caregiving for a spouse or partner with dementia. How the division of labor shapes caregiving approaches is sometimes examined, but how this might differ by sexual orientation has not been explored. Previous research has often examined physical tasks and, to a lesser degree, emotion work. Here, we focus on cognitive labor (Daminger, 2019): the mental labor involved in managing the household and social relationships, including anticipating needs, identifying ways to address these, making decisions, and monitoring outcomes. This theoretical backdrop informs our analysis of data garnered from in-depth interviews conducted among gay/lesbian and heterosexual older adults (N=57) who care for their spouses with dementia. However couples may have divided these cognitive steps previously, caregivers must perform them all alone, given care receivers' cognitive losses. Our analyses reveal that caregivers find being responsible for everything exhausting, but this manifests differently based on gender and sexual orientation. Heterosexual women, for instance, found making all the decisions problematic, whereas heterosexual men reported difficulty in anticipating needs. For the most part, the challenging issues for gay and lesbian caregivers were more varied, as their previous division of labor had been negotiated differently, without a reliance on traditional gender roles. Finally, we uncover a novel domain for cognitive labor: being the "memory keeper." Predominantly undertaken by women, this work involved maintaining important memories and markers of the care receiver's (and couple's) past.

### FEASIBILITY OF A COMMUNITY-LED VIRTUAL SELF-CARE PROGRAM FOR AFRICAN AMERICAN FAMILY CAREGIVERS

Abiola Keller[1], Nia Norris[2], Bashir Easter[3], Andrea Garr[4], Gail Morgan[5], Ramona Dicks-Williams[6], and Colleen Galambos[7], *1. Marquette University, Milwaukee, Wisconsin, United States, 2. UW-Madison Wisconsin Alzheimer's Institute Regional Milwaukee Office, Milwaukee, Wisconsin, United States, 3. University of Wisconsin Madison, Madison, Wisconsin, United States, 4. Wisconsin Department of Health Services, Milwaukee, Wisconsin, United States, 5. UW Madison School of Medicine and Public Health, Milwaukee, Wisconsin, United States, 6. Community Member, Milwaukee, Wisconsin, United States, 7. University of Wisconsin-Milwaukee, Milwaukee, Wisconsin, United States*

As the population of older adults in the U.S. increases, the need for family caregivers will mirror this growth. Despite a greater intensity of caregiving, African American caregivers are less likely to access formal support services and engage in self-care practices. To address the self-care needs of African American family caregivers, a community-engaged approach was used to develop and implement a half-day virtual self-care program. The development of the program was guided by the Individual and Family Self-Management Theory. This study evaluates experiences of program attendees. The specific aims are to examine the acceptability and practicality of the virtual self-care program. Women who self-identified as African American and a caregiver (i.e., taking care of another adult) were personally invited to attend. All attendees were emailed an electronic survey containing Likert-type and open-ended questions. Responses were examined for patterns and key content-related categories using inductive content analysis. Eleven of the sixteen attendees responded to the survey. All eleven strongly agreed (64%) or agreed (36%) that the event met their expectations and/or needs. In addition to providing opportunity to take time to engage in self-care, the event created a virtual space for women to focus on themselves. Women spoke about three distinct ways the event met their needs: 1) learning and trying new things, 2) access to resources, and 3) having a shared experience. These findings suggest that virtual programs may be used as an additional resource to support the health of African American women who care for older adults.

### EFFECTS OF PARENTING STYLE IN CHILDHOOD ON MENTAL HEALTH OUTCOMES OF CAREGIVING IN ADULTHOOD: A QUALITATIVE ANALYSIS

Abby Baumbach[1], M. Courtney Hughes[1], Lily Derain[1], and Yujun Liu[2], *1. Northern Illinois University, DeKalb, Illinois, United States, 2. Northern Illinois University, Naperville, Illinois, United States*

The life course perspective suggests that caregivers of aging parents bring their histories of relationships with aging parents, such as childhood maltreatment, to the care environment. These histories would then impact the dynamics and consequences of caregiving. However, there is a lack of research on the impact of childhood parental interactions on adult relationships with aging parents, particularly in the context of caregiving. The study aims to understand how relationships with caregivers while growing can impact an individual's role as a caregiver later in life. The qualitative study included 47 adult family caregiver survey respondents who care for their parents, with a mean age of 46.7, ranging from 20 to 79 years old. The respondents were asked to reflect on their recent experience of providing care for their loved ones and how experiences with their caregiver growing up may have influenced their current relationship dynamic. The researchers used Dedoose V.9.0.17 to perform a codebook thematic analysis. Themes from the survey analysis linking childhood to current experience included reciprocating good care, performing obligatory care, and stopping the generational transference of negative care. When focusing on specific parental genders when receiving care in childhood, levels of presence and affection stood out as important aspects of their father's caregiving. Themes associated with maternal caregiving included good relationships, strained relationships, and nurturing. Knowledge about the impact of childhood experiences can help program designers develop interventions to help lessen caregiver burdens that consider childhood care receiving experiences and the challenges and opportunities they present.

### FAMILY CAREGIVERS' PSYCHOLOGICAL WELL-BEING AND PHYSICAL HEALTH IN THE CONTEXT OF THE WORK ENVIRONMENT.

Barbara Hodgdon, and Jen Wong, *The Ohio State University, Columbus, Ohio, United States*

As working family caregivers navigate work and care responsibilities, the work environment may contribute to their psychological well-being and physical health outcomes. Working caregivers who provide multi-generational care (e.g., sandwiched caregivers) experience greater vulnerability when compared to non-sandwiched caregivers (e.g., filial caregivers). This vulnerability could be mitigated by supportive work characteristics (e.g., greater decision authority and supervisor support). Informed by the life course perspective, this study compared the psychological well-being and physical health of working sandwiched and filial caregivers, and the moderating role of work characteristics (decision authority and supervisor support). Sandwiched (n=80; Mage=46.3) and filial (n=62; Mage=55.7) caregivers from the Midlife in the United States (MIDUS-II) Survey provided information about their background, caregiving, employment, well-being, and health via a set of questionnaires and phone interview. Regression analyses showed that sandwiched caregivers exhibited lower levels of generativity than filial caregivers. Moderation analyses revealed that sandwiched caregivers with greater decision authority trended toward exhibiting greater autonomy than other caregivers. Sandwiched caregivers with greater decision authority also exhibited significantly less difficulty with instrumental activities of daily living (IADLs) than other caregivers. Finally, sandwiched caregivers with greater than average supervisor support trended toward exhibiting higher level of generativity as compared to other caregivers. Results highlight the impact of sandwiched caregiving on autonomy, generativity, and IADLs in the context of decision authority and supervisor support. Findings may inform workplace programs and policies aimed at enhancing caregivers' sense of decision making and increasing supervisor support in order to promote caregivers' psychological well-being and physical health.

### IMPACTS OF CAREGIVING ON WELL-BEING AMONG SPOUSAL CAREGIVERS TO OLDER ADULTS USING COARSENED EXACT MATCHING

Ruotong Liu[1], Iris Chi[2], and Shinyi Wu[2], *1. Oregon Health & Science University, Marina del Rey, California, United States, 2. University of Southern California, Los Angeles, California, United States*

Spousal caregivers to older adults may be at increased risks of negative health outcomes because they are also dealing with their own aging and health issues. Estimating the impacts of caregiving without controlling for caregivers' own aging and aging-related health decline could exaggerate the negative health consequences of caregiving, whereas focusing on caregivers only could face the risk of selection bias where healthier individuals enter and/or remain in caregiving. In this study, we used coarsened exact matching to compare outcomes in wellbeing between spousal caregivers and spousal non-caregivers. Pooled panel data from Wave 8 to Wave 13 in the Health and Retirement Study was utilized with two consecutive waves of data for each spousal caregiver. The sample included 203,162 person-wave observations from 5,162 unique individuals, among whom 3,577 were spousal caregivers. Variables used for matching were classified into three categories: care obligations, the willingness to provide care, and the ability to provide care. Outcome of interest was depressive symptoms measured by CES-D 8. A total of 2,741 (81.01%) spousal caregivers were matched with 18,043 observations of spousal non-caregivers. Regression analysis indicated that being a spousal caregiver was statistically significantly associated with a 0.27 unit increase in depressive symptoms in the subsequent wave, after controlling for other potentially confounding variables such as caregiver's race/ethnicity, length of current marriage, etc. Our results highlighted the elevated needs to address mental health among spousal caregivers, and indicated that long-term care program and policy should take into consideration the mental health among spousal caregivers.

### MEANING FOCUSED COPING: IMPLICATIONS FOR FAMILY CAREGIVER BURDEN AND POSITIVE ASPECTS OF CARE

JoAnna Dieker[1], and Sara Qualls[2], *1. University of Colorado Colorado Springs, San Antonio, Texas, United States, 2. University of Colorado Colorado Springs, Colorado Springs, Colorado, United States*

The Stress Process Model (Pearlin et al., 1991) posits that different styles of coping influence both positive and negative psychological outcomes in family caregivers. Meaning-focused coping is hypothesized to promote positive psychological experiences, but presently it is unclear how meaning-focused coping is related to caregiver burden. The present study tested the associations among meaning-focused coping, indicators of burden, and positive aspects of care in adult child and spousal caregivers of older adults with dementia. An online sample of caregivers ranging in age from 30-68 (n = 219) completed a measure of meaning-focused coping (COPE-PRG; Carver et al., 1989) and the Caregiver Reaction Scale (CRS; O'Malley & Qualls, 2016) which assessed overload, role captivity, personal growth, and competence. Regression analyses indicated that meaning-focused coping predicted less role captivity and predicted greater personal growth and competence. However, meaning-focused coping did not account for significant variance in perceived overload. The findings extend prior research by demonstrating that meaning-focused coping strategies may faciliate positive aspects of care during chronically stressful experiences such as caregiving. Findings highlight that meaning-focused strategies may be more helpful for coping with role captivity (e.g., feeling unable to participate in work or family roles outside of caregiving) and less helpful for coping with subjective overload (e.g., a sense of overwhelm) in caregivers. Results inform clinical interventions that promote meaning and reduce burden in caregivers.

### THE WORST PART OF THE DAY: DAILY REFLECTIONS OF DEMENTIA FAMILY CAREGIVERS

Chunhong Xiao[1], Vicki Winstead[2], and Carolyn Pickering[1], *1. The University of Alabama at Birmingham, Birmingham, Alabama, United States, 2. The University of Alabama at Birmingham, School of Nursing, Birmingham, Alabama, United States*

Family caregivers struggle with multi-dimensional demands in caring for persons living with dementia (PLWD). The challenges of caregiving combined with the requirements of daily life can impact the care of PLWD and the health and well-being of the caregivers. The purpose of this study was to describe caregivers' perceptions of the worst part of their day in the context of daily caregiving challenges. Family caregivers completed online surveys reporting on various parts of their day. The survey included an optional open-ended question: "what was the worst part of your day?" Caregivers (N=165) completed diaries for 21 days resulting in 1,773 surveys that included a response to the optional open-ended question. A subset of data was analyzed using content analysis to identify initial codes and themes; further content analysis of the complete dataset was used to confirm and refine the identified codes and themes. Final analysis revealed 6 themes describing caregivers' perceptions of the worst part of their day. These themes included days in which they had to: 1) cope with changes in their relationship with the PLWD, 2) manage their own health-related issues such as illness and lack of sleep, 3) struggle when there was a lack of help or support, 4) deal with daily life demands in the home along with the demands of caregiving, 5) cope with negative emotions such as sadness, grief, or anger over the disease process, 6) cope with physical exhaustion. The findings reflect daily stressors associated with caregiving for PLWD.

### HEALTH BEHAVIORS AND MENTAL HEALTH OUTCOMES OF INFORMAL CAREGIVERS DURING THE COVID-19 PANDEMIC

Yujun Liu[1], Yuxin Zhao[2], and M. Courtney Hughes[3], *1. Northern Illinois University, Naperville, Illinois, United States, 2. Booz Allen Hamilton, Washington, District of Columbia, United States, 3. Northern Illinois University, DeKalb, Illinois, United States*

Differences exist in health behaviors and mental health outcomes between spouse and adult children caregivers and between caregivers of individuals with or without dementia. We aimed to examine the change in health behaviors and mental health outcomes of informal caregivers of older adults during the COVID-19 pandemic and explore disparities based on caregiver relationship and care recipient. Using the 2020 National Health and Aging Trends Study (NHATS) COVID-19 supplement for Family Members and Friends, our sample included adult family or friend caregivers of Medicare beneficiaries aged 65 or older in the US (N = 2,062). We used ANOVA and two-way ANCOVA to assess the effects of the pandemic, caregiver relationship type, and care recipient condition (dementia vs. non-dementia) on seven health behaviors and two mental health outcomes. We performed all post hoc analyses using Bonferroni corrections controlling the covariates age, gender, marital status, education, and self-reported health of the caregiver. Compared to before the pandemic and to spouse caregivers, adult children caregivers reported: 1) significantly less time walking and significantly more time watching TV or online programs, and 2) a significantly higher level of emotional distress and depressive symptoms. Compared to caregivers for people without dementia, those who provide care for people with dementia experienced less sleep and a higher level of depressive symptoms during the pandemic than before the pandemic. Health professionals should consider the impact of COVID-19 and the vulnerability of adult children caregivers and caregivers of individuals with dementia when designing and delivering health and wellness programs.

### INFLUENCES ON CUSTODIAL GRANDCHILDREN'S RECEIVING TREATMENT FOR DISTRESS

Bert Hayslip[1], and Julian Montoro-Rodriguez[2], *1. University of North Texas, Denton, Texas, United States, 2. University of North Carolina at Chatlotte, Charlotte, North Carolina, United States*

Little work exists regarding the difficulties facing grandchildren raised by their grandparents, where previous research suggests such children to be especially at risk for psychological difficulties and that they often have limited access to potentially efficacious psychosocial interventions. The present study integrates these two perspectives in examining multiple sociodemographic, family-related, and grandchild-specific factors differentiating custodial grandchildren who were being treated for a variety of emotional and behavioral difficulties (N = 80) and those who were not (N = 157), as reported by the custodial grandparent. A MANOVA (F 23, 176 = 9.74, $p < .01$, eta2 = .56) indicated that custodial grandchildren who were receiving treatment came from larger grandfamilies, were older, were having a greater variety of grandparent reported psychosocial difficulties, and were experiencing more emotional, behavioral, attentional, and relationship problems. The grandparents of such grandchildren experienced more parental strain, reported less social support, were less resilient, were less satisfied with the grandparent role, and reported poorer health. They also reported being less strongly attached to the grandchild and expressed more negative affect toward him/her. However, they also reported more openness regarding a variety of difficulties that might be addressed by mental health professionals. A subsequent discriminant analysis reflecting a weighted linear combination of the above factors (X2 19 = 132.09, $p < .01$, Wilks' Lambda = .498) correctly classified 86.1% of cases based upon treatment status. These findings provide a basis for understanding the determinants of custodial grandchilden's receiving needed psychosocial interventions crucial to their well-being and adjustment.

### POOR SLEEP COMMON AMONG HOSPICE FAMILY CAREGIVERS AND ASSOCIATED WITH WORSE CAREGIVER HEALTH

Lauren Starr[1], Karla Washington[2], Kyle Pitzer[2], George Demiris[1], and Debra Parker Oliver[2], *1. University*

*of Pennsylvania, Philadelphia, Pennsylvania, United States, 2. Washington University in St. Louis, St. Louis, Missouri, United States*

Over 1.2 million hospice family caregivers in the United States are at risk for interrupted, insufficient sleep due to overnight caregiving responsibilities, anxiety or intrusive thoughts, and inadequate caregiving support. Insomnia contributes to health inequities yet is underrecognized and undertreated. The prevalence of insomnia among hospice family caregivers is not well understood. The purpose of this preliminary study was to describe insomnia prevalence among hospice family caregivers and identify factors that differentiate caregivers with sleep difficulties from caregivers without sleep difficulties. This observational study included 57 hospice family caregivers of cancer patients enrolled in a randomized clinical trial for caregivers [NCT02929108]. Results showed 49.1% of hospice family caregivers had subthreshold insomnia to severe clinical insomnia, as measured by the Insomnia Severity Index. Although social determinant of health variables did not differ based on caregiver insomnia status, caregivers with insomnia (5.0, median) self-rated their physical health significantly lower than caregivers without insomnia (8.0, median) (P< 0.001). Directionally, the distance a caregiver lived from a care-recipient also differed by insomnia status, with 70% of caregivers with insomnia co-residing compared to 46% of caregivers without insomnia. Overall, 78.9% of hospice family caregivers had no caregiving support; anxiety and depression were highly prevalent. Clinicians should screen hospice family caregivers for sleep disorders and seek to improve caregiver health by offering sleep interventions tailored to the specific needs of hospice family caregivers and connecting caregivers to health resources for their own wellbeing. Policy makers must expand hospice benefits to include additional caregiver support.

### CHANGING FAMILY DESTINIES, DIVERGENT FAMILY CAREGIVING PATTERNS: DO BIRTH COHORTS, GENDER, RACE, AND SES MATTER?

Haoshu Duan, *University of Maryland, Baltimore, Maryland, United States*

Over the past few decades, Americans have experienced a series of demographic transitions include prolonged longevity and rise in the complexities of family structures. The Baby Boomer cohort is at the forefront of these transitions, which has profound implications on their later-life family relations and practices of family caregiving. Most caregiving literature has focused on static care experiences over a short time, while neglecting long-term care experiences. Using 10 waves of longitudinal data from HRS (2000-2018) and latent profile analysis, I identified five prominent long-term caregiving patterns: light parental caregivers (44.1%), intensive spousal caregivers (5.6%), sandwiched caregivers (5.5%), light grandchildren caregivers (38.7%), and heavy grandchildren caregivers (6.2%). Further, I conduct multinominal logistic regression to investigate how birth cohorts, gender, race, and education shaped these patterns. Results suggested that later cohorts have seen a decline in intensive spousal caregivers, light and heavy grandchildren caregivers, but an increase in light parental caregivers. Women are more likely to be sandwiched caregivers than men, and black caregivers are more likely to be intensive spousal caregivers, heavy grandchildren caregivers, and sandwiched caregivers than white. By contrast, white and more educated caregivers are more likely to be light parental caregivers, and this pattern becomes more pronounced in later cohorts. The findings suggest divergent destinies of family caregiving patterns among later cohorts. More disadvantaged groups are shouldering heavier care responsibilities than advantaged groups. Targeted care services should be implemented to ease the care burdens of the vulnerable population.

### FAMILY DEPENDENCE AND ITS ASSOCIATION WITH SELF-REPORTED HEALTH AMONG OLDER ADULTS IN EASTERN NEPAL

Aman Shrestha[1], Isha Karmacharya[2], Saruna Ghimire[2], Ranju Mehta[3], and Uday Narayan Yadav[4], *1. Miami University, Cincinnati, Ohio, United States, 2. Miami University, Oxford, Ohio, United States, 3. Little Buddha College of Health Science, Kathmandu, Bagmati, Nepal, 4. The Australian National University, Canberra, Australian Capital Territory, Australia*

Historically, filial piety (caring for one's parents) has been prevalent in Nepal. The demographic transition has resulted in a burgeoning population of older adults, but limited institutional support is available to address their socio-economical and health needs. As such, the family has remained the paramount source of support to meet the health, social and economic needs of Nepali older adults. In this study, we explored the relationship between family dependency (conceptualized as the source of family support) and self-reported health among Nepali older adults. A community-based cross-sectional survey was conducted in two districts (Sunsari and Morang) of eastern Nepal. Using a multistage cluster random sampling design 847 older adults (≥ 60 years) were interviewed. Self-reported health was assessed in terms of a five-item Likert scale dichotomized into poor and good health. Two independent variables of interest were dependency on family for living and for daily activities. Binary logistic regression drew the inferences while adjusting for important confounders. About 29.4% of the participants reported poor health, 77% reported dependency on family for living and 46% reported dependency for daily activities. Those dependent on family for living had 46% higher odds of good health but the statistical significance was lost after adjusting for control variables. Likewise, those dependent on family for daily activities were three times more likely (OR: 3.22; 95%CI: 2.15 - 4.83) to report good health than their counterparts after controlling for confounders. Our findings emphasize the importance of family support for the health of older adults in Nepal.

### CAREGIVER TLC: A VIRTUAL PSYCHOEDUCATIONAL PROGRAM FOR CAREGIVERS: BASELINE PRELIMINARY DATA

Julian Montoro-Rodriguez[1], Dolores Gallagher-Thompson[2], Jennifer Ramsey[3], Ann Choryan Bilbrey[4], Bruno Kajiyama[5], Kendra Jason[1], and Larry W Thompson[4], *1. University of North Carolina at Charlotte, Charlotte, North Carolina, United States, 2. Stanford University, Stanford, California, United States, 3. Case Western Reserve University, Cleveland, Ohio, United States, 4. Optimal Aging Center, Sunnyvale, California,*

*United States, 5. Photozig, Inc., Moffett Field, California, United States*

Family caregivers are at increased risk for negative impacts on their psychological and physical health compared to non-caregivers. Virtual caregiving programs are beneficial as caregivers may not have time to devote to face-to-face programs and especially important to caregivers in the context of the COVID-19 pandemic. Our team at the University of North Carolina at Charlotte is testing the efficacy of the Caregiver Thrive, Learn & Connect Virtual Program adapted from the Coping with Caregiving evidence-based multicomponent intervention (Gallagher-Thompson et al., 2003). The program offers to registered caregivers six weekly sessions over Zoom teleconferencing in small groups led by trained professionals from community partners serving socio-demographically diverse caregivers. Sessions address stress management, mood management, resilience, self-care, coping strategies, and isolation. Preliminary baseline data on 42 participants indicates that caregivers are primarily female (87%), on average 64 years old, and from diverse racial backgrounds: white (69%), African American (29%) and Asian American (2%). Participants provide care to persons with memory troubles or dementia (66%) and chronic health conditions (34%). Baseline data on initial levels of caregivers' psychosocial outcomes indicated salient levels of mental health outcomes for burden (high = 49%; mild = 35%); anxiety (moderate = 16%; severe = 20%) and depression (mild levels = 35%; moderately and severe level of depression = 33%). Caregivers for chronic health conditions reported significantly higher anxiety compared to dementia caregivers. The Caregiver TLC program offers support to the targeted caregiver population seeking to improve their level of competence, mental health and social isolation.

### SESSION 6760 (POSTER)

### LONG-TERM CARE

### STATE HOME AND COMMUNITY-BASED SERVICES AND RESIDENTIAL CARE TRANSITIONS AMONG COMMUNITY-DWELLING OLDER ADULTS

Linda Chyr[1], Chanee Fabius[2], and Emmanuel Drabo[2], *1. Elevance Health, Baltimore, Maryland, United States, 2. Johns Hopkins Bloomberg School of Public Health, Baltimore, Maryland, United States*

Medicaid home and community-based services (HCBS) provide integral health-related and personal care to support community-dwelling older adults. Growing literature suggests that states with more generous HCBS expenditures may delay or substitute costly nursing home care, but evidence is limited on the impact of HCBS spending on transitions to residential care settings like assisted living. This study determines the association of state Medicaid HCBS generosity—HCBS spending as a percent of total long-term services and supports expenditure—on the probability of incident transitions to residential care settings or nursing homes. Publicly available HCBS expenditure data was linked to a nationally representative sample of 7,197 community-dwelling older adults participating in the National Health and Aging Trends Study from 2011-2018. A discrete-time, competing risk regression model estimated the association between HCBS generosity and transitions from community to residential care settings or nursing homes, adjusting for sociodemographic, socioeconomic, and health factors. Most older adults remained in the community (93.7%). Incident transitions into residential care settings were twice as likely to occur compared to transitions to nursing homes (4.0% vs 2.3%, respectively). Older adults residing in states with higher HCBS generosity (one percentage point increase) are less likely to transition to nursing homes (relative risk ratio [RRR], 0.24; 95% Cl, 0.05-1.10; p< 0.1). Greater HCBS generosity was not associated with transitions to residential care setting. Assessing HCBS generosity on transitions elucidates important contextual factors affecting the movement of older adults across the care continuum.

### WAYFINDING DESIGN IN LONG-TERM-CARE COMMUNITIES: EVALUATION OF COMPLEXITY

Rebecca Davis[1], and Margaret Calkins[2], *1. Grand Valley State University, Grand Rapids, Michigan, United States, 2. IDEAS Institute, Cleveland Hts, Ohio, United States*

Wayfinding, the ability to find one's way, is a significant problem for many older adults; especially those with cognitive impairment. Long term care communities (LTCC) are often fraught with challenges for wayfinding. Those who cannot find their way are at risk for decreased engagement and loss of independence. A critical need is to assess architectural and design features that promote effective wayfinding in an objective way. This study describes the results of a wayfinding design evaluation of 12 LTCC (4 Assisted living; 2 Independent Living; and 6 mixed residency). We measured space syntax axial integration (SSAI; a measure of the visual connectedness) and a Revised Wayfinding Checklist. The results showed low integration values in all communities, ranging from an R3 of 1.43-2.03, indicating low connectedness (and increased wayfinding complexity). The Checklist score totals ranged from 23 – 34 (M = 27.45) out of a possible total of 17 – 51. Results showed that the buildings were overall complex, all had long corridors (>100 feet), over half used multiple elevators to get to common areas; and over 60% had complex, multi-building layouts. Design wise, visibly accessible restrooms were not present; over half of the LTCC had insufficient lighting; and signage color/contrast and letter size was less than recommended. All sites had few directional signs at decision points. Thus, the review of these LTCC showed that most buildings were very complex, with low connectedness, and had room to improve design features like signage and lighting to support wayfinding ability for the residents.

### LONG-TERM CARE STAFF DECISION-MAKING PROCESSES IN DETERMINING DAILY WELL-BEING OF RESIDENTS

Mary (Libbey) Bowen[1], Ju Young Shin[2], and Meredeth Rowe[3], *1. University of Delaware and Department of Veterans Affairs, Newark, Delaware, United States, 2. University of Delaware, Newark, Delaware, United States, 3. University of South Florida, Tampa, Florida, United States*

Much of the care provided to residents in long term care (LTC) comes from staff with minimal training, particularly in the area of the assessment of complex clinical presentations. This study utilized semi-structured focus group techniques with 25 clinical staff to understand how they determine whether a resident is functioning within or outside their daily "norm" and how they follow-up on these determinations. The overall finding was that each staff member develops an internally stored phenotype of each resident's norm. Three primary themes emerged: 1) the staff's phenotype becomes the 'gold standard' to determine whether there is a change and/or a cause for concern; 2) staff include sleep routines, fatigue, participation in usual activities, physical and mental status, and social interactions with others in their phenotypes. Staff reflect in what seems like a random manner through these comparisons, and, from this process, 3) a judgement emerges that helps inform clinical decision-making and potential action of the staff member. In most scenarios, staff decided there was no cause for concern. The identified weakness in this strategy was that staff rarely mentioned or used complementary and confirmatory formal assessment or referral mechanisms to ensure their judgment was correct. Thus, staff utilize complex multidimensional thinking and memory in decision-making but lack the skills or institutional structure to augment this with more formal and reliable techniques. This represents an interesting opportunity to build on existing assessment skills with additional types of assessment, technology-based information and formal referral mechanisms.

### PEEKING UNDER THE COVERS: EXAMINING ETHICAL ISSUES IN ASSISTED LIVING

Kelly Turner[1], Gretchen Agans[2], Erin Donohue[3], Barqueisha Madison[2], Jason Lesandrini[2], Jennifer Morgan[3], and Candace Kemp[3], *1. Georgia State University & WellStar Health System, Atlanta, Georgia, United States, 2. Wellstar, Atlanta, Georgia, United States, 3. Georgia State University, Atlanta, Georgia, United States*

Assisted living (AL) communities comprise complex social environments composed of residents, staff, and other care partners, including external workers and residents' family and friends. Interactions among these stakeholders, especially pertaining to decisions related to resident care, frequently are fraught with value conflicts and uncertainties. Although much attention has been devoted to the ethics of aging and end-of-life in acute care settings, there is very little research on ethical issues in AL. To address this critical knowledge gap, our interdisciplinary team of gerontologists and bioethicists developed a typology of ethics issues in AL, adapting existing categorizations designed for acute care. We applied this typology to qualitative data gathered over a one-year period as part of an NIA-funded longitudinal study focused on residents with dementia living in four diverse AL communities (R01AG062310). The team coded 465 fieldnotes and interviews, analyzing data for the frequency and context of ethical issues. We found ubiquitous conflicts between residents' autonomy and care partners' obligations to maintain resident safety, as well as a high prevalence of organizational issues related to staffing and expertise. Comparing code frequency and type across the four communities, we discovered manifold ways in which facility size, organizational structure, and care practices affect the profile of value conflicts in resident care. During data analysis, we also developed new codes for ethics issues arising in AL, including, 'cognitive decline stigma' and 'uncertainty regarding professional obligations,' creating new avenues of study. We conclude our presentation by discussing implications for best practices and policy formation in AL.

### INVOLVEMENT IN CARE DECISION-MAKING AND ADVERSE OUTCOME ONSET IN COMMUNITY-DWELLING CARE RECIPIENTS IN JAPAN

Ayane Komatsu, Takeshi Nakagawa, Taiji Noguchi, and Tami Saito, *National Center for Geriatrics and Gerontology, Obu, Aichi, Japan*

The involvement of older adults in care decision-making may enhance their daily life motivation and quality of life. Furthermore, it could contribute to their better prognosis in long-term care. We examined the association between decision-making involvement and the onset of adverse outcomes, such as institutionalization and death, among older adults under long-term care. This study used two-year longitudinal survey data of Japanese community-dwelling care recipients aged 65 and above. The participants were followed regarding the onset of institutionalization and deaths. The status of involvement in decision-making was assessed based on one item and the selection among the following response options: "very much involved," "fairly involved," "not very involved," "never involved," "unclarified wishes," and "absence of person supporting decision-making." A multivariable logistic regression analysis estimated the odds ratios (OR) and 95% confidence intervals (CI) for the onset of adverse outcomes, composite of institutionalization and death. A total of 707 participants with no severe cognition disabilities (MMSE>12) and no missing variables at the baseline were included and responded to the follow-up survey. At the baseline, 36.5% reported being very much involved in decision-making. The onset of adverse outcomes was observed in 17.5% of participants (institutionalization, 5.1%; death, 12.4%). Compared to those with very high involvement in decision making, those who were not involved were more likely to have adverse events, even after adjusting for covariates (OR=2.86 [95% CI: 1.21-6.76], p=0.016). Our findings show the importance of decision-making involvement in daily care regarding better prognoses in long-term care.

### COMMUNICATION AND WORKFLOW ON A TYPICAL DAY IN NH VERSUS A DAY WITH A CHANGE IN RESIDENT CONDITION

Alyce Ashcraft, Donna Owen, John Culberson, and Huaxin Song, *Texas Tech University Health Sciences Center, Lubbock, Texas, United States*

A major reason technologic innovations for nursing home (NH) communication have not worked is because testing of technology fails to consider workflow of the environment. The aim of this observational study was to examine communication and workflow surrounding assessment of NH residents on a dementia care unit with suspected UTI to establish baseline data prior to introduction of technology to enhance NH staff communication. The flow of communication between CNAs, LVNs, and PCPs was recorded using a structured observation on an online survey platform. For a 3-month period, 3 days a week, an observation period from

7am to 11am was chosen to reflect one peak staff interaction time. Field notes describing the environment and what was being talked about during communication events were analyzed using content analysis. A total of 185 communication events were recorded by 3 trained observers, yielding 22 assessments for change in condition (pain, UTI, falls). The LVN was the center of 44% of communication. After the LVN assessed the resident, 43% of the time no further action was taken by the LVN; 17% of the time the PCP was texted about a change in condition. Types of change in condition was limited. Workflow surrounding LVN collection of information on days related to change in condition reflected focused communication among staff occurring away from resident rooms and nurses' station. The NH is a stable environment for provision of nursing care, thus suggesting communication technology can be accepted by staff and added into the care routine.

### COLLECTIVE TRAUMA AND PERSON-CENTERED PRACTICE IN NURSING HOMES

Jennifer Morgan, Yun-Zih Chen, Waqar Ahmad, and Elisabeth Burgess, *Georgia State University, Atlanta, Georgia, United States*

Nursing home staff have experienced multilayered collective trauma. Grief from experiencing numerous resident deaths, perceived threats to their safety, stress from understaffed working conditions, financial stress, and interpersonal conflict within overwhelmed teams may impact practice. COVID-19 precautions and pandemic working conditions may have provided additional barriers to implementing person-centered care. This study (N=379) descriptively analyzes data gathered from the staff of eleven Georgia nursing homes (summer 2021) to assess the perceived impact of COVID-19 on person-centered care practice. Approximately 40% of respondents agreed that 76-100% of resident members in nursing homes were infected with COVID-19. Over 75% of staff agreed that staff experienced anxiety related to COVID 19 precautions, COVID 19 precautions made it difficult for staff to give choice to residents, and the nursing home is more short-staffed. Conversely, more than 75% of staff reported that they were satisfied with their job, they received the appropriate training and support to be successful, nursing home managers treated direct care workers with respect and they were provided with the necessary PPE. Fewer staff (60-65%) reported that there never seemed to be enough time to get everything done, that COVID 19 precautions impacted their relationships with residents, they were provided with resources for emotional support or they received recognition for working through the pandemic. Future research is needed to understand the limiting and promoting factors for person-centered care as staff experience collective trauma.

### SUCCESSFUL TRANSITIONS TO LONG-TERM CARE COMMUNITIES

Daniel Koltz, and Rebecca Koltz, *Montana State University, Bozeman, Montana, United States*

Older adults who transition to independent and assisted-living communities perceive their transition experience differently. This research focused on understanding what factors were predominant for a successful transition from a long-time home to a dependent living community. A constructivist grounded theory method was used to explore the experiences of 18 older adults who had relocated within the past year. The participants of this study were aged 65-95 years and are equally represented by gender. Equal numbers of respondents transitioned into independent and assisted-living accommodations. Five factors related to a central concept of behavioral attitude were found to be key for a successful transition. When an older adult reported a successful transition, their behavioral attitude was positive about their new living environment. The five factors that contributed to their positive attitude are creating a new place, increased community integration, sense of safety and security, independence while dependent, and accepting a new life stage. The theory that emerged from the research emphasizes that when an older adult has increased awareness about the five factors associated with adapting to a dependent living community, this awareness will promote a positive behavioral attitude and increase the opportunity for success during and after a transition. Family members, LTC community administrators and social workers could all benefit from understanding these factors for a successful transition. Enhancing a positive experience for an older adult and improving their behavioral attitude toward the new transition.

### FAMILY MEMBERS' COMMUNICATION WITH LONG-TERM CARE PROVIDERS AND ITS INFLUENCE ON RESIDENT WELL-BEING

Francesca Falzarano[1], Verena Cimarolli[2], and Karen Siedlecki[3], *1. Weill Cornell Medicine, Douglaston, New York, United States, 2. LeadingAge, Washington, District of Columbia, United States, 3. Fordham University, Bronx, New York, United States*

Considerable research has examined communication dynamics among family members and staff in nursing homes (NHs) and has demonstrated that better communication is associated with more optimal psychosocial outcomes in both family caregivers and formal care providers. However, the literature on how communication dynamics influence resident functioning is limited, and it has yet to be determined how communication impacts residents across other care contexts, such as Assisted Living Facilities (ALFs). Thus, using data from the National Health and Aging Trends Study and the National Study on Caregiving, the purpose of this study was to examine family perceptions of communication with formal care providers (i.e., frequency, availability, and helpfulness of communication) and its influence on resident outcomes in two samples of long-term care residents (n=337 in ALFs, n=112 in NHs) and their family caregivers, and to compare how results differ across care setting. When examining the full sample of long-term care residents, findings showed that better communication was associated with lower depressive symptoms and negative affect. When investigating differences across care settings, we found that those residing in NHs exhibited higher levels of depressive and anxiety symptoms compared to ALF residents. Further, better communication was associated with lower levels of depressive symptoms only among ALF residents. Our findings provide insights into how interpersonal dynamics between family and formal care providers influence resident functioning and underscores the importance of enhanced communication among all members of the primary care team – that is, healthcare providers, residents, and their family members.

### STAFFS' PSYCHOSOCIAL WORK ENVIRONMENT IN RELATION TO RECIPIENT SATISFACTION IN HOME CARE SERVICES

Ingemar Kåreholt[1], Dan Lundgren[2], Zarina Nasar Kabir[3], and Anne-Marie Boström[3], *1. Jönköping University, Jönköping, Jonkopings Lan, Sweden, 2. Municipality of Värnamo, Värnamo, Jonkopings Lan, Sweden, 3. Karolinska Institutet, Huddinge, Stockholms Lan, Sweden*

In accordance with 'aging in place' policy, older persons in Sweden are increasingly encouraged to continue living at home and if necessary be supported by home care service (HCS). Studies have examined whether the work environment of staff has an impact on the experiences and the wellbeing of the older persons in nursing homes, but few have examined such associations in HCS. The setting was 16 HCS work units. Two surveys were sent, one to staff on psychosocial working conditions, one to care recipients on care satisfaction. For each work unit, data on individual recipient satisfaction was matched to average values on psychosocial work conditions. Outcomes analyzed with linear regressions were overall recipient satisfaction, based on one question, and indexes on: assessment of implementation of services, contact with staff, and sense of security. Index on treatment by staff was analyzed with ordered logistic regressions due to skewed distribution. We used cluster correlated standard errors (clustering on work units). Results showed that good working conditions are important for recipient satisfaction, specifically overall recipient satisfaction, treatment by staff, and sense of security. Psychosocial work factors most important were work group climate, overall job strain, sense of mastery, job control, frustrated empathy, balancing competing needs, balancing emotional involvement, and lack of recognition. Having more home help hours was associated to stronger relation between working conditions and recipient satisfaction, especially with overall recipient satisfaction and treatment by staff as outcomes.

### ORGANIZATIONAL SUPPORTS ASSOCIATED WITH NURSING ASSISTANTS' JOB SATISFACTION DURING COVID-19

Natasha Bryant[1], Verena Cimarolli[1], Francesca Falzarano[2], and Robyn Stone[1], *1. LeadingAge, Washington, District of Columbia, United States, 2. Weill Cornell Medicine, Douglaston, New York, United States*

The COVID-19 pandemic has disproportionately affected nursing home residents and staff, including the nursing assistants who provide critical supports for older adults and people with disabilities. The pandemic has added significant strain to an already vulnerable nursing home workforce, which has historically experienced high levels of turnover, chronic staffing shortages, and high burnout. At the same time, it has generated awareness of the value of the direct care workforce to provide care in places for those most at risk from the disease. Job satisfaction of nursing assistants is as major driver of turnover and intent to leave the job. Research has demonstrated organizational supports and job stresses that are associated with job satisfaction and turnover. However, limited research has investigated the factors associated with job satisfaction in nursing homes during the COVID-19 pandemic. Using data from an employee engagement and management system, we examined organizational supports (e.g., supervision, appreciation, safety) and work-related stresses associated with job satisfaction among nursing assistants in nursing homes (n=402). Higher quality of supervision, feeling appreciated for the job, and feeling safe and comfortable at work were associated with higher levels of job satisfaction. Work-related job stressors –increased workload demands and understaffing – were associated with lower job satisfaction. Our findings provide insights into the importance of employer supports and reducing work-related stressors in nursing assistants' job satisfaction and practical implications for nursing home leadership regarding how to support workers

### THE RELATIONSHIP BETWEEN THE FEAR OF FALLING AND QUALITY OF LIFE IN NURSING HOME RESIDENTS: THE ROLE OF ACTIVITY RESTRICTION

Dongjuan Xu[1], Yaqi Wang[2], Shanshan Zhu[2], and Kefang Wang[2], *1. Purdue University, West Lafayette, Indiana, United States, 2. Shandong University, Jinan, Shandong, China (People's Republic)*

Falls among older adults are a significant later life course event with detrimental impacts on health, quality of life (QOL), and mortality. Fear of falling (FOF) is a common problem, which can lead to physical and social activity restriction and poor QOL. However, few studies have comprehensively explored the relationships among FOF, activity restriction, and QOL among nursing home residents. This study aimed to investigate: 1) whether FOF is negatively associated with QOL; and 2) the mediating role of activity restriction in the relationship between FOF and QOL among nursing home residents in mainland China. This is a cross-sectional study. A total of 316 residents from 27 nursing homes participated in this study. The mixed-effects multivariate linear or ordinal logistic regression models were conducted. All statistical models were adjusted for resident- and facility-level variables. More than half of nursing home residents (55.4%) reported they often or always reduced their activities. As expected, FOF was directly associated with lower levels of QOL ($\beta = -0.19$, $p = 0.010$). However, the strength of this association was attenuated and no longer significant with the inclusion of activity restriction in the model. FOF was positively associated with activity restriction ($\beta = 0.33$, $p < 0.001$) and activity restriction was negatively associated with QOL (rarely/sometimes reduced activity: $\beta = -1.23$, $p = 0.386$; often/always reduced activity: $\beta = -3.85$, $p = 0.014$). Given the aging of our populations, it is important to take FOF and activity restriction into account when caring for older people.

### THE VALUE PROPOSITION FOR DIVERSITY: CREATING A PIPELINE OF DIVERSITY IN THE LTSS SECTOR

Natasha Bryant, Alexandra Hennessa, Adrienne Ruffin, and Robyn Stone, *LeadingAge, Washington, District of Columbia, United States*

The long-term services and supports (LTSS) sector is a microcosm of systemic racism that exits in our society. Nationally, half of frontline professional caregivers are nonwhite, while mid- and executive-level managers and board members are predominately white. Research has identified this lack of diversity in management and leadership as a major contributor to turnover and recruitment challenges among frontline staff. We will summarize the findings from three applied research activities: 1) DEI survey of chief executive officers (CEOs) of multi-setting LTSS organizations; 2) interviews with chief diversity officers and CEOs on current workplace DEI efforts and the challenges; and 3) interviews with leaders of color who have had experience with the LTSS sector. Not-for-profit multisite organizations and life plan communities lack diversity among the senior leadership team and board members (12% are people of color). A DEI initiative is more likely to be successful if it has buy-in from the CEO and board members, aligns with the organization's strategic plan and mission, is integrated into the organizational culture, and is assessed regularly to measure its impact and identify needed adjustments. Leaders of color in aging services acknowledge the barriers – lack of diversity among leaders and residents and the microaggressions that people of color experience - that make it challenging for people of color to work in the field. They recommend increasing diversity among senior leaders, investing in enhancing DEI, and spreading awareness of the LTSS field and its career opportunities to communities of color.

### AGE-RELATED DIFFERENCES IN HEALTH-RELATED QUALITY OF LIFE AMONG WESTERN CANADIAN NURSING HOME RESIDENTS

Bianca Shieu[1], Todd Schwartz[2], Matthias Hoben[3], Mark Toles[2], Anna Beeber[4], and Ruth Anderson[2], *1. University of Pittsburgh, Pittsburgh, Pennsylvania, United States, 2. UNC Chapel Hill, Chapel Hill, North Carolina, United States, 3. University of Alberta, Edmonton, Alberta, Canada, 4. Johns Hopkins University, Baltimore, Maryland, United States*

Nursing homes (NHs) typically focus on health-related quality of life (HRQoL) among residents aged 65 and over despite approximately 7% of NH residents are younger (aged 18-64). Research suggests that the needs of younger NH residents are not being met and they may have low HRQoL. However, differences in HRQoL of younger and older NH residents may not be apparent in studies that use HRQoL measures designed for research with older NH residents. We hypothesized that the younger residents would have lower HRQoL mean scores than the older (aged ≥ 65) residents using a HRQoL measure based on the HRQoL score derived from Resident Assessment Instrument – Minimum Data Set 2.0 items. The measure uses items that emphasize physical aspects of quality of life rather than social aspects. In a sample of 21,129 residents from 94 NHs in Western Canada, we performed descriptive analyses, t-test, chi-square test, and an adjusted propensity score (PS) analysis through retrospective cohort study from years 2016 to 2017. The HRQoL index score ranged from -.351 to .996 (Mean= 0.693, SD=0.265). In the PS model, the adjusted mean score for younger was higher than for older adults with a mean difference at 0.061 (95% CI 0.031, 0.091) ($p<.001$). Other domains such as mental health condition of quality of life must be examined in younger NH residents because it is a crucial factor influencing their daily lives, thereby we can explore a more complete set of HRQoL domains of them and redesign care for their unique needs.

### REFLECTIONS OF ADULTS TRANSITIONING TO LONG-TERM CARE FACILITIES: LOOKING BACK AND LOOKING FORWARD

Pei-Fen Chang, and Gayle Hersch, *Texas Woman's University, Houston, Texas, United States*

Making a transition to a long-term care facility (LTCF) is often difficult for older adults. This study's intent was to explore ways in which life experiences and culture of older persons facilitate a successful transition to a LTCF. Eighteen participants were interviewed using a semi-structured interview guide to understand significant life events and personal factors impacting their lives. Interview data were analyzed using NVivo software to organize and manage emerging themes. The analysis revealed three related themes: person, current environments, and lifelong occupations. Within each major theme, subthemes were identified providing greater detail of how adaptive strategies are utilized to make such transitions positive. Participants were enabled to reflect upon their strengths and strategies available to adapt to these new residential settings. These perspectives are beneficial for healthcare practitioners to understand so as to personalize care and individualize the person's adjustment to a 'new' home.

### PERCEPTIONS OF NURSES DELIVERING NURSING HOME VIRTUAL CARE SUPPORT: A QUALITATIVE PILOT STUDY

Justin Blackburn[1], Carol Mills[1], Yvette Tran[1], Valerie Yeager[1], Kathleen Unroe[2], and Ann Holmes[1], *1. IU Richard M. Fairbanks School of Public Health at IUPUI, Indianapolis, Indiana, United States, 2. Indiana University Center for Aging Research, Regenstrief Institute, Inc., Indianapolis, Indiana, United States*

Avoidable hospitalizations among nursing home residents result in poorer health outcomes and excess costs. Consequently, efforts to reduce avoidable hospitalizations have been a priority over the recent decade. However, many potential interventions are time-intensive, require dedicated clinical staff, and nursing homes are chronically understaffed. The Optimizing Patient Transfers, Impacting Medical Quality, and Improving Symptoms: Transforming Institutional Care (OPTIMISTIC) project was one of seven sites selected by CMS as "enhanced care & coordination providers" and was implemented from 2012 to 2020. A virtual program based on the principles of OPTIMISTIC was developed in the spring of 2020 with the goal of expanding the reach of the program's services. This qualitative study explores the perceptions and experiences of the nurses that piloted a virtual care support project in 11 nursing homes in a midwestern state, and identified the nurses' perceived facilitators of, and barriers to, the effectiveness of delivering a novel virtual care support program. A key finding from this analysis is that relationships, communication, and access to information were identified as common themes facilitating or impeding the perceived effectiveness of implementation of virtual care support programs within nursing homes, from the perspective of the nurses delivering the services. The experiences and recommendations of the program nurses provide insights into crucial elements important to the implementation of similar virtual care support models, and the role of telehealth in bridging healthcare workforce gaps.

### SESSION 6770 (POSTER)

### LONG-TERM CARE, PERSON-CENTERED CARE

### CHRONIC DISEASE AND LIFE EXPECTANCY DISPARITIES OF OLDER ADULTS IN SINGAPORE

Xueying Guo[1], Hwee lin Wee[2], and Cynthia Chen[3], *1. national university of singapore, Singapore, Singapore, 2. National University of Singapore, Singapore, Singapore, 3. National University of Singapore, Singapore, Singapore*

Singapore is one of the countries with longest life expectancy, also one of the most rapidly ageing societies in the world. This study investigates the future demographic transitions in Singapore, focusing on the life expectancy disparities of the older adults. We developed a dynamic Markov microsimulation model based on the Singapore Multi-Ethnic Cohort study. The model was adapted to project future trends of chronic diseases, mental illnesses as well as hospitalisation costs of Singapore older people from 2020 to 2050. The prevalences of diabetes, heart disease, hypertension, stroke, disability, dementia and depression of people aged 51 and above was projected to be increasing. Overall projected life expectancy at age 51 for male is 27.8 years, and for female is 34.5 years. Moreover, for older people with secondary or upper education, the overall projected life expectancy at age 51 is 34.1 years, active life expectancy at age 51 is 28.6 years. For older people with primary or lower education, the overall life expectancy at age 51 is projected to be 27.9 years, and active life expectancy projected to be 23.9 years. Our study has significant social and economic implications for policymakers in the way that it demonstrates disparate trends in aging and costs among different education groups in Singapore.

### HOME-BASED EXERCISE PROGRAM WITH MEAL DELIVERY INCREASES DAILY STEP COUNT IN HOMEBOUND OLDER ADULTS

Harmanpreet Kaur, and Jessica Lee, *McGovern Medical School at UTHealth, Houston, Texas, United States*

The United Nations projects adults aged 60 and older will reach 2.1 billion by 2050. With this trend, there will be an increase in older adults who are homebound, leading to more frailty with increased healthcare utilization and institutionalization. Improvements in frailty and homebound status may occur through increased physical activity, which has been shown in multiple randomized controlled trials to improve frailty. Our study aims to evaluate the effects of a home-based exercise program, administered through Meals on Wheels, on gait speed and frailty status in sixty-four homebound adults age 60 and older. All participants receive meal delivery for 12 weeks and half are randomized to receive an exercise kit with weekly exercise handouts. All participants are asked to wear an activity tracker with an additional longitudinal measure at 6 months. We now have 6 participants who have completed the study. Preliminary analysis was conducted on step count between the exercise and non-exercise group using two-sample t-tests. Average daily step count in the exercise group (n=3) was 2656 compared to 813 in the non-exercise group (n=3), $p< 0.00001$ during the first 12 weeks. The step count in the exercise group did decrease during the weeks after the intervention stopped, however they remained more active than the non-exercise group, 1112 vs 548, $p< 0.0001$. No adverse events were noted. As we continue to enroll participants, we are encouraged that the home-based exercise program with meal delivery appears to be a safe and effective way to increase physical activity in homebound older adults.

### PERCEIVED ROLES IN MEDICATION SAFETY: PATIENTS' PERSPECTIVES

Fatoumata Jallow[1], Elisa Stehling[1], Zara Sajwani[2], Kathryn Daniel[1], Yan Xiao[1], Kimberly Fulda[3], and Anna Espinoza[3], *1. University of Texas at Arlington, Arlington, Texas, United States, 2. Children's Health, Dallas, Texas, United States, 3. University of North Texas Health Science Center, Fort Worth, Texas, United States*

Community-dwelling older adults are vulnerable to medication safety-related harms. Prevention of medication-related harms in the outpatient setting starts with thorough and thoughtful medication reconciliation at each patient encounter. Comprehensive medication reconciliation is challenging for prescribers to provide in busy time-pressured practices. Older adults currently taking five or more daily prescription medications were recruited for this qualitative study. From the participants' perspective, we explored the role of the prescriber, pharmacist, and patient in medication safety. During the COVID-19 pandemic, interviews were conducted from October 2020 to June 2021. Results from these interviews suggest that older adults recognized their role in medication safety supersedes just taking the pills as prescribed. Older adults understand that they must play an essential role in the coproduction of quality health services. Subthemes that emerged from the patient's perceived role were “taking fewer medications,” “locking them up,” “keeping appointments,” and “reading the label.” Pharmacists were expected to inform participants of any changes in their medications, such as the color, shape, or dosage, and ensure no drug interactions. Primary care providers are expected to coordinate care between all specialists treating their patients and any medication prescribed by those specialists. There was a high level of trust in the provider's knowledge, skill, and experience, along with a low level of patient engagement in decision-making around deprescribing. Among older adults, self-perceptions of their role in medication safety varied widely. Educating prescribers and pharmacists about the role expectations of this vulnerable population can help improve medication safety.

### SOCIAL INTERACTION AND PSYCHOLOGICAL WELL-BEING OF PERSONS LIVING WITH DEMENTIA IN LONG-TERM CARE

Kyung Hee Lee, and Ji Yeon Lee, *Yonsei University, Seoul, Seoul-t'ukpyolsi, Republic of Korea*

Although social interaction might play a critical role to improve psychological well-being of old adults with dementia in long-term care, lack of social interaction between staff and residents has been reported. The purpose of this study was to examine whether social interaction is associated with psychological well-being of persons living with dementia during care in long-term care. We analyzed 258 videos from 30 participants with dementia. Each participant was taken nine videos at 0, 3, and 6 months at three care events (i.e., personal care, mealtime, and activity). Social interaction was assessed by quality (i.e., positive, neutral, and negative), type (i.e., verbal, nonverbal, and both), and presence of interaction (i.e., no and yes). Psychological well-being was measured by positive and negative emotional expressions. A mixed model was chosen for the data analysis since these repeatedly measured observation data were nested within subjects. Mixed models showed that positive and neutral interactions were significantly associated with positive emotional expressions of residents after controlling covariates while negative interaction was significantly associated with negative emotional expressions. There was no significance between interaction type and emotional expressions. This study highlights the importance of positive care staff interactions in dementia care. In addition, the institutional efforts to create an environment to reduce negative interactions can be of great help in promoting the psychological well-being of persons living with dementia.

### HOSPITAL TRANSFERS: PERSPECTIVES OF NURSING HOME RESIDENTS AND NURSES

Alma Ahmetovic[1], Claire Drucker[2], Lesa Huber[3], Kathleen Unroe[4], and Susan Hickman[2], *1. Indiana University School of Nursing, Bloomington, Indiana, United States, 2. Indiana University School of Nursing, Indianapolis, Indiana, United States, 3. Indiana University School of Public Health, Bloomington, Indiana, United States, 4. Indiana University Center for Aging Research, Regenstrief Institute, Inc., Indianapolis, Indiana, United States*

Between 1 million and 2.2 million nursing home residents are transferred to a hospital emergency department each year. These transfers are costly, have negative health outcomes, and can increase the morbidity and mortality of residents. The purpose of this study was to examine the transfer process between the nursing home and the hospital from the perspective of nursing home residents and nurses, focusing on how decisions were made to transfer residents. Using a qualitative descriptive method, 22 participants were recruited from four nursing homes located in rural Indiana. Purposive sampling, semi-structured interviews, and conventional content analysis were used to collect and analyze narratives obtained from residents and nurses about their experiences with a recent transfer. The participants described four aspects of the transfer process: transfer decisions, transport experiences, hospital stays, and returns to the nursing home. The most common reason for transfers was an acute exacerbation of a chronic condition, and the decision to transfer was often made by a nurse. Most residents found aspects of the transfer aversive or upsetting. The return to the nursing home was typically welcomed but often challenging due to problems with mobility, medications, and cognitive changes. Participants also provided several recommendations for avoiding potentially preventable transfers including adding "in-house" diagnostic testing and treatment equipment, improving staff competencies in managing acute exacerbations, increasing staffing, improving communication among staff, and increasing staff familiarity with resident histories and preferences. The findings have several clinical and policy implications for preventing or decreasing the negative effects of hospital transfers.

### EFFECTS OF DANCE INTERVENTIONS ON COGNITION IN OLDER ADULTS: A META-ANALYSIS

Tanapa Rittiwong[1], Nattaya Wongyara[2], and Joanne Schneider[1], *1. Saint Louis University, Saint Louis, Missouri, United States, 2. Saint Louis University, Saint louis, Missouri, United States*

Background: Dance interventions have been shown to improve cognition in older adults. However, no meta-analysis evaluates that effect. Objective: We examined the effects of dance on cognition in older adults and examined the moderating effects of participant, methods, and intervention characteristics.

Methods: We searched 14 databases using the search terms: dance AND cognition AND older adults. Researchers of primary studies compared dance with control groups of adults≥60 years old, measured cognition, and wrote in English. Two researchers independently coded studies and discussed to achieve consensus. Using the random-effects model, we computed effect sizes (ES) using Hedges' g with 95%CIs along with heterogeneity statistics and conducted moderator analyses.

Results: Twenty-five studies included 1,908 participants (70.9±5.2years old). Dance had a strong, positive effect on both global cognition (ES=0.52, 95%CI [0.25, 0.78], p=.000) and memory (ES=0.56, 95%CI [0.08, 1.04], p=.022) and a moderate effect on executive function (ES=0.35, 95%CI [0.06, 0.65], p=.019) and attention (ES=0.21, 95%CI [0.06, 0.36], p=.005). Several variables moderated the relationship including the country, dancing in pairs or not, or with partners vs. non-partners. Methodological quality indicators that moderated the relationship was computing a priori power and comparing participant characteristics at baseline. Weeks of dancing and minutes/session also moderated the relationship.

Conclusion: Dance can significantly improve cognition in older adults. Healthcare providers might use dance interventions as an alternative treatment to improve cognition in older adults. Future researchers should explore the effects of type of dances on cognition and measure outcome changes over time.

### POLYPHARMACY, ANXIETY, AND QUALITY OF LIFE IN LONG-TERM CARE RESIDENTS

Hannah Dangerfield[1], Judith Scott[2], Jennifer Zohn[2], Daniel Segal[2], and Melissa Benton[2], *1. University of Colorado Colorado Springs, Golden, Colorado, United States, 2. University of Colorado Colorado Springs, Colorado Springs, Colorado, United States*

The prevalence of polypharmacy (≥5 medications per day) is rising among older adults. Multiple medications are increasingly prescribed to treat multiple comorbidities in this growing population. An increase in anxiety and a decline in quality of life are also seen with polypharmacy, although evidence is limited. This study investigated the relationship between anxiety and quality of life in long-term care residents with polypharmacy. Sixty-nine long-term care residents who reported polypharmacy completed a questionnaire regarding anxiety and quality of life. Anxiety was measured with the Geriatric Anxiety Scale Long-term Care (GAS-LTC), health-related quality of life was measured with two questions from the RAND-36, and medication-related quality of life was measured with the Medication-related Quality of Life Scale (MRQoLS). Mean age was 80.74 ± 7.8 years. The majority were female (72%) and white (88%). The mean GAS-LTC score was 2.75 ± 2.4, indicating relatively low overall anxiety. There were moderate-strong, negative correlations between anxiety and all quality of life measures indicating that as anxiety increased, quality of life decreased. Specifically, anxiety was negatively related to general health-related quality of life ($r = -.51$, $p < 0.001$), health-related quality of life compared to one year ago ($r = -.54$, $p < 0.001$), and medication-related quality of life ($r = -.57$, $p < 0.001$). Among this group of older adults with polypharmacy residing in long-term care, those with greater anxiety reported diminished quality of life related to both overall health and use of medications. Further studies to examine mechanism and causality are needed.

### PUNCTUATED EQUILIBRIA: AN ANALYSIS OF HEALTHCARE UTILIZATION PATTERNS FOR OLDER ADULTS WITH CHRONIC DISEASE

Christopher Crowley, Amy Stuck, A. Camille McBride, and Claire Andrews, *West Health Institute, La Jolla, California, United States*

The growing population of older adults requires healthcare delivery models that are both cost effective and directed toward the higher rates of chronic conditions, frailty and functional impairment often present as one ages. Analyzing healthcare utilization patterns of older adults—especially those over the age of 75—will better inform how to re-design care delivery processes.While the presence of chronic conditions requires consistent treatment, the intensity of health services required on a day-to-day basis is highly variable. Many individuals with at-risk health status are remarkably stable, exhibiting high levels of function and low utilization of care over extended periods of time. Periodically, however, these phases are punctuated by catastrophic episodes that can lead to greater health decline, morbidity, and mortality. During cycles of health decline, a cascade of other challenges stemming from an individual's underlying risks, predispositions, and complexities can arise. This punctuated equilibrium of utilization for older adults offers insight to assess ways to improve care and lower costs. In this paper, we analyze and quantify both these states of punctuated equilibria as well as the extensive periods of stability. The associated utilization patterns are analyzed by processing Medicare claims data. Our analysis showed that over 80% of members in a group of anticipated high-risk Medicare beneficiaries over the age of 75 exhibited low utilization for 11 months out of a given year. These findings complement research exploring care redesign processes at a system level, specifically directed to high-cost episodes of care as opposed to patient-level targeting.

### HEALTHY AGING TALK AROUNDS: NEAR AND FAR

Lindsay Mullins, *Franciscan Missionaries of Our Lady University, Baton Rouge, Louisiana, United States*

BackgroundLoneliness and chronic health conditions are the two most cited ailments among older adults (OAs) that deter independent living. Community-tailored health programs increase feelings of connectivity to neighbors and link OAs to healthcare resources. MethodsDesign. The Healthy Aging Talk Around program is product of CAB formation, input, and a series of talks in-person (near) and virtually (far). Using qualitative methods, data was collected to determine significant health issues for OAs and qualitative data were collected through surveys to evaluate knowledge, access, and feelings of connectivity related to series gatherings. Setting and Population. Inner-city and rural OAs in the deep south from various communities (2 FBOs, 1 Assisted Living, 2 neighborhood groups). Series was held in community and later online. Attendance ranged between 75-130 OAs. Measures. Qualitative measures included pre and post series surveys (n=110; n = 91) with questions to determine the impact of the series. Analysis. Content analysis was conducted with the qualitative data to determine the impact of the series on 1.) knowledge of health issue(s), 2.) likelihood of accessing health resources, and 3.) feelings of connectivity and isolation. ResultsThe qualitative data analysis suggested the series as relatable because information was tailored linked to local community resources. Discussion of independent living challenges fostered connectivity with neighbors. Once virtual, knowledge and access were similar, feelings of connectivity were less prevalent. ConclusionA community-tailored health program series can increase knowledge of health issues, access to resources, and create neighborly connectivity potentially influencing ability to age in place.

### PERSPECTIVES OF UNDER-RESOURCED PATIENTS ABOUT THE MEDICARE ANNUAL WELLNESS VISIT

Christine Ferrone[1], Roberta Goldman[2], Joanne Wilkinson[3], Jeffrey Borkan[4], and Phillip Clark[1], *1. University of Rhode Island, Kingston, Rhode Island, United States, 2. Warren Alpert Medical School of Brown University, Pawtucket, Rhode Island, United States, 3. Alpert Medical School of Brown University, Pawtucket, Rhode Island, United States, 4. The Warren Alpert Medical School of Brown Univerity, Providence, Rhode Island, United States*

The Medicare Annual Wellness Visit (AWV) involves a personalized risk assessment and prevention plan to help prevent disease and disability among older adults. Though studies have shown that AWV uptake is suboptimal, little is known about the perspectives and experiences of patients completing it. Family medicine physicians are conducting AWVs at a Rhode Island pilot teaching clinic participating in the GWEP's Age-Friendly Health Systems Initiative. To evaluate perspectives of the clinic's under-resourced patients, 20 qualitative telephone interviews were conducted following their AWV. Interview recordings were analyzed for content and themes. Few patients recognized the term 'Annual Wellness Visit' or recognized it was significantly different from other visits: "I noticed it was a bit different, but not that different. I'm usually not covered for a visit like that by Medicare." Mental health, mobility and medications were discussed at most of the AWVs. Some patients noticed that a physician did the medication review instead of the nurse. Patients had varying experiences of how advance directives were discussed, and some thought it unnecessary since a family member knows their preferences. A few patients were dissatisfied that their physician did not focus on their chronic or acute health problems. However, most patients "trusted" their physicians to do "whatever is necessary" for them, and were glad to have had the AWV because "instead of talking about being sick all the time, talking about how to stay well". Recommendations for patient education to improve understanding and the experience of the AWV will be offered.

### CAN THE PUBLIC LONG-TERM CARE INSURANCE SERVICES IN JAPAN PREVENT THE DETERIORATION OF CARE LEVELS?

Aki Sugiura[1], Reiko Kanaya[2], Yasushi Takeya[1], Hiroshi Toki[3], Ryohei Yamamoto[1], and Miyae Yamakawa[1], *1. Osaka University, Suita, Osaka, Japan, 2. Osaka University, Osaka, Osaka, Japan, 3. Health and Counseling Center, Suita, Osaka, Japan*

In Japan, the public long-term care insurance system supports a super-aged society. The long-term care service (LTCS) system is designed to prevent deterioration of care levels. We conducted a retrospective cohort study to clarify whether using LTCS prevents the deterioration of care levels. We use anonymized data of the Osaka National Health Insurance Database. We analyzed 16,469 subjects who were 65–74 years of age and certified at the mild care level from 2012 to 2015 at the time of initial certification. A baseline measurement was conducted 1 year after initial certification, and observation was continued until the subject's care level deteriorated. Statistical analysis was performed using the Kaplan-Meier method and the Cox proportional hazards model. The results revealed that the period until the deterioration of care level (which occurred in 25% of subjects) was 6 months in the service use group and 22 months in the service non-use group. Although we included sex, care level, dementia, and Parkinson's disease as variables in the statistical analysis, service users were more likely to deteriorate than non-service users (Hazard Risks: 1.89; 95% confidence interval: 1.79–2.00). It is likely that other factors, such as heart disease, may also be involved, and there may be a bias related to family caregiver factors. In conclusion, the current findings suggest that using LTCS cannot prevent the deterioration of care levels. Future studies should verify the effectiveness of LTCS by adding further variables and data regarding family caregivers and changes in outcomes.

### HEALTH PROFILES OF OLDER NURSING HOME RESIDENTS BY SUICIDAL IDEATION: A LATENT CLASS ANALYSIS

Yiyang Yuan[1], Adrita Barooah[2], Deborah Mack[3], Kate Lapane[3], and Christine Ulbricht[4], *1. University of Massachusetts Chan Medical School, Burlington, Massachusetts, United States, 2. University of Massachusetts Boston, Boston, Massachusetts, United States, 3. University of Massachusetts Chan Medical School, Worcester, Massachusetts, United States, 4. National Institute of Mental Health, National Institutes of Health, Rockville, Maryland, United States*

In US nursing homes, 2% of residents have documentation of suicidal ideation (SI). Whether older residents' health profiles differ by SI is unknown. Using the 9th Patient Health Questionnaire-9 (PHQ-9) item on Minimum Data Set 3.0, we identified 15,277 older residents with and 562,184 without SI. Latent class analysis using frailty, cognitive impairment, palliative care index, pain, and remaining PHQ-9 items as indicators identified health profiles by at-admission SI and estimated the association between profiles and SI at 90 days. In residents with at-admission SI, four profiles emerged: (1) frail, intact/moderate cognitive impairment, all depressive symptoms (prevalence: 22.8%); (2) frail, moderate/severe pain, depressed mood, sleep problems, fatigue (32.2%); (3) frail, severe cognitive impairment, depressed mood, fatigue, feelings of worthlessness (22.9%); (4) prefrail, moderate/severe cognitive impairment, depressed mood (22.0%). Compared to the residents in profile 4, those in profile 1 [adjusted OR (aOR): 1.24; 95% Confidence Interval (CI):1.11-1.37] and those in profile 2 [aOR: 1.11; 95% CI: 1.01-1.22] were more likely to continue reporting SI at 90 days. In residents without at-admission SI, three profiles emerged: (1) frail, depressed mood, fatigue (33.9%); (2) frail, severe cognitive impairment (38.1%); (3) prefrail/frail (28.0%). Residents in profile 1 were more [aOR: 2.80; 95% CI: 2.60-3.00] while those in profile 2 were less [aOR: 0.79; 95% CI: 0.71-0.86] likely profile 1 residents to report SI at 90 days. Findings indicate substantial heterogeneity in the health profiles between and within older residents with and without self-reported SI.

### INTERVENTIONS TARGETING LONELINESS AND ISOLATION AMONG LONG-TERM CARE RESIDENTS: A SYSTEMATIC REVIEW

Audrieanna Raciti[1], Amy Lyons[2], and Yu-Ping Chang[2], *1. Buffalo, New York Mills, New York, United States, 2. University at Buffalo, Buffalo, New York, United States*

The impact of loneliness and social isolation on health is a significant concern among people residing in long term care. Researchers have begun to design and test interventions to lessen the burden of these impacts on the individual as well as our already strained healthcare system. The purpose of this review is to synthesize types and outcomes of interventions designed to reduce loneliness and isolation among long term care residents. Six databases: CINAHL, PUBMED, Cochrane, Web of Science, PsycInfo, and Embase were searched to identify studies leveraging the use of various interventions for alleviating feelings of loneliness and isolation within long term care residents. Multiple keywords were used including loneliness, social isolation, long-term care, nursing home, treatment, and strategies. Articles were screened if they were published between 2012 and present. Twenty studies were included in this systematic review. All studies have a small sample size. Only four studies were randomized controlled trials. Interventions included laughter therapy, adaptive sports, social robotics, horticultural therapy, spirituality, electronic cognitive behavioral therapy, music therapy, virtual reality, expressive arts, telephone discussions, video conferencing, peer mentoring, and animal assisted therapy. The majority of the interventions demonstrate a significant improvement in alleviating feelings of loneliness and social isolation, while some interventions also show improvement in quality of life and physical ability. However, some studies have a high attrition rate. Future research is needed to utilize a more rigorous design with a large sample size. Participant engagement should be considered as part of the intervention activities.

### "I EAT TO NOT DIE": DIET AND EXERCISE EXPERIENCES OF OLDER ADULTS LIVING ALONE

MoonKi Choi[1], and Juyeon Bae[2], *1. Kangwon National University, Chuncheon-si, Kangwon-do, Republic of Korea, 2. Yeoju Institute of Technology, Yeoju-si, Kyonggi-do, Republic of Korea*

Living alone is a critical health risk factor among older adults. Regular exercise and a balanced diet are important for maintaining an independent and healthy life. However, it can be more difficult for older adults living alone to maintain healthy exercise and dietary habits due to the lack of social support. This was a qualitative descriptive study to explore the life changes that accompany living alone, focusing on exercise and diet and the need to improve the nutrition and physical activity of older adults living alone. Thematic analyses were applied to analyze the qualitative data collected from semistructured in-depth interviews. Fifteen subjects, including 5 men and 10 women, participated in the interviews. The mean age was 77.73 ± 7.37 years, and the mean duration of living alone was 17.73 ± 12.89 years. Six main themes were developed: unavoidable difficulties of living alone (lack of someone to depend on and loneliness), just eating "to not die", exercise that is good for health but hard to do alone, conflict between maintaining independence and dependence on others, coping with available resources, and resources needed for regular exercise and a balanced diet. Living alone led to poor dietary habits, such as skipping meals or insufficient food intake. Food security should be ensured through the reinforcement of social support, financial assistance, and the operation of public cafeterias. In addition, a tailored group exercise program that can meet the need for social interaction and improve self-efficacy would help older adults living alone maintain regular exercise habits.

### USE OF COMPLEMENTARY HEALTH APPROACHES AND PRESCRIPTION OPIOIDS AMONG VETERANS WITH MUSCULOSKELETAL DISORDERS

Ling Han[1], Joseph Goulet[2], Melissa Skanderson[2], Doug Redd[3], Cynthia Brandt[4], and Qing Zeng[3], *1. Yale School of Medicine, New Haven, Connecticut, United States, 2. VA Connecticut Healthcare System, West Haven, Connecticut, United States, 3. George Washington University, Washington, District of Columbia, United States, 4. Yale School of Public Health, New Haven, Connecticut, United States*

Complementary and integrative health approaches (CIH) are recommended in national guidelines for managing chronic pain and de-prescribing opioids. We followed 1,993,455 opioid-naïve Veterans with musculoskeletal disorders for two years after index diagnosis during 2005-2017. CIH exposure was defined as primary care visits for acupuncture, massage and chiropractic therapy using natural language processing and administratively coded data. Opioid prescriptions dispensed during follow-up period were abstracted from Veterans Health Administration electronic pharmacy records. Propensity score (PS) was used to match one control for each CIH recipient. Overall, 140,902 (7.1%) Veterans received CIH, with those age ≥65y the lowest prevalence (2.7%). Cox proportional hazard model revealed that time to first opioid prescriptions was longer for CIH recipients than PS-matched controls (136,148 matched pairs) and varied across age (p for interaction 0.003). The adjusted Hazard Ratio (HR) was 0.48 (95% Confidence Interval (CI): 0.45-0.51) for Veterans age ≥65y, 0.44 (95% CI: 0.43-0.45) for 50-64y and 0.47 (95% CI: 0.46-0.48) for ≤49y. Restricted mean survival time (RMST) models estimated a smaller CIH benefit for Veterans ≥65y, with an average 3.3 (95% CI: 3.2-3.5) month RMST difference, in contrast to 4.2 (95% CI: 4.1-4.3) and 3.7 (95% CI: 3.6-3.8) months for younger counterparts. Sensitivity analyses in full cohort or modeling total supply and daily dose of opioid prescriptions derived consistent results. These findings suggest potential benefits of CIH in delaying and reducing opioids prescriptions for patients with chronic pain. The observations of lower rate and smaller benefit of CIH use among older Veterans warrants further investigations.

### CURRENT PRACTICE PATTERNS IN HOME HEALTH OCCUPATIONAL THERAPY

Chiung-ju Liu, Hannah Burch, Sabrina Glover, Abigail Donofrio, Gianna Oliveros, and Gianna Demeo, *University of Florida, Gainesville, Florida, United States*

Occupational therapy practitioners are essential members of the home health rehabilitation team to support patients transitioning from acute care to home. However, home health occupational therapy services have been underutilized, in part, due to limited understanding of services that the profession provides. Improving this understanding is necessary to help patients receive timely services. The purpose of the study was to describe the current practice patterns in home health occupational therapy. Fifty home health occupational therapy practitioners from 27 states completed an electronic survey between May and June 2021. The survey consisted of questions about assessment and treatment, communication with physical therapy colleagues, and the impact of the Patient-Driven Grouping Model and the coronavirus disease 2019 pandemic on service delivery. Results showed that occupational therapy assessments focused on personal care activities, mobility and balance, and cognition. The treatment content usually included training in activities of daily living, functional mobility, the application of compensatory strategies (e.g., home adaptations), and fall prevention strategies. Most respondents communicated with their physical therapy colleagues at least once a week on topics related to scheduling, changes in a patient's condition, and falls and safety concerns. Seventy percent of respondents experienced a reduction of home visits during the recent Medicare payment reform and the pandemic. These practitioners felt that some patients might be discharged prematurely because of this reduction. The survey results highlight the pertinence of occupational therapy services to support patients' independence and safety at home and the need to increase these services for home health patients.

### THE ASSOCIATION BETWEEN ACCESS TO MEDICAL CARE AND RESIDENT OUTCOMES IN NURSING HOMES

Krittika Bali, *University of Alberta, Edmonton, Alberta, Canada*

The nursing home population is vulnerable and medically complex, yet little is known about models of medical service provision and associated quality outcomes. The goal of this study is to examine the association between physician (MD) and nurse practitioner (NP) accessibility and practice sensitive outcomes. This project used data from the Translating Research in Elder Care (TREC) longitudinal study and the routinely collected Resident Assessment Instrument – Minimum Data Set version 2.0 (RAI-MDS 2.0) to test the association between the availability of MDs and NPs in nursing homes (NH) and clinically-relevant resident outcomes of antipsychotic medication (APM) use without indication of psychosis, physical restraint use, hospitalization and emergency department (ED) transfers, and polypharmacy. Eight models were created using logistic regression to test the association between the access measures of daily presence of MD or NP on unit and MDs being involved in care planning and each of the four resident outcomes. The sample consisted of 10,888 residents across 320 units in 92 facilities. Staff from 277 (86%) units reported an MD or NP visited daily and 318 (99%) units reported that the MD or NP could be reached when needed. Following adjustment for multiple confounding variables, there were no associations between either measure of access and any of the resident outcomes. Although we did not find any associations between our measures of access and resident outcomes, additional research which more directly measures physician and NP activities in the NH is required.

### FREQUENT USERS OF EMERGENCY MEDICAL SERVICES IN A METROPOLITAN CITY: INDIVIDUAL- AND SYSTEM-LEVEL CONSIDERATIONS

Beatrice Martinez[1], Maria Aranda[1], Iris Aguilar[2], Stephen Sanko[2], and William Vega[2], *1. University of Southern California, Los Angeles, California, United States, 2. USC, Los Angeles, California, United States*

Frequent 911 calls from older adults place a significant burden on emergency response systems. Drawing from key informant interviews, we explore factors implicated in repeated Emergency Medical Services (EMS) among older people in the City of Los Angeles and provide system and policy recommendations to mitigate overuse of EMS 911 calls. Los Angeles Fire Department (LAFD) dispatch call records documented 370,016 EMS encounters from 202,471 unique individuals over 50 from 2012 to 2016. Frequent 911 callers over the age of 50 (n=8), family members (n=6), Los Angeles Fire Department (LAFD) personnel (n=10), and community service providers (n=3) participated in in-depth, qualitative interviews to understand individual- and system-level factors that increase the likelihood of repeated calls. All interviews were audio recorded, transcribed, and analyzed thematically. Guided by Grounded Theory, two themes emerged across all interviewee groups pointing to factors that contributed to 911 calls: 1) The Nature of 911 Calls; 2) Barriers to Access. Patients and family members were aligned on a third theme: 3) Previous Encounters: Interactions with emergency care providers, while LAFD and service providers were in agreement on a fourth theme: 4) The Role of EMS Responders and Community Providers. All groups provided recommendations for the theme, 5) Change the System so that 911 calls do not serve as the default choice. Findings demonstrate the need to improve coordination between LAFD, medical and human service providers. Care coordination can support individual- and system-level supports for patients and families to address their needs and reduce repeated EMS use.

### LONGITUDINAL CHANGES IN LEADERSHIP, JOB STRAIN, AND SOCIAL SUPPORT OVER FIVE YEARS IN NURSING HOMES

Annica Backman[1], Marie LINDKVIST[2], Hugo Lovheim[2], and David Edvardsson[3], *1. Umea university, Umea, Vasterbottens Lan, Sweden, 2. Umea University, Umea, Vasterbottens Lan, Sweden, 3. La Trobe University, Bundoora, Victoria, Australia*

Nursing home leadership has been described as crucial for staff job strain and social support but longitudinal data are lacking. This study aimed to explore changes in nursing home managers' leadership, staff characteristics, job strain and social support by comparing matched units in a five-year follow-up study. Repeated cross-sectional, valid and reliable, measures of leadership, person-centered care, psychosocial climate and demographic variables were collected from managers and staff n=3605 in 2014 and n=2985 staff in 2019. Descriptive statistics and regression analyses with generalized estimating equations were used. The results showed that associations between leadership and staff job strain and social support increased in strength over time. This indicates that leadership has the potential to reduce staff job strain and increase the perception of social support among staff, and the significance of leadership increases over time. It was also shown that workforce characteristics had changed with higher proportion of enrolled nurses and a smaller proportion of nurse's assistants at follow-up. An increase was also shown in staff reporting that the job required too much work effort, and perceived deteriorating collegiality and work atmosphere at follow-up.

### SESSION 6780 (POSTER)

### PHYSICAL FUNCTION, MOBILITY, AND FALLS

### INTRACELLULAR WATER CONTENT IN LEAN MASS IS ASSOCIATED WITH MUSCLE FUNCTION AND PHYSICAL ACTIVITY AMONG OLDER ADULTS

Murad Taani[1], and Kimberly Dembinski[2], *1. University of Wisconsin-Milwaukee, Milwaukee, Wisconsin, United States, 2. Ovation Communities, Milwaukee, Wisconsin, United States*

High intracellular water (ICW) content in lean mass (LM) has been associated with better functional capacity and lower frailty risk in older adults and suggested to be a useful muscle quality indicator. However, limited research exists on the relationship of ICW to muscle function and physical activity among older adults. The purpose of this study is to examine the relationship between ICW/LM ratio and muscle function and physical activity among older adults. In a cross-sectional correlational study of older adults aged 70 years or older, a tetrapolar bioimpedance spectroscopy device (Impedimed SFB7, Australia) was used to assess both ICW and LM, and the ICW/LM ratio (mL/kg) was calculated. The Short Physical Performance Battery (SPPB) and Timed Up and

Go (TUG) tests were used to measure muscle function. The Actigraph GT3X+ accelerometer (Actigraph, Inc., FL, USA) was used to measure light and moderate-vigorous physical activity and steps per day. Multiple regression analyses were used. For 96 recruited participants (mean age 82.5 ± 7.4 years; 79 [82.3%] were female), mean ICW/LM ratio was 245.1 ± 28.1 mL/kg. The ICW/LM ratio was positively associated with SPPB score, gait speed, moderate-vigorous physical activity, and steps per day, after adjusting for age, sex, and LM. The ICW/LM ratio was negatively associated with TUG score, after adjusting for age, sex, and LM. The findings suggest that intracellular water content in lean mass may affect muscle function and physical activity among older adults. Further studies are needed to verify these findings and determine any potential cause-and-effect relationships.

### BRAIN RESPONSE IN THE ELDERLY AFTER TAI CHI PRACTICE ASSESSED WITH NEUROIMAGING TECHNIQUES: A LITERATURE REVIEW

Hao (Howe) Liu[1], Yasser Salem[2], and Eric Arguello[1], *1. Allen College, Waterloo, Iowa, United States, 2. Hofstra University, Hempstead, New York, United States*

Tai Chi (TC) has been often provided to older adults by rehabilitation professionals for medical dysfunction and anti-aging healthcare. In the last 10 years, there has been an increase in the number of studies examining the effects of TC on brain as assessed by neuroimaging including near infrared spectroscopy (NIRS), and structure and functional magnetic resonating imaging (sMRI & fMRI). Thus, the purpose of this literature review is to evaluate how TC practice may affect the brain in the elderly as assessed by neuroimaging techniques. A comprehensive literature search was conducted using a variety of key words with different search engines to search from the last 10 years until January 15, 2022. Studies were included if they investigated topographic brain responses after TC practice in the elderly population. A total of 12 original studies with 15 articles met the criteria and were included for the review process. The results showed increased volume of cortical grey matter, improved neural activity, and increased neural connectivity in different brain regions, including the frontal, temporal, and occipital lobes, followed by cerebellum and thalamus. Intriguingly, the longer one practices TC, the more his/her brain areas may be altered. Such neural findings after TC practice are often associated with neurobehavioral improvements in attention, cognitive execution, memory, emotion, and risk-taking behaviors. Tai Chi is a promising exercise that is able to improve morphological capability and neurofunctional activity in the brain in the elderly. These improvements seem to be associated with time-length of TC practice.

### RELATIONSHIPS OF CEREBRAL PERFUSION WITH GAIT SPEED ACROSS AGE AND SYSTOLIC BLOOD PRESSURE LEVELS

Beverly Gwen Windham[1], Michael Griswold[2], Kevin Sullivan[1], Thomas Mosley[1], Michelle Mielke[3], David Knopman[4], Ron Petersen[5], and Prashanthi Vemuri[5], *1. UMMC-The MIND Center, Jackson, Mississippi, United States, 2. The MIND Center at UMMC, Jackson, Mississippi, United States, 3. Mayo Clinic, Rochester, Minnesota, United States, 4. Department of Neurology, Mayo Clinic, Rochester, Minnesota, United States, 5. Mayo Clinic Rochester, Rochester, Minnesota, United States*

We hypothesized that poorer cerebral perfusion could impair mobility, particularly at lower systolic blood pressure (SBP). Cerebral perfusion was measured via arterial spin-labelled (ASL)-MRI in community-dwelling adults of the Mayo Clinic Study of Aging. Usual-pace gait speed was assessed over 5.6 meters on an electronic mat. Linear regression models estimated cross-sectional gait speed associations with ASL and modifying effects of age and SBP, adjusting for sex and body mass index. Results report differences in gait speed (meter/second) per standard deviation (SD) lower ASL or SBP (mean=139.1 mmHg, SD=19.7 mmHg). Among 479 participants (aged 31-94 years; mean age 67.6 years; 44% women; mean gait speed 1.17m/s), relationships of ASL with gait speed varied by age and SBP (ASL-x-age interaction: p<.007; ASL-x-SBP interaction: p<.007). At age 65, 75 or 85 years and a SBP of 120mmHg, each SD lower ASL was associated with a 0.04 m/s (0.01,0.07), 0.07 m/s (0.04,0.10), and 0.09 m/s (0.05,0.13) slower gait speed at respective ages. At age 65, 75 or 85 years with low ASL (1 SD below the mean), each SD lower SBP was associated with 0.06 (0.02,0.09) m/s, 0.03 (0.01,0.06) m/s or .01 (-0.02,0.04) m/s slower gait speed, respectively. With high ASL (mean ASL+1SD), lower SBP trended towards faster gait at older age, e.g. estimated gait speed was 0.04 m/s (0.00,0.09) faster for each SD lower SBP at age 85years. Poorer cerebral perfusion may contribute to slower gait, particularly with lower SBP. The interrelations of cerebral perfusion, SBP, and age with mobility merit further study.

### ASSOCIATION OF PERSISTENT DEPRESSIVE SYMPTOMS WITH PHYSICAL ACTIVITY IN KNEE OSTEOARTHRITIS

Rhea Mehta[1], Michelle Shardell[2], Alice Ryan[3], Yu Dong[1], Brock Beamer[3], Marc Hochberg[1], and Alan Rathbun[1], *1. University of Maryland, Baltimore, Baltimore, Maryland, United States, 2. University of Maryland School of Medicine, Baltimore, Maryland, United States, 3. University of Maryland Baltimore, Baltimore, Maryland, United States*

Comorbid depression in knee osteoarthritis (OA) is associated with declines in physical activity, but how persistent depressive symptoms impact physical activity over time remains unclear. We aimed to determine how the persistence of depressive symptoms affects physical activity in knee OA. Participants (n=2,222) from the Osteoarthritis Initiative had radiographic disease (Kellgren-Lawrence grade ≥ 2) in at least one knee. The Center for Epidemiologic Studies Depression Scale (CES-D; range = 0-60) assessed depressive symptoms from baseline through the first three annual follow-up visits, and persistence was operationalized using the cumulative average severity of symptoms over time. Self-reported physical activity was measured from the first to fourth annual follow-up visit using the Physical Activity Scale for the Elderly (PASE; range = 0-793). The primary method of analysis utilized marginal structural models and included exposure by time interactions in the structural outcome model. Baseline depressive symptoms negatively impacted physical activity at the first follow-up (β = -0.7279; 95% CI: -1.1645, -0.2912), but at later time points, effect estimates were closer to the null and not statistically significant. The association between time-averaged CES-D scores from baseline through the first follow-up and physical activity at year two was -0.1410 (95% CI: -0.7105, 0.4286); and 0.2578 (95% CI: -0.3261, 0.8415) for average CES-D scores through follow-up visit two and physical activity at year three. Thus, the negative influence of persistent depressive symptoms on physical activity decreased over time. Physical activity may not consistently decline with persistent depressive symptoms in adults with knee OA.

### SCALING UP A FALL PREVENTION PROGRAM IN RURAL COMMUNITIES: FEASIBILITY AND LESSONS LEARNED

Melissa O'Connor[1], Jane Strommen[2], Sean Brotherson[2], Philip Estepp[2], Rachel Grace[2], Megan Pedersen[2], Teri Undem[2], and Divya Saxena[2], *1. North Dakota State University, Moorhead, Minnesota, United States, 2. North Dakota State University, Fargo, North Dakota, United States*

In North Dakota, 28% of adults over age 65 report falling each year, resulting in substantial morbidity and mortality. Evidence-based fall prevention programs can benefit quality of life and maintenance of daily living activities, particularly for community-dwelling older adults. The Stepping On fall prevention program, a national evidence-based program, is designed to educate older adults about risk factors for falls, safety strategies, and coping behaviors. The program consists of seven weekly community-based workshops conducted in a small-group setting by two trained facilitators. The Stepping On program was introduced in North Dakota in March 2012. Since that time, 132 workshops have taken place in rural communities across the state, serving 1,502 participants. The number of workshops and participants steadily grew between 2012 and 2019. In the year 2012-13, 127 individuals participated in ten workshops. By 2019, before the program was suspended during the pandemic, 199 individuals participated in 16 workshops; 24 workshops had previously been held in 2018. The current study discusses the implementation process as the program scaled up and expanded to more locations. The feasibility and sustainability of conducting the program in isolated rural areas, challenges encountered, and lessons learned will also be discussed. It is important that residents in rural communities have access to evidence-based programs like Stepping On so they can experience the benefits.

### SKELETAL MUSCLE HEALTH AND LOWER URINARY TRACT SYMPTOMS IN OLDER ADULTS

Peggy Cawthon[1], Scott Bauer[2], Candace Parker-Autry[3], Kaiwei Lu[4], Steven Cummings[5], Russell T. Hepple[6], Rebecca Scherzer[4], and Kenneth Covinsky7, *1. California Pacific Medical Center Research Institute, San Francisco, California, United States, 2. UCSF and San Francisco VA, San Francisco, California, United States, 3. Wake Forest School of Medicine, Winston-Salem, North Carolina, United States, 4. San Francisco VA, San Francisco, California, United States, 5. San Francisco Coordinating Center, San Francisco, California, United States, 6. University of Florida, Gainesville, Florida, United States, 7 University of California, San Francisco, San Francisco, California, United States*

Lower urinary tract symptoms (LUTS) are associated with increased risk of mobility limitations among older adults. Our objective was to evaluate the association of muscle (D3Cr muscle mass, MRI total thigh muscle volume, Keiser extensor power, grip strength) and physical performance (400m walk, SPPB) measures with LUTS severity and bother among adults age >70 years in the Study of Muscle, Mobility and Aging (SOMMA). We used data from the first 132 women and 103 men to complete their baseline visit where LUTS were assessed using the LURN Symptom Index-10 (SI-10) plus a global urinary bother question. We calculated Spearman correlation coefficients and chi-square tests as appropriate, stratified by sex. Among women, LURN SI-10 scores were inversely correlated with D3Cr muscle mass/body weight ($\rho$=-0.217, P=0.01), peak leg power/body weight ($\rho$=-0.179,P=0.04), and SPPB ($\rho$=-0.173,P=0.047), but not 400m walk, MRI thigh muscle volume, or grip strength (P>0.1 for all). 46% of women in the lowest tertile of % muscle mass versus 38% in the highest tertile reported they were at least "somewhat bothered" by urinary symptoms (P=0.04). Among men, no muscle or physical performance measures were significantly associated with LURN SI-10 or urinary bother (P>0.2 for all). In conclusion, older women with greater muscle mass, leg power, and SPPB scores had reduced LUTS severity whereas LURN SI-10 was not significantly correlated with muscle and physical performance measures in older men. Older women with higher D3Cr muscle mass were also less bothered by urinary symptoms, supporting muscle health as a novel female LUTS mechanism.

### ASSOCIATIONS BETWEEN POSTURAL SWAY AND GAIT DOMAINS DURING WALKING WHILE TALKING

Oshadi Jayakody, Helena Blumen, Emmeline Ayers, Cuiling Wang, and Joe Verghese, *Albert Einstein College of Medicine, Bronx, New York, United States*

Poor gait performance during walking-while-talking (WWT) is associated with an increased falls risk. Whether this association between WWT and falls is moderated by balance is not known. To address this, we examined 1) cross-sectional associations between postural sway and gait domains during WWT and, 2) whether postural sway moderated the effects of gait domains on falls. Gait (using a computerized walkway) and postural sway in anteroposterior (AP) and mediolateral (ML) directions (using the SwayStar™ system) during WWT were assessed in 357 community-dwelling older adults (M Age 77.9 years; 55.5% female). Principal component analysis was used to derive distinct gait domains: pace (speed, step length), rhythm (swing, stance, step time) and variability (step length variability, double support time variability). Falls incidence over 12 months were ascertained using bi-monthly calls. Multiple linear regression (adjusted for age, sex, and education) was used to examine the associations between postural sway and gait domains. Poisson regression was used to examine the associations of number of falls with gait domains and postural sway. Greater AP- and ML-sway were associated with slower pace, greater rhythm, and greater variability (p< 0.005). Slower pace (not rhythm or variability) was associated with falls incidence (n=95) and the effect of pace on falls (although p>0.005) was reduced by the ML-sway ($\beta$ for interaction between pace and sway -0.001 95%CI -0.002, 0.0001). These findings suggest that greater sway during WWT is associated with poorer gait performance. Interventions to maintain ML-sway during WWT may assist with reducing the effects of pace on falls risk.

### ASSOCIATION BETWEEN CALF MUSCLE FATTY INFILTRATION AND PHYSICAL PERFORMANCE AMONG HEALTHY OLDER ADULTS

Miji Kim, and Chang Won Won, *Kyung Hee University, Seoul, Seoul-t'ukpyolsi, Republic of Korea*

Muscle fatty infiltration is associated with impaired physical performance. However, the association between ectopic fat, such as lower extremity fat distribution, and sex-specific differences among older adults remains to be determined. This study aimed to examine sex-specific differences in calf muscle fat infiltration and their association with physical performance among healthy older adults aged 76 to 80 years. The pilot study comprised 40 subjects (20 men; 20 women) matched by age and body mass index. The participants underwent dual-energy X-ray absorptiometry, magnetic resonance imaging (MRI), and proton magnetic resonance spectroscopy (1H-MRS) to assess body composition and lower extremity fat distribution. The tibialis anterior intramyocellular lipid (IMCL) as assessed by 1H-MRS was negatively associated with the five-times sit-to-stand test scores (rs=0.518, p=0.023) in men, while the soleus IMCL content was negatively associated with the timed up-and-go test scores (rs=0.472, p=0.048) in women. However, the soleus extramyocellular lipid (EMCL) content was positively associated with the five-times sit-to-stand test scores (rs=−0.488, p=0.040) in women, but this association was not statistically significant in men. Regarding the calf cross-sectional area (CSA) parameters assessed by MRI, calf subcutaneous fat CSA was positively associated with the usual gait speed (rs=0.447, p=0.048) in women only. To conclude, this study showed an inverse correlation between IMCL content and physical performance in healthy older individuals and calf muscle-specific IMCL based on sex differences. Furthermore, our results suggest that greater EMCL content in the soleus and calf subcutaneous fat might affect physical performance positively in older women but not older men.

### EFFECTS OF RESISTANCE EXERCISE ON MOBILITY AND LIFE SATISFACTION AMONG OLDER CHINESE AMERICANS

Mei-Lan Chen, Douglas Gardenhire, Ying-Jung Wu, and Mi-Jung Lee, *Georgia State University, Atlanta, Georgia, United States*

Physical activity interventions have shown to be potentially effective for preventing mobility impairment and improving life satisfaction in older adults. However, few studies investigated the effect of resistance exercise training on mobility and life satisfaction in older Chinese Americans. The purpose of this study was to test the effect of a 12-week resistance exercise intervention (two times weekly) on mobility (balance, gait speed, and chair stand performance) and life satisfaction in a sample of community-dwelling older Chinese Americans aged 60-89 years (N = 30; mean age 77.9 ± 5.0 years). The study was a two-group randomized controlled trial (RCT). All participants were randomly assigned into either the resistance exercise group or the wait-list control group (15 participants in each group). Participants' balance, gait speed, chair stand performance, and life satisfaction were assessed at baseline and 12 weeks. Descriptive statistics and t tests were used for data analysis. The results indicated that older adults participated in the resistance exercise program had significant improvements in balance, gait speed, chair stand performance, and life satisfaction at the end of the trial. Compared to the control group, the resistance exercise group had significantly greater improvements in balance, gait speed, chair stand performance, and life satisfaction at 12 weeks follow-up. The findings suggest that resistance exercise has beneficial effects in improving mobility and life satisfaction for older adults. This resistance exercise program provides a basis for developing larger RCTs for further determining the efficacy of resistance exercise among older Chinese Americans.

### SEDENTARY BEHAVIORS AND PHYSICAL ACTIVITY EXPERIENCES OF LATINX OLDER ADULTS: A THEMATIC CONTENT ANALYSIS

Suweena Bohplian[1], Kristy Liu[2], Cynthia Fritschi[1], and Ulf Bronas[1], *1. University of Illinois Chicago, Chicago, Illinois, United States, 2. University of Illinois at Chicago, Chicago, Illinois, United States*

Sedentary behavior (SB) accounts for ~7.5 % of cardiometabolic mortality globally. The burden of cardiometabolic disease is especially high among Latinx adults and increases with age. Physical activity (PA) is crucial for maintaining health, yet most Latinx older adults do not meet PA guidelines and spend much time in SB. The aim of this study was to use thematic content analysis to describe the meaning of SB; and the experiences, facilitators, and barriers to PA among Latinx adults. Individual interviews were conducted among 30 Latinx older adults aged 55 to 75 years (60.7 ± 5.7 years) and BMI 20.78 to 42.13 kg/m2 (29.8 ± 4.4 kg/m2) who were enrolled in a larger study of PA. Results showed major themes included meaning of SB, consequences of SB, barriers, and facilitators of PA. Meaning subthemes included sitting for leisure as different from sitting work and value statements about laziness. Three participants did not understand the word "sedentary behaviors". Consequence subthemes included effects on physical (circulation, obesity, fatigue) and mental health (cognitive and psychological). Barriers to PA included COVID-19, environment (weather, safety), culture (language, no relationships with non Latinx communities), time constraints (work), and physical conditions (pain and illness). Facilitators of PA include self-motivation, family, and community support (public parks, gyms, PA program from community organizations). These findings shed deeper meaning about SB and PA among the Latinx culture. These findings will be key in developing culturally appropriate interventions to improve physical and mental health specifically for the Latinx older adult population.

### PROTEIN SUPPLEMENTATION NEGATIVELY IMPACTS GLUCOSE HOMEOSTASIS IN A WEIGHT LOSS INTERVENTION IN OLDER ADULTS

John Batsis[1], Curtis Petersen[2], David Lynch[3], Hillary Spangler[1], and Summer Cook[4], *1. University of North Carolina at Chapel Hill, Chapel Hill, North Carolina, United States, 2. Dartmouth College, Hanover, New Hampshire, United States, 3. UNC, Chapel Hill, North*

*Carolina, United States, 4. University of New Hampshire, Durham, New Hampshire, United States*

Background: Higher dietary intake of protein has also been associated with increased risk of type 2 diabetes, yet losing weight improves glucose metabolism and homeostasis. We evaluated whether a multicomponent weight loss intervention in older persons with obesity aged 65+ years augmented with protein supplementation impacts glucose homeostasis.

Methods: A 12-week, non-randomized, parallel group intervention in 28 older rural adults with obesity (body mass index (BMI)≥30kg/m2) was conducted at a community aging center of a protein and non-protein arms. Both received individualized, weekly dietitian visits with twice weekly physical therapist-led group strength training classes; aerobic exercise was prescribed outside the classes. The protein group was provided with whey protein supplementation three times weekly post-strength training. Pre/post fasting glucose, insulin, and c-peptide levels were assessed with HOMA-IR and HOMA beta-cell function calculated.

Results: Mean age was 72.9±4.4 (86% female) and 73.0±6.3 (79% female), p=0.94, with baseline BMI of 37.6±6.9 and 36.6±5.5, in the protein and non-protein groups, respectively. Mean weight-loss was -3.45±2.86kg and -5.79±3.08kg (both p< 0.001; Δ p=0.047). Visceral fat decreased less in the protein group (-0.02mL vs. -1.02ml;p=0.007), while appendicular lean mass did not (p=0.44). There was lower fasting glucose (protein:-4mg±13.9mg/dL vs. non-protein: 12.2±25.8, effect size:-0.40;p=.10), insulin (-1.32±4.68 vs. -7.79±10.14, ES:0.82;p=0.01), and higher c-peptide (-0.02±0.67 vs. -0.69±1.25,ES:0.66;p=0.06) at follow-up. HOMA-IR also decreased less (-0.18±0.64 vs. -1.08±1.50, ES:0.78; p=0.02) and b-cell function (-3.94±22.61 vs. -16.81±33.85; ES:0.45; p=0.09]).

Conclusions: A multicomponent obesity intervention incorporating protein supplementation led to lower degree of weight loss and mitigated the benefit on glucose homeostasis.

### COGNITIVE-MOTOR GAMING IN OLDER ADULTS: FEASIBILITY OF TELEASSESSMENT AND TELEREHABILITATION ON FALL RISK

Tanvi bhatt[1], Savitha Subramaniam[1], Spyros Kitsiou[2], Lakshmi Kannan[2], and Edwina Wilson[2], *1. University of Illinois at Chicago, Chicago,, Illinois, United States, 2. University of Illinois at Chicago, Chicago, Illinois, United States*

There is limited guidance for clinically relevant tele-assessment, and access to a comprehensive physical-activity (PA) based telerehabilitation paradigm to enhance physical function, and slow progression of frailty among older adults (OA). In this we first examined the usability, safety, and feasibility of tele-assessment and telerehabilitation program on physical, and CV function. Subsequently evaluated its compliance and effectiveness. Healthy OAs (n=23,>65 years) participated a custom-designed exergaming-based tele-exercise program (EG-BTxP) delivered in home-setting for 4 weeks (3 sessions/week) consisting of 4 exercise modules: Dancing, aerobics, cognitive-motor gaming and mind-body exercises (yoga and tai-chi) in groups of 5. Pre- and post-training, a real-time online teleassessment was performed and change in lower limb strength, endurance (30-second chair stand test), static balance (one-legged stand test, Romberg test), and dynamic balance (4-step square test), aerobic endurance (2-minute step in place test) were assessed. Participants were provided with wearable sensors and education to self-monitor heart rate during exercise and report back to health coach after each module. Participants responded positively on the qualitative usability survey and there were no adverse events . All participants were able to tolerate the teleassessment and tele-intervention with a compliance of >90%. Post-training, there were improvements in 30-second chair stand test; (p< 0.01), one-legged stand test, Romberg test, and 4-step square test, (p< 0.05). Further, number of steps in the 2-minute step-in-place test increased (p < 0.05). These findings suggest that gaming-based tele-exercise programs could be safely implemented in community-based settings to increase compliance with participation for improved physical rehabilitation outcomes.

### DAILY EXERCISE DOES NOT ALTER CEREBRAL BLOOD FLOW REGULATION DURING TILT IN OLDER ADULTS AFTER TWO WEEKS OF BEDREST

Andrew Robertson[1], Eric Hedge[2], Carmelo Mastrandrea[2], Federico Granados Unger[2], and Richard Hughson[2], *1. University of Waterloo, Waterloo, Ontario, Canada, 2. Schlegel-UW Research Institute for Aging, Waterloo, Ontario, Canada*

Bedrest is associated with cardiovascular and cerebrovascular changes that are linked to a greater prevalence of orthostatic intolerance; however, much of what we know from bedrest trials comes from young adults. How older adults respond to extended periods of activity restriction is not well understood. The current work tested the hypothesis that impaired control of cerebral blood flow in older adults following bedrest could be attenuated by regular exercise. Twenty-two older adults (55-65 years, 11 women) participated in a randomized controlled trial involving 14 days of continuous 6-degree head-down bedrest. The cohort was randomized to an exercise intervention (EX), involving daily aerobic, high-intensity interval, and resistance training, or a control group (CON), involving passive manual therapy. Cerebral blood flow velocity, cardiac output, and arterial blood pressure were measured continuously during a passive 80-degree head-up tilt protocol for up to 15 minutes. Responses in the cerebral and peripheral vascular beds were quantified by vascular resistance (cerebral: resistance area product (RAP); peripheral: total peripheral resistance (TPR)). Following bedrest, both supine RAP and TPR were elevated (p< 0.01), with no effect of exercise training. In addition, the number of participants who could not complete 15 minutes of tilt increased from 3 to 15 (8 EX, 7 CON). In these non-finishers, RAP dropped ~20% and TPR dropped ~15% during the final 2 minutes of tilt, with no difference between groups. These data suggest that multimodal exercise training is insufficient to prevent changes in cardiovascular and cerebrovascular regulation in older adults observed during 14-day bedrest.

### HEALTH BENEFITS OF TAEKWONDO INTERVENTION IN OLDER ADULTS LIVING IN NURSING HOMES

Yongseop Kim[1], Junhyoung Kim[2], Dongchul Seo[2], and Sua Han[2], *1. Indiana University at Bloomington,*

*Bloomington, Indiana, United States, 2. Indiana University, Bloomington, Indiana, United States*

Physical inactivity is prevalent among nursing home residents, and it contributes to loss of lower body muscle mass, which raises the risk of falling for the older adult population. Accumulative evidence shows that engaging in leisure-time physical activity improves physical functions, mental health, and cognitive capacities and provides social benefits in older adults. Taekwondo, a form of Korean traditional martial arts, has the potential to improve the health benefits among nursing home residents in the United States. Thus, the aim of this study was to explore the health advantages for nursing home residents of participation in a Taekwondo program modified to accommodate their physical and cognitive conditions. An observational research method was incorporated to explore the health benefits of Taekwondo. 10 prospective participants (5 females, 5 males; mean age = 80 years) from the local nursing home facility participated in in-depth semi-structured interviews. The researchers employed Strauss and Corbin's (1998) grand-tour and mini-tour question strategies, as well as Creswell's (2009) five phases of data gathering and analysis. The constant comparative approach was utilized to analyze the data in this study. As a consequence of participating in Taekwondo, four key themes were recognized as health benefits: (a) supporting mental health, (b) increasing physical functions, (c) stimulating cognitive capacities, and (d) facilitating healthy social connection. The findings of this study suggest that Taekwondo might be utilized as a therapeutic intervention to improve physical activity and reduce the negative psychological effects of nursing home confinement.

### OLDER ADULT PERSPECTIVES ABOUT ONLINE EXERCISE CLASSES DURING THE COVID-19 PANDEMIC AT MULTIPLE TIME POINTS

Michelle Porter, Dallas Murphy, Nicole Dunn, Ruth Barclay, Stephen Cornish, Jacquie Ripat, Kathryn Sibley, and Sandra Webber, *University of Manitoba, Winnipeg, Manitoba, Canada*

Engaging in physical activity can bring health benefits for older adults. However, during the pandemic the availability of in-person exercise classes has been sporadic. As such, online exercise programs have become more common. This research had the goal of exploring the uptake of online exercise programs by older adults in Manitoba, Canada in the first few months in the pandemic and then more than 1.5 years into the pandemic. Older adults (65 years and older) were recruited via emails from a variety of community organizations. Participants completed anonymous online surveys in summer 2020 (n=678) and fall 2021 (n=570). Less than 50% of respondents reported participating in online exercise classes during the pandemic in both surveys. For both surveys, pre-recorded classes were the most common, however, this decreased from 80% in the first survey, to 57% in the second survey. Conversely, live classes where the instructor could see the participants increased from 17% in the 2020 survey, to 47% in the 2021 survey. Additionally, platform use shifted from YouTube as the most popular in the first survey, to Zoom in the second survey. Most of the online classes originated from their local communities. Of those who participated in online exercise early and later in the pandemic, about two thirds reported that they would continue online exercise classes outside of the pandemic. A major reason for not participating was because they enjoy the social aspect of in-person classes. The perspectives of the study participants will be valuable for policymakers, programmers, and instructors.

### SESSION 6790 (POSTER)

### SOCIAL ISOLATION AND LONELINESS (BSS)

### COMPANIONSHIP TO ADDRESS QUALITY OF LIFE AND LONELINESS AMONG OLDER ADULTS WITH SEVERE LONELINESS

Kelsey McNamara, and Ellen Rudy, *Papa Inc., Miami, Florida, United States*

Research indicates that loneliness is a powerful predictor of premature mortality. Less is known about those who experience “severe loneliness” and their responsiveness to companionship as a means to clinically improve. We sought to understand the epidemiology of a cohort of lonely older adults and the impact of companion care on severe loneliness and quality of life. Companion care was provided through Papa Inc., a national service that pairs older adults with “Papa Pals” who provide companionship and assistance with everyday tasks. Participants have free access if their Medicare Advantage plan offers it. The sample included adults ages 65+, active in Papa in 2021, and provided follow-up data (UCLA-3 Loneliness Scale; CDC's Healthy Days Measures) as of September 2021. Analysis utilized t-tests and Chi-square tests with significance set at $p< 0.05$. A total of 2650 participants were identified as lonely at baseline, 435 were successfully contacted to collect complete follow-up data. Of the follow-up cohort, 22% were classified as severely lonely at baseline reporting mean UCLA score of 8, and 15 mentally and 14 physically unhealthy days on average. Of those severely lonely, 62% were female, 11% were ages 85+. Sixty percent of severely lonely participants experienced clinical improvements and moved to a lower category of loneliness (mean change in UCLA: –3.37) and reduced their unhealthy days (–6.20 mental; –2.09 physical). This real-world evidence suggests a companionship program can improve loneliness and quality of life among severely lonely older adults. Earlier interventions to prevent chronic loneliness should be explored.

### DEVELOPING A MOBILE PHONE APPLICATION TO DETECT SOCIAL ISOLATION OVER MULTIPLE TIME POINTS

Rajlaxmi Biyani[1], Anupam Joshi[2], Gretchen Tucker[3], Dana Bradley[4], and Carmen Sceppa[5], *1. University of Maryland Baltimore County, Fairfax, Virginia, United States, 2. University of Maryland Baltimore County, Baltimore, Maryland, United States, 3. University of Maryland, Baltimore, Columbia, Maryland, United States, 4. University of Maryland, Baltimore County, Baltimore, Maryland, United States, 5. Northeastern University, Boston, Massachusetts, United States*

Social isolation is an important predictor of mortality and morbidity in older persons. The increase in social isolation is often a result of major life events such as retirement, leading to the loss of social connections. The effects of the pandemic have exacerbated the loss of social connectedness. Measures to ensure social distancing have made technologies like voice and video calling, text messaging, and other phone mediated communication important resources for connecting people. Measuring social isolation often requires periodic administration of a paper survey instrument, like the Lubben Social Network Scale (LSNS). The approach was taken by Lubben for predicting a person's social isolation level typically involves looking at their general frequency of interactions with family and friends along with the specific frequencies of private, tentative discussions happening during those interactions. This paper explores the process used to develop a tool to measure social isolation continuously and automatically by using an older person's frequency and tone of telephonic conversations. We categorized a person's family and friends into three sub-categories based on the self-reported level of closeness shared with them. We then analyzed the person's conversation (content and tones). Lastly, we trained the deep learning model (Watson) using these captured tone values to determine the social isolation index of the individual. Our preliminary results indicate that we are able to correctly predict their degree of social isolation as judged by Lubben's scale using their phone conversations. This technology may be promising for the assessment and intervention of social isolation.

### LONELINESS AND COGNITIVE FUNCTION IN OLDER ADULTS WITHOUT DEMENTIA: A SYSTEMATIC REVIEW

Karra Harrington[1], Shradha Vasan[2], Jee eun Kang[1], Martin Sliwinski[1], and Michelle Lim[2], *1. The Pennsylvania State University, University Park, Pennsylvania, United States, 2. Swinburne University of Technology, Hawthorn, Victoria, Australia*

Loneliness has consistently been associated with dementia risk. An important precursor to dementia is cognitive decline, which can begin decades prior to clinical diagnosis of dementia. Therefore, understanding about the relationship between loneliness and cognitive function in healthy older adults may inform our understanding of how loneliness contributes to dementia risk. The aim of this systematic review was to identify the extent to which loneliness affects cognition in older adults who do not have dementia. A systematic search of five databases (PubMed, PsycNET, Web of Science, EBSCOhost, Scopus) from inception to August 31st 2021 was completed, including search terms related to loneliness, aging, and cognition. A total of 4,302 unique articles were screened for inclusion, resulting in 16 studies that met full criteria (six cross-sectional and ten longitudinal). Three of the six (50%) cross-sectional studies reported significant negative associations between loneliness and cognitive function, while six of the ten (60%) longitudinal studies reported that loneliness was associated with greater cognitive decline over time. We did not find a significant relationship between loneliness and cognitive function in the rest of the studies. There was substantial variation across studies in the measures of loneliness and cognitive function. Furthermore, many studies relied on cognitive screening tools to identify cognitive outcomes, which may not be sensitive to subtle cognitive changes that precede dementia. Future studies should consider using validated and sensitive measures of loneliness and cognitive function, and examining these relationships prospectively, in order to, assess these relationships in a more robust way.

### LONELINESS AND ACTIVITIES OF DAILY LIVING PERFORMANCE IN OLDER ADULTS: EXPLORING THE MODERATING ROLE OF COGNITION

Bianca Velez, and Cynthia Dulaney, *Xavier University, Cincinnati, Ohio, United States*

Resulting from changes in the sociodemographic landscape and the global pandemic, rates of loneliness have exponentially risen (Myers & Palmarini, 2017; Piette et al., 2020). Prior research demonstrates that loneliness is associated with an increased risk of cognitive and functional impairment (Cacioppo & Hawkley, 2009; de Jong Gierveld & van Tilburg, 1995; Holmén, et al., 1992; Wilson et al., 2007) as well as depression (Cacioppo et al., 2006; Kwon et al., 2017; Purtill, 2018). Although cognition has been studied as either a predictor or outcome of loneliness, it has yet to be examined as a moderator between loneliness and functional impairment (i.e., performance of basic and instrumental activities of daily living; ADLs, IADLs). Participants (N = 106) were community and non-community dwelling adults aged 65 years and older who completed four self-report measures assessing loneliness, ADLs/IADLs, cognitive functioning and depression. Hierarchical linear regression analyses were conducted to test the moderation model. Data revealed that cognition moderated the relationship between loneliness and IADLs. Follow-up simple slope analyses revealed that high cognition and low cognition were associated with decreased IADL performance amongst individuals with loneliness, and this association was stronger when cognition was low. The current study sheds light on the social, emotional, and physical disruptions the pandemic has caused in the lives of older adults. It highlights the need for interventions to enhance the quantity and quality of social relationships, and older adults' involvement in physical and cognitive activities to uphold functional independence in later life.

### EXPECTATIONS OF RECOGNITION AND BENEFITS OF CREATIVE ACTIVITIES FOR MIDDLE AND OLDER ADULTS

Darby Mackenstadt[1], Carolyn Adams-Price[2], and Sarah Israel[1], *1. Mississippi State University, Starkville, Mississippi, United States, 2. Mississippi State University, STARKVILLE, Mississippi, United States*

Creative hobbies have been found to be beneficial for mental health, especially if an individual considers their hobby an important part of their identity (Adams-Price & Morse, 2018). Although receiving recognition from friends or family for one's creative abilities has been seen to decrease depressive symptoms for adults, these benefits may depend on expectations and type of recognition desired (Israel et al, 2020). Those who participate in creative activities as a career compared to those who participate as hobbyist may not receive the same benefits due to the stress artists endure trying to earn a living (Barker et al, 2009). This study aimed to explore the relationships between hobby recognition, hobby's impact on identity, and mental well-being. A sample of 279 primarily white (88%) female (95.3%) adults aged 40 to 84 (M = 59.9-years-old) were interviewed during the Covid-19 Pandemic. A structural equation model was developed in AMOS 28.0 to correlate these variables and do a multiple groups analysis, comparing 100 older adults (65+) and 179 middle aged adults (40-64) who all reported at least one creative hobby. Results found a negative relationship between identifying with a creative hobby and receiving recognition for the hobby, a negative relationship between mental well-being and recognition, and a positive relationship between identifying with one's hobby and mental well-being. The results suggest that recognition from others may have mixed effects on mental health but identifying with one's creative hobby may be a protective factor. Implications expectations of recognition will be discussed.

### LONELINESS AND DEPRESSION: EXAMINING THE MODERATING EFFECTS OF RESILIENCE RESOURCES

Xiaoyan Zhang, and Merril Silverstein, *Syracuse University, Syracuse, New York, United States*

Loneliness is highly prevalent among older adults and can negatively influence their mental health. However, less is known about the factors that might mitigate the effects of loneliness on mental health outcomes such as depression. We propose that resilience resources may serve as potential protective factors that buffer the impact of loneliness on depressive symptoms. This study aimed to (a) test the effects of loneliness, as well as resilience factors of perceived family support and optimism, on depressive symptoms, and (b) examine resilience factors as moderators of the relationship between loneliness and depressive symptoms. Participants derived from the Health and Retirement Study (HRS), a nationally representative sample of the population aged 50 years and older. The analytic sample was selected with baseline measurements in either 2006 or 2008 and two follow-ups across four-year intervals (N= 7,336). Structural equation modeling with latent variables and interaction terms was used to investigate study aims. Results revealed that (a) loneliness was significantly related to an increase in depressive symptoms; both perceived family support and optimism significantly reduced depressive symptoms, and (b) the link between loneliness and depressive symptoms was weaker when older adults reported higher levels of perceived family support and greater optimism. This study highlights the important protective roles played by perceived family support and optimism in reducing the adverse impact of loneliness on depression. Results suggest that strengthening family support and fostering optimism might be promising avenues for improving mental health in older adults particularly among those experiencing loneliness.

### IMPACT OF A SOCIAL CONNECTION PROGRAM ON OLDER ADULTS DURING THE COVID-19 PANDEMIC

Rachel Ungar[1], Rifky Tkatch[2], Yan Cheng[2], Sandra Kraemer[3], Michael McGinn[2], and Ellen Wicker[4], *1. Optum Labs, Oak Park, Michigan, United States, 2. Optum Labs, Minnetonka, Minnesota, United States, 3. UnitedHealthcare, Minnetonka, Minnesota, United States, 4. ASI, Inc., Washington, District of Columbia, United States*

Background: Research demonstrates social connections decrease loneliness and improves life satisfaction among older adults. However, the COVID-19 pandemic limited social connectedness, specifically for older adults. Thus, programs aimed to increase social connectedness among older adults are integral to their well-being. Purpose: The primary objective of this study was to determine if the telephonic Peer-to-Peer (P2P) program could improve social connectedness and reduce loneliness among older adults. A secondary objective was to improve life satisfaction and social support.

Methods: Eligible older adults (age 65+) were recruited via outbound calls and/or a mailer. Participants were mailed a T1 survey, completed intervention training, and matched into a dyad. The matched dyad engaged in weekly telephone calls for 12 weeks. Post 12 weeks, participants completed a T2 survey, and a T3 four weeks later.

Results: A total of 475 participants completed a T1, and 125 dyads (250 individuals) completed a T3. Older females were more likely to participate (77% female, 44% 75+ old). Individuals who were lonelier at baseline showed a significant improvement in loneliness throughout the program, as well as improvement in social support, life satisfaction, and subjective happiness.

Conclusion: Results from this program showed significant improvement in psychosocial well-being outcomes for lonely older adults. This program took place during the initial months of the COVID-19 pandemic and demonstrated success for lonely older adults with limited technology who may have been socially isolated during this time.

### LONELINESS DURING THE COVID-19 PANDEMIC AND THE EFFECT ON PSYCHOSOCIAL FACTORS AND HEALTH OUTCOMES

Veronica Dinaj[1], Rifky Tkatch[1], Yan Cheng[1], Julianne Holt-Lunstad[2], Carla Perissinotto3, Sandra Kraemer[4], James Schaeffer[1], and Charlotte Yeh[5], *1. Optum Labs, Minnetonka, Minnesota, United States, 2. Brigham Young University, Provo, Utah, United States, 3 University of California, San Francisco,San Francisco, California, United States, 4. UnitedHealthcare, Minnetonka, Minnesota, United States, 5. ASI, Inc., Washington, District of Columbia, United States*

Loneliness and social isolation have harmful impacts on health and well-being; thus, the social distancing mandates during the COVID-19 pandemic, meant to protect our most vulnerable populations including older adults, may have had unintended consequences. The current study aimed to assess changes in loneliness (prior to and during the pandemic) and its impact on outcomes such as perceived stress, resilience, purpose in life, quality of life and health outcomes. An annual survey on healthy aging was mailed to a randomly selected national sample (age >65 years) in 2018/2019. Respondents completed the survey again in 2020 (N=3,564) to measure the impact of COVID-19. Measures included well-being and various psychosocial factors. Health care claims data were also used to derive diagnoses for health conditions. Respondents were 52% female and 44% between 65-74 years old. Loneliness symptoms increased for one-fifth of the sample during the pandemic. Respondents lonely at both time 1 and time 2 had lower resilience, purpose in life, and quality of life and higher perceived stress compared to those only lonely at one time point or not lonely. Respondents lonely at both time points had higher rates of health conditions compared to non-lonely respondents. In conclusion, older adults experienced increased levels of loneliness during COVID-19. Those with chronic loneliness had worse outcomes than those who were not lonely or inconsistently lonely and they exhibit lower resilience and purpose and higher stress and health complications. These findings suggest that timing and early intervention for loneliness is important for older adults.

### SOCIAL SUPPORT AND LONELINESS AS DETERMINANTS OF THE ONSET OF DISABILITY AMONG PUERTO RICAN OLDER ADULTS

Amilcar Matos-Moreno[1], Neil Mehta[2], Eduardo Villamor[3], Lu Wang[3], and Carlos Mendes de Leon[4], *1. The Pennsylvania State University, Bayamon, Pennsylvania, Puerto Rico, 2. UTMB, Galveston, Texas, United States, 3. University of Michigan, Ann Arbor, Michigan, United States, 4. University of Michigan, Ann arbor, Michigan, United States*

Background: The effect of loneliness and social support on health is poorly understood among older Puerto Ricans. As family size continues to decrease in Puerto Rico due to out-migration, a higher number of older adults have fewer family members to rely on, which may lead to detrimental health outcomes.Method: Using both waves (2002-03 and 2006-07) of the Puerto Rican Elderly: Health Conditions database, we examined the association between social support, living alone, and incident disability among a sample of older adults over 60 years of age residing in Puerto Rico. Disability was defined as the occurrence of difficulties with Activities of Daily Living (ADLs).

Results: 13.4% of older adults in our sample developed some form of disability. Older adults who developed a disability indicated receiving higher levels of social support (2.04 vs. 1.64) and loneliness (30.7% vs. 22.8%). Using multivariate logistic regression, we found that receiving social support increased the odds of developing a disability by 17% (OR: 1.17; CI: 1.02 – 1.35). Older adults who live alone had 58% higher odds of developing a disability (OR: 1.58; CI: 1.01 – 2.46).

Conclusion: The presence of social support and loneliness was correlated with a population prone to developing disabilities. Our findings concur with the well-established literature on psychosocial determinants in late life. However, this study represents the first attempt to understand psychosocial measures and disability in Puerto Rico. Public health organizations and healthcare systems must develop new societal mechanisms of support for older adults at risk of developing disabilities.

### THE GEOGRAPHIC LAYOUTS OF OLDER EUROPEANS' SOCIAL NETWORKS AND LONELINESS IN THE COVID-19 PANDEMIC

Haosen Sun[1], and Markus Schafer[2], *1. Indiana University Bloomington, Toronto, Ontario, Canada, 2. University of Toronto, Toronto, Ontario, Canada*

Many older Europeans sustain core personal networks that are geographically dispersed. The pandemic has brought concerns about their mental health, especially during lockdown periods when travel was not permissible and casual local contact was limited. This study examines whether and how older adults' loneliness and depressed feelings vary by the prior geographic layouts of their core discussion networks. It uses a sample of community-dwelling respondents aged 50 and above (Wave 6, in 2015) from the Survey of Health, Aging, and Retirement in Europe (SHARE), with a follow-up at Wave 8 in 2020 during the height of the COVID pandemic. Latent Class Analysis and linear regression show that individuals whose networks were comprised mainly of families 5-25km and >25km away were not especially likely to feel lonely or distressed, despite typically lacking nearby confidants. We also uncover groups of people occupying more compositionally diverse networks. While these individuals were generally more likely to perceive loneliness, they tended not to attribute such feelings to the pandemic onset. The exception was individuals sustaining diverse networks comprised mainly of friends and families at intermediate distances (5-25km), who perceived heightened loneliness in the pandemic. Overall, even scattered at longer distances, family-oriented networks demonstrate good protectiveness against loneliness and depression. Meanwhile, diverse networks appeared to fall short in protectiveness under some conditions of geographic dispersion.

### THE COMBINED EFFECTS OF LONELINESS AND SOCIAL ISOLATION ON MENTAL HEALTH IN A NATIONAL SAMPLE OF OLDER ADULTS

Melissa Krook, Corey Mackenzie, Verena Menec, and Jessica Cameron, *University of Manitoba, Winnipeg, Manitoba, Canada*

Social connections are important to maintain health across adulthood. Loneliness and social isolation are global issues and are linked to negative mental health outcomes worldwide, especially among older adults. Past research focuses primarily on loneliness and isolation separately, though many older people experience them simultaneously. Also, there is a paucity of research examining mechanisms through which combinations of loneliness/isolation result in poor mental health. My first objective examined how combined loneliness/isolation affect psychological distress among a group of older adults, and how grouping this sample into four groups of loneliness (yes/no) and isolation (yes/no) may help identify which group(s) are at the greatest risk for distress. My second objective explored perceived social support and relationship satisfaction as mediators of the effects of combined loneliness/solation on distress. I addressed these objectives with a cross-sectional national sample of 2,745 Canadian older adults, aged 55 to 101 years, who completed self-report measures of loneliness, social isolation, social support, relationship satisfaction, perceived physical health, and psychological distress. Those experiencing greater combined loneliness/isolation also experienced higher levels of distress. For lonely older adults, experiencing isolation simultaneously predicted clinically significant distress, but this was not true for those who were not lonely. Participants who were both lonely/isolated had the poorest mental health because they were less satisfied with their relationships, but not because they had less perceived social support. The present study has the potential to expand what we know about pathways through which combinations of loneliness/isolation may lead to poor mental health in older adults.

### I HAVE TO COPE WITH IT: THE VOICES OF OLDER AFRICAN IMMIGRANTS EXPERIENCING SOCIAL ISOLATION AND LONELINESS IN THE US

Dolapo Adeniji[1], and Gifty Ashirifi[2], *1. IUPUI, Avon, Indiana, United States, 2. Indiana University, Indianapolis, Indiana, United States*

Social isolation and loneliness have been recognized as significant challenges in the world of older adults. For older African immigrants living with their families in the US, researchers have captured factors such as language barriers, cultural differences, and limited access to transportation to contribute their feelings of social isolation and loneliness. However, little is known about how they cope with these challenges. As the population of older African immigrants continues to increase in the US, it is pertinent to expand knowledge about their experiences for the purposes of social work practice and policy development. Using a qualitative approach, this study recruited and conducted in-depth interviews with 11 participants aged 63 -79. Four themes emerged from the data through a thematic analysis approach which includes a) Positive Self-talk: “I have to cope with it”, b) Technology/Social media: "if I cannot interact physically outside, then, I go through the social media”/Watch TV”, c) Intergenerational social engagement beyond caregiving: "They [grandchildren] are my immediate constituency”, and d) Digging deep through faith. Although the result of this study shows that older Africans immigrants are finding strategies to cope with social isolation and loneliness further support is needed specially to strengthen their coping skills and enhance their social network with people outside of their families.

### A NEW NORMAL: EXAMINING THE LINKAGES BETWEEN SOCIAL TECHNOLOGY AND LONELINESS

Yeon Ji Ryou[1], Natasha Peterson[1], Mengya Wang[1], and Joseph Svec[2], *1. Iowa State University, Ames, Iowa, United States, 2. Iowa state university, Ames, Iowa, United States*

As the COVID-19 pandemic continues to shape social landscapes, social isolation and loneliness are major issues with mental and physical ramifications. In recent years, individuals of all ages are turning to social technology (ST) to communicate with loved ones. However, the viability of ST as a substitute to in-person interaction remains hotly debated. Moreover, few studies examined how psychosocial factors interact with ST in mental health outcomes. Using the 2020 survey data (N = 1,969) from Health and Retirement Study (HRS), this research examines whether and to what extent ST ameliorates loneliness among 65+ individuals. We identify which personality dimensions moderate the relationship between ST and loneliness using the conceptual framework of the five-factor and the unified theory of acceptance and use of technology model. Linear regression analyses are conducted to determine direct and interaction effects. Results indicate that greater ST use corresponds negatively with loneliness. We also observe that particular personality traits (e.g. extraversion) are negatively associated with loneliness, while neuroticism corresponds positively with loneliness. In the interaction between neuroticism and ST, neuroticism mitigates the association between social technology and loneliness. These findings indicate that ST can be a positive source of social connectivity but the extent may be conditioned on personality profiles. Plausibly, neurotic individuals may exacerbate behavioral propensities by technology use. As evidenced by the robust independent effects of ST and conditional nature of technology connections with neurotic profiles. These findings imply that future intervention should consider individual differences when developing mental health programs using ST.

### COMMUNITY OUTCOMES FROM A PSYCHOSOCIAL INTERVENTION FOR LONELINESS AND SOCIAL ISOLATION IN OLDER ADULTS

Matthew Amick, Olivia Gartlan, Max Zubatsky, and Marla Berg-Weger, *Saint Louis University, St. Louis, Missouri, United States*

Social isolation and loneliness are quickly becoming global health epidemics, where older adults are experiencing greater chronic health conditions and other complications as a result of this trend. This prevalence is likely underreported, given that stigma and health disparities associated with social isolation and loneliness often prevent older adults from reporting this to family members and professionals. This study's aim is to determine the rates of older adult social isolation and social connections from a psychosocial group model called Circle of Friends (CoF). Circle of Friends is built on a model of group rehabilitation model, with the aim to alleviate and prevent loneliness in older adults. The group protocol consists of 6-8 older adults who self-identify as lonely or socially isolated, who meet 12 times over three months. A midwestern university conducted virtual CoF groups between 2020-2022 to underserved areas of a metropolitan city. Researchers used the UCLA Loneliness Scale and Lubben Social Support Scale to determine differences in rates of loneliness and social supports. 15 members participated in three virtual CoF groups, with over 50% of the sample reporting low socioeconomic living conditions. Preliminary findings show that group members reported a 3-point decrease in overall loneliness ($p<.05$), but no differences in frequency of social supports through friends or family. While our research group continues to collect more data on outcomes of our CoF groups, these initial results highlight the need for more community interventions and social connection resources for older adults lacking financial means for healthcare options.

### COMPARING DATA-DRIVEN AND THEORY-DRIVEN APPROACHES TO CLASSIFY SOCIAL RELATIONSHIPS ACROSS THE LIFE COURSE

Elliot Friedman[1], Elizabeth Teas[2], and Kristine Marceau[1], *1. Purdue University, West Lafayette, Indiana, United States, 2. Purdue University, Lafayette, Indiana, United States*

A life course perspective on social relationships highlights the salience of specific relationships at specific times in life, but analyses that account for life course trajectories in social relationships are rare. The present study explores the properties of two analytic approaches to classifying life course relationships: 1) a priori, or theoretical, classification strategies, and 2) latent profile analysis (LPA). Including multiple dimensions of social relationships at different time points across the life course, we determined whether theoretical or LPA profiles better predict later-life functional limitations, given that functional impairment is prevalent among middle-aged and older adults. We also assessed whether the two approaches are differentially associated with a broad set of covariates. Data were from three waves of the Midlife in the United States (MIDUS) study (n = 6,909). Relationship variables (parental affection, parental discipline, social support, social strain, and positive relations with others) were from wave 1. Functional limitations were measured at wave 3. Both the LPA and theoretical approach resulted in 4 profiles (although there were qualitative differences), and both approaches differentiated covariate values in similar ways. Loglikelihood comparisons in Mplus showed that the LPA, compared to the theoretical approach, was more sensitive to detecting differences in functional limitations. Overall, the data-driven profiles differed from theory-based profiles, which may be substantively meaningful for understanding associations between life-course relationships and health. Practical implications include development of a measure for future research to apply these novel life-course social relationship groups, particularly with smaller samples not suitable for LPA.

### EXAMINING THE CUMULATIVE EFFECTS OF SOCIAL ISOLATION AND LONELINESS IN OLDER ADULTS WITH HIV

Moka Yoo-Jeong, *Northeastern University, Melrose, Massachusetts, United States*

Social isolation exists when one has limited or lacks social contact with others and is distinct from loneliness, an affective state on the perception of isolation. Social isolation and loneliness are recognized as risks to well-being among older adults. Less in known about the cumulative effects of social isolation and loneliness in older persons with HIV (OPWH). Using cross-sectional data on OPWH (age ≥50) recruited from an outpatient HIV clinic in Atlanta, GA (N=146), we aimed to 1) describe the overlap between social isolation and loneliness and 2) examine the combined effects of social isolation and loneliness on quality-of-life (QoL), HIV-related stigma, depressive symptoms, and comorbidity burden. Loneliness and social isolation were assessed using the PROMIS-Social Isolation Scale and Social Network Index, respectively. Participants were grouped into four categories into 'lonely only,' 'isolated only,' 'both lonely and isolated,' or 'neither.' Bivariate and adjusted associations were conducted. Among participants (mean age=56.53), 26.7% (n=39) were considered 'lonely' only, 12.3% (n=18) 'isolated' only, 15.1% (n=22) 'both lonely and isolated,' and 45.9% (n=67) 'neither.' In bivariate analyses, individuals categorized as 'both lonely and isolated' were likely to have past homelessness and higher depressive symptoms, stigma, and comorbidity burden, and lower QoL. In adjusted models, 'both isolated and lonely' significantly predicted QoL, stigma, and depressive symptoms. Findings highlight the critical emphasis on targeting OPWH who are both isolated and lonely.

### COGNITIVELY ENGAGING SOLITARY ACTIVITIES: ANOTHER LAYER OF PROTECTION AGAINST LONELINESS

Haosen Sun[1], and Markus Schafer[2], *1. Indiana University Bloomington, Toronto, Ontario, Canada, 2. University of Toronto, Toronto, Ontario, Canada*

Participating in social activities and sustaining more extensive networks are beneficial in mitigating loneliness in the aging population. However, these coping strategies are subject to multiple structural and individual constraints, including neighborhood stressors, physical limitations, and social-economic strain. The present research scrutinizes cognitively engaging solitary activities as a potential alternative to alleviate loneliness. It examines their intersections with social activities and networks and how disadvantaged groups prone to loneliness can benefit from them. We use Wave 4 and 6 of the Survey of Health, Ageing and Retirement in Europe to perform linear and logistic regressions. Results show that 78% of older Europeans perform solitary activities such as reading and playing word/number games at least weekly, a much higher rate than the 29% engaged in formal social activity. Social and personal activities do not compete but instead complement each other. Older individuals performing solitary activities report significantly lower loneliness, and this engagement compensates for low social participation and small social networks. Routine solitary activities are also protective among individuals at older ages and among those lacking a partner, additional housemates, and children. Overall, the present research highlights that solitary activities, particularly the cognitively engaging ones, are a practical layer of protection against loneliness. Future research should further examine variations in the effectiveness between solitary activities and explore the possibilities and challenges in bridging them with digital media and technologies to alleviate loneliness.

### MULTIMORBIDITY AND QUALITY OF LIFE: EXAMINING THE PATHWAYS THROUGH LONELINESS, ADL, IADL, AND DEPRESSIVE SYMPTOMS

Stefan Sieber[1], Angélique Roquet[2], Charikleia Lampraki[2], and Daniela Jopp[2], *1. LIVES Centre, University of Lausanne, Lausanne, Vaud, Switzerland, 2. University of Lausanne, Lausanne, Vaud, Switzerland*

Background. Multimorbidity, defined as two or more chronic conditions, negatively affects quality of life. However, little is known about the underlying pathways leading from multimorbidity to lower quality of life (QoL). Objective. The objective of this paper was to examine multiple potential mediating factors (loneliness, Activities of Daily Living [ADL], Instrumental Activities of Daily Living [IADL], depressive symptoms) in the relationship between multimorbidity and quality of life. Furthermore, we explored moderated mediation patterns by age, gender, and education. Methods. Longitudinal data were drawn from five waves (from 2011 to 2020) of the Survey of Health, Ageing and Retirement in Europe (SHARE). We included 37,082 individuals aged 50 years and older in the analyses. The predictor (multimorbidity) and covariates were measured four and the mediators two years before the QoL outcome (CASP-12). Confounder-adjusted mediator and outcome models were fitted using mixed-effects models. The total association was decomposed into direct and indirect pathways applying causal mediation analyses with Monte-Carlo simulations. Results. Multimorbidity was associated with lower QoL four years later; 2.12%, 6.17%, and 19.90% of the association was mediated by ADL, IADL, and depressive symptoms, respectively. Mediation by loneliness was not significant. Generally, the mediation patterns were more pronounced in women and individuals with tertiary education as compared to primary and secondary. Differential mediation by age quartiles did not reveal a clear pattern. Conclusions. The results showed that depressive symptoms were the most important mediator between multimorbidity and QoL, with ADL and IADL also playing an important role.

### SESSION 6800 (POSTER)

### SOCIAL ISOLATION AND LONELINESS (SRPP)

### CHARACTERISTICS OF NORWEGIAN OLDER ADULTS LIVING ALONE OVER THREE DECADES (1995–2019)

Milan Chang Gudjonsson[1], Mona Michelet[2], and Bjørn Heine Strand[3], *1. Norwegian Institute of Public Health, Reykjavik, Not Applicable, Iceland, 2. Vestfold Hospital Trust, Oslo, Oslo, Norway, 3. Norwegian Institute of Public Health, Oslo, Oslo, Norway*

Introduction: Among older adults who are living alone, social isolation, and physical and cognitive impairment are more common than among those who do not live alone. The proportion of older adults who live alone is expected to increase further. The current study investigated the disability and formal care service use among older adults living alone between 1995-2016 in Norway.

Methods: A total of 31,263 people aged 70+ participated in the Trøndelag Health Study (HUNT) survey 2 (1995/97), 3 (2006/08), and 4 (2017/19). The study assessed the use of formal care including practical help (PH) and home nursing (HN), and disability in personal and instrumental activities of daily living (PADL:7 items and IADL: 6 items).

Results: More than 70% of older adults living alone were women (74% in HUNT2, 77% in HUNT3, 71% in HUNT4). The use of PH service among those who were living alone decreased from 33.7% to 18.6%, while HN service use increased from 10.4% to 16.4%. The percentage of PADL disability among those who lived alone was similar (10.3% to 9.1%) between 1995-2019. However, the percentage of IADL disability has decreased from 37.6% (1995-97) to 27% (2017-19) while in all cohorts IADL disability rates were significantly higher among those who were living alone than those who were living with others.

Conclusions: Although the use of HN service among older adults who were living alone decreased during 1995-2016, those who were living alone in all cohorts had higher IADL disability rates compared to those who lived with others.

### OLDER AMERICANS LIVING ALONE: AN INTERSECTIONAL ANALYSIS OF SOCIAL ISOLATION RISKS DURING THE COVID-19 PANDEMIC

Anna Bokun, Sarah Flood, and Phyllis Moen, *University of Minnesota, Minneapolis, Minnesota, United States*

More Americans aged 50-75 are living alone than ever before (about a third of adults over 60 live alone today–see Ausubel 2020; Esteve 2020), placing older adults at risk of social isolation, and especially so during COVID-19. Not only do demographers' project the rate of older adults living alone will continue to rise, but they also predict increasing racial disparities due to differential population aging rates (Verdery and Margolis 2017). We pinpoint two mediators of social isolation: employment status and living arrangements, drawing on panel data from the Current Population Survey, from January 2018 through August 2021 (N = 83,232), to investigate whether the pandemic increased disparities in vulnerabilities to social isolation across different subgroups. We use an intersectional lens to consider the experiences of population groups defined by gender, age, race/ethnicity and social class. We know that employment has important protective health benefits (Berkman, et al. 2000; Kelly, et al. 2017). Living arrangements condition social isolation, especially in terms of living alone (Cudjoe, et al. 2020). The aim of our study is twofold. First, we aim to show how the dynamics of living alone and employment participation for older adults change between the immediate pre-Covid period and Covid period, using COVID-19 as a natural experiment in precipitating change. Second, we show how demographic characteristics intersect to structure vulnerability to social isolation during the same period. Our results demonstrate the risks of social isolation are not evenly distributed, suggesting the need for policies and practices promoting social inclusion.

### NOBODY KNOWS ME HERE: LONELINESS AMONG OLDER AFRICAN IMMIGRANTS

Dolapo Adeniji[1], Gifty Ashirifi[2], and Margaret Adamek[2], *1. IUPUI, Avon, Indiana, United States, 2. Indiana University, Indianapolis, Indiana, United States*

Migration in later life is often accompanied by stressors such as acculturation and disconnection from previous social networks in one's country of origin. Loneliness emerged in previous studies as one of the challenges that negatively impact older immigrants' physical and mental health. Despite the rapid growth of this population in the United States, there is limited knowledge about loneliness experiences of older African immigrants. Thus, this study explored the loneliness experiences of older African immigrants living with their adult children in the United States. In-depth interviews were conducted with nine participants aged 69-84. The tape recordings of the interviews were transcribed verbatim for thematic analysis. Findings show that loneliness was prevalent among study respondents, despite living with their adult children's family. Four major themes emerged from the study: 1) "Nowhere to go" (loneliness experience), 2) "It makes one sick if one is doing nothing" (consequences of loneliness), 3) "My grandkid makes me happy" (protective strategies) and 4) "If they ask older people to come. I will go there" (way forward in the community). Living with adult children may not prevent loneliness for older immigrant parents. Although the participants demonstrated resilience, their coping strategies were insufficient to overcome their loneliness. To address the gap in resolving loneliness, strategies are needed to increase older African immigrants' social connectedness with their peers, family, and community. Interventions that promote social interactions and embeddedness into their local community are needed to ensure a good quality of life for older African immigrants.

### SOCIAL CONNECTIONS AND HEALTH AMONG OLDER ADULTS LIVING IN SUBSIDIZED HOUSING: LOCAL STAKEHOLDERS PERSPECTIVES

Avery Bullock[1], Joseph Gallo[2], Carl Latkin[2], Cynthia Boyd[2], and Thomas Cudjoe[3], *1. Johns Hopkins University School of Medicine, Baltimore, Maryland, United States, 2. Johns Hopkins University, Baltimore, Maryland, United States, 3. Johns Hopkins School of Medicine, Baltimore, Maryland, United States*

Social isolation among older adults living in the United States is a major public health problem that disproportionately affects those living below the poverty line. Though decades of epidemiological studies have provided important insights on the impact of social isolation on health, there have been limited qualitative studies on the dynamics of social connections among older adults in subsidized housing. This study aims to advance our understanding of the perspectives of local stakeholders in the field of subsidized housing on the social connections of older adults and how to enhance social connection. Semi-structured interviews were conducted between July 2021 and October 2021 with local stakeholders in Baltimore's subsidized housing community to better understand social connections among older adults. Stakeholders included, but were not limited to, the following positions: service coordinators, property managers, maintenance staff, and security guards. Interviews were imported and analyzed using NVIVO12 software and a qualitative content analytic approach. Preliminary themes identified included: barriers and facilitators of connection, engagement with family, and ideas about interventions. Mental and physical health challenges, access to and use of communications technology, and lack of transportation were barriers while food incentives, gift card giveaways, and resident champions were facilitators of social connection. Social isolation among older adults is an important concern among stakeholders in subsidized housing. To promote social connection in older adults, interventions must adopt a multi-pronged approach that addresses the barriers and leverage existing facilitators within subsidized housing communities.

### LONELINESS AND PSYCHOLOGICAL WELL-BEING OF THE INSTITUTIONAL OLDER ADULTS DURING THE COVID-19 PANDEMIC

Shiau-Fang Chao[1], Hui-chuan Hsu[2], and Yun-Pei Su[2], *1. National Taiwan University, Taipei, Taipei, Taiwan (Republic of China), 2. Taipei Medical University, Taipei, Taipei, Taiwan (Republic of China)*

Purpose: Institutional residents experienced more restrictions in the lockdown of covid-19. The purpose of this study was to examine the association of loneliness and lonely literacy with mental health wellbeing during covid-19 for older institutional residents.

Methods: The participants living in the 13 long-term care institutions who were aged 65 and more and able to communicate with were invited in the survey (n=143). Mental well-being was measured by depressive symptoms and life satisfaction. Loneliness was measured by the 6-item UCLA loneliness scale. In addition, demographics, health status, active and passive coping strategies, social support from family and friends, social interaction changes after covid-19, loneliness change after covid-19, and worries about covid-19 were investigated. Linear regression and logistic regression models were conducted.

Results: The mean of the loneliness score (6~24) was 9.71 (SD=4.02). Factors related to loneliness increased during covid-19 for the institutionalized residents included having more physical function difficulties (OR=1.179), feeling more lonely (OR=1.146), and having more worries for covid-19 (OR=2.317). The residents having depressive symptoms was related to have more loneliness (OR=1.269), worse self-rate health (OR=0.320), and increased more loneliness during covid-19 (OR=3.233); while having high life satisfaction was related to less loneliness (OR=0.859), less physical difficulties (OR=0.834), higher satisfaction of family support (OR=2.835), and not increasing loneliness during covid-19 (OR=0.255).

Conclusion: Loneliness during covid-19 is related to more depressive symptoms and lower life satisfaction, especially during covid-19. Learning active coping strategy and providing proactive and helpful environment for the long-term care residents is suggested during the pandemics.

### DECREASED FREQUENCY OF SMALL TALK DURING COVID-19 PANDEMIC AND MENTAL HEALTH: LONGITUDINAL SURVEYS IN JAPAN

Hiroshi Murayama[1], and Ikuko Sugawara[2], *1. Tokyo Metropolitan Institute of Gerontology, Tokyo, Tokyo, Japan, 2. Bunri University of Hospitality, Suginami-ku, Tokyo, Japan*

The coronavirus disease 2019 (COVID-19) has drastically reduced opportunities for small talk. As small talk involves socializing, such deprivation can be stressful. This study examined the association between the change in frequency of small talk before and during the pandemic and the mental health of middle-aged and older people. We conducted web-based longitudinal questionnaire surveys from March to May 2020 and from September to October 2021 among members of a Japanese social networking service. We analyzed 867 responses of people who participated in both surveys (mean age, 68.0 ± 8.0 years; men, 68.1%). This study was approved by the Research Ethics Committee of University of Tokyo. Change in small talk frequency before and during the pandemic was assessed using a single item, "Have the opportunities for small talk (including face-to-face, phone, video call, etc.) with someone other than cohabiting family members changed?" We divided the responses into "increase," "no change," and "decrease." Mental health outcomes included psychological well-being and loneliness. A total of 57.0%, 34.4%, and 8.7% reported "decrease," "no change," and "increase," respectively, from before to during the pandemic. After adjusting for potential covariates, multiple regression analyses showed that people who felt their small talk frequency decreased during the pandemic compared to pre-pandemic period had lower psychological well-being and greater loneliness than those who did not. We observed no interaction between change in small talk frequency and age/sex. Our study quantitatively revealed the importance of small talk during the pandemic in maintaining mental health.

### INTERFACE OF INFORMAL AND FORMAL SUPPORT IN THE LINKAGE BETWEEN COGNITIVE DECLINE AND LONELINESS

Soobin Park[1], Sojung Park[1], Takashi Amano[2], and BoRin Kim[3], *1. Washington University in St. Louis, St. Louis, Missouri, United States, 2. Rutgers University-Newark, Newark, New Jersey, United States, 3. University of New Hampshire, Durham, New Hampshire, United States*

Older adults with cognitive impairment are at risk of higher loneliness due to increasing challenges to retain essential social skills and decreasing social networks. This study explores ways to reduce the relationship between cognitive function and loneliness. Drawing on the cognitive discrepancy theory of loneliness, we hypothesized loneliness may be reduced by improving an individual's actual level of social interaction, creating opportunities for social relationships. This study examined complex relationships among informal (frequency of social contact with family members and friends) and formal support (use of home- and community-bound services, HCBS), and loneliness, among older adults with different levels of cognitive impairment. Data came from the Health and Retirement Study (2012) (N=651) with the sample included respondents 51+ years who completed a modified Telephone Interview for Cognitive Status. Results from hierarchical regression showed older people with a lower level of cognitive function were less likely to experience loneliness (b=-.15, p<.01). Although frequent social contact with family and friends reduces loneliness (b=-.24, p<.001), it did not moderate the relationship between cognitive function and loneliness. Interestingly, the use of HSBC also turned out to be not a significant protective factor. Our findings suggest merely increasing social interaction levels with family and friends may not necessarily curb loneliness for older adults with cognitive impairment. Given the current characteristics and nature of HCBS primarily focus on providing basic physical needs of older adults, prioritizing the development of emotional comfort and recreational activities that alleviate their psychological loneliness is essential, especially amidst the pandemic.

### SESSION 6810 (POSTER)

### SOCIAL NETWORKS AND SUPPORTS: FRIENDS, NEIGHBORS, AND FAMILIES

### BEYOND HOUSING

Marianne Raimondo, *Rhode Island College, Greenville, Rhode Island, United States*

Social isolation and loneliness in older adults are a public health challenge that has been exacerbated during the COVID pandemic. While quarantining although it was necessary to protect individuals from the virus, older adults lost their usual social supports, connectedness, and relationships. Research has shown that loneliness and social isolation have many negative effects on a person's physical and mental health. This presentation will describe an initiative of Age Friendly RI which addressed the problem of social isolation among older adults living in a congregate housing community who struggled with stress, anxiety, depression, and exacerbation of mental illness. Through a partnership between a state college and a community based mental health center, and supported with grant funding, behavioral health services and supports were embedded in the housing community. The project was multi-faceted involving community health workers and a community support professional who provided outreach and emotional support, the use of technology to help residents connect with medical providers or loved ones or enjoy music, games, movies, and students who provided outreach and taught older adults how to use technology. A social isolation scale was administered to identify residents at high risk of social isolation and targeted intervention. The evaluation of the program will be discussed in terms of the interventions implemented and the outcomes realized, including resident well-being and satisfaction. Finally, it will suggest a pathway for sustaining the integration of behavioral health services in housing to better meet the behavioral health needs of older residents beyond the pandemic.

### SOCIAL RELATIONSHIPS AND COGNITIVE IMPAIRMENT IN LATER LIFE

Patricia Thomas[1], Madison Sauerteig-Rolston[2], Shawn Bauldry[2], Samuel Nemeth[2], and Kenneth Ferraro[2], *1. Purdue University--West Lafayette, West Lafayette, Indiana, United States, 2. Purdue University, West Lafayette, Indiana, United States*

With population aging and the growing population at-risk of cognitive impairment, Alzheimer's disease, and related dementias, there is a need for a better understanding of how social factors may affect cognitive impairment. Drawing from stress process theory and social integration theory, we examine the impact of positive and negative dimensions of relationship quality with friends and family on cognitive impairment in later life. We analyze Cox proportional hazards models using nationally representative panel data from the Health and Retirement Study (2006-2016, N=10,626) to examine how the quality of family and friend relationships in adulthood may influence risk of cognitive impairment. Strain with family members and with friends was significantly related to higher risk of cognitive impairment (Hazard Ratio: 1.15 [95% CI: 1.08, 1.21] and 1.20 [95% CI: 1.13, 1.28], respectively). Support from family members was beneficial, related to lower risk of cognitive impairment (HR: 0.93, CI: .89, .99); however, support from friends was related to higher risk of cognitive impairment (HR: 1.10, CI: 1.05, 1.14). Findings reveal the implications of social support and strain in different types of relationships for cognitive health.

### THE STRUCTURAL AND COGNITIVE SOCIAL CAPITAL OF KOREAN OLDER ADULTS AND THEIR DEPRESSION TRAJECTORIES

Jinhyun Kim, Sun-Bi Um, Xiang Shi, and Hyunjung Park, *Pusan National University, Geumjeong-gu, Pusan-jikhalsi, Republic of Korea*

Despite the rapid increase of older population in South Korea, we do not understand the patterns of their social capital and relationships with depression trajectories in depth. This study aims to identify the patterns of social capital of Korean older adults aged 65 years and older based on latent class analyses and their depression trajectories by latent classes. For data analyses, 3,606 Korean older adults from the Korean welfare panel study data were selected. Latent class analyses were conducted by using various components of structural social capital such as number of contacts with family, friends, and neighbors and cognitive social capital such as trust, reciprocity, and satisfaction from social relationships. Results showed that three latent classes were found. We named the first class as a strong structural and cognitive group because it showed the highest levels of number of social contacts, trust and reciprocity. The second class was called as a weak structural and strong cognitive group because it showed lower levels of social contacts but higher levels of satisfaction from social relationships. The third class was called a weak structural and cognitive group because it showed the lowest levels of social contacts, trust, reciprocity, and satisfaction from social relationships. Results from latent growth curve modeling, the second class showed the lowest depressive symptoms but the third class showed the highest depressive symptoms at baseline. Only the third class showed significant rate of changes in depression. Differences in depression trajectories by three latent classes need to be further discussed.

### THE MENTAL HEALTH BENEFIT OF FRIEND NETWORKS IN OLDER KOREAN AMERICANS: THE CONDITIONING EFFECT OF FAMILY TYPE

Yuri Jang[1], Nan Sook Park[2], Juyoung Park[1], David Chiriboga[2], William Haley[2], and Miyong Kim[3], *1. University of Southern California, Los Angeles, California, United States, 2. University of South Florida, Tampa, Florida, United States, 3. University of Texas at Austin, Austin, Texas, United States*

Building on the importance of family and friends as sources of social connectedness in later years of life, we conducted a contextual examination of their independent and interactive roles in predicting mental health, using a compensatory social convoy model. In a sample of older Korean Americans, we anticipated that friend networks would be a more important predictor of mental distress when strong family relationships were absent. Data were from 2,140 participants in the Study of Older Korean Americans, a multi-state survey of Korean immigrants age 60 and older (Mage = 73.4, SD = 7.97). To identify family types, latent profile analysis (LPA) was performed with 17 variables assessing family networks, positive and negative interactions with family members, and incidence of mistreatment by family. Linear regression models of mental distress then examined the direct effects of family type and networks of friends, as well as their interactions. LPA on family-related items identified three family types: close-knit, mixed, and dysfunctional. Membership in the close-knit group and a larger network of friends were associated with lower levels of mental distress. In addition, a significant interaction was found between dysfunctional family type and friend network ($B = -.35$, $SE = .08$, $p < .001$), where the positive effect of friend networks was most pronounced in the context of dysfunctional family relationships. These findings support the use of a compensatory social convoy model. Substituting for family resources, friend networks yielded compensatory mental health protection for those with dysfunctional family relations.

### "STOP HASSLING ME!": HASSLES, PERCEIVED SUPPORT, AND MENTAL HEALTH OUTCOMES IN OLDER ADULTHOOD

Amy Gourley, Lucas Hamilton, Max Coleman, and Anne Krendl, *Indiana University, Bloomington, Indiana, United States*

Prior work suggests that older adults with larger social networks have better mental health, potentially due to having greater access to support from different network members. However, other research suggests that perceptions of support, which do not necessarily align with network size, predict better mental health and higher life satisfaction. It could be that, irrespective of network size, the presence of hassles – network members that are perceived as causing problems or making life difficult – is associated with lower perceptions of support, which negatively impacts mental health. We tested this possibility using social network data from 137 older adults (Mage = 74.25 years) who completed the PhenX social interview and well-validated measures of depression, perceived social support, and life satisfaction. In linear regression models controlling for other network variables, perceived support and the level of hassles independently predicted depression and life satisfaction. Testing our mediation predictions, hassles significantly mediated the effect of perceived support on depression and life satisfaction. Alternatively, perceived support only mediated the effect of hassles on life satisfaction. Taken together, these findings may indicate that the presence of hassles in one's network predicts mental health symptomology and life satisfaction, irrespective of perceptions of support or other network characteristics (e.g., network size). Thus, while perceptions of social support appear to be important predictors of life satisfaction, network characteristics better predict mental health symptomology. Future work on social engagement interventions may benefit from a targeted approach depending on the outcome of interest.

### DO I GET BY WITH A LITTLE HELP FROM MY FRIENDS? FINDINGS FROM THE IOWA UNMARRIED SURVIVORS STUDY

Robert Hensley[1], Alex Bishop[2], and Peter Martin[3], *1. Southeast Community College, Lincoln, Nebraska, United States, 2. Oklahoma State University, Stillwater, Oklahoma, United States, 3. Iowa State University, Ames, Iowa, United States*

The purpose of this study was to analyze the role that selected personality variables (Neuroticism, Agreeableness, Conscientiousness, Extroversion, and Openness), social support, and coping played in loneliness of 227 ($M = 78.2$, $SD = 8.1$) participants in the Iowa Unmarried Survivors Study. This study is a sample of never married, divorced, and widowed participants. Blocked multiple regression analyses were used in this study. In the first block, age, gender, ethnicity, past schooling, current marital status, total illnesses, and childhood poverty were included. The second block contained the aforementioned personality variables, and the third block included both social support and coping. Results indicated that both Neuroticism and Extraversion were significant predictors of loneliness, $\beta = .266$, $p < .00$, $\beta = -.294$, $p < .05$, and $\beta = .27$, $p < .00$, respectively. In short, the greater the neuroticism, the higher the score in loneliness. Moreover, the lower the level of extraversion, the higher the score in loneliness. In addition, social support served as a significant predictor of loneliness, $\beta = -.412$, $p < .00$. The more social support in the participants' lives, the lower level of loneliness. Finally, coping predicted loneliness. In essence, the more loneliness in their lives, the greater the use of coping mechanisms utilized. This model explained 58% of the variance in loneliness scores. Navigating through late adulthood as an unmarried survivor presents a host of challenges, and these results add to our understanding of the link between personality, social support, coping, and loneliness in late life.

### THE RELATIONSHIP BETWEEN SOCIAL NETWORK AND PURPOSE IN LIFE: APPLICATION OF A RANDOM INTERCEPT CROSS-LAGGED MODEL

Gina Lee, *Iowa State University, Ames, Iowa, United States*

The purpose of this study is to examine if there are reciprocal effects between social network size and purpose in life among older adults using data from the National Health and Aging Trends Study. A second aim is to assess whether there are moderated effect of gender on this relationship. The sample included 1,485 male and 2,058 female adults 65 years and older. In order to examine the reciprocal effects between social network size and purpose in life over four time points (2017, 2018, 2019, and 2020), a random intercept cross-lagged model (Model 1) was computed. Then, two multiple group RI-CLPM analyses (Model 2 and 3) were computed in order to test moderation of gender on the relationship. Model 2 estimates the cross-lagged parameters freed and model 3 estimates the cross-lagged parameters constrained. The results indicated that Model 1 fit the data well, $\chi 2(9) = 26.06$, $p = .002$. The carry-over effects of social network and purpose in life were significant. The spill-over effect from wave 3 purpose in life on wave 4 social network was significant. The variance of the random intercepts were significant, indicating that there are stable, trait-like differences between social network and purpose in life. The results of the multiple group model revealed that there were no significant differences between the freed and constrained models, indicating that lagged effect for male and female appear to be same. Future research should explore if other factors like race or education show moderated effects.

### PERSONALITY AND LONELINESS IN LATER LIFE: THE MEDIATING ROLE OF FRIENDSHIP

Emily Lim, and Jeffrey Burr, *University of Massachusetts Boston, Boston, Massachusetts, United States*

Loneliness is prevalent among American older adults and is related to poor health outcomes. Existing studies on the association between personality and loneliness are limited and no study examined the mediating role of friendship. This study investigated how personality traits are linked to loneliness, and how friendship mediated the relationship between personality and loneliness. Using three waves of the Health and Retirement Study (N=3,259), we estimated a model with personality traits in 2010 (openness to experience, extraversion, consciousness, agreeableness, and neuroticism), friendship dimensions in 2014 (number of close friends, frequency of contact with friends, positive and negative friendship qualities), and loneliness in 2018 among community-dwelling adults aged 50 years and above (M=65.54 years old, SD=8.93). Extraversion and neuroticism were negatively and positively associated with loneliness, respectively. All friendship dimensions, except negative friendship quality, were negatively associated with loneliness. Our structural equation modeling results indicated that the number of close friends negatively mediated the relationship between extraversion and loneliness. The relationships between extraversion, agreeableness, neuroticism and loneliness were negatively mediated by positive friendship quality. Negative friendship quality positively mediated the relationship between neuroticism and loneliness, and negatively mediated the consciousness-loneliness link. Friendship contact frequency negatively mediated the relationships between openness to experience, extraversion, and loneliness but positively mediated the relationship between consciousness and loneliness. These results provided insights that help us better comprehend the mechanisms leading to loneliness. Social intervention programs could be developed that tailor different personality traits and friendship dimensions to potentially reduce loneliness in later life.

### THE EFFECT OF SUBJECTIVE AND OBJECTIVE RELATIONSHIPS ON THE LIFE SATISFACTION AND HEALTH OF OLDER ADULTS

Meeryoung Kim, *Daegu University, Daegu, Kyongsang-bukto, Republic of Korea*

This study examines the effects of subjective and objective relationships among older adults on life satisfaction. Human beings are social animals and must live in relationships with people. In particular, in a situation where social networks decrease in old age, objective or subjective social relationships that can receive help when needed, and satisfaction with such relationships can improve the quality of life in old age. This study used the sixth additional wave (2016) and the seventh wave (2017) of the Korean Retirement Income Study. The subjects of this study were older adults who are aged 65 and older, and the sample size was 3,423. The number of objective relationships, subjective social support, relational satisfaction, and having a spouse and children were used as independent variables. Life satisfaction and health were used as dependent variables. Demographic variables were controlled. Multiple regression was used for data analysis. The more objective relationships there were, the higher the life satisfaction and health. Subjective social support also significantly increased life satisfaction and health. Satisfaction with interpersonal relationships also significantly increased both life satisfaction and health. Having a spouse made a significant difference in affecting life satisfaction and health. The results showed that both subjective and objective relationships in old age improved the quality of life of older adults, but the presence or absence of children made no significant difference in affecting life satisfaction or health. This implies that as you get older, you may find that having a spouse becomes very important.

### WITHIN- AND BETWEEN-PERSON EFFECTS OF HEALTH AND SOCIAL RELATIONSHIPS WITH FAMILY AND NEIGHBORS ON WELL-BEING

Midori Takayama[1], Yoshiko Ishioka[2], and Ikuko Sugawara[3], *1. Keio University, Yokohama, Kanagawa, Japan, 2. O.P. Jindal Global University, Sonipat, Haryana,*

*India, 3. Bunri University of Hospitality, Suginami-ku, Tokyo, Japan*

Good health and positive social relationships are significant resources in well-being. However, little is known about how intrapersonal changes and interpersonal differences in health and social relationships are associated with well-being in later life. This study aimed to examine within- and between-person effects of physical health and social relationships with family, friends, and neighbors (social support and companionship) on well-being. We analyzed longitudinal data from a representative sample of older Japanese adults aged 74 to 86 years (N=1388). The results from multilevel models showed that decline in health (intrapersonal changes) was associated with lower well-being. Nevertheless, there was the interaction effect of social relationships with family and neighbors (between-person differences) in the relationship between health and well-being change. Among participants with lower social support from family and neighbors, the negative association between decline of health and well-being was stronger. Conversely, participants with higher social support reported higher well-being regardless of physical health change. Contrastingly, among participants with higher companionship with family and neighbors, participants reported higher well-being at the time of maintaining or increasing physical health. For participants with lower companionship, health change was not associated with well-being. These findings suggest social support has a preventive effect on well-being in the face of declining health, while companionship has a facilitative impact on well-being in good health. The results also suggest relationships with neighbors as well as family play an important role in well-being in very late life.

### SESSION 6820 (POSTER)

### SOCIAL ROLES, PHYSICAL FUNCTION, AND THE EXPERIENCE OF DEPRESSION

### IMPACT OF BEHAVIORAL ACTIVATION ON COMORBID DEPRESSION AND TYPE 2 DIABETES IN LOW-INCOME ADULTS

Christopher Griffith, and Leilani Feliciano, *University of Colorado Colorado Springs, Colorado Springs, Colorado, United States*

Older age is a significant risk factor for type 2 diabetes mellitus (T2DM), and depression risk increases with comorbid illnesses such as T2DM. Older adults with depression have increased risk of developing somatic symptoms and decreased interest in activities, which impacts diabetes self-care. This makes depression an important target for treatment alongside T2DM. Behavioral activation (BA) is a therapeutic intervention shown to be effective in the treatment of moderate to severe depression. The current study examined the utility of brief BA treatment (once weekly for 4 weeks) across older adults aged 60-80, middle-aged adults aged 45-59, and younger adults aged 18-44. Fifty-six adults with diabetes participated in an in-home diabetes management program with BA to treat depression in low-income adults. Participants completed weekly measures of well-being and depressive symptoms. Two mixed-subjects analyses of variance (3 x 2 ANOVAs) found that BA was significantly effective at improving subjective well-being, $F(1, 53) = 38.24$, $p < .001$, $\eta p2 = .42$, and reducing depressive symptoms, $F(1, 38) = 27.25$, $p < .001$, $\eta p2 = .42$, but found no interaction effects between either age group and well-being or depressive symptoms ($p = .173$ and $.302$, respectively). Results indicate that BA is effective for treating depression in adults with comorbid T2DM regardless of age, making it a useful therapeutic tool for both the high number of older adults with T2DM and their younger counterparts. Further research should examine other outcomes (diabetes management) to evaluate the full extent of BA's effectiveness.

### UNRAVELING THE AMBIGUITY BETWEEN COGNITION AND DEPRESSION: A LONGITUDINAL ANALYSIS OF OLDER ADULTS

Meghann Roessler[1], Mohit Manchella[2], Anmoldeep Singh[3], Max Coleman[4], Heather Francis[1], Andrew Saykin[5], Shannon Risacher[5], and Liana Apostolova[5], *1. Indiana University, Indianapolis, Indiana, United States, 2. University of Southern Indiana, Carmel, Indiana, United States, 3. Indiana University, Newburgh, Indiana, United States, 4. Indiana University, Bloomington, Indiana, United States, 5. Indiana University School of Medicine, Indianapolis, Indiana, United States*

Research on directionality of the relationship between cognitive functioning and depression is ambiguous, especially when considering implications for patients with mild cognitive impairment or Alzheimer disease (AD). Previous research suggests that depression in late life could be a preclinical manifestation of AD before other cognitive symptoms are detectable. Some research supports the hypothesis that level of depression can independently predict level of cognition. Other research suggests that depression is a risk factor for developing dementia or AD late in life. Further research on the impact of subjective cognitive decline versus objective cognitive performance in association with depressive symptoms is a critical area to explore. Using data from the Social Networks in Alzheimer Disease (SNAD) study and the Indiana Alzheimer Disease Research Center (IADRC), we conducted a preliminary longitudinal analysis of 196 focal subjects (Mage = 71.6 years, Pfemale = 63%). Interviews conducted one year apart were leveraged to elucidate the bi-directional relationship between depressive symptomology and cognition. Using a lagged dependent variable approach controlling for age, sex, race, and education, the results indicate that only executive function predicts depression at timepoint 2 (-0.31 SD, $p< 0.001$). However, depression predicts focal Cognitive Change Index (CCI) (0.06 SD, $p< 0.05$), processing speed (-0.04 SD, $p< 0.05$), and episodic memory (-0.04 SD, $p< 0.05$). Other cognitive domains examined, including attention, language, visual/spatial skills, MoCA score, and informant-rated CCI, were not significant as predictors or as outcomes. These results suggest that depression may be a more robust predictor of cognition than cognition is of depression.

### DEPRESSION, DEPRESSIVE COGNITIONS, AND DEPRESSIVE SYMPTOMS IN CAREGIVING GRANDMOTHERS: A GROWTH CURVE MODEL

Christopher Burant[1], Carol Musil[2], Jaclene Zausniewski[2], and Alexandra Jeanblanc[2], *1. Case Western Reserve*

University, Parma, Ohio, United States, 2. Case Western Reserve University, Cleveland, Ohio, United States

Grandmothers caring for grandchildren have elevated levels of depressive symptoms compared to grandmothers who do not provide care. Depressive symptoms are state-like in nature and describe recent depressive symptoms. The Depressive Cognition Scale© captures changes in negative thinking patterns that often precede depressive symptoms. Depressive cognitions, according to Beck's theory of depression, are the first depressive symptoms to appear and typically lead to more serious symptoms of depression. Specifically, depressive cognitions reflect negative thinking patterns. Previously diagnosed depression may contribute to current levels of depressive cognitions and depressive symptoms. Data were collected on 342 participants in a longitudinal nationwide online research study of caregiving grandmothers. A latent growth curve model was used to track the trajectory of depressive symptoms at four time points (baseline, 2 weeks, 12 weeks, and 24 weeks). This latent growth curve model was tested to gain an understanding of how diagnosed depression and depressive cognitions impact the trajectory of depressive symptoms over time. The model fit the data well (Chi Square=24.301.; df=12; p=.019; TLI=.977; CFI=.987; RMSEA=.055). Baseline depressive cognitions strongly impacted the intercept (Standardized Beta=.65, p<.001) and the slope of depressive symptoms (Standardized Beta=-.65, p<.001), diagnosed depression predicted depressive cognitions (Standardized Beta =.32, p<.001) and the intercept of depressive symptoms (Standardized Beta=.36, p<.001). The continued impact of diagnosed depression and depressive cognitions over 24 weeks indicates the need for potential interventions to further address diagnosed depression and depressive cognitions as a way to decrease depressive symptoms in grandmother caregivers.

### THE RELATIONSHIP BETWEEN EATING DIFFICULTY AND DEPRESSIVE SYMPTOMS: MODERATING EFFECTS OF EATING AND LIVING ALONE

Junyub Lim, Yeonsoo Shin, and Giyeon Kim, *Chung-Ang University, Seoul, Seoul-t'ukpyolsi, Republic of Korea*

Background and Objectives: Previous studies report that poor oral health reduces the opportunity for social interaction and increases the risk of psychological distress and that eating alone affects depressive symptoms and its connection differs by living arrangements. The present study aims to investigate the moderating effects of eating alone and living arrangements on the relationship between difficulty eating food due to poor oral health and depressive symptoms among Korean older adults.

Methods: Data were drawn from the 2020 Survey of Living Conditions and Welfare Needs of Korean Older Persons, a nationally representative survey. A total of 9,920 participants aged 65 years and older were included in analyses.

Results: Results from hierarchical regression analyses showed that difficulty eating food was positively correlated with depressive symptoms and eating alone did not moderate the relationship between difficulty eating food and depressive symptoms (b = 1.394, p < .001; b=-0.062, p > 0.05, respectively). However, the effects of eating alone significantly differed by living arrangements (b = 1.663, p < .05). In the case of older adults who live alone, eating alone significantly moderated the relationship between difficulty eating food and depressive symptoms, whereas for those living with others, eating alone did not moderate the relationship. Discussion: Social isolation can be a risk factor that worsens the mental health of older adults who have difficulty eating due to poor oral health. Practical implications for ways to reduce loneliness are discussed.

### RELATIONSHIPS BETWEEN PHYSICAL/MENTAL MULTIMORBIDITY AND DEPRESSIVE SYMPTOMS AMONG OLDER BLACK AMERICANS

Seungjong Cho[1], and Tyrone Hamler[2], *1. Texas Tech University, Lubbock, Texas, United States, 2. Case Western Reserve University, Cleveland, Ohio, United States*

The purpose of this study was to identify the association between physical/mental health problems and depressive symptoms among older Black Americans (age ≥ 65) using the 2018 Health and Retirement Study (N = 812). People with multiple medical conditions face substantial emotional difficulties including depressive symptomatology. The study of depressive symptoms among older Black Americans is an emerging area of research. Several existing studies reported a higher prevalence of depressive symptoms among this population. They also experience a disproportionate burden of multimorbidity, both physically and mentally. For statistical analysis, depressive symptoms were measured with the eight-item CES-D scale (M = 1.514, SD = 1.916). Physical multimorbidity was measured by asking whether respondents had ever been diagnosed with seven diseases (high blood pressure, diabetes, cancer, lung disease, heart disease, stroke, and arthritis; M = 2.876, SD = 1.284). Mental multimorbidity was measured by asking whether they had ever been diagnosed with three diseases (clinical depression, Alzheimer's Disease, or other memory impairment including dementia; M = .228, SD = .467). Covariates included gender, marital status, and income (logged). A negative binomial regression showed that (a) higher numbers of physical health problems were associated with higher depressive symptoms (Incidence rate ratios [IRR] = 1.073, p = .048); (b) higher number of mental health problems were also related to higher depressive symptoms among older Black Americans (IRR = 2.077, p < .001). These findings underscore the importance of identifying and managing depressive symptoms among older Black Americans, who present with greater physical/mental health multimorbidity.

### SESSION 6830 (POSTER)

### STRESS, RESILIENCE, AND SUCCESSFUL AGING

### AGING ACROSS THE LIFE COURSE: RESEARCH COLLECTIONS AVAILABLE FROM THE NATIONAL ARCHIVE OF COMPUTERIZED DATA ON AGING

James McNally, and Kathryn Lavender, *University of Michigan, Ann Arbor, Michigan, United States*

Creating and maintaining sustainable data archives can be challenging, but it offers clear advantages. Properly curated data can be used by multiple researchers, testing a variety of hypotheses and increasing the return on investment to the expensive data collection process. An internally managed archival system also provides greater control and autonomy in the equitable distribution of data resources. This process ensures all researchers will have full use of the data for original research, teaching, and new directions once the data leaves the control of the local investigator. This poster reviews the advantages of having a local strategy to preserve and share gerontological research data. Using the National Archive of Computerized Data on Aging (NACDA) as a working example, the poster offers an overview of collections at NACDA. Using our metadata tools and variable search database, NACDA can identify studies in its collections that examine aspects of aging and health among adults during their lifecourse. Many of the studies are longitudinal or repeat measure cross-sectional studies. We can also identify studies that focus on aging not maintained by NACDA are available to interested researchers. Using a strategy of archival preservation combined with a strong focus on productive research Innovation in Aging, NACDA has amassed data and metadata covering a wide array of studies worldwide that address the aging lifecourse. Because our collections are multinational, we share these data at no cost to interested users worldwide

### THE MULTIDISCIPLINARY HISTORIES OF ACTIVE AGING IN POLAND

Jessica Robbins-Panko, *Wayne State University, Detroit, Michigan, United States*

In contemporary Poland, Universities of the Third Age are the most visible institutional forms of active aging. These lifelong-learning institutions that are specifically for retirees often cultivate ideals of independence through workshops and classes that teach new, and potentially transformative, skills and hobbies (Kobylarek, 2018). Universities of the Third Age in Poland emerged out of the fields of andragogy, pedagogy, and social work, fields that have regional intellectual roots in the late 19th/early 20th-century presocialist era, and are based on radically different ideals of personhood, relationality, and care than those of the contemporary postsocialist neoliberal era (Robbins, 2021). This paper analyzes 1) historical data from institutional archives of two Universities of the Third Age in Poland, and 2) secondary sources on histories of andragogy, pedagogy, and social work, to create a locally grounded intellectual history of active aging in central and eastern Europe. The Polish case offers an opportunity to think across divergent political-economic eras, in which assumptions about the value of a person to society have shifted. By tracing how the fields of andragogy, pedagogy, and social work have shaped active aging in Poland, this paper finds that 1) dichotomies of East/West, socialist/capitalist, and individual/collective are insufficient to explain the history of contemporary practices of active aging, and 2) intellectual history can reveal complex relations between political-economic change, and ideals and practices of aging. These findings have implications for advancing gerontological theories of 1) active aging in cross-cultural contexts, and 2) how active aging relates to sociopolitical change.

### PSYCHOLOGICAL WELL-BEING IN OLDER ADULTS AFTER MULTIPLE SEVERE WEATHER EVENTS

Katie Cherry[1], Matthew Calamia[1], Emily Elliott[1], Luke Miller[1], Laura Sampson[2], and Sandro Galea[3], *1. Louisiana State University, Baton Rouge, Louisiana, United States, 2. Harvard T.H. Chan School of Public Health, Boston, Massachusetts, United States, 3. Boston University, Boston, Massachusetts, United States*

Catastrophic hurricanes and flooding threaten health and well-being, although the long-term consequences of these events for survivors are poorly understood. In 2005, Hurricane Katrina devastated the US Gulf Coast. Many lost homes in these storms and relocated permanently inland. In August of 2016, historic flooding in Baton Rouge, Louisiana devastated a 22-parish (county) region, resulting in widespread destruction and a second round of disaster-related losses for those who relocated to Baton Rouge after Katrina. The present research is part of a larger longitudinal study on health and well-being after multiple disasters. Cherry et al. (2021) reported that greater flood damage was associated with more symptoms of depression and post-traumatic stress during the Wave 1 immediate impact phase. Here we examined symptoms of depression, anxiety and post-traumatic stress at Wave 2, a follow-up assessment that occurred 9 (+/- 3) months after Wave 1 testing. Three flood exposure groups were compared: non-flooded (controls), single disaster (flooded in 2016) and double disaster (flooded in 2005 and again in 2016). Results indicated that symptoms of depression and post-traumatic stress, which were elevated at Wave 1 for the single and double disaster groups relative to the non-flooded controls, were reduced at Wave 2 and did not differ from the controls. Correlation analyses revealed that age was negatively associated with symptoms of post-traumatic stress, depression, and anxiety, consistent with the inoculation view of post disaster psychological reactions. Implications of these data for understanding older adults' psychological health after multiple disaster exposures are discussed.

### CHALLENGES AND REWARDS EXPERIENCED IN THE VERY OLD PARENT-CHILD RELATIONSHIP: RELATIONSHIP QUALITY MATTERS

Kathrin Boerner[1], Kyungmin Kim[2], Yijung Kim[3], Elizabeth Gallagher[1], and Daniela Jopp[4], *1. University of Massachusetts Boston, Boston, Massachusetts, United States, 2. Seoul National University, Seoul, Seoul-t'ukpyolsi, Republic of Korea,*
*3. The University of Texas at Austin, Austin, Texas, United States, 4. University of Lausanne, Lausanne, Vaud, Switzerland*

Very old parents and their "old" children are a growing group in industrialized countries worldwide. However, little is known about the nature and implications of this relationship constellation, especially the challenges and/or rewards experienced within the relationship. We therefore examined factors associated with perceptions of challenge and reward among very old parents and their children. Using data from 114 very old parent-child dyads in the Boston Aging Together Study, we estimated Actor-Partner Interdependence Models to predict challenge, reward, and challenge/reward ratio outcomes of dyad members as a function of relationship quality, support exchanges, family norms, and personality. Relationship quality emerged as the most influential predictor, albeit more consistently for children than for parents. When children experienced the parent-child relationship as more positive, both children and parents experienced fewer challenges and more rewards (i.e., both actor and partner effect). Parents' experience of relationship quality was only associated with their own challenge perceptions (i.e., actor effect only). The roles of support exchanges, family norms, and personality were relatively minor, with few significant effects if at all. Given the importance of relationship quality for challenge and reward perceptions, support services or interventions targeting relationship quality could be a key pathway to minimizing challenges and maximizing rewards among very old parents and their children. Focusing on relationship perceptions of the child may be particularly critical in improving the experiences of both parent and child.

### LIVING WITH END-STAGE LIVER DISEASE: PERSPECTIVES OF YOUNGER AND OLDER PATIENT–CARE PARTNER DYADS

Lissi Hansen, Susan Rosenkranz, and Shirin Hiatt, *Oregon Health & Science University, Portland, Oregon, United States*

The uncertain trajectory of end-stage liver disease (ESLD) leaves patients and their informal care-partners to face psychological, physical, social, and financial burdens, complicating their relationship. Knowledge is lacking on how living with ESLD affects patients and informal care-partners. The study purpose was to compare how ESLD affects relationships between younger and older patient-care-partner dyads. Patients with ESLD and informal care-partners were recruited through liver clinics at two hospital settings. They completed questionnaires including two open-ended questions about how ESLD affected their relationship (R01NR016017, NINR/NIH). Patient eligibility criteria: age >21 and MELD-Na score >15. Care-partner eligibility criteria: age ≥18 and identified as patient's primary support. Conventional content and qualitative dyadic analysis were used to analyze written responses to the two questions. Data were available for 140 patient-care-partner dyads. Patients averaged 57 years old (range, 23-83 years; 69% were male; 31% with ETOH as primary etiology). Care partners averaged 57.5 years old (range, 19-85 years; 74% were female). Older dyads were more likely to report having a closer relationship, although tempered by uncertainty and symptoms limiting activities. Younger dyads emphasized managing symptoms, financial stress, caregiving burden, and lack of intimacy among spousal/partners. Diverging and overlapping themes within dyads reflected caregiving challenges, relationship strain, and support from other relationships. Clinicians should provide information and identify helpful resources that support the patient-care-partner dyad and their ability to manage ESLD. Future longitudinal dyadic studies should examine the patient-care-partner relationship at different points in the disease progression to develop interventions that help living with ESLD.

### CHANGE IN OLDER ADULTS' SELF-EFFICACY TO HANDLE DISASTERS AFTER A PREPAREDNESS PROGRAM: ROLE OF SOCIAL NETWORKS

Saida Jaboob[1], Lena Thompson[2], Abinaya Ilavarasan[2], and Sato Ashida[3], *1. University of Iowa, Coralville, Iowa, United States, 2. University of Iowa, Iowa City, Iowa, United States, 3. University of Iowa College of Public Health, Iowa City, Iowa, United States*

Social support networks provide a context within which older adults maintain their safety and well-being. Preparing for disasters and emergency situations can help older adults stay safe independent after a disaster. Self-efficacy to prepare for and handle disaster situations can influence how older adults proactively prepare and protect themselves. Fifty-four older adults 61 to 92 years of age in Eastern Iowa were interviewed before and after participating in an intervention program that helped them develop personalized disaster management plans. Participants reported seven support network members on average, ranging from one to 23. About one-third of the network members were participants' children and grandchildren, 11% were their siblings and parents, and 58% were non-family members such as friends and neighbors. Out of 549 network members identified, 245 were selected as someone participants can depend on during emergency situations; 47% were family whereas 43% were non-family members. Participants who reported higher numbers of network members whom they can depend on in emergency situations at baseline showed more increase in self-efficacy to manage disasters one-month after receiving the intervention (p=0.02). Having social support network members whom older adults feel they can turn to during emergency situations may help boost their confidence in handling and preparing for disasters through participating in a disaster preparedness program. Participants indeed identified additional members whom they could depend on in disaster situations after the intervention. Future studies may test strategies to enhance social support networks to increase confidence among older adults to prepare for and handle disasters.

### MORTALITY RISK BY STATE-LEVEL POVERTY IN COLOMBIA AT DIFFERENT AGE GROUPS

Margarita Osuna[1], Eileen Crimmins[2], and Jennifer Ailshire[2], *1. University of Southern California Leonard Davis School of Gerontology, Santa Clarita, California, United States, 2. University of Southern California, Los Angeles, California, United States*

Residence in high poverty states has been associated with increased mortality risk in the United States, but less attention has been paid to the relationship between state-level poverty and mortality in younger to older adults in Latin America. Poorer states in Colombia, one of the most populous and rapidly aging countries in Latin America, tend to report less access to healthcare, education, and economic opportunities. We examine the relationship between mortality and state-level poverty in Colombia by age and gender. We use data from the 2018 Colombian Census and Vital Statistics to calculate mortality levels and male-female ratios in mortality separately for three broad age categories: young (ages 20-39), middle-aged (40-69), and older adults (70 or older). We find an association between high poverty and high mortality risk among younger men, no association for middle-aged men, and a negative association between mortality risk and poverty among older men. We did not find any evidence for an association between state-level poverty and mortality at any ages. Our results highlight that, for men at older ages, poverty had a counterintuitive association with mortality. These results may be due to selective survival older ages in Colombia or to older adults who are aging into a social safety net that includes healthcare and income benefits.

Future research should investigate the impact that unequal access to economic resources and distribution of health care resources has on women and men across age groups in Colombia.

### CROSS-NATIONAL COMPARISONS OF STRESS AND WELL-BEING IN THE INTERNATIONAL FAMILY OF HEALTH AND RETIREMENT STUDIES

Yoobin Park[1], Alexandra Crosswell[1], and Drystan Phillips[2], *1. University of California, San Francisco, San Francisco, California, United States, 2. University of Southern California, Los Angeles, California, United States*

Strong evidence demonstrates the long-term influence of stress and well-being on psychological, social, and physical health outcomes across the lifespan. Because of this, stress and well-being measures have been added to nearly all of the International Family of Health and Retirement Studies. However, this newly available data has not been compared cross-nationally or within-country to unpack how culture influences these important predictors of healthy aging. Using the Gateway to Global Aging Data, which provides harmonized data from the Health and Retirement Study and its sibling nationally representative studies, levels of self-reported stress (e.g. job stress, discrimination, loneliness) and well-being (e.g. quality of life, life satisfaction) are compared across 30 countries. Data come from the following studies: HRS, ELSA, SHARE, TILDA, CHARLS, KLoSA, MHAS, and JSTAR. We used data from the latest study wave for which the relevant survey was implemented. Average age of participants across studies is 67 and 55% are women. Initial analyses show stressor specific findings such as participants in Korea reported greater work stress than participants in Japan, England, the United States, and across Europe, and the United States reported higher loneliness than China and England, but not higher than Ireland. Reporting cross-national and within-country variation in these measures will be generative in pointing to new research directions for understanding how culture influences health and aging trajectories.

### YOUNGER AND OLDER ADULTS PERCEPTIONS OF STRESSORS AFTER A FLOOD

Katie Cherry[1], Marlene Friis[2], Piper Bordes[1], Matthew Calamia[1], and Emily Elliott[1], *1. Louisiana State University, Baton Rouge, Louisiana, United States, 2. Tulane University School of Medicine, New Orleans, Louisiana, United States*

In August of 2016, historic flooding in Baton Rouge, Louisiana resulted in catastrophic damages and claimed 13 lives. This study is part of a larger research program on post-flood health and well-being across the adult lifespan. Participants (n=223, age range: 18-88 years) were tested during the immediate impact phase (Wave 1) and most participated in a follow-up assessment 9 (+/- 3) months later (Wave 2). In this study, we compared participants' narrative responses to an open-ended question at Wave 2 concerning the most stressful aspect of the 2016 flood. We hypothesized that older flood survivors would report stressors related to rebuilding and financial loss more often than younger survivors based on the Conservation of Resources theory (Hobfoll, 1989). Three groups were compared: non-flooded (controls), single disaster (flooded in 2016) and double disaster (flooded in 2005 and again in 2016). To create younger and older comparison groups, age was split at the median with sample sizes that ranged from 28 to 34 younger and older participants within each flood exposure group. Content analyses of responses by independent coders blind to the purpose of the study revealed that older flood victims reported greater stressors related to rebuilding flood-damaged homes and financial stressors than did their younger counterparts. In contrast, younger flood victims were more likely to report childcare issues and being displaced from their homes as stressors compared to the older victims. Implications of these data for understanding age-related vulnerabilities after severe weather events are discussed.

### SESSION 6840 (POSTER)

### INTERVENTIONS (BS)

### UROLITHIN A: GUT-BRAIN DIETARY INTERVENTION IN PARKINSON'S DISEASE

Jenny Hong Yu Ng, and Julie Andersen, *Buck Institute for Research on Aging, Novato, California, United States*

Gastrointestinal dysfunction is amongst the most common prodromal symptoms of Parkinson's disease (PD). Pathological alpha-synuclein has been detected in the intestines prior to disease onset, and a leaky gut is also implicated in its etiology. Thus, we hypothesized that modulation of the gut microbiome and intestinal immune milieu via early dietary intervention may act to mitigate PD pathogenesis. Urolithin A (UA) is a gut metabolite shown to ameliorate geriatric diseases by increasing mitophagy and dampening inflammation. The aim of our study is to elucidate its mechanism of action and therapeutic efficacy in PD, which to date is unclear. Preliminary flow cytometric data demonstrates that administration of a UA-diet significantly increased the proportion of colonic gamma-delta ($\gamma\delta$) T cells in nine-month-old Thy-1 $\alpha$-syn mice, which are downregulated relative to non-transgenics on a non-UA control diet. PD patients have been reported to have higher levels of $\gamma\delta$ T cells in their cerebrospinal fluid and, while little is known about colonic $\gamma\delta$ Ts in the context of PD, these cells are anti-inflammatory and responsible for intestinal repair in several colitis models. Our data suggests a retention of lymphocytes involved in the targeted migration from the gut to the brain, which may contribute to gut epithelial integrity. Proportion of induced regulatory T cells in peripheral blood, which are critical for immune tolerance, also increased significantly with a UA-diet. In addition, UA-fed mice showed a slight improvement in novel object recognition. Additional analyses are underway to comprehensively evaluate the impact of UA on PD pathology.

### COMBINING SCLEROSTIN AND DKK1 INHIBITORS TO IMPROVE BONE PROPERTIES IN THE AGED SKELETON

Roy Choi, and Alexander Robling, *Indiana University School of Medicine, Indianapolis, Indiana, United States*

Targeting the secreted Wnt inhibitor sclerostin has been an attractive strategy to improve skeletal health. Sclerostin antibody (romosozumab-aqqg; Evenity) was recently approved by the FDA to treat patients at increased risk of fracture. However, an increased risk of cardiovascular events was reported, resulting in issue a ‘black box warning’ requirement for romosozumab. One potential solution to lower the risk of adverse events is to reduce the medication dose. Previously, we found that dual inhibition of sclerostin and Dkk1 produced extremely potent synergistic bone anabolic effects, in both genetic and pharmacological models. While Dkk1 inhibition alone has no consistent bone-building effects, combining antibodies that target sclerostin (Scl-mAb) and Dkk1 (Dkk1-mAb) at 3:1 ratio resulted in 2-3X more bone gain as Scl-mAb alone. Further, much lower total doses of dual antibody treatment, given at optimized proportions, generated equivalent bone anabolic effects as Scl-mAb alone (at much higher doses), suggesting that a combinational strategy has obvious translational benefits. Finally, we tested whether low-dose combination therapy can maintain the same osteogenic effect as Scl-mAb in adult (6 month) and aged (20 month) mice. Outcome measures derived from radiographic, biomechanical, and histomorphometric assays revealed that a 3:1 ratio of Scl-mAb:Dkk1-mAb at 12.5mg/kg was as efficacious as 25mg/kg of Scl-mAb alone, in both age groups. Moreover, cortical porosity—a significant factor contributing to skeletal fragility in the aged skeleton—was significantly reduced by both Scl-mAb and low-dose combination treatment. In conclusion, our findings suggest that optimized low-dose combinational therapy is viable strategy for improving skeletal fragility.

### ORALLY ACTIVE, CLINICALLY TRANSLATABLE SENOLYTICS RESTORE A-KLOTHO IN MICE AND HUMANS

Yi Zhu[1], Larissa Langhi Prata[1], Erin Wissler Gerdes[1], Jair Netto[1], Tamar Pirtskhalava[1], Nino Giorgadze[2], Utkarsh Tripathi[1], and Christina Inman[1], *1. Mayo Clinic, Rochester, Minnesota, United States, 2. May Clinic, Rochester, Minnesota, United States*

Decreased α-Klotho, a geroprotective factor, and increased senescent cell burden are both associated with early onset of physical disability, cognitive impairment, and premature all-cause mortality. It has been demonstrated that eliminating senescent cells can enhance physical function, cognition, and survival in mice, as does overexpressing α-Klotho. Mice with low α-Klotho exhibit accelerated senescent cell accumulation, recombinant α-Klotho decreases senescent cell burden and restores lifespan in these mice, and senescent epidermal cells are reduced in mice overexpressing α-Klotho. Here, we tested the hypothesis that senescent cells cause decreased α-Klotho and hence that reducing senescent cells can increase α-Klotho. Senescent cell conditioned medium (CM) reduced α-Klotho in cultured non-senescent human umbilical vein endothelial cells (HUVECs), renal tubular endothelial cells, and astrocytes. These effects of senescent CM were partially attenuated by neutralizing antibodies against the senescence-associated secretory phenotype (SASP) factors, activin A and IL-1α. Transplanting senescent cells into younger mice caused decreased urine and brain α-Klotho. Genetically reducing highly p16Ink4a-expressing cells in old INK-ATTAC mice or administering the senolytics, Dasatinib plus Quercetin (D+Q) or Fisetin (F), to young mice transplanted with senescent cells, young diet-induced obese (DIO) mice, or naturally-aged mice increased urine, kidney, and/or brain α-Klotho. Treating patients with idiopathic pulmonary fibrosis (IPF), a cellular senescence-related disease, with D+Q led to increased urinary α-Klotho. Thus, targeting senescent cells causes increases in the geroprotective factor α-Klotho, potentially amplifying the beneficial effects of senolytic drugs.

### OXR1 STABILIZES THE RETROMER TO EXTEND LIFESPAN AND NEURONAL HEALTH BY DIETARY RESTRICTION

Kenneth Wilson[1], Sudipta Bar[1], Eric Dammer[2], Birgit Schilling[1], Nicholas Seyfried[2], Hugo Bellen[3], Lisa Ellerby[1], and Pankaj Kapahi[1], *1. Buck Institute for Research on Aging, Novato, California, United States, 2. Emory University School of Medicine, Atlanta, Georgia, United States, 3. Baylor College of Medicine, Houston, Texas, United States*

Dietary restriction (DR) delays aging and neurodegeneration, but the mechanisms behind this remain unclear. We reared over 150 fully sequenced fly strains from the Drosophila Genetic Reference Panel under ad libitum feeding or diet-restricted conditions and measured lifespan as well as healthspan to identify new targets for DR-mediated longevity. Through genome-wide association study, we identified genetic variants associated with influencing these traits under each dietary condition. A variant in mustard (mtd, called Oxidation resistance 1, OXR1, in humans), significantly associated with DR-specific lifespan. We demonstrate that mtd/OXR1 in neurons is necessary for DR-mediated lifespan extension. Neuronal knockdown of mtd also accelerates sensory decline, arguing for a specific role of mtd/OXR1 in neuroprotection. We show that mtd is essential for stabilizing the retromer complex, which is necessary for trafficking transmembrane proteins and lipids for reuse. As a result of OXR1 deficiency, the retromer destabilizes and lysosomes become overused. Overexpression of retromer proteins or supplementation with chaperone compound R55 rescues the lifespan defects and neurodegeneration seen in mtd-deficient flies, and R55 is capable of rescuing lysosomal aggregation and OXR1-retromer co-localization in cells from humans with OXR1 deficiency. We further show through multi-omic analyses in flies and humans that mtd/OXR1 associates with accelerated transcriptomic aging and proteins involved in neurodegenerative diseases, including Alzheimer's disease (AD). Overexpression of OXR1 and retromer proteins rescued AD-associated phenotypes in a fly model of AD. Thus, mtd/OXR1 enhances protein recycling in response to DR through the retromer, improving neuronal health and lifespan through mechanisms conserved across species.

### EXTENDING A HEALTHY LIFESPAN WITH 3-HYDROXYANTHRANILIC ACID

George Sutphin[1], Hope Dang[1], Raul Castro-Portuguez[1], Luis Espejo[1], Sam Freitas[1], and Jeremy Meyers[2], *1. University of Arizona, Tucson, Arizona, United States, 2. University of Washington, Tucson, Arizona, United States*

Metabolism of tryptophan by the kynurenine pathway is increasingly linked to aging. Kynurenine pathway enzymes and metabolites influence a range of molecular processes critical to healthy aging, including regulation of inflammatory and immune responses, cellular redox homeostasis, and energy production. Aberrant kynurenine metabolism occurs during normal aging and is implicated in many age-associated pathologies including chronic inflammation, atherosclerosis, neurodegeneration, and cancer. We and others previously identified three kynurenine pathway genes—kynu-1, tdo-2, and acsd-1—for which decreasing expression extends lifespan in invertebrates. More recently we discovered that knockdown of haao-1, a fourth kynurenine pathway gene encoding the enzyme 3-hydroxyanthranilic acid dioxygenase (HAAO), extends lifespan by ~30% and delays age-associated health decline in Caenorhabditis elegans. Lifespan extension is mediated by increased physiological levels of the HAAO substrate 3-hydroxyanthranilic acid (3HAA). Aging mice fed a diet supplemented with 3HAA are similarly long-lived. The mechanism of action liking 3HAA to aging is complex and partially overlaps with multiple pathways previously implicated in aging. We recently identified activation of the Nrf2/SKN-1 oxidative stress response and alterations to iron homeostasis as key players in the benefits 3HAA. Ongoing work explores the relationship between 3HAA, Nrf2/SKN-1, and iron in C. elegans and mammalian aging, age-associated immune decline, and cancer. This works provides a foundation for detailed examination of the molecular mechanisms underlying the benefits of 3HAA, and how these mechanisms interact with other anti-aging interventions. We anticipate that these findings will bolster growing interest in developing pharmacological strategies to target tryptophan metabolism to improve health aging.

### LEVERAGING THE NDUFS4-/- MOUSE AS A PLATFORM FOR TESTING LONGEVITY INTERVENTIONS

Alessandro Bitto, Cara Tobey, Ayush Sharma, Anthony Grillo, and Matt Kaeberlein, *University of Washington, Seattle, Washington, United States*

Mitochondrial dysfunction is one of the hallmarks of biological aging, as well as the driving factor for mitochondrial diseases. Up to 30% of mitochondrial disorders are due to mutations affecting the activity of Complex I in the electron transport chain. Loss of the Complex I subunit Ndufs4 recapitulates symptoms of Leigh Syndrome, a pediatric mitochondrial disease, in mouse. Ndufs4-/- mice suffer developmental delays, early onset of neurological symptoms and extremely reduced lifespan. Several studies have now shown that Ndufs4-/- mice are exquisitely responsive to treatments and interventions of interest in the biology of aging, such as rapamycin, NAD+ precursors, reduced oxygen tension, alpha-keto-glutarate precursors, and the antidiabetic drug acarbose. These results point to common mechanisms underlying both aging and mitochondrial disorders. To put this hypothesis to the test, we show that Ndufs4-/- mice are responsive to a wide range of longevity interventions previously tested in worms, mice, and by the National Institute on Aging's Intervention Testing Program. These observations support the hypothesis that mitochondrial and metabolic dysfunction induced by Complex I deficiency may be a key component of biological aging as well as mitochondrial disease. Furthermore, we propose that the Ndufs4-/- mice provide an affordable testing ground for candidate longevity interventions.

### DELETION OF THROMBOSPONDIN-1 PRESERVES HEMATOPOIETIC STEM CELL HEALTHSPAN DURING AGING

Pradeep Ramalingam[1], Michael Gutkin[2], Michael Poulos[2], and Jason Butler[2], *1. University of Florida, Gainesville, Florida, United States, 2. Hackensack University Medical Center, Nutley, New Jersey, United States*

Aging is associated with defects within blood stem cells, termed hematopoietic stem cells (HSC), including a loss of their self-renewal potential and a skewed differentiation towards myeloid lineages at the expense of lymphoid cells. Collectively, these HSC defects manifest as anemias, poor response to vaccines and an increased incidence of myeloid neoplasms in older adults. Unlike other somatic stem cells, aged HSCs have been shown to be refractory towards established anti-aging interventions including caloric restriction, exercise, parabiosis and plasma transfer. Thrombospondin-1 (TSP1) was initially discovered as an anti-angiogenic molecule, and recent studies have identified that TSP1 promotes age-related pathologies including chronic inflammation, reactive oxygen species (ROS) generation, and mitochondrial dysfunction. Notably, each of these TSP-1 regulated processes have been shown to critically influence HSC biology, particularly in the context of aging. However, whether TSP-1 directly regulates HSC activity remains unexplored. Here, we sought to determine whether TSP-1 is essential for HSC development, and whether blocking TSP1 signaling could ameliorate age-related HSC defects. Utilizing murine models, we demonstrate that TSP-1 is dispensable for normal HSC development and hematopoiesis. We show that deletion of TSP-1 is sufficient to preserve HSC fitness during aging, as evidenced by preservation of youthful self-renewal potential and balanced lineage reconstitution during serial HSC transplantation assays. Mechanistically, we identify that TSP-1 adversely impacts mitochondrial metabolism within HSCs, and show that loss of TSP-1 prevents the age-related decline in HSC mitochondrial membrane potential. Our findings identify TSP-1 as a pro-geronic factor that can be targeted to preserve HSC healthspan.

### A FAT-PROMOTING BOTANICAL EXTRACT ARTEMISIA SCOPARIA EXERTS GEROPROTECTION IN C. ELEGANS

Bhaswati Ghosh[1], Hayden Guidry[2], Maxwell Johnston[1], and Adam Bohnert[1], *1. Louisiana State University, BATON ROUGE, Louisiana, United States, 2. Louisiana State University, Baton Rouge, Louisiana, United States*

Like other biological processes, aging is not random but subject to molecular control. Natural products that modify core metabolic parameters, including fat content, may provide entry points to extend animal lifespan and promote healthy aging. Here, we show that a botanical extract from Artemisia scoparia (SCO), which promotes fat storage and metabolic resiliency in mice, extends the lifespan of the nematode Caenorhabditis elegans by up to 40%. Notably, this lifespan extension depends significantly on SCO's effects on fat; SCO-treated worms exhibit heightened levels of unsaturated fat, and inhibiting Δ9 desaturases, which oversee biosynthesis of monounsaturated fatty acids, prevents SCO-dependent fat accumulation and lifespan extension. At an upstream signaling level, SCO prompts changes to C. elegans fat regulation by stimulating nuclear translocation of transcription factor DAF-16/FOXO, an event that requires AMP-activated protein kinase under this condition. Importantly, animals treated with SCO are not only long lived but also show improved stress resistance in late adulthood, suggesting that this fat-promoting intervention may enhance some aspects of physiological health in older age. These findings identify SCO as a natural product that can modify fat regulation for longevity benefit and add to growing evidence indicating that elevated fat can be pro-longevity in some circumstances.

### FUCOIDANS ARE NOVEL SENOTHERAPEUTICS THAT ENHANCE SIRT6 AND DNA REPAIR ACTIVITY

Lei Zhang[1], Brian Hughes[2], Gregory Tombline[3], Luise Angelini[2], Matt Yousefzadeh[2], Vera Gorbunova[3], Laura Niedernhofer[2], and Paul Robbins[2], *1. University of Minnesota, Saint Paul, Minnesota, United States, 2. University of Minnesota, Minneapolis, Minnesota, United States, 3. University of Rochester, Rochester, New York, United States*

With age, senescent cells accumulate in various tissues where they contribute to loss of tissue homeostasis, aging, and age-related diseases through their inflammatory senescence-associated secretory phenotypes (SASPs). Senotherapeutics able to selectively eliminate senescent cells, termed senolytics, or suppress the detrimental SASPs, termed senomorphics, have been demonstrated to improve age-associated comorbidities and aging phenotypes. To discover novel senotherapeutics translatable to promote healthy longevity, we conducted a drug screening of diverse natural products based on the characteristic senescence-associated β-galactosidase activity. Several fucoidans from different brown seaweed were found to exhibit potent senotherapeutic activity. Fucoidans are long-chain sulfated polysaccharides found in various species of brown algae including seaweed. The best senomorphic fucoidan was able to suppress senescence in cultured senescent fibroblasts, in ex vivo human tissue explants, and in vivo in mouse models of natural and accelerated aging. Specifically, fucoidan reduced markers of cellular senescence and SASP in senescent mouse and human cells. Acute treatment of the fucoidan in naturally aged mice reduced tissue senescence, especially in the kidney and lung. Chronic treatment of the fucoidan in Ercc1-/Δ progeria mice attenuated composite aging symptoms and extended healthspan. Interestingly, preliminary mechanistic studies demonstrated that fucoidan can improve non-homologous end-joining-directed DNA damage repair and increase the mono-ADP-ribosylation activity of SIRT6, suggesting a relationship between cellular senescence, DNA repair, and SIRT6 signaling pathways. Collectively, fucoidans were identified as novel senotherapeutics with translational potential for reducing cellular senescence, ameliorating age-associated phenotypes, and extending healthspan as well as able improve DNA repair pathways through modulation of SIRT6 activity.

### EFFECT OF L-VALINE TREATMENT ON SIRTUIN (SIRT1 AND SIRT2) ISOFORMS

Shakshi Sharma, Xiaomin Zhang, Gohar Azhar, and Jeanne Wei, *University of Arkansas for Medical Sciences (UAMS), Little Rock, Arkansas, United States*

L-valine is one of the essential branched-chain amino acids (BCAAs) required for synthesis of proteins in human body. It promotes muscle growth and tissue repair and is important for immune function. Recent data indicate that BCAAs can activate sirtuins expression and elevate mitochondrial biogenesis and fatty acid oxidation in both adipocytes and myotubes thereby increasing life span. Sirtuins are a conserved family of proteins, play a critical role in maintaining metabolic health by deacetylating many target proteins in numerous tissues, and regulate mitochondrial function and the aging process. Due to multiple effect of sirtuins on aging, we sought to determine whether the addition of valine might enhance sirtuin gene expression. We utilized the C2C12 skeletal muscle cell line grown on physiological normal glucose (100mg/dL) media. The cells were treated with two different concentrations of valine (0.5 and 1.0mM) for different time intervals (18 and 24). Gene expression of sirtuin 1 (SIRT1) and sirtuin 2 (SIRT2) isoforms were determined by RT-PCR. The results showed increased expression of the sirtuin gene isoforms after treatment with valine. Relative expression varies with in different isoforms of SIRT1 (v1 and v2) and SIRT2 (v1, v2 and v3). Among all, SIRT1 v1 and SIRT2 v1 showed maximum expression as compared to the other isoforms used in the study. Our study showed that adequate supplementation of L-valine enhanced sirtuin gene expression, which may promote healthy muscles and healthy aging.

### LOSS OF HYPOXIA SIGNALING IMPAIRS RESPONSE TO AEROBIC EXERCISE IN AGED MICE

Indranil Sinha[1], Yori Endo[2], and Mehran Karvar[1], *1. Brigham and Women's Hospital, Boston, Massachusetts, United States, 2. Harvard Medical School, Boston, Massachusetts, United States*

To assess the differential effects of exercise with age, Young (Y, 10-12 weeks) and Old (O, 23-25 months) mice were subjected to regimented treadmill running or no regimented exercise. Y, trained mice experienced a significant increase in maximal distance running, maximal speed of running, and lean muscle mass in comparison to age-matched, untrained controls. O mice did not improve significantly in any of these measures following training. Transcriptome analysis of gastrocnemius from Y mice demonstrated differential regulation of 120 genes with exercise. None of these genes were similarly regulated in the O group. Genes most upregulated following exercise in Y mice were direct targets of the hypoxia signaling pathway. Immunoblotting demonstrated that aryl hydrocarbon receptor nuclear translocator (ARNT), a critical regulator of hypoxia signaling, increased 3-fold with exercise in Y mice, but this increase was absent in O mice following exercise. To assess whether this loss of ARNT in O muscle impaired the exercise response, we generated a mouse with inducible, skeletal muscle-specific knockout of ARNT (ARNT muscle (m) KO). Following regimented exercise, ARNT mKO mice did not improve maximal distance running, maximal running speed, or lean muscle mass in comparison to untrained ARNT mKO mice. Littermate, age-matched ARNT wild type mice increased significantly in all of these measures following training. Administration of ML228, an ARNT agonist, increased maximal running distance and speed in response to exercise training in O mice. These results suggest that restoration of ARNT and hypoxia signaling may restore the physiologic response to exercise in aging.

### LATE BREAKING ABSTRACTS

### SESSION 1470 (BIOLOGICAL SCIENCES INVITED SYMPOSIUM)

### LATE BREAKING SESSION: BIOLOGICAL SCIENCE PROGRAM

Chair: Viviana Perez

### ALTERED SUPER ENHANCER – PROMOTER INTERACTIONS MEDIATED BY YY1 UNDERLIE AGE-INDUCED TRANSCRIPTOME

Weiwei Dang, Luyang Sun, and Brenna McCauley, *Baylor College of Medicine, Houston, Texas, United States*

Significant changes in transcriptome during aging have been observed in various tissues and cell types, even at the single-cell level. However, the molecular mechanisms that underlie these changes remain poorly understood. Using an ex vivo mesenchymal stem cells (MSC) replicative aging model and Hi-C technology, we discovered that, rather than large-scale changes in chromosome conformation, alterations to super enhancer – promoter looping interactions had the best positive correlation with age-associated transcriptome. Furthermore, we found evidence that these age-induced transcriptomic changes are mediated by YY1, a key regulator of promoter-enhancer looping. This work, for the first time, unveiled a molecular mechanism elucidating the changes in transcription during aging.

### INTERMITTENT KETOGENIC DIETS EXTEND LIFESPAN AND ATTENUATE INFLAMMAGING IN MALE MICE

Zeyu Zhou, Kyoungmi Kim, Jon Ramsey, and Jennifer Rutkowsky, *University of California, Davis, Davis, California, United States*

A ketogenic diet (KD) has been shown to increase functional lifespan in male mice when fed isocalorically to a control diet (CD). However, it is not clear if intermittent ketogenic diets (IKD) that induce brief and recurring periods of ketosis would be as effective at prolonging lifespan. In the present study, 12-month-old C57BL/6JN male mice were fed an intermittent single day KD (KD fed on Mon, Wed and Fri each week, IK1) or 2-day KD (KD fed on 2 consecutive days each week, IK2). These two IKD regimes were selected to mimic the widely used alternate-day or 5:2 intermittent fasting approaches, without the caloric deficit. Our results showed that lifespan was significantly increased in both IK1 and IK2 groups, with IK1 showing a 13.6% and IK2 showing a 7.9% increase in median lifespan compared to the CD ($p < 0.01$). At 27 months of age, levels of circulating proinflammatory cytokines were significantly decreased in IK1 and IK2 mice compared to control mice. Also, levels of proinflammatory cytokines were significantly elevated in 27-month-old control mice compared to 12-month-old mice, while levels in IK1 and IK2 mice were not significantly altered with aging, and these effects were still persistent during days the animals were fed the CD. Unlike the continuous KD, health span measures were not altered in IK1 or IK2 mice at 26 months of age. In summary, IKDs that produce shorts episodes of ketosis can be effective at extending longevity in mice, possibly by attenuating systemic inflammation with aging.

### SINGLE-CELL ANALYSIS OF SKELETAL MUSCLE MACROPHAGES REVEALS AGE-ASSOCIATED FUNCTIONAL SUBPOPULATIONS

Chang-Yi Cui, Linda Krasniewski, Papiya Chakraborty, Krystyna Mazan-Mamczarz, Luigi Ferrucci, and Myriam Gorospe, *National Institute on Aging, Baltimore, Maryland, United States*

Tissue-resident macrophages represent a group of highly responsive innate immune cells that acquire diverse functions by polarizing towards distinct subpopulations. The subpopulations of macrophages that reside in skeletal muscle (SKM) and their changes during aging are poorly characterized. By single-cell transcriptomic analysis with unsupervised clustering, we found eleven distinct macrophage clusters in male mouse SKM with enriched gene expression programs linked to reparative, proinflammatory, phagocytotic, proliferative, and senescence-associated functions. Using a complementary classification, membrane markers LYVE1 and MHCII identified four macrophage subgroups: LYVE1-/MHCIIhi (M1-like, classically activated), LYVE1+/MHCIIlo (M2-like, alternatively activated), and two new subgroups, LYVE1+/MHCIIhi and LYVE1-/MHCIIlo. Notably, one new subgroup, LYVE1+/MHCIIhi, had traits of both M2 and M1 macrophages, while the other new subgroup, LYVE1-/MHCIIlo, displayed strong phagocytotic capacity. Flow cytometric analysis validated the presence of the four macrophage subgroups in SKM, and found that LYVE1- macrophages were more abundant than LYVE1+ macrophages in old SKM. A striking increase in proinflammatory markers (S100a8 and S100a9 mRNAs) and senescence-related markers (Gpnmb and Spp1 mRNAs) was evident in macrophage clusters from older mice. In sum, we have identified dynamically polarized SKM macrophages and propose that specific macrophage subpopulations contribute to the proinflammatory and senescent traits of old SKM.

### REGENERATION OF AGING LUNG TISSUE: A NOVEL WAY OF REOPENING THE PLASTICITY WINDOW OF POSTNATAL ALVEOLAR TYPE II CELLS

Hannah Hausman,[1] Hannah Golding,[2] Amitoj Sawhney,[3] and Douglas Brownfield[3], *1. St. Catherine University, St. Paul, Minnesota, United States, 2. University of Colorado Boulder, Boulder, Colorado, United States, 3. Mayo Clinic, Rochester, Minnesota, United States*

Prevalence of chronic lung diseases, such as pulmonary fibrosis and chronic obstructive pulmonary disease, have been found to increase with age. Pulmonary disease can occur from failure to maintain alveolar type (AT) 1 and alveolar type 2 cells located in the epithelium of the lung. It is currently known that AT2 cells are the facultative stem cells of the lung, and can transform into AT1 cells which make up the structure of the alveoli. This process happens throughout embryonic development, but AT2 cells can also replenish AT1 cell populations post lung injury. This research investigates whether this process that normally occurs during development can also occur in mature AT2 cells, and studies this question using genetically engineered mouse models at a postnatal stage. Using a double inhibition mechanism, we attempted to increase cellular proliferation by administering an agonist and conducted cellular lineage tracing with an SpcCreER lox system via tamoxifen-induced GFP expression. Through lineage labeling, we saw AT1 cells arising from AT2 cells that became plastic, mirroring the plasticity observed in development. Further research is needed to determine if this agonist can be used to epigenetically unlock mature AT2 cells. This result would provide a mechanism to temporarily induce plasticity in a patient's own AT2 cells in an attempt to regenerate their lungs in disease.

### THE ROLE OF THE ENDOPLASMIC RETICULUM UNFOLDED PROTEIN STRESS RESPONSES IN A C. ELEGANS MODEL OF QUIESCENCE

Arianna Pankey, *Howard University, Washington, District of Columbia, United States*

Cells devote extraordinary energy to translate and fold approximately 20,000 proteins encoded by the human genome. About one third of these proteins are transmembrane proteins—correctly folded in the endoplasmic reticulum (ER). The ER Unfolded Protein Response (UPRER) is a response to disturbances at the ER. As an organism ages, cells lose the ability to activate the UPRER. Our lab uses C. elegans—a microscopic roundworm—as a model organism to determine how loss of ER function can contribute to aging. The UPRER plays a key role in maintenance of functional quiescence. Quiescent cells exist in a reversible state of dormancy and can receive specific environmental cues to proliferate. The developmental state known as L1 arrest resembles the same features as quiescent cells. IRE-1 is required for worms to exit their quiescent state after starvation. Understanding the genetic pathways that are best at suppressing ire-1 terminal L1 arrest could indicate ways to improve functional quiescence. We forced the worms into an L1 arrest state; The unfolded protein response in the endoplasmic reticulum maintains functional quiescence within the cell. The worm's ability to develop once they exit this quiescent stage effectively, indicated the ability of the worm to maintain protein homeostasis despite non-ideal conditions. It was found that all tested mutations were unable to demonstrate improvement in ability to maintain development when arrested long-term. Our data indicates that our worms were counted prematurely. In the future, we will allow for a longer growth period as well as larger quantities of food.

### SESSION 3481 (PAPER)

### LATE BREAKING: COMMUNITY-BASED PARTICIPATORY RESEARCH APPROACHES I

### COGNITION AND HEALTH: PRELIMINARY RESULTS OF A MUSIC CREATIVITY INTERVENTION FOR ADULTS AT RISK OF MCI

E. Lim Lydia Wu-Chung,[1] Anthony Brandt,[1] Melia Bonomo,[1] Bryan Denny,[1] Christof Karmonik,[2] Jefferson Frazier,[3] Karl Blench,[1] and Christopher Fagundes[1], *1. Rice University, Houston, Texas, United States, 2. Houston Methodist Research Institute, Houston, Texas, United States, 3. Houston Methodist Hospital, Houston, Texas, United States*

As the number of people with dementia is projected to rise to 13.8 million by 2050, there is a growing need to develop interventions that prevent or slow down disease progression in at-risk individuals. Older adults are particularly vulnerable, given that increasing age is the strongest predictor of dementia. Music interventions are promising, non-pharmaceutical treatment options for slowing down cognitive decline and enhancing psychological health. However, more music-related clinical trials are needed to evaluate treatment efficacy and to identify biobehavioral mechanisms of change. Project Chroma is a Stage 1 semi-randomized clinical trial, developed to assess the effects of a novel, music creativity curriculum on cognitive, socio-emotional, neurobiological, and immunological outcomes. In this study of 58 older adults with or without MCI, we demonstrate that Project Chroma has good feasibility and acceptability: participation, retention and satisfaction rates were comparable to other similarly designed clinical trials. Preliminary analyses revealed that participants in the music condition, relative to those in the control condition, showed marked improvements in cognitive functioning. Slight changes in socioemotional well-being were observed, which may be attributed to a minimally distressed sample. This study contributes to a growing literature substantiating music interventions as effective options for curtailing cognitive decline. Forthcoming work will examine the effects of music creativity on neural and immune outcomes.

### USABILITY AND ACCEPTABILITY OF A PEER-DELIVERED TECHNOLOGY BASED MENTAL HEALTH INTERVENTION FOR FAMILY CAREGIVERS

Caroline Collins-Pisano,[1] Amanda Leggett,[2] and Karen Fortuna[3], *1. University of Colorado, Colorado Springs, Hanover, New Hampshire, United States, 2. Wayne State University, Ypsilanti, Michigan, United States, 3. Dartmouth College, Hanover, New Hampshire, United States*

Family caregivers of people with dementia are critical to the quality of life of care recipients and the sustainability of healthcare systems but face increased risk of emotional distress and negative health outcomes. The purpose of this study was to examine the usability, acceptability, and preliminary effectiveness of a technology-based and caregiver-delivered peer support program, "Caregiver Remote Education and Support" (CARES) smartphone application and to identify barriers and facilitators to the ethical use of former caregivers as technology-based interventionists. Nine family caregivers of people with dementia received the CARES intervention and three former family caregivers of people with dementia were trained to deliver the CARES system. Quantitative data were collected at baseline and at the end of the two-week field usability study. Qualitative data were also collected at the end of the two-week field usability study. The pilot study demonstrated that a two-week, peer- delivered and technology supported mental health intervention designed to improve burden, stress, and strain levels was experienced by former and current family caregivers as usable, acceptable, and ethical. CARES was associated with non-statistically significant improvements in burden, stress, and strain levels. This pre/post field usability study demonstrated it is possible to train former family caregivers of people with dementia to use technology to deliver a mental health intervention to current caregivers. Future studies would benefit from a longer trial, a larger sample size, a randomized controlled design, and a control of covariables such as stages of dementia, years providing care, and the severity of dementia symptoms.

### USING COMMUNITY-BASED PARTICIPATORY RESEARCH TO PROMOTE MENTAL HEALTH LITERACY IN OLDER CHINESE

Jessie Ho-Yin Yau, Edwin Lok Yan Wong, Hotinpo Sky Kanagawa, Nicole HL Wong, Tianyin Liu, Gloria H.Y. Wong, and Terry Lum, *1. The University of Hong Kong, Hong Kong, Hong Kong*

Whilst traditional mental health literacy programmes utilized a top-down approach, no bottom-up community-based participatory research (CBPR) model had been used to promote mental health literacy among older adults. Moreover, the existing CBPR model was developed in the West and might not be applicable in Chinese communities given different socio-cultural contexts. This research aimed to fill the gap by implementing a CBPR project to promote mental health literacy among older adults and developing a CBPR model in Chinese context. A year-long CBPR project was conducted in five Hong Kong districts from May 2021 to August 2022. A district advisory group was formed in each district, which comprised 50 community older adults, 2 NGO social workers, and 2 researchers. Each district had their own promotional activities that were initiated and designed by older adults, including street booths, art workshops, videos and photos to promote mental health and introduce relevant information and resources. Researchers recorded field observations in each district activity and conducted focus group discussions with stakeholders. Collected data suggested that specific elements are important for a CBPR model in Chinese context, including participant empowerment, technical support, stakeholder expectation management and potential community contribution. Following the implementation of district promotional activities, a territory-wide advisory group was formed to promote mental health literacy on a territory level in the coming year. This is the first large-scale CBPR project that promoted mental health literacy among older adults in Chinese context. Implications for future research and practice will be discussed.

### CAUSAL INFERENCE IN PSYCHOPATHOLOGY OF DEPRESSIVE SYMPTOMS IN MIDDLE-AGED AND OLDER ADULTS

Zheng Zhu,[1] Xiang Qi,[1] Yaolin Pei,[1] Jing Wang,[2] and Bei Wu[1], *1. New York University, New York, New York, United States, 2. The University of North Carolina at Chapel Hill, Chapel Hill, North Carolina, United States*

Most previous research studied depression as a holistic conception and ignored the complex relationships between depressive symptoms. How depressive symptoms interact with each other is still unknown. The aims of this study were to develop symptom networks of middle-aged and older adults and to explore the core symptom in the symptom networks. This study used three-wave data from the China Health and Retirement Longitudinal Study (CHARLS) in 2013 (T1), 2015 (T2), and 2018 (T3). Depressive symptoms were measured by the 10-item Epidemiological Research Center for Depression Scale (CES-D). A multilevel vector autoregression model (VAR) was used to identify 10 depressive symptoms dynamically interacting with each other over time. A total of 3558 participants were included in the final analysis. The strongest direct effects were "D10: felt fearful" -> "D6: felt everything I did was an effort" ($\beta$=0.14). "D10: felt fearful" reported the largest value of out-predictability (r=0.064) and out-strength (r=0.635). "D3: felt depressed" reported the largest value of in-predictability (r=0.077) and in-strength (r=0.545). Substantial heterogeneity in the network may stem from an individual's gender and living region. "Felt fearful" was the strongest predictor compared to the other nine depressive symptoms based on node centrality. It was also the most crucial bridge node between negative symptoms cluster and positive affects cluster. Network density and the sum of all absolute strength centrality should also be incorporated into clinical practice as key indicators of emotional vulnerability, particularly in male middle-aged and older adults.

### SESSION 3482 (PAPER)

### LATE BREAKING: INNOVATIONS IN CLINICAL PRACTICE I

### CHARACTERISTICS OF HOSPITALS AND PROVIDER MARKETS ASSOCIATED WITH INCREASES IN HOME HEALTH CARE USE

Elizabeth Watts,[1] Taylor Bucy,[1] John McHugh,[2] and Dori Cross[1], *1. University of Minnesota School of Public Health, Minneapolis, Minnesota, United States, 2. Columbia University Mailman School of Public Health, New York, New York, United States*

Many older adults require post-acute care from a nursing home or home health agency following hospitalization. Recent trends show providers are increasingly relying on home health agencies rather than institutionalized settings, with home health volume surpassing skilled nursing facility (SNF) volume since 2017. Using MedPAR patient encounter data from 2016–2019 and provider data from CMS, we analyze changes in the profile of patients receiving home health over time, showing that individuals discharged to home health are increasing in complexity based on hospital length of stay, comorbidities, and use of critical care services. Mixed effects models additionally suggest that grouping patients at the hospital and market level helps to account for unexplained variation in whether a patient is likely to receive home health versus SNF services. Examining the characteristics of hospitals and provider markets with increasing rates of home health referrals over time, we found that hospitals with increasing rates of discharge to home health were more likely to be for-profit facilities in urban areas with higher operating margins. However, this increase was not consistently tied to a corresponding decrease in rates of discharge to SNF, suggesting that hospitals are experiencing a combination of both patient-shifting across post-acute settings as well as an overall increase in baseline complexity of hospitalized patients over this time period. These results have implications for understanding the how policies currently being considered to improve value in the post-acute sector filter through heterogeneous market structures and complex organizational environments to impact patient care decisions.

### BRAIN ACTIVITY DURING DUAL-TASK STANDING IN OLDER ADULTS WITH MILD COGNITIVE IMPAIRMENT

Melike Kahya,[1] Natalia Gouskova,[2] On-Yee Lo,[3] Junhong Zhou,[4] Davide Cappon,[3] Alvaro Pascual-Leone,[2] Lewis Lipsitz,[5] and Brad Manor[6], *1. Marcus Institute for Aging Research, Harvard Medical School, Boston, Massachusetts, United States, 2. Hebrew SeniorLife, Roslindale, Massachusetts, United States, 3. Marcus Institute for Aging Research, Boston, Massachusetts, United States, 4. Harvard Medical School/Hebrew SeniorLife, Roslindale, Massachusetts, United States, 5. Hebrew SeniorLife, Brookline, Massachusetts, United States, 6. Hebrew SeniorLife/Harvard Medical School, Roslindale, Massachusetts, United States*

Performance of a cognitive task while standing disrupts balance in older adults. This disruption is exaggerated in those with mild cognitive impairment (MCI). Moreover, older adults with MCI who exhibit greater dual-task 'cost' are more likely to develop falls and dementia. EEG studies suggest that cognitive-motor dual-tasking is associated with brain activity fluctuations originating from central brain regions at specific frequencies, particularly in the alpha-band (8–13 Hz). We hypothesized that older adults with MCI would demonstrate decreased EEG alpha power during dual-task standing compared to healthy controls, and that decreased alpha power would be associated with elevated dual-task cost. We recorded postural sway and EEG in 14 participants with MCI [Montreal Cognitive Assessment (MoCA) < 25] and 16 healthy older adults [MoCA>25] as they completed trials of standing with and without serial subtractions. Postural sway metrics were derived, and from EEG we calculated absolute alpha-, theta-, and beta-band powers within a-priori defined regions-of-interest: the left and right anterior, central, and posterior regions. Repeated Measures ANOVA demonstrated that participants with MCI exhibited decreased alpha power in the central regions during dual-task standing compared to healthy controls (p= 0.01). No significant difference was observed for theta and beta-band powers between participants with MCI and healthy controls. In those with MCI, lower alpha power during dual-task standing correlated with increased dual-task cost to postural sway path (worse balance) (r=-0.4, p=0.03). These results provide preliminary evidence that specific patterns of brain activity during dual-tasking are disrupted in MCI and this is associated with elevated dual-task costs.

### UNMET TRANSITIONAL CARE NEEDS OF OLDER ADULTS DISCHARGED FROM HOME HEALTH CARE: A QUALITATIVE NEEDS ASSESSMENT

Ayomide Bankole,[1] Tyra Girdwood,[2] Jennifer Leeman,[1] Jennifer Womack,[3] and Mark Toles[1], *1. University of North Carolina-Chapel Hill, Chapel Hill, North Carolina, United States, 2. Duke University, Durham, North Carolina, United States, 3. Appalachian State University, Boone, North Carolina, United States*

Transitional care is widely recognized as a set of time-limited services to support older adults during transfers between settings and providers of care; however, little is known about services that prepare older adults and their caregivers for transitions from Home Health Care (HHC) to independent self-care at home. The objective of this study was to describe HHC patients and clinician perceptions of unmet needs after HHC discharge and recommendations to address them in future research. A multiple case-study design was used in a single large HHC organization in North Carolina. Data were collected using chart reviews of HHC records and semi-structured interviews with paired patients (or caregivers as proxy) and HHC clinicians (Nf17 pairs). A transitional care intervention model was used as a conceptual framework to guide framework analysis of interview transcripts. Most patients were White (65%) and female (53%) with mean age of 83 years. Clinicians were Registered nurses and Physical therapists. Across cases, 17 patient and clinicians pairs described three unmet needs: (1)skills for symptom management; (2)community-based support for transportation, food, social engagement, and health services; (3)in-home support to assist with safety, and activities of daily living. HHC patients recommended new services while clinicians emphasized the need for resources to improve their capacity to prepare patients for discharge. These findings will inform an innovative adaptation of transitional care and other supports to prepare older adults and caregivers for transitions from HHC to independent self-care at home. Future research should explore adding transitional care services to address these needs.

### TESTING A DYADIC SLEEP INTERVENTION FOR PERSONS LIVING WITH COGNITIVE IMPAIRMENT AND THEIR CARE PARTNERS

Glenna Brewster, *Emory University, Lithonia, Georgia, United States*

Sleep disturbance is prevalent among persons living with cognitive impairment (PLWCI) and their care partners, suggesting that dyadic sleep disturbance is an interdependent phenomenon. There is also interdependence in the dyadic sleep disturbance. Despite the pervasiveness of shared sleep disturbance among these dyads and its negative impact on dyadic health outcomes, there are remarkably few interventions for sleep disturbance in which both members of the dyad participate. This study examines sleep outcomes of PLWCI-care partner dyads with insomnia symptoms. Dyads were simultaneously participating in a cognitive behavioral therapy for insomnia intervention. The Epworth Sleepiness Scale and the Insomnia Severity Index assessed daytime sleepiness and insomnia, respectively, at baseline and immediately post-intervention. We compared pre- and post-intervention ESS and ISI scores. For the 14 participants (n=7 dyads), care partners were spouses, on average 71.6 years, female (100%) and highly educated (18.85 years). The PLWCI were on average 73.85 years with 18.28 years of education. Daytime sleepiness improved for four and worsened for two care partners. It improved for three and worsened for three PLwCI. Insomnia improved for five care partners and four PLwCI. Two dyads experienced congruent improvement of daytime sleepiness. Four dyads reported congruent improvement of insomnia symptoms. A behavioral sleep intervention simultaneously engaging both the PLWCI and the care partners resulted in a reduction in insomnia and daytime sleepiness symptoms for most of the participants. This study provides preliminary evidence that PLwCI-care partner dyads with sleep disturbance can work together to improve their sleep outcomes.

### LATE BREAKING: QUALITY IMPROVEMENT INNOVATIONS DURING THE COVID-19 PANDEMIC I

Alisha Johnson, *University of Missouri, Columbia, Missouri, United States*

### THE INFLUENCE OF TREATMENT DELAY INCREASE RISKS OF CANCER MORTALITY AMONG OLDER ADULTS: A POPULATION-BASED STUDY

Jansen Cambia,[1] Edmund Cedric Orlina,[2] and Jason Jiashuan Liu[1], *1. National Yang Ming Chiao Tung University, National Yang Ming Chiao Tung University, Taipei, Taiwan (Republic of China), 2. Department of Health - Rizal Cancer Registry, Department of Health - Rizal Cancer Registry, Rizal, Philippines*

Treatment delays during the ongoing COVID-19 pandemic has worsened oncologic outcomes, such as increasing early mortality among older adults. We investigated the association of treatment delay on the 2-year and 5-year risk of all-cause mortality, and stratified among age ≤60 and age >60 years. This was a retrospective study of cancer patients using the Department of Health-Rizal Cancer Registry (DOH-RCR) from 1971–2012. We employed Poisson regression analysis to compare the risk of death among patients with different treatment delay lengths, defined as interval from diagnosis to treatment of ≤30 days, 31–60 days, and >60 days. We included 16,472 cancer patients. After adjusting for confounding, the 2-year risk of death was significantly higher among patient with treatment delay of >60 days by IRR=1.27 [95% CI=1.2–1.3] and 5-year risk by IRR=1.21 [95% CI=1.2–1.3], compared to delay of less than 60 days. Consistent findings were observed by age-groups, revealing that delay of >60 days puts age≤60 and age>60 years at higher risk of 5-year mortality, by IRR=1.21 [95% CI=1.2–1.3] and IRR=1.20 [95% CI=1.1–1.3], respectively. Treatment delay was associated with overall cancer mortality for cancer diagnoses at any age range. However, further investigation is needed to understand the predictors of longer treatment delay, which may provide consolation to balanced care during the current pandemic.

### CARDIOVASCULAR CONSEQUENCES OF COVID-19 INFECTION IN OLDER ADULTS WITH A HISTORY OF HEART FAILURE

Claudia Beatriz Marcano Ocasio,[1] Andrew Crawford,[2] Mary Parianos,[3] Marina Stukova,[4] Alan Wong,[2] Ali Vaeli Zadeh,[5] Elias Collado,[5] and Joshua Larned[6], *1. Holy Cross Hospital - - Fort Lauderdale, F, Ford Lauderdale, Florida, United States, 2. Holy Cross Health-University of Miami, Fort Lauderdale, Florida, United States, 3. University of Miami, Fort Lauderdale, Florida, United States, 4. University of Miami at Holy Cross, Fort Lauderdale, Florida, United States, 5. Holy Cross Health-Jim Moran Cardiovascular Research Institute, Fort Lauderdale, Florida, United States, 6. Holy Cross Health-University of Miami, Miami, Florida, United States*

Background: COVID-19 frequently affects the cardiovascular system. Advanced age and pre-existing heart failure HF are considered risk factors for a poor prognosis. However, the cardiovascular consequences of COVID-19 in older adults with a history of HF have not been clearly depicted. Methods: A retrospective review was conducted using the PearlDiver database (PearlDiver Technologies, Fort Wayne, IN). Using ICD codes, patients aged 65–75 and Elixhauser Comorbidity Score(ECI)>4 with a history of HF admitted for COVID-19 were identified. This cohort was propensity score matched with a group of patients without HF by age, gender, other cardiovascular diseases, and ECI. Records from both groups were reviewed for new onset of cardiovascular-related conditions, including myocardial infarction(MI), arrhythmias, and hypertension, within one year following the admission. Pearson's chi-squared test was used to compare groups. The strength of association was reported using Risk Ratios (RR). A p-value < 0.05 was deemed significant. Results: 4,014 members in each group were identified. A history of HF was associated with an increased risk of MI(RR=1.18, CI95% =1.005–1.37, p=0.04), and arrhythmias (RR=1.17, CI95% =1.08–1.26, p < 0.00001). No differences present in risk of myocarditis(RR=0.05, CI95%=0.12–1.99, p=50) across groups. Conclusion: Older adults with a history of HF are more likely to experience MI and arrhythmias over a year after hospitalization for COVID-19.

### SUPPORT AND UTILIZATION OF ADVANCED PRACTICE NURSES IN LONG-TERM CARE TO MAXIMIZE OUTCOMES AND EMERGENCY RESPONSE

Alisha Johnson[1] and Tracie Harrison[2], *1. University of Missouri, Columbia, Missouri, United States, 2. University of Arkansas for Medical Sciences, Little Rock, Arkansas, United States*

Incorporation of Advanced Practice Registered Nurses (APRNs) into Long-Term Care (LTC) results in sustained positive outcomes and has potential to help guide LTC facilities in times of emergency such as the COVID-19 pandemic. To optimally utilize the role of an APRN in the care of older adults and people with disabilities requires understanding the needs of LTC while balancing the personal characteristics of APRNs transitioning into practice (TTP). Findings from an ethnographic dataset (participant observations, interviews, field notes) of a cohort of 9 APRNs embedded full-time into 5 LTC settings over their first year of practice (overlapping with the first 3 ½ months of the COVID-19 pandemic) were integrated to glean lessons about LTC and APRN characteristics associated with successful APRN transition and utilization during stable and emergent care. Guided by a complexity science sensitizing framework focused on emergent and holistic systems, findings lead to multiple conclusions. The ability to influence positive outcomes in routine and emergent situations must be modeled. APRNs require meaningful mentoring relationships and acceptance by administrators and practitioners to develop successful TTP. Successful TTP included their embrace of role as resident caretaker, their participation in facility quality improvement, and their positive impact in an emergency response. Utilization and transition were enabled by facilities with organized characteristics versus inhibited by chaotic ones, which was exacerbated by the pandemic. Personal role fulfillment drove many APRNs to push through adversity. Administrators must address and honor concerns and attempts at self-care during times of doubt.

### EXPLORING INTERSECTIONS OF NURSING HOME CULTURE, IMPROVEMENT, AND DOCUMENTATION-RELATED ATTITUDES

Hannah Baker, *Concordia University Wisconsin, West Bend, Wisconsin, United States*

Documentation of patient care and characteristics is an important part of nursing home (NH) operations, affecting financial aspects, quality improvement (QI) efforts, inter-provider communication, and potential for medical errors. However, relationships among NH culture, QI, and documentation-related attitudes are unclear. This study explored Wisconsin NH staff and administrator perspectives on these variables (n = 10 in 8 NHs), using interviews structured on a composite of Schein's organizational culture typology and Shortell's Quality Improvement Implementation Survey concepts. Interview questions related to perspectives and emotions about NHs' documentation quality, OC, QI efforts, and nursing staff turnover. Verbatim transcription, member checks, and team-based transcript coding and analysis ensured good data quality. Thematic analysis was used to construct a "storyline" and refine the original theoretical model. Sample data fit the model well, supporting the perception of pairwise relationships between NH OC, QI, and documentation. One of Schein's culture types was not represented in the sample. Attitudes and perceived influences on documentation-related quality were mostly negative. This research contributes to knowledge on long-term care and could point future research toward (1) similar work in dissimilar NHs to replicate results, (2) closer examination of staffing variability, (3) quantitative work on effects of education and auditing on documentation-related attitudes, and (4) strategies to improve attitudes without increasing staff stress levels.

### SESSION 4020 (PAPER)

### LATE BREAKING: INNOVATIONS IN CLINICAL PRACTICE II

### MULTI-CASE STUDY OF A NOVEL HEALTHCARE DELIVERY MODEL TO SUPPORT AGING IN PLACE IN RURAL, REMOTE, AND SMALL URBAN AREAS

Wendy Hulko,[1] Noeman MIrza,[2] Kathy Rush,[3] Dawn Hemingway,[4] Sarah de Leeuw,[4] Katrina Plamondon,[3] Emily Bartle,[1] and Lilly Chisholm[1], *1. Thompson Rivers University, Kamloops, British Columbia, Canada, 2. University of Windsor, Windsor, Ontario, Canada, 3. University of British Columbia, Kelowna, British Columbia, Canada, 4. University of Northern British Columbia, Prince George, British Columbia, Canada*

Aging in place has long been a policy objective in Canadian healthcare, with accompanying concerns about older adults in rural and remote regions, and growing interest in small urban areas as distinct from large urban ones. As one of the six health authorities in British Columbia (BC), Interior Health (IH) developed and implemented an innovative healthcare delivery model to support aging in place for older adults, while addressing system issues of cost containment and resource allocation. By transferring funds from acute care to primary care, IH created Seniors Health and Wellness Centres (SHWCs) in two small urban areas (Kelowna and Kamloops) and a third one with two rural sites (Salmon Arm and Revelstoke). Our multi-case study aimed to compare how the SHWCs are meeting their objectives, and addressing the priorities of rural and small urban older adults and their social determinants of health (SDoH). We used mixed methods of data collection and analysis, including key informant interviews (n=9), service user questionnaires (n=10), document analysis (n=19), and secondary data analysis of service usage (n=2343) to answer research questions (RQs) on the outcomes and impacts of IH's restructuring. The results show the outcomes (RQ1) to be three SHWCs which vary considerably in their design and usage, including access, quality, and continuity of care. Our findings on the impacts (RQ2) indicate the SHWCs are meeting their cost reduction aims while concentrating on some SDoH (income & dis/ability) and neglecting others (gender, racism, rurality), and progressing slowly in addressing certain priorities of older adults.

### UNDERSTANDING OLDER ADULTS' ATTITUDES TOWARD MOBILE AND WEARABLE TECHNOLOGIES TO SUPPORT HEALTH AND COGNITION

Ibukun Fowe and Walter Boot, *Florida State University, Tallahassee, Florida, United States*

Technologies that facilitate remote patient monitoring, virtual care, and telehealth are becoming increasingly prevalent, accelerated due to the challenges providing healthcare during the COVID-19 pandemic and the need for more efficient and effective methods to care for the expanding older adult population. Despite the potential benefits of these technologies, their promise can be hampered by low rates of acceptance and adoption among older adults. We assessed older adults' (N = 92) attitudes towards the use of wearable and mobile technologies for 1) predicting cognitive decline, 2) assisting with adherence to health activities, and 3) collection of self-report data to understand current health status and predict future problems. Participants generally rated hypothetical technology solutions as useful ($M = 4.2$, $SD = 0.70$, 1 = Strongly Disagree, 5 = Strongly Agree), were interested in learning more about these technologies ($M = 3.81$, $SD = 0.86$), and indicated willingness to adopt these technologies themselves ($M = 3.83$, $SD = 0.93$); however, participants did indicate some privacy concerns ($M = 2.92$, $SD = 1.02$. General technology attitudes and previous experience using mobile devices were the best predictors of acceptance and willingness to adopt these technologies ($r = 0.50$, $p < .001$; $r = 0.22$, $p < .05$, respectively). These findings suggest generally positive attitudes and highlight potential targets for intervention to increase the uptake of these technologies, maximizing their benefits.

### CAN MONITORING OLDER ADULTS' TECHNOLOGY USE PATTERNS HELP DETECT COGNITIVE DECLINE?

Shelia Cotten, Reza Ghaiumy Anarsky, and Amy Schuster, *Clemson University, Clemson, South Carolina, United States*

Approximately six million U.S. older adults are diagnosed with Alzheimer's Disease (AD), and this number is expected to more than double by mid-century. Monitoring older adults' cognitive functioning is essential for the early detection of cognitive diseases, such as AD, which can increase treatment efficacy and slow AD's progression. Cognitive functioning tests, however, have limitations as administrating them can be costly and time-consuming. This study aimed to determine whether technology use patterns could be used as an indicator of cognitive functioning. We utilized six waves (from 2015 to 2020) of data from the National Health and Aging Trends Study to assess whether stopping the use of different types of technology (using the Internet, computers or tablets, or sending texts and emails) are associated with cognitive functioning (memory, orientation, and executive functioning). We controlled for age, gender, marital status, race, education, activities of daily living, and overall health. Our analyses showed that stopping the use of going online, computers, tablets, and sending texts and emails were related to cognitive decline. These results suggest that practitioners should incorporate assessments of their patient's online activity and use as it may be an innovative approach that is economical and efficient to identify individuals at risk of cognitive decline.

### TECHNOLOGY TO PROTECT HIPS IN SERIOUS FALLS - PRELIMINARY EVIDENCE

REBECCA TARBERT,[1] and Richard Stefanacci[2], *1. Active Protective Technologies, Inc, Allentown, Pennsylvania, United States, 2. Jefferson College of Population Health, Philadelphia, Rhode Island, United States*

Major injuries from falling in the older adult population is a tragic outcome that is growing in number. There is limited evidence of methodology to avoid major injuries from falls for those most at risk. Hip fractures are a catastrophic outcome from falls that incur cost to the individual, family and healthcare system. A study is currently underway in older adult living settings designed to demonstrate the efficacy and safety of a wearable smartbelt that deploys a protective airbag around the hips prior to landing on the ground as a mitigation for fall-related major hip injuries. The smartbelt technology includes embedded sensors and WiFi connection for data sharing. The primary endpoint of the study is to examine the efficacy and safety of the smartbelt in a population of older adults with high-risk profile of falls and major hip injuries. The study will be a comparative analysis of rate of major hip injuries from falls between a retrospective control group and a selected intervention group wearing the smartbelt for 6 months with all subjects continuing to receive standard of care for fall injury mitigation. Implementation methodology of clinical study performance in the care settings and integration into plan of care will be outlined. Outcomes from the enrolled subjects to date in the study will be shared including fall risk factors, fall events and injuries from falls reported.

### PERCEPTIONS OF OLDER ADULTS AND CAREGIVERS OF AN AUTOMATED ROBOT TO FACILITATE AGING IN PLACE

Donna Case,[1] Nathan Velazquez,[1] and Tatjana Savich[2], *1. University of Michigan Flint, Flint, Michigan, United States, 2. Quality Care Rehab & Autism Center, Shelby Township, Michigan, United States*

This study explored the perceptions of forty older adults 55+ and caregivers of the usefulness of the Labrador Retriever (https://labradorsystems.com/) in facilitating aging in place. Participants consisted of older adults who are enrolled in a PACE program or reside at The Village of Oakland Woods in southeastern Michigan as well as providers and board members of organizations that provide support to these older adults. Participants viewed an in-person demonstration of the Labrador Retriever before verbally completing a thirteen question survey with answers input by research team members. The questions were a combination of multiple choice questions such as "Having seen the Labrador demonstration, do you think the Labrador Retriever System could help you in your setting?" followed by open ended questions such as "Having seen the Labrador Retriever, how might this system help you in your setting?". A total of 9 qualitative themes related to the potential uses of the Labrador Retriever System were identified as: Medications, Aging in Place, Safety and Fall Prevention, Carrying Items (i.e. laundry, food, cleaning supplies), Hydration, Independence, Reducing Caregiver Burden, Nutrition, and Cognitive Issues. These themes are consistent with the barriers to aging in place identified in the literature.

### SESSION 4021 (PAPER)

### LATE BREAKING: MOTIVATING PATIENTS FOR HEALTH BEHAVIOR CHANGE I

### MOTIVATIONAL INTERVIEWING TO MODIFY SORTING AND DISCARDING BEHAVIORS IN HOARDING DISORDER

Caitlyn Nix and Mary Dozier, *Mississippi State University, Mississippi State, Mississippi, United States*

The act of sorting and discarding possessions is the fundamental health behavior that underlies clutter, a core symptom and signifier of hoarding disorder. In older adults, excessive clutter can cause hazardous conditions in the home that increase the risk of being unable to age in place safely. Current evidence-based treatments for hoarding require lengthy treatment dosages and primarily target fears surrounding sorting/discarding. Because not all older adults with hoarding disorder experience a fear-response when sorting/discarding, other targets for hoarding treatment must be evaluated in this population, including directly targeting motivation for behavior change. The current pilot study investigated motivation to sort and discard items as a treatment target for hoarding disorder in older adults. The rationale for this treatment study was that targeting motivation to engage in regular sorting/discarding of household clutter would result in decreased hoarding severity by creating a sustainable change in behavior. Fifteen participants (age 50 and up, M = 62) engaged in six in-home sessions of a novel intervention that combined intensive motivational interviewing with sorting practice (i.e., spending time engaging in the target behavior of sorting and discarding items in the home). This presentation will discuss the applications of motivational interviewing for hoarding disorder in older adults, including skills related to engaging, focusing, evoking, and planning. Our initial treatment outcomes suggest that motivation is a worthwhile treatment target for hoarding disorder.

### THE EFFECT OF OBESITY ON COVID-19 OUTCOMES AMONG HOSPITALIZED OLDER ADULTS

Mary Parianos,[1] Ali Vaeli Zadeh,[2] Ricardo Criado Carrero,[3] Claudia Marcano,[1] Andrew Crawford,[4] Elias Collado,[2] and Joshua Larned[4], *1. University of Miami, Fort Lauderdale, Florida, United States, 2. Holy Cross Health-Jim Moran Cardiovascular Research Institute, Fort Lauderdale, Florida, United States, 3. University of Miami/Holy Cross Hospital, Fort Lauderdale, Florida, United States, 4. Holy Cross Health-University of Miami, Fort Lauderdale, Florida, United States*

Background: According to the CDC, approximately 30% of hospitalizations for COVID-19 infection between the onset of the pandemic and November 2020 were attributed to obesity. However, there is limited data on how obesity affects the overall outcome of COVID-19 in hospitalized older adults. Methods: A retrospective study was conducted using the PearlDiver database (PearlDiver Technologies, Fort Wayne, IN). Using ICD-10 codes, a cohort of patients aged 65–75 and Elixhauser Comorbidity Index (ECI) >4 with a history of obesity admitted for COVID-19 was identified. This cohort was matched with a group of patients with no history of obesity, considering age, gender, and ECI. Records from both groups were reviewed for multiple outcomes over 30 days following admission. Pearson's chi-squared was used to compare groups. The strength of association was reported using Risk Ratios (RR). A p-value < 0.05 was deemed significant. Results: There were 151,429 members in each group. Obese individuals had a higher risk of 30-day all-cause readmission (RR=1.10, CI95% 1.07–1.11, p < 0.0001), ICU admission (RR=1.11, CI95% 1.08–1.15, p < 0.0001), acute thromboembolic events (RR=1.14, CI95% 1.07–1.2, p < 0.001), and deep venous thrombosis (RR=1.21, CI95% 1.12–1.32, p < 0.00001). There was no difference in length of hospitalization. Conclusion: Obesity is a modifiable risk factor that negatively affects COVID-19 outcomes in the older population. Given the prevalence of obesity in our population, primary and secondary obesity prevention is more important than ever.

### UNDERSTANDING MOTIVATIONS FOR PARTICIPATING IN EVIDENCE-BASED FALL PREVENTION PROGRAMS ON ZOOM

Andy Rapoport and Margaret Danilovich, *CJE SeniorLife, Chicago, Illinois, United States*

The pivot to remote delivery of NCOA evidence-based programs has increased participant reach due to eliminating geographic or transportation barriers. Despite the convenience of participation in one's own home, challenges for helping patients adhere to behavior change remain. This paper will present data from one community-based organization's NCOA evidence-based falls prevention program implementation of Bingocize, Tai Chi, Otago, and SAIL on Zoom. Across all programs, only 36% of people who registered for workshops attended the first session. Participants, on average, only attended 27% of the sessions in a given workshop. To explore the barriers and facilitators to attendance in order to encourage patients to change health behavior, we used a mixed-methods approach to evaluate reasons for suboptimal adherence among n=735 participants across the 4 programs. We performed sub-group analysis to examine barriers and facilitators by the self-reported attendance rate. We conducted semi-structured interviews with n=14 participants, focusing on those who had less than 50% attendance for the workshop they registered for, and also received n=234 survey responses. For participants who attended >50% of workshop sessions, class enjoyment was the most cited facilitator of attendance. However, for those who attended < 50% of workshop sessions, email reminders were the most cited attendance facilitator. Results from this project provide critical information about specific barriers that different sub-groups of older adults face regarding participation in NCOA evidence-based programs to develop strategies to help motivate patients for health behavior change.

### ENGAGING OLDER ADULTS IN HEALTH PROMOTION: PILOT STUDY OF TEAM GAMEPLAY OF AN EDUCATIONAL EXERGAME IN A SENIOR CENTER

Laurie Ruggiero,[1] Elizabeth Orsega-Smith,[1] Amy Nichols,[1] Joshua Varghese,[2] Nancy Getchell,[1] Rachel DeLauder,[1] Reza Koiler,[1] and Roghayeh Barmaki[1], *1. University of Delaware, Newark, Delaware, United States, 2. Wilmington University, Williamstown, New Jersey, United States*

Digital health games offer one innovative approach to engage older adults to support healthy aging. Multiple reviews have described the positive impact of health games. Limited research has examined multi-focus health games implemented in senior centers. Informed by healthy aging theory and community-engaged methods, our multi-disciplinary team developed/refined an educational exergame with a combined focus on educating about healthy lifestyle behaviors (i.e., physical activity, healthy eating), stimulating cognitive functioning, and engaging movement to support healthy aging. A pilot study (Nf13; mean age = 78, 100% female) examined team gameplay (4 sessions in two weeks) in a senior center. Teams (2–3 members) worked together to answer knowledge, trivia, and cognitive challenge questions and competed for the highest score. A post-gameplay survey asked about acceptability, usability (i.e., adapted System Usability Scale), and perceived game impact. Preliminary results suggest team gameplay was engaging and nearly all (>90%) agreed/strongly agreed that they enjoyed playing with others (i.e., on teams); were comfortable doing the physical movements during gameplay; were satisfied with game educational, trivia, and cognitive questions; enjoyed the social part of team gameplay; would recommend the game to others; and the game increased their knowledge and motivation regarding physical activity and healthy eating. The System Usability Scale was above 70, on average, suggesting above average usability for the game. Findings support use of this educational exergame as an innovative way to engage older adults in health promotion. Presentation will describe game development/refinement, senior center pilot, and implications for future research and senior center translation.

### DEPLOYING IN-HOME SENSORS TO COMMUNITY-DWELLING OLDER ADULTS: ADOPTION OF TECHNOLOGY AND LESSONS LEARNED

Erin Robinson, Gashaye Melaku Tefera, Geunhye Park, Marjorie Skubic, Noah Marchal, Mihail Popescu, James Keller, and Rantz Marilyn, *University of Missouri, Columbia, Missouri, United States*

In-home, sensor technologies can promote chronic disease self-management and independence among older adults. However, the translation of these technologies from assistive living/long-term care to community-dwelling older adults is lacking. This study aimed to tailor such technologies for private use by older adults, and gather feedback about adoption/interpretation of sensor-generated health information to make informed health decisions. Participants (Nf33; 72.7% female) aged 60+ (Mean=79.5) had three types of sensors installed: 1) depth sensors that track gait parameters and falls; 2) passive infrared motion sensors that track room activity; and 3) hydraulic bed mats that track sleep, respiration, and heart rate. Participants were also offered a Garmin smartwatch/fitness tracker. A health app was developed for participants to retrieve their sensor-generated health information via an Amazon Echo Show device with touch display and voice-activated capabilities, and a web-based interface. Data were collected over two years, and feedback was solicited during four quarterly interviews and one exit interview. Thematic analysis revealed participants used the Echo Show to retrieve their health information, set medication/health appointment reminders, and for non-health related purposes (e.g. music, weather). Regular retrieval of health information was low, partly due to technology skills, lack of interest, health issues, and technical/internet issues. Despite this, participants reported a sense of security in having the sensors installed and valued the depth sensor's fall detection abilities. This study yielded many additional findings that will be presented, such as participant recommendations to facilitate greater adoption. Implications can inform technological solutions for older adult health self-management and aging-in-place.

### SESSION 4022 (PAPER)

### LATE BREAKING: APPLYING REFRAMING AGING IN YOUR WORK I

### THE RELATIONSHIP BETWEEN CHILDHOOD ADVERSITY AND MENTAL HEALTH IN OLDER ADULTS

James Lian, Kaarin Anstey, and Kim Kiely, *University of New South Wales, Sydney, New South Wales, Australia*

Background: There is a need to understand how early adversity is linked with mental health in older adults. The aim of this study was to 1) explore the optimal way to operationalise a scale of adverse childhood experiences (ACEs) and 2) examine the association between ACEs with depression and anxiety in older adulthood. Methods: Data were from Wave 1 of the Personality and Total Health (PATH) Through Life Project (N = 7485, 51% women). Older adults aged 60–65 reported their childhood experiences of domestic adversity on a 17-item scale (e.g., physical abuse, neglect, poverty). Depression and anxiety were assessed using four validated screening instruments (GDS, GAS, MCS-12, PHQ-9). Three approaches to scoring the ACE scale were compared: i) cumulative risk, ii) factor analysis, and iii) latent class analysis (LCA). Confirmatory factor analysis (CFA) of the dimensional model of adversity and psychopathology was examined. Linear regression models estimated associations between ACEs and mental health, adjusting for age, race, education, and gender. Results: Childhood adversity was associated with late life depression and anxiety using the cumulative risk approach. CFA examined latent factors of threat and deprivation in our dataset, which were highly correlated, leading to problems with multicollinearity when estimating associations. Finally, LCA revealed six classes of ACEs: high adversity, low adversity, low affection, authoritarian upbringing, high parental dysfunction, and moderate parental dysfunction. High adversity and high parental dysfunction were associated with higher levels of depression and anxiety symptoms compared to other latent classes.

### TRENDS IN MENTAL ILLNESS ACROSS ASSISTED LIVING SETTINGS: RESIDENT AND COMMUNITY FACTORS

Bryant Carlson,[1] Sarah Dys,[2] Ozcan Tunalilar,[2] and Paula Carder[2], *1. Oregon Health & Science University - Portland State University School of Public Health, Portland, Oregon, United States, 2. Portland State University, Portland, Oregon, United States*

Although rates of serious mental illness (SMI), depression, and anxiety are becoming more common among assisted living (AL) and residential care (RC) residents, few studies have investigated resident and community-level factors to ensure that the settings and resources are adequate to meet residents' mental health needs. Using a representative sample of Oregon AL/RC residents (n = 1,013), this study uses descriptive cross-sectional analysis to examine intrastate variation in the prevalence of SMI, depression, and anxiety related to: (1) resident-level characteristics (e.g., mental illness comorbidity, psychotropic medication use, and Medicaid status); (2) community-level characteristics (e.g., facility type and urban/rural geographic designation); and (3) health services utilization (e.g., emergency room visits and hospital admission). Results indicate that 12% of AL/RC residents had an SMI diagnosis, nearly half of whom also had depression and anxiety, and 80% were Medicaid eligible. One in six residents with SMI received at least three psychotropic medications in the last week. Residents with SMI were also more likely to live in RC facilities than AL facilities, in facilities with Medicaid contracts, and in urban rather than rural settings. Compared to residents without SMI, a larger share of residents with SMI utilized hospital emergency rooms (32% vs. 18%) and were admitted to the hospital overnight (15% vs. 9%). Findings underscore characteristics associated with potentially higher needs among AL/RC residents with SMI compared to their counterparts, with implications for the high quality provision of mental health services and quality of life and care in these settings.

### INTERGENERATIONAL CONNECTIONS: TURNING POINTS IN COLLEGE STUDENT ATTITUDES ABOUT OLDER ADULTS AND AGING

Wendy Watson,[1] Sandra Faulkner,[1] Madison Pollino,[1] Jaclyn Shatterly,[2] and Charlie Stelle[1], *1. Bowling Green State University, Bowling Green, Ohio, United States, 2. University of Alabama, Tuscaloosa, Alabama, United States*

Intergenerational Connections was a semester-long community-based engagement project in which thirty-four undergraduate students enrolled in a relational communication course met via Zoom with older adults in a long-term care facility for four dialogues about close relationships. The project used a Critical Interpersonal and Family Communication Pedagogy approach to have students critically engage with stereotypes and expectations about older adults through class readings, discussion, and interaction. The students wrote reflections after each dialogue and a final reflection expressing their expectations and experiences of interacting with an older adult. We analyzed the reflections using a turning point analysis and found two turning points related to Perceptions of Aging and Developmental Changes and Understanding and Expectations of Relationships. Students recognized stereotypes they held about older adults and aging and how engaging with an older adult dispelled many of those assumptions. Students were surprised by how much they had in common with their older adult partner. They learned about relationships through their dialogues with their partner and found many "words of wisdom" they wanted to incorporate into their relationships. In their final papers, students reflected on being advocates for older adults and how this project helped them understand that they can have conversations with older adults and not be afraid that they will not be able to connect. This paper will discuss how this community engagement project served to reframe aging for these young adults, and students' recognition of the role that older adults play and the value that their involvement brings to society.

### UNCOVERING THE POSITIVE ASPECTS OF CAREGIVING: A PROFILE OF CAREGIVERS' DEMOGRAPHIC AND CARE CONTEXTS

Yeonjung Lee,[1] and Alex Bierman[2], *1. Chung-Ang University, Seoul, Seoul-t'ukpyolsi, Republic of Korea, 2. University of Calgary, Calgary, Alberta, Canada*

Burden and benefits of caregiving experiences can coexist. The objective of this research is to describe and compare the predictors of the two intertwined caregiving experiences. This study examines how the variations in caregiving experiences can be explained in terms of both positive and negative aspects of caregiving, respectively by demographic characteristics and care related contexts. The Caregiving, Aging, and Financial Experiences study is a national survey intended to examine social conditions and well-being among a representative sample of 4,010 Canadians between age 65 and 85. Within the sample, 1,641 informal caregivers are the focus of the current analysis. Scales of positive and negative caregiving experiences are employed. Findings based on principal axis factor analysis shows that there is clear separate factor loadings between the positive and negative caregiving experiences. Subsequent seemingly unrelated regression analysis shows that there are similarities as well as differences in predictors between the two caregiving experiences. Lastly, the variance explained differs markedly between the two measures, with over 26% of the variance in negative caregiving accounted for by demographic and caregiving factors, but less than 4% of the variance in positive caregiving. This study demonstrates that positive aspects of caregiving is not simply the flip-side of negative caregiving. Standard predictors do not sufficiently explain positive caregiving as well as negative caregiving. Consequently, greater attention to factors that account for positive aspects of caregiving is warranted for an inclusive understanding of caregiving experiences.

### LOVE OR OBLIGATION TO "SEE THEM THROUGH:" BURDENS AND GAINS IN CAREGIVING FOR CENTENARIANS

Eric Ngai Yin Shum,[1] Bobo Hi Po Lau,[1] Peter Martin,[2] Joey Chung Yue Siu,[3] Karen Siu Lan Cheung,[4] Cecilia Lai Wan Chan,[4] Grace Man Yee Chan,[5] and James Ka Hay Luk[6], *1. Hong Kong Shue Yan University, Hong Kong, Hong Kong, 2. Iowa State University, Ames, Iowa, United States, 3. Caritas Institute of Higher Education, Hong Kong, Hong Kong, Hong Kong, 4. The University of Hong Kong, Hong Kong, Hong Kong, 5. The Hong Kong Council of Service, Hong Kong, Hong Kong, 6. TWGHs Fung Yiu King Hospital (FYKH), Hospital Authority, Hong Kong, Hong Kong*

In Hong Kong, the population of centenarians increased from about 3,000 in 2011 to over 10,000 in 2021. The growth of this population has led to challenges concerning how far family caregivers, who are usually older adults themselves, could care for their spouse or parents. In 2021, we launched the 2nd Hong Kong Centenarian Study and included the voices of family caregivers. Notwithstanding the increased difficulties of caregiving during COVID outbreaks, our interviews with 120 caregivers revealed low to moderate scores of caregiving burden and gains (measured by 4-items from the Zarit Burden Scale and 5-items from the Positive Aspects of Caregiving Scale). Female and older (aged 70 or above) caregivers reported more emotional distress, burden, and poorer self-rated health, while younger caregivers (less than 70 years old) sustained a wider social network. Financial stress was related to smaller social network size and more emotional distress. When being asked what sustained their motivation to care for their spouse or for their parents, "filial obligation to see them through" and "repaying for love" were answered as key motivators. Caregivers also derived pride and satisfaction from contributing to the remarkable longevity of their loved ones or from witnessing their loved ones recovering from life-threatening traumas (e.g., falls, hospitalization), but felt helpless when faced with escalating care needs due to their own deteriorating physical health and capacities. "Double-old caregiving" will become more common, and society will need to overhaul the care system to support these motivated families who have escalated care needs.

### SESSION 4023 (PAPER)

### LATE BREAKING: COMMUNITY-BASED PARTICIPATORY RESEARCH APPROACHES II

### AI AND AGING: IDENTIFYING IMPORTANT LIVING ACTIVITIES FOR HEALTHY AGING IN SINGAPORE LONGITUDINAL AGING COHORT

Xin Zhong,[1] Feng Yang,[2] Wangyang Hu,[3] Xinyi Gwee,[3] Denise CHUA Qian Ling,[3] and Tze Pin Ng[3], *1. A*STAR Singapore, Singapore, Singapore, 2. Institute of High Performance Computing, Singapore, Singapore, 3. National University of Singapore, Singapore, Singapore*

Population aging in Singapore has encouraged the development of ambient smart communities to support healthy aging. Poor understanding of the living activities that address both clinical and biological concerns may plague efficient aging service designs and community health programs. We have designed a new computational workflow to identify important living activities for older adults in Singapore. We investigated innovatively three pillars of human life aspects: activities, clinical health, and biological health to identify living activities that are significantly associated with both clinical health and biological health. Cross-sectional data analyses were performed on 1356 community-living Chinese older adults of 65–80 years old in the Singapore Longitudinal Aging Study II (SLASII) cohort. 7 out of 29 living activities were found significantly associated with clinically healthy aging and showed improved prediction accuracy towards health status in machining learning schemes. Furthermore, biological age has been computed by screening and modeling 66 biomarkers. 15 out of the 29 living activities were found significantly associated with biologically healthy aging. Checking the overlapping living activities, we have found that physical exercise and cognitive-simulating activities are the most important activities for healthy aging: such as jogging regularly and reading, writing, and doing puzzles often. We regroup participants into active and non-active groups according to these two activities. The Keplan-Meier survival analysis showed statistically significant differences in survival time between the active and non-active groups ($p < 0.001$) in an 8-year longitudinal study. The workflow, results and biomarkers may provide references for future health program design improvement.

### NEEDS FOR SUPPORTING PEOPLE AGING WITH HIV: RESULTS FROM A CBPR COMMUNITY-NEEDS ASSESSMENT

Andy Rapoport, Rick Guasco, Teresa Musolino, Melanie Reese, Kenneth Teasley, and Margaret Danilovich, *CJE SeniorLife, Chicago, Illinois, United States*

Nearly half of people living with HIV in the U.S. are over age 50 and are navigating age-related changes while managing the complexities and challenges of HIV. People aging with HIV are an emerging community—it is imperative to understand the key issues faced by this community so to improve health outcomes and eliminate health disparities. This paper presents a CBPR approach by the SHARE board (a community advisory group comprised of older people living with HIV) and academic researchers. SHARE members conducted a mixed-methods needs assessment using a semi-structured interview guide they had developed, as well as a survey to determine community priorities for research, healthcare, and social services for people aging with HIV. SHARE members conducted 32 semi-structured interviews with individuals from across the country. From the 216 survey responses (mean age 55 years), 35% were Black, 30% were Hispanic, 50% were male, and 43% had been living with HIV for 11–15 years. 44% were fearful that long-term HIV medications will adversely affect them with aging. Triangulating interviews and surveys, financial strains and caregiving were primary age-related concerns. Affording medication and transportation were primary barriers to accessing healthcare. Most respondents had experienced stigma due to both age and HIV status. Respondents felt the most important areas for future research were optimizing management of multiple co-morbidities including HIV, and cognitive changes with HIV. Results from this community-led needs assessment identified key areas for interventions and research to address conditions disproportionately affecting the health of those aging with HIV.

### OVERCOMING OLDER ADULT RESISTANCE TO TECHNOLOGY TO SUPPORT DIGITAL HEALTH LITERACY: A CBPR APPROACH

Paul Freddolino,[1] Fei Sun,[1] Ha-Neul Kim,[1] Dona Wishart,[2] Eileen Godek,[3] and Megan Bentley[1], *1. Michigan State University, East Lansing, Michigan, United States, 2. Administration, Otsego, Michigan, United States, 3. Otsego County Commission on Aging, Otsego, Michigan, United States*

Overall low digital use by older adults creates a barrier to tools that enable them access to basic and specialty healthcare through telehealth and address their isolation and loneliness. In particular, older adults with low-income, like low-income isolated Home Delivered Meal (HDM) program recipients, can be most vulnerable to this barrier. A university team and a local Commission on Aging jointly secured funding to pilot the Virtual Table (VT) model which builds on relationships between HDM drivers and meal recipients to overcome lack of interest and skepticism of technology like the internet and video chatting. For the pilot, 25 recipients were recruited to learn basic technology skills (e.g., email, video chat) from trusted peer tutors through weekly one-to-one sessions, in-person or by video, leading to knowledge and skills to support telehealth utilization. Twenty recipients (80%) are completing the pilot. Results to date show increased technology use and comfort, together with confidence to use telehealth. Participants valued the interactions with tutors as well as what they learned. Feedback from tutors, agency staff, and participants has been used to refine the pilot during implementation. Building on the lessons learned and feedback, the goal for the next stage of collaboration is developing a set of effective tools to implement VT in several diverse communities. Four VT 'graduates' will be actively involved as co-producers of these revised tools. In addition, older adult home-delivered-meal (HDM) recipients, agency staff, tutors, and other older adult volunteers will directly participate in ongoing evaluation of the new resources.

### INTERPROFESSIONAL COLLABORATIVE RESEARCH ASSESSING OLDER ADULT TECHNOLOGY USE (OR NONUSE)

Donna Case, Sheryl Groden, MIchelle Sahli, and Wendy Tremaine, *University of Michigan-Flint, Flint, Michigan, United States*

This interprofessional study conducted by faculty in Occupational Therapy, Social Work and Public Health explored older adult technology use (or nonuse) by 216 Genesee County Michigan residents. Three professional lenses informed survey development, implementation and data analysis. Occupational therapy emphasized accessibility, how physical or cognitive impairments hinder technology use, and role of technology to complete daily living activities. Social work focused on technology use to facilitate social connectivity, decrease risks for mental health problems, and resource access. Public health explored if technology use or nonuse impacts health. Results indicated that 14% of participants want to learn to obtain transportation (social, health) via the internet, 13% stated they wanted to learn how to use technology to access medical records, 12.5% to attend on-line appointments, 11% reported they would engage in additional technology-based social activities (e.g. communicating with family/friends), 10% to order/refill medications, and 9% of older adults surveyed had difficulty accessing technology due to a physical, cognitive or sensory impairment. Researchers found the questions provided an integrated view of factors influencing older adult use or nonuse of technology and provided a guide for designing collaborative interventions to facilitate older adult access and use of technology to result in positive holistic health outcomes.

### SESSION 4681 (PAPER)

### LATE BREAKING: COMMUNITY-BASED PARTICIPATORY RESEARCH APPROACHES III

### USING HUMAN CENTERED DESIGN TO DEVELOP TWO NEW MEASURES OF LIVING WELL WITH DEMENTIA

Sheila Molony,[1] Sam Fazio,[2] Sheryl Zimmerman,[3] Ricci Sanchez,[2] Joe Montminy,[2] Cindy Barrere,[1] Rachel Montesano,[1] and Kimberly Van Haitsma[4], *1. Quinnipiac University, Hamden, Connecticut, United States, 2. Alzheimer's Association, Chicago, Illinois, United States, 3. University of North Carolina, Chapel Hill, North Carolina, United States, 4. Pennsylvania State University, University Park, Pennsylvania, United States*

Background: New measures are needed that can provide a holistic approach to capturing salient outcomes contributing to quality of life in dementia. Purpose: To use human centered design to develop, prototype and evaluate new positive psychosocial measures for persons living with dementia Methods: This project used a conceptual framework of human centered design to obtain and analyze data from multiple sources, including individual and focus group interviews, observations in the field and related methods to enhance empathy for the lived experience of dementia, and to design prototypes for two new measures. Results: Qualitative analysis of interview data, integration of data from the theoretical and empirical literature and human centered design techniques including brainstorming and ideation were used to define constructs and create prototypes for a new Living Well with Dementia Inventory and Good Day-Bad Day assessment tool. Results of qualitative analysis, item development, and initial validity assessment will be presented. Conclusion: Human centered design is a useful method for development of person-centered measures. This method may complement traditional methods of instrument development and CBPR.

### IT'S A FAMILY AFFAIR: COMMUNITY-BASED RESEARCH PARTNERSHIP TO CREATE A FAMILY-CENTERED DEMENTIA CAREGIVER PROGRAM

Rachel O'Conor,[1] Dianne Oladejo,[1] Sarah Filec,[1] Janice Knox,[2] Angel Griffin,[2] Joyce Chapman,[3] and Tonya Roberson[4], *1. Northwestern University, Chicago, Illinois, United States, 2. Helping Communities Help Themselves, Chicago, Illinois, United States, 3. Far South Chicago Coalition, Chicago, Illinois, United States, 4. Governors State University, Chicago, Illinois, United States*

People in the Black community are two times more likely to develop dementia than white people. This racial disparity is due to both social and structural factors which shape access to power, resources and exposure to health-damaging conditions by race. Due to the significant cognitive changes that occur, Black families assume substantial caregiving responsibilities to support family members, yet few caregiving programs offer culturally tailored support and training to the preferences and familial care norms of Black caregivers. In response, individuals from two community-based organizations (CBOs) and an academic medical center partnered to develop a culturally tailored, community-based, family-centered dementia caregiving research program for individuals living in far south Chicago. A team of stakeholders identified by the CBOs convened in the Fall 2021 to discuss the need for the proposed program, and together a key informant interview guide was designed. We have conducted 16 of 30 interviews and preliminary analyses of the interviews has revealed that caregivers are largely caring for parents or grandparents and are sharing caregiving responsibilities with other family members. Caregivers endorsed a need for the program; preferred a hybrid format and the need to include content related to 1) education on dementia, 2) emotional coping, and 3) linkage to medical and community-based resources. Anticipated barriers to participation included, time, location, and care for their family member while they participated. Our stakeholder team will continue to conduct key informant interviews, review and interpret data findings, and collectively develop a community-based family-centered caregiving program.

### AFRICAN-AMERICAN ALZHEIMER'S CAREGIVER TRAINING AND SUPPORT PROJECT 2 PILOT STUDY: OUTCOMES ANALYSIS

Robert Glueckauf,[1] Nik Lampe,[2] Michelle Kazmer,[3] Alexandra Nowakowski,[4] Yuxia Wang,[1] Naomi Thelusma,[1]

Dominique Williams,[1] and Cordy McGill-Scarlett[1], *1. Florida State University College of Medicine, Tallahassee, Florida, United States, 2. Vanderbilt University, Nashville, Tennessee, United States, 3. Florida State University, Tallahassee, Florida, United States, 4. Florida State University College of Medicine - Orlando Regional Campus, Orlando, Florida, United States*

The purpose of this study was to conduct an initial evaluation of the quantitative and qualitative outcomes of the African-American Alzheimer's Caregiver Training and Support Project 2 (ACTS2). Quantitative objectives focused on assessing changes in caregiver depression and health status, as well as the severity of caregiving and self-care problems from pre- to post-intervention. Secondary quantitative analyses examined post-treatment changes in social support and caregiver burden. Qualitative objectives included examining caregivers' perceptions of the effectiveness of in-session training activities, quality of relationships among participants and their facilitator, and appraisals of spiritual elements of the program. Nine African American family caregivers of older adults with dementia completed the ACTS2 lay pastoral care facilitator-led, telephone cognitive-behavioral intervention. The twelve-week training program included 7 skills-building groups and 5 individual problem-solving sessions. Significant improvements were found on the majority of dependent measures, including caregiver depression, health status, problem severity, and social support. Qualitative analysis highlighted the value caregivers placed on relationships with their co-participants and group facilitators, the role of spirituality within the program, and the importance of goal setting in improving caregiver distress and self-care. Convergence was found between quantitative and qualitative findings, particularly improvements in the domains of caregiver distress, health status, and social support. Overall, the findings of the pilot study were promising. Replication using a randomized controlled design with a larger sample size is needed to test the reliability of the findings. Benefits of tailoring intervention to caregivers' sociocultural preferences and spiritual values are also addressed.

### REMOTELY MEASURED IN-HOME DISTANCE FROM CARE RECIPIENT PREDICTS DEMENTIA CAREGIVERS' LONELINESS

Kuan-Hua Chen,[1] Yuxuan Chen,[2] Darius Tran Levan,[1] Claire Yee,[1] Jennifer Merrilees,[3] Julian A. Scheffer,[1] Samson Chen,[4] and Robert W. Levenson[1], *1. University of California, Berkeley, Berkeley, California, United States, 2. Stanford University, Stanford, California, United States, 3. University of California, San Francisco, San Francisco, California, United States, 4. Tracmo, Inc., Taipei, Taipei, Taiwan (Republic of China)*

Caregivers (CGs) of a family member with dementia often experience increased loneliness, which can result from reduced social interactions and increased physical distancing associated with care recipient's (CR) disease progression. Through a university-industry collaboration, we developed new technology for measuring physical distance remotely. CGs and CRs wore watches that monitored their location in the home using low-energy Bluetooth technology that enabled long battery life (up to four months). Using measures of proximity of the watches to three plug-in Bluetooth receivers located in the home, we assessed CG-CR physical distance on a second-by-second basis over a six-month period. Participants were 27 CRs diagnosed with dementia or mild cognitive impairment and their co-residing familial CGs. CG loneliness was measured by questionnaire at the beginning and end of the study. Both watches and receivers were remotely installed (by CGs). Results indicated that CG-CR physical distance increased from the first three months to the last three months of the study ($t = 20.67$, $p < 0.001$). In addition, greater increases in CG-CR physical distance over the six-month period were associated with greater increases in CG loneliness ($r = 0.46$, $p = 0.03$). These results advance our understanding of how increases in physical distancing between CGs and CRs contribute to increased CG loneliness (a well-established risk factor for depression and other mental health symptoms). The study also underscores the value of remote technologies that allow for in-home long-term monitoring for research on interpersonal distance and other social behaviors in CG-CR and other aging dyads.

### SESSION 4682 (PAPER)

### LATE BREAKING: MOTIVATING PATIENTS FOR HEALTH BEHAVIOR CHANGE II

### DEVELOPMENT AND APPLICATION THE QUESTIONNAIRE FOR MEDICAL CHECKUP OF OLD-OLD (QMCOO) - TAIWAN VERSION

Jui-Ying Hung, *Chaoyang University of Technology, The National Center for Geriatrics and Gerontology, Wufeng, Taichung, Taiwan (Republic of China)*

In 2022, population over the age of 65 is currently about 3.8 million (accounting for about 16% of the total population) as an "aged society" in Taiwan. According to the report about the frailty of the elderly increases with age, especially 75 - 79 years old, are the most obvious functional decline (The Ministry of Health and Welfare, 2022). This trend that also happened in 2005, the interim report of the 3rd national health promotion measures showed an increased prevalence of diabetes mellitus and obesity in Japan. In response to this, Dr. Satake and Dr. Arai (2020) develop the questionnaire for Medical Checkup of Old-Old (QMCOO) suited measure frailty to people aged ≧75 years better and composed of 15 questions regarding the following 10 domains: health condition, mental health, eating behavior, oral function, body weight loss, physical function and falls, cognition function, smoking, social participation and social support implemented in April 2020. This study authorized by Dr. Satake et, al in August 2022, aims to translate the QMCOO in Chinese version and through expert discussions to confirm that there are no difference between the original version in Japanese/English, and the apply query specification for QMCOO in Taiwan. This study recruited 300 older adults in Taichung city to answer all the questions in QMCOO and analysis the diagnostic frailty distributed status compared to the data measure in the Kihon Checklist in the same time. This finding the cutoff scores 2/3 for the QMCOO might be effective in diagnosing frailty in Taiwan.

### LONGITUDINAL ASSOCIATIONS BETWEEN FALLS AND DEPRESSIVE SYMPTOMS AMONG CHINESE OLDER ADULTS

Nie Zuoting,[1] Eunjin Lee Tracy,[2] Yan Du,[3] Sha Li,[1] and Rumei Yang[1], *1. Nanjing Medical University School of Nursing, Nanjing, Jiangsu, China (People's Republic), 2. University of Pittsburgh School of Medicine, Pittsburgh, Pennsylvania, United States, 3. UT Health San Antonio, San Antonio, Texas, United States*

Falls and depressive symptoms are prevalent and costly to Chinese older adults. Although falls and depressive symptoms are frequently interrelated, previous studies were mainly based on cross-sectional data, and the nature of interrelationships between them were not well understood. In this study, we aimed to examine the longitudinal bidirectional relationship between falls and depressive symptoms among Chinese older adults, and whether this bidirectional relationship is different for males and females, given that females are found to suffer from falls and depressive symptoms significantly more than males. Older adults aged 60 years+ who completed all three waves of data from the China Health and Retirement Longitudinal Study on falls and depressive symptoms between 2011 (wave 1), 2013 (wave 2), and 2015 (wave 3) were included in the current analysis (Nf2,203). Data were analyzed using random intercept multilevel models that adjusted for demographic information (e.g., age, education, and marital status). Overall, there is a relatively stable rates of comorbid falls and depression (about 10%) over time among Chinese older adults. Significant bidirectional associations at between-person and within-person levels were observed between depressive symptoms and falls over time, with greater depressive symptoms associated with higher risk for falls and vice versa. However, such associations were not different between males and females. Taken together, these findings indicate the importance of addressing both depression and falls at the same time to prevent falls in the clinical practice for both males and females.

### MOTIVATING CHANGE IN OLDER ADULTS: MOTIVATIONAL CIGARETTE SMOKING CESSATION MESSAGE TESTING

Adrienne Johnson, and Jesse Kaye, *University of Wisconsin School of Medicine and Public Health - Center for Tobacco Research and Intervention, Madison, Wisconsin, United States*

Cigarette smoking remains the leading preventable cause of death and disability in the U.S., resulting in ~480,000 deaths annually. Older adults who smoke bear a disproportionate weight of the health consequences of smoking, including cancer, mortality, and the greatest health-related fear of older adults: dementia. Compared to younger adults, older adults who smoke are half as likely to make a quit attempt, but more likely to stay quit using evidence-based treatments. Research suggests the increased risk of dementia among people who currently smoke may motivate adults ages >50 to quit smoking, particularly if given a clear/actionable strategy. Research also suggests Fear-based messages may perform differently than Hope-based messages. 820 adults (ages 50–80) without dementia who smoke, completed an online survey evaluating time-matched messages (randomly assigned between-subjects: Control Nf266, Fear of dementia Nf274, Hope from quitting Nf280) on motivation and intentions to quit smoking. Participants' demographics were Mage=61.1 years (SD=7.4), 48.0% cisgender women, 66.6% White, 23.3% Black. Mann-Whitney U Tests were use to examine change scores for each variable due to non-normal distributions. Compared to control message (water ad), the Fear message showed greater increase in motivation to quit U(Ncontrol=266, Nfear=274)=30391, z=-3.33, p=.001. The Hope message did not differ from the control or Fear message (p's>.05). Intention to quit did not differ between messages (p's>.05). A Fear-based message highlighting that smoking increases the risk of developing dementia, motivated quitting more than a control message. Future work should examine the feasibility, acceptability, and behavioral impact of this motivational message in healthcare settings.

### THE IMPACTS OF LIFESTYLE ON DEPRESSION AND LIFE SATISFACTION AMONG CHINESE OLDER ADULTS: A 7-YEAR FOLLOW-UP STUDY

Man-Man Peng, *Beijing Normal University, Zhuhai, Guangdong, China (People's Republic)*

This study aims to explore the long-term effects of lifestyle-related factors and physical health on life satisfaction among older adults by transitions in mental health conditions. Using data derived from the China Health and Retirement Longitudinal Study (CHARLS), the analytic sample included 643 older adults. Linear regression analyses were used to examine the cross-sectional and longitudinal associations of lifestyle-related factors and physical health with depression risk and life satisfaction in older adults. In this study, sleep duration and multimorbidity was found to be significantly related to baseline and follow-up depressive symptoms in older adults. Compared to non-drinkers, current drinkers reported more severe depressive symptoms. More depressive symptoms were associated with worse impairment in physical function or Activities of Daily Living (IADLs). Among older adults remaining no depressive symptoms at baseline and follow-up, current drinkers tended to have lower life satisfaction than non-drinker. Shorter sleep duration showed a longitudinal correlation with lower life satisfaction. In the subgroups of emerging depression, past drinkers tended to have lower life satisfaction than non-drinkers, and baseline multimorbidity significantly predicted lower subsequent life satisfaction. In conclusion, our findings identified drinking and shorter sleep duration as the lifestyle-related detrimental factors of late-life depression and life satisfaction in Chinese community-dwelling older adults. Other physical-health-related risk factors of depression included worse impairment in physical function or IADLs, and multimorbidity. Our findings have implications for future psychosocial interventions targeted at alleviating depressive symptoms and promoting life satisfaction of the older adults based on their long-term mental and physical health conditions.

### SESSION 4683 (PAPER)

### LATE BREAKING: INNOVATIONS IN CLINICAL PRACTICE III

### VALIDATION OF GLIM CRITERIA ON MALNUTRITION IN CHINESE OLDER INPATIENTS

Tong Ji,[1] Yun Li,[2] Pan Liu,[2] Yaxin Zhang,[2] Yu Song,[2] and Lina Ma[2], *1. Capital Medical University Xuanwu Hospital, Beijing, Beijing, China (People's Republic), 2. Xuanwu Hospital Capital Medical University, Beijing, Beijing, China (People's Republic)* Backgrounds: Malnutrition is a nutritional disorder that has a high incidence and can lead to multiple poor prognoses, such as frailty. Early identification and correct evaluation of malnutrition are essential for improving clinical outcomes. Objective: Therefore, we aimed to explore the applicability and effectiveness of the Global Leadership Initiative on Malnutrition (GLIM) criterion for identifying undernutrition in older inpatients. Methods: In total, 223 participants aged ≥60 years were involved. Nutrition was evaluateded employing MNA-FF and GLIM criteria, which adopts a two-step procedure. The first step was to use three different methods for nutritional risk screening: NRS-2002, MNA-SF, and MUST. The second step is to diagnose undernutrition. The Clinical Frailty Scale was used to assess frailty. Sensitivity, specificity, Youden index, kappa values, and positive and negative predictive values were measured to test the validity of the GLIM criteria. Logistic regression models were used to assess whether there was a correlation between malnutrition and frailty. Results: We found that 32.3–49.8% of inpatients were at risk of malnutrition based on the GLIM diagnosis using the three different screening tools, 19.3–27.8% of patients were malnourished. The GLIM criteria with MNA-SF as the diagnostic validation and MNA-FF as a reference showed high consistency (k = 0.629; p < 0.001), sensitivity (90.5%), and specificity (86.4%). Logistic regression analysis showed that malnutrition, using MNA-SF with the GLIM criteria, was relevant to higher likelihood of frailty (OR=1.887; 95% CI: 1.184–2.589). Conclusions: The GLIM criteria with the MNA-SF may be a more reliable malnutrition assessment process in elderly inpatients.

### EFFECT OF CHANGE IN BODY SIZE ON MORTALITY IN THE INITIALLY HEALTHY COMMUNITY-DWELLING OLDER ADULTS

Sultana Monira Hussain,[1] Anne Newman,[2] Lawrence Beilin,[3] Andrew Tonkin,[1] Robyn Woods,[1] Johannes Neumann,[4] Mark Nelson,[5] and John McNeil[1], *1. Monash University, Melbourne, Victoria, Australia, 2. University of Pittsburgh, Pittsburgh, Pennsylvania, United States, 3. The University of Western Australia, Perth, Western Australia, Australia, 4. University Heart & Vascular Center Hamburg, Hamburg, Hamburg, Germany, 5. Menzies Research Institute, University of Tasmania, Hobart, Tasmania, Australia*

Utilising data from the ASPirin in Reducing Events in the Elderly trial participants [aged >70-years (Australians), >65-years (US)], we estimated percent change in body size (BMI: body mass index, WC: waist circumference) from baseline and the second annual visits. Both BMI and WC changes were categorised as 1) change within 5% (stable), 2) decrease by 5–10%, 3) decrease by >10%, 4) increase by 5–10%, and 5) increase by >10%. Mortality events were classified according to the underlying cause by adjudicators. Hazard ratios were calculated to compare mortality between body size change categories. Amongst 16,773 participants, 1,269 mortality were observed over an average 4.4±1.7 years. In men compared to stable BMI, a 5–10% decrease in BMI had a 35% higher (HR 1.35, 95% CI 1.08–1.67), and a >10% decrease in BMI had a 3.84-fold higher (HR 2.99, 95% CI 2.44–3.68) risk in all-cause mortality. A decrease in BMI was associated with a higher cause-specific (cancer, cardiovascular disease [CVD], and non-cancer non-CVD) mortality. A decrease in BMI was associated with higher mortality risk in women, however, the magnitude of association was weaker than men. A decrease in WC was predictive of mortality but with a much weaker relationship than a decrease in BMI. Weight gain was not predictive of mortality except for non-cancer non-CVD mortality in women. Physicians should be aware of the ominous consequence of weight loss, especially amongst older men. The risk extends beyond an increased risk of cancer, extending to CVD and a range of other life-limiting conditions.

### REVISITING ELECTRONIC FRAILTY INDEX BY ADDING A LAB-BASED MARKER OF NUTRITION: A HEART FAILURE COHORT

Seulgi Kim,[1] Ariela Orkaby,[2] Catherine Park,[3] Molly Horstman,[3] Salim Virani,[1] Orna Intrator,[4] Aanand Naik,[3] and Javad Razjouyan[3], *1. Baylor College of Medicine, Houston, Texas, United States, 2. VA Boston, Boston, Massachusetts, United States, 3. Veterans Affairs, Houston, Texas, United States, 4. Veterans Health Administration, Rochester, New York, United States*

Malnutrition is associated with worse prognosis and increased risk for adverse outcomes in patients hospitalized with heart failure (HF). The objective is to assess the utility of adding the Prognostic Nutritional Index (PNI), a validated measure of nutritional status, to the Veterans Health Administration frailty index (VA-FI) in predicting time to death in patients hospitalized with HF. We conducted a retrospective cohort study of veterans age ≥50 years hospitalized with HF as their primary diagnosis. PNI was calculated using lab values in the year prior to hospitalization with the following equation: 10 x serum albumin (g/dL) + 0.005 x total lymphocyte count (mm3). VA-FI identified five groups: robust (≤0.1), prefrail (0.1–0.2), frail (0.2–0.3), moderately frail (0.3–0.4), and severely frail (>0.4). PNI was added to VA-FI (VA-FI-Nutrition) using the same cutoffs. We identified changes in frailty status using VA-FI versus VA-FI-Nutrition by summarizing the count by each class and reported the hazard ratio (HR) for all-cause mortality in each VA-FI category based on the new VA-FI-Nutrition groups. VA-FI-Nutrition identified patients within each VA-FI class that belong to the next frailty strata: robust (20.2%), prefrail (18.3%), frail (16.7%) and moderately frail (16.7%). We observed higher mortality rates among those whose frailty class changed based on VA-FI-Nutrition compared to VA-FI: robust (HR, 1.65, 95%CI:1.38, 1.97), prefrail (HR, 1.52, 95%CI: 1.41, 1.65), frail (HR, 1.42, 95%CI: 1.33, 1.52), and moderately frail (HR, 1.33, 95%CI: 1.24, 1.43). Adding PNI to VA-FI provides a more accurate mortality assessment and may be utilized to triage high-risk patients.

### SESSION 4684 (PAPER)

### LATE BREAKING: APPLYING REFRAMING AGING IN YOUR WORK II

### A THEORETICAL EXAMINATION INTO THE CONNECTIONS BETWEEN LINKED LIVES AND LATER LIFE DISABILITIES

Akwasi Adjei Gyimah, and Samuel Van Vleet, *Miami University, Oxford, Ohio, United States*

As people age, they find themselves living similar lives to those around them. This concept is often referred to as linked lives. Linked lives explains that individual lives are often interdependent. This is due to various elements such as social and historical influences. These influences can imprint networks of shared relationships. Researchers have found that the first point of socialization as humans are social relationships with family and friends. However, while the connections of linked lives are often attributed to early life course trajectories, little research has evaluated the interdependency that is created with disabilities in later life. Life course disability research is a key factor of gerontology because a majority of older adults encounter disabilities within later life. This attributed disability can often forge bound and interdependence within people and their own social networks. This paper theoretically examines different perspectives of disabilities research and how it relates to the fundamental principles of linked lives. This paper highlights the gap in the current gerontological understanding of the later life links among older adults with disabilities. This theory paper provides the needed information for gerontologist focusing on older people with disabilities to be able to evaluate allocations needed for providing adequate social support and services tailored towards the needs of these interdependent lives.

### THE DETERMINANTS OF LIFE SATISFACTION AMONG MONGOLIAN OLDER ADULTS

Saranchuluun Otgon,[1] Khorolsuren Lkhagvasuren,[1] Yerkebulan Mukhtar,[1] Anujin Baatar,[2] Casati Fabio,[3] Agnieszka Czajka,[4] and Sugarmaa Myagmarjav[1], *1. Mongolian National University of Medical Sciences, Ulaanbaatar, Ulaanbaatar, Mongolia, 2. The International University of Ulaanbaatar, Ulaanbaatar, Ulaanbaatar, Mongolia, 3. University of Trento, Trentino-Alto Adige, Italy, 4. Polish-Japanese Academy of Information Technology, Warsaw, Kujawsko-Pomorskie, Poland*

Background: As the average life expectancy and the proportion of the elderly population increases, approaches that limit their policies, programs, and activities to health issues are changing worldwide. International organizations, such as the WHO, United Nations, have pledged their member countries to update their policies on the elderly population and increase their psychological and social participation over the next 20 years. We determined social demographic factors of life satisfaction among Mongolian older adults. Methods: A cross-sectional study recruited three hundred four older adults recruited by geriatric doctors and seniors' association units in urban and rural areas of Mongolia. The questionnaire included life satisfaction, social network, loneliness, social participation, self-rated health, and demographics. We used multiple linear regression analyses. Result: Life Satisfaction of the older adults living in urban is higher than those living in rural area ($\beta = 0.954$, $p = 0.001$). Good self-rate of health ($\beta = 1.013$ $p = 0.001$), voluntary work ($\beta = 0.847$ $p = 0.001$), and employment increase life satisfaction levels. Life satisfaction scores increased by 0.3 points for male older adults with a 1-point increase in education, 0.9 points for women living in cities ($\beta = 0.929$ $p = 0.002$), and 0.8 points for volunteering ($\beta$=0.790 p=0.006) respectively. However, when the loneliness score increases by one, the satisfaction score decreases by 0.3 points ($\beta = -0.353$ $p = 0.013$). Conclusions: The level of satisfaction of the Mongolian older adults is associated with self-rate of health, education level, loneliness, social activities, and living areas.

### THE QUALITY IN QUALITATIVE: AN EXAMINATION OF RETIREMENT INTERVIEWS

Michelle Silver, *University of Toronto, Toronto, Ontario, Canada*

Qualitative interviews are a dynamic and complex way to understand life experiences. Retirement is an ever-evolving, dynamic, and complex social construct we associate with the end of one's career. Exploring what retirement means to different people in the context of critical gerontological theories of successful aging can contribute to a better understanding of the implications of this important transition at the individual and societal level. However, sifting through participants stories is not always a straightforward endeavor, particularly in the case when participants have complex and dynamic stories. This paper examines the value of qualitative research methods in unpacking complex personal narratives. As the landscape surrounding mature workers' experiences continues to change, this paper extends policy debates about retirement, as well as scholarly conversations about the richness and complexity of qualitative research.

### FEELING VS BEING CONNECTED: DIFFERENTIATING LONELINESS AND ISOLATION IN NEAR- AND CENTENARIANS

Lian Ying Chun Pat,[1] Bobo Hi Po Lau,[1] Peter Martin,[2] Joey Chung Yue Siu,[3] Karen Siu Lan Cheung,[4] Cecilia Lai Wan Chan,[4] Grace Man Yee Chan,[5] and James Ka Hay Luk[6], *1. Hong Kong Shue Yan University, Hong Kong, Hong Kong, 2. Iowa State University, Ames, Iowa, United States, 3. Caritas Institute of Higher Education, Hong Kong, Hong Kong, Hong Kong, 4. The University of Hong Kong, Hong Kong, Hong Kong, 5. The Hong Kong Council of Service, Hong Kong, Hong Kong, 6. TWGHs Fung Yiu King Hospital (FYKH), Hospital Authority, Hong Kong, Hong Kong*

While gerotranscendence theories postulate that older adults tend to orient themselves toward solitude, activity theories highlight the importance of continuing social and meaningful engagement for well-being across lifespan. The distinction between loneliness and social isolation is particularly observable in older adults of advanced age who are often facing accelerated decline in physical and functional health, therefore restricting their opportunities to interact with others.

This has been particularly disturbing during the previous two years under COVID. This study utilized data from the 2nd Hong Kong Centenarian Study which interviewed 120 family caregivers of older adults aged 95 or above in 2021–2022 when the city experienced almost an entire year of the outbreak. Using family or friend proxy information as well as caregiver ratings of whether older adults expressed feelings of social isolation and loneliness, we found that 10.7% of older adults reported high levels of loneliness and isolation; 26.7% feeling low in both; 11.5% were isolated but not lonely, and 38.2% were lonely but not isolated. Loneliness ratings were more strongly associated with psychological well-being (Patient Health Questionnaire-4), autonomy, happiness, perceived usefulness, worries, and death anxiety than did isolation, with the latter negatively correlated with optimism. Participants rated in the low isolation/loneliness group were least (death) anxious than the other three groups. Our findings underscore the divergence of isolation and loneliness for adults of advanced age and call for psychological support for oldest-old adults who continue to face social isolation, especially when society gradually recovers from COVID.

### COMPARING SUCCESSFUL AGING OF NEAR-CENTENARIANS AND CENTENARIANS: FINDINGS FROM 2011 AND 2021/22

Eric Ngai Yin Shum,[1] Bobo Hi Po Lau,[1] Karen Siu Lan Cheung,[2] Grace Man Yee Chan,[3] Joseph Shiu Kwong Kwan,[4] James Ka Hay Luk,[5] Peter Martin,[6] and Cecilia Lai Wan Chan[2], *1. Hong Kong Shue Yan University, Hong Kong, Hong Kong, 2. The University of Hong Kong, Hong Kong, Hong Kong, 3. The Hong Kong Council of Service, Hong Kong, Hong Kong, 4. Imperial College London, London, England, United Kingdom, 5. TWGHs Fung Yiu King Hospital (FYKH), Hospital Authority, Hong Kong, Hong Kong, 6. Iowa State University, Ames, Iowa, United States*

Successful aging (SA) was proposed by Robert J Havighurst in 1961 to capture how older adults add “lives onto (their) years.” While there is a consensus regarding the multidimensionality of the concept, the set of criteria that should be applied to older adults of advanced age remain controversial. Notwithstanding their inevitable decline in physical health, adults of advanced age may still enjoy good psychosocial well-being. In this light, we compared the proportion of “successful agers” in two cohorts of adults aged 95 or above who lived with their families in 2011 (Nf77) and 2021/22 (Nf120) in Hong Kong using two models – Model A: i: Good subjective health, ii: more well-off than average, iii: as happy as young (Cho et al., 2012) and Model B: i: Weekly social activities, ii: absence of dementia, iii: intact sight and hearing ability, iv: intact mobility (Nosraty et al, 2012). Both models have been applied in adults aged 90 or above. In the 2011 cohort, 13.0% and 16.9% of our sample fulfilled the SA criteria of Model A & B respectively. The percentages fell to 1.7% and 13.7% respectively in the 2021/22 cohort. The decrease is due to less participants fulfilling the financial criterion of Model A, as well as the criteria on intact sight and hearing ability and the absence of dementia of Model B. COVID presents multidimensional challenges for adults of advanced age. Examining the dimensions that are most impacted will help orient recovery works along the direction of SA.

### SESSION 5082 (PAPER)

### LATE BREAKING: COMMUNITY-BASED PARTICIPATORY RESEARCH APPROACHES IV

### A CLINICAL TRIAL USING METHYLATION AGE TO EVALUATE CURRENT ANTIAGING PRACTICES

Josh Mitteldorf, *DataBETA Project, Philadelphia, Pennsylvania, United States*

Recent advances in the technology of “aging clocks” based on DNA methylation suggest that it may be possible to measure changes in the rate of human aging over periods as short as a year or two. To the extent that methylation (and other biomarkers) are valid surrogates for biological age, the testing of antiaging interventions has thus become radically cheaper, faster, and more practical. Together with colleagues at McGill University, I have initiated a clinical trial to evaluate some of the most popular antiaging strategies currently deployed by “early adopters” in the lay community of personal health activists. We are recruiting 5000 subjects, age 45–65, and interviewing them in detail about their diet, drugs and supplements, exercise, social, and other practices that plausibly contribute to modulate the rate of aging. They agree to submit saliva samples for analysis of methylation age at the beginning and end of a 2-year test period. Primary endpoint is the difference in methylation age over the course of 2 years. Results will be viewed as an exploratory study to identify synergistic combinations of age-retarding treatments. All data (redacted for privacy) will be open sourced, available to the scientific community and to the public.

### CHANGES IN GENERAL SELF-EFFICACY FOLLOWING THE WITS WELLNESS PROGRAM: PRELIMINARY FINDINGS FROM THE 12-WEEK RCT

Neha Gothe,[1] Molly Hofer,[2] Chelsey Byers,[3] Laura Payne,[1] and Julie Bobitt[4], *1. University of Illinois at Urbana Champaign, Urbana, Illinois, United States, 2. University of Illinois Extension, Chicago, Illinois, United States, 3. University of Illinois Extension, Champaign, Illinois, United States, 4. University of Illinois at Chicago, Chicago, Illinois, United States*

Self-efficacy among older adults has been associated with better sleep, increased vitality, decreased pain and discomfort as well as overall satisfaction with life. Interventions designed to increase general self-efficacy can therefore have wide-ranging effects on individual’s health as well as reduce the burden on healthcare systems. In this abstract we present preliminary data examining the impact of Wits Wellness on general self-efficacy among participants. Middle aged and older adults, Nf285 (Mean age 65.58 yrs, males=28) were randomized to either a Wits Wellness group or a Waitlist Control for 12-weeks. The 12-week wellness program addresses multiple factors including physical activity, stress, sleep, social isolation and diet with the goal of empowering older adults to make healthier lifestyle choices. At baseline and end of the 12-week trial, the 10-item PROMIS general self-efficacy scale was used to assess global self-efficacy which reflects a problem-solving approach despite perceived obstacles and challenges. Older participants aged 65 and above, who attended 8 or more weeks (Nf123, n=63 in Wits Wellness group) of the program demonstrated significant increases in their general self-efficacy. Results showed a significant group*time effect favoring the Wits Wellness intervention (F(1,120)=4.10, p=.045, partial eta2=.03). The intervention was most effective in boosting confidence among the older adults within the trial (>65 yrs.). These findings demonstrate preliminary efficacy of the Wits Wellness program and are significant as general self-efficacy is considered an important moderator of healthy adaptation to illness which is critical in old age. Funded by the Midwest Roybal Center grant P30AG022849.

### SESSION 5083 (PAPER)

### LATE BREAKING: INNOVATIONS IN CLINICAL PRACTICE IV

### AN OLDER ADULT FRACTURE PROGRAM EMPLOYING AGE-FRIENDLY CARE CAN BE IMPLEMENTED AT YOUR HOSPITAL AND IMPROVE OUTCOMES

Kathleen Breda, Michelle Keller, Hiroshi Gotanda, Karma McKelvey, Carol Lin, and Sonja Rosen, *Cedars-Sinai Medical Center, Los Angeles, California, United States*

Older adult fractures are expected to increase dramatically over the next several decades, with hip fractures reaching an estimated 6.26 million worldwide by 2050. A combined approach to the care of older adult fracture patients was first reported in the early 1990s, emphasizing geriatric and orthopedic co-management. Early studies showed significant improvements in complications, function, and discharge rates to nursing homes. Subsequent studies demonstrated shorter time-to-surgery, lengths of stay (LOS), lower readmission rates, reduced in-hospital mortality, and lower cost of care. The studies are from large academic centers whose physicians are employed faculty or have dedicated geriatric units. Less studied is whether similar models could be implemented in community health systems/mixed practice settings like Cedars-Sinai. Cedars-Sinai's older adult fracture program integrates inpatient and outpatient care and uses best practices of the 4Ms Framework of an Age-Friendly Health System, including What Matters Most, Mentation, Medications, and Mobility. The program was designed through the consensus of interprofessional champions and empirical evidence. Results showed enrolled patients with all fracture types had significantly lower LOS (marginal effect [ME]: -2.12, 95%CI: -2.61, -1.63), LOS index (ME: -0.33, 95%CI: -0.42, -0.25), and total direct costs (ME: -$5316.4, 95%CI: -6806.31, -3826.5). There was no significant difference in time-to-surgery. Of the 746 enrolled patients, 170 (23%) had a post-discharge visit with a participating geriatrician within 6 months. Findings indicate a systematic approach to improving care for older adults with fractures improves inpatient outcomes in a mixed practice setting. Future studies will examine the effectiveness of Cedar-Sinai's outpatient program.

### PROFILE OF OKLAHOMA CLIENTS REFERRED TO ADULT PROTECTIVE SERVICES FOR ALLEGED SELF-NEGLECT

Samantha Tuft, Farida Ejaz, Miriam Rose, and Courtney Reynolds, *Benjamin Rose Institute on Aging, Cleveland, Ohio, United States*

Self-neglect is the most commonly reported allegation of abuse to Adult Protective Service (APS) agencies nationwide. Researchers collaborated with Oklahoma APS to understand characteristics of allegedly self-neglecting clients who were reported to APS (focus of this paper), and to subsequently refer a random sub-sample to home-and community-based services and track outcomes. Clients (n = 188) were interviewed by phone; data were collected on demographic and background characteristics, physical and mental health, social support, social isolation, and the impact of COVID-19 on them. Most clients were white, 18% were American Indian, and 14% were African American. Their ages ranged from 18–92 years, 63% were women, most were low-income ($12,000-$15,000 annually), and 60% lived alone. Many used public benefits such as Medicare (75%), Social Security, and Medicaid. Based on PHQ scores, about a third had moderate to severe symptoms of depression; and 27% scored similarly on anxiety. More than a third saw family and friends less often during the pandemic. APS administrative data was also obtained on these clients and preliminary analyses indicated that the 188 study participants had a total of 205 allegations of self-neglect prior to the study, with 76% of the allegations needing a referral for community services. During the intervention phase, however, only 31 clients had any type of abuse allegations with 24 being for self-neglect. About half needed community services. It is possible that services they were referred to prior and during the study was related to the decline in self-neglect allegations during the study period.

### PLASMA HIGH DENSITY LIPOPROTEIN CHOLESTEROL AND RISK OF FRACTURES IN OLDER ADULTS

Sultana Monira Hussain,[1] Peter Ebeling,[1] Anna Barker,[1] Lawrence Beilin,[2] Andrew Tonkin,[1] and John McNeil[1], *1. Monash University, Melbourne, Victoria, Australia, 2. The University of Western Australia, Perth, Western Australia, Australia*

The ASPirin in Reducing Events in the Elderly trial participants (ASPREE, aged >70-years Australians), for whom high-density lipoprotein cholesterol (HDL-C) levels were measured were included. Fractures included were confirmed by medical imaging and included both traumatic and pathological fractures. Fractures were confirmed by an expert review panel. Cox proportional hazards regression was used to calculate hazard ratio (HR) and 95% confidence interval (CI) for associations with fractures. Of the 16262 participants who had a plasma HDL-C measurement at baseline 1,659 experienced at least one episode of fracture over a median of 3.98 years (interquartile range, 0.02, 7.0 years). In the fully adjusted model, each mmol/L increment in HDL-C was associated with a 34% (HR 1.34, 95% CI 1.20–1.50) higher risk of fractures. The results remained similar when these analyses were stratified by sex. Sensitivity analyses demonstrated that these associations persisted when the analyses were repeated including only: 1) nontraumatic fractures, 2) participants not on osteoporotic medications, 3) participants who were never-smokers and reported that they did not drink alcohol, and 4) participants who walked outside less than 30 minutes and reported no participation in moderate/vigorous physical activity. No association was observed between non-HDL-C and fractures. This prospective observational study suggests that higher levels of HDL-C are associated with higher fracture risk. This association was independent of the common risk factors of fractures.

### POTENTIALLY INAPPROPRIATE MEDICATION USE INCREASES RISK OF INCIDENT DISABILITY IN HEALTHY OLDER ADULTS

Jessica Lockery,[1] Taya Collyer,[2] Robyn Woods,[2] Suzanne Orchard,[2] Anne Murray,[3] and Michael Ernst[4], *1. RMIT University, Melbourne, Victoria, Australia, 2. Monash University, Melbourne, Victoria, Australia, 3. Hennepin Healthcare, Minneapolis, Minnesota, United States, 4. The University of Iowa, Iowa City, Iowa, United States*

Background: Efforts to minimize medication risks among older adults include avoidance of potentially inappropriate medications (PIMs). However, most PIMs research has focussed on aged or inpatient care, creating an evidence gap for community-dwelling older adults. To address this, we investigated the impact of PIMs use in the ASPREE clinical trial. Methods: ASPREE enrolled 19,114 community-dwelling participants aged 70+ years (65+ if US minorities) without a history of major cardiovascular disease, cognitive impairment, and significant physical disability. PIMs was defined according to a modified 2019 AGS Beers Criteria. Cox proportional-hazards regression models were used to estimate the association between baseline PIMs exposure, and disability-free survival, death, disability, and hospitalization, with adjustment for comorbidities including frailty. Results: At baseline, 7396 (39% of total) participants were prescribed at least one PIM. Compared with those unexposed, participants on a PIM at baseline were at an increased risk of persistent physical disability (Adjusted HR 1.47, 95%CI 1.21, 1.80) and hospitalization (Adjusted HR 1.26, 95%CI 1.20, 1.32), but had similar rates of disability free survival and death. These effects did not vary by polypharmacy status. PIMs exposure was associated with higher risk of disability followed by hospitalization (Adjusted HR 1.92, 95%CI 1.25, 2.96) as well as vice versa (Adjusted HR 1.54, 95%CI 1.15, 2.05). Conclusions: PIMs exposure is associated with increased risk of incident disability and hospitalization. Increased risk of disability prior to hospitalization suggests that PIMs use may start the disability cascade, emphasizing the importance of caution when prescribing PIMs for community-dwelling older adults.

### A STEPPED WEDGE CLUSTER RANDOMIZED TRIAL OF THE STRIDE SUPERVISED WALKING PROGRAM IN VA HOSPITALS

Susan Hastings, Karen Stechuchak, Cynthia Coffman, Ashley Choate, Courtney Van Houtven, Virginia Wang, Cassie Meyer, and Caitlin Kappler, *Durham VA Health Care System, Durham, North Carolina, United States*

In trials, hospital walking programs improved functional ability after discharge, but little evidence exists on their effectiveness under routine practice conditions or their implementation across health systems. We conducted a Type III hybrid implementation-effectiveness, stepped-wedge cluster randomized trial (SW-CRT) in 8 Veterans Affairs hospitals examining a hospital-based walking program known as STRIDE. Based on the SW-CRT design, hospitals were randomized to a sequence (timeline) for STRIDE, and additionally randomized 1:1 to receive implementation support according to the Replicating Effective Programs (REP) framework only or REP plus additional team-based communication training known as CONNECT. The study was powered to examine impact of STRIDE on discharge destination to home vs other (primary outcome); hospital length of stay (LOS) was a secondary outcome. Patient-hospitalizations in pre-STRIDE time periods (n=8167) were similar to post-STRIDE time periods (n=9070) (e.g., mean age 73, 97% male, 28–30% Black race). In adjusted models, odds of discharge to home were higher among eligible patients hospitalized in post-STRIDE time periods (OR 1.6; 95% CI 1.3–2) compared to pre-STRIDE. Findings were robust to 3 sets of sensitivity analyses. There was no difference in LOS (IRR 1.01; 95% CI 0.94,1.09). Hospitals randomized to CONNECT had higher program reach (mean 13% vs 3%) but lower daily fidelity (mean 25.7% vs 37.5%). Despite limited direct program reach, implementation of a hospital walking program was associated with higher odds of discharge to home. Implementation strategies like CONNECT that enhance teamwork and communication may improve patient access to clinical programs being implemented in new settings.

### SESSION 5084 (PAPER)

### LATE BREAKING: APPLYING REFRAMING AGING IN YOUR WORK IV

### SURVIVAL OF THE FITTEST OR FRAILEST? COMPARING THE HEALTH OF CENTENARIANS OVER A 10-YEAR PERIOD

Lian Ying Chun Pat,[1] Bobo Hi Po Lau,[1] Karen Siu Lan Cheung,[2] Grace Man Yee Chan,[3] Joseph Shiu Kwong Kwan,[4] James Ka Hay Luk,[5] Peter Martin,[6] and Cecilia Lai Wan Chan[2], *1. Hong Kong Shue Yan University, Hong Kong, Hong Kong, 2. The University of Hong Kong, Hong Kong, Hong Kong, 3. The Hong Kong Council of Service, Hong Kong, Hong Kong, 4. Imperial College London, London, England, United Kingdom, 5. TWGHs Fung Yiu King Hospital (FYKH), Hospital Authority, Hong Kong, Hong Kong, 6. Iowa State University, Ames, Iowa, United States*

The first centenarian study in Hong Kong was conducted in 2011 and examined the multidimensional health of adults aged 95 or older. The 2011 study found that, among a population of about 3,000 centenarians, a significant proportion enjoyed a high degree of autonomy in their daily functions in relatively good health. The study has been repeated in 2021/22 (i.e., born in 1926 or before) when the city had more than 11,000 centenarians. Comparison of the two samples (2011: Nf77; 2021/22: Nf120) who lived with their family shows a significant difference in functional health, but not as much for physical health, favouring the 2011 cohort. More than 75% of the 2011 cohort demonstrated autonomy in activities of daily living (Bathing: 77.9%, dressing: 85.7%, toileting: 90.9%, indoor transfer: 89.6%; continence:75.3% and feeding: 94.8%). Only about half of the 2021/22 cohort were autonomous in these areas (40.0%, 44.3%, 54.7%, 42.5%, 63.2%, 46.7%, respectively). The number of chronic illnesses between the two cohorts were comparable (Mean(SD): 2011: 2.7 (1.6); 2021: 3.26 (1.60), yet dementia and frailty were more prevalent in the 2021 cohort (dementia: 44%; frailty: 9.1%) then the 2011 cohort (41.0%; 23.4%). Our findings alert metropolitans worldwide to the fast-increasing population of adults of advanced age with significant personal care and health needs in the community. Existing care for older adults has to be reframed and overhauled to provide comprehensive home- and personal-care support which will be essential for realizing ageing-in-place for adults in advanced age, especially after social distancing policies in COVID-19.

### FRAILTY INDEX AT THE BEGINNING OF ELDERHOOD AND HEALTHCARE COSTS AND UTILIZATION OVER 10 YEARS

Jieun Jang and Dae Kim, *Hebrew SeniorLife, Harvard Medical School, Boston, Massachusetts, United States*

Background: We assessed whether the frailty index measured at the beginning of elderhood can predict healthcare costs over 10 years in a nationwide Korean population. Methods: This retrospective cohort study included 215,887 individuals who underwent a standardized comprehensive geriatric assessment at the age of 66 years as part of the National Screening Program for Transitional Ages in 2007–2009 and participants were followed up until December 31, 2019, from the Korean National Health Insurance database. Frailty status was defined based on a 39-item frailty index: robust (< 0.15), pre-frail (0.15 to < 0.25), frail (≥0.25). Generalized linear model was used to examine any changes in healthcare cost among pre-frail group, frail group following 10 years from the age of 66 years, relative to changes in healthcare cost of the robust group. This study constructed an interaction term between the frail group and age. Results: Frailty status at age 66 years was associated with an increased annual total healthcare cost per NHI beneficiary (robust * age vs frail * age: $\beta$ = 89.5, SE = 4.0, P < .0001), annual inpatient healthcare cost per NHI beneficiary (robust * age vs frail * age: $\beta$ = 70.3, SE = 4.1, P < .0001) over 10 years, but not significant in annual outpatient cost per NHI beneficiary after adjusting for frailty category, demographic factor, socioeconomic factor, and time fixed effect. Conclusions: The frailty index at the age of 66 years was associated with an accelerated increase in healthcare costs over 10 years.

### A PSYCHOMETRICALLY ROBUST LONGITUDINAL RESEARCH MEASURE OF FRAILTY: FIVE DIMENSIONS ACROSS AGE AND TIME

Stacey Voll,[1] Graciela Muniz Terrera,[2] and Scott Hofer[1], *1. University of Victoria, Victoria, British Columbia, Canada, 2. Ohio University, Athens, Ohio, United States*

The research construct of frailty in aging requires a measure with sound psychometric properties, that is stable across longitudinal points of observation. Using Exploratory Factor Analysis and Longitudinal Mixed Methods, we developed a five-factor research measures of frailty that is robust across time. Standardized regression scores for each factor allow us to estimate the change in severity of dysfunction as individuals age. We propose a system for developing research tools for the concept of frailty in large longitudinal data sets, and present our findings of five factors of frailty for females and males from the English Longitudinal Study on Ageing.

### DEVELOPING A NOVEL FRAILTY INDEX TO STUDY FRAILTY OF SEXUAL AND GENDER MINORITY OLDER ADULTS IN THE ALL OF US DATABASE

Chelsea Wong,[1] Michael Wilczek,[2] Jordon Bosse,[3] Louisa Smith,[3] Justin Manjourides,[3] Ariela Orkaby,[4] and Brianne Olivieri-Mui[3], *1. Harvard Multicampus Geriatric Medicine Fellowship Program, Brookline, Massachusetts, United States, 2. Northeastern University, Gray, Maine, United States, 3. Northeastern University, Boston, Massachusetts, United States, 4. VA Boston, Boston, Massachusetts, United States*

Prevalence of frailty among older sexual and gender minority adults (OSGM) is unknown despite disparities in mental health, medical comorbidities, and physical function. The NIH-funded All of Us Program was launched May 2018 aiming to enroll 1 million US participants focusing on those underrepresented in biomedical research, including OSGM. Using validated methods, we developed an All of Us deficit accumulation frailty index (AoU-FI) consisting of 33-items using baseline survey responses of adults aged 50+. Deficit domains include comorbidities, physical functioning, mental health, cognition, and sensory impairment. AoU-FI was valid if ≤20% of items were missing and ≤70% were comorbidities. OSGM self-identified or had discordance between gender and sex responses. OSGM (n=5,678) and non-OSGM (n=66,325), were similar in age (mean (IQR) = 66.7 (60–73) vs 66.9 (60–74)) but were more diverse (White 78% vs 82%, Black 7.5% vs. 6.5%, Hispanic/Latino 6.9% vs. 5.9%). AoU-FI had an expected gamma distribution across groups. OSGM frailty had a narrower range (0–0.67 vs. 0–0.75) and higher mean of 0.19 (sd=0.11) vs 0.17 (sd=0.1) compared to non-OSGM. To our knowledge, this is the first study of frailty among OSGM. Findings suggest OSGM experience worse frailty, highlighting the need to understand disparities in frailty, identify interventions, and develop policies to support OSGM. Additionally, our novel AoU-FI creates opportunities to apply frailty to diverse participants and types of data from digital health to genomics within the All of Us database.

### REFRAMING STUDENT EXPERIENCES AND ATTITUDES TOWARDS WORKING WITH OLDER ADULTS

Iveris Martinez, *California State University, Long Beach, Long Beach, California, United States*

We are experiencing a shortage of trained health and social service providers to meet the needs of an aging society. However, few students have positive opportunities to work with older adults in their training. If they interact with older adults it is usually in end-of-life and nursing home care settings. We therefore need to find creative ways to motivate students in these fields to choose to work with older adults. We recruited students from health and social services programs to implement four health promotion projects at an older adult low-income residential community. We asked students (Nf22) to reflect on their experiences, and analyzed responses using a grounded theory approach. Myths regarding working with older adults included that they were mean, difficult, not technologically savvy, nor physically active. Initially nervous and uncertain about working with older adult prior to their experience, students gained confidence and had fun. They reported rewarding experiences, built relationships, and learned the benefits of prevention programs for older adults, and reconsidering their career trajectories to focus on working with older adults. Encouraging positive student experiences working with older adults can help prepare to develop the health and human services workforce for an aging society.

### SESSION 5171 (PAPER)

### LATE BREAKING: MOTIVATING PATIENTS FOR HEALTH BEHAVIOR CHANGE III

### YOU DON'T KNOW WHAT YOU DON'T KNOW: BLACK/LATINX CANCER SURVIVORS' KNOWLEDGE AND USE OF SURVIVORSHIP CARE PLAN

Candidus Nwakasi, Cristy Romero, Kateri Sinkler, and Abigail Pawlowicz, *Providence College, Providence, Rhode Island, United States*

Compared to Whites, Black and Latinx cancer survivors and their families experience disproportionate adverse effects of cancer and cancer therapy. This is due to extreme psychosocial, physical, emotional, and financial challenges they experience, thus, highlighting racial/ethnic disparity in cancer survivorship. A cancer survivorship care plan (SCP) is important for improving cancer health and quality of life, but the effectiveness of SCP as a tool to address the disproportionately persistent poor health and quality of life of Blacks/Latinx compared to Whites deserve critical attention. This study is part of a larger study on evaluating cancer survivorship in Rhode Island. We explored knowledge and use of SCP among Blacks and Latinx cancer survivors in Rhode Island. The study employed a qualitative descriptive method. Semi-structured interviews in English and Spanish were conducted with a purposive sample of 12 cancer survivors, 8 Latinx and 3 Blacks (Mage = 62 years). Their responses were transcribed and analyzed for themes. The 3 major themes identified were: 1) invisibility of survivors; 2) understanding the needs of cancer survivors; and 3) issues of empowerment. Our findings draw attention to the need for tailored interventions targeting Black/Latinx cancer survivors. One of such interventions include designing programs to increase the accessibility of SCP to help improve quality of life of cancer survivors from disadvantaged population groups.

### THE IMPACT OF A DIGITAL CANCER SURVIVORSHIP PATIENT ENGAGEMENT TOOLKIT ON OLDER CANCER SURVIVORS' HEALTH OUTCOMES

Eun-Shim Nahm,[1] Mary McQuaige,[2] Katarina Steacy,[2] Nancy Corbit,[2] Shijun Zhu,[1] and Hohyun Seong[1], *1. University of Maryland School of Nursing, Baltimore, Maryland, United States, 2. University of Maryland Marlene and Stewart Greenbaum Comprehensive Cancer Center, Baltimore, Maryland, United States*

Cancer is a disease that predominantly affects older adults. The median age at diagnosis is 66 years and 62% of the 15.5 million American cancer survivors are age ≥65 years. Provision of supportive care after treatment is critical to this group due to their complex care needs; however, limited resources are available to them. As increasing numbers of older survivors adopt technology, digital health programs have significant potential to help them improve their health and communicate with their providers. Previously, we developed/tested a digital Cancer Survivorship Patient Engagement Toolkit program for older adults, CaS-PET Silver. The aim was to examine the preliminary impact of CaS-PET Silver on older survivors' health outcomes. This was a 2-armed RCT with two observations (baseline, 8 weeks) on a sample of 60 survivors age ≥65 years (mean age, 70.1±3.8), who were treated with curative intent within 12 months from enrollment (02/2020-01/2022, COVID-19 pandemic). Outcomes included health-related quality of life (HRQoL), self-efficacy for coping with cancer, symptom burden, health behaviors, and patient-provider communication. Data were analyzed using descriptive statistics, linear mixed models, and content analysis. The majority of participants were black (68.3%, n=41) and female (56.6%, n=34). At 8 weeks, CaS-PET Silver group showed significantly improved physical HRQoL ($p < .001$, ES=0.64) and symptom burden (p=.053, ES= -0.41). Self-efficacy (ES=0.56), mental HRQoL (ES=0.26), and communication (ES=0.40) showed a tendency to improve. Most participants reported benefits from the program on health management (mean, 19.41±2.6 [3–21]). Further research is needed with larger, diverse older cancer populations.

### THE RELATION OF AGE TO OUTCOMES IN THE DIABETES LIFESTYLE CHANGE PROGRAM CONDUCTED BY VIRGINIA COOPERATIVE EXTENSION

Azin Pourkhalili,[1] Carlin Rafie,[2] Arash Sarshar,[1] and Lynn Margheim[1], *1. Virginia Tech, Blacksburg, Virginia, United States, 2. Virginia Tech/Virginia Cooperative Extension, Blacksburg, Virginia, United States*

The National Diabetes Prevention Program established by the Centers for Disease Control and Prevention promotes the implementation of an evidence-based lifestyle change program (LCP) to prevent or delay the onset of diabetes. The LCP is a 12-month program with 26 lessons covering topics on healthy diets, increasing physical activity, managing stress, and coping with triggers, among others. It includes weekly goal setting, food, and physical activity tracking, and group support. The goals of the program are 5–7% sustained weight loss and 150 minutes of physical activity weekly. Little is known about the real-world effectiveness of the LCP in different age groups, particularly in older adults. The aim of this study was to evaluate the effects of age on LCP outcomes (weight loss, average physical activity, program attendance) conducted by Virginia Cooperative Extension from 2017 - 2022. Among 191 participants enrolled in the

LCP, 141 (73%) completed eight or more sessions and 56% were above 60 years of age. Results show there was a significant direct relationship (coefficient=0.05, p=0.001) between age and weight loss percentage. Participants older than 60 had significantly (p=0.03) higher average physical activity (215 min per week) compared to those under 60 years old (161 min per week). In addition, participants above 60 had significantly (p=0.03) higher attendance (22 sessions) compared to those under 60 years of age (19 sessions). These findings suggest that targeting different age groups and intervention delivery methods can improve program outcomes.

### DIABETES MELLITUS AND HARD BRAKING EVENTS IN OLDER DRIVERS

Difei Liu,[1] Stanford Chihuri,[2] Thelma Mielenz,[2] Linda Hill,[3] Carolyn DiGuiseppi,[4] David Strogatz,[5] and Guohua Li[5], *1. Columbia University Medical Center, New York, New York, United States, 2. Columbia University, New York, New York, United States, 3. University of California San Diego, La Jolla, California, United States, 4. University of Colorado Anschutz Medical Campus, Aurora, Colorado, United States, 5. Bassett Research Institute, Cooperstown, New York, United States*

There are an estimated 37 million people with diabetes mellitus (DM) in the United States, including 16 million older adults. DM can impair patients' driving safety due to diabetic peripheral neuropathy, hypoglycemia, or hyperglycemia, and eye diseases. However, few studies have examined the association between DM and driving safety based on naturalistic driving data. Data for this study came from the Longitudinal Research on Aging Drivers (LongROAD) project, a multisite naturalistic driving study of 2990 drivers aged 65–79 years at baseline. Driving data for the study participants were recorded by in-vehicle recording devices for up to 44 months. We used multivariable negative binomial modeling to estimate the incidence rate ratios (IRRs) and 95% confidence intervals (CIs) of hard braking events (i.e., proxies for unsafe driving behavior defined as maneuvers with deceleration rates ≥ 0.4 g) associated with DM. Of the 2856 study participants eligible for this study, 482 (16.9%) reported having DM at baseline. The overall incidence rate of hard braking events was 1.16 per 1000 miles. Adjusted for age, race/ethnicity, marital status, education level, annual household income, urbanicity, history of stroke, and number of medications, drivers with DM had a 10% increased rate of hard braking events compared to drivers without DM (adjusted IRR 1.10; 95% CI: 1.08, 1.12). Results of this study indicates that DM is associated with a significantly increased rate of hard braking events in older drivers, suggesting less safe driving. Driving safety should be incorporated into DM management and care programs.

### SESSION 5172 (PAPER)

### LATE BREAKING: APPLYING REFRAMING AGING IN YOUR WORK V

### APPLYING REFRAMING AGING GUIDELINES WHEN REVIEWING FOR NON-AGING JOURNALS

Zainab Suntai, *Baylor University, Waco, Texas, United States*

The Reframing Aging initiative began in 2012 with the aim of changing the way in which the public views aging, and one of the key tenets of the initiative centers around language that perpetuates negative views of aging. Despite widespread knowledge about the initiative, recent publications in high-tier journals point to a gap in adopting the Reframing Aging initiatives outside of aging journals. Terms that project a negative view of older adults are still used in manuscript titles and within abstracts and bodies of research publications in non-aging journals. As researchers who publish aging-related work, members of organizations such as the Gerontological Society of America are often solicited as reviewers for their expertise in the field of aging. While many researchers in aging may review for aging-related journals, such expertise is often needed in non-aging journals. As such, it is critical for aging researchers to continue to advance the Reframing Aging initiative when conducting reviews of manuscripts that do not adhere to the guidelines. This presentation will provide explicit examples of such publications and review specific steps that reviewers can take in addressing the Reframing Aging initiative in future reviews.

### MEASURING AGE-FRIENDLINESS OF TRANSPORTATION, MOBILITY COMMUNITY CHARACTERISTICS: A SCOPING REVIEW

Kellia Hansmann,[1] David Deemer,[2] Carolyn McAndrews,[3] and Stephanie Robert[3], *1. William S Middleton Memorial Veterans Hospital, Madison, Wisconsin, United States, 2. University of Wisconsin School of Medicine and Public Health, Madison, Wisconsin, United States, 3. University of Wisconsin Madison, Madison, Wisconsin, United States*

Age-friendly community initiatives seek to identify and develop infrastructure and services that promote active aging. Inclusive characteristics of transportation and outdoor spaces can improve everyone's mobility around their communities but are particularly important to adults who are transitioning to non-driving. In this study, we aimed to identify and describe age-friendly community indicators that evaluate characteristics most associated with community-level mobility, specifically transportation and outdoor spaces. We conducted a scoping review of peer-reviewed and gray literature to find records describing the development of age-friendly community indicators and then to identify indices with measures of transportation and outdoor spaces resources. We searched PubMed, Scopus, Ebsco, and Web of Science in August 2022 for literature published since 2005. Our search resulted in 1,012 unique records; screening for relevance by title and abstract resulted in 33 records that received full text reviews. We identified 9 final indicators for inclusion and reviewed records to describe the source of the indices, development methodology, and specific indicators used to measure transportation and outdoor spaces. Included indicators were sponsored by governmental or non-profit public health organizations (n=4) and academic researchers (n=5), including one academic partnership with a for-profit insurance company. Most indices (n=6) described measures drawn from subjective data collected during community assessments. Age-friendly indicators were not developed specifically for investigating older adults' transition to non-driving, a missing focus that represents a significant gap. Aligning indicators with this common life transition will facilitate research into the interventions promoting mobility at the community level for all.

### AN AGE-FRIENDLY COURSE ADOPTING THE 4M GERIATRIC MODEL IN A PRIMARY CARE NURSE PRACTITIONER PROGRAM

Ophelia Empleo-Frazier,[1] Ami Marshall,[1] Margaret Doyle,[2] Andrea Rink,[3] Noelle Gallant,[4] Barry Wu,[3] and Richard Marottoli[2], *1. Yale University School of Nursing, Orange, Connecticut, United States, 2. Yale University, New Haven, Connecticut, United States, 3. Yale University School of Medicine, New Haven, Connecticut, United States, 4. Yale New Haven Health System, New Haven, Connecticut, United States*

Training nurse practitioners to assume an expanded role in management of geriatric patients is key to addressing the care needs of a growing older adult population. To that end, the Yale School of Nursing revised geriatric coursework required of family nurse practitioner (FNP) and adult geriatric nurse practitioners (AGNP) to ensure the curriculum was sufficiently in-depth, engaging, and inclusive. Revisions included a new course, "Advanced Primary Care of the Older Adult" which focuses on the role of the nurse practitioner in the assessment, diagnosis, and management of primary geriatric syndromes. The 4M model (Medications, Mentation, Mobility, and what Matters most) provided the framework. Additionally, faculty added expert speakers, interactive dementia animated videos, book and movie discussions and a geriatric telehealth simulation component. Content was guided by training needs data collected from prior nurse practitioner students. At the end of each course, students were surveyed to assess attitudes towards geriatric care and satisfaction with the course. We compared survey responses pre (academic year 2019–2020, Nf18, response rate=53%) and post (academic years 2020–2021 and 2021–2022, Nf28, response rate=44%) rollout. In multivariate regression analyses adjusted for differences in age, gender, race, program (AGNP and FNP) and previous geriatric experience, we found significant improvement in measures of both confidence in providing geriatric care and satisfaction with course content. Analysis of qualitative data confirmed student satisfaction with the new course. Future work will include expansion and more rigorous evaluation of the program, including measures of student knowledge of best practices and increasing survey participation rates.

### DEVELOPMENT OF OPEN EDUCATIONAL RESOURCES FOR AN AGING AND MENTAL HEALTH COURSE

Madison Ambrose, Jocelyn McGee, Sinai Wood, and Chris Zakrzewski, *Baylor University, Waco, Texas, United States*

The financial pressures associated with a gerontological education are significant given the rising costs associated with education. This fact is particularly relevant for economically vulnerable university students. One source of stress is the cost of textbooks and other required course materials. Indeed textbook prices have increased at a rate faster than other educational expenses. Additionally, the perspectives of diverse and underrepresented scholars are often not as readily available in some textbooks. In this presentation, our process of adapting an undergraduate Aging and Mental Health course to reduce cost and also showcase diverse perspectives is described in detail. The goals of this project were to: 1) adapt a preexisting course to include low- and no-cost content through the integration of open educational resources (OER); 2) provide the most up-to-date evidence based information for content and skills development in the area of aging and mental health; and 3) highlight the perspectives of diverse or traditionally underrepresented scholars in this field of study. We will also share the process of developing an interactive Pressbooks webbook from a strengths-based perspective wholistic perspective.

### AN AGE-FRIENDLY NURSING HOME PROJECT ECHO IN RURAL COMMUNITIES

Lee Jennings, Teri Round, Diana Sturdevant, and Keith Kleszynski, *University of Oklahoma Health Sciences Center, Oklahoma City, Oklahoma, United States*

We launched an Age-Friendly Nursing Home Project ECHO in October 2021 focused on the 4Ms (mobility, medications, mentation, what matters most) with emphasis on dementia and QI in long-term care. One-hour virtual sessions included a short expert presentation and case discussion, were offered twice weekly, and recordings were posted online. Topics included person-centered care, advance care planning, LGBTQIA+ care, fall prevention, medication reduction, dementia resident activities, oral health, skin integrity, staff retention, COVID-19, PDSA cycles, root cause analysis, team huddles, among others. 235 individuals from 80 nursing homes, 19 ALFs, 6 continuing care communities, and 10 VA-affiliated sites in 5 states (OK, AR, KS, MO, CO) participated in the first four 6-week ECHO series; 27% attended ≥2 series. Most attendees were nursing home administrators (46%), directors of nursing (20%), nursing assistants (12%), or activity directors (11%). Most were female (91%) and worked in rural settings (77%). 237 attended an additional COVID-19 update session. 152 participants (65%) completed an evaluation. 91% rated the program as valuable (score≥8/10); 99% would recommend to others; 57% discussed topics with colleagues; 59% reviewed materials after sessions; 25% implemented new QI processes; and 25% made a change to resident care. Suggestions for future sessions included: active shooter training, antibiotic stewardship, team building, staff-resident communication, dementia training for non-clinical staff, weight loss, capacity determination, and family caregiver support. An age-friendly nursing home Project ECHO was well-received by health professionals in long-term care. ECHO can successfully expand the reach of training in long-term care, especially in rural areas.

### SESSION 5173 (PAPER)

### LATE BREAKING: INNOVATIONS IN CLINICAL PRACTICE V

### YES, AND...ENHANCING DEMENTIA CARE COMPETENCY THROUGH IMPROVISATIONAL THEATRE TECHNIQUES

Candace Kemp,[1] Jennifer Craft Morgan,[1] Andrea Hill,[1] Emerald Pullon,[1] Elisabeth Burgess,[1] Molly Perkins,[2] and Alexis Bender[3], *1. Georgia State University, Atlanta, Georgia, United States, 2. Emory University School of Medicine, Atlanta, Georgia, United States, 3. Emory University, Atlanta, Georgia, United States*

As the number of persons living with dementia escalates globally, innovations in clinical practice are needed to promote care interactions that lead to positive outcomes for care recipients and their care partners, including family, friends, direct care workers, and other care providers. Our ongoing NIH-funded study, focused on meaningful engagement and quality of life, points to the potential benefits of training care partners to use improvisational (improv) theatre skills in the context of dementia care. We present analysis of data derived from two one-year-long waves of qualitative data collection involving persons with dementia (n=59), their formal and informal care partners (n=165), and participant observation (over 12,000 hours) in six diverse assisted living communities. We identified a minority of care partners who, relative to others, had greater success engaging residents and eliciting positive responses. They also experienced less frustration during care encounters. In interactions with persons with dementia, these individuals routinely extended and accepted invitations to communicate and demonstrated an ability to be in the moment and meet people where they were. They accepted uncertainty about interactions and recognized the need to be flexible. These approaches parallel key improv tenets, which are teachable. We argue that improv training for care partners would enhance care competency and led to positive outcomes for care recipients and providers. Findings contribute to a small, but growing innovative body of literature from a variety of disciplines, including health care, that demonstrates the benefits of improv training in a myriad scenarios and settings, including in dementia care.

### PREVALENCE, BURDEN, AND LOCATION OF CEREBRAL MICROINFARCTS AND THEIR ASSOCIATION WITH LATE-LIFE BLOOD PRESSURE

Mo-Kyung Sin,[1] Yan Cheng,[2] Ali Ahmed,[3] Jeffrey Roseman,[4] and Edward Zamrini[5], *1. Seattle University, Seattle, Washington, United States, 2. George Washington University, Washington, District of Columbia, United States, 3. Washington DC Veterans Medical Center, Washington DC, District of Columbia, United States, 4. University of Alabama at Birmingham, Birmingham, Alabama, United States, 5. Irvine Clinical Research, Irvine, California, United States*

Little is known about the relationship of late-life blood pressure (BP) profiles (level, trend and variability) with the presence, number and regional distribution (cortical vs subcortical) of cerebral microinfarcts. We examined these associations in older adults (age ≥65) using the Adult Changes in Thought (ACT) data. 551 participants (94.6% ≥80 years, 58.6% women, 94.2% white) had complete data on microinfarcts and 4 BP measures. Late-life BP, defined by the four BP values before death (2 year-time gap between measures), was treated using 3 different approaches: (1) categories (based on mean of 4 values); (2) trend (based on the difference between values 1 and 4); and (3) variability (based on standard deviation of the mean of the changes in 4 BP values from 1 to 4). Multivariable-adjusted logistic regression models were used to examine the associations of the late-life BP with microinfarcts, adjusting for relevant covariates. Each of the 5 systolic BP categories between 120 and 169 mmHg (reference: 110–119 mmHg) had significant twice higher odds (4 times for SBP ≥170) of presence and number of microinfarcts, which was only significant for subcortical region. Similar higher odds were observed for systolic BP trend (increase/decrease/no change), which was significant for both cortical and subcortical microinfarcts. Systolic BP variability had no significant association with microinfarcts. Similar associations were observed with diastolic BP. In conclusion, the presence (especially for subcortical regions) and number of microinfarcts were more likely to be in those with higher mean BP and those whose BP changed over the study.

### CARDIOVASCULAR DISEASES PREDICT ALZHEIMER'S DISEASE ONSET IN NON-DEPRESSED ADULTS

Ginny Natale, and Sean Clouston, *Stony Brook University, Stony Brook, New York, United States*

We examined the mediating or moderating effect of stroke on the effect of cardiovascular disease (CVD) and the onset of Alzheimer's disease (AD) in a nationally-representative sample of older Americans with and without clinical depression. Diagnosis of CVD was adjudicated with the established Health and Retirement Study (1992–2016) methodology and included self-reported coronary heart disease, angina, heart failure, myocardial infarction, or other heart conditions. Probable-AD and probable-Stroke were identified inferentially using a validated pattern recognition algorithm. Analyses were stratified by a validated cutoff of the Center for Epidemiologic Studies Depression Scale (CES-D.) Participants (Nf17,154) were observed an average of 8 times over the span of 20 years (Obs=138,510). 12% of all AD cases are preventable by eliminating CVD in older adults in the US, compared to the 31% of all AD cases are preventable by eliminating stroke in the population. In non-depressed adults, risks for accelerated AD onset included: CVD, stroke, age, diabetes, and current smoking. Non-depressed females had delayed AD onset than males. The interaction of CVD x Stroke accelerated AD onset by 1.79 times (95%C.I. =1.63–1.97) in non-depressed adults. CVD was not a significant risk of AD onset in depressed adults. We conclude that 1)reducing the incidence of CVD and strokes in the US would drastically reduce the number of new cases of AD; and 2) stroke compounds the CVD hazards of accelerated AD onset; and 3) clinical depression is a key modulator of the effect of the risk factors of accelerated AD onset.

### HOPE PARTIALLY MEDIATES STRESS AND PERCEIVED BURDEN IN FAMILY CAREGIVERS OF PERSONS LIVING WITH A DEMENTIA

Jocelyn McGee, Dennis Myers, Rebecca Meraz, Clay Polson, Weiming Ke, and Angela McClellan, *Baylor University, Waco, Texas, United States*

Recent research suggests that hope may positively impact perceived burden in family caregivers of persons living with a dementia. However, there are few studies that have examined hope, as a multidimensional construct, on perceived burden. The purpose of this study was to investigate the relationship between objective and subjective stressors, hope and perceived burden in a sample of caregivers using taking into consideration multiple hope dimensions—specifically hope-agency and hope-pathway. Hierarchical multiple regression and mediation analyses were utilized in a sample of one-hundred and fifty-five family caregivers. Multiple regression analysis revealed that low hope-agency predicted high levels of perceived burden after controlling for known burden correlates. Hope-pathway did not impact perceive burden. Objective stress on perceived burden was partially mediated by hope-agency. The multi-dimensional aspects of hope should be taken into consideration when assessing this population. Hope-related psychosocial interventions aimed at bolstering multiple aspects of hope among family caregivers of persons with a dementia should be further developed and assessed in clinical intervention research.

### DATA-DRIVEN IMPROVEMENTS TO DEMENTIA DISEASE DETECTION STRATEGIES IN AN FQHC SETTING

Theresa M Sivers-Teixeira,[1] Patrick Tabon,[2] Jessie Yuan,[3] Erin Thayer,[4] Sonali Matta,[3] and Bonnie Olsen[4], *1. University of Southern California, Alhambra, California, United States, 2. University of Southern California School of Pharmacy, Los Angeles, California, United States, 3. Eisner Health, Los Angeles, California, United States, 4. Keck School of Medicine of University of Southern California, Alhambra, California, United States*

Despite higher prevalence of dementing illnesses in Latina/o populations, detection is lower compared to white populations, compounding racial/ethnic and economic disparities. Undetected illness compromises effective delivery of primary care and impedes linkages to much needed services. Development of robust detection and culturally sensitive management of cognitive impairment is critical for adequate care of this population. The Geriatric Workforce Enhancement Program at the University of Southern California (USC) partnered with Eisner Health, a Federally Qualified Health Center, serving a primarily immigrant, Spanish-speaking, uneducated, urban older adult population, to help achieve their goal of becoming an Age-Friendly Health System to prioritize identification and management of cognitive impairment. Over 14 months, a sustainable clinic workflow was developed and implemented to detect, evaluate, diagnose, and develop care plans for patients and their care partners. Staff and provider education was delivered through didactics, workshops, case reviews, and at-elbow training. Additional efforts focused on EHR optimization and alignment of existing clinical resources. Frequency data was extracted using i2iTracks software reflecting pre and post-implementation. Results show a higher percentage of patients diagnosed with cognitive impairment in the post-implementation period (7.35%) compared to pre-implementation (4.05%). Detailed data tracking the volume of patients engaged at each step of the workflow supports meaningful analysis of barriers and opportunities for optimization, such as how and where additional resources and efforts will yield the greatest effects. Data-driven strategies such as these, strengthen efficiency and effectiveness of the collaborative process and result in sustainable outcomes for this underserved population.

### SESSION 9000 (POSTER)

### LATE BREAKING POSTER SESSION I

### A QUALITY INITIATIVE TO INCREASE HOSPICE REFERRALS FOR STARS PATIENTS DURING THE SECOND COVID SURGE

Perihan El Shanawany,[1] Chris Nouryan,[2] Chris Choi,[3] Ann Flynn,[3] and Edith Burns[4], *1. Northwell Health, Plainview, New York, United States, 2. Zucker School of Medicine at Hofstra Northwell, Uniondale, New York, United States, 3. Northwell Health, Uniondale, New York, United States, 4. Zucker School of Medicine at Hofstra Northwell, Manhasset, New York, United States*

Background: CMS uses the Overall Hospital Quality Star Rating program to stratify hospitals based on specific quality criteria (e.g., 30-day readmissions of older adults with pneumonia). "STARS patients" experience more readmissions, longer LOS and often have more complex discharge plans. During the second surge of COVID we implemented a program to increase hospice referrals through early identification and implementation of goals of care (GOC) conversations. Methods: Electronic Medical Records reviewed for STARS patients from pre- (1/2019-7/2020) and post (3/2021–2/2022) program implementation. Location: 2 community-based hospitals. Data collected: demographics, hospital outcomes, discharge disposition. Data compared with Student's t tests and Chi square. Results: 459 patients, 177 pre-program and 282 post-program were included. Groups were similar in age (83.0 vs 83.6), LACE score (13.0 vs 12.8), principal diagnoses (PNA: 41.5% vs 46.0%, HF/COPD/AMI: 58.5% vs 54.0%), and mortality (3.5 vs. 4.0%). LOS increased 4.9 days vs. 6.1 days ($p < 0.01$), readmission rates unchanged: 12.6% vs 13.0% ($p=0.90$). GOC conversations increased, 48.6% to 75.0% ($p < 0.01$), DNR from 24% to 44% ($p < 0.01$), and comfort measures from 0.5% to 5% ($p < 0.01$). Hospice referrals increased from 0.5% to 11.2% ($p < 0.01$). Discussion: Early identification of STARS patients increased GOC conversations, DNR, comfort measures and hospice referral. Patients across time periods were similar in age, LACE and admitting diagnoses. LOS increased by a day, likely reflecting time needed to arrange discharge disposition. Increased hospice at end-of-life is associated with better quality care and patient/family satisfaction. This program may be adapted to larger, academic medical centers within the health system.

### 65 &THRIVE: TRANSFORMING OLDER ADULT CARE IN THE EMERGENCY DEPARTMENT

Tracey Vien,[1] and Stella Bobroff[2], *1. Kaiser Permanente Med Group/Southern California, Los Angeles, California, United States, 2. Kaiser Permanente, Los Angeles, California, United States*

Kaiser Permanente Los Angeles Medical Center's (LAMC) Utilization Management Department expanded our geriatric initiative throughout the hospital with emphasis in the

Emergency Department (ED). The ED is the first point of patient contact in the hospital, early identification and initiation of interventions needed for potential discharge barriers will assist in timely discharges. LAMC obtained Geriatric ED Accreditation (GEDA) Level 2 this year, the first in the Los Angeles County to achieve this title. This was done by implementing ten new policies with quality improvement (QI) metrics, conducting age sensitivity trainings for our staff, and enhancing our ED rooms to be geriatric friendly. Two of the ten interventions in our ED include 1) volunteers to provide support to our geriatric patients prevent functional decline, prevent delirium and readmission rates, and 2) palliative care consults initiated in the ED prevent suffering and promote the best possible quality of life for patients who are facing a serious illness. Between 2020 and 2021, there has been a 15% increase for the census of 65+ patients. 21% increase of number of older adults with ED readmissions, and a 13% increase in number of older adults staying in the ED for more than 8 hours. With the increasing older adult population, we see a rise in our admission and readmissions of older adults in our ED. This indicates a need for geriatric specialized care and Kaiser plans to obtain GEDA for all Southern California Hospitals.

### INITIAL EXPERIENCE OF THE FIRST HELPLINE DEVOTED TO SUPPORTING CONCERNED PERSONS IN THE LIVES OF ELDER ABUSE VICTIMS

E-Shien Chang,[1] Lisa Rachmuth,[1] Yuichi Seki,[1] Sandy Regenbogen-Weiss,[1] Serena Ross,[1] Lina Irias,[1] Mark Lachs,[1] and Tony Rosen[2], *1. Weill Cornell Medicine, New York, New York, United States, 2. Weill Cornell Medical College / NewYork-Presbyterian Hospital, Pelham, New York, United States*

Elder abuse is common and can deeply impact older adults who experience it. Little is known, however, about another group that may be profoundly affected: non-abusing family members, friends, and neighbors, referred to as “concerned persons”. We describe the initial experience of the first telephone helpline in the U.S. devoted to supporting these concerned persons. The New York City Elder Abuse Center developed a Concerned Persons Helpline to assist concerned persons and alleged victims in New York State. Using this frontline call data, we examined characteristics of concerned persons, circumstances surrounding alleged abuse, and interventions offered. Overall, the helpline received 864 total calls (16.6 calls per week) over a one-year period between 10/1/20 to 9/31/21. An initial subset of 149 logged calls were used for this exploratory analysis. Concerned persons most commonly reported that an older adult in their life was suffering from financial exploitation (28.9% of all callers), followed by caregiver neglect (26.2%), with 42.2% reporting poly-victimization. Relationship of callers to alleged victims was most commonly non-abusing adult child (37.6%), followed by other relatives (13.4%), friends (12.8%), and neighbors (10.0%). An adult child was also the most commonly reported perpetrator (36.9%). A large proportion of alleged victims suffered from cognitive impairment and/or physical disabilities (55.7%). Intervention provided included referrals to social services, civil and legal services, victim assistance programs, and caregiver counseling programs. Developing resources to support concerned persons should continue to be a research and practice priority in elder abuse.

### GLOBAL ELDER ABUSE: A MEGA-MAP OF SYSTEMATIC REVIEWS

Lilly Estenson,[1] Kelly Marnfeldt,[1] Yongjie Yon,[2] Christopher Mikton,[3] and Kathleen Wilber[1], *1. University of Southern California, Los Angeles, California, United States, 2. World Health Organization, Copenhagen, Hovedstaden, Denmark, 3. World Health Organization, Geneva, Geneve, Switzerland*

Elder abuse is an increasingly prevalent public health problem that requires a global political and evidence-based response. To make global evidence on elder abuse easier to locate and analyze, we constructed a mega-map – an interactive map that systematically identifies and overviews existing systematic reviews – on elder abuse prevalence, consequences, risk and protective factors, and interventions. Following Campbell Collaboration methodological guidelines, we conducted a comprehensive database and grey literature search of the global elder abuse literature (n=2,776) to identify systematic reviews that examine one or more of these four abuse domains among adults age 60+ (n=111). We then coded the reviews to identify key characteristics, including the types of abuse, settings, World Health Organization (WHO) geographic regions, and demographic subgroups represented in each review, that can be filtered in the mega-map. We also adapted the Joanna Briggs Institute Critical Appraisal Checklist for Systematic Review and Research Syntheses to appraise the quality of each review. We found that the focus of existing evidence syntheses is most commonly interventions (n=59), followed by prevalence (n=52), risk factors (n=51), consequences (n=31), and protective factors (n=16). The majority of published elder abuse studies have been conducted in the European, Western Pacific, and Region of the Americas WHO geographic regions. Few reviews examine systemic abuse and system-level interventions. Future studies on elder abuse should address these gaps in research on protective factors and systemic aspects of elder abuse, and build evidence about elder abuse in the African, South-East Asian, and Eastern Mediterranean WHO geographic regions.

### EXPLORING FRONTLINE PERSONNELS’ PERCEPTIONS OF SUPPORT, KNOWLEDGE, AND RESOURCES FOR ELDER ABUSE

Sarah Marrs, Courtney O’Hara, Catherine MacDonald, Miranda Yelvington, and Tracey Gendron, *Virginia Commonwealth University, Richmond, Virginia, United States*

In Virginia, the number of both reported and substantiated cases of abuse in later life have grown steadily over the last several years (DARS, 2021; O’Connor, 2019). Unfortunately, the resources provided to combat elder abuse have not maintained the same steady increase; in many cases, funding to agencies that work to prevent abuse in later life has even been reduced (DARS, 2021). To better understand the gap that exists between need and available resources in Virginia, we conducted an exploratory sequential mixed methods study [QUAL → quan] (Creswell & Plano Clark, 2018). In the qualitative strand, we conducted a series of interviews and focus groups with professionals who often are called upon to respond/intervene when elder abuse is suspected: aging and victim services (n = 9), healthcare providers (n = 7), law enforcement (n = 8). Three themes emerged from the qualitative data: increasing capacity, prevention/coordination, and ageism. Findings from the qualitative strand were then used to guide implementation of the quantitative strand. These participants (N = 2,891) worked in a variety of frontline settings with older adults or in aging services, mostly in health care, law enforcement, social services. Project findings point to a need for increased resources for and training on elder abuse, a shift from reaction to prevention of abuse, improved care coordination and case management, and a need to reduce harmful ageist beliefs. Moreover, our findings highlight the effect structural and societal ageism at the policy level perpetuates the prevalence of abuse in later life.

### ENGAGING IN CQI TO ENHANCE ADULT PROTECTIVE SERVICES INTAKE AND SCREENING: A QUALITATIVE ANALYSIS

Joan Davitt,[1] Jocelyn Brown,[2] Peiyuan Zhang,[2] Erin Barry-Dutro,[2] and Greg Sesek[3], *1. University of Maryland, School of Social Work, Baltimore, Maryland, United States, 2. University of Maryland, Baltimore, Baltimore, Maryland, United States, 3. Department of Human Services, Baltimore, Maryland, United States*

This qualitative evaluation study was part of a larger Continuous Quality Improvement initiative implemented by the Maryland Department of Human Services Office of Adult Services to improve the Adult Protective Services (APS) screening and intake process statewide. Researchers conducted two 90-minute focus groups via Zoom with 21 intake and screening staff from 14 counties in Maryland in the summer of 2022. Focus group participants were mostly White and African American women with an average of 6.5 years working in adult services. Interviews focused on understanding challenges in APS intake/screening and strategies to overcome those challenges. Each interview was recorded and transcribed verbatim and three researchers used open and axial coding to identify themes. The key challenge expressed by all participants was the difficulty obtaining adequate information from referral sources, in order to discern whether the adult was vulnerable and if the alleged abuse/neglect/exploitation was being committed by a trusted caregiver or fiduciary as defined by state law. Inadequate referral information results in supervisors/screeners making time-consuming follow-up calls to obtain additional information or cases inappropriately screened in for investigation. This challenge was exacerbated by intake workers inexperienced in APS, understaffing, unclear policies and screening tools for APS, and community partners' and the public's unfamiliarity with APS services and policy. Strategies to overcome these challenges included additional staff training, public education, additional resources for APS, clearer policies, and improving the APS screening tool. Quality improvement strategies developed from this study that are relevant to APS systems nationwide will be discussed.

### ELDER ABUSE SCREENING PRACTICES IN THE VETERANS HEALTH ADMINISTRATION: RESULTS FROM A NATIONWIDE EVALUATION

Lena Makaroun,[1] Jaime Halaszynski,[2] Kathrine Smith,[3] Melissa Dichter,[4] Ann-Marie Rosland,[5] Carolyn Thorpe,[6] and Tony Rosen[7], *1. VA Pittsburgh Healthcare System/University of Pittsburgh, Pittsburgh, Pennsylvania, United States, 2. VA Butler Health Care System, Butler, Pennsylvania, United States, 3. VA Fargo Health Care System, Fargo, North Dakota, United States, 4. Temple University College of Public Health, Philadelphia, Pennsylvania, United States, 5. University of Pittsburgh/VA Pittsburgh Healthcare System, Pittsburgh, Pennsylvania, United States, 6. UNC Eshelman School of Pharmacy, Chapel Hill, North Carolina, United States, 7. Weill Cornell Medical College / NewYork-Presbyterian Hospital, Pelham, New York, United States*

Elder abuse (EA) is common and has devastating health consequences yet is rarely detected by healthcare professionals. Veterans are at high risk for EA, and the Veterans Health Administration (VHA) has experience screening for complex psychosocial phenomena including intimate partner violence. While the VHA has national policy regarding mandatory reporting of EA cases, little is known about the extent to which VHA sites currently screen for EA in a standardized fashion and what approaches are used. To address this knowledge gap, we conducted a national survey of all 170 parent station VHA medical centers from January to August of 2021. Surveys were distributed electronically to the Social Work Chief at each site, as social work is responsible for interpersonal violence response in VHA. The survey assessed the presence and characteristics of EA-specific screening practices as well as general abuse/neglect screening conducted with patients of all ages, including older adults. Follow up emails were sent to sites who reported conducting screening requesting additional details not included in the initial survey. Overall, 138 sites (81%) responded to the survey. Among respondents, 3% reported screening older adults for EA using a previously published tool, while 2% reported screening for EA with an unstudied or locally-developed tool. Forty-three percent reported doing general abuse/neglect screening using unstudied questions/tools for patients of all ages, and 41% reported no EA screening at their site. The wide variability in current EA screening practices in VHA presents an important opportunity to standardize and improve EA detection practices.

### EARLY OUTCOMES OF A NATIONAL CONSORTIUM TO ADVANCE FAMILY CAREGIVERS IN NURSING EDUCATION

Hui Zhao,[1] Lindsay Mullins,[2] Andra Davis,[3] Kathryn Sexson,[4] and Sara Hart[5], *1. James Madison University, Harrisonburg, Virginia, United States, 2. Franciscan Missionaries of Our Lady University, Baton Rouge, Louisiana, United States, 3. University of Portland, Portland, Oregon, United States, 4. UC Davis, Sacramento, California, United States, 5. University of Utah, Salt Lake City, Utah, United States*

Family caregivers (FCGs) hold primary responsibility in caring for people with chronic illness often with little training and insufficient resources Standardized guidance for supporting FCGs currently does not exist. Given this fact, it is an urgent call to healthcare professionals, particularly nurses as the key members of the healthcare team, to promote education for FCGs. In April 2022, experts in the field of family caregiving from UC Davis and University of Utah schools of nursing, convened educators and champions of FCGs from across the country and formed the Family Caregiving Nursing Education Consortium to advance the inclusion of FCGs in nursing education in order to address the lack of standardized nursing competencies for family caregiving. The consortium consists of members from nine diverse universities representing member expertise in pediatrics, geriatrics, palliative care, disabilities, mental health, and underserved communities in order to address a broader range of health issues through the lifespan. Preliminary data were reviewed and identified as a framework, including Family Caregiver Competencies for Interprofessional Education (4 domains) developed by the school of nursing at UC Davis and the AACN Essentials (10 competency categories). Members mapped the family caregiving competencies to the AACN Essentials (2021) during monthly meetings. Though current work presented an early-stage outcome, it marched through crucial steps, including institutional environmental scans, needs assessments, and toolkit development, as building blocks that not only advanced the inclusion of family caregiving education in nursing curricula but also provided foundation to guide the next stage of work.

### AGEISM: AGING KNOWLEDGE AND PERSPECTIVES OF THE AGING NUTRITION NETWORK

Savannah Schultz,[1] Sarah Francis,[1] and Alexandra Bauman[2], *1. Iowa State University, Ames, Iowa, United States, 2. Iowa Department on Aging, Des Moines, Iowa, United States*

Addressing ageism is essential to support older adult well-being. The Nutrition and Aging Resource Center (NRCNA) (1) examined the general characteristics, aging perceptions, and preferred training modalities of national aging nutrition network providers and (2) measured the impact an ageism webinar had on topic familiarity, knowledge, and behavioral intent. Frequencies were run to analyze the characteristics, aging perceptions, and behavioral intent while Wilcoxon signed rank tests measured outcome changes. Ageism was assessed via the W.H.O. Ageism Quiz (AQ) while aging perceptions were measured with the Facts on Aging Quiz (FAQ) and Expectations Regarding Aging (ERA). Respondents (n=1910) were primarily female (63%), non-Hispanic (90%), and white (87%) with an average age of 50 years. At least one-half had moderate ageism and aging perspective scores [Mean scores: AQ = 4.9±1.9 (max 8); FAQ =5.9±1.8 (max 10); ERA = 54.4±18.7 (max 100)]. The most preferred continuing education format was live webinars (44.3%). In response, a one-hour webinar about unconscious aging bias was offered in June 2022. A retrospective evaluation was used to assess the webinar impact (n=130 responses). A significant increase in subject matter familiarity ($Z= -8.8$, $p < 0.0001$) and knowledge ($Z= -8.5$, $p < 0.0001$) was noted. Further, analyses revealed positive attitudes (beneficial, good idea), perceived behavior control, indirect social norms (client support), and intent to reflect on past interactions with an older adult(s) to see how age-related biases might have influenced those interactions. These results illustrate the need for and the impact of ageism awareness training among those who work with the aging population.

### AGE-INCLUSIVE PRINCIPLES ON CAMPUS: EMBRACING DIVERSITY ACROSS THE LIFESPAN

Kelly Munly,[1] and Lauren Jacobson[2], *1. Penn State - Altoona, Altoona, Pennsylvania, United States, 2. Penn State Altoona, Altoona, Pennsylvania, United States*

In this poster, researchers based at a Mid-Atlantic university campus provide an understanding of their evaluative steps informing an adaptation of the Diversity Circles program to include a multigenerational component, supporting diversity across the lifespan. Project methods and analysis have been informed by critical theoretical frameworks, including feminist gerontology, that illuminate the invisibility of age, even in the context of intersectional work. Pilot feedback from five participants from a condensed program in an Adult Development and Aging course informed the interview approach. Post-program semi-structured interviews, with program participants, including students and older adults (n=7), and community stakeholders (n=18), provided feedback on diversity needs at the campus and in the surrounding community, as well as on program content and experience and opportunity for further curriculum integration of concepts of age-friendliness, ageism, and age-awareness. Stakeholders interviewed included community practicum liaisons, university advising and student affairs staff, faculty and staff previously engaging in diversity-related activities, university administrators, university personnel attending to enrollment matters, and staff and faculty interested in student-centered curriculum design. Semi-structured interviews were chosen for data collection because of their capacity to provide saturated data from a small, purposeful sample. Focused codes emergent from the interviews included both a) suggestions for curriculum adaptation and modification as well as the value of existing content and b) issues of age-friendliness and ageism more generally. The research team looks toward incorporating suggestions within their findings in an expansion of the program on their campus, and disseminating findings for the benefit of other campuses' programs.

### 'IT RESTORED A BIT OF THEIR HUMANITY": BRIDGING THE GAP THROUGH HANDS-ON INTERGENERATIONAL LEARNING

Katherine Meszaros, and Renee Beard, *College of the Holy Cross, Worcester, Massachusetts, United States*

The Covid-19 pandemic laid bare many of the social inequities and vulnerabilities within the American nursing home industry. Intergenerational learning is thought to be an effective approach to debunking ageism and addressing social isolation, especially in institutional care settings. This study used a mixed method approach to explore the benefits of intergenerational learning, here between college-aged students and nursing home residents. We analyzed qualitative observational data, including 56 hours of participant observation during TimeSlips™ sessions with 32 students and 30 residents at a nursing home in Worcester, Massachusetts. A content analysis of 68 student reflections on the classroom community-based learning (CBL) experience was also performed. This poster reports on the findings from the students who participated in the intergenerational learning project in 2020–2022. A series of common themes emerged. Many students began their experience as skeptics, but retrospectively reported that it was transformative. Personal interactions with elders revealed to students the shared humanity that had with older generations, which then forced them to confront their own ageism and ableism. Intergenerational learning also encouraged students to reflect on their lives, ask what type of person they want to be, what world they would like to live in, and gain new life lessons and perspectives on now to age meaningfully. The value of intergenerational learning far exceeds providing nursing home residents with social stimulation. It can be just as formative to the personal philosophies and outlooks of college-aged students. Intergenerational learning provides tangible and intangible "in-the-moment" benefits to those who participate.

### A PILOT STUDY OF AN INTERGENERATIONAL BOOK CLUB: LESSONS LEARNED FOR IMPROVING FEASIBILITY

Katelyn Frey, Jennifer Sublett, Jennifer Stanley, and Harvey Stearns, *The University of Akron, Akron, Ohio, United States*

Intergenerational book clubs have been successful in reducing ageist attitudes of younger adults (YA) while increasing feelings of social connectedness among older adults (OA; Lohman et al., 2003). We implemented both in-person (Nf11: YA=4, OA=7) and virtual (Nf8: YA=5, OA=3) modalities for an intergenerational book club to increase intergenerational interactions on campus, considering aging researchers' claim of heightened ageism and exacerbated loneliness following the beginning of the COVID-19 pandemic (Ayalon et al., 2020; Brooke & Jackson, 2020). Both groups met thrice over twelve weeks and completed measures on ageist attitudes ($\alpha$=.83), engagement with the group ($\alpha$=.91), and social connectedness ($\alpha$=.91) each time. There was attrition in both groups (final meeting: Nf9 (in-person) and Nf3 (virtual)). To better understand overall motivations to participate and what factors may have contributed to attrition, we utilized content analysis with participants' feedback to examine their motivation for joining, what they enjoyed most after participating, and what they would improve. Participants were motivated to join because they enjoyed reading; comparatively, they enjoyed group discussions and hearing different perspectives. Future recommendations include meeting more often, increasing the number and size of groups, and scheduling meetings closer together. The majority wished to continue participating. The data suggest future expansion of intergenerational book clubs on campus are desired to support age-friendly interactions and needed to further examine if these interactions can curb ageism and increase social connectedness among diverse age groups. Discussion will include recommendations regarding measures, lessons learned for an optimal protocol, and next steps.

### THE RELATIONSHIP BETWEEN AGE DISCRIMINATION AND QUALITY OF LIFE OF DEPENDENT COMMUNITY DWELLING OLDER PERSONS

Felipe Sandoval Garrido, *University of Tsukuba, TSukuba, Ibaraki, Japan*

The objective of this study is to examine the age discrimination suffered by dependent older adults and its effects on their quality of life, using a large representative cohort study. Dependency is defined as having difficulties with activities of daily living, both basic (ADL) and instrumental (IADL). To identify perceived ageism, we used data collected by the English Longitudinal Study of Ageing (ELSA) in 2010–2011 (wave 5) that asked respondents about being discriminated because of their age. Quality of life was measured using the CASP-19 scale. We performed both cross-sectional and longitudinal analyses using the subsequent 2012–2013 (wave 6) and 2018–2019 (wave 9) follow-ups. Multivariable logistic regression analysis was used to estimate the odds ratios of experiencing perceived age discrimination. The results show that a quarter (22%) of all respondents experienced age discrimination. Those suffering from dependency and age discrimination had independently significant lower quality of life scores. Perceived age discrimination was cross-sectionally associated with being male, white, in poor physical and mental health, highly educated, with lower wealth. Longitudinally, with being male (odds ratio -OR-: 1.5), highly educated (OR: 1.3), and poor mental health (OR: 1.7). However, quality of life change was not statistically significant among dependent older persons in subsequent waves. Understanding ageism is important to create policies for future interventions. The present results reinforce the idea of previously documented groups at risk of age discrimination in order to protect them, but also the complex panorama of bio-psychosocial determinants involved in tackling it.

### OLDER, ENTITLED, & EXTREMELY OUT-OF-TOUCH: DOES "OK, BOOMER" SIGNIFY THE EMERGENCE OF A NEW OLDER ADULT STEREOTYPE?

Katelyn Frey, and Toni Bisconti, *University of Akron, Akron, Ohio, United States*

"OK, Boomer" is a phrase used online by younger adults to dismiss an older person for their perceived out-of-touch or offensive beliefs (Lorenz, 2019). An initial study utilizing content analysis techniques with a younger adult sample (N = 316) explored how two theoretical frameworks from the age stereotype literature, the Stereotype Content Model (Fiske et al., 2002) and Hummert et al.'s (1994) age stereotype categories, could explain this phenomenon and the enduring online references to "Boomers." We found that neither theory adequately captured how younger adults view the titular "Boomer;" the low-competence/low-warmth quadrant of the Stereotype Content Model was the only quadrant with support (N = 32), albeit not a large amount, whereas the age stereotype category data revealed that "Boomers" partially overlapped with elements of the "Shrew/Curmudgeon," the "Severely Impaired," and the "John Wayne Conservative" to varying degrees. This necessitated a second study that used an open-ended, interpretative phenomenological analysis approach to better understand younger adults' perceived age-related stereotypes of "Boomers." Results identified seven unique characteristics ascribed to "Boomers" and older people in general: closed-minded, argumentative, out-of-touch, offensive, critical, nostalgic, and/or conservative, all of which seem to possess a decidedly hostile ageist undertone. Future work should examine exactly how synonymous "Boomers" are with older adulthood, the potential underlying hostile and/or benevolent ageism associated with using this phrase, and how derogatory age-based internet jargon influences age stereotype development and intergenerational relations given the heightened ageism following the COVID-19 pandemic.

### NOT ONE AND THE SAME: VARIED EXPERIENCES OF BENEVOLENT AGEISM IN OLDER ADULTHOOD

Jennifer Sublett and Toni Bisconti, *University of Akron, Akron, Ohio, United States*

Prior work has explored benevolent and hostile attitudes toward older adults (Chasteen & Cary, 2015) and their paternalistic, mixed incompetent-warm perceptions within the Stereotype Content Model (SCM; Fiske et al., 2002); however, older adults' perspectives of these attitudes have not received similar attention. This study assessed older adults' experiences with benevolent and hostile ageism using an adapted version of the Ambivalent Ageism Scale (AAS; Cary et al., 2017; $\alpha$=.85) and using a mean split, examined if there are differences among the younger- and older-older adults. Within a sample of older adults (Nf202) that ranged in age from 65–90 (M=71.6), we found that when comparing those aged 65–70 and 71 and above, those who were older (M=1.76, SD=.44) reported more experiences with benevolent ageism than their younger counterparts (M=1.56, SD=.42), t(200)=3.30, p =.001). These groups did not significantly differ in hostile ageism, t(200)= -.05, p = .96. When participants were asked why they have been treated thusly as described by the AAS items, 18% of the participants believed it was due to their age, in comparison to those who attributed it to personality (41%), gender (9%), or unknown factors (11%). Benevolent ageism is clearly not a monolithic experience for older adults as those who are older report more patronization, and not all participants felt age was the defining reason for their treatment. Further work should assess how these experiences are qualitatively different across diverse age groups of older adults, as ageism reduction interventions may need to be tailored appropriately.

### CHALLENGING AGEISM IN HEALTHCARE THROUGH INTERPROFESSIONAL EDUCATION

Min Kyoung Park,[1] and Diane Martin[2], *1. University of Maryland, Baltimore County, Ellicott City, Maryland, United States, 2. University of Maryland, Baltimore, Baltimore, Maryland, United States*

Broadly defined as prejudice, discrimination, or stereotyping of older adults, ageism is prevalent in the healthcare system. It is implicated in the over- or under-diagnosis and/or treatments provided to older persons, resulting in decreased quality of life and increased costs of care. In Fall 2021, we developed a virtual interprofessional education (IPE) activity to increase professional health and social care trainees' awareness of ageism and to biases they may hold. During the asynchronous portion of the training, participants complete the Harvard Implicit Association Test (age), watch a video, and respond to related threads on Blackboard, which provides an opportunity to discuss experiences prior to participating in a synchronous training activity incorporating a presentation on ageism in healthcare. To date, 98 trainees have provided qualitative data on the impact of the ageism training components. Overwhelmingly, trainees indicated the value of information provided; many of whom responded that the test revealed their own biases. Participants commented that the training was an opportunity to open their eyes to ageism and that it made them more aware of the importance of understanding older adults' capabilities and limitations in a respectful way. More than half (55.86%) of threaded discussions noted that the ageism training helped them to realize their biases against older adults and understand older people's challenges. Quantitative data from pre/post IPE surveys indicated increased interest in working with older adults. Responses illustrate the need to and value of incorporating anti-ageism content into educational programs of our future health and social care workforce.

### ASSOCIATIONS BETWEEN ATTITUDES TOWARDS AGING AND CLINICAL DECISIONS AMONGST INTERNAL MEDICINE RESIDENTS

Brent Schell,[1] Megan Carr,[2] Jesse Katz,[1] and Maryam Hasan[1], *1. Baystate Medical Center, Springfield, Massachusetts, United States, 2. High Point University, High Point, North Carolina, United States*

It has been shown that physicians-in-training can develop negative attitudes towards caring for older patients. Little is known about how these attitudes develop throughout training or if they are associated with ageist clinical decisions. In this pilot study, Internal Medicine (IM) residents completed the Expectations Regarding Aging (ERA-12) survey, a validated instrument that assesses expectations regarding physical health, mental health, and cognitive function in older adults. Residents then completed a survey that included simulated clinical scenarios as part of an educational intervention on Ageism. Of the 72 residents invited to participate, 48 residents (67%) completed the ERA-12 survey. Responses were obtained from all training levels including 24 PGY-1 (50%), 10 PGY-2 (21%), and 14 PGY-3 (29%). When grouped by training year, there were no statistically significant differences found with regards to total ERA-12 scores or expectations regarding physical health, cognitive function, or mental health of older adults. When grouped by responses to clinical scenarios, residents with more biased clinical responses were associated with lower total ERA scores (73 vs. 91, p = 0.015). Those with lower total ERA scores indicated they were inclined to initiate CODE STATUS discussions earlier with older adults and were less inclined to consider coexisting depression. Despite limitations in sample size and the subjective nature of simulated clinical scenarios, this pilot study suggests a possible association between expectations regarding aging and clinical decision-making. More research is needed to assess the strength of this association and if it can be modified through educational initiatives.

### THE EFFECTS OF NEIGHBORHOOD DEPRIVATION AND PERCEIVED NEIGHBORHOOD PROBLEMS ON DEPRESSIVE SYMPTOMS

Jean Choi,[1] Elizabeth Muñoz,[1] Robin Corley,[2] Sally Wadsworth,[2] and Chandra Reynolds[3], *1. The*

*University of Texas at Austin, Austin, Texas, United States, 2. University of Colorado Boulder, Boulder, Colorado, United States, 3. University of California, Riverside, Riverside, California, United States*

The progression of detrimental mental health outcomes may begin before old age, and investigating correlates earlier in the lifespan may provide insights into prevention efforts. Neighborhoods have been linked with mental health in adulthood, but there is variation in measurement across studies. Studies have examined the independent associations between either of objective or subjective measures of neighborhoods, but few to none have examined both of these in one study. We assessed the effects of both objective (Area Deprivation Index; ADI) and subjective, or perceived, neighborhood characteristics on depressive symptoms among early midlife adults, using data from the Colorado Adoption/Twin Study of Lifespan behavioral development and cognitive aging (Nf1166, Mage=33.51 (5.07)). Participants completed self-report assessments of perceptions of their neighborhoods (e.g., perceived safety, disorder). Addresses were geocoded and linked with 2010 Census data, and we created a standardized ADI composite. To account for subject non-independence, we fitted multilevel linear regressions, controlling for relevant confounding variables. Results showed that higher ADI was linked with greater depressive symptoms (d = .04), but the effect of ADI was no longer significant and was reduced by about 30% after accounting for subjective neighborhood domains. Perceived neighborhood disorder had the largest effect (d = .19) and remained the sole predictor of depressive symptoms when all perceived domains were included in one model. Findings suggest that residents' subjective interpretations of their neighborhood surroundings may be shaped by the broader structural contexts in which they live in, revealing a potential pathway through which objective neighborhoods affect depression.

### SUPPORTING AGING IN PLACE WITH MOBILITY IMPAIRMENTS THROUGH ANNOTATED TELEPRESENCE TECHNOLOGY

Ben Thompson, Maribeth Gandy, Kala Jordan, and Laura Levy, *Georgia Institute of Technology, Atlanta, Georgia, United States*

Most smarthome and smartphone technologies are designed with younger adults in mind, even though many older adults can benefit greatly from their use. Through our examination of how to make these technologies more accessible to adults aging in place with mobility impairments, we have found remote troubleshooting to be a possible solution to many problems. However, we have found most current teleconferencing solutions to be lacking in the limited perspectives of a single webcam, as well as from a lack of ability to point out specific items and locations within a camera view. As such, Our team developed a prototype system that used multiple desk space and room space views, as well as on screen annotations, to facilitate remote troubleshooting and social interactions through a teleconferencing service. This prototype was then evaluated with adults aging in place with mobility impairments, as well as accessibility experts, in order to see what parts of the system could be utilized for troubleshooting, as well as to see where the system was lacking. The studies tested the system's ability to be used for troubleshooting, as well as their uses in games and social interactions. We have gathered positive feedback for these prototypes in the areas of troubleshooting, socialization, games, and for use in other accessibility studies. From these results, we have begun development of a mobile deployment kit for use of our teleconferencing solution in other accessibility studies.

### SUBSIDIZED SENIOR HOUSINGS (SSH) FOR LOW-INCOME OLDER ADULTS IN THE U.S.: A SCOPING REVIEW

Sojung Park,[1] Soobin Park,[2] Takashi Amano,[3] Jihye Baek,[4] BoRin Kim,[5] and Byeongju Ryu[4], *1. Washington University in Saint Louis, Saint Louis, Missouri, United States, 2. Washington University in St. Louis, Saint Louis, Missouri, United States, 3. Rutgers University-Newark, Newark, New Jersey, United States, 4. Washington University in St. Louis, St. Louis, Missouri, United States, 5. University of New Hampshire, Durham, New Hampshire, United States*

Subsidized senior housing (SSH) has been advocated as a key component of a community-based, long-term care policy for low-income older adults that links housing with health and social services to support aging-in-place (AIP). Relative to the accumulating evidence on for-profit housing models, SSH remained understudied. Guided by the Person-Environmental fit perspective, this scoping review described and synthesized existing literature on SSH to identify strengths and gaps in the literature. With a five-step scoping review method, we focused on the empirical studies published from 2010 in the U.S using four electronic databases and additional manual searching. Sixty-six articles met the inclusion criteria. Study participants were predominantly non-Hispanic White and female; most studies were quantitative surveys with a cross-sectional design. Five areas of outcomes emerged: (1) Health and well-being (45%), (2) Healthcare use and health behavior (24%), (3) Social Relations (11%), (4) Housing Relocation (12%), and (5) Technology (8%). In examining the study outcomes, the existing articles primarily focus on the personal (29%) and environmental (71%) dimensions, including 9% of the articles investigating both dimensions. For each dimension, the majority of the research tends to highlight residents' health conditions and characteristics of supports and service programs in the housing. The largest proportion of the literature studied the relationship between residents' health and the outcomes related to health and well-being. Future research needs to examine subpopulations of older residents in SSH (e.g., racial/ethnic minority, LGBTQIA, and others) as well as the current and future role of technology in SSH and community partnership.

### PERSONALITY PROFILE OF RURAL-DWELLING OLDER ADULTS IN AN ONGOING HOARDING TREATMENT STUDY

Caitlyn Nix and Mary Dozier, *Mississippi State University, Mississippi State, Mississippi, United States*

Symptoms of hoarding disorder, such as significant household clutter, difficulty discarding, and excessive acquisition tend to increase in later life. With many challenges involved in aging in place, older adults may be particularly vulnerable to adverse events occurring while living with excessive clutter

(e.g., fall risk, sanitation issues). Therefore, understanding what factors predict hoarding symptom severity is an essential step towards increasing older adults' ability to age in place while experiencing hoarding symptoms. Personality traits have been demonstrated to predict hoarding symptoms in a wide range of ages. However, a sample of older adults in this clinical population has not been evaluated. The IPIP-NEO-60 is a shortened version of a widely used open-source personality measure that utilizes the five-factor model of personality (Neuroticism, Extraversion, Openness, Agreeableness, and Conscientiousness). We used this measure to evaluate the personality profiles of rural-dwelling older adults (age 50 and up, M = 63) enrolled in an ongoing treatment study for hoarding disorder (n = 14). Compared to normative data from 910 adults aged 50 and up and matched for gender, most participants scored in the average range for Neuroticism, Extraversion, and Openness, with a nearly even split between high, low, and average scores for Agreeableness. Half of participants scored in the low range for Conscientiousness. Given the considerable proportion of participants demonstrating low Conscientiousness in this sample, screening a larger sample for beyond-average facet scores within this factor (e.g., self-efficacy, cautiousness) may assist clinicians in selecting impactful treatment targets for hoarding disorder in older adults.

### MACHINE LEARNING TO PREDICT HOMEBOUND STATUS IN OLDER ADULTS USING CANADIAN LONGITUDINAL STUDY ON AGING DATASET

Shehroz Khan, Can (Shirley) Cui, and Andrea Iaboni, *Toronto Rehabilitation Institute, Toronto, Ontario, Canada*

Individuals who are unable to leave their home or with great difficulty are considered homebound or semi-homebound. Homebound status is strongly associated with disability, social isolation, healthcare use and costs, and mortality. Most homebound older adults have multiple chronic conditions and poor health. There is not enough information about homebounded older adults in Canada. The Comprehensive cohort of the Canadian Longitudinal Study on Aging (CLSA) presents an excellent opportunity to study the complex factors associated with homebound status and the interplay between physical, social, psychological, and environmental determinants over time. This is a population-level study which makes use of provincial healthcare registration data to sample older adults across the country. We obtained the first wave of CLSA dataset containing samples from 21667 individuals over 3223 variables. We developed a definition of 'homeboundedness' in using life-space index variables present in the CLSA datatset. The dataset contained numerical, categorical and missing values. After preprocessing, we selected 1101 Homebound and 20521 Non-Homebound individuals, and 1771 variables. We showed that Random Forest classifier (with missing values) provided an AUC ROC and PR of 0.89 and 0.49. The missing value imputation did not improve the results significantly. Using feature hashing, we converted the dataset to numerical values; the AUCs improved to 0.96 and 0.71 at the cost of losing interpretation. In future, we will consult clinical experts in choosing the relevant features and use analytical methods to select features and compare. We will also test these predictive models on the next wave of this dataset.

### GEOGRAPHIC AVAILABILITY OF SUBSIDIZED SENIOR HOUSING AND NEIGHBORHOOD DISPARITIES IN MISSOURI

Byeongju Ryu,[1] Jihye Baek,[1] Sojung Park,[2] Yung Chun,[1] Takashi Amano,[3] and BoRin Kim[4], *1. Washington University in St. Louis, St. Louis, Missouri, United States, 2. Washington University in Saint Louis, Saint Louis, Missouri, United States, 3. Rutgers University-Newark, Newark, New Jersey, United States, 4. University of New Hampshire, Durham, New Hampshire, United States*

Subsidized senior housing (SSH) provides an affordable option to low-income older adults for economic stability and aging-in-place. To date, limited attention has been given to the geographic availability of SSH and its relation to neighborhood characteristics (e.g., socioeconomic characteristics and availability of supportive services). The current study aims to describe (1) the availability of government-subsidized senior housing (SSH-availability) in Missouri and examine (2) to what extent the housing availability is associated with county-level neighborhood characteristics. First, SSH-availability in each county was measured with the number of subsidized units for older adults divided by the eligible population (i.e., aged 65 or over, income below poverty level, and spending 30% or more of income on housing). The Geographic Information System (GIS) was used to examine the distribution of SSH in Missouri. Second, based on existing literature, we used principal component analysis with multiple indicators to create two neighborhood characteristics (i.e., socioeconomic deprivation and health and social service indices). Non-parametric bivariate analyses (Kruskal-Wallis tests) were conducted with the availability quartiles and each index. Our findings showed that in 66 counties (57% of entire counties in Missouri), SSH-availability was below 20%. Bivariate analyses showed SSH-availability was significantly associated with both neighborhood characteristics: counties with higher SSH-availability were more likely to be socioeconomically deprived and face a lack of health and social services. The current study suggests the government should address the geographic disparities in SSH for low-income older adults. Also, more supportive services are needed around SSH to facilitate aging-in-place of vulnerable older adults.

### COMMUNITY-DWELLING OLDER ADULTS' PERCEPTIONS OF SMART HOME SURVEILLANCE: AN INTEGRATIVE REVIEW

Shehroz Khan,[1] Jessica Campbell,[2] Jacob Buchan,[3] Andria Bianchi,[1] Jesse Hoey,[4] and Charlene Chu[3], *1. Toronto Rehabilitation Institute, Toronto, Ontario, Canada, 2. University of Victoria, Victoria, British Columbia, Canada, 3. University of Toronto, Toronto, Ontario, Canada, 4. University of Waterloo, Waterloo, Ontario, Canada*

Background: Many older adults wish to use smart homes for aging in place, health monitoring, and enhanced safety. However, concerns over privacy and security remain pressing. User perception studies can help to inform policy and design solutions. Aim: To explore community-dwelling older adults' (50+) perceptions of smart home surveillance. Methods: As part of a larger scoping review of smart home user perception based on four non-mutually exclusive categories: privacy, safety, purpose of data collection, and risk, we found

68 results. 15 studies focused on older adults exclusively and were included in this review. Results: The included studies mainly focused on smart speakers, motion sensors, or home monitoring systems. 13 studies (87%) discussed user privacy concerns in terms of data collection and access. Nine studies (60%) reported that users were enthusiastic about the potential for home safety, improved health outcomes and independent living with smart homes. Seven risk awareness studies (47%) featured a range of perspectives on sharing sensitive information due to the possibility of data breaches and third-party misuse, with some reporting a willingness to trade privacy for enhanced safety. Finally, four studies (27%) explored user knowledge of data collection purposes. While many were uncertain of the details, users were generally more comfortable sharing smart home data with healthcare professionals than others. Conclusion: This review has helped us in creating a user perception survey that is currently in the fielding stage. Given Canada's increasing aging population and technological advances, privacy regulators and designers should focus on older adults' concerns.

### RISK FACTORS FOR HAZARDOUS DRINKING AMONG DEMENTIA FAMILY CAREGIVERS

Jessica Hernandez Chilatra, Wesley Browning, Danny Wang, and Carolyn Pickering, *The University of Alabama at Birmingham, Birmingham, Alabama, United States*

Little is known about alcohol use disorder (AUD) among family caregivers of people living with dementia, even though it is suspected as a contributing cause for caregiving outcomes such as elder mistreatment. This study examines the frequency of and potential risk factors for hazardous drinking (one dimension of AUD, measured by the Alcohol Use Disorders Identification Test AUDIT-C) among a sample of dementia family caregivers (Nf165). We used data from the first wave of an ongoing longitudinal study on daily dementia caregiving experiences. We computed binary logistic regression models using SPSS version 28 to identify potential risk factors. Among our sample, 20% of caregivers identified as positive for hazardous drinking. Caregivers with greater difficulty regulating their emotions (OR= 1.046, P=0.005, [95%CI] = 1.014–1.079) had significantly greater odds for hazardous drinking, as well as those with a history of physical neglect in childhood (OR=1.176, P=0.01, [95%CI] =1.037–1.334) and those who seek social support as a coping mechanism (OR=1.119, P=0.01, [95%CI] = 1.027–1.220). Additionally, the higher number of ADLs their relative needs assistance with the lower the odds (OR=0.775, p=0.01, [95%CI] =0.64–0.94) of hazardous drinking. Demographic factors such as gender and age were not associated with hazardous drinking, though the sample was predominantly female (90%). Findings show that dementia family caregivers experience hazardous drinking at a rate higher than the general population (6–16%) suggesting family caregivers are at a uniquely higher risk for AUD. Furthermore, the potential risk factors could inform future screening efforts to identify family caregivers experiencing hazardous drinking.

### AI-ASSISTED METHODS FOR ASSESSING AFFECT AND BEHAVIORAL SYMPTOMS IN DEMENTIA: A SYSTEMATIC REVIEW

Ying-Ling Jao,[1] Yo-Jen Liao,[2] Fengpei Yuan,[3] Ziming Liu,[3] Xiaopeng Zhao,[3] Wen Liu,[4] Diane Berish,[1] and James Wang[1], *1. Pennsylvania State University, University Park, Pennsylvania, United States, 2. Pennsylvania State University, State College, Pennsylvania, United States, 3. University of Tennessee, Knoxville, Tennessee, United States, 4. University of Iowa, Iowa City, Iowa, United States*

Negative affect and neurobehavioral symptoms occur in most people with dementia and significantly impact their health outcomes and sense of wellbeing. Detecting these symptoms in this population is challenging due to associated cognitive impairment and communication difficulties. Innovative technology and artificial intelligence (AI)-assisted tools are emerging for assessing affect and neurobehavioral symptoms in individuals with dementia. This review synthesizes research evidence to identify existing AI-assisted measurement tools and evaluate their accuracy in assessing affect and symptoms in people with mild cognitive impairment and dementia. PubMed, CINAHL, Scopus, and Web of Science databases were searched. Eight articles were identified. Multiple machine learning (ML) models were developed to assess affect, apathy, anxiety, depression, agitation, and wandering. One ML model detected positive and negative affect via facial expression with an overall accuracy of 86%. One ML model detected apathy based on speech and achieved an area under curve (AUC) accuracy of 0.77–0.88. Another speech-based ML model, based on paralinguistic markers, predicted apathy, anxiety, and depression by ≥0.3 points. Another model detected wandering based on activity monitoring data and showed 98% sensitivity and specificity. Furthermore, multiple ML models were developed to detect agitation using multi-modal sensors with AUC ranging from 0.50–0.82. Findings suggest that AI-assisted tools are a promising approach to detecting affect and neurobehavioral symptoms, yet the evidence is limited. More research is needed to develop comprehensive, accurate models to detect neurobehavior symptoms. The results have significant implications for supporting research and clinical practice to promote quality of care for people with dementia.

### ADULT INFECTIONS ARE ASSOCIATED WITH HIGHER RISK OF ALZHEIMER'S DISEASE BUT LOWER RISK OF CANCER

Svetlana Ukraintseva, Vladimir Popov, Hongzhe Duan, Arseniy Yashkin, Igor Akushevich, Konstantin Arbeev, and Anatoliy Yashin, *Duke University, Durham, North Carolina, United States*

Growing evidence suggests that infections may promote Alzheimer's disease (AD). Chronic infections have also been linked to several cancers. However, there is evidence that acute febrile infections may treat some cancers, suggesting that anti-pathogen immune response could help fight off transformed cells. To explore potentially ambivalent role of infections in cancer and AD, we estimated associations between history of infection and subsequent risks of AD, cancer (all sites combined), and all-cause mortality, in sample of 270K participants of the UK Biobank. We found that prior infection is associated with 29% higher risk of AD (RR=1.29, 95% CI[1.02,1.63], both sexes). In contrast, the overall cancer risk was 49% lower in people with history of infection, compared to individuals without such history (RR=0.49, 95% CI[0.46,0.52], both sexes). History of infections was also associated with 7% reduced total survival at ages 70+ (RR=0.93, 95% CI[0.92,0.94]); however, the negative effect was less evident at younger ages. Results of our study suggest that infections may antagonistically influence chances of AD and cancer. They also show that impact of infections on survival depends on age and is more detrimental at older ages, which may reflect general decline in immune resilience due to aging, as well as the fact that cancer becomes a major contributor to mortality risk at younger ages than AD. Understanding the trade-offs between major diseases is essential for optimizing disease prevention and pro-longevity interventions, since measures aiming to prevent one disease may sometimes increase risks of other major conditions and/or all-cause mortality.

### A THEORETICAL FRAMEWORK FOR UTI PREVENTION AND MANAGEMENT IN COMMUNITY-DWELLING OLDER PERSONS WITH DEMENTIA

Kuan-Ching Wu,[1] and Oleg Zaslavsky[2], *1. University of Washington, Seattle, Washington, United States, 2. Unversity of Washington, Seattle, Washington, United States*

UTI admission rates are 3.4 times higher among persons with dementia (PWD) than among those without dementia. The hospitalizations often result in adverse health complications such as the increased risk of delirium, cognitive decline, and higher mortality. To date, several UTI guidelines exist for specific populations, including women, children, people with catheters, and long-term care patients; however, there has been little focus on non-pharmacological prevention and management in PWD. The goal of this work is to propose a theoretical framework for UTI prevention and management in community-dwelling older PWD with the following three aims: 1) identify factors and outcomes of UTIs in community-dwelling older PWD; 2) generate a conceptual framework informed by Bronfenbrenner's 1977 ecological model; 3) integrate preventive measures to the adapted framework. We conducted a scoping literature review utilizing the following keywords and concepts: risk factors, UTI management and prevention, community-dwelling older adults, and dementia. The UTIP framework was generated with four levels of risk factors (individual, interpersonal, community, and national) and four domains of affected outcomes (PWD, caregivers, PWD and their caregivers, and community/national). Additionally, seven themes of preventive measures emerged: physical activity and mobility, behavioral therapy, personal hygiene, phytotherapy, dietary, compound, and hormone. Finally, a table of care management opportunities was proposed according to dementia severity and caregivers' involvement. In conclusion, the UTIP framework synthesizes exciting and is flexible to accommodate new evidence. As such, the framework is informative for programs, practices, and policies to lower the risks of preventable admission in PWD.

### A DEMENTIA IMMERSION SIMULATION EXPERIENCE AS A TRAINING TOOL FOR HEALTH CARE PROFESSIONALS

Sandra Sanchez-Reilly,[1] Paula Reilly,[2] Elizabeth Reilly,[2] Isabella Silva,[2] Lyda Arevalo,[2] and Gabriela Zaragoza[3], *1. South Texas Veterans Health Care System and the University of Texas Health Science Center at San Antonio, San Antonio, Texas, United States, 2. South Texas Veterans Health Care System, San Antonio, Texas, United States, 3. UTHSCSA, San Antonio, Texas, United States*

Background:Dementia, a progressive, devastating and incurable disease affects millions of older Americans and their caregivers. Effective dementia-training programs for caregivers and healthcare professionals (HP) can lead to improved patient outcomes. Simulation-educational experiences are innovative with long-lasting impact. Currently, there are no simulation hands-on experiences focused on dementia care to train HP or hired caregivers (HC). Objective: To evaluate the educational effectiveness of a Dementia Immersion Simulation Experience (DISE) intervention among HP and HC. Methods: DISE is a face-to-face 2-hour intervention that includes virtual reality, hands-on simulation with multiple sensory experiences, group debriefing led by dementia-caregiving expert. Program evaluation and pre/post knowledge assessments were administered. Results: Nf110. HP, Nf72; HC, Nf25. Pre/post mean score of knowledge assessment (scale 0–12) for all was 8.3/9.7 (p < 0.0001). Participants were also grouped by whether they were "well-informed" (achieving 10 or better on knowledge assessment) or had "knowledge gap" (9 or fewer). 25% were well-informed on dementia before DISE; 63% after DISE (p < 0.0001). DISE program evaluation showed 98% participants highly rated experience across all categories. Evaluation scores further support an effective program. Furthermore, number of clinical referrals seeking expertise from a dementia specialist/team increased by 50% two months post-intervention. Conclusion: DISE is a successful tool to teach, support and empower HP and HC, effectively changing care provided to individuals with dementia by using hands-on simulation training. Further studies are needed to evaluate the effectiveness of DISE in improving behavioral symptoms, training family caregivers, and decreasing other dementia-related undesirable outcomes such as nursing home placement.

### A COMPREHENSIVE ALZHEIMER'S AND DEMENTIA CARE PROGRAM (ADCP) FOR PATIENTS AND CAREGIVERS

Madieska Ramirez,[1] Prabhash Kakarla,[2] Marzena Gieniusz,[1] Jessica Alvarez,[1] Chris Nouryan,[3] and Edith Burns[4], *1. Hofstra-Northwell, New Hyde Park, New York, United States, 2. Hofstra University, New Hyde Park, New York, United States, 3. Zucker School of Medicine at Hofstra Northwell, Uniondale, New York, United States, 4. Zucker School of Medicine at Hofstra Northwell, Manhasset, New York, United States*

Background: Alzheimer's and related dementias (ADRD) are progressive and associated with behavioral and psychological symptoms (BPSD) contributing to caregiver stress. We report initial impact of implementing UCLA-designed ADCP to support ADRD patients and caregivers. The program centralizes care, utilizing a nurse dementia-care specialist. Methods: Prospective repeated measures of ADRD patient/caregiver dyads from 3/2021–7/2022. Measures: BPSD, ADL/IADL, cognition, and caregiver burden, distress and depression. Results: Total of 154 patient/caregiver dyads enrolled to-date. 42 have been enrolled for one-year: 23 with complete data, 5 pending follow-up, 14 disenrolled (2 expired, 3 to hospice, 2 moved, 7 lost contact). For 23 patients with baseline and follow up, Neuropsychiatric Inventory Questionnaire decreased from 16.3 to 13.1 (p=0.170), Patient Health Questionnaire-9 (PHQ-9) for caregivers decreased (3.5 to 2.2, p=0.044), Montreal Cognitive Assessment decreased from 11.3 to 8.4 (p=0.045), and Dementia Burden Scale (DBS) showed nonsignificant decrease, 20.6 to 18.4 (p=0.312). Cornell Scale for Depression in Dementia was unchanged, 5.67 to 5.14 (p=0.380), and ADL and IADL decreased one year after enrollment, from 4.05 to 2.36 (p < 0.01) and 1.38 to 0.238 (p < 0.01) respectively. Conclusion: Although attrition rate was high for the small sample (1/3 of dyads enrolled in the program for a year), there were improvements in some metrics for both patients and caregivers, despite the expected decline in cognitive function and ADL/IADL. As data is accrued, we anticipate it will identify patients and support resources that will be most beneficial.

### WHAT MOTIVATES PARTICIPATION IN ALZHEIMER'S PREVENTION RESEARCH: A MIXED-METHODS STUDY

Lizbeth Vera Murillo,[1] Maria Vander Meulen,[1] Monique Villamor,[1] Angel Collie,[1] Sarah Cline,[1] Jody Nicholson,[1] and Jerri Edwards[2], *1. University of North Florida, Jacksonville, Florida, United States, 2. University of South Florida, Tampa, Florida, United States*

6.5 million Americans aged 65 and older are currently living with Alzheimer's disease or related dementias (ADRD; Alzheimer's Association, 2022). By 2060, it is estimated that this number will increase to 13.9 million (Matthews et al., 2018). Therefore, it is imperative to gain insight into participants' personal motivations and expectations of research to advance community participation. The Preventing Alzheimer's with Cognitive Training (PACT) study is a National Institute of Health, National Institute on Aging-funded, multi-site clinical trial examining the prevention of mild cognitive impairment and ADRD through computer-based cognitive training. Across 5 locations, data were collected from 2,360 cognitively normal participants (M=73.03 years, range=65–97, SD=5.04). The current project explores individuals' motivations and expectations of cognitive training (CT) utilizing a mixed-method approach by coding qualitative open-ended questions about motivation to participate and comparing how motivational themes aligned with expectations about CT from the Expectations Assessment Scale (EAS; Rabipour et al., 2018). Six themes for participant motivation emerged: direct experience with the disease (26.9%), concern about brain health and aging (23.9%), general personal interest (17.9%), general interest in research (20.1%), referral to the study (5.8%), and altruism (5.4%). After completing the initial training session, motivation themes did not differentiate satisfaction with (p=.06) or perceived success of (p=.11) the CT program. Understanding participants' motivations can further expand and optimize recruitment and retention strategies in AD prevention research. Future research will focus on how these themes influence adherence and retention and relate to participant demographic characteristics (i.e., education, gender, race, and ethnicity).

### USER PERSONAS TO GUIDE TECHNOLOGY INTERVENTION DESIGN TO SUPPORT CAREGIVER-ASSISTED MEDICATION MANAGEMENT

Anna LINDEN,[1] Priya Loganathar,[1] Richard Holden,[2] Malaz Boustani,[3] Noll Campbell,[4] Aaron Ganci,[5] and Nicole Werner[2], *1. University of Wisconsin-Madison, Madison, Wisconsin, United States, 2. Indiana University School of Public Health-Bloomington, Bloomington, Indiana, United States, 3. Indiana University School of Medicine, IU Center for Aging Research, Indianapolis, Indiana, United States, 4. Purdue University, Indianapolis, Indiana, United States, 5. IUPUI, Indianapolis, Indiana, United States*

Informal caregivers often help manage medications for people with ADRD. Caregiver-assisted medication management has the potential to optimize outcomes for caregivers and people with ADRD, but is often associated with suboptimal outcomes. We used the user-centered design persona method to represent the needs of ADRD caregivers who manage medications for people with ADRD to guide future design decisions for technology interventions. Data were collected through virtual contextual inquiry in which caregivers (Nf24) sent daily multimedia text messages depicting medication management activities for seven days each, followed by an interview that used the messages as prompts to understand medication management needs. We applied the persona development method to the data to identify distinct caregiver personas, i.e., evidence-derived groups of prospective users of a future intervention. We used team-based affinity diagramming to organize information about participants based on intragroup (dis)similarities, to create meaningful clusters representing intervention-relevant attributes. We then used group consensus discussion to create personas based on attribute clusters. The six identified attributes differentiating personas were: 1. medication acquisition, 2. medication organization, 3. medication administration, 4. monitoring symptoms, 5. care network, 6. technology preferences. Three personas were identified based on differences on those attributes: Regimented Ruth (independent, proactive, tech savvy, controls all medications), Intuitive Ian (collaborative, uses own judgment, some technology, provides some medication autonomy), Passive Pamela (reactive, easy going, technology novice, provides full medication autonomy). These personas can be used to guide technology intervention design by evaluating how well intervention designs support each of them.

### UNIQUE TRANSKINGDOM MICROBIOME SIGNATURES LINKED WITH COGNITIVE DECLINE IN OLDER ADULTS OF MIAGB CONSORTIUM COHORT

Diptaraj Chaudhari,[1] Shalini Jain,[1] Hariom Yadav,[1] Michal Masternak,[2] Peter Holland,[3] Marc Agronin,[4] and MiaGB Consortium Team[1], *1. University of South Florida, Tampa, Florida, United States, 2. University of Central Florida, Orlando, Florida, United States, 3. Florida Atlantic University, Boca Raton, Florida, United States, 4. Miami Jewish Health, Miami, Florida, United States*

The prevalence of age-related cognitive disorders is increasing. Effective prevention and treatment interventions are unavailable due to a poor understanding of aging biology. Multiple emerging evidence indicates that the gut microbiome is linked with age-related disorders; however, their clinical importance in differentiating and predicting the risk of cognitive decline or dementia is largely elusive. Utilizing samples and data of a large, multi-site clinical study across the state of Florida called Microbiome in aging Gut and Brain (MiaGB) Consortium, our whole genome microbiome sequencing revealed that the viral and archaeal population was significantly reduced in the gut of older adults with dementia (n=8) compared to those with mild cognitive impairment (MCI) (n=25) and normal cognition (n=59). Whereas the fungi were exclusively detected in the controls only. Alpha diversity of the participants with MCI and dementia was lower than the cognitively healthy controls. The abundance of Actinobacteria and Verrucomicrobia phyla was higher, and Firmicutes phylum was lower in the participants with dementia. Bacteriophages Lactobacillus prophage Lj771 and Microbacterium phage Min1 were exclusively detected in the gut of the participants with dementia. The study also identifies key metabolic pathways altered in the controls versus the cognitive impairment state. Our biomarker discovery analyses also revealed that these unique microbiome signatures and pathways might have predictive power for cognitive decline and dementia risk and offer new targets for future therapeutic interventions.

### UNDERSTANDING DEMENTIA DISCOURSE DURING ALZHEIMER'S AWARENESS MONTH IN CANADA: FIRST INSIGHTS FROM A TWITTER STUDY

Juanita-Dawne Bacsu,[1] Megan E. O'Connell,[2] Allison Cammer,[2] Mehrnoosh Azizi,[2] Soheila Ahmadi,[2] Karl Grewal,[2] Shoshana Green,[2] and Corinne Berger[2], *1. Thompson Rivers University, Kamloops, British Columbia, Canada, 2. University of Saskatchewan, Saskatoon, Saskatchewan, Canada*

Twitter has become a key platform for public health campaigns, ranging from mental health awareness week to diabetes awareness month. However, there is a paucity of knowledge about how Twitter is being used to support health campaigns, particularly for Alzheimer's Awareness Month. This presentation aims to: 1) identify how Twitter was used to share dementia discourse during Canada's Alzheimer's Awareness Month in January; and 2) explore actions to enhance dementia awareness using Twitter for future Alzheimer's Awareness Month campaigns. Tweets were collected from Twitter using the Twint application in Python from January 1 to January 31, 2022. Filters were used to exclude irrelevant tweets (5,820), and the remaining 1,289 tweets were exported to Excel. Tweets were divided among eleven coders and analyzed using inductive thematic analysis. Analysis revealed four main themes: dementia education and advocacy; fundraising and promotion; sharing experiences of dementia; and opportunities for future actions such as collaborative partnerships and educational tweets to correct stigmatizing language and dementia stereotypes. Increased educational content, collaborative partnerships, and evidence-informed research are essential to enhancing dementia awareness strategies on Twitter during Alzheimer's Awareness Month in Canada. Further research is needed to develop, implement, and evaluate methods to improve dementia awareness on Twitter during Alzheimer's Awareness Month and beyond.

### TRENDS IN OSTEOPOROSIS DRUG USE AMONG MEDICARE BENEFICIARIES WITH & WITHOUT ALZHEIMER'S DISEASE/RELATED DEMENTIAS

Peyton Armstrong,[1] Yong-Fang Kuo,[2] Jordan Westra,[1] and Mukaila Raji[2], *1. UTMB, Galveston, Texas, United States, 2. University of Texas Medical Branch at Galveston, Galveston, Texas, United States*

Background: Osteoporotic fractures are a leading cause of disability and premature death in the elderly. Patients with Alzheimer's and related dementia (ADRD) have high rates of osteoporosis (OP) and substantial risk of osteoporotic fractures. Yet research is sparse on trends and predictors of OP medication use in ADRD. Methods: Medicare beneficiaries with OP aged ≥67 years with Medicare parts A/B/D without HMO from 2016–2018. Outcome was receipt of OP medications in 2018. A multivariable logistic regression assessed association between ADRD and OP drug prescribing, adjusted for age, sex, race, region, Medicare entitlement, dual Medicaid eligibility, chronic conditions, number of provider visits/hospitalizations, and nursing home (NH) resident status. Age/ADRD and NH residency/ADRD interactions were tested. Results: Sample consisted of 47,871 people with OP and ADRD and 201,840 with OP without ADRD. OP drug use was 38.6% in ADRD patients vs. 52.7% in non-ADRD. After adjustment for demographics, chronic conditions, previous hospitalizations/physician visits, the OR for OP drug in ADRD vs. Non-ADRD was 0.85 (95% CI: 0.83–0.87). NH residents had lower odds for OP medication (OR: 0.61, 95% CI: 0.58–0.64). There were significant interactions between ADRD/age and between ADRD/NH residency. The OR for OP drug use associated with ADRD was 0.88 (95% CI: 0.86–0.90) among community-dwelling elders and 0.66 (95% CI: 0.64–0.69) among NH residents. Conclusions: ADRD patients received OP drugs at lower rates than non-ADRD counterparts. More research is needed on prescribing or deprescribing OP drugs in context of ADRD severity, patient preferences, remaining life expectancy and time-to-benefit from OP drugs.

### THE WHATMATTERS APP: CO-DESIGNING PERSON-CENTERED CARE FOR PEOPLE WITH DEMENTIA IN CARE SETTINGS

Lillian Hung,[1] Ellen Guo,[2] Mariko Sakamoto,[1] Karen Lok Yi Wong,[1] Candy Tran,[3] Jim Mann,[4] Annette Berndt,[4] and Eva Egeberg[5], *1. University of British Columbia, Vancouver, British Columbia, Canada, 2. The University of British Columbia, University Endowment Lands, British Columbia, Canada, 3. School of Nursing University of British Columbia, University Endowment Lands, British Columbia, Canada, 4. Community Engagement Advisory Network, Vancouver, British Columbia, Canada, 5. Emily Carr University, Vancouver, British Columbia, Canada*

The purpose of our study is to provide comfort through digital resources (e.g., music and visual materials) for patients/residents with dementia in hospitals and long-term care. By working with users (patients/residents, families, and staff) and using a co-design approach, we have developed a mobile app prototype called "WhatMatters" to equip staff with a useful digital tool for delivering person-centered care in hospitals and long-term care homes. Using user experience co-design methods, we conducted a series of virtual co-design workshops with acute and long-term care staff (n=10), clinical experts (n=3), residents (n=3), and patient and family partners (n=7) to understand: (a) what "comfort" means, (b) how care needs are communicated and provided for, and (c) how a mobile app may be used to support psychosocial needs of people living with dementia in hospital and long-term care settings. Thematic analysis has identified three themes to inform the development of the mobile app WhatMatters: (a) familiarity brings comfort, (b) sharing of information between staff and families allows for continuity of care and person-centered care, and (c) accessible and curated content can evoke memories and create a comforting space. Our study conclusions are: It is feasible and necessary to work with users (including clinical staff, patient, and family partners) and relevant stakeholders to co-design a mobile app, a useful tool to support the delivery of person-centered care in care settings.

### THE ROLE OF SLEEP DISTURBANCE IN COGNITIVE OUTCOMES AMONG LONELY ADULTS IN THE HEALTH AND RETIREMENT STUDY

Adrienne Grudzien, *Florida International University, Miami, Florida, United States*

Both sleep disturbance and loneliness have been linked to poorer cognitive performance and increased risk of dementia in older adults. Loneliness is believed to influence cognitive functioning directly and indirectly through the HPA axis dysfunction, which increases systemic inflammation through endocrine, immune system, and genetic pathways which produce a range of negative effects on brain tissue. Higher levels of HPA axis activity associated with lonely feelings may lead to poorer sleep quality, which decreases beta-amyloid and tau clearance, as well as memory consolidation, which may further disrupt sleep through alterations in brain tissue. Despite the growing body of research, little is known about how sleep disturbance and loneliness, both modifiable risk factors, interact to effect cognition and dementia risk over time. Secondary analysis of the 2006, 2010, 2014, and 2018 waves of the Health and Retirement Study was completed using data from 1,092 participants (mean age: 66.24; SE: .1366). Mixed effects regression modeling was used to evaluate the role of sleep disturbance in the relation between loneliness (R-UCLA loneliness scale, 11 item) and four cognitive performance measures: Immediate and Delayed Word Recall, Serial 7s, and Backward Count, and cognitive status category (cognitively normal, cognitively impaired, and dementia) across a 12-year period. Sleep disturbance moderated the relation between loneliness and Immediate Word Recall ($p=.048$) and Serial 7s ($p=.032$) outcomes even after controlling for demographic covariates and depression. Findings suggest that sleep disturbance may exacerbate the negative physiological effects of loneliness on episodic memory and executive functioning in older adults.

### THE RELATIONSHIP BETWEEN SOCIOECONOMIC DISADVANTAGE AND DEMENTIA CAREGIVER BURDEN

Alexis Beverly,[1] Christina Baucco,[1] Seho Park,[2] Matthew Schroeder,[3] Shelley Johns,[2] Katherine Judge,[4] and NIcole Fowler[5], *1. Regenstrief Institute, Indianapolis, Indiana, United States, 2. Indiana University School of Medicine, Indianapolis, Indiana, United States, 3. Regenstrief Institute, Inc., Indianapolis, Indiana, United States, 4. Cleveland State University, Cleveland, Ohio, United States, 5. Indiana University School of Medicine, IU Center for Aging Research, Indianapolis, Indiana, United States*

More than 16 million people provide unpaid care to someone with Alzheimer's disease or a related dementia (ADRD) in the United States. These caregivers frequently report experiencing psychological and physical burden. Prior research shows that socioeconomic disadvantaged neighborhoods have higher rates of ADRD, but little is known about caregiver burden. We hypothesized more socioeconomic disadvantage is associated with higher caregiver burden. We performed a secondary analysis using baseline data on dementia caregivers (n=132) enrolled in the Indiana University Telephone Acceptance and Commitment Therapy for Caregivers (TACTICs) pilot trials. Mean (SD) caregiver age was 61.6 (11.6) years; 83.3% women; 78.8% white; 19.7% black. Seventy-two percent of the caregivers lived with the care recipient and 50.8% reported moderate dementia severity. A linear regression model examined the relationship between caregiver's national Area Deprivation Index (ADI) score (ranging from 1–100 with higher scores indicating greater socioeconomic disadvantage) and caregiver burden. The following covariates were included: age, sex, race, education, shared residence with care-recipient, health status, anxiety, depression, and dementia severity of care recipient. Higher ADI was associated with lower caregiver burden ($\beta=-0.222$, $p < 0.001$). Caregiver burden has a significant negative relationship with ADI and dementia severity ($p<-.001$ and 0.046, respectively) and positive relationship with anxiety ($p=0.014$) controlling other covariates in the model. Although we found no support for the hypothesis, further research should examine these factors with how caregiver's ADI may impact other psychosocial wellbeing outcomes. Discussion will highlight the need for caregivers to have access to resources that can aid them through their experience.

### THE IMPACTS OF NURSING HOME COVID-19 SOCIAL ISOLATION POLICIES ON RESIDENTS LIVING WITH DEMENTIA

Bill Buron,[1] Tim Killian,[2] and Cassy Eng[1], *1. The University of Tulsa, Tulsa, Oklahoma, United States, 2. University of Arkansas, Fayetteville, Arkansas, United States*

Using reports from 31 nursing home activity directors (AD) from across the United States, we examined responses to COVID-19 social isolation policies/practices in relation to AD's perceptions of the impact on residents living with dementia (RLWD). AD's perceptions are particularly valuable because they are generally responsible for bolstering social connectedness. We approached this study from the sociological perspective of personhood (Buron, 2008; Kitwood, 1997) that suggests social isolation may threaten personhood status, lead to increased agitation, and other behaviors as mechanisms to preserve self. Most ADs reported that social isolation protocols began in March 2020 and were still ongoing through the beginning of 2022. Over 90% reported a 50% or more reduction in family member/guest visits, with over 30% reporting a 90% or more reduction in visits. Systemic content analyses were applied to open-ended responses. These patterns suggested ADs had concerns about (1) poor quality care as a result of a) a reduced workforce leading to caregiver burnout, and b) less accountability of caregivers (to families/visitors in their absence) for resident care, and (2) increasing signs of depression and negative behaviors among RLWD. Some ADs felt that Isolation policies disrupted routines of RLWD, leading to confusion and a greater progression of their dementia. Some ADs felt the need to "fill-in" as an attempt to make up for the loss of interactions with family members. The results of this study indicate a need to consider future pandemic policies and potential impacts on residents living with dementia in nursing home settings.

### THE IMPACT OF 12 WEEKS OF ADAPTED DANCE ON BALANCE, GAIT, AND LOWER EXTREMITY FUNCTION AMONG PERSONS WITH DEMENTIA

Keara Quijano,[1] and Crystal Bennett[2], *1. University of West Florida, Milton, Florida, United States, 2. University of West Florida, Lillian, Alabama, United States*

Person's living with dementia commonly experience difficulty with mobility. Difficulties with these activities can lead to an increased fall risk, resulting in an increased loss of independence. Dance engages various parts of the brain including the cerebellum that is primarily involved in coordinating balance, posture, body positioning, and voluntary movement. An aim of this study was to assess whether 12 weeks of adapted dance improves balance, usual walking speed, and lower extremity function among persons living with dementia. An experimental design was used to randomly assign persons with dementia to either a 12-week adapted dance or social stimulation group. The convenience sample consisted of 12 participants, ages ranging from 62–97 years. The adapted dance is low impact where one foot is always in contact with the floor and is appropriate for older adults with cognitive and physical limitations. At baseline and at 12 weeks, measures of balance, gait, and lower extremity function were assessed. From baseline to posttest, the dance group had greater increased times for maintaining tandem balance (+47.5%) and faster times for usual gait speeds (+15.1%); compared with the social stimulation group tandem balance time (+0.98%) and usual gait speed times (+10.5%). A limitation of this study is the small sample size.

### THE EFFICACY OF MUSIC AND DEMENTIA EDUCATION PROJECT ON HEALTH CARE PROFESSIONAL STUDENTS: PRELIMINARY OUTCOMES

Jiaming Shi,[1] Fei Sun,[1] Jen Hirsch,[1] Dan Cohen,[2] and Ha-Neul Kim[1], *1. Michigan State University, East Lansing, Michigan, United States, 2. Right to Music, New York City, New York, United States*

This study aims to improve dementia literacy and competence to use music in practice with people with dementia among students in health professional fields. The eight-week curriculum was developed by a gerontology social worker and a subject matter expert on personalized music use for people with dementia. Two focus groups were completed in Spring of 2022 with people living with dementia and care partners to refine the curriculum and include information that those with lived experience felt was important for future health care professionals to know. The study used 2 single group designed cohorts of students who received the 8-week educational intervention over Zoom. Each cohort included 2 persons living with dementia (PWD) as co-trainers. Pre- and post- surveys included a modified Alzheimer's Disease Knowledge Scale and Dementia Attitude Scale. Participants (n=17) included Social Work = 8, Nursing = 2, Psychology =3, and Others =4. Total of 11 student participants completed both pre-test and post-test surveys. Paired t-tests were used to compare pre-post differences. At post-test, participants reported increased dementia knowledge (M=2.45, p=.0027); increased dementia attitudes (M=13.18, p=.0057); and improved competency on using music with PWD (M=.97, p=.0045) compared to the baseline scores. The 8-week intervention showed a statistically positive impact on dementia attitudes and competency of music use. The findings of this study laid the groundwork for future studies on training current service professionals and comparing their practice after the training to non-trained professionals to measure the impact of the training in intervention.

### THE COMPARISON OF DUAL TASK TIME AMONG APOE CARRIERS AGAINST NON-APOE CARRIERS IN AGING ADULTS

Cody Diehl,[1] Anthony Campitelli,[1] Megan Jones,[1] Joshua Gills,[2] Sally Paulson,[3] Jordan Glenn,[4] Kelsey Bryk,[5] and Michelle Gray[1], *1. University of Arkansas, Fayetteville, Arkansas, United States, 2. Rutgers University-Newark, Jersey City, New Jersey, United States, 3. St. Elizabeth Healthcare, Cincinnati, Ohio, United States, 4. Neurotrack Technologies, Redwood City, California, United States, 5. Neurotrack Technologies, Inc., Redwoods City, California, United States*

No single variable has been identified as the sole factor for predicting an individuals' declining health status. In order to predict mental or physical decline in older adults, researchers have designed tasks that include both a physical and mental or cognitive demand known as dual task (DT). The purpose of implementing dual task is to assess the possible changes in accuracy when a second task gets compounded with a given single task. Our study consists of males and females aged from 45 to 75 years who have risk factors for dementia. Each subject was randomly assigned to either a health coaching (HC), or health education (HE) control group. The HC group received coaching to address the lifestyle areas known to be linked to AD risk, such as diet, exercise, sleep, stress, cognitive training, and social interaction. Blood markers of each individual have been assessed to determine carrier status of Apolipoprotein E 4 (APOE4) due to the correlation between the allele and increased risk of Alzheimer's. To be included in the study, the participants must have at least 2 positive risk factors for AD as determined by the Australian National University-Alzheimer Disease Risk Index (ANU-ADRI). Among non-APOE carriers, a 2x3 (group x time) mixed ANOVA revealed a significant interaction effect wherein the HC group improved DTC score more than the HE group from time 1 to time 3, F (2, 362) =1.253, P=0.017. No significant interaction was noted for APOE carriers.

### THE ASSOCIATION OF LIFESTYLE AND SOCIAL ENGAGEMENT WITH DEMENTIA: A NATIONWIDE POPULATION-BASED COHORT STUDY

Jiyoon Jang, Sang Hwa Lee, and Eunhee Cho, *College of Nursing, Yonsei University, Seoul, Seoul-t'ukpyolsi, Republic of Korea*

As the aging population increases, the number of people with dementia is expected to rise rapidly, resulting in a growing burden on families and society. Delaying and preventing the clinical onset of dementia are significant public health goals. Unhealthy lifestyles and social disengagement have been identified to be modifiable risk factors for all-cause dementia. However, this has not been fully examined in the Korean population. Therefore, this study aimed to determine the association of lifestyle and social engagement with dementia incidence, based on the Korean Longitudinal Study on Aging (KLoSA) database with recently added dementia-related variables (diagnosis, medication administration, and treatment). The study included 5,071 participants; cases are patients diagnosed with dementia from 2010 to 2020 and controls are older adults without dementia. Logistic regression analysis was used to examine the relationship of dementia with lifestyle factors (alcohol consumption, smoking, exercising) and social engagement. Among the lifestyle factors, the current smoking status was associated with an increased risk of dementia (OR = 2.359, $p < .05$) after controlling the health-related covariates and general characteristics. Furthermore, increased participation in social activities was significantly related with a decreased risk of dementia (OR = 0.575, $p < .01$). This study highlighted the importance of non-smoking and social engagement in reducing the risk of dementia in later life. Therefore, implementation of interventions that focus on these leading risk factors can significantly reduce the burden of dementia in Korea.

### SUPPORTING DEMENTIA CAREGIVERS DURING COVID-19 WITH CAREHEROES IT: USAGE PATTERNS AND OUTCOMES

Nicole Ruggiano,[1] Ellen Brown,[2] C. Victoria Framil,[2] Shannon Hurley,[3] Lisa Roberts,[2] Peter Clarke,[2] Sai Chaithra Allala,[2] and Jane Daquin[1], *1. University of Alabama, Tuscaloosa, Alabama, United States, 2. Florida International University, Miami, Florida, United States, 3. Florida State University, Tallahassee, Florida, United States*

Caregivers of people with dementia often experience negative physical and mental health outcomes due to the complex challenges posed by dementia symptoms and navigating care systems. The COVID-19 pandemic posed even greater hurdles for caregivers as many health care and support services transferred to virtual formats and required increased use of information technologies (IT). This research examined usage data and psychosocial outcomes of dementia caregivers who used a new mobile-enabled web-based app that was offered as an adjunct to clinical care at two memory clinics (Birmingham, AL; Miami, FL) during the pandemic. This app, called CareHeroes: (1) allows caregivers to self-assess their emotional and mental health; (2) offers a secure platform for tracking and communicating patient-related information among caregivers and providers; and (3) offers caregiver education. CareHeroes is also available in Spanish. Effort was made to recruit digitally underserved populations of caregivers (e.g., rural dwelling, underserved racial/ethnic groups). This presentation will review caregiver outcomes (e.g., burden, depression, user data) at baseline and 3-month follow-up. Among the 22 caregivers who enrolled in the study, 17 completed baseline and 3-month follow-up interviews. Over a 14-month period, participants logged onto CareHeroes 131 times, which varied by month. Caregivers most often used CareHeroes to complete assessment tools. Findings indicate that depression and burden were lower at 3-month follow-up compared to baseline, though this reduction was not statistically significant. The presentation will also review challenges of integrating a new technology intervention designed to promote telehealth during the COVID-19 pandemic.

### R1P DISPERSION IN WHITE MATTER CORRELATES WITH COGNITIVE IMPAIRMENT IN OLDER ADULTS

Fatemeh Adelnia, Taylor Davis, Lealani Mae Acosta, Amanda Puckett, Feng Wang, Zhongliang Zu, Kevin Harkins, and John Gore, *Vanderbilt University Medical Center, Nashville, Tennessee, United States*

Alzheimer's disease is the most frequent form of dementia in older adults and has a total estimated worldwide cost that rises to $2 trillion by 2030. Much previous neuroimaging research in AD has focused on the roles of amyloid and tau proteins using PET, but there have also been several studies that have implicated microvascular changes as an early indicator of damage related to later dementia. Here, we developed and refined a non-invasive 3D-R1ρ dispersion imaging technique using different locking fields to quantify microvasculature changes within brain tissues in persons with mild cognitive impairment (MCI) compared to healthy controls. The fractional difference in R1ρ comparing different locking fields provides a unique way of characterizing changes in the geometry and structure of microvasculature. After providing informed consent, 40 adults aged 62 to 82 years (n=17MCI) underwent cognitive assessments and MRI scan at 3T. We found the fractional change in R1ρ of the whole brain white matter is significantly greater in persons with MCI, and the correlation remained significant (ß=-0.4, p-value=0.01) after introducing age (ß=0.2, p-value=0.2) and sex (ß=-0.1, p-value=0.5) as covariates. The white matter hypertonicity lesion volume measured from conventional MRI was also correlated with the health status (p-value < 0.05); however, the size of the regression coefficient was substantially smaller (53% lower), and it was no longer significant (p-value=0.14) after adjusting for age and sex. This work establishes a new non-invasive method that can potentially characterize changes in microvasculature anatomy with the progression of cognitive impairment regardless of an age effect.

### PRIMARY CARE PROVIDER PERSPECTIVES ON LANGUAGE AND COMMUNICATION BARRIERS IN DEMENTIA DIAGNOSIS AND CARE

Alma Hernandez de Jesus,[1] Melissa Ma,[2] Daniel Dohan,[3] Cecilia Alagappan,[4] Katherine Rankin,[5] and Alissa Sideman[2], *1. UCSF, San Francisco, California, United*

*States, 2. University of California, San Francisco, San Francisco, California, United States, 3. UC San Francisco, San Francisco, California, United States, 4. University of California San Francisco, Memory and Aging Center, San Francisco, California, United States, 5. University of California San Francisco, San Francisco, California, United States*

Dementia diagnosis and care relies on extensive communication between a doctor, patient, and oftentimes a family caregiver. Communication is important for recognizing when there is a cognitive concern, gaining an understanding of the patient's history of cognitive decline, engaging in cognitive testing, and providing quality care post-diagnosis. We conducted a qualitative study of 35 primary care clinicians and primary care nurse practitioners working in safety net settings in California to understand facilitators and barriers to dementia diagnosis and care. Using thematic analysis, we identified similar themes to those that have been explored extensively related to language and communication in doctor-patient relationship. Topics ranging from challenges that emerge when there is language discordance, difficulties finding and using interpreters, and challenges related to communicating medical topics in a patient's native language. Specifically related to dementia, we found that language challenges emerge due to inadequate translation or availability of cognitive testing and post-diagnostic resources in multiple languages. However, we also identified unique challenges related to communication, including hearing loss, communicating about trauma during the diagnostic history interview, navigating the logistics of care, including communicating with specialists, building trust and rapport when there is language discordance, and cultural miscommunication even when the spoken language is the same. These issues affect historically marginalized individuals and communities, especially when dementia and cognitive impairment are present. We suggest new approaches and policies to enhance communication and better ways of working with patients who are hard of hearing.

### PREDICTING DYADIC RELATIONSHIP STRAIN IN INDIVIDUALS WITH DEMENTIA

Amanda MacNeil,[1] Katherine Judge,[2] and David Bass[3], *1. Cleveland State University, Fairview Park, Ohio, United States, 2. Cleveland State University, Cleveland, Ohio, United States, 3. The Benjamin Rose Institute on Aging, Cleveland, Ohio, United States*

Recent literature has addressed the perceptions of individuals with dementia to understand how they experience their illness, with evidence suggesting these perceptions are impactful. Few studies, however, have used a conceptual model to explore different aspects of the illness. One aspect to consider is dyadic relationship strain, or feelings of tension, manipulation, and stress between the individual and caregiver. Little work has addressed this strain from the perspective of the individual with dementia who may have different feelings about the quality of the relationship. Cognition and function are two hallmark symptoms in dementia, however little work has addressed how the perception of these two areas impacts the illness experience. Perceptions of difficulties in these two areas may impact dyadic relationship strain as they necessitate increased care and changes in the relationship. Guided by the Stress Process Model for Individuals with Dementia, this study assessed potential predictors of dyadic relationship strain, finding personal activities of daily living (PADLs) to be impactful. In a multiple regression, PADLS (b=.319, p = 02) predicted strain above and beyond two measures of cognition: objective cognitive impairment (b =-.011, p =.93) and perceived memory difficulty (b=.003, p=.311) suggesting that perceived function is impactful for dyadic relationship strain. Because PADLS include more hands-on assistance, the perception of difficulty may create more feelings of embarrassment or stress and impact the perception of relationship strain. Future intervention work may target perceptions of function to improve the dyadic relationship by using techniques such as open communication about difficulties.

### POSITIVE EMOTION EXPRESSION AND ADAPTIVE COPING BEHAVIORS FOR ADULT CHILDREN OF PARENTS WITH MEMORY LOSS

Dustin Gad, Amanda Piechota, and Joan Monin, *Yale School of Public Health, New Haven, Connecticut, United States*

The broaden-and-build theory suggests that experiences of positive emotion may lead to enhanced utilization of adaptive coping strategies, and a decrease in maladaptive coping strategies (Gloria & Steinhardt, 2016). This relationship between positive emotion and adaptive coping has yet to be studied directly in a sample of adult child caregivers of a parent with memory loss. As part of a larger study investigating relationship dynamics between adult children and their parents with memory loss, adult children 18 years of age and older (n= 67) responded to self-report surveys and engaged in a 6 minute, video recorded, positive interaction session, playing "name that tune" with each other. The session was observationally coded by two coders for "enjoyment/enthusiasm/fun" (k= .516), "laughter" (k= .631), and "positive affect displayed towards partner" (k=.464), using a reliable and valid support-seeking and caregiving behavior coding system (Collins & Feeney, 2000). Spearman's rank correlations between these behavior codes and the self-reported Brief COPE Inventory (BCI) scores suggested positive correlations between "laughter" and the emotional support subscale of the BCI (rs=.259, p= .034), and between "positive affect displayed towards partner" and the venting subscale of the BCI (rs= .256, p= .036). These findings suggest that the expression of positive emotions in the caregiving process might yield important psychological benefits to the caregiver, through increased utilization of specific coping mechanisms. More research in this area is needed to determine whether positive emotion expression is associated with the use of adaptive coping more so than maladaptive coping mechanisms in this population.

### PERCEIVED SOCIAL ISOLATION IS CORRELATED WITH BRAIN STRUCTURE AND COGNITIVE TRAJECTORY IN ALZHEIMER'S DISEASE

Ye Zhang,[1] Yasuko Tatewaki,[2] Yingxu Liu,[2] Naoki Tomita,[2] Yumi Takano,[2] Taizen Nakase,[2] Tatsushi Mutoh,[2] and Yasuyuki Taki[2], *1. Tohoku University, Sendai, Miyagi, Japan, 2. Institute of Development, Aging and Cancer, Tohoku University, Sendai, Miyagi, Japan*

Perceived social isolation was associated with future cognitive decline and increase risk of Alzheimer's disease (AD). However, the impacts of perceived social isolation depending on different clinical stages of AD have not been elucidated. This study was to investigate the influence of perceived social isolation, or loneliness on brain structure and future cognitive trajectories in patients who are living with or are at risk for AD. 176 patients (mean age of 78 years, 39 subjective cognitive decline [SCD], 53 mild cognitive impairment [MCI], 84 AD) underwent structural MRI and neuropsychological testing. Loneliness was measured by one binary item question "Do you often feel lonely?". Voxel-based morphometry was conducted to evaluate regional gray matter volume (rGMV) difference associated with loneliness in each group. Subgroup analysis was performed in 51 patients with AD (n=23) and pre-dementia status (SCD-MCI, n=28) using the longitudinal scores of Alzheimer's Disease Assessment Scale-cognitive component-Japanese version (ADAS-Jcog). Whole brain VBM analysis comparing lonely to non-lonely patients revealed loneliness was associated with decreased rGMV in bilateral thalamus in SCD patients, and in the left middle occipital gyrus and the cerebellar vermal lobules I-V in MCI patients. Annual change of ADAS-Jcog in patients who reported loneliness was significantly greater comparing to these non-lonely in SCD-MCI group, but not in AD group. Our results indicate that perceived social isolation, or loneliness, might be a comorbid symptom of patients with SCD or MCI, which make them more vulnerable to the neuropathology of future AD progression.

### PARTNERING WITH DEMENTIA CAREGIVERS TO DEVELOP AND REFINE THE ENACT INTERVENTION

Jacqueline Eaton,[1] Sarah Neller,[2] Moroni Fernandez Cajavilca,[3] Julene Johnson,[4] and Lee Ellington[5], *1. University of Utah, Salt Lake City, Utah, United States, 2. University of Tennessee, Knoxville, Tennessee, United States, 3. University of Utah, Bronx, New York, United States, 4. University of California, San Francisco, San Francisco, California, United States, 5. University of Utah and Huntsman Cancer Institute, Salt Lake City, Utah, United States*

The behavioral symptoms of dementia negatively affect family caregivers. Several interventions have been developed to support caregiver needs, and evidence suggests improved outcomes when interventions actively engage caregivers. However, the strategies to implement active engagement techniques have not been sufficiently studied. Enhancing Active Caregiver Training (EnACT) is an arts-based intervention aimed at actively engaging dementia caregivers as they view and interact with participant-informed vignettes that portray caregiving experiences. We conducted a series of three iterative focus groups with dementia caregivers (n=9) to identify scenarios and refine activities for inclusion within the EnACT intervention. We analyzed the data in three cycles, using structural, descriptive, and pattern coding. From the first focus group, we coded 182 items as what went well, illustrating how caregivers resonated with video content, applied scenarios to individual experiences, and appreciated the "informative" vignettes that facilitated learning, processing, and awareness of the realities of caregiving. We coded 42 items as needing to change, including considerations for caregiver impact, confusing components, accessibility, and missing content. Participants identified eight specific vignettes that resonated and focused on the topics of respite, behavioral symptoms, validation, burnout, and communication. Intervention revisions were made based on focus group feedback, including: 1) selecting video vignettes for intervention use and 2) creating an index of vignette topics to facilitate multiple discussion pathways to align with dementia caregiver training and support groups. Partnering with dementia caregivers has the potential to make intervention activities engaging and applicable to participant needs.

### NUMBER OF CHILDREN AND RISK OF ALZHEIMER'S DISEASE AND DEMENTIA: EVIDENCE FROM THE UK BIOBANK

Yan Zhang, and Jason Fletcher, *University of Wisconsin-Madison, Madison, Wisconsin, United States*

This study uses a large-scale dataset of half a million respondents aged 39–73 from the UK Biobank to examine how parity (i.e., number of children) may influence parents' risk of Alzheimer's Disease and dementia (AD/D). We use respondents' (i.e., children's) reports of their parents' dementia status as the outcome variable. 38,040 respondents in the sample reported that their mother had AD/D, and 20,304 respondents reported their fathers had AD/D. Fixed effects logistic regression models suggest that compared to parents who had one child, high parity (> 4 kids) is associated with a lower risk of AD/D for both mothers and fathers. Moreover, as the parity increases, the protective effects become larger. This study advances prior dementia literature with two contributions. Methodologically, largely representative AD/D cases can increase the power of analysis. Also, children's reported AD/D cases are very likely through the observation of parents' entire life span, which can reduce misclassification of AD/D status and measurement errors from selective samples or self-reported cases. Empirically, this study provides important evidence suggesting protective effects of high parity on the risk of AD/D for both fathers and mothers. It implies that parity, as one of the life course contexts, may link to the risk of dementia in later life. More future work is needed to explore potential mechanisms.

### LONELINESS IS INVERSELY ASSOCIATED WITH EXERCISE AND SLEEP APNEA IN OLDER WOMEN AT RISK FOR ALZHEIMER'S DISEASE

Madina Danish,[1] Alyx Shepherd,[2] Kitty Lui,[2] Atul Malhotra,[2] Sheri Hartman,[2] Ellen Lee,[2] Erin Sundermann,[2] and Sarah Banks[2], *1. University of California San Diego, San Diego, California, United States, 2. University of California, San Diego, San Diego, California, United States*

Background: Modifiable risk factors (MRF) for Alzheimer's disease (AD) include sedentary behavior, sleep apnea, and loneliness; however, how these MRFs influence one another is unclear. We examined correlations among loneliness, physical activity and sleep apnea among older women at higher risk for AD. Methods: Data were collected as part of the Women: Inflammation and Tau Study, which recruits women age 65–85 with higher AD genetic risk and mild impairment on the Montreal Cognitive Assessment. Participants completed the UCLA Loneliness Scale and home sleep tests to derive the Apnea Hypopnea Index (AHI), a measure of sleep apnea severity. Women wore ActiGraph accelerometers for one week to measure average Moderate-Vigorous Activity per day (MVPA). We used Spearman correlation to examine the relationships among AHI, MVPA and Loneliness Scale score. Results: Preliminary data were available for 12 women (mean age=72.4 [SD=2.8], 100% non-Hispanic White). MVPA ranged from 0.3–52.8 minutes (mean=9.38 [SD=14.1]; AHI ranged from 1.5–28.3 (mean=14.0 [SD=9.0] and Loneliness Scale score ranged from 0–34 (mean=9.8 [SD=x]). Significant, positive associations were observed between AHI and Loneliness (rho=.80, p=.006) and a moderate relationship at trend level was notable between higher MVPA and less Loneliness (rho=-9.53, p=.07). AHI did not relate to MVPA. Conclusion: Results suggest that loneliness independently relate to both sleep apnea severity and sedentary behavior in older women. Combined interventions that target loneliness in addition to physical activity and sleep may be important in women at-risk for AD.

### LIGHT THERAPY FOR SLEEP DISTURBANCE OF NURSING FACILITY RESIDENTS WITH DEMENTIA: A SYSTEMATIC REVIEW

Wenxin Bian,[1] Mark Toles,[2] and Rose Mary Xavier[1], *1. University of North Carolina at Chapel Hill, Chapel Hill, North Carolina, United States, 2. UNC Chapel Hill, Chapel Hill, North Carolina, United States*

Background: Bright light therapy has shown promise in addressing sleep problems in nursing facility residents with dementia. However, recent studies yielded conflicting outcomes and few studies focused on nursing facilities. The purpose of this systematic review was to describe effectiveness of light interventions in nursing facility residents with dementia. Method: We searched PubMed, CINAHL, EMBASE, PsychINFO, and Scopus using key terms "sleep", "dementia" and "residential facilities", and synthesized data with thematic analysis and vote-counting. Results: Of eight studies that met inclusion criteria, six were randomized controlled trials and 2 were quasi-experimental. Sample size ranged from 11 to 77 residents. Studies tested 3 light therapies: timed bright light (n=6), timed regular light (n=1), and variable 24hour light (n=1). Light delivery method, light exposure, and adherence to therapy protocols were not consistently reported. All studies indicated light therapy improved some resident outcomes, such as sleep efficacy and total sleep time; however, 88% of studies did not report sampling strategies or a statistical power analysis and 22% had small sample size (n < 15). Conclusion: Insufficient evidence is available to recommend light therapies for nursing facility residents with dementia. Adequately statistically powered studies that are rigorously designed with representative samples are needed for robust estimation of the effects of light therapy on sleep. Future studies must account for the unique characteristics of nursing facility residents with dementia that impact their adherence to light therapy.

### LIFE-COURSE MULTI-DISCIPLINARY PREDICTORS OF DEMENTIA AMONG OLDER ADULTS

Sayaka Kuwayama,[1] Kevin González,[1] Freddie Márquez,[1] Hector González,[1] and Wassim Tarraf[2], *1. University of California San Diego, La Jolla, California, United States, 2. Wayne State University, Detroit, Michigan, United States*

The incidence of dementia is rapidly increasing. Identifying risk factors for dementia may help improve risk assessment, increase awareness for risk reduction, and identify potential targets for interventions. We use a life-course multi-disciplinary modeling framework to examine leading predictors of incident dementia (ID). We use the Health and Retirement Study (HRS) to measure 57 exposures across 7 different domains: (1) demographic, (2) adverse childhood socioeconomic and psychosocial, (3) adverse adulthood experiences, (4) adult socioeconomic status, (5) health behaviors, (6) social connections, and (7) adult psychological conditions. Our outcome is ID (over 8-years) operationalized using Langa-Weir classification for adults aged 65+ years who meet criteria for cognitively normal at the baseline when all exposures are measured (Nf 1,622 in testing set and Nf1,460 in validation set). We compare standard methods (Logistic regression) with machine learning (ML) approaches (Lasso, Random Forest) in identifying highly predictive exposures across the risk domains of interest. Standard methods identified lower education, childhood financial duress, and pessimism as among the leading factors associated with ID. Psychological factors explained the highest variance for ID, followed by adult socioeconomic and adverse childhood factors. However, ML techniques differed in their identification of (1) predictors and (2) factors predictive importance. The findings emphasize the importance of upstream risk factors and the long-reach of childhood experiences on cognitive health. The ML approaches highlight the importance of life-course multi-disciplinary frameworks for improving dementia risk assessment. Further investigations are needed to identify how complex interactions of life-course risk factors can be addressed through interventions.

### INTERNATIONAL LONG-TERM CARE HOME QUALITY ASSURANCE: HOW DO EUROPEAN MEASURES ADDRESS DEMENTIA?

Michael Lepore,[1] Franziska Zúñiga,[2] Jos Schols,[3] Klaus Wingenfeld,[4] Theo van Achterberg,[5] and Briana Murray[1], *1. University of Maryland School of Nursing, Baltimore, Maryland, United States, 2. University of Basel, Basel, Basel-Stadt, Switzerland, 3. Maastricht University, Maastricht, Limburg, Netherlands, 4. University of Bielefeld, Bielefeld, Nordrhein-Westfalen, Germany, 5. KU Leuven, Leuven, Vlaams-Brabant, Belgium*

Alzheimer's Disease and related dementias (ADRD) are prevalent conditions in long-term care homes (LTCHs) with about 50% of LTCH residents living with ADRD in many countries. Despite the prevalence of ADRD in LTCHs, a recent examination of LTCH quality assurance programs in four countries revealed a small percentage of LTCH quality measures across those countries addressed ADRD, most commonly as a risk adjuster, and once each as an inclusion or exclusion criterion. To better understand how quality assurance programs address ADRD internationally, we examined LTCH quality measures in four European countries—The Netherlands, Switzerland, Germany, and Belgium—following procedures previously used to examine international LTCH quality assurance measures. 73 measures were examined across 4 quality assurance programs. 26% of the measures addressed ADRD. The programs addressed ADRD in starkly different ways: in The Netherlands and Belgium no measures addressed ADRD; in Germany the majority (13/15) of measures addressed ADRD as an exclusion or inclusion criterion; and in Switzerland all the measures addressed ADRD through risk adjustment. Although limited to examining measures from LTCH quality assurance programs in four European countries, this study adds evidence that ADRD tends not to be addressed by LTCH quality measures, but when ADRD is addressed, it tends to be through risk adjustment. LTCH regulators, policymakers, and providers can use this information to assess options for addressing ADRD in quality assurance programs. Future research is needed to assess how standard indicators of quality differ across quality assurance regimes and how to address dementia beyond risk adjustment.

### INCORPORATING SPIRITUALITY INTO COGNITIVE STIMULATION THERAPY GROUPS FOR PERSONS WITH DEMENTIA

Ebow Nketsiah,[1] Max Zubatsky,[1] and Marla Berg-Weger[2], *1. Saint Louis University, St. Louis, Missouri, United States, 2. Saint Louis University - St Louis, MO, Saint Louis, Missouri, United States*

With the rise in Dementia-Related Disorders globally, few non-pharmacological approaches have been developed to effectively target cognitive impairment and memory loss. Cognitive Stimulation Therapy (CST) has shown effectiveness in improving cognition and quality of life of older adults with dementia. To date, no studies have incorporated spirituality into CST for improving the cognition and quality of life of older adults with dementia. This study's aim is to evaluate the effectiveness of CST with a spirituality component compared to the traditional CST group intervention. Our team grouped participants (Nf34) into either spiritual or traditional groups based on their location of residence and level of cognition. Preliminary results showed that spiritual-themed groups improved in mental health status, mobility, and depression. The spiritual groups had a mean difference of 1.85-points improvement on mental status exam as compared to 0.2-point reduction for the traditional groups. In addition, the spiritual groups had a mean difference of 4.80-points improvement in mobility and 0.6-point improvement on depression as compared to a 5.25-points reduction in mobility and a 2.13-points reduction on depression of the traditional groups respectively. Compared to the traditional CST group, there were no significant differences noted regarding cognition and memory recall. Incorporating spirtiuality and other faith-based themes in groups may provide additional benefit for individuals with memory loss. Both healthcare and long-term care facilities may benefit from incorporating group interventions such as CST into their routine care with older adults.

### IMPLEMENTING VISUAL VIDEOS AND IMAGES WITH PEOPLE WITH DEMENTIA IN CARE SETTINGS: A SCOPING REVIEW

Karen Lok Yi Wong,[1] Mario Bayani,[2] Jim Mann,[2] Annette Berndt,[2] Lily Wong,[2] Carly Wang,[1] Diane Pan,[1] and Lillian Hung[1], *1. University of British Columbia, Vancouver, British Columbia, Canada, 2. Community Engagement Advisory Network, Vancouver, British Columbia, Canada*

There is limited literature on using visual videos and images with people with dementia in care settings. We conducted a scoping review on this topic to fill this literature gap. Our scoping review adopted the Joanna Briggs Institute scoping review methodology. We eventually included eleven papers for the review and conducted the content analysis. We found the facilitators for implementing visual videos and images with people with dementia in care settings: 1. Matching people's interests 2. Being congruent with people's cognitive abilities 3. Support from families and staff 4. Using in a group setting. We also found the barriers: 1. Staff is unwilling to support 2. Lack of resources 3. Not congruent with the cognitive or other abilities of the people. We found benefits of using visual videos and images with this population: 1. Encourage expression 2. Facilitate discussions with other people 3. Improve well-being. We also found drawbacks: the potential of arousing negative emotions and memories. We suggest future research should include the voices of people with dementia, staff should be trained to support the people in case negative memories and emotions are aroused, and there should be consideration of using visual videos and images to tackle isolation and loneliness in care settings. With these findings, this scoping review should shed light on implementing visual videos and images in care settings.

### HEALTH EXP ERIENCES OF SEXUAL AND GENDER MINORITY PEOPLE LIVING WITH DEMENTIA AND THEIR CAREGIVERS: A SCOPING REVIEW

Melissa Harris,[1] and Jennifer May[2], *1. Duke University, Durham, North Carolina, United States, 2. Duke, Durham, North Carolina, United States*

People living with dementia and older adults who identify as sexual and gender minority (SGM - lesbian, gay, bisexual, transgender, queer individuals) both represent minoritized groups of older adults, but little is known about the health experiences of older adults who are at the intersection of having dementia and also identify as SGM. This review explored the extent and nature of research focused on health and healthcare experiences of SGM people with dementia and/or their informal caregivers. A scoping review framework was used given the exploratory nature of the project's purpose. Health librarians comprehensively searched four databases (MEDLINE, CINAHL, PsycINFO, AgeLine) to identify relevant studies. A total of 8,137 unique titles and abstracts were reviewed by 2 independent reviewers. Forty citations were determined relevant and reviewed by full text. Eight studies met inclusion criteria and were analyzed thematically. Study methods were quantitative cross-sectional (n=2), qualitative exploratory (n=4), and a mixed methods case study (n=1). Studies were published from 2010–2022. Sample sizes ranged from 1 to 415 (M=113, SD=116). Four studies focused on caregivers, 2 on persons with dementia, and 1 on dyads. Themes emerged pertaining to caregiver health and well-being, identity, relationships, disclosure, discrimination, and safety. Findings highlight the significance of inclusive care that addresses intersecting psychosocial and health-related identities (e.g., cognitive status, SGM status, race/ethnicity, substance abuse history, multimorbidity) and that protects the rights of families of choice. More research is needed to better understand how sociopolitical structure influences dynamics between cognitive health and SGM status among older adults.

### GERIATRICIAN PERSPECTIVES ON THE MANAGEMENT OF MILD COGNITIVE IMPAIRMENT/ MILD ALZHEIMER'S DISEASE

Ganesh Gopalakrishna,[1] Jeremy Pruzin,[1] Susan Alford,[2] Carol Hamersky,[2] Anup Sabharwal,[2] and Stephen Brunton[3], *1. Banner Alzheimer's Institute, Phoenix, Arizona, United States, 2. Novo Nordisk, Inc., Plainsboro, New Jersey, United States, 3. Primary Care Education Consortium, Winnsboro, South Carolina, United States*

Mild cognitive impairment (MCI) and Alzheimer's disease (AD) dementia are characterized by cognitive impairment and in AD a progressive functional impairment. This study sought to understand the role of geriatricians in treating patients with MCI/mild AD dementia. An anonymous, online survey was conducted in the US with 301 healthcare professionals (HCPs), including 75 primary care providers who practice as board certified geriatricians, and treat ≥10 patients per month with MCI/mild AD dementia. Geriatricians reported that among their patients with MCI/mild AD dementia, they initially make this diagnosis 70% of the time. Geriatricians reported that the topics commonly discussed with patients at the time of diagnosis are treatment options (85%), care management strategies (81%) and disease progression (80%). When referring patients to other specialties for treatment, geriatricians most often referred to neurologists (41%). Most (83%) geriatricians considered themselves the coordinator of care for patients with MCI/mild AD dementia; however, only 5% of non-geriatrician HCP respondents view geriatricians as such. Geriatricians reported prescribing/recommending treatments for ongoing management of MCI/mild AD dementia, with the most common being mental exercises (76%), acetylcholinesterase inhibitors (75%), social interaction (73%), medications to manage comorbidities (72%), and lifestyle improvements (72%). Most (76%) geriatricians have received advanced formal training in MCI/mild AD dementia care with the majority (84%) expressing that they feel very confident in managing patients with MCI/mild AD dementia. Geriatricians are specialists that are well trained and confident in diagnosing, treating, and managing patients with MCI/mild AD dementia.

### GAIT AND/OR BALANCE DISTURBANCES AND THE ONSET OF ALZHEIMER'S DISEASE IN MILD COGNITIVE IMPAIRMENT

Sangwoo Ahn, *University of Tennessee at Knoxville, Knoxville, Tennessee, United States*

Physical frailty is one of the fundamental hallmarks of aging. Older adults with mild cognitive impairment (MCI) are more likely to exhibit some characteristics of physical frailty such as worse gait and/or balance. Gait and/or balance disturbances may precede cognitive decline among cognitively healthy older adults. The purpose of this longitudinal observational study was to identify the temporal relationship between gait and/or balance disturbances and the onset of Alzheimer's disease (AD) because it has not been well characterized among older adults with MCI. Data of 2,692 older adults with MCI (ages 74.5 ± 7.1 years, women making up 47% of the sample) were from the National Alzheimer's Coordinating Center's Uniform Data Set. Risk of incident AD by baseline gait and/or balance disturbances was examined by Cox regression models over 4.0 ± 3.0 years, adjusting for demographic characteristics, medical conditions, and study site where the data were collected. The presence of gait and/or balance disturbances predicted 38% to 55% (hazard ratios [HR] 1.38 to 1.55) increased risk of AD. Findings remained robust in sub-group analyses by sex and there were no noticeable changes compared with the original analyses. Thus, regardless of sex, gait and/or balance disturbances predicted the onset of AD among older adults with MCI. Health care providers may need to (1) catch a warning of possible cognitive decline by a frequent assessment of gait and/or balance disturbances and (2) provide effective strategies to improve gait and/or balance disturbances to potentially delay the onset of AD.

### EXPLORATION OF CARE CONTENTS AFFECTING STRESS VARIABILITY IN FAMILY CAREGIVERS OF OLDER PEOPLE WITH DEMENTIA

Sonoko Kabaya, Chieko Greiner, Masahide Nakamura, Yuko Yamaguchi, and Zhang Xuanrong, *Kobe University, Kobe City, Hyogo, Japan*

Support for family caregivers (FCs) caring for older people with dementia in the home settings is a common issue in developed countries. Previous studies have reported that the increase in the burden of FCs not only causes health problems, but also makes it difficult to continue care at home and causes serious problems such as abuse. However, it is not clear how FCs' stress, which leads to increased burden, fluctuates within a day through daily care activities. The aim of this study was to explore care activities that influence the increase in FCs' stress. We recruited one dyad of an older adult with dementia over the age of 65 and a FC. Data collection was performed for consecutive 7 days throughout 24 hours. We adopted hamon® developed by Mitsufuji Corp. Japan to measure Heart Rate Variability (HRV)-based stress. Moreover, we asked a FC to talk with Virtual Agent on a laptop freely to understand when and what kind of care activities were done and how FC's feelings were. Kruskal-Wallis's test and Bonferroni's multiple comparison test were used to compare differences in stress in the care contents. Results showed that sleeping care, which is composed of such as assistance in using the toilet and changing clothes, gives more stress to FC, compared with morning care which includes such as assistance in changing clothes, eating breakfast, and taking medicine ($p=0.042$). this finding is beneficial for considering how to reduce FC's stress on daily care activities.

### EXAMINING THE HEALTH EQUITY OF PEOPLE WITH DEMENTIA DURING THE COVID-19 PANDEMIC: FIRST INSIGHTS FROM A TWITTER STUDY

Juanita-Dawne Bacsu,[1] Megan E. O'Connell,[2] Allison Cammer,[2] Soheila Ahmadi,[2] Corinne Berger,[2] Mehrnoosh Azizi,[2] Rory Gowda-Sookochoff,[2] and Karl Grewal[2], *1. Thompson Rivers University, Kamloops,*

*British Columbia, Canada, 2. University of Saskatchewan, Saskatoon, Saskatchewan, Canada*

The COVID-19 pandemic has deepened issues of health inequity and social injustice against people with dementia. Despite having one of the highest mortality rates, little research focuses on the COVID-19 impact of people with dementia. This presentation aims to: 1) explore the COVID-19 experiences and key factors of health inequity among people with dementia during the pandemic; and 2) identify actions to improve the health equity of people with dementia in the pandemic. We collected 6,243 relevant tweets using the Twint application in Python from September 8, 2020, to December 8, 2021. Tweets were divided among eleven coders and analyzed using thematic analysis. Analysis identified three primary themes: structural inequities (e.g., restricted access to health and support services, ageism, social isolation, vaccination barriers, and inadequate staffing in care facilities); frustration and despair due to loss (e.g., loss of cognitive abilities, loss of time with loved ones, and loss of life); and resiliency and hope for the future (e.g., lifting of restrictions and COVID-19 vaccine). There is an urgent need for policymakers to improve the health equity of people with dementia in the pandemic. Tackling COVID-19 inequities requires revisiting infection control policies to improve access to health and support services, recognizing the essential role of family care partners, and providing resources to help support people with dementia during the pandemic. Moreover, it is essential that COVID-19 policy responses are informed by evidence-informed research and authentic partnerships that embrace the insight and lived experiences of people with dementia.

### ENGAGING OLDER ADULTS WITH ALZHEIMER'S DISEASE IN EXERCISE: IMPACT ON CAREGIVERS

Tai Sims,[1] Kristine Talley,[2] Joseph Gaugler,[2] Cynthia Peden-McAlpine,[2] Laura Kirk,[2] and Fang Yu[3], *1. Minnesota State University, Mankato, Mankato, Minnesota, United States, 2. University of Minnesota, Minneapolis, Minnesota, United States, 3. Arizona State University, Phoenix, Arizona, United States*

Informal caregivers provide the bulk of care for persons with Alzheimer's dementia (PWAD) living at home, resulting in increased burden, and deteriorations in wellbeing and health. The stress process model hypothesizes characteristics of PWAD influence caregiver outcomes. Few studies have identified if engaging PWAD in exercise influences caregiver outcomes. This ancillary mixed methods study of a RCT evaluated the impact of a 6-month, moderate-intensity aerobic exercise intervention for community-dwelling PWAD on family caregiver burden, wellbeing and general health. Quantitative data was collected at baseline, 3, 6, 9 and 12 months using Zarit Burden Interview, Caregiver Strain Index, Caregiver Wellbeing Scale, and 36-item Short Form Health Survey. Qualitative data was collected at 6 and 9 months using semi-structured interviews. The convergent mixed methods design included ANCOVA analyses of quantitative and content analysis of qualitative data. Participants were 25 (17 intervention, 8 control) caregivers aged 34–86 years who were primarily white, females that lived with care-recipient. Based on group assignment, quantitative and qualitative results provided mixed insight on influence of intervention on burden and wellbeing, and found no influence on general health. Quantitative findings indicated caregiver characteristics of relationship with care-recipient, gender and education influenced wellbeing, and relationship with care-recipient, co-residence and education influenced general health; and caregiver characteristics did not influence burden. Qualitative findings suggest improved burden and wellbeing were consequences of respite and social support, rather than the exercise intervention. This study provides insight that integrating family caregiver components into community-based exercise programs may benefit PWAD and their family caregivers.

### ENGAGING A DIVERSE PATIENT AND CARE PARTNER COUNCIL TO REFINE DEMENTIA CARE DIGITAL HEALTH TOOLS

Jessica Cassidy,[1] Mary Fisher,[2] Evelyn Romeo,[3] Samantha Holden,[3] Melanie Caffrey,[4] Donald Nease,[3] and Hillary Lum[2], *1. University of Texas at Arlington, Arlington, Texas, United States, 2. University of Colorado Anschutz Medical Campus, Aurora, 3. UC Health Anschutz, Aurora, Colorado, United States, 4. Colorado Springs, Colorado, United States*

Digital health tools have potential to reduce health disparities for persons with dementia and their family care partners (i.e., dementia dyads). We aimed to promote digital health equity through engaging stakeholders to identify priorities and suggestions for refinements to digital health tools. We convened a council, "Memory Research Partners in Caring and Technology" comprised of three dementia dyads, two care partners, and 10 community members with experience as research partners. The council met six times over seven months. Methods of engagement included: a) delivering preparatory educational materials and hosting guest speakers; b) World Café participatory methods and small group discussions using Google jamboards; and c) individual consultations with partners for additional input. Data was analyzed using rapid qualitative analysis and member checking with research partners. We evaluated engagement methods with a research partner survey and analyzed responses using descriptive statistics. Research partners identified key priorities for equitable refinement of digital health tools, including: 1) Communicating with dementia dyads based on their preferences; 2) Creating user-friendly patient portals; 3) Ensuring digital health tools offer dementia dyads convenience; 4) Providing technology supports; and 5) Facilitating connection to community resources. Stakeholder feedback regarding project engagement indicated majority strongly agreed their input was accurately heard (69%); communication and scheduling was accommodating (77%); they contributed to the research project (69%); and they gained new knowledge on digital tools (54%) and dementia (85%). Research partner insights and ongoing member checking will inform future next steps towards improving dementia care coordination and communication using digital tools.

### EMOTIONAL FUNCTIONING IN PEOPLE WITH DEMENTIA AND CAREGIVER AFFECT DURING DYADIC INTERACTIONS

Hannah Cozad,[1] Yuxuan Chen,[2] Casey Brown,[3] Julian A. Scheffer,[3] Kuan-Hua Chen,[3] and Robert W. Levenson[3], *1. Georgetown University, Oakland,*

*California, United States, 2. Stanford University, Stanford, California, United States, 3. University of California, Berkeley, Berkeley, California, United States*

Dementia caregivers can experience negative affect when interacting with their care recipients. However, few studies have examined the specific factors that predict caregiver negative affect in this dyadic context. We hypothesized that deficits in care recipients' emotional functioning would be associated with increased intensity of caregivers' negative affect during interactions with their care recipient. Caregivers (Nf100) reported on two aspects of their care recipients' emotional functioning: (1) emotion recognition (the ability to recognize other people's emotions), and (2) negative emotional reactivity (the ability to generate negative emotional responses). Dyads then visited the laboratory and engaged in a 10-minute conversation about an area of conflict in their relationship. Caregivers then watched recordings of their conversation while rating the valence and intensity of their experienced affect using a rating dial. We used these ratings to quantify changes in caregivers' emotional valence across the course of the conversation. Caregivers of care recipients with greater deficits in emotion recognition demonstrated greater increases in negative affect across the conversation. In contrast, care recipients' negative emotional reactivity was not related to changes in the valence of caregivers' affect across the conversation. Findings remained significant even after accounting for caregiver baseline valence ratings, biological sex, and age, as well as the care recipients' diagnosis and level of cognitive impairment. Results reveal the important role that care recipients' deficits in emotion recognition play in caregivers' emotional lives. Caregivers' negative affect may be more likely to increase when their care recipient has deficits in emotion recognition.

### DEVELOPMENT AND IMPLEMENTATION OF THE COGDRISK DEMENTIA RISK ASSESSMENT TOOL AND INTERACTIVE WEBSITE

Scherazad Kootar, Ruth Peters, Md Hamidul Huque, Ranmalee Eramudugolla, and Kaarin Anstey, *University of New South Wales, Sydney, New South Wales, Australia*

We developed a comprehensive risk assessment tool for dementia – Cognitive Health and Dementia Risk Assessment (CogDrisk) and a version specifically for Alzheimer's disease called CogDrisk-AD that could be applicable in low and high-resource settings. This tool incorporates risk and protective factors identified through systematic synthesis of observational studies that report risk ratios. Risk and protective factors included in the tool were selected on the strength of evidence as well as the availability of measures that are practicable in a range of clinical and research contexts. Seventeen risk/protective factors were identified for inclusion in the dementia algorithm to estimate the risk of dementia while sixteen factors were identified for the AD model, with an overlap in the majority of the factors. CogDrisk and the CogDrisk-AD were predictive of dementia and AD when validated across four high-quality international cohort studies. To enable the CogDrisk tool to be implemented in practice our team has developed an interactive website where individuals 18 years and above can complete the CogDrisk questionnaire, obtain a personalised risk profile, and receive feedback on their risk profile. The website was developed with the capacity to collect and store data. We anticipate that the tool can be used by members of the public, in clinical settings and as a screening or outcome measure for clinical trials.

### DESCRIBING THE EVOLUTION OF MEDICATION USE OVER TIME IN PEOPLE LIVING WITH DEMENTIA USING NETWORK ANALYSIS

Abby Emdin,[1] Alexa Boblitz,[2] Laura Maclagan,[2] Jennifer Bethell,[3] Jennifer Watt,[1] Daniel Harris,[1] Colleen Maxwell,[4] and Susan Bronskill[1], *1. University of Toronto, Toronto, Ontario, Canada, 2. ICES, Toronto, Ontario, Canada, 3. Toronto Rehabilitation Institute, Toronto, Ontario, Canada, 4. University of Waterloo, Kitchner, Ontario, Canada*

Prescribing for community-dwelling older adults living with dementia is complex. Multiple medications may be used to manage symptoms associated with dementia and/or co-existing chronic conditions, and can lead to problematic polypharmacy. Our objective was to use network analysis, a data science method, to provide a comprehensive description of co-prescribed medications in persons with dementia and describe whether these patterns change over time. We created a population-based cohort of community-dwelling older adults (aged 67+ years) in Ontario, Canada, newly diagnosed with dementia (between April 2014 and January 2019), from health administrative data, and developed medication networks at one year prior to, at, and for up to five years following dementia diagnosis. Among 136,292 individuals newly diagnosed with dementia, the mean age was 82.2 years and 59% were female. The most common medication subclasses dispensed at diagnosis were primarily cardiovascular medications: statins (45.6%), proton pump inhibitors (27.3%), beta-blockers (27.0%), calcium blockers (25.1%), and ACE inhibitors (24.6%). Similar proportions of medication subclasses were found at five years after diagnosis, except cholinesterase inhibitors (34.0% at five years were dispensed cholinesterase inhibitors compared to 16.9% at diagnosis). The most frequent co-prescribed medication pairs at diagnosis included statins and beta-blockers (16.0%), proton pump inhibitors (16.0%), and ace inhibitors (15.4%), respectively. Co-prescription was similar at five years, but also included higher frequency of co-prescribing with cholinesterase inhibitors (e.g., 19.4% were prescribed cholinesterase inhibitors and statins). Network diagrams demonstrate the complexity of prescribing in this population and highlight concurrent prescribing which may require careful monitoring or deprescribing.

### DEMENTIA-FRIENDLY IN THE CONTEXT OF HOSPITALIZATION: A CONCEPT ANALYSIS

Ellen Munsterman, Pamela Cacchione, Nancy Hodgson, and G. Adriana Perez, *University of Pennsylvania, Philadelphia, Pennsylvania, United States*

Persons living with dementia experience more and longer hospitalizations when compared to those without dementia. In response, initiatives to address the hospital experience for individuals with dementia and their families are becoming more common. The term "dementia-friendly" attached to terms such as "hospital", "ward", "initiative", "program", etc. is being used to describe this response.

Literature has begun to summarize current knowledge of dementia-friendly initiatives and define characteristics of dementia friendly hospitals, however, a formal definition of the concept of "dementia-friendly" is lacking. A concept analysis was completed using the 8-step Walker and Avant method including: selecting the concept and aim, identifying use of the concept in the literature, determining defining attributes, antecedents, consequences, and empirical referents, and developing cases that further distinguish the concept. The goal of this analysis was to clarify the definition of dementia-friendly in the context of hospitalization. Attributes of dementia-friendly in the hospital include dementia-specific staff education, use of person-centered care practices, environmental modification, and nursing-specific factors such as attitude, time to spend with patients and adapting responses based on patient need. Antecedents include recognition of the impact of hospitalization on older people with dementia. Consequences include enhanced staff knowledge and patient outcomes including decreased length of stay and decreased use of antipsychotic medication. Based on the above analysis, a conceptual definition for dementia-friendly in the context of hospitalization is proposed. This definition highlights the identified antecedents and attributes with a focus on the essential role of nursing in delivering dementia-friendly care.

### CROSS-CULTURAL COMPARISON OF A LATENT DEMENTIA INDEX IN MEXICO AND THE UNITED STATES

Joseph Saenz, and Christopher Beam, *University of Southern California, Los Angeles, California, United States*

Latent variable models have recently been used to extract latent dementia index scores using cognitive and functional ability data. Yet, no cross-cultural comparisons have been conducted. This analysis tests metric measurement invariance of a latent dementia indicator (LDI) among older adults in Mexico and the US using different statistical criteria. We then test whether demographic risk factors relate with the LDI similarly depending on criteria used to test measurement invariance. Data included the MexCog (Mexico, n=2,265) and Harmonized Cognitive Assessment Protocol (US HCAP, n=3,347). The LDI is a latent variable measured using 13 cognitive and 10 functional common items. Measurement invariance was tested in multiple group confirmatory factor analyses using both nested chi-square difference tests and comparisons of comparative fit indices (CFI). Covariances between demographic risk factors (age and education) and the LDI were compared across models achieved using different measurement invariance criteria. Full metric invariance of the LDI was achieved using CFI comparisons but not with chi-square comparisons, which required nine loadings to be freely estimated across studies to achieve partial metric invariance. Regardless of criteria used to test measurement invariance, age and education correlated with the LDI in both studies in expected directions with similar parameter estimates regardless of approach used to test measurement invariance. The LDI may be a valid tool to compare associations between risk factors and dementia in the HCAP and MexCog. The LDI related with demographic factors in expected ways and findings were robust to statistical approach used for measurement invariance testing.

### COGNITIVE STATUS AND HEALTH CARE UTILIZATION IN AN EMERGENCY DEPARTMENT: EARLY RESULTS OF A LONGITUDINAL PILOT STUDY

Walter Dawson, Natasha Spoden, Sarah Gothard, Kirsten Wright, and Jeffrey Kaye, *Oregon Health & Science University, Portland, Oregon, United States*

Many people living with Alzheimer's disease and related dementias (ADRD) never receive a formal disease diagnosis. This compromises wellbeing and increases health services utilization and care costs. Wider screening and assessment for ADRD may increase access to supportive care, improve allocation of medical care, and foster interventions that prevent or delay disease progression. A sample of Medicare-enrolled individuals 65+ (n=60) consecutively presenting to the Oregon Health & Science University (OHSU) emergency department (ED) in Portland, Oregon consented to the study and were administered the TICS, a validated tool for telephone-based assessments of cognition, post-discharge from the ED. Study participants were asked about their physical health via the modified Cumulative Illness Rating Scale (M-CIRS), and their cognitive health via the PROMIS Cognitive Measure Questions on Mental Clarity. Care utilization patterns were measured via review of participants' electronic health records (EHR) for three years prior to study enrollment focusing on total hospitalizations, ED visits, and primary care (PC) visits. Medicare-enrolled adults 65+ recently discharged from the ED were a feasible population to perform a cognitive assessment. The study enrollment rate was 24.2% (n=60). Enrollment was limited to patients with their PC affiliated with the OHSU health system, which excluded the majority (792, 73.8%) of ED patients Medicare-enrolled, 65+ from our study (1,072). This study provides preliminary evidence to support focusing on older ED patients to administer cognitive assessments, linking outcomes to the EHR, and ultimately providing a platform for future research on impacts of under-diagnosed ADRD on population-level health outcomes and care utilization.

### CO-DESIGNED PERSON-CENTERED NARRATIVES FOR PEOPLE WITH ALZHEIMER'S AND RELATED DEMENTIAS

Sarah Flessa,[1] James Harrison,[2] Tajalei Willard,[2] Michael Chandler,[1] and Stephanie Rogers[3], *1. MemoryWell, Washington, District of Columbia, United States, 2. UCSF School of Medicine, San Francisco, California, United States, 3. UCSF, San Francisco, California, United States*

MemoryWell's professional writers capture and share patient life stories with care teams and families in healthcare settings, with the goal of improving person-centered care. This mixed methods study employs participatory-design methods to assess the feasibility and acceptability of adapting this existing life story service to an acute care setting and implementing stories into the electronic health record (EHR) for older adults with Alzheimer's and Related Dementias (ADRD). Through in-depth interviews and surveys, we engage patients, family caregivers and care providers to co-design the life story process adaptation and development of EHR wireframes to iterate and pilot in the acute care setting. We will present the co-designed EHR wireframes and data analyzed using qualitative content analysis. Results describe life story utility and potential impacts on outcomes for people with ADRD. Findings from in-depth interviews with 11 providers indicated high feasibility and acceptability, e.g. describing a high likelihood of daily use. Providers shared barriers and facilitators, e.g. time and accessibility, and potential impacts on person-centered care and patient outcomes, e.g. trust, cultural competency, and delirium. Patient and caregiver data collection is underway; one patient and one caregiver have been interviewed out of ten for each respective group. Interviews with all groups have suggested that life stories would be useful for helping patients in unfamiliar and disorienting environments and learning information such as hobbies and interests would be helpful for providers to better understand and engage with patients.

### CLAIMS-BASED FRAILTY INDEX (CFI) AS A MEASURE OF DEMENTIA SEVERITY IN MEDICARE BENEFICIARIES WITH ADRD

Chan Mi Park,[1] Stephanie Denise Sison,[2] Ellen McCarthy,[1] Natalia Gouskova,[1] and Dae Kim[1], *1. Hebrew SeniorLife, Roslindale, Massachusetts, United States, 2. Beth Israel Deaconess Medical Center, Brookline, Massachusetts, United States*

Lack of a dementia severity measure in administrative claims data is a major barrier in Alzheimer's Disease and Related Dementias (ADRD) research using such data sources. A claims-based frailty index (CFI; range 0 to 1, higher scores indicating greater frailty) is a validated measure of frailty that can be calculated from administrative claims data and is correlated with functional status. We conducted a cross-sectional study to examine whether CFI can be a claims-based measure to differentiate dementia severity in 814 participants with ADRD with linked to Medicare claims in the National Health and Aging Trends Study (NHATS). We estimated the Functional Assessment Staging Test (FAST) scale (3: mild cognitive impairment; 4: mild dementia; 5: moderate dementia; 6–7 moderate-to-severe dementia) using NHATS variables and calculated CFI from Medicare claims in the prior 12 months. The prevalence of FAST stage 5 or higher was 244 (weighted percentage, 25.9%). The C-statistic of CFI to identify FAST stage 5 or higher was 0.78 [95% CI:0.73–0.82], with a CFI cut-point of 0.3 achieving the maximum sensitivity 72.5% and specificity 67.5%. The equipercentile linking procedure showed that CFI scores of 0.30, 0.35, and 0.40 corresponded to FAST stages 4, 5, and 6, respectively. Our results support the utility of a CFI as a proxy of dementia severity in administrative claims data.

### CAREGIVER USABILITY TESTING OF WEB-BASED AND VIRTUAL REALITY REMINISCENCE THERAPY FOR PERSONS WITH DEMENTIA

Winnie Sun, and Alvaro Quevedo, *Ontario Tech University, Oshawa, Ontario, Canada*

Reminiscence therapy (RT) is a multi-sensory treatment that uses a combination of sight, touch, taste, smell and sound to help persons with dementia (PWD) remember events, people and places from their past lives. Currently, digital technologies such as mobile applications and immersive solutions including virtual and augmented reality, are gaining momentum as supplementary tools for RT. This paper presents a usability study of a web-based and virtual reality application to understand the limitations and opportunities of digital platforms for facilitating engaging experiences for PWD towards recalling memories, while easing the therapy process for the caregivers. A total of ten healthcare caregivers were recruited from the Geriatric Dementia Unit and Geriatric Transitional Unit in Ontario Shores Center for Mental Health Sciences, Ontario Canada. Usability feedback from the caregivers were collected from the interviews after the completion of the System Usability Scale (SUS) questionnaire. Institutional caregivers found both web-based and virtual reality (VRRT) usable with SUS score above average (68/100), but required improvements related to the onboarding training of caregivers. The interview revealed four overarching themes related to the VRRT: (1) Ease of use; (2) Positive impact on caregiving; (3) Potential of reduction in responsive behaviors; (4) Feasibility for promoting social connection during COVID-19 pandemic. Next steps will focus on improving the user experience and expanding the application for immersive VR supporting head-mounted displays, hand tracking, and physiological measures, as well as conducting an usability study with PWD to expand our understanding of using RT digital tools with various levels of immersion.

### ARTISTIC ENGAGEMENT AT A MEMORY CARE FACILITY, VIA A TELEPRESENCE ROBOT

Amy Knepple Carney, Vanessa Hillman, Chloe Lehman, Elizabeth Holland, and Lizzie Gowin, *University of Wisconsin- Oshkosh, Oshkosh, Wisconsin, United States*

There is a growing body of research that shows participation in arts interventions (e.g., poetry, storytelling, music, and dance) can be beneficial for those living with dementia. The current study examines social interactions when the artist is not physically present, but in the room via a telepresence robot. Through the telepresence robot, dementia care residents were connected to a network of arts presenters. Using a checklist examining social behaviors (verbal and non-verbal), we assessed residents' social engagement with the artist, other residents, and staff during these creative visits. Typical activities within the care facility were also observed. The observations were conducted on 10 individuals living within the memory care facility. Although the typical facility activities elicited social verbal behaviors, they tended to be more physical in nature and had a larger number of social nonverbal behaviors. When the behavior was verbal it tended to be more noises of excitement, such as "wooo" and "oh yeah." Although the artist engagement activities elicited social nonverbal behaviors, they tended to be more verbally engaging and there were a larger number of social verbal behaviors during these activities. With the artist engagement we observed residents spontaneously singing songs, and helping to create poetry. The facility was already using activities that allowed the residents to become physically engaged and with the addition of artist visits there was an increase in verbal engagement. By having greater options for residents to create engagement we may see people who don't typically engage become more involved.

### ANXIETY, COGNITIVE FUNCTION, CHANGE, AND IMPAIRMENT AMONG DIVERSE OLDER HISPANICS/LATINOS.

Roberto Gonzalez-Prado,[1] Alejandra Morlett Paredes,[2] Wassim Tarraf,[3] Ariana Stickel,[1] Fernando Testai,[4] Linda Gallo,[1] Frank Pendedo,[5] and Hector González[2], *1. San Diego State University, San Diego, California, United States, 2. University of California, San Diego, San Diego, California, United States, 3. Wayne State University, Detroit, Michigan, United States, 4. University of Illinois, Chicago, Chicago, Illinois, United States, 5. University of Miami, Coral Gables, Florida, United States,*

Introduction: Anxiety and depression often co-occur and may be risk factors for neurodegenerative diseases such as Alzheimer's disease and related dementias. We examined associations between symptoms of anxiety and cognitive change and impairment in middle-aged and older Hispanics/Latinos. Method: 6,162 participants aged ≥50 (mean age=63.4, SD=±8.2 years) were enrolled in the Study of Latinos-Investigations of Neurocognitive Aging study (SOL-INCA), an ancillary to the Hispanic Community Health Study/Study of Latinos (HCHS/SOL). Exposures included the Spielberger State-Trait Anxiety Inventory 10-item (STAI-10) and the Center for Epidemiology Scale for Depression 10-item (CESD-10) at HCHS/SOL Visit 1 (2008–2011). Outcomes include cognitive tests at Visit 1 and 2 about 7 years later in SOL-INCA and mild cognitive impairment diagnosis (MCI; NIA-AA diagnostic criteria). Separate models were created to examine associations between cognition and anxiety adjusting for demographic variables, antianxiety medication, and depressive symptoms. Results: After adjusting for demographic variables and medication use, STAI-10 scores were associated with Visit 2 cognitive function ($p < 0.05$) and MCI ($p < 0.01$), but not with changes in cognitive function over time. After adjusting for depressive symptoms, most effects of anxiety were attenuated, with exception of B-SEVLT-Recall ($p < 0.05$). Discussion: Anxiety symptoms were associated with lower memory function 7 years later among diverse older Hispanics/Latinos. Anxiety was associated with 7-year cognitive change and MCI, but those associations were explained by depressive symptoms and other covariables. Future research should examine cognition in relation to anxiety and depression since the two commonly co-occur.

### ANTICIPATED SUICIDAL AND DEATH IDEATION IN RESPONSE TO AN IMAGINED DEMENTIA DIAGNOSIS

Molly Maxfield,[1] Allie Peckham,[1] Dara James,[2] Laura Lathrop,[3] and Amy Fiske[4], *1. Arizona State University, Phoenix, Arizona, United States, 2. University of South Alabama, Mobile, Alabama, United States, 3. University of Denver, Denver, Colorado, United States, 4. West Virginia University, Morgantown, West Virginia, United States*

Alzheimer's disease and related dementias are prevalent, incurable, and highly impactful diagnoses. Dementias are therefore feared diagnoses. Dementia-related anxiety (DRA) is anxiety about a current or future diagnosis of dementia and the associated complex symptoms. In a mixed methods study, semi-structured interviews were conducted to identify causes of DRA and revealed that numerous adults anticipated suicidal or death ideation if diagnosed with dementia. Fifty cognitively healthy, community-dwelling adults aged 58 to 89 (M = 70.92, SD = 6.08; 64% female) were recruited from a university participant registry and Memory Clinic. Among participants endorsing anticipated suicidal or death ideation, responses ranged from active plans, including interest in physician-assisted suicide, to more passive wishes to hasten death rather than continue to live with dementia. Within reports of both anticipated suicidal and death ideation, subthemes emerged, including the concern about becoming a burden to others in more advanced stages of dementia, the devaluation of life or the self with dementia, and the desire for (and anticipated thwarting of) control and independence. Statements of anticipated suicidal and death ideation were contingent on a future dementia diagnosis and may reflect errors in affective forecasting. Nevertheless, given the prevalence of dementias and older adults' elevated rates of suicide, the intersection of these two public health issues warrants greater attention and further investigation.

### TRANSLATION AND CULTURAL ADAPTATION OF THE PREFERENCES FOR EVERYDAY LIVING INVENTORY INTO KOREAN

Jenny Kwon,[1] and So Young Shin[2], *1. Miami University, Oxford, Ohio, United States, 2. Inje University, Busan, Pusan-jikhalsi, Republic of Korea*

Valid and reliable measures are necessary to provide person-centered care tailored to the individual. However, there are no such measures in Korean nursing home settings. Therefore, the purpose of this study was to translate and culturally adapt the Preferences for Everyday Living Inventory (PELI); test face validity; and finalize a Korean version of PELI (PELI-K). The translation and cultural adaptation were done according to guidelines of the International Society for Pharmacoeconomics and Outcomes. After translating, cognitively capable Korean older adults (n=10) reviewed the PELI items and completed a questionnaire. Face validity was assessed by three questions regarding grammar and wording, understandability, and cultural relevance using a 4-point Likert scale: 1 (strongly disagree) to 4 (strongly agree). Higher scores indicate better validity. The mean (±SD) age of participants was 67 (±2.50) years. The mean score of appropriateness for grammar and wording was 2.70 (±.82); understandability was 3.70 (±.48); and cultural relevance was 3.70 (±.67). Participants found the Korean version of PELI easy to understand and interpret, and culturally relevant. However, some translations have room for improvement in rephrasing sentences and using alternative wording. Based on cognitive debriefing results and several suggestions from participants, necessary changes were made before creating a final version of PELI-K. These findings suggest that PELI was successfully translated and culturally adapted to Korean. Implementing PELI-K in Korean nursing home settings will help with eliciting individual preferences and incorporating them into care delivery. Next steps can evaluate care quality improvement and increased residents' satisfaction.

### THE EFFECTS OF A HAND-BASED HOME-TRAINING PROGRAM ON FINE FORCE PERCEPTION IN OLDER ADULTS

Rachel Logue Cook, Kathy Kern, and Susan Brown,
*University of Michigan, Ann Arbor, Michigan, United States*

The ability to use and manipulate the hands is essential for completing daily activities such as dressing, food preparation, and medication adherence, but declines with age. Assessments of hand function typically include measures of maximum strength despite our recent findings that fine force control may be more indicative of function than strength. The purpose of this study was to determine the effectiveness of a hand-based intervention on improving various aspects of hand function in older adults. Eleven older adults (mean age: 77±6.8y, 7 females) completed a 6-week home-based training program consisting of a series of sensorimotor tasks. Hand assessments included tactile sensation, dexterity, maximum grip and pinch strength, and fine force control using a pinch force matching paradigm. In the fine force task, participants were given a reference force (25% MVC) and were instructed to match the force without visual feedback using either the same (ipsilateral) or opposite (contralateral) hand as the reference force. After the training program, older adults displayed a 35% reduction ($p < 0.01$) in matching error during the contralateral fine force control task. Additionally, both tactile sensation and dexterity showed improvements in the dominant hand ($p < 0.05$). No differences were observed in maximum pinch or grip strength. We observed improvements in fine force control, tactile sensibility, and dexterity, all of which contribute to hand function needed for daily activities, as a result of the home-based intervention. These results underscore the importance of measuring different aspects of hand function and the need for additional hand-based interventions in the aging population.

### BRINGING THE HAND TO THE HEAD: AGE-RELATED CHANGES IN MOVEMENT STRATEGY

Andrew Droste,[1] Stephen Cain,[2] Tanner Trenary,[3] Rachel Logue Cook,[3] Alanson Sample,[3] David Burke,[4] Paula Anne Newman-Casey,[1] and Susan Brown[3], *1. University of Michigan Medicine - Kellogg Eye Center, Ann Arbor, Michigan, United States, 2. West Virginia University, Morgantown, West Virginia, United States, 3. University of Michigan, Ann Arbor, Michigan, United States, 4. University of Michigan Medical School, Ann Arbor, Michigan, United States*

Functional independence in older adults relies heavily on the ability to perform daily activities requiring motion of the upper extremity that brings the hand to the head. Such tasks include hygiene activities, eating and drinking, adjusting hearing aids, or putting in eye drops – all of which can be challenging for the older individual. Despite the importance of these activities of daily living, little is known regarding age-related changes in control strategies that may contribute to performance impairments. Twelve older (mean age: 75±5.5y) and 12 young (mean age: 23±1.6y) participants performed arm movements that stopped in front of the ipsilateral eye. Movements were made under proprioceptive guidance by occluding visual feedback via blindfold. Participants first made a reference movement to determine final hand position and then reproduced the memory-based movement. The task was performed by each arm from a seated position. Inertial sensors attached to each wrist captured movement data, which were used to calculate movement characteristics (velocities, timing, smoothness). As expected, proprioceptively guided movements made by the young group were highly irregular, particularly during the deceleration phase, indicating intermittent proprioceptive monitoring of arm position in the absence of vision. In contrast, a clear difference in movement strategy was seen in older adults where movements were faster and smoother ($p < 0.05$), suggestive of impaired utilization of movement-related position feedback. Future studies will determine to what extent such age-related differences in control strategy contribute to functional difficulties with tasks requiring accurate placement of the hand to the head.

### VARIATION IN STATE ENFORCEMENT REGULATIONS REGARDING FINANCIAL PENALTIES AGAINST ASSISTED LIVING FACILITIES

Jacy Weems,[1] and Kali Thomas[2], *1. Brown University School of Public Health, Providence, Rhode Island, United States, 2. Brown University, Providence, Rhode Island, United States*

Provider behavior is known to be driven by market forces, including costs. Financial penalties are the primary enforcement remedy available to state agencies in deterring substandard practices in assisted living (AL) communities. States develop licensing and enforcement requirements for AL communities, and regulations vary by license. Using state regulations and statutes sourced for AL license types from Nexis Uni, we used key phrases to extract information from all 50 states and D.C. to characterize how state agencies calculate and assess financial penalties for licensed AL community violations. Fines are assessed for each day of substandard behavior or as a one-time amount; within the nursing home sector, per-day fines tend to be higher than per-instance fines as they accumulate. We found that of the states analyzed so far (n=25), ten states (40%) may impose either per-day or per-instance fines, nine (36%) can impose per-instance fines only, three (12%) may impose per-day fines only, and three (12%) incorporated facility size in assessing fine amounts. One state had no policies concerning AL fine enforcement. The median per-instance maximum fine amount is $2,000 [IQR $500-$5,000], while the median maximum per-day fine is $500 per day [IQR $150-$2,000]. Eleven states (44%) utilize tiered amount categories based on violation scope and severity. Agencies also consider characteristics such as community size and past performance when assessing fine amounts. Inconsistent penalties across states illuminate the need to assess the impact of current regulations, identify best enforcement practices to improve overall compliance, and share best practices between enforcement agencies.

### LONELINESS AND PSYCHOLOGICAL DISTRESS AMONG AFRICAN AMERICAN ASSISTED LIVING RESIDENTS APPROACHING END OF LIFE

Christopher Arthur,[1] Alexis Bender,[2] and Molly Perkins[3], *1. University of Washington, Seattle, Washington, United States, 2. Emory University, Atlanta, Georgia, United States,*

*3. Emory University School of Medicine, Atlanta, Georgia, United States*

Social isolation and loneliness are common at end of life and important contributors to psychological distress, particularly among older adults. Despite evidence that isolation and loneliness are generally prevalent in assisted living (AL), little research has examined these states within the context of end of life (EOL). This study uses secondary data from an EOL study in assisted living to qualitatively characterize the experience of isolation and loneliness in African American AL residents with varying levels of psychological distress. We performed a thematic analysis using ethnographic and interview data collected from 25 residents (64% female) in a large (90+ bed) all-African American AL community in metropolitan Atlanta. We assessed psychological distress using the PHQ-4 and used this measure in qualitative analysis to explore varying patterns of social isolation and loneliness across residents. Findings showed high variability in psychological distress based on PHQ-4 scores (range 0–12, mean = 3.5, SD = 3.75). Across themes of social isolation and loneliness, we compared experiences of residents with low and high psychological distress. The maintenance of agency in the face of isolation and loneliness was the predominant pattern among the low-distress group, while passive withdrawal was consistently identified among the high-distress group. We classified these themes in terms of individual interpersonal relationships characterized by self-isolation and negotiation of family connections, as well as AL community-level relationships characterized by the liminality of the lived AL experience. Results have important implications for multilevel interventions designed to reduce social isolation, loneliness, and psychological distress among this population.

### ASSISTED LIVING ADMINISTRATORS' JOB SATISFACTION, WORK STRESSORS, AND INTENT TO LEAVE DURING THE COVID-19 PANDEMIC

Mary McSweeney-Feld,[1] Teta Barry,[2] Bo Yang,[1] and H. Wayne Nelson[1], *1. Towson University, Towson, Maryland, United States, 2. Arcadia University, Glenside, Pennsylvania, United States*

This study examines how job satisfaction in six sub-scales and selected stressors and demographic covariates influenced assisted living administrators' (ALF) intentions to quit during the COVID-19 pandemic. Quantitative and qualitative data were collected from 103 ALF administrators as part of a national study of long-term care administrators' intent to quit during the COVID-19 pandemic funded by the Foundation of the National Association of Long-Term Care Administrator Boards in Washington, DC. Descriptive statistics were collected for the sample, and correlations between variables were examined, as well as responses from 3 open-ended questions that were coded for analysis. Although generally satisfied, roughly 41 percent of ALF administrators reported that they were intending to quit. Qualitative data suggested that job satisfaction was influenced by a more nuanced interpretation of job characteristics and work environment intrinsic factors such as adequacy of staffing and resources, changing regulations during the COVID-19 pandemic, and external supports such as family and friends. Given the limited research on the impact of the COVID-19 pandemic on assisted living communities and their administrators, the results of this study can help to inform policies and strategies for providing support for this segment of long-term services and its workforce during widespread disasters.

### FRAMEWORKS OF THE RELATIONSHIP BETWEEN HIV AND AGING AMONG HIV CLINIC PROVIDERS: A QUALITATIVE STUDY

Madeline Villalba,[1] Rachel Schenkel,[2] Michael Yin,[3] Peter Gordon,[3] and Abigail Baim-Lance[1], *1. Icahn School of Medicine at Mount Sinai, New York, New York, United States, 2. Emory University School of Medicine, Atlanta, Georgia, United States, 3. Columbia University Medical Center, New York, New York, United States*

As a result of advancements in antiretroviral therapy (ART), people living with HIV (PLWH) are living longer: it is estimated that 70% of PLWH in the US will be over the age of 50 by 2030, raising questions of what it means to age with HIV and how to care for these individuals most effectively. To qualitatively explore how providers who care for older PLWH and other comorbidities conceptualize the relationship between HIV and aging, we conducted 11 semi-structured interviews with physicians, RN case managers, and administrators at two health systems' HIV clinics in New York City and the Hudson Valley between November 2019 and July 2020. We coded the interviews, developing a codebook through an iterative process, and thematically analyzed the data. Analysis revealed a range of interrelationships between HIV, aging, and comorbidities across pathophysiological and psychosocial dimensions. Providers commonly espoused that, for older PLWH, comorbidities are comparatively more difficult to manage than HIV and constitute the primary driver of pathophysiology and/or mortality. In contrast, providers viewed patients as regarding HIV as more deadly and engaging with HIV care more diligently, which providers related to patients' long-term experiences of living through the HIV/AIDS epidemic. Providers' comorbidity-centric framings of the clinical relationship between HIV, aging, and comorbidities mark a departure from HIV-dominant forms of thinking. Our findings have important implications for interdisciplinary care management and suggest how to support the development of asset-based approaches to encourage patient engagement with comorbidity care based on fidelity to HIV regimens.

### EXAMINING THE IMPACT OF HEART DISEASE AND DIABETES ON THE MENTAL HEALTH OF OLDER MINORITY ADULTS

Sree Saroj Sainath Panchagnula, *Jackson State University, Jackson, Mississippi, United States*

As the world's population ages and healthcare costs associated with the care of the elderly rise, it is pertinent to address these concerns. This study examines how older adults' mental health is affected by chronic medical illnesses. It is hypothesized that minority older adults, on average, have worse mental and physical health outcomes than their majority counterparts. A secondary data analysis of the National Health and Aging Trends Study, revealed that older minority adults experience higher rates of depression than non-minority older adults, as measured by the Patient Health Questionnaire – 2 (PHQ-2). European-Americans significantly differ from African Americans (AA) and

Hispanics regarding their level of anxiety, as measured by the Generalized Anxiety Disorder – 2 (GAD-2) measure. AA and Hispanics are significantly more likely to exhibit anxiety than their European-American counterparts. Minority older adults with a higher rate of surgeries are not substantially more likely to be depressed, according to the PHQ-2. Minority older adults who reported having a difficult time falling asleep are considerably more depressed than older minority adults who did not endorse depressive symptomatology on the PHQ-2. Minority older adults who had two or more social supports in their lives have lower levels of anxiety compared to older minority adults who have less than two social supports in their lives. The findings from this study serves as groundwork to promote equity between majority and minority older adults, which will improve their abilities to perform independent activities of daily living successfully.

### COMPARTMENTAL FEMUR CORTICAL THICKNESS IN OLDER ADULTS DIFFERS BY DEMOGRAPHICS AND PHYSICAL FUNCTION

Justin Lam,[1] Delanie Lynch,[2] Diana Madrid Fuentes,[2] Karan Devane,[3] Marjorie Howard,[2] Kristen Beavers,[3] and Ashley Weaver[2], *1. Carnegie Mellon University, Pittsburgh, Pennsylvania, United States, 2. Wake Forest School of Medicine, Winston Salem, North Carolina, United States, 3. Wake Forest University Health Sciences, Winston-Salem, North Carolina, United States, 6. Wake Forest University, Winston-Salem, North Carolina, United States*

Previous studies have examined the relationship between cortical bone properties and fracture risk in older populations, but they have yet to examine how these properties change within the femur. Most hip fractures are classified by their location: femoral neck, intertrochanteric, or subtrochanteric fractures. Since cortical thickness can vary throughout the femur, a quantitative method of examining thickness by region was developed using a cortical mapping approach applied to computed tomography (CT) scans. Subject-specific finite element (FE) models of proximal femurs were created from baseline CT scans of 107 older adults (ages 60 – 85; 70% White; 72% Female) with obesity as classified by BMI (33.8 ± 4 kg/m2). An existing FE model was morphed to the segmented geometry of each subject's proximal femur. A nearest neighbor search assigned cortical thickness values to the nearest finite element model node. Cortical thickness was grouped into four femoral compartments: femoral head, femoral neck, intertrochanteric and subtrochanteric regions. Pairwise paired t-tests indicated that cortical thickness differed between femoral compartments ($p < 0.05$). Multivariate regression models showed greater cortical thicknesses in femoral head, neck, and intertrochanteric regions in African Americans compared to Whites ($p < 0.05$). Additionally, these models suggest an association between cortical thickness and physical function assessments, such as the timed up-and-go test and leg muscle strength test. Since cortical thickness is not constant throughout the femur, future studies can use the framework developed here to assess each compartment individually and investigate the relationships between cortical thickness, demographics, and functional assessments.

### ACCELERATED BRAIN AGING IS ASSOCIATED WITH MORTALITY ACROSS RACE

Ramon Casanova,[1] Andrea Anderson,[1] Ryan Barnard,[1] Keenan Walker,[2] Timothy Hughes,[1] Stephen Kritchevsky,[1] and Lynne Wagenknecht[1], *1. Wake Forest School of Medicine, Winston-Salem, North Carolina, United States, 2. National Institute of Health, Baltimore, Maryland, United States*

There is an increasing interest in using machine learning and artificial intelligence to estimate chronological age using neuroimaging data. The gap between chronological age and estimated brain age (brain age gap, BAG) is used as a measure of accelerated/resilient brain aging. Accelerated brain aging has been associated with increased mortality risk. However, these reports are based on cohorts mostly composed by white individuals. Here we capitalized on the racially diverse nature of the Atherosclerosis Risk in Communities Study (ARIC) cohort to investigate associations of brain across race. We used brain MRI scans from 1172 cognitively normal ARIC participants that were collected at ARIC Visit 5. Of those 772 were White and 366 were African Americans. We used Cox regression models to investigate BAG values associations with mortality. There were 163 deaths (dw = 124 and daa = 39) over 8 years of follow-up. Participants were stratified by tertiles according to BAG values. We found that, compared to those individuals with BAG scores in the highest tertile (>=1.15), those who scored in the lowest tertile (<= -1.3 years) to be associated with significantly lower mortality among the White (HR=0.41, 95% CI, [0.26–0.66], $p < 0.001$) and Black (HR=0.43, 95% CI, [0.20–0.92], $p = 0.03$) participants after adjusting for age, race-center, sex, education, diabetes, smoking and hypertension. Our analyses show that our approach to estimate chronological age using high-dimensional elastic net regression, produces BAG values which are associated with mortality not only in White individuals but also in African Americans.

### WHITE MATTER CHANGE NEAR CEREBRAL MICROBLEEDS AFTER MTBI INVOLVES AGE AND SEX DEPENDENT COGNITIVE DECLINE

ANDREI IRIMIA,[1] Van Ngo,[1] Nikhil Chaudhari,[1] Fan Zhang,[2] Shantanu Joshi,[3] Lauren O'Donnell,[2] Nasim Sheikh-Bahaei,[1] and Helena Chui[1], *1. University of Southern California, Los Angeles, California, United States, 2. Harvard Medical School, Boston, Massachusetts, United States, 3. University of California, Los Angeles, Los Angeles, California, United States*

We tested the null hypothesis that, after mild traumatic brain injury (mTBI), white matter changes near cerebral microbleeds (CMBs) are associated with cognitive decline. Magnetic resonance images were acquired from 62 adults with mTBI and from 203 matched healthy controls. A week post-injury, mTBI participants had 2.7±2.6 traumatic CMBs in WM, located 6.1±4.4 mm from the cortical mantle. About 6 months later, 97% of CMBs were associated with significant reductions (34%±11%, $q < 0.05$) in the fractional anisotropy (FA) of WM streamlines within ~1 cm of CMBs. Male sex and older age were significant risk factors for larger reductions ($q < 0.05$). CMBs in the corpus callosum, cingulum bundle, inferior and middle longitudinal fasciculi were associated with FA changes that were significantly and positively associated with changes in cognitive functions mediated by these structures (q < 0.05). These findings distinguish non-traumatic from traumatic CMBs according to CMB-related changes in surrounding WM. Our findings also challenge the assumption that traumatic CMBs are cognitively silent and identify older age and male sex as risk factors for mTBI-related cognitive decline in the presence of CMBs. In conclusion, mTBI with CMB findings on MRI can be described as a clinical endophenotype that warrants longitudinal mapping and quantification of cognitive function.

### RACIAL AND ETHNIC DIFFERENCES IN LUNG CANCER SCREENING ELIGIBILITY AND HEALTH CARE UTILIZATION AMONG OLDER SMOKERS

Jaqueline Avila,[1] Ugoji Nwanaji-Enwerem,[2] Randi Williams,[3] Hilary Robbins,[4] and Jasjit Ahluwalia[1], *1. Brown University School of Public Health, Providence, Rhode Island, United States, 2. Warren Alpert School of Medicine, Brown University, Providence, Rhode Island, United States, 3. Lombardi Comprehensive Cancer Center, Georgetown University Medical Center, Washington, District of Columbia, United States, 4. International Agency for Research on Cancer, Lyon, Rhone-Alpes, France*

The United States Preventive Task Force (USPSTF) expanded the lung cancer screening guidelines with low-dose CT scan (LDCT) to narrow racial and ethnic screening disparities. However, there is a need to examine if eligible individuals can access and use health care. The objective was to examine racial and ethnic differences in LDCT eligibility and health services access and utilization among LDCT-eligible individuals. Data comes from adults 50 to 80 years old in the 2018 Health and Retirement Study (HRS), with at least a 20 pack-year smoking history who currently smoke, or have quit < 15 years ago (n=7,624). The outcomes were LDCT eligibility, access to health care (health insurance and usual place of care), and health care use (visits to the doctor). White individuals were more likely to be LDCT eligible than Black and Hispanic individuals. Among those LDCT eligible, Hispanic individuals were less likely to have insurance (OR:0.43, [95%CI: 0.21; 0.86]) and to visit the doctor than White individuals (OR: 0.38 [0.19; 0.76]). Compared to White individuals, Black individuals were more likely to say their usual place of care was the ER or "other" place (OR: 2.65 [1.63; 3.32] and Hispanic individuals were more likely to say they do not have a usual place of care (OR: 1.94 [1.10; 3.41]). Expanding the criteria for lung cancer screening may not be enough to reduce racial and ethnic disparities. More efforts should address racial and ethnic disparities in the implementation of lung cancer screening, including access and use of health care.

### PANCREATIC CANCER PROLIFERATION IS INDUCED BY GLI2-COL1 SIGNALING IN FIBROBLASTS IN THE TUMOR MICROENVIRONMENT

Madeline Ross,[1] Renzo Vera,[2] Maite Fernandez-Barrena,[2] Murat Toruner,[2] Ezequiel Tolosa,[2] Luciana Almada,[2] Rolf Brekken,[3] and Martin Fernandez-Zapico[2], *1. St. Catherine University, St. Paul, Minnesota, United States, 2. Mayo Clinic Schulze Center for Novel Therapeutics, Rochester, Minnesota, United States, 3. UT Southwestern, Dallas, Texas, United States*

Pancreatic ductal adenocarcinoma (PDAC) affects many older adults in the United States. This aggressive type of pancreatic cancer is resistant to many chemotherapies, resulting in a poor prognosis and a five-year survival rate of less than ten percent. To study PDAC progression and develop novel therapies, it is important to better understand the tumor microenvironment (TME) and the contributing cell populations and molecular mechanisms. Despite the well-described role of soluble Type I collagen in the regulation of pancreatic cancer TME, the molecular events modulating its expression/activity remain elusive. Here, we describe a novel mechanism controlling the levels of soluble collagen in cancer associated fibroblasts (CAFs), a major producer of Type I collagen in pancreatic cancer TME. Specifically, we provide evidence that the transcription factor GLI2 is required for the expression of COL1A1, a key component of the Type I collagen fiber, stimulated by the transforming growth factor β (TGFβ). Our methodology included expression studies from patient samples, ChIP-qPCR, qPCR, RNA-seq analysis, gene set enrichment analysis, biological assays, and gene ontology analysis. Our results confirm that the TGFβ-GLI2 axis in CAFs is able to activate EGR1-proliferative signaling downstream of collagen signaling in pancreatic cancer cells contributing to our understanding of the molecular underpinnings of pancreatic cancer TME. Further studies that define the complete role of EGR1 in PDAC may lead to the development of novel therapies targeting EGR1 or CAFs to promote enhanced quality of life following a PDAC diagnosis.

### OLDER CANCER SURVIVORS' POST-TRAUMATIC GROWTH DURING THE COVID-19 PANDEMIC

Kaleigh Ligus, Emily Fritzson, Caroline Salafia, Crystal Park, and Keith Bellizzi, *University of Connecticut, Storrs, Connecticut, United States*

Older adults and immune-compromised individuals (e.g., cancer survivors) were quickly identified as high-risk groups during the COVID-19 pandemic. Research has examined adverse psychological consequences due to the pandemic in older adults with chronic conditions. However, no research has examined the potential for positive consequences of COVID-19 in older adults with cancer. The current study examined the association between COVID-19 risk, stress, exposure, and perceived post-traumatic growth (PPTG) in adults with cancer. Cancer survivors (n = 231) enrolled in an ongoing longitudinal study completed a one-time COVID-19 experience assessment, including PPTG as a result of the COVID pandemic. Participants (mean age = 68 years) had completed primary treatment for breast, prostate, or colorectal cancer within the past two years. On average, survivors reported modest PPTG related to COVID-19 (mean = 6.03, range = 0–20). Using linear regression, we found that gender, cancer type, and marital status were related to PPTG. Higher COVID-19 risk, more COVID-19 stressors, and higher illness exposure (e.g., having had COVID-19) were significantly positively associated with PPTG scores (ps < .05). In spite of the potential adverse psychological consequences of the COVID-19 pandemic, these results suggest adults with cancer, in our sample, report modest PPTG. Higher COVID-19-related perceived risk, stress, and exposure predicted higher PPTG scores. These findings are interesting in light of studies that suggest PPTG occurs only after time passes from the stressor. Future research could examine how coping with cancer may influence coping with a co-existing or subsequent stressor and its relationship to PPTG.

### CORRELATES OF CANNABIS USE AMONG MIDDLE-AGED AND OLDER ADULTS: FINDINGS FROM NESARC-III

Kevin Yang,[1] Jaclyn Bergstrom,[1] and Alison Moore[2], *1. UC San Diego School of Medicine, San Diego, California, United States, 2. University of California, San Diego, La Jolla, California, United States*

Cannabis use is increasing faster among both middle-aged and older adults compared to younger adults, but its demographic and physical health correlates comparing middle-aged and older adults need further exploration. We examined data from a US representative sample of middle-aged (50–64 years, Nf8,932) and older (65+ years, Nf5,806) adults from the 2012–2013 National Epidemiologic Survey on Alcohol and Related Conditions (NESARC-IIII). We conducted logistic regression analyses to test associations of cannabis use with demographic and past-year physical health correlates and examined differences between the two age groups. An estimated 5.6% of middle-aged and 1.3% of older adults used cannabis in the past year. Compared to middle-aged adults, older adults had higher rates of cannabis use for medical purposes (15.8% vs. 12.3%, p=0.033). Both age groups had increased odds for being male (Odds Ratio (OR)=2.32 and 2.63, for middle-aged and older adults, respectively) and residing in the West (OR=1.82 and 3.04, respectively). Middle-aged cannabis users were at decreased odds for having at least some college education (OR=0.63), income >$70,000 (OR=0.37), being married (OR=0.32), and reporting excellent/very good general health (OR=0.65). Middle-aged cannabis users were at increased odds for reporting digestive disease (OR=1.96), musculoskeletal pain (OR=1.34), and nerve pain (OR=1.77), while older cannabis users were only at increased odds for reporting digestive disease (OR=2.91). Findings indicate differences in cannabis use correlates between middle-aged and older adults. With increasing legalization of cannabis nationally, improved understanding of these correlates among more recent cohorts will assist in monitoring cannabis use among the older adult population.

### CANNABIS USE PERCEPTIONS, PATTERNS AND SYMPTOM MANAGEMENT IN OLDER VS. MIDDLE AGE AFTER A CANCER DIAGNOSIS

Margaret Fahey, and Erin McClure, *Medical University of South Carolina, Charleston, South Carolina, United States*

Prevalence of cannabis among U.S. older adults (> 65 years) is increasing and is also common among cancer patients of all ages. Less is known how cannabis beliefs and use differ in older vs. middle-age after cancer diagnosis. This NCI-funded P30 administrative supplement included a cross-sectional survey in a state with illegal access to cannabis. Participants (Nf1,038) had past cancer diagnosis (< 2 years). Comparisons were run between older (> 65 years; n=509) and middle-aged adults (45–64; n=378) relating to cannabis perceptions, patterns of use, and symptom management (among those endorsing use) with chi-square and t-test analyses. There were no age-group differences in prevalence of ever or current use, nor likelihood of discussing cannabis with provider. Use after diagnosis was less likely in older vs. middle-age (21% vs. 33%). Of those using after diagnosis, there were no age-group differences in reasons for use, but older adults reported less neuropathy relief and less digestive improvement from cannabis. When asked about potential benefits of cannabis, older adults were less likely to expect relief from neuropathy and nausea, increased appetite, decreased medication use, cancer cure, and treatment of another condition and more likely to expect improved sleep. When endorsing potential risks, older adults were more likely to report addiction and increased use of other substances and less likely to report legal reasons, job loss, and negative reactions. Findings indicate unique expectations and symptom management in older age, highlighting importance of oncology providers to discuss cannabis with this age group as rates rise.

### MEASURING SPECIALTY CARE UTILIZATION AMONG MEDICALLY FRAGILE, LOW-INCOME OLDER OR DISABLED ADULTS

Kelly Goo, Mina Silberberg, Howard Eisenson, Fred Johnson, and Sandra Stinnett, *Duke University Medical Center, Durham, North Carolina, United States*

The purpose of this research was to quantify the rates of specialty care referral completion and identify variables associated with successful completion among a population of low-income, elderly and/or disabled homebound patients with barriers to accessing office-based care. This was a cross-sectional study using descriptive and multivariate predictive analysis of specialty care referral completion, operationalized as attending an appointment of the same specialty care type within 6 months of being referred. Independent variables include patient age, sex, race, marital status, health insurance, blood pressure, and body mass index. Patient characteristics, referral information, and appointment information from July 1, 2014 to July 1, 2019 were extracted from electronic health record data of patients enrolled in the Just for Us primary care home visiting program in Durham, NC. Specialty care referrals were restricted to those for office-based consultations for chronic disease co-management originating from an outpatient primary care provider. Of 443 total referrals identified from 162 patients, 36% were successfully completed. Being married and female gender were found to be associated with successful referral completion. Of the 217 total patients in the study sample, 25% were identified as not having any referrals. No included patient characteristics were found to be significantly associated with being referred to specialty care. This study demonstrates that the specialty care needs of a medically and socially vulnerable population of homebound patients are not being adequately met.

### LESSONS FROM THE IMPLEMENTATION OF THE TRANSITIONAL CARE MODEL DURING THE COVID-19 PANDEMIC

Molly McHugh,[1] Karen Hirschman,[1] Brianna Morgan,[1] Monica Ahrens,[2] Alexandra Hanlon,[2] Onome Osokpo,[1] and Mary Naylor[1], *1. University of Pennsylvania, Philadelphia,*

*Pennsylvania, United States, 2. Virginia Polytechnic Institute and State University, Roanoke, Virginia, United States*

Multiple randomized controlled trials (RCTs) evaluating the Transitional Care Model (TCM)- an advanced practice registered nurse-led (APRN), team-based, care management strategy- have shown improved outcomes for older adults transitioning from hospital to home. In the current RCT, enrollment of older adults hospitalized with heart failure, chronic obstructive pulmonary disease, or pneumonia began in February 2020, just as the COVID-19 pandemic developed across the U.S. The COVID-19 pandemic dramatically impacted healthcare delivery across diverse local contexts. This parallel convergent mixed methods study aimed to explore the implementation of the TCM intervention as intended during Year 2 of the RCT (February 2021 to January 2022). This mixed-methods analysis presents the challenges and strategies to a key TCM component, "hospital-to-home," which focuses on delivering in-person visits to patients, as identified through a qualitative descriptive analysis of 63 clinical team and leadership meetings combined with implementation fidelity data collected simultaneously on 188 TCM participants. In Year 2 of the trial, COVID-19-specific challenges continued, including COVID-19 exposure, policy changes, patients declining services, and limited safety equipment. Some challenges to the hospital-to-home TCM component occurred regardless of COVID-19, including patient (e.g., lack of engagement), nurse provider (e.g., TCM learning curve), and system (e.g., reduced primary care access) barriers. Collectively, these challenges resulted in lower fidelity to APRNs visit patterns during TCM delivery. Strategies to address these challenges were identified. The findings provide critical insight into how to target quality improvement strategies to improve the delivery of services, such as the TCM, from hospital to home settings.

### THE EFFECTS OF TBI SEVERITY AND PTSD ON SUBJECTIVE COGNITIVE DECLINE IN OLDER VETERANS

Alin Alshaheri,[1] Monica Ly,[2] Kelsey Thomas,[2] and Lisa Delano-Wood[2], *1. San Diego State University, San Diego, California, United States, 2. VA San Diego Healthcare System, San Diego, California, United States*

Both post-traumatic stress disorder (PTSD) and traumatic brain injury (TBI) have been linked to subjective cognitive complaints; however, less is known about the unique relationships of PTSD symptoms and TBI severity with domain-specific subjective cognitive declines in older Veterans. The current study included 215 Vietnam-Era Veterans from the Department of Defense-Alzheimer's Disease Neuroimaging Initiative (age M=69.62 years, SD=4.30). General linear models, adjusting for age, education, apolipoprotein E ε4 status, self-reported health, MMSE score, and depressive symptoms, examined the unique effects of PTSD symptoms (measured using the Clinician-Administered PTSD Scale) and TBI severity (none, mild, moderate/severe) on subjective cognitive decline measured using the Everyday Cognition (ECog) measure. Greater current PTSD symptoms were associated with greater subjective cognitive declines on the ECog total score (*t*=.198, p=.009) as well as memory (*t*=.197, p=.008), language (*t*=.228, p=.004), and visuospatial (*t*=.216, p=.007) subscales. Compared with participants with no TBI, mild TBI was associated with greater subjective cognitive decline on the ECog total score (*t*=.329, p=.026) as well as the language (*t*=.334, p=.030) and divided attention (*t*=.411, p=.007) subscales, while moderate-to-severe TBI was associated with greater subjective declines in planning (*t*=.291, p=.049) and divided attention (*t*=.320, p=.032). There was no interaction between PTSD and TBI severity on ECog scores. Results suggest that PTSD symptoms and mild versus moderate/severe TBI differentially impact specific domains of subjective cognitive decline. Future work should investigate how repetitive TBI may modify these relationships, and whether PTSD and TBI severity are associated with objective cognition and quality of life in older Veterans.

### SOCIAL DETERMINANTS OF ORAL HEALTH AMONG OLDER ADULTS: RURALITY MATTERS

Aimee Ballard,[1] Maya Wetherbee,[1] Laura Bernstein,[2] and Julie Hicks Patrick[2], *1. WVU, Morgantown, West Virginia, United States, 2. West Virginia University, Morgantown, West Virginia, United States*

Oral health is often viewed as a predictor of emotional well-being. Older age and any tobacco use are among the strongest predictors of poor oral health. We sought to examine the roles of age, tobacco use, and dental health in relation to emotional well-being. Moreover, we identified unique predictors of oral health. Data were provided by 197,786 adults completing the 2020 BRFSS (mean age = 54.3 yrs; 46% men; 80% white; 15% rural; 41% had ever regularly used tobacco). Although 53.6% reported no loss of natural teeth, 29.5% reported losing between 1 and 5 teeth and 17% reported having lost 6 or more natural teeth. Hierarchical linear regression analyses revealed that younger adults, smokers, urban-dwellers, and those with more tooth loss experienced more poor mental health days, F (4, 359016) = 4307.6, p < .001, R^2= .05. Tooth loss uniquely contributed to the variance explained, above and beyond the other predictors (Step 2: F (1, 359016) = 3722.01, p, .001). We also conducted a multinomial logistic regression to test whether age, tobacco use, and rurality could differentiate among levels of tooth loss. These equations were significant, with odds ratios showing that rurality was the strongest predictor of tooth loss. Our results suggest that rurality is a strong predictor of oral health, even beyond the influence of age and tobacco use. Future interventions used to improve the oral health of people, including older adults, must consider the greater needs present to rural-dwelling Americans

### WOMEN'S HEALTH: PARADOXICAL HEALTH DISPARITIES AMONG ASIAN AMERICAN WOMEN

Janella Camp, Laura Bernstein, and Julie Hicks Patrick, *West Virginia University, Morgantown, West Virginia, United States*

Asian Americans are among the fastest growing ethnic minority groups in the U.S (Budiman & Ruiz, 2017), but women's healthcare is understudied. This may allow potential health disparities to go unnoticed. Our study aims to determine whether Asian American women are utilizing preventative health care services and to examine relations with self-reported health. We used data from a national sample AQ1 of American women (Nf 58,934; mean age = 47.3 years; range 18 to 80+) from the 2020 Behavioral Risk Factor

Surveillance System (BRFSS) data of the Centers for Disease Control and Prevention (CDC). We examined the recency of receiving a PAP smear, a mammogram, and the HPV test, along with subjective assessments of health. Asian American women reported less recent PAP smears, mammograms, and HPV tests, relative to their counterparts. However, Asian American women reported better general and physical health than non-Asian American women. To examine whether Asian American status contributed to health reports above and beyond that accounted for by the preventative tests and age, we conducted a 3-step hierarchical regression. Even after controls, Asian American status accounted for unique variance in health outcomes [F (1, 58,928) = 36.51, p < .001]. Post hoc exploratory analyses further examine the role of race in women's preventative health care. Our findings indicate that Asian American women report less use of medical services, but better general and physical health. These results suggest that further studies are needed to explore other health behaviors that may account for better health reports among Asian American women.

### HEALTH DISPARITIES RELATED TO COST OF MEDICAL COVERAGE

Nathaniel Palmer, Laura Bernstein, and Julie Hicks Patrick, *West Virginia University, Morgantown, West Virginia, United States*

Older age is often associated with challenges to functional ability. There are many government and private programs in place to help support the functioning of adults, including Medicaid, Medicare, and Tricare. These programs are not always effective, especially for middle aged and older adults. We examined whether cost was a barrier to receiving care and whether health insurance status acted as moderators of the relationship between age and functional ability. We used data from 379,606 adults who completed the 2020 Behavior Risk Factor Surveillance System (median age = 54.4 years; 54.2% women; 75.3% white non hispanic). The moderated regression equation was significant, F (7, 379598) 3275.74, p < .001, R^2 = .057. Although direct effects of age emerged as significant, the relation on functional ability was qualified by two 2-way interactions: age by insurance (b = .003) and age by prohibitive cost (b = .008). No other effects emerged as significant. Examination of the means revealed that lack of insurance was especially challenging to the functional ability of middle aged adults, whereas cost prohibitiveness was especially challenging for the functioning of older adults. Our results highlight the critical need to research the efficacy of health insurance policies amongst the middle aged and older populations in the United States

### LIVING IN HISTORICALLY REDLINED NEIGHBORHOODS AND ACCELERATED BIOLOGICAL AGING AMONG OLDER ADULTS

Jennifer Ailshire,[1] Calley Fisk,[2] and Chelsey Jones[3], *1. University of Southern California, Los Angeles, California, United States, 2. University of Southern California, Columbia, California, United States, 3. Howard University, Washington, District of Columbia, United States*

Various neighborhood factors, including greenspace availability and neighborhood socioeconomic disadvantage, are associated with accelerated biological age (aging faster than expected based on chronological age) among older adults. Neighborhoods, however, evolve overtime and historical neighborhood conditions may be influential in the aging process of older residents. In the United States, many neighborhoods were impacted by historical redlining (a federally sponsored, discriminatory program targeting communities of color throughout the 1930s), wherein neighborhoods received grades based on demographics and quality of infrastructure. Overwhelmingly, neighborhoods with more residents of color were considered less desirable or "redlined" and prior research links living in historically redlined neighborhoods to poor health across the life course and worse neighborhood conditions. We link data on redlining of U.S. census tracts from the National Community Reinvestment Coalition (NCRC) to the 2016 Health and Retirement Study Venous Blood Study (HRS-VBS) to assess whether living in historically redlined neighborhoods is associated with advanced biological aging among adults aged 55 and older. Our indicator of expanded biological age is a summary measure of 22 biomarkers associated with cardiovascular, immune, and metabolic functioning. Using multivariate linear regression models controlling for an individual's age, sex, and race/ethnicity, we found that individuals living in neighborhoods historically designated as "declining" or "hazardous" were about 2 years older biologically than individuals living in neighborhoods designated as "best/desirable". These findings suggest that residing in neighborhoods differentially shaped by historical forces impacts rates of accelerated aging. Identifying these historical neighborhood conditions may help target interventions addressing disparities in aging.

### INVESTIGATING THE ROLE OF STRESS PATHWAY ACTIVATION IN MEIOTIC DIFFERENTIATION AND REJUVENATION IN BUDDING YEAST

Akosua Biritwum,[1] and Alena Bishop[2], *1. Howard University, Bellevue, Washington, United States, 2. University of California, Berkeley, Berkeley, California, United States*

Sexually reproducing organisms undergo meiosis to produce gametes for the next generation. In budding yeast, meiosis resets replicative lifespan as gametes produced by aged progenitors are born young and devoid of any senescence-associated factors. In addition, certain transcription factors that are involved in stress response are expressed when yeast cells undergo meiosis. The overarching goal of this project is to determine the role of stress transcription factors in meiotic rejuvenation. As part of this goal, I set out to develop an assay to measure the fitness of yeast gametes that were depleted of a given stress response transcription factor. I focused on Yap1 and Skn7, two transcription factors involved in oxidative stress, and characterized gamete fitness by measuring survival in response to environmental stressors, including acid and alkaline. I found that gametes depleted for Yap1 have mild sensitivity to acid and dramatic sensitivity to alkaline. These findings implicate a role of Yap1 in gamete fitness in a physiologically relevant context since acidic and alkaline environments exist in the digestive tract of fruit flies, which are natural predators of yeast. Further investigation of how stress response transcription factors affect meiotic rejuvenation can help us develop strategies to counteract cellular aging in different contexts.

### INVESTIGATING THE NEURAL AFFECTS OF 17 ALPHA-ESTRADIOL (17A-E2) ON APOE4 PHENOTYPES

Tavia Roache,[1] Christian Pike,[2] and Cassandra McGill[3], *1. Howard University, Washington, District of Columbia, United States, 2. USC, Los Angeles, California, United States, 3. University of Southern California, Los Angeles, California, United States*

Aging and APOE4 genotype are the primary risk factors for late onset Alzheimer's disease (AD). Previous drugs trials for AD have focused on amyloid beta which has yielded minimal effects; hence a drug targeting aging could prove more efficacious. 17 alpha-estradiol (17α-E2) is an enantiomer of 17 beta-estradiol that has been shown to extend lifespan in male mice. We hypothesize that 17α-E2 will improve aging related phenotypes caused by the APOE4 genotype. We enrolled 10-month-old APOE3 or APOE4 targeted replacement mice and randomized them to either control or 17α-E2 diet for 20 weeks. At weeks 15 to 17, mice underwent various behavior assays and tissues were collected at the end of the 20 weeks. We observed behavioral improvement in APOE4 mice treated with 17α-E2 and asked whether this effect is associated with: (1) change in activity or anxiety, (2) change in neural stem cells, or (3) change in microglial activation. Activity levels but not neurogenesis of microglial burden were affected by APOE genotype and 17α-E2 treatment, suggesting a potential contribution to observed memory improvement in APOE4 mice treated with 17α-E2. Continued research on the neural effects of 17α-E2 pose potential benefits to mitigate the effects of aging.

### SESSION 9010 (POSTER)

### LATE BREAKING POSTER SESSION II

### USING VIRTUAL REALITY IN AGED CARE SETTINGS: A SCOPING REVIEW

Lillian Hung,[1] Flora To-Miles,[1] Winnie Kan,[1] Alisha Temirova,[1] Jim Mann,[2] and Christine Wallsworth[3], *1. University of British Columbia, Vancouver, British Columbia, Canada, 2. Community Engagement Advisory Network, Vancouver, British Columbia, Canada, 3 Vancouver, British Columbia, Canada*

Recent advancement of virtual reality (VR) technology has led to growing interest in using VR in aged-care settings. VR can help ameliorate experiences of loneliness and social isolation, which is especially important during the COVID-19 pandemic. As a result, increasingly more studies are being published on this topic, and a comprehensive review of studies examining the facilitators and barriers of adopting VR in these settings is needed. This scoping review reports the facilitators and barriers to implementing VR in care settings among older adults, as well as the impact on social engagement and/or loneliness. We followed the Joanna Briggs Institute scoping review methodology, and searched the following databases: CINHAL, Embase, Medline, PsycInfo, Scopus, and Web of Science. Inclusion criteria includes articles published in the last five years that focus on older adults using VR in aged-care settings. 199 articles were retrieved and 21 articles were included in our review. Most of the articles (38%) originated from Australia. Key facilitators for using VR in aged care settings are the technology being user-friendly, comfortable, and easy to clean. Barriers included: technology issues (e.g., internet connectivity), staff attitude/worries, and impact on residents, and structural considerations (e.g., lack of staff and time to assist with the VR program). VR technology can decrease loneliness and feelings of isolation, and provide opportunities to engage with others. Our review of the current evidence offers insights and recommendations for health care professionals to use VR technology in aged care settings, maximizing benefits and minimizing risks among users.

### USING MDS TO MEASURE TRANSFER TRAUMA IN NURSING HOME RESIDENTS

Ana Montoya, Pil Park, Chiang-Hua Chang, and Julie Bynum, *University of Michigan, Ann Arbor, Michigan, United States*

Transferring long-term nursing home residents between facilities has been shown to compromise their quality of life. The negative consequences associated with transfers have been termed transfer trauma and its spectrum spans from functional, cognitive, and emotional decline to hospitalizations, and even death. The objective of this study was to develop a composite measure of transfer trauma after a nursing home-to-nursing home transfer using clinically relevant events and sub-measures covering functional, cognitive, behavioral, and emotional domains based on validated scales available in the Minimum Data Set (MDS) assessments. MDS Data were used to identify long-stay nursing home residents and obtain information on transfers, clinical assessments, and facilities. This study is a cross-sectional cohort analysis of long-stay nursing home residents in the state of Michigan who had a nursing home-to-nursing home transfer during our study period (March 1, 2018-October 31, 2018). We developed a measure of transfer trauma as a composite from 5 previously validated measures: 1) Activity of Daily Living (ADL) Self-Performance Scale; 2) Cognitive Function Scale (CFS); 3) Patient Health Questionnaire-9 (PHQ-9/PHQ-9-OV); 4) Agitated Reactive Behavior Scale (ARBS); 5) falls. The distribution and frequency of each sub-measure were analyzed to assess scaling of the composite measure. Of a total of 817 residents who transferred in our study period, 549 had complete composite measures, and from those 242 (44%) met criteria for transfer trauma. These findings demonstrate the need for further research to identify underlying transfer trauma and develop interventions to ameliorate the negative effects of transfers on this vulnerable population.

### TRANSITION OF HEALTH PROFILES IN COGNITIVELY INTACT NURSING HOME RESIDENTS WITH SUICIDAL IDEATION

Yiyang Yuan,[1] Adrita Barooah,[2] Deborah Mack,[1] Kate Lapane,[1] Anthony Rothschild,[3] and Christine Ulbricht[4], *1. University of Massachusetts Chan Medical School, Worcester, Massachusetts, United States, 2. University of Massachusetts Boston, Boston, Massachusetts, United States, 3. UMass Chan Medical School, Worcester, Massachusetts, United States, 4. Takeda, Cambridge, Massachusetts, United States*

For older adults entering nursing homes with self-reported suicidal ideation (SI), little is known about how their health profiles change during the first 90 days post admission. Using the 9th Patient Health Questionnaire-9 item on Minimum Data Set 3.0, we identified 5,397 cognitively intact, older adults with SI at admission, 29% of whom continued to report SI at 90 days. Latent transition analysis identified five health profiles at admission: (1) frail, severe pain, all depressive symptoms (prevalence: 25%); (2) frail, moderate pain, depressed mood, insomnia/hypersomnia, fatigue (27%); (3) frail, no pain, depressed mood, fatigue, and worthlessness (18%); (4) pre-frail, no pain, depressed mood (15%); (5) robust, no pain, depressed mood, insomnia/hypersomnia, fatigue (15%). At 90 days, while the profiles remained mostly the same, improvement in depressive symptoms and pain were observed: (1) frail, severe pain, most depressive symptoms (17%); (2) frail, no pain, depressed mood, and fatigue (28%); (3) frail, no pain, depressed mood, fatigue, and worthlessness (15%); (4) pre-frail, no pain, no depressive symptoms (25%); (5) robust, no pain, depressed mood, and fatigue (16%). Most residents remained in the same profile over time. Of those who were in Profile (1) at admission, 20% transitioned into Profile (2), 12% to Profile (4), and 7% to Profile (5). This work highlights the need to now investigate the potential drivers for specific profile transitions among nursing home residents presenting with SI on admission, thereby informing interventions for improved identification and treatment of SI in the nursing home setting.

### TRANSFER TRAUMA AND MAJOR EVENTS AMONG NURSING HOME RESIDENTS BEFORE AND DURING THE COVID-19 PANDEMIC

Ana Montoya, Pil Park, Chiang-Hua Chang, and Julie Bynum, *University of Michigan, Ann Arbor, Michigan, United States*

The negative consequences of transfers are known as transfer trauma. Nursing home (NH)-to-NH transfers place long-term NH residents at risk for developing transfer trauma and this risk may have increased during the COVID-19 pandemic in the setting of a state policy that increased the number of residents who transferred between NHs. The objective of this cross-sectional cohort analysis was to assess the incidence of transfer trauma and major events (hospitalization/death/discharges) among long-term NH residents who transferred from one NH to another before and during the COVID-19 pandemic using a composite measure of transfer trauma based on validated scales from Minimum Data Set (MDS) assessments. A total of 750 residents transferred in the pre-COVID cohort and 795 in the COVID cohort were eligible for assessment of transfer trauma and major events. After adjusting for demographic characteristics, residents in the COVID cohort were almost twice more likely to die and almost three times more likely to discharge within 90 days compared to those in the pre-COVID cohort (AOR=1.94, 95%CI [1.15, 3.26] and AOR= 2.86, 95%CI [2.30, 3.56], respectively). Residents in the COVID cohort were less likely to experience transfer trauma compared to those in the pre-COVID cohort. In the during-COVID cohort, 26% of residents had a COVID-19 diagnosis and they were less likely to experience transfer trauma compared to residents without a COVID-19 diagnosis (AOR=0.34, 95%CI [0.23, 0.50]). It is important to note that some residents may have not stayed in the nursing home long enough to assess them for transfer trauma.

### STAFFING DIFFERENCES IN NURSING HOME SPECIAL FOCUS FACILITIES AND SPECIAL FOCUS FACILITY CANDIDATES

Annie Rhodes,[1] Faika Zanjani,[1] Thomas Caprio,[2] Tracey Gendron,[3] and Leland Waters[1], *1. Virginia Commonwealth University, Richmond, Virginia, United States, 2. University of Rochester, Rochester, New York, United States, 3. VCU, Richmond, Virginia, United States*

Introduction: Centers for Medicare and Medicaid Services designates “Special Focus Facilities” (SFF), which are nursing homes receiving special oversight for persistent quality problems. “Special Focus Facility Candidates” (SFFc) are nursing homes with quality problems that are designated as candidates to be future SFFs. Recent academic literature has not examined if SFF and SFFc have significant differences in staffing hours per resident day (HPRD). Methods: Nursing homes that were SFF or SFFc in 2020 were matched to 5-star nursing homes. Monthly staffing averages were aggregated, and two-way ANOVAs with Tukey Post Hoc were conducted to detect level differences in HPRD across the three facility groups. Gross and Case-Mix staffing HPRD were analyzed, along with daily resident census. Results: The final sample was n=197 SFFc, and n= 50 SFF, which were matched with n=247 5-star nursing homes for a total sample of Nf494. Over 2020, daily census for 5-star nursing homes was lower (M=72.36, SD=52.5) than SFFc (M=93.77, SD=68.34) or SFFs (M=97.88, SD=44.06). There was a significant difference between SFF and SFFc HPRD in Case-Mix Aide F(2, 5748)=187.6, p= < .005, and Registered Nurse (RN), F(2,5748)=323,p=.003 care. There were no significant differences between SFF and SFFc in HPRD Aide F(2,5748)=380,p=.63, Practical Nurse F(2,5748)=1.1, p=.211, Case-Mix Practical Nurse F(2,5748)=19.57,p=.39, Case-Mix RN F(2,5748)=9.51,p=.91 or Total HPRD F(2,5748)=472.6,p=.16, care. Discussion: There is only a significant difference in staffing levels observed between SFF and SFFc for Aide staffing and RN staffing. This information supports researchers and policymakers in delineating the differences and similarities between SFF and SFFcs.

### RETHINKING NURSING HOME ARCHITECTURE AND DESIGN IN THE LIGHT OF THE COVID-19 PANDEMIC

Desmond O’Neill, Tom Grey, Dimitra Xidous, Jennifer O’Donoghue, and Mehak Puntambekar, *Trinity College Dublin, Dublin, Dublin, Ireland*

Introduction: The huge death rate in nursing homes during the COVID-19 pandemic raised serious questions as to whether the built environment of nursing homes was a factor in this very high mortality, as well as a factor in quality of life. Method: We embarked on a wide-ranging study involving a review of Irish policy, stakeholder engagement, Irish case studies, literature review, and international case studies to understand the key issues that influence the planning, design, and operation of nursing home settings settings, and to identify how these shape care models and the physical environment. Results: The project generated the following key themes: a) including the voices of residents, family and staff in co-creation of design and research; b) integrating nursing homes with the overall housing spectrum; c) linking nursing homes with ageing in place policy; d) further research on optimal design; e) understanding resident diversity; f) greater inclusion of Universal Design principles; g) designing for resilience; and h) Convergence between infection control and quality of life Discussion: Our Research Findings have been developed to identify major current issues related to the built environment and its role in creating a balance between quality of life and COVID-19 infection control in Irish and international nursing home settings. These findings are relevant for a wide range of stakeholders and will be disseminated across a number of channels to continue this conversation and help to continue the evolution of nursing home design.

### POSITIVE AFFECT AND SOCIAL SUPPORT SATISFACTION AMONG NEWLY ADMITTED LONG-TERM CARE RESIDENTS

Darby Simon, Benjamin Mast, Emilee Ertle, and Suzanne Meeks, *University of Louisville, Louisville, Kentucky, United States*

Social support serves as a protective factor against depression and other negative outcomes among older adults. Satisfaction with social support among long-term care residents has not received as much attention, and the experience of support among newly admitted long-term care residents has not been investigated. The current study examines what variables influence a long-term care resident's satisfaction with their social support. This sample consists of 48 individuals newly admitted into long-term nursing home care. This study used the Social Support Questionnaire (SSQ6) to gather information on social support network size and overall satisfaction of perceived social support. Correlations were estimated between social support satisfaction, social support network size, and psychosocial factors including depression (PHQ-9), anxiety (RAID), religious involvement, cognitive status (BIMS), positive and negative affect (PANAS). Satisfaction with support was significantly correlated with social network size ($r = .328$, $n = 48$, $p < .05$) and positive affect ($r = .437$, $n = 24$, $p < .05$). In a regression model with support satisfaction as the dependent variable, both social network size and positive affect contributed significantly to model R2 $F(2,21) = 2.441$, $p = .049$ $R2 = .250$. Satisfaction with support depends upon the number of supporters available, but also on levels of positive affect.

### MEASURING WORKPLACE LEARNING CONDITIONS FOR NURSING STAFF IN LONG-TERM GERIATRIC CARE

Merel (E.A.) Van Lierop,[1] Judith Meijers,[1] Erik van Rossum,[2] and Sandra Zwakhalen[1], *1. Maastricht University, Maastricht, Limburg, Netherlands, 2. Zuyd University of Applied Sciences, School of Nursing, Heerlen, Limburg, Netherlands*

To deal with challenges in practice such as workload and complexity in long-term care delivery, learning in practice (workplace learning) is becoming increasingly important. Nursing staff needs to take the lead and learn continuously in practice using a bottom up approach to promote workplace learning. Therefore, insight in workplace learning conditions (like work culture and learning climate) in long-term care is necessary. Based on these insights, nurses can define challenges and goals on how to stimulate workplace learning. Currently, no instruments are available that measure these conditions in the long-term nursing care setting in a snap shot. Therefore, the goal of this study was to develop a quick-scan to measure workplace learning conditions for nursing staff in long-term geriatric care. This quick-scan was developed in 3 steps: (1) investigating how to measure conditions for workplace learning, (2) selecting appropriate measurement tools to measure conditions for workplace learning, and (3) testing the feasibility of the quick-scan in practice. As a result, the final feasible quick-scan measured 11 conditions regarding workplace learning: (1) Trust: values and culture, (2) Team: support and management, (3) Colleagues: support and respect, (4) Constraints in undertaking the job, (5) Urgency, (6) Learner agency, (7) Collaboration, (8) Hybrid learning, (9) Coaching, (10) Flexibility – formal and informal learning, and (11) Assessment-as-learning. Additionally, questions were added regarding availability of sources for learning, knowledge and skills, and communication. Validated questionnaires that were included were the Culture of Care Barometer and HILL model questionnaire.

### MEASURING BARRIERS TO LONG-TERM CARE RESIDENT PREFERENCES: DEVELOPMENT AND CONTENT VALIDITY TESTING

Laura Block,[1] Ella Greenhalgh,[1] and Tonya Roberts[2], *1. UW-Madison School of Nursing, Madison, Wisconsin, United States, 2. University of Wisconsin-Madison, Madison, Wisconsin, United States*

Person-centered care (PCC), or delivery of care consistent with preferences, can improve outcomes for residents in long-term care (LTC). However, PCC has not been universally adopted. Few studies focus on measuring specific barriers to meeting resident preferences, hindering identification of actionable targets to improve PCC delivery. The purpose of this study was to develop and test the content validity of a survey that measures staff barriers to fulfilling resident preferences for daily care and activities. Item sets comprised of barriers and preferences were informed by a review of literature and a descriptive, qualitative study with 19 LTC staff. An expert panel (n=8) was convened to test content validity, and content validity indices (CVI) were calculated. The initial survey consisted of 12 item sets assessing whether knowledge gaps, resident characteristics (e.g. health needs), family, facility rules/policies, availability of options, or staffing/workload functioned as barriers to meeting resident preferences for daily care and activities (CVI = 0.76 barriers; CVI = 0.89 preferences). Revisions informed by expert feedback resulted in a total of 17 items sets. Second round testing with experts (n=10) revealed overall improved item sets (CVI = 0.77 barriers; CVI = 0.96 preferences). These findings indicate that the survey items have initial content validity in measuring staff barriers to meeting resident daily care and activity preferences. Further psychometric testing with LTC staff is needed prior to implementing the survey, which will represent the first tool of its kind for supporting measurement of staff-derived targets for PCC interventions and policy change.

### INFORMATION TO BE COLLECTED IN THE ASSESSMENT FOR PARENTS AND CHILDREN WITH "8050 PROBLEMS" IN JAPAN

Takako Ayabe,[1] Yoshito Takemoto,[2] Shinichi Okada,[3] and Sadaaki Fukui[4], *1. BAIKA Women's University, Ibaraki, Osaka, Japan, 2. Okayama Prefectural University, Soja, Okayama, Japan, 3. Osaka Metropolitan University, Osaka, Osaka, Japan, 4. Indiana University, Indianapolis, Indiana, United States*

In Japan, the "8050 problems" have become a social issue, where parents and children are parents in their 80s and children in their 50s and live together. In the case of parents and children with "8050 problems," the parents are older, but the children are often dependent, withdrawn, and have financial problems. Recently, the number of such cases has increased in care management. The previous studies indicated that it was important to know what information was gathered in the assessment in such cases. The research was conducted from February to March of 2022 by self-administered questionnaires. The survey was mailed to all 1,410 care management centers in Osaka City. The response rate was 11.8%. The statistical analysis was an exploratory factor analysis. Six factors in the significant information in the assessment were extracted by the factor analysis: Physical, mental and living conditions of children; Parents' physical and mental conditions; Parents' perspectives on their lives; Parents and children's financial situations; Living environment; and Relationships between the informal and formal supports and the parents and children. The Cronbach's alpha for internal consistency was greater than 0.8 for each factor. In addition, the four factors were strongly associated among the six factors. In conclusions, the present study found that information about the physical and mental status, living conditions, community relations, and financial situations of the parents and children with "8050 problems" was important in the assessment of the parents and children, and that the information is closely related to each other.

### CALL FOR ACTION: STRENGTHENING LONG-TERM CARE IN NEPAL

Usha Dhakal, and Sara McLaughlin, *Miami University, Oxford, Ohio, United States*

This paper aims to introduce the existing long-term care (LTC) system in Nepal, identify key emerging issues, and provide possible policy recommendations for addressing weaknesses in the current system. Although the majority of older Nepali adults rely on their adult children for care, this traditional care arrangement is weakening for a variety of reasons. As a result, older Nepali adults increasingly seek care elsewhere such as "old age homes" (OAH), which generally meet only their basic needs. More recently, adult day centers have been established that enable older Nepali to receive help with personal care and instrumental tasks and/or health care needs in the absence of their families; however, they are primarily located in big cities and largely limited to those who can afford to pay. With a growing older population and societal changes that make familial care increasingly challenging for adult children, the Government of Nepal must foster the development of a sustainable system of LTC. This will necessarily involve building and sustaining a skilled geriatric workforce, capitalizing on the natural "villages" that have existed in Nepal for hundreds of years, and standardizing and monitoring the operation of LTC facilities. Additionally, public service campaigns to help destigmatize the use of OAH and day centers and integration of home and community-based services to make care arrangements locally could help make these newer LTC approaches more acceptable. Finally, nationally representative studies aimed at understanding the health and care needs of Nepal's rapidly increasing older population is of utmost priority.

### BARRIERS AND FACILITATORS FOR FOREIGN EDUCATED NURSES TO PROVIDE QUALITY LONG-TERM CARE

Roy Thompson,[1] Kirsten Corazzini,[2] Thomas Konrad,[3] Michael Cary,[4] Susan Silva,[4] and Eleanor McConnell[4], *1. University of Missouri, Colombia, Missouri, United States, 2. University of New Hampshire, Durham, New Hampshire, United States, 3. University of North Carolina at Chapel Hill, Chapel Hill, North Carolina, United States, 4. Duke University, Durham, North Carolina, United States*

Unprecedented registered nursing shortages in long-term care (LTC) threaten the provision of person-centered care for older adults in the United States (US). LTC facilities recruit Foreign Educated Nurses (FENs) to address shortages, which raises concerns about care quality due to cultural, linguistic and communication differences among nurses; yet studies have not thoroughly explored FENs' perspectives on these issues. The purpose of this study was to advance our understanding of FENs' professional experiences as they began employment in LTC by exploring factors that inhibit or facilitate their provision of quality care. This qualitative descriptive study used purposive sampling to recruit FENs through professional organizations. Eligible FENs were ≥ 18 years old, worked ≥1 year in LTC, and represented racial and ethnic minority groups from Low and Middle Income Countries. In-depth narrative interviews, ranging from 45–60 minutes, were conducted. Applying content analysis, a priori and inductive coding generated themes. Participants (n=12) interviewed were all married females. Most were 50–59 years old (41.7%), Asian (75.0%), BSN-prepared (58.3%), and reported 31–50 years of nursing experience (50%). Positive facility characteristics, acculturation, effective workplace integration and positive support from colleagues, residents, and their families facilitated the provision of quality care. Conversely, negative facility characteristics, cultural barriers, discrimination and ineffective workplace integration were barriers to providing quality care. FENs highlighted culturally-sensitive strategies such as providing structured mentorship and preceptorship programs that supported them in providing person-centered care. FENs confirmed the need to address racial and anti-immigrant discrimination for achieving more equitable and inclusive workplaces.

### VALIDITY EVIDENCE FOR THE MALE DEPRESSION RISK SCALE-22 (MDRS-22) IN YOUNGER AND OLDER ADULT MALES

Montgomery Owsiany, and Amy Fiske, *West Virginia University, Morgantown, West Virginia, United States*

Men are two times less likely to be diagnosed with major depressive disorder (MDD) than women. However, suicide rates are nearly four times higher in men than women, increasing to six times or more when comparing older men to older women. Masculine depression is characterized by symptoms that are not usually assessed by diagnostic criteria for MDD, including drug and alcohol abuse, anger and aggression, and risk-taking. Previous studies have largely neglected to consider the possibility of age-related differences in the presentation of masculine depression. Given research on age-related differences in MDD, there may be age-related differences in the presentation of masculine depression as well. The present study assessed age invariance of the MDRS-22 through a multi-group confirmatory factor analysis (CFA), evaluated validity evidence of the MDRS-22, and tested the MDRS-22's ability to assess suicidal ideation and behaviors. There was a significant difference between the configural and first-order metric models of the CFA showing that the MDRS-22 was not age invariant ($\Delta X2 = 451.47$, $\Delta df = 16$, $p < .001$). The MDRS-22 showed convergent validity evidence with assessments of MDD, alignment with masculine norms, suicidal behaviors, problematic alcohol use, and aggression. The MDRS-22 showed concurrent validity evidence with another assessment of masculine depression. Finally, MDRS-22 scores significantly predicted suicide risk above PHQ-9 scores ($F(2,440) = 138.774$, $p < .001$, $R2 = .385$). Overall, the study highlights the importance of screening males for masculine depression. Further research is needed to determine if masculine depression presents differently in younger and older males.

### THE EFFECT OF SUPPLEMENTAL NUTRITION ASSISTANCE PROGRAM GENEROSITY ON DEPRESSIVE SYMPTOMS AMONG OLDER AMERICANS

Peiyi Lu, Katrina Kezios, and Adina Zeki Al Hazzouri, *Columbia University, New York, New York, United States*

Food insecurity has been indicated to associate with more depressive symptoms. Supplemental Nutrition Assistance Program (SNAP) is an established anti-poverty government program that targets food insecurity. The goal of this study was to assess whether SNAP generosity, defined at the state level, impacts the relationship of food insecurity and depressive symptoms among older Americans. We linked state-level SNAP policy data to the individual-level data of older adults (aged 60+) from the longitudinal Health and Retirement Study (Nf12,801). Our study baseline was survey year 2000. A state is considered SNAP generous when it eliminates income/assets limit from its SNAP eligibility criteria. All HRS participants reported their food insecurity status and depressive symptoms biennially. Participants were followed until 2018. We conducted state fixed-effect models adjusted for demographic and health measures. Results indicate household food insecurity was associated with higher risk of elevated depressive symptoms (OR=1.83, 95%CI=1.61, 2.07) from a fixed effect logistic model, and this effect was stronger when living in non-generous states compared with generous states though confidence intervals were too wide to be conclusive (OR_interaction =1.09, 95% CI= 0.83, 1.42). The relative excess risk due to interaction (RERI) was 0.17 (95%CI= -0.30, 0.63). Our study finding suggested that there is no to weak evidence that state-level SNAP generosity may alleviate or offset the risk of depressive symptoms associated with food-insecurity.

### SOCIAL ENGAGEMENT IN LATER LIFE: INTERDEPENDENCE OF MARRIED COUPLE'S MENTAL HEALTH WELL-BEING.

Yulri Kim, *Iowa State University, Ames, Iowa, United States*

Social engagement in later life often provides a sense of belongings and social identity. Given its salient meanings for older adults in Korea where social ties play important roles on the mental health and well-being, different conceptualizations of social engagement have been examined, such as formal and informal social activities. As numerous studies have suggested, informal activities are associated with better well-being, however, it is unclear whether formal social engagement of husbands and wives influence the other partner's mental health outcomes. Based on the Interdependence Theory (Rusbult & Van Range, 2008), we expect that formal and informal social engagement of husbands and wives are associated with their own depressive symptoms and further those of their spouses. Using a sample of 1,195 couples (Nf 2,390) of married older adults aged 65 and above from the 8th wave of Korean Longitudinal Study of Aging (KLoSA), we tested the relationship between social engagement and depressive symptoms using actor partner interdependence model (APIM). The findings indicated that for both husbands and wives, there was a negatively significant association between one's informal social activity on their own depressive symptoms (actor effect) and their spouse's depressive symptoms (partner effect). No significant findings were observed for formal social activities. Overall, this study suggests the importance of informal social activity compared to formal social activity to lower depressive symptoms for themselves and their spouses in later life. Moving forward, incorporating a partner's informal social activity can provide useful information in predicting one's own mental health well-being.

### RESILIENCY AS A PROCESS: A QUALITATIVE INVESTIGATION INTO U.S. VETERANS' EXPERIENCES AGING WITH HIV

Kiera Chan,[1] Alexis Bender,[1] Vincent Marconi,[2] Amy Justice,[3] Theodore Johnson,[1] Keith McInnes,[4] and Molly Perkins[5], *1. Emory University, Atlanta, Georgia, United States, 2. Emory University, Decatur, Georgia, United States, 3. Yale University, New Haven, Connecticut, United States, 4. Boston University, Boston, Massachusetts, United States, 5. Emory University School of Medicine, Atlanta, Georgia, United States*

U.S. military Veterans aging with HIV represent a unique special population. The aim of this qualitative study was to investigate risk and protective factors associated with Veterans' ability to age well with HIV. Participants included 25 Veterans (≥ age 50) participating in the Veterans Aging Cohort Study who were recruited from the Atlanta Veterans Affairs (VA) Medical Center. This study conducted semi-structured interviews, social network mapping, and sociodemographic and health surveys. Participants ranged in age from 50 to 72, with a mean age of 59. Most (80%) were male, and more than half (60%) were African American. Findings showed that many participants experienced adversity in childhood or early adulthood, including military sexual violence, childhood abuse, and non-military domestic violence, as well as harassment based on sexual identity. Many also had histories of substance abuse. Although military life provided stability for some, most experienced some form of instability after leaving the service, including financial difficulties and loss of valued military ties. Timing of diagnosis, whether in the military or after the military, impacted resiliency. Receiving an HIV diagnosis was an important turning point in participants' lives characterized by either maladaptive (e.g., suicide ideation) or therapeutic (e.g., health promoting behaviors) coping strategies. Positive social support, including close relationships many developed with providers at the VA HIV clinic, was an important protective factor. The sample experienced cumulative life events that shaped their ability to age with HIV. Findings have important implications for interventions to promote Veterans' ability to age well with HIV.

### OLDER VETERANS' PREFERENCES FOR FORMAT AND DESIGN OF EDUCATIONAL MENTAL HEALTH BROCHURES

Ruifeng Cui,[1] Armando Rotondi,[2] Alaa Shalaby,[3] and Judith Callan[2], *1. VA Pittsburgh Healthcare System, Morgantown, West Virginia, United States, 2. VA Pittsburgh Health Care, Pittsburgh, Pennsylvania, United States, 3. VA Pittsburgh Healthcare System, Pittsburgh, Pennsylvania, United States*

Older Veterans may have lower mental health literacy and education than younger Veterans. The Veterans Affairs Healthcare System (VA) has created many educational brochures on mental health to facilitate service utilization by Veterans, however few studies have solicited Veteran stakeholder feedback pertaining to their design and effectiveness. The present qualitative pilot study aimed to develop a brief general mental health educational brochure for older Veterans seen in VA medical settings. The brochure aims to provide information on mental health symptoms and VA mental health resources. Participants (Nf5, mean age=67.2) were older adult White male Veterans. An iterative design process was used to develop and refine the educational brochure which consisted of qualitative interviews with Veteran stakeholders on their mental health needs and their feedback on the brochure. The brochure was then modified based on prior Veteran feedback before being tested with subsequent Veteran participants. The brochure received high ratings on clarity (8.8/10), conciseness (8.6/10), and importance (8.6/10). Qualitative interviews revealed that Veterans appreciated the simplistic presentation of the content ("It's very informative for being so simple, it caught my attention"), the generality of the content ("it is encouraging because it is a broad approach to a spectrum of mental health concerns"), and contact number content ("Direct lines, that's really nice"). These findings support the use of user-centered design methods to develop educational materials. The findings indicated that a brief educational mental health print brochure was an acceptable method to promote mental health literacy and use of VA mental health services.

### DIFFERENCES AND SIMILARITIES OF ELDERLY PERSONS IN SWEDEN WITH A DIAGNOSIS OF PSYCHOSIS OR NON-PSYCHOSIS (SMI)

Per Bülow,[1] Pia H. Bülow,[2] and Deborah Finkel[3], *1. Jönköping University, School of Health and Welfare, Jönköping, Jonkopings Lan, Sweden, 2. School of Health and Welfare, Jönköping, Jonkopings Lan, Sweden, 3. Indiana University Southeast, New Albany, Indiana, United States*

Psychiatric care in Sweden is jointly organized by psychiatric practice and municipal social services. To determine who is entitled to support from the municipalities, the concept of "psychiatric disability" was created in connection with psychiatric reform in 1995. Psychiatric disability is a poorly identified concept and in Sweden, a person has severe mental illness (SMI) if they have difficulties in carrying out activities in crucial areas of life, these difficulties are caused by a mental disorder, and they are prolonged. Internationally, SMI is often synonymous with psychosis, but in Sweden other severe psychiatric conditions are included, but not dementia. Both practically and ethically, the unclear definition of SMI is a problem because it determines whether a person is granted interventions and what forms the interventions take. We investigated similarities and differences in people defined as SMI, divided into two groups, psychosis (Nf222) and non-psychosis (Nf253). Adults with SMI aged 65 or over (in 2016) have been assessed using data from four surveys carried out between 1996 and 2011, as well data available from national registers. People with psychosis had worse functional levels on the Global Assessment of Functioning and more unmet needs, according to Camberwell Assessment of Needs. However, differences between psychosis and non-psychosis groups varied across measures (e.g., education, income, living situation) and results differed depending on age at onset, year of first admission to a mental hospital, and length of institutionalization. These variables had a greater impact on the similarities and differences between measures than the diagnosis itself.

### AFFORDABILITY, PERCEIVED RACISM, AND HEALTHCARE SYSTEM DISTRUST AMONG AFRICAN AMERICAN WOMEN AGED 45 AND OVER

Jacqueline Wiltshire,[1] Carla Sampson,[2] Keith Elder,[3] and Paul Musey[1], *1. Indiana University, Indianapolis, Indiana, United States, 2. New York University, New York, New York, United States, 3. Mississippi, Clinton, Mississippi, United States*

Unaffordable healthcare costs—a major concern for Americans--disproportionately affect African Americans (AA), who are more likely to distrust the healthcare system due to past mistreatment and discrimination. However, the relationships between affordability, mistreatment, discrimination, and healthcare system distrust are unclear. Using cross-sectional survey data from a community-based sample of 313 African American women aged 45 and over, we assessed the relationships between the ability to get needed care due to costs, negative healthcare experiences, perceived racism, and healthcare system distrust. Linear regression and mediation analyses were conducted to assess relationships. Approximately 23% of women reported the inability to get needed care because of costs, and 44% had a negative experience with a healthcare provider. Healthcare system distrust was higher among women unable to get healthcare because of costs (β = 2.66; p = 0.005) or had a negative experience with a healthcare provider (β = 3.02; p < 0.001). However, perceived racism in the healthcare system, a significant predictor of distrust (β = 0.81; p < 0.001), attenuated the relationships between inability to get health care because of costs (β = 1.74; p = 0.051) and negative experience with a healthcare provider (β = 1.79; p = 0.013). Perceived racism explains 34% and 46%, respectively, of the relationships between affordability, negative experience with healthcare provider, and healthcare system distrust. These findings are important given the relevance of building trust and understanding needs to address health inequities. Future research should explore whether these findings hold for AA men and other minoritized groups.

### A COLLABORATIVE AUTOETHNOGRAPHY OF FOREIGN-BORN FACULTY IN THE FIELD OF AGING

Chih-ling Liou,[1] and Karel Kalaw[2], *1. Kent State University, North Canton, Ohio, United States, 2. University of Central Oklahoma, Edmond, Oklahoma, United States*

We draw from our personal insights as foreign-born females in the U.S. to explore how our minority status has affected our experiences/identities as faculty in the field of aging. We practiced intersectionality by considering how our understandings of "foreignness" in academia and the aging field are intertwined with other markers of difference, including race, national origin, language, and academic programs. We also draw from tenets of collaborative autoethnography to engage two autoethnographies from two countries in Asia to pool their lived experiences and collaboratively analyze and interpret them for commonalities and differences. We begin by sharing stories about graduate study pursuits and becoming faculty in the U.S. We note how our foreignness shapes our lives as aging scholars in the U.S. academy and our personal views on aging. After exchanging our first set of writings, we identified key experiences to focus on in the subsequent writing periods. Our minority backgrounds, teaching aging subjects, and using qualitative methodology are shared identification. However, our personal stories within the U.S. society and within the academy also diverge our identities and aging experiences from each other. Moreover, the themes that deal directly with identity development and the perception of aging provide a deep understanding of aging among the non-native-born population. This study highlights the value of collaborative autoethnography as a method of inquiry and reflection. Findings demonstrate that non-native-born female faculty in the field of aging faced multi-faceted challenges in both professional and personal realms. Implications for supporting foreign-born female aging scholars are discussed.

### RACIAL-ETHNIC VARIATION IN MULTIMORBIDITY PATTERNS AND HEALTHCARE SERVICES AMONG OLDEST-OLD PATIENTS

Jinmyoung Cho,[1] Gelareh Rahimighazikalayeh,[2] and Heather Allore[3], *1. Baylor Scott & White Research Institute, Temple, Texas, United States, 2. Baylor Scott & White Research Institute, Dallas, Texas, United States, 3. Yale University, New Haven, Connecticut, United States*

Adults aged 85 years and older ("oldest-old") are perceived as survivors resilient to age-related risk factors. Although considerable heterogeneity has been often observed in this population, less is known about the unmet needs in health and healthcare service utilization for diverse patients in healthcare systems. We examined racial-ethnic variation in patterns of multimorbidity associated with emergency department (ED) and clinic visits among oldest-old patients with multiple chronic conditions (MCCs). Administrative and clinical data from an integrated healthcare system for five years included 25,801 oldest-old patients with MCCs. Hierarchical cluster analysis identified patterns of MCCs by four racial-ethnic groups (White, Black, Hispanic, & Other). Clusters associated with ED and clinic visits were analyzed using generalized estimation equations. The average of 5.79 (±2.79) MCCs ranging from 5.36 (±2.61) for Other to 5.82 (±2.79) for Hispanic patients at baseline decreased over time. Hypothyroidism, Alzheimer's Disease and related dementia, bone-and-joint, metabolism syndrome, and pulmonary-vascular clusters were commonly observed across the groups. Unique cluster patterns were identified among Black patients (e.g., renal diseases were grouped with metabolic syndrome cluster). While almost all clusters were significantly associated with ED and clinic visits among White patients, distinctive clusters were significantly related to ED and clinic visits among Hispanic patients (e.g., bone–and-joint cluster grouped with renal diseases was significantly associated with ED [RR=1.36, p < .0001] and clinic [RR=1.39, p < .0001] visits, respectively). Patterns of multimorbidity and its significant association with healthcare service utilization varied by race-ethnicity. Findings suggest a need for culturally tailored care management within integrated healthcare systems.

### NEITHER HERE NOR THERE, YOU BECOME A STRANGER FOR BOTH SIDES: SUCCESSFUL AGING IN CHINESE AMERICAN OLDER ADULTS

Mushira Khan,[1] Chien-Ching Li,[2] and Ezer Kang[3], *1. Mather Institute, Evanston, Illinois, United States, 2. Rush University, Chicago, Illinois, United States, 3. Howard University, Washington, District of Columbia, United States*

Previous studies have critiqued the inordinate emphasis on high physical and cognitive functioning and a relative absence of cultural factors in our understanding of the aging process. The multi-year Perceptions of Aging Well in Diverse Populations study explores (1) the meanings attached to aging well across cultures and (2) similarities and differences in these perceptions within diverse racial and/or ethnic groups. This presentation highlights findings from in-depth, semi-structured qualitative interviews with Chinese Americans 50 years and older (n=25; 10 male, 15 female; mean age 58.3 years). Thematic analysis of the data showed that generation status, age at arrival, density of social networks, and level of acculturation were key determinants of successful aging. Specifically, those who were born in the United States or had immigrated at an early age appeared to value their independence and were actively making retirement plans. Late-life immigrants were often lonely and more dependent on their adult children. Financial security and healthcare access were important considerations for mid-life immigrants. Several participants felt like an 'outsider' in the US but also felt like they were regarded as 'foreigners' in their country of origin, resulting in feelings of ambivalence and alienation. Many participants, particularly women, reported feeling less safe in their neighborhoods given the increasing prevalence of violence against Asian Americans in recent years and some were even contemplating moving to another country post-retirement. These findings provide insights into how aging is experienced among Chinese Americans and may help inform initiatives to support successful aging in this population sub-group.

### EXPLORING DEPRESSION AND ITS CORRELATES AMONG RESETTLED BHUTANESE OLDER ADULTS IN OHIO

Saruna Ghimire,[1] Bunsi Chapadia,[1] Isha Karmacharya,[1] Aman Shrestha,[1] Janardan Subedi,[2] and Robert Applebaum[1], *1. Miami University, Oxford, Ohio, United States, 2 Miami University, Oxford, Ohio, United States*

Following the "ethnic cleansing" by the Bhutanese government in the 1990s, Nepali-lingual Bhutanese fled from southern Bhutan and spent about two decades in refugee camps in Nepal before resettlement in the US and other countries. During post-resettlement, this population had a high rate of suicide and mental health problems. However, studies specifically among resettled older Bhutanese are lacking. This study aims to estimate the prevalence of depression and explore its correlates among resettled Bhutanese Older Adults in Ohio. A cross-sectional survey was conducted in Ohio (Columbus, Akron, Cleveland, and Cincinnati). The exploratory study surveyed 275 participants between January-June 2022, using snowball sampling, given the absence of a sampling frame. Study participants include adults 55 years and above, identified with the help of local community-based organizations. Depression was measured using the Geriatric Depression Scale, and life satisfaction, social support and resiliency were measured using validated standard tools. The prevalence of depression was 31.8%. In the adjusted model, factors associated with lower odds of depression were better self-reported health, satisfaction with life (OR=0.08, 95%CI: 0.01–0.46) and high social support (OR=0.23, 95%CI: 0.07–0.78). Resiliency was inversely associated with the odds of depression (low resiliency: OR=4.98, 95%CI: 2.02–12.28; high resiliency: OR=0.21, 95%CI: 0.07–0.64). Given the lack of a basic health profile of this population, this exploratory study is a necessary stepping stone and the first of its kind. This study will help to inform the relevant stakeholders and community leaders and provide baseline evidence for further research and programmatic actions.

### ESTABLISHING A SPANISH FOCUSED ADVANCED CARE PLANNING EDUCATIONAL SESSION FOR LATINA BREAST CANCER SURVIVORS

Moroni Fernandez Cajavilca,[1] Kara Dassel,[2] Gabriela Portugal,[3] Rebecca Utz,[2] and Katherine Supiano[2], *1. University of Utah, Bronx, New York, United States, 2. University of Utah, Salt Lake City, Utah, United States, 3. Alliance Community, Murray, Utah, United States*

In this exploratory, qualitative study, a focus group was conducted with Latina breast cancer survivors and patients to explore their feedback on how a culturally, sensitive educational session looks like, and whether a Spanish-speaking advanced care planning (ACP) educational session might be able to identify barriers for promoting awareness and discussion of ACP. First, the educational session informed participants on ACP, specifically an advanced directive (AD) and barriers that contribute to decreased rates in ACP among Latino populations. Second, a group interview was facilitated by the project lead using a semi structured interview guide. Eight Latina cancer survivors or patients participated in the educational session. Thematic analysis revealed four themes: 1) Familial Involvement 2) Need for Advanced Care Planning Education 3) Addressing Language and Cultural Barriers and 4) Culturally Sensitive and Informative Resources. A Spanish focused educational session may reduce current barriers that hinder ACP conversations, and may lead to increased rates of participants engaging in AD documentation.

### THE EFFECTS OF TYPE 2 DIABETES MELLITUS ON POSTURAL ADAPTATION DURING A VISUAL SEARCH TASK IN OLDER ADULTS

Alexander Wolfe,[1] Tongjian You,[2] Suzanne Leveille,[2] Brad Manor,[3] and Azizah Jor'dan[4], *1. University of Massachusetts Boston, Dorchester, Massachusetts, United States, 2. University of Massachusetts Boston, Boston, Massachusetts, United States, 3. Hebrew SeniorLife/ Harvard Medical School, Roslindale, Massachusetts, United States, 4. University of Massachusetts, Boston, Massachusetts, United States*

Type II diabetes mellitus (T2DM) is associated with a reduction in sensory integration capacity that often results in cognition and postural control deficits. The effects of T2DM on the ability to adapt one's postural sway while standing and performing a visual search task (VST) are unknown. Twenty-three healthy older adults (HOA) (70–90 years) and 20 older adults with T2DM (67–93 years) performed multiple trials of quiet standing with and without a VST (i.e., counting 1 target letter in a grid of random letters). Postural sway acceleration measures were jerk, velocity, range, and pathlength in the anterior-posterior (AP) and medial-lateral (ML) direction, as well as elliptical area. Postural adaptation to the VST was defined as the absolute change between conditions. VST accuracy (%) was defined as the percentage of participant-reported target letters compared to total target letters. In general, for both groups, performing the VST resulted in an average reduction in sway jerk, velocity, pathlength, and range when performing the VST, compared to control (T2DM & HOA, $p < 0.04$). VST accuracy and the magnitude of postural adaptation to the VST were similar between groups ($p>0.15$). Within the T2DM group, those who performed worse on the VST exhibited less adaptation (i.e., smaller decrease) in ML velocity ($r=0.47$, $p=0.04$). No other differences or associations were observed. Compared to healthy older adults, those with T2DM demonstrated a similar capacity to adapt their postural control in response to a VST. However, this group exhibited different characteristic changes in sway which were linked to task performance.

### EXAMINING THE IMPACT OF FAR-INFRARED TECHNOLOGY ON QUALITY OF LIFE IN OLDER ADULTS

Arianna Balingit,[1] and Joshua Guggenheimer[2], *1. St. Catherine University, St.Paul, Minnesota, United States, 2. St. Catherine University, St. Paul, Minnesota, United States*

The purpose of this study was to examine the effects of far-infrared heat (FIR) on pain management and quality of life (QOL) in older adults (OA). FIR utilizes a long-wave light that simulates dry sauna-like conditions. Examining the relationship between FIR and pain is important due to the increased prevalence of chronic pain associated with aging and the impact it has on QOL and physical performance. 9 OA completed the study, 8 of whom were women. Our intervention consisted of assigning participants to either a convective (CON) or a convective and far-infrared heat (FIR) group, with convective heat set to 60oC. Participants received 6, 30-minute heat sessions over 3 weeks. Pre- and post-test assessments included physical measures such as range of motion, gait speed, timed up-and-go, and hand grip strength. Additionally, we conducted standardized questionnaires to determine pain severity and interference on daily life, and the impact such has on overall QOL. T-tests were used to compare the groups' pre- and post-assessment responses. Results indicated that pain severity was significantly reduced (from 3.31 to 2.5, $P < 0.05$) in the FIR group from pre-to-post, and that pain inference was significantly reduced (from 1.26 to 0.43, $P < 0.05$) in the CON group from pre-to-post. There were no significant differences from pre-to-post testing for any other measures. These findings suggest that heat therapy was successful in reducing pain over time for OA, but that FIR heat specifically, was not superior to that of convective heat alone.

### DIGITAL DIVIDE AND DIFFICULTIES IN ACQUIRING HEALTH RESOURCES IN DISABLED OLDER ADULTS DURING THE PANDEMIC

Eunjin Yang,[1] Min Jung Kim,[2] and Kyung Hee Lee[3], *1. Yonsei University College of Nursing, Seoul, Seoul-t'ukpyolsi, Republic of Korea, 2. College of Nursing, Yonsei University, Seoul, Seoul-t'ukpyolsi, Republic of Korea, 3. Yonsei University, Seoul, Seoul-t'ukpyolsi, Republic of Korea*

The digital divide refers to the gap between those who are connected and those who are not connected to the internet and technologies. Although COVID-19 worsens the digital divide and health inequality, few studies have focused on the relationship between digital divide and difficulties in acquiring of health resources during the pandemic. This study aimed to identify the relationship between internet use and difficulties in acquiring health resources among older adults with disabilities during the COVID-19 pandemic. This secondary analysis study included 4,871 older adults aged 55 and older among 7,025 total responders of the 2020 survey of people with disabilities in South Korea. As the results, only 23.7% of older adults with disabilities used the internet. Non-internet users are more likely to have difficulties in acquiring COVID-19-related information (aOR 1.59) and buying and using personal protective equipment (aOR 1.36). However, difficulties in using medical services (aOR 1.21) was not statistically significant. Considering that older adults with disabilities have triple burdens from old age, disabilities, and the digital divide amid COVID-19, healthcare providers need to pay more attention to mitigate gaps between internet users and non-non users among this population. By narrowing the digital divide, decreasing health gaps and increasing well-being among older adults with disabilities will be guaranteed.

### EMERGENCY DEPARTMENT USE FOR DENTAL PROBLEMS AMONG MEDICARE FEE-FOR-SERVICE OLDER ADULTS IN THE U.S. (2016 TO 2020)

Roshani Dahal,[1] Stephanie Jarosek,[2] and Beth Virnig[3], *1. University of Minnesota, Twin Cities, Minneapolis, Minnesota, United States, 2. University of Minnesota, Minneapolis, Minnesota, United States, 3. University of Florida, Gainesville, Florida, United States*

Medicare Fee-for-Service (FFS) does not include dental coverage for older adults (65 years of age and older) but does cover emergency visits for dental problems. This study leverages Medicare Limited Data Sets to examine the use of emergency department (ED) for preventable, non-traumatic dental conditions (NTDCs) among Medicare FFS older adults from 2016 to 2020. Nationally, ~43.6 million beneficiaries sought care at the ED (average: ~8.7 million annually). Among these, ~550,000 ED visits (1.27%; ~110K annually) were for NTDCs as one of the diagnosis codes and ~200,000 ED visits (0.45%; ~40K annually) were for NTDCs as a primary diagnosis. Approximately, 5–6% of older adults with ED-NTDCs have multiple visits (94% with 1 ED-NTDC visit annually). Rates were similar in most years; however, ED use was lower in 2020 (COVID-19 pandemic). The most common diagnosis reasons include periapical abscess (tooth infection), sialadenitis, dental caries, jaw pain, and lesions of oral mucosa. Younger (65 to 74 years) and Black older adults were more likely to have primary ED-NTDC visits. Medicare paid ~$190 million for ED-NTDC visits (average: $38 million annually). Costs vary by inpatient (9%) and outpatient visits (91%). For ED-NTDCs as a primary diagnosis, the average Medicare payments for outpatient and inpatient visits were approximately $330 and $8,100, respectively. ED use for NTDCs indicates inappropriate use of valuable resources, because care provided in the EDs is incomplete (e.g., antibiotics, pain medication). Lack of follow-up with a dentist likely results in return ED visits, thus increasing costs to beneficiaries and public programs.

### ACTIVITY GOAL SETTING ON CHRONIC LOWER BACK PAIN FOR OLDER VETERANS RECEIVING CHIROPRACTIC CARE

Casey Rogers,[1] Madeleine Hackney,[2] Lisa Zubkoff,[3] and Katharina Echt[4], *1. Birmingham/Atlanta VA GRECC, Birmingham, Alabama, United States, 2. Emory School of Medicine, Atlanta, Georgia, United States, 3. GRECC, Birmingham, Alabama, United States, 4. Birmingham/Atlanta GRECC, Birmingham, Alabama, United States*

There is currently no literature addressing the impact that chiropractic services have on older adults achieving individualized specific goals throughout a course of care for chronic low back pain. This study aims to explore the impact of setting a self-determined, "what matters most" activity/goal of rehabilitation care with relevant activities as part of standard chiropractic care on the self-rated pain and disability of older Veterans. Participants were randomized into two groups. The first, an experimental group where participants identified a goal and received standard chiropractic care. The second, a control group that received standard chiropractic care only. Participants underwent six sessions of care. Outcome assessment tools were utilized at pre- and post-treatment for both groups as primary measures and an individualized goal setting measurement tool was utilized for those randomized to the experimental group. After treatment, all participants had self-reported improvement in their condition and all participants assigned to a "goal setting group" achieved their desired goal. Despite some outcome measures remaining unchanged, this self-reported improvement may be important in the future of chiropractic services for the older Veteran population suffering with chronic low back pain in achieving self-determined goals of importance.

### THE RELATION BETWEEN PROVIDER RESPONSES AND PATIENT AND CARE PARTNER SELF-EFFICACY IN PALLIATIVE CARE APPOINTMENTS

Hannah Silverstein,[1] Meghan McDarby,[2] and Brian Carpenter[3], *1. Washington University in St. Louis, St. Louis, Missouri, United States, 2. Memorial Sloan Kettering Cancer Center, New York, New York, United States, 3. Washington University in St. Louis, Saint Louis, Missouri, United States*

Person-centered palliative care (PC) fosters patient and care partner participation during medical appointments. The current study used Bandura's Social Cognitive Theory to examine whether the manner in which healthcare providers respond to patient and care partner questions during initial PC appointments is associated with their self-efficacy in question asking. We analyzed provider responses to direct and embedded questions during initial outpatient PC appointments at a large academic medical center. Provider responses were coded in terms of timeliness (number of dialogue exchanges between a question and a response) and completeness (how fully providers answered questions). Provider responses to direct questions were more timely ($M = 1.91$, $SD = 3.55$) compared to responses to embedded questions ($M = 4.05$, $SD = 6.13$), $t(127) = -3.55$, $p < 0.001$, whereas completeness was similar for both direct ($M = 1.43$, $SD = 0.71$) and embedded questions ($M = 1.59$, $SD = 0.96$), $t(127) = -1.59$, $p = 0.114$. On average, receiving more timely responses was not significantly correlated with self-efficacy, $r(49) = -0.038$, $p = 0.791$, nor was receiving more complete responses $\rho(49) = -0.178$, $p = 0.211$. These findings suggest that timeliness and completeness of responses may not be factors that affect self-efficacy, thus future research should continue to explore other factors, such as empathy present in provider responses. These findings also suggest that future research should investigate the role and effectiveness of embedded questions as an information-seeking behavior in patients and care partners given that providers took longer to respond to them.

### CHILDHOOD ADVERSITY IMPACTS PERSONALITY DEVELOPMENT ACROSS ADULTHOOD

Sarah Miller, Meredith Willard, Jacob Alderson, and Nicholas Turiano, *West Virginia University, Morgantown, West Virginia, United States*

Throughout adulthood, people's personalities typically change favorably such that agreeableness and conscientiousness increase, and neuroticism decreases. However, the impact of early life adversity on these changes in personality has yet to be examined. We utilized data from 6,382 community dwelling adults (ages 25–75) that completed 3-waves of the Midlife Development in the U.S. Study (MIDUS) in 1995–96, 2005–06, and 2013–14. Early life adversity was computed using 16 retrospective items assessing emotional and physical abuse, socioeconomic disadvantage, familial instability, and early-life poor health. Personality was assessed with the Big 5 MIDI measure. Latent growth curves confirmed there was significant variability in change over the ~20 year follow-up (all RMSEAS $< .06$; and CFI/TLI $> .96$). More specifically, higher levels of early adversity predicted higher initial levels of neuroticism, and lower levels of conscientiousness and extraversion. Additionally, higher adversity predicted a steeper decrease in neuroticism across 20-years (all p's $< .05$). Examination of specific adversity types revealed that emotional abuse and having poor health at a young age were especially detrimental to personality development. This research provides evidence that high levels of adverse experiences in childhood predict unfavorable personality development throughout adulthood. Moreover, this research demonstrates that early life adversity, especially certain types, can have lifelong detrimental effects on development. Interventions for those who have experienced adversity should be implemented as early in life as possible to prevent suboptimal psychological development trajectories. Such interventions could improve life outcomes if personality development is more normalized.

### 'THEY ASKED ME TO JOIN; I JOINED:' LESSONS FOR SENIOR CENTERS ON SUPPORTING PHYSICAL ACTIVITY

Sarah Chard,[1] and John Schumacher[2], *1. UMBC, Baltimore, Maryland, United States, 2. University of Maryland, Baltimore County, Baltimore, Maryland, United States*

Among older adults, physical activity (PA) remains a foundation for maintaining and improving health. However, over 25% of adults aged ≥ 50 engage in no physical activity outside of work hours (CDC 2016). PA does correlate with senior center participation but little is known about the social factors that contribute to sustaining senior center PA routines. We report on findings from qualitative interviews conducted with Black older adults (Nf22) on the "story" of their senior center involvement, particularly their PA participation. Interviews were recorded and transcribed verbatim; co-authors independently conducted a line-by-line analysis to identify themes. They then reconciled and sorted themes. Four themes provided insights on participants' PA engagement: 1) Retirement as opportunity; 2) Invitation and welcome; 3) Physician messaging; and 4) Sense of community. Participants strongly connected retirement with the opportunity to engage in regular PA; work and family obligations previously prevented PA in daily life. Participants also linked trying new PA programs to the senior center's culture of extending one-to-one invitations. Physician messaging specifically "to exercise" provided additional validation for prioritizing senior center PA within daily schedules. Finally, a sense of community, of being missed if not attending, provided further incentive to sustain PA routines. These findings provide guidance and simple lessons that senior centers can employ to encourage participation in their PA programming. The themes may also inform the design of survey research examining patterns of PA among older adults.

### THE DYNAMIC RELATIONSHIP BETWEEN MULTIDIMENTIONAL ENVIRONMENT AND PHYSICAL ACTIVITY PARTICIPATION

Yalu Zhang, Donghui Mei, Heming Pei, and Gong Chen, *Peking University, Beijing, Beijing, China (People's Republic)*

Objectives: Physical activity participation is a preventive factor in improving physical and cognitive functions. Both physical and social environments are essential in evaluating older adults' opportunities to participate in physical activities. This study aims at assessing the multidimensional environment in physical activity participation and examining how the change in the multidimensional environments could affect their participation in physical activities. Methods: This paper combined the nationally representative data, the China Longitudinal Aging Social Survey data 2014–2018, and the county-level administrative data extracted from various statistical yearbooks. It used the latent variable cross-lagged panel model to capture the dynamic relationship between environment and physical activities. Additionally, the dynamic analytical model considers a multidimensional environment, including indoor-, outdoor-, human-, social-environment, etc., by adding the latent variables. Results: The descriptive statistics show that older adults who are in a better environment (.147 in the indoor environment, $p < 0.001$; .389 in the outdoor environment, $p < 0.01$; .571 in family support, $p < 0.05$; .346 in childhood activity experience, $p < 0.001$) are more likely to frequently participate in physical activities (three times or more per week). The results of the latent cross-lagged panel models indicate that every one percent increase in the multidimensional environmental index is expected to cause a 4.1% increase in the likelihood of participating in physical activities among Chinese older adults. Discussion: In addition to the continuous efforts to improve the physical environment, policymakers and researchers should pay more attention to the social environment to encourage more older adults to participate in physical activities.

### PHYSICAL ACTIVITY, BALANCE, AND BICYCLING IN OLDER ADULTS

Maya Baughn, Victor Arellano, Brieanna Hawthorne-Crosby, Amanda Grimes, Joey Lightner, and Gregory King, *University of Missouri-Kansas City, Kansas City, Missouri, United States*

Falls are a critical public health issue among older adults. One notable factor contributing to falls in older adults is a deterioration of the structures supporting balance and overall balance control. Preliminary evidence suggests older adults who ride a bicycle have better balance than those who do not. Cycling may be an effective intervention to prevent falls among older adults. This study aims to objectively measure the relationship between bicycling, physical activity, and balance for older adults. Older adult cyclists (n=19) and non-cyclists (n=27) were recruited to (1) complete a survey that assessed demographics; (2) wear an accelerometer for 3 weeks to objectively assess physical activity; and (3) complete balance-related tasks on force platforms. Mann-Whitney U-tests were performed to understand differences in balance and physical activity between cyclists and non-cyclists. Cyclists are significantly more physically than non-cyclists ($U = 102.00$, $p = .015$; $U = 81.00$, $p=.002$). Cyclists had less sway velocity in eyes open conditions than non-cyclists ($U = 360.00$, $p=.009$), indicating a more tightly regulated postural control strategy that may relate to higher stability in cyclists. Task duration among cyclists in single-leg stand was longer than non-cyclists (left leg: $p < .001$ and right leg: $p < .005$) indicating increased postural stability. This study demonstrates the possible implications for cycling on balance and reducing fall risk.

### PHYSICAL ACTIVITY IN DEMENTIA PATIENTS AND CAREGIVERS

Prabhash Kakarla,[1] Madieska Ramirez,[2] Jessica Alvarez,[2] Marzena Gieniusz,[2] ann swartz,[3] sergey tarima,[4] Chris Nouryan,[5] and Edith Burns[6], *1. Hofstra University, New Hyde Park, New York, United States, 2. Hofstra-Northwell, New Hyde Park, New York, United States, 3. University of Wisconsin-Milwaukee, Milwaukee, Wisconsin, United States, 4. PhD., Milwaukee, Wisconsin, United States, 5. Zucker School of Medicine at Hofstra Northwell, Uniondale, New York, United States, 6. Zucker School of Medicine at Hofstra Northwell, Manhasset, New York, United States*

Many studies have explored the benefits of physical activity (PA) in mitigating risk for and symptoms of dementia. However, there are challenges to implementing PA with dementia patients. We conducted an acceptability/feasibility study of PA in patients with Dementia and their caregivers. A semi-structured telephone interview was conducted. Questions piloted with 2 independent faculty for content validity. A random sample of 10 participants was recruited from patient-caregiver dyads enrolled in an Alzheimer's Dementia Care program (ADCP) at an academic geriatrics practice. Responses manually recorded and personal health information omitted. Descriptive statistics generated using SPSS and response themes categorized by the authors. Major themes included lack of time for caregivers; time requirement would increase caregiver burden (50%). Caregivers of patients with home health aides were less likely to consider time a barrier. Other themes included functional status as a challenge (40%), difficulty motivating patient (40%), unsteady gait (20%), difficulty using devices for video-based exercise (20%). Caregivers expressed preference for asynchronous, in-home PA that could be adapted to their schedules and patient capabilities. Although PA is beneficial for patients with dementia, multiple challenges to PA implementation exist. Main barriers are time requirements and greater caregiver burden. Time would be required to motivate patients, assist with managing technology, and functional impairment that poses potential risk of injury (e.g., gait instability). Engaging Home Health Aides in PA programs could address some of these barriers. Alternative PA (i.e.: chair exercises) could be a solution. Responses will be utilized to modify a future PA intervention.

### OLDER ADULT ONLINE EXERCISE CLASSES DURING THE COVID-19 PANDEMIC: A SURVEY OF SERVICE PROVIDER PERSPECTIVESS

Dallas Murphy,[1] Michelle Porter,[2] Ruth Barclay,[2] Stephen Cornish,[2] Jacquie Ripat,[2] Kathryn Sibley,[2] Sandra Webber,[2] and Nicole Dunn[2], *1. University of Manitoba, WINNIPEG, Manitoba, Canada, 2. University of Manitoba, Winnipeg, Manitoba, Canada*

COVID-19 rendered the availability of exercise facilities sporadic and online exercise programs subsequently became more common. This research explored online exercise classes delivered to older adults during the pandemic from the perspective of service providers. Sixty-seven service providers completed the survey (88% female). The majority (54%) of respondents had worked in the fitness industry for greater than 10 years, and 66% were fitness class instructors, while fewer were managers (9%) and personal trainers (8%). Three participants had experience providing online exercise classes prior to the pandemic, while 43 more had experience providing online exercise classes since the pandemic began. Of these 46 service providers, 87% offered classes live through Zoom. The majority (64%) offered classes through an organization, and 61% charged a fee for participants to take part. The most common type of class was a general fitness class (63%), followed by yoga and flexibility classes (39%), and strength training (17%). Regarding equipment used, weights were most frequently required (69%), followed by resistance bands (49%) and mats (44%). Most classes lasted 40–60 minutes (59%) and were low intensity (74%). Of the 21 respondents who did not provide online exercise classes, 43% indicated this was because of a lack of interest, and 19% cited not knowing how to use technology to deliver classes online, though most (71%) indicated they would consider offering online classes in the future. This research reveals the adaptability of service providers and may serve to inform the continued development of online exercise programs for older adults.

### OBESE AFRICAN AMERICAN OLDER WOMEN'S EFFECT OF EXERCISE TRAINING ON HEALTH CAPABILITIES AND LIFE SATISFACTION

Dongwook Cho,[1] and Sung Kyeom Kim[2], *1. Alcorn State University, Lorman, Mississippi, United States, 2. Dongseo University, Busan, Pusan-jikhalsi, Republic of Korea*

The purpose of this study is to determine whether a 12-week exercise course influences obese African American older women's health capabilities and life satisfaction levels. Participants, who passed an initial screening and pre-assessment, were asked to measure current health capabilities and life satisfaction levels both prior to, and following, the 12-week exercise training. Researchers found that improvement in health capabilities, although there were no significant differences following exercise training participation, while obese African American older women had significantly higher life satisfaction levels. The findings suggest that a 12-week exercise training can be beneficial to enhance obese African American older women's health capabilities and life satisfaction levels. Professionals in the field of gerontology and exercise should provide the appropriate exercise trainings so that obese African American older women would be able to optimize their life satisfaction levels.

### MUSCLE GROUP SPECIFIC SKELETAL MUSCLE AGING: A FIVE YEAR LONGITUDINAL STUDY IN SEPTUAGENARIANS

Masatoshi Naruse, William Fountain, Alex Claiborne, Holmes Finch, Scott Trappe, and Todd Trappe, *Ball State University, Muncie, Indiana, United States*

Most of the scientific literature on human skeletal muscle aging has focused on the quadriceps femoris, with the remaining few hundred skeletal muscles receiving limited specific investigation. This longitudinal investigation compared changes in skeletal muscle size via computed tomography of the quadriceps (rectus femoris, vastus lateralis, vastus medialis, vastus intermedius), hamstrings (biceps femoris short and long heads, semitendinosus, semimembranosus), psoas, rectus abdominis, lateral abdominals (obliques and transversus abdominus), and paraspinal muscles (erector spinae and multifidi) of older individuals from the Health ABC study at baseline and 5.0±0.1 years later (n=469, 73±3y & 78±3y, 49% women, 33% black). Skeletal muscle size decreased ($p < 0.05$) in quadriceps (-3.3%), hamstrings (-5.9%), psoas (-0.4%), and rectus abdominus (-7.0%). The hamstrings and rectus abdominus atrophied approximately twice as much as the quadriceps ($p < 0.05$), while the quadriceps atrophied substantially more than the psoas ($p < 0.05$). The paraspinals (+4.3%) and lateral abdominals (+5.9%) hypertrophied ($p < 0.05$) to a similar degree ($p>0.05$) over the 5 years. These data suggest that skeletal muscle mass in older individuals changes in a muscle group specific fashion in the eighth decade, a critical time period in the aging process. A broader understanding of muscle group specific skeletal muscle aging is needed to better guide exercise programs and other interventions that mitigate decrements in physical function with aging.

### INFLUENCE OF ASPIRIN ON AGING SKELETAL MUSCLE SIZE

William Fountain, Masatoshi Naruse, Alex Claiborne, Holmes Finch, Scott Trappe, and Todd Trappe, *Ball State University, Muncie, Indiana, United States*

Aspirin is one of the most commonly consumed cyclooxygenase (COX)-inhibitors and anti-inflammatory drugs and may provide insight into the control of age-related skeletal muscle atrophy. This investigation compared skeletal muscle size (via computed tomography) of older individuals from the Health ABC study that did not consume aspirin or any other COX-inhibiting drug (non-consumers; n=1,155, 74±3y, 48% women, 45% black) to those that consumed aspirin daily (and not any other COX-inhibiting drug) and for at least one year (aspirin consumers; n=515, 74±3y, 39% women, 30% black, average aspirin consumption: 6y). In white and black men, the long-term aspirin consumers had significantly larger quadriceps muscle size compared to non-consumers ($p < 0.05$). In white men only, these differences were influenced by age ($p < 0.05$). In white and black women, quadriceps muscle size was not different between the aspirin consumers and non-consumers ($p>0.05$). In men and women of either race, there was no hamstrings muscle size difference between the aspirin consumers and non-consumers ($p>0.05$). These data suggest that long-term regulation of the COX pathway with the common over-the-counter drug aspirin may impact aging muscle mass in a sex and muscle group specific fashion, which is supported by previous mechanistic human and animal studies.

### FEASIBILITY AND ACCEPTABILITY OF A HYBRID AEROBIC EXERCISE PROGRAM FOR OLDER ADULTS DURING THE COVID-19 PANDEMIC

Emily Erlenbach,[1] Revati Malani,[2] Edward McAuley,[1] and Neha Gothe[3], *1. University of Illinois, Urbana Champaign,*

*Urbana Champaign, Illinois, United States, 2. University of Illinois--Urbana-Champaign, Urbana Champaign, Illinois, United States, 3. University of Illinois at Urbana Champaign, Urbana, Illinois, United States*

The COVID-19 pandemic and ensuing lockdowns, physical distancing and mask mandates exacerbated the challenges older adults (OAs) face towards exercise engagement. We present data on the feasibility, safety, and acceptability of a hybrid (in-person and virtual) aerobic exercise program for OAs. Nf39 (30 females, Mage=64.10) low-active OAs completed an aerobics-based exercise program as part of a larger ongoing RCT. Participants exercised 3x/week by attending one in-person and two Zoom-based exercise classes. Attendance, attrition, format preferences, and adverse events were documented. Participants also completed an anonymous survey to detail their experiences with the hybrid delivery model. Thirty participants completed the program. Total average attendance of 83.64%; 58.93% and 24.71% of the attended sessions were on Zoom and in-person, respectively. On post-program surveys, 36.67% reported preferring Zoom sessions, followed by 33.33% preferring both formats equally. No adverse events were reported. From the anonymous program feedback surveys, common reasons for preferring the Zoom sessions included convenience; not having to wear a mask; not worrying about COVID exposure; and not feeling self-conscious about exercising with others. Commonly reported reasons for preferring the in-person sessions were increased motivation from group energy; social support; more space to move around; and better engagement with instructors. Collectively, these findings show a hybrid aerobic exercise program is feasible and safe for OAs to engage in and is overall well-accepted. Findings are encouraging for the design of future exercise programs for OAs, who continue to remain a vulnerable population during the pandemic but need a structure to remain sufficiently active.

### EFFECTS OF FLAVANOL (+)-EPICATECHIN ON SKELETAL MUSCLE OF AGED RATS

Israel Ramirez-Sanchez,[1] Viridiana Navarrete,[2] Leonor Galera,[2] and Francisco Villarreal[3], *1. University of California San Diego, Mexico City, Distrito Federal, Mexico, 2. IPN, Mexico City, Distrito Federal, Mexico, 3. UCSD, San Diego, California, United States*

Sarcopenia is a progressive and generalized age-related skeletal muscle (SkM) disorder characterized by the accelerated loss of muscle mass (atrophy) and function. SkM atrophy is associated with increased incidence of falls, functional decline, frailty and mortality. In its early stage, SkM atrophy is associated with oxidative stress, increased pro-inflammatory cytokine levels and proteasome-mediated protein degradation. These processes also link to the activation of atrophy associated factors and signaling pathways which lack any approved, targeted pharmacotherapy. We have characterized the capacity of flavanols specifically, epicatechin to favorably modulate SkM mass and function in animal models of SkM disease and in proof of concept studies in humans including muscular dystrophies. In this study we wished to evaluate the capacity of this flavanol to ameliorate aging associated sarcopenia. Using 24-month-old male rats a 4 week oral administration of the flavanol (+)-epicatechin (1mg/kg/day) was implemented while control rats only received vehicle (water). SkM strength (grip) and endurance (treadmill walk), muscle size, (muscle mass, cross sectional area of fibers), ubiquitin-proteasome atrophic pathway (including myostatin, Murf1 and Atrogin1 by Western blots) and markers of oxidative stress and inflammation in gastrocnemius were quantified. We also analyzed a relevant protein synthesis pathway (IGF/AKT/mTOR). The use of (+)-epicatechin in aged rats led to significant changes as follows: 1) improved SkM mass and function, 2) downregulation of atrophic pathway, 3) reduced levels of oxidative stress and proinflammatory cytokines and, 4) upregulation of protein synthesis pathway. Given its safety and tolerance profile this agent warrant its rigorous investigation in clinical trials.

### EFFECTS OF A SIX-MONTH EXERCISE PROGRAM ON PHYSICAL ACTIVITY AMONG OLDER ADULTS WITH CHRONIC CONDITIONS

Revati Malani,[1] Emily Erlenbach,[2] Edward McAuley,[2] and Neha Gothe[3], *1. University of Illinois--Urbana-Champaign, Urbana Champaign, Illinois, United States, 2. University of Illinois, Urbana Champaign, Urbana Champaign, Illinois, United States, 3. University of Illinois at Urbana Champaign, Urbana, Illinois, United States*

Health benefits of structured physical activity (PA) for older adults have been well documented. Older adults are highly impacted with major chronic conditions, leading to limitations in daily activities. This study examined the effects of an exercise program on moderate-vigorous physical activity (MVPA) and sedentary time after six months of exercise. Participants from a larger-ongoing trial were randomized into either yoga, aerobic or stretch-tone groups. A subsample of 58 participants was classified as chronic individuals (CI) based on presence of at least one chronic condition, and 31 as healthy individuals (HI), who completed accelerometer measures before and after the exercise program. A repeated measures analysis was performed to identify changes in PA over time for HI and CI. At both time-points, MVPA was significantly higher for HI. There was a significant increase in MVPA at month six for all participants, irrespective of health status. Mean MVPA in minutes for baseline HI: 153, CI: 98 and for month 6 HI: 188 CI: 116. There were no significant differences in sedentary time after the six-months of exercise. Although there was an increase in MVPA for HI and CI after structured exercise, no change was noted for sedentary time. Increased sedentary time has been proven to be a risk factor for poor health and although meeting criteria for MVPA is important, it is essential to address sedentary time in CI by implementing educational and interventional components that focus specifically on sedentary time. Funding: This data is from NCT04323163 funded by NIH-NIA – grant-AG066630.

### DOES TAI CHI PROMOTE COGNITIVE FUNCTION WITHOUT THE EFFECTS OF PHYSICAL FUNCTION?: META-REGRESSION APPROACH

Rhayun Song,[1] Moonkyoung Park,[1] Jacqueline Shin,[2] Xianqi Gao,[1] and Yuelin Li[1], *1. Chungnam National University, Daejeon, Taejon-jikhalsi, Republic of Korea, 2. Indiana State University, Terre Haute, Indiana, United States*

This systematic review aimed to examine the effects of TCQ on cognitive and physical functions in older adults using a meta-regression approach. The systematic search in 13 electronic databases identified 19 randomized studies (n=2365, mean age=70.3 years) published in English (k=17), Korean (k=1) and Chinese (k=1). A review of bias was assessed by two raters according to Cochrane RoB 2.0, resulting in low risk (k=6), some concern (k=12), and one high-risk study. Tai Chi (k=16) and Qigong (k=3) were applied for an average duration of 20.2 weeks. The control groups received either alternative exercise (k=14) or no treatment (k=5). The results of the meta-analysis on 19 RCTs using a random-effects model showed the significant effect of TCQ on cognitive function (Hedges's g =0.32, 95% CI= 0.18, 0.46) and physical function (Hedges's g= 0.35, 95% CI= 0.21, 0.49). In addition, meta-regression was used to explore the effect size of TCQ in association with the level of physical function. The effects of TCQ on cognitive function remained significant (Q=38.86, p=.003) when controlling for the effect of physical function in this model (unexplained variance = 0.0199). The coefficient of the physical function was significant (b=0.47, p=.008), showing that 58% of heterogeneity was explained by physical function as a moderator variable. It confirmed that changes in physical function were associated with changes in cognitive function. The findings imply the potential health benefits of TCQ in promoting cognitive function among older adults directly and indirectly through improving physical function.

### CONSTRUCT VALIDITY OF EXERCISE ADHERENCE RATING SCALE IN COMMUNITY DWELLING OLDER WOMEN: PROOF OF CONCEPT

Helen Graham, Judith Scott, and Melissa Benton, *University of Colorado Colorado Springs, Colorado Springs, Colorado, United States*

Exercise is an effective health promotion strategy that has a positive effects on frailty and sarcopenia, particularly in older women, yet they do not adhere to exercise guidelines. Accurate assessment is critical for clinical management and research with this population. Interviews and exercise logs are most commonly used to assess adherence to exercise. However, they are burdensome and not highly reliable. A brief validated measure of exercise adherence for assessment is greatly needed. The six-item Exercise Adherence Rating Scale (EARS) was originally validated among a clinical orthopedic population but not been used in community-dwelling adults. Our aim was to determine the feasibility of using the EARS to assess exercise adherence among community-dwelling older women. Twelve women (age=72±7 years) completed one-time assessment for this proof-of-concept study. For the EARS, overall Cronbach's alpha was 0.78 with inter-item correlations of 0.72–0.89 for all but item three that pertains to healthcare professionals (correlation of 0.27). When item three was removed, overall Cronbach's alpha increased to 0.80. Correlation analysis with the reduced five-item scale demonstrated good construct validity. There were significant relationships with global quality of life (r=0.76, p < .01) and physical function (r=0.61, p < .05). There were also moderate to strong relationships with variables expected to be linked to exercise adherence including mobility (r=0.57), BMI (r=0.51), vigorous (r=0.40) and moderate (r=0.36) exercise, upper body strength (r=0.41) and lower body strength (r=0.30). Based on these findings use of a five-item version of the EARS is feasible and appears to be a valid measure of exercise adherence.

### THE IMPACT OF THE OLD AGE PENSION EXPANSION ON BLOOD PRESSURE AMONG OLDER MEN IN RURAL SOUTH AFRICA

Haeyoon Chang,[1] Janet Jock,[2] Molly Rosenberg,[3] and Lindsay Kobayashi[4], *1. University of Michigan-Ann Arbor/School of Public Health, Ann Arbor, Michigan, United States, 2. O'Neill School of Public and Environmental Affairs, Bloomington, Indiana, United States, 3. Indiana University/School of Public Health, Bloomington, Indiana, United States, 4. University of Michigan, Ann Arbor, Michigan, United States*

The impact of improved socioeconomic welfare after retirement on cardiovascular health among older men in low-income settings is unknown. Using natural experiment, we investigated the impact of eligibility for additional Old Age Pension (OAP) income on systolic blood pressure (SBP) among older men in rural South Africa using data from 1,208 men aged ≥60 in the population-representative "Health and Aging in Africa: A Longitudinal Study of an INDEPTH Community in Rural South Africa" (HAALSI) cohort. The South African Old Age Pension (OAP) incrementally expanded age eligibility for men from age 65 prior to 2008 to age 60 in 2010. This expansion provided exogenous variation in pension income among men, based on their year of birth. We estimated predicted SBP among the birth cohorts of men who were eligible for one through five extra years of OAP income, using a multivariable linear regression model estimated in those without access to extra pension income (over age 65 when the expansion rolled out) based on covariates. We then estimated mean SBP difference scores among men in each of these five birth cohorts, based on their observed SBP minus predicted SBP. Men in OAP expansion birth cohorts had lower SBP than those who were not exposed to extra pension, although the differences were not statistically significant (5-years estimate = -2.735 (p=0.353) vs. 1-year estimate = 1.633 (p=0.531)). This trend suggests a possible long-term benefit of blood pressure control with greater cumulative pension income among men living in rural, low-income settings.

### ENTERING MEDICARE ADVANTAGE FROM EMPLOYER-SPONSORED PLANS: CHANGE IN OUT-OF-POCKET COSTS AND UTILIZATION AT AGE 65

Alexandra Glynn Hames, and Eric Roberts, *University of Pittsburgh, Pittsburgh, Pennsylvania, United States*

An increasing proportion (nearly 40%) of older adults entering Medicare at age 65 are electing to enroll in private Medicare Advantage (MA) plans, which reflects more generous coverage relative to traditional Medicare and similarity to many features of employer sponsored plans. However, few studies have examined the impact of transitioning to MA from an employer-sponsored plan on out-of-pocket costs and health services utilization. We used longitudinal administrative data from Optum Insights between 2010 and 2021 to follow individuals from ages 62 through 67, spanning continuous enrollment in employer-sponsored insurance and MA. We employed an event-study design to estimate within-person changes in our outcomes after individuals entered to MA at age 65 (n=5,548), controlling for linear age trends. Relative to ages 62–64, MA enrollees at ages 66 and 67 had lower out-of-pocket spending on outpatient services (-$503/year, 95% CI: -$602, $404), emergency department visits (-$27/year, 95% CI: -$35, -$19), and prescription drugs (-$77/year, 95% CI: -$101, -$53). In contrast, utilization of outpatient services increased (1.4 visits/year, 95% CI: 0.8, 2.0), including physician evaluation and management visits (0.46 visits/year, 95% CI: 0.25, 0.68). After entering MA, individuals were more likely to have physician office visits with no out-of-pocket costs than at ages 62–64 (0.99 additional zero-cost visits/year, 95% CI: 0.81, 1.2). These results suggest MA may offer older adults more affordable care for routine and chronic conditions, compared to employer-sponsored insurance. These findings are timely as policymakers continue to deliberate proposals, such as expanding Medicare eligibility, to improve access to affordable insurance.

### ASSESSING THE NEW INFLATION REDUCTION ACT'S IMPACT ON EXPANDING MEDICARE PART D LOW-INCOME SUBSIDIES

Feng-Hua Loh,[1] and Bruce Stuart[2], *1. Touro College of Pharmacy, New York, New York, United States, 2. University of Maryland School of Pharmacy, Baltimore, Maryland, United States*

The recently enacted Inflation Reduction Act (IRA) of 2022 extends full low-income subsidies (LIS) to Medicare Part D recipients with limited assets and incomes up to 150% of the Federal Poverty level (FPL) beginning in 2024. Under current law, full Part D subsidies (no annual premiums and nominal cost sharing) are available only to those with incomes up to 135% of the FPL. Partial subsidies are available to those with incomes between 135% and 150% of FPL. Increasing the level of benefits to those currently only eligible for partial subsidies has the potential of significantly lowering out-of-pocket medication costs that pose a barrier to adherence for many low-income Medicare beneficiaries, but the outcome of the new policy will depend on how many enroll. Historically, enrollment among those eligible for partial subsidies has hovered around 30%, but the latest data on LIS participation rates are for 2014. We undertook this study to update LIS eligibility and enrollment numbers to 2019, with a particular emphasis on identifying characteristics of beneficiaries who may benefit from the new policy. Using data from the Medicare Current Beneficiary Survey, we determined that 73% of beneficiaries eligible for full subsidies under current law were enrolled, but only 25% eligible for partial subsidies were enrolled. We conclude that while the IRA will raise the overall level of Part D subsidies, it will be important to engage stakeholders and promote outreach to maximize participation in the leadup to implementation.

### AGE DIFFERENCES IN DISTINCT TYPES OF SOCIAL INTERACTIONS AND EMOTIONAL WELL-BEING

Yuxuan Chen,[1] Kevin Chi,[2] and Laura Carstensen[2], *1. Stanford University, Stanford, California, United States, 2. Stanford University, Palo Alto, California, United States*

Social interaction is central to emotional well-being. However, less is known about how the surroundings of social interactions are associated with emotional well-being and whether they differ by age. The present study examined age differences in the predication of emotional well-being as a function of two aspects of social interactions: (1) the extent to which individuals would rather be interacting with someone else and (2) the degree of closeness to the interactant(s). A sample of 190 people, aged 18 to 94 years, participated in an experience sampling study where they reported emotional experience and aspects of the social interaction at five randomly selected times each day across a one-week period for a total of 35 randomly selected occasions. Across age groups, the greater the extent to which people would rather be interacting with someone else was associated with higher levels of negative emotion and lower levels of positive emotion. However, these associations were more pronounced for younger adults, who compared to older adults, experienced greater increases in negative emotions and greater decreases in positive emotion in these contexts. Furthermore, younger but not older adults experienced lower levels of negative emotion when interacting with someone they knew well. Results highlight the important role that age plays in the link between daily social interactions and emotional well-being. Age differences in social interactions, emotional resilience, and situation selection will be discussed.

### VARIATIONS IN BENEFITS OF INTERGENERATIONAL TUTORING IN THE "NEW NORMAL"

Peter Sun,[1] Nancy Morrow-Howell,[2] Mary Click,[3] and Kendra Minch[3], *1. Washington University in St. Louis, Clayton, Missouri, United States, 2. Washington University in St. Louis, St. Louis, Missouri, United States, 3. The Oasis Institute, St. Louis, Missouri, United States*

This study explored variations in self-perceived benefits of intergenerational tutoring. The study's sample consisted of 329 older adults who tutored children in-person in the 2021–2022 school year. Due to the COVID-19 pandemic, some of the respondents had experienced a period in which volunteering was remote, virtual, or not possible. Demographic information was collected in a fall pre-test survey, identifying first-time volunteers (first time volunteering in ten years), caregivers (100 or more hours in the last two years helping someone who needed assistance), and male volunteers. Self-perceived benefits of tutoring (physical, emotional, and cognitive health, increased social activities, use time more productively, contribute to the well-being of children, and feel better about myself) were collected in a spring post-test survey. Being a first-time volunteer was significantly associated with improved health (X2 = 4.17, p = 0.041, Cramer's V = 0.11), even after controlling for baseline self-reported health (p = 0.020). A larger proportion of first-time volunteers (34.2%) reported improvements in at least two areas of health (physical, emotional, and cognitive) due to their involvement in the intergenerational tutoring program, compared to non-first-time volunteers (18.5%). There were no significant differences in perceived benefits for caregivers or males. These findings suggest that targeting non-volunteers for involvement in tutoring programs may maximize health benefits of engagement. Specifically targeting males and caregivers may not be necessary, given that they benefit similarly to other populations.

### TRAIT SELF-CONTROL AND ATTACHMENT ANXIETY IN RETIREES' DEPRESSIVE SYMPTOMS AND EMOTIONAL WELL-BEING.

Jensine Paoletti,[1] E. Lim Lydia Wu-Chung,[1] Ryan Brown,[2] Michelle Chen,[3] Angie LeRoy,[4] and Christopher Fagundes[1], *1. Rice University, Houston, Texas, United States, 2. Rice University, Houston, Texas, United States, 3. Northwestern University, Evanston, Illinois, United States, 4. Baylor University, Waco, Texas, United States*

Trait self-control (TSC) is a well-known predictor of well-being. TSC may reduce distraction from hedonistic pursuits and enable more effective goal-directed behavior. Attachment anxiety (AA) is a trait that predicts lower relationship quality and lower well-being; people high on AA are characterized by hyper-sensitivity, proximity-seeking and excessive rumination. In retirement, these traits may interact to predict wellbeing. Methods: 120 retired participants took part in data collection (M = 62.37 years old; SD = 10.07 years; 68% women, 70% white). Data collection included self-reported AA (Experience in Close Relationships), TSC (Brief Self-Control Scale), depressive symptoms (Center for Epidemiological Studies Depression Scale) and emotional well-being (RAND 36-Item Health Survey). We conducted hierarchical linear regressions and accounted for demographic and health-related covariates. Results: TSC was negatively related to depressive symptoms and positively related to emotional well-being. AA was positively related to depressive symptoms and negatively related to emotional well-being. We found an interaction predicting depressive symptoms such that participants with low TSC and high AA had the highest depressive symptoms ($p = .036$). This interaction was not significant for emotional well-being. Discussion: After retiring, maintaining a healthy social life may be more effortful, as there are fewer 'built-in' social interactions (i.e., with coworkers). Retirees with higher TSC may be able to maintain their social life more effectively than those with lower TSC. Similarly, people with lower AA may be able to obtain more enjoyment from their social lives. Together, lower AA and higher TSC may be protective mechanisms for depressive symptoms.

### PARKINSON'S DISEASE AND STIGMA: A MULTIPLE CASE STUDY

Beth Mastel-Smith, and Melinda Hermanns, *The University of Texas at Tyler, Tyler, Texas, United States*

Parkinson's disease (PD) is a neurodegenerative disorder caused by dopamine deficiency. PD affects emotional and physical health and causes people with PD and caregivers to experience stigma. Four types of stigma have been described: public, associated, self-, and structural. The aims of this study were to explore (a) how stigma is experienced by people with PD and caregivers, (b) whether stigma towards people with PD and caregivers is represented in the literature and social media and (c) examine whether findings support Stigma Theory and theoretical propositions. This was a multi-case qualitative study. Cases included a literature review, interviews with people with PD and caregivers, and examination of social media posts. A purposive sample included seven people with PD and five caregivers who were interviewed online using an interview guide. The study was approved by the University IRB and participants signed consent. Thematic analysis identified codes which aligned with conceptual definitions of Stigma Theory. Findings revealed that public stigma was presented in all four cases. Self- and structural stigma were represented in the literature and interviews with people with PD and caregivers. Associated stigma appeared in the literature and from caregivers' descriptions. Three of six theoretical propositions were supported. Four types of stigma toward people with PD were prevalent and interrelationships between types of stigma noted. A multi-pronged approach to reducing stigma is needed at the individual, organizational and community levels. Research regarding stigma experienced by diverse populations and the health effects of stigma for people with PD is recommended.

### CONTEMPLATING TEMPORAL LANDMARKS, SENSING TIME, AND PURSUING A MEANINGFUL LIFE IN ADULTHOOD

DaEun Kim, and Hsiao-Wen Liao, *Georgia Institute of Technology, Atlanta, Georgia, United States*

Research has shown that experiencing and anticipating temporal landmarks affect one's goal priorities and motivation. Particularly, milestone birthdays (MBD) remind people of where they stand in life timeline, prompting time experience that has consequences for pursuit of meaning. The current study examines this thesis in the context of adult development and aging. We tested whether (1) thinking about past and future MBDs (vs. a regular birthday) affects young, middle-aged, and older adults' possession of and search for meaning in life and (2) sense of time, and (3) the association between contemplating MBDs and meaning in life is, at least in part, explained by sense of time. Participants (N = 239) were randomly assigned to one of the three conditions to verbally visualize a past MBD, a future MBD, or an upcoming regular birthday. They then completed a meaning in life questionnaire, which assessed presence of and search for meaning (Steger et al., 2016). Future time perspective (Lang & Carstensen, 1996) and time savoring (Carstensen et al. in prep) were also measured. We found that thinking about past and future MBDs (vs. regular birthdays) prompted people to search for meaning. Thinking about past MBDs (vs. regular birthdays) also elicited a sense that time left in life is limited. Mediation analysis indicated that perceived future time constraints helped explain the relation of recalling past MBDs to search for meaning. Results held when age effects were considered. We draw on motivation theories of temporal landmarks and socioemotional selectivity to discuss the findings.

### BASELINE PURPOSE IN LIFE PREDICTS FUNCTIONAL HEALTH ACROSS 16 WEEKS IN OLDER ADULTS WITH PROGRESSIVE VISION LOSS

Wingyun Mak,[1] and Silvia Sörensen[2], *1. The New Jewish Home Research Institute on Aging, New York, New York, United States, 2. University of Rochester, Rochester, New York, United States*

Greater purpose in life is associated with various positive outcomes in later life, such as, lower mortality risk, protection against cognitive decline, and lower spending on medical care and emergency room visits. Recent research from the Health and Retirement Study has shown that older adults with greater purpose in life have fewer physical limitations after four years. There has been no research to investigate purpose in life in older adults with progressive vision loss. The aim of this study is to examine whether purpose in life predicts functional health (i.e., activities of daily living) across a 16-week period of time in a sample of older adults (aged 60–96) with vision loss due to macular degeneration who were enrolled in a resilience building program (Nf 122). A repeated measures GLM showed that functional health did not change across 16 weeks (ns). Age ($F(1, 112)=8.91$, $p=.003$, $\eta p2=.07$) and purpose in life ($F(1,112)=13.21$, $p < .001$, $\eta p2= .11$) were significant overall predictors of functional health across time. Gender, education, cognition, marital status, depression, and loneliness at baseline were not associated with overall functional health. People who were younger were more likely to have better functional health over 16 weeks; purpose in life added 11% of explained variance. Because purpose in life is malleable and also significantly associated with better functional health in older adults with progressive vision loss, future interventions that enhance purpose in life should be studied with this population.

### TESTING A NEW MODEL OF REABLEMENT-FOCUSED INTEGRATED CARE IN ADULT DAY SERVICE USERS IN TAIWAN: A PRELIMINARY STUDY

Yu-Ting Chang,[1] Hsiao-Wei Yu,[2] and Pay-Shin Lin[1], *1. Chang Gung University, Taoyuan, Taoyuan, Taiwan (Republic of China), 2. Chang Gung University of Science and Technology, Taoyuan City, Taoyuan, Taiwan (Republic of China)*

There is a growing consensus that implementing personal-centered therapeutic activities at adult day services (ADS) leads to functional improvement in care recipients and reduces the burden on caregivers. Taiwan has introduced reablement-focused integrated care (RFIC) model for ADS users, the effects of this new model merit investigation. This study aimed to examine whether the RFIC model promotes physical and mental function in care recipients and improves caregiver satisfaction. We recruited ADS-care recipients and their family caregivers (N = 14 dyads) through purposive sampling for a quasiexperimental study. Six dyads were assigned to the RFIC intervention group and eight dyads to the control group. The RFIC intervention consisted of ten one-hour sessions over two months. The functional abilities of care recipients and the care satisfaction of caregivers were assessed at baseline and after the intervention. The RFIC group, compared to the control group, showed an improvement in care satisfaction score, and the group difference was significant (RFIC group: mean (SD) change score = 1.17 (2.64); control group: mean (SD) change score = −3.38 (2.20); $p < 0.01$). There were no differences in physical and mental function between the RFIC and control groups. These preliminary results suggest that when reablement is used as a means of personal-centered therapeutic training for ADS users, it can help caregivers cope with care problems and improve how they appraise their mastery and satisfaction as regards care. However, the effects of RFIC on the physical and mental function of care recipients require further investigation.

### PHOTO-ELICITATION- A VIEW OF DEEP OLD AGE AND SOCIAL INCLUSION

Elaine Eliopoulos, *University College London, London, England, United Kingdom*

Photo-elicitation- A View of Deep Old Age and Social Inclusion While visual methodologies have gained prominence in aging research, focus on deep old age has been uncommon. The variety of visual representations portraying a range of ‘ageless aging’ to frail older people may not fully capture daily lived experience. The inclusion of those in deep old age to depict their aging bodies is a crucial missing element enhancing our understanding of the nuances of deep old age. This study fills that gap. Photo-elicitation, followed by semi-structured interviews, was used to examine the role of the body for participants 80 years+ in three US island contexts to understand what impact, if any, their bodies had on their ability to be socially included in ways they chose. Digital cameras were provided to participants to photograph their lived bodily experience. Those who had visual challenges were assisted in taking photographs. Recent analysis of the photographs revealed important findings that will contribute to theoretical development of deep old age: participants who took photos without assistance did not take a single photograph of their bodies. They took photos primarily of their environment and places of community connections while minimising physical challenges. Their photos contextualised their experience of the physical challenges that played a role in their sense of inclusion within their communities. This finding questions current dominant models of the third and fourth age as dichotomous formulations related to embodied agency. In doing so it opens new possibilities for theoretical reflection on deep old age.

### PERCEPTIONS OF ADRD CARE PARTNERS IN A VIRTUAL CO-DESIGN PROCESS

Nicole Werner,[1] Anna Jolliff,[1] Reid Parks,[2] Anna LINDEN,[3] Christian Elliott,[4] and Matthew Zuraw[5], *1. Indiana University School of Public Health-Bloomington, Bloomington, Indiana, United States, 2. University of Wisconsin - Madison, Madison, Wisconsin, United States, 3. University of Wisconsin-Madison, Madison, Wisconsin, United States, 4. CareVirtue, Palm Springs, California, United States, 5. DialedIn Solutions, San Diego, California, United States*

Participatory design, or co-design, is an effective method for creating useful digital products with older adults. However, little is known about older adults’ perceptions of virtual co-design. The purpose of this study was to explore ADRD care partner perceptions of their participation in virtual co-design, including the degree to which participants felt their participation influenced the design. After participating in a 5-session virtual co-design process, care partners completed individual semi-structured interviews and surveys focused on exploring their perceptions of the co-design process. Surveys used three sliding scale items and open text responses. Interview transcripts and survey data were analyzed using thematic analysis and descriptive statistics, respectively. Co-designers were Nf7 older adults (71% female) with a mean age of 65. On a scale of 1–100, co-designers rated the extent to which their own participation influenced the design process at a mean of 88, their fellow co-designers at 90, and the research team at 82. Thematic analysis identified five design process perceptions: 1) the process built community among co-designers; 2) sessions were well-facilitated and organized; 3) the prototype would be helpful to other care partners like them; 4) co-designers felt ownership over the prototype; and 5) co-designers had long-term goals for the product. Results suggest that care partners perceived themselves as having considerable influence on the final prototype. Those designing interventions for ADRD care partners should be encouraged that involving them in the design process may provide unanticipated benefits to care partners and build an intervention responsive to care partner needs.

### IMPROVING MEDICATION MANAGEMENT FOR HOME-DWELLING OLDER ADULTS WITH COGNITIVE IMPAIRMENT: END-USER INPUT

Rana Zadeh,[1] Cary Reid,[2] Elaine Wethington,[3] Sara Czaja,[2] Elizabeth Capezuti,[4] Wendy Shields,[5] and Deb Traunstein[6], *1. Cornell University, Ithaca, New York, United States, 2. Weill Cornell Medicine, Ithaca, New York, United States, 3. Cornell, Ithaca, New York, United States, 4. Hunter College of the CUNY (City University of New York), New York, New York, United States, 5. Johns Hopkins University, Baltimore, Maryland, United States, 6. Visiting Nurse Service of Ithaca and Tompkins County, Ithaca, New York, United States*

HOPES™ Program is a joint community-university partnership, intended to build capacity for improving medication adherence and reducing medication mistakes, misuse, and abuse for community-dwelling older adults with reduced cognitive functioning (CWCs) in New York State (NYS). The goal of this program is to contribute to the health and safety of older adults, empower them and their caregivers to reduce medication non-adherence consequences and risks, and enable older adults to continue living at home while managing their health conditions. This program applied a collective user-centered approach to connect resources, identify and address barriers, and reach the most vulnerable groups. One aspect of our user-centered approach included a medication management and safety training module video that was adapted from the Medication Use Safety Training for Seniors™ (MUST for Seniors™) and underwent alpha and beta testing. A qualitative analysis was also conducted on input from home-care providers, CWCs, and their designated caregivers about the current practices, challenges, and solutions related to medication management. Our preliminary findings demonstrated a need for research on reducing the steep estimated national costs of medication non-adherence. Thus, a sub-team has developed a methodology to build an economic perspective on the scope and magnitude of interventions to enhance medication management practices for CWCs. A NYS-specific survey was also developed in close collaboration with providers, university partners, and state-level advocacy organizations according to our preliminary findings to document the state and magnitude of medication non-adherence, discern risk factors and determinants of non-adherence, and explore solutions and barriers.

### CARING FOR CHILDREN WITH DISABILITIES, WORKING, AND SAVING FOR RETIREMENT OVER THE LIFE COURSE

Shirley Porterfield,[1] and Eunsun Kwon[2], *1. University of Missouri-St. Louis, Saint Louis, Missouri, United States, 2. Fairleigh Dickinson University, Florham Park, New Jersey, United States*

In the US, financial security in retirement depends on having savings and other assets accumulated during the pre-retirement years. Pension savings accrue over the working life, in defined-benefit or defined-contribution retirement plans. Mothers with children with disabilities experience different life-course work trajectories than mothers without children with disabilities, though data used in previous studies primarily examine a single point in time or a short span of years. This paper draws longitudinal data from the nationally-representative 1979 National Longitudinal Survey of Youth for the years 1987 through 2018, using a life-course perspective and sequence analysis to identify long-term work patterns among women with children who do and do not have disabilities with an explicit focus on variations in occupational class and employment status. We found a distinctive pattern of long-term work history with five types: Full-time semi-professional to not working, Constantly not working, Semi-professional full time, Professional full time, and Not working to full time work. Results from regression analyses revealed variation in mothers' household financial preparation for retirement at late mid-adulthood. Compared with mothers who held professional full-time jobs throughout their adulthood, mothers who started full time jobs in middle age and have children with disabilities were less likely to have pension plans. Mothers of children with disabilities who left the labor force in early middle age tended to have lower retirement savings. Policy interventions to address these mothers' caregiving ability to stay engaged in the workforce and prepare for their retirement need to be explored.

### ANTI-AGING COMPOUNDS METFORMIN AND THIOFLAVIN T CAN ACT ON GENETICALLY DIVERSE, PROTEOSTASIS-COMPROMISED STRAINS

Monica Driscoll,[1] Brian Onken,[2] Yunpeng Xu,[2] Yuhua Song,[2] Madhuri Achanta,[2] Christine Sedore,[3] Patrick Phillips,[3] and Gordon Lithgow[4], *1. Rutgers, The State University of New Jersey, New Brunswick, New Jersey, United States, 2. Rutgers University, Piscataway, New Jersey, United States, 3. University of Oregon, Eugene, Oregon, United States, 4. Buck Institute, Novato, California, United States*

The Caenorhabditis Intervention Testing Program (CITP) seeks to identify robust and reproducible pro-longevity interventions affecting a genetically diverse panel of representative Caenorhabditis strains from each of 3 species – C. elegans, C. briggsae, and C. tropicalis. The CITP test strains represent genetic diversity on the order of mouse to human. Anchoring principles of the CITP are that efficacious interventions that target fundamental and conserved biology will hold strong potential for translation to higher organism benefit and that such interventions can be rapidly identified using Caenorhabditis models. The CITP is creating multi-species "at risk" test sets, in which components of aging hallmarks (such as proteostasis, stress response circuits) are genetically compromised in diverse genetic backgrounds. Such genetic test sets might expand understanding of intervention action and help rank compounds for translational testing. Toward this end, we used CRISPR to generate deletion mutants of arsenite-inducible protein (C. elegans aip-1, human AIRAP/ARAPL) in which proteostasis and oxidative stress responses are compromised across phyla. All aip-1 mutants are short lived compared to their control wild type strains, highlighting the essential role of proteostasis in maintaining healthy aging. Our previous studies identified metabolic modulator Metformin and anti-amyloid Thioflavin T as effective pro-longevity, pro-healthspan interventions in multiple CITP test strains. We find that Metformin and Thioflavin T impact the aip-1 deletion strains similarly to WT variants. Together, our results demonstrate that Metformin and Thioflavin-T can positively impact lifespan in diverse genetic backgrounds in which proteostasis is compromised, suggesting wide-ranging health benefits of these potent prolongevity compounds.

### WOMEN AT RISK: SOCIOECONOMIC STATUS, LIFESTYLE FACTORS, AND BRAIN VULNERABILITY AMONG JAPANESE AND SWEDISH FEMALE

Yingxu Liu,[1] Yasuko Tatewaki,[1] Benjamin thyreau,[1] Yinghao Liu,[2] Ye Zhang,[3] Nina Karalija,[4] Carl-johan Boraxbekk,[4] and Yasuyuki Taki[1], *1. Institute of Development, Aging and Cancer, Tohoku University, Sendai, Miyagi, Japan, 2. Tohoku University, Sendai, Miyagi, Japan, 3. Tohoku University, Sendai, Miyagi, Japan, 4. Umeå University, Umeå, Vasterbottens Lan, Sweden*

Background: While multiple modifiable lifestyle factors and disease management have been highlighted for preventing dementia and ameliorating neurodegeneration, women and the disadvantaged socioeconomic status (SES) population still bear disproportionate burdens. Objective: Investigate and compare the potential pathways of SES, lifestyle factors, imaging biomarkers, and cognition in two community dwelling cohorts in Japan and Sweden. Subjects: The Kumamoto Cohort included 576 cognitively healthy females (73.66 ± 5.96 years); the Betula Cohort included 195 cognitively healthy females (63.91 ± 13.41 years). Methods: We constructed structural equational modeling by lifestyle factors including exercise, social activity, sleep, drinking, and smoking status; disease conditions included obesity, diabetes, hypertension, and depressive disorder; brain imaging biomarkers included regional gay matter volume (GMV) and cortical thickness obtained from T1 weighted magnetic resonance imaging scans and global cognition score. We also examined SES-related gray matter volume and cortical thickness map locations at the whole brain level. Results: SES was positively associated with GMV of limbic lobe (not cortical thickness), Kumamoto Cohort: standardized direct $\beta$ =0.21 (0.13;0.28); Betula Cohort: standardized direct $\beta$ =0.27 (0.13; 0.41). This SES-GMV association was mediated by disease conditions and lifestyle in Kumamoto Cohort: indirect $\beta$ =-0.013 (0.001; 0.054). We also found several regions, including the medial frontal gyrus, superior frontal gyrus, hippocampus, and thalamus, were commonly sensitive to SES status in two cohorts. Conclusions: Although the observational nature of the study precludes proof of causality, our findings suggest that promoting disease management is crucial to tackling the neurodegeneration burden in the female facing SES disparities.

### THE EFFECTS OF VISUAL SEARCH ON POSTURAL SWAY COMPLEXITY IN OLDER ADULTS WITH AND WITHOUT TYPE 2 DIABETES

Kellyn Jason,[1] Junhong Zhou,[2] Wanting Yu,[3] Brad Manor,[4] and Azizah Jor'dan[5], *1. University of Massachusetts Boston, Boston, Massachusetts, United States, 2. Harvard Medical School/Hebrew SeniorLife, Roslindale, Massachusetts, United States, 3. Hebrew SeniorLife, Roslindale, Massachusetts, United States, 4. Hebrew SeniorLife/Harvard Medical School, Roslindale, Massachusetts, United States, 5. University of Massachusetts, Boston, Massachusetts, United States*

Type II diabetes mellitus (T2DM) is linked to impairments in the interconnecting and interacting components (e.g., cognitive-motor demand) essential for standing postural control. The temporal dynamics of postural sway are complex, thus higher complexity reflects the system's capacity to adapt to cognitive-motor demands. We aimed to characterize the effects of T2DM on postural sway complexity during visual search tasks (VST). Twenty-four older adults without T2DM (OA) (age=67–93 years) and 20 older adults with T2DM (age=70–90 years) performed multiple trials of quiet standing with and without a VST. The VST consisted of low difficulty (LD) (i.e., counting the frequency of one designated letter in a random grid of letters) and high difficulty (HD) (i.e., counting two different letters). The complexity of the postural sway acceleration signals in the anterior-posterior (AP) and medial-lateral (ML) directions were quantified using multiscale entropy. Across participants, matched-pairs analyses revealed that both AP and ML sway complexity increased during the LD condition, compared to control ($p < 0.007$). During HD, only the AP sway complexity increased, compared to control ($p=0.0001$). Within the OA, both AP and ML sway complexity increased during the LD condition ($p < 0.009$), while performance of the HD task showed an increase in AP sway complexity compared to control ($p=0.0002$). Within the T2DM, AP sway complexity increased only during LD, compared to control ($p=0.03$). The multi-scale dynamics of postural control when standing while performing VSTs differ among older adults with and without T2DM, reflected by diminished capacity to increase complexity during the visual search conditions.

### NEUROFILAMENT LIGHT CHAIN (NFL) AND GENERAL COGNITIVE ABILITY IN ADULTS APPROACHING MIDLIFE

Chandra Reynolds,[1] Andrew Smolen,[2] Christopher Link,[2] Donald Evans,[2] Ryan Bruellman,[1] Luke Evans,[2] and Sally Wadsworth[2], *1. University of California, Riverside, Riverside, California, United States, 2. University of Colorado Boulder, Boulder, Colorado, United States*

Neurofilament light chain (NfL) is a biomarker indexing axonal integrity where small NfL variations may be associated with cognitive performance in early adulthood and high values associated with neurodegenerative disorders such as Alzheimer's disease. In the Colorado Adoption/Twin Study of Lifespan behavioral development and cognitive aging (CATSLife1) individuals were tested at 28–49 years (M=33.1, SD=4.9). Quanterix Simoa assays of plasma NfL (pNfL) were measured in duplicate, and we included values for 1159 individuals where 1098 had available general cognitive ability scores and sociodemographic covariates. Unadjusted NfL values were consistent with other studies of early-mid adulthood (M = 5.9, SD = 3.1, range = 1.14 – 40.1 pg/mL) and 6% showed values outside expected normal reference limits (>10 pg/mL). After adjusting for technical covariates and skew, higher natural log-transformed pNfL was associated with age (r = 0.27) and female sex (r = 0.07). Moreover, adjusting for sociodemographic covariates, higher pNfL was associated with lower general cognitive ability (GCA) (r = -.06), where associations were more pronounced above the mean pNfL value (r = -.08). Multi-level regression analyses suggested that GCA-NfL associations were modified by age, whereby the worse performance was observed at higher ages and pNfL values (p <= 0.03), accounting for sibling relatedness and sociodemographic covariates. We observed small negative associations of higher plasma NfL and lower cognitive performance, where associations may become magnified with increasing age in early- to mid-adulthood.

### EXERCISE SLOWS NEURAL STEM CELL AGING AND AD RISK WITH CLONAL SELECTION

Wang Xiang, Lei Peng, Maxwell Bay, Michelle Liu, Jonathan Levi, Tianli Ding, Fifi Pan, and Michael Bonaguidi, *University of Southern California, Los Angeles, California, United States*

Exercise acts as a countermeasure against aging and Alzheimer's disease (AD). Several lines of evidence indicate that the beneficial effects of exercise target neurogenesis and synaptic plasticity. However, how exercise shapes neural stem cell (NSC) function to maintain lifelong hippocampal plasticity remains unknown. Here, we utilize single-cell technologies to uncover exercise slows NSC aging and reduces AD risk. In vivo single-cell lineage tracing revealed that exercise promotes clonal selection by activating quiescent NSCs for self-renewal and depleting neurogenic NSCs. Interestingly, NSC symmetric self-renewal compensated for those lost to differentiation allowing for enhanced neurogenesis without prematurely depleting the overall NSC pool. Prolonged clonal tracing further showed that the selected self-renewed NSCs are more resistant to aging-related NSC depletion. Single-cell RNA-sequencing and multiple bioinformatic analyses determined that exercise slows NSC molecular aging. In particular, exercise mitigates an age-associated increase of AD risk gene expression within NSCs. Our results demonstrate a new regenerative function of exercise to promote healthier brain aging.

### EFFECTS OF REMOVING VISUAL INPUTS ON POSTURAL SWAY COMPLEXITY IN YOUNG ADULTS AND OLDER ADULTS WITH AND WITHOUT T2D

Kellyn Jason,[1] Junhong Zhou,[2] Wanting Yu,[3] Brad Manor,[4] and Azizah Jor'dan[5], *1. University of Massachusetts Boston, Boston, Massachusetts, United States, 2. Harvard Medical School/Hebrew SeniorLife, Roslindale, Massachusetts, United States, 3. Hebrew SeniorLife, Roslindale, Massachusetts, United States, 4. Hebrew SeniorLife/ Harvard Medical School, Roslindale, Massachusetts, United States, 5. University of Massachusetts, Boston, Massachusetts, United States*

Standing postural control is complex and involves sensory inputs from the visual, vestibular, and somatosensory systems. Postural sway complexity, as often assessed by multiscale entropy, has been linked to the ability of the postural control system to adapt to "stressors," including the removal of visual inputs. We aimed to determine the effects of removing visual inputs (i.e., eyes closed, EC) on postural sway complexity in young adults (YA), and older adults (OA) with and without type 2 diabetes mellitus (T2DM). Twenty-five YA (age=21–30 years), 25 OA (age=70–90 years), and 19 T2DM (age=67–93 years) stood for 30 seconds under two conditions – eyes open (EO), and EC for three trials. Postural sway acceleration was measured in the medial-lateral (ML) and anterior-posterior (AP) direction during EO and EC conditions. Sway complexity was quantified using multi-scale entropy. The YA group had a significant reduction in ML and AP sway complexity during EC when compared to EO (p=0.04 and p < 0.001, respectively). Though not significant, the OA group exhibited a trend towards increased ML sway complexity during EC compared to EO standing (p=0.07). No significant changes in AP sway complexity were observed in OA or T2DM, or in ML sway complexity in T2DM. The dynamics of postural control differ between YA, OA, and T2DM groups. There was a significant difference in postural sway complexity between the EO and EC among YA, with no differences in OA and T2DM between conditions. The lack of change suggests maladaptation to stressors, such as vision loss.

### SEX DIFFERENCES IN SKELETAL MUSCLE ENERGETICS AMONG OLDER ADULTS

Philip Kramer,[1] Giovanna Distefano,[2] Sofhia Ramos,[3] Bret Goodpaster,[2] Paul Coen,[3] David Marcinek,[4] Peggy Cawthon,[5] and Anthony Molina[6], *1. Wake Forest University School of Medicine, Winston-Salem, North Carolina, United States, 2. Translational Research Institute for Metabolism and Diabetes, AdventHealth, Orlando, Florida, United States, 3. AdventHealth, Orlando, Florida, United States, 4. University of Washington, Seattle, Washington, United States, 5. University of California, San Francisco, San Francisco, California, United States, 6. UC San Diego School of Medicine - Division of Geriatrics, Gerontology, and Palliative Care, La Jolla, California, United States*

Mobility impairment is the most common disability among older adults, with an earlier presentation, higher prevalence, and greater severity in women compared to men. A decline in skeletal muscle metabolism contributes to the loss of mobility with age. However, it is unknown if sex-specific differences in muscle energetics can explain the disparity in mobility impairment among men and women. In the Study of Muscle, Mobility, and Aging (SOMMA), muscle energetics was characterized using in vivo Phosphorus Magnetic Resonance Spectroscopy and High-Resolution Respirometry of permeabilized fiber bundles from muscle biopsies. In this analysis of 773 participants aged 70–94 years, 519 were women, of which 16% were deemed lower extremity mobility impaired based on a Short Physical Performance Battery (SPPB) score

### MUSCLE MITOCHONDRIAL ENERGETICS ARE ASSOCIATED WITH GREATER MULTIMORBIDITY IN MEN

Theresa Mau,[1] Terri Blackwell,[2] Anne Newman,[3] Peggy Cawthon,[4] Steve Cummings,[1] Philip Kramer,[5] Russell T. Hepple,[6] and Stephen Kritchevsky[7], *1. San Francisco Coordinating Center, San Francisco, California, United States, 2. California Pacific Medical Center, San Francisco, California, United States, 3. University of Pittsburgh, Pittsburgh, Pennsylvania, United States, 4. University of California, San Francisco, San Francisco, California, United States, 5. Wake Forest University School of Medicine, Winston-Salem, North Carolina, United States, 6. University of Florida, Gainesville, Florida, United States, 7. Wake Forest School of Medicine, Winston Salem, North Carolina, United States*

Older adults who have poorer muscle mitochondrial energetics tend to have lower cardiorespiratory fitness—which is associated with the risk of cardiovascular disease, cancer, and dementia. In the Study of Muscle, Mobility, and Aging (SOMMA), we tested the associations of muscle mitochondrial energetics (MME) with a multimorbidity index in this cohort of women (Nf507) and men (Nf349) 70+ years of age. The index included 13 age-related chronic conditions (arthritis, cancer, cardiac arrhythmia, chronic kidney disease, chronic obstructive pulmonary disease, coronary artery disease, congestive heart failure, dementia, depression, diabetes, osteoporosis, stroke, and aortic stenosis). Participants were grouped by 0, 1, 2, or 3+ conditions and were on average 76.3 years old, 59.2% women, 12.5% Black, 85.5% White, with 2.0% reporting other identities. MME were measured in permeabilized muscle fibers by high-resolution respirometry in vitro (Leak and MaxOXPHOS) and in vivo by 31P-magnetic resonance spectroscopy (ATPmax). The gender interaction p-value with MME was 0.04 (Leak), 0.34(MaxOXPHOS), and 0.09(ATPmax). In men, we found that for every 1 SD decrease of mitochondrial respiration, there was a significant increase of proportional odds ratio (POR) for higher multimorbidity count: POR (95%CI) for Leak was 1.47(1.18, 1.86), $p < 0.001$ and for Max OXPHOSCI+CII was 1.35(1.08,1.68), $p < 0.01$. For ATPmax, was 1.22(0.98,1.52), p=0.08. These were adjusted for age, race, site, education, smoking status, weight, height, weight*height, and self-reported physical activity. These trends were absent in women (Leak=0.97(0.79, 1.20), p=0.79, MaxOXPHOS=1.05(0.86, 1.28), p=0.66, ATPmax=0.98 (0.82, 1.17), p=0.81), and future analyses will deconstruct the multimorbidity index to determine gender-specific associations with each condition.

### IMPACT OF INTERGENERATIONAL SERVICE LEARNING ON PSYCHOLOGICAL DISTRESS FOR HOMEBOUND OLDER ADULTS DURING COVID

Keith Chan,[1] Christina Marsack-Topolewski,[2] Maggie Ratnayake,[3] Jillian Graves,[2] Sarah LaFave,[3] and Diane Fenske[2], *1. Hunter College, City University of New York, New York City, New York, United States, 2. Eastern Michigan University, Ypsilanti, Michigan, United States, 3. Lori's Hands, Newark, Delaware, United States*

More than 2 million of older adults are homebound and 5 million need help leaving their homes. They often experience social isolation, food insecurity, and lack of connection to community resources, which for many has intensified since the pandemic. To date, home-based services for those aging in place are lacking. Using newly available data, this study examined the benefits of an intergenerational home-based service learning program in reducing psychological distress for a community-based sample of 190 homebound older adults during the COVID-19 pandemic. Multivariate regression analyses were conducted to examine the association of living in one's own home, disability status, presence of child and spousal caregivers, and length of services from the program with psychological distress. Findings indicated that length of service with the intergenerational in-home support program was associated with lower psychological distress ($\beta = -0.16$, $p < 0.05$). Having a child as a caregiver was associated with lower psychological distress ($\beta = -0.15$, $p < 0.05$). Poor health status was associated with higher levels of psychological distress ($\beta = 0.16$, $p < 0.05$). Living in one's own home, having a spouse as a caregiver, disability status, and having a long-term medical condition were not associated with psychological distress in the analysis. Results from this study suggest that intergenerational in-home support services can help reduce psychological distress for homebound older adults. Policies and practice can support a pipeline of geriatric health professionals through innovative service learning models to benefit older adults, caregivers, and students.

### SLEEP AND CAREGIVER BURDEN FOR CAREGIVERS OF PERSONS WITH DEMENTIA: A SCOPING REVIEW

Meghan Mattos,[1] Veronica Bernacchi,[2] Kelly Shaffer,[3] Virginia Gallagher,[3] Shinae Seo,[1] and Carol Manning[4], *1. University of Virginia, School of Nursing, Charlottesville, Virginia, United States, 2. Penn State College of Medicine, State College, Pennsylvania, United States, 3. University of Virginia, Charlottesville, Virginia, United States, 4. UVA, Charlottesville, Virginia, United States*

Caregivers of persons with dementia report worse sleep when compared to the general population. This scoping review synthesizes evidence regarding the link between caregiver burden and dementia caregivers' sleep. A systematic search was completed in PubMed, CINAHL, PsycINFO, and Web of Science for pertinent literature published through March 2022. Included original research articles studied informal adult caregivers of persons living with mild cognitive impairment or dementia, documented the relationship between subjective caregiver burden and caregiver sleep, and were written in English. Extracted data were organized in tables, compared, and synthesized. The search yielded 540 non-duplicate articles screened by title and abstract; 118 full-text articles were reviewed; of these, 26 were included. Most studies were cross-sectional, with variable sample sizes (range n=40–669). Sleep was operationalized across the 25 quantitative studies in terms of subjective quality (n=19) and objective sleep parameters using actigraphy (n=4), and polysomnography (n=2). Of studies reporting subjective sleep quality, 16 reported a significant positive association between caregiver burden (84%) with the remaining found null results. Half of the studies that used objective measures of sleep (actigraphy and polysomnography) found a positive association between sleep and caregiver burden, while the other half did not (n=3). Results suggest that, while subjective sleep quality is commonly impacted by dementia caregiving burden, there is a lack of corresponding evidence on the relationship between burden and objective sleep metrics. Caregiver burden was also not measured consistently across studies, and future studies should focus on consistent measurement of caregiver burden and determination of directionality.

### RURAL/URBAN DIFFERENCES IN LIFETIME OCCUPATION AND ALL-CAUSE MORTALITY AMONG MEXICAN ADULTS

Mariela Gutierrez,[1] Rebeca Wong,[2] and Yong-Fang Kuo[3], *1. University of Texas Medical Branch, School for Public and Population Health, Galveston, Texas, United States, 2. University of Texas Medical Branch, Galveston, Texas, United States, 3. University of Texas Medical Branch at Galveston, Galveston, Texas, United States*

Mortality is affected by occupation dimensions, such as physical and mental demands, environmental exposures, and access to resources. Few studies have examined the relationship between occupation and mortality in countries with shifting occupation profiles, such as Mexico. We used 2001–2018 data from the Mexican Health and Aging Study to investigate the association between lifetime occupation type and all-cause mortality among participants aged 50 and older. We grouped participants' longest-held occupations into five categories: (1) no main job; (2) agriculture; (3) domestic, service; (4) administrative, professionals, sales; and (5) production, industrial, transportation. We used Cox Proportional Hazard regression to study death risk associated with occupation, adjusting models for baseline demographic, health, and job characteristics. We stratified analysis by rural and urban to account for differences in occupation composition. Our sample included 10,482 participants, with 4,147 deaths occurring during follow-up. The percentage of participants in administrative, professional or sales was higher in urban areas than rural areas (60.7% vs. 20.5%). The percentage of participants in agriculture was higher in rural areas than urban areas (81.8% vs. 18.2%). Compared to professionals, participants in agriculture, industry, or transportation had a slightly higher risk of death (HR=1.65, 95% CI 1.59 to 1.77). Agriculture workers in rural areas had a higher risk of death than non-agriculture workers in rural areas. Considering rural/urban differences in occupations and their effect on mortality can inform policy aimed at improving the working environment for Mexican adults. Understanding rural/urban differences in mortality associated with occupation can elucidate socioeconomic inequalities in mortality.

### REEXAMINING RACE AND ETHNICITY FROM A STRUCTURAL RACISM AND SOCIAL DETERMINANTS OF HEALTH LENS IN COHORT STUDIES

Shawna Follis, *Stanford University, San Francisco, California, United States*

To advance research on the role of race, ethnicity, and structural racism in health disparities, recent calls-to-action have described methods to correct race and ethnicity categorization and to measure structural and social determinants of health (SSDOH). Following the rapid advance in the understanding of race and SSDOH, long running cohort studies typically lack access to this novel data, which has limited SSDOH evidence to cross-sectional and ecological associations. We implemented methods to rectify this data gap in prospective cohort studies, using The Women's Health Initiative (WHI) cohort of women age 50–79 years at baseline as a case study. We evaluated the quality, precision, and representativeness of race, ethnicity, and SSDOH data compared with the target US population and operationalized methods to quantify structural determinants in cohort studies. The use of a revised race and ethnicity categorization aligned with theory-based recommendations and improved measurement precision, including decreasing missing data and participants reporting "other" race. The revised categorization was harmonized to the US Census permitting description of the generalizability of WHI race and ethnicity groups in relation to the target population of US women. We found that WHI women reflected broader trends to the target population in relation to structural racism between race and ethnicity groups, while mean rates of SSDOH were systematically more advantageous in WHI. These results underscore the need to implement theoretically justified race and ethnicity measurement to accurately investigate the intersectional role of SSDOH underlying racial and ethnic health disparities among older age women.

### DO ADVERSE ADULT LIFE EVENTS MODERATE THE IMPACT OF CHILDHOOD ADVERSITY ON COGNITION AFTER AGE 50?

Kimson Johnson, *University of Michigan, Ann Arbor, Michigan, United States*

Population-based studies of childhood adversity and adulthood trauma have shown varying prevalence and incidence rates due to differences in conceptualization and assessment of these events. Adverse childhood and adult events are prevalent in the general population and have deleterious effects on adult health over the life course. Exposure to adverse childhood events (ACEs) (i.e., household dysfunction) and adverse adult events (AAEs) (i.e., history of military combat or incarceration) can shape health behaviors, overall health, and disease trajectories. Despite the well-known lasting health effects of ACEs and AAEs, little is known about these events' singular and cumulative effects on cognitive health in mid to late life. The objective of this study is to examine if AAEs moderate the relationship between ACEs and cognitive functioning. Data was utilized from the 2010–2018 waves of the Health and Retirement Study (HRS) (N =3,320). Latent Growth Curve Modeling indicated that one or more ACEs were associated with a significant decrease in cognitive functioning ($\beta$=-0.19, $p < .05$). However, one or more AAEs were not significantly associated with a decrease in cognitive functioning ($\beta$=-0.19,$p>.05$). Finally, moderation analysis shows that AAEs do not significantly moderate the relationship between ACEs and cognitive functioning ($\beta$=-.32, $p>.05$). A better understanding of the timing and distribution of exposures to ACEs and AAEs can support targeting interventions and prevention screening measures. Inclusion of ACEs and AAEs in cognitive assessments could provide information about modifiable risk factors to reduce the potential negative impact of these stressors on cognitive aging trajectories in mid to late life.

### DISPARITIES IN LATE-STAGE BREAST/COLORECTAL CANCER DIAGNOSIS AMONG HISPANIC, WHITE, & BLACK TX MEDICARE PATIENTS

Mathilda Nicot-Cartsonis,[1] Mukaila Raji,[2] Yong-Fang Kuo,[2] and Biai Digbeu[2], *1. John Sealy School of Medicine, Galveston, Texas, United States, 2. University of Texas Medical Branch at Galveston, Galveston, Texas, United States*

Disparities in late-stage breast or colorectal cancer diagnosis in younger populations are associated with Social Determinants of Health-SDOH (education, poverty, housing, employment). We hypothesized that—in older Medicare beneficiaries—disparities in late-stage cancer diagnosis between Hispanics, non-Hispanic Blacks (NHB) and non-Hispanic white (NHW) would be associated with SDOH, comorbidities and primary care-PCP access. We thus used 2005–2017 Texas Cancer Registry data linked with Medicare data for patients aged ≥66 (Nf86,501). Variables included were age at diagnosis, sex, comorbidities, poverty level, education, primary care provider, and breast/colorectal cancer screening within 1 year. For breast cancer in women (Hispanic n=6380, NHW n=39, 225, non-Hispanic Black n=4055), fully adjusted model showed significantly higher odds of late-stage cancer diagnosis only in NH Black patients (OR= 1.11 95% CI= 1.01–1.22) compared with NHW; adjustment for comorbidities and SDOH partially decreased the odds of late-stage diagnosis relative to NWH. Interaction terms between race-ethnicity and poverty were not significant. For colorectal cancer, fully adjusted multivariate model showed significantly higher odds of late-stage diagnosis among Hispanics (n=6053, OR=1.07;95% CI= 1.00–1.15) and NH-Black patients (n=3318, OR=1.29 95% CI= 1.19–1.40) relative to NHW (n=27,470); adjustment for SDOH was associated with a partial decrease in the high odds of late-stage diagnosis in Hispanic and NH Black patients. Interaction terms between race-ethnicity and poverty were not significant. Racial disparities in late-stage breast and colorectal cancer diagnoses remained after adjusting for SDOH and other clinically-relevant factors, underscoring needs for studies on optimizing access to screening and timely cancer treatments in racial/ethnic minorities.

### ASSOCIATION BETWEEN LIMITED ENGLISH PROFICIENCY AND MEDICARE ENROLLMENT CHOICE

Lilly Estenson, and Mireille Jacobson, *University of Southern California, Los Angeles, California, United States*

Choosing between traditional Medicare and Medicare Advantage (MA) is an important health and financial decision that can be especially complicated for Medicare beneficiaries who have limited proficiency speaking or reading English (LEP). In this study, we used data from the 2016–2018 Medicare Current Beneficiary Survey and ordinary least squares regression to examine the association between LEP and MA enrollment. Among the 19,621 respondents (32,912 person-year observations) who answered LEP questions, 9.2% self-reported having LEP and 38% were enrolled in MA. Limited proficiency reading English was significantly associated with enrollment choice; respondents who reported reading English "not well" or "not at all" were 6.7 percentage points less likely to enroll in MA than respondents who reported reading English "well" or "very well" (SE=0.025; $p < 0.01$). However, when we stratified respondents by language spoken at home, we discovered considerable variation in this association among language subgroups (English: -6.5 percentage points, SE=0.029, $p < 0.05$; Spanish: -1.5 percentage points, SE=0.051, p=NS; other: -11.2 percentage points, SE=0.065, $p < 0.10$). Additionally, respondents with limited proficiency reading English were 8.0 percentage points less likely to positively rate their Medicare knowledge (SE=0.022, $p < 0.01$) and 6.4 percentage points less likely to review their Medicare options annually (SE=0.023, $p < 0.01$) than respondents with English reading proficiency. Limited proficiency speaking English was not significantly associated with MA enrollment, Medicare knowledge, or annual options review. These findings suggest that English literacy rather than speaking proficiency may be a crucial determinant of enrollment choices. Language access around health insurance information is critical for equitable Medicare enrollment.

### THE RISE OF LONELINESS: TRENDS AMONG A LARGE MEDICARE ADVANTAGE COHORT OVER THE COVID-19 PANDEMIC

Kelsey McNamara, and Ellen Rudy, *Papa Inc., Miami, Florida, United States*

Loneliness is an established risk factor for premature mortality, especially among older adults. National trends are indicating an increase in loneliness after the COVID-19 pandemic. We sought to explore annual trends in loneliness and quality of life among a large dataset of Medicare Advantage (MA) members. Papa Inc. is a national service that pairs older adults with "Papa Pals" who provide companionship and assistance with everyday tasks. Participants have free access if their health plan offers it. Analysis included members from MA plans that were active with Papa January 2020 through March 2022. Members (n=11,037) were assessed at enrollment screening (UCLA Three-Item Loneliness Scale, CDC's Healthy Days Measure). Analysis used Chi-square tests, significance set at $p < 0.05$. Among the sample, 81% were aged 65+, 14% were from a majority non-white community. Analysis revealed the following trends in annual prevalence rates (2020; 2021; 2022): lonely (43.2%; 39.3%; 40.9%), severely lonely (9.2%; 12.1%; 15.1%), 14+ physically unhealthy days (24.3%; 19.9%; 39.3%). In regression analysis, loneliness score increased by 0.05 (95CI: 0.02 - 0.07) per quarter over the 27 months. Our data mimics other sources highlighting a slow and steady increase in loneliness suggesting older adults' hesitancy to engage in "normal activities." Changes in unhealthy days follow COVID-19 trends with a significant increase during the height of the Omicron variant. The prolonged effects of isolation and loneliness are profound, thus greater emphasis should be placed on population level interventions, especially those connected to the healthcare system, to help address loneliness.

### PATHWAYS OF IMPROVING SOCIAL CAPABILITY OF OLDER ADULTS WITH FUNCTIONAL LIMITATIONS: FINDINGS FROM MIXED METHODS

Yalu Zhang, Yiyi Xiong, and Gong Chen, *Peking University, Beijing, Beijing, China (People's Republic)*

Objectives: Social capabilities are the opportunities to realize people's potential. Despite an established positive link between health status and social capabilities among older adults, the relationship mechanisms are understudied. Methods: Using the China Longitudinal Aging Social Survey data 2014–2018, this paper examined the possible mediating role of community participation through which older adults (aged 60 and above) in China with functional limitations improved or maintained their social capabilities. By conducting the 12 in-depth interviews, this study explores how community participation altered the negative associations between functional limitations and social capabilities among Chinese older adults. Results: Findings from the quantitative study show that both physical (-.136, $p < 0.001$) and cognitive (-.149, $p < 0.001$) functional limitations showed consistent and negative effects on the social capabilities of older adults, and the effects varied between males and females. The mediation analysis results show that community participation accounted for a substantial proportion of the impact of functional limitations (36.33%) on social capabilities. However, functional limitations still had strong, negative direct effects of their own. Findings from the qualitative narrative synthesis show that peer companionship, regular physical activities, and reduced digital obstacles to accessing online social media during social participation are the self-perceived driving force in enhancing their sense of security, freedom of expression, and sense of social cohesion. Discussion: Findings from this study highlight the need for more social policies and services to encourage community participation among older adults.

### LONELINESS AND SELF-RATED DAILY SLEEP QUALITY: RESULTS FROM THE EINSTEIN AGING STUDY

Jee eun Kang,[1] Orfeu Buxton,[1] Jinshil Hyun,[2] and Martin Sliwinski[1], *1. The Pennsylvania State University, University Park, Pennsylvania, United States, 2. Albert Einstein College of Medicine, Bronx, New York, United States*

Poor sleep quality is a common problem and has profound physical and mental effects on older adults. Loneliness, another common and harmful condition for older adults, is associated with heightened levels of vigilance and feeling unsafe, which may contribute to poor sleep. Although some studies show that trait loneliness is related to compromised sleep, there are few studies examining whether and how day-to-day variations in loneliness relate to sleep quality. The current study aimed to examine within- and between-person associations of loneliness and daily self-rated sleep quality using ecological momentary assessment (EMA) approach. Community-dwelling participants (n=317, Mage=77 (range=70–90), 56% white) reported loneliness and self-rated sleep quality during a 14-day EMA study via mobile phones. Trait loneliness was measured once prior to EMA. Unexpectedly, multilevel modeling showed that the amount of loneliness experienced on a given day did not predict sleep quality, however lower levels of prior night sleep quality predicted elevated levels of loneliness ($p < .01$). Regarding the between-person association, individual differences in trait loneliness were not related to sleep quality, but differences in average levels of EMA loneliness were related to sleep quality, such that people who felt higher levels of loneliness on average during the 14 days reported worse quality of sleep during that time period than others ($p < .0001$). These results suggest that worse sleep quality may be a factor that increases loneliness, and may not be a consequence of loneliness. Potential benefits of using EMA loneliness in research of daily sleep quality in older adults will be discussed.

### LONELINESS AMONG OLDER IMMIGRANTS LIVING IN SUBSIDIZED SENIOR HOUSING: DOES PERCEIVED SOCIAL COHESION MATTER?

Jihye Baek,[1] BoRin Kim,[2] Sojung Park,[3] and Byeongju Ryu[1], *1. Washington University in St. Louis, St. Louis, Missouri, United States, 2. University of New Hampshire, Durham, New Hampshire, United States, 3. Washington University in Saint Louis, Saint Louis, Missouri, United States*

The study compared the level of loneliness among older immigrants living in subsidized senior housing to non-immigrant residents. Also, the role of perceived social cohesion on loneliness was investigated, focusing on the differential influence among older immigrants and non-immigrants. Residents of subsidized senior housing in St. Louis and the Chicago area were recruited, and 182 responses were used in the analysis (126 immigrants and 56 non-immigrants). Descriptive analysis examined the difference in loneliness between immigrants and non-immigrants. Also, multiple regression models estimated the association between 1) immigrant status and loneliness, 2) perceived social cohesion and loneliness, and 3) the moderating role of immigrant status. Linear regression model showed that there was no significant difference between loneliness levels between immigrants and non-immigrants. However, perceived social cohesion was negatively associated with loneliness ($\beta$=-.338, SE= .025, $p < .001$). Additionally, immigration status moderated the relationship between perceived social cohesion and loneliness ($\beta$=-1.143, SE= .052, $p < .01$), which implies that immigrants may benefit more from higher perceived social cohesion in terms of loneliness compared to their non-immigrant counterparts. High perceived social cohesion might be an important community-level protective factor against loneliness in old age, especially for low-income older adults living in subsidized senior housing. Creating socially cohesive environments, particularly for this subgroup, would be crucial for mitigating loneliness and facilitating aging-in-place.

### COMPANION CARE ASSOCIATED WITH REDUCTION IN ADMISSIONS AND EMERGENCY DEPARTMENT USE AMONG OLDER ADULTS

Kelsey McNamara,[1] Ellen Rudy,[1] Anne Armao,[2] Kerri Towsley,[2] Samantha Lang,[3] and Carla Perissinotto[4], *1. Papa Inc., Miami, Florida, United States, 2. SummaCare, Akron, Ohio, United States, 3. Weill Cornell Medicine, New York, New York, United States, 4. UCSF, San Francisco, California, United States*

Older adults with unmet social needs, including social isolation and loneliness, have higher rates of hospital readmission and emergency department (ED) use. Our objective was to determine if Papa Inc., a nationwide service that pairs older adults with "Papa Pals" for companionship and assistance, was associated with reductions in readmissions and ED visits. SummaCare, a Medicare Advantage Organization in Ohio, partnered with Papa to offer free companion care to their members. We explored changes in inpatient admission rates and ED high-utilizers (defined as members with 4+ ED visits in a calendar year) from 2018 through 2021 using a claims-based analysis. Analysis included members who used 30+ minutes of Papa services in 2021 and had historical claims data from 2019 (n=1,420), matched 1:1 to a non-Papa comparison group (n=1,420) using a validated risk scoring model. Overall, average age was 78, 62% were female, 2% were ED high-utilizers in 2021. Increased enrollment in Papa occurred post hospitalization. The case-mix adjusted 30-day readmission rate in 2021 was 12.6% for Papa users (compared to 14.1% for non-Papa; and 14.5% for Papa users before enrollment), revealing a 1.5% to 2% decline in readmission rate after enrollment in Papa. Using case-mix adjusted relativities, Papa members had 0.89 readmissions for every 1.00 readmission for non-Papa users. Compared to the matched cohort, the Papa cohort had 34% fewer ED high-utilizers during the intervention year. Results provide preliminary evidence that a social companionship service may reduce readmissions and frequent ED use, and can inform future trials on companion-based interventions.

### SEX DIFFERENCES IN MITOCHONDRIAL RESILIENCE: EVIDENCE FROM BABOON HEPATOCYTES

Daniel Adekunbi,[1] Cun Li,[1] Peter Nathanielsz,[1] and Adam Salmon[2], *1. University of Wyoming, Laramie, Wyoming, United States, 2. The University of Texas Health Science Center at San Antonio, San Antonio, Texas, United States*

Events that occur in utero set the trajectory for later-life diseases and longevity. Compelling data exist for interactions between developmental programming and aging, but the underlying mechanisms are not clearly defined. Fetal exposure to glucocorticoids (GC) is associated with alteration in hepatic enzymes and metabolic function in later life. We previously reported increased hepatic lipid accumulation and obese phenotype in middle-age male baboons exposed to GC as fetuses. The mitochondria play significant roles in cellular processes including stress responses and possibly a nexus between developmental programming and aging. The present study investigated the long-term effects of in utero GC exposure on mitochondrial bioenergetics using hepatocytes derived from aging baboons (16–18 years, average lifespan 21 years). Mitochondrial bioenergetics of both left and right lobe liver hepatocytes were examined as well as potential sex differences in mitochondrial function. Cell viability following isolation was similar among sexes and liver lobes but hepatocytes from males were highly energetic compared to females. Significant bioenergetic differences were observed in hepatocytes isolated from female baboons' left and right liver lobes, with higher basal, maximal, and ATP-linked respiration in left lobe hepatocytes compared to the right lobe. These lobe-specific bioenergetic differences were absent in males. Interestingly, $H_2O_2$-induced oxidative stress significantly modified male baboon hepatocyte bioenergetics but females were unaffected, suggesting mitochondrial resilience in females compared to males. These data demonstrate that early life exposure to GC elicits a sex-specific effect on mitochondrial function. These mitochondrial differences might drive differences in cell senescence between males and females.

### SASP PROTEIN ASSOCIATIONS WITH HUMAN AGING HIGHLIGHTS THE ROLE OF GDF15, CST3, AND IGFBP2

Daniel Evans,[1] Danielle Young,[1] Toshiko Tanaka,[2] Nathan Basisty,[3] Luigi Ferrucci,[4] Judith Campisi,[5] and Birgit Schilling[5], *1. California Pacific Medical Center, San Francisco, California, United States, 2. NIA IRP, Baltimore, Maryland, United States, 3. National Institute of Aging, Baltimore, Maryland, United States, 4. National Institute on Aging, Baltimore, Maryland, United States, 5. Buck Institute for Research on Aging, Novato, California, United States*

Cellular senescence is a hallmark of aging and can result in a Senescence-Associated Secretory Phenotype (SASP). SASP is characterized by an increased production of proinflammatory cytokines and damage to surrounding tissues. There is substantial evidence from non-human models demonstrating that SASP contributes to tissue dysfunction and pathogenic effects of aging. However, there are relatively fewer studies on the relationship between SASP and aging in humans. In this report, we determined the relationship between SASP circulating proteins and a wide range of aging-related clinical traits in two human cohort studies (BLSA and InChianti) using aptamer-based proteomics. Clinical traits examined were waist circumference, glucose levels, systolic and diastolic blood pressure, C-reactive protein, Interleukin-6, alkaline phosphatase, blood urea nitrogen, gait speed, grip strength, albumin, and red blood cell distribution width. In the meta-analysis of regression model results from both studies, 38 of the 77 SASP proteins examined were significantly associated with age. Of the 38 age-associated SASP, 26 were associated with one or more age-associated clinical traits. A cluster of three SASP proteins (GDF15, CST3, and IGFBP2) showed significant associations with inflammatory markers, renal function and hematological traits. A second cluster was significantly associated with grip strength and gait speed, two measures of physical function that are both strongly associated with mortality. Taken together, these results support the relevance of SASP proteins to human aging and point to specific protein components of the SASP that should be further examined for the potential to be biomarkers of human aging.

### SALIVA BASED BIOMARKERS TO IDENTIFY COGNITIVE IMPAIRMENT IN OLDER ADULTS

Vinod Yata,[1] Shalini Jain,[2] Michal Masternak,[3] Peter Holland,[4] Marc Agronin,[5] Hariom Yadav,[2] and MiaGB Consortium Team[2], *1. Center for Excellence of Aging and Brain Repair, USF Health, Tampa, Florida, United States, 2. University of South Florida, Tampa, Florida, United States, 3. University of Central Florida, Orlando, Florida, United States, 4. Florida Atlantic University, Boca Raton, Florida, United States, 5. Miami Jewish Health, Miami, Florida, United States*

The prevalence of cognitive decline and dementia is increasing in older adults, their prognosis is poor. Multiple emerging evidence shows that early intervention can delay and /or prevent their progression, however, early-detection markers are invasive, expensive, and not easy to routinely measure. Saliva can be an attractive source of early cognitive decline markers because oral health is linked with cognitive function and oral fluids are connected with brain fluids, and abnormal brain protein markers can leak out to saliva and vice-versa. Using global unbiased LC-MS/MS-based proteomics of 22 saliva samples of cognitively impaired and 39 cognitively healthy older adults (>60 years old) from large, multi-site study called Microbiome in aging Gut and Brain (MiaGB) consortium, revealed that 22 proteins were uniquely for cognitively impaired group while 44 were unique for cognitively healthy controls. In addition, among 78 differentially abundant proteins between cognitively impaired and control groups, half (39) were upregulated, and half (39) were downregulated. Notably, unique proteins in saliva of participants with cognitive impairment were from neurological pathways like NGF signaling, mTOR signaling and LPS-stimulated MAPK signaling. In addition, differentially abundant proteins in participants with cognitive impairment enriched with glucocorticoid receptor signaling, LXR/RXR activation, and L-DOPA degradation-pathways, while they were deficient of pathways like complement C3 and lysozyme pathways. We discovered that the novel saliva proteins and their pathways could blindly differentiate cognitive impaired from healthy older adults. These data suggest that we discovered novel saliva-based proteins that can be used as biomarker to predict/diagnose cognitive-impairment in older adults.

### ROBUST LONGEVITY EFFECTS OF NOVEL COMPOUNDS TESTED BY THE CAENORHABDITIS INTERVENTION TESTING PROGRAM

Monica Driscoll,[1] Brian Onken,[2] Christine Sedore,[3] Anna Coleman-Hulbert,[3] David Hall,[4] Gordon Lithgow,[4] Patrick Phillips,[3] and Viviana Perez Montes[5], *1. Rutgers, The State University of New Jersey, New Brunswick, New Jersey, United States, 2. Rutgers University, Piscataway, New Jersey, United States, 3. University of Oregon, Eugene, Oregon, United States, 4. Buck Institute, Novato, California, United States, 5. National Institute on Aging, Bethesda, Maryland, United States*

Many efficacious interventions that promote mouse longevity (sirtuins, TOR, insulin signaling) have identification roots in invertebrate genetics. The Caenorhabditis Intervention Testing Program (CITP) was charged by the NIA to evaluate pharmacological interventions that promote healthy aging in a robust and reproducible manner across diverse genetic backgrounds of natural variant Caenorhabditis strains. The central premise of the CITP effort is that compounds that have strong effects across diverse genetic backgrounds have enhanced probability of translatability into pre-clinical research. Indeed, some CITP-verified compounds have been shown to promote healthspan and longevity in mouse models, in support of this fundamental premise. One example that supports the Geroscience hypothesis (anti-aging compounds will promote systemic health) is our finding that when HBX, the counterpart of one of the strongest C. elegans longevity interventions (ThioT), was tested in a comprehensive multi-laboratory collaboration (Buck Institute), a remarkable increase in bone health in aging mice emerged (PMID 33778327). Our current effort includes pursuing compound submissions from the larger scientific aging community, as well as identification of candidates via high-throughput screening of chemical libraries and data mining of peer-reviewed publications. We now evaluate whole-organism RNA sequence data to estimate the mode of action for successful interventions, and conduct mortality analysis from a generalized family of distributions on high-resolution automated lifespan data to determine whether a given intervention changes the rate or onset of aging. Our recent lifespan-altering discoveries include a small molecule, a proprietary compound, an isothiocyanate and a vitamin derivative. We will present on the breaking compound successes.

### FACTORS AFFECTING ANHYDROBIOSIS SURVIVAL IN EUTARDIGRADE PARAMACROBIOTUS EXPERIMENTALIS (MACROBIOTIDAE)

Amit Kumar Nagwani, *Adam Mickiewicz University, Poznan, Wielkopolskie, Poland*

Amit Kumar Nagwani, Iwona Melosik, Łukasz Kaczmarek, Hanna Kmita Faculty of Biology, Adam Mickiewicz University Poznań, Poland

Background: Tardigrades, considered among animals the most resistant to complete loss of body water (desiccation) occurring during anhydrobiosis, are also interesting because of their place between the two primary invertebrate model organisms (Caenorhabditis elegans and Drosophila melanogaster). The effect of anhydrobiosis intensification on tardigrades' age-specific survival has rarely been studied. The eutardigrade Paramacrobiotus experimenatlis is an excellent model of the study because their remarkable capacity for anhydrobiosis. This gives also possibility to apply the species in research on anhydrobiosis as an anti-aging strategy. Aim: Main goal of present study was to determine the effect of anhydrobiosis intensification, due to application of different duration and numbers of desiccation episodes, on the age-specific survival of Pam. experimenatlis. Methods: Tardigrades were cultured under laboratory conditions, divided according to their age and sex, and subjected to several short and long desiccation episodes (3 and 30 days, respectively). The revival from desiccation was evaluated after 2-48h following rehydration. Results: Individuals` age and anhydrobiosis intensity affected tardigrades` survival. Young adult animals survived the best, and the oldest ones the worst. The number of desiccation episodes affected animal survival stronger than desiccation duration. Conclusion: The results are important for anhydrobiosis study as an-anti aging strategy. The work was supported by the research grants of the National Science Centre, Poland, NCN: 2016/21/B/NZ4/00131 and 2021/41/N/NZ3/01165.

### GLYNAC SUPPLEMENTATION IN OLDER ADULTS PROTECTS FROM MEAL DRIVEN OXIDATIVE STRESS AND INFLAMMATION: RESULTS OF A RCT

Rajagopal Sekhar, George Taffet, and Premranjan Kumar, *Baylor College of Medicine, Houston, Texas, United States*

Elevated oxidative stress (OxS) and inflammation are linked to many age-associated abnormalities, but underlying contributing factors are not well understood. In a randomized clinical trial (RCT) in older adults (OA), we investigated the contribution of a single carbohydrate-meal on OxS and inflammation, and tested the protective effect of supplementing GlyNAC (combination of glycine+N-acetylcysteine) from the meal-driven rise in OxS and inflammation. We studied 20 OA and 10 YA (young-adults) to (1) compare the impact of a 75g oral glucose-meal on plasma markers of OxS (as TBARS) and inflammation (as IL-6); (2) re-assess these outcomes after supplementing OA with either GlyNAC or isonitrogenous placebo (alanine) for 16-weeks, and YA with GlyNAC for 2-weeks. We found that (1) 2-hours after a glucose-meal, compared to YA the OA had higher increases (expressed as percent-change over baseline) of OxS (TBARS –0.1 +/- 0.6 vs. 5.6 +/- 0.6, $p < 0.0000$) and inflammation (IL-6 3.5 +/- 1.2 vs. 11.2 +/- 0.7, $p < 0.0000$); (2) GlyNAC supplementation in OA protected against the glucose meal-driven increase in OxS (TBARS pre vs post: 4.8 +/- 0.9 vs. -0.4 +/- 0.8, p=0.002) and inflammation (IL-6 pre vs. post: 10.8 +/- 3.2 vs. 4.2 +/- 2.3, p=0.0001). No improvements occurred in OA receiving placebo or YA receiving GlyNAC. These findings suggest that: (1) In OA, a single carbohydrate-meal significantly increases OxS and inflammation; (2) GlyNAC supplementation protects from carbohydrate meal-driven increases in OxS and inflammation. Additional research to investigate the implications of supplementing GlyNAC on improving meal-related health in aging are warranted.

### BIOPHYTIS BIO101 IN SARCOPENIA: UPDATE ON THE SARA PROGRAM: FROM SARA-INT TOWARDS THE PHASE 3 STUDY

Cendrine Tourette,[1] Waly Dioh,[2] Carole Margalef,[2] Jean Mariani,[2] Sam Agus,[3] Rob Van Maanen,[2] and Stanislas Veillet[2], *1. Biophytis SA, Paris, Ile-de-France, France, 2. Biophytis, Paris, Ile-de-France, France, 3. Biophytis Inc., Cambridge, Massachusetts, United States*

Sarcopenia is a progressive muscle disorder increasing with age that may lead to mobility disability. SARA program strives to develop a viable option to treat community dwelling older adults suffering from sarcopenia. SARA-INT is a randomized three-arm interventional study (BIO101 175 mg bid / BIO101 350 mg bid / placebo) with treatment duration of 6 months. Eligibility criteria for sarcopenia were meeting FNIH criteria and Short Physical Performance Battery (SPPB) score ≤ 8/12 in men and women aged ≥ 65 years; primary endpoint was the 400-meter walking test (400MWT). 233 participants aged 65 years and older were randomized, 232 and 156 participants were included in the Full Analysis Set (FAS) and Per-Protocol (PP) populations, respectively. Due to COVID-19 pandemic, most end-of-treatment efficacy assessments are missing for 55% of the participants, reducing the studies' power. BIO101 350 mg bid treatment led to an improvement in the primary endpoint, the gait speed from the 400MWT of 0.07 m/s in the FAS population (not statistically significant) and of 0.09 m/s in the PP population (nominally statistically significant, p=0.008) after 6 months; this is close to MCID in sarcopenia (0.1 m/s). BIO101 350mg bid treatment effect on the 400MWT is confirmed in PP sub-populations at high risk of mobility disability. Trends were observed with other endpoints. BIO101 showed a very good safety profile at both doses. Biophytis will initiate the phase 3 program by end 2022, targeting a severe sarcopenic population. Outcomes of the interactions with regulatory agencies on study design will be presented.

### PATTERNS OF MULTIMORBIDITY IN AGING, RADIATION-EXPOSED NON-HUMAN PRIMATES

Ellen Quillen,[1] Maggie Stainback,[1] Jamie Justice,[2] John Olson,[1] George Schaaf,[1] and J. Mark Cline[1], *1. Wake Forest School of Medicine, Winston Salem, North Carolina, United States, 2. Wake Forest School of Medicine, Winston-Salem, North Carolina, United States*

Resilience to stressors is a major component of biological aging and may mediate the onset of multimorbidity in older adults. The Wake Forest Non-Human Primate Radiation Survivor Cohort (RSC) provides a novel opportunity to study aging and resilience in 250 rhesus macaques (Macaca mulatta) with single-dose radiation exposures 0–15 years prior and 50 controls with semi-annual clinical, imaging, and biomarker measurements taken over their lifespan. Multimorbidity is extremely common among irradiated animals. Only 38% of animals have none of 20 common chronic diseases, falling to 16% of animals over age 8 and 11% over age 10 (middle-aged animals). 70% of animals have 5 or more diagnoses in this oldest cohort. The presence of any one disease increases the likelihood of having a second, co-morbid condition. Nevertheless, some animals continue disease-free until late in life, highlighting substantial variability in resilience. To identify patterns of multimorbidity, survival curves for each diagnosis were generated for age and time since radiation and k-median clustered resulting in four groupings of aging-associated morbidities. Bone, brain, and gastrointestinal disorders arise 3.5 years after radiation on average, followed by skin, heart, and cataracts. At 4.65 years, animals are at increased risk of being underweight and overweight and developing diabetes, hypertension, and hepatic dysfunction. Tumor, lung, and kidney disorders arise approximately 6 years after exposure. In all cases, these age-related disorders occur significantly earlier in irradiated animals than controls. These findings highlight the clustering of multimorbidities in aging, radiation-challenged primates and the potential of the RSC in studying resilience.

### EVALUATING INSTRUMENTS FOR ASSESSING HEALTHSPAN: A MULTI-CENTER CROSS-SECTIONAL STUDY IN THE COMPANION DOG

Frances Chen,[1] Tarini Ullal,[2] Jessica Graves,[3] Ellen Ratcliff,[3] Alexander Naka,[3] Brennen McKenzie,[3] Tennery Carttar,[3] and Michael LaCroix-Fralish[4], *1. Loyal, part of Cellular Longevity Inc, San Francisco, California, United States, 2. University of CA, Davis, Davis, California, United States, 3. Cellular Longevity Inc, San Francisco, California, United States, 4. Marist College, Poughkeepsie, New York, United States*

The companion dog is an emerging model in translational geroscience and offers novel opportunities to investigate aging biology and potential gerotherapeutics. However, there is a scarcity of validated tools and clinical outcome measures to characterize and understand the impact of aging in this translational species. Here we report on a multi-center, cross sectional veterinary clinical study, where we evaluated a clinical questionnaire (Canine Frailty Index; CFI; Banzato et al., 2019) to assess frailty and an owner assessment tool (VetMetrica HRQL) to evaluate HRQL in 451 adult companion dogs. Results support evidence of validity for the tools by confirming expectations that frailty and HRQL deteriorate with age. CFI scores were significantly higher (higher frailty) and HRQL scores significantly lower (worse HRQL) in old dogs (≥ 7 years of age) compared to young dogs (≥ 2 and < 6 years of age). Body size (small < 25lbs or large > 50lbs) was not associated with CFI or total HRQL score. However, older, larger dogs showed faster age-related decline in HRQL scores specific to owner-reported activity and comfort. Findings suggest that the clinician-assessed CFI and owner-reported VetMetrica HRQL may be useful tools to evaluate two determinants of healthspan in dogs: the accumulation of frailty and the progressive decline in quality of life. Establishing validated tools that operationalize the assessment of canine healthspan is critical for the linking pathophysiological mechanisms to aging phenotypes in the companion dog and for accelerating the development of gerotherapeutics that benefit both human and veterinary medicine.

### SEX DIFFERENCES IN LIFESPAN RESPONDING TO EARLY AND LATE ONSET DIETARY RESTRICTION

Nisi Jiang, Vivian Diaz, Randy Strong, and James Nelson, *UT Health San Antonio, San Antonio, Texas, United States*

Dietary restriction (DR) extends lifespan in many animal models. In mice, a range of factors affects the magnitude of the pro-longevity effect of DR, including the strain, restriction level, sex, and age of treatment onset. Early onset DR dramatically prolongs lifespan, whereas DR started later in life has diminished or no significant effect. The lifespan responses of males and females to DR also differ. Whether there is a sex difference in the lifespan response to the age of onset of DR has not been examined. Probing the basis for the variable response to DR can help elucidate the underlying mechanisms that mediate lifespan extension. We diet restricted UM-HET3 mice of both sexes from either 5 months (early-onset) or 20 months (late-onset) of age and monitored their survival. Early onset DR significantly prolonged lifespan in both males and females, which increased median lifespans by 54% and 18.5%, respectively. By contrast, although late onset DR prolonged lifespans in both sexes, the magnitude of increase in males (1.4%) was significantly lower than in females (15.8%, p=0.0474). Moreover, the decrease in the magnitude of the response to late-onset DR compared to that of early-onset DR is more dramatic in males than females. Further analysis found a significant interaction (p=0.0001) between early and late-onset DR in males but not in females (p=0.3818). Together, these results indicate that the lifespan response to delayed onset of DR diminishes more in males than in females, providing another model to probe the underlying mechanisms of DR.

### FOOD AND SUPPLEMENT COMBINATIONS TO PREVENT OR POSTPONE AGING, MCI, AND ALZHEIMER'S DISEASE.

Alec Pruchnicki,[1] Barrie Kavasch,[2] Barrie Sachs,[3] Roseanne Schnoll,[4] and Rolf Martin[3], *1. Vista on 5th, New York, New York, United States, 2. Blueberry Study MMT Corporation, Sherman, Connecticut, United States, 3. MMT Corporation, Sherman, Connecticut, United States, 4. Brooklyn College, CUNY, Sherman, Connecticut, United States*

Evidence of many interacting causes of aging has renewed interest in food and nutrient combinations that may blunt the main causes[PMC9184560, PMC8889622] and postpone aging, mild cognitive impairment and Alzheimer's ("A-MCI-Alz") in contrast to prior failures[PMC9232186]. We present an updated list of anti-aging/pro-longevity strategies to address limitations of previous food/nutrient combinations. Our list has two unusual features: (i) all interventions should be administered concurrently so no fundamental causes of A-MCI-Alz are without remediation and can inflame and leave smoldering important causal factors, (ii) all interventions should be provided daily or weekly to help avoid re-ignition of key factors driving A-MCI-Alz. These two features can help explain past longevity-trial failures[PMC9310407]: no previous study to our knowledge has included most or all of the following interventions, so untreated causes of A-MCI-Alz likely remained active[PMC9114803]. Our recommended interventions include intermittent-or-continuous calorie restriction or fasting[PMID: 33138875, PMC9351392, PMC7956384], protein- and/or methionine-restriction[PMC9197402, PMC4023024, PMC9197406], consumption of moderately-strong metal chelators (e.g. polyphenols in blueberries[PMC1413581], other berries[PMC8271923], grapes[PMC7096489, PMC6386230], cherries[PMC8745076], fruits with hesperidin/hesperitin [PMC9310407] like oranges, soybeans with genistein [PMC4286753], ginger [PMC5852742], ginseng [PMC9358063, PMC3830124, PMC6874434], green/rooibos/oolong teas[PMC6567241, PMIDs: 33285593, 19996359], coffee[PMC4926853, PMC9145055], chocolate[PMC9287426]), antioxidant-rich foods[PMC7530501], B vitamins including vitamin B5 (pantothenic acid[PMC9232186, PMC8725342, https://doi.org/10.3181%2F00379727-99-24442]) to help metabolize partly-toxic aldehydes[PMC6802361] and improve/lower lactate/pyruvate and NAD+/NADH ratios[PMC7346061], ingesting adequate-to-high amounts of longevity-correlated tyrosine[PMC6681387], cysteine[PMID: 20191258], glutathione[PMC8912885, PMC5005830], niacin, nicotine acid and/or NADH[PMC7346061, PMC6982340], spermidine[PMC8436989], anti-inflammatory NSAIDS[PMC4270464], autophagy inducers[PMC6191153] fiber[PMC8705837] and fluids[PMID: 21585170] to help remove excess metals, toxic metabolites and damaged organelles, while limiting methionine-rich, other pro-aging foods[PMC4023024].

### INVESTIGATING MALE INFERTILITY: EDITING GENES IMPORTANT FOR SPERMATOGENESIS IN A CELL CULTURE SYSTEM

Cassandra Norbeck, and Kellie Agrimson, *Saint Catherine University, Saint Paul, Minnesota, United States*

The four levels of aging include the specific disease, the systemic complications that lead to the disease, the loss of cellular integrity that affects the system, and macromolecule malfunction that alters cellular integrity. Similarly, this project investigates infertility and testicular cancer, misregulation of the endocrine system, problems with germ and Sertoli cell maintenance, and ultimately gene expression changes that alter cellular proliferation and death. Infertility affects 4.5–6% of North American males and up to 15% of couples worldwide. Infertility may be caused by unknown genetic factors, as up to 2,300 genes are pertinent to male fertility. This research project aims to create the molecular toolbox needed to evaluate gene function in cultured male germ cells. We will use mouse primary spermatogonial stem cells and a human testicular cancer cell line to knock out the functions of genes Phosphoprotein 1 (Spp1) and Inhibitor of DNA Binding 4 (Id4) in the testis to determine the effect of the knockouts on male germ cell proliferation and cell death. We designed single guide RNAs (gRNAs) using online bioinformatic tools and amplified the genes from human and mouse genomic DNA to demonstrate the effectiveness of our gRNAs in vitro. These gRNAs and Cas9 compose the beginnings of the molecular toolbox used to electroporate cultured cells. This research will contribute to the greater scientific community by providing insight into the function of Spp1 and Id4 relating to male fertility and providing a methodology for future research in aging-related fertility diseases and testicular cancer.

### P16-EXPRESSING SENESCENT CELLS ARE A DOUBLE-EDGED SWORD IN SHAPING IMMUNE RESPONSES WITH AGE

Blake Torrance, Hunter Panier, Andreia Cadar, Dominique Martin, Erica Lorenzo, Jenna Bartley, and Laura Haynes, *UConn Health, Farmington, Connecticut, United States*

Aging results in the accumulation of senescent cells which can cause dysfunction in many contexts but the effects on immune responses remain unclear. Here, we aimed to probe the effects of clearing senescent cells in aged mice on the immune response to influenza infection. We utilized a powerful p16 trimodality reporter mouse model (p16-3MR): under the control of the p16 promoter, these mice express cassettes encoding luciferase, RFP, and herpesvirus thymidine kinase (HSV-TK). p16 is commonly upregulated in senescent cells so this model allows us to selectively delete those cells by treating with ganciclovir (GCV), which will induce apoptosis in cells expressing HSV-TK. We hypothesized that while p16-expressing senescent cells may exacerbate dysfunctional responses to a primary infection, they may play a protective role in resolving inflammation and fostering memory cell generation. We found that deletion of p16-expressing cells enhanced viral clearance and decreased infiltration of pro-inflammatory flu-specific CD8 T cells during the primary response to infection. Conversely, at 30 days post infection, there were fewer flu-specific CD8 memory T cells and lower amounts of anti-viral antibodies in the lungs of GCV treated mice. We also observed perturbations in memory T cell trafficking in GCV treated mice. Furthermore, GCV treated mice were unable to mount an effective memory response and were unable to control viral load following a heterosubtypic challenge. This suggests that targeting senescent cells may potentiate primary responses while limiting the ability to form durable and protective immune memory with age.

### ARE WE GOING TO FEEL COMFORTABLE THERE?: EMERGENT FINDINGS FROM THE DIVERSITY IN LIFE PLAN COMMUNITIES STUDY

Mushira Khan,[1] Pankaja Desai,[2] and Catherine O'Brien[3], *1. Mather Institute, Evanston, Illinois, United States, 2. Rush University Medical Center, Chicago, Illinois, United States, 3. Mather, Evanston, Illinois, United States*

Life Plan Communities (LPCs), formerly known as Continuing Care Retirement Communities, offer an array of amenities as well as a continuum of care where residents may live independently or access assisted living or skilled care if needed. Racial/ethnic minority older adults are under-represented among LPC residents. One likely contributor to this lack of diversity is that racial/ethnic minorities make up a relatively small proportion of higher income groups. However, there may be additional reasons for their relative absence in most LPCs. The purpose of the 'Diversity in Life Plan Communities' study is to (1) identify barriers that impact the decision to move into an LPC for racial/ethnic minorities, and (2) identify strategies to increase resident diversity in LPCs. In this presentation we share preliminary findings from in-depth, semi-structured, qualitative interviews with community-dwelling Black, Latinx, and South Asian adults (n=10) living in Chicagoland and case study interviews with current LPC 'resident champions' (n=3) who are actively involved in efforts to increase resident diversity at their respective LPCs. Thematic analysis of the data showed that lack of awareness about LPCs, concerns about racism, desire to age-in-place among loved ones, and perceived non-availability of culturally-congruent activities were key barriers to moving into an LPC. These findings suggest that in order to increase resident diversity within their communities, LPC operators, staff, and current residents may consider targeted outreach efforts in racial/ethnic communities, take steps to create a welcoming environment and engage in more culturally congruent activities, and involve adult children/grandchildren in the decision-making process.

### SESSION 9020 (POSTER)

### LATE BREAKING POSTER SESSION III

### ADVANCE CARE PLANNING MOTIVATED BY THE COVID-19 PANDEMIC

Karuna Thomas,[1] Fursan Sahawneh,[1] and Brian Carpenter[2], *1. Washington University in St. Louis, St. Louis, Missouri, United States, 2. Washington University in St. Louis, Saint Louis, Missouri, United States*

The COVID-19 pandemic has exposed older adults to complex healthcare situations, via personal experience or media stories about serious illness. Hearing about lengthy intubation, sedation, rapid decline, and distress at the end of life has the potential to prompt people to reevaluate their perspective on their own end-of-life care. This study explored advance care planning (ACP) among older adults and whether COVID-19 experiences altered their healthcare preferences and planning. One hundred and fifty-one respondents (M age = 71.2 yrs, range = 55–93) completed an online survey about ACP completion, ACP conversations, and life-prolonging interventions. Respondents were mainly female (78%), White (71%), and well educated (77% with at least a bachelor's degree). A substantial proportion had not completed an advance directive (31%) or chosen a medical power of attorney (33%), and 78% of them intended to complete them in the next year or had initiated ACP. Among those who had completed ACP, a quarter intended to make changes, with 13% less open to life-prolonging medical treatments and 13% more open to them. Despite these intentions, a small proportion of people had ACP conversations since the start of the pandemic with their spouse/partner (37%), children (25%), siblings, (19%), friends (27%), primary care physician (16%), or other healthcare provider (12%). Results of this study suggest that now may be a critical moment to encourage older adults to have ACP conversations, in light of how their experience during the pandemic has motivated a reconsideration of treatment preferences.

### VIEWS OF OLDER ADULTS ON SUPPORT OPTIONS TO RELIEVE THEIR WORKING INFORMAL CAREGIVERS: A STORY COMPLETION STUDY

Eline Vos,[1] Simone de Bruin,[2] Suzanne Pees,[1] Allard van der Beek,[3] and Karin Proper[1], *1. National Institute for Public Health and the Environment, Bilthoven, Utrecht, Netherlands, 2. Windesheim University of Applied Sciences, Zwolle, Overijssel, Netherlands, 3. Amsterdam Public Health research institute, Amsterdam UMC, Amsterdam, Noord-Holland, Netherlands*

Older care recipients living at home increasingly rely on support from informal caregivers, who often combine their caregiving tasks with a paid job. Because of this combination of tasks, working caregivers may become overloaded. Different caregiver support options are available, that also affect older care recipients' lives, such as respite care, technology, or home care. The aim of this qualitative study was to obtain insight into the perceptions, concerns and preferences of older informal care recipients about the use of support to relieve their working informal caregivers. We performed a story completion writing task among 23 informal care recipients aged 65 years or older, using hypothetical situations to let participants reflect on different caregiver relief support options. The task was followed-up by a story-mediated interview. We used thematic analysis to inductively analyze story completions and interviews. We found that older adults were often willing to use support to relieve their caregivers, to ensure their wellbeing. However, they were also concerned about how the use of such support may conflict with their interests, preferences and values (e.g. respect for their autonomy, privacy, having a trusting relationship with caretakers, paying attention to human aspects in care). Older adults were most hesitant to use adult day care facilities and technological support options. While these areas of tension cannot always be completely resolved, it is important to jointly identify and discuss these, and work towards solutions to balance the respective interests, values and needs of older adults and caregivers.

### USING SOCIAL MEDIA TO UNDERSTAND FAMILY CAREGIVER PERSPECTIVES ON ASSISTIVE TECHNOLOGY FOR PERSONS WITH ALS

Yong K Choi and Salhah Alomairi, *University of California Davis School of Medicine, Sacramento, California, United States*

ALS Forum (alsforums.com) is an online support group for those who have motor neuron disease or amyotrophic lateral sclerosis (ALS). It provides information, advice, connection, care, and assistance to persons living with ALS and their family caregivers. This study aimed to analyze the online discussion posts by caregivers of persons with ALS and assess their perceptions, experiences, and challenges of using assistive technologies (AT). Posts were extracted from the ALS caregiver discussion forum via web scraping using Python in March 2022. The extracted posts are dated between June 2005 and January 2022. The total number of posts and replies generated during this period was 52,292 by 2,397 unique users. Additional searches were conducted using assistive technology-related keywords to create the text dataset. A total of 263 posts by 137 unique users were identified. After the initial review, 186 posts were included in the final analysis. Thematic analysis revealed six major themes: (1) Uses of AT (automation/control, communication, mobility); 2) Barriers and Facilitators (cost, effort to learn new technology, rejection, interoperability, customer service); 3) Usability (hardware and software-related); 4) Sharing Experience on AT; 5) Giving Advice; 6) Seeking Information on AT. The findings are consistent with previous literature that online health discussion forums provide access to rich data that contains lived experiences of patients and caregivers. The findings may inform the design of future assistive technology that incorporates the patients' and caregivers' perspectives to increase the adoption and long-term sustainability of the technology for people living with ALS and their caregivers.

### OLDER ADULTS INFLUENCING THE CIVIC ENGAGEMENT OF VOLUNTEER CAREGIVERS DURING THE COVID-19 PANDEMIC

Kathleen Gale, *Eras Senior Network Inc., Waukesha, Wisconsin, United States*

Older adults who do not qualify for government entitlement programs but lack sufficient financial or other personal resources help them to age in place rely on community organizations to assist them with social determinants of health. However, these organizations struggle with high rates of volunteer caregiver turnover, a looming crisis spotlighted during the COVID-19 pandemic. At least one group of volunteer caregivers continued to serve during this period. This phenomenological study investigated the experiences of eight volunteer caregivers who served older adults through a Faith in Action model volunteer driver program during the first year of the pandemic. Findings indicate that a volunteer's commitment to older adults, awareness of the needs of older adults, and established relationships with older adults were more important than concerns about COVID-19. Study participants were alert to the needs of older adults during and between service activities, making personal sacrifices of time, money, and physical exertion to accommodate needs. Participants approached service caring for the older adult and expected reciprocal care in the form of appreciation and respect. They considered discontinuing service if efforts weren't appreciated or if deeply held values were violated. The volunteer service organization was key in mitigating conflict within the relationship of the volunteer and older adult, thereby increasing the likelihood that the volunteer would continue serving. Implications include training older adult clients to meet expectations of care and appreciation for volunteers, managing volunteers whose personal sacrifices exceed the scope of volunteer service, and supporting volunteers whose values have been violated.

### ASSOCIATION OF RELIGIOUS ATTENDANCE WITH NEUROPSYCHIATRIC SYMPTOMS, COGNITION, AND SLEEP IN COGNITIVE IMPAIRMENT

Katherine Britt,[1] Kathy Richards,[2] Shelli Kesler,[2] Gayle Acton,[2] Jill Hamilton,[3] and Kavita Radhakrishnan[2], *1. University of Pennsylvania, Austin, Texas, United States, 2. University of Texas at Austin, The, Austin, Texas, United States, 3. Emory University, Atlanta, Georgia, United States*

Neuropsychiatric symptoms (NPS), cognitive decline, and sleep disturbances are common among older adults with cognitive impairment. Religious practices may protect mental and physical health, yet few studies have been reported in older adults with cognitive impairment. Utilizing the Health and Retirement Study in 2006 and 2008 and sub study, Aging, Demographics, and Memory study in 2006–2007 and 2008–2009, we examined the association of religious attendance with NPS, cognition, and sleep disturbances controlling for social interaction in older adults with cognitive impairment (N = 63). Bootstrapped Spearman's partial Rho correlation was conducted separately for time points one (T1) and two (T2); Wilcoxon signed-rank tests were used to examine significant change over time. Mean age was 81.89(5.26) years, 65.9% were non-Hispanic White, 50.1% were female, and mean cognition (Clinical Dementia Rating) was .94(.228). Significant changes over 1.5 years were found for sleep disturbances but not for NPS and cognition. Significant associations were found for religious attendance and NPS (T1: rs (97)= - .103, 95% CI [-.108, -.098], p < .0005 and T2: -.243, 95% CI [-.246,-.239], p < .0005), cognition, (T1: rs (97) = - .119, 95% CI [-.122, -.115], p < .0005, and T2: rs (97) = -.104, 95% CI [-.107,-.102], p < .0005), and sleep disturbances, (T1: rs (97) = .028, 95% CI [.023, .033], p < .001, and T2: rs (97) = -.051, 95% CI [-.056,-.047], p < .001). Increased religious attendance was associated with lower NPS and cognition at both time points and greater sleep disturbances at T1 but lower at T2. Longitudinal studies are needed to examine associations further.

### THE EFFECTS OF ENVIRONMENT-INDUCED RELAXATION ON COGNITIVE FORAGING UNDER STRESS IN MIDLIFE AND BEYOND

Jessie Chin, Shannon Mejía, Smit Desai, Faith-Christina Washington, Durga Ramesh, Falguni Deshpande, and Sanjana Dash, *University of Illinois at Urbana-Champaign, Champaign, Illinois, United States*

We defined enrichment seeking as the capacity for adults to engage in the novel and intellectually challenging activities for exploring diverse experience. Although enrichment seeking is associated with cognitive resilience in older adulthood, the tendency for older adults to adopt explorative behavior decreases with age in concert with decline in executive control. The goal of our study was to examine to what extent older adults can adjust their cognitive foraging performance under stress by inducing relaxation through the task environment. We used cognitive foraging games (including word search puzzle games) to understand how older adults optimize their search performance by the tradeoff between exploration (seeking new information) and exploitation (staying at old information). A modified Trier paradigm, where game performance was observed by a neurologist via video conferencing, induced stress; relaxation was manipulated by turning on an electric fireplace in the study room. The study followed a 2 (relaxation) X 2 (stress) within-subjects experimental design. Sixty-one adults (34 middle-age; 27 older adults; 54% women) played games on a tablet under stress / no stress conditions with and without the fireplace in a simulated smart home environment. The interaction effect of relaxation and stress on cognitive foraging performance was significant, indicating that with the relaxation elements in the environment (i.e., a fireplace), older adults are likely to have better cognitive foraging performance under stress. The association can be in part explained by individual differences in cognitive abilities and strategies. Implications on facilitating enrichment seeking through environmental factors are discussed.

### THE EFFECT OF DEPRESSION AND SUBJECTIVE HEALTH CONDITION ON INTRA-INDIVIDUAL COGNITIVE VARIABILITY

Shayne Lin, and Rebecca Allen, *University of Alabama, Tuscaloosa, Alabama, United States*

Intra-individual cognitive variability (IICV) is defined as variability in performance across cognitive domains during neuropsychological assessment. High cognitive variability (i.e., IICV) is associated with Alzheimer's disease diagnosis and mortality among older adults (Anderson et al., 2016; Watermeyer et al., 2021; Vaughan et al., 2013). To date, no research has explored, to the author's knowledge, the effect of depression and physical health on IICV among older adults. The current investigation used the nationally represented Health and Retirement Study (HRS) data set and explored the effect of depression, measured by Center for Epidemiological Studies Depression (CESD), and subjective health, rated on a 5-point Likert scale, on IICV. Data from 39,800 participants and 11 time points were analyzed. IICV was calculated by the individual t score standard deviation divided by the individual mean t score. Dependent variables were centered before being entered into a multilevel model that took time of data collection into consideration. With multilevel modelling, after controlling for demographics (i.e., gender, race, ethnicity, and age), both depression, b = 0.003, SE = 0.0001, p < .001, and subjective health condition, b = 0.006, SE = 0.0002, p < .001, were associated with IICV. This study has implications about the use of IICV in neuropsychological assessment with older adults. In addition to one's cognitive functioning, both depression and physical health have direct effect on IICV, which may be more than merely a cognitive marker for Alzheimer's disease as researchers have previously found.

### SOCIAL ENGAGEMENT AND SUBJECTIVE COGNITIVE FUNCTION AMONG OLDER ADULTS: RURAL-URBAN DIFFERENCES

Alisha Thompson, Scott Wilks, and Michelle Livermore, *Louisiana State University, Baton Rouge, Louisiana, United States*

Increasing age significantly relates with a decline in subjective cognitive function, while age and cognitive function decline strongly links with a diagnosis under the umbrella of Alzheimer's disease and related dementias (ADRD). Recent literature points to social engagement as a potential mitigator to cognitive function decline. Social engagement often differs by geography of residence, with typically higher engagement among those in urban areas compared to rural counterparts. The purpose of this study was twofold: (a) to explore the relationship between social engagement and subjective cognitive function among community-dwelling older adults; and (b) to examine any disparity in social engagement apropos to rural vs. urban residency within this population. The theoretical framework, combining activity theory and cognitive reserve theory, guided the understanding of the relationship between social engagement and subjective cognitive function. A functionalist lens was included to explain rural-urban differences. Secondary data of community-dwelling older adults from AARP's 2016 Social Engagement and Brain Health Survey (Nf746) were utilized. Results from descriptive analyses, correlations, and several regression models were reported. Bivariate regression models examined primary predictor variables – social engagement and geography of residence. Multivariate models examined sample characteristics, engagement, and geography of residence. Neither social engagement nor rural vs. urban residence achieved statistical significance in the models. Physical health, emotional wellbeing, marital status (identifying as married), and ethnicity (identifying as Black or African American) were positively, significantly associated with subjective cognitive function. Implications for community-dwelling older adults are discussed.

### RELIABILITY AND VALIDITY OF A REDUCED SET OF NAVIGATION ITEMS IN COMMUNITY-DWELLING OLDER ADULTS

Michael Prevratil, Dorota Kossowska-Kuhn, Nicholas Gray, and Neil Charness, *Florida State University, Tallahassee, Florida, United States*

Navigation is a complex skill that is used in everyday living, whether it be to travel across a country or to travel to a local store. How one successfully navigates through their environment involves many different processes, including spatial navigation, route generation, and orientation. An issue with investigating those separate constructs within navigation is the number of questions required to assess them reliably. As a part of a larger project, a large sample of community-dwelling older adults (ages 60–90) completed an online survey answering questions related to navigation. Among those were three subscales: a general wayfinding subscale, a subscale asking how often they felt lost when moving around near and far spaces, and a subscale asking how often they needed help navigating around near and far spaces. Each of these subscales contained fewer than eight items. The goal of the analysis was to determine the reliability and validity of these subscales, and this was accomplished through calculating Cronbach's α and an exploratory factor analysis (EFA). Cronbach's α for each of the individual subscales were above 0.8, indicating high reliability. EFA results output five unique factors. Noticeably, the wayfinding subscale was broken into two factors, one for route generation ability and one for mental mapping ability. while the "Feeling Lost" and "Needing Help" subscales produced a dichotomy between nearer distances (ex. Your immediate neighborhood) and farther distances (ex. Your state). This contrast, along with its implications, are discussed further.

### RACE AND DETERMINANTS OF HEALTH MODERATE ASSOCIATIONS BETWEEN COGNITIVE DECLINE AND DRIVING MOBILITY OVER 10 YEARS

Katie Wheeler,[1] Caitlin Pope,[2] Tyler Bell,[3] Brooke Carroll,[1] Lesley Ross,[4] and Karlene Ball[1], *1. University of Alabama at Birmingham, Birmingham, Alabama, United States, 2. University of Kentucky, Lexington, Kentucky, United States, 3. University of California, San Diego, La Jolla, California, United States, 4. Clemson University, Clemson, South Carolina, United States*

The ability to drive a vehicle is an everyday function that helps older adults maintain independence. Few observational studies have examined the relationship between cognitive decline and driving mobility and in context of racial differences and social determinants of health (SDOH). To address this empirical gap, this study aimed to characterize how cognitive functioning is longitudinally associated with driving mobility (driving space, driving exposure, and driving difficulty) in older age, and how it may vary by race and SDOH. Using the control arm of the Advanced Cognitive Training in Vital Elderly study (n=581, 24.5% Black), multilevel models examined longitudinal associations between processing speed, visual attention, memory, and reasoning with driving mobility outcomes. Race and SDOH moderations were explored. Only declines in reasoning and processing speed related to driving mobility, moderated by race and SDOH. Reasoning decline related to increased driving space in White ($\beta$=-.21,p=.006) but not Black older adults (p=.286). Processing speed decline related to greater driving exposure in Black older adults ($\beta$=-.15,$p < .001$) but less driving exposure in White older adults ($\beta$=.13,p=.006). Processing speed decline related to reduced driving exposure ($\beta$=-.06,p=.001) and increased driving difficulty ($\beta$=-.35,$p < .001$), but only in people living in poorer neighborhood and built environment and poorer social community contexts, respectively. Overall, findings emphasize that relationships between cognitive decline and driving mobility are dependent on race and SDOH. Consideration of such factors may help target those in greatest need to sustain safe driving mobility and functional independence.

### PHENOME-WIDE SEARCH OF COGNITIVE RESERVE PROXIES ACROSS AGE COHORTS

Anqing Zheng,[1] Sally Wadsworth,[2] Naomi Friedman,[3] and Chandra Reynolds[4], *1. University of California, Riverside, riverside, California, United States, 2. University of Colorado Boulder, Boulder, Colorado, United States, 3. University of Colorado, Boulder, Boulder, Colorado, United*

*States, 4. University of California, Riverside, Riverside, California, United States*

Cognitive reserve (CR) refers to adaptability allowing for better cognitive outcomes given the degree of brain changes or other risk factors for cognitive decline. Despite significant research efforts, our knowledge of cognitive reserve proxies remains limited. Studies predominantly use a single sociodemographic variable (e.g., education attainment) as a proxy measure when CR can manifest in multiple domains. Studies also tend to rely on older samples, whereas adversity factors of cognitive performance may have differing onset ages, suggesting different risk or protective mechanisms at different ages. We examine two cohort datasets spanning from early adolescence (Adolescent Brain Cognitive Development study, N = 5559) up to the cusp of mid-adulthood (Colorado Adoption/Twin Study of Lifespan behavioral development and cognitive aging; CATSLife, N = 1327) to evaluate the role of CR proxies across over 100 variables. Defined as a moderator between a cognitive outcome and structural brain measure, these variables cover behavioral, environmental, physical and mental health, and psychological trait domains. Using cross-validated regularized regression, we identified dozens of factors acting as CR proxies (e.g., leisure reading as protective and screen usage as a risk factor during adolescence). We then use a within-family design to examine the moderation effect over and above genetic and environmental covariates. For example, adolescents who read more tend to show better cognitive performance given their gray matter volume (b = .093, 95%CI = [0.035, 0.152]). This study aims to identify factors that could be targeted with scalable prevention and intervention efforts earlier in life to maximize cognitive functioning.

### NEW EVIDENCE OF HEALTHIER AGING. POSITIVE COHORT EFFECT ON COGNITIVE DECLINE.

Fernando Massa,[1] Alejandra Marroig,[2] Graciela Muniz Terrera,[3] and Scott Hofer[4], *1. Universidad de la República, Montevideo, Montevideo, Uruguay, 2. Universidad de la Republica Uruguay, Montevideo, Montevideo, Uruguay, 3. Ohio University, Athens, Ohio, United States, 4. University of Victoria, Victoria, British Columbia, Canada*

Cross sectional studies have shown cohort effects in cognition, limited research exists about cohort effects on cognitive trajectories. Indeed, most longitudinal research conducted to study aging-related cognitive change focus on the association between risk factors and mean change in cognition, considering individual differences too, but longitudinal norms of cognitive function are less studied. In this study, we aim to test whether cohort effects exist across the distribution of verbal fluency trajectories, that is, whether cohort effects vary across different trajectory quantiles. With this purpose, we estimated norms using data from 9 waves of the English Longitudinal Study of Aging (ELSA). We considered the individuals born in the 1920s, 1930s, and 1940s to assess cohort effects. The methodological framework consisted of quantile mixed models where the effect of age was adjusted using splines. To test for possible cohort effects across the 5th, 50th and 90th quartiles, the coefficients associated with the splines varied among cohorts. Our results suggest that cognitive decline is less pronounced for individuals born in more recent decades ($p < 0.001$), supporting our hypothesis of cohort effects. Moreover, these results are consistent across quantiles (p-value < 0.001). Additionally, we found that quantiles of verbal fluency at a certain age is higher in participants from more recent cohorts compared to those in older cohorts. Our findings contribute to a better understanding of cognitive decline in older adults, demonstrating population changes over time at different levels of changes in verbal fluency.

### EDUCATION AND COGNITIVE ABILITY: EXPLORING DIRECT AND INDIRECT EFFECTS IN THE UNITED STATES AND MEXICO

Joseph Saenz, and Amina Khan, *University of Southern California, Los Angeles, California, United States*

Education positively relates with cognition, which may be explained by enhanced cognitive reserve. However, education may also impact cognition indirectly by improving health, health behaviors, and life-course socioeconomic status (SES). This analysis explores the associations between education and cognition in the US and Mexico and quantifies the extent to which associations are direct versus indirect through health and SES. We use data from two studies: the MexCog in Mexico (n=2,042) and US Harmonized Cognitive Assessment Protocol (HCAP, n=3,267). Cognitive domains included Memory, Executive Function, Language, Visuospatial, and Orientation. Karlson-Holm-Breen (KHB) methods were used in linear regression models to quantify how much of the associations between years of education and cognitive domains were direct versus indirect through chronic conditions, income, wealth, smoking, and exercise. In regression models, years of education related positively with all cognitive domains in both studies, even when controlling for health and SES. KHB mediation analyses suggested that most of the education-cognition association was direct. In MexCog, estimates of the percent of the education-cognition association that was indirect through health and SES ranged from 4.17% (Memory) to 5.15% (Executive Function). In HCAP, indirect effects ranged from 8.95% (Orientation) to 12.15% (Language). Education was associated with better cognitive abilities in the US and Mexico regardless of cognitive domain or adjustment for late-life health and SES. Results suggested that education primarily related with cognition directly and that effects of education on cognitive abilities are not eliminated by reducing educational disparities in the late-life health and SES factors we analyzed.

### COGNITIVE INTERFERENCE ACROSS THE LIFESPAN

Rachel Cole, Arturo Espinoza, Brooke Yeager, and Nandakumar Narayanan, *University of Iowa, Iowa City, Iowa, United States*

Mental processes that facilitate goal-directed behavior can be negatively affected by age-related biological and physiological changes. Such cognitive changes impact daily activities like driving and using the computer, which can in turn influence social relationships and capacity to work. Previous research has shown that older adults are more affected by interference than young adults. It is critical to understand how cognition changes across the adult lifespan, as cognition during middle age may be predictive of cognitive decline in older age. We evaluated performance on the MultiSource Interference Task (MSIT). The MSIT draws on Stroop, Flanker, and Simon-type tasks; it taxes interference resolution and typically results in interference-related slowing. We studied 60 individuals (32 adults 18–39 years old, 17 adults 40–59 years old, and 11 adults 60–99 years old). As expected, age had a significant effect on accuracy interference (difference in errors between interference and control trials). Surprisingly, however, young adults demonstrated the highest (worst) accuracy interference cost, while middle-aged and older adults had similar interference cost. Replicating previous findings, age group did not have an effect on reaction time interference cost (slowing of response between interference and control trials). It is notable that, in this study, older adults did not fare worse than middle-aged adults. These surprising findings challenge long-held theories that age negatively impacts interference resolution. Understanding differences (and similarities) between cognition in middle-age and older age will be critical for promoting healthy cognition throughout the lifespan, which benefits daily activities and quality of life.

### CHARACTERIZING PERFORMANCE VARIABILITY ACROSS COGNITIVE TASKS IN OLDER ADULTS WITH AND WITHOUT ADHD

Laura M. Klepacz,[1] Eric Cerino,[1] and Almar A. L. Kok[2], *1. Northern Arizona University, Flagstaff, Arizona, United States, 2. Amsterdam University Medical Center, Amsterdam, Noord-Holland, Netherlands*

Most research on cognitive performance among individuals with Attention Deficit/Hyperactivity Disorder (ADHD) focuses on younger persons and on cognitive variability within speeded response-time tasks. Dispersion, i.e., variability across a range of cognitive domains, is emerging as a promising indicator of age-related and pathological cognitive impairment. However, there has yet to be an evaluation of differences in dispersion among older adults with and without ADHD. We address this gap by assessing associations of age and ADHD status with dispersion. We hypothesize older adults will exhibit greater dispersion than comparatively younger adults and explore whether individuals with ADHD exhibit greater dispersion than individuals without ADHD. In a sample of 231 adults from the Longitudinal Aging Study Amsterdam (Average age=71.64 years, SD=7.7, 59% female), 23 individuals met DSM-IV criteria for ADHD and 208 were classified as neurotypically functioning. Participants completed 13 tasks spanning domains of attention, fluency, memory, processing speed, and reasoning. Dispersion across the tasks was calculated as an intraindividual standard deviation. We regressed dispersion on age and ADHD status and adjusted for sex. Older age was significantly associated with greater dispersion (Est =0.06, SE=0.03, p=0.01). However, dispersion profiles did not vary as a function of ADHD status (Est.=-0.25, SE=0.67, p>05). Preliminary results suggest that dispersion across cognitive tasks may not be a sensitive marker of ADHD in older adults, although statistical power to detect differences was relatively low in the current study. As expected, age was a significant predictor of increased dispersion, consistent with accounts of age-related changes in neurological integrity.

### ASSOCIATION OF COGNITIVE STATUS AND CONSUMPTION OF UNPROCESSED AND ULTRA-PROCESSED FOODS IN BRAZILIAN OLDER ADULTS

Graziele Silva,[1] Carol Freiria,[2] Tábatta Brito,[3] Flávia Arbex Silva Borim,[2] and Ligiana P. Corona[4], *1. UNICAMP, Limeira, Sao Paulo, Brazil, 2. Universidade Estadual de Campinas, Campinas, Sao Paulo, Brazil, 3. Universidade Federal de Alfenas, Alfenas, Sao Paulo, Brazil, 4. Universidade Estadual de Campinas/ Faculdade de Ciências Aplicadas, Limeira, Sao Paulo, Brazil*

The decrease in consumption of fresh or minimally processed foods and the increase in ultra-processed foods are being observed in the diet of older adults and these changes may lead to worsening health status and cognition. We aimed to evaluate the association between cognitive status and food consumption according to the level of processing in Brazilian older adults. Cross-sectional study, with a sample of 585 older adults (≥60 years). Cognition was evaluated using the Cognitive Skills Screening Instrument (CASI-S), considering cognitive deficit when scores < 23 in participants aged 60–69 and < 20 in those aged ≥70 years. Foods reported in 24-hour food recall were classified according to their processing level into four groups of NOVA proposal: 1) unprocessed/minimally processed foods, 2) culinary ingredients, 3) processed foods (products made only from groups 1 and 2); and 4) ultra-processed foods. We estimated the means of total CASI-S score and its four domains according to the quartiles of intake of each food group, and evaluated the association between cognitive decline and each food group intake using logistic models adjusted for gender, age, schooling. Individuals in the highest quartile of unprocessed/minimally processed foods intake had higher scores in temporal orientation (p=0.034), verbal fluency (p=0.002), and total CASI-S score (p=0.004). The scores did differ according to the intake of the other food groups. The ultra-processed was the only group associated with cognitive deficit (OR:1.02; p=0.002). Results suggest nutritional counselling for older adults should focus in reducing ultra-processed and increasing unprocessed foods to help preventing cognitive deficit.

### ASSOCIATION OF COGNITION AND SMARTPHONE SURVEY ATTRIBUTES IN THE ELECTRONIC FRAMINGHAM HEART STUDY

Chathurangi Pathiravasan,[1] Alexa Beiser,[1] Sudha Seshadri,[2] Claudia Satizabal,[3] Yuankai Zhang,[1] Xuzhi Wang,[4] Chunyu Liu,[1] and Joanne Murabito[5], *1. Boston University School of Public Health, Boston, Massachusetts, United States, 2. University of Texas Health Sciences Center, San Antonio, Texas, United States, 3. Glenn Biggs Institute for Alzheimer's & Neurodegenerative Diseases, University of Texas Health Sciences Center, San Antonio, Texas, United States, 4. Department of Biostatistics, Boston University School of Public Health, Boston, Massachusetts, United States, 5. Boston University, Boston, Massachusetts, United States*

Mobile technology offers a remote method to monitor health in older adults and it may provide a platform for early detection of cognitive decline. We aimed to examine attributes of smartphone survey use in the electronic Framingham Heart Study (eFHS) cohort in relation to cognitive testing performed at the time of enrollment. eFHS participants who returned smartphone surveys and underwent cognitive testing were considered in the study (n=1810). The CERAD recall score, Victoria Stroop test interference score, and dichotomous AD8 and MOCA (MOCA score ≤ 19, AD8 score ≥2) were considered as primary exposures. App-based survey adherence was defined as a dichotomous outcome based on whether at least one survey was completed at each 3-month period from baseline to 12 months. Several time attributes were considered including survey return time, touch time, step time, and question completion time. Linear mixed models (LMM for time attributes outcomes and generalized LMM for adherence outcome) were fitted for each cognitive score as the predictor adjusting for age, sex, race/ethnicity, and education level. Results suggest that higher CERAD recall scores were associated with higher odds of completing surveys. There was a significant association between all cognitive exposures and survey time attributes. Participants with poorer cognitive function (lower CERAD, higher stroop interference, MOCA score ≤ 19, AD8 score ≥2) had delayed survey return times, higher touch time, higher step time and higher question completion time. This study contributes to the growing body of evidence that smartphones may be an important tool to identify cognitive decline.

### REIMAGINING AGING: EXAMINING THE IMPACT OF LIFETIME DISCRIMINATION EXPERIENCES ON EVERYDAY METAMEMORY

Timothy Ly, Jeanne Cundiff, Jason DeCaro, and Rebecca Allen, *The University of Alabama, Tuscaloosa, Alabama, United States*

Lifetime and recent experiences of discrimination (based on race, gender, religion, sexual orientation, etc.) contribute to impaired performance on cognitive assessments. However, the underlying mechanism by which discrimination negatively impacts cognition is unclear. Recent research investigating stress-induced impairment of metamemory may address the relationship between discrimination experiences and cognitive impairment. The aim of this study was to determine the relationship between lifetime experiences of discrimination, especially recent experiences, and everyday metamemory from a lifespan perspective (ages 20–75), using data collected from the Midlife in the United States (MIDUS Refresher 1) Daily Diary Project (N = 782). Results from a 2-level multilevel model showed that the relationship between recent experiences of discrimination explained unique variance in impaired metamemory accuracy (25%; $\beta$ = .377, $\sigma$ = .052, 95% CI [.275, .479]), suggesting that individuals with recent discrimination experiences reported more cognitive complaints. Furthermore, the relationship between age, recent experiences of discrimination, and impaired metamemory showed that younger individuals reported more complaints after experiencing discrimination than older individuals ($\beta$ = .192, $\sigma$ = .092, 95% CI [.011, .373]. Individual differences accounted for 45% of the variance in the number of cognitive complaints. These findings demonstrate the need for more research into understanding metamemory accuracy as an underlying mechanism by which the psychosocial stressor of discrimination impacts cognition across the lifespan. Moreover, understanding the experiences of diverse aging populations, including experiences of discrimination, and their impact on cognition will inform research on interventions to promote positive cognitive health outcomes across the lifespan.

### PROLONGED NIGHTLY FASTING AMONG OLDER ADULTS: A PILOT STUDY EXPLORING CHANGES IN COGNITIVE FUNCTION

Dara James,[1] Dorothy Sears,[2] Linda Larkey,[2] Molly Maxfield,[2] Edward Ofori,[2] Seung Yong Han,[2] Kate Alperin,[2] and Peyton Osha[2], *1. University of South Alabama, Mobile, Alabama, United States, 2. Arizona State University, Phoenix, Arizona, United States*

Aging is significantly associated with cognitive decline. A growing number of US adults ages ≥ 65 years have neurocognitive impairment resulting in compromised immediate and/or long-term health outcomes. Interventions to mitigate cognitive decline and promote healthy aging are needed. Research in intermittent fasting (IF) suggests positive health outcomes related to improvements in circadian rhythm and metabolism, which influence cognition. IF regimens may therefore result in improved neurocognitive health. We conducted an IF single-group, pre/post pilot study to explore changes in neurocognitive. Older adults (≥65 years of age; Nf18) with self-reported memory decline engaged in an 8-week, remotely-delivered, prolonged nightly fasting (PNF) intervention (14-hour nightly fasting, 10-hour daytime eating window). Our primary exploratory outcome was 8-week change in neurocognitive function assessed via composite score of the Memory and Attention Phone Screener (MAPS). Trends in outcome change were assessed with paired t-tests. Participants were mean age 69.7 years, non-Hispanic White, predominantly female (94%), married (50%), and employed (65%). Completion defined as percentage of participants that completed the intervention from those that started the intervention; completion rate was 90%. Paired t-test indicated a significant increase in scores on a neurocognitive screen (MAPS) pre/post-intervention (p=0.02) with a medium effect size (Cohen's d=0.58). Findings suggest that PNF, a type of IF regimen targeted to align food intake with circadian rhythms, may significantly improve neurocognitive function among older adults with self-reported memory decline. These promising pilot results suggest a need for fully-powered, randomized controlled trials to test the efficacy of this non-pharmacological, low cost-to-burden ratio intervention.

### PHYSICAL ACTIVITY TOGETHER FOR COUPLES WITH MILD COGNITIVE IMPAIRMENT: A FEASIBILITY STUDY

Sangwoo Ahn,[1] Sandy Cobb,[1] Scott Crouter,[1] Chung Eun Lee,[2] and Joel Anderson[1], *1. University of Tennessee at Knoxville, Knoxville, Tennessee, United States, 2. Baruch College, New York, New York, United States*

Spouses often assume the role of informal caregivers for older adults living with mild cognitive impairment (MCI). These couples represent a population with low levels of physical activity, however, there is limited research regarding promotion of physical activity among these dyads. The purpose of this study was to evaluate the feasibility of a dyadic intervention, hypothesized to enhance physical activity via mutual support. We recruited three dyads (mean age: 66 [MCI] and 67 [spouse] years) from a local memory clinic. The dyads were asked to engage in physical activity together for 16 weeks. The weekly intervention consisted of 150 minutes of moderate-to-vigorous physical activity (MVPA) on their own and two lower body strength training sessions per week, one videoconferencing-supervised and one on their own. The adherence rate to the weekly MVPA and strength training was 88% and 83%, respectively. However, the percentage meeting the goal of 150 minutes/week of MVPA as measured using a Fitbit was 31% and 73% among those with MCI and spouses, respectively. There were no issues with the retention rate (100%) or safety; satisfaction scores were high (MCI = 93%, spouses = 98%). Exit interviews revealed three key themes: (1) value of the program (accountability, togetherness), (2) difficulties (differences in physical capacity between spouses), and (3) suggestions to improve the program (incorporating various types of strength training). Our dyadic intervention was feasible to support regular physical activity for older adults living with MCI and their spouses. Future work will incorporate participants' feedback to optimize the intervention.

### EVERYDAY FUNCTION PROFILES IN PRODROMAL STAGES OF MOTORIC COGNITIVE RISK SYNDROME: A PROSPECTIVE COHORT STUDY

Nigel Kravatz, Dristi Adhikari, Emmeline Ayers, and Joe Verghese, *Albert Einstein College of Medicine, Bronx, New York, United States*

Assessment of everyday function is crucial to diagnosing pre-dementia and dementia syndromes in older individuals. Prodromal stages of mild cognitive impairment (MCI) are associated with mild limitations in complex activities of daily living. It is unknown whether such limitations exist in the prodromal period before criteria is met for motoric cognitive risk syndrome (MCR), a pre-dementia syndrome defined by subjective cognitive complaints and slow gait speed. We compared complex everyday functional profiles at baseline in 46 community-dwelling older individuals (aged 65+) with normal cognitive performance at baseline who went on to develop incident MCR ('pre-MCR') with 265 older individuals who remained cognitively normal over follow-up. Patients diagnosed with MCI and global cognitive function scores more than 1.5 standard deviations below the mean at baseline were excluded. The mean number of limitations on complex everyday function at baseline was 3.52±3.1 in the pre-MCR cases and 1.78±2.2 in the 265 normal controls (OR 1.28; 95% CI 1.14–1.43). After adjusting for demographic factors, comorbidities, and follow-up time, the overall number of limitations remained significantly higher in the pre-MCR participants compared to the normal participants (OR 1.18; 95% CI 1.02–1.37). Of the limitations, difficulty completing hobbies and handling finances were associated with pre-MCR after adjustments. Limitations on complex everyday functions in individuals occur before they meet criteria for incident MCR on longitudinal follow-up. Such limitations over time will help clinicians identify individuals at-risk for dementia early in clinical practice.

### DEPRESSIVE SYMPTOMS AND SUBJECTIVE MEMORY AMONG MIDDLE-AGED AND OLDER MIDWESTERN COUPLES

Lisa Thrane,[1] Dan Russell,[1] Rachael Turner,[2] Peter Rendell,[3] Jyoti Savla,[4] Celinda Reese-Melancon,[2] and Jennifer Margrett[1], *1. Iowa State University, Ames, Iowa, United States, 2. Oklahoma State University, Stillwater, Oklahoma, United States, 3. Australian Catholic University, Fitzroy, Victoria, Australia, 4. Virginia Tech, Blacksburg, Virginia, United States*

Older adults face unique challenges with the COVID-19 pandemic and its effects on mental health and cognition (Lebrasseur et al., 2021). Across the age spectrum, depression is associated with self-reported forgetfulness (Loprinzi, 2018; Ronnlund et al., 2011), and this trend is consistent among older adults with mild cognitive impairment (Ryu et al., 2016). There is some suggestion of gender differences in prospective and retrospective memory ratings among couples (Smith et al., 2000). However, little is known about how depression relates to perceptions of everyday memory performance among couples. In the current study, 47 married or cohabiting couples completed a baseline survey. The mean age of both women and men was 62 years. An Actor-Partner Interdependence Model with latent variables was employed to examine depressive symptoms with an adapted version of the Center for Epidemiological Studies-Depression scale (CES-D; Radloff, 1977) and responses to the Prospective and Retrospective Memory Questionnaire (PRMQ; Smith et al., 2000). Both constructs were parceled separately by gender and summed prior to the analysis. For women, preliminary findings revealed that higher levels of depressive symptoms were related to a negative perception of their own memory ($\beta = .28$). Conversely, men's memory abilities were not associated with their depressive symptoms. Lastly, higher levels of depressive symptoms in men were associated with an increase in women's self-reported forgetfulness ($\beta = .24$). These findings are suggestive of a "couple-oriented" effect (Kenny et. al., 2006), whereas there was not a statistically significant relationship between women's depressive symptomology and men's cognitive functioning.

### COGNITIVE-FOCUSED INTERVENTIONS FOR ADULTS WITH DIABETES: AN INTEGRATIVE REVIEW

Bohyun Kim, and Jie Hu, *The Ohio State University, Columbus, Ohio, United States*

Adults with diabetes and impaired memory and executive functions are more likely to experience difficulties in diabetes self-management resulting in poor glycemic control. Cognitive-focused interventions are widely accepted as an effective way to improve cognitive function in individuals at high risk of mild to severe cognitive dysfunction. This review synthesizes the effects of cognitive-focused interventions on cognitive ability, diabetes self-management, and glycemic control in adults with diabetes. A systematic review of randomized controlled/clinical trials studies published in English between 2012–2022 was conducted using the Preferred Reporting Items for Systematic Reviews and Meta-Analysis guideline. PubMed, CINAHL, Embase, and Web of science were searched. Cochrane Collaboration's Risk of Bias tool was used to evaluate the bias and quality of the studies. Nine studies met the inclusion criteria. Cognitive ability and diabetes self-management were assessed using different measurements and glycemic control was measured with A1C. Seven studies applied cognitive training, one provided working memory training, and one used occupational therapy. Seven studies combined cognitive training with a co-intervention including self-efficacy, lifestyle management, physical training, chronic disease self-management program, square-stepping exercise, psychoeducational intervention, and empowerment. Two studies provided a cognitive-focused intervention only. All nine studies showed statistically significant improvements in at least one outcome variable. Cognitive-focused interventions have a positive effect on improving memory and executive function. However, the evidence of cognitive-focused interventions on self-management and glycemic control has not been established. Future studies to improve cognitive using effective strategies to improve cognitive function enhancing diabetes self-management behaviors and glycemic control are warranted.

### COGNITIVE FUNCTION AND LONELINESS AMONG OLDER ADULTS: DYNAMICS OF SOCIAL NETWORK AND PERCEIVED SOCIAL SUPPORT

Soobin Park,[1] Sojung Park,[2] BoRin Kim,[3] Jihye Baek,[4] and Takashi Amano[5], *1. Washington University in St. Louis, Saint Louis, Missouri, United States, 2. Washington University in Saint Louis, Saint Louis, Missouri, United States, 3. University of New Hampshire, Durham, New Hampshire, United States, 4. Washington University in St. Louis, St. Louis, Missouri, United States, 5. Rutgers University-Newark, Newark, New Jersey, United States*

As cognitive reserves diminish with age, older adults become aware of age-related losses and allocate their resources accordingly. Drawing on the selective optimization with compensation and socioemotional selectivity theory, older adults may select emotionally close social relationships to manage emotional distress caused by deterioration in cognitive functions. This study investigates whether different levels of cognitive functions [no impairment (NI), cognitive impairment no dementia (CIND), and dementia] influence the degree of loneliness among older adults, and how heterogeneous patterns of social relations affect this association. We used data from the 2018 Health and Retirement study, which included 4,500 respondents over 51 years of age who completed a modified Telephone Interview for Cognitive Status. Using comprehensive social-relational indicators for the structure and quality of four relational sources (spouse or partner, children, family, and friends), we applied latent class analysis and identified five subgroups of social relations: restricted–limited support (Nf190, 4.22%), family and friends focused–ambivalent (Nf1,252, 27.82%), diverse–positive (Nf2,203, 48.96%), negative (Nf500, 11.11%), and spouse focused–ambivalent (Nf355, 7.89%). Hierarchical regression showed that those in the CIND ($p < .032$) and dementia group ($p < .037$) were more likely to experience loneliness than those in the NI group, and those with dementia who were in the category of spouse focused–ambivalent support ($p < .001$) had relatively lower levels of loneliness. Reliance on spousal support may be due to people selecting and adapting increasingly on closer relations to counter their vulnerability and feel less lonely. Interventions to sustain this core relationship should be encouraged, including spousal-caregiving relief programs.

### COMFORT COMMUNICATION IN HOME HEALTH: FEASIBILITY, ACCEPTABILITY, AND PRELIMINARY EFFECTIVENESS

Sharon Bigger,[1] Noah Zanville,[2] Elaine Wittenberg,[3] Gail Towsley,[4] and Lee Glenn[1], *1. East Tennessee State University, Johnson City, Tennessee, United States, 2. HCA Healthcare, North Carolina Division, Asheville, North Carolina, United States, 3. California State University Los Angeles, Los Angeles, California, United States, 4. University of Utah, Salt Lake City, Utah, United States*

Home health is the fastest-growing long-term care setting in the U. S.. However, evidence on effective clinician-patient-family communication in home health is lacking. This prospective, two-arm, pre-post randomized controlled trial aimed to assess feasibility, acceptability, and preliminary effectiveness of the COMFORT (Connect, Options, Meaning-making, Family Caregiver, Openings, Relating, and Team) communication model in home health interprofessional staff (IHHS). IHHS ($n = 18$) were randomized into two groups: Group 1 (control) ($n=10$) received seven asynchronous modules, and Group 2 (intervention) ($n = 8$) received the same modules plus a 2-hour synchronous class with interactive slide presentation and exercises. Measures included completion rates, acceptability ratings, comfort with communication in palliative and end-of-life care (C-COPE), and moral distress in health professionals (MMD-HP). Regardless of group, COMFORT was highly acceptable (>4) to IHHS. COMFORT was positively correlated with improved C-COPE scores ($p = 0.037$). Moral distress scores did not differ before and after the intervention; however, baseline moral distress scores were found to be higher in IHHS when compared to an academic medical center sample from a previous study. Levels of acceptability of COMFORT were significantly related to clinician levels of considering leaving a job due to moral distress (chi square = 7.6, $p = 0.02$, Kruskal-Wallis rank sum test). Findings suggest that COMFORT training increases IHHS comfort with palliative and end-of-life communication, especially among clinicians with histories of considering leaving a job or having left a job due to moral distress.

### ADDRESSING SOCIAL ISOLATION IN NURSING HOMES DURING COVID-19: A NATIONAL SURVEY

Natalia Nielsen,[1] J. Lee Hargraves,[2] Carol Cosenza,[3] Emily Lim,[3] Adrita Barooah,[3] Emily McPhillips,[4] Catherine Dube,[4] and Kate Lapane[5], *1. University of Massachusetts Medical School, Worcester, Massachusetts, United States, 2. University of Massachusetts - Boston, Boston, Massachusetts, United States, 3. University of Massachusetts Boston, Boston, Massachusetts, United States, 4. UMass Chan Medical School, Worcester, Massachusetts, United States, 5. University of Massachusetts Chan Medical School, Worcester, Massachusetts, United States*

COVID-19 related policies introduced extraordinary social disruption in nursing homes. In response to the unprecedented COVID-19 pandemic, congregated long term care living facilities attempted and/or implemented innovative intervention strategies to alleviate loneliness in residents. We surveyed Directors of Nursing/Administrators of 1,669 homes sampled in strata defined by size (number of beds 30–99, 100+) and quality ratings (1, 2–4, 5) between February-May 2022. The response rate was 30%. Almost 2/3rds of respondents completed it online and the rest via paper. Analyses included nonresponse survey weights to provide nationally representative results. Among a list of 17 situations that occurred, staff shortages was identified as extremely stressful by the majority. Staff were extremely stressed about doing more to meet resident needs and keeping up with rapidly changing regulations which often lacked clinical sense. One third of respondents were extremely concerned about their home's ability to meet residents' social needs before vaccines, dropping to 13% after vaccines. Nursing homes tried and perceived as most useful using technology (tablets, phones, emails), assigning staff as a family contact, and staff spending more time with residents. Nearly 60% were extremely concerned about staff burnout/mental health before vaccines and 40% remained extremely concerned after vaccines. Many nursing homes attempted to mitigate the harmful effects of social isolation during the pandemic, despite the stressful circumstances in which staff worked. The extent to which various approaches were implemented varied. While concerns about social isolation reduced after vaccines were available, administrators remain extremely concerned about staff burnout and mental health.

### ADDRESSING SOCIAL ISOLATION IN NURSING HOMES DURING COVID-19 – QUALITATIVE DATA FROM A NATIONAL SURVEY

Emily McPhillips,[1] Catherine Dube,[1] Natalia Nielsen,[2] J. Lee Hargraves,[3] Carol Cosenza,[4] Emily Lim,[4] Adrita Barooah,[4] and Kate Lapane[5], *1. UMass Chan Medical School, Worcester, Massachusetts, United States, 2. University of Massachusetts Medical School, Worcester, Massachusetts, United States, 3. University of Massachusetts - Boston, Boston, Massachusetts, United States, 4. University of Massachusetts Boston, Boston, Massachusetts, United States, 5. University of Massachusetts Chan Medical School, Worcester, Massachusetts, United States*

The COVID-19 pandemic had dramatic, sometimes devastating impacts on nursing homes, residents, and staff. Rapid deployment of innovative approaches to resident care was required even while under sustained distress. We collected textual responses to open-ended questions about COVID-19 experiences through a national nursing home survey of Directors of Nursing/Administrators in February-May 2022. We employed a stratified (by size and quality ratings) sample of 1,669 nursing homes. Response rate was 30%, and 51% of responders answered > 1 open-ended question. We conducted an iterative thematic qualitative analysis yielding 10 themes. Respondents described addressing social isolation using new technology; enlisting staff from across the nursing home [beyond-the-call effort, gifting of voluntary time], and new ways for residents to safely connect with family. Respondents felt severely limited by COVID regulations that seemed to ignore residents' mental health needs. The majority of respondents felt significant professional and personal impact of the pandemic experience: "The pandemic was the most stressful situation I have encountered in 26 years of nursing" – "What a toll it took on all us emotionally, physically, and mentality" – "Every day was a challenge and I felt hopeless" – Some respondents plan to quit: "I am now seeking other employment. It has been too much for too long and has directly affected my mental health." Nursing homes reported extraordinary efforts put forth by administration and staff to meet the needs of residents. Efforts to retain nursing staff are needed given profound impacts of the pandemic on their personal and professional lives.

### WHEN THE LIGHTS WENT OUT AT ADULT DAY CENTERS: CAREGIVER WELL-BEING DURING THE COVID-19 LOCKDOWNS

Rachael Turner,[1] Celinda Reese-Melancon,[1] Erin Harrington,[2] and Micaela Andreo[3], *1. Oklahoma State University, Stillwater, Oklahoma, United States, 2. Pennsylvania State University, University Park, Pennsylvania, United States, 3. University of Texas at Dallas, Dallas, Texas, United States*

Adult Day Centers (ADC) are community-based programs that provide services to adults with dementia or other disabilities. Unfortunately, many ADCs were closed during the early stages of the COVID-19 pandemic, leaving caregivers without an important resource. The current study sought to identify novel challenges that caregivers experienced with the closure of ADCs. From May to July 2020, caregivers were recruited from ADCs across the United States and completed an online survey regarding ADC use, feelings of caregiver burden, perceived stress, and perceived change in these variables and others (i.e., time spent caregiving, caregiver and care recipient memory) resulting from the pandemic. Qualitative reports of additional pandemic-related caregiving experiences were also captured. The current work included 70 caregivers, most of whom were caring for a family member with dementia. Correlational analyses indicated that more frequent ADC use prior to the pandemic was associated with reporting an increase in the amount of time spent on caregiving tasks, an increase in caregiver burden, and increased feelings of stress since the onset of the pandemic. Prior use was not related to change in perceptions of caregiver forgetfulness or changes in care recipient memory behaviors. Prominent themes from caregivers' qualitative reports included mental health concerns, apprehension regarding the virus itself, and the need for respite. These findings support theories of caregiver stress (Pearlin et al., 1990) and provide insight into how caregivers were impacted by the loss of a key resource to support their caregiving.

### THE RISK OF COVID-19-RELATED MYOCARDITIS IN OLDER ADULTS WITH A HISTORY OF CORONARY ARTERY DISEASE

Andrew Crawford,[1] Ali Vaeli Zadeh,[2] Andres Redondo,[3] Karen Bravo-Ramirez,[4] Ana De Diego Diaz,[5] Ricardo Criado Carrero,[6] Elias Collado,[2] and Joshua Larned[7], *1. Holy Cross Health-University of Miami, Fort Lauderdale, Florida, United States, 2. Holy Cross Health-Jim Moran Cardiovascular Research Institute, Fort Lauderdale, Florida, United States, 3. University of Miami, Fort Lauderdale, Florida, United States, 4. University of Miami at Holy Cross, Miami, Florida, United States, 5. University of Miami/Holy Cross Hospital, Fort Luderdale, Florida, United States, 6. University of Miami/Holy Cross Hospital, Fort Lauderdale, Florida, United States, 7. Holy Cross Health- University of Miami, Miami, Florida, United States*

Background: Data suggest an increased incidence of myocarditis (MC) associated with the COVID-19 virus. However, the risk factors for COVID-19-related MC remains poorly understood and debated. Therefore, we sought to evaluate the correlation of a history of coronary artery disease (CAD) with MC in older adults admitted for COVID-19. Methods: Data were obtained from the PearlDiver database (PearlDiver Technologies, Fort Wayne, IN). The study included patients aged 65–75, hospitalized with a primary diagnosis of COVID-19, and Elixhauser Comorbidity index(ECI) >4. History of CAD upon admission was used to split the cohort into two propensity score-matched groups considering age, gender, other cardiovascular diseases, and ECI. Records from both groups were reviewed to identify patients diagnosed with MC during and up to one month after admission. Pearson's chi-squared test was used to compare groups. The strength of association was reported using Risk Ratios (RR). A p-value < 0.05 was deemed significant. Results: 182,556 patients with and 218,729 without a history of CAD admitted for COVID-19 were identified. Patients with a history of CAD were more likely to be male(54.7% vs. 42% p < 0.0001), older(mean age 70.62 vs. 70.30, p < 0.001), and had more comorbidities(ECI=11 vs. 8, p < 0.0001). After propensity score matching, 0.13% of patients with CAD and 0.12% without CAD developed MC within one month of admission(RR= 1.05, CI95%=0.87–1.26, p=0.61). Conclusion: One month following admission for COVID-19, the risk of MC was not significantly higher in older persons with a history of CAD.

### THE COVID-19 PANDEMICS' IMPACTS ON OLDER ADULTS' MENTAL HEALTH: THE BUFFERING EFFECTS OF NEIGHBORHOOD COHESION

Kedong Ding, *Case Western Reserve University, Cleveland, Ohio, United States*

Background: During the COVID-19 pandemic, many older adults reported less frequency of having in-person contact due to the worries of COVID -19, which raises concerns about their mental health. The past research suggests neighborhood cohesion can facilitate social participation and have positive effects on mental health. This study aims to examine the impact of the decreased social contacts due to COVID on older adults' depressive symptoms, and whether the relationship is moderated by neighborhood cohesion. Method: Data were from the 2020 waves of the Health and Retirement Study (HRS) (n=2,732). Participants included community-dwelling adults aged 65 years or older. The independent variable is the perceived changing amount of in-person contact due to the pandemic. Neighborhood cohesion was measured by four-item sales that assess the extent to which respondents feel about the neighborhoods and neighbors' trust. The depressive symptoms were measured by the 8-item CES-D scale. All models were controlled for sociodemographic characteristics and health status. Multivariate regression models were used to and an interaction term was created to examine the moderating effects of neighborhood cohesion. Results: The results from the multivariate regression model show that perceived neighborhood cohesion was associated with fewer depressive symptoms ($\beta$=-.16, p < 0.001). Not having enough in-person contact since the pandemic is associated with more depressive symptoms ($\beta$=.24, p < 0.001). High perceived neighborhood cohesion attenuates the relationship between not having enough in-person contact and depressive symptoms. Conclusion: This study demonstrates the buffering effects of neighborhood cohesion on Covid-19 impacts on older adults' in-person contact and mental health outcomes.

### TELEMEDICINE TRENDS IN THREE NURSING HOMES DURING THE COVID-19 PANDEMIC

Lindsay Sandell,[1] Tena Baar,[2] Raza Haque,[2] and Angela Zell[2], *1. Michigan State University College of Human Medicine, Grand Rapids, Michigan, United States, 2. Michigan State University, East Lansing, Michigan, United States*

Physical distancing and visiting restrictions during the COVID-19 pandemic posed significant challenges in providing timely medical care in nursing homes. The rising number of new COVID-19 cases created hardship in providing scheduled and non-urgent care visits. Virtual visits were pivotal in providing patient care. However, the additional responsibility of facilitating virtual visits for both social and clinical purposes further constrained the ability of nursing home staff to provide care. The critical deficiency of the workforce, rising numbers of new cases, and required triage of patients have been significant barriers to regularly scheduled care. We hypothesize that regular non-COVID-19 services in the evaluated nursing home facilities were lower than COVID-19 visits during this period. Our goal is to show the types of services affected during the pandemic. In this study, we analyzed 563 virtual visits during the COVID-19 pandemic in three nursing home facilities in Michigan from December 2020 through February 2022. Upon analyzing the types of services and trends, our results revealed that the number of COVID-19 related visits (68) was significantly lower than non-COVID-19 related visits (485), refuting our hypothesis. This illustrates that routine care could still be delivered during the pandemic. Additionally, the overall number of virtual visits declined steadily over the study period. This trend could suggest an increase of in-person services or a decrease in COVID-19 cases. The decline could also be related to the barriers faced by the nursing home workforce considering the time and additional responsibility of monitoring a virtual visit.

### RESILIENCE AMONG RESIDENTS, THEIR FAMILY, AND STAFF OF A CONTINUING CARE RETIREMENT COMMUNITY

Justine Sefcik, Yeji Hwang, Zachary Hathaway, Martha Coates, Shelby Hufnal, Isabella Stoll, Rose Ann DiMaria-Ghalili, and Kathleen Fisher, *Drexel University, Philadelphia, Pennsylvania, United States*

There is emerging literature about older adults' experience of loneliness during the COVID-19 pandemic in long term care (LTC) facilities due to isolation protocols. Additionally, staff challenges while providing care for older adults in LTC has also been highlighted. While the literature emphasizes negative pandemic experiences, a gap exists with understanding resilience during the pandemic in LTC settings. The aim of this qualitative descriptive study was to explore the experience of resilience in a Continuing Care Retirement Community (CCRC) among residents, their family members, and staff. We conducted 19 in-person interviews and 1 via

Zoom in fall 2021 with 5 residents (65 and older), 5 family members, and 10 staff (e.g., administrators, nurses, nursing assistants). A conventional content analysis was employed. While we did hear how the pandemic had a negative impact on everyone, the main themes of resilience identified were: 1) overcoming the pandemic together (sense of community); 2) experience and adaptation (over time being able to adapt to the disruption in their life); 3) staying safe (engaging in precautions and self-care strategies); and 4) positivity (mindset of getting through anything and relying on spirituality). Study findings can inform CCRC administrators on how to support residents, their family, and staff during future pandemics and other challenging times that may disrupt normal routines.

### RACE AND AGE DIFFERENCES IN DEPRESSION AND ANXIETY DURING COVID-19: A LONGITUDINAL STUDY

Xiaochuan Wang, Mengyu Xu, Kim Gryglewicz, and Susanny Beltran, *University of Central Florida, Orlando, Florida, United States*

The COVID-19 pandemic has negatively affected the physical and mental health of many. It remains unclear whether such impacts differ across diverse races or age groups, or along the course of the pandemic. This study assessed trends in levels of depression and anxiety symptoms among U.S. adults during COVID-19 and whether differences emerged across race and age. Data were drawn from the Understanding America Study (UAS) COVID-19 survey, a longitudinal panel survey of a nationally representative sample of over 6,000 individuals. A mixed effect linear model was conducted to assess the influence of race and age on the level of depression and anxiety over time during the pandemic, controlling for covariates such as marital status, employment status, and household income. Results indicate that greater age was associated with lower levels of depression and anxiety. Additionally, trends in levels of depression and anxiety vary across races (e.g., minority populations generally reported lower or comparable levels of depression and anxiety comparing to the White). Findings further suggest significant interactions between age and race, especially among minoritized adults. Study findings underscore the importance of future research and tailored strategies to improve culturally sensitive and age-appropriate mental health services targeting diverse populations.

### PANDEMIC RECOVERY IN NURSING HOMES: IMPACT OF A 5-WEEK CURRICULUM ON RESILIENCE AND GROWTH IN DIRECT CARE WORKERS

Susan Wehry[1] and Prakash Patel[2], *1. University of New England, Biddeford, Maine, United States, 2. University of New England College of Osteopathic Medicine, Biddeford, Maine, United States*

The COVID-19 pandemic significantly impacted people working and living in nursing homes. A virtual five-week curriculum was developed to support recovery and foster growth in direct care workers (DCWs). We hypothesized DCWs would be interested, acquire knowledge and self-care skills, and utilize a post traumatic growth (PTG) self-assessment tool for reflection. The 5 week voluntary program was offered twice. Sessions consisted of a didactic presentation, Q&A, skill practice and action commitment. Each 30 minute session was offered twice weekly, a.m. and p.m., to accommodate shift schedules. Polls were conducted within sessions. Participants who attended all sessions received a certificate of attendance and completed a 4 question, 5-point Likert scale survey and the PTG tool. 53 DCWs participated in at least one session; 30 completed the program and ranked weeks 2 (95%), 3 (65%), and 4 (60%) as most useful. Most used skills were breathing (95%), gratitude practice (91%), catch-check-change (61%), self-massage (61%), and reducing exposure to toxic news (61%). DCWs endorsed self-improvement (96%), curiosity (91%), and “having a hard time” (61%) as most common reasons for enrolling. 100% reported the course completely or almost completely met their goals. In-session polling indicated attendees “felt better or more confident” at the conclusion of each session. 100% successfully utilized the PTG tool. The curriculum was responsive to DCWs needs and interests. DCWs acquired and utilized resilience skills. Some components of the curriculum were more effective than others. Shorter staff training sessions are more attractive to staff and employers. Further study is warranted.

### OPTIMIZING MEDICATION RECONCILIATION AMONG OLDER ADULTS DURING COVID-19 INFECTION TREATMENT WITH PAXLOVID

John Chen, and Tarah Browne, *Kaiser Permanente Med Group/Southern California, Harbor City, California, United States*

COVID-19 infection treatment (CIT) has reframed optimizing medication reconciliation in older adults. In December 2021, FDA authorized Emergency Use Authorization of Paxlovid (Nirmatrelvir/Ritonavir), protease inhibitor blocking SARS-CoV-2 replication, to reduce severe COVID-19 and mortality in high-risk patients1. Pharmacodynamic and pharmacokinetic changes in geriatric population warrant a stepwise, patient-centered approach for prescribing Paxlovid by reviewing - 1) drug-drug interactions with current medications2, 2) renal dosing, 3) risks-benefits-alternatives, 4) adverse drug events (ADE), i.e. polypharmacy and prescribing cascade. Case A – Community-dwelling 65-year-old Hispanic male history of paroxysmal ventricular tachycardia and end-stage renal disease (ESRD) requiring CIT- 1) Paxlovid contraindication with Amiodarone2 2) ESRD – not candidate 3) risks outweigh benefits, 4) safer alternatives with less ADE. Case B – Long term care 80-year-old Hispanic female history of stroke, type 2 diabetes mellitus and dementia requiring CIT -1) Paxlovid contraindication with Atorvastatin2 (Atorvastatin held during Paxlovid treatment then restarted at lower dose “deprescribing”), 2) estimated glomerular filtration rate 46 with renal dosing – Paxlovid 150mg/100mg, 3) benefits outweigh risks, 4) monitored ADE in controlled setting. Optimizing medication reconciliation during CIT in older patients can decrease preventable ADE. Further research is essential to align CIT efficacy with the fundamental geriatric principles, including shared decision-making, long-term prognosis, and life care planning. 1Najjar-Debbiny R, Gronich N, Weber G, Khoury J, Amar M, Stein N, Goldstein LH, Saliba W. Effectiveness of Paxlovid in Reducing Severe COVID-19 and Mortality in High Risk Patients. Clin Infect Dis. 2022 Jun 2:ciac443.

2Lexicomp Drug Interaction Checker. Accessed August 6, 2022.

### LACKING FAMILY TIES DURING A GLOBAL PANDEMIC: OLDER ADULTS CONCEPTUALIZATIONS OF SOCIAL SUPPORT

Bryce Van Vleet, Emily Kinkade, Heather Fuller, and Andrea Huseth-Zosel, *North Dakota State University, Fargo, North Dakota, United States*

Social support from family provided benefits for coping during the COVID-19 pandemic; however, not all older adults had access to family. The present study investigates how older adults without access to traditional family ties conceptualized their social relationships throughout the first two years of the pandemic. A subsample of eight older adults without direct access to traditional family ties were identified from a larger 5-wave interview study conducted between April 2020 and June 2022. Transcripts were holistically coded and three overarching themes emerged: constraints of place, redefining family, and feelings of isolation and closeness. The first theme addressed having family members living far away and uncertainty of when they would get to see them again. However, these distance barriers could be overcome through technology. The second theme illuminated that during the pandemic, those without access to traditional family ties redefined their social relationships by developing fictive kin from neighbors, colleagues, and friends. The third theme highlighted that some older adults felt they were lacking strong social networks and were concerned they had nobody to contact if they needed help, while others felt that despite limitations, their social relationships grew closer due to connection through alternative forms of communication (e.g. texting). Results from this study clarify how traditional family ties were challenged and strengthened during physical distancing for some older adults. These findings extend the literature on how fictive kin forms in older adulthood during temporary crises and suggests potential avenues for social connection for older adults lacking traditional family support.

### INFLAMMATORY BIOMARKERS DIFFER AMONG HOSPITALIZED VETERANS INFECTED WITH COVID-19 VARIANTS

Catherine Park,[1] Benjamin Seligman,[2] Bret Hicken,[3] Shahriar Tavakoli-Tabasi,[4] Amir Sharafkhaneh,[4] Christopher Amos,[4] Andrew Chou,[4] and Javad Razjouyan[1], *1. Veterans Affairs, Houston, Texas, United States, 2. David Geffen School of Medicine at UCLA, Los Angeles, California, United States, 3. Veterans Affairs, Salt Lake City, Utah, United States, 4. Baylor College of Medicine, Houston, Texas, United States*

The SARS-CoV-2 infection pathogenesis reported a complex interplay between virus and host immune response. In this study, we hypothesized that Omicron variant causes less inflammation and cytokine release than Delta and Alpha variants as measured by the first laboratory biomarkers during hospitalization. This is a retrospective cohort study of veterans who tested positive for COVID-19 and were hospitalized at the Veterans Health Administration due to COVID-19 infection. We defined three groups of patients based on highly likely period representing variants: Alpha (12/01/2020-05/31/2021), Delta (09/01/2021-11/30/2021), and Omicron (02/01/2022-07/11/2022). The risk of abnormality levels in biomarkers (C-reactive protein [CRP], ferritin, albumin, alanine aminotransferase [ALT], aspartate transaminase [AST], and lactate), as well as in-hospital mortality, were calculated using logistic regression adjusted by age, sex, body-mass-index, race, Charlson comorbidity index, VA frailty index, and vaccine record status. Of 414,213 Veterans tested for COVID-19, 74,342 Veterans (age: 67.8±14.3 years, BMI: 29.4±7.1 kg/m2) met the criteria: Alpha, 18,159 (34.4%); Delta, 23,414 (44.4%); Omicron, 11,207 (21.2%). Compared with Omicron, we observed significantly higher odds of abnormality levels in CRP (Alpha, adjusted odds ratio (aOR)=1.38; Delta, aOR=1.74), ferritin (Alpha, aOR=2.02; Delta, aOR=2.33), albumin (Alpha, aOR=1.16; Delta, aOR=1.15), ALT (Alpha, aOR=1.13; Delta, aOR=1.12), AST (Alpha, aOR=1.31; Delta, aOR=1.57), and lactate (Alpha, aOR=1.62; Delta, aOR=2.27) as well as mortality (Alpha, aOR=2.19; Delta, aOR=2.95). Veterans infected with Omicron showed less severe biomarkers' responses compared to Alpha and Delta and lower mortality risk. Understanding the biomarkers' responses of each patient across the different variants could be used to enhance acute patients' management.

### IN THEIR VOICES: A QUALITATIVE STUDY OF OLDER ADULTS' PERSPECTIVES OF COVID-19 PANDEMIC

Chih-ling Liou, *Kent State University, North Canton, Ohio, United States*

Older adults are a very heterogeneous group and are likely to experience the pandemic in a variety of ways. This study is to give older adults voices to share their experiences of this pandemic. Data were collected in spring 2022 using semi-structured interviews with 46 adults (74% females) aged 66 and 97 residing in the Midwestern United States. Participants were asked to share how the pandemic affects them, their vision for the future, and how they cope during the pandemic. Participants expressed both positive and negative effects of pandemics. Although they shared more information and examples of the negative effects, most of them did express an optimistic view of the future. The most reported negative effects were few contacts with family and friends, isolation/loneliness, and confinement/restrictions. The most reported positive effects were having more time, getting things done, driving less, and becoming more appreciative. Their perspectives toward the COVID-19 pandemic are varied: some believed that the effects of the pandemic are just temporary, and others compared it to the wars that were much worse. Findings reveal that oldest-old (85+) and old-old (75–84) participants are better at regulating their negative effects compared to the young-old (65–74). Older adults during the pandemic are not just passive help receivers but can be proactive helpers to give advice and coping strategies as well as provide emotional support to others. Older adults' adaptability during the COVID-19 pandemic should be better understood to reverse the image of their vulnerabilities and promote late-life coping during crises.

### IMPACT OF COVID-19 PANDEMIC ON MENTAL HEALTH IN OLDER ADULTS: COMPARISON BETWEEN 2020 AND 2022

Dara Kiu Yi Leung,[1] Tianyin Liu,[1] Wen Zhang,[2] Wai-wai Kwok,[2] Lesley Sze,[2] Gloria H.Y. Wong,[2] and Terry Lum[2], *1. The University of Hong Kong, Hong Kong, Hong Kong, 2. The University of Hong Kong, Hong Kong, Hong Kong*

The COVID-19 pandemic had a major impact on older adults' mental health, but less is known about its longer-term effect. We examined changes in depressive and anxiety symptoms among older adults between the onset and two years into the pandemic. Data were drawn from two cross-sectional telephone surveys conducted with older adults aged ≥ 60 years in Hong Kong in 2020 and 2022. Respondents were screened for depression and anxiety using Patient Health Questionnaire-2 (PHQ-2) and General Anxiety Disorders-2 (GAD-2) and, if screened positive (i.e. scoring ≥ 3 in PHQ-2 or GAD-2), evaluated with PHQ-9 and GAD-7 for symptom severity. After case-control matching baseline age, gender, living districts, and pre-existing mental health conditions based on the respondent ratio between the two surveys (i.e. 2:1 ratio), 4095 and 2099 respondents from the 2020 and 2022 surveys were included in the analysis. Respondents' average baseline age was 75 years old, 77% were female, and 13% had a pre-existing mental health condition. There were significant increases in the proportion of older adults screened positive for depression (8.3% to 13.5%) and anxiety (6.9% to 11.4%) and a significant increase in depressive symptom severity (4.63 to 7.72) between 2020 and 2022 ($p < .001$). Logistic regression suggested that, over two years, older adults with pre-existing mental health conditions were 1.59 times more likely to screen positive for depression than those without such conditions. Linear regression suggested that males were associated with increased depressive (B=-2.42, p=.004) and anxiety (B=-2.49, p=.021) symptom severity than females over the years.

### HOW DOES THE COVID-19 PANDEMIC AFFECT LATE WORKING LIFE? EUROPEAN EMPLOYMENT TRAJECTORIES FROM 2011 TO 2021

Wiebke Schmitz, *Unversity of Vechta, Vechta, Niedersachsen, Germany*

Background: One of the immediate impacts of the pandemic was the increase in unemployment and the reduction of working hours - especially among women, those with lower job security and in countries with a weaker social welfare system. The aging society is at further risk, which is already challenged by a growing shortage of workers and the rising costs of pensions. To understand the long-term consequences of COVID-19 for the aging workforce, this paper advances existing research by exploring from a life-course perspective how employment after the COVID-19 outbreak is anchored in larger employment histories (2011–2021). Methods: Sequence-analyses are applied using retrospective life history data combined with both COVID-19 Surveys (2020–2021) from SHARE including respondents aged 50+. Using cluster-analyses, the resulting sequences are classified into six groups. By applying descriptive-analyses, I examine how these groups differ by gender, work quality, and country. Results: Older workers – especially men – with continuous full-time employment histories are less frequently affected by unemployment and fewer working hours during COVID-19. Whereas those – especially women – with disruptive employment and part-time work histories are more frequently affected by unemployment after the outbreak of COVID-19. Respondents in countries characterized by social democratic welfare compared to liberal welfare regimes are less likely affected by unemployment during the pandemic. Discussion: The pandemic particularly puts older workers with disruptive employment histories at additional risk of labor market exit. Therefore policymakers need to address inequalities in earlier life to prevent long-term consequences of social inequality caused by the pandemic.

### HALF OF OLDER ADULTS HOSPITALIZED WITH COVID-19 EXPERIENCE SYMPTOMS ONE YEAR LATER

Aleda Leis,[1] Sydney Fine,[2] E.J. McSpadden,[1] Carrie Karvonen-Gutierriez,[1] Emily Nichols,[3] Lois Lamerato,[4] Anurag N. Malani,[5] and Emily Martin[2], *1. University of Michigan, Ann Arbor, Michigan, United States, 2. University of Michigan School of Public Health, Ann Arbor, Michigan, United States, 3. UM Michigan Center for Respiratory Research and Response, Ann Arbor, Michigan, United States, 4. Henry Ford Health, Detroit, Michigan, United States, 5. St. Joseph Mercy Hospital, Ypsilanti, Michigan, United States*

Older adults hospitalized with severe COVID-19 are at higher risk of experiencing serious in-hospital outcomes and long-term health consequences following discharge. Declines in health and functional ability post-hospitalization are important infection-related outcomes. This study's aim was to examine functional recovery one year following COVID-19 hospitalization. Twenty-one adults ≥60 years of age hospitalized with confirmed COVID-19 infection between 3/2020–5/2020 in Southeast Michigan completed a survey 9–15 months post-discharge including items from the Fried Frailty score, Short Form 36 Physical Assessment, PROMIS Dyspnea Scale, and the World Health Organization Disability Assessment Schedule. Mean age at hospital admission was 69 (standard deviation 7). Half of participants (52%) indicated they had too little energy to do the things they wanted to do, 52% (n=11) indicated moderate to severe shortness of breath when walking up two flights of stairs, and 43% (n=9) indicated they were limited a lot in walking several blocks. Additionally, 57% (n=12) indicated they were severely or extremely emotionally affected by their health due to their COVID-19 infection. Results were similar in only those ≥70 years (n=7). Our survey indicates that half of patients hospitalized with severe COVID-19 from the first infection wave in Southeast Michigan are significantly affected up to a year or more after their initial infection, and may benefit from long-term outpatient care. More research is needed to inform development of effective treatments for the long-term emotional and physical impacts of severe COVID-19.

### FINANCIAL HARDSHIPS AND MEDICATION ADHERENCE DURING THE COVID-19 PANDEMIC

Megan Lytle,[1] Dawn Carr,[2] and Patricia Homan[2], *1. Florida State University, Tallahassee, Florida, United States, 2. Florida State University, Tallahassee, Florida, United States*

Medication nonadherence is associated with numerous negative health outcomes among older adults, including myocardial infarction, stroke, preventable hospitalizations, and increased risk of decline in self-reported health status. Maintaining continuous use and access to needed medications in later life has important implications for quality and length of life. A primary barrier shown to interfere with medication adherence in older adults is an inability to pay for medication. Relative to their younger counterparts, older adults have more financial protections that increase access to needed prescription medication through health insurance coverage. Despite these added protections, older adults are more likely to experience financial insecurity, with some evidence suggesting that COVID-19 accentuated existing vulnerabilities. Data are derived from the Health and Retirement Study (HRS), leveraging data drawn from the 2016, 2018, and 2020 study waves (n=3,185). Logistic models were used to evaluate the association between five COVID-19 related financial setbacks (i.e., inability to pay mortgage/rent, credit card bills, utility or insurance bills, medical bills, and inadequate money for food), and medication nonadherence among adults 60+. Results show that net of pre-COVID financial vulnerabilities and socioeconomic status, individuals who reported being unable to pay medical bills and those unable to pay rent/mortgage after the start of the pandemic reported higher odds (19% higher and 230% higher odds, respectively) of not taking/filling their prescription medication due to cost. Results suggest that greater financial protections for housing and medical bills among financially vulnerable older adults will increase medication adherence.

### FINANCIAL HARDSHIPS AND DEPRESSIVE SYMPTOMS DURING COVID: THE MODERATING ROLE OF RESILIENCE

Brooke Helppie-McFall,[1] Dawn Carr,[2] Miles Taylor,[2] and Amanda Sonnega[1], *1. University of Michigan, Ann Arbor, Michigan, United States, 2. Florida State University, Tallahassee, Florida, United States*

Financial hardships during COVID (FHDC) are a particularly salient stressor that older people faced during the pandemic. Financial stress is associated with increased depressive symptoms and may be especially consequential to mental health among older people who have fewer resources (i.e., financially vulnerable). Recent evidence shows that psychological resilience has important protective effects for mental health among older adults who experience major stressors. This study, based on the recently released full 2020 core wave of the Health and Retirement Study, examines the association between having experienced one or more major financial setbacks following the start of the pandemic and depressive symptoms. We evaluate the consequences of FHDC for mental health, how FHDC are associated with previous financial vulnerability, and the role of psychological resilience in shaping the effects of FHDC. Results show that having experienced FHDC is associated with an increase in depressive symptoms. In addition, reporting financial vulnerabilities four years prior to the pandemic was also associated with increased depressive symptoms. Finally, psychological resilience was associated with a significant, protective effect on depressive symptoms, and moderated the consequences of FHDC. Specifically, we find that those who had FHDC and had average or below average resilience experienced significant increases in depressive symptoms, but those with above average resilience did not experience increases in depressive symptoms despite having FHDC, accounting for the consequences of previous financial vulnerabilities. These results suggest that psychological resilience has potential to be a protective resource for mental health consequences of financial stress among older adults.

### DIFFERENTIAL ROLES OF COVID-19-RELATED STRESSORS IN MENTAL HEALTH PROBLEMS: A NETWORK APPROACH

Wen Zhang,[1] Tianyin Liu,[2] Dara Kiu Yi Leung,[2] Wai-wai Kwok,[1] Lesley Sze,[1] Gloria H.Y. Wong,[1] and Terry Lum[1], *1. The University of Hong Kong, Hong Kong, Hong Kong, 2. The University of Hong Kong, Hong Kong, Hong Kong*

COVID-19-related stress is heterogeneous and associated with increased mental health conditions in older adults. This study is to investigate relationships between different stressors and how different stressors may increase risks for mental health conditions through a network approach. A telephone survey was conducted among 4,921 older adults (age≥60) from April to June 2022 during the biggest community outbreak of COVID-19 in Hong Kong. The validated 8-item COVID-19-Related Stress Scale (CSS-old) (Cronbach's α: 0.91) was used to investigate the different stressor for older people in Hong Hong. Respondents were screened for depression using the Patient Health Questionnaire-2 (PHQ-2), anxiety using the Generalized Anxiety Disorder 2-item (GAD-2), and stressors with the CSS-old, 4708 responded to all questions. A regularized partial correlation network via graphical LASSO procedure was computed to analyze the relationship between 8 stressors; a directed acyclic graph (DAG) via a Bayesian hill-climbing algorithm was generated from CSS-old and comorbidity network with PHQ-2 and GAD-2 items. Network analyses identified CSS-old item 6 (families or friends infected), item 3 (daily life interrupted), item 5 (fear of infection affecting the family), and item 8 (worry for community's health) as the core stressors. DAG analysis found a key triggering role for item 1 (suspension of community services), and the activation of the mental health problems occurred through item 1, which bridged the COVID-19-related stress and mental health problems. These findings suggested that providing support for families with COVID-19 patients and alternative services during community service suspension may reduce mental health problems risks.

### CULTURAL AND SOCIAL FACTORS IN CARE DELIVERY BY AFRICAN AMERICAN CAREGIVERS OF ADULTS WITH DEMENTIA AND COVID-19

Idorenyin Udoh, and Elias Mpofu, *University of North Texas, Denton, Texas, United States*

African American /Black caregivers make up 13% of the total number of adult caregivers in the United States and

41% more likely to provide help greater than three activities of daily living than other ethnicities. How they perceive socio-cultural factors to influence their care giver roles is less documented. This study aims to synthesize the evidence on the role of culture and social factors in African American caregiver perceptions delivering care to older adults with dementia and COVID-19. Searches of the Ageline, Medline, PsycInfo, Academic search complete Psychology and Behavioral science collection and Google scholar for empirical study publications on socio-cultural factors for dementia care and COVID-19 among African American caregivers yielded six studies. To be included, studies met the following criteria (a) focus on African American caregivers of older adults with dementia and focus on Covid-19 (b) socio-cultural factors (c) perceptions and practices. (d)published between 2019–2022. Studies indicate compassionate care practices by African American caregivers of persons with dementia and Covid-19. African American care givers of persons with dementia and COVID19 perceive caregiving as a responsibility they owe and not a job. They also perceived to be guided by their racial identity and faith beliefs, integrating family values and culture into caregiving. African American carers of persons living with dementia and COVID-19 have compassion and resilient care self-perceptions in caregiving to people with Dementia and COVID-19. Supporting compassionate care delivery by African American carers requires understanding social and cultural factors driving their commitment to quality care.

### COVID-19 WIDOWHOOD OR PANDEMIC WIDOWHOOD: EXAMINING THE DIFFERENTIAL IMPLICATIONS FOR MENTAL HEALTH

Haowei Wang,[1] Emily Smith-Greenaway,[2] Shawn Bauldry,[3] Rachel Margolis,[4] and Ashton Verdery[5], *1. The Pennsylvania State University, Quincy, Massachusetts, United States, 2. University of Southern California, Los Angeles, California, United States, 3. Purdue University, West Lafayette, Indiana, United States, 4. Western University, London, Ontario, Canada, 5. The Pennsylvania State University, State College, Pennsylvania, United States*

Millions of COVID-19 widows worldwide face elevated mental health risks that foreshadow worsening physical health and elevated mortality. It remains unknown whether the excess mental health problems for COVID-19 widows are a result of the "bad death" experiences from COVID-19 (e.g., unexpected death and high levels of medical intervention) or pandemic-induced social changes (e.g., social isolation and limited funerals). This study examines whether older adults whose spouses died of COVID-19 disease have worse mental health (self-reported depression, loneliness, and trouble sleeping) than those whose spouses died from causes other than COVID-19 before and during the pandemic. We used Survey of Health, Ageing and Retirement in Europe data collected before (Wave 8, fielded October 2019 to March 2020) and during the pandemic (COVID-19 Supplement-2, fielded June to August 2021) to compare three groups whose spouses died (a) before the pandemic, (b) from COVID-19 during the pandemic, and (c) from non-COVID-19 causes during the pandemic. We find those spouses died from COVID-19 have higher risks of self-reported depression, loneliness, and trouble sleeping than those losing a spouse before the pandemic. However, losing a spouse due to non-COVID-19 causes during the pandemic is not significantly associated with worse mental health compared to pre-pandemic scenarios. During the pandemic, older adults whose spouses died from COVID-19 report higher risks of loneliness than those spouses died from non-COVID-19 causes. This study suggests losing a spouse due to COVID-19 presents unique mental health risks for older adults, clarifying prior theories about mental health impacts of pandemic bereavement.

### COVID-19 VACCINATIONS AMONG COMMUNITY-DWELLING PERSONS LIVING WITH DEMENTIA AND THEIR CAREGIVERS

Brandy Renee McCann,[1] Jyoti Savla,[1] Karen Roberto,[2] and Rosemary Blieszner[1], *1. Virginia Tech, Blacksburg, Virginia, United States, 2. Virginia Polytechnic Institute & State University, Blacksburg, Virginia, United States*

There is a paucity of information regarding the uptake of the COVID-19 vaccine among community-dwelling persons living with dementia and their family caregivers. We examined factors influencing COVID-19 vaccine acceptance and hesitancy among community-dwelling persons living with dementia and their family caregivers during the early months of the vaccine rollout (February-March, 2021). Data came from three waves of telephone interviews (Mtime = 38.33 minutes) with 26 family caregivers living in rural Appalachian Virginia (96% White, 81% Female, Mage= 63±12 years, 42% Spouse Caregivers). We conducted a four-stage trajectory-based thematic content analysis and used the health belief model to interpret the data. Whereas all family caregivers and their relative living with dementia were eligible for the vaccine at Wave 3, only 10 dyads received it, while 10 dyads did not. In two families, the caregivers received the vaccine but not the person living with dementia; in four families, the caregiver had not received the vaccine, but their relative did. COVID vaccination acceptance was influenced by perceived direct and indirect health risks, cues from trusted allies, and ability to overcome vaccination barriers. Homebound people living with dementia faced unique barriers including transportation to vaccine sites and providers' inability to deliver the vaccine to the home. Caregivers who refused or delayed vaccinations for themselves or their relatives discussed confusion about eligibility, low perceived risk of COVID, vaccine fear, and personal choice beliefs. Findings inform direct care delivery for interdependent family members who live in remote locations facing a public health crisis.

### COVID-19 RELATED MEDICAL CARE DELAYS IN UNDERSERVED AFRICAN AMERICAN OLDER ADULTS WITH COMORBIDITIES

Edward Adinkrah, Sharon Cobb, Lucy Kibe, Roberto Vargas, and Mohsen Bazargan, *Charles R. Drew University of Medicine and Science, Los Angeles, California, United States*

Health seeking behaviors have been negatively impacted during the COVID-19 pandemic particularly among minority older adults. There is a paucity of data concerning African American older adults who delayed, reduced, or stopped visits to primary and specialty providers. To outline the magnitude of this health disparity, this study examines the patterns and correlates of seeking preventive and specialized healthcare within this population. One-hundred and fifty (150) underserved African Americans older adults took part in this health advisor-led cross-sectional study. They completed a survey that inquired about seeking medical, dental, emergency room, and/or specialty care. We employed descriptive analyses to assess patterns of healthcare and multinomial logistic regression to document correlates of healthcare utilization. Almost one-third of participants (32%) delayed or did not get at least one type of care because of COVID-19. Dental care was the most frequent healthcare service that was missed (23%), followed by primary care (10%) and specialty care (7%). A higher level of depressive symptoms, lower level of educational attainment, and higher number of chronic conditions were associated with a higher number of delayed or cancelled medical care. Almost one-third of participants had never used any type of telehealth. Additionally, access and use of telehealth had no impact on healthcare utilization. One-year post pandemic, while vaccines are widely available, COVID-19 is disproportionately worsening healthcare disparities that exist even among African American older adults in underserved and under-resourced communities with access to healthcare. Interventional studies are urgently needed.

### CLINICIANS' PERSPECTIVES ON THE EVOLUTION OF PALLIATIVE CARE DELIVERY DURING THE COVID-19 PANDEMIC

Laura Quintero Silva,[1] Andiara Schwingel,[2] Julie Bobitt,[3] and Minakshi Raj[4], *1. University of Illinois Urbana-Champaign, Champaign, Illinois, United States, 2. University of Illinois, Urbana Champaign, Urbana Champaign, Illinois, United States, 3. University of Illinois at Chicago, Chicago, Illinois, United States, 4. University of Illinois Urbana Champaign, Champaign, Illinois, United States*

Palliative care has been primarily delivered to patients in person since its inception. During the Covid-19 pandemic, providing palliative care was especially challenging for clinicians due to public health measures to contain the virus that required them to interact virtually with patients and families. While the rapid implementation of telehealth has been examined in other clinical contexts, limited research has studied the impact of the pandemic on palliative care delivery. This study examined the experiences clinicians faced when providing palliative care to older adult patients during the pandemic. Between April 2021 and March 2022, we interviewed 29 geriatricians and palliative care specialists from 11 institutions across the US. We asked clinicians about their experiences with palliative care during the pandemic, including challenges and opportunities related to the changing nature of palliative care delivery. We analyzed interviews using reflexive thematic analysis. The following three themes emerged from the analysis: (1) Clinicians' challenges adjusting to virtual care; (2) System-level barriers, restrictions, and uncertainties about Covid-19; and (3) Older adult patients' context and vulnerability (i.e., loss of social engagement, isolation, loneliness, delayed access to care) that increased the complexity of their health conditions. In conclusion, clinicians' experiences during the pandemic shed light on the evolution of palliative care delivery and the importance of preparing them for new care models that account for virtual delivery and that address the diverse needs of older adults that emerge during public health crises.

### CHANGES IN OLDER ADULTS' LONELINESS OVER TWO YEARS OF COVID-19: A MIXED-METHODS ANALYSIS

Emily Kinkade, Heather Fuller, Bryce Van Vleet, and Andrea Huseth-Zosel, *North Dakota State University, Fargo, North Dakota, United States*

Throughout the COVID-19 pandemic, social distancing and stay-at-home behaviors have been recommended to protect older adults from severe illness. However, despite disease reduction benefits, self-isolation may place older adults at an increased risk for loneliness, another serious public health concern. This longitudinal, mixed-methods study investigates whether older adults' perceptions of loneliness changed across two years of the pandemic and how older adults described their experiences with loneliness at different points of the pandemic. Between April 2020 and June 2022, five waves of interviews were conducted with 76 Midwestern older adults between the ages of 70 and 97. At each interview, participants completed the UCLA Three-Item Loneliness Scale and answered several open-ended questions about their current daily life, social connections, and experiences and perceptions during the pandemic. A repeated-measures ANOVA indicated that participants' loneliness scores significantly decreased between the first and final interview, although there was a nonsignificant increase in loneliness at the third interview. Thematic coding of the interview transcripts identified four emergent themes related to loneliness across two years of the pandemic: 1) differentiation of isolation from loneliness, 2) acceptance of circumstances, 3) adaptation to prevent loneliness, and 4) barriers to adaptation. Findings suggest older adults had nuanced and shifting experiences of loneliness throughout the pandemic, in which they generally showed increasing acceptance and strong adaptation to feelings of loneliness. However, adaptation was also challenged as the pandemic endured. Our discussion will highlight protective and risk factors for older adults' loneliness and directions for future study and practice.

### BEFORE & AFTER THE 2020 NATIONAL SHUTDOWNS: PANDEMIC EXPERIENCES OF OLDER AMERICAN ADULTS WITH & WITHOUT A DISABILITY

Fanni Farago, *AARP, Washington D.C., District of Columbia, United States*

Since the onset of the COVID-19 pandemic, there have been drastic changes to people's day-to-day lives. Following the 2020 U.S. national shutdown, day-to-day life included increased social isolation, COVID-19 anxieties, disruptions to employment, and limited access to staple goods for many. Compared with the general population, the impacts of these changes are much less understood for the aging population, and particularly for older adults with a disability or chronic disease, who are at greater risk of adverse effects from COVID-19 and related circumstances. Drawing on the breaking results of the AARP Vital Voices national survey

(n=2071), conducted throughout the pandemic (2019 to 2021), this poster presentation sheds light on the pandemic experiences of older American adults with and without disability or chronic disease. Interesting findings are discussed across age groups and by gender. Overall, the results show notable differences in the social connection experiences of older adults with a disability or chronic disease (n=571) pre-and-post the national shutdown, along with differences in the accessibility of household items, staple foods, and medical care in the wake of the 2020 U.S. national shutdown. The implications of these findings are discussed in the context of related literature.

### ASSOCIATIONS BETWEEN COVID-19 RELATED STRESS, COVID-19 EXPERIENCES, AND MENTAL HEALTH RISKS IN OLDER PEOPLE

Tianyin Liu,[1] Dara Kiu Yi Leung,[1] Wen Zhang,[2] Wai-wai Kwok,[2] Lesley Sze,[2] Edwin Wong,[2] Gloria H.Y. Wong,[2] and Terry Lum[2], *1. The University of Hong Kong, Hong Kong, Hong Kong, 2. The University of Hong Kong, Hong Kong, Hong Kong*

There is increasing recognition of the need to understand the mechanism of psychological impact brought by COVID-19. The present research used the Delphi technique to develop a COVID-19-Related Stress Scale for older people in Hong Kong (CSS-old) (study one) and examined its associations with COVID-19 experiences and mental health risks (study two). In study one, 17 helping professionals and 20 service users co-developed an 8-item CSS-old through four rounds of Delphi. In study two, a cross-sectional telephone survey was conducted between April and June 2022 among 4,921 older people (age≥60) recruited through community centres. Respondents were assessed using Patient Health Questionnaire-2 (PHQ-2), Generalized Anxiety Disorder 2-item (GAD-2), and CSS-old; their experiences with COVID-19 (infection, close friend/family infection) and demographical information were collected. A three-factor solution of CSS-old was identified after dropping one item (X2(df) = 83.53(11), CFI=0.996, TLI=0.993, RMSEA=0.037): (1) disruption to routines; (2) fear of infecting families/friends; and (3) concern for the community's health. Structural equation modelling analyses revealed that being female (B=0.45), having close friend/family infected (B=1.10) and having a pre-existing mental health condition (B=1.87) were positively associated with COVID-19-related stress. Infection of COVID-19 (BPHQ=0.22; BGAD=0.24) and a pre-existing mental health condition (BPHQ=0.71; BGAD=0.59) had direct associations with depressive and anxiety symptoms; COVID-19-related stress mediated the relationship between close friend/family infection with depressive (B=0.20) and anxiety symptoms (B=0.21, all $p < 0.05$). These results suggest that older people's COVID-19-related stress is beyond infection of the disease, and different experiences with COVID-19 may increase depression and anxiety risks through different pathways.

### COMPARING INFORMANT BASED MEASURES OF COGNITIVE FUNCTIONING BETWEEN THE US AND MEXICO

Yuan Zhang,[1] Phillip Cantu,[2] and Joseph Saenz[3], *1. University of North Carolina at Chapel Hill, Chapel Hill, North Carolina, United States, 2. The University of Texas Medical Branch, Galveston, Texas, United States, 3. University of Southern California, Los Angeles, California, United States*

Informant reports are important for understanding cognitive impairment and dementia in older populations. Recently, several population-based aging studies have included harmonized direct and informant assessments to assess the prevalence of dementia globally as part of the international Harmonized Cognitive Assessment Protocol (HCAP). However, the social and cultural contexts of nations, sociodemographic characteristics of informants, and the subject-informant relationship may affect the meaning and accuracy of informant reports of cognitive decline and functioning. This study examines the extent to which informant characteristics and the informant-respondent relationships are associated with informant-reported cognitive function in the United States and Mexico. Data are from the 2016 US Health and Retirement Study (HRS) HCAP (Nf 2,918) and the Ancillary Study of Cognitive Aging in Mexico (Mex-Cog) (Nf 1,750). Informant-reported cognitive function was measured by the informant Community Screening Instrument for Dementia (CSI-D). Linear regression is used to assess the association of informant characteristics and the information-participant relationship with the total CSI-D score. We find older Americans have worse informant-reported cognitive functioning but higher directly-assessed cognitive functioning than Mexicans. Nearly 80% of informants lived with the subject in Mexico, compared to less than half in the US. In both countries, older informants, children and other family members (compared to spouse) report less cognitive impairment. In Mexico, female informants reported more impairment. In the US, coresident informants reported more impairment. This research shows how social environments influence informant reporting. Understanding heterogeneity in informants is vital when examining informant measures of cognitive function in cross-countries studies.

### A CROSSWALK OF COMMONLY USED FRAILTY SCALES

Stephanie Denise Sison,[1] Sandra Shi,[2] Kyung Moo Kim,[3] Gahee Oh,[2] Nessa Steinberg,[2] Sohyun Jeong,[2] Ellen McCarthy,[2] and Dae Kim[4], *1. Beth Israel Deaconess Medical Center, Brookline, Massachusetts, United States, 2. Hebrew Senior Life, Roslindale, Massachusetts, United States, 3. John A. Burns School of Medicine University of Hawaii, Honolulu, Hawaii, United States, 4. Hebrew Senior Life, Boston, Massachusetts, United States*

Several validated scales have been developed to measure frailty, yet it remains unknown how these measures are related. We used data from 7,070 community-dwelling older adults who participated in National Health and Aging Trend Study round 5 to construct a crosswalk among frailty measures. We operationalized the 60-item Frailty Index (FI), Study of Osteoporotic Fracture (SOF) Index, FRAIL Scale, Frailty Phenotype, Clinical Frailty Scale (CFS), Vulnerable Elder Survey-13 (VES-13), Tilburg Frailty Indicator (TFI), Groningen Frailty Indicator (GFI), and Edmonton Frailty Scale (EFS). Missing data, needed for the calculation of frailty scores, were imputed using multiple imputation by chained equations method. We then linked the scores of each frailty measure to FI using the equipercentile method, a statistical procedure that links different scales by equating percentile distributions. Participants considered frail on FI (cutpoint of 0.25) corresponded to the following scores on each frailty measure: SOF 1.3, FRAIL 1.7, Phenotype 1.7, CFS 5.3, VES-13 5.5, TFI 4.4, GFI 4.4, and EFS 5.8. Conversely, individuals considered frail on each frailty measure corresponded to the following FI scores: 0.37 (SOF), 0.40 (FRAIL), 0.42 (Phenotype), 0.21 (CFS), 0.19 (VES-13), 0.28 (TFI), 0.22 (GFI), and 0.37 (EFS). The CFS, VES-13, TFI and GFI each discriminates between non-frail and frail people in the pre- to mildly frail spectrum on the FI, whereas the SOF, FRAIL Scale, Phenotype, and EFS detect those in the higher frailty spectrum on the FI. Our results provide clinicians and researchers with a useful tool to convert and interpret frailty across scales.

### ULTRASOUND MUSCLE THICKNESS AS AN EASY METHOD TO ASSESS LEAN MASS AND GRIP STRENGTH IN OLDER ADULTS

Lara Vilar Fernandes,[1] Gabriela Benatti De Oliveira,[2] Ana Carolina Junqueira Vasques,[1] Flavia Andrade,[3] Xia Yu Summer Chen,[4] and Ligiana P. Corona[5], *1. University of Campinas - UNICAMP, Limeira, Sao Paulo, Brazil, 2. University of Campinas - UNICAMP, Campinas, Sao Paulo, Brazil, 3. University of Illinois--Urbana-Champaign, Urbana, Illinois, United States, 4. University of Illinois at Urbana Champaign, Urbana, Illinois, United States, 5. Universidade Estadual de Campinas/ Faculdade de Ciências Aplicadas, Limeira, Sao Paulo, Brazil*

Ultrasound is emerging as an effective method for measuring muscle mass in older adults, being more cost-effective than other image methods, such as Dual-energy X-ray absorptiometry (DXA) and computed tomography, and more accurate than bioelectrical impedance and anthropometry. We aimed to evaluate the correlation between measurements of muscle thickness (MT) measured by a portable A-mode ultrasound, the total lean mass (TLM) measured by DXA, and grip strength (GS) among community-dwelling Brazilian older adults. This is a cross-sectional pilot study with 30 participants (9 men and 21 women) aged 60–78. MT was assessed in five anatomic sites (triceps, biceps, anterior and posterior thigh, calf) using a portable A-mode ultrasound (BodyMetrix BX-2000 - IntelaMetrix, Inc.); TLM was assessed by DXA (GE Healthcare's Lunar iDXA), and GS was assessed using a Saehan dynamometer. Pearson´s test was used for correlation analysis between each site MT and TLM; linear regressions were estimated to verify the association between GS and each site MT, controlling for age. Lower limb measurements (anterior and posterior thigh, calf) were not associated with TLM and GP. Triceps MT was significantly correlated to TLM ($r=0.565$; $p=0.001$) and associated to GS ($\beta=0.699$; $p=0.004$); biceps MT was also correlated to TLM ($r=0.425$; $p=0.019$) and associated to GS ($\beta=0.652$; $p=0.012$). Our results suggest that upper limb ultrasound measurements using portable equipment can be a helpful cost-effective indicator of muscle mass and function in older adults. Further results and conclusions will be reported after completing the study with the total planned sample (n=150).

### SEX DIFFERENCES AND LONGITUDINAL QUANTILES OF FRAILTY TRAJECTORIES WITH MISSING DATA DUE TO DEATH

Alejandra Marroig,[1] Fernando Massa,[2] Scott Hofer,[3] and Graciela Muniz Terrera[4], *1. Universidad de la Republica Uruguay, Montevideo, Montevideo, Uruguay, 2. Universidad de la República, Montevideo, Montevideo, Uruguay, 3. University of Victoria, Victoria, British Columbia, Canada, 4. Ohio University, Athens, Ohio, United States*

Scarce evidence exists about frailty trajectories, but the evidence suggests that women live longer with higher levels of frailty. When progression of frailty was studied, the focus has been on mean trajectories and research has ignored death. Here, we aim to assess the role of sex, age, and education in different quantiles of the distribution of frailty trajectories. We derived a frailty index (FI, range 0–1) based on the accumulation of deficits in individuals aged 65 at baseline (n=6929) using data from the Survey of Health, Ageing, and Retirement in Europe (SHARE). We applied weighted Generalized Estimating Equations (weighted GEE) to adjust the quantiles of the FI trajectory by sex, and education. The results show that the median FI trajectory increases with age (b_age0.5=0.008, $p < 0.001$) and this increase is higher for women than men (b_age*sex0.5=0.003, $p < 0.001$), but sex differences disappear for the most frail (0.9 percentile) once the missing data process is accounted for (b_age*sex0.9=0.002, p=0.085). For the most frail the increase with age is higher than for those at the median (b_age0.9=0.018, $p < 0.001$; test diff, $p < 0.001$) and education reduces the progression of the median FI (b_age*edu0.5=-0.0003, $p < 0.001$). Our approach advances the understanding of frailty trajectories of older adults, showing differences across quantiles. Thus, this may improve the practice and design of interventions aimed at older adults. In addition, we address the process of missing information and the results show new insights, particularly for those who are most frail.

### SERUM PROGRANULIN AS A POTENTIAL BIOMARKER FOR FRAILTY IN CHINESE OLDER ADULTS

Pan Liu,[1] Yun Li,[1] Shijie Li,[1] Yaxin Zhang,[1] Yu Song,[1] Tong Ji,[2] Ying Li,[1] and Lina Ma[1], *1. Xuanwu Hospital Capital Medical University, Beijing, Beijing, China (People's Republic), 2. Capital Medical University Xuanwu Hospital, Beijing, Beijing, China (People's Republic)*

Background: Frailty increases the risk of adverse health outcomes in older adults. Progranulin is a secreted glycoprotein involved in regulating various biological processes. A few studies have investigated the relationship between progranulin and frailty in middle-aged and older populations; however, different perspectives exist. We aimed to evaluate the association of progranulin with frailty in older Chinese adults. Methods: We included 265 older in-patients who were divided into the frail (n=107) and non-frail (n=158) groups according to the FRAIL scale. Serum IL-6, CXCL-10, and progranulin levels were assayed. Spearman's correlation analysis and logistic regression models were used to analyze the association of serum biomarkers with frailty, and the ROC was used to evaluate the diagnostic progranulin value for frailty. Results: The frail group was older, had lower

BMI, higher prevalence of coronary heart disease, worse grip strength and walking speed, and higher serum levels of IL-6, CXCL-10, and progranulin, than the non-frail group. Progranulin levels were negatively correlated with grip strength and positively correlated with IL-6 and CXCL-10. The logistic regression analysis showed that IL-6, CXCL-10, and progranulin were associated with frailty, respectively. Furthermore, progranulin remained associated with frailty after adjusting for age, sex, BMI, smoking, and chronic diseases (OR=1.003, 95%CI=1.001–1.005, p=0.013). The AUC of serum progranulin levels for diagnostic frailty was 0.872 (95%CI=0.829–0.914, p < 0.001). Conclusion: High serum progranulin levels were observed in frail older in-patients and associated with worse physical function and chronic inflammation. This association indicates that progranulin may be a potential biomarker for frailty.

### PSYCHOMETRICS IN THE DEVELOPING OF LONGITUDINAL MEASURES OF FRAILTY

Stacey Voll,[1] Graciela Muniz Terrera,[2] and Scott Hofer[1], *1. University of Victoria, Victoria, British Columbia, Canada, 2. Ohio University, Athens, Ohio, United States*

Frailty has become an increasingly important aspect in the field of health research in studies of vulnerability to adverse events and individuals functioning due to accumulated health deficits. Yet, there is no agreement on how to measure frailty or identify adults as frail, resulting in high heterogeneity between estimates of frailty and identification of frail individuals. The purpose of this poster is to provide a summary of the process in the development of a frailty index with robust psychometric properties to be used in research using longitudinal health study sets.

### DEVELOPMENT OF PREDICTIVE MODELS FOR INCIDENT FRAILTY IN OLDER ADULTS: MACHINE LEARNING APPROACHES

Heeeun Jung,[1] Miji Kim,[2] Mina Park,[3] Jinhyung Ryu,[4] Kyung-Ryoul Mun,[4] and Chang Won Won[1], *1. Kyung Hee University, Dongdaemun-gu, Seoul-t'ukpyolsi, Republic of Korea, 2. Kyung Hee University, Seoul, Seoul-t'ukpyolsi, Republic of Korea, 3. Korea University, Seongbuk-gu, Seoul-t'ukpyolsi, Republic of Korea, 4. Korea Institute of Science and Technology, Seongbuk-gu, Seoul-t'ukpyolsi, Republic of Korea*

Frailty is not only a clinical symptom accompanying adverse health outcomes but also a syndrome that involves a dynamic transition between varying phases. This study aimed to develop machine learning (ML) models for predicting incident frailty over a 2-year follow-up period among Korean community-dwelling adults aged 70 years and older. We conducted prospective analyses (n=2,408) among participants who underwent measurement of frailty status at baseline and 2-year follow-up from the Korean Frailty and Aging Cohort Study. Frailty was defined as the presence of three or more of five components using the Fried frailty phenotype. A total of 2,250 participants classified as non-frail at baseline and 116 (5.2%) with incident frailty during the 2-year follow-up. 32 variables selected by stepwise analysis were used as inputs among 77 variables composed of sociodemographic and clinical characteristics. The different ML models such as Logistic Regression(LR), K-Nearest Neighbor(KNN), Gaussian Naïve Base(NB), Support Vector Machine(SVM), and Random Forest(RF) with the 10-fold cross validation were compared. A feature selection, grid search, and data resampling methods were applied to find the best inputs and models. The best performing inputs of 16 variables were found and Gaussian NB achieved the highest accuracy of 0.82 and F1-score of 0.79 in predicting frailty incidence after 2-years with selected 16 inputs, followed by RF (accuracy 0.82, F1-score 0.76) and LR (accuracy 0.80, F1-score 0.76). We developed ML models for predicting frailty and is expected to contribute to the early prediction of frail older adults and timely intervention in clinical and community settings.

### "IBASHO" AND THE OBJECTIVE WELL-BEING OF OLDER ADULTS IN A MARGINALIZED COMMUNITY.

Yuichi Watanabe, *Musashino University, Nishitokyo, Tokyo, Japan*

"IBASHO" in Japan means a place where people can feel safe, have roles, and recognize their lives as meaningful. If IBASHO helps older adults to see their lives as meaningful, implementing "IBASHO" in the community would possibly contribute to community members' objective well-being. This research examines the relationship between the activities that make up "IBASHO" and the objective well-being of older adults. Data were gathered by surveying a marginalized community every two years from 2009 to 2019 (Nf882). Multiple regression analysis was conducted. A six-point scale derived from a survey question measured the objective well-being of older adults as the dependent variable. For measuring "IBASHO," five items asked about engagement in activities such as "hosting a tea meeting" or "holding a hobby workshop," with the extent of engagement measured using a five-point scale. The total of these five items' points (Cronbach's alpha=.860) was analyzed as an independent variable. Gender, age, economic status, health status, and living alone were also included in the analysis. Results show "IBASHO" activity is a significant predictor of the objective well-being of older adults (p < .000) in the full model. Age, economic status, and health status are also significantly associated with well-being. Findings suggest that implementing "IBASHO" within community settings is associated with higher levels of well-being among older adults in the community. "IBASHO" is crucial to realizing the "no one left behind" policy in Japan.

### YOU'VE GOT A FRIEND: SOCIAL FACTORS AND DEPRESSIVE SYMPTOMS IN OLDER ADULTS

Erika Fenstermacher,[1] and Amy Fiske[2], *1. West Virginia University, Pursglove, West Virginia, United States, 2. West Virginia University, Morgantown, West Virginia, United States*

Depression in late life is associated with disability, lower quality of life, increased mortality, and risk of suicide. Research suggests that functional disability, which is often brought about by a medical condition, may precede depressive symptoms and may be a major factor for older adults developing depression. Social support can be beneficial for both emotional and physical health. Numerous studies demonstrate that social networks, perceived and subjective social support, and satisfaction with support received moderate the relation between health problems and depression. Findings are mixed regarding received and objective social support and only one study was found that examined the impact of giving support to others. The current study addressed gaps in the literature by focusing on three facets of social relationships: availability of support, participant giving help, and participant receiving help. Data from the 2011 wave of the Wisconsin Longitudinal Study were used (N = 3906; mean age of 71.27; SD = .948). We examined the relationships between medical conditions, functional impairment, depressive symptoms, and social support using regression analyses. Medical conditions and functional impairment each independently predicted depressive symptoms. Availability of support significantly predicted lower levels of depressive symptoms and amount of help received predicted higher levels of depressive symptoms, controlling for medical conditions and functional impairment. These findings suggest that simply having someone available, not necessarily interacting with them for help, could reduce depressive symptoms in older adults.

### WHAT I KNOW NOW THAT I WISH I HAD KNOWN WHEN I WAS YOUNGER: OLDER WOMEN'S RELATIONSHIP ADVICE AS SKINNY POETRY

Wendy Watson, Sandra Faulkner, and Charlie Stelle, *Bowling Green State University, Bowling Green, Ohio, United States*

Research has demonstrated that relationships are important in the lives of older women. Eighteen women between the ages of 64–84 participated in interviews about their lives and relationships across the life course. As a final question, the women were asked, "What do you know now about relationships that you wish you'd known when you were young?" Responses to that question were examined using a skinny poem. The Skinny poem is a short poetry form that portrays a vivid image in 11 "skinny" lines. It is a type of lyric poem that uses condensed images, expressions of personal feelings, and demonstrations of emotional context in musical lines. Skinnys were written by one author and analyzed by a second author. This poster will present the 18 skinny poems and the analysis of the poems. In these skinnys, one can see the relationship experiences the women had throughout their lives. There is a connection to wisdom, a recognition that they have grown and learned about relationships over time and have advice they wished their younger selves had known. Emotions range from anger and anxiety to trepidation and regret. Most of the women reflect on things they would have done differently, from how they treated others to how they felt about themselves. They recognize that relationships take work and that as older women, they still do not know everything about relationships. They also learned about themselves as individuals and wished they had not searched for their identity and self-worth in their connection to others.

### THE INFLUENCE OF MARRIAGE AND COHABITATION ON PHYSICAL ACTIVITY AMONG MIDDLE-AGED AND OLDER PEOPLE

Shuhan Yuan,[1] Kit Elam,[1] Jeanne Johnston,[2] and Angela Chow[2], *1. Indiana University, Bloomington, Bloomington, Indiana, United States, 2. Indiana University Bloomington, Bloomington, Indiana, United States*

The Behavioral Risk Factor Surveillance System (2020) indicates that 30.6% of middle-aged and older adults were physically inactive. Whereas marital status is linked to physical activity (PA), it is increasingly common for couples to cohabitate, making it important to capture these relationships. The stress/social support theory(Burman & Margolin, 1992) highlights the importance of relationship quality in long-term relationships, but this hasn't been cohesively examined relative to PA. This study investigated whether partnered living status (married/cohabitating) and partnered living quality (support/strain from partner, partner disagreements) were associated with PA in middle-aged/older adults. Data were from a nationally representative longitudinal study, Midlife in the United States(waves 1–3; Nf1113; aged 49–93). Subjects were categorized into four groups based on partnered living status over the three waves: partnered living at all waves (57.9%), non-partnered living (separated, divorced, widowed, or never married) at all waves (17.6%), change from partnered living to non-partnered living (20.8%), and change from non-partnered living to partnered living (3.8%). Regressions were conducted to test the effect of partnered living status and relationship quality on the frequency of moderate and vigorous PA at wave 3. Subjects who changed from non-partnered to partnered living had the highest moderate and vigorous PA levels. Partner support was positively associated with moderate PA ($\beta$=0.50, $p < 0.01$), and partner disagreements was negatively associated with vigorous PA ($\beta$=-0.27, $p < 0.01$). Results suggest that relationship status and quality can influence PA among the aging population. Public health educators should provide additional social support to older adults to promote PA.

### PREDICTORS OF INDIVIDUAL TRANSPORTATION ALTERNATIVES IN THE TRANSITION TO NON-DRIVING

Kellia Hansmann,[1] Carolyn McAndrews,[2] and Stephanie Robert[3], *1. William S Middleton Memorial Veterans Hospital, Madison, Wisconsin, United States, 2. University of Wisconsin Madison, Madison, Wisconsin, United States, 3. UW-Madison, Madison, Wisconsin, United States*

For older adults who transition to non-driving, successful coping includes maintaining mobility. Individual transportation alternatives, e.g. rides from friends and family, are important coping factors in the transition to non-driving. However, it is unclear to what extent social network composition predicts the accessibility of these individual transportation alternatives. We examined whether various aspects of social networks (including living with a spouse/partner; having a child; living with a child; having other household members; having anyone to talk to about important things) were associated with the actual receipt of rides from family and friends. Using the National Health and Aging Trends Study (NHATS), a nationally representative cohort of Medicare beneficiaries ages 65 and older, we conducted a cross-sectional analysis using a subsample of community-dwelling older adults whose driving status was known at the baseline interview in 2015 (n = 5,889). We used logistic regression to estimate the association between social network indicators with the odds of receiving a ride from a family member or friend. When adjusting for biopsychosocial characteristics and current driving status, we found that having a spouse or partner was associated with a significant odds of having received a ride (adjusted OR, 95% CI = 1.41, 1.05–1.90) as was being able to name at least five people the participant could talk to about important things (adjusted OR, 95% CI = 1.65, 1.14–2.38). Our findings support previous evidence that marital status and support from social network members outside one's household may be particularly important for accessible individual transportation alternatives.

### ARE OLDER ADULTS' SOCIAL PARTICIPATION AND RELATIONSHIP QUALITY RELATED TO THEIR SPOUSE'S COGNITION?

Changmin Peng,[1] Yan Lin,[2] Shan Qu,[3] and Yiyang Yuan[4], *1. University of Massachusetts Boston, Brookline, Massachusetts, United States, 2. University of Massachusetts Boston, Bedford, New Hampshire, United States, 3. University of Massachusetts Boston, Boston, Massachusetts, United States, 4. University of Massachusetts Chan Medical School, Worcester, Massachusetts, United States*

Limited studies have quantified older adults' profiles of social participation and relationship quality. How such profiles were associated with their spouse/partner's cognitive function also remains unknown. Using the Health and Retirement Study (2014/2016), we identified 3,722 community-dwelling, cognitively intact, married/partnered respondents living with their spouse/partner. Spouse/partner's cognitive function was ascertained by the Langa-Weier Classification [Intact/Cognitively Impaired but not Demented (CIND)/Demented]. Social participation was measured by the frequency of volunteer, charity, education, sport/social clubs, and non-religious organization activities. Relationship quality was measured by the perceived positive and negative support from spouse, children, relatives, and friends. Latent profile analysis identified profiles of social participation and relationship quality. Multinomial logistic regression estimated the association between spouse/partner's cognitive function and the respective profiles. Three social participation profiles were identified: (1) Limited social participation (prevalence: 69%; reference); (2) Frequent volunteer participation (10%); (3) Frequent non-volunteer participation (21%). Three relationship quality profiles were identified: (1) Positive overall support (68%; reference); (2) Positive spousal support (18%); (3) Negative spousal support with positive non-spousal support (14%). Those with a spouse/partner that were CIND or demented were significantly less likely to frequently participate in volunteer or non-volunteer activities, while more likely to perceive negative spousal support and positive non-spousal support. Given these findings and the essential role of social participation and relationship quality in older adults' well-being, programs focusing on older adults living with a spouse/partner with impaired cognition are needed to help them maintain social connectedness.

### INVOLUNTARY JOB LOSS AND GENDERED HEALTH OUTCOMES IN MIDDLE AGE AND LATER LIFE: A LIFE COURSE ANALYSIS

Qian Song,[1] and Caitlin Connelly[2], *1. Universiy of Massachusetts Boston, Boston, Massachusetts, United States, 2. UMASS Boston, Boston, Massachusetts, United States*

Job loss is associated with a range of negative health outcomes. However, the causality of job loss and poor health is unclear, due to issues of endogeneity and reverse causality (i.e., adverse health causing job loss). We also need a better understanding of the long-term effects of job loss, and how the life stage in which job loss occurred yields different health outcomes for women and men in later life. To address these gaps, we take advantage of a quasi-experimental setting — the policy-driven layoffs of the State-Owned-Enterprises (SOEs) during the 1990s – mid-2000s in urban China. Using the life history survey of the China Health and Retirement Longitudinal Study 2014 and the 2015 wave, we examine the long-term effects of job loss on self-rated health (scored 1 – 5) of individuals in middle age and later life (aged 45+). After controlling for individual childhood health, life-time work history, as well as demographics and socioeconomic status, results from linear regressions show that overall, job loss from SOEs reduces self-rated health by 0.12 ($p < .05$) for both women and men. However, job loss hurts females' self-rated health the most when job loss occurred in early-life, a life stage in which childbearing and child caregiving were most intensive (aged 35 or earlier; $\beta = -0.24$, $p < .05$), whereas males' self-rated health is hardest hit if job loss occurred in mid-life (aged 36 – 45; $\beta = -0.35$, $p < .01$). The results suggest that involuntary job loss produce gendered health outcomes due to gendered life courses.

### SHORT-TERM DIESEL EXHAUST PARTICLE NEUROTOXICITY MEDIATED BY TLR4 ACROSS AGE AND SEX

Kristina Shkirkova,[1] Hongqiao Zhang,[2] Arnold Diaz,[3] Wendy Mack,[4] Constantinos Sioutas,[2] Caleb Finch,[2] Christian Pike,[2] and William Mack[2], *1. USC Keck School of Medicine, Los Angeles, California, United States, 2. USC, Los Angeles, California, United States, 3USC, Los Angeles, Armed Forces - AP, United States, 4. USC, Los Angeles, California, United States*

Experimental animal exposures show neurotoxic effects of diesel in young mice even after short-term exposures. We hypothesize that neuroinflammatory effects of diesel exhaust particles (DEP) are mediated by toll-like receptor 4 (TLR4) activation in brain microglia. We studied both sexes of young (2 months) and middle-aged (18 months) TLR4 flx/flx (CX3CR1CreER +/-) mice, in which macrophage-specific TLR4 deletion is induced by tamoxifen. DEP from National Institute of Science and Technology (NIST 2975) was suspended in pure water and re-aerosolized for 5 hr exposure at 100 µg/m3 concentration. Experimental groups were Filtered Air+Corn Oil, DEP+Corn Oil, Filtered Air+Tamoxifen, DEP+Tamoxifen. We studied markers of inflammation, oxidative stress, and microglia activation in the white matter of corpus callosum. At baseline, middle-aged mice showed higher levels of microglia activation (Iba-1), complement activation (C5, C5a), and oxidative stress (8-OHdG, 4HNE). DEP significantly increased microglial activation, inflammation, and oxidative stress. TLR4 knockdown showed a rescue effect in DEP group for Iba-1 and 8-OHdG in young but not in middle-aged mice. Both C5a and 4HNE were rescued by TLR4 knockdown in young and middle-aged mice, possibly with less robust effects in older mice. No significant sex effects were observed. Middle-aged mice have higher levels of baseline white matter inflammation and oxidative stress. DEP exposure caused robust neuroinflammatory and oxidative responses in white matter across ages. TLR4 knockdown attenuated DEP caused neuroinflammatory and oxidative responses, suggesting that microglia play an important role in DEP neurotoxicity.

### PREDICTORS OF 400M WALKING TIME: THE STUDY OF MUSCLE MOBILITY AND AGING (SOMMA)

Steve Cummings,[1] Sheena Patel,[2] Peggy Cawthon,[3] Paul Coen,[4] Russell T. Hepple,[5] Theresa Mau,[1] and Anne Newman[6], *1. San Francisco Coordinating Center, San Francisco, California, United States, 2. CPMC Research Institute, San Francisco, California, United States, 3. University of California, San Francisco, San Francisco, California, United States, 4. AdventHealth, Orlando, Florida, United States, 5. University of Florida, Gainesville, Florida, United States, 6. University of Pittsburgh, Pittsburgh, Pennsylvania, United States*

Longer 400m walking time has been strongly associated with overall mortality and many other aging-related outcomes in prior studies. Inability to walk 400m defines 'mobility disability'. We assessed characteristics that might contribute to 400m walking time in SOMMA, a cohort of 879 men and women over age 70. We excluded people with a gait speed < 0.6 m/s. The analysis included 49 factors representing several domains: muscle mass and strength, body composition, oxygen delivery, muscle mitochondrial ATP generation, fitness by cardiopulmonary exercise testing (VO2 peak), fatigability by questionnaire, usual physical activity, depressed mood, cognitive performance, lower extremity pain, lower extremity sensation, smoking, wealth and income, and general health. We used both elastic net and forward step-wise regression to generate multivariate models. Factors associated with longer (worse) walking time in both elastic net and forward step-wise regression models were: *weaker leg power, *fatigue during treadmill testing, *higher BMI, *greater self-reported fatigability, *worse performance on the timed DSST test, *fewer hours spent walking, *taking more medications, *older age, *poorer fitness (VO2peak), *more joint stiffness, *smoking, and *reduced peripheral sensation. These results indicate that 400m walk time is influenced by numerous factors. Some are also associated with overall mortality and aging outcomes and therefore they might underlie the prognostic value of the 400m walk. Additionally, some of the predictors could be improved, such as leg strength, cardiopulmonary fitness, more time walking, quitting smoking, and treatment of joint stiffness, perhaps reducing the risk of mobility disability.

### RURAL AGE-FRIENDLY ECOSYSTEMS: A SCOPING REVIEW WITH RECOMMENDATIONS FOR AGE-FRIENDLY COMMUNITIES

Daniel Liebzeit,[1] Anna Krupp,[2] Jacinda Bunch,[2] and Harleah Buck[3], *1. University of Iowa, North Liberty, Iowa, United States, 2. University of Iowa, Iowa City, Iowa, United States, 3. Csomay Center for Gerontological Excellence, Iowa City, Iowa, United States*

The population of older adults in rural areas is rising, and they experience higher rates of poverty and chronic illness, have poorer health behaviors, and experience different challenges than those in metropolitan areas. Promoting age-friendly rural communities and healthcare is key to improving outcomes and addressing disparities. This international scoping review seeks to 1) map the state of the science of age-friendly systems in rural areas regarding structural characteristics, processes for delivering age-friendly practices, and outcomes of age-friendly systems, 2) analyze strengths, weakness, opportunities, and threats of age-friendly system implementation, and 3) make person, practice, and policy-level recommendations to support active aging. Articles were retrieved from PubMed, CINAHL, AgeLine, PsychINFO, EMBASE, Scopus, and Academic Search Elite on 10/26/21 and included if they used age-friendly framing, self-identified as rural, and reported empiric data. Data were charted across three analytic layers: socioecological model, Donabedian's framework, and SWOT analysis. While age-friendly systems in this review were heterogeneous, many utilized the World Health Organization age-friendly cities framework. Results reveal limited data on outcomes relevant to organizations. While the SWOT analysis revealed many strengths of age-friendly systems, it also revealed several weaknesses, threats, and gaps. Weaknesses included over-reliance on trained volunteers and staff, communication, and teamwork. Threats included community and health system barriers and challenges in developing regions. Despite the benefits of age-friendly systems, we must acknowledge limitations of the evidence base, pursue opportunities to examine organizational metrics to support implementation and sustainability of age-friendly systems, and leverage improvements in age-friendliness at a community level.

### AN EVIDENCE-BASED APPROACH TO TELEPHONE REASSURANCE CALLS: A RANDOMIZED CONTROLLED TRIAL PROTOCOL

Morgan Minyo,[1] David Bass,[2] Catherine McCarthy,[3] Katherine Judge,[1] Fatima Perkins,[4] and Douglas Beach[4], *1. Cleveland State University, Cleveland, Ohio, United States, 2. The Benjamin Rose Institute on Aging, Cleveland, Ohio, United States, 3. Benjamin Rose Institute on Aging, Cleveland, Ohio, United States, 4. Western Reserve Area Agency on Aging, Western Reserve Area Agency on Aging, Ohio, United States*

The COVID-19 Pandemic forced many health and community organizations to suspend some previously offered services and supports for older adults. This created heightened concerns about negative impacts of isolation and loneliness on already vulnerable older adults. In response, the Western Reserve Area Agency on Aging (WRAAA) began the TeleCare Program, providing weekly telephone reassurance calls to home and congregate meal clients to maintain contact and identify unmet service needs. Because of its success, the TeleCare Program has continued as a regularly offered service, but the WRAAA sought to improve the Program by integrating it with BRI Care ConsultationTM, an evidence-based program of the Benjamin Rose Institute on Aging (BRIA). This poster reports on initial results of a randomized controlled trial to test the efficacy and feasibility of a new protocol for delivering telephone reassurance calls. The new protocol is modeled after the Initial Assessment in BRI Care

Consultation, which is a semi-structured, telephone conversation covering a comprehensive range of potentials problems. The protocol also connects high-risk clients to existing available home- and community-based services. Results demonstrate that components of proven evidence-based interventions are feasible to integrate into existing programs and services. This includes adapting a model for problem-identification and assessment based on a consumer-driven conceptual framework, a record keeping system to enhance consistency and fidelity of program delivery, and establishing a formal referral protocol to more seamlessly link clients to other available services.

### AGE AND SITE DIFFERENCES IN PLANNED AND PERFORMED ACTIONS IN RESPONSE TO IDENTIFIED RISKS IN OLDER ADULTS

Deborah Finkel,[1] Linda Johansson,[2] Björn Westerlind,[3] Ulrika Lindmark,[4] and Marie Ernsth-Bravell[2], *1. Indiana University Southeast, New Albany, Indiana, United States, 2. Jönköping University, Jönköping, Jonkopings Lan, Sweden, 3. Linköping University, Linköping, Ostergotlands Lan, Sweden, 4. Karlstad University, Karlstad, Varmlands Lan, Sweden*

The Swedish health care system focuses on allowing older adults to “age in place”; however, that approach assumes that home health services are adequate to support health and prevent unnecessary decline. Data from the Senior Alert national quality register in Sweden were examined to compare the quality of care across care locations. First registration in Senior Alert was available for 2914 adults aged 57–109 (median age = 81): 3.6% dementia unit, 7.8% home health care, 4.4% rehabilitation unit, 62.8% hospital, 21.4% care home. There were significant differences across units in the number of identified risks in 4 categories: falls, malnutrition, oral health, and pressure ulcer. Individuals in rehabilitation units averaged 2.4 risks, individuals in dementia and care homes averaged 2.0 risks, and individuals in home health care and hospitals averaged 1.4 risks. For individuals with identified risks, the differences between planned and performed actions for each risk independently were greatest for those in home health care. Moreover, the correlation between total planned and performed actions in home health care was .79 for adults aged 65–80 years and .39 for adults aged 81 and over. The correlation did not differ across age for the other care units. Results suggest that individuals most in need of actions to address health risks (older adults in home health care) are least likely to have the actions performed. Training and support of workers responsible for home health care need to be improved if the “age in place” policy is to continue.

### TRAJECTORIES OF HEALTH RECOVERY AFTER HIP FRACTURE IN OLDER ADULTS: A SCOPING REVIEW

Tingzhong (Michelle) Xue,[1] Jiepin Cao,[2] Leila Ledbetter,[3] Margaret Graton,[3] Yan Zhan,[4] Dingyue (Demy) Wang,[3] Youran Lee,[3] and Eleanor McConnell[3], *1. Duke University School of Nursing, Durham, North Carolina, United States, 2. NYU Grossman School of Medicine, New York City, New York, United States, 3. Duke University, Durham, North Carolina, United States, 4. Yale University, Orange, Connecticut, United States*

Hip fracture recovery outcomes in older adults are characterized by high mortality, lowered functional status, and feelings of being disrupted from a normal life. Studying recovery trajectories through the lens of resilience can provide novel perspectives for developing interventions targeting to promote recovery. However, the lack of knowledge of recovery trajectories and their variations in hip fracture patients impedes such efforts. This review aims to synthesize current evidence on how multiple health domains change longitudinally after hip fracture in older adults. The Joanna Briggs Institute scoping review methodology was followed, and seven databases were searched including Medline (PubMed), EMBASE, Web of Science Core Collection, CINAHL, Proquest Dissertations and Theses, Cochrane Central Register of Controlled Trials, and Cochrane Database of Systematic Reviews. No date limits were applied, and the final search resulted in 7,515 articles. Articles in English with participants aged 60 years and above who experienced a low-energy, nonpharmacological hip fracture in any health setting were selected. Results regarding multiple domains of health outcomes will be synthesized, including physical health (e.g. functional status, pain, nutrition, and mobility/physical performance), cognition, psychosocial health (e.g. depression, anxiety, social isolation, loneliness, and behavioral and psychological symptoms of dementia when individuals with dementia were included), and multidimensional outcomes such as health-related quality of life. Methodological challenges and limitations will be discussed. This review has important implications for clinicians and researchers to improve individualized treatment plans and research methodologies by providing a comprehensive, critical review of knowledge regarding health trajectories in older adults after hip fracture.

### PHYSICAL SYMPTOM TRAJECTORIES OF OLDER ADULTS DURING THE COVID-19 PANDEMIC

Dylan Lee,[1] Soyoung Choun,[2] Hye Soo Lee,[2] Maria Kurth,[2] and Carolyn Aldwin[3], *1. Touro University Nevada, Henderson, Nevada, United States, 2. Oregon State University, Corvallis, Oregon, United States, 3. Oregon State University, Corvallis, Oregon, United States*

Older adults experience increased risk for morbidity and mortality during the COVID-19 pandemic (CDC, 2021). Social distancing and lockdown to prevent contagion may affected physical and mental health. We examined the fifteen physical symptom trajectories of older adults during the early months of the COVID-19 pandemic. We also examined age, gender, and marital status differences in each physical symptom trajectory. The sample consisted of 247 older adults (Mage = 71.1, SD = 7.3, 88.7% White, 73% women, 73.4% married), who participated in eight weekly longitudinal online surveys from April 28 to June 23, 2020. Random-effects logistic regression analysis controlling for age, gender, marital status, and depressive symptoms showed that the nine physical symptoms (headache, constipation/diarrhea, muscle soreness, shortness of breath, tightness of chest, backache, heart pounding, congestion, and sore throat) significantly decreased in the first few weeks, but then six symptoms (constipation/diarrhea, shortness of breath, tightness of chest, heart pounding, congestion, and sore throat) increased in later weeks of the study period.

Middle-aged participants reported higher number of headache, tightness of chest, heart pounding, and nausea/upset stomach than older participants. Women experienced more constipation/diarrhea, trembling/shaking, and sore throat than men. Those who were not married responded with higher number of shortness of breath, backache and poor appetite compared to married participants. Higher depressive symptoms were significantly related to each physical symptom. The results suggest that healthcare providers should evaluate physical symptoms focusing on the patients who are at greater risk of poor health during the pandemic.

### LIKELIHOOD OF ADOPTION OF CAREMOBI: ADDRESSING COMMUNICATION GAPS BETWEEN ADULT DAY HEALTH CENTERS AND PRIMARY CARE

Zuha Ali,[1] and Tina Sadarangani[2], *1. Indiana University School of Medicine, Indianapolis, Indiana, United States, 2. New York University, New York, New York, United States*

Adult Day Health Centers (ADHC) are non-residential congregate facilities that serve 250,000 cognitively and functionally impaired adults daily. These centers are effective platforms for chronic disease management and interdisciplinary care coordination, with serial observations on participants over multiple hours and days per week. However, previous research indicates that ADHCs experience challenges in communication with primary care providers (PCPs). This is because 92% of ADHCs lack interoperable EHRs (electronic health record systems) and do not have the financial and technical infrastructure to implement them. CareMOBI (Mhealth for Organizations to Bolster Interconnectedness) is a mobile application in development meant to streamline the information communication between ADHC, PCP, and family caregiver populations. The purpose of this study was to understand the likelihood of adoption of CareMOBI by primary care providers. Demographics of the study included 87.50% female and 12.5% male primary care providers with a median age of 38 years. Participants were recruited through parallel convergent design, and data was merged into a matrix defined by 4 major themes: perceived value in geriatric care, ease of use, fit within workflow, and likelihood of adoption. The results revealed that a majority percentage of providers were likely to adopt the app, saw the app as valuable, and easy to use. In response to fit within a provider's workflow, apprehensions surrounding interoperability arose. These apprehensions regarding interoperability are the most important to address through a user-centered approach and as the app is pilot tested into adult day health centers.

### INVESTIGATING OLDER ADULTS' PERSPECTIVES ON TELEHEALTH ROBOTICS

Samuel Olatunji,[1] Husna Hussaini,[2] Kenneth Blocker,[1] Naveen Uppalapati,[3] Girish Krishnan,[3] and Wendy Rogers[4], *1. University of Illinois Urbana-Champaign, Champaign, Illinois, United States, 2. University of Illinois Urbana-Champaign, Urbana Champaign, Illinois, United States, 3. University of Illinois at Urbana Champaign, Urbana Champaign, Illinois, United States, 4. University of Illinois Urbana Champaign, Champaign, Illinois, United States*

The current social distancing directives have heightened the need for older adults to seek remote healthcare solutions. Telehealth robots could support home health by carrying out on-demand tasks for diagnosis and treatment at the user's home through remote teleoperation by the healthcare provider. Acceptability of such telehealth robots by older adults is critical to their successful deployment. The overall goal of this research was to investigate older adults' perceptions and attitudes towards a telehealth robot supporting health checkups at home. Specific objectives were to explore potential healthcare use cases for the robot; identify facilitators and barriers to its use; and elicit design requirements for use in healthcare contexts. We interviewed 5 men and 5 women (ages 66–73) after they viewed a series of videos demonstrating the potential uses of the telehealth robot. The older adults had positive first impressions towards the telehealth robot and were generally open to the idea of using it for telehealth tasks. They conveyed a high level of trust with the robot, especially if it were controlled by a healthcare provider, yet expressed concerns with privacy and security of health information that would need to be addressed in the design of security protocols. They described added value of the robot's healthcare support while suggesting potential improvements to the robot. This research provided insights into older adults' perceptions and attitudes towards a telehealth robot as well as identified potential healthcare use cases that would inform the design requirements for telehealth robots in different home healthcare contexts.

### COMMUNITY-RECRUITED OLDER ADULTS DIFFER FROM PATIENT POPULATIONS

Catherine Striley, Piyush Chaudhari, Roger Fillingim, and Linda Cottler, *University of Florida, Gainesville, Florida, United States*

Researchers often conduct randomized controlled trials among patient populations that may not reflect the community in which findings will be translated. The University of Florida's community engagement program HealthStreet provides a diverse sample in which to consider differences between people 65 years of age and older who have seen a physician in the past 12 months and those who have not. This provides a conservative test of potential biases in patient-only samples. Based on a sample of 1,663 people, 65 years of age and older, who were recruited by Community Health Workers in the North Florida region from November 2011 through July 2022, 88% had seen a physician in the past 12 months. Those who had not (12%) were significantly more likely to be non-white than white, to be in good/excellent than fair/poor health, and significantly less likely to have a range of health conditions including high blood pressure, depression, heart conditions, diabetes, a digestive health condition, a dental health condition, or cancer. Yet residents who hadn't seen a doctor were just as likely to be willing to participate in a future health study (both 94%) that only asked about health, accessed medical records (both 87%) or didn't provide reimbursement (both 81%), and were not significantly different in attitudes toward participating in research in general. Recruiting of older adults should be conducted in the community, not just in patient populations, which are likely to be less diverse and sicker than those recruited through a community engagement program.

### SELF-TAILORED SUCCESSFUL AGING: OLDER ADULTS' POINTS OF VIEW

Michal Tsadok Cohen,[1] Sara Rosenblum,[1] and Sonya Meyer[2], *1. Haifa University, Haifa, Hefa, Israel, 2. Ariel University, Ariel, HaMerkaz, Israel*

The study aimed to map out the main constructs involved in aging successfully, as perceived by older adults, and highlight the importance of self-management. This qualitative study was comprised of eighteen interviewees and sixty participants across nine focus groups, including: Independent older adults aged 65 to 84 years living at home, health professionals working with older adults, and family members of older adults. Participants shared experiences concerning their everyday occupations and routines. Focus groups were recorded, transcribed verbatim, and qualitatively analyzed, using the constructivist grounded theory principles.

Results: Three successful aging theoretical categories emerged: self-management abilities, daily lifestyle, and individual differences. An example for self-management abilities can be described by a 67-year-old man's son, who said: "He is a very busy man, he doesn't have any commitments, but he always creates things for himself to do". Results revealed diversity in how older adults perceive successful aging. For example, a 68-year-old woman said: "Retirement is like heaven, I enjoy every moment, there's a lot to do", whereas a 69-year-old man claimed: "Nobody needs you anymore… I retired a year ago and I still haven't succeeded in organizing a daily schedule". Additional results and quotes reflecting the categories will be represented. Conclusion: Older adults who succeed in managing their daily life, and experience control of their own life, delineate successful aging. The diversity accentuates that each older adult needs to tailor their own self fitted "suit" to wear as they stride successfully into their aging years.

### FEASIBILITY OF INTEGRATED TECHNOLOGIES TO PROMOTE LEISURE ACTIVITY ENGAGEMENT AMONG ASSISTED LIVING RESIDENTS

Phyllis Gaspar,[1] Elena Do,[2] Junhyoung Kim,[3] Angela Chow,[4] Adam Sobol,[5] Raia Francisco,[2] and Lisa McFarland[6], *1. CareBand Inc., Sioux Falls, South Dakota, United States, 2. CareBand Inc., Chicago, Illinois, United States, 3. Indiana University, Bloomington, Indiana, United States, 4. Indiana University Bloomington, Bloomington, Indiana, United States, 5. Careband Inc., Chicago, Illinois, United States, 6. Axiatp, Zionsville, Indiana, United States*

Research suggests that advanced technologies, such as wearable technology and tablets, can serve an important role in the cost-effective, accessible delivery of health promotion that is individualized, immersive, and engaging for users. The present study was designed to test the feasibility and acceptability of two integrated technologies among assisted living facility residents: CareBand's wearable technology and Eperture's RememberStuff (R/S) tablet platform. The purpose of the qualitative component of the project was to provide preliminary information from the perspective of the assisted living facility participants on these two technologies. Another goal was to assess the challenges of their participation. Drawing on qualitative thematic analyses of data collected from semi-structured interviews with 16 participants (four staff members and 12 residents) prior to and following the 6-week integrated technology program, three themes emerged as positive outcomes of the technology experiences: (a) leisure activity engagement, (b) exploration of tablet features, and (c) cognitive stimulation. This finding suggests that these technologies may enhance leisure engagement and cognitive function. On the other hand, there were three challenges that participants experienced while participating in the program: (a) inability to grasp functionality, (b) need for continued education for the technology, and (c) negative stereotypes toward technology use. These challenges provide guidance for future protocol design in follow-up studies to further explore the integrated technology efficacy for older adult users. The practical implications of this study and suggestions for health professionals are discussed.

### COMMUNITY CENTERED APPROACHES TO INCREASE PARTICIPATION OF AFRICAN AMERICANS IN BRAIN HEALTH AND ADRD RESEARCH

Priscilla Barnes,[1] Bianca Cureton,[2] Nenette Jessup,[2] Natalie Sutton,[3] Carl Hill,[4] Patrick Shih,[5] Hugh Hendrie,[6] and Yvonne Lu[7], *1. Indiana University at Bloomington, Bloomington, Indiana, United States, 2. IU School of Nursing, Indianapolis, Indiana, United States, 3. Alzheimer's Association, Greater Indiana Chapter, Indianapolis, Indiana, United States, 4. Alzheimer's Association, Chicago, Illinois, United States, 5. Indiana University School of Informatics, Computing, and Engineering, Indianapolis, Indiana, United States, 6. Indiana University School of Medicine, Indianapolis, Indiana, United States, 7. Indiana University School of Nursing, Indianapolis, Indiana, United States*

African Americans/Blacks continue to be underrepresented as participants in Alzheimer's Disease and related dementia (ADRD) and brain research. Numerous challenges such as lack of information about the Alzheimer's Disease and related dementia (ADRD), socioeconomic barriers, historical and systemic racism, and distrust of research goals and processes persist in research participation. Research approaches tend to be more recruitment oriented rather than partnership driven that do not address these challenges. As a result, community engagement approaches are increasingly being recognized as a means of building trust and creating new pathways for participation in ADRD studies. This poster focuses on the preliminary work of the Collaborative on Aging Research and Engagement (CARE) --- a community academic partnership comprising the CARE Advisory Team (a community action team of 10 African American leaders), Alzheimer's Association, the Alzheimer's Association Greater Indiana Chapter, IU Schools of Nursing, Public Health, and Informatics, Computer Science, and Engineering, and the Indiana Alzheimer's Disease Research Center. The goal of the partnership is to facilitate active engagement of African Americans aged 45 years and older in research opportunities taking place in in Central and Northwest Indiana. Experiences and perspectives shared at the CARE Advisory Team meetings as well as memos from the researcher staff generated five lessons learned in building relationship oriented, as opposed to recruitment driven, processes. These lessons will be used to develop a community engagement framework focused on the integration of culturally relevant outreach practices in promoting ADRD research opportunities in African American/Black communities.

### THE SOCIAL COST OF MODIFIABLE RISK FACTORS IN SINGAPORE

Vanessa Tan,[1] Stefan Ma,[2] and Cynthia Chen[3], *1. National University of Singapore, Singapore, Singapore, 2. Ministry of Health Singapore, Singapore, Singapore, 3. National University of Singapore, Singapore, Singapore*

Close to half of total disease burdens are attributable to modifiable risk factors, indicating that many illnesses are preventable by modifying behaviours such as increasing physical activity levels or maintaining a healthy diet. This study measures the social costs attributable to modifiable risks in Singapore, one of the most rapidly ageing populations in the world. Measuring the social cost of modifiable risk factors can help public health policymakers prioritise public health programmes and allocate resources. Our study builds on the comparative risk assessment framework from the Global Burden of Disease study. We used a prevalence-based cost-of-illness approach to estimate the social cost attributable to modifiable risk factors. We included healthcare costs from inpatient hospitalisation and productivity losses from absenteeism and premature mortality. Our results found that metabolic risks had the highest social cost of S$2.20 billion in 2019, followed by lifestyle risks of S$1.98 billion and substance risks of S$1.56 billion. Across the risk factors, the social costs were largely driven by productivity losses, heavily skewed towards the older working-age group. For metabolic risks, approximately 80% of the total cost (S$1.82 billion) was from those aged above 45 years old. This study provides evidence of the high social cost of modifiable risks and highlights the importance of developing holistic public health promotion programmes. Our findings suggest that implementing effective population-based programmes targeting multiple modifiable risks would have a strong potential to manage rising disease burdens and healthcare costs, especially with an ageing population.

### THE EFFECTS OF LOSING A SECURE JOB ON HEALTH BEHAVIORS ACROSS THE LIFE COURSE

Qian Song,[1] Emily Lim,[2] and Jackie Zhang[3], *1. Universiy of Massachusetts Boston, Boston, Massachusetts, United States, 2. University of Massachusetts Boston, Boston, Massachusetts, United States, 3. Grand Valley State University, Allendale Charter Township, Michigan, United States*

Involuntary, no-fault job loss from a relatively secure position has become more common among workers. However, it is not clear how displaced workers cope with the stress induced by the job loss and change their health behaviors due to work precarity, especially in the long-term. To address these gaps, we take advantage of the case of policy-driven layoffs from the State-Owned Enterprises in transitional China (1990s – mid-2000s), which resembles a quasi-experimental design, and examine effects of losing a secure job on dietary diversity, drinking, and smoking behaviors. We use the China Health and Nutrition Survey (1989–2015) and fixed effects models for controlling individual confounders. The results show that job loss decreases displaced workers' dietary diversity ($\beta$=-.18, $p < .05$); however, the decline bounced back after three years after job loss. The career stage in which job loss occurred made a difference. The reduction of dietary diversity only applies to early-career job loss (aged 35 or younger, $\beta$=-.24, $p < .1$), and mid-career job loss (aged 36 – 45, $\beta$=-.37, $p < .01$). Job loss increases probability of drinking (OR=1.36, $p < .1$), but the effect is only statistically significant for late-career job loss (aged 45+, OR=1.60, $p < .1$). Risks of heavy drinking are only increased for early-career job losers (OR=2.09, $p < .05$). Risks of smoking are not impacted by job loss. The findings highlight how health behaviors serve as coping strategies for job loss, how the effects vary by the timing of job loss, and how health promotions could be helpful to address the potential risks of work displacement.

### TRUST IN GOD AND THE CHURCH, NOT NEIGHBORS: EXPLORING SOCIAL COHESION AMONG AGING HIV+ INDIVIDUALS

Thi Vu,[1] Katherine Dunham,[2] Anna Lin-Schweitzer,[2] Meron Girma,[3] Megan Lee,[4] and Yusuf Ransome[2], *1. Yale University, New Haven, Connecticut, United States, 2. Yale School of Public Health, New Haven, Connecticut, United States, 3. Yale University School of Public Health, New Haven, Connecticut, United States, 4. Yale School of Medicine, New Haven, Connecticut, United States*

Rates of new HIV diagnoses among people 55+ years old remained unchanged between 2015–2019, suggesting potential HIV prevention challenges in the aging population due to co-morbidities, social isolation, and age-related stigma. Socio-cultural factors play a strong role in influencing HIV outcomes. Social cohesion — including appraisals of trust in neighbors and levels of belongingness to a community — has been shown to be an important channel for HIV prevention in the general population. However, there is scant evidence about how social cohesion relates to HIV prevention and care engagement among older adults. We employed a convergent, parallel mixed-methods study to investigate this topic among a sample (n =17) of adults aged 50+ living with HIV in New Haven, CT. We conducted semi-structured interviews which were analyzed using thematic analysis in NVivo.v12. We also collected quantitative data in RedCap and calculated descriptive statistics in STATA.v16. Participants were on average 57 years of age (SD=4); 53% female, and 69% Black/African American. Participants' trust in their neighborhoods was low [mean=2.61 (range 1 - 5, where 5 indicates high trust)]. In interviews, trust did not significantly influence one's HIV status disclosure or care management. Instead, participants often identified their faith in God or relationship with local churches as significant sources of social and informational support they often rely on for their HIV-related needs. Our results show that investigating the impact of religious belonging on outcomes may be a fruitful path of research to improve HIV outcomes among the aging population.

### IFIT3 GENE EXPRESSION AND FUNCTION CONTRIBUTE TO HAND PATHOLOGY EVEN WITH ANTIRETROVIRAL THERAPIES (CARTS).

Ranjit Kumar Das,[1] Deepa Roy,[2] Nirakar Sahoo,[1] and Upal Roy[2], *1. The University of Texas Rio Grande Valley, Edinburg, Texas, United States, 2. The University of Texas Rio Grande Valley, Brownsville, Texas, United States*

The advent of cART has revolutionized the management of HIV-1 infection and saved the lives of millions of people worldwide. Yet, HIV-Associated Neurocognitive Disorder (HAND) continues to be clinically relevant with aging people with HIV-1 (PWH). The underlying mechanism of HAND remains poorly understood. In this regard Signal transducer and activator of transcription 1(STAT1) and interferon-induced protein with tetratricopeptide repeats 3 (IFIT3) genes have been shown to induce neuroinflammation and neuronal cell death. However, the role of IFIT3 in HAND pathology has not been well established. Therefore, the present work investigated the significant role of IFIT3 gene in the development of HAND. In the current study, we used an in-vitro model to expose the neuroblastoma cell-line SH-SY5Y with clinically relevant cARTs drug and HIV Tat protein to observe the STAT1 and IFIT3 genes dysregulation. Furthermore, study also investigates the STAT1 and IFIT3 protein dysregulation through immunocytochemistry and western blot assay. Overall observation indicated that HIV-Tat protein upregulated gene expression whereas with the cART exposure there was a downregulation of STAT1 and IFIT3 genes. We next aim to investigate the role of STAT1 and IFIT3 genes in HIV-induced neuroinflammation. The current study has established IFIT3 as a biomarker marker for the detection of HAND.

### ASSESSING THE NIH TOOLBOX EMOTION BATTERY AMONG PEOPLE AGING WITH HIV AND WHO USE METHAMPHETAMINES.

Alexis Bender,[1] Hannah LF Cooper,[1] David Moore,[2] Molly Perkins,[3] and Regine Haardoerfer[1], *1. Emory University, Atlanta, Georgia, United States, 2. University of California at San Diego, San Diego, California, United States, 3. Emory University School of Medicine, Atlanta, Georgia, United States*

Measurement of social relationships is challenging because constructs have dimensions that can be assessed at multiple levels. Consequently, researchers often operationalize social relationships differently, making it difficult to compare results with the same outcome across studies. The National Institutes of Health (NIH) commissioned the Toolbox Assessment of Neurological and Behavioral Function to create a set of harmonized measures to use across studies. The NIH Toolbox-Emotions Battery (NIHTB-EB) was normed in English and Spanish-speaking populations, which found support for a three-factor structure (negative affect, social satisfaction, and psychological well-being). Here we investigated the factor structure of the NIHTB-EB among a sample of 862 people aged 17 to 82 with and without HIV and methamphetamine use in comparison to the factor structure found in the NIH Toolbox norming project. Initial confirmatory factor analysis did not support the three-factor structure identified previously for the full sample or subsamples. Subsequent exploratory factor analyses showed a good model fit for a four-factor solution for the full sample, with slight variations in factor loading across subgroups. In addition to the three factors described in the norming project, our solution contains an additional factor, "rejection and hostility," which includes scores from social satisfaction and negative affect in the normative population. The four-factor solution will be utilized for subsequent analyses using the NIHTB-EB in these study samples. Our analyses highlight the importance of assessing measures in distinct sub-populations to understand possible challenges to measurement invariance and ultimately allow the use of NIHTB-EB across groups.

### PAY RATES AND EMOTIONAL LABOR: CONTENT ANALYSIS OF JOB ADVERTISEMENTS FOR HOME HEALTH AIDES

Brittney Pond, *University of California, San Francisco, San Francisco, California, United States*

In the United States, over 3.5 million home health aides provide care and assistance to older adults and people with disabilities living in the community (Almeida, Cohen, Stone, & Weller, 2021). These providers are often tasked with emotionally and physically demanding work and are poorly compensated for their labor. In this poster I discuss the connection between the emotional demands of home health care jobs and their compensation through a quantitative content analysis of online job advertisements for home health aide positions (n=312). This research addresses two primary research questions: 1) what are the prevailing pay rates for home health aides and how do they compare to living wages? 2) what is the relationship between emotion work requirements and pay rates in home health aide job advertisements? In about 57% of the job advertisements hourly wages were not sufficient to meet the living wage level according to the MIT Living Wage Calculator. Using linear probability models, I found that there is no statistically significant difference between pay rates listed by home health agencies requiring emotional labor and home health agencies not requiring emotional labor while holding education, experience, and licensure requirements constant. Stratifying the data by home health agency/company size (1,000 employees or less and 5,000 employees or more) yielded no statistically significant results. These findings support and expand upon existing literature that home health aides are undercompensated for their work regardless of agency size, and provide evidence for the need for fair compensation and support.

### IMPACT OF RECEIVING HOME-BASED PRIMARY CARE FOR PERSONS LIVING WITH DEMENTIA: PATIENT AND CAREGIVER PERSPECTIVES

Meghan Mattos,[1] Veronica Bernacchi,[2] Karen Duffy,[3] Laura Jepson,[4] Stephanie Gonzalez,[1] and Justin Mutter[4], *1. University of Virginia, School of Nursing, Charlottesville, Virginia, United States, 2. Penn State College of Medicine, State College, Pennsylvania, United States, 3. UVA Health, Charlottesville, Virginia, United States, 4. University of Virginia, Charlottesville, Virginia, United States*

Homebound persons living with dementia (PWD) require healthcare services that proliferate with physical and cognitive changes over time. PWD often experience fragmented care that results in poor health outcomes and delayed healthcare access. The objective of the Virginia at Home Program (VaH), a recently initiated home-based primary care (HBPC) program, is to promote long-term sustainability and proliferation of HBPC. The study purpose was to examine the impact of VaH on the overall care and outcomes of homebound PWD and their families. We used quantitative and qualitative methods to describe the impact of VaH on patient care and outcomes over six months. We compared clinical outcomes (e.g., activities of daily living, frailty, behavioral symptoms) at baseline and six months using paired t-tests. We also conducted semi-structured phone interviews at baseline and six months with PWD-caregiver dyads. Thirty PWD-caregiver dyads enrolled. Of the 17 dyads that completed both visits, PWD's physical function and caregiver's burden decreased significantly over time (ps < .05). There was a significant improvement in PWD's behavioral symptoms over the six-month period (p=.03). Three preliminary themes emerged from qualitative data: 1) participants established trusting relationships with the VaH team, 2) VaH supported caregivers in their caregiving responsibilities, and 3) participants met their pre-program goals through increased healthcare access. Findings suggest that the HBPC intervention improved care satisfaction and healthcare access and alleviated caregiver burden. Future studies should consider introducing HBPC programs in this hard-to-reach population to decrease acute care utilization and health care costs while improving care satisfaction and quality.

### PET OWNERSHIP AND MAINTENANCE OF EXECUTIVE FUNCTION IN OLDER ADULTS–EVIDENCE FROM THE BLSA

Erika Friedmann,[1] Nancy Gee,[2] Eleanor Simonsick,[3] Melissa Kitner-Triolo,[4] Erik Barr,[5] Barbara Resnick,[6] Ikmat Adesanya,[6] and Merve Gurlu[1], *1. University of Maryland Baltimore, Baltimore, Maryland, United States, 2. Virginia Commonwealth University, Richmond, Virginia, United States, 3. National Institute on Aging, Baltimore, Maryland, United States, 4. Brain Aging & Behavior Section, Baltimore, Maryland, United States, 5. University of Maryland School of Nursing, Baltimore, Maryland, United States, 6. University of Maryland, Baltimore, Maryland, United States*

Pet ownership (PO) or human-animal interaction (HAI) has been associated with better physical and mental health in individuals with existing disease or disability. In experimental studies, HAI improves some aspects of cognitive function. No research addresses HAI and longitudinal changes in cognitive function in older adults. We examined the relationship of PO to maintaining executive function (EF) within generally healthy community-dwelling older adults in the Baltimore Longitudinal Study of Aging (BLSA). We hypothesized less EF deterioration among pet owners than non-owners; and less deterioration among dog-walkers than owners who don't walk their dogs. 637 women (55.9%) and men aged 50–100 years (M=68.3, SD=9.6) completed a PO questionnaire which ascertained ownership history and comprehensive examination every 1–4 years over 1–13 years (M=7.5, SD=3.6). Linear mixed models with time varying PO examined changes in EF according to PO. Pet owners (n=185) were younger (p < 0.001) and healthier (p=0.030) than non-owners; thus, age and comorbidities were included as covariates in the longitudinal analyses. EF, assessed using the Trail-Making Test (TMT), deteriorated with age (p's < 0.001). Deterioration was less severe among pet owners than non-owners (TMT A: p < 0.001; B: p < 0.001; difference: p=0.042) and dog owners than non-owners (TMT A: p=0.025; B: p=0.009; difference: p=0.076), but not among cat owners than non-owners. Among dog owners (n=73), dog walkers experienced less deterioration than non-walkers (TMT A: p=0.156; B: p=0.001; difference: p < 0.001). This study provides the first longitudinal evidence that PO may contribute to maintaining EF among community-dwelling generally healthy older adults as they age.

### EXAMINING HUMAN-ANIMAL INTERACTIONS AND THEIR EFFECT ON FRAILTY IN LATER LIFE: A SCOPING REVIEW

Ashley Taeckens-Seabaugh, Mary Corcoran, and Kevin Morris, *University of Denver, Denver, Colorado, United States*

Research suggests that human-animal interactions (HAIs) can improve the health and well-being of humans throughout their lifespan. While HAIs may facilitate healthy aging broadly, scant research has focused on HAIs as an intervention for adults aged 50 and older as it pertains to a comprehensive perspective of frailty. Moreover, scholarly literature lacks a consistent frailty definition, resulting in a lack of cohesion when evaluating the effectiveness of frailty interventions. This scoping review research proposes a comprehensive frailty definition and explores what is known about HAI interventions available to older adults as they relate to frailty statuses. Despite broad inclusion criteria, only four articles were relevant to this literature review, confirming the scarcity of relevant completed research thus far. Thematic analysis of reported results includes dog ownership as a protective factor regarding frailty statuses, the interconnected health effects of pet ownership, and meaning and purpose implications. Future interdisciplinary research should consider HAIs outside of pet ownership as frailty interventions for older adults, be mindful of population differences as they relate to intervention effectiveness, and work towards a universal, comprehensive definition of frailty that will aid in evaluating the frailty intervention effectiveness.

### THE GIFT OF MUSIC: AN INTERGENERATIONAL CAMPUS-COMMUNITY PARTNERSHIP

Whitney Nesser, and Scott Buchanan, *Indiana State University, Terre Haute, Indiana, United States*

Intergenerational programs bring individuals together across a continuum of age to share experiences. This study was designed to engage university music students with residents at a senior living community. University music student groups performed monthly at a local senior living community and completed pre- and post- performance evaluations to assess performance expectations, level of interest in the performance, perception of factors determining performance success, and perception of performance importance for the senior residents. A total of 24 students participated in one of three musical ensembles (Choir=11; Flutes=5; Steel Drums=8) during the months of February, March, and April 2022. Across all three ensembles, 50% of the students had never performed for residents at a senior living community. On a scale of 1–5, with 1 being "Not at all" and 5 being "Very much", 21 students indicated a "4" or "5" as to the importance of the performance for the residents whereas 15 students indicated a "4" or "5" in response to the importance of the performance for themselves. Most students reported looking forward to the performance, and following the performance indicated it had been a success. Factors identified as determining performance success included comments such as: “How much the audience enjoyed themselves!” and “Performers and audience enjoyed the performance. We would love to come back!”. Our findings suggest that performing live music in an intergenerational campus-community setting is beneficial not only for students but also for senior living facility residents.

### AGING DRAG QUEENS: RISKS, REWARDS, EXPRESSION AND PERFORMANCE

Laura Donorfio, Brian Chapman, and Kathleen Henneberry, *University of Connecticut, Waterbury, Connecticut, United States*

This poster presentation will share the exploratory findings of a qualitative research study examining the lived experiences of older drag queens, age 50+. Drag queens are an understudied and underrepresented population in the social science literature (Knutson, Koch, Sneed, Lee, & Chung, 2020; O’Brien, 2018). Older drag queens have yet to be studied in the social science literature. “Drag Queens possess unique characteristics as a subpopulation of the lesbian, gay, bisexual, and transgender (LGBT) community that warrant exploration” (Knutson, Koch, Sneed, & Lee, 2018). A comprehensive literature review identified approximately a dozen research driven articles from 2004 to the present, studying such areas as motivations, obstacles and stressors in becoming and being a drag queen (Hopkins, 2004; Knutson et al., 2021), depression, substance abuse, and resilience (Knutson et al., 2019; Tillewein & Kruse-Diehr, 2021) and the role gender plays via sexuality identities (Taylor & Rupp, 2004, Levitt et al. 2017). This research seeks to better understand how drag expression is integrated with one’s persona and how it interfaces with dragism, coping, and if drag expression can be used as a tool to foster resilience. Using a semi-structured interview protocol and thematic analysis, many themes emerged including the risks and rewards associated with drag expression, the relationship between aging and being a drag performer, and the shift in traditional versus 21st century drag. Lastly, we highlight the implications for gender and LGBTQIA+ theory and plans for future research.

### THEMES FROM CONVERSATIONS WITH MEDICAL TRAINEES ON LGBTQ OLDER ADULTS

Shobhana Sandhu, Mackenzi Kim, Lynn Wilson, Nyann Biery, and Kimberly Infante, *Lehigh Valley Health Network, Allentown, Pennsylvania, United States*

Approximately 6–12% of the US population 65 or older self-identifies as LGBTQ. This population faces immense barriers when accessing care, including the bias from healthcare professionals. Efforts to combat this bias through formal education are minimal. Using a mixed methods study with one-group pretest-posttest design and focus groups, medical learners were included in sessions involving a showing of Gen Silent and a post-viewing discussion. Themes from discussion were extracted by two independent reviewers. Medical learners (Nf15) included residents and faculty of psychiatric and emergency medicine at Lehigh Valley Health Network. Themes included: recognition of the social isolation faced by LGBTQ older adults, recognition of barriers to care including stigma and bias, challenges supporting patients and enabling patients’ openness, a need for a community resource repository, opportunities for EMR optimization, and physicians as advocates. These results highlight the need for additional training for medical trainees as well as the efficacy of using a tool like Gen Silent to accomplish this.

### THE IMPACTS OF DEPRESSION AND SUICIDE ATTEMPTS AMONG OLDER MEN WHO HAVE SEX WITH MEN

Alex Siu Wing Chan,[1] and Elsie Yan[2], *1. Hong Kong Polytechnic University, Hong Kong, Hong Kong, 2. The Hong Kong Polytechnic University, Hong Kong, Hong Kong*

Suicidality among older adults has attracted much attention due to their vulnerability. Older men who have sex with men (OMSM) have rarely been studied psychologically. Study examines factors that affect the mental health of OMSM, including depression, suicidal tendency, and suicide likelihood. OMSM in the United States are analyzed using descriptive statistics for correlations between depression and suicidal tendency. A literature review helped us select scales based on the regression model we constructed. Control variables were assessed for validity and relevance. A dependent variable was depression, and a dependent variable was suicidal tendencies. Depression and suicidal tendency scores significantly differed between men who have sex with men and the general population ($t = 67.084, 58.193$, $P < 0.01$). Suicidal tendencies and depression are significantly higher among homosexuals than among general groups. The regression analysis shows older men who have sex with men are more likely to suffer from depression and suicide ($P < 0.01$). Depression and suicide rates in OMSM are higher than those in the general population. The level of depression, in the intermediary test, mediates both the effect of OMSM on individual suicidal tendency and individual suicidal behavior ($P < 0.01$). Suicidal tendencies in OMSM can be reduced through depression intervention.

### NAVIGATING DEMENTIA DURING COVID-19: THE EXPERIENCES OF GAY AND LESBIAN OLDER ADULTS

Laura Girling,[1] and Mike Splaine[2], *1. Towson University, Towson, Maryland, United States, 2. Splaine Consulting, Columbia, Maryland, United States*

By 2030, it is estimated that 30 million individuals worldwide will have Alzheimer’s disease or related-dementias (hereafter dementia). Described as a “modern epidemic of later life,” dementia research has begun to reflect the diversity of our aging society with greater attention to minority populations. Nonetheless, some marginalized dementia-affected populations remain understudied. Estimates suggest more than 1 million lesbian, gay, bisexual, and transgender older adults will have dementia by 2030. Despite sizeable predictions, dementia-affected gay and lesbian populations remain critically understudied particularly in relation to COVID-19. To date, there is scarce literature focusing on how community-dwelling gay and lesbian adults with dementia navigate the management of their condition during the COVID-19 pandemic. In order to identify how community-dwelling gay and lesbian adults manage their dementia during the COVID-19 pandemic, data were combined from two interview-based studies. Content analysis was conducted on the interview narrative of the subset of individuals with dementia identifying as gay or lesbian and their study partner(n=18). Thematic findings include: 1.triple marginalization (age, sexual orientation, cognitive status), 2.social isolation, 3.programming effects (e.g., adult day programming, ADRD support groups), 4.informal and formal care interruptions, and 5.symptom acceleration. While all members of the public have experienced difficulties since the onset of COVID-19, health threats vary depending on social circumstances and vulnerabilities. Analyses indicate community-dwelling gay and lesbians with dementia are uniquely impacted by COVID-19 and face unique challenges. Attunement to such issues are of special interest to community-level policy makers, health providers, and older LGBTQ adults themselves, requiring further attention.

### LONELINESS IN LGBTQIA+ OLDER ADULTS: A SCOPING REVIEW

Katherine Bowers,[1] Joan Carpenter,[2] Marleen Thornton,[3] Hannah Fetting,[4] and Erin Johnson[5], *1. University of Maryland, Baltimore, Maryland, United States, 2. University of Maryland School of Nursing, Baltimore, Maryland, United States, 3. Notre Dame of Maryland University, Baltimore, Maryland, United States, 4. Johns Hopkins Bayview, Baltimore, Maryland, United States, 5. Johns Hopkins Hospital, Baltimore, Maryland, United States*

Loneliness in older adulthood is responsible for poor mental and physical health, cognitive decline, and early mortality. Older adults in the LGBTQIA+ community experience additional layers of vulnerability, such as discrimination, higher rates of childlessness, health inequity, and economic disparity that place them at high risk for loneliness. The purpose of this scoping review is to explore the existing literature on loneliness in LGBTQIA+ older adults and identify knowledge gaps to inform future research recommendations. Using the Arskey & O'Malley methodological framework, our team searched PubMed, Embase, CINAHL, PsychINFO, Scopus, and Google Scholar using keywords related to older adults, sexual and gender minorities, and loneliness. We identified 61 articles from 1980 to 2022. Most of the research in this literature reflected studies using descriptive and qualitative approaches. Intervention studies were the least reported. One opinion piece is included. Initial findings highlight associations between discrimination and loneliness, poor mental health and loneliness, the challenges of HIV in older adulthood and loneliness, and loneliness experienced in caregiving and end of life in LGBTQIA+ older adults. Importantly, many studies emphasized the protective nature of strong social support and community ties against loneliness. Equally, the literature suggests that limited social support is a significant risk factor for loneliness in this population. Older gender non-binary and LGBTQIA+ people of color were underrepresented in many studies. Future research should include intervention research that targets loneliness in this population as well as studies focused on understanding loneliness more comprehensively in underrepresented LGBTQIA+ older adults.

### INTERNALIZED HOMOPHOBIA MEDIATING THE RELATIONSHIP OF SELF-CONCEALMENT ON DEPRESSION IN LGB OLDER ADULTS

Christopher Calzada, Carl St. Goar, Matthew Hollander, Vennisia Mo, Jamie Kiefer, Ben Hougaard, Nicole Carre, and Rowena Gomez, *Palo Alto University, Palo Alto, California, United States*

Previous research has demonstrated that self-concealment of sexual orientation may negatively affect the wellbeing of lesbian, gay, and bisexual (LGB) adults (Hu, Wang, & Wu, 2013). Additionally, studies have indicated that internalized homophobia significantly predicts depressive symptoms in LGB older adults (Sharma & Subramanyam, 2020). The present study examined how internalized homophobia may mediate the predictive relationship between self-concealment on depression symptoms in LGB older adults. As part of a larger study, participants (Nf301) responded to several questions and measures including the Sexual Orientation Self-Concealment Scale, The Internalized Homophobia Scale, and the Center for Epidemiological Studies Depression Scale. Using the Baron and Kenny's Method for Mediation (1986), the data analysis indicated that internalized homophobia fully mediates the relationship between self-concealment and depression severity ($ps < .05$). These results suggest that older adults who self-conceal their sexual orientation may experience an increase in depressive symptoms because of internalized homophobias. These findings may help clinicians better understand the mechanisms contributing to the high rates of depressive symptoms within the older LGB population. Furthermore, the findings suggest that cognitive restructuring of thoughts related to internalized homophobia and self-concealment may improve depressive severity in older LGB adults.

### IMPACT OF COVID-19 ON THE MENTAL HEALTH OF OLDER SEXUAL MINORITY CANADIANS: A LONGITUDINAL ANALYSIS

Alexandra Grady, and Arne Stinchcombe, *University of Ottawa, Ottawa, Ontario, Canada*

The COVID-19 pandemic and related restrictions have had negative impacts on mental health. Sexual minorities (i.e., lesbian, gay, and bisexual; LGB people) have been shown to exhibit mental health disparities stemming from minority stress experiences. The purpose of this study was to examine the mental health trajectories of older sexual minority Canadians in comparison to heterosexual Canadians before and during the pandemic. We used data from the Canadian Longitudinal Study on Aging (CLSA), where data were collected from participants at baseline (2011–2015), first follow-up (2015–2018), and two time points during the COVID-19 pandemic (April-December 2020). General Estimating Equations (GEE) were used to model changes in depression symptoms (CESD-10; n=47,728) and loneliness (UCLA 3-item loneliness scale; n=41,698), adjusting for covariates (i.e., age, sex, income, race, education). Results showed that LGB participants reported more symptoms of depression ($\beta=.595$, $p < .001$) and loneliness ($\beta=.313$, $p < .001$) in comparison to heterosexual participants. Mental health outcomes worsened over time for all participants. These findings highlight the impacts of the pandemic on mental health and the need for tailored interventions for older lesbian, gay, and bisexual individuals.

### HUMAN CENTERED DESIGN TO DEVELOP A LGBTQ+ COMMUNITY ADVISORY BOARD FOR SKILLED NURSING FACILITIES

Jennifer May,[1] Jason Wheeler,[1] Ames Simmons,[2] Mark Hensley,[3] Jerry O'Donnell,[4] Joseph Wheeler,[5] and Kirby Ellis[6], *1. Duke, Durham, North Carolina,*

United States, 2. Duke University, Washington, District of Columbia, United States, 3. AARP NC, Greensboro, North Carolina, United States, 4. Community Member, Greensboro, North Carolina, United States, 5. Carolina Aging Alliance, Inc., Raleigh, North Carolina, United States, 6. Liberty Healthcare, Pittsboro, North Carolina, United States

Lesbian, gay, bisexual, transgender, queer (LGBTQ+) adults will require inclusive skilled nursing care as they age, but there is a paucity in LGBTQ+ specific training resources for skilled nursing facility (SNF) healthcare workers. Development of community advisory boards is becoming common in research studies as funders place emphasis on stakeholder engagement, but how advisory boards are created with community member involvement at the onset remains less clear. This presentation describes the creation of a community advisory board of LGBTQ+ community members, community organizations, and SNF administrators that work together to develop LGBTQ+ resources for 25 SNFs. Advisory board members were identified using a design thinking framework and the Field Guide to Human-Centered Design. Potential board members were identified through networking with community-based organizations, existing relationships with SNFs, and by word of mouth. The advisory board consists of 3 LGBTQ+ adults, 4 SNF administrators, 1 AARP community outreach and advocacy associate director, 1 advocacy coordinator for LGBTQ+ older adults, 1 SNF physician liaison, and 1 nurse scientist. To gain buy-in, individual interviews and focus groups with potential advisory board members were conducted. Meetings provided an opportunity to describe project goals and how potential member's expertise would guide development of resources. LGBTQ+ adult community members received compensation for their participation. The advisory board is meeting monthly and approaching the design phase. Community stakeholder involvement in early stages of development will help ensure that LGBTQ+ resources are relevant and applicable to enhance health and well-being of LGBTQ+ older adults in the future.

### HEALTH INFLUENCES OF UNIQUE AND INTERSECTING IDENTITIES: AGE, SEXUAL ORIENTATION AND RACE

Laura Bernstein, and Julie Patrick, *West Virginia University, Morgantown, West Virginia, United States*

Lesbian, gay, and bisexual older adults are an understudied group with limited research using nationally representative samples. Although there are known physical health disparities based on age, sexual orientation, and race, the effects of intersecting identities have not been fully examined. We were interested in how these identities individually influence health and whether they interact to effect physical health. Using the 2020 Behavioral Risk Factor Surveillance System (M age = 54.30, SD = 17.52), we examined whether sexual orientation was related to the number of poor physical health days experienced and whether that relation was moderated by age and race. The overall model was significant (F (5, 223356) = 511.86, p < .001, R2 = .0113). Significant direct effects of sexual orientation and race emerged, but these were qualified by a significant sexual orientation by race interaction (b = -.09) A significant main effect emerged for age (b = 1.08) but the sexual orientation by age interaction failed to reach significance. There was a significant three-way interaction between age, race, and sexual orientation (F (2, 223356) = 6.34, p < .01). Investigation into the means found that multiracial adults tend to report more poor health days than White, Black, or Hispanic adults, except for bisexual older and younger adults. These results highlight the importance of examining intersecting identities when trying to understand health disparities across the lifespan. Understanding how these identities interact helps to identify specific barriers these groups face and ultimately improve health equity, specifically in an aging population.

### RISKS FOR LATER-LIFE COGNITIVE IMPAIRMENT: DISENTANGLING CHILDHOOD AND EARLY FAMILY-LIFE PREDICTORS

Lindsey Mundy,[1] Kimson Johnson,[2] and Jacqui Smith[2], *1. University of Michigan, Tuscaloosa, Alabama, United States, 2. University of Michigan, Ann Arbor, Michigan, United States*

Observing later-life health outcomes using a life-course model uncovers critical periods of influence. We investigated the relationship between five multi-component measures of childhood disadvantage (determined by Morton et al., 2022) and risk of cognitive impairment (determined by the Langa-Weir Classification) in later life. Data from the Health and Retirement Study (n= 9,509) were used. We conducted a multivariable parametric survival analysis to examine the relationships between the early life predictors of low socioeconomic status (SES), childhood impairments, risky adolescent behaviors, risky parental behaviors, and childhood chronic illness and the risk of developing later-life cognitive impairment between 2006 and 2016. We found that each additional indicator of low SES, risky adolescent behavior, and impairment in childhood increased the risk of developing cognitive impairment over the 10 years studied (HR=1.14, p < .05; HR=1.15, p < .05; HR=1.19, p < .05). Additional indicators of risky parental behaviors, however, were related to a smaller risk of developing cognitive impairment over time (HR=0.96, p < .05). Childhood chronic illness was not significantly associated with the risk of developing cognitive impairment. These results show that early-life factors might have long-term implications for the development of cognitive impairment in later life and display differential relationships with risk when parsed into competing categories. Measures of disadvantage indicating smaller risk may point to the resilience and plasticity of the brain in early life and highlight the need for the study of multiple life stages in order to track predictors of cognitive impairment throughout the life course.

### LIFECOURSE DETERMINANTS OF DEPRESSION AMONG OLDER MEXICAN ADULTS

Paige Downer,[1] John Prochaska,[2] and Rebeca Wong[3], *1. University of Texas Medical Branch, League City, Texas, United States, 2. University of Texas Medical Branch, Galeveston, Texas, United States, 3. University of Texas Medical Branch, Galveston, Texas, United States*

Older adults' exposure to poverty, poor health, and negative life events over their lifetime creates cumulative adversity, increasing their risk of depressive symptoms. We hypothesize that those with disadvantaged sociodemographic characteristics like household poverty, chronic health conditions, and negative lifecourse exposures will be more likely to report a high number of depressive symptoms in old age. Three sequential multivariable logistic regression models were estimated using Wave 3 (2012) of the Mexican Health and Aging Study (MHAS). The final sample included 5,610 respondents aged 50 and older, of which 34.3% reported depression measured by a modified 9-item CES-D scale. The sample was mostly female (63.3%) with a mean age of 69. Present-day conditions were measured by quality of the home, consumer durables, chronic health conditions, and health insurance. Early-life conditions were measured by the respondent's mother's education and exposure to poverty and illness during childhood. In 2012, older Mexican adults living in homes built with poor construction materials or homes that lack access to water and sanitation (OR=1.24) were more likely to experience high depressive symptoms. In addition, those living in homes without consumer durable goods (OR=1.23) were at increased risk. Older Mexican adults who experienced poverty (OR=1.16) or illness during childhood (OR=1.21) were more likely to report a high number of depressive symptoms in old age. In conclusion, we find evidence of a "long-arm" of childhood, whereas older Mexican adults' exposure to poverty and illness in childhood increases their likelihood of poor mental health outcomes, regardless of their present-day conditions.

### GENDER DIFFERENCES IN RURAL-URBAN MIGRATION AND ITS IMPACT ON MENTAL HEALTH IN LATER LIFE

Jingwen Zhang, James Nazroo, and Nan Zhang, *The University of Manchester, Manchester, England, United Kingdom*

Although rural-to-urban migration has been well researched, how gender shapes processes and outcomes, including later life health outcomes, has not been thoroughly investigated. Guided by a life course perspective, this study explores gender differences in rural-urban migration patterns and its association with mental health in later life among Chinese older adults. Exploiting rich life history data from the China Health and Retirement Longitudinal Study, we employ sequence analysis to identify the typical migration trajectories of Chinese older adults. Moderated mediation analysis is then used to examine gender-specific health pathways linking migration trajectories and later-life mental health. The results indicate that: men and women follow different migration trajectories across the life course. Men are more likely to migrate between rural and urban areas, and to migrate multiple times. Rural migrants who settled in urban regions have better mental health in later life than return migrants or rural non-migrants; the gender gap in mental health is marginally smaller among early urban settlers than rural non-migrants. Household income and Hukou conversion mediate the relationship between migration trajectories and later-life mental health among older people of rural origin. Household income in later life has stronger mediation effects for migrant men than for migrant women. The study suggests that a life course perspective and awareness of gender dynamics should be incorporated in policymaking to reduce the rural-urban divide and gender-based inequality in mental health.

### PERSPECTIVES OF THE FEMALE SPOUSE IN DEMENTIA CAREGIVING IN FORMAL CAREGIVING RESOURCE USE

Anna Satake, *Univerisity of California, Davis, American Canyon, California, United States*

The purpose of this qualitative descriptive study was to explore female spousal caregivers' decisions regarding the use of formal caregiving resources in caring for a partner with dementia. Participants included 11 female spouses who provided caregiving to their husbands with dementia in the home setting. Findings suggested that a caregiver's willingness or reluctance to use any resources were influenced by factors that related to them (e.g., assessment of their skills/abilities), their husband (e.g., his belief he didn't need help) and, to a lesser degree, the resource qualities (e.g., cost). Caregivers seemed to make the decision to use resources only after careful consideration of their husband's needs, his understanding of what he needed, and how well the resource would fit for him and whether he would accept that help. Interestingly, factors like cost and access were less germane to the caregiver's decision. This implies that the decision-making process for a wife caregiver in regards to the care for a partner with dementia is complex and involves their readiness and needs as the caregiver, the needs and readiness of the persons with dementia, and the qualities of the resources. These factors are all dynamic, and clinicians and formal caregiving services need to have ongoing conversations and assessments throughout the caregiving journey to assess both the caregiver's willingness and reluctance to start a specific service.

### SESSION 9030 (POSTER)

### LATE BREAKING POSTER SESSION IV

### RISK OF HEART FAILURE AFTER CATHETER ABLATION VS. MEDICAL THERAPY FOR ATRIAL FIBRILLATION IN OLDER ADULTS

Ricardo Criado Carrero,[1] Andrew Crawford,[2] Ali Vaeli Zadeh,[3] Alan Wong,[2] Alexander Fleischhacker,[1] Elias Collado,[3] and Joshua Larned[4], *1. University of Miami/Holy Cross Hospital, Fort Lauderdale, Florida, United States, 2. Holy Cross Health-University of Miami, Fort Lauderdale, Florida, United States, 3. Holy Cross Health-Jim Moran Cardiovascular Research Institute, Fort Lauderdale, Florida, United States, 4. Holy Cross Health- University of Miami, Miami, Florida, United States*

Background: Age increases the risk of atrial fibrillation (AF). Catheter ablation (CA) proved being safer and superior to anti-arrhythmic drugs (AADs) in maintaining sinus rhythm and preventing readmissions. However, data on the risk of developing heart failure (HF) following each treatment in older adults is limited. Methods: A retrospective study was conducted using the PearlDiver database (PearlDiver Technologies, Fort Wayne, IN). Using ICD 9 and 10 codes, a cohort of patients without a history of HF, aged 65 to 75, with an Elixhauser Comorbidity Index (ECI) of >4 and new-onset AF (index event) was identified. The cohort was then divided into two groups based on treatment received following the index event and matched considering age, gender, ECI, and other cardiovascular diseases. Records from both groups were reviewed for the first episode of HF over 12 months following the initiation of treatment. Pearson's chi-squared test was used to compare groups. The strength of association was reported using Risk Ratios (RR). A p-value < 0.05 was deemed significant. Results: 6,446 were included in each group. The mean age, gender and ECI were indifferent. 958 (15%) of patients treated with AADs and 987 (15.3%) of patients treated with CA had a first episode of HF over a year after the treatment which was not significantly different between groups (RR=1.03, CI95% = 0.95- 1.11, p=0.49). Conclusion: The modality of treatment after the first episode of AF in older population doesn't significantly affect the risk of HF over the first year.

### IMPACT OF OACS ON OUTCOMES OF COVID-19 INFECTION IN OLDER ADULTS WITH HISTORY OF ATRIAL FIBRILLATION

Ricardo Criado Carrero,[1] Andrew Carl Crawford,[1] Ali Vaeli Zadeh,[2] Alan Wong,[3] Ana De Diego Diaz,[4] Elias Collado,[2] and Joshua Larned[5], *1. University of Miami/Holy Cross Hospital, Fort Lauderdale, Florida, United States, 2. Holy Cross Health-Jim Moran Cardiovascular Research Institute, Fort Lauderdale, Florida, United States, 3. Holy Cross Health-University of Miami, Fort Lauderdale, Florida, United States, 4. University of Miami/Holy Cross Hospital, Fort Luderdale, Florida, United States, 5. Holy Cross Health- University of Miami, Miami, Florida, United States*

Background: Age, atrial fibrillation (AF), and COVID-19 infection predispose patients to hypercoagulability and poor outcomes. It is unclear if older adults with AF and COVID-19 infection would benefit from oral anticoagulants (OACs). Methods: A retrospective study was conducted using the PearlDiver database (PearlDiver Technologies, Fort Wayne, IN). Using ICD-10 codes, adults aged 65–75 and Elixhauser Comorbidity index(ECI) >4 with a history of AF admitted for COVID-19 were identified. The use of OACs for 6 months before the index event was used to split the cohort into two propensity score-matched groups considering age, gender, and ECI. Records from both groups were reviewed for multiple outcomes during the same admission. Pearson's chi-squared test was used to compare groups. The strength of association was reported using Risk Ratios (RR). A p-value < 0.05 was deemed significant. Results: We compared 16,967 individuals in both anticoagulated and non-anticoagulated groups. Anticoagulated patients had a lower risk of mortality (RR=0.11, p=0.026), and a higher risk of 30-day all-cause readmission(RR=1.12, p < 0.0001). However, there were no differences in ICU admission, gastrointestinal bleeding, intracranial hemorrhage, thromboembolic events, or length of hospitalization. Conclusion: Compared to non-anticoagulated patients, older adults with a history AF on chronic oral anticoagulants had a lower risk of all-cause mortality, and higher risk of 30-day all-cause readmission. This information would help clinicians decide whether to prescribe OACs to this population of patients.

### GENDER AND AGE EFFECTS ON PTG EXPLAINED BY PSYCHOLOGICAL FACTORS IN CARDIAC PATIENTS

Amy Ai, and Crim Sabuncu, *Florida State University, Tallahassee, Florida, United States*

The present study explored the role of gender on posttraumatic growth (PTG) 30-months after cardiac surgery and mediation of preoperative psychosocial factors. Two previous meta-analyses found that woman tended to have more growth than men, and that PTG was associated with improved overall health, but most studies were cross-sectional and small-scale. Using a prospective design, we followed 262 patients undergoing cardiovascular operations with surveys and objectively measured medical indices from the Society of Thoracic Surgeons' (STS) national database. Participants completed a follow-up survey on PTG 30-month after surgery. Bivariate correlations related PTG with female gender, age, minority race, marriage, and faith-based factors, but no medical or other psychosocial factors. In hierarchical regressions, gender and age were linked with PTG, alongside other demographics in Step-1. These effects sustained after entry of STS indices, in Step-2, but the gender role diminished after adding medical comorbidities, preoperative depression, optimism, hope, and social support in Step-3. No other factor in these steps had an impact. The age effect vanished when faith factors were entered, but only positive spiritual coping (PSC) was related to PTG. Finally, the role of PSC on PTG was mediated by perceived spiritual support. The final model was significant, accounting for one-third of the variance [$F(20, N = 215) = 4.896$, $p < .001$, $R2 = .334$] in PTG. This suggests that more research should aim to explore the effect of gender and age on PTG in general. For cardiac patients, care providers may encourage positive faith-based coping prior to cardiovascular surgery.

### EFFECT OF ASPIRIN IN COVID-19 OUTCOMES OF OLDER ADULTS WITH A HISTORY OF CORONARY ARTERY DISEASE

Ali Vaeli Zadeh,[1] Ricardo Criado Carrero,[2] Alan Wong,[3] Noah Mandile,[4] Marina Stukova,[5] German Lopez,[5] Elias Collado,[1] and Joshua Larned[6], *1. Holy Cross Health-Jim Moran Cardiovascular Research Institute, Fort Lauderdale, Florida, United States, 2. University of Miami/Holy Cross Hospital, Fort Lauderdale, Florida, United States, 3. Holy Cross Health-University of Miami, Fort Lauderdale, Florida, United States, 4. Jim Moran Cardiovascular Institute, Fort Lauderdale, Florida, United States, 5. University of Miami at Holy Cross, Fort Lauderdale, Florida, United States, 6. Holy Cross Health-University of Miami, Miami, Florida, United States*

Background: Advanced age and coronary artery disease (CAD) have been associated with a dismal prognosis in patients infected with COVID-19, most likely due to the virus's thrombogenic effects. Older adults with a history of CAD have routinely used low-dose Aspirin (LDA) as prevention due to their increased risk of cerebro-cardiovascular events. However, it is unclear if this population would benefit from LDA when infected with COVID-19. Methods: A retrospective study was conducted using the PearlDiver database (PearlDiver Technologies, Fort Wayne, IN). Using ICD codes, patients aged 65–75 and Elixhauser Comorbidity Score(ECI)>4 with a history of CAD admitted for COVID-19 were identified. The use of LDA for 1 month before the index event was used to split the cohort into two matched groups by age, gender, ECI, and other cardiovascular diseases. Records of groups were reviewed for multiple outcomes 30 days after admission. Pearson's chi-squared test was used to compare groups. The strength of association was reported as Risk Ratios (RR). Results: 4,017 patients with no difference in the mean age, gender, and ECI were included in each group. No differences present in 30-days all-cause readmission(RR=1.04, CI95% =0.92–1.17, p=0.49), mortality(RR=0.63, CI95%=0.30–1.29, p=0.28), ICU admission(RR=1.01, CI95%=0.89–1.15, p=0.79), gastrointestinal bleeding(RR=1.09, CI95% = 0.85–1.40, p=0.51), and intracranial hemorrhage(RR=0.69, CI95%=0.26–1.83, p=0.62) between groups. Conclusion: LDA didn't improve the evaluated outcomes in older persons 30 days after admission. A plausible explanation is that COVID-19's thrombogenic mechanism is likely atypical.

### COMORBIDITIES ASSOCIATED WITH RISK OF MYOCARDITIS IN HOSPITALIZED COVID-19 OLDER ADULTS

Andrew Crawford,[1] Andres Redondo,[2] Ali Vaeli Zadeh,[3] Alan Wong,[1] Ricardo Criado Carrero,[4] Alexander Fleischhacker,[4] Elias Collado,[3] and Joshua Larned[5], *1. Holy Cross Health-University of Miami, Fort Lauderdale, Florida, United States, 2. University of Miami, Fort Lauderdale, Florida, United States, 3. Holy Cross Health-Jim Moran Cardiovascular Research Institute, Fort Lauderdale, Florida, United States, 4. University of Miami/Holy Cross Hospital, Fort Lauderdale, Florida, United States, 5. Holy Cross Health- University of Miami, Miami, Florida, United States*

Background: Older adults are afflicted more severely by COVID-19. SARS-CoV-2 can be complicated by myocarditis (MC), and the incidence of MC has been shown to correlate linearly with severity. However, data on comorbidities associated with MC in this population is scarce. Methods: Data were obtained from the PearlDiver database (PearlDiver Technologies, Fort Wayne, IN). The study used ICD codes to include patients hospitalized with a primary diagnosis of COVID-19, aged 65–75, and Elixhauser Comorbidity index(ECI)>4. Within this cohort, we identified patients diagnosed with MC 60 days after admission and compared their baseline comorbidities upon admission to those without MC. Pearson's chi-squared test was used to compare groups. The strength of association was reported by Risk Ratios (RR). A p-value < 0.05 was deemed significant. Results: 412,582 patients admitted with COVID-19 as the primary diagnosis were identified. 0.12% of this cohort developed MC over the following 60 days. The MC group was more likely to be male(57%, p=0.0001), with similar mean age(70.4, p=0.86) and mean ECI(9.4, p=0.07) to the no-MC group. Patients who developed MC have significantly higher rates of prior heart failure(RR= 1.30, CI95%=1.07–1.57, p=0.008). There was no difference between groups in terms of history of arrhythmias(p=0.36), cerebrovascular disease(p=0.09), chronic kidney disease(p=0.13), CAD(P=0.19), diabetes(p=0.48), ischemic heart disease(p=0.06), tobacco use(p=0.39), alcohol use(p=0.17), HIV(p=0.79), and severe liver disease(p=0.14). Conclusion: A history of heart failure increased the likelihood of developing MC in older adults.

### ASSOCIATION BETWEEN STATE AND TRAIT ANXIETY AND CARDIOVASCULAR DISEASE

Ruifeng Cui,[1] Montgomery Owsiany,[2] and Alaa Shalaby[3], *1. VA Pittsburgh Healthcare System, Morgantown, West Virginia, United States, 2. West Virginia University, Morgantown, West Virginia, United States, 3. VA Pittsburgh Healthcare System, Pittsburgh, Pennsylvania, United States*

Cardiovascular disease (CVD) consistently ranks among the top ten leading causes of death, with prevalence rates being the highest among middle aged and older adults. Research has found that CVD has a negative bidirectional association with anxiety. However, the literature is limited regarding what specific aspects of anxiety, i.e., state and/or trait, are associated with CVD. The present study investigated the association between state and trait anxiety and CVD in a national sample of 640 middle aged and older adults. Participants (M age = 56.2, SD = 11.8) were from the Midlife in the United States (MIDUS) Biomarker sub-project. State anxiety was assessed with the Mood and Anxiety Symptom Questionnaire-anxiety subscale and trait anxiety was assessed with the Spielberger Trait Anxiety Inventory. In a binary logistic regression model including both state and trait anxiety, only trait anxiety significantly predicted presence of CVD (p = .05). In particular, trait anxiety was significantly associated with circulatory disease (p = .05), heart murmurs (p = .03), and blood clots (p = .03). Among middle-aged and older adults, trait anxiety is associated with having several different CVDs. These findings support assessing and addressing trait anxiety among middle aged and older adults with CVD.

### AGE DIFFERENCES IN DEPRESSION AND ANXIETY LEVELS OF OPEN HEART SURGERY PATIENTS

Crim Sabuncu, and Amy Ai, *Florida State University, Tallahassee, Florida, United States*

Age, as well as gender, are key demographic measures in cardiac patients. Further, depression and anxiety have been long established to be comorbidities of heart disease (HD). In this prospective study, to examine the impact of age and gender in preoperative depression and postoperative anxiety symptoms of HD patients receiving open heart surgery (OHS), we collected two waves of survey data along with medical indices from patients at a top-10 heart center in the USA (n = 481, mean age = 62.18 ± 12.04, female 42%). The collected survey data included socio-demographic information (age, gender, race), religious affiliation and other religious factors, general health and health behaviors, medical comorbidities, cardiovascular health indices, dispositional optimism, hope, social support, depression symptoms, and anxiety symptoms. Statistical analysis was carried out using hierarchical linear regression analyses. Findings support that higher age correlates with increased levels of cardiac symptoms. Further, younger age was associated with higher levels of postoperative depression, while older age was associated with higher levels of postoperative anxiety. Higher levels of dispositional optimism were consistently associated with lower levels of both postoperative depression and postoperative anxiety regardless of age. Female gender had an impact on depression scores but not on anxiety scores. Findings suggest that medical practitioners might benefit from being more attentive to non-medical conditions such as mood states, dispositional optimism, and general positive expectations about the future in post-OHS life. The findings also suggest that there might be age and gender differences in expected postoperative psychosocial symptomology for OHS patients.

### THE ROLE OF MEDITERRANEAN DIET IN THE PRIMARY AND SECONDARY PREVENTION OF ATRIAL FIBRILLATION: A SCOPING REVIEW

Yan Su,[1] Kuan-Ching Wu,[2] Oleg Zaslavsky,[3] and Marilyn Prasun[1], *1. Illinois State University, Normal, Illinois, United States, 2. University of Washington, Seattle, Washington, United States, 3. Unversity of Washington, Seattle, Washington, United States*

Atrial fibrillation (AF) is the most common cardiac arrhythmia, especially in patients older than 65. The prevalence of AF increased 3-fold over the last 50 years. All treatments, such as cardioversion, ablation, and anticoagulation, are associated with risks and relapses. Besides, these strategies may not apply to all patients with AF. Adherence to the Mediterranean dietary pattern is assumed as an ideal nutritional model for AF. Yet, there is a gap on 1) how adherence to the Mediterranean diet prevents AF and 2) how the Mediterranean diet affects complications in patients with AF. A scoping review was performed in June 2022 to identify studies focusing on the Mediterranean diet and AF. A total of 334 articles were retrieved and 12 met inclusion criteria. Six studies were interventions (n=6) and six were observational studies (n=6). In the six intervention studies, five were nested within the PREDIMED (Prevención con Dieta Mediterránea) study. In AF occurrence (n=6), the results were inconsistent. The Mediterranean diet plus extra virgin olive oil was significantly and favorably related to AF, however, Mediterranean diet plus nuts was not, when compared with the low-fat diet; Mediterranean diet modified how long chain acylcarnitine affected development of AF but not how tryptophan or arginine metabolites affected AF. In patients diagnosed with AF (n=6), Mediterranean diet favorably related to adverse cardiovascular events, oxidative stress, and Mediterranean diet adherence, and did not affect anticoagulation for those taking anticoagulants. Further research is needed to examine how the Mediterranean diet affects the development of AF.

### SHORT- AND LONG-TERM OUTCOMES AFTER HIP SURGERY IN OLDER ADULTS WITH AND WITHOUT HEART FAILURE

Sijia Wei,[1] Wei Pan,[2] Tingzhong (Michelle) Xue,[3] Hideyo Tsumura,[4] Chiyoung Lee,[5] and Eleanor McConnell[2], *1. Northwestern University Feinberg School of Medicine, Chicago, Illinois, United States, 2. Duke University, Durham, North Carolina, United States, 3. Duke University School of Nursing, Durham, North Carolina, United States, 4. University of Michigan School of Nursing, Durham, North Carolina, United States, 5. University of Washington Bothell, Bothell, Washington, United States*

Older adults with heart failure (HF) have a higher risk for adverse outcomes after hip fracture surgery than those without. Propensity score matching (PSM) reduces selection bias and makes a direct group comparison (older adults with and without HF) possible. Thus, this study aimed to assess the impact of HF on short-and long-term outcomes after hip fracture surgery in older adults living with and without HF. Electronic health records data of older adults (n = 1171) hospitalized for hip fracture surgery between October 2015 and December 2018 were extracted. Comparison groups (with and without HF) were identified using 1:1 ratio PSM to control for observed differences in baseline characteristics. Regression models were used to compare group differences in outcomes. Although in analyses without PSM, older adults with HF were more likely to have higher 90-day readmission, and 30-, 90-, and 365-day mortality, this association was not significant after controlling for selection bias. However, the associations between having HF with 30-day readmission and longer length of stay were significant before and after PSM. Additionally, if patients did not receive hip fracture surgery procedures within two days of admission, they had a 3.6-day longer inpatient stay (P-value < 0.0001) and were 47.8 times more likely to die during hospitalization (95%CI 4.9–482.0, P-value < 0.001). Being non-white was significantly associated with higher 90- and 365-day mortality. Future research should consider PSM approach on national representative datasets to rigorously evaluate the effects of HF on mortality and readmission following hip fracture surgery in older adults.

### DECREASING THE USE OF BENZODIAZEPINES AND SEDATIVE HYPNOTICS IN POST OPERATIVE OLDER ADULTS

Amaka Opute, *UTSW Medical Center Dallas, Dallas, Texas, United States*

Purpose: The purpose of this project was to decrease the prescribing of benzodiazepines and sedative hypnotics in post-operative older adults Background: Routine prescribing of benzodiazepines and sedative hypnotics as first line choices for sleep medication is a known contributor to prolonged hospital stays, increased fall rates, increased development of delirium, higher health care costs, and long-term cognitive decline. Methodology: Practice changes included monthly provider and nursing staff education inservices, coupled with transparent monitoring of provider prescribing practices. The outcomes measure was to decrease the prescribing of benzodiazepines and sedative hypnotics by 5% within 90 days and increase the prescribing of melatonin by 10%. Results: When comparing six months of data pre and post implementation on the unit, there was a 33% decrease in the prescribing of benzodiazepines/sedative hypnotics and a 12% increase in the prescribing of Melatonin. There was also a 28% decrease in the hours of patient safety sitter use, a marker for delirium. Implications: This quality improvement project decreased the number of benzodiazepines and sedative-hypnotics prescribed by expanding provider and nursing knowledge, which ultimately led to fewer hours spent utilizing patient safety sitters, thereby lowering healthcare costs, while concurrently improving the quality of care delivered

### DECREASING PAIN, ANXIETY, AND CONFUSION IN TERMINALLY ILL VETERANS

Denise Kresevic,[1] Muralidhar Pallaki,[2] Bridgette Wasil,[3] Marilyn Swanson,[3] and Christopher Burant[4], *1. VA Northeast Ohio Healthcare System, Sagamore Hills, Ohio, United States, 2. VA Northeast Ohio Healthcare System, Cleveland, Ohio, United States, 3. VA Northeast Ohio Healthcare System, Cleveland, Ohio, United States, 4. Case Western Reserve University, Parma, Ohio, United States*

Terminally ill patients often receive medications for pain and anxiety that result in sedation that may inhibit communication with their loved ones. Balancing comfort while maintaining meaningful communications is a common dilemma for patients, staff, and families. Many patients report confusion and a decreased ability to communicate with families and health care providers, resulting in fear, anger, and frustration. Additionally, family members (25 to 33%) also feel frustrated and angry not being able to communicate with their loved ones needs and wishes. Delirium, a distressing syndrome characterized by disturbed consciousness, reduced ability to focus attention, altered cognition is experienced by 62–88% of terminally ill patients. Unfortunately, it is often underdiagnosed and undertreated. While not all delirium can be prevented it is estimated that 50% of terminal delirium may be ameliorated. This project was designed to proactively identify terminally ill veteran preferences for nonpharmacological interventions to reduce pain, anxiety, and prevent delirium. In the sample of Veterans from an inpatient VAMC Hospice (n=31) the mean age was 79.46 (sd=10.69); 60.7% were Black and 39.3% were white; 60.9% had cancer and 27.6% had heart failure. The most common patient delirium related behaviors were verbal agitation, physical agitations, physical aggression, anxiety, and confusion. Veterans identified the following comfort priorities: decreased noise (82.6%) and lighting (82.6%), warm blankets (78.3%), music (65.2%), emotional support (73.9%), increased family involvement (43.5%) and (20.8%) going outdoors. Preventative individualized nonpharmacological interventions decreased disruptive delirium related behaviors over 95% of the time and should be incorporated into routine hospice care.

### SERVED BY SYSTEMS: AGING WITH INTELLECTUAL AND DEVELOPMENTAL DISABILITIES AND INEQUITIES IN HEALTHCARE

Danielle Waldron,[1] Emily Hartford,[2] Jacquelin Sauer,[2] and Jeffrey Stokes[3], *1. Stonehill College, Taunton, Massachusetts, United States, 2. Stonehill College, Easton, Massachusetts, United States, 3. University of Massachusetts Boston, Boston, Massachusetts, United States*

Adults and older adults with intellectual and developmental disabilities (I/DD) often experience medical complexity, accelerated aging, and shortened life expectancy. This project explores health service utilization (HSU) of adults with I/DD receiving state services in the U.S. over the adult lifespan before and after the implementation of the Affordable Care Act (ACA). Three waves of cross-sectional data (2008–2019) from the National Core Indicators-In Person Survey were analyzed using multilevel mixed effects logistic regression (n=46,284). Older adults with I/DD were more likely to receive a physical exam, flu vaccine, eye exam, hearing test, and a dental exam compared to younger individuals, although these differences were small. Compared to non-Hispanic whites, non-Hispanic blacks were less likely to receive physical exams, flu vaccines, and dental exams; non-Hispanic other were less likely to receive eye exams and dental exams; and Hispanic persons were less likely to receive eye exams, flu vaccines, and dental exams. Individuals with I/DD living in states that expanded Medicaid were at 68% greater odds of receiving a physical exam than those who did not. Our research indicates that overall adults with I/DD are not yet reaching HSU of pre-ACA times, perhaps due to the oversaturation of existing providers. National and state policies, along with individual case management each play a role in ensuring healthy aging of individuals with I/DD. A call to action to better understand and integrate these three entities may help improve the potential for healthy aging of this group.

### LOVE AMONG OLDER AFRICAN AMERICAN COUPLES: AN ACTOR PARTNER INTERDEPENDENCE MODEL ANALYSIS

Kadija Mussa,[1] and Chalandra Bryant[2], *1. University of Minnestoa, Falcon Heights, Minnesota, United States, 2. University of Minnesota, Minneapolis, Minnesota, United States*

Older women are often described as being asexual and uninterested in sex or intimacy (McHugh & Interligi, 2015). Thus, most research examining older couples describes those couples as primarily enjoying companionate or compassionate love – a type of love reflecting care and concern for another person (Allen et al., 2018). Unlike companionate or compassionate love, passionate love refers to a “state of intense longing for union with another” (Hatfield & Rapson, 1993, p. 67). Relatively little is known about passionate love and older couples (Hatfield & Rapson, 1993); moreover, far less is known about passionate love among African American older couples. Using data collected from African American couples (332 couples aged 20 to 39 and 90 couples aged 40 to 79), Actor-Partner Interdependence Models were used. For both age groups, 20 to 39 and 40 to 79, husbands’ and wives’ reports of marital quality were significantly associated with each other at Time 1. Husbands’ and wives’ reports of passionate love (assessed at Time 2) were not significantly associated with each other – for either age group under study. Cross paths (partner effects) were not significant for either of the two age groups; wives’ marital quality (Time 1) did not significantly predict husbands’ passionate love (Time 2), nor did husbands’ marital quality (Time 1) predict wives passionate love (Time 2). It is important to note that the older and younger age groups exhibited a similar pattern of results, suggesting that passion and physical intimacy may operate in similar ways for both.

### IMPACT OF A DRIVING DECISION AID ON DECISIONAL CONFLICT AMONG OLDER ADULT DRIVERS AND THEIR STUDY PARTNERS

NIcole Fowler,[1] Rachel Johnson,[2] Ryan Peterson,[3] Matthew Schroeder,[4] Carolyn DiGuiseppi,[5] Duke Han,[6] Linda Hill,[7] and Marian Betz[8], *1. Indiana University School of Medicine, IU Center for Aging Research, Indianapolis, Indiana, United States, 2. Colorado School of Public Health, Aurora, Colorado, United States, 3. University of Colorado, Aurora, Colorado, United States, 4. Regenstrief Institute, Inc., Indianapolis, Indiana, United States, 5. University of Colorado Anschutz Medical Campus, Aurora, Colorado, United States, 6. University of Southern California, Alhambra, California, United States, 7. University of California San Diego, La Jolla, California, United States, 8. University of Colorado School of Medicine, Aurora, Colorado, United States*

Forty-four million US licensed drivers are ≥65 years old and at higher crash risk. Decision-making about stopping or continuing driving is difficult and often involves family and friends. This study examines if decision conflict about changing driving habits is associated between older adult drivers and their study partners (SPs) (i.e., family member or friend). Data were from a multi-site trial assessing a driving decision aid. Decision conflict about stopping or continuing driving for drivers and their SPs were measured with the Decision Conflict Scale (DCS). Dyadic associations between drivers' and SPs' DCS scores pre- and post-decision aid implementation were analyzed using an actor-partner interdependence model. Among 228 driver-SP dyads, driver mean (SD) age was 77.1 (5.1) years; 50.0% female; 98.7% non-Hispanic; 94.7% white; and 97.8% urban-dwelling. SPs mean age was 66.1 years (13.9); 65.8% female; 95.6% non-Hispanic; 92.1% white; and commonly the driver's spouse (54.6%) or adult child (21.1%). Most drivers (71.7%) and SPs (63.3%) had baseline DCS scores < 25 (drivers mean 18.5 (SD 12.3); SPs 20.5 (16.8)), suggesting low decision conflict. DCS was correlated within dyads at baseline (r=0.18, p < 0.01), and baseline DCS was associated with post-decision aid DCS (p < 0.001 for SPs [β=0.73] and drivers [β=0.73]). While SPs' baseline DCS was not associated with drivers' post-decision aid DCS, drivers' baseline DCS and SPs' post-decision DCS were (β=0.10; p=0.036). Higher decision conflict about driving felt by older drivers is frequently shared by their SPs, in whom decision conflict may persist even after a driving decision aid intervention.

### EMPATHIC RESPONSE AND MARITAL QUALITY FOR SAME- AND DIFFERENT-SEX MIDLIFE COUPLES: MEDIATING ROLE OF DYADIC COPING

Susanna Joo,[1] Hye Won Chai,[2] Jihye Lee,[1] Hyoun K. Kim,[1] and Debra Umberson[2], *1. Yonsei University, Seoul, Seoul-t'ukpyolsi, Republic of Korea, 2. The University of Texas at Austin, Austin, Texas, United States*

Dyadic coping is a daily interpersonal process that married couples use to manage stress and maintain their marriage. However, little is known about its mediating role in the association between empathic response and marital quality among same-sex and different-sex couples. This study aimed to examine the extent to which dyadic coping mediates the association between empathic response and marital quality, focusing on middle-aged men and women in same-sex and different-sex marriages. We used dyadic data from the Health and Relationships Project (HARP), including 124 gay, 171 lesbian, and 124 straight couples. Results from the actor-partner interdependence mediation model (APIMeM) showed that dyadic coping within couples mediated the association between empathic response and marital quality for all couple types (i.e., gay, lesbian, and straight couples). More empathic response was associated with better dyadic coping, which led to higher marital quality. While such mediated paths did not differ significantly between gay and lesbian couples, direct associations between empathic responses and marital quality were only significant among lesbian couples. Additionally, there were gendered patterns within straight couples; while female spouses' empathic response was associated with their and their male spouses' marital quality through the couple's dyadic coping, such a mediated path was not significant for male spouses' empathic response. These findings suggest dyadic coping as an effective strategy for enhancing marital quality among same-sex and different-sex married couples, but the mediating role of dyadic coping is gendered in different-sex marriages.

### PERCEIVED FINANCIAL VULNERABILITY, WEALTH, AND WEALTH CHANGE: THE HEALTH AND RETIREMENT STUDY

Madison Maynard,[1] Emily Flores,[2] Daniel Paulson,[1] and Peter Lichtenberg[2], *1. University of Central Florida, Orlando, Florida, United States, 2. Wayne State University, Detroit, Michigan, United States*

Financial vulnerability among older adults is an escalating social concern given all too frequent financial exploitation of this population. The 6-item Perceived Financial Vulnerability (PFV) was derived from the 56-item Lichtenberg Financial Decision Rating Scale to examine awareness and psychological vulnerability regarding finances. It was included as an experimental module in the Health and Retirement Study (HRS) in 2018. Prior findings have identified significant associations of PFV with wealth, demographics, and health status. The goals of the current study were to examine the relationship between wealth, changes in wealth, and perceived financial vulnerability. The sample included 1156 respondents to the HRS. Respondents were 57.5% female and 77.2% Caucasian. Average age was 68.28 (SD = 10.74) and average years of education was 13.11 (SD = 2.88). The PFV demonstrated adequate internal consistency (α = .60). Total assets at baseline (2016) and change in total assets over two waves (2016 to 2018) were independently stratified into deciles and used as primary predictors of perceived financial vulnerability in 2018. A multiple linear regression model was conducted to examine these relationships. Consistent with previous findings, demographics (R2=0.04, F(5,1150)=10.07, p < .001) and baseline wealth (B= -0.20, SE=0.02, p < .001) predicted PFV scores. Subsequent addition of wealth change (B=-0.06, SE=0.02, p=.002) significantly contributed to overall variance accounted for (p < .01). Negative wealth change over two years and low baseline wealth related to higher PFV. These findings support the construct and the PFV measure as valid and informative.

### INADEQUATE PHYSICAL ACTIVITY ON HEALTHCARE EXPENDITURES AMONG MIDDLE AGE AND OLDER WORKING ADULTS IN THAILAND

Katika Akksilp,[1] Wanrudee Isaranuwatchai,[2] Yot Teerawattananon,[2] and Cynthia Chen[3], *1. National University of Singapore, Singapore, Singapore, 2. Health Intervention and Technology Assessment Program, Muang Nonthaburi, Nonthaburi, Thailand, 3. National University of Singapore, Singapore, Singapore*

Physical inactivity is a significant risk factor for developing non-communicable diseases. This study estimates the additional health costs due to physical inactivity in Thai middle age and older working adults. We included participants aged 40 years and above from the Physical Activity at Work study, who had valid physical activity data from ActiGraph. Health costs were collected using the Health and Welfare Survey and the Work Productivity and Activity Impairment Questionnaire in Thailand. Direct and indirect costs (absenteeism and presenteeism) of the most recent illness in the previous month were collected. Active participants were defined as having at least 150 minutes of moderate-intensity equivalent activity per week. Two-part models were used to compare health costs between active and non-active participants. Data from 105 participants aged 40 to 63 were included in the analysis, where 42% of participants were active. Inadequate physical activity was significantly associated with increased direct health costs of 160.5THB (95%CI: 31.3 to 289.7THB) (~7.8 times higher in inactive). Further adjustment for sex, age, education, obesity, and cardiovascular condition yielded similar findings. After incorporating these indirect costs, the societal cost of having inadequate physical activity was 791.22THB (95%CI: 115.6 to 1466.8THB) (~1.3 times higher in inactive), even after adjusting for the covariates. Obesity and the female were also associated with higher societal costs (551.9THB and 342.9THB, respectively), although these results were not significant. Increasing physical activity in middle age and older working adults to meet the current guidelines may reduce Thailand's health costs.

### FINANCIAL HARDSHIP AND INCOME SECURITY AND MENTAL HEALTH AMONG OLDER WORKERS: MULTI-CHANNEL SEQUENCE APPROACH

Oejin Shin,[1] Sojung Park,[2] Eunsun Kwon,[3] and Byeongju Ryu[4], *1. Illinois State University, Savoy, Illinois, United States, 2. Washington University in Saint Louis, Saint Louis, Missouri, United States, 3. Fairleigh Dickinson University, Florham Park, New Jersey, United States, 4. Washington University in St. Louis, St. Louis, Missouri, United States*

Drawing on the life course perspective and multi-channel sequence approach, this research identifies long-term financial hardship and income security patterns among older adults and examines their relationship with depressive symptoms. Data were from the 2010 to 2018 HRS with 3,077 older workers aged 62–71 in 2010. Four clusters of key sequences were identified: 1) High food and low medical insecurities with private pension plans (43%); 2) Moderate food and low medical insecurities with gradually acquired private pension plans (13%); 3) High food and low medical insecurities with both private and employment-based pension plans (40%); 4) High food insecurity with employment-based pension plans(3%). Regarding the association with depressive symptoms, a group of people who experienced high food and low medical insecurities with private pension plans (b= 0.14, p < 0.1) and those who experienced moderate food and low medical insecurities with gradually acquired private pension plans (b= 0.32, p < 0.01) were more likely to have depressive symptoms compared to those high food and low medical insecurities with both private and employment-based pension plans. These findings highlight the heterogeneity of long-term patterns of financial hardships and patterns of income security, including the limited accessibility for a pension plan through their occupation and that an individual pension plan may not be sufficient to ensure mental health in later life. The results imply the importance of dual security of private and employment-based pensions on their mental health and provide important insights for future tailored income support programs for older adults.

### END-OF-LIFE HOSPITALIZATION SPENDING IN THE LAST SIX MONTHS OF LIFE AMONG CANCER PATIENTS

Katika Akksilp,[1] Vanessa Tan,[1] Julian Lim,[2] Keith Lim,[3] and Cynthia Chen[4], *1. National University of Singapore, Singapore, Singapore, 2. Saw Swee Hock School of Public Health, Singapore, Singapore, 3. National University Hospital, Singapore, Singapore, 4. National University of Singapore, Singapore, Singapore*

End-of-life care may constitute a vast proportion of health care expenditure globally. In the US, one-quarter of all medicare spending goes towards healthcare for people near the end of life. This is especially true for cancer patients. Our study aimed to estimate hospital spending in cancer patients in the last six months of life. Patients with cancer who died between June 2015 to December 2016 (n=653) were extracted from Singapore's electronic medical records. A two-part model was used to estimate hospitalization spending. In the last month of life, we found that 85% of the patients did not have chemotherapy, 43% had good quality care, and 31% had palliative care. The average total hospitalization spending in the last six months of life was S$59,180. Further analysis by month from death revealed that spending increased sharply by 62%, from $7,591 in the sixth month of life to $12,315 in the last month of life. Decedents who received chemotherapy in the last month of life spent $6,925 more on hospitalization expenditure than those who did not ($17,915 vs $10,990). Among decedents seen by a palliative doctor, hospital expenditures in the last month were $8,745 higher compared to those who did not ($16,119 vs $7,374). The study depicted the huge hospitalization spending during the end-of-life for cancer patients in Singapore. Expenditure on end-of-life care can be further reduced with investment in community-based services and integrated care across the community and acute sectors, where hospital-to-home services and earlier palliative care can provide potential cost-effective solutions.

### A MODEL FOR STUDENT PARTICIPATION IN COMMUNITY-ENGAGED RESEARCH FOR OLDER ADULTS: THE ENROLL PROGRAM

Matthew Peterson,[1] Tina Newsham,[2] Alicia Sellon,[1] Madison Norris,[1] Leanza Sidey,[1] Christian Barnes,[1] William Holland,[1] and Jodi Rich[3], *1. University of North Carolina Wilmington, Wilmington, North Carolina, United States, 2. UNC Wilmington, Wilmington, North Carolina, United States, 3. New Hanover County Parks & Gardens, Wilmington, North Carolina, United States*

The current academic climate has left naturally occurring faculty-led student research experiences and mentoring a challenge. This is coupled with an already existing dearth of future scientists interested in aging-related research. The overarching goal of the Enhancing Research for OnLine Learners (EnROLL) Program is to facilitate research collaboration among undergraduate minoritized students, graduate distance learners, health sciences faculty, and academic partners. The program includes a variety of strategic initiatives, one of which includes building five inter-institutional research cores: epidemiology, preclinical, allied health, applied learning, and community engagement. Here, we present a community-based participatory project led by students and researchers in the EnROLL community engagement core in partnership with local government. Researchers at UNCW were contacted by New Hanover County Parks & Gardens staff to assist in wellness planning for area older adults. Two surveys were developed: Nf103 community members and Nf8 staff members completed separate surveys. Community respondents were 60.8 +/- 13.7 years old, 72% female, and 80% Caucasian. Approximately 58% of community respondents were at least somewhat likely to participate in wellness programming at County locations. Based on preferred days, times, and types of classes of the respondents, a comprehensive weekly schedule of wellness classes was developed and provided to County staff for implementation under the guidance of a UNCW masters student. This community engagement project, and the EnROLL program, can serve as a model for student-engaged, aging-related research in the health sciences, particularly as gerontologists work to diversify the demographics of researchers who focus on aging.

### WORKING TOWARD AN AGE-FRIENDLY UINDY: EXAMINING THE PROCESS AS IT UNFOLDS

Lisa Borrero, and Heidi Ewen, *University of Indianapolis, Indianapolis, Indiana, United States*

Universities must adapt by expanding their understanding of inclusivity in higher education, acknowledging new aging-related career opportunities for students and their role as destinations for lifelong learning and community engagement for older adults. Accordingly, nearly 100 institutions have committed to age-inclusivity on their campuses by achieving Age-Friendly University (AFU) designation. For those interested in pursuing this designation at their institutions, an important part of the process is learning from one another about strategies used at other campuses that could be tailored to their own needs. With this in mind, this poster presentation will outline the initial strategies and resulting outcomes of the path to AFU designation at the University of Indianapolis (UIndy) via a preliminary "discovery" phase. Using GSA's Tools for Advancing Age Inclusivity in Higher Education as a guide, AGHE members at the University of Indianapolis are in the beginning stages of their own process, characterized by discussions with various campus stakeholders during the recent spring semester. This "listening tour" has revealed strong support of the AFU principles and an eagerness to be part of an age-inclusive vision. These conversations have identified existing infrastructure and other strengths that support age-inclusivity, as well as current challenges to be managed and new opportunities to pursue. The authors will also describe the next phase of the process, including interviews with older UIndy alumni and retired faculty and staff about engagement with retired community members, as well as subsequent steps, such as working with colleagues to design viable age-friendly initiatives.

### TECHNOLOGY-DRIVEN INTERGENERATIONAL PROGRAM IN THE PANDEMIC

Sheryl Groden, Matthew Carpenter, Jennifer Blackwood, and Allon Goldberg, *University of Michigan-Flint, Flint, Michigan, United States*

Intergenerational programs increase interaction among groups allowing both generations to share their beliefs, talents, knowledge, and wisdom. The Senior Programming Intergenerational College Experience (SPICE) Project was designed to deliver an 11-week long intergenerational program with college-aged and older adult participants and was provided virtually during the pandemic. The purpose of this study was to describe the participation characteristics and attitudes towards aging and technology of older adults who completed the SPICE program. Older adult recruitment occurred through paper fliers posted at senior centers/housing, libraries, faith-based organizations in Genesee County, Michigan and through a large statewide electronic study recruitment portal. Older adult participants were English speaking; aged 55+ years; able to participate in live Zoom sessions once/week; and were committed to complete >75% of the SPICE program. Online surveys were completed before/after SPICE to evaluate attitudes towards aging and technophilia. Results from 15 older adults who completed >75% of SPICE sessions indicated that they were able to cope with life as they got older (60%), viewed aging as a positive experience (85.7%), were not afraid to use new technology (68.6%) and believed technology was useful/fun (88.6%). Older adults were divided on willingness to try new technology and their ability to keep up with technological progress. These data indicate that for higher education institutions focused on designing age-inclusive online opportunities, older adults who possess a positive attitude toward aging and technology may be more likely to complete intergenerational programs.

### GAUGING INTEREST AND NEEDS IN PROFESSIONAL DEVELOPMENT AND CONTINUING EDUCATION IN AGING IN MANITOBA, CANADA

Michelle Porter, William Kops, and Nicole Dunn, *University of Manitoba, Winnipeg, Manitoba, Canada*

The Centre on Aging at the University of Manitoba in Canada conducted a needs assessment on continuing education (CE) and professional development (PD) in aging with people who work and connect with older adults. During winter and spring 2022, an online survey of 35 questions asked respondents about their own or their staff and volunteer topics of interest, as well as logistics of CE/PD workshop training. A total of 146 participants responded. Respondents were from different regions of Manitoba, with the highest proportion (42%) located in the Winnipeg region. About 86% were female and 12% male, ranging in age from 18 to 75: with most falling in the 35 to 64 age range. Respondents were asked to indicate their level of interest for topics on a five-point scale from Not Interested to Very Interested. The topics Aging Through the Lifespan and Wellness received the highest number of responses, while Indigenous Aging received the least. Across the 1,712 individual ratings of suggested topics, the highest proportion were rated as interested (35%) and very interested (31%); while 19% rated level of interest as moderate, 11% minimal, and 4% not interested. When asked about potential workshop formats, the majority (63%) preferred a mix of both in-person and online delivery formats with preference for 1–2-hour workshops and a certificate as proof of completion. About two-thirds indicated a requirement to complete CE/PD credits to maintain related credentials. These findings provide considerations for higher education institutions on CE/PD for those working and connecting with older adults.

### AFRICAN-AMERICAN DEMENTIA CAREGIVER STRATEGIES INVENTORY (DCSI-A): INITIAL PSYCHOMETRIC EVALUATION

Nik Lampe,[1] Robert Glueckauf,[2] Benjamin Behers,[2] Yuxia Wang,[2] and Sarai Jean-Pierre[2], *1. Vanderbilt University, Nashville, Tennessee, United States, 2. Florida State University College of Medicine, Tallahassee, Florida, United States*

Recent studies have found promising evidence of the benefits of cognitive-behavioral interventions (CBIs) in improving depression and health status of African-American dementia caregivers (CGs). However, limited information exists about the specific CB strategies used in these investigations and which CB strategies were most effective in reducing caregiver distress and distress-related health problems. To address these shortcomings, Glueckauf and colleagues developed the African-American Dementia Caregiver Strategies Inventory (DCSI-A) using data from two CBI interventions (Nf129). The primary objectives of the current study were: (a) to identify the most frequently used categories of CBI strategies deployed by African-American CGs and (b) to conduct an initial evaluation of the psychometric properties of the DCSI-A. Descriptive statistics were calculated to assess strategy category frequency and percent of total responses. The two most frequently used strategies were: (1) CG Health-Related Intervention Strategies with 19 endorsements, 16.10% of total strategies and (2) Strategies for CG Social, Recreational, Personal Enhancement and Other Community Activities with 18 endorsements, 15.25% of total responses. These findings suggested that health-related and social, recreational, and personal enhancement strategies were the most commonly used by African-American CBI participants. Intercoder agreement was assessed across two one-month coding intervals, each of which contained 5 coding sessions. Mean percent of agreement and Kappa were .94 and .93, respectively. Both reliability measures fell within the highly acceptable range. Coder drift was minimal across the two time intervals. Future research will evaluate the psychometric properties of the DCSI-A with a larger sample of African-American CGs undergoing CBI.

### ADVANCING DIVERSITY IN ADRD/AGING RESEARCH AND CLINICAL CARE: EDUCATIONAL AND CAREER TRAJECTORIES OF MSTEM MENTEES

Sheri Thompson, *UC San Diego, La Jolla, California, United States*

As an NIA-funded mentorship program, MADURA addresses the lack of diversity among Aging and Alzheimer's Disease & Related Dementia (ADRD) researchers and clinicians. Aims include improving retention and academic success of URM MSTEM undergraduates, and increasing rates of graduate/medical school applications and entry into Aging/ADRD clinical careers. MADURA offers paid research lab experience, weekly whole-cohort research skills training, guest seminars and presentations, and weekly faculty-facilitated small group supervision, advising and support. It served 23–29 undergraduates/quarter, in its initial five quarters. The Program accepts students at varying undergraduate educational levels, particularly because students from target populations often enter as Junior-level transfers from community colleges. Thus, although the Program just completed its second year, 17 MADURA trainees have already graduated. While ongoing data collection on Program, Mentor and Student Mentee performance is beneficial, outcomes data are of utmost importance to ensuring achievement of aims. This poster will present descriptive data on MADURA graduates' immediate employment and educational activities. Notable findings include a significant proportion of trainees who take a gap year before applying to graduate or medical school (for financial and personal reasons), and others taking internships, post-baccalaureate training or research jobs, to strengthen future medical and graduate school applications. Understanding the multiple pathways of recent graduates will enable mentorship programs to help current trainees critique and optimize preparations for their selected educational and career trajectories. These findings also suggest additional mentorship program outcomes of interest (beyond a sole focus on graduate program acceptance), and the need for longer-term alumni follow-up.

### THE IHSS+ ADRD TRAINING PROJECT: BOLSTERING THE DEMENTIA CARE WORKFORCE IN CALIFORNIA

Jarmin Yeh,[1] Brittney Pond,[1] Matthew Beld,[1] Corinne Eldridge,[2] and Leslie Ross[1], *1. University of California, San Francisco, San Francisco, California, United States, 2. Center for Caregiver Advancement, Los Angeles, California, United States*

California's In-Home Supportive Services (IHSS) program provides vital home care to low-income consumers, some of whom live with Alzheimer's disease or related dementias (ADRD). Yet, most IHSS caregivers receive little or no training in dementia care. With the prevalence of ADRD among Californians age 55+ projected to increase 127% by 2040, reaching over 1.5 million people, the IHSS consumer population living with ADRD will likely increase at a similar rate, exacerbating the need for dementia-trained home care workers. This poster describes preliminary outcomes of the IHSS+ ADRD Training Project, a 10-week, competency-based training program, aiming to reach 600 IHSS caregivers in Alameda County, California, by 2024. All planned in-person activities switched to virtual strategies due to the COVID-19 pandemic. To date, 348 IHSS caregivers have been trained through 16 classes, with 9 classes offered in English, 3 classes in Spanish, and 4 classes in Cantonese. A quasi-experimental, longitudinal design was used to evaluate the project's impact through pre, post, and 3-month follow-up surveys. Caregiver outcome measures included the: 1) Dementia Knowledge Assessment Tool 2, 2) Fortinsky self-efficacy scale, 3) Caregiver Self-Assessment Questionnaire, and 4) Patient Health Questionnaire-2. Preliminary outcomes trend toward significant increases in caregivers' knowledge about ADRD and self-efficacy to maximize care they provide to consumers. While caregivers also reported slight increases in stress and depression, they expressed high levels of satisfaction with the training. Future analysis will include comparing IHSS caregiver outcomes to healthcare utilization patterns of IHSS consumers before and after their caregiver's participation in the training.

### REVISITING THE TEACHING NURSING HOME: QUALITATIVE ANALYSIS OF NURSING STUDENTS' CLINICAL EXPERIENCE

Brianna Morgan,[1] Kierra Foley,[2] Nancy Hodgson,[1] Ann Kolanowski,[3] Kim Strauch,[4] Nancy Zionts,[5] Chelsea Dickson,[6] and Howard Degenholtz[7], *1. University of Pennsylvania, Philadelphia, Pennsylvania, United States, 2. Penn Nursing, Philadelphia, Pennsylvania, United States, 3. Penn State University, State College, Pennsylvania, United States, 4. Penn School of Nursing, Philadelphia, Pennsylvania, United States, 5. Jewish Healthcare Foundation, Pittsburgh, Pennsylvania, United States, 6. University of Pittsburgh School of Public Health, Pittsburgh, Pennsylvania, United States, 7. University of Pittsburgh, Pittsburgh, Pennsylvania, United States*

Revisiting the Teaching Nursing Home is a two-year pilot project to address the long-term care workforce shortage by introducing nursing students to geriatric nursing while also improving quality of care within nursing homes. The initiative has multiple components: enhanced clinical rotations for nursing students with partner schools of nursing, implementation of the Institute for Healthcare Improvement Age-Friendly Health System "4M" quality improvement model, and an online learning network. Undergraduate and graduate nursing students at three Schools of Nursing participated in clinical experiences at regional nursing homes. Students completed an "activity feedback" form after each clinical rotation at the nursing home or related activity, such as a session about the 4Ms or quality improvement/assessment. The activity feedback form asked students to share their most important takeaway and suggestions for improvement. Data from 340 feedback forms was coded qualitatively using conventional and directed content analysis using the American Association of Colleges of Nursing (AACN) Essentials for Professional Nursing Education. Multiple coders and audit trials were used to establish rigor. Students' takeaways encompassed 7 of 8 key concepts in the AACN Essentials; Knowledge for Nursing Practice, Person-Centered Care, and Interprofessional Partnerships were most frequently mentioned. Students provided numerous suggestions for improving their clinical experiences including facilitated learning from instructors and supported engagement with nursing home staff. In conclusion, the program addressed many of the core competencies designated by AACN. One recommendation that flows from these findings is to enhance the role of clinical preceptors in the nursing home setting to facilitate mentored training.

### REVISITING THE TEACHING NURSING HOME: IMPACT ON NURSING STUDENTS' PERCEPTIONS OF WORKING IN LONG-TERM CARE

Howard Degenholtz,[1] Marie Boltz,[2] Donna Fick,[3] Nancy Hodgson,[4] Kim Strauch,[5] Jacob Kariuki,[6] Yurun Cai,[1] and Dawn Pajerski[6], *1. University of Pittsburgh, Pittsburgh, Pennsylvania, United States, 2. Penn State, State College, Pennsylvania, United States, 3. Penn State University, University Park, Pennsylvania, United States, 4. University of Pennsylvania, Philadelphia, Pennsylvania, United States, 5. Penn School of Nursing, Philadelphia, Pennsylvania, United States, 6. University of Pittsburgh School of Nursing, Pittsburgh, Pennsylvania, United States*

Revisiting the Teaching Nursing Home is a two-year pilot project to address the long-term care workforce shortage by introducing nursing students to geriatric nursing while improving quality of care within nursing homes. The initiative has multiple components: enhanced clinical rotations for nursing students with partner schools of nursing, implementation of the Institute for Healthcare Improvement Age-Friendly Health System "4M" quality improvement model, and an online learning network. Nursing students at three schools of nursing participated in the clinical rotations at regional nursing homes. The experience was limited to students in one specific course at each school of nursing. At the beginning and end of the spring 2022 semester, students rated their competence in: patient assessment, collaborating with the care team, gathering clinical information, medication review, eliciting resident values, and health promotion. Students also rated their preferences for working in long-term care and with older adults. Data from 85 responses at the start of semester and 64 responses to the end of semester survey were analyzed. Analysis of student responses found that students self-rated competencies improved in all areas except eliciting resident values. Prior to their clinical experience, students ranked working in long-term care and with older adults lower than other settings or populations. The rankings were unchanged after their clinical experiences. These initial findings suggest that although the Teaching Nursing Home Program is meeting the pedagogical goals, attitudinal shifts may require different strategies.

### RE-AIM EVALUATION OF A VIRTUAL "AGING WITH PAIN" INTEGRATIVE HEALTH EDUCATION PROGRAM DURING THE COVID-19 PANDEMIC

Jennifer Brailsford,[1] Sophia Sheikh,[2] Jason Beneciuk,[3] Monika Patel,[2] Brittany Johnson,[4] Robin Moorman Li,[5] Phyllis Hendry,[2] and Natalie Spindle[2], *1. University of Florida, Jacksonville, Florida, United States, 2. University of Florida College of Medicine – Jacksonville, Jacksonville, Florida, United States, 3. University of Florida, Gainesville, Florida, United States, 4. UF Health Jacksonville, Jacksonville, Florida, United States, 5. University of Florida College of Pharmacy, Jacksonville, Florida, United States*

Literature suggests integrative pain management strategies reduce chronic pain and opioid use. However, many older adults are unaware of these options. The Aging and Integrative Pain Assessment and Management Initiative (AI-PAMI) launched in 2020, providing webinars and recorded presentations on integrative pain management for adults > age 50, caregivers and healthcare providers. The RE-AIM (Reach, Effectiveness, Adoption, Implementation, Maintenance) framework was used to evaluate AI-PAMI via the following measures: participant demographics, survey results, program elements and qualitative findings. Reach: There have been > 20,000 views of recorded content and 48% (885/1,859) of registrants attended a live webinar. Effectiveness: Survey results demonstrate 75%

of providers and 73% of older adults/caregivers reported new knowledge gain; and 80% of providers and 60% of older adults/caregivers reported changing their pain management practice/routine. Adoption: Presentations were delivered by 33 multidisciplinary experts from 12 different institutions. Six regional stakeholders promoted AI-PAMI using their dissemination networks. Implementation: The COVID-19 pandemic changed program delivery from an in-person model to virtual. To date, AI-PAMI has delivered 17 live webinars and 25 recorded presentations. Live webinars are delivered with a didactic, Q&A discussion and follow-up email. To refine AI-PAMI, 11 healthcare providers and 16 older adults participated in focus groups or in-depth interviews. Maintenance: AI-PAMI is in its third year and will be maintained under a long-standing institution-wide program. Website content will be sustained and remain free access. AI-PAMI is a valuable educational resource for older adults, caregivers, and healthcare providers. Virtual delivery is accommodating for a post-COVID environment.

### PRACTICE CHANGE OUTCOMES OF POST-GRADUATE TRAINING IN ADVANCED CLINICAL DEMENTIA PRACTICES

Daniel Kaplan,[1] and Keith Chan[2], *1. Adelphi University, Cortlandt Manor, New York, United States, 2. Hunter College, City University of New York, New York City, New York, United States*

Neurocognitive disorder is common among older adults with often progressive symptoms impacting multiple domains of life and with major implications and challenges for family involved in care. Clinical providers including doctors, nurses, and social workers play instrumental roles in addressing health and mental health vulnerabilities associated with neurocognitive disorder as skillful interventions can effectively improve wellness and quality of life for patients and care partners. Yet, providers report needing expanded knowledge to deliver appropriate clinical dementia care. An advanced clinical dementia practice certificate program at a major university in the U.S. offers a 45-hour training that covers 35 clinical practices identified by an interprofessional advisory group. This study aims to test the learning impacts of the educational program. Since 2017, 74 participants have completed this training. Their self-reported frequency of use of each clinical practice was measured before and after the training using a 5-item rating scale ranging from “Never” to “Several times per day”. Comparisons across two time periods demonstrate significant increases in overall use of clinical practices (mean difference: 0.45), with a large mean effect size (Cohen’s $d = 0.8$). These increases were statistically significant in 30 of 35 practices ($p < 0.05$). The largest effects were observed for clinical practices emphasizing the teaching of practical care strategies and the application of person-centered interventions. Implications of this educational method for enhancing clinician practice behaviors and recommendations for quantitative evaluation of training outcomes will be presented.

### VOCATIONAL ASSESSMENT AND INDIVIDUAL DEVELOPMENT PLANNING IN DIVERSITY-FOCUSED UNDERGRADUATE MENTORSHIP

Sheri Thompson, *UC San Diego, La Jolla, California, United States*

The UC San Diego MADURA Mentorship Program, funded the National Institute on Aging, strives to improve diversity in Aging/Alzheimer’s Disease and Related Dementias (ADRD) research and clinical practice. Goals include improved academic success of undergraduate URM trainees, and facilitating graduate/medical school applications and entry into Aging/ADRD research or clinical careers. Mentees receive paid research experience, training, Program and lab-based faculty supervision, peer support, seminars, and professional development opportunities. To enhance educational and career planning, MADURA incorporated Individual Development Plan (IDP) use, in 2022. IDP training by a Career Services presenter was followed by interactive IDP completion during small group break-outs, with ongoing revisions occurring throughout the quarter. Further, all trainees were offered formal vocational assessment (Strong Interest Inventory®, College Edition). 21 trainees participated and some engaged in individual 30-minute feedback sessions. All mentors were asked to incorporate IDP and vocational assessment discussions into year-end professional development meetings with mentees. Data to be presented includes: Student evaluation of IDP training; prior vocational assessment access; student and mentor ratings of IDP and vocational assessment feasibility, utility and satisfaction; and cohort vocational profile characteristics. Discussion includes implementation challenges, benefits and limitations of IDP and vocational assessment use, and future research suggestions.

### CHANGING AGING MYTHS AND READINESS FOR INTERPROFESSIONAL EDUCATION IN FAMILY NURSE PRACTITIONER STUDENTS

David Picella,[1] and Diana Lynn Woods[2], *1. Azusa Pacific University, Diamond Bar, California, United States, 2. Azusa Pacific University, Azusa, California, United States*

The preparation of Family Nurse Practitioner (FNP) students caring for the older adult population is inadequate. The design was a quasi-experimental pilot study ($N = 30$) to assess change in myths about aging and readiness for interprofessional education (IPE) in FNP students near graduation. The Woolf Aging Quiz (WAQ) containing 24 true-false myths about older adults, and the Readiness for Interprofessional Learning Scale (RIPLS) containing 19 items rated on a 5-point Likert scale (high score indicates more readiness), were completed at baseline and post-intervention. The intervention consisted of 4-weeks of education on normal aging, agism, age friendly healthcare, and IPE using online weekly brief video presentations and peer discussion. Thirty FNP students, mean age 43.5 years, 80.6% ($N = 25$) female, 19.4% ($N = 6$) male. A paired t-test showed no significant pre-post difference in the WAQ ($t = -1.388$, $p < .05$). McNemar crosstab analysis of the WAQ items indicated 14 aging myths where students chose the wrong answer pre and post intervention. In addition, the RIPLS showed no significant change (t = .793, p < .05) from pre-intervention (mean = 35.87) to post-intervention (mean = 34.42). FNP students near graduation held strong ageist beliefs, reflected in the WAQ, and were unprepared for IPE. Both of these results were resistant to change. The data suggests that FNP programs need to address aging and IPE content earlier and provide students with exposure to older adults that addresses myths about aging affords both theoretical and practical opportunities for interprofessional learning.

### RELAXATION ELEMENTS WITHIN A HOME ENVIRONMENT AND OLDER ADULTS' SOCIOEMOTIONAL EXPERIENCES OF CHALLENGING TASKS

Shannon Mejía,[1] Jessie Chin,[1] Faith-Christina Washington,[1] Smit Desai,[1] Durga Ramesh,[1] Falguni Deshpande,[1] and Sungjae Hong[2], *1. University of Illinois at Urbana-Champaign, Champaign, Illinois, United States, 2. University of Illinois Urbana Champaign, Champaign, Illinois, United States*

Older adults protect their well-being under stressful experiences by carefully selecting their environments, social interactions, and tasks. This bias toward positive over negative experiences may reduce engagement in novel, intellectually challenging, or social complex activities that promote healthy aging over the long term. Environments that foster socioemotional well-being could provide older adults with the psychological resources to engage in novel and challenging tasks that support healthy aging. This study examined how relaxation elements within the environment, such as a fireplace, can support socioemotional experiences while engaging in complex cognitive tasks. The study employed a 2x2 (relaxation vs. not X stress vs. not) within-subject design and took place within the home office of a home simulation environment. Under each condition, 61 participants (age 50–82, 57% female) played a cognitive game on a tablet, reflected for 30 seconds, and described feelings while playing the game by telling a story and rating positive and negative adjectives. Relaxation and stress were manipulated via an electric fireplace and a modified Trier paradigm where game performance was observed remotely by a neurologist. The relaxation condition significantly differentiated the coupling of positive and negative sentiment and positive and negative affect. The negative correlation between positive and negative affect increased, whereas stories became more emotionally complex, with both positive and negative sentiment, when the fireplace was on. Our findings suggest that simple modifications to the environment can differentiate the socioemotional experiences of engaging in novel and challenging experiences. The implications for enrichment seeking in older adulthood are discussed.

### THE SENIOR COMMUNITY SERVICE EMPLOYMENT PROGRAM: NEW DATA ON OLDER ASIAN WORKERS

Patrick Ho Lam Lai,[1] and Cal Halvorsen[2], *1. Boston College, Boston, Massachusetts, United States, 2. Boston College, Allston, Massachusetts, United States*

The Senior Community Service Employment Program (SCSEP) is the only federal employment program for people aged 55 and older. While 6% of the approximately 60,000 participants are Asian and federal funds are set aside to serve Asian workers, little is known about their personal characteristics and experiences in SCSEP. Using novel data from an online and paper-based Massachusetts survey of SCSEP participants from April to August 2022 offered in multiple languages, this study aimed to describe Asian participants' characteristics and experiences in SCSEP. Respondents (Nf39) ranged in age from 58 to 73. Almost all spoke a language other than English at home, and all respondents were born outside of the U.S. Nearly half reported a high school degree or less, and none reported "excellent" health. One-third reported feeling lonely occasionally or in specific situations. While two-thirds have made recent tradeoffs in paying for important goods and services (e.g., food and health care), most reported that their personal finances, social engagement, family life, and self-confidence have improved due to SCSEP. Further, 9 in 10 agreed that their supervisors understood the goals of SCSEP and were supportive. A quarter said separately that it was very likely they would search for a paid job or volunteer role after exiting SCSEP. These results contribute to the small amount of literature on older Asian American workers while informing the work of organizations that serve older Asian workers who have experienced multiple barriers to employment relating to nativity status, language abilities, and anti-Asian discrimination.

### THE IMPACT OF RACE AND GENDER ON OVERALL CARE RATING AT THE END OF LIFE: APPLYING THE THEORY OF INTERSECTIONALITY

Zainab Suntai, *Baylor University, Waco, Texas, United States*

Research focused on the quality of care received at the end of life has advanced over the past few decades, but few studies have employed an intersectional lens to assess differences in care quality. The theory of intersectionality suggests that individuals with membership in two or more vulnerable groups may be at risk of experiencing increased hardships and stressors across the lifespan. To test the theory of intersectionality and fill the gap in research, this study aimed to assess the combined impact of race and gender on the quality of care received at the end of life among older adults. Data were derived from the combined Round 3 to Round 10 of the National Health and Aging Trends Study, an annual longitudinal panel survey of adults aged 65 and older in the United States. Chi-square tests were used for bivariate analyses and two multivariate logistic regressions were run. Results showed that White men were the most likely to have had excellent or good care at the end of life, followed by White women, Black men, and then Black women. This points to a significant disadvantage for Black women, who lag behind all other groups in other life domains such as income, education, and access to health insurance. Interventions may include intersectionality-focused cultural humility training, cultural matches between patients and providers, and the adoption of a universal health insurance plan to reduce the gaps in service quality caused by the dominance of employment-based health insurance in the United States.

### SLEEP CHARACTERISTIC OF AN OLDER END OF LIFE (EOL) CAREGIVER: A SINGLE CASE STUDY

Hyeyoung Park,[1] Sunghoon Lee,[1] Amelia Bailey,[1] Yunda Liu,[1] and Cynthia Jacelon[2], *1. University of Massachusetts Amherst, Amherst, Massachusetts, United States, 2. University of Massachusetts Amherst College of Nursing, Amherst, Massachusetts, United States*

Background: Extensive caregiving work, psychological distress, and caregiving burdens put end of life (EOL) caregivers at great risk of sleep disturbances. Methods: A single case mixed method design was used to explore older EOL caregivers' sleep characteristics. Multi-dimensional data was collected through a series of methods including questionnaires (caregiver and care recipient demographics, care recipient symptom severity, caregiver caregiving strain, depressive symptoms, and sleep quality), 14 days of National Sleep Foundation (NSF) sleep diary, 28 days of daily sleep and activity measured by wearable personal sleep monitoring devices (Garmin vivofit 4), and a semi-structured interview. Data was analyzed using thematic content analysis. Findings: A 60 year old female taking care of her 85 years old mother with dementia, kidney cancer, and several other illnesses was recruited for this case study. She showed overall poor sleep quality measured by the Pittsburgh Sleep Quality Index (PSQI, total score: 8), and she slept 6.73 (±1.27) hours per day on average with 52.96% of deep sleep, 41.63% of light sleep, and 5.41% awake times. The caregiver reported that her daily sleep quality depended on her care recipient's health condition each night. She also described others supporting for her caregiving work as a "double-edged sword," because she needed to trade some privacy for the additional help. Conclusion: Further study is needed for EOL caregivers' poor sleep quality considering external factors and sleep intervention for EOL caregivers should be developed considering these external caregiving contexts.

### MOTIVATING PATIENTS WITH PARKINSON'S DISEASE AND THEIR FAMILIES TO DISCUSS PREPARATION FOR END-OF-LIFE CARE

Jiayun Xu,[1] Susan Hickman,[2] Jessica Huber,[1] Rebecca Sudore,[3] Cleveland Shields,[1] Dilhara Moonesinghe,[1] Aubrey Parr,[1] and Taylor Kong[1], *1. Purdue University, West Lafayette, Indiana, United States, 2. Indiana University School of Nursing, Indianapolis, Indiana, United States, 3. University of California San Francisco, San Francisco, California, United States*

Parkinson's disease is the second most common neurological illness among older adults. Although most patients with Parkinson's face communication challenges in advanced illness, few talk about care preferences and are unprepared for worsening illness as a result. Thus, the purpose of the study was to explore the experience of making end-of-life decisions or the preparation of making end-of-life decisions among patients with Parkinson's, their family caregivers, and clinicians. The long-term goal of this study is to develop a medical decision-making resource for patients and families to better prepare for the end of life. We conducted one-hour semi-structured interviews with nine patients, 12 caregivers, and 12 clinicians to explore perceptions of whether end-of-life discussions may be difficult, the preferred timing of end-of-life information, and how to best motivate patients and their families to prepare. Descriptive thematic qualitative analysis methods were used to analyze the transcribed interviews. Participants were on average 59.7±15.3 years of age, 69.7% female, and 69.7% married. The predominant reasons for delaying end-of-life discussions were the unpredictability of Parkinson's disease, limited illness and prognostic awareness, and clinicians not initiating the discussion earlier in the disease course. There was a mismatch in preferences for timing of end-of-life information, with patients and family wanting end-of-life information presented earlier in the illness than clinicians. All participants desired a resource to help patients and caregivers cope emotionally, to guide future decisions, and to help prompt conversations. Findings indicate that despite provider concerns, patients and their families want early information about end-of-life issues.

### INTERVENTIONS FOR FAMILY CAREGIVERS RECEIVING PALLIATIVE/HOSPICE CARE AT HOME: A SCOPING REVIEW.

Taeyoung Park,[1] Sulaiman Alshakhs,[1] Elisabeth Sweet,[1] Meghan McDarby,[2] Sara Czaja,[3] Cary Reid,[3] Ronald Adelman,[3] and Veerawat Phongtankuel[4], *1. Weill Cornell Medicine, New York, New York, United States, 2. Memorial Sloan Kettering Cancer Center, New York, New York, United States, 3. Weill Cornell Medicine, Ithaca, New York, United States, 4. Weill Medical College of Cornell University, New York, New York, United States*

With the growth of palliative and hospice care, patients with a terminal illness and their family caregivers have opportunities to pursue care focusing on alleviation of symptoms and optimizing quality of life in the home setting. However, caregivers are often overburdened with caregiving responsibilities leading to poor physical and mental health. Given the importance of family caregivers for patients who elect to receive hospice/palliative care at home along with the negative outcomes associated with caregiving, conducting a review of caregiver interventions is critical to understand what has been studied and identify gaps in the field. A scoping review was conducted to describe interventions focused on family caregivers providing care to terminally ill patients who receive palliative/hospice care in the home setting. Variables collected included intervention delivery details, study population, participant demographics, and measured outcomes. Our search yielded 14,527 articles, of which 70 articles on 54 unique interventions were included in the final analysis. Over three-quarters (77.8%) of the interventions were conducted in North America and Europe. Interventions commonly aimed to reduce the physical burden of caregiving (53.7%), educate the caregiver (53.7%), and improve psychological well-being (48.1%). The majority (58.6%) of the articles evaluated interventions for feasibility outcomes. The lack of studies in non-Western countries points to a cultural disparity in this field. Secondly, despite the variety of interventions, religious and spiritual needs in palliative care need to be further addressed. As palliative and hospice care is an emerging area of research, most studies are still implementing interventions and assessing feasibility.

### HOW DOES THE INTERSECTION OF RACE AND GENDER AFFECT HOSPICE CARE AT THE END OF LIFE?

Zainab Suntai, *Baylor University, Waco, Texas, United States*

Research in the palliative and end-of-life-care field has shown that hospice care in the final months of life can improve outcomes such as pain, emotional well-being, and physical comfort. Yet, researchers often find significant disparities in the ability to access hospice care, with Black individuals being less likely to have hospice care at the end of life. The theory of intersectionality suggests that the combination of multiple vulnerable identities may add to the number of hardships and stressors that an individual experiences across the lifespan. To test the theory, this study aimed to access whether gender moderated the relationship between race and the receipt of hospice care at the end of life. Data were derived from Round 3 to Round 10 of the National Health and Aging Trends Study, and two multivariate regression models were used to assess the relationship between the race/gender interaction and hospice care at the end of life (Model 1: main effects, Model 2: interaction term). Results showed that the effect of race on hospice care was indeed dependent on gender, with Black women being the least likely to have hospice care at the end of life. This points to the combined disadvantages resulting from membership in two vulnerable groups (i.e., being a woman and being Black), including fewer hospice facilities in Black-populated areas, race and gender discrimination in hospice referrals, and other factors that combine to reduce hospice access for Black women. Implications for research, practice, and policy are provided.

### AN EXAMINATION OF ADVANCED CARE PLANNING IN BLACK AMERICANS: DIRECTION TO IMPROVE ENGAGEMENT

Rachel Bassett, Daniel Paulson, and Nicole Valdes McClure, *University of Central Florida, Orlando, Florida, United States*

Past studies have found that older Black Americans are less likely to establish advance care directives (ACDs) than their White age peers, and medical distrust has been identified as one possible cause. Others have suggested the formulaic approach of ACD documentation which may conflict with the spiritual and cultural nature of these decisions. The Five Wishes ACD was developed partially to address this need. This study seeks to 1) replicate past findings regarding race and ACD adoption, and test the hypotheses that 2) Race will differentially predict Advanced Care Planning (ACP) engagement scores between conditions (Five Wishes vs State Directive), and 3) level of trust in physicians will relate to ACP engagement. The sample ($N = 186$) was recruited from Amazon Mechanical Turk and Prolific. Participants ranged from 50 to 77 years, were predominantly female (56.8%), White (51.61%), married (52.6%), and college-educated (70 .6%). ANCOVA results were a nonsignificant main effect of ACP engagement by race ($F(1, 185) = 1.93$, $p = .166$) and nonsignificant interaction of race by condition ($F(1, 185) = 0.16$, $p < .69$). Trust in physician scale scores predicted ACP engagement ($F(1, 185) = 16.15$, $p < .001$). The lack of an effect of race on ACP utilization may be explained by educational and SES characteristics of the sample by contrast to prior studies. The Five Wishes and State Directive ACD documents resulted in similar ACD engagement. These results suggest that trust in physicians is a primary barrier to ACP utilization.

### VALIDATION OF THE ENVIRONMENTAL AUDIT SCORING EVALUATION (EASE) TOOL FOR LTC HOUSEHOLDS

Migette Kaup,[1] Margaret Calkins,[2] Adam Davey,[3] Robert Wrublowsky,[4] Ashlyn Zachgo,[1] Samantha Gibson,[5] and Madison Parker[6], *1. Kansas State University, Manhattan, Kansas, United States, 2. IDEAS Institute, Cleveland Hts, Ohio, United States, 3. University of Delaware, Newark, Delaware, United States, 4. MMP Architects, Inc., Winnipeg, Manitoba, Canada, 5. University of Kansas, Lawrence, Kansas, United States, 6. Wheat State Manor, Whitewater, Kansas, United States*

This poster will share the results from a research initiative funded by the National Institutes of Health to assess the validity of the Environmental Audit Scoring Evaluation (EASE) tool in its ability to distinguish between different types of skilled care models based on the environmental and operational practices that can be observed and documented. The EASE tool was compared against three existing tools; PEAP, TESS-NH, and EAT-HC. Twenty-eight living areas in nursing homes across the state of Kansas identified as a traditional, household, or hybrid model were observed. The scores of the EASE were compared against the scores of three existing tools in order to evaluate its construct validity. The EAT-HC was most closely related to the EASE, with an R-value of 0.8817. The PEAP and the TESS-NH were less correlated to the EASE, with R-values of 0.8175 and 0.7097, respectively. Results found that the EASE was able to distinguish between traditional and homelike settings, though it could not identify hybrid models with a high degree of certainty. The analysis of variance between homelike and traditional homes was significant at 0.016, while the variance between homelike and hybrid and between hybrid and traditional were not significant. Inter-rater reliability of the EASE was consistently high (.96 and above). The outcomes demonstrated the EASE tool was able to assess the homelike characteristics of the environment of nursing homes better than or equally as well as previously validated tools.

### URBAN ENVIRONMENT AND AFFECTIVE STATES IN REAL TIME: AN ECOLOGICAL MOMENTARY ASSESSMENT STUDY OF OLDER ADULTS

Peichao Liang,[1] Yingqi Guo,[2] On Fung Chan,[3] Cheryl Hiu-Kwan Chui,[3] Yuqi Liu,[4] Shiyu Lu,[5] and Terry Lum[3], *1. The University of Hong Kong, Hong Kong, Hong Kong, 2. The Hong Kong Polytechnic University, Hong Kong, Hong Kong, 3. The University of Hong Kong, Hong Kong, Hong Kong, 4. South China University of Technology, Guangzhou, Guangdong, China (People's Republic), 5. City University of Hong Kong, Kowloon, Hong Kong*

Older adults are more dependent on their surrounding environment. Extensive research has demonstrated beneficial effects of both nature and built environment on mental health of older people. However, most previous research used cross-sectional designs failed to test the intraindividual variability between environment, behavior, and mental wellness in daily life. We used ecological momentary assessment (EMA), activity sensors, and GPS tracking to examine the association between real-time environment, mobility and activity, and momentary affect among older adults in Hong Kong. Data collection and data processing was conducted from December 2021 to May 2022. 168 older adults aged 65 to 84 received seven EMA prompts per day during a fifteen-day period, and completed a total of 17,345 momentary assessments of affective states, mobility, and activities. A set of GPS-derived indicators were used to measure the real-time environment. To disaggregate the between- and within-person effects, we used multilevel models to estimate three dimensions of affect, i.e., valence, calmness, and energetic arousal, in EMA observations, nested within individual participants. Preliminary results indicate significant concurrent associations between environmental attributes and momentary affect at the within-person level, while the between-person differences appear to be either null or modest. Being out of home is associated with higher valence ratings (b=0.04, p=0.0427), while exposure to green is associated with a lower level of energetic arousal (b=-0.03, p=0.0163). Greater walkability is consistently associated with higher momentary affect ratings in three dimensions, but these associations are not statistically significant. Implications of these findings for promoting healthy aging will be discussed.

### IMPACT OF HOME AMBIENT TEMPERATURE ON SELF-REPORTED MOOD AND ATTENTION IN COMMUNITY-DWELLING OLDER ADULTS

Amir Baniassadi,[1] Wanting Yu,[2] Ryan Day,[3] Angel Wong,[3] Thomas Travison,[1] Lewis Lipsitz,[4] and Brad Manor[5], *1. Harvard Medical School, Boston, Massachusetts, United States, 2. Hebrew SeniorLife, Roslindale, Massachusetts, United States, 3. Hinda and Arthur Marcus Institute for Aging Research, Boston, Massachusetts, United States, 4. Hebrew SeniorLife, Brookline, Massachusetts, United States, 5. Hebrew SeniorLife/Harvard Medical School, Roslindale, Massachusetts, United States*

Background: Many older adults experience variations in daily mood and/or attention. Lab-based studies show that, among other variables, ambient temperature can influence both. The objective of this study was to determine if and how habitual home temperature influences self-reported mood and attention in this population. Methods: Ambient temperature and humidity data were collected from the homes of 41 community-dwelling older adults (age=78±7, 35 females) living in Boston from June 1st to Aug 15th. Participants received two time-stamped smartphone-based questionnaires each day to report their mood and attention. Results: On average, participants completed 86(±29) questionnaires. Those with most variations in subjective outcomes (top quartile of % of time reporting "feeling down/depressed" or "difficult keeping attention"), compared to the rest of the sample, tended to reside in homes with both higher mean ambient temperature (p=0.01) and greater deviation in temperature over time (p=0.10). Logistic regression analysis combining data from all participants revealed that ambient temperature at the time of response did not predict either self-reported outcome. However, within-subject analyses indicated that of the 17 participants who reported at least some variation in attention or mood, the likelihood of experiencing poor mood and/or attention was correlated with time-synced ambient temperature in six individuals. Gender, age, or housing type (affordable vs. private) did not predict the presence of such associations. Conclusion: Variations in self-reported mood and attention are at least partially explained by the home thermal environment in a non-trivial fraction of older adults.

### GREENSPACE AND COGNITION: EXPLORING SPECIFIC GREENSPACE MEASURES

Aniruddh Ajith,[1] Sara Godina,[1] Xiaonan Zhu,[1] Alyson Harding,[2] and Andrea Rosso[1], *1. University of Pittsburgh, Pittsburgh, Pennsylvania, United States, 2. University of Minnesota School of Public Health, Minneapolis, Minnesota, United States*

Increased exposure to greenspace is potentially associated with lower risk of Alzheimer's disease and related disorders (ADRD) through increased physical activity, decreased stress, cleaner air, and social cohesion. Previous studies have primarily used global measures of greenspace, thus specific aspects of different types of greenspaces are overlooked. We assess how different measures of greenspaces associate with global cognitive functioning. We examined data from year 10 of the Pittsburgh site of Health, Aging, and Body Composition Study, a longitudinal cohort study of community-dwelling, Black and White adults aged 70–79 years at baseline. Measures derived from the National Land Cover Database (NLCD) use satellite imaging and include total greenspace, forests, and an index of greenspace diversity. Measures from Google Street View virtual neighborhood audits include dedicated greenspace that promotes physical activity, abandoned buildings, and undeveloped land. We measured global cognitive functioning using the Modified Mini-Mental State Examination (3MS). We conducted cross-sectional linear regression models. Covariates included race, education, and neighborhood socioeconomic status. Age was not included as a covariate because the range was narrow, and results did not vary by age. The sample (n=584) was 82.2 years old on average, 57% female, and 35% Black. Total greenspace (F=0.46, p=0.49), forests, and greenspace diversity, dedicated greenspace for physical activity (F=0.87, p=0.35), undeveloped land (F=1.49, p=0.22), and abandoned buildings (F=3.6, p=0.058) were not associated with 3MS, although the relation with abandoned buildings approached significance. We found no association between greenspaces and ADRD, although specific measures, such as abandoned buildings, may need further investigation.

### AGE, PHYSICAL ACTIVITY AND MITOCHONDRIAL FUNCTION IN THE STUDY OF MUSCLE MOBILITY AND AGING (SOMMA)

Anne Newman,[1] Terri Blackwell,[2] Paul Coen,[3] Yujia Qiao,[4] Frederico Toledo,[5] Ezequiel Zamora,[6] and Nancy Glynn[4], *1. University of Pittsburgh, Pittsburgh, Pennsylvania, United*

*States, 2. California Pacific Medical Center, San Francisco, California, United States, 3. AdventHealth, Orlando, Florida, United States, 4. University of Pittsburgh, School of Public Health, Pittsburgh, Pennsylvania, United States, 5. University of Pittsburgh, School of Medicine, Pittsburgh, Pennsylvania, United States, 6. Wake Forest University School of Medicine, Winston Salem, North Carolina, United States,*

We studied whether lower physical activity (PA) in older adults may partly explain the age-related decline in muscle mitochondrial function in the SOMMA cohort of 879 men and women, aged 70+. PA was measured both subjectively (CHAMPS questionnaire) and objectively (wrist-worn ActiGraph Link) classifying PA levels as sedentary, light, or moderate to vigorous (MVPA, ≥3 METS). Muscle mitochondrial function was assessed ex vivo via respirometry in permeabilized fiber bundles and as in vivo by 31Phosphorus magnetic resonance spectroscopy (31P-MRS) of the maximal rate of muscle ATP regeneration (ATPMAX in the quadriceps after contraction. Of 809 with respirometry or 31P-MRS, mean age was 76.4, 58.3% were women, 12.7% Black, 85.2% White and 2.1% other race/ethnicity. Mean OXPHOS and ATPMAX values were 1.91 pmol/(s*mg) and 0.016 mM/sec lower per 5-year age increment. Men self-reported more MVPA than women, but women recorded more MVPA with actigraphy. For both self-reported and recorded activity, associations with maximal tissue oxidative phosphorylation (OXPHOS) as well as ATPMAX were stronger for MVPA than for sedentary or light activity. After adjustment for age, sex, and clinic site, OXPHOS was lower for each standard deviation) fewer minutes/week of MVPA [reported, SD=408 min/wk: -3.66 pmol/(s*mg) (95% CI -4.93, -2.39); recorded, SD=600 min/wk: -1.93 pmol/(s*mg) (95% CI -3.31, -0.55)]. Similar associations were noted for ATPMAX. MVPA attenuated the association of age with mitochondrial function by 31–50% for both OXPHOS and ATPMAX, suggesting that MVPA could offset the lower mitochondrial function with age or that lower mitochondrial function impedes MVPA at older ages.

### THE ASSOCIATION BETWEEN FRAILTY AND PERCEIVED FATIGABILITY IN THE LONG LIFE FAMILY STUDY

Benjamin Schumacher,[1] Dustin Kehler,[2] Alexander Kulminski,[3] Stacy Andersen,[4] Theresa Gmelin,[1] Kaare Christensen,[5] Mary Wojczynski,[6] and Nancy Glynn[7], *1. University of Pittsburgh, Pittsburgh, Pennsylvania, United States, 2. Dalhousie University, Halifax, Nova Scotia, Canada, 3. Duke University, Durham, North Carolina, United States, 4. Boston University School of Medicine, Boston, Massachusetts, United States, 5. Southern Denmark University, Odense, Syddanmark, Denmark, 6. Washington University, Saint Louis, Missouri, United States, 7. School of Public Health, University of Pittsburgh, Pittsburgh, Pennsylvania, United States*

Higher levels of frailty, quantified by a frailty index (FI), may be linked to fatigue severity as tasks become more physically and mentally demanding. However, the association between frailty and fatigability—quantification of vulnerability to fatigue in relation to specific intensity and duration of activities—has not been assessed. Using cross-sectional data from the Long Life Family Study Visit 2 (2014–2017; n=2,524; mean age +/- standard deviation 71.4+/-11.2 years; 55% women; 99% White), we examined the association between a 79-item FI (ratio of number of health problems reported (numerator) out of the 79 (denominator); higher percentage=greater frailty) and perceived physical and mental fatigability using the Pittsburgh Fatigability Scale (PFS) (range 0–50; higher scores=greater fatigability). Mean+/-SD FI scores were 0.08+/-0.06 and mean+/-SD PFS Physical and Mental scores were 13.7+/-9.6 (39.5% more severe, >=15) and 7.9+/-8.9 (22.8% more severe, >=13), respectively. Both PFS subscale scores were higher for each 0.10 increment in FI. Mean PFS scores were 10.7 and 34.2 (Physical) and 5.7 and 28.8 (Mental) for FI scores of < 0.10 (non-frail) and ≥0.30 (moderate-severely frail), respectively. In mixed effects models, a 0.03 higher FI score (accepted clinically meaningful increase in FI) was associated with 1.9-point higher PFS Physical (95% confidence interval (CI) 1.7–2.1) and 1.7-point higher PFS Mental (95% CI 1.5–1.9) scores after accounting for family structure and adjusting for age, sex, field center, body mass index, smoking status, education, and marital status. Individuals with higher FI scores may benefit from targeted interventions to mitigate further poor health outcomes.

### DOES MOTIVATION PROMOTE PHYSICAL ACTIVITY IN OLDER ADULTS WITH CHRONIC MUSCULOSKELETAL PAIN?

Kailyn Witonsky, Xiaonan Zhu, Andrea Rosso, and Caterina Rosano, *University of Pittsburgh, Pittsburgh, Pennsylvania, United States*

Chronic musculoskeletal pain is common and limits physical activity in older adults. Understanding why some older adults remain physically active despite chronic musculoskeletal pain, “pain resiliency”, may offer insights to promote physical activity. We investigated the cross-sectional association of three indicators of motivation or mood with physical activity in adults over the age of 65 with chronic musculoskeletal pain from the Cardiovascular Health and Cognition Study. Of 2,703 participants with brain scans, 919 (34%) reported at least one month of either foot, knee, hip, or back pain and 47% of those with pain had moderate to high levels of self-reported physical activity, defined as “pain resilient”. Indicators of motivation included: (1) self-reported motivation: a composite score of perceived effort, difficulty getting going in the morning, and difficulty concentrating, (2) social network score from the Lubben Social Network scale, and (3) a composite depression scale. Separate multivariable linear models estimated the association between physical activity and each indicator, controlling for gender, race, BMI, age, gait speed, number of medications, number of pain sites, cognitive function, as measured by the digit substitution symbol test, and brain integrity, as measured by level of white matter hyperintensities. Higher self-reported motivation (β=-0.11, p=0.02) and larger social networks (β=0.03, p=0.01) were associated with higher levels of physical activity. Better mood was not associated with higher levels of physical activity (β=-0.017, p=0.34). Albeit based on cross-sectional self-reported measures, our research suggests the need for further research investigating psychosocial contributions to pain resilience.

### CT-DERIVED PARAVERTEBRAL MUSCLE RADIOMIC FEATURES ARE ASSOCIATED WITH MORTALITY IN THE MROS STUDY

Leon Lenchik,[1] Katey Webber,[2] Eric Orwoll,[3] Jane A Cauley,[4] Peggy Cawthon,[5] and Kristine Ensrud[6], *1. Wake Forest School of Medicine, Winston Salem, North Carolina, United States, 2. UCSF, San Francisco, California, United States, 3. OHSU, Portland, Oregon, United States, 4. School of Public Health, University of Pittsburgh, Pittsburgh, Pennsylvania, United States, 5. University of California, San Francisco, San Francisco, California, United States, 6. University of Minnesota Medical School and Minneapolis VA Health Care System, Minneapolis, Minnesota, United States*

The purpose was to determine if CT-derived radiomic features (biomarkers of muscle heterogeneity) are associated with all-cause mortality (independent of muscle size and density) in 2644 men (mean age 74.0) in the MrOS Study. Our fully-automated machine learning algorithm determined paraspinous muscle area and density. We also identified 75 radiomic features of muscle texture at the level of T12 vertebra. We used factor analysis to reduce the number of radiomic features into latent variables that explain the underlying data. Association of these factors, muscle cross-sectional area, and muscle density with all-cause mortality was determined using Cox proportional hazards models adjusted for CT-derived muscle area and density CT parameters (scanner model, slice thickness, tube current), participant age, height, total body fat, grip strength, walking speed, leg power, diabetes status, physical activity, self-reported health, and number of medications. Multiple comparisons were accounted for using false discovery rate testing. After a mean 13.3 ± 5.9 years of follow-up, 1945 (73.6%) men died. Muscle area and density and all 6 radiomic factors identified were significantly different in survivors compared with deceased. In fully adjusted models, 54 of 75 (72%) individual radiomic features were significantly associated with mortality. In fully adjusted models, low muscle density (HR/SD increment = 0.85; CI = 0.75,0.99), radiomic Factor 1 (HR/SD increment = 0.76; CI = 0.62,0.95), radiomic Factor 2 (HR/SD increment = 1.47, CI =1.23, 1.76) but not muscle area (HR/SD increment: 1.18, CI = 0.94, 1.48) or the other radiomic factors identified, were independently associated with mortality.

### COMPUTING, DENTISTRY AND AGING: ORAL HEALTH, BIOLOGICAL AGE AND MORTALITY

Xin Zhong, *A*STAR Singapore, Singapore, Singapore*

Oral health is essential to general well-being across the lifespan. Recent study on number of natural teeth, denture use and mortality in non-Singaporean elderly indicated the importance of oral determinants. To the best of our knowledge, no study has envisioned the association between biological age and oral health among community-dwelling older Singaporeans. By leveraging the acquired knowledge from Singapore Longitudinal Ageing Study (SLAS), our aim is to examine the association between biological age, oral health, and mortality. Our hypothesis is that older adults with or without dental problems will have a significant difference in mortality and other health outcomes. Our methodology is to combine a more realistic AI approach and a hypothesis-driven multidisciplinary approach to examine the association between oral health and general health outcomes (mortality, hospitalization, etc). Cross-sectional data analyses were performed on 2844 community-living Chinese older adults of 65–80 years old in the Singapore Longitudinal Aging Study II (SLASII) cohort. The Keplan-Meier survival analysis showed statistically significant differences in survival time between the group with dental problems and the group without dental problems (p < 0.001) in an 8-year longitudinal study. Results also showed a significant difference between biologically younger, biologically older, and the intermediate group groups (p = 0.0012). Those groups were classified by the computed biological age. Our preliminary results will produce a plethora of new links between oral health, biological age, and mortality. The research outcome may serve as a baseline for policymakers to evaluate and promote subsidized oral health programs.

### A COMPARATIVE STUDY OF FALL RISK ASSESSMENT IN COMMUNITY DWELLING OLDER ADULTS IN INDIA

Snehal Kulkarni, *Savitribai Phule Pune University, Pune, Maharashtra, India*

Falls in older adults can be prevented with early identification. Recent research investigates the use of wearable sensors to provide quantitative data on mobility parameters to assess risk of fall. However, the efficiency of these new methods in comparison to conventional fall risk assessment tools is unknown. Therefore, the current study compares the sensor-based fall risk assessment with conventional fall risk measures. The study included 659 community-dwelling older adults (>60 years) who were followed for 12 months after baseline. The American Geriatric Society Fall Risk Assessment tool was used to assess risk of fall conventionally and Kinesis QTUG was used to assess the fall risk using sensors. Receiver operating characteristics curve (ROC) was used to compare the sensor-based method and the conventional method of assessing fall risk. Out of the 659 community-dwelling older adults, 24% (163) of older adults reported a fall in 12 months. According to the sensor-based assessment, 23% of older adults had low risk, 50% had medium risk and 27% had high risk of fall and according to the conventional method 66.1% were in low risk, 27.1% were in medium risk and 6.8% were in high risk. The ROC analysis showed that the sensor-based methods (AUC-78%; sensitivity- 93.8% specificity – 56.3%) outperformed the conventional method (AUC – 61.2%; sensitivity – 82.8% and specificity - 67.2%) of identifying older adults at risk of fall. Therefore, use of simple wearable sensors can determine the risk of future falls.

### THE MEANING OF EXPERIENCING FEAR OF FALLING AMONG OLDER ADULTS

Hanne Dolan,[1] and Natalie Pool[2], *1. Arizona State University, Phoenix, Arizona, United States, 2. University of Northern Colorado, Greeley, Colorado, United States*

Fear of falling (FOF) is prevalent among older adults. While the concept has been conceptually defined and factors associated with FOF has been extensively explored in nursing and other health sciences, the experience of this fear is often overlooked. The purpose of this study was to illuminate the phenomena of FOF using the rich descriptions elicited from four (Nf4) older adults. The meaning of these experiences was then interpreted in conjunction with the known and emergent FOF body of literature. Each participant was interviewed twice using videoconferencing as part of a larger study exploring the lived experience of being at risk for falling in the hospital. A total of eight interview transcripts were analyzed using van Manen's interpretive phenomenological methodology. The philosophical underpinning of this study is the philosophy of caring in nursing outlined by Kari Martinsen. Three major interpretive themes emerged: Loss of Self, Part of my Existence, and Remaining Safe Within the Boundaries of Fear. These themes describe how FOF is the fear of being suspended in time and space and losing connection with oneself both physically and mentally during a fall. The fear becomes a part of one's existence, ranging from worry to all-consuming panic, and the body becomes unpredictable. FOF means living within invisible boundaries of fear, where feelings of helplessness, uselessness, and isolation are common. Relationships with others can both temper and ignite the FOF, and caregivers must understand the meaning of this experience to improve support of older adults in managing this overwhelming experience.

### PHYSICAL ACTIVITY AND SEDENTARY TIME ASSOCIATIONS WITH NON-FRACTURE FALL INJURY (NFFI) AND FRACTURE

Lily Bessette,[1] Andrea LaCroix,[2] Benjamin Schumacher,[1] Michael LaMonte,[3] John Schousboe,[4] Kristine Ensrud,[5] Jane A Cauley,[6] and Elsa Strotmeyer[6], *1. University of Pittsburgh, Pittsburgh, Pennsylvania, United States, 2. University of California, San Diego, La Jolla, California, United States, 3. University at Buffalo - SUNY, BUFFALO, New York, United States, 4. HealthPartners, Inc, Park Nicollet Clinic, Minnesota, United States, 5. University of Minnesota Medical School and Minneapolis VA Health Care System, Minneapolis, Minnesota, United States, 6. School of Public Health, University of Pittsburgh, Pittsburgh, Pennsylvania, United States*

Understanding fall injury risk factors and circumstances may lead to better prevention. However, many fall injury studies focus on fractures from hospitalizations or emergency events, rather than non-fracture fall injuries (NFFI), which comprise >50% of fall injuries in older adults. We hypothesized that risk factors were differently associated with NFFI vs. fracture fall injuries in a community-based cohort of ambulatory women with fall injuries in the Objective Physical Activity and Cardiovascular Disease Health in Older Women (OPACH) ancillary of the Women's Health Initiative Long Life Study. Women with daily fall calendars over 1-year follow-up completed telephone interviews regarding fall injury circumstances (Nf662; mean 79.6 + 6.7 years; 73.3% White). Risk factors and fall circumstances were assessed with first reported fall injury (NFFI vs. fracture) using univariate and multivariate logistic regression. Participants with NFFI vs. fracture were more likely to be non-white, less likely to seek clinical treatment or need help up from fall (all $p < 0.05$). Adjusting for age, race, and BMI, NFFI vs. fracture were more likely to report >=6 hours sitting (OR=1.72, 95% CI=1.07–2.73) and less likely to report weekly moderate exercise (OR=0.61, 95% CI=0.38–0.96), though total accelerometer-measured sedentary time and physical activity (PA) were not significant. Self-reported PA level at the time of fall (OR=1.31, 95% CI=0.82–2.09) and walking outside for >10 minutes >= 1/week (OR=1.24, 95% CI=0.77–1.98) were not different for NFFI vs. fractures. Older women with NFFI vs. fracture had more sedentary time and less moderate exercise, which may have implications for fall injury severity.

### OLDER PRIMARY CARE PATIENTS' ENGAGEMENT WITH STEADI FALL PREVENTION RECOMMENDATIONS

Hiroko Kiyoshi-Teo,[1] Bryanna De Lima,[1] Debbie Cohen,[1] Kerri Winters-Stone,[2] and Elizabeth Eckstrom[1], *1. Oregon Health & Science University, Portland, Oregon, United States, 2. Oregon Health & Science University, Porltand, Oregon, United States*

The Stopping Elderly Accidents, Deaths, and Injuries (STEADI) initiative is a CDC-disseminated strategy to provide fall prevention recommendations to high risk patients. However, whether older patients engage with fall prevention recommendations is less clear. The aims of this study were to: 1) describe STEADI fall prevention recommendations provided to eligible older patients; and 2) understand patients' attitudes, confidence and adherence to recommendations. Fall prevention recommendations were identified by chart review (age ≥65 years old; STEADI score ≥4 (high risk); free of severe cognitive impairment). For those who consented to the study, questionnaires measured recollection (Yes/No), feelings (Positive/Negative), overall confidence to reduce fall risks through the recommendations (0–10 scale; 10 = most confident), and adherence to the specific recommendations (Yes/No). Results showed that 30% (n = 458) of eligible older adults received fall prevention recommendations. Among study participants (n = 182; 69% female, age 80.2 ± 7.7 years, STEADI score 7.0 ± 2.6), 73% reported positive reactions to recommendations. Overall confidence to reduce gait/balance-related fall risks through recommendations was 7.52 (n = 137) with adherence to physical therapy at 70% and tai chi at 38%. Overall confidence to reduce blood pressure-related fall risks through recommendations was 7.45 (n = 55) with adherence to adjusting medications at 89% compared to 69% for compression socks. Participants reported varying levels of adherence despite positive reactions and moderate confidence to reduce their fall risks. Additional research is needed to identify barriers and facilitators to improve adherence.

### FEASIBILITY OF FALLS PREVENTION PROGRAMS IMPLEMENTATION BASED ON ACADEMIC-COMMUNITY PARTNERSHIPS

Uma Kelekar,[1] Cathy Elrod,[1] Patricia Heyn,[2] and Rita Wong[3], *1. Marymount University, Arlington, Virginia, United States, 2. Center for Optimal Aging, Marymount University, Fairfax Station, Virginia, United States, 3. Marymount University, Arlington, Virginia, United States*

The Northern Virginia (NoVa) region is diverse in age, race, ethnicity, economic status, health status, culture, and language. Based on state data, it is estimated that 28.7%

of the 75,000 older adults in NoVa will experience at least one fall annually. Since 2014, the Administration for Community Living (ACL) has sponsored implementation of evidence-based fall prevention programs (EBFPPs). The aims of this study are to 1) describe the EBFPP participants and delivery sites and 2) report initial effectiveness. Marymount University (MU) received two cooperative agreement grants (2016, 2018) from the ACL to implement in NoVa three EBFPPs: 1) Stay Active and Independent for Life (SAIL), 2) A Matter of Balance (MOB), and 3) Otago Exercise Program (OEP). The Northern Virginia Falls Prevention Alliance was formed and a Regional Training Office was built. The data source is the ACL national falls database. From 2016–2022, over 5,000 older adults were reached, over 400 EBFPP leaders were trained, and programs were delivered at 60 sites. Data analysis indicates that falls decreased from 20% to 15%. Those participating in MOB showed the greatest decline in falls (from 34% to 14%). Fall injuries declined from 11% to 7% and ED visits decreased from 3% to 1%. Preliminary analysis indicates beneficial impact: this academic-community partnership has reached people at low risk of falling (SAIL), and had an impactful decline in falls for individuals at moderate to high risk (MOB). Future research will investigate program outcome differences based on other demographics and delivery models.

### FACTORS ASSOCIATED WITH FRID USE IN OLDER BLACK AND WHITE MEN AND WOMEN: THE HEALTH ABC STUDY

Jimmie Roberts, obert M. Boudreau, Lingshu Xue, Jane A Cauley, and Elsa Strotmeyer, *School of Public Health, University of Pittsburgh, Pittsburgh, Pennsylvania, United States*

Medications that increase falls, fall-risk increasing drugs (FRIDs), are common in older adults. Two FRID definitions, the CDC Steadi-Rx and Swedish National Board of Health and Welfare, are widely accepted. We hypothesized that FRID use risk factors vary by definition in 1,352 community-dwelling older adults in the Health, Aging, and Body Composition Study (Health ABC; 2007–2008 clinic visit; 83.4±2.8 years; 54.1% women; 34.9% Black). FRID use by either definition was associated with chronic health conditions, medical care including non-FRID use, higher BMI and depression scores, and less walking exercise (all $p < 0.05$). Steadi-Rx FRID use was also associated with more falls, ADL difficulty, and better cognitive scores. Using stepwise multivariate Poisson regression adjusting for demographics, lifestyle/behavior factors, and comorbidity, a 1-unit increase in BMI and depression score was associated with an approximately 2% mean FRID count increase for both definitions and 7% mean FRID count increase per 1-unit increase in non-FRID count. Both definitions had a 40% and 15% lower mean FRID count, respectively, with hypertension and cardiovascular disease (CVD) history. Better cognitive scores were associated with 1% mean increase in Steadi-Rx FRID count and a mean decrease of 13% and 33% FRID count, respectively, with cancer history and having primary healthcare. In identically adjusted logistic regression, FRID use (yes/no) was associated in a consistent direction with BMI, depression score, non-FRID count, hypertension, CVD, having primary healthcare, and also less likely with low-vs-high income (OR=0.18[0.06–0.50]). Risk factors differ by FRID definition, with Steadi-Rx identifying more predictors than the Swedish definition.

### BURDEN OF FALL-RISK INCREASING DRUGS IN OLDER ADULTS PRESENTING WITH FALLS TO THE EMERGENCY ROOM

Martin Casey,[1] Joshua Niznik,[1] Greta Anton,[2] Michelle Meyer,[1] Casey Kelley,[3] Jan Busby-Whitehead,[1] Kathleen Davenport,[4] and Ellen Roberts[1], *1. University of North Carolina at Chapel Hill, Chapel Hill, North Carolina, United States, 2. University of North Carolina Medical Center, Chapel Hill, North Carolina, United States, 3. Unicersity of North Carolina at Chapel Hill, Chapel Hill, North Carolina, United States, 4. Univerisity of North Carolina at Chapel Hill, Chapel Hill, North Carolina, United States*

Prescribing of fall-risk increasing drugs (FRIDs) may be an important driver of falls in older adults. Understanding the types and frequencies of FRIDs prescribed to older adults presenting with falls to an emergency department (ED) may help identify opportunities for deprescribing. We performed a cross sectional analysis of data collected from a pharmacist-led fall-prevention program focused on older adults presenting with a fall to an academic ED in the southeastern United States between August 2020 – December 2021. ED pharmacists identified older adults (≥65 years old) presenting with a chief complaint of 'fall' and then performed a medication reconciliations to verify and obtain data on outpatient prescription drug use. FRIDs were identified in accord with the 2019 American Geriatrics Society Beers Criteria and the Centers for Disease Control and Prevention Stopping Elderly Accidents, Deaths, & Injuries (STEADI-Rx) list. The ED pharmacists performed medication reconciliations on 424 unique older adults presenting with a fall. The cohort had a mean age of 81.3 years and were mostly female (63.3%) and white (84.9%). Prescription use of FRIDs were identified in 45.8% (194/424) of older adults presenting with a fall. An estimated 25.5% (108/424) of the subjects were prescribed 2 or more FRIDs. The most common FRIDs identified were antidepressants (25.9%), anticonvulsants (18.6%), opioids (12.7%), benzodiazepines (8.5%), and antipyschotics (3.1%). . Prescription use of FRIDs, including use of 2 or more FRIDs, was common in older adults presenting with a fall to the ED.

### THE IMPACT OF TECHNOLOGY USE ON CUSTODIAL GRANDPARENTS' DEPRESSION

Sarah Pace, Yanfeng Xu, and Theresa Harrison, *University of South Carolina, Columbia, South Carolina, United States*

During COVID-19, custodial grandparents experienced symptoms of depression due to social isolation, and technology use may reduce depression. This study examines the association between level of comfort of using technology and depression among custodial grandparents. Cross-sectional survey data (N = 287) were collected via multiple sources, including state agencies, local non-profit organizations serving kinship families, foster parent associations, schools, and Qualtrics Panels between March 2021 and March 2022. The average age of grandparents was 55 years. Over half were female (71.43%), had some college education (68.75%), and were White (55.12%). Depression was measured using the CES-D 10 scale. This variable was dummy coded using 8 as the cutoff score (Andresen et al., 1994). The level of comfort using technology was measured by combining four survey questions. Grandparent age, race, gender, marital status, home ownership, geographic area, disability, employment, education, physical health, telehealth, and telemental health were controlled. The logistic regression model revealed that those who had a higher comfort level with technology had significantly lower odds of having depression (OR = .543, p = < .001). Those with physical health had significantly lower odds of having depression (OR = .727, p = .044). Grandparents who needed telehealth (OR = 2.81, p =.005) and telemental health services (OR = 2.93, p =.003) had significantly higher odds of having depression. This research implies that custodial grandparents' use of technology, particularly their comfort levels with technology may reduce depression among grandparents. This will inform practice and policy for those who work with custodial grandparents.

### THE RECIPROCAL RELATIONSHIP OF SELF-RATED HEALTH AND INSTRUMENTAL HELP FROM CHILDREN: EVIDENCE FROM GERMANY

Lisa Jessee, *University of Cologne, Cologne, Nordrhein-Westfalen, Germany*

Prior research suggests that receiving support from children may promote health among ageing parents. Poor health, however, often precedes the need for support. Yet, research linking instrumental support and self-rated health (SRH) has been deficient in two regards: First, little research has addressed the relationship between instrumental support and SRH simultaneously using longitudinal data. Second, only few studies, have accounted for unobserved confounders (e.g. within-person effects) and potential reverse causality. Recently developed dynamic panel models with fixed effects provide an opportunity to address these methodological issues. Using four waves of the German Ageing Survey (DEAS) among 4,496 adults aged 40 years and older, the current study investigates the potential bidirectional relationship between instrumental support and SRH. I find that prior receipt of instrumental support from children is not a significant predictor of future reported poor SRH. Similarly, previous poor SRH does not significantly predict the likelihood of receiving instrumental support from children at follow-up. Independent of older adults' SRH, however, prior receipt of instrumental support is a statistically significant predictor of receiving support at follow-up. Regardless of receiving children's instrumental support, poor SRH in the past predicts poor SRH in the future. The results shed new light on prior bidirectional associations between SRH and instrumental support in the German context. Previous results may be partly explained by a lack of controls for unobserved individual characteristics. The findings suggest that policy interventions may not exclusively rely on older adults' receipt of support from children in the event of health declines.

### INTERGENERATIONAL RELATIONSHIPS OF RUSSIAN SPEAKERS IN ISRAEL IN THE SHADOW OF PROPAGANDA: FACEBOOK POSTS ANALYSIS

Ksenya Shulyaev, and Tova Band-Winterstein, *University of Haifa, Haifa, Hefa, Israel*

Part of the elderly Russian-speaking community in Israel is exposed to the TV channels of the Russian government. These channels are impacted by Russian propaganda and, as such, use false claims and manipulative techniques. The aim of this presentation is to shed light on the impact of Russian media on intergenerational relationships of Russian-speaker families in Israel. We conducted a preliminary qualitative study based on a thematic analysis of Facebook posts (Nf34) from March to June 2022. Analysis revealed four key themes: (1) "Tragedy of many families", the importance of unity of opinion and the problem of difference, a key element in the emotional well-being (or its lack) of Russian-speaking families at the moment; (2) "It's useless to explain something", the perception of older parents who support Putin as victims of "brainwashing", with comments on "zombies", totalitarian sects, and psychotropic drugs; (3) "The truth will destroy them", the perception of disbelief in war as a defensive reaction necessary for the elderly, because the reality may destroy them; (4) "A topic that is not talked about", a refusal to discuss Ukraine seen as the only way to save the relationship, speaking instead about health issues, daily life issues, and the weather. The findings underline that the Russian-Ukrainian war conflict impacts intergenerational solidarity and closeness, and calls for an intervention that provides support for both the elderly and their adult children.

### GRANDPARENT-PARENT RELATIONSHIP, CAREGIVING MEANING AND FAMILY WELLBEING: AN APIM STUDY OF 200 DYADS

Xu Liao, *City University of Hong Kong, Hong Kong, Hong Kong*

While many older persons contribute to their families by providing care to their grandchildren (GC), the role transition could bring challenges to both grandparents (GP) and parents (P) especially for those who evenly share caregiving duties. Despite growing research on grandparenthood, a knowledge gap remains in the study of GP-P partnership; it is essential to examine GP and P as a unit and learn how the dyadic relationship influences their family. The purpose of this study is to gain a further understanding of GP-P relationship, meaning of caregiving, and family well-being (FWB). Data from 200 GP-P dyads in China were collected by a questionnaire survey. The study participants were chosen from families with one GC aged 0–8 who is cared for by GP and P together, and identified by the GC's teachers. The actor-partner interdependence model (APIM) was adopted in data analysis. Results showed that for co-(grand)parenting relationship and FWB, the actor effect was statistically significant for both GP and P; so was the partner effect from P to GP. Likewise, for meaning of caregiving and FWB, the actor effect was statistically significant for both GP and P; so was the partner effect from GP to P. The results advance knowledge about GP-P partnership and how feelings and actions could impact the overall family well-being. The findings can also guide the development of practices and services that enhance the function of GP-P collaboration and their family well-being.

### FRAGMENTED FAMILIES OF OLDER FORCED UKRAINIAN MIGRANTS: PRELIMINARY DATA FROM A HELP CENTER IN NORTHERN ISRAEL

Ksenya Shulyaev,[1] Yulia Macarenko,[2] and Tova Band-Winterstein[1], *1. University of Haifa, Haifa, Hefa,*

*Israel, 2. Volunteer Help Center for Refugees from Ukraine, Haifa, Hefa, Israel*

Since the beginning of the Russia-Ukrainian war, more than 50,000 people have come to Israel from Ukraine as "repatriates" (able to acquire citizenship) or "tourists" (with a right to live in Israel during the war, without citizenship), about 30% are older adults, for whom forced migration poses serious challenges. The aim of this presentation is to describe the socio-demographic characteristics of older migrants' fragmented families, who turn to one of the Volunteer Help Center in Northern Israel. Descriptive statistics on older adults from the database of about 900 families who received support during April-June 2022 were performed. Data revealed: 189 migrants aged 55+ (M(SD)=69.7(8.2); range 55–89); 148 (78.3%) were female; 106 (56%) had Israeli citizenship or were in the process of receiving it; 75 (40 %) were "tourists". Among "tourists", about half (Nf30) came to visit their adult children. "Repatriates" and "tourists" did not differ in age (F(159)=1.574, p=ns). As a rule, older adults came with family members (Nf136(72.7%)). However among "tourists" 41.9% came alone and only 17.1% among "repatriates" (X=13.374, p < 0.001). Older adults were mostly accompanied by daughters (Nf80(58.8%)), usually without husbands, in 127 fragmented families (78.2%) were children under 18. Only 15% came with a spouse (half of the men, 5.5% of women), and five came with mothers. This preliminary data draws attention to the unique family fragmented structure of older displaced people, as this should be considered when developing an assistance program.

### ACCEPTABILITY DATA OF A TECHNOLOGY-ENABLED CARE COACHING SERVICE FOR CAREGIVERS OF INDIVIDUALS WITH DEMENTIA

Katherine Judge,[1] Amelia Thomley,[2] Lauren Stratton,[3] Emily Silver,[2] Kerry Finegan,[3] Monica Moreno,[3] Sam Fazio,[3] and Joe Chung[2], *1. Cleveland State University, Cleveland, Ohio, United States, 2. Kinto, Cambridge, Massachusetts, United States, 3. Alzheimer's Association, Chicago, Illinois, United States*

This poster presents acceptability data from the Kinto Care Coaching Intervention, an innovative technology-enabled care coaching service for caregivers of individuals with dementia. The 30-day program provides caregiver support and financial information through: 1) an initial one-on-one care coaching meeting; 2) interactive and on-going support and educational resources through an app; 3) access to support groups; and 4) if needed, additional meetings with their care coach. The solution is funded through the NIH's Small Business Innovation and Research (SBIR) program, with the specific goals of developing mobile technology that is: 1) acceptable and usable for caregivers of all ages and 2) supports cost-effective deployment of the coaching intervention at scale. To assess program acceptability, participants completed a survey after their one-on-one care coaching meeting and after the 30-day program. On average, participants (n=32) were M=51.94 (SD=12.07) years old; 68.8% female; 71.9% White; 75% married; and 56.3% worked full-time. Using a 5-point Likert scale, nearly all participants (96.6%) indicated they 'agreed' or 'strongly agreed' that their care coach was: helpful in explaining the program; provided useful information; assisted with developing goals; and were supportive. Participants rated the program resources and technology as very helpful with mean ratings ranging from 4.41 to 4.69. When asked about the overall program acceptability, participants indicated they were extremely satisfied with the program (M=4.81; SD=.40), with 100% of participants 'agreed' or 'strongly agreed' that they were satisfied. Discussion will highlight key program components along with next steps in testing program efficacy.

### A LATENT CLASS ANALYSIS OF STRESSORS AND RESOURCES AMONG SPOUSAL CAREGIVERS TO OLDER ADULTS IN THE UNITED STATES

Ruotong Liu,[1] Iris Chi,[2] and Shinyi Wu[2], *1. Oregon Health & Science University, Marina del Rey, California, United States, 2. University of Southern California, Los Angeles, California, United States*

Spousal caregivers to older adults experience differing intensities of co-occurring caregiving stressors and resources. Analyzing their heterogeneous profiles can improve recognition of spousal caregivers needing support and caregiving impact on their health. This study aims to identify latent classes among spousal caregivers to older adults and to explore the associated background characteristics and health outcomes. Using pooled data from Round 5 and Round 7 of National Study of Caregiving and the linked National Health and Aging Trends Study, eight indicators for stressors and three indicators for resources are included for latent class analysis to identify distinguishable spousal caregiver subgroups. ANOVA test or Chi-square test are used to understand the different characteristics across spousal caregiver classes. Multivariate regression is conducted to examine associations between class membership and health outcomes. Three latent classes are identified among the 793 included spousal caregivers: low-stress low-support class (39%), medium-stress high-support class (36%), and high-stress medium-support class (25%). Compared to the other two classes, high-stress medium-support class includes more female than male, medium-stress high-support class has greater portions of non-Hispanic Black individuals, and caregivers in low-stress low-support class have fewer comorbidities. In addition, compared to low-stress low-support class, spousal caregivers in high-stress medium-support class reported lower (0.42 unit) level of self-rated health and higher (1.87 unit) level of depressive symptoms. These results confirmed the heterogeneity among spousal caregivers, and highlighted the elevated needs to address the physical and mental health needs among spousal caregivers whose levels of resources could not offset the caregiving stress they experience.

### THE RELATIONSHIP BETWEEN ATTACHMENT, RELATIONSHIP QUALITY, AND DAILY STRESS AMONG DEMENTIA CAREGIVERS

Paulina Wuestefeld, Paige Goodwin, and Robert Intrieri, *Western Illinois University, Macomb, Illinois, United States*

The current study assesses the relationship between attachment style, relationship quality, and daily stress among family caregivers of persons with dementia. The study incorporates a longitudinal burst design. In an initial meeting with the investigator, participants completed measures of attachment style (Experiences in Close Relationships Questionnaire Revised), past and current relationship quality (RQ), emotion regulation (Emotion Regulation Questionnaire), and caregiver burden (Zarit Burden Interview). For the next 14 days, participants were sent a link to a brief survey that included the Positive Affect and Negative Affect Schedule and the Perceived Stress Scale. They were also asked to estimate what proportion of their daily stress did they attribute to caregiving. There are significant positive correlations between caregiver burden and current negative RQ, r = 0.70, p = .008, as well as between attachment anxiety and current negative RQ, r = 0.60, p = .029. According to regression analyses, attachment anxiety predicts negative current RQ, R2 = .37, F(1, 11) = 6.32, p = .029. Current negative RQ predicts caregiver burden, R2 = .49, F(1, 11) = 10.55, p = .008. Additional analyses will explore the link between attachment style, emotion regulation, burden, relationship quality, and longitudinal assessments of daily stress and affect. Though preliminary, study results suggest that beyond caregiver burden, daily stress and well-being of caregivers is impacted by the relationship between attachment, relationship quality, and emotion regulation.

### STUDENT CARE PARTNERING AMONG STUDENTS AT A HBCU: EXPLORING THE POTENTIAL IMPACT ON STUDENT PERSISTENCE

Claudia Thorne, *Coppin State University, Baltimore, Maryland, United States*

The number of students enrolled in higher education who are taking care of older adults, many of whom may be suffering from an illness, is expanding. The numerous factors in care partnering can compromise student success and persistence towards graduation. While most care partners experience challenges, the care partnering experience of Black students is magnified because Historically Black Colleges and Universities (HBCUs) have a higher enrollment of nontraditional and first-generation college students. Students may be primary care partners who provide most of the care, or they may provide supportive care as secondary, tertiary, and auxiliary care partners within an extended family or kinship network. Aging in place, within the context of family and community, emerges from African culture and tradition deeply grounded in filial loyalty. Care partnering is nuanced in the interaction of race, ethnicity, gender, family relationships, and student status; however, little is known about the lived experience of Black student care partners and how to support them to facilitate their persistence towards graduation. This poster describes initial efforts to understand Black student experiences in care partnering in the context of a Mid-Atlantic HBCU and suggests the next steps in a multi-year research program.

### INCREASING MEDICATION SAFETY AWARENESS IN RURAL OLDER ADULTS

Kara Haughtigan,[1] Susan Jones,[2] Eve Main,[2] Elizabeth Groves,[2] and Melinda Joyce[3], *1. School of Nursing and Allied Health, Western Kentucky University, Bowling Green, Kentucky, United States, 2. Western Kentucky University, Bowling Green, Kentucky, United States, 3. WKHL/Med Center Health Research Foundation, Bowling Green, Kentucky, United States*

Compared to urban populations, rural residents have a higher incidence of chronic diseases and poorer health outcomes. Most medications in the United States are consumed by older adults who are more susceptible to adverse drug events due to the presence of multiple chronic conditions and physiologic changes in the body. This community-based project evaluated the impact of an interdisciplinary medication education intervention on medication knowledge and adherence to medications and refills. The study was a quasi-experimental pretest/posttest design with a convenience sample. The project was marketed via a multi-media approach. Most participants reported learning of the event at a senior center. Each participant received a private educational session for their specific medications with a nurse practitioner, pharmacist, or pharmacy resident. Forty-nine older adults participated in the study with 48 (97.9%) completing both the pretest and posttest. The average age of participants was 71.4 years and the average number of medications per participant was 5.4. There was a statistically significant difference in the adherence to medication and refills subscale score (p = .003). There was no statistically significant difference in the medication knowledge subscale (p = .192), however, the scores did trend upward indicating an increase in medication knowledge. Forty-four (89.8%) participants reported they were more comfortable understanding their medication since participating in the program and 48 (98%) reported they would recommend the program to others. The results of this study suggest community-based outreach medication educational programs can increase adherence to medication and refills for older adults residing in rural areas.

### RELATIONSHIPS AMONG ATTITUDES TOWARD DEMENTIA, QUALITY OF LIFE, AND MIDLIFE WOMEN'S SYMPTOMS IN FAMILY CAREGIVERS

Heejung Kim,[1] Seo Yun Kim,[2] Roger Yau,[2] Glenna Brewster,[2] Wonshik Chee,[2] and Eun Ok Im[2], *1. Yonsei University, Seoul, Kyonggi-do, Republic of Korea, 2. Emory University, Atlanta, Georgia, United States*

Attitudes towards dementia and caregiving differ by family caregivers' racial/ethnic backgrounds. However, there is a gap in the literature on midlife women family caregivers' attitudes toward Alzheimer's Disease (AD) and family caregiving. The study purposes were to (1) explore racial/ethnic variations in midlife women family caregivers' attitudes toward AD and family caregiving and (2) examine the relationships among their attitudes towards dementia and caregiving, quality of life, and physical and psychological symptoms. This cross-sectional study was conducted through an online survey among 36 Whites, 41 African Americans, 40 Hispanics, and 55 Asians. The structured measures consisted of two types of attitudes (Attitude toward AD and Related Dementias Scale and Questions on Attitudes toward AD Caregiving), health-related quality of life (EQ-5D-5L), and multidimensional symptoms (Midlife Women's Symptom Index). The data were analyzed using one-way ANOVA and multiple linear regression analyses with SPSS 26. Asian caregivers perceived the care recipients' symptoms as more bothersome than White caregivers

(p = .039). Asian caregivers reported lower levels of behavioral skills and shared responsibility compared with other racial/ethnic groups of caregivers (p < .01). African Americans showed more positive attitudes toward family caregiving compared with Hispanics and Asians (p = .001). The regression analyses indicated that more positive attitudes toward family caregiving were significantly related to a better quality of life and fewer symptoms (both physical and psychological symptoms; p < .05). Culturally tailored interventions that incorporate caregivers' attitudes are needed to improve midlife women family caregivers' quality of life and symptoms.

### PRELIMINARY FINDINGS OF A STUDY ON LONGITUDINAL CHANGES IN EXPRESSED EMOTION IN FAMILY CAREGIVERS WITH DEMENTIA

Xiaoji Liu,[1] Reiko Kanaya,[1] Kazue Shigenobu,[1] Keigo Takiue,[2] and Miyae Yamakawa[1], *1. Osaka University, suita, Osaka, Japan, 2. Gifu University, Gifu, Gifu, Japan*

Purpose: The aim of this study was to explore and describe changes in expressed emotion (EE) among family caregivers of dementia and its associated factors at different time points over 3 months. Method: A longitudinal observation study using a 3-month survey was conducted in home care settings, Japan. We collected data on the demographic details of family caregivers currently providing care, which included care burden (Zarit Burden Inventory, ZBI), closeness of the caregiving relationship (Relationship Closeness Scale, RCS), depression (Geriatric Depression Scale, GDS). We used the validated Family Attitude Scale (FAS) to assess EE, in which higher scores indicate a greater intensity of emotional expression. Results: To date, 45 family caregivers completed the present study. FAS scores were 39.67 ± 24.90 at month 1 (T1), 38.64 ± 23.28 at month 2 (T2), and 39.69 ± 24.52 at month 3 (T3). The change in FAS scores did not show a statistical difference over the three-month period (p=0.816). Multiple regression analysis of FAS at T1 showed that the main influencing factors were gender, marriage, RCS, care burden, and depression ($R2=0.763$, p < 0.001); the main influencing factors at T2 were marriage and care burden ($R2=0.584$, p < 0.001); the main influencing factors at T3 were care burden and depression ($R2=0.674$, p < 0.001). Conclusions: Early identification of risk factors may help in the development of interventions to prevent high levels of EE in family caregivers. We plan to continue refining our analyses to test the validity of our hypothesis.

### PREDICTING CARE-RECIPIENTS' WELLBEING BASED ON SPOUSAL CAREGIVERS' CO-OCCURRING STRESSORS AND RESOURCES

Ruotong Liu,[1] Iris Chi,[2] and Shinyi Wu[2], *1. Oregon Health & Science University, Marina del Rey, California, United States, 2. University of Southern California, Los Angeles, California, United States*

Besides caregivers, care-recipients' health outcomes can be impacted by caregiving as well. The aim of the current study is to explore whether care-recipients' longitudinal health outcomes differ by caregivers' profiles characterized by the co-occurring and relative intensities of caregiving stressors and resources. Data from Round 5 and Round 7 of National Study of Caregiving and Round 5 to Round 8 of the linked National Health and Aging Trends Study are utilized. After multiple imputation, sample characteristics are described and compared among the 639 unique care-recipients by caregivers' latent classes. Generalized estimating equations (GEE) with first-order autoregressive covariance are estimated to examine the differences in care recipients' health outcomes at baseline and rates of change across groups. The average self-rated health at baseline is 2.73 (SD=1.02), and the average baseline depressive symptoms are 2.65 (SD=2.76). GEE results indicate that compared to care-recipients cared by low-stress low-support spousal caregivers, those cared by medium-stress high-support and high-stress medium-support partners score 0.20 and 0.35 unit lower in self-rated health at baseline, respectively, and those cared by high-stress medium-support caregivers on average score 1.21 unit higher in depressive symptoms at baseline. No statistical differences in rate of change are detected for both self-rated health and depressive symptoms across groups. Despite different levels of health at baseline, our research does not find care-recipients' rates of change in health different across groups over one-year. Future studies are needed to further explore the longer-term differences in rates of change and to better understand the caregiving dyads.

### OLDER ADULT CAREGIVERS' THOUGHTS ON WOUND CARE RESOURCES

Kristen Swartzell,[1] Janet Fulton,[2] and Jane Von Gaudecker[2], *1. IU School of Medicine, Indianapolis, Indiana, United States, 2. Indiana University, Indianapolis, Indiana, United States*

As healthcare increasingly shifts to home and community-based settings, informal caregiver responsibilities are increasing beyond assistance with activities of daily living to include complex care procedures previously performed by licensed caregivers in clinical settings. With an aging population, increasing numbers of older adults are assuming a caregiving role and these older adult caregivers are performing complex care procedures such as wound care. The negative physical and mental health consequences of caregiving for older adult caregivers are well documented in the literature. However, access to and use of resources are associated with better physical and mental health. Past research on caregiving resources has utilized pre-determined resource variables. Little is known about older adult caregivers' salient thoughts on resources important to caregiving and performing complex care procedures. This study utilized thematic analysis of qualitative interview data to identify themes and patterns related to resources as described by older adult caregivers. The following seven themes related to resources needed or utilized were identified: 1) expert guidance from healthcare professionals; 2) written instructions; 3) relationships with healthcare professionals for obtaining wound care supplies; 4) additional durable medical equipment; 5) financial resources; 6) coverage for caregiver personal time; and 7) select persons for social and emotional support. Older adult caregivers need and use a variety of resources when providing wound care. As increasing numbers of older adults choose to 'age in place', the importance of adequate resources to sustain care recipients and their caregivers in the home setting is critical.

### INSOMNIA SYMPTOM TRAJECTORIES OF SPOUSE CAREGIVERS OF OLDER ADULTS WITH FUNCTIONAL LIMITATIONS

Dexia Kong,[1] and Peiyi Lu[2], *1. The Chinese University of Hong Kong, Hong Kong, Hong Kong, 2. Columbia University, New York, New York, United States*

Background: Studies have suggested older spouse caregivers experience burden-related adverse health outcomes compared to non-caregivers. However, potential causal inferences remain unclear. This study examined the effect of caregiving on insomnia symptoms of spouse caregivers over time, compared to non-caregiver samples matched by propensity score (PS). Methods: Longitudinal data from the Health and Retirement Study from 2006 to 2018 were used. Caregivers (Nf403) were respondents (aged 50+) who assisted their heterosexual spouses in performing (instrumental) activities of daily living at baseline. PS-matching was used to match non-caregivers based on sociodemographic, household, and health-related characteristics. Symptoms of insomnia were evaluated every four years for both groups. The link between caregiver status and number/severity of insomnia symptoms over time was assessed using a Poisson mixed-effects model. Results: The propensity-matched sample achieved a satisfactory covariate balance. There was no statistically significant difference between caregivers and non-caregivers in the number of insomnia symptoms at baseline (β_caregiver=0.092, 95% CI = -0.017, 0.201). However, caregivers reported a slower increase in insomnia symptoms compared to non-caregivers (β_caregiver×time = -0.012, 95% CI = -0.021, -0.003). Results were cross-validated in modelling the severity of insomnia symptoms. Conclusion: There is weak evidence that a spouse's role as a caregiver may be beneficial for his/her sleep health over time. The negative effects of caring on older individuals' sleep may vary depending on the caregiving context. The potential health benefits of informal spousal caring and their underlying mechanisms warrant further investigations.

### INFORMAL CAREGIVING BURNOUT AMONG THE SANDWICH GENERATION

Erika Fenstermacher, Montgomery Owsiany, and Barry Edelstein, *West Virginia University, Morgantown, West Virginia, United States*

Twenty-nine percent of U.S. adults care for children. Of those adults, 12% are multigenerational caregivers who also provide unpaid care for one or more adults. Many multigenerational caregivers are considered members of the "sandwich generation," which is a term for multigenerational caregivers who provide care, financial support, and emotional support for both their children and parents. Approximately 71% of this generation is between the ages of 40 and 59, and approximately 10% are 60 or older. The sandwich generation is largely understudied and presents challenges including informal caregiving burnout and depression. Informal caregiving burnout is a syndrome that results from the stress of providing care. Researchers have not yet investigated burnout in the sandwich generation. The present study examined how sandwich generation caregivers differed from caregivers of children and caregivers of parents regarding depression and burnout. Two measures of burnout were used (non-caregiving burnout = degree of physical and psychological fatigue and exhaustion; informal caregiving burnout = degree of physical and psychological fatigue and exhaustion related to caregiving). We found that sandwich generation caregivers and caregivers of parents scored significantly higher than caregivers of children on informal caregiving burnout. Caregiving burnout and non-caregiving burnout were significantly correlated with depression ($r = .496$, $p < .001$ and $r = .773$, $p < .001$, respectively). Burnout is higher in sandwich generation caregivers and those who care for parents than burnout among those who care only for children. This study is unique in its investigation of informal caregiving burnout among the sandwich generation.

### IDENTIFYING FACTORS ASSOCIATED WITH CAREGIVER NEGLECT IN OLDER PERSONS WITH DEMENTIA

E-Shien Chang,[1] Sara Czaja,[1] Tony Rosen,[2] and Jerad Moxley[1], *1. Weill Cornell Medicine, New York, New York, United States, 2. Weill Cornell Medical College / NewYork-Presbyterian Hospital, Pelham, New York, United States*

Caregiver neglect is common among older persons with dementia and is associated with significant morbidity and mortality. Despite this, little empirical research exists examining factors that contribute or prevent neglect. Our goal was to identify caregiving factors that may be associated with caregiver neglect. Data were drawn from the baseline information of 240 caregivers enrolled in the Caring for the Caregiver Network study who provided care to a family member with dementia. Caregiver depression, burden, social support, perceived mutuality with care-recipients, positive aspects of caregiving, and self-perceived caregiving preparedness, were measured using validated scales. To maximize sensitivity in our measure of caregiver neglect, we operationalized caregiver neglect as consisting of: 1) caregivers' failure to meet care-recipients' needs, using 8-item instrumental activities of daily living (IADL) and 6-item ADL scales, and 2) caregivers not receiving additional formal services (e.g., visiting nurses; home care aides) to address care-recipients' unmet needs. Selection of independent variables into multivariate regression models examining predictors of neglect was based on significance in bivariate analysis ($p < .10$). Caregiver neglect was found in 29.2% of caregivers. The caregiver being male was significantly associated with greater risk of neglect. In the final adjusted model, only caregiver preparedness was found to be a significant risk factor for neglect ($\beta = -.16$ $SE=.10$, $p < .05$). This analysis provides the first evidence of the association between caregiver preparedness and risk of caregiver neglect. Future research should examine possible psycho-social mechanism linking preparedness and neglect to inform neglect prevention and intervention programming.

### HEALTHY CAREGIVER SELECTION AMONG DEMENTIA CAREGIVERS: THE ROLE OF SOCIAL SUPPORT

Hantao Jiang,[1] and Xueqing Wang[2], *1. Columbia University, Princeton, New Jersey, United States, 2. Princeton University, Princeton, New Jersey, United States*

As of 2019, more than 4 million older adults aged 65+ in the United States are cognitively impaired, including the diagnoses of mild cognitive impairment (MCI) and dementia.

Caregivers to these older adults bear significant burden, reflected as high prevalence of chronic stress and mental health problems among the caregiver population. It is thus crucial to understand the wellbeing of the caregiver population to design effective policies. Previous studies have documented survival advantage of dementia caregivers compared to non-caregiving individuals as well as other types of caregivers, namely caregivers to persons with MCI or other types of chronic conditions. However, it remains less clear how the role of social support explains dementia caregiver's survival advantage. In this paper, we directly compare the level and type of social support between different types of caregivers, and examine to what extent the difference in social support explains the survival and health advantage of dementia caregivers compared to caregivers to persons with MCI, non-cognitive impairment chronic conditions and non-caregiving individuals. We use the 12 waves of the Health and Retirement Study and apply multivariate and survival analysis to calculate difference in age-specific hazard ratios. Our preliminary results show that dementia caregivers tend to secure stronger support from family members than caregivers to persons with MCI. Our results have potential to shed light on the empirical puzzle of healthy caregiver selection effect and have direct implications for designing effective intervention to improve health of the caregiver population.

### HEALTHCARE LITERACY AMONG INFORMAL CAREGIVERS: THE ROLE OF SOCIAL SUPPORT SYSTEM AND BURNOUT

Robyn Medcalf,[1] Jessika Dayrit,[2] Shirley Salom-Bail,[1] Cesz Islaya,[1] Shelly Foutz-Lowe,[1] and Renuka Alladi[1], *1. Department of Veterans Affairs, Palo Alto Health Care System, Palo Alto, California, United States, 2. Washington University in St. Louis, St. Louis, Missouri, United States*

Informal caregivers are integral components in the lives of individuals with health problems. However, little is known if the number of hours of informal care correlates with healthcare literacy levels. Thus, using the Center for Disease Control (CDC) Behavioral Risk Factor Surveillance (BRFSS) 2016 public dataset, the aim of this study examines the association between informal caregivers' hours of care and their health literacy level, by controlling their social/emotional support, type of care they provide, and socioeconomic level. Data was weighted for complex sampling design and logistic regression to examine the data (Nf3,249). The study's results show that being full-time or part-time caregiver has no association to healthcare literacy in verbal (OR=0.99, 95% CI=0.57–1.71) and written (OR=1.21, 95% CI=0.78–1.85) comprehensions. Further, caregiver's type of care does not correlate to healthcare literacy level in verbal (OR=0.87, 95% CI=0.58–1.32; OR=0.93, 95% CI=0.59–1.45) and written (OR=0.91, 95% CI=0.66–1.27; OR=0.81, 95% CI=0.57–1.16) comprehensions. However, caregivers who rarely-to-never receive social/emotional support are four times more likely to have difficulties understanding verbal health information than those who have always-to-usually receive social/emotional support (OR=4.02, 95% CI=2.45–6.61). Caregivers who rarely-to-never receive social/emotional support are two times more likely to have difficulties understanding written health information compared to those that have always-to-usually receive social/emotional support (OR=2.30, 95% CI=1.51–3.51). Caregivers with lower socioeconomic backgrounds are at higher probability of low healthcare literacy level. Our study suggests further longitudinal and qualitative research, to understand healthcare literacy level among informal caregivers, and how social support systems correlates to healthcare literacy level.

### FACTORS ASSOCIATED WITH VOLUNTEERING AMONG FAMILY CAREGIVERS OF OLDER ADULTS

Sol Baik,[1] Rachel Coleman,[2] and Jennifer Crittenden[2], *1. University of Virginia Weldon Cooper Center for Public Service, Charlottesville, Virginia, United States, 2. University of Maine, Bangor, Maine, United States*

Family caregiving has been linked to an increased risk of poor mental health, poor physical health, and higher rates of perceived social isolation among caregivers. Despite the connection between caregiving and negative outcomes, or perhaps because of this connection, caregivers seek out and enjoy other life roles and activities including formal volunteerism. To explore the connection between informal caregiving and volunteering and establish the representative prevalence of formal volunteering among caregivers, descriptive and multivariate logistic regression analyses were carried out with data from 1,745 caregivers in the National Study of Caregiving (NSOC) (2017). Utilizing social capital theory four models were constructed with salient demographic characteristics associated with volunteering (model 1), caregiving context (model 2), caregiver physical and mental health (model 3), along with participation in informal and formal social networks (model 4). About a quarter of the sample participated in volunteering (26%). The average age was 60.5 years (SD = 14.3) and more than half consisted of female caregivers (67.11%). Non-Hispanic Whites (62.9%) were the majority of the sample, followed by non-Hispanic Blacks (28%), Hispanic (6.5%), and caregivers in other racial/ethic groups (2.6%). Gender, educational achievement, caregiving for a spouse, coresiding with care recipient, caregiving for multiple care recipients, quality of relationship with care recipient, caregiver psychological well-being, having emotional/physical support, attending religious services, and group activity participation were all significant indicators for caregiver volunteerism. Findings support the importance of both human and social capital in volunteering among caregivers.

### EXERGAME INTERVENTION TO PROMOTE FAMILY CAREGIVERS' SOCIAL SUPPORT, PHYSICAL ACTIVITY, AND WELL-BEING

Xin Yao Lin, and Margie Lachman, *Brandeis University, Waltham, Massachusetts, United States*

Family caregivers often experience high stress, social isolation, poor mental and physical health, and have a sedentary lifestyle. The current study was a randomized trial (Nf76) comparing the effectiveness of Go&Grow (social vs non-social exergame app) to promote well-being through increased social support and physical activity for family caregivers over a 6-week intervention. Both groups received daily reminders to use the app. Findings showed the treatment group increased significantly more than the control group in well-being (management of distress) and social support (satisfaction with contact quality). There was an indirect effect of change in social support in which the treatment group increased more than the control group in satisfaction with contact quality, which in turn led to more positive affect and less loneliness. The increase in steps ranged from 3% to 12% across conditions. Social support moderated the relationship between condition and step changes, such that those in the treatment group who increased more in overall social support and knowing others' experiences increased steps more than those with less support, while change in steps for the control group was not related to support level. In the treatment group, those who used the social features of the app more frequently had a greater increase in steps compared to those who used the social features less often. These findings can offer new insights into implementing behavior change mechanisms using exergames for sedentary and socially isolated family caregivers to improve their health with modifiable behavioral factors such as physical activity and social support.

### CAREGIVING BURDENS OF TASK TIME AND TASK DIFFICULTY AMONG PAID AND UNPAID CAREGIVERS OF PERSONS LIVING WITH DEMENTIA

Matthew Smith,[1] Chung Lin Kew,[1] Gang Han,[1] Lucas Wilson,[1] Shinduk Lee,[2] John Fitch,[1] and Marcia Ory[1], *1. Texas A&M University, College Station, Texas, United States, 2. University of Utah, Salt Lake City, Utah, United States*

Demands of caregivers of persons living with dementia (PLWD) are often influenced by the context of their caregiving situation. This study examines factors associated with caregiving burden in terms of task time and task difficulty among paid and unpaid caregivers of PLWD. Cross-sectional baseline survey data were analyzed from 110 paid and unpaid caregivers of PLWD participating in a larger NIH-funded study assessing the feasibility of using a novel in-situ sensor system. Oberst Caregiving Burden Scale constructs of task time and task difficulty served as dependent variables. Two least squares regression models were fitted, controlling for contextual items related to the caregiver, care recipient, and caregiving logistics. Caregivers whose care recipients were female (B=-0.29, P=0.006), had more chronic conditions (B=0.31, P=0.011), and had lower Mini-Mental State Exam scores (B=-0.20, P=0.015) reported higher task time burdens. Caregivers whose care recipients had other paid caregivers (B=0.30, P=0.031) and spent more months/years caring for their care recipients (B=0.28, P=0.004) reported higher task time burdens. Caregivers' task time burden was positively associated with their emotional stress level (B=0.30, P=0.020). Caregivers' task difficulty burden was positively associated with their emotional stress (B=0.30, P=0.029) and depressive symptomatology (B=0.32, P=0.002). Results reinforce the relationship between caregiver burden and mental health. While the care recipient's disease profile and needs were drivers of task time burden, which may also require coordination with other paid caregivers, task difficulty was emotionally driven. Findings highlight the importance of caregiver support services and programming for mental health.

### THE ROLE OF PAID CAREGIVERS IN HOME-BASED DEMENTIA CARE: FAMILY, PAID CAREGIVER, AND GERIATRICIAN PERSPECTIVES

Deborah Watman, and Jennifer Reckrey, *1. Icahn School of Medicine at Mount Sinai, New York City, New York, United States*

Paid caregivers (e.g., personal care aides, home health aides, other direct care workers) provide essential care that allows people with dementia to remain living at home, yet little is known about the lived experience of this care. This project uses multiple perspective, qualitative longitudinal interviews to explore paid caregiver role in home-based dementia care. We conducted one-on-one interviews via telephone or zoom with the family caregiver, paid caregiver, and geriatrician of an individual person with moderate or severe dementia (n=9) living at home in New York City. After an initial interview, up to 2 additional interviews (at 3 and 6 month intervals) were also conducted for a total of 75 interviews with 29 unique respondents. Interviews were audio-recorded, transcribed, and analyzed using the framework method of analysis. Interviews revealed nuanced care arrangements, but paid caregiver role in care remained largely stable over time. Key findings include: (1) Family caregivers played a primary role in determining overall paid caregiver role in care, (2) Paid caregivers describe the emotional components of caregiving (e.g. being "like family", having patience) more frequently than families or doctors, and (3) Doctors rarely engage with paid caregivers unless family involvement is limited. The unique structure of each triad emphasizes the importance of person-centered dementia home care. Formal care plans may not reflect the nuances of care arrangements and responsibilities. Rather than prescriptive standards for home care, improved communication and clear expectation setting may help meet the complex needs of people with dementia and their families.

### THE POTENTIAL CLINICAL AND ECONOMIC IMPACTS OF OVER-THE-COUNTER HEARING AIDS IN THE UNITED STATES

Ethan Borre,[1] Mohini Johri,[1] Judy Dubno,[2] Juliessa Pavon,[3] Evan Myers,[1] Howard Francis,[1] Osondu Ogbuoji,[1] and Gillian Sanders Schmidler[1], *1. Duke University, Durham, North Carolina, United States, 2. Medical University of South Carolina, Charleston, South Carolina, United States, 3. Duke University School of Medicine, Durham, North Carolina, United States*

Food and Drug Administration regulations (released 8/16/22) for over-the-counter (OTC) hearing aids (HAs) allow the purchase of OTC HAs for adults with perceived mild-to-moderate hearing loss. Given this new development, the quality-of-life benefits and costs of OTC devices are now relevant but also unknown. We sought to project the population-level costs and effectiveness of US OTC HA provision. We used a microsimulation of hearing loss (DeciBHAL-US) to simulate: 1) traditional HA provision validated to published estimates of HA uptake (10-year delay), and 2) OTC HA provision for persons with perceived mild-to-moderate hearing loss at increased uptake rates (5-year delay) across quality-of-life and cost combinations. Simulated adults experienced probabilities of hearing loss (0.1–10%/year), subsequent age- and severity-dependent HA uptake (traditional HAs: 0.5–8%/year; OTC HAs: 1–16%/year) and discontinuation (4–13%/year). Utility benefit for traditional HAs was +0.11, and was +0.05–0.11 for OTC HAs. Costs included traditional HA uptake ($3,690; maintenance=$1,000/year) and OTC HA uptake ($600-$2,000; maintenance=$220–500/year). Our model projected lifetime discounted quality-adjusted-life-years (QALYs) for traditional HAs of 18.140/person, and 18.139–18.174/person for OTC HAs varying with utility benefit (+0.05–0.11). As long as OTC HA utility benefit was >+0.05, increased access resulted in higher QALYs and was cost-effective ($4,000/QALY-$92,500/QALY, varying with HA cost and utility benefit). Our results were sensitive to variations in OTC HA uptake rates. In conclusion, OTC HAs may increase population health and access to hearing healthcare in a cost-effective manner, and our model may continue to explore the costs and benefits of OTC HAs as pricing and evidence evolves.

### PERSONAL STRATEGIES FOR MANAGING EVERYDAY ACTIVITY CHALLENGES AMONG ADULTS AGING WITH VISION LOSS

Kara Mumma,[1] Elena Remillard,[1] and Wendy Rogers[2], *1. Georgia Institute of Technology, Atlanta, Georgia, United States, 2. University of Illinois Urbana Champaign, Champaign, Illinois, United States*

An estimated 6% of adults in the United States ages 65 and older are living with vision loss and may experience other age-related declines and conditions co-morbidly (e.g., arthritis, hypertension, hearing loss). As such, they are likely to experience challenges in performing everyday activities, but little is known about their personal strategies or innovative solutions for managing activity challenges. The Aging Concerns, Challenges, and Everyday Solution Strategies (ACCESS) study explored everyday activity challenges and strategies among older adults (Nf60; ages 60–79) with long-term vision loss (prior to age 50). The current analysis focused on understanding participants' own methods for managing activity challenges, or things they have come up with, beyond traditional methods, such as getting help from other people (e.g., caregiver) or using assistive devices (e.g., white cane, screen reader). We coded response strategies among participants' own methods, and the most frequently reported methods included: learning/familiarity/organization, planning ahead/prioritizing, and perseverance/patience/assertiveness. Response codes were classified using the Selection, Optimization, and Compensation model to better understand how these strategies are being used to adapt to losses. The most common strategies were classified as elective selection with optimization, which are strategies that entail continued performance of challenging activities through behavioral methods, without bringing in any new means to assist. Illustrative example quotes are featured to provide insight on the context of personal strategies. With greater understanding of solutions people aging with vision loss are employing to manage activity challenges, we can increase awareness about effective solutions and identify opportunities for supportive technology solutions.

### USING THE COLLECTIVE IMPACT FRAMEWORK TO REFRAME AGING IN MISSISSIPPI

Kina White,[1] Kaye Bender,[2] and David Buys[3], *1. Mississippi State Department of Health, Ridgeland, Mississippi, United States, 2. Mississippi Public Health Association, Jackson, Mississippi, United States, 3. Mississippi State University, Ms State, Mississippi, United States*

Trust for America's Health (TFAH), the Mississippi State Department of Health (MSDH), and the Mississippi Public Health Association (MPHA) partnered to organize the Mississippi Age-Friendly Public Health System (AFPHS) Work Plan. Built on the foundation of Reframing Aging training offered by the Gerontological Society of America in May 2021, the Work Plan aims to reframe how Mississippi's public health system addresses aging using the AFPHS 6Cs. Developed by the MS AFPHS Advisory Committee using a collective impact framework, the Work Plan has now been opened for public comment prior to being finalized for adoption across partner agencies. An AFPHS focuses on promoting health; preventing injury; managing chronic conditions; optimizing physical, cognitive, and mental health; and facilitating social engagement for older adults. The Work Plan's purpose is to guide MSDH and partner agencies in implementing an AFPHS for Mississippi. This is Phase One of a comprehensive age-friendly movement for Mississippi, with the next phase being an age-friendly state. Much of the work that MSDH will accomplish under this plan lays the foundation for the state to then have many age and dementia friendly communities. This presentation will discuss how Mississippi is reframing aging through collective impact and AFPHS 6Cs frameworks; the results of the public vetting of the Work Plan; and the final Work Plan components based on the public vetting. The process and its results can be replicated in other rural states where resources for aging populations are limited and historical perspectives on healthy aging are open to change.

### GLOBAL CLINICIAN PERSPECTIVES ON NON-OPERATIVE HIP FRACTURE MANAGEMENT DURING COVID-19

Lucille Xiang,[1] Mriganka Singh,[1] Lynn McNicoll,[1] and Iain Moppett[2], *1. Brown University, Providence, Rhode Island, United States, 2. University of Nottingham, Nottingham, England, United Kingdom*

There are currently no guidelines regarding clinician decision making in the type of hip fracture management among older adults. Cultural, social, structural and economic differences between global healthcare systems may result in differing approaches. This study's objectives were to identify possible factors influencing clinicians' decision to undertake a non-operative hip fracture management approach among older adults, and to determine whether there is global heterogeneity regarding these factors between high income countries (HIC), and low- and middle-income countries (LMIC) clinicians. A SurveyMonkey questionnaire was distributed to clinicians through the Fragility Fracture Network's Perioperative Special Interest Group and clinicians' personal networks between

May 24 and July 25, 2021. 406 respondents from 51 countries returned the questionnaire, of which 225 respondents came from HIC and 180 from LMIC. Clinicians from HIC reported a greater median [IQR] estimated proportion of admitted patients with hip fracture undergoing surgery (96% [95–99]) than those from LMIC (85% [75–95]) of mean (SD) at 94% (8) compared to 81% (16) among LMIC clinicians (p=2.94e-23). Several factors seemed to influence the clinician hip fracture management decision making process. Global heterogeneity seems to exist between HIC and LMIC clinicians regarding factors such as anticipated life expectancy, ability to pay, treatment costs, insufficient resources, and perception of risk in hip fracture management decision-making. This is the first international sampling of clinician perspectives regarding hip fracture management. Further research is necessary for the development of best practice guidelines to improve hip fracture management decision-making and quality of hip fracture care among older adults.

### GIDDY UP! LESSONS LEARNED FROM INTRODUCING AN ONLINE, NATURE-BASED INTERVENTION TO ASSISTED LIVING

Megan Westmore,[1] Keith Anderson,[2] and Rebecca Mauldin[1], *1. University of Texas at Arlington, Arlington, Texas, United States, 2. University of Mississippi, Oxford, Mississippi, United States*

The processes involved with developing, funding, and conducting intervention studies in long-term care present a unique set of challenges for researchers. In this presentation, we explore the experiences of researchers evaluating an online nature-based intervention in assisted living. The first challenge lies in creating or finding a novel intervention specific to the setting and population. In this case, the researchers found and tailored an existing online, interactive program that focused on ranch life in rural Montana – Days at Dunrovin. In partnership with the developer of Days at Dunrovin, a group intervention was developed specifically for long-term care settings. The second challenge is finding a facility that has the desire, environment, and staffing to support the intervention. In this case, the researchers were able to partner with an assisted living facility that had an owner who was interested in research, an environment that matched the experimental design of the study, and a staff that was eager to find new ways for residents to engage with the world, particularly during the COVID-19 pandemic. The final challenge involves selecting a funding mechanism that best fits the needs of the study. In this case, the researchers targeted a private foundation that provided ample funding along with the latitude and flexibility that allowed the study to evolve and pivot as necessary. This presentation concludes with recommendations for both budding and seasoned researchers who seek to introduce creative interventions into long-term care settings.

### BARRIERS AND STRATEGIES TO ENGAGE AND RETAIN DIVERSE RURAL DYADS IN PSYCHOSOCIAL INTERVENTIONS

Michael McCarthy, Y. Evie Garcia, Mara Cassady, Neshay Mall, Pamela Bosch, and Steven Barger, *Northern Arizona University, Flagstaff, Arizona, United States*

It is often challenging to engage and retain rural Latinx and Native Americans in psychosocial interventions after stroke, despite the fact that these populations tend to experience poorer health outcomes. Although strategies for effective patient-provider communication exist, few studies focus on the cultural aspects of engagement and retention of diverse dyads in a rural context. The aim of this study is to describe barriers to engagement and retention of diverse rural dyads in post-stroke psychosocial interventions, as well as to elucidate specific strategies to address these barriers. Semi-structured interviews were conducted with Nf14 clinical providers with extensive experience serving diverse rural couples and families. Qualitative data were analyzed using interpretive description methods. Seven key barriers were identified, grouped broadly into: 1. Those presented by providers (1a. Lack of time and attention given to dyads “telling their story”; 1b. Lack of culturally appropriate interpersonal skills; 1c. Incongruence between provider-patient values) and 2. Those existing within dyads themselves (2a. Low priority given to interpersonal and emotional issues; 2b. Concern about confidentiality in small communities and mistrust of those in power due to current or historical experiences with dominant cultures; 2c. Perception of stigma surrounding interpersonal and emotional issues; 2d. Tendency to be polite, accommodating, or feign investment). Providers offered specific culturally-informed strategies to overcome barriers which in turn may increase engagement and minimize attrition. Providers and researchers may benefit from these insights as they work to communicate with, build trust, and meet the unique needs of these populations.

### LINKS BETWEEN LONELINESS AND SLEEP AMONG PARTNERED OLDER ADULTS: AFFECTIONATE TOUCH AS A MODERATOR

Carly Lawrence, and Christina Marini, *Adelphi University, Garden City, New York, United States*

Sleep problems and loneliness both increase with age and undercut older adults’ health and well-being. Loneliness is a predictor of sleep problems, however, research in this area often fails to also consider the role of relationship quality. The current study therefore focuses on spousal relationship quality as it has been shown to have a proximal influence on sleep. We examined relative effects of physical and emotional facets of relationship quality–namely affectionate touch and emotional support (in addition to loneliness). We further examined the potential for relationship quality to buffer effects of loneliness on sleep. We utilized data from a nationally representative sample of older adults from the National Social Life Health and Aging Project (NSHAP). Participants were partnered (N = 559) and completed a novel sleep module that included subjective (i.e., insomnia symptoms) and objective (i.e., Wake After Sleep Onset; WASO) markers of sleep. Upon controlling for demographics and mental and physical health, a different pattern of findings emerged for each facet of sleep. For subjective sleep, older adults who were more lonely reported more insomnia symptoms, but only when spousal emotional support was low. For objective sleep, older adults who reported more affectionate touch experienced less WASO. These findings suggest that loneliness that occurs in the context of low emotional support from spouses is particularly damaging for older adults’ subjective sleep quality. Further, affectionate touch from spouses may represent an important intervention target for promoting felt security that helps older adults stay asleep throughout the night.

### FRAILTY AND DEPRESSIVE SYMPTOMS MEDIATE THE IMPACTS OF FOOD INSECURITY ON SLEEP DEFICIENCY AMONG OLDER FILIPINOS

Tuo-Yu Chen,[1] Grace Cruz,[2] and Yasuhiko Saito[3], *1. Taipei Medical University, Taipei City, Taipei, Taiwan (Republic of China), 2. University of Philippines, Diliman, Quezon, Philippines, 3. Nihon University, Chiyoda-ku, Tokyo, Japan*

Although research has shown that food insecurity may lead to sleep problems due to mental illness, it remains unclear whether physical health also mediates this relationship in addition to poor mental health. We investigated whether frailty and depressive symptoms mediated the association of food insecurity with sleep deficiency (i.e., inadequate and poor sleep) among community-dwelling older adults. We analyzed the baseline data of the Longitudinal Study of Ageing and Health in the Philippines, a study with national representative sample of adults aged 60 years or older (Nf5,187). Sleep deficiency was conceptualized as self-reported sleeping less than 6 hours, complaining about trouble with falling asleep and staying asleep, and/or having non-restorative sleep. Food insecurity was defined as being hungry and not having enough food in the past three months in the household. Frailty was operationalized using modified definitions under Fried's criteria. Depressive symptoms were assessed using the Center for Epidemiological Studies-Depression Scale. Covariates included sociodemographics (age,sex,education,wealth,insurance,liv ing arrangement,urbanity), health (pain,chronic diseases), and lifestyles (smoking,drinking). Mediation analysis was performed using PROCESS macro. The results from bootstrapping showed that the indirect effects from food insecurity through frailty (b=.024,SE=.009,95%CI=.009-.043) and depressive symptoms (b =.118,SE=.022,95% CI=.078-.166) onto sleep deficiency were significant, but the direct effect from food insecurity to sleep deficiency was not (b=.243,SE=.134,95% CI=-.018-.505). The results suggested that the effects of food insecurity on sleep deficiency were fully mediated through frailty and depressive symptoms among community-dwelling older Filipinos. Preventing frailty and depression may help improve sleep health among individuals being food insecure.

### USING STORYTELLING TO ASSESS NURSE'S KNOWLEDGE IN CARING FOR OLDER ADULTS

Michele Murphy-Boucher,[1] and Diana Lynn Woods[2], *1. Providence Holy Cross Medical Center/Azusa Pacific University, Mission Hills, California, United States, 2. Azusa Pacific University, Azusa, California, United States*

Continuing education, knowledge acquisition, and competence is required of all professional Registered Nurses. Remaining current in care delivery trends based on evidence practice is the responsibility of nurses supported by professional development specialists and educators. This project was conducted as a quality improvement project, using the evidence-based teaching modality of storytelling to educate practitioners in caring for older adults. This project addresses the question: For leaders of professional development and education focused on acute care and ambulatory practice, who have not had specialty geriatric training, how does using case-based/story-telling education for the care of patients 65 years and older affect confidence levels of the 4 M's (mentation, mobility, what matters and medication) of elder care? A convenience sample of 12 Directors of Professional Development participated, and a validated comparison of responses to the Gerontological Nursing Competence Questionnaire (GNCQ) and the Facts on Aging (2015 version) (FAQ) were used for collecting data of pre intervention and post intervention. An eight-minute video was the educational intervention. GNCQ responses for confidence in knowledge and confidence in teaching were statistically significant. Although an increase in the mean score for interest in additional training, it was not statistically significant. The FAQ showed no statistical significance for the pre and post results. Findings indicate that storytelling is a viable teaching modality for increasing nurse's knowledge of caring for older adults based on the concepts of the 4 Ms of age-friendly care. Keywords: storytelling, case studies, nurse education, professional development, narratives, and adult learning.

### POSITIVE AGEING AND DEATH OR DYING: A SCOPING REVIEW

Robin Otto,[1] Noelle Fields,[2] Keith Anderson,[3] and Michael Bennett[1], *1. University of Texas at Arlington, Arlington, Texas, United States, 2. The University of Texas at Arlington, Arlington, Texas, United States, 3. University of Mississippi, Oxford, Mississippi, United States*

Ageing world populations and increases in life expectancy have facilitated an interest in developing ageing models that promote inclusivity and positive perceptions of ageing. This scoping review examines how and to what extent research that utilizes successful, active, productive, and healthy ageing framework(s) include death or dying. The study followed Joanna Briggs Institute's methodological standard for scoping reviews and PRISMA guidelines and conformed to Arskey and O'Malley's five-stage framework. A thematic analysis was used to identify how research utilizes the concepts of death and dying in the context of positive ageing models. The analysis identified five core themes: (a) the critique that death and dying dimensions in positive ageing models are absent; (b) older adults' outlooks on death and dying while ageing well; (c) religious and spiritual dimensions of ageing well; (d) negative consequences of positive ageing models without death and dying dimensions; and (e) the future of death and dying in positive ageing models. The results bolster support for a paradigm shift that redefines what it means to age successfully without denying death. Incorporating the topics of death and dying into ageing conversations encourages individuals to ponder their end-of-life preferences and proactively participate in their advanced care plans. Death and dying conversations help care providers support people to live and die in a manner that is meaningful to them and inspire those receiving care to live fully and deeply and to think about the legacy that they want to leave.

### HOW TO ENGAGE SENIORS AS VOLUNTEERS IN SOCIAL CARE SECTORS: A CASE STUDY OF A TIMEBANK IN HONG KONG

Shiyu Lu, *City University of Hong Kong, Kowloon, Hong Kong*

Objective: Exploring the roles of older adults as volunteers in social care settings has attracted wide attention to facilitate healthy aging. However, knowledge of engaging older adults as volunteers in social care sectors remains scant. This study explores theoretical mechanisms for promoting volunteer engagement among older adults in the social care sector. Method: This study used a time bank program called Good Hands in Hong Kong as a case study. Good Hands was established in 2018 and engaged almost 200 older adult volunteers to provide buddying and accompany (e.g., home visits and medical escort services) to over 390 frail peers in the community. This study adopted a qualitative method. Three semi-structured focus group interviews with 18 participants, including senior volunteers and the timebank staff, were conducted in January – August 2021. The thematic analysis. Stakeholder checks were conducted in July 2022 to enhance the credibility of the findings. Results: Three emergent themes were identified as critical components to facilitate engagement among senior volunteers: (1) strong cross-sector collaboration, (2) meaningfulness in voluntary work comprising four subthemes, including capacity optimization, care capacity enhancement, belonging cultivation, and value recognition, and (3) a co-producing environment. In addition, this study also identified the challenges related to the sustainability of the timebank program. Conclusion. This study is the first to explore mechanisms for promoting volunteer engagement among older adults in the social care sector. The findings provide theoretical and practical implications for the roles of older adults in social welfare production for our society.

### COPING AND LIFE SATISFACTION OF OLDER PEOPLE DURING THE COVID-19 PANDEMIC

Dongjuan Xu, Nasreen Lalani, and Gregory Arling, *Purdue University, West Lafayette, Indiana, United States*

Covid-19 put older individuals at high risk for increased morbidity and mortality, isolation, reduced coping and life satisfaction. Optimism, sense of mastery and closeness with family and friends can enhance coping and life satisfaction among older adults. No such studies were found during the pandemic. Our study examined the associations between optimism, sense of mastery, closeness with spouse, family, and friends, physical and psychological functioning and its effects on coping and life satisfaction. A national representative sample of 1,890 community dwelling older adults was obtained from the 2020 Health and Retirement Study COVID-19 data during March 2020-June 2021. A structural equation modeling approach used to test the associations and their direct and indirect effects on life satisfaction. Coping was seen as a mediator affecting these relationships and their effects on life satisfaction. Optimism ($\beta = .318$, $p < .001$), mastery ($\beta = .195$, $p < .001$) closeness with spouse/partner ($\beta = .199$, $p < .001$), closeness with children (($\beta = .075$, $p < .010$), friends ($\beta = .086$, $p < .001$), had significant positive direct and indirect effects on life satisfaction. Frailty ($\beta = -.137$, $p < .001$), comorbidities ($\beta = -.057$, $p < .050$), and IADL limitations ($\beta = -.118$, $p < .001$) had negative direct effects on life satisfaction. Optimism, sense of mastery and closeness with family/friends promotes coping and life satisfaction whereas frailty and comorbidities negatively influence coping and life satisfaction of the older adults. Community interventions should target coping strategies that enhances optimism, mastery, and interpersonal closeness among older adults during pandemic.

### A PILOT ASSESSMENT OF A TABLET-BASED INTERVENTION FOR HOMEBOUND OR SOCIALLY ISOLATED OLDER ADULTS WITH DEPRESSION

Caroline Galo,[1] Natalie Benda,[1] Isabel Olivia Rollandi,[2] Sara Czaja,[3] Marco Ceruso,[1] and Jo Anne Sirey[2], *1. Weill Cornell Medicine, New York, New York, United States, 2. Weill Cornell Medicine, White Plains, New York, United States, 3. Weill Cornell Medicine, Ithaca, New York, United States*

Older adults who are homebound or socially isolated have high rates of loneliness and depression with fewer opportunities for treatment. Our team extended an existing psychotherapy intervention (Engage & Connect) to improve access to mental health care for older adults who are homebound. We iteratively created a tablet-based application (Engage PRISM) leveraging a user-centered design approach to provide older adults, particularly those with limited technology experience, an easy-to-use application to support social reward. Engage PRISM connects clients with the psychotherapy intervention and additional features to increase social reward exposure virtually. All eligible participants received a K92 ZTE tablet, equipped with the Engage PRISM application, internet service, Zoom, and access to Selfhelp's Virtual Senior Center. Participants were then enrolled in the 9-week Engage & Connect intervention delivered by a licensed mental health counselor via Zoom on the tablet each week. We evaluated feasibility of the intervention and preliminary effect on depressive symptoms through a weekly PHQ-9. Feasibility was assessed through participants' ability to use the tablet to access mental health treatment. We provided tablets to eight participants ages 67 to 84; participants demonstrated 100% feasibility of use of the tablet intervention. We dropped two participants from the study due to a greater level of care needed. All participants were provided with referrals prior to ending the study. Preliminary evidence indicates that four of the six remaining participants had experienced a reduction in depressive symptoms (i.e., had lower PHQ-9 scores) three weeks into the study reporting over a 30% reduction on average.

### IS THERE A CAUSAL RELATIONSHIP BETWEEN ONLINE HEALTH INFORMATION SEEKING AND ANXIETY AMONG OLDER ADULTS?

Shelia Cotten,[1] Reza Ghaiumy Anarsky,[1] and Amy Schuster[2], *1. Clemson University, Clemson, South Carolina, United States, 2. Clemson University, Seneca, South Carolina, United States*

The growth of the Internet has provided users unlimited access to an abundance of information, including health information. The Internet is typically the first source searched by U.S. adults (18 years and older) when seeking health information. Factors such as age and anxiety are associated with online health information seeking (OHIS). Older adults (65 years and older) are increasing their Internet use and OHIS, although OHIS decreases with age. Importantly, OHIS can lead to improved health outcomes for older adults. The relationship between OHIS and anxiety is less clear, with some studies finding individuals with higher anxiety are more likely to search for online health information while others find the reverse pattern. Anxiety affects up to 20% of older adults and is often unrecognized and untreated. Given the lack of research examining the relationship between OHIS and anxiety among older adults and the proportion of older adults who experience anxiety, we analyzed six waves (2015- 2020) of data from the National Health and Aging Trends Study to assess the causal relationship between anxiety and OHIS using a Random Intercept Cross-lagged Panel Model framework, a statistical approach to assess causal relationships. These results suggest that anxiety is leading to OHIS in the next wave, rather than the reverse; however, OHIS was not related to anxiety in the next wave. This suggests that OHIS is not capable of mitigating older adults' anxiety. Practitioners working with older adults should be aware that older adults may need additional support to manage their anxiety.

### HOW TO PROMOTE INTERGENERATIONAL INTEGRATION THROUGH ONLINE COMMUNICATION: A MODERATED MEDIATION MODEL

Yeseul Lee, Miseon Kang, Susanna Joo, and Hyoun K. Kim, *Yonsei University, Seoul, Seoul-t'ukpyolsi, Republic of Korea*

The present study examined the extent to which online communication skills led to the degree of intergenerational integration mediated through loneliness, focusing on whether such a mediating path would be moderated by age groups. Intergenerational integration is a concept that emphasizes acceptance and flexibility to recognize diversity based on each other's values across multiple generations or groups of varying ages. The older group included 315 Koreans aged 65 or above, and the younger group included 322 Koreans aged 19 to 29. Both groups completed an online survey conducted from December 2021 through January 2022. The results showed loneliness significantly mediated the association between online communication skills and the degree of intergenerational integration among both older and young adults; the higher the level of online communication skills, the lower the level of loneliness, leading to high levels of intergenerational integration. However, the mediation path was moderated by age; the association between online communication skills and loneliness was stronger for young adults compared to older adults. Additionally, the direct path was moderated by age: the association between online communication skills and intergenerational integration was stronger for older adults compared to young adults. The findings indicate that programs aimed at improving online communication skills (e.g., SNS or video calls) can reduce loneliness and thus promote intergenerational integration in both younger and older adults. This study highlights the importance of promoting online communication ability to increase intergenerational integration of both older and young adults.

### EXPANDING WORLDS: ASSESSING STRATEGIES TO IMPLEMENTING A VIRTUAL REALITY PROGRAM IN RESPITE CARE

Emily Quinones-Visot,[1] Louanne Bakk,[1] Katy Allen,[2] Mary Milnamow,[1] and Jason Dauenhauer[3], *1. State University of New York, University at Buffalo, Buffalo, New York, United States, 2. Lifespan of Rochester, Rochester, New York, United States, 3. SUNY College at Brockport, Brockport, New York, United States*

Virtual reality (VR) is an immersive experience that simulates real or imagined environments and provides an innovative solution to social isolation and loneliness. Implementation science was used to identify barriers and facilitators to implementing a novel VR intervention as part of a community-based participatory research project at Lifespan of Rochester within their Partners in Care (PIC) volunteer respite program. The VR intervention will be facilitated by PIC staff and volunteers with caregivers of community-dwelling persons living with dementia (PLwD) receiving respite services. A mixed-methods design was used to identify perceived barriers and facilitators to implementation. Focus groups and self-administered surveys were completed by PIC staff (Nf10) to assess their thoughts and concerns related to program implementation. Surveys included validated measures to assess self-rated ability to perform VR functional components, attitudes toward VR, beliefs on the acceptability, appropriateness, and feasibility of the VR program, effectiveness of improving comfort, confidence, and preparedness of facilitators. Identified barriers included lack of VR knowledge, skills, and confidence in capabilities; concerns related to safety, confusion, and triggers for PLwD; and the need to strengthen buy-in from staff. Implementation strategies were selected from the Expert Recommendations for Change project and tailored to support effective uptake and behavior change among PIC staff and volunteers. Identified evidence-based strategies included training and shadowing opportunities, identifying champions, and developing a trauma-informed approach manual. These findings illustrate the need to carefully examine organizational factors that can impede the implementation. The benefits of this approach and the lessons learned will be discussed.

### BARRIERS AND FACILITATING FACTORS TO HEALTH MANAGEMENT TECH ADOPTION IN A RURAL MEALS ON WHEELS SAMPLE

David Wihry,[1] Jennifer Jain,[1] Jennifer Crittenden,[1] Lenard Kaye,[1] and Tabatha Caso[2], *1. University of Maine, Bangor, Maine, United States, 2. Eastern Area Agency on Aging, Brewer, Maine, United States*

Meals on Wheels clients served through the aging network represent a significant population of high need older adults who could benefit from health management technology. This poster presents the results of a study targeting Meals on Wheels clients in four rural U.S. counties on opportunities and barriers to adoption of technology-based health management interventions. Paper surveys were provided to 690 Meals on Wheels clients and 154 were returned (22.3% response rate). The mean age of respondents was 71.7 years and the sample was composed of 39% men and 61% women. When asked to select the most valuable features of home health management technology, 42% indicated vital signs monitoring, followed by remote access to classes and events at Area Agencies on Aging (21%), and video visits with medical providers (20%). When asked about current technology usage, 48% indicated never having engaged in a telehealth visit over a computer, smartphone or tablet. Top ranking barriers to health management technology utilization were affordability and privacy concerns. A majority (61%) of the sample disagreed or strongly disagreed that they have the money to afford a computer, smart phone, or tablet and 69% agreed or strongly agreed that they are concerned about privacy or scams when using the internet. Approximately four in ten (38%) are interested in using technology to manage their health. Additionally, 45% reported using a smartphone. The poster will also explore demographic differences in older consumer perceived barriers and opportunities and programmatic implications for expanding health management technology adoption.

### USING DIGITAL TECHNOLOGIES TO IMPROVE READMISSION AND ADHERENCE AMONG OLDER ADULTS WITH HEART FAILURE

Melissa O'Connor,[1] Nilmini Wickramasinghe,[2] Vijay Gehlot,[3] Elliot Sloane,[4] Suresh Chalasani,[5] Ramona Wallace,[6] Patricia G Wisk,[7] and Stefan Gravenstein[8], *1. Villanova University, M. Louise Fitzpatrick College of Nursing, Villanova, Pennsylvania, United States, 2. Swinburne University of Technology & Epworth HealthCare, Hawthorne, Victoria, Australia, 3. Villanova University, Villanova, Pennsylvania, United States, 4. Villanova University, Villanova, Pennsylvania, United States, 5. University of Wisconsin-Parkside, Kenosha, Wisconsin, United States, 6. Western Michigan University, Kalamazoo, Michigan, United States, 7. University of Illinois at Chicago, Holland, Michigan, United States, 8. Brown University, Providence, Rhode Island, United States*

Heart failure (HF) affects approximately 26 million people globally resulting in morbidity, mortality, and rehospitalization. With 30-day rehospitalization rates ranging from 19% to 25%, HF is burdensome for patients and challenging for clinicians to manage. HF costs in the U.S. exceeded $30 billion in 2012, projected to exceed $70 billion by 2030, and produce estimated social care costs of 12 billion in 2020. Therefore, reducing HF readmissions should be a global priority. A technology-based platform may assist in the goal of reducing readmissions. This platform may accomplish this goal by providing several digital tools that can assist the clinician in the care process, while simultaneously enabling patients with self-care through more effective communication in all care settings. Digital twins (DT) and the nursing Avatar (NA) are two such tools. The DT is a virtual, real-time digital representation of a physical object/process. It originated from NASA to improve physical model simulation of spacecraft in 2010 (Wickramasinghe et al., 2021). It is possible to achieve similar benefits in healthcare by developing suitable DT of patients in specific clinical contexts (Wickramasinghe et al., 2021; Lui et al., 2019). So to, the NA was studied by Bickmore, et al (2209) and found ultimately to be the preferred discharge communication method. As such, we proffer the development of digital twins and NA for HF patients to support better provider decision and patient communication. This will result in HF care being more precise, consistent, reproducible, and personalized resulting in improved diagnosis, treatment, and care outcomes.

### SIMPLE SOLUTIONS TO A COMPLEX PROBLEM: LISTENING TO DIRECT CARE WORKERS' PERCEPTIONS ABOUT RETENTION

Isha Karmacharya, and Leah Janssen, *Miami University, Oxford, Ohio, United States*

Direct care workers (DCWs) play a crucial role in delivering long-term services in nursing homes, assisted living, and in-home care. The work is physically and emotionally demanding and generally low-paid, which makes retaining and managing the workforce extremely challenging and has resulted in an unending workforce crisis. This study provides data on retention-promoting factors from the perspective of DCWs working in high-performing facilities and home care agencies that were identified through national and state retention data and reviews from case management agencies. Qualitative interviews were conducted in the spring of 2022 with a purposive sample of DCWs (n=16). Interviews were transcribed, checked for accuracy, and coded via thematic analysis. Three overarching themes emerged: work culture, appreciation, and monetary benefits. Retention was supported by a work culture that emphasizes person-centered care, family atmosphere, relationship building, staff empowerment, coaching supervision, participative leadership, effective communication, and flexible working conditions. DCWs experienced appreciation from employers, supervisors, and clients, which they perceived to promote retention. This theme includes valuing, respecting, recognizing, and acknowledging the efforts of DCWs. Monetary benefits promoting retention were seen as regular wage raises, employee assistance funds, gift cards, bonuses, paid time off, health insurance benefits, and travel reimbursement. This study demonstrates that while worker retention is a crisis, there are modifiable aspects of the job that can promote workers' retention. DCWs' retention can be impacted by work culture and appreciation practices. Study findings have implications for facility policy and practice pertaining to DCW's retention.

### JOB CHARACTERISTICS ASSOCIATED WITH INTENT TO QUIT AMONG NURSING HOME EMPLOYEES AND MANAGERS

Katherine Kennedy,[1] and David Mohr[2], *1. Providence VA Medical Center, Providence, Rhode Island, United States, 2. VA Boston Healthcare System, Boston, Massachusetts, United States*

High turnover and recruitment challenges of nursing home employees and managers is an ongoing concern. This study's objective was to examine intent to quit among all staff and assess the roles of job characteristics and job satisfaction. Employees and managers within one nursing home chain working in direct patient care or nursing were compared. Data came from the Work, Family, Health Network 18-month follow-up survey in 2012 (Total = 1,000, Managers = 101, Employees = 899). A cumulative logit model controlling for demographics was estimated for lower intent to quit. Herzberg's Two-Factor Theory of Work Motivation guided the study. Employees scored significantly lower on family-supportive supervisor behaviors (FSSBs), schedule control (SC), and decision authority (DA) than managers. Employees and managers did not differ on job satisfaction, intent to quit, or job demands. Satisfied workers had 5.88 times greater odds of lower intent to quit compared to workers who were neutral or disagreed ($p < .0001$). DA (OR=0.29) and SC (OR=0.19) were related to higher intent to quit. In contrast, FSSBs (OR = 1.45), safety compliance (OR=1.41), and the combination of high DA with high SC (OR = 1.4) were related to lower intent to quit. Among nursing home staff, lower intent to quit may be achieved through improving job satisfaction, safety culture, the quality of supervision, and job enrichment through more schedule control and decision-making power. It is also imperative to develop strategies to retain those with more education through further research.

### INTEGRATED FAITH BASED CURRICULUM FOR COMMUNITY-BASED OUTREACH WORKERS: ADDRESSING INEQUITIES AMONG OLDER ADULTS

Karen Bullock,[1] Kim Stansbury,[2] and Terrance Ruth[2], *1. Boston College, Chestnut Hill, Massachusetts, United States, 2. NC State University School of Social Work, Raleigh, North Carolina, United States*

Older Latino and Black Americans experience health inequities and a lack of culturally competent clinician may influence this outcome. Community-Based Outreach (CBO) has proven to be effective in addressing such challenges. CBO is an effective approach to engaging communities that have been historically marginalized and are less likely to utilize hospice and palliative than other racial/ethnic groups. Yet, few models and modules for training are available in the literature to prepare CBO workers. This presentation describes the development, coordination, content and implementation of an Interprofessional Educational (IPE) training and curricular innovation to meet needs of diverse older adults diagnosed with a serious illness.

Methods: A community-based outreach approach that integrated faith-based/spiritual care curriculum to train African American and Latino outreach workers to support older adults with serious illnesses, with a focus on advanced care planning (ACP) that explored potential barriers to equitable care. A collaboration with faith-based community representatives to design the intervention research protocol was essential. Results: The curriculum consisted of three parts: (1) a 36-hour classroom component delivered in three modules organized around eight themes: what is hospice and palliative care, spirituality and the meaning of death, understanding the dying process and decision making, goals of care, culturally responsive care; (2) weekly visits; and (3) field education/workforce development. At the end of the training, learners were able to: (a) express knowledge about culture differences; (b) demonstrate skills – listening, identifying meaning in the decision making, convey health information using patient-centered language; (c) understand cultural influencing decision making.

### Participant Index

Arabic numbers refer to session numbers.
Duplicate numbers indicate the participant is listed as an author on multiple abstracts within a session.

**A**
Aartsen, Marja J.: 2410
Abadir, Peter M.: 6411
Abah, Theresa L.: 6350
Abbasi, Khuzeman: 2510, 6240
Abbate, Lauren M.: 4260
Abbott, Katherine M.: 2340, 3180, 3520, 4270, 4360, 4610, 6130
Abboud, Anissa: 3360
Abe, Koji: 6210
Abenojar, John Paul T.: 3950
Abe, Takumi: 6320, 6710
Abraham, Sitara J.: 4150
Abrams, Leah: 4110
Abshire Saylor, Martha: 3330
Abudu-Birresborn, Diana: 1420
Abujarad, Fuad: 5100
Acevedo-Fontanez, Adrianna I.: 6450
Achanta, Madhuri: 9010
Acharya, Geeta: 3690
Acosta, Lealani Mae: 4461
Acton, Gayle: 9020
Adamek, Margaret E.: 6330, 6630, 6800
Adamsen, Collette: 6180, 6280
Adams, Mariah: 6270
Adams, Mary Anne: 1400
Adams, Peter: 1200
Adams, Michaela: 6210
Adams-Price, Carolyn: 3380, 6280, 6790
Adams-Price, Carolyn E.: 4660
Adaralegbe, Adeleye A.: 2510, 6240
Addington, Elizabeth: 2300
Addison, Odessa: 5050
Adebusoye, Lawrence A.: 3900
Adekunbi, Daniel: 5001, 6010, 9010
Adelman, Ronald: 6530, 9030
Adelnia, Fatemeh: 4461
Adena, Maja: 2410
Adeniji, Dolapo O.: 6790, 6800
Adepoju, Omolola E.: 3540
Adesanya, Ikmat: 9020
Adeyombo, Adewale A.: 6500
Adhikari, Bandita: 6010
Adhikari, Dristi: 9020
Adinkrah, Edward: 9020
Adjei Gyimah, Akwai: 4684
Adler-Milstein, Julia: 1510, 6270
Adler, Nancy: 3070
Adrian, Shayle: 1250
Afaya, Agani: 6320
Aflatoony, Leila: 4030
Afsar Ali, Afnaan F.: 1570
Agans, Gretchen: 6760
Agarwal, Arunika: 4690
Agerholm, Janne: 6200
Aggarwal, Neelum: 6160
Agrawal, Yuri: 6740
Agrimson, Kellie S.: 9010
Agronin, Marc: 9000, 9010
Aguilan, Jennifer: 1200
Aguilar, Iris: 6770
Aguilar, Kaysey: 6710
Aguirre, Annabella S.: 2290
Agus, Sam: 9010
Agyemang, Lillian: 6390
Ahles, Tim A.: 1100, 3400
Ahluwalia, Jasjit: 9000
Ahmadi, Soheila: 9000
Ahmad, Omar: 3750
Ahmad, Waqar: 6760
Ahmed, Ali: 5173
Ahmed, Courtney: 3000
Ahmetovic, Alma: 6770
Ahn, Hyochol: 4490
Ahn, Roy: 4240
Ahn, Sangwoo: 9000, 9020
Ahrens, Monica L.: 9000
Ai, Amy: 9030
Aichele, Stephen R.: 3115, 3250
Aiello, Allison E.: 3430, 6050
Aiken Morgan, Adrienne T.: 2380
Aikens, James: 3130, 6060
Ailshire, Jennifer A.: 1410, 4140, 4690, 5000, 5130, 6310, 6710, 6830, 9000
Aitken, Sarah: 2090, 6280
Aizenstein, Howard: 1050
Ajith, Aniruddh: 9030
Ajrouch, Kristine J.: 1240, 3430, 5170
Akahane, Manabu: 2580
Aker, Narin: 3555
Akincigil, Ayse: 1170
Akishita, Masahiro: 6240
Akksilp, Katika: 9030
Akosah, Kwame O.: 1480
Akosionu, Odichinma: 3910
Akre, Ellesse-Roselee: 1320
Akushevich, Igor: 1000, 1390, 3720, 4290, 6060, 6110, 6411, 9000
Al Snih, Soham: 6300
Alagappan, Cecilia: 9000
Alamer, Khalid A.: 6510
Alam, Md Towfiqul: 2170
Alaniz, Amber: 4830
Alappan, Narendrakumar: 6270
Alarid-Escudero, Fernando: 6230
Albala, Cecilia: 6320
Albarran, Marz: 3000
Albers, Elizabeth A.: 1480, 4060
Albert, Jeffrey: 6200
Albert, Steven M.: 2330, 3880, 6130
Albrecht, Jennifer: 6740
Alcantar, Sandy: 3750
Alcorn, Edwin: 6420
Alderson, Jacob E.: 2140, 4620, 6550, 9010
AlDossary, Heba: 1530
Aldridge, Melissa D.: 5030
Aldwin, Carolyn M.: 2470, 5140, 6210, 9020
Alexander, Anne: 3460
Alexander, Bruce: 6100
Alexander, Gregory L.: 1510
Alexander, Karah: 4030
Alexander-Miller, Martha A.: 6100
Alford, Susan: 9000
Alireza, Salami: 3590
Ali, Sumbleen: 1290
Ali, Talha: 3360
Ali, Zuha: 9020
Alladi, Renuka: 9030
Allaire, Jason C.: 2380, 3270
Allala, Sai Chaithra: 9000
Allan, Alexa C.: 2380
Allemand, Mathias: 4620
Allen, Julie Ober: 4290
Allen, Katy: 9030
Allen, Laura D.: 1400
Allen, Lauren: 6210
Allen, Rebecca S.: 2030, 3251, 6100, 9020
Allen, Tammy D.: 2620, 6600
Allgood, Sallie: 4360
Allison, Matthew: 4330
Allison, Theresa A.: 3050
Allore, Heather G.: 3110, 6340, 6450, 6740, 9010
Allsopp, Richard: 3580, 6010
Almada, Luciana: 9000
Almeida, David M.: 2430, 2470, 2620, 3730, 3770, 4080, 5060, 6260, 6580, 6600
AlObaidi, Alya: 6641
Alomairi, Salhah A.: 9030
Alpass, Fiona: 3900
Alperin, Kate: 9020
Alpert, Jordan: 6510
Alshaheri, Alin: 9000
Alshakhs, Sulaiman: 9030
Alsulaily, Amjed: 4100
Altukhys, Abdulmajeed: 6420
Alvarado, Angela: 1310
Alvarez, Jessica: 9000, 9010
Alvarez, Valerie: 1310
Alves de Lima, Khalil: 4461
Alwan, Elizabeth: 6210
Amano, Takashi: 5130, 6060, 6800, 9000, 9020
Ambrose, Madison: 5172
Amestoy, Maya E.: 6440
Amick, K. Allison: 4461, 6010
Amick, Matthew E.: 6790
Amini, Reza: 6220
Amos, Christopher: 9020
Amstrong, Naomi: 6130
Ananthasubramaniam, Aparna: 4630

© The Author(s) 2022. Published by Oxford University Press on behalf of The Gerontological Society of America.

Anastasia, Trena: 3460
Andel, Ross: 2620, 4080, 4465, 6290, 6600
Andersen, Julie: 6840
Andersen, Stacy: 2501, 4810, 4870, 9030
Anderson, Andrea: 9000
Anderson, Brianna L.: 6240
Anderson, Hannah L.: 3520
Anderson, Joel: 2390, 9020
Anderson, Jon: 2060
Anderson, Katie: 6411
Anderson, Keith A.: 3000, 9030
Anderson, Loretta: 1030, 3140
Anderson, Nicole: 6380
Anderson, Rozalyn: 1140, 2180, 2330
Anderson, Ruth A.: 1540, 4210, 6760
Anderson, Sharon D.: 1420
Andes, Laura: 2690
Andrade, Flavia: 4370, 6650, 9020
Andrasfay, Theresa: 3260, 6710
Andreoletti, Carrie: 1070, 3800
Andreo, Micaela: 9020
Andrew, Nyche T.: 4710
Andrews, Claire: 6770
Anetzberger, Georgia J.: 3920, 4440, 4830
Angel, Barbara: 6320
Angelini, Luise: 2501, 6840
Angel, Jacqueline L.: 3020, 3240
Ang, Gregory: 3620, 4220
Ang, Shannon: 6710
Anilkumar Hemalatha, Sruthi: 2150, 6610
An, Jaesung: 4190, 6170
Ankuda, Claire K.: 5070
Anstey, Kaarin J.: 4022, 9000
Antczak, Radoslaw: 4690
Antic, Mina: 2620
Anton, Greta: 9030
Antonucci, Toni C.: 1240, 1290, 2440, 3430, 5170
An, Yang: 6740
Apostolova, Liana G.: 3570, 6820
Applebaum, Robert: 1530, 3180, 9010
Appleby, James: 4400
Apsley, Abner T.: 5060
Aranda, Maria P.: 2420, 4420, 6180, 6770
Arbeev, Konstantin: 1000, 1390, 4290, 4810, 6010, 6110, 6411, 9000
Arbex Silva Borim, Flávia: 9020
Ardakani, Azam: 6210
Aree-Ue, suparb: 3840
Arellano, Victor: 9010
Arevalo, Lyda: 9000
Argawal, Shailesh: 5001
Arguello, Eric: 6780
Arias-Merino, Elva Dolores: 6290, 6380
Arieli, Rotem: 4140
Arisoyin, Abimbola E.: 2510, 6240
Arling, Gregory: 9030
Armah, Henrietta Q.: 6590
Armao, Anne: 9010
Armstrong, Peyton A.: 9000
Arnold, Matthew: 1140
Arnold, Robin: 6220
Arora, Kanika: 2245
Arpawong, Thalida: 5140
Arpino, Bruno: 4690
Arrasmith, Chloe: 2340
Arroyo-Miranda, Mirna L.: 1440
Arshad, Faaizah: 3560
Ars, Joan: 6450
Arthur, Christopher B.: 9000
Aryal, Subhash: 6510, 6600
Asana, Ventra: 3860
Asante, Samuel: 6350
Asghari, Mehran: 6510
Ashby, Joan L.: 6110
Ashcraft, Alyce S.: 6760
Ashebir, Sophia: 3260, 4090
Ashe, Maureen C.: 3150, 6580
Ashida, Sato: 3240, 6830
Ashirifi, Gifty: 6790, 6800
Asquith-Heinz, Zayla: 3790, 4710
Assi, Lama: 6710
Astell, Arlene: 2460, 6380
Atherton, Olivia E.: 2620
Atkins, Ghislaine C.: 6750
Atkinson, Elizabeth: 3241
Atkinson, Taylor J.: 6290
Auerbach, Samantha: 6510
Ausserhofer, Dietmar: 1530
Austad, Steven N.: 2501, 3001, 4410
Austin, Thomas R.: 3400
Au-Yeung, Wan-Tai M.: 6080
Avent, Elizabeth S.: 3010
Aversa, Zaira: 3241
Avila, Jaqueline C.: 9000
Avila-Rieger, Justina: 2270
Avni, Elinore: 1450
Avtzi, Stella: 6450
Awata, shuichi: 3010
Ayabe, Takako: 9010
Ayalon, Liat: 1400
Ayasoufi, Katayoun: 6010
Ayele, Roman: 6210
Ayers, Emmeline: 6780, 9020
Azar, Martina: 6260
Azer, Lilian: 4691
Azhar, Gohar: 6130, 6180, 6840
Azizi, Mehrnoosh: 9000
Azman, Nur Diyana: 2150
Azuero, Andres: 6390

**B**

Bédard, Michel: 6170
Béland, François: 2580
Bülow, Pia H.: 9010
Baar, Tena: 9020
Baatar, Anujin: 4684
Babitzke, Jennifer: 2720
Bacsu, Juanita-Dawne R.: 9000
Badawi, Sarah: 6140
Bae, Harold: 4810, 6010
Baehr, Laura: 1120
Baek, Jihye: 4370, 5150, 9000, 9010, 9020
Baek, Jiwon: 6400
Baer-Benson, Henry: 3390
Baggett, Sharon: 1190
Bagley, Olivia: 1390, 6411
Baier, Rosa R.: 3310
Baik, Dawon: 6280
Baik, Sol: 4510, 9030
Bailey, Amelia: 9030
Bailey, Dionne J.: 6360
Baillargeon, Emma M.: 4340
Baim-Lance, Abigail: 1160, 3660, 6320, 6610, 9000
Baird, Lola: 2660, 6220
Baird, Sean: 6390
Bair, Heather: 2520
Bai, Xue: 1400
Bai, Yi-dong: 2501
Bakeman, Roger: 2520
Baker, Elaina: 5070, 6310
Baker, Emma: 4240
Baker, Hannah: 3483
Baker, Julia: 2340
Baker, Tamara: 2360, 3420, 6180
Baker, Zachary G.: 2730, 3310, 6070
Bakker, Franka C.: 4550
Bakk, Louanne: 6390, 9030
Bakulski, Kelly M.: 3430
Balaswamy, Shantha: 6090
Balbim, Guilherme M.: 6570
Baldwin, Megan: 3360
Bali, Krittika: 6770
Balingit, Arianna R.: 9010
Ballard, Aimee: 9000
Ballard, Erin E.: 6590
Ballew, Shoshana A.: 4330
Ball, Karlene: 9020
Balmuth, Alexa: 3260, 6310
Balubaid, Afnan: 6280
Banchereau, Jacques: 3001
Bandeen-Roche, Karen: 1010, 2200, 3110
Band-Winterstein, Tova: 9030
Banerjee, Sreya: 4190
Banerjee, Subhranshu: 6290
Bangerter, Lauren R.: 2160, 6110, 6530
Baniassadi, Amir: 9030
Baniya, Ganesh: 6690
Bankole, Ayomide O.: 1160, 3482
Banks, Sarah: 6100
Banks, Sarah J.: 9000
Banner, Davina: 2210
Banwo Fatai, Khadijat: 6430
Barbakoff, Daniel: 6140
Barbeau, Sean: 3540
Barbee, Harry: 1320
Barber, David: 6150
Barber, Justin: 4870, 6720
Barber, Sarah J.: 3190, 6250
Barbieri, Steven: 4270
Barbour, Elizabeth: 6300
Barbut, Mariana: 6380
Barclay, Ruth: 6780, 9010
Barczi, Steven: 6420
Barger, Steven: 9030
Barinas-Mitchell, Emma: 4810
Barker, Anna: 5083
Barlow, Meaghan: 2620, 6580
Barmaki, Roghayeh: 4021
Barmon, Christina: 1070
Barnard, Ryan: 9000
Barnes, Christian: 9030
Barnes, Deborah E.: 3050
Barnes, Jessica T.: 3190
Barnes, Lisa L.: 2450, 3290, 3600, 6290
Barnes, Priscilla A.: 9020
Barnes, Stacy L.: 6280
Barnes, Timothy: 6580
Barnet, Jodi H.: 4560
Barnett, Michael D.: 6050, 6410
Barooah, Adrita: 2400, 6770, 9010, 9020
Barrón, Yolanda: 6100

Barragan, Cassandra: 6000
Barragan, Noel: 6060, 6220, 6280
Barrere, Cindy: 4681
Barr, Erik: 9020
Barreto, Manuela: 2690
Barrett, Anne: 4030, 6190, 6250, 6470, 6520
Barrett, Kendall: 2260, 6280
Barrett, Matthew: 6320
Barrett, Rod: 1280
Barry-Dutro, Erin: 9000
Barry, Lisa C.: 2510, 5120
Barry, Lisa: 3500
Barry, Teta: 9000
Bar, Sudipta: 6010, 6840
Barta, Hayley: 5001
Bartholomae, Suzanne: 4510
Bartle, Emily: 4020
Bartlett, Sara P.: 4750
Bartley, Emily J.: 2300
Bartley, Jenna M.: 9010
Bartley, Mairead: 6110
Bartoldus, Victoria: 5010
Bartone, Michael: 1070
Bartres-Faz, David: 3510
Barve, Shirish: 6290
Barzilai, Nir: 6010
Basaran Sahin, Duygu: 6640
Bashian, Hannah M.: 2030, 2660, 6220
Basic, Ajla: 6180
Basisty, Nathan: 9010
Basque, Shalane: 5000
Bass, Danica: 6050, 6410
Bass, David: 6530, 9000, 9020
Bass, Ellen J.: 5020
Bassett, Rachel: 9030
Bast, Elizabeth: 6420
Batsis, John A.: 2080, 4640, 6780
Battle, Brian: 2510
Baucco, Christina M.: 9000
Baucom, Brian: 1320, 2540
Bauer, Scott R.: 6320, 6780
Baughn, Maya: 9010
Bauldry, Shawn: 6810, 9020
Bauman, Alexandra: 9000
Baumann, Sara: 6130
Baumbach, Abby: 6750
Baum, Carolyn: 4790
Baumgart, Matthew: 1560, 2490
Bayani, Mario: 9000
Bayliss, Elizabeth: 3360
Bay, Maxwell: 9010
Bayne, Alycia: 3100, 4240
Bazargan, Mohsen: 9020
Beach, Brian: 3210
Beach, Douglas: 9020
Beach, Scott R.: 4090
Beachy, Tim: 5010
Beam, Christopher R.: 6290, 9000
Beamer, Brock: 6780
Bean, Jonathan: 5050
Beard, Renee L.: 9000
Beard, Sheriden: 3000
Bear, Todd M.: 2590
Beato, Morris: 6700
Beattie, Zachary: 2450
Beaulaurier, Richard: 6420
Beaulen, Audrey: 1160
Beaulieu, Celeste M.: 3800, 6000
Beauregard, Lisa: 1260
Beavers, Daniel: 3510
Beavers, Kristen: 6641, 9000
Beazley, Anthony: 6000
Becker, James T.: 1340
Becker, Melanie: 6380
Becker, Todd D.: 1390, 2700, 5150, 6720
Beck, Jennifer: 6010
Beck, Samuel: 3810
Beeber, Anna: 3170, 4210, 5010, 6450, 6760
Beech, Bettina M.: 6180
Behers, Benjamin J.: 9030
Behrens, Liza L.: 3520
Beigay, Teresa: 2590
Beilin, Lawrence: 4683, 5083
Beiser, Alexa S.: 9020
Belanger, Emma: 3200, 4820, 5081
Beld, Matthew: 9030
Belinka, Benjamin: 6411
Bellah, Sydney: 6620
Bellantoni, Michele: 1110
Bell, Bethany A.: 2300, 4730
Bellelli, Giuseppe: 5010
Bellen, Hugo: 6840
Belli, Hayley M.: 3060
Bellingtier, Jennifer A.: 2470, 2750
Bellizzi, Keith: 9000
Bell, Mallory: 6380
Bello, Ghalib: 1350
Bell, Sue Anne: 4465
Bell, Tyler R.: 3000, 4225, 6050, 6200, 9020
Belsky, Daniel: 3115, 5060
Beltran, Susanny J.: 6090, 6350, 6700, 9020
Belza, Basia: 5010
Benatti De Oliveira, Gabriela: 9020
Benayoun, Bérénice A.: 3001
Benda, Natalie C.: 9030
Benden, Mark: 6710
Bender, Alexis A.: 1170, 3760, 5173, 9000, 9010, 9020
Bender, Andrew R.: 4040
Bender, Anna E.: 1530
Bender, Emma N.: 6160
Bender, Kaye: 9030
Beneciuk, Jason: 9030
Benedetti, Kelli: 6411
Ben-Eliezer, Daniel: 6420
Benett, Jenil: 6220
Benjamin, Cynthia: 3050
Bennett, Crystal: 9000
Bennett, David A.: 2140, 4620
Bennett, Dayna S.: 3180
Bennett, Michael: 9030
Benson, Clark: 2160, 6310
Benson, Jacquelyn J.: 1300
Bentley, Megan: 4023
Benton, Donna: 4510, 6310
Benton, Melissa J.: 6770, 9010
Benton, Nathan: 3610, 6380
Benz, Hannah: 6140
Berbiche, Djamal: 6740
Berdai Chaouni, Saloua: 3610
Berdychevsky, Liza: 3260, 6390
Berg-Carramusa, Cara: 1280
Bergeman, Cindy S.: 4555, 6380
Bergen, Gwen: 4100
Berger, Corinne: 9000
Berger, Ann: 6280
Bergeron, Caroline: 2250, 6710
Berghoff, Ashley: 6280
Berg, Kristen: 4100
Bergmann, Johannes-Michael: 3090
Bergman, Sky: 4520, 4750
Bergman, Yoav S.: 3490, 6480
Bergstrom, Jaclyn: 4461, 6010, 9000
Bergstrom, Kelly A.: 3850
Berg-Weger, Marla: 1110, 6720, 6790, 9000
Berish, Diane: 1110, 2530, 3720, 9000
Berkman, Lisa F.: 3040
Berlinger, Nancy: 3585, 4380
Berman, Rebecca: 4270, 4590
Bernacchi, Veronica: 9010, 9020
Berna, Renee: 6560
Bernays, Florence: 5100
Berndt, Annette: 9000
Bern-Klug, Mercedes E.: 4830
Bernstein, Laura: 9000, 9020
Berridge, Clara: 3670, 6260
Berry, Sarah: 2290
Berry-Stoelzle, Maresi: 6100
Berta, Whitney: 4210
Bertoni, Alain: 3400
Bertram, Lars: 1010, 6010, 6641
Besnier, Florent: 6570
Besser, Lilah: 1050
Bessette, Lily G.: 9030
Bessette, Lily G: 6641
Best, Irene: 2230
Best, John R.: 6290
Best, Ryan: 6440
Bethea, Traci N.: 3400
Bethell, Jennifer: 6270, 9000
Betley, Wendy: 6280
Betrisey, Carine: 6140
Betts Adams, Kathryn: 6250
Betzel, Richard: 6290
Betz, Joshua: 3560
Betz, Marian E.: 6470, 9030
Beverly, Alexis R.: 9000
Beydoun, Safa: 6010
Bhagianadh, Divya: 2245
Bharadwaj, Mythili: 6000
Bhat, Aarti: 3770, 5060, 6580
Bhat, Ajay: 6010
Bhattacharjya, Sutanuka: 4160
Bhattacharyya, Kallol Kumar: 4465, 6540
Bhatta, Tirth: 4905, 6170, 6200
Bhatti, Danish: 6420
Bhatti, Usama: 2160
Bhatt, Jem: 3610
Bhatt, Tanvi: 4800, 6420, 6570, 6780
Bherer, Louis: 6570, 6740
Bianchi, Andria: 9000
Bian, Wenxin: 9000
Bibbo, Jessica: 1190, 3530
Bielak, Allison A M: 1100
Bierman, Alex: 4022
Biery, Nyann: 9020
Biese, Kevin: 4390
Biesmans, Jesper: 6270
Biggar, Virginia S.: 2160
Bigger, Sharon: 9020
Biggs, Mary L.: 3400
Bijou, Christina: 4225, 6200
Bilbrey, Ann Choryan: 6750

Bildtgård, Torbjörn M.: 1300
Billings, Pamela: 6360
Binford, Sasha: 6460
Birditt, Kira: 1450, 2440, 3480, 3730
Biritwum, Akosua B.: 9000
Birkeland, Robyn W.: 4220
Bisconti, Toni L.: 4730, 9000
Bishnoi, Alka: 6740
Bishop, Alena: 9000
Bishop, Alex: 6180, 6620, 6810
Bishop, Christine E.: 6360, 6540
Bishop, Nicholas J.: 1090
Bitto, Alessandro: 6840
Biyani, Rajlaxmi M.: 6790
Bjore, Emma: 3460
Bjork, Lara: 3100
Blackberry, Irene D.: 3550
Blackburn, Justin: 1190, 4100, 6760
Black, Kathy: 4465
Blackshear, Chad: 3110, 4200
Blackwell, Terri: 9010, 9030
Blackwood, Jennifer: 6220, 6450, 6510, 9030
Blair, Elizabeth: 2020
Blanche, Erna I.: 6740
Blanc, Judite: 6680
Blaskewicz Boron, Julie: 2140, 6090, 6170, 6290, 6390
Bleijlevens, Michel H.: 5100, 6270, 6560
Blench, Karl: 3481
Blieszner, Rosemary: 3240, 9020
Blinka, Marcela D.: 2690
Blocker, Kenneth A.: 3410, 4160, 9020
Block, Laura: 2160, 3750, 9010
Bloom, David E.: 4690
Bluck, Susan: 4555, 6300, 6340, 6380
Blumen, Helena: 6780
Boafo, Jonelle: 3060
Boakye, Maxwell: 6110
Bobitt, Julie: 2245, 5082, 5130, 9020
Boblitz, Alexa: 9000
Bobo, Michelle: 6740
Bobroff, Stella: 9000
Bochnak, Paula: 3620
Bodenheimer, Lisa: 6560
Boelen, Paul A.: 4460
Boerner, Kathrin: 1320, 2440, 2720, 4460, 6480, 6830
Boger, Jennifer: 2460
Bohnert, Adam: 2501, 6840
Bohplian, Suweena: 6780
Bokka, Anirudh: 6411
Bokun, Anna: 6800
Boland, Angela K.: 6290
Boland, Elizabeth M.: 6470
Bolkan, Cory: 4900, 6610
Bollens-Lund, Evan: 4530, 5030
Bolton, Cherish A.: 6120
Bolt, Sascha R.: 2090, 3555, 6270
Boltz, Marie: 2230, 2530, 2680, 3310, 3520, 4300, 4760, 6420, 9030
Bonaguidi, Michael: 9010
Bonazinga, Lindsay: 3460
Bonds Johnson, Kalisha: 1440, 3240, 4610, 4850
Bonewald, Lynda: 5001
Bonifas, Robin P.: 6060
Bonner, Gloria: 6160
Bonomo, Melia: 3481
Bons, Joanna: 3241
Boockvar, Kenneth S.: 5030
Boot, Walter R.: 1570, 3410, 3670, 4020, 4190, 6290
Borasio, Gian Domenico: 2680, 3720, 4480, 6730
Boraxbekk, Carl-johan: 9010
Borcar, Apruva: 6641
Bordes, Piper J.: 6830
Borek, Pamela: 4150
Boren, Hannah: 6270
Borgmann, Lisa: 2750
Boring, Michael A.: 4120
Borkan, Jeffrey: 6770
Boron, Julie B.: 2140
Boroumand, Shahdokht: 6230
Borrat-Besson, Carmen: 2680
Borre, Ethan D.: 9030
Borrero, Lisa: 2350, 3780, 9030
Borson, Soo: 1150
Bos, Angelo Jose G.: 3620
Boscardin, W. John: 2510, 3690, 4460, 5030
Boscart, Veronique: 6270
Bosch, Pamela: 9030
Bosse, Jordon D.: 5084
Boström, Anne-Marie: 6760
Botoseneanu, Anda: 6290, 6340, 6450, 6740
Botsch, Meghan: 2160
Bouchard, Elizabeth G.: 6430
Bouchard, Lauren M.: 6580
Boudreau, Jacqueline H.: 1040
Boudreau, Robert M.: 9030
Bouldin, Erin D.: 1330, 3780, 6280, 6310, 6530
Bourassa, Kyle: 5050
Boustani, Malaz A.: 6580, 9000
Bowblis, John: 3180, 3520, 3950, 6540
Bo, Wei: 6280
Bowen, Lauren M.: 3800, 6000
Bowen, Mary (Libbey) E.: 6760
Bowen, Pamela G.: 6750
Bowers, Katherine: 3140, 9020
Bowles, Kathryn H.: 6100
Boxer, Rebecca: 3360
Box, Helen: 6280
Boyd, Cynthia: 2690, 6800
Boyd, Kelly: 6220
Boyko, Edward: 3400
Boyle, Joseph: 6070
Boyle, Julia T.: 2030
Brabham, Deborah D.: 1420
Bradley, Dana B.: 1250, 1400, 3420, 6790
Bradshaw, Spencer: 6410
Brailsford, Jennifer: 9030
Brandt, Anthony: 3481
Brandt, Cynthia: 6770
Brandt, Martina: 6300
Brandt, Nicole: 3650, 6350, 6560
Brasher, Melanie: 6400
Brauer, Simon G.: 1240
Braun, Kathryn L.: 3220, 6180
Bravata, Dawn M.: 6390
Bravo-Ramirez, Karen: 9020
Bravo, Rosana: 6140
Breda, Kathleen M.: 5083
Breitfelder, Laura K.: 1020
Brekken, Rolf: 9000
Brem, Rachel: 6010
Brennan-Ing, Mark: 3760
Brenner, Rachel: 1330
Brenowitz, Willa D.: 2670, 3560
Breton, Juliana: 6570
Brewster, Glenna S.: 3482, 6600, 9030
Brewster, Katharine K.: 3600
Brickman, Adam: 4470
Bridges, Katherine: 3700
Bridges, Preston B.: 6020
Brinson, Jessica: 3560
Brintz, Benjamin J.: 1330
Brisendine, Matt: 5091
Bristol, Alycia: 3500
Brito, Tábatta: 9020
Brittany, Dennis: 4190
Britt, Katherine C.: 6260, 9020
Brodrick, Marisa: 6310
Brody, Abraham: 2130, 3060, 6110
Brody, Lilla: 2000, 6310, 6530
Brody, Rebecca: 4680
Broen, Tiana: 6390
Brom, Heather: 1530
Bronas, Ulf G.: 4800, 6411, 6780
Bronskill, Susan: 9000
Brooks Carthon, Margo: 1530
Brooks, Amber K.: 2300, 6180
Brosius, Tracy: 3460
Brothers, Allyson: 5110
Brotherson, Sean: 6780
Browdie, Richard: 3180
Brown-Borg, Holly: 2180
Brown, Casey L.: 4850, 9000
Brown, Cynthia J.: 3350
Browne, Julia: 6170
Brown, Ellen L.: 9000
Browne, Tarah B.: 9020
Brownfield, Douglas: 1470
Brown-Hughes, Travonia: 2070
Browning, Wesley R.: 6750, 9000
Brown, J. Scott: 6520
Brown, Jocelyn: 9000
Brown, Lana M.: 6220, 6360
Brown, Lauren: 6200
Brown, Lisa M.: 4465, 6010
Brown, Maria T.: 3000, 6300
Brown, Monique J.: 3760
Brown, Patrick J.: 3600
Brown, Rebecca: 6140
Brown, Ryan: 9010
Brown, Shelby: 6540
Brown, Susan L.: 1300, 4070
Brown, Susan H.: 9000
Brown, Teressa: 1420, 6740
Brown, Tyson: 6180
Bruce, Marino A.: 6180
Bruefach, Tyler: 2200
Bruellman, Ryan: 9010

Brunton, Stephen: 9000
Brush, David M.: 4480
Bruzios, Kathryn: 4900
Bryan, Stirling: 4720
Bryant, Chalandra: 9030
Bryant, Natasha S.: 2720, 6210, 6760
Bryanton, Olive: 6250
Bryk, Kelsey: 6450, 9000
Bubu, Omonigho M.: 6680
Buchanan, Scott: 9020
Buchan, Jacob: 9000
Buchmann, Nikolaus: 1010
Buchwalter, Abigail: 5091
Buck, Harleah: 1180, 3910, 3930, 4650, 9020
Buckley, Thomas D.: 2700, 6610, 6680
Buckwalter, Kathleen C.: 3680
Bucy, Taylor: 3130, 3482, 3660, 3910, 5130, 6540
Budhathoki, Chakra: 4640
Budhwani, Hina: 6740
Bueno, Michael V.: 2250
Bui, Cindy N.: 2440
Bui, Nhat Minh: 6270
Bulanda, Jennifer R.: 6260, 6520
Bulgarelli, Alexandre F.: 6100
Bull, Amanda: 6230
Bullock, Avery L.: 6800
Bullock, Karen: 9030
Bülow, Per: 9010
Bunch, Jacinda: 9020
Bunker, Jennifer: 3830
Burack, Orah R.: 6100
Burant, Christopher J.: 3115, 5140, 6090, 6160, 6820, 9030
Burch, Hannah: 6770
Burgdorf, Julia G.: 1180, 4640
Burek, Sam: 5001
Burgess, Elisabeth: 5173, 6760
Burgos Zuniga, Claudia: 6000, 6220
Burke, David T.: 9000
Burke, Emily S.: 6100, 6280
Burke, Robert E.: 3130
Burk-Leaver, Erin Y.: 3760
Burman, Bonnie K.: 3530
Burnes, David: 4090, 6480
Burnette, Denise: 2700, 6610
Burnett, Jason: 4890
Burnham, Dyanna: 6430
Burns, Annette: 2690
Burns, Catherine: 2460
Burns, Edith: 4700, 9000, 9010
Buron, Bill: 9000
Burr, Jeffrey A.: 2040, 3220, 6670, 6810
Burrow, Anthony L.: 2430
Burston, Betty: 1070, 6090
Burton, Cassandra: 3700
Burton, Ellen: 1190
Busby-Whitehead, Jan: 9030
Buse, Caroline: 6420
Buta, Brian: 1010
Butler, Brenda: 3860
Butler, Jason: 6840
Butler, Jorie: 4010
Buxton, Orfeu M.: 2620, 4080, 6600, 9010
Buys, David R.: 6340, 9030
Buysse, Daniel J.: 4080
Bybee, Sara: 1320, 2540
Byers, Amy L.: 2510
Byers, Chelsey: 5082, 5130, 6000
Bylund, Peggy R.: 6590
Bynum, Julie: 3130, 6060, 9010
Byrd, DeAnnah R.: 6290

C

Cabrero Castro, Jose Eduardo: 2580
Cacchione, Pamela: 9000
Cadar, Andreia N.: 9010
Cadmus, Eniola O.: 3900, 3920
Caffrey, Christine: 6100, 6540
Caffrey, Melanie: 9000
Cagle, John: 1390, 4510, 6730
Cagney, Kathleen A.: 6550
Cagnin, Annachiara: 1030
Cain, Stephen M.: 9000
Cai, Yurun: 1280, 4040, 4120, 6740, 9030
Calamia, Matthew R.: 6830
Calasanti, Toni: 6210, 6750
Caldera, Selena: 2650
Calfee, Kristin: 4080
Calkins, Margaret: 6760, 9030
Callahan, Christopher: 4280, 6220
Callan, Judith: 9010
Calo, William A.: 3520
Calzada, Christopher: 9020
Camacho, David: 2700, 4225, 5150, 6180
Camacho, Simone: 6070, 6720
Cambia, Jansen M.: 3483
Cameron, Jessica: 6790
Cammer, Allison: 9000
Campbell, Jessica: 9000
Campbell, Kaitlyn M.: 2520
Campbell, Laura M.: 2450
Campbell, Margaret: 4160
Campbell, Noll: 6510, 6590, 9000
Campetti, Randi: 4890
Campisi, Judith: 3241, 9010
Campitelli, Anthony: 6450, 6510, 9000
Camp, Janella: 6200, 9000
Camp, Kathlene: 1250
Canell, Anastasia E.: 6130, 6310
Cannon, Rachel: 6530
Can, Reyyan: 3490
Cantu, Phillip A.: 3020, 6210, 6300, 9020
Cao, Jiepin: 9020
Cao, Katy (Qiuchang): 4370, 5080
Cao, Kevin: 3910
Cao, Yuxin: 3450
Capezuti, Elizabeth: 9010
Capolino, Alexandria C.: 3780, 6400
Cappon, Davide: 3482
Caprio, Thomas V.: 1110, 4580, 6140, 9010
Carbonell, Ellen: 2560
Cardenas, Valeria L.: 6120
Carder, Paula: 3200, 3910, 4022, 6540
Carey, Rebecca: 3460
Carino, James: 2460
Carling, Stacy: 3460
Carlson, Bryant: 4022
Carlson, Michelle C.: 3690, 3750
Carman, Katherine: 6440
Carmichael, Alicia G.: 2420
Carnahan, Jennifer L.: 4100
Carneiro, Francis: 6180
Carpenter, Brian D.: 2030, 3420, 4480, 6160, 6520, 6730, 9010, 9020
Carpenter, Joan G.: 3520, 9020
Carpenter, Matthew: 9030
Carr, Alan: 4225, 6580
Carrasquillo-Sánchez, Kiara: 2700
Carr, Dawn: 1230, 2200, 9020
Carr, Deborah: 1450, 1490, 3070
Carre, Nicole: 9020
Carrico, Catherine: 3460
Carrillo, Alejandro: 2220, 6640
Carrion, Iraida V.: 3550
Carr, Megan: 9000
Carroll, Brooke: 9020
Carroll, Judith E.: 1100, 3400, 3440, 4560
Carroll, Simon: 2600
Carstensen, Laura: 3190, 6170, 9010
Carter, Kara: 1090
Carter, Lorraine: 6000
Carttar, Tennery: 9010
Carver, Chase M.: 3241, 6010, 6411
Cary, Michael P.: 9010
Casanova, Giuliana: 6620
Casanova, Ramon: 9000
Case, Donna J.: 4020, 4023
Case, Sami L.: 6411
Casey, Martin F.: 9030
Cash, Antonia: 6680
Caskie, Grace I. L.: 6130, 6310
Caso, Tabatha: 9030
Cassady, Mara: 9030
Cassatt, Sarah: 5050
Cassidy, Jessica: 6530, 9000
Castagna, Nicolette: 6420
Castaneda-Avila, Maira A.: 2640
Castaneda, Salvador: 2560
Castle, Steven: 4260
Castro-Portuguez, Raul: 6840
Castro, Saul: 4060
Cattaneo, Gabriele: 3510
Cauley, Jane A.: 2290, 4080, 4120, 6650
Cauley, Jane A: 9030
Cavalcante, Ricardo: 6100
Cavalli, Stefano: 6380
Cawthon, Peggy M.: 2290, 3340, 3690, 4080, 4120, 4600, 6200, 6320, 6780, 9010, 9020, 9030
Cazet, Jack F.: 3810
Celardo, Caitlin: 3130
Cene, Crystal: 2200
Cenko, Erta: 6510
Centi, Sophia: 6280
Cenzer, Irena: 5030
Cerbone, Felicia G.: 4240
Cerino, Eric S.: 2430, 2470, 9020
Cerino, Lauren: 6310
Ceruso, Marco: 9030
Cervantes Navarrete, Matilde: 2600
Cervantes, Diana E.: 1110
Cetkowski, Richard: 6560

Chai, Hye Won: 6190, 9030
Chakraborty, Papiya: 1470
Chakravarty, Esha: 6430
Chakravarty, Indrani: 6430
Chalasani, Suresh B.: 9030
Chamberlain, Stephanie: 4210, 5010
Chambers, Kevin: 3100
Chamut, Steffany: 6230
Chan, Alex Siu Wing: 9020
Chan, Angelique: 4170
Chan, Athena Chung Yin: 6310
Chan, Cecilia Lai Wan: 4022, 4684, 5084
Chandler, Michael: 9000
Chandra, Rashmi: 6411
Chane, Samson: 6330
Chaney, Kimberly E.: 5000
Chang Gudjonsson, Milan: 1190, 6220, 6800
Chang, Chiang-Hua: 9010
Chang, Da Jung: 6220
Chang, E-Shien: 4900, 9000, 9030
Chang, Haeyoon: 9010
Chang, Juliet: 3750
Chang, Jun Ha: 6420
Chang, Ling-Hui: 4450, 6350
Chang, Megan: 6740
Chang, Pei-Fen: 6760
Chan, Grace Man Yee: 4022, 4684, 5084
Chang, Sun Ju: 6320, 6510, 6650
Chang, Yu-Chien: 6540
Chang, Yu-Ping: 6110, 6280, 6510, 6770
Chang, Yu-Ting: 9010
Chan, Keith T.: 3860, 9010, 9030
Chan, Kiera: 9010
Chan, On Fung: 9030
Chanti-Ketterl, Marianne: 4420
Chao, Linda: 3050
Chao, Shiau-Fang: 6240, 6700, 6800
Chao, Tony C.: 1120
Chao, Ying-Yu: 4700, 6480
Chapadia, Bunsi: 9010
Chaperon, Claudia: 6280
Chapman, Brian: 9020
Chapman, Joyce: 4681
Chapman, Karen R.: 1120
Chapman, Silvia: 4470, 6260
Chappell, Amanda N.: 6210
Chappell, Richard J.: 2670
Charara, Rima: 6220
Chard, Sarah E.: 9010
Charles, Doreek: 1310
Charles, Susan T.: 2430, 2470, 3730
Charness, Neil: 3410, 4400, 6290, 9020
Chase, Jo-Ana: 2250
Cha, Seung-Eun: 6750
Chasteen, Alison: 5000, 6640
Chataut, Akankshya: 6390
Chaudhari, Diptaraj S.: 9000
Chaudhari, Nikhil: 9000
Chaudhari, Piyush V.: 9020
Chaudhury, Habib: 3450, 4090, 6500
Chaves, Paulo H M.: 4490
Cheek, Dana: 4830
Chee, Kyong Hee: 4660
Chee, Wonshik: 9030
Chei, Choy Lye: 6290
Chen, Cynthia: 3390, 3620, 3820, 4220, 6330, 6770, 9020, 9030
Chen, Danica: 4001
Chen, Diefei: 4870
Chen-Edinboro, Lenis P.: 1030, 6350
Chen, Frances L.: 9010
Cheng, Grand: 4170
Cheng, Kent Jason G.: 6520
Cheng, Mengling: 6200
Chen, Gong: 9010
Chen, Guangzhou: 3270
Cheng, Yan: 6790, 5173
Cheng, Zhaowen: 6170
Chen, Honglei: 2670
Chen, Hongtu: 4700
Chen, Huashuai: 1100
Chen, Jen-Hao: 3040
Chen, Jiachen: 6450, 6740
Chen, Jiu-Chiuan: 3510
Chen, John S.: 9020
Chen, Kuan-Hua: 4681, 4850, 9000
Chen, Lei: 3770
Chen, Li: 6200
Chen, Lu: 3020
Chen, Mei-Lan: 6780
Chen, Michelle: 9010
Chen, Ping: 1280
Chen, Randi: 3580, 6010
Chen, Ruijia: 4690
Chen, Samson: 4681
Chen, Siting: 6450
Chen, Tuo-Yu: 6330, 6600, 9030
Chen, Xi: 1000, 5010, 6450
Chen, Xia Yu Summer: 9020
Chen, Xiaomeng: 4600
Chen, Xiayu S.: 4370, 6650
Chen, Ya-Mei: 6090, 6350, 6540
Chen, Yanjun: 2670
Chen, Yun-Zih: 6760
Chen, Yuxuan: 4681, 4850, 9000, 9010
Cherry, Katie E.: 6830
Chesney, Margaret A.: 3050
Cheung, Ethan Siu Leung: 4240, 6230, 6500
Cheung, Karen Siu Lan: 4022, 4684, 5084
Cheung, Krystal: 6430
Chew, Hui Jun: 2210
Chia, Ngee Choon: 3820
Chidebe, Runcie: 6430
Chien, Jessie: 6290
Chien, Shao-Yun: 6420
Chihuri, Stanford: 5171
Chi, Iris: 1500, 1540, 3870, 6240, 6680, 6750, 9030
Chikama, Hitomi: 6450
Chi, Kevin: 9010
Childs, Elisa: 6720
Chima, Emmanuel: 4770
Chin, Janecca: 1450
Chin, Jessie: 9020, 9030
Chintapanyakun, Teepatad: 3840
Chippendale, Tracy: 3880, 6110
Chiriboga, David: 6490, 6810
Chiriboga, Germán: 6140
Chisholm, Lilly: 4020
Chittams, Jesse: 1530
Chiu, Chi-Tsun: 4420
Chiu, Venus: 4330
Chiu, Wan-Yu: 6350
Chmelka, Gina: 3130
Choate, Ashley: 5083
Cho, Dongwook: 9010
Chodosh, Joshua: 1560
Cho, Eunhee: 6080, 6270, 6300, 6320, 9000
Cho, HanNah: 6390, 6730
Cho, Hyunkeun: 3240
Choi-Allum, Lona: 6480
Choi, Bomi: 6390
Choi, Catherine: 6010
Choi, Chris: 9000
Choi, Eunyoung: 3220, 4690, 5000
Choi, Ga-Young: 6480
Choi, Gi Won: 6320, 6510
Choi, HwaJung: 3130
Choi, Hyo: 6010
Choi, Hyojin: 4510
Choi, Jean: 9000
Choi, JiYeon: 6400
Choi, Mi Sun: 6500
Choi, Namkee G.: 2240, 6350
Choi, Roy B.: 6840
Choi, Seongmi: 6400
Choi, Seonhwa: 6270
Choi, Won: 2550
Choi, Yeon Jin: 6180, 6240, 6310
Choi, Yong K: 9020
Choi, Yoonseok: 6210, 6390, 6580
Cho, Jinmyoung: 6100, 9010
Cho, Joonyoung: 6090
Chong, Natalie: 5070
Chong, Wayne F.: 2290, 3840
Choo, Esther: 5100
Chopik, William J.: 1240, 2220, 2280, 4010, 6640
Chopra, Anita: 6560
Cho, Seungjong: 6820
Cho, Sunghwan: 2700
Cho, Susie: 1150
Cho, Tsai-Chin: 2270, 3130
Chou, Andrew: 9020
Choula, Rita B.: 2650
Choun, Soyoung: 5140, 6210, 9020
Chow, Angela: 9020
Chow, Esther O.: 6350, 6530
Chow, Natalie: 6390
Chow, Stephanie W.: 2730, 4250, 6320
Cho, Youngmin: 5010, 6450
Cho, Youngshin: 6420
Christensen, Kaare: 4810, 6010, 9030
Christian, Thomas: 1310, 6720
Christman, Eva-Marie A.: 4260
Christy, Jacob: 1540
Chua Qian Ling, Denise: 4023
Chubinski, Jennifer: 3160
Chu, Charlene H.: 1420, 6640, 9000
Chuengsatiansup, Komatra: 4700
Chui, Cheryl Hiu-Kwan: 9030
Chui, Helena: 9000
Chu, Li: 3190, 6170
Chun, Alice: 3390
Chunga, Richard E.: 2720
Chung, Edwin: 6390
Chung, Edwin K. H.: 3630
Chung, Gawon: 6060

Chung, Jane: 3670, 6070
Chung, Joe: 9030
Chung, Misook: 6280
Chung, Shang-En: 5110
Chung, Soondool: 6490
Chung, Tammy: 3860
Chung, Wonock: 6710
Chun, Sanghee: 6380
Chun, Yung: 9000
Churchill, Linda C.: 2290
Chyr, Linda C.: 6760
Chyu, Joanna: 6210
Ciaralli, Spencier R.: 6430
Ciccone, Isabella S.: 6110
Cicero, Ethan: 2390, 3290
Cichy, Kelly E.: 2440
Cidav, Tom: 4500
Cigolle, Christine: 6420
Cimarolli, Verena R.: 2720, 6210, 6760
Cinader-Galvan, Morgan: 6420
Ciolfi, Mary Lou: 6540
Cipher, Daisha M.: 1080, 6060, 6630
Claiborne, Alex: 9010
Clair, Catherine A.: 3330
Clark, Callahan: 6110
Clark, Daniel: 6270
Clarke, Anthony J.: 2290
Clarke, Barry: 1180
Clarke, Peter J.: 9000
Clarke, Philip B.: 4060, 6250
Clarke, Philippa: 1050, 2570, 3690
Clark, Jaylyn F.: 6180
Clark, Jeff: 3460
Clark, Josef P.: 1140
Clark, Katherine: 2610
Clark, Phillip G.: 6770
Clarkson, Benjamin: 3241
Clark, Sunday: 4900
Clay, Olivio J.: 6750
Cleary, Jennifer: 1290
Clem, Sarah E.: 6340
Clevenger, Carolyn: 5160
Click, Mary: 9010
Cline, J. Mark: 9010
Cline, Sarah M.: 9000
Cloud, Leslie J.: 6420
Clouston, Sean: 3440, 3510, 4700, 5173, 6200, 6440, 6680
Cloyes, Kristin G.: 1320, 2070, 2310, 2540
Coates, Emma: 6110
Coates, Martha C.: 5020, 9020
Cobb, Sandy: 9020
Cobb, Sharon: 9020
Coccia, Kathryn: 6720
Cochrane, Barbara: 1150
Cochran, Lelah: 4160
Coen, Paul M.: 3340, 4600, 9010, 9020, 9030
Coffman, Cynthia J.: 5083
Cohen Quay, Lisa: 6500
Cohen, Alan: 6140
Cohen, Dan: 4270, 9000
Cohen, Debbie: 9030
Cohen, Harvey J.: 3400
Cohen, Iris: 6070
Cohen, Ken: 6110
Cohen, Marc: 1260, 3320, 4270, 6140
Cohen, Wendy: 6060
Cohen, Yafit: 1300
Cohn-Schwartz, Ella: 2480
Colón, Lisa: 5150
Coldiron, Allyson M.: 6050, 6410
Coleman, Carissa K.: 2520, 2530, 4030, 5010
Coleman-Hulbert, Anna: 9010
Coleman, Max E.: 6810, 6820
Coleman, Rachel: 3540, 6270, 9030
Cole, Rachel C.: 9020
Cole, Robert N.: 6411
Cole, Steve: 3440, 4560
Coletta, Giulia: 3620
Colichino, Elena: 1350
Collado, Elias: 3483, 4021, 6740, 9020, 9030
Collie, Angel: 9000
Collins-Pisano, Caroline: 3481
Collyer, Taya: 5083
Compernolle, Ellen: 1230, 5150, 6440
Cong, Zhen: 1540
Coniff, Joshua: 4220
Connell, Cathleen: 2420, 3130
Connelly, Caitlin: 9020
Conner, Norma: 6700
Connolly, Marie-Therese: 4090
Conrad, Karen: 4900
Conrad, Ken: 4900
Conroy, Molly B.: 1120
Conti, Jennifer L.: 1040
Convery, Kimberly: 2130, 6110
Conway, Adam: 1320
Conway, Cathy: 6680
Conwell, Yeates: 4580, 6270
Conyers, Christian: 1170
Coogle, Constance L.: 2260
Cook, Diane J.: 6420
Cook, Summer B.: 6780
Coon, David W.: 2250
Cooper, Hannah LF: 9020
Cooper, Jasmine: 1240
Cooper, Lisa A.: 4680
Cooper, Lujira J.: 2540
Copeland, Molly: 1230
Cope, Marie T.: 6520
Corazzini, Kirsten N.: 1060, 3170, 6110, 6280, 9010
Corbit, Nancy: 5171
Corcoran, Mary: 9020
Cordell, Ashlee: 6150, 6370, 6390
Coresh, Josef: 2670
Corley, Connie S.: 6350
Corley, Robin: 9000
Cornell, David J.: 2610
Cornell, Portia Y.: 3130, 5081
Cornish, Stephen: 6780, 9010
Coromac-Medrano, Juliamaria: 6730
Cortes, Constanza J.: 5091
Cortes, Tiffany M.: 6140, 6320
Cosarderelioglu, Caglar: 6411
Cosentino, Stephanie: 4470, 6260
Cosenza, Carol: 9020
Costantini, Hiroko: 6310
Costello, Emily: 6120
Cotten, Shelia R.: 4020, 9030
Cottler, Linda B.: 9020
Cotton, Quinton D.: 3750
Cotton, Sam: 1110
Coughlin, Joseph: 3260, 3450, 4090, 6310
Coulourides Kogan, Alexis M.: 3390
Coulson, Alison: 2160
Coursaris, Constantinos: 4770
Covinsky, Ken E.: 5030
Covinsky, Kenneth e.: 5030, 6110, 6320, 6780
Cox Sullivan, Sheila: 6360
Cox, Brian: 6100
Cox, Keri: 6570
Cox, Nicholas: 2060
Coyle, Caitlin: 1170, 2010, 6090, 6700
Coyle, Caitlyn: 2050
Cozad, Aisha Bonner: 3700
Cozad, Hannah O.: 9000
Crabtree-Ide, Christina R.: 6430
Craft Morgan, Jennifer: 5173
Crandall, Carolyn J.: 4080
Crandall, Jason: 6570
Crane, Breanna M.: 3690, 3750
Crane, Michael: 1350
Cranley, Lisa: 1420
Cranston, Teresa A.: 6100
Crary, John F.: 6010
Crawford, Andrew: 3483, 4021, 6740, 9020, 9030
Crawford, Andrew Carl: 9030
Cremer, Svenja: 6270
Crespo-Fierro, Michele: 2130
Crews, Deidra C.: 2510, 4680
Criado Carrero, Ricardo: 4021, 9020, 9030
Crimmins, Eileen: 2080, 3260, 3440, 4560, 5000, 6010, 6710, 6830
Crittenden, Jennifer: 3540, 6270, 6540, 9030
Crivello, David A.: 4260
Croft, Janet: 3510, 4120, 6280
Crosby, Paris: 5140
Cross, Dori A.: 3130, 3482, 3660
Crosswell, Alexandra D.: 6830
Crouch, Adele: 3680
Crouch, Maria C.: 1380, 4710
Crouter, Scott: 9020
Crowder, Kim D.: 1420
Crowe, Michael: 4225, 6200, 6590, 6750
Crowley, Christopher: 6770
Crowley, Max: 4060
Cruickshanks, Karen J.: 2670
Cruz, Grace: 6600, 9030
Cruz-Oliver, Dulce: 6140
Cudjoe, Thomas KM: 2530, 2690, 3130, 6800
Cuellar, Alison E.: 3130
Cui, Can (Shirley): 9000
Cui, Chang-Yi: 1470
Cui, Ruifeng: 9010, 9030
Culberson, John: 6760
Cullen, Jennifer: 6510
Cullen, John: 2090
Culminskaya, Irina: 6010
Cummings, Steve: 3340, 6780, 9010, 9020
Cummings, Steven R.: 4600

Cummins, Phyllis: 4430, 4555, 6130, 6670, 6710
Cundiff, Jeanne M.: 9020
Cunich, Michelle: 6280
Cunningham, Chris: 2410, 3770
Cureton, Bianca R.: 9020
Curl, Angela L.: 5150, 6260, 6470
Currie, Katherine L.: 4150
Curtis, Brenda: 6170
Curtis, Danny: 6740
Curtis, Laura: 1030
Cutchin, Malcolm: 6650
Cutler, Ronald: 1200
Cvejkus, Ryan K.: 3400, 4600, 4810, 6450
Czaja, Sara: 2000, 3410, 4090, 6480, 6530, 9010, 9030
Czajka, Agnieszka: 4684
Düzel, Sandra: 1010

**D**

Dabelko-Schoeny, Holly: 2010, 3770, 4370
Dabiri, Sanaz: 6170
Dabrowski, Barbara: 3460
Dahal, Poshan: 6690
Dahal, Roshani: 9010
Daks, Jennifer: 2660
Dalal, Yamini: 1200
Dale, William: 2550
Dallas, Mark: 5001
Dallas, Sarah: 5001
D'Ambrosio, Lisa: 3260, 3450, 4090, 6310
D'Amico, Danielle: 4490, 6440
Damier, Sashah: 6220
Dammer, Eric: 6840
Damush, Teresa M.: 6390
Dandrea, Kaye R.: 6100
Danesh, Valerie: 6100
Dangerfield, Hannah J.: 6770
Dang, Hope: 6840
Dang, Linh: 1160, 4630
Dang, Stuti: 1330, 4010, 6420
Dang, Weiwei: 1470
Daniel, George: 6170
Daniel, Kathryn M.: 1080, 6630, 6770
Danielson, Ramona A.: 6280
Danilovich, Margaret: 4021, 4023, 4270
Danish, Madina: 9000
D'Antonio, Patricia M.: 2100, 3120, 4400, 4691
Daquin, Jane C.: 9000
Darby, Nina: 6530
Darling, Nancy: 6520
Darvishpoor Kakhki, Ali: 3620
Dash, Kim: 4890
Das, Sai Krupa: 2180
Dash, Sanjana: 9020
Das, Ranjit Kumar: 9020
Dassel, Kara B.: 3030, 3710, 9010
Dauenhauer, Jason: 2350, 9030
Daugherty, Ana M.: 6290
Daum, Christine H.: 6390, 6560, 6680
Davagnino, Judith: 6420
Davatzikos, Christos: 4200
Davenport, Chad: 1120
Davenport, Kathleen: 9030
Davey, Adam: 9030
Davidson, Patricia: 6140
Davidson, Patrick: 6290
Davies, Nathan: 3555
Davila, Heather: 4770
Davis, Amanda: 1180
Davis, Andra: 9000
Davis, Elizabeth: 2560
Davis, Jamaine: 1440
Davis, Kimberly D.: 2260
Davis, Linda: 4660
Davis, Rebecca L.: 6760
Davis, Shelly: 6180, 6280
Davis, Taylor: 4461
Davitt, Joan K.: 9000
Davlyatov, Ganisher: 3660, 4820
Davy, Philip MC: 3580
Dawkins, Emily: 6270
Dawson, Walter: 4790, 9000
Dayama, Neeraj: 6420
Dayanidhi, Sudarshan: 2500
Dayrit, Jessika G.: 6210, 9030
Day, Ryan: 9030
de Boer, Bram: 1160, 2090, 3555, 4300
de Bruin, Simone: 4550, 9020
De Diego Diaz, Ana: 9020, 9030
De Fries, Carson M.: 4240, 4430, 6500
De Geest, Sabina: 6270
de Klerk-Rubin, Vicki: 6000
de Leeuw, Sarah: 4020
De Lima, Bryanna: 9030
De Main, Atami: 6260
de Man-van Ginkel, Janneke: 6270
de Medeiros, Kate B.: 3585, 4180, 4670
de Paulo Couto, Clara: 2480
de Rosa, Cristina: 6280
de Vries, Brian: 6750
De Vries, Erica: 1160
Deal, Jennifer A.: 2270, 2530, 2670, 3560, 4050, 4330
DeAnda, Jaden: 6490
Dean, Elizabeth: 6010
DeAngelis, Reed: 6180
Dearborn, Lynne M.: 4170
Deardorff, W. James: 5030
Deason, Karley: 6000
Deason, Rebecca G.: 6160
DeCarli, Charles S.: 3600
DeCaro, Jason A.: 9020
DeCherrie, Linda: 6610
Decker, Autumn E.: 4900
Decker, Elyse: 4710
Deemer, David A.: 5172
Degenholtz, Howard B.: 1400, 2590, 3660, 9030
DeGennaro, Jennifer: 6560
DeGroot, Lyndsay: 3330
Deichert, Jerome: 6700
Deimling, Gary T.: 2640, 6430
del Pino, Homero E.: 3750
Delaney, Colin: 1200
Delano-Wood, Lisa: 9000
DeLauder, Rachel M.: 4021
Delbaere, Kim: 4340
Delgado, JoJo: 6100
Delgado-Rodriguez, Annaliet: 4750
Delhaes, Flavien: 6380
DeLiema, Marguerite: 3160, 4900
DeLiema, Marti: 6360
Deller, Juergen: 6700
Delvecchio, Julia: 3620
DeMarshall, Cassandra: 6411
Dembinski, Kimberly: 6780
Demeo, Gianna: 6770
Demeter, Naor: 1300
Demiris, George: 3670, 6390, 6730
Demuth, Ilja: 1010, 3070, 4040, 6010, 6641
Denhaerynck, Kris: 6270
Denning, Emily C.: 6340
Dennison, Christopher: 4070
Denny, Bryan: 3481
Denson, Kathryn: 6280
Depp, Colin A.: 2450
Derain, Lily: 6750
Desai, Pankaja: 9010
Desai, Shreya: 1330, 6430, 6530
Desai, Smit: 9020, 9030
DeSanto-Madeya, Susan: 6140
Deshmukh, Maya: 6110
Deshpande, Falguni: 9020, 9030
Desir, Marianne E.: 1330
Dessale, Shambel: 6330
Deters, Kacie D.: 1440
Devanand, Davangere P.: 2160
Devane, Karan S.: 9000
DeVengencie, Faith: 2340
Devereaux Melillo, Karen: 2610
Dey, Judith: 3640, 4350
Dey, Judy: 3640
Dezine, Marie L.: 6220
Dhakal, Usha: 9010
Di Gessa, Giorgio: 3210, 6310
Diane Ploch, Emily: 6310
Diaz, Arnold: 9020
Diaz, Fernando Erra: 3001
Diaz, Jose A.: 3770
Diaz-Ramirez, L. Grisell: 3690
Díaz-Santos, Mirella: 3750
Diaz-Valdes, Antonia E.: 6350
Diaz, Vivian: 9010
DiBello, Julia R.: 3230
Dichter, Melissa E.: 9000
DiCicco, Heather: 1340
Dickerson, Anne E.: 6470
Dickman Portz, Jennifer: 2280, 3360, 3930, 4650
Dickson, Chelsea: 9030
Dicks-Williams, Ramona: 6750
Dictus, Cassandra M.: 4360, 5010
Dieciuc, Michael: 1570
Diefenbach, Michael: 4700
Diehl, Christin: 1250
Diehl, Cody L.: 9000
Diehl, Manfred K.: 5110
Diekemper, Karla A.: 6220
Dieker, JoAnna L.: 6750
Dierckx de Casterlé, Bernadette: 6560
Dietrich, Mary: 1180
Digbeu, Biai: 9010
DiGirolamo, Grace: 5091
DiGuiseppi, Carolyn: 5171, 9030
Dikhtyar, Oksana: 3820, 4430
Dilanchian, Andrew T.: 1570, 3410
Dillon, Ellis: 1310

DiMaria-Ghalili, Rose Ann: 1120, 2610, 4870, 5020, 9020
Dinaj, Veronica: 6790
Ding, Hui: 6530
Ding, Jingzhong: 2241, 3400
Ding, Kedong: 9020
Ding, Tianli: 9010
DiNitto, Diana M.: 6350
Dinman, Michele: 3800
Dioh, Waly: 9010
Dionne-Odom, J. Nicholas: 6390
DiScala, Sandra: 6000
Disini, Bridget: 6500
Distefano, Giovanna: 3340, 9010
Dixon, Bradley J.: 6450
DJousse, Luc: 3600
Đoàn, Lan N.: 1480
Dobalian, Aram: 2660
Dobbins, Jessica: 3540
Dobbs, Debra: 2720, 3910, 4130, 4465, 4650, 4820, 6070
Dodge, Hiroko H.: 2450, 3580, 4870
Do, Elena: 9020
Dohan, Daniel: 9000
Doherty, Caroline: 3100
Dokos, Malinda: 6560
Dolan, Hanne R.: 9030
Dolansky, Mary: 1530
Dolbin-MacNab, Megan L.: 4060
Domingue, Ben: 3000
Domoto-Reilly, Kimiko: 6420
Donath, Carolin: 4300
Donehower, Allison K.: 1300
Dominguez, Mauricio: 5001
Dong, Fanghong: 6510
Dong, Juan: 6641
Dong, Linh: 6290
Dong, XinQi: 3870, 4700, 6480
Dong, Yu: 6780
Donlon, Timothy: 3580, 6010
Donnellan, Kelsey M.: 3010, 6060
Donnelly, Rachel: 4460
Donofrio, Abigail: 6770
D'Onofrio, Gail: 5100
Donohoe, Maria: 3240
Donohue, Erin: 6760
Donorfio, Laura KM: 3030, 9020
Dooley, Erin E.: 4050
Doraiswamy, P. Murali: 2241
Doran, Kevin: 1260
Dorr, David: 6340, 6740
Dorris, Jennie L.: 1340
Dorsey, Susan G.: 6641
Dos Santos, Camila Mello: 6100
Dosa, David M.: 3200, 4465, 4820
Douglas, Jason A.: 3440
Douglas, Natalie F.: 1270, 2340, 3780
Douglas, Sara: 6510
Dowd, Jennifer B.: 6050
Downer, Brian G.: 2580, 4225, 6300
Downer, Paige B.: 9020
Downey, Laura: 6280
Doyle, Cassidy: 1100, 3540, 6070
Doyle, Margaret F.: 6450, 6740
Doyle, Margaret: 5172
Doza, Adit: 4040
Dozier, Mary E.: 4021, 9000
Drabo, Emmanuel: 6760
Dragan, Deanna: 5082
Drake, Joshua: 5091
Drance, Elisabeth: 1420
Drazich, Brittany F.: 3750, 4760
Dreer, Laura: 6750
Drewelies, Johanna: 1010, 3070, 3150, 6641
Dreyer, Jan: 3090
Driesse, Tiffany M.: 4640
Driessnack, Martha: 3240
Driggers, Amelia: 6340
Driscoll, Monica: 3810, 9010
Driver, Jane A.: 3600
Dronamraju, Neha: 6270
Droste, Andrew P.: 9000
Drost, Jennifer: 1190
Drucker, Claire: 6770
Drummond, Neil: 6150
Dryden, Eileen M.: 1040
Duangjina, Thitinan: 3500
Duan, Haoshu: 6750
Duan, Hongzhe: 1390, 4290, 9000
Duan, Yinfei: 4210
Duara, Ranjan: 6140
Duarte, Andres: 6140
Dubal, Dena: 3001
Dube, Catherine E.: 9020
Dube, Jeanné: 6050, 6410
Duberstein, Paul R.: 4500
Dube, Victoria P.: 6090
Dubno, Judy R.: 9030
Duchesneau, Emilie: 4320
Duchowny, Kate: 3690
Duezel, Sandra: 1010, 3070, 4040, 6641
Duffy, Karen: 9020
Dufour, Alyssa B.: 3600
Dugan, Beth: 1090, 3100, 6090, 6200, 6510
Duggan, Katherine: 6580
Duggan, Mariu: 6270
Duggleby, Wendy: 1420
Duke Boynton, Felicia D.: 6010
Dulaney, Cynthia L.: 6790
Dulaney, Sarah: 6270
Dunbar, Annie Zean R.: 4430
Dunham, Katherine: 9020
Dunkle, Ruth E.: 6090, 6230
Dunlap, Janette: 6420
Dunlap, Pamela: 3690
Dunning, Lauren: 3540
Dunn, Nicole J.: 6780, 9010, 9030
Duodu, Vennesa: 6140
Dupre, Matthew E.: 1540, 5140
Dupuy, Emma G.: 6570
Durduran, Turgut: 6450
Durga, Aditi: 2130, 3060, 6110
Du, Shufa: 2080
Duthie, Edmund: 6280
Du, Yan: 4190, 4682
Dvorak, Katy: 3550
Dvorak, Robert: 4480
Dye, Cheryl: 4500
Dykstra, Taitea: 4461
Dys, Sarah: 2570, 3200, 4022
Dzierzewski, Joseph M.: 2030
Dzikowski, Natalie: 4620
Dziura, James: 5100

### E

E Kheirbek, Raya: 6350
Easter, Bashir: 6750
Eastman, Marisa R.: 4110
Easton, Scott: 4730
Eaton, Jacqueline: 4520, 9000
Ebeling, Peter: 5083
Ebili, Ujunwa: 2510, 6240
Eby, David W.: 3000, 6470
Echt, Katharina: 9010
Eckstrom, Elizabeth: 9030
Edelman, Linda S.: 2060, 2310
Edelstein, Barry: 9030
Edland, Steven: 6411
Edney, Sarah: 3620
Edwards, Chelsea: 5100
Edwards, Jerri D.: 9000
Egbuchiem, Henry: 2510, 6240
Egeberg, Eva: 9000
Egert, Michal: 1300
Ehlman, Katie: 6220
Ehrlich, Joshua: 3690, 4690, 6680
Eich, Teal: 6290
Eid, Fatima: 5060
Eifler, Morgan: 1030
Einstadter, Douglas: 4100
Eisenson, Howard: 9000
Eisenstein, Amy: 1080, 4590
Ejaz, Farida K.: 3920, 4770, 4900, 5083, 6150, 6370, 6390
Ekerdt, David J.: 6710
Ekram, A R M Saifuddin: 4320, 6320
El Shanawany, Perihan: 9000
El Sharu, Husam: 6590
Elam, Kit: 9020
Elayoubi, Joanne: 6290
Elbogen, Eric B.: 6170
Eldadah, Basil: 2160, 3080
Elder, Keith: 9010
Eldridge, Corinne: 9030
Elfenbein, Pamela: 2610
Eliopoulos, Elaine M.: 9010
Ellerby, Lisa: 6840
Ellington, Lee: 1320, 2070, 2540, 4530, 6310, 9000
Elliott, Christian: 9010
Elliott, Emily M.: 6830
Elliott, Michael: 2080, 6140
Ellis, Julie M.: 2160
Ellis, Julie: 4250
Ellis, Katrina: 4010
Ellis, Kirby B.: 9020
Elman, Alyssa: 4900
Elman, Jeremy A.: 6050, 6490
Elmore, Catherine E.: 3500, 6310
Elrod, Cathy: 9030
Elrod, Leigh: 2060
Elswick, R.K.: 6420
Elugbadebo, Olufisayo: 6100
Emdin, Abby: 9000
Emery-Tiburcio, Erin: 2030, 2560
Emlet, Charles A.: 4540
Empleo-Frazier, Ophelia: 5172
Emrani, Sheina: 6411

Endo, Yori: 5001, 6840
Eng, Cassy: 9000
Engeland, Christopher G.: 4610, 4620, 6290
Engel, Ilana J.: 6650, 6730
Engelman, Corinne D.: 2670
Engelman, Michal: 6710
Engel, Rafael: 6090
English, Tammy: 2470, 4570, 6170
Enguidanos, Susan: 3390, 4100, 6120, 6720
Ensrud, Kristine: 2290, 4080, 6320, 9030
Eoff, Elizabeth: 6130
Epel, Elissa: 3070, 3440
Epps, Fayron: 1170, 1380, 3240, 5160
Epstein, Mara M.: 2640
Eramudugolla, Ranmalee: 9000
Erich, Michele: 6350
Ericson, Tracy: 6540
Erlenbach, Emily: 9010
Ernsth-Bravell, Marie: 9020
Ernst, Joy S.: 6000
Ernst, Michael E.: 4320, 5083, 6320
Ersek, Mary: 1390, 1460
Ertle, Emilee: 6290, 9010
Erus, Guray: 4200
Escorcia, Wilber: 5001
Esiaka, Darlingtina: 6070, 6200, 6430
Eskandari, Anahita: 6170
Esmaeili, Roghayeh: 3620
Espejo, Edie: 4460, 5030
Espejo, Luis: 6840
Espeland, Mark A.: 3400, 3510
Espinosa, Oscar: 6120
Espinoza, Anna: 6770
Espinoza, Arturo I.: 9020
Espinoza, Sara E.: 4320, 6020, 6140, 6320, 6390
Esposito, Michael H.: 4110
Estabrooks, Carole A.: 2090, 4210, 4720
Estenson, Lilly: 9000, 9010
Estenson, Lilly H.: 3010
Estepp, Philip: 6780
Estus, Erica N.: 3780, 6400
Etchin, Anna: 2660, 6220
Evans, Daniel S.: 9010
Evans, Donald: 9010
Evans, Luke: 9010
Evans, Michele K.: 5060
Evans, Nate: 6740
Evelyn-Gustave, Allyson: 4680
Ewen, Heidi: 4520, 9030
Eyler, Lisa T.: 6050
Ezeamama, Amara E.: 4700

**F**

Fabelo, Humberto E.: 2700
Fabio, Casati: 4684
Fabius, Chanee: 4640, 6760
Fadel, Meghan: 6060
Fagundes, Christopher: 3481, 9010
Fahey, Margaret C.: 9000
Fairchild, Kaci: 6010
Fairchild, Thomas J.: 1250
Fairchild, Vickie: 4790
Fakha, Amal: 2090
Falkovic, Margaret B.: 1540
Falvey, Jason R.: 2580, 4220
Falzarano, Francesca: 2000, 6210, 6530, 6760
Fan, Erica K.: 3690
Fanfan, Dany: 4650
Fang, Tyson: 6310
Fang, Yuan: 6450, 6740
Farago, Fanni: 9020
Farah, Fatima: 6411
Farah, Kimberly: 2350, 3800
Farhat, Farah: 4340
Farina, Mateo: 1410, 1440, 2080, 3040
Farmer, Heather R.: 3440, 6290
Farnell, Yuhua Z.: 6010
Farrell, Barb: 6270
Farrell, Kurt: 6010
Farsijani, Samaneh: 3830
Fashaw-Walters, Shekinah: 2650, 4780, 6540
Fassi, Janelle E.: 1260, 6000
Faul, Anna: 1110
Faul, Jessica: 3000, 6010
Faulkner, Sandra: 4022, 9020
Faurot, Keturah R.: 4320
Fausto, Bernadette: 6070, 6200
Fauth, Elizabeth Braungart: 6410, 6560
Favez, Lauriane: 1530, 2090
Fazio, Sam: 4010, 4681, 6130, 9030
Federman, Alex: 6610
Fehland, Jess: 2160
Feitosa, Mary: 4600, 4810, 6010
Felber, Nadine Andrea: 6400
Felderhoff, Brandi J.: 1310
Feliciano, Leilani: 3115, 6170, 6820
Felix, Ashley: 2200
Felix, Holly: 6420
Felts, Adam: 6310
Feng, Guanggang: 6480
Feng, Qiushi: 1100
Feng, Xin: 6641
Feng, Zhanlian: 4350
Fennell, Gillian: 4580, 6440
Fennema-Notestine, Christine: 6050, 6490
Fenske, Diane L.: 9010
Fenstermacher, Erika A.: 9020, 9030
Fenton, Laura: 6580
Ferdows, Nasim B.: 4420
Ferguson, Giselle: 6550
Ferguson, Verna: 6720
Fernandez Cajavilca, Moroni: 9000, 9010
Fernandez-Barrena, Maite: 9000
Fernandez-Zapico, Martin: 9000
Ferraguzzi, Gaia: 5010
Ferraro, Kenneth F.: 4230, 5060, 6290, 6380, 6810
Ferreira Leitao Azevedo, Renato: 4190
Ferreira, Katelyn: 1350
Ferris, Chloe: 3400
Ferrone, Christine F.: 6770
Ferrucci, Luigi: 1470, 4200, 4230, 4340, 4600, 9010
Fetting, Hannah: 9020
Fick, Donna M.: 3720, 6270, 9030
Fielding, Roger A.: 3830
Field, Noelle: 2010
Fields, Beth: 1340, 4310, 6530
Fields, Noelle L.: 1080, 6060, 6530, 6630, 9030
Fiestosa, Mary F.: 4810
Filec, Sarah: 4681
Fillingim, Roger: 6510, 9020
Finan, Julia M.: 2050
Finch, Caleb: 9020
Finch, Holmes: 9010
Finch, Laura: 1230
Finegan, Kerry: 9030
Fine, Sydney: 9020
Fingerman, Karen L.: 1450, 2440, 3730, 6170, 6460
Finkel, Deborah G.: 3440, 6050, 9010, 9020
Finlay, Jessica M.: 1050, 2570, 3495, 4110
Finley, Erin: 1330
Finley, Margaret A.: 1120
Finnie, Dawne M.: 2160
Finsel, Julia: 6700
Fiocco, Alexandra J.: 4490, 6440
Fiori, Katherine L.: 3480, 6170
Fischer, Karen: 6110
Fisher, Abbey: 3520
Fisher, Kathleen: 1120, 5020, 9020
Fisher, Lauren: 6280
Fisher, Mary: 9000
Fish, Laura J.: 1540
Fisk, Calley: 9000
Fiske, Amy: 9000, 9010, 9020
Fiske, Sarah: 6740
Fiskum, Gary: 6641
Fitch, John: 6310, 9030
Fitzpatrick, Annette L.: 5010
Flaherty, Ellen: 4390
Flanagan, Jane: 2230
Flanagan, Margaret E.: 6411
Flatt, Jason: 2390, 3290, 3950
Fleischhacker, Alexander: 9030
Flessa, Sarah: 9000
Fletcher, Jason: 6110, 9000
Flett, Gordon L.: 4630
Flieger, Signe P.: 6360
Flood, Sarah: 6800
Flores, Eileen S.: 4691
Flores, Elena: 6180
Flores, Emily V.: 6290, 9030
Flores-Villavicencio, Maria Elena: 6290, 6380
Floyd, Alex: 1390
Flynn, Ann: 9000
Fogle, Stephen John: 1070, 2260
Foley, Kierra: 9030
Follis, Shawna: 9010
Fong, Joelle: 1100
Fong, Mei-Chia: 1190
Foppa Pedretti, Nicolo: 1350
Foradori, Megan: 1530
Ford, Ariel: 2130, 3060
Forman, Daniel E.: 4600
Forrester, Sarah: 1090
Fortinsky, Richard H.: 2150, 2510, 3310, 3680, 4280
Fortuna, Karen L.: 3481
Foster, D. Brian: 6411
Foster, Kristie: 4390
Fountain, William: 9010
Foutz-Lowe, Shelly: 9030
Fowe, Ibukun E.: 4020
Fowler, Michael: 4640

Fowler, NIcole R.: 6220, 6390, 6450, 6470, 6580, 9000, 9030
Fox, Aimee: 2250, 2280, 3930
Fox, Jessica: 4240
Framil, C. Victoria: 9000
Francisco, Raia: 9020
Francis, Erica: 3520
Francis, Heather: 6820
Francis, Howard W.: 9030
Francis, Jess: 1240
Francis, Sarah L.: 9000
Frangiosa, Theresa: 2160
Franke, Kevin: 3460
Frank, Robynn: 1480
Franz, Carol E.: 6050, 6490
Franzosa, Emily: 1040, 1160, 3660, 6530, 6610
Frase, Robert T.: 1290, 6520
Fraser, Sarah: 4340
Fratoni Souza, Kristin N.: 2350, 3780
Fratoni-Souza, Kristin: 6400
Frazier, Cleothia: 6600
Frazier, Hilaree: 6411
Frazier, Jefferson: 3481
Frechman, Erica: 1180
Freddolino, Paul P.: 4023, 4770, 6060
Fredericksen, Jessica: 6280
Fredrickson, Barbara: 4850
Fredrika, Opur: 1170
Fredriksen Goldsen, Karen I.: 1320, 4540
Freedman, Vicki A.: 4700, 5070
Freeman, Shannon: 2210, 2600, 3450, 6270
Freiria, Carol: 9020
Freitas, Sam: 6840
Frey, Joseph J.: 6120
Frey, Katelyn T.: 9000
Frey-Law, Laura: 6220
Friberg Felsted, Katarina: 1370, 4520
Frichembruder, Karla: 6100
Friedman, Elliot: 6080, 6790
Friedman, Esther: 6690
Friedman, Naomi: 9020
Friedmann, Erika: 9020
Friedrich, Anna-Carina: 4300
Fried, Terri: 3360
Friis, Marlene: 6830
Fritschi, Cynthia: 6080, 6300, 6780
Fritz, Heather A.: 6650
Fritz, Julie: 1120
Fritz, Michele C.: 4770
Fritz, Roschelle L.: 6420
Fritzson, Emily: 9000
Froberg, Ruthann: 4890
Fruhauf, Christine A.: 2250, 2280, 3710, 3930
Fry, Liam: 6260
Fuentes-Casiano, Edna: 6160
Fugate-Whitlock, Elizabeth: 4520, 6640
Fujiwara, Yoshinori: 6320, 6710
Fukui, Chie: 4440
Fukui, Sadaaki: 9010
Fulda, Kimberly: 6770
Fullen, Matthew C.: 4060, 6250
Fuller, Heather: 3480, 6230, 9020
Fuller, Kristi: 6090
Fuller-Thomson, Esme R.: 4225, 6200
Fulmer, Terry: 4890
Fulop, Tamas: 6140
Fulton, Janet: 9030
Fu, Mingqi: 6450
Funderburg, Allesia: 3550
Fung, Nicole Long Ki: 2480
Fung, Celeste: 1510
Fung, Helene H.: 2480, 3250, 6250
Fung, John Chye: 3450
Fung, Sai-fu: 6350, 6530
Fu, Rong: 6350
Fu, Xiaoyu: 6520

G

G. Cuevas, Pearl Ed: 3310
Gabor, Helena E.: 1220
Gabriel, Kelley Pettee: 2290
Gad, Dustin: 9000
Gadkari, Gauri: 5081
Gagnon, Christine: 6570
Gaillard, Trudy: 6140
Galambos, Colleen: 1460, 4250, 4830, 6750
Galea, Sandro: 6830
Gale, Kathleen S.: 9020
Galera, Leonor: 9010
Galik, Elizabeth: 3650, 3830, 4760, 6560
Gallagher, Elizabeth A.: 6480, 6830
Gallagher-Thompson, Dolores: 1330, 6430, 6750
Gallagher, Virginia T.: 9010
Gallant, Noelle: 5172
Galligan, Catherine J.: 2710
Gallo, Joseph: 6800
Gallo, Linda: 9000
Galloway, Rebecca: 4150
Galo, Caroline: 9030
Galucia, Natalie: 3800
Galvin, Angeline: 4810
Galvin, James E.: 1030, 6070, 6720
Gamaldo, Alyssa A.: 2380, 3270
Ganci, Aaron: 9000
Gan, Daniel Rong Yao: 3450, 4590, 4860, 6290, 6500
Gandy, Maribeth: 9000
Gan, Siqi: 4460, 5030
Gao, Ran: 6110
Gao, shuhong: 6110
Gao, Sibo: 6460
Gao, Xianqi: 9010
Gao, Xiaojun: 6430
Garbarino, Jason: 1070
Garbarino, Lisa: 2490, 6060
Garcia Morales, Emmanuel: 3560, 4050, 6710
Garcia, Catherine: 1480, 4225, 6200
Garcia, Giovanni: 6140
Garcia, Linda: 6150, 6210
Garcia, Marc A.: 2510, 4420
Garcia Morales, Emmanuel: 4050
Garcia, Odalis: 6580
Garcia, Patricia: 6140
Garcia, Sandra: 1330, 6420
Garcia, Y. Evie: 9030
Gardenhire, Douglas S.: 6780
Gardier, Dawn: 6140
Gardiola, Andrea: 6230
Garell, Catia: 6230
Garr, Andrea: 6750
Garren, Patrik A.: 6100
Garrido, Melissa: 3200
Garrison, Betsy: 6200
Garrity, Katherine: 6270
Garron, Angelina: 4570
Gartlan, Olivia: 6790
Garvan, Cynthia: 2300
Gary, Katharine: 6060
Gasdaska, Angela: 3160, 3640, 4350
Gaspar, Phyllis M.: 9020
Gasser, Susan M.: 1200
Gassoumis, Zach: 3010
Gaster, Barak: 5010
Gately, Megan E.: 1040, 6420
Gatz, Margaret: 3510, 6050
Gaugler, Joseph E.: 1050, 1170, 1480, 1560, 2130, 2160, 3020, 3310, 4060, 4220, 5160, 6060, 6070, 6540, 9000
Gaurke, MaryKatherine A.: 4180
Gautam, Ramraj: 2610
Gayda, Mathieu: 6570
Gay, Emma: 4600
Gaziano, J Michael: 3600
Geary, Carol R.: 4590
Gebeyaw, Getachew: 6330
Gee, Gilbert: 4780
Gee, Nancy R.: 9020
Gehlot, Vijay: 9030
Geissal, Erik: 6610
Geisser, Sophia R.: 6110
Gellert, Paul: 6110
Gellis, Zvi: 4750
Gendron, Tracey: 3670, 9000, 9010
Geng, Jing: 6210, 6750
George, Joanna: 6390
George, Kristen M.: 3600
George, Mollie J.: 6090
George, Rosmy: 4870
Gepner, Adam D.: 5050
Gerhardus, Ansgar: 5170
Gerhart, Olga: 4660
Gershon, Robyn: 6090
Gerstorf, Denis: 1010, 1020, 3070, 3150, 4570, 6210, 6390, 6580, 6641
Getchell, Nancy: 4021
Getz, Marjorie A.: 6000
Ghaemmaghami, Sina: 5091
Ghaiumy Anarsky, Reza: 4020, 9030
Ghanbari, Mohsen: 5170
Ghattas, Catherine: 6641
Ghazi Saidi, Ladan: 1270, 3780
Ghiasi, Akbar: 4820
Ghimire, Saruna: 6750, 9010
Ghosh, Bhaswati: 2501, 6840
Ghosh, Dibendu: 6350
Ghosh, Ritam: 5090
Giasson, Hannah L.: 2220, 6640
Gibbons, Laura E.: 3510
Gibbs, Lisa: 2070
Gibson, Allison: 1380, 2460, 4790, 4870, 6060, 6720
Gibson, Regina V.: 6130, 6180
Gibson, Samantha: 9030
Gieniusz, Marzena: 9000, 9010
Giffuni, Jamie: 5050
Giles, Sadie: 6210, 6750

Gillespie, Suzanne: 2660
Gilligan, Megan: 1290, 2440, 4070, 4460, 6520
Gills, Joshua: 6450, 6510, 9000
Gill, Thomas M.: 2760, 5100
Gilmartin-Thomas, Julia F.: 6320
Gilmore-Bykovskyi, Andrea L.: 2160, 3750, 4610, 6310
Gilpin Macfoy, Fiona: 6170
Gilsanz, Paola: 3600
Gimenez-Santana, Alejandro: 6200
Gimie, Afnan M.: 4220
Gimm, Gilbert: 3130, 3870, 4510
Gincel, Dan: 6320
Ginsburg, Liane: 4720
Giordani, Bruno: 4700, 6290
Giorgadze, Nino: 6840
Girdwood, Tyra: 3482
Girling, Laura: 9020
Girma, Meron: 9020
Girouard, helene: 6740
Gitlin, Laura N.: 2150, 2420, 2610, 3310, 4870
Giuliani, Cristina: 6010
Gladyshev, Vadim: 1140
Glaser, Kathryn: 6430
Glasheen, William: 3540
Glassner, Ashlie A.: 2420
Gleason, Kathy: 3360
Gleason, Shayna: 3100, 6700
Gleissner, Laura: 3610, 6380
Glenn, Jordan: 6450, 6510, 9000
Glenn, Lee: 9020
Glicksman, Allen: 4360
Glover, Sabrina: 6770
Gluck, Mark: 6070, 6200
Glueckauf, Robert L.: 4681, 9030
Glymour, Maria: 3510, 3690
Glynn Hames, Alexandra: 9010
Glynn, Nancy W.: 4120, 4600, 4810, 9030
Gmelin, Theresa: 9030
Gnanasekaran, Gowrishankar: 6270
Gobbens, Robbert: 4550
Godde Chrisco, Kanya: 6080
Godek, Eileen: 4023
Godina, Sara: 1050, 9030
Godsey, George A.: 6411
Goebel, Jan: 3070
Goetz, Vanaditz: 1270
Goh, Kim Huat: 4220
Goh, Veronica Shimin: 2150
Goidel, Kirby: 2250
Goldberg, Allon: 6510, 9030
Goldberg, Anne: 6210
Goldberg, Jaime: 6520
Golden, Jeannette: 2690
Golden, Robyn L.: 2560
Golden, Shelley: 5060
Golding, Hannah: 1470
Goldman, Alyssa: 5150
Goldman, Roberta E.: 6770
Goldstein, Daniel R.: 3241
Goldstein, Jenna: 4690
Goler, Timothy: 6200
Golub, Justin S.: 3600
Gomes Da Rocha, Carla: 6380
Gomez, Rowena: 6010, 6170, 9020
Gonyea, Judith G.: 4430, 6730
González, cesar: 6330
González, Hector: 9000
González, Kevin: 9000
Gonzales, Ernest: 3890, 6630, 6680
Gonzalez Bautista, Emmanuel: 4290
Gonzalez, Alexander: 4510
Gonzalez, Joanna: 6140
Gonzalez-Lopez, Paloma M.: 2160
Gonzalez-Prado, Roberto: 9000
Gonzalez, Richard: 2420, 2440, 4010, 4700
Gonzalez, Stephanie: 9020
Goode, Collette: 6270
Goodman, Michael: 3290
Goodpaster, Bret: 3340, 4340, 4600, 9010
Goodwin, Paige: 9030
Goodwin, Rebecca M.: 4530, 6310
Goodyear, Kimberly: 4580
Goo, Kelly: 9000
Gooneratne, Nalaka: 6600
Gopalakrishna, Ganesh: 9000
Gopalakrishnan, Chandrasekar: 6110
Gopalan, Shyamie: 4620
Gopher, Daniel: 6420
Gorbenko, Ksenia: 1160, 3660, 6610
Gorbunova, Galina: 1000
Gorbunova, Vera: 2501, 6840
Gordon, Barbara: 1110, 1320
Gordon, Kristina C.: 5090
Gordon-Larsen, Penny: 2080
Gordon, Peter: 9000
Gordon, Stacey: 6630
Gore, Janelle: 1170
Gore, John: 4461
Gore, Rebecca J.: 2710
Gorospe, Myriam: 1470
Goss, David: 4790
Gossner, Jacob: 6410, 6560
Gotanda, Hiroshi: 5083
Gothard, Sarah: 4790, 6080, 9000
Gothelf, Isabel: 1320
Gothe, Neha P.: 5082, 5130, 9010
Goto, Jun: 6240
Gottesman, Elaine: 4900
Gottesman, Eve: 6220
Gough Courtney, Margaret: 6080
Gould, Christine: 6310
Goulet, Joseph: 6770
Gourley, Amy N.: 6810
Gouskova, Natalia: 3482, 3510, 9000
Gowda-Sookochoff, Rory E.: 9000
Gowin, Lizzie: 9000
Goyal, Parul: 6740
Gozalo, Pedro: 3200
Gräßel, Elmar: 4300
Grünjes, Carlotta: 1020
Graber, Meghan: 5001
Grace, Rachel: 6780
Grady, Alexandra: 9020
Grady, James: 2150
Graf, Allyson S.: 4520
Graff, Sarah: 1200
Graf, Sylvie: 2480
Graham, David: 1280
Graham, Eileen K.: 2140, 2620, 4620
Graham-Engeland, Jennifer E.: 4620, 6290
Graham, Helen L.: 9010
Graham, Lyndsey N.: 2140
Graham, Robert J.: 2020
Granados Unger, Federico: 4120, 6780
Grant, Claire: 4010, 6130, 6310
Granville, Lisa: 6420
Graton, Margaret: 9020
Graupner, Jeffrey: 6220
Gravenstein, Stefan: 9030
Graves, Jessica: 9010
Graves, Jillian: 9010
Gray, Ja'Lynn: 3330
Gray, Michelle: 6450, 6510, 9000
Gray, Nicholas: 6290, 9020
Gray, Taylor R.: 6120
Greaney, Jody: 3730
Green, Aval-Na'Ree S.: 4150
Greenberg, Phyllis A.: 6000, 6220
Greenberg, Sherry A.: 1120, 1250, 1530
Greenberg, Steven: 3580
Greenfield, Emily: 1170, 2010, 2570, 4860, 5080
Green, Geoff: 6320
Greenhalgh, Ella: 9010
Green-Harris, Gina: 3750
Green, Shoshana: 9000
Greer, Faith: 4520
Gregor, Neil: 4260
Greiner, Chieko: 6100, 6110, 9000
Grelle, Kaitlin: 6160
Grenier, Amanda: 6640
Grewal et al., Karl S.: 9000
Grey, Tom: 9010
Grieb, Suzanne: 2690
Griffin, Angel: 4681
Griffin, Joan M.: 2000, 2160, 3390, 6530
Griffin, Meridith: 3620
Griffin, Patrick: 1140
Griffin, Shannon: 2020
Griffin, Terry: 1420
Griffith, Christopher X.: 6820
Griffith, Eric E.: 6210
Griggs, Stephanie: 2200
Grillo, Anthony: 6840
Grimes, Amanda: 9010
Grimm, Kevin E.: 6490
Griswold, Michael: 2670, 3110, 4200, 6780
Grošelj, Darja: 1210
Groarke, John: 3830
Groden, Sheryl: 4023, 9030
Groen-van de Ven, Leontine: 4550
Groenvynck, Lindsay: 1160
Gross, Alden L.: 2200, 2270, 3510, 6740
Grossberg, George T.: 6260
Gross, David: 1380
Gross, Evan: 6480
Grossman, Robin: 4225, 6200
Groulx, Mark: 3450
Groves, Elizabeth: 9030
Growdon, Matthew E.: 5030, 6110
Growney, Claire M.: 2470, 6170

Grudzien, Adrienne: 9000
Gruenewald, Tara L.: 6670
Grühn, Daniel: 3190
Grund, Ethan: 3241
Gruneberg, Audrey: 5000
Gruss, Valerie: 3500
Gryglewicz, Kim: 6350, 9020
Guasco, Rick: 4023
Guerbaai, Raphaelle Ashley: 6270
Guest, Aaron: 3420, 3710
Gugala, Zbigniew: 1120
Guggenheimer, Joshua D.: 9010
Gugliucci, Marilyn R.: 1370, 3800, 6100, 6270
Guidry, Hayden: 2501, 6840
Guillory, Logan A.: 6410
Gu, Lin: 5050
Gunn, Grayson: 1170
Guo, Ellen: 9000
Guo, Fangqi: 3440
Guo, Man: 1090, 1130, 6500
Guo, Xueying: 6770
Guo, Yi: 4040
Guo, Yingqi: 9030
Gupta, Riddhi: 2550, 6440
Gurinovich, Anastasia: 2501, 4810, 6010
Gurlu, Merve: 9020
Gur-Yaish, Nurit: 1160, 2680
Gusmano, Michael K.: 1260, 2040, 4500
Gustafson, Deborah: 3760
Gustavson, Allison: 3310
Gutierrez, Angela: 6140
Gutierrez, J Antonio T.: 5050
Gutierrez, Mariela: 4225, 6300, 9010
Gutierrez-Ramirez, Paulina: 4190
Gutkin, Michael: 6840
Gutman, Gloria M.: 1210
Gu, Xiuzhu: 6700
Guzzardo, Mariana T.: 6210
Gwee, Xinyi: 4023

**H**

Haapala, Heidi: 2500
Haardoerfer, Regine: 9020
Haas, Allen: 5100
Haase, Claudia: 3150, 4570, 4850
Haas, Steven A.: 1090
Habegger, Alex: 6740
Habermann, Elizabeth: 6450
Habiballa, Leena: 3241
Habib, Semper: 4555, 6380
Hackett, Sara E.: 1490, 2070, 2720, 4465
Hackney, Madeleine: 9010
Haddad, Yara: 4100
Hadley, Evan: 4810
Hado, Edem: 2650
Hageman, Sally: 6400
Hagen, Erika W.: 4560
Hager, David: 6140
Haggar, Katherine: 4860
Hägg, Sara: 3590
Haghighat, Misha D.: 4170
Hagler, Donald J.: 6050
Hahne, Jessica: 4480, 6520
Haibach-Beach, Pamela: 2530
Haileyesus, Tadesse: 4100
Hajdar, Melisa: 6230
Hakimjavadi, Ramtin: 1510
Halaszynski, Jaime J.: 9000
Haley, William E.: 2720, 3910, 4650, 6810
Halladay, Christopher: 3130
Hall, Charles: 4700
Hall, David: 9010
Hall, Katherine S.: 2660, 4260, 5050, 6170
Hall, LaToya N.: 6480
Hall, Marissa: 5060
Halpin, Sean N.: 2640, 3550
Halter, Colt M.: 6290
Haltermann, William: 3640
Halvorsen, Cal J.: 1080, 9030
Ham, Carolyn R.: 1060
Hamel, Kate: 6250
Hamermesh, Daniel: 2410
Hamers, Jan P.: 1160, 2090, 5100, 6560
Hamersky, Carol: 9000
Hamilton, Jill: 9020
Hamilton, Lucas J.: 2400, 3570, 6810
Hamilton, Tonua: 1120
Hamlat, Elissa: 3440
Hamler, Tyrone: 6820
Hamm, Callie: 6570
Hamm, Jeremy: 6580
Hammond, Robert: 3540
Han, Benjamin H.: 2245, 2760
Hancock, Cynthia R.: 4520
Hancock, David W.: 6480
Hancock, Laura M.: 2670
Handing, Elizabeth: 3115, 3250
Handlery, Reed: 6740
Han, Duke: 1430, 4070, 6580, 9030
Handzo, George: 6270
Hanes, Douglas W.: 6200, 6680
Han, Gang: 6310, 9030
Han, Gyounghae: 2440, 3220, 6520, 6670
Han, Hae-Ra: 3330, 3560, 4680
Han, Heather: 4520
Hanis, Jen: 6440
Han, Ling: 6770
Hanlon, Alexandra L.: 9000
Hanlon, Samuel: 6270
Han, Molly: 1570
Hannan, Marian T.: 2290
Hannan, Mary: 6411
Han, Sae Hwang: 1100, 3730, 6170, 6520
Hansen, Jared: 1330
Hansen, Lissi: 6830
Hansen, Thomas: 2410
Han, Seung Yong: 9020
Hansmann, Kellia J.: 5172, 9020
Hanson, Andrew: 6450
Hanson, Heidi A.: 4530
Hanson, Paige: 2520
Hansson, Isabelle: 4620
Han, Sua: 6170, 6780
Hantke, Nathan: 1340
Happ, Mary Beth: 3360, 5090, 6310
Haque, Raza: 2210, 9020
Hardiman, Madison: 2501
Harding, Alyson: 3690, 9030
Hardin-Sigler, Kristen: 6160
Hardman, Alisha: 6280
Hare, Joshua M.: 6320
Hargraves, J. Lee: 9020
Haring, Nicole: 4670
Haring, Rodney: 6430
Harkins, Kevin: 4461
Harlow, Sioban: 2080, 6140
Harnish, Autumn: 4510
Harrington, Candace C.: 2150
Harrington, Erin E.: 6290, 9020
Harrington, Karra D.: 3250, 6790
Harris, Daniel: 9000
Harris, Jordan: 6090
Harris, Maurita T.: 3670, 6300
Harris, Melissa L.: 6420, 9000
Harris, Melodee: 6180
Harrison, James: 9000
Harrison, Krista L.: 5030
Harrison, Sean L.: 6390
Harrison, Stephanie L.: 2290, 4080
Harrison, Theresa: 3740, 9030
Harrison, Tracie C.: 3483
Harris, Rebekah: 4260, 5050
Harris, RianSimone: 1450
Hartford, Emily: 9030
Hartman, Mikael: 3390
Hartmann, Christine W.: 1040
Hartman, Sheri J.: 9000
Hart, Sara: 9000
Harvey, Idethia S.: 6650
Harzelak, Jaroslaw: 4600
Hasan, Danish: 6180
Hasan, Maryam: 9000
Hasche, Leslie: 2310, 4240, 4430
Hasdemir, Dilara: 1150
Hashemi, Mahdi: 3200
Hasper, John: 5091
Hassan, Mahdi: 6740
Hass, Zach: 4900, 6630
Hastings, Susan N.: 5083
Hata, Toshiki: 6710
Hathaway, Zachary: 5020, 9020
Haughtigan, Kara: 9030
Hauser, Elliott: 1570
Hausman, Hannah: 1470
Haverhals, Leah M.: 2660, 2720, 6210
Hawes, Frances M.: 5140, 6140
Hawkley, Louise: 1230, 6440
Hawley, Chelsea: 4770
Hawthorne-Crosby, Brieanna: 9010
Hayashida, Cullen T.: 6580
Hayat, Shabina: 2270
Hayden, Kathleen M.: 2670
Hayduk, Leslie: 4210
Hayes, Emily J.: 6580
Hayes, Michael: 3160
Hayes, Susan L.: 5081
Haynes, Laura: 9010
Hays, Garrett: 5091
Hayslip, Bert: 6750
Hazimeh, Fatima: 3860
Hazuda, Helen: 6140
Hazzan, Afeez A.: 2350, 2530, 6280
Hazzan, Joyce: 6280
Head, Elizabeth N.: 2490
Head, Robert: 6170
Heatwole Shank, Kendra: 6300
Heavner, Mojdeh: 6350

He, Daqing: 6150
Hedge, Eric: 4120, 6780
Heffernan, Kristin: 2350
Heffner, Michelle: 6090
Hege, Adam: 4820
Heid, Allison R.: 6130
Heideman, Kristin: 6210, 6411
Heij, Ward: 1120
Heilbrunn, Emily S.: 3520
Heilman, Keri: 1550
Heimler, Stephanie R.: 4461, 6010
Hein, Maria: 2520, 5010
Heintz, Hannah: 2030, 2660
Heinz, Melinda S.: 3610, 6380
Heisel, Marnin J.: 4630
Heislbetz, Claus: 1270
He, Linchen: 4330
Helppie-McFall, Brooke: 9020
Helsinger, Abigail L.: 4555, 6130, 6670, 6710
Hemingway, Dawn: 4020
Henage, Cris: 1110
Henderson, Charles R.: 4225, 6180
Hendricksen, Meghan: 1090, 1530
Hendrie, Hugh C.: 9020
Hendrix Rogers, Anita: 1530
Hendry, Phyllis: 9030
Henegan, James: 3110, 4200
Henley, Laura: 4790
Henneberry, Kathleen: 9020
Henne, Jeff: 1320
Hennessa, Alexandra: 2010, 6760
Henrich, Christina M.: 3115
Hensley, Mark C.: 9020
Hensley, Robert B.: 6810
Heo, Jinmoo: 6380
Hepburn, Kenneth: 3240, 4030, 5160
Hepple, Russell T.: 3340, 6780, 9010, 9020
Heppner, Alexandra: 2340, 4360, 6130
Heppner, Marie-Marleen: 1270
Herbert, Brittney-Shea: 6220
Herbert, Christopher: 3900
Herd, Pamela: 2400
Hermanns, Melinda: 9010
Herman, William: 2080
Hernandez Chilatra, Jessica A.: 9000
Hernandez de Jesus, Alma V.: 9000
Hernandez, Manuel E.: 6740
Hernandez, Olivia: 5020
Hernandez-Swift, Karla: 4010
Herr, Keela: 6310
Herrmann, Francois: 6380
Herrmann, Lynn K.: 6160
Hersch, Gayle I.: 6760
Hertzog, Christopher: 6710
Herz, Jasmin: 4461
Heslop, Thomas: 3000
Hess, Rachel: 1120
Hess, Thomas M.: 2480
Heston-Mullins, Jennifer: 3180
Heussner, Pia: 5110
Heyn, Patricia C.: 1060, 3350, 3410, 4400, 4860, 9030
Hiatt, Shirin O.: 4790, 6830
Hicken, Bret: 9020
Hickman, Louis: 2280
Hickman, Ronald: 2200
Hickman, Susan: 4100, 6770, 9030
Hicks Patrick, Julie: 6140, 6200, 9000
Hicks, Hilary J.: 6570
Hiddemann, Wolfgang: 5110
Higbie, Anna: 6390
Higgins-Chen, Albert T.: 1140
Hill, Andrea M.: 5173
Hill, Carl V.: 9020
Hillhouse, Andrew: 6010
Hill, Linda: 5171, 9030
Hillman, Vanessa: 9000
Hill, Patrick L.: 2430, 4620, 6580
Hilmer, Sarah: 2090
Hilsabeck, Tyler AU: 6010
Hines, Anika L.: 4680
Hinrichsen, Gregory: 4250
Hinton, Ladson: 3310, 4700
Hinyard, Leslie: 6720
Hiroto, Kimberly: 3420
Hirsch, Jen: 4270, 9000
Hirschman, Karen: 6200, 6280, 9000
Hirst, Roxana: 4150
Hisamatsu, Yasushi: 6450
Hitchcock Noel, Polly: 1330
Hladek, Melissa: 2200, 4680
Hoben, Matthias: 4210, 4720, 5010, 6760
Hobgood, Sarah: 6320
Hochberg, Marc: 6780
Hochgraeber, Iris: 3090
Hodes, Richard: 3080, 3280
Hodgdon, Barbara T.: 6750
Hodgson, Nancy: 3310, 3680, 3930, 6110, 6300, 6390, 6510, 6600, 9000, 9030
Hoe, Deborah: 3390
Hoey, Jesse: 9000
Hofer, Molly: 5082, 5130
Hofer, Scott M.: 1020, 2140, 3440, 3510, 3590, 5084, 6290, 6440, 6580, 9020
Hoffman, Adar: 6580
Hoffmann, Anna-Louisa: 3090
Hofmann, Brittany: 3480
Hoft, Daniel: 3600
Hogans, Beth B.: 6220
Hogentogler, Ellie: 3520
Holaday, Louisa: 4130
Holden, Richard J.: 3060, 6270, 9000
Holden, Samantha: 9000
Holland, Diane: 3390, 6110
Holland, Elizabeth: 9000
Hollander, Matthew: 9020
Holland, Peter: 9000, 9010
Holland, William: 9030
Holle, Bernhard: 3090
Holley, Lyn M.: 4710, 6700
Hollingshaus, Michael: 4530
Hollingsworth, Emily: 6270
Holloway, Jeremy C.: 6370
Holmes, Ann: 6760
Holmes, Ashleigh: 6280
Holmes, Rachel: 4290
Holstad, Marcia: 3760
Holtgrave, Peter: 6060
Holt, Heidi L.: 2490, 6060
Holt-Lunstad, Julianne: 6790
Holtzer, Roee: 3250, 4340
Homan, Patricia: 6180, 9020
Honda, Miwako: 6000
Hong, Chelin: 6420
Hong, Joanna H.: 3730
Hong, Michin: 3220, 6120, 6160
Hong, Seunghye: 3220
Hong, Soyun: 6400
Hong, Sungjae: 1240, 9030
Hood, Nancy: 3520
Hoogeboom, Thomas: 1120
Hooker, Karen: 1080, 2280, 3490, 5000
Hooper, Savannah: 6020
Hooyman, Nancy: 6260
Hoppmann, Christiane: 1020, 2480, 3150, 6210, 6390, 6580
Hoque, Masudul: 3720, 6060
Horhota, Michelle: 6640
Hornbuckle, Lyndsey: 4030
Hornstein, Erika: 4680
Horstman, Molly J.: 4683, 6320
Hossain, Lubna: 3240
Hosseini, Maryam A.: 3880
Hosseini, Shera: 6000
Hostetler, Andrew J.: 2610
Hougaard, Ben: 9020
Hougaard, Benjamin T.: 6170
Hough, Katrina: 2690
Hou, Su-I: 1480, 2290, 4450
Hou, Yifei: 6520
Howard, annie Green: 2080
Howard, Marjorie: 9000
Howard, Michelle: 6000
Howe, Judith L.: 3420, 6220
Howell, Abigail: 1380
Howell, Britteny M.: 2260, 6270
Howington, Marshall: 6010
Hoy-Ellis, Charles: 4540
Hoyle, Jessica N.: 2050
Hryhorenko, Jennifer: 5091
Hsieh, Jessica: 6230, 6480, 6690
Hsu, Hui-chuan: 6800
Hsu, Jean W.: 2241
Hua, Cassandra L.: 4465, 5081
Huang, Alison: 2530
Huang, Jing: 4080
Huang, Liming: 6200, 6300
Huang, Pai--Chuan: 4450
Huang, Shijiao: 6010
Huang, Shuo: 4730
Huang, Weiliang: 6641
Huang, Wenxuan: 3260, 6730
Huang, Xiuxiu: 6150
Huang, Ying: 3550
Hubbard, Jennifer L.: 1110
Hubbard, Zachary L.: 3690
Huber, Jessica: 9030
Huber, Lesa: 6770
Hubner, Sarah: 6090, 6390
Hudgins, Kristen: 2020
Hueluer, Gizem: 1020
Huffman, Kim: 2241
Huffmyer, Mark: 6060
Hufnal, Shelby: 5020, 9020
Huggins, Madison L.: 3500
Hughes, Anne K.: 1400, 4770
Hughes, Brian P.: 2501, 6270
Hughes, Brian: 6840
Hughes, Jaime M.: 4770
Hughes, Kelly L.: 4350
Hughes, M. Courtney: 6750
Hughes, MacKenzie L.: 6710

Hughes, Matthew L.: 2750
Hughes, Robin E.: 1530
Hughes, Susan: 4800
Hughes, Tiffany F.: 1280
Hughes, Timothy: 9000
Hughson, Richard: 4120, 6780
Huh, Jimi: 5140
Huis in het Veld, Judith: 4550
Hu, Jie: 9020
Hu, Jinxiang: 4030
Hulko, Wendy: 4020
Hull, James: 6690
Hu, Luxin D.: 4060
Humber, Marika B.: 1330, 6310
Hu, Mei-Chen: 3600
Hu, Mengyao: 4700, 5070, 1540
Hung, Chang-Chiao: 6140
Hung, Jui-Ying: 4682
Hung, Lillian: 1550, 3450, 4590, 9000, 9010
Hung, William: 1040, 1350, 6320
Hunt, Angela: 6540
Hunter, Melissa: 3640, 4350
Hunt, Lauren: 3680, 4460, 5030
Huo, Meng: 3730, 4170, 4460
Huppert, Theodore J.: 4340
Huque, Md Hamidul: 9000
Hu, Rita X.: 1290, 2220, 6230
Hurley, Shannon: 9000
Huseth-Zosel, Andrea: 3480, 9020
Hussaini, Husna: 4160, 9020
Hussain, Sultana Monira: 4683, 5083
Hu, Wangyang: 4023
Hu, Xujing: 6110
Huynh, Grace: 4680
Hu, Yuanyuan: 6230
Hu, Yue: 3250, 6250
Hwang, Ann: 6140
Hwang, Charles: 5001
Hwang, Ula: 5100
Hwang, Woosang: 1450, 6520
Hwang, Yeji: 5020, 6300, 6470, 9020
Hynes, Mary T.: 6380
Hyun, Jinshil: 2140, 9010

I

Iaboni, Andrea: 9000
Iacob, Eli: 3500
Iaconi, Alba: 4210
Ibrahim-Bacha, Kareem: 6140
Ibuka, Yoko: 6300
Ickert, Edmund: 1280
Igarashi, Heidi: 6210
Iglesias, Pablo: 6411
Ihara, Emily S.: 2260, 3130, 4510, 6280
Iijima, Katsuya: 6240
Ikeda, Naoki: 4440
Ikeuchi, Tomoko: 6390, 6400, 6580
Ikram, Arfan: 5170
Ilardo, Joan: 2210, 6180
Ilavarasan, Abinaya: 6830
Ilori, Folasade: 6170
Imamura, Tomoaki: 2580
Im, Eun Ok: 9030
Imus, Philip: 1010
Infante, Kimberly: 9020
Infurna, Frank: 4060, 6490
Ingeborg Berg, Anne: 4620
Ingle, Pilar: 4240, 4650, 6720
Ingram, Cory: 3390
Ingvalson, Stephanie N.: 4220
Inman, Christina: 6840
Inman, Morgan: 6250
Inoue, Megumi: 2260, 3200, 4510
Insel, Kathleen C.: 4190
Intharakasem, Sirirat: 3840
Intrator, Orna: 1330, 6320, 6420, 4683
Intrieri, Robert C.: 9030
Iny, Nicole: 6100
Inzitari, Marco: 6450
Iob, Eleonora: 3210
Irani, Elliane: 2200, 3630, 6410
Irimia, Andrei: 9000
IRIMIA, ANDREI: 9000
Isaacowitz, Derek: 2330
Isaacs, Lauren: 4240
Isaranuwatchai, Wanrudee: 9030
Ishado, Emily: 1150
Ishioka, Yoshiko: 6580, 6810
Ishitake, Tatsuya: 6450
Islam, Farhana: 3260
Islaya, Cesz: 6210, 9030
Israel, Sarah: 6790
Issakainen, Mervi: 2460
Itoh, Sakiko: 6390, 6400
Ittermann, Till: 1010
Iveniuk, James: 2550, 6440, 6550
Iwamoto, Miyuki: 6000
Iwarsson, Susanne: 4220
Iwasaki, Yoshitaka: 6170
Iyer, Lakshmi: 4820
Iyer, Sowmya S.: 6310

J

Jablonski, Rita: 1390
Jaboob, Saida M.: 6830
Jacelon, Cynthia: 6740, 9030
Jack, Jr., Clifford: 2670
Jackson, Eva M J: 2490, 3510, 4120, 6060, 6280
Jackson, Kathleen: 2330
Jackson, Kathryn: 4620
Jackson, Piper: 2210
Jackson, Rebecca: 2200
Jacobs Dodson, Lindsey: 6220
Jacobs, Christine: 3600
Jacobs, Jeremy M.: 6610
Jacobs, Josephine C.: 1330
Jacobs, Mary Lindsey: 2030
Jacobson, Lauren: 9000
Jacobson, Mireille: 3200, 9010
Jahn, Georg: 6250
Jahoor, Farook: 2241
Jain, Akanksha: 6430
Jain, Felipe A.: 4190
Jain, Jennifer: 3540, 9030
Jain, Mohit: 4461, 6010
Jain, Shalini: 9000, 9010
Jain-Washburn, Ethan: 6010
Jajodia, Anushka: 3330
Jaldin, Michelle: 6570
Jallow, Fatoumata: 6770
James, Bryan D.: 2140, 4620
James, Dara: 9000, 9020
James, Dawn: 6740
James, Jennifer: 5120
Jang, Jae Young: 6450, 6510, 6740
Jang, Jieun: 5084
Jang, Jiyoon: 9000
Jang, Yuri: 2420, 3160, 4370, 5000, 6180, 6490, 6810
Janke, Megan C.: 3270
Jankowski, Thomas B.: 4590, 6400
Jansen, Carl-Philipp: 2450
Jansen, Taylor: 3100, 3320, 6090, 6510
Janssen, Daisy: 6270
Janssen, Leah: 9030
Jao, Ying-Ling: 2530, 3830, 6110, 9000
Jarosek, Stephanie: 9010
Jarrín, Olga F.: 4500
Jarrott, Shannon E.: 1080, 2350, 3780, 3890, 5000
Jarvis, Megan: 6110
Jason, Kellyn: 9010
Jason, Kendra: 6180, 6750
Javeed, Ashir: 3590
Javier, Noelle Marie: 4250
Jayakody, Oshadi: 6780
Jayani, Reena V.: 6740
Jeamjitvibool, Thanakrit: 6411
Jeanblanc, Alexandra: 3115, 5140, 6090, 6820
Jean-Louis, Girardin: 6680
Jean-Pierre, Sarai N.: 9030
Jeffrey, Kullgren: 3495
Jenkins, Emerald: 4680
Jenkins, Hailey-Thomas: 6500
Jennings, Lee A.: 5172
Jennings, Stephen: 5050
Jen, Sarah: 2540, 6070, 6190
Jensen, Adelaide: 6290
Jensen, Christine J.: 6070
Jeong, Chung Hyeon: 4370, 5150
Jeong, Ha Na: 6320
Jeong, Mijin: 6070
Jeong, Sohyun: 9020
Jepson, Laura: 9020
Jessee, Lisa: 9030
Jessup, Nenette M.: 9020
Jeste, Dilip V.: 3760, 6200
Jester, Dylan J.: 4465, 6200
Jiang, Hantao: 9030
Jiang, Jingjing: 3200
Jiang, Kening: 2670, 3560, 4050
Jiang, Nisi: 9010
Jiang, Shumin: 6480
Jiang, Xin: 4040
Jiang, Yanping: 3860, 6200, 6290
Jiao, Yuqin: 3115
Jia, Yuane: 5130
Jicha, Gregory: 1380, 4790, 4870, 6060, 6720
Jimenez Zambrano, Joyce: 6720
Jimenez, Daniel E.: 6170
Jing, Bocheng: 5030
Jin, Yichen: 4200
Ji, Tong: 4683, 9020
Jiwani, Rozmin B.: 6390
Ji, Yuelyu: 6150
Jock, Janet: 9010
Johanessen, Aud: 3610
Johanning, Jason: 6740
Johansson, Linda: 9020
John, Karen: 1480

John, Oliver P.: 2620
John, Samantha: 6060
Johnson, Adrienne L.: 4682
Johnson, Alisha H.: 3483
Johnson, Brittany: 9030
Johnson, Daniel: 6350
Johnson, Elma B.: 3310
Johnson, Erin: 9020
Johnson, Erin P.: 3500
Johnson, F. Bradley: 6010
Johnson, Florence U.: 2420, 6310
Johnson, Fred: 9000
Johnson, Gerhardt: 6420
Johnson, Haley: 6510
Johnson, Ian: 4380
Johnson, Julene K.: 9000
Johnson, Justin: 1190
Johnson, Kim G.: 6450
Johnson, Kimson E.: 9010, 9020
Johnson, Mark: 5001
Johnson, Matilda: 3260
Johnson, Rachel: 6470, 9030
Johnson, Theodore M.: 9010
Johns, Shelley A.: 9000
Johnston, Daphne M.: 1380
Johnston, Jeanne: 9020
Johnston, Julia: 1380, 6060
Johnston, Maxwell: 2501, 6840
Johri, Mohini: 9030
Jolliff, Anna: 9010
Jones, Brian D.: 4160
Jones, Brittany: 4540
Jones, Chelsey: 9000
Jones, Clifford: 6100
Jones, Faith M.: 3460
Jones-Harden, Brenda J: 4225, 6180
Jones, Megan: 6450, 6510, 9000
Jones, Miranda: 2070
Jones, Patricia L.: 1440, 3080
Jones, Randy: 2070
Jones, Rebecca: 2540
Jones, Richard: 3510
Jones, Susan: 9030
Jones, Tessa: 2130, 6110
Jonsson-Funk, Michele L.: 4320
Joo, Heesoo: 6400
Joo, Susanna: 6390, 9030
Jopp, Daniela S.: 2440, 6200, 6380, 6480, 6580, 6790, 6830
Jor'dan, Azizah: 9010
Jordan, Kala v.: 9000
Jordan, Sarah R.: 4260
Jordan, Tamara: 3770
Joseph KJ, Vinod: 1060
Joseph, Carly A.: 4110
Joshi, Anupam: 6790
Joshi, Shantanu: 9000
Jox, Ralf J.: 2680, 3720, 6730
Joyce, Jillian L.: 6260
Joyce, Melinda: 9030
Jubanyik, Karen: 5100
Ju, Catherine H.: 6520
Judd, Suzanne: 1050
Judge, Katherine: 4010, 6130, 6310, 6410, 9000, 9020, 9030
Ju, Eunae: 2070
Juliano, Celina: 3810
Julian, Vallejo: 5001
June, Andrea: 1070, 1420, 3800
Jung, Hailey H.: 4540
Jung, Heeeun: 9020
Jung, Hye-Young: 2090
Jung, Jong Hyun: 6240
Jung, Karen: 2330
Jung, Woosub: 6420
Junqueira Vasques, Ana Carolina: 9020
Jurgens, David: 4630
Juris, Jill: 2350, 3270, 3780
Ju, Sehyun: 6240
Justice, Amy C.: 9010
Justice, Jamie N.: 2241, 2760, 3400, 4691, 9010
Justinas, Narbutas: 3590
Jutkowitz, Eric: 4580, 5130, 6230, 6360, 6540
Kühn, Simone: 1010, 4040

**K**

Kaasalainen, Sharon: 3555
Kabaya, Sonoko: 9000
Kabeto, Mohammed U.: 3040
Kabudula, Chodziwadziwa Whiteson: 3040
Kadey, Kylie: 6450
Kado, Deborah: 2290
Kaeberlein, Matt: 6840
Kahana, Eva: 2640, 4905, 6170, 6200, 6390, 6690
Kahana, Jeffrey S.: 6390
Kahn, Kathleen: 3040
Kahya, Melike: 3482
Kai, Ichiro: 3010
Kajiyama, Bruno: 6750
Kakarla, Prabhash: 9000, 9010
Kakinuma, Tomohiro: 2580
Kalaw, Karel: 9010
Kales, Helen: 2420, 2530
Kalesnikava, Viktoryia A.: 5060
Kalies, Christian H.: 6010
Kallianpur, Kalpana J.: 3580
Kallmi, Selmi: 3850
Kalvesmaki, Andrea: 4010
Kalyani, Rita R.: 1010
Kamal, Arif: 6390
Kamalyan, Lily: 2450
Kamkhoad, Donruedee: 6450
Kanagawa, Hotinpo Sky: 3481
Kanaya, Reiko: 6110, 6450, 6770, 9030
Kane, Alice E.: 1140
Kane, Kevin: 2290
Kane, Maureen A.: 6641
Kang, Bada: 6110, 6280
Kang, Chaehyeon: 6420
Kang, Ezer: 9010
Kang, Hyun: 6280
Kang, Jee eun: 6790, 9010
Kang, Karmeet Kaur: 3310
Kang, Miseon: 9030
Kang, Suk-Young: 6400
Kannan, Lakshmi: 4800, 6780
Kann, Michael R.: 6260
Kan, Winnie: 9010
Kao, Hsueh-Fen S.: 6140
Kao, Yu Hsiang: 4060
Kapahi, Pankaj: 6010, 6840
Kapasi, Alifiya: 2140
Kaplan, Daniel B.: 9030
Kaplan, Robert: 4330
Kapoor, Shaunik: 3670
Kappari, Sai Raj: 3260
Kappler, Caitlin B.: 5083
Karakaslar, Onur: 3001
Karakida, Maki: 6200
Karalija, Nina: 9010
Karam, Rita: 4555, 6130, 6670, 6710
Karasik, David: 3400
Karasik, Rona J.: 3030, 3420, 6000, 6220
Karbakhsh, Mojgan M.: 1210
Kåreholt, Ingemar S.: 3440, 6200, 6450, 6760
Karel, Michele J.: 6110
Karia, Francis: 6450
Karikari, Grace: 6350
Karim, Saleema: 6420
Kari, Nitya: 1320
Kariuki, Jacob K.: 9030
Karki, Dinesh: 6390
Karlamangla, Arun S.: 6440, 6580
Karlawish, Jason: 3680, 3930
Karmacharya, Isha: 2020, 6750, 9010, 9030
Karmonik, Christof: 3481
Karnati, Yashaswi: 6510
Karraker, Amelia W.: 2550
Karr, Justin E.: 6590
Karvar, Mehran: 6840
Karvonen-Gutierriez, Carrie: 2080, 6140, 6650, 9020
Kaskie, Brian P.: 2245, 3660
Kasler, Kamryn: 2340, 4360
Kasseye, Eyayu: 6330
Kassim, Adetola: 6740
Kastner, Keri: 2590
Kastner, Monika: 6380
Katagiri, Keiko: 6700
Katsev, Danielle: 1120
Katsumata, Asako: 3920, 4440, 6630
Kattari, Leo: 1400
Katz, Benjamin: 2580, 6290, 6440
Katzel, Leslie: 5050, 6220
Katz, Gabrielle R.: 6360
Katz, Jesse: 9000
Katz, Maya: 2720
Katz, Mindy J.: 6290
Katzorreck, Martin: 1020
Kaufman, Brystana G.: 3390
Kaufman, Jennifer e.: 3760
Kaufmann, Christopher N.: 2170, 4080, 4560, 4840
Kaup, Migette L.: 9030
Kaur, Harmanpreet: 6770
Kaushal, Navin: 3630, 6080, 6570, 6740
Kavalieratos, Dio: 4530
Kavanagh, Colleen: 6430
Kavasch, Barrie: 9010
Kavcic, Voyko: 6090, 6290
Kayali, Nahla: 3430
Kaye, Jeffrey: 1340, 2450, 4790, 6080, 6450, 9000
Kaye, Jesse T.: 4682
Kaye, Lenard: 3540, 6540, 9030
Kayser, Jay R.: 6580
Kazembe, Neo: 6240
Kazmer, Michelle M.: 4681
Kearney, Leanne: 6280
Kearns, William D.: 3540
Keatings, Margaret: 6270
Keefe, Bronwyn: 6130
Keel, Pamela K.: 6020
Keenan, Teresa A.: 3495
Keesee, Francesca: 6280
Kehler, Dustin S.: 9030
Keilman, Linda: 1400
Keith, NiCole R.: 6080

Kelekar, Uma: 9030
Kelfve, Susanne: 6200
Kellar, Garrett: 1280
Keller, Abiola: 6750
Keller, James: 4021
Keller, Krystyna R.: 6170
Keller, Michelle S.: 5083
Kelley, Amy S.: 4250, 5030, 5070
Kelley, Amy: 3080, 3280
Kelley, Casey J.: 9030
Kelley, Jessica A.: 4070
Kelley, Lynette: 6420
Kelley, Megan: 2340, 6130
Kelly-Adams, Deirdre: 6230, 6690
KELLY, AMBER L.: 5060
Kelly, Brenna C.: 4530
Kelly, Christopher M.: 6700
Kemp, Candace L.: 5173, 6760
Kendrick, Alexis: 6740
Kennedy, David: 6690
Kennedy, Katherine A.: 3950, 5081, 9030
Kennedy, Meaghan A.: 1040
Kennedy, Richard E.: 6340
Kennedy, Teri: 6120
Kenny, Brooks: 6140
Kerdic, Richard: 2090
Kern, Kathy L.: 9000
Kerns, Lucy: 1280
Kero, Katherine: 6090, 6290
Kerr, Margaret: 4240
Kesler, Shelli R.: 9020
Kessler, Jamie: 3040
Kew, Chung Lin: 2250, 6310, 6710, 9030
Ke, Weiming: 5173
Key, Kent: 6180
Kezios, Katrina: 9010
Khalil, Hussein M.: 6510
Khamzina, Madina: 2050, 6390
Khan, Amina: 9020
Khan, Amna: 3480
Khan, Anam M.: 3690
Khan, Arooj: 4080
Khan, Mushira: 6180, 9010
Khan, Nibraas: 5090
Khan, Shehroz: 6640, 9000
Khavjou, Olga A.: 3640
Khemai, Chandni: 6270
Khumrungsee, Montri: 2610
Khyzr, Maryam: 5001
Kiasat, Shadi: 6450
Kibe, Lucy: 9020
Kidana, Kiwami: 6240
Kiecolt-Glaser, Janice K.: 3150, 4470
Kiefer, Jamie: 9020
Kiekhofer, Rachel: 6270
Kiel, Douglas: 3400
Kiely, Kim: 4022
Kietzman, Kathryn G.: 3770
Killian, Tim S.: 6200, 9000
Kilpela, Lisa S.: 6020
Kim, Hyunji: 3190
Kimball, Patricia F.: 4090
Kim, Boeun: 2510
Kim, Bohyun: 9020
Kim, Bon: 3220, 6670
Kim, BoRin: 4370, 5150, 6800, 9000, 9010, 9020
Kim, Catherine S.: 3140
Kim, Chang Oh: 6320
Kim, Christina: 4240
Kim, Dae H.: 5084, 6110, 6641, 9000, 9020
Kim, DaEun: 9010
Kim, Dahye: 4170
Kim, Eun-Kyeong: 2450
Kim, Eunkyo: 6270
Kim, Eunkyung: 6400
Kim, Eunyoung: 6280
Kim, Giyeon: 6240, 6340, 6820
Kim, Gwang Suk: 6320
Kim, Ha-Neul: 1170, 4023, 4270, 6060, 9000
Kim, Harper S.: 2501
Kim, Heejung: 6400, 6420, 9030
Kim, Hongjik: 6240
Kim, Hyeon Jung: 6170, 6290
Kim, Hyoun K.: 6390, 9030
Kim, Hyunjee: 5081
Kim, Hyun-Jun: 4540
Kim, Jeehoon: 6400
Kim, Jeungkun: 6400
Kim, Jinhyun: 6810
Kim, Jiwon: 3020
Kim, Joo Ri: 6320
Kim, Joohyun: 2440
Kim, Jung Ki: 2080, 6710
Kim, Junhyoung: 3220, 6170, 6780, 9020
Kim-Knauss, Yaeji: 2480
Kim, Kyeongmo: 6120, 6610
Kim, Kyoungmi: 1470
Kim, Kyung Moo: 9020
Kim, Kyungmin: 1320, 2040, 2160, 2440, 3220, 4170, 4460, 6480, 6520, 6670, 6750, 6830
Kim, Laurie: 6420
Kim, Mackenzi: 9020
Kim, Meeryoung: 6670, 6810
Kim, Miji: 6450, 6510, 6690, 6740, 6780, 9020
Kim, Min Jung: 6080, 6300, 9010
Kim, Minji: 6320
Kim, Miyong T.: 6810
Kim, Narae: 3200
Kim, Patricia: 2300
Kim, Patricia S.: 6530
Kim, Seiyoun: 2090
Kim, Seo Yun: 9030
Kim, Seon: 2700, 6610
Kim, Seoyoun: 3160, 4660
Kim, Seulgi E.: 4683
Kim, Sohyun: 4870
Kim, Sooyoung: 5090
Kim, Steffi M.: 1380, 3790, 4710
Kim, Su Yeong: 4730, 6180
Kim, Sue: 6320
Kim, Sung Kyeom: 9010
Kim, Yeoju: 6240
Kim, Yesol: 6400
Kim, Yi Hyun: 6420
Kim, Yijung K.: 2440, 3730, 6480, 6830
Kim, Yongseop: 6780
Kim, Yoon-Myung: 6390
Kim, Youn Kyoung (Lily): 6500
Kim, Yulri: 9010
Kincaid, Reilly: 6520
Kindratt, Tiffany B.: 6310
King, Barbara: 6641
King, Ben: 3540
King, Christina: 3241
King, Gregory: 9010
King, Jennifer: 6430
King, Sharla: 6390
King, Sina J.: 4620
Kinkade, Emily E.: 6230, 6640, 9020
Kinney, Jennifer M.: 2440
Kinosian, Bruce: 1330
Kinzig, Elizabeth: 3530
Kipnis, Jonathan: 4461
Kirch, Matthias: 3495
Kirkegaard, Allison: 6690
Kirk, Jennifer M.: 6740
Kirkland, James L.: 2760
Kirk, Laura: 9000
Kishimoto, Kyoko: 3420
Kitamura, Akihiko: 6710
Kitase, Yukiko: 5001
Kitner-Triolo, Melissa: 9020
Kitsiou, Spyros: 6780
Kittle, Krystal R.: 1320, 2390
Kivi, Marie: 4620
Kiyoshi-Teo, Hiroko: 9030
Klaiber, Patrick: 2430
Klassen-Ross, Tammy: 2210
Klee, Matthias: 6150
Kleineidam, Luca: 2140
Klepacz, Laura M.: 2470, 9020
Kleszynski, Keith: 3610, 5172
Kline, Danielle: 4650
Klinedinst, Jennifer: 6641
Klinger, Christopher: 6230, 6480, 6690
Klopack, Eric T.: 3440, 4560, 6010
Knebl, Janice A.: 1110, 1250
Knebusch, Clare: 6160
Knepple Carney, Amy: 9000
Knight, Jamie: 2140, 6290
Knight, Rebekah: 3190
Knimbeng, manka: 3310
Knopman, David S.: 3560, 6780
Knopp-Sihota, Jennifer A.: 6270
Knox, Janice: 4681
Knox, Katie: 2520
Knurek, Sean: 6180
Kobayashi, Lindsay C.: 1050, 2270, 3040, 3495, 4110, 9010
Kobayashi, Masaki: 6000
Kodali, Maneetha: 6180
Koehler, Kerstin: 3090
Koehly, Laura M.: 1480, 3240, 6170
Koerner, Susan S.: 6240
Ko, Eunjung OS: 5090
Koeuth, Sokha: 3310
Koffer, Rachel E.: 2220, 3070
Ko, Fred: 1350, 6320
Kohanski, Ronald A.: 3080
Koh, Eun: 6480
Koh, Jemima: 3820, 6330
Kohn, Jordan N.: 6200
Koi, Janice Jue Xin: 6411
Koiler, Reza: 4021
Koirala, Binu: 6140
Koizumi, Naoru: 3200
Kok, Almar A. L.: 9020
Kolanowski, Ann: 2160, 3310, 3720, 9030
Kolodisner, Jude G.: 2250
Kolodziejczak, Karolina: 3150
Kolodziejczyk, Ida: 6510
Kolpakov Nikitin, Stanislav: 6411
Koltermann, Ken: 6420
Koltz, Daniel J.: 6760
Koltz, Rebecca L.: 6760

Komalasari, Renata: 6110
Komatsu, Ayane: 6210, 6250, 6760
Kong, Dexia: 3500, 9030
Kong, Jooyoung: 4730, 6520
Kong, Lan: 3520
Kong, Taylor: 9030
Konomos, Michael: 2640, 3550
Konrad, Thomas R.: 9010
Kontra, Joseph: 6100
Koo, Hyun Jung: 2420, 5070
Kooiman, Nia: 3520
Koon, Lyndsie M.: 4160
Kootar, Scherazad: 9000
Kopera-Frye, Karen: 1480, 6100
Kops, William: 9030
Koren, Chaya: 1300
Koren, Yael: 4640
Koreshi, Shanika Y.: 3900
Kornadt, Anna E.: 2750, 3490
Kossowska-Kuhn, Dorota: 9020
Kostyniuk, Lidia P.: 3000, 6470
Koszalinski, Rebecca: 1060
Kotecho, Messay G.: 6330, 6630
Kotwal, Ashwin A.: 2550, 2690
Koufacos, Nicholas: 6220
Koumoutzis, Athena: 2440, 3180, 4010
Kovaleva, Mariya: 5160
Kovtun, Mikhail: 6110
Kowalchik, Kalei H.: 3520
Kozlov, Elissa: 1310, 6390
Kraemer, Sandra: 6170, 6580, 6790
Krakauer, John W.: 3750
Kramer, Jenna: 4555, 6130, 6670, 6710
Kramer, Philip A.: 9010
Kraschnewski, Jennifer L.: 3520
Krasnansky, Katie: 4680
Krasniewski, Linda: 1470
Krassikova, Alexandra: 6270
Kratzer, André: 4300
Kraus, William E.: 2241
Kravatz, Nigel L.: 9020
Kravchenko, Julia: 1000, 3720, 6110
Kremen, William S.: 6050, 6490
Krendl, Anne C.: 3570, 6290, 6810
Kresevic, Denise M.: 9030
Kriebernegg, Ulla: 3585
Krishnan, Girish: 9020
Kritchevsky, Stephen B.: 2241, 2300, 2530, 3340, 3400, 4120, 4600, 6641, 9000, 9010
Kroeber, Maria M.: 6000, 6220
Krok-Schoen, Jessica L.: 2200, 3550
Krook, Melissa: 6790
Kropp, Denise: 3530
Krupp, Anna: 9020
Kryscio, Richard: 4870
Kubala, Andrew G.: 4080
Kuball, Tim: 6250
Kube, Erin R.: 2030
Kubzansky, Laura D.: 4690
Kuchel, George A.: 3001, 4410
Kuipers, Allison L.: 4810, 6450
Kulbok, Pamela: 1030
Kulkarni, Rajendra: 3200
Kulkarni, Snehal S.: 9030
Kullgren, Jeffrey: 3495
Kulminski, Alexander: 6010, 6411, 9030
Kumar, Amit: 4780
Kumar, Premranjan: 2241, 9010
Kumar, Priyanka: 3560
Kumar, Raj G.: 5030
Kumar, Sharma: 4190
Kumar, Sidharth: 6390
Kunkel, Miranda C.: 1530, 2340, 4360
Kunkel, Suzanne: 2010, 2020
Kunkle, Rachel: 6280
Kunz Lomelin, Alan Adolfo: 6630
Kunzmann, Ute: 1020
Kuo, Tony: 6060, 6220, 6280
Kuo, TsuAnn: 4440, 4450
Kuo, Yong-Fang: 5100, 9000, 9010
Kurazume, Ryo: 6000
Kurth, Maria: 5140, 6210, 9020
Kushnir Groman, Tamara: 3390
Kusmaul, Nancy: 4510, 4830, 6720
Kusters, Cynthia D.: 4560
Kutcher, Katie: 4520
Kuwayama, Sayaka: 9000
Kuy, Kakada: 6200
Kuzmik, Ashley: 2230, 4760
Kwak, Jung: 6270
Kwan, Joseph Shiu Kwong: 4684, 5084
Kwan, Rick Yiu Cho: 3840
Kwekkeboom, Kristine: 6280
Kwok, Wai-wai: 9020
Kwon, Eunsun: 9010, 9030
Kwon, Jenny: 9000
Kwon, Simona C.: 1480
Kwon, Soonhyung: 4190
Kyriakos, Markides: 6210
Löckenhoff, Corinna: 6210, 6690
López-Anuarbe, Mónika: 6310
López-Ortega, Mariana: 3020

L

Lach, Helen W.: 6100
Lachman, Margie E.: 2140, 2430, 6440, 9030
Lachs, Mark: 4090, 6480, 9000
LaCroix, Andrea Z.: 4560, 6020, 9030
LaCroix-Fralish, Michael: 9010
Ladda, Shawn: 3680
Ladd, Ilene G.: 4150
Laditka, James N.: 2050
Laditka, Sarah B.: 2050
LaFave, Sarah: 9010
Laffer, Alex: 6570
Lafronza, Vincent: 6120
Lai, Daniel WL: 4090
Laine, Abby J.: 3850
Lai, Patrick Ho Lam: 9030
Lakadamyali, Melike: 1200
Lalani, Nasreen: 9030
Lalji, Kahir: 2600
Lamba, Shiv: 6420
Lambert, Dawn: 1310
Lamerato, Lois: 9020
Lam, Justin W.: 9000
LaMonte, Michael J.: 9030
Lampe, Nik M.: 2390, 4681, 9030
Lampkin, Cheryl: 3495
Lampraki, Charikleia: 6380, 6580, 6790
Landes, Scott: 2660
Landi, Francesco: 3830
Landman, Bennett A.: 4200
Landreth, Gary: 6220
Lane, Nancy E.: 6200
Lane, Sandi J.: 4820, 6090
Langa, Kenneth M.: 1050, 2270, 3000, 3130, 4690, 6150
Langan, Erica: 3390
Lange-Maia, Brittney: 6650
Lang, Frieder R.: 2480, 2750
Langhi Prata, Larissa: 6840
Lang, Samantha: 9010
Lanham, Holly: 4210
Lanigan, Kerry: 4010, 6130
Lanternier, Paula: 6090
Lapane, Kate: 2640, 6770, 9010, 9020
Lapid, Maria I.: 2160
LaPierre, Tracey: 2720
Laporte-Uribe, Franziska: 3610
Laraia, Barbara: 3070, 3440
Larkey, Linda K.: 9020
Larkina, Marina: 2220, 6050
Larned, Joshua: 3483, 4021, 6740, 9020, 9030
Laryionava, Katsiaryna: 5110
Lassell, Rebecca KF: 2130, 6110
Latham-Mintus, Kenzie: 5080
Lathrop, Laura: 9000
Latimer, Abigail: 3930
Latimer, Nia: 3140
Latkin, Carl: 2690, 6800
Latshaw, Emily: 5090
Lauber, Mckenzie: 6280
Lau, Bobo Hi Po: 4022, 4684, 5084
Lauderdale, diane: 1230
Lau, Jackson: 6641
Laurant, Miranda: 4720
Lavalley, Ryan: 4360
LaValley, Susan A.: 6430
Lavender, Kathryn: 6830
Lavery, Angela: 3610
Lawhorne, Larry: 1060
Lawlor, Brian: 2690
Lawrence, Carly W.: 9030
Law, Royal: 4100
Lawson, Kimberly: 4650
Lawson, Olivia: 6270
Layman, Shannon: 4510
Leach, Carrie: 4590, 6400
Leach, Heather J.: 2280, 3930
Leahy, Suzanne: 6220
Leal, Isabel: 6180
Leaphart, Desirae: 4240
Leavey, Gerry: 2690
LeBlanc, Brian: 4270
LeBlanc, David J.: 3000
LeBrasseur, Nathan: 2241, 3241
Lecchino, Catia: 6570

Lech, Sonia: 6110
Ledbetter, Leila: 9020
Le, Dung D.: 6300
Lee, Alexandra K.: 6110
Lee, Amanda: 3050
Lee, Anna K.: 2380
Lee, Bih-O: 6140
Lee, Cathy C.: 4260
Lee, Chae Man: 3100, 6090, 6510
Lee, Changhan D.: 4001
Lee, Changmin: 6390
Lee, Chiyoung: 9030
Lee, Chung Eun: 9020
Lee, Daehyun: 6450, 6510, 6740
Leedahl, Skye N.: 2350, 3780, 6400
Lee, Deborah: 3450
Lee, Dylan: 6210, 9020
Lee, Edward B.: 6010
Lee, Ellen E.: 9000
Lee, Eun Jeong: 6480
Lee, Gina: 4140, 6810
Lee, Haena: 6240
Lee, Hannah: 5070, 6310
Lee, Harold: 4690
Lee, Hee Y.: 3220, 6100, 6180
Lee, Hye Soo: 6210, 9020
Lee, Hyo Jung: 6620
Lee, Hyunmin: 6500
Lee, Jane S.: 6690
Lee, Janet Lok Chun: 3840
Lee, Jeannie K.: 4190
Lee, Jennifer: 3050
Lee, Jeong Eun: 2200
Lee, Jessica: 6770
Lee, Ji Hyun: 1240
Lee, Ji Yeon: 6320, 6770
Lee, Jihye: 9030
Lee, Jinkook: 2270
Lee, JuHee: 6280
Lee, JuHyeong: 6240
Lee, Jung-Ah: 2070
Lee, Jungjoo: 6170
Lee, Kathy: 6530
Lee, Kelsey: 6010
Lee, Kyung Hee: 6320, 6390, 6770, 9010
Lee, Kyungmi: 6390
Lee, Lewina O.: 2620
Lee, Lewis H.: 3220
Leeman, Jennifer: 3482
Lee, Megan: 9020
Lee, Mi-Jung: 6780
Lee, Minhong: 6120
Lee, Mo-Yee: 4370
Lee, Rain: 6420
Lees Haggerty, Kristin: 4890
Lee, Sang E.: 6160
Lee, Sang Hwa: 9000
Lee, Sanghee R.: 6380
Lee, Sei: 6110
Lee, Seoyeon: 6750
Lee, Shinduk: 6310, 9030
Lee, Soo-Jeong: 2710
Lee, Soomi: 1100, 2620, 4080, 4560, 6200, 6600, 6710
Lee, Sophia: 3260
Lee, Sun Ah: 5060
Lee, Sunghee: 6420
Lee, Sunghoon I.: 9030
Lee, Susan: 4160
Lee, Trevor: 1330
Lee, Yeonjung: 4022, 4250, 4750
Lee, Yeonjung Jane: 3220, 6180
Lee, Yeseul: 9030
Lee, Yookyong: 3290
Lee, Yooumi: 3220
Lee, Youjung: 3740
Lee, Youran: 9020
Leff, Bruce A.: 1160, 5030
Legault, Véronique: 6140
Leger, Kate A.: 2430, 3730
Leggett, Amanda N.: 2420, 3481, 5070, 6310
Lehman, Chloe: 9000
Lehning, Amanda: 4510
Leipzig, Rosanne: 4250
Leis, Aleda: 6650, 9020
Leiser, Scott: 6010
Leist, Anja K.: 6150
Leist, Christine P.: 6350
Leithaus, Merel: 2090
Le, Joie: 3260
Lekhak, Nirmala: 4905, 6170, 6200
Lemaster, Philip: 6490
Lemieux, Mackenzie: 4461
Lenart-Bugla, Marta: 5170
Lenchik, Leon A.: 9030
Lendon, Jessica P.: 6100, 6540
Leng, Iris: 2241
Lenoir, Kristin M.: 4150
Lensing, Shelly Y.: 6360
Lenze, Eric: 3850
Lenzen, Natalie: 4050
Le, Ocean: 6530
Leonard, Natalie: 2420
Leoutsakos, Jeannie-Marie: 4050
Lepore, Michael J.: 6110, 9000
Lera, Lydia: 6320
Lerner, Alan: 6160
LeRoy, Angie: 9010
Lesandrini, Jason: 6760
Leshchyk, Anastasia: 2501, 4810, 6010
Leslie, Kathleen: 6640
Lessem, Rachel N.: 3390, 4270, 4590
L'Etoile, Noelle: 6411
Letts, Elyse: 6560, 6680
Leung, Dara Kiu Yi: 9020
Leung, Kevin: 6100
Levasseur, Melanie: 6140
Leveille, Suzanne: 1280, 4640, 6200, 9010
Levenson, Robert W.: 4681, 4850, 9000
Levi, Jonathan: 9010
Levine, David: 3660
Levine, Hannah: 1550
Levine, Morgan: 1140
Levin, Michael: 6410
Levkoff, Sue E.: 4700
Levy, AnneMarie: 2460
Levy, Becca: 3890, 5110
Levy, Joseph F.: 2580
Levy, Laura: 9000
Levy, Shellie-Anne: 3251
Lewin, Peter A.: 5020
Lewinson, Terri: 2570, 4380
Lewis, Jordan P.: 2310, 3790, 4010, 4710
Lewis, Nathan A.: 2140, 6290, 6580
Lewis, Stuart: 4090
Lewis, Tene: 2080
Leykum, Luci K.: 1330, 4010
L'Heureux, Tanya: 1420
Li, Angela: 6120
Liang, Jiaming: 1500, 2420
Liang, Peichao: 9030
Liang, Xiaohui: 4640
Lian, James: 4022
Liao, Hsiao-Wen: 9010
Liao, Xu: 9030
Liao, Yo-Jen: 2530, 6110, 9000
Libon, David J.: 6411
Li, Chengdong: 4190
Li, Chien-Ching: 6430, 9010
Li, Chin-Shang: 6110, 6280, 6510
Li, Choi Ha: 1500
Lichtenberg, Peter A.: 3251, 3800, 4090, 4230, 4400, 4870, 6290, 6480, 6580, 9030
Lichtenstein, Maya L.: 4150
Li, Cun: 5001, 9010
Liddy, Clare: 1510
Lieberman, Lauren: 2530
Liebman, Michael: 4360
Liebzeit, Daniel J.: 9020
Liechty, Toni: 3270, 6170, 6710
Li, Fanfan: 1130, 6450
Lighthall, Nichole: 2290, 3840
Light, Michael A.: 4380
Lightner, Joey: 9010
Li, Grace: 6210
Li, Guohua: 5171
Ligus, Kaleigh: 9000
Li, Hui-Ling: 6530
Li, Jie (Jenni): 3230
Li, Jing: 5030
Li, Jingyi: 1150
Li, Juan: 1130, 6140, 6450
LI, Junxin: 3330, 4080, 4640, 6600
Li, Kaigang: 5110
Li, Ke: 4240, 6680
Likos, Kelly: 2390
Li, Lun: 4250, 4750, 6530
Li, Lydia: 3010
Lima Passos, Valeria: 5100, 6560
Lim, Aaron: 6580
Lim, Emily: 6810, 9020
Li, Mengchi: 4080, 4640
Li, Meng-Hao: 3200
Li, Mengting: 3860
Li, Mengze: 4810, 6010
Li, Minghui: 3230
Li, Mingyang: 3540
Li, Mirandy: 4060
Lim, Jue Tao: 3390
Lim, Julian: 9030
Lim, Junyub: 6820
Lim, Keith: 9030
Lim, Ki: 4160
Lim, Kia Yee: 3820
Lim, Michelle H.: 6790
Lim, Miranda: 2450
Lim, Nicole: 3620
Lim-Soh, Jeremy: 4170, 6710
Lim, Swee Ho: 3390
Lin Gomez, Scarlett: 3690
Lin, Carol: 5083
Lincoln, Karen D.: 3420, 3890
Lindauer, Allison: 4790, 6080
Lindberg, Brian W.: 1110, 2740, 3120, 3300, 3495, 3960
Lindberg, John E.: 2710
Linden, Anna: 6310, 9000, 9010
Lindenberger, Ulman: 1010, 3070, 4040

Linden-Carmichael, Ashley: 6260
Lind, Kimberly A.: 6430
Lind, Majse: 6300
Lindmark, Ulrika: 9020
Lindquist, Lee: 1030
Lindsey, Ronald W.: 1120
Lin, Frances: 4241
Lin, Frank R.: 2670, 3560, 4050, 4680
Lin, Haiqun F.: 4500
Lin, I-Fen: 1300, 1450, 4070
Lin, Jielu: 1480, 6170
Lin, Joshua K: 6641
Link, Christopher D.: 9010
Links, Paul S.: 4630
Lin, Pay-Shin: 9010
Lin, Rachel R.: 6140
Lin, Ruei-Lung: 6411
Lin-Schweitzer, Anna: 9020
Lin, Shayne S.: 9020
Lin, Shih-Yin: 2130, 3060, 6110
Lin, Sunny: 6540
Lin, Tianze: 4040
Lin, Xin Yao: 9030
Lin, Yan: 3320, 9020
Lin, Yen: 3120
Lin, Yi Chun: 5090
Lin, Yuxin: 6130
Lin, Zhixuan: 6250
Lin, Zhiyong: 4010, 4460, 6750
Lin, Zhuoer: 5010, 6450
Liotta, Madeleine M.: 4240
Liou, Chih-ling: 9010, 9020
Liou, Shyhnan: 2480
Lipnicki, Darren M.: 4340
Lipsitz, Lewis A.: 3482, 3510, 3600, 9030
Lipton, Richard B.: 6290
Li, Qinghua: 4350
Li, Qiwei: 4640
Li, Sha: 4682
Li, Shijie: 9020
Li, Shiyu: 4190
Lithgow, Gordon: 9010
Little, Kristina: 6500
Little, Milta O.: 6450
Litzelman, Kristin: 4240, 4510, 6280
Liu, Chang: 1400
Liu, Chiung-ju: 6740, 6770
Liu, Chun: 2241
Liu, Chunyu: 9020
Liu, Darren: 1070, 6090
Liu, Difei: 5171
Liu, Fangyu: 4120, 4330
Liu, Haiyong: 3100
Liu, Hao (Howe): 6780
Liu, Hui: 1230, 2400
Liu, Huiying: 6530
Liu, Jason Jiashuan: 3483
Liu, Jinyu: 3010, 4240, 6230, 6720
Liu, Kristy: 6780
Liu, Li-Chuan: 6000
Liu, Lili: 6390, 6560, 6680
Liu, Liu: 3670
Liu, Michelle: 9010
Liu, Mingxin: 6140
Liu, Pan: 4290, 4683, 9020
Liu, Pi Ju: 6630
Liu, Pi-Ju: 4900
Liu, Rui: 3290
Liu, Ruotong: 6750, 9030
Liu, Sizhe: 1080
Liu, Tianyin: 3481, 9020
Liu, Wei-Cheng: 6700
Liu, Wen: 2520, 2530, 3450, 3830, 4870, 9000
Liu, Xiaoji: 9030
Liu, Yongmei: 3400
Liu, Xinran: 6130
Liu, Yin: 4560
Liu, Yinghao: 9010
Liu, Yingxu: 9000, 9010
Liu, yongmei: 3400
Liu, Yujun: 6750
Liu, Yunda: 9030
Liu, Yuqi: 9030
Liu, Zhijian: 1130, 6140
Liu, Zhong-xu: 6290
Liu, Ziming: 9000
Liu, Zuyun: 6411
Livermore, Michelle M.: 9020
Li, Wenjun: 2290
Li, Xiaoli: 3540, 6540
Li, Xiaoqian: 6411
Li, Ying: 9020
Li, Yixia: 2510
Li, Yue: 4580, 6140
Li, Yuelin: 9010
Li, Yun: 4290, 4683, 9020
Li, Zhuoying: 6641
Lkhagvasuren, Khorolsuren: 4684
Llaneza, Danielle H.: 4691, 6390
Lobchuk, Michelle M.: 1420
Lockery, Jessica: 5083
Lock, Sarah L.: 1430
Loeb, Susan: 6700
Loerzel, Victoria: 2290, 3840
Loewenstein, David: 4090
Loganathar, Priya: 9000
Logan, Paige E.: 3570
Logsdon, Rebecca: 3000
Logue Cook, Rachel N.: 9000
Logue, Melissa: 6740
Loh, Feng-Hua: 9010
Lohman, Kurt: 3400
Lohman, Matthew: 6320
Loika, Yury: 6010
Loiko, Elena: 6010
Lokanathan, Tharshini: 2290, 3840
Lomeli, Sindy: 3390, 6120
Longo, Valter: 2180
Lo, On-Yee: 3482
Lopes Deliveira, Thais: 3590
Lopes, Maria: 2620
Lopez Mendez, Mauricio: 6360
Lopez Paz, Cinthie: 6060
Lopez, Acacia: 3740, 6520, 6580
Lopez, German: 9030
Lopez, Maria J.: 4500
Lopez, Noelle: 3190
Lord, Justin: 3660, 4820
Lorenz, Karl: 6430, 6530
Lorenzo, Erica C.: 9010
Lorenz, Rebecca A.: 6100, 6510
Lorraine, Mion C.: 5090
Losinski, Genna: 6570
Lou, Vivian Wei Qun: 3840
Louras, Peter: 6010
Lou, Vivian Wei Qun: 3840, 4090, 6530
Louwagie, Katie W.: 4220, 6070
Lou, Yifan: 3010, 4240, 6720
Love, Catherine: 2410, 3770
Lowers, Jane: 4530
Lowther, Johnna: 1420
Lox, Dennis M.: 6641
Lucas, Kaitlyn: 6410
Lucchini, Roberto: 1350
Lu, Chi-pang: 4450
Luchsinger, Jose A.: 2160
Luciano, Michelle: 6450
Lücke, Anna Jori: 1020
Łuc, Mateusz: 5170
Ludlow, Jared A.: 6510
Luezas-Shamakian, Lorella: 4100
Luft, Benjamin: 4700
Lui, Kitty K.: 9000
Lui, Li-Yung: 4120
Lujan, Irene: 3460
Lu, Kaiwei: 6320, 6780
Lu, Kevin: 3230
Luk, James Ka Hay: 4022, 4684, 5084
Lukkahatai, Nada: 3330, 4080
Lum, Hillary: 4280, 6210, 6420, 9000
Lum, Terry Y.: 3481, 9020, 9030
Lundgren, Dan: 6760
Lund, Jennifer L: 4320
Lunetta, Kathryn L.: 3400, 6450, 6740
Lun, Janetta: 1220
Luo, Hao: 6110
Luo, Huabin: 3870
Luo, Jiangtao: 3540
Luo, Jing: 2620, 4620
Luo, Liying: 6610
Luo, Minxia: 1020, 2450
Luong, Gloria: 2280, 2470
Luo, Yan: 6100, 6180
Luo, Ye: 2080
Lu, Peiyi: 3500, 9010, 9030
Lu, Shiyu: 9030
Lustgarten, Michael: 4810
Lu, Yi: 6210
Lu, Yvonne Y.: 9020
Lu, Zhaohui: 6100
Lydon, Elizabeth A.: 6290, 6300, 6390
Lyketsos, Constantine G.: 4050
Lyles, Courtney: 6270
Ly, Monica: 9000
Lynch, David H.: 2080, 4640, 6780
Lynch, Delanie: 9000
Lynch, Kristine E.: 6320
Lynch, Morgan E.: 6290
Lynott, Jana: 3100
Lyons, Amy: 6770
Lyons, Karen S.: 3240, 6520
Ly, Timothy K.: 9020
Lytle, Megan N.: 9020
Márquez, Freddie: 9000
Mäki-Petäjä-Leinonen, Anna: 2460

**M**

Macarenko, Yulia: 9030
MacCarthy, Daniel: 6320
MacCormack, Jennifer K.: 4570
Macdonald, Birthe: 1020
MacDonald, Catherine: 9000
MacDonald, Stuart: 6590
MacGregor, Tara: 6270
Ma, Chenjuan: 3950, 4370
Mack, Deborah: 6770, 9010
Mackenstadt, Darby: 6790
Mackenzie, Corey: 6790
Mackiewicz, Marissa K.: 6000, 6320
Mack, Wendy: 9020

Mack, William: 9020
Maclagan, Laura: 9000
MacLean, Alyssa: 6440
MacLean, Maria C.: 6250, 6640
MacNeil, Amanda M.: 9000
MacNeil, Andie: 4090, 4225, 6200, 6480
Madarasmi, Saira: 4190
Madden, Kenneth M.: 3150, 6580
Madera, Sonia: 6420
Madero, Erica: 6510
Madison, Annelise A.: 4470
Madison, Barqueisha: 6760
Madrid Fuentes, Diana A.: 9000
Maeda, Harumi: 4470
Maercker, Andreas: 5100
Ma, Flora: 2030, 3420
Magana, Maribel: 3750
Magee, Catherine: 6280
Mage, Susanna M.: 4510, 6310
Magnuson, Justin: 1110
Mahmood, Atiya N.: 6500
Mahmoudi, Elham: 3130, 4040, 6060
Mahon, Elizabeth D.: 6440
Mahoney, Martin C.: 6430
Maierhofer, Roberta: 4670
Mailhot, Tanya: 3720
Main, Eve: 9030
Mair, Christine A.: 4690, 4860
Majid, Marjan: 4050
Makaroun, Lena K.: 4900, 9000
Makhnevich, Alex: 4700
Mak, Wingyun: 6100, 9010
Malani, Anurag N.: 9020
Malani, Preeti: 3495
Malani, Revati N.: 9010
Malatyali, Ayse: 4500
Malhotra, Atul: 9000
Malhotra, Rahul: 2150, 3160, 4170, 6710
Ma, Lina: 4290, 4683, 9020
Maliski, Sally L.: 6120
Mall, Neshay: 9030
Malone, Michael: 6280
Malone, Susan K.: 1130
Ma, Luyan: 6170
Maly, Monica: 3760
Ma, Melissa: 9000
Mamo, Sara K.: 6270
Manalel, Jasmine: 1290, 3760
Manchella, Mohit K.: 3570, 4860, 6820
Mandelblatt, Jeanne S.: 1100, 3400
Mandile, Noah: 9030
Manella, Kathleen J.: 2060
Manggaard, Jennifer: 6110
Manheim, Chelsea: 2660, 2720
Manietta, Christina: 3090
Manini, Todd M.: 2170, 4840, 6320, 6510
Manisha, Sengupta: 6100, 6540
Manja, Veena: 6530
Manjourides, Justin: 5084
Manly, Jennifer: 4470
Mann, Frank D.: 3510, 6440
Manning, Carol: 1030, 9010
Manning, Kenneth: 5050
Manning, Lydia k.: 6580
Mann, Jim: 4860, 9000, 9010
Mann-Krzisnik, Dylan: 3630
Manoharan, Sneha: 6390
Manor, Brad: 3482, 3510, 9010, 9030
Manrique Espinoza, Betty Soledad: 4290
Mansion, Natalie: 3670
Mansoor, Marrium: 6440
Mao, Hui-Fen: 6350
Mao, Weiyu: 1540, 3870
Mao, Ziling: 3830
Marano, Thomas: 6100
Marashi, Sheida: 2460
Marasinghe, Himasara: 6150
Maras, Jessica: 2430
Marcano Ocasio, Claudia Beatriz: 3483
Marcano, Claudia: 4021
Marceau, Kristine: 6790
Marchal, Noah: 4021
Marches, Radu: 3001
Marcinek, David: 9010
Marconi, Vincent C.: 9010
Marcus, Michael S.: 4880
Mardini, Mamoun T.: 4840, 6320, 6510
Margalef, Carole: 9010
Margheim, Lynn: 5171
Margolis, Rachel: 4070, 6300, 6310, 9020
Margrett, Jennifer: 9020
Mariani, Diane: 2560
Mariani, Jean: 9010
Marilyn, Rantz: 1460, 4021
Marinez, Julia: 3010
Marini, Christina M.: 2660, 3480, 6170, 6210, 9030
Marino, Francesca: 4330, 4600
Marino, Victoria R.: 2620
Markert, Dana: 1170
Markides, Kyriakos S.: 3240, 6300
Markids, Kyriakos: 3020
Markkanen, Pia K.: 2710
Markus, Marcello: 1010
Markwardt, Sheila: 6740
Marmo, Suzanne: 3290
Marnfeldt, Kelly A.: 3010, 9000
Marottoli, Richard: 5100, 5172
Marquez, Carlos: 6320
Marquez, David X.: 4800, 6570
Marquez, Eladio: 3001
Marroig, Alejandra: 9020
Marrone, Nicole: 3560
Marroquin-Serrano, Madeleine: 6000
Marrs, Sarah: 2260, 9000
Marsack-Topolewski, Christina: 3860, 9010
Marshall, Ami: 5172
Marshall, Gillian L.: 6180
Marshall, Lynn M.: 6320
Marshall, Shawn: 6170
Marshall, Victoria: 6020
Marsh, Courtney A.: 6120
Martens, PhD, Nancy L.: 6420
Martí Haidar, Yamile M.: 2700
Martin, Damali N.: 3430
Martin, Diane J.: 1370, 3140, 9000
Martin, Dominique E.: 9010
Martin, Emily T.: 9020
Martinez Lenis, Vanessa: 6250
Martinez-Amezcua, Pablo: 4050
Martinez, Beatrice: 6770
Martinez, Iveris L.: 5084
Martinez, Sujey: 3750
Martin, Mike: 2450, 6380
Martino, Alexa: 1240
Martin, Peter: 3580, 4022, 4140, 4684, 5084, 6810
Martin, Rolf J.: 9010
Martins-Klein, Bruna: 6210, 6411
Martire, Lynn: 6210, 6250
Martorella, Geraldine: 4490
Marx, Katherine: 3310
Marziliano, Allison: 4700
Masaki, Kamal H.: 3580
Maslow, Katie: 4150
Mason, Aleigha: 1530
Mason, Lisa Reyes: 6500
Mason, Tyler: 6580
Masoud, Sara: 2420
Massa, Fernando F.: 9020
Masse, Sybil: 1160, 3660, 6610
Massihzadegan, Setarreh: 6700
Massimo, Lauren M.: 1270, 3680, 3930, 5030, 6280
Mast, Benjamin T.: 3251, 6290, 9010
Ma, Stefan: 9020
Mastel-Smith, Beth: 9010
Masternak, Michal: 9000, 9010
Masterova, Kseniya S.: 6740
Mastrandrea, Carmelo: 4120, 6780
Mathavan, Nandini: 6590
Mathis, Kenneth: 4490
Ma, Tianzhou: 2300
Matos-Moreno, Amilcar: 4905, 6790
Matsumoto, Yoshiko: 4470
Matsushita, Kunihiro: 4330
Matta, Sonali: 5173
Matta, Tara D.: 3360
Mattek, Nora: 4790
Matteson, Tyler J.: 3190
Matthews, Alicia K.: 6430
Matthews, Karen A.: 3070
Mattos, Meghan: 9010, 9020
Matusovsky, Bri: 2690
Mauldin, Rebecca: 9030
Maung, Kenwin: 6330
Maurer, Jürgen: 2680, 3720, 4480, 6550, 6730
Mauss, Iris B.: 2620
Mau, Theresa: 3340, 9010, 9020
Maxfield, Molly: 9000, 9020
Maxi, Andrea: 6420
Maxwell, Cathy A.: 1180, 3450
Maxwell, Christopher D.: 1390
Maxwell, Colleen: 9000
Mayeda, Elizabeth R.: 3510, 3600
Ma, Ying: 6500
May, Jennifer: 9000, 9020
Maynard, Madison H.: 9030
May, Peter: 3200
Ma, Yu: 6100
Mazan-Mamczarz, Krystyna: 1470
Mazzola, Paolo: 5010

Mbao, Mbita: 6130
McAdams-DeMarco, Mara: 4680
McAlister, Jennifer: 6280
McAndrews, Carolyn: 5172, 9020
McAtee, Robin: 6000, 6130, 6210
McAuley, Edward: 9010
McBride, A. Camille: 6770
McCabe, Donna: 2130
Mccaffery, Jeanne: 3400
McCain, Dextiny: 2380
McCall, Stephen: 2710
McCann, Brandy Renee: 9020
McCarthy, Catherine: 9020
McCarthy, Ellen: 9000, 9020
McCarthy, Michael J.: 9030
McCauley, Brenna: 1470
McClain-Moss, Lisa: 6320
McClean Cooke, Wendy: 4030
McCleary, Lynn: 1420
McClellan, Angela: 5173
McClintock, Martha K.: 1230, 6110
McCloskey, Rose: 3555
McClure, Erin A.: 9000
McClure, Leslie: 4870
McConnell, Eleanor S.: 1130, 6110, 6280, 6450, 9010, 9020, 9030
McConnell, William R.: 4860
McCray, Rachel: 6680
McCulloch, Charles E.: 6320
McCurry, Darcy S.: 4150
McDaniel, Jodi: 5090
McDaniel, Kennedy A.: 4680
McDarby, Meghan: 2030, 6730, 9010, 9030
McDowell, Cynthia: 6590
McDuffie, Danielle L.: 1490
McEniry, Mary: 4225, 6200
McFarland, Lisa: 9020
McGaffigan, Erin: 4270, 6120
McGarry, Brian E.: 6140
McGee, Jocelyn S.: 5172, 5173, 6650
McGill, Cassandra: 9000
McGill-Scarlett, Cordy: 4681
McGilton, Katherine: 3170, 6270
McGinn, Michael: 6790
McGovern, Ian: 6100
McGowan, Deric: 4200
McGrew, Kathryn: 4430
McGuire, Lisa C.: 1560, 2490, 3120, 3510, 4120, 5010, 6060, 6280
McGuire, Patricia: 3930
McHugh, John P.: 3130, 3482
McHugh, Molly: 9000
McInnes, Keith: 9010
McIntyre, Alissa C.: 6100
McKay, Tara: 1320
McKelvey, Karma: 5083
McKenzie, Brennen: 9010
McKibbin, Christine: 3460
McKiddy, Sarah: 1150
McKinney, Brandee: 6410
McLaren, Jaye: 1040
McLaughlin, Jessica K.: 2720
McLaughlin, Sara: 9010
McLennan, Lauren K.: 6680
McLeod, Don: 4720
McLeskey, Nanci K.: 2060
McMahan, Christopher: 4500
McManus, Benjamin: 3000
McMillan, Corey T.: 6010, 6140
McMillan, Ryan: 5091
McMurray, Josephine J.: 2460
McNair, Bryan: 6280
McNally, James: 6830
McNamara, Kelsey C.: 6790, 9010
McNamara, Maeve: 1140
McNeil, John J.: 4320, 4683, 5083
McNicoll, Lynn: 9030
McPhee, Patrick: 2500
McPherson, Rachel: 6170
McPhillips, Emily: 9020
McPhillips, Miranda: 3310, 4080, 6300, 6600
McQuade, Laura: 3380
McQuaid, Tina A.: 6130
McQuaige, Mary: 5171
McQuarrie, Angelica: 3620
McRae, Thomas D.: 3830
McSpadden, E.J.: 9020
McSweeney-Feld, Mary H.: 9000
Meador, Lauren: 6470
Medcalf, Robyn: 9030
Medina, Luis D.: 1440, 6060
Meeks, Huong: 4530
Meeks, Suzanne: 2330, 3910, 9010
Meer, Margarita: 1140
Mehegan, Laura: 1430, 6150
Mehling, Wolf: 3050
Mehrabi, Fereshteh: 2580
Mehranfard, Danial: 6320
Mehta, Jade S.: 6590
Mehta, Neil: 4905, 6210, 6330, 6790
Mehta, Ranju: 6750
Mehta, Rhea: 6780
Mei, Donghui: 9010
Meier, Clément: 2680, 3720, 6550, 6730
Meier, Helen: 3430, 6010
Meier, Tabea: 3150, 4570
Meijers, Judith: 1160, 3555, 4720, 6270, 9010
Meinertz, Naomi R.: 2440
Meinow, Bettina: 6200
Mejía, Shannon T.: 1240, 2050, 2220, 3490, 9020, 9030
Mejia Arango, Silvia: 4225, 6300
Melendez, Robert: 2570
Melgar Castillo, Andrea I.: 4220
Melis, Rene: 5170
Mellencamp, Kagan A.: 1300
Melters, Daniel: 1200
Melton, Colleen C.: 6500
Meltzer, Gabriella Y.: 4110, 6090
Melvin, Tonisha: 3330
Mendenhall, Alexander R.: 3810
Mendes de Leon, Carlos: 4905, 6790
Mendez, Jennifer C.: 6220
Mendez, Nicole: 6720
Mendiratta, Priya: 6180
Mendoza Ruvalcaba, Neyda Ma.: 6290, 6380
Mendoza, Lisandra: 6140
Mendoza, Mayra: 2420
Mendoza, Nancy: 3740
Menec, Verena: 6790
Mengel-From, Jonas: 4810, 6411
Menges, Carolyn: 6420
Meng, Hongdao: 3540, 3910, 4650, 6070, 6290
Menne, Heather: 4770
Menon, Shreya: 6460
Meraz, Rebecca: 5173, 6650
Merchant, Anwar: 6320
Mercury, Monica: 2410
Merdjanoff, Alexis A.: 6090
Merianos, Ashley L.: 2250, 6710
Merickel, Jennifer: 6420
Merkin, Sharon Stein: 6440
Mernitz, Sara E.: 6190
Merrilees, Jennifer: 4681, 4850
Merten, Natascha: 2670
Meszaros, Katherine: 9000
Metcalf, Emily E.: 1040, 6420
Metcalf, Judith: 3540
Methot, Stephen: 1200
Metzelthin, Silke F.: 6270
Meyer, Anthon: 6270
Meyer, Cassie: 5083
Meyer, Kayla: 6570
Meyer, Kylie: 2070, 2420, 4510, 4790
Meyer, Michelle L.: 9030
Meyers, Jeremy: 6840
Meyer, Sonya: 9020
Mezuk, Briana: 1160, 4630, 5060
Miao, Hongyu: 4490
Michael, Cherish: 6250, 6470
Michael, Esposito: 1050
Michalek, Joel: 6320
Michelet, Mona: 1190, 6800
Mickle, Angela: 2300
Mielenz, Thelma: 5171
Mielke, Michelle: 6450, 6780
Migliaccio, John N.: 4880
Migneault, Deborah: 1310
Mignot, Emmanuel: 4560
Migovich, Miroslava: 5090
Miguel-Cruz, Antonio: 6390, 6560
Mikton, Christopher: 9000
Milani, Sadaf A.: 6710
Miles, Toni: 4460
Milidonis, Mary: 6420
Milisen, Koen: 6560
Miljkovic, Iva: 4340, 6450
Millar, Roberto J.: 1250
Millenbah, Ashley N.: 6070
Miller, III, Edgar R.: 4680
Miller, Carrie A.: 6650
Miller, Edward A.: 1260, 2040, 3320, 6620
Miller, Emika S.: 3360, 4650, 5090
Miller, Erin: 6220
Miller, Hillary: 6010
Miller, James W.: 2280
Miller, Julie: 3450, 4090, 6310
Miller, Katherine: 6110
Miller, Luke R.: 6830
Miller, Lyndsey: 4790, 6080, 6520

Miller, Matthew: 3050
Miller, Maura: 6000
Miller, Michael E.: 2530
Miller, Sally M.: 3450
Miller, Sara: 6260
Miller, Sarah J.: 9010
Miller, Vivian J.: 6090
Mills, Carol: 6760
Mills, Sarah: 5060
Millstein, Joshua: 3510
Milnamow, Mary: 6390, 9030
Mimbs, Hope: 6250
Minard, Charles G.: 2241
Minch, Kendra: 9010
Min, Deborah: 3330
Mindlis, Irina: 3115
Mingo, Chivon A.: 4555, 6180
Min, Judy: 1320
Min, Jungkyung: 6650
Minor, Lisa: 6360
Mintus, Kenzie: 3690
Mintzer, Jacobo': 2245
Minyo, Morgan J.: 9020
Miranda, Fabio: 3880
Mirza, Noeman: 4020
Mirza, Raza: 6230, 6480, 6690
Mitchell, Barbara: 6530
Mitchell, Brendan A.: 4200
Mitchell, Jamie: 6180
Mitchell, Kimberly M.: 1250
Mitchell, Lauren L.: 4220
Mitchell, Susan: 1530
Mitin, Natalia: 2241
Mitteldorf, Josh: 5082
Mitzner, Tracy L.: 4160
Miwa, Hiroyasu: 6390, 6400
Miyawaki, Christina E.: 6530
Mizuki, Maiko: 6240
Mlinac, Michelle E.: 2030
Mobley, James: 2501
Mock, Colleen: 6550
Mockler, David: 3200
Moeller, Stacey: 6060
Moen, Phyllis: 6800
Moermans, Vincent R.: 6560
Moffat, Scott D.: 6710
Moffit, Reagan: 4600
Mogle, Jacqueline: 2430, 2470, 2530, 3520, 3730, 6260, 6290
Mohr, David C.: 9030
Mois, George: 1570, 3410, 6000, 6390
Molina, Anthony J.: 4461, 6010, 9010
Molinari, Victor: 6540
Molinsky, Jennifer: 3900, 4380
Moll, Allison C.: 6440
Molnar, Lisa J.: 3000, 6470
Molony, Sheila L.: 4681
Monaghan-Geernaert, Pamela: 2210
Monahan, Patrick O.: 6280
Monaro, Sue R.: 2090, 6280
Monheit, Alan C.: 1260
Monin, Joan K.: 3360, 4850, 9000
Monroe-Lord, Lillie: 6210
Monroe, Todd B.: 3360, 4650
Montano, Anna Rae: 4770
Montano, Monty: 6641
Montelpare, William: 6250
Montepare, Joann M.: 1070, 2350, 3420, 3800, 6000
Montesano, Rachel: 4681
Montgomery, Lucas: 5080
Monti, Stefano: 2501
Montminy, Joe: 4681
Montoro-Rodriguez, Julian: 6750
Montoya, Ana: 9010
Moo, Lauren R.: 1040, 6420
Moone, Rajean: 1320, 2060, 6360
Moonesinghe, Dilhara: 9030
Mooney, Kathi: 1320, 2540
Moon, Heehyul: 4420
Moore, Alison M.: 9000
Moore, Ann Z.: 4200
Moore, Carley: 6230, 6690
Moore, David J.: 3760, 9020
Moored, Kyle D.: 3690, 3750, 4600
Moore, Jade T.: 6360
Moore, Miranda: 1170
Moore, Raeanne C.: 2450
Moore, Reese: 2340
Moorman Li, Robin: 9030
Moorman, Sara M.: 2400, 3570
Moppett, Iain: 9030
Moradoghli, Fereshteh: 3620
Morales Mejia, Yenifer L.: 6050, 6410
Morales, Knashawn H.: 6180
Moray, Juno: 6480
Moreland, Briana: 4100
Morelli, Alexandra: 2350
Moreno, Monica: 4010, 6130, 9030
Moreno, Oscar: 6140
Morey, Miriam C.: 4260, 5050, 6170
Morgan, Amanda: 6300
Morgan, Brianna: 1440, 3680, 3930, 4610, 9000, 9030
Morgan, Damian: 2460
Morgan, Gail D.: 6750
Morgan, Jennifer C.: 6760
Morgan, Kerri: 2050
Morgan, Matthew R.: 2700
Morissette, Lee F.: 4390
Morlett Paredes, Alejandra: 9000
Morley, John: 3600
Moro, Tatiana: 6740
Moro, Teresa: 2560
Morrill, Kristin A.: 6430
Morris, Brian J.: 3580
Morris, Emily P.: 1240
Morris, Kevin: 9020
Morris, Michael: 6420
Morrison, Sean: 4460, 5030
Morrow-Howell, Nancy: 3800, 9010
Morse, Janice M.: 1120
Mortensen, W Ben: 6500
Mortman, Nadav J.: 6641
Morton, Patricia M.: 5060
Mor, Vincent: 4780
Moskowitz, Judy: 2300
Mosley, Thomas: 2670, 3110, 3560, 6780
Mosqueda, Laura: 5100, 6580
Moss, Karen O.: 3360, 4650, 5090
Mossman, Audrey: 4680
Mota, Miriam: 6450
Motel-Klingebiel, Andreas: 2410
Moulder, Robert Glenn: 1020
Mouton, Charles: 5100
Mo, Vennisia: 6170, 9020
Moxley, Jerad: 3410, 9030
Moye, Jennifer: 2030, 2660, 3850, 6220
Moyo, Patience: 4580
Mpofu, Elias: 1090, 6110, 9020
Mroczek, Daniel K.: 2140, 2620, 4620
Mroz, Emily L.: 1490, 3360, 6340
Mroz, Tracy: 6260
Muñoz, Elizabeth: 3890, 4730, 6180, 9000
Muñoz-Lobato, Fernando: 6411
Mu, Christina X.: 4080, 4560, 6200, 6600
Mudar, Raksha A.: 3410, 6290, 6300, 6390
Mueller, Christine: 3170, 1060, 1460
Mueller, Kimberly D.: 2160
Mueser, Kim: 6170
Mui, Ada C.: 6500
Mukhi, Priya: 6310
Mukhtar, Yerkebulan: 4684
Mulcahy, John: 5130, 6540
Mulhorn, Kristine A.: 2260, 2680, 6220
Mullen, Nadia: 6170
Mullholand, Mary Ellen: 6390
Mullin, Donncha S.: 6450
Mullins, C. Daniel: 4220
Mullins, Lindsay: 6770, 9000
Mumma, Kara T.: 4160, 9030
Mundadan, Reanne G.: 6460
Mundy, Lindsey M.: 1240, 9020
Mungas, Dan M.: 3510, 3600
Muniz Terrera, Graciela: 2140, 3510, 5084, 6440, 6450, 9020
Munkácsy, Erin: 2501
Mun, Kyung-Ryoul: 9020
Munly, Kelly A.: 2050, 9000
Munoz, Richard A.: 1330
Munsterman, Ellen: 9000
Murabito, Joanne M.: 3400, 6450, 6740, 9020
Muramatsu, Naoko: 3890
Murata, Chiyoe: 6250
Murayama, Hiroshi: 6280, 6800
Murff, Harvey: 6740
Murillo, Fernando: 5120
Murillo, Rosenda: 2170
Murphey, Yi Lu: 4700
Murphy-Boucher, Michele: 9030
Murphy, Dallas J.: 6780, 9010
Murphy, Ian E.: 2010
Murphy, James D.: 6200
Murphy, Kyle A.: 4160
Murphy, Sara: 1250
Murphy, Terrence E.: 3110
Murray, Anne: 5083
Murray, Briana: 9000
Murray, Laura: 6160, 6460
Murray, Sarah: 6100
Musallam, Noura: 6210

Musey, Paul: 9010
Musich, Shirley: 6170
Musil, Carol M.: 3115, 4060, 5140, 6090, 6820
Musi, Nicolas: 6020, 6320
Musolino, Teresa: 4023
Mussa, Kadija: 9030
Muss, Hyman: 6420
Mustafa, Muhammad Ibrar: 6690
Mutambudzi, Miriam: 6300
Mutchler, Jan: 6090, 6700
Mutoh, Tatsushi: 9000
Muto, Yoko: 6280
Mutter, Justin: 9020
Mwendwa, Denee: 6170
Myagmarjav, Sugarmaa: 4684
Myck, Michal: 2410
Myers, Dennis: 5173
Myers, Dillon: 6390
Myers, Evan R.: 9030
Myers, Jennifer: 6450, 6510
Myers, John: 1130
Myers, Julie: 1060
Myers, Sara: 6740

**N**

Na, Annalisa: 1120, 4870
Nabe-Nielsen, Kirsten: 6200
Nabors, Emily: 3900, 6230
Nadash, Pamela: 1260, 2040, 3320, 3940, 4130
Nadorff, Danielle K.: 2040, 3740, 6520, 6580
Naganathan, Vasi: 2090, 6280
Nagel, Corey L.: 6340, 6450, 6740
Nagele, Robert G.: 6411
Naglie, Gary: 6170
Nagwani, Amit Kumar: 9010
Nahabedian, Anissa: 6350
Nahm, Eun-Shim: 5171
Nah, Suyoung: 6210, 6250
Naik, Aanand D.: 4683, 6320
Naioti, Eric: 6320
Najafzadeh, Mehdi: 6641
Najarian, Cyrus: 3130, 6060
Naka, Alexander: 9010
Nakagawa, Kazuma: 3580
Nakagawa, Takeshi: 6210, 6250, 6760
Nakamoto, Isuzu: 6280
Nakamura, Masahide: 9000
Nakanishi, Yasuhiro: 2580
Nakase, Taizen: 9000
Nakashima, Taeko: 6120
Nakayama, Riko: 6240
Nakazawa, Atsushi: 6000
Nalawade, Vinit: 1390
Nam, Youngwon: 6520
Naranjo Rincon, Samuel D.: 6290
Narayanan, Nandakumar: 9020
Narine, Ameesha: 4555, 6380
Narine, Donnette: 4555, 6130, 6670, 6710
Narita, Miki: 6320
Naruse, Masatoshi: 9010
Nasar Kabir, Zarina: 6760
Nassauer, Joan I.: 6490
Natale, Ginny: 5173
Nathanielsz, Peter: 5001, 9010
Nathan, Kobi: 6270
Naughton, Michelle: 2200
Navarrete, Viridiana: 9010
Nawaz, Huma: 6320
Naylor, Mary: 6200, 9000
Nazroo, James: 9020
Nderitu, Sara: 3160
Nearing, Kathryn A.: 4260, 6420
Nease, Donald: 9000
Nebenzahl, Paul: 6540
Nedlund, Ann-Charlotte: 2460
Neelamegam, Malinee: 3550
Neff, Donna F.: 6140
Nehrkorn-Bailey, Abigail M.: 2040, 5110
Nekolaichuk, Erica: 4090
Neller, Sarah: 2310, 9000
Nelligan, Labrini: 6270
Nelson, Christi L.: 4730
Nelson, Christi: 4540
Nelson, Christopher: 6010
Nelson, Ehlana: 3040
Nelson, Eve-Lynn: 6650
Nelson, H. Wayne: 9000
Nelson, Ian M.: 5081
Nelson, James: 9010
Nelson, Katie: 3330, 6140
Nelson, Mark R.: 4683, 6320
Nelson, Matt: 1530, 3180
Nelson, Monica E.: 6600
Nelson, Niccole A.: 4555, 6380
Nelson, Sarah: 6270
Nelson, Toben F.: 6510
Nemati, Donya: 3630, 6080
Nemeth, Samuel: 6810
Neppl, Ronald: 5001
Nesser, Whitney: 9020
Netter, Molly: 1060
Netto, Jair: 6840
Netzer, Giora: 6350
Neugroschl, Judith: 5100
Neumann, Johannes: 4683
Neupert, Shevaun D.: 1290, 2140, 2470, 2750, 3490, 6710
Newman, Anne: 3340, 3400, 3830, 4120, 4340, 4410, 4683, 4810, 9010, 9020, 9030
Newman-Casey, Paula Anne: 9000
Newman, David N.: 4220
Newman, Jasmine: 3520
Newman, John C.: 4410
Newmyer, Lauren: 4070
Newsham, Tina: 4520, 6350, 9030
Newsom, Jason T.: 6340, 6450, 6740
Newton, Nicky: 3480
Ng, Crystal: 1100, 1450, 3730
Ng, Derek K.: 2270
Ng, Jenny Hong Yu: 6840
Ng, Kwun Kei: 6411
Ng, Michelle: 4570
Ngo, Van: 9000
Ngo, Victoria: 1330, 6310
Ng, Ted Kheng Siang: 2050, 3115
Ng, Tze Pin: 3450, 4023
Nguyen, An T.: 3750
Nguyen, Andrew H.: 1040
Nguyen, Annie: 4070, 6580
Nguyen, Duy: 3290
Nguyen, Giang: 3770
Nguyen, Uyen-Sa: 2080
Ng, Weiwen: 6540
Ng, Yan Er: 3241
Ng, Zoe Ziyi: 3250, 6210
Nichenko, Anna S.: 5091
Nicholas, Jennifer S.: 4190
Nichols, Amy: 4021
Nichols, Emily: 9020
Nichols, Emma: 2270
Nichols, Eunice Lin: 1080
Nichols, Madeline J.: 6520, 6580
Nicholson, Jody: 9000
Nicklas, Barbara J.: 2241, 4600, 6641
Nicolay, Sarah: 3530
Nicosia, Francesca M.: 3050
Nicot-Cartsonis, Mathilda: 9010
Nicotera, Nicole: 4430
Niedernhofer, Laura: 2501, 6840
Nielsen, Lisbeth: 3080
Nielsen, Natalia: 9020
Nieman, Carrie L.: 3560, 4050, 4680
Nijhuis-van der Sanden, Ria: 1120
Nikitin, Jana: 2480, 2750
Nikolich-Žugich, Janko: 6010
Nikpour, Jacqueline A.: 1530
Nilsen, Charlotta: 3440, 6200, 6450
Nimrod, Galit: 6390
Ninteau, Kacy: 6540
Nishioka, Yuichi: 2580
Nishizuka, Chiharu: 6220
Nitanai, Ryoichi: 6240
Nix, Caitlyn A.: 4021, 9000
Niznik, Joshua: 9030
Nketsiah, Ebow T.: 9000
Nkimbeng, Manka: 1050, 1480, 3310, 6540
Nkwata, Allan K.: 1410, 4700
Noblitt, Jessica A.: 6190, 6250, 6520
Noblitt, Paul: 6650
Noda, Tatsuya: 2580
Noel, Polly H.: 1330, 4010
Noel, Sabrina E.: 2610
Nofuji, Yu: 6320, 6710
Noguchi, Taiji: 6210, 6250, 6760
Noh, Hyunjin: 3220
Noh, Ji Yeon: 6010
Nohria, Raman: 1540
Nolan, Timiya: 2200
Nolte, Julia: 6520
Noppert, Grace A.: 6050
Norbeck, Cassandra S.: 9010
Nordheim, Johanna: 6110
Noriega de la Colina, Adrian: 3630, 6740
Norris, Haley: 1320
Norris, Madison: 9030
Norris, Nia C.: 6750
Norton, Edward: 3130
Norton, Peter G.: 4210, 4720
Nouryan, Chris: 9000, 9010
Novak, Joshua: 4850, 6410
Nowaczynski, Aliya: 4090
Nowakowski, Alexandra C.: 4681
Nowels, Molly: 1310
Numata, Hanako: 4440
Nuno, Miriam: 6110
Nuspl, Megan: 6270
Nwakasi, Candidus: 5171, 6430

Nwanaji-Enwerem, Ugoji: 9000
Nyamathi, Adey: 2070
Nyberg, Lars: 3590
Nygaard, Marianne: 6010
Nygard, Louise: 2460
Nyman, Rosita: 6640
Nyquist, Linda: 6470
Nyrop, Kirsten: 6420
Nyrup, Rune: 6640
O'Connor, Melissa: 6100, 9030
O'Donnell, Lauren: 9000

**O**

O'Barr, Gregory W.: 3460
Öberg, Peter: 1300
Obiagwu, Joseph: 4560
Obomsawin, Anik: 4490
O'Brien, Catherine: 9010
O'Brien, Christopher T.: 6640
O'Brien, Emma M.: 6280
O'Brien, Erica: 2470
O'Brien, Kelly M.: 2490
Occean, James: 1200
Ocegueda, Omar: 6010
Ochoa-Lopez, Andrea P.: 1440
O'Connell, Denise: 6270
O'Connell, Megan E.: 9000
O'Connell, Tom: 5001
O'Connor, Daniel: 6210
O'Connor, Deborah: 4860
O'Connor, Maureen K.: 1040
O'Connor, Melissa L.: 6780
O'Conor, Rachel: 1030, 4681
Oczkowska, Monika: 2410
Odden, Michelle: 1000, 3400
Odd, Kaleena: 2140
O'Dea, Eireann: 4860, 6500
Odgen, Lydia: 2050
Odom, Erica L.: 4120
Odom, Teresa L.: 6360
O'Donnell, Arden E.: 4430, 6730
O'Donnell, Jerry: 9020
O'Donoghue, Jennifer: 9010
Ofori, Edward: 9020
Ofotokun, Ighovwerha: 3760
Ogawa, Elisa: 5050
Ogawa, Kenichi: 6450
Ogbuoji, Osondu: 9030
Ogino, Ryogo: 6240
Ogle, Destiny: 6520
Ogletree, Aaron M.: 2580
O'Grady, Thomas: 6090
Ogunjesa, Babatope: 6530
Ogunrinde, Joyce O.: 6300
O'Hara, Courtney: 9000
O'Hare Kelly, Erin: 6420
Oh, Esther S.: 4050
Oh, Gahee: 9020
Oh, Janet: 1080
Oh, Jeewon: 1240, 2280
Oh, Minsuk: 6510, 6650
Oh, Sungho: 6100, 6390
OKADA, Shinichi: 9010
Okahashi, Sayaka: 6210
Okeahialam-Iheanyi, Chidi: 6390
O'Keefe, Patrick: 3440, 3510, 6440
Okoye, Safiyyah M.: 2580
Okuzono, Sakurako S.: 4690
Oladejo, Dianne: 4681
Olanisa, Olawale: 2510, 6240
Olanrewaju, Sherif A.: 6700
Olatunji, Samuel A.: 9020
Olds, Danielle: 2720
Oleary, Bryant: 1280
Oliva, Anthony A.: 6320
Olivari, Benjamin: 3510, 4120, 5010, 6280, 6680
Oliveira, Eduardo: 6700
Oliveira, Iara R.: 3640, 4350
Oliver, JoAnn S.: 6100
Oliveros, Gianna: 6770
Olivia, Landon: 1300
Oliviera, Iara: 4350
Olivieri-Mui, Brianne: 5084
Olmos, Brenda: 1060
Oloo, Alice: 6530
Oloruntoba, Oluyomi: 6710
Olsen, Bonnie: 4890, 5173
Olson, John D.: 9010
Olson, Marin G.: 3740, 6520, 6580
O'Malley, Kelly: 2660, 4770, 6220
Omar, Zaliha: 1060
Omenyi, Chiazam L.: 4680
Omeragic, Faris: 6470
Omobowale, Olubukola C.: 6100
O'Muircheartaigh, Colm: 2550
Omura, John D.: 2490, 4120, 6280
O'Neill, Desmond: 3380, 3585, 9010
O'Neil, Moira: 2100
Ong, Anthony D.: 2300, 3730, 4620
Ong, Lydia Q.: 2430
Onken, Brian: 9010
Ono, Mayuko: 6580
Opsasnick, Lauren: 1030
Opute, Amaka E.: 9030
Orange, Joseph: 6160, 6460
Orchard, Suzanne: 5083
Orekoya, Afolabi: 2510, 6240
Orewa, Gregory N.: 3660, 4820
Orji, Gloria: 6430
Orkaby, Ariela R.: 3600, 4683, 5084, 6320
Orlina, Edmund Cedric A.: 3483
Orlovsky, Irina: 6210, 6411
Ornstein, Katherine A.: 1160, 1190, 1350, 4460, 4530, 4860, 5030, 5070, 6310, 6530
Orom, Heather: 6510
Orsega-Smith, Elizabeth: 4021
Orsulic-Jeras, Silvia: 6150, 6370, 6390
Ortega, María: 1550
Ortenberg, Elia K.: 1220
Orth, Jessica: 1390
Ortiz, Cristina: 6100
Ortiz, Kasim: 2390
Ortmeyer, Heidi K.: 5050
Orwoll, Eric: 9030
Ory, Marcia: 6310, 9030
Osada, Hisao: 6580
Ó Súilleabháin, Páraic S.: 4620
Oshana, Jodie: 6210
Osha, Peyton: 9020
O'Shea, Brendan Q.: 2270, 4110
Osiecka, Zuzanna: 6070, 6200
Osokpo, Onome H.: 6200, 9000
Osorio Castaneda, Camilo A.: 6090
Ospina-Romero, Monica: 1050
Østbye, Truls: 1540
Osteen, Kathryn: 6650
O'Sullivan, Julie L.: 6110
O'Sullivan, Roger: 1270, 2690, 3380
Osuna, Margarita M.: 6830
Osvold, Lisa: 3460
Oswald, Austin: 2390, 2540
Otgon, Saranchuluun: 4684
Otto, Robin B.: 9030
Overbeck, Kevin: 6560
Owen, Donna: 6760
Owoaje, Eme T.: 3900
Owsiany, Montgomery T.: 9010, 9030
Owusu, Cynthia: 6510
Oya, Akari: 3480
Oyebode, Abiodun O.: 6500
Oyebode, Jan: 1270
Oyinlola, Oluwagbemiga: 6100
Ozdemir Van Dyk, Semra: 3160
Ozgur, Tosun: 6530

**P**

P. Corona, Ligiana: 9020
Pace, Sarah: 9030
Pacheco, Kelly: 5150
Packard, Benjamin: 6270
Padeken, Jan: 1200
Padron, Dilianna: 6140
Pageau, Félix: 6400
Pagedar, Nitin A.: 3550
Pagoto, Sherry: 6450, 6650
Pai, Manacy: 2400
Pajerski, Dawn: 9030
Pajewski, Nicholas M.: 4150
Pak, Karla: 6480
Palacio, Ana: 6420, 6140
Palan Lopez, Ruth P.: 1530
Palen, Ted: 3360
Palgi, Yuval: 1160
Pallaki, Muralidhar: 9030
Palmer, Janice L.: 6100
Palmer, Lauren: 3160
Palmer, Nathaniel: 9000
Palm, Rebecca: 3090
Palms, Jordan D.: 1240
Palta, Priya: 4050
Panchagnula, Sree Saroj Sainath: 9000
Pan, Diane: 9000
Pandya, Naushira: 6220
Pan, Fifi: 9010
Pang, Celeste: 6480
Pangle, Amanda: 6130
Panicker, Amina: 1120
Panier, Hunter A.: 9010
Pankey, Arianna: 1470
Pankow, James S.: 2670, 3400
Pan, Men-An: 4450
Pan, Stephanie: 3400
Pantalon, Michael: 5100
Pantleay, Harveen: 4510
Pan, Wei: 6110, 9030
Pan, Xi: 2080
Pan, Yiming: 4290
Paoletti, Jensine: 9010
Paolillo, Emily W.: 2450
Paone, Deborah: 4310
Papa, Carol-Lynne: 4890
Pappadis, Monique R.: 5100

Pardasani, Manoj: 3290
Parekh, Rupal M.: 6250
Parham, Cheryl: 4260
Parianos, Mary: 3483, 4021
Parinandi, Narasimham: 5090
Parish Johnson, Julie: 1330
Park, Amy: 1350, 6320
Park, Catherine: 4683, 6320, 9020
Park, Chan Mi: 9000
Park, Cherrie: 3740
Park, Crystal: 9000
Parker Oliver, Debra: 6730
Parker, Andrew: 6440
Parker-Autry, Candace: 6780
Parker, Daniel: 2241
Parker, Danitra: 2501
Parker, Elizabeth A.: 5050
Parker, Lauren J.: 3310
Parker, Madison: 9030
Parker, Martha: 6710
Parkes, David: 1430
Park, Geunhye: 1210, 4021
Park, Grace: 1180
Park, Hayoung: 6390
Park, Hong-Jae: 4430
Park, Hyeyoung K.: 9030
Park, Hyunjung: 6810
Park, Jeongsoo: 2480
Park, Jihye: 4320
Park, Jina: 4370, 5150
Park, Joon-Hyuk: 2290, 3840
Park, JuYoung: 1030, 1550, 2210
Park, Juyoung: 6180, 6810
Park, Ki Tae: 3220
Park, Mei Linn N.: 3220
Park, Min Kyoung: 9000
Park, Mina: 9020
Park, Moonkyoung: 6320, 9010
Park, Nan Sook: 3220, 6490, 6810
Park, Pil: 9010
Park, Seho: 6580, 9000
Park, Sojung: 4370, 5150, 6240, 6800, 9000, 9010, 9020, 9030
Park, Soobin: 6800, 9000, 9020
Parks, Reid: 9010
Park, Taeyoung: 6690, 9030
Park, Yoobin: 6830
Parmar, Jasneet D.: 1420
Parnia, Sam: 2160
Parraga, Carla B.: 6140
Parr, Aubrey: 9030
Parslow, Chad: 3330
Parsons, Kelly: 1380, 6060, 6720
Parsons, Pamela L.: 2260
PART Working Group: 6010
Partap, Angie: 3000
Parthasarathy, Sairam: 6510
Pasatiempo, Ana Maria: 6210
Pascual-Leone, Alvaro: 3482, 3510
Pasquini, Giancarlo: 6550
Passos, Joao: 3241
Patel, Darpan: 6390
Patel, Monika: 9030
Patel, Prakash: 9020
Patel, Sandip Kumar: 3241
Patel, Sanjay R.: 2620, 4080
Patel, Sheena: 9020
Patel, Susan: 5050
Patel, Sweta: 3830, 4465
Pate, Melannie: 6640
Pathiravasan, Chathurangi H.: 9020
Pat, Lian Ying Chun: 4684, 5084
Patorno, Elisabetta: 6110, 6641
Patrick, Julie H.: 2040, 9020
Patskanick, Taylor: 3260, 3450, 4090
Patterson, Sarah E.: 5070, 6310
Patti, Gary J.: 4810
Paudel, Anju: 2230
Paul, Constanca: 6560
Paulsen, Adam J.: 2670
Paulson, Daniel: 4480, 9030
Paulson, Sally: 6450, 6510, 9000
Paul, Sudeshna: 6310
Pauly, Theresa: 3150, 6210, 6390
Pavlidis, George: 2410
Pavlovic, Noelle: 3330
Pavon, Juliessa: 9030
Pawlowicz, Abigail: 5171
Payne-Foster, Pamela: 6100
Payne, Laura: 5082, 5130, 6170
Peña, Miguel: 6510
Peach, Laura: 6270
Pearce, Rahul C.: 6050
Pearsall, Vesselina: 3241
Pearson, Megan: 4260, 5050
Peckham, Allie: 9000
Peden-McAlpine, Cynthia: 9000
Pedersen, Ingeborg: 4300
Pedersen, Megan: 6780
Peeler, Anna: 6140
Peeri, Noah: 2080
Pees, Suzanne: 9020
Pei, Heming: 9010
Pei, Yaolin: 1130, 1540, 3481
Pelton, Leslie: 2560
Pencina, Karol: 6641
Pendedo, Frank: 9000
Pendergrast, Claire: 4770
Peng, Chagmin: 2040
Peng, Changmin: 9020
Peng, Lei: 9010
Peng, Man-Man: 4682
Peng, Siyu: 6690
Peng, Siyun: 3570, 6220
Penna, Victoria: 6470
Penney, Lauren S.: 1330, 4010, 6390, 6420
Peppard, Paul E.: 4560
Perales Puchalt, Jaime: 2070
Perez Montes, Viviana: 9010
Perez, Emily M.: 2260
Perez, G. Adriana: 3060, 6300, 9000
Perez, Hector: 6390, 6560
Perez, Janelle: 4210
Perez, Viviana I.: 1470
Perianayagam, Arokiasamy: 1060
Perissinotto, Carla: 2690, 6790, 9010
Perissi, Valentina: 4001
Perkhounkova, Yelena: 2520, 5010
Perkins, Anthony: 6580
Perkins, Fatima: 9020
Perkins, Molly M.: 3290, 3760, 4030, 5173, 9000, 9010, 9020
Perls, Thomas T.: 2501, 4810, 6010
Perone, Angela: 2730, 4820, 6060
Perreault, Hannah: 4340
Perre, Taylor: 6720
Perry, Brea L.: 3570, 4860, 6220
Perry, Katrice M.: 6180
Perry, Marieke: 5170
Perry, Tam E.: 3860, 4380, 6180
Perweiler, Elyse: 6560
Perzynski, Adam T.: 2640, 4100
Pessin, Lea: 4070
Pestine-Stevens, Althea: 2010
Pesut, Barb: 3500
Peszka, Jennifer: 6210
Peters-Beumer, Lisa: 3000
Petersen, Curtis: 6780
Petersen, Ron: 6780
Peters-Nehrenheim, Viktoria: 3610
Peterson, Andrew: 2160
Peterson, Colleen M.: 4220, 6510
Peterson, Donna: 6280
Peterson, Jennifer: 2260
Peterson, Lindsay J.: 2720, 4465
Peterson, Mark: 2500
Peterson, Matthew: 1060, 9030
Peterson, Natasha L.: 2200, 4905, 6790
Peterson, Rachel L.: 3600
Peterson, Ryan A.: 6470, 9030
Peters, Ruth: 9000
Petkus, Andrew J.: 3510
Petrillo, Kelsi: 6510
Petrovčič, Andraž: 1210
Petrovsky, Darina: 6110, 6600
Pfeffer, Bruce: 6100
Pfund, Gabrielle N.: 2430, 4620
Pham, Catherine: 3050
Phelan, Elizabeth: 6260
Philipp, Ian: 6010
Phillips, Drystan: 6830
Phillips, Karon L.: 3120, 6350
Phillips, Kristin: 6420
Phillips, Patrick: 9010
Phillips, Stuart: 3620
Phillips, Will: 4520
Phinney, Alison: 4860
Phongtankuel, Veerawat: 9030
Piazza, Jennifer R.: 2430, 2470, 2660, 3730
Picard, Martin: 4001
Picchiello, Matthew C.: 6160
Piccinin, Andrea: 2140, 6290
Picella, David V.: 9030
Pickering, Andrew M.: 2501, 4461, 5091
Pickering, Carolyn E.: 1390, 2250, 6110, 6390, 6590, 6750, 9000
Piechota, Amanda: 3360, 4850, 9000
Piedra, Lissette M.: 2550
Piercy, Cameron W.: 1570
Pierett, Jason: 3540
Piersol, Catherine: 2150
Pike, Christian: 9000, 9020
Pilla, Scott: 3400

Pillemer, Karl A.: 2000, 4090, 6310, 6480, 6520, 6530, 6690
Pimentel, Camilla M.: 1040, 4770
Ping, Yongjing: 1000
Pinheiro, Cristina N.: 6590
Pini, Lorenzo: 1030
Pinkert, Christiane: 3090
Pinto, Jayant M.: 1230, 3560, 6110
Pipinos, Iraklis: 6740
Pirtskhalava, Tamar: 6840
Pivodic, Lara: 1270
Plamondon, Katrina: 4020
Plante, David T.: 4560
Plassman, Brenda L.: 4420
Plaza-Rodriguez, Alma: 6411
Pless Kaiser, Anica: 2660, 6220
Plotzke, Michael R.: 1310, 6720
Plude, Dana: 1220
Plummer, Sherry: 4890
Plys, Evan: 4650
Pluth, Jack: 5001
Pocnet, Cornelia: 6380
Podgorski, Carol: 4860
Podlesek, Anja: 6090
Poe, Abigail: 2250, 6170
Poghosyan, Lusine: 1530
Pohl, Janet S.: 2250
Pohnert, Anne: 1530
Polivka, Larry: 4130
Polk, Thad A.: 4040
Pollino, Madison: 4022
Polson, Clay: 5173
Pomeroy, Mary Louise: 3130
Ponce, Julian: 6350
Pond, Brittney L.: 9020, 9030
Pondikos, Maria: 6420
Pond, Richard S.: 6350
Pongan, Lauren: 6530
Pool, Natalie: 9030
Poon, Cecilia: 2030, 3850
Poon, Lydia: 6390
Pope, Caitlin N.: 2450, 3000, 4225, 6060, 6200, 9020
Popejoy, Lori: 3170, 6270
Pope, Natalie E.: 5080
Popescu, Mihail: 4021
Poplawski, Antonina: 6400
Popov, Vladimir: 9000
Porterfield, Shirley L.: 9010
Porter, Kristie A.: 3640, 4350
Porter, Michelle M.: 6780, 9010, 9030
Portugal, Gabriela: 9010
Possin, Katherine: 6270
Post, Wendy: 3400
Poteat, Tonia: 1400
Potempa, Kathleen: 4870
Poulos, Michael: 6840
Poulsen, Rachel A.: 6420
Pourkhalili, Azin: 5171
Powell, Danielle: 2670
Powell, Nathaniel: 2560
Powers, Becky: 6320
Powers, Daniel: 6260
Powers, John David: 3360
Powers, Sara M.: 6150, 6370, 6390, 6530
Pradhan, Rohit: 3660, 6420
Prasad, Anyah: 2400
Prasun, Marilyn: 9030
Preddie, Alaina: 6390
Preisser, John: 1160
Prendergast, Alice: 6090
Prentice, Kailee: 6050, 6410
Prescott, Mike: 6500
Pressman, Peter: 4850
Presti, Peter: 4160
Pretzer-Aboff, Ingrid A.: 6420
Prevodnik, Katja: 1210
Prevratil, Michael: 9020
Price, Ashley N.: 6710
Price, Catherine C.: 4840, 6320
Price, Debora: 6310
Price, Elvin: 6320
Prideaux, Matt: 5001
Prigerson, Holly G.: 1490
Primack, Abby S.: 3810
Prina, Matthew: 1000
Priyambada, Priya: 6180
Prochaska, John: 9020
Procter-Gray, Elizabeth: 2290
PROMISE Study Group: 3160
Proper, Karin: 9020
Prost, Stephanie Grace: 5120
Proulx, Christine M.: 6680
Pruchnicki, Alec: 9010
Prusaczyk, Beth: 3310
Pruzin, Jeremy: 9000
Prvu Bettger, Janet: 4260
Puca, Annibale: 6010
Puckett, Amanda: 4461
Puerto, Geraldine: 6140
Puga, Frank: 2250, 6170, 6390
Pugh, Jacqueline A.: 1330
Pugh, Kelly: 6740
Pugh, Kendra: 1040
Pugh, Mary Jo: 1330
Pukhraj, Ambri: 6430, 6530
Pullen, Carly: 6140
Pullon, Emerald: 5173
Punke, Elizabeth: 3460
Punksungka, Wonmai: 4555, 6130, 6670, 6710
Puntambekar, Mehak: 9010
Purol, Mariah: 2280
Purwins, Daniel: 3090
Putnam, Michelle: 2050
Puts, Martine: 1420
Puurveen, Gloria: 3500
Pyles, Karly: 6580
Pynoos, Jon: 3900, 6230

**Q**

Qiao, Yujia: 4600, 9030
Qin, Tian: 4700
Qin, Weidi: 1160, 3860, 6730
Qin, Yongmei: 6490
Qin, Yue: 6710
Qiu, Paddy: 6120
Qiu, Xiao: 4820
Qi, Xiang: 1130, 1540, 3115, 3481, 3870
Quach, Emma D.: 1040
Quach, Lien: 2080
Qualls, Sara: 6750
Qualter, Pamela: 2690
Quashie, Nekehia T.: 4690, 6300
Quevedo, Alvaro: 9000
Quijano, Keara: 9000
Quillen, Ellen E.: 9010
Quinn, Margaret M.: 2710
Quinn, Natalie: 4520
Quiñones, Ana R.: 1090, 2300, 3310, 6340, 6450, 6740
Quinones-Visot, Emily: 6390, 9030
Quintero Silva, Laura: 9020
Quintero, Yadira Y.: 6080
Qu, Shan: 1070, 9020

**R**

Rababa, Mohammad: 6640
Rabinowitz, Jill A.: 4330
Rachmuth, Lisa: 9000
Raciti, Audrieanna E.: 6770
Radhakrishnan, Kavita: 6260, 9020
Rados, Robert: 6090
Raetz, Jaqueline G.: 5010
Rafford, Meghan: 2250
Rafie, Carlin: 5171
Rahemi, Zahra: 4500
Rahimighazikalayeh, Gelareh: 9010
Rahman, Anna: 3390
Rahman, Hafizur: 6740
Rahman, Momotazur: 4780, 5081
Raimondo, Marianne: 6810
Rainville, Chuck: 6150, 6480
Rait, Greta: 3555
Rajagopalan, Subashini: 5070
Rajagopal, Shilpa: 6270
Raji, Mukaila: 9000, 9010
Raj, Minakshi: 9020
Ramachandran, Vasan S.: 3400
Ramadhani, Widya A.: 4170, 6240, 6300
Ramalingam, Pradeep: 6840
Ramasamy, Ramalakshmi: 6641
Ramasubramanian, Ramya: 6010
Ramaswamy, Ravishankar: 4250
Ramdas, Kevin N.: 6320
Ramesh, Durga: 9020, 9030
Ramirez Gomez, Liliana A.: 4190
Ramirez, Javier E.: 6420
Ramirez, Madieska: 9000, 9010
Ramirez, Mildred: 2160
Ramirez-Ruiz, Mara: 6170
Ramirez-Sanchez, Israel: 9010
Ramirez, Valerie: 4190
Ram, Nilam: 3150, 4570, 6170
Ramos, Katherine: 2300
Ramos, S. Raquel: 2130
Ramos, Sofhia V.: 9010
Ramsdale, Erika: 6270
Ramsey, Jennifer: 6750
Ramsey, Jon: 1470
Randa, Cari: 3450
Randall, George K.: 6620
Randhawa, Paavan: 1550
Rangghran, Parisa: 6641
Ranka, Sanjay: 6510
Rankin, Katherine P.: 9000
Ransome, Yusuf: 9020
Rao, Sunil V.: 5050
Rapoport, Andy S.: 4021, 4023, 4270
Rapoport, Mark: 6170
Rasekaba, Tshepo D.: 3550
Ratcliff, Ellen: 9010
Rathbun, Alan: 6780
Rathfon, Megan: 6460
Ratnayake, Maggie: 9010
Rauer, Amy: 3480, 4030, 6170
Rauktis, Mary: 6680
Rausenberger, Alex: 5001
Ravichandran, Sathyabaarathi: 3001
Ravitch, Sharon: 3680, 3930

Raymond, Jennifer: 4390
Razavi, Marianne: 2550
Razjouyan, Javad: 4683, 6320, 9020
Reaves, Steven: 2560
Reblin, Maija: 2070, 4510
Rebok, George: 2530, 4680, 5110
Reckrey, Jennifer: 1180, 1190, 5030, 5070, 6530, 9030
Reczek, Rin: 4225, 6200
Redd, Doug: 6770
Redfern, Mark S.: 3690
Redman, Leanne: 2180
Redmond, Leslie C.: 6270
Redondo, Andres: 9020, 9030
Reed, Nia: 6280
Reed, Nicholas S.: 2530, 2670, 3560, 4050, 6710
Reel, Candice D.: 2030
Reese, Jordan: 4150
Reese-Melancon, Celinda: 9020
Reese, Melanie: 4023
Reeves, Alexis: 2080, 6140
Reeves, Jennifer: 6270
Reeves, Mathew: 4770
Regenbogen-Weiss, Sandy: 9000
Rehman, Aziz: 6600
Reid, Cary: 2300, 5150, 6310, 9010, 9030
Reid, Christopher M.: 4320
Reider, Lisa: 2580
Reid, Kerry: 4620
Reilly, Dermot: 2690
Reilly, Elizabeth: 9000
Reilly, Paula: 9000
Reinhardt, Joann P.: 6100
Reisdorf, Bianca C.: 1210
Reither, Eric N.: 4560
Remillard, Elena T.: 4160, 9030
Rendell, Peter: 9020
Renna, Megan E.: 4470
Rennke, Stephanie: 6460
Rentscher, Kelly E.: 3400
Reres, Adam: 6700
Resciniti, Nicholas V.: 1000, 6320
Resnick, Barbara: 2230, 3090, 3650, 3830, 4760, 6170, 6350, 9020
Resnick, Neil: 3350
Resnick, Susan M.: 3510, 4200, 4330, 4340
Reuben, David B.: 4150
Revenson, Tracey: 3115
Reyes, Adriana M.: 4420
Reyes, Laurent: 3290, 5080
Reyes, Mariana: 6060, 6220, 6280
Reynolds, Addam: 5130
Reynolds, Chandra A.: 9000, 9010, 9020
Reynolds, Christina: 2450
Reynolds, Courtney: 4770, 4900, 5083
Rhoads, Timothy W.: 1140
Rhodes, Annie S.: 1110, 2510, 6140, 9010
Rhodes, Shanae: 2420
Rhodus, Elizabeth: 1380, 4790, 4870, 6060
Rhoten, Bethany: 1180
Riaz, Muhammad: 6280
Richards, Bradley: 1310
Richards, Kathy: 6260, 9020
Richards, Nicole E.: 3730
Rich, Jodi: 9030
Richman, Alice R.: 3100
Richmond, Therese S.: 6390
Rich, Stacey D.: 3550
Rick, Chelsea: 6270
Rickenbach, Elizabeth: 1260
Riediger, Michaela: 2470
Riemenschneider, Falk: 3620
Riffin, Catherine: 2000, 6310, 6530, 6690
Rikhtehgaran, Farinaz: 6500
Riley, Alicia R.: 4480
Ring, Lauren: 4360
Rink, Andrea: 5172
Ripat, Jacquie: 6780, 9010
Risacher, Shannon L.: 3570, 6820
Risbud, Rashmi D.: 1330, 6430, 6530
Ritche, Christine S.: 5070
Ritchie, Christine S.: 5030
Ritter, Ashley Z.: 4360
Rittiwong, Tanapa: 6770
Rivera Almaraz, Ana: 4290
Rivera-Hernandez, Maricruz: 1090, 4780
Rivera, Rebecca L.: 6270
Rivera-Torres, Solymar: 6130
Rizer, Sandra: 6260
Rizzo, Matthew: 6420
Rizzuto, Debora: 3590
Roach, Ashley: 1530
Roache, Tavia: 9000
Roach, James: 1250
Roach, Lauren: 4225, 6200
Robbins, Hilary: 9000
Robbins, Nathaniel M.: 2670
Robbins-Panko, Jessica C.: 6370, 6830
Robbins, Paul: 2501, 6840
Roberson, Tonya: 4681
Roberto, Karen A.: 1220, 3240, 9020
Roberts, Amy R.: 4830, 6260
Roberts, Ellen: 1110, 9030
Roberts, Eric: 9010
Roberts, Jimmie: 9030
Roberts, Josephine: 6080
Roberts, LeAnna: 6180
Roberts, Lisa: 9000
Roberts, Mitchell: 6390
Robertson, Andrew D.: 4120, 6780
Robertson, Anna C.: 6170
Roberts, Shelby: 6060
Robert, Stephanie A.: 5172, 9020
Roberts, Tonya J.: 3520, 9010
Robinette, Jennifer W.: 3440
Robinson-Ector, Kaitlynn: 2300
Robinson, Erin L.: 1210, 3760, 4021
Robinson, Kristen: 3160
Robinson-Lane, Sheria G.: 2420, 3495, 3890
Robison, Julie: 1310, 2040, 2150
Robison, Raele: 3830
Robitaille, Annie: 6150, 6210
Robling, Alexander G.: 6840
Röcke, Christina: 1020, 2450, 3150
Rockwood, Kenneth: 1180
Rodakowski, Juleen: 1340
Rodgers, Joeseph: 3440, 3510, 6440
Rodin, Rebecca: 4460, 5030
Rodi, Scott: 4390
Rodríguez Díaz, Melina: 6290, 6380
Rodriguez, Diana: 5110
Rodriguez, Elisa M.: 6430
Rodriguez-Gonzalez, Maribel: 6000
Rodriguez, Gustavo: 6140
Rodriguez, Miriam J.: 6140
Rodriguez, Misha: 4360
Rodriguez, Sonia L.: 6010
Roe, Lauren S.: 2290
Roes, Martina: 1270, 3090, 3610, 4270, 4610
Roessler, Meghann E.H: 6820
Rogers, Casey: 9010
Rogers, Geoff: 4090
Rogers, Stephanie: 6270, 9000
Rogers, Wendy A.: 1570, 2050, 3410, 3670, 4160, 4190, 6000, 6240, 6300, 6390, 9020, 9030
Rogozinski, Amos: 2680
Rogina, Blanka: 5001
Rogus-Pulia, Nicole: 3830
Rohner, Ronald P.: 1290
Rohner, Shauna L.: 4225, 6580, 5100
Roldan, Lanyce: 6100
Rollandi, Isabel Olivia: 9030
Romanelli, Meghan: 4540
Roman, Youssef: 6320
Romeo, Evelyn: 9000
Romero, Cristy: 5171
Romero, Ryan: 6140
Rommerskirch-Manietta, Mike: 3090
Romo, Rafael: 1030
Ronneberg, Corina R.: 6620
Rooks, Ronica N.: 2730, 3430
Roopsawang, Inthira: 3840
Roose, Steven P.: 3600
Root, Eve: 6130
Roquet, Angélique: 6380, 6580, 6790
Rorai, Vanessa: 6180
Rosaldo, Manuel: 6700
Rosano, Caterina: 1050, 3250, 4340, 6450, 9030
Rose, Andrew: 6620
Rosebush, Christina E.: 1480, 4060, 6540
Rose, Jacob: 3241
Rose, Johnie: 6510
Rose, Karen M.: 3360, 4650, 5090
Roseman, Jeffrey: 5173
Rose, Miriam: 4770, 4900, 5083, 6370
Rosemond, Cherie: 4360
Rosenberg, Molly: 3040, 9010
Rosenberg, Walter: 2560
Rosenblum, Sara: 9020
Rosendaal, Nicole: 3200, 5081
Rosenkranz, Susan: 6830
Rosen, Sonja: 5083
Rosenstein, Olivia: 1120
Rosen, Tony: 4900, 9000, 9030
Rosich, Rosellen M.: 1380, 4710
Rosko, Ashley: 3550
Rosland, Ann-Marie: 9000
Ross, Clifford: 4225, 6200

Rosselli, Monica: 4220
Ross, Lesley: 3270, 9020
Ross, Leslie: 6530, 9030
Ross, Madeline: 9000
Rosso, Andrea L.: 1050, 3250, 3690, 4340, 4600, 6450, 9030
Ross, Sarah E.: 1110, 1250
Ross, Serena: 9000
Ross, Webster: 3580
Rosteius, Katharina: 4300
Rote, Sunshine: 3020, 3240, 4420
Roth, Adam R.: 4860
Roth, David L.: 2630, 5110
Rothermund, Klaus: 2480
Rothschild, Anthony J.: 9010
Rotondi, Armando: 9010
Round, Teri: 5172
Rowan, Noell L.: 1320, 6350
Rowe, John W.: 6330
Rowe, Meredeth: 6760
Roy, Deepa: 9020
Roy, Indrakshi: 4780
Roy, Upal: 9020
Rozario, Philip A.: 2240
Rubinstein, Stephanie R.: 6440
Rubtsova, Anna: 3760
Rudolph, James: 3830, 3130
Rudroff, Thorsten: 2245
Rudy, Ellen T.: 6790, 9010
Ruffin, Adrienne: 6760
Ruggeri, Siena: 3940
Ruggiano, Nicole: 6750, 9000
Ruggiero, Laurie: 4021
Ruiz, Martha: 6510
Rule, Payton: 6160
Runge, Katherine: 2690
Rupper, Randall: 1330
Rupprecht, Fiona S.: 2480, 2750
Rurka, Marissa: 4070
Rusch, Amber V.: 6200
Rush, Jonathan: 2430, 2470, 3730, 6290, 6580
Rush, Kathy: 4020
Russell, Andrea: 2280
Russell, Dan: 9020
Russell, David: 1190
Russel, Wynfred: 1050, 1480, 6540
Russ, Tom: 6450
Rutherford, Bret R.: 3600
Ruth, Terrance: 9030
Rutkowsky, Jennifer: 1470
Rutledge, Emily: 6390, 6560
Ryan, Alice: 6780
Ryan, Cayley E.: 6610
Ryan, Joanne: 4320, 6320
Ryan, Lindsay H.: 3430, 3480
Rydberg Sterner, Therese: 3590
Ryd, Charlotta: 2460
Rymaszewska, Joanna: 5170
Ryou, Yeon Ji: 4140, 4905, 6790
Ryser, Valerie-Anne: 4480, 6550
Ryskina, Kira: 2090
Ryu, Ahyun: 6320
Ryu, Byeongju: 4370, 5150, 6240, 9000, 9010, 9030
Ryu, Jinhyung: 9020
Ryuno, Hirochika: 6100, 6110
Sörensen, Silvia: 9010

S

Sabat, Steven R.: 1380
Sabharwal, Anup: 9000
Sabino-Laughlin, Eilleen: 2070
Sabra, Ahmad: 1350
Sabuncu, Crim: 9030
Sacco, Paul: 4510
Sachdev, Perminder S.: 4340
Sachs, Barrie: 9010
Sachs, Greg: 4100, 6280
Sadak, Tatiana: 1150, 6260
Sadarangani, Tina: 2130, 3060, 9020
Sadeq, Nasreen: 6000
Sadowski, Cheryl: 6270
Saeidzadeh, Seyedehtanaz: 4720
Saenz, Joseph: 3040, 4420, 9000, 9020
Sagong, Hae: 6180
Saguez, Rodrigo: 6320
Sahawneh, Fursan: 9020
Sahli, MIchelle W.: 4023
Sahoo, Nirakar: 9020
Sahoo, Shalini: 6490
Saifuddin, Fatema: 6411
Saini, Manik: 2600
Saito, Tami: 6210, 6250, 6280, 6760
Saito, Yasuhiko: 6290, 6600, 9030
Sajatovic, Martha: 6160, 6510
Sajwani, Zara: 6770
Sakamoto, Mariko: 4860, 9000
Salafia, Caroline: 9000
Salas, Aileen: 4225, 6580
Salas, Joanne: 3600
Saleem, Ahsan: 6270
Saleem, Zainab: 6640
Salem, Yasser: 6780
Saliba, Debra: 1460
Salinas Rodríguez, Aarón: 4290
Salinger, Maggie R.: 5030
Salmon, Adam: 5001, 6010, 9010
Salom-Bail, Shirley: 6210, 9030
Saloniki, Eirini-Christina: 6700
Salvador, Andrea Francesca: 4461
Salvo, Erin: 4090
Salzman, Talia: 4340
Samant, Rahul S.: 5091
Sama, Susan R.: 2710
Samper-Ternent, Rafael: 3020, 4150, 6330
Sample, Alanson P.: 9000
Sampson, Carla: 9010
Sampson, Elizabeth l.: 3555
Sampson, Joshua: 4560
Sampson, Laura: 6830
Sampson, Natalie: 6490
Samuel, Laura J.: 2510, 3560
Samuel, Raja: 6090
Samus, Quincy: 4280
Sanchez-Reilly, Sandra: 9000
Sanchez, Ricci: 4681
Sandell, Lindsay L.: 9020
Sanders Schmidler, Gillian D.: 9030
Sanders, Beth: 6150, 6390
Sanders, Bethany E.: 3770
Sanders, Edie C.: 3670
Sanders, Jason: 3400
Sanders, John W.: 6100
Sanders, Lisbeth: 6370
Sanders, Margaret: 1190, 3530
Sandhu, Shobhana: 9020
Sandoval Garrido, Felipe A.: 9000
Sands, Bryan: 3810
Sands, Talyah: 3010, 6060
Sanford, Angela: 6260
Sanford, Jon A.: 4160
Sang, Elaine: 6100
Sanko, Stephen: 6770
Sanmartin Berglund, Johan: 3590
Sanner, Caroline: 1300
Sanni, Mubarak: 2300
Santana, Leticia: 2560
Santanasto, Adam J.: 3400, 4120, 4340, 4600, 4810
Santora, Kenneth: 3080, 3280
Santos, Flavia H.: 1060
Sanz Simon, Sharon: 6420
Sapozhnikova, Anna: 4850
Sappington, Erica: 6390
Saraboon, Yuwadee: 3840
Sardina, Angie L.: 3270, 6350
Sargent, Lana: 6140, 6320
Sarif, Nawaj: 2150, 6610
Sarkar, Abhirup: 6411
Sarkar, Nilanjan: 5090
Sarker, Ananya: 6390
Sarna, Kaylee: 6160
Sarnevitz, Janelle: 1070
Sarshar, Arash: 5171
Sass, Alexandra: 6180
Satake, Anna: 9020
Satizabal, Claudia L.: 9020
Satoh, Kana: 4440
Sauer, Jacquelin: 9030
Sauerteig-Rolston, Madison R.: 6290, 6810
Saunders, Michele J.: 3870
Saunders, Pamela A.: 4180
Savage, Kirstie M.: 2060
Savary, Patricia: 6130
Savich, Tatjana T.: 4020
Saviers, Brittany: 1420
Savla, Jyoti: 3240, 9020
Savundranayagam, Marie: 2520, 5000, 6160, 6460
Sawhney, Amitoj: 1470
Sawyer, Linda M.: 6420
Saxena, Divya: 6780
Sayed, Linda: 3430
Sayer, Tina B.: 3000
Saykin, Andrew J.: 3570, 6820
Scales, Kezia: 2710
Scanland, Susan: 6260
Sceppa, Carmen: 6790
Schönstein, Anton D.: 5110
Schaaf, George W.: 9010
Schaefer, Sarah K.: 5110
Schaeffer, James: 6170, 6580, 6790
Schafer, Marissa J.: 3241, 6010, 6411
Schafer, Markus H.: 6790
Schaff, Branden: 6220
Schapira, Lidia: 6430, 6530
Scheckler, Samara: 3900, 4380
Scheel, Jennifer: 4300
Scheffer, Julian A.: 4681, 9000
Schell, Brent R.: 9000
Schenck, Sabine: 3460
Schenkel, Rachel: 9000

Schepens Niemiec, Stacey L.: 4420
Scher, Clara: 1170, 5130
Scherer, Danette: 6000
Scherer, Hailey: 6140
Scherer, Philipp E.: 4241
Scherrer, Jeffrey: 3600
Scherzer, Rebecca: 6320, 6780
Schiaffino, Melody K.: 3670, 3750
Schiller, Gabrielle S.: 1160, 3660, 6320, 6610
Schilling, Birgit: 3241, 6840, 9010
Schilling, Oliver K.: 1020
Schiltz, Nicholas K.: 6510
Schilz, Matthew: 4240
Schipf, Sabine: 1010
Schmid, Arlene A.: 2280, 3930
Schmidt, Annika: 4300
Schmidt, Laura I.: 2750
Schmidt, Nicholas E.: 2030, 3850
Schmitz, Wiebke: 9020
Schneider, Haley: 3240
Schneider, Joanne: 6770
Schnoll, Roseanne: 9010
Schoenborn, Nancy: 2200
Schols, Jos: 6270, 9000
Schousboe, John T.: 9030
Schrack, Jennifer A.: 4050, 4120, 4330, 4600, 6740
Schriner, Sam: 6010
Schroeder, Matthew W.: 6390, 6450, 6580, 6650, 9000, 9030
Schroepfer, Tracy: 6720
Schubert, Carla R.: 2670
Schubert, Cathy C.: 6390
Schukle, Jamie S.: 4260
Schulman-Green, Dena: 1540
Schulte, Phillip: 6450
Schultz Lee, Kristen: 4070
Schultz, Linda: 6100
Schultz, Michael: 6641
Schultz, Rebecka: 6560
Schultz, Savannah R.: 9000
Schulz, Marc: 2620
Schulz, Richard: 4090
Schumacher, Benjamin T.: 9030
Schumacher, John G.: 6350, 9010
Schuman, Donna: 6250
Schumm, L.Philip: 2550
Schupf, Nicole: 6010
Schurman, Charles: 3241
Schuster, Amy M.: 4020, 9030
Schut, Bibi: 4460
Schut, Henk A.W.: 4460
Schutte, Brian C.: 4470
Schutte, Debra L.: 4470, 6420
Schwab, Delphine Roulet: 6400
Schwartz, Abby J.: 3100
Schwartzkopf, Ashley: 6390
Schwartz, Todd: 6760
Schwarzberg, Arielle: 1100
Schwingel, Andiara: 6530, 9020
Scott, Adrienne W.: 3560
Scott, Judith M.: 6770, 9010
Scott, Mallary: 3100
Scott, Rachel K.: 3740, 6520, 6580
Scott, Stacey B.: 3250, 3440, 4620, 6550
Scrivano, Rachel: 2350, 3780
Seale Reiff, jenni: 4680
Seaman, Aaron T.: 3550
Sears, Dorothy D.: 9020
Sears, Jeanelle: 4420
Sebastiani, Paola: 2501, 4810, 6010
Seckin, Gul: 6430
Sedore, Christine: 9010
Seeman, Teresa E.: 3440, 4560, 6440, 6580
Seetharaman, Kishore: 3450, 6500
Sefcik, Justine S.: 1120, 3310, 5020, 6110, 6390, 6600, 9020
Segal, Daniel L.: 6550, 6770
Segelman, Micah: 3640, 4350
Segerstrom, Suzanne C.: 5140, 6590
Sehgal, Raghav: 1140
Seifert, Allison M.: 1420
Seifert, Imke: 5170
Seino, Satoshi: 6320, 6710
Seixas, Azizi: 6680
Sejdic, Ervin: 3690, 4340
Sekhar, Rajagopal V.: 2241, 9010
Seki, Yuichi: 9000
Seligman, Benjamin: 4690, 6320, 9020
Sellers, Christina: 6350
Sellon, Alicia: 9030
Semelka, Charles T.: 6100
Semprini, Jason: 3660
Sen, Payel: 1200
Seo, Dongchul: 6780
Seo, Jin Young: 6480
Seo, Jisu: 6320
Seong, Hohyun: 5171
Seo, Shinae: 9010
Serdaly, Christine: 2090
Serra, Monica C.: 4260, 6390
Serrao Hill, Melanie M.Y: 6580
Servat, Caroline: 3540
Sesek, Greg: 9000
Seshadri, Sudha: 9020
Sesso, Howard D.: 3600
Severance, Jennifer J.: 1110, 1250, 6130
Sexson, Kathryn: 9000
Seyfried, Nicholas: 6840
Seyyedmirza, Paria: 6310
Shaaban, C. Elizabeth: 1050
Shadel, Doug: 6480
Shadmi, Efrat: 1160
Shadyab, Aladdin: 2200, 4560
Shaffer, Kelly: 9010
Shagalow, Shaina: 6260
Shah, Avani A.: 6180
Shah, Avantika: 4580, 6270
Shah, Priya: 3010, 6060
Shah, Raj C.: 6320
Shah, Rishabh: 6430
Shah, Sheetal: 6180
Shake, Matthew: 6570
Shakya, Iju: 4100
Shalaby, Alaa: 9010, 9030
Shalev, Idan: 5060
Shambley-Ebron, Donna: 6140
Shanbhag, Meera L.: 4680
Shang, Erhua: 6250
Sharafkhaneh, Amir: 9020
Shardell, Michelle: 3110, 6780
Sharit, Joseph: 3410
Sharma, Ayush: 6840
Sharma, Hari: 3950, 6360
Sharma, Shakshi: 6840
Sharma, Shubam: 3360, 4555, 6340, 6380
Sharp, Julia L.: 2250, 2280, 3930
Sharrett, Richey A.: 3560
Shatterly, Jaclyn: 4022
Shavit, Yochai Z.: 3190, 6170
Shaw, Clarissa: 2520, 2530, 5010
Shaw, Philip: 6170
Shawver, Zared: 3440
Shay, Kimberly A.: 2310
Shayya, Ashley: 6090
Shean, John: 3010, 6060, 6680
Shea, Steven: 3400
Sheck, Isabelle N.: 6350
Sheftel, Mara G.: 6300
Sheikh-Bahaei, Nasim: 9000
Sheikh, Sophia: 9030
Sheils, Natalie: 6110
Sheldon, Marisa: 3770
Shelley, Mack: 3500
Shellito, Natalie: 3320
Shende, Shraddha: 6390
Sheng, Chen: 6200
Shen, Huei-Wern: 6120
Shen, Jenny Y.: 4580, 6270
Shepherd, Alyx L.: 9000
Shepherd-Banigan, Megan: 6110
Sheppard, Christine: 4090
Sheppard, Luvon: 6280
Sherman, Aurora M.: 3480, 6580
Sherman, Ledric: 2250
Sherwood, Laura: 6350
Shewmaker, Peter: 6230, 6360
Shewokis, Patricia: 5020
Shi, Changyou: 1200
Shields, Cleveland: 9030
Shields, Pamela D.: 5090
Shields, Wendy: 9010
Shieu, Bianca: 6760
Shifflett, Benjamin: 6411
Shigenobu, Kazue: 9030
Shih, Patrick: 9020
Shi, Jiaming: 9000
Shi, Changyou: 1200
Shi, Ling: 1280
Shimabukuro, Michio: 6010
Shimada, Chiho: 6580
Shin, Donghee: 6240
Shin, Hyung Eun: 6450, 6510, 6740
Shin, Jacqueline C.: 9010
Shin, Jinhee: 6320
Shin, JiWon: 5090
Shin, John J.: 3560
Shin, Ju Young: 6760
Shinkai, Shoji: 6320, 6710
Shin, Marlena: 4770
Shin, Oejin: 9030
Shin, So Young: 6270, 9000
Shin, Yeonsoo: 6820
Shippee, Nathan: 6540
Shippee, Tetyana P.: 1050, 1480, 2650, 3310, 3910, 4130, 5130, 6540
Shirk, Steven D.: 1040
Shiroma, Kristina: 6730
Shirsat, Nikita: 3050
Shi, Sandra M.: 9020

Shi, Xiang: 6810
Shi, Ying: 6110
Shkirkova, Kristina: 9020
Shmuel, Shahar: 4320
Shofner, Sabrina J.: 2200
Shreeniwas, Sudha: 2730
Shrestha, Aman: 6750, 9010
Shrestha, Laxman: 4250
Shrestha, Srishti: 2670
Shrira, Amit: 3490
Shrout, M. Rosie: 3150, 4470
Shugrue, Noreen: 1310
Shulyaev, Ksenya: 1160, 2680, 9030
Shuman, Stephen K.: 3870
Shum, Eric Ngai Yin: 4022, 4684
Siaton, Bernadette: 6220
Sibille, Kimberly: 2300
Sibley, Kathryn: 6780, 9010
Sidani, Souraya: 6270
Siddiq, Hafifa: 3430
Sideman, Alissa: 9000
Sidey, Leanza: 9030
Sidhu, Amat: 6220
Sidoli, Simone: 1200
Sieber, Frederick: 1010
Sieber, Stefan: 6580, 6790
Siebert, Jelena S.: 5110
Siedlecki, Karen: 6760
Siegel, Elena O.: 2680
Siegler, Eugenia L.: 3000
Sieverding, Monika: 2750
Sigurdardottir, Sigurveig H.: 6220
Sihapark, Siranee: 4700
Sikder, Abu: 4190
Silberberg, Mina: 9000
Silbersack, Johanna: 3910
Silbert, Lisa C.: 2450, 4870
Sillner, Andrea: 2680, 3720, 6420
Silva, Graziele: 9020
Silva, Isabella: 9000
Silva, Jennifer: 3130
Silva-Smith, Amy L.: 6650
Silva, Susan G.: 9010
Silver, Emily: 9030
Silverman, Michael Alan: 6000
Silver, Michelle: 4684
Silverstein, Hannah I.: 6730, 9010
Silverstein, Merril: 1450, 3500, 6520, 6790
Silverstein, Nina M.: 3100, 3800, 6000, 6090, 6510
Silver, Stephanie A.: 2160
Simcox, Judith: 4241
Simmons, Ames: 9020
Simmons, Sandra F.: 4580, 6270
Simning, Adam: 6140
Simon, Darby M.: 6290, 9010
Simone, Abigail: 6450, 6510
Simon, Lois: 2020
Simon, Michael: 2090, 6270
Simonsick, Eleanor M.: 4120, 4200, 4330, 4600, 6740, 9020
Simpson, Deborah: 6280
Simpson, Elizabeth: 2040, 3320
Simpson, Felicia: 3400
Simpson, Luke: 6210
Sims, Tai: 9000
Sims, Wynton: 6460
Sinah, Indranil: 5001
Sinclair, Christian T.: 6730
Sinclair, David A.: 1140, 6641
Sindi, Shireen: 3440, 6450
Singer, Dianne: 3495
Singer, Katelyn J.: 6290
Singer, Rachel: 4240
SIngh, Abanish: 1000
Singh, Anmoldeep: 3570, 6820
Singh, Mriganka: 9030
Singh, Nainwant: 6530
Singh, Priyanka: 6100
Singh, Roshni D.: 6140
Singh, Rufus: 6090
Singleton, Meki: 1400
Sinha, Indranil: 6840
Sinkler, Kateri: 5171
Sin, Mo-kyung: 5173
Sin, Nancy L.: 2430
Sinvani, Liron: 4700
Sioutas, Constantinos: 9020
Sirey, Jo Anne: 9030
Sison, Stephanie Denise M.: 6641, 9000, 9020
Siu, Albert: 3660
Siu, Joey Chung Yue: 4022, 4684
Sivakolunthuvel, Balamurugan: 6100
Sivers-Teixeira, Theresa M: 4890, 5173
Skanderson, Melissa: 6770
Skarphol, Tricia: 3910
Skemp, Eleanor A.: 6520
Skemp, Lisa: 1180
Skinner, Kelly: 6270
Skinner, Robbie: 2020
Skipper, Antonius D.: 6620
Skoblow, Hanamori F.: 4691, 6680
Skoog, Ingmar: 3590
Skow, Laura: 4120
Skubic, Marjorie: 4021, 6390
Slattum, Patricia W.: 2260, 3230, 6000, 6320
Slaug, Bjorn: 4220
Slaven, James E.: 6100, 6280, 6390
Slayday, Riki E.: 3250
Sliwinski, Martin J.: 3250, 6290, 6550, 6790, 9010
Sloan, Allyson E.: 6210
Sloane, Elliot: 9030
Sloane, Philip D.: 3910
Sloane, Richard: 5050
Sluys, Erica: 2300
Smail, Emily J.: 2170, 4840, 6510
Small, Brent J.: 1100, 3400, 6180
Smichenko, Juliana: 1160
Smirnov, Igor: 4461
Smith, Alex: 4460
Smith, Alexander k.: 5030
Smith, Claire E.: 1100, 2620, 4080
Smith, Emily: 6570
Smith-Greenaway, Emily: 9020
Smith, Gregory C.: 4060
Smith, Jacqui: 1410, 2220, 6050, 6580, 9020
Smith, Jason R.: 3510
Smith, Jennifer L.: 4060, 6250
Smith, Jessica: 6010
Smith, Kathrine A.: 9000
Smith, Kelly: 6210
Smith, Ken R.: 4530, 6310
Smith, Kevin M.: 4830
Smith, Lenora: 4870
Smith, Lindsey: 3200, 3910, 5081
Smith, Louisa H.: 5084
Smith, Matthew L.: 2250, 6310, 6710, 9030
Smith, Patsy: 1060
Smith, Phillip: 6641
Smith-ray, Renae: 6100
Smith, Rebecca: 6500
Smith, Sherri: 5140
Smith, Thomas: 4200
Smith, Thomas J.: 6710
Smit, Kirsten: 3555
Smits, Carolien: 3610
Smolen, Andrew: 9010
Smoyer, Karen E.: 3830
Sneed, Rodlescia S.: 1160, 3450, 3770, 6310
Snider, Christina A.: 6740
Snyder, Rani: 2560, 3940
Snyder, Yonda: 1190
Sobol, Adam: 9020
So, Christine: 3480, 6170
Sok, An: 6120
Sol, Ketlyne: 1240
Solomon, Phyllis: 4750
Solway, Erica: 3495
Somers, Virend: 6740
Somerville, Ceara: 1170, 2010, 6090
Sommerer, Yasmine: 1010, 6010
Sommet, Nicolas: 6200
Song, Huaxin: 6760
Song, Hwasung: 6670
Song, Jiaxiu: 6180
Song, Mi-Kyung: 6310
Song, MinKyoung: 3240
Song, Qian: 3040, 6200, 9020
Song, Rhayun: 6320, 9010
Song, Xiao: 4700
Song, Xiaowei: 1180
Song, Yu: 4683, 9020
Song, Yuhua: 9010
Song, Yuting: 2090, 4210
Song, Zeyuan: 2501, 4810, 6010
Son, Julie S.: 3270
Sonnega, Amanda J.: 9020
Sorkin, John: 6220
Soto, Elizabeth: 4470
Sotres-Alvarez, Daniela: 4330
Sousa, Susana: 6560
Spangler, Hillary B.: 4640, 6780
Spang, Robert P.: 6110
Spaulding, Trent: 4820
Spechler, Lily: 6210
Spetz, Joanne: 3950, 6530
Spindle, Natalie: 9030
Spini, Dario: 6200
Spinner, Ruth: 6100
Spira, Adam P.: 4330
Spira, Dominik: 6010, 6641
Spiro, Avron: 2620
Splaine, Mike: 9020
Splitstone, Matthew J.: 6000
Spoden, Natasha: 9000
Spradley, Laura: 6210
Sprague, Briana N.: 4340
Sprang, Rob: 1380
Springfield, Sparkle: 2200
Springstein, Tabea: 4570
Squillace, Marie R.: 3640, 4350
Sreenivas, Ramavarapu S.: 4160
Sreevalsan, Kavya: 6320

Srinivasan, Erica G.: 6000
Sritan, Sarawut: 2610
St. Goar, Carl: 9020
St. Louis, Renee M.: 6470
Stürmer, Til: 4320
Staal, Bart: 1120
Staben, Omar: 6490
Stachler, Eva: 6510
Stacke, Tobias: 3090
Stadler, Gertraud: 3070
Stainback, Maggie: 9010
Stallard, Eric: 6010, 6411
Stallings, Devita T.: 6100
Stanback, Brianne M.: 6000
Standridge, Natalie: 6670
Stanfield, Alyssa: 6000
Stange, Kurt: 2640
Stankovic, John: 5090
Stanley, Jennifer: 9000
Stansbury, Kim: 9030
Stark, Emma: 4650
Staton, Lori: 6280
Stavrinos, Despina: 3000
Stawnychy, Michael A.: 6100
Stawski, Robert S.: 6520, 6580
Steacy, Katarina: 5171
Stearns, Harvey L.: 9000
Stebbins, Rebecca: 6050
Stechuchak, Karen M.: 5083
Steckenrider, Janie S.: 6190
Steele, Jenessa: 6140
Steele, Joel S.: 4790
Steer, Christopher: 3550
Steering Committee, Brain Health in the Community (BHIC): 4860
Stefanacci, Richard: 4020
Stefanacci, Richard G: 6260
Steffen, Ann M.: 2030, 3251, 3850
Steffen, Melissa: 6100
Stehling, Elisa: 6770
Steinberg, Nessa: 9020
Steinhagen-Thiessen, Elisabeth: 1010, 3070, 4040, 6641
Steinman, Bernard A.: 3460, 6140
Steinman, Michael A.: 5030
Stelle, Charlie: 4022, 9020
Stenhaug, Ben: 3000
Stephan, Abigail T.: 3270
Stephens, Caroline E.: 4530, 6310
Stephens, Jacquelyn E.: 3150, 4570
Stepniak, Catherine: 1290, 6520
Steptoe, Andrew: 2270, 3210
Sterling, Madeline: 2710
Sterns, Harvey L.: 6000
Stern, Yaakov: 6420
Stessman, Jochanan: 6610
Stevens, Alan B.: 4150, 6100
Stevens, Erin: 3550
Stevens, Jen: 6280
Steward, Andrew T.: 2310, 4240, 4430
Stewart Steffensmeier, Kenda: 6100
Stewart, Heather G.: 1210
Stickel, Ariana: 9000
Stiefler, Susanne: 4300
Stiles, Robert: 6650
Stinchcombe, Arne: 6170, 9020
Stinnett, Sandra: 9000
Stirland, Lucy: 6450
Stoeckle, Rebecca J.: 4890
Stokes, Jeffrey E.: 1090, 2400, 4460, 6200, 9030
Stolfa, Claire: 6180
Stoll, Isabella: 5020, 9020
Stone, Arianna: 6720
Stone, Katie L.: 4080, 4560, 6200
Stone, Lisa E.: 6550
Stone, Robyn I.: 2010, 2720, 3640, 4360, 6210, 6760
Storms, Aaron D.: 6120
Stout, Jeffrey R.: 2290, 3840
Straker, Jane: 3520
Strand, Bjørn Heine: 1190, 6800
Stransky, Stephanie: 1200
Stratton, Lauren: 4010, 6130, 9030
Stratton, Sara K.: 4900
Strauch, Kim: 9030
Strauss, Anna Wiersma: 1450
Strayer, Andrea L.: 6641
Street, Lara: 6140
Striley, Catherine L.: 9020
Strobl, Carolin: 3115
Stroebel, Armin M.: 3090
Stroebe, Margaret S.: 4460
Strogatz, David: 5171
Strommen, Jane: 6780
Strong, Jessica V.: 6250, 6440, 6640
Strong, Randy: 9010
Strotmeyer, Elsa S.: 2670, 3690, 4120, 6650, 9030
Strough, JoNell: 6210
Struble, Laura M.: 4870
Stuart, Bruce C.: 9010
Stuart, Robin K.: 3670
Stuck, Amy: 6770
Stukova, Marina: 3483, 9030
Sturdevant, Diana: 1060, 5172
Subedi, Janardan: 9010
Sublett, Jennifer F.: 9000
Subramaniam, Savitha: 6420, 6570, 6780
Suda, Hitoshi: 4440
Sudore, Rebecca: 3050, 9030
Suen, Jonathan J.: 2530, 3560, 4680
Sugawara, Ikuko: 6580, 6800, 6810
Sugg, Maggie: 4820
Sugiura, Aki: 6770
Suitor, J Jill: 1290, 2440, 4070, 4460, 6520
Sul, Hei Sook: 4241
Suliburk, James: 2241
Sullivan, Darcy: 2720
Sullivan, Dennis: 6420
Sullivan, Jennifer L.: 4770
Sullivan, Kevin: 2670, 6780
Sullivan, Marlysa: 1550
Sullivan, Suzanne S.: 6110, 6280
Sundermann, Erin E.: 9000
Sun, Fei: 1170, 1500, 4023, 4270, 5150, 6060, 9000
Sung, Liwen: 4450
Sung, Pildoo: 3160, 4170
Sun, Haosen: 6790
Sun, Luyang: 1470
Sun, Minglong: 6420
Sun, Na: 4820
Sun, Peter: 6500, 9010
Sun, Rongjun: 1100
Suntai, Zainab: 5172, 9030
Sun, Winnie: 6270, 9000
Sun, Xiaoyi: 6411
Sun, Yuxiang: 6010
Super, Nora: 1560, 3540
Supiano, Katherine: 1320, 2540, 9010
Suppiah, Sumithra: 3160
Surachman, Agus: 3070, 3440
Surage, Jayshree: 1550
Sur, Aparajita: 3160
Suriaga, Armiel A.: 4580
Suri, Anisha: 3690, 4340
Su, Tai-Te: 2050, 2220
Sutphin, George L.: 6840
Sutton, MaryAnn: 6130
Sutton, Natalie: 9020
Su, Wei-Hsuan: 6010
Su, Yan: 9030
Su, YanJhu: 1070, 3100, 6090, 6450, 6510
Su, Yun-Pei: 6800
Svec, Joseph: 4905, 6790
Swagger, Phildra: 6140
Swanson, Marilyn: 9030
Swartz, Ann: 9010
Swartzell, Kristen: 9030
Swartzendruber, Lora L.: 2720
Sweeder, Logan A.: 2680, 3720, 6420
Sweeny, Brian: 4360
Sweerts, Lieke: 1120
Sweet, Elisabeth: 9030
Swenor, Bonnielin K.: 2530, 3560, 4690
Swierenga, Sarah: 4770
Swisa, Orel: 3490
Syed, Sumaiyah U.: 5060
Sylvers, Dominique: 6310
Sylvester, Donna: 6220
Syme, Robin: 2600
Synder, Joel: 4905, 6170
Szanton, Sarah L.: 2510, 3330, 3560, 4310, 4640, 4680
Szcześniak, Dorota: 5170
Sze, Lesley: 9020
Szto, Peter: 6350
Szymkowski, Amanda: 6280

### T

T.V., Sekher: 2150
Taani, Murad H.: 6780
Tabler, Jennifer: 3460
Tabon, Patrick K.: 5173
Tabuchi, Takahiro: 6280
Tadros, Clarissa: 6670
Taeckens-Seabaugh, Ashley: 2730, 9020
Taffet, George E.: 2241, 2740, 9010
Tahija, Nina: 6530
Taitel, Michael: 6100
Takagi, Emiko: 6000
Takahashi, Paul: 6110
Takano, Yumi: 9000
Takase, Mai: 6240, 6280
Takayama, Midori: 6580, 6810
Takemoto, Yoshito: 9010
Takeya, Yasushi: 6110, 6450, 6770
Takiue, Keigo: 9030
Taki, Yasuyuki: 9000, 9010
Talbot, C. Conover: 6411
Talegawkar, Sameera: 4200
Taler, Vanessa: 6290
Talley, Kristine: 9000
Talley, Susan A.: 6510

Talmage, Lexi: 2340
Tamariz, Leonardo: 6140
Tamburri, Nicholas B.: 6590
Tamla Rai, Vijaya: 6690
Tanaka, Toshiko: 4200, 9010
Tan, Alai: 5090
Tan, Chenxin: 3870
Tan, Chuen Seng: 3620
Tan, Ern Yu: 3390
Tang, Ada: 3620
Tan, Gamaliel: 4220
Tang, Bin: 2450
Tan, Genesis: 6070, 6200
Tang, Fengyan: 4240, 6680
Tang, Jennifer Y.M.: 6110
Tang, Junhan: 2170, 4320
Tangpricha, Vin: 3290
Tang, Weizhou: 6540
Taniguchi, Yu: 6320
Tan, Jaron: 6580
Tan, Jeremy: 3620
Tanner, Jackie: 6230
Tan, Ngiap Chuan: 3160
Tan, Sharon: 6480
Tan, Shyuan Ching: 3270
Tan, Su Ming: 3390
Tan, Tira: 3390
Tan, Vanessa: 4220, 9020, 9030
Tan, Veronique Kiak Mien: 3390
Tan, Xiao-Di: 6010
Tan, Yi Wen: 3160
Tappeiner, Waltraud: 1530
Tappen, Ruth M.: 4220, 4580
Tarabochia, Dawn: 1280
Tarasenko, Lisa: 3830
Tarbert, Rebecca J.: 4020
tarima, sergey: 9010
Tarraf, Wassim: 2510, 3430, 4040, 4420, 9000
Tarr, Julie E.: 6090, 6230
Tate, Ashley: 6210, 6250
Tate, Judith A.: 5090
Tate, Rio S.: 6180
Tatewaki, Yasuko: 9000, 9010
Tavakoli-Tabasi, Shahriar: 9020
Tavares, Jane L.: 3320, 5140, 6140
Tay, Djin L.: 4530, 6310
Taylor, Britain M.: 6590
Taylor, Catherine: 6540
Taylor, Janiece L.: 3330, 3750, 4680
Taylor, Miles: 2200, 9020
Taylor, Philip: 2460
Taylor, Tonya: 3760
Taylor, Tracy A.: 6100, 6280
Tay, Sarah Siew Cheng: 3160
Team, MiaGB Consortium: 9000, 9010
Teas, Elizabeth: 6080, 6790
Teasley, Kenneth: 4023
Teaster, Pamela B. Teaster B.: 3920, 4130
Tecson, Kristen: 6100
Teerawattananon, Yot: 9030
Tefera, Gashaye Melaku: 1210, 4021
Teixeira, Laetitia: 6560
Tell, Eileen J.: 1260, 3940
Telonidis, Jacqueline: 2060
Temirova, Alisha: 9010
Templin, Thomas: 4470, 6420
Teno, Joan: 3200, 3830
Teresi, Jeanne A.: 2160
Terhune, Katherina N.: 4520
Teri, Linda: 4540
Tesema, Naomi: 6110
Teshale, Salom: 3940
Teshome, Abraham Z.: 6630
Testai, Fernando: 9000
Teupen, Sonja: 1270
Tewary, Sweta: 6220
Tezcan-Guentekin, Huerrem: 3610
Thackeray, Anne: 1120
Thaitrong, Phatt: 4680
Thangwaritorn, Pilar: 6570
Thanik, Erin: 1350
Tharpe, Rita: 6160
Tha, Soe Han A.: 2690
Thayer, Erin K.: 4890, 5173
Thelusma, Naomi: 4681
Thiamwong, Ladda: 2290, 3840
Thibault, Olivier: 6411
Thielke, Stephen: 6450
Thierry, Amy: 6290
Thillainadesan, Janani: 2090, 6280
Thoma, Myriam V.: 4225, 5100, 6580
Thomas Hebdon, Megan C.: 2070
Thomas, Ariane: 6060, 6220, 6280
Thomas, Duncan: 6580
Thomas, Kali: 3130, 3200, 3910, 4465, 4580, 4780, 4820, 5081, 9000
Thomas, Karuna S.: 9020
Thomas, Kelsey: 9000
Thomas, Kristi: 6730
Thomas, Michael L.: 6200
Thomas, Patricia A.: 6290, 6810
Thomeer, Mieke B.: 4225, 6200
Thomley, Amelia: 9030
Thompson, Alisha R.: 6500, 9020
Thompson, Amber: 4480
Thompson, Atalie C.: 2530
Thompson, Ben: 9000
Thompson, Katherine: 6220
Thompson, Larry W: 6750
Thompson, Lena: 3240, 6830
Thompson, Roy A.: 9010
Thompson, Sheri: 9030
Thomson, Cynthia: 6430
Thomson, Donna: 6000
Thorne, Claudia: 1250, 9030
Thorne, Trina: 2090
Thornton, Marleen: 9020
Thorpe, Jr., Roland J.: 2380, 2510, 3330, 3440, 3560, 6180, 6290
Thorpe, Carolyn: 9000
Thorvaldsson, Valgeier: 4620
Thrane, Lisa: 9020
Thurston, Rebecca C.: 3070
Thyagarajan, Bharat: 4810, 6010
Thygesen, Lau C.: 4530
Thyreau, Benjamin: 9010
Tian, Chenxin: 6740
Tian, Junyan: 3270
Tian, Qu: 4120, 4200, 4330, 4340
Tian, Yi Jiao (Angelina): 6400
Tibirica, Lize: 6200
Tiecker, Ana Paula: 3620
Tien, Hsiao-Chuan: 2080
Tiernan, Chad: 6510
Tighe, Patrick J.: 4840, 6320
Timinski, John: 6170
Tingvold, Maiken: 2750
Tissot, Juliana T.: 6240
Tjia, Jennifer: 6140
Tkatch, Rifky: 6580, 6790
Tobey, Cara: 6840
Tobey, Leah: 6000
Tobin, Courtney Sinclair S.: 3440
Tobon Vallejo, Diana: 4340
Todorova, Irina: 6210
Toki, Hiroshi: 6110, 6450, 6770
Tolea, Magdalena I.: 6070, 6720
Toledo, Frederico G.: 4600, 9030
Toles, Mark: 1160, 3482, 6760, 9000
Tollman, Stephen M.: 3040
Tolosa, Ezequiel: 9000
Tombline, Gregory: 2501, 6840
To-Miles, Flora: 9010
Tomioka, Michiyo: 6580
Tomita, Naoki: 9000
Tompkins, Catherine: 2260, 4510, 6280
Tonkikh, Orly: 2680
Tonkin, Andrew M.: 4320, 4683, 5083
Toof, Robin: 2610
Toosizadeh, Nima: 6510
Toplyn, Lavona: 6560
Torgé, Cristina Joy: 6640
Torioge, Trevor: 6010
Torke, Alexia M.: 6100, 6280
Torrance, Blake L.: 9010
Torres-Gil, Fernando: 4450, 3770
Torres, Sandra: 2040
Torres, Stacy: 6200
Torrisan, Maral: 6740
Toruner, Murat: 9000
Toto, Pamela E.: 4310, 4600
Tourette, Cendrine: 9010
Touron, Dayna R.: 2750
Townsend, Michelly: 2610
Towsley, Gail L.: 2060, 2310, 9020
Towsley, Kerri: 9010
Toyama, Itsuko K.: 6120
Toyama, Masahiro: 6290
Toyokawa, Noriko: 6520
Toyokawa, Teru: 6520
Tracy, Elizabeth: 3115
Tracy, Eunjin Lee: 4682
Tracy, Karen: 5010, 6260
Trainum, Katie: 1570
Tran Levan, Darius: 4681
Tran, Candy: 9000
Tran, Elizabeth: 6570
Tran, Isabelle: 2080
Tran, Nathaniel: 1320
Tran, Sydney J.: 6580
Tran, Thanh: 6641
Tran, Yvette: 6760
Trappe, Scott: 9010
Trappe, Todd: 9010
Traunstein, Deb: 9010
Traver, Anthony C.: 2010, 3770
Travers, Jasmine: 1460, 2650, 3170, 4130

Travison, Thomas: 3110, 3600, 9030
Traywick, LaVona S.: 1420, 6740
Tremaine, Wendy: 4023
Trenary, Tanner T.: 9000
Trepanier, Jessica: 6100
Trinh, Mimi: 1570
Trinh-Shevrin, Chau: 1480
Trinkoff, Alison: 6560
Tripathi, Utkarsh: 6840
Trivedi, Ranak: 1330, 4010, 6310, 6430, 6530
Troutman, Brooke: 1080, 6630
Trowbridge, Kimberly S.: 6590
Truitt, William: 6220
Trujillo Tanner, Corinna M.: 6580, 6680
Trujillo, Miguel: 4430
Tsadok Cohen, Michal: 9020
Tsai, Pao-Feng: 6180
Tsai, Su-Ling: 6140
Tse, Dwight Cheuk Kit: 2480
Tse, Hin Wing (Florence): 4730, 6180
Tseng, Han-Yun (Heidi): 5110
Tseng, Tung-Sung: 4060
Tsoi, Yuet Yan: 3820
Tsugihashi, Yukio: 2580
Tsukada, Noriko: 3920, 4440, 6630
Tsumura, Hideyo: 9030
Tucker, Gretchen G.: 1250, 6790
Tucker, Julia E.: 1100, 6170
Tucker-Roghi, Gina C.: 2060
Tucker-Seeley, Reginald: 4510, 6540
Tuft, Samantha E.: 5083
Tunalilar, Ozcan: 3200, 4022, 6540
Tung, Yi-Hsuan: 1500, 6240
Turan, Bulent: 6590
Turfah, Ali: 6220
Turiano, Nicholas A.: 2140, 4620, 6550, 9010
Turkelson, Angela: 2440, 3480
Turner, Arlener D.: 6680
Turner, Kelly: 6760
Turner, Natalie: 3670
Turner, Rachael: 9020
Turner, Shelbie: 1080, 2280, 5000
Turney, Indira: 4470
Tu, Wanzhu: 1390, 6270
Tu, Xin: 6490
Twal, Riad: 1250
Tyler, Denise A.: 3640, 4350
Tyler, Janice: 4360
Tyrrell, Daniel: 3241

U

Ucar, Duygu: 3001
Udina Argilaga, Cristina: 6450
Udoh, Idorenyin Imoh: 9020
Ugiliweneza, Beatrice: 6110
Uittenhove, Kim: 6380, 6580
Ukraintseva, Svetlana: 1000, 1390, 4290, 4810, 6411, 9000
Ulbricht, Christine: 6770, 9010
Ullal, Tarini: 9010
Umberson, Debra J.: 4460, 6190, 9030
Um, Sun-Bi: 6810
Undem, Teri: 6780
Une, Saki: 6000
Ungar, Lyle: 6170
Ungar, Rachel: 6790
Unroe, Kathleen T.: 1390, 3170, 4100, 6760, 6770
Uppalapati, Naveen: 9020
Ursal, DJ: 4190
Utz, Rebecca L.: 4480, 4530, 6310, 9010
Uva, Katherine: 3780
Vélez Ortiz, Daniel: 5150

V

Vadean, Florin: 6700
Vaeli Zadeh, Ali: 3483, 4021, 6740, 9020, 9030
Valadares, Kevin J.: 6220
Valdes McClure, Nicole: 9030
Vale, Michael T.: 4730
Van Vleet, Samuel C.: 4684, 6050
van Achterberg, Theo: 1160, 2090, 9000
van Antwerp, Kodi: 6300
Van Bogart, Karina: 6290
Van Den Heever, Carmen: 4890
van der Beek, Allard: 9020
van der Kaaij, Serena: 4550
van der Steen, Jenny T.: 3555, 6270
van der Velpen, Isabelle F.: 5170
van der Wees, Philip: 1120
van der Willik, Odette: 2300
Van Haitsma, Kimberly: 2340, 2680, 3310, 3520, 4681, 6130, 6420
van Heumen, Lieke: 2050
Van Houtven, Courtney H.: 5083
van Leeuwen, Theo T.: 4550
Van Lierop, Merel (E.A.): 4720, 9010
Van Maanen, Rob: 9010
Van Puymbroeck, Marieke: 2280, 3930
van Rossum, Erik: 4720, 9010
Van Vleet, Bryce: 3480, 9020
Van Vught, Anneke: 4720
van Zyl, Carin: 6120
Vander Meulen, Maria E.: 9000
Vander Wyk, Brent: 5100
Vanderboom, Catherine E.: 3390
VanderWerf, Jessica A.: 6000, 6220
VandeWeerd, Carla: 6390
VanHaitsma, Kimberly: 4360
VanHoven, Miri: 6411
Vanias, Kaitlyn J.: 6530
VanSwearingen, Jessie: 3690
Varadaraj, Varshini: 3560
Varadhan, Ravi: 1010
Varela, Alberto: 2060
Varela, Antonio: 6740
Vargas, Laura X.: 4225, 6180
Vargas, Roberto: 9020
Varghese, Joshua: 4021
Varner Perez, Shelley: 6100
Varteresian, Taya: 3750
Vasan, Shradha: 6790
Vasiliadis, Helen-Maria: 6740
Vasquez, Elizabeth: 2170
Vazquez, Julia: 2700, 4225, 6180
Veal, Britney: 6290
Vecchione, Katia: 4030
Vedder, Anneke: 4460
Veeramah, Krishna: 4620
Vega, William: 6770
Veillet, Stanislas: 9010
Velasquez, Alfredo J.: 3440
Velazquez, Nathan: 4020
Velez, Bianca: 6790
Veliz, Phillip: 4690
Vemuri, Prashanthi: 6780
Vendittelli, Rebecca: 6290
Venema, Dawn: 6170
Venetto, Angelina: 4080
Vera Murillo, Lizbeth C.: 9000
Vera, Daniel: 1140
Vera, Renzo: 9000
Verbeek, Frank: 4720
Verbeek, Hilde: 1160, 2090, 3555, 4300, 6560
Verble, Danielle: 2390
Verdery, Ashton: 4070, 6300, 6310, 9020
Vergara, Lizandra G.: 1570, 6240
Verghese, Joe: 4340, 6780, 9020
Vernooij-Dassen, Myrra: 5170
Vernooij, Meike: 5170
Versey, H. Shellae: 3290, 5080, 6350
Verspoor, Eline: 5170
Vetter, Valentin M.: 1010, 6010, 6641
Vickers, Jasmine K.: 6110, 6590
Vickerstaff, Victoria: 3555
Victor, Christina: 2690
Viejo, Henry: 6650
Vien, Tracey L.: 9000
Vilar Fernandes, Lara: 9020
Villalba, Madeline: 9000
Villamor, Eduardo: 4905, 6790
Villamor, Monique V.: 9000
Villarreal, Francisco: 9010
Villeneuve, Evan: 3860
Vilpert, Sarah: 2680, 3720, 4480, 6550, 6730
Vincent, David: 3290
Virani, Salim: 4683
Virnig, Beth A.: 9010
Visaria, Abhijit: 2150
Visser, Geraldine: 4550
Vivek, Sithara: 6010
Viviano, Jeffrey: 6411
Vivoda, Jonathon: 6230
Voelkner, Abigail R.: 6130
Vogel, Carlyn E.: 3910, 4465, 4650
Vogel, David: 1140
Vogelsmeier, Amy: 3170, 4210
Vogle, Sophie: 6210
Vogt, Jax-Patrick: 6290
Voigt-Antons, Jan-Niklas: 6110
Voll, Stacey: 3440, 3510, 5084, 6440, 9020
Volpi, Elena: 4150, 6740
Von Ah, Diane: 2640
Von Gaudecker, Jane: 9030
von Gunten, Armin: 6380

von Humboldt, Sofia: 6180
Vonk, Jet: 4470
Vos, Eline: 9020
Voss, Lauren: 6080
Vu, Christine: 2080
Vu, Long: 6510
Vu, Nguyen: 6290, 6600
Vu, Thi: 4850, 9020
Vydiswaran, Vinod: 6060
Wöhrmann, Anne: 6700

**W**

Wacker, Michael: 5001
Wada, Tadashi: 4440
Wade, Gina: 4241
Wade, Kate: 4520
Wadhwa, Rohan: 6070
Wadsworth, Sally J.: 9000, 9010, 9020
Wagenknecht, Lynne: 9000
Wagner, Gert G.: 2470
Wagner, Jennifer: 6090
Wagster, Molly: 3080
Wahl, Hans-Werner: 2450, 2750, 5110
Waite, Linda J.: 1230, 2550
Wakefield, Dorothy: 1310, 2150
Wakita, Anna: 6180
Wakui, Tomoko: 3010, 6390, 6400
Walcott, Quinnlyn: 6120
Waldegrave, Charles: 2410, 3770
Waldinger, Robert J.: 2620
Waldron, Danielle: 9030
Waldrop, Deborah: 3930
Waldrop, Drenna: 4030
Walker, Jacob: 6220
Walker, Keenan: 9000
Walker, Leah: 6420
Walker, Ruth: 6640
Wallace, Andrea: 3500
Wallace, Cara L.: 6720
Wallace, Lauren A.: 2050
Wallace, Meredith L.: 2620, 4080
Wallace, Ramona: 9030
Wallace, Sarah: 6310
Waller, Dylan E.: 1040, 6420
Wallhagen, Margaret: 4050, 6460
Wall, Melanie W.: 3600
Wallsworth, Christine: 9010
Walsemann, Katrina M.: 1410
Walsh, Christine E.: 5060
Walsh, Sarah: 3160, 6000
Walston, Jeremy: 1010, 4680, 6411
Waltrip, Leah: 6260
Wan, Emily: 4470
Wang, Carly: 9000
Wang, Chen-pin: 6320
Wang, Claire: 4860, 6500
Wang, Cuiling: 6780
Wang, Danny: 1390, 2250, 6110, 6590, 9000
Wang, Dingyue (Demy): 9020
Wang, Ethan B.: 3560
Wang, Fei: 2200, 3630, 6410
Wang, Feng: 4461
Wang, Haowei: 6310, 9020
Wang, Hongning: 5090
Wang, Hongying: 6010
Wang, Huijun: 2080
Wang, James: 9000
Wang, Jiangxia: 3560
Wang, Jing: 1540, 4190, 1130, 4210, 6140, 6450, 3481
Wang, Jinjiao: 4580, 6140, 6270
Wang, Kaipeng: 1540, 4700, 5150
Wang, Kefang: 6760
Wang, Li: 6500
Wang, Lin: 1200
Wang, Lu: 4905, 6790
Wang, Mengya: 4510, 4905, 6790
Wangmo, Tenzin: 6400
Wang, Pengcheng: 6110
Wang, Qin: 6140
Wang, Qiong (Annabel): 4241
Wang, Sammy: 1130
Wang, Shaohung: 6170
Wang, Shuangshuang: 6530, 6720
Wang, Siqi: 2560
Wang, Song: 4700
Wang, Virginia: 5083
Wang, Weijun: 6280
Wang, Xiao (Joyce): 3200, 4820, 5081
Wang, Xiaochuan: 6090, 6350, 9020
Wang, Xinhui: 3510
Wang, Xueqing: 9030
Wang, Xuzhi: 9020
Wang, Yaqi: 6760
Wang, Yi: 1090, 6120, 6500
Wang, Yihan: 5030
Wang, Yiwen: 6290
Wang, YuHsuan (Olivia): 6720
Wang, Yuxia: 4681, 9030
Wang, Zhendong: 6150
Wanigatunga, Amal A.: 4120, 4330, 4600, 6740
Wanigatunga, Sarah K.: 4330
Wan, Qiaoqin: 6150
Wänström, Linda: 6440
Wan, Yihan: 6010
Waring, Molly E.: 6450
Warner, David F.: 6510
Warner, David: 6450
Warner, Nafisseh: 6450
Warren-Findlow, Jan: 2050
Warren, Keith: 4370
Warren, Rachel: 6460
Warren, Stacie: 6010
Washington, Faith-Christina: 2220, 9020, 9030
Washington, Karla T.: 6720, 6730
Washington, Tiffany R.: 2250, 2630
Wasil, Bridgette: 9030
Wastesson, Jonas: 6200
Watanabe, Kentaro: 6390, 6400
Watanabe, Yuichi: 9020
Waters, Leland: 1110, 2260, 6000, 9010
Watkinson, Erica: 2520
Watkins, Yashika: 6160
Watman, Deborah: 9030
Watson, Amanda A.: 6420
Watson, Wendy: 4022, 9020
Watt, Jennifer: 9000
Watts, Amber: 6190, 6570, 6650, 6730
Watts, Chelsea D.: 3230
Watts, Elizabeth E.: 3482
Waziry, Reem: 2241
Weaver, Ashley A.: 9000
Weaver, France: 3160
Weaver, Raven H.: 4900, 6610
Webb, Christopher: 2530
Webber, Anna: 6310
Webber, Katey: 9030
Webber, Sandra: 6780, 9010
Webber, Sophia: 6120
Webb, Fern J.: 6140
Webb, Nicole: 3550
Webster, Britney: 4060
Webster, Katelyn E.: 4320
Webster, Noah J.: 6310, 6490
Weech-Maldonado, Robert: 3660, 4820
Wee, Hwee lin: 6770
Weeks, Delaney: 2520
Weems, Jacy: 9000
Wehry, Susan: 3540, 6700, 9020
Weibel, Robert: 2450
Weidmann, Rebekka: 2280
Wei, Jeanne: 6130, 6180, 6840
Weil, Joyce: 3610
Wei, Melissa Y.: 2300
Weingrad, Zach: 5091
Weir, David R.: 3000
Wei, Sijia: 6280, 9030
Weiskittle, Rachel E.: 6220
Weissberger, Gali: 6480, 6580
Weiss, David: 2750
Welch, Sarah: 6270
Welle, Kevin: 5091
Wellens, Nathalie: 6270
Wendel-Hummell, Carrie L.: 2720
Wennberg, Alexandra: 1030
Werner, Nicole E.: 9000, 9010
Wesley, Alan: 5050
West, Brady: 3000
Westerhof, Gerben: 5110
Westerlind, Björn: 9020
West, Jessica S.: 5140
Westmore, Megan R.: 3000, 9030
Westra, Jordan: 9000
Westrick, Ashly C.: 1050, 2270, 4110
West, Stephanie T.: 3270
Wetherbee, Maya: 9000
Wethington, Elaine: 2300, 5150, 9010
Whalen, Laura: 2490
Wharton, Tracy C.: 6120
Wharton, Whitney: 2390
Wheaton, Felicia V.: 3260
Wheeler, David: 6070
Wheeler, Jason: 9020
Wheeler, Joseph E.: 9020
Wheeler, Kara: 6270
Wheeler, Katie: 9020
Whetung, Cliff: 5130, 6680
Whitbourne, Susan K.: 3800, 6000
White, Avalon: 6070, 6580, 6680
White, Carole L.: 2420, 4790
White, Elizabeth L.: 5081
Whitehead, Brenda: 6290
White, Justin: 6520
White, Katie: 2010, 3770
White, Kellee: 2300, 4730
White, Kina: 9030
White, Lon R.: 6411
White, Renee: 6720

White, Weyling: 3100
Whitfield, Keith E.: 2380, 3270, 4400, 6290
Whitmer, Rachel A.: 3600
Whitson, Heather: 2670, 4290, 4690
Whitten, Justin M.: 1280
Whitt-Glover, Melicia: 6180
Whitworth, Tanya: 1450
Wicker, Ellen: 6170, 6790
Wickramasinghe, Nilmini: 9030
Wickson-Griffiths, Abigail: 3555
Wickwire, Emerson: 6740
Widman, Christy A.: 6430
Wiechart, Kelly A.: 3540, 6070
Wiegelmann, Henrik: 5170
Wiemers, Emily E.: 1450
Wiemken, Timothy: 3600
Wiese, Lisa A.: 2210
Wieser, Heike: 1530
Wihry, David C.: 3540, 6270, 6540, 6700, 9030
Wikiera, John: 3000
Wilber, Kathleen: 3010, 4370, 4510, 6310, 9000
Wilckens, Max: 6700
Wilcock, Donna: 4870
Wilczek, Michael P.: 5084
Wilder, Jocelyn: 1230
Wiley, Jessica: 4520
Wilk, Christa: 6150, 6370, 6390
Wilkie, Rachel: 5130
Wilkinson, Anna V.: 1480
Wilkinson, Joanne: 6770
Wilks, Scott E.: 9020
Willard, Meredith: 4620, 6550, 9010
Willard, Tajalei: 9000
Willcox, Bradley J.: 3580, 6010
Willcox, D. Craig: 6010
Willems, Jules: 5100
Williams, Alice: 4150
Williams, Alicia R.: 6480
Williams, Brie: 2510, 5120
Williams, Dominique: 4681
Williams-Farrelly, Monica: 1410
Williams, Ishan C.: 1030, 2070, 2260, 6060
Williams, Kai: 2060
Williams, Karen: 3180
Williams, Kristine N.: 2520, 2530, 3240, 4030, 5010
Williams, Kyreon: 4160
Williamson, Jeff D.: 2530, 4150
Williams, Randi: 9000
Williams, Teresa: 6100
Williman, Martha R.: 3530
Willis, David: 6350
Willroth, Emily C.: 2140, 2620
Wills, Aria: 6270
Wills, Celia E.: 3360, 4650
Wilmoth, Janet M.: 2660
Wiloughby, Orion: 5091
Wilroth, Emily: 4620
Wilson, Deborah H.: 2200, 4680
Wilson, Edwina: 6420, 6780
Wilson, Jeffrey S.: 5080
Wilson, Kenneth A.: 6010, 6840
Wilson, Lara: 6350
Wilson-Lawson, Kimberly S.: 3360
Wilson, Loretta: 6100
Wilson, Lucas: 6310, 9030
Wilson, Lucy: 6270
Wilson, Lynn: 9020
Wilson, Megan E.: 2430
Wilson, Paige: 2530
Wilson, Stephanie J.: 3150, 4470, 5060
Wilson, Steven: 6700
Wilson, Traci L.: 2020
Wiltshire, Jacqueline C.: 9010
Windham, Beverly Gwen: 2670, 3110, 3600, 6780
Wingenfeld, Klaus: 9000
Wingo, Anna: 6720
Wingood, Gina: 3760
Winkler, Eva: 5110
Winship, Jodi: 3670
Winstead, Vicki: 6750
Winters-Stone, Kerri: 9030
Wise-Swanson, Breanne: 6610
Wishart, Dona J.: 4023
Wisk, Patricia G F.: 9030
Wissler Gerdes, Erin: 6840
Wister, Andrew: 6500, 6530
Witonsky, Kailyn: 9030
Witt, Dean D.: 2150
Wittenberg, Elaine: 9020
Witter, Julie: 6220
Witt, Paul: 4900
Witzel, Dakota D.: 5140, 6520, 6580
Wladkowski, Stephanie P.: 3930, 6000, 6720
Wojczynski, Mary K.: 4600, 4810, 9030
Wolfe, Alexander: 9010
Wolfe, Megan: 3120, 6350
Wolff, Jennifer: 4640, 4680
Wolf, Katie E.: 5150
Wolf, Michael: 1030
Wolf-Ostermann, Karin: 5170, 4300
Wolk, David A.: 6010
Womack, Jennifer L.: 3482
Won, Chang Won: 6450, 6510, 6690, 6740, 6780, 9020
Wong, Alan: 3483, 6740, 9030
Wong, Alyssa: 6110
Wong, Angel: 9030
Wong, Bob: 2310
Wong, Chelsea N.: 5084
Wong, Edwin: 9020
Wong, Edwin Lok Yan: 3481
Wong, Gloria H.Y.: 3481, 6110, 9020
Wong, Jen: 3270, 6750
Wong, Karen Lok Yi: 9000
Wong, Lily: 9000
Wong, Nicole HL: 3481
Wong, Rebeca: 2580, 4225, 6300, 6330, 6710, 9010, 9020
Wong, Rita: 9030
Wongsin, Utoomporn: 6330
Wongyara, Nattaya: 6770
Woodard, John L.: 6290, 6440, 6450
Woodard, LeChauncy: 3540
Wood, Elizabeth Geneva: 3870
Woodhouse, Mark: 6540
Wood, Leila: 5100
Wood, Rachel: 3670
Woods, Diana Lynn: 9030
Wood, Sinai: 5172
Woods, Robyn L.: 4320, 4683, 5083, 6320
Woods, Tonja: 3460
Woodward, Amanda T.: 4770
Woo, Jean: 3500
Woo, Jihee: 6090
Wooten, Karen: 3510
Working Group, Part: 6010
Worthington, Amber K.: 6270
Wretman, Christopher J.: 3910
Wright, Gus A.: 6010
Wright, Kathy D.: 3360, 4650, 5090
Wright, Kirsten: 1340, 9000
Wright, Regina S.: 2380, 6170
Wroblewski, Kristen E.: 1230, 6110
Wrosch, Carsten: 2620
Wrublowsky, Robert: 9030
Wrzus, Cornelia: 1020, 2470, 6590
Wu, Barry: 5172
Wu, Bei: 1080, 1130, 1540, 3115, 3481, 3870, 4240, 4500, 6140, 6450
Wu, Chao-Yi: 1340, 2450, 4790, 4870
Wu, Chenkai: 1000, 2170, 4040, 4320, 6200
Wu-Chung, E. Lim Lydia: 3481, 9010
Wu, Deqing: 6411
Wuestefeld, Paulina: 9030
Wuestney, Katherine: 6420
Wu, Heng: 6350
Wu, Jia-Rong: 3930
Wu, Kuan-Ching: 6420, 9000, 9030
Wu, Li-Xue: 4450
Wu, Lizi: 6580
Wu, Qiao: 2080
Wurm, Annika I.: 6050
Wurm, Susanne: 5110
Wurtz, Heather: 1160, 3660, 6610
Wu, Shih-Cyuan: 6540
Wu, Shinyi: 1500, 6240, 6750, 9030
Wu, Shwu-Chong: 6350
Wu, Tsung-Chin: 6490
Wu, Wenbo: 3130, 6060
Wu, Wenmiao: 6530
Wu, Yanyan: 6180
Wu, Ying-Jung: 6780
Wyatt-Brown, Anne M.: 4660
Wylie, Molly: 3320
Wyte-Lake, Tamar: 2660, 2720

### X

Xanthakis, Vanessa: 3400
Xavier, Rose Mary: 9000
Xiang, Lucille: 9030
Xiang, Qingyan: 2501
Xiang, Wang: 9010
Xiao, Chunhong: 6750
Xiao, Qian: 4560
Xiao, Yan: 6770
Xidous, Dimitra: 9010
Xie, Bo: 1570, 6150, 6260, 6730
Xie, Rui: 2290, 3840
Xie, Sharon: 6140
Xie, Yixia: 5001
Xiong, Yiyi: 9010
Xuanrong, Zhang: 9000
Xu, Dongjuan: 6760, 9030
Xue, Lingshu: 9030
Xu, Emily L.: 6530
Xue, Qian-Li: 1010, 3110, 4680

Xue, Tingzhong (Michelle): 9020, 9030
Xue, Wei-Lin: 6630
Xu, Hanzhang: 1130, 1540, 6450
Xu, Hongwei: 3010
Xu, Jiayun: 9030
Xu, Lili: 3950, 6360
Xu, Ling: 1080, 6060, 6630
Xu, Mengyu: 9020
Xu, Qi: 6350
Xu, Qingwen: 6230
Xu, Shu: 3100, 6090, 6450, 6510
Xu, Wenyao: 6280
Xu, Yanfeng: 3740, 9030
Xu, Yunpeng: 9010

**Y**

Yadav, Hariom: 9000, 9010
Yadav, Uday Narayan: 6750
Yaffe, Kristine: 1430, 2670, 3560, 4560, 5030, 6110
Yahr, Andrea: 6530
Yakong, Vida N.: 1420
Yamaga, Ryonosuke: 6240
Yamaguchi, Yuko: 9000
Yamakawa, Miyae: 6110, 6450, 6770, 9030
Yamamoto, Hiroshi: 6450
Yamamoto-Mitani, Noriko: 4440
Yamamoto, Ryohei: 6110, 6450, 6770
Yamanaka, Takashi: 6240
Yamashita, Takashi: 4555, 6130, 6520, 6670, 6710
Yamazaki, Sachiko: 6580
Yam, Hawking: 1480, 6540
Yancura, Loriena: 3740
Yan, Elsie: 4090, 9020
Yang, Bo K.: 9000
Yang, Eunjin: 6390, 6460, 6650, 9010
Yang, Feng: 4023
Yang, Iris: 5060
Yang, Jiue-An: 2450
Yang, Kevin H.: 9000
Yang, Laiji: 3600
Yang, Luyan: 1500
Yang, Mia: 4150
Yang, Na: 1200
Yang, Rumei: 4682
Yang, Wei: 3870
Yang, Yang C.: 3115, 5060
Yang, Yulin: 6200
Yan, Jinjin: 4730
Yankeelov, Pamela: 1110
Yan, Mengzhao: 3900, 4370
Yannatos, Isabel: 6140
Yao, Jacqueline: 6010
Yao, Nengliang: 6530, 6720
Yaqub, Amber: 5170
Yashin, Anatoliy: 1000, 1390, 4290, 6010, 6110, 6411, 9000
Yashkin, Arseniy: 1000, 1390, 4290, 6110, 9000
Yata, Vinod K.: 9010
Yauch, John: 2590
Yau, Jessie Ho-Yin: 3481
Yauk, Jessica: 4650, 6290
Yau, Roger: 9030
Yeager, Brooke: 9020
Yeager, Valerie: 6760
Yee, Claire: 4681, 4850
Yefimova, Maria: 1390
Yeh, An-Yun: 4700
Yeh, Charlotte: 6170, 6580, 6790
Yeh, Jarmin C.: 3290, 5080, 9030
Yellseagle, Debara: 1060
Yelvington, Miranda: 9000
Ye, Minzhi: 2640
Yentes, Jennifer: 6170
Yeo, Hyesu: 6700, 6720
Yeow, Adrian: 4220
Yeung, Dannii: 3630, 6390
Ye, Wei: 6700
Yi, Eun-Hye G.: 6120
Yi, Julie: 4680
Yin, Cheng: 1090, 3540
Yin, Michael: 9000
Yi, Stella S.: 1480
Ylitalo, Kelly: 6510, 6650
Yoder, Karmen: 6220
Yokoyama, Yuri: 6320, 6710
Yonashiro-Cho, Jeanine: 3010
Yoneda, Tomiko: 2140, 6290
Yon, Yongjie: 9000
Yoo-Jeong, Moka: 4905, 6790
Yoo, Ji Won: 6420
Yoon, Hyun Woo: 3160
Yoon, Joonsik: 1450
Yoon, Jung Min: 6560
Yoon, Si On: 3830
Yoon, YeaSeul: 6270
Yoo, Subin: 6280
Yoquinto, Lucas: 3450
Yorgason, Jeremy B.: 2660, 6070, 6580, 6680
Yoshikawa, Aya: 6310
Yoshinaga, Shushi: 2680
Yoshino Benavente, Julia: 1030
Yost, Elizabeth: 6220
Young, Danielle: 9010
Young, Heather: 2680
Young, Kim: 3550
Young, Yuchi: 6090, 6720
Yousefzadeh, Matt: 2501, 3241, 6840
You, Suha: 6340
You, Tongjian: 1280, 9010
Yow, W. Quin: 2290, 3250, 3840, 6210, 6411
Yuan, 3. Jessie: 5173
Yuan, Fengpei: 9000
Yuan, Jing: 4640
Yuan, Keming: 4100
Yuan, Shuhan: 9020
Yuan, Yiyang: 6770, 9010, 9020
Yue, Lirong: 6530
Yu, Fang: 4580, 6270, 9000
Yu, Hsiao-Wei: 9010
Yu, Jiao: 3260, 6730
Yu, Kexin: 1500, 2570, 4870
Yu, Lei: 4820
Yung, Raymond: 6470
Yun, Ji-Young: 3330
Yun, Soo R.: 3810
Yu, Wanting: 3510, 9010, 9030
Yu, Wenshan: 1410
Yu, Xinfang: 6620
Yu, Xuexin: 2270, 3040

**Z**

Zaccaria, Daniele: 6380
Zachgo, Ashlyn: 9030
Zadeh, Rana: 9010
Žádník, Štěpán: 1210
Zafar, Anum: 4500
Zafar, Mateeha: 6060
Zaheed, Afsara: 1240
Zahodne, Laura B.: 1240, 1410, 2360, 3510, 4470, 4700
Zajdel, Melissa: 6170
Zakoul, Heidi: 6641
Zakrajsek, Andrea G.: 6000
Zakrajsek, Jennifer S.: 3000, 6470
Zakrzewski, Chris: 5172
Zambrano Garza, Elizabeth: 6210, 6390
Zammit, Andrea R.: 4700
Zamora, Ezequiel: 9030
Zamrini, Edward: 5173
Zanetti, Mariella: 5010
Zang, Emma: 1540
Zang, Ying: 1160
Zanier, Nicole: 3000, 6470
Zaninotto, Paola: 3210
Zanjani, Faika A.: 2510, 6140, 9010
Zannas, Anthony: 3440
Zanville, Noah: 9020
Zanwar, Preeti: 1060, 2640, 3870
Zúñiga, Franziska: 1530, 2090, 6270, 9000
Zaragoza, Gabriela: 4510, 9000
Zarit, Steven H.: 1450
Zarow, Chris: 6411
Zarowitz, Barbara J.: 3650
Zaslavsky, Oleg: 6420, 9000, 9030
Zausniewski, Jaclene: 3115, 5140, 6090, 6820
Zavala, Daisy V.: 4620
Zazdravnykh, Evguenii: 1060
Zazueta-Borboa, Jesús-Daniel Z.: 6330
Zeki Al Hazzouri, Adina: 9010
Zelin, Alexandra: 6640
Zelinski, Elizabeth M.: 5000, 6440
Zell, Angela: 2210, 9020
Zeng, Qing: 6770
Zhai, Wanting: 3400
Zhang, Alice: 6650
Zhang, Amanda: 1230
Zhang, Bing: 2080
Zhang, Bo: 4620
Zhang, Fan: 9000
Zhang, Hongqiao: 9020
Zhang, Jackie: 9020
Zhang, Jingwen: 9020
Zhang, Jingyun: 6411
Zhang, Keqing: 1080
Zhang, Lei: 2501, 6840
Zhang, Lingling: 2080
Zhang, Meng: 6530
Zhang, Ming: 4700
Zhang, Mutian: 1500, 3390, 6540
Zhang, Nan: 9020
Zhang, Peiyuan: 1390, 6730, 9000
Zhang, Shenghao: 1570, 6290
Zhang, Shifang: 6150
Zhang, Shiyang: 1450, 6460
Zhang, Tan: 6641
Zhang, Wei: 1080, 1100
Zhang, Weiwei: 4691
Zhang, Wen: 9020
Zhang, Xiaomin: 6840
Zhang, Xiaoyan: 6790
Zhang, Xinyao: 2080
Zhang, Xu: 3241
Zhang, Yalu: 9010
Zhang, Yan: 2400, 4730, 9000
Zhang, Yaxin: 4683, 9020
Zhang, Ye: 9000, 9010
Zhang, Yingyang: 6110

Zhang, Yuan S.: 2270, 3040, 9020
Zhang, Yuankai: 9020
Zhang, Yun: 3440, 3510
Zhang, Yuteng: 5001
zhang, Zhenmei: 3010
Zhang, Ziying: 6500
Zhan, Rong-Fang: 1090
Zhan, Yan: 9020
Zhao, Dandan: 2080
Zhao, Duzhi: 4530, 5070
Zhao, Erfei: 5000
Zhao, Hui: 1030, 9000
Zhao, Xiaopeng: 1250, 9000
Zhao, Xiaoyan: 6150
Zhao, Yuxin: 6750
Zhao, Zixuan: 4700
Zha, Peijia: 4700
Zheng, Anqing: 9020
Zhong, Jie: 3060
Zhong, Lily: 2150
Zhong, Xin: 4023, 9030
Zhong, Zi-Ting: 6540
Zhou, Gang: 6420
Zhou, Juan Helen: 6411
Zhou, Junhong: 3482, 3510, 4040, 9010
Zhou, Rui: 6720
Zhou, Shuai: 1400, 3820
Zhou, Weijiao: 4320
Zhou, Yuanjin: 1150, 4010, 6260
Zhou, Zehui L.: 4680
Zhou, Zexi: 1450, 6170
Zhou, Zeyu: 1470
Zhu, Huanzhi: 6140, 6450
Zhu, Jingmin: 3210
Zhu, Shanshan: 6760
Zhu, Shijun: 5171
Zhu, Xiaonan: 3250, 3690, 4340, 9030
Zhu, Xiaoqian: 2670
Zhu, Yating: 4430
Zhu, Yi: 6840
Zhu, Yujun: 4100
Zhu, Zheng: 3481
Ziade, Garnelle: 6380
Zigman Suchsland, Monica: 5010
Zilcha-Mano, Sigal: 3600
Zilioli, Samuele: 6200, 6290
Zimmerman, Kristin M.: 2260
Zimmerman, Sheryl: 3910, 4681
Zingmark, Magnus: 4220
Zionts, Nancy B.: 9030
Zisberg, Anna: 1160, 2680
Zmora, Rachel: 4060
Zmuda, Joseph M.: 3400, 4810, 6450
Zohn, Jennifer: 6770
Zonderman, Alan B.: 5060
Zou, Ning: 6150
Zubatsky, Max: 6790, 9000
Zubkoff, Lisa: 9010
Zucchero, Renee' A.: 4750
Zullo, Andrew: 4580
Zuoting, Nie: 4682
Zuraw, Matthew: 9010
Zurlo, Karen A.: 4380
Zu, Zhongliang: 4461
Zwakhalen, Sandra M.: 4720, 6270, 9010
